# Global, regional, and national age-sex specific mortality for 264 causes of death, 1980–2016: a systematic analysis for the Global Burden of Disease Study 2016

**DOI:** 10.1016/S0140-6736(17)32152-9

**Published:** 2017-09-16

**Authors:** GBD 2016 Causes of Death Collaborators, Amanuel Alemu Abajobir, Cristiana Abbafati, Kaja M Abbas, Foad Abd-Allah, Semaw Ferede Abera, Victor Aboyans, Olatunji Adetokunboh, Ashkan Afshin, Anurag Agrawal, Alireza Ahmadi, Muktar Beshir Ahmed, Amani Nidhal Aichour, Miloud Taki Eddine Aichour, Ibtihel Aichour, Sneha Aiyar, Fares Alahdab, Ziyad Al-Aly, Khurshid Alam, Noore Alam, Tahiya Alam, Kefyalew Addis Alene, Ayman Al-Eyadhy, Syed Danish Ali, Reza Alizadeh-Navaei, Juma M Alkaabi, Ala'a Alkerwi, François Alla, Peter Allebeck, Christine Allen, Rajaa Al-Raddadi, Ubai Alsharif, Khalid A Altirkawi, Nelson Alvis-Guzman, Azmeraw T Amare, Erfan Amini, Walid Ammar, Yaw Ampem Amoako, Nahla Anber, Hjalte H Andersen, Catalina Liliana Andrei, Sofia Androudi, Hossein Ansari, Carl Abelardo T Antonio, Palwasha Anwari, Johan Ärnlöv, Megha Arora, Al Artaman, Krishna Kumar Aryal, Hamid Asayesh, Solomon W Asgedom, Tesfay Mehari Atey, Leticia Avila-Burgos, Euripide Frinel G Avokpaho, Ashish Awasthi, Tesleem Kayode Babalola, Umar Bacha, Kalpana Balakrishnan, Aleksandra Barac, Miguel A Barboza, Suzanne L Barker-Collo, Simon Barquera, Lars Barregard, Lope H Barrero, Bernhard T Baune, Neeraj Bedi, Ettore Beghi, Yannick Béjot, Bayu Begashaw Bekele, Michelle L Bell, James R Bennett, Isabela M Bensenor, Adugnaw Berhane, Eduardo Bernabé, Balem Demtsu Betsu, Mircea Beuran, Samir Bhatt, Sibhatu Biadgilign, Kelly Bienhoff, Boris Bikbov, Donal Bisanzio, Rupert R A Bourne, Nicholas J K Breitborde, Lemma Negesa Bulto Bulto, Blair R Bumgarner, Zahid A Butt, Lucero Cahuana-Hurtado, Ewan Cameron, Julio Cesar Campuzano, Josip Car, Rosario Cárdenas, Juan Jesus Carrero, Austin Carter, Daniel C Casey, Carlos A Castañeda-Orjuela, Ferrán Catalá-López, Fiona J Charlson, Chioma Ezinne Chibueze, Odgerel Chimed-Ochir, Vesper Hichilombwe Chisumpa, Abdulaal A Chitheer, Devasahayam Jesudas Christopher, Liliana G Ciobanu, Massimo Cirillo, Aaron J Cohen, Danny Colombara, Cyrus Cooper, Benjamin C Cowie, Michael H Criqui, Lalit Dandona, Rakhi Dandona, Paul I Dargan, José das Neves, Dragos V Davitoiu, Kairat Davletov, Barbora de Courten, Barthelemy Kuate Defo, Louisa Degenhardt, Selina Deiparine, Kebede Deribe, Amare Deribew, Subhojit Dey, Daniel Dicker, Eric L Ding, Shirin Djalalinia, Huyen Phuc Do, David Teye Doku, Dirk Douwes-Schultz, Tim R Driscoll, Manisha Dubey, Bruce Bartholow Duncan, Michelle Echko, Ziad Ziad El-Khatib, Christian Lycke Ellingsen, Ahmadali Enayati, Holly E Erskine, Sharareh Eskandarieh, Alireza Esteghamati, Kara Estep, Carla Sofia e Sa Farinha, André Faro, Farshad Farzadfar, Valery L Feigin, Seyed-Mohammad Fereshtehnejad, João C Fernandes, Alize J Ferrari, Tesfaye Regassa Feyissa, Irina Filip, Samuel Finegold, Florian Fischer, Christina Fitzmaurice, Abraham D Flaxman, Nataliya Foigt, Tahvi Frank, Maya Fraser, Nancy Fullman, Thomas Fürst, Joao M Furtado, Emmanuela Gakidou, Alberto L Garcia-Basteiro, Teshome Gebre, Gebremedhin Berhe Gebregergs, Tsegaye Tewelde Gebrehiwot, Delelegn Yilma Gebremichael, Johanna M Geleijnse, Ricard Genova-Maleras, Hailay Abrha Gesesew, Peter W Gething, Richard F Gillum, Maurice Giroud, Giorgia Giussani, William W Godwin, Audra L Gold, Ellen M Goldberg, Philimon N Gona, Sameer Vali Gopalani, Hebe N Gouda, Alessandra Carvalho Goulart, Max Griswold, Rajeev Gupta, Tanush Gupta, Vipin Gupta, Juanita A Haagsma, Nima Hafezi-Nejad, Alemayehu Desalegne Hailu, Gessessew Bugssa Hailu, Randah Ribhi Hamadeh, Mitiku Teshome Hambisa, Samer Hamidi, Mouhanad Hammami, Jamie Hancock, Alexis J Handal, Graeme J Hankey, Yuantao Hao, Hilda L Harb, Habtamu Abera Hareri, Mohammad Sadegh Hassanvand, Rasmus Havmoeller, Simon I Hay, Fei He, Mohammad T Hedayati, Nathaniel J Henry, Ileana Beatriz Heredia-Pi, Claudiu Herteliu, Hans W Hoek, Masako Horino, Nobuyuki Horita, H Dean Hosgood, Sorin Hostiuc, Peter J Hotez, Damian G Hoy, Chantal Huynh, Kim Moesgaard Iburg, Chad Ikeda, Bogdan Vasile Ileanu, Asnake Ararsa Irenso, Caleb Mackay Salpeter Irvine, Sheikh Mohammed Shariful Islam, Kathryn H Jacobsen, Nader Jahanmehr, Mihajlo B Jakovljevic, Mehdi Javanbakht, Sudha P Jayaraman, Panniyammakal Jeemon, Vivekanand Jha, Denny John, Catherine O Johnson, Sarah Charlotte Johnson, Jost B Jonas, Mikk Jürisson, Zubair Kabir, Rajendra Kadel, Amaha Kahsay, Ritul Kamal, André Karch, Seyed M Karimi, Chante Karimkhani, Amir Kasaeian, Nigussie Assefa Kassaw, Nicholas J Kassebaum, Srinivasa Vittal Katikireddi, Norito Kawakami, Peter Njenga Keiyoro, Laura Kemmer, Chandrasekharan Nair Kesavachandran, Yousef Saleh Khader, Ejaz Ahmad Khan, Young-Ho Khang, Abdullah Tawfih Abdullah Khoja, Mohammad Hossein Khosravi, Ardeshir Khosravi, Jagdish Khubchandani, Aliasghar Ahmad Kiadaliri, Christian Kieling, Daniel Kievlan, Yun Jin Kim, Daniel Kim, Ruth W Kimokoti, Yohannes Kinfu, Niranjan Kissoon, Mika Kivimaki, Ann Kristin Knudsen, Jacek A Kopec, Soewarta Kosen, Parvaiz A Koul, Ai Koyanagi, Xie Rachel Kulikoff, G Anil Kumar, Pushpendra Kumar, Michael Kutz, Hmwe H Kyu, Dharmesh Kumar Lal, Ratilal Lalloo, Tea Lallukka Nkurunziza Lambert, Qing Lan, Van C Lansingh, Anders Larsson, Paul H Lee, James Leigh, Janni Leung, Miriam Levi, Yongmei Li, Darya Li Kappe, Xiaofeng Liang, Misgan Legesse Liben, Stephen S Lim, Patrick Y Liu, Angela Liu, Yang Liu, Rakesh Lodha, Giancarlo Logroscino, Alan D Lopez, Stefan Lorkowski, Paulo A Lotufo, Rafael Lozano, Timothy C D Lucas, Stefan Ma, Erlyn Rachelle King Macarayan, Emilie R Maddison, Mohammed Magdy Abd El Razek, Marek Majdan, Reza Majdzadeh, Azeem Majeed, Reza Malekzadeh, Rajesh Malhotra, Deborah Carvalho Malta, Helena Manguerra, Tsegahun Manyazewal, Chabila C Mapoma, Laurie B Marczak, Desalegn Markos, Jose Martinez-Raga, Francisco Rogerlândio Martins-Melo, Ira Martopullo, Colm McAlinden, Madeline McGaughey, John J McGrath, Suresh Mehata, Toni Meier, Kidanu Gebremariam Meles, Peter Memiah, Ziad A Memish, Melkamu Merid Mengesha, Desalegn Tadese Mengistu, Bereket Gebremichael Menota, George A Mensah, Tuomo J Meretoja, Atte Meretoja, Anoushka Millear, Ted R Miller, Shawn Minnig, Mojde Mirarefin, Erkin M Mirrakhimov, Awoke Misganaw, Shiva Raj Mishra, Ibrahim Abdelmageem Mohamed, Karzan Abdulmuhsin Mohammad, Alireza Mohammadi, Shafiu Mohammed, Ali H Mokdad, Glen Liddell D Mola, Sarah K Mollenkopf, Mariam Molokhia, Lorenzo Monasta, Julio C Montañez, Marcella Montico, Meghan D Mooney, Maziar Moradi-Lakeh, Paula Moraga, Lidia Morawska, Chloe Morozoff, Shane D Morrison, Cliff Mountjoy-Venning, Kalayu Birhane Mruts, Kate Muller, Christopher J L Murray, Gudlavalleti Venkata Satyanarayana Murthy, Kamarul Imran Musa, Jean B Nachega, Mohsen Naghavi, Aliya Naheed, Luigi Naldi, Vinay Nangia, Bruno Ramos Nascimento, Jamal T Nasher, Gopalakrishnan Natarajan, Ionut Negoi, Josephine Wanjiku Ngunjiri, Cuong Tat Nguyen, Quyen Le Nguyen, Trang Huyen Nguyen, Grant Nguyen, Minh Nguyen, Emma Nichols, Dina Nur Anggraini Ningrum, Vuong Minh Nong, Jean Jacques N Noubiap, Felix Akpojene Ogbo, In-Hwan Oh, Anselm Okoro, Andrew Toyin Olagunju, Helen E Olsen, Bolajoko Olubukunola Olusanya, Jacob Olusegun Olusanya, Kanyin Ong, John Nelson Opio, Eyal Oren, Alberto Ortiz, Majdi Osman, Erika Ota, Mahesh PA, Rosana E Pacella, Smita Pakhale, Adrian Pana, Basant Kumar Panda, Songhomitra Panda-Jonas, Christina Papachristou, Eun-Kee Park, Scott B Patten, George C Patton, Deepak Paudel, Katherine Paulson, David M Pereira, Fernando Perez-Ruiz, Norberto Perico, Aslam Pervaiz, Max Petzold, Michael Robert Phillips, David M Pigott, Christine Pinho, Dietrich Plass, Martin A Pletcher, Suzanne Polinder, Maarten J Postma, Farshad Pourmalek, Caroline Purcell, Mostafa Qorbani, Beatriz Paulina Ayala Quintanilla, Amir Radfar, Vafa Rahimi-Movaghar, Mohammad Hifz Ur Rahman, Mahfuzar Rahman, Rajesh Kumar Rai, Chhabi Lal Ranabhat, Zane Rankin, Puja C Rao, Goura Kishor Rath, Salman Rawaf, Sarah E Ray, Jürgen Rehm, Robert C Reiner, Marissa B Reitsma, Giuseppe Remuzzi, Satar Rezaei, Mohammad Sadegh Rezai, Mohammad Bagher Rokni, Luca Ronfani, Gholamreza Roshandel, Gregory A Roth, Dietrich Rothenbacher, George Mugambage Ruhago, Rizwan SA, Soheil Saadat, Perminder S Sachdev, Nafis Sadat, Mahdi Safdarian, Sare Safi, Saeid Safiri, Rajesh Sagar, Ramesh Sahathevan, Joseph Salama, Payman Salamati, Joshua A Salomon, Abdallah M Samy, Juan Ramon Sanabria, Maria Dolores Sanchez-Niño, Damian Santomauro, Itamar S Santos, Milena M Santric Milicevic, Benn Sartorius, Maheswar Satpathy, Maria Inês Schmidt, Ione J C Schneider, Sam Schulhofer-Wohl, Aletta E Schutte, David C Schwebel, Falk Schwendicke, Sadaf G Sepanlou, Edson E Servan-Mori, Katya Anne Shackelford, Saeid Shahraz, Masood Ali Shaikh, Mansour Shamsipour, Morteza Shamsizadeh, Jayendra Sharma, Rajesh Sharma, Jun She, Sara Sheikhbahaei, Muki Shey, Chloe Shields, Mika Shigematsu, Rahman Shiri, Shreya Shirude, Ivy Shiue, Haitham Shoman, Mark G Shrime, Inga Dora Sigfusdottir, Naris Silpakit, João Pedro Silva, Jasvinder A Singh, Abhishek Singh, Eirini Skiadaresi, Amber Sligar, David L Smith, Alison Smith, Mari Smith, Badr H A Sobaih, Samir Soneji, Reed J D Sorensen, Joan B Soriano, Chandrashekhar T Sreeramareddy, Vinay Srinivasan, Jeffrey D Stanaway, Vasiliki Stathopoulou, Nicholas Steel, Dan J Stein, Caitlyn Steiner, Sabine Steinke, Mark Andrew Stokes, Mark Strong, Bryan Strub, Michelle Subart, Muawiyyah Babale Sufiyan, Bruno F Sunguya, Patrick J Sur, Soumya Swaminathan, Bryan L Sykes, Rafael Tabarés-Seisdedos, Santosh Kumar Tadakamadla, Ken Takahashi, Jukka S Takala, Roberto Tchio Talongwa, Mohammed Rasoul Tarawneh, Mohammad Tavakkoli, Nuno Taveira, Teketo Kassaw Tegegne, Arash Tehrani-Banihashemi, Mohamad-Hani Temsah, Abdullah Sulieman Terkawi, J S Thakur, Ornwipa Thamsuwan, Kavumpurathu Raman Thankappan, Katie E Thomas, Alex H Thompson, Alan J Thomson, Amanda G Thrift, Ruoyan Tobe-Gai, Roman Topor-Madry, Anna Torre, Miguel Tortajada, Jeffrey Allen Towbin, Bach Xuan Tran, Christopher Troeger, Thomas Truelsen, Derrick Tsoi, Stefanos Tyrovolas, Kingsley N Ukwaja, Eduardo A Undurraga, Rachel Updike, Olalekan A Uthman, Benjamin S Chudi Uzochukwu, Job F M van Boven, Tommi Vasankari, Narayanaswamy Venketasubramanian, Francesco S Violante, Vasiliy Victorovich Vlassov, Stein Emil Vollset, Theo Vos, Tolassa Wakayo, Mitchell T Wallin, Yuan-Pang Wang, Elisabete Weiderpass, Robert G Weintraub, Daniel J Weiss, Andrea Werdecker, Ronny Westerman, Brian Whetter, Harvey A Whiteford, Tissa Wijeratne, Charles Shey Wiysonge, Belete Getahun Woldeyes, Charles D A Wolfe, Rachel Woodbrook, Abdulhalik Workicho, Denis Xavier, Qingyang Xiao, Gelin Xu, Mohsen Yaghoubi, Bereket Yakob, Yuichiro Yano, Mehdi Yaseri, Hassen Hamid Yimam, Naohiro Yonemoto, Seok-Jun Yoon, Marcel Yotebieng, Mustafa Z Younis, Zoubida Zaidi, Maysaa El Sayed Zaki, Elias Asfaw Zegeye, Zerihun Menlkalew Zenebe, Taddese Alemu Zerfu, Anthony Lin Zhang, Xueying Zhang, Ben Zipkin, Sanjay Zodpey, Ababi Zergaw Giref, Anwar Rafay, Emin Murat Tuzcu, Sergey Petrovich Ermakov, Peilin Shi, Parkash C Gupta

## Abstract

**Background:**

Monitoring levels and trends in premature mortality is crucial to understanding how societies can address prominent sources of early death. The Global Burden of Disease 2016 Study (GBD 2016) provides a comprehensive assessment of cause-specific mortality for 264 causes in 195 locations from 1980 to 2016. This assessment includes evaluation of the expected epidemiological transition with changes in development and where local patterns deviate from these trends.

**Methods:**

We estimated cause-specific deaths and years of life lost (YLLs) by age, sex, geography, and year. YLLs were calculated from the sum of each death multiplied by the standard life expectancy at each age. We used the GBD cause of death database composed of: vital registration (VR) data corrected for under-registration and garbage coding; national and subnational verbal autopsy (VA) studies corrected for garbage coding; and other sources including surveys and surveillance systems for specific causes such as maternal mortality. To facilitate assessment of quality, we reported on the fraction of deaths assigned to GBD Level 1 or Level 2 causes that cannot be underlying causes of death (major garbage codes) by location and year. Based on completeness, garbage coding, cause list detail, and time periods covered, we provided an overall data quality rating for each location with scores ranging from 0 stars (worst) to 5 stars (best). We used robust statistical methods including the Cause of Death Ensemble model (CODEm) to generate estimates for each location, year, age, and sex. We assessed observed and expected levels and trends of cause-specific deaths in relation to the Socio-demographic Index (SDI), a summary indicator derived from measures of average income per capita, educational attainment, and total fertility, with locations grouped into quintiles by SDI. Relative to GBD 2015, we expanded the GBD cause hierarchy by 18 causes of death for GBD 2016.

**Findings:**

The quality of available data varied by location. Data quality in 25 countries rated in the highest category (5 stars), while 48, 30, 21, and 44 countries were rated at each of the succeeding data quality levels. Vital registration or verbal autopsy data were not available in 27 countries, resulting in the assignment of a zero value for data quality. Deaths from non-communicable diseases (NCDs) represented 72·3% (95% uncertainty interval [UI] 71·2–73·2) of deaths in 2016 with 19·3% (18·5–20·4) of deaths in that year occurring from communicable, maternal, neonatal, and nutritional (CMNN) diseases and a further 8·43% (8·00–8·67) from injuries. Although age-standardised rates of death from NCDs decreased globally between 2006 and 2016, total numbers of these deaths increased; both numbers and age-standardised rates of death from CMNN causes decreased in the decade 2006–16—age-standardised rates of deaths from injuries decreased but total numbers varied little. In 2016, the three leading global causes of death in children under-5 were lower respiratory infections, neonatal preterm birth complications, and neonatal encephalopathy due to birth asphyxia and trauma, combined resulting in 1·80 million deaths (95% UI 1·59 million to 1·89 million). Between 1990 and 2016, a profound shift toward deaths at older ages occurred with a 178% (95% UI 176–181) increase in deaths in ages 90–94 years and a 210% (208–212) increase in deaths older than age 95 years. The ten leading causes by rates of age-standardised YLL significantly decreased from 2006 to 2016 (median annualised rate of change was a decrease of 2·89%); the median annualised rate of change for all other causes was lower (a decrease of 1·59%) during the same interval. Globally, the five leading causes of total YLLs in 2016 were cardiovascular diseases; diarrhoea, lower respiratory infections, and other common infectious diseases; neoplasms; neonatal disorders; and HIV/AIDS and tuberculosis. At a finer level of disaggregation within cause groupings, the ten leading causes of total YLLs in 2016 were ischaemic heart disease, cerebrovascular disease, lower respiratory infections, diarrhoeal diseases, road injuries, malaria, neonatal preterm birth complications, HIV/AIDS, chronic obstructive pulmonary disease, and neonatal encephalopathy due to birth asphyxia and trauma. Ischaemic heart disease was the leading cause of total YLLs in 113 countries for men and 97 countries for women. Comparisons of observed levels of YLLs by countries, relative to the level of YLLs expected on the basis of SDI alone, highlighted distinct regional patterns including the greater than expected level of YLLs from malaria and from HIV/AIDS across sub-Saharan Africa; diabetes mellitus, especially in Oceania; interpersonal violence, notably within Latin America and the Caribbean; and cardiomyopathy and myocarditis, particularly in eastern and central Europe. The level of YLLs from ischaemic heart disease was less than expected in 117 of 195 locations. Other leading causes of YLLs for which YLLs were notably lower than expected included neonatal preterm birth complications in many locations in both south Asia and southeast Asia, and cerebrovascular disease in western Europe.

**Interpretation:**

The past 37 years have featured declining rates of communicable, maternal, neonatal, and nutritional diseases across all quintiles of SDI, with faster than expected gains for many locations relative to their SDI. A global shift towards deaths at older ages suggests success in reducing many causes of early death. YLLs have increased globally for causes such as diabetes mellitus or some neoplasms, and in some locations for causes such as drug use disorders, and conflict and terrorism. Increasing levels of YLLs might reflect outcomes from conditions that required high levels of care but for which effective treatments remain elusive, potentially increasing costs to health systems.

**Funding:**

Bill & Melinda Gates Foundation.

## Introduction

Tracking age-sex-specific death rates by cause is an essential component of health surveillance. Recent health challenges such as the emergence of Zika and Ebola viruses, or the ongoing challenges of interpersonal violence, conflict, drug deaths, and natural disasters, affect health-system decision making.^1^, ^2^ Rapid progress to reduce mortality is possible for some causes, as evidenced by previously documented declines in central Europe for cardiovascular disease death rates or decreasing mortality from malaria in eastern sub-Saharan Africa.^3^ Trends in cause-specific mortality can inform decision makers about what programmes might be working, where progress lags behind, and the emergence of new or unexpected health challenges. The broader health agenda of the Sustainable Development Goals (SDGs) requires expanded tracking of a number of non-communicable diseases (NCDs) and injuries. Support for this expanded agenda in a world of complex health changes requires comprehensive, comparable, and timely estimates of causes of death by cause and by age, sex, location, and year.

**Research in context**

**Evidence before this study**

This paper builds on the Global Burden of Disease Study 2015 (GBD 2015). GBD 2015 provided estimates on 249 causes of death for 195 countries and territories, including subnational assessments for 11 countries from 1980 to 2015. GBD 2015 also provided analyses of causes of death in relation to the Socio-demographic Index (SDI)—a measure of per capita income, education, and total fertility. In addition, periodically updated estimates of causes of death are produced by WHO for a broad list of causes for all age groups, for cancers by the International Agency for Research on Cancer, and for child causes by the Maternal and Child Epidemiology Estimation group. Many groups also publish periodically on specific causes for a subset of locations. The GBD study remains the only peer-reviewed, comprehensive, and annual assessment of mortality by age, sex, cause, and location for a long time series that complies with the GATHER guidelines.

**Added value of this study**

GBD 2016 both provides estimates for 2016 and updates the entire time series from 1980 produced for GBD 2015. This update advances the measurement of deaths and years of life lost (YLLs) in several ways. First, greater data availability or policy interest supported several causes being removed from broader residual categories and separately assessed in the GBD cause hierarchy, including multidrug and extensively drug-resistant tuberculosis, alcoholic cardiomyopathy, urogenital congenital anomalies, and self-harm by firearm. Second, the terminal age group in all previous GBD analyses was 80 years and older; this age group has been separated into 80–84 years, 85–89 years, 90–94 years, and age 95 years and older. Third, we added 169 country-years of vital registration (VR) data at the national level and 24 verbal autopsy studies. Fourth, the verbal autopsy (VA) data collected through the Sample Registration System for the period 2004–13 were shared by the Government of India with the Indian Council of Medical Research for inclusion in the GBD analysis; these data included detailed International Classification of Diseases codes for deaths in each state, stratified by urban and rural residence. Fifth, we included data and expanded estimation to the level of local government areas for England and provinces in Indonesia. Sixth, we analysed and report on the fraction of deaths captured by VR systems that are assigned to major garbage codes. Seventh, we created a star rating system for the overall quality of cause of death data for each location in each year; this system represents VR completeness, percentage of deaths coded to causes that cannot be true underlying causes of death (garbage codes), detail of the cause list and age groups, and time periods covered. Eighth, we modelled antiretroviral therapy (ART) coverage for each location-year by CD4 count at initiation, age, and sex based on household survey data; this was a revision to the UNAIDS model assumption of ART coverage being highest among populations most in need. Ninth, important model improvements were implemented for malaria, tuberculosis, HIV/AIDS, and cancers. Tenth, we provide more exploration of the patterns of changing YLLs for SDI quintiles as assessed in 2016. Last, we explore the relation between rates of change and levels of age-standardised YLL rates.

**Implications of all the available evidence**

Quality and coverage of cause of death data are slowly improving, strengthening the basis for cause of death estimation; improved and sustained use and collection of data is an important contribution of the GBD study. Globally, age-standardised YLL rates have declined since 1980—particularly for communicable, maternal, neonatal, and nutritional diseases. However, age-standardised rates significantly increased by 2016 for some locations and a few causes, highlighting emerging challenges. Overall, global progress has generally been faster for the largest causes of YLLs than causes resulting in fewer YLLs, suggesting future shifts in the relative ranking of causes of premature mortality.

Several episodic efforts to estimate global and national mortality from specific diseases exist, as well as more limited efforts to estimate mortality from a comprehensive set of causes.^4^, ^5^, ^6^, ^7^, ^8^, ^9^, ^10^, ^11^, ^12^, ^13^, ^14^, ^15^, ^16^, ^17^ The latest assessment from the Maternal and Child Epidemiology Estimation (MCEE) group reports estimates for 15 cause groups of child death for 194 countries for the period 2000–15,^18^ while the Global Health Estimates (GHE) programme through WHO recently published estimates for 176 causes of death for 183 countries from 2000 to 2015.^19^ The Global Burden of Disease (GBD) study, however, provides the only annual, comprehensive assessment of a detailed set of underlying causes disaggregated by age, sex, location, and year, enhancing opportunities to make comparisons across time and between locations.

The primary objective of this study was to estimate mortality for 264 causes by sex for 23 age groups in 195 countries or territories from 1980 to 2016. This GBD cycle incorporates seven notable updates or changes: (1) new data sources released since GBD 2015; (2) data sources from earlier years that were published in the past year; (3) further disaggregation of national or subnational units for selected locations; (4) further disaggregation of residual causes into individual causes, particularly those of policy interest; (5) improved data-processing methods such as the redistribution of deaths assigned to International Classification of Diseases (ICD) codes that cannot be underlying causes of death (garbage codes); (6) model improvements for synthesising different sources of data and filling in data gaps; and (7) novel ways to visualise, summarise, or analyse results, such as by development status. These advances stem from both published critiques and recommendations from the extensive GBD network of 2518 collaborators from 133 countries and three territories. As with each annual cycle of GBD, the entire time series was re-estimated to ensure that all comparisons are made using a consistent dataset and methods; these results, therefore, supersede all previously published GBD cause of death estimates.

## Methods

### Overview

The GBD study provides a highly standardised approach to dealing with the multiple measurement challenges in cause of death assessment, including variable completeness of vital registration (VR) data, levels and trends in the fraction of deaths assigned to garbage codes, the use of verbal autopsy (VA) studies in locations with incomplete VR, and overall data missingness. Here we provide a general description, organised in 12 sections; detail is provided in the methods appendix (appendix 1 p 288). Statistical code used in estimation is available through an online repository; analyses were done using Python version 2.7.12 and 2.7.3, Stata version 13.1, and R version 3.2.2. As in GBD 2015, we follow the Guidelines for Accurate and Transparent Health Estimates Reporting (GATHER) for the development and documentation of GBD 2016 (appendix 1 p 292).

### Geographical units and time periods

The GBD geographical hierarchy includes 195 countries and territories grouped within 21 regions and seven GBD super-regions (appendix 1 p 460). For the GBD 2016 estimation, new subnational assessments were developed for Indonesia by province and for England by local government area. In this publication, we present subnational estimates for all countries with a population greater than 200 million in 2016: Brazil, China, India, Indonesia, and the USA. The likelihood of substantial geographical heterogeneity in these large populations is high, requiring disaggregated assessments to be policy relevant. Due to space limitations, we only provide these subnational estimates in maps; detailed subnational assessments will be provided in separate publications.

Cause-specific estimation for GBD 2016 covers the years 1980 to 2016. For a subset of analyses in this paper, we focus on the past decade, from 2006 to 2016, to address more current policy priorities. GBD 2016 results for all years and by location can be explored further with dynamic data visualisations.

### GBD cause list

For GBD, each death is attributed to a single underlying cause—the cause that initiated the series of events leading to death—in accordance with ICD principles. This categorical attribution of causes of death differs from the counterfactual approach, which calculates how many deaths would not have occurred in the absence of disease. GBD also differs from approaches involving excess mortality in people with disease monitored through cohort or other studies. Deaths in such studies might be assigned as the underlying cause, be causally related to the disease, or include deaths with confounding diagnoses.^3^

The GBD cause list is organised as a hierarchy (appendix 1 p 477), with each level composed of causes of death that are mutually exclusive and collectively exhaustive. The GBD cause hierarchy, with corresponding ICD9 and ICD10 codes, is detailed in appendix 1 (p 300). GBD Level 1 causes are grouped as three broad categories: communicable, maternal, neonatal, and nutritional (CMNN) diseases; NCDs; and injuries. Level 2 causes contain 21 cause groups, including subsets of CMNN causes, cancers, cardiovascular diseases, and types of injuries (eg, transport injuries, self-harm, and interpersonal violence). Individual causes are primarily recorded at Level 3 (eg, malaria, asthma, and road injuries), while a subset of Level 3 causes are disaggregated further to Level 4 causes (eg, four sub-causes within chronic kidney disease).

For GBD 2016, we disaggregated some Level 3 causes to expand the cause hierarchy used for GBD 2015 by 18 causes of death. GBD cause list expansion was motivated by two main factors: inclusion of causes that result in substantial burden and inclusion of causes that are of high policy relevance. New causes for GBD 2016 included Zika virus disease, congenital musculoskeletal anomalies, urogenital congenital anomalies, and digestive congenital anomalies. Other leukaemia was added as a Level 4 subcause to leukaemia rather than being estimated in the Level 3 residual category of other neoplasms. The Level 3 cause of collective violence and legal intervention was separated into “executions and police conflict” and “conflict and terrorism”. Disaggregation of existing Level 3 causes resulted in the addition of 11 detailed causes at Level 4 of the cause hierarchy: drug-susceptible tuberculosis, multidrug-resistant tuberculosis, and extensively drug-resistant tuberculosis; drug-susceptible HIV–tuberculosis, multidrug-resistant HIV–tuberculosis, and extensively drug-resistant HIV–tuberculosis; alcoholic cardiomyopathy, myocarditis, and other cardiomyopathy; and self-harm by firearm, and self-harm by other means. Within each level of the hierarchy the number of collectively exhaustive and mutually exclusive causes for which the GBD study estimates fatal outcomes is three at Level 1, 21 at Level 2, 145 at Level 3, and 212 at Level 4. For GBD 2016, separate estimates were developed for a total of 264 unique causes and cause aggregates.

### Sources of cause of death data

The GBD study combines multiple data types to assemble a comprehensive cause of death database. Sources of data included VR and VA data; cancer registries; surveillance data for maternal mortality, injuries, and child death; census and survey data for maternal mortality and injuries; and police records for interpersonal violence and transport injuries. Since GBD 2015, 24 new VA studies and 169 new country-years of VR data at the national level have been added. Six new surveillance country-years, 106 new census or survey country-years, and 528 new cancer-registry country-years were also added. An important development has been the release of the Sample Registration System (SRS) VA data by the Government of India for use in GBD. This includes cause of death data for 455 460 deaths covered by SRS from 2004–06, 2007–09, and 2010–13 across all Indian states and union territories. For this analysis, we established 2005, 2008, and 2012 as midpoint years for these three periods. The SRS in India is operated by the Office of the Registrar General of India working under the Ministry of Home Affairs, Government of India. Using the 2001 census, 7597 geographical units, 4433 (58·4%) of which were rural, were sampled for the 2004–13 SRS, ultimately covering a population of 6·7 million across all states and union territories.^20^ The inclusion of SRS for GBD 2016 offers a comprehensive picture of causes of death in India, particularly in rural areas. For a subset of causes, we used the India Medical Certification of Cause of Death (MCCD) data source or Survey of Causes of Death (SCD) data rather than SRS. The decision to use MCCD and SCD data in addition to SRS was limited to causes for which we had clear evidence of time trends not reflected by using the three SRS midpoint years alone (eg, maternal mortality). The Office of the Registrar General of India is not involved with the production of the GBD modelled estimates, and as a result their estimates might differ from those presented here. Methods for standardisation or correction of data sources are described in detail in appendix 1 (p 14).

### Socio-demographic Index (SDI) and epidemiological transition analysis

The SDI was developed for GBD 2015 to provide an interpretable synthesis of overall development, measured by the geometric mean of scores on relative scales of lag-dependent income per capita (LDI), average educational attainment in the population aged older than 15 years, and total fertility rates (TFR).^3^ For GBD 2016, the SDI was slightly revised; the correlation of the GBD 2015 and GBD 2016 versions of SDI is 0·977 (p<0·0001)—see Wang and colleagues^21^ for details on the changes. We estimated the relationship between SDI and each age-sex-cause death rate using Gaussian process regression (appendix 1 p 282). These relationships were used to estimate deaths and YLLs expected on the basis of SDI alone for each age-sex-location-year.

### Cause of death data standardisation and processing

Crucial steps in the standardisation of cause of death data include dealing with the small fraction of deaths that are not assigned an age or sex; deaths assigned to broad age groups not 5-year age groups; and various revisions of the ICD and national variants of the ICD. Details on the standardised protocols for these cases are provided in appendix 1 (p 9). A key step to the GBD cause of death database development is identifying and redistributing deaths assigned to ICD codes that cannot be underlying causes of death (eg, senility or low back pain); are intermediate causes of death rather than the underlying cause (eg, sepsis and heart failure); or lack specificity in coding (eg, unspecified cancer or unspecified cardiovascular disease). These so-called garbage codes are redistributed using the GBD method established by Naghavi and colleagues^22^ and explained in greater detail in appendix 1 (p 19). In brief, deaths coded in this manner were reassigned to likely causes of death using four approaches: proportional reassignment, regression models, fractional reassignment of a death assigned multiple causes, and redistribution based on fixed proportions. For each approach, garbage codes were redistributed by age, sex, location, and year.

**Figure 1:**
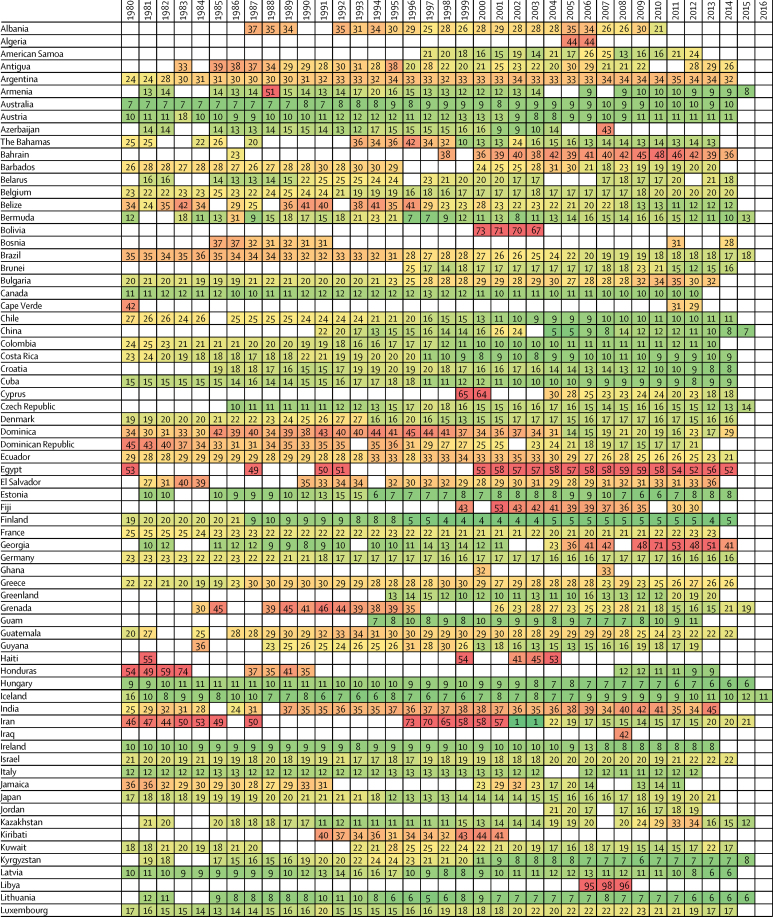

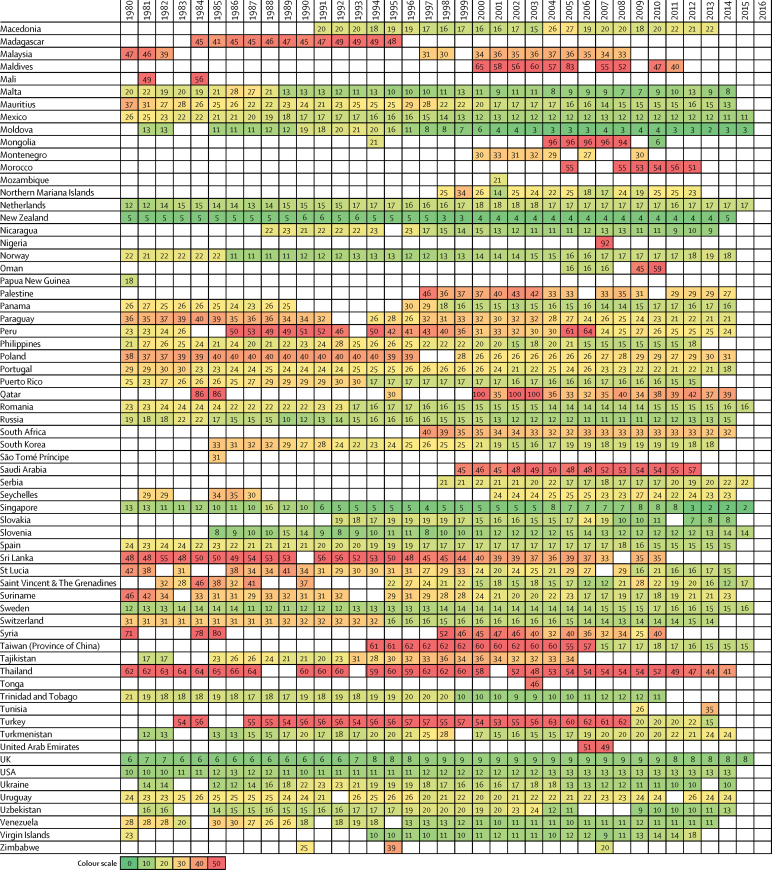
Percent of garbage coded deaths in GBD levels 1 and 2 for all ages by country and year, 1980–2016 Cells are colour-coded by percent of data redistributed in a given country-year from garbage coding to a likely underlying cause of death. Blank white cells indicate lack of vital registration. Major garbage codes are causes of death that are redistributed onto Level 1 or 2 of the GBD cause hierarchy. Countries without vital registration for 1980–2016 are not listed. High percentages of vaguely coded deaths highlight the importance of improvements in accurate reporting of mortality data to avoid redistribution of deaths to broad level causes.

The GBD cause hierarchy allows redistribution of garbage codes across different levels of specificity. For example, the garbage code “cancer, unspecified” contains sufficient detail to be redistributed across all cancers (at Level 3 of the cause hierarchy). We distinguish four levels of garbage codes based on the levels of the GBD cause hierarchy across which they are redistributed. Major garbage codes are those that are redistributed across causes that span Levels 1 and 2 of the GBD cause hierarchy such as heart failure or sepsis. Figure 1 shows the proportion of major garbage codes in VR data by location-year. The fraction of deaths assigned to major garbage codes varies widely, even across high SDI countries. Because of the potential for bias, data sources with location-years with more than 50% of deaths assigned to major garbage codes were excluded from the GBD analysis. Additional details on garbage code redistribution can be found in appendix 1 (p 19).

### Data completeness assessment

We assessed VR completeness by location-year as part of the GBD 2016 all-cause mortality analysis.^21^ Due to the potential for selection bias in incomplete VR, we excluded VR sources that were less than 50% complete in any given location. We also characterised sources as non-representative if they were estimated to be 50–70% complete. We used completeness estimates to inform variance of our statistical models, with lower completeness resulting in higher variance. Ultimately, all included sources were adjusted to 100% completeness by multiplying the cause fraction for a given location-age-sex-year by the estimated all-cause mortality for that location-age-sex-year. Appendix 1 (p 291) shows VA and VR availability and completeness by country from 1980 to 2016.

For GBD 2016, we developed a rating system that applies a level of 0 to 5 stars to describe the quality of data available for each country over the full time series from 1980 to 2016. These ratings were not used to directly adjust estimates; instead they provide context for interpreting the overall reliability of cause of death estimation for a location. Ratings were based on the fraction of deaths “well certified” in each location and time period; the latter was defined by six 5-year intervals and a terminal interval of seven years from 2000 to 2016. To qualify as well certified for each interval, we multiplied three measures: (1) completeness of death registration; (2) fraction of deaths not assigned to major garbage codes; and (3) fraction of deaths assigned to detailed GBD causes. Subnational VA data were multiplied by 0·10 because they might differ substantially from national results if they were available. VA data were further adjusted by 0·64, or the published chance-corrected concordance for physician-certified VA compared with medical certification of death.^23^ The percent of data well certified by location is provided in table 1; additional details on the selection of adjustment factors are in appendix 1 (p 31). By location and time interval, we assigned the following stars using bins that were arbitrarily selected but meant to capture a range of quality from highest to lowest: 5 stars if percent of data well certified equaled or exceeded 85%; 4 stars for 65% to less than 85%; 3 stars for 35% to less than 65%; 2 stars for 10% to less than 35%; 1 star for greater than 0% to less than 10%; and 0 stars for 0%. More detail on the calculations is provided in appendix 1 (p 31).

**Table 1:** Data quality rating from 0 to 5 stars, maximum percent well certified per 5-year interval and percent well certified across time series by country, 1980–2016

	**Data quality rating**	**1980–84**	**1985–89**	**1990–94**	**1995–99**	**2000–04**	**2005–09**	**2010–16**	**1980–2016**
Afghanistan	✭✩✩✩✩	0·0	0·0	0·0	0·0	4·6	33·5	0·0	5·4
Albania	✭✭✭✩✩	0·0	65·9	67·0	71·3	65·8	56·8	45·0	53·1
Algeria	✭✩✩✩✩	0·0	0·0	0·0	0·0	0·0	16·8	0·0	2·4
American Samoa	✭✭✭✩✩	0·0	0·0	0·0	78·6	81·0	83·7	71·0	44·9
Andorra	✩✩✩✩✩	0·0	0·0	0·0	0·0	0·0	0·0	0·0	0·0
Angola	✭✩✩✩✩	0·0	0·0	0·0	0·0	0·0	0·0	4·3	0·6
Antigua and Barbuda	✭✭✭✭✩	51·8	71·4	72·3	80·0	79·8	79·2	73·6	72·6
Argentina	✭✭✭✭✩	76·5	69·8	68·5	67·6	66·7	65·6	67·8	68·9
Armenia	✭✭✭✭✩	69·9	76·4	82·1	81·8	87·4	90·8	91·9	82·9
Australia	✭✭✭✭✭	93·1	93·1	92·4	92·4	91·3	90·5	90·3	91·9
Austria	✭✭✭✭✭	89·5	90·6	89·3	88·6	91·9	90·8	89·2	90·0
Azerbaijan	✭✭✭✩✩	71·7	74·0	79·7	74·3	73·2	42·9	0·0	59·4
The Bahamas	✭✭✭✭✩	74·6	79·7	63·8	78·0	80·2	79·8	77·6	76·3
Bahrain	✭✭✭✩✩	0·0	76·5	0·0	62·2	55·0	51·8	63·8	44·2
Bangladesh	✭✭✩✩✩	2·8	4·4	23·6	4·1	10·2	6·3	38·6	12·9
Barbados	✭✭✭✭✩	72·6	73·6	72·5	70·7	75·8	82·1	81·4	75·5
Belarus	✭✭✭✭✩	81·4	86·6	77·1	79·9	83·0	82·7	82·6	81·9
Belgium	✭✭✭✭✩	77·0	77·2	81·1	84·1	83·1	83·0	80·2	80·8
Belize	✭✭✭✭✩	54·0	56·9	46·8	76·9	71·6	80·7	84·7	67·4
Benin	✭✩✩✩✩	0·0	0·6	0·0	0·0	0·0	0·0	0·0	0·1
Bermuda	✭✭✭✭✭	89·0	86·5	84·7	90·9	89·4	86·4	90·5	88·2
Bhutan	✩✩✩✩✩	0·0	0·0	0·0	0·0	0·0	0·0	0·0	0·0
Bolivia	✭✩✩✩✩	0·0	0·0	0·0	0·0	12·4	0·0	0·0	1·8
Bosnia and Herzegovina	✭✭✩✩✩	0·0	64·4	64·5	0·0	0·0	0·0	68·8	28·3
Botswana	✩✩✩✩✩	0·0	0·0	0·0	0·0	0·0	0·0	0·0	0·0
Brazil	✭✭✭✭✩	58·3	62·4	65·0	69·8	75·0	80·4	82·7	70·5
Brunei	✭✭✭✩✩	0·0	0·0	0·0	85·4	82·9	81·9	81·8	47·4
Bulgaria	✭✭✭✭✩	80·4	80·7	79·7	76·0	71·8	73·5	70·3	76·1
Burkina Faso	✭✩✩✩✩	0·2	0·0	0·0	4·6	5·6	4·6	0·3	2·2
Burundi	✭✩✩✩✩	0·0	0·0	2·3	0·0	0·0	0·0	0·0	0·3
Cambodia	✭✩✩✩✩	0·0	0·0	0·0	0·0	1·6	3·5	0·0	0·7
Cameroon	✩✩✩✩✩	0·0	0·0	0·0	0·0	0·0	0·0	0·0	0·0
Canada	✭✭✭✭✭	88·6	89·8	88·3	88·2	89·6	90·1	90·1	89·3
Cape Verde	✭✭✩✩✩	58·3	0·0	0·1	0·0	0·0	0·0	69·7	18·3
Central African Republic	✩✩✩✩✩	0·0	0·0	0·0	0·0	0·0	0·0	0·0	0·0
Chad	✩✩✩✩✩	0·0	0·0	0·0	0·0	0·0	0·0	0·0	0·0
Chile	✭✭✭✭✩	75·5	75·1	76·6	84·8	90·9	90·3	90·0	83·3
China	✭✭✭✩✩	0·0	0·0	71·7	70·5	73·0	72·6	69·3	51·0
Colombia	✭✭✭✭✩	71·7	73·3	75·3	84·5	86·0	86·3	87·8	80·7
Comoros	✩✩✩✩✩	0·0	0·0	0·0	0·0	0·0	0·0	0·0	0·0
Congo (Brazzaville)	✩✩✩✩✩	0·0	0·0	0·0	0·0	0·0	0·0	0·0	0·0
Costa Rica	✭✭✭✭✭	79·8	81·8	80·2	91·2	91·8	89·8	90·8	86·5
Côte d'Ivoire	✭✩✩✩✩	0·0	1·0	1·0	0·0	0·0	0·2	0·2	0·4
Croatia	✭✭✭✭✩	0·0	82·7	83·7	80·7	84·1	86·5	87·9	72·2
Cuba	✭✭✭✭✭	84·6	84·6	83·2	88·3	90·1	91·0	91·5	87·6
Cyprus	✭✭✩✩✩	0·0	0·0	0·0	28·7	58·3	66·7	66·5	31·5
Czech Republic	✭✭✭✭✩	0·0	90·3	89·4	84·8	85·1	84·8	87·8	74·6
Democratic Republic of the Congo	✭✩✩✩✩	0·0	2·3	2·9	0·0	0·0	0·0	0·0	0·7
Denmark	✭✭✭✭✩	80·6	78·8	84·0	86·7	85·3	84·1	84·6	83·5
Djibouti	✩✩✩✩✩	0·0	0·0	0·0	0·0	0·0	0·0	0·0	0·0
Dominica	✭✭✭✭✩	70·4	61·5	62·1	62·9	69·5	85·3	83·6	70·7
Dominican Republic	✭✭✭✩✩	56·3	56·3	45·8	54·0	58·9	58·2	67·2	56·7
Ecuador	✭✭✭✭✩	71·6	68·1	67·7	63·7	61·6	66·4	68·2	66·8
Egypt	✭✭✭✩✩	33·3	46·9	43·7	0·0	42·9	40·6	48·4	36·5
El Salvador	✭✭✭✩✩	72·8	0·0	57·8	63·4	65·6	66·6	64·0	55·7
Equatorial Guinea	✩✩✩✩✩	0·0	0·0	0·0	0·0	0·0	0·0	0·0	0·0
Eritrea	✩✩✩✩✩	0·0	0·0	0·0	0·0	0·0	0·0	0·0	0·0
Estonia	✭✭✭✭✭	89·0	90·9	93·7	93·0	92·0	93·8	93·8	92·3
Ethiopia	✭✭✩✩✩	0·0	1·1	2·3	0·6	4·8	46·6	45·5	14·4
Federated States of Micronesia	✩✩✩✩✩	0·0	0·0	0·0	0·0	0·0	0·0	0·0	0·0
Fiji	✭✭✩✩✩	0·0	0·0	0·0	33·2	56·6	58·8	63·4	30·3
Finland	✭✭✭✭✭	81·1	90·5	91·6	95·7	95·7	94·5	95·6	92·1
France	✭✭✭✭✩	76·2	78·0	78·1	78·7	79·1	79·4	77·9	78·2
Gabon	✩✩✩✩✩	0·0	0·0	0·0	0·0	0·0	0·0	0·0	0·0
Georgia	✭✭✭✭✩	85·9	83·2	78·0	74·2	77·6	51·2	58·7	72·7
Germany	✭✭✭✭✩	77·5	78·2	83·1	83·9	83·2	83·6	84·0	81·9
Ghana	✭✩✩✩✩	0·0	0·1	1·6	0·9	8·6	20·8	0·5	4·6
Greece	✭✭✭✭✩	79·7	81·1	71·3	71·9	72·2	76·5	74·1	75·3
Greenland	✭✭✭✩✩	0·0	0·0	0·0	90·2	89·7	89·7	87·8	51·1
Grenada	✭✭✭✭✩	69·9	61·4	62·0	60·7	77·3	76·3	83·8	70·2
Guam	✭✭✭✩✩	0·0	0·0	89·0	85·9	77·1	71·8	66·1	55·7
Guatemala	✭✭✭✭✩	79·2	70·5	71·5	70·8	67·9	70·7	73·4	72·0
Guinea	✭✩✩✩✩	0·0	0·0	0·0	3·3	0·0	0·0	0·0	0·5
Guinea-Bissau	✭✩✩✩✩	0·0	0·0	0·1	1·1	0·0	0·0	0·0	0·2
Guyana	✭✭✭✭✩	51·5	71·7	64·0	66·2	79·0	77·7	73·5	69·1
Haiti	✭✩✩✩✩	19·3	1·4	1·1	10·6	4·6	0·0	0·0	5·3
Honduras	✭✭✩✩✩	31·7	36·9	35·6	0·4	0·0	12·4	13·9	18·7
Hungary	✭✭✭✭✭	90·6	89·3	89·9	90·8	92·6	93·3	93·6	91·4
Iceland	✭✭✭✭✭	91·3	92·8	94·0	94·1	93·5	92·8	91·4	92·8
India	✭✭✩✩✩	3·6	3·5	3·7	4·9	5·2	52·8	49·1	17·5
Indonesia	✭✭✩✩✩	0·1	0·0	1·3	0·4	0·1	42·8	56·7	14·5
Iran	✭✭✭✩✩	13·3	13·0	0·0	31·3	91·5	60·7	71·7	40·2
Iraq	✭✩✩✩✩	0·0	0·0	0·0	0·0	0·0	32·2	0·0	4·6
Ireland	✭✭✭✭✭	90·1	91·1	91·5	90·7	90·6	92·5	92·4	91·3
Israel	✭✭✭✭✩	80·9	81·7	82·8	83·3	81·8	80·2	79·0	81·4
Italy	✭✭✭✭✭	88·5	87·8	87·7	87·3	88·2	88·7	87·7	88·0
Jamaica	✭✭✭✩✩	64·6	66·1	55·8	0·0	68·4	77·2	75·7	58·3
Japan	✭✭✭✭✩	82·5	80·8	80·5	87·6	84·9	84·3	81·2	83·1
Jordan	✭✭✩✩✩	0·0	0·0	0·0	1·0	68·2	76·3	64·2	30·0
Kazakhstan	✭✭✭✭✩	76·3	81·5	89·5	89·0	82·2	77·8	86·1	83·2
Kenya	✭✩✩✩✩	0·0	2·8	0·0	0·5	5·1	5·4	0·8	2·1
Kiribati	✭✭✩✩✩	0·0	0·0	43·7	69·1	34·4	0·0	0·0	21·0
Kuwait	✭✭✭✭✩	81·5	82·0	75·6	78·1	83·4	85·0	83·5	81·3
Kyrgyzstan	✭✭✭✭✩	71·0	76·4	71·0	73·0	85·9	87·7	90·9	79·4
Laos	✭✩✩✩✩	0·0	1·3	0·0	0·0	0·0	0·0	0·0	0·2
Latvia	✭✭✭✭✭	90·6	91·4	87·9	92·0	91·1	89·2	93·8	90·8
Lebanon	✭✩✩✩✩	0·0	2·2	0·0	0·0	0·0	0·0	0·0	0·3
Lesotho	✩✩✩✩✩	0·0	0·0	0·0	0·0	0·0	0·0	0·0	0·0
Liberia	✭✩✩✩✩	2·2	2·3	3·6	0·0	0·0	0·0	0·0	1·2
Libya	✭✩✩✩✩	0·0	0·0	0·0	0·0	0·0	3·6	0·0	0·5
Lithuania	✭✭✭✭✭	87·6	92·2	91·7	94·7	92·6	93·1	94·4	92·3
Luxembourg	✭✭✭✭✩	86·4	86·7	85·3	84·9	82·2	78·2	82·0	83·7
Macedonia	✭✭✭✩✩	0·0	0·0	80·1	81·5	81·6	78·9	74·6	56·7
Madagascar	✭✩✩✩✩	2·7	3·3	2·3	2·2	0·0	0·0	0·0	1·5
Malawi	✭✩✩✩✩	0·0	2·8	0·0	0·6	2·2	3·8	0·4	1·4
Malaysia	✭✭✩✩✩	19·3	0·0	0·0	32·0	36·5	40·8	0·0	18·4
Maldives	✭✭✩✩✩	0·0	0·0	0·0	0·0	44·1	48·4	60·2	21·8
Mali	✭✩✩✩✩	4·3	0·0	0·1	0·0	0·0	0·0	0·0	0·6
Malta	✭✭✭✭✭	81·0	84·5	88·4	90·0	89·0	93·0	90·9	88·1
Marshall Islands	✩✩✩✩✩	0·0	0·0	0·0	0·0	0·0	0·0	0·0	0·0
Mauritania	✩✩✩✩✩	0·0	0·0	0·0	0·0	0·0	0·0	0·0	0·0
Mauritius	✭✭✭✭✩	73·8	78·5	78·7	78·2	83·0	84·7	85·3	80·3
Mexico	✭✭✭✭✩	65·2	71·9	72·7	76·7	79·4	81·7	88·1	76·5
Moldova	✭✭✭✭✭	83·9	87·1	77·2	84·8	90·0	89·6	90·3	86·1
Mongolia	✭✭✩✩✩	0·0	0·0	62·9	0·0	3·3	4·6	81·4	21·8
Montenegro	✭✭✩✩✩	0·0	0·0	0·0	0·0	70·6	72·9	0·0	20·5
Morocco	✭✩✩✩✩	0·0	17·0	0·0	0·0	0·0	37·9	14·3	9·9
Mozambique	✭✩✩✩✩	0·0	0·0	0·0	0·1	7·0	56·6	0·0	9·1
Myanmar	✭✩✩✩✩	0·0	0·0	0·0	0·0	0·0	2·8	0·0	0·4
Namibia	✩✩✩✩✩	0·0	0·0	0·0	0·0	0·0	0·0	0·0	0·0
Nepal	✭✩✩✩✩	2·9	2·7	0·0	0·6	0·6	8·9	0·0	2·2
Netherlands	✭✭✭✭✩	88·2	85·8	84·9	84·0	82·3	83·3	83·3	84·5
New Zealand	✭✭✭✭✭	95·2	95·0	94·7	96·7	96·4	96·3	95·7	95·7
Nicaragua	✭✭✭✩✩	0·0	55·8	59·4	66·1	71·7	78·7	84·9	59·5
Niger	✭✩✩✩✩	0·0	0·0	0·0	0·0	0·0	35·9	0·0	5·1
Nigeria	✭✩✩✩✩	0·0	0·0	4·0	0·0	0·0	0·1	3·8	1·1
North Korea	✩✩✩✩✩	0·0	0·0	0·0	0·0	0·0	0·0	0·0	0·0
Northern Mariana Islands	✭✭✭✩✩	0·0	0·0	0·0	75·3	75·3	72·3	55·2	39·7
Norway	✭✭✭✭✭	78·6	89·2	88·4	88·3	86·4	84·2	83·0	85·4
Oman	✭✭✩✩✩	0·0	0·0	0·0	0·0	0·0	71·0	33·0	14·9
Pakistan	✭✩✩✩✩	0·0	2·9	1·4	0·0	0·8	11·5	0·0	2·4
Palestine	✭✭✩✩✩	0·0	0·0	0·0	29·0	29·1	28·2	29·7	16·6
Panama	✭✭✭✭✩	69·2	71·6	0·0	79·0	82·2	84·1	84·1	67·2
Papua New Guinea	✭✩✩✩✩	8·2	3·4	0·0	0·0	0·0	0·0	0·0	1·7
Paraguay	✭✭✭✩✩	55·1	51·4	59·0	62·6	60·0	62·6	65·7	59·5
Peru	✭✭✭✩✩	58·9	34·4	36·5	48·2	60·3	60·2	60·4	51·3
Philippines	✭✭✭✭✩	71·7	73·8	65·8	65·9	72·6	72·4	71·8	70·6
Poland	✭✭✭✭✩	62·5	60·3	60·4	71·6	74·2	73·6	71·9	67·8
Portugal	✭✭✭✭✩	76·8	77·1	76·1	74·2	78·8	77·5	79·8	77·2
Puerto Rico	✭✭✭✭✩	77·1	74·6	79·9	83·4	84·0	84·0	84·7	81·1
Qatar	✭✭✩✩✩	8·4	10·0	0·0	51·6	48·2	56·2	44·0	31·2
Romania	✭✭✭✭✩	77·4	78·5	83·3	84·8	85·5	86·2	85·5	83·0
Russia	✭✭✭✭✭	81·6	88·4	87·8	84·6	87·6	88·9	88·4	86·8
Rwanda	✭✩✩✩✩	0·0	0·0	0·0	0·0	0·0	2·5	0·0	0·4
Saint Lucia	✭✭✭✭✩	69·3	66·2	70·6	72·5	79·2	78·4	85·2	74·5
Saint Vincent and the Grenadines	✭✭✭✭✩	71·6	61·1	58·6	79·0	81·0	83·0	87·5	74·5
Samoa	✩✩✩✩✩	0·0	0·0	0·0	0·0	0·0	0·0	0·0	0·0
Saõ Tomé and Príncipe	✭✩✩✩✩	0·0	69·0	0·0	0·0	0·0	0·0	0·0	9·9
Saudi Arabia	✭✭✩✩✩	0·0	0·0	0·0	26·3	31·7	34·6	34·5	18·2
Senegal	✭✩✩✩✩	2·0	2·4	2·6	2·5	0·0	0·0	0·0	1·4
Serbia	✭✭✭✩✩	0·0	0·0	0·0	73·1	75·1	79·7	77·9	43·7
Seychelles	✭✭✭✩✩	69·9	63·6	0·0	0·0	75·9	77·0	78·1	52·1
Sierra Leone	✭✩✩✩✩	0·0	0·0	3·8	0·0	0·0	0·0	0·0	0·5
Singapore	✭✭✭✭✭	89·1	89·6	95·0	95·3	95·1	92·5	97·8	93·5
Slovakia	✭✭✭✩✩	0·0	0·0	82·4	82·7	85·2	90·3	92·9	61·9
Slovenia	✭✭✭✭✩	0·0	89·4	91·1	88·8	88·3	87·4	87·3	76·0
Solomon Islands	✩✩✩✩✩	0·0	0·0	0·0	0·0	0·0	0·0	0·0	0·0
Somalia	✩✩✩✩✩	0·0	0·0	0·0	0·0	0·0	0·0	0·0	0·0
South Africa	✭✭✩✩✩	0·0	0·0	0·8	45·2	51·9	52·6	57·0	29·6
South Korea	✭✭✭✩✩	0·0	57·8	74·6	75·3	84·6	81·5	80·9	65·0
South Sudan	✩✩✩✩✩	0·0	0·0	0·0	0·0	0·0	0·0	0·0	0·0
Spain	✭✭✭✭✩	76·7	78·9	80·1	83·3	83·2	84·0	85·4	81·7
Sri Lanka	✭✭✭✩✩	51·8	50·9	46·5	55·5	63·6	67·4	65·5	57·3
Sudan	✩✩✩✩✩	0·0	0·0	0·0	0·0	0·0	0·0	0·0	0·0
Suriname	✭✭✭✩✩	59·7	62·1	58·6	58·5	66·0	64·9	65·1	62·1
Swaziland	✭✩✩✩✩	0·0	0·0	0·0	0·0	0·0	0·0	0·0	0·0
Sweden	✭✭✭✭✭	87·6	88·4	88·0	87·0	85·9	85·4	84·8	86·7
Switzerland	✭✭✭✭✩	69·3	69·2	68·3	84·6	84·4	86·6	86·1	78·4
Syria	✭✭✭✩✩	29·2	15·8	0·0	54·5	59·2	70·0	59·6	41·2
Taiwan (province of China)	✭✭✭✩✩	0·0	0·0	37·2	37·3	39·4	83·9	84·5	40·3
Tajikistan	✭✭✭✩✩	67·1	61·0	68·8	53·7	46·4	47·7	0·0	49·2
Tanzania	✭✩✩✩✩	0·0	3·1	1·9	1·8	4·9	2·6	0·0	2·1
Thailand	✭✭✭✩✩	28·4	27·1	33·9	47·7	47·7	52·0	57·5	42·1
The Gambia	✭✩✩✩✩	3·2	2·6	2·5	1·1	0·9	1·3	0·0	1·7
Timor-Leste	✩✩✩✩✩	0·0	0·0	0·0	0·0	0·0	0·0	0·0	0·0
Togo	✩✩✩✩✩	0·0	0·0	0·0	0·0	0·0	0·0	0·0	0·0
Tonga	✭✩✩✩✩	0·0	0·0	0·0	0·0	53·6	0·0	0·0	7·7
Trinidad and Tobago	✭✭✭✭✭	79·2	80·3	81·4	89·6	90·5	89·6	89·0	85·7
Tunisia	✭✩✩✩✩	0·0	0·0	0·0	0·0	0·0	28·8	24·7	7·6
Turkey	✭✭✭✩✩	16·9	20·7	22·1	24·9	37·4	72·8	84·4	39·9
Turkmenistan	✭✭✭✭✩	83·9	86·0	79·7	74·1	65·5	66·8	70·6	75·2
Uganda	✭✩✩✩✩	0·0	0·0	0·0	0·0	0·0	2·7	0·0	0·4
Ukraine	✭✭✭✭✭	84·7	87·8	81·0	83·5	83·8	89·0	90·4	85·7
United Arab Emirates	✭✩✩✩✩	0·0	0·0	0·0	0·0	0·0	36·5	0·0	5·2
UK	✭✭✭✭✭	93·1	93·9	93·9	91·9	91·4	91·4	91·3	92·4
Northern Ireland	✭✭✭✭✭	91·5	93·6	93·8	93·6	91·7	91·9	92·5	92·6
Scotland	✭✭✭✭✭	94·3	93·9	93·1	92·4	93·7	93·4	93·0	93·4
Wales	✭✭✭✭✭	90·2	93·5	92·5	93·2	92·0	91·9	92·2	92·2
England	✭✭✭✭✭	93·4	94·0	94·0	91·7	91·1	91·2	91·9	92·5
USA	✭✭✭✭✭	90·3	89·0	89·5	88·8	88·0	87·3	86·9	88·5
Uruguay	✭✭✭✭✩	76·3	75·6	77·2	79·1	79·2	78·6	75·7	77·4
Uzbekistan	✭✭✭✭✩	82·6	85·2	80·0	72·1	61·1	63·0	65·3	72·8
Vanuatu	✩✩✩✩✩	0·0	0·0	0·0	0·0	0·0	0·0	0·0	0·0
Venezuela	✭✭✭✭✩	79·2	74·3	81·9	87·8	89·9	89·5	89·0	84·5
Vietnam	✭✩✩✩✩	0·0	0·5	0·1	0·4	0·0	44·1	3·4	6·9
Virgin Islands	✭✭✭✩✩	73·2	0·0	81·6	84·9	72·0	67·9	60·5	62·9
Yemen	✩✩✩✩✩	0·0	0·0	0·0	0·0	0·0	0·0	0·0	0·0
Zambia	✭✩✩✩✩	0·0	0·0	0·0	0·0	0·0	5·4	5·5	1·6
Zimbabwe	✭✭✩✩✩	0·0	0·0	32·5	35·3	0·0	23·8	0·0	13·1

Maximum values of percent well certified within each 5-year interval, as well as a data quality rating from 0 to 5 stars and the percent well certified over the entire time series (1980–2016) are shown for each country. “Percent well certified” is calculated as described in appendix 1 (p 31). Values of 0 indicate no vital registration or verbal autopsy data with sufficient detail for the 5-year interval. Countries are given 0 to 5 stars depending on the percent well certified for the full time series (1980–2016). Classification is as follows: 85–100%, 5 stars; 65–84%, 4 stars; 35–64%, 3 stars; 10–34%, 2 stars; >0–9%, 1 star; 0%, 0 stars. Instances in the table that show 1 star despite all zeros in percent well certified are a result of very small values that round to 0 at one decimal place.

### Cause of death estimation

In GBD, the vast majority of cause of death estimates are modelled using the Cause of Death Ensemble model (CODEm). Due to their unique epidemiology or known biases, a subset of causes of death are modelled using alternative estimation strategies: negative binomial models for relatively rare causes, incidence and case fatality models, subcause proportion models, and prevalence-based models. The estimation of HIV/AIDS also requires a different modelling approach;^21^ and in previous publications.^3^, ^21^, ^24^ Due to lags in reporting, estimates for the most recent years rely more on the modelling process. Additional details on CODEm and all alternative estimation strategies are provided below and in appendix 1 (p 33 and p 35).

Major methodological changes from GBD 2015 were made for several models in GBD 2016: the distribution of antiretroviral therapies (ART) in countries with high HIV/AIDS prevalence were modelled based on an empirical pattern derived from household studies rather than on the assumption that ART was allocated to those individuals most in need; tuberculosis was modelled for prevalence of disease and then for prevalence of latent infection, which were then used as covariates for the CODEm model; malaria in high-endemicity Africa was estimated using a pixel-level geospatial model, while malaria outside of Africa was estimated using a new suite of spatiotemporal covariates in CODEm; and cancer mortality-to-incidence data inclusion and modelling were revised to better capture the likely effects of worse access to treatment in lower-SDI settings.

#### CODEm

CODEm, used for 177 causes of death for GBD 2016, is the GBD cause of death estimation approach in which a large number of model specifications are systematically tested in terms of functional forms and permutations of relevant covariates which are subsequently used to predict true levels for each cause of death.^25^, ^26^ CODEm uses multiple iterations of cross-validation tests to evaluate the out-of-sample predictive validity of model variants that met predetermined requirements for direction and significance of regression coefficients. These models were then combined into a weighted ensemble model, with models performing best on out-of-sample prediction error of both levels and trends weighted highest. Additional details of the methods used to develop these ensemble models are provided in appendix 1 (p 33). Independent CODEm models were run for each cause of death by sex, and separately for countries with and without extensive complete VR data. All data were included in models for countries without extensive VR coverage to enhance predictive validity; data from countries without extensive VR coverage were excluded from models for countries with this coverage to avoid inflation of uncertainty.

#### Negative binomial models

We used negative binomial models for nine causes of death (other intestinal infectious diseases; upper respiratory infections; diphtheria; varicella and herpes zoster; schistosomiasis; cysticercosis; cystic echinococcosis; ascariasis; and iodine deficiency) for which death counts are typically very low, or might frequently have zero counts in high-SDI countries.

#### Incidence and case fatality models

For causes in locations with insufficient data from VR or VA data, we used incidence and case fatality models—also known as natural history models—separately estimating incidence and case fatality rates and then combining them to produce estimates of cause-specific mortality. We used incidence and case fatality models for 14 causes: measles; visceral leishmaniasis; African trypanosomiasis; yellow fever; syphilis (congenital); typhoid fever; paratyphoid fever; whooping cough; Zika virus disease; and acute hepatitis A, B, C, and E. We also used an incidence and case fatality model for malaria incidence in sub-Saharan Africa as produced by the Malaria Atlas Project and age-sex-specific case fatality rates from available data.^27^

#### Subcause proportion models

For some causes—meningitis, maternal disorders, liver cancer, cirrhosis, and chronic kidney disease—data other than VR data provide considerable additional detail (eg, end-stage renal disease registries), or data are reported in too few places to be modelled directly in the CODEm framework. In these cases, we first estimated the parent cause using CODEm and then estimated subcauses by each age-sex-location-year using the Bayesian meta-regression tool DisMod-MR 2.1, developed for the GBD studies.^21^, ^26^, ^28^

#### Prevalence-based models

An increased likelihood of reporting Alzheimer's disease and other dementias, Parkinson's disease, and atrial fibrillation and flutter as underlying causes of death on death certificates has resulted in an apparent large increase in death rates associated with these diseases. The absence of a parallel increase of the same magnitude in reported rates of age-specific prevalence of these diseases supports the view that these changes are reporting artefacts rather than true changes in epidemiology. Because the redistribution algorithms used to build the cause of death database for previous iterations of GBD did not seem to adequately capture this trend in death certification over time for these causes, estimates for these three causes for GBD 2016 were derived from prevalence surveys and from estimates of excess mortality based on deaths certified in countries with the greatest proportion of deaths allocated to the correct underlying cause of death in recent years. The derivation of cause-specific mortality rates from prevalence and excess mortality models was completed in DisMod-MR 2.1.

### CoDCorrect algorithm for combining estimates

After generating underlying cause of death estimates and accompanying uncertainty, we combined these models into estimates that are consistent with the levels of all-cause mortality estimated for each age-sex-year-location group using a cause of death correction procedure (CoDCorrect). Using 1000 draws from the posterior distribution of each cause and 1000 draws from the posterior distribution of the estimation of all-cause mortality, we used CoDCorrect to rescale the sum of cause-specific estimates to equal the draws from the all-cause distribution (appendix 1 p 280). We introduced a change in the CoDCorrect algorithm to take into account that deaths from Alzheimer's disease and Parkinson's diseases are more likely miscoded to lower respiratory infections, protein-energy malnutrition, other nutritional deficiencies, cerebrovascular disease, interstitial nephritis and urinary tract infections, decubitus ulcer, and pulmonary aspiration and foreign body in airway than other causes (see appendix 1 p 279 for details).^29^, ^30^, ^31^

Fatal discontinuities occur when events such as military operations or terrorism, natural disasters, major transportation accidents, or large infectious disease outbreaks lead to abrupt departures from expected mortality rates in a given location. To capture these events, we used VR data for locations assigned a 4-star or 5-star data quality rating over the period from 1980 to 2016. For locations with a 3-star rating or lower (122 of 195 locations), we used the Uppsala Conflict Data Program for military operations and terrorism;^14^ the Centre for Research on the Epidemiology of Disasters' International Emergency Disasters Database for natural disasters, transport accidents, fires, exposure to mechanical forces (eg, building collapses, explosions), and famine;^32^ and the Global Infectious Diseases and Epidemiology Network for cholera and meningococcal meningitis. The latter two infectious diseases were included as fatal discontinuities for GBD 2016 because CODEm smooths year-to-year irregularities in deaths from these causes and thus risks underestimating their effects. There is frequently a lag in reporting and data publishing for the most recent years, so we used supplementary data sources, including news reports, when gaps existed for known fatal discontinuities. Detail on the data and analytic approaches used for fatal discontinuities is available in appendix 1 (p 39).

### YLL computation

As for GBD 2015, we calculated the years of life lost (YLLs)—a measure of premature mortality—from the sum of each death multiplied by the standard life expectancy at each age. For GBD 2016, the standard life expectancy at birth was 86·6 years, derived from the lowest observed risk of death for each 5-year age group; to avoid problems associated with small numbers, we restricted this to all populations greater than 5 million individuals in 2016. Age-standardised mortality rates and YLL rates were computed using the world standard population developed for the GBD study,^3^ which is a time-invariant standard. Details of these calculations are available in appendix 1 (p 281).

### Uncertainty analysis

Point estimates for each quantity of interest were derived from the mean of the draws, while 95% uncertainty intervals (UIs) were derived from the 2·5th and 97·5th percentiles. Uncertainty in the estimation is attributable to sample size variability within data sources, different availability of data by age, sex, year, or location, and cause-specific model specifications. We determined UIs for components of cause-specific estimation based on 1000 draws from the posterior distribution of cause-specific mortality by age, sex, and location for each year included in the GBD 2016 analysis. In this way, uncertainty could be quantified and propagated into the final quantities of interest. Limits on computational resources mean we do not propagate uncertainty in the covariates used by cause of death models. We remain unable to incorporate uncertainty from garbage code redistribution algorithms into our final estimates. When measuring changes over time, the change was considered statistically significant if the posterior probability of an increase (or decrease) was at least 95%—ie, if the mortality rate increased (or decreased) in at least 95% of the draws. Future methodological improvements that allowed the incorporation of more sources of uncertainty could result in currently marginally significant results no longer being significant within our definition.

### Role of the funding source

The funder of the study had no role in the study design, data collection, data analysis, data interpretation, or the writing of the report. All authors had full access to the data in the study and had final responsibility for the decision to submit for publication.

## ResultsData quality rating

**Figure 2:**
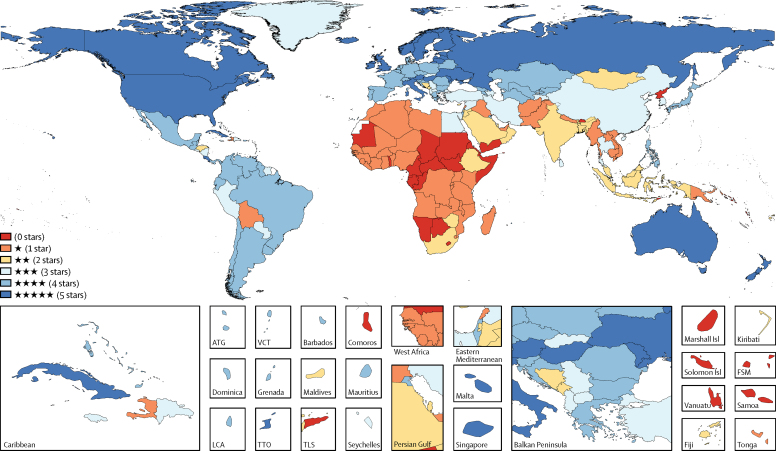
Classification of national time series of vital registration and verbal autopsy data, 1980–2016, on the basis of the fraction of deaths well certified and assigned to a detailed GBD cause Only vital registration data and verbal autopsy data were considered for this metric, and a country with no data in this form received 0 stars. Verbal autopsy data were down-weighted as a whole, to represent lower accuracy in cause of death ascertainment, and studies which were not nationally representative were significantly down-weighted for the star rating. Stars were assigned in proportion to completeness, percentage of deaths assigned to major garbage codes, time series availability, age and sex coverage, and geographical coverage. GBD=Global Burden of Disease. ATG=Antigua and Barbuda. FSM=Federated States of Micronesia. LCA=Saint Lucia. TLS=Timor-Leste. TTO=Trinidad and Tobago. VCT=Saint Vincent and the Grenadines.

We applied a rating system scored with stars to describe the quality of data available by locations over the full time series from 1980 through 2016. Using this rating system, 25 countries were assigned 5 stars, 48 countries had 4 stars, 30 countries had 3 stars, 21 countries had 2 stars, and 44 countries were assigned 1 star (figure 2). While most countries with a 5-star time series rating were high-SDI countries, some high-SDI countries were rated at 4 stars, such as France, Poland, and Puerto Rico. Some high-middle-SDI countries such as Argentina, Brazil, and Israel also received data quality ratings of 4 stars. A rating of 0 stars was assigned to 27 countries where no VA or VR data were available over the period from 1980 to2016.

### Global causes of death

Cause-specific mortality estimates in each year of the GBD estimation period 1980–2016 by age and sex are available through an online results tool and through the previously mentioned data visualisation tool. Global estimates of total deaths and YLLs and age-standardised death and YLL rates by cause across all levels of the GBD cause hierarchy for the years 2006 and 2016, as well as the percentage change in mortality over that time period, are shown in table 2. Globally, CMNN causes resulted in 19·3% (95% UI 18·5–20·4) of the total deaths in 2016 (10·6 million [10·1 million to 11·1 million]). NCDs accounted for 72·3% (95% UI 71·2–73·2) of global deaths in 2016, or 39·5 million deaths (38·8 million to 40·3 million), and injuries caused 8·43% (8·00–8·67) of global deaths that year, or 4·61 million deaths (4·36 million to 4·77 million). Both the total number of deaths and age-standardised rates from CMNN causes decreased from 2006 to 2016; total CMNN deaths decreased by 23·9% (95% UI 21·6–26·1), while age-standardised death rates decreased by 32·3% (30·3–34·2). While total NCD deaths increased from 2006 to 2016, rising 16·1% (95% UI 14·2–18·0)—an additional 5·47 million deaths—the global age-standardised NCD death rate decreased 12·1% (10·6–13·4), to 614·1 deaths (603·0–625·3) per 100 000 in 2016. Total deaths due to injuries varied minimally between 2006 and 2016, rising from 4·59 million (95% UI 4·35 million to 4·71 million) to 4·61 million deaths (4·36 million to 4·77 million); at the same time, age-standardised injury death rates decreased by 14·4% (12·0–16·5) to 64·4 deaths (60·7–66·6) per 100 000 in 2016.

**Table 2:** Global deaths, age-standardised death rates per 100 000, YLL numbers, and age-standardised YLL rates per 100 000, and percent change between 2006 and 2016 for both sexes combined for all GBD causes and levels 1 through 4 of the cause hierarchy

					**All age deaths (thousands)**		**Age–standardised death rate (per 100 000)**		**All age YLLs (thousands)**		**Age–standardised YLL rate (per 100 000)**	
					2016	Percent change 2006–16	2016	Percent change 2006–16	2016	Percent change 2006–16	2016	Percent change 2006–16
**All causes**					**54 698·6 (54 028·7 to 55 514·9)**	4·1 (2·8 to 5·6)*	**832·7 (822·7 to 845·0)**	–16·8 (−17·9 to −15·7)*	**1 585 865·0 (1 559 573·0 to 1 613 799·5)**	–11·8 (−13·4 to −10·2)*	**22 562·3 (22 192·0 to 22 966·1)**	–23·0 (−24·4 to −21·6)*
	**Communicable, maternal, neonatal, and nutritional disorders**				**10 558·0 (10 097·7 to 11 143·4)**	–23·9 (−26·1 to −21·6)*	**154·1 (147·1 to 163·1)**	–32·3 (−34·2 to −30·3)*	**566 351·5 (544 844·2 to 589 177·0)**	–31·1 (−33·5 to −28·7)*	**8021·0 (7714·9 to 8343·2)**	–35·1 (−37·3 to −32·8)*
		**HIV/AIDS and tuberculosis**			**2246·8 (2172·8 to 2314·5)**	–34·7*(−36·5 to −32·9)	**31·0 (30·0 to 31·9)**	–44·8 (−46·3 to −43·3)*	**94 262·2 (91 006·5 to 97 422·8)**	–38·0 (−39·4 to −36·3)*	**1263·3 (1220·8 to 1304·4)**	–45·8 (−47·1 to −44·4)*
			Tuberculosis		1213·1 (1161·5 to 1265·4)	–20·9 (−24·5 to −17·9)*	17·3 (16·5 to 18·1)	–36·1 (−39·2 to −33·8)*	40 718·8 (38 983·5 to 42 538·2)	–24·9 (−27·7 to −22·0)*	554·3 (530·4 to 579·1)	–36·5 (−38·9 to −34·1)*
				Drug–susceptible tuberculosis	1105·9 (1055·6 to 1158·5)	–20·6 (−24·1 to −17·7)*	15·8 (15·1 to 16·5)	–35·9 (−38·8 to −33·5)*	37 134·8 (35 422·4 to 38 932·7)	–24·5 (−27·3 to −21·4)*	505·7 (482·1 to 530·0)	–36·2 (−38·6 to −33·6)*
				Multidrug–resistant tuberculosis without extensive drug resistance	96·2 (80·0 to 113·3)	–28·9 (−35·6 to −21·5)*	1·4 (1·1 to 1·6)	–42·4 (−47·9 to −36·5)*	3221·6 (2688·6 to 3805·5)	–32·7 (−39·1 to −25·4)*	43·8 (36·5 to 51·8)	–43·2 (−48·6 to −37·0)*
				Extensively drug–resistant tuberculosis	10·9 (8·9 to 13·2)	67·6 (45·9 to 92·7)*	0·2 (0·1 to 0·2)	36·4 (19·1 to 56·3)*	362·4 (294·9 to 439·4)	56·1 (34·8 to 80·7)*	4·9 (4·0 to 5·9)	31·4 (13·7 to 52·2)*
			HIV/AIDS		1033·8 (987·4 to 1081·6)	–45·8 (−47·7 to −43·7)*	13·7 (13·1 to 14·3)	–52·8 (−54·4 to −51·0)*	53 543·4 (50 984·7 to 56 292·0)*	–45·2 (−47·0 to −43·2)*	708·9 (675·5 to 743·9)	–51·3 (−52·9 to −49·5)*
				Drug–susceptible HIV/AIDS - tuberculosis	215·7 (148·7 to 288·6)	–52·7 (−55·4 to −50·0)*	2·9 (2·0 to 3·8)	–59·1 (−61·5 to −56·9)*	11 308·6 (7797·9 to 15 096·2)*	–52·0 (−54·7 to −49·4)*	150·0 (103·5 to 200·3)	–57·4 (−59·8 to −55·1)*
				Multidrug–resistant HIV/AIDS - tuberculosis without extensive drug resistance	18·4 (11·2 to 27·7)	–53·5 (−62·2 to −43·7)*	0·2 (0·1 to 0·4)	–59·9 (−67·5 to −51·2)*	967·9 (586·3 to 1472·2)*	–52·5 (−61·9 to −41·4)*	12·8 (7·8 to 19·6)	–57·8 (−66·2 to −47·9)*
				Extensively drug–resistant HIV/AIDS - tuberculosis	1·2 (0·7 to 1·8)	44·7 (26·4 to 67·0)*	0·0 (0·0 to 0·0)	25·7 (9·7 to 45·1)*	56·8 (34·0 to 88·7)*	43·0 (25·1 to 64·9)*	0·7 (0·4 to 1·2)	26·3 (10·4 to 45·8)*
				HIV/AIDS resulting in other diseases	798·5 (713·4 to 890·1)	–43·4 (−46·1 to −40·5)*	10·6 (9·4 to 11·7)	–50·6 (−53·0 to −48·1)*	41 210·1 (36 586·4 to 46 337·7)	–42·8 (−45·4 to −40·1)*	545·3 (484·2 to 612·9)	–49·2 (−51·4 to −46·8)*
		**Diarrhoea, lower respiratory infections, and other common infectious diseases**			**4805·2 (4381·2 to 5480·6)**	–18·4 (−21·9 to −14·2)*	**72·7 (66·2 to 83·1)**	–29·6 (−32·4 to −26·0)*	**209 304·9 (195 330·8 to 228 343·1)**	–34·1 (−37·7 to −30·1)*	**2994·1 (2796·5 to 3264·0)**	–38·4 (−41·6 to −34·8)*
			Diarrhoeal diseases		1655·9 (1244·1 to 2366·6)	–24·2 (−32·2 to −14·2)*	25·1 (18·8 to 36·0)	–35·9 (−42·4 to −27·2)*	66 908·7 (56 202·7 to 85 858·5)	–37·4 (−43·7 to −30·4)*	959·5 (806·3 to 1230·0)	–42·2 (−47·6 to −36·2)*
			Intestinal infectious diseases		155·4 (87·6 to 255·4)	–14·7 (−22·0 to −8·9)*	2·1 (1·2 to 3·5)	–20·5 (−27·3 to −14·9)*	10 476·5 (5926·7 to 17 188·5)	–17·3 (−25·2 to −10·8)*	142·6 (80·7 to 233·5)	–21·8 (−29·5 to −15·5)*
				Typhoid fever	128·2 (70·1 to 210·2)	–15·7 (−22·8 to −10·0)*	1·7 (1·0 to 2·9)	–21·1 (−27·8 to −15·9)*	8729·6 (4775·3 to 14 334·4)	–18·1 (−25·8 to −12·0)*	118·9 (65·1 to 195·4)	–22·5 (−29·8 to −16·5)*
				Paratyphoid fever	25·2 (11·8 to 49·2)	–6·6 (−14·1 to 0·3)	0·3 (0·2 to 0·7)	–14·0 (−20·7 to −7·8)*	1596·6 (750·5 to 3096·7)	–9·4 (−17·9 to −1·8)*	21·6 (10·1 to 41·8)	–15·1 (−23·0 to −8·0)*
				Other intestinal infectious diseases	2·1 (0·6 to 5·5)	–37·3 (−80·5 to 102·0)	0·0 (0·0 to 0·1)	–41·0 (−81·4 to 85·8)	150·3 (40·8 to 410·6)	–40·6 (−84·9 to 130·3)	2·1 (0·6 to 5·9)	–42·9 (−85·4 to 118·9)
			Lower respiratory infections		2377·7 (2145·6 to 2512·8)	–8·2 (−12·4 to −3·9)*	36·8 (33·2 to 38·9)	–22·1 (−25·3 to −18·9)*	91 363·1 (84 223·2 to 97 870·3)	–30·1 (−34·6 to −25·5)*	1319·8 (1215·4 to 1412·3)	–34·5 (−38·7 to −30·3)*
			Upper respiratory infections		2·3 (2·0 to 2·7)	–26·2 (−37·4 to −12·1)*	0·0 (0·0 to 0·0)	–34·9 (−44·7 to −22·9)*	126·7 (105·2 to 154·9)	–32·6 (−47·5 to −13·5)*	1·8 (1·5 to 2·2)	–37·2 (−50·9 to −19·5)*
			Otitis media		1·1 (0·8 to 1·5)	–41·1 (−53·8 to −22·1)*	0·0 (0·0 to 0·0)	–49·2 (−59·4 to −33·5)*	50·4 (37·5 to 72·4)	–47·2 (−61·8 to −27·3)*	0·7 (0·5 to 1·0)	–51·9 (−65·0 to −33·7)*
			Meningitis		318·4 (265·2 to 408·7)	–8·4 (−19·3 to 9·5)	4·5 (3·8 to 5·8)	–16·3 (−26·0 to 0·2)	20 383·0 (16 781·5 to 26 724·1)	–13·7 (−25·7 to 7·5)	286·3 (235·7 to 377·5)	–18·2 (−29·7 to 2·3)
				Pneumococcal meningitis	23·1 (18·7 to 30·9)	0·2 (−10·6 to 16·1)	0·3 (0·3 to 0·4)	–12·0 (−21·1 to 1·5)	1268·4 (996·2 to 1721·5)	–6·1 (−19·0 to 13·2)	17·7 (13·9 to 24·1)	–13·0 (−24·9 to 5·1)
				*Haemophilus influenzae* type B meningitis	31·4 (25·4 to 41·4)	–6·9 (−20·2 to 14·0)	0·4 (0·4 to 0·6)	–13·6 (−25·6 to 5·7)	2177·5 (1723·9 to 2955·2)	–10·7 (−24·8 to 12·0)	30·8 (24·4 to 42·0)	–14·7 (−28·2 to 7·1)
				Meningococcal infection	127·4 (105·4 to 164·0)	–22·9 (−32·2 to −8·7)*	1·8 (1·5 to 2·3)	–29·3 (−37·6 to −16·6)*	8159·6 (6630·4 to 10 743·5)	–28·5 (−38·3 to −12·5)*	114·4 (92·7 to 150·7)	–32·2 (−41·6 to −17·0)*
				Other meningitis	136·4 (112·7 to 178·0)	8·7 (−4·7 to 32·5)	1·9 (1·6 to 2·5)	–0·9 (−12·7 to 21·3)	8777·6 (7123·5 to 11 853·7)	4·2 (−10·8 to 32·9)	123·4 (100·0 to 167·7)	–1·2 (−15·7 to 26·3)
			Encephalitis		102·9 (83·9 to 138·4)	–1·9 (−19·1 to 20·9)	1·5 (1·2 to 2·0)	–14·1 (−28·3 to 5·0)	5053·3 (4020·1 to 6845·0)	–13·2 (−32·0 to 12·2)	70·6 (56·2 to 95·5)	–19·7 (−36·9 to 3·3)
			Diphtheria		1·1 (0·8 to 1·5)	–66·4 (−77·7 to −48·3)*	0·0 (0·0 to 0·0)	–68·2 (−78·9 to −50·8)*	86·9 (62·4 to 123·4)	–67·0 (−78·6 to −47·7)*	1·2 (0·9 to 1·7)	–68·6 (−79·7 to −49·7)*
			Whooping cough		73·0 (38·9 to 126·1)	–36·3 (−63·5 to 17·6)	1·0 (0·6 to 1·8)	–38·3 (−64·7 to 13·7)	6170·8 (3287·6 to 10 666·2)	–36·2 (−63·5 to 17·6)	88·3 (47·1 to 152·6)	–38·3 (−64·7 to 13·8)
			Tetanus		36·7 (22·2 to 47·2)	–59·5 (−65·5 to −52·9)*	0·5 (0·3 to 0·7)	–62·4 (−67·8 to −56·3)*	2362·8 (1440·7 to 3057·9)	–62·7 (−68·9 to −55·5)*	33·5 (20·2 to 43·4)	–64·1 (−70·0 to −57·1)*
			Measles		68·1 (25·5 to 146·1)	–72·5 (−77·0 to −67·8)*	1·0 (0·4 to 2·1)	–73·7 (−77·9 to −69·1)*	5702·6 (2133·9 to 12 239·1)	–72·5 (−77·0 to −67·7)*	81·0 (30·3 to 173·9)	–73·6 (−77·9 to −69·0)*
			Varicella and herpes zoster		12·5 (11·4 to 13·9)	–15·2 (−22·2 to −7·9)*	0·2 (0·2 to 0·2)	–28·9 (−34·4 to −23·2)*	620·0 (557·0 to 693·3)	–21·7 (−31·9 to −11·7)*	8·8 (7·9 to 9·9)	–27·0 (−36·3 to −17·8)*
		**Neglected tropical diseases and malaria**			**843·6 (708·0 to 989·0)**	–24·7 (−38·7 to −7·6)*	**11·9 (10·0 to 14·0)**	–29·9 (−42·9 to −14·3)*	**61 330·0 (50 832·0 to 73 173·5)**	–26·9 (−41·9 to −8·1)*	**866·1 (715·6 to 1035·1)**	–30·6 (−44·9 to −12·6)*
			Malaria		719·6 (594·6 to 863·0)	–25·9 (−41·4 to −6·1)*	10·2 (8·4 to 12·3)	–30·5 (−45·3 to −12·0)*	54 460·5 (44 151·0 to 66 240·1)	–27·7 (−44·1 to −6·6)*	771·1 (622·8 to 939·3)	–31·1 (−46·8 to −10·9)*
			Chagas disease		7·1 (6·7 to 7·8)	1·4 (−4·9 to 9·5)	0·1 (0·1 to 0·1)	–21·4 (−26·3 to −15·2)*	156·1 (146·2 to 168·7)	–7·3 (−13·3 to 0·2)	2·2 (2·1 to 2·4)	–26·4 (−31·2 to −20·5)*
			Leishmaniasis		13·7 (7·7 to 23·0)	–54·1 (−57·9 to −49·8)*	0·2 (0·1 to 0·3)	–58·9 (−62·1 to −55·1)*	705·8 (398·3 to 1204·2)	–58·0 (−61·9 to −53·9)*	9·7 (5·5 to 16·6)	–61·1 (−64·7 to −57·2)*
				Visceral leishmaniasis	13·7 (7·7 to 23·0)	–54·1 (−57·9 to −49·8)*	0·2 (0·1 to 0·3)	–58·9 (−62·1 to −55·1)*	705·8 (398·3 to 1204·2)	–58·0 (−61·9 to −53·9)*	9·7 (5·5 to 16·6)	–61·1 (−64·7 to −57·2)*
			African trypanosomiasis		2·3 (1·2 to 3·8)	–76·3 (−83·3 to −66·1)*	0·0 (0·0 to 0·1)	–79·0 (−85·0 to −70·1)*	126·5 (63·5 to 212·1)	–76·2 (−83·3 to −65·9)*	1·7 (0·8 to 2·9)	–78·2 (−84·7 to −68·8)*
			Schistosomiasis		10·1 (9·3 to 11·0)	–22·1 (−28·7 to −14·5)*	0·1 (0·1 to 0·2)	–36·9 (−42·2 to −30·7)*	367·4 (333·9 to 401·8)	–23·9 (−31·1 to −15·6)*	5·0 (4·6 to 5·5)	–35·3 (−41·5 to −28·2)*
			Cysticercosis		1·0 (0·9 to 1·2)	–17·8 (−21·5 to −14·6)*	0·0 (0·0 to 0·0)	–30·2 (−33·2 to −27·5)*	47·2 (39·8 to 56·3)	–21·0 (−25·5 to −17·3)*	0·6 (0·5 to 0·8)	–30·1 (−33·9 to −26·8)*
			Cystic echinococcosis		1·0 (0·8 to 1·2)	–36·6 (−40·5 to −33·1)*	0·0 (0·0 to 0·0)	–46·3 (−49·3 to −43·4)*	46·0 (36·8 to 57·8)	–42·1 (−46·9 to −37·7)*	0·6 (0·5 to 0·8)	–48·4 (−52·5 to −44·4)*
			Dengue		37·8 (10·9 to 52·7)	81·8 (42·3 to 132·6)*	0·5 (0·2 to 0·7)	60·9 (26·1 to 105·8)*	1975·1 (619·3 to 2751·8)	59·8 (24·7 to 106·9)*	27·0 (8·5 to 37·7)	46·8 (14·9 to 89·8)*
			Yellow fever		5·8 (1·2 to 16·7)	–11·8 (−25·9 to 6·4)	0·1 (0·0 to 0·2)	–18·4 (−31·4 to −1·8)*	373·9 (80·8 to 1074·9)	–11·9 (−26·9 to 7·4)	5·0 (1·1 to 14·5)	–17·0 (−31·3 to 1·3)
			Rabies		13·3 (7·2 to 19·1)	–47·4 (−56·2 to −35·3)*	0·2 (0·1 to 0·3)	–53·4 (−61·2 to −42·7)*	744·2 (383·7 to 1106·2)	–48·7 (−58·9 to −34·1)*	10·1 (5·2 to 15·1)	–52·9 (−62·4 to −39·5)*
			Intestinal nematode infections		4·9 (4·0 to 6·1)	–39·2 (−50·0 to −24·6)*	0·1 (0·1 to 0·1)	–42·9 (−53·0 to −29·3)*	385·3 (309·2 to 484·2)	–40·0 (−51·2 to −24·7)*	5·4 (4·4 to 6·8)	–43·3 (−53·9 to −28·7)*
				Ascariasis	4·9 (4·0 to 6·1)	–39·2 (−50·0 to −24·6)*	0·1 (0·1 to 0·1)	–42·9 (−53·0 to −29·3)*	385·3 (309·2 to 484·2)	–40·0 (−51·2 to −24·7)*	5·4 (4·4 to 6·8)	–43·3 (−53·9 to −28·7)*
			Ebola virus disease		0·0 (0·0 to 0·0)	··	0·0 (0·0 to 0·0)	··	0·2 (0·2 to 0·2)	··	0·0 (0·0 to 0·0)	··
			Zika virus disease		0·0 (0·0 to 0·1)	··	0·0 (0·0 to 0·0)	··	1·0 (0·2 to 3·2)	··	0·0 (0·0 to 0·0)	··
			Other neglected tropical diseases		27·1 (19·2 to 34·0)	3·1 (−23·2 to 34·3)	0·4 (0·3 to 0·5)	–4·8 (−28·3 to 23·4)	1940·7 (1328·9 to 2505·5)	2·2 (−26·1 to 38·1)	27·5 (18·8 to 35·5)	–2·7 (−29·7 to 31·7)
		**Maternal disorders**			**230·6 (212·5 to 253·4)**	–23·6 (−29·3 to −16·7)*	**3·0 (2·8 to 3·3)**	–30·5 (−35·7 to −24·2)*	**12 817·8 (11 808·4 to 14 106·4)**	–24·7 (−30·4 to −17·9)*	**166·7 (153·6 to 183·5)**	–31·1 (−36·3 to −24·8)*
			Maternal haemorrhage		72·4 (58·5 to 89·1)	–23·8 (−31·6 to −15·2)*	0·9 (0·8 to 1·2)	–30·7 (−37·7 to −23·1)*	4018·5 (3248·0 to 4975·8)	–25·0 (−32·9 to −16·3)*	52·2 (42·2 to 64·6)	–31·5 (−38·7 to −23·6)*
			Maternal sepsis and other pregnancy related infections		19·5 (14·3 to 26·2)	–26·7 (−35·6 to −17·5)*	0·3 (0·2 to 0·3)	–33·2 (−41·3 to −25·0)*	1093·6 (789·3 to 1478·2)	–27·9 (−36·8 to −18·6)*	14·2 (10·3 to 19·2)	–33·8 (−41·9 to −25·3)*
			Maternal hypertensive disorders		31·6 (24·5 to 39·8)	–20·8 (−28·4 to −12·4)*	0·4 (0·3 to 0·5)	–27·6 (−34·7 to −20·1)*	1780·8 (1360·7 to 2265·9)	–21·8 (−29·5 to −13·7)*	23·2 (17·8 to 29·4)	–28·0 (−35·1 to −20·7)*
			Maternal obstructed labour and uterine rupture		10·3 (6·8 to 14·6)	–22·2 (−31·8 to −12·4)*	0·1 (0·1 to 0·2)	–29·9 (−38·7 to −21·2)*	553·7 (369·6 to 798·4)	–23·0 (−32·2 to −13·5)*	7·2 (4·8 to 10·4)	–30·1 (−38·8 to −21·8)*
			Maternal abortion, miscarriage, and ectopic pregnancy		19·7 (14·6 to 26·1)	–22·0 (−31·1 to −12·3)*	0·3 (0·2 to 0·3)	–29·3 (−37·6 to −20·7)*	1081·9 (796·2 to 1466·6)	–22·9 (−32·0 to −13·1)*	14·1 (10·4 to 19·0)	–29·6 (−37·9 to −20·7)*
			Indirect maternal deaths		35·7 (26·4 to 46·8)	–21·6 (−28·8 to −13·3)*	0·5 (0·3 to 0·6)	–28·9 (−35·5 to −21·3)*	1987·9 (1463·8 to 2619·8)	–22·9 (−30·1 to −14·7)*	25·8 (19·1 to 34·0)	–29·6 (−36·2 to −22·1)*
			Late maternal deaths		4·1 (2·5 to 6·6)	–22·3 (−28·9 to −15·1)*	0·1 (0·0 to 0·1)	–29·4 (−35·0 to −23·2)*	228·5 (134·5 to 370·9)	–23·4 (−29·9 to −16·2)*	3·0 (1·8 to 4·8)	–29·9 (−35·4 to −23·5)*
			Maternal deaths aggravated by HIV/AIDS		2·0 (1·3 to 2·7)	–14·3 (−24·6 to −1·6)*	0·0 (0·0 to 0·0)	–23·1 (−32·4 to −11·5)*	105·4 (66·7 to 142·9)	–17·9 (−27·8 to −6·0)*	1·4 (0·9 to 1·9)	–26·1 (−35·0 to −15·3)*
			Other maternal disorders		35·3 (26·8 to 45·2)	–27·4 (−34·8 to −19·2)*	0·5 (0·3 to 0·6)	–33·8 (−40·8 to −26·4)*	1967·5 (1475·0 to 2540·0)	–28·5 (−36·2 to −20·3)*	25·6 (19·2 to 33·1)	–34·4 (−41·4 to −26·8)*
		**Neonatal disorders**			**1731·0 (1644·1 to 1822·9)**	–25·3 (−29·3 to −21·3)*	**25·2 (23·9 to 26·5)**	–25·0 (−29·0 to −21·0)*	**149 832·2 (142 306·5 to 157 780·0)**	–25·3 (−29·3 to −21·3)*	**2179·4 (2069·9 to 2295·0)**	–25·0 (−29·0 to −21·0)*
			Neonatal preterm birth complications		620·4 (568·7 to 674·7)	–27·5 (−33·7 to −21·5)*	9·0 (8·3 to 9·8)	–27·3 (−33·5 to −21·2)*	53 703·1 (49 224·8 to 58 402·3)	–27·5 (−33·7 to −21·5)*	781·1 (716·0 to 849·5)	–27·3 (−33·5 to −21·2)*
			Neonatal encephalopathy due to birth asphyxia and trauma		524·9 (466·7 to 576·2)	–23·1 (−30·3 to −15·6)*	7·6 (6·8 to 8·4)	–22·8 (−30·0 to −15·2)*	45 435·3 (40 397·0 to 49 877·4)	–23·1 (−30·3 to −15·6)*	660·8 (587·5 to 725·3)	–22·8 (−30·0 to −15·2)*
			Neonatal sepsis and other neonatal infections		243·0 (205·0 to 317·7)	–11·8 (−21·9 to 1·5)	3·5 (3·0 to 4·6)	–11·5 (−21·7 to 1·8)	21 029·1 (17 740·3 to 27 500·0)	–11·8 (−21·9 to 1·5)	306·0 (258·1 to 400·1)	–11·5 (−21·7 to 1·8)
			Haemolytic disease and other neonatal jaundice		49·2 (42·6 to 57·0)	–42·8 (−50·7 to −34·4)*	0·7 (0·6 to 0·8)	–42·7 (−50·5 to −34·2)*	4258·6 (3689·2 to 4937·3)	–42·8 (−50·7 to −34·4)*	62·0 (53·7 to 71·8)	–42·7 (−50·5 to −34·2)*
			Other neonatal disorders		293·6 (265·6 to 322·8)	–29·7 (−36·6 to −21·3)*	4·3 (3·9 to 4·7)	–29·4 (−36·3 to −21·1)*	25 406·1 (22 984·9 to 27 937·5)	–29·7 (−36·6 to −21·3)*	369·5 (334·3 to 406·4)	–29·5 (−36·3 to −21·1)*
		**Nutritional deficiencies**			**368·1 (334·0 to 422·7)**	–12·9 (−21·7 to −2·1)*	**5·5 (5·0 to 6·3)**	–23·7 (−30·8 to −15·4)*	**19 504·7 (17 125·0 to 22 894·2)**	–24·1 (−35·0 to −10·5)*	**278·9 (245·0 to 327·0)**	–28·6 (−38·6 to −16·1)*
			Protein-energy malnutrition		308·4 (276·9 to 355·8)	–15·4 (−24·9 to −3·7)*	4·6 (4·2 to 5·3)	–24·9 (−32·6 to −15·6)*	17 514·0 (15 224·7 to 20 732·3)	–25·3 (−36·5 to −11·1)*	251·0 (218·4 to 296·9)	–29·2 (−39·8 to −16·1)*
			Iodine deficiency		2·2 (1·6 to 3·1)	0·6 (−34·4 to 48·1)	0·0 (0·0 to 0·0)	–13·9 (−42·1 to 24·6)	102·6 (66·1 to 168·8)	–8·9 (−49·2 to 66·6)	1·4 (0·9 to 2·4)	–16·7 (−53·4 to 49·7)
			Iron-deficiency anaemia		3·0 (2·5 to 3·8)	8·9 (−9·1 to 27·2)	0·0 (0·0 to 0·1)	–11·6 (−27·8 to 4·9)	114·4 (101·1 to 134·8)	5·6 (−9·1 to 22·3)	1·6 (1·4 to 1·9)	–5·9 (−21·0 to 9·0)
			Other nutritional deficiencies		54·5 (46·0 to 65·0)	2·6 (−4·8 to 11·1)	0·8 (0·7 to 1·0)	–17·2 (−22·9 to −10·7)*	1773·8 (1481·2 to 2040·8)	–12·6 (−21·7 to 0·1)	24·9 (20·8 to 28·6)	–22·9 (−30·4 to −12·6)*
		**Other communicable, maternal, neonatal, and nutritional diseases**			**332·7 (281·0 to 395·8)**	–15·2 (−21·4 to −7·9)*	**4·8 (4·0 to 5·7)**	–23·4 (−28·5 to −17·1)*	**19 299·6 (14 992·7 to 24 689·3)**	–22·7 (−29·6 to −14·4)*	**272·4 (210·4 to 350·1)**	–26·9 (−33·3 to −19·1)*
			Sexually transmitted diseases excluding HIV		115·8 (69·9 to 177·0)	–26·0 (−34·8 to −15·0)*	1·7 (1·0 to 2·5)	–28·1 (−36·6 to −17·7)*	9470·1 (5539·1 to 14 702·0)	–26·8 (−35·8 to −15·6)*	136·1 (79·4 to 211·4)	–28·3 (−37·1 to −17·5)*
				Syphilis	109·6 (63·5 to 170·8)	–27·0 (−36·0 to −15·6)*	1·6 (0·9 to 2·5)	–28·5 (−37·3 to −17·4)*	9228·2 (5288·0 to 14 456·1)	–27·2 (−36·4 to −15·7)*	132·8 (76·1 to 208·1)	–28·5 (−37·5 to −17·2)*
				Chlamydial infection	1·2 (1·0 to 1·3)	–4·5 (−11·7 to 12·2)	0·0 (0·0 to 0·0)	–20·7 (−26·5 to −7·6)*	46·8 (39·3 to 53·4)	–9·2 (−16·8 to 8·0)	0·6 (0·5 to 0·7)	–21·0 (−27·4 to −6·3)*
				Gonococcal infection	3·4 (2·8 to 3·8)	–4·1 (−11·1 to 12·5)	0·0 (0·0 to 0·1)	–20·9 (−26·5 to −7·9)*	127·4 (105·6 to 144·5)	–8·2 (−15·3 to 8·9)	1·7 (1·4 to 1·9)	–20·9 (−26·9 to −6·4)*
				Other sexually transmitted diseases	1·6 (1·4 to 1·8)	–5·9 (−13·0 to 11·2)	0·0 (0·0 to 0·0)	–21·0 (−26·8 to −7·2)*	67·7 (56·5 to 76·9)	–9·2 (−16·4 to 8·1)	0·9 (0·8 to 1·0)	–20·5 (−26·8 to −5·7)*
			Hepatitis		134·0 (127·8 to 140·0)	–13·3 (−16·9 to −9·4)*	1·9 (1·8 to 2·0)	–26·1 (−29·1 to −22·9)*	5497·9 (5228·7 to 5778·4)	–25·3 (−29·5 to −21·1)*	74·8 (71·1 to 78·6)	–33·5 (−37·1 to −29·7)*
				Acute hepatitis A	5·2 (4·3 to 6·2)	–45·4 (−56·7 to −30·3)*	0·1 (0·1 to 0·1)	–48·0 (−58·7 to −34·1)*	378·9 (302·7 to 458·9)	–49·4 (−61·4 to −33·1)*	5·4 (4·3 to 6·6)	–50·8 (−62·5 to −35·1)*
				Hepatitis B	100·3 (94·0 to 106·3)	–4·5 (−9·0 to 0·2)	1·4 (1·3 to 1·5)	–21·1 (−24·8 to −17·5)*	3658·4 (3417·2 to 3917·8)	–12·4 (−17·6 to −7·0)*	49·4 (46·1 to 52·9)	–24·8 (−29·0 to −20·2)*
				Hepatitis C	2·5 (1·9 to 3·2)	–0·7 (−13·2 to 13·7)	0·0 (0·0 to 0·0)	–20·5 (−30·8 to −8·8)*	77·2 (60·9 to 97·7)	–8·8 (−19·5 to 2·7)	1·1 (0·8 to 1·3)	–23·5 (−32·1 to −13·4)*
				Acute hepatitis E	26·1 (22·1 to 30·4)	–30·5 (−37·5 to −23·0)*	0·4 (0·3 to 0·4)	–36·5 (−42·5 to −29·9)*	1383·4 (1195·8 to 1570·3)	–41·3 (−47·8 to −34·1)*	18·9 (16·4 to 21·5)	–44·9 (−50·9 to −38·2)*
			Other infectious diseases		82·9 (56·2 to 103·7)	1·9 (−13·0 to 22·5)	1·2 (0·8 to 1·5)	–10·4 (−23·0 to 7·7)	4331·6 (2650·2 to 5920·9)	–7·0 (−27·5 to 22·8)	61·6 (37·4 to 84·5)	–12·6 (−31·5 to 15·4)
	**Non-communicable diseases**				**39 529·6 (38 805·4 to 40 253·2)**	16·1 (14·2 to 18·0)*	**614·1 (603·0 to 625·3)**	–12·1 (−13·4 to −10·6)*	**819 437·1 (804 360·1 to 836 584·8)**	7·5 (5·5 to 9·5)*	**11 850·1 (11 633·5 to 12 096·5)**	–13·7 (−15·2 to −12·2)*
		**Neoplasms**			**8927·4 (8755·0 to 9089·2)**	17·8 (15·8 to 19·9)*	**133·9 (131·3 to 136·3)**	–9·4 (−10·8 to −7·8)*	**208 041·2 (203 600·0 to 212 089·6)**	12·4 (10·4 to 14·6)*	**2949·0 (2886·0 to 3005·5)**	–10·7 (−12·3 to −9·0)*
			Lip and oral cavity cancer		176·5 (169·2 to 183·0)	30·9 (25·8 to 35·5)*	2·6 (2·5 to 2·7)	0·7 (−3·1 to 4·2)	4492·6 (4287·5 to 4678·0)	26·2 (20·6 to 31·4)*	62·4 (59·5 to 64·9)	–0·4 (−4·6 to 3·7)
			Nasopharynx cancer		63·7 (60·6 to 67·0)	12·7 (6·2 to 18·9)*	0·9 (0·9 to 0·9)	–11·1 (−16·1 to −6·2)*	1866·4 (1770·5 to 1967·2)	6·7 (0·2 to 13·1)*	25·5 (24·2 to 26·8)	–13·6 (−18·8 to −8·5)*
			Other pharynx cancer		118·6 (109·3 to 125·1)	30·7 (19·8 to 39·2)*	1·7 (1·6 to 1·8)	1·1 (−7·3 to 7·5)	3151·7 (2896·0 to 3333·6)	26·7 (15·6 to 34·8)*	43·6 (40·1 to 46·1)	–0·3 (−8·8 to 6·1)
			Oesophageal cancer		414·9 (404·4 to 427·2)	3·7 (0·9 to 7·0)*	6·2 (6·1 to 6·4)	–20·7 (−22·8 to −18·2)*	9164·6 (8913·5 to 9444·1)	0·7 (−2·3 to 4·2)	131·0 (127·5 to 135·1)	–22·0 (−24·3 to −19·3)*
			Stomach cancer		834·2 (813·5 to 855·5)	0·9 (−1·6 to 3·4)	12·6 (12·3 to 12·9)	–22·5 (−24·5 to −20·7)*	18 045·3 (17 580·1 to 18 535·0)	–4·0 (−6·5 to −1·5)*	258·4 (251·8 to 265·2)	–24·7 (−26·7 to −22·8)*
			Colon and rectum cancer		829·6 (797·3 to 860·4)	21·2 (15·7 to 25·8)*	12·8 (12·3 to 13·2)	–8·5 (−12·5 to −5·2)*	16 597·9 (15 919·5 to 17 213·7)	17·0 (11·1 to 21·7)*	239·9 (230·2 to 248·7)	–8·9 (−13·4 to −5·3)*
			Liver cancer		828·9 (796·2 to 858·0)	21·0 (17·1 to 25·2)*	12·1 (11·6 to 12·5)	–5·9 (−8·8 to −2·7)*	20 915·7 (20 029·1 to 21 731·0)	15·1 (11·2 to 19·6)*	291·9 (279·8 to 303·0)	–8·4 (−11·5 to −4·9)*
				Liver cancer due to hepatitis B	349·5 (302·0 to 391·8)	16·5 (12·1 to 21·7)*	5·0 (4·3 to 5·6)	–8·5 (−11·9 to −4·5)*	9704·0 (8495·1 to 10 846·8)	10·7 (6·1 to 16·1)*	133·3 (117·0 to 149·0)	–11·1 (−14·6 to −6·7)*
				Liver cancer due to hepatitis C	159·7 (143·4 to 176·1)	24·8 (20·7 to 28·2)*	2·4 (2·2 to 2·7)	–5·2 (−8·2 to −2·7)*	3267·8 (2889·5 to 3621·5)	20·8 (16·7 to 24·7)*	47·2 (41·9 to 52·3)	–6·7 (−9·9 to −3·9)*
				Liver cancer due to alcohol use	129·2 (109·8 to 150·5)	30·4 (22·6 to 40·3)*	1·9 (1·6 to 2·2)	0·0 (−6·0 to 7·3)	2892·1 (2438·2 to 3361·4)	28·0 (20·3 to 38·4)*	41·3 (34·9 to 47·9)	–0·7 (−6·8 to 7·1)
				Liver cancer due to other causes	190·5 (169·7 to 214·6)	20·6 (15·6 to 25·4)*	2·8 (2·5 to 3·1)	–5·4 (−9·1 to −1·9)*	5051·8 (4479·8 to 5703·8)	13·8 (8·3 to 18·9)*	70·0 (62·1 to 79·1)	–8·5 (−12·6 to −4·7)*
			Gallbladder and biliary tract cancer		161·6 (148·7 to 171·0)	17·7 (13·3 to 22·3)*	2·5 (2·3 to 2·6)	–11·0 (−14·2 to −7·7)*	3269·8 (2965·9 to 3487·8)	14·7 (9·6 to 19·7)*	47·2 (43·0 to 50·3)	–11·3 (−15·1 to −7·5)*
			Pancreatic cancer		405·5 (394·4 to 416·0)	30·2 (26·2 to 33·7)*	6·2 (6·0 to 6·4)	–1·5 (−4·5 to 1·1)	8145·0 (7933·7 to 8359·2)	26·7 (22·6 to 30·4)*	118·2 (115·1 to 121·2)	–2·2 (−5·2 to 0·7)
			Larynx cancer		111·0 (107·6 to 114·6)	13·2 (9·5 to 17·0)*	1·6 (1·6 to 1·7)	–13·0 (−15·8 to −10·1)*	2674·7 (2586·8 to 2767·6)	9·4 (5·6 to 13·3)*	37·6 (36·4 to 38·9)	–14·8 (−17·7 to −11·9)*
			Tracheal, bronchus, and lung cancer		1706·9 (1659·4 to 1753·4)	18·3 (15·0 to 21·5)*	25·8 (25·1 to 26·5)	–9·3 (−11·8 to −6·9)*	35 966·8 (34 937·6 to 36 979·0)	13·5 (9·9 to 16·8)*	519·0 (504·5 to 533·5)	–11·9 (−14·6 to −9·3)*
			Malignant skin melanoma		61·7 (54·4 to 66·6)	25·9 (19·4 to 31·7)*	0·9 (0·8 to 1·0)	–2·9 (−7·9 to 1·5)	1460·7 (1302·0 to 1614·1)	18·6 (12·7 to 24·6)*	20·5 (18·3 to 22·7)	–5·0 (−9·7 to −0·3)*
			Non-melanoma skin cancer		53·1 (51·1 to 55·2)	27·3 (23·3 to 32·4)*	0·8 (0·8 to 0·9)	–4·8 (−7·6 to −1·1)*	991·7 (953·5 to 1031·3)	18·7 (14·3 to 24·3)*	14·4 (13·9 to 15·0)	–7·0 (−10·3 to −2·8)*
				Non-melanoma skin cancer (squamous-cell carcinoma)	53·1 (51·1 to 55·2)	27·3 (23·3 to 32·4)*	0·8 (0·8 to 0·9)	–4·8 (−7·6 to −1·1)*	991·7 (953·5 to 1031·3)	18·7 (14·3 to 24·3)*	14·4 (13·9 to 15·0)	–7·0 (−10·3 to −2·8)*
			Breast cancer		545·6 (516·5 to 581·7)	17·0 (9·5 to 23·9)*	7·9 (7·5 to 8·5)	–9·9 (−15·4 to −4·9)*	14 368·9 (13 568·9 to 15 369·7)	13·8 (5·6 to 21·9)*	198·0 (187·0 to 211·7)	–9·5 (−15·9 to −3·5)*
			Cervical cancer		247·2 (204·1 to 263·5)	7·5 (1·2 to 15·5)*	3·5 (2·9 to 3·7)	–16·0 (−20·7 to −9·8)*	7204·1 (5855·6 to 7673·4)	4·9 (−1·4 to 13·1)	98·1 (79·9 to 104·5)	–15·8 (−20·9 to −9·3)*
			Uterine cancer		87·5 (83·1 to 92·0)	11·4 (5·4 to 19·3)*	1·3 (1·2 to 1·4)	–14·9 (−19·4 to −9·0)*	1973·2 (1875·5 to 2070·1)	6·8 (0·9 to 14·6)*	28·0 (26·6 to 29·4)	–16·6 (−21·2 to −10·6)*
			Ovarian cancer		165·0 (156·7 to 172·7)	22·1 (15·5 to 28·0)*	2·4 (2·3 to 2·5)	–6·6 (−11·5 to −2·1)*	4141·9 (3927·5 to 4340·6)	20·8 (13·8 to 27·0)*	57·7 (54·7 to 60·5)	–5·1 (−10·4 to −0·2)*
			Prostate cancer		380·9 (320·8 to 412·9)	30·8 (24·5 to 36·6)*	6·1 (5·2 to 6·7)	–3·1 (−7·6 to 1·3)	5540·6 (4536·2 to 5992·1)	26·5 (19·3 to 32·2)*	85·6 (70·4 to 92·8)	–4·1 (−9·4 to 0·4)
			Testicular cancer		8·7 (8·3 to 9·0)	2·9 (−1·1 to 7·0)	0·1 (0·1 to 0·1)	–12·3 (−15·5 to −8·8)*	368·1 (350·8 to 386·9)	–1·8 (−6·0 to 2·6)	4·9 (4·6 to 5·1)	–13·5 (−17·1 to −9·6)*
			Kidney cancer		131·8 (127·3 to 136·2)	27·4 (23·2 to 31·5)*	2·0 (1·9 to 2·1)	–2·8 (−5·9 to 0·3)	2910·0 (2799·9 to 3016·4)	21·9 (17·3 to 26·4)*	41·7 (40·2 to 43·3)	–3·8 (−7·4 to −0·3)*
			Bladder cancer		186·2 (180·5 to 191·7)	23·4 (19·4 to 27·0)*	2·9 (2·9 to 3·0)	–7·7 (−10·6 to −5·1)*	3150·2 (3043·9 to 3242·0)	18·0 (13·2 to 21·9)*	46·9 (45·4 to 48·3)	–9·4 (−12·9 to −6·5)*
			Brain and nervous system cancer		227·0 (204·8 to 241·3)	21·6 (17·3 to 28·9)*	3·2 (2·9 to 3·4)	–1·8 (−5·4 to 4·2)	7554·1 (6820·7 to 8181·2)	13·5 (9·1 to 20·5)*	103·6 (93·5 to 111·8)	–3·9 (−7·6 to 2·1)
			Thyroid cancer		42·9 (41·2 to 44·7)	19·8 (14·4 to 26·2)*	0·6 (0·6 to 0·7)	–7·6 (−11·5 to −2·7)*	1043·2 (998·7 to 1088·0)	15·0 (9·0 to 21·7)*	14·6 (14·0 to 15·3)	–8·2 (−12·9 to −2·8)*
			Mesothelioma		30·2 (28·3 to 31·9)	28·9 (23·1 to 33·9)*	0·5 (0·4 to 0·5)	–0·9 (−5·3 to 3·0)	649·6 (610·1 to 688·0)	23·4 (17·8 to 28·1)*	9·3 (8·8 to 9·9)	–2·8 (−7·1 to 0·9)
			Hodgkin's lymphoma		28·7 (24·6 to 33·8)	–6·2 (−9·8 to −3·1)*	0·4 (0·3 to 0·5)	–22·4 (−25·3 to −19·9)*	1097·7 (916·4 to 1300·8)	–11·7 (−14·9 to −8·3)*	14·9 (12·4 to 17·6)	–23·1 (−25·9 to −20·4)*
			Non-Hodgkin lymphoma		239·6 (221·2 to 247·9)	27·3 (21·5 to 31·0)*	3·6 (3·3 to 3·7)	–0·6 (−5·1 to 2·1)	6636·0 (6030·0 to 6928·7)	22·3 (15·5 to 26·8)*	92·8 (84·5 to 96·8)	1·2 (−4·4 to 4·8)
			Multiple myeloma		98·4 (87·4 to 109·8)	28·7 (24·5 to 33·9)*	1·5 (1·3 to 1·7)	–2·6 (−5·7 to 1·2)	2044·4 (1839·2 to 2262·6)	26·5 (22·1 to 32·2)*	29·5 (26·6 to 32·7)	–1·8 (−5·2 to 2·5)
			Leukaemia		310·2 (286·1 to 324·4)	8·7 (5·5 to 12·3)*	4·6 (4·2 to 4·8)	–11·9 (−14·4 to −9·2)*	9990·0 (9167·1 to 10 596·1)	–2·4 (−6·6 to 1·9)	138·4 (127·1 to 146·9)	–15·2 (−18·7 to −11·6)*
				Acute lymphoid leukaemia	50·9 (46·2 to 55·6)	12·5 (1·7 to 18·0)*	0·7 (0·6 to 0·8)	–2·9 (−11·8 to 1·7)	2391·0 (2182·1 to 2644·5)	3·4 (−8·0 to 9·6)	32·6 (29·7 to 36·0)	–6·0 (−16·1 to −0·4)*
				Chronic lymphoid leukaemia	35·4 (33·1 to 40·1)	18·0 (13·6 to 22·9)*	0·6 (0·5 to 0·6)	–11·6 (−14·7 to −8·1)*	645·9 (602·6 to 738·7)	12·2 (7·7 to 17·2)*	9·5 (8·9 to 10·8)	–12·2 (−15·6 to −8·5)*
				Acute myeloid leukaemia	85·3 (78·4 to 89·7)	22·1 (17·7 to 25·6)*	1·3 (1·2 to 1·3)	–2·2 (−5·4 to 0·5)	2622·6 (2419·5 to 2809·8)	13·8 (8·6 to 18·0)*	36·5 (33·7 to 38·9)	–3·0 (−7·1 to 0·5)
				Chronic myeloid leukaemia	21·9 (20·2 to 23·8)	–2·8 (−6·7 to 0·9)	0·3 (0·3 to 0·3)	–24·0 (−27·1 to −21·1)*	598·0 (538·8 to 661·3)	–7·1 (−11·3 to −2·4)*	8·2 (7·4 to 9·1)	–23·7 (−27·0 to −20·2)*
				Other leukaemia	116·6 (103·3 to 123·0)	–0·9 (−6·0 to 4·5)	1·7 (1·5 to 1·8)	–18·6 (−22·5 to −14·4)*	3732·4 (3252·4 to 3950·1)	–15·1 (−20·1 to −9·6)*	51·7 (45·1 to 54·6)	–25·5 (−29·7 to −20·9)*
			Other neoplasms		431·3 (392·7 to 443·8)	30·0 (24·5 to 33·0)*	6·4 (5·8 to 6·5)	2·8 (−1·8 to 5·1)	12 626·3 (11 487·3 to 13 043·6)	21·5 (16·8 to 24·9)*	175·3 (159·3 to 181·1)	2·2 (−1·8 to 4·9)
		**Cardiovascular diseases**			**17 646·6 (17 281·7 to 18 071·1)**	14·5 (12·1 to 17·1)*	**277·9 (272·1 to 284·6)**	–14·5 (−16·2 to −12·5)*	**319 638·7 (312 436·7 to 327 187·0)**	8·0 (5·7 to 10·7)*	**4683·9 (4580·4 to 4794·3)**	–15·7 (−17·5 to −13·6)*
			Rheumatic heart disease		314·6 (302·3 to 328·7)	–7·4 (−13·5 to 0·7)	4·7 (4·5 to 4·9)	–26·9 (−31·6 to −20·8)*	8347·6 (7957·2 to 8806·0)	–16·5 (−22·3 to −10·0)*	116·5 (111·1 to 122·7)	–30·1 (−34·9 to −24·6)*
			Ischaemic heart disease		9480·5 (9230·5 to 9757·7)	19·0 (16·2 to 22·1)*	149·7 (145·8 to 154·1)	–11·6 (−13·6 to −9·3)*	167 695·2 (163 400·6 to 172 479·7)	13·2 (10·1 to 16·2)*	2461·1 (2398·0 to 2530·4)	–12·3 (−14·6 to −10·0)*
			Cerebrovascular disease		5528·2 (5334·6 to 5734·7)	5·1 (2·7 to 7·9)*	86·5 (83·3 to 89·9)	–21·0 (−22·9 to −19·0)*	101 992·8 (99 104·6 to 105 018·7)	0·9 (−1·5 to 3·4)	1496·3 (1451·8 to 1542·8)	–21·5 (−23·4 to −19·6)*
				Ischaemic stroke	2690·2 (2571·8 to 2817·6)	9·3 (5·9 to 12·9)*	43·4 (41·4 to 45·5)	–19·3 (−21·7 to −16·7)*	40 095·1 (38 501·7 to 41 842·1)	5·0 (1·8 to 8·5)*	611·6 (587·1 to 638·0)	–20·1 (−22·6 to −17·4)*
				Haemorrhagic stroke	2838·1 (2748·6 to 2934·1)	1·5 (−0·7 to 3·7)	43·1 (41·7 to 44·7)	–22·7 (−24·4 to −21·1)*	61 897·6 (60 240·2 to 63 722·7)	–1·6 (−3·8 to 0·6)	884·7 (860·4 to 910·9)	–22·5 (−24·3 to −20·8)*
			Hypertensive heart disease		893·7 (698·6 to 982·9)	28·7 (14·5 to 42·9)*	14·3 (11·0 to 15·7)	–4·4 (−14·8 to 5·7)	14 955·0 (12 105·8 to 16 330·6)	19·4 (8·5 to 33·0)*	221·7 (178·6 to 241·8)	–7·2 (−15·9 to 3·0)
			Cardiomyopathy and myocarditis		339·5 (282·6 to 371·1)	13·1 (5·1 to 23·9)*	5·2 (4·3 to 5·7)	–13·0 (−19·0 to −4·7)*	8159·0 (7052·7 to 9049·7)	0·7 (−8·5 to 12·6)	114·7 (99·2 to 126·6)	–16·5 (−23·7 to −7·0)*
				Myocarditis	46·5 (35·8 to 51·1)	8·8 (−0·7 to 24·8)	0·7 (0·6 to 0·8)	–17·1 (−24·7 to −2·4)*	1234·4 (992·2 to 1358·3)	–5·4 (−14·5 to 5·0)	17·3 (13·9 to 19·0)	–18·1 (−26·0 to −8·5)*
				Alcoholic cardiomyopathy	83·3 (67·2 to 102·9)	–4·6 (−21·3 to 17·0)	1·2 (1·0 to 1·4)	–24·0 (−36·8 to −7·5)*	2494·3 (1967·4 to 3151·6)	–10·9 (−28·8 to 14·2)	33·8 (26·8 to 42·6)	–27·0 (−41·3 to −7·7)*
				Other cardiomyopathy	209·7 (170·3 to 224·7)	23·4 (16·5 to 31·4)*	3·3 (2·7 to 3·5)	–7·1 (−12·6 to −1·1)*	4430·4 (3771·6 to 4730·0)	10·7 (3·4 to 19·4)*	63·5 (53·9 to 67·8)	–9·2 (−15·0 to −2·6)*
			Atrial fibrillation and flutter		239·2 (188·7 to 293·6)	42·8 (39·0 to 46·6)*	4·0 (3·2 to 5·0)	–1·1 (−3·2 to 1·0)	2336·9 (1890·4 to 2827·8)	35·7 (32·2 to 39·5)*	37·8 (30·5 to 45·8)	–1·0 (−3·3 to 1·1)
			Aortic aneurysm		166·6 (162·0 to 171·6)	20·5 (17·0 to 24·9)*	2·6 (2·6 to 2·7)	–10·1 (−12·6 to −7·0)*	2881·8 (2800·9 to 2975·5)	14·8 (10·7 to 20·1)*	42·6 (41·4 to 43·9)	–10·9 (−13·9 to −7·1)*
			Peripheral vascular disease		60·7 (45·4 to 89·5)	37·4 (26·0 to 51·3)*	1·0 (0·8 to 1·5)	–2·6 (−10·5 to 6·9)	715·5 (544·4 to 1007·0)	28·5 (17·9 to 42·5)*	11·2 (8·5 to 15·9)	–4·2 (−12·1 to 5·7)
			Endocarditis		96·0 (82·1 to 112·8)	29·2 (24·0 to 33·5)*	1·5 (1·3 to 1·7)	–1·1 (−5·2 to 2·8)	2329·1 (2067·1 to 2756·1)	18·5 (11·8 to 23·9)*	33·0 (29·2 to 39·1)	–1·3 (−6·7 to 3·0)
			Other cardiovascular and circulatory diseases		527·5 (493·1 to 627·5)	20·5 (16·6 to 26·1)*	8·4 (7·8 to 9·9)	–9·8 (−12·8 to −5·9)*	10 225·8 (9436·4 to 12 584·1)	10·0 (6·0 to 15·8)*	149·1 (137·9 to 182·7)	–11·2 (−14·2 to −6·6)*
		**Chronic respiratory diseases**			**3542·3 (3403·6 to 3739·6)**	5·6 (2·8 to 9·1)*	**56·0 (53·8 to 59·2)**	–20·6 (−22·7 to −18·0)*	**61 574·6 (59 099·4 to 65 209·2)**	**–0·2 (−2·9 to 3·8)**	**917·9 (881·0 to 971·6)**	–22·2 (−24·4 to −19·3)*
			Chronic obstructive pulmonary disease		2934·3 (2817·2 to 3120·4)	5·5 (2·4 to 9·5)*	46·8 (45·0 to 49·8)	–23·4 (−23·4 to −18·2)*	47 146·2 (44 992·8 to 50 032·3)	0·5 (−2·5 to 4·9)	712·5 (681·0 to 755·9)	–22·7 (−24·9 to −19·4)*
			Pneumoconiosis		21·5 (20·4 to 23·0)	2·0 (−5·1 to 7·6)	0·3 (0·3 to 0·4)	–21·8 (−27·1 to −17·6)*	414·9 (391·5 to 450·2)	–5·1 (−13·1 to 0·7)	6·0 (5·7 to 6·6)	–25·1 (−31·2 to −20·7)*
				Silicosis	10·4 (9·6 to 11·7)	–1·6 (−14·7 to 5·5)	0·2 (0·1 to 0·2)	–24·3 (−34·1 to −19·0)*	210·2 (194·3 to 230·8)	–7·6 (−21·7 to −0·1)*	3·0 (2·8 to 3·3)	–27·1 (−38·0 to −21·2)*
				Asbestosis	3·5 (2·4 to 4·1)	21·0 (13·3 to 30·9)*	0·1 (0·0 to 0·1)	–7·9 (−13·7 to −0·4)*	61·0 (46·3 to 71·9)	10·9 (4·4 to 19·6)*	0·9 (0·7 to 1·1)	–11·9 (−17·3 to −4·3)*
				Coal workers pneumoconiosis	2·7 (1·8 to 3·1)	–11·3 (−19·5 to 0·4)	0·0 (0·0 to 0·0)	–32·6 (−38·8 to −23·9)*	46·6 (30·4 to 54·2)	–16·6 (−24·6 to −6·6)*	0·7 (0·5 to 0·8)	–35·2 (−41·3 to −27·5)*
				Other pneumoconiosis	4·9 (4·2 to 6·6)	7·3 (−1·3 to 15·9)	0·1 (0·1 to 0·1)	–17·5 (−24·0 to −11·0)*	97·2 (81·9 to 127·9)	–2·0 (−9·5 to 6·0)	1·4 (1·2 to 1·9)	–22·2 (−28·3 to −15·6)*
			Asthma		420·0 (338·8 to 517·7)	–2·6 (−10·0 to 4·1)	6·3 (5·1 to 7·8)	–24·3 (−30·3 to −19·1)*	10 499·3 (8643·2 to 12 621·2)	–9·1 (−15·5 to −2·6)*	148·5 (122·1 to 178·6)	–26·0 (−31·3 to −21·0)*
			Interstitial lung disease and pulmonary sarcoidosis		127·5 (90·8 to 147·7)	40·4 (25·9 to 51·8)*	2·0 (1·4 to 2·3)	5·9 (−4·3 to 13·9)	2305·4 (1695·6 to 2717·0)	33·3 (17·2 to 46·1)*	34·1 (25·0 to 40·0)	4·3 (−7·6 to 14·0)
			Other chronic respiratory diseases		39·0 (27·3 to 45·6)	24·3 (15·6 to 33·4)*	0·6 (0·4 to 0·7)	–1·1 (−7·6 to 5·5)	1208·8 (847·4 to 1438·7)	12·6 (2·9 to 23·8)*	16·8 (11·8 to 20·0)	–3·6 (−11·5 to 5·2)
		**Cirrhosis and other chronic liver diseases**			**1256·9 (1197·1 to 1376·9)**	12·4 (7·2 to 18·4)*	**18·0 (17·1 to 19·6)**	–11·1 (−15·2 to −6·5)*	**37 283·1 (35 413·3 to 41 443·0)**	7·1 (1·8 to 13·4)*	**509·4 (483·8 to 565·8)**	–12·5 (−16·8 to −7·5)*
			Cirrhosis and other chronic liver diseases due to hepatitis B		365·6 (330·8 to 422·6)	12·0 (6·3 to 19·1)*	5·2 (4·7 to 6·0)	–11·4 (−15·9 to −6·0)*	10 846·5 (9787·9 to 12 777·4)	7·8 (1·9 to 15·0)*	147·9 (133·4 to 173·8)	–12·3 (−17·0 to −6·4)*
			Cirrhosis and other chronic liver diseases due to hepatitis C		326·8 (295·1 to 365·0)	15·5 (10·0 to 22·0)*	4·7 (4·2 to 5·2)	–9·3 (−13·7 to −4·3)*	9455·5 (8516·3 to 10 669·0)	11·8 (6·2 to 18·3)*	129·1 (116·5 to 145·1)	–9·8 (−14·3 to −4·6)*
			Cirrhosis and other chronic liver diseases due to alcohol use		334·9 (306·5 to 371·9)	13·7 (8·8 to 19·6)*	4·8 (4·4 to 5·3)	–11·0 (−14·6 to −6·5)*	9440·3 (8601·0 to 10 523·6)	9·4 (4·2 to 15·7)*	129·1 (117·6 to 143·7)	–12·1 (−16·1 to −7·1)*
			Cirrhosis and other chronic liver diseases due to other causes		229·6 (206·2 to 258·1)	7·4 (2·2 to 13·1)*	3·3 (3·0 to 3·7)	–13·5 (−17·4 to −9·4)*	7540·8 (6769·2 to 8562·4)	–1·7 (−7·0 to 4·4)	103·2 (92·9 to 117·4)	–16·3 (−20·7 to −11·5)*
		**Digestive diseases**			**1092·3 (1042·8 to 1177·8)**	9·0 (5·8 to 13·3)*	**16·7 (15·9 to 17·9)**	–15·7 (−18·2 to −12·3)*	**27 082·1 (25 736·0 to 29 026·4)**	**–0·8 (−4·7 to 3·1)**	**383·8 (364·7 to 411·3)**	–17·6 (−20·7 to −14·6)*
			Peptic ulcer disease		246·7 (230·1 to 272·7)	–7·6 (−11·8 to −3·9)*	3·7 (3·5 to 4·1)	–28·7 (−31·9 to −25·8)*	5742·3 (5308·9 to 6470·0)	–14·6 (−18·4 to −11·1)*	81·3 (75·3 to 91·4)	–30·8 (−33·9 to −27·9)*
			Gastritis and duodenitis		43·0 (39·3 to 47·7)	5·2 (−0·6 to 12·3)	0·7 (0·6 to 0·7)	–19·3 (−23·9 to −14·0)*	1017·4 (930·6 to 1148·4)	–1·1 (−8·1 to 7·6)	14·4 (13·2 to 16·2)	–19·7 (−24·7 to −13·3)*
			Appendicitis		50·2 (45·0 to 57·4)	–3·3 (−10·1 to 4·3)	0·7 (0·6 to 0·8)	–19·7 (−25·9 to −13·7)*	1886·6 (1684·3 to 2200·2)	–13·2 (−21·3 to −4·6)*	25·8 (23·0 to 30·1)	–23·3 (−30·3 to −16·0)*
			Paralytic ileus and intestinal obstruction		254·6 (213·3 to 280·8)	17·2 (11·3 to 25·2)*	3·9 (3·2 to 4·2)	–7·3 (−11·9 to −1·7)*	7572·5 (6329·3 to 8263·9)	3·2 (−2·9 to 10·5)	107·2 (89·8 to 116·8)	–10·6 (−15·5 to −5·0)*
			Inguinal, femoral, and abdominal hernia		43·7 (35·6 to 52·1)	6·1 (−1·3 to 12·9)	0·7 (0·6 to 0·8)	–19·0 (−24·8 to −14·0)*	954·6 (739·8 to 1139·7)	–3·4 (−8·8 to 1·4)	13·7 (10·6 to 16·3)	–21·3 (−26·0 to −17·6)*
			Inflammatory bowel disease		41·6 (34·5 to 45·1)	13·4 (1·6 to 26·8)*	0·6 (0·5 to 0·7)	–14·1 (−22·0 to −3·5)*	981·6 (819·4 to 1144·7)	6·0 (−10·3 to 18·7)	13·9 (11·6 to 16·2)	–12·9 (−24·8 to −3·0)*
			Vascular intestinal disorders		100·9 (92·9 to 113·7)	22·4 (16·2 to 28·6)*	1·6 (1·5 to 1·8)	–9·0 (−13·6 to −4·5)*	1671·0 (1537·0 to 1908·2)	16·6 (10·2 to 23·5)*	25·0 (23·0 to 28·6)	–9·5 (−14·4 to −4·7)*
			Gallbladder and biliary diseases		101·8 (96·1 to 118·1)	21·6 (16·9 to 27·3)*	1·6 (1·5 to 1·9)	–9·0 (−12·6 to −4·6)*	1866·8 (1758·6 to 2184·2)	11·6 (7·8 to 16·3)*	27·3 (25·7 to 31·9)	–11·5 (−14·6 to −7·7)*
			Pancreatitis		112·0 (97·4 to 124·6)	15·4 (9·3 to 21·8)*	1·6 (1·4 to 1·8)	–8·7 (−13·3 to −3·9)*	3274·2 (2832·8 to 3650·6)	9·5 (2·4 to 16·5)*	44·7 (38·7 to 49·9)	–9·7 (−15·4 to −4·1)*
			Other digestive diseases		97·7 (88·9 to 108·1)	15·9 (10·9 to 21·4)*	1·5 (1·4 to 1·7)	–12·4 (−15·7 to −8·7)*	2115·0 (1937·5 to 2386·8)	4·3 (−4·2 to 14·2)	30·4 (27·9 to 34·2)	–14·5 (−20·5 to −7·8)*
		**Neurological disorders**			**2825·8 (2497·0 to 3217·6)**	40·5 (38·0 to 42·9)*	**47·6 (42·0 to 54·2)**	**–0·4 (−1·7 to 1·0)**	**34 154·5 (30 976·2 to 38 350·7)**	24·1 (21·1 to 27·3)*	**532·3 (479·5 to 601·2)**	–2·9 (−4·8 to −0·9)*
			Alzheimer's disease and other dementias		2382·1 (2060·4 to 2777·6)	44·7 (41·9 to 47·4)*	40·8 (35·4 to 47·5)	0·1 (−1·3 to 1·6)	22 348·8 (19 381·8 to 26 349·2)	37·4 (34·7 to 39·8)*	365·6 (317·0 to 431·4)	0·1 (−1·5 to 1·6)
			Parkinson's disease		211·3 (167·8 to 265·2)	40·1 (36·6 to 43·6)*	3·5 (2·7 to 4·4)	2·6 (0·5 to 4·7)*	2528·1 (1992·3 to 3147·4)	35·4 (32·4 to 38·5)*	40·4 (31·7 to 50·2)	2·6 (0·5 to 4·7)*
			Epilepsy		126·1 (118·6 to 135·5)	–1·4 (−7·5 to 7·4)	1·7 (1·6 to 1·9)	–14·2 (−19·2 to −6·9)*	5945·4 (5555·1 to 6409·6)	–8·5 (−15·3 to 1·3)	80·0 (74·7 to 86·3)	–16·9 (−23·1 to −8·1)*
			Multiple sclerosis		18·9 (16·6 to 21·0)	17·1 (5·4 to 22·5)*	0·3 (0·2 to 0·3)	–7·3 (−16·1 to −3·1)*	567·4 (517·3 to 646·9)	12·4 (2·2 to 19·0)*	7·7 (7·0 to 8·8)	–8·9 (−16·6 to −3·6)*
			Motor neuron disease		34·3 (33·1 to 35·4)	25·3 (20·4 to 28·7)*	0·5 (0·5 to 0·5)	–2·7 (−6·7 to 0·0)*	855·9 (819·4 to 883·3)	19·4 (14·2 to 22·4)*	12·2 (11·6 to 12·6)	–4·0 (−8·0 to −1·5)*
			Other neurological disorders		53·1 (50·9 to 55·4)	24·5 (18·9 to 29·2)*	0·8 (0·7 to 0·8)	2·1 (−1·8 to 5·5)	1908·8 (1775·7 to 2020·3)	13·9 (7·1 to 20·1)*	26·4 (24·5 to 27·9)	0·4 (−4·9 to 5·3)
		**Mental and substance use disorders**			**318·3 (283·2 to 343·7)**	7·0 (0·3 to 15·0)*	**4·3 (3·9 to 4·7)**	–11·4 (−17·0 to −5·0)*	**12 033·7 (10 748·3 to 13 076·4)**	**1·9 (−5·2 to 10·5)**	**159·5 (142·4 to 173·2)**	–13·3 (−19·4 to −6·2)*
			Alcohol use disorders		173·9 (145·5 to 190·9)	1·1 (−7·1 to 10·5)	2·4 (2·0 to 2·6)	–17·6 (−24·3 to −10·2)*	6214·0 (5164·1 to 6877·8)	–3·2 (−11·5 to 6·8)	82·6 (68·7 to 91·3)	–19·3 (−26·2 to −11·2)*
			Drug use disorders		143·8 (130·3 to 158·8)	15·2 (4·8 to 26·4)*	2·0 (1·8 to 2·2)	–2·6 (−11·3 to 6·7)	5787·3 (5264·5 to 6426·7)	8·0 (−2·1 to 20·4)	76·5 (69·6 to 84·9)	–5·9 (−14·5 to 4·8)
				Opioid use disorders	86·2 (72·7 to 94·7)	15·2 (2·2 to 30·7)*	1·2 (1·0 to 1·3)	–1·5 (−12·8 to 11·3)	3656·9 (3098·2 to 4048·4)	8·1 (−4·2 to 24·3)	48·2 (40·8 to 53·3)	–5·1 (−15·8 to 8·9)
				Cocaine use disorders	8·8 (7·1 to 11·3)	6·8 (−1·1 to 16·9)	0·1 (0·1 to 0·2)	–10·6 (−17·1 to −2·0)*	357·0 (289·0 to 463·9)	2·6 (−5·3 to 13·4)	4·7 (3·8 to 6·1)	–12·0 (−18·8 to −2·8)*
				Amphetamine use disorders	5·2 (4·3 to 6·9)	16·7 (5·3 to 32·3)*	0·1 (0·1 to 0·1)	–1·2 (−10·7 to 12·4)	224·2 (185·2 to 300·3)	12·1 (0·5 to 28·2)*	2·9 (2·4 to 3·9)	–2·8 (−12·8 to 11·4)
				Other drug use disorders	43·5 (39·4 to 52·9)	16·9 (6·9 to 25·6)*	0·6 (0·6 to 0·7)	–3·2 (−11·5 to 3·8)	1549·2 (1395·6 to 1961·5)	8·3 (−1·7 to 17·7)	20·7 (18·7 to 26·0)	–6·6 (−15·2 to 1·4)
			Eating disorders		0·6 (0·5 to 0·7)	4·6 (−0·7 to 10·9)	0·0 (0·0 to 0·0)	–6·4 (−11·2 to −1·1)*	32·4 (28·8 to 36·1)	3·0 (−2·4 to 9·6)	0·4 (0·4 to 0·5)	–6·9 (−11·8 to −1·3)*
				Anorexia nervosa	0·5 (0·5 to 0·6)	2·9 (−2·7 to 9·2)	0·0 (0·0 to 0·0)	–7·8 (−12·7 to −2·5)*	29·2 (25·8 to 32·4)	1·5 (−4·2 to 7·9)	0·4 (0·3 to 0·4)	–8·1 (−13·1 to −2·4)*
				Bulimia nervosa	0·1 (0·1 to 0·1)	21·8 (10·4 to 33·0)*	0·0 (0·0 to 0·0)	6·4 (−3·7 to 16·0)	3·2 (2·8 to 4·0)	19·7 (8·2 to 30·7)*	0·0 (0·0 to 0·1)	5·7 (−4·5 to 15·3)
		**Diabetes, urogenital, blood, and endocrine diseases**			**3191·1 (3112·9 to 3271·9)**	28·4 (26·3 to 30·4)*	**49·1 (47·9 to 50·3)**	–1·6 (−3·2 to −0·2)*	**71 460·5 (69 629·0 to 73 928·8)**	16·6 (14·7 to 18·8)*	**1023·2 (997·4 to 1057·7)**	–4·9 (−6·4 to −3·1)*
			Diabetes mellitus		1437·7 (1402·7 to 1471·0)	31·1 (28·9 to 33·4)*	22·1 (21·6 to 22·7)	–0·9 (−2·5 to 0·8)	28 650·0 (27 998·1 to 29 279·4)	25·3 (23·2 to 27·7)*	415·4 (405·9 to 424·6)	–2·1 (−3·8 to −0·3)*
			Acute glomerulonephritis		11·0 (10·5 to 11·5)	10·4 (4·9 to 15·6)*	0·2 (0·2 to 0·2)	–10·8 (−15·2 to −6·4)*	320·4 (305·4 to 337·8)	–3·7 (−8·7 to 1·4)	4·5 (4·2 to 4·7)	–17·5 (−21·6 to −13·2)*
			Chronic kidney disease		1186·6 (1150·7 to 1236·6)	28·8 (25·5 to 31·4)*	18·2 (17·7 to 19·0)	–1·5 (−3·9 to 0·4)	26 260·5 (25 371·0 to 27 674·3)	16·9 (13·9 to 20·0)*	373·9 (361·5 to 393·3)	–5·0 (−7·4 to −2·7)*
				Chronic kidney disease due to diabetes mellitus	500·8 (452·4 to 544·0)	30·1 (26·2 to 32·8)*	7·6 (6·9 to 8·3)	–0·6 (−3·4 to 1·3)	10 965·2 (9948·0 to 11 927·8)	22·4 (18·7 to 25·4)*	156·2 (141·9 to 169·7)	–3·3 (−6·0 to −1·0)*
				Chronic kidney disease due to hypertension	299·7 (268·2 to 335·5)	34·7 (30·5 to 38·0)*	4·8 (4·3 to 5·4)	–1·0 (−4·0 to 1·0)	4927·1 (4406·7 to 5548·1)	25·1 (21·1 to 28·6)*	73·0 (65·5 to 82·3)	–2·9 (−5·8 to −0·6)*
				Chronic kidney disease due to glomerulonephritis	150·1 (133·2 to 168·9)	17·3 (13·8 to 20·7)*	2·2 (2·0 to 2·5)	–6·3 (−8·5 to −4·2)*	4453·8 (3958·4 to 5035·2)	5·0 (1·7 to 8·7)*	61·5 (54·8 to 69·5)	–9·9 (−12·3 to −7·0)*
				Chronic kidney disease due to other causes	236·0 (207·0 to 266·4)	27·1 (23·3 to 30·9)*	3·6 (3·2 to 4·1)	–0·9 (−3·5 to 1·4)	5914·5 (5263·1 to 6715·1)	11·2 (7·4 to 15·4)*	83·2 (74·2 to 94·3)	–6·3 (−8·9 to −3·3)*
			Urinary diseases and male infertility		275·2 (267·0 to 284·1)	30·8 (25·8 to 34·5)*	4·3 (4·2 to 4·5)	–1·0 (−4·6 to 1·6)	5825·7 (5620·2 to 6028·5)	14·5 (8·6 to 18·8)*	84·1 (81·2 to 87·0)	–5·1 (−9·8 to −1·6)*
				Interstitial nephritis and urinary tract infections	203·5 (193·7 to 213·9)	38·8 (31·8 to 45·2)*	3·3 (3·1 to 3·4)	3·4 (−1·4 to 7·8)	4040·9 (3794·0 to 4296·0)	24·1 (15·6 to 31·3)*	58·8 (55·2 to 62·4)	1·9 (−4·6 to 7·7)
				Urolithiasis	18·7 (15·9 to 25·8)	17·4 (7·6 to 40·1)*	0·3 (0·2 to 0·4)	–9·0 (−16·5 to 7·9)	415·1 (351·4 to 568·3)	4·8 (−4·9 to 26·9)	5·9 (5·0 to 8·1)	–13·9 (−21·6 to 3·9)
				Other urinary diseases	52·9 (45·3 to 59·3)	10·8 (−0·4 to 25·3)	0·8 (0·7 to 0·9)	–13·2 (−22·2 to −2·2)*	1369·8 (1187·7 to 1559·0)	–4·6 (−12·8 to 9·0)	19·4 (16·8 to 22·0)	–19·3 (−26·5 to −8·2)*
			Gynaecological diseases		8·3 (7·4 to 9·0)	13·6 (3·6 to 23·8)*	0·1 (0·1 to 0·1)	–7·7 (−16·4 to 0·4)	265·5 (239·1 to 289·1)	2·8 (−4·6 to 12·5)	3·6 (3·2 to 3·9)	–12·8 (−19·1 to −4·6)*
				Uterine fibroids	2·9 (2·0 to 3·6)	15·8 (−3·0 to 37·2)	0·0 (0·0 to 0·1)	–7·2 (−22·9 to 9·8)	87·9 (59·0 to 109·9)	6·9 (−8·2 to 28·2)	1·2 (0·8 to 1·5)	–12·2 (−25·1 to 5·0)
				Polycystic ovarian syndrome	0·4 (0·2 to 0·8)	–13·2 (−25·1 to 9·3)	0·0 (0·0 to 0·0)	–26·5 (−36·5 to −7·8)*	18·7 (7·0 to 35·8)	–14·7 (−26·6 to 7·9)	0·2 (0·1 to 0·5)	–27·0 (−37·0 to −8·6)*
				Endometriosis	0·1 (0·0 to 0·1)	21·3 (−12·0 to 60·8)	0·0 (0·0 to 0·0)	3·7 (−24·8 to 37·6)	3·1 (1·2 to 4·5)	18·6 (−13·8 to 57·4)	0·0 (0·0 to 0·1)	2·9 (−25·6 to 36·3)
				Genital prolapse	0·9 (0·5 to 1·3)	4·0 (−13·4 to 37·7)	0·0 (0·0 to 0·0)	–21·8 (−35·2 to 2·5)	14·4 (7·5 to 20·7)	–14·0 (−27·5 to 18·8)	0·2 (0·1 to 0·3)	–31·8 (−42·4 to −5·1)*
				Other gynaecological diseases	4·0 (2·9 to 5·0)	18·2 (6·0 to 37·3)*	0·1 (0·0 to 0·1)	–1·4 (−11·7 to 14·4)	141·4 (101·4 to 171·9)	4·9 (−5·7 to 21·9)	1·9 (1·4 to 2·3)	–8·2 (−17·2 to 6·8)
			Haemoglobinopathies and haemolytic anaemias		128·0 (113·1 to 149·1)	3·0 (−1·7 to 10·1)	1·9 (1·7 to 2·2)	–12·3 (−15·8 to −7·2)*	5749·2 (5096·8 to 6685·3)	–7·4 (−14·0 to 2·7)	80·0 (70·9 to 93·4)	–14·5 (−20·6 to −5·3)*
				Thalassaemias	6·3 (5·4 to 7·7)	–33·0 (−39·6 to −18·9)*	0·1 (0·1 to 0·1)	–35·6 (−42·0 to −21·7)*	493·3 (422·8 to 608·9)	–34·0 (−40·9 to −19·3)*	6·9 (5·9 to 8·6)	–36·4 (−43·2 to −22·0)*
				Sickle cell disorders	55·3 (48·1 to 65·8)	–1·0 (−9·7 to 11·2)	0·8 (0·7 to 0·9)	–6·5 (−14·7 to 5·5)	3800·6 (3296·5 to 4494·7)	–4·1 (−13·5 to 9·8)	52·2 (45·2 to 62·0)	–8·4 (−17·6 to 5·4)
				G6PD deficiency	17·9 (15·3 to 21·6)	18·6 (14·3 to 23·3)*	0·2 (0·2 to 0·3)	1·2 (−2·6 to 5·0)	711·8 (610·6 to 850·2)	4·7 (0·7 to 9·9)*	9·6 (8·2 to 11·4)	–7·6 (−11·1 to −3·0)*
				Other haemoglobinopathies and haemolytic anaemias	48·6 (42·7 to 56·9)	10·5 (6·9 to 14·7)*	0·8 (0·7 to 0·9)	–17·3 (−19·8 to −14·5)*	743·6 (651·5 to 875·2)	–9·1 (−12·2 to −5·9)*	11·2 (9·8 to 13·2)	–26·5 (−28·8 to −23·8)*
			Endocrine, metabolic, blood, and immune disorders		144·3 (122·6 to 153·6)	24·4 (17·3 to 29·0)*	2·2 (1·8 to 2·3)	–0·6 (−5·9 to 2·8)	4389·1 (3902·7 to 4910·9)	7·6 (1·8 to 13·6)*	61·8 (54·9 to 68·8)	–6·2 (−11·0 to −1·1)*
		**Musculoskeletal disorders**			**89·2 (78·9 to 98·1)**	16·3 (11·7 to 20·4)*	**1·4 (1·2 to 1·5)**	–10·2 (−13·5 to −7·1)*	**2198·2 (1965·6 to 2494·1)**	6·8 (1·6 to 11·5)*	**30·8 (27·6 to 34·9)**	–11·0 (−15·0 to −7·4)*
			Rheumatoid arthritis		31·0 (26·5 to 35·8)	8·5 (3·3 to 14·9)*	0·5 (0·4 to 0·6)	–17·7 (−21·7 to −13·1)*	574·2 (487·6 to 669·0)	0·6 (−4·6 to 7·8)	8·5 (7·2 to 9·8)	–20·6 (−24·4 to −15·4)*
			Other musculoskeletal disorders		58·2 (51·1 to 64·6)	20·9 (13·8 to 25·7)*	0·9 (0·8 to 1·0)	–5·5 (−10·8 to −1·1)*	1624·1 (1432·7 to 1864·5)	9·2 (2·1 to 13·7)*	22·4 (19·7 to 25·6)	–6·7 (−12·4 to −3·1)*
		**Other non–communicable diseases**			**639·7 (576·4 to 703·6)**	–10·5 (−17·9 to −1·7)*	**9·3 (8·4 to 10·3)**	–15·0 (−21·5 to −7·1)*	**45 970·6 (40 880·9 to 50 868·2)**	–15·8 (−23·1 to −7·4)*	**660·3 (587·0 to 731·0)**	–17·7 (−24·7 to −9·3)*
			Congenital anomalies		498·9 (440·2 to 556·6)	–16·5 (−24·5 to −7·2)*	7·1 (6·3 to 8·0)	–18·4 (−26·1 to −9·2)*	40 707·2 (35 761·9 to 45 627·6)	–17·6 (−25·5 to −8·1)*	584·3 (512·8 to 655·0)	–18·9 (−26·6 to −9·6)*
				Neural tube defects	40·1 (27·9 to 60·5)	–26·4 (−37·6 to −13·8)*	0·6 (0·4 to 0·9)	–27·1 (−38·1 to −14·5)*	3407·5 (2362·1 to 5150·3)	–26·7 (−37·9 to −14·1)*	49·3 (34·2 to 74·5)	–27·2 (−38·3 to −14·6)*
				Congenital heart anomalies	221·3 (197·7 to 253·8)	–18·8 (−26·4 to −6·5)*	3·1 (2·8 to 3·6)	–21·0 (−28·3 to −8·9)*	17 809·2 (15 807·2 to 20 444·4)	–19·9 (−27·5 to −7·3)*	254·4 (225·6 to 292·1)	–21·5 (−28·9 to −9·2)*
				Orofacial clefts	2·2 (1·2 to 3·7)	–30·2 (−49·3 to −10·1)*	0·0 (0·0 to 0·1)	–30·3 (−49·4 to −10·3)*	192·2 (107·0 to 316·4)	–30·2 (−49·3 to −10·2)*	2·8 (1·6 to 4·6)	–30·4 (−49·4 to −10·4)*
				Down's syndrome	14·8 (12·8 to 18·4)	–1·3 (−27·5 to 18·5)	0·2 (0·2 to 0·3)	–9·4 (−32·5 to 8·1)	981·2 (850·2 to 1274·6)	–8·3 (−33·7 to 13·5)	13·8 (11·9 to 18·0)	–12·8 (−36·5 to 7·6)
				Other chromosomal abnormalities	17·5 (13·2 to 24·6)	6·5 (−12·5 to 25·1)	0·3 (0·2 to 0·4)	5·0 (−13·7 to 23·0)	1457·9 (1090·9 to 2066·9)	5·8 (−13·3 to 24·7)	21·0 (15·7 to 29·9)	4·8 (−14·1 to 23·4)
				Congenital musculoskeletal and limb anomalies	8·8 (6·3 to 15·7)	–17·0 (−30·3 to 0·4)	0·1 (0·1 to 0·2)	–18·2 (−31·4 to −1·1)*	722·0 (519·7 to 1318·6)	–17·7 (−31·2 to 0·0)	10·4 (7·5 to 19·0)	–18·4 (−31·7 to −0·8)*
				Urogenital congenital anomalies	12·1 (9·6 to 14·8)	–8·0 (−19·4 to 7·2)	0·2 (0·1 to 0·2)	–12·3 (−23·0 to 1·4)	896·5 (698·4 to 1105·0)	–11·8 (−23·0 to 3·4)	12·9 (10·0 to 15·9)	–13·7 (−24·7 to 1·0)
				Digestive congenital anomalies	34·3 (26·5 to 53·2)	–18·2 (−30·5 to −2·9)*	0·5 (0·4 to 0·8)	–19·0 (−31·3 to −3·7)*	2915·7 (2252·5 to 4540·7)	–18·6 (−30·8 to −3·3)*	42·2 (32·6 to 65·8)	–19·1 (−31·4 to −3·8)*
				Other congenital anomalies	147·9 (104·1 to 205·0)	–13·1 (−25·1 to 3·3)	2·1 (1·5 to 2·9)	–14·6 (−26·4 to 1·6)	12 324·9 (8589·3 to 17 181·3)	–13·7 (−25·7 to 2·6)	177·5 (123·6 to 247·5)	–14·9 (−26·7 to 1·4)
			Skin and subcutaneous diseases		111·7 (71·8 to 144·4)	36·1 (26·8 to 47·9)*	1·7 (1·1 to 2·3)	3·5 (−3·0 to 12·5)	2759·2 (1738·6 to 3611·7)	25·3 (11·9 to 41·8)*	39·7 (25·3 to 52·1)	5·7 (−5·0 to 19·4)
				Cellulitis	18·9 (10·4 to 25·5)	58·3 (46·5 to 76·9)*	0·3 (0·2 to 0·4)	20·3 (12·1 to 33·0)*	437·3 (235·6 to 565·9)	44·9 (30·4 to 69·8)*	6·2 (3·4 to 8·1)	19·2 (8·1 to 38·7)*
				Pyoderma	62·0 (39·0 to 83·2)	36·0 (23·2 to 49·4)*	0·9 (0·6 to 1·3)	7·1 (−2·4 to 17·1)	1827·5 (1139·9 to 2485·2)	23·3 (6·9 to 41·8)*	26·0 (16·2 to 35·3)	6·6 (−7·1 to 22·0)
				Decubitus ulcer	26·4 (16·9 to 36·0)	23·7 (16·8 to 34·6)*	0·4 (0·3 to 0·6)	–11·4 (−16·7 to −2·3)*	380·6 (245·0 to 516·8)	17·1 (11·1 to 27·5)*	5·8 (3·7 to 7·8)	–9·0 (−13·9 to −0·2)*
				Other skin and subcutaneous diseases	4·3 (3·0 to 6·3)	36·3 (27·5 to 48·2)*	0·1 (0·0 to 0·1)	4·6 (−2·4 to 14·8)	113·8 (77·8 to 160·5)	22·4 (12·1 to 36·2)*	1·6 (1·1 to 2·3)	5·5 (−2·8 to 17·2)
			Sudden infant death syndrome		29·1 (23·4 to 34·9)	–17·4 (−33·2 to −1·1)*	0·4 (0·3 to 0·5)	–18·0 (−33·7 to −1·8)*	2504·2 (2015·2 to 3003·1)	–17·4 (−33·2 to −1·2)*	36·4 (29·3 to 43·6)	–18·0 (−33·7 to −1·9)*
		**Injuries**			**4611·0 (4364·8 to 4768·9)**	**0·5 (−2·0 to 3·3)**	**64·4 (60·7 to 66·6)**	–14·4 (−16·5 to −12·0)*	**200 076·3 (191 347·7 to 207 066·5)**	–6·7 (−9·5 to −3·6)*	**2691·2 (2570·7 to 2786·3)**	–16·6 (−19·1 to −13·8)*
		**Transport injuries**			**1437·3 (1400·0 to 1492·5)**	**–1·7 (−4·0 to 0·5)**	**19·6 (19·1 to 20·3)**	–15·2 (−17·2 to −13·4)*	**65 706·9 (63 870·9 to 68 591·2)**	–7·4 (−9·7 to −5·1)*	**874·6 (850·1 to 912·9)**	–17·2 (−19·2 to −15·1)*
			Road injuries		1342·3 (1307·6 to 1393·7)	–1·9 (−4·1 to 0·3)	18·3 (17·8 to 19·0)	–15·4 (−17·3 to −13·6)*	61 412·1 (59 638·9 to 64 244·1)	–7·7 (−9·9 to −5·5)*	817·4 (793·6 to 854·7)	–17·4 (−19·3 to −15·5)*
				Pedestrian road injuries	514·3 (485·8 to 546·7)	–3·1 (−8·5 to −0·1)*	7·1 (6·7 to 7·6)	–17·8 (−22·3 to −15·3)*	21 741·0 (20 466·4 to 23 243·5)	–10·8 (−15·9 to −7·5)*	292·1 (275·1 to 312·3)	–20·9 (−25·3 to −18·1)*
				Cyclist road injuries	74·7 (68·5 to 83·5)	0·2 (−4·6 to 7·1)	1·0 (0·9 to 1·1)	–15·1 (−19·2 to −9·2)*	3095·5 (2811·6 to 3487·7)	–6·7 (−11·8 to 0·1)	41·4 (37·6 to 46·7)	–17·8 (−22·3 to −11·5)*
				Motorcyclist road injuries	251·3 (227·0 to 269·9)	–0·8 (−5·6 to 3·1)	3·3 (3·0 to 3·6)	–12·2 (−16·4 to −8·9)*	12 601·4 (11 425·9 to 13 642·7)	–5·3 (−9·7 to −1·3)*	165·5 (150·0 to 179·3)	–14·2 (−18·1 to −10·7)*
				Motor vehicle road injuries	488·7 (454·6 to 549·4)	–1·5 (−5·1 to 4·8)	6·6 (6·1 to 7·4)	–14·4 (−17·5 to −9·2)*	23 391·2 (21 813·5 to 26 453·9)	–6·0 (−9·5 to 0·3)	310·6 (289·7 to 351·2)	–15·4 (−18·5 to −9·8)*
				Other road injuries	13·2 (12·2 to 16·4)	–0·9 (−7·4 to 9·4)	0·2 (0·2 to 0·2)	–15·3*(−20·8 to −6·3)	582·9 (535·1 to 725·8)	–7·4 (−13·8 to 2·1)	7·8 (7·2 to 9·7)	–17·2 (−22·9 to −8·8)*
			Other transport injuries		95·0 (88·8 to 106·6)	1·8 (−4·4 to 11·0)	1·3 (1·2 to 1·5)	–12·6 (−17·7 to −4·9)*	4294·8 (3991·3 to 4796·1)	–3·8 (−10·4 to 5·4)	57·2 (53·1 to 63·9)	–14·1 (−19·9 to −6·1)*
		**Unintentional injuries**			**1803·9 (1588·0 to 1889·3)**	**0·0 (−3·1 to 3·2)**	**26·3 (23·1 to 27·6)**	–16·1 (−18·6 to −13·6)*	**69 727·1 (62 737·6 to 73 048·2)**	–13·4 (−16·3 to −9·8)*	**961·0 (864·1 to 1007·6)**	–22·3 (−24·8 to −19·2)*
			Falls		678·5 (559·2 to 719·3)	20·0 (13·1 to 25·1)*	10·5 (8·6 to 11·1)	–6·6 (−11·7 to −2·8)*	16 827·4 (14 325·0 to 17 828·3)	3·2 (−4·3 to 9·1)	238·3 (201·6 to 252·8)	–12·2 (−18·4 to −7·7)*
			Drowning		302·9 (272·7 to 322·4)	–19·0 (−22·2 to −13·3)*	4·2 (3·8 to 4·5)	–27·4 (−30·2 to −22·3)*	16 575·7 (15 016·4 to 17 803·4)	–26·9 (−30·4 to −20·3)*	226·0 (204·4 to 243·0)	–32·3 (−35·7 to −26·2)*
			Fire, heat, and hot substances		132·1 (110·1 to 141·6)	–8·6 (−12·9 to −3·4)*	1·9 (1·6 to 2·0)	–22·9 (−26·1 to −19·1)*	5696·0 (4651·7 to 6188·5)	–15·7 (−21·1 to −7·8)*	78·0 (63·9 to 84·8)	–24·2 (−29·0 to −17·4)*
			Poisonings		57·1 (42·4 to 63·6)	–13·9 (−23·9 to −0·8)*	0·8 (0·6 to 0·9)	–25·0 (−33·6 to −13·7)*	2851·0 (2118·6 to 3240·5)	–18·8 (−29·3 to −4·9)*	38·9 (28·8 to 44·4)	–25·9 (−35·6 to −13·3)*
			Exposure to mechanical forces		154·8 (124·0 to 165·1)	–7·9 (−13·8 to −4·5)*	2·1 (1·7 to 2·3)	–19·7 (−24·9 to −16·9)*	7509·6 (6132·2 to 8051·9)	–14·8 (−19·9 to −11·1)*	101·8 (83·0 to 109·2)	–22·6 (−27·1 to −19·3)*
				Unintentional firearm injuries	23·0 (18·2 to 24·8)	–4·9 (−11·4 to 0·3)	0·3 (0·3 to 0·3)	–17·5 (−22·8 to −13·2)*	1123·7 (881·7 to 1233·1)	–8·7 (−16·2 to −3·2)*	15·0 (11·8 to 16·5)	–17·3 (−24·0 to −12·4)*
				Unintentional suffocation	22·6 (17·4 to 26·0)	–11·9 (−22·2 to −3·2)*	0·3 (0·2 to 0·4)	–18·1 (−27·6 to −10·4)*	1474·7 (1151·6 to 1717·1)	–20·2 (−28·0 to −12·0)*	20·8 (16·2 to 24·3)	–23·4 (−30·9 to −15·4)*
				Other exposure to mechanical forces	109·3 (84·2 to 115·9)	–7·6 (−13·1 to −4·3)*	1·5 (1·2 to 1·6)	–20·5 (−25·2 to −17·7)*	4911·2 (3856·7 to 5218·2)	–14·4 (−19·0 to −10·7)*	65·9 (51·8 to 70·1)	–23·5 (−27·5 to −20·2)*
			Adverse effects of medical treatment		126·7 (109·3 to 140·5)	9·1 (5·0 to 14·3)*	1·9 (1·6 to 2·1)	–10·8 (−14·2 to −7·5)*	4602·0 (3861·1 to 5157·1)	–2·7 (−9·2 to 7·1)	64·1 (53·8 to 71·9)	–13·9 (−19·1 to −6·5)*
			Animal contact		91·6 (68·8 to 102·2)	–3·3 (−9·8 to 6·9)	1·3 (1·0 to 1·4)	–16·9 (−22·3 to −8·3)*	4268·9 (3176·5 to 4791·5)	–9·9 (−16·6 to 0·5)	58·1 (43·2 to 65·2)	–18·8 (−24·7 to −9·2)*
				Venomous animal contact	78·8 (56·8 to 89·4)	–3·6 (−10·7 to 7·0)	1·1 (0·8 to 1·2)	–17·0 (−23·0 to −8·0)*	3662·0 (2606·9 to 4190·1)	–10·5 (−17·4 to 0·1)	49·8 (35·4 to 57·0)	–19·3 (−25·5 to −9·7)*
				Non-venomous animal contact	12·8 (10·3 to 17·4)	–1·5 (−9·5 to 8·0)	0·2 (0·1 to 0·2)	–15·8 (−22·3 to −8·4)*	606·9 (479·7 to 841·7)	–6·2 (−15·6 to 5·1)	8·3 (6·6 to 11·6)	–15·3 (−23·9 to −5·2)*
			Foreign body		106·3 (92·5 to 114·9)	6·7 (−0·7 to 17·0)	1·6 (1·4 to 1·7)	–10·2 (−15·4 to −2·0)*	4703·0 (4114·4 to 5317·9)	–3·0 (−12·8 to 9·3)	66·0 (57·6 to 74·8)	–11·3 (−19·6 to −0·7)*
				Pulmonary aspiration and foreign body in airway	95·9 (82·5 to 104·5)	7·5 (−0·2 to 18·8)	1·4 (1·2 to 1·5)	–9·8 (−15·2 to −0·9)*	4203·2 (3638·9 to 4809·7)	–2·5 (−12·7 to 11·3)	59·2 (51·0 to 67·7)	–10·8 (−19·5 to 1·1)
				Foreign body in other body part	10·3 (7·9 to 12·0)	–0·3 (−7·8 to 8·8)	0·1 (0·1 to 0·2)	–13·7 (−19·5 to −7·1)*	499·8 (373·0 to 586·3)	–7·7 (−16·3 to 1·5)	6·8 (5·1 to 8·0)	–15·9 (−23·3 to −8·1)*
			Environmental heat and cold exposure		55·6 (36·4 to 71·5)	–12·4 (−24·4 to −2·6)*	0·8 (0·5 to 1·0)	–28·3 (−38·3 to −20·7)*	1921·0 (1216·2 to 2408·6)	–19·8 (−31·0 to −10·0)*	26·3 (16·6 to 32·7)	–31·1 (−41·3 to −22·8)*
			Other unintentional injuries		98·3 (84·2 to 102·8)	–12·3 (−16·2 to −8·6)*	1·3 (1·1 to 1·4)	–23·1 (−26·5 to −20·0)*	4772·4 (4186·1 to 5000·1)	–17·5 (−21·1 to −13·8)*	63·4 (55·6 to 66·5)	–25·6 (−28·8 to −22·4)*
		**Self-harm and interpersonal violence**			**1207·9 (1108·8 to 1291·0)**	**–2·7 (−6·7 to 2·3)**	**16·3 (15·0 to 17·5)**	–16·5 (−20·0 to −12·3)*	**54 833·9 (50 105·6 to 58 459·5)**	–5·6 (−9·4 to −0·7)*	**724·5 (662·3 to 771·8)**	–16·1 (−19·5 to −11·8)*
			Self-harm		817·1 (762·0 to 883·7)	–3·0 (−7·4 to 2·3)	11·2 (10·4 to 12·1)	–18·0 (−21·6 to −13·6)*	34 621·4 (32 412·0 to 37 408·6)	–6·5 (−10·7 to −1·1)*	458·4 (428·7 to 495·4)	–17·8 (−21·4 to −13·0)*
				Self-harm by firearm	67·5 (55·4 to 84·1)	4·3 (−2·5 to 14·1)	0·9 (0·8 to 1·1)	–11·6 (−17·0 to −3·9)*	2840·1 (2373·7 to 3578·9)	–0·8 (−7·6 to 9·7)	37·6 (31·4 to 47·4)	–12·6 (−18·4 to −3·9)*
				Self-harm by other specified means	749·6 (700·9 to 812·6)	–3·6 (−8·2 to 2·0)	10·2 (9·6 to 11·1)	–18·5 (−22·2 to −13·9)*	31 781·4 (29 699·5 to 34 445·4)	–7·0 (−11·2 to −1·4)*	420·8 (393·4 to 455·5)	–18·2 (−21·9 to −13·4)*
			Interpersonal violence		390·8 (320·8 to 453·7)	–1·9 (−6·9 to 4·8)	5·2 (4·3 to 6·0)	–13·3 (−17·8 to −7·3)*	20 212·5 (16 632·1 to 23 093·9)	–3·9 (−9·0 to 2·5)	266·1 (219·0 to 304·0)	–13·0 (−17·5 to −7·3)*
				Physical violence by firearm	161·0 (107·2 to 182·5)	5·7 (1·1 to 10·6)*	2·1 (1·4 to 2·4)	–5·3 (−9·5 to −0·8)*	8615·9 (5744·5 to 9727·9)	3·9 (−0·8 to 9·0)	112·9 (75·2 to 127·4)	–5·2 (−9·7 to −0·5)*
				Physical violence by sharp object	97·4 (78·1 to 128·5)	–9·4 (−15·9 to 1·4)	1·3 (1·0 to 1·7)	–20·4 (−26·1 to −10·6)*	4876·5 (3900·9 to 6470·2)	–11·6 (−17·9 to −1·1)*	63·9 (51·1 to 84·7)	–20·5 (−26·3 to −11·1)*
				Physical violence by other means	132·4 (111·3 to 168·4)	–4·4 (−13·5 to 6·7)	1·8 (1·5 to 2·3)	–16·3 (−24·3 to −6·4)*	6720·1 (5734·0 to 8489·4)	–7·1 (−16·3 to 3·2)	89·3 (76·2 to 112·5)	–16·2 (−24·5 to −6·9)*
		**Forces of nature, conflict and terrorism, and executions and police conflict**			**161·9 (112·6 to 215·1)**	99·8 (26·8 to 228·2)*	**2·2 (1·5 to 2·9)**	80·6 (15·3 to 193·7)*	**9808·4 (6797·5 to 13 037·7)**	101·8 (27·5 to 238·4)*	**131·2 (90·9 to 174·4)**	87·4 (18·6 to 213·8)*
			Exposure to forces of nature		7·1 (4·2 to 10·1)	–49·2 (−63·0 to −35·2)*	0·1 (0·1 to 0·1)	–55·2 (−67·3 to −43·1)*	357·6 (217·9 to 507·7)	–52·4 (−64·8 to −39·4)*	4·8 (2·9 to 6·8)	–56·4 (−67·7 to −44·7)*
			Conflict and terrorism		150·5 (101·5 to 202·7)	143·3 (42·6 to 370·6)*	2·0 (1·4 to 2·7)	122·4 (30·5 to 328·2)*	9226·0 (6241·2 to 12 407·4)	140·8 (41·9 to 372·1)*	123·4 (83·5 to 166·0)	124·7 (32·4 to 339·7)*
			Executions and police conflict		4·4 (2·3 to 5·0)	–17·0 (−25·7 to −6·6)*	0·1 (0·0 to 0·1)	–26·5 (−33·9 to −17·6)*	224·8 (119·7 to 261·6)	–19·2 (−27·7 to −8·2)*	3·0 (1·6 to 3·4)	–26·5 (−34·1 to −16·7)*

95% UIs are in parentheses. Asterisks denote statistically significant increases or decreases (p<0·05). YLL=years of life lost. GBD=Global Burden of Disease. UI=uncertainty interval.

**Figure 3:**
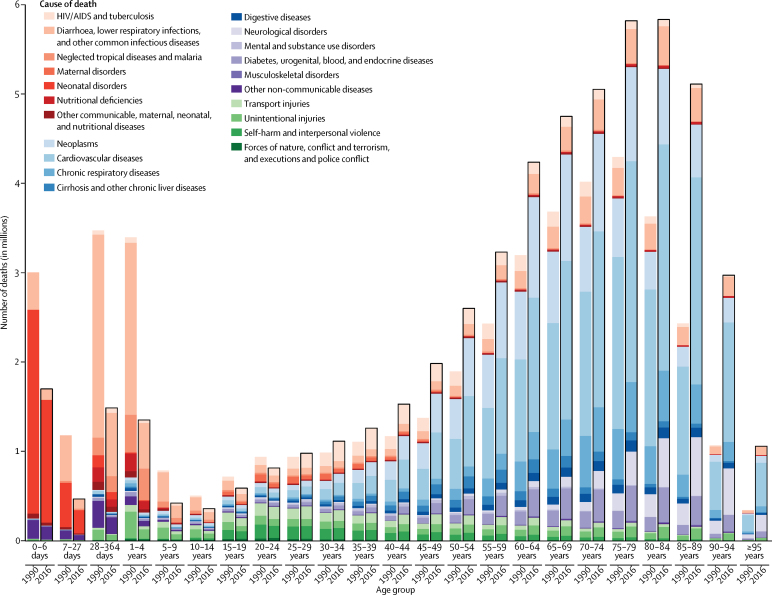
Global composition of number of deaths by Level 2 causes for 23 GBD age groups, both sexes combined, 1990 versus 2016 Composition of Level 2 causes of death globally for males and females combined, by age group, showing difference in composition between 1990 and 2016. Number of total deaths due to Level 2 causes is indicated by height of bar; causes are colour-coded to highlight the relative number of total deaths due to a specific cause. GBD=Global Burden of Disease.

Figure 3 shows the number of deaths in 1990 and 2016 by GBD age group for the 21 GBD Level 2 causes. Total deaths declined in the age group intervals of 0–6 days, 7–27 days, 28–364 days, 1–4 years, 5–9 years, 10–14 years, 15–19 years, and 20–24 years, and increased by more than 60% in age groups 80–84 years, 85–89 years, 90–94 years, and 95 years and older. Shifts at age 90 and older were the most substantial, with a 17·8% (95 UI 176–181) increase in the number of deaths in the 90–94 age group and 210% (208–212) in age 95 years and older, illustrating a profound shift toward deaths at older ages since 1990. Between 1990 and 2016, the global number of deaths from cardiovascular diseases for people aged older than 70 years increased by 53·7% (95% UI 49·3–57·8) to 11·1 million deaths (10·9 million to 11·4 million). Notably, deaths from neoplasms also increased for older ages, rising 86·3% (95% UI 81·0–90·5) to 3·93 million deaths (3·85 million to 4·01 million) for age 70 years and older in 2016. Causes of deaths for those aged older than age 70 years that increased by more than 90% were neurological disorders; diabetes, urogenital, blood, and endocrine diseases; unintentional injuries; other non-communicable diseases; musculoskeletal disorders; and mental and substance use disorders.

### Communicable, maternal, neonatal, and nutritional diseases

Generally, communicable diseases decreased as a leading source of death, and much of this decrease was driven by reductions in large contributors to global mortality, including HIV/AIDS, malaria, tuberculosis, and diarrhoeal diseases (table 2). Overall, HIV/AIDS deaths decreased by 45·8% (95% UI 43·7–47·7) from 1·91 million deaths (1·81–2·00) in 2006 to 1·03 million deaths (987 000 to 1·08 million) in 2016. This decrease in absolute mortality level was accompanied by a large decrease in the global age-standardised HIV/AIDS death rate, which dropped 52·8% (95% UI 51·0–54·4) from 29·0 deaths (27·6–30·3) per 100 000 in 2006 to 13·7 deaths (13·1–14·3) per 100 000 in 2016. HIV/AIDS mortality peaked in 2005 globally, with declining death rates probably reflecting the successful expansion of ART programmes and programmes focused on the prevention of mother-to-child transmission (PMTCT). An estimated 1·21 million deaths (95% UI 1·16 million to 1·27 million) were caused by tuberculosis in 2016, a decrease of 20·9% (17·9–24·5) since 2006. Among subcauses of tuberculosis, drug-susceptible tuberculosis deaths composed the largest portion (91·2% [95% UI 90·9–91·6]) of overall tuberculosis deaths; the fastest decrease from 2006 to 2016 occurred for deaths from multidrug-resistant tuberculosis (28·9% [21·5–35·6]). Both total deaths and age-standardised death rates from diarrhoeal diseases fell between 2006 and 2016; total deaths decreased by 24·2% (95% UI 14·2–32·2) from 2·18 million deaths (1·72 million to 3·01 million) in 2006 to 1·66 million deaths (1·24 million to 2·37 million) in 2016, while age-standardised rates dropped by 35·9% (27·2–42·4) from 39·2 deaths per 100 000 (30·1 to 55·0) in 2006 to 25·1 deaths (18·8–36·0) per 100 000 in 2016. Other communicable diseases that decreased in terms of total deaths included malaria, measles, leishmaniasis, and intestinal infectious diseases, which decreased by 25·9% (95% UI 6·13–41·4), 72·5% (67·8–77·0), 54·1% (49·8–57·9), and 14·7% (8·87–22·0), respectively, from 2006 to 2016.

Progress in lowering mortality levels and rates was notably slower from 2006 to 2016 for some communicable diseases. For several causes, changes from 2006 to 2016 in global numbers of deaths were not significant: Chagas disease (increase of 1·42% [95% UI −4·88 to 9·49), yellow fever (decrease of 11·8% [–25·9 to 6·44]), and other neglected tropical diseases (NTD; an increase of 3·06% [–23·2 to 34·3]). Dengue was the only NTD with a significant increase in cause-specific mortality, with an 81·8% (95% UI 42·3–132·6) increase in total deaths, from 20 800 deaths (6000–26 500) in 2006 to 37 800 (10 900–52 700) deaths in 2016, while age-standardised rates increased from 0·3 deaths per 100 000 (0·01–0·4) in 2006 to 0·5 (0·2–0·7) deaths per 100 000 in 2016. The global number of deaths from Zika virus disease—newly estimated for GBD 2016— was two deaths (95% UI 1–5) in 2015 and 19 deaths (4–57) in 2016.

All maternal and neonatal causes of death decreased globally in terms of both total deaths and age-standardised death rates between 2006 and 2016. The largest decrease in deaths from maternal disorders were for other maternal disorders (35 300 deaths [95% UI 26 800–45 200] in 2016), maternal sepsis and other maternal infections (19 500 deaths [14 300–26 200] in 2016), and maternal haemorrhage (72 400 deaths [58 500–89 100] in 2016), which represented decreases of 27·4% (19·2–34·8), 26·7% (17·5–35·6), and 23·8% (15·2–31·6), respectively, from 2006. Total deaths from maternal disorders decreased by 23·6% (95% UI 16·7–29·3), while age-standardised death rates across maternal disorders decreased by 30·5% (24·2–35·7) to 3·0 (2·8–3·3) per 100 000 in 2016. Neonatal disorders decreased by 25·3% (95% UI 21·3–29·3) for total deaths, declining from 2·32 million deaths (2·24 million to 2·42 million) in 2006 to 1·73 million deaths (1·64 million to 1·82 million) in 2016, and by 25·0% (21·0–29·0) for age-standardised death rates (33·6 deaths [32·4–35·1] per 100 000 in 2006 to 25·2 deaths [23·9–26·5] per 100 000 in 2016). The largest decrease for neonatal disorders was for haemolytic disease and other neonatal jaundice, which caused 36 900 fewer deaths in 2016 than in 2006 (a reduction of 42·8% [95% UI 34·4–50·7]).

Deaths from nutritional deficiencies constituted 3·49% (95% UI 3·31–3·79) of total deaths due to CMNN causes, resulting in 368 100 (334 000–422 700) in 2016. Protein-energy malnutrition caused the largest number of deaths for nutritional deficiencies with 308 000 deaths (95% UI 277 000–356 000) in 2016, followed by other nutritional deficiencies, which caused 54 500 deaths (46 000–65 000). Progress toward reducing mortality rates associated with nutritional deficiencies was similar to maternal and neonatal disorders: age-standardised mortality rates for all nutritional deficiencies decreased by 23·7% (95% UI 15·4–30·8) from 7·26 deaths (6·75–7·86) per 100 000 in 2006 to 5·54 deaths (5·04–6·34) per 100 000 in 2016.

### Non-communicable diseases

For NCDs in 2016, the largest number of deaths at Level 2 were caused by cardiovascular diseases (17·6 million deaths [95% UI 17·3 million to 18·1 million]) followed by neoplasms (8·93 million deaths [8·75 million to 9·09 million]), and chronic respiratory diseases (3·54 million deaths [3·40 million to 3·74 million]; table 2).

Globally, deaths from cardiovascular disease increased by 14·5% (95% UI 12·1–17·1) between 2006 and 2016, though age-standardised death rates from cardiovascular disease decreased by 14·5% (12·5–16·2) over this same time period. Ischaemic heart disease and cerebrovascular disease (stroke) combined accounted for more than 85·1% of all cardiovascular disease deaths in 2016. Total deaths from ischaemic heart disease rose by 19·0% (16·2–22·1), increasing from 7·96 million deaths (7·81 million to 8·12 million) in 2006 to 9·48 million deaths (9·23 million to 9·76 million) in 2016, which largely accounts for the overall increase in total deaths from cardiovascular diseases. Declines in age-standardised cardiovascular disease mortality rates were primarily driven by declines in cerebrovascular disease death rates, which decreased 21·0% (95% UI 19·0–22·9) between 2006 and 2016, from an age-standardised death rate of 110 deaths per 100 000 (106–113) in 2006 to 86·5 deaths (83·3–89·9) per 100 000 in 2016. The absolute number of deaths and the total YLLs from diabetes both increased between 2006 and 2016 by 31·1% (95% UI 28·9–33·4) and 25·3% (23·2–27·7), respectively, while age-standardised YLL rates decreased 2·12% (0·29–3·81) over the same time period.

Deaths from neoplasms increased globally by 17·8% (95% UI 15·8–19·9), rising from 7·58 million deaths (7·46 million to 7·67 million) in 2006 to 8·93 million deaths (8·75 million to 9·09 million) in 2016. Over the same time period, the overall age-standardised neoplasm death rate fell by 9·38% (7·78–10·8) from 147·7 deaths per 100 000 (145·4 to 149·5) in 2006 to 133·9 deaths (131·3–136·3) per 100 000 in 2016. From 2006 to 2016, increases of greater than 30% occurred for several neoplasms that were large causes of deaths (greater than 200 000 in 2016): prostate cancer (30·8% [95% UI 24·5–36·6], to 381 000 deaths [321 000–413 000]); pancreatic cancer (30·2% [26·2–33·7], to 405 000 deaths [394 000–416 000]); and other neoplasms (30·0% [24·5–33·0], to 431 000 deaths [393 000–444 000]). Total global deaths decreased significantly for only one type of neoplasm from 2006 to 2016: Hodgkin's lymphoma (decreased 6·24% [95% UI 3·07–9·75]). Age-standardised death rates fell across most neoplasms, most notably for stomach cancer (decrease of 22·5% [95% UI 20·7–24·5], to 12·6 deaths [12·3–12·9] per 100 000 in 2016) and Hodgkin's lymphoma (decrease of 22·4% [19·9–25·3], to 0·4 deaths [0·4–0·5] per 100 000 in 2016). Both lung cancer and breast cancer deaths increased from 2006 to 2016, from 1·44 million deaths (95% UI 1·42 million to 1·47 million) to 1·71 million deaths (1·66 million to 1·75 million) for lung cancer and from 466 000 deaths (451 000–486 000) to 546 000 deaths (517 000–582 000) for breast cancer, but age-standardised mortality rates for these causes decreased by 9·31% (6·9–11·8) and 9·92% (4·87–15·4), respectively, over the same time period.

Chronic respiratory diseases contributed 8·96% of NCD deaths in 2016, with chronic obstructive pulmonary disease (COPD) leading to the most deaths from these conditions (2·93 million deaths [95% UI 2·82 million to 3·12 million]). Since 2006, age-standardised death rates from COPD significantly decreased (21·1% [18·2–23·3]), to 46·8 deaths (45·0–49·8) per 100 000. Age standardised rates for asthma also decreased (24·3% [19·1–30·3] to 6·29 deaths [5·08–7·77] per 100 000), but increased, although not significantly, for interstitial lung disease and pulmonary sarcoidosis (5·85% [–4·31 to 13·9], to 2·0 deaths [1·42–2·31] per 100 000). Among NCD causes included in the expansion of the GBD cause hierarchy for GBD 2016, age-standardised death rates fell globally between 2006 and 2016 for alcoholic cardiomyopathy, digestive congenital anomalies, and congenital musculoskeletal and limb anomalies, by 24·0% (95% UI 7·52–36·8), 19·0% (3·73–31·3), and 18·2% (1·11–31·4), respectively, while rates did not change significantly on a global scale for amphetamine use disorders, opioid use disorders, or other drug use disorders over that time period.

### Injuries

Although global age-standardised death rates fell across all injuries by 14·4% (95% UI 12·0–16·5) from 75·3 deaths (71·0–77·3) per 100 000 in 2006 to 64·4 deaths (60·7–66·6) per 100 000 in 2016, total injury deaths were largely unchanged from levels in 2006 (table 2). Unintentional injuries accounted for the most injury deaths in 2016, with 1·80 million deaths (95% UI 1·59 million to 1·89 million) composed mainly of deaths from falls (678 000 deaths [559 000–719 000]), drowning (302 000 deaths [273 000–322 000]), and exposure to mechanical forces (155 000 deaths [124 000–165 000]). Age-standardised rates for unintentional injuries overall decreased 16·1% (13·6–18·6) from 2006 to 2016 from 31·4 deaths (27·6–32·7) per 100 000 to 26·3 deaths (23·1–27·6) per 100 000. In terms of number of deaths in 2016, unintentional injuries were followed by transport injuries (1·44 million deaths [95% UI 1·40 million to 1·49 million]), and self-harm and interpersonal violence (1·21 million deaths [1·11 million to 1·29 million]). Deaths from physical violence by firearm were the largest portion (41·2%) of overall interpersonal violence in 2016; globally, age-standardised rates for deaths and for YLLs of physical violence by firearm decreased from 2006 to 2016 by 5·34% (95% UI 0·80–9·52) and 5·19% (0·51–9·71), respectively. Self-harm by firearm constituted 8·26% of global deaths from self-harm; age-standardised rates of both deaths and YLLs from self-harm by firearm decreased from 2006 to 2016 by 11·6% (95% UI 3·96–17·0) and 12·6% (3·87–18·4), respectively. The largest decreases in injury deaths from 2006 to 2016 occurred for exposure to forces of nature (49·2% [95% UI 35·2–63·2], to 7060 deaths [4220–10 100]) and drowning (19·0% [13·3–22·2], to 303 000 deaths [273 000–322 000]), while the largest increase was for conflict and terrorism at 143·3% (42·6–370·6), from 61,900 deaths (33 ,100 to 91 ,000) deaths to 150 000 deaths [101 400–202 700]).

**Figure 4:**
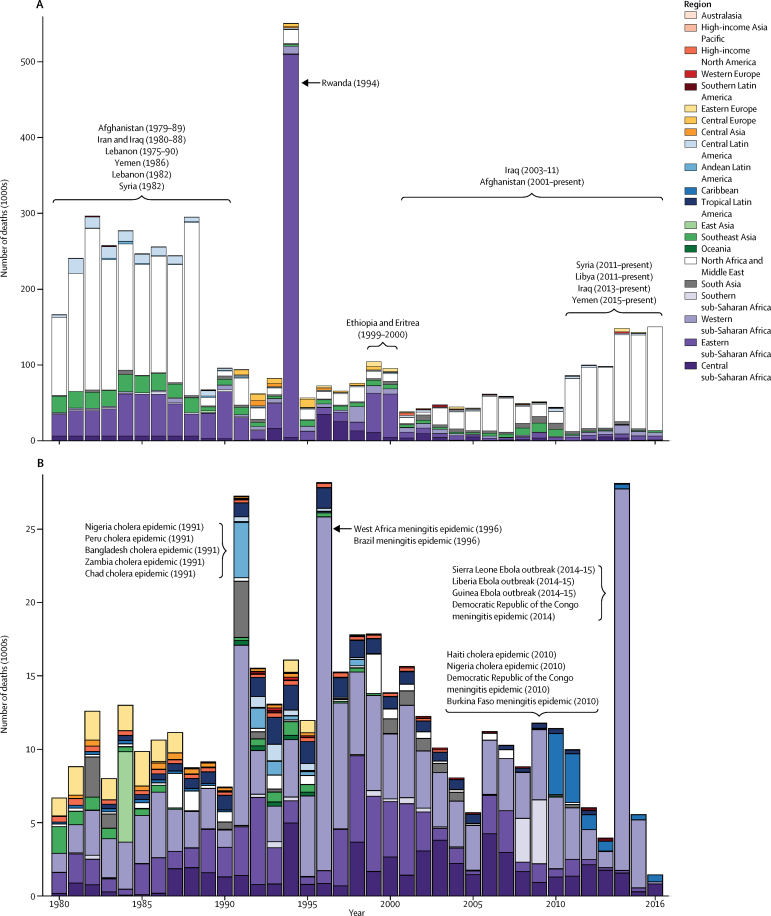

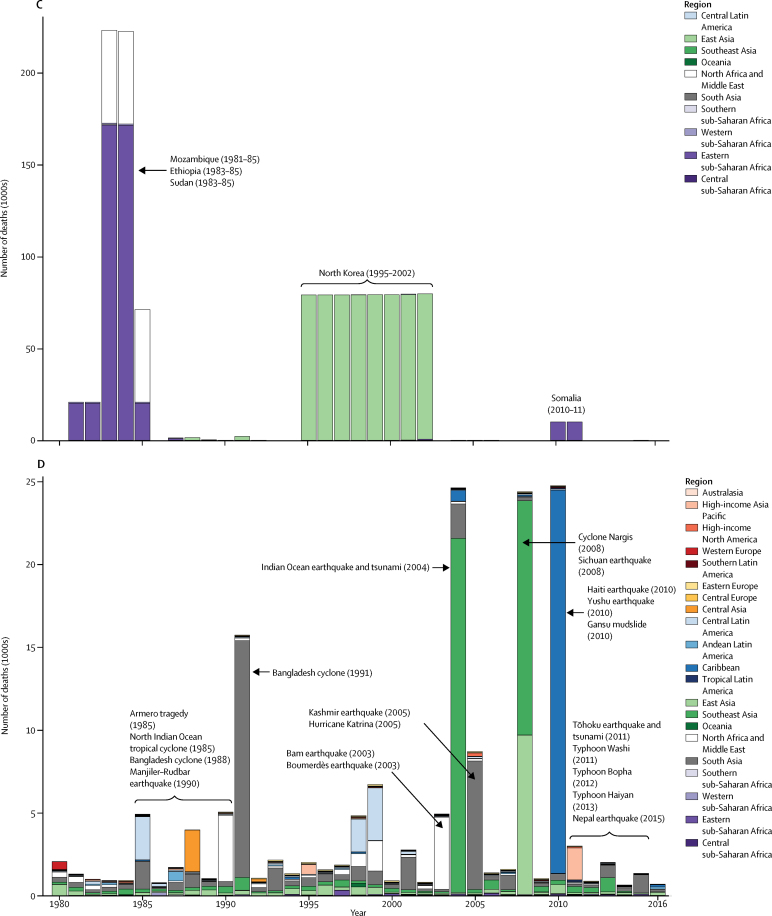

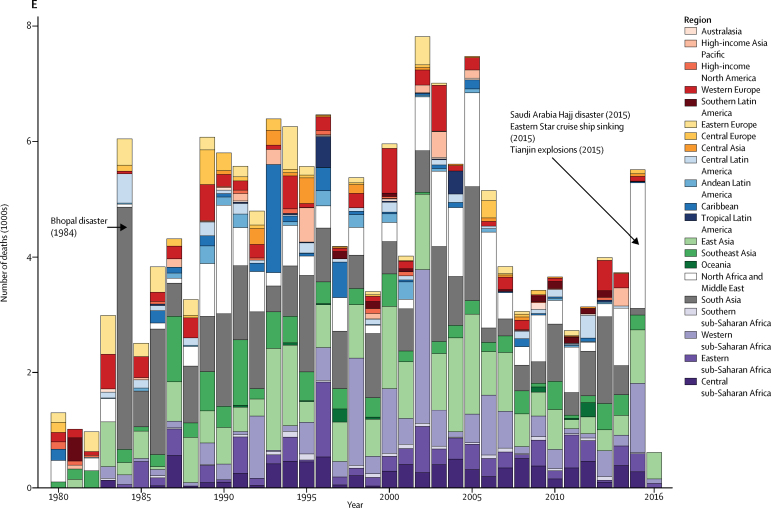
Deaths due to fatal discontinuities by category (A, conflict and terrorism; B, epidemics; C, famine; D, natural disasters; E, other injuries) and region from 1980 to 2016, both sexes combined Number of deaths due to fatal discontinuities are presented by region for each of the shock cause groups. Results are shown every year from 1980 to 2016; regions are colour-coded by super-region. Regions for which data were unavailable for specific causes are not presented. Specific events that cause a disproportionate number of deaths are identified. Conflict and terrorism includes military operations, civil conflicts, and terrorist attacks. Epidemics include outbreaks of cholera, meningococcal meningitis, and Ebola virus disease. Famine includes deaths due to protein-energy malnutrition. Natural disasters include exposure to forces of nature. Other injuries includes other transport injuries, fire, heat, and hot substances, poisonings, and other exposure to mechanical forces.

Large, abrupt changes in mortality levels can result from a number of causes and these stochastic events are separately modelled as fatal discontinuities in the GBD study due to their departure from typically observed demographic or epidemiological trends (figure 4). From 1980 to 1988, conflict and terrorism resulted in 2·28 million deaths (95% UI 1·55 million to 3·11 million) worldwide (figure 4A). In 1994, deaths in Rwanda (504 000 deaths [95% UI 180 000– 826 000]) dominated the highest single-year death toll from conflict and terrorism worldwide. The recent increase in global deaths from conflict and terrorism (2011–16) was dominated by mortality in North Africa and the Middle East which ranged from a low of 77·2% (95% UI 63·0–83·9) in 2014 to a high of 90·9% (85·8–94·3) in 2016 of global conflict and terrorism deaths during this time period. Epidemic-prone infectious diseases also resulted in fatal discontinuities from 1980 to 2016 (figure 4B) with peak years in 1991, 1996, and 2014 dominated by mortality from infectious disease outbreaks in western sub-Saharan Africa. Protein-energy malnutrition resulted in 1·22 million (95% UI 670 000 to 1·81 million) deaths globally from 1980 to 2016 (figure 4C), a total that is largely composed of mortality in eastern sub-Saharan Africa and North Africa and the Middle East during the 1980s, and in countries in ast Asia from 1995 through 2002.

Natural disasters—categorised as exposure to forces of nature—were large contributors to fatal discontinuities between 1980 and 2016 (figure 4D). Generally reflecting single large events within regions, high mortality levels from exposure to forces of nature were estimated for the years 1991 (143 000 [95% UI 65 000–222 000] deaths in South Asia), 2004 (214 000 [127 000–297 000] deaths in Southeast Asia), 2008 (141 000 [73 400–209 000] deaths in Southeast Asia, and 96 300 [15 300–185 000] deaths in East Asia), and 2010 (231 000 [36 700–443 000] deaths in the Caribbean). Fatal discontinuities from other injuries were more evenly distributed between regions, and total mortality in each year represents the accumulation of many smaller events relative to other forms of fatal discontinuity (figure 4E). Additional details on fatal discontinuities by location can be found in the additional supplemental results (appendix 2 p 1297).

### Causes of child death

Table 3 shows the major causes of under-5 deaths within each Level 1 cause grouping for 2006 and 2016 in addition to the median percent change over that time period. In 2016 there were 5·00 million deaths (95% UI 4·78 million to 5·23 million) in children under 5, a decrease of 33·0% (29·7–36·2) from 2006, when 7·46 million (7·27 million to 7·66 million) children died. Deaths in neonates (0–27 days of age) composed the largest proportion, 43·3% (95% UI 42·7–43·8), of total under-5 deaths in 2016. Deaths in this group decreased by 28·9% (95% UI 25·5–32·1) from 2006 to 2016, from 3·04 million (2·97 million to 3·12 million) to 2·16 million (2·06 million to 2·27 million); these decreases occurred across all mortality sources, although differences were not significant for neonatal sepsis and other neonatal infections. The largest percent change for neonates was in deaths due to tetanus, which decreased 67·6% (95% UI 57·9–75·3) from 46 300 deaths (24 000–62 100) in 2006 to 15 000 deaths (8330–20 400) in 2016, followed by diarrhoeal diseases, which decreased 58·3% (51·8–63·6) from 88 400 deaths (79 200–99 600) to 36 900 deaths (32 700–41 300), then by lower respiratory infections, which decreased 44·5% (38·9–49·6) from 278 000 deaths (245 000–305 000) to 154 000 deaths (133 000–172 000).

**Table 3:** Selected causes of global neonatal, childhood, and under-5 deaths in 2016 with mean percent change between 2006 and 2016 for both sexes combined

					**Neonates aged 0–27 days**		**Post-neonates aged 28–364 days**		**Children aged 1–4 years**		**Under-5 totals**	
					2016 (thousands)	Percent change 2006–16	2016 (thousands)	Percent change 2006–16	2016 (thousands)	Percent change 2006–16	2016 (thousands)	Percent change 2006–16
**All causes**					**2163·4 (2064·2 to 2265·4)**	–28·9 (−32·1 to −25·5)*	**1485·2 (1420·1 to 1550·8)**	–34·5 (−37·4 to −31·3)*	**1350·6 (1280·0 to 1428·9)**	–37·2 (−40·5 to −33·3)*	**4999·3 (4775·5 to 5234·7)**	–33·0 (−36·2 to −29·7)*
	**Communicable, maternal, neonatal, and nutritional disorders**				**1929·9 (1838·0 to 2027·2)**	–29·8 (−32·9 to −26·3)*	**1163·2 (1103·1 to 1225·2)**	–37·9 (−40·9 to −34·6)*	**1036·9 (970·7 to 1111·8)**	–40·3 (−44·0 to −35·9)*	**4130·1 (3921·6 to 4343·0)**	–35·0 (−38·2 to −31·5)*
		**HIV/AIDS and tuberculosis**			**··**	**··**	**54·6 (49·8 to 60·2)**	–55·3 (−59·1 to −50·6)*	**33·1 (29·8 to 37·0)**	–60·2 (−64·3 to −54·5)*	**87·7 (80·3 to 96·4)**	–57·2 (−60·9 to −52·8)*
			HIV/AIDS		··	··	43·2 (39·2 to 48·0)	–58·6 (−62·4 to −53·9)*	18·5 (16·8 to 20·4)	–67·1 (−69·8 to −63·9)*	61·7 (56·0 to 68·0)	–61·6 (−64·8 to −57·6)*
		**Diarrhoea, lower respiratory infections, and other common infectious diseases**			**227·0 (201·7 to 248·6)**	–48·3 (−52·3 to −43·9)*	**709·1 (657·6 to 763·9)**	–42·2 (−46·4 to −37·7)*	**510·3 (456·8 to 569·6)**	–46·4 (−52·2 to −39·3)*	**1446·5 (1339·9 to 1564·0)**	–44·7 (−49·0 to −39·9)*
			Diarrhoeal diseases		··	··	1·1 (0·3 to 3·4)	–34·4 (−86·5 to 235·6)	22·6 (11·0 to 41·2)	–20·8 (−34·5 to −6·0)*	23·7 (11·8 to 42·2)	–21·6 (−38·0 to −5·1)*
			Intestinal infectious diseases		154·2 (132·9 to 171·9)	–44·5 (−49·6 to −38·9)*	337·7 (306·4 to 377·3)	–40·4 (−45·5 to −34·5)*	160·7 (137·5 to 186·9)	–44·8 (−53·1 to −35·9)*	652·6 (586·5 to 720·6)	–42·5 (−47·8 to −37·1)*
			Lower respiratory infections		19·1 (14·4 to 26·6)	–22·3 (−35·4 to −3·7)*	70·8 (55·0 to 104·5)	–15·9 (−32·3 to 13·5)	56·4 (42·3 to 88·6)	–24·7 (−43·4 to 10·6)	146·3 (114·6 to 216·4)	–20·3 (−35·4 to 8·3)
			Meningitis		··	··	34·3 (18·2 to 59·6)	–35·5 (−63·5 to 19·7)	33·6 (18·0 to 57·6)	–36·4 (−64·0 to 20·1)	68·0 (36·3 to 117·3)	–35·9 (−63·4 to 18·0)
			Whooping cough		15·0 (8·3 to 20·4)	–67·6 (−75·3 to −57·9)*	2·5 (1·4 to 3·9)	–65·5 (−77·7 to −47·2)*	1·3 (0·6 to 2·3)	–61·4 (−75·6 to −39·5)*	18·8 (10·6 to 24·9)	–67·0 (−73·8 to −57·9)*
			Tetanus		··	··	17·7 (6·5 to 39·3)	–72·0 (−76·5 to −66·6)*	41·3 (15·7 to 87·3)	–72·0 (−76·9 to −66·7)*	59·0 (22·1 to 126·0)	–72·0 (−76·6 to −66·9)*
			Measles		17·2 (12·4 to 23·4)	–40·1 (−59·1 to −12·4)*	178·1 (134·5 to 228·3)	–26·3 (−47·2 to 2·0)	355·0 (284·5 to 4 31·9)	–29·7 (−46·2 to −8·2)*	550·2 (433·1 to 679·9)	–29·0 (−46·9 to −5·4)*
		**Neglected tropical diseases and malaria**			**15·4 (10·7 to 21·4)**	–42·5 (−62·4 to −13·5)*	**167·3 (123·2 to 217·4)**	**–27·4 (−49·1 to 2·8)**	**334·3 (261·4 to 410·3)**	–30·0 (−47·6 to −6·8)*	**516·9 (398·2 to 647·9)**	–29·7 (−48·4 to −4·6)*
			Malaria		1639·1 (1556·1 to 1726·2)	–25·9 (−29·7 to −22·0)*	82·2 (70·4 to 93·1)	–15·0 (−30·4 to −0·2)*	9·7 (7·7 to 11·4)	–8·0 (−31·8 to 11·2)	1731·0 (1644·1 to 1822·9)	–25·3 (−29·3 to −21·3)*
		**Neonatal disorders**			**590·6 (541·3 to 643·4)**	–27·9 (−33·7 to −22·1)*	**27·4 (22·0 to 32·0)**	–19·3 (−37·8 to −1·8)*	**2·4 (1·7 to 3·0)**	**–8·8 (−40·0 to 18·3)**	**620·4 (568·7 to 674·7)**	–27·5 (−33·7 to −21·5)*
			Neonatal preterm birth complications		504·2 (449·2 to 552·2)	–23·5 (−30·7 to −15·7)*	16·5 (12·3 to 20·5)	–15·0 (−32·7 to 6·8)	4·1 (3·2 to 4·9)	–7·9 (−28·4 to 11·3)	524·9 (466·7 to 576·2)	–23·1 (−30·3 to −15·6)*
			Neonatal encephalopathy due to birth asphyxia and trauma		224·9 (190·1 to 298·3)	–12·5 (−22·6 to 0·7)	17·2 (12·9 to 21·8)	–1·3 (−24·8 to 23·2)	0·9 (0·5 to 1·3)	11·0 (−29·8 to 71·6)	243·0 (205·0 to 317·7)	–11·8 (−21·9 to 1·5)
			Neonatal sepsis and other neonatal infections		45·8 (39·7 to 53·5)	–43·1 (−50·8 to −34·8)*	3·3 (2·4 to 4·4)	–39·6 (−55·9 to −17·6)*	0·2 (0·1 to 0·2)	–39·5 (−57·4 to −14·1)*	49·2 (42·6 to 57·0)	–42·8 (−50·7 to −34·4)*
			Haemolytic disease and other neonatal jaundice		273·6 (246·8 to 301·2)	–30·7 (−37·4 to −22·4)*	17·8 (14·4 to 20·9)	–12·9 (−32·9 to 7·0)	2·1 (1·8 to 2·5)	–9·9 (−36·4 to 13·9)	293·6 (265·6 to 322·8)	–29·7 (−36·6 to −21·3)*
			Other neonatal disorders		273·6 (246·8 to 301·2)	–30·7 (−37·4 to −22·4)	17·8 (14·4 to 20·9)	–12·9 (−32·9 to 7·0)	2·1 (1·8 to 2·5)	–9·9 (−36·4 to 13·9)	293·6 (265·6 to 322·8)	–29·7 (−36·6 to −21·3)
		**Nutritional deficiencies**			**··**	**··**	**80·6 (69·6 to 95·5)**	–26·5 (−37·1 to −11·3)*	**91·8 (74·9 to 114·0)**	–31·0 (−46·2 to −10·7)*	**172·5 (147·7 to 204·5)**	–29·0 (−41·2 to −13·3)*
		**Other communicable maternal neonatal and nutritional diseases**			**46·6 (29·0 to 69·3)**	–31·7 (−40·6 to −20·6)*	**58·6 (38·8 to 82·8)**	–21·7 (−34·3 to −4·0)*	**37·0 (26·2 to 50·5)**	–29·8 (−42·3 to −11·9)*	**142·2 (95·4 to 201·8)**	–27·3 (−36·7 to −15·8)*
			Sexually transmitted diseases excluding HIV		38·4 (21·6 to 60·5)	–33·9 (−42·8 to −22·9)*	39·5 (22·2 to 6 2·2)	–21·2 (−32·2 to −7·8)*	21·7 (11·9 to 34·0)	–24·7 (−37·6 to −9·8)*	99·6 (56·4 to 156·5)	–27·3 (−36·9 to −15·5)*
				Syphilis	38·4 (21·6 to 60·5)	–33·9 (−42·8 to −22·9)*	39·5 (22·2 to 62·2)	–21·2 (−32·2 to −7·8)*	21·7 (11·9 to 34·0)	–24·7 (−37·6 to −9·8)*	99·6 (56·4 to 156·5)	–27·3 (−36·9 to −15·5)*
	**Non-communicable diseases**				**216·8 (191·7 to 239·3)**	–20·6 (−28·2 to −10·7)*	**249·0 (224·6 to 273·8)**	–18·5 (−25·3 to −11·0)*	**153·9 (131·5 to 175·4)**	–21·2 (−29·5 to −12·2)*	**619·6 (555·4 to 680·8)**	–19·9 (−26·7 to −12·9)*
		Other non-communicable diseases			202·8 (178·5 to 224·6)	–20·3 (−28·1 to −9·7)*	187·9 (163·9 to 211·7)	–17·1 (−24·9 to −8·3)*	60·9 (45·2 to 75·3)	–17·0 (−28·7 to −4·3)*	451·6 (393·6 to 505·7)	–18·6 (−26·1 to −9·8)*
				Congenital birth defects	200·0 (175·9 to 221·9)	–20·2 (−28·2 to −9·6)*	156·8 (135·4 to 178·3)	–18·0 (−26·8 to −7·3)*	58·4 (42·9 to 72·5)	–17·9 (−29·8 to −5·0)*	415·2 (360·0 to 465·5)	–19·0 (−27·0 to −9·5)*
				Sudden infant death syndrome	2·8 (2·2 to 3·3)	–25·0 (−40·2 to −12·1)*	26·3 (21·2 to 31·7)	–16·5 (−32·8 to 1·1)	··	··	29·1 (23·4 to 34·9)	–17·4 (−33·2 to −1·1)*
	**Injuries**				**16·8 (14·4 to 18·3)**	–25·3 (−32·0 to −17·1)*	**73·0 (64·9 to 80·3)**	–19·9 (−26·9 to −12·1)*	**159·8 (145·8 to 175·3)**	–27·4 (−34·7 to −17·6)*	**249·6 (227·3 to 270·5)**	–25·2 (−32·0 to −17·2)*
			Transport injuries		2·2 (1·9 to 2·7)	–37·8 (−48·2 to −23·5)*	9·7 (8·6 to 11·3)	–26·4 (−36·3 to −12·8)*	32·7 (28·9 to 36·8)	–27·2 (−36·4 to −16·3)*	44·6 (40·3 to 50·0)	–27·6 (−36·0 to −18·0)*
				Road injuries	1·9 (1·7 to 2·4)	–37·5 (−47·9 to −23·7)*	8·9 (7·9 to 10·3)	–26·5 (−36·5 to −13·4)*	30·9 (27·3 to 34·9)	–27·2 (−35·9 to −16·5)*	41·7 (37·4 to 46·7)	–27·6 (−35·8 to −18·0)*
			Unintentional injuries		11·4 (9·3 to 12·6)	–28·5 (−35·4 to −20·2)*	58·3 (50·3 to 65·3)	–20·0 (−27·9 to −11·7)*	107·3 (95·9 to 119·7)	–33·3 (−40·3 to −23·5)*	177·0 (157·1 to 196·1)	–29·1 (−35·8 to −20·8)*
				Drowning	0·6 (0·5 to 0·7)	–27·8 (−37·0 to −16·3)*	5·5 (4·6 to 6·6)	–35·1 (−42·8 to −19·1)*	44·6 (38·8 to 50·7)	–44·4 (−51·3 to −32·8)*	50·7 (44·0 to 57·3)	–43·4 (−50·2 to −31·4)*

Asterisks denote statistically significant changes. Data in parenthesis are 95% uncertainty intervals. This table shows major causes of death within each Level 1 group that accounted for deaths in children younger than 5 years. Under 5=between birth and age 5 years.

Total deaths among post-neonates (aged 28–364 days) and children aged 1–4 years decreased between 2006 and 2016 by 34·5% (95% UI 31·3–37·4) to 1·49 million deaths (1·42 million to 1·55 million) and 37·2% (33·3–40·5) to 1·35 million deaths (1·28 million to 1·43 million), respectively. Among post-neonates, half of deaths in 2016 were caused by lower respiratory infections (22·7% [95% UI 20·7–25·3], 338 000 deaths [306 000–377 000]), diarrhoeal diseases (15·5% [13·7–17·5], 231 000 deaths [202 000–263 000]), and malaria (11·3% [8·35–14·53], 167 000 deaths [123 000–217 000]). Injuries contributed relatively more to mortality among children aged 1–4 years compared with children younger than 1 year, accounting for 160 000 deaths in 2016 [146 000–175 000]. Deaths from measles decreased the most from 2006 to 2016 among both post-neonates and children aged 1–4 years, dropping by 72·0% (95% UI 66·6–76·5) from 63 100 deaths (23 700 to 140 000) to 17 700 deaths (6470–39 300) and by 72·0% (66·7–76·9) from 147 000 deaths (59 600 to 302 000) to 41 300 (15 700–87 300), respectively.

### Global YLLs (by cause)

**Figure 5:**
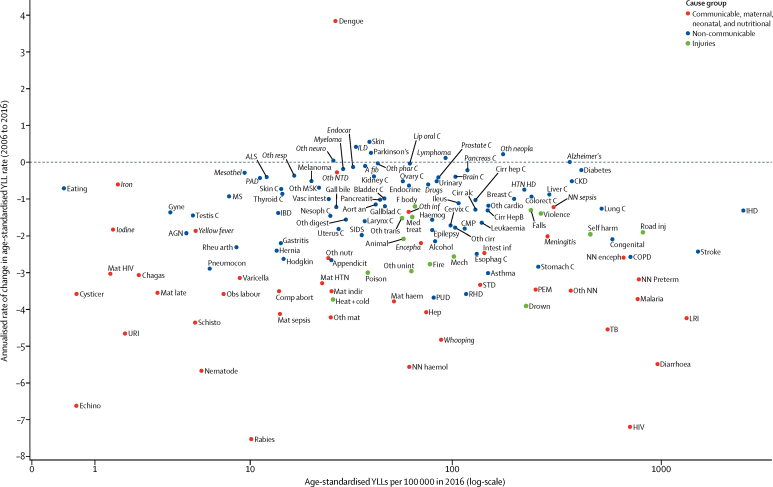
Annualised rate of change in age-standardised YLLs from 2006 to 2016 versus global age-standardised YLLs per 100 000 due to each Level 3 cause Causes with 100 000 YLLs or more are presented. YLLs are represented on a logarithmic scale. Italicised causes highlight changes that are not statistically significant. Level 3 causes related to fatal discontinuities (conflict and terrorism, executions and police conflict, and exposures to forces of nature) are excluded. Not shown in figure: Afr tryp: YLL=1·69 ARC=-15·23, Measles: YLL=80·99 ARC=–13·32, Zika virus disease: YLL=0·01 ARC=48·47, Ebola virus disease: YLL=0·003 ARC=32·57, Diphtheria: YLL=1·23 ARC=-11·58, Otitus: YLL=0·7 ARC=–7·32, tetanus: YLL=33·52 ARC=–10·24, Leish: YLL=9·73 ARC=–9·44. ARC=annualised rate of change. A fib=atrial fibrillation and flutter. Afr tryp=African trypanosomiasis. AGN=acute glomerulonephritis. Alcohol=alcohol use disorders. ALS=motor neuron disease. Alzheimer=Alzheimer's disease and other dementias. Animal=animal contact. Aort an=aortic aneurysm. Appendicit=appendicitis. Bladder C=bladder cancer. Brain C=brain and nervous system cancer. Breast C=breast cancer. Cervix C=cervical cancer. Chagas=Chagas disease. Cirr alc=cirrhosis and other chronic liver diseases due to alcohol use. Cirr HepB=cirrhosis and other chronic liver diseases due to hepatitis B. Cirr hep C=cirrhosis and other chronic liver diseases due to hepatitis C. CKD=chronic kidney disease. CMP=cardiomyopathy and myocarditis. Colorect C=colon and rectum cancer. Comp abort=maternal abortion, miscarriage, and ectopic pregnancy. Congenital=congenital birth defects. COPD=chronic obstructive pulmonary disease. Cysticer=cysticercosis. Diabetes=diabetes mellitus. Diarrhoea=diarrhoeal diseases. Disaster=exposure to forces of nature. Drown=drowning. Drugs=drug use disorders. Eating=eating disorders. Echino=cystic echinococcosis. Encepha=encephalitis. Endocar=endocarditis. Endocrine=endocrine, metabolic, blood, and immune disorders. Oesophag C=oesophageal cancer. F body=foreign body. Fire=fire, heat, and hot substances. Gall bile=gallbladder and biliary diseases. Gallblad C=gallbladder and biliary tract cancer. Gastritis=gastritis and duodenitis. Gyne=Gynecological diseases. Heat + cold=environmental heat and cold exposure. Haemog=haemoglobinopathies and haemolytic anaemias. Hep=hepatitis. Hernia=inguinal, femoral, and abdominal hernia. HIV=HIV/AIDS. Hodgkin=Hodgkin's lymphoma. HTN HD=hypertensive heart disease. IBD=inflammatory bowel disease. IHD=ischaemic heart disease. ILD=interstitial lung disease and pulmonary sarcoidosis. Ileus=paralytic ileus and intestinal obstruction. Intest inf=intestinal infectious diseases. Iodine=iodine deficiency. Iron=iron-deficiency anaemia. Kidney C=kidney cancer. Larynx C=larynx cancer. Leish=leishmaniasis. Lip oral C=lip and oral cavity cancer. Liver C=liver cancer. LRI=lower respiratory infections. Lung C=tracheal, bronchus, and lung cancer. Lymphoma=non-Hodgkin lymphoma. Mat haem=maternal haemorrhage. Mat HIV=maternal deaths aggravated by HIV/AIDS. Mat HTN=maternal hypertensive disorders. Mat indir=indirect maternal deaths. Mat late=late maternal deaths. Mat sepsis=maternal sepsis and other maternal infections. Mech=exposure to mechanical forces. Med treat=adverse effects of medical treatment. Melanoma=malignant skin melanoma. Mesothel=mesothelioma. MS=multiple sclerosis. Myeloma=multiple myeloma. Nasoph C=nasopharynx cancer. Nematode=intestinal nematode infections. NN enceph=neonatal encephalopathy due to birth asphyxia and trauma. NN haemol=haemolytic disease and other neonatal jaundice. NN Preterm=neonatal preterm birth complications. NN sepsis=neonatal sepsis and other neonatal infections. Obst labor=maternal obstructed labor and uterine ruptures. Oth cardio=other cardiovascular and circulatory diseases. Oth cirr=cirrhosis and other chronic liver diseases due to other causes. Other digest=other digestive diseases. Oth inf=other infectious diseases. Oth mat=other maternal disorders. Oth MSK=other musculoskeletal disorders. Oth neopla=other neoplasms. Oth neuro=other neurological disorders. Oth NN=other neonatal disorders. Oth NTD=other neglected tropical diseases. Oth nutr =other nutritional deficiencies. Oth phar C=other pharynx cancer. Oth resp=other chronic respiratory diseases. Oth trans=other transport injuries. Oth unint=other unintentional injuries. Otitis=otitis media. Ovary C=ovarian cancer. PAD=peripheral artery disease. Pancreas C=pancreatic cancer. Pancreatit=pancreatitis. Parkinson's=Parkinson's disease. PEM=protein-energy malnutrition. Pneumocon=pneumoconiosis. Poison=poisonings. Prostate C=prostate cancer. PUD=peptic ulcer disease. RHD=rheumatic heart disease. Rheu arth=rheumatoid arthritis. Road inj=road injuries. Schisto=schistosomiasis. SIDS=sudden infant death syndrome. Skin=skin and subcutaneous diseases. Skin C=non-melanoma skin cancer. State viol=executions and police conflict. STD=sexually transmitted diseases excluding HIV. Stomach C=stomach cancer. Stroke=cerebrovascular disease. TB=tuberculosis. Testis C=testicular cancer. Thyroid C=thyroid cancer. URI=upper respiratory infections. Urinary=urinary diseases and male infertility. Uterus C=uterine cancer. Varicella=varicella and herpes zoster. Vasc intest=vascular intestinal disorders. Violence=interpersonal violence. Whooping=whooping cough. YLLs=years of life lost.

Figure 5 shows both the level of age-standardised YLL rates for each cause and the trend since 2006 represented as the annualised rate of change in the age-standardised YLL rate. Two causes had statistically significant, positive annualised rates of change in age-standardised YLL rates since 2006: dengue (3·8% [95% UI 1·4–6·4]); and Parkinson's disease (0·25% [0·054–0·46]). Among the leading ten causes of YLLs, the median rate of change was a decrease of 2·89%, higher than the median rate of change (decrease of 1·59%) for causes below the leading ten for YLLs.

**Figure 6:**
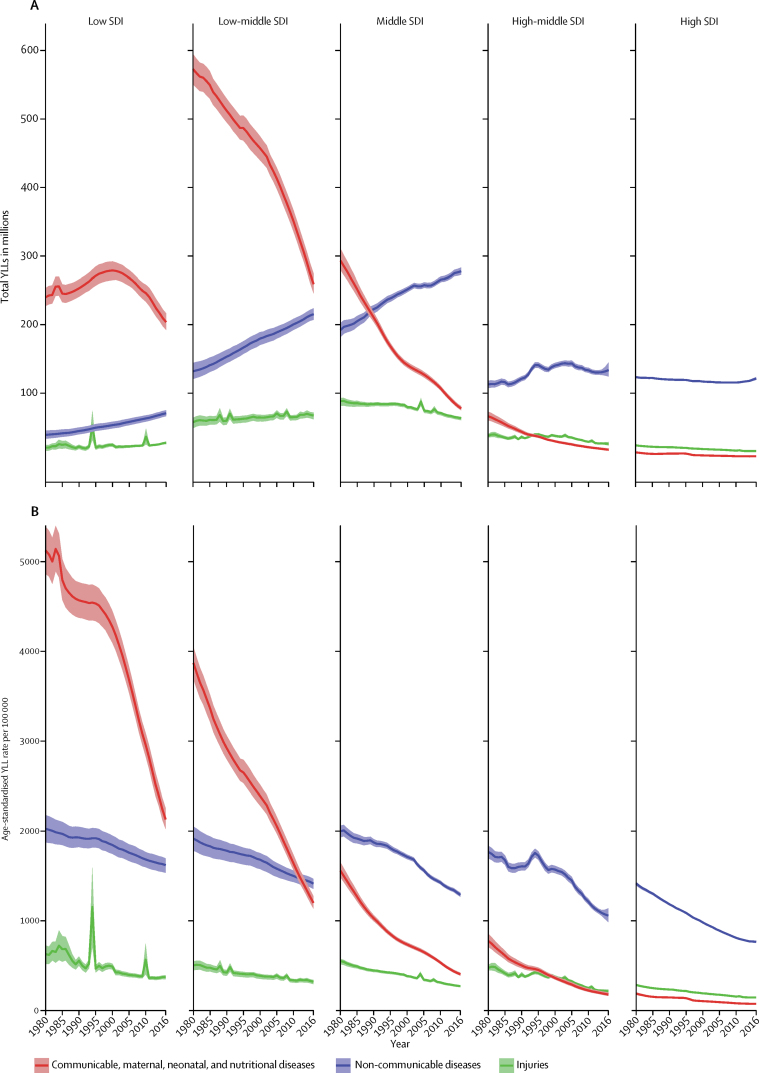
Trends of (A) total YLLs and (B) age-standardised YLL rates from 1980 to 2016, by GBD Level 1 cause, by SDI quintile Shaded areas show 95% uncertainty intervals. GBD=Global Burden of Disease. SDI=Socio-demographic Index. YLLs=years of life lost.

Total YLLs by SDI quintile for Level 1 causes within the GBD cause hierarchy are shown in figure 6A. The greatest total burden of YLLs for CMNN causes in 2016 were in low and low-middle SDI, at 204 million (95% UI 193 million to 217 million) and 259 million (246 million to 274 million), respectively. The largest decrease in total YLLs from 1990 to 2016 was for CMNN causes in middle SDI, which decreased by 36·9% (95% UI 34·7–39·2). The largest increase in total YLLs was for injuries in low SDI 21·2% (95% UI 11·7–31·9).

Trends in age-standardised YLL rates (figure 6B) further illustrate the epidemiological transition within and across locations by SDI quintiles. Across quintiles, the greatest difference between SDI quintiles in YLL age-standardised rates in each time period was for CMNN causes, which ranged from 1875·4 (95% UI 1793·1–2032·5) per 100 000 in high SDI to 51 247·8 (48 640·9–53 887·2) per 100 000 in low SDI in 1980, and in 2016 from 739·0 (707·7–761·7) per 100 000 in high SDI to 21 299·6 (20 220·9–22 549·4) per 100 000 in low SDI. Declines in age-standardised YLL rates for CMNN causes over the 37 years examined ranged from 60·6% (95% UI 58·2–64·7) for high SDI locations, 77·0% (73·4–79·9) for high-middle SDI, 74·1% (72·3–76·0) for middle SDI, 69·0% (66·9–70·9) for low-middle SDI, and 58·5% (55·6–61·0) for low SDI.

Although not as large as the gradient between SDI quintiles observed for CMNN causes, age-standardised YLL rates for NCDs were generally higher at lower increments of SDI in each year examined. In all quintiles, age-standardised rates for NCDs have decreased; by contrast with trends for CMNN causes, the pace of decline for NCD rates was slowest in low SDI and fastest in high SDI. Age-standardised YLL rates due to injuries varied the least across quintiles and over time but were highest in the low-SDI quintile. The primary exception was the large increase in age-standardised YLL rates from injuries among low-SDI locations in 1994, a finding driven by deaths from conflict and terror in Rwanda. Reflecting the availability of data for causes of death, the UIs for each of the cause groupings are larger in the lower SDI quintiles.

**Figure 7:**
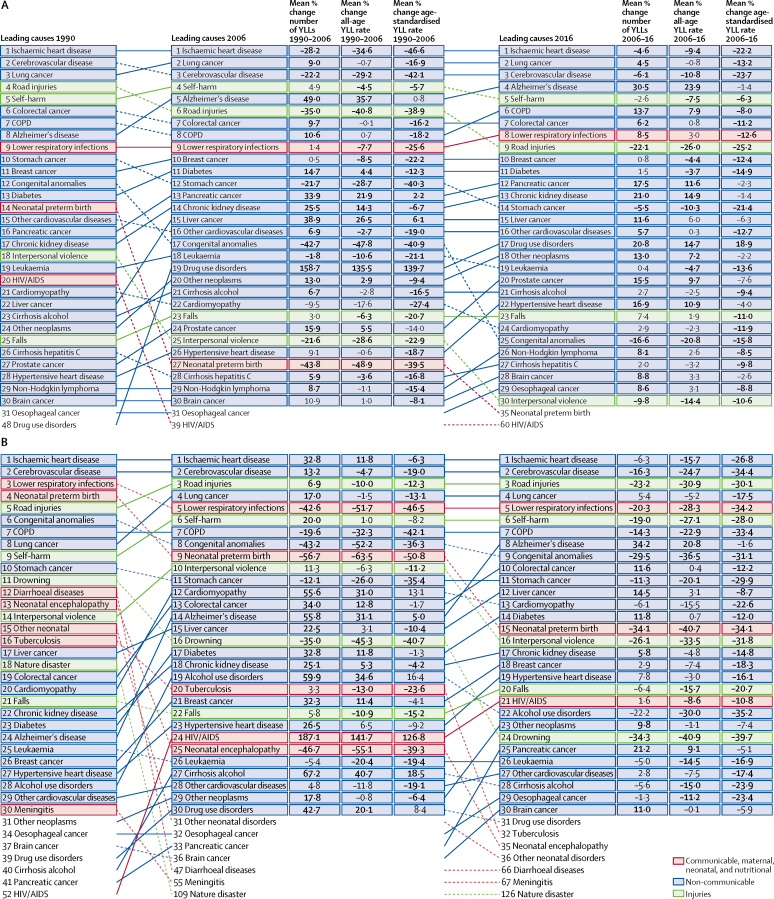

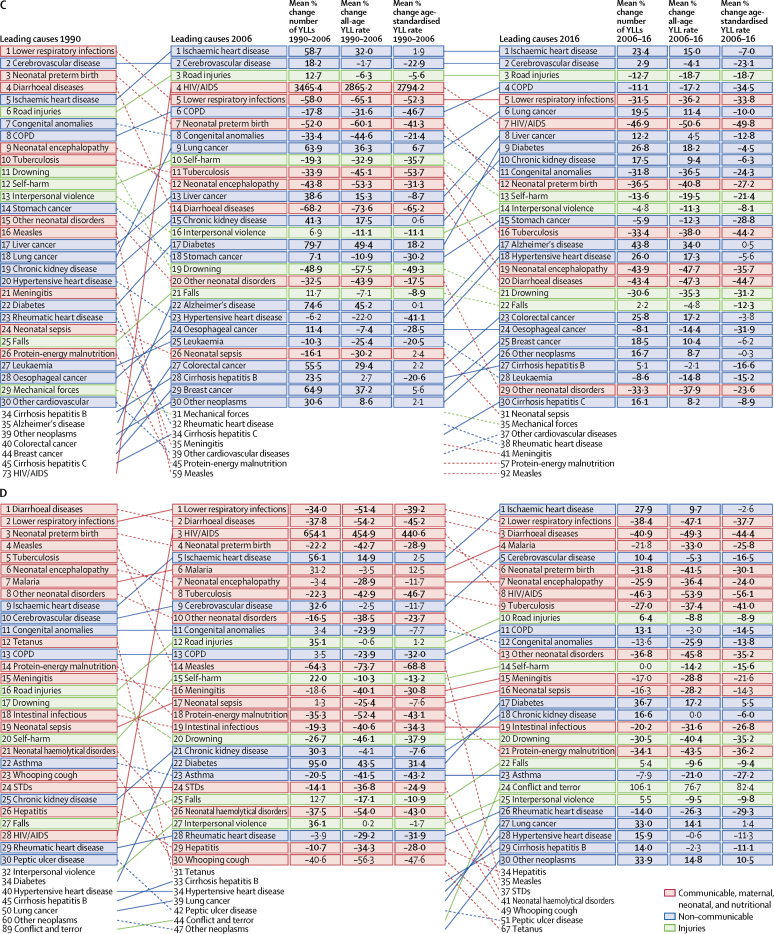

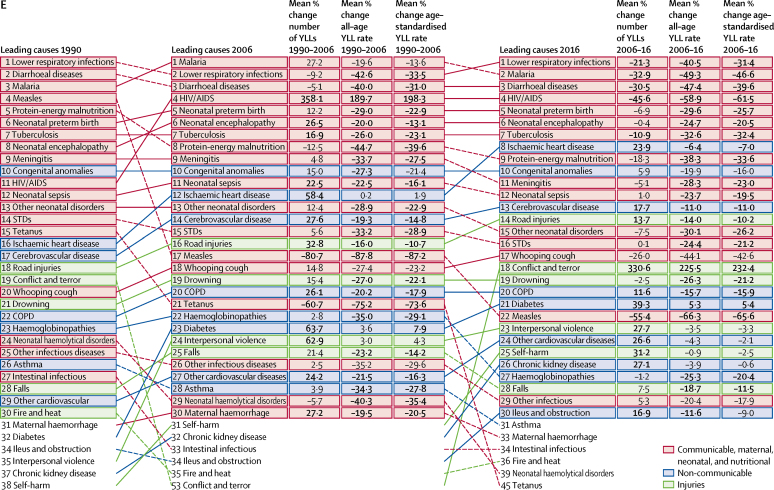
Leading 30 Level 3 causes of total YLLs by SDI grouping (high, A; high-middle, B; middle, C; low-middle, D; low, E) for 1990, 2006, and 2016, with percent change in number of YLLs, and all-age and age-standardised rates Causes are connected by arrows between time periods; solid lines are increases and dashed lines are decreases. For the time period 1990–2006 and for 2006–16, three measures of change are shown: percent change in the number of YLLs, percent change in the all-age YLL rate, and percent change in the age-standardised YLL rate. Statistically significant changes are shown in bold. COPD=chronic obstructive pulmonary disease. STDs=sexually transmitted diseases. SDI=Socio-demographic Index. YLLs=years of life lost.

The number of communicable diseases in the leading 30 causes of YLLs in each quintile of SDI decreased with increasing SDI, reflecting the ongoing epidemiological transition (figure 7). In 2016, the leading 30 causes of all-age YLLs for high-SDI locations were predominantly from NCDs and injuries, with one CMNN cause—lower respiratory infections—within the leading 30 causes (figure 7A). There was no change from 2006 to 2016 in the leading three causes of YLLs for high-SDI, high-middle-SDI, and middle-SDI locations, although age-standardised YLL rates decreased for each of these causes (figure 7A–C). The largest reduction in age-standardised YLL rates for high-SDI locations came from road injuries, which fell by 25·2% (95% UI 22·5–27·6), while the largest increase was for drug use disorders (rising by 18·9% [13·8–23·3]). For both low-middle-SDI and low-SDI quintiles, age-standardised rates decreased from 2006 to 2016 for all causes ranked higher than the tenth leading cause (figure 7D, E) with the exception of ischaemic heart disease at low-middle SDI, for which the increase was non-significant. In low-SDI locations, shifts in cause rankings by YLLs from 2006 to 2016 occurred beyond the top ten causes, with many CMNN causes decreasing in rank, surpassed by NCD causes and injuries.

### Country-specific findings

In 2016, the burden of all-cause YLLs ranged from an age-standardised rate of 6834·9 (95% UI 6702·8–6974·3) per 100 000 in Japan to 80 115·5 (68 631·5–92 475·5) per 100 000 in the Central African Republic, however, the causes that contributed the most YLLs varied with location—although regional patterns and patterns associated with SDI were also evident. The global shift toward NCDs for both sexes has been driven by the effects of population growth, ageing, and the epidemiological transition;^3^, ^25^ the leading cause in each location provides a very high-level view of how these factors have affected patterns of premature mortality across the world. Detailed location-specific findings on YLLs are available online.

### Leading causes of YLLs

**Figure 8:**
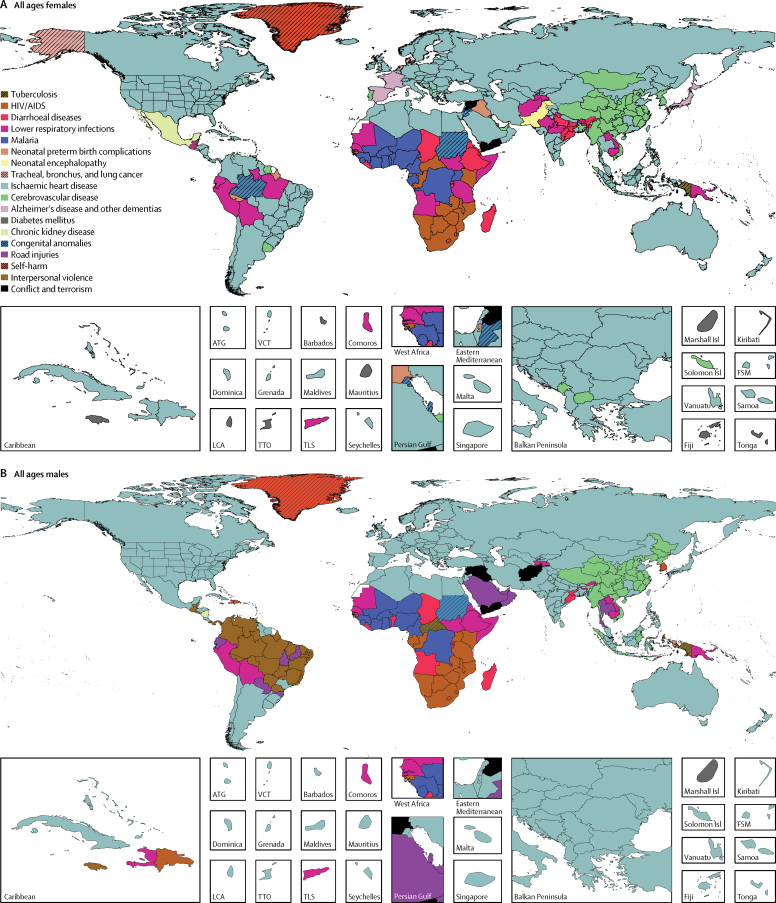
Leading Level 3 causes of total YLLs by country, for all ages, females (A) and males (B) ATG=Antigua and Barbuda. FSM=Federated States of Micronesia. Marshall Isl=Marshall Islands. Solomon Isl=Solomon Islands. LCA=Saint Lucia. TLS=Timor-Leste. TTO=Trinidad and Tobago. VCT=Saint Vincent and the Grenadines. YLLs=years of life lost.

Figure 8 maps the leading Level 3 causes of YLLs in 2016 in 195 countries and territories by sex. Ischaemic heart disease was the leading cause of YLLs for men in 113 countries and for 97 countries for women, spanning both high-SDI and high-middle SDI locations as well as many lower SDI locations, such as Kyrgyzstan. For both sexes, cerebrovascular disease was the leading cause of YLLs for many countries in Southeast and East Asia. Interpersonal violence was the leading cause for men in a corridor that runs from Central America through Tropical Latin America. In India, ischaemic heart disease was the leading cause for men and women. The leading cause of YLLs in China was cerebrovascular disease for both men and women. Across much of sub-Saharan Africa, the leading cause for both men and women varied between HIV/AIDS, malaria, diarrhoeal diseases, and lower respiratory infections; however, for men in the Central African Republic, tuberculosis was the leading cause. A few leading causes stand out for their departure from global or regional patterns; for men these included conflict in Syria, Yemen, and Afghanistan, and self-harm in Greenland and South Korea. For women, the general patterns were similar with the exception of a higher ranking for Alzheimer's disease as compared to men, in France, Spain, and Japan. Also notable were the high levels of YLLs for women from chronic kidney disease in Mexico, neonatal encephalopathy in Pakistan, cerebrovascular disease in Uruguay, and neonatal preterm birth complications in Iraq. Maternal disorders were not the leading cause of YLLs in any location.

### Observed YLLs compared to expected YLLs

**Figure 9:**
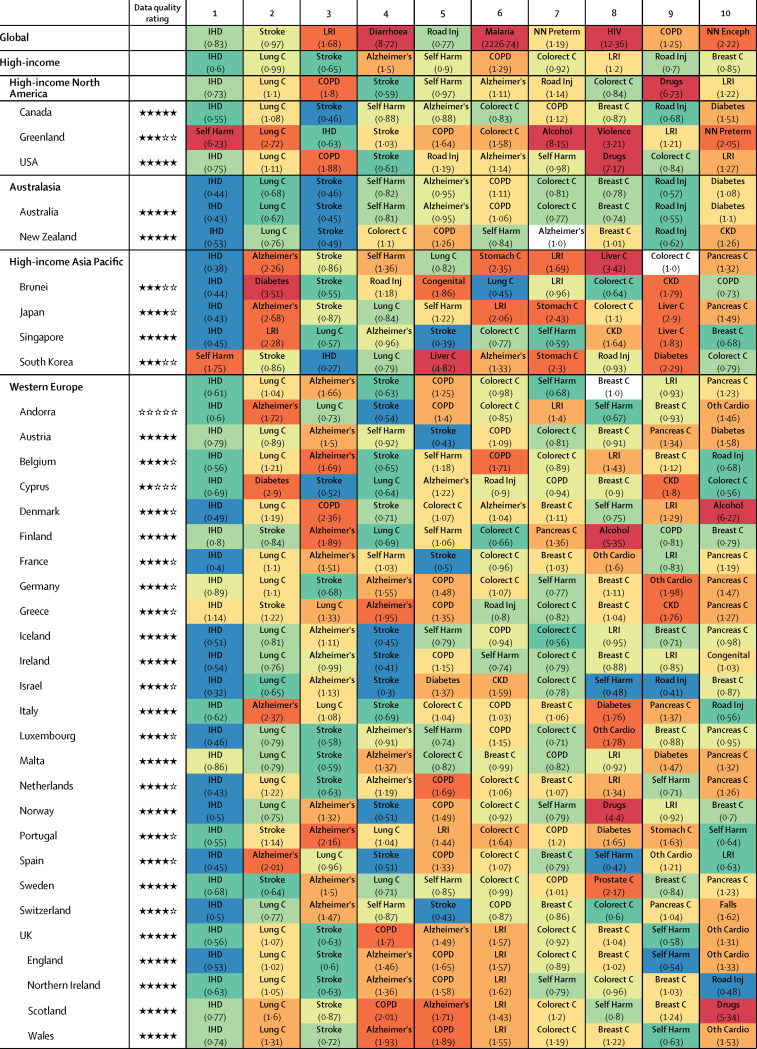

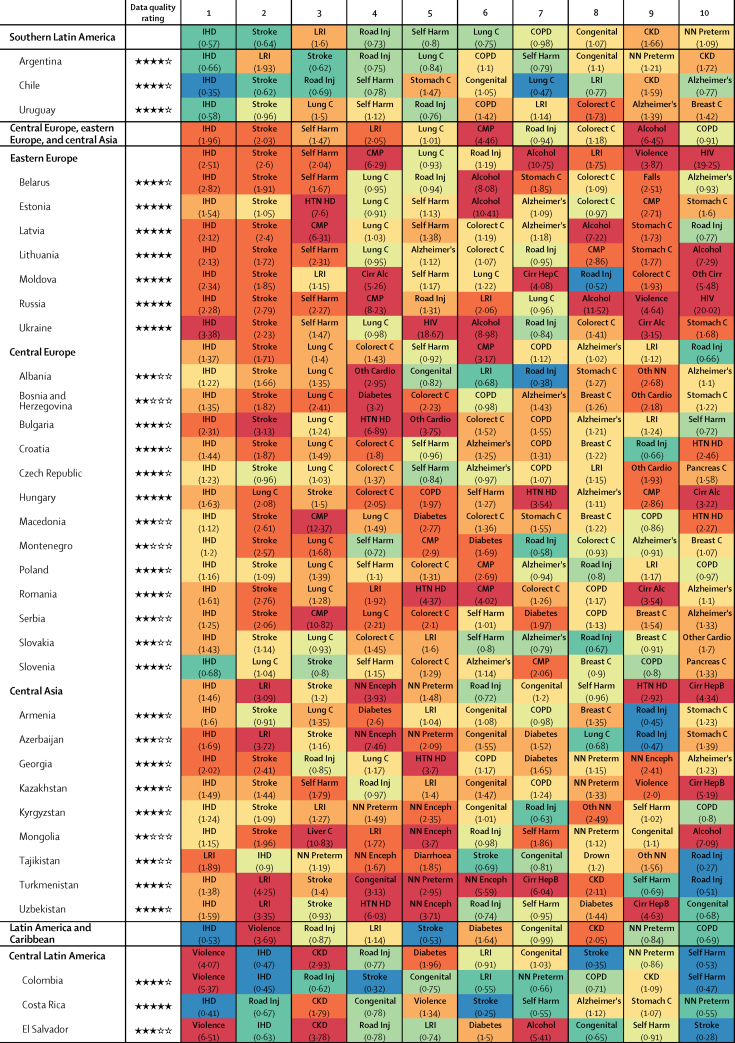

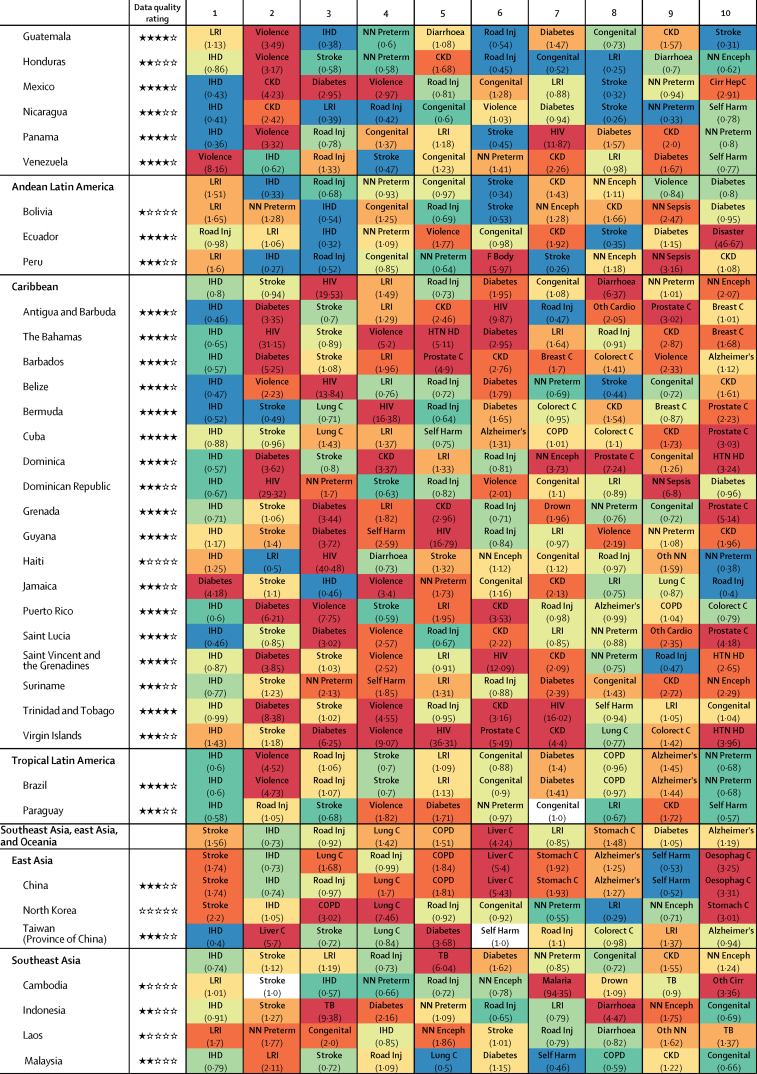

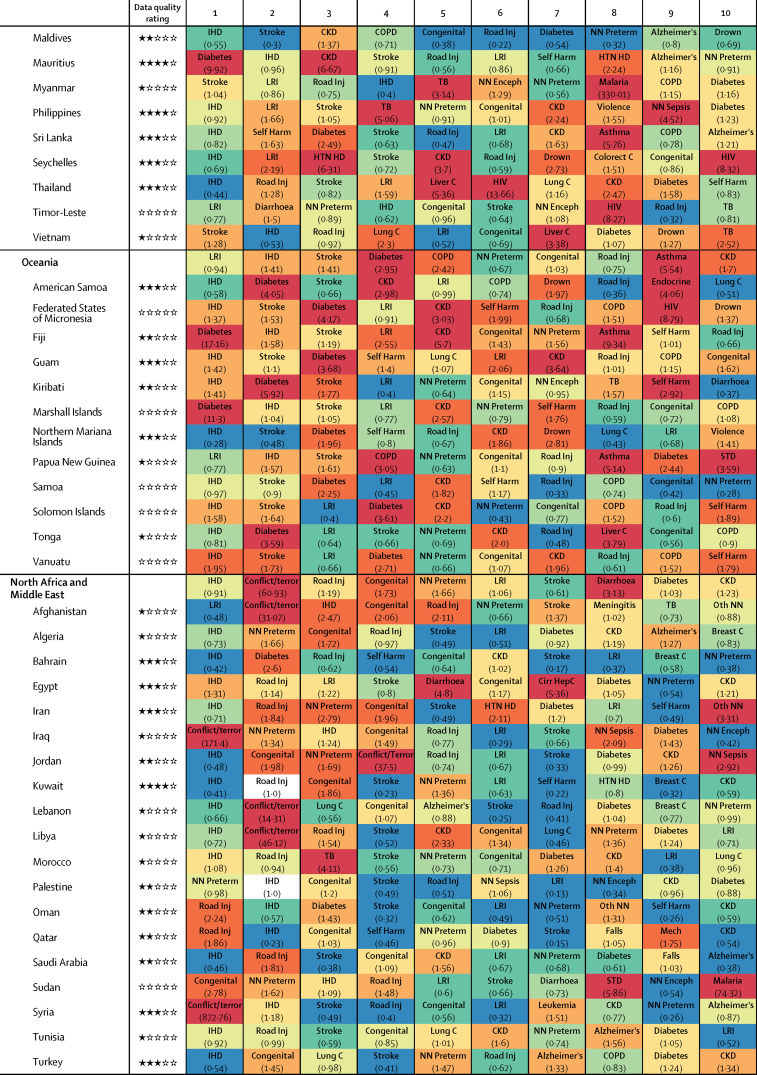

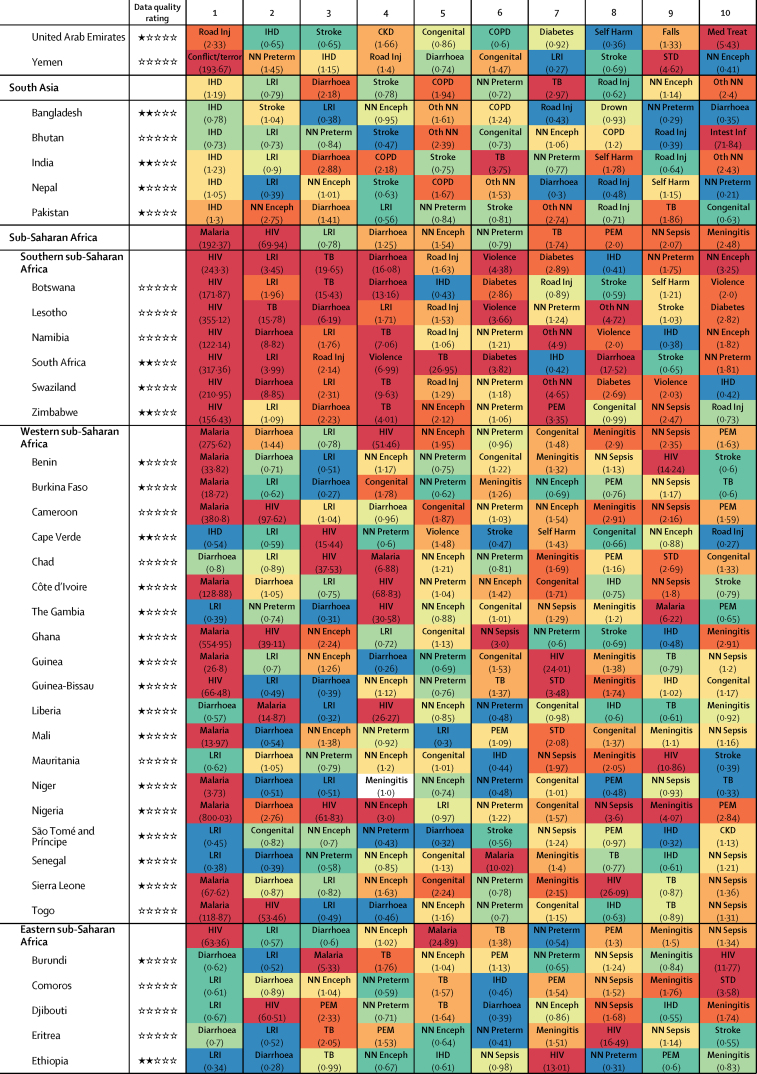

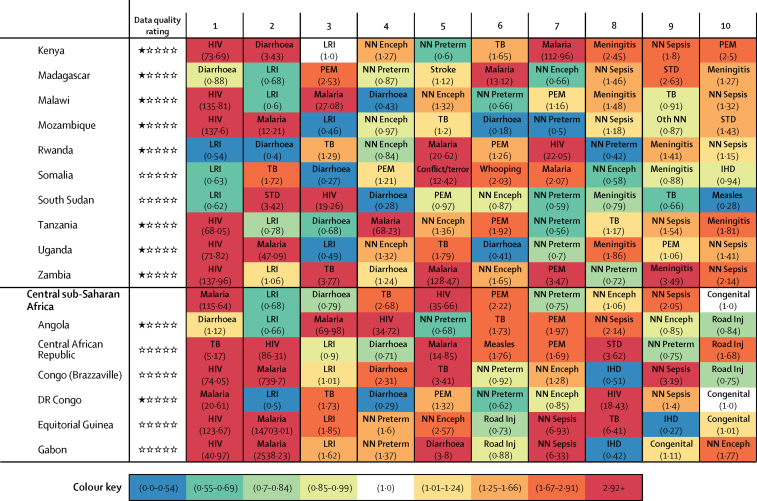
Leading 10 causes of total YLLs with the ratio of observed YLLs to YLLs expected on the basis of SDI in 2016, by location, with data quality rating Values shown in brackets represent the ratio of observed YLLs to predicted YLLs on the basis of SDI, rounded to two digits. Colour ranges were calculated to place a roughly equal number of cells into each bin. Alcohol=alcohol use disorders. Alzheimer=Alzheimer's disease and other dementias. Asthma=asthma. Breast C=breast cancer. Cirr Alc=cirrhosis and other chronic liver diseases due to alcohol use. Cirr HepB=cirrhosis and other chronic liver diseases due to hepatitis B. Cirr HepC=cirrhosis and other chronic liver diseases due to hepatitis C. CKD=chronic kidney disease. CMP=cardiomyopathy and myocarditis. Colorect C=colon and rectum cancer. Conflict terror=conflict and terrorism. Congenital=congenital birth defects. COPD=chronic obstructive pulmonary disease. Diabetes=diabetes mellitus. Diarrhoea=diarrhoeal diseases. Disaster=exposure to forces of nature. Drown=drowning. Drugs=drug use disorders. Endocrine=endocrine, metabolic, blood, and immune disorders. F Body=foreign body. HIV=HIV/AIDS. HTN HD=hypertensive heart disease. IHD=ischaemic heart disease. Intest Inf=intestinal infectious diseases. Leukemia=leukaemia. Liver C=liver cancer. LRI=lower respiratory infections. Lung C=tracheal, bronchus, and lung cancer. Mech=exposure to mechanical forces. NN Enceph=neonatal encephalopathy due to birth asphyxia and trauma. NN Preterm=neonatal preterm birth complications. NN Sepsis=neonatal sepsis and other neonatal infections. Oth Cardio=other cardiovascular and circulatory diseases. Oesophag C=oesophageal cancer. Oth Cirr=cirrhosis and other chronic liver diseases due to other causes. Oth Inf=other infectious diseases. Oth NN=other neonatal disorders. Pancreas C=pancreatic cancer. PEM=protein-energy malnutrition. Prostate C=prostate cancer. Road Inj=road injuries. SDI=Socio-demographic Index. Self Harm=self-harm. STD=sexually transmitted diseases excluding HIV. Stomach C=stomach cancer. Stroke=cerebrovascular disease. TB=tuberculosis. Violence=interpersonal violence. YLL=year of life lost.

The leading ten causes of YLLs by location and the ratio of the observed and expected YLLs on the basis of SDI alone in 2016 are detailed in figure 9. A variety of patterns emerge from this comparison, beginning with variation in leading causes by location. Globally, ischaemic heart disease and cerebrovascular disease were the leading causes of YLLs for both sexes for 123 countries in 2016, including in China (cerebrovascular disease [stroke]) and India (ischaemic heart disease). Countries where other causes ranked higher were primarily found in three GBD super-regions: sub-Saharan Africa, where HIV/AIDS was the leading cause of YLLs for 16 of 46 locations; North Africa and the Middle East where conflict and terrorism was the leading cause in three of 21 locations; and Latin America and the Caribbean, where either interpersonal violence or lower respiratory infections were the leading causes of YLLs in six of 32 locations. Nonetheless, ischaemic heart disease was common among countries of Latin America and the Caribbean, ranking in the leading three causes in all countries in the region, and lower respiratory infections were also commonly within the leading five causes of YLLs in South Asia and sub-Saharan Africa. For locations in the high-income GBD super-region, cancers, particularly the cause grouping of tracheal, bronchus, and lung cancer, ranked within the leading five causes of YLLs in 32 of 34 locations. In 2016, of the ten leading causes of YLLs globally, ischaemic heart disease was the cause for which YLLs were most often lower than expected on the basis of SDI alone (in 117 locations globally). This was commonly the case in the region of North Africa and the Middle East, where Saudi Arabia, Bahrain, Kuwait, and Qatar in particular had observed-to-expected ratios of YLLs for ischaemic heart disease below 0·50. Other leading causes for which observed YLLs were notably lower than expected included neonatal preterm birth complications in many countries of both south Asia and southeast Asia, and cerebrovascular disease in western Europe. The opposite, where observed levels were much higher than expected on the basis of SDI, was most commonly noted in the case of HIV/AIDS and malaria in sub-Saharan Africa; diabetes mellitus, especially in Oceania; and cardiomyopathy and myocarditis, particularly in eastern and central Europe. In China, a number of cancers—lung cancer, liver cancer, stomach cancer, and oesophageal cancer—were within the leading ten causes of YLLs, and each of these caused higher than expected levels of YLLs on the basis of SDI; from 1·70 times higher for lung cancer to 5·43 times higher for liver cancer. For India, YLLs from tuberculosis, diarrhoeal diseases, COPD, and the residual category of other neonatal causes were more than twice as high as expected in 2016 based on SDI alone (3·75, 2·88, 2·18, and 2·43 times higher, respectively).

## Discussion

### Main findings

The quality of data available for estimating the global causes of death has improved; with the new rating system reported here, 50 countries had a higher star rating in the most recent period (2010–16) compared with their overall rating since 1980. The global transition from a pattern of premature mortality dominated by CMNN diseases to one dominated by NCDs and injuries can be seen in the 37 years of data examined for GBD 2016. This transition was characterised by declining rates of CMNN diseases in all SDI quintiles, with faster rates of decline in lower-SDI quintiles. NCDs surpassed CMNN diseases in terms of global all-ages YLL rate in 2009 and earlier—in 1992—for age-standardised rates. NCD age-standardised YLL rates also declined for each SDI quintile from 1980 to 2016, but rates of decline were slowest at low SDI and fastest for high SDI. Injury age-standardised YLL rates declined in each SDI quintile, although this was punctuated by large increases in some years due to fatal discontinuities from conflict and terrorism, disasters, and epidemics. The impact of these fatal discontinuities was more noticeable in lower-SDI quintiles. While declines in age-standardised YLL rates are an indicator of progress, the absolute numbers of YLLs increased for NCDs in all but the high-SDI quintile; these increases in absolute YLLs from NCDs occurred in tandem with steadily increasing life expectancy in most locations worldwide.^21^ Increasing numbers of YLLs and the associated higher prevalence of chronic diseases will drive rising needs for health service provision and pose substantial financial, workforce capacity, and managerial challenges for health systems. Declining age-standardised rates, but rising numbers of YLLs for non-communicable diseases, such as breast cancer, oesophageal cancer, and ischaemic stroke, are fundamentally driven by population growth and rising average population age. Between the three broad Level 1 cause groups, there was greater heterogeneity in rates of change for age-standardised YLLs for CMNN causes compared with non-communicable causes, while the least variation occurred for injuries. At a more detailed cause level, there is even greater heterogeneity in trends. Over the 37-year period analysed here, global deaths due to the HIV/AIDS epidemic rose from 4224 deaths (95% UI 2842–6274) in 1980, peaked in 2005 at 1·91 million (1·81 million to 2·01 million), and declined to 1·03 million (0·99 million to 1·08 million) in 2016. Since 2006, statistically significant increases in YLL rates occurred for opioid use, amphetamine use, and other drug use disorders in some locations—particularly at high-SDI. Globally, progress has been neither universal nor uniform. For SDI quintiles, and by GBD regions or locations, there was considerably more heterogeneity in trends—eg, 36 countries had significant increases in age-standardised YLLs across 3 or more of Level 3 causes.

### Cross cutting themes

At the global level, significant declines from 2006 to 2016 in age-standardised YLL rates occurred for the leading ten causes by number of global YLLs. The median annualised rate of change for this set of leading causes of YLLs was a decrease of 2·89%, compared with 1·59% for the remainder of causes in the hierarchy. Generally, the findings suggest that we have observed faster rates of decline for causes with larger initial YLL rates. If annualised rates of decline are compared to levels of age-standardised YLLs in 2006 rather than 2016, these observations also hold true. This phenomenon of greater progress on average for larger problems^33^ is also seen in each SDI quintile. The consistency of this finding suggests that it is unlikely to be due to chance alone. One alternative explanation is that through investments in research and development, national and global policy, and strategic allocation of resources, a more concerted and sustained effort has been made to tackle the leading causes in each location. This hypothesis provides an optimistic view of society's potential to take on new challenges as they emerge; however, confirming that this is the best explanation for the pattern we observe is challenging. There are specific supporting examples such as the rise of the HIV/AIDS epidemic in some countries, followed by the development and mass rollouts of PMTCT and ART,^34^ with subsequent declines in YLL rates. Another example is the population-change-driven rise of malaria, followed by the emergence of drug resistance, then subsequent decline in malaria death rates traced to insecticide-treated bednet scale-up and artemisinin combination therapy.^27^ The rise and now apparent fall in alcohol-related mortality in countries in Eastern Europe might be yet another example.^35^, ^36^, ^37^, ^38^ Further research on the broader drivers of these patterns that transcend the details of specific causes is warranted.

Since 1980, annualised rates of decline for NCDs have been faster for high-SDI quintiles than low-SDI quintiles—a sharp contrast with the rapid reductions in CMNN diseases achieved by lower-SDI locations. This trend might reflect combinations of funding priorities, international programmes, and social determinants of health and behaviours, as well as the crucial role of access and quality of both primary and secondary personal health-care in preventing deaths from a number of NCDs, and point to where gaps persist in providing high-quality health services to properly address these conditions. Past studies show the effect of access to high-quality personal health care on both communicable and non-communicable diseases, highlighting the importance of prioritising personal health-care access and quality for all populations across the development spectrum.^39^, ^40^, ^41^, ^42^ Our findings here correspond with a GBD 2015 analysis of personal health care access and quality,^43^ wherein absolute levels of and progress on NCDs amenable to personal health care were greater among higher-SDI locations than those of lower SDI. These differences are probably driven by myriad factors, including access to effective pre-hospital care, differences in primary and secondary care services; improved diagnosis and management of many conditions; availability and staffing of specialised health units and related equipment required for more complex disease management or surgery; and financing structures.^42^, ^44^

International declarations and agreements for development and health have attracted political actions at the highest level but have also generally focused on indicators related to CMNN causes. This focus is reflected in the rapid reductions in CMNN diseases achieved by lower-SDI locations over the past four decades. However, until the 2015 adoption of the SDGs, NCDs were not strong priorities in these declarations and did not receive equivalent levels of political commitment in many locations.^45^, ^46^, ^47^ By contrast, many high-SDI locations have national health priorities and policies that focus on NCDs, risk factors, or behavioural interventions, and have invested in these programmes outside of the explicit support of international declarations. In addition to the role of health care, improvements to the broader social determinants of health might be less developed in low-SDI locations—eg, low-income and middle-income countries lag behind in implementing evidence-based tobacco control regulation.^48^ Variations in age-standardised YLL rates by cause and over time might provide insight into how health care or other determinants of health evolve alongside development. We can further examine locations that have attained better health outcomes than expected on the basis of SDI to identify potential avenues for accelerating risk modification programmes, development of regulations, health-care access, or health-care quality in places lacking this success.

Seven SDG indicators are based on measures of cause-specific mortality beyond the Millennium Development Goal agenda, specifically death rates due to natural disasters (SDG 1.5.1, 11.5.1, and 13.1.2); cardiovascular disease, cancer, diabetes, and chronic respiratory disease among 30–70-year-olds (SDG 3.4.1); self-harm (SDG 3.5.1); road injuries (SDG 3.6.1); unintentional poisonings (SDG 3.9.3); interpersonal violence (SDG 16.1.1); and conflict (SDG 16.1.2).^17^ The inclusion of several high-priority NCDs and injuries for the post-2015 agenda has been widely lauded, but critiques of current indicators and the omission of particular causes or health areas are comparably prevalent. Two causes—Alzheimer's disease and other dementias, and chronic kidney disease, ranked fourth and 11th, respectively, among the leading causes of death globally—are increasing sources of health burden, particularly for low-SDI to middle-SDI locations; however, the SDG agenda offers at best a minimal platform for drawing attention to the health care and monitoring needs of these conditions. Globally, reductions in age-standardised death rates due to hepatitis C have largely stagnated since 2010 even though a highly effective cure is available—and in the USA, total deaths from hepatitis C have now surpassed deaths from all other notifiable infectious diseases,^49^ yet the SDGs are focused on tracking hepatitis B incidence (SDG 3.3.4). Several SDG indicator revisions and potential additions were recently proposed to the Inter-Agency and Expert Group on Sustainable Development Goal Indicators, suggesting that an opportunity might exist to better align the sustainable development agenda with the world's most pressing causes of untimely death.^4^

The accelerated declines in cause-specific YLLs rates for nearly all causes is occurring despite threats to human health such as climate change, antimicrobial resistance, obesity, emerging infectious diseases, and conflict.^6^, ^50^, ^51^, ^52^, ^53^, ^54^, ^55^, ^56^, ^57^, ^58^, ^59^, ^60^ The debate over whether progress in human health can continue through some combination of innovation and a societal focus on leading problems despite the advent of these risks in some ways parallels an explanation for the well-known environmentalist's paradox in which human so-called well-being has continued to improve globally even as resources are depleted and many ecosystems show signs of degradation.^53^ An alternative explanation is that these threats have substantial time lags so that health consequences of climate change, for example, might be major in the future even if not notable to date. The continued global increase and expansion of dengue and its four serotypes is a potential indicator of the complex changes that are underway and might be partly related to changes in climate in addition to other factors.^61^ Other challenges, such as conflict and terrorism, are clearly causing reversals in some locations such as Syria and Yemen. The apparent reversal in progress in survival in the USA is a complex phenomenon whose causes and magnitude remain contested, but recent research has pointed to rising mortality among some groups, especially non-Hispanic whites, from increased deaths from drugs, alcohol, and suicide, coupled with slower progress in reducing deaths from cardiovascular disease and cancer, and rising levels of obesity and associated disease.^57^, ^62^ These threats are substantial and deserving of policy attention and response. Given the gulf between a future driven by a continuation of the trends we have observed in the last 37 years and one dominated by emerging risks, close monitoring of patterns in health outcomes will be essential. Further work to identify specific health outcomes in particular locations might be sentinel markers of the effect of these threats might also improve our capability for early detection of changes in trends in certain locations.

### Changes in GBD 2016 compared with GBD 2015

A strength of the GBD study is the re-analysis of the entire time series using continually improving methods and newly available data sources. Estimates for a given cause, location, or year are not necessarily constant between GBD iterations as new techniques or data sources improve model validity and decrease uncertainty from various sources. The magnitude of differences in estimation between GBD 2016 and GBD 2015 is presented in the appendix (2 p 26); specific method or data changes underlying several notable differences in estimation are discussed in greater detail below.

One of the most important changes in GBD 2016 was the release of the SRS VA data to the India GBD collaboration by the Government of India. These detailed ICD code data were for the period 2004 to 2013, and were disaggregated by urban and rural areas in each state. The inclusion of the detailed SRS VA data in GBD 2016 substantially changed estimates for multiple causes. In some cases, the new SRS data over a 10-year period not only changed results for India but, through changing coefficients in regression models, also modified estimates for other locations that were lower in the stars system of data quality rankings developed for GBD 2016. These data are a tremendous resource and it is a welcome development that the data have been shared with the Indian Council of Medical Research, providing the opportunity for their inclusion in GBD. Nevertheless, these data are still based on VA, which in rigorous validation tests performs well for some causes and not others.^63^

An important emphasis in GBD 2016 has been reporting by location-year on the extent of garbage coding on death certificates. By introducing the concept of levels of garbage coding we have focused attention on those deaths assigned to garbage codes that get redistributed across the three large cause groups in GBD or the 21 Level 2 cause groups that have the largest effect on cause of death patterns. Reporting on the fraction of deaths assigned to major garbage codes by location-year provides a tool for national statistical authorities to track progress in improving the quality of death certification. We hope that annual reporting on this quantity might encourage policies and programmes to improve the quality of death certification. Countries such as Finland, Moldova, New Zealand, Singapore, and that have less than 5% of deaths on average since 2010 assigned to major garbage codes strongly imply that high-quality certification is possible at the national level. Even in the population aged older than 80 years, these countries have kept major garbage coding less than 20%. By contrast, some systems, such as those in Egypt, Thailand, and Turkey, have more than 50% of deaths assigned to major garbage categories. Given that registration systems are recording these deaths, which requires considerable institutional development and system infrastructure, the marginal value of intervening to improve death certification quality would be great. Initiatives such as the Bloomberg Data for Health Initiative will hopefully lead to improvements in cause of death certification and coding.^64^ At the global level, the fraction of all registered deaths assigned to major garbage codes decreased over the past 37 years, owing to ongoing improvements in data collection and recording. Because there is a strong relationship between garbage coding and age, and important demographic shifts are underway, reporting of garbage codes should perhaps in the future be age standardised.

In the interest of providing more guidance about the quality of the data used for estimating causes of death, we have introduced a scoring system for the overall quality of the time series estimates for a location ranging from 5 stars (best) to 0 stars (worst). The quality rating of the time series has been assigned on the basis of the fraction of well certified deaths. In 2010–16, we reported more countries with 4-star or 5-star ratings. The improvements in data quality in some large countries, such as Brazil, China, and India, is particularly encouraging. The goal of GBD is to generate unbiased estimates for all locations with 95% UIs that reflect sampling error, non-sampling error, and modelling error based on the available evidence. These UIs are meant to also communicate to the user the strength of the evidence supporting each cause-specific estimate. In settings with five stars, the results in future iterations of GBD are unlikely to change even if the model life tables improve or there are changes in how garbage codes are redistributed. Because of the weaker empirical basis, results for locations with lower star ratings are more subject to change in how data are processed or models estimated; however, even for locations with lower-quality data, our methods support the generation of unbiased estimates and as such we do not expect deaths in these locations to be systematically higher or lower than estimated. For GBD 2016, we have opted to develop a relatively simple system and accommodated the system to settings where only VA studies are available. In future GBD updates, we might improve on this first iteration of the quality rating system.

Updates in the data collated and improvements to the methodological approach for malaria mortality estimation in the GBD 2016 iteration have resulted in changes in estimates of both contemporary mortality and trends through time. At the global level, estimates for malaria in the most recent years are slightly higher compared with those in GBD 2015, but earlier years were estimated to be lower, including the years of peak global malaria mortality in the early 2000s. As such, the proportional decline in malaria deaths between 2006 and 2015 is now estimated to be 27·9% (95% UI 8·71–42·8), which is smaller than the WHO estimated decline of 42% (33–55).^7^ These changes were driven primarily by updates to estimates for sub-Saharan Africa, where the slightly slower rate of decline since 2006 reflects the inclusion of geospatial predictions of new cross-sectional household surveys in the Malaria Atlas Project, reporting higher infection prevalence or lower coverage of malaria control interventions, both of which translate into larger mortality estimates. Changes to estimates in the earlier part of the time series reflect mainly refined covariates, including access to and efficacy of antimalarial drugs. Outside sub-Saharan Africa, changes were driven mainly by (1) a modified mortality model that is informed by the newly developed Malaria Atlas Project estimates of clinical incidence through time for each country, which in turn draw upon a new assembly of routine case surveillance data; and (2) new adoption of notification data. These modifications led to different changes in different countries, but most notably overall declines in predicted malaria deaths in Myanmar and India. Changes in India were most pronounced and driven by inclusion of the much-improved SRS mortality data, which lowered estimates in all years, but particularly earlier years.

We changed the modelling strategy of tuberculosis in GBD 2016 by first modelling prevalence of disease and prevalence of latent infection, which were then used as covariates for the CODEm model. This, together with the addition of SRS data for India and changes to the mortality envelope, has not resulted in major changes to our general conclusions on the global epidemiology of tuberculosis, although the number of deaths at the global level was slightly higher than that of GBD 2015 for all years. The estimated number of deaths in several African countries have significantly increased; Nigeria and Zambia were notable with more than twice the number of estimated deaths in 2015. In addition, for the first time, we have estimated multidrug-resistant tuberculosis and extensively drug-resistant tuberculosis (8·8% [95% UI 7·4–10·4] of all tuberculosis deaths) from the global tuberculosis envelope. Due to the different composition of drug-resistant types, these numbers are lower than the 13·9% of drug-resistant tuberculosis deaths (multidrug-resistant and rifampicin-resistant tuberculosis combined) among all tuberculosis deaths reported by WHO for the same year.^6^

As in previous iterations of GBD, cancer mortality was estimated using mortality data from VR system data and VA studies, as well as cancer incidence data from cancer registries that were transformed to mortality estimates using separately modelled mortality-to-incidence ratios (MIR). GBD 2015 estimated MIRs did not capture the likely effects of worse access to treatment in lower-SDI settings; we have revised the MIR data-inclusion and modelling approach to better capture the relationship observed in high-quality registry data between MIR (and implicitly 5-year survival) and health-system access and quality of care. The changes in MIR modelling as well as the changes in data led to some shifts in cancer mortality. For example, estimates of deaths from other pharynx cancers in 2010 at the global level increased by 72·3% for men and by 73·8% for women; most of this increase comes from India. Estimates for deaths from Hodgkin's lymphoma in 2010 increased by 28·1% for men and by 16·8% for women compared with GBD 2015 at the global level. This difference comes mainly from an increase in the mortality estimates for sub-Saharan Africa, where the inclusion of more registry data for Nigeria, Uganda, and other locations changed the estimates. Deaths estimated from lip and oral cavity cancer in 2010 increased by 17·0% for both sexes compared with GBD 2015, which again was mainly caused by a large increase in deaths estimated for India due to the addition of SRS as well as an increase in the MIR estimate. Compared with in GBD 2015, estimated deaths in 2010 due to other neoplasms increased by 20·3% for men and by 5·1% for women. This increase was mainly due to a change in redistribution of myelodysplastic syndrome, which was redistributed to the leukaemia subcauses for GBD 2015, and to other neoplasms for GBD 2016.

In view of the large outbreak of Zika virus disease across the Americas, WHO's declaration of the outbreak as a Public Health Emergency of International Concern, and broad concern about the disease, we added Zika virus disease to the GBD cause list. Although Zika virus infection is primarily associated with non-fatal outcomes (eg, fever, rash, Guillain-Barré syndrome, and congenital outcomes), a small number of deaths have been reported, and these are captured within GBD 2016. The global number of Zika virus deaths was comparatively small—estimated at two (95% UI 1–5) in 2015 and 19 (4–57) in 2016. Given trends in the disease, we do not expect the number of Zika virus disease deaths to increase substantially in the coming years.

We have explored alternative data processing and modelling for neonatal causes. Even in countries with 5-star cause of death quality ratings, there is remarkable variation in neonatal cause of death patterns. Western Europe is an example where the overall death rate due to neonatal disorders in the early neonatal period is quite similar across the region, but there is as much as a three-fold difference in death rates within Level 3 causes (eg, neonatal preterm birth complications, cardiovascular diseases).^65^, ^66^ There is no reason to believe that these large differences in the causes of deaths in the first month of life between countries with nearly the same SDI in the same GBD region are real; rather, we strongly suspect there is variation in medical culture in how neonatal deaths are assigned.

For example, in some locations all deaths in premature infants might be assigned to prematurity as a cause and not only those where inadequate development of organ systems is the underlying cause of death. As a concrete clinical example, particular locations might be predisposed to assign deaths following clinical events, such as intraventricular haemorrhage and necrotising enterocolitis to cardiovascular diseases and neonatal sepsis, respectively, instead of preterm birth complications or congenital birth defects. We tested alternative modelling strategies, but improvements in the assignment of neonatal deaths to specific causes might require improvements in the fundamental quality of the data, reconsideration of GBD cause classification in these age groups, or considering alternative age-specific data redistribution approaches.

### Comparison of GBD 2016 to other estimates

WHO has produced cause of death Global Health Estimates (GHE) at the country level with the most recent spanning from 2000 to 2015 for 183 countries and 176 causes of death.^19^ These estimates combined GBD 2015 estimates, International Agency for Research on Cancer (IARC) cancer estimates, UN Population Division life tables, vital registration data for 70 countries, and selected cause-specific and country-specific adjustments. Cause-specific comparisons of these estimates to GBD 2015 and GBD 2016 are provided in appendix 2 (pp 25–26). Uncertainty bounds for GHE 2015 cause-specific estimates are available from WHO online sources.^19^ The GBD study, as recognised in the GHE 2015 technical paper, remains the “only source of comprehensive uncertainty estimates for mortality by cause.”^19^

The Globocan project at IARC produces estimates of major cancer types on a periodic basis for 184 countries; IARC estimates do not currently meet GATHER guidelines.^15^ We believe that the IARC estimates of MIR in the lower three SDI quintiles are low for a number of important cancers, particularly where there is clear evidence of the impact of access and quality of care, and empirical evidence of this gradient from recent analyses employing the Healthcare Access and Quality Index.^43^ Due to the complexities of the IARC estimates, if the bias in their estimates from underestimated MIRs primarily affects their estimates of incidence or the site-specific mortality estimates is unclear. Given the push to accelerate declines in under-5 deaths, several efforts are underway to quantify deaths by cause in children.^67^ The MCEE, WHO, and GBD each produce estimates of child deaths by age and cause. WHO uses MCEE results for the limited causes included in that analysis and supplements for other child causes using the GBD 2015 results.

### Limitations

GBD 2016 has made a number of advances in methodology to address the unique difficulties of estimating cause-specific mortality; at the same time, we recognise that limitations remain. Limitations that reflect aspects of specific causes—such as inconsistencies between cause of death and prevalence data for select causes, complexities around including mental health disorders as risks for death for many causes, efficiency in capturing the effects of differential use of ART for younger age groups or by sex, the lack of detail available for causes such as drug use disorders, or the effects of mass migrations on estimation—are described in greater detail in appendix 1 (p 39). Here, we identify cross-cutting limitations applicable across many causes. First, the newly developed data quality ratings by location do not incorporate the extent of redistribution for miscoded causes of death or other sources of error that might affect the accuracy of estimation based on those data. Second, both VR data and VA data sources depend on how accurately underlying cause of death is assigned and this is complicated by multimorbidities. Through correction for under-registration and garbage code redistribution algorithms, we have made substantial efforts to enhance the comparability of results; systematic problems in selected locations might still remain and affect the estimated time trends. Third, in estimating fatal discontinuities for countries with a 3-star data quality rating or lower, we primarily relied on international organisations that collate these data, and thus our results are subject to the limitations in data coverage or representativeness of those sources; details of adjustments for known data issues are in appendix (1 p 273). Fourth, in adjusting VA studies relative to medical certification, we rely on the single available study on this comparison;^68^ of necessity this is a limited basis for the adjustment. Fifth, sources of VA data vary substantially in terms of the training provided and the instrument used in collecting the data, which might reduce the comparability of cause of death data between locations. Sixth, our approach to garbage code redistribution is vital to the results presented in GBD 2016—although our methods of redistribution could theoretically contribute bias, we have identified no evidence of this.^3^ Seventh, a low level of identified garbage coding for a given location does not necessarily indicate quality or accuracy in cause of death certification. Eighth, while some causes use negative binomial modelling approaches to improve estimation with over-dispersed data, we have not yet developed a standardised empirical approach for selecting causes to use this method. Ninth, we have not been able to systematically carry uncertainty from the statistical models used for many of the garbage code redistribution algorithms through to our final estimates due to limitations in computational requirements and storage needed; we are exploring ways this can be accommodated in future GBD iterations. Tenth, additional sources of uncertainty might not be captured, such as for the covariates used in the models with the exception of the HIV crude death rate. Finally, GBD results are necessarily a combination of data and estimation. Due to lags in reporting, estimates for the most recent years rely more on the modelling process—evidenced by larger median UI by year between 2012 and 2016— as do estimates for locations with low levels of data completeness.

### Future directions

Based on feedback from across the GBD collaboration, the Independent Advisory Committee to the GBD study, we have identified a number of areas where GBD 2017 and subsequent GBD updates can improve the estimation of causes of death. First, we would like to be able to capture uncertainty in garbage code assignment into the final UIs for our estimates. Given the computational requirements for CODEm, propagating uncertainty in the primary data used for modelling will require major changes in data storage and computational capacity or substantial changes to the CODEm model pool. Second, as spatially explicit analyses become available for more causes, such as diarrhoeal diseases, lower respiratory infections, tuberculosis, HIV, and many NTDs, these more granular assessments should affect the GBD estimates. Third, the Child Health and Mortality Prevention Surveillance study—funded by the Bill & Melinda Gates Foundation—might provide important high-quality data on the underlying causes of death in a sample of deaths in low-SDI settings based on minimally invasive tissue sampling. This could change our understanding of the leading causes of death in some groups such as neonatal sepsis. Fourth, we will continue the push toward more subnational assessments in future iterations, targeting the countries with the largest populations and those that are about to surpass the 200 million population mark.

## Conclusion

Patterns of global health are clearly changing, with more rapid declines in CMNN conditions than for other diseases and injuries. This is a laudable and welcome reflection of the intense focus of the global health community over the past several decades on improving child survival and reducing pregnancy-related risks. The impact of the mass scale-up of interventions funded through development assistance on mortality from diseases such as HIV/AIDS, malaria, and measles is best measured by comprehensive annual cause of death assessments as reported in this paper. These data also point to the much slower declines in mortality for major NCDs and injuries, suggesting that these conditions, which cause very substantial mortality in young and middle-aged adults, need to receive much greater policy priority given the compelling evidence from some countries that bold public policies to reduce avoidable mortality from these causes are effective. Moreover, it is much less a matter of financing such policy initiatives than sustained government commitment toward them that will ensure they have the impact that is intended. The true value of timely, comprehensive, and annual mortality data in informing policy dialogue depends greatly on their diagnostic accuracy and completeness; as this study demonstrates, the majority of countries still lack good-quality vital registration systems to adequately support public policy, mandating that future global health development strategies include improvements for these systems.

Correspondence to: Prof Christopher J L Murray, Institute for Health Metrics and Evaluation, Seattle, WA 98121, USA cjlm@uw.edu

For the **online repository** see https://github.com/ihmeuw/ihme-modeling

To download the data in this table, please visit the **Global Health Data Exchange** (GHDx) at: http://ghdx.healthdata.org/node/311076

For the **International Disaster Database** see http://www.emdat.be/database

To download the data in this table, please visit the **Global Health Data Exchange (GHDx)** at: http://ghdx.healthdata.org/node/311076

To download the data in this table, please visit the **Global Health Data Exchange (GHDx)** at: http://ghdx.healthdata.org/node/311076

For the **data visualtion tool** see https://vizhub.healthdata.org/gbd-compare
