# Department of Error

**DOI:** 10.1016/S0140-6736(17)32646-6

**Published:** 2017-10-28

*GBD 2016 Causes of Death Collaborators. Global, regional, and national age-sex specific mortality for 264 causes of death, 1980–2016: a systematic analysis for the Global Burden of Disease Study 2016.* Lancet *2017; **390**: 1151–1210*—The full-text version of this Article has been updated so that the list of authors is displayed in the correct order, in line with the pdf version, rather than in alphabetical order. This correction has been made to the online version as of Oct 12, 2017.

