# Supplementary material for: Global, regional, and national age-sex specific mortality for 264 causes of death, 1980–2016: a systematic analysis for the Global Burden of Disease Study 2016
Source: Lancet. 2017 Sep 16;390(10100):1151–210. doi: 10.1016/S0140-6736(17)32152-9 (PMC5605883; doi:10.1016/S0140-6736(17)32152-9)
Supplement: Supplementary appendix 2 [file mmc2.pdf]

THE LANCET

Supplementary appendix 2

This appendix formed part of the original submission and has been peer reviewed. We post it as supplied by the authors.

Supplement to: GBD 2016 Causes of Death Collaborators. Global, regional, and national age-sex specific mortality for 264 causes of death, 1980–2016: a systematic analysis for the Global Burden of Disease Study 2016. *Lancet* 2017; **390:** 1151–210.

Appendix 2: Supplemental Results

Supplementary Results for "Global, regional, and national age-sex specific mortality for 264 causes of death, 1980–2016: a systematic analysis for the Global Burden of Disease Study 2016"

This supplement provides additional figures and more detailed results for causes of death, and years of life lost.

Complete results from the Global Burden of Disease Study (GBD 2016) are now measured in terabytes. These results are available in an interactive data downloading tool in the Global Health Data exchange (GHDx). The tool contains the complete set of results from all GBD 2016 papers; however, specialized tables from the papers will available as separate entries in the GHDx, as was made available in GBD 2015. Access the results and tables through the GHDx at http://ghdx.healthdata.org/gbd-results-tool.

Supplementary Results Figures and Tables:

SR Figure 1a-e. Trends from 1980 to 2016, by GBD Level 1 cause, in YLLs (I, in millions), crude rates (II, per 100,000 people), and age−standardized rates (III, per 100,000 people) by SDI quintile. YLL=year of life lost……. 3

SR Figure 2a-e. Annualized rate of change in age-standardized YLL rate from 2006 to 2016 versus global age-standardized YLLs per 100,000 due to Level 3 causes, by SDI quintile. YLL=year of life lost……. 8

SR Figure 3. Annualized rate of change in all-age YLL rate from 2006 to 2016 versus global all-age YLL rate per 100,000 for each Level 3 cause. YLL=year of life lost……. 13

SR Figure 4a-j. Leading 30 GBD cause hierarchy Level 3 causes of YLLs by SDI grouping and sex for 1990, 2006, and 2016, with percent change in number of YLLs, and all-age and age-standardised rates……. 14

SR Figure 5. Difference between GBD 2016 and WHO 2015 deaths for selected causes, all ages, by sex, 1990, 2005, 2010, 2015……. 24

SR Figure 6. Difference between GBD 2015 and WHO 2015 deaths for selected causes, all ages, by sex, 1990, 2005, 2010, 2015……. 25

SR Figure 7. Difference between GBD 2016 and GBD 2015 deaths by cause and sex for all ages, 1990, 2005, 2010, 2015……. 26

SR Table 1. Global deaths and YLL numbers, age-standardized rates per 100,000, and percent change between 2006 and 2016 for females for all GBD causes and Levels 1 through 4 of the cause hierarchy. 95% UIs are in parentheses. UI=uncertainty interval. YLL=year of life lost……. 27

SR Table 2. Global deaths and YLL numbers, age-standardized rates per 100,000, and percent change between 2006 and 2016 for males for all GBD causes and Levels 1 through 4 of the cause hierarchy. 95% UIs are in parentheses. UI=uncertainty interval. YLL=year of life lost……. 32

SR Table 3. Global deaths by cause and age group for females (with 95% uncertainty intervals), 1990, 1995, 2000, 2006, 2010, and 2016……. 37

SR Table 4. Global deaths by cause and age group for males (with 95% uncertainty intervals), 1990, 1995, 2000, 2006, 2010, and 2016…………………………………………………………………………………………........... 55

SR Table 5. Global deaths by cause and age group for both sexes combined (with 95% uncertainty intervals), 1990, 1995, 2000, 2006, 2010, and 2016……………………………………………………………………………… 73

SR Table 6. Global YLLs by cause and age group for females (with 95% uncertainty intervals), 1990, 1995, 2000, 2006, 2010, and 2016. YLL=year of life lost…………………………………………………………………………….... 91

SR Table 7. Global YLLs by cause and age group for males (with 95% uncertainty intervals), 1990, 1995, 2000, 2006, 2010, and 2016. YLL=year of life lost……………………………………………………………………………. 109

SR Table 8. Global YLLs by cause and age group for both sexes combined (with 95% uncertainty intervals), 1990, 1995, 2000, 2006, 2010, and 2016. YLL=year of life lost…………………………………………… 127

SR Table 9. Deaths by location, cause, and age group for females (with 95% uncertainty intervals), 2016………. 145

SR Table 10. Deaths by location, cause, and age group for males (with 95% uncertainty 2016.. 529

SR Table 11. Deaths by location, cause, and age group for both sexes combined (with 95% uncertainty intervals), 2016……… 913

SR Table 12a-e. Deaths due to fatal discontinuities by country, all ages, for both sexes combined, 1980-2016……… 1297

SR Table 13. Comparison of GBD 2016 and GLOBOCAN estimated cancer mortality by cause and sex, 2012……… 1312

SR Figure 1a: Trends from 1980 to 2016, by GBD level 1 cause, in YLLs (I, in millions), crude rates (II, per 100,000 people), and age − standardized rates (III, per 100,000) for High SDI from 1980 to 2016. YLL=year of life lost.

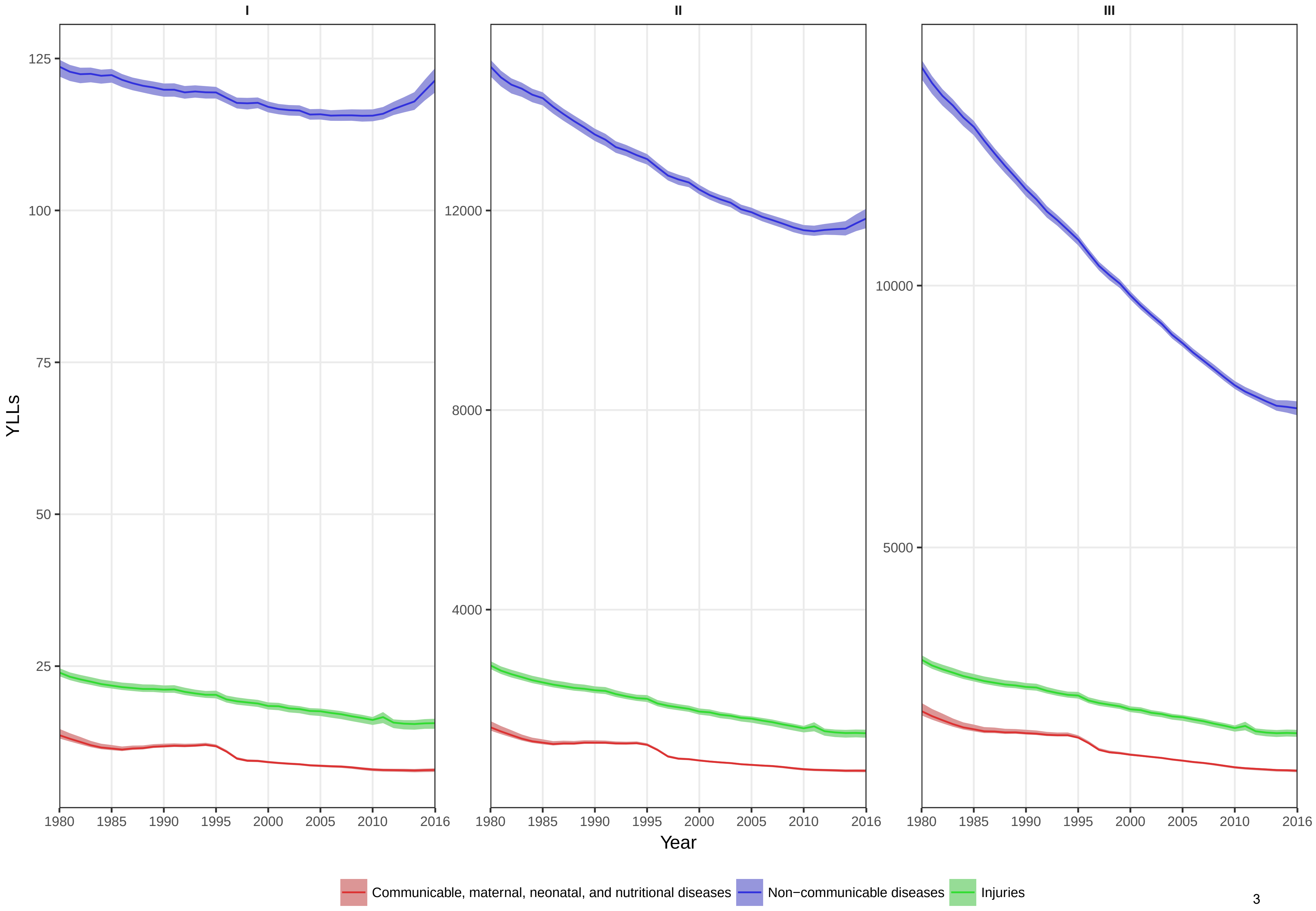

SR Figure 1b: Trends from 1980 to 2016, by GBD level 1 cause, in YLLs (I, in millions), crude rates (II, per 100,000 people), and age − standardized rates (III, per 100,000) for High−middle SDI from 1980 to 2016. YLL=year of life lost.

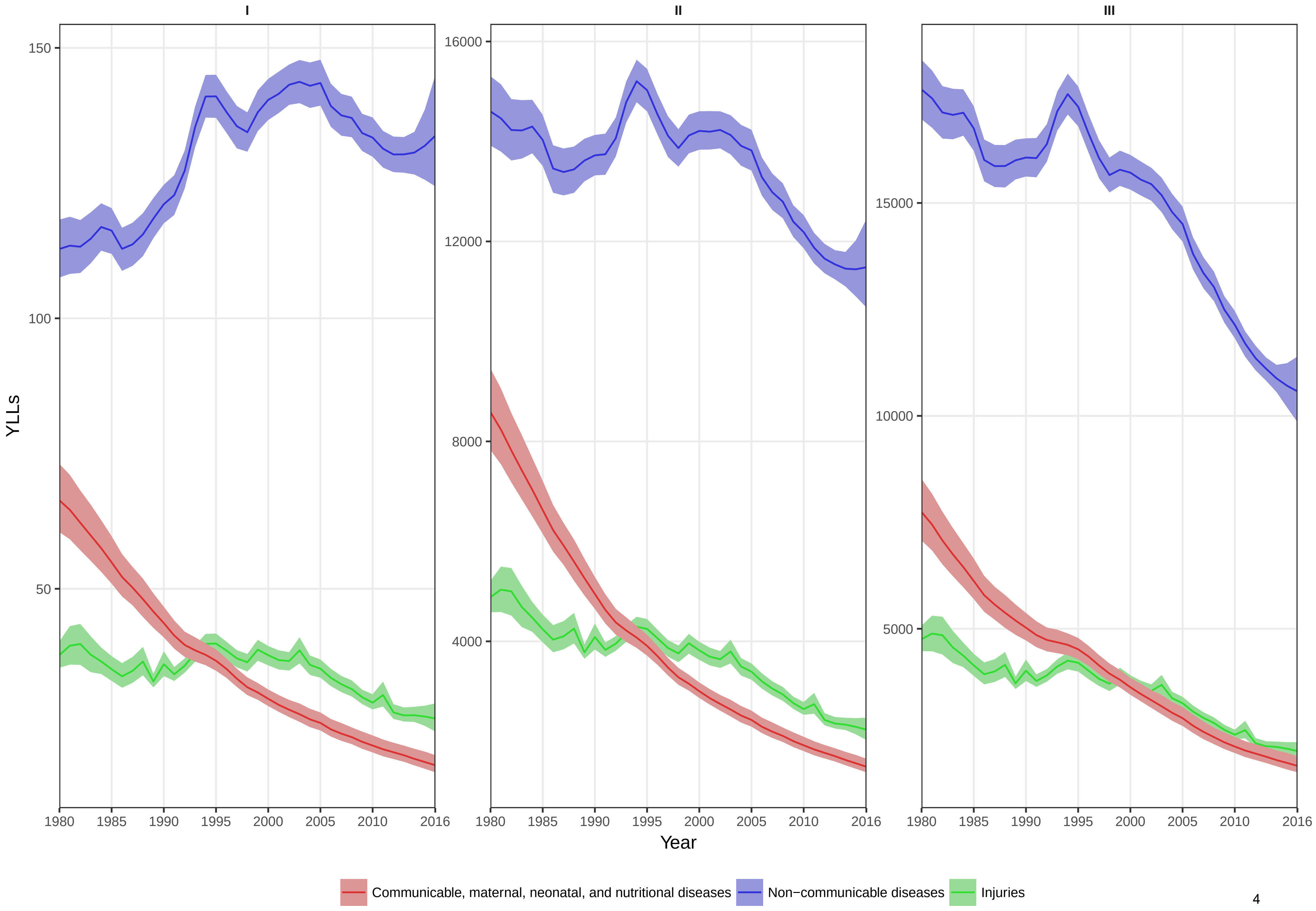

SR Figure 1c: Trends from 1980 to 2016, by GBD level 1 cause, in YLLs (I, in millions), crude rates (II, per 100,000 people), and age − standardized rates (III, per 100,000) for Middle SDI from 1980 to 2016. YLL=year of life lost.

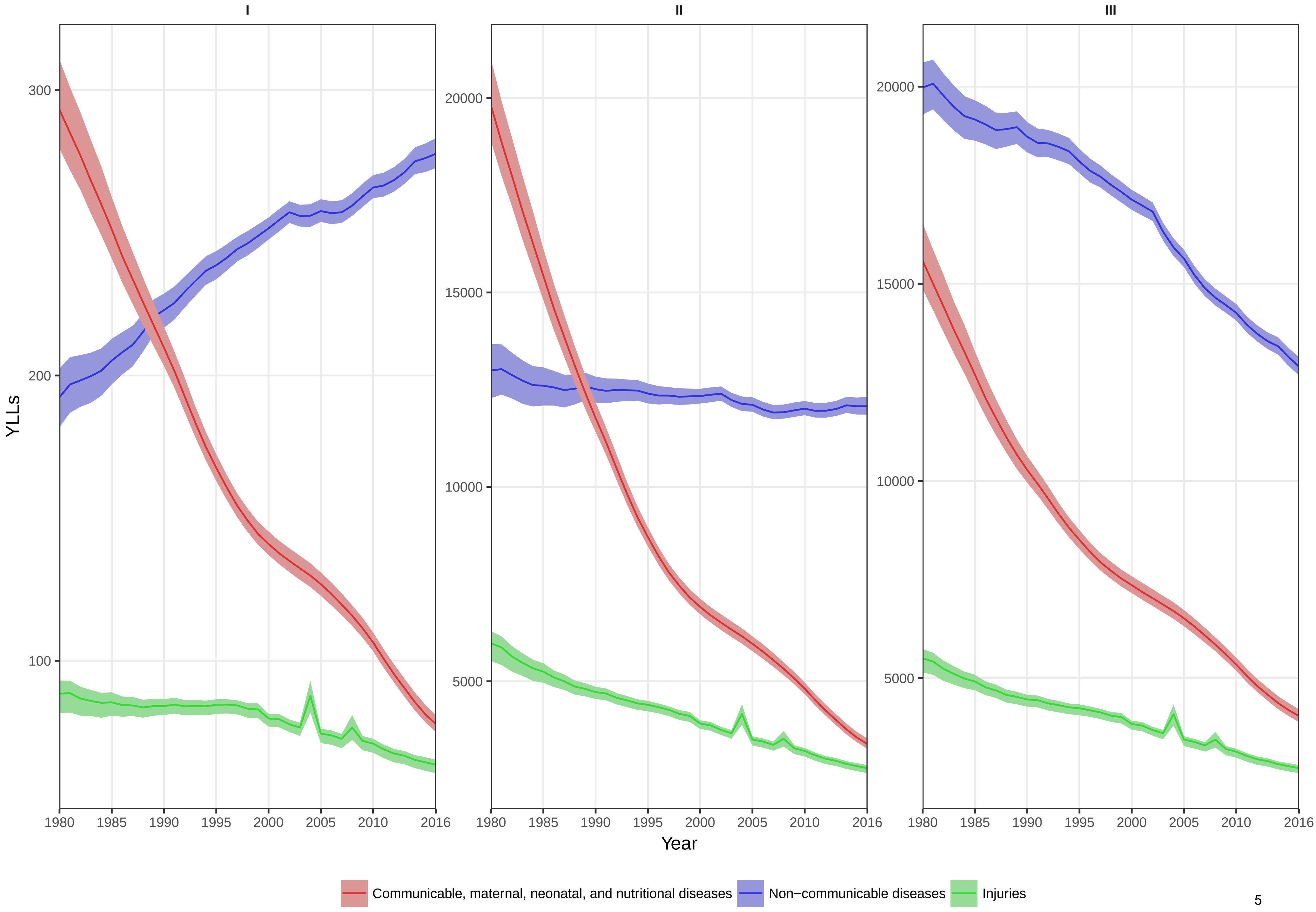

SR Figure 1d: Trends from 1980 to 2016, by GBD level 1 cause, in YLLs (I, in millions), crude rates (II, per 100,000 people), and age − standardized rates (III, per 100,000) for Low−middle SDI from 1980 to 2016. YLL=year of life lost.

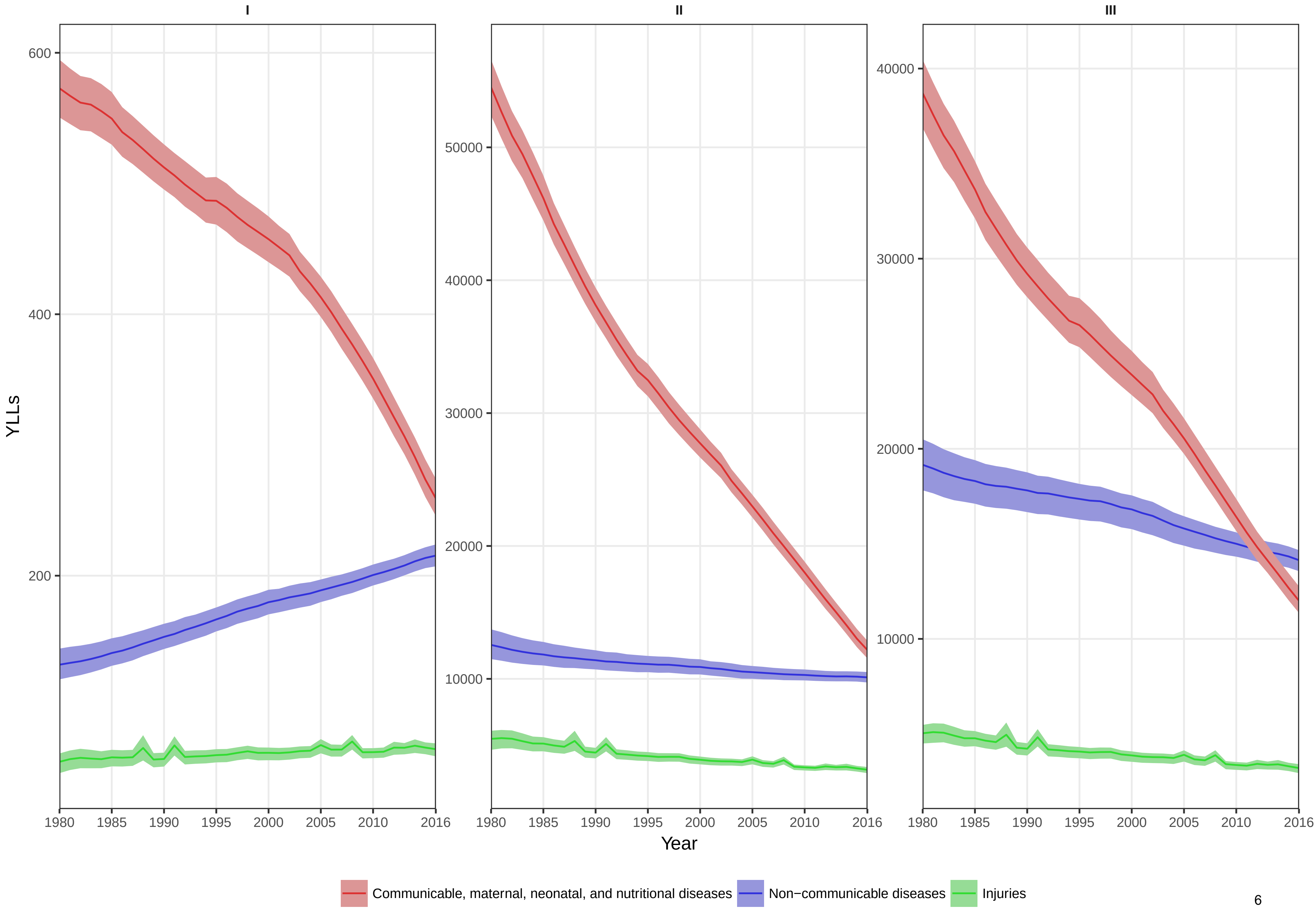

SR Figure 1e: Trends from 1980 to 2016, by GBD level 1 cause, in YLLs (I, in millions), crude rates (II, per 100,000 people), and age − standardized rates (III, per 100,000) for Low SDI from 1980 to 2016. YLL=year of life lost.

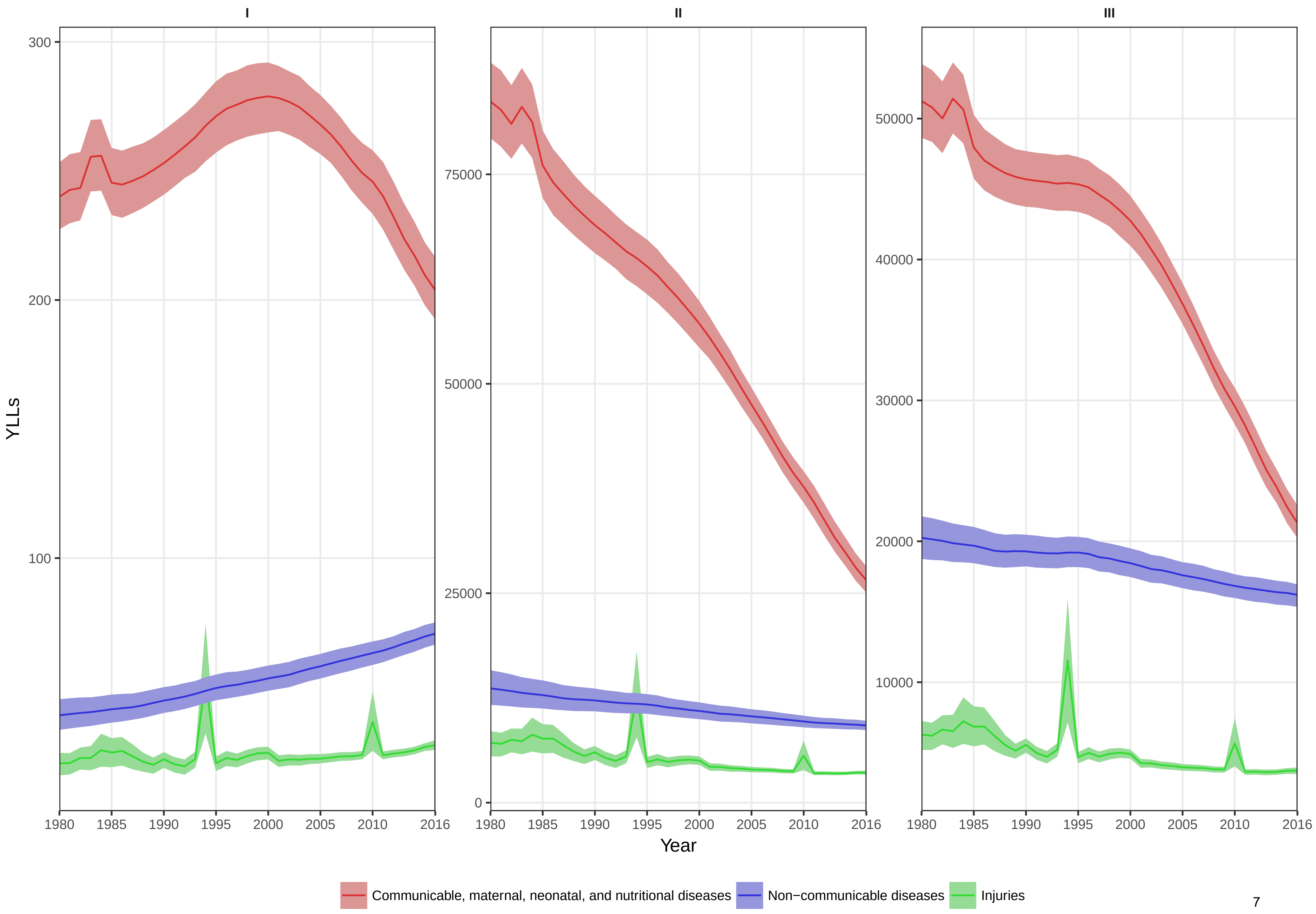

SR Figure 2a: Annualised rate of change in age-standardised YLL rate from 2006-2016 versus age-standardised YLLs (per 100,000) due to level 3 causes for High SDI locations, both sexes combined

Not shown in figure: Yellow Fev: YLL = 0 ARC = 0, Dengue: YLL = 0.08 ARC = 12.17, Schisto: YLL = 0 ARC = -43.98, Cysticer: YLL = 0.05 ARC = -4.32, Malaria: YLL = 0.01 ARC = -6.44, Echino: YLL = 0.03 ARC = -7.23, Nematode: YLL = 0 ARC = -7.97, Zika: YLL = 0.025 ARC = 55.35

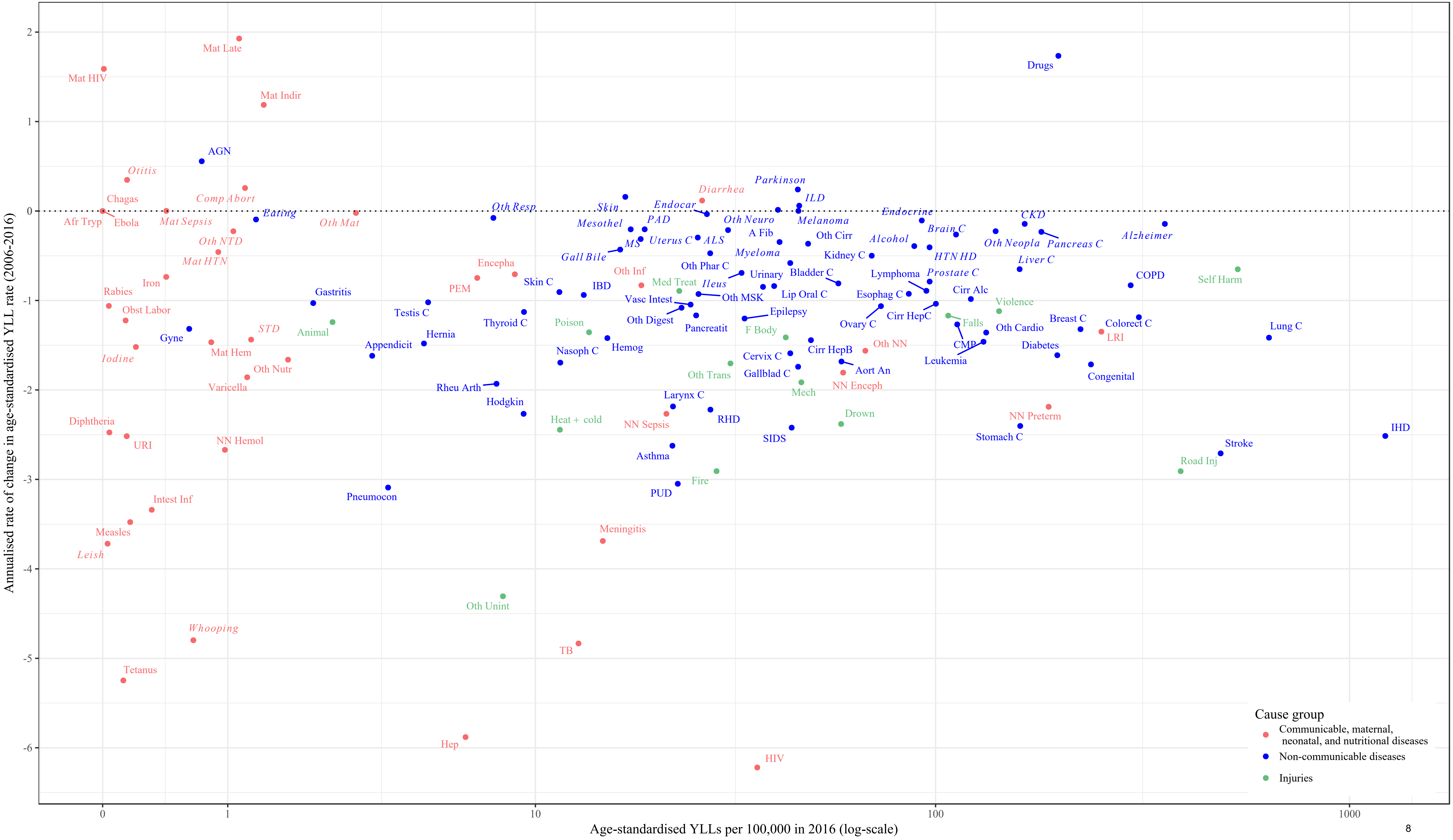

SR Figure 2b: Annualised rate of change in age-standardised YLL rate from 2006-2016 versus age-standardised YLLs (per 100,000) due to level 3 causes for High-middle SDI locations, both sexes combined

Not shown in figure: Diabetes: YLL = 238.11 ARC = -1.28, Afr Tryp: YLL = 0 ARC = 0, Dengue: YLL = 8.15 ARC = 4.68, Rabies: YLL = 0.78 ARC = -12.69, Leish: YLL = 0.49 ARC = -13.48, Tetanus: YLL = 1.15 ARC = -11.25, Measles: YLL = 3.26 ARC = -11.95, Zika: YLL = 0.014 ARC = 49.52

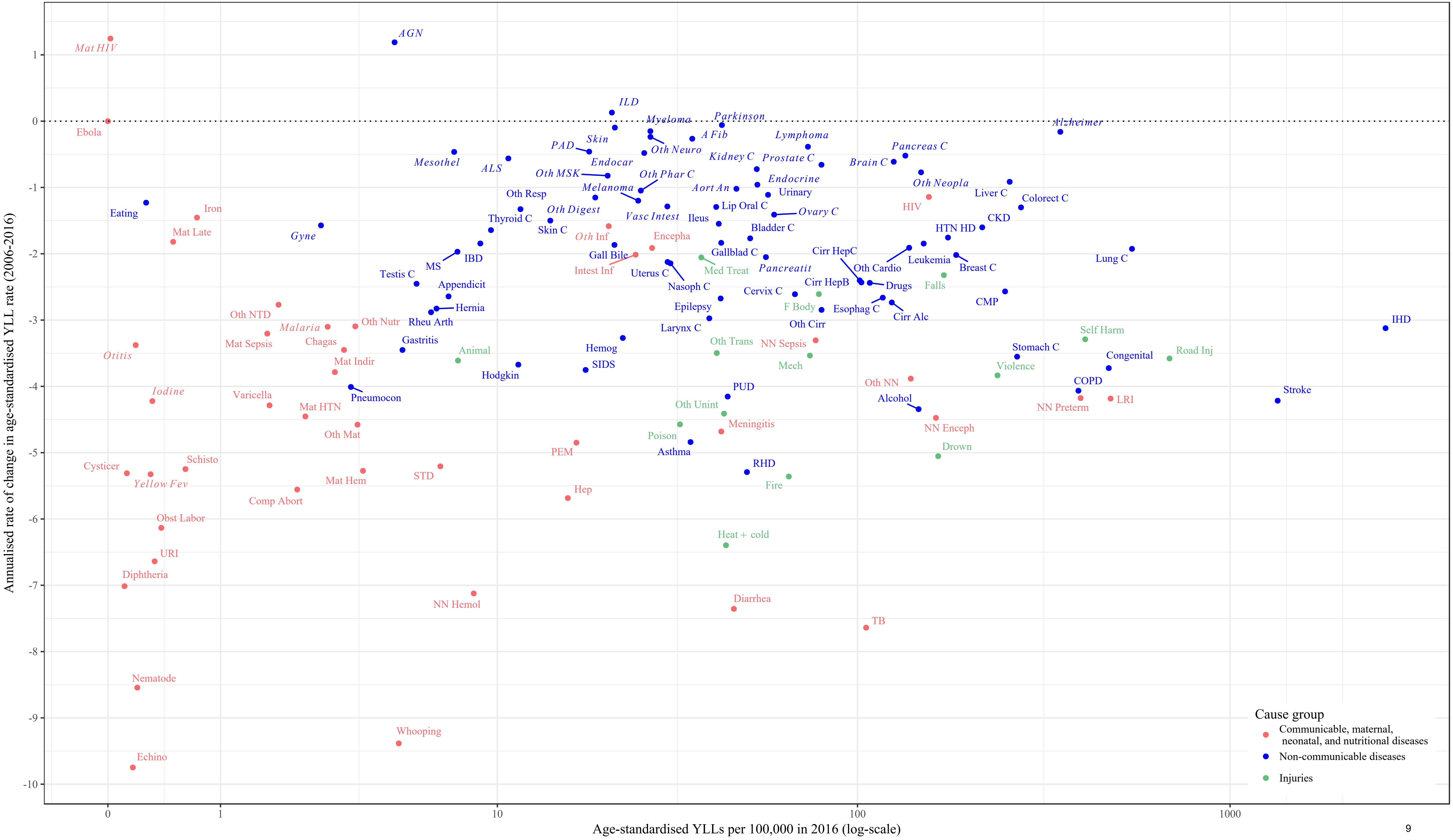

SR Figure 2c: Annualised rate of change in age-standardised YLL rate from 2006-2016 versus age-standardised YLLs (per 100,000) due to level 3 causes for Middle SDI locations, both sexes combined

Not shown in figure: Afr Tryp: YLL = 0 ARC = -57.84, Dengue: YLL = 41.51 ARC = 5.05, Measles: YLL = 21.79 ARC = -10.16, Schisto: YLL = 1.47 ARC = -9.45, rabies: YLL = ARC = , Tetanus: YLL = 8.81 ARC = -9.95, Zika: YLL = 0.02 ARC = 53.06

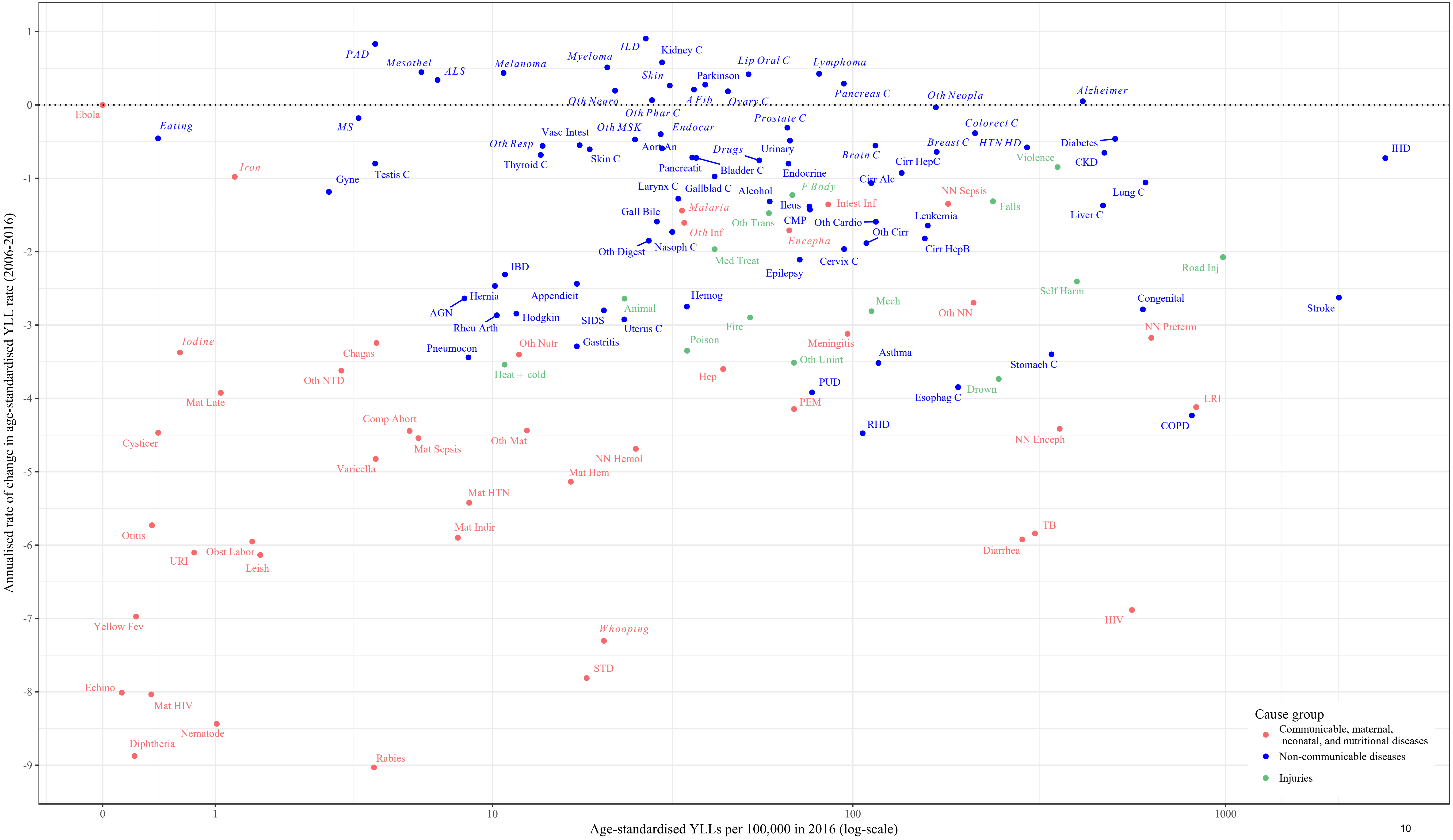

SR Figure 2d: Annualised rate of change in age-standardised YLL rate from 2006-2016 versus age-standardised YLLs (per 100,000) due to level 3 causes for Low-middle SDI locations, both sexes combined

Not shown in figure: Afr Tryp: YLL = 0.26 ARC = -14.66, Measles: YLL = 114.43 ARC = -16.36, Otitis: YLL = 0.99 ARC = -10.78, Diphtheria: YLL = 1.8 ARC = -11.38, Leish: YLL = 10.46 ARC = -11.06, Rabies: YLL = 16.15 ARC = -9.58, Tetanus: YLL = 50.64 ARC = -11.87, Zika: YLL = 0.004 ARC = 36.66

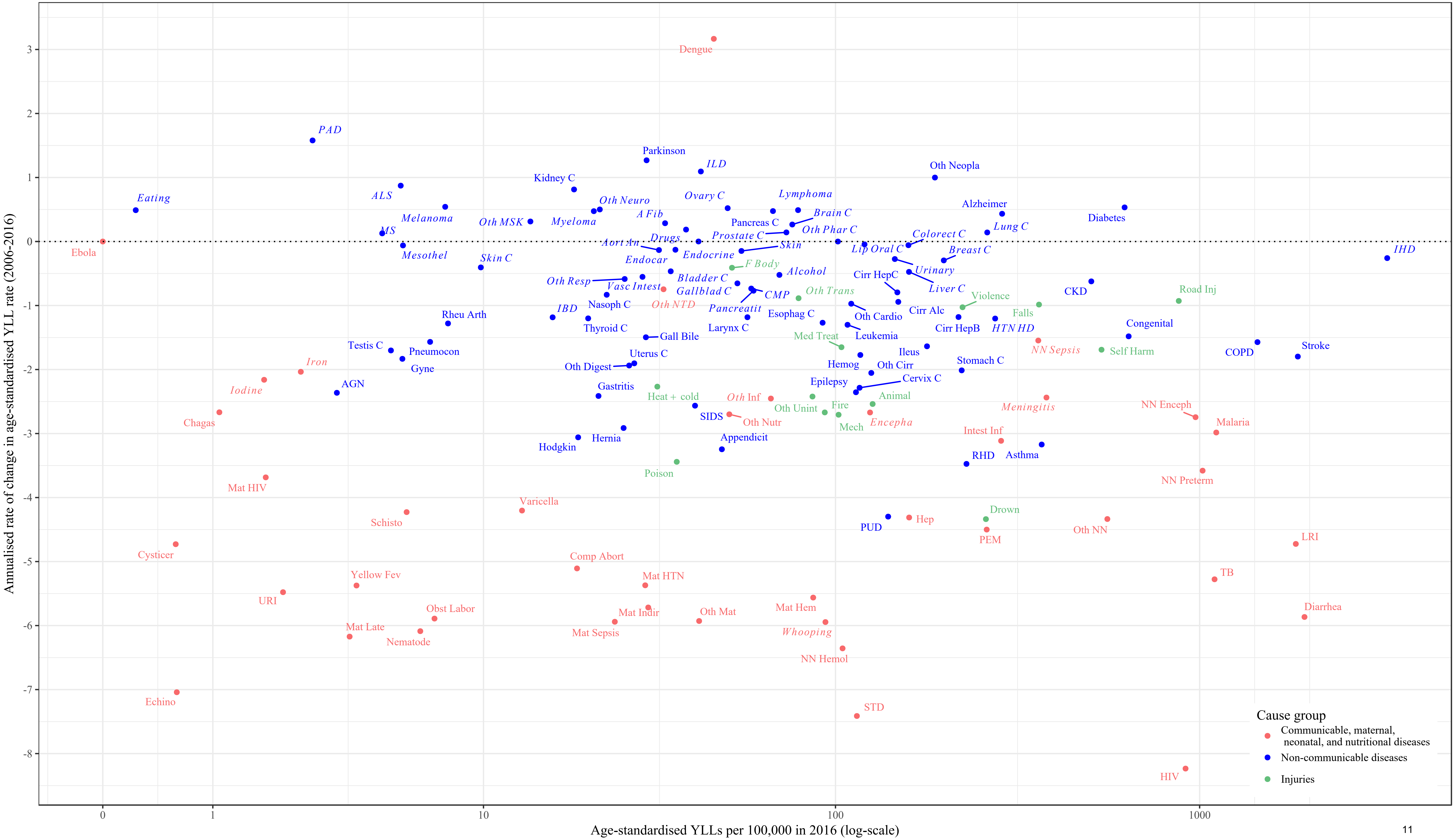

SR Figure 2e: Annualised rate of change in age-standardised YLL rate from 2006-2016 versus age-standardised YLLs (per 100,000) due to level 3 causes for Low SDI locations, both sexes combined

Not shown in figure: Afr Tryp: YLL = 16.21 ARC = -17.4, Diphtheria: YLL = 2.99 ARC = -14.22, Measles: YLL = 181.39 ARC = -10.68, Ebola: YLL = 0.03 ARC = 57.74, Leish: YLL = 60.82 ARC = -10.18, Tetanus: YLL = 98.57 ARC = -9.31, HIV: YLL = 2559.05 ARC = -9.55, Zika: YLL = 0.001 ARC = 17.05

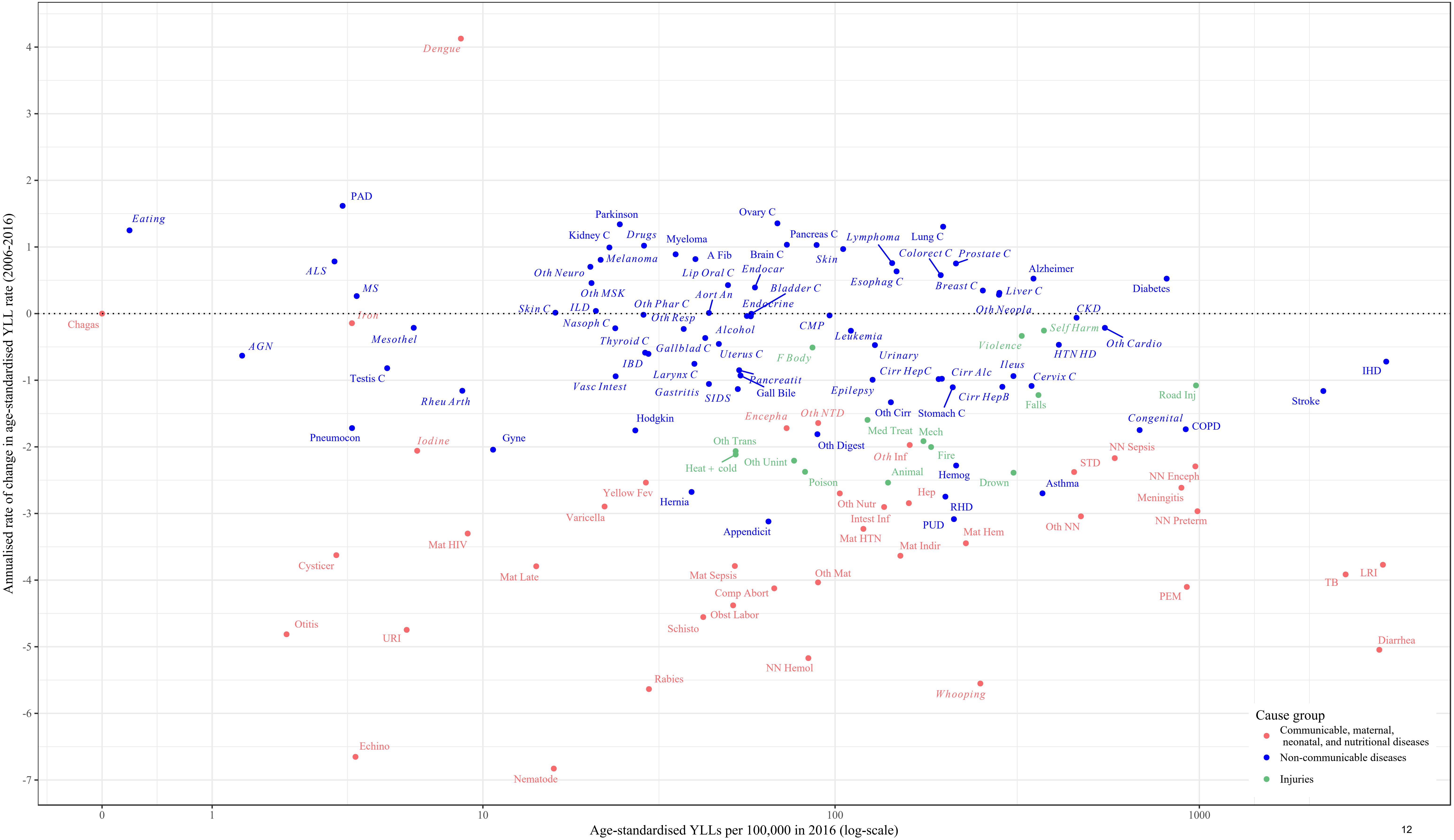

SR Figure 3: Annualised rate of change in all-age YLL rate from 2006 to 2016 versus global all-age YLL rate per 100,000 for each Level 3 cause

Not shown in figure: Afr Tryp: YLL = 1.71 ARC = -15.53, Measles: YLL = 77.14 ARC = -14.08, Zika: YLL = 0.01 ARC = 48.62, Ebola: YLL = 0.003 ARC = 32.67, Diphtheria: YLL = 1.18 ARC = -12.27, Otitis: YLL = 0.68 ARC = -7.57, Tetanus: YLL = 31.96 ARC = -11.04, Leish: YLL = 9.55 ARC = -9.84

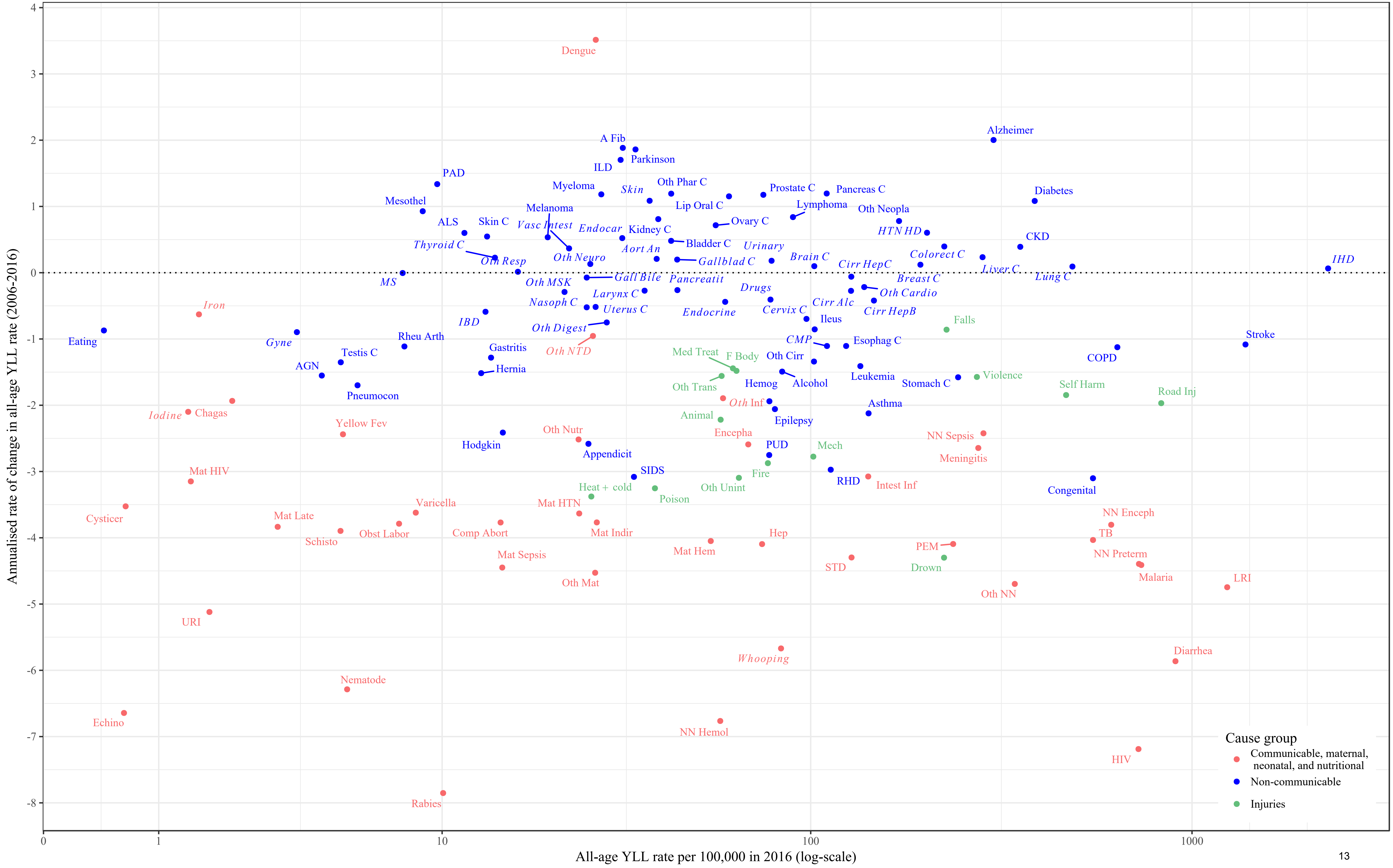

SR Figure 4a: Leading 30 GBD cause hierarchy level 3 causes of High SDI YLLs for males for 1990, 2006 and 2016.

Causes are connected by arrows between time periods. Communicable maternal, neonatal and nutritional disorders are shown in red, non-communicable causes in blue and injuries in green. For the time period 1990 to 2006 and for 2006 to 2016, three measures of change are shown: percent change in the number of YLLs, percent change in the all-age YLL rate and percent change in the age-standardized YLL rate. Numbers in bold highlight statistically significant changes.

Leading causes 1990	Leading causes 2006	Mean % change number of YLLs 1990-2006	Mean % change all-age YLL rate 1990-2006	Mean % change age-standardized YLL rate 1990-2006	Leading causes 2016	Mean % change number of YLLs 2006-2016	Mean % change all-age YLL rate 2006-2016	Mean % change age-standardized YLL rate 2006-2016
1 Ischaemic heart disease	1 Ischaemic heart disease	**-27.2**	**-33.8**	**-46.9**	1 Ischaemic heart disease	**-4.1**	**-9.0**	**-22.7**
2 Lung cancer	2 Lung cancer	-1.4	**-10.4**	**-27.1**	2 Lung cancer	0.5	**-4.7**	**-18.2**
3 Cerebrovascular disease	3 Cerebrovascular disease	**-18.3**	**-25.7**	**-40.4**	3 Cerebrovascular disease	**-4.8**	**-9.7**	**-23.8**
4 Road injuries	4 Self-harm	5.6	-4.1	**-5.7**	4 Self-harm	-2.6	**-7.7**	**-6.7**
5 Self-harm	5 Road injuries	**-35.6**	**-41.5**	**-39.4**	5 COPD	**12.2**	**6.4**	**-12.8**
6 COPD	6 Colorectal cancer	**16.9**	**6.2**	**-13.9**	6 Colorectal cancer	**8.2**	2.6	**-11.5**
7 Colorectal cancer	7 COPD	-1.8	**-10.8**	**-30.2**	7 Road injuries	**-22.6**	**-26.6**	**-25.7**
8 Stomach cancer	8 Lower respiratory infect	**2.7**	**-6.7**	**-25.4**	8 Alzheimer disease	**37.2**	**30.1**	-1.1
9 Lower respiratory infect	9 Alzheimer disease	**54.0**	**39.9**	1.0	9 Lower respiratory infect	**10.0**	4.3	**-13.6**
10 Congenital anomalies	10 Stomach cancer	**-18.8**	**-26.2**	**-39.8**	10 Liver cancer	**11.7**	5.9	-7.2
11 HIV/AIDS	11 Diabetes	**31.0**	**19.0**	-2.5	11 Diabetes	**5.4**	-0.1	**-12.9**
12 Neonatal preterm birth	12 Liver cancer	**39.6**	**26.8**	**4.8**	12 Prostate cancer	**15.5**	**9.5**	**-11.6**
13 Interpersonal violence	13 Prostate cancer	**15.9**	**5.3**	**-19.1**	13 Stomach cancer	-4.6	**-9.6**	**-22.2**
14 Prostate cancer	14 Pancreatic cancer	**36.1**	**23.7**	0.7	14 Pancreatic cancer	**17.8**	**11.7**	-3.3
15 Alzheimer disease	15 Chronic kidney disease	**26.9**	**15.3**	**-7.1**	15 Chronic kidney disease	**21.9**	**15.6**	**-3.1**
16 Diabetes	16 Cirrhosis alcohol	**7.2**	-2.6	**-17.8**	16 Drug use disorders	**16.6**	**10.6**	**15.0**
17 Liver cancer	17 Drug use disorders	**139.7**	**117.8**	**124.6**	17 Cirrhosis alcohol	1.5	-3.7	**-10.7**
18 Cirrhosis alcohol	18 Interpersonal violence	**-21.2**	**-28.4**	**-22.2**	18 Oesophageal cancer	**9.8**	4.1	**-9.0**
19 Cardiomyopathy	19 Leukaemia	0.8	**-8.4**	**-19.9**	19 Other cardiovascular	**6.9**	1.4	**-12.9**
20 Leukaemia	20 Oesophageal cancer	**23.5**	**12.2**	**-8.2**	20 Leukaemia	2.9	-2.4	**-12.9**
21 Pancreatic cancer	21 Cirrhosis hepatitis C	**6.3**	**-3.4**	**-18.1**	21 Falls	4.7	-0.7	**-12.2**
22 Falls	22 Other cardiovascular	**3.8**	**-5.7**	**-22.6**	22 Other neoplasms	**13.0**	**7.1**	-3.4
23 Chronic kidney disease	23 Congenital anomalies	**-42.5**	**-47.8**	**-40.6**	23 Cirrhosis hepatitis C	0.5	-4.7	**-11.5**
24 Other cardiovascular	24 Falls	2.3	**-7.1**	**-19.3**	24 Cardiomyopathy	2.2	-3.1	**-12.4**
25 Cirrhosis hepatitis C	25 Cardiomyopathy	-5.3	-14.0	**-25.8**	25 Interpersonal violence	**-10.3**	**-15.0**	**-11.1**
26 Oesophageal cancer	26 Other neoplasms	**12.5**	2.2	**-9.9**	26 Alcohol use disorders	2.2	-3.0	**-5.7**
27 Other neoplasms	27 Alcohol use disorders	**20.7**	**9.7**	-2.4	27 Non-Hodgkin's lymphoma	**9.7**	4.0	-8.4
28 Drowning	28 Neonatal preterm birth	**-45.3**	**-50.3**	**-41.0**	28 Congenital anomalies	**-16.3**	**-20.6**	**-15.3**
29 Alcohol use disorders	29 Non-Hodgkin's lymphoma	**9.4**	-0.6	**-15.7**	29 Brain cancer	9.2	3.6	-2.8
30 Non-Hodgkin's lymphoma	30 Brain cancer	12.4	2.1	-8.1	30 Hypertensive heart disease	**19.8**	**13.6**	-1.6
31 Brain cancer	31 Hypertensive heart disease				35 Neonatal preterm birth			
35 Hypertensive heart disease	37 HIV/AIDS				40 Drowning			
38 Drug use disorders	38 Drowning				50 HIV/AIDS			

Legend:

Communicable, maternal, neonatal and nutritional

Non-communicable

Injuries

SR Figure 4b: Leading 30 GBD cause hierarchy level 3 causes of High-middle SDI YLLs for males for 1990, 2006 and 2016.

Causes are connected by arrows between time periods. Communicable maternal, neonatal and nutritional disorders are shown in red, non-communicable causes in blue and injuries in green. For the time period 1990 to 2006 and for 2006 to 2016, three measures of change are shown: percent change in the number of YLLs, percent change in the all-age YLL rate and percent change in the age-standardized YLL rate. Numbers in bold highlight statistically significant changes.

Leading causes 1990
1 Ischaemic heart disease
2 Cerebrovascular disease
3 Road injuries
4 Lower respiratory infect
5 Neonatal preterm birth
6 Congenital anomalies
7 Lung cancer
8 COPD
9 Self-harm
10 Drowning
11 Interpersonal violence
12 Stomach cancer
13 Neonatal encephalopathy
14 Diarrhoeal diseases
15 Liver cancer
16 Tuberculosis
17 Falls
18 Other neonatal
19 Nature disaster
20 Cardiomyopathy
21 Alcohol use disorders
22 Colorectal cancer
23 Chronic kidney disease
24 Leukaemia
25 Mechanical forces
26 Oesophageal cancer
27 Meningitis
28 Diabetes
29 Drug use disorders
30 Other cardiovascular
35 Cirrhosis alcohol
36 Hypertensive heart disease
37 Alzheimer disease
39 Cirrhosis hepatitis B
42 Cirrhosis hepatitis C
43 Pancreatic cancer
44 HIV/AIDS

Leading causes 2006	Mean % change number of YLLs 1990-2006	Mean % change all-age YLL rate 1990-2006	Mean % change age-standardized YLL rate 1990-2006
1 Ischaemic heart disease	**39.7**	**16.9**	-4.8
2 Cerebrovascular disease	**24.1**	3.8	**-14.7**
3 Road injuries	**11.2**	**-7.0**	**-10.3**
4 Lung cancer	**12.8**	-5.6	**-17.5**
5 Self-harm	**32.3**	10.7	-0.6
6 Lower respiratory infect	**-33.1**	**-44.0**	**-38.0**
7 COPD	**-13.4**	**-27.5**	**-39.4**
8 Interpersonal violence	11.4	-6.8	-11.8
9 Congenital anomalies	**-42.7**	**-52.1**	**-36.4**
10 Neonatal preterm birth	**-56.2**	**-63.4**	**-50.6**
11 Stomach cancer	**-9.1**	**-24.0**	**-34.6**
12 Cardiomyopathy	**65.0**	**38.0**	16.2
13 Liver cancer	**25.2**	4.8	**-10.2**
14 Drowning	**-31.1**	**-42.4**	**-38.4**
15 Alcohol use disorders	**55.5**	30.1	12.7
16 Tuberculosis	**12.2**	-6.1	**-20.6**
17 Colorectal cancer	**42.9**	**19.6**	2.2
18 Falls	**12.4**	-6.0	**-10.9**
19 HIV/AIDS	**158.1**	**115.9**	**100.8**
20 Chronic kidney disease	**26.9**	**6.2**	-4.5
21 Diabetes	**45.0**	**21.3**	**4.8**
22 Drug use disorders	**45.7**	**21.9**	9.4
23 Cirrhosis alcohol	**65.3**	**38.2**	**13.3**
24 Alzheimer disease	**65.6**	**38.5**	**4.8**
25 Neonatal encephalopathy	**-45.2**	**-54.2**	**-38.1**
26 Oesophageal cancer	**24.6**	4.3	**-9.9**
27 Cirrhosis hepatitis B	**63.1**	**36.5**	**13.3**
28 Leukaemia	0.6	**-15.8**	**-15.0**
29 Cirrhosis hepatitis C	**70.5**	**42.6**	**18.7**
30 Hypertensive heart disease	**32.4**	10.8	-7.8
31 Other cardiovascular			
33 Mechanical forces			
34 Other neonatal			
35 Pancreatic cancer			
50 Diarrhoeal diseases			
57 Meningitis			
98 Nature disaster			

Leading causes 2016	Mean % change number of YLLs 2006-2016	Mean % change all-age YLL rate 2006-2016	Mean % change age-standardized YLL rate 2006-2016
1 Ischaemic heart disease	-6.5	**-16.5**	**-26.8**
2 Cerebrovascular disease	**-12.1**	**-21.5**	**-31.5**
3 Road injuries	**-22.3**	**-30.7**	**-30.0**
4 Lung cancer	2.6	-8.4	**-20.5**
5 Self-harm	-17.1	**-26.0**	**-27.6**
6 Lower respiratory infect	**-20.0**	**-28.6**	**-33.2**
7 COPD	**-12.1**	**-21.5**	**-32.4**
8 Liver cancer	**17.3**	4.7	-7.2
9 Interpersonal violence	**-26.1**	**-34.0**	**-32.6**
10 Stomach cancer	-8.9	**-18.6**	**-28.8**
11 Cardiomyopathy	-8.0	-17.9	**-24.1**
12 Congenital anomalies	**-29.0**	**-36.6**	**-30.5**
13 Colorectal cancer	**16.1**	3.6	**-9.9**
14 Neonatal preterm birth	**-34.1**	**-41.2**	**-33.9**
15 Alcohol use disorders	-20.7	**-29.2**	**-34.4**
16 Alzheimer disease	**38.7**	**23.9**	-0.6
17 Falls	-6.8	**-16.8**	**-20.7**
18 Chronic kidney disease	**8.1**	-3.5	**-13.4**
19 Diabetes	**17.9**	5.2	**-8.3**
20 HIV/AIDS	-1.0	**-11.6**	**-13.7**
21 Drowning	**-33.9**	**-41.0**	**-39.6**
22 Oesophageal cancer	5.5	-5.8	**-19.1**
23 Cirrhosis alcohol	-6.0	**-16.1**	**-24.7**
24 Drug use disorders	-10.0	-19.6	-22.3
25 Hypertensive heart disease	12.5	0.4	**-12.7**
26 Leukaemia	-0.9	**-11.6**	**-14.0**
27 Cirrhosis hepatitis B	-2.6	**-13.0**	**-21.3**
28 Tuberculosis	**-46.3**	**-52.1**	**-55.3**
29 Pancreatic cancer	**21.8**	8.7	-5.7
30 Cirrhosis hepatitis C	-2.6	-13.1	**-21.8**
33 Other cardiovascular			
35 Neonatal encephalopathy			
36 Mechanical forces			
37 Other neonatal			
63 Meningitis			
65 Diarrhoeal diseases			
115 Nature disaster			

Legend:

Communicable, maternal, neonatal and nutritional

Non-communicable

Injuries

SR Figure 4c: Leading 30 GBD cause hierarchy level 3 causes of Middle SDI YLLs for males for 1990, 2006 and 2016.

Causes are connected by arrows between time periods. Communicable maternal, neonatal and nutritional disorders are shown in red, non-communicable causes in blue and injuries in green. For the time period 1990 to 2006 and for 2006 to 2016, three measures of change are shown: percent change in the number of YLLs, percent change in the all-age YLL rate and percent change in the age-standardized YLL rate. Numbers in bold highlight statistically significant changes.

Leading causes 1990
1 Lower respiratory infect
2 Cerebrovascular disease
3 Neonatal preterm birth
4 Road injuries
5 Ischaemic heart disease
6 Diarrhoeal diseases
7 Congenital anomalies
8 COPD
9 Neonatal encephalopathy
10 Tuberculosis
11 Drowning
12 Self-harm
13 Interpersonal violence
14 Liver cancer
15 Stomach cancer
16 Lung cancer
17 Other neonatal
18 Measles
19 Chronic kidney disease
20 Falls
21 Oesophageal cancer
22 Mechanical forces
23 Meningitis
24 Neonatal sepsis
25 Hypertensive heart disease
26 Leukaemia
27 Diabetes
28 Cirrhosis hepatitis B
29 Protein-energy malnutrition
30 Rheumatic heart disease
35 Cirrhosis hepatitis C
38 Other neoplasms
42 Colorectal cancer
43 Cirrhosis alcohol
50 Alzheimer disease
70 HIV/AIDS

Leading causes 2006	Mean % change number of YLLs 1990-2006	Mean % change all-age YLL rate 1990-2006	Mean % change age-standardized YLL rate 1990-2006
1 Ischaemic heart disease	**64.1**	**36.6**	5.5
2 Cerebrovascular disease	**23.4**	2.7	**-19.3**
3 Road injuries	**19.8**	-0.3	-0.7
4 HIV/AIDS	**2,568.3**	**2,121.0**	**2,044.1**
5 Lower respiratory infect	**-54.8**	**-62.3**	**-48.6**
6 COPD	**-14.2**	**-28.6**	**-44.2**
7 Neonatal preterm birth	**-50.9**	**-59.1**	**-40.1**
8 Congenital anomalies	**-33.2**	**-44.4**	**-21.4**
9 Lung cancer	**65.3**	**37.6**	**8.1**
10 Liver cancer	**43.1**	**19.1**	-5.1
11 Interpersonal violence	**11.9**	**-6.9**	**-8.0**
12 Tuberculosis	**-29.9**	**-41.6**	**-51.5**
13 Self-harm	-0.2	-16.9	**-22.2**
14 Neonatal encephalopathy	**-43.0**	**-52.6**	**-30.5**
15 Drowning	**-43.5**	**-53.0**	**-44.9**
16 Stomach cancer	**10.6**	**-8.0**	**-27.9**
17 Diarrhoeal diseases	**-69.1**	**-74.3**	**-66.1**
18 Chronic kidney disease	**44.4**	**20.2**	2.8
19 Diabetes	**83.4**	**52.7**	**20.3**
20 Falls	17.2	-2.4	-5.5
21 Oesophageal cancer	**17.4**	-2.3	**-24.2**
22 Other neonatal	**-34.9**	**-45.8**	**-20.7**
23 Cirrhosis hepatitis B	**30.2**	**8.3**	**-15.6**
24 Mechanical forces	-9.1	**-24.4**	**-19.3**
25 Hypertensive heart disease	-4.9	-20.8	**-40.1**
26 Neonatal sepsis	**-18.9**	**-32.5**	-1.4
27 Leukaemia	-1.5	**-18.0**	**-13.3**
28 Cirrhosis hepatitis C	**37.7**	**14.6**	**-11.5**
29 Colorectal cancer	**63.9**	**36.4**	**8.4**
30 Alzheimer disease	**79.9**	**49.7**	**3.0**
32 Other neoplasms			
33 Cirrhosis alcohol			
35 Meningitis			
39 Rheumatic heart disease			
49 Protein-energy malnutrition			
60 Measles			

Leading causes 2016	Mean % change number of YLLs 2006-2016	Mean % change all-age YLL rate 2006-2016	Mean % change age-standardized YLL rate 2006-2016
1 Ischaemic heart disease	**28.2**	**19.7**	-2.2
2 Cerebrovascular disease	**9.6**	2.3	**-17.5**
3 Road injuries	**-10.3**	**-16.3**	**-16.4**
4 COPD	**-5.5**	**-11.8**	**-30.1**
5 Lung cancer	**21.0**	**12.9**	**-8.7**
6 Lower respiratory infect	**-29.3**	**-34.0**	**-31.1**
7 Liver cancer	**13.9**	**6.4**	**-10.6**
8 HIV/AIDS	**-40.8**	**-44.7**	**-44.5**
9 Interpersonal violence	-2.2	**-8.7**	-5.9
10 Self-harm	-4.6	**-10.9**	**-13.9**
11 Neonatal preterm birth	**-35.9**	**-40.2**	**-26.3**
12 Chronic kidney disease	**20.8**	**12.7**	-2.7
13 Congenital anomalies	**-32.1**	**-36.6**	**-24.4**
14 Diabetes	**33.5**	**24.6**	1.6
15 Stomach cancer	-2.1	**-8.6**	**-26.0**
16 Tuberculosis	**-32.2**	**-36.7**	**-43.7**
17 Drowning	**-29.4**	**-34.1**	**-29.9**
18 Falls	2.8	-4.0	**-10.0**
19 Neonatal encephalopathy	**-44.1**	**-47.8**	**-35.6**
20 Oesophageal cancer	-1.3	**-7.8**	**-26.3**
21 Hypertensive heart disease	**36.1**	**27.1**	3.2
22 Cirrhosis hepatitis B	7.6	0.5	**-13.6**
23 Alzheimer disease	**44.3**	**34.7**	0.9
24 Colorectal cancer	**33.2**	**24.3**	2.2
25 Diarrhoeal diseases	**-45.2**	**-48.9**	**-45.5**
26 Cirrhosis hepatitis C	**16.9**	**9.2**	**-7.3**
27 Other neoplasms	**18.7**	**10.8**	1.5
28 Leukaemia	**-4.3**	**-10.6**	**-10.9**
29 Cirrhosis alcohol	**16.1**	**8.4**	**-7.7**
30 Mechanical forces	**-21.9**	**-27.1**	**-25.9**
31 Other neonatal			
32 Neonatal sepsis			
44 Meningitis			
45 Rheumatic heart disease			
58 Protein-energy malnutrition			
87 Measles			

Legend:
Communicable, maternal, neonatal and nutritional
Non-communicable
Injuries

SR Figure 4d: Leading 30 GBD cause hierarchy level 3 causes of Low-middle SDI YLLs for males for 1990, 2006 and 2016.

Causes are connected by arrows between time periods. Communicable maternal, neonatal and nutritional disorders are shown in red, non-communicable causes in blue and injuries in green. For the time period 1990 to 2006 and for 2006 to 2016, three measures of change are shown: percent change in the number of YLLs, percent change in the all-age YLL rate and percent change in the age-standardized YLL rate. Numbers in bold highlight statistically significant changes.

Leading causes 1990
1 Lower respiratory infect
2 Diarrhoeal diseases
3 Neonatal preterm birth
4 Measles
5 Tuberculosis
6 Neonatal encephalopathy
7 Other neonatal
8 Ischaemic heart disease
9 Malaria
10 Road injuries
11 Cerebrovascular disease
12 Tetanus
13 Congenital anomalies
14 COPD
15 Drowning
16 Meningitis
17 Protein-energy malnutrition
18 Neonatal sepsis
19 Intestinal infectious
20 Self-harm
21 Neonatal hemolytic
22 Asthma
23 STDs
24 HIV/AIDS
25 Whooping cough
26 Chronic kidney disease
27 Falls
28 Hepatitis
29 Interpersonal violence
30 Peptic ulcer disease
35 Cirrhosis hepatitis B
38 Diabetes
43 Lung cancer
46 Cirrhosis hepatitis C
49 Cirrhosis alcohol
57 Other neoplasms
81 Conflict & terror

Leading causes 2006	Mean % change number of YLLs 1990-2006	Mean % change all-age YLL rate 1990-2006	Mean % change age-standardized YLL rate 1990-2006
1 Lower respiratory infect	**-32.6**	**-50.4**	**-37.5**
2 Diarrhoeal diseases	**-34.6**	**-51.8**	**-43.3**
3 Ischaemic heart disease	**65.5**	**21.9**	**9.3**
4 Neonatal preterm birth	**-23.1**	**-43.4**	**-30.2**
5 HIV/AIDS	**482.9**	**329.4**	**306.8**
6 Neonatal encephalopathy	-3.5	**-28.9**	-12.4
7 Malaria	30.6	-3.8	11.3
8 Tuberculosis	**-17.4**	**-39.1**	**-43.7**
9 Road injuries	**42.1**	4.6	**5.6**
10 Cerebrovascular disease	**39.5**	2.8	**-5.9**
11 Other neonatal	**-20.2**	**-41.2**	**-27.6**
12 Congenital anomalies	3.3	-23.9	-8.1
13 COPD	5.2	**-22.5**	**-30.4**
14 Measles	**-64.5**	**-73.9**	**-69.2**
15 Self-harm	**32.6**	-2.4	-7.1
16 Neonatal sepsis	0.2	**-26.2**	-9.2
17 Meningitis	-18.5	**-40.0**	**-29.9**
18 Drowning	**-23.9**	**-44.0**	**-36.2**
19 Intestinal infectious	**-17.5**	**-39.2**	**-33.4**
20 Chronic kidney disease	**37.6**	1.4	-2.8
21 Protein-energy malnutrition	-33.4	**-51.0**	**-41.4**
22 Interpersonal violence	**45.6**	7.2	3.1
23 Falls	**18.7**	-12.6	-7.5
24 Asthma	**-21.7**	**-42.3**	**-43.7**
25 Diabetes	**99.5**	**46.9**	**34.3**
26 STDs	**-15.6**	**-37.8**	**-26.1**
27 Neonatal hemolytic	**-40.6**	**-56.3**	**-46.3**
28 Cirrhosis hepatitis B	**49.3**	**9.9**	-1.8
29 Hepatitis	**-12.9**	**-35.9**	**-29.3**
30 Lung cancer	**57.4**	**16.0**	5.2
31 Tetanus			
33 Whooping cough			
35 Conflict & terror			
39 Cirrhosis hepatitis C			
43 Cirrhosis alcohol			
44 Peptic ulcer disease			
46 Other neoplasms			

Leading causes 2016	Mean % change number of YLLs 2006-2016	Mean % change all-age YLL rate 2006-2016	Mean % change age-standardized YLL rate 2006-2016
1 Ischaemic heart disease	**30.0**	**11.6**	-0.5
2 Lower respiratory infect	**-35.4**	**-44.6**	**-34.4**
3 Diarrhoeal diseases	**-39.5**	**-48.0**	**-43.0**
4 Road injuries	**10.4**	**-5.3**	**-5.8**
5 Cerebrovascular disease	**13.4**	-2.7	**-13.8**
6 Neonatal preterm birth	**-31.2**	**-40.9**	**-29.4**
7 Neonatal encephalopathy	**-25.7**	**-36.3**	**-23.8**
8 Malaria	-22.7	**-33.6**	-26.6
9 Tuberculosis	**-25.6**	**-36.2**	**-40.1**
10 HIV/AIDS	**-42.5**	**-50.7**	**-52.8**
11 COPD	**14.3**	-1.9	**-13.4**
12 Congenital anomalies	-14.8	**-26.9**	-14.9
13 Other neonatal	**-38.7**	**-47.4**	**-37.1**
14 Self-harm	10.6	-5.1	-7.6
15 Chronic kidney disease	**18.4**	1.6	-4.5
16 Meningitis	-15.1	**-27.2**	-19.4
17 Neonatal sepsis	-17.8	**-29.5**	-15.8
18 Diabetes	**38.5**	**18.8**	**7.0**
19 Interpersonal violence	**9.0**	-6.4	-7.5
20 Drowning	**-26.1**	**-36.6**	**-31.6**
21 Intestinal infectious	**-18.9**	**-30.4**	**-25.7**
22 Falls	6.9	-8.2	-7.7
23 Conflict & terror	109.0	79.4	82.6
24 Asthma	-8.3	**-21.3**	**-27.3**
25 Cirrhosis hepatitis B	**16.5**	0.0	-8.4
26 Protein-energy malnutrition	**-30.7**	**-40.5**	**-32.9**
27 Lung cancer	**31.6**	**13.0**	1.3
28 Cirrhosis hepatitis C	**21.6**	4.3	-5.0
29 Other neoplasms	**35.5**	**16.3**	**12.4**
30 Cirrhosis alcohol	**21.0**	3.9	-6.0
35 Hepatitis			
40 Measles			
42 Neonatal hemolytic			
46 Peptic ulcer disease			
47 STDs			
55 Whooping cough			
67 Tetanus			

Legend:
Communicable, maternal, neonatal and nutritional
Non-communicable
Injuries

SR Figure 4e: Leading 30 GBD cause hierarchy level 3 causes of Low SDI YLLs for males for 1990, 2006 and 2016.

Causes are connected by arrows between time periods. Communicable maternal, neonatal and nutritional disorders are shown in red, non-communicable causes in blue and injuries in green. For the time period 1990 to 2006 and for 2006 to 2016, three measures of change are shown: percent change in the number of YLLs, percent change in the all-age YLL rate and percent change in the age-standardized YLL rate. Numbers in bold highlight statistically significant changes.

Leading causes 1990	Leading causes 2006	Mean % change number of YLLs 1990-2006	Mean % change all-age YLL rate 1990-2006	Mean % change age-standardized YLL rate 1990-2006	Leading causes 2016	Mean % change number of YLLs 2006-2016	Mean % change all-age YLL rate 2006-2016	Mean % change age-standardized YLL rate 2006-2016
1 Lower respiratory infect	1 Malaria	25.6	-20.9	-14.1	1 Lower respiratory infect	**-19.5**	**-39.2**	**-29.5**
2 Diarrhoeal diseases	2 Lower respiratory infect	-8.1	**-42.1**	**-32.2**	2 Malaria	**-33.0**	**-49.4**	**-46.7**
3 Malaria	3 Diarrhoeal diseases	-1.3	**-37.8**	**-29.5**	3 Diarrhoeal diseases	**-28.4**	**-45.9**	**-38.7**
4 Measles	4 HIV/AIDS	**231.2**	**108.6**	**106.3**	4 HIV/AIDS	**-40.8**	**-55.3**	**-57.7**
5 Neonatal preterm birth	5 Neonatal preterm birth	12.7	**-29.0**	**-22.6**	5 Neonatal encephalopathy	1.3	**-23.5**	**-19.1**
6 Protein-energy malnutrition	6 Neonatal encephalopathy	**30.1**	**-18.0**	-10.6	6 Neonatal preterm birth	-6.0	**-29.0**	**-25.0**
7 Tuberculosis	7 Tuberculosis	**17.8**	**-25.8**	**-23.3**	7 Tuberculosis	**-7.9**	**-30.5**	**-30.6**
8 Neonatal encephalopathy	8 Protein-energy malnutrition	-11.1	**-44.0**	**-38.3**	8 Ischaemic heart disease	**26.3**	-4.7	-5.2
9 Meningitis	9 Meningitis	1.3	**-36.2**	**-28.3**	9 Protein-energy malnutrition	-14.5	**-35.5**	**-30.6**
10 HIV/AIDS	10 Neonatal sepsis	24.2	-21.8	-15.0	10 Road injuries	**16.8**	**-11.8**	**-8.8**
11 Neonatal sepsis	11 Ischaemic heart disease	**60.3**	1.0	2.8	11 Neonatal sepsis	-1.5	**-25.7**	**-21.6**
12 Other neonatal	12 Congenital anomalies	19.8	-24.6	-18.0	12 Meningitis	-5.2	**-28.5**	**-21.3**
13 Congenital anomalies	13 Road injuries	**37.7**	**-13.3**	-8.0	13 Congenital anomalies	3.5	-21.9	-17.8
14 Road injuries	14 Other neonatal	8.7	**-31.6**	**-25.5**	14 Other neonatal	-10.3	**-32.2**	**-28.4**
15 Tetanus	15 STDs	4.2	**-34.4**	**-29.8**	15 Cerebrovascular disease	**21.5**	**-8.3**	**-8.2**
16 Conflict & terror	16 Cerebrovascular disease	**30.6**	**-17.8**	**-12.8**	16 STDs	-0.3	**-24.7**	**-21.3**
17 STDs	17 Measles	**-81.1**	**-88.1**	**-87.4**	17 Conflict & terror	**347.4**	**237.8**	**239.8**
18 Ischaemic heart disease	18 Whooping cough	12.8	-29.0	-24.6	18 Drowning	-0.8	**-25.1**	**-20.0**
19 Cerebrovascular disease	19 Drowning	17.7	**-25.9**	**-20.8**	19 Interpersonal violence	**30.5**	-1.5	-2.3
20 Whooping cough	20 Interpersonal violence	**69.6**	6.8	6.8	20 Whooping cough	-26.0	-44.2	-42.6
21 Drowning	21 Tetanus	**-59.7**	**-74.6**	**-72.9**	21 Self-harm	**39.8**	5.5	3.9
22 Other infectious	22 COPD	**17.4**	**-26.1**	**-24.4**	22 Diabetes	**41.7**	**7.0**	**6.5**
23 COPD	23 Other infectious	1.3	-36.2	-30.3	23 COPD	10.4	**-16.6**	**-16.8**
24 Hemoglobinopathies	24 Self-harm	**61.6**	1.8	-0.1	24 Other infectious	7.4	-18.9	-15.3
25 Neonatal hemolytic	25 Falls	**22.3**	**-23.0**	**-14.5**	25 Measles	**-55.4**	**-66.3**	**-65.6**
26 Interpersonal violence	26 Mechanical forces	**20.4**	**-24.1**	**-19.0**	26 Chronic kidney disease	**28.5**	-3.0	-0.3
27 Falls	27 Diabetes	**62.4**	2.3	6.1	27 Falls	12.3	**-15.2**	-9.8
28 Mechanical forces	28 Hemoglobinopathies	-0.5	**-37.3**	**-31.1**	28 Other cardiovascular	**26.2**	-4.7	-1.9
29 Intestinal infectious	29 Neonatal hemolytic	-4.3	**-39.7**	**-34.5**	29 Ileus & obstruction	**19.9**	-9.5	-7.4
30 Asthma	30 Chronic kidney disease	**47.6**	-7.1	1.7	30 Mechanical forces	5.4	**-20.4**	**-17.5**
32 Other cardiovascular	31 Ileus & obstruction				32 Hemoglobinopathies			
33 Ileus & obstruction	32 Other cardiovascular				34 Intestinal infectious			
34 Self-harm	33 Intestinal infectious				37 Asthma			
36 Chronic kidney disease	34 Asthma				38 Neonatal hemolytic			
37 Diabetes	43 Conflict & terror				39 Tetanus			

Legend:

Communicable, maternal, neonatal and nutritional

Non-communicable

Injuries

SR Figure 4f: Leading 30 GBD cause hierarchy level 3 causes of High SDI YLLs for females for 1990, 2006 and 2016.

Causes are connected by arrows between time periods. Communicable maternal, neonatal and nutritional disorders are shown in red, non-communicable causes in blue and injuries in green. For the time period 1990 to 2006 and for 2006 to 2016, three measures of change are shown: percent change in the number of YLLs, percent change in the all-age YLL rate and percent change in the age-standardized YLL rate. Numbers in bold highlight statistically significant changes.

Leading causes 1990	Leading causes 2006	Mean % change number of YLLs 1990-2006	Mean % change all-age YLL rate 1990-2006	Mean % change age-standardized YLL rate 1990-2006	Leading causes 2016	Mean % change number of YLLs 2006-2016	Mean % change all-age YLL rate 2006-2016	Mean % change age-standardized YLL rate 2006-2016
1 Ischaemic heart disease	1 Ischaemic heart disease	**-29.6**	**-35.8**	**-48.0**	1 Ischaemic heart disease	**-5.3**	**-10.1**	**-22.8**
2 Cerebrovascular disease	2 Cerebrovascular disease	**-26.2**	**-32.7**	**-44.9**	2 Alzheimer disease	**26.9**	**20.5**	-1.3
3 Breast cancer	3 Alzheimer disease	**46.4**	**33.6**	0.7	3 Cerebrovascular disease	**-7.5**	**-12.1**	**-24.3**
4 Alzheimer disease	4 Lung cancer	**37.9**	**25.8**	**7.8**	4 Lung cancer	**12.5**	**6.9**	**-5.0**
5 Lung cancer	5 Breast cancer	0.3	**-8.5**	**-21.0**	5 Breast cancer	0.7	**-4.3**	**-11.6**
6 Colorectal cancer	6 Colorectal cancer	1.6	**-7.3**	**-20.7**	6 COPD	**15.6**	**9.8**	**-3.7**
7 Road injuries	7 COPD	**32.8**	**21.1**	0.7	7 Colorectal cancer	3.5	-1.6	**-11.8**
8 Lower respiratory infect	8 Lower respiratory infect	-0.3	**-9.1**	**-27.2**	8 Lower respiratory infect	**6.5**	1.2	**-13.0**
9 COPD	9 Self-harm	2.8	**-6.2**	**-6.8**	9 Self-harm	-2.4	**-7.2**	-5.7
10 Congenital anomalies	10 Diabetes	-0.2	**-8.9**	**-22.6**	10 Diabetes	-3.2	**-8.0**	**-18.2**
11 Diabetes	11 Road injuries	**-33.1**	**-39.0**	**-37.8**	11 Pancreatic cancer	**17.2**	**11.3**	-1.4
12 Self-harm	12 Pancreatic cancer	**31.4**	**19.8**	**3.0**	12 Chronic kidney disease	**20.0**	**14.1**	-0.7
13 Stomach cancer	13 Chronic kidney disease	**24.0**	**13.1**	**-7.4**	13 Ovarian cancer	5.1	-0.1	**-9.1**
14 Neonatal preterm birth	14 Ovarian cancer	**6.6**	**-2.8**	**-16.2**	14 Other cardiovascular	4.6	-0.6	**-12.7**
15 Ovarian cancer	15 Other cardiovascular	**10.0**	0.3	**-16.0**	15 Road injuries	**-20.6**	**-24.6**	**-24.1**
16 Other cardiovascular	16 Stomach cancer	**-26.7**	**-33.2**	**-43.0**	16 Other neoplasms	**13.0**	**7.4**	-1.2
17 Chronic kidney disease	17 Congenital anomalies	**-42.8**	**-47.8**	**-41.1**	17 Stomach cancer	**-7.3**	**-11.9**	**-21.3**
18 Pancreatic cancer	18 Other neoplasms	**13.6**	**3.6**	**-8.7**	18 Hypertensive heart disease	**14.2**	8.5	-6.4
19 Leukaemia	19 Hypertensive heart disease	3.8	-5.4	**-23.3**	19 Leukaemia	-3.1	**-7.9**	**-15.4**
20 Cervical cancer	20 Leukaemia	**-5.3**	**-13.6**	**-23.3**	20 Congenital anomalies	**-16.9**	**-21.1**	**-16.2**
21 Hypertensive heart disease	21 Cervical cancer	**-16.5**	**-23.8**	**-32.2**	21 Liver cancer	**11.5**	5.9	-5.9
22 Cardiomyopathy	22 Liver cancer	**37.2**	**25.1**	5.9	22 Drug use disorders	**31.0**	**24.5**	**28.6**
23 Other neoplasms	23 Neonatal preterm birth	**-41.7**	**-46.8**	**-37.4**	23 Cervical cancer	**-5.0**	**-9.7**	**-14.0**
24 Non-Hodgkin's lymphoma	24 Cardiomyopathy	-15.8	-23.2	**-31.8**	24 Cardiomyopathy	4.1	-1.1	**-11.9**
25 Falls	25 Non-Hodgkin's lymphoma	**7.7**	-1.8	**-15.4**	25 Falls	**12.3**	6.7	**-8.9**
26 Brain cancer	26 Brain cancer	9.0	-0.5	**-8.8**	26 Brain cancer	8.3	2.9	-2.5
27 Interpersonal violence	27 Falls	4.2	**-4.9**	**-22.6**	27 Non-Hodgkin's lymphoma	**5.8**	0.5	**-9.5**
28 Cirrhosis alcohol	28 Drug use disorders	**220.9**	**192.7**	**189.2**	28 Endo/metab/blood/immune	10.2	4.7	-2.2
29 Gallbladder cancer	29 Cirrhosis alcohol	**5.6**	**-3.7**	**-15.8**	29 Cirrhosis alcohol	**5.6**	0.3	**-6.5**
30 Liver cancer	30 Endo/metab/blood/immune	**41.0**	**28.6**	16.2	30 Atrial fibrillation	**21.8**	**15.7**	**-4.3**
36 Endo/metab/blood/immune	31 Gallbladder cancer				31 Neonatal preterm birth			
38 Atrial fibrillation	32 Atrial fibrillation				34 Gallbladder cancer			
62 Drug use disorders	35 Interpersonal violence				40 Interpersonal violence			

Legend:

Communicable, maternal, neonatal and nutritional

Non-communicable

Injuries

SR Figure 4g: Leading 30 GBD cause hierarchy level 3 causes of High-middle SDI YLLs for females for 1990, 2006 and 2016.

Causes are connected by arrows between time periods. Communicable maternal, neonatal and nutritional disorders are shown in red, non-communicable causes in blue and injuries in green. For the time period 1990 to 2006 and for 2006 to 2016, three measures of change are shown: percent change in the number of YLLs, percent change in the all-age YLL rate and percent change in the age-standardized YLL rate. Numbers in bold highlight statistically significant changes.

Leading causes 1990
1 Ischaemic heart disease
2 Cerebrovascular disease
3 Lower respiratory infect
4 Neonatal preterm birth
5 Congenital anomalies
6 COPD
7 Road injuries
8 Breast cancer
9 Diarrhoeal diseases
10 Stomach cancer
11 Self-harm
12 Neonatal encephalopathy
13 Alzheimer disease
14 Colorectal cancer
15 Diabetes
16 Lung cancer
17 Drowning
18 Other neonatal
19 Nature disaster
20 Chronic kidney disease
21 Leukaemia
22 Cervical cancer
23 Hypertensive heart disease
24 Rheumatic heart disease
25 Cardiomyopathy
26 Other cardiovascular
27 Interpersonal violence
28 Tuberculosis
29 Liver cancer
30 Other neoplasms
32 Falls
33 Ovarian cancer
35 Brain cancer
38 Pancreatic cancer
41 Cirrhosis other
48 Cirrhosis alcohol
76 HIV/AIDS

Leading causes 2006	Mean % change number of YLLs 1990-2006	Mean % change all-age YLL rate 1990-2006	Mean % change age-standardized YLL rate 1990-2006
1 Ischaemic heart disease	**23.7**	4.7	**-10.4**
2 Cerebrovascular disease	3.3	**-12.5**	**-24.3**
3 Lower respiratory infect	**-53.9**	**-60.9**	**-56.1**
4 Congenital anomalies	**-43.8**	**-52.4**	**-36.3**
5 Road injuries	-5.7	**-20.1**	**-19.6**
6 Breast cancer	**31.9**	**11.7**	-3.7
7 Alzheimer disease	**50.6**	**27.6**	**5.2**
8 COPD	**-29.3**	**-40.1**	**-48.0**
9 Neonatal preterm birth	**-57.3**	**-63.9**	**-51.1**
10 Lung cancer	**34.2**	**13.7**	0.3
11 Colorectal cancer	**24.8**	5.7	**-7.1**
12 Diabetes	**23.4**	4.5	**-6.5**
13 Self-harm	**-10.3**	**-24.0**	**-29.9**
14 Stomach cancer	**-17.1**	**-29.8**	**-38.3**
15 Chronic kidney disease	**23.0**	4.2	-5.2
16 Hypertensive heart disease	**21.4**	2.8	-11.2
17 Cardiomyopathy	**37.5**	16.5	3.2
18 Cervical cancer	**9.3**	**-7.4**	**-20.0**
19 Interpersonal violence	11.0	-5.9	-10.2
20 Neonatal encephalopathy	**-48.7**	**-56.5**	**-41.2**
21 Leukaemia	**-12.7**	**-26.1**	**-25.2**
22 Liver cancer	**15.0**	-2.6	-14.0
23 Other cardiovascular	-1.1	-16.2	**-22.8**
24 Other neoplasms	**16.5**	-1.3	**-6.6**
25 Ovarian cancer	**23.3**	4.4	-8.6
26 Other neonatal	**-35.6**	**-45.5**	-26.2
27 Pancreatic cancer	**45.4**	**23.1**	**8.9**
28 Brain cancer	**14.2**	-3.2	-6.1
29 Drowning	**-46.5**	**-54.7**	**-48.0**
30 Cirrhosis other	**47.5**	**25.0**	**15.3**
31 Tuberculosis			
32 Falls			
33 HIV/AIDS			
34 Cirrhosis alcohol			
36 Rheumatic heart disease			
41 Diarrhoeal diseases			
112 Nature disaster			

Leading causes 2016	Mean % change number of YLLs 2006-2016	Mean % change all-age YLL rate 2006-2016	Mean % change age-standardized YLL rate 2006-2016
1 Ischaemic heart disease	-6.2	-14.9	**-26.9**
2 Cerebrovascular disease	**-21.0**	**-28.3**	**-37.7**
3 Alzheimer disease	**31.5**	**19.4**	-1.9
4 Breast cancer	2.7	-6.8	**-17.4**
5 Lower respiratory infect	**-20.9**	**-28.2**	**-35.4**
6 Road injuries	**-26.2**	**-33.0**	**-32.5**
7 Lung cancer	**15.0**	4.4	**-8.7**
8 Congenital anomalies	**-30.0**	**-36.5**	**-31.8**
9 COPD	**-18.6**	**-26.1**	**-36.7**
10 Colorectal cancer	6.2	-3.6	**-15.4**
11 Diabetes	6.3	-3.5	**-15.6**
12 Neonatal preterm birth	**-34.1**	**-40.2**	**-34.4**
13 Chronic kidney disease	3.0	-6.5	**-17.1**
14 Stomach cancer	**-15.7**	**-23.5**	**-32.4**
15 Hypertensive heart disease	3.4	-6.1	**-19.2**
16 Self-harm	**-25.8**	**-32.6**	**-32.2**
17 Cardiomyopathy	-1.8	-10.9	-20.1
18 Cervical cancer	-4.6	-13.4	**-21.9**
19 Other neoplasms	9.1	-1.0	-7.4
20 Liver cancer	6.3	-3.5	-15.0
21 Other cardiovascular	6.7	-3.2	**-14.4**
22 Ovarian cancer	9.5	-0.6	-12.0
23 Pancreatic cancer	**20.4**	9.3	-4.6
24 Leukaemia	-10.7	**-18.9**	**-21.4**
25 Brain cancer	9.1	-1.0	-6.7
26 HIV/AIDS	**8.6**	-1.5	-3.8
27 Interpersonal violence	-26.1	**-32.9**	**-32.1**
28 Falls	-5.1	**-13.9**	**-21.8**
29 Cirrhosis other	-9.3	-17.7	**-24.8**
30 Cirrhosis alcohol	-4.6	-13.4	**-23.0**
31 Neonatal encephalopathy			
32 Other neonatal			
34 Drowning			
39 Rheumatic heart disease			
40 Tuberculosis			
55 Diarrhoeal diseases			
125 Nature disaster			

Legend:
Communicable, maternal, neonatal and nutritional
Non-communicable
Injuries

SR Figure 4h: Leading 30 GBD cause hierarchy level 3 causes of Middle SDI YLLs for females for 1990, 2006 and 2016.

Causes are connected by arrows between time periods. Communicable maternal, neonatal and nutritional disorders are shown in red, non-communicable causes in blue and injuries in green. For the time period 1990 to 2006 and for 2006 to 2016, three measures of change are shown: percent change in the number of YLLs, percent change in the all-age YLL rate and percent change in the age-standardized YLL rate. Numbers in bold highlight statistically significant changes.

Leading causes 1990
1 Lower respiratory infect
2 Cerebrovascular disease
3 Neonatal preterm birth
4 Diarrhoeal diseases
5 Ischaemic heart disease
6 Congenital anomalies
7 COPD
8 Neonatal encephalopathy
9 Self-harm
10 Road injuries
11 Tuberculosis
12 Drowning
13 Measles
14 Other neonatal
15 Chronic kidney disease
16 Rheumatic heart disease
17 Stomach cancer
18 Diabetes
19 Hypertensive heart disease
20 Protein-energy malnutrition
21 Meningitis
22 Lung cancer
23 Leukaemia
24 Breast cancer
25 Liver cancer
26 Other cardiovascular
27 Neonatal sepsis
28 Whooping cough
29 Interpersonal violence
30 Cervical cancer
31 Alzheimer disease
32 Falls
38 Colorectal cancer
40 Other neoplasms
87 HIV/AIDS

Leading causes 2006	Mean % change number of YLLs 1990-2006	Mean % change all-age YLL rate 1990-2006	Mean % change age-standardized YLL rate 1990-2006
1 Ischaemic heart disease	**51.2**	**25.6**	-2.7
2 Cerebrovascular disease	**11.4**	**-7.5**	**-27.4**
3 HIV/AIDS	**5,499.3**	**4,552.7**	**4,566.7**
4 Lower respiratory infect	**-61.5**	**-68.0**	**-56.1**
5 COPD	-22.5	**-35.6**	**-49.8**
6 Neonatal preterm birth	**-53.5**	**-61.3**	**-42.9**
7 Congenital anomalies	**-33.7**	**-44.9**	**-21.3**
8 Road injuries	-5.4	**-21.4**	**-17.8**
9 Diabetes	**76.5**	**46.6**	**16.2**
10 Diarrhoeal diseases	**-67.1**	**-72.7**	**-64.3**
11 Self-harm	**-37.4**	**-48.0**	**-49.0**
12 Neonatal encephalopathy	**-44.9**	**-54.2**	**-32.4**
13 Chronic kidney disease	**37.7**	**14.4**	-2.2
14 Tuberculosis	**-40.5**	**-50.6**	**-57.5**
15 Lung cancer	**60.6**	**33.5**	4.0
16 Breast cancer	**64.6**	**36.8**	4.6
17 Alzheimer disease	**71.1**	**42.2**	-1.4
18 Stomach cancer	0.9	**-16.1**	**-34.2**
19 Liver cancer	**26.7**	5.2	-16.5
20 Hypertensive heart disease	-7.6	-23.2	**-42.0**
21 Cervical cancer	**31.2**	9.0	**-16.8**
22 Other neonatal	**-28.9**	**-41.0**	-12.8
23 Drowning	**-59.9**	**-66.7**	**-58.6**
24 Rheumatic heart disease	**-36.5**	**-47.2**	**-55.4**
25 Leukaemia	**-19.7**	**-33.3**	**-28.3**
26 Colorectal cancer	**46.2**	**21.5**	-4.7
27 Neonatal sepsis	-11.8	**-26.7**	8.1
28 Interpersonal violence	-11.9	-26.8	-22.7
29 Falls	-0.2	**-17.0**	**-15.2**
30 Other neoplasms	**27.4**	5.9	-0.1
33 Meningitis			
35 Other cardiovascular			
38 Protein-energy malnutrition			
56 Measles			
64 Whooping cough			

Leading causes 2016	Mean % change number of YLLs 2006-2016	Mean % change all-age YLL rate 2006-2016	Mean % change age-standardized YLL rate 2006-2016
1 Ischaemic heart disease	**16.2**	**8.0**	**-13.5**
2 Cerebrovascular disease	**-6.7**	**-13.3**	**-30.7**
3 Lower respiratory infect	**-34.3**	**-38.9**	**-36.7**
4 COPD	**-19.3**	**-25.0**	**-40.7**
5 Diabetes	**21.0**	**12.5**	**-9.6**
6 HIV/AIDS	**-53.6**	**-56.8**	**-55.4**
7 Road injuries	**-20.4**	**-26.0**	**-26.2**
8 Congenital anomalies	**-31.4**	**-36.3**	**-24.2**
9 Chronic kidney disease	**13.4**	**5.4**	**-10.5**
10 Neonatal preterm birth	**-37.3**	**-41.7**	**-28.4**
11 Alzheimer disease	**43.5**	**33.3**	0.4
12 Breast cancer	**17.7**	9.4	-7.0
13 Lung cancer	**16.2**	8.0	**-12.6**
14 Self-harm	**-27.0**	**-32.2**	**-32.6**
15 Hypertensive heart disease	15.6	7.5	-14.0
16 Liver cancer	7.0	-0.6	**-18.2**
17 Diarrhoeal diseases	**-41.5**	**-45.6**	**-43.9**
18 Neonatal encephalopathy	**-43.6**	**-47.6**	**-35.7**
19 Tuberculosis	**-35.7**	**-40.3**	**-45.4**
20 Stomach cancer	**-13.4**	**-19.5**	**-34.1**
21 Cervical cancer	4.1	-3.3	**-18.0**
22 Colorectal cancer	**16.6**	8.4	**-11.2**
23 Other neoplasms	**14.1**	**6.1**	-2.5
24 Other neonatal	**-31.2**	**-36.0**	**-21.5**
25 Falls	0.7	-6.4	**-16.7**
26 Leukaemia	**-14.2**	**-20.2**	**-20.7**
27 Rheumatic heart disease	**-25.0**	**-30.3**	**-38.7**
28 Drowning	**-33.8**	**-38.4**	**-34.7**
29 Interpersonal violence	-17.1	**-22.9**	-19.4
30 Neonatal sepsis	**-21.3**	**-26.9**	-10.3
32 Other cardiovascular			
38 Meningitis			
49 Protein-energy malnutrition			
86 Whooping cough			
90 Measles			

Legend:
Communicable, maternal, neonatal and nutritional
Non-communicable
Injuries

SR Figure 4i: Leading 30 GBD cause hierarchy level 3 causes of Low-middle SDI YLLs for females for 1990, 2006 and 2016.

Causes are connected by arrows between time periods. Communicable maternal, neonatal and nutritional disorders are shown in red, non-communicable causes in blue and injuries in green. For the time period 1990 to 2006 and for 2006 to 2016, three measures of change are shown: percent change in the number of YLLs, percent change in the all-age YLL rate and percent change in the age-standardized YLL rate. Numbers in bold highlight statistically significant changes.

Leading causes 1990	Leading causes 2006	Mean % change number of YLLs 1990-2006	Mean % change all-age YLL rate 1990-2006	Mean % change age-standardized YLL rate 1990-2006	Leading causes 2016	Mean % change number of YLLs 2006-2016	Mean % change all-age YLL rate 2006-2016	Mean % change age-standardized YLL rate 2006-2016
1 Diarrhoeal diseases	1 Diarrhoeal diseases	**-40.5**	**-56.3**	**-47.0**	1 Diarrhoeal diseases	**-42.2**	**-50.5**	**-45.7**
2 Lower respiratory infect	2 Lower respiratory infect	**-35.3**	**-52.5**	**-40.7**	2 Lower respiratory infect	**-41.4**	**-49.8**	**-40.9**
3 Neonatal preterm birth	3 HIV/AIDS	**976.5**	**691.3**	**721.3**	3 Ischaemic heart disease	**24.6**	6.7	-5.5
4 Measles	4 Malaria	31.7	-3.2	13.8	4 Malaria	-21.0	**-32.3**	-25.0
5 Neonatal encephalopathy	5 Neonatal preterm birth	**-21.0**	**-41.9**	**-27.3**	5 Cerebrovascular disease	6.8	**-8.5**	**-19.4**
6 Tuberculosis	6 Ischaemic heart disease	**42.8**	5.0	-6.3	6 Neonatal preterm birth	**-32.6**	**-42.2**	**-31.0**
7 Malaria	7 Neonatal encephalopathy	-3.2	**-28.8**	-11.0	7 Neonatal encephalopathy	**-26.1**	**-36.7**	**-24.3**
8 Other neonatal	8 Cerebrovascular disease	**24.9**	**-8.2**	**-17.6**	8 HIV/AIDS	**-50.1**	**-57.3**	**-59.5**
9 Ischaemic heart disease	9 Other neonatal	-11.5	**-35.0**	-18.7	9 COPD	**11.4**	-4.6	**-16.0**
10 Cerebrovascular disease	10 Tuberculosis	**-29.5**	**-48.2**	**-51.2**	10 Congenital anomalies	-12.2	**-24.8**	-12.5
11 Congenital anomalies	11 Congenital anomalies	3.4	**-24.0**	-7.3	11 Tuberculosis	**-29.4**	**-39.5**	**-42.3**
12 Protein-energy malnutrition	12 Measles	**-64.0**	**-73.6**	**-68.3**	12 Other neonatal	**-34.4**	**-43.8**	**-32.9**
13 Tetanus	13 COPD	1.2	**-25.6**	**-33.8**	13 Self-harm	**-11.7**	**-24.4**	**-24.8**
14 COPD	14 Self-harm	11.9	**-17.7**	**-19.5**	14 Diabetes	**34.9**	**15.6**	4.0
15 Meningitis	15 Meningitis	-18.8	**-40.3**	**-31.7**	15 Neonatal sepsis	-14.4	**-26.7**	-12.4
16 Intestinal infectious	16 Protein-energy malnutrition	**-36.9**	**-53.6**	**-44.4**	16 Meningitis	-19.2	-30.7	-24.0
17 Self-harm	17 Neonatal sepsis	2.8	**-24.4**	-5.6	17 Road injuries	-6.3	**-19.7**	**-18.4**
18 Drowning	18 Road injuries	**16.9**	**-14.1**	**-9.8**	18 Breast cancer	**26.5**	8.4	-3.4
19 Neonatal sepsis	19 Intestinal infectious	**-21.3**	**-42.1**	**-35.4**	19 Intestinal infectious	**-21.8**	**-33.0**	**-28.0**
20 Maternal haemorrhage	20 Drowning	**-31.1**	**-49.4**	**-40.7**	20 Protein-energy malnutrition	**-37.1**	**-46.1**	**-39.2**
21 Whooping cough	21 Diabetes	**91.0**	**40.4**	**28.5**	21 Chronic kidney disease	**13.7**	-2.6	**-8.4**
22 Road injuries	22 STDs	**-12.6**	**-35.8**	**-23.7**	22 Asthma	-7.4	**-20.7**	**-27.0**
23 STDs	23 Maternal haemorrhage	**-25.2**	**-45.0**	**-48.4**	23 Falls	2.9	**-11.8**	**-11.6**
24 Asthma	24 Asthma	-19.0	**-40.4**	**-42.5**	24 Rheumatic heart disease	-14.4	**-26.6**	**-30.1**
25 Neonatal hemolytic	25 Chronic kidney disease	**19.9**	**-11.8**	**-14.0**	25 Hypertensive heart disease	**12.3**	-3.8	-14.7
26 Rheumatic heart disease	26 Breast cancer	**54.5**	13.6	0.3	26 Maternal haemorrhage	**-32.7**	**-42.3**	**-42.6**
27 Hepatitis	27 Rheumatic heart disease	-4.7	**-30.0**	**-32.7**	27 Drowning	**-37.8**	**-46.7**	**-41.3**
28 Other maternal disorders	28 Whooping cough	-40.5	-56.2	-47.0	28 Cervical cancer	4.4	**-10.5**	**-20.6**
29 Chronic kidney disease	29 Neonatal hemolytic	**-33.2**	**-50.9**	**-38.7**	29 Conflict & terror	99.8	71.2	81.5
30 Falls	30 Hepatitis	-8.1	**-32.5**	**-26.4**	30 Alzheimer disease	**43.2**	**22.7**	2.9
32 HIV/AIDS	31 Falls				34 STDs			
34 Breast cancer	32 Cervical cancer				36 Hepatitis			
36 Diabetes	33 Hypertensive heart disease				37 Measles			
37 Cervical cancer	34 Tetanus				38 Whooping cough			
39 Hypertensive heart disease	38 Other maternal disorders				43 Neonatal hemolytic			
60 Alzheimer disease	45 Alzheimer disease				49 Other maternal disorders			
102 Conflict & terror	55 Conflict & terror				72 Tetanus			

Legend:
Communicable, maternal, neonatal and nutritional
Non-communicable
Injuries

SR Figure 4j: Leading 30 GBD cause hierarchy level 3 causes of Low SDI YLLs for females for 1990, 2006 and 2016.

Causes are connected by arrows between time periods. Communicable maternal, neonatal and nutritional disorders are shown in red, non-communicable causes in blue and injuries in green. For the time period 1990 to 2006 and for 2006 to 2016, three measures of change are shown: percent change in the number of YLLs, percent change in the all-age YLL rate and percent change in the age-standardized YLL rate. Numbers in bold highlight statistically significant changes.

Leading causes 1990
1 Lower respiratory infect
2 Diarrhoeal diseases
3 Malaria
4 Measles
5 Protein-energy malnutrition
6 Neonatal preterm birth
7 Tuberculosis
8 Neonatal encephalopathy
9 Meningitis
10 Congenital anomalies
11 STDs
12 Other neonatal
13 Neonatal sepsis
14 Cerebrovascular disease
15 Ischaemic heart disease
16 HIV/AIDS
17 Tetanus
18 Whooping cough
19 Conflict & terror
20 Road injuries
21 Maternal haemorrhage
22 Hemoglobinopathies
23 COPD
24 Maternal indirect
25 Cervical cancer
26 Drowning
27 Asthma
28 Neonatal hemolytic
29 Intestinal infectious
30 Other cardiovascular
31 Maternal hypertension
33 Falls
34 Diabetes
41 Chronic kidney disease
43 Breast cancer

Leading causes 2006	Mean % change number of YLLs 1990-2006	Mean % change all-age YLL rate 1990-2006	Mean % change age-standardized YLL rate 1990-2006
1 Malaria	28.9	-18.2	-13.0
2 Lower respiratory infect	-10.4	**-43.1**	**-34.9**
3 Diarrhoeal diseases	-9.0	**-42.2**	**-32.5**
4 HIV/AIDS	**605.7**	**348.2**	**405.6**
5 Neonatal preterm birth	11.7	**-29.1**	**-23.2**
6 Neonatal encephalopathy	**21.6**	**-22.7**	-16.4
7 Tuberculosis	15.8	**-26.4**	**-23.0**
8 Protein-energy malnutrition	-14.1	**-45.4**	**-41.1**
9 Congenital anomalies	10.6	-29.8	-24.6
10 Meningitis	9.3	**-30.6**	**-26.5**
11 Ischaemic heart disease	**56.2**	-0.8	0.8
12 Cerebrovascular disease	**25.3**	**-20.4**	**-16.1**
13 Neonatal sepsis	20.0	**-23.8**	-17.7
14 Other neonatal	17.2	**-25.6**	-19.5
15 STDs	7.0	**-32.0**	**-28.1**
16 Measles	**-80.3**	**-87.5**	**-86.9**
17 Whooping cough	16.5	-26.0	-22.0
18 Road injuries	**21.7**	**-22.7**	**-17.5**
19 Maternal haemorrhage	**27.2**	**-19.2**	**-19.8**
20 COPD	**35.9**	-13.7	-10.6
21 Cervical cancer	28.5	**-18.4**	-14.9
22 Maternal indirect	**19.0**	**-24.4**	**-25.8**
23 Hemoglobinopathies	6.0	**-32.7**	**-27.2**
24 Diabetes	**65.1**	4.9	**9.5**
25 Drowning	10.6	-29.8	-25.2
26 Asthma	13.2	**-28.1**	**-20.8**
27 Maternal hypertension	**31.7**	**-16.4**	**-17.9**
28 Other cardiovascular	**23.1**	**-21.8**	**-17.9**
29 Tetanus	**-62.2**	**-76.0**	**-74.7**
30 Falls	20.2	-23.6	-14.0
31 Breast cancer			
32 Chronic kidney disease			
33 Neonatal hemolytic			
34 Intestinal infectious			
67 Conflict & terror			

Leading causes 2016	Mean % change number of YLLs 2006-2016	Mean % change all-age YLL rate 2006-2016	Mean % change age-standardized YLL rate 2006-2016
1 Lower respiratory infect	**-23.3**	**-42.0**	**-33.4**
2 Malaria	**-32.9**	**-49.2**	**-46.6**
3 Diarrhoeal diseases	**-32.7**	**-49.1**	**-40.7**
4 HIV/AIDS	**-49.9**	**-62.1**	**-65.1**
5 Neonatal preterm birth	-8.1	**-30.4**	**-26.6**
6 Neonatal encephalopathy	-2.8	**-26.4**	**-22.4**
7 Tuberculosis	**-15.0**	**-35.7**	**-34.9**
8 Congenital anomalies	8.4	-18.0	-14.3
9 Ischaemic heart disease	**21.0**	**-8.4**	**-8.9**
10 Protein-energy malnutrition	**-22.9**	**-41.7**	**-37.3**
11 Meningitis	-4.9	-28.0	**-24.9**
12 Cerebrovascular disease	**14.7**	**-13.2**	**-13.1**
13 Neonatal sepsis	4.7	**-20.7**	-16.5
14 Other neonatal	-4.3	**-27.6**	**-23.6**
15 STDs	0.5	**-24.0**	**-21.0**
16 Whooping cough	-26.1	-44.0	-42.6
17 Road injuries	5.8	**-19.9**	**-14.3**
18 Maternal haemorrhage	-4.9	**-28.0**	**-29.0**
19 COPD	12.6	**-14.8**	**-15.0**
20 Cervical cancer	**20.3**	-8.9	-10.1
21 Diabetes	**37.0**	3.7	4.3
22 Measles	**-55.4**	**-66.3**	**-65.6**
23 Other cardiovascular	**26.9**	-4.0	-2.4
24 Conflict & terror	**298.3**	**201.4**	**213.7**
25 Maternal indirect	-6.8	**-29.5**	**-30.3**
26 Hemoglobinopathies	-0.6	**-24.8**	**-20.6**
27 Breast cancer	**39.7**	5.7	3.9
28 Asthma	0.0	**-24.3**	**-21.8**
29 Chronic kidney disease	**25.4**	-5.1	-1.0
30 Drowning	-6.3	-29.1	-24.0
31 Maternal hypertension			
33 Falls			
35 Intestinal infectious			
42 Neonatal hemolytic			
57 Tetanus			

Legend:
Communicable, maternal, neonatal and nutritional
Non-communicable
Injuries

SR Figure 5. Difference between GBD 2016 and WHO 2015 deaths for selected causes, all ages, by sex, 1990, 2005, 2010, 2015

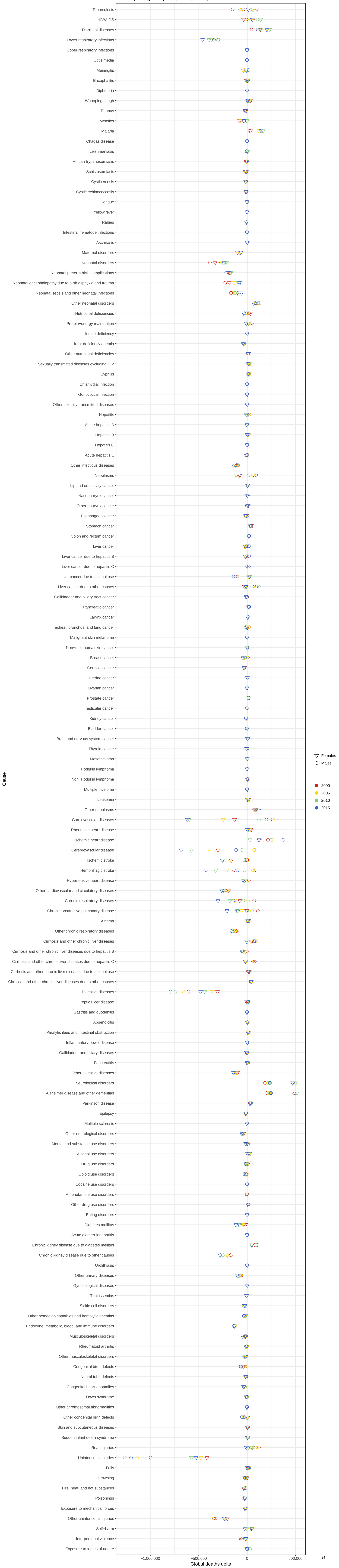

SR Figure 6. Difference between GBD 2015 and WHO 2015 deaths for selected causes, all ages, by sex, 1990, 2005, 2010, 2015

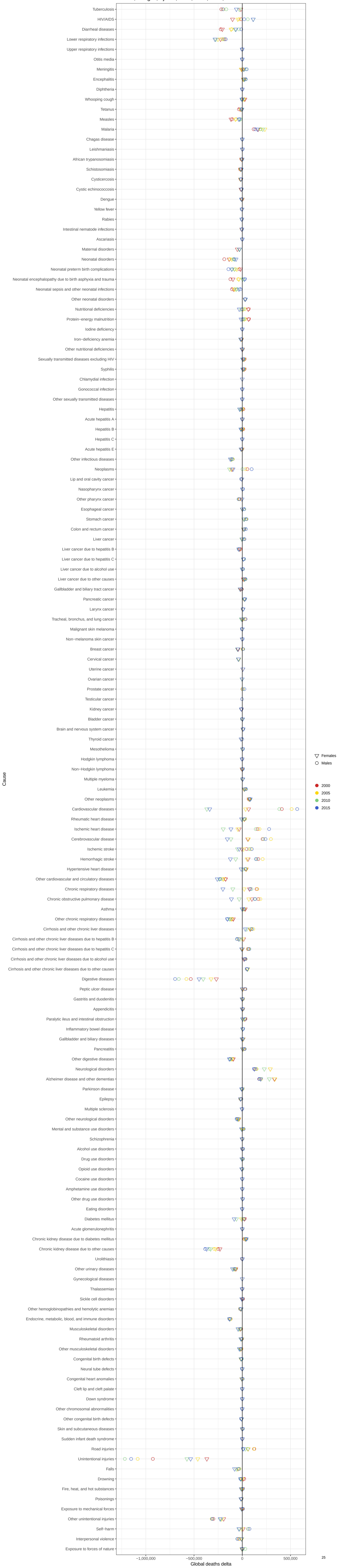

SR Figure 7. Difference between GBD 2016 and GBD 2015 deaths by cause and sex for all ages, 1990, 2005, 2010, 2015

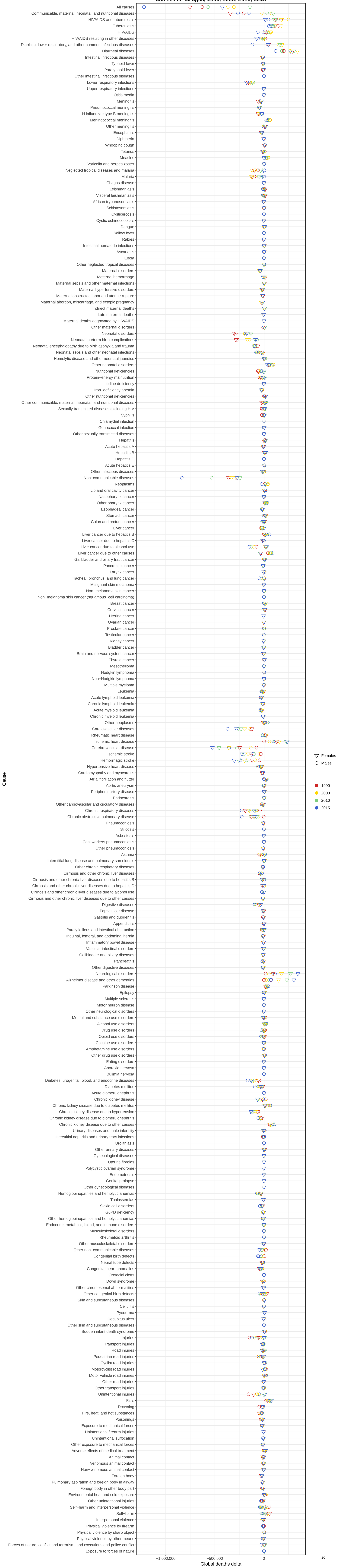

SR Table 1: Global deaths and YLL numbers, age-standardized rates per 100,000, and percent change between 2006 and 2016 for females for all GBD causes and Levels 1 through 4 of the cause hierarchy. 95% UIs are in parentheses. UI=uncertainty interval. YLL=year of life lost.

	All Age Deaths (thousands)		Age-standardized Death Rate (per 100,000)		All Age YLLs (thousands)		Age-standardized YLL Rate (per 100,000)	
	2016	Percent Change 2006-2016	2016	Percent Change 2006-2016	2016	Percent Change 2006-2016	2016	Percent Change 2006-2016
**All causes**	24,703·3 (24,253·3 to 25,268·5)	1·9 (0·0 to 4·4)	689·6 (677·1 to 705·3)	-19·2 (-20·7 to -17·2)	660,461·1 (647,528·0 to 674,537·3)	-15·4 (-17·2 to -13·5)	18,572·7 (18,204·4 to 18,969·1)	-26·1 (-27·7 to -24·5)
**Communicable, maternal, neonatal, and nutritional disorders**	4,970·3 (4,649·4 to 5,443·9)	-25·5 (-27·9 to -22·4)	141·2 (132·2 to 154·3)	-34·1 (-36·0 to -31·7)	261,410·3 (250,599·0 to 273,523·0)	-33·0 (-35·3 to -30·3)	7,536·9 (7,219·6 to 7,888·2)	-36·9 (-39·0 to -34·4)
**HIV/AIDS and tuberculosis**	897·4 (856·0 to 937·8)	-40·3 (-42·2 to -38·0)	24·4 (23·2 to 25·5)	-49·0 (-50·7 to -47·1)	38,717·6 (36,973·6 to 40,493·8)	-43·3 (-45·1 to -41·3)	1,047·9 (1,000·8 to 1,095·1)	-49·9 (-51·5 to -48·2)
Tuberculosis	448·1 (411·8 to 475·6)	-21·7 (-25·8 to -16·5)	12·3 (11·3 to 13·1)	-36·3 (-39·7 to -32·0)	14,688·1 (13,549·3 to 15,633·8)	-26·6 (-30·2 to -22·5)	399·6 (368·2 to 425·6)	-37·3 (-40·4 to -33·6)
Drug-susceptible tuberculosis	410·3 (375·1 to 437·4)	-21·6 (-25·7 to -16·5)	11·3 (10·3 to 12·0)	-36·2 (-39·7 to -31·9)	13,442·0 (12,368·1 to 14,316·4)	-26·4 (-30·1 to -22·3)	365·8 (336·6 to 390·1)	-37·1 (-40·3 to -33·5)
Multidrug-resistant tuberculosis without extensive drug resistance	34·5 (28·1 to 41·2)	-27·3 (-35·6 to -18·1)	0·9 (0·8 to 1·1)	-40·7 (-47·7 to -33·0)	1,137·9 (932·7 to 1,361·9)	-32·1 (-39·6 to -23·8)	30·9 (25·3 to 37·0)	-41·9 (-48·4 to -34·8)
Extensively drug-resistant tuberculosis	3·4 (2·7 to 4·2)	78·7 (52·9 to 110·4)	0·1 (0·1 to 0·1)	45·4 (24·0 to 71·4)	108·2 (88·3 to 132·3)	65·0 (41·0 to 94·7)	2·9 (2·4 to 3·6)	40·5 (20·0 to 65·7)
HIV/AIDS	449·3 (424·3 to 474·7)	-51·7 (-54·0 to -49·2)	12·0 (11·4 to 12·7)	-57·7 (-59·6 to -55·5)	24,029·4 (22,596·7 to 25,532·9)	-50·2 (-52·4 to -48·0)	648·3 (610·0 to 688·2)	-55·5 (-57·4 to -53·5)
Drug-susceptible HIV/AIDS - Tuberculosis	93·6 (63·4 to 126·9)	-56·2 (-58·8 to -53·5)	2·5 (1·7 to 3·4)	-61·9 (-64·1 to -59·6)	5,036·7 (3,401·5 to 6,837·2)	-55·0 (-57·6 to -52·2)	136·3 (92·1 to 184·9)	-59·8 (-62·1 to -57·4)
Multidrug-resistant HIV/AIDS - Tuberculosis without extensive drug resistance	7·5 (4·5 to 11·5)	-57·4 (-65·9 to -47·3)	0·2 (0·1 to 0·3)	-62·9 (-70·4 to -54·1)	409·4 (244·3 to 630·4)	-55·6 (-64·9 to -44·6)	11·1 (6·6 to 17·2)	-60·2 (-68·7 to -50·3)
Extensively drug-resistant HIV/AIDS - Tuberculosis	0·4 (0·2 to 0·6)	38·3 (19·9 to 58·5)	0·0 (0·0 to 0·0)	20·3 (4·2 to 37·9)	18·7 (11·3 to 29·2)	39·0 (20·6 to 59·3)	0·5 (0·3 to 0·8)	23·5 (7·3 to 41·4)
HIV/AIDS resulting in other diseases	347·8 (306·9 to 391·2)	-50·2 (-53·3 to -47·0)	9·3 (8·2 to 10·5)	-56·3 (-58·9 to -53·4)	18,564·7 (16,329·2 to 21,009·1)	-48·7 (-51·6 to -45·6)	500·4 (440·2 to 565·6)	-54·0 (-56·6 to -51·3)
**Diarrhoea, lower respiratory infections, and other common infectious diseases**	2,352·8 (2,020·0 to 2,892·6)	-19·7 (-24·4 to -13·5)	66·9 (57·4 to 82·0)	-31·7 (-35·5 to -26·7)	97,620·0 (87,534·5 to 110,930·1)	-36·3 (-40·8 to -30·9)	2,822·2 (2,536·2 to 3,193·4)	-40·7 (-44·9 to -35·9)
Diarrhoeal diseases	884·6 (562·7 to 1,482·1)	-23·8 (-34·3 to -10·2)	25·1 (16·1 to 41·8)	-36·3 (-44·5 to -25·2)	32,580·0 (24,611·3 to 47,025·8)	-38·9 (-47·3 to -29·2)	938·1 (714·8 to 1,339·5)	-44·1 (-51·3 to -36·1)
Intestinal infectious diseases	70·6 (39·7 to 114·8)	-15·7 (-24·4 to -7·6)	2·0 (1·1 to 3·2)	-21·4 (-29·6 to -13·7)	4,725·2 (2,651·9 to 7,717·0)	-18·6 (-28·0 to -9·7)	132·5 (74·4 to 216·2)	-23·0 (-32·1 to -14·1)
Typhoid fever	57·8 (31·5 to 94·5)	-16·7 (-24·9 to -9·5)	1·6 (0·9 to 2·6)	-22·1 (-29·8 to -15·3)	3,909·8 (2,130·3 to 6,464·6)	-19·3 (-28·4 to -11·8)	109·7 (59·7 to 181·7)	-23·6 (-32·0 to -16·2)
Paratyphoid fever	11·5 (5·4 to 22·5)	-7·0 (-15·7 to 1·3)	0·3 (0·1 to 0·6)	-14·5 (-22·3 to -7·0)	721·6 (338·1 to 1,401·1)	-10·3 (-19·5 to -1·2)	20·0 (9·4 to 38·9)	-15·8 (-24·3 to -7·3)
Other intestinal infectious diseases	1·3 (0·3 to 4·1)	-36·6 (-86·9 to 191·5)	0·0 (0·0 to 0·1)	-40·6 (-87·4 to 167·3)	93·7 (18·8 to 327·4)	-39·8 (-89·9 to 257·0)	2·8 (0·5 to 9·7)	-42·2 (-90·6 to 237·5)
Lower respiratory infections	1,111·4 (970·6 to 1,204·5)	-10·4 (-15·9 to -4·6)	31·6 (27·6 to 34·3)	-25·0 (-29·4 to -20·7)	41,395·1 (35,842·7 to 45,698·6)	-33·0 (-38·7 to -26·6)	1,202·1 (1,039·1 to 1,329·0)	-37·6 (-42·7 to -31·6)
Upper respiratory infections	1·0 (0·8 to 1·2)	-28·5 (-44·1 to -6·2)	0·0 (0·0 to 0·0)	-37·0 (-50·8 to -17·1)	55·9 (43·1 to 74·3)	-35·0 (-55·2 to -4·6)	1·6 (1·2 to 2·2)	-39·3 (-58·4 to -10·5)
Otitis media	0·5 (0·4 to 0·7)	-45·3 (-61·4 to -25·1)	0·0 (0·0 to 0·0)	-53·2 (-66·4 to -36·2)	22·9 (16·5 to 32·3)	-53·2 (-69·9 to -29·0)	0·6 (0·5 to 0·9)	-57·4 (-72·5 to -35·6)
Meningitis	141·5 (115·8 to 196·1)	-11·3 (-23·4 to 15·6)	4·0 (3·3 to 5·7)	-18·8 (-30·1 to 7·1)	9,128·3 (7,215·8 to 13,797·9)	-14·8 (-29·8 to 18·9)	264·1 (207·3 to 403·1)	-19·1 (-33·5 to 14·1)
Pneumococcal meningitis	8·7 (7·2 to 11·7)	-9·8 (-19·3 to 9·2)	0·2 (0·2 to 0·3)	-20·9 (-29·1 to -2·4)	468·1 (362·9 to 690·0)	-14·7 (-27·3 to 13·8)	13·4 (10·3 to 19·9)	-20·7 (-32·8 to 7·3)
H influenzae type B meningitis	11·6 (9·1 to 16·9)	-16·2 (-29·6 to 13·7)	0·3 (0·3 to 0·5)	-22·3 (-34·9 to 6·4)	796·9 (604·4 to 1,261·5)	-19·0 (-34·4 to 16·3)	23·2 (17·5 to 37·0)	-22·6 (-37·4 to 12·1)
Meningococcal infection	49·9 (41·3 to 67·9)	-28·0 (-37·5 to -7·3)	1·4 (1·2 to 1·9)	-34·1 (-42·8 to -14·5)	3,188·8 (2,522·0 to 4,670·4)	-32·5 (-43·3 to -7·2)	92·0 (72·5 to 136·0)	-35·9 (-46·3 to -11·3)
Other meningitis	71·3 (56·9 to 102·6)	7·0 (-8·9 to 42·2)	2·0 (1·6 to 2·9)	-1·9 (-16·4 to 32·0)	4,674·5 (3,577·4 to 7,239·8)	4·8 (-14·8 to 49·4)	135·5 (103·4 to 211·6)	-0·3 (-19·2 to 43·1)
Encephalitis	48·7 (38·1 to 69·7)	-2·0 (-20·1 to 24·5)	1·4 (1·1 to 2·0)	-14·7 (-30·3 to 8·0)	2,331·0 (1,776·9 to 3,363·6)	-15·0 (-35·0 to 14·4)	66·3 (50·5 to 95·6)	-21·4 (-39·9 to 5·5)
Diphtheria	0·5 (0·3 to 0·8)	-67·3 (-81·8 to -41·9)	0·0 (0·0 to 0·0)	-69·0 (-82·9 to -44·6)	40·3 (25·7 to 63·6)	-67·7 (-82·6 to -40·6)	1·2 (0·7 to 1·9)	-69·2 (-83·5 to -43·2)
Whooping cough	39·7 (21·6 to 69·9)	-36·3 (-63·7 to 18·7)	1·2 (0·6 to 2·1)	-38·5 (-65·0 to 14·5)	3,360·5 (1,823·8 to 5,901·4)	-36·3 (-63·7 to 18·6)	99·3 (53·9 to 174·3)	-38·5 (-65·0 to 14·6)
Tetanus	14·3 (8·9 to 20·2)	-63·0 (-69·6 to -55·5)	0·4 (0·3 to 0·6)	-65·9 (-71·8 to -59·0)	891·1 (555·9 to 1,212·4)	-66·5 (-73·3 to -58·5)	26·0 (16·4 to 35·2)	-67·8 (-74·3 to -60·1)
Measles	33·3 (12·3 to 71·9)	-72·7 (-77·5 to -67·5)	1·0 (0·4 to 2·1)	-73·9 (-78·4 to -68·9)	2,787·9 (1,032·6 to 6,005·6)	-72·7 (-77·5 to -67·5)	81·8 (30·3 to 176·1)	-73·8 (-78·4 to -68·8)
Varicella and herpes zoster	6·5 (5·6 to 7·9)	-14·6 (-23·0 to -5·2)	0·2 (0·2 to 0·2)	-28·5 (-35·5 to -20·6)	301·9 (264·0 to 351·3)	-22·0 (-33·5 to -7·8)	8·7 (7·6 to 10·2)	-27·3 (-38·2 to -13·8)
**Neglected tropical diseases and malaria**	408·5 (318·5 to 505·4)	-24·7 (-42·8 to -1·3)	11·8 (9·1 to 14·7)	-30·0 (-46·7 to -8·1)	29,706·7 (22,674·6 to 37,363·4)	-27·1 (-46·6 to -1·1)	865·1 (657·4 to 1,092·9)	-30·8 (-49·5 to -5·6)
Malaria	356·2 (272·4 to 449·9)	-25·3 (-45·1 to 3·3)	10·3 (7·9 to 13·0)	-30·0 (-48·8 to -3·2)	26,865·6 (19,852·7 to 34,464·8)	-27·2 (-48·4 to 3·3)	784·6 (578·4 to 1,008·3)	-30·7 (-51·0 to -1·4)
Chagas disease	3·1 (2·8 to 3·7)	7·5 (-2·1 to 25·3)	0·1 (0·1 to 0·1)	-16·9 (-24·5 to -3·2)	62·6 (56·8 to 74·8)	-2·5 (-11·7 to 13·8)	1·7 (1·6 to 2·1)	-22·8 (-30·1 to -9·9)
Leishmaniasis	6·4 (3·2 to 11·4)	-54·0 (-58·8 to -49·0)	0·2 (0·1 to 0·3)	-59·0 (-63·2 to -54·6)	327·1 (162·8 to 593·3)	-58·2 (-62·8 to -53·3)	9·2 (4·6 to 16·6)	-61·3 (-65·6 to -56·7)
Visceral leishmaniasis	6·4 (3·2 to 11·4)	-54·0 (-58·8 to -49·0)	0·2 (0·1 to 0·3)	-59·0 (-63·2 to -54·6)	327·1 (162·8 to 593·3)	-58·2 (-62·8 to -53·3)	9·2 (4·6 to 16·6)	-61·3 (-65·6 to -56·7)
African trypanosomiasis	1·1 (0·5 to 1·9)	-76·5 (-87·6 to -57·1)	0·0 (0·0 to 0·1)	-79·1 (-89·1 to -61·9)	57·9 (25·4 to 105·5)	-76·5 (-88·5 to -54·9)	1·6 (0·7 to 2·9)	-78·4 (-89·5 to -58·9)
Schistosomiasis	4·6 (4·0 to 5·1)	-24·1 (-33·7 to -13·7)	0·1 (0·1 to 0·1)	-38·5 (-46·3 to -29·9)	154·6 (136·1 to 173·7)	-27·0 (-37·1 to -15·7)	4·2 (3·7 to 4·7)	-38·1 (-46·8 to -28·5)
Cysticercosis	0·5 (0·4 to 0·6)	-21·6 (-25·7 to -17·5)	0·0 (0·0 to 0·0)	-33·3 (-36·9 to -29·8)	22·1 (18·4 to 26·8)	-25·5 (-30·8 to -20·7)	0·6 (0·5 to 0·7)	-33·9 (-38·7 to -29·6)
Cystic echinococcosis	0·5 (0·4 to 0·6)	-37·6 (-41·7 to -33·2)	0·0 (0·0 to 0·0)	-47·3 (-50·9 to -43·7)	20·2 (15·8 to 26·0)	-44·1 (-49·3 to -38·8)	0·6 (0·4 to 0·7)	-50·2 (-54·9 to -45·5)
Dengue	15·8 (2·9 to 23·7)	73·2 (17·7 to 131·2)	0·4 (0·1 to 0·7)	51·9 (3·5 to 102·5)	809·9 (155·9 to 1,226·2)	48·3 (-0·6 to 101·1)	22·6 (4·4 to 34·2)	36·0 (-9·2 to 85·4)
Yellow fever	1·6 (0·3 to 4·7)	-11·1 (-30·7 to 14·6)	0·0 (0·0 to 0·1)	-17·7 (-35·9 to 6·2)	102·4 (21·1 to 299·4)	-11·1 (-31·6 to 16·3)	2·8 (0·6 to 8·3)	-16·0 (-36·0 to 9·7)
Rabies	4·7 (2·1 to 8·1)	-51·1 (-60·8 to -35·0)	0·1 (0·1 to 0·2)	-57·2 (-65·9 to -43·2)	247·7 (101·2 to 436·3)	-53·3 (-64·2 to -33·4)	6·9 (2·8 to 12·1)	-57·5 (-67·6 to -39·1)
Intestinal nematode infections	2·7 (2·1 to 3·5)	-40·9 (-54·9 to -22·0)	0·1 (0·1 to 0·1)	-44·6 (-57·8 to -26·7)	214·0 (163·3 to 281·0)	-41·8 (-56·1 to -21·7)	6·2 (4·8 to 8·2)	-44·9 (-58·6 to -25·9)
Ascariasis	2·7 (2·1 to 3·5)	-40·9 (-54·9 to -22·0)	0·1 (0·1 to 0·1)	-44·6 (-57·8 to -26·7)	214·0 (163·3 to 281·0)	-41·8 (-56·1 to -21·7)	6·2 (4·8 to 8·2)	-44·9 (-58·6 to -25·9)
Ebola	0·0 (0·0 to 0·0)	0·0 (0·0 to 0·0)	0·0 (0·0 to 0·0)	0·0 (0·0 to 0·0)	0·2 (0·2 to 0·2)	0·0 (0·0 to 0·0)	0·0 (0·0 to 0·0)	0·0 (0·0 to 0·0)
Zika virus	0·0 (0·0 to 0·0)	0·0 (0·0 to 0·0)	0·0 (0·0 to 0·0)	0·0 (0·0 to 0·0)	0·6 (0·1 to 1·9)	0·0 (0·0 to 0·0)	0·0 (0·0 to 0·1)	0·0 (0·0 to 0·0)
Other neglected tropical diseases	11·3 (5·7 to 16·5)	-8·1 (-38·5 to 35·8)	0·3 (0·2 to 0·5)	-14·5 (-42·7 to 26·5)	821·9 (390·6 to 1,227·7)	-9·4 (-41·7 to 41·8)	24·0 (11·3 to 36·0)	-13·5 (-44·4 to 35·5)
**Maternal disorders**	230·6 (212·5 to 253·4)	-23·6 (-29·3 to -16·7)	6·1 (5·6 to 6·7)	-30·3 (-35·5 to -24·0)	12,817·8 (11,808·4 to 14,106·4)	-24·7 (-30·4 to -17·9)	340·5 (313·7 to 374·7)	-30·8 (-36·1 to -24·6)
Maternal haemorrhage	72·4 (58·5 to 89·1)	-23·8 (-31·6 to -15·2)	1·9 (1·6 to 2·4)	-30·5 (-37·5 to -22·8)	4,018·5 (3,248·0 to 4,975·8)	-25·0 (-32·9 to -16·3)	106·6 (86·1 to 131·9)	-31·2 (-38·4 to -23·3)
Maternal sepsis and other pregnancy related infections	19·5 (14·3 to 26·2)	-26·7 (-35·6 to -17·5)	0·5 (0·4 to 0·7)	-32·9 (-41·2 to -24·7)	1,093·6 (789·3 to 1,478·2)	-27·9 (-36·8 to -18·6)	29·1 (21·0 to 39·4)	-33·5 (-41·6 to -25·0)
Maternal hypertensive disorders	31·6 (24·5 to 39·8)	-20·8 (-28·4 to -12·4)	0·8 (0·7 to 1·1)	-27·3 (-34·4 to -19·8)	1,780·8 (1,360·7 to 2,265·9)	-21·8 (-29·5 to -13·7)	47·4 (36·3 to 60·2)	-27·7 (-34·8 to -20·3)
Maternal obstructed labour and uterine rupture	10·3 (6·8 to 14·6)	-22·2 (-31·8 to -12·4)	0·3 (0·2 to 0·4)	-29·6 (-38·5 to -21·0)	553·7 (369·6 to 798·4)	-23·0 (-32·2 to -13·5)	14·7 (9·8 to 21·2)	-29·8 (-38·5 to -21·5)
Maternal abortion, miscarriage, and ectopic pregnancy	19·7 (14·6 to 26·1)	-22·0 (-31·1 to -12·3)	0·5 (0·4 to 0·7)	-29·0 (-37·3 to -20·3)	1,081·9 (796·2 to 1,466·6)	-22·9 (-32·0 to -13·1)	28·7 (21·2 to 38·9)	-29·3 (-37·5 to -20·4)
Indirect maternal deaths	35·7 (26·4 to 46·8)	-21·6 (-28·8 to -13·3)	0·9 (0·7 to 1·2)	-28·7 (-35·2 to -21·0)	1,987·9 (1,463·8 to 2,619·8)	-22·9 (-30·1 to -14·7)	52·7 (39·0 to 69·5)	-29·3 (-35·9 to -21·7)

SR Table 1: Global deaths and YLL numbers, age-standardized rates per 100,000, and percent change between 2006 and 2016 for females for all GBD causes and Levels 1 through 4 of the cause hierarchy. 95% UIs are in parentheses. UI=uncertainty interval. YLL=year of life lost.

Late maternal deaths	4·1 (2·5 to 6·6)	-22·3 (-28·9 to -15·1)	0·1 (0·1 to 0·2)	-29·1 (-34·8 to -22·7)	228·5 (134·5 to 370·9)	-23·4 (-29·9 to -16·2)	6·1 (3·6 to 9·8)	-29·6 (-35·2 to -23·2)
Maternal deaths aggravated by HIV/AIDS	2·0 (1·3 to 2·7)	-14·3 (-24·6 to -1·6)	0·1 (0·0 to 0·1)	-23·0 (-32·3 to -11·4)	105·4 (66·7 to 142·9)	-17·9 (-27·8 to -6·0)	2·8 (1·8 to 3·8)	-26·0 (-34·9 to -15·2)
Other maternal disorders	35·3 (26·8 to 45·2)	-27·4 (-34·8 to -19·2)	0·9 (0·7 to 1·2)	-33·6 (-40·6 to -26·1)	1,967·5 (1,475·0 to 2,540·0)	-28·5 (-36·2 to -20·3)	52·3 (39·3 to 67·6)	-34·1 (-41·1 to -26·5)
**Neonatal disorders**	748·4 (709·8 to 789·6)	-25·2 (-29·3 to -21·2)	22·5 (21·3 to 23·7)	-25·1 (-29·2 to -21·1)	64,780·6 (61,434·4 to 68,344·2)	-25·2 (-29·3 to -21·2)	1,946·2 (1,845·7 to 2,053·4)	-25·1 (-29·2 to -21·1)
Neonatal preterm birth complications	266·1 (237·3 to 299·7)	-28·4 (-34·5 to -21·0)	8·0 (7·1 to 9·0)	-28·3 (-34·4 to -20·9)	23,033·5 (20,544·4 to 25,946·3)	-28·4 (-34·5 to -21·0)	692·0 (617·3 to 779·6)	-28·3 (-34·4 to -20·9)
Neonatal encephalopathy due to birth asphyxia and trauma	218·3 (187·4 to 245·9)	-23·7 (-32·4 to -12·7)	6·6 (5·6 to 7·4)	-23·6 (-32·3 to -12·6)	18,892·0 (16,223·7 to 21,282·1)	-23·7 (-32·4 to -12·7)	567·5 (487·4 to 639·4)	-23·6 (-32·3 to -12·6)
Neonatal sepsis and other neonatal infections	105·4 (82·3 to 156·3)	-9·4 (-23·7 to 5·5)	3·2 (2·5 to 4·7)	-9·3 (-23·7 to 5·6)	9,123·2 (7,123·5 to 13,528·4)	-9·4 (-23·7 to 5·5)	274·1 (214·1 to 406·5)	-9·3 (-23·7 to 5·6)
Hemolytic disease and other neonatal jaundice	21·6 (18·0 to 26·2)	-43·0 (-52·9 to -30·7)	0·6 (0·5 to 0·8)	-42·9 (-52·9 to -30·7)	1,866·4 (1,554·0 to 2,266·2)	-43·0 (-52·9 to -30·7)	56·1 (46·7 to 68·1)	-42·9 (-52·9 to -30·7)
Other neonatal disorders	137·1 (118·8 to 154·5)	-27·3 (-36·9 to -16·1)	4·1 (3·6 to 4·6)	-27·2 (-36·8 to -16·0)	11,865·4 (10,285·1 to 13,369·2)	-27·3 (-36·9 to -16·1)	356·5 (309·0 to 401·6)	-27·2 (-36·8 to -16·0)
**Nutritional deficiencies**	184·3 (168·1 to 204·8)	-15·1 (-23·7 to -4·7)	5·3 (4·8 to 5·9)	-26·6 (-34·2 to -17·7)	9,182·3 (7,904·5 to 10,789·4)	-28·1 (-39·3 to -14·0)	267·1 (229·3 to 314·6)	-32·6 (-43·3 to -19·1)
Protein-energy malnutrition	149·8 (134·3 to 171·0)	-18·0 (-28·1 to -6·3)	4·3 (3·8 to 4·9)	-28·3 (-37·0 to -18·2)	8,060·7 (6,816·8 to 9,676·4)	-29·6 (-41·8 to -14·1)	235·9 (199·2 to 283·5)	-33·5 (-44·9 to -19·0)
Iodine deficiency	1·2 (0·8 to 2·0)	-2·2 (-42·2 to 65·0)	0·0 (0·0 to 0·1)	-16·4 (-50·4 to 39·5)	55·4 (31·7 to 107·7)	-13·0 (-59·9 to 92·3)	1·6 (0·9 to 3·1)	-20·4 (-63·4 to 79·3)
Iron-deficiency anaemia	1·6 (1·3 to 2·1)	7·8 (-16·1 to 35·2)	0·0 (0·0 to 0·1)	-12·3 (-31·7 to 10·9)	56·8 (46·5 to 74·1)	4·2 (-17·7 to 34·0)	1·6 (1·3 to 2·1)	-6·8 (-26·6 to 21·0)
Other nutritional deficiencies	31·7 (27·6 to 35·9)	0·5 (-8·4 to 10·4)	0·9 (0·8 to 1·0)	-18·6 (-25·7 to -10·7)	1,009·5 (839·5 to 1,177·7)	-16·1 (-26·5 to -5·9)	28·1 (23·2 to 32·9)	-26·1 (-35·1 to -16·9)
**Other communicable, maternal, neonatal, and nutritional diseases**	148·2 (123·1 to 181·2)	-17·8 (-24·4 to -10·5)	4·2 (3·5 to 5·2)	-26·1 (-31·7 to -19·8)	8,585·4 (6,499·9 to 11,213·7)	-25·3 (-32·7 to -17·3)	247·9 (186·2 to 325·3)	-29·5 (-36·5 to -21·9)
Sexually transmitted diseases excluding HIV	61·9 (38·1 to 92·9)	-25·1 (-34·4 to -13·6)	1·8 (1·1 to 2·7)	-28·1 (-37·1 to -17·0)	4,922·5 (2,903·9 to 7,559·6)	-26·4 (-35·9 to -14·4)	145·3 (85·3 to 223·6)	-28·5 (-37·7 to -16·8)
Syphilis	55·9 (32·1 to 86·7)	-26·8 (-36·5 to -14·5)	1·7 (0·9 to 2·6)	-28·7 (-38·2 to -16·8)	4,688·1 (2,659·3 to 7,324·1)	-27·1 (-36·9 to -14·6)	139·0 (78·8 to 217·4)	-28·8 (-38·4 to -16·6)
Chlamydial infection	1·2 (1·0 to 1·3)	-4·5 (-11·7 to 12·2)	0·0 (0·0 to 0·0)	-20·2 (-26·2 to -6·7)	46·8 (39·3 to 53·4)	-9·2 (-16·8 to 8·0)	1·2 (1·0 to 1·4)	-20·6 (-27·1 to -5·8)
Gonococcal infection	3·3 (2·7 to 3·7)	-3·7 (-11·0 to 12·5)	0·1 (0·1 to 0·1)	-20·3 (-26·2 to -7·3)	122·4 (101·6 to 139·7)	-8·1 (-15·4 to 8·9)	3·3 (2·7 to 3·7)	-20·5 (-26·8 to -6·1)
Other sexually transmitted diseases	1·6 (1·3 to 1·8)	-5·6 (-12·9 to 11·1)	0·0 (0·0 to 0·0)	-20·3 (-26·5 to -6·6)	65·1 (54·3 to 74·7)	-9·0 (-16·6 to 7·6)	1·7 (1·5 to 2·0)	-20·1 (-26·6 to -5·6)
Hepatitis	56·3 (51·5 to 60·2)	-15·6 (-22·5 to -10·3)	1·6 (1·4 to 1·7)	-28·0 (-34·1 to -23·5)	2,299·6 (2,093·6 to 2,475·7)	-28·8 (-35·5 to -23·3)	63·4 (57·7 to 68·3)	-36·2 (-42·3 to -31·3)
Acute hepatitis A	2·6 (2·0 to 3·2)	-45·0 (-59·5 to -25·2)	0·1 (0·1 to 0·1)	-47·9 (-61·8 to -28·6)	188·5 (142·3 to 240·8)	-48·6 (-64·4 to -28·0)	5·6 (4·2 to 7·1)	-50·2 (-65·6 to -30·0)
Hepatitis B	40·3 (36·4 to 43·8)	-5·1 (-12·9 to 2·5)	1·1 (1·0 to 1·2)	-21·6 (-27·8 to -15·2)	1,434·2 (1,284·0 to 1,570·1)	-14·1 (-22·4 to -5·2)	38·8 (34·8 to 42·5)	-25·9 (-32·9 to -18·4)
Hepatitis C	1·1 (0·8 to 1·5)	-4·0 (-20·8 to 17·4)	0·0 (0·0 to 0·0)	-23·1 (-36·3 to -5·6)	34·7 (26·4 to 44·7)	-12·9 (-26·7 to 4·0)	0·9 (0·7 to 1·2)	-26·7 (-38·2 to -12·3)
Acute hepatitis E	12·2 (10·2 to 14·5)	-32·9 (-42·0 to -23·4)	0·3 (0·3 to 0·4)	-39·4 (-47·6 to -30·9)	642·2 (548·5 to 746·7)	-44·4 (-52·7 to -35·7)	18·0 (15·4 to 20·9)	-47·8 (-55·5 to -39·4)
Other infectious diseases	30·1 (22·7 to 37·0)	-3·1 (-14·4 to 16·1)	0·9 (0·6 to 1·0)	-17·2 (-27·3 to -0·7)	1,363·3 (1,024·3 to 1,754·9)	-13·5 (-25·7 to 11·4)	39·2 (29·3 to 50·6)	-19·9 (-31·1 to 3·1)
**Non-communicable diseases**	18,337·4 (17,800·5 to 18,878·1)	13·7 (11·0 to 17·2)	510·1 (495·1 to 525·0)	-13·9 (-15·9 to -11·3)	344,035·3 (333,777·6 to 354,437·9)	4·9 (2·0 to 8·0)	9,523·2 (9,244·8 to 9,812·4)	-15·6 (-17·8 to -13·1)
**Neoplasms**	3,755·2 (3,644·6 to 3,862·1)	14·9 (12·0 to 18·3)	103·8 (100·8 to 106·8)	-11·0 (-13·2 to -8·4)	88,060·8 (85,384·6 to 90,579·7)	10·1 (7·2 to 13·6)	2,399·1 (2,325·4 to 2,467·4)	-11·8 (-14·2 to -9·1)
Lip and oral cavity cancer	58·9 (55·8 to 62·0)	32·0 (26·9 to 37·7)	1·6 (1·5 to 1·7)	1·2 (-2·7 to 5·5)	1,372·3 (1,298·6 to 1,444·7)	27·6 (22·1 to 34·0)	37·2 (35·2 to 39·1)	0·8 (-3·6 to 5·7)
Nasopharynx cancer	16·5 (15·9 to 17·2)	3·2 (-1·5 to 8·6)	0·4 (0·4 to 0·5)	-18·3 (-22·0 to -14·0)	488·9 (470·0 to 511·6)	-1·8 (-6·5 to 3·6)	13·1 (12·6 to 13·7)	-20·1 (-23·9 to -15·7)
Other pharynx cancer	31·9 (29·3 to 34·3)	20·6 (13·4 to 28·5)	0·9 (0·8 to 0·9)	-6·0 (-11·7 to 0·2)	850·2 (784·6 to 916·9)	16·8 (9·5 to 25·1)	22·9 (21·2 to 24·8)	-7·0 (-12·8 to -0·5)
Oesophageal cancer	108·5 (104·9 to 112·4)	-10·4 (-13·7 to -6·7)	3·0 (2·9 to 3·1)	-31·2 (-33·8 to -28·3)	2,231·3 (2,152·6 to 2,315·3)	-13·0 (-16·4 to -9·4)	61·3 (59·2 to 63·6)	-32·2 (-34·9 to -29·4)
Stomach cancer	298·0 (287·8 to 309·6)	-3·3 (-6·7 to 0·8)	8·3 (8·0 to 8·6)	-25·5 (-28·1 to -22·4)	6,307·1 (6,088·4 to 6,563·8)	-8·0 (-11·3 to -4·1)	172·4 (166·4 to 179·4)	-27·2 (-29·8 to -24·1)
Colon and rectum cancer	379·8 (361·6 to 398·6)	16·1 (9·5 to 22·7)	10·5 (10·0 to 11·1)	-11·9 (-16·8 to -6·8)	7,205·5 (6,847·4 to 7,573·8)	12·0 (4·8 to 19·1)	197·6 (187·7 to 207·9)	-12·3 (-17·9 to -6·8)
Liver cancer	238·7 (218·4 to 263·5)	16·6 (5·9 to 31·1)	6·6 (6·1 to 7·3)	-9·5 (-17·8 to 1·5)	5,336·7 (4,900·4 to 5,914·8)	11·1 (0·2 to 27·5)	145·9 (133·9 to 161·6)	-12·0 (-20·6 to 0·9)
Liver cancer due to hepatitis B	83·2 (67·2 to 100·1)	3·6 (-7·4 to 21·1)	2·3 (1·8 to 2·8)	-18·7 (-27·3 to -5·3)	2,143·5 (1,762·3 to 2,573·7)	-1·7 (-12·7 to 16·6)	58·0 (47·3 to 69·5)	-21·0 (-29·6 to -6·7)
Liver cancer due to hepatitis C	61·1 (54·4 to 68·1)	20·1 (11·8 to 26·6)	1·7 (1·5 to 1·9)	-8·3 (-14·8 to -3·3)	1,133·0 (979·1 to 1,267·2)	16·0 (6·6 to 23·4)	31·3 (27·1 to 35·0)	-9·9 (-17·1 to -4·0)
Liver cancer due to alcohol use	72·0 (59·4 to 85·3)	33·7 (19·5 to 56·0)	2·0 (1·7 to 2·4)	3·1 (-8·0 to 20·0)	1,538·8 (1,250·5 to 1,857·6)	32·0 (17·5 to 57·2)	42·3 (34·5 to 51·0)	2·5 (-9·0 to 21·6)
Liver cancer due to other causes	22·5 (19·2 to 25·9)	14·3 (4·6 to 24·1)	0·6 (0·5 to 0·7)	-10·7 (-18·3 to -3·4)	521·4 (447·6 to 599·4)	8·3 (-2·1 to 19·8)	14·3 (12·2 to 16·3)	-12·7 (-20·9 to -3·9)
Gallbladder and biliary tract cancer	94·8 (90·2 to 99·0)	14·9 (9·8 to 19·6)	2·6 (2·5 to 2·7)	-12·4 (-16·3 to -8·9)	1,914·0 (1,792·4 to 2,009·6)	12·9 (6·9 to 18·1)	52·4 (49·1 to 55·0)	-12·2 (-16·7 to -8·1)
Pancreatic cancer	192·1 (185·4 to 199·6)	28·2 (23·1 to 33·4)	5·4 (5·2 to 5·6)	-2·4 (-6·3 to 1·5)	3,545·7 (3,425·9 to 3,683·2)	25·5 (20·1 to 31·1)	98·1 (94·8 to 102·0)	-2·7 (-6·9 to 1·6)
Larynx cancer	15·8 (15·2 to 16·5)	5·5 (1·0 to 10·1)	0·4 (0·4 to 0·5)	-18·1 (-21·7 to -14·6)	380·1 (364·1 to 396·6)	1·9 (-2·7 to 6·7)	10·3 (9·9 to 10·8)	-19·4 (-23·1 to -15·7)
Tracheal, bronchus, and lung cancer	530·3 (509·6 to 546·6)	21·8 (17·2 to 25·9)	14·8 (14·2 to 15·2)	-6·3 (-9·8 to -3·1)	10,865·1 (10,429·9 to 11,227·5)	17·2 (12·1 to 21·6)	298·8 (286·9 to 308·8)	-8·4 (-12·3 to -5·0)
Malignant skin melanoma	27·6 (21·7 to 32·2)	19·5 (8·3 to 28·8)	0·8 (0·6 to 0·9)	-7·7 (-16·3 to -0·5)	626·9 (505·5 to 737·6)	13·1 (2·7 to 23·1)	17·0 (13·7 to 20·0)	-9·2 (-17·5 to -1·3)
Non-melanoma skin cancer	17·9 (17·2 to 18·8)	29·5 (22·5 to 37·5)	0·5 (0·5 to 0·5)	-3·7 (-8·9 to 2·2)	301·5 (287·5 to 317·1)	20·1 (12·4 to 29·0)	8·3 (7·9 to 8·7)	-5·5 (-11·4 to 1·6)
Non-melanoma skin cancer (squamous-cell carcinoma)	17·9 (17·2 to 18·8)	29·5 (22·5 to 37·5)	0·5 (0·5 to 0·5)	-3·7 (-8·9 to 2·2)	301·5 (287·5 to 317·1)	20·1 (12·4 to 29·0)	8·3 (7·9 to 8·7)	-5·5 (-11·4 to 1·6)
Breast cancer	535·3 (506·4 to 573·1)	16·6 (9·1 to 23·8)	14·6 (13·8 to 15·6)	-9·3 (-15·1 to -3·8)	14,124·7 (13,325·8 to 15,178·6)	13·5 (5·3 to 21·8)	379·4 (357·8 to 408·1)	-9·3 (-15·8 to -2·9)
Cervical cancer	247·2 (204·1 to 263·5)	7·5 (1·2 to 15·5)	6·7 (5·6 to 7·2)	-15·4 (-20·1 to -9·1)	7,204·1 (5,855·6 to 7,673·4)	4·9 (-1·4 to 13·1)	192·8 (156·8 to 205·3)	-15·5 (-20·6 to -9·0)
Uterine cancer	87·5 (83·1 to 92·0)	11·4 (5·4 to 19·3)	2·4 (2·3 to 2·6)	-14·1 (-18·8 to -8·1)	1,973·2 (1,875·5 to 2,070·1)	6·8 (0·9 to 14·6)	53·8 (51·1 to 56·5)	-16·3 (-20·9 to -10·2)
Ovarian cancer	165·0 (156·7 to 172·7)	22·1 (15·5 to 28·0)	4·5 (4·3 to 4·7)	-5·4 (-10·5 to -0·8)	4,141·9 (3,927·5 to 4,340·6)	20·8 (13·8 to 27·0)	112·0 (106·2 to 117·4)	-4·4 (-9·9 to 0·5)
Prostate cancer	-	-	-	-	-	-	-	-
Testicular cancer	-	-	-	-	-	-	-	-
Kidney cancer	45·7 (43·9 to 48·0)	22·3 (16·9 to 28·8)	1·3 (1·2 to 1·3)	-6·1 (-10·2 to -1·0)	959·3 (919·2 to 1,008·9)	16·9 (10·9 to 23·8)	26·5 (25·4 to 27·9)	-6·6 (-11·4 to -1·1)
Bladder cancer	48·3 (46·0 to 50·5)	18·7 (12·0 to 25·1)	1·3 (1·3 to 1·4)	-11·0 (-16·1 to -6·3)	769·8 (733·3 to 803·6)	13·9 (6·6 to 20·7)	21·3 (20·3 to 22·3)	-11·7 (-17·4 to -6·5)
Brain and nervous system cancer	98·7 (85·9 to 106·6)	20·1 (15·1 to 26·0)	2·7 (2·4 to 2·9)	-2·9 (-7·0 to 1·8)	3,186·0 (2,780·0 to 3,512·1)	12·2 (7·3 to 18·3)	86·8 (75·7 to 95·6)	-4·9 (-9·1 to 0·0)
Thyroid cancer	25·9 (24·6 to 27·3)	13·5 (6·9 to 21·7)	0·7 (0·7 to 0·8)	-11·7 (-16·8 to -5·4)	621·3 (583·5 to 656·8)	9·4 (2·2 to 18·0)	16·9 (15·9 to 17·9)	-12·3 (-18·0 to -5·3)
Mesothelioma	8·1 (7·8 to 8·6)	23·0 (14·7 to 29·4)	0·2 (0·2 to 0·2)	-4·3 (-10·5 to 0·4)	181·7 (171·2 to 195·8)	17·2 (8·0 to 24·0)	5·0 (4·7 to 5·3)	-5·9 (-13·1 to -0·6)
Hodgkin lymphoma	10·0 (7·8 to 11·9)	-8·5 (-12·1 to -4·7)	0·3 (0·2 to 0·3)	-24·3 (-27·3 to -21·2)	362·4 (270·8 to 424·5)	-13·6 (-17·6 to -9·3)	9·8 (7·3 to 11·5)	-24·8 (-28·2 to -21·2)

SR Table 1: Global deaths and YLL numbers, age-standardized rates per 100,000, and percent change between 2006 and 2016 for females for all GBD causes and Levels 1 through 4 of the cause hierarchy. 95% UIs are in parentheses. UI=uncertainty interval. YLL=year of life lost.

Non-Hodgkin's lymphoma	100·5 (95·7 to 104·1)	24·1 (18·8 to 28·6)	2·8 (2·7 to 2·9)	-2·8 (-6·8 to 0·6)	2,605·0 (2,426·6 to 2,734·2)	20·5 (14·1 to 26·5)	71·5 (66·6 to 75·1)	-0·3 (-5·5 to 4·6)
Multiple myeloma	47·4 (40·5 to 54·9)	25·5 (20·1 to 31·3)	1·3 (1·1 to 1·5)	-4·2 (-8·3 to 0·3)	950·5 (818·0 to 1,074·3)	24·4 (17·6 to 31·0)	26·2 (22·5 to 29·7)	-3·1 (-8·3 to 2·2)
Leukaemia	129·7 (113·0 to 139·3)	3·0 (-3·0 to 9·9)	3·6 (3·1 to 3·8)	-16·4 (-21·2 to -10·9)	4,083·5 (3,477·4 to 4,487·3)	-8·2 (-14·8 to -0·2)	112·1 (95·6 to 123·4)	-20·2 (-25·8 to -13·4)
Acute lymphoid leukaemia	20·4 (16·8 to 24·3)	5·5 (-10·9 to 16·3)	0·6 (0·5 to 0·7)	-9·1 (-22·3 to 0·1)	934·3 (782·6 to 1,143·1)	-4·4 (-20·1 to 6·7)	25·8 (21·7 to 31·6)	-13·1 (-27·0 to -3·0)
Chronic lymphoid leukaemia	14·5 (13·2 to 15·8)	10·9 (4·1 to 20·0)	0·4 (0·4 to 0·4)	-16·4 (-21·4 to -9·7)	256·4 (229·6 to 288·8)	5·3 (-2·8 to 15·3)	7·1 (6·3 to 8·0)	-16·8 (-22·8 to -9·1)
Acute myeloid leukaemia	36·1 (31·8 to 39·0)	17·4 (10·6 to 23·0)	1·0 (0·9 to 1·1)	-5·3 (-10·6 to -0·7)	1,085·0 (913·1 to 1,215·9)	9·0 (0·5 to 15·8)	29·8 (25·1 to 33·4)	-6·7 (-13·4 to -0·9)
Chronic myeloid leukaemia	9·6 (8·1 to 11·2)	-6·5 (-12·2 to -0·9)	0·3 (0·2 to 0·3)	-26·6 (-30·9 to -22·2)	254·0 (206·1 to 309·0)	-10·8 (-17·4 to -3·9)	6·8 (5·6 to 8·3)	-26·4 (-31·6 to -21·0)
Other leukaemia	49·2 (40·2 to 53·1)	-6·4 (-14·2 to 2·8)	1·4 (1·1 to 1·5)	-23·4 (-29·5 to -16·3)	1,553·7 (1,226·8 to 1,682·4)	-20·1 (-28·3 to -8·9)	42·6 (33·7 to 46·2)	-30·2 (-37·2 to -20·8)
Other neoplasms	194·8 (182·1 to 201·4)	27·0 (22·8 to 31·3)	5·4 (5·0 to 5·6)	0·6 (-2·7 to 4·0)	5,472·2 (5,071·0 to 5,670·9)	19·2 (14·0 to 24·1)	149·6 (138·6 to 155·0)	0·3 (-3·9 to 4·4)
**Cardiovascular diseases**	8,376·8 (8,090·7 to 8,710·2)	10·7 (7·2 to 15·2)	233·3 (225·2 to 242·6)	-17·3 (-19·9 to -13·9)	131,150·5 (126,440·5 to 136,246·6)	3·6 (0·3 to 7·9)	3,632·3 (3,500·8 to 3,773·7)	-19·1 (-21·6 to -15·7)
Rheumatic heart disease	183·1 (173·0 to 195·9)	-8·5 (-17·6 to 2·9)	5·1 (4·8 to 5·4)	-28·0 (-35·1 to -19·3)	4,601·2 (4,279·0 to 5,002·8)	-17·5 (-26·7 to -7·6)	125·4 (116·6 to 136·1)	-31·3 (-39·0 to -23·1)
Ischaemic heart disease	4,370·3 (4,175·9 to 4,567·3)	16·0 (11·5 to 21·2)	121·7 (116·3 to 127·2)	-13·9 (-17·2 to -10·1)	64,539·6 (61,710·3 to 67,531·1)	9·8 (5·2 to 15·0)	1,789·5 (1,710·2 to 1,873·0)	-15·0 (-18·5 to -11·0)
Cerebrovascular disease	2,596·5 (2,456·7 to 2,744·6)	-0·3 (-4·6 to 4·7)	72·5 (68·6 to 76·5)	-24·8 (-28·1 to -21·1)	42,898·8 (41,000·7 to 44,913·3)	-5·0 (-9·2 to -0·5)	1,188·8 (1,136·1 to 1,245·2)	-25·9 (-29·1 to -22·3)
Ischaemic stroke	1,346·7 (1,263·0 to 1,446·5)	3·7 (-1·9 to 10·0)	37·7 (35·3 to 40·5)	-22·9 (-27·0 to -18·1)	17,888·3 (16,855·2 to 19,130·5)	-1·5 (-7·0 to 4·6)	501·7 (472·7 to 536·6)	-24·4 (-28·6 to -19·7)
Hemorrhagic stroke	1,249·8 (1,191·8 to 1,312·4)	-4·3 (-8·2 to -0·6)	34·8 (33·1 to 36·5)	-26·8 (-29·7 to -24·0)	25,010·6 (24,003·8 to 26,161·1)	-7·4 (-11·3 to -3·7)	687·1 (659·5 to 718·6)	-26·9 (-29·9 to -24·0)
Hypertensive heart disease	495·7 (366·5 to 587·5)	22·9 (7·6 to 41·5)	13·8 (10·2 to 16·4)	-8·4 (-19·4 to 4·9)	7,541·6 (5,756·3 to 8,785·8)	13·4 (1·7 to 32·1)	208·8 (159·3 to 243·6)	-11·8 (-20·9 to 2·5)
Cardiomyopathy and myocarditis	149·9 (117·5 to 168·8)	14·7 (5·2 to 27·1)	4·1 (3·2 to 4·6)	-13·5 (-20·6 to -4·0)	2,915·6 (2,404·5 to 3,340·8)	-0·1 (-11·1 to 15·0)	80·0 (66·0 to 91·4)	-17·6 (-26·3 to -5·4)
Myocarditis	25·1 (17·5 to 28·1)	6·3 (-6·1 to 27·1)	0·7 (0·5 to 0·8)	-20·0 (-29·3 to -3·3)	550·7 (399·9 to 623·6)	-8·7 (-21·5 to 2·0)	15·2 (11·0 to 17·2)	-21·6 (-32·2 to -11·9)
Alcoholic cardiomyopathy	22·1 (15·7 to 30·3)	-9·7 (-33·2 to 26·6)	0·6 (0·4 to 0·8)	-29·2 (-47·5 to -1·2)	588·6 (410·8 to 845·2)	-14·4 (-39·4 to 25·4)	15·8 (11·0 to 22·6)	-30·4 (-50·6 to 1·9)
Other cardiomyopathy	102·7 (80·7 to 112·9)	24·3 (15·5 to 34·4)	2·8 (2·2 to 3·1)	-7·3 (-14·1 to 0·2)	1,776·2 (1,487·6 to 1,939·8)	9·1 (-1·2 to 22·3)	49·1 (41·1 to 53·5)	-10·9 (-18·9 to -0·8)
Atrial fibrillation and flutter	147·7 (116·7 to 182·7)	39·4 (34·6 to 44·4)	4·1 (3·2 to 5·0)	-1·6 (-4·7 to 1·6)	1,352·1 (1,091·2 to 1,653·2)	33·1 (28·0 to 38·2)	37·6 (30·3 to 45·7)	-1·4 (-4·8 to 2·0)
Aortic aneurysm	62·9 (60·2 to 65·5)	24·4 (18·3 to 30·2)	1·7 (1·7 to 1·8)	-7·4 (-11·9 to -3·2)	931·8 (890·4 to 981·7)	18·0 (10·7 to 24·9)	25·8 (24·6 to 27·2)	-8·0 (-13·6 to -2·8)
Peripheral vascular disease	32·5 (17·8 to 57·8)	40·5 (22·7 to 59·0)	0·9 (0·5 to 1·6)	-0·8 (-13·6 to 12·0)	315·8 (174·4 to 547·2)	32·8 (14·9 to 51·3)	8·8 (4·9 to 15·2)	-1·2 (-14·7 to 12·6)
Endocarditis	51·0 (40·7 to 62·3)	26·4 (19·3 to 31·9)	1·4 (1·1 to 1·7)	-3·6 (-9·1 to 1·3)	1,074·3 (929·7 to 1,291·9)	15·0 (6·1 to 21·3)	29·6 (25·7 to 35·6)	-4·4 (-11·7 to 0·9)
Other cardiovascular and circulatory diseases	287·4 (263·6 to 365·5)	19·9 (14·2 to 28·5)	8·0 (7·3 to 10·2)	-10·2 (-14·3 to -3·9)	4,979·7 (4,466·6 to 6,603·3)	10·3 (4·0 to 19·4)	138·0 (123·9 to 182·8)	-11·1 (-16·1 to -3·9)
**Chronic respiratory diseases**	1,512·4 (1,399·1 to 1,711·6)	2·7 (-2·2 to 10·5)	42·4 (39·2 to 48·0)	-22·2 (-25·9 to -16·2)	25,273·1 (23,312·2 to 28,802·2)	-2·6 (-7·1 to 6·6)	704·8 (649·9 to 803·2)	-23·5 (-27·1 to -16·4)
Chronic obstructive pulmonary disease	1,236·8 (1,145·0 to 1,403·9)	2·0 (-3·3 to 9·8)	34·7 (32·2 to 39·4)	-23·2 (-27·1 to -17·3)	18,900·4 (17,326·2 to 21,511·1)	-2·6 (-7·8 to 5·6)	529·3 (485·5 to 602·6)	-24·5 (-28·4 to -18·1)
Pneumoconiosis	2·5 (2·2 to 2·9)	9·9 (2·8 to 22·0)	0·1 (0·1 to 0·1)	-15·8 (-21·2 to -6·5)	44·6 (39·1 to 52·9)	-0·4 (-6·8 to 12·3)	1·2 (1·1 to 1·5)	-20·4 (-25·6 to -10·3)
Silicosis	0·6 (0·5 to 0·8)	4·7 (-9·9 to 22·8)	0·0 (0·0 to 0·0)	-19·9 (-31·1 to -6·1)	10·2 (8·5 to 12·7)	-4·3 (-19·9 to 14·6)	0·3 (0·2 to 0·4)	-24·6 (-36·8 to -9·6)
Asbestosis	0·6 (0·5 to 0·7)	10·8 (2·2 to 22·6)	0·0 (0·0 to 0·0)	-14·6 (-21·2 to -5·2)	12·4 (10·6 to 15·3)	0·3 (-7·1 to 11·6)	0·3 (0·3 to 0·4)	-18·5 (-24·6 to -8·9)
Coal workers pneumoconiosis	0·3 (0·2 to 0·4)	0·1 (-11·0 to 16·5)	0·0 (0·0 to 0·0)	-23·8 (-32·2 to -12·0)	4·1 (3·0 to 5·5)	-11·0 (-21·6 to 7·2)	0·1 (0·1 to 0·2)	-29·8 (-38·1 to -15·9)
Other pneumoconiosis	1·0 (0·9 to 1·3)	15·7 (5·9 to 29·7)	0·0 (0·0 to 0·0)	-11·3 (-18·8 to -0·3)	18·0 (15·2 to 22·7)	4·4 (-3·5 to 17·8)	0·5 (0·4 to 0·6)	-16·4 (-22·9 to -5·9)
Asthma	206·7 (168·1 to 261·7)	-0·9 (-12·3 to 12·7)	5·7 (4·7 to 7·3)	-22·5 (-31·5 to -12·1)	5,017·1 (4,122·4 to 6,397·9)	-7·8 (-16·8 to 7·2)	137·8 (113·3 to 175·7)	-24·7 (-32·2 to -12·6)
Interstitial lung disease and pulmonary sarcoidosis	55·8 (36·9 to 67·1)	35·6 (16·8 to 50·3)	1·6 (1·0 to 1·9)	3·2 (-10·2 to 14·4)	985·0 (669·3 to 1,215·3)	28·5 (8·7 to 45·8)	27·3 (18·6 to 33·7)	1·6 (-13·5 to 14·9)
Other chronic respiratory diseases	10·6 (6·4 to 13·9)	27·5 (18·0 to 41·9)	0·3 (0·2 to 0·4)	2·2 (-5·6 to 14·0)	326·1 (203·9 to 441·8)	13·9 (6·2 to 30·7)	9·1 (5·7 to 12·3)	-0·6 (-7·4 to 14·2)
**Cirrhosis and other chronic liver diseases**	400·0 (376·1 to 421·6)	8·8 (1·7 to 14·8)	11·0 (10·4 to 11·6)	-14·4 (-20·0 to -9·8)	10,489·3 (9,811·3 to 11,106·4)	2·1 (-5·4 to 8·6)	284·8 (266·4 to 301·5)	-16·7 (-22·8 to -11·4)
Cirrhosis and other chronic liver diseases due to hepatitis B	93·8 (84·4 to 103·9)	6·9 (-1·0 to 12·9)	2·6 (2·3 to 2·9)	-16·2 (-22·3 to -11·5)	2,387·0 (2,138·2 to 2,647·1)	2·5 (-5·8 to 8·9)	64·7 (58·0 to 71·8)	-17·5 (-24·3 to -12·4)
Cirrhosis and other chronic liver diseases due to hepatitis C	90·2 (80·7 to 100·8)	13·3 (5·7 to 20·1)	2·5 (2·2 to 2·8)	-11·7 (-17·6 to -6·5)	2,243·4 (2,003·9 to 2,505·4)	9·1 (1·1 to 16·2)	60·8 (54·4 to 67·6)	-12·6 (-18·9 to -7·0)
Cirrhosis and other chronic liver diseases due to alcohol use	101·0 (91·7 to 111·2)	11·4 (4·6 to 18·3)	2·8 (2·5 to 3·1)	-13·4 (-18·7 to -8·2)	2,474·4 (2,242·8 to 2,746·8)	6·6 (-0·9 to 14·6)	67·0 (60·7 to 74·4)	-15·1 (-21·0 to -8·9)
Cirrhosis and other chronic liver diseases due to other causes	115·1 (103·8 to 127·4)	5·1 (-2·2 to 10·8)	3·2 (2·9 to 3·5)	-15·9 (-21·5 to -11·5)	3,384·5 (3,052·0 to 3,725·2)	-5·1 (-13·1 to 1·4)	92·4 (83·2 to 101·5)	-19·7 (-26·2 to -14·6)
**Digestive diseases**	518·5 (480·8 to 549·8)	7·8 (3·7 to 12·0)	14·4 (13·3 to 15·3)	-16·9 (-20·0 to -13·7)	11,486·6 (10,469·0 to 12,328·2)	-2·9 (-8·5 to 2·7)	317·6 (289·3 to 341·1)	-19·4 (-23·9 to -14·9)
Peptic ulcer disease	105·5 (96·7 to 117·9)	-8·6 (-13·1 to -3·5)	2·9 (2·7 to 3·3)	-29·6 (-33·0 to -25·8)	2,207·3 (2,004·4 to 2,492·3)	-16·1 (-20·7 to -10·9)	60·7 (55·1 to 68·5)	-32·0 (-35·6 to -27·8)
Gastritis and duodenitis	18·0 (16·0 to 21·2)	-0·1 (-7·8 to 13·2)	0·5 (0·4 to 0·6)	-23·5 (-29·3 to -13·6)	364·4 (319·5 to 423·2)	-8·6 (-16·7 to 7·7)	10·0 (8·8 to 11·6)	-25·9 (-32·1 to -13·1)
Appendicitis	26·5 (21·8 to 32·5)	-5·8 (-14·8 to 3·9)	0·7 (0·6 to 0·9)	-21·8 (-29·0 to -14·0)	982·7 (804·2 to 1,220·2)	-16·1 (-28·0 to -3·6)	26·9 (22·0 to 33·5)	-25·7 (-36·1 to -14·9)
Paralytic ileus and intestinal obstruction	123·7 (95·2 to 138·6)	16·3 (8·3 to 23·6)	3·4 (2·7 to 3·9)	-8·3 (-14·5 to -2·8)	3,388·2 (2,587·0 to 3,832·4)	1·5 (-6·8 to 10·0)	94·8 (72·6 to 107·6)	-12·2 (-19·3 to -5·3)
Inguinal, femoral, and abdominal hernia	17·1 (11·9 to 23·2)	5·0 (-2·7 to 14·0)	0·5 (0·3 to 0·6)	-21·0 (-26·8 to -14·4)	303·2 (210·9 to 421·1)	-3·5 (-10·4 to 4·5)	8·4 (5·8 to 11·7)	-23·4 (-28·9 to -17·1)
Inflammatory bowel disease	22·1 (16·5 to 25·0)	10·7 (-0·4 to 23·7)	0·6 (0·5 to 0·7)	-15·7 (-23·4 to -5·7)	471·9 (352·9 to 590·1)	3·9 (-13·0 to 16·8)	13·0 (9·7 to 16·3)	-14·4 (-27·2 to -4·2)
Vascular intestinal disorders	55·3 (50·5 to 61·3)	19·6 (12·5 to 28·8)	1·5 (1·4 to 1·7)	-10·4 (-15·6 to -3·7)	812·4 (732·4 to 914·7)	14·6 (7·7 to 24·6)	22·7 (20·5 to 25·5)	-10·8 (-16·3 to -3·3)
Gallbladder and biliary diseases	58·1 (53·4 to 66·5)	17·0 (11·9 to 22·5)	1·6 (1·5 to 1·8)	-12·1 (-15·9 to -8·1)	1,008·3 (914·4 to 1,185·1)	6·9 (1·8 to 12·4)	27·8 (25·1 to 32·6)	-15·1 (-19·1 to -10·8)
Pancreatitis	42·8 (34·8 to 51·1)	13·0 (5·1 to 24·4)	1·2 (1·0 to 1·4)	-11·8 (-17·9 to -3·2)	1,037·0 (818·2 to 1,262·1)	6·2 (-2·2 to 19·8)	28·2 (22·2 to 34·3)	-13·6 (-20·2 to -2·8)
Other digestive diseases	49·5 (45·7 to 53·5)	14·1 (9·2 to 21·1)	1·4 (1·3 to 1·5)	-14·0 (-17·6 to -9·1)	911·2 (820·2 to 1,025·0)	1·8 (-8·5 to 13·4)	25·3 (22·7 to 28·5)	-16·9 (-24·6 to -8·2)
**Neurological disorders**	1,733·4 (1,525·7 to 1,973·0)	39·1 (36·0 to 42·1)	48·0 (42·3 to 54·6)	-0·6 (-2·4 to 1·3)	18,395·4 (16,467·7 to 20,903·6)	24·7 (21·1 to 29·0)	512·8 (460·0 to 582·5)	-3·2 (-5·7 to -0·2)
Alzheimer's disease and other dementias	1,539·4 (1,325·2 to 1,781·3)	41·9 (38·6 to 45·2)	42·6 (37·0 to 49·2)	-0·1 (-1·9 to 1·9)	13,543·8 (11,717·6 to 15,865·4)	34·9 (31·8 to 38·2)	378·6 (328·4 to 445·2)	-0·1 (-2·2 to 2·1)
Parkinson's disease	93·5 (73·7 to 118·4)	35·3 (30·7 to 40·3)	2·6 (2·1 to 3·3)	0·7 (-2·4 to 4·1)	1,062·7 (834·4 to 1,331·3)	30·9 (26·5 to 35·8)	30·1 (23·6 to 37·7)	0·8 (-2·4 to 4·4)
Epilepsy	51·0 (45·0 to 56·0)	-2·4 (-10·5 to 12·0)	1·4 (1·2 to 1·5)	-15·3 (-22·3 to -3·0)	2,315·5 (2,065·6 to 2,552·2)	-11·2 (-20·0 to 4·8)	63·5 (56·6 to 70·1)	-19·1 (-27·1 to -4·5)
Multiple sclerosis	11·2 (9·4 to 12·6)	17·1 (1·2 to 26·1)	0·3 (0·3 to 0·3)	-7·0 (-18·9 to -0·1)	332·9 (293·0 to 387·4)	12·6 (-1·8 to 23·4)	8·9 (7·8 to 10·3)	-8·6 (-19·5 to -0·1)
Motor neuron disease	15·0 (14·4 to 15·7)	24·0 (18·3 to 28·3)	0·4 (0·4 to 0·4)	-3·3 (-7·7 to 0·1)	355·3 (337·2 to 374·7)	19·4 (13·5 to 23·4)	9·8 (9·3 to 10·4)	-4·2 (-8·9 to -0·9)
Other neurological disorders	23·3 (21·9 to 24·5)	23·4 (19·0 to 29·2)	0·6 (0·6 to 0·7)	0·3 (-2·8 to 4·2)	785·2 (696·4 to 861·1)	12·6 (7·1 to 21·3)	21·7 (19·2 to 23·9)	-1·7 (-6·0 to 4·7)

SR Table 1: Global deaths and YLL numbers, age-standardized rates per 100,000, and percent change between 2006 and 2016 for females for all GBD causes and Levels 1 through 4 of the cause hierarchy. 95% UIs are in parentheses. UI=uncertainty interval. YLL=year of life lost.

**Mental and substance use disorders**	70·5 (64·8 to 77·5)	9·0 (-0·9 to 20·6)	1·9 (1·7 to 2·1)	-10·7 (-18·7 to -1·2)	2,447·5 (2,247·2 to 2,715·0)	2·0 (-7·7 to 14·0)	65·2 (59·9 to 72·3)	-13·2 (-21·5 to -3·2)
Alcohol use disorders	26·3 (23·1 to 30·8)	-5·8 (-18·3 to 12·1)	0·7 (0·6 to 0·8)	-24·2 (-34·2 to -10·0)	853·0 (736·9 to 1,015·2)	-11·1 (-24·3 to 6·9)	22·7 (19·6 to 27·0)	-26·6 (-37·4 to -11·8)
Drug use disorders	43·7 (40·3 to 47·2)	20·3 (8·6 to 30·3)	1·2 (1·1 to 1·3)	-0·2 (-9·8 to 8·1)	1,570·9 (1,447·9 to 1,714·2)	10·8 (-0·6 to 21·4)	41·9 (38·6 to 45·7)	-3·9 (-13·7 to 5·2)
Opioid use disorders	23·0 (18·3 to 25·5)	19·3 (6·8 to 30·7)	0·6 (0·5 to 0·7)	0·4 (-10·2 to 9·8)	884·0 (720·1 to 979·0)	9·8 (-2·5 to 20·9)	23·5 (19·2 to 26·0)	-4·1 (-14·8 to 5·6)
Cocaine use disorders	2·1 (1·7 to 3·3)	22·7 (5·8 to 59·1)	0·1 (0·0 to 0·1)	3·3 (-10·9 to 34·1)	85·4 (69·0 to 138·3)	17·4 (0·6 to 53·3)	2·3 (1·8 to 3·7)	1·3 (-13·1 to 32·7)
Amphetamine use disorders	1·3 (1·1 to 2·2)	36·5 (13·8 to 94·3)	0·0 (0·0 to 0·1)	16·4 (-2·6 to 66·1)	55·8 (46·0 to 95·9)	29·5 (6·4 to 86·2)	1·5 (1·2 to 2·5)	13·8 (-6·2 to 64·8)
Other drug use disorders	17·4 (15·4 to 20·3)	20·2 (9·9 to 29·3)	0·5 (0·4 to 0·5)	-2·2 (-10·5 to 5·1)	545·6 (479·0 to 665·1)	9·9 (0·0 to 19·1)	14·6 (12·8 to 17·7)	-5·7 (-14·2 to 2·1)
Eating disorders	0·4 (0·4 to 0·5)	9·2 (2·2 to 16·6)	0·0 (0·0 to 0·0)	-1·8 (-7·9 to 4·7)	23·7 (20·2 to 27·0)	7·7 (0·9 to 14·9)	0·6 (0·5 to 0·7)	-2·1 (-8·2 to 4·3)
Anorexia nervosa	0·4 (0·3 to 0·5)	7·6 (0·5 to 14·7)	0·0 (0·0 to 0·0)	-3·1 (-9·4 to 3·3)	21·5 (18·3 to 24·5)	6·3 (-0·7 to 13·8)	0·6 (0·5 to 0·7)	-3·2 (-9·5 to 3·4)
Bulimia nervosa	0·0 (0·0 to 0·1)	26·7 (13·9 to 40·3)	0·0 (0·0 to 0·0)	12·4 (1·0 to 24·4)	2·2 (1·7 to 2·7)	23·5 (10·9 to 37·2)	0·1 (0·0 to 0·1)	10·7 (-0·4 to 22·8)
**Diabetes, urogenital, blood, and endocrine diseases**	1,600·1 (1,546·0 to 1,650·8)	25·6 (22·8 to 28·4)	44·5 (43·0 to 45·9)	-3·5 (-5·6 to -1·5)	33,501·6 (32,318·9 to 34,767·3)	13·8 (10·8 to 17·1)	924·2 (891·5 to 960·2)	-7·2 (-9·5 to -4·7)
Diabetes mellitus	760·1 (732·7 to 785·4)	27·3 (23·7 to 30·9)	21·2 (20·4 to 21·9)	-3·0 (-5·7 to -0·3)	14,239·5 (13,760·5 to 14,711·0)	21·4 (17·8 to 24·8)	393·0 (379·8 to 406·1)	-4·9 (-7·6 to -2·2)
Acute glomerulonephritis	4·6 (4·3 to 4·8)	3·5 (-4·0 to 11·3)	0·1 (0·1 to 0·1)	-16·5 (-22·5 to -10·3)	131·6 (123·0 to 141·4)	-8·7 (-15·7 to -1·2)	3·6 (3·4 to 3·9)	-21·7 (-27·7 to -15·1)
Chronic kidney disease	540·8 (522·9 to 559·2)	26·2 (22·4 to 29·5)	15·0 (14·5 to 15·5)	-3·5 (-6·3 to -1·1)	10,996·5 (10,602·3 to 11,459·0)	13·9 (10·0 to 17·4)	301·4 (290·6 to 314·0)	-7·9 (-11·0 to -5·0)
Chronic kidney disease due to diabetes mellitus	212·7 (191·3 to 232·1)	26·9 (22·8 to 30·5)	5·9 (5·3 to 6·4)	-2·9 (-6·0 to -0·3)	4,337·7 (3,922·9 to 4,739·6)	18·8 (14·4 to 22·7)	118·7 (107·3 to 129·4)	-6·3 (-9·6 to -3·5)
Chronic kidney disease due to hypertension	145·6 (130·3 to 161·9)	31·3 (27·0 to 35·5)	4·0 (3·6 to 4·5)	-2·9 (-5·9 to -0·1)	2,180·9 (1,947·2 to 2,434·7)	20·9 (16·5 to 25·0)	60·2 (53·6 to 67·4)	-6·0 (-9·3 to -3·1)
Chronic kidney disease due to glomerulonephritis	74·7 (66·0 to 84·3)	16·5 (12·1 to 20·6)	2·1 (1·8 to 2·3)	-7·6 (-10·6 to -4·9)	1,997·1 (1,768·8 to 2,235·5)	3·2 (-1·1 to 7·3)	54·5 (48·4 to 60·8)	-12·2 (-15·4 to -9·0)
Chronic kidney disease due to other causes	107·8 (95·1 to 123·0)	25·5 (21·2 to 29·9)	3·0 (2·6 to 3·4)	-2·7 (-5·7 to 0·0)	2,480·8 (2,206·8 to 2,790·3)	9·4 (4·8 to 13·8)	67·9 (60·5 to 76·6)	-8·5 (-12·0 to -5·1)
Urinary diseases and male infertility	138·1 (131·6 to 144·4)	32·7 (25·4 to 37·3)	3·8 (3·7 to 4·0)	0·4 (-5·2 to 3·8)	2,672·1 (2,523·1 to 2,808·3)	16·8 (7·1 to 21·9)	73·8 (69·6 to 77·6)	-3·6 (-11·6 to 0·7)
Interstitial nephritis and urinary tract infections	115·1 (109·0 to 123·1)	38·5 (30·3 to 46·2)	3·2 (3·0 to 3·4)	3·8 (-2·1 to 9·3)	2,127·0 (1,997·2 to 2,304·7)	26·0 (14·3 to 35·2)	58·8 (55·1 to 63·7)	3·2 (-6·2 to 10·5)
Urolithiasis	8·9 (7·4 to 11·7)	25·3 (10·0 to 48·8)	0·2 (0·2 to 0·3)	-3·2 (-14·7 to 14·4)	187·1 (153·0 to 236·3)	10·1 (-5·2 to 35·7)	5·1 (4·2 to 6·5)	-9·4 (-21·8 to 11·0)
Other urinary diseases	14·1 (8·9 to 17·3)	2·0 (-20·9 to 21·8)	0·4 (0·2 to 0·5)	-19·6 (-37·5 to -4·3)	358·1 (242·4 to 461·4)	-16·8 (-32·5 to -2·2)	9·9 (6·7 to 12·7)	-29·2 (-42·2 to -17·2)
Gynecological diseases	8·3 (7·4 to 9·0)	13·6 (3·6 to 23·8)	0·2 (0·2 to 0·2)	-7·7 (-15·8 to 0·6)	265·5 (239·1 to 289·1)	2·8 (-4·6 to 12·5)	7·1 (6·4 to 7·7)	-12·7 (-18·9 to -4·5)
Uterine fibroids	2·9 (2·0 to 3·6)	15·8 (-3·0 to 37·2)	0·1 (0·1 to 0·1)	-7·3 (-22·7 to 10·1)	87·9 (59·0 to 109·9)	6·9 (-8·2 to 28·2)	2·3 (1·6 to 2·9)	-12·2 (-24·9 to 5·0)
Polycystic ovarian syndrome	0·4 (0·2 to 0·8)	-13·2 (-25·1 to 9·3)	0·0 (0·0 to 0·0)	-26·4 (-36·4 to -7·7)	18·7 (7·0 to 35·8)	-14·7 (-26·6 to 7·9)	0·5 (0·2 to 0·9)	-26·9 (-36·9 to -8·4)
Endometriosis	0·1 (0·0 to 0·1)	21·3 (-12·0 to 60·8)	0·0 (0·0 to 0·0)	3·9 (-24·7 to 37·9)	3·1 (1·2 to 4·5)	18·6 (-13·8 to 57·4)	0·1 (0·0 to 0·1)	3·1 (-25·4 to 36·8)
Genital prolapse	0·9 (0·5 to 1·3)	4·0 (-13·4 to 37·7)	0·0 (0·0 to 0·0)	-21·9 (-35·1 to 3·5)	14·4 (7·5 to 20·7)	-14·0 (-27·5 to 18·8)	0·4 (0·2 to 0·6)	-32·1 (-42·7 to -5·0)
Other gynecological diseases	4·0 (2·9 to 5·0)	18·2 (6·0 to 37·3)	0·1 (0·1 to 0·1)	-1·6 (-12·1 to 14·4)	141·4 (101·4 to 171·9)	4·9 (-5·7 to 21·9)	3·8 (2·7 to 4·6)	-8·2 (-17·2 to 6·9)
Hemoglobinopathies and hemolytic anaemias	73·6 (61·7 to 88·1)	4·1 (-2·5 to 13·8)	2·0 (1·7 to 2·4)	-11·9 (-17·2 to -4·2)	3,116·9 (2,573·6 to 3,698·6)	-7·0 (-16·3 to 9·7)	87·5 (72·3 to 104·0)	-14·2 (-22·8 to 1·1)
Thalassemias	3·2 (2·7 to 4·1)	-32·1 (-39·7 to -10·5)	0·1 (0·1 to 0·1)	-34·9 (-42·3 to -13·9)	247·0 (206·4 to 319·4)	-33·6 (-41·3 to -11·0)	7·1 (5·9 to 9·3)	-36·1 (-43·6 to -14·0)
Sickle cell disorders	31·5 (25·4 to 37·4)	-0·9 (-12·2 to 18·7)	0·9 (0·7 to 1·0)	-6·5 (-17·4 to 12·6)	2,119·0 (1,679·7 to 2,518·5)	-4·0 (-16·4 to 18·5)	59·8 (47·2 to 71·0)	-8·2 (-20·6 to 14·3)
G6PD deficiency	7·4 (6·0 to 9·2)	27·6 (20·2 to 35·8)	0·2 (0·2 to 0·2)	8·9 (2·5 to 15·7)	285·6 (230·7 to 348·6)	10·1 (3·4 to 20·2)	7·7 (6·2 to 9·4)	-2·8 (-8·9 to 5·8)
Other hemoglobinopathies and hemolytic anaemias	31·4 (25·9 to 38·9)	10·7 (5·7 to 15·6)	0·9 (0·7 to 1·1)	-17·2 (-21·2 to -13·7)	465·3 (387·7 to 578·5)	-9·5 (-13·3 to -5·4)	12·9 (10·8 to 16·1)	-27·3 (-30·2 to -24·1)
Endocrine, metabolic, blood, and immune disorders	74·7 (59·5 to 81·6)	21·3 (13·0 to 27·3)	2·1 (1·7 to 2·3)	-3·8 (-10·0 to 0·7)	2,079·4 (1,781·9 to 2,380·3)	4·2 (-4·7 to 16·6)	57·8 (49·6 to 66·2)	-9·6 (-17·1 to 0·7)
**Musculoskeletal disorders**	61·6 (52·2 to 70·6)	14·1 (8·2 to 19·0)	1·7 (1·4 to 1·9)	-10·9 (-15·3 to -7·2)	1,514·2 (1,275·6 to 1,788·0)	5·0 (-1·8 to 10·4)	41·2 (34·6 to 48·5)	-11·8 (-17·4 to -7·5)
Rheumatoid arthritis	21·1 (17·3 to 26·3)	6·4 (-0·5 to 13·6)	0·6 (0·5 to 0·7)	-18·6 (-23·6 to -13·3)	380·2 (310·3 to 479·3)	-0·8 (-8·1 to 6·7)	10·5 (8·6 to 13·3)	-21·6 (-26·8 to -15·8)
Other musculoskeletal disorders	40·5 (33·4 to 45·8)	18·5 (10·7 to 23·9)	1·1 (0·9 to 1·3)	-6·2 (-12·2 to -1·6)	1,134·0 (952·5 to 1,328·0)	7·1 (-1·2 to 12·5)	30·6 (25·7 to 35·8)	-7·8 (-14·7 to -3·3)
**Other non-communicable diseases**	308·7 (274·8 to 356·9)	-8·7 (-20·1 to 3·3)	9·0 (8·0 to 10·4)	-14·2 (-24·5 to -3·7)	21,716·3 (18,899·9 to 25,021·1)	-14·4 (-25·7 to -2·4)	641·4 (557·3 to 740·2)	-16·6 (-27·5 to -5·1)
Congenital anomalies	235·9 (202·2 to 272·8)	-15·0 (-26·9 to -2·3)	7·0 (6·0 to 8·1)	-17·1 (-28·6 to -4·8)	19,277·2 (16,441·9 to 22,446·7)	-15·9 (-27·7 to -3·0)	570·9 (486·3 to 665·4)	-17·5 (-29·1 to -4·8)
Neural tube defects	16·7 (10·5 to 24·5)	-24·7 (-37·8 to -10·3)	0·5 (0·3 to 0·7)	-25·6 (-38·6 to -11·3)	1,420·5 (889·8 to 2,084·7)	-25·0 (-38·1 to -10·5)	42·4 (26·6 to 62·2)	-25·7 (-38·8 to -11·3)
Congenital heart anomalies	101·8 (82·1 to 130·4)	-17·0 (-28·6 to 4·8)	3·0 (2·4 to 3·8)	-19·4 (-30·6 to 1·7)	8,207·6 (6,583·8 to 10,604·5)	-17·9 (-29·4 to 4·5)	241·8 (193·8 to 312·8)	-19·7 (-31·0 to 2·2)
Orofacial clefts	1·1 (0·5 to 1·9)	-23·2 (-47·7 to 4·5)	0·0 (0·0 to 0·1)	-23·6 (-48·1 to 4·0)	96·6 (44·2 to 163·0)	-23·3 (-47·7 to 4·4)	2·9 (1·3 to 4·9)	-23·6 (-48·1 to 4·0)
Down's syndrome	7·0 (5·7 to 9·7)	8·0 (-6·8 to 22·0)	0·2 (0·2 to 0·3)	-1·6 (-14·4 to 11·3)	456·6 (367·7 to 681·2)	2·1 (-12·5 to 18·6)	13·2 (10·6 to 19·8)	-3·4 (-17·5 to 12·3)
Other chromosomal abnormalities	10·0 (6·9 to 17·2)	8·9 (-14·8 to 28·1)	0·3 (0·2 to 0·5)	7·4 (-15·8 to 26·2)	837·5 (573·9 to 1,458·9)	8·4 (-15·2 to 27·8)	25·0 (17·1 to 43·6)	7·2 (-16·1 to 26·4)
Congenital musculoskeletal and limb anomalies	4·0 (2·7 to 7·7)	-16·1 (-34·2 to 4·1)	0·1 (0·1 to 0·2)	-17·6 (-35·3 to 2·1)	331·4 (222·2 to 637·0)	-16·9 (-34·9 to 3·6)	9·8 (6·6 to 19·0)	-17·9 (-35·7 to 2·4)
Urogenital congenital anomalies	5·0 (3·4 to 7·3)	-13·2 (-32·9 to 10·0)	0·1 (0·1 to 0·2)	-17·5 (-36·0 to 3·4)	367·8 (243·4 to 542·0)	-16·9 (-36·3 to 6·8)	10·8 (7·2 to 16·1)	-18·9 (-37·8 to 3·9)
Digestive congenital anomalies	13·9 (9·7 to 23·4)	-11·7 (-25·8 to 3·4)	0·4 (0·3 to 0·7)	-12·8 (-26·8 to 2·2)	1,177·1 (819·4 to 1,993·1)	-12·1 (-26·0 to 3·1)	35·2 (24·5 to 59·6)	-12·9 (-26·8 to 2·2)
Other congenital anomalies	76·4 (44·8 to 117·2)	-14·5 (-29·9 to 11·2)	2·3 (1·3 to 3·5)	-16·1 (-31·3 to 9·1)	6,382·1 (3,712·3 to 9,857·5)	-15·0 (-30·4 to 10·6)	189·8 (110·3 to 293·3)	-16·4 (-31·5 to 9·1)
Skin and subcutaneous diseases	60·9 (34·2 to 90·3)	33·6 (25·8 to 42·1)	1·7 (1·0 to 2·5)	2·2 (-3·6 to 9·4)	1,405·1 (760·1 to 2,048·7)	22·8 (10·0 to 36·4)	39·5 (21·3 to 57·8)	4·5 (-6·1 to 16·1)
Cellulitis	9·3 (4·4 to 15·6)	51·8 (41·5 to 69·6)	0·3 (0·1 to 0·4)	15·5 (8·1 to 29·2)	198·3 (90·4 to 297·8)	39·0 (24·6 to 65·6)	5·5 (2·5 to 8·3)	15·0 (3·7 to 36·4)
Pyoderma	33·1 (17·6 to 51·2)	35·7 (24·4 to 47·1)	0·9 (0·5 to 1·4)	7·2 (-1·4 to 15·5)	944·3 (489·2 to 1,442·5)	21·9 (5·1 to 39·0)	26·7 (13·8 to 40·9)	6·3 (-8·0 to 20·7)
Decubitus ulcer	15·9 (8·7 to 24·6)	21·3 (11·6 to 33·5)	0·4 (0·2 to 0·7)	-12·3 (-19·3 to -2·6)	200·5 (107·7 to 308·0)	14·7 (6·1 to 24·4)	5·6 (3·0 to 8·6)	-10·4 (-17·2 to -1·7)
Other skin and subcutaneous diseases	2·5 (1·5 to 4·3)	34·0 (23·2 to 47·1)	0·1 (0·0 to 0·1)	3·4 (-4·7 to 15·3)	62·0 (35·1 to 100·6)	19·6 (6·7 to 31·9)	1·7 (1·0 to 2·8)	3·2 (-7·4 to 14·4)
Sudden infant death syndrome	12·0 (9·2 to 15·8)	-20·2 (-37·3 to 0·8)	0·4 (0·3 to 0·5)	-21·0 (-37·9 to -0·1)	1,033·9 (790·0 to 1,363·3)	-20·2 (-37·3 to 0·8)	31·0 (23·7 to 40·9)	-21·0 (-37·9 to -0·2)
**Injuries**	1,395·6 (1,277·5 to 1,467·2)	-2·1 (-5·0 to 1·2)	38·4 (35·1 to 40·4)	-17·8 (-20·2 to -15·0)	55,015·5 (51,097·0 to 57,833·5)	-11·9 (-15·4 to -7·8)	1,512·6 (1,404·6 to 1,591·2)	-20·9 (-24·0 to -17·2)
**Transport injuries**	336·7 (323·0 to 351·8)	-7·1 (-11·6 to -3·7)	9·2 (8·8 to 9·6)	-20·7 (-24·6 to -17·9)	14,419·3 (13,778·6 to 15,195·5)	-14·6 (-19·4 to -11·2)	392·9 (375·6 to 414·1)	-23·5 (-27·8 to -20·5)
Road injuries	318·1 (305·4 to 333·0)	-7·4 (-11·7 to -4·1)	8·7 (8·3 to 9·1)	-21·0 (-24·6 to -18·3)	13,644·9 (13,037·8 to 14,338·4)	-14·8 (-19·3 to -11·4)	371·8 (355·3 to 390·5)	-23·7 (-27·7 to -20·6)
Pedestrian road injuries	142·3 (135·1 to 150·8)	-9·5 (-15·1 to -3·8)	3·9 (3·7 to 4·1)	-23·8 (-28·4 to -19·2)	5,668·9 (5,368·7 to 6,056·3)	-18·2 (-24·4 to -12·6)	155·1 (146·9 to 166·0)	-27·4 (-32·8 to -22·6)

SR Table 1: Global deaths and YLL numbers, age-standardized rates per 100,000, and percent change between 2006 and 2016 for females for all GBD causes and Levels 1 through 4 of the cause hierarchy. 95% UIs are in parentheses. UI=uncertainty interval. YLL=year of life lost.

Cyclist road injuries	15·4 (13·4 to 18·8)	-12·3 (-17·6 to -1·4)	0·4 (0·4 to 0·5)	-25·5 (-30·0 to -16·2)	631·0 (545·1 to 792·7)	-19·5 (-24·7 to -8·0)	17·1 (14·7 to 21·5)	-28·7 (-33·2 to -18·4)
Motorcyclist road injuries	35·8 (32·2 to 38·6)	-10·9 (-17·9 to -4·5)	1·0 (0·9 to 1·0)	-22·1 (-28·1 to -16·7)	1,693·0 (1,519·6 to 1,834·1)	-17·8 (-24·6 to -10·8)	45·7 (41·1 to 49·6)	-25·7 (-31·8 to -19·5)
Motor vehicle road injuries	121·3 (113·7 to 130·6)	-3·3 (-8·1 to 1·9)	3·3 (3·1 to 3·6)	-16·6 (-20·7 to -12·1)	5,504·7 (5,151·1 to 5,940·1)	-9·5 (-14·5 to -3·9)	149·8 (140·2 to 161·8)	-18·3 (-22·8 to -13·2)
Other road injuries	3·3 (2·8 to 4·8)	4·2 (-5·9 to 30·0)	0·1 (0·1 to 0·1)	-10·5 (-19·2 to 11·6)	147·2 (122·6 to 213·5)	-2·5 (-13·8 to 23·5)	4·0 (3·3 to 5·8)	-11·8 (-22·3 to 11·7)
Other transport injuries	18·6 (16·6 to 21·2)	-1·5 (-14·1 to 7·4)	0·5 (0·5 to 0·6)	-16·3 (-26·9 to -9·0)	774·4 (691·5 to 887·3)	-10·8 (-25·2 to -1·3)	21·2 (18·9 to 24·3)	-19·9 (-32·8 to -11·5)
**Unintentional injuries**	680·1 (559·5 to 729·2)	3·0 (-1·0 to 7·2)	18·9 (15·6 to 20·3)	-15·9 (-19·0 to -12·6)	22,711·5 (18,932·4 to 24,669·4)	-14·3 (-18·6 to -7·9)	635·3 (529·8 to 689·7)	-23·3 (-27·0 to -17·6)
Falls	294·5 (226·3 to 317·3)	21·7 (13·2 to 28·6)	8·2 (6·3 to 8·8)	-7·6 (-14·1 to -2·4)	5,539·1 (4,265·5 to 6,032·6)	2·2 (-8·3 to 10·5)	154·8 (119·1 to 169·0)	-14·5 (-23·1 to -7·7)
Drowning	87·8 (66·4 to 103·9)	-21·9 (-28·1 to -4·6)	2·5 (1·9 to 2·9)	-30·7 (-36·3 to -15·6)	4,674·4 (3,558·2 to 5,606·7)	-31·5 (-38·5 to -13·5)	131·7 (100·4 to 158·2)	-36·7 (-43·2 to -19·9)
Fire, heat, and hot substances	64·7 (48·5 to 71·5)	-8·2 (-13·4 to -3·0)	1·8 (1·3 to 2·0)	-22·2 (-26·4 to -17·8)	2,768·9 (1,998·6 to 3,109·4)	-15·8 (-23·2 to -7·4)	76·7 (55·5 to 86·2)	-23·5 (-30·3 to -16·0)
Poisonings	21·0 (14·5 to 23·6)	-15·4 (-25·8 to -5·0)	0·6 (0·4 to 0·7)	-25·9 (-35·1 to -16·3)	1,052·1 (726·9 to 1,232·7)	-20·9 (-33·2 to -5·4)	29·5 (20·3 to 34·7)	-27·0 (-38·6 to -12·4)
Exposure to mechanical forces	38·4 (29·9 to 42·4)	-1·8 (-10·0 to 4·8)	1·1 (0·8 to 1·2)	-15·3 (-22·0 to -9·8)	1,831·1 (1,409·3 to 2,041·4)	-12·1 (-19·6 to -3·4)	51·4 (39·5 to 57·3)	-19·2 (-26·0 to -11·4)
Unintentional firearm injuries	5·2 (4·4 to 5·5)	2·4 (-2·2 to 7·5)	0·1 (0·1 to 0·2)	-14·9 (-18·6 to -10·9)	199·8 (179·7 to 218·6)	-7·4 (-12·9 to -0·8)	5·5 (4·9 to 6·0)	-17·1 (-22·0 to -11·3)
Unintentional suffocation	7·5 (4·9 to 9·1)	-13·0 (-26·5 to 1·0)	0·2 (0·1 to 0·3)	-18·2 (-31·1 to -4·9)	532·2 (341·1 to 651·4)	-18·7 (-31·1 to -4·5)	15·6 (9·9 to 19·1)	-21·6 (-33·8 to -7·8)
Other exposure to mechanical forces	25·7 (19·0 to 28·7)	1·1 (-10·8 to 8·8)	0·7 (0·5 to 0·8)	-14·4 (-24·1 to -8·0)	1,099·1 (823·3 to 1,225·7)	-9·3 (-20·6 to 1·0)	30·4 (22·9 to 33·9)	-18·3 (-28·1 to -9·0)
Adverse effects of medical treatment	60·8 (50·7 to 70·4)	6·2 (1·7 to 11·8)	1·7 (1·4 to 2·0)	-13·7 (-17·2 to -10·1)	2,039·1 (1,638·0 to 2,353·3)	-6·9 (-13·6 to 3·6)	56·7 (45·7 to 65·5)	-18·0 (-23·7 to -9·6)
Animal contact	38·8 (15·9 to 47·1)	-0·7 (-9·5 to 14·8)	1·1 (0·4 to 1·3)	-14·4 (-21·9 to -1·0)	1,818·8 (753·8 to 2,227·3)	-7·7 (-18·6 to 10·1)	50·4 (20·9 to 61·7)	-16·4 (-26·3 to 0·0)
Venomous animal contact	34·1 (12·6 to 42·3)	-1·6 (-10·2 to 14·2)	0·9 (0·3 to 1·2)	-15·2 (-22·7 to -1·5)	1,584·1 (584·1 to 1,983·2)	-8·8 (-19·1 to 9·1)	43·8 (16·2 to 54·8)	-17·5 (-26·7 to -1·4)
Non-venomous animal contact	4·7 (2·6 to 8·2)	5·9 (-11·3 to 20·4)	0·1 (0·1 to 0·2)	-8·4 (-22·5 to 3·7)	234·6 (129·4 to 417·7)	0·0 (-23·5 to 19·1)	6·6 (3·6 to 11·7)	-8·6 (-29·9 to 8·3)
Foreign body	41·5 (34·0 to 46·4)	10·0 (1·1 to 20·1)	1·2 (1·0 to 1·3)	-8·4 (-14·8 to 0·3)	1,731·0 (1,437·0 to 2,085·8)	-2·1 (-13·3 to 12·2)	49·6 (41·1 to 59·9)	-9·8 (-19·6 to 3·1)
Pulmonary aspiration and foreign body in airway	37·1 (29·9 to 41·7)	11·7 (3·0 to 23·1)	1·0 (0·8 to 1·2)	-7·4 (-14·0 to 2·4)	1,532·6 (1,241·7 to 1,855·8)	-0·6 (-12·6 to 15·2)	44·1 (35·7 to 53·5)	-8·3 (-19·2 to 6·0)
Foreign body in other body part	4·3 (3·2 to 6·0)	-2·9 (-14·1 to 8·1)	0·1 (0·1 to 0·2)	-16·3 (-25·1 to -7·4)	198·4 (152·2 to 293·4)	-12·7 (-24·2 to -0·5)	5·5 (4·2 to 8·1)	-20·2 (-30·4 to -9·0)
Environmental heat and cold exposure	15·4 (7·9 to 19·0)	-9·3 (-25·1 to 5·2)	0·4 (0·2 to 0·5)	-27·5 (-40·7 to -15·7)	462·8 (235·1 to 578·6)	-19·6 (-33·0 to -5·7)	12·8 (6·4 to 16·0)	-30·9 (-43·1 to -19·2)
Other unintentional injuries	17·2 (12·3 to 18·6)	-16·3 (-22·4 to -8·5)	0·5 (0·3 to 0·5)	-27·4 (-32·6 to -20·7)	794·2 (589·0 to 872·4)	-25·1 (-30·8 to -16·4)	21·9 (16·3 to 24·0)	-31·8 (-36·9 to -23·8)
**Self-harm and interpersonal violence**	330·7 (308·7 to 353·7)	-12·6 (-17·8 to -7·5)	8·9 (8·3 to 9·6)	-24·7 (-29·2 to -20·4)	14,837·8 (13,769·5 to 15,916·3)	-15·3 (-20·8 to -9·9)	398·9 (369·8 to 428·3)	-23·9 (-28·8 to -19·0)
Self-harm	257·6 (241·3 to 273·8)	-13·7 (-19·2 to -7·6)	7·0 (6·5 to 7·4)	-26·1 (-30·7 to -20·9)	11,188·9 (10,385·4 to 11,956·8)	-16·3 (-22·3 to -9·5)	300·0 (278·3 to 320·7)	-25·1 (-30·3 to -19·0)
Self-harm by firearm	10·0 (8·8 to 11·2)	-3·0 (-14·4 to 12·0)	0·3 (0·2 to 0·3)	-16·6 (-27·2 to -4·5)	439·0 (374·5 to 491·8)	-7·7 (-18·1 to 8·8)	11·7 (10·0 to 13·2)	-17·0 (-27·3 to -2·7)
Self-harm by other specified means	247·6 (231·5 to 263·6)	-14·1 (-19·6 to -8·0)	6·7 (6·2 to 7·1)	-26·4 (-31·1 to -21·2)	10,750·0 (9,958·9 to 11,491·3)	-16·6 (-22·6 to -9·7)	288·2 (266·8 to 308·4)	-25·3 (-30·6 to -19·2)
Interpersonal violence	73·1 (57·4 to 91·8)	-8·4 (-18·6 to 13·7)	2·0 (1·6 to 2·5)	-19·4 (-28·5 to 0·1)	3,648·9 (2,848·6 to 4,569·1)	-12·1 (-22·2 to 10·7)	99·0 (77·1 to 124·0)	-20·0 (-29·3 to 0·7)
Assault by firearm	17·3 (11·2 to 21·2)	3·2 (-5·5 to 23·6)	0·5 (0·3 to 0·6)	-8·4 (-16·4 to 9·5)	880·4 (577·1 to 1,084·3)	0·2 (-9·0 to 21·1)	23·7 (15·5 to 29·3)	-8·6 (-17·0 to 10·5)
Assault by sharp object	18·9 (13·9 to 24·8)	-8·8 (-19·7 to 23·8)	0·5 (0·4 to 0·7)	-20·5 (-30·1 to 7·4)	888·1 (650·6 to 1,175·8)	-12·4 (-23·2 to 20·9)	23·8 (17·5 to 31·5)	-21·3 (-31·0 to 8·5)
Assault by other means	36·9 (29·3 to 47·6)	-12·8 (-24·8 to 5·1)	1·0 (0·8 to 1·3)	-23·1 (-33·7 to -7·1)	1,880·5 (1,484·5 to 2,476·6)	-16·7 (-28·7 to 2·5)	51·4 (40·5 to 67·8)	-23·9 (-34·9 to -5·9)
**Forces of nature, conflict and terrorism, and executions and police conflict**	48·0 (33·4 to 63·8)	95·2 (23·4 to 221·8)	1·3 (0·9 to 1·8)	78·0 (12·9 to 191·9)	3,046·9 (2,106·9 to 4,051·7)	98·8 (24·9 to 237·2)	85·4 (59·0 to 113·6)	86·9 (17·6 to 216·3)
Forces of nature, conflict and terrorism, and state actor violence	2·7 (1·6 to 3·9)	-49·0 (-63·1 to -34·6)	0·1 (0·0 to 0·1)	-55·3 (-67·5 to -43·2)	133·3 (81·6 to 188·9)	-52·6 (-65·1 to -39·5)	3·7 (2·3 to 5·2)	-56·7 (-67·9 to -44·8)
Conflict and terrorism	44·7 (30·2 to 60·0)	139·5 (41·0 to 367·0)	1·2 (0·8 to 1·7)	120·8 (30·2 to 328·9)	2,882·6 (1,959·6 to 3,865·9)	136·1 (38·9 to 364·8)	80·9 (55·0 to 108·5)	122·7 (31·2 to 337·5)
Executions and police conflict	0·6 (0·4 to 0·8)	1·7 (-12·4 to 18·1)	0·0 (0·0 to 0·0)	-13·3 (-25·7 to 1·0)	31·0 (22·2 to 38·4)	3·2 (-15·7 to 23·4)	0·9 (0·6 to 1·1)	-6·4 (-23·8 to 12·9)

SR Table 2: Global deaths and YLL numbers, age-standardized rates per 100,000, and percent change between 2006 and 2016 for males for all GBD causes and Levels 1 through 4 of the cause hierarchy. 95% UIs are in parentheses. UI=uncertainty interval. YLL=year of life lost.

	All Age Deaths (thousands)		Age-standardized Death Rate (per 100,000)		All Age YLLs (thousands)		Age-standardized YLL Rate (per 100,000)	
	2016	Percent Change 2006-2016	2016	Percent Change 2006-2016	2016	Percent Change 2006-2016	2016	Percent Change 2006-2016
**All causes**	29,995·3 (29,538·0 to 30,504·0)	6·0 (4·3 to 8·0)	1,001·5 (987·2 to 1,017·0)	-15·2 (-16·5 to -13·8)	925,403·8 (907,497·0 to 943,821·3)	-9·1 (-10·8 to -7·1)	26,762·4 (26,255·6 to 27,273·8)	-20·7 (-22·2 to -19·0)
**Communicable, maternal, neonatal, and nutritional disorders**	5,587·7 (5,324·1 to 5,946·1)	-22·5 (-24·8 to -20·0)	170·0 (161·2 to 182·4)	-30·8 (-32·9 to -28·7)	304,941·2 (292,852·0 to 317,877·4)	-29·4 (-31·8 to -26·9)	8,512·2 (8,171·4 to 8,881·8)	-33·4 (-35·7 to -31·1)
**HIV/AIDS and tuberculosis**	1,349·4 (1,305·7 to 1,397·4)	-30·4 (-32·8 to -28·2)	38·2 (36·9 to 39·6)	-41·8 (-44·0 to -39·9)	55,544·7 (53,711·8 to 57,661·8)	-33·6 (-35·5 to -31·7)	1,483·6 (1,435·4 to 1,538·8)	-42·4 (-44·1 to -40·9)
Tuberculosis	764·9 (724·8 to 805·1)	-20·5 (-25·4 to -16·5)	22·9 (21·7 to 24·1)	-36·5 (-40·6 to -33·3)	26,030·7 (24,704·0 to 27,465·1)	-23·9 (-28·0 to -20·1)	713·8 (677·4 to 753·2)	-36·3 (-39·7 to -33·2)
Drug-susceptible tuberculosis	695·6 (657·9 to 735·0)	-20·0 (-24·7 to -15·9)	20·8 (19·7 to 22·0)	-36·1 (-40·0 to -32·8)	23,692·8 (22,365·2 to 25,105·6)	-23·4 (-27·3 to -19·6)	649·8 (614·1 to 688·1)	-35·8 (-39·2 to -32·7)
Multidrug-resistant tuberculosis without extensive drug resistance	61·7 (51·1 to 73·2)	-29·7 (-36·2 to -22·1)	1·8 (1·5 to 2·2)	-43·6 (-48·8 to -37·5)	2,083·7 (1,729·3 to 2,464·6)	-33·0 (-39·7 to -25·6)	57·1 (47·4 to 67·6)	-44·0 (-49·5 to -37·9)
Extensively drug-resistant tuberculosis	7·5 (6·1 to 9·2)	63·0 (39·8 to 90·2)	0·2 (0·2 to 0·3)	32·5 (14·1 to 53·5)	254·2 (205·4 to 311·3)	52·6 (29·5 to 79·7)	6·9 (5·6 to 8·5)	27·8 (8·9 to 50·0)
HIV/AIDS	584·5 (559·5 to 612·8)	-40·1 (-42·0 to -38·1)	15·3 (14·7 to 16·0)	-48·2 (-49·8 to -46·4)	29,514·0 (28,145·9 to 31,039·1)	-40·3 (-42·0 to -38·3)	769·8 (734·9 to 809·0)	-47·2 (-48·7 to -45·4)
Drug-susceptible HIV/AIDS - Tuberculosis	122·2 (85·1 to 161·5)	-49·5 (-52·7 to -46·8)	3·2 (2·2 to 4·2)	-56·7 (-59·4 to -54·2)	6,271·9 (4,376·6 to 8,275·8)	-49·3 (-52·3 to -46·5)	163·6 (114·2 to 216·0)	-55·2 (-58·0 to -52·8)
Multidrug-resistant HIV/AIDS - Tuberculosis without extensive drug resistance	10·9 (6·7 to 16·3)	-50·5 (-59·3 to -40·5)	0·3 (0·2 to 0·4)	-57·5 (-65·2 to -48·9)	558·5 (340·8 to 848·8)	-49·9 (-59·1 to -39·1)	14·6 (8·9 to 22·1)	-55·8 (-64·0 to -46·2)
Extensively drug-resistant HIV/AIDS - Tuberculosis	0·8 (0·5 to 1·2)	47·9 (27·6 to 71·8)	0·0 (0·0 to 0·0)	28·4 (11·0 to 49·0)	38·1 (22·8 to 59·8)	45·0 (25·3 to 68·3)	1·0 (0·6 to 1·6)	27·8 (10·5 to 48·3)
HIV/AIDS resulting in other diseases	450·7 (404·7 to 501·4)	-36·7 (-39·4 to -33·9)	11·8 (10·6 to 13·1)	-45·1 (-47·4 to -42·6)	22,645·5 (20,198·3 to 25,422·7)	-37·0 (-39·6 to -34·3)	590·6 (526·8 to 661·4)	-44·2 (-46·5 to -41·9)
**Diarrhoea, lower respiratory infections, and other common infectious diseases**	2,452·4 (2,183·0 to 2,859·0)	-17·1 (-21·4 to -12·5)	80·7 (71·7 to 95·0)	-27·8 (-31·6 to -24·2)	111,684·9 (102,680·2 to 123,342·4)	-32·2 (-36·8 to -27·3)	3,175·4 (2,919·2 to 3,514·3)	-36·3 (-40·4 to -31·9)
Diarrhoeal diseases	771·4 (526·1 to 1,200·8)	-24·7 (-34·7 to -13·9)	25·3 (16·8 to 39·8)	-36·2 (-44·6 to -26·9)	34,328·7 (27,456·8 to 45,446·9)	-36·0 (-43·9 to -26·6)	978·9 (778·5 to 1,299·0)	-40·5 (-47·5 to -32·6)
Intestinal infectious diseases	84·8 (47·9 to 140·3)	-13·9 (-20·7 to -7·8)	2·3 (1·3 to 3·7)	-19·7 (-26·0 to -14·2)	5,751·3 (3,253·8 to 9,455·0)	-16·2 (-23·7 to -10·0)	152·1 (86·2 to 249·7)	-20·9 (-28·1 to -14·8)
Typhoid fever	70·4 (38·9 to 115·2)	-14·9 (-21·7 to -9·1)	1·9 (1·0 to 3·1)	-20·4 (-26·9 to -15·0)	4,819·7 (2,649·6 to 7,893·0)	-17·1 (-24·5 to -10·9)	127·5 (70·0 to 208·9)	-21·6 (-28·7 to -15·6)
Paratyphoid fever	13·7 (6·3 to 26·8)	-6·2 (-13·4 to 0·7)	0·4 (0·2 to 0·7)	-13·7 (-20·3 to -7·2)	875·0 (405·3 to 1,713·2)	-8·7 (-16·7 to -0·9)	23·0 (10·6 to 45·0)	-14·5 (-22·0 to -7·2)
Other intestinal infectious diseases	0·8 (0·2 to 2·4)	-38·5 (-84·7 to 206·5)	0·0 (0·0 to 0·1)	-42·0 (-84·6 to 168·8)	56·6 (12·4 to 188·9)	-41·9 (-88·2 to 261·3)	1·6 (0·3 to 5·2)	-44·1 (-88·7 to 248·0)
Lower respiratory infections	1,266·3 (1,117·9 to 1,393·6)	-6·1 (-11·6 to -1·1)	43·8 (38·4 to 48·0)	-19·7 (-24·3 to -16·3)	49,968·0 (45,945·7 to 54,317·3)	-27·5 (-32·9 to -21·3)	1,449·9 (1,334·1 to 1,577·3)	-31·8 (-36·9 to -26·4)
Upper respiratory infections	1·3 (1·1 to 1·5)	-24·2 (-38·4 to -5·7)	0·0 (0·0 to 0·0)	-33·3 (-44·8 to -18·0)	70·8 (57·7 to 90·0)	-30·6 (-48·2 to -5·3)	2·0 (1·6 to 2·5)	-35·5 (-51·8 to -12·9)
Otitis media	0·6 (0·4 to 0·9)	-36·6 (-50·6 to -1·4)	0·0 (0·0 to 0·0)	-44·6 (-56·0 to -15·1)	27·5 (17·9 to 47·1)	-41·0 (-58·0 to -8·9)	0·8 (0·5 to 1·3)	-46·1 (-61·3 to -16·5)
Meningitis	176·9 (134·8 to 245·4)	-6·0 (-18·6 to 9·6)	5·0 (3·8 to 6·9)	-14·0 (-25·1 to -0·4)	11,254·8 (8,814·1 to 15,100·4)	-12·8 (-26·0 to 4·0)	307·8 (241·5 to 411·9)	-17·5 (-29·8 to -2·0)
Pneumococcal meningitis	14·4 (10·7 to 21·5)	7·5 (-6·7 to 24·8)	0·4 (0·3 to 0·6)	-5·7 (-17·4 to 8·2)	800·3 (595·4 to 1,106·2)	-0·2 (-15·7 to 20·5)	21·9 (16·4 to 30·1)	-7·6 (-21·3 to 10·9)
H influenzae type B meningitis	19·8 (15·2 to 27·9)	-0·4 (-16·0 to 20·1)	0·6 (0·4 to 0·8)	-7·5 (-21·8 to 11·1)	1,380·6 (1,065·9 to 1,865·0)	-5·0 (-21·5 to 17·3)	38·0 (29·4 to 51·2)	-9·2 (-25·0 to 12·0)
Meningococcal infection	77·5 (58·4 to 110·8)	-19·2 (-30·6 to -5·4)	2·2 (1·6 to 3·1)	-25·6 (-35·7 to -14·0)	4,970·8 (3,825·2 to 6,706·0)	-25·7 (-37·4 to -11·3)	135·7 (104·8 to 182·3)	-29·6 (-40·7 to -16·1)
Other meningitis	65·2 (49·0 to 90·3)	10·5 (-4·3 to 29·5)	1·8 (1·4 to 2·6)	0·5 (-12·1 to 16·6)	4,103·1 (3,165·3 to 5,479·0)	3·6 (-11·2 to 24·1)	112·1 (86·9 to 149·7)	-2·2 (-16·1 to 17·1)
Encephalitis	54·1 (44·1 to 77·4)	-1·9 (-19·5 to 20·0)	1·6 (1·3 to 2·3)	-14·1 (-28·3 to 3·6)	2,722·4 (2,181·0 to 3,869·7)	-11·7 (-31·3 to 11·9)	75·0 (60·2 to 106·1)	-18·3 (-35·9 to 3·0)
Diphtheria	0·6 (0·4 to 1·0)	-65·6 (-80·8 to -38·9)	0·0 (0·0 to 0·0)	-67·5 (-82·0 to -41·5)	46·6 (30·1 to 76·9)	-66·4 (-82·1 to -37·3)	1·3 (0·8 to 2·1)	-68·0 (-83·0 to -40·0)
Whooping cough	33·3 (17·4 to 57·3)	-36·2 (-64·0 to 21·4)	0·9 (0·5 to 1·6)	-38·1 (-65·2 to 17·6)	2,810·4 (1,468·3 to 4,847·3)	-36·1 (-64·0 to 21·3)	78·0 (40·8 to 134·5)	-38·1 (-65·1 to 17·6)
Tetanus	22·4 (11·8 to 30·1)	-56·9 (-64·8 to -46·5)	0·6 (0·3 to 0·8)	-59·9 (-67·0 to -50·5)	1,471·7 (761·7 to 2,026·7)	-60·0 (-68·8 to -48·5)	40·7 (21·0 to 56·1)	-61·4 (-69·9 to -50·5)
Measles	34·8 (13·1 to 74·8)	-72·4 (-77·2 to -67·3)	1·0 (0·4 to 2·1)	-73·4 (-78·1 to -68·6)	2,914·7 (1,097·4 to 6,264·2)	-72·3 (-77·2 to -67·2)	80·3 (30·2 to 172·5)	-73·4 (-78·1 to -68·5)
Varicella and herpes zoster	6·0 (5·4 to 6·8)	-15·9 (-24·9 to -4·9)	0·2 (0·2 to 0·2)	-30·2 (-36·9 to -22·2)	318·1 (278·9 to 366·7)	-21·5 (-33·1 to -6·6)	8·9 (7·8 to 10·3)	-26·9 (-37·5 to -13·3)
**Neglected tropical diseases and malaria**	435·1 (351·1 to 537·4)	-24·6 (-42·7 to -1·5)	12·1 (9·8 to 14·9)	-29·8 (-46·8 to -8·7)	31,623·3 (25,003·1 to 40,037·0)	-26·8 (-46·6 to -1·7)	867·6 (685·7 to 1,102·7)	-30·4 (-49·4 to -6·7)
Malaria	363·3 (281·8 to 463·8)	-26·4 (-47·3 to 2·1)	10·1 (7·8 to 12·9)	-31·0 (-50·5 to -5·0)	27,594·9 (20,952·6 to 36,113·2)	-28·1 (-49·5 to 2·1)	758·6 (572·8 to 994·7)	-31·4 (-52·0 to -2·7)
Chagas disease	4·0 (3·7 to 4·4)	-2·9 (-10·4 to 5·6)	0·1 (0·1 to 0·1)	-25·1 (-30·8 to -18·9)	93·5 (86·6 to 101·9)	-10·3 (-17·8 to -2·2)	2·8 (2·6 to 3·0)	-28·8 (-34·7 to -22·6)
Leishmaniasis	7·3 (4·4 to 11·6)	-54·2 (-58·3 to -49·5)	0·2 (0·1 to 0·3)	-58·9 (-62·5 to -54·7)	378·7 (227·8 to 598·7)	-57·8 (-61·9 to -53·2)	10·3 (6·2 to 16·2)	-60·9 (-64·7 to -56·8)
Visceral leishmaniasis	7·3 (4·4 to 11·6)	-54·2 (-58·3 to -49·5)	0·2 (0·1 to 0·3)	-58·9 (-62·5 to -54·7)	378·7 (227·8 to 598·7)	-57·8 (-61·9 to -53·2)	10·3 (6·2 to 16·2)	-60·9 (-64·7 to -56·8)
African trypanosomiasis	1·2 (0·5 to 2·3)	-76·1 (-87·8 to -56·6)	0·0 (0·0 to 0·1)	-78·9 (-89·2 to -62·7)	68·7 (29·6 to 126·9)	-76·0 (-88·2 to -54·5)	1·8 (0·8 to 3·3)	-78·1 (-89·2 to -58·1)
Schistosomiasis	5·5 (4·9 to 6·2)	-20·3 (-29·4 to -9·9)	0·2 (0·1 to 0·2)	-35·9 (-43·0 to -27·8)	212·8 (188·3 to 241·3)	-21·4 (-31·4 to -9·7)	5·8 (5·1 to 6·5)	-33·3 (-41·7 to -23·5)
Cysticercosis	0·5 (0·4 to 0·6)	-14·1 (-17·8 to -10·2)	0·0 (0·0 to 0·0)	-27·1 (-30·2 to -24·0)	25·2 (21·3 to 29·9)	-16·5 (-21·0 to -12·1)	0·7 (0·6 to 0·8)	-26·3 (-30·4 to -22·6)
Cystic echinococcosis	0·5 (0·5 to 0·7)	-35·7 (-40·0 to -31·2)	0·0 (0·0 to 0·0)	-45·5 (-48·9 to -42·1)	25·8 (20·7 to 32·1)	-40·5 (-45·9 to -35·2)	0·7 (0·6 to 0·9)	-47·0 (-51·6 to -42·5)
Dengue	21·9 (4·8 to 31·1)	88·5 (31·6 to 158·5)	0·6 (0·1 to 0·9)	67·6 (17·5 to 128·5)	1,165·2 (280·3 to 1,676·9)	68·9 (17·1 to 134·9)	31·2 (7·6 to 44·9)	55·3 (7·5 to 116·1)
Yellow fever	4·2 (0·9 to 12·3)	-12·1 (-29·9 to 10·2)	0·1 (0·0 to 0·3)	-19·0 (-35·2 to 1·3)	271·5 (57·7 to 773·7)	-12·2 (-31·6 to 13·2)	7·1 (1·5 to 20·3)	-17·6 (-36·0 to 6·1)
Rabies	8·5 (3·3 to 11·8)	-45·2 (-57·1 to -30·1)	0·2 (0·1 to 0·3)	-51·1 (-62·1 to -38·2)	496·5 (187·8 to 728·4)	-46·1 (-58·9 to -27·8)	13·2 (5·0 to 19·4)	-50·3 (-62·1 to -33·3)
Intestinal nematode infections	2·2 (1·7 to 2·8)	-36·8 (-52·3 to -17·7)	0·1 (0·0 to 0·1)	-40·7 (-55·2 to -22·9)	171·3 (129·6 to 220·9)	-37·7 (-53·8 to -17·5)	4·7 (3·5 to 6·1)	-41·1 (-56·3 to -21·8)
Ascariasis	2·2 (1·7 to 2·8)	-36·8 (-52·3 to -17·7)	0·1 (0·0 to 0·1)	-40·7 (-55·2 to -22·9)	171·3 (129·6 to 220·9)	-37·7 (-53·8 to -17·5)	4·7 (3·5 to 6·1)	-41·1 (-56·3 to -21·8)
Ebola	0·0 (0·0 to 0·0)	0·0 (0·0 to 0·0)	0·0 (0·0 to 0·0)	0·0 (0·0 to 0·0)	0·0 (0·0 to 0·0)	0·0 (0·0 to 0·0)	0·0 (0·0 to 0·0)	0·0 (0·0 to 0·0)
Zika virus	0·0 (0·0 to 0·0)	0·0 (0·0 to 0·0)	0·0 (0·0 to 0·0)	0·0 (0·0 to 0·0)	0·4 (0·1 to 1·3)	0·0 (0·0 to 0·0)	0·0 (0·0 to 0·0)	0·0 (0·0 to 0·0)
Other neglected tropical diseases	15·7 (10·1 to 21·7)	13·0 (-21·1 to 53·1)	0·4 (0·3 to 0·6)	3·3 (-26·5 to 39·2)	1,118·8 (612·8 to 1,611·2)	12·8 (-24·3 to 60·5)	30·8 (16·9 to 44·4)	7·0 (-27·9 to 52·0)
**Maternal disorders**	-	-	-	-	-	-	-	-
Maternal haemorrhage	-	-	-	-	-	-	-	-
Maternal sepsis and other pregnancy related infections	-	-	-	-	-	-	-	-
Maternal hypertensive disorders	-	-	-	-	-	-	-	-
Maternal obstructed labour and uterine rupture	-	-	-	-	-	-	-	-
Maternal abortion, miscarriage, and ectopic pregnancy	-	-	-	-	-	-	-	-
Indirect maternal deaths	-	-	-	-	-	-	-	-
Late maternal deaths	-	-	-	-	-	-	-	-
Maternal deaths aggravated by HIV/AIDS	-	-	-	-	-	-	-	-
Other maternal disorders	-	-	-	-	-	-	-	-
**Neonatal disorders**	982·6 (928·3 to 1,036·0)	-25·4 (-29·7 to -20·8)	27·7 (26·2 to 29·2)	-25·0 (-29·3 to -20·2)	85,051·7 (80,350·3 to 89,667·6)	-25·4 (-29·7 to -20·8)	2,398·1 (2,265·6 to 2,528·2)	-25·0 (-29·3 to -20·3)
Neonatal preterm birth complications	354·3 (321·2 to 389·1)	-26·9 (-34·6 to -19·2)	10·0 (9·1 to 11·0)	-26·4 (-34·2 to -18·7)	30,669·6 (27,801·9 to 33,683·1)	-26·9 (-34·6 to -19·2)	864·7 (783·9 to 949·7)	-26·4 (-34·2 to -18·7)

SR Table 2: Global deaths and YLL numbers, age-standardized rates per 100,000, and percent change between 2006 and 2016 for males for all GBD causes and Levels 1 through 4 of the cause hierarchy. 95% UIs are in parentheses. UI=uncertainty interval. YLL=year of life lost.

Neonatal encephalopathy due to birth asphyxia and trauma	306·6 (263·1 to 348·8)	-22·7 (-31·8 to -13·4)	8·6 (7·4 to 9·8)	-22·2 (-31·3 to -12·8)	26,543·3 (22,778·3 to 30,196·5)	-22·7 (-31·8 to -13·4)	748·3 (642·1 to 851·3)	-22·2 (-31·3 to -12·8)
Neonatal sepsis and other neonatal infections	137·6 (107·9 to 175·8)	-13·5 (-26·6 to 0·8)	3·9 (3·0 to 5·0)	-13·0 (-26·2 to 1·3)	11,905·9 (9,337·4 to 15,220·1)	-13·5 (-26·6 to 0·8)	335·9 (263·5 to 429·4)	-13·1 (-26·2 to 1·3)
Hemolytic disease and other neonatal jaundice	27·6 (22·9 to 33·3)	-42·7 (-53·8 to -32·1)	0·8 (0·6 to 0·9)	-42·4 (-53·5 to -31·7)	2,392·2 (1,982·1 to 2,881·2)	-42·7 (-53·8 to -32·1)	67·5 (55·9 to 81·3)	-42·4 (-53·5 to -31·7)
Other neonatal disorders	156·5 (137·5 to 176·9)	-31·7 (-40·0 to -22·4)	4·4 (3·9 to 5·0)	-31·3 (-39·6 to -21·9)	13,540·7 (11,898·1 to 15,312·1)	-31·7 (-40·0 to -22·4)	381·8 (335·5 to 431·7)	-31·3 (-39·6 to -21·9)
**Nutritional deficiencies**	183·8 (156·4 to 240·1)	-10·5 (-24·0 to 11·4)	5·9 (5·1 to 7·6)	-21·1 (-31·3 to -6·6)	10,322·4 (8,455·7 to 13,600·4)	-20·2 (-36·3 to 7·8)	290·8 (238·9 to 382·2)	-24·7 (-39·5 to 0·6)
Protein-energy malnutrition	158·6 (133·6 to 205·4)	-12·7 (-27·1 to 11·2)	5·1 (4·3 to 6·4)	-22·0 (-33·2 to -4·9)	9,453·3 (7,673·4 to 12,456·5)	-21·3 (-38·1 to 7·7)	266·0 (216·5 to 349·8)	-25·3 (-40·9 to 1·2)
Iodine deficiency	1·0 (0·6 to 1·6)	4·4 (-39·0 to 84·5)	0·0 (0·0 to 0·0)	-10·8 (-47·5 to 48·8)	47·2 (26·7 to 87·5)	-3·5 (-54·7 to 111·4)	1·3 (0·7 to 2·4)	-12·1 (-57·6 to 91·1)
Iron-deficiency anaemia	1·4 (1·1 to 1·9)	10·3 (-6·1 to 27·2)	0·0 (0·0 to 0·1)	-10·5 (-28·0 to 5·5)	57·6 (45·7 to 71·3)	7·0 (-6·6 to 22·3)	1·6 (1·2 to 1·9)	-4·8 (-19·6 to 9·5)
Other nutritional deficiencies	22·9 (16·3 to 33·4)	5·6 (-4·6 to 21·8)	0·8 (0·6 to 1·1)	-16·2 (-24·6 to -3·5)	764·3 (536·3 to 1,115·5)	-7·4 (-20·4 to 14·3)	21·9 (15·4 to 31·8)	-18·8 (-29·3 to -1·9)
**Other communicable, maternal, neonatal, and nutritional diseases**	184·5 (154·6 to 217·9)	-13·0 (-20·4 to -3·5)	5·4 (4·5 to 6·3)	-21·1 (-27·6 to -13·1)	10,714·3 (8,352·7 to 13,511·8)	-20·4 (-29·0 to -8·6)	296·7 (230·3 to 375·0)	-24·7 (-32·6 to -13·4)
Sexually transmitted diseases excluding HIV	53·9 (30·7 to 84·0)	-27·1 (-36·7 to -15·6)	1·5 (0·9 to 2·4)	-28·3 (-37·6 to -17·0)	4,547·6 (2,565·9 to 7,095·1)	-27·3 (-37·0 to -15·7)	127·2 (71·7 to 198·4)	-28·2 (-37·7 to -16·6)
Syphilis	53·7 (30·6 to 83·9)	-27·1 (-36·7 to -15·6)	1·5 (0·9 to 2·3)	-28·3 (-37·7 to -16·9)	4,540·0 (2,558·4 to 7,082·2)	-27·3 (-37·0 to -15·7)	127·0 (71·5 to 198·1)	-28·2 (-37·8 to -16·6)
Chlamydial infection	0·0 (0·0 to 0·0)	0·0 (0·0 to 0·0)	0·0 (0·0 to 0·0)	0·0 (0·0 to 0·0)	0·0 (0·0 to 0·0)	0·0 (0·0 to 0·0)	0·0 (0·0 to 0·0)	0·0 (0·0 to 0·0)
Gonococcal infection	0·1 (0·1 to 0·1)	-12·6 (-30·0 to 12·1)	0·0 (0·0 to 0·0)	-27·9 (-42·2 to -7·0)	5·0 (3·5 to 5·9)	-11·7 (-29·9 to 11·3)	0·1 (0·1 to 0·2)	-21·8 (-38·3 to -1·9)
Other sexually transmitted diseases	0·1 (0·0 to 0·1)	-12·8 (-28·9 to 12·1)	0·0 (0·0 to 0·0)	-30·5 (-43·2 to -10·1)	2·6 (1·8 to 3·1)	-12·6 (-29·3 to 10·9)	0·1 (0·0 to 0·1)	-24·3 (-39·3 to -3·6)
Hepatitis	77·8 (73·2 to 81·7)	-11·5 (-15·7 to -6·8)	2·2 (2·1 to 2·4)	-24·8 (-28·3 to -20·9)	3,198·3 (3,006·4 to 3,391·4)	-22·6 (-27·1 to -17·4)	86·4 (81·3 to 91·5)	-31·4 (-35·2 to -26·9)
Acute hepatitis A	2·7 (2·1 to 3·2)	-45·8 (-59·4 to -23·7)	0·1 (0·1 to 0·1)	-48·1 (-61·0 to -28·0)	190·3 (144·7 to 239·5)	-50·0 (-64·7 to -27·1)	5·3 (4·0 to 6·7)	-51·4 (-65·6 to -29·1)
Hepatitis B	60·0 (55·8 to 64·1)	-4·0 (-9·7 to 2·0)	1·7 (1·6 to 1·8)	-20·8 (-25·5 to -16·0)	2,224·2 (2,053·9 to 2,394·6)	-11·3 (-18·0 to -4·0)	60·0 (55·6 to 64·5)	-24·1 (-29·7 to -18·4)
Hepatitis C	1·3 (1·0 to 1·8)	2·4 (-15·3 to 23·8)	0·0 (0·0 to 0·1)	-18·3 (-32·8 to -1·3)	42·5 (32·6 to 55·5)	-5·2 (-19·9 to 13·0)	1·2 (0·9 to 1·5)	-20·8 (-33·2 to -6·0)
Acute hepatitis E	13·8 (11·6 to 16·3)	-28·2 (-36·4 to -18·5)	0·4 (0·3 to 0·5)	-33·7 (-41·1 to -25·4)	741·2 (633·3 to 851·0)	-38·3 (-45·9 to -29·5)	19·9 (17·0 to 22·9)	-42·2 (-49·2 to -34·3)
Other infectious diseases	52·8 (31·8 to 70·2)	5·0 (-16·4 to 37·1)	1·6 (1·0 to 2·1)	-5·7 (-22·3 to 20·9)	2,968·3 (1,524·3 to 4,344·1)	-3·6 (-30·7 to 38·9)	83·0 (42·5 to 121·8)	-8·8 (-34·0 to 31·2)
**Non-communicable diseases**	21,192·2 (20,722·1 to 21,674·0)	18·2 (16·0 to 20·7)	740·3 (724·6 to 756·0)	-10·9 (-12·3 to -9·2)	475,401·8 (463,874·5 to 488,096·6)	9·5 (7·1 to 12·2)	14,404·1 (14,061·1 to 14,781·0)	-12·5 (-14·2 to -10·4)
**Neoplasms**	5,172·2 (5,053·6 to 5,288·9)	20·0 (17·4 to 22·5)	171·9 (167·9 to 175·7)	-8·8 (-10·6 to -7·0)	119,980·3 (116,967·1 to 122,781·1)	14·1 (11·6 to 16·7)	3,580·8 (3,492·1 to 3,662·6)	-10·1 (-12·1 to -8·2)
Lip and oral cavity cancer	117·6 (111·5 to 123·5)	30·3 (23·1 to 36·8)	3·7 (3·5 to 3·8)	0·0 (-5·3 to 4·7)	3,120·3 (2,944·4 to 3,295·2)	25·5 (17·7 to 32·5)	89·1 (84·2 to 93·9)	-1·1 (-7·0 to 4·2)
Nasopharynx cancer	47·2 (44·3 to 50·3)	16·5 (7·4 to 24·2)	1·4 (1·3 to 1·5)	-8·6 (-15·5 to -2·7)	1,377·4 (1,289·0 to 1,475·0)	10·1 (1·1 to 18·3)	38·5 (36·0 to 41·1)	-11·1 (-18·2 to -4·8)
Other pharynx cancer	86·7 (78·6 to 92·4)	34·9 (21·0 to 45·9)	2·6 (2·4 to 2·8)	3·5 (-7·1 to 12·1)	2,301·5 (2,079·5 to 2,457·3)	30·8 (16·8 to 41·4)	65·6 (59·3 to 70·0)	2·2 (-8·6 to 10·5)
Oesophageal cancer	306·4 (296·2 to 318·2)	9·9 (5·8 to 14·7)	9·9 (9·6 to 10·3)	-16·7 (-19·6 to -13·1)	6,933·3 (6,693·1 to 7,212·6)	6·0 (1·9 to 10·9)	207·4 (200·3 to 215·7)	-18·3 (-21·3 to -14·6)
Stomach cancer	536·2 (520·2 to 553·4)	3·4 (-0·1 to 6·9)	17·9 (17·3 to 18·4)	-21·3 (-23·9 to -18·8)	11,738·3 (11,373·0 to 12,140·9)	-1·7 (-5·2 to 1·8)	355·0 (344·3 to 367·2)	-23·5 (-26·2 to -20·9)
Colon and rectum cancer	449·8 (429·6 to 469·5)	25·8 (19·4 to 31·2)	15·5 (14·8 to 16·2)	-6·0 (-10·6 to -2·1)	9,392·4 (8,946·9 to 9,779·0)	21·1 (14·5 to 26·3)	288·1 (274·5 to 300·0)	-6·5 (-11·4 to -2·4)
Liver cancer	590·2 (563·4 to 614·0)	22·9 (16·8 to 27·9)	18·3 (17·5 to 19·0)	-4·7 (-9·2 to -1·1)	15,579·0 (14,799·0 to 16,280·6)	16·5 (10·1 to 21·9)	446·0 (424·3 to 465·0)	-7·2 (-12·0 to -3·0)
Liver cancer due to hepatitis B	266·4 (232·5 to 296·4)	21·3 (14·5 to 27·2)	8·0 (7·0 to 8·9)	-5·0 (-9·9 to -0·6)	7,560·5 (6,646·6 to 8,372·2)	14·7 (7·5 to 20·7)	212·2 (186·9 to 235·4)	-7·8 (-13·2 to -3·1)
Liver cancer due to hepatitis C	98·6 (88·3 to 109·2)	27·8 (21·3 to 32·5)	3·3 (2·9 to 3·6)	-3·6 (-8·4 to -0·2)	2,134·8 (1,889·2 to 2,369·1)	23·5 (16·7 to 28·4)	64·6 (57·6 to 71·7)	-5·2 (-10·1 to -1·5)
Liver cancer due to alcohol use	57·2 (48·8 to 66·0)	26·5 (19·9 to 31·9)	1·8 (1·6 to 2·1)	-3·7 (-8·6 to 0·4)	1,353·3 (1,151·0 to 1,570·5)	23·7 (17·0 to 29·5)	40·1 (34·1 to 46·3)	-4·1 (-9·1 to 0·0)
Liver cancer due to other causes	168·0 (150·0 to 190·0)	21·5 (15·2 to 27·1)	5·2 (4·6 to 5·8)	-5·3 (-9·9 to -1·3)	4,530·4 (4,006·9 to 5,123·0)	14·5 (7·9 to 20·4)	129·1 (114·4 to 145·9)	-8·2 (-13·2 to -3·8)
Gallbladder and biliary tract cancer	66·8 (54·3 to 75·4)	22·0 (15·2 to 28·5)	2·3 (1·9 to 2·6)	-8·8 (-13·5 to -4·1)	1,355·8 (1,072·1 to 1,541·1)	17·3 (10·0 to 23·7)	41·9 (33·3 to 47·6)	-9·8 (-15·3 to -5·0)
Pancreatic cancer	213·4 (206·2 to 219·6)	32·0 (27·7 to 35·9)	7·1 (6·9 to 7·3)	-0·7 (-3·9 to 2·1)	4,599·4 (4,445·3 to 4,741·7)	27·7 (23·5 to 31·6)	139·7 (135·1 to 144·1)	-1·8 (-5·0 to 1·2)
Larynx cancer	95·2 (91·8 to 98·8)	14·6 (10·3 to 18·9)	3·0 (2·9 to 3·1)	-12·9 (-16·2 to -9·7)	2,294·6 (2,209·5 to 2,387·4)	10·8 (6·3 to 15·2)	67·2 (64·7 to 69·9)	-14·4 (-17·8 to -11·0)
Tracheal, bronchus, and lung cancer	1,176·6 (1,134·8 to 1,215·9)	16·9 (12·2 to 20·9)	39·1 (37·7 to 40·4)	-11·4 (-14·8 to -8·4)	25,101·7 (24,108·4 to 25,951·3)	11·9 (7·1 to 16·1)	765·8 (735·7 to 791·4)	-13·7 (-17·3 to -10·6)
Malignant skin melanoma	34·1 (29·9 to 36·9)	31·6 (21·8 to 39·2)	1·1 (1·0 to 1·2)	1·2 (-6·4 to 6·7)	833·8 (740·3 to 937·9)	23·1 (13·9 to 31·5)	24·4 (21·7 to 27·1)	-1·6 (-9·0 to 4·9)
Non-melanoma skin cancer	35·1 (33·7 to 36·6)	26·2 (21·6 to 31·2)	1·3 (1·2 to 1·3)	-6·5 (-9·6 to -3·1)	690·2 (660·8 to 721·1)	18·2 (13·5 to 23·5)	21·6 (20·6 to 22·5)	-8·3 (-11·7 to -4·5)
Non-melanoma skin cancer (squamous-cell carcinoma)	35·1 (33·7 to 36·6)	26·2 (21·6 to 31·2)	1·3 (1·2 to 1·3)	-6·5 (-9·6 to -3·1)	690·2 (660·8 to 721·1)	18·2 (13·5 to 23·5)	21·6 (20·6 to 22·5)	-8·3 (-11·7 to -4·5)
Breast cancer	10·2 (7·3 to 11·1)	40·3 (23·0 to 49·1)	0·3 (0·2 to 0·4)	5·0 (-7·4 to 11·3)	244·2 (171·1 to 265·9)	36·5 (17·8 to 45·7)	7·2 (5·1 to 7·8)	5·9 (-8·1 to 12·9)
Cervical cancer	-	-	-	-	-	-	-	-
Uterine cancer	-	-	-	-	-	-	-	-
Ovarian cancer	-	-	-	-	-	-	-	-
Prostate cancer	380·9 (320·8 to 412·9)	30·8 (24·5 to 36·6)	14·9 (12·7 to 16·2)	-6·4 (-10·5 to -2·1)	5,540·6 (4,536·2 to 5,992·1)	26·5 (19·3 to 32·2)	192·7 (159·2 to 208·2)	-6·3 (-11·4 to -2·1)
Testicular cancer	8·7 (8·3 to 9·0)	2·9 (-1·1 to 7·0)	0·2 (0·2 to 0·3)	-12·6 (-15·8 to -9·2)	368·1 (350·8 to 386·9)	-1·8 (-6·0 to 2·6)	9·7 (9·2 to 10·2)	-13·9 (-17·5 to -10·0)
Kidney cancer	86·1 (82·5 to 89·4)	30·3 (25·0 to 35·6)	2·9 (2·8 to 3·0)	-1·6 (-5·5 to 2·2)	1,950·8 (1,862·6 to 2,041·7)	24·5 (18·6 to 30·3)	58·6 (56·1 to 61·2)	-2·7 (-7·1 to 1·6)
Bladder cancer	137·9 (133·1 to 142·4)	25·2 (20·8 to 29·1)	5·1 (4·9 to 5·3)	-7·9 (-11·0 to -5·1)	2,380·4 (2,292·4 to 2,462·5)	19·3 (14·4 to 23·6)	77·5 (74·6 to 80·1)	-9·6 (-13·0 to -6·5)
Brain and nervous system cancer	128·3 (111·4 to 140·6)	22·7 (16·5 to 32·4)	3·8 (3·3 to 4·1)	-1·2 (-6·3 to 6·8)	4,368·0 (3,776·1 to 4,858·8)	14·5 (8·8 to 23·3)	120·7 (104·5 to 133·7)	-3·2 (-8·1 to 4·6)
Thyroid cancer	17·0 (16·0 to 17·8)	30·7 (23·9 to 36·9)	0·6 (0·5 to 0·6)	0·1 (-4·9 to 4·8)	421·9 (394·6 to 443·1)	24·5 (17·2 to 30·8)	12·3 (11·5 to 12·9)	-1·2 (-6·8 to 3·8)
Mesothelioma	22·1 (20·2 to 23·8)	31·2 (23·6 to 37·5)	0·7 (0·7 to 0·8)	-0·8 (-6·5 to 4·0)	468·0 (429·6 to 501·0)	25·9 (18·3 to 31·9)	14·3 (13·1 to 15·2)	-2·3 (-8·0 to 2·3)
Hodgkin lymphoma	18·7 (15·5 to 23·4)	-5·0 (-9·6 to -0·5)	0·5 (0·5 to 0·7)	-21·5 (-25·3 to -18·0)	735·3 (594·7 to 932·7)	-10·7 (-15·2 to -6·5)	19·9 (16·1 to 25·1)	-22·3 (-26·0 to -18·8)
Non-Hodgkin's lymphoma	139·1 (122·6 to 145·9)	29·7 (21·3 to 34·2)	4·5 (4·0 to 4·7)	0·4 (-6·1 to 3·7)	4,031·0 (3,512·3 to 4,259·3)	23·4 (15·8 to 28·5)	115·2 (100·3 to 121·5)	1·9 (-4·8 to 5·7)
Multiple myeloma	51·0 (42·3 to 57·9)	31·9 (26·0 to 38·3)	1·7 (1·4 to 2·0)	-1·5 (-5·6 to 3·1)	1,093·8 (910·2 to 1,242·9)	28·4 (21·2 to 35·1)	33·3 (27·7 to 37·9)	-1·0 (-6·2 to 4·1)
Leukaemia	180·4 (165·5 to 194·3)	13·2 (10·6 to 15·9)	5·8 (5·3 to 6·2)	-8·9 (-10·9 to -6·8)	5,906·5 (5,351·2 to 6,424·0)	2·1 (-1·5 to 5·4)	166·3 (151·1 to 180·7)	-11·6 (-14·5 to -8·9)
Acute lymphoid leukaemia	30·6 (27·9 to 34·1)	17·6 (10·5 to 24·0)	0·9 (0·8 to 1·0)	1·5 (-3·9 to 6·3)	1,456·7 (1,324·4 to 1,627·8)	9·1 (-0·7 to 17·6)	39·2 (35·7 to 43·7)	-0·9 (-9·3 to 6·7)
Chronic lymphoid leukaemia	20·9 (19·2 to 24·8)	23·5 (18·2 to 29·2)	0·8 (0·7 to 0·9)	-8·8 (-12·7 to -4·5)	389·6 (358·8 to 471·6)	17·3 (12·0 to 22·7)	12·4 (11·4 to 15·0)	-9·6 (-13·6 to -5·4)
Acute myeloid leukaemia	49·2 (43·7 to 53·8)	25·8 (20·5 to 30·7)	1·6 (1·4 to 1·7)	-0·2 (-4·2 to 3·4)	1,537·6 (1,386·6 to 1,726·5)	17·4 (11·4 to 22·5)	43·6 (39·2 to 48·6)	-0·6 (-5·5 to 3·5)
Chronic myeloid leukaemia	12·4 (11·3 to 13·8)	0·3 (-3·9 to 5·5)	0·4 (0·4 to 0·5)	-22·4 (-25·6 to -18·5)	343·9 (311·4 to 390·8)	-4·1 (-8·4 to 1·8)	9·8 (8·9 to 11·0)	-21·8 (-25·3 to -17·5)
Other leukaemia	67·4 (58·8 to 73·8)	3·6 (0·0 to 8·1)	2·2 (1·9 to 2·3)	-15·0 (-17·8 to -11·7)	2,178·7 (1,870·2 to 2,368·7)	-11·0 (-15·0 to -6·3)	61·4 (52·8 to 66·8)	-21·8 (-25·1 to -18·0)
Other neoplasms	236·5 (204·7 to 246·0)	32·6 (25·0 to 36·6)	7·5 (6·6 to 7·8)	4·3 (-2·1 to 7·4)	7,154·0 (6,185·5 to 7,479·2)	23·4 (17·9 to 27·7)	202·7 (175·7 to 211·7)	3·5 (-1·4 to 6·9)
**Cardiovascular diseases**	9,269·8 (9,019·3 to 9,518·9)	18·1 (15·4 to 21·1)	330·5 (321·9 to 338·7)	-12·2 (-14·0 to -10·1)	188,488·2 (183,067·8 to 194,328·5)	11·3 (8·4 to 14·7)	5,824·0 (5,659·6 to 5,997·2)	-13·3 (-15·5 to -10·9)

SR Table 2: Global deaths and YLL numbers, age-standardized rates per 100,000, and percent change between 2006 and 2016 for males for all GBD causes and Levels 1 through 4 of the cause hierarchy. 95% UIs are in parentheses. UI=uncertainty interval. YLL=year of life lost.

Rheumatic heart disease	131·5 (124·7 to 138·8)	-5·8 (-13·0 to 2·5)	4·4 (4·1 to 4·6)	-25·4 (-30·6 to -19·3)	3,746·4 (3,525·9 to 3,965·3)	-15·2 (-22·6 to -7·3)	107·1 (101·0 to 113·2)	-28·5 (-34·4 to -22·0)
Ischaemic heart disease	5,110·3 (4,953·2 to 5,270·6)	21·8 (18·6 to 25·3)	182·8 (177·4 to 188·3)	-9·8 (-12·0 to -7·4)	103,155·6 (99,825·9 to 106,713·2)	15·4 (12·0 to 19·4)	3,186·4 (3,084·5 to 3,295·5)	-10·7 (-13·2 to -7·8)
Cerebrovascular disease	2,931·8 (2,831·8 to 3,029·3)	10·5 (7·3 to 13·8)	103·2 (99·4 to 106·9)	-17·7 (-20·0 to -15·4)	59,094·0 (57,176·0 to 61,000·6)	5·7 (2·5 to 9·1)	1,834·8 (1,774·7 to 1,892·7)	-18·2 (-20·7 to -15·7)
Ischaemic stroke	1,343·5 (1,280·6 to 1,405·5)	15·5 (11·4 to 19·6)	50·2 (47·9 to 52·6)	-15·7 (-18·5 to -12·9)	22,206·9 (21,146·4 to 23,259·0)	11·0 (6·6 to 15·3)	736·1 (700·9 to 770·6)	-16·4 (-19·5 to -13·2)
Hemorrhagic stroke	1,588·3 (1,528·3 to 1,648·1)	6·6 (3·6 to 9·9)	53·0 (50·9 to 55·1)	-19·6 (-21·8 to -17·3)	36,887·1 (35,556·7 to 38,233·2)	2·7 (-0·3 to 6·0)	1,098·7 (1,059·6 to 1,139·9)	-19·5 (-21·8 to -17·0)
Hypertensive heart disease	398·1 (299·6 to 439·1)	36·7 (19·4 to 46·6)	14·7 (10·9 to 16·2)	1·1 (-12·3 to 7·9)	7,413·4 (5,803·5 to 8,297·2)	26·3 (13·6 to 36·1)	235·1 (181·5 to 262·8)	-1·9 (-12·6 to 5·4)
Cardiomyopathy and myocarditis	189·6 (154·6 to 213·5)	12·0 (1·3 to 25·3)	6·3 (5·1 to 7·0)	-12·5 (-20·0 to -3·0)	5,243·5 (4,406·7 to 6,012·5)	1·1 (-10·5 to 16·0)	150·1 (125·4 to 171·5)	-16·0 (-25·2 to -4·3)
Myocarditis	21·4 (16·3 to 23·9)	11·8 (1·1 to 25·5)	0·7 (0·6 to 0·8)	-12·9 (-22·0 to 0·8)	683·6 (538·7 to 787·9)	-2·5 (-12·6 to 11·5)	19·4 (15·1 to 22·1)	-14·9 (-23·8 to -2·9)
Alcoholic cardiomyopathy	61·3 (46·6 to 79·3)	-2·7 (-23·1 to 24·6)	1·8 (1·4 to 2·3)	-21·5 (-37·0 to -1·6)	1,905·7 (1,428·5 to 2,525·2)	-9·7 (-31·1 to 19·9)	52·4 (39·5 to 69·2)	-25·7 (-42·8 to -2·1)
Other cardiomyopathy	106·9 (85·1 to 115·5)	22·5 (14·5 to 31·5)	3·8 (3·0 to 4·1)	-7·3 (-13·2 to 0·0)	2,654·1 (2,196·1 to 2,936·0)	11·8 (3·0 to 21·0)	78·4 (64·4 to 86·0)	-8·3 (-15·0 to -0·9)
Atrial fibrillation and flutter	91·5 (73·0 to 113·1)	48·5 (44·5 to 52·5)	4·0 (3·2 to 5·0)	0·2 (-1·9 to 2·3)	984·8 (798·9 to 1,195·3)	39·5 (35·9 to 43·2)	37·9 (30·7 to 46·0)	-0·3 (-2·5 to 1·7)
Aortic aneurysm	103·7 (99·7 to 108·5)	18·3 (13·3 to 24·8)	3·8 (3·6 to 3·9)	-13·0 (-16·4 to -8·4)	1,950·0 (1,870·0 to 2,044·6)	13·3 (7·9 to 20·3)	61·8 (59·3 to 64·7)	-13·0 (-17·0 to -8·0)
Peripheral vascular disease	28·2 (21·5 to 38·7)	33·9 (21·7 to 49·5)	1·1 (0·9 to 1·6)	-4·7 (-12·7 to 5·4)	399·7 (295·0 to 523·4)	25·3 (12·3 to 41·9)	13·9 (10·4 to 18·5)	-6·7 (-15·6 to 4·8)
Endocarditis	45·0 (37·3 to 56·4)	32·6 (27·5 to 37·8)	1·5 (1·2 to 1·9)	2·5 (-1·1 to 6·0)	1,254·7 (1,042·5 to 1,601·6)	21·6 (15·5 to 27·6)	36·2 (30·1 to 46·2)	1·7 (-3·1 to 6·3)
Other cardiovascular and circulatory diseases	240·1 (208·3 to 303·7)	21·2 (17·3 to 26·9)	8·7 (7·7 to 10·9)	-9·1 (-12·0 to -5·4)	5,246·2 (4,473·8 to 6,783·7)	9·7 (5·3 to 16·3)	160·7 (137·4 to 206·9)	-11·1 (-14·4 to -6·5)
**Chronic respiratory diseases**	2,029·9 (1,963·3 to 2,091·7)	7·8 (4·9 to 10·9)	74·7 (72·3 to 76·9)	-20·3 (-22·4 to -18·1)	36,301·5 (35,009·8 to 37,509·7)	1·5 (-1·4 to 4·6)	1,175·0 (1,133·5 to 1,213·5)	-21·8 (-24·0 to -19·5)
Chronic obstructive pulmonary disease	1,697·5 (1,613·7 to 1,777·5)	8·2 (5·3 to 11·5)	63·5 (60·6 to 66·3)	-20·5 (-22·6 to -18·2)	28,245·8 (26,478·4 to 29,826·0)	2·7 (-0·6 to 6·2)	935·7 (882·4 to 985·7)	-21·9 (-24·2 to -19·4)
Pneumoconiosis	19·0 (18·0 to 20·6)	1·1 (-6·7 to 7·3)	0·7 (0·6 to 0·7)	-23·7 (-28·8 to -19·3)	370·3 (348·2 to 404·9)	-5·7 (-14·1 to 0·6)	11·6 (10·9 to 12·6)	-26·3 (-32·6 to -21·7)
Silicosis	9·8 (9·0 to 10·9)	-2·0 (-15·3 to 5·5)	0·3 (0·3 to 0·4)	-25·9 (-35·1 to -20·6)	200·0 (183·9 to 220·5)	-7·7 (-22·0 to -0·1)	6·1 (5·7 to 6·8)	-27·9 (-38·6 to -22·0)
Asbestosis	2·9 (1·8 to 3·4)	23·4 (13·6 to 34·5)	0·1 (0·1 to 0·1)	-8·4 (-15·7 to -0·4)	48·5 (33·8 to 58·6)	14·0 (5·3 to 24·1)	1·6 (1·1 to 1·9)	-11·3 (-18·2 to -3·0)
Coal workers pneumoconiosis	2·4 (1·5 to 2·8)	-12·4 (-20·9 to -0·2)	0·1 (0·1 to 0·1)	-34·9 (-41·0 to -25·9)	42·5 (25·5 to 50·3)	-17·1 (-25·9 to -6·3)	1·4 (0·8 to 1·6)	-36·5 (-43·1 to -28·2)
Other pneumoconiosis	3·9 (3·2 to 5·5)	5·3 (-4·8 to 15·6)	0·1 (0·1 to 0·2)	-19·9 (-27·9 to -12·2)	79·2 (64·8 to 108·7)	-3·4 (-12·3 to 6·2)	2·5 (2·0 to 3·4)	-23·7 (-31·1 to -16·0)
Asthma	213·4 (156·4 to 290·7)	-4·2 (-12·7 to 5·6)	7·0 (5·2 to 9·6)	-26·5 (-33·5 to -18·8)	5,482·3 (4,058·8 to 7,118·0)	-10·2 (-17·2 to -2·0)	160·5 (118·7 to 210·6)	-27·4 (-33·3 to -20·6)
Interstitial lung disease and pulmonary sarcoidosis	71·7 (46·9 to 85·6)	44·5 (21·9 to 59·7)	2·6 (1·7 to 3·1)	6·9 (-8·9 to 17·5)	1,320·5 (886·1 to 1,609·4)	37·1 (15·6 to 53·7)	42·2 (28·0 to 51·1)	5·7 (-10·4 to 17·8)
Other chronic respiratory diseases	28·3 (18·7 to 35·1)	23·2 (13·6 to 33·9)	0·9 (0·6 to 1·2)	-3·6 (-9·9 to 3·0)	882·7 (578·9 to 1,109·0)	12·1 (0·4 to 26·4)	25·0 (16·5 to 31·3)	-5·1 (-14·0 to 5·6)
**Cirrhosis and other chronic liver diseases**	856·8 (804·7 to 967·6)	14·2 (7·5 to 21·9)	25·5 (23·9 to 28·5)	-9·8 (-15·0 to -3·9)	26,793·8 (25,106·4 to 30,695·5)	9·1 (2·4 to 17·0)	739·0 (692·9 to 845·0)	-10·8 (-16·3 to -4·6)
Cirrhosis and other chronic liver diseases due to hepatitis B	271·8 (243·2 to 323·3)	13·9 (6·3 to 22·4)	8·1 (7·2 to 9·5)	-9·9 (-15·7 to -3·1)	8,459·5 (7,579·8 to 10,116·8)	9·4 (1·5 to 17·8)	233·2 (209·6 to 278·1)	-10·8 (-17·1 to -3·8)
Cirrhosis and other chronic liver diseases due to hepatitis C	236·6 (213·8 to 267·9)	16·3 (9·5 to 24·2)	7·0 (6·4 to 7·9)	-8·8 (-14·2 to -2·8)	7,212·1 (6,472·9 to 8,179·7)	12·6 (5·8 to 20·4)	199·4 (179·2 to 225·9)	-9·1 (-14·5 to -3·1)
Cirrhosis and other chronic liver diseases due to alcohol use	233·9 (211·7 to 263·0)	14·7 (8·4 to 22·2)	7·0 (6·4 to 7·8)	-10·2 (-15·0 to -4·4)	6,965·9 (6,268·5 to 7,878·4)	10·4 (4·1 to 18·4)	193·2 (174·5 to 219·5)	-11·1 (-16·1 to -4·9)
Cirrhosis and other chronic liver diseases due to other causes	114·6 (100·6 to 133·6)	9·8 (2·5 to 18·1)	3·4 (3·0 to 3·9)	-10·8 (-16·3 to -4·6)	4,156·3 (3,633·0 to 4,920·1)	1·3 (-6·4 to 9·6)	113·2 (99·2 to 133·8)	-13·3 (-19·4 to -6·7)
**Digestive diseases**	573·8 (540·8 to 653·4)	10·1 (6·1 to 16·5)	19·3 (18·2 to 22·1)	-14·8 (-17·7 to -9·7)	15,595·5 (14,681·4 to 17,434·1)	0·8 (-3·8 to 6·4)	452·7 (426·8 to 507·0)	-16·4 (-19·9 to -12·1)
Peptic ulcer disease	141·2 (128·4 to 164·7)	-6·9 (-11·8 to -1·3)	4·7 (4·3 to 5·4)	-28·4 (-32·2 to -24·1)	3,535·1 (3,181·2 to 4,172·0)	-13·6 (-18·3 to -8·4)	103·4 (93·2 to 121·9)	-30·2 (-33·9 to -26·0)
Gastritis and duodenitis	24·9 (22·2 to 28·5)	9·5 (2·3 to 17·8)	0·8 (0·7 to 0·9)	-16·6 (-22·2 to -10·6)	653·0 (580·0 to 753·9)	3·7 (-5·3 to 13·2)	19·0 (16·9 to 21·8)	-16·1 (-22·2 to -9·0)
Appendicitis	23·7 (20·9 to 28·6)	-0·2 (-7·7 to 8·0)	0·7 (0·6 to 0·9)	-17·6 (-24·7 to -10·9)	903·9 (794·8 to 1,092·1)	-9·8 (-16·1 to -1·5)	24·7 (21·7 to 29·7)	-20·6 (-26·2 to -13·6)
Paralytic ileus and intestinal obstruction	130·9 (109·9 to 163·2)	18·0 (11·4 to 26·5)	4·3 (3·7 to 5·4)	-6·5 (-11·2 to -0·7)	4,184·3 (3,462·9 to 5,010·8)	4·6 (-3·0 to 12·7)	120·1 (99·7 to 144·3)	-9·3 (-15·4 to -2·9)
Inguinal, femoral, and abdominal hernia	26·6 (20·1 to 33·2)	6·9 (-1·7 to 14·5)	0·9 (0·7 to 1·2)	-18·5 (-25·7 to -12·6)	651·4 (463·5 to 798·7)	-3·3 (-9·2 to 3·3)	19·4 (13·8 to 23·7)	-20·6 (-26·1 to -15·4)
Inflammatory bowel disease	19·6 (16·3 to 21·5)	16·6 (3·2 to 31·8)	0·7 (0·6 to 0·7)	-12·0 (-20·5 to -0·2)	509·8 (424·4 to 583·2)	8·0 (-8·7 to 22·4)	14·9 (12·4 to 16·9)	-11·4 (-23·4 to -0·3)
Vascular intestinal disorders	45·6 (39·3 to 57·2)	26·0 (17·6 to 35·8)	1·7 (1·4 to 2·1)	-6·7 (-12·5 to 0·6)	858·6 (751·3 to 1,090·4)	18·6 (9·0 to 29·3)	27·3 (23·8 to 34·8)	-8·1 (-15·0 to -0·4)
Gallbladder and biliary diseases	43·8 (39·8 to 55·4)	28·3 (21·7 to 37·4)	1·6 (1·5 to 2·1)	-4·9 (-9·7 to 2·4)	858·4 (782·9 to 1,077·7)	17·7 (12·0 to 24·4)	26·9 (24·5 to 33·9)	-7·0 (-11·5 to -1·7)
Pancreatitis	69·3 (57·7 to 79·7)	17·0 (8·8 to 26·7)	2·1 (1·7 to 2·4)	-6·6 (-13·0 to 0·9)	2,237·2 (1,855·0 to 2,555·1)	11·1 (2·6 to 20·8)	61·4 (51·0 to 70·0)	-7·8 (-14·7 to 0·1)
Other digestive diseases	48·2 (40·5 to 57·4)	17·9 (10·6 to 26·4)	1·7 (1·4 to 2·0)	-10·5 (-15·3 to -5·4)	1,203·7 (1,053·3 to 1,448·5)	6·2 (-4·1 to 19·2)	35·7 (31·1 to 42·9)	-12·6 (-19·8 to -4·0)
**Neurological disorders**	1,092·4 (966·8 to 1,245·8)	42·8 (40·0 to 45·5)	46·1 (40·7 to 52·6)	0·6 (-0·8 to 1·9)	15,759·1 (14,392·6 to 17,521·6)	23·3 (20·1 to 27·0)	547·0 (494·8 to 612·7)	-2·5 (-4·3 to -0·4)
Alzheimer's disease and other dementias	842·8 (720·4 to 1,000·8)	50·0 (46·8 to 53·1)	37·5 (32·1 to 44·2)	1·3 (-0·1 to 2·8)	8,805·0 (7,558·1 to 10,493·9)	41·3 (38·5 to 44·2)	344·8 (296·1 to 410·3)	0·9 (-0·6 to 2·5)
Parkinson's disease	117·8 (93·7 to 147·6)	44·2 (40·3 to 47·9)	4·7 (3·8 to 5·9)	2·2 (-0·2 to 4·3)	1,465·4 (1,163·2 to 1,824·5)	38·9 (35·6 to 42·0)	53·9 (42·6 to 66·9)	2·4 (0·1 to 4·5)
Epilepsy	75·1 (70·3 to 81·0)	-0·8 (-6·1 to 7·1)	2·1 (2·0 to 2·2)	-13·6 (-18·0 to -7·0)	3,629·9 (3,375·5 to 3,936·2)	-6·6 (-12·6 to 2·3)	96·1 (89·4 to 104·1)	-15·5 (-20·8 to -7·5)
Multiple sclerosis	7·7 (6·5 to 8·9)	17·0 (7·3 to 23·5)	0·2 (0·2 to 0·3)	-7·5 (-14·9 to -2·8)	234·5 (205·5 to 281·8)	12·0 (2·0 to 19·4)	6·5 (5·6 to 7·7)	-9·2 (-17·0 to -3·4)
Motor neuron disease	19·3 (18·3 to 20·1)	26·4 (20·3 to 30·9)	0·6 (0·6 to 0·6)	-2·6 (-7·3 to 0·9)	500·6 (473·1 to 519·7)	19·4 (13·3 to 23·4)	14·6 (13·8 to 15·2)	-3·9 (-8·6 to -0·7)
Other neurological disorders	29·8 (27·6 to 31·2)	25·4 (17·6 to 30·8)	0·9 (0·8 to 1·0)	3·1 (-2·7 to 7·2)	1,123·7 (1,059·1 to 1,174·1)	14·8 (6·1 to 21·0)	31·1 (29·3 to 32·4)	1·7 (-5·4 to 7·0)
**Mental and substance use disorders**	247·8 (211·7 to 271·7)	6·5 (-1·9 to 16·2)	6·9 (5·9 to 7·5)	-11·7 (-18·4 to -3·9)	9,586·2 (8,255·1 to 10,556·5)	1·9 (-6·6 to 11·8)	253·6 (218·1 to 279·0)	-13·5 (-20·7 to -5·1)
Alcohol use disorders	147·6 (120·1 to 164·3)	2·4 (-6·9 to 13·0)	4·1 (3·3 to 4·5)	-16·4 (-23·8 to -8·0)	5,361·0 (4,350·2 to 6,003·0)	-1·8 (-11·3 to 9·0)	142·9 (116·0 to 159·9)	-18·1 (-26·0 to -9·2)
Drug use disorders	100·0 (86·3 to 114·9)	13·1 (-1·1 to 28·3)	2·8 (2·4 to 3·2)	-3·7 (-16·4 to 8·8)	4,216·5 (3,669·8 to 4,858·0)	6·9 (-6·3 to 22·3)	110·6 (96·0 to 127·3)	-6·7 (-18·4 to 6·6)
Opioid use disorders	63·2 (51·3 to 72·5)	13·7 (-3·3 to 34·1)	1·7 (1·4 to 2·0)	-2·1 (-16·9 to 14·4)	2,772·9 (2,269·2 to 3,192·2)	7·6 (-8·2 to 28·3)	72·3 (59·2 to 83·2)	-5·6 (-19·5 to 12·3)
Cocaine use disorders	6·7 (4·6 to 8·5)	2·7 (-6·9 to 14·9)	0·2 (0·1 to 0·2)	-14·2 (-22·1 to -4·1)	271·6 (184·1 to 349·4)	-1·3 (-10·8 to 10·6)	7·1 (4·9 to 9·1)	-15·6 (-23·7 to -5·5)
Amphetamine use disorders	3·9 (2·9 to 5·2)	11·4 (-1·3 to 26·2)	0·1 (0·1 to 0·1)	-6·0 (-16·5 to 6·3)	168·4 (126·8 to 223·8)	7·3 (-6·0 to 21·9)	4·4 (3·3 to 5·8)	-7·3 (-18·2 to 5·3)
Other drug use disorders	26·2 (22·6 to 34·3)	14·8 (0·8 to 28·1)	0·8 (0·7 to 1·0)	-4·0 (-15·8 to 6·8)	1,003·6 (860·6 to 1,339·7)	7·4 (-5·6 to 20·0)	26·7 (23·0 to 35·6)	-7·1 (-18·5 to 3·7)
Eating disorders	0·2 (0·1 to 0·2)	-5·8 (-10·6 to 8·0)	0·0 (0·0 to 0·0)	-16·4 (-20·6 to -4·2)	8·7 (7·3 to 10·1)	-7·8 (-12·6 to 6·4)	0·2 (0·2 to 0·3)	-17·3 (-21·5 to -4·7)
Anorexia nervosa	0·1 (0·1 to 0·2)	-8·3 (-12·9 to 6·5)	0·0 (0·0 to 0·0)	-18·1 (-22·2 to -4·8)	7·7 (6·5 to 8·9)	-10·1 (-14·8 to 4·5)	0·2 (0·2 to 0·2)	-18·9 (-23·1 to -5·5)
Bulimia nervosa	0·0 (0·0 to 0·0)	13·4 (-0·1 to 24·9)	0·0 (0·0 to 0·0)	-3·4 (-14·4 to 6·3)	1·1 (0·8 to 1·4)	12·5 (-1·4 to 24·0)	0·0 (0·0 to 0·0)	-3·1 (-14·7 to 6·6)
**Diabetes, urogenital, blood, and endocrine diseases**	1,591·0 (1,539·7 to 1,646·7)	31·3 (28·7 to 34·0)	55·1 (53·3 to 56·9)	-0·4 (-2·3 to 1·6)	37,958·9 (36,688·7 to 39,675·5)	19·1 (16·7 to 22·2)	1,133·5 (1,095·6 to 1,182·6)	-3·0 (-4·9 to -0·6)
Diabetes mellitus	677·6 (657·1 to 696·1)	35·7 (32·8 to 38·6)	23·3 (22·6 to 24·0)	1·3 (-0·9 to 3·4)	14,410·5 (13,986·7 to 14,806·3)	29·4 (26·5 to 32·4)	439·8 (427·3 to 452·0)	0·6 (-1·6 to 2·9)
Acute glomerulonephritis	6·5 (6·1 to 6·9)	15·8 (8·4 to 23·3)	0·2 (0·2 to 0·2)	-6·4 (-12·6 to -0·2)	188·8 (177·4 to 201·6)	0·1 (-6·7 to 6·3)	5·4 (5·1 to 5·7)	-14·3 (-19·9 to -8·9)
Chronic kidney disease	645·7 (605·3 to 684·2)	31·1 (26·5 to 35·4)	22·5 (21·2 to 23·8)	-0·7 (-4·2 to 2·2)	15,264·0 (14,296·6 to 16,263·0)	19·2 (15·4 to 24·5)	454·1 (425·4 to 482·4)	-3·3 (-6·3 to 0·6)
Chronic kidney disease due to diabetes mellitus	288·0 (257·8 to 315·7)	32·5 (27·4 to 36·7)	9·7 (8·7 to 10·7)	0·1 (-3·6 to 3·1)	6,627·5 (5,934·7 to 7,260·8)	24·8 (20·3 to 29·5)	197·4 (176·3 to 215·7)	-1·6 (-5·0 to 1·8)

SR Table 2: Global deaths and YLL numbers, age-standardized rates per 100,000, and percent change between 2006 and 2016 for males for all GBD causes and Levels 1 through 4 of the cause hierarchy. 95% UIs are in parentheses. UI=uncertainty interval. YLL=year of life lost.

Chronic kidney disease due to hypertension	154·1 (136·2 to 174·1)	38·1 (32·1 to 42·8)	5·9 (5·2 to 6·7)	-0·6 (-5·0 to 2·4)	2,746·2 (2,416·3 to 3,153·9)	28·6 (23·1 to 33·7)	88·2 (78·1 to 100·6)	-1·0 (-5·1 to 2·5)
Chronic kidney disease due to glomerulonephritis	75·5 (66·3 to 86·5)	18·1 (14·0 to 23·5)	2·4 (2·1 to 2·8)	-5·5 (-8·3 to -2·1)	2,456·6 (2,150·3 to 2,836·4)	6·5 (2·3 to 12·7)	68·6 (60·6 to 78·5)	-8·1 (-11·3 to -3·5)
Chronic kidney disease due to other causes	128·1 (111·6 to 147·3)	28·4 (23·8 to 34·6)	4·4 (3·9 to 5·1)	0·0 (-3·6 to 3·6)	3,433·7 (3,034·6 to 3,918·3)	12·4 (8·0 to 19·2)	99·9 (88·2 to 114·0)	-4·9 (-8·3 to 0·4)
Urinary diseases and male infertility	137·1 (132·4 to 142·2)	29·0 (24·9 to 33·8)	5·1 (4·9 to 5·3)	-2·9 (-5·7 to 0·4)	3,153·6 (3,033·0 to 3,287·7)	12·6 (7·9 to 18·0)	95·9 (92·3 to 99·8)	-6·5 (-10·0 to -2·4)
Interstitial nephritis and urinary tract infections	88·5 (79·6 to 95·1)	39·3 (32·2 to 46·8)	3·4 (3·1 to 3·6)	3·2 (-1·5 to 8·2)	1,914·0 (1,699·0 to 2,083·6)	22·0 (13·5 to 30·3)	59·2 (53·0 to 64·3)	0·6 (-5·5 to 6·8)
Urolithiasis	9·8 (8·1 to 14·9)	11·1 (-0·2 to 36·5)	0·3 (0·3 to 0·5)	-14·7 (-23·2 to 4·2)	227·9 (186·8 to 354·0)	0·8 (-10·4 to 24·9)	6·8 (5·6 to 10·4)	-17·8 (-26·8 to 1·8)
Other urinary diseases	38·9 (32·9 to 45·2)	14·3 (5·9 to 29·4)	1·4 (1·2 to 1·6)	-12·5 (-19·4 to -1·8)	1,011·7 (848·2 to 1,170·7)	0·6 (-6·8 to 16·0)	29·8 (25·0 to 34·6)	-15·7 (-21·7 to -3·6)
Gynecological diseases	-	-	-	-	-	-	-	-
Uterine fibroids	-	-	-	-	-	-	-	-
Polycystic ovarian syndrome	-	-	-	-	-	-	-	-
Endometriosis	-	-	-	-	-	-	-	-
Genital prolapse	-	-	-	-	-	-	-	-
Other gynecological diseases	-	-	-	-	-	-	-	-
Hemoglobinopathies and hemolytic anaemias	54·4 (46·9 to 68·8)	1·7 (-3·9 to 8·5)	1·7 (1·5 to 2·1)	-13·1 (-17·1 to -8·0)	2,632·3 (2,261·7 to 3,305·5)	-7·8 (-15·7 to 1·9)	72·5 (62·5 to 90·9)	-14·8 (-21·8 to -6·1)
Thalassemias	3·1 (2·5 to 3·8)	-33·8 (-41·7 to -18·7)	0·1 (0·1 to 0·1)	-36·3 (-43·9 to -21·5)	246·4 (201·7 to 311·6)	-34·4 (-42·4 to -18·5)	6·7 (5·5 to 8·6)	-36·6 (-44·5 to -21·1)
Sickle cell disorders	23·8 (20·2 to 31·1)	-1·3 (-12·1 to 10·3)	0·6 (0·5 to 0·8)	-6·5 (-16·7 to 4·8)	1,681·5 (1,418·5 to 2,178·1)	-4·2 (-15·7 to 9·2)	45·1 (37·9 to 58·1)	-8·4 (-19·4 to 5·2)
G6PD deficiency	10·4 (8·5 to 13·7)	12·9 (8·9 to 17·3)	0·3 (0·2 to 0·4)	-4·0 (-7·3 to -0·4)	426·2 (347·8 to 542·8)	1·3 (-2·8 to 6·1)	11·4 (9·3 to 14·6)	-10·7 (-14·1 to -6·4)
Other hemoglobinopathies and hemolytic anaemias	17·1 (14·5 to 21·8)	10·1 (5·3 to 16·4)	0·7 (0·6 to 0·9)	-18·2 (-21·9 to -13·6)	278·3 (232·2 to 354·4)	-8·4 (-11·9 to -3·8)	9·3 (7·8 to 11·8)	-25·8 (-28·6 to -22·0)
Endocrine, metabolic, blood, and immune disorders	69·6 (56·7 to 77·6)	28·0 (20·4 to 33·2)	2·3 (1·8 to 2·6)	3·3 (-2·9 to 7·1)	2,309·7 (1,881·1 to 2,694·3)	10·9 (3·5 to 18·7)	65·8 (53·2 to 76·7)	-2·7 (-8·8 to 3·5)
**Musculoskeletal disorders**	27·6 (24·4 to 31·1)	21·6 (17·4 to 26·9)	1·0 (0·9 to 1·1)	-7·1 (-10·2 to -3·3)	684·1 (604·6 to 786·7)	11·0 (6·1 to 16·3)	20·2 (17·8 to 23·1)	-8·5 (-12·1 to -4·1)
Rheumatoid arthritis	9·9 (7·7 to 11·1)	13·1 (8·0 to 19·5)	0·4 (0·3 to 0·4)	-14·8 (-18·5 to -10·4)	194·0 (150·8 to 225·0)	3·4 (-2·0 to 11·1)	6·1 (4·8 to 7·0)	-18·0 (-21·8 to -12·7)
Other musculoskeletal disorders	17·7 (15·6 to 20·9)	26·9 (20·7 to 33·2)	0·6 (0·5 to 0·7)	-1·9 (-6·4 to 3·1)	490·1 (437·1 to 586·8)	14·4 (7·9 to 19·8)	14·1 (12·5 to 16·7)	-3·6 (-8·7 to 1·2)
**Other non-communicable diseases**	330·9 (289·6 to 370·0)	-12·1 (-20·7 to -0·9)	9·6 (8·3 to 10·7)	-15·5 (-23·3 to -5·3)	24,254·3 (21,238·7 to 27,360·6)	-17·0 (-25·5 to -5·7)	678·4 (593·5 to 764·4)	-18·6 (-26·9 to -7·5)
Congenital anomalies	263·1 (229·8 to 300·3)	-17·8 (-26·8 to -5·1)	7·3 (6·4 to 8·3)	-19·4 (-28·3 to -7·1)	21,429·9 (18,608·1 to 24,602·9)	-19·0 (-28·0 to -6·2)	596·9 (518·1 to 685·2)	-20·1 (-29·0 to -7·5)
Neural tube defects	23·3 (12·6 to 41·9)	-27·6 (-41·1 to -9·5)	0·7 (0·4 to 1·2)	-28·1 (-41·6 to -10·0)	1,986·9 (1,068·1 to 3,576·2)	-27·8 (-41·2 to -9·6)	55·7 (30·0 to 100·2)	-28·2 (-41·6 to -10·0)
Congenital heart anomalies	119·4 (97·3 to 144·3)	-20·3 (-31·4 to -3·1)	3·3 (2·7 to 4·0)	-22·2 (-33·0 to -5·4)	9,601·6 (7,742·0 to 11,674·9)	-21·6 (-32·4 to -4·1)	266·2 (214·2 to 323·9)	-23·0 (-33·6 to -5·9)
Orofacial clefts	1·1 (0·5 to 2·3)	-36·0 (-56·5 to -5·0)	0·0 (0·0 to 0·1)	-36·0 (-56·6 to -5·0)	95·6 (45·7 to 196·6)	-36·0 (-56·6 to -5·0)	2·7 (1·3 to 5·5)	-36·0 (-56·6 to -5·0)
Down's syndrome	7·8 (6·1 to 11·5)	-8·4 (-39·3 to 18·2)	0·2 (0·2 to 0·3)	-15·1 (-42·5 to 7·9)	524·6 (399·4 to 837·7)	-15·7 (-44·7 to 13·3)	14·4 (11·0 to 23·1)	-19·5 (-46·5 to 7·6)
Other chromosomal abnormalities	7·5 (5·6 to 11·4)	3·4 (-21·1 to 32·5)	0·2 (0·2 to 0·3)	1·9 (-22·1 to 30·3)	620·4 (460·6 to 955·0)	2·5 (-22·1 to 32·2)	17·3 (12·9 to 26·8)	1·6 (-22·8 to 31·0)
Congenital musculoskeletal and limb anomalies	4·7 (3·4 to 8·3)	-17·7 (-34·6 to 1·6)	0·1 (0·1 to 0·2)	-18·6 (-35·2 to 0·5)	390·6 (280·0 to 697·0)	-18·4 (-35·1 to 1·4)	10·9 (7·8 to 19·5)	-18·9 (-35·5 to 0·8)
Urogenital congenital anomalies	7·1 (5·3 to 9·3)	-4·0 (-20·4 to 15·1)	0·2 (0·1 to 0·3)	-8·3 (-23·4 to 9·2)	528·7 (390·2 to 699·4)	-7·8 (-23·8 to 11·9)	14·8 (10·9 to 19·5)	-9·6 (-25·1 to 9·7)
Digestive congenital anomalies	20·4 (14·8 to 30·8)	-22·1 (-35·7 to -4·8)	0·6 (0·4 to 0·9)	-22·6 (-36·1 to -5·4)	1,738·7 (1,252·0 to 2,628·6)	-22·5 (-35·9 to -5·1)	48·9 (35·2 to 73·8)	-22·8 (-36·3 to -5·4)
Other congenital anomalies	71·6 (52·3 to 95·7)	-11·5 (-23·6 to 8·7)	2·0 (1·5 to 2·7)	-12·8 (-24·8 to 6·6)	5,942·8 (4,317·1 to 8,003·4)	-12·3 (-24·3 to 7·6)	166·0 (120·5 to 223·7)	-13·2 (-25·2 to 6·4)
Skin and subcutaneous diseases	50·8 (28·3 to 67·5)	39·1 (26·0 to 55·3)	1·8 (1·0 to 2·4)	5·3 (-3·5 to 17·1)	1,354·1 (758·6 to 1,854·3)	28·0 (12·0 to 48·2)	40·0 (22·4 to 54·2)	6·9 (-5·6 to 23·7)
Cellulitis	9·6 (4·3 to 14·3)	65·2 (45·1 to 98·9)	0·3 (0·2 to 0·5)	25·8 (10·7 to 49·3)	239·0 (110·5 to 349·8)	50·1 (29·4 to 86·7)	7·1 (3·3 to 10·4)	23·1 (6·6 to 53·0)
Pyoderma	28·9 (15·9 to 38·3)	36·4 (21·0 to 54·6)	1·0 (0·5 to 1·3)	6·8 (-4·3 to 20·3)	883·2 (469·7 to 1,236·2)	24·9 (7·2 to 47·2)	25·6 (13·8 to 35·3)	6·7 (-7·9 to 25·6)
Decubitus ulcer	10·5 (6·0 to 15·4)	27·4 (18·1 to 40·3)	0·4 (0·2 to 0·6)	-9·6 (-16·5 to 0·9)	180·1 (104·9 to 271·7)	19·7 (10·1 to 32·5)	5·9 (3·4 to 8·8)	-7·3 (-14·6 to 3·2)
Other skin and subcutaneous diseases	1·8 (1·1 to 2·9)	39·8 (28·1 to 58·1)	0·1 (0·0 to 0·1)	6·6 (-2·7 to 21·7)	51·8 (31·2 to 81·7)	25·9 (12·6 to 48·5)	1·5 (0·9 to 2·4)	8·3 (-2·6 to 28·3)
Sudden infant death syndrome	17·1 (12·4 to 21·0)	-15·3 (-34·3 to 6·9)	0·5 (0·3 to 0·6)	-15·7 (-34·7 to 6·4)	1,470·3 (1,069·9 to 1,809·1)	-15·3 (-34·3 to 6·9)	41·4 (30·2 to 51·0)	-15·7 (-34·7 to 6·3)
**Injuries**	3,215·4 (3,061·2 to 3,340·8)	1·7 (-1·4 to 5·0)	91·2 (86·4 to 94·8)	-13·0 (-15·7 to -10·3)	145,060·8 (139,220·1 to 150,547·0)	-4·6 (-7·6 to -1·1)	3,846·1 (3,691·1 to 3,987·9)	-14·9 (-17·6 to -11·9)
**Transport injuries**	1,100·6 (1,067·8 to 1,147·2)	0·1 (-2·4 to 2·8)	30·1 (29·3 to 31·4)	-13·6 (-15·8 to -11·4)	51,287·6 (49,578·1 to 53,848·8)	-5·2 (-7·8 to -2·6)	1,344·9 (1,300·6 to 1,411·0)	-15·4 (-17·7 to -13·1)
Road injuries	1,024·1 (992·8 to 1,067·0)	-0·1 (-2·7 to 2·5)	28·0 (27·2 to 29·2)	-13·8 (-15·9 to -11·6)	47,767·2 (46,177·4 to 50,128·2)	-5·4 (-8·1 to -2·9)	1,252·5 (1,211·3 to 1,313·4)	-15·5 (-17·9 to -13·3)
Pedestrian road injuries	372·0 (342·3 to 405·3)	-0·5 (-7·4 to 3·8)	10·5 (9·6 to 11·4)	-15·4 (-21·2 to -11·8)	16,072·0 (14,709·0 to 17,605·6)	-7·8 (-14·5 to -3·8)	427·2 (390·7 to 467·9)	-18·4 (-24·1 to -14·8)
Cyclist road injuries	59·4 (53·7 to 66·1)	4·1 (-2·6 to 13·3)	1·7 (1·5 to 1·8)	-12·2 (-17·7 to -4·5)	2,464·5 (2,209·1 to 2,779·3)	-2·7 (-9·7 to 6·8)	65·6 (58·9 to 73·9)	-14·6 (-20·6 to -6·1)
Motorcyclist road injuries	215·5 (191·3 to 234·3)	1·1 (-4·7 to 5·5)	5·7 (5·0 to 6·1)	-10·5 (-15·6 to -6·5)	10,908·5 (9,710·8 to 11,924·7)	-3·0 (-8·7 to 1·4)	281·4 (250·2 to 307·7)	-12·4 (-17·5 to -8·4)
Motor vehicle road injuries	367·4 (332·0 to 426·6)	-0·9 (-5·4 to 7·4)	9·9 (9·0 to 11·5)	-14·0 (-17·8 to -6·9)	17,886·5 (16,236·3 to 20,833·3)	-4·9 (-9·1 to 3·2)	466·8 (423·6 to 543·4)	-14·7 (-18·5 to -7·6)
Other road injuries	9·9 (8·7 to 12·3)	-2·5 (-9·8 to 8·0)	0·3 (0·2 to 0·3)	-17·1 (-23·2 to -7·7)	435·7 (382·3 to 540·9)	-9·0 (-16·1 to -0·3)	11·6 (10·2 to 14·4)	-19·1 (-25·3 to -11·3)
Other transport injuries	76·4 (70·4 to 86·3)	2·6 (-3·8 to 12·7)	2·1 (1·9 to 2·4)	-11·9 (-17·2 to -3·4)	3,520·4 (3,233·4 to 3,945·1)	-2·1 (-8·9 to 7·7)	92·4 (84·9 to 103·6)	-13·0 (-18·8 to -4·4)
**Unintentional injuries**	1,123·7 (1,006·8 to 1,176·1)	-1·7 (-5·7 to 2·1)	34·2 (30·3 to 35·8)	-16·2 (-19·5 to -13·1)	47,015·6 (43,005·2 to 49,165·3)	-13·0 (-16·3 to -9·3)	1,281·6 (1,170·0 to 1,339·6)	-21·8 (-24·7 to -18·6)
Falls	384·0 (318·3 to 408·9)	18·8 (10·2 to 24·7)	13·0 (10·7 to 13·8)	-5·9 (-12·2 to -1·3)	11,288·3 (9,651·6 to 11,988·9)	3·7 (-5·1 to 10·2)	322·4 (274·0 to 343·2)	-11·1 (-18·3 to -5·9)
Drowning	215·1 (200·0 to 225·8)	-17·8 (-20·9 to -14·1)	5·9 (5·5 to 6·2)	-25·9 (-28·7 to -22·8)	11,901·3 (11,097·8 to 12,569·5)	-24·8 (-28·4 to -21·0)	316·6 (295·4 to 334·2)	-30·5 (-33·8 to -26·9)
Fire, heat, and hot substances	67·4 (57·0 to 75·6)	-8·9 (-14·7 to -0·1)	2·0 (1·7 to 2·3)	-23·5 (-27·9 to -17·0)	2,927·2 (2,439·4 to 3,302·3)	-15·6 (-22·3 to -3·5)	79·8 (66·6 to 89·9)	-24·8 (-30·7 to -15·1)
Poisonings	36·1 (24·5 to 41·4)	-13·0 (-24·2 to 5·5)	1·0 (0·7 to 1·2)	-24·6 (-34·7 to -9·2)	1,799·0 (1,204·8 to 2,097·4)	-17·5 (-28·7 to 0·2)	48·2 (32·2 to 56·6)	-25·3 (-35·7 to -9·6)
Exposure to mechanical forces	116·4 (91·5 to 123·8)	-9·7 (-15·9 to -6·1)	3·3 (2·6 to 3·5)	-21·3 (-26·5 to -18·2)	5,678·6 (4,591·6 to 6,099·4)	-15·7 (-20·8 to -11·8)	150·9 (122·2 to 162·1)	-23·8 (-28·1 to -20·3)
Unintentional firearm injuries	17·8 (13·4 to 19·5)	-6·8 (-14·8 to -0·3)	0·5 (0·4 to 0·5)	-18·8 (-25·3 to -13·3)	923·9 (692·1 to 1,030·2)	-9·0 (-17·9 to -2·1)	24·2 (18·1 to 26·9)	-17·7 (-25·5 to -11·5)
Unintentional suffocation	15·1 (11·5 to 17·7)	-11·4 (-22·1 to -1·1)	0·5 (0·3 to 0·5)	-18·1 (-27·2 to -9·6)	942·5 (735·4 to 1,132·7)	-21·1 (-29·7 to -11·1)	26·0 (20·1 to 31·3)	-24·3 (-32·5 to -14·8)
Other exposure to mechanical forces	83·6 (62·9 to 88·8)	-10·0 (-16·0 to -6·0)	2·3 (1·7 to 2·5)	-22·4 (-27·7 to -18·9)	3,812·1 (2,930·1 to 4,070·3)	-15·7 (-20·5 to -11·8)	100·7 (77·4 to 107·5)	-25·0 (-29·2 to -21·5)
Adverse effects of medical treatment	65·9 (53·9 to 76·4)	11·8 (6·4 to 17·7)	2·1 (1·7 to 2·4)	-8·2 (-12·9 to -3·3)	2,562·9 (2,080·2 to 2,985·2)	0·9 (-7·2 to 10·7)	71·5 (58·3 to 83·4)	-10·4 (-16·8 to -3·2)
Animal contact	52·8 (48·7 to 58·4)	-5·1 (-12·4 to 8·4)	1·5 (1·4 to 1·7)	-18·9 (-25·2 to -7·4)	2,450·2 (2,263·4 to 2,705·6)	-11·5 (-19·5 to 1·8)	65·9 (60·9 to 72·7)	-20·5 (-27·5 to -8·6)
Venomous animal contact	44·7 (40·5 to 50·0)	-5·0 (-13·1 to 9·2)	1·3 (1·1 to 1·4)	-18·7 (-25·6 to -6·3)	2,077·9 (1,873·2 to 2,339·7)	-11·8 (-19·8 to 2·1)	55·9 (50·4 to 62·9)	-20·8 (-27·9 to -8·3)
Non-venomous animal contact	8·1 (6·6 to 10·0)	-5·3 (-13·7 to 6·2)	0·2 (0·2 to 0·3)	-20·1 (-27·1 to -10·9)	372·3 (292·0 to 460·3)	-9·7 (-20·2 to 3·6)	10·0 (7·9 to 12·4)	-19·4 (-28·2 to -7·9)
Foreign body	64·8 (54·9 to 73·6)	4·6 (-3·8 to 15·5)	2·0 (1·7 to 2·3)	-11·5 (-17·1 to -3·3)	2,972·0 (2,498·6 to 3,469·9)	-3·6 (-14·2 to 8·2)	82·4 (69·1 to 95·8)	-12·3 (-21·1 to -2·4)
Pulmonary aspiration and foreign body in airway	58·8 (49·6 to 68·5)	4·9 (-3·7 to 17·0)	1·8 (1·5 to 2·1)	-11·5 (-17·1 to -2·4)	2,670·5 (2,245·9 to 3,189·0)	-3·5 (-14·2 to 9·8)	74·3 (62·3 to 88·4)	-12·3 (-21·3 to -1·1)

SR Table 2: Global deaths and YLL numbers, age-standardized rates per 100,000, and percent change between 2006 and 2016 for males for all GBD causes and Levels 1 through 4 of the cause hierarchy. 95% UIs are in parentheses. UI=uncertainty interval. YLL=year of life lost.

Foreign body in other body part	6·0 (3·9 to 7·4)	1·5 (-6·9 to 12·7)	0·2 (0·1 to 0·2)	-12·3 (-18·2 to -3·6)	301·5 (184·5 to 378·5)	-4·1 (-15·1 to 7·4)	8·1 (5·0 to 10·2)	-12·9 (-22·0 to -3·0)
Environmental heat and cold exposure	40·2 (25·0 to 57·1)	-13·5 (-26·5 to 1·0)	1·2 (0·7 to 1·7)	-28·5 (-38·9 to -17·5)	1,458·1 (905·7 to 1,987·1)	-19·8 (-32·2 to -6·5)	39·9 (24·7 to 54·5)	-31·3 (-42·2 to -20·3)
Other unintentional injuries	81·1 (70·3 to 85·4)	-11·3 (-15·9 to -7·2)	2·2 (1·9 to 2·3)	-22·2 (-26·2 to -18·6)	3,978·1 (3,548·3 to 4,189·0)	-15·8 (-20·0 to -11·8)	103·9 (92·6 to 109·3)	-24·4 (-28·1 to -20·8)
**Self-harm and interpersonal violence**	877·2 (785·8 to 952·0)	1·7 (-4·4 to 8·2)	24·0 (21·5 to 26·1)	-13·1 (-18·3 to -7·5)	39,996·1 (35,668·4 to 43,376·9)	-1·4 (-7·0 to 4·8)	1,044·5 (932·9 to 1,132·3)	-13·0 (-17·9 to -7·5)
Self-harm	559·5 (492·3 to 617·4)	2·8 (-5·3 to 10·4)	15·6 (13·7 to 17·2)	-13·7 (-20·5 to -7·5)	23,432·5 (20,838·9 to 25,838·5)	-1·0 (-8·8 to 6·7)	617·3 (546·8 to 681·8)	-13·9 (-20·6 to -7·3)
Self-harm by firearm	57·5 (45·2 to 73·9)	5·7 (-2·6 to 15·9)	1·6 (1·3 to 2·1)	-11·3 (-17·9 to -3·7)	2,401·1 (1,922·7 to 3,117·8)	0·6 (-7·7 to 11·4)	63·3 (50·5 to 82·0)	-12·0 (-19·0 to -2·9)
Self-harm by other specified means	502·0 (447·2 to 556·9)	2·5 (-5·8 to 10·3)	14·0 (12·4 to 15·5)	-14·0 (-20·8 to -7·3)	21,031·4 (18,715·6 to 23,356·0)	-1·2 (-9·2 to 6·8)	553·9 (492·1 to 615·7)	-14·1 (-21·0 to -7·1)
Interpersonal violence	317·7 (251·5 to 375·0)	-0·3 (-5·2 to 8·1)	8·3 (6·6 to 9·9)	-12·0 (-16·3 to -4·6)	16,563·6 (13,203·6 to 19,225·1)	-1·9 (-6·6 to 5·8)	427·3 (340·5 to 496·4)	-11·6 (-15·8 to -4·6)
Assault by firearm	143·7 (91·5 to 165·0)	6·0 (0·5 to 11·7)	3·7 (2·4 to 4·3)	-5·3 (-10·4 to -0·1)	7,735·5 (4,933·9 to 8,848·6)	4·4 (-1·3 to 10·1)	198·5 (126·6 to 227·0)	-5·2 (-10·4 to 0·1)
Assault by sharp object	78·5 (58·3 to 108·6)	-9·5 (-16·6 to 2·6)	2·1 (1·5 to 2·9)	-20·6 (-26·7 to -9·4)	3,988·4 (2,987·2 to 5,511·4)	-11·4 (-18·3 to 0·0)	102·7 (76·8 to 141·8)	-20·6 (-26·8 to -10·1)
Assault by other means	95·6 (75·7 to 129·1)	-0·7 (-11·1 to 13·9)	2·5 (2·0 to 3·4)	-13·5 (-22·5 to -0·3)	4,839·7 (3,906·5 to 6,463·5)	-2·8 (-13·1 to 10·3)	126·1 (101·6 to 168·3)	-12·9 (-22·1 to -1·1)
**Forces of nature, conflict and terrorism, and executions and police conflict**	113·9 (79·0 to 151·2)	101·8 (28·3 to 232·1)	3·0 (2·1 to 4·0)	80·8 (15·7 to 194·3)	6,761·5 (4,684·5 to 8,985·1)	103·2 (28·7 to 238·5)	175·0 (121·3 to 232·6)	87·0 (18·7 to 210·7)
Forces of nature, conflict and terrorism, and state actor violence	4·3 (2·6 to 6·2)	-49·3 (-63·0 to -35·7)	0·1 (0·1 to 0·2)	-55·1 (-67·1 to -43·1)	224·3 (136·2 to 318·5)	-52·2 (-64·6 to -39·4)	5·9 (3·6 to 8·4)	-56·3 (-67·6 to -44·7)
Conflict and terrorism	105·8 (71·3 to 142·6)	144·9 (43·5 to 372·1)	2·8 (1·9 to 3·7)	121·8 (30·3 to 326·4)	6,343·4 (4,283·2 to 8,542·7)	143·0 (43·1 to 374·1)	164·1 (110·8 to 221·1)	124·8 (32·5 to 337·6)
Executions and police conflict	3·7 (1·8 to 4·4)	-19·6 (-29·5 to -8·0)	0·1 (0·0 to 0·1)	-28·5 (-37·4 to -18·8)	193·8 (97·2 to 230·3)	-21·9 (-31·3 to -10·3)	5·0 (2·5 to 5·9)	-29·3 (-37·8 to -18·7)

SR Table 3: Global deaths by cause and age group for females (with 95% uncertainty intervals), 1990

Cause	Early Neonatal	Late Neonatal	Post Neonatal	1 to 4	5 to 9	10 to 14	15 to 19	20 to 24	25 to 29	30 to 34	35 to 39	40 to 44	45 to 49	50 to 54	55 to 59	60 to 64	65 to 69	70 to 74	75 to 79	80 to 84	85 to 89	90 to 94	95 plus
All causes	1,275,613 (1,248,891-1,300,549)	528,146 (517,545-539,428)	1,661,643 (1,627,314-1,691,515)	1,722,435 (1,681,985-1,762,508)	363,761 (359,650-367,810)	224,812 (221,837-227,795)	315,871 (310,652-321,206)	389,975 (383,103-396,959)	372,951 (366,148-379,662)	378,899 (372,404-385,355)	420,831 (413,309-428,593)	445,055 (438,178-452,501)	525,283 (517,616-533,171)	733,932 (723,651-744,525)	959,671 (946,756-973,104)	1,293,936 (1,277,419-1,311,446)	1,618,095 (1,598,016-1,638,795)	1,868,031 (1,844,378-1,891,694)	2,188,513 (2,162,431-2,214,331)	2,070,305 (2,051,409-2,090,032)	1,521,900 (1,511,441-1,533,207)	721,483 (717,821-725,107)	250,741 (249,639-251,888)
Communicable, maternal, neonatal, and nutritional disorders	1,169,870 (1,132,009-1,206,626)	473,245 (451,564-491,075)	1,401,520 (1,332,732-1,460,679)	1,389,816 (1,311,968-1,471,851)	228,599 (213,744-244,308)	109,456 (101,153-118,864)	130,162 (121,722-140,214)	175,579 (167,283-184,920)	170,152 (162,902-178,485)	157,502 (150,564-166,005)	147,152 (140,317-155,956)	119,547 (112,395-128,303)	112,961 (103,666-126,366)	125,875 (111,787-146,180)	146,574 (126,992-174,737)	176,497 (147,980-219,726)	207,694 (173,965-261,828)	247,402 (201,437-313,685)	235,811 (192,113-298,520)	217,596 (179,924-265,455)	149,849 (125,467-177,477)	64,480 (55,151-74,356)	23,376 (20,615-26,278)
HIV/AIDS and tuberculosis	-	-	20,796 (17,685-24,001)	28,288 (19,491-35,874)	10,364 (8,058-12,946)	9,780 (8,269-11,341)	22,971 (20,660-25,401)	39,393 (35,978-42,597)	48,019 (44,039-51,831)	48,507 (44,489-52,266)	50,084 (45,110-55,044)	43,800 (39,494-48,280)	46,333 (40,841-52,387)	54,488 (47,373-61,710)	60,473 (51,818-69,417)	62,190 (52,068-72,847)	63,846 (52,328-75,255)	66,105 (53,220-79,355)	52,183 (42,786-62,560)	38,757 (30,508-48,657)	19,811 (15,564-25,680)	5,936 (4,624-7,960)	1,437 (1,109-1,926)
Tuberculosis	-	-	12,194 (9,324-15,199)	22,102 (13,716-29,586)	9,713 (7,462-12,326)	9,584 (8,056-11,142)	20,810 (18,445-23,141)	31,033 (27,848-33,775)	35,159 (32,066-38,449)	36,549 (32,897-39,760)	40,389 (35,732-44,947)	36,953 (32,861-41,499)	41,194 (35,617-47,184)	50,982 (43,949-58,213)	57,900 (49,261-66,968)	60,364 (50,098-71,036)	62,562 (51,000-74,026)	65,544 (52,646-78,781)	51,900 (42,497-62,270)	38,694 (30,443-48,591)	19,784 (15,538-25,654)	5,927 (4,616-7,952)	1,433 (1,105-1,922)
Drug-susceptible tuberculosis	-	-	12,135 (9,274-15,102)	21,983 (13,627-29,451)	9,665 (7,418-12,276)	9,524 (8,006-11,072)	20,690 (18,321-23,031)	30,828 (27,644-33,566)	34,951 (31,882-38,233)	36,328 (32,705-39,506)	40,129 (35,505-44,674)	36,715 (32,618-41,252)	40,949 (35,348-46,882)	50,676 (43,611-57,865)	57,530 (48,946-66,553)	59,919 (49,755-70,589)	62,076 (50,601-73,428)	64,987 (52,288-78,135)	51,415 (42,040-61,727)	38,350 (30,157-48,236)	19,597 (15,399-25,403)	5,871 (4,557-7,890)	1,418 (1,092-1,904)
Multidrug-resistant tuberculosis without extensive drug resistance	-	-	60 (43-79)	120 (77-165)	48 (36-62)	61 (47-77)	120 (94-149)	205 (161-255)	207 (162-258)	221 (175-276)	259 (204-325)	239 (184-303)	244 (190-306)	305 (234-387)	371 (289-469)	444 (342-576)	486 (365-627)	556 (416-726)	486 (367-636)	344 (250-497)	188 (132-289)	57 (37-93)	15 (9-27)
Extensively drug-resistant tuberculosis	-	-	0 (0-0)	0 (0-0)	0 (0-0)	0 (0-0)	0 (0-0)	0 (0-0)	0 (0-0)	0 (0-0)	0 (0-0)	0 (0-0)	0 (0-0)	0 (0-0)	0 (0-0)	0 (0-0)	0 (0-0)	0 (0-0)	0 (0-0)	0 (0-0)	0 (0-0)	0 (0-0)	0 (0-0)
HIV/AIDS	-	-	8,601 (7,712-9,709)	6,186 (5,435-7,256)	652 (547-800)	195 (165-239)	2,160 (1,752-2,646)	8,360 (7,036-9,971)	12,860 (10,615-15,323)	11,958 (10,195-13,900)	9,695 (8,182-11,474)	6,847 (5,847-8,033)	5,139 (4,314-6,112)	3,506 (2,962-4,184)	2,573 (2,123-3,094)	1,826 (1,539-2,199)	1,284 (1,057-1,552)	561 (436-710)	283 (225-355)	63 (49-83)	26 (21-34)	9 (7-11)	4 (4-4)
Drug-susceptible HIV/AIDS - Tuberculosis	-	-	3,142 (2,517-3,660)	2,330 (1,884-2,767)	218 (171-273)	49 (35-64)	762 (600-962)	2,786 (2,124-3,534)	3,975 (2,915-5,117)	3,742 (2,779-4,699)	3,110 (2,342-3,906)	2,322 (1,787-2,841)	1,812 (1,384-2,235)	1,267 (1,006-1,552)	919 (724-1,142)	642 (506-786)	444 (347-555)	197 (144-255)	98 (75-125)	18 (13-25)	7 (5-10)	2 (2-3)	1 (0-1)
Multidrug-resistant HIV/AIDS - Tuberculosis without extensive drug resistance	-	-	13 (8-21)	10 (6-15)	1 (1-2)	0 (0-1)	3 (2-5)	12 (7-19)	19 (12-30)	20 (12-31)	16 (10-26)	12 (7-19)	9 (6-14)	7 (4-10)	5 (3-8)	4 (2-6)	3 (2-4)	1 (1-2)	1 (0-1)	0 (0-0)	0 (0-0)	0 (0-0)	0 (0-0)
Extensively drug-resistant HIV/AIDS - Tuberculosis	-	-	0 (0-0)	0 (0-0)	0 (0-0)	0 (0-0)	0 (0-0)	0 (0-0)	0 (0-0)	0 (0-0)	0 (0-0)	0 (0-0)	0 (0-0)	0 (0-0)	0 (0-0)	0 (0-0)	0 (0-0)	0 (0-0)	0 (0-0)	0 (0-0)	0 (0-0)	0 (0-0)	0 (0-0)
HIV/AIDS resulting in other diseases	-	-	5,446 (4,793-6,435)	3,846 (3,310-4,740)	432 (360-549)	146 (123-179)	1,395 (1,096-1,785)	5,562 (4,472-7,142)	8,866 (6,966-11,198)	8,196 (6,710-10,251)	6,569 (5,306-8,285)	4,513 (3,721-5,650)	3,318 (2,727-4,184)	2,233 (1,859-2,816)	1,649 (1,333-2,127)	1,181 (968-1,503)	838 (681-1,040)	363 (271-484)	184 (144-242)	45 (35-61)	19 (15-25)	7 (5-8)	3 (3-4)
Diarrhoea, lower respiratory infections, and other common infectious diseases	183,158 (155,988-216,419)	231,511 (203,475-253,603)	1,103,495 (1,027,722-1,179,675)	1,008,798 (894,222-1,137,596)	165,117 (140,002-192,935)	74,770 (62,550-87,709)	50,662 (40,240-65,732)	41,136 (31,861-56,126)	35,058 (26,808-48,672)	33,100 (24,947-46,771)	32,467 (24,628-46,499)	31,580 (24,204-45,205)	37,999 (28,023-56,106)	52,978 (38,070-80,670)	68,849 (49,189-104,789)	95,168 (66,778-147,521)	123,760 (88,063-188,069)	162,342 (115,273-243,077)	164,542 (120,058-236,613)	163,654 (123,809-219,746)	118,240 (93,109-150,984)	52,359 (43,249-63,476)	19,206 (16,450-22,340)
Diarrhoeal diseases	32,556 (27,177-38,714)	64,807 (53,149-75,782)	393,379 (339,228-442,117)	306,008 (238,078-370,174)	32,893 (13,215-64,213)	16,464 (6,677-32,499)	15,972 (6,718-32,753)	16,007 (7,010-32,816)	13,824 (5,745-28,824)	13,889 (5,792-28,708)	13,950 (5,757-28,655)	13,454 (5,970-28,341)	17,748 (7,602-37,532)	26,160 (10,711-55,321)	36,726 (16,139-76,114)	53,678 (23,116-111,352)	69,568 (29,899-140,675)	94,435 (41,123-183,072)	90,444 (41,564-170,228)	86,008 (41,858-146,498)	54,251 (28,933-86,836)	20,383 (10,753-31,458)	5,839 (3,316-8,869)
Intestinal infectious diseases	-	-	1,694 (219-7,098)	16,594 (7,724-31,637)	33,633 (17,902-56,996)	19,076 (10,037-30,570)	9,380 (4,686-16,571)	4,325 (2,129-7,588)	3,228 (1,644-5,518)	2,582 (1,353-4,354)	2,071 (1,116-3,386)	1,699 (910-2,782)	1,531 (814-2,579)	1,323 (683-2,283)	1,066 (511-1,936)	908 (440-1,617)	786 (435-1,318)	611 (347-1,005)	431 (243-710)	248 (139-415)	122 (68-211)	38 (20-66)	10 (5-20)
Typhoid fever	-	-	84 (39-167)	14,016 (6,029-28,453)	29,754 (15,309-51,465)	16,419 (8,417-26,753)	8,251 (4,087-15,058)	3,708 (1,814-6,564)	2,630 (1,329-4,592)	2,013 (1,031-3,447)	1,590 (837-2,687)	1,288 (674-2,165)	1,137 (594-1,929)	1,004 (507-1,730)	827 (385-1,521)	727 (349-1,312)	651 (343-1,081)	507 (282-818)	341 (192-558)	196 (110-315)	89 (49-143)	26 (14-42)	6 (3-10)
Paratyphoid fever	-	-	12 (5-26)	1,317 (511-2,935)	3,760 (1,620-7,677)	2,619 (1,174-5,180)	1,092 (457-2,235)	585 (252-1,224)	571 (250-1,176)	530 (233-1,040)	442 (205-843)	376 (175-739)	353 (159-715)	302 (131-610)	219 (91-457)	158 (65-327)	109 (49-214)	74 (34-142)	48 (22-88)	28 (13-53)	13 (6-24)	4 (2-7)	1 (0-2)
Other intestinal infectious diseases	-	-	1,598 (134-6,947)	1,260 (86-5,639)	119 (11-541)	37 (4-167)	37 (3-173)	32 (2-150)	27 (2-124)	38 (3-187)	39 (4-170)	35 (3-183)	41 (4-192)	17 (2-86)	20 (2-93)	23 (2-94)	26 (3-119)	30 (3-126)	42 (4-194)	24 (3-94)	20 (2-83)	8 (1-29)	4 (1-13)
Lower respiratory infections	105,133 (87,760-132,380)	116,627 (101,033-136,324)	484,201 (419,208-555,251)	303,496 (238,809-374,013)	27,115 (20,628-33,877)	11,637 (9,261-13,691)	8,795 (7,190-10,299)	8,958 (7,100-10,613)	8,797 (7,202-10,261)	8,374 (6,786-9,789)	9,287 (7,747-11,019)	9,561 (7,812-11,106)	10,955 (9,035-12,712)	16,289 (13,483-19,098)	21,987 (17,810-25,754)	31,969 (25,648-37,345)	42,974 (35,067-50,595)	56,910 (46,582-66,290)	65,285 (53,927-75,977)	71,005 (60,691-81,818)	60,442 (52,927-68,773)	30,670 (26,772-35,074)	12,937 (11,189-14,708)
Upper respiratory infections	55 (35-87)	28 (16-47)	572 (382-843)	511 (298-853)	35 (21-57)	22 (13-34)	45 (30-64)	39 (26-58)	38 (26-55)	37 (25-53)	42 (29-60)	39 (26-55)	45 (32-65)	52 (36-73)	59 (39-84)	68 (46-96)	63 (43-90)	55 (38-77)	56 (38-81)	37 (26-55)	26 (18-37)	10 (7-13)	2 (2-4)
Otitis media	8 (6-11)	47 (30-76)	348 (216-633)	492 (218-1,009)	298 (159-600)	207 (132-330)	114 (72-192)	58 (35-103)	81 (48-148)	79 (47-155)	66 (40-128)	117 (67-214)	85 (51-151)	89 (56-148)	184 (112-327)	130 (83-200)	201 (122-305)	163 (99-264)	115 (68-179)	80 (47-129)	38 (24-58)	15 (10-22)	4 (3-6)
Meningitis	3,610 (2,204-4,708)	7,725 (4,007-9,905)	46,026 (22,903-58,346)	48,815 (18,794-66,765)	12,973 (9,067-15,888)	8,113 (6,097-9,660)	6,515 (4,859-7,900)	5,066 (4,023-6,273)	4,180 (3,134-5,216)	3,571 (2,759-4,527)	3,406 (2,694-4,195)	2,924 (2,345-3,789)	3,424 (2,699-4,437)	3,781 (2,974-5,026)	4,135 (3,299-5,263)	4,053 (3,232-5,339)	4,315 (3,419-5,481)	4,642 (3,688-5,858)	3,043 (2,430-3,731)	2,609 (2,078-3,363)	1,310 (1,038-1,686)	446 (359-569)	134 (112-163)
Pneumococcal meningitis	155 (92-219)	338 (187-463)	2,299 (1,268-3,081)	2,395 (936-3,576)	672 (448-919)	422 (293-598)	329 (225-451)	240 (168-330)	233 (160-320)	259 (173-362)	293 (193-429)	294 (195-454)	368 (231-562)	397 (273-596)	455 (320-637)	468 (343-644)	496 (361-677)	539 (376-748)	382 (286-511)	305 (221-425)	154 (121-196)	57 (46-71)	18 (14-24)
H influenzae type B meningitis	403 (238-545)	858 (427-1,143)	4,978 (2,318-6,631)	4,817 (1,724-6,926)	1,069 (714-1,413)	565 (412-731)	402 (283-536)	306 (227-405)	269 (194-354)	243 (178-330)	238 (176-326)	205 (148-282)	242 (171-341)	263 (193-370)	289 (207-387)	282 (206-377)	288 (206-393)	311 (235-419)	213 (154-286)	179 (132-239)	90 (66-119)	30 (21-41)	9 (6-13)
Meningococcal infection	1,465 (898-1,923)	3,213 (1,689-4,204)	19,816 (10,260-25,446)	22,779 (9,166-31,301)	6,480 (4,446-8,181)	3,950 (2,899-4,831)	3,159 (2,375-3,914)	2,395 (1,888-2,960)	1,892 (1,415-2,359)	1,521 (1,159-1,956)	1,395 (1,069-1,753)	1,230 (958-1,553)	1,422 (1,123-1,798)	1,610 (1,227-2,100)	1,761 (1,369-2,278)	1,724 (1,328-2,308)	1,783 (1,364-2,344)	1,820 (1,395-2,418)	1,250 (938-1,582)	1,034 (770-1,336)	543 (408-712)	188 (142-251)	55 (40-72)
Other meningitis	1,586 (956-2,158)	3,316 (1,649-4,387)	18,933 (9,094-24,630)	18,824 (7,025-26,779)	4,752 (3,284-6,169)	3,176 (2,378-3,960)	2,626 (1,922-3,222)	2,124 (1,602-2,632)	1,787 (1,328-2,285)	1,547 (1,187-1,979)	1,479 (1,140-1,887)	1,195 (952-1,570)	1,393 (1,074-1,846)	1,511 (1,171-2,029)	1,630 (1,273-2,121)	1,579 (1,204-2,104)	1,749 (1,332-2,300)	1,972 (1,509-2,604)	1,198 (924-1,547)	1,090 (829-1,420)	522 (411-700)	172 (134-226)	52 (40-68)
Encephalitis	397 (209-541)	858 (408-1,268)	8,046 (3,855-12,565)	12,312 (4,948-23,248)	4,503 (2,404-7,523)	2,716 (1,596-4,436)	1,698 (1,091-2,599)	1,518 (955-2,244)	1,145 (726-1,710)	1,104 (725-1,697)	988 (656-1,493)	986 (634-1,436)	1,103 (715-1,659)	1,270 (869-1,875)	1,509 (1,042-2,312)	1,807 (1,232-2,668)	1,914 (1,209-2,754)	1,964 (1,196-2,780)	1,661 (1,045-2,229)	1,361 (851-1,860)	703 (447-918)	199 (134-257)	54 (38-67)
Diphtheria	-	-	1,202 (736-1,794)	2,838 (1,684-4,773)	417 (242-665)	170 (107-261)	77 (49-113)	43 (27-66)	37 (23-55)	32 (20-49)	45 (29-66)	44 (28-67)	51 (34-77)	64 (43-95)	50 (32-75)	-	-	-	-	-	-	-	-
Whooping cough	-	-	41,465 (19,123-80,131)	44,803 (20,463-87,446)	4,961 (2,125-10,024)	911 (388-1,896)	325 (135-698)	247 (102-531)	145 (58-317)	100 (40-219)	79 (31-175)	63 (25-140)	60 (23-131)	64 (25-142)	67 (27-144)	-	-	-	-	-	-	-	-
Tetanus	41,330 (22,294-72,220)	41,183 (23,718-60,467)	12,349 (7,515-21,670)	5,724 (2,549-11,736)	1,770 (704-3,738)	2,711 (749-5,977)	3,092 (1,136-6,564)	2,476 (751-5,839)	2,258 (819-4,818)	2,458 (1,049-5,563)	1,965 (943-4,538)	2,345 (1,146-5,311)	2,722 (1,328-6,801)	3,660 (1,572-8,828)	2,845 (1,258-6,675)	2,408 (1,084-5,668)	3,664 (1,458-8,671)	3,300 (1,148-7,608)	3,081 (1,093-6,887)	1,681 (560-3,729)	660 (229-1,393)	193 (64-432)	50 (16-107)
Measles	-	-	112,482 (42,836-230,523)	265,005 (112,897-501,878)	45,531 (19,085-89,754)	12,441 (4,542-26,752)	4,523 (1,560-10,738)	2,254 (762-5,348)	1,149 (386-2,752)	689 (232-1,670)	413 (138-1,003)	255 (85-615)	162 (54-392)	124 (41-297)	96 (32-230)	-	-	-	-	-	-	-	-
Varicella and herpes zoster	69 (53-88)	235 (182-295)	1,732 (1,325-2,216)	2,200 (1,573-2,901)	989 (733-1,314)	302 (234-389)	126 (98-162)	146 (116-182)	174 (138-219)	188 (151-234)	154 (125-191)	94 (75-117)	113 (90-143)	101 (79-128)	125 (97-157)	148 (118-188)	275 (216-353)	262 (204-331)	426 (334-539)	625 (488-827)	690 (531-909)	405 (305-549)	176 (125-255)
Neglected tropical diseases and malaria	497 (215-694)	9,198 (6,145-13,131)	94,086 (65,647-124,998)	210,066 (149,496-275,381)	33,176 (21,634-46,128)	16,020 (11,189-21,119)	8,174 (5,945-10,476)	7,767 (5,786-10,014)	5,911 (4,448-7,852)	5,610 (4,308-7,503)	6,582 (5,108-8,613)	6,424 (4,919-8,341)	6,622 (5,053-8,731)	7,699 (5,975-10,374)	7,318 (5,814-9,594)	8,421 (6,408-10,771)	7,849 (6,130-10,217)	6,665 (5,152-8,802)	5,062 (3,853-6,697)	2,770 (2,122-3,539)	1,588 (1,166-2,095)	348 (249-454)	169 (103-298)
Malaria	-	8,801 (5,774-12,737)	87,620 (58,457-118,720)	189,447 (129,091-255,195)	22,999 (13,032-34,843)	10,293 (6,259-14,850)	4,076 (2,625-5,611)	3,863 (2,595-5,325)	2,564 (1,738-3,645)	2,216 (1,503-3,176)	2,692 (1,850-3,751)	2,617 (1,776-3,680)	2,520 (1,725-3,594)	3,044 (2,038-4,391)	3,280 (2,166-4,555)	4,798 (3,216-6,804)	3,971 (2,606-5,803)	3,415 (2,234-5,047)	2,606 (1,718-3,805)	1,532 (994-2,173)	914 (558-1,357)	172 (93-265)	114 (54-239)
Chagas disease	-	-	3 (3-4)	2 (2-2)	2 (2-2)	4 (3-4)	10 (8-11)	23 (20-26)	48 (41-55)	86 (74-99)	126 (111-144)	177 (157-200)	233 (205-265)	317 (282-360)	374 (330-425)	418 (373-471)	414 (370-459)	392 (360-426)	347 (313-384)	242 (220-267)	115 (104-127)	40 (36-45)	11 (10-12)
Leishmaniasis	-	-	1,689 (785-3,327)	2,802 (1,212-5,523)	1,559 (711-3,087)	958 (454-1,832)	1,134 (539-2,091)	1,157 (552-2,209)	911 (440-1,685)	845 (403-1,574)	1,066 (497-2,012)	1,108 (517-2,102)	1,097 (515-2,052)	1,123 (541-2,104)	857 (398-1,629)	713 (328-1,358)	773 (375-1,471)	838 (397-1,577)	680 (315-1,278)	351 (163-673)	220 (98-408)	43 (17-80)	15 (5-29)
Visceral leishmaniasis	-	-	1,689 (785-3,327)	2,802 (1,212-5,523)	1,559 (711-3,087)	958 (454-1,832)	1,134 (539-2,091)	1,157 (552-2,209)	911 (440-1,685)	845 (403-1,574)	1,066 (497-2,012)	1,108 (517-2,102)	1,097 (515-2,052)	1,123 (541-2,104)	857 (398-1,629)	713 (328-1,358)	773 (375-1,471)	838 (397-1,577)	680 (315-1,278)	351 (163-673)	220 (98-408)	43 (17-80)	15 (5-29)
African trypanosomiasis	-	-	-	314 (52-1,151)	697 (151-1,923)	768 (190-1,940)	903 (222-2,082)	883 (231-2,133)	818 (193-2,123)	747 (191-1,971)	673 (173-1,835)	621 (132-1,782)	589 (134-1,831)	534 (125-1,678)	526 (101-1,708)	409 (86-1,379)	326 (67-1,062)	234 (47-940)	150 (25-574)	71 (12-287)	25 (4-109)	6 (1-23)	1 (0-5)
Schistosomiasis	-	-	0 (0-0)	434 (252-727)	168 (103-265)	212 (139-311)	183 (124-260)	217 (150-306)	251 (178-358)	284 (197-401)	358 (251-499)	390 (274-565)	442 (311-626)	567 (394-809)	702 (492-978)	736 (525-1,039)	803 (560-1,161)	717 (508-1,031)	499 (362-685)	240 (174-333)	105 (76-140)	27 (18-38)	5 (2-8)
Cysticercosis	0 (0-0)	0 (0-0)	0 (0-0)	97 (62-143)	56 (41-75)	38 (29-47)	31 (24-39)	42 (33-53)	49 (40-59)	57 (46-70)	48 (39-58)	44 (37-54)	43 (35-52)	54 (45-64)	55 (47-66)	43 (36-51)	41 (34-50)	25 (21-30)	16 (13-19)	9 (7-11)	4 (3-5)	1 (1-2)	0 (0-1)
Cystic echinococcosis	-	-	-	266 (170-401)	91 (60-133)	48 (33-69)	24 (17-33)	30 (21-41)	41 (30-56)	41 (29-54)	48 (36-64)	40 (30-52)	48 (36-64)	52 (40-67)	58 (44-76)	60 (46-76)	59 (46-76)	47 (37-61)	46 (36-57)	29 (22-36)	16 (12-19)	5 (4-6)	1 (1-2)
Dengue	-	-	644 (97-1,749)	1,134 (189-2,890)	534 (87-1,433)	351 (47-956)	226 (33-518)	207 (24-597)	145 (19-367)	111 (13-306)	95 (14-258)	88 (11-234)	112 (16-277)	132 (16-359)	138 (20-356)	126 (18-339)	114 (18-276)	51 (10-102)	43 (9-84)	33 (7-65)	21 (5-37)	8 (2-15)	2 (0-3)
Yellow fever	-	2 (0-6)	35 (7-116)	300 (57-914)	554 (103-1,724)	489 (96-1,468)	416 (79-1,272)	323 (66-955)	255 (48-823)	201 (41-645)	164 (32-497)	140 (27-405)	124 (23-393)	108 (21-342)	91 (18-287)	69 (13-219)	52 (10-161)	33 (6-110)	18 (3-54)	7 (1-21)	2 (0-8)	1 (0-2)	0 (0-0)
Rabies	-	-	618 (322-1,713)	3,242 (1,418-6,527)	4,748 (1,781-8,891)	2,310 (655-3,829)	890 (324-1,415)	799 (275-1,267)	634 (317-1,211)	845 (365-1,754)	1,126 (412-1,894)	1,005 (487-2,014)	1,225 (642-2,779)	1,512 (941-3,838)	973 (550-2,246)	754 (344-1,425)	997 (398-1,908)	620 (355-1,524)	421 (226-1,209)	108 (37-234)	90 (22-232)	17 (4-43)	7 (2-19)
Intestinal nematode infections	-	-	525 (402-675)	6,038 (4,390-7,970)	877 (664-1,142)	160 (124-208)	49 (37-62)	29 (22-38)	22 (16-29)	28 (21-36)	26 (20-33)	18 (13-23)	21 (15-27)	34 (25-43)	31 (23-40)	37 (28-48)	28 (21-36)	42 (32-54)	33 (25-42)	20 (14-27)	7 (5-10)	4 (3-6)	1 (1-2)
Ascariasis	-	-	525 (402-675)	6,038 (4,390-7,970)	877 (664-1,142)	160 (124-208)	49 (37-62)	29 (22-38)	22 (16-29)	28 (21-36)	26 (20-33)	18 (13-23)	21 (15-27)	34 (25-43)	31 (23-40)	37 (28-48)	28 (21-36)	42 (32-54)	33 (25-42)	20 (14-27)	7 (5-10)	4 (3-6)	1 (1-2)
Ebola	0 (0-0)	0 (0-0)	0 (0-0)	0 (0-0)	0 (0-0)	0 (0-0)	0 (0-0)	0 (0-0)	0 (0-0)	0 (0-0)	0 (0-0)	0 (0-0)	0 (0-0)	0 (0-0)	0 (0-0)	0 (0-0)	0 (0-0)	0 (0-0)	0 (0-0)	0 (0-0)	0 (0-0)	0 (0-0)	0 (0-0)
Zika virus	0 (0-0)	0 (0-0)	0 (0-0)	0 (0-0)	0 (0-0)	0 (0-0)	0 (0-0)	0 (0-0)	0 (0-0)	0 (0-0)	0 (0-0)	0 (0-0)	0 (0-0)	0 (0-0)	0 (0-0)	0 (0-0)	0 (0-0)	0 (0-0)	0 (0-0)	0 (0-0)	0 (0-0)	0 (0-0)	0 (0-0)
Other neglected tropical diseases	497 (215-694)	394 (181-567)	2,952 (1,097-4,595)	5,991 (1,416-11,748)	892 (350-1,522)	388 (181-645)	233 (113-358)	195 (85-268)	174 (79-237)	148 (64-217)	160 (71-209)	176 (73-231)	167 (75-218)	223 (99-300)	233 (99-309)	260 (103-352)	274 (124-367)	250 (105-347)	204 (98-291)	128 (68-198)	68 (38-108)	24 (13-37)	11 (6-17)
Maternal disorders	-	-	-	-	-	1,794 (1,504-2,098)	40,639 (36,516-44,207)	78,643 (72,401-84,330)	73,366 (67,588-78,150)	63,143 (58,440-67,241)	51,051 (46,906-54,780)	30,664 (28,025-33,420)	15,791 (13,943-17,866)	2,578 (2,115-3,144)	-	-	-	-	-	-	-	-	-
Maternal haemorrhage	-	-	-	-	-	372 (253-536)	11,747 (7,886-15,627)	27,212 (20,647-33,850)	26,349 (19,674-33,383)	22,528 (16,946-28,246)	17,950 (13,306-22,975)	9,679 (6,732-13,100)	3,933 (2,719-5,435)	462 (268-735)	-	-	-	-	-	-	-	-	-
Maternal sepsis and other pregnancy related infections	-	-	-	-	-	212 (139-302)	5,111 (3,338-7,624)	7,834 (4,822-11,874)	6,939 (4,654-9,953)	5,836 (3,802-8,286)	4,823 (3,210-7,038)	3,062 (1,970-4,538)	1,708 (1,106-2,593)	286 (178-450)	-	-	-	-	-	-	-	-	-
Maternal hypertensive disorders	-	-	-	-	-	293 (181-418)	5,207 (3,316-7,287)	9,073 (6,128-12,799)	8,019 (5,231-11,590)	6,749 (4,298-10,060)	5,556 (3,708-8,032)	3,316 (1,991-5,134)	1,558 (957-2,373)	234 (121-392)	-	-	-	-	-	-	-	-	-
Maternal obstructed labour and uterine rupture	-	-	-	-	-	60 (31-105)	1,177 (600-2,001)	2,681 (1,597-4,169)	2,805 (1,636-4,417)	2,803 (1,594-4,303)	2,633 (1,581-4,297)	1,738 (950-2,970)	942 (492-1,560)	145 (62-277)	-	-	-	-	-	-	-	-	-
Maternal abortion, miscarriage, and ectopic pregnancy	-	-	-	-	-	162 (98-242)	3,102 (1,935-4,669)	5,861 (3,689-8,930)	5,343 (3,505-8,130)	5,085 (3,298-7,326)	4,679 (2,885-6,718)	3,239 (2,147-4,695)	2,106 (1,374-3,075)	321 (203-479)	-	-	-	-	-	-	-	-	-
Indirect maternal deaths	-	-	-	-	-	251 (158-368)	4,995 (3,082-7,443)	9,792 (6,397-14,250)	9,840 (6,382-14,171)	8,463 (5,182-12,485)	6,056 (3,900-8,703)	3,668 (2,222-5,630)	2,239 (1,310-3,320)	490 (261-768)	-	-	-	-	-	-	-	-	-
Late maternal deaths	-	-	-	-	-	42 (21-76)	775 (446-1,273)	1,405 (775-2,391)	1,344 (745-2,310)	1,156 (660-1,912)	905 (490-1,514)	565 (276-958)	305 (141-504)	53 (22-110)	-	-	-	-	-	-	-	-	-
Maternal deaths aggravated by HIV/AIDS	-	-	-	-	-	0 (0-0)	38 (21-57)	129 (74-185)	164 (97-231)	142 (78-205)	100 (58-146)	53 (29-79)	24 (13-37)	4 (2-6)	-	-	-	-	-	-	-	-	-
Other maternal disorders	-	-	-	-	-	403 (282-574)	8,485 (5,697-12,391)	14,656 (9,621-21,018)	12,564 (8,515-17,441)	10,381 (6,767-14,733)	8,350 (5,577-12,370)	5,344 (3,329-7,914)	2,976 (1,938-4,293)	584 (396-828)	-	-	-	-	-	-	-	-	-
Neonatal disorders	962,723 (916,844-1,010,966)	222,396 (198,556-258,554)	61,411 (50,219-81,144)	5,578 (4,044-8,602)	-	-	-	-	-	-	-	-	-	-	-	-	-	-	-	-	-	-	-
Neonatal preterm birth complications	430,666 (386,089-475,480)	79,610 (68,041-103,927)	26,096 (21,232-36,976)	1,610 (1,154-2,555)	-	-	-	-	-	-	-	-	-	-	-	-	-	-	-	-	-	-	-
Neonatal encephalopathy due to birth asphyxia and trauma	283,480 (249,785-323,465)	33,124 (27,407-42,930)	11,333 (8,451-16,250)	2,062 (1,419-3,402)	-	-	-	-	-	-	-	-	-	-	-	-	-	-	-	-	-	-	-
Neonatal sepsis and other neonatal infections	58,664 (31,307-85,336)	46,521 (27,165-66,280)	7,366 (3,939-11,822)	388 (94-795)	-	-	-	-	-	-	-	-	-	-	-	-	-	-	-	-	-	-	-
Hemolytic disease and other neonatal jaundice	31,082 (23,366-41,699)	22,426 (16,542-30,281)	4,788 (3,462-6,893)	280 (181-422)	-	-	-	-	-	-	-	-	-	-	-	-	-	-	-	-	-	-	-
Other neonatal disorders	158,831 (129,418-196,297)	40,715 (32,271-56,725)	11,828 (8,841-18,281)	1,238 (807-2,237)	-	-	-	-	-	-	-	-	-	-	-	-	-	-	-	-	-	-	-
Nutritional deficiencies	-	-	82,779 (71,644-95,471)	101,596 (76,332-131,271)	7,471 (5,885-9,107)	3,102 (2,575-3,616)	2,520 (2,241-2,825)	2,787 (2,506-3,110)	2,915 (2,613-3,239)	2,613 (2,372-2,906)	2,448 (2,230-2,695)	2,869 (2,619-3,173)	2,329 (2,112-2,630)	3,192 (2,873-3,625)	4,145 (3,707-4,739)	5,059 (4,512-5,743)	6,390 (5,717-7,291)	6,950 (6,261-7,848)	9,331 (8,417-10,501)	9,262 (8,463-10,385)	8,471 (7,796-9,436)	5,165 (4,767-5,669)	2,346 (2,181-2,557)
Protein-energy malnutrition	-	-	79,209 (68,249-91,378)	96,780 (72,071-125,566)	6,040 (4,712-7,445)	2,351 (1,937-2,758)	1,636 (1,404-1,894)	1,539 (1,313-1,783)	1,770 (1,517-2,043)	1,479 (1,271-1,698)	1,415 (1,219-1,622)	1,876 (1,658-2,115)	1,208 (1,029-1,411)	1,731 (1,484-2,014)	2,747 (2,394-3,144)	3,455 (3,043-3,951)	4,283 (3,777-4,897)	5,091 (4,532-5,712)	7,262 (6,511-8,142)	7,321 (6,589-8,216)	6,937 (6,235-7,752)	4,340 (3,872-4,842)	1,988 (1,717-2,204)
Iodine deficiency	-	-	346 (87-974)	283 (52-922)	92 (26-257)	20 (4-56)	33 (9-90)	37 (8-107)	37 (10-97)	42 (11-113)	37 (10-95)	28 (9-76)	25 (7-76)	40 (10-115)	47 (15-122)	49 (15-125)	71 (24-178)	67 (23-148)	65 (23-147)	48 (16-116)	26 (7-64)	9 (2-29)	4 (1-15)
Iron-deficiency anaemia	-	-	361 (50-719)	111 (53-224)	121 (41-213)	99 (43-180)	99 (46-162)	104 (49-169)	86 (40-128)	86 (47-117)	101 (45-144)	97 (73-135)	79 (57-119)	85 (60-176)	76 (55-111)	91 (66-126)	92 (62-133)	174 (103-279)	190 (106-343)	156 (89-260)	91 (35-160)	48 (22-81)	39 (21-59)
Other nutritional deficiencies	-	-	2,864 (2,017-3,731)	4,422 (1,802-7,149)	1,217 (853-1,729)	631 (476-830)	753 (611-925)	1,108 (919-1,324)	1,022 (853-1,214)	1,005 (840-1,218)	895 (744-1,073)	867 (706-1,054)	1,016 (837-1,250)	1,336 (1,070-1,650)	1,274 (999-1,634)	1,464 (1,147-1,881)	1,945 (1,502-2,505)	1,618 (1,283-2,121)	1,814 (1,440-2,473)	1,738 (1,379-2,459)	1,417 (1,115-2,280)	768 (584-1,370)	315 (231-651)
Other communicable, maternal, neonatal, and nutritional diseases	23,493 (13,838-36,738)	10,140 (6,235-15,370)	38,954 (26,517-56,859)	35,490 (24,257-49,838)	12,470 (8,804-17,405)	3,991 (3,295-4,686)	5,196 (4,605-5,810)	5,853 (5,302-6,450)	4,884 (4,375-5,407)	4,529 (4,074-5,019)	4,521 (4,122-4,978)	4,210 (3,849-4,625)	3,887 (3,521-4,309)	4,940 (4,446-5,572)	5,789 (5,232-6,496)	5,659 (5,064-6,403)	5,849 (5,154-6,752)	5,341 (4,663-6,349)	4,694 (4,104-5,563)	3,153 (2,662-4,026)	1,738 (1,447-2,213)	673 (544-841)	217 (168-261)
Sexually transmitted diseases excluding HIV	21,763 (12,043-34,978)	8,347 (4,685-13,532)	27,089 (14,702-44,635)	19,995 (10,694-33,461)	6,793 (3,721-11,488)	108 (59-151)	635 (394-829)	762 (492-975)	860 (563-1,094)	682 (443-845)	726 (480-884)	684 (453-829)	625 (422-770)	653 (395-828)	838 (482-1,073)	652 (333-862)	531 (326-665)	365 (239-450)	420 (265-521)	256 (177-308)	156 (113-187)	71 (52-85)	24 (17-29)
Syphilis	21,763 (12,043-34,978)	8,347 (4,685-13,532)	27,089 (14,702-44,635)	19,995 (10,694-33,461)	6,793 (3,721-11,488)	59 (32-82)	179 (111-234)	213 (138-273)	176 (115-224)	140 (91-174)	89 (59-108)	74 (49-90)	61 (41-75)	124 (75-157)	195 (112-250)	157 (80-207)	136 (83-170)	110 (72-136)	111 (70-138)	66 (46-80)	35 (25-42)	15 (11-18)	4 (3-5)
Chlamydial infection	-	-	-	-	-	14 (8-19)	82 (51-108)	165 (106-211)	144 (94-184)	72 (47-89)	117 (77-142)	128 (85-155)	110 (74-135)	101 (61-128)	129 (74-166)	102 (52-135)	49 (30-61)	43 (28-52)	54 (34-67)	46 (31-55)	29 (21-34)	13 (9-16)	4 (3-5)
Gonococcal infection	-	-	-	-	-	24 (13-34)	203 (126-265)	238 (153-304)	375 (245-476)	306 (198-379)	321 (213-391)	318 (211-386)	293 (198-361)	290 (176-368)	340 (196-435)	244 (125-323)	265 (163-332)	158 (103-194)	201 (127-250)	113 (78-136)	72 (52-86)	34 (25-41)	12 (8-14)
Other sexually transmitted diseases	-	-	-	-	-	11 (6-16)	171 (106-223)	146 (94-187)	165 (108-210)	164 (107-203)	199 (132-242)	163 (108-198)	161 (109-198)	138 (83-175)	174 (100-223)	149 (76-197)	81 (50-101)	55 (36-68)	54 (34-67)	31 (21-37)	20 (15-24)	9 (7-11)	4 (2-4)
Hepatitis	-	-	6,989 (6,051-7,973)	10,277 (7,917-12,600)	3,826 (3,017-4,687)	2,896 (2,386-3,439)	3,599 (3,172-4,046)	4,305 (3,850-4,828)	3,349 (2,991-3,744)	3,132 (2,813-3,507)	3,186 (2,885-3,536)	2,888 (2,637-3,217)	2,554 (2,298-2,846)	3,190 (2,877-3,583)	3,773 (3,405-4,194)	3,488 (3,143-3,911)	3,444 (3,072-3,869)	3,176 (2,831-3,607)	2,566 (2,288-2,904)	1,456 (1,267-1,688)	648 (567-759)	186 (165-214)	41 (36-48)
Acute hepatitis A	-	-	4,521 (3,349-5,740)	1,240 (682-1,914)	95 (58-142)	47 (29-70)	65 (40-100)	78 (40-138)	44 (20-80)	32 (13-62)	29 (13-50)	28 (13-52)	26 (11-48)	35 (17-63)	44 (21-77)	50 (24-88)	51 (26-86)	51 (26-87)	47 (25-79)	28 (15-46)	12 (7-18)	4 (2-5)	1 (0-1)
Hepatitis B	-	-	2,468 (1,424-3,643)	1,370 (759-2,170)	365 (194-601)	472 (249-766)	1,394 (928-1,932)	2,864 (2,105-3,655)	2,710 (2,208-3,203)	2,774 (2,380-3,186)	2,907 (2,558-3,269)	2,634 (2,323-2,967)	2,323 (2,048-2,639)	2,893 (2,534-3,305)	3,398 (2,932-3,842)	3,095 (2,658-3,551)	3,004 (2,581-3,473)	2,718 (2,317-3,145)	2,134 (1,751-2,533)	1,074 (830-1,355)	399 (276-527)	92 (54-135)	17 (8-26)
Hepatitis C	-	-	-	-	97 (63-143)	35 (22-52)	45 (28-67)	52 (27-87)	33 (15-59)	27 (11-50)	32 (14-57)	34 (16-61)	40 (17-72)	69 (31-124)	109 (50-197)	112 (51-200)	102 (50-178)	85 (38-153)	69 (34-119)	40 (20-68)	19 (11-30)	6 (3-9)	1 (1-2)
Acute hepatitis E	-	-	-	7,667 (5,687-9,674)	3,269 (2,527-4,061)	2,342 (1,838-2,838)	2,096 (1,544-2,606)	1,311 (677-1,979)	562 (263-899)	299 (129-532)	218 (102-390)	191 (78-369)	165 (70-307)	194 (82-354)	221 (92-457)	231 (93-449)	287 (127-531)	322 (145-590)	317 (133-595)	314 (145-511)	219 (121-338)	85 (49-125)	22 (13-32)
Other infectious diseases	1,730 (1,014-2,124)	1,793 (947-2,596)	4,876 (3,334-6,015)	5,218 (3,149-8,348)	1,851 (1,237-2,502)	987 (694-1,270)	962 (678-1,205)	786 (566-965)	675 (469-798)	715 (491-874)	610 (450-745)	637 (458-795)	708 (528-889)	1,096 (842-1,470)	1,178 (918-1,560)	1,519 (1,150-2,121)	1,875 (1,410-2,593)	1,800 (1,341-2,565)	1,707 (1,281-2,353)	1,441 (1,063-2,178)	934 (682-1,361)	416 (300-572)	151 (107-192)
Non-communicable diseases	97,470 (72,243-125,753)	48,765 (35,376-65,760)	194,756 (151,628-250,248)	156,100 (115,806-201,124)	57,159 (49,893-65,112)	54,696 (50,032-59,814)	81,613 (76,018-85,918)	98,161 (92,970-102,427)	110,317 (105,644-114,162)	144,498 (139,106-149,353)	203,709 (196,424-210,023)	268,075 (260,545-275,238)	361,388 (349,410-371,421)	552,611 (532,963-568,102)	757,358 (730,642-777,870)	1,058,273 (1,019,187-1,087,280)	1,349,670 (1,297,436-1,387,265)	1,560,657 (1,499,319-1,607,654)	1,889,968 (1,829,380-1,935,691)	1,798,319 (1,753,504-1,840,589)	1,332,678 (1,305,043-1,358,297)	638,913 (629,112-649,734)	221,265 (218,147-224,513)
Neoplasms	517 (438-565)	340 (289-387)	5,081 (3,930-6,270)	16,014 (12,318-22,123)	14,152 (12,210-16,485)	13,464 (11,871-15,561)	17,333 (15,668-18,627)	21,063 (19,691-22,195)	28,013 (26,648-29,287)	45,912 (43,590-47,995)	74,969 (71,047-78,570)	104,317 (99,379-108,553)	136,521 (130,641-142,101)	194,216 (186,568-201,509)	241,973 (232,994-251,896)	297,998 (289,117-309,549)	325,793 (316,556-337,535)	301,760 (292,722-316,480)	289,826 (281,422-302,535)	204,855 (198,456-213,942)	118,328 (114,642-123,111)	44,510 (42,949-46,307)	11,926 (11,440-12,370)
Lip and oral cavity cancer	-	-	-	-	-	-	134 (123-149)	233 (211-265)	311 (278-355)	499 (452-572)	759 (686-872)	1,298 (1,165-1,470)	1,922 (1,747-2,137)	2,695 (2,455-2,952)	3,244 (2,958-3,576)	3,998 (3,639-4,359)	3,736 (3,449-4,037)	3,129 (2,860-3,374)	2,976 (2,754-3,171)	2,438 (2,265-2,616)	1,596 (1,487-1,706)	695 (646-744)	209 (195-221)
Nasopharynx cancer	-	-	-	-	74 (62-90)	130 (106-169)	272 (232-321)	364 (321-413)	419 (376-474)	594 (529-670)	935 (836-1,050)	1,193 (1,054-1,375)	1,485 (1,306-1,744)	1,952 (1,692-2,320)	2,286 (1,983-2,711)	1,988 (1,708-2,351)	1,616 (1,409-1,857)	1,331 (1,157-1,517)	889 (784-1,000)	491 (428-562)	216 (190-245)	69 (62-77)	17 (15-18)
Other pharynx cancer	-	-	-	-	-	-	160 (133-188)	198 (165-235)	298 (250-344)	427 (355-494)	620 (520-722)	973 (830-1,115)	1,616 (1,400-1,830)	2,378 (2,064-2,705)	2,639 (2,306-2,954)	3,043 (2,674-3,411)	2,629 (2,336-2,939)	2,249 (1,967-2,523)	1,338 (1,176-1,514)	871 (759-987)	433 (388-482)	144 (132-158)	35 (33-38)
Oesophageal cancer	-	-	-	-	-	-	213 (193-237)	361 (336-389)	444 (408-484)	718 (666-773)	1,340 (1,247-1,438)	2,372 (2,202-2,560)	4,011 (3,750-4,310)	7,017 (6,587-7,568)	10,988 (10,262-11,895)	15,105 (14,022-16,599)	17,603 (16,333-19,412)	17,899 (16,359-20,109)	14,430 (13,362-16,168)	8,800 (8,190-9,786)	4,672 (4,392-5,110)	1,545 (1,451-1,675)	342 (321-368)
Stomach cancer	-	-	-	-	-	-	701 (644-753)	1,717 (1,586-1,844)	2,958 (2,712-3,178)	5,533 (5,153-5,863)	8,895 (8,309-9,423)	11,311 (10,566-11,963)	13,803 (12,951-14,639)	19,901 (18,766-21,149)	26,596 (25,160-28,364)	35,985 (33,977-38,610)	41,667 (39,292-44,797)	41,614 (39,170-45,080)	41,181 (39,017-44,072)	29,433 (27,964-31,436)	16,636 (15,815-17,664)	5,827 (5,560-6,175)	1,414 (1,344-1,486)
Colon and rectum cancer	-	-	-	-	-	-	378 (334-430)	923 (817-1,040)	1,473 (1,343-1,637)	2,795 (2,535-3,121)	4,244 (3,923-4,657)	5,917 (5,545-6,435)	8,910 (8,365-9,680)	14,263 (13,455-15,360)	19,455 (18,421-20,831)	26,465 (25,230-27,945)	31,885 (30,513-33,438)	32,598 (31,211-34,125)	38,645 (37,098-40,261)	31,305 (30,097-32,535)	20,784 (19,964-21,582)	8,818 (8,457-9,190)	2,480 (2,360-2,586)
Liver cancer	-	-	-	-	244 (162-328)	316 (203-402)	566 (355-699)	1,069 (659-1,352)	1,604 (1,045-1,909)	2,662 (1,689-3,214)	4,419 (2,761-5,265)	6,149 (3,875-7,183)	7,841 (5,277-9,146)	11,577 (8,229-13,397)	15,378 (11,254-17,866)	18,590 (14,721-21,394)	20,803 (16,720-23,567)	19,681 (16,560-22,049)	16,771 (14,975-18,422)	10,384 (9,515-11,660)	5,111 (4,776-5,680)	1,551 (1,455-1,719)	333 (311-363)
Liver cancer due to hepatitis B	-	-	-	-	102 (58-153)	172 (99-238)	351 (205-459)	764 (454-990)	1,192 (770-1,468)	1,952 (1,204-2,451)	3,094 (1,867-3,844)	3,920 (2,417-4,853)	4,445 (2,829-5,573)	5,790 (3,866-7,256)	6,791 (4,281-9,040)	7,131 (4,560-9,377)	7,068 (4,654-9,224)	6,317 (4,116-8,339)	4,962 (3,332-6,660)	2,729 (1,797-3,780)	1,252 (903-1,668)	351 (247-479)	71 (43-103)
Liver cancer due to hepatitis C	-	-	-	-	10 (7-14)	16 (11-22)	28 (19-38)	62 (42-86)	108 (67-164)	197 (126-275)	343 (227-493)	614 (426-837)	1,045 (727-1,401)	1,889 (1,392-2,466)	2,891 (2,188-3,655)	4,011 (3,190-4,993)	4,698 (3,857-5,617)	4,666 (3,898-5,579)	4,779 (4,075-5,647)	3,635 (3,107-4,296)	2,039 (1,789-2,358)	694 (596-788)	161 (136-189)
Liver cancer due to alcohol use	-	-	-	-	-	-	4 (2-7)	23 (13-37)	60 (29-103)	190 (111-302)	525 (275-889)	1,027 (596-1,548)	1,626 (884-2,480)	2,867 (1,738-4,099)	4,416 (2,626-6,523)	5,904 (4,032-8,222)	7,328 (5,494-9,477)	7,052 (5,232-9,105)	5,468 (4,147-6,945)	2,958 (2,117-3,860)	1,286 (931-1,655)	340 (235-457)	64 (37-93)
Liver cancer due to other causes	-	-	-	-	132 (90-189)	128 (85-178)	183 (120-255)	219 (137-309)	245 (152-367)	323 (198-466)	456 (278-651)	588 (367-823)	724 (455-993)	1,031 (699-1,397)	1,281 (869-1,734)	1,544 (1,166-1,989)	1,709 (1,337-2,128)	1,647 (1,272-2,076)	1,562 (1,230-1,948)	1,063 (839-1,350)	534 (434-654)	166 (133-207)	36 (27-48)
Gallbladder and biliary tract cancer	-	-	-	-	-	-	47 (42-53)	82 (72-93)	160 (138-181)	388 (337-440)	789 (684-905)	1,475 (1,287-1,666)	2,437 (2,178-2,735)	4,062 (3,665-4,471)	5,669 (5,194-6,181)	8,199 (7,694-8,816)	9,737 (9,157-10,475)	9,467 (8,906-10,254)	10,584 (10,129-11,256)	8,087 (7,780-8,588)	4,495 (4,314-4,718)	1,611 (1,543-1,693)	395 (376-414)
Pancreatic cancer	-	-	-	-	-	-	69 (64-76)	135 (125-148)	236 (222-253)	426 (403-455)	881 (839-935)	1,580 (1,524-1,649)	2,612 (2,525-2,702)	4,791 (4,632-4,954)	7,420 (7,182-7,665)	11,743 (11,303-12,131)	14,922 (14,444-15,386)	15,214 (14,724-15,709)	16,476 (15,919-17,038)	12,240 (11,755-12,716)	7,212 (6,927-7,495)	2,681 (2,566-2,792)	672 (640-702)
Larynx cancer	-	-	-	-	-	-	73 (66-81)	113 (100-126)	124 (108-145)	231 (199-272)	366 (334-401)	592 (537-659)	906 (824-1,001)	1,350 (1,238-1,495)	1,709 (1,569-1,890)	1,981 (1,815-2,180)	2,116 (1,946-2,346)	1,949 (1,776-2,186)	1,435 (1,316-1,588)	930 (852-1,034)	438 (408-483)	137 (129-149)	31 (29-33)
Tracheal, bronchus, and lung cancer	-	-	-	-	-	-	343 (245-391)	637 (466-711)	1,207 (952-1,319)	2,438 (2,057-2,674)	4,712 (4,308-5,108)	7,899 (7,372-8,382)	11,677 (11,030-12,314)	18,774 (17,810-19,762)	27,187 (25,876-28,577)	37,937 (36,244-39,691)	44,991 (43,225-47,239)	42,803 (41,063-45,077)	37,645 (36,130-39,486)	22,816 (21,762-24,081)	11,601 (11,057-12,205)	3,773 (3,578-3,979)	924 (873-972)
Malignant skin melanoma	-	-	-	-	-	-	105 (82-133)	191 (154-244)	317 (251-384)	483 (382-570)	692 (546-828)	868 (670-1,050)	943 (740-1,136)	1,266 (1,012-1,568)	1,408 (1,167-1,775)	1,817 (1,544-2,298)	1,935 (1,605-2,504)	1,733 (1,447-2,312)	1,936 (1,644-2,594)	1,598 (1,376-2,217)	978 (812-1,341)	416 (347-579)	136 (114-184)
Non-melanoma skin cancer	-	-	-	-	-	-	70 (63-80)	85 (75-95)	102 (92-110)	126 (113-138)	188 (167-206)	234 (212-254)	320 (291-344)	429 (392-461)	557 (510-599)	734 (679-791)	856 (796-916)	966 (897-1,038)	1,106 (1,046-1,170)	1,281 (1,205-1,352)	1,142 (1,082-1,204)	637 (604-672)	287 (271-304)
Non-melanoma skin cancer (squamous-cell carcinoma)	-	-	-	-	-	-	70 (63-80)	85 (75-95)	102 (92-110)	126 (113-138)	188 (167-206)	234 (212-254)	320 (291-344)	429 (392-461)	557 (510-599)	734 (679-791)	856 (796-916)	966 (897-1,038)	1,106 (1,046-1,170)	1,281 (1,205-1,352)	1,142 (1,082-1,204)	637 (604-672)	287 (271-304)
Breast cancer	-	-	-	-	-	-	259 (184-327)	806 (640-1,017)	2,625 (2,186-3,161)	7,790 (6,721-9,108)	16,139 (14,365-18,605)	23,505 (21,288-26,732)	29,565 (26,849-33,256)	37,784 (34,887-41,706)	40,443 (37,689-44,937)	40,205 (37,988-43,977)	38,101 (36,323-41,326)	30,158 (28,816-32,901)	28,579 (27,348-31,014)	20,838 (19,911-22,261)	13,036 (12,436-13,863)	5,352 (5,096-5,665)	1,671 (1,593-1,766)
Cervical cancer	-	-	-	-	-	-	271 (207-318)	943 (746-1,155)	2,580 (2,187-3,117)	6,186 (5,218-7,563)	11,201 (9,574-13,808)	15,939 (13,288-20,339)	19,714 (16,525-24,808)	23,693 (20,010-30,256)	25,319 (21,667-32,297)	24,649 (21,502-30,966)	21,581 (18,991-26,849)	17,046 (15,198-21,001)	12,861 (11,877-14,679)	8,252 (7,633-9,723)	4,010 (3,735-4,631)	1,366 (1,284-1,527)	370 (349-407)
Uterine cancer	-	-	-	-	-	-	118 (104-132)	285 (252-313)	437 (391-481)	726 (649-791)	1,364 (1,212-1,478)	1,997 (1,817-2,152)	3,252 (2,936-3,498)	5,234 (4,818-5,613)	6,810 (6,232-7,303)	9,072 (8,501-9,628)	9,619 (9,055-10,165)	7,894 (7,493-8,326)	7,054 (6,717-7,409)	4,689 (4,464-4,907)	2,462 (2,353-2,575)	895 (853-942)	235 (222-247)
Ovarian cancer	-	-	-	-	-	-	470 (398-526)	663 (570-739)	904 (802-982)	1,425 (1,275-1,545)	2,544 (2,275-2,754)	4,258 (3,862-4,479)	6,540 (6,073-6,869)	10,454 (9,831-10,944)	11,762 (11,160-12,221)	14,004 (13,271-14,628)	13,959 (13,365-14,529)	11,374 (10,956-11,800)	10,107 (9,708-10,528)	6,585 (6,304-6,891)	3,392 (3,246-3,538)	1,177 (1,127-1,228)	274 (260-287)
Prostate cancer	-	-	-	-	-	-	-	-	-	-	-	-	-	-	-	-	-	-	-	-	-	-	-
Testicular cancer	-	-	-	-	-	-	-	-	-	-	-	-	-	-	-	-	-	-	-	-	-	-	-
Kidney cancer	17 (16-20)	9 (8-11)	142 (118-182)	634 (475-898)	346 (304-412)	151 (138-171)	100 (94-109)	122 (115-134)	159 (151-172)	213 (201-230)	347 (330-372)	549 (528-573)	861 (829-897)	1,503 (1,446-1,563)	2,232 (2,146-2,321)	3,210 (3,088-3,335)	3,715 (3,595-3,841)	3,459 (3,346-3,572)	3,675 (3,535-3,825)	2,719 (2,605-2,825)	1,572 (1,504-1,641)	585 (557-616)	142 (135-150)
Bladder cancer	-	-	-	-	-	-	52 (46-59)	71 (63-80)	104 (93-116)	176 (158-199)	296 (266-334)	492 (447-551)	670 (611-748)	1,212 (1,117-1,337)	1,730 (1,612-1,880)	2,695 (2,537-2,888)	3,760 (3,560-3,994)	4,339 (4,111-4,577)	5,606 (5,357-5,844)	4,920 (4,721-5,124)	3,394 (3,247-3,545)	1,486 (1,418-1,554)	431 (410-452)
Brain and nervous system cancer	68 (43-86)	46 (29-58)	729 (382-986)	2,687 (1,656-3,589)	3,095 (2,234-3,577)	2,395 (1,807-2,718)	1,755 (1,327-2,005)	1,745 (1,321-1,982)	1,874 (1,497-2,086)	2,229 (1,781-2,462)	2,893 (2,318-3,199)	3,157 (2,581-3,495)	3,491 (2,886-3,833)	4,795 (3,961-5,226)	5,687 (4,903-6,221)	6,375 (5,629-7,018)	6,374 (5,709-7,035)	4,874 (4,392-5,417)	3,945 (3,596-4,481)	2,105 (1,895-2,442)	929 (839-1,056)	256 (230-287)	55 (49-61)
Thyroid cancer	-	-	-	-	-	87 (69-106)	199 (155-238)	316 (254-373)	367 (308-423)	437 (365-502)	526 (440-603)	749 (640-856)	967 (829-1,103)	1,521 (1,310-1,727)	1,915 (1,695-2,139)	2,198 (1,983-2,423)	2,382 (2,174-2,614)	2,193 (2,016-2,380)	2,057 (1,930-2,216)	1,463 (1,380-1,564)	808 (763-881)	273 (258-294)	58 (54-62)
Mesothelioma	-	-	-	-	-	-	45 (34-67)	63 (43-94)	89 (62-130)	96 (70-140)	160 (121-228)	250 (200-347)	205 (170-274)	344 (298-438)	476 (415-604)	614 (551-745)	691 (631-820)	647 (597-761)	826 (761-961)	514 (475-561)	253 (237-272)	90 (84-96)	22 (21-24)
Hodgkin lymphoma	4 (1-5)	2 (1-3)	70 (20-116)	257 (76-426)	390 (175-592)	520 (261-689)	794 (470-953)	891 (583-1,041)	882 (598-1,021)	803 (563-911)	775 (516-876)	722 (479-841)	641 (430-738)	830 (530-960)	898 (597-1,041)	1,083 (711-1,265)	1,159 (803-1,338)	940 (670-1,085)	837 (639-958)	525 (412-599)	234 (188-266)	62 (52-70)	13 (11-15)
Non-Hodgkin's lymphoma	25 (19-28)	19 (15-23)	340 (224-460)	1,153 (806-1,733)	1,096 (819-1,382)	979 (796-1,164)	1,015 (866-1,120)	1,181 (1,003-1,304)	1,207 (1,071-1,316)	1,320 (1,163-1,429)	1,600 (1,441-1,716)	1,947 (1,755-2,101)	2,166 (2,020-2,350)	3,090 (2,883-3,409)	3,959 (3,733-4,323)	5,717 (5,410-6,207)	6,900 (6,571-7,464)	6,788 (6,476-7,398)	6,561 (6,247-7,099)	4,967 (4,710-5,513)	2,870 (2,711-3,126)	1,019 (962-1,112)	253 (237-271)
Multiple myeloma	-	-	-	-	-	-	117 (79-144)	140 (96-173)	122 (90-146)	135 (104-163)	251 (192-302)	479 (379-563)	800 (637-935)	1,499 (1,212-1,760)	2,126 (1,759-2,407)	3,256 (2,592-3,840)	4,069 (3,433-4,884)	3,647 (3,094-4,601)	3,921 (3,348-5,179)	2,957 (2,553-3,928)	1,669 (1,438-2,284)	575 (496-814)	119 (100-162)
Leukaemia	152 (96-181)	119 (72-150)	2,696 (1,416-3,662)	8,152 (4,829-13,008)	6,675 (4,951-8,951)	6,328 (4,944-8,550)	6,097 (4,679-7,814)	4,987 (3,852-6,275)	4,212 (3,276-5,064)	4,010 (3,128-4,894)	4,509 (3,492-5,398)	4,301 (3,558-5,107)	4,517 (3,835-5,359)	5,493 (4,770-6,498)	6,525 (5,815-7,770)	7,872 (7,250-9,327)	8,587 (7,963-9,956)	8,075 (7,558-9,396)	8,341 (7,918-9,293)	6,258 (5,976-6,924)	3,877 (3,688-4,217)	1,607 (1,523-1,725)	470 (445-502)
Acute lymphoid leukaemia	34 (25-51)	28 (19-50)	544 (359-1,073)	2,220 (1,482-4,864)	2,381 (1,794-3,741)	1,989 (1,579-3,087)	1,353 (1,073-2,184)	890 (715-1,468)	754 (607-1,177)	542 (438-871)	602 (485-920)	543 (444-788)	558 (453-776)	629 (522-858)	801 (661-1,110)	831 (699-1,161)	759 (647-1,090)	644 (548-900)	540 (475-683)	348 (312-445)	183 (166-224)	66 (60-79)	18 (16-21)
Chronic lymphoid leukaemia	-	-	-	-	-	-	135 (91-247)	130 (87-232)	126 (90-210)	139 (100-222)	197 (143-315)	214 (165-304)	195 (154-288)	351 (293-473)	518 (443-660)	964 (854-1,166)	1,198 (1,078-1,405)	1,165 (1,063-1,346)	1,555 (1,429-1,713)	1,303 (1,221-1,415)	956 (892-1,029)	420 (393-453)	135 (125-145)
Acute myeloid leukaemia	19 (14-27)	15 (10-23)	245 (159-425)	892 (585-1,664)	730 (522-1,032)	886 (681-1,235)	895 (731-1,206)	725 (601-985)	700 (591-916)	705 (599-899)	823 (692-1,070)	848 (735-1,042)	982 (867-1,219)	1,130 (1,003-1,344)	1,311 (1,184-1,542)	1,675 (1,549-1,898)	1,863 (1,747-2,092)	1,795 (1,695-2,000)	1,864 (1,761-2,081)	1,274 (1,202-1,413)	673 (632-738)	250 (236-271)	64 (59-68)
Chronic myeloid leukaemia	-	-	-	-	-	-	285 (189-394)	304 (210-405)	426 (306-568)	456 (340-579)	490 (375-608)	607 (484-739)	637 (517-767)	817 (667-953)	918 (785-1,060)	1,067 (945-1,222)	1,126 (1,009-1,277)	1,018 (917-1,159)	1,081 (989-1,188)	895 (817-990)	546 (492-602)	240 (215-266)	74 (66-80)
Other leukaemia	99 (53-126)	76 (39-106)	1,907 (820-2,807)	5,039 (2,359-8,172)	3,564 (2,221-4,974)	3,453 (2,300-4,598)	3,430 (2,233-4,242)	2,938 (1,911-3,629)	2,206 (1,505-2,615)	2,169 (1,482-2,609)	2,397 (1,658-2,856)	2,089 (1,583-2,451)	2,144 (1,656-2,541)	2,565 (2,108-3,097)	2,976 (2,490-3,580)	3,336 (2,941-4,056)	3,640 (3,248-4,254)	3,454 (3,135-4,212)	3,302 (3,056-3,855)	2,438 (2,273-2,856)	1,519 (1,421-1,728)	630 (585-721)	180 (167-202)
Other neoplasms	251 (224-273)	145 (121-171)	1,103 (863-1,468)	3,130 (2,465-4,766)	2,232 (1,936-2,673)	2,559 (2,274-2,876)	2,909 (2,690-3,137)	2,743 (2,533-2,936)	2,799 (2,608-3,011)	3,045 (2,823-3,272)	3,522 (3,313-3,755)	4,110 (3,866-4,440)	4,653 (4,405-5,003)	6,307 (5,948-6,795)	7,555 (7,169-8,182)	9,462 (8,990-10,134)	10,398 (9,903-11,107)	9,692 (9,244-10,373)	10,042 (9,539-10,715)	7,389 (7,012-7,898)	4,509 (4,285-4,817)	1,861 (1,768-1,971)	538 (509-567)
Cardiovascular diseases	2,567 (2,359-2,824)	1,982 (1,675-2,315)	17,801 (15,592-20,477)	16,640 (13,866-19,767)	8,228 (7,089-9,388)	9,191 (8,297-10,057)	20,310 (18,839-21,598)	28,315 (26,559-29,840)	33,766 (31,970-35,309)	45,514 (43,195-47,745)	64,867 (61,719-67,739)	90,031 (86,372-93,609)	127,583 (122,093-133,524)	208,850 (199,737-219,312)	307,516 (293,629-323,983)	465,694 (444,842-490,868)	639,624 (612,134-675,977)	785,331 (750,917-833,370)	1,042,452 (1,003,493-1,093,710)	1,059,850 (1,024,212-1,109,368)	799,762 (775,668-835,951)	380,686 (367,641-396,607)	139,716 (134,007-144,867)
Rheumatic heart disease	-	-	-	4,763 (3,571-6,216)	2,500 (1,963-3,081)	3,678 (3,058-4,207)	5,734 (4,688-6,709)	6,408 (5,223-7,541)	6,790 (5,308-8,170)	7,600 (5,962-9,215)	9,530 (7,574-11,329)	11,026 (8,704-13,108)	11,528 (9,026-13,720)	16,082 (12,751-18,936)	18,565 (15,006-21,842)	21,686 (17,651-25,306)	23,274 (18,983-27,149)	24,368 (19,854-29,101)	22,589 (18,881-26,968)	17,014 (14,548-20,164)	10,388 (9,019-12,327)	4,284 (3,804-4,950)	1,272 (1,145-1,439)
Ischaemic heart disease	-	-	1,108 (838-1,469)	1,070 (762-1,691)	645 (504-872)	651 (539-817)	5,895 (5,199-6,983)	8,483 (7,671-9,668)	10,990 (9,903-12,494)	16,636 (15,185-18,754)	22,933 (21,246-25,420)	32,660 (30,456-35,578)	49,618 (46,693-53,185)	82,170 (77,425-87,234)	127,114 (120,381-134,520)	203,974 (193,343-215,442)	286,296 (272,924-301,763)	351,537 (333,579-371,395)	508,884 (489,454-533,072)	543,997 (527,377-566,958)	434,403 (422,486-452,146)	222,005 (215,676-231,335)	87,713 (85,284-90,722)
Cerebrovascular disease	1,175 (1,012-1,381)	882 (695-1,128)	7,398 (5,708-9,533)	5,059 (3,796-7,241)	2,690 (2,237-3,334)	2,605 (2,268-3,079)	4,779 (4,321-5,404)	8,057 (7,331-9,027)	9,840 (8,929-10,919)	13,973 (12,813-15,426)	22,733 (21,111-24,760)	33,767 (31,525-36,204)	49,815 (46,773-53,251)	84,097 (78,960-90,333)	123,491 (116,250-132,772)	186,208 (175,369-201,657)	255,374 (239,388-278,023)	314,454 (292,711-347,579)	384,967 (359,261-421,740)	363,593 (336,752-397,936)	242,780 (222,968-268,366)	97,949 (88,433-108,911)	32,051 (28,449-35,357)

SR Table 3: Global deaths by cause and age group for females (with 95% uncertainty intervals), 1990

Cause	Early Neonatal	Late Neonatal	Post Neonatal	1 to 4
Ischaemic stroke	-	-	448 (331-595)	297 (229-414)
Hemorrhagic stroke	1,175 (1,012-1,381)	882 (695-1,128)	6,949 (5,349-8,919)	4,762 (3,564-6,815)
Hypertensive heart disease	-	-	312 (106-450)	228 (67-350)
Cardiomyopathy and myocarditis	394 (266-489)	331 (183-449)	3,015 (1,871-4,613)	2,044 (1,164-3,282)
Myocarditis	85 (52-134)	85 (47-160)	1,154 (609-2,182)	864 (480-1,751)
Alcoholic cardiomyopathy	-	-	-	-
Other cardiomyopathy	309 (202-396)	246 (119-359)	1,861 (955-3,046)	1,181 (559-2,141)
Atrial fibrillation and flutter	-	-	-	-
Aortic aneurysm	-	-	-	-
Peripheral vascular disease	-	-	-	-
Endocarditis	67 (40-85)	52 (33-81)	1,021 (611-1,630)	527 (303-1,091)
Other cardiovascular and circulatory diseases	931 (681-1,101)	717 (464-915)	4,946 (2,896-6,340)	2,948 (1,448-4,374)
Chronic respiratory diseases	0 (0-0)	0 (0-0)	0 (0-0)	17,757 (13,280-21,513)
Chronic obstructive pulmonary disease	-	-	0 (0-0)	4,617 (2,981-7,552)
Pneumoconiosis	-	-	-	-
Silicosis	-	-	-	-
Asbestosis	-	-	-	-
Coal workers pneumoconiosis	-	-	-	-
Other pneumoconiosis	-	-	-	-
Asthma	-	-	-	11,624 (6,059-15,155)
Interstitial lung disease and pulmonary sarcoidosis	-	-	-	382 (93-1,142)
Other chronic respiratory diseases	0 (0-0)	0 (0-0)	0 (0-0)	1,134 (634-2,434)
Cirrhosis and other chronic liver diseases	-	-	-	7,786 (5,994-9,802)
Cirrhosis and other chronic liver diseases due to hepatitis B	-	-	-	29 (20-41)
Cirrhosis and other chronic liver diseases due to hepatitis C	-	-	-	24 (15-34)
Cirrhosis and other chronic liver diseases due to alcohol use	-	-	-	-
Cirrhosis and other chronic liver diseases due to other causes	-	-	-	7,734 (5,957-9,731)
Digestive diseases	1,308 (779-1,673)	1,516 (940-2,019)	13,084 (9,146-17,820)	17,588 (10,637-27,007)
Peptic ulcer disease	-	-	-	3,721 (2,105-6,150)
Gastritis and duodenitis	-	-	-	571 (193-1,096)
Appendicitis	-	-	-	3,280 (1,182-5,865)
Paralytic ileus and intestinal obstruction	1,308 (779-1,673)	1,516 (940-2,019)	13,084 (9,146-17,820)	2,855 (1,282-4,467)
Inguinal, femoral, and abdominal hernia	-	-	-	368 (110-779)
Inflammatory bowel disease	-	-	-	1,246 (328-3,765)
Vascular intestinal disorders	-	-	-	156 (51-233)
Gallbladder and biliary diseases	-	-	-	750 (255-1,209)
Pancreatitis	-	-	-	158 (59-281)
Other digestive diseases	-	-	-	4,484 (2,266-7,238)
Neurological disorders	-	-	4,764 (3,426-6,093)	5,582 (3,311-8,030)
Alzheimer's disease and other dementias	-	-	-	-
Parkinson's disease	-	-	-	-
Epilepsy	-	-	3,101 (1,653-4,086)	4,145 (2,197-6,051)
Multiple sclerosis	-	-	-	-
Motor neuron disease	4 (4-6)	5 (4-6)	520 (338-731)	237 (120-358)
Other neurological disorders	-	-	1,144 (704-1,556)	1,200 (588-1,795)
Mental and substance use disorders	6 (3-9)	16 (11-23)	0 (0-0)	0 (0-0)
Alcohol use disorders	-	-	-	-
Drug use disorders	6 (3-9)	16 (11-23)	0 (0-0)	0 (0-0)
Opioid use disorders	6 (3-9)	16 (11-23)	0 (0-0)	0 (0-0)
Cocaine use disorders	0 (0-0)	0 (0-0)	0 (0-0)	0 (0-0)
Amphetamine use disorders	0 (0-0)	0 (0-0)	0 (0-0)	0 (0-0)
Other drug use disorders	0 (0-0)	0 (0-0)	0 (0-0)	0 (0-0)
Eating disorders	-	-	-	-
Anorexia nervosa	-	-	-	-
Bulimia nervosa	-	-	-	-
Diabetes, urogenital, blood, and endocrine diseases	2,234 (1,590-2,678)	1,693 (1,200-2,038)	15,729 (12,643-18,424)	24,068 (15,312-31,145)
Diabetes mellitus	33 (30-35)	75 (65-86)	720 (622-843)	837 (684-1,010)
Acute glomerulonephritis	-	-	176 (129-238)	290 (205-410)
Chronic kidney disease	-	-	2,650 (2,276-3,081)	5,092 (4,154-6,112)
Chronic kidney disease due to diabetes mellitus	-	-	66 (43-98)	127 (77-193)
Chronic kidney disease due to hypertension	-	-	19 (13-28)	53 (34-77)
Chronic kidney disease due to glomerulonephritis	-	-	1,127 (826-1,417)	2,199 (1,614-2,884)
Chronic kidney disease due to other causes	-	-	1,437 (1,110-1,793)	2,714 (2,036-3,530)
Urinary diseases and male infertility	139 (124-165)	183 (145-239)	2,036 (1,757-2,494)	2,202 (1,734-2,922)
Interstitial nephritis and urinary tract infections	58 (38-79)	79 (46-117)	1,185 (866-1,545)	1,206 (637-1,895)
Urolithiasis	-	-	-	-
Other urinary diseases	81 (67-102)	104 (76-147)	852 (644-1,122)	997 (641-1,549)
Gynecological diseases	-	-	-	-
Uterine fibroids	-	-	-	-
Polycystic ovarian syndrome	-	-	-	-
Endometriosis	-	-	-	-
Genital prolapse	-	-	-	-
Other gynecological diseases	-	-	-	-
Hemoglobinopathies and hemolytic anaemias	1,129 (689-1,538)	653 (358-825)	5,385 (2,597-7,354)	12,097 (3,573-18,319)
Thalassemias	380 (227-546)	215 (108-306)	1,880 (810-2,801)	3,302 (1,195-5,287)
Sickle cell disorders	659 (378-1,037)	414 (215-539)	3,332 (1,637-4,592)	8,457 (2,130-13,312)
G6PD deficiency	16 (10-23)	10 (5-14)	74 (42-108)	178 (79-278)
Other hemoglobinopathies and hemolytic anaemias	73 (49-99)	14 (8-19)	99 (55-142)	159 (80-232)
Endocrine, metabolic, blood, and immune disorders	933 (527-1,110)	782 (378-1,047)	4,762 (2,598-6,275)	3,549 (1,864-5,026)
Musculoskeletal disorders	-	-	-	-
Rheumatoid arthritis	-	-	-	-
Other musculoskeletal disorders	-	-	-	-
Other non-communicable diseases	90,833 (66,095-118,957)	43,213 (30,678-59,817)	138,297 (98,527-189,682)	50,664 (28,509-82,337)
Congenital anomalies	90,833 (66,095-118,957)	40,962 (28,870-57,011)	118,094 (81,108-166,049)	49,618 (27,425-81,270)
Neural tube defects	17,517 (10,517-22,302)	6,653 (3,420-9,149)	14,958 (7,588-21,986)	4,392 (1,855-8,576)
Congenital heart anomalies	27,345 (15,772-35,004)	15,572 (9,183-22,733)	54,269 (32,195-80,817)	23,568 (12,458-40,980)
Orofacial clefts	1,004 (491-1,708)	489 (241-769)	714 (319-1,142)	111 (26-252)
Down's syndrome	770 (474-1,622)	408 (244-1,046)	1,935 (1,162-4,638)	818 (455-2,548)
Other chromosomal abnormalities	2,365 (1,306-5,646)	1,445 (789-3,943)	3,146 (1,595-8,841)	519 (228-2,134)
Congenital musculoskeletal and limb anomalies	2,343 (1,219-4,705)	709 (324-1,676)	1,384 (605-3,545)	498 (175-1,762)
Urogenital congenital anomalies	2,388 (1,175-3,851)	957 (401-1,866)	1,575 (598-3,406)	422 (137-1,055)
Digestive congenital anomalies	6,378 (4,511-11,188)	2,958 (2,073-5,732)	7,037 (4,459-17,734)	1,418 (758-5,671)
Other congenital anomalies	30,723 (13,625-52,873)	11,771 (3,760-23,361)	33,074 (9,851-67,327)	17,872 (3,824-43,712)
Skin and subcutaneous diseases	-	-	2,213 (814-3,272)	1,046 (380-1,619)
Cellulitis	-	-	103 (22-200)	91 (20-189)
Pyoderma	-	-	2,056 (745-3,082)	855 (302-1,381)
Decubitus ulcer	-	-	-	38 (17-71)
Other skin and subcutaneous diseases	-	-	53 (23-101)	62 (30-111)
Sudden infant death syndrome	-	2,251 (1,468-3,768)	17,990 (11,822-29,465)	-
Injuries	8,274 (6,292-9,693)	6,137 (4,576-7,378)	65,366 (53,259-78,650)	176,519 (133,259-210,126)
Transport injuries	720 (539-941)	894 (595-1,164)	9,258 (7,306-11,002)	33,462 (24,428-40,292)
Road injuries	636 (478-824)	836 (548-1,078)	8,494 (6,443-9,982)	31,507 (22,046-37,439)
Pedestrian road injuries	296 (208-393)	405 (259-605)	4,093 (3,172-5,439)	19,410 (14,934-25,310)
Cyclist road injuries	-	-	-	812 (306-1,252)
Motorcyclist road injuries	127 (75-199)	90 (45-149)	1,367 (781-1,874)	2,916 (1,155-4,228)
Motor vehicle road injuries	203 (137-279)	336 (194-490)	2,937 (1,841-4,006)	8,201 (4,358-12,078)
Other road injuries	10 (6-16)	6 (3-9)	98 (48-187)	168 (74-339)
Other transport injuries	84 (40-136)	58 (30-107)	764 (459-1,360)	1,955 (1,158-3,743)
Unintentional injuries	5,487 (3,804-6,655)	4,406 (3,007-5,549)	51,054 (39,409-63,737)	128,057 (91,252-159,257)
Falls	1,180 (582-1,821)	527 (257-797)	7,204 (3,732-10,673)	12,297 (6,510-18,339)
Drowning	251 (193-282)	198 (134-238)	6,755 (4,177-8,190)	70,688 (45,129-87,205)
Fire, heat, and hot substances	197 (139-231)	311 (171-405)	4,688 (2,698-5,955)	13,284 (6,720-18,930)
Poisonings	224 (111-370)	189 (108-270)	3,190 (1,232-5,278)	6,866 (3,275-10,519)
Exposure to mechanical forces	1,201 (615-1,611)	1,191 (588-1,635)	9,370 (5,637-12,614)	6,269 (3,860-8,744)
Unintentional firearm injuries	34 (26-38)	26 (19-32)	291 (214-359)	433 (283-591)
Unintentional suffocation	986 (439-1,398)	1,090 (491-1,523)	7,452 (3,864-10,706)	2,272 (1,107-3,881)
Other exposure to mechanical forces	181 (119-221)	76 (48-93)	1,627 (930-2,165)	3,564 (1,767-5,217)
Adverse effects of medical treatment	952 (598-1,144)	789 (432-1,034)	6,183 (2,931-8,271)	4,936 (1,784-7,857)
Animal contact	131 (45-178)	152 (45-227)	1,763 (619-2,596)	4,297 (1,342-8,284)
Venomous animal contact	85 (22-130)	98 (24-155)	1,353 (415-2,017)	3,432 (969-6,384)
Non-venomous animal contact	46 (17-79)	53 (18-107)	410 (154-927)	865 (287-3,214)
Foreign body	871 (475-1,077)	804 (428-1,117)	9,201 (4,947-13,571)	5,003 (2,394-8,871)
Pulmonary aspiration and foreign body in airway	763 (426-945)	728 (397-1,020)	7,789 (4,436-11,617)	3,802 (1,896-7,019)
Foreign body in other body part	109 (26-151)	76 (14-113)	1,412 (274-2,116)	1,201 (312-2,096)
Environmental heat and cold exposure	205 (82-269)	122 (44-173)	1,419 (455-1,961)	918 (351-1,403)
Other unintentional injuries	274 (215-344)	123 (90-157)	1,280 (901-1,635)	3,500 (1,996-4,936)
Self-harm and interpersonal violence	1,295 (770-1,617)	365 (205-465)	3,207 (1,547-4,137)	5,352 (2,698-8,069)

Cause	5 to 9	10 to 14	15 to 19	20 to 24	25 to 29	30 to 34	35 to 39	40 to 44	45 to 49	50 to 54	55 to 59	60 to 64	65 to 69	70 to 74	75 to 79	80 to 84	85 to 89	90 to 94	95 plus
Ischaemic stroke	242 (197-304)	200 (165-243)	775 (686-889)	1,483 (1,308-1,668)	1,786 (1,590-1,994)	2,190 (1,949-2,427)	3,332 (2,991-3,645)	5,007 (4,552-5,401)	8,976 (8,162-9,619)	17,616 (16,227-19,004)	29,737 (27,305-32,074)	72,021 (67,070-77,390)	108,191 (100,797-116,209)	140,192 (130,917-152,323)	219,726 (205,399-236,832)	224,001 (207,535-241,353)	159,129 (146,600-173,906)	66,237 (60,031-73,002)	22,600 (20,133-24,884)
Hemorrhagic stroke	2,447 (2,041-3,035)	2,406 (2,093-2,851)	4,003 (3,613-4,536)	6,574 (6,000-7,375)	8,054 (7,306-8,936)	11,782 (10,800-13,037)	19,402 (17,951-21,150)	28,761 (26,857-30,854)	40,839 (38,289-43,742)	66,480 (62,311-71,720)	93,754 (87,520-101,337)	114,187 (106,284-125,463)	147,183 (136,069-162,761)	174,262 (160,121-196,917)	165,241 (152,141-187,141)	139,592 (127,501-157,558)	83,650 (75,919-95,171)	31,712 (28,475-36,099)	9,451 (8,343-10,624)
Hypertensive heart disease	144 (62-205)	123 (59-173)	910 (504-1,176)	1,257 (662-1,558)	1,459 (852-1,817)	2,232 (1,296-2,728)	3,372 (1,997-4,118)	4,887 (3,155-5,873)	7,689 (4,946-9,148)	12,876 (8,771-15,423)	19,460 (13,543-23,277)	26,252 (17,541-31,425)	37,350 (26,059-44,632)	47,690 (31,815-56,213)	57,650 (41,844-69,562)	52,834 (39,814-64,567)	38,742 (28,560-48,971)	18,913 (14,393-22,768)	6,280 (4,566-8,211)
Cardiomyopathy and myocarditis	765 (582-1,107)	742 (606-1,027)	905 (758-1,290)	1,109 (929-1,532)	1,354 (1,131-1,717)	1,572 (1,324-1,887)	2,003 (1,675-2,367)	2,463 (2,053-2,859)	2,511 (2,037-2,956)	4,198 (3,435-5,084)	5,262 (4,315-6,128)	6,445 (5,350-7,591)	7,330 (5,796-8,165)	7,901 (5,912-8,775)	12,897 (8,291-14,919)	16,553 (9,177-19,924)	16,584 (8,389-20,423)	8,675 (4,321-10,718)	3,106 (1,555-3,801)
Myocarditis	353 (251-560)	300 (227-456)	415 (319-631)	503 (397-777)	462 (364-669)	385 (301-547)	451 (354-606)	464 (361-615)	325 (252-431)	420 (318-550)	539 (420-685)	503 (371-627)	653 (474-798)	829 (603-998)	1,437 (854-1,771)	1,981 (1,034-2,536)	2,227 (1,074-2,956)	1,342 (608-1,796)	556 (252-765)
Alcoholic cardiomyopathy	-	-	73 (51-110)	118 (86-169)	243 (185-321)	434 (321-558)	685 (496-921)	939 (694-1,239)	1,001 (711-1,334)	1,988 (1,406-2,692)	2,081 (1,531-2,806)	2,418 (1,829-3,380)	1,948 (1,547-2,623)	1,401 (1,103-1,928)	1,737 (1,327-2,267)	1,461 (1,024-1,905)	1,076 (692-1,437)	536 (316-713)	217 (130-285)
Other cardiomyopathy	412 (280-609)	442 (337-616)	417 (330-584)	488 (383-674)	648 (510-836)	753 (593-947)	868 (713-1,086)	1,060 (883-1,291)	1,186 (945-1,375)	1,790 (1,462-2,106)	2,642 (2,114-2,991)	3,523 (2,745-3,950)	4,729 (3,413-5,303)	5,670 (3,921-6,475)	9,723 (5,737-11,608)	13,112 (6,727-16,355)	13,281 (6,035-16,895)	6,796 (3,070-8,750)	2,333 (1,088-2,945)
Atrial fibrillation and flutter	-	-	-	-	-	12 ([illegible])	48 ([illegible])	100 ([illegible])	172 ([illegible])	316 ([illegible])	578 ([illegible])	1,272 ([illegible])	2,630 ([illegible])	4,880 ([illegible])	8,977 ([illegible])	17,937 ([illegible])	19,734 ([illegible])	9,802 ([illegible])	2,875 ([illegible])
Aortic aneurysm	-	-	[illegible]	[illegible]	[illegible]	[illegible]	[illegible]	[illegible]	[illegible]	[illegible]	[illegible]	[illegible]	[illegible]	[illegible]	[illegible]	[illegible]	[illegible]	[illegible]	[illegible]
Peripheral vascular disease	-	-	[illegible]	[illegible]	[illegible]	[illegible]	[illegible]	[illegible]	[illegible]	[illegible]	[illegible]	[illegible]	[illegible]	[illegible]	[illegible]	[illegible]	[illegible]	[illegible]	[illegible]
Endocarditis	237 (168-331)	245 (192-308)	[illegible]	[illegible]	[illegible]	[illegible]	[illegible]	[illegible]	[illegible]	[illegible]	[illegible]	[illegible]	[illegible]	[illegible]	[illegible]	[illegible]	[illegible]	[illegible]	[illegible]
Other cardiovascular and circulatory diseases	1,246 (757-1,551)	1,148 (726-1,374)	[illegible]	[illegible]	[illegible]	[illegible]	[illegible]	[illegible]	[illegible]	[illegible]	[illegible]	[illegible]	[illegible]	[illegible]	[illegible]	[illegible]	[illegible]	[illegible]	[illegible]
Chronic respiratory diseases	4,273 (3,430-5,070)	3,587 (2,896-4,152)	[illegible]	[illegible]	[illegible]	[illegible]	[illegible]	[illegible]	[illegible]	[illegible]	[illegible]	[illegible]	[illegible]	[illegible]	[illegible]	[illegible]	[illegible]	[illegible]	[illegible]
Chronic obstructive pulmonary disease	[illegible]	[illegible]	[illegible]	[illegible]	[illegible]	[illegible]	[illegible]	[illegible]	[illegible]	[illegible]	[illegible]	[illegible]	[illegible]	[illegible]	[illegible]	[illegible]	[illegible]	[illegible]	[illegible]
Pneumoconiosis	[illegible]	[illegible]	[illegible]	[illegible]	[illegible]	[illegible]	[illegible]	[illegible]	[illegible]	[illegible]	[illegible]	[illegible]	[illegible]	[illegible]	[illegible]	[illegible]	[illegible]	[illegible]	[illegible]
Silicosis	[illegible]	[illegible]	[illegible]	[illegible]	[illegible]	[illegible]	[illegible]	[illegible]	[illegible]	[illegible]	[illegible]	[illegible]	[illegible]	[illegible]	[illegible]	[illegible]	[illegible]	[illegible]	[illegible]
Asbestosis	[illegible]	[illegible]	[illegible]	[illegible]	[illegible]	[illegible]	[illegible]	[illegible]	[illegible]	[illegible]	[illegible]	[illegible]	[illegible]	[illegible]	[illegible]	[illegible]	[illegible]	[illegible]	[illegible]
Coal workers pneumoconiosis	[illegible]	[illegible]	[illegible]	[illegible]	[illegible]	[illegible]	[illegible]	[illegible]	[illegible]	[illegible]	[illegible]	[illegible]	[illegible]	[illegible]	[illegible]	[illegible]	[illegible]	[illegible]	[illegible]
Other pneumoconiosis	[illegible]	[illegible]	[illegible]	[illegible]	[illegible]	[illegible]	[illegible]	[illegible]	[illegible]	[illegible]	[illegible]	[illegible]	[illegible]	[illegible]	[illegible]	[illegible]	[illegible]	[illegible]	[illegible]
Asthma	[illegible]	[illegible]	[illegible]	[illegible]	[illegible]	[illegible]	[illegible]	[illegible]	[illegible]	[illegible]	[illegible]	[illegible]	[illegible]	[illegible]	[illegible]	[illegible]	[illegible]	[illegible]	[illegible]
Interstitial lung disease and pulmonary sarcoidosis	[illegible]	[illegible]	[illegible]	[illegible]	[illegible]	[illegible]	[illegible]	[illegible]	[illegible]	[illegible]	[illegible]	[illegible]	[illegible]	[illegible]	[illegible]	[illegible]	[illegible]	[illegible]	[illegible]
Other chronic respiratory diseases	[illegible]	[illegible]	[illegible]	[illegible]	[illegible]	[illegible]	[illegible]	[illegible]	[illegible]	[illegible]	[illegible]	[illegible]	[illegible]	[illegible]	[illegible]	[illegible]	[illegible]	[illegible]	[illegible]
Cirrhosis and other chronic liver diseases	[illegible]	[illegible]	[illegible]	[illegible]	[illegible]	[illegible]	[illegible]	[illegible]	[illegible]	[illegible]	[illegible]	[illegible]	[illegible]	[illegible]	[illegible]	[illegible]	[illegible]	[illegible]	[illegible]
Cirrhosis and other chronic liver diseases due to hepatitis B	[illegible]	[illegible]	[illegible]	[illegible]	[illegible]	[illegible]	[illegible]	[illegible]	[illegible]	[illegible]	[illegible]	[illegible]	[illegible]	[illegible]	[illegible]	[illegible]	[illegible]	[illegible]	[illegible]
Cirrhosis and other chronic liver diseases due to hepatitis C	[illegible]	[illegible]	[illegible]	[illegible]	[illegible]	[illegible]	[illegible]	[illegible]	[illegible]	[illegible]	[illegible]	[illegible]	[illegible]	[illegible]	[illegible]	[illegible]	[illegible]	[illegible]	[illegible]
Cirrhosis and other chronic liver diseases due to alcohol use	[illegible]	[illegible]	[illegible]	[illegible]	[illegible]	[illegible]	[illegible]	[illegible]	[illegible]	[illegible]	[illegible]	[illegible]	[illegible]	[illegible]	[illegible]	[illegible]	[illegible]	[illegible]	[illegible]
Cirrhosis and other chronic liver diseases due to other causes	[illegible]	[illegible]	[illegible]	[illegible]	[illegible]	[illegible]	[illegible]	[illegible]	[illegible]	[illegible]	[illegible]	[illegible]	[illegible]	[illegible]	[illegible]	[illegible]	[illegible]	[illegible]	[illegible]
Digestive diseases	[illegible]	[illegible]	[illegible]	[illegible]	[illegible]	[illegible]	[illegible]	[illegible]	[illegible]	[illegible]	[illegible]	[illegible]	[illegible]	[illegible]	[illegible]	[illegible]	[illegible]	[illegible]	[illegible]
Peptic ulcer disease	[illegible]	[illegible]	[illegible]	[illegible]	[illegible]	[illegible]	[illegible]	[illegible]	[illegible]	[illegible]	[illegible]	[illegible]	[illegible]	[illegible]	[illegible]	[illegible]	[illegible]	[illegible]	[illegible]
Gastritis and duodenitis	[illegible]	[illegible]	[illegible]	[illegible]	[illegible]	[illegible]	[illegible]	[illegible]	[illegible]	[illegible]	[illegible]	[illegible]	[illegible]	[illegible]	[illegible]	[illegible]	[illegible]	[illegible]	[illegible]
Appendicitis	[illegible]	[illegible]	[illegible]	[illegible]	[illegible]	[illegible]	[illegible]	[illegible]	[illegible]	[illegible]	[illegible]	[illegible]	[illegible]	[illegible]	[illegible]	[illegible]	[illegible]	[illegible]	[illegible]
Paralytic ileus and intestinal obstruction	[illegible]	[illegible]	[illegible]	[illegible]	[illegible]	[illegible]	[illegible]	[illegible]	[illegible]	[illegible]	[illegible]	[illegible]	[illegible]	[illegible]	[illegible]	[illegible]	[illegible]	[illegible]	[illegible]
Inguinal, femoral, and abdominal hernia	[illegible]	[illegible]	[illegible]	[illegible]	[illegible]	[illegible]	[illegible]	[illegible]	[illegible]	[illegible]	[illegible]	[illegible]	[illegible]	[illegible]	[illegible]	[illegible]	[illegible]	[illegible]	[illegible]
Inflammatory bowel disease	[illegible]	[illegible]	[illegible]	[illegible]	[illegible]	[illegible]	[illegible]	[illegible]	[illegible]	[illegible]	[illegible]	[illegible]	[illegible]	[illegible]	[illegible]	[illegible]	[illegible]	[illegible]	[illegible]
Vascular intestinal disorders	[illegible]	[illegible]	[illegible]	[illegible]	[illegible]	[illegible]	[illegible]	[illegible]	[illegible]	[illegible]	[illegible]	[illegible]	[illegible]	[illegible]	[illegible]	[illegible]	[illegible]	[illegible]	[illegible]
Gallbladder and biliary diseases	[illegible]	[illegible]	[illegible]	[illegible]	[illegible]	[illegible]	[illegible]	[illegible]	[illegible]	[illegible]	[illegible]	[illegible]	[illegible]	[illegible]	[illegible]	[illegible]	[illegible]	[illegible]	[illegible]
Pancreatitis	[illegible]	[illegible]	[illegible]	[illegible]	[illegible]	[illegible]	[illegible]	[illegible]	[illegible]	[illegible]	[illegible]	[illegible]	[illegible]	[illegible]	[illegible]	[illegible]	[illegible]	[illegible]	[illegible]
Other digestive diseases	[illegible]	[illegible]	[illegible]	[illegible]	[illegible]	[illegible]	[illegible]	[illegible]	[illegible]	[illegible]	[illegible]	[illegible]	[illegible]	[illegible]	[illegible]	[illegible]	[illegible]	[illegible]	[illegible]
Neurological disorders	[illegible]	[illegible]	[illegible]	[illegible]	[illegible]	[illegible]	[illegible]	[illegible]	[illegible]	[illegible]	[illegible]	[illegible]	[illegible]	[illegible]	[illegible]	[illegible]	[illegible]	[illegible]	[illegible]
Alzheimer's disease and other dementias	[illegible]	[illegible]	[illegible]	[illegible]	[illegible]	[illegible]	[illegible]	[illegible]	[illegible]	[illegible]	[illegible]	[illegible]	[illegible]	[illegible]	[illegible]	[illegible]	[illegible]	[illegible]	[illegible]
Parkinson's disease	[illegible]	[illegible]	[illegible]	[illegible]	[illegible]	[illegible]	[illegible]	[illegible]	[illegible]	[illegible]	[illegible]	[illegible]	[illegible]	[illegible]	[illegible]	[illegible]	[illegible]	[illegible]	[illegible]
Epilepsy	[illegible]	[illegible]	[illegible]	[illegible]	[illegible]	[illegible]	[illegible]	[illegible]	[illegible]	[illegible]	[illegible]	[illegible]	[illegible]	[illegible]	[illegible]	[illegible]	[illegible]	[illegible]	[illegible]
Multiple sclerosis	[illegible]	[illegible]	[illegible]	[illegible]	[illegible]	[illegible]	[illegible]	[illegible]	[illegible]	[illegible]	[illegible]	[illegible]	[illegible]	[illegible]	[illegible]	[illegible]	[illegible]	[illegible]	[illegible]
Motor neuron disease	[illegible]	[illegible]	[illegible]	[illegible]	[illegible]	[illegible]	[illegible]	[illegible]	[illegible]	[illegible]	[illegible]	[illegible]	[illegible]	[illegible]	[illegible]	[illegible]	[illegible]	[illegible]	[illegible]
Other neurological disorders	[illegible]	[illegible]	[illegible]	[illegible]	[illegible]	[illegible]	[illegible]	[illegible]	[illegible]	[illegible]	[illegible]	[illegible]	[illegible]	[illegible]	[illegible]	[illegible]	[illegible]	[illegible]	[illegible]
Mental and substance use disorders	[illegible]	[illegible]	[illegible]	[illegible]	[illegible]	[illegible]	[illegible]	[illegible]	[illegible]	[illegible]	[illegible]	[illegible]	[illegible]	[illegible]	[illegible]	[illegible]	[illegible]	[illegible]	[illegible]
Alcohol use disorders	[illegible]	[illegible]	[illegible]	[illegible]	[illegible]	[illegible]	[illegible]	[illegible]	[illegible]	[illegible]	[illegible]	[illegible]	[illegible]	[illegible]	[illegible]	[illegible]	[illegible]	[illegible]	[illegible]
Drug use disorders	[illegible]	[illegible]	[illegible]	[illegible]	[illegible]	[illegible]	[illegible]	[illegible]	[illegible]	[illegible]	[illegible]	[illegible]	[illegible]	[illegible]	[illegible]	[illegible]	[illegible]	[illegible]	[illegible]
Opioid use disorders	[illegible]	[illegible]	[illegible]	[illegible]	[illegible]	[illegible]	[illegible]	[illegible]	[illegible]	[illegible]	[illegible]	[illegible]	[illegible]	[illegible]	[illegible]	[illegible]	[illegible]	[illegible]	[illegible]
Cocaine use disorders	[illegible]	[illegible]	[illegible]	[illegible]	[illegible]	[illegible]	[illegible]	[illegible]	[illegible]	[illegible]	[illegible]	[illegible]	[illegible]	[illegible]	[illegible]	[illegible]	[illegible]	[illegible]	[illegible]
Amphetamine use disorders	[illegible]	[illegible]	[illegible]	[illegible]	[illegible]	[illegible]	[illegible]	[illegible]	[illegible]	[illegible]	[illegible]	[illegible]	[illegible]	[illegible]	[illegible]	[illegible]	[illegible]	[illegible]	[illegible]
Other drug use disorders	[illegible]	[illegible]	[illegible]	[illegible]	[illegible]	[illegible]	[illegible]	[illegible]	[illegible]	[illegible]	[illegible]	[illegible]	[illegible]	[illegible]	[illegible]	[illegible]	[illegible]	[illegible]	[illegible]
Eating disorders	[illegible]	[illegible]	[illegible]	[illegible]	[illegible]	[illegible]	[illegible]	[illegible]	[illegible]	[illegible]	[illegible]	[illegible]	[illegible]	[illegible]	[illegible]	[illegible]	[illegible]	[illegible]	[illegible]
Anorexia nervosa	[illegible]	[illegible]	[illegible]	[illegible]	[illegible]	[illegible]	[illegible]	[illegible]	[illegible]	[illegible]	[illegible]	[illegible]	[illegible]	[illegible]	[illegible]	[illegible]	[illegible]	[illegible]	[illegible]
Bulimia nervosa	[illegible]	[illegible]	[illegible]	[illegible]	[illegible]	[illegible]	[illegible]	[illegible]	[illegible]	[illegible]	[illegible]	[illegible]	[illegible]	[illegible]	[illegible]	[illegible]	[illegible]	[illegible]	[illegible]
Diabetes, urogenital, blood, and endocrine diseases	[illegible]	[illegible]	[illegible]	[illegible]	[illegible]	[illegible]	[illegible]	[illegible]	[illegible]	[illegible]	[illegible]	[illegible]	[illegible]	[illegible]	[illegible]	[illegible]	[illegible]	[illegible]	[illegible]
Diabetes mellitus	[illegible]	[illegible]	[illegible]	[illegible]	[illegible]	[illegible]	[illegible]	[illegible]	[illegible]	[illegible]	[illegible]	[illegible]	[illegible]	[illegible]	[illegible]	[illegible]	[illegible]	[illegible]	[illegible]
Acute glomerulonephritis	[illegible]	[illegible]	[illegible]	[illegible]	[illegible]	[illegible]	[illegible]	[illegible]	[illegible]	[illegible]	[illegible]	[illegible]	[illegible]	[illegible]	[illegible]	[illegible]	[illegible]	[illegible]	[illegible]
Chronic kidney disease	[illegible]	[illegible]	[illegible]	[illegible]	[illegible]	[illegible]	[illegible]	[illegible]	[illegible]	[illegible]	[illegible]	[illegible]	[illegible]	[illegible]	[illegible]	[illegible]	[illegible]	[illegible]	[illegible]
Chronic kidney disease due to diabetes mellitus	[illegible]	[illegible]	[illegible]	[illegible]	[illegible]	[illegible]	[illegible]	[illegible]	[illegible]	[illegible]	[illegible]	[illegible]	[illegible]	[illegible]	[illegible]	[illegible]	[illegible]	[illegible]	[illegible]
Chronic kidney disease due to hypertension	[illegible]	[illegible]	[illegible]	[illegible]	[illegible]	[illegible]	[illegible]	[illegible]	[illegible]	[illegible]	[illegible]	[illegible]	[illegible]	[illegible]	[illegible]	[illegible]	[illegible]	[illegible]	[illegible]
Chronic kidney disease due to glomerulonephritis	[illegible]	[illegible]	[illegible]	[illegible]	[illegible]	[illegible]	[illegible]	[illegible]	[illegible]	[illegible]	[illegible]	[illegible]	[illegible]	[illegible]	[illegible]	[illegible]	[illegible]	[illegible]	[illegible]
Chronic kidney disease due to other causes	[illegible]	[illegible]	[illegible]	[illegible]	[illegible]	[illegible]	[illegible]	[illegible]	[illegible]	[illegible]	[illegible]	[illegible]	[illegible]	[illegible]	[illegible]	[illegible]	[illegible]	[illegible]	[illegible]
Urinary diseases and male infertility	[illegible]	[illegible]	[illegible]	[illegible]	[illegible]	[illegible]	[illegible]	[illegible]	[illegible]	[illegible]	[illegible]	[illegible]	[illegible]	[illegible]	[illegible]	[illegible]	[illegible]	[illegible]	[illegible]
Interstitial nephritis and urinary tract infections	[illegible]	[illegible]	[illegible]	[illegible]	[illegible]	[illegible]	[illegible]	[illegible]	[illegible]	[illegible]	[illegible]	[illegible]	[illegible]	[illegible]	[illegible]	[illegible]	[illegible]	[illegible]	[illegible]
Urolithiasis	[illegible]	[illegible]	[illegible]	[illegible]	[illegible]	[illegible]	[illegible]	[illegible]	[illegible]	[illegible]	[illegible]	[illegible]	[illegible]	[illegible]	[illegible]	[illegible]	[illegible]	[illegible]	[illegible]
Other urinary diseases	[illegible]	[illegible]	[illegible]	[illegible]	[illegible]	[illegible]	[illegible]	[illegible]	[illegible]	[illegible]	[illegible]	[illegible]	[illegible]	[illegible]	[illegible]	[illegible]	[illegible]	[illegible]	[illegible]
Gynecological diseases	[illegible]	[illegible]	[illegible]	[illegible]	[illegible]	[illegible]	[illegible]	[illegible]	[illegible]	[illegible]	[illegible]	[illegible]	[illegible]	[illegible]	[illegible]	[illegible]	[illegible]	[illegible]	[illegible]
Uterine fibroids	[illegible]	[illegible]	[illegible]	[illegible]	[illegible]	[illegible]	[illegible]	[illegible]	[illegible]	[illegible]	[illegible]	[illegible]	[illegible]	[illegible]	[illegible]	[illegible]	[illegible]	[illegible]	[illegible]
Polycystic ovarian syndrome	[illegible]	[illegible]	[illegible]	[illegible]	[illegible]	[illegible]	[illegible]	[illegible]	[illegible]	[illegible]	[illegible]	[illegible]	[illegible]	[illegible]	[illegible]	[illegible]	[illegible]	[illegible]	[illegible]
Endometriosis	[illegible]	[illegible]	[illegible]	[illegible]	[illegible]	[illegible]	[illegible]	[illegible]	[illegible]	[illegible]	[illegible]	[illegible]	[illegible]	[illegible]	[illegible]	[illegible]	[illegible]	[illegible]	[illegible]
Genital prolapse	[illegible]	[illegible]	[illegible]	[illegible]	[illegible]	[illegible]	[illegible]	[illegible]	[illegible]	[illegible]	[illegible]	[illegible]	[illegible]	[illegible]	[illegible]	[illegible]	[illegible]	[illegible]	[illegible]
Other gynecological diseases	[illegible]	[illegible]	[illegible]	[illegible]	[illegible]	[illegible]	[illegible]	[illegible]	[illegible]	[illegible]	[illegible]	[illegible]	[illegible]	[illegible]	[illegible]	[illegible]	[illegible]	[illegible]	[illegible]
Hemoglobinopathies and hemolytic anaemias	[illegible]	[illegible]	[illegible]	[illegible]	[illegible]	[illegible]	[illegible]	[illegible]	[illegible]	[illegible]	[illegible]	[illegible]	[illegible]	[illegible]	[illegible]	[illegible]	[illegible]	[illegible]	[illegible]
Thalassemias	[illegible]	[illegible]	[illegible]	[illegible]	[illegible]	[illegible]	[illegible]	[illegible]	[illegible]	[illegible]	[illegible]	[illegible]	[illegible]	[illegible]	[illegible]	[illegible]	[illegible]	[illegible]	[illegible]
Sickle cell disorders	[illegible]	[illegible]	[illegible]	[illegible]	[illegible]	[illegible]	[illegible]	[illegible]	[illegible]	[illegible]	[illegible]	[illegible]	[illegible]	[illegible]	[illegible]	[illegible]	[illegible]	[illegible]	[illegible]
G6PD deficiency	[illegible]	[illegible]	[illegible]	[illegible]	[illegible]	[illegible]	[illegible]	[illegible]	[illegible]	[illegible]	[illegible]	[illegible]	[illegible]	[illegible]	[illegible]	[illegible]	[illegible]	[illegible]	[illegible]
Other hemoglobinopathies and hemolytic anaemias	[illegible]	[illegible]	[illegible]	[illegible]	[illegible]	[illegible]	[illegible]	[illegible]	[illegible]	[illegible]	[illegible]	[illegible]	[illegible]	[illegible]	[illegible]	[illegible]	[illegible]	[illegible]	[illegible]
Endocrine, metabolic, blood, and immune disorders	[illegible]	[illegible]	[illegible]	[illegible]	[illegible]	[illegible]	[illegible]	[illegible]	[illegible]	[illegible]	[illegible]	[illegible]	[illegible]	[illegible]	[illegible]	[illegible]	[illegible]	[illegible]	[illegible]
Musculoskeletal disorders	[illegible]	[illegible]	[illegible]	[illegible]	[illegible]	[illegible]	[illegible]	[illegible]	[illegible]	[illegible]	[illegible]	[illegible]	[illegible]	[illegible]	[illegible]	[illegible]	[illegible]	[illegible]	[illegible]
Rheumatoid arthritis	[illegible]	[illegible]	[illegible]	[illegible]	[illegible]	[illegible]	[illegible]	[illegible]	[illegible]	[illegible]	[illegible]	[illegible]	[illegible]	[illegible]	[illegible]	[illegible]	[illegible]	[illegible]	[illegible]
Other musculoskeletal disorders	[illegible]	[illegible]	[illegible]	[illegible]	[illegible]	[illegible]	[illegible]	[illegible]	[illegible]	[illegible]	[illegible]	[illegible]	[illegible]	[illegible]	[illegible]	[illegible]	[illegible]	[illegible]	[illegible]
Other non-communicable diseases	[illegible]	[illegible]	[illegible]	[illegible]	[illegible]	[illegible]	[illegible]	[illegible]	[illegible]	[illegible]	[illegible]	[illegible]	[illegible]	[illegible]	[illegible]	[illegible]	[illegible]	[illegible]	[illegible]
Congenital anomalies	[illegible]	[illegible]	[illegible]	[illegible]	[illegible]	[illegible]	[illegible]	[illegible]	[illegible]	[illegible]	[illegible]	[illegible]	[illegible]	[illegible]	[illegible]	[illegible]	[illegible]	[illegible]	[illegible]
Neural tube defects	[illegible]	[illegible]	[illegible]	[illegible]	[illegible]	[illegible]	[illegible]	[illegible]	[illegible]	[illegible]	[illegible]	[illegible]	[illegible]	[illegible]	[illegible]	[illegible]	[illegible]	[illegible]	[illegible]
Congenital heart anomalies	[illegible]	[illegible]	[illegible]	[illegible]	[illegible]	[illegible]	[illegible]	[illegible]	[illegible]	[illegible]	[illegible]	[illegible]	[illegible]	[illegible]	[illegible]	[illegible]	[illegible]	[illegible]	[illegible]
Orofacial clefts	[illegible]	[illegible]	[illegible]	[illegible]	[illegible]	[illegible]	[illegible]	[illegible]	[illegible]	[illegible]	[illegible]	[illegible]	[illegible]	[illegible]	[illegible]	[illegible]	[illegible]	[illegible]	[illegible]
Down's syndrome	[illegible]	[illegible]	[illegible]	[illegible]	[illegible]	[illegible]	[illegible]	[illegible]	[illegible]	[illegible]	[illegible]	[illegible]	[illegible]	[illegible]	[illegible]	[illegible]	[illegible]	[illegible]	[illegible]
Other chromosomal abnormalities	[illegible]	[illegible]	[illegible]	[illegible]	[illegible]	[illegible]	[illegible]	[illegible]	[illegible]	[illegible]	[illegible]	[illegible]	[illegible]	[illegible]	[illegible]	[illegible]	[illegible]	[illegible]	[illegible]
Congenital musculoskeletal and limb anomalies	[illegible]	[illegible]	[illegible]	[illegible]	[illegible]	[illegible]	[illegible]	[illegible]	[illegible]	[illegible]	[illegible]	[illegible]	[illegible]	[illegible]	[illegible]	[illegible]	[illegible]	[illegible]	[illegible]
Urogenital congenital anomalies	[illegible]	[illegible]	[illegible]	[illegible]	[illegible]	[illegible]	[illegible]	[illegible]	[illegible]	[illegible]	[illegible]	[illegible]	[illegible]	[illegible]	[illegible]	[illegible]	[illegible]	[illegible]	[illegible]
Digestive congenital anomalies	[illegible]	[illegible]	[illegible]	[illegible]	[illegible]	[illegible]	[illegible]	[illegible]	[illegible]	[illegible]	[illegible]	[illegible]	[illegible]	[illegible]	[illegible]	[illegible]	[illegible]	[illegible]	[illegible]
Other congenital anomalies	[illegible]	[illegible]	[illegible]	[illegible]	[illegible]	[illegible]	[illegible]	[illegible]	[illegible]	[illegible]	[illegible]	[illegible]	[illegible]	[illegible]	[illegible]	[illegible]	[illegible]	[illegible]	[illegible]
Skin and subcutaneous diseases	[illegible]	[illegible]	[illegible]	[illegible]	[illegible]	[illegible]	[illegible]	[illegible]	[illegible]	[illegible]	[illegible]	[illegible]	[illegible]	[illegible]	[illegible]	[illegible]	[illegible]	[illegible]	[illegible]
Cellulitis	[illegible]	[illegible]	[illegible]	[illegible]	[illegible]	[illegible]	[illegible]	[illegible]	[illegible]	[illegible]	[illegible]	[illegible]	[illegible]	[illegible]	[illegible]	[illegible]	[illegible]	[illegible]	[illegible]
Pyoderma	[illegible]	[illegible]	[illegible]	[illegible]	[illegible]	[illegible]	[illegible]	[illegible]	[illegible]	[illegible]	[illegible]	[illegible]	[illegible]	[illegible]	[illegible]	[illegible]	[illegible]	[illegible]	[illegible]
Decubitus ulcer	[illegible]	[illegible]	[illegible]	[illegible]	[illegible]	[illegible]	[illegible]	[illegible]	[illegible]	[illegible]	[illegible]	[illegible]	[illegible]	[illegible]	[illegible]	[illegible]	[illegible]	[illegible]	[illegible]
Other skin and subcutaneous diseases	[illegible]	[illegible]	[illegible]	[illegible]	[illegible]	[illegible]	[illegible]	[illegible]	[illegible]	[illegible]	[illegible]	[illegible]	[illegible]	[illegible]	[illegible]	[illegible]	[illegible]	[illegible]	[illegible]
Sudden infant death syndrome	[illegible]	[illegible]	[illegible]	[illegible]	[illegible]	[illegible]	[illegible]	[illegible]	[illegible]	[illegible]	[illegible]	[illegible]	[illegible]	[illegible]	[illegible]	[illegible]	[illegible]	[illegible]	[illegible]
Injuries	[illegible]	[illegible]	[illegible]	[illegible]	[illegible]	[illegible]	[illegible]	[illegible]	[illegible]	[illegible]	[illegible]	[illegible]	[illegible]	[illegible]	[illegible]	[illegible]	[illegible]	[illegible]	[illegible]
Transport injuries	[illegible]	[illegible]	[illegible]	[illegible]	[illegible]	[illegible]	[illegible]	[illegible]	[illegible]	[illegible]	[illegible]	[illegible]	[illegible]	[illegible]	[illegible]	[illegible]	[illegible]	[illegible]	[illegible]
Road injuries	[illegible]	[illegible]	[illegible]	[illegible]	[illegible]	[illegible]	[illegible]	[illegible]	[illegible]	[illegible]	[illegible]	[illegible]	[illegible]	[illegible]	[illegible]	[illegible]	[illegible]	[illegible]	[illegible]
Pedestrian road injuries	[illegible]	[illegible]	[illegible]	[illegible]	[illegible]	[illegible]	[illegible]	[illegible]	[illegible]	[illegible]	[illegible]	[illegible]	[illegible]	[illegible]	[illegible]	[illegible]	[illegible]	[illegible]	[illegible]
Cyclist road injuries	[illegible]	[illegible]	[illegible]	[illegible]	[illegible]	[illegible]	[illegible]	[illegible]	[illegible]	[illegible]	[illegible]	[illegible]	[illegible]	[illegible]	[illegible]	[illegible]	[illegible]	[illegible]	[illegible]
Motorcyclist road injuries	[illegible]	[illegible]	[illegible]	[illegible]	[illegible]	[illegible]	[illegible]	[illegible]	[illegible]	[illegible]	[illegible]	[illegible]	[illegible]	[illegible]	[illegible]	[illegible]	[illegible]	[illegible]	[illegible]
Motor vehicle road injuries	[illegible]	[illegible]	[illegible]	[illegible]	[illegible]	[illegible]	[illegible]	[illegible]	[illegible]	[illegible]	[illegible]	[illegible]	[illegible]	[illegible]	[illegible]	[illegible]	[illegible]	[illegible]	[illegible]
Other road injuries	[illegible]	[illegible]	[illegible]	[illegible]	[illegible]	[illegible]	[illegible]	[illegible]	[illegible]	[illegible]	[illegible]	[illegible]	[illegible]	[illegible]	[illegible]	[illegible]	[illegible]	[illegible]	[illegible]
Other transport injuries	[illegible]	[illegible]	[illegible]	[illegible]	[illegible]	[illegible]	[illegible]	[illegible]	[illegible]	[illegible]	[illegible]	[illegible]	[illegible]	[illegible]	[illegible]	[illegible]	[illegible]	[illegible]	[illegible]
Unintentional injuries	[illegible]	[illegible]	[illegible]	[illegible]	[illegible]	[illegible]	[illegible]	[illegible]	[illegible]	[illegible]	[illegible]	[illegible]	[illegible]	[illegible]	[illegible]	[illegible]	[illegible]	[illegible]	[illegible]
Falls	[illegible]	[illegible]	[illegible]	[illegible]	[illegible]	[illegible]	[illegible]	[illegible]	[illegible]	[illegible]	[illegible]	[illegible]	[illegible]	[illegible]	[illegible]	[illegible]	[illegible]	[illegible]	[illegible]
Drowning	[illegible]	[illegible]	[illegible]	[illegible]	[illegible]	[illegible]	[illegible]	[illegible]	[illegible]	[illegible]	[illegible]	[illegible]	[illegible]	[illegible]	[illegible]	[illegible]	[illegible]	[illegible]	[illegible]
Fire, heat, and hot substances	[illegible]	[illegible]	[illegible]	[illegible]	[illegible]	[illegible]	[illegible]	[illegible]	[illegible]	[illegible]	[illegible]	[illegible]	[illegible]	[illegible]	[illegible]	[illegible]	[illegible]	[illegible]	[illegible]
Poisonings	[illegible]	[illegible]	[illegible]	[illegible]	[illegible]	[illegible]	[illegible]	[illegible]	[illegible]	[illegible]	[illegible]	[illegible]	[illegible]	[illegible]	[illegible]	[illegible]	[illegible]	[illegible]	[illegible]
Exposure to mechanical forces	[illegible]	[illegible]	[illegible]	[illegible]	[illegible]	[illegible]	[illegible]	[illegible]	[illegible]	[illegible]	[illegible]	[illegible]	[illegible]	[illegible]	[illegible]	[illegible]	[illegible]	[illegible]	[illegible]
Unintentional firearm injuries	[illegible]	[illegible]	[illegible]	[illegible]	[illegible]	[illegible]	[illegible]	[illegible]	[illegible]	[illegible]	[illegible]	[illegible]	[illegible]	[illegible]	[illegible]	[illegible]	[illegible]	[illegible]	[illegible]
Unintentional suffocation	[illegible]	[illegible]	[illegible]	[illegible]	[illegible]	[illegible]	[illegible]	[illegible]	[illegible]	[illegible]	[illegible]	[illegible]	[illegible]	[illegible]	[illegible]	[illegible]	[illegible]	[illegible]	[illegible]
Other exposure to mechanical forces	[illegible]	[illegible]	[illegible]	[illegible]	[illegible]	[illegible]	[illegible]	[illegible]	[illegible]	[illegible]	[illegible]	[illegible]	[illegible]	[illegible]	[illegible]	[illegible]	[illegible]	[illegible]	[illegible]
Adverse effects of medical treatment	[illegible]	[illegible]	[illegible]	[illegible]	[illegible]	[illegible]	[illegible]	[illegible]	[illegible]	[illegible]	[illegible]	[illegible]	[illegible]	[illegible]	[illegible]	[illegible]	[illegible]	[illegible]	[illegible]
Animal contact	[illegible]	[illegible]	[illegible]	[illegible]	[illegible]	[illegible]	[illegible]	[illegible]	[illegible]	[illegible]	[illegible]	[illegible]	[illegible]	[illegible]	[illegible]	[illegible]	[illegible]	[illegible]	[illegible]
Venomous animal contact	[illegible]	[illegible]	[illegible]	[illegible]	[illegible]	[illegible]	[illegible]	[illegible]	[illegible]	[illegible]	[illegible]	[illegible]	[illegible]	[illegible]	[illegible]	[illegible]	[illegible]	[illegible]	[illegible]
Non-venomous animal contact	[illegible]	[illegible]	[illegible]	[illegible]	[illegible]	[illegible]	[illegible]	[illegible]	[illegible]	[illegible]	[illegible]	[illegible]	[illegible]	[illegible]	[illegible]	[illegible]	[illegible]	[illegible]	[illegible]
Foreign body	[illegible]	[illegible]	[illegible]	[illegible]	[illegible]	[illegible]	[illegible]	[illegible]	[illegible]	[illegible]	[illegible]	[illegible]	[illegible]	[illegible]	[illegible]	[illegible]	[illegible]	[illegible]	[illegible]
Pulmonary aspiration and foreign body in airway	[illegible]	[illegible]	[illegible]	[illegible]	[illegible]	[illegible]	[illegible]	[illegible]	[illegible]	[illegible]	[illegible]	[illegible]	[illegible]	[illegible]	[illegible]	[illegible]	[illegible]	[illegible]	[illegible]
Foreign body in other body part	[illegible]	[illegible]	[illegible]	[illegible]	[illegible]	[illegible]	[illegible]	[illegible]	[illegible]	[illegible]	[illegible]	[illegible]	[illegible]	[illegible]	[illegible]	[illegible]	[illegible]	[illegible]	[illegible]
Environmental heat and cold exposure	[illegible]	[illegible]	[illegible]	[illegible]	[illegible]	[illegible]	[illegible]	[illegible]	[illegible]	[illegible]	[illegible]	[illegible]	[illegible]	[illegible]	[illegible]	[illegible]	[illegible]	[illegible]	[illegible]
Other unintentional injuries	[illegible]	[illegible]	[illegible]	[illegible]	[illegible]	[illegible]	[illegible]	[illegible]	[illegible]	[illegible]	[illegible]	[illegible]	[illegible]	[illegible]	[illegible]	[illegible]	[illegible]	[illegible]	[illegible]
Self-harm and interpersonal violence	[illegible]	[illegible]	[illegible]	[illegible]	[illegible]	[illegible]	[illegible]	[illegible]	[illegible]	[illegible]	[illegible]	[illegible]	[illegible]	[illegible]	[illegible]	[illegible]	[illegible]	[illegible]	[illegible]

SR Table 3: Global deaths by cause and age group for females (with 95% uncertainty intervals), 1990

Cause	Early Neonatal	Late Neonatal	Post Neonatal	1 to 4	5 to 9	10 to 14	15 to 19	20 to 24	25 to 29	30 to 34	35 to 39	40 to 44	45 to 49	50 to 54	55 to 59	60 to 64	65 to 69	70 to 74	75 to 79	80 to 84	85 to 89	90 to 94	95 plus
Self-harm	-	-	-	-	-	6,774<br>(5,419-8,151)	39,590<br>(34,122-44,580)	52,458<br>(47,558-57,165)	40,246<br>(36,958-43,531)	28,968<br>(26,315-31,552)	25,634<br>(23,282-27,980)	18,616<br>(17,116-20,005)	15,560<br>(14,384-16,759)	16,167<br>(14,946-17,355)	14,690<br>(13,476-15,897)	13,898<br>(12,853-15,025)	13,504<br>(12,516-14,620)	12,187<br>(11,198-13,387)	10,558<br>(9,792-11,554)	7,129<br>(6,657-7,770)	3,416<br>(3,192-3,694)	977<br>(915-1,051)	193<br>(182-206)
Self-harm by firearm	-	-	-	-	-	216<br>(125-361)	1,297<br>(683-2,022)	1,495<br>(810-2,046)	1,162<br>(749-1,502)	973<br>(719-1,248)	849<br>(644-1,095)	687<br>(550-875)	574<br>(466-742)	505<br>(404-679)	461<br>(371-634)	404<br>(328-556)	391<br>(308-577)	315<br>(235-492)	293<br>(220-463)	221<br>(175-322)	137<br>(117-177)	62<br>(55-77)	22<br>(19-25)
Self-harm by other specified means	-	-	-	-	-	6,558<br>(5,252-7,868)	38,293<br>(33,191-42,942)	50,963<br>(46,296-55,316)	39,084<br>(35,963-42,210)	27,994<br>(25,493-30,527)	24,786<br>(22,536-27,017)	17,930<br>(16,503-19,300)	14,986<br>(13,838-16,189)	15,662<br>(14,485-16,870)	14,229<br>(13,041-15,423)	13,493<br>(12,475-14,616)	13,112<br>(12,128-14,203)	11,872<br>(10,893-13,057)	10,265<br>(9,517-11,250)	6,908<br>(6,434-7,542)	3,280<br>(3,064-3,538)	915<br>(857-983)	171<br>(161-183)
Interpersonal violence	1,295<br>(770-1,617)	365<br>(205-465)	3,207<br>(1,547-4,137)	5,352<br>(2,698-8,069)	3,453<br>(2,033-4,598)	3,095<br>(2,092-3,976)	6,285<br>(4,403-7,891)	8,422<br>(5,854-10,594)	7,591<br>(5,427-9,087)	7,174<br>(5,016-8,632)	6,058<br>(4,277-7,013)	4,559<br>(3,263-5,392)	3,355<br>(2,441-3,960)	3,075<br>(2,346-3,805)	2,401<br>(1,801-2,965)	2,228<br>(1,720-2,837)	1,829<br>(1,417-2,422)	1,519<br>(1,178-1,943)	1,467<br>(1,130-1,870)	558<br>(437-739)	256<br>(192-331)	85<br>(70-118)	29<br>(22-39)
Assault by firearm	47<br>(26-70)	22<br>(9-31)	251<br>(124-392)	367<br>(206-671)	346<br>(205-514)	488<br>(329-681)	1,480<br>(945-1,899)	1,855<br>(1,179-2,204)	1,825<br>(1,112-2,151)	1,580<br>(929-1,877)	1,350<br>(768-1,617)	990<br>(588-1,195)	723<br>(440-894)	599<br>(355-740)	444<br>(273-568)	382<br>(244-507)	302<br>(197-408)	256<br>(168-356)	214<br>(141-305)	42<br>(24-59)	21<br>(12-30)	6<br>(4-10)	2<br>(1-4)
Assault by sharp object	70<br>(42-104)	42<br>(15-63)	247<br>(93-356)	515<br>(188-861)	493<br>(230-703)	581<br>(313-766)	1,417<br>(840-1,882)	2,296<br>(1,367-2,990)	2,144<br>(1,355-2,673)	2,143<br>(1,368-2,612)	1,811<br>(1,115-2,198)	1,441<br>(902-1,764)	992<br>(633-1,211)	857<br>(579-1,067)	645<br>(411-792)	598<br>(415-769)	426<br>(290-533)	401<br>(284-506)	377<br>(259-480)	140<br>(106-201)	57<br>(43-84)	21<br>(18-34)	7<br>(5-11)
Assault by other means	1,178<br>(690-1,495)	302<br>(174-393)	2,709<br>(1,330-3,583)	4,470<br>(2,251-6,809)	2,613<br>(1,552-3,501)	2,026<br>(1,355-2,748)	3,388<br>(2,327-4,473)	4,271<br>(3,030-5,824)	3,622<br>(2,651-4,627)	3,451<br>(2,461-4,426)	2,897<br>(2,068-3,590)	2,128<br>(1,576-2,687)	1,641<br>(1,215-2,051)	1,618<br>(1,228-2,036)	1,313<br>(1,030-1,758)	1,248<br>(948-1,665)	1,100<br>(864-1,512)	862<br>(647-1,129)	876<br>(685-1,165)	376<br>(292-502)	178<br>(128-233)	58<br>(43-79)	20<br>(14-27)
Forces of nature, conflict and terrorism, and executions and police conflict	772<br>(666-874)	471<br>(401-543)	1,847<br>(1,473-2,232)	9,648<br>(7,628-11,705)	6,219<br>(4,464-7,980)	5,220<br>(3,676-6,796)	4,936<br>(3,815-6,076)	3,999<br>(3,100-4,893)	2,936<br>(2,157-3,691)	2,576<br>(1,894-3,251)	2,109<br>(1,528-2,676)	1,646<br>(1,190-2,093)	1,402<br>(1,027-1,770)	1,375<br>(976-1,769)	1,339<br>(918-1,762)	1,251<br>(838-1,678)	1,149<br>(771-1,527)	824<br>(555-1,096)	581<br>(405-759)	376<br>(276-478)	205<br>(138-274)	62<br>(38-87)	30<br>(23-38)
Forces of nature, conflict and terrorism, and state actor violence	16<br>(6-27)	62<br>(23-102)	512<br>(185-837)	2,736<br>(962-4,487)	2,581<br>(897-4,253)	2,330<br>(835-3,809)	1,594<br>(600-2,577)	1,284<br>(499-2,060)	1,174<br>(457-1,884)	1,032<br>(401-1,658)	900<br>(352-1,444)	719<br>(290-1,149)	589<br>(243-935)	632<br>(257-1,009)	672<br>(266-1,079)	665<br>(262-1,068)	597<br>(235-960)	450<br>(191-713)	314<br>(146-484)	186<br>(93-280)	114<br>(52-179)	41<br>(18-65)	12<br>(5-18)
Conflict and terrorism	755<br>(651-855)	408<br>(352-462)	1,315<br>(1,133-1,490)	6,858<br>(5,887-7,794)	3,599<br>(3,085-4,084)	2,847<br>(2,434-3,238)	3,296<br>(2,816-3,750)	2,685<br>(2,289-3,064)	1,714<br>(1,455-1,959)	1,519<br>(1,290-1,734)	1,183<br>(1,007-1,349)	904<br>(772-1,028)	791<br>(675-900)	724<br>(616-825)	644<br>(550-733)	556<br>(472-636)	528<br>(448-603)	344<br>(292-394)	251<br>(213-289)	163<br>(138-188)	70<br>(59-82)	11<br>(9-12)	16<br>(13-19)
Executions and police conflict	-	-	20<br>(12-30)	54<br>(23-122)	39<br>(30-55)	43<br>(33-63)	45<br>(30-62)	30<br>(25-39)	48<br>(26-70)	25<br>(21-34)	25<br>(19-40)	22<br>(16-37)	21<br>(14-36)	19<br>(14-29)	23<br>(13-39)	30<br>(13-52)	24<br>(12-43)	29<br>(11-54)	16<br>(9-23)	26<br>(10-44)	21<br>(4-39)	10<br>(5-18)	3<br>(1-5)

SR Table 3: Global deaths by cause and age group for females (with 95% uncertainty intervals), 1995

Cause	Early Neonatal	Late Neonatal	Post Neonatal	1 to 4	5 to 9	10 to 14	15 to 19	20 to 24	25 to 29	30 to 34	35 to 39
All causes	1,179,767 (1,153,729-1,205,638)	449,241 (438,378-459,213)	1,478,048 (1,443,956-1,511,530)	1,521,266 (1,478,458-1,561,288)	352,736 (348,482-356,773)	221,960 (219,280-224,608)	304,817 (299,575-310,145)	404,070 (396,746-411,528)	434,967 (426,379-444,869)	443,684 (435,831-452,269)	474,047 (465,027-483,243)
Communicable, maternal, neonatal, and nutritional disorders	1,080,341 (1,042,617-1,112,709)	401,917 (384,330-416,264)	1,249,792 (1,189,121-1,301,766)	1,244,675 (1,179,519-1,311,192)	223,093 (208,922-238,083)	109,223 (101,207-118,168)	128,429 (120,490-138,789)	187,212 (178,614-197,585)	205,401 (196,028-216,427)	195,965 (187,211-206,218)	179,501 (170,885-189,548)
HIV/AIDS and tuberculosis	-	-	37,335 (34,227-40,777)	37,091 (30,088-43,614)	12,568 (10,542-14,844)	9,824 (8,435-11,180)	24,174 (21,813-26,626)	55,029 (50,578-60,264)	83,398 (75,750-91,352)	85,840 (79,020-92,884)	80,354 (74,022-87,123)
Tuberculosis	-	-	10,963 (8,423-13,787)	18,656 (12,278-24,630)	8,624 (6,727-10,865)	8,912 (7,562-10,304)	19,385 (17,124-21,606)	29,001 (26,179-31,558)	33,757 (30,865-36,376)	35,274 (31,864-38,097)	38,731 (34,394-42,581)
Drug-susceptible tuberculosis	-	-	10,586 (8,083-13,380)	18,015 (11,803-23,767)	8,292 (6,458-10,452)	8,559 (7,229-9,904)	18,612 (16,439-20,758)	27,717 (24,890-30,200)	32,247 (29,498-34,890)	33,734 (30,374-36,465)	37,063 (32,992-40,689)
Multidrug-resistant tuberculosis without extensive drug resistance	-	-	373 (260-504)	635 (394-886)	328 (241-439)	349 (272-447)	764 (608-934)	1,266 (1,019-1,556)	1,488 (1,193-1,830)	1,517 (1,219-1,852)	1,641 (1,302-2,050)
Extensively drug-resistant tuberculosis	-	-	3 (2-4)	6 (4-8)	3 (3-5)	4 (3-5)	9 (7-11)	18 (14-22)	21 (16-27)	24 (18-31)	27 (21-35)
HIV/AIDS	-	-	26,372 (24,499-28,489)	18,435 (16,803-20,423)	3,944 (3,531-4,485)	912 (766-1,120)	4,789 (3,985-5,857)	26,028 (22,301-30,461)	49,641 (42,646-57,197)	50,566 (44,796-56,758)	41,623 (36,501-47,226)
Drug-susceptible HIV/AIDS - Tuberculosis	-	-	8,963 (6,882-10,553)	6,532 (5,112-7,704)	1,320 (1,036-1,550)	283 (215-359)	1,532 (1,170-1,951)	7,107 (5,058-9,233)	11,658 (8,102-15,793)	11,937 (8,234-16,110)	10,528 (7,247-13,972)
Multidrug-resistant HIV/AIDS - Tuberculosis without extensive drug resistance	-	-	277 (175-422)	192 (122-291)	36 (22-57)	8 (5-12)	58 (37-87)	239 (151-354)	376 (238-570)	386 (244-592)	336 (213-505)
Extensively drug-resistant HIV/AIDS - Tuberculosis	-	-	1 (1-2)	1 (1-1)	0 (0-1)	0 (0-0)	0 (0-1)	1 (1-2)	3 (2-4)	4 (2-6)	3 (2-5)
HIV/AIDS resulting in other diseases	-	-	17,130 (15,256-19,755)	11,710 (10,301-13,866)	2,588 (2,259-3,088)	621 (515-789)	3,199 (2,537-4,156)	18,680 (15,052-23,418)	37,604 (30,539-45,640)	38,239 (32,119-45,388)	30,755 (25,562-37,014)
Diarrhoea, lower respiratory infections, and other common infectious diseases	150,898 (126,668-173,606)	178,901 (159,859-194,796)	929,274 (860,070-996,999)	843,961 (744,088-967,407)	154,880 (131,931-181,137)	72,857 (61,300-86,616)	49,878 (39,814-64,786)	41,241 (32,289-56,757)	35,707 (27,580-49,255)	33,904 (25,844-48,099)	33,586 (25,667-48,151)
Diarrhoeal diseases	24,926 (21,184-29,456)	49,652 (42,463-56,896)	317,503 (272,443-361,596)	246,967 (190,874-300,901)	31,245 (12,814-62,030)	16,357 (6,599-32,402)	16,262 (6,877-33,492)	16,190 (7,159-32,869)	14,025 (6,056-29,273)	14,255 (5,940-29,174)	14,372 (5,937-29,515)
Intestinal infectious diseases	-	-	1,453 (187-6,578)	16,322 (7,759-30,579)	32,828 (17,659-54,580)	18,662 (9,797-29,912)	9,236 (4,658-16,185)	4,485 (2,174-7,989)	3,328 (1,703-5,764)	2,691 (1,421-4,577)	2,190 (1,170-3,607)
Typhoid fever	-	-	87 (40-169)	13,916 (5,970-27,088)	28,916 (15,153-48,614)	15,865 (8,066-25,583)	8,051 (3,965-14,518)	3,808 (1,807-6,751)	2,674 (1,340-4,696)	2,060 (1,049-3,587)	1,646 (864-2,819)
Paratyphoid fever	-	-	13 (5-27)	1,341 (524-2,866)	3,800 (1,659-7,650)	2,754 (1,246-5,279)	1,149 (479-2,335)	644 (274-1,308)	629 (274-1,274)	593 (262-1,176)	500 (235-935)
Other intestinal infectious diseases	-	-	1,354 (96-6,468)	1,065 (67-4,898)	112 (8-513)	42 (3-207)	35 (2-171)	33 (3-165)	26 (2-120)	38 (4-157)	44 (3-206)
Lower respiratory infections	92,889 (75,352-114,700)	95,330 (82,315-110,240)	414,801 (359,599-478,688)	249,673 (197,785-306,776)	26,543 (20,621-32,688)	11,827 (9,510-13,875)	8,839 (7,183-10,400)	9,248 (7,402-11,070)	9,421 (7,702-11,169)	9,003 (7,279-10,644)	10,087 (8,447-11,978)
Upper respiratory infections	51 (31-80)	23 (13-40)	505 (329-758)	444 (262-739)	33 (20-54)	21 (12-32)	41 (27-61)	39 (26-56)	41 (27-59)	39 (26-57)	42 (28-61)
Otitis media	7 (5-9)	39 (22-70)	255 (132-525)	433 (189-932)	284 (145-603)	195 (117-355)	93 (58-160)	52 (30-94)	76 (43-147)	62 (36-115)	52 (31-92)
Meningitis	3,726 (2,261-4,886)	7,036 (3,976-9,071)	43,338 (23,350-54,226)	44,358 (20,165-60,762)	12,643 (9,119-15,620)	8,271 (6,408-10,018)	6,721 (5,119-8,227)	5,309 (4,304-6,526)	4,517 (3,422-5,632)	3,836 (2,986-4,847)	3,638 (2,907-4,538)
Pneumococcal meningitis	154 (94-220)	293 (173-407)	2,051 (1,198-2,759)	2,126 (940-3,148)	651 (440-898)	423 (294-599)	332 (225-463)	249 (172-348)	249 (169-344)	274 (184-392)	308 (201-456)
H influenzae type B meningitis	414 (244-567)	777 (420-1,019)	4,666 (2,347-6,166)	4,359 (1,808-6,275)	1,043 (719-1,391)	573 (417-758)	411 (290-555)	319 (238-428)	290 (209-383)	261 (191-350)	254 (187-345)
Meningococcal infection	1,502 (936-1,974)	2,942 (1,709-3,804)	18,708 (10,629-23,884)	20,597 (9,729-28,501)	6,335 (4,538-7,864)	4,043 (3,067-4,992)	3,274 (2,538-4,108)	2,512 (2,009-3,143)	2,046 (1,546-2,596)	1,640 (1,269-2,105)	1,489 (1,147-1,861)
Other meningitis	1,655 (992-2,238)	3,024 (1,646-3,967)	17,914 (9,360-23,202)	17,277 (7,445-24,147)	4,614 (3,327-5,917)	3,232 (2,477-4,031)	2,704 (2,021-3,339)	2,229 (1,710-2,777)	1,933 (1,443-2,458)	1,661 (1,281-2,140)	1,587 (1,227-2,023)
Encephalitis	387 (214-521)	807 (411-1,141)	7,815 (3,970-11,297)	11,380 (5,257-20,788)	4,667 (2,661-7,601)	2,915 (1,805-4,590)	1,800 (1,208-2,649)	1,648 (1,077-2,389)	1,254 (825-1,850)	1,219 (829-1,832)	1,084 (751-1,602)
Diphtheria	-	-	922 (522-1,531)	2,117 (1,151-3,782)	335 (191-562)	141 (86-221)	66 (40-104)	37 (22-58)	32 (19-50)	27 (16-44)	39 (24-61)
Whooping cough	-	-	36,709 (17,320-70,432)	39,224 (18,250-74,202)	4,523 (1,911-9,543)	839 (360-1,767)	287 (122-596)	221 (93-459)	135 (56-278)	94 (38-198)	71 (29-153)
Tetanus	28,851 (15,117-47,449)	25,807 (14,668-38,241)	8,014 (4,524-13,746)	3,924 (1,763-8,154)	1,305 (538-2,835)	2,188 (696-4,852)	2,429 (948-4,703)	1,903 (667-4,288)	1,704 (716-3,493)	1,892 (925-3,921)	1,502 (786-3,357)
Measles	-	-	96,399 (36,156-204,933)	227,144 (95,196-445,161)	39,541 (16,121-78,108)	11,149 (4,053-24,118)	3,985 (1,352-9,421)	1,970 (655-4,712)	1,003 (338-2,426)	600 (202-1,450)	360 (119-875)
Varicella and herpes zoster	62 (48-79)	207 (162-265)	1,560 (1,205-2,005)	1,975 (1,465-2,592)	933 (692-1,237)	293 (224-375)	119 (93-154)	138 (110-170)	172 (136-211)	187 (150-234)	149 (120-186)
Neglected tropical diseases and malaria	532 (228-756)	10,288 (7,001-14,524)	104,615 (75,700-140,544)	226,756 (163,580-299,209)	35,265 (22,589-48,454)	17,489 (11,961-23,074)	8,437 (6,206-10,963)	8,158 (5,980-10,721)	6,252 (4,722-8,555)	5,901 (4,457-8,127)	6,832 (5,223-9,234)
Malaria	-	9,909 (6,627-14,141)	98,999 (70,489-135,849)	209,123 (148,367-280,319)	26,140 (15,284-38,538)	12,255 (7,423-17,351)	4,796 (3,215-6,818)	4,619 (3,171-6,528)	3,114 (2,130-4,416)	2,679 (1,841-3,891)	3,290 (2,260-4,824)
Chagas disease	-	-	2 (2-3)	2 (1-2)	1 (1-2)	3 (3-3)	9 (7-10)	18 (16-21)	36 (31-41)	64 (56-74)	101 (88-115)
Leishmaniasis	-	-	932 (433-1,785)	1,477 (628-2,934)	809 (366-1,638)	517 (232-999)	594 (281-1,120)	632 (301-1,206)	533 (248-1,011)	486 (216-920)	584 (269-1,097)
Visceral leishmaniasis	-	-	932 (433-1,785)	1,477 (628-2,934)	809 (366-1,638)	517 (232-999)	594 (281-1,120)	632 (301-1,206)	533 (248-1,011)	486 (216-920)	584 (269-1,097)
African trypanosomiasis	-	-	-	339 (55-1,161)	941 (191-2,757)	1,013 (250-2,529)	1,173 (296-2,963)	1,152 (243-3,073)	1,028 (273-2,816)	966 (239-2,859)	876 (192-2,624)
Schistosomiasis	-	-	0 (0-0)	374 (218-644)	155 (95-237)	205 (133-307)	177 (119-256)	214 (145-307)	257 (181-370)	286 (202-399)	354 (244-517)
Cysticercosis	0 (0-0)	0 (0-0)	0 (0-0)	89 (57-133)	52 (38-71)	39 (30-49)	29 (23-38)	38 (29-48)	52 (42-63)	62 (50-75)	50 (40-61)
Cystic echinococcosis	-	-	-	231 (149-352)	84 (55-122)	48 (32-68)	24 (16-33)	29 (20-40)	41 (29-56)	39 (28-53)	50 (37-66)
Dengue	-	-	719 (138-1,843)	1,172 (315-2,592)	656 (142-1,465)	387 (78-947)	252 (46-589)	234 (33-603)	167 (27-386)	137 (19-374)	116 (20-283)
Yellow fever	-	1 (0-4)	24 (5-79)	207 (38-621)	414 (77-1,226)	389 (76-1,163)	329 (66-1,014)	254 (51-754)	197 (40-595)	154 (30-496)	129 (25-392)
Rabies	-	-	498 (272-1,313)	2,615 (1,178-5,248)	4,366 (1,431-8,439)	2,083 (537-3,497)	770 (288-1,209)	709 (223-1,046)	587 (298-1,141)	829 (359-1,740)	1,063 (399-1,768)
Intestinal nematode infections	-	-	473 (361-619)	5,318 (3,889-6,940)	807 (603-1,047)	152 (116-191)	46 (35-59)	29 (21-38)	23 (17-30)	29 (22-37)	27 (20-35)
Ascariasis	-	-	473 (361-619)	5,318 (3,889-6,940)	807 (603-1,047)	152 (116-191)	46 (35-59)	29 (21-38)	23 (17-30)	29 (22-37)	27 (20-35)
Ebola	0 (0-0)	0 (0-0)	2 (2-3)	7 (5-9)	7 (5-9)	3 (2-4)	6 (4-7)	26 (19-33)	28 (21-36)	17 (12-21)	35 (26-44)
Zika virus	0 (0-0)	0 (0-0)	0 (0-0)	0 (0-0)	0 (0-0)	0 (0-0)	0 (0-0)	0 (0-0)	0 (0-0)	0 (0-0)	0 (0-0)
Other neglected tropical diseases	532 (228-756)	377 (181-544)	2,965 (1,184-4,703)	5,801 (1,412-12,813)	833 (359-1,438)	395 (192-669)	233 (121-376)	205 (92-306)	189 (85-263)	155 (71-225)	158 (76-210)
Maternal disorders	-	-	-	-	-	1,668 (1,395-1,976)	38,146 (34,248-41,472)	73,897 (68,065-79,185)	71,693 (66,133-76,330)	62,539 (57,694-66,554)	51,026 (47,007-54,706)
Maternal haemorrhage	-	-	-	-	-	350 (236-498)	10,870 (7,365-14,521)	25,270 (19,289-31,558)	26,103 (19,254-33,230)	22,557 (16,759-28,160)	17,989 (13,235-23,276)
Maternal sepsis and other pregnancy related infections	-	-	-	-	-	191 (121-276)	4,711 (2,965-7,138)	7,384 (4,561-11,167)	6,603 (4,405-9,449)	5,596 (3,566-8,050)	4,577 (2,993-6,704)
Maternal hypertensive disorders	-	-	-	-	-	293 (178-427)	5,184 (3,237-7,300)	8,907 (6,015-12,527)	8,024 (5,113-11,703)	6,913 (4,337-10,313)	5,817 (3,847-8,358)
Maternal obstructed labour and uterine rupture	-	-	-	-	-	56 (29-102)	1,076 (539-1,840)	2,212 (1,260-3,516)	2,461 (1,415-3,943)	2,507 (1,408-3,895)	2,451 (1,450-4,112)
Maternal abortion, miscarriage, and ectopic pregnancy	-	-	-	-	-	126 (75-200)	2,787 (1,697-4,279)	5,412 (3,420-8,151)	5,140 (3,354-7,803)	4,829 (3,098-7,118)	4,381 (2,642-6,486)
Indirect maternal deaths	-	-	-	-	-	244 (153-363)	4,925 (3,062-7,253)	9,640 (6,335-14,041)	9,876 (6,349-14,306)	8,581 (5,209-12,611)	6,384 (4,090-9,185)
Late maternal deaths	-	-	-	-	-	40 (17-77)	723 (366-1,301)	1,286 (615-2,406)	1,245 (614-2,349)	1,098 (570-1,946)	891 (440-1,571)
Maternal deaths aggravated by HIV/AIDS	-	-	-	-	-	0 (0-0)	73 (41-105)	291 (172-396)	397 (243-533)	363 (219-497)	273 (165-378)
Other maternal disorders	-	-	-	-	-	369 (248-548)	7,796 (5,197-11,374)	13,495 (8,754-19,396)	11,845 (7,934-16,710)	10,096 (6,496-14,401)	8,264 (5,452-12,389)
Neonatal disorders	904,491 (865,762-948,326)	202,623 (186,960-225,362)	54,852 (46,199-68,647)	5,277 (4,014-7,504)	-	-	-	-	-	-	-
Neonatal preterm birth complications	387,779 (350,357-432,609)	68,684 (59,967-87,352)	22,126 (18,392-30,477)	1,478 (1,112-2,176)	-	-	-	-	-	-	-
Neonatal encephalopathy due to birth asphyxia and trauma	272,469 (243,350-307,339)	30,274 (25,490-37,195)	10,327 (7,795-14,315)	1,985 (1,439-2,991)	-	-	-	-	-	-	-
Neonatal sepsis and other neonatal infections	60,955 (32,972-90,065)	46,411 (27,880-62,608)	7,384 (4,275-11,361)	400 (99-818)	-	-	-	-	-	-	-
Hemolytic disease and other neonatal jaundice	28,258 (21,558-36,325)	18,713 (13,991-24,358)	4,011 (2,884-5,570)	230 (151-351)	-	-	-	-	-	-	-
Other neonatal disorders	155,030 (130,018-186,470)	38,541 (31,565-50,536)	11,003 (8,440-15,778)	1,183 (824-1,988)	-	-	-	-	-	-	-
Nutritional deficiencies	-	-	84,976 (69,525-101,601)	97,474 (74,265-125,832)	7,806 (6,184-9,588)	3,433 (2,821-4,108)	2,690 (2,330-3,068)	3,111 (2,650-3,619)	3,350 (2,864-3,880)	3,042 (2,638-3,491)	2,995 (2,502-3,507)
Protein-energy malnutrition	-	-	81,988 (66,729-98,324)	93,149 (70,713-121,074)	6,441 (4,993-8,019)	2,688 (2,134-3,300)	1,792 (1,502-2,113)	1,827 (1,422-2,266)	2,150 (1,696-2,618)	1,839 (1,450-2,243)	1,887 (1,425-2,397)
Iodine deficiency	-	-	290 (72-872)	241 (37-869)	82 (22-258)	19 (4-56)	32 (8-88)	37 (9-107)	39 (9-105)	42 (11-118)	40 (11-117)
Iron-deficiency anaemia	-	-	188 (38-300)	75 (41-155)	107 (43-169)	89 (43-152)	93 (47-156)	102 (51-164)	96 (43-130)	95 (51-120)	110 (46-156)
Other nutritional deficiencies	-	-	2,510 (1,764-3,303)	4,009 (1,728-6,522)	1,176 (814-1,662)	637 (484-828)	774 (632-942)	1,145 (973-1,341)	1,065 (896-1,269)	1,066 (888-1,261)	958 (805-1,139)
Other communicable, maternal, neonatal, and nutritional diseases	24,420 (14,384-38,907)	10,105 (6,292-15,211)	38,741 (25,476-56,095)	34,117 (23,084-48,276)	12,573 (8,926-17,717)	3,952 (3,310-4,563)	5,103 (4,567-5,654)	5,776 (5,245-6,295)	5,001 (4,525-5,479)	4,740 (4,322-5,204)	4,707 (4,323-5,137)
Sexually transmitted diseases excluding HIV	22,805 (12,746-37,075)	8,543 (4,797-13,668)	28,090 (15,583-45,521)	20,484 (10,814-34,017)	7,028 (3,836-11,793)	107 (64-149)	610 (414-778)	757 (511-958)	902 (619-1,132)	704 (484-868)	735 (522-879)
Syphilis	22,805 (12,746-37,075)	8,543 (4,797-13,668)	28,090 (15,583-45,521)	20,484 (10,814-34,017)	7,028 (3,836-11,793)	58 (35-81)	172 (117-219)	212 (143-268)	185 (127-232)	145 (100-179)	90 (64-108)
Chlamydial infection	-	-	-	-	-	14 (8-19)	79 (54-101)	164 (111-207)	151 (104-190)	74 (51-91)	118 (84-142)
Gonococcal infection	-	-	-	-	-	24 (14-33)	195 (132-248)	236 (160-299)	393 (269-493)	316 (217-389)	326 (231-389)
Other sexually transmitted diseases	-	-	-	-	-	11 (7-16)	164 (111-209)	145 (98-183)	173 (119-217)	170 (117-209)	202 (143-241)
Hepatitis	-	-	6,280 (5,465-7,202)	8,964 (6,994-11,037)	3,819 (3,001-4,692)	2,915 (2,388-3,403)	3,598 (3,158-4,016)	4,284 (3,834-4,725)	3,460 (3,128-3,806)	3,348 (3,008-3,693)	3,385 (3,052-3,733)
Acute hepatitis A	-	-	4,069 (3,000-5,161)	1,052 (563-1,648)	91 (56-135)	47 (31-69)	66 (40-95)	82 (44-136)	50 (24-93)	37 (16-68)	34 (16-63)
Hepatitis B	-	-	2,210 (1,311-3,282)	1,104 (635-1,760)	318 (165-522)	426 (222-680)	1,288 (865-1,843)	2,756 (2,017-3,488)	2,754 (2,230-3,200)	2,933 (2,543-3,344)	3,059 (2,697-3,422)
Hepatitis C	-	-	-	-	93 (60-141)	35 (22-51)	45 (29-67)	54 (29-89)	36 (17-64)	30 (14-55)	36 (15-66)
Acute hepatitis E	-	-	-	6,808 (5,166-8,575)	3,316 (2,573-4,116)	2,407 (1,930-2,863)	2,199 (1,678-2,680)	1,391 (805-2,041)	620 (321-1,030)	348 (154-624)	255 (113-448)
Other infectious diseases	1,615 (957-1,950)	1,563 (870-2,182)	4,371 (3,127-5,245)	4,669 (2,963-6,998)	1,726 (1,248-2,226)	930 (707-1,151)	895 (675-1,084)	735 (566-883)	638 (486-738)	687 (527-818)	587 (472-731)
Non-communicable diseases	92,197 (68,042-116,780)	42,344 (32,297-54,439)	172,446 (137,032-216,760)	133,754 (101,280-169,588)	56,736 (50,376-63,861)	55,606 (50,950-60,325)	79,131 (74,419-83,018)	101,407 (96,661-105,349)	125,917 (120,643-129,943)	162,072 (156,284-167,117)	220,398 (213,130-226,677)
Neoplasms	460 (399-498)	291 (255-326)	4,262 (3,458-5,091)	13,983 (11,550-17,784)	14,744 (13,041-16,799)	13,865 (12,544-15,613)	16,367 (15,164-17,378)	21,950 (20,679-23,145)	32,652 (31,071-34,086)	51,910 (49,498-54,130)	80,740 (76,952-84,292)
Lip and oral cavity cancer	-	-	-	-	-	-	136 (123-151)	251 (224-288)	357 (319-413)	581 (521-671)	874 (781-1,009)
Nasopharynx cancer	-	-	-	-	74 (64-86)	122 (101-153)	229 (202-267)	347 (310-386)	495 (445-548)	653 (590-714)	882 (804-968)
Other pharynx cancer	-	-	-	-	-	-	169 (144-196)	211 (179-246)	319 (276-359)	467 (402-527)	678 (582-768)
Oesophageal cancer	-	-	-	-	-	-	176 (162-192)	372 (343-397)	543 (499-589)	832 (771-892)	1,407 (1,312-1,502)
Stomach cancer	-	-	-	-	-	-	599 (555-643)	1,678 (1,565-1,783)	3,369 (3,163-3,584)	5,920 (5,595-6,260)	8,698 (8,246-9,185)
Colon and rectum cancer	-	-	-	-	-	-	360 (323-404)	955 (853-1,059)	1,701 (1,533-1,864)	3,196 (2,900-3,490)	4,582 (4,239-4,997)
Liver cancer	-	-	-	-	262 (184-324)	313 (218-372)	497 (344-564)	1,089 (676-1,291)	2,072 (1,277-2,454)	3,265 (2,034-3,757)	4,588 (2,959-5,249)
Liver cancer due to hepatitis B	-	-	-	-	115 (69-158)	169 (106-218)	295 (186-360)	777 (464-949)	1,565 (960-1,917)	2,406 (1,453-2,891)	3,152 (1,936-3,813)
Liver cancer due to hepatitis C	-	-	-	-	10 (8-14)	16 (11-22)	27 (19-35)	66 (45-88)	131 (81-197)	231 (151-321)	370 (247-521)
Liver cancer due to alcohol use	-	-	-	-	-	-	4 (2-7)	26 (14-41)	81 (39-144)	252 (141-408)	603 (316-1,040)
Liver cancer due to other causes	-	-	-	-	136 (97-183)	128 (89-168)	171 (118-223)	221 (142-299)	296 (176-443)	376 (232-529)	462 (291-657)
Gallbladder and biliary tract cancer	-	-	-	-	-	-	45 (41-50)	85 (75-96)	183 (158-207)	437 (380-495)	873 (753-1,000)
Pancreatic cancer	-	-	-	-	-	-	67 (63-73)	144 (132-158)	283 (260-314)	492 (458-536)	977 (917-1,053)
Larynx cancer	-	-	-	-	-	-	62 (56-68)	107 (96-120)	132 (117-150)	239 (208-277)	375 (342-409)
Tracheal, bronchus, and lung cancer	-	-	-	-	-	-	324 (246-355)	676 (509-759)	1,471 (1,120-1,645)	2,905 (2,412-3,194)	5,170 (4,724-5,634)
Malignant skin melanoma	-	-	-	-	-	-	106 (85-128)	208 (165-258)	343 (272-412)	532 (423-635)	793 (622-945)
Non-melanoma skin cancer	-	-	-	-	-	-	62 (57-67)	88 (78-99)	127 (114-142)	150 (135-166)	202 (183-224)
Non-melanoma skin cancer (squamous-cell carcinoma)	-	-	-	-	-	-	62 (57-67)	88 (78-99)	127 (114-142)	150 (135-166)	202 (183-224)
Breast cancer	-	-	-	-	-	-	294 (208-354)	921 (723-1,106)	3,094 (2,511-3,621)	8,943 (7,710-10,186)	18,182 (16,264-20,551)
Cervical cancer	-	-	-	-	-	-	243 (185-279)	950 (747-1,119)	2,791 (2,405-3,266)	6,626 (5,710-7,801)	11,970 (10,332-14,043)
Uterine cancer	-	-	-	-	-	-	99 (90-109)	289 (253-317)	543 (477-598)	843 (750-920)	1,451 (1,283-1,568)
Ovarian cancer	-	-	-	-	-	-	485 (428-546)	717 (623-816)	1,031 (904-1,163)	1,631 (1,482-1,819)	2,859 (2,609-3,187)
Prostate cancer	-	-	-	-	-	-	-	-	-	-	-
Testicular cancer	-	-	-	-	-	-	-	-	-	-	-
Kidney cancer	15 (14-17)	8 (7-9)	115 (98-142)	534 (419-706)	335 (300-385)	150 (137-166)	96 (90-105)	120 (111-133)	167 (155-183)	228 (215-249)	378 (355-409)
Bladder cancer	-	-	-	-	-	-	47 (43-52)	70 (64-78)	120 (108-132)	195 (176-216)	314 (283-349)
Brain and nervous system cancer	62 (41-75)	40 (27-49)	633 (356-824)	2,431 (1,578-3,111)	3,354 (2,486-3,833)	2,560 (1,969-2,868)	1,724 (1,338-1,922)	1,901 (1,415-2,134)	2,275 (1,724-2,541)	2,659 (2,076-2,934)	3,203 (2,585-3,501)
Thyroid cancer	-	-	-	-	-	89 (70-109)	213 (163-263)	338 (268-394)	411 (345-468)	489 (408-556)	579 (486-661)
Mesothelioma	-	-	-	-	-	-	45 (35-65)	67 (46-96)	105 (73-148)	109 (81-154)	176 (136-242)
Hodgkin lymphoma	2 (1-3)	1 (1-2)	51 (16-77)	196 (66-297)	368 (171-544)	521 (264-692)	749 (459-890)	878 (581-1,036)	911 (601-1,070)	831 (577-947)	785 (534-897)
Non-Hodgkin's lymphoma	24 (19-27)	18 (15-22)	313 (216-406)	1,108 (799-1,543)	1,213 (934-1,479)	1,096 (889-1,290)	1,064 (915-1,177)	1,307 (1,103-1,477)	1,440 (1,260-1,614)	1,569 (1,344-1,751)	1,849 (1,644-2,023)
Multiple myeloma	-	-	-	-	-	-	116 (82-137)	159 (108-193)	155 (107-181)	164 (123-193)	293 (222-343)
Leukaemia	126 (85-148)	96 (65-119)	2,159 (1,280-2,753)	6,803 (4,448-9,722)	6,768 (5,214-8,701)	6,250 (5,054-8,099)	5,349 (4,284-6,534)	5,053 (3,878-6,177)	5,009 (3,697-5,960)	4,512 (3,486-5,494)	4,703 (3,660-5,499)
Acute lymphoid leukaemia	31 (23-45)	25 (18-42)	492 (342-857)	2,047 (1,476-3,896)	2,463 (1,949-3,702)	2,101 (1,713-3,088)	1,332 (1,084-2,006)	946 (775-1,464)	882 (708-1,375)	624 (508-987)	667 (550-987)
Chronic lymphoid leukaemia	-	-	-	-	-	-	116 (84-189)	132 (90-226)	153 (107-262)	158 (116-247)	215 (159-326)
Acute myeloid leukaemia	18 (13-25)	14 (10-20)	236 (162-360)	876 (600-1,465)	790 (576-1,063)	982 (765-1,313)	940 (765-1,209)	804 (663-1,079)	795 (654-1,050)	800 (684-1,020)	948 (803-1,203)
Chronic myeloid leukaemia	-	-	-	-	-	-	272 (184-369)	309 (208-409)	456 (321-613)	481 (357-620)	517 (394-642)
Other leukaemia	77 (45-96)	57 (33-76)	1,430 (693-1,999)	3,880 (2,024-5,767)	3,516 (2,322-4,589)	3,167 (2,231-4,005)	2,690 (1,867-3,212)	2,863 (1,828-3,482)	2,724 (1,694-3,304)	2,450 (1,653-2,929)	2,356 (1,630-2,772)
Other neoplasms	229 (205-251)	128 (112-148)	992 (795-1,297)	2,912 (2,366-4,211)	2,370 (2,091-2,775)	2,764 (2,474-3,126)	3,010 (2,770-3,250)	2,972 (2,728-3,211)	3,204 (2,974-3,469)	3,441 (3,175-3,706)	3,899 (3,649-4,196)
Cardiovascular diseases	2,188 (2,019-2,375)	1,525 (1,337-1,735)	14,504 (12,878-16,263)	13,312 (11,256-15,395)	7,683 (6,813-8,617)	8,792 (7,973-9,573)	18,962 (17,617-20,061)	28,199 (26,459-29,725)	37,870 (35,785-39,711)	50,167 (47,510-52,554)	69,686 (66,382-72,844)
Rheumatic heart disease	-	-	-	3,578 (2,746-4,639)	2,267 (1,810-2,785)	3,485 (2,919-4,005)	5,235 (4,299-6,135)	5,857 (4,824-6,906)	7,124 (5,807-8,494)	7,826 (6,347-9,392)	9,237 (7,528-10,983)
Ischaemic heart disease	-	-	916 (720-1,175)	813 (602-1,243)	602 (488-791)	631 (530-777)	5,748 (5,115-6,657)	8,737 (8,012-9,843)	12,509 (11,469-14,051)	18,671 (17,315-20,753)	25,716 (23,994-28,282)
Cerebrovascular disease	946 (823-1,110)	635 (518-788)	5,748 (4,611-7,131)	3,941 (3,013-5,466)	2,361 (2,029-2,823)	2,353 (2,078-2,745)	4,077 (3,723-4,576)	8,002 (7,298-8,921)	11,154 (10,249-12,434)	15,331 (14,132-16,795)	23,820 (22,287-25,896)

Cause	40 to 44	45 to 49	50 to 54	55 to 59	60 to 64	65 to 69	70 to 74	75 to 79	80 to 84	85 to 89	90 to 94	95 plus
All causes	551,418 (541,982-560,182)	624,098 (614,346-633,251)	750,391 (739,301-761,260)	1,033,841 (1,017,994-1,048,880)	1,366,160 (1,347,257-1,384,536)	1,781,625 (1,756,507-1,805,519)	2,156,211 (2,127,665-2,183,656)	2,188,571 (2,162,347-2,213,967)	2,338,039 (2,313,375-2,361,634)	1,831,331 (1,817,927-1,844,380)	930,167 (925,375-935,000)	322,949 (321,558-324,415)
Communicable, maternal, neonatal, and nutritional disorders	150,685 (142,662-161,702)	132,723 (122,824-147,507)	134,262 (120,382-156,080)	154,487 (134,820-186,868)	186,882 (157,802-235,318)	219,202 (183,726-281,359)	261,229 (214,489-333,209)	244,867 (200,836-315,005)	238,064 (197,685-289,019)	175,058 (148,058-206,438)	82,394 (71,351-94,710)	31,678 (27,920-35,595)
HIV/AIDS and tuberculosis	69,360 (63,987-74,616)	62,896 (57,408-68,360)	62,247 (55,894-67,929)	64,714 (56,941-71,679)	65,104 (55,854-73,621)	65,197 (54,765-74,753)	64,873 (53,474-75,812)	50,118 (42,002-57,883)	38,777 (31,366-46,712)	20,627 (16,723-25,282)	6,480 (5,218-8,248)	1,622 (1,277-2,080)
Tuberculosis	37,944 (34,080-41,599)	39,398 (34,724-43,835)	46,549 (40,342-52,073)	54,140 (46,395-61,015)	57,761 (48,629-66,180)	60,133 (49,682-69,628)	62,444 (50,780-73,336)	48,992 (40,801-56,776)	38,470 (31,009-46,421)	20,504 (16,585-25,149)	6,444 (5,183-8,215)	1,611 (1,266-2,065)
Drug-susceptible tuberculosis	36,192 (32,529-39,763)	37,683 (33,159-41,987)	44,606 (38,653-49,904)	51,801 (44,385-58,572)	55,113 (46,478-63,203)	57,316 (47,449-66,319)	59,507 (48,660-70,198)	46,546 (38,683-54,118)	36,502 (29,344-44,025)	19,471 (15,671-23,914)	6,129 (4,906-7,822)	1,533 (1,207-1,973)
Multidrug-resistant tuberculosis without extensive drug resistance	1,723 (1,370-2,135)	1,689 (1,332-2,108)	1,919 (1,486-2,410)	2,306 (1,804-2,855)	2,615 (2,014-3,351)	2,782 (2,114-3,560)	2,902 (2,230-3,723)	2,419 (1,847-3,067)	1,945 (1,440-2,592)	1,020 (739-1,404)	310 (220-453)	77 (53-117)
Extensively drug-resistant tuberculosis	29 (22-37)	26 (20-33)	24 (19-31)	33 (25-42)	32 (25-41)	36 (27-46)	35 (26-44)	28 (21-35)	24 (18-31)	13 (9-17)	4 (3-5)	1 (1-1)
HIV/AIDS	31,417 (28,161-35,357)	23,498 (20,646-26,664)	15,697 (13,959-17,817)	10,575 (9,176-12,268)	7,343 (6,340-8,481)	5,064 (4,305-5,953)	2,429 (1,962-2,964)	1,125 (910-1,375)	307 (237-408)	124 (97-165)	37 (29-50)	11 (9-13)
Drug-susceptible HIV/AIDS - Tuberculosis	8,705 (6,054-11,181)	7,046 (5,015-8,895)	5,166 (3,849-6,310)	3,516 (2,720-4,368)	2,474 (1,915-3,018)	1,710 (1,326-2,117)	828 (610-1,036)	383 (286-490)	98 (74-131)	38 (29-52)	11 (8-15)	2 (2-3)
Multidrug-resistant HIV/AIDS - Tuberculosis without extensive drug resistance	277 (176-411)	226 (145-334)	169 (107-251)	119 (76-177)	83 (53-123)	56 (36-84)	27 (17-41)	13 (8-20)	5 (3-7)	2 (1-3)	1 (0-1)	0 (0-0)
Extensively drug-resistant HIV/AIDS - Tuberculosis	2 (2-4)	2 (1-3)	1 (1-2)	1 (1-1)	1 (0-1)	0 (0-0)	0 (0-0)	0 (0-0)	0 (0-0)	0 (0-0)	0 (0-0)	0 (0-0)
HIV/AIDS resulting in other diseases	22,432 (18,992-26,786)	16,224 (13,688-19,746)	10,361 (8,845-12,553)	6,939 (5,773-8,717)	4,785 (3,967-5,915)	3,297 (2,703-4,099)	1,574 (1,230-2,030)	729 (570-933)	204 (152-279)	83 (64-115)	26 (20-35)	8 (7-10)
Diarrhoea, lower respiratory infections, and other common infectious diseases	34,690 (26,891-49,651)	39,893 (30,069-58,542)	52,561 (38,058-79,984)	70,948 (51,271-108,651)	100,316 (70,730-155,964)	131,177 (93,303-199,476)	173,969 (125,668-257,378)	172,461 (126,549-249,427)	179,992 (138,455-239,782)	138,447 (110,429-174,623)	66,469 (55,501-79,991)	25,623 (22,142-29,604)
Diarrhoeal diseases	14,538 (6,458-30,335)	18,035 (7,803-37,971)	25,470 (10,591-54,956)	37,089 (16,590-76,559)	55,699 (24,038-116,061)	72,556 (31,180-146,727)	97,933 (43,374-188,013)	94,184 (43,670-178,751)	91,831 (46,026-156,332)	61,040 (32,637-97,818)	24,049 (12,956-36,883)	6,888 (3,960-10,574)
Intestinal infectious diseases	1,895 (1,039-3,154)	1,576 (851-2,620)	1,301 (672-2,260)	1,084 (519-1,996)	941 (454-1,688)	833 (459-1,365)	651 (365-1,056)	460 (259-753)	270 (149-443)	135 (75-236)	42 (23-76)	12 (6-26)
Typhoid fever	1,403 (733-2,361)	1,143 (587-1,925)	966 (486-1,686)	820 (389-1,504)	737 (354-1,326)	678 (357-1,125)	533 (296-853)	364 (204-593)	210 (118-335)	96 (53-153)	28 (15-45)	6 (3-10)
Paratyphoid fever	457 (212-876)	388 (174-777)	317 (138-648)	244 (100-508)	182 (75-371)	127 (58-250)	86 (39-162)	56 (27-104)	33 (16-61)	15 (7-29)	5 (2-9)	1 (0-2)
Other intestinal infectious diseases	36 (3-169)	45 (4-223)	18 (2-85)	19 (2-87)	23 (2-103)	28 (3-132)	33 (3-157)	41 (5-183)	27 (3-132)	24 (3-105)	10 (1-35)	5 (1-19)
Lower respiratory infections	11,234 (9,196-13,141)	12,692 (10,539-14,634)	17,469 (14,462-20,490)	24,124 (19,556-27,737)	35,233 (27,777-40,646)	47,705 (38,516-55,212)	64,996 (53,098-74,378)	69,794 (57,045-80,453)	81,294 (69,214-93,663)	73,560 (64,838-83,331)	40,936 (35,899-46,590)	18,229 (15,779-20,609)
Upper respiratory infections	45 (31-65)	49 (33-71)	50 (35-71)	59 (41-84)	68 (46-97)	66 (45-94)	57 (41-80)	57 (39-80)	41 (29-57)	29 (21-40)	12 (9-17)	3 (2-4)
Otitis media	102 (62-187)	67 (43-119)	62 (39-103)	147 (91-262)	110 (70-176)	177 (109-287)	135 (86-208)	94 (61-142)	68 (43-102)	33 (22-49)	14 (10-21)	4 (3-6)
Meningitis	3,341 (2,683-4,324)	3,768 (2,997-4,826)	3,922 (3,099-5,148)	4,406 (3,538-5,483)	4,361 (3,498-5,737)	4,666 (3,716-5,981)	5,159 (4,128-6,450)	3,236 (2,588-3,993)	2,946 (2,316-3,752)	1,527 (1,224-1,953)	544 (442-683)	166 (139-196)
Pneumococcal meningitis	329 (219-515)	400 (253-613)	410 (279-613)	478 (333-673)	494 (357-683)	522 (375-721)	595 (414-832)	386 (283-522)	331 (238-450)	175 (138-223)	68 (56-84)	22 (17-29)
H influenzae type B meningitis	234 (171-322)	265 (187-371)	270 (195-388)	306 (222-409)	302 (221-404)	311 (224-420)	345 (263-457)	223 (163-293)	200 (149-261)	104 (77-138)	36 (26-48)	11 (7-16)
Meningococcal infection	1,419 (1,123-1,800)	1,563 (1,238-1,957)	1,651 (1,291-2,167)	1,879 (1,489-2,415)	1,856 (1,445-2,475)	1,943 (1,491-2,524)	2,019 (1,573-2,679)	1,354 (1,024-1,707)	1,189 (881-1,522)	643 (491-834)	232 (178-295)	69 (51-87)
Other meningitis	1,359 (1,070-1,764)	1,540 (1,193-2,033)	1,591 (1,242-2,106)	1,743 (1,363-2,220)	1,709 (1,310-2,251)	1,890 (1,473-2,451)	2,200 (1,700-2,861)	1,274 (983-1,615)	1,226 (943-1,546)	606 (483-781)	207 (166-268)	64 (49-82)
Encephalitis	1,201 (803-1,691)	1,247 (844-1,817)	1,280 (899-1,869)	1,652 (1,176-2,443)	1,999 (1,395-2,891)	2,148 (1,430-3,112)	2,234 (1,445-3,089)	1,851 (1,212-2,393)	1,589 (1,032-2,153)	879 (580-1,121)	265 (182-342)	72 (51-89)
Diphtheria	39 (24-61)	45 (27-72)	53 (34-82)	44 (27-68)	-	-	-	-	-	-	-	-
Whooping cough	62 (24-136)	55 (22-117)	56 (23-122)	60 (25-130)	-	-	-	-	-	-	-	-
Tetanus	1,906 (1,060-3,930)	2,109 (1,189-4,746)	2,701 (1,273-5,860)	2,083 (1,060-4,860)	1,761 (919-3,981)	2,756 (1,318-6,104)	2,543 (1,093-5,592)	2,372 (1,031-4,850)	1,327 (535-2,659)	517 (209-899)	153 (58-272)	42 (15-81)
Measles	228 (75-554)	139 (47-337)	104 (34-250)	81 (27-197)	-	-	-	-	-	-	-	-
Varicella and herpes zoster	99 (79-125)	112 (88-143)	94 (73-116)	118 (91-149)	144 (112-185)	269 (212-340)	260 (200-330)	413 (326-523)	627 (485-811)	725 (561-964)	455 (337-637)	206 (143-315)
Neglected tropical diseases and malaria	6,756 (5,283-8,877)	6,746 (5,194-9,439)	7,743 (6,016-10,932)	7,606 (5,837-10,248)	9,119 (6,963-12,089)	8,436 (6,465-11,237)	7,165 (5,472-9,481)	5,410 (4,144-7,261)	3,114 (2,395-4,053)	1,800 (1,317-2,408)	411 (291-540)	206 (118-376)
Malaria	3,263 (2,220-4,741)	3,023 (2,038-4,439)	3,618 (2,419-5,414)	3,845 (2,604-5,612)	5,703 (3,791-8,432)	4,799 (3,172-7,154)	4,214 (2,712-6,326)	3,200 (2,122-4,752)	1,927 (1,280-2,757)	1,146 (730-1,688)	223 (117-341)	150 (68-320)
Chagas disease	150 (134-169)	207 (184-235)	276 (245-309)	358 (318-405)	387 (349-429)	423 (381-465)	395 (361-431)	345 (311-383)	264 (239-290)	144 (131-158)	51 (47-57)	13 (12-14)
Leishmaniasis	624 (286-1,178)	626 (278-1,218)	642 (314-1,212)	526 (241-985)	431 (198-815)	464 (214-892)	499 (238-935)	395 (179-734)	220 (100-411)	137 (61-262)	30 (11-56)	10 (2-20)
Visceral leishmaniasis	624 (286-1,178)	626 (278-1,218)	642 (314-1,212)	526 (241-985)	431 (198-815)	464 (214-892)	499 (238-935)	395 (179-734)	220 (100-411)	137 (61-262)	30 (11-56)	10 (2-20)
African trypanosomiasis	779 (181-2,516)	771 (146-2,654)	709 (141-2,233)	680 (144-2,438)	534 (94-1,829)	455 (75-1,668)	309 (56-1,109)	175 (27-713)	86 (15-330)	33 (5-140)	9 (1-34)	2 (0-8)
Schistosomiasis	400 (280-567)	455 (320-649)	570 (398-820)	691 (494-989)	744 (543-1,047)	799 (545-1,110)	723 (511-1,023)	516 (380-707)	261 (191-353)	116 (84-158)	30 (20-42)	5 (3-9)
Cysticercosis	50 (41-60)	49 (40-59)	57 (47-70)	59 (50-71)	47 (39-57)	44 (36-53)	28 (23-34)	17 (13-20)	10 (8-12)	5 (4-6)	2 (1-2)	1 (0-1)
Cystic echinococcosis	44 (32-57)	47 (35-63)	49 (37-63)	60 (45-78)	57 (43-74)	60 (47-78)	56 (43-70)	40 (31-50)	35 (27-44)	19 (15-24)	6 (4-8)	1 (1-2)
Dengue	116 (18-281)	134 (21-323)	150 (20-373)	167 (28-404)	156 (26-398)	143 (25-322)	63 (13-120)	52 (12-102)	42 (10-79)	28 (7-51)	13 (3-22)	3 (1-5)
Yellow fever	106 (21-331)	90 (19-277)	81 (15-264)	66 (13-202)	52 (10-158)	38 (7-114)	25 (5-78)	14 (3-41)	6 (1-19)	2 (0-7)	1 (0-2)	0 (0-0)
Rabies	980 (418-1,713)	1,114 (567-2,308)	1,317 (807-3,392)	884 (488-1,987)	693 (301-1,255)	905 (359-1,636)	560 (317-1,378)	410 (221-1,217)	104 (34-220)	87 (21-223)	16 (4-44)	8 (2-19)
Intestinal nematode infections	20 (15-26)	21 (16-28)	33 (25-42)	31 (23-40)	38 (29-48)	29 (22-38)	44 (33-55)	34 (26-44)	22 (16-28)	8 (6-11)	5 (4-7)	2 (1-3)
Ascariasis	20 (15-26)	21 (16-28)	33 (25-42)	31 (23-40)	38 (29-48)	29 (22-38)	44 (33-55)	34 (26-44)	22 (16-28)	8 (6-11)	5 (4-7)	2 (1-3)
Ebola	27 (20-34)	38 (28-48)	28 (21-35)	20 (15-25)	19 (14-25)	5 (4-6)	4 (3-5)	0 (0-0)	0 (0-0)	0 (0-0)	0 (0-0)	0 (0-0)
Zika virus	0 (0-0)	0 (0-0)	0 (0-0)	0 (0-0)	0 (0-0)	0 (0-0)	0 (0-0)	0 (0-0)	0 (0-0)	0 (0-0)	0 (0-0)	0 (0-0)
Other neglected tropical diseases	197 (85-260)	172 (81-225)	213 (103-304)	219 (103-300)	257 (111-351)	271 (134-368)	246 (112-347)	211 (109-306)	137 (76-220)	74 (45-121)	26 (16-42)	12 (8-20)
Maternal disorders	31,844 (29,093-34,451)	16,032 (14,215-18,009)	2,772 (2,270-3,411)	-	-	-	-	-	-	-	-	-
Maternal haemorrhage	10,176 (7,121-13,686)	4,051 (2,767-5,585)	521 (310-825)	-	-	-	-	-	-	-	-	-
Maternal sepsis and other pregnancy related infections	3,102 (1,967-4,711)	1,665 (1,048-2,573)	284 (170-455)	-	-	-	-	-	-	-	-	-
Maternal hypertensive disorders	3,562 (2,166-5,535)	1,669 (1,040-2,553)	274 (141-463)	-	-	-	-	-	-	-	-	-
Maternal obstructed labour and uterine rupture	1,754 (971-2,966)	1,003 (540-1,632)	166 (71-324)	-	-	-	-	-	-	-	-	-
Maternal abortion, miscarriage, and ectopic pregnancy	3,299 (2,229-4,715)	2,034 (1,332-2,971)	307 (191-472)	-	-	-	-	-	-	-	-	-
Indirect maternal deaths	3,954 (2,402-6,036)	2,415 (1,416-3,553)	567 (316-893)	-	-	-	-	-	-	-	-	-
Late maternal deaths	556 (214-988)	306 (124-541)	59 (22-125)	-	-	-	-	-	-	-	-	-
Maternal deaths aggravated by HIV/AIDS	148 (86-209)	71 (41-102)	10 (6-16)	-	-	-	-	-	-	-	-	-
Other maternal disorders	5,292 (3,259-7,893)	2,818 (1,767-4,145)	584 (362-855)	-	-	-	-	-	-	-	-	-
Neonatal disorders	-	-	-	-	-	-	-	-	-	-	-	-
Neonatal preterm birth complications	-	-	-	-	-	-	-	-	-	-	-	-
Neonatal encephalopathy due to birth asphyxia and trauma	-	-	-	-	-	-	-	-	-	-	-	-
Neonatal sepsis and other neonatal infections	-	-	-	-	-	-	-	-	-	-	-	-
Hemolytic disease and other neonatal jaundice	-	-	-	-	-	-	-	-	-	-	-	-
Other neonatal disorders	-	-	-	-	-	-	-	-	-	-	-	-
Nutritional deficiencies	3,343 (2,957-3,761)	2,994 (2,484-3,611)	3,994 (3,259-4,767)	5,222 (4,252-6,204)	6,372 (5,229-7,546)	8,069 (6,791-9,453)	9,158 (7,568-10,845)	11,817 (9,800-13,949)	12,579 (10,570-14,680)	12,142 (10,180-14,217)	8,215 (6,783-9,832)	3,959 (3,258-4,726)
Protein-energy malnutrition	2,224 (1,870-2,578)	1,792 (1,299-2,320)	2,476 (1,790-3,195)	3,726 (2,864-4,594)	4,634 (3,637-5,715)	5,765 (4,600-6,997)	7,097 (5,637-8,695)	9,603 (7,763-11,500)	10,387 (8,505-12,368)	10,314 (8,438-12,275)	7,181 (5,721-8,675)	3,494 (2,792-4,280)
Iodine deficiency	34 (10-88)	30 (8-92)	44 (12-139)	52 (16-154)	53 (17-146)	78 (26-185)	78 (29-193)	70 (26-164)	56 (18-131)	31 (9-77)	11 (2-35)	6 (1-21)
Iron-deficiency anaemia	108 (84-136)	82 (64-110)	82 (61-162)	81 (61-106)	98 (66-126)	105 (71-149)	188 (124-288)	207 (131-349)	177 (111-271)	113 (38-189)	58 (26-93)	49 (29-74)
Other nutritional deficiencies	977 (819-1,166)	1,090 (918-1,296)	1,392 (1,131-1,668)	1,363 (1,105-1,682)	1,588 (1,273-1,992)	2,121 (1,665-2,664)	1,795 (1,433-2,272)	1,937 (1,548-2,474)	1,959 (1,585-2,694)	1,684 (1,350-2,639)	965 (747-1,716)	409 (305-854)
Other communicable, maternal, neonatal, and nutritional diseases	4,692 (4,332-5,087)	4,161 (3,840-4,522)	4,945 (4,524-5,468)	5,996 (5,533-6,651)	5,972 (5,426-6,698)	6,324 (5,686-7,209)	6,064 (5,399-7,001)	5,062 (4,477-5,906)	3,602 (3,101-4,458)	2,042 (1,762-2,541)	819 (694-995)	269 (221-317)
Sexually transmitted diseases excluding HIV	721 (511-863)	640 (466-769)	596 (396-740)	793 (511-993)	581 (349-738)	505 (351-616)	364 (263-440)	393 (272-477)	252 (186-298)	164 (127-193)	79 (61-93)	28 (21-33)
Syphilis	78 (55-94)	63 (46-75)	113 (75-140)	185 (119-231)	140 (84-177)	129 (90-157)	110 (79-133)	104 (72-126)	65 (48-77)	37 (29-44)	16 (13-19)	5 (4-6)
Chlamydial infection	135 (96-162)	113 (82-135)	92 (61-114)	122 (79-153)	91 (55-116)	46 (32-57)	42 (31-51)	50 (35-61)	45 (33-53)	30 (23-36)	14 (11-17)	5 (4-6)
Gonococcal infection	335 (238-401)	300 (218-360)	265 (176-329)	322 (207-403)	218 (131-276)	252 (175-308)	157 (114-190)	188 (130-229)	111 (82-131)	76 (59-89)	37 (29-44)	13 (10-16)
Other sexually transmitted diseases	172 (122-206)	165 (120-198)	126 (84-156)	165 (106-206)	133 (80-168)	77 (53-94)	55 (40-66)	50 (35-61)	30 (23-36)	21 (16-25)	10 (8-12)	4 (3-5)
Hepatitis	3,304 (3,039-3,596)	2,808 (2,584-3,061)	3,334 (3,019-3,679)	4,059 (3,712-4,478)	3,902 (3,541-4,316)	3,944 (3,576-4,379)	3,819 (3,415-4,240)	3,012 (2,695-3,356)	1,882 (1,664-2,143)	882 (787-1,011)	273 (246-310)	63 (57-71)
Acute hepatitis A	38 (18-66)	35 (17-60)	44 (20-75)	53 (26-94)	64 (32-108)	68 (35-113)	74 (39-121)	64 (34-105)	43 (24-67)	19 (12-29)	6 (4-9)	1 (1-2)
Hepatitis B	2,983 (2,646-3,299)	2,532 (2,254-2,815)	2,987 (2,611-3,365)	3,623 (3,184-4,078)	3,418 (2,978-3,876)	3,385 (2,898-3,875)	3,207 (2,728-3,716)	2,457 (2,011-2,881)	1,358 (1,022-1,685)	523 (352-703)	130 (75-192)	24 (12-39)
Hepatitis C	42 (19-76)	48 (24-88)	80 (37-146)	127 (60-228)	139 (66-248)	131 (62-218)	117 (53-201)	89 (44-150)	57 (29-94)	28 (16-44)	9 (5-15)	2 (1-4)
Acute hepatitis E	241 (103-473)	193 (88-345)	224 (102-403)	255 (105-482)	282 (112-534)	360 (161-616)	422 (202-775)	401 (161-723)	425 (214-683)	312 (175-471)	128 (72-183)	35 (21-48)
Other infectious diseases	667 (520-814)	713 (577-868)	1,014 (827-1,317)	1,144 (935-1,468)	1,489 (1,179-2,020)	1,876 (1,474-2,485)	1,881 (1,477-2,567)	1,657 (1,293-2,223)	1,468 (1,120-2,183)	995 (769-1,427)	467 (355-627)	178 (135-220)
Non-communicable diseases	329,126 (320,202-337,207)	429,497 (416,881-439,938)	560,131 (540,661-574,588)	817,549 (787,792-838,372)	1,115,093 (1,069,738-1,147,021)	1,494,357 (1,437,720-1,534,776)	1,825,316 (1,755,066-1,877,337)	1,879,381 (1,813,549-1,928,252)	2,038,828 (1,988,432-2,085,893)	1,609,716 (1,577,299-1,638,529)	824,848 (811,989-837,228)	283,216 (278,953-287,169)
Neoplasms	128,172 (122,690-133,323)	164,405 (158,345-170,517)	198,376 (191,552-205,564)	260,415 (251,821-270,616)	311,406 (302,210-324,595)	358,795 (348,857-372,837)	365,602 (355,134-382,255)	294,732 (285,792-309,257)	240,183 (232,653-251,724)	148,893 (143,813-155,314)	60,199 (58,100-62,852)	16,412 (15,720-17,034)
Lip and oral cavity cancer	1,549 (1,393-1,746)	2,187 (1,992-2,440)	2,861 (2,602-3,190)	3,597 (3,267-3,984)	4,418 (4,003-4,876)	4,232 (3,883-4,578)	3,749 (3,420-4,046)	3,159 (2,881-3,395)	2,857 (2,652-3,067)	2,009 (1,880-2,145)	927 (871-989)	284 (267-301)
Nasopharynx cancer	1,436 (1,295-1,591)	1,649 (1,491-1,839)	1,860 (1,660-2,130)	2,206 (1,959-2,556)	2,038 (1,789-2,342)	1,664 (1,495-1,854)	1,393 (1,253-1,547)	926 (841-1,031)	568 (510-626)	261 (238-285)	93 (85-101)	23 (21-25)
Other pharynx cancer	1,137 (1,000-1,279)	1,763 (1,568-1,954)	2,447 (2,179-2,716)	2,852 (2,541-3,144)	3,275 (2,902-3,614)	2,874 (2,558-3,185)	2,603 (2,299-2,892)	1,446 (1,260-1,631)	1,018 (896-1,151)	549 (492-611)	192 (176-209)	47 (44-50)
Oesophageal cancer	3,002 (2,792-3,222)	4,859 (4,581-5,193)	7,097 (6,708-7,601)	11,201 (10,567-12,064)	15,997 (14,944-17,356)	19,078 (17,860-20,880)	19,859 (18,479-21,864)	16,389 (15,125-18,247)	10,945 (10,201-12,130)	5,884 (5,540-6,432)	2,091 (1,977-2,267)	468 (442-501)
Stomach cancer	13,361 (12,756-14,004)	15,810 (15,032-16,604)	18,334 (17,434-19,356)	26,441 (25,184-28,009)	35,245 (33,485-37,766)	43,248 (41,034-46,011)	45,389 (43,190-48,728)	38,489 (36,651-41,269)	31,732 (30,266-33,932)	19,004 (18,082-20,149)	7,295 (6,967-7,694)	1,822 (1,737-1,910)
Colon and rectum cancer	7,293 (6,822-7,838)	10,815 (10,189-11,569)	14,299 (13,509-15,263)	21,251 (20,089-22,566)	26,947 (25,771-28,322)	34,896 (33,416-36,588)	40,475 (38,792-42,348)	36,923 (35,562-38,416)	35,428 (34,078-36,789)	25,566 (24,558-26,746)	11,728 (11,266-12,234)	3,379 (3,228-3,525)
Liver cancer	8,284 (5,188-9,437)	9,918 (6,642-11,148)	12,146 (8,847-13,404)	16,400 (12,359-18,047)	21,143 (16,845-23,357)	24,359 (19,795-26,458)	23,785 (20,067-26,184)	19,160 (16,672-20,906)	13,190 (12,171-14,515)	6,857 (6,458-7,495)	2,254 (2,125-2,435)	497 (467-533)
Liver cancer due to hepatitis B	5,236 (3,126-6,339)	5,509 (3,487-6,951)	5,917 (4,015-7,329)	7,043 (4,646-9,111)	7,981 (5,186-10,446)	8,095 (5,312-10,382)	7,212 (4,691-9,436)	5,662 (3,773-7,632)	3,458 (2,302-4,720)	1,663 (1,201-2,198)	512 (362-684)	106 (64-151)
Liver cancer due to hepatitis C	774 (526-1,051)	1,267 (875-1,669)	1,992 (1,472-2,580)	3,084 (2,322-3,865)	4,500 (3,573-5,553)	5,589 (4,655-6,599)	6,004 (5,083-7,041)	5,343 (4,573-6,267)	4,535 (3,908-5,284)	2,731 (2,422-3,087)	1,005 (875-1,134)	244 (208-286)
Liver cancer due to alcohol use	1,536 (868-2,385)	2,290 (1,228-3,490)	3,193 (1,965-4,512)	4,950 (3,104-6,939)	6,966 (4,812-9,384)	8,720 (6,692-10,856)	8,595 (6,542-10,747)	6,429 (4,867-8,122)	3,885 (2,796-4,989)	1,762 (1,301-2,259)	500 (354-664)	94 (55-137)
Liver cancer due to other causes	738 (460-1,035)	853 (541-1,164)	1,043 (716-1,412)	1,323 (919-1,740)	1,694 (1,300-2,188)	1,954 (1,533-2,401)	1,973 (1,543-2,441)	1,726 (1,361-2,137)	1,311 (1,044-1,650)	701 (575-856)	237 (189-295)	54 (40-71)
Gallbladder and biliary tract cancer	1,780 (1,561-2,007)	2,847 (2,552-3,163)	4,103 (3,706-4,517)	6,016 (5,490-6,554)	8,332 (7,807-8,895)	10,502 (9,887-11,245)	11,272 (10,672-12,004)	9,658 (9,197-10,356)	8,736 (8,384-9,265)	5,409 (5,183-5,710)	2,113 (2,017-2,229)	542 (514-567)
Pancreatic cancer	1,970 (1,868-2,098)	3,251 (3,107-3,444)	5,003 (4,819-5,253)	8,203 (7,885-8,631)	12,345 (11,881-12,911)	16,553 (15,991-17,273)	18,999 (18,390-19,858)	16,550 (16,002-17,267)	14,235 (13,705-14,808)	9,225 (8,876-9,624)	3,690 (3,546-3,856)	943 (903-978)
Larynx cancer	675 (617-744)	983 (903-1,070)	1,314 (1,210-1,440)	1,716 (1,586-1,885)	2,022 (1,855-2,219)	2,198 (2,029-2,411)	2,132 (1,962-2,348)	1,463 (1,348-1,599)	1,072 (989-1,186)	531 (497-576)	179 (169-193)	41 (38-43)
Tracheal, bronchus, and lung cancer	10,224 (9,518-10,979)	14,762 (13,988-15,624)	19,848 (18,918-20,763)	28,907 (27,723-30,268)	40,331 (38,674-42,335)	50,849 (49,018-53,277)	53,916 (51,890-56,802)	43,014 (41,349-45,315)	29,947 (28,623-31,582)	16,100 (15,333-16,875)	5,605 (5,335-5,879)	1,401 (1,327-1,469)
Malignant skin melanoma	1,065 (834-1,293)	1,237 (956-1,472)	1,313 (1,057-1,589)	1,663 (1,358-2,021)	1,881 (1,579-2,323)	2,219 (1,797-2,813)	2,315 (1,876-3,011)	1,888 (1,539-2,405)	1,873 (1,559-2,477)	1,252 (1,007-1,664)	573 (465-768)	188 (152-253)
Non-melanoma skin cancer	305 (278-335)	404 (366-440)	437 (402-469)	601 (559-641)	771 (715-829)	929 (865-995)	1,133 (1,059-1,220)	1,101 (1,043-1,178)	1,456 (1,376-1,549)	1,388 (1,323-1,456)	830 (788-875)	377 (358-397)
Non-melanoma skin cancer (squamous-cell carcinoma)	305 (278-335)	404 (366-440)	437 (402-469)	601 (559-641)	771 (715-829)	929 (865-995)	1,133 (1,059-1,220)	1,101 (1,043-1,178)	1,456 (1,376-1,549)	1,388 (1,323-1,456)	830 (788-875)	377 (358-397)
Breast cancer	29,128 (26,549-32,403)	36,792 (33,625-40,543)	40,304 (37,449-44,203)	46,247 (43,245-50,675)	42,694 (40,307-46,791)	42,528 (40,414-46,235)	38,819 (37,012-41,930)	28,598 (27,327-31,516)	24,337 (23,264-26,010)	16,570 (15,880-17,613)	7,299 (6,974-7,715)	2,323 (2,217-2,439)
Cervical cancer	18,627 (16,097-22,242)	22,162 (19,096-26,803)	23,751 (20,250-29,034)	26,403 (22,769-32,106)	25,248 (22,109-30,304)	22,988 (20,459-27,319)	19,519 (17,581-22,885)	12,421 (11,442-13,959)	9,287 (8,670-10,536)	4,791 (4,494-5,366)	1,717 (1,623-1,867)	461 (435-496)
Uterine cancer	2,541 (2,259-2,745)	3,929 (3,546-4,217)	5,321 (4,811-5,711)	7,402 (6,761-7,925)	9,499 (8,789-10,148)	10,670 (9,962-11,322)	9,578 (9,049-10,118)	6,923 (6,575-7,290)	5,377 (5,152-5,633)	3,002 (2,876-3,133)	1,158 (1,106-1,215)	301 (284-317)
Ovarian cancer	5,162 (4,799-5,654)	8,026 (7,566-8,691)	10,513 (9,930-11,360)	13,073 (12,420-13,857)	14,311 (13,724-15,162)	15,309 (14,638-16,115)	14,600 (13,991-15,294)	9,998 (9,611-10,508)	7,812 (7,500-8,185)	4,387 (4,207-4,591)	1,632 (1,560-1,709)	384 (365-403)
Prostate cancer	-	-	-	-	-	-	-	-	-	-	-	-
Testicular cancer	-	-	-	-	-	-	-	-	-	-	-	-
Kidney cancer	656 (625-695)	1,055 (1,009-1,112)	1,515 (1,453-1,587)	2,432 (2,324-2,563)	3,276 (3,162-3,415)	4,149 (3,996-4,314)	4,567 (4,406-4,755)	3,643 (3,512-3,777)	3,323 (3,189-3,452)	2,112 (2,015-2,209)	829 (787-875)	203 (192-214)
Bladder cancer	586 (527-650)	792 (722-871)	1,261 (1,153-1,385)	1,863 (1,731-2,019)	2,797 (2,618-2,996)	4,062 (3,828-4,319)	5,232 (4,952-5,520)	5,489 (5,235-5,751)	5,652 (5,414-5,889)	4,257 (4,057-4,456)	1,999 (1,907-2,102)	590 (560-618)
Brain and nervous system cancer	4,054 (3,339-4,403)	4,447 (3,706-4,795)	4,908 (4,109-5,312)	6,343 (5,461-6,848)	6,889 (6,016-7,583)	7,313 (6,485-8,085)	6,253 (5,607-6,831)	4,313 (3,799-4,792)	2,701 (2,386-3,001)	1,277 (1,103-1,394)	373 (318-405)	78 (68-85)
Thyroid cancer	896 (768-1,009)	1,100 (942-1,245)	1,580 (1,370-1,788)	2,063 (1,822-2,303)	2,355 (2,118-2,600)	2,670 (2,434-2,923)	2,626 (2,426-2,844)	2,066 (1,931-2,234)	1,645 (1,554-1,761)	983 (934-1,065)	361 (342-386)	81 (76-86)
Mesothelioma	302 (245-408)	243 (205-314)	335 (292-426)	506 (446-618)	611 (552-730)	744 (684-861)	778 (720-884)	752 (691-882)	556 (518-606)	297 (279-320)	110 (103-117)	27 (25-29)
Hodgkin lymphoma	815 (533-944)	706 (483-809)	777 (497-900)	881 (609-1,039)	1,065 (690-1,238)	1,173 (818-1,351)	1,015 (749-1,142)	774 (581-877)	570 (452-635)	273 (222-302)	76 (65-84)	16 (14-18)
Non-Hodgkin's lymphoma	2,453 (2,174-2,665)	2,717 (2,486-2,936)	3,422 (3,124-3,693)	4,507 (4,212-4,834)	6,292 (5,988-6,858)	7,898 (7,573-8,473)	8,863 (8,516-9,536)	7,091 (6,803-7,652)	6,301 (5,993-6,829)	3,972 (3,777-4,274)	1,505 (1,429-1,604)	371 (349-394)
Multiple myeloma	603 (468-688)	995 (789-1,131)	1,616 (1,307-1,844)	2,398 (1,964-2,676)	3,488 (2,767-4,013)	4,606 (3,798-5,348)	4,722 (3,874-5,770)	4,051 (3,259-4,955)	3,555 (2,928-4,449)	2,206 (1,785-2,839)	813 (659-1,070)	169 (140-219)
Leukaemia	5,278 (4,355-6,149)	5,383 (4,600-6,294)	5,533 (4,789-6,361)	6,960 (6,228-8,043)	8,154 (7,472-9,592)	9,426 (8,771-10,794)	9,979 (9,384-11,297)	8,238 (7,800-9,217)	7,200 (6,879-7,907)	4,864 (4,628-5,273)	2,166 (2,060-2,323)	641 (608-685)
Acute lymphoid leukaemia	674 (562-967)	673 (551-925)	644 (536-859)	884 (746-1,180)	894 (765-1,246)	866 (750-1,220)	781 (684-1,064)	539 (480-705)	394 (354-522)	218 (200-266)	86 (79-101)	24 (22-29)
Chronic lymphoid leukaemia	264 (207-378)	240 (193-341)	352 (290-456)	581 (496-714)	1,010 (867-1,214)	1,358 (1,191-1,594)	1,520 (1,385-1,702)	1,525 (1,397-1,697)	1,536 (1,428-1,654)	1,244 (1,162-1,332)	593 (555-639)	194 (181-207)
Acute myeloid leukaemia	1,053 (923-1,303)	1,235 (1,101-1,523)	1,213 (1,090-1,446)	1,511 (1,387-1,761)	1,798 (1,670-2,061)	2,159 (2,021-2,449)	2,422 (2,289-2,687)	1,990 (1,883-2,222)	1,562 (1,479-1,733)	889 (833-984)	350 (329-380)	89 (83-96)
Chronic myeloid leukaemia	690 (543-849)	727 (591-871)	801 (642-944)	963 (820-1,108)	1,041 (909-1,183)	1,177 (1,034-1,313)	1,266 (1,144-1,415)	972 (895-1,057)	981 (898-1,065)	664 (606-727)	313 (286-340)	95 (86-103)
Other leukaemia	2,597 (1,928-3,064)	2,508 (1,915-2,920)	2,523 (2,028-2,959)	3,020 (2,495-3,571)	3,410 (2,986-4,114)	3,865 (3,467-4,530)	3,990 (3,629-4,772)	3,211 (2,974-3,773)	2,727 (2,557-3,162)	1,848 (1,731-2,081)	824 (772-941)	238 (223-268)
Other neoplasms	4,993 (4,694-5,364)	5,571 (5,280-5,910)	6,478 (6,164-6,909)	8,283 (7,870-8,984)	9,983 (9,520-10,609)	11,658 (11,134-12,441)	12,030 (11,533-12,802)	10,211 (9,767-10,933)	8,809 (8,420-9,372)	5,869 (5,615-6,245)	2,592 (2,472-2,728)	757 (720-799)
Cardiovascular diseases	109,965 (105,367-114,284)	150,983 (144,338-156,524)	208,597 (199,864-217,635)	332,992 (318,634-347,674)	485,066 (462,763-510,596)	711,940 (681,552-746,964)	921,368 (884,067-967,706)	994,250 (954,207-1,044,630)	1,169,607 (1,130,898-1,224,105)	945,679 (916,230-985,957)	480,409 (464,679-499,081)	173,296 (166,390-179,329)
Rheumatic heart disease	12,102 (10,029-14,297)	12,124 (10,047-14,114)	14,019 (11,459-16,487)	17,777 (14,848-20,624)	20,661 (17,325-23,955)	23,243 (19,531-26,703)	24,766 (21,000-28,970)	21,862 (18,762-25,781)	18,310 (16,240-21,340)	11,864 (10,629-13,661)	5,352 (4,880-6,036)	1,658 (1,531-1,830)
Ischaemic heart disease	40,862 (38,454-44,389)	59,679 (56,464-63,518)	84,738 (80,121-90,208)	143,123 (135,302-150,633)	213,836 (203,128-224,561)	321,871 (307,102-336,413)	422,952 (403,802-442,003)	469,515 (450,369-490,250)	586,239 (568,074-608,985)	505,225 (491,794-525,719)	274,218 (267,009-284,970)	105,324 (102,364-109,247)
Cerebrovascular disease	41,276 (38,865-44,131)	59,027 (55,842-62,765)	83,350 (78,715-88,702)	130,850 (123,851-139,317)	194,494 (183,449-209,079)	286,106 (269,217-307,099)	365,199 (343,657-396,719)	378,025 (353,312-412,918)	413,601 (384,082-451,872)	293,672 (270,959-322,506)	127,638 (115,829-140,801)	41,203 (36,830-45,434)

SR Table 3: Global deaths by cause and age group for females (with 95% uncertainty intervals), 1995

Cause	Early Neonatal	Late Neonatal	Post Neonatal	1 to 4	5 to 9	10 to 14	15 to 19	20 to 24	25 to 29	30 to 34	35 to 39
Ischaemic stroke	-	-	355 (272-457)	217 (174-289)	205 (172-251)	180 (150-217)	707 (627-807)	1,477 (1,296-1,665)	1,976 (1,786-2,188)	2,377 (2,142-2,617)	3,467 (3,138-3,800)
Hemorrhagic stroke	946 (823-1,110)	635 (518-788)	5,393 (4,326-6,702)	3,724 (2,827-5,168)	2,156 (1,857-2,578)	2,173 (1,920-2,537)	3,370 (3,078-3,777)	6,525 (5,963-7,298)	9,179 (8,404-10,239)	12,954 (11,902-14,198)	20,353 (18,963-22,154)
Hypertensive heart disease	-	-	250 (112-338)	174 (68-249)	135 (70-187)	123 (68-167)	883 (570-1,122)	1,243 (750-1,536)	1,618 (966-1,993)	2,412 (1,514-2,897)	3,513 (2,232-4,295)
Cardiomyopathy and myocarditis	372 (243-449)	276 (163-359)	2,587 (1,651-3,802)	1,763 (1,071-2,583)	783 (629-1,097)	769 (649-1,008)	927 (796-1,215)	1,226 (1,044-1,611)	1,617 (1,312-2,042)	1,918 (1,614-2,262)	2,619 (2,126-3,035)
Myocarditis	79 (47-124)	69 (39-118)	952 (532-1,669)	733 (438-1,367)	367 (268-553)	300 (236-430)	405 (328-559)	548 (432-788)	581 (451-824)	460 (375-637)	516 (411-670)
Alcoholic cardiomyopathy	-	-	-	-	-	-	78 (58-117)	143 (107-195)	291 (220-372)	582 (435-740)	1,052 (772-1,367)
Other cardiomyopathy	293 (185-368)	207 (110-288)	1,635 (889-2,538)	1,030 (516-1,745)	415 (297-597)	470 (373-628)	445 (361-578)	535 (407-717)	745 (549-964)	876 (669-1,080)	1,051 (844-1,268)
Atrial fibrillation and flutter	-	-	-	-	-	-	-	-	-	14 (10-20)	53 (39-71)
Aortic aneurysm	-	-	-	-	-	-	126 (106-158)	187 (161-224)	249 (214-300)	328 (280-395)	453 (390-549)
Peripheral vascular disease	-	-	-	-	-	-	-	-	-	-	-
Endocarditis	63 (39-83)	46 (30-71)	967 (623-1,527)	507 (311-987)	246 (184-340)	263 (211-325)	458 (376-550)	585 (488-698)	806 (690-950)	732 (621-850)	870 (718-1,019)
Other cardiovascular and circulatory diseases	806 (575-934)	568 (394-702)	4,036 (2,618-5,068)	2,537 (1,353-3,569)	1,289 (794-1,553)	1,168 (777-1,379)	1,508 (1,059-1,814)	2,361 (1,571-2,769)	2,793 (1,834-3,300)	2,934 (2,101-3,465)	3,405 (2,503-4,054)
Chronic respiratory diseases	0 (0-0)	0 (0-0)	0 (0-0)	14,810 (11,675-17,857)	4,022 (3,248-4,773)	3,504 (2,920-4,043)	5,367 (4,494-5,955)	6,684 (5,643-7,334)	8,564 (7,200-9,254)	10,507 (8,685-11,363)	14,060 (11,608-15,154)
Chronic obstructive pulmonary disease	-	-	0 (0-0)	3,676 (2,425-5,929)	992 (632-1,396)	726 (467-931)	2,227 (1,605-2,703)	3,270 (2,447-3,743)	4,776 (3,505-5,494)	6,026 (4,524-6,886)	7,669 (5,795-8,829)
Pneumoconiosis	-	-	-	-	-	-	24 (16-30)	36 (23-45)	43 (28-53)	58 (39-75)	43 (31-54)
Silicosis	-	-	-	-	-	-	2 (1-3)	4 (2-6)	7 (3-13)	15 (8-23)	7 (4-10)
Asbestosis	-	-	-	-	-	-	10 (7-13)	16 (10-20)	17 (11-21)	18 (13-23)	16 (12-21)
Coal workers pneumoconiosis	-	-	-	-	-	-	2 (1-4)	2 (1-4)	3 (1-5)	7 (2-11)	3 (1-5)
Other pneumoconiosis	-	-	-	-	-	-	9 (6-12)	15 (9-19)	16 (11-21)	19 (13-26)	17 (12-23)
Asthma	-	-	-	9,688 (5,557-12,363)	2,462 (1,825-3,098)	2,385 (1,919-2,893)	2,862 (2,302-3,457)	3,074 (2,421-3,825)	3,366 (2,622-4,116)	3,972 (3,024-4,753)	5,683 (4,394-6,690)
Interstitial lung disease and pulmonary sarcoidosis	-	-	-	339 (96-924)	145 (66-328)	79 (46-152)	112 (58-229)	147 (76-284)	206 (116-384)	284 (158-494)	440 (255-765)
Other chronic respiratory diseases	0 (0-0)	0 (0-0)	0 (0-0)	1,106 (628-2,285)	423 (273-636)	314 (206-437)	142 (88-207)	156 (101-218)	173 (105-235)	167 (95-225)	225 (124-305)
Cirrhosis and other chronic liver diseases	-	-	-	6,669 (5,240-8,201)	2,543 (2,126-3,044)	2,628 (2,269-2,958)	3,880 (3,511-4,246)	5,550 (5,106-6,061)	6,441 (5,999-6,939)	9,002 (8,385-9,638)	12,515 (11,785-13,277)
Cirrhosis and other chronic liver diseases due to hepatitis B	-	-	-	25 (17-35)	24 (18-33)	50 (36-67)	127 (84-178)	723 (509-965)	1,485 (1,099-1,924)	2,529 (2,081-3,077)	3,713 (3,076-4,419)
Cirrhosis and other chronic liver diseases due to hepatitis C	-	-	-	20 (12-31)	20 (13-27)	39 (27-55)	96 (62-139)	479 (334-668)	919 (641-1,241)	1,679 (1,333-2,050)	2,647 (2,052-3,321)
Cirrhosis and other chronic liver diseases due to alcohol use	-	-	-	-	-	-	29 (16-45)	246 (173-342)	549 (393-743)	1,506 (1,236-1,781)	3,010 (2,459-3,622)
Cirrhosis and other chronic liver diseases due to other causes	-	-	-	6,623 (5,206-8,144)	2,499 (2,088-2,992)	2,539 (2,197-2,862)	3,628 (3,289-3,979)	4,102 (3,563-4,622)	3,487 (2,901-4,065)	3,288 (2,662-3,940)	3,145 (2,384-3,939)
Digestive diseases	1,156 (730-1,472)	1,172 (786-1,500)	11,051 (7,992-14,926)	13,477 (8,391-20,713)	5,163 (4,191-6,265)	4,589 (3,954-5,323)	6,587 (5,540-7,300)	7,776 (6,625-8,565)	8,632 (7,377-9,444)	8,863 (7,778-9,743)	9,886 (8,547-10,761)
Peptic ulcer disease	-	-	-	2,885 (1,702-4,773)	1,425 (1,031-1,938)	1,505 (1,195-1,925)	1,991 (1,616-2,433)	2,767 (2,302-3,236)	2,829 (2,365-3,267)	3,036 (2,596-3,562)	3,388 (2,843-3,916)
Gastritis and duodenitis	-	-	-	453 (173-875)	186 (106-289)	129 (85-182)	186 (127-252)	275 (187-357)	378 (259-492)	432 (289-567)	462 (325-588)
Appendicitis	-	-	-	2,773 (1,021-4,924)	1,137 (745-1,481)	1,063 (792-1,303)	1,796 (1,303-2,159)	1,437 (996-1,708)	1,473 (1,033-1,746)	986 (734-1,184)	996 (729-1,187)
Paralytic ileus and intestinal obstruction	1,156 (730-1,472)	1,172 (786-1,500)	11,051 (7,992-14,926)	2,678 (1,306-4,307)	1,056 (589-1,371)	977 (588-1,224)	1,480 (853-1,861)	1,391 (812-1,706)	1,513 (873-1,866)	1,360 (819-1,685)	1,537 (947-1,889)
Inguinal, femoral, and abdominal hernia	-	-	-	289 (100-610)	133 (75-192)	88 (56-121)	98 (55-129)	97 (55-126)	90 (53-117)	109 (64-139)	147 (80-189)
Inflammatory bowel disease	-	-	-	923 (302-2,666)	171 (89-326)	106 (66-177)	162 (101-283)	273 (165-475)	324 (207-542)	355 (220-558)	366 (246-539)
Vascular intestinal disorders	-	-	-	119 (41-177)	25 (16-33)	23 (16-29)	37 (24-47)	92 (55-116)	118 (72-150)	167 (114-205)	231 (165-281)
Gallbladder and biliary diseases	-	-	-	511 (227-806)	212 (151-291)	170 (135-229)	193 (156-244)	344 (267-406)	486 (373-564)	639 (492-749)	831 (648-971)
Pancreatitis	-	-	-	122 (51-218)	119 (59-189)	128 (75-202)	289 (189-436)	546 (340-826)	727 (467-1,095)	1,028 (697-1,459)	1,163 (841-1,606)
Other digestive diseases	-	-	-	2,725 (1,337-4,263)	699 (522-925)	401 (320-517)	354 (273-451)	554 (436-681)	696 (562-841)	751 (609-898)	765 (616-933)
Neurological disorders	-	-	4,514 (3,406-5,640)	5,154 (3,302-7,193)	3,337 (2,503-4,204)	4,206 (3,164-5,034)	5,367 (4,227-6,159)	5,424 (4,156-6,122)	5,137 (3,911-5,744)	5,109 (4,002-5,770)	4,571 (3,817-5,120)
Alzheimer's disease and other dementias	-	-	-	-	-	-	-	-	-	-	-
Parkinson's disease	-	-	-	-	-	-	-	0 (0-1)	7 (3-13)	24 (12-43)	49 (25-86)
Epilepsy	-	-	2,982 (1,633-3,785)	3,813 (2,194-5,338)	2,764 (1,878-3,509)	3,545 (2,375-4,253)	4,793 (3,533-5,528)	4,924 (3,604-5,601)	4,531 (3,259-5,076)	4,250 (3,152-4,817)	3,327 (2,601-3,725)
Multiple sclerosis	-	-	-	-	-	-	-	83 (57-141)	193 (131-306)	329 (238-473)	582 (433-795)
Motor neuron disease	4 (3-5)	5 (4-6)	442 (288-598)	211 (111-314)	26 (20-33)	26 (23-32)	21 (18-25)	21 (17-25)	32 (27-41)	56 (49-68)	102 (94-122)
Other neurological disorders	-	-	1,091 (688-1,472)	1,130 (591-1,669)	547 (397-689)	635 (460-812)	553 (395-709)	396 (319-464)	374 (315-424)	450 (397-496)	510 (466-547)
Mental and substance use disorders	5 (3-7)	13 (10-18)	0 (0-0)	0 (0-0)	7 (5-10)	9 (6-12)	2,527 (1,652-2,975)	3,997 (2,359-4,624)	4,854 (2,916-5,546)	4,896 (3,573-5,482)	5,601 (4,607-6,205)
Alcohol use disorders	-	-	-	-	-	-	266 (240-293)	423 (377-470)	795 (698-892)	1,401 (1,215-1,606)	2,500 (2,152-2,860)
Drug use disorders	5 (3-7)	13 (10-18)	0 (0-0)	0 (0-0)	0 (0-0)	0 (0-0)	2,233 (1,364-2,667)	3,530 (1,885-4,142)	4,011 (2,041-4,678)	3,449 (2,167-3,965)	3,059 (2,090-3,462)
Opioid use disorders	5 (3-7)	13 (10-18)	0 (0-0)	0 (0-0)	0 (0-0)	0 (0-0)	1,387 (865-1,705)	2,177 (1,202-2,637)	2,461 (1,307-3,021)	1,966 (1,272-2,360)	1,757 (1,188-2,088)
Cocaine use disorders	0 (0-0)	0 (0-0)	0 (0-0)	0 (0-0)	0 (0-0)	0 (0-0)	74 (22-133)	132 (41-223)	155 (50-268)	195 (81-299)	170 (86-270)
Amphetamine use disorders	0 (0-0)	0 (0-0)	0 (0-0)	0 (0-0)	0 (0-0)	0 (0-0)	79 (22-207)	157 (37-468)	174 (42-519)	98 (31-256)	71 (29-166)
Other drug use disorders	0 (0-0)	0 (0-0)	0 (0-0)	0 (0-0)	0 (0-0)	0 (0-0)	693 (366-886)	1,065 (530-1,336)	1,220 (583-1,517)	1,190 (658-1,447)	1,060 (667-1,272)
Eating disorders	-	-	-	-	7 (5-10)	9 (6-12)	28 (21-35)	45 (35-57)	48 (38-60)	45 (39-54)	43 (36-51)
Anorexia nervosa	-	-	-	-	7 (4-9)	9 (6-11)	27 (21-35)	44 (35-56)	47 (37-59)	44 (38-52)	41 (34-49)
Bulimia nervosa	-	-	-	-	0 (0-1)	0 (0-1)	0 (0-1)	0 (0-1)	1 (0-1)	1 (1-2)	2 (1-2)
Diabetes, urogenital, blood, and endocrine diseases	2,169 (1,535-2,624)	1,508 (1,094-1,781)	13,879 (11,320-16,112)	21,308 (13,610-27,575)	8,087 (6,517-9,600)	9,773 (8,158-11,131)	14,050 (11,996-15,526)	15,746 (14,030-17,111)	16,126 (14,879-17,260)	17,366 (16,165-18,590)	19,278 (18,260-20,373)
Diabetes mellitus	26 (24-28)	53 (47-62)	592 (521-680)	709 (584-843)	650 (565-741)	1,056 (939-1,160)	1,795 (1,666-1,922)	2,598 (2,432-2,756)	2,987 (2,829-3,147)	3,656 (3,459-3,854)	5,016 (4,764-5,278)
Acute glomerulonephritis	-	-	120 (93-159)	198 (153-258)	183 (150-231)	171 (136-225)	151 (128-177)	269 (225-325)	343 (281-415)	296 (250-349)	317 (276-369)
Chronic kidney disease	-	-	2,184 (1,909-2,464)	4,231 (3,496-4,973)	2,180 (1,877-2,486)	2,607 (2,295-2,891)	3,686 (3,416-4,001)	5,622 (5,227-6,054)	6,674 (6,238-7,187)	7,490 (6,985-8,051)	8,541 (8,001-9,107)
Chronic kidney disease due to diabetes mellitus	-	-	54 (36-78)	105 (64-159)	69 (39-112)	110 (67-174)	253 (151-393)	803 (505-1,159)	1,549 (1,090-2,060)	2,279 (1,665-2,916)	3,095 (2,387-3,865)
Chronic kidney disease due to hypertension	-	-	16 (10-22)	43 (29-61)	36 (23-55)	72 (47-108)	154 (104-222)	343 (235-474)	530 (369-722)	715 (516-947)	961 (724-1,258)
Chronic kidney disease due to glomerulonephritis	-	-	937 (707-1,174)	1,832 (1,348-2,396)	929 (657-1,212)	1,166 (836-1,517)	1,706 (1,250-2,170)	2,469 (1,800-3,155)	2,532 (1,790-3,298)	2,347 (1,648-3,104)	2,225 (1,522-3,011)
Chronic kidney disease due to other causes	-	-	1,178 (935-1,444)	2,251 (1,742-2,888)	1,146 (873-1,467)	1,258 (917-1,625)	1,572 (1,123-2,043)	2,006 (1,361-2,646)	2,064 (1,431-2,815)	2,149 (1,499-2,865)	2,260 (1,609-2,941)
Urinary diseases and male infertility	136 (122-157)	164 (132-207)	1,920 (1,665-2,290)	2,035 (1,620-2,586)	694 (577-841)	587 (498-688)	1,213 (1,071-1,422)	1,455 (1,318-1,671)	1,297 (1,197-1,463)	1,490 (1,373-1,678)	1,630 (1,517-1,816)
Interstitial nephritis and urinary tract infections	62 (42-82)	77 (50-111)	1,162 (898-1,476)	1,184 (718-1,770)	335 (233-437)	271 (196-344)	806 (644-991)	954 (760-1,140)	780 (608-925)	929 (762-1,086)	1,085 (899-1,248)
Urolithiasis	-	-	-	-	93 (49-132)	89 (48-124)	106 (62-145)	130 (68-179)	120 (63-163)	131 (68-167)	150 (93-183)
Other urinary diseases	74 (60-93)	87 (63-121)	758 (573-1,007)	850 (551-1,320)	267 (187-397)	227 (161-337)	301 (208-478)	371 (260-596)	396 (286-591)	430 (318-615)	395 (281-599)
Gynecological diseases	-	-	-	-	-	-	244 (188-280)	331 (251-384)	366 (293-416)	470 (383-529)	581 (488-670)
Uterine fibroids	-	-	-	-	-	-	16 (8-34)	22 (12-41)	37 (18-63)	75 (30-135)	169 (66-255)
Polycystic ovarian syndrome	-	-	-	-	-	-	3 (0-10)	28 (6-85)	71 (18-150)	60 (13-155)	76 (18-169)
Endometriosis	-	-	-	-	-	-	5 (1-10)	3 (1-6)	3 (1-7)	6 (2-12)	5 (2-7)
Genital prolapse	-	-	-	-	-	-	15 (4-48)	15 (3-34)	24 (8-45)	27 (4-48)	9 (3-18)
Other gynecological diseases	-	-	-	-	-	-	205 (144-247)	263 (177-329)	230 (129-313)	302 (179-407)	323 (191-469)
Hemoglobinopathies and hemolytic anaemias	1,117 (676-1,537)	588 (348-760)	4,978 (2,605-6,670)	11,129 (3,670-16,527)	3,180 (1,840-4,444)	4,416 (2,950-5,474)	6,012 (4,026-7,280)	4,285 (2,675-5,425)	3,099 (1,954-3,851)	2,581 (1,769-3,270)	1,806 (1,296-2,278)
Thalassemias	320 (207-456)	175 (103-252)	1,557 (787-2,257)	2,625 (1,189-4,095)	1,242 (756-1,814)	673 (500-934)	397 (287-544)	256 (171-361)	170 (116-213)	84 (62-108)	49 (38-62)
Sickle cell disorders	700 (399-1,115)	388 (221-542)	3,278 (1,706-4,330)	8,237 (2,205-12,483)	1,759 (886-2,410)	3,408 (2,185-4,407)	4,918 (3,148-6,058)	3,227 (1,907-4,104)	2,006 (1,197-2,560)	1,531 (987-1,971)	851 (575-1,071)
G6PD deficiency	16 (10-22)	9 (5-12)	70 (41-100)	172 (81-256)	92 (62-129)	175 (118-228)	340 (237-448)	396 (268-552)	485 (338-640)	431 (307-576)	378 (284-491)
Other hemoglobinopathies and hemolytic anaemias	81 (53-108)	16 (10-21)	73 (42-101)	96 (55-134)	87 (62-123)	161 (116-202)	358 (262-468)	406 (282-574)	438 (305-574)	536 (393-711)	528 (391-670)
Endocrine, metabolic, blood, and immune disorders	890 (507-1,046)	702 (361-906)	4,083 (2,411-5,174)	3,007 (1,818-4,113)	1,200 (914-1,432)	937 (724-1,067)	949 (768-1,080)	1,185 (926-1,290)	1,361 (1,089-1,578)	1,384 (1,131-1,659)	1,388 (1,170-1,686)
Musculoskeletal disorders	-	-	-	-	443 (301-628)	803 (585-1,090)	1,371 (995-1,755)	1,884 (1,295-2,451)	2,032 (1,495-2,718)	1,811 (1,384-2,357)	1,764 (1,404-2,201)
Rheumatoid arthritis	-	-	-	-	97 (52-191)	94 (57-176)	92 (58-179)	134 (79-289)	153 (94-313)	186 (123-338)	235 (155-384)
Other musculoskeletal disorders	-	-	-	-	346 (231-508)	710 (525-942)	1,279 (924-1,632)	1,750 (1,217-2,285)	1,879 (1,378-2,492)	1,625 (1,255-2,132)	1,529 (1,222-1,867)
Other non-communicable diseases	86,216 (62,800-110,703)	37,830 (28,504-49,701)	124,235 (92,612-165,559)	45,041 (27,339-69,538)	10,707 (8,470-14,386)	7,436 (6,140-9,410)	4,652 (3,858-5,657)	4,197 (3,500-5,132)	3,608 (3,093-4,319)	2,442 (2,054-2,917)	2,295 (1,907-2,745)
Congenital anomalies	86,216 (62,800-110,703)	35,826 (26,695-47,460)	105,762 (77,104-142,517)	43,915 (26,122-68,565)	10,392 (8,153-14,133)	7,126 (5,873-9,051)	4,202 (3,510-5,161)	3,847 (3,213-4,727)	3,269 (2,806-3,904)	2,052 (1,750-2,468)	1,825 (1,553-2,185)
Neural tube defects	15,206 (9,174-19,204)	4,994 (2,768-6,804)	11,377 (6,080-16,652)	3,392 (1,420-6,549)	753 (507-1,024)	463 (325-591)	211 (150-275)	172 (122-236)	97 (72-135)	78 (60-105)	55 (42-76)
Congenital heart anomalies	25,864 (15,178-32,561)	14,088 (8,994-18,852)	49,865 (31,848-69,866)	21,314 (12,987-34,239)	6,164 (4,531-7,912)	4,601 (3,523-5,661)	2,707 (2,083-3,137)	2,738 (2,183-3,223)	2,432 (1,993-2,819)	1,393 (1,147-1,617)	1,284 (1,065-1,504)
Orofacial clefts	884 (415-1,483)	404 (193-629)	593 (265-902)	101 (23-233)	-	-	-	-	-	-	-
Down's syndrome	763 (492-1,562)	389 (247-892)	1,876 (1,180-4,207)	794 (473-2,160)	154 (101-277)	131 (92-212)	115 (85-192)	113 (87-188)	131 (105-203)	133 (106-195)	123 (100-178)
Other chromosomal abnormalities	2,457 (1,429-5,565)	1,409 (828-3,307)	3,074 (1,759-8,061)	508 (254-1,765)	131 (82-256)	80 (55-130)	62 (43-99)	38 (28-56)	38 (27-54)	26 (18-36)	19 (13-25)
Congenital musculoskeletal and limb anomalies	2,229 (1,192-4,512)	623 (314-1,326)	1,231 (589-3,076)	428 (176-1,407)	145 (86-278)	127 (81-207)	87 (55-135)	57 (34-89)	49 (29-73)	34 (21-47)	25 (18-33)
Urogenital congenital anomalies	2,244 (1,143-3,517)	843 (388-1,525)	1,398 (587-2,812)	364 (139-852)	178 (102-298)	164 (96-255)	65 (42-99)	58 (37-85)	56 (33-81)	46 (28-64)	49 (29-68)
Digestive congenital anomalies	6,071 (4,336-10,662)	2,617 (1,881-4,820)	6,384 (4,341-14,860)	1,260 (741-4,770)	140 (102-254)	76 (59-117)	49 (37-76)	34 (26-49)	19 (16-26)	17 (14-23)	16 (13-19)
Other congenital anomalies	30,497 (14,051-51,733)	10,457 (3,715-19,570)	29,964 (10,027-58,475)	15,754 (3,918-37,317)	2,728 (1,204-5,077)	1,484 (747-2,497)	906 (467-1,500)	636 (307-1,086)	448 (229-742)	324 (182-509)	253 (151-387)
Skin and subcutaneous diseases	-	-	2,347 (848-3,465)	1,126 (401-1,748)	314 (115-493)	310 (126-435)	451 (175-626)	350 (151-465)	339 (162-474)	390 (197-584)	470 (236-689)
Cellulitis	-	-	116 (26-219)	101 (22-210)	38 (11-67)	36 (12-62)	43 (16-64)	45 (18-62)	32 (15-49)	51 (25-83)	55 (25-92)
Pyoderma	-	-	2,173 (787-3,245)	921 (315-1,502)	227 (76-375)	219 (79-324)	334 (120-472)	230 (92-317)	236 (100-327)	244 (114-369)	327 (151-461)
Decubitus ulcer	-	-	-	41 (17-77)	23 (12-39)	27 (15-47)	38 (19-64)	39 (19-61)	44 (24-71)	64 (33-107)	60 (32-103)
Other skin and subcutaneous diseases	-	-	58 (24-108)	64 (31-113)	27 (13-44)	28 (14-45)	36 (18-56)	37 (19-59)	27 (15-42)	31 (17-49)	28 (16-46)
Sudden infant death syndrome	-	2,004 (1,346-3,187)	16,126 (10,680-25,398)	-	-	-	-	-	-	-	-
Injuries	7,229 (5,314-8,488)	4,981 (3,724-5,896)	55,810 (45,789-66,589)	142,837 (111,952-169,581)	72,907 (64,019-80,433)	57,131 (52,037-61,183)	97,257 (91,308-102,592)	115,450 (110,015-120,418)	103,649 (98,939-107,883)	85,648 (81,210-89,321)	74,148 (70,067-77,358)
Transport injuries	742 (537-1,006)	722 (539-890)	8,459 (7,017-9,761)	27,973 (21,935-32,902)	23,440 (20,841-26,079)	16,102 (14,688-17,412)	26,560 (24,986-28,031)	28,561 (27,024-30,167)	24,821 (23,420-26,250)	21,955 (20,768-23,192)	20,248 (19,186-21,344)
Road injuries	655 (477-872)	679 (502-835)	7,763 (6,196-8,857)	26,302 (20,014-30,620)	22,533 (20,048-25,138)	15,194 (13,862-16,497)	24,923 (23,446-26,450)	27,229 (25,753-28,837)	23,586 (22,218-25,156)	20,868 (19,734-22,142)	19,281 (18,265-20,422)
Pedestrian road injuries	304 (203-424)	313 (220-436)	3,662 (2,892-4,711)	15,836 (12,625-19,921)	13,760 (11,836-15,887)	7,863 (6,931-8,898)	8,527 (7,594-9,575)	9,638 (8,521-10,963)	8,219 (7,197-9,410)	7,923 (7,022-8,887)	7,885 (7,094-8,817)
Cyclist road injuries	-	-	-	717 (331-1,061)	835 (582-1,037)	954 (742-1,126)	1,119 (871-1,332)	1,029 (708-1,261)	926 (659-1,134)	1,005 (750-1,184)	859 (644-1,020)
Motorcyclist road injuries	133 (74-215)	68 (41-99)	1,210 (785-1,618)	2,369 (1,133-3,432)	1,538 (1,150-1,939)	1,535 (1,244-1,861)	4,065 (3,488-4,760)	5,001 (4,260-5,858)	4,231 (3,620-4,965)	3,711 (3,213-4,286)	2,756 (2,378-3,179)
Motor vehicle road injuries	207 (134-282)	293 (196-392)	2,791 (1,854-3,714)	7,217 (4,323-10,166)	6,230 (4,956-7,548)	4,721 (4,036-5,454)	10,981 (9,946-12,222)	11,341 (10,185-12,719)	10,054 (9,054-11,261)	8,046 (7,232-8,936)	7,605 (6,869-8,343)
Other road injuries	11 (7-17)	6 (3-9)	100 (54-192)	163 (83-319)	170 (113-243)	121 (90-179)	231 (169-382)	220 (162-345)	158 (117-251)	183 (135-302)	177 (133-273)
Other transport injuries	87 (40-151)	44 (26-69)	696 (438-1,163)	1,670 (1,067-2,877)	907 (670-1,255)	908 (709-1,132)	1,637 (1,274-1,957)	1,332 (996-1,602)	1,234 (928-1,461)	1,087 (839-1,287)	967 (775-1,131)
Unintentional injuries	4,932 (3,423-5,965)	3,738 (2,623-4,565)	43,756 (34,191-53,960)	106,097 (78,501-130,119)	43,350 (35,549-49,066)	28,821 (24,268-31,600)	24,531 (20,420-26,818)	24,725 (21,273-26,691)	22,689 (19,679-24,378)	21,350 (18,302-23,028)	19,381 (16,978-20,774)
Falls	1,126 (569-1,726)	449 (234-681)	6,223 (3,448-8,786)	9,997 (5,738-14,676)	4,164 (3,292-5,056)	2,581 (2,132-3,013)	2,851 (2,411-3,251)	2,877 (2,498-3,274)	2,695 (2,341-3,038)	2,780 (2,388-3,152)	3,085 (2,735-3,481)
Drowning	219 (168-245)	159 (111-188)	5,595 (3,591-6,703)	57,443 (36,082-70,118)	21,966 (15,570-25,351)	13,260 (9,790-14,840)	7,466 (5,469-8,177)	5,868 (4,397-6,445)	5,135 (3,797-5,638)	4,264 (3,135-4,672)	3,581 (2,685-3,922)
Fire, heat, and hot substances	175 (125-202)	261 (156-328)	3,990 (2,397-5,018)	11,202 (6,149-15,483)	3,758 (2,726-4,578)	2,728 (1,945-3,173)	4,861 (3,347-5,674)	5,682 (3,802-6,584)	4,609 (3,212-5,288)	4,280 (3,087-4,927)	3,416 (2,565-3,826)
Poisonings	158 (89-223)	138 (89-183)	2,507 (1,087-4,151)	5,452 (2,796-8,460)	1,950 (1,567-2,624)	1,960 (1,650-2,646)	1,576 (1,360-2,095)	1,982 (1,684-2,736)	1,721 (1,479-2,490)	1,366 (1,179-1,949)	1,230 (1,059-1,621)
Exposure to mechanical forces	1,061 (521-1,381)	1,002 (477-1,391)	7,476 (4,750-9,411)	5,273 (3,413-7,122)	2,935 (2,381-3,605)	1,713 (1,466-2,088)	1,723 (1,343-1,984)	1,617 (1,308-1,830)	1,926 (1,521-2,221)	1,667 (1,416-1,886)	1,534 (1,253-1,720)
Unintentional firearm injuries	30 (23-34)	22 (16-26)	245 (182-297)	366 (251-482)	302 (248-355)	238 (200-267)	304 (263-343)	322 (277-361)	375 (311-422)	395 (343-442)	272 (225-302)
Unintentional suffocation	855 (353-1,170)	916 (408-1,297)	5,753 (3,048-7,686)	1,805 (946-2,937)	719 (551-1,135)	507 (423-823)	144 (116-207)	165 (125-226)	169 (125-229)	113 (74-136)	98 (73-130)
Other exposure to mechanical forces	176 (114-212)	64 (42-78)	1,478 (859-1,924)	3,102 (1,632-4,342)	1,914 (1,383-2,481)	968 (728-1,184)	1,275 (895-1,539)	1,130 (815-1,337)	1,383 (993-1,650)	1,159 (913-1,386)	1,164 (889-1,342)
Adverse effects of medical treatment	844 (545-1,017)	670 (395-846)	5,442 (2,743-7,207)	4,374 (1,678-6,957)	1,588 (987-2,060)	1,089 (787-1,312)	1,211 (883-1,388)	2,170 (1,574-2,511)	2,172 (1,538-2,530)	1,868 (1,324-2,186)	1,978 (1,469-2,279)
Animal contact	125 (43-166)	137 (43-198)	1,712 (615-2,476)	4,103 (1,343-7,555)	3,541 (1,300-5,440)	2,824 (994-3,984)	2,123 (775-2,835)	1,794 (645-2,393)	1,625 (599-2,141)	2,040 (656-2,807)	1,471 (512-1,986)
Venomous animal contact	83 (21-122)	92 (23-141)	1,342 (417-1,966)	3,294 (977-5,885)	3,050 (1,048-4,691)	2,560 (819-3,609)	1,925 (651-2,634)	1,577 (504-2,164)	1,421 (488-1,912)	1,872 (567-2,627)	1,316 (422-1,813)
Non-venomous animal contact	42 (16-78)	45 (16-91)	370 (144-850)	810 (285-2,937)	491 (220-1,112)	264 (126-523)	198 (108-384)	218 (109-411)	204 (103-365)	168 (84-336)	156 (78-284)
Foreign body	831 (454-1,020)	728 (408-968)	8,645 (4,931-12,469)	4,531 (2,363-7,751)	1,403 (934-1,890)	858 (595-1,047)	697 (496-864)	853 (593-1,045)	834 (583-989)	822 (612-954)	878 (679-1,009)
Pulmonary aspiration and foreign body in airway	747 (415-920)	669 (379-896)	7,568 (4,358-11,071)	3,590 (1,898-6,264)	1,046 (667-1,400)	645 (449-776)	366 (267-426)	423 (316-506)	448 (333-533)	535 (395-613)	582 (441-677)
Foreign body in other body part	84 (23-114)	59 (12-82)	1,077 (247-1,517)	941 (281-1,577)	357 (173-532)	213 (105-292)	331 (190-464)	431 (228-583)	386 (204-505)	287 (155-374)	296 (174-375)
Environmental heat and cold exposure	164 (66-215)	100 (36-141)	1,109 (378-1,503)	788 (302-1,192)	418 (202-558)	206 (118-255)	368 (178-453)	355 (178-441)	392 (204-503)	617 (306-818)	846 (432-1,146)
Other unintentional injuries	230 (189-274)	94 (73-115)	1,057 (769-1,309)	2,934 (1,835-3,952)	1,627 (1,288-2,004)	1,603 (1,254-1,877)	1,655 (1,222-1,911)	1,525 (1,185-1,746)	1,579 (1,256-1,811)	1,646 (1,318-1,884)	1,362 (1,092-1,532)
Self-harm and interpersonal violence	1,229 (709-1,471)	330 (197-405)	2,884 (1,534-3,495)	4,893 (2,788-6,584)	3,666 (2,249-4,499)	9,970 (8,763-11,443)	43,774 (39,960-47,913)	60,117 (55,746-64,275)	54,610 (51,156-58,092)	40,951 (38,248-43,790)	33,324 (31,093-35,722)

Cause	40 to 44	45 to 49	50 to 54	55 to 59	60 to 64	65 to 69	70 to 74	75 to 79	80 to 84	85 to 89	90 to 94	95 plus
Ischaemic stroke	6,120 (5,621-6,595)	10,702 (9,881-11,418)	17,108 (15,751-18,357)	32,849 (30,455-35,196)	74,626 (69,509-79,859)	124,728 (116,653-132,621)	169,000 (158,598-180,296)	208,030 (194,174-222,930)	251,417 (234,165-270,096)	191,802 (177,552-207,745)	85,973 (77,916-93,839)	28,829 (25,733-31,683)
Hemorrhagic stroke	35,156 (33,049-37,588)	48,325 (45,571-51,418)	66,242 (62,224-70,819)	98,001 (92,068-104,908)	119,868 (111,544-131,158)	161,378 (150,378-176,788)	196,199 (182,629-217,430)	169,995 (156,725-191,572)	162,184 (148,830-182,463)	101,870 (92,929-115,660)	41,665 (37,496-47,154)	12,374 (10,928-13,890)
Hypertensive heart disease	5,755 (3,939-6,867)	8,596 (5,722-10,150)	12,685 (8,868-15,334)	20,097 (14,620-24,173)	27,093 (19,223-32,654)	39,648 (29,258-47,653)	51,650 (37,449-61,945)	57,501 (42,656-68,526)	58,705 (45,511-73,004)	45,810 (34,157-58,799)	24,155 (18,518-29,277)	8,359 (5,979-10,623)
Cardiomyopathy and myocarditis	3,538 (2,870-4,101)	3,822 (3,114-4,502)	3,975 (3,263-4,624)	6,597 (5,555-7,645)	6,530 (5,424-7,594)	8,354 (6,839-9,398)	10,141 (7,596-11,319)	11,295 (7,810-12,742)	16,747 (10,157-19,621)	18,676 (10,096-22,612)	10,549 (5,598-12,785)	4,014 (2,115-4,827)
Myocarditis	594 (478-771)	407 (322-536)	424 (332-540)	578 (457-722)	536 (396-653)	719 (526-878)	1,021 (726-1,226)	1,303 (857-1,552)	2,118 (1,201-2,640)	2,644 (1,365-3,495)	1,672 (833-2,217)	730 (354-962)
Alcoholic cardiomyopathy	1,583 (1,145-1,994)	1,867 (1,344-2,415)	1,638 (1,269-2,063)	3,036 (2,360-3,853)	2,283 (1,784-3,018)	2,399 (1,868-3,353)	1,868 (1,464-2,600)	1,405 (1,131-1,871)	1,531 (1,144-1,983)	1,288 (843-1,709)	692 (407-912)	247 (154-313)
Other cardiomyopathy	1,360 (1,110-1,627)	1,548 (1,225-1,782)	1,913 (1,508-2,177)	2,983 (2,355-3,379)	3,711 (2,871-4,184)	5,236 (3,840-5,867)	7,252 (4,951-8,380)	8,587 (5,564-9,957)	13,098 (7,403-15,807)	14,745 (7,311-18,527)	8,184 (4,050-10,361)	3,037 (1,490-3,830)
Atrial fibrillation and flutter	125 (91-164)	210 (152-278)	331 (245-436)	641 (371-966)	1,371 (895-1,957)	2,973 (1,814-4,514)	6,001 (4,254-8,393)	8,909 (5,644-13,298)	20,477 (15,223-26,437)	24,272 (18,221-31,566)	12,870 (9,816-16,644)	3,788 (2,921-4,898)
Aortic aneurysm	604 (534-708)	838 (750-977)	1,134 (1,030-1,309)	1,823 (1,660-2,098)	2,278 (2,153-2,488)	3,773 (3,574-4,087)	5,584 (5,326-5,964)	6,585 (6,310-6,963)	7,689 (7,363-8,073)	6,201 (5,962-6,514)	3,104 (2,992-3,244)	950 (915-989)
Peripheral vascular disease	59 (34-77)	84 (42-121)	130 (65-202)	268 (138-420)	488 (254-779)	950 (474-1,558)	1,457 (688-2,431)	2,062 (1,035-3,489)	3,794 (1,939-6,768)	3,930 (2,028-7,560)	2,618 (1,311-4,657)	1,164 (552-2,057)
Endocarditis	1,116 (896-1,325)	961 (753-1,126)	1,063 (827-1,266)	1,435 (1,088-1,727)	2,024 (1,519-2,466)	2,660 (1,941-3,275)	3,283 (2,374-4,031)	3,651 (2,553-4,601)	4,393 (2,896-5,620)	3,738 (2,368-4,674)	2,171 (1,336-2,772)	785 (486-997)
Other cardiovascular and circulatory diseases	4,528 (3,257-5,336)	5,641 (4,128-6,726)	7,172 (5,299-8,514)	10,381 (7,417-12,224)	16,292 (11,592-18,942)	22,360 (16,162-25,504)	30,335 (22,077-33,997)	34,843 (25,761-39,024)	39,651 (31,531-44,876)	32,290 (27,148-35,487)	17,734 (15,686-19,426)	6,051 (5,608-6,643)
Chronic respiratory diseases	20,780 (16,654-22,376)	31,493 (23,808-34,099)	48,338 (35,619-52,902)	81,413 (59,912-89,252)	134,386 (97,288-147,420)	189,162 (140,484-207,267)	256,796 (193,350-280,026)	271,188 (205,661-294,589)	249,386 (188,307-271,139)	166,032 (130,332-179,446)	69,879 (55,749-75,470)	20,204 (16,845-21,593)
Chronic obstructive pulmonary disease	12,403 (9,176-14,226)	19,787 (13,779-23,182)	33,456 (23,095-39,014)	59,678 (41,789-68,986)	107,143 (74,261-121,404)	156,806 (111,569-175,667)	223,070 (164,053-245,755)	239,504 (178,124-261,870)	224,188 (166,468-244,580)	150,770 (116,782-163,370)	64,083 (50,708-69,327)	18,615 (15,307-19,906)
Pneumoconiosis	66 (48-85)	62 (44-79)	77 (54-99)	125 (88-164)	168 (119-215)	215 (161-276)	295 (225-364)	292 (225-361)	249 (199-298)	148 (124-174)	56 (49-65)	18 (16-21)
Silicosis	17 (10-25)	18 (11-28)	18 (11-28)	35 (19-51)	50 (30-75)	62 (37-95)	106 (68-151)	101 (66-141)	83 (59-115)	46 (35-64)	18 (14-23)	5 (4-7)
Asbestosis	20 (15-26)	19 (14-26)	24 (17-33)	35 (24-48)	40 (25-55)	40 (26-53)	51 (30-68)	50 (29-68)	38 (23-50)	24 (15-30)	10 (6-12)	3 (2-4)
Coal workers pneumoconiosis	8 (3-12)	5 (2-8)	8 (3-12)	15 (7-24)	19 (9-29)	34 (17-51)	42 (22-64)	46 (25-70)	29 (17-42)	20 (12-28)	7 (5-10)	3 (2-4)
Other pneumoconiosis	22 (16-31)	19 (13-26)	26 (18-35)	40 (27-57)	59 (39-85)	79 (54-111)	96 (68-134)	95 (68-134)	98 (74-130)	57 (46-75)	22 (18-28)	7 (6-9)
Asthma	7,429 (5,708-8,768)	10,631 (7,942-12,736)	13,382 (9,669-16,713)	19,547 (13,980-24,332)	24,244 (17,069-31,541)	28,229 (20,136-36,527)	28,631 (20,732-37,388)	26,684 (19,760-34,885)	20,563 (15,468-27,959)	12,032 (9,353-16,580)	4,436 (3,491-6,183)	1,169 (951-1,581)
Interstitial lung disease and pulmonary sarcoidosis	581 (330-992)	814 (462-1,384)	1,151 (626-2,070)	1,703 (944-3,130)	2,435 (1,329-4,310)	3,378 (1,996-5,701)	4,175 (2,723-6,585)	4,015 (2,778-5,879)	3,594 (2,683-4,915)	2,408 (1,824-3,239)	979 (796-1,415)	302 (246-437)
Other chronic respiratory diseases	301 (170-410)	199 (104-267)	271 (147-362)	359 (190-489)	396 (212-550)	535 (285-726)	626 (318-830)	694 (344-950)	791 (339-1,069)	674 (261-914)	325 (127-439)	100 (41-133)
Cirrhosis and other chronic liver diseases	17,839 (16,903-18,861)	21,077 (20,008-22,209)	26,160 (24,716-27,531)	32,796 (31,056-34,358)	37,083 (35,045-39,037)	40,047 (37,913-42,321)	38,414 (36,097-40,692)	29,570 (27,887-31,243)	23,289 (21,983-24,737)	14,094 (13,402-14,944)	5,678 (5,389-6,001)	1,829 (1,718-1,939)
Cirrhosis and other chronic liver diseases due to hepatitis B	5,281 (4,501-6,113)	5,617 (4,666-6,662)	6,694 (5,481-8,008)	8,349 (6,925-9,954)	9,893 (8,134-12,032)	10,737 (8,834-12,811)	10,384 (8,487-12,466)	8,170 (6,749-9,585)	5,766 (4,689-6,913)	3,015 (2,522-3,520)	1,028 (829-1,249)	284 (203-375)
Cirrhosis and other chronic liver diseases due to hepatitis C	3,759 (3,034-4,567)	4,589 (3,574-5,619)	5,783 (4,549-7,035)	6,992 (5,690-8,359)	7,851 (6,174-9,724)	8,738 (7,169-10,387)	8,560 (7,040-10,258)	6,151 (4,899-7,473)	5,009 (3,959-6,161)	3,165 (2,567-3,768)	1,297 (1,003-1,601)	408 (281-538)
Cirrhosis and other chronic liver diseases due to alcohol use	4,811 (4,122-5,536)	6,022 (5,002-7,040)	7,403 (6,234-8,631)	8,908 (7,362-10,516)	9,527 (7,659-11,582)	10,362 (8,585-12,143)	9,709 (8,063-11,535)	7,280 (6,111-8,467)	5,947 (4,889-6,989)	3,770 (3,261-4,282)	1,618 (1,354-1,880)	548 (437-664)
Cirrhosis and other chronic liver diseases due to other causes	3,987 (3,076-5,001)	4,849 (3,730-6,110)	6,280 (4,901-7,802)	8,547 (6,690-10,619)	9,812 (7,537-12,351)	10,210 (7,862-12,736)	9,761 (7,540-12,272)	7,969 (6,240-9,689)	6,567 (5,209-8,126)	4,145 (3,511-4,895)	1,734 (1,419-2,086)	589 (446-746)
Digestive diseases	11,751 (10,110-12,641)	14,484 (12,485-15,715)	18,548 (15,999-20,163)	27,201 (23,325-29,657)	34,329 (29,249-37,793)	43,598 (37,498-47,647)	49,331 (43,537-53,368)	50,027 (44,655-53,855)	51,384 (46,524-55,564)	40,020 (36,827-42,896)	20,427 (18,843-21,825)	6,707 (6,245-7,111)
Peptic ulcer disease	4,227 (3,570-4,755)	5,033 (4,226-5,672)	7,113 (6,048-8,078)	9,151 (7,784-10,404)	12,317 (10,486-13,827)	15,397 (13,038-17,269)	16,646 (14,253-18,565)	16,663 (14,252-18,502)	15,186 (13,302-16,705)	11,061 (9,909-12,115)	5,208 (4,705-5,690)	1,603 (1,467-1,748)
Gastritis and duodenitis	582 (416-714)	745 (555-915)	913 (683-1,131)	1,177 (855-1,461)	1,550 (1,170-1,939)	1,915 (1,413-2,407)	1,995 (1,473-2,524)	2,239 (1,708-2,816)	2,292 (1,793-2,930)	1,656 (1,324-2,093)	828 (680-1,040)	265 (219-325)
Appendicitis	778 (575-928)	1,014 (721-1,228)	943 (706-1,151)	1,908 (1,388-2,313)	1,725 (1,233-2,168)	1,714 (1,253-2,203)	1,489 (1,123-1,889)	1,333 (1,000-1,706)	1,006 (748-1,293)	678 (514-862)	309 (232-398)	91 (68-118)
Paralytic ileus and intestinal obstruction	2,022 (1,234-2,508)	2,453 (1,486-2,992)	3,168 (2,002-3,885)	5,030 (3,242-6,155)	5,509 (3,563-6,766)	6,706 (4,446-8,089)	7,107 (5,231-8,498)	7,066 (5,415-8,538)	7,395 (5,713-9,030)	6,339 (4,941-7,565)	3,599 (2,782-4,310)	1,235 (935-1,478)
Inguinal, femoral, and abdominal hernia	211 (131-281)	292 (174-372)	554 (327-726)	1,042 (566-1,306)	1,424 (758-1,881)	1,776 (968-2,264)	2,028 (1,082-2,573)	1,899 (1,055-2,386)	2,441 (1,410-3,062)	1,833 (1,097-2,310)	941 (569-1,225)	288 (183-388)
Inflammatory bowel disease	384 (266-536)	503 (348-687)	513 (374-674)	756 (572-998)	896 (651-1,128)	1,130 (866-1,375)	1,562 (1,215-1,870)	1,658 (1,209-1,913)	1,931 (1,432-2,159)	1,605 (1,195-1,786)	889 (641-997)	324 (240-367)
Vascular intestinal disorders	330 (256-390)	544 (432-649)	735 (599-872)	1,246 (1,013-1,465)	2,073 (1,747-2,418)	3,512 (3,039-4,005)	5,040 (4,480-5,600)	5,739 (5,215-6,316)	6,547 (6,044-7,103)	5,080 (4,680-5,523)	2,355 (2,151-2,587)	674 (604-743)
Gallbladder and biliary diseases	929 (749-1,061)	1,205 (952-1,379)	1,684 (1,345-1,943)	2,469 (1,981-2,808)	3,450 (2,726-3,975)	4,733 (3,826-5,422)	5,428 (4,423-6,183)	5,703 (4,725-6,643)	6,115 (5,278-7,026)	4,931 (4,313-5,509)	2,679 (2,386-2,975)	924 (832-1,013)
Pancreatitis	1,423 (1,081-1,956)	1,709 (1,307-2,406)	1,713 (1,310-2,435)	2,567 (1,944-3,524)	2,657 (2,042-3,597)	3,267 (2,573-4,393)	3,371 (2,681-4,356)	2,785 (2,222-3,607)	2,719 (2,261-3,469)	1,865 (1,547-2,330)	826 (692-996)	251 (201-295)
Other digestive diseases	865 (722-1,044)	986 (830-1,208)	1,211 (1,021-1,509)	1,856 (1,554-2,319)	2,727 (2,308-3,421)	3,448 (2,917-4,279)	4,665 (4,064-5,598)	4,943 (4,294-5,866)	5,753 (5,161-6,625)	4,971 (4,497-5,594)	2,794 (2,535-3,083)	1,051 (953-1,151)
Neurological disorders	4,635 (3,992-5,124)	5,069 (4,540-5,689)	6,958 (6,026-8,095)	10,108 (8,310-12,256)	17,872 (14,571-22,257)	33,948 (26,766-42,701)	67,387 (52,131-85,752)	117,419 (91,412-148,565)	188,757 (149,174-239,039)	206,928 (164,963-253,008)	143,701 (119,410-170,084)	49,050 (39,357-60,882)
Alzheimer's disease and other dementias	44 (20-85)	867 (523-1,301)	2,585 (1,740-3,642)	5,183 (3,390-7,233)	11,843 (8,591-16,221)	26,042 (19,232-34,926)	56,094 (41,048-74,914)	104,696 (77,653-135,098)	173,179 (134,289-221,988)	195,581 (154,107-242,216)	139,241 (114,597-165,814)	47,931 (38,297-59,911)
Parkinson's disease	82 (45-141)	119 (65-207)	194 (110-312)	419 (246-627)	1,013 (686-1,459)	2,567 (1,800-3,452)	5,736 (4,002-7,768)	8,939 (6,441-11,919)	12,516 (9,303-16,692)	9,400 (7,150-12,484)	3,755 (2,806-4,919)	918 (661-1,228)
Epilepsy	2,846 (2,253-3,149)	1,976 (1,604-2,189)	1,985 (1,603-2,186)	1,808 (1,413-2,015)	2,002 (1,559-2,228)	1,910 (1,469-2,136)	2,090 (1,644-2,367)	1,139 (947-1,268)	949 (821-1,057)	706 (612-783)	248 (223-273)	69 (62-76)
Multiple sclerosis	809 (597-1,048)	926 (707-1,159)	844 (690-1,091)	901 (749-1,240)	801 (672-1,093)	786 (687-1,047)	677 (559-869)	426 (347-543)	314 (241-381)	149 (107-170)	43 (32-49)	9 (7-10)
Motor neuron disease	219 (204-258)	385 (364-447)	565 (534-644)	816 (778-905)	1,115 (1,068-1,194)	1,405 (1,348-1,483)	1,466 (1,409-1,533)	1,069 (1,025-1,114)	703 (670-741)	312 (295-329)	76 (72-80)	14 (13-14)
Other neurological disorders	635 (593-666)	796 (735-847)	786 (752-834)	981 (935-1,034)	1,099 (1,050-1,160)	1,237 (1,188-1,300)	1,325 (1,272-1,381)	1,149 (1,091-1,210)	1,096 (1,034-1,166)	781 (733-833)	338 (317-362)	110 (104-117)
Mental and substance use disorders	6,226 (5,289-6,859)	5,704 (5,022-6,324)	4,736 (4,189-5,213)	5,613 (4,987-6,281)	4,183 (3,608-4,567)	4,029 (3,573-4,401)	2,680 (2,288-2,929)	2,331 (2,007-2,543)	1,599 (1,452-1,737)	1,004 (911-1,091)	450 (402-484)	146 (133-156)
Alcohol use disorders	3,292 (2,880-3,756)	3,532 (3,098-4,052)	3,105 (2,779-3,480)	3,840 (3,339-4,431)	2,558 (2,304-2,858)	2,279 (2,047-2,529)	1,129 (1,050-1,218)	890 (810-984)	504 (468-544)	293 (275-315)	120 (112-130)	41 (38-43)
Drug use disorders	2,891 (2,065-3,260)	2,131 (1,578-2,393)	1,631 (1,212-1,833)	1,773 (1,356-2,018)	1,625 (1,152-1,824)	1,750 (1,330-1,954)	1,551 (1,154-1,745)	1,441 (1,122-1,612)	1,095 (937-1,212)	711 (607-784)	329 (280-359)	105 (91-115)
Opioid use disorders	1,675 (1,160-1,969)	1,221 (895-1,445)	926 (659-1,095)	1,051 (763-1,259)	897 (632-1,070)	978 (704-1,167)	629 (442-790)	556 (419-724)	379 (274-497)	245 (181-309)	116 (82-142)	36 (25-44)
Cocaine use disorders	152 (77-230)	105 (53-164)	63 (30-91)	61 (31-91)	61 (25-92)	63 (30-94)	15 (8-26)	13 (7-21)	4 (2-5)	3 (2-4)	2 (1-2)	1 (0-1)
Amphetamine use disorders	65 (28-144)	48 (22-102)	32 (14-69)	33 (14-73)	33 (12-78)	32 (13-71)	11 (4-26)	11 (5-22)	2 (1-3)	2 (1-2)	1 (0-1)	0 (0-0)
Other drug use disorders	999 (618-1,189)	757 (513-889)	610 (411-716)	628 (450-738)	634 (418-745)	676 (462-803)	896 (652-1,058)	860 (627-993)	710 (595-807)	461 (386-528)	211 (179-240)	68 (59-76)
Eating disorders	43 (37-51)	41 (35-49)	0 (0-0)	0 (0-0)	-	-	-	-	-	-	-	-
Anorexia nervosa	39 (34-47)	37 (33-45)	0 (0-0)	0 (0-0)	-	-	-	-	-	-	-	-
Bulimia nervosa	4 (3-5)	3 (2-4)	0 (0-0)	0 (0-0)	-	-	-	-	-	-	-	-
Diabetes, urogenital, blood, and endocrine diseases	25,636 (24,335-26,990)	32,492 (30,870-34,273)	44,338 (42,162-46,729)	62,132 (59,131-65,425)	85,136 (81,144-90,433)	105,844 (100,723-112,182)	116,448 (110,618-124,271)	111,928 (106,099-119,226)	106,186 (100,888-112,683)	79,674 (76,230-83,819)	39,649 (37,925-41,597)	13,703 (13,078-14,320)
Diabetes mellitus	8,487 (8,032-8,880)	12,647 (11,995-13,260)	20,016 (18,988-20,978)	31,417 (29,849-33,008)	47,486 (45,134-50,200)	60,087 (56,992-63,554)	64,855 (61,304-68,593)	60,359 (56,811-64,050)	54,368 (51,208-57,768)	35,761 (33,789-37,986)	15,357 (14,555-16,249)	4,398 (4,142-4,645)
Acute glomerulonephritis	370 (325-423)	340 (293-396)	262 (230-301)	371 (324-427)	494 (428-569)	548 (478-641)	580 (500-689)	529 (454-639)	424 (365-503)	259 (224-310)	86 (76-100)	29 (25-34)
Chronic kidney disease	10,646 (10,014-11,298)	12,656 (11,864-13,449)	16,415 (15,395-17,559)	21,642 (20,356-23,132)	27,074 (25,482-29,306)	32,084 (30,311-34,516)	35,473 (33,465-38,422)	36,039 (33,948-38,746)	34,742 (33,040-37,178)	28,194 (26,820-29,816)	15,209 (14,410-15,958)	5,609 (5,292-5,894)
Chronic kidney disease due to diabetes mellitus	4,410 (3,412-5,377)	5,616 (4,405-6,933)	7,570 (5,732-9,302)	10,402 (8,145-12,545)	13,351 (10,729-15,925)	15,233 (11,648-18,422)	15,724 (12,380-19,160)	14,515 (11,139-17,615)	11,604 (8,577-14,670)	7,939 (5,733-10,435)	4,012 (2,805-5,352)	1,505 (995-2,028)
Chronic kidney disease due to hypertension	1,445 (1,103-1,876)	1,951 (1,500-2,488)	2,871 (2,181-3,723)	4,195 (3,247-5,391)	5,772 (4,545-7,357)	7,494 (5,805-9,714)	9,207 (7,049-11,702)	10,755 (8,435-13,543)	11,410 (9,212-14,072)	10,960 (8,842-13,331)	6,582 (5,438-7,761)	2,521 (2,006-3,060)
Chronic kidney disease due to glomerulonephritis	2,302 (1,551-3,080)	2,312 (1,523-3,169)	2,552 (1,696-3,599)	2,952 (2,001-4,035)	3,224 (2,109-4,554)	3,823 (2,575-5,426)	4,261 (2,933-5,972)	4,363 (2,948-6,298)	4,837 (3,401-6,477)	3,484 (2,464-4,835)	1,607 (1,120-2,136)	545 (330-864)
Chronic kidney disease due to other causes	2,489 (1,724-3,360)	2,777 (1,871-3,869)	3,422 (2,231-4,815)	4,092 (2,805-5,760)	4,728 (3,139-6,808)	5,534 (3,707-7,852)	6,281 (4,284-8,556)	6,406 (4,418-8,911)	6,892 (4,882-9,213)	5,811 (4,009-7,866)	3,008 (2,169-3,994)	1,038 (656-1,493)
Urinary diseases and male infertility	1,897 (1,777-2,113)	2,209 (2,063-2,422)	2,880 (2,660-3,202)	4,244 (3,896-4,664)	5,121 (4,737-5,566)	6,682 (6,175-7,202)	7,526 (6,962-8,118)	7,867 (7,373-8,433)	9,038 (8,577-9,561)	8,337 (7,955-8,754)	5,359 (5,107-5,607)	2,343 (2,230-2,454)
Interstitial nephritis and urinary tract infections	1,311 (1,120-1,484)	1,520 (1,266-1,710)	2,024 (1,707-2,305)	2,916 (2,418-3,303)	3,445 (2,816-3,879)	4,820 (4,150-5,344)	5,708 (5,032-6,235)	6,034 (5,341-6,565)	7,296 (6,653-7,804)	7,035 (6,519-7,507)	4,636 (4,315-4,942)	2,086 (1,936-2,206)
Urolithiasis	162 (91-198)	222 (136-270)	268 (162-326)	384 (252-474)	469 (323-565)	620 (434-787)	648 (459-802)	598 (432-770)	606 (436-777)	424 (293-559)	240 (155-319)	77 (50-107)
Other urinary diseases	424 (305-628)	468 (341-720)	588 (403-943)	944 (655-1,486)	1,207 (848-1,875)	1,242 (853-1,944)	1,170 (849-1,834)	1,234 (891-1,935)	1,137 (865-1,771)	878 (655-1,414)	484 (357-777)	180 (131-305)
Gynecological diseases	587 (490-672)	790 (674-905)	639 (529-728)	474 (391-547)	276 (231-320)	419 (352-492)	238 (200-282)	165 (143-207)	348 (277-425)	239 (196-290)	100 (83-122)	34 (29-41)
Uterine fibroids	220 (109-310)	367 (166-509)	346 (150-472)	226 (86-335)	105 (45-172)	147 (67-243)	60 (29-104)	41 (25-62)	88 (31-178)	49 (19-101)	20 (10-37)	6 (3-11)
Polycystic ovarian syndrome	73 (18-148)	112 (27-234)	177 (53-337)	-	-	-	-	-	-	-	-	-
Endometriosis	7 (4-12)	11 (5-19)	8 (3-14)	-	-	-	-	-	-	-	-	-
Genital prolapse	9 (3-16)	10 (3-18)	16 (4-39)	102 (16-189)	85 (22-147)	133 (37-222)	94 (39-148)	52 (30-77)	146 (48-245)	93 (38-155)	37 (17-58)	11 (6-17)
Other gynecological diseases	277 (167-418)	291 (144-512)	91 (37-204)	146 (74-255)	85 (46-150)	139 (75-227)	85 (54-133)	72 (53-97)	115 (66-203)	97 (64-157)	42 (30-66)	16 (12-22)
Hemoglobinopathies and hemolytic anaemias	2,182 (1,655-2,924)	2,120 (1,630-2,874)	2,103 (1,633-2,838)	1,756 (1,379-2,320)	1,853 (1,478-2,486)	2,418 (1,929-3,233)	3,724 (2,997-4,952)	3,133 (2,478-3,994)	3,300 (2,463-3,994)	3,750 (2,729-4,591)	1,841 (1,250-2,222)	635 (418-759)
Thalassemias	36 (28-48)	28 (23-37)	18 (14-25)	12 (10-19)	6 (5-9)	3 (3-6)	2 (1-2)	1 (0-1)	0 (0-0)	0 (0-0)	0 (0-0)	0 (0-0)
Sickle cell disorders	1,043 (764-1,374)	888 (648-1,182)	634 (460-867)	280 (212-370)	102 (80-139)	64 (52-85)	35 (27-46)	14 (9-18)	6 (4-8)	2 (1-3)	1 (0-1)	0 (0-0)
G6PD deficiency	405 (307-550)	424 (319-602)	403 (301-545)	297 (219-414)	221 (166-306)	196 (145-279)	183 (136-265)	95 (71-129)	46 (34-62)	28 (21-38)	7 (5-10)	1 (1-2)
Other hemoglobinopathies and hemolytic anaemias	699 (541-960)	781 (609-1,087)	1,048 (827-1,422)	1,167 (923-1,548)	1,524 (1,209-2,017)	2,155 (1,733-2,859)	3,504 (2,833-4,641)	3,024 (2,393-3,855)	3,247 (2,422-3,927)	3,720 (2,704-4,549)	1,834 (1,244-2,211)	633 (416-756)
Endocrine, metabolic, blood, and immune disorders	1,468 (1,203-1,665)	1,730 (1,432-1,970)	2,024 (1,615-2,237)	2,229 (1,827-2,521)	2,832 (2,361-3,296)	3,606 (2,984-4,199)	4,053 (3,375-4,696)	3,836 (3,106-4,481)	3,966 (3,295-4,692)	3,133 (2,619-3,597)	1,697 (1,368-1,972)	655 (532-760)
Musculoskeletal disorders	1,940 (1,529-2,370)	1,870 (1,531-2,231)	1,848 (1,482-2,189)	2,302 (1,909-2,654)	2,998 (2,526-3,524)	3,809 (3,223-4,292)	4,510 (3,798-4,999)	4,563 (3,843-5,169)	4,568 (3,936-5,157)	3,676 (3,043-4,087)	1,939 (1,528-2,125)	727 (578-802)
Rheumatoid arthritis	363 (249-532)	523 (366-693)	719 (508-882)	1,149 (836-1,368)	1,698 (1,270-1,985)	2,285 (1,771-2,669)	2,784 (2,198-3,263)	2,754 (2,118-3,185)	2,427 (1,902-2,926)	1,577 (1,231-1,998)	637 (486-800)	178 (137-237)
Other musculoskeletal disorders	1,577 (1,246-1,902)	1,347 (1,098-1,632)	1,130 (919-1,341)	1,153 (973-1,392)	1,300 (1,077-1,602)	1,524 (1,287-1,873)	1,727 (1,382-2,113)	1,809 (1,438-2,166)	2,141 (1,683-2,520)	2,099 (1,585-2,369)	1,302 (955-1,451)	549 (425-603)
Other non-communicable diseases	2,182 (1,782-2,634)	1,920 (1,500-2,371)	2,231 (1,598-2,944)	2,577 (1,851-3,327)	2,632 (1,831-3,610)	3,186 (2,128-4,610)	2,780 (1,574-4,518)	3,373 (2,038-5,483)	3,869 (2,196-6,241)	3,716 (2,003-5,812)	2,519 (1,227-3,788)	1,143 (502-1,692)
Congenital anomalies	1,600 (1,374-1,988)	1,155 (971-1,357)	1,001 (840-1,182)	1,028 (875-1,221)	913 (772-1,117)	808 (700-971)	-	-	-	-	-	-
Neural tube defects	47 (36-62)	36 (28-46)	30 (22-37)	31 (23-39)	26 (19-32)	27 (19-37)	-	-	-	-	-	-
Congenital heart anomalies	1,137 (953-1,376)	558 (458-653)	427 (353-512)	423 (350-500)	356 (300-445)	341 (292-424)	-	-	-	-	-	-
Orofacial clefts	-	-	-	-	-	-	-	-	-	-	-	-
Down's syndrome	131 (109-177)	209 (165-274)	200 (162-265)	197 (165-265)	148 (123-212)	66 (55-96)	-	-	-	-	-	-
Other chromosomal abnormalities	19 (12-26)	16 (9-18)	17 (10-20)	18 (10-22)	13 (6-15)	12 (8-16)	-	-	-	-	-	-
Congenital musculoskeletal and limb anomalies	23 (15-29)	25 (15-31)	23 (15-29)	25 (17-30)	23 (13-28)	26 (14-31)	-	-	-	-	-	-
Urogenital congenital anomalies	46 (29-60)	66 (40-86)	86 (52-113)	110 (72-139)	123 (79-163)	141 (98-185)	-	-	-	-	-	-
Digestive congenital anomalies	14 (11-18)	18 (14-22)	22 (17-27)	25 (19-31)	28 (22-34)	33 (25-39)	-	-	-	-	-	-
Other congenital anomalies	183 (114-266)	226 (142-323)	195 (120-289)	199 (126-296)	198 (128-282)	162 (108-225)	-	-	-	-	-	-
Skin and subcutaneous diseases	582 (302-872)	766 (398-1,147)	1,231 (638-1,864)	1,549 (812-2,334)	1,719 (912-2,639)	2,377 (1,319-3,768)	2,780 (1,574-4,518)	3,373 (2,038-5,483)	3,869 (2,196-6,241)	3,716 (2,003-5,812)	2,519 (1,227-3,788)	1,143 (502-1,692)
Cellulitis	62 (32-117)	86 (38-148)	147 (64-243)	189 (87-328)	238 (102-393)	290 (136-515)	338 (152-618)	371 (173-709)	416 (201-885)	361 (178-742)	240 (99-510)	108 (46-231)
Pyoderma	399 (190-569)	538 (253-822)	882 (416-1,346)	1,075 (532-1,664)	1,120 (547-1,806)	1,436 (742-2,374)	1,496 (808-2,514)	1,598 (904-2,743)	1,416 (785-2,547)	1,232 (659-2,100)	774 (398-1,275)	331 (158-534)
Decubitus ulcer	84 (44-145)	107 (58-183)	162 (78-287)	233 (118-411)	300 (157-511)	560 (280-934)	847 (463-1,424)	1,278 (679-2,127)	1,870 (971-2,978)	1,967 (932-2,844)	1,395 (614-2,027)	659 (255-950)
Other skin and subcutaneous diseases	36 (20-61)	35 (20-61)	39 (22-66)	52 (29-87)	62 (35-107)	92 (51-152)	100 (56-177)	127 (70-218)	168 (86-288)	156 (74-265)	110 (49-176)	44 (17-71)
Sudden infant death syndrome	-	-	-	-	-	-	-	-	-	-	-	-
Injuries	71,606 (67,675-74,439)	61,878 (58,356-64,294)	55,999 (52,408-58,358)	61,805 (57,729-64,576)	64,185 (59,178-67,400)	68,065 (62,379-71,941)	69,666 (63,522-73,849)	64,323 (58,373-68,488)	61,147 (55,297-65,629)	46,557 (40,932-49,957)	22,925 (19,496-24,647)	8,055 (6,703-8,685)
Transport injuries	21,028 (19,997-22,239)	17,443 (16,524-18,454)	15,954 (15,013-17,034)	17,192 (16,084-18,481)	17,161 (16,001-18,695)	15,790 (14,745-17,066)	15,722 (14,648-16,983)	12,255 (11,391-13,278)	8,797 (8,261-9,565)	4,544 (4,263-4,929)	1,656 (1,557-1,808)	496 (461-540)
Road injuries	19,955 (18,954-21,127)	16,451 (15,584-17,515)	15,061 (14,174-16,074)	16,417 (15,329-17,641)	16,191 (15,093-17,672)	14,910 (13,943-16,127)	14,910 (13,886-16,114)	11,565 (10,780-12,545)	8,276 (7,771-9,021)	4,258 (3,982-4,624)	1,538 (1,443-1,676)	450 (417-493)
Pedestrian road injuries	8,365 (7,541-9,349)	7,248 (6,549-8,030)	6,678 (6,062-7,307)	7,878 (7,156-8,731)	8,864 (8,107-9,805)	8,422 (7,773-9,294)	8,698 (7,935-9,595)	7,199 (6,611-7,929)	5,252 (4,900-5,746)	2,583 (2,401-2,863)	841 (779-958)	225 (204-272)
Cyclist road injuries	1,028 (780-1,201)	1,001 (783-1,157)	819 (674-948)	912 (758-1,062)	853 (712-991)	843 (717-967)	797 (664-957)	397 (305-478)	185 (138-234)	97 (74-139)	35 (26-56)	0 (0-1)
Motorcyclist road injuries	2,956 (2,544-3,436)	2,121 (1,847-2,498)	1,994 (1,718-2,357)	1,873 (1,600-2,243)	1,304 (1,092-1,595)	998 (841-1,219)	777 (627-974)	405 (321-529)	216 (170-296)	92 (72-124)	30 (24-44)	11 (9-16)
Motor vehicle road injuries	7,441 (6,664-8,274)	5,887 (5,330-6,578)	5,411 (4,882-6,096)	5,559 (4,965-6,296)	5,097 (4,556-5,947)	4,572 (4,124-5,320)	4,570 (4,080-5,327)	3,427 (3,046-3,993)	2,537 (2,297-3,000)	1,439 (1,282-1,683)	608 (523-706)	204 (167-236)
Other road injuries	165 (124-257)	194 (145-293)	159 (118-251)	195 (141-308)	73 (54-100)	75 (56-110)	68 (50-98)	136 (98-221)	85 (59-137)	48 (32-76)	23 (14-36)	9 (6-15)
Other transport injuries	1,073 (854-1,252)	992 (798-1,156)	893 (701-1,047)	775 (633-904)	970 (786-1,128)	879 (730-1,025)	812 (682-944)	691 (568-801)	521 (434-601)	286 (241-331)	118 (100-136)	46 (40-52)
Unintentional injuries	20,081 (17,392-21,447)	19,431 (16,839-20,853)	19,156 (16,285-20,835)	23,937 (20,777-25,944)	27,953 (23,558-30,462)	33,622 (27,945-36,815)	36,990 (30,951-40,527)	39,142 (32,724-42,732)	42,833 (36,361-46,705)	37,180 (31,377-40,252)	19,733 (16,192-21,343)	7,198 (5,836-7,808)
Falls	3,292 (2,935-3,705)	3,755 (3,239-4,176)	4,512 (3,800-5,026)	7,080 (5,769-7,992)	10,326 (8,201-11,746)	14,606 (11,362-16,780)	18,398 (14,503-20,838)	22,316 (18,027-25,042)	26,220 (21,516-29,339)	24,572 (20,324-27,073)	13,321 (10,785-14,779)	4,729 (3,730-5,365)
Drowning	3,467 (2,701-3,793)	3,050 (2,387-3,339)	2,825 (2,129-3,094)	3,107 (2,468-3,418)	3,284 (2,556-3,638)	3,521 (2,790-3,949)	3,453 (2,765-3,844)	3,184 (2,487-3,567)	2,464 (2,011-2,769)	1,311 (1,093-1,464)	414 (346-462)	95 (80-105)
Fire, heat, and hot substances	2,791 (2,160-3,127)	2,755 (2,096-3,091)	2,484 (1,899-2,765)	2,789 (2,185-3,098)	2,830 (2,250-3,228)	3,548 (2,831-4,017)	3,663 (2,947-4,192)	3,733 (2,971-4,205)	4,017 (3,116-4,563)	3,090 (2,324-3,479)	1,559 (1,109-1,743)	619 (425-683)
Poisonings	1,294 (1,133-1,659)	1,008 (866-1,327)	975 (842-1,265)	1,039 (902-1,332)	967 (842-1,282)	1,057 (904-1,349)	1,045 (916-1,344)	749 (647-1,011)	659 (580-898)	378 (336-514)	158 (141-212)	53 (44-71)
Exposure to mechanical forces	1,671 (1,379-1,865)	1,461 (1,204-1,624)	1,136 (942-1,269)	1,611 (1,288-1,810)	1,404 (1,095-1,608)	1,359 (1,066-1,534)	1,232 (1,019-1,369)	1,064 (894-1,173)	1,149 (963-1,267)	947 (778-1,046)	515 (412-574)	201 (161-224)
Unintentional firearm injuries	290 (247-325)	269 (234-304)	184 (151-205)	216 (189-246)	178 (151-200)	200 (172-223)	208 (178-233)	259 (225-292)	276 (232-313)	236 (192-268)	141 (114-162)	56 (44-67)
Unintentional suffocation	93 (59-113)	132 (110-187)	140 (118-209)	182 (152-294)	67 (46-93)	77 (51-101)	90 (61-121)	78 (46-101)	81 (51-102)	61 (37-76)	25 (18-34)	8 (6-11)
Other exposure to mechanical forces	1,288 (1,015-1,480)	1,059 (813-1,208)	812 (640-929)	1,213 (899-1,407)	1,159 (860-1,350)	1,081 (792-1,241)	934 (737-1,056)	727 (583-815)	793 (632-886)	650 (504-735)	349 (260-398)	137 (102-155)
Adverse effects of medical treatment	2,067 (1,537-2,373)	2,367 (1,800-2,680)	1,950 (1,521-2,208)	2,531 (1,963-2,916)	3,528 (2,809-4,054)	3,864 (3,040-4,495)	3,964 (3,159-4,678)	3,624 (2,793-4,224)	3,670 (2,726-4,271)	3,098 (2,153-3,617)	1,689 (1,138-1,994)	635 (426-748)
Animal contact	2,089 (730-2,763)	1,791 (681-2,321)	2,242 (874-2,955)	1,669 (695-2,156)	1,945 (714-2,614)	1,912 (735-2,547)	1,785 (700-2,429)	1,139 (486-1,508)	696 (327-915)	334 (175-430)	118 (68-146)	37 (24-45)
Venomous animal contact	1,921 (641-2,578)	1,582 (555-2,094)	2,016 (744-2,714)	1,434 (554-1,900)	1,767 (617-2,406)	1,733 (634-2,354)	1,596 (563-2,217)	993 (374-1,355)	570 (240-777)	258 (118-347)	86 (44-110)	24 (14-30)
Non-venomous animal contact	168 (83-295)	209 (102-350)	225 (115-381)	235 (127-395)	178 (82-297)	179 (92-303)	189 (93-327)	146 (77-235)	126 (70-176)	76 (47-111)	32 (21-44)	13 (9-18)
Foreign body	1,053 (828-1,209)	1,012 (786-1,134)	965 (737-1,100)	1,372 (1,087-1,566)	1,305 (1,002-1,463)	1,413 (1,079-1,574)	1,546 (1,099-1,745)	1,649 (1,165-1,894)	2,041 (1,379-2,380)	1,864 (1,199-2,242)	1,086 (662-1,327)	481 (299-591)
Pulmonary aspiration and foreign body in airway	795 (620-922)	838 (648-960)	800 (602-914)	1,166 (901-1,364)	1,151 (884-1,309)	1,262 (959-1,420)	1,357 (972-1,540)	1,465 (1,035-1,683)	1,817 (1,213-2,136)	1,663 (1,075-2,006)	983 (589-1,210)	444 (276-549)
Foreign body in other body part	258 (149-333)	174 (93-216)	165 (100-210)	206 (124-266)	154 (85-197)	150 (77-183)	188 (89-232)	184 (73-229)	224 (90-282)	201 (70-258)	102 (37-131)	37 (14-47)
Environmental heat and cold exposure	1,130 (609-1,551)	1,252 (654-1,728)	1,089 (612-1,600)	1,711 (1,008-2,580)	1,553 (875-2,249)	1,427 (811-2,142)	1,332 (748-1,865)	1,187 (619-1,527)	1,408 (800-1,886)	1,214 (650-1,628)	677 (325-898)	266 (114-321)
Other unintentional injuries	1,227 (1,009-1,399)	981 (823-1,113)	979 (730-1,144)	1,029 (832-1,192)	811 (665-953)	917 (713-1,101)	572 (483-666)	497 (402-596)	509 (411-611)	372 (305-448)	197 (158-236)	82 (65-98)
Self-harm and interpersonal violence	29,455 (27,511-31,418)	24,062 (22,494-25,587)	20,048 (18,860-21,221)	19,785 (18,456-20,941)	18,158 (17,080-19,274)	17,756 (16,628-18,849)	16,211 (15,181-17,438)	12,383 (11,619-13,406)	9,062 (8,480-9,960)	4,543 (4,280-4,903)	1,446 (1,360-1,563)	310 (291-335)

SR Table 3: Global deaths by cause and age group for females (with 95% uncertainty intervals), 1995

Cause	Early Neonatal	Late Neonatal	Post Neonatal	1 to 4	5 to 9	10 to 14	15 to 19	20 to 24	25 to 29	30 to 34	35 to 39	40 to 44	45 to 49	50 to 54	55 to 59	60 to 64	65 to 69	70 to 74	75 to 79	80 to 84	85 to 89	90 to 94	95 plus
Self-harm	-	-	-	-	-	6,661<br>(5,548-7,833)	36,948<br>(32,844-40,920)	50,786<br>(46,366-54,828)	45,738<br>(42,110-49,402)	32,328<br>(29,592-34,925)	25,852<br>(23,787-27,895)	23,019<br>(21,244-24,691)	19,194<br>(17,935-20,431)	16,687<br>(15,672-17,752)	16,427<br>(15,431-17,401)	15,509<br>(14,534-16,574)	15,304<br>(14,418-16,365)	14,128<br>(13,202-15,346)	10,788<br>(10,043-11,802)	8,342<br>(7,779-9,190)	4,200<br>(3,952-4,564)	1,328<br>(1,246-1,438)	273<br>(257-292)
Self-harm by firearm	-	-	-	-	-	230<br>(144-371)	1,358<br>(743-2,071)	1,544<br>(853-2,068)	1,169<br>(747-1,565)	996<br>(722-1,311)	938<br>(718-1,286)	802<br>(639-1,078)	684<br>(550-911)	557<br>(441-774)	487<br>(381-701)	408<br>(310-614)	407<br>(304-627)	357<br>(259-566)	308<br>(219-508)	252<br>(198-373)	166<br>(141-211)	82<br>(72-98)	29<br>(26-34)
Self-harm by other specified means	-	-	-	-	-	6,430<br>(5,365-7,539)	35,590<br>(31,798-39,203)	49,242<br>(45,002-52,994)	44,569<br>(41,049-48,062)	31,332<br>(28,658-33,826)	24,914<br>(22,960-26,922)	22,217<br>(20,517-23,880)	18,510<br>(17,251-19,712)	16,130<br>(15,107-17,193)	15,940<br>(14,919-16,905)	15,102<br>(14,116-16,108)	14,898<br>(14,015-15,923)	13,771<br>(12,832-14,950)	10,480<br>(9,708-11,497)	8,089<br>(7,507-8,941)	4,035<br>(3,785-4,391)	1,246<br>(1,171-1,346)	243<br>(229-261)
Interpersonal violence	1,229<br>(709-1,471)	330<br>(197-405)	2,884<br>(1,534-3,495)	4,893<br>(2,788-6,584)	3,666<br>(2,249-4,499)	3,309<br>(2,251-3,943)	6,826<br>(4,692-8,021)	9,331<br>(6,320-11,042)	8,872<br>(6,078-10,163)	8,622<br>(5,780-9,867)	7,472<br>(4,897-8,508)	6,436<br>(4,222-7,409)	4,868<br>(3,180-5,634)	3,361<br>(2,303-3,869)	3,358<br>(2,174-3,908)	2,649<br>(1,784-3,069)	2,451<br>(1,728-2,883)	2,083<br>(1,483-2,411)	1,595<br>(1,121-1,833)	720<br>(506-856)	342<br>(238-409)	118<br>(93-156)	37<br>(28-50)
Assault by firearm	51<br>(26-67)	22<br>(10-31)	254<br>(128-340)	390<br>(231-595)	380<br>(228-481)	577<br>(348-700)	1,792<br>(1,043-2,092)	2,088<br>(1,262-2,407)	2,019<br>(1,167-2,358)	1,842<br>(1,034-2,166)	1,664<br>(867-2,002)	1,282<br>(706-1,548)	942<br>(531-1,111)	690<br>(394-824)	544<br>(306-652)	444<br>(266-551)	362<br>(222-465)	327<br>(203-427)	243<br>(152-329)	49<br>(27-66)	26<br>(14-36)	8<br>(5-13)	3<br>(2-6)
Assault by sharp object	69<br>(40-92)	42<br>(16-61)	235<br>(97-321)	486<br>(197-706)	556<br>(274-723)	613<br>(340-762)	1,459<br>(870-1,761)	2,535<br>(1,470-3,100)	2,615<br>(1,523-3,154)	2,640<br>(1,584-3,154)	2,232<br>(1,265-2,692)	2,091<br>(1,187-2,536)	1,488<br>(849-1,809)	928<br>(549-1,108)	916<br>(497-1,124)	728<br>(432-883)	598<br>(350-726)	568<br>(366-689)	417<br>(261-505)	183<br>(127-238)	77<br>(55-103)	30<br>(25-46)	9<br>(7-14)
Assault by other means	1,109<br>(643-1,341)	267<br>(165-332)	2,394<br>(1,293-2,948)	4,017<br>(2,276-5,591)	2,730<br>(1,677-3,471)	2,118<br>(1,477-2,731)	3,575<br>(2,467-4,464)	4,708<br>(3,243-6,087)	4,239<br>(2,983-5,257)	4,140<br>(2,824-4,950)	3,576<br>(2,433-4,218)	3,063<br>(2,068-3,668)	2,438<br>(1,668-2,902)	1,743<br>(1,220-2,038)	1,899<br>(1,267-2,247)	1,477<br>(983-1,739)	1,491<br>(1,076-1,827)	1,188<br>(835-1,423)	935<br>(667-1,110)	488<br>(343-588)	239<br>(159-286)	79<br>(56-103)	25<br>(17-33)
Forces of nature, conflict and terrorism, and executions and police conflict	326<br>(248-405)	190<br>(144-237)	711<br>(534-884)	3,874<br>(2,908-4,865)	2,452<br>(1,830-3,100)	2,238<br>(1,660-2,840)	2,392<br>(1,781-3,027)	2,048<br>(1,513-2,611)	1,529<br>(1,126-1,958)	1,392<br>(1,019-1,791)	1,195<br>(867-1,536)	1,042<br>(742-1,368)	942<br>(650-1,251)	841<br>(578-1,124)	891<br>(609-1,202)	912<br>(612-1,237)	898<br>(601-1,224)	743<br>(474-1,038)	543<br>(325-781)	456<br>(265-667)	291<br>(174-422)	90<br>(49-133)	51<br>(34-69)
Forces of nature, conflict and terrorism, and state actor violence	4<br>(3-5)	15<br>(10-20)	125<br>(82-166)	653<br>(429-869)	642<br>(424-861)	653<br>(431-877)	509<br>(335-696)	475<br>(298-667)	456<br>(286-645)	415<br>(263-586)	376<br>(235-532)	391<br>(228-569)	369<br>(201-556)	353<br>(191-534)	387<br>(208-580)	410<br>(214-627)	398<br>(205-611)	381<br>(188-593)	316<br>(146-505)	270<br>(118-437)	158<br>(66-257)	58<br>(23-96)	15<br>(6-25)
Conflict and terrorism	322<br>(246-399)	175<br>(133-217)	571<br>(435-708)	3,170<br>(2,413-3,961)	1,770<br>(1,348-2,211)	1,539<br>(1,167-1,927)	1,844<br>(1,405-2,301)	1,542<br>(1,181-1,917)	1,020<br>(785-1,264)	948<br>(725-1,184)	790<br>(604-986)	624<br>(480-777)	549<br>(426-678)	468<br>(364-576)	479<br>(364-598)	472<br>(360-589)	475<br>(357-599)	331<br>(250-416)	211<br>(158-266)	160<br>(116-207)	112<br>(82-144)	20<br>(15-26)	33<br>(26-42)
Executions and police conflict	-	-	15<br>(10-24)	51<br>(23-128)	40<br>(32-54)	45<br>(35-63)	39<br>(30-52)	31<br>(26-41)	53<br>(27-74)	28<br>(23-37)	28<br>(20-45)	27<br>(19-42)	24<br>(16-39)	19<br>(14-30)	25<br>(14-42)	30<br>(13-51)	25<br>(11-44)	30<br>(11-59)	16<br>(8-25)	26<br>(10-42)	21<br>(4-39)	11<br>(5-19)	3<br>(1-6)

SR Table 3: Global deaths by cause and age group for females (with 95% uncertainty intervals), 2000

Cause	Early Neonatal	Late Neonatal	Post Neonatal	1 to 4	5 to 9	10 to 14	15 to 19	20 to 24	25 to 29	30 to 34	35 to 39
All causes	1,086,122 (1,063,422-1,109,177)	386,740 (378,859-394,832)	1,294,074 (1,260,302-1,324,466)	1,318,032 (1,272,819-1,361,913)	312,915 (308,955-316,816)	217,329 (214,494-220,314)	309,587 (303,258-315,417)	395,175 (385,876-404,145)	467,013 (453,708-480,242)	501,301 (490,675-512,603)	535,568 (524,157-547,515)
Communicable, maternal, neonatal, and nutritional disorders	990,992 (957,623-1,020,685)	344,397 (331,457-355,674)	1,090,927 (1,039,244-1,135,241)	1,086,387 (1,026,195-1,145,617)	202,224 (190,652-214,037)	106,474 (98,825-114,609)	127,896 (119,715-138,248)	193,461 (183,585-204,481)	247,180 (234,308-262,562)	249,911 (238,396-263,392)	231,049 (219,082-243,939)
HIV/AIDS and tuberculosis	-	-	54,904 (51,457-58,341)	44,824 (39,241-50,586)	18,139 (16,295-20,173)	13,674 (12,226-15,158)	26,571 (24,169-29,159)	67,929 (61,781-74,950)	131,639 (120,368-144,604)	144,078 (134,190-154,206)	133,651 (123,592-144,279)
Tuberculosis	-	-	10,115 (7,858-12,922)	15,947 (10,916-20,795)	7,235 (5,749-9,033)	8,133 (6,911-9,370)	19,250 (17,015-21,493)	27,862 (25,131-30,241)	32,451 (29,656-35,117)	34,043 (31,087-36,806)	37,445 (33,581-40,981)
Drug-susceptible tuberculosis	-	-	9,383 (7,246-12,015)	14,714 (10,136-19,366)	6,605 (5,234-8,237)	7,401 (6,290-8,531)	17,549 (15,520-19,557)	25,407 (22,788-27,676)	29,624 (26,966-32,139)	31,058 (28,237-33,597)	34,196 (30,608-37,431)
Multidrug-resistant tuberculosis without extensive drug resistance	-	-	725 (521-1,006)	1,219 (786-1,750)	621 (448-847)	720 (550-907)	1,672 (1,323-2,099)	2,404 (1,962-2,910)	2,764 (2,257-3,319)	2,919 (2,370-3,527)	3,175 (2,601-3,869)
Extensively drug-resistant tuberculosis	-	-	8 (6-11)	14 (9-19)	9 (7-12)	12 (9-15)	28 (22-35)	51 (41-62)	63 (50-77)	65 (51-80)	73 (59-90)
HIV/AIDS	-	-	44,789 (42,465-47,193)	28,877 (26,929-30,853)	10,904 (10,063-11,893)	5,540 (4,983-6,255)	7,322 (6,191-8,888)	40,067 (34,403-47,473)	99,189 (87,546-112,435)	110,036 (100,377-120,298)	96,207 (86,159-106,562)
Drug-susceptible HIV/AIDS - Tuberculosis	-	-	14,811 (11,319-17,706)	10,022 (7,899-11,779)	3,622 (2,859-4,269)	1,829 (1,505-2,159)	2,361 (1,810-2,977)	11,193 (7,614-14,932)	21,898 (14,099-30,566)	23,749 (14,993-33,372)	21,960 (14,197-30,219)
Multidrug-resistant HIV/AIDS - Tuberculosis without extensive drug resistance	-	-	1,086 (683-1,616)	724 (446-1,114)	241 (152-363)	121 (75-186)	192 (127-287)	835 (516-1,264)	1,557 (924-2,443)	1,691 (981-2,645)	1,560 (919-2,406)
Extensively drug-resistant HIV/AIDS - Tuberculosis	-	-	5 (4-8)	3 (2-5)	1 (1-2)	1 (1-1)	2 (1-2)	6 (4-9)	14 (8-21)	16 (10-26)	15 (9-24)
HIV/AIDS resulting in other diseases	-	-	28,887 (25,609-33,079)	18,128 (15,998-20,983)	7,040 (6,212-8,182)	3,589 (3,131-4,272)	4,767 (3,906-6,152)	28,033 (22,362-36,034)	75,720 (61,768-91,622)	84,579 (71,854-98,949)	72,671 (60,485-86,481)
Diarrhoea, lower respiratory infections, and other common infectious diseases	123,617 (102,951-144,550)	139,088 (125,597-152,453)	761,761 (705,912-817,929)	682,182 (595,574-785,108)	130,975 (112,802-153,139)	66,481 (55,888-78,978)	49,737 (40,017-64,948)	41,121 (32,255-56,987)	35,536 (27,452-49,516)	33,718 (25,793-47,702)	33,740 (25,920-48,508)
Diarrhoeal diseases	18,754 (16,021-21,865)	37,696 (32,890-43,436)	251,447 (213,763-290,434)	200,610 (156,973-248,950)	27,428 (12,054-54,197)	15,218 (6,741-30,721)	16,477 (7,191-33,873)	16,372 (7,331-33,761)	14,069 (6,115-28,640)	14,144 (6,006-28,545)	14,315 (6,064-29,575)
Intestinal infectious diseases	-	-	1,184 (168-4,458)	14,906 (6,959-27,596)	28,310 (15,639-46,797)	17,136 (9,037-27,211)	9,340 (4,714-16,376)	4,707 (2,307-8,366)	3,418 (1,774-5,860)	2,745 (1,448-4,664)	2,259 (1,221-3,751)
Typhoid fever	-	-	88 (41-170)	12,713 (5,556-24,926)	24,832 (13,102-41,660)	14,489 (7,340-23,443)	8,093 (3,996-14,335)	3,960 (1,892-7,154)	2,702 (1,364-4,730)	2,068 (1,070-3,538)	1,684 (881-2,921)
Paratyphoid fever	-	-	13 (5-29)	1,268 (502-2,707)	3,388 (1,528-6,895)	2,613 (1,178-5,013)	1,217 (514-2,444)	719 (300-1,495)	692 (298-1,389)	642 (284-1,271)	539 (249-1,018)
Other intestinal infectious diseases	-	-	1,083 (79-4,372)	925 (56-4,112)	90 (7-432)	33 (3-150)	30 (2-133)	28 (3-113)	25 (2-124)	34 (3-164)	37 (3-175)
Lower respiratory infections	79,033 (62,795-96,896)	76,643 (64,604-88,160)	342,702 (300,026-394,238)	196,971 (158,320-243,573)	22,997 (17,965-28,035)	11,469 (9,122-13,350)	9,094 (7,506-10,917)	9,304 (7,384-11,220)	9,621 (7,905-11,531)	9,331 (7,619-11,154)	10,567 (8,791-12,555)
Upper respiratory infections	46 (28-76)	19 (11-34)	435 (273-703)	379 (208-666)	27 (16-44)	19 (12-30)	41 (27-61)	35 (23-55)	38 (25-56)	37 (25-54)	41 (28-60)
Otitis media	4 (3-7)	26 (14-52)	169 (78-367)	292 (130-703)	194 (87-442)	141 (77-269)	64 (37-119)	38 (21-79)	52 (28-105)	31 (16-72)	31 (17-60)
Meningitis	3,808 (2,415-5,044)	6,488 (4,167-8,561)	40,251 (24,305-50,882)	39,432 (20,596-53,479)	11,207 (8,520-14,001)	7,998 (6,248-9,748)	7,090 (5,377-8,709)	5,439 (4,436-6,786)	4,594 (3,441-5,779)	3,931 (3,085-5,022)	3,706 (2,959-4,762)
Pneumococcal meningitis	152 (90-218)	259 (166-366)	1,786 (1,106-2,446)	1,838 (923-2,730)	560 (389-777)	401 (279-574)	345 (231-486)	250 (166-353)	246 (166-341)	275 (182-397)	309 (202-459)
H influenzae type B meningitis	419 (253-573)	704 (431-936)	4,279 (2,425-5,625)	3,827 (1,888-5,428)	912 (636-1,209)	549 (397-731)	427 (303-579)	321 (241-429)	292 (211-394)	266 (198-362)	257 (191-354)
Meningococcal infection	1,544 (998-2,049)	2,765 (1,786-3,558)	17,693 (11,169-22,623)	18,366 (10,083-25,058)	5,616 (4,204-7,171)	3,921 (2,978-4,900)	3,460 (2,674-4,332)	2,583 (2,058-3,286)	2,073 (1,590-2,674)	1,670 (1,302-2,145)	1,507 (1,174-1,973)
Other meningitis	1,694 (1,035-2,340)	2,759 (1,683-3,727)	16,493 (9,529-21,422)	15,402 (7,701-21,938)	4,120 (2,990-5,362)	3,127 (2,376-3,898)	2,857 (2,148-3,558)	2,285 (1,744-2,848)	1,983 (1,488-2,504)	1,721 (1,306-2,212)	1,633 (1,270-2,111)
Encephalitis	359 (217-465)	733 (419-1,000)	7,420 (4,270-10,248)	10,123 (5,431-16,879)	4,220 (2,655-6,471)	2,905 (1,961-4,444)	1,975 (1,394-2,852)	1,780 (1,243-2,537)	1,352 (956-1,957)	1,304 (948-1,965)	1,165 (867-1,745)
Diphtheria	-	-	784 (432-1,408)	1,834 (863-3,664)	276 (155-474)	123 (67-210)	61 (36-96)	33 (19-56)	28 (17-44)	25 (14-41)	34 (21-54)
Whooping cough	-	-	35,334 (16,720-66,164)	36,997 (17,437-69,661)	4,145 (1,866-8,419)	818 (363-1,673)	289 (125-600)	215 (92-448)	131 (55-277)	92 (38-196)	70 (29-150)
Tetanus	21,558 (10,664-36,294)	17,304 (9,659-26,561)	5,696 (3,112-9,862)	2,731 (1,152-6,140)	895 (387-2,035)	1,609 (511-3,331)	1,933 (757-3,741)	1,464 (541-3,198)	1,262 (547-2,762)	1,419 (696-2,945)	1,119 (578-2,506)
Measles	-	-	74,958 (28,060-159,080)	176,178 (72,843-343,623)	30,468 (12,321-61,147)	8,776 (3,145-19,220)	3,260 (1,134-7,740)	1,604 (551-3,816)	809 (281-1,933)	479 (163-1,148)	286 (98-691)
Varicella and herpes zoster	54 (42-69)	178 (138-228)	1,380 (1,060-1,773)	1,731 (1,276-2,278)	807 (592-1,066)	270 (209-352)	115 (90-145)	130 (102-164)	161 (128-201)	179 (144-221)	145 (114-182)
Neglected tropical diseases and malaria	554 (231-820)	11,714 (8,193-16,259)	116,795 (84,875-154,680)	241,041 (179,486-313,537)	35,152 (24,074-46,613)	17,979 (12,879-23,360)	9,021 (6,676-11,835)	8,662 (6,437-11,487)	6,458 (4,736-8,701)	6,023 (4,543-8,085)	6,904 (5,258-9,266)
Malaria	-	11,354 (7,835-15,887)	111,744 (79,574-149,672)	226,219 (163,400-300,809)	28,266 (18,188-39,899)	13,848 (9,123-19,462)	5,874 (3,956-8,273)	5,705 (3,740-8,075)	3,875 (2,577-5,625)	3,287 (2,208-4,823)	3,923 (2,683-5,727)
Chagas disease	-	-	2 (1-2)	1 (1-1)	1 (1-1)	2 (2-2)	6 (6-7)	13 (12-15)	26 (22-29)	44 (39-51)	75 (66-86)
Leishmaniasis	-	-	665 (309-1,289)	953 (406-1,877)	499 (235-1,024)	330 (151-641)	396 (183-768)	432 (201-811)	356 (163-666)	320 (149-601)	388 (178-745)
Visceral leishmaniasis	-	-	665 (309-1,289)	953 (406-1,877)	499 (235-1,024)	330 (151-641)	396 (183-768)	432 (201-811)	356 (163-666)	320 (149-601)	388 (178-745)
African trypanosomiasis	-	-	-	321 (62-1,303)	786 (168-2,438)	877 (229-2,331)	1,044 (257-2,881)	1,044 (253-2,938)	888 (209-2,311)	837 (181-2,331)	772 (153-2,230)
Schistosomiasis	-	-	0 (0-0)	308 (175-537)	134 (82-206)	186 (119-278)	164 (112-235)	193 (131-276)	230 (160-323)	261 (186-372)	321 (225-452)
Cysticercosis	0 (0-0)	0 (0-0)	0 (0-0)	78 (49-116)	46 (33-64)	35 (27-44)	31 (24-39)	37 (29-47)	50 (40-62)	58 (47-71)	48 (39-59)
Cystic echinococcosis	-	-	-	190 (123-291)	74 (49-106)	45 (31-63)	23 (16-31)	25 (17-34)	35 (25-49)	36 (26-49)	44 (32-59)
Dengue	-	-	669 (134-1,653)	1,097 (285-2,433)	629 (145-1,433)	367 (81-894)	259 (50-635)	237 (36-655)	175 (30-459)	150 (22-434)	129 (24-324)
Yellow fever	-	1 (0-3)	20 (4-61)	176 (34-524)	357 (71-1,072)	329 (60-963)	282 (57-876)	222 (43-679)	163 (34-508)	128 (25-402)	105 (21-316)
Rabies	-	-	371 (213-967)	1,951 (818-3,711)	2,927 (997-5,877)	1,433 (420-2,414)	655 (231-1,035)	526 (176-794)	453 (234-835)	709 (283-1,449)	906 (340-1,564)
Intestinal nematode infections	-	-	400 (304-527)	4,392 (3,221-5,848)	660 (500-848)	132 (103-169)	43 (32-55)	26 (19-34)	21 (15-27)	27 (20-35)	25 (18-33)
Ascariasis	-	-	400 (304-527)	4,392 (3,221-5,848)	660 (500-848)	132 (103-169)	43 (32-55)	26 (19-34)	21 (15-27)	27 (20-35)	25 (18-33)
Ebola	0 (0-0)	0 (0-0)	2 (1-2)	15 (11-19)	5 (4-7)	5 (4-6)	7 (5-9)	8 (6-10)	9 (7-12)	14 (11-18)	14 (11-18)
Zika virus	0 (0-0)	0 (0-0)	0 (0-0)	0 (0-0)	0 (0-0)	0 (0-0)	0 (0-0)	0 (0-0)	0 (0-0)	0 (0-0)	0 (0-0)
Other neglected tropical diseases	554 (231-820)	358 (183-528)	2,923 (1,190-4,599)	5,340 (1,435-11,693)	769 (351-1,394)	390 (191-674)	238 (125-406)	195 (98-303)	175 (89-258)	150 (74-222)	153 (81-217)
Maternal disorders	-	-	-	-	-	1,521 (1,281-1,791)	34,773 (31,200-38,086)	67,347 (61,023-72,331)	65,674 (60,228-70,061)	58,467 (53,912-62,663)	49,140 (45,376-52,745)
Maternal haemorrhage	-	-	-	-	-	313 (209-446)	9,648 (6,562-12,999)	21,746 (16,338-27,577)	22,219 (16,154-28,571)	20,039 (14,885-25,275)	16,922 (12,434-21,860)
Maternal sepsis and other pregnancy related infections	-	-	-	-	-	160 (101-230)	4,085 (2,567-6,119)	6,478 (3,937-9,876)	5,872 (3,880-8,431)	5,038 (3,220-7,295)	4,200 (2,774-6,127)
Maternal hypertensive disorders	-	-	-	-	-	285 (172-414)	5,034 (3,180-7,056)	8,683 (5,940-12,105)	7,856 (5,162-11,370)	6,843 (4,287-10,237)	5,913 (3,962-8,493)
Maternal obstructed labour and uterine rupture	-	-	-	-	-	56 (28-100)	1,064 (534-1,825)	2,166 (1,247-3,445)	2,415 (1,374-3,859)	2,485 (1,414-3,838)	2,417 (1,411-3,995)
Maternal abortion, miscarriage, and ectopic pregnancy	-	-	-	-	-	113 (67-186)	2,668 (1,610-4,065)	5,088 (3,188-7,698)	4,946 (3,287-7,393)	4,596 (3,020-6,649)	4,166 (2,586-5,966)
Indirect maternal deaths	-	-	-	-	-	237 (152-343)	4,870 (2,990-7,274)	9,701 (6,482-14,128)	10,154 (6,569-14,418)	8,812 (5,344-12,757)	6,445 (4,193-9,272)
Late maternal deaths	-	-	-	-	-	36 (16-73)	654 (329-1,192)	1,188 (575-2,224)	1,149 (562-2,155)	1,032 (545-1,865)	871 (428-1,526)
Maternal deaths aggravated by HIV/AIDS	-	-	-	-	-	1 (0-1)	81 (46-117)	354 (215-483)	559 (359-729)	524 (327-696)	407 (255-553)
Other maternal disorders	-	-	-	-	-	319 (213-468)	6,670 (4,464-9,893)	11,943 (7,866-17,115)	10,505 (7,146-14,545)	9,096 (5,957-12,967)	7,798 (5,120-11,476)
Neonatal disorders	845,331 (810,376-886,011)	184,893 (172,955-201,431)	49,087 (41,467-59,745)	5,014 (4,042-6,538)	-	-	-	-	-	-	-
Neonatal preterm birth complications	346,130 (314,567-392,865)	58,425 (50,872-74,121)	18,295 (15,289-24,907)	1,358 (1,077-1,838)	-	-	-	-	-	-	-
Neonatal encephalopathy due to birth asphyxia and trauma	260,025 (231,317-288,456)	27,580 (23,100-33,145)	9,554 (7,276-12,907)	1,964 (1,537-2,670)	-	-	-	-	-	-	-
Neonatal sepsis and other neonatal infections	62,489 (35,117-93,926)	45,968 (29,226-59,905)	7,551 (4,615-11,056)	364 (100-708)	-	-	-	-	-	-	-
Hemolytic disease and other neonatal jaundice	25,565 (20,100-32,010)	15,852 (12,064-20,485)	3,386 (2,427-4,900)	193 (130-292)	-	-	-	-	-	-	-
Other neonatal disorders	151,122 (128,937-178,933)	37,068 (30,929-45,791)	10,301 (7,967-14,190)	1,134 (837-1,725)	-	-	-	-	-	-	-
Nutritional deficiencies	-	-	74,071 (60,544-87,887)	83,667 (63,745-106,522)	7,013 (5,553-8,599)	3,211 (2,653-3,811)	2,664 (2,287-3,051)	2,835 (2,469-3,241)	3,204 (2,740-3,679)	3,066 (2,562-3,597)	2,860 (2,389-3,343)
Protein-energy malnutrition	-	-	71,521 (57,685-85,423)	79,975 (60,468-102,575)	5,867 (4,532-7,297)	2,519 (2,020-3,051)	1,721 (1,403-2,068)	1,491 (1,199-1,801)	1,970 (1,551-2,408)	1,850 (1,378-2,320)	1,741 (1,315-2,198)
Iodine deficiency	-	-	246 (60-727)	196 (30-740)	68 (15-193)	19 (4-60)	33 (9-94)	38 (8-106)	40 (10-113)	43 (11-114)	42 (11-126)
Iron-deficiency anaemia	-	-	66 (33-139)	54 (31-127)	83 (39-139)	71 (43-123)	78 (48-142)	80 (52-135)	69 (43-103)	63 (47-81)	70 (47-102)
Other nutritional deficiencies	-	-	2,238 (1,572-2,958)	3,443 (1,553-5,562)	995 (722-1,334)	603 (469-773)	832 (684-987)	1,226 (1,048-1,422)	1,126 (951-1,317)	1,109 (938-1,289)	1,008 (846-1,171)
Other communicable, maternal, neonatal, and nutritional diseases	21,491 (12,797-33,960)	8,702 (5,488-12,989)	34,310 (22,934-49,337)	29,658 (20,298-41,859)	10,945 (7,835-15,338)	3,608 (3,008-4,106)	5,129 (4,567-5,641)	5,566 (5,039-6,079)	4,668 (4,244-5,061)	4,559 (4,157-4,958)	4,754 (4,361-5,192)
Sexually transmitted diseases excluding HIV	19,908 (11,252-32,295)	7,222 (4,007-11,576)	24,848 (13,474-40,028)	17,997 (9,602-29,733)	6,238 (3,438-10,271)	103 (69-136)	589 (431-737)	724 (521-877)	883 (639-1,080)	689 (500-822)	725 (550-848)
Syphilis	19,908 (11,252-32,295)	7,222 (4,007-11,576)	24,848 (13,474-40,028)	17,997 (9,602-29,733)	6,238 (3,438-10,271)	56 (37-74)	166 (122-208)	203 (146-246)	181 (131-221)	142 (103-169)	89 (67-104)
Chlamydial infection	-	-	-	-	-	13 (9-17)	77 (56-96)	157 (113-190)	148 (107-181)	72 (53-86)	117 (88-136)
Gonococcal infection	-	-	-	-	-	23 (15-30)	188 (138-235)	226 (163-274)	384 (278-470)	309 (224-368)	321 (243-375)
Other sexually transmitted diseases	-	-	-	-	-	11 (7-14)	158 (116-198)	139 (100-168)	170 (123-207)	166 (120-198)	199 (151-232)
Hepatitis	-	-	5,366 (4,659-6,104)	7,308 (5,782-8,865)	3,184 (2,512-3,899)	2,624 (2,137-3,028)	3,640 (3,194-4,061)	4,111 (3,674-4,540)	3,161 (2,841-3,474)	3,185 (2,880-3,520)	3,441 (3,096-3,798)
Acute hepatitis A	-	-	3,430 (2,449-4,460)	838 (457-1,314)	75 (43-119)	43 (26-63)	65 (40-95)	75 (39-125)	44 (21-78)	36 (17-64)	37 (17-64)
Hepatitis B	-	-	1,936 (1,082-2,937)	928 (525-1,467)	262 (139-439)	392 (208-646)	1,285 (852-1,823)	2,594 (1,889-3,308)	2,495 (2,069-2,928)	2,774 (2,406-3,170)	3,099 (2,717-3,503)
Hepatitis C	-	-	-	-	88 (58-131)	31 (20-47)	44 (27-66)	51 (27-84)	33 (15-59)	29 (13-53)	38 (17-70)
Acute hepatitis E	-	-	-	5,542 (4,229-6,961)	2,759 (2,133-3,415)	2,158 (1,715-2,536)	2,245 (1,731-2,714)	1,391 (804-2,049)	589 (298-971)	345 (166-575)	267 (127-478)
Other infectious diseases	1,583 (958-1,916)	1,480 (840-2,037)	4,095 (3,027-4,905)	4,353 (2,814-6,070)	1,523 (1,106-1,948)	881 (657-1,075)	901 (687-1,063)	730 (558-853)	624 (480-720)	686 (535-794)	588 (484-709)
Non-communicable diseases	88,410 (65,317-112,647)	37,980 (30,179-48,120)	154,965 (125,786-191,430)	112,917 (86,126-141,841)	48,964 (43,744-54,704)	55,027 (50,898-59,406)	82,099 (77,327-86,193)	96,464 (92,178-100,051)	121,893 (117,145-125,976)	165,044 (158,894-169,993)	228,105 (221,111-234,572)
Neoplasms	416 (369-449)	258 (232-284)	3,559 (3,019-4,118)	12,121 (10,411-14,589)	12,957 (11,512-14,480)	14,518 (13,119-15,785)	17,334 (16,013-18,313)	20,600 (19,376-21,620)	31,987 (30,286-33,405)	54,142 (51,595-56,349)	84,964 (81,175-88,569)
Lip and oral cavity cancer	-	-	-	-	-	-	147 (132-164)	261 (231-296)	383 (341-430)	649 (583-737)	1,012 (905-1,152)
Nasopharynx cancer	-	-	-	-	54 (48-62)	107 (92-129)	214 (191-242)	267 (240-294)	406 (367-444)	625 (574-683)	843 (780-912)
Other pharynx cancer	-	-	-	-	-	-	183 (157-213)	232 (200-265)	341 (302-378)	496 (437-550)	737 (648-825)
Oesophageal cancer	-	-	-	-	-	-	175 (161-189)	330 (305-352)	520 (479-570)	859 (797-923)	1,474 (1,375-1,579)
Stomach cancer	-	-	-	-	-	-	602 (558-647)	1,419 (1,318-1,511)	2,954 (2,777-3,138)	5,631 (5,328-5,923)	8,361 (7,930-8,781)
Colon and rectum cancer	-	-	-	-	-	-	373 (335-412)	870 (784-958)	1,662 (1,515-1,808)	3,339 (3,084-3,602)	4,816 (4,496-5,149)
Liver cancer	-	-	-	-	213 (163-257)	324 (244-372)	500 (371-567)	881 (624-1,012)	1,904 (1,303-2,197)	3,536 (2,419-3,967)	4,958 (3,443-5,535)
Liver cancer due to hepatitis B	-	-	-	-	88 (58-119)	175 (119-218)	290 (198-357)	604 (414-732)	1,421 (964-1,717)	2,635 (1,764-3,086)	3,468 (2,347-4,079)
Liver cancer due to hepatitis C	-	-	-	-	9 (7-12)	16 (12-22)	28 (20-36)	60 (44-81)	130 (82-192)	255 (169-350)	413 (283-572)
Liver cancer due to alcohol use	-	-	-	-	-	-	4 (2-6)	20 (12-31)	68 (34-118)	237 (142-375)	569 (308-964)
Liver cancer due to other causes	-	-	-	-	116 (88-149)	133 (98-171)	178 (126-231)	196 (140-258)	284 (182-420)	410 (270-577)	508 (344-722)
Gallbladder and biliary tract cancer	-	-	-	-	-	-	47 (42-52)	82 (73-92)	188 (162-213)	472 (411-533)	976 (843-1,117)
Pancreatic cancer	-	-	-	-	-	-	73 (67-79)	132 (121-143)	285 (263-312)	534 (502-574)	1,080 (1,023-1,152)
Larynx cancer	-	-	-	-	-	-	61 (56-67)	97 (87-109)	120 (104-138)	235 (204-273)	372 (339-407)
Tracheal, bronchus, and lung cancer	-	-	-	-	-	-	340 (268-374)	603 (507-671)	1,405 (1,154-1,560)	3,096 (2,677-3,374)	5,584 (5,174-5,989)
Malignant skin melanoma	-	-	-	-	-	-	110 (90-131)	199 (168-250)	330 (269-403)	505 (415-609)	779 (633-951)
Non-melanoma skin cancer	-	-	-	-	-	-	67 (62-73)	80 (73-89)	125 (113-139)	167 (153-183)	223 (203-243)
Non-melanoma skin cancer (squamous-cell carcinoma)	-	-	-	-	-	-	67 (62-73)	80 (73-89)	125 (113-139)	167 (153-183)	223 (203-243)
Breast cancer	-	-	-	-	-	-	336 (247-389)	971 (792-1,136)	3,211 (2,638-3,653)	9,391 (8,241-10,529)	19,154 (17,342-21,303)
Cervical cancer	-	-	-	-	-	-	249 (184-277)	929 (697-1,072)	2,831 (2,364-3,158)	6,827 (5,779-7,717)	12,417 (10,736-13,987)
Uterine cancer	-	-	-	-	-	-	100 (90-111)	243 (215-266)	480 (429-526)	852 (771-920)	1,530 (1,374-1,647)
Ovarian cancer	-	-	-	-	-	-	513 (462-570)	687 (628-775)	1,059 (959-1,182)	1,703 (1,567-1,878)	3,029 (2,802-3,336)
Prostate cancer	-	-	-	-	-	-	-	-	-	-	-
Testicular cancer	-	-	-	-	-	-	-	-	-	-	-
Kidney cancer	15 (14-16)	7 (6-8)	109 (94-132)	503 (410-628)	307 (275-346)	160 (148-174)	102 (95-110)	123 (114-133)	176 (163-190)	241 (227-258)	393 (372-419)
Bladder cancer	-	-	-	-	-	-	48 (43-53)	61 (56-68)	103 (94-113)	193 (174-213)	320 (290-352)
Brain and nervous system cancer	56 (39-67)	35 (25-42)	543 (336-679)	2,099 (1,491-2,583)	2,965 (2,302-3,343)	2,731 (2,181-3,037)	1,887 (1,473-2,107)	1,836 (1,422-2,032)	2,301 (1,799-2,562)	2,861 (2,273-3,147)	3,545 (2,850-3,850)
Thyroid cancer	-	-	-	-	-	81 (67-97)	204 (160-247)	317 (260-367)	380 (321-432)	468 (401-530)	567 (484-641)
Mesothelioma	-	-	-	-	-	-	49 (38-67)	65 (47-90)	106 (78-143)	116 (90-156)	185 (148-243)
Hodgkin lymphoma	2 (1-2)	1 (1-1)	30 (13-41)	130 (56-194)	286 (144-406)	472 (246-616)	706 (441-836)	794 (547-965)	827 (565-992)	755 (532-867)	740 (503-854)
Non-Hodgkin's lymphoma	23 (18-26)	17 (15-20)	281 (211-358)	1,041 (800-1,384)	1,216 (940-1,459)	1,207 (969-1,397)	1,153 (993-1,274)	1,288 (1,138-1,434)	1,502 (1,312-1,672)	1,700 (1,470-1,866)	2,019 (1,806-2,190)
Multiple myeloma	-	-	-	-	-	-	128 (93-151)	154 (112-187)	161 (116-189)	182 (139-211)	332 (255-380)
Leukaemia	105 (75-123)	80 (59-97)	1,716 (1,116-2,111)	5,666 (3,939-7,275)	5,705 (4,414-6,906)	6,409 (5,240-7,773)	5,549 (4,344-6,421)	4,518 (3,449-5,098)	4,716 (3,572-5,347)	4,765 (3,657-5,467)	5,045 (3,884-5,672)
Acute lymphoid leukaemia	28 (21-39)	22 (17-36)	435 (318-685)	1,868 (1,432-3,089)	2,217 (1,813-3,115)	2,165 (1,804-3,084)	1,429 (1,191-2,041)	932 (773-1,320)	909 (748-1,307)	690 (570-1,015)	750 (620-1,078)
Chronic lymphoid leukaemia	-	-	-	-	-	-	117 (87-186)	115 (82-176)	145 (108-239)	167 (129-252)	229 (177-346)
Acute myeloid leukaemia	16 (12-22)	13 (10-18)	218 (157-306)	835 (601-1,261)	736 (553-941)	1,029 (824-1,363)	1,022 (837-1,294)	836 (684-1,069)	836 (677-1,075)	857 (724-1,068)	1,063 (872-1,304)
Chronic myeloid leukaemia	-	-	-	-	-	-	271 (183-369)	279 (189-379)	426 (300-569)	448 (328-585)	490 (368-615)
Other leukaemia	60 (37-75)	44 (29-57)	1,062 (570-1,416)	2,963 (1,653-4,182)	2,752 (1,817-3,422)	3,215 (2,253-3,849)	2,710 (1,817-3,215)	2,356 (1,543-2,817)	2,400 (1,547-2,900)	2,602 (1,752-3,002)	2,514 (1,719-2,875)
Other neoplasms	215 (194-235)	118 (105-133)	881 (741-1,144)	2,681 (2,238-3,720)	2,213 (1,952-2,556)	3,027 (2,740-3,358)	3,420 (3,131-3,710)	3,162 (2,891-3,416)	3,510 (3,204-3,799)	3,944 (3,561-4,253)	4,472 (4,140-4,783)
Cardiovascular diseases	1,913 (1,762-2,076)	1,195 (1,063-1,358)	12,023 (10,727-13,329)	10,383 (9,009-11,743)	6,248 (5,541-6,933)	8,269 (7,545-8,952)	19,245 (17,793-20,475)	26,311 (24,548-27,740)	35,507 (33,532-37,096)	49,672 (47,013-51,802)	70,517 (67,257-73,530)
Rheumatic heart disease	-	-	-	2,752 (2,143-3,544)	1,836 (1,482-2,233)	3,213 (2,740-3,691)	5,298 (4,367-6,151)	5,481 (4,517-6,476)	6,285 (5,124-7,504)	7,253 (5,833-8,642)	8,667 (7,057-10,323)
Ischaemic heart disease	-	-	753 (604-935)	574 (438-824)	478 (390-609)	580 (497-698)	5,913 (5,287-6,839)	8,484 (7,781-9,577)	12,079 (11,177-13,458)	18,968 (17,605-20,881)	26,788 (25,025-29,184)
Cerebrovascular disease	782 (677-924)	466 (382-573)	4,374 (3,556-5,386)	2,983 (2,341-4,008)	1,892 (1,647-2,232)	2,181 (1,948-2,483)	3,928 (3,591-4,386)	7,115 (6,518-7,965)	10,131 (9,338-11,216)	14,988 (13,918-16,383)	23,790 (22,312-25,749)

Cause	40 to 44	45 to 49	50 to 54	55 to 59	60 to 64	65 to 69	70 to 74	75 to 79	80 to 84	85 to 89	90 to 94	95 plus
All causes	597,505 (588,186-607,617)	724,248 (713,188-735,424)	848,078 (836,145-860,233)	1,011,972 (997,492-1,026,842)	1,425,049 (1,405,984-1,444,873)	1,830,629 (1,807,392-1,854,728)	2,311,248 (2,280,212-2,341,571)	2,494,636 (2,465,741-2,522,391)	2,285,266 (2,264,009-2,305,681)	2,032,138 (2,016,986-2,046,401)	1,127,024 (1,121,121-1,132,461)	412,656 (410,904-414,274)
Communicable, maternal, neonatal, and nutritional disorders	193,798 (184,783-205,386)	169,642 (158,872-185,127)	156,367 (143,393-177,641)	162,433 (144,461-193,143)	194,476 (166,614-241,890)	232,742 (196,901-296,675)	276,589 (229,002-354,341)	263,154 (217,648-336,094)	250,335 (208,082-308,411)	191,423 (161,325-227,448)	97,323 (84,669-112,147)	40,537 (35,980-45,521)
HIV/AIDS and tuberculosis	112,709 (105,901-119,571)	97,353 (90,959-104,415)	83,125 (77,425-88,954)	74,313 (67,475-80,900)	70,657 (62,442-77,873)	71,092 (61,649-79,521)	68,006 (57,965-77,385)	52,241 (44,744-59,358)	39,091 (32,563-46,138)	20,890 (17,375-24,680)	6,858 (5,723-8,285)	1,778 (1,466-2,169)
Tuberculosis	36,665 (33,038-40,210)	40,336 (35,859-44,692)	45,163 (39,943-50,251)	51,365 (44,764-57,282)	55,050 (47,185-61,963)	60,165 (50,871-68,405)	62,149 (51,824-71,252)	49,568 (42,050-56,700)	38,320 (31,814-45,394)	20,585 (17,076-24,352)	6,774 (5,645-8,210)	1,758 (1,446-2,149)
Drug-susceptible tuberculosis	33,410 (30,031-36,696)	36,825 (32,553-41,021)	41,324 (36,475-46,027)	47,061 (40,770-52,664)	50,332 (43,168-56,880)	55,048 (46,594-62,895)	56,864 (47,597-65,581)	45,415 (38,427-52,152)	35,070 (29,138-41,486)	18,890 (15,684-22,539)	6,253 (5,206-7,565)	1,628 (1,336-1,988)
Multidrug-resistant tuberculosis without extensive drug resistance	3,178 (2,579-3,822)	3,436 (2,751-4,220)	3,766 (2,988-4,648)	4,231 (3,329-5,296)	4,625 (3,613-5,801)	5,020 (3,878-6,387)	5,185 (3,989-6,640)	4,072 (3,134-5,178)	3,185 (2,393-4,158)	1,657 (1,259-2,168)	509 (382-688)	126 (92-178)
Extensively drug-resistant tuberculosis	76 (60-93)	76 (60-92)	73 (59-89)	74 (59-91)	93 (73-115)	97 (75-121)	101 (78-126)	82 (63-103)	65 (49-85)	38 (29-50)	13 (10-17)	3 (3-5)
HIV/AIDS	76,044 (69,982-82,139)	57,017 (51,996-62,670)	37,962 (35,015-41,328)	22,948 (20,524-25,697)	15,608 (13,752-17,642)	10,927 (9,595-12,374)	5,857 (4,998-6,710)	2,673 (2,224-3,129)	771 (617-949)	305 (246-374)	84 (69-102)	20 (17-23)
Drug-susceptible HIV/AIDS - Tuberculosis	18,915 (12,556-25,293)	15,851 (10,933-20,477)	11,819 (8,604-14,522)	7,575 (5,851-9,231)	5,260 (4,155-6,407)	3,740 (2,997-4,501)	2,019 (1,575-2,452)	926 (713-1,133)	268 (202-343)	103 (78-133)	27 (21-35)	5 (4-7)
Multidrug-resistant HIV/AIDS - Tuberculosis without extensive drug resistance	1,348 (811-2,038)	1,145 (711-1,722)	855 (542-1,273)	540 (343-812)	371 (240-553)	260 (168-387)	139 (87-207)	64 (40-97)	20 (12-30)	8 (5-12)	2 (1-3)	0 (0-1)
Extensively drug-resistant HIV/AIDS - Tuberculosis	12 (8-19)	10 (6-14)	5 (4-8)	3 (2-5)	2 (2-3)	1 (1-2)	1 (0-1)	0 (0-0)	0 (0-0)	0 (0-0)	0 (0-0)	0 (0-0)
HIV/AIDS resulting in other diseases	55,770 (47,364-65,050)	40,011 (34,014-47,228)	25,282 (21,881-29,917)	14,830 (12,659-17,820)	9,975 (8,393-12,044)	6,925 (5,921-8,308)	3,698 (3,044-4,485)	1,682 (1,343-2,082)	483 (382-606)	194 (155-241)	55 (44-66)	14 (12-16)
Diarrhoea, lower respiratory infections, and other common infectious diseases	34,754 (27,067-49,154)	42,182 (31,981-61,893)	53,606 (39,966-80,402)	69,325 (50,521-105,374)	101,577 (72,474-158,239)	137,189 (99,182-210,356)	184,437 (133,759-273,043)	186,477 (138,262-266,880)	190,668 (147,004-255,023)	153,165 (122,962-193,282)	80,007 (67,451-95,941)	33,534 (29,251-38,626)
Diarrhoeal diseases	14,449 (6,345-30,096)	18,774 (8,248-39,667)	24,933 (10,730-52,845)	35,320 (16,194-73,689)	54,960 (23,882-117,194)	74,763 (33,358-153,525)	102,502 (46,331-197,475)	99,298 (46,435-188,429)	98,284 (50,295-167,849)	66,218 (36,233-106,862)	27,474 (15,331-42,749)	8,253 (4,946-12,517)
Intestinal infectious diseases	1,960 (1,063-3,220)	1,720 (940-2,857)	1,307 (679-2,263)	1,045 (500-1,908)	948 (461-1,692)	886 (487-1,440)	709 (398-1,148)	513 (289-844)	293 (165-460)	141 (80-239)	46 (25-82)	14 (7-33)
Typhoid fever	1,437 (749-2,457)	1,231 (627-2,112)	954 (481-1,679)	777 (361-1,419)	730 (345-1,316)	716 (379-1,203)	578 (321-926)	402 (227-649)	231 (128-369)	102 (57-162)	30 (17-49)	7 (4-12)
Paratyphoid fever	488 (224-957)	450 (202-886)	337 (146-674)	250 (102-516)	196 (81-406)	144 (66-287)	99 (45-188)	66 (30-121)	38 (18-70)	17 (8-32)	5 (2-10)	1 (1-2)
Other intestinal infectious diseases	35 (3-163)	39 (4-185)	16 (1-65)	17 (1-71)	22 (2-97)	26 (2-121)	32 (3-168)	45 (5-193)	24 (3-108)	23 (3-88)	11 (1-38)	6 (1-25)
Lower respiratory infections	11,762 (9,689-13,735)	14,098 (11,794-16,390)	19,464 (16,195-22,769)	25,013 (20,064-28,604)	37,596 (29,720-43,128)	51,793 (41,670-59,752)	70,960 (57,001-81,235)	78,756 (63,892-89,759)	85,472 (71,942-98,512)	82,999 (72,509-94,065)	50,933 (44,588-58,077)	24,705 (21,379-27,854)
Upper respiratory infections	42 (28-61)	52 (35-73)	52 (36-76)	56 (38-78)	68 (46-98)	66 (46-95)	58 (40-83)	59 (40-82)	40 (29-56)	31 (22-44)	14 (10-19)	4 (2-5)
Otitis media	52 (28-107)	35 (20-61)	31 (20-51)	90 (49-167)	79 (45-146)	135 (77-240)	100 (61-165)	69 (45-108)	47 (30-76)	24 (16-36)	11 (7-16)	4 (3-5)
Meningitis	3,388 (2,740-4,437)	4,012 (3,208-5,093)	4,051 (3,252-5,322)	4,345 (3,473-5,448)	4,361 (3,536-5,796)	4,771 (3,869-6,288)	5,302 (4,287-6,778)	3,336 (2,714-4,282)	3,064 (2,432-4,008)	1,588 (1,280-2,086)	584 (478-735)	182 (153-219)
Pneumococcal meningitis	331 (214-529)	421 (263-654)	421 (288-629)	470 (327-659)	488 (347-682)	525 (373-754)	595 (407-839)	390 (283-541)	320 (223-461)	172 (132-223)	70 (56-88)	23 (17-31)
H influenzae type B meningitis	236 (172-327)	282 (200-388)	278 (203-395)	300 (218-404)	302 (222-415)	318 (228-427)	355 (268-472)	229 (169-312)	206 (152-273)	108 (81-148)	39 (28-53)	12 (8-18)
Meningococcal infection	1,424 (1,134-1,830)	1,661 (1,345-2,100)	1,691 (1,308-2,282)	1,820 (1,444-2,378)	1,845 (1,459-2,504)	1,965 (1,522-2,664)	2,059 (1,608-2,753)	1,365 (1,058-1,834)	1,228 (932-1,647)	671 (521-926)	252 (195-329)	77 (58-98)
Other meningitis	1,397 (1,100-1,817)	1,648 (1,270-2,154)	1,661 (1,309-2,200)	1,755 (1,352-2,237)	1,726 (1,311-2,305)	1,963 (1,542-2,598)	2,292 (1,760-2,964)	1,351 (1,055-1,759)	1,309 (1,002-1,697)	636 (504-828)	223 (179-286)	70 (53-90)
Encephalitis	1,283 (923-1,851)	1,454 (1,070-2,136)	1,383 (1,072-2,047)	1,622 (1,243-2,481)	2,119 (1,582-3,200)	2,332 (1,705-3,481)	2,517 (1,772-3,577)	2,098 (1,495-2,801)	1,781 (1,254-2,473)	1,010 (729-1,330)	320 (238-439)	92 (70-120)
Diphtheria	36 (22-57)	42 (25-64)	49 (29-80)	39 (23-61)	-	-	-	-	-	-	-	-
Whooping cough	60 (24-128)	56 (23-120)	55 (23-116)	56 (24-119)	-	-	-	-	-	-	-	-
Tetanus	1,446 (780-2,968)	1,712 (966-3,762)	2,109 (993-4,511)	1,568 (773-3,480)	1,312 (671-2,901)	2,180 (1,119-4,666)	2,037 (937-4,084)	1,937 (898-3,787)	1,067 (464-1,983)	406 (161-671)	121 (48-204)	33 (13-62)
Measles	182 (62-433)	114 (39-271)	82 (28-194)	63 (22-148)	-	-	-	-	-	-	-	-
Varicella and herpes zoster	95 (75-121)	113 (90-143)	91 (71-116)	109 (86-138)	134 (106-170)	262 (206-332)	251 (197-320)	410 (321-527)	620 (484-794)	747 (567-996)	504 (353-677)	249 (161-364)
Neglected tropical diseases and malaria	6,775 (5,129-9,078)	6,796 (5,250-9,181)	7,601 (5,761-10,200)	7,574 (5,671-10,203)	9,471 (6,969-12,889)	9,028 (6,850-12,244)	7,695 (5,587-10,571)	5,934 (4,392-8,059)	3,421 (2,533-4,523)	2,079 (1,483-2,840)	470 (329-611)	242 (137-468)
Malaria	3,878 (2,593-5,626)	3,639 (2,401-5,447)	4,134 (2,667-6,095)	4,367 (2,920-6,334)	6,490 (4,200-9,753)	5,714 (3,622-8,625)	5,048 (3,120-7,695)	3,853 (2,477-5,790)	2,295 (1,492-3,411)	1,431 (878-2,174)	268 (142-405)	183 (81-408)
Chagas disease	117 (105-133)	171 (153-192)	238 (212-266)	304 (269-345)	356 (321-396)	385 (345-426)	406 (370-444)	360 (327-397)	280 (255-310)	168 (152-184)	71 (65-78)	17 (15-18)
Leishmaniasis	409 (186-778)	399 (181-765)	409 (192-761)	342 (162-650)	316 (143-590)	391 (180-751)	361 (165-687)	301 (137-560)	176 (79-326)	118 (47-223)	21 (6-39)	8 (1-17)
Visceral leishmaniasis	409 (186-778)	399 (181-765)	409 (192-761)	342 (162-650)	316 (143-590)	391 (180-751)	361 (165-687)	301 (137-560)	176 (79-326)	118 (47-223)	21 (6-39)	8 (1-17)
African trypanosomiasis	668 (152-1,941)	635 (133-2,001)	593 (120-1,958)	561 (121-2,001)	450 (85-1,770)	393 (58-1,542)	250 (50-970)	155 (26-655)	72 (12-309)	30 (4-115)	7 (1-29)	1 (0-6)
Schistosomiasis	362 (254-517)	429 (312-596)	547 (382-775)	656 (468-909)	688 (499-968)	768 (552-1,064)	685 (492-956)	507 (372-669)	265 (194-356)	121 (87-161)	32 (21-46)	6 (3-9)
Cysticercosis	50 (42-61)	47 (39-57)	58 (48-69)	56 (47-68)	45 (37-54)	44 (37-54)	28 (23-34)	19 (15-24)	11 (8-15)	5 (4-6)	2 (1-2)	1 (0-1)
Cystic echinococcosis	41 (30-54)	44 (33-59)	47 (35-60)	51 (39-68)	55 (42-71)	59 (45-76)	51 (40-65)	45 (35-56)	27 (21-35)	19 (15-24)	8 (5-11)	2 (1-2)
Dengue	130 (21-344)	155 (25-423)	159 (26-432)	182 (30-471)	178 (31-474)	167 (29-417)	74 (16-163)	64 (15-140)	49 (12-106)	34 (8-75)	16 (5-32)	3 (1-7)
Yellow fever	87 (17-283)	75 (15-227)	66 (13-215)	56 (11-167)	43 (8-133)	32 (6-99)	21 (4-64)	12 (2-38)	5 (1-15)	2 (0-6)	0 (0-1)	0 (0-0)
Rabies	834 (359-1,519)	995 (444-1,798)	1,106 (603-2,665)	764 (368-1,647)	579 (238-968)	787 (306-1,362)	490 (286-1,128)	374 (199-1,058)	83 (27-170)	66 (19-143)	12 (3-28)	6 (1-14)
Intestinal nematode infections	18 (14-24)	22 (16-29)	32 (24-42)	29 (21-36)	36 (27-46)	29 (22-38)	43 (33-55)	34 (26-43)	22 (16-28)	8 (6-11)	6 (4-8)	2 (1-3)
Ascariasis	18 (14-24)	22 (16-29)	32 (24-42)	29 (21-36)	36 (27-46)	29 (22-38)	43 (33-55)	34 (26-43)	22 (16-28)	8 (6-11)	6 (4-8)	2 (1-3)
Ebola	13 (10-17)	15 (11-18)	6 (5-8)	8 (6-11)	5 (4-7)	4 (3-5)	2 (1-2)	0 (0-1)	0 (0-0)	0 (0-0)	0 (0-0)	0 (0-0)
Zika virus	0 (0-0)	0 (0-0)	0 (0-0)	0 (0-0)	0 (0-0)	0 (0-0)	0 (0-0)	0 (0-0)	0 (0-0)	0 (0-0)	0 (0-0)	0 (0-0)
Other neglected tropical diseases	166 (85-232)	170 (89-244)	207 (106-295)	199 (103-288)	231 (112-332)	255 (141-355)	237 (118-355)	210 (118-322)	137 (81-228)	77 (49-133)	28 (18-47)	13 (9-24)
Maternal disorders	31,239 (28,711-33,953)	15,916 (14,285-17,668)	2,895 (2,388-3,521)	-	-	-	-	-	-	-	-	-
Maternal haemorrhage	9,868 (6,915-13,254)	4,009 (2,795-5,489)	549 (331-869)	-	-	-	-	-	-	-	-	-
Maternal sepsis and other pregnancy related infections	2,814 (1,762-4,320)	1,565 (979-2,420)	279 (162-461)	-	-	-	-	-	-	-	-	-
Maternal hypertensive disorders	3,703 (2,274-5,635)	1,712 (1,073-2,576)	294 (153-494)	-	-	-	-	-	-	-	-	-
Maternal obstructed labour and uterine rupture	1,777 (999-3,000)	1,006 (533-1,658)	179 (79-342)	-	-	-	-	-	-	-	-	-
Maternal abortion, miscarriage, and ectopic pregnancy	3,111 (2,087-4,458)	2,005 (1,346-2,848)	310 (200-462)	-	-	-	-	-	-	-	-	-
Indirect maternal deaths	4,077 (2,456-6,088)	2,493 (1,481-3,690)	621 (351-987)	-	-	-	-	-	-	-	-	-
Late maternal deaths	561 (214-1,003)	302 (117-547)	64 (23-134)	-	-	-	-	-	-	-	-	-
Maternal deaths aggravated by HIV/AIDS	225 (135-308)	103 (61-145)	15 (8-23)	-	-	-	-	-	-	-	-	-
Other maternal disorders	5,102 (3,130-7,611)	2,720 (1,746-3,976)	584 (365-859)	-	-	-	-	-	-	-	-	-
Neonatal disorders	-	-	-	-	-	-	-	-	-	-	-	-
Neonatal preterm birth complications	-	-	-	-	-	-	-	-	-	-	-	-
Neonatal encephalopathy due to birth asphyxia and trauma	-	-	-	-	-	-	-	-	-	-	-	-
Neonatal sepsis and other neonatal infections	-	-	-	-	-	-	-	-	-	-	-	-
Hemolytic disease and other neonatal jaundice	-	-	-	-	-	-	-	-	-	-	-	-
Other neonatal disorders	-	-	-	-	-	-	-	-	-	-	-	-
Nutritional deficiencies	3,566 (2,990-4,183)	2,858 (2,451-3,281)	3,937 (3,223-4,657)	5,218 (4,203-6,197)	6,631 (5,304-8,003)	8,625 (7,127-10,172)	9,743 (7,924-11,635)	12,722 (10,505-14,985)	13,127 (10,819-15,417)	12,886 (10,805-15,210)	8,987 (7,484-10,532)	4,649 (3,816-5,568)
Protein-energy malnutrition	2,437 (1,912-3,024)	1,600 (1,237-1,974)	2,383 (1,720-3,079)	3,706 (2,810-4,605)	4,837 (3,620-6,119)	6,134 (4,790-7,613)	7,512 (5,809-9,265)	10,273 (8,182-12,411)	10,782 (8,643-13,046)	10,890 (8,847-13,095)	7,807 (6,298-9,351)	4,100 (3,264-5,019)
Iodine deficiency	35 (11-90)	33 (8-106)	47 (11-164)	56 (16-171)	57 (18-156)	89 (27-255)	89 (30-208)	82 (30-180)	58 (20-133)	35 (11-84)	13 (2-48)	7 (1-24)
Iron-deficiency anaemia	63 (49-118)	53 (43-87)	62 (45-162)	54 (39-88)	58 (41-93)	65 (45-110)	134 (80-266)	154 (90-320)	135 (82-244)	94 (44-166)	55 (27-89)	54 (33-80)
Other nutritional deficiencies	1,030 (875-1,194)	1,172 (992-1,363)	1,445 (1,183-1,706)	1,401 (1,136-1,680)	1,680 (1,375-2,038)	2,338 (1,860-2,903)	2,008 (1,623-2,445)	2,214 (1,813-2,772)	2,152 (1,754-2,818)	1,867 (1,521-2,809)	1,112 (874-1,907)	488 (372-1,005)
Other communicable, maternal, neonatal, and nutritional diseases	4,755 (4,390-5,122)	4,537 (4,222-4,869)	5,203 (4,783-5,665)	6,003 (5,502-6,521)	6,139 (5,611-6,776)	6,808 (6,142-7,499)	6,708 (6,055-7,462)	5,779 (5,182-6,407)	4,029 (3,540-4,735)	2,403 (2,140-2,786)	999 (875-1,129)	334 (283-372)
Sexually transmitted diseases excluding HIV	714 (538-838)	665 (509-776)	563 (399-669)	725 (499-866)	524 (355-634)	487 (342-580)	363 (270-428)	402 (298-468)	261 (189-304)	184 (144-211)	95 (75-108)	35 (27-39)
Syphilis	78 (58-91)	65 (50-76)	107 (76-127)	169 (116-201)	126 (85-152)	124 (87-148)	110 (81-129)	106 (79-124)	68 (49-79)	42 (32-47)	20 (16-23)	6 (5-7)
Chlamydial infection	134 (101-157)	117 (90-137)	87 (62-104)	112 (77-134)	82 (56-99)	45 (31-53)	42 (31-50)	52 (38-60)	46 (34-54)	34 (26-39)	17 (14-20)	6 (5-7)
Gonococcal infection	332 (250-390)	312 (238-364)	250 (177-298)	294 (202-351)	196 (133-237)	244 (171-290)	157 (116-185)	193 (143-224)	115 (83-134)	85 (67-97)	45 (36-51)	17 (13-19)
Other sexually transmitted diseases	170 (128-200)	171 (131-200)	119 (84-141)	151 (104-180)	120 (81-145)	74 (52-88)	55 (41-64)	52 (38-60)	32 (23-37)	24 (19-27)	12 (10-14)	5 (4-6)
Hepatitis	3,375 (3,079-3,654)	3,106 (2,873-3,354)	3,583 (3,277-3,948)	4,154 (3,780-4,539)	4,102 (3,744-4,494)	4,395 (3,975-4,828)	4,357 (3,919-4,772)	3,514 (3,173-3,868)	2,193 (1,960-2,447)	1,104 (994-1,222)	355 (323-396)	85 (77-95)
Acute hepatitis A	43 (21-75)	48 (25-81)	55 (27-94)	62 (32-101)	74 (37-127)	80 (41-132)	91 (49-149)	85 (44-136)	52 (30-83)	27 (16-39)	8 (5-13)	2 (1-3)
Hepatitis B	3,033 (2,674-3,383)	2,778 (2,481-3,060)	3,198 (2,829-3,574)	3,677 (3,219-4,102)	3,558 (3,050-4,034)	3,744 (3,193-4,227)	3,613 (2,990-4,155)	2,819 (2,294-3,298)	1,562 (1,205-1,909)	639 (428-848)	166 (98-240)	32 (15-51)
Hepatitis C	46 (22-83)	58 (27-104)	91 (44-161)	140 (72-239)	157 (75-276)	153 (79-254)	144 (74-245)	116 (57-200)	70 (38-113)	38 (22-57)	13 (8-21)	3 (2-6)
Acute hepatitis E	253 (108-473)	222 (106-395)	240 (111-431)	275 (112-519)	313 (122-592)	418 (179-729)	509 (239-911)	494 (201-873)	509 (266-824)	400 (223-595)	168 (97-236)	48 (30-66)
Other infectious diseases	666 (539-803)	766 (630-924)	1,057 (877-1,303)	1,124 (943-1,369)	1,513 (1,248-1,943)	1,926 (1,536-2,387)	1,987 (1,609-2,552)	1,862 (1,497-2,303)	1,575 (1,269-2,169)	1,115 (896-1,445)	549 (432-664)	214 (162-248)
Non-communicable diseases	332,908 (323,604-340,729)	484,785 (470,360-495,658)	628,595 (610,044-642,677)	791,069 (761,989-810,252)	1,163,596 (1,116,623-1,193,017)	1,526,851 (1,463,423-1,566,635)	1,958,968 (1,884,221-2,014,713)	2,157,865 (2,087,869-2,208,439)	1,973,080 (1,914,626-2,018,985)	1,788,490 (1,749,042-1,819,895)	1,001,774 (985,845-1,016,235)	361,646 (356,142-366,751)
Neoplasms	130,924 (125,730-135,874)	188,557 (182,303-194,498)	230,507 (223,497-237,450)	258,926 (251,162-267,392)	328,339 (319,285-338,932)	368,810 (359,646-380,397)	396,504 (385,706-409,567)	356,747 (346,938-368,802)	245,834 (238,393-254,629)	176,208 (170,465-182,696)	77,827 (75,082-80,904)	22,534 (21,624-23,354)
Lip and oral cavity cancer	1,755 (1,590-1,966)	2,576 (2,365-2,813)	3,235 (2,964-3,546)	3,802 (3,455-4,192)	4,943 (4,448-5,404)	4,677 (4,245-5,084)	4,282 (3,906-4,641)	3,832 (3,516-4,089)	3,116 (2,870-3,348)	2,413 (2,260-2,574)	1,206 (1,136-1,281)	388 (367-410)
Nasopharynx cancer	1,261 (1,157-1,365)	1,782 (1,655-1,925)	1,996 (1,825-2,193)	2,098 (1,916-2,333)	1,971 (1,784-2,171)	1,726 (1,591-1,873)	1,452 (1,331-1,588)	969 (897-1,065)	610 (556-666)	312 (289-337)	119 (111-129)	32 (30-34)
Other pharynx cancer	1,227 (1,098-1,363)	2,033 (1,844-2,222)	2,647 (2,399-2,893)	2,957 (2,670-3,239)	3,536 (3,149-3,865)	3,106 (2,781-3,432)	2,890 (2,573-3,181)	1,717 (1,507-1,909)	1,128 (980-1,273)	650 (582-720)	245 (224-267)	62 (58-66)
Oesophageal cancer	2,842 (2,664-3,025)	5,503 (5,186-5,876)	8,047 (7,615-8,510)	11,000 (10,419-11,675)	15,725 (14,831-16,788)	20,065 (18,919-21,340)	21,026 (19,699-22,590)	17,920 (16,794-19,283)	11,904 (11,213-12,803)	6,943 (6,574-7,426)	2,555 (2,431-2,719)	613 (583-645)
Stomach cancer	12,229 (11,682-12,756)	16,942 (16,141-17,771)	20,017 (19,084-20,984)	23,875 (22,761-25,132)	34,395 (32,747-36,377)	41,863 (40,038-44,110)	46,369 (44,262-48,997)	42,127 (40,321-44,419)	29,903 (28,486-31,554)	20,797 (19,826-21,889)	8,765 (8,390-9,169)	2,400 (2,288-2,505)
Colon and rectum cancer	7,393 (6,941-7,877)	12,335 (11,720-13,073)	16,587 (15,817-17,498)	20,351 (19,372-21,458)	28,259 (27,138-29,596)	34,453 (33,086-35,979)	43,099 (41,457-44,942)	45,243 (43,630-46,964)	34,066 (32,831-35,274)	29,188 (28,039-30,387)	14,833 (14,220-15,480)	4,553 (4,352-4,736)
Liver cancer	7,727 (5,453-8,619)	11,992 (8,557-13,185)	14,692 (10,975-16,131)	16,935 (13,011-18,488)	22,054 (18,041-23,983)	27,344 (22,179-29,729)	27,520 (23,311-29,592)	23,061 (20,173-24,766)	15,092 (13,513-16,145)	8,791 (8,051-9,340)	3,108 (2,896-3,286)	745 (686-791)
Liver cancer due to hepatitis B	4,946 (3,314-5,907)	6,958 (4,797-8,480)	7,488 (5,235-9,225)	7,639 (5,081-9,805)	8,719 (5,697-11,284)	9,578 (6,342-12,353)	8,720 (5,652-11,305)	6,859 (4,589-9,111)	4,336 (2,885-5,842)	2,321 (1,674-3,023)	749 (533-988)	169 (104-238)
Liver cancer due to hepatitis C	808 (572-1,091)	1,528 (1,074-2,036)	2,392 (1,778-3,083)	3,285 (2,490-4,114)	4,800 (3,819-5,925)	6,303 (5,238-7,519)	7,171 (6,056-8,455)	7,033 (6,050-8,159)	5,208 (4,504-6,055)	3,499 (3,125-3,923)	1,404 (1,233-1,576)	370 (319-426)
Liver cancer due to alcohol use	1,246 (735-1,918)	2,454 (1,352-3,795)	3,535 (2,140-5,083)	4,588 (2,787-6,778)	6,690 (4,494-9,275)	9,217 (6,726-11,796)	9,270 (6,912-11,825)	6,974 (5,163-8,906)	4,005 (2,799-5,279)	2,059 (1,479-2,670)	623 (431-834)	125 (71-187)
Liver cancer due to other causes	728 (495-1,015)	1,053 (698-1,443)	1,277 (900-1,760)	1,423 (994-1,882)	1,844 (1,425-2,397)	2,246 (1,757-2,788)	2,359 (1,846-2,930)	2,194 (1,767-2,689)	1,543 (1,222-1,926)	912 (750-1,107)	331 (264-411)	81 (60-106)
Gallbladder and biliary tract cancer	1,975 (1,719-2,248)	3,326 (2,953-3,710)	4,639 (4,231-5,106)	5,841 (5,343-6,355)	8,501 (7,946-9,032)	10,175 (9,586-10,807)	11,408 (10,799-12,084)	11,170 (10,671-11,746)	8,065 (7,737-8,456)	6,036 (5,776-6,332)	2,680 (2,551-2,819)	755 (714-791)
Pancreatic cancer	2,148 (2,045-2,273)	3,882 (3,733-4,064)	6,163 (5,942-6,452)	8,501 (8,174-8,916)	13,611 (13,113-14,203)	17,433 (16,860-18,195)	21,272 (20,609-22,096)	21,087 (20,376-21,855)	14,583 (14,065-15,121)	11,114 (10,705-11,563)	4,873 (4,674-5,079)	1,313 (1,255-1,364)
Larynx cancer	669 (611-735)	1,055 (976-1,147)	1,362 (1,262-1,482)	1,642 (1,516-1,787)	2,027 (1,857-2,219)	2,176 (2,005-2,369)	2,190 (2,020-2,382)	1,539 (1,434-1,654)	1,080 (1,003-1,174)	583 (548-627)	213 (201-227)	50 (48-53)
Tracheal, bronchus, and lung cancer	10,776 (9,994-11,534)	17,800 (17,040-18,631)	24,348 (23,340-25,409)	30,387 (29,195-31,931)	43,290 (41,581-45,138)	53,803 (51,830-56,360)	61,097 (59,002-63,787)	54,151 (52,130-56,402)	35,001 (33,348-36,652)	21,387 (20,431-22,381)	7,978 (7,586-8,330)	2,106 (1,991-2,208)
Malignant skin melanoma	1,079 (877-1,347)	1,353 (1,086-1,649)	1,568 (1,260-1,908)	1,552 (1,259-1,848)	2,052 (1,683-2,459)	2,138 (1,736-2,610)	2,507 (1,954-3,118)	2,434 (1,902-2,964)	1,813 (1,468-2,308)	1,469 (1,135-1,863)	749 (591-968)	258 (201-341)
Non-melanoma skin cancer	309 (284-338)	502 (462-541)	550 (513-589)	616 (574-661)	859 (799-923)	1,049 (975-1,122)	1,335 (1,256-1,425)	1,398 (1,328-1,481)	1,507 (1,431-1,584)	1,628 (1,552-1,705)	1,083 (1,029-1,134)	532 (503-560)
Non-melanoma skin cancer (squamous-cell carcinoma)	309 (284-338)	502 (462-541)	550 (513-589)	616 (574-661)	859 (799-923)	1,049 (975-1,122)	1,335 (1,256-1,425)	1,398 (1,328-1,481)	1,507 (1,431-1,584)	1,628 (1,552-1,705)	1,083 (1,029-1,134)	532 (503-560)
Breast cancer	30,390 (27,756-33,330)	41,600 (38,489-45,710)	47,162 (44,219-50,872)	46,504 (43,571-50,579)	46,643 (44,248-51,226)	43,190 (41,084-46,553)	41,393 (39,623-44,644)	35,682 (34,248-38,503)	23,637 (22,680-25,129)	19,024 (18,267-20,005)	9,337 (8,943-9,814)	3,134 (3,000-3,272)
Cervical cancer	19,215 (16,644-21,819)	24,904 (21,756-28,819)	26,243 (22,403-30,394)	26,043 (22,403-30,632)	26,003 (22,929-30,081)	23,136 (20,241-27,020)	20,555 (18,519-23,370)	14,144 (12,971-15,418)	9,307 (8,513-10,434)	5,402 (5,052-5,903)	2,074 (1,953-2,225)	563 (532-600)
Uterine cancer	2,640 (2,388-2,843)	4,651 (4,264-4,958)	6,253 (5,746-6,671)	7,283 (6,680-7,772)	10,176 (9,507-10,836)	10,891 (10,198-11,508)	10,314 (9,755-10,872)	8,347 (7,943-8,759)	5,391 (5,173-5,613)	3,474 (3,336-3,626)	1,454 (1,387-1,522)	391 (370-411)
Ovarian cancer	5,442 (5,050-5,929)	9,189 (8,682-9,852)	12,378 (11,735-13,224)	12,700 (12,120-13,524)	15,475 (14,834-16,296)	15,271 (14,625-16,067)	15,785 (15,166-16,526)	12,865 (12,395-13,492)	7,831 (7,542-8,162)	5,235 (5,033-5,455)	2,131 (2,039-2,236)	527 (502-551)
Prostate cancer	-	-	-	-	-	-	-	-	-	-	-	-
Testicular cancer	-	-	-	-	-	-	-	-	-	-	-	-
Kidney cancer	680 (650-712)	1,176 (1,124-1,230)	1,767 (1,695-1,843)	2,292 (2,202-2,385)	3,491 (3,359-3,624)	4,150 (4,006-4,290)	5,025 (4,827-5,227)	4,816 (4,648-5,003)	3,329 (3,204-3,455)	2,579 (2,463-2,695)	1,122 (1,067-1,178)	293 (278-308)
Bladder cancer	597 (544-661)	869 (800-947)	1,414 (1,300-1,538)	1,831 (1,695-1,980)	2,872 (2,699-3,080)	3,972 (3,737-4,226)	5,397 (5,138-5,691)	6,440 (6,144-6,738)	5,478 (5,275-5,677)	4,887 (4,673-5,105)	2,535 (2,411-2,660)	800 (761-837)
Brain and nervous system cancer	4,277 (3,532-4,625)	5,409 (4,460-5,836)	6,093 (5,042-6,591)	6,460 (5,437-6,983)	7,594 (6,617-8,294)	7,924 (6,799-8,547)	7,184 (6,156-7,727)	5,628 (4,714-6,043)	3,101 (2,518-3,379)	1,682 (1,298-1,837)	525 (406-572)	115 (91-126)
Thyroid cancer	881 (767-998)	1,174 (1,029-1,318)	1,625 (1,436-1,822)	1,982 (1,760-2,217)	2,381 (2,164-2,607)	2,732 (2,503-2,981)	2,812 (2,613-3,029)	2,444 (2,292-2,615)	1,624 (1,539-1,740)	1,105 (1,046-1,188)	449 (426-477)	110 (104-116)
Mesothelioma	310 (258-402)	274 (238-339)	378 (338-456)	475 (425-574)	647 (594-748)	736 (683-842)	833 (778-929)	903 (845-1,008)	543 (512-586)	350 (330-371)	142 (135-151)	37 (35-40)
Hodgkin lymphoma	755 (507-878)	713 (508-834)	787 (525-924)	772 (544-950)	988 (668-1,157)	1,079 (755-1,272)	964 (733-1,146)	794 (641-934)	515 (408-584)	281 (235-308)	85 (75-95)	19 (17-22)
Non-Hodgkin's lymphoma	2,663 (2,322-2,873)	3,196 (2,915-3,451)	4,063 (3,697-4,359)	4,781 (4,364-5,050)	6,923 (6,466-7,414)	8,336 (7,894-8,869)	9,685 (9,298-10,276)	9,056 (8,638-9,593)	6,683 (6,377-7,028)	4,922 (4,665-5,215)	2,045 (1,926-2,166)	522 (490-553)
Multiple myeloma	668 (522-757)	1,179 (948-1,325)	1,935 (1,557-2,206)	2,483 (2,059-2,783)	3,831 (3,012-4,366)	4,857 (3,973-5,504)	5,208 (4,209-6,194)	5,225 (4,095-6,229)	3,649 (2,891-4,373)	2,682 (2,079-3,256)	1,084 (844-1,368)	239 (188-297)
Leukaemia	5,353 (4,390-5,952)	6,254 (5,191-7,042)	6,481 (5,473-7,183)	6,917 (6,019-7,774)	8,733 (7,803-9,837)	9,848 (8,908-10,972)	10,958 (10,193-12,136)	10,422 (9,888-11,281)	7,317 (7,009-7,959)	5,771 (5,501-6,168)	2,802 (2,664-2,991)	859 (815-911)
Acute lymphoid leukaemia	740 (619-986)	823 (683-1,111)	787 (638-1,010)	923 (779-1,178)	1,006 (879-1,320)	950 (835-1,317)	914 (812-1,203)	677 (610-838)	418 (380-548)	255 (234-306)	110 (101-128)	33 (30-39)
Chronic lymphoid leukaemia	277 (221-377)	275 (230-378)	404 (344-508)	540 (463-673)	1,066 (930-1,263)	1,316 (1,169-1,529)	1,631 (1,501-1,807)	1,956 (1,792-2,130)	1,499 (1,402-1,603)	1,479 (1,372-1,570)	790 (736-842)	264 (242-282)
Acute myeloid leukaemia	1,170 (1,000-1,408)	1,471 (1,280-1,754)	1,512 (1,331-1,746)	1,602 (1,425-1,839)	2,084 (1,893-2,319)	2,389 (2,206-2,662)	2,846 (2,697-3,080)	2,785 (2,650-3,042)	1,758 (1,669-1,937)	1,180 (1,108-1,292)	489 (461-530)	129 (120-138)
Chronic myeloid leukaemia	666 (521-820)	734 (582-898)	825 (652-975)	830 (687-981)	1,003 (864-1,142)	1,058 (896-1,185)	1,219 (1,061-1,358)	1,113 (1,025-1,205)	822 (762-894)	696 (634-759)	360 (325-391)	112 (101-122)
Other leukaemia	2,501 (1,832-2,859)	2,951 (2,177-3,326)	2,952 (2,297-3,320)	3,022 (2,399-3,471)	3,574 (2,981-4,122)	4,135 (3,476-4,635)	4,348 (3,829-4,977)	3,891 (3,536-4,329)	2,820 (2,649-3,214)	2,161 (2,030-2,392)	1,053 (985-1,166)	320 (300-356)
Other neoplasms	5,661 (5,304-6,040)	6,886 (6,546-7,253)	8,076 (7,697-8,494)	8,847 (8,421-9,324)	11,358 (10,855-11,991)	12,681 (12,149-13,325)	13,943 (13,351-14,635)	13,333 (12,795-13,970)	9,561 (9,164-9,983)	7,504 (7,175-7,876)	3,633 (3,467-3,808)	1,117 (1,064-1,169)
Cardiovascular diseases	109,093 (104,556-113,451)	168,413 (161,505-174,423)	229,896 (220,963-238,633)	311,662 (297,477-324,033)	505,320 (484,185-528,579)	712,720 (682,213-744,978)	988,932 (947,310-1,031,267)	1,143,155 (1,101,821-1,186,629)	1,076,649 (1,041,313-1,124,012)	1,018,647 (987,948-1,056,130)	567,443 (548,181-587,394)	213,383 (204,816-220,533)
Rheumatic heart disease	10,726 (8,778-12,706)	12,228 (10,186-14,203)	13,848 (11,423-16,131)	15,401 (12,737-17,819)	19,858 (16,667-22,795)	23,015 (19,358-26,182)	24,910 (21,187-28,344)	22,614 (19,636-25,574)	18,168 (16,123-20,602)	13,343 (12,091-14,911)	6,540 (6,036-7,167)	2,196 (2,055-2,367)
Ischaemic heart disease	42,431 (40,028-45,784)	68,218 (64,952-72,152)	94,321 (89,738-100,259)	135,751 (128,528-143,524)	228,396 (217,941-239,255)	320,505 (305,612-335,583)	459,696 (439,805-479,747)	553,743 (532,203-575,301)	522,616 (504,190-543,313)	531,634 (517,215-550,943)	317,778 (309,421-329,078)	125,838 (122,468-130,461)
Cerebrovascular disease	39,833 (37,673-42,451)	65,547 (62,066-69,257)	92,343 (87,548-97,372)	121,830 (115,512-129,023)	200,772 (190,246-212,805)	289,233 (273,406-307,686)	393,646 (371,691-417,961)	428,749 (401,898-457,844)	390,681 (363,635-420,947)	325,754 (301,436-353,434)	154,281 (140,664-169,017)	52,651 (46,971-57,862)

SR Table 3: Global deaths by cause and age group for females (with 95% uncertainty intervals), 2000

Cause	Early Neonatal	Late Neonatal	Post Neonatal	1 to 4	5 to 9	10 to 14	15 to 19	20 to 24	25 to 29	30 to 34	35 to 39
Ischaemic stroke	-	-	273 (210-352)	156 (127-201)	157 (134-188)	160 (136-188)	671 (594-764)	1,331 (1,169-1,502)	1,807 (1,628-2,010)	2,279 (2,063-2,492)	3,415 (3,112-3,713)
Hemorrhagic stroke	782 (677-924)	466 (382-573)	4,100 (3,349-5,058)	2,827 (2,210-3,808)	1,735 (1,514-2,047)	2,021 (1,808-2,304)	3,256 (2,972-3,629)	5,784 (5,273-6,489)	8,324 (7,650-9,228)	12,709 (11,806-13,905)	20,375 (19,025-22,074)
Hypertensive heart disease	-	-	193 (107-258)	123 (64-175)	99 (62-130)	111 (70-150)	869 (610-1,119)	1,100 (770-1,398)	1,490 (1,005-1,860)	2,363 (1,627-2,853)	3,559 (2,416-4,361)
Cardiomyopathy and myocarditis	374 (227-451)	245 (155-304)	2,480 (1,526-3,131)	1,571 (981-2,070)	745 (572-889)	846 (714-1,001)	1,148 (871-1,340)	1,353 (1,033-1,556)	1,860 (1,372-2,168)	2,056 (1,630-2,335)	2,783 (2,190-3,168)
Myocarditis	80 (47-125)	59 (36-92)	896 (578-1,455)	654 (440-1,127)	350 (264-446)	352 (280-437)	536 (372-654)	591 (420-725)	672 (469-842)	564 (420-709)	646 (447-788)
Alcoholic cardiomyopathy	-	-	-	-	-	-	84 (65-113)	159 (119-204)	330 (260-413)	532 (419-657)	977 (751-1,208)
Other cardiomyopathy	294 (175-367)	186 (109-243)	1,584 (856-2,273)	918 (489-1,449)	395 (283-540)	494 (401-633)	528 (396-663)	603 (430-762)	858 (589-1,097)	960 (709-1,178)	1,159 (895-1,378)
Atrial fibrillation and flutter	-	-	-	-	-	-	-	-	-	16 (11-21)	59 (43-78)
Aortic aneurysm	-	-	-	-	-	-	135 (115-164)	176 (152-209)	239 (207-285)	335 (289-397)	477 (417-563)
Peripheral vascular disease	-	-	-	-	-	-	-	-	-	-	-
Endocarditis	61 (38-80)	42 (28-61)	895 (618-1,376)	453 (298-824)	234 (179-310)	273 (225-331)	497 (417-605)	625 (525-749)	871 (736-1,044)	791 (682-924)	936 (800-1,084)
Other cardiovascular and circulatory diseases	696 (508-814)	442 (347-551)	3,329 (2,380-4,157)	1,927 (1,191-2,680)	965 (720-1,188)	1,064 (789-1,293)	1,456 (1,134-1,914)	1,978 (1,573-2,550)	2,551 (1,927-3,175)	2,904 (2,282-3,687)	3,457 (2,803-4,317)
Chronic respiratory diseases	0 (0-0)	0 (0-0)	0 (0-0)	11,337 (8,948-14,189)	3,175 (2,679-3,805)	3,073 (2,654-3,659)	5,167 (4,527-6,096)	6,161 (5,433-6,992)	7,845 (6,896-8,706)	9,962 (8,605-10,937)	13,563 (11,817-14,806)
Chronic obstructive pulmonary disease	-	-	0 (0-0)	2,631 (1,823-4,116)	697 (503-965)	582 (427-738)	2,038 (1,586-2,472)	2,766 (2,256-3,235)	4,079 (3,193-4,710)	5,471 (4,422-6,265)	7,041 (5,684-8,132)
Pneumoconiosis	-	-	-	-	-	-	22 (16-28)	29 (21-35)	37 (27-46)	55 (41-69)	44 (34-54)
Silicosis	-	-	-	-	-	-	1 (1-2)	2 (1-3)	5 (3-9)	13 (8-20)	6 (4-9)
Asbestosis	-	-	-	-	-	-	10 (8-13)	13 (10-17)	15 (11-19)	18 (14-23)	17 (13-22)
Coal workers pneumoconiosis	-	-	-	-	-	-	2 (1-3)	2 (1-2)	2 (1-4)	6 (2-9)	3 (1-4)
Other pneumoconiosis	-	-	-	-	-	-	9 (6-12)	12 (8-15)	14 (10-18)	19 (14-25)	18 (13-23)
Asthma	-	-	-	7,463 (4,463-10,061)	1,950 (1,506-2,537)	2,093 (1,727-2,636)	2,827 (2,356-3,509)	3,037 (2,460-3,815)	3,320 (2,637-4,123)	3,950 (3,075-4,792)	5,746 (4,542-6,860)
Interstitial lung disease and pulmonary sarcoidosis	-	-	-	276 (95-679)	125 (62-256)	79 (49-138)	125 (66-237)	163 (87-297)	224 (130-390)	308 (177-509)	496 (291-807)
Other chronic respiratory diseases	0 (0-0)	0 (0-0)	0 (0-0)	967 (565-1,916)	403 (262-565)	320 (211-440)	155 (95-229)	167 (109-234)	185 (113-251)	177 (103-239)	236 (136-321)
Cirrhosis and other chronic liver diseases	-	-	-	5,382 (4,259-6,491)	2,200 (1,839-2,577)	2,504 (2,184-2,777)	3,990 (3,624-4,326)	5,565 (5,108-6,045)	6,419 (5,981-6,842)	9,144 (8,526-9,749)	12,836 (12,102-13,568)
Cirrhosis and other chronic liver diseases due to hepatitis B	-	-	-	20 (13-28)	21 (15-28)	47 (34-63)	126 (84-176)	707 (493-946)	1,443 (1,058-1,865)	2,560 (2,111-3,081)	3,792 (3,115-4,523)
Cirrhosis and other chronic liver diseases due to hepatitis C	-	-	-	17 (11-25)	18 (12-25)	39 (27-54)	102 (67-146)	518 (361-710)	945 (662-1,270)	1,711 (1,359-2,086)	2,754 (2,138-3,460)
Cirrhosis and other chronic liver diseases due to alcohol use	-	-	-	-	-	-	30 (17-46)	255 (179-354)	562 (407-764)	1,518 (1,256-1,800)	3,075 (2,512-3,716)
Cirrhosis and other chronic liver diseases due to other causes	-	-	-	5,346 (4,234-6,437)	2,161 (1,808-2,529)	2,418 (2,115-2,686)	3,731 (3,392-4,049)	4,085 (3,556-4,609)	3,468 (2,893-4,033)	3,355 (2,703-4,025)	3,216 (2,431-4,047)
Digestive diseases	1,037 (669-1,314)	923 (662-1,151)	9,599 (7,290-12,429)	10,454 (6,553-16,667)	4,274 (3,513-5,284)	4,307 (3,730-5,023)	6,847 (5,793-7,684)	7,732 (6,685-8,517)	8,499 (7,387-9,341)	8,843 (7,909-9,917)	10,045 (8,847-11,091)
Peptic ulcer disease	-	-	-	1,870 (1,180-3,096)	866 (640-1,227)	1,055 (827-1,374)	1,464 (1,153-1,850)	2,148 (1,749-2,572)	2,216 (1,865-2,621)	2,500 (2,115-2,976)	2,918 (2,472-3,390)
Gastritis and duodenitis	-	-	-	318 (135-608)	143 (89-221)	117 (82-166)	171 (119-241)	251 (182-326)	347 (249-456)	428 (309-571)	449 (331-579)
Appendicitis	-	-	-	2,438 (939-4,393)	1,081 (742-1,440)	1,137 (869-1,443)	2,177 (1,636-2,645)	1,706 (1,194-2,033)	1,694 (1,245-2,036)	1,131 (849-1,379)	1,157 (856-1,395)
Paralytic ileus and intestinal obstruction	1,037 (669-1,314)	923 (662-1,151)	9,599 (7,290-12,429)	2,557 (1,306-4,138)	1,097 (655-1,424)	1,130 (714-1,396)	1,876 (1,107-2,299)	1,768 (1,072-2,153)	1,817 (1,091-2,198)	1,673 (1,026-2,052)	1,861 (1,161-2,257)
Inguinal, femoral, and abdominal hernia	-	-	-	223 (94-448)	129 (76-188)	92 (62-136)	114 (65-150)	113 (64-150)	103 (61-140)	124 (74-169)	164 (93-221)
Inflammatory bowel disease	-	-	-	708 (290-2,071)	152 (86-292)	114 (72-183)	181 (113-318)	298 (183-523)	358 (228-597)	399 (251-610)	418 (280-601)
Vascular intestinal disorders	-	-	-	98 (35-155)	22 (14-30)	24 (17-30)	41 (25-53)	103 (60-134)	129 (80-165)	179 (122-222)	256 (186-318)
Gallbladder and biliary diseases	-	-	-	414 (205-685)	193 (137-278)	182 (145-256)	196 (164-263)	334 (278-426)	476 (390-581)	651 (533-793)	850 (696-1,034)
Pancreatitis	-	-	-	101 (43-187)	106 (56-180)	135 (85-221)	315 (209-491)	576 (376-873)	756 (508-1,147)	1,083 (751-1,551)	1,254 (902-1,727)
Other digestive diseases	-	-	-	1,728 (688-3,012)	485 (339-673)	320 (254-422)	313 (235-411)	435 (329-549)	603 (460-742)	675 (533-829)	718 (573-865)
Neurological disorders	-	-	4,202 (3,261-5,185)	4,580 (3,086-6,126)	2,942 (2,285-3,600)	4,040 (3,282-4,754)	5,561 (4,543-6,350)	5,244 (4,206-5,886)	5,051 (4,013-5,574)	5,214 (4,303-5,784)	4,828 (4,164-5,335)
Alzheimer's disease and other dementias	-	-	-	-	-	-	-	-	-	-	-
Parkinson's disease	-	-	-	-	-	-	-	1 (0-1)	8 (3-15)	28 (14-52)	60 (31-107)
Epilepsy	-	-	2,811 (1,650-3,520)	3,391 (2,072-4,558)	2,427 (1,732-3,019)	3,350 (2,506-3,949)	4,926 (3,834-5,620)	4,730 (3,633-5,336)	4,421 (3,343-4,893)	4,317 (3,360-4,803)	3,500 (2,791-3,884)
Multiple sclerosis	-	-	-	-	-	-	-	86 (62-141)	193 (142-292)	315 (246-439)	578 (470-768)
Motor neuron disease	4 (3-5)	5 (4-6)	361 (250-497)	183 (104-267)	23 (19-29)	26 (23-31)	23 (20-26)	20 (17-24)	33 (29-40)	59 (54-71)	113 (105-132)
Other neurological disorders	-	-	1,029 (694-1,358)	1,006 (555-1,456)	492 (368-618)	664 (495-845)	613 (437-793)	407 (333-472)	398 (338-448)	495 (441-541)	576 (530-615)
Mental and substance use disorders	6 (3-9)	13 (10-17)	0 (0-0)	0 (0-0)	8 (6-10)	11 (7-13)	2,420 (1,880-2,778)	3,201 (2,503-3,574)	4,051 (3,150-4,467)	4,600 (3,788-4,984)	5,446 (4,828-5,878)
Alcohol use disorders	-	-	-	-	-	-	263 (238-289)	404 (355-453)	818 (717-929)	1,269 (1,132-1,417)	2,265 (2,012-2,546)
Drug use disorders	6 (3-9)	13 (10-17)	0 (0-0)	0 (0-0)	0 (0-0)	0 (0-0)	2,124 (1,585-2,463)	2,748 (2,033-3,102)	3,179 (2,273-3,579)	3,278 (2,456-3,619)	3,127 (2,495-3,417)
Opioid use disorders	6 (3-9)	13 (10-17)	0 (0-0)	0 (0-0)	0 (0-0)	0 (0-0)	1,349 (1,000-1,610)	1,750 (1,259-2,047)	1,990 (1,377-2,350)	1,879 (1,349-2,149)	1,775 (1,302-2,017)
Cocaine use disorders	0 (0-0)	0 (0-0)	0 (0-0)	0 (0-0)	0 (0-0)	0 (0-0)	65 (29-115)	93 (48-152)	117 (63-189)	185 (110-277)	187 (123-281)
Amphetamine use disorders	0 (0-0)	0 (0-0)	0 (0-0)	0 (0-0)	0 (0-0)	0 (0-0)	68 (32-121)	101 (49-190)	124 (57-234)	90 (48-150)	77 (45-122)
Other drug use disorders	0 (0-0)	0 (0-0)	0 (0-0)	0 (0-0)	0 (0-0)	0 (0-0)	641 (410-807)	804 (543-971)	948 (616-1,143)	1,123 (776-1,331)	1,087 (844-1,276)
Eating disorders	-	-	-	-	8 (6-10)	11 (7-13)	32 (24-40)	49 (39-60)	55 (44-65)	53 (45-61)	54 (45-62)
Anorexia nervosa	-	-	-	-	7 (5-9)	10 (7-12)	31 (24-39)	47 (37-58)	52 (42-63)	50 (42-57)	50 (42-58)
Bulimia nervosa	-	-	-	-	0 (0-1)	0 (0-1)	1 (1-2)	2 (2-3)	2 (2-3)	3 (2-4)	4 (3-5)
Diabetes, urogenital, blood, and endocrine diseases	2,050 (1,465-2,516)	1,318 (985-1,557)	12,089 (9,986-14,125)	18,098 (11,694-23,507)	7,039 (5,771-8,456)	9,640 (8,137-10,954)	14,911 (12,712-16,549)	15,735 (14,191-17,166)	16,709 (15,439-17,911)	18,736 (17,523-20,029)	21,308 (20,235-22,467)
Diabetes mellitus	20 (18-22)	38 (32-44)	476 (420-540)	596 (503-697)	576 (501-654)	1,076 (967-1,179)	1,945 (1,794-2,092)	2,645 (2,460-2,818)	3,194 (3,003-3,384)	3,959 (3,730-4,186)	5,652 (5,348-5,968)
Acute glomerulonephritis	-	-	89 (69-119)	144 (114-180)	142 (117-176)	167 (136-208)	143 (124-168)	194 (164-230)	278 (228-341)	279 (236-328)	294 (255-339)
Chronic kidney disease	-	-	1,834 (1,642-2,056)	3,429 (2,884-4,008)	1,898 (1,653-2,153)	2,551 (2,283-2,808)	3,944 (3,639-4,261)	5,535 (5,128-5,911)	6,810 (6,335-7,286)	8,089 (7,555-8,626)	9,360 (8,812-9,929)
Chronic kidney disease due to diabetes mellitus	-	-	44 (30-64)	85 (52-128)	58 (32-94)	109 (66-172)	274 (163-427)	719 (457-1,026)	1,523 (1,074-2,032)	2,536 (1,864-3,194)	3,453 (2,651-4,293)
Chronic kidney disease due to hypertension	-	-	13 (8-18)	34 (22-48)	30 (20-46)	70 (45-103)	166 (111-237)	335 (231-464)	541 (369-739)	786 (575-1,044)	1,073 (810-1,405)
Chronic kidney disease due to glomerulonephritis	-	-	788 (601-972)	1,488 (1,088-1,928)	806 (580-1,049)	1,139 (821-1,477)	1,815 (1,340-2,293)	2,440 (1,803-3,100)	2,593 (1,846-3,381)	2,470 (1,731-3,308)	2,373 (1,619-3,198)
Chronic kidney disease due to other causes	-	-	989 (808-1,212)	1,823 (1,415-2,317)	1,003 (775-1,273)	1,232 (902-1,598)	1,688 (1,210-2,191)	2,042 (1,390-2,694)	2,153 (1,506-2,903)	2,297 (1,599-3,036)	2,462 (1,726-3,207)
Urinary diseases and male infertility	129 (116-148)	145 (120-182)	1,796 (1,585-2,066)	1,797 (1,478-2,191)	638 (536-769)	599 (518-692)	1,378 (1,225-1,577)	1,633 (1,486-1,808)	1,465 (1,351-1,600)	1,685 (1,562-1,842)	1,869 (1,746-2,024)
Interstitial nephritis and urinary tract infections	69 (47-88)	79 (53-108)	1,175 (917-1,463)	1,142 (747-1,594)	338 (248-429)	302 (224-366)	953 (776-1,137)	1,127 (911-1,305)	937 (743-1,072)	1,100 (895-1,252)	1,296 (1,084-1,457)
Urolithiasis	-	-	-	-	93 (51-131)	103 (57-139)	138 (74-192)	157 (80-220)	141 (71-190)	165 (85-212)	183 (110-225)
Other urinary diseases	60 (48-80)	67 (47-96)	621 (456-864)	655 (402-1,065)	207 (144-333)	193 (135-314)	287 (193-497)	349 (237-594)	387 (281-609)	421 (313-660)	390 (284-637)
Gynecological diseases	-	-	-	-	-	-	238 (192-269)	325 (260-373)	369 (312-416)	474 (391-533)	605 (517-690)
Uterine fibroids	-	-	-	-	-	-	15 (7-33)	21 (12-38)	37 (18-61)	75 (32-131)	172 (72-261)
Polycystic ovarian syndrome	-	-	-	-	-	-	3 (0-9)	26 (6-79)	68 (18-149)	55 (12-151)	71 (19-155)
Endometriosis	-	-	-	-	-	-	4 (1-10)	3 (1-7)	3 (1-7)	6 (2-11)	5 (2-8)
Genital prolapse	-	-	-	-	-	-	14 (4-45)	13 (3-29)	20 (7-40)	25 (4-45)	8 (3-16)
Other gynecological diseases	-	-	-	-	-	-	203 (143-241)	262 (180-318)	240 (141-316)	312 (190-417)	349 (216-491)
Hemoglobinopathies and hemolytic anaemias	1,086 (656-1,529)	514 (327-692)	4,455 (2,574-5,867)	9,613 (3,440-14,335)	2,752 (1,694-3,816)	4,341 (2,954-5,423)	6,269 (4,115-7,697)	4,253 (2,717-5,376)	3,089 (2,031-3,838)	2,643 (1,854-3,387)	1,895 (1,400-2,390)
Thalassemias	276 (188-389)	145 (95-208)	1,270 (746-1,804)	1,993 (1,078-2,955)	986 (670-1,391)	623 (483-857)	404 (294-546)	201 (145-279)	159 (108-198)	92 (70-118)	51 (40-64)
Sickle cell disorders	734 (410-1,157)	351 (212-508)	3,067 (1,759-4,071)	7,385 (2,233-11,309)	1,635 (868-2,300)	3,409 (2,223-4,424)	5,178 (3,311-6,482)	3,347 (2,039-4,300)	2,069 (1,272-2,646)	1,597 (1,046-2,056)	906 (631-1,148)
G6PD deficiency	17 (10-24)	8 (5-12)	66 (42-94)	157 (85-237)	84 (59-117)	173 (120-221)	376 (267-484)	385 (277-529)	484 (343-639)	488 (350-648)	416 (317-540)
Other hemoglobinopathies and hemolytic anaemias	59 (39-79)	9 (6-12)	51 (32-70)	78 (48-107)	46 (35-64)	136 (102-169)	311 (226-404)	319 (231-450)	376 (273-485)	467 (352-618)	522 (405-662)
Endocrine, metabolic, blood, and immune disorders	815 (494-941)	621 (349-792)	3,438 (2,324-4,236)	2,519 (1,686-3,429)	1,035 (852-1,278)	906 (753-1,088)	994 (849-1,182)	1,151 (962-1,314)	1,505 (1,187-1,734)	1,608 (1,284-1,906)	1,633 (1,337-1,930)
Musculoskeletal disorders	-	-	-	-	405 (293-545)	859 (660-1,098)	1,510 (1,118-1,871)	1,766 (1,344-2,153)	2,082 (1,609-2,648)	2,035 (1,614-2,483)	2,012 (1,621-2,415)
Rheumatoid arthritis	-	-	-	-	77 (47-145)	87 (60-157)	83 (55-160)	102 (66-205)	136 (92-260)	186 (135-328)	243 (175-375)
Other musculoskeletal disorders	-	-	-	-	328 (234-454)	772 (597-979)	1,427 (1,049-1,776)	1,664 (1,276-2,054)	1,946 (1,501-2,457)	1,849 (1,453-2,260)	1,768 (1,405-2,126)
Other non-communicable diseases	82,985 (60,525-106,948)	34,268 (26,895-44,259)	113,493 (87,277-146,975)	40,564 (25,648-62,112)	9,716 (8,009-12,646)	7,805 (6,646-9,420)	5,114 (4,280-6,112)	4,149 (3,546-4,998)	3,742 (3,276-4,376)	2,695 (2,310-3,168)	2,586 (2,164-3,013)
Congenital anomalies	82,985 (60,525-106,948)	32,488 (25,304-42,193)	96,560 (73,329-127,196)	39,385 (24,465-60,891)	9,400 (7,629-12,309)	7,460 (6,331-9,094)	4,591 (3,877-5,489)	3,755 (3,192-4,557)	3,353 (2,936-3,920)	2,239 (1,958-2,600)	2,023 (1,766-2,347)
Neural tube defects	12,067 (7,372-15,893)	3,440 (2,030-4,811)	7,735 (4,138-11,766)	2,492 (939-5,108)	453 (297-653)	312 (223-419)	156 (111-209)	110 (78-164)	72 (52-108)	61 (48-83)	47 (36-65)
Congenital heart anomalies	25,446 (14,930-32,531)	13,300 (8,934-17,211)	47,561 (31,613-65,000)	19,442 (12,363-31,872)	5,689 (4,242-7,199)	5,014 (4,024-5,945)	3,013 (2,327-3,472)	2,677 (2,169-3,159)	2,498 (2,097-2,831)	1,537 (1,310-1,750)	1,426 (1,220-1,658)
Orofacial clefts	791 (375-1,259)	344 (166-558)	481 (217-711)	84 (19-178)	-	-	-	-	-	-	-
Down's syndrome	801 (519-1,533)	386 (259-786)	1,799 (1,199-3,653)	774 (479-1,897)	161 (106-258)	144 (101-212)	135 (100-211)	130 (100-199)	149 (119-220)	155 (123-218)	149 (120-201)
Other chromosomal abnormalities	2,666 (1,629-5,754)	1,422 (872-3,006)	3,093 (1,936-7,034)	508 (284-1,529)	136 (95-237)	92 (66-137)	77 (56-117)	46 (34-63)	45 (31-59)	33 (21-43)	27 (17-32)
Congenital musculoskeletal and limb anomalies	2,179 (1,181-4,414)	579 (309-1,235)	1,104 (555-2,651)	363 (163-1,151)	126 (78-236)	129 (87-202)	93 (58-149)	53 (34-81)	46 (29-68)	35 (22-48)	26 (19-34)
Urogenital congenital anomalies	2,136 (1,085-3,244)	771 (388-1,285)	1,240 (565-2,291)	315 (134-689)	161 (97-262)	161 (100-244)	68 (45-103)	57 (38-83)	56 (35-82)	50 (31-68)	53 (32-72)
Digestive congenital anomalies	5,928 (4,140-10,443)	2,493 (1,787-4,440)	5,883 (4,158-13,024)	1,147 (700-3,951)	134 (100-230)	80 (63-116)	54 (41-82)	36 (28-50)	21 (17-27)	18 (15-24)	17 (14-21)
Other congenital anomalies	30,971 (15,029-51,876)	9,753 (3,944-17,595)	27,663 (10,254-52,327)	14,260 (3,878-33,155)	2,539 (1,193-4,578)	1,528 (829-2,438)	994 (538-1,595)	647 (325-1,096)	467 (249-767)	351 (205-539)	278 (172-411)
Skin and subcutaneous diseases	-	-	2,435 (928-3,568)	1,179 (428-1,832)	317 (117-478)	345 (139-489)	523 (202-734)	394 (170-527)	389 (184-532)	455 (220-667)	563 (276-803)
Cellulitis	-	-	133 (30-243)	114 (25-223)	42 (13-70)	43 (15-70)	55 (18-82)	59 (22-84)	44 (18-60)	69 (29-99)	78 (31-114)
Pyoderma	-	-	2,243 (865-3,329)	952 (336-1,550)	222 (76-352)	237 (85-349)	378 (134-542)	245 (98-341)	260 (112-371)	274 (128-414)	379 (175-535)
Decubitus ulcer	-	-	-	47 (19-84)	24 (12-38)	33 (17-50)	47 (23-72)	47 (23-71)	53 (27-81)	76 (37-119)	72 (36-117)
Other skin and subcutaneous diseases	-	-	60 (25-114)	66 (30-116)	29 (13-49)	33 (16-53)	44 (21-69)	42 (21-63)	31 (17-49)	37 (20-57)	34 (19-54)
Sudden infant death syndrome	-	1,780 (1,219-2,803)	14,498 (9,861-21,926)	-	-	-	-	-	-	-	-
Injuries	6,719 (5,165-7,697)	4,363 (3,411-4,994)	48,183 (40,315-57,094)	118,729 (96,262-139,031)	61,727 (54,349-67,947)	55,828 (50,872-59,581)	99,592 (93,121-104,760)	105,250 (99,408-110,162)	97,941 (93,186-102,368)	86,346 (81,527-89,957)	76,414 (71,895-79,513)
Transport injuries	709 (502-921)	584 (476-693)	7,416 (6,418-8,448)	23,028 (18,797-26,544)	19,930 (17,818-22,160)	15,833 (14,524-17,140)	27,657 (26,027-29,252)	28,186 (26,559-29,627)	26,491 (24,865-28,010)	24,532 (23,233-25,755)	22,677 (21,579-23,843)
Road injuries	627 (461-814)	549 (448-646)	6,805 (5,750-7,713)	21,670 (17,315-24,923)	19,167 (17,141-21,253)	14,934 (13,740-16,158)	25,865 (24,368-27,351)	26,902 (25,348-28,370)	25,241 (23,705-26,706)	23,392 (22,156-24,643)	21,661 (20,587-22,781)
Pedestrian road injuries	284 (191-380)	239 (183-310)	3,074 (2,429-3,876)	12,641 (10,334-15,708)	11,352 (9,838-13,051)	7,635 (6,838-8,489)	8,797 (7,870-9,737)	9,160 (8,204-10,237)	8,673 (7,723-9,679)	9,040 (8,169-9,987)	8,913 (8,101-9,816)
Cyclist road injuries	-	-	-	620 (347-885)	732 (563-892)	1,031 (852-1,206)	1,264 (1,032-1,532)	1,041 (796-1,261)	1,080 (843-1,277)	1,312 (1,038-1,515)	1,127 (886-1,301)
Motorcyclist road injuries	131 (73-202)	54 (36-75)	1,066 (750-1,391)	1,935 (1,075-2,689)	1,266 (982-1,564)	1,413 (1,198-1,656)	4,093 (3,600-4,725)	4,738 (4,131-5,443)	4,449 (3,839-5,196)	4,208 (3,704-4,754)	3,100 (2,717-3,509)
Motor vehicle road injuries	201 (132-264)	252 (192-316)	2,569 (1,874-3,274)	6,320 (4,234-8,505)	5,655 (4,654-6,702)	4,732 (4,132-5,353)	11,465 (10,463-12,693)	11,733 (10,656-13,088)	10,872 (9,926-12,124)	8,637 (7,828-9,597)	8,326 (7,532-9,189)
Other road injuries	11 (7-18)	5 (3-8)	97 (58-182)	153 (89-280)	162 (115-226)	122 (96-171)	246 (185-393)	230 (179-351)	167 (130-250)	196 (153-301)	194 (152-279)
Other transport injuries	82 (36-140)	34 (23-49)	611 (412-1,014)	1,358 (924-2,257)	763 (589-997)	899 (723-1,072)	1,792 (1,432-2,113)	1,284 (1,049-1,513)	1,250 (1,013-1,453)	1,140 (933-1,302)	1,015 (847-1,168)
Unintentional injuries	4,292 (3,071-5,123)	3,120 (2,255-3,701)	37,228 (29,496-45,624)	84,773 (64,525-102,919)	34,915 (28,558-39,659)	27,341 (22,826-29,893)	25,014 (20,469-27,353)	23,698 (19,924-25,761)	22,459 (19,006-24,229)	21,647 (18,287-23,396)	19,810 (17,074-21,303)
Falls	1,004 (550-1,504)	376 (213-543)	5,250 (3,232-7,428)	7,804 (4,741-11,562)	3,400 (2,750-4,076)	2,480 (2,069-2,836)	3,005 (2,535-3,400)	2,799 (2,377-3,161)	2,751 (2,295-3,074)	2,870 (2,487-3,199)	3,240 (2,822-3,641)
Drowning	191 (145-214)	129 (94-152)	4,693 (3,090-5,672)	44,817 (26,908-54,233)	16,915 (11,936-19,581)	12,400 (9,395-13,733)	7,305 (5,387-8,040)	5,113 (3,845-5,593)	4,793 (3,543-5,260)	4,308 (3,092-4,704)	3,652 (2,692-3,990)
Fire, heat, and hot substances	152 (109-177)	215 (136-263)	3,312 (2,058-4,040)	9,019 (5,107-12,211)	3,052 (2,230-3,691)	2,494 (1,788-2,893)	4,995 (3,298-5,894)	5,731 (3,692-6,694)	4,626 (3,081-5,383)	4,244 (2,974-4,875)	3,414 (2,485-3,854)
Poisonings	119 (75-167)	106 (76-138)	2,049 (971-3,332)	4,203 (2,385-6,419)	1,471 (1,187-1,865)	1,901 (1,609-2,394)	1,593 (1,313-1,957)	1,763 (1,421-2,168)	1,659 (1,353-2,093)	1,422 (1,188-1,836)	1,291 (1,036-1,510)
Exposure to mechanical forces	878 (447-1,117)	820 (410-1,102)	5,913 (3,889-7,380)	4,390 (2,955-5,898)	2,522 (2,054-3,018)	1,665 (1,439-2,000)	1,838 (1,421-2,092)	1,566 (1,248-1,745)	1,952 (1,521-2,182)	1,749 (1,480-1,926)	1,623 (1,325-1,797)
Unintentional firearm injuries	25 (20-28)	17 (14-20)	187 (149-224)	282 (198-369)	257 (215-302)	209 (182-237)	294 (257-334)	303 (261-348)	367 (307-416)	392 (337-439)	270 (226-303)
Unintentional suffocation	681 (288-912)	740 (335-1,026)	4,329 (2,420-5,752)	1,397 (782-2,294)	471 (389-760)	582 (445-902)	148 (117-210)	151 (111-199)	161 (118-212)	113 (74-134)	98 (73-128)
Other exposure to mechanical forces	171 (113-204)	62 (45-75)	1,397 (883-1,765)	2,711 (1,575-3,683)	1,684 (1,232-2,062)	985 (769-1,164)	1,396 (975-1,655)	1,112 (796-1,289)	1,424 (998-1,656)	1,245 (994-1,425)	1,255 (973-1,421)
Adverse effects of medical treatment	731 (495-873)	550 (360-673)	4,655 (2,582-6,138)	3,734 (1,543-5,719)	1,357 (891-1,740)	1,046 (779-1,255)	1,246 (930-1,435)	2,210 (1,637-2,580)	2,222 (1,616-2,565)	1,933 (1,393-2,235)	2,091 (1,565-2,410)
Animal contact	116 (40-153)	121 (39-169)	1,648 (565-2,309)	3,791 (1,269-6,489)	3,252 (1,253-4,743)	2,857 (1,019-3,928)	2,325 (866-3,082)	1,936 (701-2,575)	1,744 (663-2,295)	2,143 (700-2,899)	1,572 (552-2,116)
Venomous animal contact	80 (20-117)	85 (21-126)	1,331 (398-1,913)	3,089 (914-5,173)	2,851 (1,008-4,124)	2,605 (844-3,600)	2,120 (737-2,859)	1,724 (559-2,339)	1,549 (535-2,078)	1,979 (612-2,726)	1,419 (454-1,959)
Non-venomous animal contact	36 (15-67)	36 (14-73)	317 (130-745)	702 (254-2,396)	402 (194-905)	252 (124-513)	205 (112-416)	213 (111-419)	195 (106-360)	165 (85-334)	153 (79-279)
Foreign body	772 (434-947)	645 (382-818)	7,950 (4,801-11,204)	3,928 (2,253-6,455)	1,261 (891-1,685)	837 (617-1,006)	707 (518-870)	829 (613-1,032)	827 (609-997)	808 (631-960)	866 (702-1,008)
Pulmonary aspiration and foreign body in airway	723 (399-889)	617 (360-779)	7,392 (4,392-10,486)	3,324 (1,855-5,514)	981 (652-1,292)	651 (466-773)	385 (284-442)	431 (327-509)	468 (352-561)	537 (409-624)	579 (459-673)
Foreign body in other body part	49 (20-68)	28 (11-40)	558 (220-785)	604 (246-1,054)	280 (157-429)	186 (107-255)	322 (194-457)	399 (236-557)	359 (214-482)	271 (158-371)	287 (181-388)
Environmental heat and cold exposure	132 (52-177)	83 (29-119)	871 (307-1,189)	668 (252-1,022)	351 (163-464)	185 (109-233)	366 (175-457)	328 (163-411)	361 (187-450)	538 (272-672)	718 (374-951)
Other unintentional injuries	196 (162-229)	75 (62-91)	888 (690-1,069)	2,417 (1,608-3,193)	1,334 (1,073-1,602)	1,475 (1,187-1,686)	1,634 (1,140-1,870)	1,421 (1,092-1,609)	1,524 (1,197-1,740)	1,632 (1,318-1,828)	1,342 (1,046-1,501)
Self-harm and interpersonal violence	1,032 (662-1,218)	279 (184-332)	2,248 (1,465-2,704)	4,097 (2,667-5,261)	3,065 (2,056-3,678)	9,428 (8,436-10,587)	43,214 (39,409-47,071)	50,437 (46,424-54,310)	47,057 (44,013-50,525)	38,482 (35,949-41,008)	32,562 (30,223-34,547)

Cause	40 to 44	45 to 49	50 to 54	55 to 59	60 to 64	65 to 69	70 to 74	75 to 79	80 to 84	85 to 89	90 to 94	95 plus
Ischaemic stroke	5,856 (5,391-6,298)	11,646 (10,777-12,429)	18,605 (17,267-19,810)	28,570 (26,537-30,537)	77,767 (72,192-82,394)	120,876 (113,310-128,263)	183,042 (172,584-193,789)	240,353 (225,490-255,126)	225,444 (210,420-241,284)	206,447 (191,438-223,447)	102,475 (93,384-111,902)	36,496 (32,582-40,160)
Hemorrhagic stroke	33,977 (32,016-36,255)	53,901 (50,991-57,004)	73,738 (69,715-77,829)	93,260 (87,896-98,873)	123,005 (115,472-131,808)	168,357 (157,526-181,206)	210,604 (197,516-226,175)	188,396 (176,168-203,975)	165,236 (152,582-181,343)	119,307 (109,456-131,547)	51,807 (46,979-57,712)	16,155 (14,330-17,957)
Hypertensive heart disease	5,650 (4,178-6,840)	9,446 (6,662-11,287)	13,561 (9,911-16,659)	19,243 (14,353-23,219)	27,267 (20,156-33,205)	40,279 (30,229-48,376)	53,316 (40,648-64,296)	61,344 (47,384-74,792)	57,413 (44,933-69,346)	50,886 (38,048-63,660)	29,528 (22,286-34,978)	11,070 (7,672-13,517)
Cardiomyopathy and myocarditis	3,870 (3,107-4,431)	4,436 (3,638-5,162)	5,045 (4,137-5,799)	5,455 (4,540-6,174)	7,029 (6,035-8,460)	7,918 (6,477-8,829)	10,704 (8,365-11,798)	13,978 (9,882-15,721)	14,789 (9,645-16,843)	18,096 (11,062-21,134)	11,995 (6,983-14,265)	4,937 (2,810-5,814)
Myocarditis	717 (512-883)	535 (383-643)	554 (398-667)	635 (464-756)	624 (425-738)	842 (574-995)	1,187 (807-1,385)	1,738 (1,058-2,063)	2,205 (1,290-2,694)	3,285 (1,723-4,256)	2,532 (1,166-3,366)	1,233 (529-1,634)
Alcoholic cardiomyopathy	1,630 (1,237-2,060)	2,118 (1,610-2,661)	2,224 (1,741-2,737)	1,873 (1,499-2,329)	2,573 (2,060-3,559)	1,957 (1,586-2,730)	1,959 (1,570-2,872)	1,710 (1,398-2,373)	1,188 (933-1,574)	1,183 (804-1,509)	696 (441-878)	255 (168-316)
Other cardiomyopathy	1,523 (1,197-1,784)	1,784 (1,438-2,062)	2,267 (1,827-2,597)	2,947 (2,340-3,327)	3,832 (3,089-4,433)	5,120 (3,904-5,740)	7,559 (5,463-8,600)	10,531 (7,048-12,286)	11,396 (7,189-13,464)	13,627 (8,050-16,520)	8,767 (5,090-11,177)	3,449 (1,992-4,517)
Atrial fibrillation and flutter	134 (98-176)	253 (183-335)	393 (290-516)	654 (382-976)	1,489 (974-2,107)	3,138 (1,933-4,766)	6,645 (4,704-9,251)	10,829 (6,896-16,132)	20,077 (14,977-25,913)	27,350 (20,664-35,507)	15,743 (12,008-20,417)	4,867 (3,716-6,251)
Aortic aneurysm	644 (576-741)	981 (884-1,122)	1,337 (1,226-1,514)	1,894 (1,737-2,155)	2,470 (2,339-2,673)	3,883 (3,686-4,180)	5,958 (5,691-6,315)	7,922 (7,608-8,318)	7,863 (7,575-8,206)	7,257 (6,990-7,561)	3,982 (3,831-4,169)	1,297 (1,247-1,357)
Peripheral vascular disease	67 (39-92)	101 (53-146)	173 (87-266)	269 (144-417)	576 (295-916)	926 (474-1,485)	1,677 (790-2,597)	2,770 (1,413-4,733)	3,573 (1,827-6,165)	4,745 (2,393-8,926)	3,523 (1,748-6,350)	1,625 (758-2,912)
Endocarditis	1,215 (1,037-1,458)	1,107 (908-1,326)	1,203 (964-1,450)	1,433 (1,146-1,746)	2,166 (1,704-2,647)	2,820 (2,179-3,437)	3,590 (2,694-4,439)	4,381 (3,127-5,593)	4,464 (3,086-5,664)	4,291 (2,803-5,415)	2,785 (1,714-3,495)	1,087 (669-1,356)
Other cardiovascular and circulatory diseases	4,523 (3,588-5,602)	6,095 (4,866-7,685)	7,672 (6,097-9,514)	9,732 (7,652-12,342)	15,295 (12,130-19,159)	21,003 (16,642-26,223)	28,788 (23,086-34,243)	36,826 (30,477-42,534)	37,005 (31,324-44,402)	35,290 (31,406-39,899)	21,287 (19,770-23,732)	7,816 (7,368-8,660)
Chronic respiratory diseases	18,990 (16,138-20,343)	32,411 (26,323-34,831)	48,262 (38,024-51,947)	75,891 (59,092-82,518)	129,552 (99,854-140,966)	189,863 (148,751-205,384)	255,063 (205,829-275,479)	270,569 (221,274-290,686)	248,574 (200,675-267,135)	183,410 (152,960-195,920)	82,602 (70,087-88,101)	26,312 (23,259-27,783)
Chronic obstructive pulmonary disease	10,504 (8,383-12,081)	19,873 (15,234-23,105)	33,246 (24,852-38,169)	55,184 (41,159-63,176)	102,043 (75,668-115,211)	156,639 (118,684-173,282)	219,480 (174,851-239,214)	236,015 (190,276-253,935)	222,503 (178,601-239,662)	166,433 (137,315-177,733)	75,592 (63,926-80,753)	24,263 (21,311-25,692)
Pneumoconiosis	61 (47-76)	72 (56-89)	88 (67-109)	125 (91-162)	183 (137-228)	242 (187-304)	339 (272-407)	352 (286-413)	289 (242-345)	201 (177-228)	87 (79-98)	30 (28-34)
Silicosis	13 (9-20)	20 (14-29)	20 (14-29)	33 (19-48)	50 (32-71)	66 (41-102)	110 (75-155)	107 (75-148)	85 (63-121)	55 (44-79)	24 (20-33)	8 (7-11)
Asbestosis	20 (15-26)	23 (18-30)	28 (21-37)	35 (25-48)	44 (29-58)	43 (28-57)	60 (36-78)	67 (41-85)	51 (29-64)	37 (21-46)	17 (9-21)	6 (3-7)
Coal workers pneumoconiosis	6 (3-9)	6 (3-9)	9 (4-12)	15 (7-22)	20 (10-30)	38 (18-55)	47 (26-66)	52 (31-73)	34 (21-47)	26 (18-35)	12 (8-15)	5 (4-6)
Other pneumoconiosis	22 (16-29)	23 (17-30)	31 (23-40)	43 (30-58)	69 (49-94)	95 (68-133)	122 (93-163)	126 (96-166)	119 (94-156)	82 (68-100)	35 (30-43)	12 (10-14)
Asthma	7,437 (5,786-8,789)	11,250 (8,588-13,358)	13,248 (9,833-16,179)	18,362 (13,283-22,703)	24,097 (17,378-30,670)	28,487 (20,712-36,132)	29,464 (21,679-37,684)	27,911 (21,044-36,067)	20,473 (15,597-28,286)	12,656 (9,990-17,031)	5,022 (4,016-6,716)	1,402 (1,155-1,838)
Interstitial lung disease and pulmonary sarcoidosis	677 (392-1,079)	997 (584-1,592)	1,383 (781-2,241)	1,882 (1,047-3,232)	2,818 (1,546-4,670)	3,950 (2,229-6,378)	5,132 (3,203-7,590)	5,517 (3,566-7,457)	4,570 (2,998-5,654)	3,442 (2,253-4,104)	1,532 (1,063-1,884)	493 (340-616)
Other chronic respiratory diseases	311 (184-429)	218 (128-294)	297 (177-409)	338 (199-474)	411 (234-586)	545 (317-757)	648 (363-896)	774 (424-1,042)	739 (380-1,016)	678 (317-932)	369 (164-502)	124 (57-171)
Cirrhosis and other chronic liver diseases	18,289 (17,377-19,261)	23,328 (22,265-24,451)	28,938 (27,443-30,343)	31,586 (29,865-33,016)	38,780 (36,617-40,575)	41,069 (38,783-43,134)	41,152 (38,727-43,448)	33,587 (31,850-35,134)	23,488 (22,142-24,760)	14,947 (14,239-15,771)	6,517 (6,165-6,855)	2,228 (2,104-2,340)
Cirrhosis and other chronic liver diseases due to hepatitis B	5,184 (4,407-6,023)	6,070 (5,079-7,202)	7,208 (5,926-8,620)	8,052 (6,710-9,573)	10,137 (8,346-12,329)	11,159 (9,258-13,274)	11,016 (9,072-13,249)	8,787 (7,265-10,340)	5,911 (4,845-7,026)	3,263 (2,751-3,776)	1,192 (960-1,440)	356 (256-467)
Cirrhosis and other chronic liver diseases due to hepatitis C	4,038 (3,289-4,901)	5,104 (4,019-6,208)	6,488 (5,148-7,864)	6,859 (5,638-8,195)	8,298 (6,554-10,303)	8,982 (7,437-10,639)	9,266 (7,687-11,104)	7,273 (5,840-8,883)	5,003 (3,974-6,190)	3,257 (2,696-3,876)	1,463 (1,141-1,807)	486 (341-639)
Cirrhosis and other chronic liver diseases due to alcohol use	5,013 (4,308-5,801)	6,693 (5,586-7,798)	8,185 (6,913-9,524)	8,442 (6,983-9,989)	10,030 (8,079-12,167)	10,369 (8,572-12,193)	10,290 (8,513-12,241)	8,525 (7,099-9,968)	5,798 (4,774-6,840)	4,004 (3,475-4,529)	1,887 (1,570-2,188)	683 (540-832)
Cirrhosis and other chronic liver diseases due to other causes	4,053 (3,129-5,050)	5,460 (4,165-6,856)	7,058 (5,518-8,754)	8,233 (6,469-10,227)	10,315 (7,961-12,960)	10,558 (8,094-13,108)	10,580 (8,229-13,146)	9,001 (7,098-10,924)	6,776 (5,344-8,397)	4,423 (3,739-5,227)	1,975 (1,623-2,383)	702 (530-895)
Digestive diseases	11,860 (10,397-12,859)	15,592 (13,840-16,965)	19,486 (17,164-21,153)	26,334 (22,706-28,714)	35,159 (30,316-38,138)	44,904 (38,774-48,867)	52,435 (46,376-56,464)	56,791 (50,824-60,795)	51,252 (46,154-55,144)	44,539 (41,135-47,509)	24,659 (22,934-26,232)	8,578 (7,902-9,071)
Peptic ulcer disease	3,653 (3,106-4,187)	4,683 (3,994-5,364)	6,441 (5,511-7,295)	7,660 (6,439-8,790)	10,959 (9,403-12,242)	14,264 (12,078-16,077)	15,999 (13,672-17,832)	16,741 (14,339-18,575)	14,250 (12,580-15,574)	11,050 (9,925-12,151)	5,565 (5,071-6,117)	1,805 (1,652-1,998)
Gastritis and duodenitis	523 (382-651)	739 (575-911)	877 (677-1,066)	1,061 (815-1,291)	1,470 (1,147-1,786)	1,970 (1,521-2,408)	2,070 (1,632-2,549)	2,325 (1,877-2,848)	2,434 (2,005-2,986)	1,884 (1,565-2,356)	971 (816-1,192)	327 (279-398)
Appendicitis	896 (667-1,080)	1,231 (899-1,514)	1,084 (821-1,310)	2,119 (1,562-2,591)	1,990 (1,418-2,442)	2,024 (1,452-2,554)	1,748 (1,311-2,184)	1,625 (1,244-2,034)	1,148 (868-1,472)	810 (637-1,024)	388 (298-493)	119 (92-155)
Paralytic ileus and intestinal obstruction	2,443 (1,523-2,944)	2,962 (1,829-3,571)	3,773 (2,452-4,517)	5,753 (3,769-6,942)	6,651 (4,297-8,004)	8,199 (5,257-9,823)	8,628 (6,199-10,226)	9,111 (6,855-10,823)	8,086 (6,260-9,606)	7,625 (5,921-9,068)	4,665 (3,568-5,455)	1,686 (1,251-1,964)
Inguinal, femoral, and abdominal hernia	233 (148-329)	340 (208-450)	612 (378-861)	958 (542-1,226)	1,451 (820-1,851)	1,709 (1,003-2,171)	2,106 (1,217-2,612)	2,128 (1,307-2,689)	2,234 (1,361-2,817)	1,905 (1,221-2,499)	1,072 (675-1,469)	351 (233-490)
Inflammatory bowel disease	431 (304-596)	608 (421-816)	634 (459-816)	817 (595-1,083)	1,031 (740-1,301)	1,276 (925-1,590)	1,828 (1,352-2,206)	2,115 (1,484-2,469)	2,118 (1,464-2,360)	2,014 (1,410-2,262)	1,207 (815-1,374)	470 (323-543)
Vascular intestinal disorders	375 (295-444)	655 (532-778)	897 (745-1,051)	1,305 (1,064-1,553)	2,376 (2,019-2,750)	3,717 (3,226-4,271)	5,778 (5,139-6,494)	7,468 (6,755-8,228)	6,499 (5,982-7,125)	6,045 (5,590-6,610)	3,095 (2,835-3,423)	925 (834-1,034)
Gallbladder and biliary diseases	928 (789-1,097)	1,310 (1,093-1,540)	1,828 (1,532-2,114)	2,325 (1,954-2,718)	3,489 (2,879-4,037)	4,783 (3,904-5,640)	5,696 (4,777-6,521)	6,350 (5,454-7,304)	5,991 (5,196-6,957)	5,491 (4,845-6,110)	3,269 (2,933-3,652)	1,226 (1,109-1,349)
Pancreatitis	1,564 (1,193-2,130)	2,053 (1,557-2,829)	2,091 (1,624-2,908)	2,575 (1,893-3,562)	3,067 (2,357-4,151)	3,567 (2,711-4,856)	3,957 (3,109-5,100)	3,550 (2,862-4,555)	2,818 (2,293-3,546)	2,188 (1,838-2,718)	1,041 (882-1,264)	323 (266-391)
Other digestive diseases	813 (663-975)	1,010 (842-1,196)	1,250 (1,063-1,510)	1,762 (1,502-2,166)	2,675 (2,284-3,208)	3,395 (2,886-4,084)	4,625 (4,053-5,429)	5,378 (4,787-6,225)	5,674 (5,113-6,507)	5,527 (5,058-6,118)	3,386 (3,097-3,701)	1,347 (1,227-1,492)
Neurological disorders	4,846 (4,251-5,280)	5,994 (5,427-6,685)	8,384 (7,283-9,693)	10,601 (8,697-12,807)	19,839 (16,210-24,631)	37,490 (29,607-47,035)	77,360 (60,286-98,293)	144,950 (113,922-182,821)	196,660 (156,866-246,862)	242,455 (195,889-294,697)	183,196 (153,196-214,744)	66,461 (54,004-81,499)
Alzheimer's disease and other dementias	50 (22-95)	1,061 (645-1,573)	3,140 (2,142-4,399)	5,389 (3,550-7,589)	13,015 (9,473-17,751)	28,666 (21,293-38,344)	63,887 (46,959-85,087)	127,538 (95,865-164,478)	179,755 (139,603-230,223)	228,525 (181,700-281,293)	177,319 (146,905-208,664)	64,930 (52,524-80,087)
Parkinson's disease	102 (56-172)	172 (95-298)	281 (162-450)	502 (300-747)	1,320 (900-1,872)	3,080 (2,165-4,166)	7,276 (5,059-9,829)	12,436 (8,976-16,679)	13,587 (10,177-18,095)	11,558 (8,814-15,324)	4,956 (3,760-6,474)	1,260 (914-1,692)
Epilepsy	2,915 (2,367-3,197)	2,277 (1,869-2,489)	2,195 (1,805-2,386)	1,830 (1,425-2,015)	2,145 (1,667-2,372)	2,130 (1,640-2,360)	2,380 (1,859-2,668)	1,438 (1,207-1,575)	1,101 (942-1,217)	922 (811-1,013)	361 (327-390)	105 (95-113)
Multiple sclerosis	831 (653-1,030)	1,055 (830-1,263)	1,070 (843-1,294)	953 (763-1,197)	898 (746-1,158)	810 (676-1,010)	728 (587-862)	574 (435-679)	326 (236-367)	177 (122-198)	56 (40-63)	12 (9-13)
Motor neuron disease	239 (225-275)	459 (436-526)	705 (669-779)	880 (843-980)	1,226 (1,176-1,310)	1,484 (1,427-1,568)	1,649 (1,583-1,715)	1,523 (1,457-1,593)	819 (783-858)	420 (399-442)	110 (104-115)	20 (19-21)
Other neurological disorders	711 (671-743)	971 (906-1,029)	993 (957-1,041)	1,047 (1,009-1,099)	1,235 (1,186-1,300)	1,319 (1,270-1,382)	1,439 (1,382-1,499)	1,440 (1,371-1,513)	1,072 (1,009-1,140)	853 (796-910)	395 (370-422)	134 (126-143)
Mental and substance use disorders	6,281 (5,710-6,780)	6,480 (5,930-7,024)	5,775 (5,287-6,267)	4,372 (3,963-4,771)	4,768 (4,309-5,228)	3,922 (3,550-4,215)	2,982 (2,670-3,196)	2,760 (2,490-2,970)	1,730 (1,605-1,846)	1,289 (1,203-1,383)	629 (583-673)	221 (207-234)
Alcohol use disorders	3,176 (2,822-3,578)	3,787 (3,397-4,262)	3,712 (3,316-4,148)	2,800 (2,512-3,130)	3,032 (2,724-3,421)	2,168 (1,980-2,377)	1,295 (1,196-1,404)	1,144 (1,054-1,248)	560 (520-601)	374 (350-399)	167 (156-178)	59 (55-63)
Drug use disorders	3,053 (2,575-3,317)	2,640 (2,263-2,858)	2,062 (1,767-2,248)	1,573 (1,265-1,721)	1,736 (1,403-1,920)	1,754 (1,378-1,937)	1,687 (1,361-1,873)	1,616 (1,379-1,766)	1,170 (1,039-1,272)	915 (835-993)	463 (419-499)	162 (148-174)
Opioid use disorders	1,743 (1,321-1,971)	1,472 (1,153-1,677)	1,151 (894-1,323)	887 (683-1,021)	979 (739-1,146)	986 (748-1,181)	696 (502-836)	627 (475-769)	377 (274-484)	294 (216-362)	146 (100-178)	48 (33-59)
Cocaine use disorders	184 (117-262)	157 (111-233)	95 (62-133)	67 (41-99)	69 (39-104)	70 (39-110)	18 (11-30)	16 (9-25)	5 (3-7)	5 (2-7)	3 (1-4)	1 (0-2)
Amphetamine use disorders	74 (45-109)	68 (44-98)	44 (28-65)	35 (19-55)	35 (19-59)	34 (17-59)	12 (6-21)	12 (8-20)	2 (1-3)	3 (2-4)	2 (1-2)	1 (0-1)
Other drug use disorders	1,052 (817-1,235)	944 (754-1,111)	772 (594-903)	583 (434-682)	653 (480-798)	664 (478-786)	961 (754-1,131)	962 (766-1,102)	786 (668-882)	614 (532-706)	312 (278-359)	112 (100-128)
Eating disorders	52 (45-62)	52 (45-62)	0 (0-0)	0 (0-0)	-	-	-	-	-	-	-	-
Anorexia nervosa	47 (41-55)	47 (40-56)	0 (0-0)	0 (0-0)	-	-	-	-	-	-	-	-
Bulimia nervosa	6 (4-7)	5 (4-7)	0 (0-0)	0 (0-0)	-	-	-	-	-	-	-	-
Diabetes, urogenital, blood, and endocrine diseases	28,082 (26,743-29,450)	39,369 (37,566-41,267)	52,371 (49,942-54,834)	66,414 (63,346-69,558)	95,527 (90,980-100,569)	120,334 (114,900-126,633)	136,225 (129,865-144,326)	139,376 (132,468-146,126)	119,596 (113,862-126,636)	97,985 (93,844-102,817)	53,027 (50,744-55,478)	19,304 (18,434-20,083)
Diabetes mellitus	9,573 (9,069-10,040)	15,447 (14,700-16,164)	23,766 (22,678-24,891)	33,650 (31,973-35,121)	53,009 (50,432-55,658)	68,074 (64,929-71,454)	76,084 (72,098-80,063)	76,122 (71,930-79,953)	62,217 (58,613-66,405)	44,650 (42,447-47,109)	21,094 (20,050-22,248)	6,385 (6,023-6,726)
Acute glomerulonephritis	299 (265-337)	353 (308-403)	277 (245-314)	303 (269-342)	445 (392-507)	527 (465-613)	565 (500-651)	491 (426-572)	426 (369-492)	287 (251-327)	92 (82-105)	33 (29-38)
Chronic kidney disease	11,487 (10,810-12,107)	15,500 (14,646-16,339)	19,528 (18,438-20,638)	23,645 (22,309-24,982)	31,009 (29,282-32,991)	37,743 (35,780-40,181)	42,019 (39,895-45,039)	44,118 (41,778-46,538)	38,478 (36,611-40,952)	33,877 (32,359-35,546)	19,722 (18,800-20,674)	7,686 (7,268-8,039)
Chronic kidney disease due to diabetes mellitus	4,770 (3,708-5,764)	7,022 (5,548-8,560)	9,119 (6,993-11,115)	11,481 (9,027-13,781)	15,212 (12,341-18,113)	17,992 (13,849-21,581)	18,583 (14,634-22,403)	17,474 (13,462-21,143)	12,991 (9,623-16,245)	9,626 (7,019-12,668)	5,211 (3,698-6,928)	2,041 (1,360-2,751)
Chronic kidney disease due to hypertension	1,557 (1,187-2,014)	2,400 (1,862-3,073)	3,369 (2,577-4,382)	4,602 (3,577-5,912)	6,541 (5,142-8,270)	8,992 (6,999-11,422)	10,964 (8,504-13,699)	12,917 (10,205-16,003)	12,949 (10,542-15,806)	13,151 (10,722-15,822)	8,505 (7,056-9,957)	3,469 (2,789-4,200)
Chronic kidney disease due to glomerulonephritis	2,463 (1,653-3,284)	2,766 (1,838-3,788)	3,081 (2,097-4,312)	3,147 (2,154-4,371)	3,842 (2,572-5,372)	4,390 (2,991-6,215)	5,039 (3,503-7,014)	5,508 (3,874-7,646)	4,918 (3,528-6,522)	3,982 (2,878-5,405)	1,984 (1,447-2,561)	702 (443-1,086)
Chronic kidney disease due to other causes	2,697 (1,874-3,639)	3,313 (2,233-4,666)	3,959 (2,595-5,580)	4,415 (3,010-6,233)	5,414 (3,625-7,742)	6,368 (4,302-8,998)	7,432 (5,135-10,047)	8,220 (5,772-11,212)	7,620 (5,396-10,135)	7,118 (4,920-9,525)	4,022 (2,952-5,252)	1,474 (934-2,086)
Urinary diseases and male infertility	2,122 (1,993-2,286)	2,653 (2,493-2,857)	3,334 (3,101-3,611)	4,091 (3,783-4,470)	5,724 (5,302-6,141)	7,044 (6,506-7,609)	8,704 (8,085-9,333)	10,049 (9,461-10,644)	10,361 (9,852-10,903)	10,725 (10,263-11,203)	7,381 (7,029-7,704)	3,306 (3,158-3,452)
Interstitial nephritis and urinary tract infections	1,529 (1,295-1,698)	1,885 (1,604-2,086)	2,428 (2,093-2,703)	2,846 (2,377-3,215)	3,982 (3,316-4,405)	5,174 (4,422-5,694)	6,784 (6,010-7,353)	7,997 (7,170-8,605)	8,595 (7,909-9,155)	9,270 (8,621-9,791)	6,539 (6,108-6,901)	2,998 (2,804-3,164)
Urolithiasis	183 (105-223)	280 (168-338)	343 (203-419)	387 (240-475)	543 (360-661)	644 (434-785)	737 (526-924)	745 (544-984)	645 (480-832)	533 (392-709)	314 (220-423)	106 (78-154)
Other urinary diseases	410 (297-654)	488 (361-794)	563 (397-954)	857 (600-1,422)	1,200 (863-1,933)	1,226 (873-2,021)	1,183 (888-1,928)	1,306 (973-2,076)	1,121 (861-1,758)	922 (689-1,471)	529 (404-847)	202 (151-335)
Gynecological diseases	633 (540-715)	875 (765-982)	682 (578-771)	485 (413-555)	305 (262-346)	469 (393-537)	272 (230-316)	197 (173-236)	388 (313-465)	273 (223-324)	121 (103-145)	44 (37-51)
Uterine fibroids	230 (126-318)	416 (210-557)	381 (186-503)	233 (98-339)	121 (57-185)	171 (81-271)	74 (41-119)	53 (36-74)	102 (38-203)	56 (22-114)	24 (12-44)	8 (4-13)
Polycystic ovarian syndrome	69 (18-137)	105 (26-220)	168 (52-322)	-	-	-	-	-	-	-	-	-
Endometriosis	9 (4-14)	15 (7-25)	10 (5-17)	-	-	-	-	-	-	-	-	-
Genital prolapse	9 (4-16)	10 (4-18)	17 (5-38)	96 (17-173)	85 (25-151)	138 (41-230)	101 (42-158)	58 (35-82)	157 (51-260)	103 (41-170)	43 (20-67)	14 (8-20)
Other gynecological diseases	316 (199-453)	329 (172-553)	107 (48-223)	157 (82-268)	99 (56-166)	160 (88-256)	97 (61-152)	87 (65-115)	129 (73-228)	114 (77-180)	54 (38-81)	22 (16-30)
Hemoglobinopathies and hemolytic anaemias	2,295 (1,757-3,048)	2,403 (1,888-3,196)	2,267 (1,790-3,061)	1,792 (1,417-2,381)	1,909 (1,539-2,536)	2,561 (2,068-3,410)	4,066 (3,317-5,474)	3,568 (2,851-4,583)	3,288 (2,607-4,158)	4,185 (3,242-5,118)	2,243 (1,592-2,665)	839 (566-980)
Thalassemias	34 (27-47)	28 (23-39)	21 (18-33)	12 (9-19)	7 (5-11)	4 (3-7)	2 (2-4)	1 (1-1)	0 (0-0)	0 (0-0)	0 (0-0)	0 (0-0)
Sickle cell disorders	1,144 (838-1,513)	1,044 (779-1,398)	741 (558-1,004)	306 (236-408)	121 (97-170)	74 (60-105)	46 (38-65)	18 (14-25)	6 (5-9)	3 (2-3)	1 (0-1)	0 (0-0)
G6PD deficiency	461 (351-625)	510 (397-720)	487 (366-647)	321 (242-443)	272 (207-374)	252 (189-354)	279 (209-400)	126 (94-168)	59 (44-79)	39 (30-53)	12 (9-16)	2 (2-3)
Other hemoglobinopathies and hemolytic anaemias	656 (521-894)	822 (658-1,122)	1,018 (823-1,371)	1,153 (923-1,541)	1,509 (1,223-1,995)	2,231 (1,798-2,968)	3,738 (3,044-5,004)	3,423 (2,735-4,397)	3,223 (2,553-4,072)	4,144 (3,207-5,067)	2,230 (1,581-2,647)	836 (564-977)
Endocrine, metabolic, blood, and immune disorders	1,674 (1,388-1,880)	2,138 (1,758-2,392)	2,517 (1,979-2,725)	2,449 (2,016-2,764)	3,126 (2,662-3,635)	3,917 (3,283-4,539)	4,516 (3,792-5,188)	4,831 (3,867-5,622)	4,438 (3,522-5,228)	3,988 (3,143-4,443)	2,372 (1,788-2,686)	1,012 (743-1,122)
Musculoskeletal disorders	2,098 (1,685-2,505)	2,282 (1,891-2,688)	2,278 (1,820-2,675)	2,419 (1,980-2,780)	3,243 (2,720-3,817)	4,065 (3,412-4,611)	4,929 (4,092-5,461)	5,597 (4,660-6,215)	4,841 (4,053-5,436)	4,408 (3,567-4,888)	2,551 (1,949-2,794)	1,037 (796-1,131)
Rheumatoid arthritis	349 (260-502)	610 (457-803)	838 (626-1,048)	1,144 (867-1,386)	1,768 (1,355-2,151)	2,384 (1,870-2,870)	2,934 (2,351-3,482)	3,157 (2,534-3,756)	2,451 (1,979-3,027)	1,754 (1,416-2,331)	751 (600-1,031)	226 (183-326)
Other musculoskeletal disorders	1,750 (1,400-2,051)	1,672 (1,362-1,978)	1,440 (1,146-1,668)	1,276 (1,019-1,473)	1,475 (1,183-1,770)	1,681 (1,356-1,938)	1,995 (1,489-2,314)	2,440 (1,813-2,752)	2,390 (1,800-2,640)	2,654 (1,927-2,916)	1,799 (1,239-1,970)	811 (593-881)
Other non-communicable diseases	2,445 (2,010-2,871)	2,358 (1,824-2,829)	2,699 (1,925-3,438)	2,863 (2,025-3,644)	3,069 (2,074-4,023)	3,674 (2,345-5,142)	3,386 (1,813-5,178)	4,333 (2,487-6,783)	4,456 (2,468-6,912)	4,602 (2,419-7,009)	3,322 (1,594-4,905)	1,588 (680-2,296)
Congenital anomalies	1,749 (1,517-2,086)	1,391 (1,182-1,630)	1,215 (1,004-1,433)	1,101 (905-1,259)	1,021 (878-1,210)	863 (738-1,003)	-	-	-	-	-	-
Neural tube defects	37 (29-51)	32 (26-41)	27 (22-34)	24 (19-30)	20 (16-24)	21 (16-27)	-	-	-	-	-	-
Congenital heart anomalies	1,245 (1,057-1,462)	673 (545-771)	520 (425-603)	426 (350-497)	389 (332-471)	358 (301-424)	-	-	-	-	-	-
Orofacial clefts	-	-	-	-	-	-	-	-	-	-	-	-
Down's syndrome	158 (128-207)	273 (214-338)	275 (209-333)	260 (201-309)	199 (155-246)	89 (65-107)	-	-	-	-	-	-
Other chromosomal abnormalities	25 (14-32)	21 (11-24)	23 (13-28)	21 (12-26)	14 (8-18)	14 (9-17)	-	-	-	-	-	-
Congenital musculoskeletal and limb anomalies	23 (17-29)	27 (18-34)	25 (18-32)	23 (17-29)	21 (14-25)	21 (15-24)	-	-	-	-	-	-
Urogenital congenital anomalies	48 (33-65)	79 (49-103)	97 (65-125)	114 (78-142)	134 (90-173)	159 (117-210)	-	-	-	-	-	-
Digestive congenital anomalies	15 (12-19)	21 (16-26)	27 (21-32)	26 (20-32)	30 (24-37)	34 (27-39)	-	-	-	-	-	-
Other congenital anomalies	197 (129-279)	266 (179-364)	222 (148-312)	207 (135-301)	214 (146-290)	169 (116-227)	-	-	-	-	-	-
Skin and subcutaneous diseases	696 (352-1,016)	967 (480-1,373)	1,484 (753-2,117)	1,762 (916-2,575)	2,048 (1,051-2,967)	2,811 (1,490-4,287)	3,386 (1,813-5,178)	4,333 (2,487-6,783)	4,456 (2,468-6,912)	4,602 (2,419-7,009)	3,322 (1,594-4,905)	1,588 (680-2,296)
Cellulitis	93 (41-144)	137 (50-198)	230 (87-333)	250 (101-389)	346 (125-493)	387 (154-603)	482 (187-736)	544 (234-951)	527 (237-1,035)	495 (231-950)	342 (137-708)	158 (67-332)
Pyoderma	461 (220-669)	653 (305-988)	1,018 (483-1,534)	1,202 (598-1,823)	1,302 (635-2,004)	1,688 (845-2,667)	1,815 (950-2,951)	2,032 (1,108-3,362)	1,719 (912-2,859)	1,603 (816-2,570)	1,067 (517-1,669)	476 (211-737)
Decubitus ulcer	98 (49-161)	133 (71-220)	187 (90-317)	251 (124-434)	329 (165-540)	631 (304-1,027)	972 (503-1,556)	1,595 (796-2,530)	2,029 (1,024-3,046)	2,308 (1,080-3,264)	1,766 (767-2,508)	892 (345-1,245)
Other skin and subcutaneous diseases	43 (23-70)	45 (25-73)	49 (27-82)	59 (33-96)	71 (39-115)	106 (56-168)	117 (64-200)	163 (88-276)	182 (95-313)	196 (93-340)	147 (66-244)	63 (25-106)
Sudden infant death syndrome	-	-	-	-	-	-	-	-	-	-	-	-
Injuries	70,799 (66,253-73,548)	69,822 (65,458-72,657)	63,116 (58,817-65,782)	58,470 (54,077-61,169)	66,977 (61,149-70,420)	71,036 (64,135-75,051)	75,692 (68,117-80,214)	73,618 (66,159-78,137)	61,851 (54,972-66,218)	52,226 (46,583-55,708)	27,928 (24,105-29,856)	10,473 (8,748-11,239)
Transport injuries	22,487 (21,388-23,583)	21,312 (20,220-22,447)	18,958 (17,993-20,042)	17,624 (16,561-18,647)	18,837 (17,597-20,195)	17,437 (16,335-18,673)	17,954 (16,654-19,225)	14,520 (13,581-15,476)	9,441 (8,871-10,252)	5,530 (5,208-5,950)	2,185 (2,054-2,347)	696 (651-749)
Road injuries	21,381 (20,346-22,456)	20,127 (19,085-21,220)	17,893 (16,956-18,911)	16,848 (15,824-17,828)	17,733 (16,565-19,020)	16,456 (15,418-17,668)	16,992 (15,772-18,241)	13,679 (12,791-14,644)	8,869 (8,319-9,668)	5,169 (4,868-5,557)	2,023 (1,903-2,179)	629 (587-680)
Pedestrian road injuries	8,778 (7,991-9,626)	8,891 (8,140-9,732)	7,986 (7,294-8,689)	8,022 (7,331-8,767)	9,690 (8,894-10,582)	9,345 (8,621-10,221)	9,959 (9,143-10,903)	8,429 (7,774-9,101)	5,550 (5,167-6,041)	3,095 (2,890-3,416)	1,088 (1,006-1,254)	311 (282-374)
Cyclist road injuries	1,174 (948-1,349)	1,390 (1,138-1,577)	1,079 (914-1,244)	996 (841-1,147)	960 (813-1,140)	983 (832-1,142)	918 (761-1,122)	508 (377-608)	219 (162-278)	128 (96-179)	48 (36-73)	0 (0-1)
Motorcyclist road injuries	3,121 (2,768-3,514)	2,567 (2,256-2,959)	2,262 (1,988-2,610)	1,924 (1,683-2,261)	1,434 (1,237-1,692)	1,163 (1,001-1,378)	917 (773-1,112)	489 (406-613)	264 (217-338)	119 (97-152)	41 (34-56)	16 (13-22)
Motor vehicle road injuries	8,131 (7,326-9,003)	7,052 (6,355-7,844)	6,387 (5,776-7,096)	5,713 (5,151-6,406)	5,569 (5,014-6,322)	4,887 (4,390-5,607)	5,123 (4,599-5,895)	4,089 (3,696-4,636)	2,739 (2,476-3,188)	1,768 (1,566-2,016)	816 (704-916)	289 (233-324)
Other road injuries	177 (138-267)	228 (178-335)	178 (138-272)	193 (147-304)	79 (62-106)	79 (60-110)	77 (58-106)	164 (123-261)	97 (68-154)	59 (40-91)	30 (19-46)	13 (8-21)
Other transport injuries	1,106 (910-1,258)	1,184 (982-1,350)	1,065 (871-1,220)	776 (650-901)	1,105 (908-1,263)	981 (820-1,131)	961 (816-1,107)	841 (714-962)	572 (483-657)	361 (312-414)	161 (140-184)	67 (60-76)
Unintentional injuries	19,919 (17,092-21,375)	21,677 (18,657-23,276)	21,273 (17,998-22,948)	22,630 (19,179-24,519)	29,662 (24,538-32,281)	35,752 (28,894-39,128)	40,699 (33,419-44,423)	45,502 (37,768-49,411)	43,636 (36,725-47,582)	41,523 (35,706-44,720)	23,968 (20,103-25,802)	9,340 (7,620-10,072)
Falls	3,346 (2,929-3,729)	4,352 (3,670-4,748)	5,157 (4,302-5,670)	7,224 (5,719-8,124)	11,253 (8,818-12,660)	16,364 (12,347-18,499)	20,878 (15,975-23,503)	26,594 (21,012-29,399)	27,325 (21,847-30,343)	28,004 (23,518-30,763)	16,655 (13,383-18,245)	6,348 (4,922-7,045)
Drowning	3,305 (2,613-3,582)	3,368 (2,634-3,642)	3,120 (2,406-3,377)	2,947 (2,267-3,211)	3,437 (2,637-3,740)	3,708 (2,886-4,070)	3,777 (2,968-4,117)	3,666 (2,786-4,068)	2,773 (2,134-3,075)	1,627 (1,314-1,788)	529 (438-579)	127 (105-139)
Fire, heat, and hot substances	2,740 (2,057-3,068)	2,978 (2,204-3,330)	2,664 (2,000-2,958)	2,458 (1,861-2,737)	2,894 (2,273-3,241)	3,415 (2,698-3,796)	3,718 (2,981-4,204)	3,973 (3,242-4,498)	3,692 (2,979-4,137)	3,124 (2,436-3,452)	1,699 (1,239-1,873)	712 (499-779)
Poisonings	1,219 (1,021-1,440)	1,120 (908-1,358)	1,069 (883-1,321)	974 (791-1,177)	960 (816-1,207)	1,093 (878-1,304)	1,097 (902-1,323)	827 (667-995)	676 (567-828)	422 (360-524)	178 (159-232)	61 (54-85)
Exposure to mechanical forces	1,703 (1,380-1,887)	1,702 (1,396-1,878)	1,330 (1,094-1,462)	1,578 (1,262-1,761)	1,514 (1,169-1,717)	1,478 (1,139-1,659)	1,379 (1,118-1,525)	1,234 (1,027-1,347)	1,170 (980-1,284)	1,065 (879-1,164)	614 (500-673)	258 (209-283)
Unintentional firearm injuries	290 (249-323)	290 (253-327)	200 (165-223)	197 (176-224)	182 (155-203)	198 (170-215)	221 (188-242)	286 (242-315)	277 (224-306)	267 (204-295)	166 (131-182)	70 (54-79)
Unintentional suffocation	85 (54-102)	149 (122-205)	163 (136-230)	174 (149-264)	65 (44-88)	80 (52-104)	88 (61-117)	72 (46-92)	79 (52-98)	67 (41-83)	29 (20-37)	10 (7-13)
Other exposure to mechanical forces	1,328 (1,017-1,507)	1,263 (972-1,426)	966 (756-1,080)	1,207 (888-1,390)	1,266 (932-1,459)	1,200 (872-1,370)	1,070 (823-1,203)	876 (685-974)	814 (649-911)	731 (575-813)	419 (324-468)	177 (133-198)
Adverse effects of medical treatment	2,182 (1,631-2,507)	2,657 (2,044-3,024)	2,100 (1,666-2,400)	2,509 (1,938-2,885)	3,720 (2,996-4,314)	4,049 (3,193-4,748)	4,233 (3,420-5,045)	4,138 (3,279-5,017)	3,496 (2,756-4,282)	3,192 (2,468-4,036)	1,869 (1,423-2,489)	736 (567-975)
Animal contact	2,176 (780-2,867)	2,026 (769-2,598)	2,382 (934-3,091)	1,694 (701-2,162)	2,049 (762-2,712)	2,139 (812-2,814)	1,987 (774-2,693)	1,285 (538-1,672)	781 (359-1,024)	384 (205-483)	143 (85-173)	48 (32-57)
Venomous animal contact	2,019 (690-2,690)	1,797 (642-2,350)	2,141 (775-2,843)	1,473 (569-1,925)	1,877 (650-2,545)	1,961 (703-2,626)	1,788 (639-2,474)	1,129 (428-1,508)	649 (271-875)	297 (137-389)	102 (54-128)	31 (18-37)
Non-venomous animal contact	157 (82-290)	229 (116-382)	240 (126-402)	220 (118-391)	172 (82-288)	178 (88-308)	199 (99-349)	155 (85-247)	131 (70-188)	87 (54-132)	41 (27-54)	17 (12-22)
Foreign body	1,080 (879-1,243)	1,117 (892-1,256)	1,141 (892-1,289)	1,184 (952-1,327)	1,404 (1,099-1,579)	1,390 (1,057-1,536)	1,676 (1,219-1,888)	2,000 (1,403-2,297)	2,073 (1,411-2,426)	2,247 (1,432-2,718)	1,460 (871-1,788)	721 (419-897)
Pulmonary aspiration and foreign body in airway	825 (661-954)	933 (735-1,082)	965 (744-1,105)	1,006 (787-1,149)	1,251 (973-1,417)	1,254 (952-1,400)	1,494 (1,069-1,701)	1,827 (1,263-2,110)	1,874 (1,261-2,210)	2,055 (1,296-2,503)	1,359 (797-1,670)	680 (393-855)
Foreign body in other body part	255 (161-345)	184 (110-235)	176 (114-236)	178 (114-249)	153 (94-209)	136 (75-165)	182 (100-221)	173 (83-212)	198 (93-246)	192 (81-239)	102 (46-125)	41 (19-50)
Environmental heat and cold exposure	1,016 (547-1,359)	1,302 (678-1,802)	1,295 (727-1,920)	1,148 (651-1,588)	1,644 (911-2,328)	1,212 (667-1,660)	1,378 (751-1,871)	1,253 (676-1,654)	1,167 (693-1,549)	1,091 (659-1,631)	625 (376-991)	248 (140-386)
Other unintentional injuries	1,151 (892-1,282)	1,056 (853-1,168)	1,016 (747-1,161)	914 (719-1,044)	788 (635-897)	905 (683-1,053)	576 (494-660)	532 (431-614)	484 (392-569)	367 (314-428)	196 (163-224)	81 (67-92)
Self-harm and interpersonal violence	27,278 (25,314-28,942)	25,831 (24,121-27,335)	21,954 (20,552-23,157)	17,428 (16,320-18,389)	17,697 (16,508-18,672)	17,121 (16,126-18,129)	16,449 (15,458-17,491)	13,168 (12,290-14,004)	8,476 (7,952-9,067)	5,004 (4,727-5,308)	1,730 (1,641-1,847)	406 (383-437)

SR Table 3: Global deaths by cause and age group for females (with 95% uncertainty intervals), 2000

Cause	Early Neonatal	Late Neonatal	Post Neonatal	1 to 4	5 to 9	10 to 14	15 to 19	20 to 24
Self-harm	-	-	-	-	-	6,154<br>(5,323-7,048)	35,807<br>(31,828-39,522)	40,903<br>(36,933-44,602)
Self-harm by firearm	-	-	-	-	-	205<br>(129-319)	1,329<br>(736-2,068)	1,486<br>(840-2,028)
Self-harm by other specified means	-	-	-	-	-	5,949<br>(5,179-6,771)	34,479<br>(30,690-37,843)	39,417<br>(35,522-42,736)
Interpersonal violence	1,032<br>(662-1,218)	279<br>(184-332)	2,248<br>(1,465-2,704)	4,097<br>(2,667-5,261)	3,065<br>(2,056-3,678)	3,274<br>(2,308-3,857)	7,406<br>(5,080-8,463)	9,534<br>(6,665-11,023)
Assault by firearm	48<br>(26-61)	20<br>(10-27)	224<br>(126-295)	371<br>(233-526)	358<br>(220-438)	549<br>(327-659)	1,948<br>(1,140-2,248)	2,276<br>(1,358-2,606)
Assault by sharp object	63<br>(41-81)	38<br>(17-53)	186<br>(92-236)	414<br>(205-553)	439<br>(238-540)	617<br>(372-764)	1,622<br>(962-1,911)	2,497<br>(1,505-2,927)
Assault by other means	920<br>(584-1,093)	220<br>(152-267)	1,838<br>(1,216-2,256)	3,313<br>(2,201-4,375)	2,268<br>(1,510-2,829)	2,109<br>(1,523-2,663)	3,837<br>(2,654-4,688)	4,761<br>(3,351-6,013)
Forces of nature, conflict and terrorism, and executions and police conflict	687<br>(598-767)	380<br>(330-425)	1,291<br>(1,115-1,449)	6,831<br>(5,923-7,670)	3,816<br>(3,270-4,320)	3,226<br>(2,758-3,667)	3,708<br>(3,205-4,189)	2,928<br>(2,518-3,313)
Forces of nature, conflict and terrorism, and state actor violence	2<br>(1-3)	8<br>(5-11)	66<br>(44-88)	336<br>(222-450)	324<br>(216-435)	348<br>(232-466)	268<br>(178-357)	224<br>(150-299)
Conflict and terrorism	685<br>(597-765)	372<br>(324-415)	1,211<br>(1,057-1,351)	6,450<br>(5,645-7,187)	3,455<br>(3,020-3,850)	2,831<br>(2,474-3,160)	3,399<br>(2,971-3,791)	2,670<br>(2,330-2,985)
Executions and police conflict	-	-	14<br>(9-22)	45<br>(23-97)	37<br>(30-48)	47<br>(37-64)	41<br>(32-56)	34<br>(27-42)

Cause	25 to 29	30 to 34	35 to 39	40 to 44	45 to 49	50 to 54	55 to 59	60 to 64
Self-harm	37,906<br>(34,485-41,130)	29,771<br>(27,544-31,944)	24,903<br>(23,276-26,640)	20,668<br>(19,366-21,981)	20,248<br>(19,100-21,476)	17,824<br>(16,890-18,753)	14,447<br>(13,636-15,253)	14,576<br>(13,724-15,406)
Self-harm by firearm	1,109<br>(709-1,417)	936<br>(681-1,213)	940<br>(727-1,258)	852<br>(694-1,108)	756<br>(619-985)	620<br>(504-841)	497<br>(391-717)	405<br>(302-618)
Self-harm by other specified means	36,797<br>(33,549-39,864)	28,836<br>(26,720-30,942)	23,962<br>(22,350-25,707)	19,816<br>(18,508-21,133)	19,492<br>(18,370-20,725)	17,203<br>(16,280-18,132)	13,950<br>(13,116-14,776)	14,171<br>(13,293-15,016)
Interpersonal violence	9,151<br>(6,359-10,527)	8,710<br>(5,914-9,959)	7,659<br>(5,157-8,658)	6,610<br>(4,365-7,566)	5,583<br>(3,695-6,380)	4,131<br>(2,781-4,699)	2,981<br>(1,953-3,403)	3,120<br>(2,012-3,602)
Assault by firearm	2,147<br>(1,230-2,528)	1,930<br>(1,084-2,265)	1,774<br>(960-2,089)	1,419<br>(811-1,662)	1,109<br>(638-1,303)	817<br>(467-958)	568<br>(326-671)	503<br>(293-609)
Assault by sharp object	2,700<br>(1,613-3,203)	2,696<br>(1,662-3,136)	2,348<br>(1,397-2,738)	2,175<br>(1,270-2,598)	1,766<br>(1,057-2,106)	1,190<br>(706-1,426)	814<br>(446-968)	882<br>(509-1,065)
Assault by other means	4,305<br>(3,042-5,261)	4,084<br>(2,856-5,009)	3,537<br>(2,471-4,198)	3,017<br>(2,096-3,586)	2,707<br>(1,861-3,197)	2,123<br>(1,424-2,464)	1,599<br>(1,098-1,849)	1,735<br>(1,099-2,037)
Forces of nature, conflict and terrorism, and executions and police conflict	1,934<br>(1,648-2,199)	1,686<br>(1,436-1,922)	1,366<br>(1,160-1,567)	1,116<br>(945-1,288)	1,002<br>(849-1,157)	930<br>(790-1,070)	787<br>(664-908)	781<br>(647-911)
Forces of nature, conflict and terrorism, and state actor violence	217<br>(145-288)	202<br>(136-267)	187<br>(126-247)	182<br>(123-240)	165<br>(113-218)	146<br>(100-191)	138<br>(94-181)	159<br>(109-209)
Conflict and terrorism	1,678<br>(1,463-1,876)	1,452<br>(1,267-1,629)	1,146<br>(1,000-1,292)	904<br>(785-1,025)	808<br>(701-916)	762<br>(663-864)	624<br>(545-705)	592<br>(505-683)
Executions and police conflict	39<br>(27-50)	32<br>(25-42)	32<br>(22-49)	30<br>(20-48)	29<br>(19-44)	22<br>(16-32)	26<br>(14-44)	30<br>(13-51)

Cause	65 to 69	70 to 74	75 to 79	80 to 84	85 to 89	90 to 94	95 plus
Self-harm	14,629<br>(13,770-15,565)	13,922<br>(13,100-14,838)	11,132<br>(10,463-11,934)	7,816<br>(7,311-8,403)	4,618<br>(4,352-4,914)	1,585<br>(1,502-1,687)	360<br>(340-382)
Self-harm by firearm	381<br>(269-626)	347<br>(240-577)	332<br>(228-558)	239<br>(178-372)	172<br>(145-219)	92<br>(83-109)	36<br>(33-41)
Self-harm by other specified means	14,248<br>(13,357-15,193)	13,576<br>(12,702-14,495)	10,800<br>(10,122-11,571)	7,577<br>(7,081-8,181)	4,447<br>(4,185-4,733)	1,493<br>(1,413-1,589)	324<br>(306-344)
Interpersonal violence	2,492<br>(1,720-2,836)	2,527<br>(1,725-2,859)	2,037<br>(1,349-2,302)	660<br>(478-777)	386<br>(265-455)	144<br>(113-190)	46<br>(36-63)
Assault by firearm	396<br>(236-497)	391<br>(233-475)	306<br>(180-384)	51<br>(28-69)	29<br>(17-42)	10<br>(7-17)	4<br>(3-7)
Assault by sharp object	610<br>(347-730)	706<br>(432-840)	546<br>(320-644)	180<br>(124-225)	93<br>(64-118)	40<br>(33-58)	12<br>(10-19)
Assault by other means	1,486<br>(1,053-1,745)	1,430<br>(961-1,637)	1,185<br>(798-1,382)	430<br>(307-512)	264<br>(176-309)	94<br>(67-122)	29<br>(22-42)
Forces of nature, conflict and terrorism, and executions and police conflict	727<br>(604-845)	590<br>(481-701)	428<br>(348-510)	299<br>(243-356)	168<br>(129-209)	46<br>(35-58)	31<br>(24-39)
Forces of nature, conflict and terrorism, and state actor violence	143<br>(99-188)	145<br>(100-191)	114<br>(79-149)	81<br>(56-106)	49<br>(34-65)	18<br>(12-23)	4<br>(3-6)
Conflict and terrorism	557<br>(476-640)	413<br>(340-487)	295<br>(243-347)	190<br>(158-223)	96<br>(73-119)	16<br>(12-20)	24<br>(18-30)
Executions and police conflict	26<br>(12-45)	32<br>(11-59)	19<br>(9-29)	28<br>(10-43)	23<br>(4-44)	12<br>(5-20)	3<br>(1-6)

SR Table 3: Global deaths by cause and age group for females (with 95% uncertainty intervals), 2006

Cause	Early Neonatal	Late Neonatal	Post Neonatal	1 to 4	5 to 9	10 to 14	15 to 19	20 to 24	25 to 29	30 to 34	35 to 39	40 to 44	45 to 49	50 to 54	55 to 59	60 to 64	65 to 69	70 to 74	75 to 79	80 to 84	85 to 89	90 to 94	95 plus
All causes	985,587<br>(962,839-1,010,706)	330,259<br>(321,828-339,076)	1,087,382<br>(1,060,007-1,116,356)	1,067,771<br>(1,029,965-1,106,690)	254,638<br>(251,072-258,470)	189,447<br>(186,826-192,469)	289,549<br>(284,174-295,571)	369,434<br>(360,486-378,777)	442,518<br>(427,544-457,821)	507,845<br>(495,951-520,005)	568,744<br>(555,469-582,878)	636,198<br>(626,730-645,637)	748,031<br>(737,719-760,086)	954,905<br>(942,294-968,335)	1,139,047<br>(1,123,858-1,155,411)	1,325,560<br>(1,308,705-1,343,419)	1,823,027<br>(1,799,924-1,847,867)	2,279,731<br>(2,252,185-2,309,432)	2,628,366<br>(2,597,192-2,662,660)	2,691,937<br>(2,667,297-2,717,482)	2,088,575<br>(2,075,551-2,101,623)	1,286,137<br>(1,279,953-1,291,957)	539,393<br>(537,167-541,427)
Communicable, maternal, neonatal, and nutritional disorders	894,320<br>(863,704-926,417)	290,889<br>(280,638-302,051)	905,493<br>(866,701-943,508)	876,039<br>(828,565-928,212)	168,600<br>(160,116-177,143)	99,056<br>(92,766-106,134)	119,820<br>(112,638-129,705)	179,501<br>(169,800-191,378)	250,940<br>(235,015-269,097)	278,471<br>(264,905-292,878)	263,763<br>(252,656-281,964)	226,085<br>(217,487-237,886)	193,357<br>(182,571-208,784)	173,702<br>(161,465-195,077)	167,015<br>(150,983-198,946)	182,823<br>(159,147-227,080)	222,507<br>(188,908-284,867)	278,083<br>(230,370-359,784)	267,948<br>(221,116-346,509)	268,526<br>(223,576-333,564)	203,456<br>(170,567-247,732)	108,987<br>(94,542-126,948)	51,235<br>(45,227-57,854)
HIV/AIDS and tuberculosis	-	-	58,739<br>(55,391-62,179)	41,628<br>(38,007-45,346)	22,518<br>(21,168-23,922)	22,403<br>(20,871-24,074)	29,884<br>(27,356-32,477)	62,706<br>(56,405-70,461)	144,600<br>(130,005-161,276)	181,442<br>(169,683-193,359)	175,850<br>(163,345-188,452)	149,678<br>(143,050-156,627)	123,882<br>(116,932-131,489)	99,417<br>(94,265-104,217)	79,423<br>(73,781-84,620)	66,755<br>(60,940-71,821)	65,074<br>(57,949-70,706)	64,220<br>(55,841-70,267)	48,821<br>(42,145-53,963)	37,333<br>(31,540-42,202)	20,059<br>(17,064-22,588)	6,622<br>(5,693-7,492)	1,822<br>(1,563-2,097)
Tuberculosis	-	-	8,593<br>(6,613-10,965)	12,608<br>(9,614-15,982)	5,489<br>(4,419-6,557)	6,317<br>(5,383-7,263)	15,854<br>(14,042-17,664)	23,966<br>(21,802-26,098)	27,142<br>(24,757-29,233)	28,651<br>(26,228-30,875)	31,255<br>(28,422-33,742)	30,826<br>(28,194-33,142)	34,718<br>(31,316-37,773)	40,879<br>(36,707-44,463)	45,343<br>(39,759-49,712)	45,327<br>(39,656-50,052)	50,945<br>(43,756-56,320)	55,718<br>(47,427-61,683)	44,962<br>(38,218-50,113)	36,070<br>(30,372-40,887)	19,587<br>(16,560-22,142)	6,491<br>(5,560-7,354)	1,793<br>(1,533-2,068)
Drug-susceptible tuberculosis	-	-	7,990<br>(6,108-10,264)	11,646<br>(8,886-14,812)	5,020<br>(4,047-5,970)	5,754<br>(4,902-6,636)	14,409<br>(12,675-16,108)	21,770<br>(19,782-23,702)	24,694<br>(22,523-26,655)	26,052<br>(23,804-28,119)	28,442<br>(25,861-30,791)	27,975<br>(25,571-30,269)	31,613<br>(28,395-34,606)	37,269<br>(33,323-40,729)	41,443<br>(36,357-45,583)	41,544<br>(36,441-45,876)	46,635<br>(40,027-51,769)	50,983<br>(43,457-56,492)	41,211<br>(35,220-46,021)	33,064<br>(27,879-37,577)	18,004<br>(15,218-20,477)	5,984<br>(5,141-6,781)	1,660<br>(1,419-1,919)
Multidrug-resistant tuberculosis without extensive drug resistance	-	-	591<br>(431-784)	942<br>(668-1,278)	457<br>(338-608)	547<br>(425-690)	1,399<br>(1,114-1,729)	2,111<br>(1,724-2,552)	2,345<br>(1,952-2,804)	2,489<br>(2,062-2,999)	2,690<br>(2,246-3,193)	2,719<br>(2,279-3,223)	2,969<br>(2,469-3,527)	3,465<br>(2,854-4,179)	3,759<br>(3,053-4,585)	3,655<br>(2,945-4,495)	4,154<br>(3,256-5,100)	4,569<br>(3,607-5,674)	3,610<br>(2,802-4,527)	2,886<br>(2,205-3,627)	1,516<br>(1,172-1,906)	483<br>(376-609)	126<br>(96-165)
Extensively drug-resistant tuberculosis	-	-	12<br>(9-15)	19<br>(14-25)	12<br>(9-17)	17<br>(13-21)	45<br>(36-56)	85<br>(69-103)	103<br>(84-125)	111<br>(91-134)	123<br>(101-150)	132<br>(110-160)	137<br>(112-165)	145<br>(118-178)	141<br>(115-172)	129<br>(103-161)	156<br>(123-195)	166<br>(131-208)	141<br>(111-179)	119<br>(92-151)	67<br>(52-86)	24<br>(19-32)	7<br>(6-10)
HIV/AIDS	-	-	50,146<br>(47,522-52,795)	29,021<br>(27,429-30,637)	17,030<br>(16,168-17,963)	16,086<br>(14,907-17,403)	14,030<br>(12,303-15,918)	38,740<br>(32,794-46,547)	117,458<br>(102,773-134,055)	152,791<br>(140,691-165,050)	144,595<br>(131,935-157,213)	118,852<br>(112,344-125,534)	89,163<br>(82,503-96,226)	58,538<br>(55,210-62,057)	34,080<br>(31,445-36,739)	21,428<br>(19,205-23,599)	14,128<br>(12,718-15,725)	8,503<br>(7,474-9,587)	3,859<br>(3,325-4,415)	1,263<br>(1,021-1,511)	472<br>(386-558)	131<br>(108-154)	29<br>(25-33)
Drug-susceptible HIV/AIDS - Tuberculosis	-	-	15,367<br>(11,488-19,249)	9,384<br>(7,202-11,504)	5,206<br>(3,995-6,345)	4,714<br>(3,460-5,762)	4,248<br>(3,222-5,261)	10,507<br>(7,366-14,017)	23,913<br>(14,727-34,370)	28,791<br>(17,091-41,373)	26,212<br>(15,708-38,139)	23,722<br>(14,540-33,339)	19,331<br>(12,431-26,640)	15,687<br>(10,749-20,481)	10,299<br>(7,459-12,717)	6,852<br>(5,134-8,355)	4,638<br>(3,680-5,567)	2,871<br>(2,282-3,446)	1,304<br>(1,025-1,584)	449<br>(348-574)	164<br>(128-208)	44<br>(35-55)	8<br>(7-10)
Multidrug-resistant HIV/AIDS - Tuberculosis without extensive drug resistance	-	-	1,319<br>(847-1,944)	791<br>(510-1,157)	401<br>(265-583)	351<br>(221-528)	333<br>(213-502)	942<br>(593-1,389)	1,975<br>(1,101-3,041)	2,324<br>(1,284-3,602)	2,094<br>(1,176-3,275)	1,908<br>(1,083-2,967)	1,598<br>(940-2,460)	1,336<br>(811-1,979)	886<br>(564-1,288)	580<br>(376-826)	387<br>(252-552)	238<br>(155-338)	108<br>(69-158)	38<br>(23-62)	14<br>(9-23)	4<br>(2-6)	1<br>(0-1)
Extensively drug-resistant HIV/AIDS - Tuberculosis	-	-	13<br>(8-19)	7<br>(4-10)	4<br>(2-5)	3<br>(2-4)	4<br>(2-5)	14<br>(9-21)	34<br>(21-54)	43<br>(26-68)	39<br>(23-61)	32<br>(19-49)	26<br>(16-41)	19<br>(12-30)	12<br>(8-18)	6<br>(4-9)	4<br>(3-6)	2<br>(1-3)	1<br>(1-1)	0<br>(0-1)	0<br>(0-0)	0<br>(0-0)	0<br>(0-0)
HIV/AIDS resulting in other diseases	-	-	33,447<br>(29,123-38,368)	18,839<br>(16,443-21,572)	11,419<br>(10,056-12,945)	11,018<br>(9,578-12,753)	9,445<br>(7,860-11,426)	27,277<br>(21,532-35,197)	91,536<br>(74,731-110,556)	121,632<br>(104,538-139,907)	116,250<br>(98,947-134,071)	93,190<br>(81,101-105,914)	68,208<br>(58,225-78,356)	41,495<br>(35,637-48,076)	22,883<br>(19,889-26,982)	13,990<br>(11,815-16,646)	9,100<br>(7,829-10,724)	5,392<br>(4,592-6,367)	2,447<br>(2,025-2,884)	775<br>(625-933)	294<br>(239-352)	83<br>(68-99)	20<br>(17-23)
Diarrhoea, lower respiratory infections, and other common infectious diseases	91,807<br>(74,953-105,086)	102,073<br>(90,947-111,265)	593,460<br>(548,672-635,863)	483,816<br>(429,407-542,920)	95,675<br>(82,013-111,987)	52,492<br>(44,734-62,773)	43,124<br>(35,125-56,978)	38,179<br>(30,054-53,023)	32,808<br>(25,595-46,020)	31,329<br>(24,158-43,847)	31,701<br>(24,551-45,491)	32,892<br>(25,714-45,857)	40,570<br>(31,240-59,258)	54,884<br>(41,757-79,954)	69,808<br>(52,416-105,500)	95,672<br>(70,303-146,052)	134,738<br>(98,884-204,288)	191,251<br>(139,925-284,131)	196,544<br>(146,653-281,827)	211,536<br>(164,276-282,593)	167,228<br>(133,417-214,919)	92,611<br>(77,894-111,251)	44,327<br>(38,494-50,871)
Diarrhoeal diseases	13,186<br>(11,361-15,479)	27,370<br>(23,875-31,444)	208,027<br>(177,467-238,538)	166,390<br>(134,097-203,598)	23,133<br>(11,192-45,407)	12,926<br>(6,008-26,231)	14,682<br>(6,765-30,076)	15,294<br>(7,020-31,113)	13,153<br>(5,971-26,770)	13,063<br>(5,871-26,321)	13,245<br>(5,914-27,527)	13,318<br>(6,056-26,915)	17,540<br>(8,064-37,340)	24,624<br>(11,299-51,650)	33,578<br>(15,792-71,685)	49,799<br>(23,159-104,276)	71,158<br>(33,864-145,412)	104,485<br>(48,993-204,705)	103,248<br>(51,460-195,789)	106,217<br>(56,232-185,404)	74,100<br>(42,163-123,655)	31,508<br>(18,159-49,784)	10,067<br>(6,466-15,487)
Intestinal infectious diseases	-	-	962<br>(153-3,633)	13,467<br>(6,580-25,103)	22,938<br>(12,834-37,530)	14,582<br>(7,703-23,179)	12,038<br>(4,323-15,274)	4,877<br>(2,404-8,587)	3,431<br>(1,780-5,902)	2,664<br>(1,393-4,587)	2,224<br>(1,211-3,690)	1,959<br>(1,068-3,268)	1,760<br>(944-2,967)	1,422<br>(725-2,503)	1,036<br>(496-1,898)	893<br>(440-1,602)	903<br>(493-1,483)	785<br>(439-1,247)	592<br>(343-949)	345<br>(194-558)	159<br>(89-255)	50<br>(28-90)	15<br>(7-29)
Typhoid fever	-	-	89<br>(42-167)	11,568<br>(5,111-21,928)	20,042<br>(10,716-33,025)	12,278<br>(6,219-19,916)	7,528<br>(3,652-13,254)	4,073<br>(1,892-7,282)	2,679<br>(1,354-4,703)	1,983<br>(1,020-3,433)	1,657<br>(865-2,865)	1,446<br>(750-2,500)	1,265<br>(655-2,166)	1,030<br>(515-1,826)	762<br>(354-1,405)	683<br>(325-1,240)	725<br>(385-1,220)	640<br>(356-1,022)	471<br>(264-760)	275<br>(152-441)	118<br>(66-190)	34<br>(19-55)	8<br>(4-13)
Paratyphoid fever	-	-	13<br>(5-29)	1,172<br>(480-2,520)	2,823<br>(1,268-5,643)	2,278<br>(1,027-4,344)	1,170<br>(489-2,375)	780<br>(327-1,603)	732<br>(313-1,511)	656<br>(279-1,305)	539<br>(251-1,030)	488<br>(224-965)	465<br>(211-931)	379<br>(165-765)	258<br>(105-546)	193<br>(80-407)	157<br>(71-306)	118<br>(53-225)	83<br>(39-151)	47<br>(22-88)	20<br>(9-37)	6<br>(3-11)	1<br>(1-2)
Other intestinal infectious diseases	-	-	859<br>(70-3,527)	728<br>(36-3,731)	73<br>(4-369)	27<br>(2-113)	24<br>(1-113)	23<br>(2-108)	19<br>(1-79)	25<br>(2-129)	28<br>(2-133)	24<br>(2-110)	29<br>(3-132)	13<br>(1-60)	16<br>(1-79)	17<br>(1-81)	21<br>(2-97)	27<br>(3-115)	38<br>(4-157)	23<br>(3-97)	21<br>(3-73)	11<br>(2-42)	6<br>(1-20)
Lower respiratory infections	63,259<br>(48,611-75,696)	59,438<br>(49,507-67,411)	276,850<br>(244,702-316,874)	152,154<br>(124,269-183,009)	19,726<br>(15,100-23,243)	10,230<br>(7,984-11,812)	8,601<br>(7,045-10,229)	9,279<br>(7,407-11,053)	9,380<br>(7,664-11,119)	9,422<br>(7,676-11,174)	10,669<br>(8,697-12,435)	12,075<br>(9,856-13,954)	14,757<br>(12,240-16,861)	21,838<br>(18,030-24,804)	28,070<br>(22,441-31,929)	38,133<br>(29,819-43,059)	54,288<br>(42,793-61,061)	76,481<br>(59,680-86,371)	85,406<br>(67,273-95,906)	98,250<br>(81,001-111,777)	89,083<br>(76,078-101,647)	59,456<br>(51,212-67,771)	33,627<br>(28,860-38,145)
Upper respiratory infections	38<br>(23-61)	15<br>(8-25)	365<br>(231-578)	300<br>(167-520)	21<br>(12-34)	15<br>(9-24)	35<br>(23-52)	30<br>(20-44)	31<br>(20-47)	30<br>(21-44)	36<br>(25-53)	37<br>(26-54)	45<br>(31-63)	50<br>(35-70)	55<br>(38-81)	59<br>(40-87)	60<br>(42-86)	54<br>(37-76)	57<br>(40-79)	42<br>(29-57)	31<br>(22-42)	14<br>(11-20)	4<br>(3-6)
Otitis media	3<br>(2-4)	13<br>(7-25)	80<br>(37-179)	132<br>(63-283)	89<br>(40-236)	68<br>(37-129)	31<br>(17-61)	21<br>(12-41)	31<br>(16-59)	18<br>(9-39)	18<br>(10-40)	33<br>(17-69)	22<br>(13-44)	22<br>(13-38)	58<br>(31-113)	47<br>(27-86)	88<br>(51-165)	68<br>(42-117)	48<br>(33-74)	38<br>(25-64)	21<br>(14-31)	9<br>(6-12)	3<br>(3-4)
Meningitis	3,848<br>(2,464-5,312)	5,950<br>(4,137-8,205)	38,296<br>(26,258-49,468)	35,627<br>(23,665-47,530)	9,312<br>(7,314-11,793)	6,923<br>(5,450-8,490)	6,449<br>(4,901-7,946)	5,118<br>(4,108-6,445)	4,162<br>(3,139-5,223)	3,630<br>(2,825-4,539)	3,401<br>(2,751-4,348)	3,132<br>(2,525-4,103)	3,769<br>(3,001-4,886)	3,927<br>(3,171-5,273)	4,135<br>(3,367-5,355)	3,858<br>(3,207-5,323)	4,281<br>(3,588-6,048)	5,001<br>(4,110-6,799)	3,118<br>(2,585-4,372)	3,119<br>(2,578-4,258)	1,613<br>(1,346-2,260)	597<br>(502-799)	196<br>(169-249)
[illegible]	[illegible]	[illegible]	[illegible]	[illegible]	[illegible]	[illegible]	[illegible]	[illegible]	[illegible]	[illegible]	[illegible]	[illegible]	[illegible]	[illegible]	[illegible]	[illegible]	[illegible]	[illegible]	[illegible]	[illegible]	[illegible]	[illegible]	[illegible]

SR Table 3: Global deaths by cause and age group for females (with 95% uncertainty intervals), 2006

Cause	Early Neonatal	Late Neonatal	Post Neonatal	1 to 4	5 to 9	10 to 14	15 to 19	20 to 24	25 to 29	30 to 34	35 to 39	40 to 44	45 to 49	50 to 54	55 to 59	60 to 64	65 to 69	70 to 74	75 to 79	80 to 84	85 to 89	90 to 94	95 plus
Ischaemic stroke	-	-	213 (167-268)	122 (103-155)	119 (102-139)	128 (110-148)	642 (579-721)	1,260 (1,127-1,394)	1,572 (1,428-1,725)	2,106 (1,922-2,286)	3,463 (3,198-3,740)	5,990 (5,586-6,423)	11,440 (10,706-12,183)	20,946 (19,699-22,122)	33,180 (31,159-35,173)	66,874 (62,773-70,669)	120,325 (113,346-126,621)	170,485 (160,593-179,607)	251,585 (234,891-266,330)	263,910 (244,124-281,217)	189,909 (174,313-205,740)	108,772 (99,431-119,475)	45,956 (40,661-50,640)
Hemorrhagic stroke	643 (550-749)	371 (309-447)	3,107 (2,554-3,755)	2,257 (1,783-3,018)	1,333 (1,180-1,529)	1,628 (1,463-1,831)	2,961 (2,750-3,238)	5,056 (4,689-5,519)	6,614 (6,142-7,189)	11,062 (10,369-11,964)	19,666 (18,539-21,202)	33,233 (31,694-35,205)	50,099 (47,766-52,633)	78,970 (75,664-82,626)	100,528 (95,577-105,440)	103,943 (98,368-109,225)	151,180 (142,416-159,814)	190,095 (178,845-200,590)	180,978 (168,963-192,055)	173,435 (160,031-185,090)	113,596 (104,153-123,170)	55,177 (50,142-60,453)	19,945 (17,587-22,069)
Hypertensive heart disease	-	-	148 (90-205)	93 (55-128)	68 (50-86)	93 (66-121)	793 (591-1,012)	1,014 (757-1,287)	1,325 (995-1,624)	2,162 (1,602-2,588)	3,458 (2,539-4,190)	5,594 (4,274-6,722)	9,292 (7,027-11,225)	14,698 (11,040-17,898)	20,593 (15,775-24,629)	24,264 (18,615-29,541)	37,978 (29,607-45,924)	50,943 (39,330-61,137)	62,294 (48,674-76,769)	65,259 (49,304-79,583)	53,016 (38,585-64,436)	34,827 (24,459-39,711)	15,507 (9,579-17,677)
Cardiomyopathy and myocarditis	340 (216-405)	213 (146-259)	2,144 (1,410-2,615)	1,358 (917-1,712)	608 (486-703)	733 (609-827)	1,248 (887-1,447)	1,480 (1,075-1,697)	2,001 (1,459-2,329)	2,385 (1,858-2,739)	3,062 (2,305-3,529)	4,211 (3,289-4,803)	5,042 (4,052-5,823)	6,184 (5,007-7,068)	7,177 (6,042-8,205)	5,830 (4,963-6,881)	8,016 (6,824-9,511)	10,047 (7,949-11,092)	14,451 (10,639-16,082)	18,568 (12,169-20,977)	16,936 (11,539-19,040)	12,338 (7,988-14,150)	6,379 (3,928-7,436)
Myocarditis	70 (41-103)	50 (33-72)	731 (510-1,071)	539 (403-847)	259 (193-313)	280 (204-333)	599 (372-741)	612 (385-757)	611 (398-782)	565 (380-699)	730 (456-889)	814 (540-1,007)	577 (382-673)	701 (468-833)	803 (561-935)	618 (395-722)	862 (565-1,009)	1,246 (802-1,457)	1,839 (1,101-2,157)	2,840 (1,619-3,424)	3,270 (1,900-3,947)	3,062 (1,544-3,921)	1,955 (842-2,594)
Alcoholic cardiomyopathy	-	-	-	-	-	-	83 (66-107)	187 (137-232)	449 (316-564)	783 (562-972)	1,086 (760-1,381)	1,766 (1,322-2,211)	2,532 (1,865-3,147)	2,889 (2,148-3,508)	2,917 (2,310-3,580)	1,616 (1,340-2,075)	2,194 (1,779-3,037)	1,663 (1,374-2,256)	2,061 (1,673-2,608)	1,877 (1,215-2,382)	1,216 (692-1,564)	783 (381-1,079)	346 (176-452)
Other cardiomyopathy	270 (170-332)	162 (107-206)	1,414 (840-1,903)	818 (485-1,174)	349 (269-439)	452 (376-550)	566 (415-683)	681 (485-816)	941 (649-1,143)	1,037 (778-1,235)	1,246 (964-1,468)	1,631 (1,300-1,889)	1,933 (1,616-2,225)	2,595 (2,183-3,081)	3,457 (2,961-4,053)	3,596 (3,086-4,224)	4,961 (4,126-5,697)	7,138 (5,448-7,983)	10,551 (7,561-11,961)	13,852 (9,155-15,987)	12,450 (8,455-14,362)	8,493 (5,820-10,541)	4,078 (2,751-5,404)
Atrial fibrillation and flutter	-	-	-	-	-	-	-	-	-	16 (11-21)	64 (47-84)	149 (109-195)	274 (199-359)	478 (358-624)	804 (479-1,196)	1,514 (999-2,147)	3,378 (2,099-5,039)	7,043 (4,991-9,779)	12,108 (7,789-17,915)	25,716 (19,630-32,795)	29,326 (22,551-37,578)	18,470 (14,366-23,858)	6,585 (5,139-8,394)
Aortic aneurysm	-	-	-	-	-	-	132 (116-155)	166 (147-194)	219 (194-256)	310 (273-364)	467 (414-544)	668 (609-756)	1,049 (964-1,180)	1,563 (1,448-1,729)	2,229 (2,062-2,473)	2,394 (2,272-2,561)	3,950 (3,750-4,221)	5,688 (5,423-5,999)	7,988 (7,632-8,363)	9,366 (9,014-9,712)	7,710 (7,440-7,960)	4,742 (4,571-4,941)	1,879 (1,803-1,976)
Peripheral vascular disease	-	-	-	-	-	-	-	-	-	-	-	72 (44-104)	115 (62-185)	209 (114-345)	367 (201-598)	554 (293-895)	1,054 (558-1,724)	1,598 (794-2,595)	3,042 (1,617-5,245)	4,810 (2,597-8,566)	4,840 (2,580-9,033)	4,196 (2,220-7,905)	2,264 (1,137-4,336)
Endocarditis	54 (35-71)	37 (26-51)	793 (581-1,203)	414 (291-711)	222 (179-284)	267 (222-316)	500 (428-595)	651 (546-775)	886 (751-1,035)	806 (701-948)	970 (839-1,143)	1,292 (1,122-1,573)	1,210 (995-1,467)	1,403 (1,145-1,694)	1,664 (1,347-2,069)	2,208 (1,754-2,699)	2,981 (2,377-3,680)	3,809 (2,961-4,767)	4,826 (3,548-6,153)	5,605 (3,901-7,254)	4,799 (3,166-5,978)	3,376 (2,115-4,241)	1,564 (943-1,940)
Other cardiovascular and circulatory diseases	563 (452-677)	346 (278-432)	2,528 (2,024-3,174)	1,496 (1,120-2,094)	734 (601-1,012)	814 (675-1,137)	1,311 (1,109-1,852)	1,747 (1,488-2,448)	2,185 (1,857-3,046)	2,558 (2,196-3,505)	3,182 (2,718-4,364)	4,314 (3,722-5,766)	5,748 (5,033-7,659)	7,811 (6,836-10,294)	9,915 (8,523-13,367)	13,180 (11,502-18,180)	18,139 (15,858-24,530)	24,821 (21,887-32,980)	32,970 (28,011-41,911)	39,175 (35,886-49,267)	33,378 (31,036-40,563)	22,600 (21,250-26,345)	10,116 (9,516-11,421)
Chronic respiratory diseases	0 (0-0)	0 (0-0)	0 (0-0)	7,848 (6,202-11,666)	2,194 (1,860-3,037)	2,329 (2,005-3,109)	4,151 (3,686-5,520)	5,190 (4,701-6,562)	6,170 (5,654-7,580)	8,050 (7,354-9,652)	11,704 (10,650-14,011)	16,503 (15,226-19,289)	27,150 (24,827-31,251)	45,225 (40,154-49,907)	70,320 (62,133-75,294)	106,136 (92,095-114,404)	162,677 (142,600-175,688)	224,759 (202,308-245,316)	243,690 (221,209-272,108)	237,275 (218,572-262,129)	173,012 (161,205-188,981)	87,439 (82,264-93,952)	31,122 (29,331-33,616)
Chronic obstructive pulmonary disease	-	-	0 (0-0)	1,609 (1,091-2,558)	393 (301-568)	373 (297-500)	1,579 (1,317-2,027)	2,161 (1,871-2,635)	2,805 (2,409-3,457)	4,037 (3,466-4,786)	5,843 (5,126-6,890)	8,817 (7,715-10,232)	15,914 (13,851-18,365)	30,830 (26,705-34,723)	51,019 (43,581-57,155)	82,195 (69,452-91,865)	132,167 (113,692-145,527)	190,865 (171,526-208,462)	209,494 (189,883-234,132)	209,182 (193,695-231,698)	155,155 (144,732-170,013)	79,589 (75,032-86,326)	28,527 (26,840-30,885)
Pneumoconiosis	-	-	-	-	-	-	18 (14-24)	21 (17-27)	22 (18-28)	38 (31-48)	39 (32-48)	55 (45-69)	61 (50-74)	90 (73-109)	127 (99-157)	157 (125-195)	223 (178-278)	325 (271-388)	364 (308-437)	345 (301-408)	223 (199-257)	111 (101-125)	45 (42-50)
Silicosis	-	-	-	-	-	-	1 (1-1)	1 (1-2)	2 (2-3)	7 (5-11)	5 (4-7)	12 (9-16)	15 (12-20)	20 (15-29)	32 (22-44)	41 (29-57)	57 (39-86)	103 (79-149)	103 (80-149)	89 (71-133)	56 (46-86)	28 (23-41)	10 (9-16)
Asbestosis	-	-	-	-	-	-	9 (7-11)	10 (8-13)	10 (8-13)	14 (12-18)	16 (13-20)	19 (15-25)	21 (16-27)	30 (23-40)	35 (26-47)	39 (27-51)	42 (30-56)	61 (38-77)	75 (46-92)	76 (41-91)	52 (27-62)	26 (13-31)	11 (5-13)
Coal workers pneumoconiosis	-	-	-	-	-	-	1 (1-1)	1 (0-1)	1 (1-1)	3 (2-4)	2 (1-2)	4 (2-6)	4 (3-6)	7 (4-10)	13 (8-20)	16 (10-24)	31 (19-44)	41 (27-59)	50 (36-71)	36 (26-52)	28 (20-40)	14 (10-20)	7 (5-10)

SR Table 3: Global deaths by cause and age group for females (with 95% uncertainty intervals), 2006

Cause	Early Neonatal	Late Neonatal	Post Neonatal	1 to 4	5 to 9	10 to 14	15 to 19	20 to 24	25 to 29
Self-harm	-	-	-	-	-	4,548<br>(4,051-5,055)	32,625<br>(29,592-35,488)	36,875<br>(34,090-39,456)	30,550<br>(28,414-32,502)
Self-harm by firearm	-	-	-	-	-	163<br>(110-247)	1,140<br>(674-1,740)	1,395<br>(828-1,882)	1,055<br>(714-1,329)
Self-harm by other specified means	-	-	-	-	-	4,385<br>(3,921-4,845)	31,486<br>(28,576-34,286)	35,480<br>(32,885-37,911)	29,494<br>(27,363-31,322)
Interpersonal violence	815<br>(598-992)	231<br>(169-283)	1,666<br>(1,304-2,130)	3,346<br>(2,521-4,349)	2,312<br>(1,744-2,873)	2,712<br>(2,062-3,273)	6,820<br>(4,970-7,842)	9,185<br>(6,867-10,950)	8,139<br>(6,187-9,696)
Assault by firearm	43<br>(27-60)	18<br>(11-24)	188<br>(122-265)	356<br>(244-492)	335<br>(219-423)	515<br>(323-623)	1,836<br>(1,133-2,118)	2,285<br>(1,446-2,600)	2,084<br>(1,323-2,366)
Assault by sharp object	54<br>(41-77)	33<br>(17-44)	137<br>(88-170)	339<br>(217-455)	278<br>(197-359)	476<br>(323-612)	1,500<br>(1,014-1,827)	2,380<br>(1,621-2,871)	2,266<br>(1,559-2,812)
Assault by other means	718<br>(518-874)	179<br>(136-229)	1,341<br>(1,072-1,745)	2,650<br>(1,971-3,507)	1,698<br>(1,254-2,209)	1,720<br>(1,310-2,198)	3,484<br>(2,530-4,411)	4,521<br>(3,376-5,878)	3,789<br>(2,931-4,786)
Forces of nature, conflict and terrorism, and executions and police conflict	374<br>(197-554)	215<br>(117-314)	776<br>(453-1,100)	4,211<br>(2,432-6,015)	2,549<br>(1,544-3,569)	2,222<br>(1,374-3,063)	2,499<br>(1,496-3,509)	2,135<br>(1,291-2,985)	1,520<br>(937-2,100)
Forces of nature, conflict and terrorism, and state actor violence	3<br>(2-4)	12<br>(8-15)	95<br>(64-125)	485<br>(324-640)	456<br>(309-603)	488<br>(327-646)	400<br>(274-528)	348<br>(236-459)	324<br>(221-428)
Conflict and terrorism	371<br>(194-551)	203<br>(106-301)	667<br>(348-992)	3,682<br>(1,917-5,473)	2,058<br>(1,070-3,062)	1,689<br>(878-2,515)	2,056<br>(1,071-3,056)	1,748<br>(923-2,585)	1,158<br>(605-1,716)
Executions and police conflict	-	-	14<br>(9-22)	44<br>(24-101)	35<br>(29-45)	44<br>(35-61)	43<br>(35-54)	39<br>(30-47)	38<br>(27-46)

Cause	30 to 34	35 to 39	40 to 44	45 to 49	50 to 54	55 to 59	60 to 64
Self-harm	24,434<br>(22,934-25,761)	23,698<br>(22,299-25,037)	20,477<br>(19,480-21,474)	19,171<br>(18,289-20,002)	19,989<br>(19,084-20,905)	16,886<br>(16,059-17,634)	13,645<br>(12,895-14,386)
Self-harm by firearm	847<br>(642-1,072)	844<br>(668-1,108)	831<br>(695-1,060)	819<br>(698-1,013)	694<br>(586-891)	566<br>(469-752)	417<br>(325-598)
Self-harm by other specified means	23,588<br>(22,114-24,894)	22,854<br>(21,527-24,129)	19,646<br>(18,643-20,674)	18,352<br>(17,490-19,206)	19,295<br>(18,337-20,204)	16,320<br>(15,478-17,073)	13,228<br>(12,493-13,946)
Interpersonal violence	7,855<br>(5,941-9,480)	6,839<br>(5,130-7,990)	6,054<br>(4,502-7,015)	5,272<br>(3,857-6,085)	4,370<br>(3,206-5,065)	3,420<br>(2,448-3,876)	2,434<br>(1,767-2,810)
Assault by firearm	1,761<br>(1,119-2,072)	1,582<br>(986-1,860)	1,357<br>(860-1,585)	1,123<br>(703-1,347)	906<br>(560-1,093)	643<br>(399-792)	485<br>(317-617)
Assault by sharp object	2,394<br>(1,683-3,039)	2,115<br>(1,423-2,524)	1,998<br>(1,323-2,356)	1,651<br>(1,109-1,949)	1,284<br>(849-1,518)	954<br>(582-1,105)	671<br>(441-799)
Assault by other means	3,700<br>(2,782-4,839)	3,142<br>(2,338-3,929)	2,699<br>(2,058-3,387)	2,498<br>(1,870-3,040)	2,181<br>(1,585-2,616)	1,822<br>(1,324-2,171)	1,278<br>(903-1,485)
Forces of nature, conflict and terrorism, and executions and police conflict	1,360<br>(844-1,866)	1,132<br>(718-1,529)	880<br>(594-1,164)	768<br>(533-998)	775<br>(528-1,017)	635<br>(448-819)	572<br>(403-739)
Forces of nature, conflict and terrorism, and state actor violence	310<br>(209-409)	295<br>(196-387)	287<br>(192-374)	262<br>(178-341)	258<br>(175-337)	239<br>(162-312)	219<br>(147-287)
Conflict and terrorism	1,013<br>(526-1,502)	797<br>(418-1,179)	555<br>(309-803)	471<br>(275-663)	488<br>(274-702)	366<br>(220-511)	327<br>(190-461)
Executions and police conflict	37<br>(26-48)	39<br>(25-55)	37<br>(23-55)	35<br>(20-51)	29<br>(19-39)	30<br>(16-45)	26<br>(13-39)

Cause	65 to 69	70 to 74	75 to 79	80 to 84	85 to 89	90 to 94	95 plus
Self-harm	14,488<br>(13,700-15,270)	13,784<br>(13,052-14,549)	11,633<br>(11,022-12,301)	8,736<br>(8,264-9,215)	4,704<br>(4,462-4,957)	1,862<br>(1,759-1,967)	492<br>(466-517)
Self-harm by firearm	364<br>(266-580)	319<br>(219-530)	316<br>(217-529)	259<br>(191-396)	171<br>(142-222)	101<br>(90-116)	46<br>(42-50)
Self-harm by other specified means	14,124<br>(13,303-14,895)	13,465<br>(12,696-14,225)	11,316<br>(10,682-12,015)	8,477<br>(8,012-8,949)	4,532<br>(4,300-4,769)	1,761<br>(1,662-1,862)	447<br>(422-469)
Interpersonal violence	2,518<br>(1,835-3,015)	2,316<br>(1,673-2,667)	2,159<br>(1,568-2,487)	777<br>(606-989)	332<br>(262-435)	156<br>(130-217)	57<br>(48-84)
Assault by firearm	410<br>(260-536)	401<br>(252-522)	343<br>(212-442)	57<br>(35-84)	31<br>(20-48)	12<br>(8-20)	5<br>(4-10)
Assault by sharp object	602<br>(381-716)	647<br>(433-767)	581<br>(378-680)	220<br>(162-289)	88<br>(68-122)	48<br>(40-68)	16<br>(13-26)
Assault by other means	1,505<br>(1,109-1,808)	1,268<br>(904-1,462)	1,235<br>(877-1,447)	500<br>(390-647)	213<br>(165-285)	97<br>(75-135)	35<br>(28-54)
Forces of nature, conflict and terrorism, and executions and police conflict	566<br>(393-733)	479<br>(339-618)	380<br>(273-487)	296<br>(215-378)	166<br>(120-213)	52<br>(39-66)	35<br>(25-45)
Forces of nature, conflict and terrorism, and state actor violence	217<br>(146-282)	206<br>(139-270)	175<br>(119-228)	130<br>(88-169)	69<br>(46-91)	26<br>(18-35)	7<br>(5-10)
Conflict and terrorism	325<br>(187-461)	244<br>(140-347)	186<br>(108-264)	136<br>(78-195)	74<br>(42-106)	14<br>(8-19)	24<br>(15-32)
Executions and police conflict	25<br>(12-38)	30<br>(12-50)	19<br>(10-27)	30<br>(10-46)	23<br>(4-39)	12<br>(5-19)	3<br>(1-6)

SR Table 3: Global deaths by cause and age group for females (with 95% uncertainty intervals), 2010

Cause	Early Neonatal	Late Neonatal	Post Neonatal	1 to 4	5 to 9	10 to 14	15 to 19	20 to 24	25 to 29	30 to 34	35 to 39	40 to 44	45 to 49	50 to 54	55 to 59	60 to 64	65 to 69	70 to 74	75 to 79	80 to 84	85 to 89	90 to 94	95 plus
All causes	907,345 (879,744-934,928)	291,976 (283,006-301,130)	959,377 (927,784-991,765)	915,274 (874,608-959,258)	236,251 (227,884-245,428)	187,246 (178,776-196,870)	266,357 (258,266-275,215)	343,917 (334,507-354,555)	412,712 (398,438-428,950)	462,709 (450,006-476,698)	527,058 (513,957-539,776)	610,458 (600,414-621,046)	740,870 (729,134-753,068)	939,509 (925,679-954,006)	1,198,025 (1,181,065-1,215,745)	1,447,714 (1,428,029-1,467,911)	1,710,743 (1,687,746-1,734,614)	2,297,033 (2,266,037-2,327,483)	2,598,053 (2,566,461-2,628,745)	2,874,590 (2,845,705-2,901,760)	2,457,826 (2,440,383-2,474,214)	1,355,409 (1,348,855-1,361,444)	642,660 (640,045-645,053)
Communicable, maternal, neonatal, and nutritional disorders	819,269 (787,638-850,477)	253,814 (244,063-264,214)	784,092 (745,417-823,458)	733,529 (690,687-784,190)	147,027 (139,912-154,803)	93,622 (88,098-100,597)	112,886 (106,559-121,586)	156,676 (148,139-166,909)	218,252 (204,386-234,752)	245,268 (233,069-258,607)	240,649 (228,101-254,313)	205,014 (197,380-214,933)	174,881 (164,910-189,693)	160,199 (148,584-179,850)	159,585 (143,645-189,575)	175,074 (153,052-215,214)	204,991 (175,606-260,356)	266,316 (221,120-345,200)	266,056 (220,152-341,287)	279,877 (230,827-350,109)	223,336 (188,080-272,614)	117,655 (101,745-137,539)	60,699 (52,910-68,783)
HIV/AIDS and tuberculosis	-	-	47,193 (44,255-50,470)	31,413 (28,788-34,182)	20,753 (19,660-21,986)	24,842 (23,424-26,262)	33,625 (31,118-36,317)	52,217 (46,857-58,331)	119,016 (106,083-133,771)	155,603 (144,301-166,876)	155,645 (144,367-166,432)	132,668 (127,675-138,057)	107,958 (101,782-115,288)	87,434 (83,129-91,398)	70,585 (65,571-75,534)	58,387 (53,452-62,793)	54,606 (48,751-59,369)	56,373 (49,485-61,933)	45,059 (39,420-49,761)	36,069 (30,907-39,994)	19,970 (17,256-22,242)	6,680 (5,805-7,519)	1,850 (1,614-2,112)
Tuberculosis	-	-	7,710 (6,064-10,055)	10,290 (8,252-12,738)	4,395 (3,554-5,296)	5,263 (4,449-6,074)	12,817 (11,305-14,402)	19,952 (18,113-21,696)	23,731 (21,551-25,646)	24,427 (22,294-26,504)	27,264 (24,910-29,633)	26,641 (24,500-28,704)	31,046 (28,371-33,644)	37,575 (34,256-40,740)	43,021 (38,533-47,232)	41,757 (36,957-45,762)	44,472 (38,631-49,074)	50,350 (43,410-55,638)	42,093 (36,327-46,701)	35,079 (29,950-39,013)	19,598 (16,885-21,839)	6,578 (5,707-7,406)	1,825 (1,588-2,089)
Drug-susceptible tuberculosis	-	-	7,190 (5,634-9,407)	9,536 (7,637-11,843)	4,038 (3,226-4,893)	4,812 (4,084-5,554)	11,677 (10,274-13,135)	18,134 (16,372-19,811)	21,588 (19,579-23,341)	22,190 (20,169-24,107)	24,805 (22,602-27,056)	24,210 (22,217-26,149)	28,311 (25,808-30,773)	34,288 (31,170-37,271)	39,288 (35,086-43,188)	38,221 (33,841-41,958)	40,796 (35,210-45,145)	46,081 (39,695-50,970)	38,605 (33,274-42,848)	32,119 (27,431-35,895)	17,991 (15,447-20,065)	6,056 (5,272-6,830)	1,685 (1,461-1,923)
Multidrug-resistant tuberculosis without extensive drug resistance	-	-	506 (374-660)	733 (541-979)	344 (258-446)	432 (334-545)	1,088 (873-1,335)	1,712 (1,391-2,059)	2,007 (1,667-2,388)	2,091 (1,737-2,504)	2,295 (1,931-2,722)	2,264 (1,891-2,681)	2,555 (2,136-3,015)	3,089 (2,556-3,707)	3,528 (2,904-4,255)	3,348 (2,728-4,042)	3,492 (2,794-4,289)	4,043 (3,250-4,970)	3,296 (2,615-4,048)	2,784 (2,177-3,477)	1,501 (1,179-1,888)	484 (380-615)	128 (98-170)
Extensively drug-resistant tuberculosis	-	-	14 (11-18)	20 (16-27)	13 (10-17)	19 (14-23)	51 (42-64)	106 (87-127)	135 (113-163)	146 (122-175)	165 (139-199)	167 (141-200)	179 (149-214)	198 (164-239)	205 (169-249)	188 (153-231)	184 (147-227)	225 (182-277)	192 (153-241)	177 (139-225)	106 (83-140)	38 (28-51)	12 (9-17)
HIV/AIDS	-	-	39,483 (37,056-42,137)	21,123 (19,706-22,575)	16,358 (15,630-17,152)	19,579 (18,464-20,798)	20,808 (18,703-23,014)	32,266 (27,220-38,696)	95,285 (82,392-109,877)	131,176 (119,522-142,855)	128,381 (116,805-139,263)	106,027 (101,205-111,351)	76,912 (71,220-83,316)	49,859 (46,919-52,972)	27,564 (25,434-29,634)	16,630 (15,079-18,296)	10,133 (9,060-11,258)	6,023 (5,295-6,795)	2,966 (2,581-3,403)	990 (813-1,196)	372 (306-445)	102 (85-121)	25 (22-29)
Drug-susceptible HIV/AIDS - Tuberculosis	-	-	11,080 (8,149-13,972)	6,273 (4,775-7,685)	4,647 (3,514-5,752)	5,237 (3,723-6,621)	5,789 (4,124-7,290)	8,279 (5,851-10,943)	19,006 (11,916-27,158)	22,052 (13,354-31,972)	20,869 (12,805-30,157)	17,183 (10,895-23,707)	15,125 (9,914-21,161)	12,060 (8,123-16,532)	7,736 (5,550-9,602)	4,927 (3,730-5,984)	3,080 (2,400-3,711)	1,908 (1,507-2,299)	948 (745-1,156)	341 (263-435)	124 (97-158)	32 (25-41)	6 (5-8)
Multidrug-resistant HIV/AIDS - Tuberculosis without extensive drug resistance	-	-	990 (630-1,453)	545 (350-805)	368 (237-528)	417 (257-611)	473 (290-701)	737 (454-1,105)	1,610 (917-2,514)	1,822 (1,003-2,898)	1,680 (935-2,661)	1,396 (788-2,174)	1,257 (731-1,965)	1,034 (610-1,548)	686 (424-974)	432 (276-618)	265 (171-379)	163 (104-235)	82 (51-121)	29 (17-45)	11 (6-17)	3 (2-4)	1 (0-1)
Extensively drug-resistant HIV/AIDS - Tuberculosis	-	-	15 (9-22)	7 (4-10)	5 (3-7)	5 (3-8)	7 (4-10)	17 (11-26)	45 (27-71)	58 (35-91)	51 (31-81)	41 (24-63)	32 (19-49)	24 (14-37)	15 (10-24)	8 (5-12)	4 (3-6)	2 (1-3)	1 (1-2)	0 (0-1)	0 (0-0)	0 (0-0)	0 (0-0)
HIV/AIDS resulting in other diseases	-	-	27,399 (23,714-31,306)	14,299 (12,457-16,471)	11,338 (10,095-12,753)	13,920 (12,206-15,873)	14,540 (12,241-17,320)	23,232 (18,521-29,398)	74,624 (60,615-91,161)	107,244 (92,529-122,962)	105,781 (91,382-120,170)	87,407 (79,053-96,390)	60,499 (51,661-69,197)	36,741 (31,231-42,232)	19,126 (16,701-22,324)	11,263 (9,731-13,237)	6,785 (5,819-7,929)	3,950 (3,304-4,670)	1,935 (1,594-2,285)	619 (503-750)	237 (195-286)	66 (55-79)	18 (16-21)
Diarrhoea, lower respiratory infections, and other common infectious diseases	73,681 (58,171-85,972)	80,253 (70,880-87,596)	502,454 (466,096-540,701)	386,582 (342,451-436,198)	81,484 (70,959-95,347)	46,953 (40,034-56,557)	38,138 (31,106-50,058)	35,084 (27,860-48,647)	31,394 (24,808-43,173)	29,440 (23,029-40,819)	30,293 (23,816-42,894)	31,141 (24,654-43,234)	39,084 (30,502-56,368)	53,636 (41,704-77,198)	71,086 (54,666-105,745)	96,445 (72,784-142,112)	128,267 (95,195-190,480)	187,685 (139,376-273,901)	198,050 (149,161-280,514)	222,735 (172,227-299,737)	185,280 (148,708-237,477)	100,293 (83,769-121,278)	52,942 (45,408-61,119)
Diarrhoeal diseases	9,579 (8,260-11,091)	20,602 (17,937-23,673)	171,415 (145,833-196,952)	132,719 (108,676-163,309)	20,057 (10,194-38,812)	11,390 (5,472-22,104)	12,701 (5,902-25,860)	13,652 (6,514-28,112)	12,241 (5,653-24,874)	12,097 (5,489-24,236)	12,389 (5,681-25,155)	12,276 (5,598-24,929)	16,232 (7,595-34,631)	22,903 (10,836-48,281)	32,513 (15,577-68,130)	46,850 (21,920-95,973)	64,265 (31,495-131,959)	97,638 (48,170-187,561)	100,020 (50,539-187,262)	108,248 (57,867-190,972)	78,767 (45,890-132,997)	34,062 (19,730-55,509)	11,270 (7,330-17,724)
Intestinal infectious diseases	-	-	943 (148-4,378)	12,207 (5,867-22,533)	19,925 (11,036-32,444)	13,230 (6,936-20,916)	8,041 (4,004-13,992)	4,811 (2,307-8,582)	3,511 (1,816-6,081)	2,681 (1,409-4,651)	2,228 (1,198-3,689)	1,957 (1,057-3,237)	1,802 (970-3,037)	1,460 (748-2,556)	1,101 (523-2,022)	913 (444-1,636)	902 (498-1,475)	825 (470-1,331)	652 (371-1,043)	392 (224-636)	184 (104-308)	56 (32-89)	17 (8-36)
Typhoid fever	-	-	92 (44-173)	10,581 (4,726-19,948)	17,398 (9,404-29,028)	11,160 (5,688-17,933)	6,924 (3,382-12,523)	3,999 (1,853-7,159)	2,719 (1,363-4,759)	1,976 (1,011-3,469)	1,643 (863-2,846)	1,432 (739-2,440)	1,284 (664-2,197)	1,047 (517-1,826)	803 (373-1,486)	693 (332-1,271)	719 (387-1,197)	672 (374-1,062)	519 (291-837)	313 (173-510)	136 (76-218)	39 (22-64)	9 (5-15)
Paratyphoid fever	-	-	14 (5-29)	1,092 (451-2,299)	2,464 (1,100-4,885)	2,047 (923-3,919)	1,098 (458-2,223)	792 (324-1,647)	777 (329-1,616)	683 (291-1,362)	560 (255-1,075)	503 (229-983)	492 (223-988)	400 (175-831)	284 (114-599)	204 (83-433)	162 (74-319)	130 (60-248)	95 (45-173)	56 (26-104)	23 (11-43)	6 (3-13)	1 (1-3)
Other intestinal infectious diseases	-	-	837 (47-4,228)	535 (38-2,186)	63 (4-348)	23 (2-122)	19 (1-91)	20 (1-108)	15 (1-71)	21 (2-99)	25 (2-119)	21 (2-89)	27 (2-121)	13 (1-52)	14 (1-59)	17 (1-75)	21 (2-122)	24 (2-110)	37 (4-192)	24 (3-95)	25 (3-102)	10 (2-36)	7 (1-24)
Lower respiratory infections	53,261 (39,283-64,118)	48,194 (39,280-54,772)	235,808 (205,186-269,170)	121,989 (98,431-148,420)	18,191 (13,786-21,608)	9,704 (7,584-11,324)	7,849 (6,389-9,122)	8,818 (6,958-10,427)	9,258 (7,444-10,828)	8,975 (7,209-10,357)	10,489 (8,488-12,115)	11,785 (9,473-13,373)	14,922 (12,173-16,974)	22,633 (18,550-25,440)	30,383 (24,391-34,485)	41,830 (32,907-47,121)	55,309 (43,566-62,450)	80,026 (61,515-90,101)	90,139 (69,699-101,835)	106,940 (87,082-121,577)	102,065 (85,715-116,909)	64,470 (54,682-73,734)	40,967 (34,708-46,723)
Upper respiratory infections	33 (19-54)	13 (7-22)	322 (191-538)	245 (132-451)	17 (10-29)	13 (8-21)	28 (18-44)	26 (16-39)	27 (17-40)	26 (17-39)	31 (21-47)	33 (23-47)	41 (29-57)	45 (32-66)	52 (36-74)	56 (40-80)	52 (37-74)	48 (34-67)	52 (38-73)	41 (30-58)	32 (23-45)	14 (11-19)	5 (3-7)
Otitis media	2 (1-3)	8 (4-15)	51 (25-103)	76 (36-156)	45 (22-105)	38 (21-70)	19 (10-36)	14 (7-30)	24 (13-48)	12 (7-26)	13 (7-28)	24 (12-54)	18 (10-37)	18 (11-30)	41 (24-78)	31 (19-55)	56 (34-97)	50 (32-93)	38 (26-60)	33 (22-53)	19 (14-27)	8 (6-11)	4 (3-4)
Meningitis	3,800 (2,460-5,557)	5,670 (3,948-8,741)	38,059 (27,214-54,908)	33,290 (23,550-49,921)	8,279 (6,509-10,497)	6,377 (5,049-8,101)	5,817 (4,451-7,235)	4,740 (3,802-5,986)	3,982 (3,035-4,981)	3,449 (2,691-4,389)	3,254 (2,623-4,180)	2,989 (2,384-3,879)	3,665 (2,884-4,716)	3,879 (3,086-5,197)	4,193 (3,411-5,429)	3,908 (3,238-5,363)	4,142 (3,442-5,816)	4,962 (4,126-6,744)	3,186 (2,622-4,376)	3,367 (2,756-4,501)	1,784 (1,489-2,440)	655 (547-873)	221 (192-277)
Pneumococcal meningitis	149 (90-226)	223 (145-344)	1,609 (1,071-2,371)	1,526 (926-2,507)	409 (277-596)	315 (213-453)	278 (184-403)	216 (144-306)	208 (138-295)	235 (151-346)	268 (167-404)	289 (182-462)	382 (236-599)	401 (271-604)	453 (313-649)	442 (318-645)	453 (318-670)	543 (365-809)	354 (251-519)	339 (234-499)	183 (141-246)	73 (57-95)	28 (21-39)
H influenzae type B meningitis	416 (260-609)	617 (413-961)	4,062 (2,741-6,064)	3,203 (2,030-4,970)	656 (479-901)	426 (309-566)	344 (243-474)	274 (205-356)	247 (179-324)	228 (166-313)	221 (161-307)	205 (148-285)	254 (179-346)	264 (193-374)	286 (211-393)	267 (198-371)	271 (201-382)	327 (246-443)	216 (159-309)	225 (168-307)	120 (91-168)	43 (32-61)	14 (10-22)
Meningococcal infection	1,445 (949-2,125)	2,286 (1,630-3,368)	15,670 (11,237-22,279)	14,527 (10,034-22,219)	3,919 (2,996-5,018)	2,995 (2,312-3,867)	2,749 (2,120-3,451)	2,203 (1,732-2,813)	1,752 (1,342-2,299)	1,419 (1,103-1,848)	1,282 (1,022-1,693)	1,224 (954-1,579)	1,467 (1,186-1,885)	1,587 (1,268-2,113)	1,711 (1,348-2,309)	1,583 (1,264-2,249)	1,637 (1,304-2,421)	1,860 (1,464-2,723)	1,269 (980-1,835)	1,313 (1,006-1,834)	740 (588-1,040)	283 (228-388)	92 (71-122)
Other meningitis	1,791 (1,119-2,659)	2,543 (1,693-4,060)	16,718 (11,521-24,462)	14,034 (9,491-21,504)	3,295 (2,395-4,327)	2,641 (2,012-3,326)	2,446 (1,810-3,062)	2,047 (1,546-2,521)	1,775 (1,294-2,208)	1,567 (1,180-1,981)	1,483 (1,127-1,890)	1,271 (1,000-1,611)	1,563 (1,179-2,007)	1,628 (1,257-2,177)	1,743 (1,353-2,274)	1,617 (1,253-2,166)	1,780 (1,393-2,385)	2,231 (1,726-2,890)	1,347 (1,045-1,809)	1,491 (1,157-1,912)	740 (593-980)	256 (202-341)	87 (67-111)
Encephalitis	303 (216-375)	590 (449-808)	6,431 (4,970-8,651)	7,507 (5,694-11,549)	2,816 (2,112-4,289)	2,148 (1,635-3,359)	1,614 (1,290-2,494)	1,690 (1,377-2,488)	1,321 (1,074-2,007)	1,270 (1,033-1,953)	1,152 (928-1,803)	1,287 (1,041-1,947)	1,497 (1,218-2,294)	1,595 (1,254-2,483)	1,942 (1,505-3,074)	2,238 (1,766-3,490)	2,480 (1,935-3,814)	3,051 (2,369-4,557)	2,701 (2,123-3,774)	2,593 (1,951-3,628)	1,531 (1,192-2,114)	498 (388-705)	156 (125-213)
Diphtheria	-	-	230 (121-425)	479 (215-996)	73 (39-133)	35 (21-56)	15 (9-23)	9 (6-13)	8 (5-12)	7 (4-11)	11 (7-16)	11 (7-16)	14 (9-20)	17 (11-24)	13 (9-19)	-	-	-	-	-	-	-	-
Whooping cough	-	-	23,829 (11,400-42,940)	24,803 (11,696-44,054)	2,546 (1,183-4,853)	500 (221-973)	171 (78-331)	132 (59-249)	79 (35-152)	54 (24-104)	41 (18-80)	35 (15-69)	33 (15-65)	34 (15-68)	36 (16-70)	-	-	-	-	-	-	-	-
Tetanus	6,665 (3,478-9,070)	5,054 (3,041-6,559)	1,914 (1,132-2,885)	910 (417-1,933)	343 (161-851)	632 (227-1,322)	758 (308-1,448)	546 (237-962)	522 (225-977)	561 (270-957)	470 (240-957)	607 (314-1,126)	731 (369-1,311)	946 (449-1,687)	699 (341-1,372)	515 (261-992)	862 (451-1,578)	880 (435-1,403)	918 (417-1,591)	557 (227-1,068)	198 (73-302)	60 (21-96)	16 (6-25)
Measles	-	-	22,392 (8,453-49,723)	51,035 (21,030-103,277)	8,630 (3,444-17,959)	2,696 (986-5,977)	1,043 (366-2,436)	547 (188-1,293)	296 (102-705)	176 (61-426)	105 (36-256)	64 (22-155)	40 (14-97)	30 (10-72)	23 (8-56)	-	-	-	-	-	-	-	-
Varicella and herpes zoster	37 (28-48)	123 (95-158)	1,059 (826-1,351)	1,320 (998-1,726)	559 (425-744)	190 (143-249)	83 (66-107)	100 (79-127)	124 (97-156)	132 (103-169)	111 (87-137)	73 (57-92)	89 (69-111)	76 (60-96)	92 (72-117)	104 (83-131)	198 (153-256)	204 (158-263)	344 (270-438)	563 (428-722)	701 (523-929)	470 (334-650)	286 (168-457)
Neglected tropical diseases and malaria	531 (225-795)	9,658 (6,394-14,015)	108,864 (77,516-144,675)	225,445 (169,201-282,412)	31,373 (22,475-40,988)	15,391 (10,757-20,689)	7,964 (5,996-10,142)	7,910 (5,881-10,169)	6,009 (4,556-7,774)	5,446 (4,153-7,008)	6,241 (4,749-8,006)	6,160 (4,712-7,901)	6,431 (4,951-8,202)	7,327 (5,533-9,512)	7,610 (5,778-9,654)	9,563 (7,151-12,630)	8,940 (6,754-11,697)	8,155 (6,115-10,936)	6,596 (5,001-8,595)	4,173 (3,137-5,387)	2,669 (1,972-3,541)	722 (502-938)	335 (207-608)
Malaria	-	9,345 (6,105-13,660)	103,003 (71,352-138,463)	212,829 (155,085-270,471)	26,484 (17,962-36,050)	12,328 (8,233-17,437)	5,445 (3,868-7,576)	5,465 (3,815-7,679)	3,845 (2,689-5,482)	3,335 (2,341-4,755)	3,896 (2,742-5,626)	3,793 (2,643-5,289)	3,771 (2,580-5,515)	4,350 (2,929-6,303)	4,646 (3,198-6,672)	6,756 (4,492-9,720)	5,973 (3,936-8,759)	5,639 (3,684-8,264)	4,419 (2,926-6,402)	2,715 (1,789-3,916)	1,755 (1,120-2,542)	373 (233-530)	234 (120-501)
Chagas disease	-	-	1 (1-1)	1 (1-1)	1 (1-1)	1 (1-2)	4 (3-4)	7 (6-8)	15 (13-17)	27 (24-31)	47 (41-54)	77 (69-87)	121 (107-137)	184 (165-205)	262 (232-297)	306 (274-346)	352 (314-397)	401 (363-450)	401 (359-455)	356 (321-397)	230 (205-264)	110 (99-123)	30 (27-34)
Leishmaniasis	-	-	1,176 (560-2,186)	1,702 (747-3,219)	755 (352-1,479)	529 (252-991)	688 (326-1,241)	759 (373-1,408)	636 (296-1,194)	567 (259-1,053)	680 (315-1,279)	688 (320-1,262)	669 (314-1,244)	687 (339-1,265)	549 (256-1,024)	475 (224-891)	551 (261-1,056)	553 (258-1,052)	451 (210-853)	257 (116-495)	168 (73-306)	32 (14-62)	12 (4-24)
Visceral leishmaniasis	-	-	1,176 (560-2,186)	1,702 (747-3,219)	755 (352-1,479)	529 (252-991)	688 (326-1,241)	759 (373-1,408)	636 (296-1,194)	567 (259-1,053)	680 (315-1,279)	688 (320-1,262)	669 (314-1,244)	687 (339-1,265)	549 (256-1,024)	475 (224-891)	551 (261-1,056)	553 (258-1,052)	451 (210-853)	257 (116-495)	168 (73-306)	32 (14-62)	12 (4-24)
African trypanosomiasis	-	-	-	81 (15-292)	226 (48-785)	267 (59-847)	289 (58-1,056)	260 (49-954)	232 (46-876)	203 (39-738)	178 (36-639)	156 (32-589)	160 (27-667)	143 (27-569)	135 (23-487)	115 (18-428)	90 (15-364)	64 (10-257)	40 (6-159)	20 (3-73)	8 (1-35)	2 (0-8)	0 (0-2)
Schistosomiasis	-	-	0 (0-0)	226 (126-391)	108 (68-171)	148 (97-216)	121 (83-172)	141 (100-192)	168 (120-229)	195 (138-268)	244 (174-335)	282 (208-391)	339 (247-465)	442 (322-609)	561 (416-764)	581 (409-813)	625 (446-862)	578 (426-773)	460 (339-621)	264 (194-350)	127 (94-169)	39 (28-54)	7 (4-11)
Cysticercosis	0 (0-0)	0 (0-0)	0 (0-0)	54 (34-80)	32 (23-44)	25 (19-32)	19 (15-25)	25 (19-32)	33 (26-41)	38 (30-47)	36 (29-44)	32 (26-39)	33 (27-41)	43 (36-53)	44 (37-53)	35 (29-42)	32 (26-38)	21 (17-25)	13 (11-17)	8 (6-10)	4 (3-5)	1 (1-2)	1 (0-1)
Cystic echinococcosis	-	-	-	112 (75-165)	45 (30-65)	27 (18-40)	12 (8-17)	15 (10-21)	24 (17-33)	25 (18-34)	29 (21-38)	26 (19-34)	32 (23-42)	36 (27-46)	42 (31-55)	40 (31-53)	40 (31-52)	38 (29-49)	33 (25-41)	27 (21-34)	20 (16-26)	7 (5-10)	2 (1-3)
Dengue	-	-	1,214 (282-2,009)	2,107 (541-3,344)	1,345 (245-2,238)	848 (197-1,458)	669 (125-1,146)	680 (100-1,207)	586 (90-980)	495 (63-863)	451 (76-779)	489 (70-835)	589 (82-1,057)	655 (84-1,147)	725 (101-1,236)	763 (84-1,321)	653 (92-1,174)	370 (54-716)	342 (53-673)	306 (46-584)	216 (38-419)	109 (21-221)	24 (6-50)
Yellow fever	-	1 (0-3)	16 (3-49)	150 (30-457)	294 (56-933)	262 (49-776)	207 (38-635)	161 (31-499)	127 (24-383)	99 (19-314)	79 (16-240)	63 (12-193)	54 (11-166)	48 (9-151)	39 (7-125)	29 (5-88)	22 (4-65)	14 (3-45)	9 (2-27)	4 (1-13)	1 (0-5)	0 (0-1)	0 (0-0)
Rabies	-	-	134 (82-437)	839 (336-1,583)	1,000 (338-2,018)	506 (156-958)	270 (95-486)	207 (77-353)	180 (104-384)	308 (122-635)	444 (164-764)	401 (193-789)	498 (240-1,047)	525 (301-1,250)	394 (172-694)	244 (95-400)	357 (130-653)	224 (128-498)	187 (103-523)	44 (15-81)	38 (11-67)	8 (2-16)	5 (1-9)
Intestinal nematode infections	-	-	274 (206-357)	2,803 (2,032-3,759)	393 (304-500)	82 (64-106)	26 (19-34)	16 (12-22)	13 (9-18)	17 (12-22)	17 (12-21)	13 (9-17)	15 (11-20)	24 (18-31)	22 (16-29)	25 (19-33)	19 (14-25)	30 (23-39)	26 (19-33)	17 (13-23)	7 (5-9)	5 (3-6)	2 (1-3)
Ascariasis	-	-	274 (206-357)	2,803 (2,032-3,759)	393 (304-500)	82 (64-106)	26 (19-34)	16 (12-22)	13 (9-18)	17 (12-22)	17 (12-21)	13 (9-17)	15 (11-20)	24 (18-31)	22 (16-29)	25 (19-33)	19 (14-25)	30 (23-39)	26 (19-33)	17 (13-23)	7 (5-9)	5 (3-6)	2 (1-3)
Ebola	0 (0-0)	0 (0-0)	0 (0-0)	0 (0-0)	0 (0-0)	0 (0-0)	0 (0-0)	0 (0-0)	0 (0-0)	0 (0-0)	0 (0-0)	0 (0-0)	0 (0-0)	0 (0-0)	0 (0-0)	0 (0-0)	0 (0-0)	0 (0-0)	0 (0-0)	0 (0-0)	0 (0-0)	0 (0-0)	0 (0-0)
Zika virus	0 (0-0)	0 (0-0)	0 (0-0)	0 (0-0)	0 (0-0)	0 (0-0)	0 (0-0)	0 (0-0)	0 (0-0)	0 (0-0)	0 (0-0)	0 (0-0)	0 (0-0)	0 (0-0)	0 (0-0)	0 (0-0)	0 (0-0)	0 (0-0)	0 (0-0)	0 (0-0)	0 (0-0)	0 (0-0)	0 (0-0)
Other neglected tropical diseases	531 (225-795)	313 (159-514)	3,045 (1,304-4,629)	4,542 (1,453-9,334)	690 (307-1,393)	367 (174-667)	214 (110-402)	175 (98-279)	150 (84-226)	135 (71-222)	140 (81-207)	141 (80-207)	149 (84-223)	190 (106-287)	192 (106-294)	196 (109-313)	226 (130-349)	223 (121-357)	215 (124-341)	156 (98-252)	95 (58-161)	36 (23-58)	19 (13-34)
Maternal disorders	-	-	-	-	-	1,336 (1,098-1,611)	27,035 (24,175-29,741)	54,455 (49,858-59,071)	55,251 (50,786-59,282)	48,609 (44,960-52,139)	42,205 (39,024-45,392)	28,104 (25,815-30,419)	14,915 (13,318-16,642)	3,448 (2,765-4,333)	-	-	-	-	-	-	-	-	-
Maternal haemorrhage	-	-	-	-	-	251 (164-373)	7,505 (5,518-9,773)	18,134 (14,674-21,918)	18,852 (14,837-23,195)	16,654 (12,865-20,425)	14,782 (11,259-18,596)	9,128 (6,550-12,006)	3,897 (2,804-5,303)	685 (390-1,097)	-	-	-	-	-	-	-	-	-
Maternal sepsis and other pregnancy related infections	-	-	-	-	-	127 (80-187)	2,694 (1,815-3,911)	4,416 (2,927-6,366)	4,524 (3,164-6,239)	3,877 (2,637-5,480)	3,205 (2,182-4,621)	2,250 (1,429-3,473)	1,366 (874-2,153)	285 (159-483)	-	-	-	-	-	-	-	-	-
Maternal hypertensive disorders	-	-	-	-	-	280 (170-406)	4,361 (2,897-5,964)	7,651 (5,449-10,118)	7,113 (4,862-9,853)	6,055 (4,038-8,640)	5,324 (3,645-7,487)	3,516 (2,183-5,235)	1,672 (1,060-2,430)	379 (196-658)	-	-	-	-	-	-	-	-	-
Maternal obstructed labour and uterine rupture	-	-	-	-	-	51 (26-89)	910 (450-1,538)	1,809 (1,043-2,840)	2,051 (1,181-3,279)	2,146 (1,233-3,257)	2,137 (1,280-3,507)	1,618 (897-2,647)	909 (492-1,525)	206 (86-387)	-	-	-	-	-	-	-	-	-
Maternal abortion, miscarriage, and ectopic pregnancy	-	-	-	-	-	123 (72-197)	2,303 (1,385-3,425)	4,167 (2,713-6,115)	4,112 (2,822-5,926)	3,744 (2,509-5,422)	3,472 (2,179-4,915)	2,759 (1,881-3,979)	1,901 (1,313-2,668)	366 (241-527)	-	-	-	-	-	-	-	-	-
Indirect maternal deaths	-	-	-	-	-	226 (143-331)	4,043 (2,658-5,925)	8,077 (5,700-11,110)	8,819 (5,986-12,167)	7,642 (4,758-10,884)	5,658 (3,757-8,002)	3,744 (2,309-5,626)	2,352 (1,443-3,499)	788 (445-1,222)	-	-	-	-	-	-	-	-	-
Late maternal deaths	-	-	-	-	-	30 (13-62)	493 (235-919)	930 (439-1,728)	973 (488-1,815)	859 (449-1,578)	738 (340-1,342)	495 (167-918)	279 (106-501)	77 (28-166)	-	-	-	-	-	-	-	-	-
Maternal deaths aggravated by HIV/AIDS	-	-	-	-	-	3 (2-4)	65 (37-92)	280 (171-381)	482 (311-632)	508 (322-667)	431 (272-576)	267 (164-367)	134 (83-185)	22 (13-33)	-	-	-	-	-	-	-	-	-
Other maternal disorders	-	-	-	-	-	246 (163-356)	4,661 (3,329-6,477)	8,989 (6,674-11,964)	8,326 (6,166-11,023)	7,124 (5,079-9,669)	6,458 (4,484-9,232)	4,327 (2,731-6,345)	2,405 (1,539-3,535)	639 (400-991)	-	-	-	-	-	-	-	-	-
Neonatal disorders	728,505 (697,098-761,474)	157,134 (148,101-167,247)	43,751 (38,270-49,850)	4,708 (4,069-5,428)	-	-	-	-	-	-	-	-	-	-	-	-	-	-	-	-	-	-	-
Neonatal preterm birth complications	281,285 (257,420-319,824)	45,945 (39,632-54,160)	14,595 (12,257-17,925)	1,191 (949-1,451)	-	-	-	-	-	-	-	-	-	-	-	-	-	-	-	-	-	-	-
Neonatal encephalopathy due to birth asphyxia and trauma	233,376 (206,580-259,124)	23,335 (18,711-28,048)	9,169 (6,927-12,276)	1,963 (1,669-2,326)	-	-	-	-	-	-	-	-	-	-	-	-	-	-	-	-	-	-	-
Neonatal sepsis and other neonatal infections	61,974 (40,046-102,783)	44,648 (33,606-59,479)	7,951 (5,432-11,275)	339 (123-565)	-	-	-	-	-	-	-	-	-	-	-	-	-	-	-	-	-	-	-
Hemolytic disease and other neonatal jaundice	19,824 (16,490-24,244)	10,979 (8,688-13,858)	2,282 (1,574-3,496)	109 (76-169)	-	-	-	-	-	-	-	-	-	-	-	-	-	-	-	-	-	-	-
Other neonatal disorders	132,046 (115,016-149,572)	32,227 (27,366-37,680)	9,755 (7,927-12,230)	1,105 (885-1,457)	-	-	-	-	-	-	-	-	-	-	-	-	-	-	-	-	-	-	-
Nutritional deficiencies	-	-	51,898 (45,487-59,762)	60,426 (47,013-78,306)	5,036 (4,176-6,004)	2,356 (2,028-2,726)	1,904 (1,698-2,128)	2,137 (1,923-2,380)	2,366 (2,140-2,595)	2,170 (1,985-2,373)	2,068 (1,876-2,265)	2,643 (2,434-2,861)	2,275 (2,094-2,472)	3,173 (2,900-3,475)	4,332 (3,960-4,721)	5,161 (4,640-5,623)	7,047 (6,396-7,669)	7,878 (7,238-8,510)	10,900 (9,972-11,732)	12,271 (11,472-13,045)	12,603 (11,762-13,493)	8,754 (8,257-9,258)	5,118 (4,787-5,448)
Protein-energy malnutrition	-	-	50,073 (43,713-57,749)	57,521 (44,382-75,132)	4,343 (3,533-5,191)	1,848 (1,566-2,154)	1,155 (1,007-1,341)	957 (828-1,112)	1,205 (1,058-1,370)	1,041 (920-1,196)	1,026 (906-1,181)	1,566 (1,410-1,744)	1,072 (939-1,227)	1,602 (1,386-1,828)	2,727 (2,419-3,056)	3,314 (2,933-3,715)	4,495 (4,019-5,009)	5,416 (4,949-5,965)	8,045 (7,362-8,810)	9,331 (8,614-10,118)	10,030 (9,161-10,928)	7,244 (6,598-7,814)	4,304 (3,682-4,688)
Iodine deficiency	-	-	198 (39-620)	193 (24-849)	48 (11-169)	15 (3-50)	28 (6-86)	36 (8-107)	38 (9-117)	39 (10-111)	40 (10-118)	33 (9-99)	32 (9-111)	51 (13-149)	58 (18-159)	61 (19-169)	89 (27-247)	93 (32-224)	93 (31-234)	75 (26-174)	43 (12-113)	15 (3-48)	10 (1-41)
Iron-deficiency anaemia	-	-	33 (15-86)	63 (32-172)	71 (35-157)	59 (38-111)	64 (45-100)	76 (57-108)	67 (45-109)	61 (47-83)	66 (46-104)	56 (45-92)	50 (40-82)	59 (42-129)	54 (38-82)	46 (31-74)	49 (32-85)	118 (70-233)	153 (90-302)	140 (88-248)	109 (39-190)	74 (27-116)	81 (38-120)
Other nutritional deficiencies	-	-	1,594 (1,061-2,140)	2,649 (1,215-4,535)	574 (434-762)	434 (336-563)	656 (537-778)	1,068 (919-1,246)	1,056 (895-1,243)	1,028 (880-1,196)	936 (801-1,086)	988 (859-1,129)	1,121 (953-1,292)	1,461 (1,252-1,697)	1,493 (1,258-1,743)	1,741 (1,439-2,010)	2,413 (2,034-2,879)	2,251 (1,860-2,608)	2,608 (2,153-3,090)	2,725 (2,241-3,335)	2,422 (2,010-3,173)	1,421 (1,153-2,111)	723 (564-1,387)
Other communicable, maternal, neonatal, and nutritional diseases	16,553 (9,970-25,734)	6,768 (4,390-10,004)	29,932 (19,879-43,402)	24,956 (17,370-35,190)	8,382 (5,931-11,983)	2,744 (2,324-3,131)	4,220 (3,736-4,691)	4,873 (4,389-5,318)	4,216 (3,842-4,587)	4,000 (3,648-4,337)	4,197 (3,815-4,583)	4,297 (3,955-4,630)	4,218 (3,885-4,535)	5,182 (4,737-5,577)	5,971 (5,473-6,423)	5,517 (4,994-5,972)	6,133 (5,456-6,685)	6,226 (5,546-6,794)	5,450 (4,797-5,936)	4,629 (4,041-5,079)	2,814 (2,406-3,073)	1,205 (1,003-1,307)	454 (374-495)
Sexually transmitted diseases excluding HIV	15,154 (8,564-24,308)	5,492 (2,994-8,673)	21,781 (12,058-34,882)	15,890 (8,598-25,701)	5,264 (2,989-8,576)	80 (61-105)	437 (349-535)	570 (471-682)	734 (591-842)	572 (456-649)	608 (503-709)	618 (499-689)	612 (518-683)	531 (396-602)	673 (481-763)	414 (329-470)	416 (311-474)	338 (267-378)	370 (299-416)	281 (223-309)	212 (179-232)	112 (95-122)	52 (44-55)
Syphilis	15,154 (8,564-24,308)	5,492 (2,994-8,673)	21,781 (12,058-34,882)	15,890 (8,598-25,701)	5,264 (2,989-8,576)	44 (33-57)	123 (98-151)	160 (132-191)	150 (121-172)	118 (94-134)	74 (62-87)	67 (54-75)	60 (51-67)	101 (75-114)	157 (112-177)	100 (79-113)	106 (79-121)	102 (81-114)	98 (79-110)	73 (58-80)	48 (40-52)	23 (20-26)	10 (8-10)
Chlamydial infection	-	-	-	-	-	10 (8-13)	57 (45-70)	123 (102-147)	123 (99-141)	60 (48-68)	98 (81-114)	116 (94-129)	108 (91-120)	82 (61-93)	104 (74-118)	65 (52-74)	38 (29-44)	39 (31-44)	47 (38-53)	50 (40-55)	39 (33-43)	20 (17-22)	9 (8-10)
Gonococcal infection	-	-	-	-	-	18 (14-24)	140 (112-171)	178 (147-213)	319 (257-367)	256 (204-291)	269 (223-314)	287 (232-321)	287 (243-320)	236 (176-268)	273 (195-309)	155 (123-176)	208 (155-237)	146 (115-163)	177 (143-199)	124 (98-137)	98 (83-107)	53 (45-58)	25 (21-27)
Other sexually transmitted diseases	-	-	-	-	-	8 (6-11)	117 (94-144)	109 (90-130)	141 (114-162)	138 (110-156)	167 (138-194)	147 (119-165)	158 (133-176)	112 (84-127)	140 (100-158)	95 (75-107)	63 (47-72)	51 (40-57)	47 (38-53)	34 (27-37)	27 (23-30)	15 (12-16)	8 (6-8)
Hepatitis	-	-	4,391 (3,837-5,030)	5,254 (4,329-6,264)	2,070 (1,680-2,508)	2,012 (1,662-2,374)	3,090 (2,704-3,506)	3,673 (3,263-4,053)	2,945 (2,643-3,232)	2,849 (2,557-3,142)	3,073 (2,749-3,404)	3,087 (2,811-3,373)	2,878 (2,623-3,141)	3,553 (3,216-3,875)	4,054 (3,703-4,396)	3,568 (3,235-3,897)	3,866 (3,450-4,295)	3,890 (3,443-4,312)	3,117 (2,743-3,455)	2,390 (2,040-2,688)	1,150 (1,005-1,285)	380 (336-418)	96 (85-105)
Acute hepatitis A	-	-	2,776 (1,844-3,734)	579 (293-954)	47 (25-75)	31 (17-48)	52 (28-85)	66 (32-111)	41 (18-77)	31 (13-59)	29 (13-54)	33 (14-61)	34 (16-59)	44 (20-79)	50 (24-84)	51 (25-90)	54 (26-97)	60 (29-102)	53 (27-93)	42 (22-70)	20 (12-31)	6 (4-10)	2 (1-3)
Hepatitis B	-	-	1,614 (840-2,695)	637 (356-1,023)	167 (88-286)	281 (143-450)	1,004 (652-1,444)	2,178 (1,592-2,808)	2,234 (1,791-2,641)	2,426 (2,080-2,785)	2,719 (2,353-3,090)	2,732 (2,389-3,060)	2,543 (2,225-2,851)	3,144 (2,760-3,535)	3,572 (3,124-3,989)	3,090 (2,644-3,483)	3,270 (2,767-3,756)	3,201 (2,642-3,716)	2,499 (2,027-2,932)	1,675 (1,252-2,091)	648 (436-872)	176 (100-260)	36 (17-58)
Hepatitis C	-	-	-	-	59 (38-91)	23 (14-35)	34 (21-52)	43 (23-70)	29 (14-50)	24 (11-42)	31 (14-57)	38 (17-65)	48 (24-85)	83 (37-144)	131 (64-224)	128 (59-221)	128 (62-223)	121 (56-219)	94 (48-162)	71 (36-119)	35 (21-55)	12 (7-19)	3 (2-6)
Acute hepatitis E	-	-	-	4,038 (3,190-4,996)	1,797 (1,429-2,180)	1,677 (1,331-2,009)	2,000 (1,551-2,463)	1,386 (810-1,975)	641 (337-1,040)	368 (177-609)	294 (131-515)	285 (129-532)	252 (121-444)	283 (133-487)	302 (128-572)	298 (130-573)	414 (200-734)	508 (233-920)	471 (198-878)	603 (317-980)	448 (259-666)	186 (110-263)	55 (33-76)
Other infectious diseases	1,398 (852-1,753)	1,277 (724-1,764)	3,760 (2,798-4,580)	3,812 (2,642-4,768)	1,047 (758-1,300)	652 (490-776)	693 (535-825)	630 (494-733)	537 (432-616)	578 (450-669)	515 (424-606)	593 (498-701)	728 (583-824)	1,098 (879-1,256)	1,244 (991-1,412)	1,535 (1,197-1,738)	1,850 (1,402-2,080)	1,998 (1,550-2,271)	1,964 (1,476-2,218)	1,958 (1,475-2,173)	1,451 (1,054-1,621)	713 (520-790)	307 (233-341)
Non-communicable diseases	83,166 (65,899-99,883)	34,679 (29,327-39,895)	134,822 (113,812-159,898)	90,473 (71,802-110,273)	37,659 (34,066-41,863)	43,971 (40,524-47,782)	68,104 (64,295-71,635)	88,681 (84,877-92,487)	107,480 (103,046-111,382)	144,158 (139,524-149,156)	214,489 (207,079-221,189)	332,983 (323,974-341,344)	494,238 (481,263-505,134)	706,457 (687,311-721,812)	962,654 (934,945-983,772)	1,198,869 (1,157,871-1,226,831)	1,432,206 (1,378,511-1,466,807)	1,947,390 (1,876,811-1,997,574)	2,246,630 (2,171,139-2,301,353)	2,511,130 (2,442,345-2,564,888)	2,164,013 (2,114,527-2,203,629)	1,199,854 (1,178,229-1,220,005)	564,012 (554,642-573,328)
Neoplasms	389 (346-415)	242 (222-262)	2,614 (2,413-2,934)	9,993 (9,055-11,518)	10,632 (9,604-11,632)	11,840 (10,803-12,787)	15,477 (14,275-16,304)	20,377 (18,983-21,230)	28,415 (26,782-29,606)	46,864 (44,661-48,575)	81,098 (78,117-83,733)	134,045 (129,832-137,770)	195,003 (189,717-199,798)	266,596 (259,738-273,031)	336,615 (328,193-344,108)	368,321 (358,777-376,244)	371,650 (362,110-379,806)	416,909 (405,928-426,753)	393,127 (380,876-403,531)	339,770 (328,594-349,773)	237,040 (228,405-245,311)	102,636 (98,382-106,688)	40,267 (38,400-41,778)
Lip and oral cavity cancer	-	-	-	-	-	-	153 (139-169)	292 (265-320)	412 (370-464)	666 (607-729)	1,128 (1,026-1,243)	2,036 (1,855-2,234)	3,154 (2,932-3,392)	4,198 (3,891-4,539)	5,115 (4,768-5,459)	6,032 (5,540-6,534)	5,483 (5,105-5,844)	5,270 (4,846-5,617)	4,887 (4,521-5,188)	4,672 (4,326-4,966)	3,565 (3,320-3,800)	1,764 (1,643-1,877)	710 (667-750)
Nasopharynx cancer	-	-	-	-	37 (33-41)	68 (60-77)	151 (138-166)	198 (185-213)	260 (243-281)	386 (360-416)	652 (613-698)	1,144 (1,075-1,226)	1,570 (1,483-1,658)	1,966 (1,863-2,075)	2,400 (2,277-2,528)	1,901 (1,789-2,012)	1,491 (1,410-1,578)	1,411 (1,328-1,505)	1,024 (963-1,085)	715 (670-757)	371 (350-391)	147 (139-156)	51 (48-53)
Other pharynx cancer	-	-	-	-	-	-	162 (139-185)	231 (202-263)	355 (314-398)	504 (452-559)	764 (681-850)	1,279 (1,153-1,415)	2,238 (2,059-2,408)	3,168 (2,929-3,401)	3,576 (3,287-3,845)	3,927 (3,564-4,213)	3,399 (3,077-3,656)	3,405 (3,027-3,666)	2,107 (1,867-2,297)	1,620 (1,403-1,784)	944 (833-1,030)	360 (326-388)	106 (99-113)
Oesophageal cancer	-	-	-	-	-	-	116 (107-126)	260 (241-282)	378 (343-424)	599 (552-651)	1,173 (1,094-1,252)	2,384 (2,231-2,548)	4,174 (3,964-4,409)	6,793 (6,516-7,097)	10,574 (10,121-11,054)	13,051 (12,462-13,638)	14,833 (14,121-15,555)	16,727 (15,980-17,552)	16,180 (15,456-16,958)	12,923 (12,257-13,569)	7,668 (7,335-8,031)	3,027 (2,886-3,167)	902 (861-941)
Stomach cancer	-	-	-	-	-	-	432 (396-468)	1,119 (1,051-1,196)	1,961 (1,850-2,073)	3,713 (3,525-3,913)	6,569 (6,265-6,887)	10,661 (10,224-11,106)	14,006 (13,511-14,531)	18,790 (18,110-19,488)	25,832 (24,879-26,835)	30,426 (29,361-31,618)	32,873 (31,712-34,141)	39,735 (38,385-41,230)	39,864 (38,312-41,498)	34,766 (33,363-36,194)	23,139 (22,195-24,081)	10,117 (9,681-10,575)	3,950 (3,759-4,120)
Colon and rectum cancer	-	-	-	-	-	-	322 (295-355)	838 (778-903)	1,428 (1,353-1,511)	2,839 (2,704-2,994)	4,652 (4,453-4,845)	7,734 (7,440-8,040)	13,164 (12,720-13,632)	19,676 (19,029-20,328)	26,908 (26,051-27,848)	31,318 (30,410-32,389)	33,984 (32,890-35,097)	44,327 (42,839-45,913)	48,047 (46,295-49,723)	46,547 (44,564-48,496)	37,530 (35,795-39,263)	18,228 (17,369-19,148)	7,760 (7,367-8,102)
Liver cancer	-	-	-	-	152 (134-174)	220 (187-246)	388 (352-454)	770 (692-866)	1,295 (1,154-1,441)	2,261 (1,953-2,462)	4,046 (3,595-4,369)	7,382 (6,416-8,087)	10,792 (9,629-11,499)	15,609 (13,651-16,725)	22,163 (19,422-23,659)	24,915 (21,919-26,384)	26,509 (23,313-28,165)	29,609 (26,120-31,539)	27,695 (25,023-29,404)	21,577 (19,467-22,936)	12,668 (11,688-13,478)	4,588 (4,282-4,900)	1,447 (1,354-1,542)
Liver cancer due to hepatitis B	-	-	-	-	55 (39-74)	102 (77-130)	246 (159-263)	503 (428-583)	893 (722-1,054)	1,560 (1,285-1,820)	2,696 (2,175-3,139)	4,525 (3,584-5,278)	5,789 (4,363-7,012)	7,407 (5,377-9,241)	9,506 (6,609-12,179)	9,437 (6,210-12,090)	9,067 (6,037-11,477)	9,325 (6,102-12,075)	8,201 (5,564-10,825)	5,861 (3,931-7,948)	3,190 (2,302-4,182)	1,097 (784-1,459)	296 (181-421)
Liver cancer due to hepatitis C	-	-	-	-	7 (5-9)	13 (9-16)	23 (17-30)	58 (44-75)	115 (73-170)	216 (150-289)	395 (284-541)	860 (650-1,136)	1,646 (1,225-2,105)	2,854 (2,183-3,574)	4,286 (3,357-5,260)	5,557 (4,478-6,794)	6,342 (5,187-7,568)	7,773 (6,549-9,067)	8,522 (7,362-9,860)	7,806 (6,740-8,989)	5,267 (4,698-5,866)	2,128 (1,893-2,387)	774 (672-881)
Liver cancer due to alcohol use	-	-	-	-	-	-	3 (2-5)	20 (12-30)	55 (30-89)	175 (112-259)	507 (291-811)	1,281 (827-1,910)	2,362 (1,462-3,495)	3,942 (2,602-5,442)	6,534 (4,367-9,312)	7,813 (5,522-10,653)	8,801 (6,733-11,124)	9,869 (7,615-12,320)	8,277 (6,247-10,471)	5,663 (4,064-7,337)	2,879 (2,132-3,642)	873 (630-1,154)	221 (135-321)
Liver cancer due to other causes	-	-	-	-	90 (73-109)	105 (82-128)	156 (120-196)	189 (147-241)	233 (158-326)	309 (224-416)	447 (335-609)	716 (539-961)	995 (749-1,309)	1,406 (1,040-1,890)	1,837 (1,384-2,403)	2,107 (1,629-2,710)	2,298 (1,773-2,838)	2,641 (2,066-3,289)	2,695 (2,133-3,341)	2,248 (1,801-2,811)	1,332 (1,093-1,599)	489 (397-604)	156 (117-203)
Gallbladder and biliary tract cancer	-	-	-	-	-	-	43 (40-47)	84 (77-90)	186 (167-205)	479 (431-525)	1,080 (950-1,202)	2,304 (2,012-2,556)	3,879 (3,515-4,218)	5,723 (5,165-6,172)	7,457 (6,772-7,984)	9,365 (8,716-9,874)	10,048 (9,397-10,576)	11,502 (10,832-12,163)	11,479 (10,886-12,045)	10,281 (9,751-10,774)	7,505 (7,097-7,916)	3,419 (3,231-3,611)	1,463 (1,384-1,540)
Pancreatic cancer	-	-	-	-	-	-	66 (62-70)	131 (125-139)	271 (258-285)	518 (497-542)	1,150 (1,107-1,201)	2,470 (2,386-2,577)	4,592 (4,458-4,740)	8,127 (7,890-8,374)	12,586 (12,223-12,983)	17,528 (17,052-18,011)	20,234 (19,720-20,740)	25,336 (24,522-26,153)	25,373 (24,501-26,246)	22,390 (21,473-23,271)	16,388 (15,626-17,103)	6,844 (6,535-7,142)	2,524 (2,390-2,635)
Larynx cancer	-	-	-	-	-	-	46 (43-50)	76 (69-85)	91 (79-108)	188 (160-223)	299 (275-325)	560 (517-608)	931 (873-997)	1,326 (1,254-1,402)	1,710 (1,612-1,805)	1,874 (1,772-1,975)	1,823 (1,721-1,935)	2,028 (1,906-2,149)	1,568 (1,483-1,643)	1,286 (1,212-1,357)	697 (658-736)	260 (246-274)	76 (72-80)
Tracheal, bronchus, and lung cancer	-	-	-	-	-	-	335 (290-362)	661 (606-714)	1,203 (1,110-1,296)	2,651 (2,365-2,929)	5,066 (4,819-5,347)	10,959 (10,472-11,490)	18,975 (18,404-19,646)	31,095 (30,139-32,243)	45,128 (43,696-46,755)	55,582 (53,741-57,266)	60,881 (59,165-63,043)	69,880 (67,469-72,109)	66,562 (64,010-69,055)	54,033 (51,258-56,665)	33,580 (31,790-35,209)	12,943 (12,142-13,594)	4,403 (4,153-4,624)
Malignant skin melanoma	-	-	-	-	-	-	99 (86-121)	199 (172-248)	323 (274-391)	476 (407-590)	740 (615-915)	1,060 (865-1,312)	1,444 (1,153-1,757)	1,881 (1,533-2,245)	2,179 (1,732-2,539)	2,452 (1,942-2,798)	2,289 (1,757-2,674)	2,750 (2,065-3,256)	2,693 (2,003-3,073)	2,708 (2,037-3,117)	2,073 (1,467-2,362)	983 (735-1,154)	462 (336-577)
Non-melanoma skin cancer	-	-	-	-	-	-	64 (60-69)	82 (77-87)	100 (95-105)	127 (120-135)	217 (204-233)	358 (336-382)	543 (512-575)	684 (648-718)	952 (905-999)	1,142 (1,085-1,197)	1,230 (1,168-1,293)	1,625 (1,548-1,705)	1,686 (1,614-1,767)	2,175 (2,076-2,276)	2,202 (2,103-2,300)	1,455 (1,387-1,527)	993 (937-1,043)
Non-melanoma skin cancer (squamous-cell carcinoma)	-	-	-	-	-	-	64 (60-69)	82 (77-87)	100 (95-105)	127 (120-135)	217 (204-233)	358 (336-382)	543 (512-575)	684 (648-718)	952 (905-999)	1,142 (1,085-1,197)	1,230 (1,168-1,293)	1,625 (1,548-1,705)	1,686 (1,614-1,767)	2,175 (2,076-2,276)	2,202 (2,103-2,300)	1,455 (1,387-1,527)	993 (937-1,043)
Breast cancer	-	-	-	-	-	-	341 (269-381)	1,049 (917-1,158)	3,252 (2,892-3,519)	8,879 (8,313-9,491)	19,095 (18,088-20,850)	31,761 (30,136-34,395)	44,504 (42,282-48,297)	55,262 (52,933-58,208)	61,262 (58,531-64,917)	54,085 (52,108-57,741)	46,245 (44,524-49,833)	45,108 (43,428-47,808)	38,550 (37,059-41,060)	33,070 (31,630-34,791)	25,219 (24,093-26,508)	11,700 (11,172-12,323)	5,339 (5,074-5,596)
Cervical cancer	-	-	-	-	-	-	201 (145-218)	851 (614-925)	2,605 (2,024-2,799)	6,151 (4,948-6,606)	11,940 (9,516-12,781)	19,147 (15,552-20,410)	25,518 (21,209-27,048)	29,564 (24,005-31,452)	31,304 (25,871-33,585)	27,146 (22,800-29,105)	22,447 (18,752-24,033)	20,666 (17,909-22,267)	14,666 (12,803-15,514)	11,798 (10,394-12,522)	6,686 (6,043-7,096)	2,507 (2,312-2,649)	864 (810-911)
Uterine cancer	-	-	-	-	-	-	79 (72-87)	208 (193-225)	345 (324-369)	642 (602-681)	1,387 (1,300-1,477)	2,613 (2,459-2,765)	4,534 (4,313-4,744)	6,896 (6,553-7,241)	9,255 (8,822-9,719)	11,225 (10,719-11,732)	10,964 (10,498-11,456)	11,020 (10,589-11,459)	9,077 (8,734-9,451)	7,400 (7,081-7,751)	4,565 (4,353-4,786)	1,826 (1,744-1,920)	656 (621-692)
Ovarian cancer	-	-	-	-	-	-	509 (467-553)	766 (715-818)	1,140 (1,060-1,206)	1,747 (1,644-1,842)	3,279 (3,069-3,456)	6,083 (5,763-6,387)	10,568 (10,019-11,033)	15,543 (14,762-16,188)	17,620 (16,766-18,234)	18,278 (17,562-18,937)	16,422 (15,857-16,949)	17,469 (16,786-18,112)	14,032 (13,472-14,554)	11,101 (10,624-11,602)	7,038 (6,672-7,377)	2,689 (2,568-2,821)	910 (865-951)
Prostate cancer	-	-	-	-	-	-	-	-	-	-	-	-	-	-	-	-	-	-	-	-	-	-	-
Testicular cancer	-	-	-	-	-	-	-	-	-	-	-	-	-	-	-	-	-	-	-	-	-	-	-
Kidney cancer	15 (14-16)	7 (7-8)	108 (101-119)	544 (491-603)	301 (278-325)	167 (156-180)	115 (109-121)	160 (153-169)	207 (198-216)	266 (254-278)	445 (426-466)	735 (711-760)	1,304 (1,257-1,358)	2,142 (2,072-2,223)	3,177 (3,066-3,314)	4,102 (3,962-4,257)	4,306 (4,173-4,437)	5,526 (5,314-5,749)	5,566 (5,339-5,792)	5,281 (5,038-5,525)	3,891 (3,696-4,091)	1,607 (1,531-1,691)	599 (566-630)
Bladder cancer	-	-	-	-	-	-	44 (41-49)	57 (53-61)	80 (75-86)	167 (156-178)	296 (276-317)	596 (557-636)	923 (873-973)	1,637 (1,546-1,727)	2,299 (2,188-2,405)	3,131 (3,005-3,257)	3,913 (3,742-4,074)	5,316 (5,112-5,512)	6,631 (6,385-6,882)	7,200 (6,861-7,522)	6,280 (5,967-6,577)	3,185 (3,022-3,341)	1,405 (1,332-1,473)
Brain and nervous system cancer	48 (36-55)	31 (23-36)	411 (321-476)	1,768 (1,457-2,095)	2,464 (2,051-2,813)	2,220 (1,825-2,564)	1,716 (1,429-1,963)	1,914 (1,580-2,155)	2,121 (1,775-2,384)	2,525 (2,158-2,844)	3,564 (3,059-4,033)	4,724 (4,115-5,397)	5,873 (5,144-6,727)	7,379 (6,440-8,477)	9,110 (7,919-10,340)	9,218 (8,112-10,352)	8,654 (7,460-9,545)	8,488 (7,056-9,183)	7,158 (5,578-7,725)	4,931 (3,656-5,399)	2,623 (1,864-2,893)	802 (567-883)	228 (155-254)
Thyroid cancer	-	-	-	-	-	64 (53-78)	168 (134-216)	296 (253-351)	355 (314-400)	427 (386-468)	542 (488-598)	888 (810-967)	1,241 (1,139-1,346)	1,852 (1,706-2,012)	2,351 (2,187-2,522)	2,530 (2,372-2,698)	2,755 (2,591-2,939)	3,027 (2,866-3,184)	2,748 (2,610-2,905)	2,230 (2,115-2,359)	1,476 (1,402-1,563)	601 (568-637)	214 (201-225)
Mesothelioma	-	-	-	-	-	-	45 (38-56)	63 (52-79)	92 (76-114)	104 (88-128)	180 (154-218)	312 (276-372)	299 (274-343)	454 (419-517)	627 (585-702)	748 (706-822)	835 (792-905)	988 (942-1,053)	1,032 (982-1,096)	894 (847-945)	555 (524-586)	211 (201-223)	76 (72-80)
Hodgkin lymphoma	1 (0-1)	1 (0-1)	15 (10-18)	78 (45-116)	165 (96-232)	320 (182-428)	490 (316-610)	617 (442-768)	653 (468-788)	583 (431-694)	580 (415-710)	614 (439-751)	585 (443-745)	699 (501-899)	744 (573-992)	824 (598-1,020)	824 (622-1,042)	781 (643-1,000)	694 (588-866)	572 (484-678)	313 (272-354)	93 (83-105)	27 (25-30)
Non-Hodgkin's lymphoma	21 (18-23)	16 (14-18)	250 (221-285)	1,132 (949-1,335)	1,388 (1,093-1,663)	1,266 (1,024-1,457)	1,161 (1,026-1,276)	1,401 (1,285-1,521)	1,572 (1,421-1,699)	1,751 (1,536-1,879)	2,182 (1,989-2,317)	3,029 (2,712-3,216)	3,681 (3,369-3,890)	5,032 (4,593-5,340)	6,315 (5,820-6,625)	8,220 (7,745-8,647)	8,944 (8,514-9,340)	10,489 (10,019-10,990)	9,804 (9,351-10,301)	9,178 (8,706-9,649)	6,897 (6,476-7,284)	2,825 (2,648-2,998)	966 (894-1,025)
Multiple myeloma	-	-	-	-	-	-	116 (98-148)	162 (130-187)	156 (127-181)	184 (148-209)	361 (292-406)	763 (632-850)	1,366 (1,151-1,592)	2,430 (2,023-2,854)	3,368 (2,882-3,957)	4,597 (3,857-5,451)	5,312 (4,433-6,244)	5,694 (4,661-6,887)	5,741 (4,531-6,940)	5,117 (3,977-6,065)	3,672 (2,838-4,436)	1,411 (1,113-1,756)	427 (330-525)
Leukaemia	83 (61-94)	64 (50-73)	1,009 (817-1,170)	3,764 (3,132-4,600)	3,969 (3,255-4,767)	4,446 (3,712-5,272)	4,339 (3,547-4,942)	4,077 (3,220-4,463)	3,595 (2,890-4,010)	3,639 (2,905-4,035)	4,434 (3,480-4,955)	5,323 (4,368-5,777)	6,190 (5,181-6,702)	7,209 (5,905-7,791)	8,780 (7,258-9,447)	9,552 (8,158-10,225)	9,790 (8,514-10,450)	11,562 (10,243-12,276)	11,394 (10,295-12,052)	10,172 (9,449-10,720)	7,741 (7,331-8,141)	3,513 (3,333-3,696)	1,436 (1,361-1,513)
Acute lymphoid leukaemia	24 (19-31)	18 (15-26)	324 (266-444)	1,529 (1,299-2,110)	1,766 (1,501-2,298)	1,761 (1,504-2,290)	1,322 (1,091-1,686)	1,026 (798-1,255)	892 (723-1,123)	672 (537-824)	807 (632-992)	878 (676-1,054)	978 (754-1,177)	1,025 (775-1,197)	1,335 (1,041-1,558)	1,250 (1,009-1,462)	1,060 (861-1,316)	1,089 (901-1,288)	842 (711-976)	621 (548-744)	376 (341-427)	161 (147-181)	61 (55-68)
Chronic lymphoid leukaemia	-	-	-	-	-	-	91 (73-126)	110 (83-147)	110 (90-152)	131 (106-178)	217 (174-301)	289 (240-365)	289 (251-362)	458 (390-546)	667 (580-793)	1,099 (936-1,246)	1,241 (1,111-1,413)	1,581 (1,445-1,726)	1,970 (1,778-2,130)	2,018 (1,875-2,162)	1,874 (1,741-1,996)	927 (869-993)	431 (394-463)
Acute myeloid leukaemia	15 (11-18)	12 (10-15)	176 (144-229)	745 (596-965)	636 (496-777)	891 (718-1,119)	960 (786-1,171)	904 (728-1,070)	882 (696-1,049)	893 (739-1,041)	1,121 (897-1,305)	1,294 (1,071-1,492)	1,741 (1,486-1,967)	1,952 (1,629-2,161)	2,283 (1,941-2,502)	2,664 (2,341-2,880)	2,842 (2,529-3,033)	3,500 (3,219-3,707)	3,526 (3,294-3,789)	2,872 (2,687-3,080)	1,915 (1,796-2,042)	739 (694-793)	253 (234-271)
Chronic myeloid leukaemia	-	-	-	-	-	-	186 (133-242)	208 (142-277)	333 (238-439)	349 (258-458)	370 (268-482)	532 (395-681)	601 (456-776)	750 (547-965)	784 (619-972)	795 (645-942)	802 (643-923)	976 (819-1,097)	839 (742-923)	869 (794-958)	722 (654-784)	339 (311-368)	146 (131-160)
Other leukaemia	44 (28-53)	33 (23-40)	508 (348-623)	1,490 (1,072-1,957)	1,567 (1,152-1,868)	1,794 (1,301-2,159)	1,781 (1,310-2,017)	1,829 (1,290-2,053)	1,378 (956-1,548)	1,594 (1,156-1,779)	1,919 (1,383-2,148)	2,330 (1,705-2,576)	2,582 (1,952-2,847)	3,025 (2,286-3,300)	3,711 (2,791-4,044)	3,744 (2,977-4,066)	3,844 (3,073-4,112)	4,415 (3,628-4,731)	4,218 (3,531-4,495)	3,791 (3,312-4,067)	2,855 (2,630-3,054)	1,347 (1,254-1,448)	545 (506-594)
Other neoplasms	221 (194-229)	124 (110-138)	822 (732-1,001)	2,706 (2,372-3,455)	2,157 (1,932-2,389)	3,069 (2,701-3,377)	3,776 (3,364-4,112)	3,813 (3,430-4,072)	3,977 (3,577-4,247)	4,393 (3,901-4,717)	5,277 (4,782-5,587)	7,123 (6,460-7,481)	8,954 (8,267-9,334)	11,462 (10,669-11,920)	13,824 (12,939-14,331)	15,152 (13,970-15,784)	15,166 (14,326-15,772)	17,168 (16,281-17,892)	16,871 (15,991-17,537)	15,134 (14,320-15,797)	11,754 (11,173-12,312)	5,530 (5,262-5,788)	2,274 (2,145-2,389)
Cardiovascular diseases	1,445 (1,337-1,563)	893 (804-1,005)	8,611 (7,845-9,462)	7,245 (6,502-8,006)	4,354 (3,954-4,797)	6,132 (5,614-6,624)	15,275 (14,334-16,327)	23,110 (21,840-24,398)	30,150 (28,813-31,391)	42,970 (41,132-44,842)	65,488 (62,686-67,920)	107,901 (104,121-111,343)	168,598 (163,070-173,165)	251,159 (242,514-258,223)	368,513 (356,018-378,582)	494,870 (476,761-509,388)	639,229 (615,451-658,764)	954,207 (920,347-983,718)	1,148,628 (1,108,558-1,181,558)	1,354,892 (1,310,952-1,394,677)	1,166,238 (1,127,970-1,203,363)	629,756 (607,902-651,545)	311,600 (298,981-322,351)
Rheumatic heart disease	-	-	-	1,585 (1,284-1,976)	1,048 (868-1,247)	2,171 (1,870-2,495)	3,685 (3,070-4,244)	4,129 (3,478-4,742)	4,498 (3,727-5,340)	5,127 (4,247-6,143)	6,621 (5,574-7,889)	8,746 (7,423-10,112)	9,467 (8,090-10,853)	11,708 (9,992-13,313)	14,017 (12,281-15,675)	15,717 (13,760-17,479)	17,428 (15,147-19,359)	20,680 (18,264-22,906)	19,738 (17,718-21,649)	18,267 (16,698-19,828)	14,336 (13,315-15,412)	7,555 (7,132-8,030)	3,384 (3,228-3,554)
Ischaemic heart disease	-	-	518 (426-639)	357 (285-465)	319 (277-373)	426 (371-494)	4,929 (4,525-5,356)	8,062 (7,526-8,639)	11,105 (10,417-11,878)	17,750 (16,679-18,889)	26,810 (25,302-28,349)	45,614 (43,478-47,823)	75,481 (72,140-78,648)	114,267 (109,307-119,356)	173,948 (166,296-180,034)	241,558 (231,849-249,679)	314,166 (300,855-325,593)	482,960 (463,672-500,575)	591,378 (568,279-610,564)	711,075 (688,313-736,288)	629,060 (611,565-650,345)	353,068 (342,777-365,554)	177,802 (172,584-185,078)
Cerebrovascular disease	545 (462-640)	326 (272-397)	2,832 (2,303-3,441)	2,086 (1,653-2,687)	1,358 (1,215-1,544)	1,651 (1,488-1,845)	3,000 (2,784-3,227)	5,892 (5,501-6,306)	7,833 (7,357-8,314)	11,929 (11,265-12,678)	20,952 (19,898-22,248)	37,519 (35,902-39,342)	60,725 (58,180-63,128)	92,343 (88,722-96,030)	134,551 (129,169-139,941)	181,491 (173,420-188,604)	235,378 (223,676-245,536)	343,196 (325,728-360,228)	396,746 (371,816-420,841)	441,566 (407,573-470,547)	338,841 (309,072-370,122)	161,216 (145,832-177,493)	76,222 (67,122-84,262)

SR Table 3: Global deaths by cause and age group for females (with 95% uncertainty intervals), 2010

Cause	Early Neonatal	Late Neonatal	Post Neonatal	1 to 4	5 to 9	10 to 14	15 to 19	20 to 24	25 to 29	30 to 34	35 to 39
Ischaemic stroke	-	-	181 (140-227)	103 (87-126)	113 (98-131)	119 (103-139)	536 (486-593)	1,180 (1,058-1,295)	1,519 (1,397-1,651)	1,946 (1,778-2,120)	3,159 (2,916-3,413)
Hemorrhagic stroke	545 (462-640)	326 (272-397)	2,650 (2,160-3,221)	1,983 (1,569-2,568)	1,246 (1,112-1,418)	1,531 (1,379-1,706)	2,463 (2,284-2,644)	4,712 (4,395-5,060)	6,314 (5,922-6,753)	9,984 (9,383-10,665)	17,793 (16,875-18,929)
Hypertensive heart disease	-	-	133 (88-177)	82 (54-109)	66 (48-79)	90 (63-114)	716 (539-896)	989 (744-1,227)	1,324 (996-1,595)	2,084 (1,556-2,463)	3,358 (2,466-3,941)
Cardiomyopathy and myocarditis	309 (207-366)	197 (142-236)	1,938 (1,357-2,341)	1,258 (917-1,546)	614 (492-699)	725 (591-816)	1,102 (788-1,268)	1,476 (1,054-1,672)	2,016 (1,463-2,321)	2,346 (1,845-2,657)	3,023 (2,306-3,453)
Myocarditis	62 (38-87)	46 (32-62)	643 (453-876)	492 (373-726)	269 (203-318)	276 (197-324)	518 (312-651)	622 (389-769)	619 (400-795)	535 (351-680)	712 (438-885)
Alcoholic cardiomyopathy	-	-	-	-	-	-	65 (53-80)	159 (120-198)	397 (297-499)	736 (535-917)	1,027 (747-1,278)
Other cardiomyopathy	247 (164-299)	151 (106-187)	1,295 (839-1,682)	766 (495-1,061)	346 (272-435)	449 (365-541)	519 (393-604)	695 (495-815)	1,000 (697-1,191)	1,075 (812-1,247)	1,285 (1,008-1,472)
Atrial fibrillation and flutter	-	-	-	-	-	-	-	-	-	16 (11-21)	65 (48-86)
Aortic aneurysm	-	-	-	-	-	-	120 (107-138)	163 (147-185)	224 (203-254)	311 (280-356)	474 (430-542)
Peripheral vascular disease	-	-	-	-	-	-	-	-	-	-	-
Endocarditis	52 (35-70)	37 (27-51)	778 (586-1,170)	413 (300-696)	219 (179-280)	271 (222-323)	488 (423-578)	662 (561-791)	924 (805-1,068)	834 (727-962)	1,009 (875-1,191)
Other cardiovascular and circulatory diseases	539 (442-642)	334 (273-426)	2,412 (1,980-2,979)	1,464 (1,123-2,002)	729 (604-976)	799 (663-1,093)	1,234 (1,052-1,739)	1,737 (1,507-2,468)	2,224 (1,921-3,076)	2,573 (2,244-3,513)	3,175 (2,744-4,358)
Chronic respiratory diseases	0 (0-0)	0 (0-0)	0 (0-0)	6,700 (5,525-9,876)	1,892 (1,603-2,634)	2,137 (1,842-2,917)	3,530 (3,128-4,763)	4,740 (4,287-6,027)	5,837 (5,330-7,311)	7,301 (6,669-8,851)	10,628 (9,713-13,023)
Chronic obstructive pulmonary disease	-	-	0 (0-0)	1,342 (945-2,149)	327 (255-493)	326 (260-447)	1,296 (1,067-1,703)	1,972 (1,710-2,441)	2,603 (2,228-3,285)	3,542 (3,029-4,289)	5,016 (4,349-6,098)
Pneumoconiosis	-	-	-	-	-	-	14 (11-18)	20 (17-25)	21 (17-26)	29 (25-37)	33 (27-41)
Silicosis	-	-	-	-	-	-	1 (0-1)	1 (1-1)	2 (1-2)	5 (4-6)	4 (3-5)
Asbestosis	-	-	-	-	-	-	7 (5-9)	10 (8-13)	10 (8-12)	12 (10-15)	14 (11-18)
Coal workers pneumoconiosis	-	-	-	-	-	-	0 (0-1)	1 (0-1)	1 (1-1)	1 (1-2)	1 (1-2)
Other pneumoconiosis	-	-	-	-	-	-	6 (4-8)	9 (7-11)	8 (7-11)	11 (9-14)	14 (11-17)
Asthma	-	-	-	4,377 (3,117-6,764)	1,115 (909-1,684)	1,422 (1,167-2,087)	1,945 (1,631-2,784)	2,382 (1,983-3,341)	2,766 (2,283-3,678)	3,217 (2,640-4,260)	4,796 (3,893-6,417)
Interstitial lung disease and pulmonary sarcoidosis	-	-	-	175 (89-396)	86 (55-148)	64 (44-94)	118 (72-180)	172 (108-260)	239 (153-363)	327 (207-468)	515 (333-734)
Other chronic respiratory diseases	0 (0-0)	0 (0-0)	0 (0-0)	806 (496-1,324)	364 (250-481)	325 (210-431)	158 (96-218)	194 (122-263)	208 (125-277)	186 (109-253)	268 (154-365)
Cirrhosis and other chronic liver diseases	-	-	-	3,436 (2,906-3,971)	1,452 (1,246-1,666)	1,837 (1,624-2,070)	3,152 (2,886-3,432)	5,088 (4,695-5,542)	6,251 (5,848-6,672)	8,742 (8,238-9,291)	12,252 (11,645-12,904)
Cirrhosis and other chronic liver diseases due to hepatitis B	-	-	-	14 (10-19)	14 (10-19)	36 (26-48)	103 (69-146)	643 (448-860)	1,311 (954-1,734)	2,258 (1,853-2,698)	3,442 (2,814-4,097)
Cirrhosis and other chronic liver diseases due to hepatitis C	-	-	-	12 (8-18)	14 (9-19)	33 (22-46)	91 (59-133)	541 (377-743)	1,032 (720-1,396)	1,797 (1,441-2,192)	2,744 (2,141-3,416)
Cirrhosis and other chronic liver diseases due to alcohol use	-	-	-	-	-	-	24 (13-37)	232 (164-319)	578 (416-783)	1,539 (1,296-1,815)	2,993 (2,459-3,587)
Cirrhosis and other chronic liver diseases due to other causes	-	-	-	3,410 (2,889-3,940)	1,424 (1,220-1,637)	1,768 (1,563-1,987)	2,934 (2,690-3,187)	3,672 (3,230-4,135)	3,330 (2,798-3,823)	3,148 (2,616-3,715)	3,073 (2,367-3,840)
Digestive diseases	835 (573-1,064)	653 (495-780)	7,755 (5,911-9,924)	7,920 (5,662-12,563)	2,977 (2,513-3,688)	3,309 (2,884-3,870)	5,481 (4,657-6,168)	6,787 (6,003-7,515)	7,659 (6,852-8,485)	7,783 (7,024-8,776)	9,167 (8,266-10,312)
Peptic ulcer disease	-	-	-	1,028 (650-1,718)	434 (328-619)	619 (495-825)	822 (668-1,049)	1,394 (1,162-1,719)	1,503 (1,285-1,797)	1,681 (1,424-2,086)	2,079 (1,788-2,530)
Gastritis and duodenitis	-	-	-	173 (104-295)	81 (60-118)	77 (58-104)	110 (80-150)	194 (160-239)	250 (203-317)	297 (243-384)	346 (282-434)
Appendicitis	-	-	-	1,839 (890-3,462)	747 (590-1,018)	884 (715-1,131)	1,712 (1,364-2,157)	1,515 (1,112-1,884)	1,598 (1,230-1,969)	1,040 (808-1,331)	1,090 (868-1,361)
Paralytic ileus and intestinal obstruction	835 (573-1,064)	653 (495-780)	7,755 (5,911-9,924)	2,462 (1,427-3,683)	920 (625-1,156)	1,063 (741-1,291)	1,842 (1,192-2,209)	1,899 (1,303-2,227)	2,013 (1,346-2,360)	1,879 (1,281-2,222)	2,077 (1,415-2,468)
Inguinal, femoral, and abdominal hernia	-	-	-	141 (73-231)	83 (56-121)	71 (51-111)	92 (60-132)	105 (67-148)	98 (63-146)	115 (74-169)	151 (95-223)
Inflammatory bowel disease	-	-	-	596 (276-1,460)	117 (77-197)	93 (64-140)	164 (112-261)	304 (208-482)	370 (259-563)	398 (277-574)	421 (309-576)
Vascular intestinal disorders	-	-	-	69 (32-104)	17 (12-22)	19 (14-25)	37 (25-47)	105 (67-132)	137 (93-173)	178 (129-221)	262 (196-322)
Gallbladder and biliary diseases	-	-	-	268 (175-392)	146 (107-222)	141 (113-205)	165 (140-222)	317 (270-405)	444 (381-558)	597 (512-767)	807 (694-1,018)
Pancreatitis	-	-	-	75 (38-126)	78 (47-121)	117 (77-164)	294 (206-398)	617 (423-809)	781 (564-1,043)	1,086 (803-1,422)	1,324 (976-1,686)
Other digestive diseases	-	-	-	1,270 (610-2,358)	354 (261-478)	226 (183-295)	242 (197-301)	337 (279-413)	464 (386-555)	510 (425-615)	608 (508-727)
Neurological disorders	-	-	3,513 (3,020-4,039)	3,753 (2,917-4,677)	2,166 (1,881-2,531)	3,065 (2,684-3,540)	4,599 (4,151-5,244)	4,864 (4,270-5,353)	4,405 (3,905-4,836)	4,470 (4,029-5,047)	4,549 (4,141-5,031)
Alzheimer's disease and other dementias	-	-	-	-	-	-	-	-	-	-	-
Parkinson's disease	-	-	-	-	-	-	-	1 (0-1)	9 (4-18)	32 (17-59)	74 (38-132)
Epilepsy	-	-	2,293 (1,738-2,621)	2,678 (2,099-3,261)	1,722 (1,425-2,008)	2,396 (2,058-2,754)	3,965 (3,517-4,546)	4,292 (3,672-4,745)	3,730 (3,196-4,094)	3,571 (3,084-4,114)	3,171 (2,745-3,495)
Multiple sclerosis	-	-	-	-	-	-	-	92 (73-131)	194 (158-259)	304 (258-399)	543 (466-728)
Motor neuron disease	4 (3-5)	5 (4-6)	275 (215-336)	159 (103-208)	21 (18-24)	23 (21-25)	22 (20-23)	23 (21-25)	35 (32-40)	61 (58-69)	124 (117-140)
Other neurological disorders	-	-	945 (706-1,184)	915 (569-1,270)	424 (342-508)	646 (486-818)	612 (455-768)	457 (392-519)	437 (383-489)	502 (460-540)	637 (598-674)
Mental and substance use disorders	4 (3-6)	16 (12-19)	0 (0-0)	0 (0-0)	9 (6-11)	11 (8-13)	1,825 (1,662-2,091)	2,944 (2,764-3,350)	3,575 (3,366-3,875)	4,218 (3,939-4,498)	5,102 (4,811-5,420)
Alcohol use disorders	-	-	-	-	-	-	193 (180-207)	336 (311-364)	730 (658-812)	1,151 (1,027-1,281)	1,813 (1,635-2,001)
Drug use disorders	4 (3-6)	16 (12-19)	0 (0-0)	0 (0-0)	0 (0-0)	0 (0-0)	1,599 (1,442-1,856)	2,553 (2,380-2,960)	2,785 (2,595-3,083)	3,008 (2,792-3,233)	3,226 (2,989-3,449)
Opioid use disorders	4 (3-6)	16 (12-19)	0 (0-0)	0 (0-0)	0 (0-0)	0 (0-0)	1,021 (862-1,211)	1,611 (1,366-1,847)	1,725 (1,381-1,957)	1,700 (1,309-1,890)	1,785 (1,312-1,959)
Cocaine use disorders	0 (0-0)	0 (0-0)	0 (0-0)	0 (0-0)	0 (0-0)	0 (0-0)	55 (35-95)	97 (68-165)	106 (81-172)	187 (146-286)	209 (164-331)
Amphetamine use disorders	0 (0-0)	0 (0-0)	0 (0-0)	0 (0-0)	0 (0-0)	0 (0-0)	60 (42-99)	109 (85-183)	120 (83-194)	96 (74-152)	102 (73-158)
Other drug use disorders	0 (0-0)	0 (0-0)	0 (0-0)	0 (0-0)	0 (0-0)	0 (0-0)	463 (321-600)	736 (588-932)	834 (672-1,090)	1,025 (870-1,314)	1,130 (981-1,458)
Eating disorders	-	-	-	-	9 (6-11)	11 (8-13)	33 (26-41)	55 (44-65)	60 (48-71)	58 (50-66)	63 (54-72)
Anorexia nervosa	-	-	-	-	8 (6-10)	11 (8-13)	32 (24-39)	51 (41-61)	55 (44-66)	53 (45-61)	57 (49-66)
Bulimia nervosa	-	-	-	-	0 (0-1)	0 (0-1)	2 (1-2)	4 (3-5)	4 (4-6)	5 (4-6)	5 (4-7)
Diabetes, urogenital, blood, and endocrine diseases	1,785 (1,325-2,272)	1,054 (844-1,274)	10,348 (8,806-12,482)	14,603 (10,163-18,831)	5,490 (4,630-6,636)	8,137 (6,884-9,298)	12,591 (11,095-13,832)	14,667 (13,451-16,155)	15,694 (14,644-16,869)	17,352 (16,388-18,561)	21,365 (20,352-22,495)
Diabetes mellitus	14 (13-15)	24 (20-28)	292 (260-330)	447 (386-516)	409 (363-460)	891 (800-987)	1,656 (1,538-1,787)	2,508 (2,354-2,665)	3,234 (3,045-3,449)	3,917 (3,712-4,149)	5,932 (5,628-6,243)
Acute glomerulonephritis	-	-	55 (40-81)	78 (65-96)	82 (70-101)	98 (81-128)	88 (79-99)	138 (122-155)	121 (107-138)	129 (115-145)	176 (158-199)
Chronic kidney disease	-	-	1,474 (1,328-1,645)	2,544 (2,239-2,910)	1,415 (1,253-1,595)	2,008 (1,818-2,211)	3,346 (3,138-3,607)	5,186 (4,873-5,537)	6,132 (5,785-6,550)	7,115 (6,682-7,603)	9,104 (8,609-9,601)
Chronic kidney disease due to diabetes mellitus	-	-	35 (23-50)	64 (41-97)	44 (25-70)	83 (51-130)	222 (133-350)	634 (397-916)	1,144 (812-1,542)	1,943 (1,440-2,514)	3,272 (2,513-4,069)
Chronic kidney disease due to hypertension	-	-	10 (7-14)	25 (17-36)	22 (14-33)	54 (36-82)	145 (97-208)	325 (226-445)	508 (358-690)	706 (519-943)	1,069 (808-1,373)
Chronic kidney disease due to glomerulonephritis	-	-	642 (494-795)	1,106 (825-1,424)	603 (436-788)	889 (641-1,148)	1,530 (1,109-1,953)	2,293 (1,710-2,937)	2,431 (1,751-3,165)	2,298 (1,624-3,028)	2,322 (1,571-3,079)
Chronic kidney disease due to other causes	-	-	788 (638-957)	1,348 (1,064-1,665)	746 (579-942)	982 (732-1,257)	1,449 (1,040-1,876)	1,934 (1,344-2,540)	2,050 (1,415-2,748)	2,168 (1,512-2,883)	2,441 (1,732-3,177)
Urinary diseases and male infertility	108 (97-123)	117 (99-141)	1,570 (1,381-1,765)	1,384 (1,177-1,639)	492 (419-572)	485 (426-548)	1,260 (1,141-1,399)	1,677 (1,535-1,831)	1,516 (1,412-1,614)	1,712 (1,602-1,821)	1,994 (1,877-2,111)
Interstitial nephritis and urinary tract infections	68 (53-80)	72 (56-92)	1,112 (935-1,314)	980 (774-1,231)	305 (249-361)	275 (227-323)	925 (810-1,059)	1,228 (1,087-1,386)	1,031 (912-1,169)	1,196 (1,075-1,348)	1,471 (1,319-1,620)
Urolithiasis	-	-	-	-	59 (45-77)	72 (54-98)	106 (76-144)	138 (94-192)	117 (79-156)	128 (87-170)	166 (125-220)
Other urinary diseases	41 (30-53)	45 (32-65)	457 (321-605)	403 (259-615)	128 (92-183)	138 (102-203)	229 (148-343)	311 (207-460)	368 (254-493)	387 (258-530)	358 (245-510)
Gynecological diseases	-	-	-	-	-	-	183 (160-203)	273 (236-303)	337 (299-374)	437 (391-486)	606 (537-676)
Uterine fibroids	-	-	-	-	-	-	11 (7-20)	21 (15-33)	34 (21-55)	66 (36-102)	163 (92-225)
Polycystic ovarian syndrome	-	-	-	-	-	-	2 (0-5)	18 (5-55)	53 (17-114)	41 (11-109)	54 (18-113)
Endometriosis	-	-	-	-	-	-	3 (1-7)	3 (1-6)	4 (1-7)	6 (2-12)	5 (2-7)
Genital prolapse	-	-	-	-	-	-	7 (3-20)	6 (2-13)	11 (5-21)	15 (3-25)	5 (2-9)
Other gynecological diseases	-	-	-	-	-	-	161 (127-182)	225 (166-261)	234 (153-283)	309 (210-371)	379 (261-479)
Hemoglobinopathies and hemolytic anaemias	1,009 (608-1,500)	417 (273-601)	4,060 (2,745-5,586)	8,029 (3,837-11,521)	2,202 (1,463-3,118)	3,860 (2,671-4,842)	5,153 (3,628-6,163)	3,636 (2,555-4,563)	2,695 (1,923-3,344)	2,369 (1,739-3,032)	1,759 (1,367-2,193)
Thalassemias	198 (138-269)	104 (72-147)	847 (571-1,195)	1,227 (866-1,720)	601 (451-831)	382 (296-492)	304 (233-392)	143 (110-192)	90 (71-118)	60 (49-82)	43 (37-59)
Sickle cell disorders	757 (439-1,204)	300 (189-454)	3,120 (2,080-4,280)	6,626 (2,835-9,877)	1,506 (913-2,159)	3,257 (2,163-4,197)	4,324 (2,975-5,220)	2,914 (1,965-3,659)	1,951 (1,332-2,467)	1,580 (1,111-2,033)	911 (675-1,157)
G6PD deficiency	17 (11-25)	7 (5-10)	66 (46-97)	136 (93-197)	71 (50-99)	151 (108-190)	325 (242-421)	342 (258-455)	453 (332-568)	459 (339-583)	409 (314-502)
Other hemoglobinopathies and hemolytic anaemias	37 (26-49)	5 (3-7)	27 (19-38)	39 (28-54)	24 (18-32)	70 (51-84)	200 (151-259)	237 (183-330)	201 (153-250)	271 (213-351)	397 (316-490)
Endocrine, metabolic, blood, and immune disorders	654 (491-778)	497 (351-659)	2,897 (2,429-3,560)	2,123 (1,738-2,804)	889 (767-1,086)	795 (681-964)	906 (808-1,100)	1,249 (1,081-1,446)	1,659 (1,298-1,911)	1,674 (1,320-1,927)	1,794 (1,462-2,096)
Musculoskeletal disorders	-	-	-	-	350 (260-454)	732 (581-903)	1,334 (1,047-1,676)	1,751 (1,427-2,138)	1,811 (1,445-2,170)	1,776 (1,443-2,118)	1,996 (1,595-2,415)
Rheumatoid arthritis	-	-	-	-	54 (38-92)	59 (43-100)	57 (42-92)	70 (53-115)	86 (67-132)	114 (91-174)	188 (143-272)
Other musculoskeletal disorders	-	-	-	-	297 (220-385)	673 (520-815)	1,278 (1,002-1,595)	1,682 (1,374-2,051)	1,725 (1,364-2,062)	1,662 (1,367-1,974)	1,808 (1,428-2,176)
Other non-communicable diseases	78,703 (61,922-95,397)	31,815 (26,701-36,866)	101,981 (82,433-124,522)	36,823 (24,863-52,325)	8,337 (7,118-9,823)	6,771 (5,987-7,565)	4,840 (4,265-5,423)	4,352 (3,860-4,828)	3,684 (3,231-4,127)	2,683 (2,349-3,144)	2,844 (2,440-3,334)
Congenital anomalies	78,703 (61,922-95,397)	30,354 (25,252-35,458)	86,526 (68,993-106,977)	35,519 (23,470-51,184)	8,048 (6,849-9,541)	6,427 (5,668-7,270)	4,283 (3,714-4,830)	3,897 (3,462-4,335)	3,237 (2,877-3,663)	2,164 (1,944-2,458)	2,182 (1,933-2,524)
Neural tube defects	9,368 (5,816-12,877)	2,254 (1,451-3,126)	5,363 (3,023-8,374)	1,990 (664-4,650)	282 (177-412)	191 (132-256)	108 (81-139)	90 (69-123)	69 (53-97)	55 (44-71)	44 (35-56)
Congenital heart anomalies	22,741 (15,182-30,990)	12,678 (9,538-16,103)	41,726 (31,329-57,662)	16,893 (11,548-28,372)	4,852 (3,904-6,190)	4,285 (3,560-5,074)	2,780 (2,339-3,210)	2,766 (2,364-3,136)	2,330 (2,022-2,630)	1,427 (1,255-1,627)	1,528 (1,336-1,757)
Orofacial clefts	620 (314-1,060)	289 (153-487)	366 (161-641)	69 (19-151)	-	-	-	-	-	-	-
Down's syndrome	838 (562-1,427)	404 (292-670)	1,759 (1,323-3,067)	800 (543-1,632)	156 (113-217)	141 (109-190)	146 (113-197)	156 (124-207)	175 (144-232)	176 (145-237)	180 (144-227)
Other chromosomal abnormalities	3,170 (2,061-6,648)	1,627 (1,075-3,041)	3,605 (2,345-7,350)	621 (368-1,626)	153 (110-235)	105 (75-143)	90 (68-127)	63 (42-79)	63 (37-78)	42 (24-51)	32 (18-37)
Congenital musculoskeletal and limb anomalies	2,233 (1,249-4,859)	577 (338-1,224)	1,021 (579-2,360)	325 (160-1,044)	101 (70-184)	99 (71-159)	78 (56-122)	54 (35-76)	44 (29-60)	33 (22-45)	27 (19-33)
Urogenital congenital anomalies	2,134 (1,156-3,457)	751 (431-1,203)	1,140 (605-1,858)	279 (143-502)	135 (88-205)	146 (96-209)	71 (48-101)	66 (44-84)	56 (36-75)	50 (34-64)	57 (35-73)
Digestive congenital anomalies	5,919 (3,786-10,892)	2,559 (1,809-4,599)	5,493 (3,891-11,042)	1,172 (710-3,900)	126 (91-196)	80 (60-113)	56 (44-81)	42 (33-56)	24 (19-30)	20 (16-25)	19 (15-22)
Other congenital anomalies	31,678 (18,485-48,252)	9,215 (4,886-14,502)	26,054 (11,940-46,203)	13,370 (4,409-28,294)	2,242 (1,272-3,632)	1,381 (873-1,976)	954 (629-1,327)	660 (410-952)	476 (298-676)	360 (237-492)	295 (201-390)
Skin and subcutaneous diseases	-	-	2,840 (1,192-4,136)	1,305 (562-1,911)	289 (122-435)	344 (150-494)	557 (233-794)	455 (208-645)	447 (229-679)	519 (272-810)	663 (337-995)
Cellulitis	-	-	169 (47-298)	128 (38-236)	42 (15-71)	46 (17-75)	64 (23-92)	81 (33-114)	57 (26-84)	92 (43-137)	103 (46-155)
Pyoderma	-	-	2,614 (1,100-3,832)	1,044 (447-1,585)	191 (78-296)	227 (91-328)	387 (153-574)	266 (116-404)	296 (143-463)	308 (153-510)	445 (219-673)
Decubitus ulcer	-	-	-	65 (24-128)	25 (12-39)	33 (17-52)	52 (25-83)	55 (28-86)	53 (30-89)	74 (37-120)	73 (38-116)
Other skin and subcutaneous diseases	-	-	57 (24-110)	68 (32-121)	31 (14-57)	38 (19-63)	55 (27-87)	53 (26-81)	40 (22-66)	45 (25-74)	41 (24-69)
Sudden infant death syndrome	-	1,461 (1,097-2,018)	12,615 (9,291-18,027)	-	-	-	-	-	-	-	-
Injuries	4,910 (3,909-5,504)	3,483 (2,787-3,925)	40,463 (34,504-46,659)	91,271 (77,035-105,588)	51,565 (42,585-60,912)	49,652 (40,803-59,040)	85,366 (77,158-93,411)	98,560 (90,799-106,152)	86,980 (79,941-93,775)	73,284 (67,008-78,953)	71,920 (66,351-76,654)
Transport injuries	422 (347-503)	404 (348-470)	5,524 (4,899-6,172)	17,647 (15,000-20,182)	14,660 (13,263-16,232)	12,067 (11,011-13,109)	22,029 (20,851-23,319)	26,486 (25,261-27,763)	22,670 (21,443-23,781)	20,121 (19,115-21,067)	21,449 (20,477-22,417)
Road injuries	380 (313-455)	379 (328-445)	5,095 (4,489-5,701)	16,679 (14,083-18,961)	14,125 (12,777-15,662)	11,391 (10,429-12,327)	20,549 (19,478-21,673)	25,314 (24,112-26,498)	21,605 (20,442-22,709)	19,181 (18,215-20,077)	20,549 (19,623-21,462)
Pedestrian road injuries	157 (125-191)	147 (122-180)	2,029 (1,708-2,414)	8,929 (7,580-10,719)	7,820 (6,935-8,887)	5,550 (4,987-6,186)	6,868 (6,331-7,487)	8,555 (7,937-9,281)	6,914 (6,391-7,456)	6,936 (6,440-7,468)	8,554 (7,976-9,158)
Cyclist road injuries	-	-	-	490 (321-721)	534 (431-724)	732 (590-1,028)	990 (834-1,348)	1,008 (879-1,225)	833 (719-1,017)	956 (808-1,250)	992 (782-1,099)
Motorcyclist road injuries	65 (45-87)	38 (28-51)	803 (627-997)	1,473 (990-1,995)	897 (746-1,062)	1,060 (919-1,210)	3,268 (2,902-3,605)	4,179 (3,701-4,571)	3,545 (3,168-3,897)	3,406 (3,034-3,741)	3,020 (2,681-3,276)
Motor vehicle road injuries	148 (109-186)	190 (159-229)	2,176 (1,789-2,547)	5,634 (4,313-7,019)	4,723 (4,105-5,398)	3,935 (3,514-4,428)	9,188 (8,504-10,031)	11,324 (10,532-12,440)	10,144 (9,438-11,152)	7,695 (7,064-8,387)	7,774 (7,155-8,455)
Other road injuries	9 (6-15)	5 (3-8)	87 (60-154)	153 (103-262)	151 (118-213)	114 (91-158)	235 (182-392)	247 (197-388)	168 (137-257)	189 (153-297)	208 (171-305)
Other transport injuries	42 (27-58)	24 (19-31)	429 (338-577)	968 (746-1,397)	535 (456-664)	676 (584-802)	1,481 (1,285-1,737)	1,171 (1,040-1,364)	1,065 (937-1,227)	940 (826-1,079)	900 (806-1,043)
Unintentional injuries	3,470 (2,525-4,039)	2,498 (1,847-2,864)	30,997 (25,480-36,756)	57,922 (47,843-67,835)	23,518 (19,830-26,271)	19,253 (15,811-21,164)	19,688 (15,726-21,714)	21,207 (17,209-23,215)	19,765 (15,931-21,653)	18,662 (14,960-20,414)	18,609 (15,261-20,152)
Falls	874 (496-1,235)	292 (176-399)	4,351 (2,862-5,886)	5,632 (3,699-8,006)	2,507 (2,055-2,868)	1,954 (1,606-2,166)	2,465 (2,060-2,698)	2,685 (2,178-2,902)	2,516 (1,967-2,709)	2,626 (2,100-2,837)	3,346 (2,697-3,605)
Drowning	152 (117-176)	96 (73-112)	3,490 (2,470-4,146)	26,427 (18,385-31,167)	10,047 (7,511-11,463)	7,791 (5,965-8,680)	5,320 (3,918-5,852)	4,135 (3,098-4,510)	3,547 (2,641-3,916)	3,080 (2,227-3,367)	3,135 (2,227-3,421)
Fire, heat, and hot substances	125 (84-148)	162 (102-198)	2,763 (1,820-3,392)	6,986 (4,492-8,806)	2,187 (1,624-2,595)	1,778 (1,262-2,052)	3,878 (2,456-4,644)	5,115 (3,110-6,088)	4,398 (2,695-5,230)	3,880 (2,458-4,598)	3,066 (2,092-3,517)
Poisonings	106 (59-153)	97 (63-128)	1,863 (933-3,393)	3,150 (1,988-4,587)	969 (724-1,131)	1,199 (902-1,349)	1,191 (897-1,330)	1,529 (1,115-1,691)	1,222 (885-1,338)	1,016 (727-1,113)	1,146 (767-1,257)
Exposure to mechanical forces	695 (349-868)	696 (326-903)	4,739 (3,023-5,688)	3,632 (2,738-4,744)	1,954 (1,583-2,221)	1,361 (1,123-1,534)	1,604 (1,213-1,801)	1,560 (1,159-1,748)	1,832 (1,297-2,084)	1,576 (1,197-1,742)	1,701 (1,200-1,925)
Unintentional firearm injuries	19 (16-21)	13 (11-15)	134 (113-161)	206 (160-263)	186 (163-218)	165 (146-192)	253 (224-292)	282 (243-325)	333 (290-373)	364 (312-415)	244 (212-280)
Unintentional suffocation	525 (211-690)	631 (262-837)	3,311 (1,659-4,150)	1,213 (699-2,058)	478 (347-678)	399 (313-565)	138 (97-167)	154 (100-177)	146 (94-166)	96 (56-112)	103 (61-118)
Other exposure to mechanical forces	151 (100-180)	52 (40-61)	1,294 (914-1,579)	2,213 (1,480-2,840)	1,290 (961-1,498)	798 (591-913)	1,213 (824-1,402)	1,124 (741-1,299)	1,353 (852-1,593)	1,116 (773-1,261)	1,354 (875-1,565)
Adverse effects of medical treatment	555 (401-666)	407 (299-491)	3,791 (2,409-4,935)	2,907 (1,464-4,421)	1,016 (746-1,312)	826 (645-1,005)	1,037 (814-1,216)	2,064 (1,595-2,432)	2,140 (1,632-2,494)	1,790 (1,368-2,118)	2,037 (1,553-2,335)
Animal contact	97 (35-126)	96 (36-125)	1,586 (606-2,128)	3,491 (1,416-4,876)	2,556 (1,148-3,313)	2,399 (932-3,143)	2,074 (832-2,733)	1,895 (730-2,427)	1,783 (708-2,247)	2,128 (772-2,790)	1,612 (585-2,106)
Venomous animal contact	67 (18-97)	68 (19-96)	1,291 (409-1,824)	2,848 (985-3,974)	2,221 (864-2,945)	2,184 (792-2,926)	1,883 (668-2,539)	1,680 (586-2,232)	1,596 (561-2,067)	1,975 (641-2,638)	1,463 (486-1,957)
Non-venomous animal contact	30 (14-53)	28 (13-52)	295 (132-646)	643 (264-1,622)	334 (182-654)	215 (113-405)	191 (110-369)	215 (123-413)	188 (113-347)	154 (89-283)	149 (83-266)
Foreign body	620 (414-730)	531 (375-630)	7,086 (5,060-9,592)	3,481 (2,352-5,516)	1,114 (845-1,450)	750 (591-902)	641 (502-781)	805 (649-999)	807 (658-987)	780 (654-956)	817 (709-999)
Pulmonary aspiration and foreign body in airway	594 (388-697)	516 (362-610)	6,830 (4,816-9,270)	3,109 (2,015-4,981)	928 (684-1,196)	619 (473-749)	372 (290-437)	443 (357-535)	483 (383-580)	544 (444-651)	566 (477-694)
Foreign body in other body part	26 (16-42)	15 (9-23)	256 (164-480)	372 (210-705)	186 (129-304)	131 (88-191)	269 (179-383)	363 (236-530)	324 (220-459)	235 (162-352)	252 (183-374)
Environmental heat and cold exposure	95 (32-134)	64 (19-93)	681 (226-989)	530 (211-779)	258 (120-338)	134 (77-166)	282 (127-366)	258 (120-324)	276 (135-343)	442 (202-556)	536 (265-679)
Other unintentional injuries	151 (129-178)	55 (47-65)	645 (521-775)	1,687 (1,220-2,156)	909 (692-1,041)	1,060 (788-1,207)	1,197 (760-1,366)	1,161 (798-1,289)	1,244 (874-1,391)	1,343 (946-1,495)	1,213 (826-1,350)
Self-harm and interpersonal violence	721 (537-931)	205 (156-270)	1,496 (1,196-2,013)	3,010 (2,287-3,991)	2,179 (1,675-2,764)	6,748 (6,002-7,576)	33,959 (31,297-36,528)	42,168 (39,176-45,049)	36,586 (34,321-38,808)	27,989 (26,316-29,810)	26,505 (24,860-28,175)

Cause	40 to 44	45 to 49	50 to 54	55 to 59	60 to 64	65 to 69	70 to 74	75 to 79	80 to 84	85 to 89	90 to 94	95 plus
Ischaemic stroke	5,751 (5,356-6,180)	11,261 (10,565-12,057)	19,512 (18,289-20,785)	33,616 (31,760-35,687)	72,449 (68,357-76,462)	102,781 (96,791-108,648)	166,765 (157,031-176,441)	228,444 (213,119-242,566)	269,390 (248,984-287,504)	216,332 (197,651-235,993)	106,439 (96,329-116,976)	53,204 (46,941-58,930)
Hemorrhagic stroke	31,768 (30,345-33,423)	49,464 (47,412-51,625)	72,831 (69,727-75,856)	100,935 (96,612-105,546)	109,042 (103,705-113,759)	132,597 (125,018-139,891)	176,430 (166,420-186,166)	168,302 (156,553-179,570)	172,176 (157,928-183,997)	122,509 (111,380-134,121)	54,777 (49,311-60,671)	23,018 (20,186-25,454)
Hypertensive heart disease	5,554 (4,170-6,513)	9,630 (7,141-11,431)	15,044 (10,965-17,956)	22,303 (17,076-26,454)	26,864 (20,412-32,061)	36,206 (27,352-43,041)	52,499 (39,805-63,061)	64,807 (48,038-78,932)	73,498 (52,679-87,280)	65,593 (43,884-77,053)	38,817 (26,319-43,698)	19,621 (11,430-22,650)
Cardiomyopathy and myocarditis	3,872 (3,066-4,381)	4,567 (3,742-5,231)	5,892 (4,990-6,870)	7,468 (6,415-8,776)	6,981 (6,102-8,590)	6,998 (5,998-8,266)	10,296 (8,363-11,790)	14,051 (10,512-15,539)	19,921 (13,095-22,594)	20,840 (14,421-23,344)	12,277 (8,431-13,757)	7,241 (4,742-8,300)
Myocarditis	835 (558-1,041)	589 (400-706)	667 (461-809)	849 (603-997)	679 (455-805)	786 (533-915)	1,180 (803-1,378)	1,759 (1,138-2,038)	2,769 (1,701-3,254)	3,656 (2,378-4,300)	2,723 (1,664-3,258)	2,147 (1,073-2,729)
Alcoholic cardiomyopathy	1,385 (1,052-1,716)	1,983 (1,509-2,440)	2,534 (1,971-3,148)	2,863 (2,252-3,639)	2,180 (1,764-2,944)	1,444 (1,192-1,955)	1,896 (1,557-2,508)	1,817 (1,375-2,281)	2,064 (1,199-2,710)	1,416 (783-1,894)	635 (340-849)	376 (177-514)
Other cardiomyopathy	1,652 (1,326-1,900)	1,995 (1,694-2,325)	2,691 (2,337-3,256)	3,755 (3,339-4,469)	4,122 (3,677-5,033)	4,767 (4,087-5,550)	7,221 (5,746-8,229)	10,475 (7,739-11,717)	15,088 (9,919-17,231)	15,769 (10,659-17,980)	8,918 (6,225-10,367)	4,718 (3,327-5,894)
Atrial fibrillation and flutter	159 (117-209)	301 (219-396)	509 (381-663)	933 (562-1,380)	1,804 (1,198-2,550)	3,442 (2,141-5,113)	7,678 (5,466-10,636)	12,717 (8,210-18,665)	28,297 (21,508-35,991)	36,199 (27,758-46,293)	19,876 (15,426-25,556)	8,008 (6,245-10,206)
Aortic aneurysm	685 (636-761)	1,098 (1,025-1,220)	1,640 (1,539-1,800)	2,425 (2,271-2,663)	2,763 (2,622-2,929)	3,823 (3,621-4,035)	5,865 (5,580-6,163)	7,893 (7,531-8,234)	9,837 (9,442-10,207)	9,183 (8,809-9,512)	5,120 (4,925-5,319)	2,370 (2,257-2,511)
Peripheral vascular disease	70 (45-106)	120 (69-194)	227 (127-383)	412 (233-692)	709 (388-1,178)	990 (543-1,627)	1,816 (931-3,027)	2,957 (1,615-5,051)	5,328 (2,922-9,613)	6,279 (3,311-12,086)	4,382 (2,391-8,304)	2,821 (1,455-5,529)
Endocarditis	1,344 (1,179-1,656)	1,287 (1,090-1,576)	1,515 (1,267-1,836)	1,848 (1,518-2,310)	2,492 (2,014-3,105)	3,050 (2,463-3,776)	4,048 (3,249-5,126)	5,076 (3,879-6,504)	6,132 (4,388-7,883)	5,899 (3,932-7,361)	3,719 (2,344-4,647)	1,954 (1,170-2,431)
Other cardiovascular and circulatory diseases	4,338 (3,772-5,787)	5,922 (5,179-7,973)	8,016 (7,114-10,674)	10,609 (9,199-14,472)	14,492 (12,848-19,807)	17,749 (15,365-24,232)	25,169 (22,199-33,391)	33,266 (29,930-42,745)	40,971 (37,602-50,972)	40,008 (37,262-47,950)	23,726 (22,350-27,789)	12,178 (11,500-13,716)
Chronic respiratory diseases	15,352 (14,207-18,389)	26,167 (24,034-31,107)	42,891 (39,010-48,823)	70,254 (63,406-77,798)	107,665 (95,562-118,596)	149,459 (132,518-166,846)	210,205 (189,886-240,001)	233,025 (213,279-270,534)	239,965 (221,396-277,417)	184,486 (172,124-210,781)	90,455 (84,960-101,658)	36,348 (34,217-40,369)
Chronic obstructive pulmonary disease	7,978 (7,028-9,427)	15,051 (13,270-17,713)	28,367 (24,929-32,526)	50,317 (44,816-56,474)	83,114 (73,239-93,193)	120,243 (106,605-133,381)	176,523 (159,185-201,165)	198,519 (182,110-232,669)	209,988 (193,711-244,869)	164,191 (152,748-188,124)	81,793 (76,503-93,228)	33,241 (31,183-37,233)
Pneumoconiosis	51 (42-63)	60 (51-75)	84 (68-104)	133 (108-163)	168 (136-209)	205 (165-256)	314 (262-381)	359 (306-431)	378 (331-451)	270 (243-312)	121 (110-138)	58 (53-63)
Silicosis	10 (8-13)	15 (11-19)	17 (13-23)	34 (24-46)	44 (33-61)	53 (37-78)	95 (73-131)	104 (83-144)	96 (77-141)	65 (54-98)	30 (25-42)	13 (11-20)
Asbestosis	18 (15-25)	21 (17-28)	29 (23-40)	38 (29-51)	41 (31-55)	39 (29-52)	59 (39-76)	72 (47-89)	83 (47-100)	67 (35-81)	29 (15-34)	14 (7-17)
Coal workers pneumoconiosis	3 (2-4)	4 (2-5)	6 (4-9)	13 (8-18)	16 (10-25)	26 (17-39)	36 (25-56)	46 (35-69)	37 (27-53)	30 (23-44)	15 (11-21)	9 (6-12)
Other pneumoconiosis	20 (16-26)	21 (17-27)	32 (25-40)	49 (38-63)	66 (51-89)	87 (67-116)	124 (98-171)	136 (109-185)	162 (133-217)	107 (92-140)	47 (41-62)	22 (19-28)
Asthma	6,231 (5,040-8,293)	9,620 (7,578-12,558)	12,285 (9,449-15,641)	16,821 (12,739-21,392)	20,339 (15,246-26,370)	23,687 (17,572-31,073)	26,194 (19,365-34,940)	25,873 (19,726-33,807)	21,139 (16,554-28,195)	13,270 (10,702-17,545)	5,381 (4,459-6,889)	1,780 (1,558-2,162)
Interstitial lung disease and pulmonary sarcoidosis	730 (474-1,000)	1,160 (778-1,596)	1,759 (1,130-2,451)	2,517 (1,619-3,555)	3,533 (2,218-4,979)	4,703 (2,903-6,490)	6,416 (4,053-8,622)	7,329 (4,662-9,044)	7,455 (4,616-8,833)	5,932 (3,388-6,855)	2,725 (1,616-3,115)	1,080 (628-1,215)
Other chronic respiratory diseases	362 (214-499)	275 (155-366)	395 (234-526)	465 (267-644)	510 (287-694)	620 (361-830)	758 (432-1,047)	946 (529-1,267)	1,005 (540-1,382)	824 (436-1,122)	435 (223-581)	189 (93-248)
Cirrhosis and other chronic liver diseases	17,722 (16,991-18,514)	23,964 (22,991-24,943)	32,906 (31,582-34,278)	38,817 (37,189-40,474)	40,454 (38,749-42,217)	39,110 (37,499-40,874)	42,607 (40,458-44,732)	37,085 (35,414-38,966)	30,168 (28,701-31,746)	19,062 (18,092-20,110)	8,158 (7,737-8,633)	3,454 (3,260-3,628)
Cirrhosis and other chronic liver diseases due to hepatitis B	4,895 (4,129-5,718)	5,736 (4,733-6,848)	7,455 (6,061-8,893)	9,026 (7,564-10,713)	9,843 (8,094-11,822)	9,914 (8,171-11,866)	10,904 (8,897-13,112)	9,425 (7,847-11,068)	7,068 (5,721-8,456)	3,993 (3,380-4,601)	1,516 (1,234-1,832)	559 (407-723)
Cirrhosis and other chronic liver diseases due to hepatitis C	4,068 (3,335-4,901)	5,662 (4,523-6,815)	7,788 (6,195-9,389)	8,810 (7,240-10,484)	9,293 (7,457-11,408)	9,262 (7,715-11,009)	10,163 (8,371-12,028)	8,552 (6,780-10,454)	6,967 (5,556-8,618)	4,400 (3,651-5,256)	1,894 (1,495-2,312)	802 (577-1,044)
Cirrhosis and other chronic liver diseases due to alcohol use	4,816 (4,138-5,552)	7,000 (5,910-8,153)	9,514 (8,059-11,040)	10,587 (8,793-12,461)	10,483 (8,480-12,649)	9,929 (8,290-11,648)	10,570 (8,850-12,406)	9,147 (7,683-10,635)	7,536 (6,227-8,820)	5,028 (4,365-5,728)	2,229 (1,869-2,583)	1,029 (813-1,249)
Cirrhosis and other chronic liver diseases due to other causes	3,943 (3,071-4,911)	5,566 (4,285-6,881)	8,148 (6,418-10,042)	10,394 (8,196-12,771)	10,834 (8,364-13,521)	10,005 (7,775-12,399)	10,970 (8,525-13,636)	9,961 (7,895-12,110)	8,597 (6,789-10,555)	5,642 (4,801-6,606)	2,520 (2,078-3,020)	1,064 (803-1,369)
Digestive diseases	11,219 (10,106-12,368)	15,607 (14,064-17,131)	20,756 (18,950-22,900)	28,830 (26,033-31,883)	34,388 (30,692-37,314)	42,262 (37,269-46,188)	52,521 (47,378-56,404)	59,195 (53,121-63,214)	63,825 (58,099-67,658)	54,734 (50,840-58,120)	29,547 (27,612-31,528)	13,391 (12,444-14,141)
Peptic ulcer disease	2,752 (2,386-3,267)	3,712 (3,205-4,302)	5,493 (4,804-6,411)	6,534 (5,671-7,767)	8,549 (7,429-9,868)	10,611 (9,240-12,289)	13,086 (11,433-14,823)	14,523 (12,670-16,357)	14,381 (12,763-15,706)	11,037 (10,069-12,412)	5,393 (4,914-6,031)	2,159 (1,967-2,435)
Gastritis and duodenitis	418 (348-520)	585 (488-720)	767 (632-929)	971 (813-1,205)	1,236 (1,052-1,468)	1,608 (1,344-1,933)	1,889 (1,611-2,223)	2,242 (1,913-2,642)	2,492 (2,148-2,939)	1,917 (1,659-2,369)	1,059 (921-1,348)	425 (375-607)
Appendicitis	885 (697-1,093)	1,290 (990-1,617)	1,210 (950-1,500)	2,265 (1,766-2,918)	1,993 (1,539-2,480)	2,047 (1,568-2,526)	1,843 (1,416-2,229)	1,820 (1,400-2,220)	1,457 (1,122-1,792)	1,072 (872-1,337)	516 (414-660)	183 (149-250)
Paralytic ileus and intestinal obstruction	2,738 (1,873-3,210)	3,468 (2,329-4,110)	4,634 (3,295-5,497)	7,166 (4,913-8,347)	7,692 (5,165-9,024)	9,597 (6,308-11,209)	10,457 (7,586-12,219)	11,285 (8,561-13,314)	11,563 (8,966-13,653)	10,658 (8,226-12,382)	6,191 (4,670-7,129)	2,850 (2,095-3,303)
Inguinal, femoral, and abdominal hernia	212 (142-323)	329 (215-467)	646 (429-992)	983 (622-1,419)	1,271 (824-1,773)	1,467 (944-2,032)	1,940 (1,243-2,639)	2,040 (1,371-2,792)	2,679 (1,733-3,489)	2,281 (1,460-3,045)	1,248 (776-1,665)	531 (339-727)
Inflammatory bowel disease	462 (340-608)	698 (506-907)	800 (593-981)	1,071 (817-1,335)	1,223 (890-1,482)	1,397 (1,001-1,688)	2,071 (1,504-2,447)	2,391 (1,665-2,722)	2,862 (1,888-3,187)	2,691 (1,778-3,068)	1,565 (1,019-1,792)	820 (523-963)
Vascular intestinal disorders	405 (331-482)	768 (636-919)	1,139 (970-1,318)	1,746 (1,479-2,031)	2,768 (2,395-3,202)	4,020 (3,490-4,626)	6,586 (5,865-7,443)	8,475 (7,650-9,424)	9,451 (8,520-10,348)	7,903 (7,216-8,682)	3,749 (3,424-4,159)	1,512 (1,355-1,711)
Gallbladder and biliary diseases	923 (810-1,144)	1,364 (1,174-1,652)	2,034 (1,782-2,478)	2,702 (2,392-3,322)	3,506 (3,076-4,224)	4,499 (3,868-5,720)	5,598 (4,925-6,653)	6,775 (5,985-7,958)	7,893 (7,078-8,902)	7,330 (6,672-8,331)	4,416 (4,035-4,996)	2,284 (2,063-2,579)
Pancreatitis	1,667 (1,261-2,136)	2,388 (1,778-3,036)	2,673 (2,036-3,402)	3,445 (2,583-4,332)	3,472 (2,583-4,497)	3,851 (2,819-4,966)	4,555 (3,427-5,539)	4,162 (3,233-5,089)	4,079 (3,222-5,005)	2,974 (2,382-3,566)	1,342 (1,110-1,612)	538 (433-640)
Other digestive diseases	756 (648-876)	1,006 (882-1,182)	1,361 (1,207-1,556)	1,947 (1,722-2,226)	2,677 (2,382-2,984)	3,164 (2,756-3,538)	4,496 (3,976-4,976)	5,482 (4,908-6,031)	6,968 (6,332-7,650)	6,871 (6,287-7,562)	4,069 (3,712-4,437)	2,090 (1,907-2,362)
Neurological disorders	4,968 (4,601-5,409)	6,691 (6,099-7,375)	10,575 (9,212-12,346)	15,437 (12,655-18,757)	25,671 (21,004-31,937)	43,898 (34,996-54,997)	94,847 (74,124-121,319)	182,649 (142,682-230,672)	286,908 (229,207-357,565)	340,883 (276,592-412,908)	251,812 (212,060-292,260)	117,614 (95,780-143,197)
Alzheimer's disease and other dementias	55 (24-108)	1,252 (751-1,862)	4,045 (2,756-5,754)	8,075 (5,347-11,400)	16,952 (12,388-23,081)	33,559 (24,959-44,555)	78,631 (57,784-104,690)	161,159 (121,806-207,230)	261,263 (202,272-333,217)	320,782 (254,055-394,514)	243,699 (203,614-283,829)	114,902 (93,042-140,727)
Parkinson's disease	137 (76-228)	235 (131-410)	424 (247-681)	848 (511-1,265)	1,796 (1,223-2,543)	3,654 (2,566-4,937)	8,910 (6,202-12,062)	15,501 (11,026-20,817)	20,546 (15,200-27,798)	16,527 (12,555-22,128)	6,829 (5,185-8,925)	2,223 (1,607-3,000)
Epilepsy	2,882 (2,514-3,170)	2,350 (2,030-2,517)	2,558 (2,181-2,801)	2,301 (1,867-2,472)	2,342 (1,959-2,532)	2,234 (1,793-2,428)	2,756 (2,270-3,015)	1,794 (1,534-1,955)	1,727 (1,527-1,878)	1,497 (1,344-1,632)	556 (507-601)	221 (203-239)
Multiple sclerosis	777 (682-992)	1,083 (964-1,320)	1,255 (1,056-1,511)	1,336 (1,061-1,564)	1,224 (881-1,423)	1,008 (728-1,137)	860 (614-959)	656 (445-729)	492 (321-552)	263 (163-299)	80 (51-90)	22 (15-25)
Motor neuron disease	279 (265-308)	563 (538-614)	948 (904-1,017)	1,307 (1,255-1,398)	1,701 (1,645-1,793)	1,843 (1,773-1,930)	1,978 (1,894-2,066)	1,851 (1,769-1,942)	1,309 (1,240-1,377)	665 (628-702)	165 (157-173)	43 (41-45)
Other neurological disorders	837 (804-871)	1,207 (1,137-1,270)	1,344 (1,297-1,396)	1,570 (1,513-1,630)	1,656 (1,586-1,732)	1,600 (1,531-1,677)	1,713 (1,633-1,798)	1,688 (1,601-1,776)	1,572 (1,474-1,673)	1,149 (1,076-1,229)	484 (456-514)	203 (191-215)
Mental and substance use disorders	5,885 (5,587-6,175)	6,981 (6,646-7,372)	7,224 (6,846-7,624)	6,394 (5,992-6,802)	4,815 (4,501-5,110)	3,782 (3,448-3,997)	3,291 (2,903-3,495)	3,286 (2,936-3,491)	2,654 (2,336-2,810)	1,934 (1,748-2,059)	934 (871-995)	431 (402-458)
Alcohol use disorders	2,283 (2,100-2,495)	3,092 (2,834-3,370)	3,677 (3,390-4,007)	3,447 (3,140-3,780)	2,615 (2,424-2,834)	1,924 (1,800-2,061)	1,342 (1,251-1,443)	1,297 (1,198-1,390)	798 (747-850)	542 (509-575)	263 (247-280)	120 (113-126)
Drug use disorders	3,534 (3,319-3,752)	3,819 (3,624-4,058)	3,547 (3,328-3,757)	2,947 (2,696-3,137)	2,200 (1,954-2,353)	1,858 (1,546-1,993)	1,949 (1,547-2,095)	1,989 (1,653-2,157)	1,855 (1,539-1,998)	1,392 (1,205-1,501)	672 (612-724)	312 (288-338)
Opioid use disorders	1,950 (1,396-2,135)	2,037 (1,341-2,266)	1,865 (1,231-2,062)	1,598 (1,164-1,773)	1,214 (947-1,366)	1,063 (814-1,209)	823 (604-952)	820 (596-955)	620 (418-738)	451 (307-528)	213 (151-256)	89 (63-108)
Cocaine use disorders	236 (175-361)	274 (218-424)	220 (167-342)	167 (128-247)	113 (82-175)	87 (61-135)	25 (16-41)	22 (14-35)	9 (6-11)	9 (4-11)	5 (2-6)	3 (1-4)
Amphetamine use disorders	114 (86-176)	128 (89-194)	106 (78-163)	87 (65-139)	58 (43-89)	44 (32-70)	18 (12-27)	19 (13-29)	5 (3-7)	6 (3-8)	4 (1-5)	3 (1-4)
Other drug use disorders	1,234 (1,063-1,657)	1,380 (1,197-1,908)	1,357 (1,196-1,860)	1,096 (944-1,393)	815 (664-999)	663 (503-808)	1,083 (826-1,270)	1,128 (907-1,319)	1,221 (1,027-1,380)	927 (790-1,047)	450 (403-512)	216 (195-251)
Eating disorders	67 (59-79)	70 (62-83)	0 (0-0)	0 (0-0)	-	-	-	-	-	-	-	-
Anorexia nervosa	58 (51-68)	61 (54-74)	0 (0-0)	0 (0-0)	-	-	-	-	-	-	-	-
Bulimia nervosa	9 (7-12)	8 (6-11)	0 (0-0)	0 (0-0)	-	-	-	-	-	-	-	-
Diabetes, urogenital, blood, and endocrine diseases	30,801 (29,630-32,075)	45,859 (43,995-47,694)	68,041 (65,314-70,354)	90,450 (86,914-93,447)	114,938 (110,146-118,758)	134,179 (128,688-139,210)	163,280 (156,096-170,108)	177,960 (169,669-185,009)	180,201 (171,195-187,965)	147,405 (141,123-153,758)	78,859 (75,367-82,437)	36,639 (34,900-38,206)
Diabetes mellitus	10,963 (10,471-11,521)	18,913 (18,074-19,749)	31,626 (30,295-32,907)	45,487 (43,711-47,220)	62,860 (60,097-65,353)	74,658 (71,199-77,739)	89,513 (85,277-93,579)	96,330 (91,361-100,696)	93,743 (88,293-98,541)	65,266 (61,788-68,365)	29,798 (28,230-31,386)	11,222 (10,609-11,791)
Acute glomerulonephritis	215 (194-238)	225 (204-248)	219 (201-239)	303 (274-335)	348 (316-385)	363 (329-403)	442 (406-483)	425 (387-468)	381 (345-420)	275 (250-300)	122 (112-133)	54 (49-60)
Chronic kidney disease	12,320 (11,732-12,953)	17,461 (16,715-18,258)	25,107 (24,048-26,202)	32,685 (31,262-34,153)	38,354 (36,706-40,140)	43,212 (41,336-45,037)	52,151 (49,823-54,368)	57,655 (54,922-60,106)	58,267 (55,486-60,907)	52,797 (50,315-55,447)	30,896 (29,459-32,398)	15,675 (14,872-16,398)
Chronic kidney disease due to diabetes mellitus	5,253 (4,077-6,358)	8,097 (6,370-9,852)	12,160 (9,445-14,735)	16,493 (13,095-19,547)	19,576 (15,817-23,058)	21,262 (16,450-25,311)	23,861 (18,998-28,232)	23,725 (18,649-28,600)	20,011 (15,380-24,564)	15,571 (11,519-20,216)	8,744 (6,343-11,455)	4,470 (3,105-5,902)
Chronic kidney disease due to hypertension	1,721 (1,307-2,230)	2,679 (2,070-3,421)	4,218 (3,231-5,461)	6,128 (4,810-7,839)	7,760 (6,146-9,842)	9,895 (7,770-12,542)	13,426 (10,593-16,621)	16,967 (13,483-21,000)	19,260 (15,792-23,310)	20,358 (16,716-24,507)	13,245 (11,058-15,566)	6,807 (5,495-8,275)
Chronic kidney disease due to glomerulonephritis	2,528 (1,681-3,415)	3,031 (1,966-4,218)	3,847 (2,613-5,472)	4,314 (2,970-6,011)	4,603 (3,013-6,492)	4,945 (3,234-7,087)	6,047 (4,187-8,482)	6,859 (4,717-9,658)	7,672 (5,588-10,041)	6,074 (4,383-8,141)	2,870 (2,086-3,760)	1,366 (884-2,081)
Chronic kidney disease due to other causes	2,818 (1,938-3,814)	3,654 (2,437-5,152)	4,882 (3,227-6,916)	5,751 (3,909-8,161)	6,415 (4,276-9,114)	7,110 (4,760-10,137)	8,818 (6,114-12,028)	10,104 (7,034-13,756)	11,325 (8,126-14,978)	10,796 (7,484-14,502)	6,037 (4,417-7,964)	3,032 (1,925-4,228)
Urinary diseases and male infertility	2,344 (2,220-2,475)	3,050 (2,893-3,204)	4,288 (4,057-4,532)	5,570 (5,229-5,903)	6,735 (6,330-7,136)	8,115 (7,589-8,584)	10,744 (10,040-11,349)	13,128 (12,339-13,794)	16,085 (15,277-16,848)	16,567 (15,752-17,342)	11,122 (10,597-11,692)	6,140 (5,839-6,447)
Interstitial nephritis and urinary tract infections	1,788 (1,623-1,941)	2,325 (2,104-2,507)	3,284 (3,021-3,574)	4,107 (3,712-4,522)	4,907 (4,406-5,406)	6,195 (5,672-6,812)	8,612 (7,926-9,294)	10,643 (9,813-11,453)	13,544 (12,694-14,419)	14,510 (13,659-15,504)	9,973 (9,386-10,612)	5,659 (5,335-5,985)
Urolithiasis	174 (131-231)	255 (193-339)	372 (273-469)	471 (353-599)	540 (415-690)	622 (483-826)	811 (645-1,123)	906 (735-1,294)	1,004 (835-1,428)	791 (644-1,160)	445 (358-669)	198 (158-322)
Other urinary diseases	383 (259-528)	470 (321-665)	632 (398-878)	992 (655-1,355)	1,287 (868-1,732)	1,298 (805-1,712)	1,320 (810-1,727)	1,579 (976-2,028)	1,537 (956-1,986)	1,266 (711-1,620)	704 (394-896)	283 (159-370)
Gynecological diseases	685 (612-759)	980 (850-1,065)	804 (716-882)	594 (522-648)	377 (333-411)	533 (469-588)	365 (316-399)	287 (247-311)	607 (493-698)	451 (364-518)	200 (162-229)	75 (61-85)
Uterine fibroids	244 (162-316)	465 (297-579)	476 (286-578)	311 (189-386)	177 (118-222)	223 (141-296)	132 (93-167)	107 (84-128)	179 (89-281)	103 (50-174)	44 (24-71)	14 (8-22)
Polycystic ovarian syndrome	53 (18-100)	83 (27-164)	146 (55-267)	-	-	-	-	-	-	-	-	-
Endometriosis	9 (4-14)	19 (8-33)	13 (6-19)	-	-	-	-	-	-	-	-	-
Genital prolapse	7 (3-12)	7 (4-12)	13 (6-26)	71 (23-120)	63 (28-106)	106 (46-170)	93 (46-141)	55 (36-75)	197 (77-314)	143 (59-224)	62 (28-95)	20 (11-30)
Other gynecological diseases	371 (254-469)	406 (254-576)	156 (80-260)	212 (134-305)	137 (89-193)	205 (129-288)	140 (90-192)	126 (94-150)	231 (146-354)	205 (140-294)	94 (65-130)	40 (29-52)
Hemoglobinopathies and hemolytic anaemias	2,325 (1,805-3,014)	2,560 (1,985-3,273)	2,580 (2,009-3,298)	2,084 (1,673-2,685)	1,975 (1,646-2,578)	2,489 (2,028-3,298)	4,483 (3,649-5,871)	3,996 (3,201-5,154)	4,189 (3,436-5,508)	5,618 (4,481-6,879)	2,873 (2,251-3,627)	1,322 (965-1,605)
Thalassemias	35 (29-51)	27 (23-40)	21 (17-35)	15 (12-27)	9 (7-17)	4 (3-9)	2 (2-5)	1 (1-2)	0 (0-1)	0 (0-0)	0 (0-0)	0 (0-0)
Sickle cell disorders	1,195 (908-1,574)	1,179 (897-1,517)	970 (745-1,259)	431 (336-572)	187 (150-264)	105 (87-150)	60 (49-90)	23 (19-39)	9 (8-16)	4 (3-7)	1 (1-1)	0 (0-0)
G6PD deficiency	489 (367-624)	579 (439-756)	614 (444-776)	447 (340-592)	364 (281-485)	313 (235-424)	383 (286-518)	217 (158-273)	102 (76-132)	83 (61-107)	20 (15-25)	4 (3-5)
Other hemoglobinopathies and hemolytic anaemias	606 (484-804)	776 (612-1,001)	975 (780-1,238)	1,191 (952-1,508)	1,415 (1,172-1,825)	2,066 (1,686-2,742)	4,039 (3,292-5,269)	3,754 (3,024-4,852)	4,077 (3,343-5,367)	5,530 (4,412-6,777)	2,852 (2,236-3,600)	1,318 (961-1,600)
Endocrine, metabolic, blood, and immune disorders	1,948 (1,553-2,140)	2,670 (2,118-2,944)	3,417 (2,669-3,696)	3,727 (2,854-4,018)	4,288 (3,432-4,797)	4,809 (3,858-5,461)	5,581 (4,409-6,282)	6,139 (4,566-6,733)	6,930 (4,963-7,583)	6,432 (4,336-7,000)	3,848 (2,472-4,202)	2,152 (1,364-2,379)
Musculoskeletal disorders	2,272 (1,868-2,755)	2,515 (2,119-2,924)	2,708 (2,221-3,254)	3,243 (2,677-3,790)	3,733 (3,159-4,440)	4,104 (3,474-4,899)	5,095 (4,193-5,854)	5,952 (5,007-6,748)	6,210 (5,214-6,999)	5,674 (4,638-6,294)	3,195 (2,567-3,511)	1,690 (1,390-1,841)
Rheumatoid arthritis	313 (236-438)	549 (432-729)	836 (650-1,082)	1,346 (1,042-1,699)	1,751 (1,422-2,174)	2,145 (1,720-2,704)	2,807 (2,246-3,467)	3,176 (2,543-3,847)	2,952 (2,349-3,646)	2,171 (1,723-2,821)	958 (722-1,233)	358 (272-505)
Other musculoskeletal disorders	1,960 (1,592-2,346)	1,965 (1,617-2,301)	1,872 (1,512-2,227)	1,898 (1,546-2,244)	1,983 (1,615-2,387)	1,959 (1,611-2,303)	2,288 (1,789-2,685)	2,776 (2,162-3,241)	3,258 (2,499-3,630)	3,503 (2,657-3,848)	2,236 (1,706-2,457)	1,332 (1,062-1,449)
Other non-communicable diseases	2,819 (2,375-3,353)	2,852 (2,227-3,442)	3,600 (2,576-4,558)	4,100 (2,851-5,126)	4,014 (2,734-5,288)	4,534 (2,867-6,387)	4,430 (2,355-6,708)	5,722 (3,302-8,934)	6,538 (3,638-10,180)	6,557 (3,581-10,226)	4,500 (2,375-7,094)	2,578 (1,205-4,175)
Congenital anomalies	1,972 (1,731-2,291)	1,626 (1,385-1,883)	1,563 (1,278-1,731)	1,571 (1,278-1,691)	1,322 (1,073-1,435)	994 (824-1,104)	-	-	-	-	-	-
Neural tube defects	36 (30-47)	36 (31-44)	33 (27-39)	29 (24-35)	21 (17-25)	20 (16-24)	-	-	-	-	-	-
Congenital heart anomalies	1,390 (1,200-1,624)	719 (590-821)	617 (517-723)	568 (477-676)	451 (380-556)	370 (323-443)	-	-	-	-	-	-
Orofacial clefts	-	-	-	-	-	-	-	-	-	-	-	-
Down's syndrome	201 (164-259)	370 (290-447)	401 (303-461)	431 (290-482)	329 (208-372)	137 (86-154)	-	-	-	-	-	-
Other chromosomal abnormalities	34 (18-40)	30 (15-36)	34 (20-44)	34 (17-44)	23 (11-31)	20 (12-24)	-	-	-	-	-	-
Congenital musculoskeletal and limb anomalies	23 (17-27)	29 (22-38)	29 (22-38)	29 (23-39)	24 (18-31)	21 (17-27)	-	-	-	-	-	-
Urogenital congenital anomalies	53 (37-68)	94 (59-120)	126 (87-165)	155 (107-202)	165 (113-207)	186 (132-236)	-	-	-	-	-	-
Digestive congenital anomalies	16 (13-20)	25 (20-31)	35 (27-43)	38 (29-47)	39 (30-48)	39 (30-46)	-	-	-	-	-	-
Other congenital anomalies	218 (157-279)	322 (247-383)	288 (217-348)	287 (217-355)	270 (209-321)	201 (152-239)	-	-	-	-	-	-
Skin and subcutaneous diseases	847 (442-1,311)	1,227 (642-1,828)	2,038 (1,053-2,972)	2,529 (1,305-3,611)	2,692 (1,434-3,975)	3,540 (1,885-5,390)	4,430 (2,355-6,708)	5,722 (3,302-8,934)	6,538 (3,638-10,180)	6,557 (3,581-10,226)	4,500 (2,375-7,094)	2,578 (1,205-4,175)
Cellulitis	121 (60-195)	195 (80-291)	367 (149-525)	448 (181-670)	522 (201-769)	553 (232-871)	708 (279-1,040)	822 (346-1,356)	955 (422-1,785)	897 (417-1,662)	610 (251-1,234)	344 (145-706)
Pyoderma	565 (285-882)	818 (401-1,297)	1,370 (682-2,124)	1,675 (848-2,571)	1,686 (851-2,610)	2,174 (1,100-3,492)	2,482 (1,275-4,013)	2,883 (1,592-4,865)	2,730 (1,409-4,511)	2,587 (1,312-4,113)	1,651 (830-2,725)	860 (395-1,422)
Decubitus ulcer	107 (53-177)	154 (82-250)	232 (108-381)	319 (156-526)	391 (196-625)	690 (328-1,091)	1,090 (561-1,756)	1,811 (910-2,899)	2,584 (1,310-3,951)	2,802 (1,415-4,138)	2,043 (985-3,123)	1,260 (551-2,013)
Other skin and subcutaneous diseases	53 (30-87)	60 (34-97)	68 (37-112)	86 (48-138)	92 (51-152)	124 (69-209)	148 (82-241)	206 (118-351)	269 (151-492)	272 (148-529)	196 (101-389)	115 (52-245)
Sudden infant death syndrome	-	-	-	-	-	-	-	-	-	-	-	-
Injuries	72,461 (66,745-77,144)	71,751 (66,125-76,198)	72,853 (66,682-77,283)	75,787 (69,239-80,411)	73,771 (66,081-78,537)	73,546 (64,321-78,728)	83,326 (72,138-89,170)	85,368 (73,098-91,257)	83,583 (70,438-89,461)	70,477 (58,858-75,637)	37,901 (30,949-40,668)	17,948 (14,385-19,306)
Transport injuries	23,699 (22,757-24,653)	22,617 (21,663-23,610)	22,962 (21,950-23,952)	23,341 (22,234-24,429)	21,109 (19,986-22,184)	17,598 (16,515-18,484)	18,406 (17,268-19,379)	15,347 (14,467-16,140)	11,665 (10,986-12,270)	6,941 (6,565-7,312)	2,848 (2,698-3,004)	1,103 (1,032-1,175)
Road injuries	22,604 (21,707-23,526)	21,419 (20,532-22,396)	21,737 (20,776-22,690)	22,373 (21,314-23,413)	19,940 (18,863-20,953)	16,606 (15,644-17,468)	17,418 (16,359-18,347)	14,453 (13,648-15,239)	10,909 (10,316-11,484)	6,461 (6,107-6,805)	2,615 (2,474-2,761)	995 (929-1,061)
Pedestrian road injuries	9,601 (8,941-10,264)	9,222 (8,639-9,882)	9,463 (8,806-10,103)	10,795 (10,091-11,514)	10,723 (10,065-11,406)	9,171 (8,467-9,794)	10,065 (9,396-10,712)	8,711 (8,177-9,187)	6,740 (6,307-7,137)	3,787 (3,556-4,067)	1,393 (1,295-1,524)	491 (448-577)
Cyclist road injuries	1,333 (1,085-1,491)	1,433 (1,232-1,627)	1,305 (1,123-1,471)	1,450 (1,232-1,632)	1,146 (935-1,313)	1,004 (849-1,179)	955 (805-1,217)	605 (486-741)	296 (225-364)	184 (142-273)	74 (58-117)	1 (1-1)
Motorcyclist road injuries	3,450 (3,059-3,731)	2,965 (2,652-3,253)	2,999 (2,710-3,296)	2,586 (2,339-2,821)	1,767 (1,531-1,964)	1,276 (1,127-1,431)	1,077 (949-1,219)	654 (568-738)	381 (329-428)	185 (164-210)	71 (63-81)	29 (26-35)
Motor vehicle road injuries	8,025 (7,196-8,801)	7,530 (6,727-8,274)	7,743 (7,018-8,421)	7,290 (6,605-7,990)	6,210 (5,630-6,832)	5,068 (4,617-5,576)	5,231 (4,747-5,815)	4,284 (3,874-4,746)	3,361 (3,084-3,756)	2,218 (1,985-2,424)	1,033 (908-1,120)	452 (374-491)
Other road injuries	196 (164-289)	269 (221-406)	227 (186-351)	251 (207-388)	94 (78-125)	87 (71-118)	90 (73-129)	200 (160-311)	132 (99-206)	86 (63-130)	44 (32-66)	22 (15-33)
Other transport injuries	1,095 (973-1,323)	1,198 (1,036-1,387)	1,225 (1,044-1,423)	968 (874-1,140)	1,169 (996-1,344)	992 (842-1,134)	988 (849-1,115)	894 (767-1,025)	756 (651-866)	479 (409-557)	233 (204-268)	108 (98-124)
Unintentional injuries	19,584 (15,937-21,136)	21,407 (17,589-22,935)	23,480 (19,167-25,191)	27,717 (22,588-29,825)	31,383 (25,146-33,908)	36,944 (28,374-40,261)	45,809 (35,811-49,960)	53,817 (42,118-58,458)	60,127 (47,084-65,391)	56,926 (45,510-61,634)	32,649 (25,565-35,257)	16,074 (12,443-17,379)
Falls	3,653 (2,875-3,949)	4,657 (3,690-4,996)	6,188 (4,877-6,664)	9,366 (6,967-10,199)	12,837 (9,693-14,075)	18,007 (12,907-19,825)	25,240 (18,269-27,979)	33,196 (24,467-36,426)	39,620 (29,114-43,411)	40,403 (30,573-43,939)	23,935 (17,504-25,999)	11,378 (8,002-12,306)
Drowning	3,135 (2,266-3,400)	3,105 (2,330-3,365)	3,240 (2,371-3,521)	3,521 (2,533-3,843)	3,509 (2,512-3,839)	3,533 (2,562-3,889)	3,776 (2,805-4,097)	4,133 (3,000-4,528)	3,456 (2,564-3,758)	2,072 (1,623-2,252)	748 (589-806)	216 (175-232)
Fire, heat, and hot substances	2,378 (1,707-2,678)	2,727 (1,925-3,071)	2,662 (1,899-2,986)	2,662 (1,922-2,990)	2,629 (1,977-2,922)	3,040 (2,332-3,407)	3,490 (2,740-3,925)	3,822 (3,076-4,317)	4,170 (3,333-4,630)	3,318 (2,660-3,653)	1,753 (1,330-1,956)	912 (646-999)
Poisonings	1,237 (829-1,365)	1,022 (697-1,129)	1,112 (787-1,215)	1,175 (794-1,307)	979 (673-1,078)	975 (656-1,072)	1,035 (734-1,132)	873 (591-968)	786 (562-852)	477 (363-520)	210 (174-251)	88 (77-129)
Exposure to mechanical forces	1,954 (1,347-2,200)	1,833 (1,339-2,031)	1,586 (1,178-1,740)	2,073 (1,542-2,298)	1,669 (1,199-1,883)	1,575 (1,122-1,791)	1,547 (1,151-1,727)	1,444 (1,081-1,597)	1,595 (1,186-1,759)	1,379 (1,059-1,514)	762 (602-832)	412 (334-451)
Unintentional firearm injuries	266 (233-298)	282 (243-316)	214 (180-241)	225 (202-261)	172 (151-190)	177 (155-192)	214 (183-228)	296 (249-322)	338 (265-363)	324 (233-351)	213 (151-232)	119 (81-129)
Unintentional suffocation	95 (50-112)	156 (113-184)	194 (137-233)	239 (175-312)	74 (40-90)	82 (45-100)	97 (55-119)	89 (43-110)	107 (52-129)	93 (41-115)	49 (23-59)	19 (10-22)
Other exposure to mechanical forces	1,593 (1,015-1,825)	1,395 (920-1,581)	1,179 (805-1,325)	1,609 (1,081-1,836)	1,423 (965-1,633)	1,316 (872-1,524)	1,237 (862-1,406)	1,059 (741-1,194)	1,150 (816-1,295)	962 (714-1,071)	500 (385-554)	275 (208-307)
Adverse effects of medical treatment	2,199 (1,664-2,527)	2,830 (2,174-3,219)	2,401 (1,930-2,782)	2,985 (2,316-3,418)	4,002 (3,176-4,573)	4,270 (3,308-4,884)	4,700 (3,734-5,462)	4,663 (3,697-5,529)	4,576 (3,640-5,622)	4,012 (3,252-5,190)	2,139 (1,777-3,007)	1,093 (913-1,605)
Animal contact	2,195 (809-2,847)	2,087 (827-2,590)	2,721 (1,057-3,426)	1,971 (833-2,460)	2,158 (821-2,774)	2,302 (877-2,978)	2,319 (900-2,981)	1,583 (650-2,018)	1,037 (471-1,318)	520 (275-638)	206 (118-243)	80 (54-91)
Venomous animal contact	2,030 (708-2,677)	1,852 (651-2,339)	2,452 (878-3,153)	1,711 (653-2,197)	1,987 (716-2,599)	2,133 (760-2,805)	2,099 (760-2,743)	1,407 (525-1,828)	873 (351-1,142)	408 (188-519)	148 (74-182)	49 (29-57)
Non-venomous animal contact	165 (92-288)	234 (124-408)	269 (146-462)	260 (144-446)	170 (86-295)	169 (87-294)	220 (104-389)	176 (93-275)	164 (93-243)	111 (71-168)	58 (37-78)	31 (20-40)
Foreign body	1,030 (888-1,255)	1,123 (980-1,385)	1,289 (1,103-1,592)	1,534 (1,340-1,877)	1,505 (1,233-1,738)	1,412 (1,100-1,571)	1,811 (1,332-2,051)	2,259 (1,618-2,628)	2,889 (1,954-3,438)	3,138 (2,009-3,857)	2,046 (1,236-2,541)	1,478 (839-1,863)
Pulmonary aspiration and foreign body in airway	793 (669-981)	951 (805-1,205)	1,096 (916-1,377)	1,328 (1,116-1,643)	1,370 (1,090-1,608)	1,305 (1,012-1,471)	1,652 (1,183-1,891)	2,104 (1,465-2,469)	2,695 (1,790-3,228)	2,955 (1,863-3,670)	1,935 (1,140-2,417)	1,421 (798-1,798)
Foreign body in other body part	236 (167-338)	173 (118-249)	193 (143-280)	205 (156-319)	135 (95-206)	106 (67-138)	159 (105-200)	156 (91-194)	194 (118-238)	183 (104-220)	111 (62-133)	57 (31-69)
Environmental heat and cold exposure	700 (361-880)	1,006 (502-1,267)	1,176 (627-1,689)	1,343 (721-1,830)	1,315 (665-1,683)	942 (445-1,189)	1,310 (605-1,663)	1,269 (592-1,603)	1,391 (741-1,737)	1,159 (668-1,667)	612 (370-987)	314 (190-518)
Other unintentional injuries	1,103 (712-1,207)	1,017 (693-1,103)	1,106 (702-1,219)	1,088 (737-1,191)	780 (547-855)	888 (598-991)	581 (436-640)	575 (418-627)	606 (458-660)	449 (367-489)	237 (178-258)	104 (86-113)
Self-harm and interpersonal violence	24,010 (22,706-25,390)	22,976 (21,708-24,251)	21,980 (20,746-23,047)	20,303 (19,191-21,248)	17,310 (16,353-18,142)	15,175 (14,273-15,997)	15,470 (14,533-16,314)	13,373 (12,555-14,094)	9,748 (9,165-10,360)	5,600 (5,309-5,924)	2,078 (1,958-2,200)	670 (629-722)

SR Table 3: Global deaths by cause and age group for females (with 95% uncertainty intervals), 2010

Cause	Early Neonatal	Late Neonatal	Post Neonatal	1 to 4	5 to 9	10 to 14	15 to 19	20 to 24	25 to 29	30 to 34	35 to 39	40 to 44	45 to 49	50 to 54	55 to 59	60 to 64	65 to 69	70 to 74	75 to 79	80 to 84	85 to 89	90 to 94	95 plus
Self-harm	-	-	-	-	-	4,126 (3,690-4,585)	27,974 (25,531-30,393)	33,522 (31,198-35,918)	28,731 (26,971-30,493)	20,931 (19,848-22,120)	20,294 (19,287-21,297)	18,609 (17,808-19,502)	18,059 (17,345-18,809)	17,812 (17,077-18,569)	16,852 (16,115-17,611)	14,578 (13,885-15,261)	13,018 (12,286-13,736)	13,062 (12,362-13,769)	11,337 (10,676-11,975)	8,943 (8,402-9,456)	5,210 (4,935-5,482)	1,918 (1,810-2,026)	604 (569-636)
Self-harm by firearm	-	-	-	-	-	147 (104-211)	989 (625-1,490)	1,277 (811-1,783)	1,027 (739-1,252)	819 (670-970)	794 (661-961)	757 (650-901)	799 (702-934)	730 (645-853)	596 (518-721)	453 (382-589)	366 (283-531)	315 (228-485)	309 (211-507)	269 (199-406)	195 (164-251)	109 (97-125)	56 (51-61)
Self-harm by other specified means	-	-	-	-	-	3,979 (3,557-4,429)	26,986 (24,673-29,300)	32,245 (30,108-34,428)	27,703 (25,925-29,436)	20,112 (19,051-21,258)	19,500 (18,547-20,483)	17,852 (17,070-18,690)	17,260 (16,549-18,006)	17,083 (16,353-17,837)	16,256 (15,515-16,998)	14,125 (13,459-14,807)	12,652 (11,935-13,359)	12,747 (12,041-13,487)	11,027 (10,374-11,652)	8,674 (8,150-9,181)	5,014 (4,742-5,280)	1,809 (1,705-1,907)	548 (517-578)
Interpersonal violence	721 (537-931)	205 (156-270)	1,496 (1,196-2,013)	3,010 (2,287-3,991)	2,179 (1,675-2,764)	2,622 (2,012-3,191)	5,985 (4,560-7,103)	8,646 (6,619-10,562)	7,855 (6,171-9,577)	7,058 (5,585-8,649)	6,211 (4,800-7,471)	5,401 (4,236-6,598)	4,917 (3,774-5,989)	4,168 (3,234-5,095)	3,451 (2,666-4,108)	2,732 (2,098-3,322)	2,157 (1,658-2,679)	2,408 (1,803-2,907)	2,037 (1,526-2,450)	805 (676-1,105)	391 (326-541)	160 (136-222)	66 (57-101)
Assault by firearm	40 (25-60)	17 (10-23)	181 (116-256)	356 (234-505)	326 (206-413)	517 (321-615)	1,772 (1,093-2,047)	2,213 (1,425-2,585)	2,093 (1,397-2,467)	1,761 (1,140-2,127)	1,542 (981-1,855)	1,309 (836-1,603)	1,144 (721-1,393)	951 (614-1,174)	698 (446-891)	555 (372-741)	422 (271-554)	429 (276-569)	360 (231-478)	60 (38-92)	35 (23-57)	13 (9-22)	7 (4-12)
Assault by sharp object	52 (39-78)	29 (17-40)	123 (79-164)	318 (207-434)	262 (189-353)	451 (314-587)	1,269 (884-1,654)	2,230 (1,636-2,835)	2,154 (1,570-2,760)	2,020 (1,535-2,647)	1,856 (1,341-2,371)	1,756 (1,287-2,255)	1,535 (1,099-1,924)	1,214 (878-1,515)	970 (645-1,181)	759 (539-992)	482 (338-625)	680 (481-832)	555 (383-707)	237 (184-334)	107 (88-156)	52 (43-74)	20 (16-31)
Assault by other means	629 (470-808)	158 (125-215)	1,192 (965-1,617)	2,337 (1,770-3,110)	1,591 (1,206-2,115)	1,654 (1,278-2,136)	2,945 (2,240-3,841)	4,203 (3,234-5,521)	3,608 (2,880-4,660)	3,277 (2,553-4,312)	2,813 (2,200-3,607)	2,336 (1,867-3,043)	2,238 (1,767-2,898)	2,002 (1,546-2,514)	1,783 (1,370-2,271)	1,418 (1,050-1,752)	1,253 (937-1,575)	1,299 (957-1,599)	1,122 (852-1,373)	508 (425-708)	248 (207-355)	95 (77-135)	40 (33-64)
Forces of nature, conflict and terrorism, and executions and police conflict	297 (226-371)	377 (168-598)	2,446 (795-4,233)	12,692 (4,208-21,814)	11,209 (3,173-20,037)	11,584 (3,099-20,954)	9,689 (2,989-17,045)	8,699 (2,662-15,347)	7,959 (2,193-14,339)	6,512 (1,881-11,637)	5,356 (1,546-9,578)	5,168 (1,402-9,352)	4,751 (1,302-8,596)	4,431 (1,234-7,995)	4,427 (1,182-8,040)	3,969 (1,066-7,208)	3,828 (985-6,988)	3,641 (899-6,690)	2,830 (702-5,191)	2,042 (534-3,714)	1,010 (287-1,818)	326 (91-591)	100 (48-156)
Forces of nature, conflict and terrorism, and state actor violence	66 (13-125)	250 (48-475)	2,014 (386-3,820)	10,284 (1,967-19,510)	9,852 (1,868-18,703)	10,411 (1,973-19,766)	8,246 (1,566-15,653)	7,440 (1,411-14,122)	7,091 (1,345-13,461)	5,730 (1,108-10,859)	4,731 (929-8,955)	4,670 (917-8,839)	4,290 (846-8,117)	3,994 (795-7,549)	4,037 (794-7,638)	3,626 (719-6,855)	3,522 (685-6,671)	3,411 (673-6,454)	2,652 (528-5,013)	1,892 (387-3,569)	920 (195-1,729)	299 (64-562)	67 (16-124)
Conflict and terrorism	231 (183-277)	126 (100-152)	418 (330-501)	2,361 (1,867-2,828)	1,321 (1,045-1,582)	1,130 (891-1,356)	1,401 (1,097-1,695)	1,217 (950-1,478)	828 (641-1,013)	746 (572-917)	589 (451-725)	462 (351-572)	428 (323-530)	410 (305-514)	360 (266-452)	321 (233-408)	286 (211-360)	204 (151-256)	161 (117-203)	123 (89-158)	70 (50-91)	14 (10-19)	30 (21-40)
Executions and police conflict	-	-	14 (9-24)	47 (25-101)	35 (30-46)	43 (34-61)	42 (33-53)	43 (32-51)	40 (29-51)	36 (26-47)	36 (25-49)	36 (24-51)	32 (22-46)	27 (19-34)	30 (18-42)	23 (15-30)	20 (12-29)	26 (12-42)	18 (9-25)	27 (10-42)	20 (5-34)	12 (5-18)	4 (1-6)

SR Table 3: Global deaths by cause and age group for females (with 95% uncertainty intervals), 2016

Cause	Early Neonatal	Late Neonatal	Post Neonatal	1 to 4	5 to 9	10 to 14	15 to 19	20 to 24	25 to 29	30 to 34	35 to 39
All causes	718,441 (686,770-750,644)	216,042 (206,692-225,640)	707,028 (676,490-738,005)	649,977 (611,748-692,244)	184,940 (180,662-189,422)	151,918 (149,087-154,927)	226,851 (221,691-232,391)	283,579 (276,512-291,201)	343,831 (333,711-354,766)	392,031 (381,362-404,093)	443,942 (432,251-456,618)
Communicable, maternal, neonatal, and nutritional disorders	643,759 (613,042-676,765)	184,631 (176,143-193,603)	557,183 (525,757-587,979)	506,258 (468,579-549,785)	112,738 (106,109-119,678)	75,924 (70,969-81,743)	97,414 (91,290-104,928)	125,351 (118,680-133,838)	158,334 (149,515-169,815)	174,141 (165,094-184,986)	170,951 (162,820-180,783)
HIV/AIDS and tuberculosis	-	-	26,792 (24,210-30,263)	16,441 (14,274-19,468)	13,744 (12,857-14,770)	19,271 (18,307-20,290)	31,903 (29,580-34,391)	40,505 (36,649-45,159)	73,813 (65,968-82,710)	91,849 (84,665-99,579)	91,373 (85,486-97,721)
Tuberculosis	-	-	5,916 (4,491-8,158)	7,032 (5,182-9,783)	3,176 (2,492-3,957)	3,932 (3,325-4,586)	9,577 (8,315-11,033)	14,909 (13,320-16,693)	18,718 (16,936-20,550)	20,875 (18,999-22,824)	23,709 (21,745-25,869)
Drug-susceptible tuberculosis	-	-	5,533 (4,172-7,698)	6,535 (4,803-9,057)	2,923 (2,289-3,665)	3,608 (3,042-4,228)	8,755 (7,586-10,051)	13,597 (12,059-15,203)	17,032 (15,375-18,835)	18,980 (17,276-20,716)	21,597 (19,697-23,561)
Multidrug-resistant tuberculosis without extensive drug resistance	-	-	368 (257-531)	476 (321-762)	238 (172-330)	304 (230-392)	766 (588-988)	1,202 (948-1,496)	1,522 (1,224-1,852)	1,708 (1,377-2,082)	1,902 (1,569-2,295)
Extensively drug-resistant tuberculosis	-	-	15 (11-20)	21 (16-30)	15 (11-21)	20 (15-27)	56 (43-73)	110 (87-136)	165 (129-208)	187 (146-241)	209 (163-266)
HIV/AIDS	-	-	20,876 (18,948-23,211)	9,409 (8,505-10,461)	10,568 (9,994-11,171)	15,339 (14,562-16,188)	22,326 (20,209-24,606)	25,597 (22,214-29,786)	55,095 (47,411-63,808)	70,974 (64,076-78,556)	67,665 (62,308-73,656)
Drug-susceptible HIV/AIDS - Tuberculosis	-	-	4,691 (3,126-6,132)	2,435 (1,683-3,115)	2,739 (1,940-3,491)	3,678 (2,530-4,798)	5,194 (3,514-6,943)	6,046 (4,132-8,051)	10,757 (7,139-14,848)	13,116 (8,590-18,201)	12,512 (8,243-17,332)
Multidrug-resistant HIV/AIDS - Tuberculosis without extensive drug resistance	-	-	453 (245-741)	223 (124-361)	225 (135-359)	295 (172-469)	420 (244-653)	508 (302-767)	868 (509-1,333)	1,043 (618-1,594)	962 (576-1,456)
Extensively drug-resistant HIV/AIDS - Tuberculosis	-	-	13 (8-21)	6 (4-10)	5 (3-9)	7 (4-11)	11 (6-17)	18 (11-28)	46 (27-72)	65 (39-103)	57 (35-89)
HIV/AIDS resulting in other diseases	-	-	15,718 (13,244-18,549)	6,744 (5,654-8,022)	7,600 (6,684-8,620)	11,359 (10,014-12,834)	16,701 (14,070-19,659)	19,025 (15,437-23,680)	43,424 (35,390-52,406)	56,751 (48,647-65,644)	54,134 (46,706-61,636)
Diarrhoea, lower respiratory infections, and other common infectious diseases	47,926 (35,951-57,871)	48,569 (42,396-54,569)	338,682 (306,867-371,885)	248,343 (212,495-284,477)	62,863 (53,867-74,320)	38,462 (32,445-46,628)	32,827 (26,427-42,647)	30,510 (24,086-42,098)	28,669 (22,732-39,412)	28,645 (22,535-39,530)	29,846 (23,579-40,963)
Diarrhoeal diseases	4,950 (4,288-5,834)	11,084 (9,343-13,220)	106,994 (89,902-125,091)	83,678 (65,460-106,135)	15,677 (7,591-30,320)	9,135 (4,256-17,971)	10,652 (4,955-21,281)	11,588 (5,244-23,678)	10,725 (4,950-21,330)	11,229 (5,126-22,554)	11,701 (5,383-23,091)
Intestinal infectious diseases	-	-	676 (129-2,688)	10,484 (5,243-19,162)	16,761 (9,404-27,212)	11,407 (6,103-18,157)	7,425 (3,715-13,000)	4,631 (2,226-8,213)	3,451 (1,774-6,014)	2,769 (1,437-4,836)	2,279 (1,211-3,838)
Typhoid fever	-	-	85 (40-163)	9,130 (4,213-17,157)	14,460 (7,819-23,895)	9,481 (4,766-15,272)	6,345 (3,065-11,193)	3,833 (1,796-6,915)	2,670 (1,359-4,710)	2,050 (1,031-3,593)	1,694 (869-2,913)
Paratyphoid fever	-	-	13 (5-29)	999 (412-2,137)	2,260 (1,017-4,507)	1,910 (870-3,599)	1,065 (444-2,147)	786 (317-1,586)	768 (326-1,595)	699 (302-1,411)	564 (255-1,084)
Other intestinal infectious diseases	-	-	577 (42-2,630)	355 (23-1,562)	41 (3-179)	16 (1-72)	15 (1-90)	13 (1-62)	13 (1-62)	20 (2-93)	21 (2-87)
Lower respiratory infections	35,972 (24,974-45,048)	29,740 (23,026-34,593)	162,168 (136,678-190,672)	78,934 (61,987-98,078)	15,662 (11,690-19,042)	8,635 (6,791-10,342)	6,999 (5,621-8,313)	7,764 (6,077-9,432)	8,764 (7,082-10,417)	9,185 (7,426-10,763)	10,772 (8,876-12,585)
Upper respiratory infections	23 (14-39)	8 (4-14)	234 (134-387)	169 (89-316)	12 (7-20)	10 (6-16)	22 (14-35)	20 (13-32)	23 (15-35)	23 (16-34)	27 (18-40)
Otitis media	1 (1-2)	5 (3-10)	33 (17-68)	51 (23-111)	33 (15-75)	28 (15-60)	13 (7-25)	10 (5-20)	20 (11-44)	10 (6-22)	11 (6-21)
Meningitis	3,469 (2,161-5,579)	4,726 (3,151-8,770)	34,489 (23,451-62,696)	27,874 (17,931-54,816)	6,739 (5,103-9,136)	5,521 (4,404-7,236)	5,218 (3,915-6,507)	4,237 (3,392-5,386)	3,758 (2,834-4,659)	3,482 (2,730-4,358)	3,325 (2,628-4,348)
Pneumococcal meningitis	136 (80-225)	188 (118-348)	1,460 (923-2,716)	1,258 (741-2,620)	330 (222-495)	267 (177-387)	243 (157-357)	188 (125-273)	192 (127-275)	232 (151-338)	266 (168-404)
H influenzae type B meningitis	357 (218-572)	492 (320-913)	3,539 (2,364-6,395)	2,559 (1,553-4,962)	520 (369-747)	359 (263-500)	300 (210-429)	238 (173-315)	226 (165-296)	223 (163-304)	220 (163-303)
Meningococcal infection	1,002 (631-1,562)	1,494 (1,014-2,689)	11,371 (7,806-20,026)	9,604 (6,179-18,556)	2,675 (2,048-3,736)	2,210 (1,707-2,965)	2,118 (1,598-2,744)	1,709 (1,359-2,318)	1,453 (1,109-1,893)	1,259 (969-1,627)	1,151 (903-1,526)
Other meningitis	1,974 (1,212-3,300)	2,553 (1,645-4,975)	18,119 (11,992-33,356)	14,453 (8,857-28,581)	3,215 (2,371-4,436)	2,685 (2,039-3,502)	2,556 (1,892-3,235)	2,101 (1,605-2,667)	1,886 (1,400-2,367)	1,768 (1,329-2,238)	1,689 (1,295-2,146)
Encephalitis	252 (180-322)	469 (354-653)	5,516 (4,184-7,598)	6,106 (4,215-9,727)	2,396 (1,561-3,997)	1,894 (1,266-3,206)	1,481 (1,062-2,391)	1,575 (1,158-2,499)	1,324 (991-2,141)	1,345 (1,019-2,135)	1,211 (929-1,960)
Diphtheria	-	-	130 (65-258)	252 (116-527)	40 (20-76)	20 (11-32)	9 (5-13)	5 (3-8)	5 (3-8)	5 (3-7)	7 (4-10)
Whooping cough	-	-	18,241 (9,648-32,168)	18,785 (10,214-32,524)	1,880 (947-3,344)	370 (184-673)	130 (65-241)	101 (51-183)	60 (29-111)	42 (21-79)	32 (16-59)
Tetanus	3,230 (1,765-4,481)	2,443 (1,546-3,450)	953 (559-1,568)	463 (212-1,126)	211 (96-495)	412 (142-897)	480 (187-995)	323 (140-593)	339 (148-626)	374 (187-647)	346 (175-655)
Measles	-	-	8,342 (2,996-18,444)	20,404 (7,744-43,193)	2,979 (1,054-6,566)	871 (305-2,008)	327 (114-765)	169 (60-393)	91 (32-212)	57 (21-133)	34 (12-80)
Varicella and herpes zoster	29 (22-37)	94 (72-124)	905 (688-1,174)	1,144 (859-1,526)	473 (346-628)	158 (122-210)	71 (55-95)	85 (66-110)	111 (86-141)	123 (97-157)	100 (77-127)
Neglected tropical diseases and malaria	468 (200-746)	7,005 (4,329-10,342)	89,579 (60,203-123,445)	175,525 (127,733-227,854)	25,766 (18,264-35,090)	13,146 (9,143-17,780)	7,117 (5,204-9,307)	7,120 (5,042-9,285)	5,295 (3,771-6,853)	5,032 (3,690-6,427)	5,870 (4,329-7,610)
Malaria	-	6,735 (4,066-10,051)	84,537 (54,749-117,956)	166,148 (118,433-218,932)	22,269 (14,930-31,530)	10,764 (7,181-15,476)	5,228 (3,707-7,331)	5,218 (3,582-7,223)	3,645 (2,516-5,254)	3,342 (2,298-4,778)	4,004 (2,829-5,790)
Chagas disease	-	-	1 (0-1)	1 (0-1)	1 (0-1)	1 (1-1)	3 (3-4)	6 (5-7)	11 (10-14)	23 (19-28)	44 (37-56)
Leishmaniasis	-	-	645 (304-1,225)	787 (348-1,500)	302 (137-608)	215 (96-420)	297 (134-553)	360 (166-677)	291 (130-552)	258 (119-498)	313 (140-582)
Visceral leishmaniasis	-	-	645 (304-1,225)	787 (348-1,500)	302 (137-608)	215 (96-420)	297 (134-553)	360 (166-677)	291 (130-552)	258 (119-498)	313 (140-582)
African trypanosomiasis	-	-	-	38 (5-174)	93 (15-363)	105 (18-380)	114 (19-417)	102 (19-416)	88 (15-326)	86 (16-312)	71 (13-284)
Schistosomiasis	-	-	0 (0-0)	143 (79-277)	76 (45-122)	112 (71-177)	93 (60-138)	107 (72-153)	131 (92-184)	161 (109-230)	206 (146-297)
Cysticercosis	0 (0-0)	0 (0-0)	0 (0-0)	39 (24-59)	25 (18-35)	21 (16-27)	16 (12-21)	20 (16-26)	29 (23-36)	35 (28-43)	31 (24-38)
Cystic echinococcosis	-	-	-	66 (43-101)	27 (18-40)	18 (12-27)	8 (5-12)	10 (7-14)	16 (12-22)	18 (12-25)	23 (17-31)
Dengue	-	-	1,068 (225-1,863)	1,855 (450-3,221)	1,150 (182-2,127)	890 (149-1,706)	751 (122-1,360)	817 (106-1,596)	692 (103-1,180)	650 (76-1,216)	615 (96-1,103)
Yellow fever	-	1 (0-2)	16 (3-53)	147 (27-430)	266 (50-783)	243 (49-727)	198 (38-591)	155 (28-483)	120 (22-343)	100 (19-307)	79 (15-245)
Rabies	-	-	70 (39-237)	535 (186-1,181)	636 (177-1,439)	344 (91-687)	184 (57-363)	140 (45-250)	112 (61-230)	205 (78-413)	326 (109-589)
Intestinal nematode infections	-	-	203 (151-275)	1,904 (1,351-2,664)	282 (213-368)	63 (48-80)	20 (14-27)	13 (9-17)	10 (7-14)	14 (10-19)	14 (10-19)
Ascariasis	-	-	203 (151-275)	1,904 (1,351-2,664)	282 (213-368)	63 (48-80)	20 (14-27)	13 (9-17)	10 (7-14)	14 (10-19)	14 (10-19)
Ebola	0 (0-0)	0 (0-0)	0 (0-0)	0 (0-0)	0 (0-0)	0 (0-0)	0 (0-0)	1 (1-1)	0 (0-0)	0 (0-0)	2 (2-2)
Zika virus	0 (0-0)	0 (0-0)	0 (0-1)	0 (0-3)	1 (0-3)	1 (0-4)	1 (0-5)	1 (0-6)	1 (0-8)	1 (0-7)	1 (0-6)
Other neglected tropical diseases	468 (200-746)	269 (136-502)	3,038 (1,331-5,301)	3,862 (1,429-7,330)	638 (279-1,342)	368 (168-726)	204 (101-396)	169 (89-290)	148 (83-229)	138 (74-234)	142 (78-216)
Maternal disorders	-	-	-	-	-	1,059 (858-1,303)	20,593 (18,205-23,483)	41,368 (36,979-46,486)	44,722 (40,454-49,535)	42,727 (38,743-46,817)	37,739 (34,334-41,531)
Maternal haemorrhage	-	-	-	-	-	202 (126-295)	5,395 (3,439-7,844)	12,802 (9,001-17,118)	14,607 (10,244-19,416)	14,166 (9,951-18,356)	12,900 (9,282-16,730)
Maternal sepsis and other pregnancy related infections	-	-	-	-	-	94 (57-146)	2,039 (1,189-3,198)	3,716 (2,117-5,855)	3,751 (2,315-5,600)	3,426 (2,057-5,289)	2,965 (1,867-4,619)
Maternal hypertensive disorders	-	-	-	-	-	233 (141-345)	3,558 (2,242-5,053)	6,053 (4,058-8,517)	5,990 (3,850-8,753)	5,531 (3,459-8,318)	4,966 (3,350-7,120)
Maternal obstructed labour and uterine rupture	-	-	-	-	-	40 (20-70)	728 (347-1,261)	1,464 (816-2,416)	1,754 (959-2,897)	1,919 (1,046-3,093)	1,877 (1,022-3,158)
Maternal abortion, miscarriage, and ectopic pregnancy	-	-	-	-	-	95 (53-153)	1,774 (974-2,761)	3,372 (2,048-5,377)	3,453 (2,132-5,382)	3,459 (2,096-5,215)	3,163 (1,824-4,692)
Indirect maternal deaths	-	-	-	-	-	184 (109-278)	3,248 (1,970-4,986)	6,320 (4,071-9,368)	7,266 (4,617-10,562)	6,866 (4,183-9,945)	5,255 (3,403-7,520)
Late maternal deaths	-	-	-	-	-	23 (10-49)	387 (173-733)	724 (331-1,377)	791 (387-1,489)	757 (390-1,376)	659 (293-1,206)
Maternal deaths aggravated by HIV/AIDS	-	-	-	-	-	2 (1-3)	44 (24-65)	180 (106-255)	357 (226-484)	456 (293-607)	441 (282-592)
Other maternal disorders	-	-	-	-	-	186 (120-282)	3,421 (2,131-5,274)	6,737 (4,250-9,884)	6,753 (4,502-9,629)	6,148 (3,907-8,858)	5,513 (3,504-8,356)
Neonatal disorders	581,806 (551,030-615,162)	123,610 (115,582-132,614)	38,623 (32,632-45,190)	4,410 (3,424-5,406)	-	-	-	-	-	-	-
Neonatal preterm birth complications	218,494 (194,307-246,824)	34,261 (27,914-40,103)	12,238 (9,633-14,898)	1,104 (716-1,422)	-	-	-	-	-	-	-
Neonatal encephalopathy due to birth asphyxia and trauma	190,200 (164,565-213,973)	18,224 (13,409-22,172)	8,041 (5,468-10,924)	1,794 (1,369-2,188)	-	-	-	-	-	-	-
Neonatal sepsis and other neonatal infections	56,999 (38,432-100,215)	40,113 (32,652-54,602)	7,940 (5,129-11,359)	370 (141-609)	-	-	-	-	-	-	-
Hemolytic disease and other neonatal jaundice	13,457 (10,790-16,849)	6,478 (5,063-8,467)	1,556 (1,026-2,456)	76 (51-121)	-	-	-	-	-	-	-
Other neonatal disorders	102,656 (89,063-117,105)	24,534 (19,537-28,997)	8,849 (6,822-11,246)	1,067 (851-1,353)	-	-	-	-	-	-	-
Nutritional deficiencies	-	-	37,930 (32,140-45,721)	42,220 (32,180-56,176)	3,841 (3,055-4,703)	1,965 (1,635-2,315)	1,653 (1,441-1,884)	1,846 (1,636-2,093)	2,123 (1,889-2,369)	2,080 (1,892-2,278)	2,031 (1,832-2,245)
Protein-energy malnutrition	-	-	36,564 (31,019-44,284)	40,035 (30,163-54,204)	3,348 (2,642-4,112)	1,568 (1,284-1,895)	1,023 (873-1,223)	831 (707-997)	1,072 (926-1,268)	968 (849-1,136)	957 (832-1,117)
Iodine deficiency	-	-	172 (32-531)	143 (17-676)	37 (8-113)	12 (2-42)	26 (5-76)	31 (7-93)	35 (8-103)	40 (9-115)	41 (10-117)
Iron-deficiency anaemia	-	-	34 (16-92)	72 (33-203)	59 (35-126)	54 (36-95)	62 (45-93)	78 (60-111)	69 (50-107)	69 (55-98)	76 (52-135)
Other nutritional deficiencies	-	-	1,160 (784-1,594)	1,970 (952-3,258)	398 (290-548)	331 (254-428)	542 (436-648)	906 (761-1,067)	947 (787-1,123)	1,003 (854-1,158)	956 (813-1,113)
Other communicable, maternal, neonatal, and nutritional diseases	13,559 (8,187-20,757)	5,447 (3,445-8,097)	25,577 (16,472-37,026)	19,319 (12,929-27,835)	6,523 (4,529-9,116)	2,022 (1,731-2,338)	3,320 (2,884-3,782)	4,001 (3,576-4,492)	3,712 (3,326-4,086)	3,807 (3,444-4,208)	4,092 (3,670-4,526)
Sexually transmitted diseases excluding HIV	12,341 (7,090-19,423)	4,357 (2,396-7,086)	19,616 (10,806-30,975)	13,543 (7,425-21,667)	4,381 (2,453-6,869)	78 (61-101)	402 (318-500)	522 (431-652)	691 (573-804)	570 (475-671)	603 (504-720)
Syphilis	12,341 (7,090-19,423)	4,357 (2,396-7,086)	19,616 (10,806-30,975)	13,543 (7,425-21,667)	4,381 (2,453-6,869)	42 (33-55)	113 (90-141)	146 (121-183)	142 (117-165)	117 (98-138)	74 (62-88)
Chlamydial infection	-	-	-	-	-	10 (8-13)	52 (41-65)	113 (93-141)	116 (96-135)	60 (50-71)	97 (81-116)
Gonococcal infection	-	-	-	-	-	17 (14-23)	129 (101-160)	163 (135-204)	301 (249-350)	256 (213-301)	267 (223-319)
Other sexually transmitted diseases	-	-	-	-	-	8 (6-11)	108 (85-134)	100 (83-125)	133 (110-154)	137 (114-161)	165 (138-197)
Hepatitis	-	-	2,517 (2,150-2,914)	2,515 (2,051-2,999)	1,271 (1,022-1,586)	1,378 (1,133-1,667)	2,324 (1,967-2,679)	2,927 (2,571-3,301)	2,521 (2,225-2,800)	2,676 (2,377-3,007)	2,997 (2,631-3,368)
Acute hepatitis A	-	-	1,603 (1,087-2,175)	293 (146-487)	32 (16-54)	22 (12-34)	39 (20-64)	51 (25-88)	34 (13-64)	27 (12-52)	27 (11-49)
Hepatitis B	-	-	914 (444-1,452)	328 (185-514)	110 (57-181)	206 (110-336)	811 (547-1,166)	1,807 (1,314-2,359)	1,967 (1,591-2,348)	2,316 (1,952-2,665)	2,683 (2,323-3,044)
Hepatitis C	-	-	-	-	40 (24-62)	17 (11-26)	27 (17-42)	35 (18-59)	25 (11-45)	23 (10-42)	30 (13-56)
Acute hepatitis E	-	-	-	1,894 (1,500-2,367)	1,090 (853-1,375)	1,133 (895-1,409)	1,447 (1,086-1,796)	1,034 (586-1,540)	496 (243-796)	309 (143-563)	257 (119-462)
Other infectious diseases	1,219 (719-1,712)	1,090 (629-1,685)	3,445 (2,484-4,833)	3,260 (2,154-4,668)	871 (637-1,183)	566 (429-734)	594 (449-793)	553 (442-721)	500 (393-628)	561 (436-702)	492 (395-606)
Non-communicable diseases	70,279 (57,263-82,627)	28,609 (25,733-32,081)	116,348 (102,213-133,820)	75,379 (62,496-90,811)	33,638 (30,286-37,521)	39,620 (36,288-43,296)	60,051 (56,443-63,678)	77,808 (74,032-81,802)	106,351 (102,148-111,302)	148,585 (142,711-155,042)	209,668 (201,639-219,405)
Neoplasms	345 (303-374)	218 (197-239)	2,393 (2,156-2,610)	9,180 (8,279-10,112)	10,130 (9,108-11,083)	11,015 (10,072-11,841)	13,995 (12,852-14,808)	18,449 (17,033-19,375)	29,878 (27,881-31,184)	49,650 (46,983-51,707)	79,177 (75,856-82,437)
Lip and oral cavity cancer	-	-	-	-	-	-	155 (138-175)	295 (263-332)	449 (401-512)	756 (686-848)	1,260 (1,147-1,396)
Nasopharynx cancer	-	-	-	-	36 (32-41)	59 (52-67)	126 (112-143)	163 (150-179)	266 (243-295)	383 (351-422)	542 (506-582)
Other pharynx cancer	-	-	-	-	-	-	148 (123-175)	214 (181-253)	353 (307-415)	540 (474-615)	827 (735-934)
Oesophageal cancer	-	-	-	-	-	-	89 (81-100)	211 (190-234)	359 (320-408)	590 (540-650)	1,077 (999-1,172)
Stomach cancer	-	-	-	-	-	-	329 (298-365)	857 (795-935)	1,828 (1,722-1,949)	3,452 (3,264-3,694)	5,524 (5,233-5,858)
Colon and rectum cancer	-	-	-	-	-	-	286 (255-323)	745 (674-838)	1,500 (1,385-1,625)	2,985 (2,779-3,191)	4,466 (4,210-4,722)
Liver cancer	-	-	-	-	144 (124-168)	201 (172-231)	350 (303-419)	638 (561-748)	1,423 (1,237-1,678)	2,358 (2,074-2,727)	3,474 (3,098-3,940)
Liver cancer due to hepatitis B	-	-	-	-	51 (36-68)	90 (67-115)	176 (131-228)	400 (326-488)	980 (777-1,214)	1,592 (1,298-1,943)	2,199 (1,765-2,658)
Liver cancer due to hepatitis C	-	-	-	-	6 (5-8)	11 (8-15)	21 (15-28)	52 (38-70)	122 (76-180)	233 (159-315)	381 (271-534)
Liver cancer due to alcohol use	-	-	-	-	-	-	3 (1-4)	18 (11-27)	63 (33-105)	197 (123-297)	479 (283-747)
Liver cancer due to other causes	-	-	-	-	86 (70-105)	100 (77-124)	150 (112-192)	168 (127-216)	258 (175-365)	336 (237-452)	415 (308-556)
Gallbladder and biliary tract cancer	-	-	-	-	-	-	40 (37-43)	74 (68-80)	189 (174-204)	508 (459-546)	1,113 (988-1,227)
Pancreatic cancer	-	-	-	-	-	-	62 (58-68)	121 (112-131)	302 (282-324)	580 (549-615)	1,170 (1,115-1,231)
Larynx cancer	-	-	-	-	-	-	41 (38-45)	64 (57-73)	89 (77-105)	190 (161-225)	291 (267-322)
Tracheal, bronchus, and lung cancer	-	-	-	-	-	-	313 (272-342)	625 (563-683)	1,350 (1,221-1,478)	2,946 (2,527-3,347)	4,465 (4,133-4,786)
Malignant skin melanoma	-	-	-	-	-	-	90 (78-114)	187 (159-237)	339 (287-420)	509 (430-631)	744 (623-935)
Non-melanoma skin cancer	-	-	-	-	-	-	52 (49-56)	67 (63-71)	103 (98-110)	125 (119-133)	189 (177-202)
Non-melanoma skin cancer (squamous-cell carcinoma)	-	-	-	-	-	-	52 (49-56)	67 (63-71)	103 (98-110)	125 (119-133)	189 (177-202)
Breast cancer	-	-	-	-	-	-	339 (270-391)	1,058 (903-1,216)	3,530 (3,108-3,936)	9,721 (8,998-10,552)	19,682 (18,457-21,934)
Cervical cancer	-	-	-	-	-	-	165 (118-183)	727 (523-819)	2,500 (1,907-2,710)	6,167 (4,886-6,684)	11,551 (9,083-12,516)
Uterine cancer	-	-	-	-	-	-	65 (59-73)	165 (149-184)	334 (307-362)	608 (565-662)	1,183 (1,097-1,279)
Ovarian cancer	-	-	-	-	-	-	500 (444-561)	761 (682-834)	1,259 (1,153-1,364)	1,971 (1,823-2,117)	3,520 (3,269-3,755)
Prostate cancer	-	-	-	-	-	-	-	-	-	-	-
Testicular cancer	-	-	-	-	-	-	-	-	-	-	-
Kidney cancer	13 (12-14)	6 (6-7)	101 (90-112)	520 (448-607)	299 (265-333)	162 (150-175)	108 (101-116)	148 (139-159)	230 (216-244)	293 (278-310)	459 (435-485)
Bladder cancer	-	-	-	-	-	-	43 (39-48)	53 (47-60)	83 (74-92)	181 (164-200)	304 (279-331)
Brain and nervous system cancer	43 (33-50)	28 (21-32)	385 (295-446)	1,678 (1,387-1,941)	2,430 (2,024-2,820)	2,139 (1,796-2,488)	1,591 (1,337-1,844)	1,764 (1,454-2,004)	2,353 (1,966-2,676)	2,780 (2,358-3,147)	3,590 (3,051-4,125)
Thyroid cancer	-	-	-	-	-	57 (47-69)	149 (115-198)	277 (231-344)	357 (307-424)	451 (404-511)	559 (507-619)
Mesothelioma	-	-	-	-	-	-	42 (35-49)	58 (48-68)	96 (82-110)	111 (95-129)	183 (157-213)
Hodgkin lymphoma	1 (0-1)	1 (0-1)	13 (9-17)	64 (38-102)	132 (79-194)	275 (153-376)	413 (272-512)	519 (371-663)	604 (424-729)	572 (414-680)	558 (388-673)
Non-Hodgkin's lymphoma	19 (17-21)	14 (12-17)	254 (215-296)	1,200 (861-1,495)	1,475 (1,116-1,802)	1,356 (1,096-1,595)	1,159 (1,016-1,310)	1,381 (1,239-1,533)	1,750 (1,569-1,912)	2,021 (1,773-2,210)	2,374 (2,108-2,552)
Multiple myeloma	-	-	-	-	-	-	109 (93-144)	157 (123-184)	179 (141-207)	217 (171-249)	399 (319-449)
Leukaemia	67 (51-76)	53 (43-62)	821 (668-955)	3,075 (2,647-3,765)	3,469 (2,953-4,248)	3,752 (3,223-4,422)	3,534 (2,883-4,146)	3,337 (2,660-3,765)	3,688 (2,938-4,214)	3,668 (2,924-4,092)	4,005 (3,231-4,591)
Acute lymphoid leukaemia	20 (16-25)	15 (13-21)	274 (225-369)	1,298 (1,084-1,723)	1,607 (1,349-2,152)	1,563 (1,342-2,020)	1,150 (954-1,472)	912 (709-1,111)	962 (755-1,152)	719 (565-868)	781 (628-958)
Chronic lymphoid leukaemia	-	-	-	-	-	-	70 (58-95)	88 (68-113)	116 (93-146)	133 (110-175)	203 (163-276)
Acute myeloid leukaemia	13 (10-16)	11 (9-14)	157 (128-206)	674 (532-871)	613 (478-777)	840 (668-1,047)	891 (730-1,099)	866 (697-1,055)	954 (738-1,131)	992 (808-1,166)	1,185 (935-1,377)
Chronic myeloid leukaemia	-	-	-	-	-	-	149 (107-194)	165 (117-216)	304 (222-388)	341 (250-438)	353 (243-465)
Other leukaemia	34 (22-40)	27 (18-31)	390 (266-448)	1,103 (820-1,327)	1,249 (977-1,483)	1,350 (1,011-1,545)	1,274 (977-1,445)	1,305 (953-1,468)	1,353 (939-1,531)	1,484 (1,106-1,648)	1,483 (1,114-1,687)
Other neoplasms	202 (182-222)	116 (103-130)	819 (723-945)	2,643 (2,327-3,030)	2,145 (1,920-2,395)	3,013 (2,665-3,326)	3,698 (3,287-4,095)	3,781 (3,350-4,164)	4,363 (3,901-4,731)	4,965 (4,424-5,370)	5,669 (5,119-6,046)
Cardiovascular diseases	1,088 (995-1,185)	678 (593-796)	6,528 (5,906-7,353)	5,263 (4,689-5,942)	3,455 (3,071-3,933)	5,010 (4,575-5,609)	12,745 (11,756-13,919)	19,207 (17,861-20,647)	28,301 (26,739-30,044)	42,472 (40,279-44,674)	62,318 (59,445-65,358)
Rheumatic heart disease	-	-	-	956 (775-1,197)	724 (591-878)	1,623 (1,379-1,938)	2,806 (2,374-3,241)	3,193 (2,726-3,640)	3,744 (3,261-4,285)	4,571 (4,005-5,249)	5,745 (5,076-6,621)
Ischaemic heart disease	-	-	334 (279-438)	241 (195-306)	249 (207-307)	346 (297-402)	4,131 (3,723-4,666)	6,755 (6,222-7,391)	10,658 (9,962-11,492)	17,905 (16,824-19,051)	26,105 (24,675-27,625)
Cerebrovascular disease	380 (326-453)	220 (185-274)	1,947 (1,612-2,360)	1,410 (1,157-1,727)	1,025 (913-1,176)	1,307 (1,189-1,451)	2,434 (2,251-2,637)	4,705 (4,375-5,097)	7,232 (6,784-7,716)	11,462 (10,810-12,118)	19,035 (17,944-20,196)

Cause	40 to 44	45 to 49	50 to 54	55 to 59	60 to 64	65 to 69	70 to 74	75 to 79	80 to 84	85 to 89	90 to 94	95 plus
All causes	538,645 (526,568-552,828)	709,873 (695,953-727,112)	961,794 (942,043-986,977)	1,208,618 (1,181,341-1,243,604)	1,638,121 (1,603,930-1,681,313)	1,936,610 (1,896,582-1,989,405)	2,181,546 (2,142,519-2,232,141)	2,704,327 (2,623,944-2,811,966)	2,928,052 (2,869,163-3,002,711)	2,901,815 (2,842,626-2,974,765)	1,907,249 (1,884,812-1,932,224)	768,095 (762,494-774,517)
Communicable, maternal, neonatal, and nutritional disorders	147,359 (140,207-156,035)	131,503 (123,125-143,273)	130,720 (120,365-148,540)	138,378 (123,155-164,738)	167,445 (146,242-205,212)	196,447 (168,572-247,616)	242,400 (201,621-310,425)	256,784 (212,565-330,591)	284,531 (235,816-358,139)	246,937 (209,025-304,943)	148,200 (128,372-173,940)	72,878 (62,843-83,739)
HIV/AIDS and tuberculosis	78,749 (74,706-82,772)	67,719 (63,398-72,388)	59,711 (55,811-63,818)	50,792 (46,861-55,259)	45,679 (42,068-49,668)	43,702 (39,562-47,817)	44,777 (39,852-49,198)	38,351 (34,235-42,459)	32,994 (28,995-36,667)	19,960 (17,543-22,241)	7,235 (6,468-8,184)	2,056 (1,811-2,377)
Tuberculosis	23,082 (21,223-24,883)	26,903 (24,663-29,033)	33,018 (30,150-35,867)	38,218 (34,567-41,906)	38,405 (35,012-42,133)	39,413 (35,326-43,412)	42,526 (37,566-47,001)	37,182 (33,050-41,284)	32,573 (28,563-36,232)	19,763 (17,318-22,047)	7,175 (6,413-8,127)	2,037 (1,793-2,359)
Drug-susceptible tuberculosis	21,027 (19,236-22,719)	24,639 (22,506-26,718)	30,194 (27,541-32,857)	34,981 (31,570-38,510)	35,131 (31,931-38,711)	36,127 (32,283-39,751)	39,016 (34,336-43,230)	34,131 (30,445-37,932)	29,836 (26,188-33,144)	18,124 (15,976-20,099)	6,606 (5,874-7,430)	1,882 (1,659-2,176)
Multidrug-resistant tuberculosis without extensive drug resistance	1,844 (1,517-2,212)	2,053 (1,695-2,485)	2,566 (2,091-3,073)	2,957 (2,394-3,564)	2,985 (2,395-3,640)	3,011 (2,404-3,704)	3,234 (2,592-4,030)	2,782 (2,226-3,424)	2,486 (1,952-3,121)	1,466 (1,144-1,846)	502 (388-661)	134 (98-191)
Extensively drug-resistant tuberculosis	212 (166-269)	212 (171-265)	257 (204-320)	280 (222-346)	289 (232-357)	275 (220-342)	276 (218-350)	269 (215-339)	251 (192-332)	173 (129-237)	67 (48-100)	21 (14-34)
HIV/AIDS	55,666 (51,973-59,431)	40,816 (36,913-44,938)	26,694 (24,093-29,634)	12,574 (11,118-14,322)	7,273 (6,369-8,327)	4,289 (3,732-4,927)	2,251 (1,897-2,637)	1,169 (975-1,382)	421 (339-523)	197 (158-242)	60 (49-72)	18 (16-21)
Drug-susceptible HIV/AIDS - Tuberculosis	10,782 (7,225-14,732)	8,424 (5,754-11,423)	6,281 (4,361-8,272)	3,060 (2,177-3,947)	1,760 (1,283-2,239)	1,019 (752-1,285)	592 (436-747)	315 (233-398)	116 (84-156)	52 (37-70)	14 (10-19)	3 (2-4)
Multidrug-resistant HIV/AIDS - Tuberculosis without extensive drug resistance	821 (494-1,260)	648 (390-995)	495 (304-746)	245 (150-367)	143 (90-214)	81 (51-123)	46 (28-70)	24 (15-37)	9 (5-14)	4 (2-6)	1 (1-2)	0 (0-0)
Extensively drug-resistant HIV/AIDS - Tuberculosis	47 (28-74)	34 (21-53)	25 (15-39)	13 (8-21)	8 (5-13)	4 (3-7)	2 (1-3)	1 (1-1)	0 (0-1)	0 (0-0)	0 (0-0)	0 (0-0)
HIV/AIDS resulting in other diseases	44,016 (38,696-49,306)	31,709 (26,896-36,745)	19,893 (16,747-23,371)	9,256 (7,724-11,255)	5,362 (4,484-6,495)	3,184 (2,671-3,841)	1,611 (1,317-1,988)	829 (674-1,038)	296 (235-378)	140 (112-180)	44 (36-55)	15 (13-17)
Diarrhoea, lower respiratory infections, and other common infectious diseases	30,649 (24,648-41,650)	37,649 (29,866-52,421)	52,203 (41,501-72,575)	69,604 (53,870-100,313)	100,608 (77,324-142,510)	129,997 (99,737-185,609)	175,979 (132,944-249,194)	195,057 (149,157-274,118)	228,994 (177,852-307,038)	206,193 (166,208-266,781)	126,903 (107,244-153,736)	63,610 (53,896-74,732)
Diarrhoeal diseases	11,506 (5,202-23,131)	14,736 (6,941-30,493)	20,561 (9,900-41,794)	29,310 (14,194-61,427)	45,024 (21,903-90,354)	59,074 (28,921-118,462)	84,483 (42,495-161,758)	92,380 (46,022-173,410)	106,001 (56,654-188,386)	82,298 (45,767-144,597)	38,820 (23,014-65,623)	12,970 (8,021-21,191)
Intestinal infectious diseases	1,950 (1,066-3,274)	1,805 (964-3,059)	1,502 (773-2,598)	1,151 (547-2,095)	1,024 (495-1,831)	966 (527-1,576)	843 (487-1,341)	719 (418-1,149)	452 (257-734)	219 (131-360)	71 (39-116)	20 (10-41)
Typhoid fever	1,436 (740-2,447)	1,295 (667-2,215)	1,086 (540-1,879)	844 (392-1,566)	777 (370-1,419)	770 (416-1,284)	685 (387-1,085)	572 (320-922)	361 (204-592)	163 (93-263)	49 (27-80)	11 (6-19)
Paratyphoid fever	494 (225-946)	484 (216-969)	406 (173-824)	295 (118-622)	233 (95-503)	178 (80-358)	137 (63-266)	111 (52-205)	68 (32-125)	29 (14-56)	8 (4-16)	2 (1-4)
Other intestinal infectious diseases	19 (2-88)	25 (2-118)	11 (1-46)	13 (1-58)	14 (1-69)	19 (2-101)	21 (2-94)	36 (3-161)	24 (3-102)	26 (3-105)	14 (2-53)	7 (1-28)
Lower respiratory infections	12,230 (10,077-14,104)	15,146 (12,515-17,564)	23,620 (19,847-27,161)	32,048 (25,922-36,407)	47,127 (37,594-53,200)	61,836 (49,338-70,078)	81,569 (62,916-93,837)	94,502 (72,668-108,010)	114,651 (93,093-131,579)	118,517 (99,717-136,527)	85,799 (72,256-98,876)	49,781 (41,948-57,485)
Upper respiratory infections	29 (20-40)	37 (26-54)	42 (30-60)	47 (33-67)	55 (39-77)	50 (36-71)	42 (30-59)	48 (35-67)	39 (29-53)	35 (25-48)	18 (13-24)	5 (4-7)
Otitis media	20 (10-44)	16 (9-33)	17 (11-29)	37 (22-70)	29 (18-50)	49 (29-91)	42 (26-73)	34 (23-53)	31 (21-48)	20 (15-29)	10 (8-14)	4 (3-5)
Meningitis	3,025 (2,376-3,882)	3,707 (2,834-4,702)	3,964 (3,120-5,257)	4,273 (3,449-5,431)	4,160 (3,505-5,649)	4,341 (3,590-6,119)	5,027 (4,157-6,809)	3,278 (2,690-4,454)	3,638 (3,000-4,827)	2,129 (1,779-2,853)	833 (696-1,120)	276 (237-348)
Pneumococcal meningitis	283 (176-456)	369 (225-573)	395 (265-582)	447 (309-633)	454 (324-659)	465 (324-701)	534 (355-799)	355 (250-518)	349 (241-509)	206 (156-278)	93 (73-120)	33 (24-45)
H influenzae type B meningitis	203 (146-279)	251 (177-341)	264 (191-371)	285 (207-390)	278 (207-386)	276 (201-392)	316 (241-425)	216 (161-309)	235 (176-320)	140 (105-193)	55 (40-77)	18 (12-26)
Meningococcal infection	1,116 (860-1,441)	1,316 (1,051-1,704)	1,463 (1,159-1,967)	1,563 (1,232-2,082)	1,537 (1,218-2,207)	1,528 (1,210-2,296)	1,614 (1,270-2,411)	1,196 (939-1,742)	1,270 (993-1,742)	820 (656-1,140)	336 (268-469)	110 (85-149)
Other meningitis	1,423 (1,113-1,791)	1,771 (1,340-2,257)	1,843 (1,408-2,450)	1,979 (1,536-2,533)	1,891 (1,463-2,542)	2,072 (1,615-2,757)	2,562 (1,994-3,287)	1,511 (1,191-1,992)	1,784 (1,383-2,257)	963 (770-1,277)	350 (282-470)	116 (90-149)
Encephalitis	1,348 (1,048-2,091)	1,582 (1,234-2,475)	1,759 (1,343-2,811)	2,127 (1,592-3,467)	2,735 (2,059-4,293)	2,912 (2,179-4,599)	3,243 (2,504-4,930)	3,150 (2,488-4,563)	3,245 (2,487-4,528)	2,102 (1,634-2,863)	743 (578-1,018)	229 (182-311)
Diphtheria	7 (5-11)	9 (7-13)	12 (8-16)	10 (7-13)	-	-	-	-	-	-	-	-
Whooping cough	26 (13-49)	25 (13-46)	26 (13-48)	28 (15-51)	-	-	-	-	-	-	-	-
Tetanus	422 (227-782)	491 (256-868)	619 (299-1,060)	480 (237-896)	351 (179-620)	583 (313-1,104)	548 (264-878)	626 (283-1,076)	404 (159-719)	159 (51-240)	52 (17-83)	15 (5-25)
Measles	21 (8-49)	13 (5-31)	10 (4-23)	8 (3-18)	-	-	-	-	-	-	-	-
Varicella and herpes zoster	65 (51-84)	79 (63-100)	69 (55-88)	84 (67-110)	102 (80-131)	186 (146-233)	183 (143-237)	319 (249-407)	533 (415-692)	715 (537-924)	557 (383-795)	309 (189-491)
Neglected tropical diseases and malaria	5,703 (4,161-7,420)	5,909 (4,178-7,669)	6,851 (4,933-8,884)	7,334 (5,297-9,368)	9,544 (6,741-12,603)	8,740 (6,235-11,374)	7,648 (5,478-10,241)	6,454 (4,677-8,471)	4,237 (3,083-5,576)	2,870 (2,017-3,845)	872 (575-1,164)	439 (247-919)
Malaria	3,854 (2,626-5,391)	3,731 (2,527-5,405)	4,316 (2,897-6,152)	4,689 (3,248-6,605)	6,808 (4,497-9,881)	5,854 (3,818-8,534)	5,483 (3,557-8,080)	4,436 (2,938-6,499)	2,680 (1,752-3,845)	1,786 (1,131-2,639)	407 (234-596)	302 (137-766)
Chagas disease	70 (60-87)	106 (90-130)	177 (153-218)	264 (222-322)	325 (282-403)	384 (325-470)	414 (358-510)	437 (377-542)	402 (355-488)	273 (237-335)	141 (122-170)	41 (36-50)
Leishmaniasis	319 (144-589)	301 (135-561)	310 (147-574)	264 (115-496)	295 (131-566)	426 (196-809)	331 (143-625)	300 (132-551)	187 (86-353)	137 (62-256)	18 (6-36)	9 (3-19)
Visceral leishmaniasis	319 (144-589)	301 (135-561)	310 (147-574)	264 (115-496)	295 (131-566)	426 (196-809)	331 (143-625)	300 (132-551)	187 (86-353)	137 (62-256)	18 (6-36)	9 (3-19)
African trypanosomiasis	63 (11-277)	64 (10-274)	53 (9-199)	53 (9-212)	47 (7-227)	35 (5-143)	23 (3-101)	15 (2-61)	8 (1-33)	3 (0-12)	1 (0-3)	0 (0-1)
Schistosomiasis	236 (166-326)	284 (196-407)	362 (256-516)	456 (328-628)	477 (335-671)	518 (361-717)	465 (332-646)	368 (259-512)	217 (153-307)	113 (82-152)	34 (23-49)	7 (4-11)
Cysticercosis	31 (26-38)	33 (27-40)	43 (36-52)	43 (36-52)	34 (28-41)	31 (26-37)	20 (16-24)	13 (11-16)	8 (7-11)	5 (4-6)	2 (1-2)	1 (1-1)
Cystic echinococcosis	21 (15-28)	26 (19-35)	29 (22-38)	34 (25-44)	35 (27-45)	35 (27-45)	29 (22-37)	27 (21-34)	20 (16-25)	17 (13-21)	7 (5-8)	2 (1-3)
Dengue	625 (90-1,143)	781 (103-1,415)	924 (114-1,686)	999 (157-1,771)	1,090 (126-1,965)	923 (133-1,571)	475 (72-893)	480 (71-904)	468 (73-901)	357 (64-710)	190 (34-396)	42 (11-84)
Yellow fever	64 (12-205)	52 (10-161)	44 (8-141)	36 (7-107)	27 (5-85)	19 (4-57)	13 (2-40)	8 (1-24)	3 (1-12)	1 (0-4)	0 (0-1)	0 (0-0)
Rabies	272 (125-509)	369 (155-684)	377 (199-821)	290 (113-538)	176 (62-258)	263 (90-445)	152 (77-329)	130 (66-337)	67 (11-118)	62 (9-104)	21 (2-37)	8 (1-15)
Intestinal nematode infections	11 (8-15)	13 (10-18)	22 (16-28)	20 (15-26)	24 (17-31)	18 (13-23)	26 (20-34)	22 (17-28)	16 (12-22)	7 (5-10)	5 (4-7)	2 (1-3)
Ascariasis	11 (8-15)	13 (10-18)	22 (16-28)	20 (15-26)	24 (17-31)	18 (13-23)	26 (20-34)	22 (17-28)	16 (12-22)	7 (5-10)	5 (4-7)	2 (1-3)
Ebola	0 (0-0)	0 (0-0)	0 (0-0)	0 (0-0)	0 (0-0)	0 (0-0)	0 (0-0)	0 (0-0)	0 (0-0)	0 (0-0)	0 (0-0)	0 (0-0)
Zika virus	1 (0-7)	1 (0-5)	1 (0-4)	1 (0-3)	0 (0-2)	0 (0-2)	0 (0-1)	0 (0-1)	0 (0-0)	0 (0-0)	0 (0-0)	0 (0-0)
Other neglected tropical diseases	137 (77-206)	149 (81-222)	192 (108-296)	186 (101-283)	206 (115-313)	235 (132-373)	217 (113-341)	218 (125-346)	160 (99-260)	110 (65-184)	46 (28-70)	24 (16-42)
Maternal disorders	25,437 (22,818-28,372)	13,699 (11,816-15,843)	3,269 (2,448-4,286)	-	-	-	-	-	-	-	-	-
Maternal haemorrhage	8,120 (5,377-11,206)	3,552 (2,432-5,022)	655 (352-1,059)	-	-	-	-	-	-	-	-	-
Maternal sepsis and other pregnancy related infections	2,054 (1,216-3,378)	1,229 (725-1,960)	261 (141-469)	-	-	-	-	-	-	-	-	-
Maternal hypertensive disorders	3,324 (2,005-5,029)	1,592 (994-2,353)	357 (171-622)	-	-	-	-	-	-	-	-	-
Maternal obstructed labour and uterine rupture	1,440 (753-2,487)	836 (434-1,438)	193 (72-367)	-	-	-	-	-	-	-	-	-
Maternal abortion, miscarriage, and ectopic pregnancy	2,431 (1,517-3,650)	1,634 (1,060-2,403)	316 (192-485)	-	-	-	-	-	-	-	-	-
Indirect maternal deaths	3,567 (2,193-5,362)	2,268 (1,320-3,364)	770 (430-1,230)	-	-	-	-	-	-	-	-	-
Late maternal deaths	443 (142-808)	254 (86-466)	75 (26-167)	-	-	-	-	-	-	-	-	-
Maternal deaths aggravated by HIV/AIDS	326 (199-442)	180 (113-258)	31 (18-47)	-	-	-	-	-	-	-	-	-
Other maternal disorders	3,733 (2,192-5,820)	2,154 (1,329-3,179)	611 (366-986)	-	-	-	-	-	-	-	-	-
Neonatal disorders	-	-	-	-	-	-	-	-	-	-	-	-
Neonatal preterm birth complications	-	-	-	-	-	-	-	-	-	-	-	-
Neonatal encephalopathy due to birth asphyxia and trauma	-	-	-	-	-	-	-	-	-	-	-	-
Neonatal sepsis and other neonatal infections	-	-	-	-	-	-	-	-	-	-	-	-
Hemolytic disease and other neonatal jaundice	-	-	-	-	-	-	-	-	-	-	-	-
Other neonatal disorders	-	-	-	-	-	-	-	-	-	-	-	-
Nutritional deficiencies	2,623 (2,413-2,856)	2,263 (2,050-2,499)	3,194 (2,910-3,501)	4,404 (4,002-4,787)	5,434 (4,920-5,915)	7,282 (6,653-7,911)	7,669 (7,078-8,266)	11,100 (10,209-11,952)	12,874 (12,003-13,753)	14,232 (13,329-15,162)	11,385 (10,657-12,128)	6,132 (5,734-6,544)
Protein-energy malnutrition	1,538 (1,374-1,734)	1,039 (895-1,208)	1,557 (1,357-1,818)	2,724 (2,432-3,119)	3,384 (3,020-3,830)	4,533 (4,075-5,114)	5,227 (4,768-5,789)	8,093 (7,371-8,870)	9,638 (8,886-10,560)	11,146 (10,317-12,125)	9,390 (8,480-10,166)	5,135 (4,546-5,574)
Iodine deficiency	34 (9-96)	32 (9-99)	48 (14-144)	65 (19-193)	68 (20-191)	97 (31-256)	96 (31-270)	103 (34-260)	82 (25-212)	53 (15-137)	22 (4-72)	12 (2-43)
Iron-deficiency anaemia	59 (49-88)	55 (46-79)	63 (47-117)	60 (43-83)	56 (41-78)	57 (38-83)	106 (60-193)	142 (78-277)	132 (81-234)	120 (46-207)	97 (32-167)	90 (40-141)
Other nutritional deficiencies	991 (843-1,139)	1,136 (963-1,321)	1,526 (1,298-1,750)	1,556 (1,291-1,833)	1,926 (1,582-2,258)	2,595 (2,132-3,047)	2,240 (1,861-2,632)	2,762 (2,280-3,262)	3,021 (2,447-3,651)	2,912 (2,401-3,617)	1,876 (1,467-2,757)	894 (685-1,568)
Other communicable, maternal, neonatal, and nutritional diseases	4,198 (3,828-4,605)	4,264 (3,911-4,642)	5,492 (4,957-6,012)	6,243 (5,673-6,798)	6,180 (5,600-6,781)	6,726 (5,983-7,466)	6,327 (5,610-7,020)	5,822 (5,077-6,466)	5,432 (4,545-6,110)	3,682 (2,982-4,170)	1,806 (1,397-2,050)	641 (497-718)
Sexually transmitted diseases excluding HIV	588 (487-680)	599 (510-677)	543 (413-635)	682 (496-804)	450 (360-512)	441 (331-514)	327 (259-374)	379 (315-440)	294 (236-332)	247 (204-277)	150 (126-165)	64 (54-69)
Syphilis	64 (53-74)	59 (50-66)	103 (78-120)	159 (115-187)	108 (86-123)	113 (85-131)	99 (78-113)	100 (83-116)	76 (61-86)	56 (46-62)	31 (26-35)	12 (10-13)
Chlamydial infection	110 (91-128)	105 (90-119)	84 (64-98)	105 (76-124)	71 (56-80)	41 (30-47)	38 (30-44)	49 (40-56)	52 (42-59)	45 (38-51)	27 (23-30)	12 (10-13)
Gonococcal infection	274 (227-316)	281 (239-317)	241 (184-282)	277 (201-326)	169 (135-192)	220 (165-257)	141 (112-162)	182 (151-211)	130 (104-147)	114 (94-128)	72 (60-79)	31 (26-34)
Other sexually transmitted diseases	140 (116-162)	154 (131-174)	115 (87-134)	142 (103-167)	103 (82-117)	67 (50-78)	49 (39-56)	49 (40-56)	36 (29-40)	32 (26-36)	20 (17-22)	9 (8-10)
Hepatitis	3,039 (2,744-3,356)	2,959 (2,670-3,257)	3,850 (3,458-4,240)	4,294 (3,882-4,678)	4,082 (3,662-4,482)	4,294 (3,803-4,788)	3,985 (3,532-4,435)	3,391 (2,995-3,748)	2,901 (2,507-3,299)	1,579 (1,381-1,783)	608 (528-696)	155 (135-178)
Acute hepatitis A	30 (12-55)	31 (15-57)	44 (20-81)	51 (24-88)	55 (26-103)	58 (26-100)	57 (27-98)	54 (26-97)	47 (24-82)	24 (13-40)	10 (5-17)	2 (1-4)
Hepatitis B	2,721 (2,357-3,072)	2,649 (2,324-2,974)	3,428 (2,992-3,887)	3,798 (3,280-4,271)	3,560 (3,024-4,027)	3,664 (3,124-4,267)	3,320 (2,776-3,831)	2,743 (2,192-3,234)	2,046 (1,509-2,526)	898 (579-1,203)	275 (150-413)	57 (26-95)
Hepatitis C	37 (17-64)	48 (22-88)	88 (40-155)	137 (63-244)	139 (65-252)	135 (65-230)	117 (58-203)	97 (48-165)	83 (42-139)	46 (27-74)	19 (11-31)	5 (2-9)
Acute hepatitis E	251 (109-490)	231 (108-410)	290 (129-524)	308 (121-591)	329 (129-671)	437 (204-792)	491 (231-888)	497 (188-939)	725 (371-1,202)	611 (343-940)	305 (184-440)	91 (55-128)
Other infectious diseases	571 (460-695)	706 (561-857)	1,100 (871-1,316)	1,267 (990-1,519)	1,647 (1,275-1,953)	1,991 (1,516-2,374)	2,015 (1,545-2,395)	2,051 (1,485-2,444)	2,237 (1,521-2,651)	1,855 (1,186-2,241)	1,048 (658-1,251)	422 (282-489)
Non-communicable diseases	327,114 (316,667-338,559)	510,137 (496,015-525,086)	758,961 (735,937-782,014)	998,524 (965,254-1,032,635)	1,389,308 (1,343,141-1,435,317)	1,659,750 (1,602,449-1,715,772)	1,860,405 (1,790,771-1,920,307)	2,360,549 (2,266,234-2,470,493)	2,556,207 (2,470,032-2,639,235)	2,571,494 (2,493,193-2,654,324)	1,705,300 (1,670,968-1,738,541)	673,356 (661,091-686,083)
Neoplasms	132,540 (128,208-136,746)	205,867 (199,213-211,904)	291,011 (281,917-300,309)	351,162 (339,573-363,241)	436,009 (421,944-450,967)	449,512 (434,383-464,464)	416,981 (402,912-430,837)	417,080 (398,782-436,034)	355,214 (340,618-369,233)	278,891 (266,576-290,733)	148,180 (141,357-155,190)	48,330 (45,849-50,495)
Lip and oral cavity cancer	2,269 (2,060-2,520)	3,614 (3,346-3,942)	4,913 (4,501-5,393)	5,973 (5,542-6,461)	7,488 (6,922-8,062)	6,724 (6,274-7,187)	5,785 (5,384-6,179)	5,640 (5,246-6,011)	5,469 (5,059-5,870)	4,591 (4,252-4,923)	2,600 (2,421-2,800)	924 (861-986)
Nasopharynx cancer	1,004 (943-1,072)	1,645 (1,560-1,737)	2,089 (1,975-2,218)	2,327 (2,219-2,460)	2,233 (2,094-2,382)	1,759 (1,641-1,887)	1,366 (1,275-1,467)	1,037 (971-1,106)	763 (708-820)	454 (426-484)	203 (190-215)	64 (60-68)
Other pharynx cancer	1,377 (1,246-1,521)	2,376 (2,176-2,582)	3,538 (3,284-3,832)	4,007 (3,681-4,358)	4,743 (4,317-5,147)	3,969 (3,610-4,322)	3,644 (3,273-3,988)	2,391 (2,109-2,628)	1,914 (1,681-2,106)	1,198 (1,055-1,312)	513 (467-553)	138 (127-148)
Oesophageal cancer	2,177 (2,023-2,357)	4,086 (3,860-4,327)	6,772 (6,475-7,128)	9,197 (8,787-9,672)	13,567 (12,850-14,305)	15,724 (14,890-16,665)	14,305 (13,584-15,142)	14,538 (13,745-15,300)	12,232 (11,559-12,919)	8,559 (8,107-9,020)	3,905 (3,714-4,115)	1,093 (1,032-1,149)
Stomach cancer	9,292 (8,892-9,753)	13,585 (13,015-14,216)	18,797 (18,024-19,639)	23,641 (22,608-24,886)	33,321 (31,887-35,069)	36,249 (34,641-38,196)	34,119 (32,691-35,705)	38,387 (36,279-40,923)	33,722 (32,059-35,393)	26,380 (24,937-27,766)	13,828 (13,163-14,545)	4,652 (4,411-4,875)
Colon and rectum cancer	7,619 (7,212-8,030)	13,857 (13,174-14,537)	21,309 (20,157-22,424)	28,173 (26,678-29,861)	37,003 (34,831-39,342)	41,196 (38,720-43,874)	42,631 (40,027-45,234)	51,415 (47,846-55,610)	47,551 (44,740-50,729)	43,560 (41,083-46,223)	26,384 (24,899-27,968)	9,100 (8,559-9,589)
Liver cancer	6,744 (5,928-7,672)	11,786 (10,751-13,221)	17,497 (15,848-19,822)	21,986 (19,664-24,937)	31,097 (27,586-35,097)	33,238 (30,234-37,168)	30,479 (27,030-34,180)	29,418 (26,507-32,555)	23,627 (21,384-25,721)	15,773 (14,451-17,074)	6,668 (6,166-7,206)	1,843 (1,708-1,999)
Liver cancer due to hepatitis B	3,923 (3,004-4,851)	6,070 (4,468-7,583)	7,901 (5,709-10,063)	8,734 (5,996-11,439)	11,277 (7,450-14,806)	10,869 (7,300-14,060)	9,024 (5,907-11,891)	8,136 (5,521-10,845)	5,998 (3,851-8,318)	3,871 (2,760-5,141)	1,479 (1,025-1,997)	384 (231-553)
Liver cancer due to hepatitis C	851 (632-1,130)	1,756 (1,276-2,258)	3,154 (2,442-3,949)	4,559 (3,592-5,589)	6,482 (5,245-7,956)	7,541 (6,224-8,994)	8,127 (6,809-9,543)	8,927 (7,633-10,436)	8,496 (7,220-9,780)	6,318 (5,617-7,090)	3,095 (2,727-3,484)	958 (832-1,098)
Liver cancer due to alcohol use	1,292 (843-1,879)	2,890 (1,800-4,262)	4,896 (3,265-6,724)	6,839 (4,879-9,247)	10,820 (8,011-14,261)	12,050 (9,536-15,060)	10,604 (8,303-13,280)	9,510 (7,326-11,778)	6,701 (4,973-8,463)	3,962 (2,999-4,938)	1,390 (1,015-1,805)	306 (189-435)
Liver cancer due to other causes	678 (511-902)	1,070 (790-1,418)	1,546 (1,146-2,052)	1,855 (1,409-2,402)	2,518 (1,936-3,245)	2,778 (2,168-3,453)	2,725 (2,153-3,383)	2,845 (2,251-3,524)	2,432 (1,926-3,043)	1,622 (1,333-1,964)	704 (567-880)	196 (146-256)
Gallbladder and biliary tract cancer	2,377 (2,052-2,589)	4,101 (3,724-4,416)	6,273 (5,613-6,755)	7,917 (7,192-8,392)	10,978 (10,259-11,562)	11,690 (11,035-12,289)	11,361 (10,786-11,930)	11,965 (11,312-12,693)	10,780 (10,185-11,399)	8,765 (8,258-9,366)	4,898 (4,603-5,221)	1,778 (1,673-1,891)
Pancreatic cancer	2,561 (2,455-2,673)	5,009 (4,835-5,214)	9,236 (8,877-9,592)	13,828 (13,299-14,409)	21,478 (20,632-22,497)	25,684 (24,537-27,004)	26,695 (25,390-27,996)	28,366 (26,869-30,035)	24,084 (22,671-25,470)	19,627 (18,541-20,693)	10,254 (9,754-10,803)	3,056 (2,889-3,212)
Larynx cancer	552 (508-603)	963 (899-1,033)	1,411 (1,335-1,495)	1,744 (1,651-1,836)	2,160 (2,050-2,267)	2,093 (1,980-2,215)	1,977 (1,860-2,096)	1,610 (1,526-1,694)	1,367 (1,285-1,448)	836 (786-886)	361 (342-382)	94 (89-99)
Tracheal, bronchus, and lung cancer	9,892 (9,289-10,503)	20,057 (18,949-21,028)	33,785 (32,198-35,220)	46,625 (44,418-48,704)	66,448 (63,113-69,640)	75,885 (72,284-79,232)	73,461 (69,890-76,432)	70,097 (66,279-73,598)	58,665 (54,717-62,211)	41,223 (38,613-43,685)	18,682 (17,259-19,842)	5,760 (5,315-6,166)
Malignant skin melanoma	1,064 (871-1,314)	1,447 (1,155-1,773)	1,998 (1,607-2,383)	2,356 (1,871-2,766)	2,891 (2,290-3,379)	2,891 (2,183-3,362)	2,735 (2,032-3,238)	3,052 (2,243-3,538)	2,796 (2,092-3,185)	2,454 (1,746-2,801)	1,488 (1,103-1,758)	542 (398-671)
Non-melanoma skin cancer	324 (304-347)	590 (553-631)	760 (716-804)	954 (896-1,016)	1,438 (1,331-1,556)	1,593 (1,468-1,742)	1,634 (1,506-1,762)	1,808 (1,676-1,959)	2,273 (2,114-2,431)	2,688 (2,531-2,871)	2,124 (2,008-2,250)	1,224 (1,146-1,293)
Non-melanoma skin cancer (squamous-cell carcinoma)	324 (304-347)	590 (553-631)	760 (716-804)	954 (896-1,016)	1,438 (1,331-1,556)	1,593 (1,468-1,742)	1,634 (1,506-1,762)	1,808 (1,676-1,959)	2,273 (2,114-2,431)	2,688 (2,531-2,871)	2,124 (2,008-2,250)	1,224 (1,146-1,293)
Breast cancer	32,709 (30,831-35,807)	47,279 (44,468-52,316)	61,029 (57,510-66,137)	65,937 (61,796-72,045)	63,813 (59,645-70,062)	56,104 (52,358-61,065)	45,578 (42,545-49,820)	42,174 (39,517-46,006)	34,039 (31,995-36,446)	28,961 (27,283-30,983)	17,238 (16,292-18,369)	6,148 (5,800-6,518)
Cervical cancer	18,871 (14,963-20,242)	26,450 (21,119-28,294)	31,434 (24,558-33,741)	32,325 (26,265-34,864)	31,339 (25,609-34,010)	25,643 (20,971-27,780)	20,341 (17,136-22,228)	15,237 (13,190-16,468)	12,215 (10,637-13,178)	7,744 (6,884-8,372)	3,429 (3,107-3,681)	1,021 (934-1,089)
Uterine cancer	2,289 (2,142-2,450)	4,401 (4,159-4,654)	7,026 (6,613-7,460)	9,183 (8,648-9,814)	12,708 (11,885-13,535)	12,941 (12,131-13,856)	10,590 (9,972-11,275)	9,744 (9,035-10,660)	7,547 (7,095-8,069)	5,326 (4,999-5,689)	2,629 (2,477-2,791)	787 (741-834)
Ovarian cancer	6,533 (6,034-6,919)	11,476 (10,722-12,090)	17,630 (16,531-18,566)	19,706 (18,453-20,868)	22,323 (21,014-23,596)	20,722 (19,447-21,978)	18,272 (17,133-19,261)	15,689 (14,643-16,825)	11,637 (10,908-12,359)	8,063 (7,532-8,548)	3,940 (3,712-4,163)	1,040 (983-1,097)
Prostate cancer	-	-	-	-	-	-	-	-	-	-	-	-
Testicular cancer	-	-	-	-	-	-	-	-	-	-	-	-
Kidney cancer	766 (733-807)	1,394 (1,335-1,461)	2,353 (2,244-2,497)	3,468 (3,271-3,723)	4,958 (4,676-5,346)	5,497 (5,181-5,925)	5,538 (5,238-5,861)	6,221 (5,771-6,768)	5,497 (5,165-5,850)	4,568 (4,299-4,824)	2,447 (2,292-2,605)	703 (661-746)
Bladder cancer	614 (571-664)	971 (920-1,034)	1,778 (1,672-1,898)	2,463 (2,344-2,609)	3,643 (3,436-3,849)	4,653 (4,390-4,918)	5,399 (5,091-5,705)	7,057 (6,601-7,519)	7,520 (6,991-8,076)	7,254 (6,755-7,749)	4,655 (4,325-4,981)	1,644 (1,543-1,739)
Brain and nervous system cancer	4,826 (4,186-5,609)	6,503 (5,604-7,633)	8,507 (7,362-9,938)	9,917 (8,636-11,534)	11,613 (10,110-13,237)	11,227 (9,631-12,415)	9,047 (7,565-9,911)	8,059 (6,267-8,841)	5,506 (4,097-6,114)	3,254 (2,376-3,649)	1,204 (855-1,355)	296 (209-336)
Thyroid cancer	906 (822-994)	1,309 (1,198-1,423)	2,010 (1,841-2,177)	2,548 (2,366-2,728)	2,959 (2,770-3,161)	3,162 (2,945-3,386)	2,999 (2,806-3,221)	2,934 (2,743-3,209)	2,351 (2,211-2,546)	1,752 (1,641-1,886)	856 (800-919)	262 (244-279)
Mesothelioma	320 (284-366)	318 (292-351)	497 (460-550)	661 (615-721)	835 (788-890)	1,034 (980-1,102)	951 (906-1,002)	1,126 (1,065-1,200)	898 (848-959)	621 (586-659)	305 (289-322)	86 (82-91)
Hodgkin lymphoma	594 (415-716)	562 (421-709)	701 (493-912)	730 (557-966)	896 (649-1,118)	900 (687-1,136)	722 (590-928)	687 (583-868)	560 (465-661)	336 (290-385)	124 (111-140)	31 (28-35)
Non-Hodgkin's lymphoma	3,335 (2,969-3,548)	4,160 (3,762-4,405)	5,883 (5,348-6,243)	7,153 (6,557-7,544)	10,254 (9,556-10,807)	11,323 (10,689-11,911)	11,444 (10,792-12,112)	10,863 (10,192-11,587)	9,852 (9,230-10,533)	7,956 (7,434-8,504)	4,088 (3,777-4,381)	1,163 (1,067-1,254)
Multiple myeloma	846 (694-956)	1,537 (1,302-1,776)	2,831 (2,371-3,267)	3,840 (3,211-4,477)	5,673 (4,703-6,708)	6,736 (5,625-7,914)	6,141 (5,011-7,460)	6,398 (5,061-7,712)	5,489 (4,310-6,453)	4,235 (3,354-5,132)	2,102 (1,674-2,637)	509 (402-639)
Leukaemia	5,015 (4,092-5,566)	6,435 (5,250-7,082)	7,779 (6,312-8,464)	9,041 (7,419-9,724)	11,168 (9,239-12,176)	11,656 (9,958-12,656)	11,509 (10,022-12,394)	12,014 (10,668-13,046)	10,327 (9,430-11,133)	8,620 (8,006-9,163)	5,055 (4,748-5,379)	1,632 (1,530-1,729)
Acute lymphoid leukaemia	888 (687-1,038)	1,082 (830-1,293)	1,170 (852-1,331)	1,478 (1,132-1,692)	1,566 (1,207-1,786)	1,337 (1,038-1,598)	1,148 (922-1,354)	938 (765-1,069)	689 (579-796)	464 (407-515)	229 (204-254)	78 (70-87)
Chronic lymphoid leukaemia	282 (231-351)	306 (260-370)	495 (408-571)	697 (596-804)	1,274 (1,045-1,449)	1,470 (1,287-1,674)	1,478 (1,330-1,638)	2,091 (1,843-2,345)	1,956 (1,772-2,172)	2,036 (1,859-2,224)	1,346 (1,235-1,470)	478 (437-519)
Acute myeloid leukaemia	1,385 (1,117-1,584)	1,881 (1,558-2,124)	2,188 (1,780-2,423)	2,540 (2,139-2,784)	3,176 (2,712-3,457)	3,539 (3,108-3,825)	3,746 (3,358-3,989)	3,878 (3,528-4,178)	3,020 (2,782-3,278)	2,169 (2,010-2,338)	1,073 (997-1,157)	297 (275-325)
Chronic myeloid leukaemia	510 (377-660)	579 (428-752)	762 (546-991)	786 (618-993)	851 (678-1,032)	864 (701-1,021)	896 (738-1,036)	830 (732-940)	811 (724-915)	732 (657-813)	474 (422-539)	148 (132-168)
Other leukaemia	1,950 (1,521-2,179)	2,586 (2,016-2,856)	3,164 (2,411-3,477)	3,542 (2,688-3,891)	4,302 (3,316-4,800)	4,446 (3,479-4,970)	4,241 (3,426-4,704)	4,277 (3,581-4,753)	3,851 (3,319-4,251)	3,219 (2,901-3,492)	1,932 (1,772-2,110)	631 (585-685)
Other neoplasms	7,666 (6,920-8,096)	9,958 (9,008-10,457)	13,175 (12,113-13,768)	15,460 (14,569-16,261)	18,982 (17,440-19,996)	19,217 (18,002-20,113)	18,257 (17,121-19,113)	19,154 (17,933-20,316)	16,533 (15,530-17,477)	14,092 (13,268-14,942)	8,200 (7,730-8,644)	2,739 (2,568-2,916)
Cardiovascular diseases	102,799 (98,638-107,118)	169,751 (163,917-176,056)	263,566 (253,965-273,751)	372,376 (357,823-387,639)	559,852 (537,417-583,154)	720,069 (689,842-751,764)	858,999 (823,369-894,013)	1,192,890 (1,129,791-1,270,013)	1,320,781 (1,263,926-1,386,787)	1,377,707 (1,314,234-1,444,451)	888,336 (854,725-922,813)	362,598 (346,630-376,641)
Rheumatic heart disease	7,645 (6,889-8,736)	8,663 (7,886-9,764)	11,271 (10,356-12,598)	12,620 (11,564-13,894)	16,275 (15,058-17,798)	18,055 (16,683-19,645)	18,372 (16,870-19,935)	18,780 (17,385-20,192)	18,104 (16,877-19,343)	16,002 (15,021-16,982)	9,971 (9,380-10,581)	3,937 (3,691-4,204)
Ischaemic heart disease	44,648 (42,615-46,766)	77,336 (73,844-80,629)	122,329 (116,513-127,846)	181,646 (173,197-189,684)	278,286 (265,446-291,482)	358,529 (340,872-377,389)	438,918 (417,068-462,339)	635,955 (596,998-681,637)	700,014 (666,184-737,970)	756,289 (719,954-795,288)	503,092 (485,585-524,538)	206,500 (199,599-214,433)
Cerebrovascular disease	33,984 (32,380-35,582)	60,461 (57,880-62,981)	94,964 (90,690-99,016)	128,818 (122,780-135,369)	199,165 (190,020-209,112)	257,460 (243,938-271,694)	294,529 (277,431-312,396)	385,081 (352,257-421,386)	406,027 (369,144-444,100)	382,841 (343,442-422,670)	214,162 (191,516-239,001)	87,813 (76,694-97,592)

SR Table 3: Global deaths by cause and age group for females (with 95% uncertainty intervals), 2016

Cause	Early Neonatal	Late Neonatal	Post Neonatal	1 to 4	5 to 9	10 to 14	15 to 19	20 to 24	25 to 29	30 to 34	35 to 39	40 to 44	45 to 49	50 to 54	55 to 59	60 to 64	65 to 69	70 to 74	75 to 79	80 to 84	85 to 89	90 to 94	95 plus
Ischaemic stroke	-	-	130 (101-168)	67 (58-81)	85 (73-101)	94 (81-110)	443 (400-491)	963 (854-1,087)	1,421 (1,299-1,581)	1,920 (1,745-2,125)	2,954 (2,686-3,263)	5,328 (4,912-5,782)	11,336 (10,501-12,254)	20,390 (18,930-21,889)	32,402 (30,153-34,969)	81,152 (75,959-87,132)	114,559 (107,031-123,060)	141,429 (131,621-152,256)	229,738 (207,914-255,994)	247,352 (224,382-272,295)	249,590 (222,706-277,142)	143,844 (129,025-160,451)	61,510 (53,767-68,402)
Hemorrhagic stroke	380 (326-453)	220 (185-274)	1,817 (1,505-2,210)	1,343 (1,095-1,652)	940 (836-1,080)	1,213 (1,102-1,344)	1,991 (1,838-2,161)	3,742 (3,473-4,062)	5,811 (5,442-6,197)	9,543 (8,984-10,083)	16,081 (15,137-17,091)	28,656 (27,274-30,061)	49,125 (46,908-51,338)	74,573 (71,092-77,921)	96,416 (91,922-101,322)	118,014 (112,183-124,057)	142,901 (134,759-151,550)	153,099 (143,650-162,603)	155,343 (143,096-168,570)	158,675 (143,868-172,649)	133,252 (119,126-147,052)	70,318 (62,576-78,340)	26,303 (22,965-29,350)
Hypertensive heart disease	-	-	80 (61-113)	58 (41-74)	55 (42-67)	79 (60-98)	618 (497-793)	843 (645-1,068)	1,250 (955-1,553)	2,110 (1,619-2,506)	3,365 (2,526-4,029)	5,561 (4,148-6,533)	9,913 (7,275-11,737)	16,082 (11,817-19,320)	23,407 (18,243-27,974)	31,226 (23,475-37,279)	41,569 (30,776-51,076)	50,463 (37,491-61,318)	68,842 (49,905-85,697)	78,661 (54,669-95,251)	80,683 (51,864-98,356)	57,036 (35,910-66,962)	23,759 (14,250-28,099)
Cardiomyopathy and myocarditis	233 (172-269)	153 (121-183)	1,450 (1,124-1,710)	963 (792-1,158)	517 (448-598)	627 (538-705)	952 (694-1,088)	1,246 (886-1,410)	1,986 (1,436-2,339)	2,424 (1,845-3,072)	3,057 (2,258-3,979)	3,970 (3,019-5,090)	4,151 (3,259-5,216)	5,806 (4,651-7,301)	7,843 (6,565-9,740)	8,264 (7,053-10,369)	8,855 (7,593-10,824)	9,743 (7,989-11,130)	15,312 (11,574-17,816)	20,048 (13,755-22,951)	25,060 (17,075-28,836)	19,046 (13,040-21,697)	8,225 (5,601-9,243)
Myocarditis	45 (29-60)	35 (25-46)	455 (332-591)	365 (287-478)	214 (168-251)	222 (158-261)	438 (267-547)	523 (318-648)	623 (408-781)	549 (363-672)	687 (424-857)	816 (550-1,007)	608 (416-729)	698 (499-864)	813 (611-979)	776 (546-926)	943 (654-1,114)	1,139 (780-1,352)	1,797 (1,188-2,137)	2,778 (1,794-3,283)	4,217 (2,853-5,073)	4,094 (2,512-5,074)	2,277 (1,296-2,818)
Alcoholic cardiomyopathy	-	-	-	-	-	-	50 (40-61)	108 (78-145)	348 (227-547)	704 (414-1,166)	987 (558-1,664)	1,354 (833-2,202)	1,481 (969-2,269)	2,164 (1,396-3,303)	2,834 (1,906-4,361)	2,556 (1,773-3,743)	1,930 (1,366-2,897)	1,348 (1,023-1,810)	2,144 (1,451-3,137)	1,461 (894-2,047)	1,462 (790-2,186)	781 (443-1,108)	369 (197-514)
Other cardiomyopathy	188 (136-220)	118 (94-144)	995 (754-1,199)	598 (462-779)	303 (257-369)	406 (351-476)	464 (359-555)	614 (445-721)	1,015 (706-1,187)	1,171 (881-1,386)	1,382 (1,070-1,652)	1,800 (1,418-2,137)	2,063 (1,710-2,443)	2,943 (2,545-3,537)	4,197 (3,666-5,118)	4,932 (4,404-6,176)	5,981 (5,148-7,132)	7,256 (5,942-8,317)	11,372 (8,417-13,039)	15,809 (10,824-18,196)	19,382 (12,952-22,545)	14,172 (9,725-16,496)	5,579 (3,968-6,660)
Atrial fibrillation and flutter	-	-	-	-	-	-	-	-	-	17 (12-23)	66 (49-88)	166 (122-219)	339 (250-449)	597 (442-781)	1,060 (631-1,567)	2,322 (1,536-3,311)	4,468 (2,802-6,606)	8,294 (6,001-11,348)	14,712 (9,397-21,997)	31,260 (23,668-39,614)	44,754 (34,330-56,973)	29,905 (22,971-38,441)	9,725 (7,615-12,393)
Aortic aneurysm	-	-	-	-	-	-	114 (102-128)	150 (137-164)	230 (212-251)	335 (306-370)	498 (454-554)	711 (657-778)	1,163 (1,088-1,269)	1,827 (1,712-1,992)	2,723 (2,553-2,942)	3,294 (3,076-3,511)	4,783 (4,473-5,095)	6,137 (5,763-6,471)	8,813 (8,252-9,375)	10,654 (10,114-11,184)	11,003 (10,492-11,464)	7,507 (7,185-7,838)	2,924 (2,795-3,065)
Peripheral vascular disease	-	-	-	-	-	-	-	-	-	-	-	77 (51-119)	126 (74-204)	257 (147-439)	486 (268-834)	902 (492-1,541)	1,367 (743-2,293)	1,836 (922-3,114)	3,688 (1,897-6,380)	5,528 (3,047-10,078)	7,864 (4,256-15,461)	7,034 (3,771-13,718)	3,311 (1,741-6,503)
Endocarditis	43 (30-59)	32 (24-44)	686 (541-998)	373 (288-591)	210 (173-265)	267 (227-319)	489 (423-591)	671 (580-819)	960 (844-1,131)	912 (806-1,055)	1,108 (964-1,336)	1,472 (1,295-1,813)	1,386 (1,200-1,713)	1,707 (1,455-2,087)	2,128 (1,785-2,675)	3,048 (2,534-3,849)	3,716 (3,068-4,662)	4,397 (3,583-5,612)	5,651 (4,423-7,320)	6,804 (5,010-8,819)	7,038 (4,855-8,976)	5,506 (3,527-7,094)	2,377 (1,509-2,992)
Other cardiovascular and circulatory diseases	432 (371-519)	274 (224-363)	2,032 (1,685-2,525)	1,261 (1,013-1,668)	675 (565-955)	761 (641-1,087)	1,201 (1,021-1,734)	1,645 (1,431-2,335)	2,240 (1,945-3,151)	2,736 (2,383-3,705)	3,340 (2,899-4,621)	4,564 (3,960-6,264)	6,213 (5,315-8,517)	8,727 (7,654-11,697)	11,646 (10,179-16,086)	17,070 (15,106-23,443)	21,267 (18,517-28,878)	26,310 (23,248-34,648)	36,055 (32,031-46,249)	43,680 (39,553-54,423)	46,173 (42,400-56,523)	35,076 (32,722-40,745)	14,025 (13,238-16,021)
Chronic respiratory diseases	0 (0-0)	0 (0-0)	0 (0-0)	5,073 (4,080-6,882)	1,540 (1,269-2,156)	1,848 (1,566-2,535)	3,005 (2,605-4,072)	4,061 (3,584-5,208)	5,367 (4,814-6,657)	7,123 (6,419-8,670)	10,200 (9,162-12,532)	14,607 (13,353-17,464)	25,948 (23,725-30,962)	44,198 (40,394-51,086)	70,213 (63,996-79,503)	118,934 (106,585-135,441)	163,727 (147,542-185,279)	200,529 (181,484-229,622)	227,112 (207,217-264,926)	240,148 (220,982-280,001)	208,202 (192,785-241,607)	116,695 (108,989-131,933)	43,884 (40,997-49,323)
Chronic obstructive pulmonary disease	-	-	0 (0-0)	984 (701-1,552)	249 (187-384)	266 (209-373)	1,089 (902-1,450)	1,648 (1,393-2,151)	2,387 (2,024-3,102)	3,421 (2,878-4,379)	4,641 (3,975-5,860)	7,344 (6,430-9,028)	15,040 (13,193-18,227)	29,333 (25,599-34,686)	49,698 (44,058-57,738)	91,965 (81,502-106,416)	132,411 (117,506-151,674)	167,442 (151,260-193,429)	191,502 (174,810-226,934)	207,949 (190,179-246,221)	184,394 (170,346-215,289)	105,122 (98,074-120,442)	39,959 (37,322-45,396)
Pneumoconiosis	-	-	-	-	-	-	10 (8-13)	15 (13-20)	20 (17-25)	28 (24-34)	28 (24-36)	46 (38-58)	62 (54-78)	90 (74-112)	135 (110-169)	191 (160-237)	234 (195-289)	294 (249-358)	372 (321-452)	400 (347-475)	321 (287-371)	174 (158-195)	72 (66-80)
Silicosis	-	-	-	-	-	-	0 (0-1)	1 (0-1)	2 (1-2)	4 (3-5)	3 (2-3)	8 (5-10)	15 (11-19)	18 (13-23)	31 (23-42)	49 (35-66)	58 (43-79)	86 (69-118)	104 (83-140)	101 (82-142)	76 (64-107)	41 (34-59)	15 (13-22)
Asbestosis	-	-	-	-	-	-	5 (4-7)	8 (6-10)	9 (8-12)	12 (10-15)	13 (10-16)	18 (14-24)	22 (18-30)	32 (25-44)	41 (32-56)	47 (36-63)	45 (34-60)	56 (40-71)	72 (49-91)	87 (50-106)	76 (41-93)	45 (23-54)	17 (9-21)
Coal workers pneumoconiosis	-	-	-	-	-	-	0 (0-0)	0 (0-0)	1 (0-1)	1 (1-2)	1 (1-1)	2 (1-3)	4 (2-5)	6 (4-9)	12 (8-18)	18 (11-26)	28 (19-42)	34 (25-50)	47 (35-70)	39 (29-54)	35 (26-51)	21 (14-28)	11 (8-15)
Other pneumoconiosis	-	-	-	-	-	-	4 (3-6)	6 (5-9)	8 (7-10)	11 (9-14)	12 (10-15)	18 (15-24)	22 (18-28)	34 (27-43)	50 (39-65)	77 (61-102)	102 (81-135)	117 (94-162)	150 (120-203)	174 (141-231)	134 (114-174)	67 (59-89)	29 (25-36)
Asthma	-	-	-	3,170 (2,516-4,428)	864 (687-1,268)	1,193 (978-1,771)	1,642 (1,363-2,345)	2,038 (1,701-2,825)	2,483 (2,060-3,280)	3,096 (2,565-4,104)	4,669 (3,869-6,160)	6,019 (4,932-8,016)	9,265 (7,441-12,176)	12,281 (9,633-15,875)	16,887 (13,162-21,525)	21,713 (16,692-28,121)	24,370 (18,705-32,078)	24,623 (18,974-32,504)	25,882 (20,155-34,133)	22,159 (17,279-29,584)	15,249 (12,355-19,858)	6,850 (5,706-8,452)	2,202 (1,897-2,640)
Interstitial lung disease and pulmonary sarcoidosis	-	-	-	149 (83-291)	77 (52-117)	60 (42-83)	113 (74-162)	169 (113-238)	253 (176-344)	368 (249-497)	565 (382-759)	796 (541-1,035)	1,280 (895-1,682)	2,045 (1,416-2,649)	2,957 (2,014-3,969)	4,423 (2,907-5,817)	5,928 (3,918-7,655)	7,334 (4,889-9,213)	8,269 (5,356-10,163)	8,480 (5,386-10,068)	7,192 (4,167-8,371)	3,928 (2,355-4,520)	1,402 (801-1,594)
Other chronic respiratory diseases	0 (0-0)	0 (0-0)	0 (0-0)	771 (471-1,275)	350 (243-477)	329 (214-448)	152 (94-214)	190 (119-266)	225 (133-309)	210 (123-291)	297 (169-412)	403 (238-556)	300 (170-405)	449 (268-601)	536 (305-752)	642 (361-879)	784 (446-1,049)	836 (469-1,129)	1,087 (601-1,494)	1,159 (619-1,577)	1,045 (551-1,410)	621 (316-825)	249 (124-339)
Cirrhosis and other chronic liver diseases	-	-	-	2,538 (2,108-3,019)	1,197 (997-1,426)	1,583 (1,352-1,825)	2,807 (2,413-3,176)	4,553 (3,989-5,133)	6,035 (5,404-6,667)	8,993 (8,109-9,978)	12,221 (11,120-13,427)	17,599 (16,201-19,104)	23,441 (21,938-25,083)	33,934 (31,750-36,186)	40,709 (38,175-43,583)	46,536 (43,570-49,422)	45,594 (42,626-48,427)	42,441 (39,548-45,414)	39,092 (36,615-41,498)	32,262 (30,161-34,385)	22,712 (21,200-24,165)	11,445 (10,700-12,188)	4,357 (4,048-4,619)
Cirrhosis and other chronic liver diseases due to hepatitis B	-	-	-	10 (7-15)	12 (9-16)	32 (22-42)	92 (61-132)	577 (398-807)	1,281 (916-1,734)	2,317 (1,890-2,805)	3,376 (2,733-4,084)	4,821 (4,029-5,677)	5,782 (4,675-6,931)	7,812 (6,327-9,397)	9,161 (7,569-10,946)	11,273 (9,214-13,693)	11,359 (9,291-13,672)	10,734 (8,725-13,059)	9,807 (8,050-11,592)	7,616 (6,110-9,135)	4,890 (4,128-5,684)	2,118 (1,700-2,566)	733 (530-940)
Cirrhosis and other chronic liver diseases due to hepatitis C	-	-	-	9 (6-13)	11 (7-16)	28 (18-40)	82 (51-122)	490 (320-685)	1,011 (695-1,379)	1,878 (1,482-2,325)	2,821 (2,159-3,548)	4,109 (3,313-4,971)	5,541 (4,448-6,720)	8,075 (6,405-9,855)	9,561 (7,880-11,397)	10,507 (8,407-12,932)	10,816 (8,887-12,943)	10,360 (8,489-12,443)	8,841 (7,128-10,905)	7,331 (5,885-9,092)	5,082 (4,180-6,084)	2,626 (2,055-3,241)	985 (709-1,282)
Cirrhosis and other chronic liver diseases due to alcohol use	-	-	-	-	-	-	21 (12-33)	208 (142-290)	554 (398-763)	1,589 (1,308-1,949)	3,037 (2,440-3,744)	4,845 (4,099-5,714)	6,751 (5,633-7,947)	9,758 (8,240-11,495)	11,194 (9,127-13,395)	12,199 (9,887-14,833)	11,713 (9,761-13,697)	10,560 (8,790-12,498)	9,988 (8,364-11,627)	8,156 (6,800-9,597)	5,967 (5,142-6,825)	3,229 (2,679-3,763)	1,251 (1,000-1,512)
Cirrhosis and other chronic liver diseases due to other causes	-	-	-	2,519 (2,091-2,993)	1,173 (979-1,399)	1,523 (1,303-1,756)	2,612 (2,254-2,942)	3,279 (2,795-3,769)	3,188 (2,641-3,740)	3,209 (2,592-3,921)	2,988 (2,279-3,840)	3,824 (2,891-4,861)	5,367 (4,129-6,708)	8,289 (6,451-10,307)	10,793 (8,470-13,405)	12,558 (9,660-15,771)	11,706 (9,050-14,507)	10,788 (8,304-13,431)	10,456 (8,336-12,741)	9,159 (7,244-11,423)	6,773 (5,722-7,930)	3,471 (2,849-4,209)	1,388 (1,049-1,777)
Digestive diseases	690 (474-909)	498 (382-632)	6,331 (4,566-8,642)	6,429 (4,415-10,288)	2,429 (2,012-3,017)	2,811 (2,432-3,269)	4,769 (4,085-5,469)	5,965 (5,225-6,777)	7,158 (6,368-8,101)	7,862 (7,097-8,903)	9,207 (8,241-10,339)	11,212 (10,063-12,397)	15,738 (14,152-17,418)	21,832 (19,776-24,150)	30,455 (27,543-33,836)	38,796 (35,071-42,423)	47,377 (42,305-51,680)	51,486 (46,072-55,895)	62,441 (56,010-67,929)	65,804 (59,859-70,621)	62,573 (57,809-67,284)	41,106 (38,280-44,023)	15,571 (14,360-16,667)
Peptic ulcer disease	-	-	-	688 (417-1,243)	317 (243-445)	479 (383-626)	624 (520-792)	1,095 (926-1,351)	1,268 (1,099-1,524)	1,529 (1,330-1,911)	1,864 (1,628-2,247)	2,470 (2,175-2,912)	3,411 (2,985-3,979)	5,299 (4,669-6,184)	6,243 (5,535-7,336)	8,750 (7,745-10,074)	10,714 (9,475-12,450)	11,645 (10,082-13,317)	13,966 (12,234-15,594)	13,975 (12,432-15,391)	11,930 (10,771-13,348)	6,863 (6,246-7,698)	2,418 (2,198-2,741)
Gastritis and duodenitis	-	-	-	125 (82-195)	65 (47-94)	63 (48-86)	89 (63-127)	158 (123-209)	226 (186-282)	293 (245-364)	336 (281-413)	394 (337-483)	589 (496-706)	801 (682-979)	939 (799-1,142)	1,369 (1,171-1,628)	1,772 (1,477-2,136)	1,834 (1,522-2,178)	2,247 (1,884-2,663)	2,583 (2,189-3,042)	2,261 (1,960-2,791)	1,382 (1,201-1,749)	520 (458-765)
Appendicitis	-	-	-	1,386 (732-2,756)	580 (462-786)	711 (572-921)	1,403 (1,106-1,812)	1,289 (959-1,633)	1,415 (1,118-1,784)	1,010 (806-1,323)	1,070 (866-1,380)	878 (700-1,100)	1,280 (1,001-1,634)	1,271 (1,015-1,616)	2,360 (1,832-3,153)	2,192 (1,753-2,842)	2,170 (1,730-2,801)	1,822 (1,437-2,262)	1,897 (1,504-2,394)	1,562 (1,226-1,953)	1,263 (1,032-1,626)	684 (552-906)	230 (192-317)
Paralytic ileus and intestinal obstruction	690 (474-909)	498 (382-632)	6,331 (4,566-8,642)	2,341 (1,338-3,910)	793 (565-1,017)	992 (720-1,212)	1,769 (1,209-2,136)	1,828 (1,331-2,161)	2,042 (1,403-2,416)	2,053 (1,432-2,460)	2,289 (1,615-2,695)	2,995 (2,116-3,502)	3,766 (2,597-4,494)	5,209 (3,764-6,240)	8,104 (5,618-9,421)	9,226 (6,347-10,878)	11,276 (7,607-13,135)	11,273 (8,318-13,354)	12,647 (9,611-14,932)	12,734 (9,775-14,905)	12,592 (9,847-14,768)	8,904 (6,728-10,251)	3,345 (2,520-3,896)
Inguinal, femoral, and abdominal hernia	-	-	-	103 (56-180)	64 (43-95)	60 (44-94)	78 (51-112)	90 (59-126)	91 (61-136)	115 (75-169)	150 (94-221)	208 (143-320)	318 (205-455)	652 (431-1,019)	999 (633-1,441)	1,373 (910-1,984)	1,545 (1,055-2,255)	1,781 (1,147-2,460)	2,069 (1,431-2,937)	2,642 (1,726-3,486)	2,498 (1,662-3,452)	1,669 (1,065-2,303)	603 (383-826)
Inflammatory bowel disease	-	-	-	496 (243-1,065)	101 (72-152)	82 (59-117)	152 (106-224)	286 (203-425)	375 (273-531)	431 (309-595)	443 (333-590)	480 (361-614)	734 (551-937)	876 (649-1,060)	1,173 (906-1,444)	1,418 (1,068-1,679)	1,646 (1,211-1,931)	2,131 (1,580-2,462)	2,520 (1,820-2,832)	2,847 (1,982-3,203)	2,856 (1,993-3,207)	2,160 (1,440-2,482)	853 (606-978)
Vascular intestinal disorders	-	-	-	48 (30-74)	13 (11-17)	16 (13-20)	32 (24-40)	94 (66-118)	131 (97-165)	186 (145-231)	274 (216-338)	420 (358-501)	793 (675-951)	1,240 (1,074-1,451)	1,920 (1,665-2,238)	3,286 (2,889-3,793)	4,857 (4,282-5,651)	6,429 (5,690-7,362)	9,527 (8,351-10,949)	9,574 (8,496-10,765)	9,233 (8,274-10,216)	5,511 (4,977-6,149)	1,717 (1,526-1,948)
Gallbladder and biliary diseases	-	-	-	182 (125-262)	125 (92-192)	116 (93-167)	147 (121-196)	285 (240-361)	426 (364-531)	616 (525-781)	837 (714-1,023)	940 (817-1,156)	1,415 (1,206-1,716)	2,192 (1,894-2,678)	2,897 (2,525-3,528)	4,010 (3,547-4,911)	5,154 (4,470-6,450)	5,505 (4,844-6,444)	7,126 (6,299-8,380)	8,303 (7,372-9,446)	8,650 (7,758-9,888)	6,347 (5,763-7,269)	2,797 (2,518-3,156)
Pancreatitis	-	-	-	51 (30-82)	60 (40-90)	94 (64-127)	252 (181-337)	534 (377-692)	751 (550-990)	1,107 (838-1,412)	1,324 (974-1,696)	1,655 (1,250-2,098)	2,400 (1,762-3,073)	2,842 (2,158-3,687)	3,698 (2,782-4,673)	4,090 (3,070-5,255)	4,532 (3,418-5,832)	4,403 (3,286-5,375)	4,563 (3,516-5,524)	4,291 (3,338-5,262)	3,547 (2,810-4,262)	1,944 (1,607-2,328)	641 (513-784)
Other digestive diseases	-	-	-	1,007 (587-1,918)	311 (251-405)	198 (166-251)	225 (186-279)	304 (258-368)	434 (374-519)	521 (444-627)	620 (527-735)	771 (668-892)	1,033 (905-1,203)	1,451 (1,286-1,645)	2,123 (1,894-2,443)	3,084 (2,752-3,438)	3,711 (3,219-4,127)	4,664 (4,072-5,185)	5,879 (5,238-6,523)	7,292 (6,490-8,010)	7,743 (7,007-8,458)	5,642 (5,124-6,256)	2,447 (2,198-2,788)
Neurological disorders	-	-	2,916 (2,588-3,344)	3,125 (2,674-3,703)	1,935 (1,687-2,242)	2,793 (2,457-3,274)	4,131 (3,678-4,820)	4,330 (3,809-4,906)	4,439 (4,020-5,043)	4,734 (4,308-5,372)	4,603 (4,178-5,093)	5,121 (4,761-5,597)	7,138 (6,495-7,921)	11,982 (10,431-14,077)	17,132 (14,053-20,837)	32,404 (26,280-40,409)	56,324 (44,611-70,980)	103,576 (80,699-132,342)	207,701 (161,559-263,741)	318,853 (254,853-399,114)	422,011 (340,356-511,261)	371,527 (310,645-433,405)	146,666 (119,024-178,347)
Alzheimer's disease and other dementias	-	-	-	-	-	-	-	-	-	-	-	55 (24-107)	1,332 (797-2,000)	4,673 (3,177-6,664)	8,964 (5,925-12,514)	21,791 (15,788-29,763)	43,224 (31,998-57,711)	85,992 (63,205-114,812)	182,949 (137,048-237,530)	290,700 (225,422-372,342)	397,129 (313,754-487,203)	359,271 (298,168-420,886)	143,285 (115,358-175,311)
Parkinson's disease	-	-	-	-	-	-	-	1 (0-1)	10 (4-20)	36 (19-66)	80 (41-142)	151 (83-252)	276 (154-484)	514 (296-834)	965 (576-1,443)	2,365 (1,599-3,351)	4,890 (3,398-6,635)	9,578 (6,655-13,005)	18,187 (12,868-24,497)	22,688 (16,550-30,903)	20,683 (15,469-28,034)	10,288 (7,729-13,607)	2,800 (2,009-3,818)
Epilepsy	-	-	1,812 (1,470-2,213)	2,158 (1,827-2,535)	1,503 (1,257-1,779)	2,085 (1,770-2,471)	3,471 (3,007-4,066)	3,777 (3,263-4,388)	3,702 (3,214-4,273)	3,723 (3,212-4,266)	3,147 (2,700-3,535)	2,930 (2,560-3,237)	2,462 (2,116-2,706)	2,813 (2,434-3,124)	2,479 (2,019-2,726)	2,782 (2,330-3,115)	2,565 (2,085-2,868)	2,936 (2,439-3,306)	1,925 (1,638-2,166)	1,853 (1,614-2,073)	1,767 (1,572-1,956)	855 (766-948)	248 (227-270)
Multiple sclerosis	-	-	-	-	-	-	-	87 (70-118)	204 (170-273)	332 (281-442)	568 (483-759)	802 (692-1,039)	1,100 (967-1,357)	1,335 (1,142-1,643)	1,462 (1,167-1,735)	1,413 (1,035-1,665)	1,274 (899-1,415)	966 (657-1,084)	737 (491-836)	517 (335-589)	305 (195-346)	119 (75-135)	28 (18-31)
Motor neuron disease	4 (3-5)	4 (4-6)	238 (187-287)	135 (88-169)	19 (16-22)	21 (19-23)	19 (17-21)	21 (19-22)	39 (35-43)	69 (64-75)	128 (119-140)	287 (272-311)	613 (584-659)	1,082 (1,029-1,153)	1,471 (1,405-1,562)	2,000 (1,918-2,100)	2,306 (2,196-2,413)	2,192 (2,077-2,305)	1,984 (1,869-2,100)	1,353 (1,266-1,433)	742 (694-788)	240 (225-255)	52 (48-54)
Other neurological disorders	-	-	865 (671-1,056)	832 (558-1,125)	413 (342-487)	687 (509-894)	641 (463-818)	445 (378-509)	484 (421-545)	575 (518-630)	681 (624-734)	894 (844-944)	1,355 (1,252-1,459)	1,566 (1,491-1,637)	1,791 (1,709-1,873)	2,054 (1,964-2,147)	2,065 (1,970-2,160)	1,912 (1,808-2,020)	1,919 (1,798-2,050)	1,742 (1,613-1,864)	1,386 (1,286-1,492)	755 (702-811)	253 (236-271)
Mental and substance use disorders	3 (3-4)	15 (12-18)	0 (0-0)	0 (0-0)	8 (6-10)	10 (8-13)	1,601 (1,374-1,919)	2,602 (2,346-3,044)	3,923 (3,519-4,480)	4,719 (4,220-5,392)	5,206 (4,654-6,023)	5,909 (5,387-6,634)	6,586 (6,074-7,247)	7,423 (6,760-8,288)	6,842 (6,029-7,881)	5,955 (5,292-6,779)	4,952 (4,424-5,546)	3,252 (2,802-3,593)	3,941 (3,467-4,349)	2,964 (2,556-3,201)	2,553 (2,255-2,750)	1,462 (1,349-1,584)	569 (523-613)
Alcohol use disorders	-	-	-	-	-	-	155 (143-168)	267 (240-302)	730 (608-914)	1,184 (952-1,496)	1,835 (1,481-2,384)	2,288 (1,929-2,821)	2,769 (2,413-3,299)	3,560 (3,054-4,243)	3,495 (2,864-4,287)	2,991 (2,543-3,612)	2,318 (2,046-2,706)	1,176 (1,062-1,332)	1,495 (1,342-1,662)	863 (790-930)	659 (609-707)	380 (352-410)	151 (139-162)
Drug use disorders	3 (3-4)	15 (12-18)	0 (0-0)	0 (0-0)	0 (0-0)	0 (0-0)	1,414 (1,193-1,730)	2,280 (2,039-2,723)	3,130 (2,783-3,658)	3,472 (3,123-3,933)	3,308 (3,011-3,678)	3,549 (3,282-3,881)	3,740 (3,456-4,063)	3,863 (3,552-4,185)	3,347 (3,003-3,689)	2,964 (2,578-3,281)	2,634 (2,191-2,918)	2,077 (1,600-2,317)	2,446 (2,016-2,762)	2,102 (1,692-2,316)	1,894 (1,598-2,080)	1,082 (974-1,183)	418 (376-456)
Opioid use disorders	3 (3-4)	15 (12-18)	0 (0-0)	0 (0-0)	0 (0-0)	0 (0-0)	914 (756-1,119)	1,437 (1,234-1,667)	1,929 (1,553-2,272)	1,958 (1,543-2,266)	1,843 (1,381-2,090)	1,983 (1,469-2,237)	2,004 (1,402-2,242)	2,036 (1,433-2,292)	1,810 (1,310-2,068)	1,651 (1,298-1,911)	1,528 (1,203-1,783)	911 (639-1,068)	1,065 (786-1,274)	754 (503-912)	678 (470-797)	355 (258-421)	128 (95-154)
Cocaine use disorders	0 (0-0)	0 (0-0)	0 (0-0)	0 (0-0)	0 (0-0)	0 (0-0)	52 (33-99)	92 (67-174)	134 (100-263)	232 (181-386)	221 (171-350)	239 (186-385)	272 (216-458)	244 (192-407)	199 (154-308)	164 (121-279)	135 (93-224)	30 (20-53)	29 (20-49)	11 (7-15)	10 (6-14)	8 (4-11)	4 (2-6)
Amphetamine use disorders	0 (0-0)	0 (0-0)	0 (0-0)	0 (0-0)	0 (0-0)	0 (0-0)	59 (39-115)	108 (83-191)	155 (113-321)	122 (96-224)	112 (83-180)	123 (99-201)	137 (111-223)	125 (99-211)	108 (83-186)	88 (68-156)	73 (53-128)	22 (15-43)	27 (19-46)	6 (4-9)	8 (5-10)	7 (3-9)	4 (1-5)
Other drug use disorders	0 (0-0)	0 (0-0)	0 (0-0)	0 (0-0)	0 (0-0)	0 (0-0)	389 (276-514)	643 (516-822)	912 (731-1,209)	1,160 (982-1,492)	1,132 (965-1,466)	1,204 (1,039-1,598)	1,327 (1,153-1,775)	1,458 (1,247-1,939)	1,230 (1,048-1,547)	1,060 (838-1,309)	898 (662-1,108)	1,114 (834-1,330)	1,325 (1,027-1,572)	1,331 (1,078-1,539)	1,198 (1,011-1,373)	713 (629-816)	283 (246-324)
Eating disorders	-	-	-	-	8 (6-10)	10 (8-13)	32 (25-40)	55 (44-66)	63 (50-74)	62 (52-72)	63 (53-72)	72 (63-86)	77 (66-95)	0 (0-0)	0 (0-0)	-	-	-	-	-	-	-	-
Anorexia nervosa	-	-	-	-	8 (6-10)	10 (7-12)	30 (24-38)	51 (41-62)	58 (46-69)	56 (47-65)	57 (48-66)	62 (54-73)	67 (58-82)	0 (0-0)	0 (0-0)	-	-	-	-	-	-	-	-
Bulimia nervosa	-	-	-	-	1 (0-1)	0 (0-1)	2 (1-2)	4 (3-5)	5 (4-6)	6 (5-8)	6 (5-7)	10 (8-14)	10 (7-14)	0 (0-0)	0 (0-0)	-	-	-	-	-	-	-	-
Diabetes, urogenital, blood, and endocrine diseases	1,538 (1,160-2,098)	863 (705-1,102)	9,037 (7,665-11,034)	12,005 (8,912-15,662)	4,909 (4,147-6,077)	7,666 (6,514-8,803)	11,445 (10,172-12,795)	13,195 (12,168-14,540)	15,461 (14,484-16,726)	18,255 (17,271-19,660)	21,893 (20,882-23,024)	32,101 (30,793-33,585)	49,878 (47,853-51,921)	77,905 (74,876-80,752)	101,460 (97,682-105,172)	141,340 (135,642-146,891)	161,453 (155,517-167,733)	172,703 (165,819-180,062)	197,252 (188,379-205,300)	205,992 (195,604-215,294)	182,214 (173,925-190,313)	115,445 (109,832-121,091)	46,125 (43,746-48,222)
Diabetes mellitus	10 (9-11)	17 (14-21)	208 (181-241)	351 (301-409)	332 (288-390)	801 (720-892)	1,407 (1,291-1,529)	2,121 (1,968-2,284)	3,253 (3,036-3,495)	4,221 (3,977-4,485)	6,359 (6,055-6,693)	11,998 (11,422-12,621)	21,193 (20,221-22,225)	37,001 (35,340-38,604)	52,139 (50,036-54,390)	77,562 (74,320-80,833)	89,252 (85,435-93,321)	94,543 (90,180-99,005)	107,303 (101,896-112,604)	109,676 (103,240-115,997)	82,262 (77,730-86,543)	43,954 (41,517-46,537)	14,110 (13,282-14,934)
Acute glomerulonephritis	-	-	62 (38-118)	66 (54-83)	76 (62-99)	91 (71-123)	67 (57-78)	97 (84-116)	142 (117-174)	133 (112-158)	125 (108-144)	166 (147-187)	240 (209-270)	245 (220-274)	256 (227-287)	426 (374-478)	453 (403-517)	441 (397-496)	443 (394-502)	415 (366-468)	359 (321-400)	176 (159-196)	84 (75-94)
Chronic kidney disease	-	-	1,229 (1,089-1,416)	1,966 (1,700-2,295)	1,221 (1,070-1,414)	1,712 (1,541-1,934)	2,920 (2,694-3,218)	4,554 (4,234-4,933)	5,971 (5,590-6,454)	7,338 (6,896-7,922)	8,962 (8,444-9,575)	12,268 (11,656-12,974)	18,616 (17,724-19,675)	28,187 (26,917-29,683)	35,577 (34,021-37,292)	46,827 (44,940-48,958)	52,016 (49,818-54,345)	54,802 (52,613-57,566)	62,747 (59,762-65,857)	64,499 (61,415-67,571)	64,296 (61,043-67,444)	45,281 (42,923-47,594)	19,829 (18,610-20,817)
Chronic kidney disease due to diabetes mellitus	-	-	29 (19-43)	50 (31-75)	38 (21-61)	68 (42-110)	184 (111-290)	521 (325-760)	1,115 (782-1,501)	1,932 (1,409-2,540)	2,985 (2,252-3,754)	4,993 (3,846-6,116)	8,564 (6,742-10,468)	13,550 (10,600-16,466)	17,886 (14,057-21,311)	23,944 (19,560-28,260)	25,632 (20,061-30,345)	25,154 (19,909-29,967)	25,915 (20,213-31,256)	22,389 (17,099-27,647)	19,127 (14,082-25,009)	12,776 (9,374-16,666)	5,867 (4,054-7,648)
Chronic kidney disease due to hypertension	-	-	8 (5-11)	19 (13-27)	19 (12-29)	46 (30-68)	126 (83-184)	287 (200-396)	490 (345-669)	719 (529-964)	1,025 (768-1,329)	1,664 (1,266-2,164)	2,860 (2,186-3,697)	4,724 (3,609-6,167)	6,531 (5,078-8,381)	9,502 (7,542-11,936)	11,917 (9,370-15,159)	14,009 (10,919-17,586)	18,111 (14,290-22,272)	21,278 (17,393-26,006)	24,706 (20,208-30,209)	18,909 (15,658-22,358)	8,622 (6,871-10,540)
Chronic kidney disease due to glomerulonephritis	-	-	541 (416-690)	858 (644-1,110)	520 (371-687)	754 (543-983)	1,332 (957-1,711)	2,023 (1,489-2,604)	2,364 (1,686-3,091)	2,393 (1,692-3,154)	2,384 (1,617-3,209)	2,623 (1,734-3,586)	3,202 (2,078-4,482)	4,270 (2,864-6,138)	4,713 (3,166-6,524)	5,499 (3,553-7,781)	5,892 (3,831-8,442)	6,233 (4,228-8,820)	7,540 (5,109-10,624)	8,205 (5,767-11,006)	7,233 (5,018-9,993)	4,443 (3,196-5,792)	1,659 (1,019-2,620)
Chronic kidney disease due to other causes	-	-	651 (525-811)	1,038 (811-1,290)	645 (492-823)	843 (626-1,081)	1,278 (913-1,651)	1,724 (1,204-2,269)	2,002 (1,368-2,688)	2,294 (1,605-3,043)	2,568 (1,836-3,327)	2,988 (2,076-4,020)	3,990 (2,654-5,700)	5,642 (3,721-7,980)	6,447 (4,343-9,171)	7,882 (5,258-11,349)	8,575 (5,731-12,353)	9,405 (6,450-12,863)	11,180 (7,762-15,414)	12,627 (8,910-16,895)	13,230 (9,010-17,930)	9,153 (6,706-12,093)	3,681 (2,347-5,221)
Urinary diseases and male infertility	85 (75-96)	87 (71-109)	1,298 (1,122-1,496)	1,083 (903-1,312)	437 (369-520)	430 (368-507)	1,208 (1,060-1,381)	1,624 (1,452-1,812)	1,557 (1,420-1,694)	1,894 (1,750-2,046)	2,186 (2,046-2,352)	2,556 (2,401-2,721)	3,336 (3,128-3,522)	5,036 (4,730-5,348)	6,563 (6,094-7,047)	8,703 (8,117-9,271)	10,383 (9,721-11,079)	11,732 (10,959-12,473)	15,435 (14,397-16,403)	18,463 (17,409-19,537)	20,194 (19,135-21,356)	16,020 (15,166-16,979)	7,758 (7,330-8,197)
Interstitial nephritis and urinary tract infections	60 (51-71)	61 (49-77)	1,031 (888-1,220)	849 (687-1,068)	291 (241-353)	260 (219-311)	939 (816-1,089)	1,260 (1,112-1,463)	1,130 (1,008-1,294)	1,400 (1,266-1,595)	1,679 (1,510-1,865)	2,021 (1,851-2,201)	2,632 (2,418-2,850)	3,996 (3,681-4,353)	5,089 (4,599-5,604)	6,648 (6,025-7,333)	8,280 (7,621-9,072)	9,767 (9,031-10,552)	12,786 (11,813-13,915)	15,727 (14,577-17,008)	17,662 (16,465-19,092)	14,386 (13,483-15,486)	7,136 (6,700-7,641)
Urolithiasis	-	-	-	-	53 (40-72)	67 (48-94)	100 (68-144)	134 (92-189)	123 (85-165)	142 (100-188)	179 (133-239)	185 (141-245)	280 (213-376)	441 (329-563)	535 (405-688)	704 (529-925)	821 (643-1,124)	808 (634-1,134)	1,112 (860-1,635)	1,159 (931-1,680)	1,087 (866-1,578)	696 (556-1,044)	273 (219-428)
Other urinary diseases	25 (19-34)	26 (18-39)	267 (180-390)	234 (152-348)	93 (67-136)	103 (75-155)	169 (109-265)	230 (151-348)	304 (202-420)	352 (228-496)	328 (213-467)	350 (233-476)	425 (277-590)	599 (363-837)	938 (595-1,281)	1,350 (856-1,819)	1,282 (766-1,721)	1,157 (674-1,560)	1,536 (879-2,011)	1,577 (888-2,039)	1,445 (728-1,913)	937 (459-1,245)	349 (172-457)
Gynecological diseases	-	-	-	-	-	-	160 (138-182)	243 (210-276)	308 (271-356)	431 (385-481)	606 (539-685)	691 (610-780)	1,012 (889-1,125)	851 (755-947)	606 (537-682)	437 (382-487)	598 (533-669)	368 (324-412)	304 (252-340)	723 (588-841)	589 (453-680)	300 (239-352)	108 (84-127)
Uterine fibroids	-	-	-	-	-	-	9 (6-15)	16 (11-25)	32 (22-50)	60 (37-90)	150 (86-208)	231 (154-304)	463 (311-571)	502 (323-606)	304 (191-376)	210 (153-259)	255 (178-324)	132 (95-165)	108 (82-131)	215 (107-318)	138 (69-212)	64 (35-98)	21 (12-31)
Polycystic ovarian syndrome	-	-	-	-	-	-	1 (0-3)	14 (4-40)	46 (17-92)	37 (11-97)	52 (18-108)	50 (18-93)	78 (28-148)	143 (63-253)	-	-	-	-	-	-	-	-	-
Endometriosis	-	-	-	-	-	-	3 (1-6)	3 (1-6)	4 (1-8)	8 (2-13)	6 (2-8)	10 (4-14)	20 (7-33)	14 (7-20)	-	-	-	-	-	-	-	-	-
Genital prolapse	-	-	-	-	-	-	4 (2-11)	5 (2-11)	9 (4-17)	13 (3-21)	5 (2-8)	6 (3-10)	7 (3-10)	12 (6-21)	63 (25-105)	59 (31-98)	99 (49-152)	83 (44-122)	52 (33-68)	209 (93-326)	167 (76-259)	83 (40-131)	26 (14-40)
Other gynecological diseases	-	-	-	-	-	-	143 (116-164)	205 (156-242)	217 (145-265)	313 (221-378)	394 (276-495)	394 (274-493)	444 (289-592)	180 (94-276)	239 (152-326)	168 (108-228)	244 (158-324)	154 (100-202)	143 (106-169)	299 (188-444)	284 (193-392)	152 (108-204)	61 (43-78)
Hemoglobinopathies and hemolytic anaemias	881 (541-1,431)	327 (221-540)	3,636 (2,495-5,335)	6,710 (3,978-9,995)	2,004 (1,422-2,922)	3,872 (2,689-4,913)	4,870 (3,521-5,971)	3,488 (2,462-4,438)	2,650 (1,934-3,334)	2,445 (1,833-3,110)	1,776 (1,396-2,224)	2,351 (1,839-3,062)	2,665 (2,055-3,367)	2,794 (2,160-3,571)	2,183 (1,771-2,785)	2,258 (1,862-2,923)	2,800 (2,312-3,697)	4,591 (3,773-5,962)	4,245 (3,406-5,470)	4,553 (3,719-5,921)	6,842 (5,368-8,454)	4,141 (3,217-5,192)	1,508 (1,138-1,834)
Thalassemias	157 (107-221)	74 (54-116)	618 (440-900)	862 (637-1,202)	479 (368-675)	285 (231-374)	250 (196-321)	126 (98-168)	90 (70-114)	61 (51-83)	43 (37-61)	41 (34-63)	40 (34-59)	28 (23-46)	21 (16-37)	13 (10-23)	9 (7-18)	5 (3-9)	2 (1-3)	1 (1-2)	0 (0-0)	0 (0-0)	0 (0-0)
Sickle cell disorders	697 (407-1,199)	247 (157-440)	2,948 (1,984-4,343)	5,730 (3,055-8,853)	1,452 (954-2,155)	3,417 (2,302-4,420)	4,210 (2,996-5,255)	2,923 (2,009-3,724)	1,959 (1,395-2,513)	1,689 (1,229-2,173)	1,038 (789-1,324)	1,363 (1,031-1,801)	1,371 (1,046-1,758)	1,199 (903-1,513)	599 (469-783)	286 (234-394)	216 (180-304)	130 (106-189)	33 (27-52)	15 (12-26)	6 (5-10)	2 (1-3)	0 (0-0)
G6PD deficiency	13 (9-20)	5 (3-7)	49 (33-77)	94 (64-143)	56 (41-81)	123 (89-157)	286 (211-374)	312 (240-419)	409 (306-519)	471 (354-603)	462 (358-568)	586 (452-758)	756 (572-980)	751 (548-937)	613 (475-805)	521 (405-684)	563 (430-754)	697 (530-932)	287 (208-359)	179 (132-231)	140 (103-180)	38 (28-48)	6 (4-8)
Other hemoglobinopathies and hemolytic anaemias	14 (9-19)	1 (1-2)	21 (15-31)	24 (18-35)	17 (13-22)	47 (36-58)	124 (95-161)	128 (103-179)	191 (147-242)	224 (178-295)	234 (187-293)	360 (288-485)	499 (392-646)	816 (655-1,031)	951 (765-1,203)	1,439 (1,190-1,838)	2,012 (1,656-2,634)	3,760 (3,093-4,853)	3,923 (3,155-5,035)	4,358 (3,568-5,714)	6,696 (5,256-8,248)	4,101 (3,190-5,149)	1,501 (1,132-1,824)
Endocrine, metabolic, blood, and immune disorders	562 (465-704)	431 (326-571)	2,604 (2,199-3,356)	1,829 (1,587-2,423)	838 (733-1,063)	760 (654-926)	813 (708-1,007)	1,067 (956-1,307)	1,580 (1,337-1,961)	1,793 (1,467-2,052)	1,879 (1,537-2,119)	2,071 (1,630-2,253)	2,816 (2,310-3,163)	3,791 (3,027-4,176)	4,136 (3,232-4,532)	5,128 (4,159-5,848)	5,951 (4,762-6,705)	6,225 (4,886-7,016)	6,776 (5,037-7,457)	7,662 (5,402-8,430)	7,671 (5,274-8,385)	5,574 (3,568-6,207)	2,728 (1,700-3,053)
Musculoskeletal disorders	-	-	-	-	328 (254-424)	660 (526-819)	1,155 (939-1,488)	1,488 (1,254-1,833)	1,906 (1,525-2,316)	1,870 (1,536-2,286)	1,896 (1,576-2,317)	2,249 (1,850-2,719)	2,673 (2,215-3,142)	2,989 (2,451-3,565)	3,445 (2,856-4,046)	4,493 (3,763-5,449)	5,077 (4,246-6,058)	5,378 (4,416-6,176)	6,368 (5,249-7,317)	6,581 (5,448-7,443)	6,517 (5,321-7,190)	4,557 (3,625-5,033)	1,981 (1,672-2,187)
Rheumatoid arthritis	-	-	-	-	45 (33-74)	49 (36-78)	42 (30-65)	49 (37-78)	76 (56-116)	100 (78-150)	146 (108-213)	263 (201-368)	522 (403-699)	830 (627-1,071)	1,289 (991-1,637)	1,973 (1,583-2,474)	2,488 (1,979-3,153)	2,771 (2,217-3,450)	3,237 (2,575-3,976)	3,034 (2,358-3,733)	2,468 (1,942-3,151)	1,289 (960-1,675)	433 (324-583)
Other musculoskeletal disorders	-	-	-	-	283 (217-363)	611 (493-759)	1,112 (902-1,421)	1,439 (1,205-1,781)	1,830 (1,455-2,232)	1,770 (1,442-2,137)	1,750 (1,432-2,083)	1,986 (1,615-2,398)	2,151 (1,738-2,563)	2,159 (1,729-2,579)	2,156 (1,742-2,536)	2,520 (2,067-3,042)	2,590 (2,111-3,059)	2,608 (2,058-3,092)	3,131 (2,468-3,627)	3,546 (2,708-3,956)	4,050 (3,089-4,460)	3,268 (2,486-3,646)	1,549 (1,279-1,705)
Other non-communicable diseases	66,611 (53,757-79,048)	26,333 (23,410-29,802)	89,143 (75,503-105,284)	31,765 (23,171-43,182)	7,707 (6,682-8,939)	6,225 (5,384-7,052)	4,399 (3,781-4,984)	3,958 (3,507-4,502)	3,883 (3,435-4,420)	2,907 (2,523-3,419)	2,946 (2,486-3,474)	2,976 (2,449-3,604)	3,118 (2,409-3,814)	4,120 (2,922-5,280)	4,729 (3,253-6,051)	4,988 (3,355-6,586)	5,664 (3,592-8,018)	5,061 (2,734-7,823)	6,672 (3,838-10,437)	7,608 (4,251-11,889)	8,113 (4,530-12,660)	6,546 (3,451-10,266)	3,274 (1,608-5,309)
Congenital anomalies	66,611 (53,757-79,048)	25,171 (22,370-28,725)	75,223 (63,477-89,521)	30,386 (21,936-41,716)	7,421 (6,404-8,689)	5,876 (5,121-6,767)	3,806 (3,325-4,348)	3,469 (3,152-3,912)	3,390 (2,998-3,915)	2,309 (2,087-2,673)	2,194 (1,958-2,566)	2,009 (1,751-2,344)	1,728 (1,460-1,987)	1,749 (1,452-1,952)	1,725 (1,414-1,876)	1,590 (1,303-1,778)	1,214 (1,016-1,356)	-	-	-	-	-	-
Neural tube defects	7,740 (4,891-10,893)	1,743 (1,185-2,468)	4,621 (2,654-7,040)	1,665 (615-3,691)	257 (169-373)	172 (121-232)	94 (72-120)	79 (61-104)	67 (52-90)	59 (49-75)	45 (37-57)	38 (31-48)	39 (33-47)	37 (31-44)	32 (26-40)	25 (21-31)	23 (19-28)	-	-	-	-	-	-
Congenital heart anomalies	18,731 (13,491-24,284)	10,506 (8,370-13,194)	35,353 (28,072-46,865)	14,517 (10,672-22,862)	4,396 (3,494-5,485)	3,838 (3,135-4,627)	2,376 (1,942-2,823)	2,391 (2,038-2,766)	2,438 (2,078-2,810)	1,489 (1,276-1,748)	1,498 (1,280-1,790)	1,384 (1,192-1,638)	725 (609-857)	658 (565-785)	576 (497-708)	522 (447-649)	437 (381-542)	-	-	-	-	-	-
Orofacial clefts	487 (231-869)	234 (108-423)	331 (128-629)	67 (18-150)	-	-	-	-	-	-	-	-	-	-	-	-	-	-	-	-	-	-	-
Down's syndrome	764 (543-1,214)	371 (284-574)	1,656 (1,282-2,714)	775 (568-1,438)	161 (123-218)	145 (113-195)	147 (117-201)	162 (130-212)	197 (164-259)	207 (173-269)	202 (165-255)	226 (189-289)	417 (322-508)	473 (352-540)	510 (344-574)	407 (258-465)	184 (111-212)	-	-	-	-	-	-
Other chromosomal abnormalities	3,118 (2,081-5,645)	1,599 (1,089-2,497)	3,769 (2,467-6,837)	651 (410-1,515)	173 (124-246)	116 (83-154)	97 (73-132)	68 (46-85)	72 (43-88)	52 (29-64)	36 (19-42)	40 (21-48)	34 (17-40)	42 (25-54)	41 (22-54)	28 (15-37)	30 (18-37)	-	-	-	-	-	-
Congenital musculoskeletal and limb anomalies	1,863 (1,158-3,768)	467 (310-901)	858 (556-1,728)	266 (152-717)	94 (68-154)	91 (68-144)	68 (50-110)	44 (31-62)	43 (29-56)	35 (24-46)	28 (20-35)	23 (17-27)	31 (23-41)	32 (24-43)	32 (25-43)	28 (21-36)	27 (21-34)	-	-	-	-	-	-
Urogenital congenital anomalies	1,741 (1,021-2,853)	598 (383-949)	952 (580-1,466)	232 (143-380)	122 (83-183)	133 (89-199)	65 (44-92)	63 (42-80)	59 (39-75)	54 (36-68)	58 (36-75)	53 (36-67)	101 (63-131)	139 (98-180)	169 (121-224)	202 (141-258)	221 (158-286)	-	-	-	-	-	-
Digestive congenital anomalies	4,992 (3,328-8,399)	2,195 (1,602-3,538)	5,015 (3,591-8,622)	1,075 (682-2,730)	122 (89-168)	78 (56-101)	54 (41-76)	41 (30-52)	25 (19-30)	22 (17-28)	20 (15-24)	17 (13-21)	26 (20-33)	39 (30-49)	42 (31-52)	46 (36-57)	48 (35-56)	-	-	-	-	-	-
Other congenital anomalies	27,175 (17,277-40,198)	7,458 (4,794-10,533)	22,667 (11,891-37,722)	11,139 (4,508-22,728)	2,097 (1,335-3,168)	1,303 (906-1,755)	905 (641-1,176)	621 (415-845)	490 (336-644)	391 (282-495)	308 (218-385)	228 (169-280)	355 (279-416)	330 (256-387)	323 (243-381)	331 (261-382)	244 (192-276)	-	-	-	-	-	-
Skin and subcutaneous diseases	-	-	3,083 (1,395-4,738)	1,379 (631-2,104)	286 (125-429)	349 (161-530)	593 (259-881)	489 (234-723)	493 (263-783)	598 (322-961)	752 (393-1,168)	967 (517-1,513)	1,390 (744-2,111)	2,372 (1,257-3,500)	3,003 (1,577-4,339)	3,398 (1,800-5,049)	4,450 (2,405-6,808)	5,061 (2,734-7,823)	6,672 (3,838-10,437)	7,608 (4,251-11,889)	8,113 (4,530-12,660)	6,546 (3,451-10,266)	3,274 (1,608-5,309)
Cellulitis	-	-	186 (59-325)	135 (45-242)	44 (16-77)	50 (19-79)	69 (27-103)	90 (39-128)	66 (32-104)	114 (56-184)	126 (59-200)	147 (74-244)	227 (99-357)	454 (193-686)	568 (238-873)	704 (282-1,037)	776 (328-1,194)	838 (350-1,295)	1,035 (452-1,711)	1,153 (518-2,139)	1,165 (550-2,153)	921 (383-1,863)	459 (195-910)
Pyoderma	-	-	2,843 (1,308-4,428)	1,104 (494-1,692)	185 (81-290)	225 (99-347)	411 (170-642)	286 (130-445)	328 (163-530)	356 (181-612)	507 (253-776)	652 (332-1,026)	934 (467-1,518)	1,588 (809-2,504)	1,988 (1,025-3,067)	2,120 (1,067-3,305)	2,715 (1,401-4,339)	2,918 (1,525-4,755)	3,494 (1,926-5,785)	3,369 (1,720-5,431)	3,382 (1,731-5,324)	2,523 (1,235-4,094)	1,152 (538-1,914)
Decubitus ulcer	-	-	-	74 (27-148)	25 (13-40)	33 (17-54)	54 (27-89)	57 (29-91)	54 (31-92)	76 (40-126)	73 (37-117)	109 (55-180)	160 (84-260)	251 (119-409)	347 (169-573)	461 (230-732)	804 (388-1,276)	1,136 (594-1,803)	1,904 (955-3,084)	2,770 (1,426-4,262)	3,231 (1,660-4,895)	2,797 (1,381-4,311)	1,522 (711-2,407)
Other skin and subcutaneous diseases	-	-	54 (23-101)	66 (31-122)	32 (15-60)	40 (19-67)	59 (29-98)	56 (29-89)	45 (25-76)	52 (29-87)	46 (27-77)	59 (33-99)	68 (39-115)	79 (45-134)	101 (55-164)	113 (63-190)	155 (86-260)	169 (95-287)	239 (138-411)	316 (174-564)	334 (179-652)	306 (159-614)	142 (69-307)
Sudden infant death syndrome	-	1,163 (904-1,505)	10,837 (8,212-14,405)	-	-	-	-	-	-	-	-	-	-	-	-	-	-	-	-	-	-	-	-
Injuries	4,403 (3,540-5,005)	2,802 (2,352-3,143)	33,497 (28,669-37,608)	68,340 (59,523-76,472)	38,564 (34,519-42,507)	36,373 (33,159-39,516)	69,387 (64,245-74,023)	80,420 (74,859-85,141)	79,146 (73,921-83,591)	69,304 (64,520-73,444)	63,323 (58,953-66,879)	64,173 (59,741-67,564)	68,232 (63,388-71,537)	72,112 (66,700-75,840)	71,716 (66,521-75,519)	81,368 (73,198-85,966)	80,414 (70,300-85,165)	78,740 (67,778-84,153)	86,994 (74,368-93,308)	87,314 (72,710-93,354)	83,384 (69,279-89,579)	53,749 (43,857-58,134)	21,861 (17,284-23,779)
Transport injuries	308 (254-373)	307 (259-373)	4,325 (3,833-4,872)	13,550 (11,899-15,653)	12,236 (10,983-13,846)	10,158 (9,297-11,229)	17,803 (16,559-19,163)	21,027 (19,684-22,324)	21,736 (20,354-23,067)	19,711 (18,609-21,008)	18,979 (17,933-20,069)	21,557 (20,466-22,657)	23,111 (21,992-24,221)	24,354 (23,128-25,640)	23,132 (21,940-24,430)	24,836 (23,453-26,231)	20,269 (19,014-21,493)	17,895 (16,802-18,983)	15,746 (14,741-16,711)	12,217 (11,453-12,921)	8,190 (7,696-8,722)	3,929 (3,685-4,218)	1,351 (1,250-1,457)
Road injuries	278 (229-340)	288 (242-351)	3,990 (3,528-4,512)	12,843 (11,248-14,879)	11,787 (10,595-13,341)	9,577 (8,764-10,565)	16,471 (15,349-17,685)	19,989 (18,723-21,242)	20,630 (19,326-21,880)	18,723 (17,683-19,966)	18,121 (17,123-19,173)	20,479 (19,465-21,573)	21,846 (20,814-22,900)	23,007 (21,805-24,243)	22,138 (21,023-23,379)	23,423 (22,101-24,760)	19,097 (17,917-20,239)	16,927 (15,917-18,015)	14,772 (13,866-15,686)	11,367 (10,695-12,005)	7,578 (7,125-8,049)	3,605 (3,372-3,866)	1,204 (1,111-1,300)
Pedestrian road injuries	109 (88-136)	105 (87-131)	1,489 (1,276-1,764)	6,427 (5,579-7,603)	6,099 (5,379-7,049)	4,376 (3,962-4,944)	5,110 (4,668-5,665)	6,262 (5,735-6,803)	6,645 (6,099-7,198)	6,509 (5,990-7,044)	6,978 (6,411-7,547)	8,139 (7,423-8,764)	9,343 (8,726-10,002)	9,815 (9,122-10,545)	10,169 (9,490-10,891)	12,413 (11,584-13,361)	10,385 (9,640-11,172)	9,422 (8,772-10,111)	8,612 (8,048-9,202)	6,962 (6,454-7,452)	4,452 (4,149-4,830)	1,916 (1,764-2,141)	599 (540-699)
Cyclist road injuries	-	-	-	398 (282-572)	454 (363-654)	589 (468-866)	742 (589-1,129)	750 (624-972)	837 (698-1,017)	898 (743-1,237)	695 (596-834)	1,074 (907-1,249)	1,469 (1,246-1,704)	1,370 (1,182-1,609)	1,314 (1,124-1,527)	1,366 (1,114-1,598)	1,173 (991-1,389)	945 (788-1,218)	646 (531-817)	316 (253-384)	222 (176-329)	101 (80-157)	1 (1-1)
Motorcyclist road injuries	40 (31-51)	27 (21-38)	569 (470-705)	1,052 (843-1,382)	705 (593-861)	831 (716-961)	2,503 (2,190-2,824)	3,203 (2,805-3,597)	3,167 (2,825-3,547)	3,166 (2,833-3,488)	2,496 (2,171-2,779)	2,982 (2,621-3,268)	3,035 (2,673-3,327)	3,249 (2,898-3,589)	2,592 (2,327-2,864)	2,152 (1,786-2,416)	1,462 (1,274-1,644)	1,083 (951-1,222)	695 (593-790)	417 (361-471)	225 (198-253)	94 (82-106)	39 (34-45)
Motor vehicle road injuries	121 (95-153)	151 (126-185)	1,854 (1,597-2,147)	4,830 (3,959-5,863)	4,391 (3,873-5,029)	3,672 (3,315-4,196)	7,904 (7,266-8,679)	9,547 (8,836-10,485)	9,812 (9,091-10,829)	7,955 (7,332-8,708)	7,741 (7,156-8,510)	8,092 (7,351-8,971)	7,716 (6,929-8,559)	8,326 (7,615-9,077)	7,792 (7,127-8,666)	7,383 (6,710-8,159)	5,977 (5,480-6,601)	5,385 (4,940-6,122)	4,604 (4,229-5,113)	3,530 (3,236-3,914)	2,576 (2,351-2,819)	1,434 (1,271-1,556)	538 (463-590)
Other road injuries	7 (5-12)	4 (3-7)	78 (54-131)	136 (96-216)	138 (104-199)	108 (87-150)	212 (155-366)	226 (172-376)	169 (135-267)	195 (155-309)	212 (169-321)	192 (158-290)	284 (232-430)	247 (202-378)	271 (218-418)	109 (91-149)	101 (82-139)	93 (75-135)	214 (170-330)	143 (112-227)	103 (78-160)	61 (45-90)	27 (20-43)
Other transport injuries	30 (21-38)	19 (16-24)	335 (279-416)	707 (573-904)	449 (374-559)	581 (490-704)	1,332 (1,097-1,623)	1,038 (886-1,225)	1,106 (955-1,313)	988 (852-1,162)	857 (753-978)	1,078 (928-1,277)	1,265 (1,104-1,510)	1,347 (1,158-1,598)	994 (882-1,154)	1,412 (1,194-1,675)	1,172 (991-1,359)	968 (833-1,112)	975 (838-1,133)	849 (721-988)	612 (522-723)	323 (283-379)	147 (131-173)
Unintentional injuries	2,806 (2,046-3,316)	1,938 (1,512-2,237)	26,535 (21,903-30,544)	44,029 (36,887-50,552)	19,323 (16,449-21,789)	15,924 (13,225-17,701)	16,505 (13,119-18,434)	17,809 (14,189-19,740)	18,772 (14,919-20,796)	18,580 (14,732-20,625)	17,581 (14,363-19,393)	18,786 (15,204-20,579)	21,652 (17,571-23,583)	24,610 (19,959-26,839)	28,599 (23,380-31,065)	36,969 (29,320-40,400)	42,944 (33,041-46,838)	46,834 (36,405-51,604)	58,399 (46,056-63,624)	66,054 (51,647-71,701)	68,819 (54,563-74,794)	47,041 (37,121-51,295)	19,627 (15,006-21,483)
Falls	751 (404-1,118)	217 (133-315)	3,664 (2,257-5,250)	4,855 (2,920-6,979)	2,172 (1,790-2,530)	1,688 (1,412-1,941)	2,116 (1,787-2,377)	2,220 (1,845-2,455)	2,497 (1,945-2,761)	2,658 (2,167-2,928)	3,185 (2,618-3,498)	3,542 (2,794-3,896)	4,955 (3,884-5,400)	6,850 (5,317-7,411)	10,025 (7,504-11,016)	15,683 (11,853-17,535)	21,383 (15,338-23,930)	26,540 (19,157-29,908)	36,706 (27,501-40,698)	44,438 (32,442-48,853)	49,470 (36,953-54,237)	34,733 (25,290-38,146)	14,150 (9,872-15,520)
Drowning	124 (98-153)	72 (57-94)	2,635 (1,951-3,578)	17,613 (13,338-21,859)	7,665 (5,890-9,378)	6,097 (4,774-7,305)	4,339 (3,192-5,202)	3,405 (2,494-4,127)	3,422 (2,451-4,095)	3,032 (2,177-3,607)	2,843 (1,988-3,324)	2,880 (2,067-3,311)	3,163 (2,292-3,617)	3,430 (2,468-3,958)	3,508 (2,503-4,067)	4,090 (2,838-4,765)	4,099 (2,893-4,835)	3,712 (2,705-4,349)	4,212 (3,028-4,947)	3,646 (2,696-4,160)	2,551 (1,963-2,898)	1,014 (793-1,122)	284 (225-311)
Fire, heat, and hot substances	100 (71-120)	120 (83-150)	2,378 (1,694-3,053)	5,655 (4,172-6,977)	1,851 (1,429-2,183)	1,507 (1,104-1,757)	3,203 (2,042-3,892)	4,304 (2,623-5,202)	3,969 (2,455-4,781)	3,837 (2,411-4,600)	3,016 (2,041-3,495)	2,332 (1,638-2,676)	2,609 (1,832-2,996)	2,638 (1,860-3,027)	2,657 (1,921-3,042)	2,879 (2,161-3,234)	3,315 (2,544-3,726)	3,287 (2,522-3,667)	3,932 (3,159-4,423)	4,117 (3,289-4,609)	3,695 (2,989-4,118)	2,298 (1,719-2,564)	1,010 (735-1,129)
Poisonings	91 (49-139)	72 (50-94)	1,709 (831-2,988)	2,588 (1,622-3,613)	789 (604-936)	893 (694-1,054)	854 (676-1,005)	1,093 (835-1,252)	1,122 (808-1,247)	931 (652-1,035)	882 (628-980)	1,037 (701-1,163)	1,026 (647-1,152)	1,123 (756-1,242)	1,087 (725-1,224)	1,110 (709-1,251)	1,097 (711-1,233)	977 (657-1,096)	857 (565-966)	766 (535-848)	544 (404-605)	272 (221-322)	103 (89-149)
Exposure to mechanical forces	523 (275-668)	535 (279-717)	3,915 (2,570-4,682)	3,072 (2,391-4,032)	1,705 (1,427-1,934)	1,214 (1,022-1,361)	1,397 (1,059-1,605)	1,318 (977-1,497)	1,840 (1,289-2,124)	1,600 (1,199-1,794)	1,544 (1,091-1,760)	1,876 (1,281-2,132)	1,951 (1,392-2,186)	1,711 (1,239-1,911)	2,145 (1,577-2,398)	2,034 (1,418-2,322)	1,877 (1,318-2,163)	1,587 (1,149-1,804)	1,556 (1,139-1,753)	1,745 (1,279-1,954)	1,683 (1,267-1,870)	1,119 (865-1,244)	483 (394-533)
Unintentional firearm injuries	15 (14-17)	10 (9-12)	114 (97-138)	176 (147-218)	165 (145-195)	150 (131-175)	234 (201-274)	261 (220-308)	325 (279-369)	377 (321-429)	249 (212-287)	275 (240-308)	290 (253-329)	221 (187-252)	234 (208-268)	196 (173-219)	197 (172-217)	205 (178-220)	310 (262-335)	353 (284-380)	385 (278-419)	294 (213-325)	147 (103-163)
Unintentional suffocation	385 (165-525)	482 (228-659)	2,663 (1,409-3,358)	1,095 (654-1,875)	450 (334-633)	373 (284-522)	123 (85-149)	131 (81-151)	151 (89-178)	101 (56-122)	93 (53-110)	93 (46-114)	158 (106-182)	203 (137-236)	242 (174-309)	100 (45-127)	110 (50-143)	114 (53-148)	111 (42-151)	129 (52-165)	136 (48-177)	72 (28-91)	27 (12-33)
Other exposure to mechanical forces	122 (82-145)	43 (34-50)	1,138 (873-1,361)	1,800 (1,377-2,214)	1,090 (833-1,273)	690 (532-789)	1,040 (708-1,231)	927 (600-1,088)	1,364 (845-1,633)	1,123 (760-1,291)	1,203 (777-1,407)	1,508 (957-1,747)	1,503 (966-1,732)	1,288 (854-1,479)	1,669 (1,108-1,920)	1,738 (1,159-2,013)	1,570 (1,037-1,847)	1,269 (876-1,469)	1,135 (780-1,299)	1,263 (872-1,442)	1,162 (838-1,307)	753 (565-848)	309 (239-347)
Adverse effects of medical treatment	426 (321-508)	300 (240-373)	3,114 (2,229-4,048)	2,321 (1,438-3,409)	888 (706-1,144)	733 (589-917)	931 (737-1,098)	1,881 (1,488-2,236)	2,069 (1,603-2,463)	1,843 (1,441-2,224)	2,096 (1,607-2,435)	2,233 (1,688-2,588)	2,890 (2,218-3,330)	2,563 (2,024-3,005)	3,197 (2,484-3,667)	4,630 (3,680-5,320)	4,840 (3,758-5,584)	4,802 (3,828-5,577)	5,045 (3,995-6,042)	4,938 (3,966-6,034)	4,667 (3,827-6,009)	3,147 (2,597-4,427)	1,269 (1,060-1,847)
Animal contact	77 (31-101)	72 (31-97)	1,359 (569-1,850)	3,034 (1,381-4,042)	2,204 (1,026-2,940)	2,095 (847-2,819)	1,893 (768-2,547)	1,753 (689-2,301)	1,734 (700-2,238)	2,232 (799-2,969)	1,667 (606-2,212)	2,261 (814-2,929)	2,155 (832-2,725)	2,864 (1,092-3,711)	2,060 (838-2,576)	2,492 (900-3,188)	2,534 (958-3,286)	2,412 (917-3,124)	1,714 (693-2,182)	1,195 (513-1,527)	645 (325-792)	273 (158-325)	101 (67-116)
Venomous animal contact	51 (15-73)	49 (16-70)	1,076 (380-1,552)	2,434 (965-3,445)	1,883 (779-2,566)	1,883 (705-2,593)	1,696 (604-2,362)	1,533 (518-2,080)	1,532 (544-2,031)	2,056 (664-2,785)	1,504 (488-2,051)	2,082 (700-2,765)	1,894 (679-2,456)	2,564 (873-3,399)	1,781 (664-2,285)	2,287 (775-2,983)	2,334 (831-3,058)	2,178 (772-2,857)	1,518 (565-1,970)	1,013 (387-1,334)	511 (219-650)	194 (94-241)	64 (37-76)
Non-venomous animal contact	26 (12-41)	23 (11-36)	283 (137-514)	599 (267-1,190)	321 (169-584)	212 (110-368)	197 (114-359)	220 (128-413)	202 (120-373)	176 (95-328)	162 (88-299)	179 (99-316)	260 (136-449)	300 (159-539)	279 (149-492)	205 (100-352)	200 (103-358)	234 (108-427)	197 (104-323)	182 (101-279)	134 (82-207)	79 (52-108)	37 (25-49)
Foreign body	533 (378-617)	466 (355-545)	6,735 (5,177-8,753)	3,262 (2,360-4,856)	1,125 (877-1,455)	750 (610-923)	601 (482-735)	755 (624-931)	821 (690-1,011)	839 (713-1,032)	848 (741-1,059)	1,096 (948-1,344)	1,141 (977-1,406)	1,362 (1,166-1,664)	1,668 (1,438-2,101)	1,800 (1,458-2,120)	1,769 (1,379-2,006)	1,893 (1,394-2,165)	2,485 (1,796-2,915)	3,116 (2,154-3,720)	3,658 (2,389-4,497)	2,986 (1,789-3,749)	1,747 (1,018-2,209)
Pulmonary aspiration and foreign body in airway	513 (356-596)	454 (343-535)	6,528 (4,931-8,536)	2,955 (2,063-4,424)	957 (726-1,255)	634 (500-797)	363 (288-436)	431 (352-527)	506 (409-623)	598 (490-737)	593 (493-738)	856 (720-1,099)	968 (798-1,219)	1,159 (966-1,451)	1,445 (1,198-1,842)	1,636 (1,297-1,939)	1,643 (1,282-1,882)	1,742 (1,259-2,012)	2,316 (1,632-2,738)	2,903 (1,951-3,483)	3,430 (2,165-4,256)	2,828 (1,671-3,570)	1,670 (966-2,121)
Foreign body in other body part	21 (14-32)	13 (8-18)	207 (144-368)	307 (192-549)	169 (118-262)	117 (80-173)	238 (163-340)	324 (213-476)	315 (219-448)	241 (170-355)	255 (184-375)	240 (168-348)	174 (123-257)	203 (156-300)	223 (169-353)	164 (117-252)	126 (83-165)	151 (98-196)	169 (99-214)	213 (129-262)	228 (131-279)	158 (88-190)	77 (42-92)
Environmental heat and cold exposure	67 (24-96)	42 (14-62)	515 (181-762)	415 (184-569)	218 (109-284)	123 (71-149)	246 (109-328)	204 (88-265)	246 (118-337)	411 (180-572)	483 (226-714)	633 (313-923)	813 (376-1,129)	999 (523-1,536)	1,241 (637-1,830)	1,414 (670-1,923)	1,062 (508-1,427)	1,099 (464-1,444)	1,312 (608-1,742)	1,373 (679-1,766)	1,290 (721-1,765)	838 (494-1,357)	332 (200-548)
Other unintentional injuries	115 (97-139)	42 (35-52)	511 (422-625)	1,214 (952-1,515)	704 (572-814)	824 (619-959)	923 (608-1,099)	877 (613-1,006)	1,049 (719-1,208)	1,197 (850-1,384)	1,017 (704-1,169)	897 (626-1,016)	948 (644-1,060)	1,070 (691-1,200)	1,012 (694-1,132)	838 (585-939)	967 (640-1,105)	526 (381-584)	580 (419-649)	720 (482-820)	617 (418-710)	362 (238-413)	147 (103-168)
Self-harm and interpersonal violence	568 (429-792)	154 (118-223)	1,257 (943-1,869)	2,468 (1,692-3,799)	1,904 (1,420-2,492)	5,792 (5,050-6,531)	29,552 (26,423-32,591)	37,144 (33,764-40,455)	35,681 (32,732-38,685)	28,283 (25,975-30,557)	24,579 (22,689-26,636)	22,198 (20,729-23,958)	21,986 (20,629-23,448)	21,758 (20,482-23,305)	18,782 (17,603-20,116)	18,454 (17,162-19,884)	16,283 (15,114-17,580)	13,309 (12,228-14,376)	12,301 (11,319-13,432)	8,636 (7,985-9,281)	6,124 (5,742-6,594)	2,715 (2,540-2,911)	807 (752-878)

SR Table 3: Global deaths by cause and age group for females (with 95% uncertainty intervals), 2016

Cause	Early Neonatal	Late Neonatal	Post Neonatal	1 to 4	5 to 9	10 to 14	15 to 19	20 to 24
Self-harm	-	-	-	-	-	3,443<br>(2,935-3,973)	24,269<br>(21,474-27,295)	29,624<br>(26,668-32,689)
Self-harm by firearm	-	-	-	-	-	132<br>(101-174)	866<br>(574-1,225)	1,175<br>(849-1,543)
Self-harm by other specified means	-	-	-	-	-	3,311<br>(2,815-3,833)	23,403<br>(20,617-26,370)	28,449<br>(25,542-31,501)
Interpersonal violence	568<br>(429-792)	154<br>(118-223)	1,257<br>(943-1,869)	2,468<br>(1,692-3,799)	1,904<br>(1,420-2,492)	2,349<br>(1,793-2,907)	5,283<br>(3,874-6,495)	7,519<br>(5,539-9,526)
Assault by firearm	36<br>(21-61)	14<br>(9-23)	169<br>(97-281)	335<br>(190-567)	305<br>(186-402)	472<br>(292-574)	1,691<br>(1,041-2,034)	2,149<br>(1,334-2,649)
Assault by sharp object	45<br>(33-79)	22<br>(13-34)	107<br>(66-161)	291<br>(171-477)	236<br>(171-337)	405<br>(288-553)	1,092<br>(724-1,479)	1,806<br>(1,251-2,492)
Assault by other means	486<br>(366-674)	117<br>(89-173)	982<br>(751-1,474)	1,842<br>(1,274-2,831)	1,363<br>(1,000-1,830)	1,472<br>(1,116-1,902)	2,500<br>(1,897-3,413)	3,564<br>(2,729-4,856)
Forces of nature, conflict and terrorism, and executions and police conflict	721<br>(492-963)	403<br>(276-537)	1,380<br>(955-1,822)	8,293<br>(5,712-11,051)	5,102<br>(3,511-6,796)	4,499<br>(3,087-6,012)	5,527<br>(3,744-7,418)	4,441<br>(3,096-5,875)
Forces of nature, conflict and terrorism, and state actor violence	1<br>(1-2)	5<br>(3-7)	39<br>(25-53)	205<br>(131-283)	197<br>(127-273)	209<br>(134-289)	171<br>(108-237)	161<br>(99-226)
Conflict and terrorism	720<br>(491-961)	398<br>(272-532)	1,328<br>(907-1,773)	8,041<br>(5,499-10,754)	4,868<br>(3,304-6,551)	4,248<br>(2,849-5,744)	5,316<br>(3,554-7,205)	4,239<br>(2,908-5,665)
Executions and police conflict	-	-	13<br>(8-23)	47<br>(26-103)	37<br>(31-49)	42<br>(34-62)	41<br>(32-55)	41<br>(32-53)

Cause	25 to 29	30 to 34	35 to 39	40 to 44	45 to 49	50 to 54	55 to 59	60 to 64
Self-harm	27,970<br>(25,456-30,465)	20,843<br>(19,138-22,628)	18,293<br>(16,984-19,812)	16,722<br>(15,727-17,959)	17,118<br>(16,154-18,148)	17,409<br>(16,538-18,574)	15,165<br>(14,212-16,252)	15,273<br>(14,164-16,410)
Self-harm by firearm	1,001<br>(802-1,183)	867<br>(752-1,011)	815<br>(704-957)	753<br>(664-873)	748<br>(667-857)	749<br>(669-862)	655<br>(581-757)	499<br>(424-605)
Self-harm by other specified means	26,969<br>(24,475-29,458)	19,976<br>(18,308-21,738)	17,478<br>(16,172-19,004)	15,970<br>(14,993-17,179)	16,370<br>(15,412-17,388)	16,660<br>(15,808-17,853)	14,510<br>(13,578-15,586)	14,774<br>(13,699-15,906)
Interpersonal violence	7,711<br>(5,904-9,682)	7,440<br>(5,840-9,151)	6,286<br>(4,780-7,910)	5,476<br>(4,206-6,801)	4,868<br>(3,707-6,084)	4,349<br>(3,377-5,413)	3,617<br>(2,788-4,422)	3,181<br>(2,465-4,039)
Assault by firearm	2,093<br>(1,346-2,586)	1,885<br>(1,188-2,305)	1,649<br>(1,039-1,996)	1,355<br>(863-1,634)	1,167<br>(733-1,458)	1,038<br>(658-1,292)	790<br>(490-1,020)	655<br>(435-865)
Assault by sharp object	2,132<br>(1,539-2,918)	2,142<br>(1,585-2,830)	1,825<br>(1,270-2,422)	1,769<br>(1,290-2,350)	1,526<br>(1,086-1,989)	1,272<br>(895-1,615)	1,019<br>(694-1,321)	895<br>(647-1,243)
Assault by other means	3,487<br>(2,734-4,667)	3,413<br>(2,630-4,500)	2,812<br>(2,197-3,705)	2,353<br>(1,849-3,071)	2,176<br>(1,690-2,872)	2,039<br>(1,563-2,695)	1,808<br>(1,398-2,331)	1,631<br>(1,212-2,062)
Forces of nature, conflict and terrorism, and executions and police conflict	2,957<br>(2,087-3,892)	2,730<br>(1,886-3,641)	2,184<br>(1,494-2,927)	1,631<br>(1,130-2,174)	1,483<br>(1,018-1,980)	1,390<br>(942-1,855)	1,202<br>(824-1,598)	1,109<br>(770-1,466)
Forces of nature, conflict and terrorism, and state actor violence	166<br>(101-237)	158<br>(96-225)	140<br>(85-199)	148<br>(85-216)	148<br>(83-222)	145<br>(81-215)	147<br>(81-219)	147<br>(83-218)
Conflict and terrorism	2,748<br>(1,889-3,658)	2,531<br>(1,691-3,411)	2,005<br>(1,316-2,732)	1,445<br>(939-1,993)	1,297<br>(842-1,790)	1,213<br>(780-1,673)	1,022<br>(657-1,405)	935<br>(606-1,284)
Executions and police conflict	43<br>(32-57)	41<br>(29-51)	39<br>(26-53)	39<br>(25-57)	38<br>(24-56)	32<br>(22-41)	34<br>(20-51)	27<br>(18-36)

Cause	65 to 69	70 to 74	75 to 79	80 to 84	85 to 89	90 to 94	95 plus
Self-harm	13,688<br>(12,699-14,778)	11,066<br>(10,234-12,025)	10,037<br>(9,221-10,952)	7,857<br>(7,268-8,474)	5,638<br>(5,279-6,070)	2,483<br>(2,324-2,665)	727<br>(677-779)
Self-harm by firearm	419<br>(338-554)	330<br>(245-475)	317<br>(220-499)	272<br>(198-402)	222<br>(186-277)	145<br>(129-163)	67<br>(60-74)
Self-harm by other specified means	13,268<br>(12,280-14,361)	10,736<br>(9,922-11,672)	9,720<br>(8,937-10,642)	7,584<br>(7,017-8,195)	5,416<br>(5,071-5,833)	2,339<br>(2,188-2,506)	660<br>(616-711)
Interpersonal violence	2,595<br>(2,008-3,327)	2,244<br>(1,672-2,751)	2,264<br>(1,726-2,842)	780<br>(659-1,086)	486<br>(400-704)	232<br>(196-322)	80<br>(68-119)
Assault by firearm	518<br>(315-685)	465<br>(284-606)	405<br>(249-538)	63<br>(39-95)	40<br>(27-63)	18<br>(12-31)	8<br>(5-15)
Assault by sharp object	591<br>(416-803)	640<br>(437-811)	620<br>(435-810)	241<br>(182-345)	137<br>(110-198)	76<br>(63-110)	24<br>(20-37)
Assault by other means	1,486<br>(1,124-1,920)	1,139<br>(841-1,451)	1,239<br>(960-1,559)	476<br>(394-671)	309<br>(255-458)	138<br>(111-197)	48<br>(39-73)
Forces of nature, conflict and terrorism, and executions and police conflict	918<br>(656-1,202)	701<br>(497-917)	547<br>(384-724)	407<br>(288-537)	251<br>(175-337)	64<br>(46-84)	77<br>(47-108)
Forces of nature, conflict and terrorism, and state actor violence	124<br>(70-185)	123<br>(68-183)	108<br>(59-163)	86<br>(47-130)	57<br>(31-85)	19<br>(10-30)	7<br>(4-10)
Conflict and terrorism	771<br>(513-1,046)	554<br>(359-762)	421<br>(263-586)	295<br>(180-416)	175<br>(101-253)	32<br>(18-46)	66<br>(36-97)
Executions and police conflict	23<br>(14-31)	24<br>(12-42)	18<br>(9-27)	26<br>(10-40)	20<br>(5-35)	13<br>(5-21)	4<br>(1-7)

SR Table 4: Global deaths by cause and age group for males (with 95% uncertainty intervals), 1990

Cause	Early Neonatal	Late Neonatal	Post Neonatal	1 to 4	5 to 9	10 to 14	15 to 19	20 to 24	25 to 29	30 to 34	35 to 39	40 to 44	45 to 49	50 to 54	55 to 59	60 to 64	65 to 69	70 to 74	75 to 79	80 to 84	85 to 89	90 to 94	95 plus
All causes	1,727,412 (1,691,079-1,762,091)	649,616 (636,037-663,571)	1,810,014 (1,772,654-1,846,568)	1,674,995 (1,634,637-1,714,193)	420,983 (416,466-425,417)	278,895 (275,636-282,331)	400,003 (394,453-405,600)	547,133 (539,630-554,834)	563,482 (555,114-572,292)	605,618 (596,806-614,720)	683,609 (673,199-694,993)	723,931 (713,284-735,359)	847,766 (835,498-860,424)	1,160,928 (1,143,414-1,179,649)	1,469,147 (1,450,047-1,489,485)	1,901,750 (1,878,851-1,927,011)	2,062,375 (2,041,006-2,084,675)	2,147,862 (2,126,628-2,171,090)	2,106,188 (2,088,798-2,125,133)	1,558,631 (1,548,931-1,568,563)	909,599 (905,426-913,560)	346,078 (344,972-347,174)	90,373 (90,103-90,654)
Communicable, maternal, neonatal, and nutritional disorders	1,587,030 (1,536,007-1,639,628)	579,463 (555,249-605,327)	1,501,351 (1,442,311-1,565,368)	1,299,498 (1,240,907-1,377,054)	223,268 (206,783-240,585)	107,556 (98,396-116,824)	92,272 (84,574-101,557)	115,594 (108,094-125,447)	141,867 (133,720-152,811)	158,988 (150,577-170,232)	166,213 (157,161-178,964)	160,962 (152,127-173,236)	168,188 (157,846-182,518)	183,295 (171,098-202,600)	200,415 (185,451-224,841)	229,356 (207,775-262,028)	235,849 (211,626-274,788)	255,999 (226,524-302,674)	228,543 (200,609-278,724)	176,695 (154,572-212,710)	110,067 (94,874-130,710)	42,901 (36,761-50,421)	12,289 (10,769-14,063)
HIV/AIDS and tuberculosis	-	-	24,517 (21,388-27,445)	32,243 (24,574-38,884)	8,878 (7,378-10,343)	8,421 (7,266-9,557)	20,987 (18,915-23,410)	50,301 (46,692-55,322)	82,566 (76,362-91,314)	99,899 (93,104-108,969)	103,008 (95,757-113,751)	99,403 (92,756-110,414)	99,644 (92,632-111,934)	105,499 (98,012-119,430)	109,564 (102,013-125,137)	112,248 (103,964-129,122)	102,590 (94,239-119,467)	97,647 (88,497-113,539)	70,553 (63,594-83,378)	41,892 (37,198-52,639)	19,421 (17,207-24,954)	5,529 (4,884-7,247)	1,194 (1,053-1,574)
Tuberculosis	-	-	15,041 (12,031-17,770)	25,983 (18,193-32,575)	8,159 (6,700-9,628)	8,157 (7,011-9,294)	18,827 (16,776-21,196)	37,597 (34,375-42,602)	52,187 (47,730-59,915)	64,048 (59,127-73,583)	70,317 (64,527-81,613)	72,978 (67,580-83,864)	81,429 (74,819-93,701)	94,546 (87,178-108,448)	101,712 (94,383-116,001)	106,788 (98,380-123,699)	98,867 (90,597-115,626)	95,515 (86,231-111,647)	69,394 (62,470-82,140)	41,715 (36,998-52,418)	19,353 (17,129-24,862)	5,511 (4,870-7,231)	1,189 (1,049-1,570)
Drug-susceptible tuberculosis	-	-	14,957 (11,946-17,680)	25,864 (18,106-32,450)	8,102 (6,648-9,576)	8,100 (6,954-9,240)	18,702 (16,649-21,046)	37,340 (34,112-42,342)	51,863 (47,425-59,572)	63,638 (58,702-73,168)	69,818 (64,021-81,007)	72,455 (67,018-83,338)	80,874 (74,256-93,085)	93,777 (86,457-107,675)	100,863 (93,647-115,093)	105,816 (97,489-122,734)	97,967 (89,747-114,568)	94,618 (85,389-110,873)	68,720 (61,766-81,527)	41,348 (36,674-51,998)	19,188 (16,970-24,677)	5,466 (4,823-7,171)	1,180 (1,040-1,558)
Multidrug-resistant tuberculosis without extensive drug resistance	-	-	84 (64-106)	119 (86-154)	57 (45-74)	57 (44-74)	125 (99-156)	257 (203-321)	324 (254-410)	411 (323-523)	500 (395-636)	523 (405-669)	555 (442-700)	769 (603-1,002)	849 (666-1,077)	971 (764-1,212)	900 (713-1,129)	897 (714-1,132)	673 (530-852)	367 (282-484)	164 (125-223)	45 (34-63)	10 (7-14)
Extensively drug-resistant tuberculosis	-	-	0 (0-0)	0 (0-0)	0 (0-0)	0 (0-0)	0 (0-0)	0 (0-0)	0 (0-0)	0 (0-0)	0 (0-0)	0 (0-0)	0 (0-0)	0 (0-0)	0 (0-0)	0 (0-0)	0 (0-0)	0 (0-0)	0 (0-0)	0 (0-0)	0 (0-0)	0 (0-0)	0 (0-0)
HIV/AIDS	-	-	9,476 (8,492-10,764)	6,260 (5,502-7,306)	719 (613-869)	263 (231-304)	2,160 (1,938-2,473)	12,703 (10,898-15,018)	30,379 (25,891-35,324)	35,851 (31,499-40,534)	32,690 (28,918-37,165)	26,425 (23,537-29,994)	18,215 (15,884-21,107)	10,953 (9,583-12,540)	7,852 (6,749-9,266)	5,461 (4,610-6,514)	3,722 (3,107-4,474)	2,132 (1,740-2,576)	1,160 (961-1,387)	177 (135-236)	68 (53-90)	18 (14-23)	4 (4-5)
Drug-susceptible HIV/AIDS - Tuberculosis	-	-	3,443 (2,777-4,037)	2,351 (1,908-2,786)	236 (184-293)	64 (46-82)	796 (687-936)	4,018 (3,048-5,113)	7,808 (5,634-10,370)	8,628 (6,265-11,341)	7,925 (5,742-10,255)	6,911 (5,058-8,852)	5,292 (3,928-6,761)	3,416 (2,643-4,191)	2,536 (1,966-3,125)	1,823 (1,387-2,282)	1,277 (979-1,591)	784 (616-982)	426 (340-523)	56 (41-76)	21 (15-28)	5 (4-7)	1 (1-1)
Multidrug-resistant HIV/AIDS - Tuberculosis without extensive drug resistance	-	-	15 (9-24)	10 (6-15)	1 (1-2)	0 (0-1)	3 (2-5)	18 (11-28)	44 (27-70)	63 (38-104)	61 (36-99)	48 (30-78)	35 (21-56)	24 (14-38)	17 (10-26)	11 (7-18)	7 (4-11)	4 (2-6)	2 (1-4)	0 (0-1)	0 (0-0)	0 (0-0)	0 (0-0)
Extensively drug-resistant HIV/AIDS - Tuberculosis	-	-	0 (0-0)	0 (0-0)	0 (0-0)	0 (0-0)	0 (0-0)	0 (0-0)	0 (0-0)	0 (0-0)	0 (0-0)	0 (0-0)	0 (0-0)	0 (0-0)	0 (0-0)	0 (0-0)	0 (0-0)	0 (0-0)	0 (0-0)	0 (0-0)	0 (0-0)	0 (0-0)	0 (0-0)
HIV/AIDS resulting in other diseases	-	-	6,018 (5,291-7,111)	3,899 (3,363-4,790)	482 (407-601)	199 (171-236)	1,361 (1,186-1,634)	8,667 (7,079-11,016)	22,526 (18,319-27,536)	27,160 (23,141-31,840)	24,704 (21,065-29,682)	19,467 (16,610-23,379)	12,888 (10,862-15,679)	7,513 (6,438-9,011)	5,300 (4,529-6,486)	3,626 (3,030-4,448)	2,438 (2,018-3,069)	1,344 (1,075-1,719)	732 (598-915)	120 (90-171)	47 (36-66)	12 (9-17)	3 (3-4)
Diarrhoea, lower respiratory infections, and other common infectious diseases	233,407 (191,152-275,178)	276,842 (219,037-313,941)	1,169,893 (1,100,304-1,253,924)	918,986 (815,763-1,061,447)	161,975 (138,070-189,255)	73,284 (61,997-85,767)	52,238 (43,093-63,874)	44,639 (35,856-57,294)	42,120 (33,586-55,565)	41,593 (33,160-54,794)	45,048 (36,125-61,183)	44,021 (35,093-59,433)	50,470 (39,883-68,662)	60,062 (46,775-84,696)	72,259 (55,375-101,062)	97,805 (74,490-137,471)	113,897 (86,096-159,577)	139,616 (104,994-194,771)	141,590 (109,630-200,457)	123,877 (99,026-165,193)	83,137 (66,883-106,614)	33,982 (27,622-42,252)	9,919 (8,235-11,877)
Diarrhoeal diseases	30,871 (26,109-37,412)	64,350 (53,322-76,960)	407,595 (356,689-456,101)	255,341 (200,108-306,838)	30,933 (14,928-56,339)	14,404 (7,205-27,310)	14,706 (7,506-27,339)	15,711 (8,515-29,598)	16,259 (8,382-31,225)	15,847 (7,378-30,385)	17,902 (9,383-36,108)	17,256 (8,724-34,140)	20,680 (10,506-40,021)	25,153 (12,416-52,584)	31,857 (15,688-61,970)	45,508 (23,135-88,175)	55,603 (28,573-104,857)	70,110 (35,352-130,686)	65,850 (34,056-129,066)	55,869 (30,534-100,585)	36,258 (20,168-61,810)	14,407 (8,223-23,213)	3,961 (2,406-6,092)
Intestinal infectious diseases	-	-	1,025 (179-4,019)	18,719 (8,623-35,866)	35,835 (19,476-59,730)	21,666 (11,661-34,156)	12,090 (6,198-20,841)	5,810 (2,917-10,057)	4,403 (2,297-7,584)	3,415 (1,814-5,836)	2,575 (1,386-4,287)	1,879 (1,019-3,171)	1,604 (862-2,672)	1,380 (716-2,350)	1,058 (509-1,921)	915 (447-1,669)	752 (427-1,237)	568 (333-907)	360 (212-568)	191 (110-303)	93 (54-149)	28 (16-44)	6 (4-11)
Typhoid fever	-	-	100 (46-192)	16,503 (7,211-32,581)	31,719 (16,750-53,135)	18,647 (9,809-29,823)	10,669 (5,327-18,849)	5,001 (2,491-8,875)	3,606 (1,849-6,329)	2,686 (1,404-4,634)	1,988 (1,053-3,461)	1,436 (753-2,478)	1,206 (641-2,058)	1,055 (529-1,868)	829 (390-1,510)	740 (355-1,337)	632 (341-1,041)	481 (277-775)	298 (169-480)	159 (89-256)	75 (42-118)	22 (12-35)	5 (3-8)
Paratyphoid fever	-	-	14 (6-31)	1,547 (612-3,453)	4,041 (1,820-7,962)	2,994 (1,360-5,628)	1,400 (603-2,797)	785 (338-1,588)	776 (338-1,558)	699 (312-1,390)	555 (260-1,064)	418 (195-794)	367 (166-711)	313 (137-607)	216 (89-447)	161 (68-330)	106 (49-203)	70 (35-133)	42 (21-77)	23 (11-42)	11 (5-20)	3 (1-6)	1 (0-1)
Other intestinal infectious diseases	-	-	911 (70-3,896)	669 (45-3,280)	74 (7-329)	24 (2-115)	21 (2-107)	24 (2-120)	20 (2-87)	29 (3-136)	32 (4-135)	25 (2-113)	31 (3-139)	12 (1-56)	13 (1-53)	14 (2-58)	15 (2-64)	16 (2-71)	20 (2-88)	10 (1-45)	8 (1-29)	3 (0-11)	1 (0-4)
Lower respiratory infections	135,368 (119,577-154,557)	140,319 (118,156-162,255)	495,246 (440,182-546,579)	254,565 (205,896-303,287)	27,559 (21,187-33,265)	12,371 (9,857-14,434)	9,784 (7,576-11,591)	10,661 (8,354-12,524)	11,036 (8,808-13,308)	12,223 (9,966-14,212)	14,250 (11,654-16,919)	15,398 (12,891-17,819)	18,195 (15,072-21,527)	23,466 (19,254-27,700)	29,115 (23,965-34,252)	42,101 (34,093-48,475)	48,941 (39,884-57,388)	61,324 (50,642-70,902)	69,592 (58,804-79,146)	63,627 (55,115-71,929)	44,556 (39,285-50,637)	18,782 (16,534-21,587)	5,743 (5,073-6,486)
Upper respiratory infections	75 (47-111)	36 (20-62)	635 (411-958)	500 (301-858)	39 (24-61)	26 (16-40)	56 (39-80)	58 (40-82)	58 (40-83)	56 (39-78)	64 (45-91)	58 (41-82)	67 (47-94)	72 (51-101)	80 (55-111)	89 (62-126)	73 (53-100)	59 (40-83)	52 (37-73)	28 (20-38)	16 (12-22)	5 (4-7)	1 (1-1)
Otitis media	7 (3-10)	22 (13-36)	291 (177-489)	395 (165-763)	191 (85-307)	193 (96-321)	83 (45-123)	63 (34-92)	43 (24-63)	37 (20-53)	46 (25-71)	53 (26-85)	135 (55-250)	151 (61-291)	75 (28-117)	62 (28-102)	82 (32-149)	73 (30-115)	49 (24-75)	42 (20-67)	22 (10-33)	9 (4-15)	3 (1-4)
Meningitis	7,350 (4,603-10,419)	12,255 (8,901-15,670)	65,696 (50,350-81,223)	58,500 (41,161-75,108)	11,698 (5,553-16,872)	7,012 (3,702-9,932)	6,026 (3,488-8,610)	5,410 (3,110-7,737)	4,635 (2,784-6,726)	4,446 (2,558-6,645)	4,363 (2,665-6,398)	3,574 (2,226-5,234)	3,897 (2,372-5,655)	3,907 (2,393-5,753)	3,866 (2,357-5,622)	3,874 (2,401-5,754)	3,744 (2,319-5,303)	2,961 (1,748-4,206)	2,268 (1,398-3,195)	1,598 (976-2,228)	807 (495-1,108)	253 (153-347)	59 (37-77)
Pneumococcal meningitis	387 (239-582)	646 (437-889)	3,742 (2,737-4,985)	3,402 (2,178-4,904)	674 (316-1,065)	399 (205-625)	341 (192-525)	301 (163-476)	304 (174-484)	362 (212-540)	431 (245-672)	417 (240-662)	487 (284-785)	476 (292-738)	483 (296-721)	510 (325-750)	489 (306-707)	392 (237-582)	308 (187-439)	213 (130-300)	100 (64-136)	31 (20-42)	7 (5-11)
H influenzae type B meningitis	1,024 (631-1,466)	1,671 (1,133-2,192)	8,383 (6,201-11,083)	6,906 (4,513-9,735)	1,087 (486-1,638)	542 (273-798)	407 (219-615)	359 (202-550)	336 (186-529)	337 (186-525)	339 (197-504)	281 (162-428)	312 (179-481)	311 (184-469)	307 (179-462)	313 (182-486)	289 (164-443)	223 (127-333)	170 (103-250)	123 (70-178)	61 (38-86)	19 (11-27)	4 (2-6)
Meningococcal infection	3,686 (2,299-5,360)	6,148 (4,333-7,962)	32,272 (24,470-40,688)	31,172 (21,374-41,038)	6,439 (3,111-9,145)	3,796 (2,041-5,352)	3,302 (1,925-4,728)	2,913 (1,652-4,165)	2,388 (1,401-3,452)	2,115 (1,227-3,198)	2,024 (1,200-3,036)	1,645 (984-2,515)	1,779 (1,008-2,639)	1,804 (1,068-2,693)	1,770 (1,028-2,585)	1,805 (1,047-2,742)	1,722 (1,014-2,518)	1,340 (746-1,951)	1,014 (581-1,451)	699 (401-999)	369 (214-520)	117 (67-166)	27 (15-36)
Other meningitis	2,253 (1,366-3,268)	3,790 (2,662-4,893)	21,299 (15,750-27,127)	17,020 (11,618-22,868)	3,499 (1,541-5,213)	2,275 (1,153-3,286)	1,977 (1,097-2,858)	1,837 (984-2,661)	1,606 (906-2,281)	1,631 (902-2,398)	1,568 (878-2,304)	1,231 (702-1,762)	1,319 (782-1,918)	1,315 (790-2,009)	1,306 (772-1,921)	1,246 (766-1,829)	1,244 (735-1,813)	1,006 (596-1,428)	776 (481-1,094)	563 (352-816)	277 (178-396)	86 (55-121)	20 (13-28)
Encephalitis	595 (281-850)	1,095 (479-1,794)	9,790 (4,905-15,223)	11,432 (5,238-20,211)	4,231 (2,451-6,116)	2,950 (1,868-3,961)	2,073 (1,421-2,655)	1,922 (1,382-2,482)	1,724 (1,203-2,144)	1,828 (1,297-2,248)	1,799 (1,320-2,292)	1,438 (1,059-1,815)	1,423 (1,044-1,771)	1,466 (1,099-1,852)	1,709 (1,265-2,089)	1,928 (1,436-2,412)	2,103 (1,479-2,551)	1,984 (1,314-2,444)	1,820 (1,167-2,300)	1,189 (761-1,523)	589 (374-751)	171 (113-213)	38 (26-47)
Diphtheria	-	-	1,342 (853-1,977)	2,841 (1,576-4,911)	432 (256-659)	185 (120-280)	85 (56-125)	57 (38-85)	51 (34-75)	46 (30-68)	64 (42-92)	61 (40-92)	68 (45-102)	78 (53-112)	56 (38-83)	-	-	-	-	-	-	-	-
Whooping cough	-	-	38,261 (16,725-75,994)	34,554 (14,996-66,699)	3,562 (1,570-7,085)	746 (315-1,561)	354 (142-764)	243 (97-521)	141 (53-314)	110 (41-253)	75 (28-174)	91 (35-209)	72 (27-165)	57 (21-130)	81 (31-182)	-	-	-	-	-	-	-	-
Tetanus	59,065 (25,144-94,862)	58,502 (23,764-85,848)	18,435 (7,255-32,650)	6,775 (2,174-12,394)	3,740 (1,376-6,044)	2,057 (906-3,127)	2,629 (1,138-4,294)	2,558 (1,135-4,001)	2,691 (1,296-4,031)	2,844 (1,365-4,281)	3,411 (1,695-5,012)	3,879 (1,874-5,695)	4,033 (1,960-5,861)	4,090 (1,903-5,822)	4,085 (1,884-5,929)	3,185 (1,463-5,048)	2,347 (1,152-3,573)	2,306 (945-3,352)	1,252 (502-1,849)	897 (378-1,385)	361 (147-573)	101 (46-162)	27 (12-43)
Measles	-	-	129,721 (49,359-265,638)	273,143 (114,544-509,536)	42,799 (17,257-84,746)	11,371 (4,071-24,887)	4,210 (1,421-9,819)	1,967 (661-4,701)	869 (293-2,046)	517 (174-1,235)	316 (107-745)	232 (79-541)	180 (63-419)	140 (47-329)	154 (52-363)	-	-	-	-	-	-	-	-
Varicella and herpes zoster	75 (58-95)	263 (201-339)	1,856 (1,465-2,393)	2,222 (1,636-2,968)	955 (723-1,244)	304 (245-382)	141 (111-175)	179 (142-224)	211 (171-260)	224 (182-278)	182 (149-225)	102 (82-128)	119 (95-144)	102 (83-128)	124 (100-153)	144 (115-179)	252 (201-314)	231 (185-289)	347 (280-428)	437 (342-550)	435 (343-555)	226 (179-280)	82 (64-104)
Neglected tropical diseases and malaria	525 (227-798)	12,332 (8,243-17,694)	102,438 (73,501-138,011)	211,438 (151,058-278,927)	35,354 (23,468-45,754)	18,933 (12,911-24,193)	11,289 (8,325-14,385)	11,989 (9,134-15,045)	8,763 (6,620-11,241)	8,453 (6,296-10,592)	8,861 (6,501-11,301)	8,317 (6,396-10,315)	8,334 (6,056-10,301)	7,587 (5,959-9,253)	7,668 (6,048-9,396)	7,622 (6,149-9,297)	7,075 (5,698-8,791)	6,053 (4,885-7,564)	4,495 (3,549-5,653)	1,989 (1,586-2,474)	1,237 (952-1,604)	229 (178-285)	82 (62-107)
Malaria	-	11,780 (7,633-17,181)	95,694 (66,177-131,049)	192,819 (131,772-261,108)	23,619 (13,708-33,394)	11,841 (6,838-17,207)	4,768 (3,096-6,486)	4,840 (3,152-6,644)	3,918 (2,496-5,249)	3,660 (2,257-5,064)	3,882 (2,398-5,601)	3,468 (2,295-4,747)	3,192 (1,947-4,505)	2,928 (1,939-4,037)	2,903 (1,973-4,053)	3,437 (2,370-4,813)	3,360 (2,247-4,911)	2,696 (1,767-3,937)	1,997 (1,288-2,949)	834 (553-1,224)	680 (445-1,000)	100 (65-142)	44 (29-66)
Chagas disease	-	-	4 (4-5)	3 (2-3)	3 (3-4)	7 (6-8)	19 (14-24)	45 (34-55)	91 (69-108)	181 (148-212)	282 (238-327)	382 (328-438)	470 (412-540)	586 (509-671)	618 (531-725)	781 (700-873)	720 (654-791)	666 (610-721)	385 (341-440)	238 (213-264)	101 (92-113)	29 (25-33)	7 (6-8)
Leishmaniasis	-	-	1,976 (1,102-3,383)	2,989 (1,486-5,304)	1,505 (821-2,575)	984 (546-1,642)	1,352 (761-2,171)	1,614 (928-2,638)	1,329 (755-2,131)	1,233 (706-2,004)	1,437 (818-2,353)	1,393 (768-2,262)	1,326 (743-2,151)	1,331 (750-2,141)	986 (542-1,654)	795 (452-1,357)	832 (465-1,365)	928 (522-1,521)	740 (412-1,227)	387 (213-638)	236 (131-379)	48 (23-85)	16 (6-29)
Visceral leishmaniasis	-	-	1,976 (1,102-3,383)	2,989 (1,486-5,304)	1,505 (821-2,575)	984 (546-1,642)	1,352 (761-2,171)	1,614 (928-2,638)	1,329 (755-2,131)	1,233 (706-2,004)	1,437 (818-2,353)	1,393 (768-2,262)	1,326 (743-2,151)	1,331 (750-2,141)	986 (542-1,654)	795 (452-1,357)	832 (465-1,365)	928 (522-1,521)	740 (412-1,227)	387 (213-638)	236 (131-379)	48 (23-85)	16 (6-29)
African trypanosomiasis	-	-	362 (60-1,340)	[illegible]	710 (164-2,008)	797 (196-1,953)	1,042 (268-2,413)	1,129 (326-2,751)	1,015 (261-2,721)	840 (216-2,228)	779 (177-2,180)	674 (150-1,964)	581 (126-1,711)	490 (101-1,400)	432 (99-1,343)	361 (70-1,238)	254 (54-833)	182 (30-666)	100 (18-385)	45 (7-173)	16 (3-58)	4 (1-17)	1 (0-3)
Schistosomiasis	-	-	0 (0-0)	468 (273-798)	207 (128-331)	256 (172-363)	229 (160-326)	312 (225-441)	351 (255-481)	378 (272-512)	455 (325-622)	493 (354-702)	527 (380-732)	606 (435-825)	693 (510-941)	716 (527-969)	699 (506-943)	596 (428-802)	388 (282-532)	165 (120-223)	66 (48-90)	15 (9-22)	2 (1-4)
Cysticercosis	0 (0-0)	0 (0-0)	0 (0-0)	75 (49-110)	47 (34-62)	36 (28-44)	33 (26-42)	50 (41-62)	62 (51-74)	65 (53-77)	56 (47-68)	50 (42-59)	47 (39-57)	53 (45-62)	48 (42-56)	43 (35-51)	31 (27-37)	18 (15-22)	11 (9-13)	5 (4-6)	2 (2-2)	1 (0-1)	0 (0-0)
Cystic echinococcosis	-	-	-	291 (188-443)	106 (70-152)	59 (40-84)	27 (19-37)	36 (25-49)	48 (35-65)	50 (37-66)	60 (45-77)	52 (40-67)	56 (42-72)	68 (54-84)	70 (55-88)	74 (59-92)	58 (45-72)	46 (36-58)	41 (32-52)	19 (14-24)	8 (6-10)	2 (2-3)	0 (0-1)
Dengue	-	-	523 (115-1,292)	1,084 (210-2,902)	578 (99-1,418)	301 (54-688)	294 (41-719)	279 (45-625)	224 (32-544)	169 (24-440)	176 (26-416)	134 (19-330)	143 (21-338)	139 (23-334)	162 (23-401)	153 (23-366)	150 (25-377)	57 (11-124)	41 (9-86)	30 (6-71)	18 (5-39)	5 (2-10)	1 (1-2)
Yellow fever	-	5 (1-17)	90 (18-275)	793 (142-2,501)	1,466 (291-4,540)	1,242 (257-3,767)	1,139 (221-3,397)	997 (207-3,106)	741 (153-2,309)	559 (113-1,676)	441 (89-1,367)	347 (70-1,002)	278 (53-872)	220 (44-652)	174 (33-545)	132 (25-408)	95 (19-294)	59 (12-184)	31 (6-101)	11 (2-34)	4 (1-12)	1 (0-3)	0 (0-0)
Rabies	-	-	654 (257-1,036)	3,011 (1,191-4,847)	5,381 (2,161-8,428)	2,911 (1,129-4,353)	2,085 (1,043-3,118)	2,377 (1,214-3,674)	687 (315-986)	1,029 (432-1,477)	974 (402-1,392)	1,043 (444-1,403)	1,407 (535-2,124)	832 (360-1,078)	1,207 (405-1,822)	795 (302-1,103)	555 (228-790)	498 (222-742)	547 (300-867)	145 (79-291)	52 (28-129)	6 (3-17)	2 (1-6)
Intestinal nematode infections	-	-	398 (303-510)	4,332 (3,169-5,748)	674 (527-847)	130 (104-160)	41 (32-53)	30 (22-38)	22 (17-29)	28 (21-36)	26 (20-34)	17 (13-22)	19 (14-24)	28 (21-35)	24 (18-32)	29 (22-36)	19 (14-24)	27 (21-34)	19 (14-24)	10 (7-13)	3 (2-4)	2 (1-2)	0 (0-1)
Ascariasis	-	-	398 (303-510)	4,332 (3,169-5,748)	674 (527-847)	130 (104-160)	41 (32-53)	30 (22-38)	22 (17-29)	28 (21-36)	26 (20-34)	17 (13-22)	19 (14-24)	28 (21-35)	24 (18-32)	29 (22-36)	19 (14-24)	27 (21-34)	19 (14-24)	10 (7-13)	3 (2-4)	2 (1-2)	0 (0-1)
Ebola	0 (0-0)	0 (0-0)	0 (0-0)	0 (0-0)	0 (0-0)	0 (0-0)	0 (0-0)	0 (0-0)	0 (0-0)	0 (0-0)	0 (0-0)	0 (0-0)	0 (0-0)	0 (0-0)	0 (0-0)	0 (0-0)	0 (0-0)	0 (0-0)	0 (0-0)	0 (0-0)	0 (0-0)	0 (0-0)	0 (0-0)
Zika virus	0 (0-0)	0 (0-0)	0 (0-0)	0 (0-0)	0 (0-0)	0 (0-0)	0 (0-0)	0 (0-0)	0 (0-0)	0 (0-0)	0 (0-0)	0 (0-0)	0 (0-0)	0 (0-0)	0 (0-0)	0 (0-0)	0 (0-0)	0 (0-0)	0 (0-0)	0 (0-0)	0 (0-0)	0 (0-0)	0 (0-0)
Other neglected tropical diseases	525 (227-798)	547 (251-913)	3,099 (1,262-5,117)	5,211 (1,461-13,545)	1,060 (486-2,001)	369 (229-561)	259 (138-373)	280 (147-397)	276 (136-376)	261 (112-341)	293 (134-369)	262 (133-341)	288 (126-375)	307 (147-387)	352 (179-444)	306 (170-400)	301 (159-393)	279 (174-373)	196 (114-258)	101 (66-145)	51 (33-72)	17 (12-24)	7 (4-9)
Maternal disorders	-	-	-	-	-	-	-	-	-	-	-	-	-	-	-	-	-	-	-	-	-	-	-
Maternal haemorrhage	-	-	-	-	-	-	-	-	-	-	-	-	-	-	-	-	-	-	-	-	-	-	-
Maternal sepsis and other pregnancy related infections	-	-	-	-	-	-	-	-	-	-	-	-	-	-	-	-	-	-	-	-	-	-	-
Maternal hypertensive disorders	-	-	-	-	-	-	-	-	-	-	-	-	-	-	-	-	-	-	-	-	-	-	-
Maternal obstructed labour and uterine rupture	-	-	-	-	-	-	-	-	-	-	-	-	-	-	-	-	-	-	-	-	-	-	-
Maternal abortion, miscarriage, and ectopic pregnancy	-	-	-	-	-	-	-	-	-	-	-	-	-	-	-	-	-	-	-	-	-	-	-
Indirect maternal deaths	-	-	-	-	-	-	-	-	-	-	-	-	-	-	-	-	-	-	-	-	-	-	-
Late maternal deaths	-	-	-	-	-	-	-	-	-	-	-	-	-	-	-	-	-	-	-	-	-	-	-
Maternal deaths aggravated by HIV/AIDS	-	-	-	-	-	-	-	-	-	-	-	-	-	-	-	-	-	-	-	-	-	-	-
Other maternal disorders	-	-	-	-	-	-	-	-	-	-	-	-	-	-	-	-	-	-	-	-	-	-	-
Neonatal disorders	1,319,860 (1,262,515-1,401,980)	274,514 (240,737-346,436)	73,633 (60,435-98,482)	7,168 (5,139-11,460)	-	-	-	-	-	-	-	-	-	-	-	-	-	-	-	-	-	-	-
Neonatal preterm birth complications	577,088 (532,936-636,056)	93,222 (76,309-133,868)	31,747 (25,075-45,291)	2,110 (1,446-3,735)	-	-	-	-	-	-	-	-	-	-	-	-	-	-	-	-	-	-	-
Neonatal encephalopathy due to birth asphyxia and trauma	394,965 (343,936-455,115)	40,258 (33,874-50,581)	11,666 (9,474-15,067)	2,552 (1,844-3,778)	-	-	-	-	-	-	-	-	-	-	-	-	-	-	-	-	-	-	-
Neonatal sepsis and other neonatal infections	85,844 (53,241-109,207)	61,049 (41,504-90,854)	10,016 (5,743-16,711)	601 (172-1,257)	-	-	-	-	-	-	-	-	-	-	-	-	-	-	-	-	-	-	-
Hemolytic disease and other neonatal jaundice	41,886 (32,052-57,815)	31,387 (23,706-42,199)	5,152 (3,722-6,982)	358 (219-584)	-	-	-	-	-	-	-	-	-	-	-	-	-	-	-	-	-	-	-
Other neonatal disorders	220,077 (189,471-254,298)	48,597 (36,923-80,408)	15,051 (11,332-23,610)	1,546 (986-3,344)	-	-	-	-	-	-	-	-	-	-	-	-	-	-	-	-	-	-	-
Nutritional deficiencies	-	-	77,949 (59,319-94,477)	96,179 (55,964-135,011)	7,856 (6,127-9,752)	2,690 (2,226-3,235)	2,116 (1,755-2,696)	2,167 (1,809-2,769)	1,914 (1,583-2,516)	2,049 (1,730-2,680)	2,151 (1,820-2,850)	2,218 (1,860-2,963)	2,458 (2,062-3,408)	3,093 (2,580-4,205)	3,806 (3,203-5,070)	4,545 (3,832-6,021)	5,695 (4,717-7,738)	6,966 (5,886-9,119)	7,357 (6,271-9,562)	6,406 (5,542-8,135)	5,023 (4,400-6,177)	2,755 (2,426-3,291)	984 (879-1,160)
Protein-energy malnutrition	-	-	75,420 (57,739-91,615)	93,467 (54,892-131,417)	6,908 (5,367-8,699)	2,265 (1,852-2,760)	1,524 (1,273-1,911)	1,537 (1,293-1,886)	1,351 (1,110-1,698)	1,478 (1,263-1,821)	1,548 (1,328-1,970)	1,617 (1,378-2,045)	1,657 (1,411-2,122)	2,197 (1,867-2,791)	2,714 (2,323-3,411)	3,241 (2,820-4,299)	4,160 (3,530-5,446)	5,403 (4,617-6,791)	5,849 (5,058-7,215)	5,228 (4,543-6,285)	4,106 (3,565-4,778)	2,310 (2,019-2,634)	836 (722-948)
Iodine deficiency	-	-	209 (45-612)	181 (31-721)	59 (17-175)	14 (3-36)	33 (9-92)	34 (8-122)	37 (10-117)	35 (9-117)	33 (9-95)	26 (7-78)	23 (6-75)	32 (9-102)	39 (13-103)	37 (12-95)	45 (15-106)	48 (18-109)	40 (14-103)	23 (7-53)	10 (3-28)	2 (1-8)	1 (0-3)
Iron-deficiency anaemia	-	-	53 (14-93)	69 (30-110)	76 (38-116)	62 (35-90)	83 (53-120)	70 (52-96)	54 (43-71)	62 (46-82)	47 (37-65)	43 (32-63)	47 (35-77)	50 (37-76)	66 (46-130)	71 (50-117)	102 (66-166)	125 (67-206)	97 (58-154)	72 (46-125)	34 (21-71)	22 (11-44)	11 (6-19)
Other nutritional deficiencies	-	-	2,266 (1,360-3,085)	2,463 (842-3,938)	813 (539-1,174)	350 (263-481)	476 (349-701)	526 (374-865)	472 (331-790)	474 (328-816)	523 (373-866)	532 (355-925)	731 (503-1,323)	814 (542-1,509)	987 (680-1,650)	1,105 (796-1,779)	1,388 (955-2,251)	1,389 (959-2,328)	1,372 (895-2,393)	1,083 (714-1,861)	870 (540-1,524)	421 (256-709)	137 (80-232)
Other communicable, maternal, neonatal, and nutritional diseases	33,237 (20,241-50,630)	15,776 (9,448-23,589)	52,921 (34,541-76,036)	33,483 (21,206-50,457)	9,204 (6,833-12,000)	4,229 (3,577-4,978)	5,642 (4,953-6,459)	6,499 (5,818-7,237)	6,504 (5,838-7,255)	6,995 (6,382-7,663)	7,145 (6,534-7,807)	7,002 (6,358-7,719)	7,281 (6,608-8,031)	7,054 (6,461-7,711)	7,118 (6,579-7,787)	7,134 (6,501-7,767)	6,594 (6,017-7,184)	5,717 (5,144-6,297)	4,547 (4,049-4,981)	2,531 (2,187-2,846)	1,250 (1,063-1,417)	406 (332-477)	110 (87-130)
Sexually transmitted diseases excluding HIV	27,355 (14,964-43,932)	11,619 (6,510-19,087)	28,603 (15,566-46,902)	11,739 (6,089-19,934)	3,771 (2,006-6,337)	106 (60-177)	187 (83-312)	180 (79-299)	255 (107-428)	386 (168-631)	257 (127-433)	249 (113-383)	220 (109-331)	271 (168-459)	390 (231-660)	236 (135-332)	225 (136-310)	249 (169-395)	280 (121-414)	138 (70-199)	75 (38-102)	29 (15-43)	13 (7-20)
Syphilis	27,355 (14,964-43,932)	11,619 (6,510-19,087)	28,603 (15,566-46,902)	11,739 (6,089-19,934)	3,771 (2,006-6,337)	90 (51-151)	158 (70-264)	155 (68-258)	213 (89-358)	327 (142-535)	228 (113-384)	226 (103-348)	199 (98-299)	252 (156-427)	361 (214-611)	220 (126-310)	211 (128-291)	247 (138-365)	257 (111-380)	126 (64-182)	67 (34-90)	25 (13-37)	10 (5-16)
Chlamydial infection	-	-	-	-	-	0 (0-0)	0 (0-0)	0 (0-0)	0 (0-0)	0 (0-0)	0 (0-0)	0 (0-0)	0 (0-0)	0 (0-0)	0 (0-0)	0 (0-0)	0 (0-0)	0 (0-0)	0 (0-0)	0 (0-0)	0 (0-0)	0 (0-0)	0 (0-0)
Gonococcal infection	-	-	-	-	-	10 (6-17)	21 (9-36)	16 (7-27)	35 (15-59)	43 (19-70)	15 (7-26)	14 (6-21)	9 (5-14)	9 (5-15)	14 (8-23)	9 (5-13)	7 (4-10)	15 (8-22)	15 (6-22)	8 (4-11)	5 (2-6)	2 (1-3)	1 (1-2)
Other sexually transmitted diseases	-	-	-	-	-	5 (3-9)	7 (3-12)	9 (4-15)	7 (3-12)	15 (7-25)	14 (7-23)	9 (4-14)	12 (6-18)	10 (6-17)	16 (9-26)	7 (4-10)	6 (4-9)	6 (4-9)	9 (4-13)	5 (2-7)	4 (2-5)	2 (1-2)	2 (1-3)
Hepatitis	-	-	9,125 (7,742-10,635)	10,673 (8,097-13,198)	3,622 (2,881-4,423)	2,987 (2,372-3,508)	3,998 (3,440-4,744)	4,988 (4,436-5,668)	5,074 (4,489-5,737)	5,315 (4,791-5,895)	5,535 (5,026-6,119)	5,735 (5,145-6,405)	5,901 (5,306-6,567)	5,535 (5,029-6,076)	5,350 (4,895-5,884)	5,031 (4,604-5,545)	4,507 (4,116-4,937)	3,679 (3,331-4,071)	2,742 (2,431-3,030)	1,346 (1,184-1,501)	573 (505-642)	138 (123-155)	27 (24-30)
Acute hepatitis A	-	-	5,614 (3,699-7,705)	1,168 (693-1,774)	84 (53-128)	38 (22-63)	52 (29-89)	65 (32-117)	53 (24-98)	44 (20-81)	46 (22-80)	49 (23-93)	53 (25-103)	56 (27-102)	60 (29-103)	60 (28-104)	56 (29-93)	49 (26-83)	40 (21-68)	21 (11-33)	9 (5-13)	2 (1-3)	0 (0-1)
Hepatitis B	-	-	3,512 (1,868-5,664)	1,706 (950-2,661)	434 (232-706)	536 (288-879)	1,500 (1,001-2,109)	3,069 (2,299-3,946)	4,009 (3,263-4,725)	4,691 (4,071-5,270)	5,080 (4,518-5,677)	5,320 (4,676-6,009)	5,468 (4,802-6,156)	5,103 (4,543-5,667)	4,890 (4,366-5,447)	4,544 (4,001-5,085)	4,027 (3,553-4,497)	3,220 (2,802-3,646)	2,325 (1,943-2,675)	1,031 (809-1,250)	364 (257-472)	72 (42-102)	11 (6-18)
Hepatitis C	-	-	-	-	106 (65-161)	33 (21-48)	40 (24-58)	46 (25-76)	38 (18-66)	35 (16-67)	41 (20-73)	54 (25-97)	72 (33-136)	94 (44-177)	123 (56-222)	136 (63-252)	111 (52-198)	85 (41-157)	65 (32-113)	32 (16-56)	15 (8-24)	4 (2-6)	1 (0-1)
Acute hepatitis E	-	-	-	7,799 (5,673-9,951)	2,998 (2,342-3,703)	2,280 (1,819-2,816)	2,407 (1,788-3,137)	1,808 (1,079-2,620)	973 (497-1,571)	544 (260-945)	368 (176-652)	312 (135-650)	307 (131-579)	282 (130-530)	277 (114-525)	290 (107-612)	314 (140-565)	326 (146-583)	312 (125-607)	262 (127-428)	186 (96-289)	60 (34-90)	14 (8-20)
Other infectious diseases	5,882 (1,373-12,064)	4,157 (1,308-7,495)	15,193 (3,879-28,880)	11,071 (3,445-24,381)	1,812 (1,289-2,365)	1,236 (950-1,597)	1,457 (1,125-1,848)	1,331 (1,009-1,659)	1,176 (930-1,395)	1,294 (1,026-1,574)	1,353 (1,083-1,592)	1,018 (838-1,197)	1,160 (937-1,383)	1,249 (1,044-1,437)	1,378 (1,122-1,557)	1,868 (1,555-2,268)	1,862 (1,547-2,187)	1,770 (1,421-2,103)	1,525 (1,235-1,778)	1,048 (819-1,276)	601 (458-738)	238 (176-305)	70 (51-87)
Non-communicable diseases	128,071 (86,389-161,523)	61,378 (40,928-79,321)	230,203 (183,037-275,650)	151,941 (119,737-183,616)	65,645 (58,422-72,920)	59,063 (54,484-63,183)	96,369 (91,506-100,362)	130,031 (125,044-134,930)	152,797 (146,588-158,727)	211,086 (203,578-218,732)	303,970 (293,947-314,147)	398,065 (386,529-408,175)	538,885 (524,740-551,886)	839,059 (818,990-857,861)	1,144,979 (1,119,425-1,167,681)	1,561,880 (1,527,362-1,591,864)	1,734,911 (1,694,287-1,767,461)	1,815,223 (1,767,310-1,852,148)	1,810,813 (1,762,608-1,844,060)	1,336,610 (1,302,315-1,362,200)	773,349 (753,253-789,559)	293,441 (286,051-300,058)	75,522 (73,814-77,072)
Neoplasms	583 (470-640)	416 (354-460)	4,496 (3,943-5,167)	16,278 (13,723-20,530)	18,730 (16,880-20,851)	17,370 (16,015-18,666)	24,116 (22,405-25,337)	26,782 (25,208-28,012)	30,363 (28,755-31,717)	44,578 (42,589-46,215)	71,065 (68,264-73,665)	102,686 (98,351-105,976)	151,516 (146,516-156,006)	251,574 (243,757-258,689)	353,526 (344,350-362,393)	462,034 (450,767-473,837)	474,090 (462,803-484,303)	420,782 (410,607-430,401)	362,360 (354,346-370,484)	217,144 (211,481-222,953)	102,533 (99,613-105,719)	31,525 (30,423-32,678)	6,615 (6,335-6,998)
Lip and oral cavity cancer	-	-	-	-	-	-	187 (167-212)	346 (315-387)	596 (537-667)	1,070 (978-1,184)	1,824 (1,676-2,011)	3,212 (2,980-3,536)	4,929 (4,627-5,375)	7,622 (7,136-8,349)	9,095 (8,615-9,838)	10,034 (9,468-10,797)	8,436 (7,979-9,057)	6,137 (5,788-6,633)	4,739 (4,475-5,053)	2,862 (2,686-3,043)	1,388 (1,296-1,483)	458 (426-488)	106 (99-113)
Nasopharynx cancer	-	-	-	-	157 (125-190)	258 (215-301)	432 (378-485)	479 (417-546)	578 (503-656)	909 (806-1,013)	1,551 (1,389-1,726)	2,172 (1,933-2,405)	2,869 (2,582-3,170)	3,994 (3,617-4,347)	4,671 (4,240-5,074)	4,445 (4,054-4,808)	3,661 (3,361-3,971)	2,701 (2,488-2,942)	1,596 (1,486-1,713)	837 (779-892)	318 (299-337)	97 (91-102)	21 (20-22)
Other pharynx cancer	-	-	-	-	-	-	98 (84-121)	159 (138-193)	203 (173-245)	447 (394-531)	961 (859-1,114)	2,093 (1,893-2,394)	3,525 (3,221-3,978)	5,729 (5,281-6,426)	6,870 (6,320-7,696)	7,325 (6,665-8,294)	6,049 (5,493-6,820)	4,183 (3,734-4,811)	2,724 (2,436-3,077)	1,479 (1,282-1,689)	573 (497-654)	146 (128-165)	24 (22-27)
Oesophageal cancer	-	-	-	-	-	-	226 (199-256)	373 (342-411)	474 (442-509)	1,109 (1,033-1,195)	2,670 (2,503-2,870)	5,973 (5,568-6,410)	11,265 (10,620-11,979)	19,930 (18,866-20,975)	29,702 (28,091-31,529)	37,141 (35,127-39,323)	36,514 (34,456-38,571)	32,287 (30,168-34,359)	21,522 (20,404-22,681)	10,649 (10,042-11,275)	4,350 (4,126-4,609)	1,102 (1,041-1,176)	197 (186-211)
Stomach cancer	-	-	-	-	-	-	787 (731-862)	1,582 (1,468-1,734)	2,559 (2,365-2,766)	5,255 (4,914-5,602)	9,399 (8,839-9,958)	14,360 (13,498-15,181)	22,396 (21,183-23,596)	39,292 (37,523-41,056)	56,974 (54,624-59,389)	75,165 (71,931-78,420)	75,017 (71,989-77,768)	69,661 (66,898-72,504)	55,797 (53,904-57,622)	30,740 (29,749-31,759)	13,603 (13,185-14,082)	3,860 (3,734-3,989)	805 (776-831)
Colon and rectum cancer	-	-	-	-	-	-	662 (594-758)	1,332 (1,231-1,494)	1,882 (1,758-2,080)	3,126 (2,933-3,411)	4,638 (4,395-5,007)	6,143 (5,861-6,534)	9,356 (8,971-9,912)	14,992 (14,441-15,698)	22,438 (21,579-23,525)	32,354 (31,274-33,704)	36,732 (35,624-38,167)	34,630 (33,671-35,889)	35,251 (34,197-36,409)	24,238 (23,395-25,051)	12,360 (11,917-12,804)	3,936 (3,804-4,082)	825 (795-856)
Liver cancer	-	-	-	-	412 (362-541)	565 (506-697)	1,138 (1,022-1,312)	2,421 (2,178-2,743)	4,419 (3,956-4,947)	8,438 (7,685-9,342)	16,119 (14,831-17,642)	20,805 (19,114-22,596)	26,647 (24,356-29,142)	35,648 (32,826-38,670)	43,329 (40,372-46,767)	46,517 (43,794-49,778)	41,218 (38,868-44,033)	33,376 (31,368-35,587)	22,323 (21,155-23,606)	10,953 (10,441-11,498)	4,252 (4,064-4,433)	1,057 (1,008-1,105)	179 (170-188)
Liver cancer due to hepatitis B	-	-	-	-	105 (72-149)	210 (151-277)	528 (395-673)	1,323 (1,093-1,594)	2,642 (2,119-3,137)	5,147 (4,220-5,984)	9,677 (7,868-11,177)	12,139 (9,916-13,999)	14,851 (11,961-17,175)	18,631 (14,732-21,991)	20,876 (16,347-24,520)	20,399 (15,326-23,911)	16,908 (13,025-19,895)	13,283 (10,025-15,702)	7,866 (6,137-9,407)	3,389 (2,486-4,177)	1,234 (960-1,485)	296 (226-371)	51 (35-68)
Liver cancer due to hepatitis C	-	-	-	-	6 (4-8)	10 (8-14)	21 (16-27)	57 (45-73)	135 (96-183)	306 (235-392)	673 (509-875)	1,189 (945-1,519)	2,181 (1,706-2,716)	4,095 (3,276-4,947)	6,689 (5,650-7,988)	8,544 (7,235-10,148)	7,794 (6,711-9,106)	6,517 (5,646-7,625)	5,218 (4,480-6,098)	3,084 (2,636-3,589)	1,336 (1,177-1,514)	361 (313-410)	63 (53-74)
Liver cancer due to alcohol use	-	-	-	-	-	-	2 (1-4)	18 (11-25)	56 (33-87)	205 (148-280)	634 (452-856)	1,157 (869-1,546)	1,699 (1,211-2,266)	2,667 (1,941-3,479)	3,591 (2,604-4,763)	4,775 (3,555-6,129)	5,227 (4,132-6,376)	4,172 (3,320-5,129)	3,103 (2,466-3,757)	1,558 (1,164-1,938)	582 (432-716)	129 (92-169)	18 (11-25)
Liver cancer due to other causes	-	-	-	-	302 (252-402)	345 (278-435)	587 (458-755)	1,024 (821-1,259)	1,587 (1,231-2,082)	2,779 (2,220-3,553)	5,136 (4,126-6,648)	6,320 (5,055-8,193)	7,917 (6,329-10,187)	10,256 (8,255-13,056)	12,173 (9,757-15,491)	12,798 (10,464-16,160)	11,289 (9,283-13,826)	9,404 (7,771-11,675)	6,135 (5,119-7,401)	2,921 (2,408-3,508)	1,100 (941-1,283)	271 (225-322)	47 (36-59)
Gallbladder and biliary tract cancer	-	-	-	-	-	-	47 (37-60)	76 (61-98)	124 (102-162)	318 (259-418)	607 (494-792)	957 (797-1,200)	1,465 (1,228-1,817)	2,699 (2,263-3,253)	3,969 (3,391-4,792)	5,444 (4,747-6,425)	5,976 (5,232-7,090)	5,889 (5,233-7,129)	5,871 (5,282-6,756)	3,867 (3,541-4,370)	1,851 (1,700-2,086)	565 (514-637)	114 (104-132)
Pancreatic cancer	-	-	-	-	-	-	93 (88-100)	189 (177-204)	325 (308-345)	806 (765-847)	1,630 (1,554-1,702)	2,890 (2,719-3,027)	4,563 (4,373-4,737)	8,305 (7,829-8,669)	12,210 (11,789-12,595)	16,444 (15,783-17,030)	17,605 (17,049-18,079)	15,659 (15,256-16,071)	13,829 (13,435-14,232)	8,222 (7,977-8,468)	3,767 (3,653-3,889)	1,113 (1,074-1,147)	219 (211-226)
Larynx cancer	-	-	-	-	-	-	114 (104-126)	142 (131-153)	191 (179-203)	426 (397-454)	1,027 (961-1,093)	2,359 (2,216-2,493)	4,342 (4,108-4,596)	8,280 (7,820-8,751)	10,734 (10,203-11,274)	13,041 (12,424-13,705)	11,726 (11,192-12,308)	8,495 (8,061-8,912)	6,168 (5,874-6,484)	3,402 (3,226-3,611)	1,395 (1,320-1,471)	374 (356-395)	67 (64-71)
Tracheal, bronchus, and lung cancer	-	-	-	-	-	-	670 (596-755)	1,295 (1,179-1,433)	2,131 (1,970-2,315)	4,189 (3,922-4,525)	8,834 (8,346-9,381)	16,697 (15,843-17,651)	30,429 (29,135-32,177)	60,985 (58,496-64,184)	92,802 (89,470-96,953)	130,226 (125,807-136,202)	134,015 (130,107-138,656)	110,353 (107,253-114,224)	90,816 (88,393-93,607)	47,019 (45,507-48,493)	18,564 (17,979-19,145)	4,463 (4,305-4,622)	746 (717-773)
Malignant skin melanoma	-	-	-	-	-	-	117 (96-140)	208 (174-246)	333 (288-379)	590 (500-647)	874 (732-962)	1,136 (967-1,237)	1,158 (994-1,257)	1,591 (1,392-1,741)	1,829 (1,659-2,087)	1,961 (1,805-2,207)	1,944 (1,792-2,269)	1,616 (1,484-1,867)	1,474 (1,387-1,764)	983 (920-1,210)	507 (473-618)	172 (161-212)	41 (38-50)
Non-melanoma skin cancer	-	-	-	-	-	-	121 (109-130)	167 (153-177)	199 (184-213)	318 (295-337)	414 (388-438)	554 (519-587)	681 (640-715)	1,080 (1,005-1,155)	1,500 (1,405-1,587)	2,509 (2,362-2,653)	2,773 (2,616-2,926)	2,662 (2,523-2,791)	2,585 (2,450-2,715)	1,947 (1,856-2,042)	1,195 (1,132-1,258)	534 (504-565)	189 (178-200)
Non-melanoma skin cancer (squamous-cell carcinoma)	-	-	-	-	-	-	121 (109-130)	167 (153-177)	199 (184-213)	318 (295-337)	414 (388-438)	554 (519-587)	681 (640-715)	1,080 (1,005-1,155)	1,500 (1,405-1,587)	2,509 (2,362-2,653)	2,773 (2,616-2,926)	2,662 (2,523-2,791)	2,585 (2,450-2,715)	1,947 (1,856-2,042)	1,195 (1,132-1,258)	534 (504-565)	189 (178-200)
Breast cancer	-	-	-	-	-	-	30 (28-31)	28 (27-31)	31 (29-35)	51 (43-62)	97 (77-115)	141 (109-164)	268 (207-309)	387 (310-443)	499 (401-568)	611 (537-737)	628 (561-759)	548 (506-678)	412 (381-485)	328 (286-373)	176 (150-196)	64 (55-74)	16 (14-19)
Cervical cancer	-	-	-	-	-	-	-	-	-	-	-	-	-	-	-	-	-	-	-	-	-	-	-
Uterine cancer	-	-	-	-	-	-	-	-	-	-	-	-	-	-	-	-	-	-	-	-	-	-	-
Ovarian cancer	-	-	-	-	-	-	-	-	-	-	-	-	-	-	-	-	-	-	-	-	-	-	-
Prostate cancer	-	-	-	-	-	-	64 (50-75)	76 (59-89)	86 (69-102)	108 (86-125)	162 (128-185)	345 (274-398)	966 (771-1,121)	2,902 (2,346-3,247)	6,979 (5,606-7,890)	15,985 (12,900-17,987)	25,670 (21,240-28,320)	33,131 (28,258-36,169)	41,654 (36,780-46,447)	33,472 (28,712-36,805)	20,331 (18,047-22,431)	7,909 (7,137-8,946)	1,850 (1,690-2,204)
Testicular cancer	-	-	-	-	-	-	507 (465-558)	1,013 (944-1,105)	1,228 (1,150-1,329)	1,195 (1,119-1,279)	967 (907-1,037)	609 (570-657)	462 (429-507)	428 (392-481)	339 (314-369)	389 (356-433)	347 (321-379)	316 (289-350)	267 (247-293)	197 (181-216)	91 (85-99)	31 (29-34)	8 (7-9)
Kidney cancer	20 (16-23)	12 (9-14)	153 (119-193)	678 (513-911)	310 (268-360)	119 (107-132)	102 (94-109)	141 (128-151)	196 (178-210)	301 (277-322)	581 (532-623)	1,055 (953-1,136)	1,790 (1,659-1,895)	3,293 (3,030-3,514)	4,765 (4,471-5,040)	6,076 (5,703-6,426)	6,178 (5,876-6,464)	5,081 (4,854-5,307)	4,952 (4,731-5,179)	3,077 (2,947-3,214)	1,444 (1,376-1,512)	435 (413-460)	84 (79-88)
Bladder cancer	-	-	-	-	-	-	72 (64-81)	124 (115-135)	155 (143-167)	313 (289-341)	591 (549-649)	1,002 (924-1,091)	1,756 (1,644-1,885)	3,488 (3,244-3,747)	5,849 (5,516-6,219)	10,088 (9,428-10,650)	12,363 (11,798-12,965)	13,050 (12,568-13,648)	14,308 (13,774-14,873)	10,656 (10,242-11,051)	5,775 (5,565-5,970)	1,960 (1,883-2,034)	432 (414-449)
Brain and nervous system cancer	83 (46-104)	58 (32-74)	859 (450-1,124)	3,036 (1,953-3,993)	3,795 (2,846-4,347)	3,109 (2,367-3,446)	2,561 (1,980-2,887)	2,366 (1,838-2,660)	2,620 (2,058-2,964)	3,172 (2,538-3,521)	4,041 (3,223-4,502)	4,521 (3,499-5,030)	4,909 (3,954-5,414)	6,567 (5,293-7,202)	7,791 (6,510-8,470)	8,544 (7,101-9,271)	7,454 (6,478-8,148)	5,354 (4,658-5,901)	3,735 (3,385-4,181)	1,723 (1,534-1,952)	620 (540-703)	150 (126-171)	28 (24-31)
Thyroid cancer	-	-	-	-	-	66 (46-83)	107 (85-122)	158 (133-175)	182 (158-196)	188 (165-203)	242 (214-261)	347 (295-374)	536 (456-578)	852 (750-914)	1,123 (1,027-1,193)	1,145 (1,073-1,216)	1,139 (1,075-1,208)	980 (921-1,054)	960 (916-1,038)	587 (556-647)	253 (239-277)	74 (69-80)	15 (14-16)
Mesothelioma	-	-	-	-	-	-	50 (43-66)	56 (48-73)	85 (71-112)	98 (84-130)	170 (145-223)	249 (215-318)	430 (377-541)	751 (650-891)	1,252 (1,091-1,457)	1,491 (1,300-1,829)	1,779 (1,564-2,243)	1,652 (1,463-2,026)	1,683 (1,495-2,037)	1,075 (958-1,285)	444 (399-512)	114 (103-128)	20 (18-22)
Hodgkin lymphoma	21 (10-28)	6 (3-9)	129 (57-164)	610 (286-822)	1,503 (786-2,020)	1,303 (797-1,587)	1,277 (862-1,562)	1,243 (860-1,488)	1,269 (922-1,489)	1,308 (966-1,560)	1,300 (947-1,532)	1,199 (842-1,420)	1,341 (938-1,607)	1,529 (1,103-1,832)	1,935 (1,374-2,285)	2,014 (1,435-2,359)	1,891 (1,382-2,200)	1,520 (1,127-1,764)	855 (654-978)	449 (349-510)	184 (143-210)	46 (37-54)	7 (6-9)
Non-Hodgkin's lymphoma	36 (26-40)	28 (20-36)	334 (236-424)	1,566 (1,073-2,205)	2,046 (1,537-2,479)	1,775 (1,433-2,036)	1,900 (1,604-2,116)	1,992 (1,784-2,300)	1,916 (1,701-2,153)	2,224 (1,939-2,451)	2,607 (2,251-2,831)	3,084 (2,590-3,364)	3,609 (3,098-3,976)	4,946 (4,348-5,513)	6,329 (5,691-7,242)	7,861 (7,263-8,825)	8,397 (7,686-9,644)	7,559 (6,967-8,735)	6,851 (6,359-8,320)	4,355 (4,048-5,432)	2,075 (1,914-2,573)	614 (563-796)	120 (109-157)
Multiple myeloma	-	-	-	-	-	-	113 (88-139)	131 (103-158)	123 (98-154)	243 (199-296)	403 (338-487)	709 (574-828)	919 (753-1,079)	1,639 (1,380-1,943)	2,507 (2,147-3,035)	3,358 (2,777-4,072)	3,836 (3,168-4,836)	3,589 (3,041-4,501)	3,417 (2,946-4,478)	2,308 (1,986-3,082)	1,132 (985-1,507)	317 (281-437)	56 (46-72)
Leukaemia	159 (123-175)	148 (127-180)	1,814 (1,519-2,398)	6,798 (5,444-9,902)	7,902 (6,966-9,704)	7,225 (6,555-8,345)	7,861 (6,840-8,803)	6,692 (5,550-7,376)	5,044 (4,207-5,489)	4,606 (3,900-4,965)	4,900 (4,145-5,292)	4,790 (4,007-5,122)	4,971 (4,362-5,279)	6,387 (5,703-6,811)	7,998 (7,130-8,484)	9,924 (9,133-10,471)	10,723 (10,073-11,295)	9,777 (9,193-10,492)	9,439 (9,025-9,995)	6,214 (5,956-6,533)	3,150 (3,012-3,312)	1,022 (976-1,068)	227 (215-236)
Acute lymphoid leukaemia	33 (27-44)	29 (20-46)	380 (246-672)	1,989 (1,269-3,721)	2,772 (2,222-3,908)	2,363 (2,055-3,028)	1,999 (1,769-2,387)	1,338 (1,178-1,609)	896 (776-1,036)	776 (678-900)	722 (636-834)	686 (583-776)	676 (579-752)	868 (741-979)	1,004 (852-1,117)	1,075 (905-1,205)	1,012 (860-1,121)	868 (738-954)	488 (412-539)	277 (237-302)	128 (111-140)	38 (33-41)	8 (7-9)
Chronic lymphoid leukaemia	-	-	-	-	-	-	149 (128-211)	143 (121-195)	108 (90-129)	92 (76-109)	104 (88-124)	151 (124-178)	239 (205-281)	509 (433-605)	840 (703-1,034)	1,378 (1,183-1,670)	1,723 (1,474-2,058)	1,713 (1,468-2,013)	1,801 (1,554-2,155)	1,386 (1,153-1,693)	787 (664-946)	269 (232-318)	63 (55-74)
Acute myeloid leukaemia	21 (14-28)	22 (15-33)	237 (162-395)	1,078 (696-2,052)	1,029 (750-1,403)	948 (769-1,201)	1,059 (899-1,286)	928 (768-1,132)	828 (684-978)	837 (711-992)	920 (786-1,072)	986 (844-1,149)	1,056 (926-1,236)	1,464 (1,299-1,722)	1,820 (1,601-2,159)	2,233 (1,991-2,687)	2,519 (2,283-3,085)	2,222 (2,023-2,821)	2,021 (1,827-2,637)	1,212 (1,095-1,636)	538 (479-735)	152 (133-213)	28 (25-41)
Chronic myeloid leukaemia	-	-	-	-	-	-	275 (237-352)	377 (316-471)	538 (458-623)	630 (528-712)	698 (601-782)	692 (594-768)	769 (671-852)	899 (784-1,000)	1,062 (911-1,175)	1,171 (1,007-1,291)	1,261 (1,081-1,376)	1,060 (902-1,154)	1,310 (1,118-1,419)	886 (764-961)	438 (377-475)	147 (121-161)	34 (28-37)
Other leukaemia	105 (70-119)	98 (70-120)	1,196 (868-1,601)	3,730 (2,834-5,453)	4,102 (3,366-4,944)	3,914 (3,233-4,416)	4,378 (3,508-4,934)	3,906 (2,967-4,424)	2,675 (2,075-3,031)	2,271 (1,775-2,524)	2,455 (1,884-2,738)	2,275 (1,736-2,536)	2,232 (1,804-2,473)	2,647 (2,165-2,908)	3,273 (2,727-3,566)	4,067 (3,459-4,430)	4,208 (3,615-4,558)	3,913 (3,387-4,219)	3,819 (3,364-4,095)	2,453 (2,139-2,636)	1,259 (1,107-1,358)	416 (363-447)	94 (82-101)
Other neoplasms	264 (212-288)	164 (126-196)	1,208 (914-1,587)	3,590 (2,875-5,304)	2,604 (2,301-3,056)	2,949 (2,700-3,256)	4,779 (4,390-5,165)	3,996 (3,638-4,333)	3,413 (3,052-3,706)	3,773 (3,408-4,074)	4,456 (4,040-4,785)	5,284 (4,795-5,677)	5,934 (5,397-6,369)	8,255 (7,466-8,867)	10,038 (9,204-10,837)	11,942 (10,935-13,075)	12,020 (11,083-12,975)	10,574 (9,783-11,434)	9,131 (8,510-10,054)	5,504 (5,119-6,106)	2,732 (2,535-3,042)	914 (839-1,005)	218 (201-237)
Cardiovascular diseases	3,896 (3,547-4,318)	2,604 (2,283-2,904)	21,181 (18,953-23,589)	16,833 (14,156-19,342)	8,695 (7,729-9,625)	9,273 (8,589-9,999)	23,961 (22,615-25,644)	38,364 (36,408-41,167)	48,021 (45,151-52,162)	72,257 (68,439-79,137)	110,590 (105,054-119,078)	153,612 (146,870-163,169)	216,549 (208,324-229,594)	350,718 (338,784-366,371)	483,381 (470,029-500,982)	677,935 (660,939-696,959)	775,368 (757,003-796,404)	832,307 (812,616-852,428)	884,804 (864,691-905,785)	694,637 (678,218-710,817)	410,899 (400,737-421,522)	158,656 (154,631-162,782)	43,537 (42,275-44,747)
Rheumatic heart disease	-	-	-	4,073 (2,983-5,666)	2,304 (1,880-2,803)	3,209 (2,812-3,636)	5,807 (5,098-6,539)	6,251 (5,356-7,131)	5,664 (4,689-6,474)	6,055 (4,950-7,024)	6,850 (5,774-7,831)	7,508 (6,208-8,609)	8,238 (6,887-9,371)	10,867 (9,187-12,320)	12,588 (10,846-14,195)	14,544 (12,575-16,333)	15,502 (13,445-17,335)	16,050 (14,006-17,862)	13,458 (11,995-14,827)	9,122 (8,117-9,990)	5,035 (4,517-5,519)	1,908 (1,729-2,078)	513 (471-554)
Ischaemic heart disease	-	-	1,327 (1,067-1,711)	1,086 (781-1,485)	578 (482-693)	578 (508-661)	6,573 (6,073-7,187)	13,113 (12,275-14,262)	20,058 (18,694-21,849)	35,756 (33,615-39,351)	58,198 (54,932-62,630)	83,268 (79,492-88,574)	119,787 (115,234-126,529)	189,785 (182,625-199,061)	255,414 (247,200-265,447)	352,324 (341,960-364,737)	394,111 (383,343-407,062)	402,743 (390,736-418,193)	452,758 (441,827-467,831)	375,591 (366,675-388,162)	232,312 (226,691-240,367)	95,366 (93,095-99,017)	28,046 (27,432-29,003)
Cerebrovascular disease	2,006 (1,684-2,388)	1,299 (1,046-1,612)	9,264 (7,395-11,875)	5,609 (4,451-7,392)	3,170 (2,760-3,731)	3,014 (2,741-3,372)	6,572 (6,067-7,173)	11,910 (11,035-12,987)	13,608 (12,504-15,204)	19,705 (18,278-22,213)	30,431 (28,364-33,730)	43,392 (40,598-47,701)	65,338 (61,695-71,382)	113,485 (107,474-122,424)	166,268 (158,995-176,879)	243,637 (233,867-256,992)	286,177 (274,185-302,110)	322,940 (308,779-341,829)	319,342 (303,937-338,537)	226,828 (213,626-241,006)	120,092 (112,250-129,229)	40,186 (37,229-43,518)	9,713 (8,799-10,507)

SR Table 4: Global deaths by cause and age group for males (with 95% uncertainty intervals), 1990

Cause	Early Neonatal	Late Neonatal	Post Neonatal	1 to 4	5 to 9	10 to 14	15 to 19	20 to 24	25 to 29	30 to 34	35 to 39
Ischaemic stroke	-	-	330 (263-423)	330 (258-439)	212 (182-250)	209 (185-239)	773 (694-852)	1,523 (1,366-1,676)	1,828 (1,630-2,038)	2,591 (2,329-2,885)	4,070 (3,677-4,475)
Hemorrhagic stroke	2,006 (1,684-2,388)	1,299 (1,046-1,612)	8,934 (7,114-11,454)	5,279 (4,175-6,952)	2,959 (2,577-3,477)	2,805 (2,546-3,142)	5,800 (5,357-6,355)	10,387 (9,599-11,431)	11,780 (10,809-13,308)	17,114 (15,851-19,423)	26,362 (24,504-29,335)
Hypertensive heart disease	-	-	390 (198-530)	167 (70-242)	119 (63-153)	136 (65-182)	874 (519-1,067)	1,474 (922-1,793)	1,805 (1,211-2,149)	2,534 (1,780-3,035)	4,032 (2,856-4,809)
Cardiomyopathy and myocarditis	592 (372-757)	404 (252-557)	3,001 (1,930-4,249)	1,923 (1,174-2,782)	773 (591-1,027)	784 (662-1,042)	1,656 (1,437-2,153)	2,022 (1,754-2,498)	2,789 (2,388-3,425)	3,666 (3,076-4,348)	5,290 (4,329-6,467)
Myocarditis	139 (64-200)	133 (60-200)	1,336 (670-2,196)	928 (402-1,547)	337 (231-503)	306 (243-458)	640 (507-945)	661 (517-965)	709 (525-1,094)	527 (405-790)	628 (475-926)
Alcoholic cardiomyopathy	-	-	-	-	-	-	129 (100-185)	264 (197-340)	713 (509-954)	1,540 (1,109-2,050)	2,607 (1,895-3,582)
Other cardiomyopathy	453 (298-593)	271 (178-391)	1,665 (1,142-2,525)	995 (674-1,658)	436 (332-596)	478 (398-612)	887 (740-1,129)	1,096 (940-1,383)	1,368 (1,134-1,682)	1,600 (1,343-1,963)	2,055 (1,640-2,509)
Atrial fibrillation and flutter	-	-	-	-	-	-	-	-	-	14 (10-19)	53 (39-71)
Aortic aneurysm	-	-	-	-	-	-	237 (201-275)	312 (273-355)	474 (399-552)	620 (540-710)	975 (860-1,107)
Peripheral vascular disease	-	-	-	-	-	-	-	-	-	-	-
Endocarditis	86 (44-122)	51 (26-75)	1,027 (626-1,390)	365 (221-499)	155 (105-190)	217 (156-273)	453 (340-617)	680 (525-942)	841 (673-1,174)	898 (701-1,194)	1,049 (813-1,357)
Other cardiovascular and circulatory diseases	1,211 (934-1,538)	850 (586-1,032)	6,173 (4,504-8,245)	3,610 (2,155-5,005)	1,595 (1,054-2,002)	1,336 (912-1,602)	1,788 (1,249-2,240)	2,601 (1,861-3,367)	2,780 (2,067-3,577)	3,009 (2,321-3,736)	3,711 (2,710-4,430)
Chronic respiratory diseases	0 (0-0)	0 (0-0)	0 (0-0)	20,137 (15,720-24,789)	4,827 (4,166-5,505)	4,299 (3,855-4,762)	6,914 (6,465-7,521)	9,495 (8,902-10,404)	11,028 (10,334-11,998)	13,416 (12,701-14,310)	18,870 (17,857-20,220)
Chronic obstructive pulmonary disease	-	-	0 (0-0)	4,723 (2,609-10,193)	1,143 (780-1,787)	786 (566-1,104)	3,156 (2,520-3,794)	4,453 (3,564-5,313)	4,953 (3,912-6,046)	6,866 (5,462-8,251)	10,113 (8,247-12,071)
Pneumoconiosis	-	-	-	-	-	-	43 (32-73)	77 (53-147)	166 (107-299)	294 (206-486)	604 (437-1,016)
Silicosis	-	-	-	-	-	-	8 (5-14)	24 (15-41)	83 (48-148)	190 (127-326)	441 (309-738)
Asbestosis	-	-	-	-	-	-	14 (11-22)	18 (14-29)	19 (14-31)	21 (16-33)	24 (18-37)
Coal workers pneumoconiosis	-	-	-	-	-	-	2 (1-4)	5 (2-9)	19 (9-36)	19 (10-36)	42 (21-73)
Other pneumoconiosis	-	-	-	-	-	-	19 (13-39)	30 (17-70)	44 (22-100)	63 (34-129)	96 (52-199)
Asthma	-	-	-	12,782 (7,684-17,585)	2,872 (2,097-3,696)	2,978 (2,408-3,564)	3,209 (2,431-3,978)	4,272 (3,218-5,259)	5,255 (3,854-6,432)	5,334 (3,725-6,770)	7,058 (4,782-9,082)
Interstitial lung disease and pulmonary sarcoidosis	-	-	-	222 (71-602)	76 (37-167)	43 (22-82)	89 (45-171)	133 (72-245)	181 (97-339)	258 (143-463)	378 (214-688)
Other chronic respiratory diseases	0 (0-0)	0 (0-0)	0 (0-0)	2,410 (595-4,910)	735 (349-1,191)	493 (234-817)	417 (244-631)	560 (346-859)	474 (266-738)	665 (385-1,021)	716 (402-1,059)
Cirrhosis and other chronic liver diseases	-	-	-	8,248 (5,273-11,045)	2,678 (2,107-3,521)	2,584 (2,187-3,078)	4,151 (3,664-4,625)	7,950 (7,092-9,564)	12,993 (11,729-14,388)	23,068 (21,006-25,556)	35,400 (32,488-38,163)
Cirrhosis and other chronic liver diseases due to hepatitis B	-	-	-	94 (55-146)	79 (55-115)	136 (96-186)	370 (253-520)	2,121 (1,585-2,817)	4,758 (3,734-5,905)	8,670 (7,301-10,203)	13,085 (10,891-15,284)
Cirrhosis and other chronic liver diseases due to hepatitis C	-	-	-	73 (44-113)	60 (41-89)	107 (74-150)	255 (169-369)	1,274 (922-1,686)	2,928 (2,240-3,772)	5,889 (4,983-7,043)	9,368 (7,610-11,558)
Cirrhosis and other chronic liver diseases due to alcohol use	-	-	-	-	-	-	69 (40-108)	580 (411-800)	1,490 (1,088-2,001)	4,437 (3,703-5,288)	8,843 (7,301-10,687)
Cirrhosis and other chronic liver diseases due to other causes	-	-	-	8,081 (5,164-10,817)	2,538 (2,003-3,342)	2,341 (1,990-2,774)	3,456 (3,029-3,885)	3,974 (3,193-4,843)	3,817 (2,850-4,936)	4,071 (2,954-5,282)	4,104 (2,876-5,554)
Digestive diseases	1,468 (1,041-1,740)	2,031 (1,378-2,700)	15,328 (10,863-20,398)	15,909 (10,242-21,946)	6,428 (5,136-7,604)	4,921 (4,020-5,577)	7,024 (5,765-7,907)	8,957 (7,615-10,059)	12,591 (10,434-14,068)	14,078 (11,825-15,576)	18,215 (15,443-20,205)
Peptic ulcer disease	-	-	-	3,078 (1,779-4,506)	1,572 (1,178-2,027)	1,515 (1,222-1,814)	2,549 (2,061-3,018)	3,780 (3,161-4,334)	4,693 (3,886-5,355)	5,662 (4,696-6,418)	7,141 (6,004-8,077)
Gastritis and duodenitis	-	-	-	502 (184-1,089)	162 (97-267)	98 (70-140)	212 (167-266)	286 (234-345)	452 (371-557)	665 (542-794)	813 (664-974)
Appendicitis	-	-	-	2,355 (1,268-3,463)	1,385 (1,009-1,779)	1,060 (803-1,282)	1,174 (921-1,507)	910 (733-1,186)	1,106 (869-1,459)	724 (579-941)	909 (716-1,219)
Paralytic ileus and intestinal obstruction	1,468 (1,041-1,740)	2,031 (1,378-2,700)	15,328 (10,863-20,398)	2,609 (1,352-3,682)	1,585 (933-2,037)	1,012 (628-1,243)	1,428 (904-1,769)	1,556 (1,012-1,946)	2,354 (1,456-2,853)	1,939 (1,263-2,323)	2,854 (1,763-3,438)
Inguinal, femoral, and abdominal hernia	-	-	-	1,176 (411-2,056)	316 (173-434)	270 (128-380)	318 (174-430)	413 (224-573)	519 (286-684)	508 (237-691)	546 (317-705)
Inflammatory bowel disease	-	-	-	1,062 (347-2,876)	178 (87-335)	126 (75-207)	145 (91-186)	237 (150-300)	344 (234-436)	353 (242-450)	391 (272-492)
Vascular intestinal disorders	-	-	-	478 (105-965)	60 (27-92)	32 (23-38)	54 (42-63)	98 (73-117)	152 (110-173)	150 (109-175)	267 (210-310)
Gallbladder and biliary diseases	-	-	-	377 (179-595)	135 (88-175)	127 (83-158)	186 (121-225)	269 (190-323)	322 (226-385)	423 (307-498)	636 (447-752)
Pancreatitis	-	-	-	151 (70-253)	91 (59-120)	95 (67-116)	315 (214-406)	806 (617-1,015)	1,844 (1,466-2,296)	2,616 (2,114-3,191)	3,393 (2,752-4,157)
Other digestive diseases	-	-	-	4,121 (2,090-6,914)	945 (630-1,382)	587 (414-843)	644 (497-816)	602 (475-759)	805 (625-1,029)	1,036 (812-1,315)	1,264 (999-1,587)
Neurological disorders	-	-	4,801 (3,661-6,171)	6,689 (4,177-9,311)	3,721 (3,017-4,459)	4,971 (4,340-5,649)	8,943 (8,216-9,558)	8,604 (8,009-9,230)	6,778 (6,313-7,194)	6,138 (5,772-6,449)	6,673 (6,262-7,032)
Alzheimer's disease and other dementias	-	-	-	-	-	-	-	-	-	-	-
Parkinson's disease	-	-	-	-	-	-	-	1 (0-2)	10 (4-20)	36 (19-65)	75 (39-131)
Epilepsy	-	-	3,100 (2,231-3,864)	5,534 (3,290-7,548)	3,208 (2,576-3,805)	4,084 (3,492-4,671)	7,036 (6,303-7,628)	7,189 (6,638-7,775)	5,910 (5,466-6,299)	5,241 (4,919-5,525)	5,440 (5,059-5,781)
Multiple sclerosis	-	-	-	-	-	-	-	48 (41-56)	106 (84-135)	229 (178-291)	372 (288-459)
Motor neuron disease	4 (4-5)	7 (5-7)	628 (415-775)	196 (113-247)	26 (21-30)	51 (44-57)	91 (83-99)	74 (68-81)	59 (55-63)	118 (110-127)	231 (217-252)
Other neurological disorders	-	-	1,073 (828-1,702)	959 (660-1,787)	487 (402-695)	836 (751-993)	1,816 (1,712-1,930)	1,292 (1,205-1,369)	692 (632-735)	515 (475-547)	555 (508-589)
Mental and substance use disorders	13 (6-22)	14 (9-18)	0 (0-0)	0 (0-0)	9 (5-11)	5 (3-7)	4,201 (2,916-5,024)	9,015 (6,434-10,408)	12,416 (9,806-13,779)	16,380 (13,305-18,118)	18,421 (15,334-20,339)
Alcohol use disorders	-	-	-	-	-	-	979 (777-1,217)	2,323 (1,870-2,803)	4,483 (3,609-5,188)	7,931 (6,272-9,149)	10,552 (8,633-12,187)
Drug use disorders	13 (6-22)	14 (9-18)	0 (0-0)	0 (0-0)	0 (0-0)	0 (0-0)	3,211 (1,949-4,008)	6,676 (4,108-8,083)	7,918 (5,654-9,165)	8,434 (6,252-9,752)	7,855 (6,056-8,985)
Opioid use disorders	13 (6-22)	14 (9-18)	0 (0-0)	0 (0-0)	0 (0-0)	0 (0-0)	2,283 (1,379-2,963)	4,792 (2,809-5,980)	5,791 (3,895-6,922)	5,922 (4,147-7,113)	5,548 (4,081-6,567)
Cocaine use disorders	0 (0-0)	0 (0-0)	0 (0-0)	0 (0-0)	0 (0-0)	0 (0-0)	150 (69-206)	302 (149-412)	353 (200-472)	550 (329-714)	489 (304-645)
Amphetamine use disorders	0 (0-0)	0 (0-0)	0 (0-0)	0 (0-0)	0 (0-0)	0 (0-0)	106 (49-148)	202 (99-284)	223 (124-299)	274 (149-376)	251 (143-347)
Other drug use disorders	0 (0-0)	0 (0-0)	0 (0-0)	0 (0-0)	0 (0-0)	0 (0-0)	672 (298-877)	1,379 (710-1,714)	1,551 (949-1,842)	1,688 (1,131-2,019)	1,568 (1,053-1,871)
Eating disorders	-	-	-	-	9 (5-11)	5 (3-7)	11 (7-15)	16 (10-21)	15 (11-20)	16 (12-21)	14 (11-18)
Anorexia nervosa	-	-	-	-	8 (5-11)	5 (3-6)	11 (7-15)	16 (10-21)	14 (11-19)	15 (11-19)	13 (10-17)
Bulimia nervosa	-	-	-	-	0 (0-1)	0 (0-1)	0 (0-1)	1 (0-1)	1 (0-1)	1 (1-2)	1 (1-1)
Diabetes, urogenital, blood, and endocrine diseases	2,753 (1,971-3,176)	2,822 (2,008-3,436)	22,033 (18,256-25,100)	23,163 (16,598-28,358)	9,380 (7,878-10,719)	8,452 (7,050-9,548)	11,238 (10,145-12,401)	16,108 (14,996-17,906)	15,075 (13,994-16,191)	18,076 (16,918-19,342)	21,780 (20,524-23,306)
Diabetes mellitus	53 (49-58)	104 (89-121)	719 (642-830)	581 (480-693)	566 (483-651)	621 (559-686)	1,046 (965-1,141)	1,970 (1,854-2,140)	2,429 (2,303-2,592)	3,910 (3,729-4,148)	5,516 (5,296-5,783)
Acute glomerulonephritis	-	-	167 (134-206)	271 (205-344)	239 (191-307)	224 (174-291)	299 (235-384)	359 (290-448)	310 (254-375)	349 (293-417)	435 (357-539)
Chronic kidney disease	-	-	4,372 (3,360-5,083)	5,018 (3,548-6,028)	2,939 (2,269-3,370)	2,570 (2,137-2,877)	4,975 (4,430-5,680)	8,056 (6,906-9,158)	8,137 (7,121-9,137)	9,507 (8,439-10,715)	11,436 (10,039-12,880)
Chronic kidney disease due to diabetes mellitus	-	-	122 (77-179)	151 (90-230)	120 (66-192)	147 (89-234)	460 (277-710)	1,408 (910-1,928)	2,067 (1,512-2,708)	3,147 (2,357-3,967)	4,601 (3,507-5,713)
Chronic kidney disease due to hypertension	-	-	32 (21-48)	52 (32-78)	53 (33-80)	76 (49-114)	227 (147-322)	537 (356-743)	669 (453-917)	931 (674-1,252)	1,312 (977-1,720)
Chronic kidney disease due to glomerulonephritis	-	-	1,690 (1,184-2,176)	1,971 (1,308-2,676)	1,164 (773-1,565)	1,089 (759-1,445)	2,130 (1,510-2,795)	3,222 (2,305-4,246)	2,909 (2,007-3,857)	2,767 (1,916-3,791)	2,620 (1,741-3,632)
Chronic kidney disease due to other causes	-	-	2,527 (1,867-3,142)	2,843 (1,980-3,641)	1,603 (1,170-2,041)	1,258 (890-1,628)	2,158 (1,535-2,796)	2,889 (2,007-3,846)	2,492 (1,686-3,409)	2,662 (1,817-3,657)	2,903 (2,037-3,957)
Urinary diseases and male infertility	298 (259-344)	612 (452-845)	4,412 (3,795-5,065)	2,590 (2,058-3,205)	881 (705-1,080)	617 (538-687)	1,056 (952-1,156)	1,398 (1,291-1,500)	1,390 (1,272-1,501)	1,642 (1,528-1,752)	1,841 (1,713-1,960)
Interstitial nephritis and urinary tract infections	113 (76-158)	275 (152-422)	2,679 (1,942-3,390)	1,231 (659-1,915)	328 (213-438)	276 (204-337)	477 (367-593)	648 (500-801)	615 (492-770)	704 (572-871)	804 (645-979)
Urolithiasis	-	-	-	-	176 (75-268)	106 (47-146)	177 (90-243)	198 (102-269)	191 (93-265)	209 (106-285)	261 (145-341)
Other urinary diseases	186 (137-229)	337 (204-503)	1,732 (1,072-2,445)	1,359 (692-2,054)	377 (225-527)	234 (147-310)	402 (273-558)	552 (377-753)	583 (399-777)	729 (527-916)	777 (555-997)
Gynecological diseases	-	-	-	-	-	-	-	-	-	-	-
Uterine fibroids	-	-	-	-	-	-	-	-	-	-	-
Polycystic ovarian syndrome	-	-	-	-	-	-	-	-	-	-	-
Endometriosis	-	-	-	-	-	-	-	-	-	-	-
Genital prolapse	-	-	-	-	-	-	-	-	-	-	-
Other gynecological diseases	-	-	-	-	-	-	-	-	-	-	-
Hemoglobinopathies and hemolytic anaemias	1,409 (919-1,730)	1,199 (705-1,525)	6,637 (4,283-8,450)	11,354 (5,804-15,395)	3,442 (2,486-4,367)	3,366 (2,216-4,225)	2,983 (2,070-3,875)	3,387 (2,645-4,785)	1,702 (1,218-2,187)	1,252 (917-1,601)	1,142 (867-1,459)
Thalassemias	419 (327-558)	409 (242-577)	2,036 (1,294-2,678)	3,172 (1,902-4,298)	1,186 (903-1,566)	470 (371-670)	260 (197-348)	156 (108-196)	63 (45-82)	32 (25-38)	21 (17-24)
Sickle cell disorders	846 (446-1,090)	728 (395-995)	4,221 (2,552-5,581)	7,525 (3,409-10,928)	1,913 (1,269-2,532)	2,445 (1,483-3,254)	2,054 (1,365-2,684)	2,225 (1,739-3,230)	866 (598-1,119)	536 (390-697)	433 (310-555)
G6PD deficiency	40 (26-56)	33 (20-46)	247 (167-345)	509 (309-737)	250 (184-333)	309 (221-407)	418 (306-562)	619 (461-855)	461 (326-604)	449 (325-579)	395 (294-508)
Other hemoglobinopathies and hemolytic anaemias	104 (74-135)	29 (19-38)	133 (92-179)	148 (95-204)	94 (76-120)	142 (112-186)	251 (192-336)	386 (296-527)	312 (238-415)	236 (182-306)	293 (229-371)
Endocrine, metabolic, blood, and immune disorders	993 (600-1,173)	907 (549-1,143)	5,728 (4,118-7,468)	3,350 (2,318-4,420)	1,314 (1,064-1,767)	1,055 (854-1,347)	878 (715-1,025)	938 (742-1,124)	1,108 (858-1,286)	1,416 (1,143-1,687)	1,410 (1,157-1,714)
Musculoskeletal disorders	-	-	-	-	415 (286-554)	451 (358-569)	465 (401-575)	564 (469-676)	522 (445-602)	512 (424-584)	609 (509-694)
Rheumatoid arthritis	-	-	-	-	106 (55-186)	101 (62-157)	86 (62-117)	105 (74-147)	89 (61-115)	81 (59-103)	104 (76-131)
Other musculoskeletal disorders	-	-	-	-	309 (222-433)	350 (285-446)	378 (326-486)	459 (382-552)	433 (369-503)	431 (359-493)	506 (426-579)
Other non-communicable diseases	119,355 (78,180-153,090)	53,485 (33,290-71,457)	162,363 (117,220-207,979)	44,683 (26,276-68,588)	10,764 (7,487-14,924)	6,736 (4,810-8,837)	5,356 (3,981-6,833)	4,192 (3,206-5,389)	3,012 (2,430-3,885)	2,583 (1,993-3,039)	2,346 (1,799-2,796)
Congenital anomalies	119,355 (78,180-153,090)	50,530 (30,416-68,594)	136,188 (90,989-182,769)	43,927 (25,699-67,678)	10,533 (7,310-14,741)	6,503 (4,655-8,697)	4,931 (3,699-6,519)	3,779 (2,899-4,994)	2,673 (2,176-3,634)	1,893 (1,581-2,538)	1,788 (1,443-2,349)
Neural tube defects	24,690 (13,636-40,012)	8,591 (4,037-15,678)	18,189 (8,542-34,302)	4,941 (1,820-12,188)	1,045 (608-1,750)	618 (384-957)	375 (243-554)	251 (160-383)	152 (98-221)	105 (68-152)	81 (54-116)
Congenital heart anomalies	41,146 (21,798-59,302)	22,602 (11,061-34,276)	69,656 (40,138-109,668)	23,764 (11,824-41,291)	6,559 (4,198-9,916)	4,204 (2,911-5,844)	3,319 (2,435-4,379)	2,652 (2,016-3,619)	1,955 (1,575-2,701)	1,319 (1,088-1,766)	1,301 (1,042-1,747)
Orofacial clefts	1,155 (340-1,981)	407 (130-759)	1,106 (365-2,038)	115 (24-265)	-	-	-	-	-	-	-
Down's syndrome	1,473 (514-4,840)	962 (286-3,683)	3,713 (1,383-12,530)	1,754 (486-7,629)	237 (121-617)	156 (88-331)	138 (90-244)	117 (82-197)	103 (74-165)	111 (78-179)	98 (69-158)
Other chromosomal abnormalities	2,241 (1,053-5,299)	1,127 (453-2,662)	2,236 (894-6,718)	466 (159-1,165)	92 (46-200)	53 (28-100)	57 (33-103)	45 (28-74)	32 (19-47)	22 (13-30)	21 (11-28)
Congenital musculoskeletal and limb anomalies	2,926 (1,773-6,435)	836 (422-2,266)	1,695 (864-4,771)	473 (207-1,660)	151 (90-343)	111 (61-178)	121 (72-175)	57 (33-77)	40 (27-55)	30 (19-40)	24 (16-33)
Urogenital congenital anomalies	3,315 (1,890-5,269)	927 (491-1,544)	1,599 (815-2,674)	327 (151-597)	136 (81-216)	118 (77-177)	56 (41-83)	53 (39-79)	47 (34-64)	46 (32-64)	46 (32-65)
Digestive congenital anomalies	11,661 (7,959-21,070)	5,780 (3,290-12,259)	12,168 (6,488-26,262)	2,169 (907-6,663)	234 (152-439)	102 (75-155)	48 (34-63)	29 (21-40)	27 (20-35)	20 (15-26)	21 (13-25)
Other congenital anomalies	30,747 (16,746-52,540)	9,297 (4,076-18,174)	25,826 (12,543-46,322)	9,917 (4,383-20,110)	2,079 (1,300-3,352)	1,141 (747-1,681)	817 (567-1,113)	575 (390-804)	318 (235-429)	240 (180-318)	196 (147-257)
Skin and subcutaneous diseases	-	-	1,478 (562-2,104)	756 (293-1,143)	230 (99-333)	234 (107-331)	426 (201-592)	413 (186-565)	340 (171-448)	689 (323-922)	558 (276-752)
Cellulitis	-	-	51 (15-79)	55 (12-89)	35 (11-58)	37 (16-55)	39 (20-59)	41 (20-59)	30 (16-48)	59 (26-89)	71 (32-115)
Pyoderma	-	-	1,374 (528-2,006)	611 (244-956)	152 (66-223)	153 (64-225)	298 (124-414)	296 (118-408)	243 (106-332)	521 (216-706)	375 (157-523)
Decubitus ulcer	-	-	-	35 (9-63)	22 (11-35)	25 (13-38)	59 (33-90)	49 (26-73)	47 (27-72)	88 (52-133)	92 (50-152)
Other skin and subcutaneous diseases	-	-	52 (19-94)	54 (17-100)	21 (11-32)	19 (11-31)	30 (19-52)	27 (14-42)	19 (12-31)	22 (13-36)	19 (12-31)
Sudden infant death syndrome	-	2,955 (2,182-4,356)	24,697 (18,502-36,574)	-	-	-	-	-	-	-	-
Injuries	12,311 (9,988-14,291)	8,774 (6,612-11,427)	78,460 (65,694-91,103)	223,557 (181,663-254,668)	132,070 (120,454-142,195)	112,276 (106,356-118,784)	211,362 (204,075-218,417)	301,507 (292,861-309,593)	268,817 (260,263-275,817)	235,544 (227,003-242,537)	213,426 (204,240-221,003)
Transport injuries	2,703 (1,876-4,140)	1,652 (1,262-2,159)	11,622 (9,602-14,523)	42,860 (35,148-50,525)	40,033 (36,136-43,516)	31,289 (29,277-33,421)	79,495 (76,030-84,624)	113,096 (108,527-120,250)	97,617 (93,603-103,505)	80,824 (77,676-85,147)	73,178 (70,404-76,995)
Road injuries	2,228 (1,564-3,410)	1,377 (1,054-1,824)	10,639 (8,781-13,396)	40,639 (33,335-48,423)	38,247 (34,416-41,642)	29,426 (27,565-31,477)	74,552 (71,233-79,690)	106,591 (101,868-114,471)	91,092 (86,720-97,335)	75,371 (72,079-79,873)	67,759 (64,935-71,635)
Pedestrian road injuries	949 (620-1,680)	551 (372-898)	4,679 (3,497-6,905)	23,564 (18,438-31,237)	22,596 (19,715-27,182)	14,752 (13,162-17,799)	19,816 (17,334-24,195)	27,546 (24,118-33,591)	24,401 (21,004-29,714)	23,066 (20,263-27,927)	22,598 (20,054-27,265)
Cyclist road injuries	-	-	-	1,210 (712-1,796)	1,975 (1,616-2,477)	2,278 (1,947-2,660)	3,134 (2,679-3,580)	3,013 (2,511-3,470)	2,701 (2,296-3,071)	2,683 (2,233-3,045)	2,589 (2,151-2,968)
Motorcyclist road injuries	162 (97-312)	181 (101-349)	1,143 (806-1,770)	2,467 (1,624-4,530)	2,209 (1,754-2,821)	3,135 (2,663-3,745)	20,330 (18,212-24,024)	28,706 (25,337-34,154)	22,815 (19,660-27,223)	16,366 (14,127-19,392)	14,106 (12,067-16,892)
Motor vehicle road injuries	1,070 (571-1,777)	621 (310-904)	4,574 (2,440-6,284)	12,920 (6,147-17,210)	11,030 (7,333-14,185)	8,848 (6,354-10,990)	30,636 (24,602-35,614)	46,367 (37,379-53,657)	40,350 (32,723-47,248)	32,683 (26,326-38,363)	27,915 (22,350-32,530)
Other road injuries	46 (22-94)	24 (14-45)	242 (153-403)	479 (267-765)	436 (314-600)	413 (298-575)	636 (459-880)	959 (676-1,263)	826 (591-1,090)	573 (409-770)	551 (390-715)
Other transport injuries	476 (239-878)	275 (134-441)	983 (591-1,424)	2,221 (1,066-3,189)	1,786 (1,312-2,270)	1,863 (1,522-2,261)	4,943 (3,913-5,618)	6,505 (5,112-7,392)	6,525 (5,266-7,388)	5,453 (4,443-6,189)	5,419 (4,430-6,112)
Unintentional injuries	8,073 (6,224-9,435)	6,553 (4,651-9,007)	62,315 (50,817-73,950)	167,361 (131,475-194,226)	81,712 (73,665-89,429)	62,386 (58,408-67,142)	62,528 (58,907-66,618)	72,758 (68,588-77,598)	63,335 (59,502-67,564)	60,960 (57,478-64,275)	60,757 (56,857-64,383)
Falls	1,593 (901-2,375)	702 (323-981)	6,909 (3,671-9,521)	13,713 (6,445-19,073)	6,551 (4,928-8,059)	6,245 (4,899-7,283)	7,240 (6,305-8,235)	10,746 (9,402-12,124)	10,710 (9,260-11,990)	11,611 (10,085-12,890)	12,760 (11,189-14,437)
Drowning	514 (378-612)	263 (181-320)	7,986 (6,525-9,192)	95,595 (74,576-110,495)	49,467 (44,301-54,362)	34,821 (32,258-37,639)	28,364 (26,356-31,032)	24,319 (22,514-27,193)	16,618 (15,375-18,216)	14,484 (13,464-15,918)	12,705 (11,791-13,797)
Fire, heat, and hot substances	374 (266-434)	327 (178-402)	5,002 (3,049-6,009)	13,755 (6,876-17,975)	4,090 (2,971-4,814)	2,324 (1,790-2,667)	2,760 (2,076-3,260)	4,397 (3,306-5,232)	4,308 (3,361-4,983)	4,258 (3,474-4,828)	4,256 (3,489-4,804)
Poisonings	571 (194-1,164)	310 (134-518)	4,858 (1,784-8,855)	9,120 (4,282-14,380)	2,961 (1,944-3,878)	2,807 (1,851-3,846)	2,976 (2,000-4,241)	4,331 (2,935-6,159)	4,006 (2,764-5,633)	3,357 (2,377-4,547)	3,242 (2,294-4,332)
Exposure to mechanical forces	1,942 (1,451-2,573)	2,167 (1,247-3,867)	13,157 (9,853-19,404)	10,710 (6,322-15,075)	5,705 (4,793-7,082)	4,672 (4,237-5,832)	7,519 (6,874-9,130)	10,896 (9,917-12,996)	10,106 (9,189-11,946)	9,289 (8,528-10,868)	9,473 (8,605-11,419)
Unintentional firearm injuries	114 (56-140)	64 (35-87)	750 (434-999)	1,233 (621-1,848)	918 (706-1,108)	1,112 (905-1,304)	2,174 (1,794-2,565)	3,072 (2,181-3,640)	2,796 (2,026-3,348)	2,128 (1,521-2,472)	1,805 (1,237-2,092)
Unintentional suffocation	1,393 (937-2,028)	1,889 (985-3,552)	10,094 (7,082-16,027)	3,429 (1,984-6,567)	1,358 (1,035-2,341)	1,164 (933-1,900)	348 (282-543)	450 (364-703)	452 (369-687)	329 (269-503)	288 (232-407)
Other exposure to mechanical forces	436 (347-500)	213 (141-262)	2,313 (1,656-2,889)	6,049 (3,288-8,286)	3,429 (2,769-4,216)	2,397 (2,148-2,910)	4,997 (4,464-6,628)	7,373 (6,583-10,011)	6,858 (6,124-8,977)	6,831 (6,150-8,775)	7,380 (6,674-9,570)
Adverse effects of medical treatment	1,025 (612-1,228)	783 (383-1,076)	6,383 (3,083-8,573)	5,318 (1,904-8,505)	1,784 (1,104-2,283)	1,301 (922-1,505)	1,469 (997-1,787)	2,186 (1,457-2,667)	1,991 (1,352-2,409)	1,968 (1,305-2,368)	2,039 (1,402-2,454)
Animal contact	399 (300-474)	413 (258-629)	2,251 (1,617-3,097)	5,315 (3,315-9,716)	4,651 (3,360-6,134)	4,215 (3,130-5,123)	3,322 (2,502-3,913)	3,501 (2,685-4,102)	3,422 (2,615-4,026)	3,249 (2,486-3,832)	3,235 (2,438-3,824)
Venomous animal contact	331 (240-403)	355 (211-547)	1,792 (1,232-2,474)	4,310 (2,582-7,753)	3,820 (2,647-5,093)	3,696 (2,708-4,560)	2,658 (1,924-3,235)	2,701 (2,021-3,247)	2,704 (2,052-3,283)	2,686 (2,018-3,255)	2,658 (1,985-3,237)
Non-venomous animal contact	68 (53-83)	58 (39-86)	459 (331-660)	1,005 (530-2,167)	831 (619-1,131)	519 (392-654)	665 (475-813)	801 (590-995)	718 (527-915)	563 (428-724)	577 (426-745)
Foreign body	931 (568-1,141)	1,133 (607-1,697)	11,882 (7,051-17,499)	7,526 (3,579-13,941)	2,443 (1,616-3,255)	1,793 (1,326-2,100)	1,263 (952-1,447)	1,854 (1,416-2,143)	1,960 (1,519-2,301)	2,522 (1,950-2,962)	2,809 (2,196-3,313)
Pulmonary aspiration and foreign body in airway	854 (518-1,052)	1,059 (579-1,602)	10,315 (6,352-16,214)	5,715 (2,928-11,270)	1,849 (1,209-2,547)	1,407 (1,029-1,709)	1,018 (801-1,217)	1,526 (1,214-1,842)	1,637 (1,308-1,976)	1,970 (1,533-2,372)	2,204 (1,668-2,719)
Foreign body in other body part	77 (22-105)	73 (16-104)	1,566 (306-2,259)	1,810 (368-3,239)	594 (280-773)	385 (174-506)	246 (83-356)	328 (130-468)	323 (129-458)	552 (272-750)	604 (293-805)
Environmental heat and cold exposure	394 (171-490)	307 (104-431)	2,437 (897-3,137)	1,662 (717-2,311)	660 (387-808)	539 (310-636)	1,116 (677-1,377)	1,631 (935-2,050)	1,862 (1,105-2,485)	2,645 (1,549-3,600)	3,175 (1,941-4,434)
Other unintentional injuries	330 (285-386)	148 (118-179)	1,451 (1,128-1,810)	4,645 (2,861-5,951)	3,399 (2,871-4,094)	3,670 (3,210-4,226)	6,497 (5,768-7,861)	8,897 (7,780-11,107)	8,352 (7,366-10,040)	7,575 (6,736-9,053)	7,064 (6,305-8,319)
Self-harm and interpersonal violence	1,129 (813-1,399)	302 (206-380)	3,309 (2,372-4,346)	5,470 (3,745-7,313)	3,998 (2,842-4,950)	11,954 (9,516-13,544)	56,616 (48,852-61,484)	95,968 (83,388-103,050)	93,477 (81,204-100,147)	83,360 (72,378-89,508)	71,815 (62,266-77,998)

Cause	40 to 44	45 to 49	50 to 54	55 to 59	60 to 64	65 to 69	70 to 74	75 to 79	80 to 84	85 to 89	90 to 94	95 plus
Ischaemic stroke	6,518 (5,860-7,126)	13,197 (11,848-14,426)	27,252 (24,537-29,517)	44,797 (41,271-48,001)	95,780 (89,759-101,277)	121,043 (114,198-127,186)	141,616 (134,604-149,126)	172,906 (164,090-182,199)	134,064 (126,087-142,019)	75,123 (70,171-80,443)	25,822 (23,914-27,876)	6,442 (5,843-6,973)
Hemorrhagic stroke	36,874 (34,434-40,857)	52,141 (48,902-57,565)	86,232 (81,168-94,225)	121,471 (115,238-131,313)	147,858 (140,512-159,906)	165,134 (156,879-179,679)	181,324 (171,925-195,242)	146,436 (138,439-158,976)	92,764 (86,852-100,193)	44,969 (41,761-49,104)	14,364 (13,251-15,766)	3,271 (2,960-3,590)
Hypertensive heart disease	5,667 (4,016-6,773)	8,120 (5,813-9,751)	13,236 (9,231-15,765)	19,321 (12,997-23,124)	26,918 (17,963-32,022)	32,072 (20,942-38,318)	38,086 (23,799-45,085)	38,338 (25,227-45,395)	28,816 (19,465-34,045)	17,139 (11,647-20,360)	6,991 (4,921-8,291)	1,800 (1,258-2,156)
Cardiomyopathy and myocarditis	6,615 (5,407-8,125)	6,672 (5,377-7,950)	10,617 (8,464-13,548)	11,170 (9,006-13,274)	12,506 (9,989-14,728)	10,928 (8,214-12,140)	9,454 (6,523-10,537)	11,291 (7,298-12,863)	10,540 (5,561-12,501)	7,713 (3,724-9,378)	3,076 (1,468-3,737)	828 (431-992)
Myocarditis	672 (485-989)	502 (376-678)	556 (384-751)	723 (498-976)	657 (438-887)	683 (459-865)	745 (505-928)	906 (595-1,143)	842 (513-1,111)	715 (432-993)	371 (215-508)	134 (73-187)
Alcoholic cardiomyopathy	3,405 (2,446-4,648)	3,507 (2,574-4,571)	6,167 (4,477-8,826)	5,496 (4,165-7,363)	5,749 (4,368-7,716)	3,426 (2,702-4,362)	2,130 (1,492-2,619)	1,909 (1,268-2,333)	1,213 (677-1,527)	573 (258-761)	199 (93-261)	66 (30-85)
Other cardiomyopathy	2,537 (1,981-3,114)	2,664 (2,106-3,184)	3,894 (3,090-4,777)	4,951 (3,759-5,805)	6,100 (4,430-6,880)	6,820 (4,688-7,680)	6,579 (4,310-7,494)	8,476 (4,956-9,883)	8,485 (4,230-10,360)	6,425 (2,861-7,989)	2,507 (1,118-3,133)	628 (304-796)
Atrial fibrillation and flutter	115 (84-154)	201 (145-271)	359 (261-471)	638 (374-969)	1,295 (843-1,849)	2,357 (1,444-3,568)	3,860 (2,719-5,369)	5,908 (3,690-9,025)	9,725 (7,271-12,559)	8,731 (6,616-11,458)	3,489 (2,637-4,571)	784 (598-1,011)
Aortic aneurysm	1,217 (1,102-1,348)	1,610 (1,473-1,765)	2,513 (2,333-2,717)	4,096 (3,835-4,366)	6,914 (6,506-7,313)	9,558 (9,074-10,028)	10,680 (10,193-11,175)	11,860 (11,353-12,404)	8,901 (8,496-9,326)	4,813 (4,582-5,043)	1,647 (1,562-1,727)	343 (324-361)
Peripheral vascular disease	85 (51-111)	145 (82-187)	374 (220-507)	666 (403-864)	1,294 (801-1,695)	1,565 (1,055-2,069)	1,834 (1,226-2,461)	2,488 (1,736-3,500)	2,442 (1,712-3,414)	1,623 (1,200-2,353)	730 (524-1,034)	238 (167-334)
Endocarditis	1,146 (886-1,472)	1,029 (813-1,349)	1,353 (1,063-1,796)	1,488 (1,151-1,941)	1,831 (1,458-2,471)	2,010 (1,593-2,639)	1,958 (1,535-2,542)	2,141 (1,687-2,810)	1,653 (1,338-2,218)	986 (781-1,355)	400 (322-544)	109 (89-157)
Other cardiovascular and circulatory diseases	4,600 (3,403-5,450)	5,408 (3,965-6,352)	8,128 (5,963-9,287)	11,732 (8,481-13,388)	16,673 (11,911-18,640)	21,087 (14,915-23,745)	24,702 (16,183-28,556)	27,220 (19,589-30,794)	21,016 (16,199-23,527)	12,455 (9,955-13,902)	4,862 (4,012-5,418)	1,162 (990-1,275)
Chronic respiratory diseases	24,616 (23,307-25,922)	38,079 (36,159-40,040)	72,726 (69,364-76,204)	119,486 (113,884-125,010)	204,136 (193,987-212,161)	261,256 (247,319-271,826)	334,960 (315,992-349,022)	314,702 (297,659-325,786)	216,163 (203,890-225,992)	116,146 (109,400-121,754)	39,204 (37,043-41,243)	9,142 (8,646-9,640)
Chronic obstructive pulmonary disease	14,974 (12,296-17,468)	25,308 (21,115-28,912)	52,879 (45,510-59,150)	92,235 (80,761-101,411)	166,366 (151,126-180,754)	220,411 (201,371-235,351)	293,404 (271,775-311,965)	277,867 (259,748-293,697)	192,889 (180,812-203,853)	104,618 (97,924-110,970)	35,542 (33,509-37,722)	8,362 (7,875-8,868)
Pneumoconiosis	576 (445-1,027)	673 (521-1,177)	1,041 (836-1,692)	1,461 (1,201-2,320)	2,345 (1,956-3,600)	3,028 (2,585-4,265)	3,239 (2,786-4,501)	3,001 (2,645-3,778)	1,786 (1,603-2,119)	809 (724-965)	226 (202-276)	49 (44-58)
Silicosis	394 (291-759)	429 (310-815)	641 (486-1,172)	880 (688-1,553)	1,340 (1,073-2,215)	1,776 (1,445-2,779)	1,785 (1,465-2,567)	1,625 (1,372-2,122)	912 (768-1,134)	375 (315-461)	86 (71-105)	14 (12-18)
Asbestosis	27 (19-42)	26 (17-41)	45 (30-65)	71 (39-105)	119 (71-202)	171 (102-350)	201 (132-401)	214 (139-482)	153 (106-329)	75 (54-165)	22 (15-46)	4 (3-8)
Coal workers pneumoconiosis	61 (27-101)	92 (40-155)	151 (76-226)	232 (126-327)	424 (222-577)	652 (307-864)	762 (382-1,021)	719 (348-892)	494 (274-596)	224 (119-272)	56 (29-69)	9 (5-11)
Other pneumoconiosis	94 (55-181)	125 (72-233)	204 (125-370)	278 (175-499)	463 (292-801)	428 (260-773)	492 (307-881)	443 (290-741)	227 (153-400)	134 (94-231)	62 (47-103)	22 (18-30)
Asthma	7,777 (5,036-10,392)	10,451 (6,431-14,248)	16,499 (9,900-23,972)	22,438 (13,373-32,271)	30,964 (18,410-45,374)	32,462 (18,972-48,161)	32,615 (19,105-50,416)	28,226 (17,416-43,439)	17,242 (11,371-27,542)	8,374 (5,651-12,449)	2,595 (1,742-4,177)	550 (376-858)
Interstitial lung disease and pulmonary sarcoidosis	510 (285-939)	730 (406-1,316)	1,260 (724-2,230)	1,956 (1,142-3,321)	2,849 (1,696-4,690)	3,720 (2,442-5,971)	4,021 (2,686-6,438)	4,187 (2,970-6,599)	3,038 (2,202-4,851)	1,567 (1,144-2,522)	504 (365-817)	99 (72-154)
Other chronic respiratory diseases	778 (438-1,116)	917 (508-1,308)	1,048 (580-1,429)	1,397 (810-1,923)	1,612 (968-2,174)	1,636 (901-2,193)	1,682 (991-2,208)	1,422 (779-1,816)	1,208 (658-1,538)	778 (438-994)	337 (193-429)	82 (51-104)
Cirrhosis and other chronic liver diseases	43,745 (40,513-46,733)	51,789 (48,092-55,402)	61,685 (57,816-66,650)	65,145 (60,865-69,142)	66,407 (62,361-71,725)	54,938 (51,315-58,596)	41,422 (38,410-44,129)	31,355 (29,113-33,158)	17,995 (16,372-19,255)	8,605 (7,659-9,244)	2,649 (2,306-2,862)	638 (546-689)
Cirrhosis and other chronic liver diseases due to hepatitis B	15,139 (12,959-17,406)	16,572 (13,782-19,478)	19,098 (15,999-22,374)	20,501 (17,310-24,300)	21,143 (17,779-25,158)	17,608 (14,824-21,186)	14,025 (11,551-16,935)	10,212 (8,343-11,900)	5,246 (4,178-6,308)	2,242 (1,868-2,638)	611 (476-753)	133 (94-177)
Cirrhosis and other chronic liver diseases due to hepatitis C	12,065 (10,216-14,267)	14,521 (11,970-17,566)	17,334 (14,306-20,831)	18,251 (15,245-21,546)	18,449 (15,218-22,728)	15,526 (13,070-18,258)	11,515 (9,768-13,493)	8,434 (6,872-10,198)	5,106 (4,116-6,217)	2,554 (2,036-3,019)	801 (587-980)	188 (131-245)
Cirrhosis and other chronic liver diseases due to alcohol use	12,241 (10,539-14,267)	15,424 (12,963-18,152)	18,609 (15,667-21,764)	18,908 (15,668-22,234)	19,104 (15,453-22,986)	15,764 (13,291-18,357)	11,225 (9,382-13,267)	9,049 (7,617-10,609)	5,362 (4,450-6,310)	2,574 (2,242-2,924)	812 (693-933)	205 (166-244)
Cirrhosis and other chronic liver diseases due to other causes	4,299 (3,106-5,832)	5,272 (3,697-7,153)	6,645 (4,779-9,012)	7,485 (5,431-10,095)	7,710 (5,518-10,528)	6,040 (4,356-7,986)	4,656 (3,347-6,266)	3,660 (2,633-4,811)	2,281 (1,649-2,990)	1,235 (936-1,526)	425 (314-555)	112 (77-154)
Digestive diseases	21,279 (17,545-23,568)	24,622 (19,919-27,173)	30,422 (24,913-33,558)	36,709 (29,922-40,318)	44,158 (36,741-48,911)	46,999 (38,765-51,886)	47,442 (39,395-52,270)	46,042 (38,756-50,527)	32,901 (28,182-36,310)	19,276 (16,448-21,237)	7,445 (6,465-8,237)	1,864 (1,614-2,060)
Peptic ulcer disease	8,874 (7,327-9,920)	11,000 (8,947-12,206)	14,395 (11,628-15,980)	17,351 (13,814-19,217)	20,603 (16,909-22,936)	21,022 (16,894-23,405)	21,083 (17,219-23,691)	19,046 (15,925-21,290)	12,403 (10,253-13,981)	6,915 (5,762-7,737)	2,427 (2,044-2,720)	564 (484-631)
Gastritis and duodenitis	899 (728-1,073)	1,116 (889-1,326)	1,378 (1,074-1,654)	1,742 (1,270-2,086)	1,967 (1,513-2,324)	1,882 (1,425-2,222)	1,984 (1,517-2,359)	1,787 (1,352-2,150)	1,176 (876-1,439)	711 (541-878)	293 (215-361)	80 (62-97)
Appendicitis	1,013 (765-1,354)	871 (637-1,179)	1,000 (710-1,360)	1,095 (802-1,467)	1,265 (924-1,728)	1,139 (862-1,566)	1,160 (880-1,597)	1,005 (766-1,371)	681 (514-944)	373 (292-516)	137 (108-189)	34 (28-46)
Paralytic ileus and intestinal obstruction	3,072 (1,764-3,781)	3,596 (2,127-4,387)	3,889 (2,512-4,707)	5,003 (3,265-6,027)	5,636 (3,790-6,743)	6,699 (4,770-7,907)	6,223 (4,482-7,615)	5,999 (4,595-7,489)	4,724 (3,651-5,780)	3,030 (2,305-3,826)	1,253 (977-1,549)	315 (246-408)
Inguinal, femoral, and abdominal hernia	831 (501-1,062)	811 (514-1,061)	1,164 (732-1,551)	1,441 (947-1,935)	1,736 (1,151-2,330)	2,159 (1,366-2,932)	2,345 (1,447-3,171)	2,620 (1,709-3,519)	1,944 (1,305-2,545)	1,237 (847-1,609)	532 (369-688)	152 (103-193)
Inflammatory bowel disease	481 (337-581)	494 (341-603)	608 (436-705)	748 (531-865)	892 (675-1,008)	980 (744-1,114)	1,044 (778-1,187)	1,130 (881-1,252)	931 (715-1,042)	578 (420-648)	240 (179-267)	63 (47-71)
Vascular intestinal disorders	428 (335-502)	609 (468-698)	1,040 (811-1,275)	1,598 (1,321-1,964)	2,463 (2,004-2,963)	3,133 (2,574-3,776)	3,411 (2,717-4,038)	3,894 (3,152-4,637)	2,960 (2,271-3,414)	1,644 (1,172-1,883)	615 (465-706)	143 (108-172)
Gallbladder and biliary diseases	767 (560-914)	890 (612-1,049)	1,276 (862-1,520)	1,713 (1,192-2,000)	2,545 (1,825-2,985)	3,088 (2,268-3,578)	3,365 (2,600-3,836)	3,929 (3,038-4,444)	3,232 (2,500-3,678)	2,028 (1,642-2,292)	823 (684-929)	200 (164-228)
Pancreatitis	3,529 (2,899-4,399)	3,517 (2,791-4,457)	3,502 (2,846-4,519)	3,382 (2,737-4,302)	3,414 (2,795-4,380)	3,060 (2,400-3,947)	2,478 (1,915-3,147)	2,398 (1,828-3,049)	1,463 (1,115-1,820)	767 (562-946)	246 (174-305)	62 (43-75)
Other digestive diseases	1,385 (1,090-1,768)	1,717 (1,366-2,192)	2,170 (1,738-2,762)	2,637 (2,126-3,304)	3,637 (2,931-4,586)	3,838 (3,114-4,724)	4,349 (3,479-5,306)	4,234 (3,440-5,120)	3,387 (2,708-4,088)	1,992 (1,562-2,434)	879 (688-1,073)	250 (193-300)
Neurological disorders	5,544 (5,218-5,851)	5,926 (5,496-6,392)	7,709 (6,846-8,815)	10,716 (9,032-12,713)	16,526 (13,751-20,249)	26,545 (21,292-32,969)	43,867 (34,245-56,433)	76,980 (60,484-97,438)	88,025 (70,313-111,386)	72,945 (58,342-89,868)	36,840 (29,612-43,871)	9,146 (7,203-11,540)
Alzheimer's disease and other dementias	34 (14-68)	647 (373-996)	2,392 (1,595-3,437)	4,763 (3,143-6,682)	9,807 (7,070-13,546)	18,452 (13,465-24,785)	34,146 (24,709-46,312)	63,028 (46,661-82,269)	74,931 (57,027-98,077)	65,511 (50,767-82,171)	34,527 (27,587-41,602)	8,697 (6,720-11,137)
Parkinson's disease	110 (60-184)	159 (87-278)	297 (170-480)	573 (341-853)	1,393 (961-1,964)	2,960 (2,094-3,959)	5,758 (4,010-7,746)	10,609 (7,651-14,129)	10,979 (8,132-14,769)	6,460 (4,908-8,591)	2,031 (1,521-2,690)	386 (271-532)
Epilepsy	3,939 (3,665-4,182)	3,390 (3,161-3,626)	2,763 (2,594-2,944)	2,617 (2,437-2,781)	2,206 (2,045-2,358)	1,928 (1,764-2,099)	1,391 (1,279-1,508)	1,048 (954-1,136)	707 (627-791)	345 (306-386)	116 (104-129)	30 (27-33)
Multiple sclerosis	503 (394-621)	504 (407-615)	619 (528-756)	618 (517-775)	596 (516-746)	498 (436-619)	334 (288-419)	246 (209-304)	125 (106-147)	45 (34-51)	11 (8-12)	2 (1-2)
Motor neuron disease	353 (333-392)	505 (479-560)	711 (673-787)	1,037 (986-1,121)	1,301 (1,237-1,396)	1,466 (1,401-1,565)	1,133 (1,085-1,200)	914 (865-969)	492 (461-523)	176 (166-186)	31 (29-32)	5 (5-5)
Other neurological disorders	606 (537-646)	721 (645-768)	927 (822-994)	1,107 (1,005-1,174)	1,222 (1,131-1,289)	1,241 (1,153-1,304)	1,106 (1,028-1,164)	1,136 (1,062-1,189)	790 (742-835)	408 (381-430)	124 (114-132)	26 (24-28)
Mental and substance use disorders	17,537 (14,733-19,755)	15,459 (13,144-17,530)	17,492 (14,449-20,325)	14,225 (11,829-16,229)	12,103 (9,995-13,982)	7,256 (6,070-7,874)	4,496 (3,618-4,867)	3,333 (2,755-3,613)	1,566 (1,364-1,688)	759 (655-822)	256 (210-280)	69 (58-75)
Alcohol use disorders	11,507 (9,395-13,495)	11,733 (9,889-13,863)	13,148 (10,629-15,857)	10,884 (9,022-12,747)	8,932 (7,440-10,580)	5,214 (4,452-5,798)	2,804 (2,414-3,220)	1,993 (1,697-2,283)	849 (742-941)	365 (315-410)	105 (88-119)	29 (24-32)
Drug use disorders	6,012 (4,665-6,902)	3,708 (2,745-4,307)	4,345 (3,400-5,038)	3,341 (2,394-3,988)	3,170 (2,191-3,767)	2,042 (1,336-2,429)	1,693 (994-2,049)	1,340 (902-1,561)	717 (544-815)	394 (303-445)	151 (115-171)	41 (32-45)
Opioid use disorders	4,268 (3,161-5,056)	2,474 (1,836-2,966)	2,985 (2,142-3,591)	2,188 (1,458-2,716)	2,064 (1,305-2,520)	1,251 (744-1,569)	723 (406-916)	614 (371-754)	301 (207-353)	174 (117-202)	70 (47-81)	20 (14-22)
Cocaine use disorders	357 (222-471)	266 (155-358)	358 (221-473)	308 (171-415)	275 (153-371)	211 (113-284)	125 (61-172)	89 (47-117)	12 (7-16)	6 (3-8)	2 (1-3)	1 (0-1)
Amphetamine use disorders	181 (108-243)	132 (76-188)	139 (78-187)	126 (62-176)	114 (57-159)	88 (43-123)	39 (18-55)	28 (13-39)	6 (3-8)	3 (1-4)	1 (1-1)	0 (0-0)
Other drug use disorders	1,206 (777-1,427)	837 (483-1,033)	863 (549-1,084)	719 (409-939)	718 (445-928)	491 (281-626)	805 (416-1,012)	609 (376-736)	398 (295-461)	212 (160-243)	78 (56-90)	20 (14-23)
Eating disorders	19 (15-24)	17 (13-21)	0 (0-0)	0 (0-0)	-	-	-	-	-	-	-	-
Anorexia nervosa	16 (13-21)	12 (9-15)	0 (0-0)	0 (0-0)	-	-	-	-	-	-	-	-
Bulimia nervosa	2 (2-4)	5 (4-8)	0 (0-0)	0 (0-0)	-	-	-	-	-	-	-	-
Diabetes, urogenital, blood, and endocrine diseases	26,203 (24,838-27,910)	32,368 (30,550-34,384)	44,122 (41,948-47,132)	58,612 (55,895-62,352)	74,740 (71,580-79,319)	84,253 (80,211-89,804)	86,083 (81,937-91,895)	86,955 (82,911-92,572)	64,855 (61,917-69,217)	39,869 (37,966-42,695)	15,843 (15,045-17,114)	4,215 (4,003-4,527)
Diabetes mellitus	8,092 (7,782-8,463)	11,564 (11,094-12,014)	17,780 (17,140-18,480)	25,674 (24,821-26,728)	34,416 (33,206-35,751)	39,106 (37,607-40,638)	39,001 (37,449-40,804)	38,246 (36,572-39,867)	26,073 (24,749-27,330)	13,587 (12,874-14,257)	4,559 (4,330-4,784)	979 (931-1,032)
Acute glomerulonephritis	373 (302-460)	442 (374-515)	532 (451-626)	678 (596-777)	676 (571-805)	707 (610-817)	726 (620-866)	600 (510-703)	321 (275-374)	165 (138-195)	58 (49-69)	21 (17-28)
Chronic kidney disease	12,831 (11,311-14,548)	14,726 (12,884-16,567)	19,555 (17,820-22,685)	24,996 (22,737-28,642)	30,181 (27,819-34,680)	33,275 (30,389-38,959)	34,211 (31,735-39,851)	34,672 (32,380-40,305)	27,139 (25,432-31,520)	18,138 (16,989-20,920)	7,627 (7,078-8,855)	2,140 (1,978-2,476)
Chronic kidney disease due to diabetes mellitus	5,796 (4,389-7,187)	7,395 (5,855-9,016)	10,146 (7,744-12,374)	13,248 (10,373-15,933)	16,269 (13,200-19,507)	17,253 (13,318-21,007)	16,642 (13,013-20,214)	15,146 (11,767-18,809)	9,962 (7,519-12,494)	5,598 (4,113-7,329)	2,196 (1,522-2,879)	622 (409-845)
Chronic kidney disease due to hypertension	1,707 (1,298-2,198)	2,219 (1,679-2,890)	3,243 (2,414-4,295)	4,681 (3,594-6,112)	6,145 (4,797-7,872)	7,581 (5,866-9,742)	8,813 (6,844-11,249)	9,603 (7,401-12,249)	8,423 (6,672-10,527)	6,764 (5,424-8,421)	3,226 (2,591-3,939)	945 (741-1,164)
Chronic kidney disease due to glomerulonephritis	2,354 (1,494-3,306)	2,075 (1,313-2,963)	2,410 (1,592-3,453)	2,526 (1,703-3,591)	2,704 (1,735-3,885)	2,849 (1,820-4,176)	2,907 (1,880-4,285)	3,471 (2,300-5,100)	3,017 (2,063-4,152)	1,786 (1,222-2,585)	654 (438-904)	181 (107-300)
Chronic kidney disease due to other causes	2,975 (2,040-4,060)	3,037 (1,996-4,394)	3,755 (2,400-5,481)	4,541 (3,009-6,634)	5,063 (3,263-7,452)	5,592 (3,728-8,027)	5,849 (3,909-8,221)	6,452 (4,386-8,939)	5,737 (4,025-7,738)	3,990 (2,716-5,441)	1,552 (1,096-2,132)	392 (246-574)
Urinary diseases and male infertility	2,155 (2,010-2,282)	2,581 (2,377-2,765)	3,331 (3,107-3,522)	3,891 (3,651-4,098)	5,756 (5,411-6,082)	6,989 (6,520-7,341)	7,675 (7,164-8,082)	8,418 (7,939-8,799)	7,371 (6,991-7,728)	5,196 (4,928-5,445)	2,472 (2,355-2,586)	770 (735-804)
Interstitial nephritis and urinary tract infections	924 (735-1,124)	1,061 (828-1,306)	1,550 (1,230-1,861)	1,822 (1,467-2,172)	2,834 (2,245-3,351)	3,433 (2,798-4,056)	3,900 (3,221-4,565)	4,821 (4,119-5,461)	4,280 (3,727-4,800)	3,195 (2,792-3,556)	1,554 (1,336-1,724)	528 (471-576)
Urolithiasis	355 (208-470)	427 (230-562)	569 (326-730)	584 (350-747)	875 (536-1,128)	1,134 (660-1,439)	1,170 (642-1,476)	1,141 (688-1,391)	804 (501-972)	490 (292-605)	195 (115-241)	50 (32-65)
Other urinary diseases	876 (605-1,141)	1,093 (746-1,425)	1,212 (809-1,646)	1,485 (1,036-1,946)	2,047 (1,418-2,803)	2,422 (1,697-3,323)	2,606 (1,905-3,498)	2,456 (1,883-3,323)	2,287 (1,826-2,943)	1,511 (1,194-1,940)	723 (580-940)	191 (152-251)
Gynecological diseases	-	-	-	-	-	-	-	-	-	-	-	-
Uterine fibroids	-	-	-	-	-	-	-	-	-	-	-	-
Polycystic ovarian syndrome	-	-	-	-	-	-	-	-	-	-	-	-
Endometriosis	-	-	-	-	-	-	-	-	-	-	-	-
Genital prolapse	-	-	-	-	-	-	-	-	-	-	-	-
Other gynecological diseases	-	-	-	-	-	-	-	-	-	-	-	-
Hemoglobinopathies and hemolytic anaemias	1,372 (1,101-1,867)	1,597 (1,311-2,375)	1,262 (1,007-1,668)	1,390 (1,125-1,812)	1,514 (1,241-1,989)	1,735 (1,492-2,265)	2,026 (1,726-2,684)	2,512 (2,158-3,251)	1,872 (1,554-2,233)	1,449 (1,172-1,692)	571 (458-656)	149 (120-171)
Thalassemias	18 (14-21)	17 (13-19)	15 (11-17)	9 (7-11)	3 (2-3)	1 (1-1)	0 (0-0)	0 (0-0)	0 (0-0)	0 (0-0)	0 (0-0)	0 (0-0)
Sickle cell disorders	459 (365-659)	461 (347-758)	168 (134-241)	73 (59-106)	36 (31-48)	26 (21-31)	12 (9-13)	6 (4-7)	2 (1-2)	1 (0-1)	0 (0-0)	0 (0-0)
G6PD deficiency	463 (357-629)	653 (514-967)	468 (358-628)	435 (333-593)	339 (257-474)	240 (188-344)	171 (133-248)	142 (110-202)	50 (38-67)	26 (20-35)	8 (6-11)	1 (1-1)
Other hemoglobinopathies and hemolytic anaemias	432 (347-571)	465 (381-653)	611 (487-786)	872 (712-1,128)	1,136 (926-1,474)	1,469 (1,263-1,914)	1,843 (1,572-2,422)	2,363 (2,029-3,061)	1,821 (1,520-2,161)	1,423 (1,147-1,661)	563 (450-645)	148 (119-170)
Endocrine, metabolic, blood, and immune disorders	1,380 (1,116-1,638)	1,459 (1,186-1,778)	1,662 (1,319-1,895)	1,983 (1,613-2,369)	2,197 (1,877-2,644)	2,441 (2,021-3,002)	2,443 (2,011-2,927)	2,507 (2,005-2,992)	2,079 (1,501-2,417)	1,334 (970-1,521)	556 (419-637)	156 (123-181)
Musculoskeletal disorders	650 (534-749)	751 (623-872)	842 (691-967)	1,094 (914-1,235)	1,459 (1,203-1,618)	1,756 (1,479-1,951)	1,912 (1,598-2,103)	2,008 (1,691-2,226)	1,462 (1,237-1,624)	944 (810-1,053)	370 (317-415)	99 (88-110)
Rheumatoid arthritis	154 (112-187)	209 (153-253)	346 (248-407)	516 (387-592)	779 (601-890)	986 (765-1,118)	1,127 (865-1,276)	1,116 (888-1,265)	720 (568-830)	395 (301-461)	131 (97-153)	31 (23-36)
Other musculoskeletal disorders	496 (413-574)	542 (461-642)	496 (411-590)	578 (484-680)	680 (566-800)	770 (643-902)	786 (647-925)	892 (730-1,068)	742 (603-870)	550 (469-646)	239 (204-276)	68 (58-77)
Other non-communicable diseases	2,193 (1,678-2,642)	1,827 (1,335-2,209)	1,768 (1,298-2,145)	2,085 (1,475-2,521)	2,382 (1,644-2,902)	2,451 (1,656-3,092)	1,952 (1,095-2,647)	2,272 (1,285-3,182)	1,860 (1,058-2,583)	1,373 (759-1,902)	653 (367-911)	198 (110-277)
Congenital anomalies	1,581 (1,252-2,078)	991 (798-1,308)	891 (758-1,196)	845 (729-1,098)	895 (765-1,238)	719 (610-944)	-	-	-	-	-	-
Neural tube defects	65 (42-97)	54 (37-76)	48 (32-69)	44 (29-69)	35 (22-59)	30 (20-49)	-	-	-	-	-	-
Congenital heart anomalies	1,186 (921-1,558)	453 (362-581)	392 (332-504)	355 (304-457)	348 (290-466)	273 (224-356)	-	-	-	-	-	-
Orofacial clefts	-	-	-	-	-	-	-	-	-	-	-	-
Down's syndrome	82 (59-125)	167 (115-277)	149 (114-254)	145 (111-243)	125 (95-220)	53 (41-94)	-	-	-	-	-	-
Other chromosomal abnormalities	15 (8-20)	15 (9-24)	20 (13-32)	18 (11-25)	16 (10-22)	10 (7-16)	-	-	-	-	-	-
Congenital musculoskeletal and limb anomalies	17 (11-23)	19 (12-26)	20 (13-26)	16 (9-20)	23 (13-28)	28 (13-37)	-	-	-	-	-	-
Urogenital congenital anomalies	47 (31-65)	74 (46-106)	83 (54-115)	101 (66-135)	131 (89-187)	151 (103-203)	-	-	-	-	-	-
Digestive congenital anomalies	17 (11-21)	25 (16-32)	23 (15-29)	25 (17-30)	36 (26-48)	31 (22-40)	-	-	-	-	-	-
Other congenital anomalies	153 (119-202)	184 (133-257)	157 (119-213)	141 (110-189)	181 (140-254)	143 (112-208)	-	-	-	-	-	-
Skin and subcutaneous diseases	612 (328-823)	837 (456-1,184)	877 (469-1,174)	1,240 (653-1,658)	1,487 (804-2,023)	1,731 (976-2,364)	1,952 (1,095-2,647)	2,272 (1,285-3,182)	1,860 (1,058-2,583)	1,373 (759-1,902)	653 (367-911)	198 (110-277)
Cellulitis	81 (39-148)	100 (48-177)	144 (58-221)	195 (72-294)	194 (89-319)	197 (75-313)	207 (88-353)	245 (100-414)	196 (81-370)	135 (57-247)	64 (28-120)	18 (7-36)
Pyoderma	422 (199-567)	583 (288-819)	574 (283-788)	809 (381-1,108)	973 (457-1,290)	1,048 (517-1,431)	1,088 (538-1,464)	1,132 (580-1,514)	709 (384-1,004)	502 (265-705)	228 (124-331)	65 (35-94)
Decubitus ulcer	89 (51-140)	130 (78-216)	132 (81-215)	201 (123-329)	276 (168-438)	431 (269-676)	602 (356-877)	815 (470-1,211)	875 (463-1,262)	675 (348-948)	334 (182-463)	106 (54-149)
Other skin and subcutaneous diseases	20 (11-35)	25 (15-42)	27 (15-49)	35 (20-59)	44 (25-77)	55 (33-96)	56 (31-96)	81 (46-129)	80 (42-124)	61 (31-94)	29 (14-46)	9 (4-15)
Sudden infant death syndrome	-	-	-	-	-	-	-	-	-	-	-	-
Injuries	164,904 (157,893-171,388)	140,692 (133,908-146,425)	138,573 (131,358-146,146)	123,753 (116,827-129,807)	110,514 (103,583-116,304)	91,614 (84,126-96,336)	76,639 (69,132-81,128)	66,832 (60,095-70,602)	45,326 (40,436-48,057)	26,184 (23,204-28,109)	9,736 (8,595-10,535)	2,562 (2,309-2,769)
Transport injuries	54,822 (52,658-57,682)	45,250 (43,390-47,475)	42,939 (41,130-44,856)	38,209 (36,680-39,809)	32,495 (31,108-34,017)	26,537 (25,350-27,833)	21,052 (20,058-22,179)	16,638 (15,959-17,406)	9,554 (9,120-10,074)	4,370 (4,159-4,650)	1,296 (1,226-1,376)	301 (283-324)
Road injuries	50,489 (48,216-53,596)	41,533 (39,719-43,799)	39,298 (37,527-41,279)	35,074 (33,613-36,794)	30,116 (28,772-31,731)	24,689 (23,547-25,976)	19,696 (18,724-20,738)	15,538 (14,903-16,316)	8,971 (8,566-9,486)	4,084 (3,872-4,335)	1,190 (1,118-1,270)	269 (251-291)
Pedestrian road injuries	17,256 (15,034-20,669)	16,142 (14,295-19,328)	16,339 (14,257-19,452)	15,063 (13,270-17,841)	14,468 (12,875-16,850)	12,190 (10,878-14,080)	10,135 (9,076-11,759)	8,677 (7,907-9,827)	4,962 (4,453-5,622)	2,344 (2,089-2,626)	647 (569-740)	152 (134-176)
Cyclist road injuries	2,414 (2,046-2,752)	2,339 (1,969-2,668)	2,629 (2,213-2,995)	2,342 (1,961-2,676)	2,241 (1,880-2,568)	1,897 (1,621-2,159)	1,526 (1,305-1,742)	1,063 (924-1,199)	579 (501-662)	205 (178-240)	46 (40-56)	1 (1-1)
Motorcyclist road injuries	9,833 (8,305-11,582)	7,037 (6,049-8,251)	5,674 (4,800-6,791)	4,719 (4,021-5,804)	2,736 (2,300-3,491)	2,098 (1,777-2,543)	1,459 (1,232-1,785)	827 (709-956)	425 (361-511)	142 (114-179)	35 (27-48)	8 (6-11)
Motor vehicle road injuries	20,527 (16,612-24,040)	15,515 (12,127-18,366)	14,097 (11,035-16,972)	12,488 (9,658-14,755)	10,319 (7,896-12,348)	8,140 (6,204-9,732)	6,276 (4,771-7,599)	4,719 (3,669-5,631)	2,848 (2,250-3,463)	1,317 (1,039-1,611)	436 (346-513)	101 (80-120)
Other road injuries	460 (334-590)	500 (361-649)	559 (403-776)	462 (330-618)	353 (251-494)	364 (257-512)	300 (208-420)	252 (172-390)	157 (106-258)	75 (50-126)	26 (17-48)	7 (5-14)
Other transport injuries	4,333 (3,502-4,908)	3,717 (2,967-4,250)	3,641 (2,886-4,195)	3,135 (2,500-3,582)	2,378 (1,880-2,749)	1,848 (1,463-2,122)	1,355 (1,073-1,571)	1,099 (876-1,279)	583 (491-698)	286 (238-355)	106 (87-136)	32 (27-40)
Unintentional injuries	50,326 (46,970-53,547)	47,729 (44,628-50,901)	50,124 (46,355-54,332)	47,159 (43,835-50,736)	45,812 (42,327-49,433)	40,323 (36,411-43,429)	35,896 (31,897-38,792)	34,592 (30,788-37,404)	26,579 (23,698-28,718)	17,602 (15,345-19,342)	7,286 (6,324-8,025)	2,018 (1,789-2,214)
Falls	11,885 (10,611-13,757)	11,938 (10,392-13,743)	13,149 (11,899-14,959)	14,186 (12,775-16,203)	15,052 (13,438-17,180)	15,592 (13,645-17,512)	15,662 (13,197-17,640)	17,500 (14,950-19,544)	14,519 (12,621-16,559)	10,525 (8,915-11,813)	4,553 (3,823-5,144)	1,223 (1,062-1,433)
Drowning	9,608 (8,941-10,550)	7,826 (7,287-8,666)	8,086 (7,557-8,894)	6,625 (6,185-7,324)	5,980 (5,594-6,631)	4,612 (4,286-5,209)	3,825 (3,542-4,301)	2,648 (2,458-3,008)	1,650 (1,505-1,889)	799 (737-926)	217 (201-250)	49 (46-56)
Fire, heat, and hot substances	3,418 (2,896-3,917)	3,193 (2,737-3,665)	3,543 (3,091-4,111)	3,255 (2,787-3,813)	3,461 (2,941-4,039)	2,966 (2,464-3,429)	2,905 (2,394-3,318)	2,799 (2,331-3,194)	2,372 (1,965-2,689)	1,544 (1,293-1,737)	636 (534-714)	187 (161-208)
Poisonings	2,859 (1,999-3,848)	2,462 (1,680-3,292)	2,488 (1,802-3,383)	2,233 (1,623-2,998)	2,137 (1,461-2,835)	1,595 (1,112-2,242)	1,393 (914-1,943)	937 (625-1,317)	571 (393-817)	280 (205-406)	95 (71-133)	29 (22-39)
Exposure to mechanical forces	6,999 (6,280-8,368)	6,737 (6,032-7,752)	6,395 (5,596-7,409)	5,163 (4,635-5,932)	3,871 (3,454-4,478)	2,955 (2,618-3,350)	2,225 (1,971-2,523)	1,932 (1,736-2,192)	1,230 (1,109-1,433)	791 (711-952)	359 (318-445)	123 (111-160)
Unintentional firearm injuries	1,259 (881-1,483)	1,231 (852-1,461)	1,131 (694-1,406)	757 (535-900)	603 (474-732)	490 (376-595)	389 (289-463)	364 (277-435)	229 (163-273)	139 (91-168)	56 (39-70)	17 (13-21)
Unintentional suffocation	241 (197-360)	176 (141-248)	171 (137-257)	155 (117-210)	74 (47-96)	79 (50-105)	74 (44-102)	255 (197-391)	249 (194-421)	202 (156-334)	109 (86-184)	43 (34-77)
Other exposure to mechanical forces	5,499 (4,876-7,092)	5,329 (4,820-6,475)	5,093 (4,539-6,134)	4,251 (3,811-5,050)	3,193 (2,824-3,801)	2,386 (2,089-2,758)	1,761 (1,564-2,044)	1,313 (1,151-1,546)	752 (660-895)	451 (396-542)	193 (167-234)	63 (56-78)
Adverse effects of medical treatment	1,929 (1,362-2,308)	1,921 (1,326-2,304)	2,023 (1,366-2,455)	2,405 (1,689-2,882)	3,144 (2,107-3,808)	3,569 (2,408-4,326)	3,085 (2,181-3,735)	2,953 (2,059-3,491)	2,157 (1,498-2,603)	1,306 (893-1,557)	516 (336-610)	139 (91-165)
Animal contact	2,853 (2,218-3,334)	3,581 (2,774-4,160)	2,844 (2,209-3,277)	3,252 (2,461-3,794)	2,884 (2,200-3,330)	2,671 (2,031-3,129)	2,144 (1,622-2,501)	1,502 (1,144-1,782)	844 (638-1,012)	364 (274-443)	100 (76-120)	30 (23-35)
Venomous animal contact	2,329 (1,769-2,789)	2,980 (2,254-3,615)	2,240 (1,715-2,653)	2,658 (1,996-3,166)	2,323 (1,730-2,746)	2,189 (1,620-2,631)	1,758 (1,307-2,126)	1,167 (866-1,439)	689 (510-850)	287 (209-358)	73 (53-91)	19 (14-23)
Non-venomous animal contact	524 (382-684)	602 (436-797)	603 (465-767)	594 (441-769)	561 (415-713)	483 (375-602)	385 (293-483)	335 (249-426)	155 (114-205)	78 (57-102)	27 (20-36)	11 (8-14)
Foreign body	2,712 (2,075-3,221)	2,309 (1,811-2,747)	3,079 (2,404-3,846)	2,809 (2,206-3,456)	2,937 (2,340-3,608)	2,219 (1,769-2,684)	1,815 (1,441-2,097)	1,905 (1,443-2,229)	1,626 (1,203-1,863)	1,017 (750-1,184)	423 (311-505)	130 (99-160)
Pulmonary aspiration and foreign body in airway	2,191 (1,684-2,701)	2,068 (1,620-2,517)	2,805 (2,169-3,584)	2,570 (2,049-3,230)	2,670 (2,111-3,394)	2,035 (1,619-2,547)	1,633 (1,328-1,988)	1,699 (1,308-2,058)	1,490 (1,111-1,748)	934 (690-1,117)	390 (292-477)	119 (90-152)
Foreign body in other body part	521 (250-704)	242 (107-328)	274 (130-375)	239 (107-319)	268 (136-356)	184 (76-244)	181 (67-249)	206 (83-278)	136 (55-183)	83 (35-112)	33 (13-44)	11 (5-15)
Environmental heat and cold exposure	3,337 (2,009-5,102)	3,227 (2,014-5,010)	4,808 (2,916-8,751)	4,087 (2,638-6,564)	3,913 (2,537-6,420)	2,522 (1,591-3,755)	1,875 (1,178-2,576)	1,683 (1,010-2,304)	1,161 (691-1,660)	739 (429-1,035)	289 (171-412)	78 (43-107)
Other unintentional injuries	4,726 (4,255-5,509)	4,535 (4,072-5,193)	3,710 (3,321-4,251)	3,144 (2,808-3,569)	2,433 (2,171-2,778)	1,621 (1,448-1,915)	969 (874-1,141)	733 (662-851)	449 (403-527)	237 (212-277)	98 (85-120)	31 (27-38)
Self-harm and interpersonal violence	54,966 (48,189-62,426)	44,211 (38,708-49,943)	42,720 (37,341-48,755)	36,053 (30,859-40,370)	30,267 (25,114-33,252)	23,230 (18,223-25,447)	18,593 (13,782-20,442)	14,854 (10,893-16,202)	8,736 (6,126-9,577)	4,010 (2,698-4,406)	1,089 (763-1,195)	220 (168-241)

SR Table 4: Global deaths by cause and age group for males (with 95% uncertainty intervals), 1990

Cause	Early Neonatal	Late Neonatal	Post Neonatal	1 to 4	5 to 9	10 to 14	15 to 19	20 to 24	25 to 29	30 to 34	35 to 39	40 to 44
Self-harm	-	-	-	-	-	6,320<br>(4,479-7,495)	30,057<br>(25,136-33,393)	52,897<br>(44,454-58,522)	52,385<br>(45,226-57,723)	48,334<br>(42,704-53,644)	44,313<br>(37,156-49,510)	35,737<br>(31,913-42,006)
Self-harm by firearm	-	-	-	-	-	423<br>(285-611)	4,145<br>(3,160-5,673)	7,053<br>(5,362-9,583)	6,726<br>(5,146-8,931)	6,007<br>(4,562-7,910)	4,963<br>(3,922-6,703)	4,096<br>(3,417-5,875)
Self-harm by other specified means	-	-	-	-	-	5,897<br>(4,072-7,112)	25,913<br>(21,169-29,233)	45,844<br>(36,870-51,166)	45,658<br>(38,682-50,474)	42,326<br>(36,754-47,184)	39,350<br>(32,518-43,867)	31,640<br>(27,756-37,013)
Interpersonal violence	1,129<br>(813-1,399)	302<br>(206-380)	3,309<br>(2,372-4,346)	5,470<br>(3,745-7,313)	3,998<br>(2,842-4,950)	5,635<br>(4,103-6,953)	26,559<br>(20,687-30,769)	43,071<br>(33,387-49,165)	41,092<br>(30,960-48,011)	35,026<br>(26,255-42,220)	27,502<br>(19,902-33,194)	19,230<br>(13,763-24,589)
Assault by firearm	123<br>(80-200)	37<br>(24-58)	445<br>(279-779)	675<br>(426-1,311)	636<br>(426-1,077)	1,600<br>(1,053-2,350)	12,106<br>(8,361-14,908)	19,860<br>(13,884-24,764)	18,762<br>(12,849-23,576)	14,851<br>(9,550-19,122)	11,531<br>(7,527-14,807)	7,627<br>(4,742-10,548)
Assault by sharp object	100<br>(61-165)	29<br>(16-43)	332<br>(171-520)	847<br>(461-1,415)	760<br>(412-1,036)	1,034<br>(601-1,363)	7,398<br>(5,071-9,326)	12,144<br>(8,495-15,588)	11,933<br>(8,090-15,519)	10,236<br>(6,667-13,859)	7,867<br>(5,026-10,844)	5,373<br>(3,534-7,623)
Assault by other means	907<br>(603-1,127)	237<br>(150-296)	2,532<br>(1,661-3,260)	3,948<br>(2,370-5,200)	2,603<br>(1,706-3,229)	3,001<br>(2,070-3,695)	7,055<br>(4,787-8,709)	11,067<br>(7,322-13,480)	10,398<br>(6,842-12,741)	9,939<br>(6,355-12,621)	8,104<br>(5,339-10,330)	6,229<br>(4,059-8,378)
Forces of nature, conflict and terrorism, and executions and police conflict	405<br>(352-461)	266<br>(216-319)	1,214<br>(837-1,602)	7,866<br>(5,941-9,790)	6,328<br>(4,320-8,352)	6,647<br>(4,330-8,978)	12,723<br>(10,149-15,306)	19,684<br>(16,560-22,748)	14,389<br>(11,703-16,721)	10,400<br>(8,484-12,259)	7,676<br>(6,171-9,282)	4,790<br>(3,827-5,794)
Forces of nature, conflict and terrorism, and state actor violence	31<br>(11-50)	68<br>(25-111)	567<br>(206-926)	2,740<br>(967-4,490)	2,986<br>(1,038-4,921)	3,525<br>(1,260-5,764)	3,510<br>(1,325-5,674)	3,069<br>(1,203-4,913)	2,706<br>(1,067-4,330)	2,269<br>(886-3,642)	1,971<br>(772-3,163)	1,426<br>(575-2,279)
Conflict and terrorism	375<br>(323-424)	198<br>(171-225)	601<br>(517-681)	5,066<br>(4,346-5,757)	3,261<br>(2,794-3,703)	3,050<br>(2,605-3,470)	8,533<br>(7,294-9,706)	13,903<br>(11,843-15,881)	9,341<br>(7,929-10,676)	6,433<br>(5,454-7,352)	4,296<br>(3,657-4,901)	2,804<br>(2,386-3,194)
Executions and police conflict	-	-	46<br>(32-75)	59<br>(43-80)	80<br>(59-101)	72<br>(53-90)	680<br>(234-930)	2,712<br>(765-3,706)	2,341<br>(611-3,214)	1,699<br>(516-2,228)	1,409<br>(408-1,914)	559<br>(219-758)

Cause	45 to 49	50 to 54	55 to 59	60 to 64	65 to 69	70 to 74	75 to 79	80 to 84	85 to 89	90 to 94	95 plus
Self-harm	30,666<br>(26,985-34,547)	31,416<br>(27,405-36,023)	27,846<br>(23,179-31,895)	24,114<br>(19,666-26,622)	19,195<br>(14,519-21,326)	15,933<br>(11,375-17,691)	13,048<br>(9,144-14,365)	8,218<br>(5,608-9,047)	3,791<br>(2,527-4,183)	1,025<br>(702-1,128)	183<br>(134-201)
Self-harm by firearm	3,387<br>(2,791-4,964)	3,222<br>(2,715-4,799)	2,855<br>(2,319-4,049)	2,606<br>(1,965-3,564)	2,349<br>(1,731-2,996)	1,985<br>(1,396-2,354)	1,799<br>(1,182-2,011)	1,242<br>(793-1,390)	575<br>(353-656)	177<br>(115-204)	34<br>(24-40)
Self-harm by other specified means	27,280<br>(23,130-30,590)	28,194<br>(23,796-32,276)	24,990<br>(20,212-28,133)	21,508<br>(16,744-23,838)	16,846<br>(12,607-18,828)	13,948<br>(9,583-15,558)	11,248<br>(7,725-12,474)	6,976<br>(4,669-7,728)	3,216<br>(2,085-3,561)	848<br>(558-938)	149<br>(105-165)
Interpersonal violence	13,545<br>(9,690-17,685)	11,304<br>(7,988-15,561)	8,208<br>(5,849-11,310)	6,153<br>(4,260-8,342)	4,035<br>(2,844-5,469)	2,661<br>(1,839-3,556)	1,806<br>(1,282-2,475)	518<br>(349-729)	219<br>(144-319)	64<br>(43-96)	37<br>(24-53)
Assault by firearm	4,980<br>(2,950-6,984)	3,699<br>(2,219-5,504)	2,574<br>(1,536-3,873)	1,767<br>(1,050-2,568)	1,124<br>(623-1,707)	719<br>(416-1,108)	455<br>(265-701)	100<br>(47-164)	40<br>(18-65)	12<br>(6-20)	8<br>(4-12)
Assault by sharp object	3,843<br>(2,446-5,481)	3,099<br>(1,934-4,658)	2,193<br>(1,307-3,398)	1,766<br>(1,050-2,654)	1,089<br>(665-1,626)	689<br>(406-1,008)	436<br>(255-695)	139<br>(84-239)	54<br>(32-90)	19<br>(12-33)	9<br>(5-14)
Assault by other means	4,722<br>(3,254-6,390)	4,506<br>(2,990-6,524)	3,440<br>(2,349-4,817)	2,620<br>(1,806-3,896)	1,822<br>(1,264-2,502)	1,253<br>(889-1,712)	915<br>(627-1,249)	279<br>(173-402)	125<br>(80-184)	33<br>(20-50)	20<br>(13-30)
Forces of nature, conflict and terrorism, and executions and police conflict	3,502<br>(2,718-4,276)	2,790<br>(2,061-3,513)	2,331<br>(1,674-3,005)	1,940<br>(1,319-2,560)	1,524<br>(1,022-2,035)	1,098<br>(748-1,450)	749<br>(524-977)	457<br>(319-592)	202<br>(140-262)	65<br>(48-82)	22<br>(17-27)
Forces of nature, conflict and terrorism, and state actor violence	1,170<br>(480-1,862)	1,157<br>(467-1,853)	1,047<br>(401-1,687)	978<br>(368-1,580)	785<br>(287-1,274)	557<br>(213-899)	369<br>(149-591)	221<br>(89-353)	97<br>(38-155)	25<br>(10-40)	6<br>(2-10)
Conflict and terrorism	1,886<br>(1,609-2,142)	1,336<br>(1,138-1,519)	1,132<br>(965-1,286)	855<br>(726-974)	667<br>(567-760)	497<br>(424-565)	361<br>(307-411)	217<br>(184-248)	94<br>(78-108)	34<br>(28-40)	13<br>(11-16)
Executions and police conflict	447<br>(152-595)	297<br>(98-380)	153<br>(78-191)	108<br>(44-136)	72<br>(27-92)	44<br>(18-56)	19<br>(10-24)	20<br>(8-29)	11<br>(4-19)	6<br>(2-10)	3<br>(1-4)

SR Table 4: Global deaths by cause and age group for males (with 95% uncertainty intervals), 1995

Cause	Early Neonatal	Late Neonatal	Post Neonatal	1 to 4	5 to 9	10 to 14	15 to 19	20 to 24	25 to 29	30 to 34	35 to 39	40 to 44	45 to 49	50 to 54	55 to 59	60 to 64	65 to 69	70 to 74	75 to 79	80 to 84	85 to 89	90 to 94	95 plus
All causes	1,595,607 (1,562,041-1,633,249)	541,480 (529,126-554,324)	1,612,118 (1,571,604-1,652,265)	1,496,328 (1,450,832-1,538,561)	414,967 (410,792-419,171)	273,454 (270,910-276,040)	393,800 (388,338-399,069)	579,506 (571,200-587,975)	662,154 (651,105-673,948)	739,366 (726,688-752,019)	816,381 (800,608-831,899)	964,420 (946,189-982,068)	1,051,770 (1,032,581-1,070,690)	1,183,088 (1,165,712-1,200,370)	1,614,482 (1,587,449-1,639,629)	2,033,471 (2,007,490-2,058,874)	2,395,659 (2,365,873-2,424,299)	2,457,138 (2,433,775-2,479,831)	2,178,289 (2,159,821-2,197,425)	1,811,740 (1,800,355-1,822,759)	1,110,125 (1,105,177-1,114,765)	441,667 (440,264-443,041)	112,038 (111,690-112,379)
Communicable, maternal, neonatal, and nutritional disorders	1,464,359 (1,417,524-1,515,115)	482,272 (462,775-501,496)	1,340,575 (1,286,969-1,397,114)	1,180,850 (1,123,926-1,244,529)	215,547 (200,070-231,584)	107,633 (98,835-117,029)	91,838 (84,618-101,326)	128,336 (120,495-138,772)	182,311 (171,555-195,722)	220,123 (208,640-233,490)	223,995 (212,283-239,372)	223,395 (212,779-237,641)	208,623 (197,636-222,519)	198,548 (186,306-217,204)	214,734 (199,860-239,001)	241,883 (220,373-275,926)	252,890 (227,729-294,139)	271,269 (240,434-320,194)	241,671 (211,914-295,127)	198,508 (174,779-237,787)	128,813 (112,138-153,020)	53,403 (46,464-62,525)	15,578 (13,695-17,794)
HIV/AIDS and tuberculosis	-	-	41,493 (38,214-44,911)	39,871 (33,995-45,940)	11,065 (9,832-12,281)	8,489 (7,387-9,556)	20,465 (18,578-22,609)	60,925 (56,637-66,196)	119,759 (110,154-130,679)	156,685 (147,553-167,946)	155,937 (146,253-167,585)	151,958 (144,137-162,667)	133,300 (125,824-143,415)	119,152 (111,908-130,272)	117,999 (110,574-130,716)	116,975 (108,935-129,349)	106,965 (98,698-119,219)	98,551 (90,470-111,592)	71,711 (65,095-82,347)	44,651 (39,932-53,501)	21,186 (19,007-25,726)	6,263 (5,597-7,776)	1,381 (1,228-1,703)
Tuberculosis	-	-	12,689 (10,241-15,262)	21,919 (16,057-27,478)	6,975 (5,811-8,105)	7,466 (6,375-8,503)	17,719 (15,876-19,822)	36,873 (33,599-41,153)	52,596 (48,389-58,884)	65,457 (60,840-73,039)	72,174 (67,117-82,310)	82,279 (76,556-92,102)	82,948 (76,954-92,510)	89,140 (82,859-100,097)	98,844 (91,813-110,930)	103,587 (95,534-116,099)	97,869 (89,601-109,934)	93,166 (85,335-106,172)	68,652 (61,963-79,128)	44,022 (39,255-52,899)	20,948 (18,752-25,478)	6,200 (5,534-7,710)	1,367 (1,215-1,689)
Drug-susceptible tuberculosis	-	-	12,230 (9,877-14,729)	21,216 (15,549-26,712)	6,659 (5,545-7,760)	7,145 (6,107-8,145)	16,955 (15,170-18,945)	35,192 (32,068-39,309)	50,193 (46,150-56,347)	62,415 (58,019-70,048)	68,720 (63,849-78,239)	78,129 (72,587-87,981)	78,855 (73,135-88,330)	84,929 (78,728-95,678)	93,886 (87,098-105,347)	98,228 (90,654-110,268)	92,739 (84,824-104,658)	88,413 (80,740-101,130)	65,063 (58,507-75,440)	41,772 (37,177-50,274)	19,936 (17,832-24,327)	5,915 (5,267-7,354)	1,308 (1,162-1,624)
Multidrug-resistant tuberculosis without extensive drug resistance	-	-	454 (340-583)	697 (486-929)	313 (242-399)	318 (244-430)	753 (592-974)	1,642 (1,295-2,136)	2,345 (1,866-2,983)	2,958 (2,388-3,738)	3,333 (2,699-4,193)	3,999 (3,232-4,950)	3,948 (3,206-4,968)	4,096 (3,317-5,165)	4,801 (3,885-5,925)	5,241 (4,247-6,548)	5,027 (4,037-6,273)	4,689 (3,764-6,022)	3,547 (2,844-4,530)	2,222 (1,764-2,866)	1,000 (788-1,323)	281 (220-376)	58 (44-81)
Extensively drug-resistant tuberculosis	-	-	4 (3-5)	6 (4-8)	3 (3-4)	3 (2-4)	11 (8-14)	39 (28-54)	58 (41-78)	84 (61-116)	121 (86-167)	151 (108-206)	145 (106-198)	115 (85-155)	156 (115-210)	118 (91-154)	103 (79-132)	64 (50-81)	42 (34-53)	27 (22-35)	12 (10-16)	3 (3-5)	1 (1-1)
HIV/AIDS	-	-	28,804 (26,739-31,242)	17,952 (16,358-19,815)	4,090 (3,672-4,648)	1,022 (872-1,222)	2,746 (2,444-3,149)	24,052 (21,005-27,572)	67,164 (59,044-76,002)	91,227 (83,088-100,489)	83,763 (76,143-92,471)	69,679 (64,010-75,931)	50,352 (45,438-56,125)	30,012 (27,365-33,211)	19,156 (17,184-21,504)	13,388 (11,684-15,189)	9,096 (7,922-10,504)	5,385 (4,646-6,228)	3,058 (2,624-3,501)	630 (520-841)	238 (198-320)	63 (53-84)	14 (12-17)
Drug-susceptible HIV/AIDS - Tuberculosis	-	-	9,829 (7,562-11,585)	6,406 (5,074-7,537)	1,357 (1,072-1,595)	297 (225-374)	860 (682-1,057)	7,052 (5,211-8,975)	15,618 (10,937-20,981)	18,761 (13,073-25,676)	17,725 (12,186-24,011)	15,833 (11,099-21,017)	12,644 (8,762-16,623)	8,637 (6,205-10,780)	5,793 (4,348-7,105)	4,232 (3,201-5,226)	2,947 (2,270-3,613)	1,846 (1,465-2,259)	1,068 (850-1,290)	209 (163-268)	76 (59-99)	20 (16-26)	4 (3-5)
Multidrug-resistant HIV/AIDS - Tuberculosis without extensive drug resistance	-	-	304 (192-459)	186 (119-279)	38 (23-59)	8 (5-13)	37 (24-54)	262 (171-382)	554 (355-832)	684 (438-1,040)	654 (424-987)	577 (375-869)	449 (284-685)	306 (193-462)	213 (137-320)	150 (95-219)	101 (65-148)	60 (38-90)	36 (22-53)	9 (6-14)	3 (2-5)	1 (1-1)	0 (0-0)
Extensively drug-resistant HIV/AIDS - Tuberculosis	-	-	2 (1-2)	1 (1-1)	1 (0-1)	0 (0-0)	0 (0-1)	2 (1-3)	6 (4-10)	14 (8-24)	16 (9-27)	11 (6-18)	6 (3-10)	3 (2-5)	2 (1-4)	1 (1-2)	1 (0-1)	0 (0-0)	0 (0-0)	0 (0-0)	0 (0-0)	0 (0-0)	0 (0-0)
HIV/AIDS resulting in other diseases	-	-	18,669 (16,651-21,570)	11,359 (9,965-13,415)	2,695 (2,360-3,213)	716 (608-885)	1,849 (1,584-2,213)	16,736 (13,905-20,509)	50,984 (42,601-61,016)	71,767 (62,212-81,855)	65,367 (56,179-75,627)	53,258 (46,064-61,263)	37,254 (32,009-43,829)	21,065 (18,250-24,912)	13,148 (11,371-15,549)	9,005 (7,667-10,756)	6,048 (5,108-7,249)	3,478 (2,912-4,239)	1,954 (1,636-2,337)	411 (327-595)	158 (127-215)	42 (34-57)	10 (8-13)
Diarrhoea, lower respiratory infections, and other common infectious diseases	197,804 (162,551-230,649)	211,052 (173,146-236,285)	989,013 (930,065-1,057,350)	783,649 (688,940-909,213)	150,858 (128,933-175,393)	71,669 (60,961-83,562)	51,973 (43,011-63,928)	46,122 (37,404-59,430)	44,070 (35,732-57,814)	44,223 (35,722-57,914)	48,570 (39,608-65,609)	51,369 (42,034-68,452)	55,507 (44,788-74,109)	60,500 (47,902-84,044)	76,342 (59,594-105,049)	103,103 (79,901-144,169)	123,672 (94,953-171,601)	151,513 (116,020-210,566)	151,095 (118,087-213,348)	140,391 (114,114-184,543)	98,147 (80,647-124,275)	42,730 (35,630-52,534)	12,617 (10,761-15,014)
Diarrhoeal diseases	25,791 (21,954-30,982)	51,188 (43,295-59,628)	335,505 (292,352-376,927)	218,730 (172,432-262,895)	28,826 (14,291-53,271)	14,183 (7,143-27,105)	14,798 (7,708-28,059)	16,004 (8,485-30,437)	16,451 (8,594-31,903)	16,176 (7,882-31,121)	18,346 (9,616-36,876)	18,893 (9,708-37,226)	20,808 (10,664-40,451)	24,276 (12,235-50,834)	31,398 (15,499-61,778)	45,854 (23,669-89,983)	57,072 (29,733-108,303)	72,322 (37,018-137,110)	69,025 (36,465-134,827)	59,956 (33,558-107,492)	39,648 (22,221-68,109)	16,490 (9,598-26,897)	4,607 (2,844-7,068)
Intestinal infectious diseases	-	-	909 (170-3,513)	18,321 (8,424-33,643)	34,195 (19,011-55,733)	21,218 (11,544-33,600)	11,929 (6,101-20,635)	6,061 (3,008-10,526)	4,523 (2,352-7,806)	3,533 (1,869-6,025)	2,700 (1,481-4,530)	2,130 (1,158-3,557)	1,634 (867-2,712)	1,348 (702-2,299)	1,057 (507-1,936)	931 (463-1,694)	795 (445-1,304)	608 (354-968)	389 (226-621)	209 (121-328)	100 (58-164)	31 (18-49)	7 (4-13)
Typhoid fever	-	-	101 (47-196)	16,152 (6,925-31,232)	30,167 (16,382-49,939)	18,093 (9,359-29,081)	10,456 (5,277-18,299)	5,175 (2,523-9,291)	3,656 (1,863-6,417)	2,731 (1,408-4,736)	2,052 (1,082-3,599)	1,590 (823-2,751)	1,202 (626-2,014)	1,006 (511-1,760)	810 (377-1,465)	739 (352-1,351)	659 (360-1,105)	510 (292-822)	319 (181-516)	171 (96-275)	79 (45-126)	24 (13-39)	5 (3-8)
Paratyphoid fever	-	-	15 (6-32)	1,569 (604-3,531)	3,967 (1,810-7,667)	3,102 (1,427-5,809)	1,453 (604-2,897)	863 (367-1,707)	845 (364-1,663)	769 (338-1,523)	616 (290-1,176)	512 (236-1,009)	403 (182-792)	329 (143-651)	235 (97-481)	178 (75-360)	121 (57-236)	81 (40-153)	49 (24-91)	27 (13-49)	13 (6-24)	4 (2-7)	1 (0-2)
Other intestinal infectious diseases	-	-	793 (68-3,420)	600 (37-2,777)	61 (5-288)	23 (2-102)	19 (2-78)	23 (2-106)	22 (2-114)	33 (3-152)	32 (3-126)	28 (2-131)	28 (3-126)	13 (1-61)	11 (1-53)	15 (1-65)	15 (2-69)	17 (2-78)	21 (3-88)	11 (1-51)	9 (1-38)	3 (0-13)	1 (0-5)
Lower respiratory infections	122,139 (108,408-136,939)	111,604 (97,190-126,178)	425,907 (381,291-468,231)	212,789 (173,041-251,382)	27,099 (20,678-32,214)	12,555 (10,011-14,587)	10,038 (7,912-11,946)	11,574 (9,142-13,863)	12,550 (10,122-15,289)	14,217 (11,750-16,653)	17,172 (14,497-20,034)	20,279 (17,314-23,309)	23,240 (19,810-27,209)	25,726 (21,371-30,362)	34,198 (28,819-39,703)	47,232 (38,516-54,188)	56,956 (46,457-66,244)	70,841 (58,852-80,904)	75,684 (63,154-86,234)	75,612 (65,135-84,881)	55,892 (49,420-62,611)	25,319 (22,487-28,781)	7,758 (6,861-8,687)
Upper respiratory infections	69 (42-107)	29 (17-50)	554 (356-866)	440 (257-744)	38 (23-58)	24 (16-36)	52 (35-73)	58 (40-83)	64 (44-89)	63 (44-88)	67 (47-92)	70 (48-98)	73 (51-101)	69 (49-97)	79 (56-112)	90 (64-128)	78 (56-106)	60 (43-81)	53 (38-72)	32 (23-44)	19 (13-25)	6 (5-8)	1 (1-2)
Otitis media	5 (2-7)	13 (7-24)	173 (86-305)	300 (118-579)	159 (71-275)	169 (81-280)	67 (35-106)	51 (28-80)	37 (20-59)	32 (16-48)	43 (22-67)	51 (24-84)	143 (58-276)	152 (62-276)	59 (24-91)	57 (24-100)	75 (27-141)	64 (28-100)	44 (22-66)	40 (18-64)	22 (10-33)	10 (4-16)	3 (1-4)
Meningitis	7,291 (4,644-10,200)	10,720 (8,112-13,554)	59,249 (46,900-73,551)	52,127 (37,119-66,779)	11,841 (6,043-17,057)	7,415 (4,173-10,572)	6,496 (3,943-9,479)	6,013 (3,498-8,707)	5,197 (3,117-7,679)	5,046 (2,963-7,712)	4,948 (3,115-7,284)	4,381 (2,805-6,444)	4,493 (2,841-6,581)	4,139 (2,581-6,143)	4,268 (2,674-6,358)	4,234 (2,660-6,379)	4,221 (2,685-6,047)	3,338 (2,029-4,834)	2,523 (1,550-3,721)	1,908 (1,178-2,731)	975 (604-1,369)	313 (194-441)	73 (46-100)
Pneumococcal meningitis	383 (238-578)	556 (375-779)	3,265 (2,376-4,336)	3,024 (1,924-4,438)	675 (331-1,043)	414 (219-649)	364 (209-578)	333 (185-549)	336 (190-538)	400 (233-610)	477 (274-753)	487 (282-789)	552 (329-900)	504 (310-804)	517 (324-790)	543 (347-822)	535 (336-785)	440 (271-664)	328 (197-480)	245 (148-360)	119 (76-165)	38 (25-51)	9 (6-13)
H influenzae type B meningitis	1,038 (642-1,503)	1,478 (1,045-1,956)	7,637 (5,593-9,951)	6,228 (4,169-8,700)	1,106 (530-1,645)	576 (305-857)	442 (244-684)	403 (228-634)	378 (213-611)	385 (215-603)	386 (228-576)	339 (195-516)	356 (211-558)	330 (198-504)	337 (201-509)	342 (199-542)	326 (190-515)	252 (147-383)	189 (115-286)	147 (83-215)	74 (46-105)	23 (14-34)	5 (3-8)
Meningococcal infection	3,708 (2,340-5,286)	5,437 (4,038-6,993)	29,514 (22,807-37,121)	28,070 (19,637-36,946)	6,551 (3,408-9,389)	4,060 (2,317-5,873)	3,608 (2,158-5,377)	3,264 (1,890-4,737)	2,689 (1,581-3,928)	2,422 (1,429-3,690)	2,308 (1,398-3,455)	2,039 (1,252-3,126)	2,063 (1,224-3,072)	1,931 (1,166-2,909)	1,975 (1,204-2,915)	1,996 (1,196-3,060)	1,966 (1,196-2,868)	1,514 (880-2,240)	1,157 (676-1,724)	854 (489-1,232)	451 (266-648)	146 (85-211)	33 (19-46)
Other meningitis	2,162 (1,325-3,150)	3,249 (2,373-4,184)	18,832 (14,331-23,594)	14,805 (10,304-19,570)	3,509 (1,672-5,233)	2,364 (1,282-3,436)	2,081 (1,196-3,080)	2,013 (1,103-3,019)	1,793 (1,015-2,582)	1,839 (1,018-2,737)	1,778 (1,036-2,594)	1,517 (889-2,173)	1,522 (952-2,246)	1,374 (840-2,134)	1,439 (876-2,121)	1,353 (834-2,036)	1,394 (842-2,054)	1,132 (684-1,650)	850 (524-1,236)	662 (418-1,002)	332 (213-486)	106 (67-151)	25 (16-35)
Encephalitis	568 (283-808)	964 (486-1,463)	9,322 (5,021-13,748)	10,788 (5,462-17,902)	4,345 (2,692-5,984)	3,143 (2,088-4,099)	2,210 (1,549-2,792)	2,077 (1,519-2,686)	1,901 (1,326-2,338)	2,027 (1,442-2,482)	2,000 (1,482-2,510)	1,790 (1,339-2,283)	1,620 (1,217-2,004)	1,482 (1,122-1,938)	1,857 (1,405-2,263)	2,066 (1,564-2,605)	2,361 (1,734-2,852)	2,245 (1,541-2,753)	2,036 (1,343-2,528)	1,398 (920-1,765)	710 (466-888)	218 (148-269)	49 (34-61)
Diphtheria	-	-	989 (557-1,597)	2,107 (1,067-4,041)	340 (195-575)	151 (92-245)	72 (44-111)	48 (29-76)	42 (27-63)	39 (24-58)	55 (35-81)	56 (35-83)	58 (38-89)	63 (41-97)	48 (31-71)	-	-	-	-	-	-	-	-
Whooping cough	-	-	33,639 (15,659-66,267)	30,218 (14,069-59,292)	3,265 (1,460-6,646)	689 (306-1,426)	312 (134-644)	217 (92-451)	129 (53-273)	102 (41-217)	68 (27-149)	90 (35-195)	65 (26-142)	49 (19-109)	71 (29-152)	-	-	-	-	-	-	-	-
Tetanus	41,875 (16,331-67,851)	36,305 (14,025-57,280)	11,729 (4,488-23,361)	4,732 (1,649-8,389)	2,828 (1,066-4,535)	1,663 (744-2,565)	2,181 (1,005-3,513)	2,131 (989-3,230)	2,226 (1,131-3,254)	2,324 (1,155-3,384)	2,722 (1,370-3,872)	3,312 (1,600-4,660)	3,107 (1,529-4,521)	2,983 (1,463-4,187)	3,063 (1,411-4,395)	2,500 (1,197-3,796)	1,867 (942-2,768)	1,809 (765-2,548)	995 (410-1,405)	777 (337-1,166)	319 (135-486)	94 (45-145)	25 (12-39)
Measles	-	-	109,384 (41,258-225,763)	231,082 (96,597-434,124)	37,009 (14,663-72,924)	10,164 (3,596-22,058)	3,687 (1,262-8,551)	1,720 (580-4,044)	745 (250-1,767)	443 (148-1,050)	271 (91-646)	208 (71-494)	152 (52-356)	116 (39-278)	128 (44-301)	-	-	-	-	-	-	-	-
Varicella and herpes zoster	67 (53-84)	228 (178-287)	1,654 (1,310-2,098)	2,015 (1,522-2,664)	913 (715-1,170)	296 (231-381)	132 (104-165)	170 (137-211)	205 (164-258)	221 (181-272)	177 (142-221)	108 (87-134)	116 (93-145)	95 (76-119)	116 (93-143)	138 (111-171)	246 (200-301)	226 (183-278)	345 (280-431)	458 (362-570)	462 (370-587)	249 (199-310)	93 (72-118)
Neglected tropical diseases and malaria	554 (243-852)	13,584 (9,253-19,248)	110,410 (78,618-149,022)	226,604 (162,194-297,375)	36,488 (25,313-48,518)	20,348 (14,411-26,490)	11,575 (8,870-14,645)	12,306 (9,353-15,449)	9,170 (7,000-11,988)	8,989 (6,727-11,580)	9,177 (6,683-11,852)	8,731 (6,710-11,169)	8,334 (6,297-10,678)	7,425 (5,898-9,628)	7,689 (6,034-9,932)	7,988 (6,352-10,145)	7,602 (6,042-9,607)	6,374 (5,082-8,074)	4,823 (3,794-6,197)	2,224 (1,775-2,782)	1,428 (1,100-1,838)	266 (210-331)	94 (70-125)
Malaria	-	13,047 (8,731-18,694)	104,781 (73,207-142,762)	210,863 (146,625-282,937)	25,893 (15,939-37,230)	13,727 (8,449-19,474)	5,644 (3,731-7,645)	5,770 (3,977-7,756)	4,671 (3,235-6,094)	4,461 (2,973-6,054)	4,769 (2,985-6,864)	4,217 (2,844-5,729)	3,661 (2,444-5,001)	3,355 (2,258-4,545)	3,357 (2,314-4,623)	4,128 (2,831-5,689)	4,136 (2,760-5,784)	3,363 (2,198-4,859)	2,544 (1,633-3,690)	1,127 (759-1,604)	905 (599-1,305)	139 (92-193)	60 (40-88)
Chagas disease	-	-	3 (2-4)	2 (1-2)	2 (2-2)	6 (5-6)	15 (12-18)	35 (28-41)	65 (52-77)	133 (109-158)	215 (182-248)	310 (270-354)	408 (354-470)	496 (434-566)	565 (483-652)	698 (631-777)	710 (643-783)	660 (601-721)	363 (321-414)	247 (223-276)	127 (114-141)	34 (30-39)	8 (7-9)
Leishmaniasis	-	-	1,060 (584-1,739)	1,555 (787-2,710)	769 (412-1,321)	522 (289-881)	714 (396-1,173)	883 (496-1,469)	766 (431-1,248)	692 (382-1,127)	772 (431-1,270)	770 (419-1,296)	744 (405-1,243)	744 (418-1,218)	598 (331-1,010)	479 (264-828)	499 (275-822)	552 (307-909)	434 (249-742)	245 (130-407)	149 (78-247)	34 (16-59)	10 (3-21)
Visceral leishmaniasis	-	-	1,060 (584-1,739)	1,555 (787-2,710)	769 (412-1,321)	522 (289-881)	714 (396-1,173)	883 (496-1,469)	766 (431-1,248)	692 (382-1,127)	772 (431-1,270)	770 (419-1,296)	744 (405-1,243)	744 (418-1,218)	598 (331-1,010)	479 (264-828)	499 (275-822)	552 (307-909)	434 (249-742)	245 (130-407)	149 (78-247)	34 (16-59)	10 (3-21)
African trypanosomiasis	-	-	-	397 (69-1,449)	966 (239-2,818)	1,073 (255-2,842)	1,336 (342-3,350)	1,475 (365-4,132)	1,310 (324-3,474)	1,186 (267-3,460)	991 (223-2,953)	873 (190-2,660)	782 (155-2,689)	712 (135-2,505)	551 (105-2,129)	455 (81-1,617)	335 (64-1,199)	231 (38-895)	143 (22-546)	59 (11-191)	18 (3-70)	4 (1-15)	1 (0-3)
Schistosomiasis	-	-	0 (0-0)	394 (222-657)	190 (120-309)	244 (162-363)	224 (155-320)	316 (221-429)	363 (258-495)	392 (285-536)	456 (330-630)	508 (374-687)	532 (388-715)	584 (426-791)	661 (485-892)	711 (520-954)	689 (505-915)	590 (426-802)	414 (304-562)	187 (136-252)	72 (52-100)	16 (10-25)	2 (1-4)
Cysticercosis	0 (0-0)	0 (0-0)	0 (0-0)	69 (43-100)	44 (33-60)	35 (28-43)	33 (27-42)	49 (39-60)	63 (52-76)	71 (59-85)	57 (47-68)	54 (46-65)	48 (40-57)	54 (45-63)	50 (43-58)	40 (33-47)	33 (28-38)	20 (17-24)	12 (10-16)	6 (5-7)	2 (2-3)	1 (0-1)	0 (0-0)
Cystic echinococcosis	-	-	-	248 (162-374)	96 (65-138)	56 (38-80)	26 (18-35)	35 (25-49)	49 (36-66)	51 (38-67)	64 (49-82)	54 (40-69)	59 (44-76)	53 (41-67)	69 (54-85)	69 (54-86)	58 (46-72)	44 (35-56)	36 (28-46)	22 (16-28)	10 (8-14)	2 (2-3)	0 (0-1)
Dengue	-	-	610 (177-1,291)	1,156 (337-2,570)	694 (206-1,507)	377 (99-754)	364 (68-799)	337 (64-703)	278 (44-632)	207 (37-480)	222 (37-488)	188 (31-422)	174 (29-373)	163 (29-368)	197 (30-464)	190 (34-414)	187 (35-433)	73 (16-143)	55 (15-105)	41 (10-84)	26 (8-52)	7 (2-15)	2 (1-3)
Yellow fever	-	4 (1-11)	59 (12-181)	542 (108-1,687)	1,035 (213-3,303)	962 (206-2,816)	911 (177-2,774)	780 (159-2,311)	575 (118-1,676)	447 (90-1,376)	352 (65-1,111)	276 (55-841)	213 (42-679)	172 (32-541)	134 (24-398)	103 (19-317)	71 (14-214)	47 (9-147)	26 (5-82)	11 (2-34)	4 (1-11)	1 (0-3)	0 (0-0)
Rabies	-	-	565 (230-860)	2,570 (1,006-4,008)	5,112 (2,078-8,182)	2,849 (1,023-4,198)	2,005 (1,006-2,910)	2,293 (1,177-3,440)	683 (324-927)	1,015 (434-1,398)	925 (394-1,272)	1,135 (491-1,509)	1,376 (533-2,024)	760 (320-979)	1,135 (372-1,698)	771 (308-1,091)	553 (234-768)	485 (218-699)	568 (310-914)	158 (90-292)	57 (31-139)	6 (4-17)	2 (1-6)
Intestinal nematode infections	-	-	354 (268-462)	3,798 (2,744-5,061)	626 (485-789)	122 (95-154)	40 (31-51)	30 (23-39)	24 (18-31)	30 (23-38)	27 (20-34)	19 (14-24)	19 (14-25)	27 (20-34)	24 (18-30)	28 (21-36)	20 (14-25)	27 (21-35)	20 (15-25)	11 (8-14)	4 (3-5)	2 (1-3)	1 (0-1)
Ascariasis	-	-	354 (268-462)	3,798 (2,744-5,061)	626 (485-789)	122 (95-154)	40 (31-51)	30 (23-39)	24 (18-31)	30 (23-38)	27 (20-34)	19 (14-24)	19 (14-25)	27 (20-34)	24 (18-30)	28 (21-36)	20 (14-25)	27 (21-35)	20 (15-25)	11 (8-14)	4 (3-5)	2 (1-3)	1 (0-1)
Ebola	0 (0-0)	0 (0-0)	6 (5-8)	4 (3-5)	5 (4-7)	3 (2-4)	9 (6-11)	9 (7-12)	30 (22-38)	31 (23-40)	43 (32-55)	33 (24-42)	24 (18-31)	17 (13-22)	16 (12-20)	13 (10-16)	7 (5-8)	0 (0-1)	0 (0-0)	0 (0-0)	0 (0-0)	0 (0-0)	0 (0-0)
Zika virus	0 (0-0)	0 (0-0)	0 (0-0)	0 (0-0)	0 (0-0)	0 (0-0)	0 (0-0)	0 (0-0)	0 (0-0)	0 (0-0)	0 (0-0)	0 (0-0)	0 (0-0)	0 (0-0)	0 (0-0)	0 (0-0)	0 (0-0)	0 (0-0)	0 (0-0)	0 (0-0)	0 (0-0)	0 (0-0)	0 (0-0)
Other neglected tropical diseases	554 (243-852)	533 (270-861)	2,973 (1,320-4,693)	5,005 (1,496-13,319)	1,055 (501-1,922)	373 (235-602)	255 (151-396)	294 (169-430)	293 (154-404)	273 (126-361)	286 (146-354)	293 (160-371)	294 (140-380)	288 (156-356)	333 (197-416)	303 (188-386)	305 (180-393)	282 (197-366)	207 (128-271)	111 (76-156)	55 (38-79)	19 (14-26)	7 (5-11)
Maternal disorders	-	-	-	-	-	-	-	-	-	-	-	-	-	-	-	-	-	-	-	-	-	-	-
Maternal haemorrhage	-	-	-	-	-	-	-	-	-	-	-	-	-	-	-	-	-	-	-	-	-	-	-
Maternal sepsis and other pregnancy related infections	-	-	-	-	-	-	-	-	-	-	-	-	-	-	-	-	-	-	-	-	-	-	-
Maternal hypertensive disorders	-	-	-	-	-	-	-	-	-	-	-	-	-	-	-	-	-	-	-	-	-	-	-
Maternal obstructed labour and uterine rupture	-	-	-	-	-	-	-	-	-	-	-	-	-	-	-	-	-	-	-	-	-	-	-
Maternal abortion, miscarriage, and ectopic pregnancy	-	-	-	-	-	-	-	-	-	-	-	-	-	-	-	-	-	-	-	-	-	-	-
Indirect maternal deaths	-	-	-	-	-	-	-	-	-	-	-	-	-	-	-	-	-	-	-	-	-	-	-
Late maternal deaths	-	-	-	-	-	-	-	-	-	-	-	-	-	-	-	-	-	-	-	-	-	-	-
Maternal deaths aggravated by HIV/AIDS	-	-	-	-	-	-	-	-	-	-	-	-	-	-	-	-	-	-	-	-	-	-	-
Other maternal disorders	-	-	-	-	-	-	-	-	-	-	-	-	-	-	-	-	-	-	-	-	-	-	-
Neonatal disorders	1,232,141 (1,178,700-1,303,823)	242,185 (217,461-290,651)	64,508 (53,910-83,012)	6,695 (5,088-9,769)	-	-	-	-	-	-	-	-	-	-	-	-	-	-	-	-	-	-	-
Neonatal preterm birth complications	518,439 (477,474-577,246)	78,314 (66,205-105,587)	26,354 (21,087-36,101)	1,892 (1,360-3,044)	-	-	-	-	-	-	-	-	-	-	-	-	-	-	-	-	-	-	-
Neonatal encephalopathy due to birth asphyxia and trauma	376,112 (333,313-424,546)	36,497 (30,872-44,834)	10,663 (8,553-13,507)	2,483 (1,867-3,492)	-	-	-	-	-	-	-	-	-	-	-	-	-	-	-	-	-	-	-
Neonatal sepsis and other neonatal infections	88,919 (55,418-113,415)	59,270 (41,308-81,311)	9,589 (5,704-15,174)	601 (175-1,247)	-	-	-	-	-	-	-	-	-	-	-	-	-	-	-	-	-	-	-
Hemolytic disease and other neonatal jaundice	37,863 (29,976-49,088)	24,262 (19,168-30,778)	4,301 (3,130-5,879)	290 (185-447)	-	-	-	-	-	-	-	-	-	-	-	-	-	-	-	-	-	-	-
Other neonatal disorders	210,809 (183,937-240,987)	43,843 (34,928-66,359)	13,602 (10,628-20,195)	1,429 (961-2,755)	-	-	-	-	-	-	-	-	-	-	-	-	-	-	-	-	-	-	-
Nutritional deficiencies	-	-	84,416 (64,264-104,261)	93,360 (59,674-127,054)	8,213 (6,468-10,208)	2,997 (2,418-3,662)	2,318 (1,879-3,057)	2,678 (2,091-3,440)	2,599 (1,957-3,439)	2,682 (2,118-3,464)	2,988 (2,219-3,985)	2,930 (2,342-3,902)	3,471 (2,564-4,762)	4,364 (3,111-6,050)	5,307 (3,808-7,211)	5,995 (4,577-8,047)	7,087 (5,687-9,584)	8,512 (6,944-11,114)	9,054 (7,518-11,765)	8,208 (7,014-10,292)	6,550 (5,670-7,888)	3,645 (3,206-4,251)	1,353 (1,189-1,576)
Protein-energy malnutrition	-	-	82,261 (62,452-101,816)	91,044 (58,749-123,809)	7,367 (5,716-9,310)	2,583 (2,045-3,210)	1,728 (1,350-2,245)	2,018 (1,496-2,581)	2,014 (1,462-2,651)	2,075 (1,577-2,651)	2,355 (1,631-3,172)	2,266 (1,745-2,907)	2,620 (1,824-3,524)	3,466 (2,337-4,766)	4,200 (2,851-5,729)	4,753 (3,506-6,282)	5,474 (4,266-7,189)	6,866 (5,458-8,763)	7,468 (6,157-9,297)	6,913 (5,888-8,300)	5,510 (4,780-6,372)	3,118 (2,731-3,512)	1,174 (1,013-1,351)
Iodine deficiency	-	-	167 (38-494)	146 (23-561)	53 (15-147)	13 (3-37)	32 (8-92)	36 (8-121)	40 (10-128)	40 (10-128)	35 (10-113)	30 (9-92)	26 (7-87)	31 (8-105)	42 (12-135)	38 (12-98)	49 (17-114)	53 (19-120)	44 (15-110)	27 (9-62)	12 (3-30)	3 (1-10)	1 (0-4)
Iron-deficiency anaemia	-	-	42 (13-71)	64 (29-104)	77 (40-115)	65 (36-95)	85 (54-121)	75 (56-100)	59 (48-76)	68 (52-88)	52 (42-69)	52 (40-83)	51 (41-78)	49 (38-75)	66 (50-122)	72 (54-110)	108 (75-166)	133 (75-200)	103 (67-163)	82 (57-136)	46 (27-80)	27 (14-49)	13 (7-22)
Other nutritional deficiencies	-	-	1,946 (1,221-2,653)	2,106 (820-3,254)	717 (497-1,007)	335 (257-450)	474 (353-706)	549 (394-907)	486 (344-807)	499 (350-844)	545 (402-896)	582 (404-1,024)	773 (540-1,393)	818 (549-1,512)	999 (711-1,685)	1,131 (845-1,827)	1,457 (1,018-2,400)	1,460 (1,032-2,479)	1,439 (982-2,534)	1,185 (815-2,018)	982 (635-1,655)	496 (318-803)	164 (100-282)
Other communicable, maternal, neonatal, and nutritional diseases	33,860 (20,281-51,910)	15,452 (9,389-22,582)	50,735 (33,406-72,562)	30,672 (20,151-46,115)	8,922 (6,644-11,903)	4,131 (3,531-4,813)	5,507 (4,874-6,160)	6,304 (5,746-6,955)	6,712 (6,093-7,393)	7,544 (6,895-8,184)	7,323 (6,781-7,981)	8,407 (7,817-9,074)	8,010 (7,369-8,709)	7,107 (6,594-7,705)	7,397 (6,895-7,961)	7,823 (7,222-8,412)	7,564 (6,977-8,084)	6,319 (5,756-6,810)	4,988 (4,499-5,433)	3,034 (2,683-3,347)	1,502 (1,309-1,667)	499 (425-570)	133 (110-154)
Sexually transmitted diseases excluding HIV	28,656 (16,069-45,675)	11,762 (6,676-18,772)	29,426 (15,844-47,914)	11,954 (6,251-20,014)	3,846 (2,047-6,468)	109 (63-184)	185 (84-291)	168 (82-276)	261 (115-452)	427 (195-653)	269 (150-438)	255 (133-348)	242 (123-339)	268 (171-433)	436 (262-679)	247 (141-345)	257 (156-344)	287 (158-410)	288 (119-435)	155 (74-228)	85 (41-123)	33 (17-46)	15 (8-21)
Syphilis	28,656 (16,069-45,675)	11,762 (6,676-18,772)	29,426 (15,844-47,914)	11,954 (6,251-20,014)	3,846 (2,047-6,468)	93 (54-156)	156 (71-246)	145 (71-238)	218 (96-377)	362 (166-554)	239 (133-389)	231 (121-316)	219 (111-306)	250 (159-403)	403 (243-629)	230 (131-321)	241 (147-323)	265 (146-378)	264 (109-399)	141 (67-208)	75 (36-109)	29 (14-40)	11 (6-17)
Chlamydial infection	-	-	-	-	-	0 (0-0)	0 (0-0)	0 (0-0)	0 (0-0)	0 (0-0)	0 (0-0)	0 (0-0)	0 (0-0)	0 (0-0)	0 (0-0)	0 (0-0)	0 (0-0)	0 (0-0)	0 (0-0)	0 (0-0)	0 (0-0)	0 (0-0)	0 (0-0)
Gonococcal infection	-	-	-	-	-	11 (6-18)	21 (10-33)	15 (7-25)	36 (16-62)	48 (22-73)	16 (9-26)	14 (7-19)	10 (5-14)	9 (5-14)	15 (9-24)	10 (6-14)	8 (5-11)	16 (9-22)	15 (6-23)	8 (4-12)	5 (3-8)	3 (1-3)	1 (1-2)
Other sexually transmitted diseases	-	-	-	-	-	6 (3-9)	7 (3-12)	8 (4-13)	7 (3-12)	17 (8-26)	15 (8-24)	9 (5-13)	13 (7-18)	10 (6-16)	17 (10-26)	7 (4-10)	7 (4-10)	7 (4-10)	9 (4-13)	5 (2-8)	4 (2-6)	2 (1-3)	2 (1-3)
Hepatitis	-	-	8,050 (6,854-9,347)	9,090 (6,926-11,238)	3,396 (2,729-4,161)	2,849 (2,422-3,372)	3,934 (3,444-4,456)	4,890 (4,427-5,425)	5,320 (4,776-5,950)	5,834 (5,325-6,404)	5,704 (5,261-6,251)	7,061 (6,494-7,695)	6,577 (6,009-7,221)	5,656 (5,233-6,143)	5,608 (5,168-6,081)	5,735 (5,314-6,180)	5,391 (4,957-5,793)	4,188 (3,855-4,525)	3,191 (2,884-3,484)	1,789 (1,592-1,960)	776 (691-853)	195 (176-215)	38 (34-41)
Acute hepatitis A	-	-	4,954 (3,511-6,471)	960 (550-1,500)	79 (51-114)	38 (24-59)	52 (30-85)	64 (34-104)	60 (28-105)	54 (24-99)	53 (24-92)	71 (33-129)	72 (34-123)	66 (32-115)	70 (35-117)	76 (36-132)	77 (38-129)	64 (34-105)	51 (26-86)	31 (17-51)	13 (7-19)	3 (2-5)	1 (0-1)
Hepatitis B	-	-	3,096 (1,876-4,585)	1,370 (768-2,191)	382 (197-617)	493 (263-794)	1,395 (931-1,987)	2,905 (2,169-3,709)	4,098 (3,332-4,855)	5,059 (4,454-5,687)	5,169 (4,594-5,743)	6,491 (5,823-7,172)	6,035 (5,406-6,724)	5,164 (4,597-5,695)	5,079 (4,532-5,588)	5,143 (4,542-5,665)	4,766 (4,211-5,270)	3,627 (3,177-4,070)	2,686 (2,251-3,078)	1,354 (1,047-1,602)	488 (337-617)	99 (60-141)	15 (8-24)
Hepatitis C	-	-	-	-	104 (67-155)	33 (21-49)	40 (25-59)	46 (23-75)	44 (20-79)	42 (19-73)	47 (21-87)	73 (32-130)	91 (43-161)	105 (49-189)	139 (65-244)	167 (81-305)	145 (66-252)	105 (50-179)	80 (39-132)	46 (22-81)	21 (12-32)	6 (3-9)	1 (1-2)
Acute hepatitis E	-	-	-	6,760 (5,006-8,602)	2,831 (2,232-3,495)	2,284 (1,850-2,792)	2,447 (1,881-3,060)	1,874 (1,154-2,601)	1,118 (593-1,799)	679 (335-1,144)	435 (195-801)	425 (181-826)	380 (168-710)	321 (146-607)	320 (146-604)	349 (141-670)	403 (169-736)	392 (180-716)	374 (150-713)	358 (173-621)	254 (136-393)	87 (49-128)	20 (12-29)
Other infectious diseases	5,204 (1,312-10,629)	3,690 (1,305-6,221)	13,259 (3,639-24,472)	9,628 (3,172-22,303)	1,681 (1,176-2,196)	1,173 (905-1,511)	1,388 (1,125-1,744)	1,247 (996-1,528)	1,131 (937-1,321)	1,283 (1,052-1,520)	1,350 (1,122-1,590)	1,091 (922-1,250)	1,191 (1,014-1,392)	1,183 (1,016-1,356)	1,353 (1,129-1,494)	1,840 (1,569-2,195)	1,916 (1,629-2,226)	1,844 (1,531-2,160)	1,508 (1,248-1,740)	1,090 (878-1,318)	642 (510-791)	271 (210-336)	81 (62-98)
Non-communicable diseases	120,070 (83,283-149,338)	51,966 (36,415-65,263)	204,185 (165,761-239,790)	132,404 (105,120-158,188)	67,877 (61,447-74,725)	60,105 (55,926-64,041)	95,685 (90,991-99,775)	137,742 (132,690-143,308)	177,337 (171,147-184,808)	248,310 (240,631-257,811)	346,449 (336,051-358,519)	521,322 (506,644-535,087)	663,513 (647,246-680,085)	843,206 (823,731-860,908)	1,255,952 (1,227,217-1,281,831)	1,670,137 (1,631,505-1,698,775)	2,032,793 (1,982,420-2,068,045)	2,096,531 (2,039,364-2,132,188)	1,865,892 (1,813,137-1,897,923)	1,559,937 (1,519,680-1,585,456)	948,996 (924,866-965,291)	375,589 (366,958-382,550)	93,149 (91,032-95,004)
Neoplasms	518 (435-560)	357 (315-390)	3,894 (3,532-4,462)	14,967 (12,915-18,206)	20,458 (18,516-22,573)	18,226 (16,708-19,446)	23,674 (22,151-24,821)	28,018 (26,836-29,391)	35,287 (33,990-37,042)	52,143 (50,394-54,223)	76,296 (73,976-78,859)	132,021 (127,891-136,101)	185,087 (180,012-189,711)	246,210 (239,972-251,783)	378,988 (369,908-388,124)	489,396 (479,277-499,834)	561,105 (549,794-572,144)	507,655 (497,921-516,893)	377,675 (369,314-384,979)	261,574 (255,599-267,750)	132,387 (128,390-136,007)	43,212 (41,810-44,680)	8,838 (8,457-9,206)
Lip and oral cavity cancer	-	-	-	-	-	-	202 (182-227)	386 (351-428)	685 (620-760)	1,254 (1,151-1,370)	2,087 (1,926-2,254)	3,995 (3,738-4,284)	5,943 (5,633-6,309)	7,696 (7,275-8,208)	10,225 (9,732-10,785)	10,705 (10,202-11,322)	10,038 (9,605-10,600)	7,506 (7,155-7,937)	4,934 (4,684-5,213)	3,339 (3,169-3,510)	1,746 (1,646-1,841)	606 (571-639)	138 (131-146)
Nasopharynx cancer	-	-	-	-	167 (141-196)	257 (218-296)	402 (355-446)	486 (427-545)	709 (630-803)	1,104 (1,000-1,236)	1,592 (1,458-1,735)	2,871 (2,641-3,108)	3,348 (3,100-3,600)	3,932 (3,631-4,227)	4,827 (4,485-5,163)	4,827 (4,489-5,162)	4,196 (3,935-4,465)	3,072 (2,878-3,261)	1,756 (1,656-1,868)	1,050 (989-1,113)	408 (387-429)	134 (127-140)	29 (28-31)
Other pharynx cancer	-	-	-	-	-	-	105 (91-125)	177 (155-211)	230 (200-269)	514 (457-590)	1,094 (986-1,236)	2,616 (2,401-2,895)	4,247 (3,952-4,666)	5,815 (5,386-6,426)	7,771 (7,243-8,502)	7,978 (7,362-8,802)	7,281 (6,692-8,039)	5,177 (4,694-5,781)	2,976 (2,666-3,324)	1,780 (1,559-1,990)	726 (636-810)	196 (175-217)	32 (29-35)
Oesophageal cancer	-	-	-	-	-	-	204 (184-225)	381 (350-419)	562 (524-615)	1,289 (1,196-1,389)	2,760 (2,613-2,949)	7,660 (7,209-8,120)	13,662 (12,983-14,406)	20,020 (19,132-21,051)	31,413 (29,876-33,084)	41,025 (39,010-43,104)	43,147 (41,097-45,316)	36,520 (34,753-38,329)	25,115 (23,826-26,423)	13,771 (13,141-14,442)	5,557 (5,318-5,795)	1,443 (1,389-1,506)	253 (241-265)
Stomach cancer	-	-	-	-	-	-	671 (630-726)	1,494 (1,410-1,611)	2,857 (2,692-3,056)	5,763 (5,477-6,093)	9,303 (8,871-9,788)	17,694 (16,887-18,493)	25,500 (24,331-26,599)	34,489 (33,164-35,849)	56,063 (53,942-58,209)	75,366 (72,689-78,175)	83,497 (80,872-86,319)	74,832 (72,488-77,211)	54,743 (53,051-56,520)	34,807 (33,772-35,843)	16,170 (15,708-16,644)	4,858 (4,721-5,012)	990 (957-1,019)
Colon and rectum cancer	-	-	-	-	-	-	667 (601-752)	1,429 (1,323-1,570)	2,220 (2,042-2,445)	3,705 (3,452-4,001)	5,184 (4,878-5,565)	7,819 (7,454-8,243)	11,563 (11,109-12,060)	15,317 (14,826-15,908)	25,008 (24,107-25,971)	35,117 (33,989-36,272)	44,992 (43,537-46,703)	44,239 (43,035-45,549)	35,410 (34,489-36,322)	28,574 (27,679-29,449)	15,790 (15,274-16,310)	5,396 (5,224-5,568)	1,106 (1,072-1,141)
Liver cancer	-	-	-	-	501 (447-611)	607 (555-692)	1,070 (973-1,200)	2,573 (2,320-2,908)	5,568 (5,084-6,253)	10,682 (9,742-11,769)	17,244 (15,939-18,723)	28,802 (26,809-31,107)	34,016 (31,725-36,360)	38,061 (35,777-40,773)	48,100 (45,326-50,986)	54,997 (52,171-57,959)	52,559 (49,963-55,035)	41,464 (39,499-43,583)	27,111 (25,821-28,528)	15,025 (14,327-15,748)	5,982 (5,751-6,222)	1,536 (1,473-1,598)	260 (248-271)
Liver cancer due to hepatitis B	-	-	-	-	132 (90-180)	226 (165-292)	492 (378-624)	1,416 (1,157-1,706)	3,350 (2,641-3,994)	6,563 (5,381-7,637)	10,422 (8,520-12,046)	17,000 (13,933-19,535)	19,109 (15,626-22,059)	20,084 (16,011-23,235)	23,594 (18,475-27,480)	24,558 (18,526-28,751)	21,489 (16,574-25,164)	15,820 (12,110-18,658)	10,104 (7,830-11,995)	4,917 (3,667-6,005)	1,787 (1,390-2,138)	436 (334-542)	74 (50-97)
Liver cancer due to hepatitis C	-	-	-	-	6 (5-8)	11 (8-15)	21 (16-28)	63 (49-81)	167 (118-229)	381 (292-493)	745 (567-964)	1,585 (1,256-1,995)	2,781 (2,183-3,457)	4,367 (3,534-5,270)	7,111 (5,932-8,513)	10,027 (8,494-11,975)	10,361 (8,904-12,007)	8,746 (7,595-10,168)	6,094 (5,284-7,103)	4,084 (3,520-4,750)	1,864 (1,641-2,107)	526 (457-599)	93 (78-110)
Liver cancer due to alcohol use	-	-	-	-	-	-	2 (1-4)	22 (14-32)	70 (42-110)	264 (187-360)	768 (545-1,049)	1,578 (1,178-2,078)	2,241 (1,615-3,026)	2,930 (2,113-3,889)	4,146 (3,027-5,473)	5,495 (4,103-7,179)	6,709 (5,344-8,190)	5,805 (4,610-7,107)	3,283 (2,632-3,998)	1,967 (1,489-2,459)	797 (594-991)	186 (131-241)	26 (16-38)
Liver cancer due to other causes	-	-	-	-	363 (306-455)	370 (303-443)	554 (441-683)	1,072 (872-1,304)	1,980 (1,540-2,601)	3,475 (2,773-4,433)	5,309 (4,277-6,799)	8,640 (6,967-11,249)	9,884 (7,998-12,647)	10,680 (8,594-13,542)	13,249 (10,759-16,562)	14,918 (12,222-18,763)	14,000 (11,600-16,873)	11,093 (9,215-13,599)	7,630 (6,372-9,216)	4,057 (3,356-4,924)	1,534 (1,307-1,799)	388 (322-463)	66 (51-83)
Gallbladder and biliary tract cancer	-	-	-	-	-	-	44 (37-56)	76 (63-101)	135 (110-179)	351 (289-466)	649 (538-847)	1,140 (961-1,472)	1,690 (1,441-2,086)	2,650 (2,241-3,222)	4,151 (3,614-5,064)	5,645 (4,983-6,632)	6,891 (6,155-8,253)	6,975 (6,265-8,379)	5,756 (5,184-6,756)	4,444 (4,066-5,079)	2,333 (2,128-2,622)	766 (706-848)	156 (143-176)
Pancreatic cancer	-	-	-	-	-	-	92 (87-99)	197 (186-213)	360 (341-388)	904 (862-956)	1,762 (1,686-1,847)	3,539 (3,397-3,698)	5,635 (5,456-5,854)	8,188 (7,942-8,464)	13,443 (12,992-13,906)	17,304 (16,840-17,812)	20,794 (20,185-21,413)	19,307 (18,830-19,780)	14,008 (13,665-14,361)	9,697 (9,416-10,017)	4,797 (4,647-4,965)	1,499 (1,452-1,545)	286 (277-295)
Larynx cancer	-	-	-	-	-	-	107 (97-117)	146 (135-157)	222 (206-236)	480 (447-511)	1,120 (1,052-1,194)	2,976 (2,804-3,137)	5,359 (5,057-5,662)	7,812 (7,417-8,223)	11,903 (11,294-12,512)	13,553 (12,939-14,172)	14,016 (13,362-14,681)	10,283 (9,849-10,703)	6,265 (5,990-6,537)	3,949 (3,751-4,154)	1,726 (1,644-1,810)	483 (461-506)	85 (82-89)
Tracheal, bronchus, and lung cancer	-	-	-	-	-	-	638 (575-705)	1,336 (1,216-1,487)	2,545 (2,328-2,787)	4,942 (4,620-5,297)	9,406 (8,944-9,963)	21,745 (20,816-22,660)	37,606 (36,151-39,238)	56,920 (54,979-59,041)	98,579 (95,089-102,619)	132,412 (128,617-136,723)	158,168 (153,787-162,911)	136,087 (132,952-139,503)	93,841 (91,553-96,412)	57,244 (55,497-59,019)	24,281 (23,526-25,067)	6,192 (6,006-6,383)	998 (964-1,030)
Malignant skin melanoma	-	-	-	-	-	-	121 (105-141)	226 (196-265)	357 (313-408)	639 (551-706)	962 (843-1,051)	1,363 (1,187-1,471)	1,495 (1,312-1,601)	1,681 (1,488-1,821)	2,115 (1,951-2,310)	2,098 (1,952-2,300)	2,371 (2,187-2,647)	2,182 (2,043-2,384)	1,628 (1,538-1,777)	1,274 (1,199-1,420)	709 (668-795)	250 (235-283)	56 (53-65)
Non-melanoma skin cancer	-	-	-	-	-	-	115 (105-123)	169 (155-183)	225 (206-245)	352 (329-372)	436 (411-463)	674 (644-705)	808 (770-841)	1,067 (1,018-1,112)	1,610 (1,538-1,682)	2,642 (2,530-2,749)	3,176 (3,033-3,322)	3,126 (3,005-3,245)	2,617 (2,511-2,721)	2,237 (2,148-2,321)	1,467 (1,408-1,526)	684 (656-712)	231 (221-241)
Non-melanoma skin cancer (squamous-cell carcinoma)	-	-	-	-	-	-	115 (105-123)	169 (155-183)	225 (206-245)	352 (329-372)	436 (411-463)	674 (644-705)	808 (770-841)	1,067 (1,018-1,112)	1,610 (1,538-1,682)	2,642 (2,530-2,749)	3,176 (3,033-3,322)	3,126 (3,005-3,245)	2,617 (2,511-2,721)	2,237 (2,148-2,321)	1,467 (1,408-1,526)	684 (656-712)	231 (221-241)
Breast cancer	-	-	-	-	-	-	30 (29-32)	31 (29-33)	36 (33-39)	62 (52-73)	122 (91-142)	190 (140-218)	337 (253-381)	428 (337-482)	574 (452-638)	687 (592-800)	750 (666-875)	695 (640-817)	434 (384-493)	395 (331-437)	231 (190-252)	91 (74-99)	22 (19-25)
Cervical cancer	-	-	-	-	-	-	-	-	-	-	-	-	-	-	-	-	-	-	-	-	-	-	-
Uterine cancer	-	-	-	-	-	-	-	-	-	-	-	-	-	-	-	-	-	-	-	-	-	-	-
Ovarian cancer	-	-	-	-	-	-	-	-	-	-	-	-	-	-	-	-	-	-	-	-	-	-	-
Prostate cancer	-	-	-	-	-	-	65 (53-76)	83 (66-97)	100 (80-119)	127 (101-149)	190 (151-218)	438 (351-509)	1,204 (978-1,371)	3,148 (2,549-3,516)	8,019 (6,492-8,974)	17,668 (14,215-19,825)	30,267 (25,421-33,202)	41,148 (35,477-44,726)	43,205 (37,405-47,469)	39,948 (33,501-43,718)	26,826 (22,613-28,880)	11,187 (9,699-12,233)	2,571 (2,242-2,848)
Testicular cancer	-	-	-	-	-	-	501 (463-549)	1,038 (968-1,135)	1,274 (1,193-1,386)	1,273 (1,200-1,364)	1,043 (984-1,109)	742 (700-787)	535 (503-572)	410 (377-449)	361 (335-388)	404 (376-434)	414 (385-444)	361 (338-385)	266 (250-283)	224 (210-240)	110 (104-117)	39 (36-41)	10 (9-11)
Kidney cancer	19 (15-21)	10 (8-12)	133 (111-166)	618 (487-806)	315 (281-361)	124 (114-137)	103 (96-113)	146 (137-160)	214 (201-234)	335 (317-358)	661 (627-702)	1,309 (1,241-1,393)	2,311 (2,201-2,437)	3,387 (3,242-3,535)	5,464 (5,224-5,758)	6,545 (6,307-6,851)	7,723 (7,406-8,075)	6,983 (6,742-7,229)	5,121 (4,950-5,318)	3,851 (3,712-4,017)	2,004 (1,922-2,098)	636 (610-665)	117 (112-123)
Bladder cancer	-	-	-	-	-	-	68 (62-76)	129 (120-140)	176 (163-190)	359 (333-390)	649 (603-704)	1,249 (1,173-1,324)	2,175 (2,062-2,301)	3,394 (3,252-3,561)	6,320 (6,056-6,600)	10,308 (9,935-10,713)	14,684 (14,063-15,288)	16,197 (15,669-16,767)	14,044 (13,555-14,487)	12,431 (12,014-12,851)	7,398 (7,151-7,652)	2,680 (2,587-2,772)	569 (548-589)
Brain and nervous system cancer	75 (42-92)	52 (31-64)	774 (427-1,004)	2,845 (1,909-3,613)	4,378 (3,171-5,012)	3,361 (2,583-3,707)	2,528 (1,994-2,794)	2,554 (2,005-2,793)	3,138 (2,465-3,436)	3,789 (3,060-4,095)	4,588 (3,740-4,976)	5,926 (4,764-6,457)	6,315 (5,153-6,841)	6,829 (5,661-7,366)	8,882 (7,474-9,561)	9,488 (7,927-10,170)	9,174 (7,840-9,802)	6,820 (5,879-7,327)	4,249 (3,718-4,603)	2,276 (1,966-2,460)	858 (720-931)	211 (180-231)	37 (32-41)
Thyroid cancer	-	-	-	-	-	70 (49-86)	111 (88-128)	169 (142-189)	207 (178-225)	214 (189-233)	274 (245-298)	434 (377-469)	639 (551-688)	877 (783-935)	1,240 (1,154-1,318)	1,247 (1,171-1,326)	1,359 (1,284-1,450)	1,197 (1,134-1,289)	1,014 (971-1,103)	709 (675-785)	326 (311-356)	100 (95-109)	20 (19-21)
Mesothelioma	-	-	-	-	-	-	52 (45-67)	62 (53-79)	102 (84-133)	118 (99-153)	197 (168-254)	314 (274-390)	525 (468-632)	796 (704-927)	1,372 (1,221-1,562)	1,564 (1,397-1,787)	1,935 (1,737-2,277)	2,070 (1,870-2,446)	1,652 (1,504-1,894)	1,234 (1,122-1,436)	553 (505-637)	149 (136-169)	25 (23-28)
Hodgkin lymphoma	16 (8-20)	4 (2-6)	97 (48-119)	495 (262-678)	1,425 (773-1,929)	1,294 (808-1,577)	1,254 (880-1,534)	1,229 (886-1,446)	1,291 (957-1,517)	1,343 (1,003-1,628)	1,328 (1,007-1,557)	1,372 (995-1,580)	1,476 (1,081-1,723)	1,408 (1,036-1,697)	1,913 (1,431-2,213)	1,962 (1,418-2,258)	2,005 (1,506-2,288)	1,643 (1,275-1,885)	813 (606-930)	486 (374-544)	209 (165-234)	54 (44-60)	9 (7-10)
Non-Hodgkin's lymphoma	34 (25-38)	26 (20-32)	312 (231-385)	1,502 (1,091-2,023)	2,243 (1,713-2,687)	1,962 (1,591-2,249)	2,026 (1,709-2,234)	2,211 (1,982-2,514)	2,199 (1,988-2,426)	2,605 (2,254-2,833)	3,006 (2,556-3,226)	3,847 (3,280-4,220)	4,469 (3,823-4,864)	5,374 (4,664-5,895)	7,145 (6,278-8,061)	8,723 (7,982-9,726)	10,126 (9,120-11,424)	9,810 (8,678-10,964)	7,573 (6,811-8,577)	5,639 (4,934-6,408)	2,880 (2,601-3,253)	890 (831-1,050)	164 (153-202)
Multiple myeloma	-	-	-	-	-	-	107 (87-131)	145 (112-175)	159 (128-195)	295 (245-361)	473 (392-564)	900 (740-1,050)	1,162 (966-1,352)	1,770 (1,477-2,056)	2,834 (2,406-3,353)	3,702 (2,978-4,380)	4,567 (3,710-5,579)	4,697 (3,833-5,668)	3,645 (2,989-4,377)	2,891 (2,341-3,589)	1,551 (1,257-1,940)	462 (381-594)	78 (61-95)
Leukaemia	135 (111-151)	121 (107-146)	1,501 (1,287-1,988)	6,136 (5,088-8,535)	8,605 (7,616-10,707)	7,398 (6,736-8,360)	7,407 (6,598-8,349)	6,850 (5,968-7,815)	5,859 (5,254-6,670)	5,342 (4,707-5,969)	5,224 (4,670-5,727)	5,961 (5,225-6,364)	5,966 (5,432-6,351)	6,406 (5,880-6,941)	8,706 (7,952-9,429)	10,579 (9,947-11,492)	12,640 (12,033-13,595)	12,323 (11,794-13,301)	9,667 (9,278-10,326)	7,421 (7,156-7,913)	4,086 (3,931-4,356)	1,413 (1,357-1,485)	305 (291-319)
Acute lymphoid leukaemia	31 (25-42)	26 (19-39)	354 (244-617)	1,909 (1,324-3,300)	2,945 (2,397-4,155)	2,553 (2,248-3,206)	2,079 (1,877-2,503)	1,449 (1,309-1,787)	1,012 (910-1,268)	892 (804-1,093)	807 (732-979)	850 (768-967)	824 (738-931)	883 (781-969)	1,129 (993-1,263)	1,150 (1,004-1,268)	1,223 (1,080-1,347)	1,084 (955-1,186)	513 (451-561)	328 (288-358)	161 (142-177)	51 (45-55)	11 (10-11)
Chronic lymphoid leukaemia	-	-	-	-	-	-	139 (122-189)	149 (128-212)	127 (109-178)	112 (95-148)	111 (97-139)	191 (166-238)	299 (259-363)	501 (436-606)	952 (794-1,182)	1,414 (1,246-1,704)	2,061 (1,805-2,504)	2,243 (1,948-2,648)	1,770 (1,516-2,075)	1,674 (1,389-2,006)	1,053 (858-1,250)	385 (327-446)	86 (75-99)
Acute myeloid leukaemia	20 (14-27)	20 (15-29)	224 (158-370)	1,064 (723-1,880)	1,135 (829-1,557)	1,053 (849-1,336)	1,154 (1,002-1,428)	1,049 (898-1,292)	947 (828-1,147)	981 (840-1,175)	1,064 (923-1,257)	1,229 (1,080-1,446)	1,339 (1,184-1,560)	1,572 (1,425-1,839)	2,116 (1,857-2,529)	2,499 (2,256-2,971)	3,128 (2,835-3,742)	3,021 (2,743-3,758)	2,214 (2,015-2,768)	1,543 (1,395-2,033)	733 (658-980)	217 (193-302)	39 (35-57)
Chronic myeloid leukaemia	-	-	-	-	-	-	273 (235-345)	383 (329-472)	569 (497-684)	665 (568-765)	737 (646-838)	783 (692-885)	885 (798-984)	875 (777-978)	1,122 (981-1,246)	1,195 (1,063-1,316)	1,435 (1,263-1,568)	1,317 (1,155-1,425)	1,245 (1,102-1,341)	1,002 (891-1,083)	554 (496-601)	198 (172-215)	42 (36-45)
Other leukaemia	84 (60-97)	74 (56-90)	922 (725-1,202)	3,163 (2,480-4,317)	4,526 (3,734-5,437)	3,792 (3,042-4,169)	3,762 (3,063-4,181)	3,821 (3,053-4,302)	3,203 (2,713-3,618)	2,692 (2,197-2,984)	2,505 (2,056-2,736)	2,908 (2,322-3,164)	2,620 (2,179-2,864)	2,574 (2,164-2,826)	3,388 (2,869-3,658)	4,321 (3,787-4,828)	4,793 (4,240-5,187)	4,657 (4,171-5,035)	3,925 (3,538-4,298)	2,873 (2,604-3,115)	1,585 (1,439-1,764)	562 (501-603)	126 (113-135)
Other neoplasms	239 (198-263)	144 (118-168)	1,078 (850-1,395)	3,372 (2,754-4,759)	2,824 (2,492-3,289)	3,153 (2,910-3,477)	4,983 (4,657-5,367)	4,297 (4,028-4,612)	3,859 (3,615-4,123)	4,303 (4,007-4,604)	4,942 (4,649-5,247)	6,444 (6,059-6,828)	7,103 (6,717-7,541)	8,335 (7,858-9,034)	10,950 (10,311-11,868)	12,851 (12,199-14,154)	14,334 (13,631-15,525)	12,938 (12,332-13,987)	9,833 (9,339-10,796)	6,877 (6,546-7,570)	3,662 (3,477-4,020)	1,276 (1,209-1,387)	291 (276-316)
Cardiovascular diseases	3,427 (3,117-3,747)	2,093 (1,879-2,301)	17,990 (16,339-19,767)	14,158 (12,119-16,073)	8,760 (7,925-9,618)	8,862 (8,266-9,529)	22,713 (21,394-24,419)	39,512 (37,703-42,091)	55,687 (53,148-59,418)	85,164 (81,368-90,955)	128,463 (123,000-135,217)	204,901 (197,469-213,649)	272,534 (263,622-283,138)	355,848 (346,087-368,207)	543,470 (527,994-559,536)	727,577 (709,950-743,612)	920,795 (896,924-942,355)	962,239 (940,717-982,636)	879,793 (857,557-899,298)	784,344 (765,286-800,364)	492,115 (480,073-504,110)	198,274 (193,351-203,230)	51,971 (50,422-53,344)
Rheumatic heart disease	-	-	-	3,316 (2,514-4,462)	2,199 (1,836-2,609)	3,055 (2,697-3,444)	5,464 (4,802-6,104)	5,917 (5,081-6,719)	5,893 (4,937-6,643)	6,447 (5,371-7,345)	7,018 (5,942-7,970)	8,847 (7,528-10,047)	8,827 (7,579-9,912)	9,595 (8,235-10,941)	12,345 (10,735-13,860)	14,152 (12,338-15,867)	15,912 (14,131-17,713)	15,639 (13,836-17,327)	13,307 (12,013-14,501)	10,198 (9,280-11,092)	5,797 (5,327-6,262)	2,322 (2,149-2,496)	620 (580-660)
Ischaemic heart disease	-	-	1,132 (928-1,390)	893 (672-1,173)	562 (477-674)	543 (481-616)	6,358 (5,912-6,905)	13,981 (13,175-15,085)	23,572 (22,296-25,560)	42,469 (40,225-45,662)	68,684 (65,245-72,725)	111,137 (106,018-116,893)	152,482 (146,332-159,421)	195,407 (188,574-202,354)	294,778 (284,636-305,227)	374,227 (362,479-385,331)	470,629 (457,326-483,874)	478,033 (464,455-493,022)	431,395 (418,911-444,912)	408,038 (397,682-420,739)	272,340 (266,023-281,203)	117,047 (114,600-121,354)	32,533 (31,811-33,629)
Cerebrovascular disease	1,730 (1,482-2,011)	1,013 (843-1,218)	7,586 (6,290-9,477)	4,628 (3,755-5,927)	3,127 (2,761-3,668)	2,760 (2,533-3,071)	5,740 (5,336-6,280)	11,916 (11,203-12,963)	15,991 (15,027-17,677)	23,221 (21,856-25,469)	34,177 (32,302-36,930)	58,271 (55,506-62,054)	80,448 (76,927-84,916)	114,629 (109,903-120,862)	181,959 (174,513-191,477)	267,917 (258,203-279,912)	343,903 (331,393-359,879)	365,259 (351,033-383,757)	333,296 (317,809-352,103)	269,145 (254,158-284,682)	148,330 (138,889-158,871)	51,581 (47,953-55,604)	12,083 (10,973-13,052)

SR Table 4: Global deaths by cause and age group for males (with 95% uncertainty intervals), 1995

Cause	Early Neonatal	Late Neonatal	Post Neonatal	1 to 4	5 to 9	10 to 14	15 to 19	20 to 24	25 to 29	30 to 34	35 to 39
Ischaemic stroke	-	-	253 (211-314)	254 (205-332)	202 (176-235)	185 (165-210)	704 (635-776)	1,523 (1,388-1,675)	2,064 (1,891-2,263)	3,020 (2,789-3,283)	4,588 (4,225-4,941)
Hemorrhagic stroke	1,730 (1,482-2,011)	1,013 (843-1,218)	7,334 (6,062-9,163)	4,374 (3,540-5,623)	2,924 (2,584-3,435)	2,575 (2,364-2,868)	5,036 (4,685-5,506)	10,394 (9,752-11,371)	13,927 (13,058-15,463)	20,201 (18,976-22,278)	29,590 (27,882-32,089)
Hypertensive heart disease	-	-	337 (196-441)	136 (70-193)	125 (71-159)	128 (72-172)	823 (542-992)	1,487 (988-1,766)	2,040 (1,428-2,377)	2,903 (2,053-3,425)	4,409 (3,237-5,172)
Cardiomyopathy and myocarditis	575 (353-722)	357 (230-466)	2,788 (1,811-3,695)	1,751 (1,145-2,381)	877 (679-1,127)	832 (723-1,046)	1,806 (1,591-2,242)	2,388 (2,086-2,852)	3,400 (2,916-4,061)	4,714 (3,921-5,497)	7,461 (5,994-8,980)
Myocarditis	132 (57-189)	111 (55-161)	1,212 (616-1,844)	815 (393-1,306)	403 (265-575)	314 (255-441)	665 (546-927)	737 (610-1,005)	896 (731-1,301)	664 (549-941)	745 (607-1,016)
Alcoholic cardiomyopathy	-	-	-	-	-	-	160 (119-236)	385 (254-505)	915 (608-1,169)	2,155 (1,539-2,745)	4,177 (3,027-5,338)
Other cardiomyopathy	443 (286-577)	246 (166-334)	1,576 (1,094-2,284)	936 (668-1,449)	474 (365-637)	518 (436-643)	980 (813-1,216)	1,265 (1,078-1,559)	1,588 (1,312-1,922)	1,895 (1,597-2,327)	2,539 (2,051-3,160)
Atrial fibrillation and flutter	-	-	-	-	-	-	-	-	-	16 (11-21)	59 (43-78)
Aortic aneurysm	-	-	-	-	-	-	257 (222-295)	352 (311-396)	558 (477-640)	735 (645-830)	1,137 (1,010-1,270)
Peripheral vascular disease	-	-	-	-	-	-	-	-	-	-	-
Endocarditis	87 (43-122)	49 (26-72)	1,047 (650-1,374)	386 (238-511)	177 (116-215)	246 (177-308)	509 (382-691)	769 (581-1,050)	971 (763-1,344)	1,092 (833-1,410)	1,323 (964-1,648)
Other cardiovascular and circulatory diseases	1,035 (815-1,307)	674 (491-825)	5,100 (3,836-6,796)	3,047 (1,889-4,200)	1,693 (1,129-2,074)	1,298 (927-1,582)	1,755 (1,302-2,220)	2,701 (2,017-3,619)	3,262 (2,380-4,215)	3,568 (2,697-4,431)	4,195 (3,112-5,121)
Chronic respiratory diseases	0 (0-0)	0 (0-0)	0 (0-0)	16,453 (12,940-20,810)	4,453 (3,908-5,057)	4,123 (3,693-4,563)	6,545 (6,087-7,088)	9,461 (8,958-10,228)	12,117 (11,457-13,104)	14,865 (14,143-15,882)	19,870 (18,786-21,103)
Chronic obstructive pulmonary disease	-	-	0 (0-0)	3,594 (2,045-7,800)	1,054 (755-1,536)	681 (508-939)	2,771 (2,204-3,356)	4,371 (3,612-5,230)	5,729 (4,664-6,895)	7,864 (6,610-9,257)	10,656 (8,786-12,576)
Pneumoconiosis	-	-	-	-	-	-	32 (25-47)	67 (52-107)	207 (139-356)	361 (259-593)	542 (437-848)
Silicosis	-	-	-	-	-	-	5 (3-8)	19 (13-28)	100 (62-164)	231 (161-377)	380 (299-587)
Asbestosis	-	-	-	-	-	-	12 (10-18)	18 (15-27)	23 (18-36)	26 (20-40)	29 (21-43)
Coal workers pneumoconiosis	-	-	-	-	-	-	1 (1-2)	4 (2-6)	25 (13-45)	23 (12-37)	39 (21-58)
Other pneumoconiosis	-	-	-	-	-	-	14 (10-23)	26 (18-49)	59 (31-127)	81 (47-157)	95 (62-167)
Asthma	-	-	-	10,500 (6,617-14,233)	2,582 (1,900-3,297)	2,890 (2,377-3,415)	3,185 (2,463-3,897)	4,251 (3,231-5,168)	5,432 (4,091-6,546)	5,580 (3,977-6,960)	7,381 (5,055-9,421)
Interstitial lung disease and pulmonary sarcoidosis	-	-	-	198 (73-496)	80 (42-163)	45 (25-81)	101 (54-181)	156 (89-271)	213 (119-376)	307 (178-525)	461 (275-792)
Other chronic respiratory diseases	0 (0-0)	0 (0-0)	0 (0-0)	2,161 (616-4,262)	736 (383-1,162)	507 (258-808)	455 (265-668)	616 (382-918)	537 (299-824)	753 (423-1,145)	830 (457-1,207)
Cirrhosis and other chronic liver diseases	-	-	-	7,103 (4,826-9,669)	2,678 (2,176-3,501)	2,701 (2,314-3,229)	4,422 (3,946-5,127)	8,618 (7,767-10,480)	15,178 (13,752-16,992)	27,158 (25,118-30,337)	40,881 (37,976-44,242)
Cirrhosis and other chronic liver diseases due to hepatitis B	-	-	-	80 (49-128)	80 (55-116)	143 (102-199)	397 (269-555)	2,300 (1,745-3,084)	5,714 (4,501-7,059)	10,453 (8,929-12,128)	15,036 (12,583-17,461)
Cirrhosis and other chronic liver diseases due to hepatitis C	-	-	-	63 (40-98)	59 (40-87)	112 (77-163)	278 (183-401)	1,372 (989-1,833)	3,226 (2,448-4,147)	6,700 (5,660-8,033)	10,743 (8,771-13,190)
Cirrhosis and other chronic liver diseases due to alcohol use	-	-	-	-	-	-	77 (44-121)	645 (460-900)	1,689 (1,228-2,301)	5,121 (4,302-6,213)	10,388 (8,583-12,613)
Cirrhosis and other chronic liver diseases due to other causes	-	-	-	6,959 (4,740-9,488)	2,540 (2,058-3,316)	2,447 (2,092-2,913)	3,670 (3,261-4,205)	4,301 (3,439-5,339)	4,549 (3,356-5,816)	4,884 (3,583-6,279)	4,714 (3,350-6,343)
Digestive diseases	1,222 (957-1,447)	1,543 (1,117-1,965)	12,656 (9,241-16,834)	12,706 (8,392-17,361)	6,094 (5,009-7,193)	4,898 (4,109-5,561)	6,996 (5,941-7,785)	9,248 (8,098-10,236)	13,567 (11,611-14,785)	15,533 (13,397-16,880)	20,023 (17,304-21,844)
Peptic ulcer disease	-	-	-	2,369 (1,478-3,383)	1,353 (1,040-1,764)	1,401 (1,135-1,685)	2,346 (1,911-2,763)	3,617 (3,015-4,116)	4,723 (3,957-5,362)	5,879 (4,951-6,644)	7,302 (6,167-8,236)
Gastritis and duodenitis	-	-	-	438 (179-920)	162 (102-263)	103 (77-146)	221 (177-278)	305 (254-371)	512 (426-626)	775 (651-925)	897 (748-1,093)
Appendicitis	-	-	-	1,876 (1,039-2,677)	1,254 (957-1,587)	1,070 (845-1,307)	1,218 (988-1,552)	968 (809-1,224)	1,210 (981-1,529)	799 (664-987)	997 (800-1,278)
Paralytic ileus and intestinal obstruction	1,222 (957-1,447)	1,543 (1,117-1,965)	12,656 (9,241-16,834)	2,477 (1,402-3,423)	1,650 (1,031-2,044)	1,108 (725-1,351)	1,537 (1,017-1,887)	1,756 (1,179-2,181)	2,633 (1,695-3,134)	2,226 (1,485-2,667)	3,280 (2,093-3,873)
Inguinal, femoral, and abdominal hernia	-	-	-	969 (352-1,662)	295 (163-397)	276 (134-388)	341 (187-454)	458 (248-626)	562 (310-716)	567 (258-763)	621 (360-782)
Inflammatory bowel disease	-	-	-	878 (326-2,250)	193 (97-349)	131 (82-202)	150 (97-189)	253 (162-323)	394 (267-522)	409 (282-535)	453 (320-585)
Vascular intestinal disorders	-	-	-	422 (108-822)	60 (29-91)	33 (24-40)	59 (48-73)	110 (83-131)	168 (127-196)	170 (129-204)	314 (250-379)
Gallbladder and biliary diseases	-	-	-	269 (151-409)	128 (94-166)	136 (91-173)	176 (130-216)	272 (215-323)	349 (269-410)	465 (364-543)	671 (517-789)
Pancreatitis	-	-	-	127 (66-204)	89 (62-117)	97 (72-118)	334 (237-423)	924 (730-1,137)	2,141 (1,760-2,578)	3,110 (2,601-3,780)	4,173 (3,451-5,105)
Other digestive diseases	-	-	-	2,880 (1,354-4,728)	912 (638-1,305)	542 (392-780)	613 (481-777)	584 (476-751)	874 (695-1,110)	1,133 (935-1,446)	1,316 (1,075-1,718)
Neurological disorders	-	-	4,365 (3,484-5,425)	6,023 (4,089-8,092)	3,856 (3,270-4,528)	5,116 (4,584-5,740)	9,080 (8,453-9,699)	9,099 (8,552-9,692)	7,697 (7,218-8,193)	7,055 (6,671-7,425)	7,517 (7,099-7,905)
Alzheimer's disease and other dementias	-	-	-	-	-	-	-	-	-	-	-
Parkinson's disease	-	-	-	-	-	-	-	1 (0-2)	12 (5-24)	42 (22-76)	87 (46-151)
Epilepsy	-	-	2,798 (2,173-3,362)	4,933 (3,256-6,416)	3,306 (2,799-3,827)	4,168 (3,690-4,705)	7,150 (6,553-7,736)	7,573 (7,052-8,123)	6,709 (6,247-7,143)	6,017 (5,668-6,344)	6,087 (5,718-6,447)
Multiple sclerosis	-	-	-	-	-	-	-	54 (46-63)	117 (94-150)	257 (207-328)	424 (338-519)
Motor neuron disease	4 (3-4)	6 (5-7)	540 (359-668)	173 (102-217)	28 (23-33)	52 (45-58)	87 (80-94)	81 (75-89)	70 (64-75)	142 (132-152)	283 (267-304)
Other neurological disorders	-	-	1,027 (817-1,562)	917 (663-1,606)	522 (436-730)	896 (811-1,057)	1,843 (1,740-1,972)	1,389 (1,317-1,475)	790 (751-838)	596 (567-626)	637 (604-667)
Mental and substance use disorders	12 (5-21)	12 (8-16)	0 (0-0)	0 (0-0)	10 (6-12)	5 (3-7)	4,592 (3,442-5,227)	10,842 (8,144-12,284)	15,415 (12,203-17,402)	20,777 (17,326-23,062)	24,448 (21,606-27,479)
Alcohol use disorders	-	-	-	-	-	-	1,035 (867-1,279)	2,804 (2,407-3,425)	5,389 (4,673-6,273)	10,141 (8,771-11,801)	14,803 (12,889-17,212)
Drug use disorders	12 (5-21)	12 (8-16)	0 (0-0)	0 (0-0)	0 (0-0)	0 (0-0)	3,546 (2,442-4,137)	8,020 (5,302-9,360)	10,006 (6,875-11,565)	10,616 (7,702-12,088)	9,629 (7,576-10,869)
Opioid use disorders	12 (5-21)	12 (8-16)	0 (0-0)	0 (0-0)	0 (0-0)	0 (0-0)	2,656 (1,801-3,184)	5,943 (3,715-7,088)	7,351 (4,708-8,725)	7,513 (4,945-8,843)	6,932 (4,966-8,060)
Cocaine use disorders	0 (0-0)	0 (0-0)	0 (0-0)	0 (0-0)	0 (0-0)	0 (0-0)	133 (69-176)	318 (167-418)	425 (241-558)	668 (404-852)	571 (377-768)
Amphetamine use disorders	0 (0-0)	0 (0-0)	0 (0-0)	0 (0-0)	0 (0-0)	0 (0-0)	93 (49-125)	212 (112-288)	280 (160-374)	342 (188-472)	287 (179-392)
Other drug use disorders	0 (0-0)	0 (0-0)	0 (0-0)	0 (0-0)	0 (0-0)	0 (0-0)	663 (356-797)	1,547 (862-1,836)	1,950 (1,181-2,316)	2,093 (1,382-2,463)	1,840 (1,321-2,194)
Eating disorders	-	-	-	-	10 (6-12)	5 (3-7)	11 (8-14)	18 (11-23)	20 (14-26)	21 (16-26)	16 (13-21)
Anorexia nervosa	-	-	-	-	9 (5-12)	5 (3-6)	10 (7-13)	18 (11-22)	19 (14-25)	19 (14-24)	15 (12-20)
Bulimia nervosa	-	-	-	-	0 (0-1)	0 (0-1)	0 (0-1)	1 (0-1)	1 (1-2)	2 (1-3)	1 (1-2)
Diabetes, urogenital, blood, and endocrine diseases	2,665 (1,879-3,030)	2,410 (1,772-2,838)	19,617 (16,637-22,129)	20,429 (14,876-24,995)	9,435 (8,123-10,988)	8,662 (7,243-9,749)	11,773 (10,597-12,988)	17,719 (16,568-19,634)	18,143 (16,762-19,347)	21,906 (20,411-23,288)	25,512 (24,050-27,096)
Diabetes mellitus	46 (43-50)	81 (70-92)	630 (568-719)	503 (428-584)	578 (503-652)	670 (609-740)	1,177 (1,090-1,281)	2,322 (2,199-2,500)	3,092 (2,938-3,296)	4,984 (4,748-5,304)	6,869 (6,596-7,188)
Acute glomerulonephritis	-	-	113 (93-136)	179 (143-223)	220 (182-266)	171 (143-202)	209 (176-249)	322 (274-389)	374 (323-439)	408 (351-473)	410 (351-478)
Chronic kidney disease	-	-	3,715 (2,951-4,214)	4,208 (3,143-5,002)	2,884 (2,298-3,257)	2,571 (2,174-2,895)	5,090 (4,599-5,758)	8,681 (7,502-9,686)	9,706 (8,507-10,687)	11,341 (9,999-12,511)	13,008 (11,518-14,427)
Chronic kidney disease due to diabetes mellitus	-	-	103 (65-151)	126 (76-192)	118 (67-189)	139 (84-224)	424 (259-650)	1,466 (953-2,020)	2,652 (1,958-3,382)	3,887 (2,911-4,921)	5,169 (3,944-6,458)
Chronic kidney disease due to hypertension	-	-	27 (18-38)	43 (27-63)	51 (33-77)	75 (48-112)	228 (145-324)	579 (387-801)	824 (563-1,132)	1,146 (829-1,531)	1,504 (1,123-1,974)
Chronic kidney disease due to glomerulonephritis	-	-	1,444 (1,022-1,866)	1,658 (1,145-2,229)	1,145 (772-1,542)	1,080 (758-1,434)	2,180 (1,549-2,868)	3,504 (2,509-4,602)	3,367 (2,358-4,482)	3,232 (2,228-4,411)	3,045 (2,051-4,169)
Chronic kidney disease due to other causes	-	-	2,141 (1,614-2,637)	2,380 (1,729-3,063)	1,568 (1,151-1,983)	1,278 (916-1,655)	2,258 (1,633-2,949)	3,133 (2,168-4,125)	2,863 (1,960-3,916)	3,076 (2,079-4,282)	3,291 (2,287-4,431)
Urinary diseases and male infertility	276 (242-315)	502 (383-658)	4,026 (3,521-4,543)	2,322 (1,854-2,826)	883 (733-1,055)	653 (582-720)	1,192 (1,084-1,298)	1,632 (1,509-1,755)	1,644 (1,524-1,767)	1,999 (1,876-2,127)	2,268 (2,112-2,410)
Interstitial nephritis and urinary tract infections	114 (79-155)	246 (152-352)	2,545 (1,902-3,150)	1,134 (637-1,723)	354 (244-455)	306 (240-369)	581 (453-697)	820 (651-972)	775 (630-929)	926 (763-1,097)	1,092 (900-1,279)
Urolithiasis	-	-	-	-	167 (82-246)	105 (53-140)	186 (106-248)	211 (120-275)	217 (112-281)	240 (129-313)	284 (168-360)
Other urinary diseases	162 (120-199)	257 (164-368)	1,481 (923-2,101)	1,188 (638-1,771)	362 (231-501)	242 (160-314)	426 (303-583)	602 (431-816)	652 (476-856)	833 (624-1,034)	892 (666-1,135)
Gynecological diseases	-	-	-	-	-	-	-	-	-	-	-
Uterine fibroids	-	-	-	-	-	-	-	-	-	-	-
Polycystic ovarian syndrome	-	-	-	-	-	-	-	-	-	-	-
Endometriosis	-	-	-	-	-	-	-	-	-	-	-
Genital prolapse	-	-	-	-	-	-	-	-	-	-	-
Other gynecological diseases	-	-	-	-	-	-	-	-	-	-	-
Hemoglobinopathies and hemolytic anaemias	1,409 (870-1,704)	1,033 (619-1,288)	6,084 (4,057-7,731)	10,305 (5,644-14,117)	3,471 (2,604-4,510)	3,490 (2,299-4,391)	3,186 (2,160-4,110)	3,704 (2,832-5,162)	1,989 (1,404-2,539)	1,443 (1,037-1,832)	1,265 (956-1,610)
Thalassemias	363 (276-458)	331 (205-445)	1,722 (1,201-2,222)	2,641 (1,785-3,530)	1,219 (965-1,634)	417 (326-585)	234 (169-301)	171 (110-227)	95 (58-132)	39 (29-48)	21 (17-25)
Sickle cell disorders	911 (481-1,173)	639 (357-850)	4,030 (2,529-5,292)	7,096 (3,475-10,180)	1,926 (1,328-2,588)	2,642 (1,627-3,484)	2,370 (1,568-3,076)	2,497 (1,941-3,609)	998 (687-1,284)	613 (443-798)	508 (360-653)
G6PD deficiency	41 (26-56)	29 (19-40)	233 (162-325)	474 (299-671)	254 (191-335)	307 (220-400)	423 (307-558)	649 (477-877)	620 (430-772)	552 (386-701)	478 (353-606)
Other hemoglobinopathies and hemolytic anaemias	94 (68-121)	34 (23-43)	98 (71-129)	94 (63-127)	71 (57-91)	124 (97-160)	158 (121-210)	387 (293-524)	276 (206-364)	240 (181-307)	259 (204-329)
Endocrine, metabolic, blood, and immune disorders	933 (556-1,090)	794 (510-963)	5,049 (3,755-6,593)	2,912 (2,119-3,809)	1,400 (1,128-1,804)	1,107 (926-1,440)	919 (759-1,086)	1,056 (835-1,249)	1,338 (985-1,527)	1,731 (1,365-2,002)	1,692 (1,352-1,986)
Musculoskeletal disorders	-	-	-	-	434 (311-573)	458 (380-577)	461 (408-580)	607 (501-726)	629 (514-724)	611 (504-695)	704 (582-792)
Rheumatoid arthritis	-	-	-	-	112 (61-191)	95 (65-139)	73 (54-96)	100 (70-139)	100 (69-134)	91 (65-121)	104 (78-133)
Other musculoskeletal disorders	-	-	-	-	322 (238-438)	363 (306-458)	387 (342-502)	507 (422-609)	529 (434-610)	520 (429-594)	600 (501-678)
Other non-communicable diseases	112,223 (76,069-141,270)	45,545 (30,650-58,700)	145,663 (109,046-180,195)	40,566 (26,141-60,055)	11,698 (8,687-15,411)	7,052 (5,362-8,909)	5,429 (4,357-6,627)	4,620 (3,751-5,707)	3,617 (2,979-4,541)	3,098 (2,413-3,604)	2,735 (2,141-3,201)
Congenital anomalies	112,223 (76,069-141,270)	42,957 (28,382-56,033)	122,381 (87,619-159,166)	39,755 (25,281-59,123)	11,441 (8,490-15,192)	6,786 (5,182-8,651)	4,926 (4,004-6,292)	4,134 (3,370-5,325)	3,209 (2,655-4,256)	2,261 (1,967-2,914)	2,057 (1,686-2,612)
Neural tube defects	21,471 (11,904-35,031)	6,376 (3,237-11,656)	13,905 (6,945-26,352)	4,079 (1,365-10,690)	1,039 (670-1,621)	556 (376-838)	310 (222-447)	245 (176-351)	193 (140-261)	129 (94-178)	89 (67-119)
Congenital heart anomalies	38,424 (21,711-53,688)	19,544 (11,080-27,747)	63,698 (41,142-94,126)	21,483 (12,570-34,851)	7,208 (5,026-10,186)	4,401 (3,266-5,844)	3,303 (2,618-4,321)	2,891 (2,333-3,821)	2,331 (1,958-3,173)	1,558 (1,328-2,047)	1,486 (1,224-1,897)
Orofacial clefts	1,061 (326-1,707)	343 (115-562)	969 (333-1,685)	101 (22-230)	-	-	-	-	-	-	-
Down's syndrome	1,408 (537-4,368)	795 (285-2,678)	3,372 (1,397-10,833)	1,543 (503-6,358)	238 (133-562)	173 (106-350)	154 (108-268)	135 (99-217)	124 (93-198)	137 (102-213)	121 (89-189)
Other chromosomal abnormalities	2,263 (1,162-5,223)	1,026 (484-2,289)	2,118 (944-6,183)	450 (184-1,121)	102 (59-198)	63 (35-113)	64 (41-112)	52 (35-85)	40 (24-55)	27 (16-34)	26 (14-32)
Congenital musculoskeletal and limb anomalies	2,796 (1,685-5,868)	720 (396-1,790)	1,522 (834-4,043)	417 (201-1,519)	160 (94-332)	116 (67-178)	121 (75-171)	62 (34-88)	47 (32-67)	37 (21-51)	28 (18-39)
Urogenital congenital anomalies	3,143 (1,859-4,898)	841 (489-1,369)	1,509 (819-2,493)	305 (160-546)	143 (92-216)	126 (89-182)	59 (45-91)	60 (46-91)	57 (41-76)	57 (39-75)	54 (37-73)
Digestive congenital anomalies	11,016 (7,593-19,024)	4,962 (3,046-9,547)	11,044 (6,337-22,967)	1,956 (908-5,682)	246 (171-424)	107 (82-158)	50 (37-64)	33 (25-44)	32 (24-41)	24 (19-31)	24 (16-29)
Other congenital anomalies	30,641 (17,173-51,033)	8,351 (4,172-15,140)	24,244 (12,134-42,100)	9,420 (4,438-18,280)	2,305 (1,454-3,526)	1,242 (859-1,746)	864 (641-1,146)	655 (456-879)	386 (279-499)	291 (217-373)	230 (180-292)
Skin and subcutaneous diseases	-	-	1,515 (619-2,135)	812 (312-1,204)	257 (113-355)	267 (125-362)	503 (236-680)	485 (218-640)	408 (198-539)	837 (386-1,101)	677 (338-894)
Cellulitis	-	-	55 (19-84)	62 (15-99)	40 (13-65)	45 (20-66)	48 (25-72)	53 (26-72)	39 (19-56)	81 (36-113)	101 (47-147)
Pyoderma	-	-	1,411 (564-2,009)	657 (254-995)	169 (75-236)	174 (73-248)	355 (150-483)	347 (138-464)	291 (127-392)	628 (268-841)	450 (197-606)
Decubitus ulcer	-	-	-	39 (11-70)	25 (12-37)	26 (14-40)	64 (36-94)	55 (28-79)	55 (30-82)	102 (57-148)	105 (56-168)
Other skin and subcutaneous diseases	-	-	49 (20-83)	54 (17-95)	23 (12-35)	22 (12-34)	35 (21-58)	31 (16-47)	23 (13-35)	26 (16-42)	22 (13-36)
Sudden infant death syndrome	-	2,588 (1,931-3,601)	21,767 (16,840-30,806)	-	-	-	-	-	-	-	-
Injuries	11,178 (8,895-13,232)	7,243 (5,523-9,089)	67,358 (56,459-77,570)	183,073 (151,820-206,980)	131,543 (121,347-141,006)	105,716 (99,970-111,630)	206,276 (198,751-212,455)	313,428 (303,700-321,348)	302,506 (291,870-310,399)	270,933 (260,098-279,299)	245,937 (233,852-255,169)
Transport injuries	2,707 (1,913-4,013)	1,354 (1,062-1,733)	9,784 (8,215-11,945)	35,687 (29,352-41,694)	40,428 (37,037-43,689)	31,039 (29,136-33,302)	79,692 (76,209-84,758)	120,379 (115,616-128,188)	114,443 (110,020-121,633)	95,452 (91,949-100,407)	84,302 (81,059-88,137)
Road injuries	2,282 (1,631-3,344)	1,141 (904-1,489)	8,928 (7,464-11,007)	33,755 (27,821-39,406)	38,522 (35,194-41,742)	29,089 (27,229-31,295)	74,466 (71,084-79,645)	113,335 (108,712-121,660)	106,976 (102,646-114,080)	89,185 (85,827-94,144)	78,142 (75,104-82,079)
Pedestrian road injuries	957 (617-1,665)	449 (314-673)	3,813 (2,911-5,362)	19,250 (15,306-24,590)	23,082 (20,482-26,768)	14,369 (12,937-16,885)	19,091 (16,762-22,653)	29,437 (26,052-35,047)	30,318 (26,902-36,084)	28,370 (25,417-33,539)	26,197 (23,385-30,577)
Cyclist road injuries	-	-	-	1,058 (683-1,474)	2,077 (1,710-2,508)	2,335 (2,017-2,682)	3,208 (2,778-3,609)	3,423 (2,924-3,897)	3,498 (3,031-3,967)	3,460 (2,962-3,905)	3,174 (2,692-3,615)
Motorcyclist road injuries	171 (103-325)	149 (84-270)	953 (695-1,462)	2,057 (1,431-3,459)	2,172 (1,721-2,721)	2,901 (2,466-3,438)	20,024 (17,999-23,474)	31,148 (27,985-36,157)	28,443 (25,080-33,230)	20,235 (17,868-23,651)	16,463 (14,569-19,217)
Motor vehicle road injuries	1,104 (622-1,773)	522 (298-712)	3,940 (2,259-5,292)	10,978 (5,979-14,573)	10,767 (7,733-13,687)	9,083 (7,106-11,269)	31,510 (26,003-37,220)	48,331 (40,118-56,576)	43,801 (36,577-52,266)	36,472 (30,465-42,948)	31,694 (26,566-36,922)
Other road injuries	50 (24-102)	21 (13-36)	222 (142-344)	413 (239-638)	423 (326-562)	400 (312-560)	633 (496-842)	997 (779-1,295)	915 (707-1,179)	648 (502-828)	614 (474-771)
Other transport injuries	425 (218-696)	212 (114-324)	856 (558-1,189)	1,931 (1,046-2,756)	1,906 (1,455-2,351)	1,950 (1,634-2,313)	5,227 (4,310-5,849)	7,044 (5,823-7,834)	7,468 (6,161-8,339)	6,266 (5,216-6,891)	6,160 (5,177-6,807)
Unintentional injuries	7,252 (5,541-8,498)	5,515 (3,991-7,214)	54,155 (43,729-63,835)	139,295 (112,240-159,971)	84,285 (76,586-91,482)	59,403 (55,505-63,853)	58,749 (55,375-62,178)	74,399 (70,562-79,240)	70,898 (66,766-75,202)	69,420 (65,592-73,238)	69,497 (65,232-73,027)
Falls	1,506 (861-2,169)	578 (307-768)	6,151 (3,572-8,127)	11,706 (6,220-15,879)	6,882 (5,426-8,223)	6,342 (5,076-7,297)	6,966 (6,112-7,869)	11,269 (10,037-12,808)	12,695 (11,070-14,267)	13,639 (11,979-15,010)	14,647 (12,838-16,337)
Drowning	463 (343-542)	208 (151-255)	6,596 (5,496-7,514)	77,567 (62,484-88,424)	52,087 (46,849-56,470)	32,106 (29,789-34,618)	25,651 (23,956-27,847)	24,843 (23,231-27,338)	18,870 (17,699-20,545)	16,999 (15,856-18,478)	15,120 (14,101-16,331)
Fire, heat, and hot substances	354 (253-408)	285 (169-347)	4,242 (2,708-5,099)	11,661 (6,402-15,070)	4,019 (2,977-4,741)	2,323 (1,822-2,646)	2,749 (2,163-3,140)	4,573 (3,592-5,193)	4,766 (3,790-5,310)	4,921 (4,107-5,539)	5,223 (4,404-5,850)
Poisonings	405 (162-800)	229 (109-358)	3,724 (1,531-6,869)	6,826 (3,468-10,446)	2,839 (1,939-3,719)	2,452 (1,783-3,302)	2,495 (1,830-3,376)	3,930 (2,928-5,341)	4,045 (3,073-5,402)	3,357 (2,570-4,501)	3,209 (2,447-4,161)
Exposure to mechanical forces	1,727 (1,198-2,252)	1,821 (1,013-3,042)	11,308 (8,545-14,965)	9,214 (5,805-12,477)	5,514 (4,687-6,883)	4,476 (4,068-5,573)	7,031 (6,464-8,258)	10,882 (10,035-12,732)	11,032 (10,224-13,094)	10,284 (9,533-12,015)	10,258 (9,263-11,814)
Unintentional firearm injuries	91 (47-110)	50 (31-67)	588 (377-794)	986 (551-1,489)	832 (631-1,010)	993 (844-1,196)	2,046 (1,773-2,495)	3,044 (2,233-3,550)	2,906 (2,059-3,354)	2,226 (1,590-2,517)	1,842 (1,283-2,084)
Unintentional suffocation	1,232 (758-1,748)	1,594 (803-2,790)	8,711 (6,020-12,202)	3,022 (1,921-5,263)	1,323 (1,024-2,237)	1,177 (943-1,949)	360 (294-555)	495 (405-778)	510 (427-792)	384 (319-581)	333 (266-480)
Other exposure to mechanical forces	404 (318-462)	176 (122-212)	2,009 (1,473-2,453)	5,206 (2,979-6,979)	3,358 (2,759-4,097)	2,305 (2,074-2,674)	4,626 (4,204-5,701)	7,343 (6,700-9,448)	7,616 (6,960-9,905)	7,674 (7,053-9,734)	8,084 (7,339-9,741)
Adverse effects of medical treatment	920 (565-1,107)	657 (359-864)	5,827 (2,973-7,787)	4,996 (1,879-7,997)	1,814 (1,150-2,294)	1,339 (967-1,538)	1,565 (1,092-1,865)	2,368 (1,650-2,800)	2,254 (1,563-2,606)	2,253 (1,545-2,609)	2,329 (1,692-2,712)
Animal contact	367 (287-437)	363 (255-502)	2,065 (1,575-2,643)	5,001 (3,366-8,223)	4,712 (3,589-5,888)	4,315 (3,314-5,210)	3,303 (2,634-3,887)	3,508 (2,900-4,033)	3,563 (2,913-4,100)	3,445 (2,842-3,969)	3,381 (2,744-3,966)
Venomous animal contact	308 (235-376)	313 (211-436)	1,671 (1,238-2,130)	4,053 (2,632-6,502)	3,795 (2,762-4,802)	3,799 (2,868-4,659)	2,675 (2,061-3,221)	2,712 (2,159-3,197)	2,734 (2,150-3,261)	2,783 (2,231-3,296)	2,794 (2,180-3,365)
Non-venomous animal contact	59 (47-72)	50 (35-70)	395 (295-538)	947 (521-1,954)	917 (724-1,184)	516 (397-637)	627 (467-773)	796 (617-955)	829 (649-999)	662 (521-821)	587 (461-730)
Foreign body	887 (525-1,096)	1,016 (576-1,422)	11,035 (6,704-15,809)	6,904 (3,518-12,128)	2,478 (1,703-3,209)	1,832 (1,377-2,124)	1,291 (1,000-1,472)	2,015 (1,540-2,301)	2,106 (1,612-2,436)	2,794 (2,149-3,272)	3,294 (2,505-3,900)
Pulmonary aspiration and foreign body in airway	824 (490-1,017)	960 (552-1,353)	9,810 (6,151-14,496)	5,372 (2,909-10,150)	1,883 (1,287-2,499)	1,453 (1,097-1,770)	1,062 (849-1,319)	1,679 (1,326-2,040)	1,751 (1,375-2,206)	2,192 (1,693-2,642)	2,651 (1,986-3,329)
Foreign body in other body part	63 (19-85)	57 (14-77)	1,226 (273-1,718)	1,532 (351-2,690)	596 (265-761)	379 (174-496)	229 (86-322)	336 (143-455)	354 (148-473)	602 (319-781)	643 (342-819)
Environmental heat and cold exposure	336 (144-413)	243 (87-324)	2,001 (773-2,568)	1,492 (657-2,071)	673 (382-808)	538 (306-633)	1,125 (676-1,376)	1,774 (1,018-2,155)	2,196 (1,282-2,724)	3,195 (1,911-4,154)	4,127 (2,579-5,824)
Other unintentional injuries	287 (253-331)	116 (94-137)	1,205 (967-1,499)	3,928 (2,559-4,983)	3,266 (2,809-3,920)	3,681 (3,251-4,155)	6,574 (5,856-7,583)	9,237 (8,306-10,877)	9,371 (8,530-11,192)	8,533 (7,744-9,731)	7,908 (7,158-8,863)
Self-harm and interpersonal violence	1,051 (721-1,260)	272 (181-331)	2,983 (2,087-3,697)	5,014 (3,454-6,192)	4,388 (2,958-5,208)	12,551 (9,864-13,914)	61,475 (52,200-66,029)	106,979 (91,468-114,409)	107,672 (90,857-115,128)	99,013 (84,137-106,366)	87,146 (73,520-95,585)

Cause	40 to 44	45 to 49	50 to 54	55 to 59	60 to 64	65 to 69	70 to 74	75 to 79	80 to 84	85 to 89	90 to 94	95 plus
Ischaemic stroke	8,836 (8,239-9,396)	16,560 (15,345-17,633)	26,914 (24,839-28,532)	50,963 (47,539-54,224)	103,617 (98,361-108,535)	150,262 (143,240-157,516)	164,624 (157,511-172,148)	172,603 (164,167-180,748)	153,035 (144,397-161,568)	91,401 (85,390-97,688)	32,891 (30,597-35,326)	7,947 (7,213-8,573)
Hemorrhagic stroke	49,436 (46,917-52,918)	63,888 (60,981-68,248)	87,714 (83,639-93,995)	130,996 (125,178-140,003)	164,300 (157,087-174,941)	193,642 (185,046-206,509)	200,635 (191,119-214,340)	160,693 (152,433-173,033)	116,109 (109,155-124,683)	56,929 (53,029-61,533)	18,691 (17,240-20,377)	4,136 (3,757-4,511)
Hypertensive heart disease	7,089 (5,147-8,250)	9,358 (6,938-11,013)	12,889 (9,336-15,041)	19,971 (14,421-23,421)	28,182 (19,649-32,533)	35,323 (24,547-41,170)	40,159 (27,508-46,220)	40,298 (27,247-46,349)	34,020 (24,064-39,066)	20,596 (14,631-23,781)	8,645 (6,339-9,973)	2,237 (1,579-2,655)
Cardiomyopathy and myocarditis	10,351 (8,276-12,435)	10,956 (8,790-13,105)	10,002 (8,212-11,747)	14,347 (11,862-16,841)	13,109 (10,627-15,174)	13,799 (10,878-15,352)	12,265 (8,430-13,605)	10,478 (7,033-11,644)	11,178 (6,326-13,063)	9,014 (4,629-10,728)	3,874 (1,944-4,638)	1,062 (557-1,262)
Myocarditis	902 (706-1,237)	645 (498-838)	562 (414-720)	799 (579-1,024)	744 (519-920)	833 (566-1,007)	940 (631-1,124)	903 (628-1,092)	986 (615-1,288)	906 (552-1,228)	488 (290-657)	175 (95-242)
Alcoholic cardiomyopathy	6,069 (4,388-7,744)	6,712 (5,018-8,501)	5,401 (4,230-6,898)	7,943 (6,284-9,942)	5,932 (4,758-7,530)	5,093 (4,218-6,613)	2,913 (2,033-3,459)	1,701 (1,127-1,988)	1,359 (751-1,682)	719 (313-926)	263 (113-339)	77 (35-99)
Other cardiomyopathy	3,380 (2,705-4,298)	3,599 (2,858-4,448)	4,038 (3,197-4,700)	5,605 (4,372-6,856)	6,433 (4,755-7,208)	7,872 (5,622-8,724)	8,411 (5,506-9,540)	7,874 (4,940-8,933)	8,833 (4,723-10,584)	7,388 (3,593-9,006)	3,123 (1,485-3,859)	810 (398-1,020)
Atrial fibrillation and flutter	139 (102-183)	243 (175-326)	381 (281-499)	704 (413-1,064)	1,410 (922-1,986)	2,764 (1,706-4,178)	4,744 (3,387-6,605)	6,066 (3,838-9,188)	11,364 (8,534-14,622)	10,850 (8,189-14,215)	4,543 (3,438-5,944)	993 (759-1,277)
Aortic aneurysm	1,554 (1,408-1,704)	2,009 (1,855-2,169)	2,701 (2,531-2,894)	4,490 (4,235-4,769)	7,312 (6,910-7,748)	10,658 (10,184-11,127)	12,996 (12,464-13,583)	12,046 (11,562-12,567)	10,458 (10,053-10,934)	6,133 (5,891-6,429)	2,232 (2,145-2,334)	446 (428-466)
Peripheral vascular disease	113 (67-147)	205 (114-272)	365 (216-470)	825 (476-1,075)	1,352 (838-1,743)	2,072 (1,330-2,665)	2,463 (1,663-3,306)	2,406 (1,595-3,142)	2,883 (1,990-3,873)	2,140 (1,516-3,003)	1,015 (719-1,377)	310 (217-434)
Endocarditis	1,566 (1,126-1,963)	1,360 (1,021-1,694)	1,438 (1,114-1,889)	1,763 (1,338-2,220)	2,045 (1,588-2,715)	2,435 (1,889-3,145)	2,452 (1,927-3,138)	2,377 (1,839-3,046)	2,086 (1,647-2,686)	1,313 (997-1,726)	562 (435-733)	149 (117-200)
Other cardiovascular and circulatory diseases	5,835 (4,306-6,871)	6,646 (4,890-7,734)	8,441 (6,349-9,600)	12,289 (9,188-14,119)	17,871 (12,614-19,747)	23,301 (16,664-25,774)	28,228 (19,694-31,251)	28,124 (19,418-31,539)	24,974 (18,562-27,618)	15,602 (12,535-16,891)	6,453 (5,471-7,000)	1,538 (1,337-1,693)
Chronic respiratory diseases	29,753 (28,246-31,278)	41,657 (39,761-43,541)	68,946 (65,255-72,137)	120,167 (114,536-124,964)	210,970 (201,025-219,270)	286,630 (273,547-297,376)	355,855 (336,198-368,669)	341,798 (324,562-353,897)	265,440 (251,777-275,590)	144,182 (136,127-150,314)	50,369 (47,829-52,668)	11,462 (10,858-12,025)
Chronic obstructive pulmonary disease	18,568 (15,567-21,383)	28,099 (24,148-31,770)	49,880 (42,969-55,918)	92,562 (82,301-101,475)	172,617 (158,010-186,846)	242,508 (223,690-258,679)	310,553 (290,072-328,230)	303,603 (285,478-319,613)	239,329 (226,271-251,177)	130,694 (122,986-137,733)	45,826 (43,372-48,314)	10,468 (9,889-11,050)
Pneumoconiosis	770 (616-1,227)	764 (620-1,259)	908 (733-1,402)	1,334 (1,120-2,061)	2,263 (1,916-3,443)	3,098 (2,692-4,332)	3,543 (3,107-4,678)	2,926 (2,592-3,791)	2,082 (1,893-2,468)	1,019 (923-1,197)	299 (268-354)	63 (58-74)
Silicosis	535 (417-947)	485 (372-860)	542 (420-960)	756 (602-1,288)	1,278 (1,048-2,062)	1,815 (1,501-2,736)	1,949 (1,653-2,683)	1,585 (1,359-2,082)	1,080 (932-1,331)	478 (410-573)	115 (97-138)	20 (17-24)
Asbestosis	36 (25-54)	34 (23-50)	47 (29-67)	83 (45-118)	133 (76-208)	207 (119-370)	262 (168-515)	241 (154-475)	205 (138-404)	112 (77-225)	34 (23-67)	6 (4-12)
Coal workers pneumoconiosis	73 (38-113)	96 (51-143)	128 (65-190)	197 (111-280)	360 (194-495)	560 (280-748)	761 (382-968)	619 (321-774)	511 (296-617)	257 (142-307)	70 (35-84)	13 (6-15)
Other pneumoconiosis	126 (83-214)	148 (98-258)	190 (123-332)	298 (201-496)	492 (326-815)	515 (347-847)	571 (386-946)	482 (325-791)	285 (203-481)	171 (126-282)	79 (60-123)	25 (20-35)
Asthma	8,781 (5,835-11,689)	10,813 (6,983-14,525)	15,694 (9,538-22,872)	22,494 (13,929-31,846)	31,118 (18,774-44,818)	34,578 (21,051-50,698)	34,586 (21,618-52,477)	28,947 (17,989-43,893)	18,697 (12,642-29,304)	9,323 (6,434-13,470)	3,054 (2,111-4,648)	665 (470-1,027)
Interstitial lung disease and pulmonary sarcoidosis	673 (391-1,163)	925 (554-1,518)	1,405 (825-2,352)	2,265 (1,347-3,681)	3,270 (1,995-5,136)	4,618 (3,050-6,951)	5,273 (3,574-7,757)	4,856 (3,346-6,937)	3,942 (2,886-5,743)	2,203 (1,595-3,262)	760 (564-1,143)	155 (117-227)
Other chronic respiratory diseases	960 (531-1,359)	1,056 (583-1,465)	1,060 (579-1,419)	1,512 (850-2,067)	1,701 (998-2,317)	1,828 (1,033-2,415)	1,900 (1,123-2,494)	1,466 (848-1,876)	1,390 (775-1,764)	942 (530-1,196)	431 (247-549)	110 (68-139)
Cirrhosis and other chronic liver diseases	57,187 (53,541-61,105)	62,868 (59,260-66,769)	65,185 (61,724-71,373)	72,534 (68,614-77,700)	73,379 (69,404-80,113)	64,328 (60,651-68,954)	49,223 (46,177-52,268)	33,478 (30,853-35,569)	21,078 (19,093-22,362)	10,356 (9,387-11,014)	3,265 (2,913-3,491)	787 (695-845)
Cirrhosis and other chronic liver diseases due to hepatitis B	20,084 (17,460-22,602)	19,905 (16,720-23,198)	20,005 (16,728-23,570)	22,743 (19,391-26,724)	23,913 (20,231-28,437)	20,849 (17,663-24,925)	16,216 (13,389-19,716)	11,581 (9,657-13,397)	6,542 (5,318-7,801)	2,803 (2,369-3,268)	769 (609-930)	167 (120-220)
Cirrhosis and other chronic liver diseases due to hepatitis C	15,305 (12,940-17,943)	17,559 (14,489-21,087)	18,397 (15,249-22,142)	20,129 (16,848-23,788)	20,127 (16,619-24,539)	18,045 (15,238-21,183)	13,886 (11,726-16,198)	8,832 (7,269-10,725)	5,756 (4,704-7,004)	2,945 (2,375-3,426)	939 (714-1,147)	225 (158-291)
Cirrhosis and other chronic liver diseases due to alcohol use	15,910 (13,798-18,388)	18,794 (15,807-22,040)	19,706 (16,614-23,070)	21,309 (17,658-25,208)	20,779 (16,759-24,934)	18,335 (15,477-21,368)	13,695 (11,509-16,165)	9,050 (7,641-10,551)	6,017 (5,028-7,019)	3,096 (2,700-3,506)	1,034 (882-1,190)	258 (209-309)
Cirrhosis and other chronic liver diseases due to other causes	5,887 (4,272-7,920)	6,610 (4,700-8,937)	7,076 (5,124-9,465)	8,353 (6,086-11,232)	8,561 (6,097-11,702)	7,100 (5,115-9,339)	5,425 (3,950-7,284)	4,014 (2,891-5,216)	2,764 (1,996-3,623)	1,513 (1,166-1,880)	523 (397-675)	137 (97-187)
Digestive diseases	25,389 (21,614-27,373)	27,481 (23,080-29,577)	29,450 (24,774-31,965)	38,392 (32,427-41,569)	45,677 (38,791-49,353)	51,863 (43,911-56,142)	52,286 (44,894-56,390)	46,537 (39,947-50,404)	37,234 (32,635-40,224)	22,972 (19,969-24,841)	9,344 (8,244-10,114)	2,301 (2,032-2,514)
Peptic ulcer disease	10,204 (8,619-11,299)	11,548 (9,627-12,582)	13,309 (10,970-14,564)	17,167 (13,972-18,814)	20,555 (17,132-22,417)	22,029 (17,962-24,118)	21,738 (18,153-23,918)	18,800 (15,934-20,728)	13,587 (11,526-15,012)	7,834 (6,697-8,610)	2,893 (2,470-3,203)	668 (580-744)
Gastritis and duodenitis	1,110 (927-1,299)	1,262 (1,029-1,486)	1,424 (1,146-1,685)	1,867 (1,423-2,217)	2,196 (1,740-2,583)	2,130 (1,671-2,454)	2,183 (1,752-2,545)	2,058 (1,612-2,411)	1,477 (1,161-1,749)	876 (691-1,046)	361 (280-426)	98 (78-115)
Appendicitis	1,194 (946-1,551)	951 (733-1,225)	988 (759-1,314)	1,144 (910-1,500)	1,303 (1,008-1,735)	1,260 (1,013-1,644)	1,280 (1,022-1,698)	1,014 (840-1,356)	755 (613-1,014)	434 (351-580)	167 (139-219)	41 (35-54)
Paralytic ileus and intestinal obstruction	3,579 (2,151-4,286)	4,020 (2,537-4,785)	4,112 (2,721-4,987)	5,579 (3,760-6,632)	6,293 (4,282-7,472)	7,761 (5,679-9,072)	7,401 (5,466-9,020)	6,465 (5,058-8,174)	5,627 (4,473-6,921)	3,807 (2,926-4,827)	1,645 (1,282-2,039)	411 (319-526)
Inguinal, femoral, and abdominal hernia	961 (586-1,206)	893 (575-1,143)	1,181 (755-1,554)	1,534 (1,036-2,006)	1,831 (1,206-2,395)	2,384 (1,579-3,111)	2,651 (1,716-3,457)	2,657 (1,754-3,519)	2,085 (1,462-2,670)	1,412 (1,031-1,790)	646 (470-809)	181 (130-224)
Inflammatory bowel disease	605 (430-752)	616 (426-756)	633 (461-739)	866 (623-1,014)	984 (745-1,139)	1,190 (894-1,354)	1,282 (968-1,444)	1,231 (945-1,357)	1,142 (865-1,259)	743 (535-828)	325 (241-360)	83 (61-92)
Vascular intestinal disorders	554 (437-667)	780 (608-926)	1,035 (818-1,248)	1,885 (1,594-2,355)	2,593 (2,145-3,164)	3,923 (3,319-4,963)	4,343 (3,558-5,300)	3,841 (3,147-4,502)	3,437 (2,643-3,957)	2,105 (1,499-2,370)	841 (625-946)	189 (140-223)
Gallbladder and biliary diseases	893 (718-1,031)	996 (747-1,137)	1,205 (894-1,405)	1,722 (1,292-1,958)	2,528 (1,914-2,907)	3,406 (2,640-3,922)	3,755 (3,047-4,232)	3,756 (3,051-4,225)	3,563 (2,911-4,015)	2,403 (2,034-2,734)	1,031 (875-1,159)	248 (207-284)
Pancreatitis	4,699 (3,965-5,855)	4,550 (3,816-5,736)	3,468 (2,905-4,372)	4,008 (3,413-5,036)	3,665 (3,039-4,660)	3,737 (3,000-4,736)	3,085 (2,439-3,807)	2,446 (1,900-3,096)	1,714 (1,328-2,088)	977 (721-1,182)	328 (233-394)	76 (57-94)
Other digestive diseases	1,591 (1,332-1,999)	1,865 (1,543-2,282)	2,094 (1,719-2,595)	2,619 (2,181-3,206)	3,728 (3,093-4,590)	4,044 (3,373-4,899)	4,569 (3,822-5,491)	4,268 (3,511-5,127)	3,846 (3,149-4,626)	2,381 (1,864-2,933)	1,109 (871-1,382)	307 (237-374)
Neurological disorders	6,929 (6,597-7,243)	7,188 (6,725-7,683)	8,076 (7,217-9,217)	11,824 (9,993-14,003)	18,181 (15,062-22,219)	31,188 (25,019-38,628)	52,901 (41,579-67,509)	80,950 (64,004-102,062)	104,397 (83,930-131,869)	91,324 (73,232-112,011)	47,898 (38,858-56,986)	11,665 (9,185-14,703)
Alzheimer's disease and other dementias	40 (17-79)	777 (448-1,197)	2,499 (1,690-3,581)	5,188 (3,421-7,326)	10,861 (7,840-14,992)	21,653 (15,758-29,080)	40,573 (29,367-54,969)	66,218 (49,310-86,696)	88,623 (68,300-115,650)	81,843 (62,847-102,588)	44,847 (35,822-53,768)	11,093 (8,620-14,183)
Parkinson's disease	143 (81-237)	204 (114-351)	327 (192-521)	664 (399-987)	1,551 (1,072-2,175)	3,612 (2,561-4,830)	7,372 (5,163-9,850)	11,192 (8,115-14,816)	13,211 (9,924-17,631)	8,219 (6,280-10,757)	2,666 (2,005-3,505)	486 (345-671)
Epilepsy	4,880 (4,623-5,104)	4,003 (3,775-4,225)	2,854 (2,694-3,029)	2,890 (2,726-3,048)	2,440 (2,282-2,585)	2,220 (2,048-2,399)	1,637 (1,517-1,756)	1,140 (1,048-1,224)	867 (780-947)	445 (403-487)	156 (143-170)	40 (37-44)
Multiple sclerosis	633 (527-779)	643 (527-767)	632 (534-763)	683 (582-857)	617 (529-770)	569 (493-684)	426 (364-514)	247 (204-299)	148 (121-166)	58 (42-64)	15 (11-16)	2 (2-2)
Motor neuron disease	470 (447-504)	655 (625-699)	791 (754-853)	1,164 (1,115-1,246)	1,410 (1,353-1,482)	1,704 (1,640-1,794)	1,494 (1,435-1,567)	978 (936-1,020)	613 (581-639)	238 (226-248)	46 (43-48)	7 (7-7)
Other neurological disorders	763 (712-804)	904 (841-954)	974 (890-1,028)	1,234 (1,139-1,303)	1,303 (1,223-1,366)	1,430 (1,347-1,491)	1,398 (1,315-1,466)	1,175 (1,101-1,230)	937 (887-985)	520 (492-547)	169 (159-178)	36 (34-38)
Mental and substance use disorders	27,171 (24,162-30,494)	23,903 (21,271-26,816)	18,279 (16,402-20,338)	19,933 (17,408-22,974)	14,369 (12,521-15,918)	10,203 (8,797-11,045)	5,709 (4,745-6,116)	3,663 (3,012-3,951)	1,974 (1,719-2,120)	1,000 (861-1,069)	349 (290-378)	93 (79-99)
Alcohol use disorders	18,255 (15,919-21,106)	18,117 (16,105-20,769)	14,227 (12,611-16,285)	15,562 (13,559-18,290)	10,894 (9,621-12,308)	7,345 (6,470-8,079)	3,705 (3,260-4,108)	2,119 (1,783-2,481)	1,007 (882-1,129)	455 (398-510)	134 (115-152)	36 (31-40)
Drug use disorders	8,890 (6,961-9,991)	5,763 (4,532-6,533)	4,053 (3,081-4,570)	4,370 (3,333-5,093)	3,475 (2,409-3,988)	2,858 (2,030-3,257)	2,003 (1,299-2,299)	1,544 (1,042-1,760)	967 (746-1,074)	545 (424-608)	215 (163-240)	56 (45-62)
Opioid use disorders	6,385 (4,606-7,424)	4,009 (2,967-4,678)	2,674 (1,816-3,165)	3,001 (2,096-3,629)	2,264 (1,410-2,700)	1,850 (1,178-2,193)	880 (534-1,056)	683 (425-810)	395 (270-462)	234 (156-271)	98 (64-112)	26 (18-29)
Cocaine use disorders	521 (343-719)	366 (246-474)	345 (215-438)	344 (209-464)	285 (161-375)	249 (147-325)	132 (70-176)	100 (57-131)	16 (9-22)	8 (5-11)	3 (2-4)	1 (1-1)
Amphetamine use disorders	258 (161-359)	176 (111-248)	141 (85-185)	145 (80-202)	123 (69-167)	105 (58-142)	41 (23-57)	32 (17-44)	7 (4-10)	4 (2-5)	1 (1-2)	0 (0-0)
Other drug use disorders	1,725 (1,164-1,986)	1,212 (816-1,411)	893 (585-1,040)	881 (569-1,065)	804 (501-959)	654 (399-781)	950 (549-1,119)	729 (437-855)	550 (403-618)	299 (226-335)	113 (84-127)	29 (22-32)
Eating disorders	26 (21-32)	22 (18-27)	0 (0-0)	0 (0-0)	-	-	-	-	-	-	-	-
Anorexia nervosa	23 (18-29)	16 (12-19)	0 (0-0)	0 (0-0)	-	-	-	-	-	-	-	-
Bulimia nervosa	3 (2-5)	7 (5-10)	0 (0-0)	0 (0-0)	-	-	-	-	-	-	-	-
Diabetes, urogenital, blood, and endocrine diseases	34,296 (32,565-36,342)	39,608 (37,595-41,664)	48,386 (46,111-51,095)	67,061 (64,525-70,561)	86,305 (82,555-91,117)	101,761 (96,931-107,835)	106,022 (101,137-111,926)	97,250 (92,863-103,147)	79,835 (76,238-84,507)	51,664 (49,241-54,724)	21,448 (20,454-22,787)	5,627 (5,366-5,974)
Diabetes mellitus	11,071 (10,645-11,539)	14,883 (14,336-15,429)	20,687 (19,966-21,441)	30,731 (29,701-31,848)	41,469 (40,068-42,896)	49,045 (47,222-50,724)	50,614 (48,616-52,592)	44,814 (42,972-46,541)	34,059 (32,357-35,553)	19,040 (18,092-19,927)	6,685 (6,365-6,979)	1,418 (1,351-1,483)
Acute glomerulonephritis	498 (423-580)	506 (443-577)	447 (387-512)	641 (572-712)	679 (598-779)	750 (674-848)	689 (604-785)	636 (555-731)	391 (346-445)	197 (172-227)	65 (56-75)	23 (19-30)
Chronic kidney disease	16,449 (14,891-18,093)	17,338 (15,511-19,204)	20,578 (18,783-23,485)	27,430 (25,116-30,704)	33,719 (31,404-38,094)	38,729 (35,830-44,638)	40,221 (37,428-46,030)	37,738 (35,199-43,483)	32,256 (30,336-36,740)	22,573 (21,187-25,507)	9,996 (9,324-11,363)	2,809 (2,610-3,160)
Chronic kidney disease due to diabetes mellitus	7,485 (5,737-9,115)	8,669 (6,894-10,512)	10,662 (8,179-13,024)	14,391 (11,288-17,124)	18,166 (14,809-21,647)	20,067 (15,692-24,287)	19,522 (15,344-23,564)	16,588 (12,885-20,610)	11,881 (8,927-14,872)	6,985 (5,132-9,142)	2,903 (2,034-3,811)	826 (550-1,109)
Chronic kidney disease due to hypertension	2,218 (1,691-2,874)	2,577 (1,953-3,318)	3,429 (2,580-4,511)	5,066 (3,898-6,671)	6,943 (5,474-8,895)	8,751 (6,807-11,163)	10,092 (7,904-12,824)	10,837 (8,380-13,780)	10,300 (8,192-12,917)	8,494 (6,834-10,443)	4,218 (3,419-5,148)	1,237 (976-1,519)
Chronic kidney disease due to glomerulonephritis	3,037 (1,931-4,253)	2,565 (1,668-3,609)	2,523 (1,665-3,594)	2,942 (2,022-4,122)	3,005 (1,958-4,313)	3,415 (2,236-4,960)	3,568 (2,353-5,163)	3,530 (2,329-5,202)	3,472 (2,408-4,756)	2,211 (1,533-3,136)	858 (585-1,169)	235 (139-381)
Chronic kidney disease due to other causes	3,709 (2,547-5,053)	3,527 (2,320-5,050)	3,963 (2,567-5,766)	5,031 (3,351-7,394)	5,606 (3,609-8,236)	6,497 (4,331-9,317)	7,040 (4,776-9,767)	6,783 (4,634-9,424)	6,602 (4,666-8,858)	4,883 (3,360-6,651)	2,017 (1,434-2,750)	512 (327-748)
Urinary diseases and male infertility	2,834 (2,662-2,983)	3,173 (2,967-3,375)	3,482 (3,259-3,692)	4,536 (4,278-4,792)	6,333 (5,962-6,680)	8,431 (7,940-8,870)	9,125 (8,570-9,570)	8,844 (8,323-9,264)	8,579 (8,104-8,957)	6,430 (6,117-6,706)	3,206 (3,055-3,349)	965 (924-1,006)
Interstitial nephritis and urinary tract infections	1,347 (1,122-1,577)	1,476 (1,188-1,741)	1,669 (1,323-1,975)	2,326 (1,904-2,686)	3,217 (2,581-3,745)	4,467 (3,698-5,176)	4,952 (4,161-5,662)	5,144 (4,390-5,784)	5,123 (4,503-5,687)	4,133 (3,636-4,536)	2,101 (1,834-2,293)	684 (611-738)
Urolithiasis	450 (278-573)	485 (292-611)	550 (335-683)	618 (390-763)	892 (550-1,112)	1,265 (789-1,564)	1,226 (732-1,483)	1,089 (670-1,322)	862 (554-1,037)	543 (337-668)	228 (140-279)	58 (37-76)
Other urinary diseases	1,038 (759-1,339)	1,213 (881-1,586)	1,263 (894-1,685)	1,592 (1,169-2,071)	2,224 (1,609-3,028)	2,699 (1,980-3,691)	2,947 (2,243-3,925)	2,611 (2,052-3,501)	2,595 (2,098-3,312)	1,755 (1,401-2,260)	878 (717-1,136)	223 (182-292)
Gynecological diseases	-	-	-	-	-	-	-	-	-	-	-	-
Uterine fibroids	-	-	-	-	-	-	-	-	-	-	-	-
Polycystic ovarian syndrome	-	-	-	-	-	-	-	-	-	-	-	-
Endometriosis	-	-	-	-	-	-	-	-	-	-	-	-
Genital prolapse	-	-	-	-	-	-	-	-	-	-	-	-
Other gynecological diseases	-	-	-	-	-	-	-	-	-	-	-	-
Hemoglobinopathies and hemolytic anaemias	1,651 (1,316-2,262)	1,789 (1,489-2,634)	1,283 (1,029-1,695)	1,432 (1,171-1,888)	1,607 (1,302-2,126)	1,912 (1,642-2,513)	2,301 (1,971-3,029)	2,558 (2,188-3,400)	2,082 (1,766-2,493)	1,742 (1,418-2,042)	744 (587-848)	197 (155-223)
Thalassemias	18 (16-23)	17 (15-21)	14 (12-18)	8 (7-10)	3 (3-4)	1 (1-1)	0 (0-1)	0 (0-0)	0 (0-0)	0 (0-0)	0 (0-0)	0 (0-0)
Sickle cell disorders	576 (454-842)	537 (405-864)	196 (155-281)	81 (65-119)	43 (35-57)	30 (25-37)	18 (14-21)	7 (5-7)	2 (2-3)	1 (0-1)	0 (0-0)	0 (0-0)
G6PD deficiency	642 (490-864)	781 (622-1,136)	536 (411-719)	484 (370-659)	428 (323-595)	305 (240-438)	246 (192-355)	171 (132-249)	62 (48-84)	31 (23-41)	7 (5-9)	1 (1-2)
Other hemoglobinopathies and hemolytic anaemias	414 (329-549)	454 (368-631)	536 (427-692)	859 (708-1,117)	1,133 (912-1,488)	1,575 (1,353-2,071)	2,037 (1,749-2,666)	2,380 (2,035-3,161)	2,017 (1,712-2,409)	1,710 (1,391-2,002)	737 (580-839)	195 (153-221)
Endocrine, metabolic, blood, and immune disorders	1,792 (1,432-2,077)	1,919 (1,542-2,245)	1,911 (1,552-2,173)	2,291 (1,872-2,666)	2,498 (2,123-2,961)	2,894 (2,409-3,532)	3,073 (2,590-3,686)	2,661 (2,239-3,239)	2,468 (1,912-2,976)	1,681 (1,309-1,966)	753 (603-872)	215 (175-253)
Musculoskeletal disorders	833 (693-949)	915 (775-1,046)	867 (718-961)	1,166 (989-1,299)	1,571 (1,305-1,718)	2,005 (1,696-2,204)	2,234 (1,887-2,426)	2,142 (1,804-2,355)	1,750 (1,489-1,923)	1,202 (1,052-1,333)	507 (421-563)	134 (118-148)
Rheumatoid arthritis	189 (134-232)	234 (172-283)	336 (242-392)	519 (384-589)	827 (641-941)	1,087 (834-1,235)	1,243 (963-1,404)	1,167 (913-1,338)	835 (675-962)	469 (361-552)	162 (121-192)	39 (30-45)
Other musculoskeletal disorders	643 (544-742)	681 (588-794)	530 (455-610)	647 (559-755)	744 (622-869)	919 (774-1,084)	991 (811-1,151)	975 (796-1,147)	915 (734-1,047)	733 (610-839)	345 (281-388)	96 (81-107)
Other non-communicable diseases	2,841 (2,205-3,291)	2,273 (1,711-2,725)	1,959 (1,441-2,368)	2,418 (1,691-2,883)	2,711 (1,855-3,265)	2,915 (1,975-3,632)	2,408 (1,362-3,206)	2,606 (1,491-3,573)	2,310 (1,321-3,200)	1,794 (1,007-2,476)	906 (502-1,232)	271 (147-374)
Congenital anomalies	2,036 (1,628-2,571)	1,235 (1,016-1,559)	942 (801-1,193)	953 (832-1,181)	973 (843-1,282)	826 (702-1,055)	-	-	-	-	-	-
Neural tube defects	89 (64-121)	64 (48-86)	48 (36-65)	44 (31-66)	34 (24-56)	32 (24-48)	-	-	-	-	-	-
Congenital heart anomalies	1,511 (1,211-1,931)	573 (465-708)	398 (337-500)	394 (336-492)	361 (302-465)	304 (251-389)	-	-	-	-	-	-
Orofacial clefts	-	-	-	-	-	-	-	-	-	-	-	-
Down's syndrome	112 (84-166)	211 (158-326)	173 (140-270)	177 (143-267)	149 (120-238)	65 (53-109)	-	-	-	-	-	-
Other chromosomal abnormalities	19 (11-24)	19 (13-29)	21 (15-34)	20 (13-28)	16 (10-24)	11 (8-19)	-	-	-	-	-	-
Congenital musculoskeletal and limb anomalies	22 (13-30)	24 (15-32)	21 (14-27)	17 (8-20)	25 (13-30)	32 (13-39)	-	-	-	-	-	-
Urogenital congenital anomalies	62 (41-82)	87 (57-121)	88 (60-121)	111 (77-145)	147 (101-206)	174 (124-232)	-	-	-	-	-	-
Digestive congenital anomalies	23 (14-27)	32 (22-40)	24 (16-29)	29 (20-34)	39 (29-52)	36 (26-45)	-	-	-	-	-	-
Other congenital anomalies	198 (151-253)	224 (168-296)	168 (129-221)	161 (130-208)	204 (160-277)	171 (136-241)	-	-	-	-	-	-
Skin and subcutaneous diseases	805 (417-1,047)	1,037 (565-1,421)	1,017 (538-1,346)	1,464 (771-1,905)	1,739 (936-2,308)	2,089 (1,174-2,816)	2,408 (1,362-3,206)	2,606 (1,491-3,573)	2,310 (1,321-3,200)	1,794 (1,007-2,476)	906 (502-1,232)	271 (147-374)
Cellulitis	124 (57-200)	148 (72-249)	187 (75-269)	263 (100-377)	251 (114-372)	265 (98-406)	285 (125-487)	302 (122-489)	262 (107-482)	191 (83-348)	95 (44-180)	27 (11-53)
Pyoderma	546 (257-717)	711 (352-935)	659 (327-902)	946 (457-1,250)	1,141 (536-1,498)	1,278 (631-1,702)	1,343 (674-1,751)	1,356 (700-1,778)	903 (491-1,277)	672 (356-920)	319 (172-450)	90 (46-125)
Decubitus ulcer	111 (61-174)	148 (88-244)	143 (86-235)	217 (130-353)	300 (178-475)	483 (297-748)	712 (425-1,051)	866 (510-1,314)	1,052 (547-1,458)	856 (434-1,169)	454 (237-614)	143 (71-194)
Other skin and subcutaneous diseases	24 (14-41)	30 (18-52)	29 (16-52)	38 (22-63)	48 (27-83)	63 (39-108)	68 (38-118)	83 (48-135)	92 (50-147)	75 (40-117)	39 (19-60)	13 (6-20)
Sudden infant death syndrome	-	-	-	-	-	-	-	-	-	-	-	-
Injuries	219,704 (207,825-229,324)	179,634 (169,656-187,589)	141,334 (132,020-147,389)	143,796 (134,548-149,935)	121,451 (113,257-126,787)	109,975 (100,957-114,700)	89,337 (81,042-93,628)	70,726 (63,513-74,463)	53,296 (47,571-56,167)	32,316 (28,536-34,321)	12,675 (11,111-13,551)	3,311 (2,941-3,530)
Transport injuries	73,477 (70,707-76,439)	56,717 (54,579-58,989)	44,919 (43,205-46,807)	42,934 (41,240-44,519)	36,246 (34,829-37,733)	31,503 (30,224-32,974)	24,821 (23,702-25,999)	18,064 (17,221-18,841)	11,405 (10,880-11,976)	5,493 (5,241-5,767)	1,682 (1,600-1,772)	388 (366-414)
Road injuries	67,853 (65,278-70,776)	52,121 (50,198-54,329)	41,143 (39,520-42,912)	39,334 (37,725-40,879)	33,603 (32,198-35,015)	29,244 (27,997-30,647)	23,209 (22,170-24,285)	16,859 (16,093-17,656)	10,711 (10,227-11,294)	5,138 (4,898-5,429)	1,545 (1,465-1,626)	346 (326-370)
Pedestrian road injuries	24,031 (21,223-27,834)	20,616 (18,402-23,582)	17,064 (15,011-19,391)	16,955 (14,946-19,250)	16,231 (14,621-18,501)	14,499 (13,046-16,280)	11,735 (10,578-13,276)	9,543 (8,766-10,696)	5,968 (5,443-6,625)	2,931 (2,657-3,223)	825 (737-930)	192 (173-219)
Cyclist road injuries	3,517 (2,976-4,017)	3,130 (2,697-3,540)	2,986 (2,567-3,382)	2,778 (2,394-3,140)	2,674 (2,280-3,009)	2,360 (2,033-2,683)	1,858 (1,618-2,103)	1,101 (965-1,233)	673 (592-768)	265 (235-318)	66 (57-79)	1 (1-1)
Motorcyclist road injuries	13,569 (11,914-15,453)	8,734 (7,694-9,965)	6,068 (5,313-7,108)	5,232 (4,593-6,176)	3,093 (2,686-3,838)	2,455 (2,121-2,945)	1,689 (1,490-2,046)	904 (779-1,031)	554 (485-642)	195 (163-238)	47 (39-63)	11 (9-15)
Motor vehicle road injuries	26,162 (21,771-30,624)	19,064 (15,635-22,356)	14,454 (11,803-17,179)	13,858 (11,292-16,375)	11,223 (9,003-13,301)	9,503 (7,587-11,234)	7,571 (6,097-8,936)	5,045 (4,072-5,883)	3,332 (2,750-3,916)	1,654 (1,372-1,955)	573 (476-661)	133 (111-152)
Other road injuries	573 (443-710)	577 (451-725)	571 (438-749)	512 (393-659)	382 (286-500)	427 (325-571)	356 (270-478)	265 (192-389)	184 (132-289)	92 (66-144)	34 (24-58)	9 (6-16)
Other transport injuries	5,624 (4,731-6,216)	4,595 (3,847-5,093)	3,777 (3,097-4,269)	3,600 (3,009-4,035)	2,643 (2,150-2,971)	2,260 (1,861-2,569)	1,612 (1,299-1,832)	1,205 (962-1,413)	694 (581-825)	355 (296-439)	137 (115-172)	41 (35-51)
Unintentional injuries	67,022 (63,004-71,188)	60,269 (56,503-64,033)	49,525 (45,978-52,512)	54,826 (51,148-58,852)	49,696 (45,888-53,115)	48,076 (43,825-51,328)	41,711 (37,158-44,626)	36,738 (32,445-39,528)	31,157 (27,566-33,399)	21,620 (18,735-23,341)	9,469 (8,157-10,249)	2,607 (2,285-2,812)
Falls	15,872 (14,289-18,096)	14,756 (13,099-16,827)	13,453 (12,161-14,771)	15,939 (14,280-17,692)	16,590 (14,526-18,265)	18,229 (16,020-20,077)	18,493 (15,760-20,564)	18,765 (15,789-20,787)	17,014 (14,676-18,929)	12,965 (10,936-14,272)	5,955 (4,955-6,566)	1,596 (1,355-1,775)
Drowning	13,194 (12,324-14,186)	10,250 (9,515-10,994)	8,247 (7,663-8,948)	8,050 (7,540-8,706)	6,805 (6,364-7,351)	5,663 (5,305-6,234)	4,449 (4,133-4,851)	2,971 (2,754-3,272)	2,127 (1,933-2,349)	1,057 (985-1,173)	295 (277-326)	65 (61-71)
Fire, heat, and hot substances	4,952 (4,265-5,596)	4,562 (3,968-5,080)	3,772 (3,270-4,219)	4,320 (3,720-4,825)	3,987 (3,456-4,482)	3,737 (3,222-4,215)	3,330 (2,833-3,772)	2,857 (2,373-3,248)	2,697 (2,242-2,974)	1,813 (1,530-2,008)	791 (657-859)	229 (188-249)
Poisonings	3,322 (2,551-4,261)	2,736 (2,037-3,470)	2,217 (1,701-2,867)	2,242 (1,783-2,935)	2,097 (1,515-2,711)	1,706 (1,254-2,232)	1,384 (995-1,793)	925 (650-1,238)	626 (454-871)	307 (238-416)	105 (83-139)	30 (25-40)
Exposure to mechanical forces	8,906 (7,975-10,438)	7,965 (7,055-8,789)	6,183 (5,414-6,824)	5,537 (4,898-6,185)	3,968 (3,549-4,481)	3,358 (2,933-3,690)	2,494 (2,172-2,744)	2,029 (1,794-2,253)	1,445 (1,264-1,637)	971 (850-1,125)	455 (391-549)	153 (132-187)
Unintentional firearm injuries	1,466 (1,015-1,680)	1,342 (942-1,554)	1,064 (652-1,311)	765 (557-887)	597 (479-704)	538 (418-635)	417 (314-484)	365 (266-423)	260 (182-299)	162 (105-189)	66 (47-77)	20 (16-23)
Unintentional suffocation	330 (278-492)	232 (191-325)	164 (132-243)	181 (139-242)	84 (50-103)	92 (55-116)	82 (44-108)	295 (215-419)	320 (229-489)	268 (188-407)	149 (108-228)	56 (40-86)
Other exposure to mechanical forces	7,109 (6,379-9,010)	6,391 (5,742-7,499)	4,955 (4,466-5,791)	4,591 (4,093-5,317)	3,287 (2,915-3,872)	2,728 (2,339-3,064)	1,995 (1,736-2,219)	1,370 (1,213-1,557)	865 (748-1,007)	540 (466-630)	240 (203-286)	77 (67-94)
Adverse effects of medical treatment	2,407 (1,779-2,787)	2,270 (1,663-2,643)	2,027 (1,437-2,364)	2,624 (1,941-3,045)	3,319 (2,327-3,905)	4,016 (2,856-4,762)	3,613 (2,637-4,302)	3,079 (2,223-3,595)	2,476 (1,754-2,931)	1,589 (1,114-1,882)	668 (453-777)	180 (122-211)
Animal contact	3,443 (2,858-3,974)	3,842 (3,139-4,475)	2,837 (2,297-3,255)	3,412 (2,734-3,921)	3,113 (2,542-3,574)	2,969 (2,372-3,433)	2,360 (1,904-2,738)	1,679 (1,347-1,956)	992 (785-1,171)	437 (347-522)	124 (98-146)	37 (30-43)
Venomous animal contact	2,786 (2,211-3,332)	3,160 (2,488-3,786)	2,255 (1,781-2,669)	2,783 (2,146-3,292)	2,500 (1,963-2,959)	2,420 (1,881-2,873)	1,939 (1,506-2,307)	1,312 (1,022-1,561)	803 (621-966)	341 (262-414)	90 (68-110)	23 (18-29)
Non-venomous animal contact	657 (507-793)	683 (511-843)	582 (460-715)	629 (495-768)	612 (496-746)	550 (443-656)	421 (337-508)	368 (283-466)	188 (146-240)	96 (74-121)	34 (26-43)	13 (11-17)
Foreign body	3,516 (2,715-4,197)	3,173 (2,477-3,787)	2,764 (2,151-3,325)	3,493 (2,694-4,208)	2,999 (2,403-3,615)	2,868 (2,271-3,457)	2,243 (1,735-2,544)	1,969 (1,474-2,227)	1,971 (1,411-2,247)	1,342 (924-1,551)	598 (413-695)	183 (131-213)
Pulmonary aspiration and foreign body in airway	2,886 (2,142-3,604)	2,883 (2,226-3,579)	2,522 (1,979-3,093)	3,234 (2,495-4,028)	2,726 (2,155-3,376)	2,652 (2,092-3,279)	2,047 (1,607-2,410)	1,744 (1,313-2,039)	1,803 (1,294-2,053)	1,239 (857-1,436)	559 (383-652)	169 (121-198)
Foreign body in other body part	630 (333-809)	290 (148-375)	242 (123-315)	259 (138-332)	273 (147-353)	216 (100-276)	197 (77-258)	225 (90-298)	167 (65-219)	103 (43-133)	40 (17-51)	13 (6-17)
Environmental heat and cold exposure	5,189 (3,204-7,411)	5,260 (3,384-8,077)	4,376 (2,751-6,886)	5,663 (3,645-9,625)	4,188 (2,714-7,063)	3,563 (2,366-5,608)	2,208 (1,397-3,159)	1,731 (1,023-2,262)	1,310 (783-1,788)	852 (506-1,174)	353 (210-494)	93 (53-121)
Other unintentional injuries	6,221 (5,656-6,988)	5,454 (4,941-6,052)	3,650 (3,302-4,057)	3,547 (3,201-3,953)	2,631 (2,368-2,941)	1,966 (1,754-2,214)	1,137 (1,027-1,269)	733 (659-825)	499 (450-567)	287 (254-328)	125 (107-147)	40 (32-48)
Self-harm and interpersonal violence	75,749 (63,492-83,398)	60,195 (50,813-66,005)	45,168 (37,516-49,359)	44,506 (36,028-48,656)	34,150 (27,026-37,090)	29,312 (22,686-31,837)	22,048 (16,797-23,902)	15,418 (11,620-16,613)	10,355 (7,487-11,244)	4,990 (3,468-5,453)	1,443 (1,007-1,571)	287 (214-315)

SR Table 4: Global deaths by cause and age group for males (with 95% uncertainty intervals), 1995

Cause	Early Neonatal	Late Neonatal	Post Neonatal	1 to 4	5 to 9	10 to 14	15 to 19	20 to 24	25 to 29	30 to 34	35 to 39	40 to 44	45 to 49
Self-harm	-	-	-	-	-	6,500<br>(4,810-7,485)	30,758<br>(26,538-33,515)	56,991<br>(47,926-61,851)	59,963<br>(51,488-65,129)	56,614<br>(48,683-61,386)	52,408<br>(44,176-57,013)	48,400<br>(41,105-53,571)	41,075<br>(34,628-45,297)
Self-harm by firearm	-	-	-	-	-	438<br>(311-618)	4,357<br>(3,337-5,890)	7,158<br>(5,493-9,674)	6,651<br>(5,003-8,645)	6,196<br>(4,587-8,139)	5,372<br>(4,206-7,075)	4,805<br>(3,858-6,650)	4,145<br>(3,312-5,999)
Self-harm by other specified means	-	-	-	-	-	6,062<br>(4,350-7,019)	26,401<br>(22,106-29,015)	49,833<br>(41,415-54,514)	53,312<br>(45,587-57,892)	50,418<br>(42,957-55,046)	47,036<br>(39,170-50,987)	43,595<br>(36,394-48,482)	36,930<br>(30,481-40,816)
Interpersonal violence	1,051<br>(721-1,260)	272<br>(181-331)	2,983<br>(2,087-3,697)	5,014<br>(3,454-6,192)	4,388<br>(2,958-5,208)	6,051<br>(4,284-7,062)	30,717<br>(23,129-34,315)	49,988<br>(37,651-55,924)	47,710<br>(35,054-54,378)	42,399<br>(30,303-49,198)	34,738<br>(24,215-40,352)	27,349<br>(18,656-32,961)	19,120<br>(12,700-23,488)
Assault by firearm	122<br>(75-187)	34<br>(21-51)	407<br>(252-655)	641<br>(413-1,094)	630<br>(403-974)	1,786<br>(1,066-2,410)	15,094<br>(9,800-17,192)	23,432<br>(15,508-27,703)	21,320<br>(14,212-25,803)	17,491<br>(10,810-21,965)	13,977<br>(8,820-17,717)	9,795<br>(5,903-13,213)	6,151<br>(3,601-8,655)
Assault by sharp object	95<br>(56-149)	25<br>(15-36)	292<br>(146-423)	740<br>(406-1,076)	897<br>(454-1,154)	1,073<br>(630-1,372)	8,097<br>(5,436-10,348)	14,206<br>(9,540-18,154)	14,519<br>(9,540-18,436)	13,133<br>(8,172-17,143)	10,694<br>(6,665-14,394)	8,636<br>(5,420-11,796)	6,011<br>(3,612-8,473)
Assault by other means	835<br>(530-992)	213<br>(129-260)	2,284<br>(1,478-2,804)	3,633<br>(2,215-4,539)	2,861<br>(1,774-3,392)	3,192<br>(2,149-3,779)	7,527<br>(5,099-9,135)	12,350<br>(7,983-14,901)	11,871<br>(7,355-14,278)	11,775<br>(7,236-14,490)	10,066<br>(6,014-12,592)	8,917<br>(5,049-11,412)	6,958<br>(4,040-8,813)
Forces of nature, conflict and terrorism, and executions and police conflict	168<br>(128-208)	103<br>(77-128)	436<br>(328-543)	3,078<br>(2,303-3,873)	2,442<br>(1,828-3,089)	2,722<br>(2,001-3,469)	6,360<br>(4,876-7,919)	11,671<br>(9,065-14,255)	9,493<br>(7,048-11,533)	7,049<br>(5,268-8,536)	4,992<br>(3,741-6,084)	3,457<br>(2,613-4,288)	2,454<br>(1,824-3,102)
Forces of nature, conflict and terrorism, and state actor violence	8<br>(5-10)	17<br>(11-23)	141<br>(93-187)	666<br>(436-890)	748<br>(496-1,006)	987<br>(650-1,326)	1,126<br>(739-1,541)	1,167<br>(734-1,643)	1,083<br>(677-1,537)	919<br>(581-1,300)	813<br>(508-1,153)	768<br>(446-1,123)	714<br>(386-1,079)
Conflict and terrorism	160<br>(122-198)	86<br>(65-106)	263<br>(201-326)	2,364<br>(1,804-2,947)	1,610<br>(1,230-2,004)	1,670<br>(1,271-2,087)	4,766<br>(3,647-5,933)	8,197<br>(6,302-10,164)	5,738<br>(4,418-7,103)	4,191<br>(3,214-5,225)	2,911<br>(2,234-3,626)	1,947<br>(1,503-2,413)	1,270<br>(990-1,564)
Executions and police conflict	-	-	32<br>(25-57)	47<br>(38-58)	84<br>(57-101)	65<br>(53-78)	468<br>(200-591)	2,307<br>(660-2,938)	2,671<br>(670-3,498)	1,938<br>(569-2,433)	1,268<br>(374-1,639)	741<br>(270-928)	470<br>(167-591)

Cause	50 to 54	55 to 59	60 to 64	65 to 69	70 to 74	75 to 79	80 to 84	85 to 89	90 to 94	95 plus
Self-harm	32,527<br>(27,279-35,583)	33,442<br>(27,320-36,562)	26,788<br>(21,092-29,150)	23,833<br>(18,082-25,868)	18,682<br>(13,605-20,230)	13,414<br>(9,720-14,532)	9,729<br>(6,889-10,591)	4,709<br>(3,186-5,165)	1,360<br>(921-1,486)	242<br>(173-264)
Self-harm by firearm	3,364<br>(2,830-4,777)	3,039<br>(2,495-4,220)	2,571<br>(1,950-3,498)	2,485<br>(1,843-3,209)	2,281<br>(1,614-2,780)	1,737<br>(1,212-1,985)	1,385<br>(898-1,573)	713<br>(436-810)	233<br>(146-264)	44<br>(29-51)
Self-harm by other specified means	29,163<br>(23,818-31,835)	30,403<br>(24,312-33,246)	24,218<br>(18,476-26,506)	21,348<br>(15,860-23,245)	16,400<br>(11,753-17,843)	11,677<br>(8,224-12,692)	8,344<br>(5,791-9,128)	3,997<br>(2,692-4,391)	1,128<br>(747-1,237)	198<br>(139-216)
Interpersonal violence	12,641<br>(8,351-16,010)	11,064<br>(7,191-13,978)	7,362<br>(4,738-9,102)	5,479<br>(3,639-7,101)	3,366<br>(2,236-4,434)	2,004<br>(1,393-2,593)	626<br>(414-845)	281<br>(185-396)	83<br>(55-121)	46<br>(30-62)
Assault by firearm	4,137<br>(2,424-6,019)	2,944<br>(1,778-4,328)	1,944<br>(1,174-2,719)	1,297<br>(726-1,954)	849<br>(484-1,297)	503<br>(297-752)	114<br>(56-179)	50<br>(23-76)	16<br>(7-24)	11<br>(5-15)
Assault by sharp object	3,707<br>(2,196-5,193)	3,294<br>(1,760-4,989)	2,262<br>(1,268-3,220)	1,576<br>(909-2,378)	904<br>(511-1,328)	492<br>(285-732)	179<br>(104-295)	73<br>(43-118)	26<br>(16-42)	11<br>(7-18)
Assault by other means	4,797<br>(2,830-6,070)	4,826<br>(2,779-6,076)	3,156<br>(1,873-4,043)	2,605<br>(1,629-3,310)	1,614<br>(1,073-2,105)	1,009<br>(662-1,281)	333<br>(207-460)	158<br>(99-225)	41<br>(26-63)	23<br>(15-34)
Forces of nature, conflict and terrorism, and executions and police conflict	1,722<br>(1,251-2,230)	1,530<br>(1,081-2,014)	1,358<br>(935-1,810)	1,084<br>(738-1,458)	758<br>(510-1,027)	506<br>(329-697)	379<br>(241-528)	212<br>(142-286)	81<br>(58-105)	29<br>(22-37)
Forces of nature, conflict and terrorism, and state actor violence	630<br>(334-957)	569<br>(306-862)	558<br>(288-857)	457<br>(230-705)	353<br>(178-546)	259<br>(125-406)	191<br>(85-307)	86<br>(38-138)	24<br>(10-38)	6<br>(2-9)
Conflict and terrorism	831<br>(651-1,012)	818<br>(633-1,013)	693<br>(537-857)	553<br>(425-684)	361<br>(284-440)	228<br>(179-279)	165<br>(129-203)	113<br>(90-137)	50<br>(40-60)	21<br>(17-25)
Executions and police conflict	261<br>(89-320)	143<br>(77-174)	108<br>(44-132)	74<br>(29-91)	44<br>(20-54)	19<br>(10-23)	23<br>(9-30)	13<br>(4-22)	8<br>(2-14)	3<br>(1-4)

SR Table 4: Global deaths by cause and age group for males (with 95% uncertainty intervals), 2000

Cause	Early Neonatal	Late Neonatal	Post Neonatal	1 to 4	5 to 9	10 to 14	15 to 19	20 to 24	25 to 29	30 to 34	35 to 39	40 to 44	45 to 49	50 to 54	55 to 59	60 to 64	65 to 69	70 to 74	75 to 79	80 to 84	85 to 89	90 to 94	95 plus
All causes	1,480,722 (1,445,569-1,515,982)	455,787 (445,008-466,568)	1,413,116 (1,372,448-1,448,868)	1,311,627 (1,268,027-1,351,427)	371,224 (367,384-375,340)	285,359 (282,765-288,083)	409,982 (404,590-415,253)	580,223 (571,691-588,910)	690,336 (677,507-704,307)	800,078 (786,564-813,417)	900,027 (883,811-916,455)	1,036,701 (1,019,040-1,054,475)	1,253,102 (1,232,572-1,274,057)	1,358,676 (1,338,157-1,379,678)	1,532,897 (1,513,588-1,552,899)	2,106,725 (2,078,889-2,134,650)	2,465,263 (2,438,347-2,493,246)	2,768,291 (2,739,022-2,798,523)	2,479,841 (2,460,451-2,500,104)	1,869,299 (1,858,260-1,880,954)	1,287,366 (1,281,403-1,293,269)	543,078 (541,301-544,947)	140,910 (140,462-141,343)
Communicable, maternal, neonatal, and nutritional disorders	1,355,046 (1,310,572-1,400,088)	404,251 (389,161-419,073)	1,171,438 (1,123,534-1,219,113)	1,046,298 (994,968-1,101,801)	200,176 (188,057-213,479)	108,217 (100,019-116,721)	94,172 (86,580-104,616)	132,236 (124,252-142,606)	209,649 (197,494-224,992)	261,875 (249,399-276,669)	275,579 (260,966-293,120)	270,164 (258,271-285,527)	259,489 (246,935-277,065)	225,671 (213,833-244,913)	215,243 (200,961-238,342)	246,031 (224,672-279,470)	259,951 (235,687-303,033)	284,551 (252,855-336,324)	255,705 (226,268-311,001)	209,805 (184,164-252,841)	144,040 (126,398-170,691)	62,919 (55,427-73,406)	19,178 (17,001-21,963)
HIV/AIDS and tuberculosis	-	-	60,014 (56,433-63,537)	45,975 (41,350-50,978)	16,967 (15,669-18,351)	12,669 (11,559-13,903)	22,049 (20,105-24,016)	65,048 (60,554-70,624)	146,895 (135,798-158,663)	197,938 (186,235-210,485)	206,060 (193,884-218,809)	196,805 (187,807-206,991)	176,681 (167,815-186,830)	140,899 (133,921-149,948)	120,378 (113,639-129,794)	117,281 (110,129-125,761)	107,919 (100,481-116,957)	100,650 (92,831-110,997)	72,423 (66,145-80,650)	46,009 (41,392-52,933)	22,669 (20,524-26,228)	6,880 (6,177-8,085)	1,579 (1,411-1,853)
Tuberculosis	-	-	10,985 (9,040-13,264)	18,539 (14,035-23,154)	5,854 (4,884-6,756)	7,002 (6,058-7,903)	18,090 (16,224-20,080)	36,967 (33,924-40,649)	52,917 (48,691-58,020)	65,433 (60,960-71,896)	73,054 (67,834-79,642)	82,960 (77,116-89,502)	90,703 (84,665-98,695)	89,513 (82,907-97,598)	91,360 (84,465-100,485)	97,698 (90,449-106,062)	94,310 (86,899-103,345)	92,102 (84,356-102,803)	67,472 (61,205-75,578)	44,860 (40,249-51,745)	22,241 (20,089-25,805)	6,768 (6,060-7,981)	1,557 (1,390-1,830)
Drug-susceptible tuberculosis	-	-	10,188 (8,353-12,313)	17,185 (13,000-21,461)	5,371 (4,503-6,220)	6,399 (5,499-7,254)	16,567 (14,765-18,404)	33,749 (30,930-37,326)	48,203 (44,237-53,215)	59,488 (55,328-65,473)	66,192 (61,328-72,472)	74,822 (69,357-80,928)	81,544 (75,749-89,178)	80,737 (74,707-88,178)	82,691 (76,620-90,868)	88,050 (81,274-95,879)	85,314 (78,769-93,409)	83,495 (75,844-93,359)	61,385 (55,546-68,799)	40,917 (36,631-47,471)	20,342 (18,340-23,612)	6,221 (5,563-7,288)	1,438 (1,282-1,693)
Multidrug-resistant tuberculosis without extensive drug resistance	-	-	788 (585-1,081)	1,340 (921-1,954)	474 (370-606)	592 (468-759)	1,494 (1,210-1,882)	3,122 (2,554-3,845)	4,565 (3,784-5,571)	5,774 (4,805-6,933)	6,636 (5,557-8,037)	7,845 (6,523-9,476)	8,828 (7,336-10,717)	8,487 (6,965-10,339)	8,440 (6,951-10,352)	9,372 (7,714-11,567)	8,780 (7,117-10,799)	8,411 (6,831-10,400)	5,961 (4,799-7,479)	3,862 (3,090-4,904)	1,858 (1,474-2,383)	535 (420-698)	117 (90-155)
Extensively drug-resistant tuberculosis	-	-	10 (8-12)	15 (11-19)	9 (7-11)	10 (8-13)	29 (23-36)	96 (77-119)	148 (119-181)	171 (138-208)	225 (183-277)	294 (239-359)	331 (267-412)	290 (234-360)	229 (186-279)	276 (225-337)	216 (176-265)	195 (158-240)	126 (101-158)	80 (64-102)	41 (32-52)	12 (10-16)	3 (2-3)
HIV/AIDS	-	-	49,029 (46,473-51,761)	27,436 (25,693-29,265)	11,113 (10,235-12,117)	5,667 (5,086-6,426)	3,959 (3,474-4,563)	28,081 (24,436-32,520)	93,978 (83,888-105,123)	132,505 (122,245-143,577)	133,006 (121,178-145,161)	113,846 (106,359-121,216)	85,978 (79,222-93,453)	51,386 (47,992-55,256)	29,018 (26,631-31,561)	19,583 (17,646-21,537)	13,609 (12,226-14,988)	8,549 (7,657-9,493)	4,951 (4,445-5,460)	1,149 (1,016-1,302)	428 (379-485)	112 (99-128)	22 (19-24)
Drug-susceptible HIV/AIDS - Tuberculosis	-	-	16,272 (12,584-19,445)	9,585 (7,732-11,256)	3,720 (2,987-4,382)	1,866 (1,546-2,199)	1,246 (984-1,495)	8,530 (6,322-10,753)	23,782 (15,999-31,956)	30,824 (20,173-41,988)	30,969 (20,346-42,015)	28,327 (18,841-37,385)	23,412 (16,091-29,849)	15,451 (11,403-18,783)	9,213 (7,202-11,019)	6,330 (4,948-7,608)	4,496 (3,554-5,413)	2,897 (2,323-3,463)	1,710 (1,407-2,028)	392 (322-471)	145 (119-174)	37 (30-45)	7 (5-8)
Multidrug-resistant HIV/AIDS - Tuberculosis without extensive drug resistance	-	-	1,194 (752-1,770)	687 (428-1,055)	247 (157-372)	124 (77-192)	112 (73-163)	724 (466-1,053)	1,900 (1,181-2,867)	2,495 (1,520-3,796)	2,490 (1,519-3,762)	2,238 (1,364-3,354)	1,846 (1,152-2,747)	1,200 (766-1,773)	695 (442-1,030)	469 (301-701)	322 (208-485)	202 (131-307)	118 (76-175)	28 (18-41)	10 (7-15)	3 (2-4)	0 (0-1)
Extensively drug-resistant HIV/AIDS - Tuberculosis	-	-	6 (4-9)	3 (2-5)	1 (1-2)	1 (1-2)	1 (1-2)	8 (5-12)	27 (17-42)	48 (29-75)	51 (31-81)	38 (23-59)	25 (15-38)	13 (8-19)	7 (4-10)	5 (3-7)	2 (2-4)	1 (1-2)	1 (0-1)	0 (0-0)	0 (0-0)	0 (0-0)	0 (0-0)
HIV/AIDS resulting in other diseases	-	-	31,556 (27,941-36,213)	17,160 (15,209-19,716)	7,145 (6,316-8,282)	3,676 (3,204-4,395)	2,600 (2,223-3,146)	18,820 (15,510-23,430)	68,269 (56,474-82,232)	99,138 (84,770-115,139)	99,495 (83,929-115,756)	83,243 (71,443-96,542)	60,695 (52,470-71,485)	34,722 (30,505-39,900)	19,103 (16,704-22,192)	12,779 (11,116-14,860)	8,789 (7,618-10,242)	5,448 (4,690-6,438)	3,122 (2,714-3,643)	728 (624-867)	273 (233-326)	72 (62-85)	15 (13-17)
Diarrhoea, lower respiratory infections, and other common infectious diseases	169,561 (140,158-197,141)	161,180 (138,513-178,769)	816,115 (764,215-872,365)	645,116 (565,789-741,542)	131,056 (112,003-151,781)	67,577 (57,303-79,374)	52,057 (42,933-63,961)	45,971 (37,334-59,357)	44,404 (36,170-57,722)	44,534 (36,172-58,118)	49,792 (40,910-67,157)	52,864 (43,395-70,864)	61,673 (50,204-82,260)	65,117 (52,897-88,556)	74,392 (58,695-102,113)	106,068 (83,188-145,685)	128,118 (99,647-174,936)	161,412 (126,542-222,242)	163,275 (129,769-227,135)	149,339 (121,679-196,832)	110,487 (92,005-139,512)	50,863 (42,916-62,014)	15,781 (13,535-18,722)
Diarrhoeal diseases	21,596 (18,335-25,335)	40,327 (34,566-46,799)	269,039 (230,791-303,751)	185,068 (144,849-223,596)	25,898 (13,246-48,463)	13,380 (6,875-25,739)	14,880 (7,825-28,224)	15,842 (8,362-30,401)	16,310 (8,518-31,041)	15,924 (7,958-30,666)	18,224 (9,594-36,634)	18,751 (10,008-36,712)	22,066 (11,262-43,407)	23,794 (12,104-49,225)	29,857 (15,065-59,155)	44,554 (23,042-87,036)	56,845 (30,020-109,168)	73,311 (37,457-139,754)	70,979 (38,157-137,564)	63,896 (36,614-116,122)	42,421 (24,665-73,472)	17,947 (10,667-29,690)	5,259 (3,321-8,184)
Intestinal infectious diseases	-	-	751 (142-3,514)	16,617 (7,827-30,656)	30,649 (17,144-50,166)	19,909 (10,810-31,467)	11,791 (6,001-20,650)	6,155 (3,048-10,713)	4,510 (2,349-7,724)	3,520 (1,883-5,952)	2,779 (1,504-4,613)	2,202 (1,188-3,660)	1,861 (999-3,047)	1,365 (717-2,336)	1,031 (492-1,875)	929 (450-1,685)	832 (467-1,363)	656 (387-1,035)	430 (249-688)	233 (133-368)	108 (63-169)	33 (19-53)	8 (4-13)
Typhoid fever	-	-	102 (47-197)	14,659 (6,405-28,266)	26,955 (14,735-44,364)	16,892 (8,809-27,221)	10,262 (5,143-18,119)	5,206 (2,537-9,332)	3,596 (1,830-6,257)	2,684 (1,397-4,699)	2,096 (1,114-3,645)	1,637 (849-2,832)	1,342 (697-2,266)	1,001 (507-1,750)	778 (362-1,434)	729 (347-1,336)	685 (370-1,153)	548 (313-876)	352 (201-567)	192 (108-310)	85 (49-136)	25 (14-40)	6 (3-9)
Paratyphoid fever	-	-	15 (6-33)	1,458 (585-3,174)	3,640 (1,659-7,110)	2,995 (1,388-5,674)	1,509 (641-2,966)	926 (387-1,872)	897 (387-1,823)	808 (357-1,600)	650 (306-1,237)	541 (249-1,039)	485 (221-961)	353 (154-705)	243 (100-495)	188 (81-383)	133 (62-257)	92 (45-170)	57 (28-104)	31 (15-57)	14 (7-26)	4 (2-8)	1 (0-2)
Other intestinal infectious diseases	-	-	634 (46-3,403)	500 (36-2,369)	54 (4-239)	23 (2-104)	20 (2-98)	24 (2-115)	17 (2-71)	28 (2-137)	33 (2-143)	24 (2-105)	34 (3-168)	11 (1-58)	11 (1-51)	13 (1-61)	14 (1-67)	16 (2-74)	21 (2-91)	10 (1-43)	9 (1-41)	3 (0-14)	1 (0-5)
Lower respiratory infections	108,714 (96,006-122,249)	86,957 (76,648-98,009)	357,824 (320,859-392,792)	173,747 (140,924-205,459)	23,809 (18,448-28,576)	12,717 (10,141-15,056)	10,485 (8,315-12,610)	11,877 (9,385-14,325)	13,202 (10,736-16,015)	14,943 (12,313-17,739)	18,463 (15,358-21,832)	22,215 (18,899-25,470)	27,448 (23,530-31,883)	30,935 (26,284-35,702)	34,602 (28,790-40,115)	51,853 (42,363-59,305)	61,662 (50,296-72,048)	79,592 (66,400-90,967)	85,633 (71,461-97,434)	80,324 (68,153-90,587)	65,222 (57,054-74,014)	31,897 (28,222-36,099)	10,237 (8,920-11,504)
Upper respiratory infections	63 (39-99)	22 (12-38)	470 (307-764)	377 (206-692)	32 (19-51)	24 (16-36)	52 (36-73)	54 (36-77)	60 (42-85)	62 (45-86)	68 (49-95)	68 (48-95)	81 (56-113)	73 (51-102)	74 (53-101)	88 (63-121)	78 (56-110)	63 (45-87)	55 (41-74)	32 (23-44)	21 (15-28)	7 (5-10)	1 (1-2)
Otitis media	3 (1-5)	8 (4-14)	107 (44-213)	204 (81-371)	109 (51-183)	126 (61-216)	51 (25-90)	38 (17-66)	29 (13-48)	25 (12-42)	37 (16-62)	42 (17-77)	149 (60-287)	141 (58-254)	45 (16-76)	49 (17-86)	63 (19-123)	50 (21-83)	40 (18-61)	34 (15-53)	19 (8-29)	9 (3-15)	3 (1-4)
Meningitis	7,215 (4,694-10,027)	9,382 (7,256-11,821)	52,271 (42,147-64,860)	45,479 (33,912-58,771)	11,177 (6,235-16,376)	7,665 (4,540-11,440)	7,034 (4,372-10,651)	6,386 (3,810-9,434)	5,545 (3,431-8,383)	5,351 (3,175-8,355)	5,324 (3,326-7,897)	4,635 (3,020-7,061)	5,105 (3,255-7,735)	4,498 (2,892-6,893)	4,242 (2,716-6,643)	4,370 (2,802-6,892)	4,428 (2,862-6,625)	3,551 (2,263-5,473)	2,716 (1,782-4,243)	2,090 (1,321-3,217)	1,114 (710-1,664)	363 (233-544)	86 (58-126)
Pneumococcal meningitis	375 (232-588)	475 (320-701)	2,742 (1,942-3,690)	2,629 (1,697-3,865)	642 (343-1,046)	426 (231-701)	391 (227-645)	353 (198-586)	356 (204-589)	422 (245-671)	514 (289-835)	515 (294-849)	615 (365-1,010)	547 (342-892)	523 (329-832)	557 (364-885)	558 (349-862)	459 (287-736)	349 (222-540)	255 (152-402)	131 (85-190)	43 (30-62)	10 (7-15)
H influenzae type B meningitis	1,030 (631-1,521)	1,290 (934-1,712)	6,731 (5,096-8,920)	5,483 (3,777-7,727)	1,058 (552-1,612)	601 (328-925)	485 (271-755)	435 (248-704)	408 (238-677)	413 (233-663)	422 (254-649)	365 (216-577)	407 (246-661)	361 (220-562)	342 (205-547)	359 (214-589)	347 (205-573)	271 (163-435)	204 (131-323)	162 (93-254)	85 (55-126)	27 (17-41)	6 (4-9)
Meningococcal infection	3,740 (2,426-5,288)	4,869 (3,739-6,384)	26,780 (21,350-34,112)	25,057 (18,198-33,359)	6,337 (3,617-9,116)	4,262 (2,567-6,255)	3,966 (2,421-6,089)	3,532 (2,137-5,228)	2,915 (1,755-4,393)	2,599 (1,537-4,072)	2,510 (1,530-3,816)	2,165 (1,368-3,440)	2,356 (1,428-3,618)	2,105 (1,315-3,247)	1,982 (1,205-3,091)	2,071 (1,294-3,340)	2,066 (1,285-3,206)	1,613 (986-2,544)	1,240 (772-1,974)	952 (565-1,472)	525 (321-804)	172 (106-263)	40 (24-60)
Other meningitis	2,070 (1,293-2,952)	2,748 (2,072-3,560)	16,018 (12,435-20,397)	12,310 (8,904-16,297)	3,140 (1,642-4,787)	2,376 (1,323-3,486)	2,192 (1,308-3,366)	2,067 (1,179-3,137)	1,866 (1,080-2,751)	1,917 (1,066-2,932)	1,877 (1,071-2,809)	1,590 (957-2,349)	1,727 (1,082-2,649)	1,485 (941-2,352)	1,396 (847-2,194)	1,383 (892-2,178)	1,457 (889-2,244)	1,209 (759-1,878)	922 (596-1,418)	721 (456-1,167)	374 (244-584)	121 (78-180)	30 (20-44)
Encephalitis	518 (285-708)	825 (491-1,123)	8,777 (5,252-12,088)	9,782 (5,755-14,563)	4,058 (2,701-5,347)	3,248 (2,298-4,140)	2,397 (1,717-3,029)	2,142 (1,624-2,806)	1,989 (1,457-2,476)	2,130 (1,573-2,670)	2,167 (1,656-2,820)	1,931 (1,500-2,555)	1,944 (1,533-2,525)	1,643 (1,314-2,239)	1,824 (1,466-2,333)	2,167 (1,764-2,865)	2,456 (1,933-3,117)	2,470 (1,859-3,134)	2,260 (1,634-2,762)	1,593 (1,128-2,003)	810 (583-994)	253 (191-319)	61 (46-78)
Diphtheria	-	-	825 (454-1,415)	1,779 (853-3,452)	295 (157-512)	138 (78-231)	68 (41-109)	44 (26-71)	39 (24-59)	35 (21-54)	49 (32-76)	49 (32-75)	55 (36-87)	57 (37-86)	41 (26-62)	-	-	-	-	-	-	-	-
Whooping cough	-	-	31,930 (14,749-60,639)	28,567 (13,276-53,412)	3,072 (1,407-6,098)	684 (302-1,396)	313 (137-652)	208 (92-433)	124 (51-261)	99 (40-212)	67 (27-143)	86 (35-181)	68 (27-149)	48 (19-103)	66 (27-140)	-	-	-	-	-	-	-	-
Tetanus	31,392 (11,804-51,405)	23,464 (9,410-37,681)	8,437 (3,055-15,961)	3,358 (1,222-5,824)	2,015 (786-3,466)	1,277 (591-2,040)	1,852 (808-3,084)	1,706 (820-2,702)	1,812 (903-2,628)	1,885 (960-2,705)	2,225 (1,115-3,097)	2,615 (1,241-3,605)	2,649 (1,328-3,582)	2,381 (1,167-3,260)	2,404 (1,159-3,270)	1,928 (943-2,874)	1,520 (745-2,162)	1,497 (629-2,109)	824 (341-1,152)	676 (297-1,016)	283 (121-429)	86 (42-130)	24 (12-37)
Measles	-	-	84,217 (31,894-175,014)	178,359 (75,127-336,494)	29,133 (11,205-59,520)	8,130 (2,853-17,672)	3,009 (1,035-6,783)	1,363 (459-3,176)	593 (203-1,379)	350 (119-808)	216 (75-500)	166 (57-385)	127 (44-292)	91 (31-208)	100 (34-231)	-	-	-	-	-	-	-	-
Varicella and herpes zoster	59 (45-77)	195 (152-248)	1,465 (1,150-1,863)	1,777 (1,315-2,323)	810 (613-1,054)	279 (219-351)	127 (101-161)	155 (123-200)	190 (151-236)	210 (169-260)	173 (143-215)	104 (84-128)	120 (98-149)	91 (73-111)	106 (87-131)	128 (104-161)	234 (190-290)	223 (178-275)	338 (275-420)	461 (371-585)	489 (385-629)	267 (211-340)	103 (80-131)
Neglected tropical diseases and malaria	566 (275-865)	15,246 (10,475-21,581)	119,021 (86,524-159,577)	241,131 (179,214-311,982)	36,702 (26,345-49,021)	21,171 (15,345-27,645)	12,268 (9,465-15,582)	12,775 (9,673-16,256)	9,568 (7,108-12,195)	9,184 (6,821-11,992)	9,398 (6,902-12,413)	8,844 (6,665-11,634)	8,478 (6,397-10,757)	7,338 (5,649-9,551)	7,568 (5,909-9,690)	8,154 (6,356-10,331)	8,139 (6,322-10,645)	6,728 (5,292-8,664)	5,150 (3,964-6,771)	2,445 (1,922-3,133)	1,722 (1,304-2,295)	312 (249-392)	113 (85-151)
Malaria	-	14,733 (9,948-21,060)	114,056 (81,024-154,433)	227,776 (166,352-298,516)	27,620 (17,647-39,470)	15,297 (10,019-21,776)	6,955 (4,804-9,622)	7,140 (4,869-9,856)	5,671 (3,884-7,827)	5,301 (3,511-7,461)	5,594 (3,582-7,960)	4,998 (3,385-6,956)	4,264 (2,829-5,966)	3,825 (2,606-5,448)	3,839 (2,615-5,471)	4,715 (3,167-6,840)	4,901 (3,239-7,263)	3,957 (2,579-5,869)	3,022 (1,954-4,451)	1,372 (914-2,031)	1,169 (754-1,707)	169 (110-243)	76 (50-112)
Chagas disease	-	-	2 (2-2)	1 (1-1)	2 (1-2)	4 (3-4)	11 (9-14)	27 (23-34)	45 (38-55)	93 (79-111)	158 (135-181)	234 (202-268)	334 (296-375)	425 (376-479)	480 (410-553)	636 (572-709)	627 (570-687)	653 (593-720)	378 (331-431)	259 (231-290)	155 (141-171)	51 (45-57)	10 (9-12)
Leishmaniasis	-	-	732 (409-1,235)	1,001 (527-1,766)	497 (275-849)	339 (186-576)	466 (250-781)	583 (323-994)	494 (279-805)	440 (246-731)	498 (274-839)	496 (266-828)	472 (257-760)	468 (259-780)	383 (208-652)	345 (187-576)	409 (221-686)	391 (217-651)	324 (176-541)	191 (102-312)	125 (63-214)	23 (9-41)	8 (2-17)
Visceral leishmaniasis	-	-	732 (409-1,235)	1,001 (527-1,766)	497 (275-849)	339 (186-576)	466 (250-781)	583 (323-994)	494 (279-805)	440 (246-731)	498 (274-839)	496 (266-828)	472 (257-760)	468 (259-780)	383 (208-652)	345 (187-576)	409 (221-686)	391 (217-651)	324 (176-541)	191 (102-312)	125 (63-214)	23 (9-41)	8 (2-17)
African trypanosomiasis	-	-	-	331 (57-1,273)	818 (178-2,438)	938 (230-2,527)	1,195 (296-3,014)	1,256 (306-3,309)	1,115 (266-3,124)	1,022 (217-2,944)	900 (195-2,667)	760 (135-2,339)	642 (133-2,103)	567 (108-1,958)	481 (86-1,991)	385 (72-1,419)	299 (57-1,124)	198 (36-765)	113 (20-448)	49 (9-190)	15 (3-59)	4 (1-11)	1 (0-2)
Schistosomiasis	-	-	0 (0-0)	323 (186-546)	164 (102-257)	229 (153-335)	223 (155-310)	314 (221-439)	358 (255-485)	389 (283-527)	447 (329-600)	481 (353-648)	528 (392-704)	578 (428-770)	639 (465-836)	669 (494-904)	686 (510-926)	578 (419-778)	405 (302-533)	197 (148-264)	80 (58-107)	19 (12-28)	3 (1-4)
Cysticercosis	0 (0-0)	0 (0-0)	0 (0-0)	60 (39-88)	39 (28-53)	32 (25-41)	32 (25-40)	48 (38-59)	62 (51-76)	68 (56-82)	56 (46-67)	54 (45-65)	50 (42-60)	56 (47-65)	49 (42-57)	38 (32-46)	35 (30-43)	21 (17-26)	11 (9-14)	6 (4-7)	3 (2-3)	1 (1-1)	0 (0-0)
Cystic echinococcosis	-	-	-	205 (133-309)	83 (56-121)	52 (35-74)	25 (17-34)	35 (25-48)	48 (35-65)	48 (35-62)	56 (43-73)	51 (38-65)	60 (45-75)	56 (43-70)	54 (42-69)	63 (50-79)	57 (45-71)	44 (34-54)	36 (29-46)	18 (14-23)	12 (9-17)	6 (3-10)	1 (0-1)
Dengue	-	-	629 (160-1,398)	1,097 (287-2,323)	654 (181-1,426)	396 (103-868)	419 (83-988)	379 (77-849)	310 (51-728)	227 (42-536)	261 (41-617)	210 (34-496)	218 (37-543)	179 (33-424)	213 (32-546)	215 (37-504)	212 (41-510)	86 (19-189)	67 (18-139)	51 (11-116)	33 (10-71)	10 (3-21)	2 (1-5)
Yellow fever	-	3 (1-9)	48 (9-154)	442 (82-1,319)	896 (175-2,747)	811 (167-2,542)	779 (161-2,224)	681 (141-2,061)	510 (98-1,600)	378 (74-1,172)	296 (58-907)	230 (45-709)	178 (35-525)	139 (26-427)	112 (22-351)	84 (18-271)	61 (12-192)	38 (7-123)	22 (4-70)	9 (2-28)	3 (1-10)	1 (0-2)	0 (0-0)
Rabies	-	-	445 (206-681)	2,108 (792-3,138)	4,391 (1,809-7,060)	2,578 (968-3,718)	1,857 (963-2,794)	1,976 (1,053-2,948)	625 (286-859)	904 (398-1,246)	802 (369-1,100)	1,014 (442-1,322)	1,385 (537-1,942)	721 (302-915)	967 (319-1,410)	677 (272-923)	522 (217-698)	440 (208-647)	539 (313-864)	160 (94-305)	60 (35-137)	7 (4-17)	3 (2-6)
Intestinal nematode infections	-	-	293 (225-374)	3,151 (2,283-4,143)	514 (391-646)	111 (87-140)	39 (29-49)	28 (21-36)	22 (17-29)	29 (22-37)	26 (19-33)	18 (14-23)	21 (16-27)	26 (20-33)	24 (18-31)	26 (20-33)	19 (14-24)	27 (20-33)	19 (14-24)	11 (8-14)	4 (3-5)	2 (2-3)	1 (0-1)
Ascariasis	-	-	293 (225-374)	3,151 (2,283-4,143)	514 (391-646)	111 (87-140)	39 (29-49)	28 (21-36)	22 (17-29)	29 (22-37)	26 (19-33)	18 (14-23)	21 (16-27)	26 (20-33)	24 (18-31)	26 (20-33)	19 (14-24)	27 (20-33)	19 (14-24)	11 (8-14)	4 (3-5)	2 (2-3)	1 (0-1)
Ebola	0 (0-0)	0 (0-0)	2 (1-2)	15 (11-20)	6 (4-7)	5 (4-6)	6 (4-7)	8 (6-10)	10 (7-12)	16 (12-20)	14 (10-18)	15 (11-19)	16 (12-20)	7 (6-9)	10 (7-13)	6 (4-7)	4 (3-5)	2 (1-2)	1 (0-1)	0 (0-0)	0 (0-0)	0 (0-0)	0 (0-0)
Zika virus	0 (0-0)	0 (0-0)	0 (0-0)	0 (0-0)	0 (0-0)	0 (0-0)	0 (0-0)	0 (0-0)	0 (0-0)	0 (0-0)	0 (0-0)	0 (0-0)	0 (0-0)	0 (0-0)	0 (0-0)	0 (0-0)	0 (0-0)	0 (0-0)	0 (0-0)	0 (0-0)	0 (0-0)	0 (0-0)	0 (0-0)
Other neglected tropical diseases	566 (275-865)	511 (284-771)	2,815 (1,394-4,365)	4,621 (1,642-11,825)	1,018 (484-1,899)	379 (236-626)	261 (154-402)	301 (184-447)	298 (172-422)	269 (141-365)	291 (165-364)	282 (172-362)	313 (165-406)	289 (172-361)	317 (211-402)	294 (203-381)	307 (195-400)	293 (213-382)	214 (145-284)	121 (85-171)	64 (44-91)	22 (16-30)	8 (6-14)
Maternal disorders	-	-	-	-	-	-	-	-	-	-	-	-	-	-	-	-	-	-	-	-	-	-	-
Maternal haemorrhage	-	-	-	-	-	-	-	-	-	-	-	-	-	-	-	-	-	-	-	-	-	-	-
Maternal sepsis and other pregnancy related infections	-	-	-	-	-	-	-	-	-	-	-	-	-	-	-	-	-	-	-	-	-	-	-
Maternal hypertensive disorders	-	-	-	-	-	-	-	-	-	-	-	-	-	-	-	-	-	-	-	-	-	-	-
Maternal obstructed labour and uterine rupture	-	-	-	-	-	-	-	-	-	-	-	-	-	-	-	-	-	-	-	-	-	-	-
Maternal abortion, miscarriage, and ectopic pregnancy	-	-	-	-	-	-	-	-	-	-	-	-	-	-	-	-	-	-	-	-	-	-	-
Indirect maternal deaths	-	-	-	-	-	-	-	-	-	-	-	-	-	-	-	-	-	-	-	-	-	-	-
Late maternal deaths	-	-	-	-	-	-	-	-	-	-	-	-	-	-	-	-	-	-	-	-	-	-	-
Maternal deaths aggravated by HIV/AIDS	-	-	-	-	-	-	-	-	-	-	-	-	-	-	-	-	-	-	-	-	-	-	-
Other maternal disorders	-	-	-	-	-	-	-	-	-	-	-	-	-	-	-	-	-	-	-	-	-	-	-
Neonatal disorders	1,155,302 (1,109,025-1,211,296)	214,625 (196,589-245,403)	56,830 (48,125-70,541)	6,239 (4,996-8,334)	-	-	-	-	-	-	-	-	-	-	-	-	-	-	-	-	-	-	-
Neonatal preterm birth complications	464,500 (425,509-520,001)	65,101 (56,206-84,197)	21,852 (17,750-28,976)	1,688 (1,290-2,431)	-	-	-	-	-	-	-	-	-	-	-	-	-	-	-	-	-	-	-
Neonatal encephalopathy due to birth asphyxia and trauma	364,570 (327,077-406,141)	33,401 (28,548-40,206)	10,292 (8,302-12,985)	2,487 (1,964-3,193)	-	-	-	-	-	-	-	-	-	-	-	-	-	-	-	-	-	-	-
Neonatal sepsis and other neonatal infections	91,304 (56,394-116,195)	57,755 (42,782-74,967)	9,375 (6,150-13,643)	542 (176-1,070)	-	-	-	-	-	-	-	-	-	-	-	-	-	-	-	-	-	-	-
Hemolytic disease and other neonatal jaundice	34,568 (28,015-43,200)	18,851 (15,434-23,307)	3,563 (2,598-4,868)	226 (152-343)	-	-	-	-	-	-	-	-	-	-	-	-	-	-	-	-	-	-	-
Other neonatal disorders	200,360 (175,250-227,940)	39,516 (32,835-54,210)	11,748 (9,259-16,523)	1,297 (941-2,153)	-	-	-	-	-	-	-	-	-	-	-	-	-	-	-	-	-	-	-
Nutritional deficiencies	-	-	75,700 (59,013-92,303)	81,931 (55,171-110,298)	7,633 (6,108-9,451)	2,937 (2,366-3,694)	2,410 (1,926-3,238)	2,531 (2,031-3,313)	2,598 (1,957-3,547)	2,900 (2,181-3,869)	2,999 (2,253-4,077)	3,474 (2,496-4,691)	3,423 (2,699-4,737)	4,461 (3,274-6,167)	5,482 (3,928-7,585)	6,695 (4,783-9,280)	7,781 (6,037-10,632)	9,000 (7,307-11,955)	9,489 (7,928-12,367)	8,668 (7,425-11,020)	7,407 (6,375-8,986)	4,272 (3,769-4,999)	1,546 (1,372-1,784)
Protein-energy malnutrition	-	-	73,798 (57,147-89,973)	79,954 (54,086-107,455)	6,827 (5,348-8,619)	2,506 (1,971-3,225)	1,756 (1,348-2,362)	1,800 (1,401-2,300)	1,963 (1,386-2,640)	2,238 (1,572-2,995)	2,301 (1,641-3,106)	2,756 (1,859-3,728)	2,451 (1,832-3,289)	3,482 (2,440-4,708)	4,340 (2,914-5,995)	5,381 (3,598-7,447)	6,041 (4,540-8,126)	7,207 (5,731-9,353)	7,734 (6,391-9,738)	7,254 (6,187-8,916)	6,236 (5,338-7,332)	3,670 (3,196-4,191)	1,341 (1,175-1,524)
Iodine deficiency	-	-	151 (31-513)	131 (21-486)	50 (11-157)	14 (3-41)	35 (8-106)	39 (7-138)	42 (9-134)	42 (11-121)	37 (10-106)	33 (9-101)	31 (8-102)	37 (10-121)	41 (13-118)	41 (14-102)	53 (19-140)	60 (21-135)	49 (16-122)	29 (11-67)	14 (4-34)	4 (1-12)	1 (0-5)
Iron-deficiency anaemia	-	-	34 (12-59)	57 (28-90)	70 (38-103)	65 (39-89)	90 (59-126)	81 (61-105)	62 (52-79)	70 (55-90)	54 (43-72)	52 (40-93)	52 (41-96)	44 (34-76)	54 (39-123)	54 (40-118)	82 (53-185)	103 (60-192)	84 (53-176)	69 (45-154)	40 (25-82)	22 (13-40)	10 (6-20)
Other nutritional deficiencies	-	-	1,717 (1,102-2,345)	1,789 (813-2,779)	685 (499-947)	352 (274-455)	529 (402-801)	611 (441-1,008)	530 (382-896)	549 (389-925)	607 (442-994)	633 (452-1,085)	888 (612-1,578)	897 (617-1,628)	1,047 (744-1,822)	1,218 (930-1,969)	1,605 (1,163-2,617)	1,629 (1,192-2,736)	1,622 (1,130-2,779)	1,316 (925-2,276)	1,117 (767-1,859)	576 (385-932)	194 (126-327)
Other communicable, maternal, neonatal, and nutritional diseases	29,617 (17,621-45,370)	13,200 (8,263-19,121)	43,758 (29,541-61,897)	25,907 (17,164-36,731)	7,819 (5,854-10,352)	3,863 (3,285-4,440)	5,388 (4,803-6,031)	5,911 (5,388-6,466)	6,185 (5,650-6,781)	7,319 (6,757-7,889)	7,320 (6,808-7,902)	8,176 (7,617-8,787)	9,233 (8,560-10,029)	7,857 (7,379-8,364)	7,424 (6,928-7,985)	7,834 (7,261-8,400)	7,993 (7,416-8,547)	6,761 (6,235-7,260)	5,368 (4,931-5,782)	3,344 (2,972-3,675)	1,756 (1,565-1,931)	591 (517-661)	158 (136-178)
Sexually transmitted diseases excluding HIV	25,058 (13,884-39,919)	9,855 (5,536-15,423)	25,683 (14,096-41,820)	10,396 (5,533-17,189)	3,430 (1,848-5,661)	90 (62-143)	137 (83-209)	113 (72-164)	178 (105-284)	295 (172-427)	221 (136-344)	164 (118-225)	160 (119-213)	215 (147-341)	290 (199-460)	164 (122-229)	168 (127-237)	198 (147-292)	181 (115-245)	107 (66-144)	67 (41-88)	28 (17-37)	13 (8-18)
Syphilis	25,058 (13,884-39,919)	9,855 (5,536-15,423)	25,683 (14,096-41,820)	10,396 (5,533-17,189)	3,430 (1,848-5,661)	77 (52-122)	116 (71-177)	98 (62-141)	149 (88-237)	250 (146-362)	196 (120-305)	149 (107-204)	144 (107-192)	200 (137-317)	269 (184-426)	153 (113-214)	158 (119-223)	183 (136-269)	166 (105-225)	98 (60-131)	60 (37-78)	25 (15-32)	10 (6-14)
Chlamydial infection	-	-	-	-	-	0 (0-0)	0 (0-0)	0 (0-0)	0 (0-0)	0 (0-0)	0 (0-0)	0 (0-0)	0 (0-0)	0 (0-0)	0 (0-0)	0 (0-0)	0 (0-0)	0 (0-0)	0 (0-0)	0 (0-0)	0 (0-0)	0 (0-0)	0 (0-0)
Gonococcal infection	-	-	-	-	-	9 (6-14)	16 (10-24)	10 (6-15)	24 (14-39)	33 (19-48)	13 (8-20)	9 (7-12)	7 (5-9)	7 (5-11)	10 (7-16)	6 (5-9)	5 (4-8)	11 (8-16)	9 (6-13)	6 (4-8)	4 (3-5)	2 (1-3)	1 (1-2)
Other sexually transmitted diseases	-	-	-	-	-	5 (3-7)	5 (3-8)	6 (4-8)	5 (3-8)	12 (7-17)	12 (7-19)	6 (4-8)	9 (6-12)	8 (5-13)	11 (8-18)	5 (4-7)	5 (4-7)	5 (3-7)	6 (4-8)	4 (2-5)	4 (2-5)	2 (1-2)	2 (1-2)
Hepatitis	-	-	6,671 (5,669-7,759)	7,237 (5,554-8,907)	2,879 (2,300-3,516)	2,612 (2,209-3,059)	3,820 (3,379-4,332)	4,578 (4,169-5,040)	4,887 (4,435-5,398)	5,741 (5,288-6,248)	5,736 (5,303-6,237)	6,906 (6,383-7,478)	7,761 (7,141-8,445)	6,378 (5,949-6,823)	5,818 (5,393-6,282)	5,806 (5,404-6,233)	5,866 (5,433-6,294)	4,569 (4,243-4,899)	3,504 (3,199-3,813)	2,041 (1,822-2,241)	938 (846-1,027)	236 (212-258)	47 (43-51)
Acute hepatitis A	-	-	4,044 (2,747-5,436)	751 (421-1,159)	66 (39-98)	36 (22-52)	49 (30-75)	57 (29-95)	53 (26-87)	52 (23-94)	53 (26-91)	74 (36-128)	100 (49-167)	91 (46-159)	81 (41-138)	81 (40-139)	85 (43-141)	75 (40-124)	62 (33-101)	36 (20-57)	16 (9-24)	4 (2-6)	1 (0-1)
Hepatitis B	-	-	2,626 (1,454-4,062)	1,112 (616-1,783)	325 (172-543)	460 (259-716)	1,371 (943-1,913)	2,764 (2,070-3,506)	3,770 (3,092-4,454)	4,986 (4,326-5,567)	5,202 (4,710-5,748)	6,346 (5,721-6,957)	7,101 (6,370-7,877)	5,798 (5,216-6,362)	5,247 (4,658-5,797)	5,195 (4,615-5,694)	5,182 (4,561-5,734)	3,925 (3,443-4,388)	2,916 (2,446-3,341)	1,535 (1,211-1,831)	586 (409-760)	119 (72-168)	19 (10-30)
Hepatitis C	-	-	-	-	83 (52-127)	31 (19-45)	38 (23-58)	42 (22-71)	39 (18-68)	40 (18-73)	48 (23-93)	77 (35-137)	114 (55-202)	127 (59-233)	150 (69-276)	171 (81-308)	160 (77-273)	122 (60-226)	95 (46-160)	54 (27-93)	26 (15-42)	7 (4-11)	2 (1-3)
Acute hepatitis E	-	-	-	5,373 (4,078-6,715)	2,406 (1,886-2,944)	2,086 (1,702-2,535)	2,362 (1,829-2,906)	1,714 (1,055-2,353)	1,025 (542-1,627)	663 (304-1,132)	432 (199-746)	410 (178-779)	445 (198-792)	361 (160-688)	339 (148-650)	359 (149-694)	439 (192-788)	447 (203-772)	431 (169-839)	417 (217-695)	310 (167-478)	106 (61-156)	26 (15-36)
Other infectious diseases	4,558 (1,298-9,215)	3,346 (1,320-5,517)	11,405 (3,538-22,867)	8,274 (3,010-16,966)	1,509 (1,094-1,983)	1,161 (894-1,464)	1,431 (1,180-1,738)	1,220 (971-1,504)	1,119 (921-1,305)	1,283 (1,034-1,508)	1,373 (1,155-1,611)	1,106 (946-1,270)	1,313 (1,114-1,504)	1,265 (1,082-1,432)	1,316 (1,111-1,448)	1,864 (1,608-2,176)	1,959 (1,697-2,228)	1,993 (1,687-2,272)	1,684 (1,441-1,931)	1,196 (986-1,447)	750 (624-879)	326 (264-386)	98 (79-116)
Non-communicable diseases	115,391 (83,069-142,210)	45,381 (34,127-55,216)	183,480 (150,753-213,176)	113,052 (90,109-135,105)	59,955 (54,256-65,663)	63,654 (59,472-67,525)	99,608 (94,911-104,094)	134,750 (129,694-140,685)	177,803 (171,554-185,265)	259,754 (251,812-268,615)	366,592 (355,994-378,114)	537,637 (524,103-551,159)	780,525 (762,210-798,260)	966,161 (945,891-986,692)	1,183,244 (1,158,993-1,203,899)	1,729,921 (1,692,434-1,759,406)	2,089,210 (2,041,297-2,123,703)	2,380,124 (2,320,642-2,419,733)	2,141,784 (2,085,976-2,175,695)	1,602,949 (1,559,440-1,631,035)	1,105,136 (1,077,651-1,122,955)	464,304 (454,145-472,067)	117,456 (114,774-119,664)
Neoplasms	469 (409-502)	310 (283-337)	3,269 (2,985-3,807)	13,375 (11,678-16,095)	18,329 (16,704-20,105)	20,271 (18,700-21,579)	25,270 (23,663-26,491)	26,961 (25,820-28,184)	35,069 (33,681-36,470)	56,359 (54,466-58,291)	82,424 (79,846-84,866)	131,006 (127,515-134,310)	218,192 (213,045-223,325)	286,882 (280,480-292,851)	358,979 (351,025-366,127)	506,092 (494,991-515,307)	584,838 (574,059-595,831)	592,384 (580,611-602,948)	462,944 (453,427-471,282)	279,024 (271,113-285,497)	162,470 (157,598-166,688)	56,713 (54,637-58,462)	11,917 (11,332-12,338)
Lip and oral cavity cancer	-	-	-	-	-	-	230 (206-258)	423 (383-468)	765 (690-847)	1,425 (1,305-1,574)	2,384 (2,205-2,594)	4,419 (4,137-4,732)	6,994 (6,653-7,358)	8,973 (8,573-9,443)	9,969 (9,517-10,488)	11,460 (10,931-12,035)	10,427 (9,980-10,974)	8,729 (8,322-9,164)	5,976 (5,696-6,253)	3,554 (3,331-3,736)	2,040 (1,919-2,141)	762 (718-800)	176 (167-185)
Nasopharynx cancer	-	-	-	-	132 (112-153)	261 (226-297)	403 (362-445)	435 (389-485)	639 (574-720)	1,190 (1,082-1,314)	1,753 (1,610-1,900)	2,789 (2,602-2,993)	4,093 (3,812-4,368)	4,533 (4,222-4,838)	4,795 (4,473-5,127)	4,995 (4,669-5,303)	4,657 (4,392-4,948)	3,552 (3,363-3,758)	2,086 (1,979-2,202)	1,179 (1,119-1,246)	527 (500-554)	178 (170-187)	40 (39-42)
Other pharynx cancer	-	-	-	-	-	-	120 (104-139)	197 (173-226)	256 (222-292)	575 (518-641)	1,219 (1,105-1,351)	2,848 (2,622-3,092)	5,066 (4,736-5,446)	6,832 (6,394-7,346)	7,708 (7,237-8,299)	8,827 (8,209-9,508)	7,846 (7,314-8,563)	6,166 (5,697-6,710)	3,744 (3,436-4,073)	2,048 (1,811-2,256)	908 (804-995)	257 (232-279)	43 (39-47)
Oesophageal cancer	-	-	-	-	-	-	209 (192-229)	354 (327-384)	554 (515-593)	1,361 (1,271-1,457)	2,903 (2,752-3,066)	7,160 (6,831-7,514)	16,041 (15,298-16,854)	23,227 (22,256-24,290)	30,758 (29,494-32,124)	42,108 (40,287-44,079)	46,725 (44,496-48,942)	42,672 (40,572-44,635)	29,071 (27,767-30,424)	16,283 (15,559-17,033)	7,386 (7,077-7,710)	1,902 (1,834-1,977)	335 (321-349)
Stomach cancer	-	-	-	-	-	-	668 (628-713)	1,289 (1,220-1,371)	2,565 (2,433-2,724)	5,675 (5,376-5,973)	9,215 (8,835-9,603)	15,921 (15,253-16,593)	28,703 (27,576-29,818)	37,668 (36,333-39,097)	48,609 (46,858-50,353)	72,934 (70,406-75,429)	84,624 (81,674-87,409)	85,162 (82,332-87,840)	61,481 (59,639-63,568)	35,757 (34,580-36,838)	19,106 (18,524-19,720)	6,069 (5,890-6,268)	1,268 (1,226-1,308)
Colon and rectum cancer	-	-	-	-	-	-	723 (655-804)	1,416 (1,314-1,547)	2,178 (2,032-2,342)	3,890 (3,660-4,128)	5,587 (5,324-5,915)	8,093 (7,782-8,441)	13,478 (13,025-13,969)	18,046 (17,544-18,598)	24,266 (23,580-25,010)	37,056 (35,992-38,225)	46,341 (45,013-47,781)	51,870 (50,433-53,517)	44,456 (43,331-45,593)	28,938 (28,122-29,759)	18,836 (18,280-19,437)	7,052 (6,824-7,284)	1,510 (1,461-1,555)
Liver cancer	-	-	-	-	421 (377-508)	694 (634-811)	1,128 (1,026-1,263)	2,327 (2,086-2,641)	5,545 (5,047-6,124)	12,309 (11,424-13,389)	19,575 (18,306-21,016)	28,045 (26,405-29,674)	43,321 (41,004-46,035)	46,516 (43,996-48,962)	50,247 (47,773-52,879)	58,869 (56,412-61,655)	60,905 (58,446-63,639)	52,488 (50,330-54,792)	34,572 (33,233-35,919)	18,494 (17,669-19,312)	8,376 (8,037-8,715)	2,212 (2,130-2,304)	387 (369-403)
Liver cancer due to hepatitis B	-	-	-	-	106 (71-145)	258 (187-335)	511 (387-642)	1,271 (1,022-1,542)	3,322 (2,655-3,947)	7,532 (6,225-8,675)	11,768 (9,691-13,461)	16,375 (13,537-18,655)	24,331 (19,704-27,815)	24,399 (19,562-28,043)	24,713 (19,415-28,668)	26,647 (20,293-31,034)	25,382 (19,887-29,522)	20,029 (15,501-23,471)	12,321 (9,478-14,552)	6,423 (4,764-7,774)	2,680 (2,112-3,193)	650 (506-804)	113 (77-146)
Liver cancer due to hepatitis C	-	-	-	-	6 (4-8)	12 (9-16)	23 (17-30)	62 (48-81)	175 (125-242)	440 (340-561)	858 (656-1,110)	1,640 (1,294-2,069)	3,367 (2,661-4,176)	5,295 (4,286-6,358)	7,270 (6,048-8,649)	10,170 (8,525-12,116)	11,695 (10,089-13,572)	11,274 (9,837-13,039)	8,236 (7,130-9,607)	4,786 (4,118-5,527)	2,501 (2,215-2,803)	753 (656-854)	139 (117-164)
Liver cancer due to alcohol use	-	-	-	-	-	-	3 (1-4)	25 (15-37)	80 (47-124)	301 (213-415)	868 (616-1,189)	1,694 (1,242-2,277)	2,740 (1,991-3,730)	3,543 (2,588-4,672)	4,138 (2,979-5,616)	5,745 (4,269-7,486)	7,165 (5,659-8,835)	6,961 (5,502-8,496)	4,440 (3,550-5,397)	2,047 (1,588-2,550)	964 (738-1,192)	246 (176-320)	35 (22-51)
Liver cancer due to other causes	-	-	-	-	309 (262-381)	424 (344-521)	591 (474-729)	969 (789-1,159)	1,969 (1,541-2,575)	4,036 (3,223-5,098)	6,081 (4,866-7,845)	8,336 (6,824-10,740)	12,883 (10,483-16,262)	13,278 (10,764-16,677)	14,125 (11,454-17,783)	16,308 (13,455-20,442)	16,663 (13,724-20,298)	14,224 (11,871-17,411)	9,575 (8,056-11,484)	5,238 (4,353-6,403)	2,231 (1,909-2,608)	563 (467-673)	99 (76-125)
Gallbladder and biliary tract cancer	-	-	-	-	-	-	46 (38-57)	72 (59-92)	133 (106-175)	371 (296-481)	698 (547-894)	1,209 (962-1,510)	1,957 (1,589-2,394)	3,000 (2,464-3,605)	4,027 (3,380-4,849)	5,862 (5,020-6,976)	7,111 (6,137-8,556)	8,028 (7,124-9,402)	6,949 (6,147-7,886)	4,556 (4,083-5,162)	2,812 (2,525-3,130)	1,011 (907-1,107)	214 (195-235)
Pancreatic cancer	-	-	-	-	-	-	102 (96-109)	197 (187-212)	366 (349-389)	959 (920-1,007)	1,897 (1,830-1,977)	3,685 (3,542-3,824)	6,629 (6,423-6,840)	9,909 (9,630-10,207)	13,200 (12,877-13,538)	18,765 (18,225-19,340)	21,859 (21,288-22,412)	22,738 (22,187-23,349)	17,654 (17,213-18,103)	10,289 (10,002-10,567)	5,886 (5,705-6,091)	1,970 (1,907-2,034)	386 (373-399)
Larynx cancer	-	-	-	-	-	-	109 (98-119)	136 (125-146)	210 (194-223)	481 (447-512)	1,080 (1,008-1,150)	2,790 (2,638-2,946)	5,721 (5,415-5,990)	8,531 (8,080-8,934)	10,132 (9,667-10,602)	13,568 (12,942-14,217)	13,585 (12,969-14,188)	11,524 (11,059-11,981)	7,374 (7,046-7,668)	4,058 (3,849-4,249)	1,988 (1,899-2,077)	595 (570-620)	108 (103-112)
Tracheal, bronchus, and lung cancer	-	-	-	-	-	-	661 (602-719)	1,218 (1,103-1,344)	2,444 (2,264-2,646)	5,271 (4,929-5,641)	9,664 (9,210-10,157)	20,811 (19,868-21,830)	42,804 (41,186-44,496)	65,254 (63,168-67,504)	87,387 (84,403-90,527)	133,404 (129,275-138,015)	158,559 (153,881-163,648)	158,043 (153,781-162,831)	117,315 (114,453-120,344)	62,230 (60,547-63,969)	30,503 (29,598-31,450)	8,305 (8,050-8,556)	1,385 (1,337-1,427)
Malignant skin melanoma	-	-	-	-	-	-	126 (112-149)	221 (199-270)	348 (315-409)	622 (547-699)	969 (857-1,104)	1,412 (1,261-1,540)	1,670 (1,495-1,805)	2,065 (1,859-2,234)	2,155 (1,992-2,359)	2,348 (2,178-2,542)	2,494 (2,272-2,732)	2,601 (2,413-2,795)	2,203 (1,998-2,350)	1,434 (1,301-1,554)	918 (818-991)	352 (313-386)	79 (72-87)
Non-melanoma skin cancer	-	-	-	-	-	-	123 (113-131)	161 (148-174)	217 (201-234)	365 (343-387)	463 (437-490)	701 (669-732)	954 (916-991)	1,279 (1,223-1,328)	1,599 (1,534-1,659)	2,891 (2,772-3,001)	3,457 (3,322-3,577)	3,738 (3,617-3,854)	3,253 (3,139-3,358)	2,421 (2,320-2,518)	1,793 (1,723-1,858)	888 (855-921)	295 (284-306)
Non-melanoma skin cancer (squamous-cell carcinoma)	-	-	-	-	-	-	123 (113-131)	161 (148-174)	217 (201-234)	365 (343-387)	463 (437-490)	701 (669-732)	954 (916-991)	1,279 (1,223-1,328)	1,599 (1,534-1,659)	2,891 (2,772-3,001)	3,457 (3,322-3,577)	3,738 (3,617-3,854)	3,253 (3,139-3,358)	2,421 (2,320-2,518)	1,793 (1,723-1,858)	888 (855-921)	295 (284-306)
Breast cancer	-	-	-	-	-	-	34 (32-35)	32 (31-33)	39 (36-42)	70 (58-82)	132 (105-148)	209 (159-233)	404 (313-445)	510 (401-561)	606 (471-659)	801 (637-881)	860 (720-964)	865 (728-958)	579 (476-630)	424 (347-464)	284 (224-304)	119 (94-129)	31 (25-34)
Cervical cancer	-	-	-	-	-	-	-	-	-	-	-	-	-	-	-	-	-	-	-	-	-	-	-
Uterine cancer	-	-	-	-	-	-	-	-	-	-	-	-	-	-	-	-	-	-	-	-	-	-	-
Ovarian cancer	-	-	-	-	-	-	-	-	-	-	-	-	-	-	-	-	-	-	-	-	-	-	-
Prostate cancer	-	-	-	-	-	-	70 (57-81)	84 (66-98)	107 (84-126)	139 (107-161)	212 (166-241)	487 (383-557)	1,428 (1,155-1,610)	3,754 (3,024-4,138)	8,280 (6,566-9,136)	19,406 (15,464-21,476)	31,988 (26,101-34,860)	46,267 (39,083-50,340)	52,311 (45,042-57,632)	40,227 (34,181-44,030)	31,563 (26,349-34,055)	14,402 (12,121-15,575)	3,424 (2,890-3,749)
Testicular cancer	-	-	-	-	-	-	492 (456-543)	984 (918-1,075)	1,202 (1,124-1,297)	1,205 (1,138-1,289)	1,024 (965-1,089)	742 (701-787)	588 (553-626)	428 (401-464)	305 (286-325)	379 (355-408)	381 (358-405)	381 (357-406)	278 (263-294)	217 (202-233)	123 (116-130)	47 (44-50)	13 (12-13)
Kidney cancer	18 (15-20)	10 (8-11)	128 (109-156)	598 (491-738)	305 (273-342)	142 (131-157)	114 (107-124)	149 (140-163)	222 (210-240)	362 (344-385)	726 (684-767)	1,446 (1,371-1,535)	2,784 (2,646-2,925)	4,323 (4,139-4,537)	5,484 (5,270-5,702)	7,310 (6,983-7,681)	8,078 (7,785-8,393)	8,464 (8,163-8,778)	6,952 (6,723-7,201)	4,090 (3,952-4,233)	2,583 (2,477-2,697)	894 (857-936)	168 (161-175)
Bladder cancer	-	-	-	-	-	-	70 (64-77)	126 (117-136)	168 (156-180)	368 (345-395)	686 (639-737)	1,309 (1,238-1,388)	2,492 (2,366-2,626)	3,968 (3,794-4,166)	5,806 (5,605-6,037)	10,539 (10,143-10,953)	14,228 (13,755-14,722)	18,237 (17,595-18,867)	17,170 (16,641-17,691)	12,372 (11,973-12,753)	8,732 (8,448-9,028)	3,467 (3,354-3,578)	766 (739-796)
Brain and nervous system cancer	67 (40-81)	45 (29-54)	659 (413-842)	2,519 (1,833-3,083)	3,946 (2,975-4,483)	3,862 (2,894-4,284)	2,689 (2,127-2,964)	2,462 (1,959-2,679)	3,163 (2,506-3,432)	4,173 (3,284-4,523)	5,094 (4,109-5,525)	6,287 (5,115-6,787)	7,601 (6,239-8,167)	8,268 (6,870-8,859)	8,868 (7,397-9,488)	10,250 (8,610-10,991)	10,020 (8,370-10,661)	8,206 (7,000-8,763)	5,496 (4,560-5,904)	2,668 (2,169-2,876)	1,154 (897-1,245)	295 (238-318)	53 (44-57)
Thyroid cancer	-	-	-	-	-	72 (53-86)	115 (95-132)	164 (145-182)	206 (184-223)	225 (205-243)	291 (263-313)	459 (404-497)	753 (673-806)	998 (920-1,061)	1,235 (1,154-1,310)	1,316 (1,245-1,403)	1,443 (1,372-1,543)	1,391 (1,320-1,500)	1,244 (1,193-1,352)	785 (748-864)	412 (393-446)	136 (130-147)	27 (26-29)
Mesothelioma	-	-	-	-	-	-	57 (50-71)	64 (55-79)	107 (90-134)	131 (111-164)	221 (189-277)	351 (310-427)	635 (572-758)	964 (879-1,096)	1,451 (1,331-1,636)	1,710 (1,564-1,889)	2,057 (1,896-2,254)	2,299 (2,133-2,491)	2,125 (1,982-2,351)	1,272 (1,182-1,406)	670 (622-752)	195 (181-218)	34 (31-38)
Hodgkin lymphoma	12 (7-14)	3 (1-3)	65 (38-79)	383 (234-553)	1,186 (689-1,574)	1,242 (794-1,524)	1,232 (878-1,497)	1,115 (835-1,358)	1,178 (901-1,430)	1,237 (920-1,557)	1,253 (951-1,524)	1,296 (943-1,525)	1,558 (1,138-1,816)	1,451 (1,109-1,759)	1,667 (1,254-1,975)	1,815 (1,386-2,168)	1,891 (1,446-2,183)	1,708 (1,359-1,985)	882 (690-1,002)	463 (355-520)	230 (181-256)	63 (52-70)	10 (9-12)
Non-Hodgkin's lymphoma	32 (24-36)	23 (20-27)	281 (227-342)	1,416 (1,088-1,783)	2,212 (1,725-2,577)	2,188 (1,751-2,481)	2,220 (1,857-2,445)	2,308 (1,998-2,575)	2,281 (2,029-2,511)	2,770 (2,382-2,973)	3,247 (2,803-3,474)	4,139 (3,568-4,407)	5,223 (4,621-5,656)	6,368 (5,664-6,971)	7,440 (6,604-8,150)	9,481 (8,717-10,438)	10,978 (9,821-12,159)	11,433 (10,050-12,579)	9,747 (8,470-10,633)	6,203 (5,263-6,857)	3,681 (3,127-3,981)	1,205 (1,062-1,332)	230 (210-261)
Multiple myeloma	-	-	-	-	-	-	118 (96-141)	146 (112-171)	166 (132-199)	334 (271-403)	534 (445-639)	997 (826-1,161)	1,386 (1,161-1,604)	2,142 (1,818-2,517)	2,956 (2,510-3,469)	4,024 (3,234-4,735)	4,875 (3,978-5,788)	5,445 (4,414-6,342)	4,751 (3,737-5,491)	3,043 (2,441-3,689)	1,914 (1,515-2,259)	619 (491-766)	108 (80-126)
Leukaemia	115 (101-135)	98 (89-120)	1,170 (1,003-1,617)	5,251 (4,463-7,154)	7,448 (6,628-9,167)	8,245 (7,444-9,497)	7,764 (6,775-8,634)	6,339 (5,465-7,093)	5,825 (5,044-6,401)	5,925 (4,989-6,410)	5,904 (5,025-6,324)	6,185 (5,335-6,593)	7,174 (6,387-7,599)	7,657 (6,871-8,197)	8,687 (7,728-9,405)	11,471 (10,482-12,311)	13,426 (12,562-14,551)	14,444 (13,648-15,483)	12,424 (11,910-13,144)	7,885 (7,584-8,363)	5,023 (4,817-5,305)	1,850 (1,782-1,954)	405 (388-426)
Acute lymphoid leukaemia	29 (24-40)	24 (19-33)	320 (237-541)	1,792 (1,337-2,848)	2,783 (2,328-3,785)	2,781 (2,417-3,516)	2,284 (2,063-2,705)	1,507 (1,367-1,808)	1,100 (983-1,316)	1,020 (901-1,222)	952 (868-1,128)	961 (856-1,092)	1,037 (918-1,160)	1,098 (966-1,200)	1,169 (1,012-1,292)	1,322 (1,149-1,439)	1,341 (1,179-1,482)	1,333 (1,181-1,482)	648 (579-711)	361 (323-397)	198 (177-219)	66 (58-71)	15 (13-16)
Chronic lymphoid leukaemia	-	-	-	-	-	-	147 (129-192)	138 (120-189)	133 (115-173)	138 (116-168)	131 (115-158)	201 (176-246)	348 (308-415)	595 (523-716)	888 (765-1,109)	1,548 (1,370-1,877)	2,070 (1,829-2,544)	2,585 (2,276-3,144)	2,309 (2,027-2,784)	1,695 (1,421-2,036)	1,264 (1,034-1,489)	506 (431-590)	113 (99-133)
Acute myeloid leukaemia	19 (13-26)	19 (14-26)	206 (155-330)	1,020 (723-1,668)	1,086 (811-1,466)	1,172 (938-1,507)	1,293 (1,121-1,569)	1,121 (949-1,369)	1,034 (888-1,238)	1,105 (932-1,306)	1,234 (1,048-1,445)	1,398 (1,216-1,637)	1,625 (1,400-1,880)	1,979 (1,762-2,270)	2,277 (1,950-2,641)	2,902 (2,534-3,331)	3,529 (3,117-4,047)	3,774 (3,296-4,451)	3,112 (2,727-3,718)	1,795 (1,580-2,184)	998 (854-1,237)	310 (281-411)	56 (50-78)
Chronic myeloid leukaemia	-	-	-	-	-	-	270 (233-333)	349 (297-427)	542 (454-636)	638 (527-725)	732 (618-829)	763 (669-864)	938 (816-1,045)	937 (822-1,038)	996 (871-1,107)	1,158 (1,019-1,271)	1,318 (1,179-1,427)	1,356 (1,195-1,466)	1,462 (1,314-1,584)	903 (812-984)	600 (549-659)	231 (205-252)	49 (44-53)
Other leukaemia	68 (52-78)	56 (46-66)	644 (533-862)	2,438 (2,011-3,282)	3,578 (2,982-4,302)	4,292 (3,489-4,817)	3,769 (3,005-4,167)	3,223 (2,530-3,606)	3,015 (2,419-3,297)	3,024 (2,335-3,323)	2,855 (2,210-3,104)	2,863 (2,259-3,091)	3,226 (2,595-3,500)	3,047 (2,518-3,298)	3,356 (2,772-3,605)	4,540 (3,878-4,987)	5,168 (4,532-5,606)	5,396 (4,827-5,909)	4,892 (4,457-5,346)	3,131 (2,878-3,417)	1,963 (1,804-2,175)	737 (670-793)	172 (156-183)
Other neoplasms	225 (187-250)	132 (112-151)	965 (800-1,241)	3,209 (2,662-4,422)	2,680 (2,380-3,107)	3,564 (3,238-3,950)	5,648 (5,201-6,078)	4,542 (4,218-4,836)	4,185 (3,798-4,458)	4,926 (4,421-5,277)	5,692 (5,160-6,020)	7,216 (6,541-7,623)	8,736 (8,147-9,195)	10,216 (9,573-10,722)	11,342 (10,693-11,852)	14,503 (13,687-15,300)	16,021 (15,302-16,747)	15,936 (15,241-16,726)	12,849 (12,323-13,538)	8,135 (7,736-8,599)	5,025 (4,775-5,330)	1,867 (1,766-1,979)	422 (401-449)
Cardiovascular diseases	3,107 (2,822-3,386)	1,670 (1,534-1,806)	14,880 (13,689-16,156)	11,376 (9,944-12,723)	7,196 (6,550-7,803)	9,050 (8,444-9,640)	22,728 (21,518-24,227)	37,504 (35,695-39,867)	55,344 (53,013-58,838)	88,617 (85,266-93,328)	134,669 (129,739-141,302)	213,329 (206,521-221,508)	321,028 (311,355-332,258)	408,975 (397,427-421,698)	503,624 (491,778-516,208)	764,036 (745,974-781,809)	935,222 (914,048-954,711)	1,103,026 (1,077,319-1,125,891)	1,006,293 (983,062-1,026,649)	774,880 (754,707-792,395)	554,650 (541,300-568,033)	238,921 (232,842-244,547)	63,579 (61,640-65,246)
Rheumatic heart disease	-	-	-	2,651 (2,051-3,448)	1,806 (1,542-2,097)	2,964 (2,620-3,296)	5,314 (4,664-5,978)	5,321 (4,550-6,051)	5,388 (4,505-6,054)	6,128 (5,123-6,892)	6,702 (5,684-7,623)	8,044 (6,800-9,125)	9,108 (7,851-10,214)	9,603 (8,317-10,886)	10,590 (9,230-11,839)	13,301 (11,666-14,870)	15,360 (13,653-17,062)	15,936 (14,131-17,413)	13,168 (11,888-14,214)	10,022 (9,124-10,798)	6,545 (6,059-6,990)	2,760 (2,572-2,932)	767 (724-810)
Ischaemic heart disease	-	-	943 (785-1,119)	704 (549-890)	468 (405-550)	562 (502-626)	6,622 (6,147-7,186)	13,874 (13,113-14,849)	24,233 (22,938-26,042)	44,746 (42,838-47,618)	71,691 (68,568-75,609)	116,347 (111,751-121,937)	178,448 (172,204-185,828)	226,864 (219,509-235,421)	272,932 (264,985-281,319)	400,045 (387,867-411,955)	467,889 (454,656-481,272)	549,396 (533,689-564,137)	505,207 (491,271-518,285)	390,737 (379,939-402,250)	297,659 (290,513-306,887)	138,576 (135,611-142,941)	39,012 (38,133-40,267)
Cerebrovascular disease	1,524 (1,294-1,775)	779 (669-931)	5,910 (4,971-7,232)	3,633 (2,975-4,562)	2,551 (2,236-2,938)	2,852 (2,605-3,132)	5,459 (5,120-5,903)	10,709 (10,125-11,537)	15,144 (14,297-16,380)	24,656 (23,431-26,414)	37,512 (35,681-39,681)	61,537 (58,783-64,399)	98,319 (94,416-102,566)	130,883 (126,187-136,361)	171,694 (165,576-178,265)	280,115 (270,863-290,004)	364,021 (351,937-376,624)	429,477 (413,374-445,452)	376,667 (361,002-393,686)	277,867 (262,394-292,208)	175,645 (164,825-186,794)	64,187 (59,821-69,124)	15,293 (13,933-16,488)

SR Table 4: Global deaths by cause and age group for males (with 95% uncertainty intervals), 2000

Cause	Early Neonatal	Late Neonatal	Post Neonatal	1 to 4	5 to 9	10 to 14	15 to 19	20 to 24	25 to 29	30 to 34	35 to 39	40 to 44	45 to 49	50 to 54	55 to 59	60 to 64	65 to 69	70 to 74	75 to 79	80 to 84	85 to 89	90 to 94	95 plus
Ischaemic stroke	-	-	186 (158-229)	190 (154-243)	159 (140-184)	193 (172-216)	658 (599-730)	1,396 (1,267-1,535)	1,973 (1,805-2,154)	3,126 (2,883-3,383)	4,825 (4,420-5,189)	8,912 (8,279-9,481)	19,371 (18,103-20,610)	30,164 (28,169-31,981)	44,171 (41,487-46,491)	108,191 (102,962-113,577)	150,372 (143,882-156,873)	194,573 (185,901-202,880)	197,386 (188,297-206,462)	150,188 (141,124-158,045)	103,582 (97,062-110,201)	40,198 (37,357-43,177)	9,935 (9,032-10,726)
Hemorrhagic stroke	1,524 (1,294-1,775)	779 (669-931)	5,725 (4,802-7,016)	3,442 (2,811-4,324)	2,392 (2,093-2,766)	2,659 (2,428-2,927)	4,800 (4,510-5,186)	9,313 (8,775-10,011)	13,171 (12,412-14,302)	21,529 (20,455-23,126)	32,686 (31,094-34,739)	52,625 (50,304-55,198)	78,949 (75,808-82,791)	100,719 (96,787-105,534)	127,523 (122,712-133,482)	171,924 (165,449-180,155)	213,649 (205,893-223,584)	234,904 (225,041-246,506)	179,282 (171,121-188,471)	127,679 (120,536-135,019)	72,062 (67,492-76,836)	23,989 (22,283-25,936)	5,359 (4,866-5,793)
Hypertensive heart disease	-	-	274 (186-367)	102 (62-142)	97 (66-121)	134 (82-176)	785 (586-971)	1,337 (990-1,632)	1,889 (1,435-2,267)	2,888 (2,125-3,452)	4,476 (3,497-5,309)	7,048 (5,432-8,396)	10,614 (8,189-13,004)	13,832 (10,617-16,275)	18,152 (13,718-21,522)	26,957 (20,555-31,867)	34,691 (25,827-40,285)	41,845 (30,699-48,583)	41,385 (30,020-47,067)	34,827 (26,290-40,477)	24,011 (17,640-28,122)	10,503 (7,964-12,074)	2,787 (2,020-3,327)
Cardiomyopathy and myocarditis	567 (350-711)	322 (224-402)	2,671 (1,763-3,399)	1,573 (1,087-2,017)	822 (616-987)	975 (828-1,174)	2,011 (1,704-2,390)	2,602 (2,125-2,947)	3,874 (3,136-4,398)	4,623 (3,841-5,365)	7,281 (6,049-8,573)	10,814 (8,836-12,906)	12,539 (10,201-14,766)	12,638 (10,633-15,215)	11,140 (9,346-13,050)	14,000 (11,758-16,814)	13,105 (10,569-14,470)	13,737 (10,157-15,064)	13,066 (8,860-14,546)	10,500 (6,421-11,921)	9,209 (5,206-10,685)	4,549 (2,494-5,327)	1,318 (718-1,539)
Myocarditis	124 (55-179)	95 (53-133)	1,124 (604-1,552)	710 (384-1,020)	368 (238-470)	400 (305-530)	769 (614-977)	770 (615-966)	1,016 (797-1,305)	785 (621-1,016)	866 (675-1,108)	1,004 (759-1,295)	802 (590-992)	685 (497-840)	776 (545-973)	821 (557-990)	957 (629-1,138)	1,172 (749-1,404)	1,231 (770-1,507)	1,094 (661-1,378)	1,196 (679-1,610)	758 (405-1,033)	271 (131-370)
Alcoholic cardiomyopathy	-	-	-	-	-	-	170 (131-252)	453 (281-588)	1,086 (694-1,376)	1,897 (1,366-2,344)	3,804 (2,945-4,822)	6,256 (4,737-8,010)	7,765 (6,079-9,767)	7,395 (5,897-9,429)	5,345 (4,269-6,699)	6,965 (5,670-9,037)	4,689 (3,774-5,842)	3,883 (2,809-4,738)	2,360 (1,449-2,719)	1,301 (688-1,563)	811 (366-1,008)	314 (145-390)	94 (44-115)
Other cardiomyopathy	443 (288-577)	227 (158-294)	1,546 (1,026-2,133)	863 (611-1,255)	454 (344-582)	575 (477-700)	1,072 (867-1,304)	1,378 (1,111-1,595)	1,772 (1,409-2,081)	1,941 (1,618-2,279)	2,611 (2,089-3,096)	3,553 (2,824-4,502)	3,971 (3,250-5,000)	4,559 (3,750-5,562)	5,019 (4,054-6,088)	6,214 (4,902-7,299)	7,458 (5,691-8,198)	8,682 (6,232-9,672)	9,474 (6,164-10,757)	8,105 (4,891-9,353)	7,202 (4,002-8,679)	3,477 (1,840-4,308)	954 (520-1,211)
Atrial fibrillation and flutter	-	-	-	-	-	-	-	-	-	17 (12-23)	65 (47-86)	149 (108-197)	285 (205-378)	448 (332-580)	727 (428-1,095)	1,520 (998-2,159)	2,939 (1,817-4,404)	5,461 (3,900-7,596)	7,417 (4,705-11,122)	11,823 (8,845-15,231)	12,648 (9,566-16,527)	5,623 (4,267-7,321)	1,259 (968-1,621)
Aortic aneurysm	-	-	-	-	-	-	266 (234-301)	359 (320-398)	577 (503-649)	792 (706-883)	1,267 (1,147-1,394)	1,735 (1,594-1,875)	2,436 (2,269-2,610)	3,268 (3,095-3,448)	4,566 (4,340-4,814)	7,639 (7,265-8,042)	10,595 (10,126-11,054)	13,718 (13,185-14,305)	14,164 (13,636-14,775)	10,531 (10,146-10,971)	7,323 (7,062-7,643)	2,872 (2,774-2,991)	578 (557-602)
Peripheral vascular disease	-	-	-	-	-	-	-	-	-	-	-	126 (75-165)	253 (145-329)	491 (289-631)	734 (442-907)	1,578 (946-2,015)	2,058 (1,309-2,533)	3,065 (2,080-3,935)	3,135 (2,123-4,010)	2,826 (1,930-3,642)	2,565 (1,789-3,488)	1,335 (936-1,806)	413 (292-575)
Endocarditis	80 (44-109)	44 (26-61)	982 (661-1,246)	369 (247-460)	169 (116-203)	266 (194-334)	550 (410-743)	810 (614-1,097)	1,063 (838-1,450)	1,135 (874-1,530)	1,343 (1,074-1,816)	1,594 (1,280-2,144)	1,554 (1,234-2,045)	1,673 (1,325-2,260)	1,716 (1,380-2,242)	2,228 (1,766-2,992)	2,621 (2,063-3,463)	2,865 (2,267-3,699)	2,960 (2,272-3,781)	2,365 (1,843-2,994)	1,677 (1,219-2,114)	761 (556-945)	208 (157-265)
Other cardiovascular and circulatory diseases	936 (712-1,168)	525 (404-649)	4,100 (3,198-5,419)	2,345 (1,598-3,354)	1,283 (945-1,682)	1,298 (976-1,687)	1,721 (1,343-2,299)	2,492 (2,096-3,567)	3,175 (2,492-4,426)	3,632 (2,880-4,787)	4,332 (3,320-5,536)	5,934 (4,741-7,409)	7,471 (5,784-9,255)	9,274 (7,422-11,124)	11,374 (9,056-13,883)	16,652 (12,810-19,325)	21,944 (16,725-25,279)	27,583 (21,308-31,731)	29,124 (23,223-32,782)	23,383 (18,332-26,256)	17,368 (14,577-19,493)	7,754 (6,931-8,734)	1,944 (1,760-2,186)
Chronic respiratory diseases	0 (0-0)	0 (0-0)	0 (0-0)	12,179 (9,613-15,232)	3,560 (3,099-4,038)	3,758 (3,389-4,158)	6,265 (5,832-6,749)	8,612 (8,119-9,231)	11,395 (10,823-12,172)	14,579 (13,891-15,461)	20,042 (19,000-21,239)	28,615 (27,232-30,017)	45,562 (43,523-47,775)	70,227 (66,845-73,443)	107,603 (102,531-112,306)	198,855 (189,808-206,522)	281,790 (268,308-292,629)	368,208 (350,067-382,002)	349,367 (330,526-362,104)	274,812 (259,917-285,338)	168,713 (160,371-175,411)	60,552 (57,677-63,146)	14,171 (13,483-14,832)
Chronic obstructive pulmonary disease	-	-	0 (0-0)	2,455 (1,408-4,921)	745 (531-1,101)	609 (457-832)	2,550 (2,034-3,088)	3,734 (3,050-4,501)	5,197 (4,173-6,267)	7,608 (6,363-8,983)	10,608 (8,872-12,576)	17,312 (14,805-20,002)	30,561 (26,335-34,550)	50,685 (44,500-56,352)	81,919 (72,725-90,185)	161,564 (147,611-174,552)	238,249 (220,280-254,252)	320,556 (301,655-338,053)	308,062 (290,083-323,414)	247,463 (233,554-259,196)	153,583 (145,553-160,720)	55,202 (52,513-58,004)	12,950 (12,298-13,645)
Pneumoconiosis	-	-	-	-	-	-	28 (23-36)	48 (40-62)	153 (122-217)	361 (276-510)	548 (460-757)	629 (534-828)	873 (735-1,239)	955 (804-1,327)	1,129 (945-1,599)	1,986 (1,696-2,745)	2,912 (2,546-3,859)	3,604 (3,212-4,446)	3,264 (2,962-3,947)	2,110 (1,928-2,455)	1,266 (1,159-1,458)	388 (354-446)	81 (75-93)
Silicosis	-	-	-	-	-	-	4 (2-6)	12 (9-15)	68 (50-96)	226 (166-311)	375 (309-511)	409 (341-558)	549 (446-848)	569 (467-871)	630 (515-946)	1,058 (875-1,581)	1,674 (1,415-2,339)	1,959 (1,685-2,486)	1,757 (1,545-2,097)	1,073 (936-1,311)	604 (523-714)	153 (130-179)	26 (22-30)
Asbestosis	-	-	-	-	-	-	11 (9-16)	16 (13-21)	22 (18-32)	29 (22-43)	33 (23-47)	40 (27-59)	44 (28-64)	57 (36-78)	83 (46-116)	145 (85-211)	233 (132-358)	338 (201-538)	361 (217-616)	274 (168-426)	181 (110-300)	59 (35-99)	11 (7-19)
Coal workers pneumoconiosis	-	-	-	-	-	-	1 (1-1)	2 (1-3)	18 (10-27)	21 (12-33)	38 (21-52)	61 (32-86)	104 (59-143)	120 (65-172)	159 (86-224)	298 (165-410)	482 (247-632)	653 (339-829)	589 (323-718)	445 (278-536)	277 (164-329)	82 (43-97)	16 (8-19)
Other pneumoconiosis	-	-	-	-	-	-	12 (10-17)	19 (15-27)	45 (32-78)	85 (53-153)	103 (76-163)	118 (85-168)	177 (127-271)	209 (146-326)	257 (173-410)	485 (334-747)	522 (365-822)	654 (474-1,017)	557 (402-863)	317 (232-502)	205 (154-325)	94 (73-146)	28 (23-41)
Asthma	-	-	-	7,752 (5,097-10,633)	2,069 (1,554-2,606)	2,594 (2,132-3,045)	3,073 (2,395-3,725)	3,989 (2,991-4,877)	5,223 (3,908-6,263)	5,461 (3,977-6,800)	7,457 (5,295-9,489)	8,847 (5,991-11,671)	11,729 (7,734-15,915)	15,636 (9,795-22,311)	20,507 (12,648-29,106)	29,690 (18,346-42,553)	33,306 (20,480-47,730)	35,191 (22,757-51,396)	29,641 (18,840-44,018)	18,829 (12,943-30,252)	9,710 (6,826-13,955)	3,302 (2,356-5,039)	756 (548-1,154)
Interstitial lung disease and pulmonary sarcoidosis	-	-	-	167 (69-389)	70 (38-133)	47 (27-78)	116 (64-195)	176 (100-288)	245 (139-408)	349 (202-573)	535 (311-901)	796 (465-1,310)	1,190 (699-1,859)	1,767 (1,054-2,734)	2,538 (1,489-3,896)	3,804 (2,315-5,762)	5,403 (3,355-7,664)	6,778 (4,322-9,107)	6,728 (4,410-8,724)	4,965 (3,248-6,318)	3,114 (2,021-3,979)	1,144 (774-1,512)	245 (170-321)
Other chronic respiratory diseases	0 (0-0)	0 (0-0)	0 (0-0)	1,805 (591-3,395)	676 (371-1,040)	508 (275-787)	497 (293-712)	664 (417-952)	577 (330-859)	800 (448-1,209)	894 (494-1,297)	1,031 (576-1,452)	1,209 (691-1,672)	1,184 (668-1,606)	1,510 (860-2,066)	1,810 (1,105-2,425)	1,921 (1,092-2,543)	2,080 (1,264-2,761)	1,671 (1,002-2,137)	1,444 (881-1,832)	1,039 (627-1,282)	516 (304-653)	139 (89-175)
Cirrhosis and other chronic liver diseases	-	-	-	5,899 (4,228-8,703)	2,481 (2,035-3,329)	2,801 (2,428-3,454)	4,856 (4,383-5,777)	9,119 (8,233-11,464)	15,787 (14,394-18,060)	28,882 (26,804-32,605)	44,262 (41,405-49,627)	60,957 (57,330-68,159)	74,971 (70,909-81,660)	74,912 (71,463-82,410)	71,800 (67,969-79,351)	77,017 (73,123-86,783)	67,745 (64,353-73,578)	55,503 (52,288-60,050)	38,774 (36,552-42,193)	22,157 (20,267-23,613)	11,611 (10,781-12,398)	3,870 (3,544-4,147)	951 (872-1,017)
Cirrhosis and other chronic liver diseases due to hepatitis B	-	-	-	68 (40-110)	76 (52-113)	150 (109-207)	434 (299-619)	2,393 (1,807-3,257)	5,825 (4,600-7,303)	11,221 (9,630-13,204)	16,559 (13,898-19,382)	21,164 (18,174-24,293)	23,944 (20,168-28,127)	22,709 (19,165-27,081)	22,631 (19,230-26,792)	25,074 (21,153-30,063)	22,537 (19,148-26,761)	18,474 (15,385-22,277)	12,957 (10,844-15,248)	7,148 (5,827-8,543)	3,303 (2,807-3,824)	943 (758-1,129)	208 (152-272)
Cirrhosis and other chronic liver diseases due to hepatitis C	-	-	-	52 (32-82)	55 (37-83)	115 (78-167)	307 (205-449)	1,495 (1,082-2,011)	3,439 (2,587-4,417)	7,048 (5,936-8,512)	11,530 (9,277-14,237)	16,509 (13,842-19,532)	20,705 (17,025-24,798)	21,314 (17,700-25,732)	20,064 (16,968-23,682)	21,074 (17,453-25,525)	18,844 (15,926-22,231)	15,599 (13,225-18,200)	10,534 (8,652-12,747)	5,979 (4,852-7,237)	3,145 (2,569-3,701)	1,079 (834-1,311)	262 (187-338)
Cirrhosis and other chronic liver diseases due to alcohol use	-	-	-	-	-	-	86 (49-138)	713 (507-1,010)	1,829 (1,316-2,515)	5,406 (4,548-6,570)	11,087 (9,116-13,590)	17,220 (14,903-19,903)	22,389 (18,889-26,237)	22,771 (19,327-26,728)	20,875 (17,379-24,699)	21,900 (17,773-26,345)	18,839 (15,883-21,887)	15,272 (12,870-18,029)	10,701 (9,002-12,618)	6,047 (5,057-7,075)	3,435 (3,000-3,893)	1,229 (1,048-1,409)	319 (258-382)
Cirrhosis and other chronic liver diseases due to other causes	-	-	-	5,780 (4,150-8,537)	2,351 (1,922-3,133)	2,536 (2,191-3,108)	4,029 (3,593-4,748)	4,519 (3,622-5,610)	4,695 (3,512-5,996)	5,207 (3,857-6,681)	5,085 (3,577-6,812)	6,065 (4,371-8,129)	7,933 (5,695-10,683)	8,118 (6,038-10,866)	8,230 (5,990-11,043)	8,970 (6,460-12,201)	7,526 (5,439-9,974)	6,158 (4,478-8,258)	4,581 (3,339-5,955)	2,984 (2,197-3,931)	1,729 (1,351-2,159)	620 (473-798)	163 (115-224)
Digestive diseases	1,061 (880-1,293)	1,212 (922-1,487)	11,107 (8,233-14,595)	10,056 (6,536-13,879)	5,144 (4,277-6,009)	4,783 (4,097-5,411)	7,131 (6,251-7,989)	9,097 (8,138-10,035)	13,402 (11,738-14,717)	15,442 (13,710-17,018)	20,343 (18,219-22,653)	25,587 (22,777-28,086)	30,141 (26,842-32,478)	31,065 (27,697-33,500)	35,395 (31,053-38,238)	45,269 (40,625-48,679)	51,659 (45,652-56,337)	56,358 (50,284-60,708)	51,320 (46,462-55,594)	37,851 (34,507-40,969)	26,053 (23,358-27,965)	11,244 (10,148-12,101)	2,862 (2,568-3,122)
Peptic ulcer disease	-	-	-	1,541 (990-2,252)	882 (676-1,137)	1,072 (861-1,338)	1,868 (1,507-2,250)	2,914 (2,375-3,379)	3,821 (3,142-4,428)	5,068 (4,288-5,779)	6,432 (5,499-7,393)	8,824 (7,586-9,919)	11,259 (9,584-12,450)	12,322 (10,766-13,598)	13,957 (11,873-15,339)	17,979 (15,630-19,718)	19,717 (16,782-21,747)	20,827 (17,883-22,763)	18,143 (15,800-19,911)	12,644 (11,029-13,898)	7,910 (6,943-8,677)	3,057 (2,690-3,371)	736 (646-815)
Gastritis and duodenitis	-	-	-	366 (150-789)	132 (87-217)	103 (78-146)	227 (181-284)	299 (245-371)	506 (421-621)	834 (707-1,022)	955 (811-1,179)	1,129 (962-1,351)	1,430 (1,212-1,706)	1,530 (1,270-1,761)	1,803 (1,466-2,114)	2,183 (1,840-2,536)	2,247 (1,912-2,572)	2,432 (2,062-2,801)	2,235 (1,882-2,581)	1,711 (1,429-1,975)	1,102 (915-1,270)	444 (369-509)	120 (102-138)
Appendicitis	-	-	-	1,478 (749-2,199)	1,137 (894-1,446)	1,143 (911-1,403)	1,425 (1,164-1,770)	1,097 (915-1,377)	1,367 (1,118-1,691)	897 (733-1,092)	1,168 (932-1,466)	1,414 (1,134-1,749)	1,191 (939-1,482)	1,161 (919-1,474)	1,244 (994-1,614)	1,475 (1,170-1,901)	1,420 (1,160-1,825)	1,541 (1,228-1,992)	1,247 (1,035-1,647)	863 (698-1,168)	529 (432-693)	210 (178-267)	54 (46-67)
Paralytic ileus and intestinal obstruction	1,061 (880-1,293)	1,212 (922-1,487)	11,107 (8,233-14,595)	2,368 (1,440-3,289)	1,632 (1,079-1,982)	1,232 (832-1,491)	1,815 (1,257-2,219)	2,064 (1,428-2,568)	2,956 (2,006-3,521)	2,524 (1,770-3,039)	3,731 (2,492-4,386)	4,085 (2,543-4,865)	4,705 (3,124-5,486)	4,748 (3,312-5,643)	6,109 (4,369-7,377)	7,196 (5,121-8,613)	8,917 (6,712-10,775)	8,957 (6,802-11,221)	8,148 (6,495-10,520)	6,390 (5,059-7,915)	4,724 (3,687-5,988)	2,145 (1,667-2,605)	551 (421-690)
Inguinal, femoral, and abdominal hernia	-	-	-	824 (321-1,404)	278 (158-371)	284 (139-396)	389 (224-504)	519 (284-683)	606 (342-750)	588 (288-765)	675 (407-839)	1,048 (652-1,281)	1,044 (695-1,290)	1,304 (876-1,667)	1,556 (1,010-2,034)	1,965 (1,351-2,548)	2,525 (1,727-3,227)	2,946 (1,996-3,793)	3,059 (2,109-3,922)	2,159 (1,548-2,790)	1,571 (1,156-1,957)	766 (597-930)	218 (165-273)
Inflammatory bowel disease	-	-	-	720 (314-1,844)	172 (92-298)	147 (93-223)	169 (112-212)	260 (172-331)	416 (294-537)	456 (308-589)	505 (360-642)	685 (499-846)	772 (542-940)	789 (584-910)	910 (662-1,044)	1,136 (860-1,294)	1,340 (1,017-1,510)	1,579 (1,188-1,780)	1,579 (1,206-1,747)	1,307 (950-1,449)	973 (691-1,085)	448 (316-501)	117 (83-132)
Vascular intestinal disorders	-	-	-	380 (100-768)	58 (30-89)	34 (25-41)	65 (54-83)	120 (93-146)	183 (142-220)	181 (141-226)	346 (280-430)	624 (512-752)	960 (765-1,166)	1,297 (1,058-1,599)	1,809 (1,576-2,253)	2,940 (2,514-3,792)	4,080 (3,529-5,204)	5,382 (4,562-6,836)	4,999 (4,158-6,054)	3,534 (2,701-4,222)	2,537 (1,816-2,847)	1,105 (829-1,239)	258 (190-302)
Gallbladder and biliary diseases	-	-	-	226 (125-361)	111 (86-156)	162 (109-207)	201 (156-253)	296 (247-369)	371 (303-449)	505 (418-610)	742 (595-912)	962 (824-1,155)	1,157 (952-1,362)	1,371 (1,101-1,590)	1,638 (1,361-1,918)	2,521 (2,105-2,891)	3,404 (2,892-4,043)	4,223 (3,607-4,933)	4,362 (3,752-5,034)	3,621 (3,171-4,255)	2,835 (2,463-3,313)	1,307 (1,148-1,485)	330 (283-383)
Pancreatitis	-	-	-	109 (61-177)	83 (60-111)	110 (85-139)	369 (280-472)	1,009 (815-1,247)	2,377 (1,960-2,875)	3,294 (2,773-3,988)	4,495 (3,694-5,489)	5,325 (4,505-6,538)	5,666 (4,748-6,905)	4,371 (3,711-5,367)	3,934 (3,268-4,821)	4,254 (3,597-5,352)	4,080 (3,264-5,168)	3,826 (3,063-4,706)	3,157 (2,540-3,968)	1,838 (1,443-2,229)	1,174 (880-1,408)	417 (308-505)	98 (78-127)
Other digestive diseases	-	-	-	2,044 (762-3,737)	659 (418-1,037)	497 (349-722)	603 (471-767)	518 (418-702)	800 (629-1,056)	1,094 (897-1,438)	1,294 (1,075-1,716)	1,492 (1,254-1,915)	1,956 (1,660-2,416)	2,173 (1,853-2,675)	2,435 (2,066-2,982)	3,620 (3,036-4,435)	3,929 (3,356-4,725)	4,648 (3,864-5,642)	4,392 (3,712-5,414)	3,784 (3,139-4,592)	2,697 (2,144-3,356)	1,345 (1,065-1,705)	381 (295-480)
Neurological disorders	-	-	3,902 (3,234-4,725)	5,245 (3,820-6,804)	3,416 (2,970-3,940)	5,355 (4,827-5,966)	9,479 (8,830-10,081)	8,777 (8,247-9,340)	7,545 (7,146-7,979)	7,292 (6,951-7,645)	8,088 (7,702-8,495)	7,418 (7,088-7,763)	8,660 (8,164-9,264)	9,748 (8,714-11,065)	12,434 (10,542-14,701)	20,263 (16,881-24,861)	35,131 (28,336-43,557)	64,133 (50,717-81,396)	101,485 (80,652-126,510)	115,075 (92,491-144,279)	111,723 (90,142-136,636)	62,184 (50,516-73,636)	15,692 (12,455-19,699)
Alzheimer's disease and other dementias	-	-	-	-	-	-	-	-	-	-	-	44 (18-86)	933 (537-1,447)	2,983 (2,000-4,253)	5,427 (3,635-7,721)	11,930 (8,595-16,400)	24,143 (17,609-32,361)	48,444 (35,253-65,402)	81,413 (61,157-105,803)	96,894 (74,403-126,772)	99,663 (77,050-125,083)	58,111 (46,848-69,503)	14,919 (11,671-18,988)
Parkinson's disease	-	-	-	-	-	-	-	1 (0-2)	13 (6-27)	50 (26-92)	107 (56-185)	174 (97-284)	287 (159-493)	457 (268-724)	812 (496-1,201)	2,037 (1,411-2,862)	4,534 (3,218-6,117)	9,883 (6,965-13,164)	15,501 (11,266-20,526)	15,372 (11,534-20,444)	10,502 (8,068-13,691)	3,569 (2,718-4,663)	660 (474-907)
Epilepsy	-	-	2,454 (1,967-2,888)	4,255 (3,018-5,336)	2,912 (2,521-3,319)	4,320 (3,857-4,827)	7,437 (6,862-7,976)	7,255 (6,761-7,798)	6,511 (6,133-6,918)	6,178 (5,852-6,489)	6,525 (6,172-6,916)	5,157 (4,916-5,394)	4,780 (4,549-4,982)	3,336 (3,183-3,480)	2,957 (2,813-3,097)	2,646 (2,494-2,780)	2,469 (2,298-2,638)	1,929 (1,799-2,039)	1,402 (1,309-1,483)	992 (897-1,073)	571 (523-616)	208 (192-224)	55 (50-59)
Multiple sclerosis	-	-	-	-	-	-	-	53 (46-62)	116 (98-151)	249 (205-331)	410 (347-532)	647 (560-820)	723 (604-865)	765 (629-902)	690 (565-847)	670 (563-822)	591 (496-705)	484 (404-577)	321 (253-366)	157 (121-172)	71 (50-79)	20 (14-21)	3 (2-3)
Motor neuron disease	4 (3-4)	5 (4-6)	452 (314-563)	151 (96-188)	24 (20-27)	58 (50-65)	87 (81-93)	77 (72-83)	70 (65-75)	151 (141-162)	323 (305-343)	537 (512-564)	812 (775-856)	988 (946-1,051)	1,250 (1,205-1,327)	1,546 (1,489-1,631)	1,876 (1,809-1,973)	1,821 (1,750-1,898)	1,401 (1,340-1,459)	726 (695-756)	325 (308-339)	66 (63-69)	9 (9-10)
Other neurological disorders	-	-	996 (828-1,409)	840 (626-1,402)	480 (405-653)	976 (887-1,133)	1,955 (1,828-2,094)	1,391 (1,314-1,466)	834 (793-875)	664 (633-695)	724 (689-755)	860 (805-902)	1,125 (1,054-1,179)	1,219 (1,125-1,284)	1,297 (1,199-1,364)	1,434 (1,333-1,502)	1,518 (1,427-1,593)	1,573 (1,482-1,652)	1,448 (1,367-1,515)	932 (877-973)	592 (562-622)	210 (199-220)	46 (43-48)
Mental and substance use disorders	12 (5-23)	10 (7-14)	0 (0-0)	0 (0-0)	10 (6-12)	7 (5-8)	4,829 (3,831-5,454)	11,261 (9,226-12,598)	16,162 (13,408-18,034)	20,706 (17,047-22,504)	24,485 (21,206-26,634)	28,414 (24,875-31,165)	28,584 (25,052-31,421)	22,565 (19,754-24,880)	16,537 (14,331-18,178)	16,352 (14,197-18,047)	10,654 (9,178-11,466)	7,181 (6,158-7,667)	4,468 (3,827-4,765)	2,259 (1,928-2,402)	1,305 (1,115-1,396)	474 (397-509)	127 (109-136)
Alcohol use disorders	-	-	-	-	-	-	1,037 (841-1,243)	2,835 (2,369-3,362)	5,607 (4,742-6,541)	9,426 (7,924-10,862)	13,858 (11,874-15,610)	18,447 (15,961-20,954)	20,819 (18,109-23,462)	17,118 (14,669-19,233)	12,876 (11,166-14,267)	12,492 (10,828-14,062)	7,686 (6,660-8,382)	4,698 (4,052-5,113)	2,704 (2,249-3,075)	1,113 (930-1,230)	556 (479-618)	174 (149-195)	48 (40-52)
Drug use disorders	12 (5-23)	10 (7-14)	0 (0-0)	0 (0-0)	0 (0-0)	0 (0-0)	3,780 (2,830-4,376)	8,409 (6,469-9,645)	10,534 (8,070-11,994)	11,255 (8,382-12,624)	10,606 (8,457-11,746)	9,936 (8,236-11,100)	7,733 (6,410-8,667)	5,446 (4,581-6,057)	3,662 (2,844-4,099)	3,860 (3,114-4,332)	2,968 (2,243-3,356)	2,483 (1,870-2,809)	1,764 (1,342-1,962)	1,147 (901-1,257)	750 (596-825)	299 (237-331)	79 (65-86)
Opioid use disorders	12 (5-23)	10 (7-14)	0 (0-0)	0 (0-0)	0 (0-0)	0 (0-0)	2,830 (2,053-3,359)	6,260 (4,348-7,373)	7,643 (5,428-8,946)	7,607 (5,115-8,833)	7,212 (5,150-8,293)	6,759 (4,888-7,819)	5,077 (3,752-5,894)	3,447 (2,483-3,992)	2,290 (1,635-2,690)	2,512 (1,812-2,919)	1,884 (1,270-2,217)	1,134 (776-1,344)	789 (534-931)	446 (315-523)	312 (204-357)	130 (84-149)	35 (24-39)
Cocaine use disorders	0 (0-0)	0 (0-0)	0 (0-0)	0 (0-0)	0 (0-0)	0 (0-0)	138 (84-183)	311 (193-435)	438 (287-563)	748 (475-929)	696 (487-915)	658 (469-896)	562 (390-747)	507 (346-639)	351 (218-448)	321 (208-426)	275 (169-359)	163 (99-218)	113 (70-147)	19 (12-26)	11 (7-15)	5 (3-6)	1 (1-1)
Amphetamine use disorders	0 (0-0)	0 (0-0)	0 (0-0)	0 (0-0)	0 (0-0)	0 (0-0)	99 (59-137)	210 (135-281)	295 (189-384)	405 (249-527)	368 (249-502)	328 (230-450)	270 (187-360)	201 (137-260)	149 (88-198)	138 (84-179)	117 (69-158)	51 (30-68)	36 (20-50)	9 (6-13)	5 (3-7)	2 (1-3)	0 (0-1)
Other drug use disorders	0 (0-0)	0 (0-0)	0 (0-0)	0 (0-0)	0 (0-0)	0 (0-0)	713 (478-846)	1,627 (1,133-2,029)	2,158 (1,545-2,528)	2,494 (1,783-2,847)	2,330 (1,764-2,807)	2,190 (1,643-2,667)	1,825 (1,403-2,218)	1,291 (995-1,570)	872 (613-1,019)	889 (644-1,082)	691 (466-811)	1,135 (798-1,306)	826 (597-949)	672 (506-755)	422 (333-471)	163 (129-180)	42 (34-48)
Eating disorders	-	-	-	-	10 (6-12)	7 (5-8)	12 (9-15)	18 (12-22)	22 (16-26)	25 (20-30)	21 (17-26)	31 (26-37)	32 (27-37)	0 (0-0)	0 (0-0)	-	-	-	-	-	-	-	-
Anorexia nervosa	-	-	-	-	10 (6-12)	7 (4-8)	12 (9-14)	17 (11-21)	21 (16-25)	23 (18-28)	20 (16-25)	28 (23-33)	23 (18-28)	0 (0-0)	0 (0-0)	-	-	-	-	-	-	-	-
Bulimia nervosa	-	-	-	-	0 (0-1)	0 (0-1)	0 (0-1)	1 (0-1)	1 (1-2)	2 (2-4)	1 (1-2)	4 (3-6)	9 (7-12)	0 (0-0)	0 (0-0)	-	-	-	-	-	-	-	-
Diabetes, urogenital, blood, and endocrine diseases	2,536 (1,789-2,906)	2,032 (1,528-2,341)	17,300 (14,626-19,481)	17,370 (13,174-21,321)	8,445 (7,366-9,754)	9,001 (7,747-10,242)	12,582 (11,450-14,105)	18,023 (16,827-20,114)	18,629 (17,321-19,958)	23,663 (22,235-25,213)	28,271 (26,707-29,888)	38,014 (36,047-40,023)	49,342 (46,949-51,755)	58,246 (55,887-61,238)	72,911 (69,906-76,165)	97,166 (93,188-101,626)	116,612 (111,977-121,964)	127,850 (122,617-134,322)	121,279 (116,423-126,963)	92,275 (87,803-97,587)	64,873 (61,871-68,275)	28,478 (27,301-30,041)	7,600 (7,249-7,995)
Diabetes mellitus	41 (37-44)	64 (57-73)	547 (497-614)	438 (375-501)	547 (475-614)	715 (650-782)	1,309 (1,212-1,418)	2,435 (2,293-2,623)	3,255 (3,099-3,459)	5,438 (5,197-5,744)	7,637 (7,342-7,984)	12,614 (12,145-13,138)	18,823 (18,221-19,452)	25,126 (24,354-25,995)	33,886 (32,842-34,997)	46,957 (45,280-48,531)	56,674 (54,735-58,618)	61,744 (59,264-64,075)	57,493 (55,206-59,628)	40,954 (38,718-42,846)	24,927 (23,702-26,081)	9,283 (8,867-9,678)	2,014 (1,916-2,103)
Acute glomerulonephritis	-	-	82 (68-99)	134 (109-163)	179 (152-216)	196 (164-235)	195 (168-229)	247 (212-289)	320 (277-369)	390 (342-445)	376 (331-432)	386 (331-447)	488 (436-548)	432 (381-485)	512 (460-565)	621 (551-703)	739 (661-830)	715 (636-807)	590 (524-670)	411 (362-464)	249 (219-285)	72 (64-82)	25 (20-31)
Chronic kidney disease	-	-	3,244 (2,662-3,695)	3,534 (2,789-4,117)	2,617 (2,084-2,945)	2,763 (2,371-3,126)	5,501 (4,977-6,241)	8,790 (7,555-9,813)	9,988 (8,641-10,893)	12,331 (10,669-13,560)	14,526 (12,765-15,794)	18,080 (15,986-19,773)	21,736 (19,333-23,703)	24,798 (22,382-27,746)	30,091 (27,453-33,017)	38,284 (35,707-42,610)	45,081 (41,454-50,750)	48,612 (45,169-54,425)	46,286 (43,499-51,691)	36,637 (34,385-41,292)	27,813 (26,044-30,940)	13,076 (12,179-14,467)	3,741 (3,486-4,120)
Chronic kidney disease due to diabetes mellitus	-	-	89 (58-131)	106 (66-162)	104 (58-165)	154 (93-247)	447 (272-687)	1,378 (874-1,911)	2,606 (1,890-3,370)	4,321 (3,248-5,428)	5,908 (4,549-7,364)	8,319 (6,344-10,086)	11,118 (8,846-13,404)	13,019 (10,068-15,685)	16,067 (12,631-18,845)	20,755 (16,797-24,505)	23,627 (18,396-28,025)	23,754 (18,831-28,354)	20,249 (15,731-24,673)	13,684 (10,371-16,980)	8,748 (6,486-11,382)	3,823 (2,734-4,946)	1,098 (737-1,469)
Chronic kidney disease due to hypertension	-	-	23 (15-33)	35 (23-51)	45 (28-68)	79 (50-118)	242 (153-347)	566 (384-788)	820 (564-1,135)	1,240 (894-1,656)	1,686 (1,259-2,199)	2,387 (1,823-3,095)	3,199 (2,437-4,164)	4,002 (2,985-5,265)	5,524 (4,215-7,277)	7,705 (6,043-9,785)	10,201 (7,978-13,061)	12,077 (9,447-15,422)	12,967 (10,177-16,393)	11,915 (9,509-14,685)	10,494 (8,482-12,873)	5,481 (4,455-6,604)	1,639 (1,296-1,992)
Chronic kidney disease due to glomerulonephritis	-	-	1,262 (909-1,622)	1,399 (968-1,876)	1,037 (698-1,393)	1,163 (811-1,554)	2,342 (1,649-3,066)	3,582 (2,557-4,755)	3,525 (2,455-4,664)	3,434 (2,337-4,731)	3,285 (2,201-4,540)	3,277 (2,111-4,556)	3,101 (2,046-4,327)	3,071 (2,086-4,363)	3,065 (2,100-4,318)	3,486 (2,321-4,950)	3,844 (2,540-5,539)	4,306 (2,878-6,198)	4,432 (3,017-6,369)	3,620 (2,538-4,943)	2,583 (1,828-3,593)	1,094 (769-1,467)	303 (185-488)
Chronic kidney disease due to other causes	-	-	1,870 (1,439-2,273)	1,994 (1,466-2,509)	1,432 (1,056-1,816)	1,367 (970-1,764)	2,471 (1,782-3,211)	3,264 (2,285-4,277)	3,038 (2,088-4,149)	3,336 (2,273-4,616)	3,647 (2,546-4,905)	4,096 (2,831-5,587)	4,319 (2,809-6,191)	4,707 (3,074-6,825)	5,436 (3,624-7,955)	6,338 (4,139-9,236)	7,410 (5,021-10,582)	8,475 (5,783-11,570)	8,637 (6,007-11,833)	7,417 (5,262-9,948)	5,988 (4,131-8,149)	2,678 (1,946-3,588)	701 (452-1,006)
Urinary diseases and male infertility	255 (223-288)	406 (321-518)	3,694 (3,265-4,162)	2,083 (1,679-2,531)	835 (699-994)	698 (628-771)	1,339 (1,220-1,448)	1,772 (1,642-1,899)	1,790 (1,665-1,924)	2,179 (2,050-2,308)	2,467 (2,319-2,612)	3,106 (2,930-3,258)	3,760 (3,551-3,968)	3,980 (3,770-4,188)	4,394 (4,156-4,599)	6,856 (6,481-7,183)	8,899 (8,417-9,320)	10,673 (10,072-11,161)	10,643 (10,081-11,105)	9,623 (9,074-10,063)	7,986 (7,593-8,312)	4,214 (4,017-4,384)	1,285 (1,229-1,336)
Interstitial nephritis and urinary tract infections	121 (82-162)	221 (145-300)	2,468 (1,874-2,991)	1,058 (632-1,559)	376 (262-486)	353 (277-422)	700 (536-823)	950 (748-1,108)	900 (717-1,062)	1,046 (840-1,223)	1,222 (993-1,429)	1,563 (1,250-1,798)	1,850 (1,466-2,161)	2,056 (1,643-2,379)	2,266 (1,779-2,615)	3,717 (2,933-4,270)	4,870 (3,934-5,641)	6,245 (5,195-6,992)	6,680 (5,659-7,358)	6,028 (5,183-6,585)	5,426 (4,773-5,857)	2,962 (2,609-3,189)	961 (872-1,028)
Urolithiasis	-	-	-	-	134 (82-194)	101 (63-134)	184 (126-245)	195 (133-250)	196 (129-258)	230 (151-294)	272 (187-342)	412 (306-515)	508 (364-636)	549 (393-664)	519 (368-632)	825 (584-996)	1,130 (805-1,370)	1,223 (863-1,479)	1,085 (792-1,331)	820 (603-1,001)	574 (419-707)	249 (176-310)	65 (47-82)
Other urinary diseases	134 (97-166)	185 (118-270)	1,226 (749-1,771)	1,024 (559-1,521)	326 (207-446)	244 (166-314)	456 (332-632)	627 (461-847)	694 (522-897)	903 (702-1,114)	974 (757-1,234)	1,130 (861-1,446)	1,401 (1,068-1,802)	1,375 (1,030-1,780)	1,610 (1,215-2,087)	2,314 (1,707-3,144)	2,899 (2,168-3,899)	3,205 (2,507-4,288)	2,878 (2,295-3,830)	2,775 (2,281-3,535)	1,986 (1,643-2,594)	1,003 (835-1,336)	259 (214-343)
Gynecological diseases	-	-	-	-	-	-	-	-	-	-	-	-	-	-	-	-	-	-	-	-	-	-	-
Uterine fibroids	-	-	-	-	-	-	-	-	-	-	-	-	-	-	-	-	-	-	-	-	-	-	-
Polycystic ovarian syndrome	-	-	-	-	-	-	-	-	-	-	-	-	-	-	-	-	-	-	-	-	-	-	-
Endometriosis	-	-	-	-	-	-	-	-	-	-	-	-	-	-	-	-	-	-	-	-	-	-	-
Genital prolapse	-	-	-	-	-	-	-	-	-	-	-	-	-	-	-	-	-	-	-	-	-	-	-
Other gynecological diseases	-	-	-	-	-	-	-	-	-	-	-	-	-	-	-	-	-	-	-	-	-	-	-
Hemoglobinopathies and hemolytic anaemias	1,387 (844-1,691)	866 (530-1,059)	5,447 (3,591-6,863)	8,713 (5,156-12,103)	3,034 (2,350-4,051)	3,456 (2,403-4,354)	3,302 (2,346-4,364)	3,769 (2,967-5,375)	1,980 (1,459-2,604)	1,496 (1,092-1,951)	1,334 (1,028-1,745)	1,742 (1,396-2,405)	2,104 (1,741-3,135)	1,417 (1,149-1,890)	1,421 (1,168-1,896)	1,636 (1,327-2,173)	1,991 (1,679-2,657)	2,483 (2,109-3,348)	2,867 (2,472-3,855)	1,980 (1,678-2,513)	1,862 (1,572-2,245)	868 (720-1,011)	244 (198-277)
Thalassemias	325 (243-409)	268 (174-349)	1,417 (1,027-1,814)	2,064 (1,523-2,705)	993 (791-1,316)	476 (361-667)	215 (158-280)	138 (94-175)	79 (54-103)	40 (30-49)	22 (18-27)	17 (14-22)	16 (14-21)	16 (14-22)	9 (8-12)	3 (3-5)	1 (1-2)	1 (1-1)	0 (0-0)	0 (0-0)	0 (0-0)	0 (0-0)	0 (0-0)
Sickle cell disorders	950 (505-1,228)	557 (320-730)	3,742 (2,350-4,909)	6,148 (3,163-9,032)	1,761 (1,248-2,426)	2,548 (1,676-3,272)	2,521 (1,768-3,435)	2,698 (2,096-4,015)	1,080 (782-1,457)	658 (490-886)	542 (402-721)	646 (516-956)	672 (504-1,102)	234 (188-344)	99 (79-145)	48 (39-67)	33 (29-42)	20 (18-24)	9 (7-10)	3 (2-3)	1 (1-1)	0 (0-0)	0 (0-0)
G6PD deficiency	45 (29-61)	28 (18-38)	219 (150-301)	422 (283-587)	225 (168-299)	335 (239-435)	451 (333-596)	671 (503-909)	630 (449-806)	597 (424-769)	515 (381-668)	699 (535-947)	932 (748-1,355)	649 (507-872)	583 (452-801)	520 (398-733)	413 (327-594)	358 (282-517)	247 (191-363)	87 (68-120)	47 (36-65)	12 (10-16)	2 (1-3)
Other hemoglobinopathies and hemolytic anaemias	68 (47-89)	13 (9-17)	69 (50-91)	78 (55-104)	55 (44-68)	98 (75-127)	115 (88-152)	262 (201-355)	190 (144-252)	202 (151-262)	255 (201-330)	379 (301-497)	485 (393-670)	517 (412-668)	730 (600-949)	1,064 (851-1,393)	1,543 (1,298-2,070)	2,104 (1,786-2,809)	2,611 (2,244-3,494)	1,891 (1,599-2,391)	1,814 (1,534-2,176)	855 (709-996)	242 (196-274)
Endocrine, metabolic, blood, and immune disorders	853 (529-1,003)	695 (476-828)	4,287 (3,287-5,697)	2,470 (1,909-3,359)	1,232 (1,038-1,658)	1,173 (1,001-1,553)	937 (803-1,171)	1,010 (854-1,271)	1,296 (1,015-1,532)	1,829 (1,450-2,210)	1,932 (1,548-2,305)	2,086 (1,702-2,425)	2,431 (1,969-2,844)	2,493 (2,032-2,798)	2,607 (2,165-3,033)	2,813 (2,405-3,285)	3,228 (2,725-3,962)	3,623 (3,081-4,389)	3,400 (2,894-4,175)	2,669 (2,217-3,329)	2,035 (1,662-2,399)	965 (817-1,158)	292 (247-346)
Musculoskeletal disorders	-	-	-	-	383 (289-495)	489 (408-609)	481 (430-600)	581 (486-686)	623 (514-708)	648 (534-736)	767 (640-853)	890 (742-1,012)	1,095 (929-1,259)	1,002 (850-1,102)	1,160 (977-1,316)	1,607 (1,373-1,784)	2,092 (1,778-2,301)	2,470 (2,090-2,683)	2,495 (2,131-2,729)	1,851 (1,543-2,025)	1,423 (1,256-1,559)	634 (536-697)	176 (159-191)
Rheumatoid arthritis	-	-	-	-	90 (54-147)	98 (67-142)	68 (52-90)	81 (58-109)	86 (60-118)	89 (62-119)	107 (81-141)	178 (128-219)	273 (202-334)	380 (277-440)	514 (384-577)	835 (640-951)	1,147 (874-1,297)	1,356 (1,046-1,534)	1,291 (1,049-1,482)	892 (715-1,008)	562 (439-675)	196 (151-237)	48 (37-57)
Other musculoskeletal disorders	-	-	-	-	293 (225-388)	391 (331-490)	413 (369-527)	501 (419-595)	537 (445-612)	558 (457-635)	660 (553-742)	712 (597-820)	822 (710-968)	623 (538-721)	647 (559-774)	771 (651-924)	945 (800-1,149)	1,114 (926-1,309)	1,204 (973-1,387)	959 (778-1,100)	861 (720-987)	438 (366-491)	128 (109-142)
Other non-communicable diseases	108,202 (76,233-134,652)	40,141 (29,107-49,911)	133,023 (101,814-162,099)	37,551 (24,315-53,676)	10,990 (8,604-13,788)	8,140 (6,417-9,912)	5,987 (4,926-7,073)	4,814 (3,932-5,624)	3,847 (3,203-4,570)	3,567 (2,785-4,040)	3,239 (2,503-3,691)	3,407 (2,582-3,865)	2,950 (2,154-3,414)	2,539 (1,810-2,912)	2,799 (1,886-3,239)	3,266 (2,169-3,866)	3,466 (2,246-4,162)	3,011 (1,656-3,854)	3,360 (1,906-4,529)	2,765 (1,554-3,714)	2,315 (1,274-3,116)	1,233 (657-1,619)	381 (200-504)
Congenital anomalies	108,202 (76,233-134,652)	37,863 (27,198-47,711)	111,963 (81,382-141,050)	36,694 (23,415-52,782)	10,728 (8,452-13,499)	7,832 (6,132-9,641)	5,384 (4,498-6,578)	4,257 (3,492-5,204)	3,372 (2,786-4,193)	2,569 (2,217-3,142)	2,402 (1,979-2,882)	2,380 (1,901-2,826)	1,557 (1,284-1,868)	1,198 (1,002-1,425)	1,057 (893-1,204)	1,131 (968-1,397)	921 (776-1,135)	-	-	-	-	-	-
Neural tube defects	18,058 (9,890-30,954)	4,727 (2,406-8,922)	10,417 (4,664-21,104)	3,673 (800-10,371)	678 (406-1,206)	500 (335-776)	233 (169-352)	167 (122-261)	137 (104-197)	108 (80-152)	77 (60-104)	70 (54-100)	64 (50-84)	46 (34-62)	36 (25-62)	27 (19-51)	27 (21-43)	-	-	-	-	-	-
Congenital heart anomalies	37,212 (21,918-50,019)	17,362 (11,375-23,165)	59,194 (41,232-81,925)	19,255 (12,367-30,423)	6,851 (5,009-9,095)	5,178 (4,035-6,594)	3,656 (2,964-4,559)	3,007 (2,476-3,749)	2,493 (2,087-3,177)	1,804 (1,523-2,222)	1,754 (1,446-2,148)	1,791 (1,425-2,141)	708 (574-855)	496 (412-595)	402 (339-493)	398 (334-501)	319 (273-406)	-	-	-	-	-	-
Orofacial clefts	981 (336-1,542)	288 (106-471)	820 (323-1,415)	86 (20-208)	-	-	-	-	-	-	-	-	-	-	-	-	-	-	-	-	-	-	-
Down's syndrome	1,385 (587-4,013)	684 (298-1,988)	3,006 (1,394-8,742)	1,383 (512-5,426)	238 (141-508)	188 (124-336)	177 (130-283)	154 (118-226)	140 (108-208)	164 (125-236)	148 (111-209)	141 (107-190)	285 (217-390)	251 (199-327)	240 (184-289)	205 (164-269)	88 (68-121)	-	-	-	-	-	-
Other chromosomal abnormalities	2,436 (1,377-4,923)	1,011 (554-2,099)	2,143 (1,125-5,401)	469 (211-1,175)	110 (68-189)	80 (44-121)	86 (51-127)	70 (42-91)	53 (27-66)	39 (19-47)	39 (17-47)	30 (13-36)	31 (16-37)	33 (19-41)	26 (15-32)	20 (13-26)	14 (10-21)	-	-	-	-	-	-
Congenital musculoskeletal and limb anomalies	2,743 (1,640-5,475)	647 (384-1,430)	1,387 (797-3,363)	367 (186-1,218)	144 (93-278)	131 (76-206)	128 (86-176)	59 (35-81)	47 (33-64)	41 (23-55)	30 (19-40)	23 (14-30)	28 (18-36)	24 (16-29)	15 (9-18)	20 (13-25)	21 (14-26)	-	-	-	-	-	-
Urogenital congenital anomalies	3,092 (1,920-4,747)	793 (493-1,233)	1,480 (854-2,474)	294 (168-518)	141 (94-210)	140 (102-192)	64 (50-98)	61 (48-93)	59 (44-79)	62 (44-81)	62 (43-81)	70 (48-92)	114 (76-153)	109 (76-141)	124 (84-154)	171 (117-224)	207 (149-268)	-	-	-	-	-	-
Digestive congenital anomalies	10,799 (7,450-17,797)	4,403 (2,783-7,597)	10,165 (6,010-19,184)	1,857 (883-5,063)	241 (171-380)	125 (93-175)	57 (41-70)	36 (27-48)	35 (26-43)	27 (20-33)	28 (18-33)	27 (17-33)	40 (28-48)	31 (20-36)	30 (20-35)	44 (33-55)	40 (27-48)	-	-	-	-	-	-
Other congenital anomalies	31,497 (18,387-50,377)	7,948 (4,447-13,539)	23,350 (12,609-38,193)	9,310 (4,556-17,616)	2,324 (1,521-3,431)	1,488 (1,013-2,012)	984 (752-1,280)	703 (496-915)	409 (304-518)	324 (240-404)	264 (208-326)	228 (176-281)	288 (220-365)	209 (162-262)	184 (143-230)	246 (195-310)	204 (160-269)	-	-	-	-	-	-
Skin and subcutaneous diseases	-	-	1,522 (648-2,081)	857 (351-1,227)	262 (118-353)	308 (144-411)	602 (277-816)	556 (252-727)	474 (228-600)	998 (453-1,296)	838 (409-1,088)	1,027 (492-1,281)	1,393 (725-1,812)	1,341 (702-1,652)	1,743 (875-2,183)	2,135 (1,111-2,702)	2,545 (1,358-3,263)	3,011 (1,656-3,854)	3,360 (1,906-4,529)	2,765 (1,554-3,714)	2,315 (1,274-3,116)	1,233 (657-1,619)	381 (200-504)
Cellulitis	-	-	66 (21-101)	72 (17-117)	46 (15-72)	57 (24-81)	63 (30-87)	74 (32-98)	62 (23-83)	120 (40-166)	156 (55-208)	205 (70-275)	256 (98-340)	315 (109-418)	354 (120-479)	383 (149-503)	369 (126-504)	424 (167-631)	443 (177-707)	354 (142-624)	281 (119-489)	147 (66-273)	42 (18-81)
Pyoderma	-	-	1,408 (589-1,959)	683 (279-988)	165 (75-226)	192 (82-269)	424 (181-577)	389 (161-512)	325 (145-419)	730 (316-972)	533 (238-716)	661 (303-840)	916 (442-1,195)	814 (412-1,058)	1,103 (518-1,445)	1,361 (631-1,764)	1,548 (757-2,008)	1,679 (831-2,150)	1,751 (908-2,256)	1,150 (613-1,535)	909 (467-1,185)	450 (233-604)	130 (63-172)
Decubitus ulcer	-	-	-	44 (13-74)	26 (13-38)	32 (17-47)	73 (40-109)	58 (31-84)	61 (33-90)	117 (62-166)	123 (63-181)	131 (69-196)	182 (103-286)	176 (97-270)	243 (137-379)	335 (188-516)	554 (319-826)	826 (483-1,203)	1,059 (593-1,566)	1,156 (606-1,641)	1,030 (513-1,425)	584 (294-763)	192 (90-252)
Other skin and subcutaneous diseases	-	-	48 (20-80)	58 (18-96)	25 (13-37)	26 (14-39)	42 (25-69)	35 (19-55)	27 (15-40)	31 (18-49)	27 (15-41)	29 (16-46)	38 (22-61)	37 (20-60)	43 (24-70)	57 (31-90)	74 (44-121)	83 (45-138)	108 (61-173)	105 (56-164)	95 (51-148)	53 (25-83)	18 (8-27)
Sudden infant death syndrome	-	2,279 (1,652-2,972)	19,538 (15,308-26,705)	-	-	-	-	-	-	-	-	-	-	-	-	-	-	-	-	-	-	-	-
Injuries	10,285 (8,184-11,791)	6,155 (4,813-7,313)	58,198 (49,343-67,442)	152,276 (128,265-171,625)	111,093 (102,311-118,755)	113,488 (107,383-118,886)	216,202 (208,482-222,684)	313,238 (303,518-320,608)	302,884 (292,219-310,537)	278,449 (266,759-285,828)	257,856 (245,479-265,690)	228,900 (216,943-237,069)	213,089 (200,959-221,758)	166,844 (155,035-173,667)	134,409 (125,143-139,416)	130,773 (120,784-136,519)	116,102 (106,525-120,741)	103,615 (94,185-108,132)	82,352 (74,223-86,151)	56,546 (50,585-59,617)	38,189 (33,910-40,289)	15,856 (13,814-16,793)	4,276 (3,765-4,534)
Transport injuries	2,448 (1,798-3,334)	1,092 (882-1,341)	8,150 (6,881-9,776)	29,236 (24,259-34,537)	34,706 (31,832-37,424)	32,716 (30,860-34,976)	83,621 (79,966-88,339)	121,218 (116,524-128,364)	116,951 (112,457-123,288)	104,053 (100,451-109,190)	93,923 (90,685-97,925)	79,565 (76,707-82,514)	71,393 (68,666-74,180)	53,857 (51,964-55,858)	43,230 (41,594-44,873)	39,386 (37,816-40,978)	35,040 (33,610-36,440)	29,171 (28,038-30,342)	21,551 (20,691-22,460)	12,646 (12,104-13,273)	6,726 (6,430-7,059)	2,161 (2,069-2,261)	509 (482-536)
Road injuries	2,086 (1,542-2,883)	926 (755-1,152)	7,401 (6,221-8,971)	27,629 (22,867-33,075)	33,049 (30,340-35,732)	30,634 (28,894-32,799)	77,732 (74,181-82,544)	113,712 (109,527-121,094)	108,954 (104,707-115,645)	97,355 (93,880-102,475)	87,096 (83,986-91,468)	73,444 (70,847-76,394)	65,720 (63,268-68,442)	49,262 (47,492-51,224)	39,518 (38,010-41,228)	36,398 (34,909-37,975)	32,483 (31,154-33,773)	27,160 (26,114-28,327)	20,056 (19,245-20,948)	11,839 (11,338-12,445)	6,284 (6,000-6,591)	1,983 (1,892-2,081)	452 (428-477)
Pedestrian road injuries	859 (566-1,429)	360 (267-525)	3,017 (2,307-4,151)	15,330 (12,333-19,471)	19,278 (17,177-21,931)	15,266 (13,838-17,474)	19,951 (17,701-23,318)	28,910 (25,587-33,687)	30,728 (27,298-35,563)	31,601 (28,700-36,430)	29,392 (26,361-33,514)	25,322 (22,668-28,852)	25,884 (23,258-29,157)	20,345 (18,192-22,953)	16,624 (14,868-18,668)	17,388 (15,699-19,486)	16,094 (14,643-18,018)	13,757 (12,533-15,233)	11,245 (10,382-12,401)	6,650 (6,115-7,329)	3,606 (3,303-3,974)	1,054 (952-1,177)	249 (226-280)
Cyclist road injuries	-	-	-	897 (605-1,243)	1,865 (1,559-2,206)	2,688 (2,355-3,092)	3,577 (3,114-4,048)	3,545 (3,050-3,975)	3,755 (3,249-4,165)	4,055 (3,495-4,526)	3,769 (3,252-4,232)	3,998 (3,484-4,498)	4,244 (3,706-4,771)	3,777 (3,326-4,197)	2,973 (2,591-3,334)	3,116 (2,718-3,505)	2,860 (2,508-3,229)	2,335 (2,068-2,625)	1,362 (1,206-1,528)	705 (628-796)	324 (288-384)	89 (78-109)	1 (1-2)
Motorcyclist road injuries	172 (104-295)	123 (76-204)	813 (606-1,217)	1,729 (1,215-2,804)	1,924 (1,548-2,336)	3,133 (2,711-3,604)	21,349 (19,110-24,678)	32,176 (28,769-36,541)	29,878 (26,487-34,068)	23,678 (20,390-26,946)	19,798 (17,270-22,393)	15,545 (13,825-17,334)	11,896 (10,423-13,318)	7,544 (6,657-8,627)	5,692 (5,044-6,697)	3,562 (3,111-4,254)	2,927 (2,532-3,401)	2,057 (1,819-2,395)	1,101 (952-1,254)	678 (583-773)	268 (228-314)	68 (57-84)	16 (14-20)
Motor vehicle road injuries	1,004 (601-1,499)	425 (274-572)	3,375 (2,219-4,430)	9,329 (5,795-12,436)	9,610 (7,324-12,003)	9,151 (7,550-11,320)	32,219 (27,579-38,314)	48,117 (41,194-56,338)	43,710 (37,881-51,902)	37,345 (32,040-44,744)	33,488 (28,738-39,170)	27,972 (24,048-32,506)	23,017 (19,548-26,808)	16,965 (14,430-20,206)	13,737 (11,643-16,240)	11,932 (9,921-14,130)	10,139 (8,444-12,007)	8,603 (7,206-10,053)	6,037 (5,120-6,985)	3,601 (3,063-4,190)	1,978 (1,690-2,314)	730 (623-831)	173 (147-195)
Other road injuries	50 (26-95)	18 (12-28)	196 (129-303)	342 (214-518)	373 (306-484)	396 (328-540)	635 (519-815)	964 (789-1,230)	883 (724-1,094)	676 (549-834)	650 (540-803)	607 (497-740)	679 (560-836)	631 (519-792)	492 (398-622)	400 (325-507)	463 (371-581)	409 (330-534)	311 (245-433)	206 (162-316)	109 (84-158)	42 (31-67)	12 (9-20)
Other transport injuries	363 (201-561)	166 (101-237)	748 (500-1,038)	1,608 (935-2,292)	1,657 (1,317-2,025)	2,082 (1,753-2,425)	5,889 (4,891-6,663)	7,406 (6,389-8,271)	7,997 (6,774-8,982)	6,698 (5,728-7,383)	6,826 (5,870-7,547)	6,121 (5,184-6,746)	5,673 (4,816-6,207)	4,595 (3,998-5,068)	3,712 (3,121-4,087)	2,988 (2,499-3,302)	2,557 (2,144-2,854)	2,011 (1,654-2,253)	1,495 (1,268-1,707)	807 (687-944)	441 (376-521)	179 (155-212)	56 (49-66)
Unintentional injuries	6,566 (4,944-7,687)	4,642 (3,421-5,713)	46,918 (38,148-55,387)	113,609 (92,045-130,776)	69,015 (62,759-74,960)	63,844 (59,821-67,956)	58,305 (55,081-61,520)	69,108 (65,508-72,780)	67,630 (64,151-71,141)	68,241 (64,805-71,494)	69,671 (65,717-73,105)	67,075 (62,541-70,731)	69,266 (64,669-73,110)	56,870 (52,442-60,446)	48,995 (45,211-51,643)	53,258 (48,604-56,671)	50,074 (44,723-53,015)	47,927 (42,389-50,873)	42,749 (37,677-45,509)	33,227 (29,095-35,646)	25,531 (22,029-27,338)	11,870 (10,164-12,720)	3,384 (2,936-3,622)
Falls	1,384 (839-1,976)	467 (299-595)	5,385 (3,488-7,014)	9,704 (5,718-12,927)	5,891 (4,748-7,047)	6,492 (5,461-7,540)	6,953 (6,297-8,028)	10,358 (9,382-12,020)	12,084 (10,791-13,715)	13,813 (12,301-15,726)	15,257 (13,655-17,208)	16,391 (14,667-18,359)	17,825 (15,616-19,844)	15,829 (13,916-17,104)	15,645 (13,712-16,978)	18,089 (15,542-19,561)	19,991 (17,106-21,727)	21,601 (17,980-23,627)	22,595 (18,782-24,712)	18,699 (15,599-20,508)	15,735 (13,083-17,119)	7,704 (6,276-8,360)	2,149 (1,762-2,325)
Drowning	415 (316-484)	167 (126-209)	5,437 (4,640-6,190)	61,655 (50,111-69,463)	41,405 (36,873-44,753)	36,563 (33,681-38,673)	25,060 (23,488-26,946)	22,470 (21,148-24,416)	17,495 (16,410-18,870)	16,054 (15,138-17,172)	14,653 (13,837-15,644)	13,114 (12,322-13,979)	11,718 (10,951-12,444)	9,440 (8,801-10,046)	7,201 (6,797-7,662)	7,458 (6,964-7,930)	5,979 (5,615-6,402)	5,330 (4,952-5,647)	3,476 (3,193-3,732)	2,392 (2,179-2,594)	1,326 (1,227-1,436)	382 (360-413)	87 (82-93)
Fire, heat, and hot substances	329 (237-379)	245 (153-296)	3,461 (2,296-4,159)	9,379 (5,485-11,975)	3,355 (2,520-3,975)	2,237 (1,741-2,581)	2,673 (2,142-3,039)	4,210 (3,396-4,794)	4,390 (3,606-4,967)	4,456 (3,806-5,136)	4,935 (4,135-5,515)	4,976 (4,219-5,554)	5,206 (4,372-5,784)	4,466 (3,715-4,977)	3,598 (2,975-3,970)	4,434 (3,651-4,899)	3,676 (3,109-4,090)	3,696 (3,173-4,173)	3,012 (2,578-3,394)	2,541 (2,158-2,827)	1,898 (1,639-2,106)	871 (748-959)	260 (221-281)
Poisonings	312 (138-636)	186 (89-315)	3,020 (1,329-5,629)	5,060 (2,816-7,950)	2,140 (1,494-2,735)	2,447 (1,787-3,124)	2,299 (1,718-2,986)	3,344 (2,528-4,312)	3,633 (2,798-4,545)	3,210 (2,384-4,139)	3,050 (2,276-3,799)	3,074 (2,368-3,709)	2,945 (2,142-3,564)	2,360 (1,826-2,854)	1,941 (1,489-2,353)	2,063 (1,522-2,512)	1,705 (1,229-2,015)	1,457 (1,072-1,771)	934 (656-1,129)	635 (458-809)	344 (262-430)	113 (94-148)	31 (27-43)
Exposure to mechanical forces	1,608 (1,026-2,041)	1,538 (833-2,299)	10,059 (7,000-12,168)	8,176 (5,439-10,611)	4,746 (4,020-5,818)	4,455 (4,048-5,475)	7,072 (6,493-7,989)	10,296 (9,451-11,316)	10,723 (9,947-12,165)	10,438 (9,712-11,661)	10,577 (9,641-11,694)	8,806 (7,896-9,797)	9,061 (8,126-9,792)	6,840 (6,063-7,410)	5,001 (4,499-5,491)	4,067 (3,603-4,488)	3,403 (2,969-3,693)	2,762 (2,386-3,004)	2,289 (1,984-2,502)	1,551 (1,323-1,705)	1,167 (971-1,279)	571 (461-647)	195 (157-220)
Unintentional firearm injuries	65 (40-80)	41 (27-55)	463 (300-651)	801 (437-1,233)	623 (493-797)	841 (721-1,030)	1,908 (1,652-2,337)	2,805 (2,059-3,318)	2,665 (1,981-3,160)	1,959 (1,476-2,281)	1,665 (1,219-1,891)	1,305 (966-1,493)	1,345 (964-1,564)	1,018 (683-1,234)	645 (481-757)	581 (461-688)	507 (391-602)	438 (333-509)	370 (287-434)	247 (182-285)	167 (115-190)	69 (54-80)	21 (19-25)
Unintentional suffocation	1,167 (605-1,592)	1,345 (639-2,081)	7,792 (4,728-9,799)	2,835 (1,800-4,330)	1,152 (890-1,942)	1,175 (958-1,927)	387 (325-580)	499 (403-769)	518 (436-783)	381 (312-566)	335 (271-476)	323 (273-483)	264 (215-367)	191 (158-287)	148 (117-199)	75 (50-91)	89 (59-116)	77 (49-102)	349 (228-441)	397 (248-524)	376 (226-481)	214 (130-269)	81 (47-101)
Other exposure to mechanical forces	376 (283-427)	152 (112-181)	1,804 (1,352-2,164)	4,540 (2,690-5,957)	2,971 (2,474-3,511)	2,439 (2,195-2,759)	4,777 (4,300-5,476)	6,992 (6,412-8,107)	7,540 (6,980-8,956)	8,098 (7,480-9,445)	8,578 (7,809-9,672)	7,178 (6,416-8,354)	7,452 (6,728-8,289)	5,631 (5,045-6,337)	4,208 (3,792-4,745)	3,411 (3,018-3,832)	2,807 (2,425-3,064)	2,247 (1,907-2,464)	1,570 (1,339-1,744)	907 (776-1,026)	624 (531-703)	287 (239-336)	93 (80-112)
Adverse effects of medical treatment	810 (519-967)	539 (336-677)	5,097 (2,836-6,774)	4,516 (1,822-7,173)	1,656 (1,105-2,093)	1,356 (999-1,586)	1,660 (1,176-1,960)	2,442 (1,736-2,871)	2,315 (1,624-2,685)	2,356 (1,636-2,743)	2,496 (1,825-2,906)	2,608 (1,945-3,012)	2,681 (1,966-3,090)	2,276 (1,672-2,600)	2,544 (1,912-2,944)	3,510 (2,529-4,083)	4,206 (3,086-4,893)	3,970 (3,043-4,756)	3,586 (2,697-4,196)	2,528 (1,914-3,082)	1,743 (1,325-2,146)	774 (583-938)	215 (162-270)
Animal contact	328 (262-385)	314 (240-398)	1,835 (1,427-2,287)	4,493 (3,081-6,894)	4,166 (3,278-5,115)	4,287 (3,399-5,121)	3,350 (2,732-3,890)	3,306 (2,797-3,807)	3,367 (2,798-3,897)	3,372 (2,840-3,837)	3,400 (2,882-3,891)	3,342 (2,846-3,818)	4,259 (3,594-4,883)	2,931 (2,465-3,322)	3,300 (2,787-3,752)	3,130 (2,653-3,555)	3,114 (2,606-3,534)	2,543 (2,129-2,891)	1,787 (1,470-2,048)	1,099 (904-1,272)	499 (409-581)	145 (118-168)	45 (37-52)
Venomous animal contact	282 (221-340)	272 (204-350)	1,539 (1,184-1,943)	3,699 (2,472-5,490)	3,470 (2,654-4,295)	3,784 (2,937-4,563)	2,760 (2,194-3,260)	2,687 (2,209-3,178)	2,689 (2,148-3,196)	2,785 (2,278-3,256)	2,867 (2,352-3,361)	2,806 (2,313-3,304)	3,545 (2,902-4,180)	2,342 (1,924-2,737)	2,730 (2,249-3,187)	2,544 (2,084-2,941)	2,566 (2,081-2,972)	2,095 (1,708-2,426)	1,407 (1,123-1,648)	898 (722-1,060)	388 (309-461)	104 (82-125)	28 (23-34)
Non-venomous animal contact	46 (35-57)	41 (30-56)	296 (214-412)	795 (426-1,624)	695 (536-940)	502 (390-627)	590 (452-715)	619 (473-746)	678 (514-815)	587 (458-717)	534 (416-656)	536 (401-663)	713 (520-893)	589 (471-708)	571 (442-698)	586 (472-718)	548 (446-659)	447 (354-540)	380 (285-484)	201 (155-252)	112 (87-138)	41 (32-50)	16 (13-20)
Foreign body	844 (496-1,041)	899 (543-1,177)	10,022 (6,475-14,023)	5,943 (3,380-10,167)	2,204 (1,610-2,789)	1,780 (1,405-2,071)	1,275 (1,025-1,475)	1,968 (1,571-2,292)	2,061 (1,655-2,411)	2,618 (2,099-3,002)	3,113 (2,495-3,693)	3,496 (2,784-4,134)	3,480 (2,777-4,142)	3,298 (2,607-3,965)	2,698 (2,141-3,183)	3,244 (2,636-3,874)	2,758 (2,220-3,314)	2,642 (2,064-2,987)	2,348 (1,732-2,659)	2,052 (1,460-2,350)	1,627 (1,097-1,903)	796 (522-945)	256 (168-303)
Pulmonary aspiration and foreign body in airway	798 (464-985)	865 (521-1,140)	9,363 (6,004-13,202)	4,996 (2,884-8,821)	1,746 (1,224-2,282)	1,424 (1,138-1,723)	1,068 (876-1,337)	1,676 (1,354-2,085)	1,749 (1,409-2,187)	2,040 (1,668-2,457)	2,487 (1,943-3,115)	2,897 (2,280-3,624)	3,178 (2,526-3,903)	3,044 (2,421-3,738)	2,494 (1,978-3,022)	2,983 (2,380-3,650)	2,563 (2,050-3,169)	2,447 (1,907-2,867)	2,136 (1,577-2,442)	1,893 (1,364-2,172)	1,517 (1,011-1,784)	752 (483-895)	242 (155-287)
Foreign body in other body part	45 (18-60)	34 (12-44)	659 (241-923)	947 (315-1,771)	458 (244-505)	355 (175-456)	206 (87-290)	293 (138-393)	312 (145-411)	578 (329-751)	626 (351-788)	599 (351-755)	302 (170-382)	254 (144-322)	205 (106-261)	262 (158-340)	195 (100-245)	195 (93-245)	211 (105-268)	159 (73-204)	110 (55-138)	44 (22-54)	14 (8-18)
Environmental heat and cold exposure	281 (123-350)	190 (74-250)	1,567 (650-2,007)	1,340 (552-1,848)	564 (328-690)	540 (300-637)	1,098 (660-1,333)	1,567 (919-1,904)	2,011 (1,257-2,531)	2,786 (1,716-3,483)	3,627 (2,303-5,096)	4,809 (2,970-6,908)	5,619 (3,578-8,606)	5,304 (3,392-8,617)	3,922 (2,540-5,915)	4,571 (2,982-7,606)	3,266 (2,094-4,744)	2,631 (1,669-3,918)	1,887 (1,137-2,487)	1,224 (766-1,645)	882 (575-1,232)	374 (246-550)	100 (63-143)
Other unintentional injuries	256 (228-293)	97 (80-116)	1,034 (839-1,243)	3,343 (2,301-4,245)	2,890 (2,517-3,332)	3,688 (3,340-4,083)	6,865 (6,182-7,673)	9,146 (8,351-10,199)	9,550 (8,799-10,714)	9,138 (8,422-10,071)	8,562 (7,878-9,363)	6,458 (5,963-7,061)	6,473 (5,956-7,061)	4,126 (3,805-4,485)	3,146 (2,866-3,413)	2,691 (2,464-2,967)	1,976 (1,803-2,157)	1,294 (1,183-1,419)	834 (760-924)	507 (460-560)	310 (277-345)	140 (122-159)	46 (38-52)
Self-harm and interpersonal violence	925 (655-1,093)	230 (160-279)	2,480 (1,789-3,076)	4,260 (3,019-5,128)	3,800 (2,652-4,401)	13,282 (10,306-14,618)	64,555 (54,607-69,006)	106,995 (91,522-113,937)	106,735 (90,922-113,574)	97,965 (82,869-104,182)	88,655 (74,815-95,215)	78,551 (66,385-85,534)	69,827 (58,539-75,654)	54,318 (44,823-58,866)	40,821 (33,541-43,910)	36,977 (29,320-39,932)	30,090 (23,635-32,519)	25,853 (20,147-27,839)	17,597 (13,626-18,924)	10,341 (7,830-11,136)	5,767 (4,235-6,217)	1,765 (1,273-1,916)	363 (282-393)

SR Table 4: Global deaths by cause and age group for males (with 95% uncertainty intervals), 2000

Cause	Early Neonatal	Late Neonatal	Post Neonatal	1 to 4	5 to 9	10 to 14	15 to 19	20 to 24
Self-harm	-	-	-	-	-	6,992 (5,146-7,912)	31,645 (27,515-34,320)	55,178 (47,409-59,324)
Self-harm by firearm	-	-	-	-	-	379 (269-531)	4,067 (3,121-5,597)	6,710 (5,095-9,075)
Self-harm by other specified means	-	-	-	-	-	6,614 (4,703-7,495)	27,578 (23,111-30,105)	48,468 (40,733-52,665)
Interpersonal violence	925 (655-1,093)	230 (160-279)	2,480 (1,789-3,076)	4,260 (3,019-5,128)	3,800 (2,652-4,401)	6,290 (4,274-7,214)	32,909 (25,150-36,386)	51,817 (39,683-57,465)
Assault by firearm	117 (69-173)	30 (19-45)	345 (212-530)	591 (379-919)	572 (357-851)	1,645 (933-2,130)	16,613 (10,519-18,825)	25,956 (16,686-29,496)
Assault by sharp object	84 (50-123)	21 (13-30)	226 (126-322)	591 (345-803)	714 (385-889)	1,204 (669-1,470)	8,630 (5,943-11,022)	13,902 (9,710-18,637)
Assault by other means	725 (488-862)	179 (118-219)	1,909 (1,327-2,339)	3,078 (2,001-3,775)	2,514 (1,580-2,937)	3,441 (2,333-4,034)	7,666 (5,485-9,793)	11,959 (8,394-14,529)
Forces of nature, conflict and terrorism, and executions and police conflict	345 (300-385)	191 (165-214)	651 (555-741)	5,171 (4,450-5,826)	3,572 (3,048-4,053)	3,646 (3,093-4,166)	9,722 (8,464-10,912)	16,017 (13,991-17,858)
Forces of nature, conflict and terrorism, and state actor violence	4 (3-5)	9 (6-12)	74 (49-99)	340 (224-455)	376 (249-507)	522 (347-700)	583 (387-777)	537 (354-717)
Conflict and terrorism	341 (297-380)	182 (158-203)	557 (486-622)	4,797 (4,196-5,346)	3,142 (2,747-3,501)	3,063 (2,677-3,418)	8,749 (7,645-9,770)	14,148 (12,339-15,811)
Executions and police conflict	-	-	20 (15-43)	34 (29-43)	54 (46-62)	61 (52-70)	391 (185-480)	1,332 (557-1,662)

Cause	25 to 29	30 to 34	35 to 39	40 to 44	45 to 49	50 to 54	55 to 59	60 to 64
Self-harm	58,478 (51,501-63,317)	55,964 (49,011-60,222)	53,447 (44,770-57,663)	50,070 (42,664-55,569)	47,851 (39,886-51,785)	39,175 (33,020-42,499)	30,995 (25,102-33,359)	28,767 (22,823-30,916)
Self-harm by firearm	6,215 (4,609-8,095)	5,652 (4,213-7,323)	5,064 (3,922-6,686)	4,765 (3,826-6,534)	4,401 (3,450-6,192)	3,745 (3,060-5,383)	2,886 (2,289-4,007)	2,503 (1,954-3,432)
Self-harm by other specified means	52,263 (45,572-56,888)	50,312 (43,230-54,730)	48,383 (39,943-52,541)	45,304 (38,337-49,547)	43,450 (35,725-47,007)	35,430 (28,871-38,397)	28,109 (22,369-30,152)	26,264 (20,462-28,340)
Interpersonal violence	48,257 (35,989-54,621)	42,001 (30,960-48,390)	35,208 (24,866-40,289)	28,482 (19,538-33,584)	21,976 (14,458-26,482)	15,143 (9,858-18,415)	9,826 (6,648-12,098)	8,210 (5,150-9,790)
Assault by firearm	22,589 (14,598-26,052)	18,210 (11,057-21,692)	15,010 (9,180-18,080)	10,878 (6,369-13,985)	7,294 (4,127-9,625)	4,858 (2,699-6,760)	3,103 (1,860-4,292)	2,138 (1,255-2,843)
Assault by sharp object	14,137 (9,626-18,001)	12,560 (8,435-16,115)	10,463 (6,724-13,702)	8,719 (5,413-11,610)	6,896 (4,132-9,206)	4,487 (2,670-6,034)	2,759 (1,598-3,890)	2,531 (1,430-3,412)
Assault by other means	11,531 (7,889-14,182)	11,230 (7,419-14,179)	9,735 (6,326-11,964)	8,885 (5,331-11,296)	7,786 (4,801-9,594)	5,799 (3,398-7,330)	3,964 (2,498-4,866)	3,541 (2,034-4,468)
Forces of nature, conflict and terrorism, and executions and police conflict	11,569 (10,082-12,917)	8,189 (7,065-9,186)	5,608 (4,813-6,292)	3,709 (3,174-4,179)	2,603 (2,227-2,945)	1,799 (1,554-2,045)	1,363 (1,171-1,547)	1,152 (977-1,328)
Forces of nature, conflict and terrorism, and state actor violence	501 (333-667)	432 (287-574)	391 (263-518)	345 (233-457)	311 (211-409)	250 (171-329)	194 (131-257)	199 (135-261)
Conflict and terrorism	9,541 (8,328-10,668)	6,318 (5,510-7,089)	4,180 (3,639-4,716)	2,753 (2,388-3,124)	1,874 (1,629-2,125)	1,334 (1,162-1,509)	1,069 (938-1,203)	873 (752-998)
Executions and police conflict	1,527 (567-1,896)	1,440 (539-1,759)	1,037 (371-1,285)	611 (234-753)	418 (186-505)	214 (94-257)	99 (68-118)	79 (43-93)

Cause	65 to 69	70 to 74	75 to 79	80 to 84	85 to 89	90 to 94	95 plus
Self-harm	24,580 (18,810-26,464)	21,765 (16,341-23,424)	15,266 (11,484-16,448)	9,722 (7,268-10,510)	5,461 (3,955-5,904)	1,666 (1,175-1,815)	308 (228-333)
Self-harm by firearm	2,266 (1,704-3,001)	2,250 (1,666-2,884)	1,921 (1,406-2,339)	1,301 (945-1,581)	776 (508-898)	269 (179-317)	51 (35-62)
Self-harm by other specified means	22,314 (17,003-24,070)	19,515 (14,542-21,115)	13,345 (9,780-14,427)	8,421 (6,117-9,183)	4,685 (3,321-5,086)	1,397 (956-1,526)	257 (185-278)
Interpersonal violence	5,510 (3,694-6,779)	4,088 (2,645-5,237)	2,331 (1,600-2,968)	619 (437-833)	306 (208-424)	98 (67-140)	55 (37-75)
Assault by firearm	1,375 (783-1,958)	978 (542-1,428)	596 (330-859)	126 (62-188)	60 (27-85)	21 (9-30)	14 (7-19)
Assault by sharp object	1,569 (941-2,156)	1,112 (615-1,560)	574 (328-817)	171 (108-256)	81 (48-127)	30 (19-51)	14 (8-23)
Assault by other means	2,566 (1,658-3,172)	1,998 (1,305-2,573)	1,161 (774-1,481)	322 (216-449)	165 (110-247)	47 (31-75)	27 (18-39)
Forces of nature, conflict and terrorism, and executions and police conflict	897 (766-1,025)	664 (559-770)	455 (386-522)	331 (282-386)	165 (139-197)	60 (47-84)	21 (18-25)
Forces of nature, conflict and terrorism, and state actor violence	155 (104-203)	134 (92-176)	90 (60-117)	57 (39-75)	26 (18-35)	7 (5-9)	2 (1-2)
Conflict and terrorism	685 (597-775)	492 (420-565)	345 (301-390)	241 (213-271)	118 (102-135)	41 (34-47)	17 (14-19)
Executions and police conflict	58 (29-70)	38 (21-46)	20 (11-27)	32 (9-62)	20 (5-42)	12 (3-33)	3 (1-4)

SR Table 4: Global deaths by cause and age group for males (with 95% uncertainty intervals), 2006

Cause	Early Neonatal	Late Neonatal	Post Neonatal	1 to 4	5 to 9	10 to 14	15 to 19	20 to 24	25 to 29	30 to 34	35 to 39	40 to 44	45 to 49	50 to 54	55 to 59	60 to 64	65 to 69	70 to 74	75 to 79	80 to 84	85 to 89	90 to 94	95 plus
All causes	1,346,365 (1,309,971-1,385,397)	380,421 (371,434-390,399)	1,181,489 (1,151,679-1,210,672)	1,083,359 (1,042,546-1,127,160)	310,782 (307,005-314,804)	247,985 (245,242-250,703)	411,125 (404,505-417,658)	574,043 (564,223-584,682)	661,354 (648,848-675,696)	796,970 (784,131-811,079)	944,199 (928,331-959,700)	1,114,039 (1,097,614-1,131,120)	1,291,553 (1,270,400-1,313,763)	1,610,176 (1,585,179-1,635,342)	1,791,464 (1,766,965-1,817,082)	1,935,251 (1,913,040-1,957,046)	2,480,291 (2,450,606-2,509,056)	2,782,885 (2,754,867-2,810,653)	2,804,679 (2,778,654-2,830,540)	2,282,231 (2,266,930-2,296,774)	1,418,337 (1,411,505-1,424,924)	660,631 (658,152-662,854)	185,537 (184,940-186,086)
Communicable, maternal, neonatal, and nutritional disorders	1,227,528 (1,186,133-1,268,010)	334,455 (323,485-346,735)	966,680 (932,132-1,003,681)	859,703 (815,239-910,945)	177,789 (167,493-188,204)	105,081 (97,326-112,978)	93,949 (86,894-103,892)	123,863 (116,257-134,796)	201,988 (189,400-218,420)	270,376 (258,066-284,896)	295,989 (281,460-312,393)	294,700 (283,932-308,639)	276,000 (263,111-292,667)	250,268 (238,333-269,835)	221,852 (208,501-244,558)	224,321 (205,759-255,163)	246,765 (223,678-288,320)	278,899 (246,730-332,037)	265,706 (233,662-322,618)	234,168 (206,147-281,829)	158,997 (139,173-190,237)	74,270 (65,181-86,835)	24,248 (21,579-27,776)
HIV/AIDS and tuberculosis	-	-	63,266 (59,913-66,527)	41,528 (38,269-44,887)	22,138 (21,020-23,338)	21,850 (20,433-23,403)	26,678 (24,498-28,693)	57,325 (53,220-62,134)	140,397 (129,337-152,143)	207,769 (195,972-220,018)	228,137 (215,053-240,775)	222,788 (214,390-230,345)	194,390 (185,533-203,481)	158,565 (151,822-165,192)	121,657 (115,752-127,245)	102,235 (96,609-108,544)	95,255 (89,206-100,940)	90,492 (83,572-97,051)	67,101 (61,755-72,016)	45,133 (40,869-49,243)	22,655 (20,608-24,890)	7,124 (6,416-7,840)	1,695 (1,527-1,875)
Tuberculosis	-	-	8,863 (7,341-10,626)	14,308 (11,550-17,247)	4,711 (4,077-5,370)	5,551 (4,900-6,297)	15,599 (14,138-17,209)	33,047 (30,447-35,978)	47,490 (44,259-51,401)	58,582 (54,917-62,326)	66,195 (62,040-70,353)	74,613 (70,106-79,095)	81,575 (76,605-86,856)	88,690 (82,958-94,102)	84,604 (79,286-89,837)	79,933 (74,517-85,716)	80,736 (75,066-86,500)	80,960 (74,228-87,398)	61,767 (56,446-66,898)	43,641 (39,460-47,646)	22,117 (20,076-24,313)	6,984 (6,276-7,702)	1,668 (1,501-1,847)
Drug-susceptible tuberculosis	-	-	8,236 (6,844-9,904)	13,275 (10,709-15,996)	4,346 (3,742-4,967)	5,093 (4,483-5,790)	14,285 (12,908-15,736)	30,145 (27,626-32,801)	43,119 (40,053-46,820)	53,034 (49,592-56,705)	59,721 (55,840-63,829)	66,882 (62,659-71,257)	72,728 (67,814-77,412)	79,221 (74,030-84,677)	76,020 (71,257-80,878)	72,306 (67,377-77,759)	73,019 (67,685-78,327)	73,523 (67,556-79,452)	56,210 (51,176-61,022)	39,850 (36,003-43,786)	20,256 (18,291-22,291)	6,414 (5,764-7,080)	1,541 (1,386-1,712)
Multidrug-resistant tuberculosis without extensive drug resistance	-	-	614 (467-808)	1,013 (750-1,377)	353 (283-442)	444 (353-553)	1,270 (1,036-1,549)	2,766 (2,276-3,314)	4,139 (3,457-4,897)	5,257 (4,415-6,211)	6,124 (5,182-7,258)	7,274 (6,144-8,590)	8,285 (7,006-9,822)	8,906 (7,473-10,569)	8,130 (6,780-9,761)	7,302 (5,998-8,893)	7,367 (6,013-8,955)	7,129 (5,838-8,679)	5,324 (4,351-6,524)	3,635 (2,948-4,507)	1,785 (1,424-2,234)	545 (434-685)	122 (97-152)
Extensively drug-resistant tuberculosis	-	-	13 (10-17)	20 (15-26)	11 (9-15)	14 (11-18)	44 (36-54)	136 (112-164)	233 (192-280)	291 (239-349)	349 (289-419)	457 (379-556)	563 (464-677)	563 (458-682)	453 (373-549)	325 (264-399)	350 (286-431)	308 (250-380)	232 (188-287)	156 (125-197)	76 (60-96)	25 (20-31)	6 (4-7)
HIV/AIDS	-	-	54,403 (51,531-57,277)	27,220 (25,743-28,748)	17,428 (16,546-18,389)	16,299 (15,069-17,647)	11,078 (9,603-12,620)	24,278 (21,022-28,371)	92,907 (82,303-104,668)	149,187 (137,938-160,602)	161,942 (149,784-174,492)	148,175 (140,705-154,847)	112,814 (105,190-120,870)	69,875 (66,168-73,483)	37,053 (34,619-39,446)	22,302 (20,427-24,096)	14,520 (13,220-15,795)	9,532 (8,635-10,413)	5,334 (4,858-5,843)	1,491 (1,330-1,659)	538 (482-597)	140 (126-155)	27 (24-30)
Drug-susceptible HIV/AIDS - Tuberculosis	-	-	16,711 (12,486-21,028)	8,845 (6,938-10,802)	5,377 (4,184-6,520)	4,877 (3,719-5,923)	3,416 (2,651-4,173)	6,742 (4,978-8,594)	21,681 (14,711-29,408)	32,730 (21,399-44,114)	34,732 (22,434-47,251)	33,315 (21,687-44,076)	27,266 (18,274-36,034)	18,973 (13,771-23,518)	10,680 (8,188-13,037)	6,749 (5,269-8,153)	4,452 (3,510-5,311)	3,042 (2,451-3,582)	1,719 (1,397-2,041)	499 (415-582)	178 (149-210)	46 (38-54)	8 (7-10)
Multidrug-resistant HIV/AIDS - Tuberculosis without extensive drug resistance	-	-	1,436 (919-2,112)	737 (483-1,067)	411 (274-597)	363 (233-548)	261 (166-398)	677 (435-985)	2,105 (1,286-3,161)	3,112 (1,876-4,714)	3,180 (1,914-4,809)	2,990 (1,813-4,484)	2,479 (1,521-3,667)	1,751 (1,122-2,514)	976 (642-1,407)	601 (392-859)	386 (257-560)	257 (174-369)	143 (97-204)	42 (27-61)	15 (10-22)	4 (3-6)	1 (0-1)
Extensively drug-resistant HIV/AIDS - Tuberculosis	-	-	14 (9-21)	6 (4-9)	4 (3-5)	3 (2-5)	3 (2-5)	16 (10-24)	62 (37-97)	105 (63-166)	100 (61-157)	82 (50-128)	59 (37-91)	36 (23-55)	19 (12-28)	10 (6-14)	6 (4-9)	4 (2-5)	2 (1-3)	1 (0-1)	0 (0-0)	0 (0-0)	0 (0-0)
HIV/AIDS resulting in other diseases	-	-	36,242 (31,537-41,574)	17,632 (15,489-20,061)	11,635 (10,237-13,128)	11,055 (9,588-12,757)	7,399 (6,217-8,888)	16,843 (13,873-20,729)	69,058 (57,422-83,313)	113,240 (98,509-130,814)	123,930 (107,013-142,612)	111,788 (99,099-127,423)	83,011 (72,608-95,202)	49,114 (43,713-55,767)	25,378 (22,514-28,708)	14,943 (13,125-17,099)	9,675 (8,475-11,022)	6,230 (5,419-7,162)	3,470 (3,050-3,987)	950 (830-1,088)	344 (302-392)	90 (79-103)	18 (16-20)
Diarrhoea, lower respiratory infections, and other common infectious diseases	130,285 (112,189-146,412)	115,279 (104,662-126,148)	633,655 (593,192-675,555)	467,489 (414,821-524,534)	103,188 (89,198-120,265)	56,521 (47,605-66,910)	47,526 (39,167-59,515)	44,913 (36,908-57,623)	43,484 (35,304-55,969)	43,983 (36,240-57,093)	48,633 (39,862-64,974)	52,104 (43,607-68,125)	61,648 (51,655-81,268)	71,813 (59,053-95,048)	80,334 (65,777-107,366)	101,686 (81,507-137,779)	129,121 (103,444-174,103)	166,433 (131,148-227,685)	178,426 (143,188-241,474)	173,539 (144,036-225,203)	124,958 (103,541-158,432)	61,535 (52,107-74,717)	20,552 (17,847-24,357)
Diarrhoeal diseases	17,236 (14,908-20,298)	30,635 (26,320-36,086)	224,641 (195,260-257,852)	164,279 (133,798-202,236)	23,588 (12,884-43,438)	11,914 (6,331-22,393)	13,685 (7,184-25,469)	15,238 (8,188-29,140)	15,687 (8,320-29,387)	15,326 (7,847-29,157)	17,291 (9,156-34,426)	17,791 (9,236-35,764)	20,776 (10,943-40,746)	24,361 (12,280-49,550)	28,863 (15,066-56,940)	40,873 (21,125-80,038)	53,754 (28,831-103,277)	72,069 (38,284-135,925)	73,022 (40,461-138,409)	69,834 (40,891-124,358)	47,608 (28,275-84,400)	20,288 (12,350-33,989)	5,988 (3,891-9,488)
Intestinal infectious diseases	-	-	731 (137-3,227)	15,043 (7,127-27,960)	26,912 (15,355-43,865)	17,623 (9,522-28,128)	11,006 (5,616-18,901)	6,408 (3,168-11,117)	4,520 (2,347-7,746)	3,424 (1,798-5,823)	2,704 (1,440-4,539)	2,203 (1,176-3,659)	1,914 (1,048-3,172)	1,540 (799-2,644)	1,041 (491-1,878)	891 (436-1,610)	868 (487-1,420)	730 (431-1,160)	512 (301-822)	285 (162-459)	125 (73-194)	36 (21-58)	8 (5-14)
Typhoid fever	-	-	103 (47-197)	13,342 (5,915-25,757)	23,560 (12,993-38,062)	14,857 (7,736-24,201)	9,539 (4,707-16,720)	5,386 (2,495-9,541)	3,558 (1,802-6,280)	2,573 (1,332-4,568)	2,030 (1,061-3,573)	1,637 (853-2,876)	1,386 (724-2,342)	1,121 (570-1,968)	777 (358-1,439)	691 (325-1,280)	708 (381-1,195)	608 (345-975)	421 (240-680)	236 (133-381)	99 (56-158)	28 (15-45)	6 (3-10)
Paratyphoid fever	-	-	15 (6-33)	1,341 (544-2,797)	3,310 (1,528-6,596)	2,750 (1,271-5,181)	1,451 (609-2,943)	1,003 (419-2,037)	946 (409-1,891)	828 (362-1,624)	651 (302-1,233)	544 (250-1,067)	502 (226-989)	409 (177-816)	256 (104-526)	189 (80-389)	147 (69-287)	108 (52-202)	71 (34-131)	39 (19-72)	16 (8-30)	5 (2-9)	1 (0-2)
Other intestinal infectious diseases	-	-	613 (37-3,094)	360 (26-1,702)	43 (3-209)	16 (1-69)	16 (1-65)	20 (2-91)	16 (1-74)	23 (2-119)	24 (2-103)	22 (2-103)	26 (2-117)	10 (1-43)	9 (1-45)	10 (1-44)	12 (1-55)	14 (1-68)	20 (2-87)	10 (1-41)	9 (1-41)	3 (1-14)	1 (0-6)
Lower respiratory infections	89,792 (79,081-101,281)	65,074 (58,904-72,856)	289,404 (266,511-312,939)	139,066 (119,199-160,248)	21,257 (16,216-25,035)	11,628 (9,232-13,683)	10,518 (8,305-12,670)	12,334 (9,745-14,771)	13,744 (11,010-16,594)	15,900 (13,156-18,599)	19,101 (15,556-22,731)	23,340 (19,429-26,886)	29,670 (24,873-34,315)	36,986 (30,871-42,116)	42,021 (35,352-48,447)	52,157 (42,133-59,825)	66,230 (53,689-76,415)	86,074 (70,646-96,843)	98,400 (80,871-110,751)	97,994 (81,821-109,573)	74,183 (63,037-83,815)	40,098 (35,146-45,368)	14,245 (12,401-15,895)
Upper respiratory infections	54 (32-88)	17 (9-30)	390 (244-625)	310 (169-582)	25 (15-41)	19 (12-30)	48 (33-69)	50 (35-71)	54 (38-77)	55 (38-78)	65 (45-90)	66 (47-90)	74 (54-102)	77 (55-108)	75 (54-104)	75 (53-104)	71 (51-97)	59 (42-80)	56 (40-76)	35 (26-48)	22 (16-30)	8 (6-12)	2 (1-2)
Otitis media	2 (1-4)	4 (2-8)	63 (32-140)	131 (57-262)	66 (36-114)	78 (44-139)	26 (15-47)	23 (13-43)	19 (10-30)	16 (9-27)	24 (13-40)	27 (13-49)	85 (41-160)	93 (48-167)	32 (14-51)	32 (13-53)	38 (16-72)	32 (18-54)	31 (17-44)	29 (15-41)	18 (8-25)	9 (3-13)	2 (1-4)
Meningitis	6,853 (4,352-9,375)	7,877 (6,079-10,030)	45,910 (38,055-55,702)	39,324 (30,753-48,921)	10,917 (6,466-16,115)	7,401 (4,653-11,244)	7,160 (4,652-11,188)	6,749 (4,168-10,298)	5,850 (3,732-9,153)	5,570 (3,414-9,089)	5,550 (3,540-8,676)	4,800 (3,129-7,575)	5,240 (3,589-8,722)	5,009 (3,419-8,284)	4,541 (3,081-7,460)	4,272 (2,899-7,319)	4,445 (3,096-7,388)	3,629 (2,508-6,344)	2,918 (2,063-5,059)	2,390 (1,589-3,983)	1,285 (866-2,143)	433 (296-718)	101 (73-165)
Pneumococcal meningitis	360 (214-541)	403 (270-574)	2,395 (1,702-3,201)	2,278 (1,488-3,328)	634 (348-1,022)	419 (237-719)	402 (236-686)	374 (211-660)	377 (217-641)	444 (264-732)	538 (309-920)	536 (306-917)	633 (368-1,099)	600 (372-1,044)	558 (362-940)	556 (361-956)	561 (356-950)	463 (290-827)	369 (246-637)	288 (179-485)	143 (96-232)	49 (34-80)	12 (8-19)
H influenzae type B meningitis	987 (609-1,415)	1,101 (799-1,478)	6,033 (4,689-7,915)	4,790 (3,412-6,599)	1,046 (589-1,645)	585 (334-927)	497 (293-825)	463 (279-787)	436 (263-745)	434 (254-714)	442 (272-716)	382 (236-645)	423 (262-753)	402 (259-669)	366 (234-611)	354 (219-631)	351 (224-618)	278 (181-505)	222 (149-392)	186 (112-313)	98 (65-158)	32 (21-53)	7 (4-12)
Meningococcal infection	3,503 (2,249-4,933)	4,012 (3,081-5,217)	23,065 (18,443-28,956)	21,263 (15,951-27,617)	6,126 (3,598-9,046)	4,078 (2,573-6,213)	3,985 (2,540-6,310)	3,688 (2,264-5,777)	3,049 (1,930-4,846)	2,670 (1,646-4,384)	2,585 (1,614-4,129)	2,216 (1,407-3,721)	2,382 (1,560-4,010)	2,308 (1,508-3,867)	2,074 (1,379-3,507)	2,005 (1,333-3,615)	2,056 (1,382-3,598)	1,638 (1,089-2,976)	1,326 (889-2,357)	1,073 (678-1,797)	603 (400-1,036)	205 (136-352)	46 (31-80)
Other meningitis	2,002 (1,234-2,731)	2,361 (1,806-3,038)	14,418 (11,288-18,068)	10,993 (8,250-14,200)	3,111 (1,760-4,821)	2,320 (1,370-3,555)	2,276 (1,392-3,556)	2,224 (1,330-3,437)	1,988 (1,209-3,096)	2,022 (1,187-3,257)	1,984 (1,186-3,089)	1,666 (1,020-2,585)	1,803 (1,176-2,948)	1,698 (1,129-2,874)	1,543 (1,018-2,569)	1,356 (882-2,323)	1,478 (986-2,444)	1,249 (832-2,135)	1,001 (698-1,700)	842 (557-1,448)	440 (297-755)	146 (100-241)	36 (25-58)
Encephalitis	464 (287-613)	671 (493-844)	7,704 (5,573-9,782)	8,280 (6,097-11,492)	3,409 (2,684-4,583)	2,880 (2,323-3,879)	2,288 (1,829-3,123)	2,095 (1,757-3,023)	1,912 (1,578-2,617)	2,075 (1,718-2,831)	2,155 (1,808-3,043)	1,935 (1,639-2,822)	1,942 (1,637-2,838)	1,897 (1,599-2,898)	1,974 (1,654-2,831)	2,097 (1,752-3,071)	2,587 (2,157-3,667)	2,678 (2,203-3,690)	2,631 (2,093-3,383)	2,033 (1,545-2,605)	1,033 (786-1,322)	327 (254-434)	79 (63-109)
Diphtheria	-	-	401 (219-714)	873 (370-1,830)	164 (83-308)	74 (42-127)	36 (22-57)	25 (15-41)	21 (14-34)	19 (12-30)	28 (17-42)	26 (17-38)	30 (20-46)	33 (22-49)	23 (16-35)	-	-	-	-	-	-	-	-
Whooping cough	-	-	25,213 (12,134-46,031)	23,070 (10,856-40,934)	2,471 (1,127-4,630)	528 (245-1,002)	238 (109-455)	163 (76-313)	92 (42-181)	73 (33-148)	50 (23-100)	65 (29-131)	50 (23-103)	38 (17-79)	50 (22-101)	-	-	-	-	-	-	-	-
Tetanus	15,836 (6,483-23,269)	10,846 (4,831-16,324)	4,278 (1,740-6,728)	1,902 (725-3,137)	1,195 (512-1,968)	751 (396-1,191)	1,164 (528-1,940)	1,109 (547-1,714)	1,166 (599-1,675)	1,193 (646-1,697)	1,420 (718-1,886)	1,682 (836-2,244)	1,703 (850-2,238)	1,647 (795-2,176)	1,570 (777-2,066)	1,179 (606-1,587)	927 (470-1,261)	963 (443-1,303)	546 (231-714)	488 (230-668)	208 (99-290)	66 (34-92)	19 (10-26)
Measles	-	-	33,679 (12,554-74,245)	73,688 (30,223-149,540)	12,501 (4,724-26,707)	3,392 (1,205-7,624)	1,249 (434-2,871)	585 (203-1,366)	260 (92-603)	157 (54-365)	97 (34-229)	75 (26-175)	59 (21-138)	45 (16-105)	46 (16-107)	-	-	-	-	-	-	-	-
Varicella and herpes zoster	47 (36-62)	155 (122-198)	1,241 (978-1,566)	1,523 (1,148-2,003)	682 (521-907)	232 (181-300)	107 (84-136)	134 (105-172)	159 (127-199)	174 (139-214)	148 (120-187)	92 (74-115)	104 (84-128)	87 (68-108)	96 (76-120)	110 (88-138)	201 (163-248)	198 (157-247)	311 (248-388)	451 (360-571)	476 (375-609)	272 (213-349)	108 (82-140)
Neglected tropical diseases and malaria	578 (292-922)	15,604 (10,668-21,986)	121,258 (89,872-159,030)	254,027 (196,289-318,410)	38,492 (27,836-49,232)	20,821 (15,364-26,735)	12,371 (9,565-15,608)	13,435 (10,138-16,843)	10,223 (7,675-13,006)	9,662 (7,296-12,425)	9,378 (7,231-12,709)	9,378 (7,190-12,031)	8,719 (6,661-10,952)	8,117 (6,276-10,287)	8,081 (6,341-10,156)	8,591 (6,723-11,203)	8,798 (6,751-11,723)	7,463 (5,792-9,939)	5,778 (4,370-7,692)	2,875 (2,261-3,781)	2,070 (1,557-2,826)	407 (323-521)	147 (110-200)
Malaria	-	15,109 (10,135-21,472)	115,430 (84,000-153,216)	240,565 (181,137-304,942)	30,523 (19,956-40,986)	15,926 (10,494-21,738)	7,501 (5,269-10,427)	8,207 (5,725-11,471)	6,431 (4,566-8,910)	6,088 (4,206-8,650)	6,177 (4,073-8,789)	5,631 (3,885-7,930)	4,793 (3,295-6,767)	4,490 (3,122-6,391)	4,410 (3,035-6,343)	5,392 (3,637-7,850)	5,628 (3,683-8,348)	4,664 (3,026-6,995)	3,546 (2,238-5,376)	1,658 (1,103-2,457)	1,427 (927-2,143)	221 (149-323)	98 (64-149)
Chagas disease	-	-	1 (1-2)	1 (1-1)	1 (1-1)	3 (2-3)	7 (6-10)	19 (15-25)	32 (28-40)	66 (56-79)	118 (103-135)	177 (154-203)	257 (227-291)	357 (315-403)	428 (373-499)	553 (499-611)	586 (533-647)	605 (556-659)	400 (350-460)	284 (253-322)	167 (152-184)	62 (55-69)	13 (12-15)
Leishmaniasis	-	-	1,386 (783-2,374)	2,142 (1,131-3,675)	1,087 (621-1,858)	731 (412-1,210)	969 (551-1,570)	1,109 (622-1,844)	904 (504-1,474)	825 (464-1,373)	980 (541-1,627)	930 (507-1,539)	879 (483-1,454)	878 (495-1,442)	636 (341-1,089)	494 (269-821)	501 (279-837)	592 (332-1,001)	468 (260-789)	237 (130-398)	144 (77-245)	31 (12-55)	10 (3-20)
Visceral leishmaniasis	-	-	1,386 (783-2,374)	2,142 (1,131-3,675)	1,087 (621-1,858)	731 (412-1,210)	969 (551-1,570)	1,109 (622-1,844)	904 (504-1,474)	825 (464-1,373)	980 (541-1,627)	930 (507-1,539)	879 (483-1,454)	878 (495-1,442)	636 (341-1,089)	494 (269-821)	501 (279-837)	592 (332-1,001)	468 (260-789)	237 (130-398)	144 (77-245)	31 (12-55)	10 (3-20)
African trypanosomiasis	-	-	-	164 (31-579)	378 (89-1,151)	435 (99-1,285)	586 (130-1,885)	659 (137-2,088)	547 (124-1,722)	477 (101-1,587)	383 (78-1,138)	335 (71-1,153)	283 (53-965)	231 (49-891)	194 (36-729)	163 (33-600)	122 (22-450)	79 (15-279)	53 (8-199)	24 (4-108)	9 (2-27)	2 (0-7)	0 (0-1)
Schistosomiasis	-	-	0 (0-0)	265 (154-451)	147 (94-237)	202 (128-322)	211 (146-294)	299 (213-422)	340 (243-467)	367 (266-503)	422 (316-561)	459 (343-600)	485 (360-641)	566 (422-754)	631 (453-839)	627 (462-829)	633 (458-850)	562 (418-749)	407 (305-538)	201 (147-269)	85 (61-114)	23 (15-34)	3 (2-5)
Cysticercosis	0 (0-0)	0 (0-0)	0 (0-0)	48 (31-71)	32 (23-43)	25 (20-32)	27 (21-34)	41 (33-52)	51 (42-63)	59 (48-71)	47 (39-56)	45 (38-54)	42 (35-51)	49 (41-58)	42 (37-50)	33 (27-40)	27 (22-32)	17 (14-20)	11 (8-14)	6 (5-9)	2 (2-3)	1 (1-1)	0 (0-0)
Cystic echinococcosis	-	-	-	156 (104-232)	68 (45-99)	43 (28-62)	21 (15-30)	29 (20-40)	41 (30-56)	42 (31-56)	50 (38-65)	46 (35-59)	53 (40-67)	50 (39-62)	49 (39-63)	47 (37-59)	49 (38-60)	38 (30-49)	32 (25-39)	21 (16-28)	9 (7-12)	3 (2-4)	1 (0-1)
Dengue	-	-	1,010 (248-1,646)	1,812 (561-2,769)	1,128 (398-1,866)	724 (213-1,127)	797 (152-1,291)	754 (132-1,141)	664 (101-1,062)	501 (77-791)	602 (81-922)	488 (71-751)	523 (81-786)	475 (78-717)	535 (72-838)	440 (69-702)	513 (84-810)	227 (37-407)	180 (34-299)	146 (24-273)	88 (21-154)	27 (8-49)	7 (3-10)
Yellow fever	-	2 (0-7)	38 (7-121)	394 (76-1,203)	799 (153-2,491)	687 (127-2,158)	639 (128-1,829)	556 (111-1,697)	428 (84-1,400)	317 (61-981)	235 (44-706)	181 (35-520)	142 (27-436)	109 (20-345)	86 (16-275)	65 (13-201)	45 (9-133)	30 (6-92)	17 (3-53)	7 (1-22)	3 (0-9)	1 (0-2)	0 (0-0)
Rabies	-	-	267 (136-392)	1,510 (602-2,197)	2,856 (1,129-4,697)	1,579 (625-2,293)	1,313 (674-1,944)	1,425 (757-2,090)	460 (191-603)	643 (273-833)	606 (269-779)	788 (341-962)	1,012 (397-1,343)	596 (251-705)	730 (236-970)	475 (168-598)	385 (150-488)	324 (150-446)	419 (244-665)	138 (87-261)	56 (36-123)	7 (5-14)	3 (2-6)
Intestinal nematode infections	-	-	226 (174-289)	2,440 (1,798-3,249)	401 (316-508)	85 (68-108)	32 (24-40)	23 (17-30)	18 (13-24)	23 (17-31)	22 (16-28)	16 (11-20)	17 (12-21)	24 (18-30)	19 (14-24)	20 (15-26)	15 (11-19)	22 (16-28)	16 (12-21)	10 (7-13)	3 (2-5)	2 (1-3)	1 (0-1)
Ascariasis	-	-	226 (174-289)	2,440 (1,798-3,249)	401 (316-508)	85 (68-108)	32 (24-40)	23 (17-30)	18 (13-24)	23 (17-31)	22 (16-28)	16 (11-20)	17 (12-21)	24 (18-30)	19 (14-24)	20 (15-26)	15 (11-19)	22 (16-28)	16 (12-21)	10 (7-13)	3 (2-5)	2 (1-3)	1 (0-1)
Ebola	0 (0-0)	0 (0-0)	0 (0-0)	0 (0-0)	0 (0-0)	0 (0-0)	0 (0-0)	0 (0-0)	0 (0-0)	0 (0-0)	0 (0-0)	0 (0-0)	0 (0-0)	0 (0-0)	0 (0-0)	0 (0-0)	0 (0-0)	0 (0-0)	0 (0-0)	0 (0-0)	0 (0-0)	0 (0-0)	0 (0-0)
Zika virus	0 (0-0)	0 (0-0)	0 (0-0)	0 (0-0)	0 (0-0)	0 (0-0)	0 (0-0)	0 (0-0)	0 (0-0)	0 (0-0)	0 (0-0)	0 (0-0)	0 (0-0)	0 (0-0)	0 (0-0)	0 (0-0)	0 (0-0)	0 (0-0)	0 (0-0)	0 (0-0)	0 (0-0)	0 (0-0)	0 (0-0)
Other neglected tropical diseases	578 (292-922)	493 (283-701)	2,899 (1,471-4,183)	4,531 (1,789-10,608)	1,072 (562-2,021)	381 (242-611)	269 (162-412)	313 (194-481)	305 (195-441)	255 (153-343)	282 (176-356)	282 (186-368)	294 (176-387)	293 (197-362)	319 (227-409)	281 (208-367)	295 (210-378)	302 (222-394)	230 (162-305)	142 (101-195)	77 (55-104)	28 (21-37)	11 (8-18)
Maternal disorders	-	-	-	-	-	-	-	-	-	-	-	-	-	-	-	-	-	-	-	-	-	-	-
Maternal haemorrhage	-	-	-	-	-	-	-	-	-	-	-	-	-	-	-	-	-	-	-	-	-	-	-
Maternal sepsis and other pregnancy related infections	-	-	-	-	-	-	-	-	-	-	-	-	-	-	-	-	-	-	-	-	-	-	-
Maternal hypertensive disorders	-	-	-	-	-	-	-	-	-	-	-	-	-	-	-	-	-	-	-	-	-	-	-
Maternal obstructed labour and uterine rupture	-	-	-	-	-	-	-	-	-	-	-	-	-	-	-	-	-	-	-	-	-	-	-
Maternal abortion, miscarriage, and ectopic pregnancy	-	-	-	-	-	-	-	-	-	-	-	-	-	-	-	-	-	-	-	-	-	-	-
Indirect maternal deaths	-	-	-	-	-	-	-	-	-	-	-	-	-	-	-	-	-	-	-	-	-	-	-
Late maternal deaths	-	-	-	-	-	-	-	-	-	-	-	-	-	-	-	-	-	-	-	-	-	-	-
Maternal deaths aggravated by HIV/AIDS	-	-	-	-	-	-	-	-	-	-	-	-	-	-	-	-	-	-	-	-	-	-	-
Other maternal disorders	-	-	-	-	-	-	-	-	-	-	-	-	-	-	-	-	-	-	-	-	-	-	-
Neonatal disorders	1,068,954 (1,026,046-1,115,795)	191,370 (178,825-207,713)	51,702 (45,366-60,665)	5,845 (4,958-7,071)	-	-	-	-	-	-	-	-	-	-	-	-	-	-	-	-	-	-	-
Neonatal preterm birth complications	410,123 (378,893-460,159)	54,699 (48,228-65,060)	18,544 (15,607-23,035)	1,460 (1,181-1,887)	-	-	-	-	-	-	-	-	-	-	-	-	-	-	-	-	-	-	-
Neonatal encephalopathy due to birth asphyxia and trauma	353,664 (317,509-392,882)	30,434 (25,632-35,622)	10,062 (8,046-12,483)	2,518 (2,070-3,007)	-	-	-	-	-	-	-	-	-	-	-	-	-	-	-	-	-	-	-
Neonatal sepsis and other neonatal infections	92,323 (59,338-119,711)	56,564 (43,635-69,907)	9,709 (6,711-13,239)	479 (197-918)	-	-	-	-	-	-	-	-	-	-	-	-	-	-	-	-	-	-	-
Hemolytic disease and other neonatal jaundice	30,514 (25,691-38,348)	14,776 (12,268-17,765)	2,823 (2,039-3,909)	160 (110-233)	-	-	-	-	-	-	-	-	-	-	-	-	-	-	-	-	-	-	-
Other neonatal disorders	182,330 (163,305-205,226)	34,898 (30,265-43,133)	10,564 (8,631-13,637)	1,228 (945-1,757)	-	-	-	-	-	-	-	-	-	-	-	-	-	-	-	-	-	-	-
Nutritional deficiencies	-	-	55,644 (47,790-64,310)	67,008 (49,984-86,949)	6,502 (5,169-8,241)	2,366 (1,925-3,156)	2,145 (1,777-3,045)	2,261 (1,869-3,168)	2,055 (1,683-2,934)	2,148 (1,799-3,025)	2,328 (1,953-3,367)	2,556 (2,161-3,653)	2,783 (2,337-4,156)	3,397 (2,849-4,907)	4,096 (3,459-5,879)	4,717 (3,969-7,002)	6,385 (5,454-9,007)	7,945 (6,855-10,902)	8,793 (7,700-11,684)	8,779 (7,782-11,121)	7,360 (6,657-8,850)	4,504 (4,142-5,157)	1,663 (1,537-1,860)
Protein-energy malnutrition	-	-	53,892 (46,192-62,488)	65,247 (48,813-84,685)	5,742 (4,545-7,380)	1,946 (1,565-2,701)	1,436 (1,150-2,089)	1,465 (1,195-1,993)	1,380 (1,105-1,966)	1,433 (1,186-1,965)	1,562 (1,281-2,297)	1,791 (1,494-2,526)	1,741 (1,446-2,504)	2,300 (1,897-3,349)	2,830 (2,365-3,988)	3,362 (2,793-5,055)	4,521 (3,802-6,395)	5,966 (5,082-8,016)	6,787 (5,900-8,889)	7,063 (6,211-8,721)	5,979 (5,274-6,906)	3,759 (3,326-4,190)	1,408 (1,229-1,555)
Iodine deficiency	-	-	142 (26-435)	123 (17-451)	45 (10-145)	11 (2-36)	35 (8-102)	40 (7-144)	46 (9-159)	42 (10-136)	40 (10-123)	32 (9-89)	30 (8-100)	39 (11-111)	47 (15-144)	41 (12-99)	54 (19-136)	65 (21-154)	56 (19-132)	35 (11-80)	15 (4-39)	5 (1-15)	2 (0-7)
Iron-deficiency anaemia	-	-	25 (12-43)	55 (29-91)	68 (37-101)	59 (34-81)	94 (65-123)	88 (66-113)	66 (54-85)	76 (59-95)	57 (47-77)	54 (42-99)	53 (42-101)	45 (35-88)	54 (40-136)	47 (33-120)	69 (45-187)	89 (51-180)	78 (51-190)	70 (44-180)	42 (24-94)	26 (13-49)	13 (6-24)
Other nutritional deficiencies	-	-	1,585 (1,051-2,148)	1,583 (821-2,365)	647 (478-859)	350 (271-451)	580 (441-897)	669 (475-1,124)	563 (407-922)	597 (407-998)	669 (470-1,082)	679 (455-1,172)	958 (647-1,699)	1,013 (699-1,762)	1,163 (835-2,021)	1,268 (962-2,073)	1,742 (1,293-2,716)	1,825 (1,337-2,965)	1,872 (1,332-3,068)	1,610 (1,184-2,587)	1,323 (954-2,124)	715 (516-1,083)	240 (167-397)
Other communicable, maternal, neonatal, and nutritional diseases	27,711 (16,465-42,009)	12,202 (7,895-17,649)	41,156 (28,129-57,772)	23,806 (16,352-32,405)	7,469 (5,588-9,740)	3,524 (3,006-4,068)	5,229 (4,639-5,809)	5,928 (5,408-6,452)	5,829 (5,354-6,369)	6,813 (6,224-7,408)	6,968 (6,460-7,502)	7,874 (7,281-8,544)	8,401 (7,719-9,115)	8,376 (7,760-9,003)	7,685 (7,149-8,242)	7,092 (6,567-7,614)	7,206 (6,676-7,713)	6,567 (6,025-7,064)	5,608 (5,161-6,096)	3,843 (3,430-4,269)	1,954 (1,730-2,143)	700 (621-781)	191 (167-212)
Sexually transmitted diseases excluding HIV	23,591 (13,067-38,185)	9,136 (5,137-14,716)	25,351 (14,165-41,039)	10,508 (5,706-17,151)	3,488 (1,894-5,602)	69 (52-94)	98 (74-131)	91 (64-120)	114 (83-149)	209 (145-284)	155 (104-202)	106 (84-165)	95 (75-142)	157 (115-238)	224 (162-345)	74 (58-134)	95 (74-152)	101 (77-197)	97 (75-168)	63 (50-99)	44 (33-59)	22 (16-29)	11 (7-14)
Syphilis	23,591 (13,067-38,185)	9,136 (5,137-14,716)	25,351 (14,165-41,039)	10,508 (5,706-17,151)	3,488 (1,894-5,602)	58 (45-80)	83 (62-111)	78 (55-103)	95 (69-125)	178 (123-241)	137 (92-179)	97 (76-150)	86 (68-129)	146 (107-221)	207 (150-320)	69 (54-125)	89 (69-142)	93 (71-181)	89 (69-154)	57 (46-90)	39 (29-52)	19 (14-25)	9 (6-11)
Chlamydial infection	-	-	-	-	-	0 (0-0)	0 (0-0)	0 (0-0)	0 (0-0)	0 (0-0)	0 (0-0)	0 (0-0)	0 (0-0)	0 (0-0)	0 (0-0)	0 (0-0)	0 (0-0)	0 (0-0)	0 (0-0)	0 (0-0)	0 (0-0)	0 (0-0)	0 (0-0)
Gonococcal infection	-	-	-	-	-	7 (5-9)	11 (8-15)	8 (6-11)	16 (11-20)	23 (16-32)	9 (6-12)	6 (5-9)	4 (3-6)	5 (4-8)	8 (6-12)	3 (2-5)	3 (2-5)	6 (4-11)	5 (4-9)	3 (3-5)	3 (2-4)	2 (1-2)	1 (1-1)
Other sexually transmitted diseases	-	-	-	-	-	4 (3-5)	4 (3-5)	4 (3-6)	3 (2-4)	8 (6-11)	8 (6-11)	4 (3-6)	5 (4-8)	6 (4-9)	9 (6-13)	2 (2-4)	3 (2-4)	2 (2-5)	3 (2-5)	2 (2-3)	2 (2-3)	1 (1-2)	1 (1-2)
Hepatitis	-	-	5,646 (4,881-6,610)	5,994 (4,670-7,304)	2,553 (2,077-3,103)	2,351 (1,987-2,754)	3,697 (3,239-4,213)	4,606 (4,195-5,044)	4,606 (4,202-5,087)	5,334 (4,872-5,838)	5,436 (5,001-5,916)	6,601 (6,013-7,260)	6,885 (6,245-7,549)	6,741 (6,216-7,341)	5,926 (5,463-6,415)	5,084 (4,730-5,486)	5,029 (4,645-5,475)	4,310 (3,949-4,676)	3,571 (3,242-3,913)	2,250 (1,987-2,511)	984 (866-1,101)	267 (237-295)	55 (49-60)
Acute hepatitis A	-	-	3,407 (2,190-4,733)	611 (342-970)	55 (33-84)	30 (18-44)	46 (27-71)	56 (29-92)	47 (22-79)	45 (20-84)	44 (20-78)	57 (25-103)	70 (35-123)	76 (38-139)	72 (36-127)	62 (31-107)	63 (31-110)	57 (28-100)	52 (26-91)	33 (17-55)	14 (8-21)	4 (2-6)	1 (0-1)
Hepatitis B	-	-	2,239 (1,159-3,599)	891 (503-1,460)	270 (144-455)	391 (207-629)	1,273 (862-1,790)	2,653 (1,960-3,383)	3,436 (2,735-4,088)	4,536 (3,886-5,141)	4,879 (4,341-5,422)	6,042 (5,395-6,715)	6,268 (5,528-6,967)	6,130 (5,481-6,786)	5,349 (4,793-5,867)	4,538 (4,051-5,008)	4,426 (3,929-4,947)	3,714 (3,205-4,174)	2,983 (2,488-3,418)	1,684 (1,326-2,013)	609 (420-791)	136 (80-189)	23 (11-35)
Hepatitis C	-	-	-	-	71 (44-112)	26 (17-39)	36 (23-54)	43 (22-71)	36 (17-61)	37 (16-67)	42 (20-75)	66 (29-115)	94 (43-169)	121 (56-225)	143 (69-258)	145 (67-258)	133 (63-227)	107 (49-184)	91 (46-164)	58 (28-100)	26 (15-42)	8 (4-12)	2 (1-3)
Acute hepatitis E	-	-	-	4,492 (3,428-5,587)	2,156 (1,716-2,630)	1,904 (1,540-2,297)	2,342 (1,833-2,847)	1,855 (1,220-2,537)	1,087 (595-1,678)	716 (341-1,210)	470 (221-825)	436 (185-804)	453 (202-812)	414 (202-731)	363 (153-699)	338 (133-671)	407 (179-750)	431 (195-800)	445 (173-859)	475 (230-765)	335 (188-518)	120 (69-177)	30 (18-42)
Other infectious diseases	4,120 (1,303-8,541)	3,065 (1,404-4,998)	10,160 (3,678-20,454)	7,303 (3,133-13,643)	1,428 (999-1,914)	1,104 (823-1,399)	1,435 (1,114-1,724)	1,231 (941-1,489)	1,110 (888-1,310)	1,269 (958-1,472)	1,378 (1,110-1,571)	1,166 (983-1,296)	1,421 (1,161-1,587)	1,477 (1,218-1,626)	1,535 (1,268-1,682)	1,934 (1,645-2,197)	2,083 (1,783-2,315)	2,156 (1,782-2,454)	1,941 (1,681-2,191)	1,529 (1,301-1,803)	926 (781-1,056)	410 (347-478)	125 (105-145)
Non-communicable diseases	110,387 (85,515-130,426)	41,114 (33,206-47,511)	165,107 (138,819-185,572)	98,242 (80,028-114,374)	50,198 (45,927-54,845)	54,184 (50,717-57,916)	100,349 (95,826-105,372)	135,074 (129,909-141,734)	170,936 (164,875-178,228)	254,519 (247,168-263,629)	380,328 (370,169-394,061)	572,489 (559,075-587,727)	794,508 (776,149-812,443)	1,156,454 (1,133,233-1,179,274)	1,403,196 (1,378,319-1,428,400)	1,587,202 (1,555,136-1,613,255)	2,108,729 (2,064,176-2,143,823)	2,392,161 (2,339,369-2,431,864)	2,440,429 (2,385,374-2,480,022)	1,975,003 (1,928,584-2,008,284)	1,213,994 (1,184,831-1,236,165)	565,807 (553,681-575,109)	155,336 (151,908-158,201)
Neoplasms	451 (391-478)	295 (269-314)	2,908 (2,696-3,289)	12,539 (11,203-14,599)	15,523 (14,209-16,877)	17,370 (16,031-18,473)	26,633 (25,133-28,122)	27,729 (26,518-28,974)	31,641 (30,378-32,934)	51,552 (49,811-53,129)	84,524 (82,140-87,356)	138,556 (135,052-142,147)	208,615 (202,995-213,232)	340,573 (333,026-347,476)	435,915 (427,697-443,595)	480,816 (469,845-489,265)	604,638 (591,335-615,729)	625,566 (611,200-636,111)	554,936 (542,559-565,098)	371,809 (360,671-380,730)	188,855 (182,414-193,836)	72,961 (70,267-75,251)	16,913 (16,082-17,487)
Lip and oral cavity cancer	-	-	-	-	-	-	248 (225-276)	481 (443-527)	868 (783-962)	1,575 (1,444-1,729)	2,756 (2,547-3,001)	4,913 (4,583-5,283)	7,547 (7,199-7,924)	10,957 (10,470-11,501)	12,295 (11,797-12,823)	11,494 (11,014-12,010)	11,685 (11,173-12,199)	9,684 (9,202-10,108)	7,363 (6,991-7,682)	4,723 (4,441-4,974)	2,440 (2,288-2,562)	959 (906-1,005)	240 (228-252)
Nasopharynx cancer	-	-	-	-	96 (83-111)	217 (191-243)	400 (364-439)	413 (375-455)	515 (471-564)	969 (896-1,057)	1,828 (1,680-1,984)	3,054 (2,849-3,292)	3,875 (3,617-4,120)	5,565 (5,218-5,928)	5,621 (5,303-5,955)	4,758 (4,457-5,065)	4,713 (4,445-4,986)	3,787 (3,589-4,000)	2,407 (2,288-2,524)	1,467 (1,396-1,539)	596 (568-624)	220 (210-230)	56 (53-58)
Other pharynx cancer	-	-	-	-	-	-	131 (116-148)	230 (204-256)	296 (263-332)	650 (592-708)	1,380 (1,267-1,511)	3,114 (2,868-3,377)	5,523 (5,174-5,885)	8,538 (8,064-9,010)	9,665 (9,157-10,149)	9,249 (8,637-9,829)	9,053 (8,468-9,593)	7,152 (6,621-7,625)	4,791 (4,440-5,088)	2,911 (2,614-3,156)	1,207 (1,069-1,314)	355 (321-380)	64 (58-68)
Oesophageal cancer	-	-	-	-	-	-	221 (203-242)	365 (341-391)	498 (467-534)	1,210 (1,142-1,297)	2,831 (2,674-3,001)	7,144 (6,759-7,492)	13,567 (13,070-14,099)	26,170 (25,186-27,173)	34,926 (33,668-36,152)	37,824 (36,337-39,339)	44,994 (43,285-46,779)	43,609 (41,736-45,451)	33,576 (32,227-34,915)	20,291 (19,462-21,117)	8,639 (8,300-8,959)	2,570 (2,481-2,663)	472 (455-491)
Stomach cancer	-	-	-	-	-	-	654 (616-693)	1,188 (1,128-1,265)	2,016 (1,917-2,141)	4,550 (4,354-4,771)	8,628 (8,305-9,009)	15,726 (15,180-16,322)	24,277 (23,477-25,094)	41,703 (40,358-43,036)	54,073 (52,509-55,699)	60,300 (58,448-62,110)	79,005 (76,722-81,397)	83,088 (80,848-85,542)	69,902 (67,766-71,900)	43,775 (42,560-45,074)	20,301 (19,711-20,944)	7,434 (7,189-7,675)	1,724 (1,661-1,780)
Colon and rectum cancer	-	-	-	-	-	-	809 (736-898)	1,619 (1,488-1,772)	2,236 (2,091-2,406)	3,917 (3,706-4,144)	6,062 (5,784-6,378)	9,041 (8,672-9,413)	14,169 (13,733-14,622)	22,125 (21,537-22,732)	30,908 (30,094-31,739)	36,584 (35,668-37,489)	49,680 (48,302-51,131)	54,610 (53,211-56,066)	53,973 (52,554-55,428)	39,458 (38,349-40,596)	21,411 (20,769-22,069)	8,850 (8,564-9,132)	2,156 (2,079-2,228)
Liver cancer	-	-	-	-	295 (262-340)	526 (477-589)	1,144 (1,049-1,264)	2,232 (2,003-2,537)	4,082 (3,711-4,610)	9,724 (9,103-10,526)	19,361 (18,204-20,817)	29,388 (27,819-31,261)	37,939 (36,104-39,736)	56,366 (54,050-59,132)	60,280 (57,630-62,971)	57,061 (54,771-59,222)	61,265 (59,129-63,457)	58,105 (56,065-60,233)	43,516 (42,019-45,155)	24,990 (24,065-25,978)	10,362 (9,962-10,756)	3,106 (2,983-3,238)	576 (549-602)
Liver cancer due to hepatitis B	-	-	-	-	65 (44-90)	184 (134-240)	508 (384-636)	1,202 (962-1,457)	2,431 (1,918-2,947)	5,884 (4,904-6,763)	11,501 (9,500-13,141)	16,999 (13,815-19,291)	20,857 (16,721-23,792)	29,414 (23,710-33,859)	29,145 (22,846-33,767)	25,556 (19,283-29,797)	25,554 (20,022-29,759)	22,413 (17,239-26,422)	15,381 (11,989-18,263)	8,212 (6,053-10,068)	3,285 (2,590-3,961)	922 (712-1,131)	161 (108-210)
Liver cancer due to hepatitis C	-	-	-	-	5 (4-6)	10 (8-14)	23 (17-30)	63 (48-83)	149 (103-214)	388 (293-491)	889 (681-1,151)	1,746 (1,387-2,220)	3,254 (2,571-4,074)	6,274 (5,054-7,577)	8,898 (7,412-10,687)	9,944 (8,420-11,788)	11,396 (9,742-13,352)	12,207 (10,632-14,174)	10,571 (9,189-12,299)	6,873 (5,898-7,902)	3,139 (2,792-3,522)	1,067 (933-1,203)	220 (188-254)
Liver cancer due to alcohol use	-	-	-	-	-	-	3 (2-4)	27 (18-40)	83 (50-129)	289 (206-399)	864 (607-1,194)	1,725 (1,280-2,305)	2,723 (1,969-3,649)	4,230 (3,059-5,584)	5,003 (3,599-6,798)	5,534 (4,059-7,291)	7,384 (5,799-9,122)	7,461 (5,893-9,163)	5,442 (4,384-6,619)	2,959 (2,285-3,697)	1,137 (890-1,403)	315 (234-403)	52 (33-74)
Liver cancer due to other causes	-	-	-	-	225 (192-269)	332 (274-397)	611 (486-741)	939 (765-1,132)	1,420 (1,121-1,854)	3,163 (2,514-3,977)	6,107 (4,933-7,819)	8,919 (7,238-11,418)	11,105 (9,075-13,946)	16,448 (13,364-20,690)	17,234 (14,080-21,640)	16,026 (13,206-19,965)	16,932 (14,159-20,570)	16,025 (13,445-19,429)	12,121 (10,277-14,492)	6,946 (5,800-8,391)	2,802 (2,396-3,255)	802 (668-953)	142 (110-179)
Gallbladder and biliary tract cancer	-	-	-	-	-	-	48 (40-57)	75 (59-89)	130 (98-158)	372 (277-451)	755 (557-924)	1,339 (1,009-1,609)	2,086 (1,606-2,414)	3,611 (2,781-4,165)	4,860 (3,910-5,622)	5,849 (4,714-6,757)	7,525 (6,202-8,692)	8,603 (7,226-9,792)	8,442 (7,209-9,374)	6,135 (5,315-6,768)	3,231 (2,771-3,513)	1,321 (1,120-1,433)	318 (281-345)
Pancreatic cancer	-	-	-	-	-	-	110 (104-116)	211 (200-223)	354 (338-376)	962 (921-1,012)	2,020 (1,952-2,096)	4,011 (3,867-4,167)	7,163 (6,949-7,409)	12,165 (11,852-12,484)	17,277 (16,810-17,709)	19,623 (19,178-20,082)	24,948 (24,297-25,568)	25,558 (24,986-26,187)	22,228 (21,678-22,839)	14,579 (14,152-14,980)	7,177 (6,956-7,409)	2,640 (2,557-2,726)	569 (548-590)
Larynx cancer	-	-	-	-	-	-	108 (99-118)	134 (125-143)	187 (175-199)	445 (414-478)	1,032 (958-1,102)	2,635 (2,469-2,786)	5,294 (5,039-5,564)	9,343 (8,918-9,726)	11,474 (10,982-11,925)	11,792 (11,300-12,320)	13,379 (12,809-13,956)	11,068 (10,649-11,479)	8,198 (7,864-8,476)	4,977 (4,757-5,174)	2,190 (2,089-2,276)	706 (676-733)	141 (135-147)
Tracheal, bronchus, and lung cancer	-	-	-	-	-	-	711 (657-766)	1,200 (1,098-1,315)	2,032 (1,903-2,187)	4,562 (4,313-4,833)	9,697 (9,170-10,239)	21,622 (20,528-22,729)	40,924 (39,567-42,336)	77,835 (75,408-80,479)	109,120 (106,038-112,402)	125,929 (122,857-128,776)	165,094 (161,024-169,325)	168,397 (164,318-172,335)	143,655 (139,609-146,949)	85,726 (83,382-88,071)	36,998 (35,929-38,115)	11,291 (10,936-11,648)	2,047 (1,977-2,115)
Malignant skin melanoma	-	-	-	-	-	-	133 (121-163)	230 (211-292)	344 (316-409)	605 (542-700)	953 (863-1,111)	1,448 (1,317-1,678)	1,756 (1,616-1,982)	2,366 (2,181-2,673)	2,810 (2,610-3,059)	2,535 (2,295-2,742)	2,944 (2,681-3,173)	2,923 (2,609-3,105)	2,818 (2,401-2,983)	2,173 (1,803-2,313)	1,232 (975-1,305)	509 (394-540)	125 (100-134)
Non-melanoma skin cancer	-	-	-	-	-	-	130 (122-139)	161 (150-173)	187 (175-200)	337 (316-359)	476 (451-503)	748 (711-786)	977 (937-1,010)	1,521 (1,466-1,571)	1,967 (1,894-2,031)	2,821 (2,700-2,942)	3,653 (3,509-3,803)	3,957 (3,806-4,111)	3,898 (3,761-4,032)	3,295 (3,162-3,421)	2,132 (2,046-2,219)	1,149 (1,110-1,190)	418 (404-433)
Non-melanoma skin cancer (squamous-cell carcinoma)	-	-	-	-	-	-	130 (122-139)	161 (150-173)	187 (175-200)	337 (316-359)	476 (451-503)	748 (711-786)	977 (937-1,010)	1,521 (1,466-1,571)	1,967 (1,894-2,031)	2,821 (2,700-2,942)	3,653 (3,509-3,803)	3,957 (3,806-4,111)	3,898 (3,761-4,032)	3,295 (3,162-3,421)	2,132 (2,046-2,219)	1,149 (1,110-1,190)	418 (404-433)
Breast cancer	-	-	-	-	-	-	37 (36-39)	35 (33-37)	43 (37-46)	79 (62-87)	156 (117-172)	248 (182-272)	469 (343-511)	686 (497-743)	801 (586-859)	900 (660-964)	1,025 (768-1,096)	1,008 (778-1,072)	703 (545-746)	588 (457-632)	336 (265-360)	148 (118-160)	43 (35-47)
Cervical cancer	-	-	-	-	-	-	-	-	-	-	-	-	-	-	-	-	-	-	-	-	-	-	-
Uterine cancer	-	-	-	-	-	-	-	-	-	-	-	-	-	-	-	-	-	-	-	-	-	-	-
Ovarian cancer	-	-	-	-	-	-	-	-	-	-	-	-	-	-	-	-	-	-	-	-	-	-	-
Prostate cancer	-	-	-	-	-	-	76 (61-87)	90 (71-103)	111 (88-129)	147 (114-166)	233 (179-259)	541 (423-607)	1,584 (1,272-1,754)	4,523 (3,634-4,927)	10,359 (8,211-11,207)	20,400 (15,783-22,134)	35,151 (28,573-37,993)	49,394 (41,089-53,518)	60,037 (51,380-66,355)	51,268 (45,080-56,463)	35,077 (29,868-37,695)	17,642 (15,006-19,004)	4,687 (3,887-5,081)
Testicular cancer	-	-	-	-	-	-	450 (413-495)	930 (864-1,015)	1,109 (1,037-1,200)	1,091 (1,031-1,165)	959 (909-1,020)	711 (674-752)	572 (542-605)	470 (442-498)	333 (315-353)	317 (299-338)	352 (331-374)	364 (343-388)	296 (280-311)	257 (240-277)	128 (121-134)	55 (52-58)	17 (16-18)
Kidney cancer	19 (16-21)	10 (8-11)	130 (116-149)	642 (557-739)	298 (271-327)	148 (136-159)	141 (128-153)	181 (168-195)	245 (227-260)	417 (384-439)	830 (777-871)	1,611 (1,535-1,691)	3,134 (2,972-3,286)	5,327 (5,078-5,585)	7,415 (7,100-7,719)	7,417 (7,120-7,680)	9,109 (8,716-9,454)	9,151 (8,785-9,473)	8,877 (8,550-9,218)	6,215 (5,955-6,438)	3,224 (3,084-3,348)	1,243 (1,194-1,296)	268 (256-280)
Bladder cancer	-	-	-	-	-	-	69 (64-75)	129 (120-139)	155 (143-168)	349 (326-373)	684 (639-730)	1,352 (1,270-1,435)	2,552 (2,425-2,679)	4,568 (4,379-4,762)	7,013 (6,758-7,288)	9,650 (9,307-9,948)	14,374 (13,902-14,878)	17,874 (17,297-18,444)	19,639 (19,020-20,285)	16,405 (15,836-16,929)	9,865 (9,509-10,200)	4,389 (4,231-4,547)	1,104 (1,060-1,150)
Brain and nervous system cancer	58 (39-68)	39 (28-44)	532 (404-635)	2,209 (1,817-2,629)	3,160 (2,668-3,597)	3,173 (2,624-3,549)	2,796 (2,297-3,090)	2,519 (2,083-2,809)	2,865 (2,406-3,182)	3,970 (3,305-4,354)	5,257 (4,436-5,834)	6,740 (5,732-7,512)	7,698 (6,686-8,494)	9,895 (8,656-10,801)	10,959 (9,482-11,855)	10,265 (8,784-11,391)	10,802 (9,257-11,806)	9,172 (7,746-9,887)	6,841 (5,514-7,346)	3,695 (2,879-3,998)	1,441 (1,100-1,563)	411 (316-446)	79 (61-86)
Thyroid cancer	-	-	-	-	-	67 (50-80)	119 (100-138)	169 (152-187)	200 (183-215)	227 (211-245)	310 (286-333)	502 (454-543)	807 (733-863)	1,229 (1,138-1,301)	1,525 (1,433-1,609)	1,332 (1,255-1,438)	1,573 (1,492-1,694)	1,538 (1,456-1,674)	1,581 (1,512-1,664)	1,076 (1,028-1,154)	503 (479-540)	183 (175-196)	40 (38-43)
Mesothelioma	-	-	-	-	-	-	62 (55-74)	69 (61-82)	108 (95-131)	135 (118-163)	241 (212-292)	397 (353-471)	693 (631-809)	1,115 (1,022-1,246)	1,738 (1,609-1,925)	1,779 (1,642-1,951)	2,317 (2,169-2,480)	2,566 (2,407-2,744)	2,564 (2,410-2,751)	1,871 (1,765-2,004)	848 (796-907)	268 (251-288)	51 (48-56)
Hodgkin lymphoma	7 (5-10)	2 (1-2)	41 (30-57)	287 (201-440)	916 (597-1,221)	1,017 (707-1,328)	1,091 (811-1,391)	997 (766-1,260)	1,036 (815-1,310)	1,069 (838-1,416)	1,120 (877-1,453)	1,184 (888-1,505)	1,399 (1,107-1,746)	1,474 (1,211-1,898)	1,692 (1,384-2,110)	1,467 (1,210-1,878)	1,679 (1,389-2,063)	1,530 (1,282-1,915)	891 (745-1,061)	521 (432-607)	234 (194-267)	70 (61-81)	12 (11-15)
Non-Hodgkin's lymphoma	29 (24-32)	21 (19-25)	251 (222-298)	1,447 (1,177-1,674)	2,325 (1,826-2,761)	2,213 (1,783-2,567)	2,382 (2,003-2,644)	2,488 (2,132-2,762)	2,289 (2,012-2,505)	2,833 (2,410-3,080)	3,507 (3,004-3,796)	4,622 (3,994-4,941)	5,674 (4,980-6,131)	7,721 (6,822-8,423)	9,170 (8,158-10,172)	9,875 (9,033-10,863)	11,832 (10,615-13,051)	12,330 (11,034-13,577)	11,409 (10,031-12,574)	8,396 (7,011-9,080)	4,472 (3,708-4,725)	1,612 (1,364-1,742)	336 (295-371)
Multiple myeloma	-	-	-	-	-	-	134 (108-161)	153 (122-182)	155 (127-186)	348 (285-422)	586 (482-709)	1,113 (936-1,314)	1,519 (1,282-1,779)	2,625 (2,231-3,078)	3,714 (3,203-4,425)	4,243 (3,414-4,913)	5,306 (4,373-6,295)	5,855 (4,663-6,710)	5,573 (4,338-6,349)	4,175 (3,270-4,928)	2,239 (1,758-2,620)	795 (623-968)	153 (114-179)
Leukaemia	109 (90-121)	91 (80-104)	1,045 (937-1,270)	4,701 (4,131-6,019)	5,885 (5,322-7,005)	6,498 (5,865-7,431)	7,935 (7,122-9,141)	6,225 (5,421-7,054)	5,051 (4,351-5,490)	5,432 (4,656-5,883)	6,159 (5,314-6,754)	6,721 (5,941-7,273)	7,279 (6,466-7,761)	9,191 (8,165-10,018)	10,738 (9,503-11,511)	11,475 (10,152-12,055)	14,584 (13,334-15,407)	15,573 (14,363-16,684)	15,026 (14,197-15,682)	10,869 (10,388-11,361)	5,907 (5,669-6,181)	2,349 (2,267-2,466)	571 (549-604)
Acute lymphoid leukaemia	29 (24-38)	23 (20-30)	317 (265-449)	1,763 (1,445-2,535)	2,565 (2,253-3,295)	2,589 (2,320-3,116)	2,431 (2,209-2,929)	1,645 (1,475-1,969)	1,135 (1,006-1,326)	1,079 (948-1,267)	1,079 (978-1,295)	1,118 (1,015-1,301)	1,179 (1,038-1,312)	1,394 (1,224-1,545)	1,513 (1,313-1,681)	1,386 (1,172-1,508)	1,554 (1,342-1,697)	1,507 (1,314-1,727)	843 (750-935)	497 (449-553)	243 (220-271)	82 (75-91)	20 (18-22)
Chronic lymphoid leukaemia	-	-	-	-	-	-	165 (146-218)	145 (130-189)	118 (103-143)	130 (110-155)	151 (132-189)	224 (201-282)	369 (328-440)	699 (628-853)	1,117 (975-1,415)	1,480 (1,316-1,778)	2,251 (1,996-2,789)	2,679 (2,388-3,291)	2,758 (2,432-3,397)	2,365 (2,016-2,871)	1,446 (1,190-1,705)	638 (551-748)	163 (143-189)
Acute myeloid leukaemia	19 (14-25)	19 (15-24)	205 (162-279)	1,026 (772-1,517)	1,015 (785-1,281)	1,081 (889-1,336)	1,405 (1,220-1,714)	1,240 (1,057-1,489)	1,092 (930-1,291)	1,189 (1,003-1,392)	1,385 (1,169-1,629)	1,606 (1,400-1,866)	1,861 (1,590-2,134)	2,461 (2,179-2,793)	2,994 (2,542-3,396)	3,209 (2,719-3,570)	4,206 (3,591-4,666)	4,419 (3,673-4,935)	4,106 (3,382-4,606)	2,737 (2,271-3,120)	1,325 (1,063-1,523)	443 (387-549)	87 (79-115)
Chronic myeloid leukaemia	-	-	-	-	-	-	242 (211-305)	311 (264-388)	489 (417-568)	549 (452-636)	638 (525-742)	678 (586-784)	846 (722-956)	918 (789-1,043)	1,001 (869-1,125)	922 (804-1,022)	1,181 (1,042-1,301)	1,158 (1,035-1,265)	1,452 (1,312-1,582)	1,073 (976-1,187)	582 (534-641)	249 (227-273)	61 (55-68)
Other leukaemia	61 (45-68)	49 (38-55)	524 (439-634)	1,912 (1,612-2,505)	2,306 (1,874-2,604)	2,828 (2,333-3,135)	3,691 (3,103-4,276)	2,885 (2,298-3,201)	2,216 (1,788-2,409)	2,485 (1,957-2,712)	2,907 (2,338-3,199)	3,095 (2,502-3,373)	3,024 (2,471-3,290)	3,719 (3,021-4,023)	4,112 (3,342-4,400)	4,478 (3,773-4,823)	5,393 (4,635-5,786)	5,811 (5,072-6,349)	5,867 (5,360-6,421)	4,197 (3,888-4,582)	2,311 (2,144-2,564)	936 (866-1,018)	240 (221-260)
Other neoplasms	229 (186-255)	133 (115-149)	908 (807-1,124)	3,252 (2,761-4,376)	2,547 (2,281-2,835)	3,511 (3,117-3,802)	6,495 (5,779-6,966)	5,209 (4,643-5,535)	4,526 (3,907-4,856)	5,577 (4,675-5,977)	6,701 (5,631-7,102)	8,631 (7,270-9,103)	10,137 (8,923-10,675)	13,486 (11,848-14,150)	15,184 (13,543-15,928)	15,877 (14,330-16,686)	18,592 (17,010-19,404)	18,670 (17,240-19,475)	16,736 (15,688-17,333)	11,974 (11,126-12,480)	6,667 (6,273-6,954)	2,690 (2,571-2,812)	644 (610-675)
Cardiovascular diseases	2,668 (2,420-2,927)	1,359 (1,259-1,472)	11,763 (10,981-12,632)	9,259 (8,408-10,054)	5,470 (5,024-5,932)	7,252 (6,803-7,733)	22,428 (21,342-23,858)	36,881 (35,358-39,087)	53,959 (51,848-56,909)	89,563 (86,664-93,333)	142,604 (137,857-148,107)	230,324 (223,581-237,232)	334,234 (324,681-344,060)	496,213 (483,486-509,001)	602,604 (590,067-617,445)	683,746 (667,651-699,325)	952,212 (932,375-974,413)	1,096,186 (1,071,183-1,120,640)	1,157,146 (1,130,716-1,182,935)	955,630 (931,388-978,704)	593,144 (577,061-609,316)	283,881 (276,181-291,248)	82,553 (79,941-84,720)
Rheumatic heart disease	-	-	-	1,988 (1,602-2,528)	1,366 (1,170-1,586)	2,415 (2,149-2,669)	4,706 (4,178-5,252)	4,763 (4,153-5,378)	4,657 (3,921-5,243)	5,314 (4,530-6,053)	6,148 (5,293-6,972)	7,403 (6,333-8,364)	7,831 (6,732-8,880)	9,841 (8,646-11,028)	10,395 (9,270-11,564)	10,619 (9,361-11,901)	13,331 (11,974-14,834)	14,373 (12,902-15,767)	12,853 (11,764-13,867)	10,652 (9,837-11,397)	6,741 (6,282-7,193)	3,238 (3,055-3,415)	959 (914-1,005)
Ischaemic heart disease	-	-	779 (669-904)	568 (466-697)	382 (330-447)	437 (388-487)	7,061 (6,620-7,634)	14,346 (13,625-15,294)	24,767 (23,508-26,469)	47,142 (45,291-49,634)	77,512 (74,470-81,059)	126,920 (122,238-131,640)	192,462 (186,031-199,370)	281,026 (272,691-289,802)	338,111 (328,812-347,466)	370,961 (361,215-380,465)	504,156 (491,031-517,540)	562,544 (546,808-577,222)	605,205 (589,482-620,610)	503,574 (490,182-517,072)	323,322 (315,401-333,490)	164,416 (160,636-169,465)	50,142 (49,030-51,815)
Cerebrovascular disease	1,283 (1,118-1,483)	621 (540-723)	4,466 (3,834-5,283)	2,957 (2,474-3,619)	1,906 (1,686-2,140)	2,253 (2,048-2,438)	5,245 (4,933-5,613)	10,012 (9,485-10,733)	13,420 (12,739-14,356)	23,021 (22,074-24,379)	39,537 (38,021-41,298)	67,270 (64,999-69,731)	96,403 (93,019-100,114)	155,960 (151,281-161,271)	196,196 (190,021-202,589)	240,145 (232,401-248,435)	349,761 (338,899-360,784)	416,505 (401,905-430,673)	420,275 (402,421-438,433)	326,592 (307,887-344,246)	182,728 (170,661-194,428)	76,184 (70,877-82,001)	19,998 (18,040-21,609)

SR Table 4: Global deaths by cause and age group for males (with 95% uncertainty intervals), 2006

Cause	Early Neonatal	Late Neonatal	Post Neonatal	1 to 4	5 to 9	10 to 14	15 to 19	20 to 24	25 to 29	30 to 34	35 to 39
Ischaemic stroke	-	-	143 (125-171)	158 (132-198)	116 (103-131)	145 (131-163)	662 (606-724)	1,403 (1,271-1,533)	1,945 (1,771-2,144)	3,162 (2,910-3,440)	5,328 (4,903-5,761)
Hemorrhagic stroke	1,283 (1,118-1,483)	621 (540-723)	4,323 (3,708-5,127)	2,799 (2,338-3,437)	1,790 (1,582-2,017)	2,108 (1,916-2,283)	4,582 (4,312-4,898)	8,609 (8,157-9,247)	11,475 (10,865-12,330)	19,860 (19,027-21,010)	34,208 (32,883-35,884)
Hypertensive heart disease	-	-	218 (159-292)	79 (55-106)	73 (57-93)	106 (74-143)	747 (596-982)	1,295 (1,038-1,678)	1,775 (1,400-2,191)	2,793 (2,119-3,453)	4,562 (3,619-5,436)
Cardiomyopathy and myocarditis	520 (331-639)	284 (206-336)	2,248 (1,591-2,752)	1,396 (995-1,714)	663 (518-784)	808 (675-932)	2,184 (1,781-2,492)	2,875 (2,209-3,208)	4,590 (3,408-5,219)	5,791 (4,392-6,675)	7,836 (6,199-8,998)
Myocarditis	105 (51-152)	80 (48-105)	861 (538-1,114)	587 (356-792)	250 (180-313)	296 (229-355)	856 (629-1,038)	803 (601-977)	1,014 (752-1,264)	789 (602-957)	969 (709-1,186)
Alcoholic cardiomyopathy	-	-	-	-	-	-	164 (129-238)	502 (307-637)	1,437 (814-1,827)	2,694 (1,760-3,376)	3,990 (2,969-4,969)
Other cardiomyopathy	415 (276-521)	204 (149-248)	1,387 (987-1,777)	809 (587-1,100)	413 (316-501)	512 (420-601)	1,164 (928-1,362)	1,570 (1,203-1,805)	2,139 (1,561-2,492)	2,308 (1,784-2,643)	2,877 (2,232-3,283)
Atrial fibrillation and flutter	-	-	-	-	-	-	-	-	-	17 (12-23)	70 (51-93)
Aortic aneurysm	-	-	-	-	-	-	266 (240-297)	367 (334-406)	585 (524-651)	820 (743-897)	1,384 (1,269-1,510)
Peripheral vascular disease	-	-	-	-	-	-	-	-	-	-	-
Endocarditis	72 (44-96)	41 (26-55)	928 (667-1,193)	365 (257-463)	162 (117-200)	268 (202-344)	584 (436-797)	879 (666-1,189)	1,178 (928-1,598)	1,249 (966-1,692)	1,454 (1,165-1,998)
Other cardiovascular and circulatory diseases	792 (607-960)	414 (322-516)	3,123 (2,493-4,149)	1,906 (1,352-2,755)	918 (740-1,326)	964 (799-1,348)	1,634 (1,365-2,305)	2,344 (1,992-3,524)	2,987 (2,505-4,375)	3,415 (2,931-4,885)	4,101 (3,352-5,777)
Chronic respiratory diseases	0 (0-0)	0 (0-0)	0 (0-0)	7,883 (6,427-9,740)	2,567 (2,281-2,871)	2,799 (2,533-3,084)	5,096 (4,753-5,481)	7,190 (6,791-7,678)	9,134 (8,624-9,703)	11,750 (11,237-12,399)	17,398 (16,582-18,290)
Chronic obstructive pulmonary disease	-	-	0 (0-0)	1,427 (894-2,628)	456 (340-639)	379 (291-519)	2,085 (1,730-2,493)	2,985 (2,453-3,522)	3,930 (3,171-4,752)	5,903 (4,983-6,897)	9,167 (7,882-10,757)
Pneumoconiosis	-	-	-	-	-	-	25 (21-30)	38 (32-46)	81 (71-97)	202 (178-234)	494 (433-596)
Silicosis	-	-	-	-	-	-	3 (2-4)	8 (6-9)	29 (25-35)	113 (96-132)	327 (280-399)
Asbestosis	-	-	-	-	-	-	10 (9-13)	14 (12-18)	19 (15-25)	25 (19-35)	34 (24-47)
Coal workers pneumoconiosis	-	-	-	-	-	-	1 (0-1)	1 (1-2)	7 (4-9)	11 (6-14)	31 (18-40)
Other pneumoconiosis	-	-	-	-	-	-	11 (9-14)	15 (12-19)	26 (21-36)	52 (44-76)	101 (83-144)
Asthma	-	-	-	4,930 (3,500-6,574)	1,468 (1,157-1,792)	1,914 (1,583-2,227)	2,380 (1,882-2,827)	3,278 (2,527-3,925)	4,294 (3,276-5,077)	4,470 (3,295-5,410)	6,285 (4,550-7,875)
Interstitial lung disease and pulmonary sarcoidosis	-	-	-	131 (64-260)	56 (33-93)	41 (25-61)	115 (71-175)	177 (109-264)	250 (153-386)	351 (220-532)	542 (340-845)
Other chronic respiratory diseases	0 (0-0)	0 (0-0)	0 (0-0)	1,395 (564-2,476)	588 (361-847)	465 (273-665)	491 (307-686)	712 (467-971)	579 (332-810)	824 (490-1,189)	910 (513-1,293)
Cirrhosis and other chronic liver diseases	-	-	-	4,659 (3,380-7,564)	2,159 (1,771-3,199)	2,456 (2,122-3,374)	4,974 (4,519-5,953)	9,724 (8,843-12,308)	16,862 (15,359-19,301)	30,449 (28,471-34,714)	47,977 (45,218-54,407)
Cirrhosis and other chronic liver diseases due to hepatitis B	-	-	-	55 (33-94)	70 (47-105)	136 (97-195)	455 (314-647)	2,543 (1,908-3,467)	5,965 (4,638-7,654)	11,421 (9,687-13,391)	17,993 (15,173-21,248)
Cirrhosis and other chronic liver diseases due to hepatitis C	-	-	-	40 (25-67)	50 (33-78)	105 (71-160)	320 (214-468)	1,636 (1,186-2,197)	3,888 (2,915-5,010)	7,697 (6,448-9,276)	12,479 (10,013-15,482)
Cirrhosis and other chronic liver diseases due to alcohol use	-	-	-	-	-	-	89 (51-142)	774 (543-1,092)	2,107 (1,516-2,861)	6,001 (5,017-7,143)	12,035 (9,900-14,733)
Cirrhosis and other chronic liver diseases due to other causes	-	-	-	4,563 (3,316-7,383)	2,039 (1,673-3,010)	2,214 (1,922-2,983)	4,110 (3,669-4,998)	4,770 (3,869-6,000)	4,903 (3,776-6,245)	5,331 (4,014-6,867)	5,471 (3,898-7,344)
Digestive diseases	985 (808-1,219)	992 (767-1,194)	9,984 (7,697-12,824)	8,849 (6,257-11,927)	4,452 (3,841-5,199)	4,297 (3,763-4,890)	6,813 (6,092-7,735)	9,026 (8,176-10,139)	12,998 (11,683-14,287)	14,876 (13,507-16,864)	20,042 (18,474-22,991)
Peptic ulcer disease	-	-	-	1,010 (696-1,430)	570 (437-753)	743 (593-946)	1,418 (1,142-1,795)	2,339 (1,884-2,850)	2,922 (2,392-3,546)	4,018 (3,343-4,833)	5,344 (4,511-6,478)
Gastritis and duodenitis	-	-	-	343 (149-764)	100 (70-152)	86 (68-119)	214 (172-266)	284 (233-355)	451 (374-556)	788 (659-980)	936 (789-1,186)
Appendicitis	-	-	-	1,294 (730-1,832)	1,015 (814-1,303)	1,101 (903-1,358)	1,450 (1,169-1,783)	1,178 (979-1,440)	1,431 (1,209-1,758)	920 (775-1,126)	1,260 (1,035-1,530)
Paralytic ileus and intestinal obstruction	985 (808-1,219)	992 (767-1,194)	9,984 (7,697-12,824)	2,443 (1,587-3,244)	1,645 (1,175-1,992)	1,269 (914-1,541)	1,954 (1,393-2,410)	2,357 (1,692-2,980)	3,219 (2,338-3,980)	2,771 (2,043-3,477)	4,072 (2,836-4,984)
Inguinal, femoral, and abdominal hernia	-	-	-	699 (295-1,141)	237 (138-308)	258 (125-361)	349 (220-448)	514 (304-648)	576 (354-692)	519 (297-639)	609 (424-833)
Inflammatory bowel disease	-	-	-	691 (330-1,578)	146 (85-232)	133 (87-189)	180 (124-220)	275 (191-338)	411 (313-517)	472 (340-608)	534 (399-678)
Vascular intestinal disorders	-	-	-	342 (100-679)	55 (30-82)	33 (25-41)	68 (57-86)	129 (105-164)	194 (157-247)	193 (154-245)	376 (302-478)
Gallbladder and biliary diseases	-	-	-	200 (122-314)	91 (71-134)	157 (113-204)	204 (169-283)	308 (263-426)	375 (320-485)	496 (430-652)	757 (655-1,026)
Pancreatitis	-	-	-	106 (67-162)	79 (56-102)	109 (78-135)	401 (293-500)	1,152 (888-1,365)	2,688 (2,135-3,102)	3,698 (3,002-4,306)	4,936 (4,005-5,825)
Other digestive diseases	-	-	-	1,722 (723-2,917)	514 (327-758)	409 (302-586)	574 (461-727)	490 (402-629)	730 (598-947)	1,000 (832-1,273)	1,219 (1,041-1,590)
Neurological disorders	-	-	3,470 (3,053-4,014)	4,675 (3,753-5,743)	2,856 (2,549-3,182)	4,583 (4,203-4,976)	9,646 (9,014-10,252)	8,908 (8,341-9,428)	7,348 (6,936-7,754)	7,084 (6,783-7,418)	8,478 (8,089-8,912)
Alzheimer's disease and other dementias	-	-	-	-	-	-	-	-	-	-	-
Parkinson's disease	-	-	-	-	-	-	-	1 (0-2)	15 (6-29)	54 (29-100)	125 (67-219)
Epilepsy	-	-	2,123 (1,838-2,386)	3,725 (2,960-4,431)	2,394 (2,135-2,658)	3,630 (3,288-3,978)	7,434 (6,875-8,002)	7,258 (6,749-7,760)	6,294 (5,917-6,692)	5,927 (5,659-6,231)	6,792 (6,438-7,171)
Multiple sclerosis	-	-	-	-	-	-	-	55 (49-64)	113 (99-151)	244 (214-328)	399 (345-531)
Motor neuron disease	4 (3-4)	6 (5-6)	394 (309-460)	137 (97-163)	20 (18-22)	45 (42-49)	97 (91-104)	81 (76-86)	68 (64-72)	157 (148-166)	352 (331-371)
Other neurological disorders	-	-	953 (825-1,252)	813 (631-1,302)	442 (378-579)	907 (844-986)	2,115 (1,943-2,260)	1,513 (1,385-1,602)	859 (793-905)	701 (657-737)	810 (767-844)
Mental and substance use disorders	9 (4-18)	9 (7-12)	0 (0-0)	0 (0-0)	8 (6-9)	6 (4-7)	4,485 (3,815-4,978)	11,069 (9,523-12,108)	16,220 (14,141-17,827)	21,050 (18,240-22,737)	24,191 (21,035-26,135)
Alcohol use disorders	-	-	-	-	-	-	1,084 (808-1,215)	2,912 (2,295-3,308)	5,875 (4,909-6,701)	10,147 (8,220-11,501)	13,741 (11,184-15,232)
Drug use disorders	9 (4-18)	9 (7-12)	0 (0-0)	0 (0-0)	0 (0-0)	0 (0-0)	3,388 (2,867-3,835)	8,140 (6,951-9,058)	10,326 (8,822-11,651)	10,879 (9,295-11,976)	10,427 (9,020-11,382)
Opioid use disorders	9 (4-18)	9 (7-12)	0 (0-0)	0 (0-0)	0 (0-0)	0 (0-0)	2,487 (1,986-2,883)	5,866 (4,575-6,650)	7,314 (5,465-8,498)	7,094 (5,389-8,119)	6,722 (5,221-7,618)
Cocaine use disorders	0 (0-0)	0 (0-0)	0 (0-0)	0 (0-0)	0 (0-0)	0 (0-0)	136 (93-193)	335 (229-498)	461 (313-667)	799 (548-1,075)	769 (564-1,040)
Amphetamine use disorders	0 (0-0)	0 (0-0)	0 (0-0)	0 (0-0)	0 (0-0)	0 (0-0)	105 (72-155)	245 (171-339)	331 (229-448)	465 (337-617)	454 (330-615)
Other drug use disorders	0 (0-0)	0 (0-0)	0 (0-0)	0 (0-0)	0 (0-0)	0 (0-0)	660 (530-879)	1,694 (1,364-2,317)	2,220 (1,778-2,881)	2,522 (2,013-3,173)	2,481 (2,056-3,181)
Eating disorders	-	-	-	-	8 (6-9)	6 (4-7)	13 (10-15)	17 (13-20)	19 (15-23)	24 (20-29)	23 (19-28)
Anorexia nervosa	-	-	-	-	7 (6-9)	5 (4-6)	12 (10-15)	16 (13-19)	18 (14-22)	21 (18-26)	21 (18-26)
Bulimia nervosa	-	-	-	-	0 (0-1)	0 (0-1)	1 (0-1)	1 (1-1)	1 (1-2)	2 (2-4)	1 (1-2)
Diabetes, urogenital, blood, and endocrine diseases	2,320 (1,661-2,694)	1,683 (1,315-1,901)	15,460 (13,454-17,313)	15,155 (12,337-18,955)	7,338 (6,525-8,641)	7,934 (6,949-9,161)	13,126 (12,043-14,803)	18,747 (17,393-21,116)	18,347 (17,197-19,669)	23,793 (22,403-25,250)	30,660 (29,067-32,444)
Diabetes mellitus	37 (34-40)	53 (47-59)	469 (427-520)	406 (355-459)	525 (462-589)	676 (618-739)	1,397 (1,300-1,509)	2,591 (2,444-2,775)	3,404 (3,237-3,600)	5,725 (5,466-6,037)	8,465 (8,112-8,812)
Acute glomerulonephritis	-	-	57 (46-70)	90 (77-109)	102 (87-121)	109 (94-130)	183 (161-208)	176 (155-196)	171 (154-192)	239 (215-268)	300 (265-341)
Chronic kidney disease	-	-	2,916 (2,377-3,291)	3,121 (2,579-3,551)	2,283 (1,853-2,579)	2,510 (2,157-2,824)	6,007 (5,311-6,639)	9,290 (7,956-10,284)	9,746 (8,534-10,597)	12,100 (10,708-13,300)	15,656 (14,078-17,198)
Chronic kidney disease due to diabetes mellitus	-	-	79 (52-116)	95 (60-144)	85 (49-136)	125 (76-201)	476 (285-731)	1,352 (866-1,877)	2,193 (1,582-2,897)	3,926 (2,953-4,993)	6,400 (4,986-7,940)
Chronic kidney disease due to hypertension	-	-	20 (14-29)	31 (20-45)	39 (24-58)	70 (44-104)	270 (171-385)	631 (430-864)	847 (588-1,155)	1,272 (922-1,700)	1,914 (1,456-2,504)
Chronic kidney disease due to glomerulonephritis	-	-	1,134 (825-1,436)	1,236 (876-1,618)	892 (611-1,195)	1,034 (714-1,369)	2,513 (1,780-3,288)	3,798 (2,716-5,019)	3,612 (2,555-4,777)	3,540 (2,463-4,776)	3,489 (2,326-4,836)
Chronic kidney disease due to other causes	-	-	1,683 (1,305-2,033)	1,759 (1,354-2,180)	1,267 (949-1,611)	1,281 (926-1,633)	2,748 (1,999-3,547)	3,509 (2,437-4,647)	3,094 (2,141-4,215)	3,362 (2,331-4,623)	3,852 (2,715-5,145)
Urinary diseases and male infertility	208 (185-231)	303 (256-368)	3,246 (2,908-3,631)	1,835 (1,542-2,196)	767 (643-910)	630 (570-690)	1,371 (1,253-1,481)	1,864 (1,719-1,992)	1,851 (1,712-2,004)	2,217 (2,084-2,337)	2,609 (2,457-2,749)
Interstitial nephritis and urinary tract infections	106 (76-134)	172 (122-222)	2,187 (1,683-2,628)	916 (557-1,360)	353 (253-453)	328 (260-393)	721 (567-847)	1,000 (812-1,167)	940 (751-1,114)	1,083 (882-1,248)	1,305 (1,068-1,504)
Urolithiasis	-	-	-	-	104 (70-154)	79 (59-105)	161 (119-225)	172 (131-239)	156 (116-230)	176 (135-256)	240 (189-334)
Other urinary diseases	101 (76-126)	132 (92-180)	1,059 (677-1,540)	919 (505-1,330)	309 (206-405)	223 (155-283)	489 (367-636)	692 (529-870)	755 (582-921)	958 (777-1,130)	1,064 (856-1,261)
Gynecological diseases	-	-	-	-	-	-	-	-	-	-	-
Uterine fibroids	-	-	-	-	-	-	-	-	-	-	-
Polycystic ovarian syndrome	-	-	-	-	-	-	-	-	-	-	-
Endometriosis	-	-	-	-	-	-	-	-	-	-	-
Genital prolapse	-	-	-	-	-	-	-	-	-	-	-
Other gynecological diseases	-	-	-	-	-	-	-	-	-	-	-
Hemoglobinopathies and hemolytic anaemias	1,335 (797-1,670)	722 (455-868)	4,941 (3,401-6,188)	7,401 (4,948-10,803)	2,574 (2,024-3,691)	2,963 (2,174-3,911)	3,180 (2,368-4,339)	3,750 (3,009-5,629)	1,878 (1,438-2,559)	1,427 (1,077-1,940)	1,335 (1,047-1,806)
Thalassemias	278 (202-356)	208 (140-265)	1,108 (814-1,408)	1,556 (1,194-1,989)	697 (549-922)	297 (227-405)	205 (151-274)	108 (80-140)	47 (36-59)	30 (24-39)	23 (19-30)
Sickle cell disorders	962 (517-1,258)	480 (281-611)	3,575 (2,325-4,623)	5,410 (3,223-8,462)	1,647 (1,234-2,566)	2,348 (1,706-3,142)	2,432 (1,803-3,411)	2,777 (2,134-4,258)	1,130 (869-1,620)	699 (538-993)	570 (450-804)
G6PD deficiency	45 (29-63)	25 (16-35)	213 (145-296)	393 (270-535)	201 (149-270)	262 (190-348)	447 (328-599)	677 (514-922)	535 (387-706)	549 (399-722)	553 (413-732)
Other hemoglobinopathies and hemolytic anaemias	51 (34-67)	8 (6-11)	44 (31-59)	42 (31-55)	28 (22-37)	55 (43-70)	97 (73-126)	189 (147-257)	166 (125-216)	149 (112-193)	188 (147-240)
Endocrine, metabolic, blood, and immune disorders	740 (510-891)	605 (457-725)	3,831 (3,146-5,142)	2,302 (1,868-3,064)	1,086 (943-1,460)	1,046 (925-1,414)	989 (862-1,266)	1,076 (934-1,383)	1,297 (1,054-1,575)	2,086 (1,561-2,393)	2,296 (1,688-2,615)
Musculoskeletal disorders	-	-	-	-	315 (259-409)	420 (358-514)	500 (442-620)	580 (492-688)	571 (485-654)	628 (529-724)	788 (674-894)
Rheumatoid arthritis	-	-	-	-	63 (45-94)	75 (56-101)	66 (49-87)	70 (51-92)	63 (47-83)	73 (54-95)	104 (80-137)
Other musculoskeletal disorders	-	-	-	-	252 (207-333)	344 (298-430)	434 (383-553)	511 (431-608)	508 (431-589)	555 (468-641)	685 (585-782)
Other non-communicable diseases	103,950 (79,364-123,704)	36,770 (29,125-43,090)	121,522 (97,685-141,131)	35,224 (23,049-47,744)	9,510 (7,926-11,429)	7,068 (5,808-8,233)	6,648 (5,657-7,652)	5,221 (4,380-5,864)	3,856 (3,253-4,506)	3,774 (3,014-4,221)	3,666 (2,918-4,084)
Congenital anomalies	103,950 (79,364-123,704)	34,692 (27,438-41,080)	101,889 (78,982-121,868)	34,305 (22,079-46,656)	9,255 (7,726-11,162)	6,756 (5,543-7,978)	5,977 (5,101-7,015)	4,594 (3,856-5,359)	3,345 (2,856-3,996)	2,638 (2,261-3,124)	2,710 (2,257-3,245)
Neural tube defects	15,422 (8,477-26,748)	3,625 (1,876-6,794)	8,475 (3,529-17,057)	3,351 (591-8,920)	434 (217-908)	268 (165-501)	158 (112-272)	112 (78-217)	74 (53-133)	65 (47-109)	50 (38-74)
Congenital heart anomalies	34,690 (22,042-44,485)	15,873 (11,591-19,793)	53,244 (38,790-68,094)	17,302 (11,577-26,449)	5,812 (4,475-7,448)	4,512 (3,695-5,578)	4,106 (3,483-4,905)	3,255 (2,751-3,845)	2,492 (2,141-3,060)	1,872 (1,600-2,228)	2,005 (1,676-2,461)
Orofacial clefts	793 (343-1,317)	231 (105-407)	641 (298-1,272)	67 (18-181)	-	-	-	-	-	-	-
Down's syndrome	1,267 (623-3,109)	580 (327-1,331)	2,561 (1,453-6,211)	1,234 (548-4,333)	219 (144-409)	179 (125-284)	195 (153-295)	174 (136-245)	152 (119-212)	180 (142-239)	173 (132-232)
Other chromosomal abnormalities	2,669 (1,622-4,752)	1,053 (642-1,980)	2,258 (1,320-4,587)	507 (252-1,188)	115 (72-179)	84 (45-116)	110 (60-142)	93 (50-114)	66 (29-81)	49 (20-59)	51 (20-62)
Congenital musculoskeletal and limb anomalies	2,806 (1,700-5,656)	620 (397-1,174)	1,299 (803-2,864)	338 (183-958)	121 (86-212)	100 (71-144)	137 (95-187)	63 (40-82)	45 (33-60)	36 (23-47)	31 (21-41)
Urogenital congenital anomalies	3,207 (2,060-4,585)	808 (544-1,156)	1,517 (960-2,348)	299 (180-485)	135 (95-196)	133 (102-181)	74 (56-105)	69 (54-96)	60 (45-79)	64 (48-84)	70 (48-89)
Digestive congenital anomalies	10,459 (7,131-16,225)	4,060 (2,752-6,288)	9,174 (5,757-16,041)	1,786 (880-4,674)	216 (158-323)	110 (86-149)	62 (46-77)	39 (30-49)	35 (27-43)	28 (22-35)	31 (20-37)
Other congenital anomalies	32,638 (20,810-47,798)	7,842 (4,826-11,818)	22,719 (13,576-35,012)	9,420 (4,810-16,298)	2,202 (1,512-3,118)	1,370 (1,003-1,782)	1,135 (894-1,441)	788 (582-975)	420 (321-528)	342 (267-425)	300 (243-374)
Skin and subcutaneous diseases	-	-	1,575 (749-2,096)	918 (409-1,262)	255 (124-343)	312 (153-418)	671 (331-962)	627 (296-829)	511 (256-664)	1,136 (537-1,513)	956 (471-1,236)
Cellulitis	-	-	71 (25-118)	79 (24-120)	50 (18-75)	64 (29-91)	70 (38-102)	88 (41-122)	70 (28-96)	150 (50-203)	180 (69-233)
Pyoderma	-	-	1,457 (691-1,955)	732 (337-1,011)	153 (77-209)	189 (85-257)	469 (210-652)	432 (185-563)	348 (163-452)	828 (380-1,112)	607 (270-809)
Decubitus ulcer	-	-	-	48 (16-79)	26 (14-39)	31 (16-45)	81 (46-126)	64 (36-97)	62 (36-93)	121 (66-176)	137 (69-197)
Other skin and subcutaneous diseases	-	-	47 (22-79)	58 (22-95)	26 (14-40)	29 (16-43)	51 (29-86)	42 (22-68)	30 (18-46)	37 (21-60)	32 (18-50)
Sudden infant death syndrome	-	2,078 (1,484-2,589)	18,058 (14,185-23,203)	-	-	-	-	-	-	-	-
Injuries	8,450 (6,912-9,523)	4,852 (3,975-5,526)	49,702 (43,068-56,731)	125,414 (108,926-140,418)	82,796 (76,889-89,197)	88,720 (83,701-93,264)	216,826 (208,507-223,900)	315,107 (304,204-324,472)	288,431 (277,218-297,062)	272,076 (260,246-279,611)	267,882 (256,522-275,453)
Transport injuries	1,731 (1,361-2,227)	793 (661-967)	6,980 (5,888-8,352)	25,524 (21,847-29,538)	27,175 (25,164-29,368)	27,065 (25,401-29,090)	89,800 (85,989-95,227)	129,787 (125,241-137,353)	115,271 (111,079-121,451)	105,210 (101,914-110,210)	105,610 (102,274-109,821)
Road injuries	1,480 (1,162-1,919)	678 (564-846)	6,331 (5,330-7,686)	24,139 (20,641-27,800)	25,818 (23,935-27,955)	25,213 (23,668-27,209)	83,787 (80,003-89,011)	122,144 (117,596-129,907)	107,319 (103,230-113,408)	98,708 (95,441-104,003)	98,555 (95,286-103,060)
Pedestrian road injuries	597 (428-878)	267 (206-365)	2,494 (1,986-3,182)	13,006 (10,729-16,293)	13,958 (12,657-15,733)	11,928 (10,877-13,643)	22,370 (19,999-25,300)	30,524 (26,988-34,148)	28,322 (25,287-31,926)	30,930 (28,166-34,867)	33,865 (30,566-37,728)
Cyclist road injuries	-	-	-	810 (585-1,116)	1,528 (1,277-1,827)	2,186 (1,911-2,557)	4,151 (3,594-4,755)	3,900 (3,370-4,443)	3,713 (3,176-4,214)	4,135 (3,604-4,713)	4,429 (3,854-4,982)
Motorcyclist road injuries	132 (89-213)	86 (61-127)	719 (552-1,028)	1,548 (1,118-2,390)	1,585 (1,309-1,899)	2,614 (2,271-2,953)	24,182 (21,521-27,135)	36,142 (32,030-40,765)	30,374 (26,777-34,407)	24,767 (21,299-28,056)	23,864 (20,450-26,339)
Motor vehicle road injuries	718 (482-980)	312 (223-408)	2,953 (2,125-3,958)	8,480 (5,946-10,867)	8,415 (6,738-10,230)	8,107 (6,843-9,767)	32,411 (28,604-38,908)	50,560 (45,002-59,328)	44,028 (39,165-51,810)	38,207 (33,771-45,286)	35,685 (30,973-41,982)
Other road injuries	34 (19-60)	14 (10-21)	164 (115-249)	296 (203-440)	332 (276-434)	378 (311-498)	673 (567-850)	1,017 (844-1,271)	883 (739-1,081)	670 (561-804)	713 (596-851)
Other transport injuries	251 (163-366)	115 (80-153)	649 (481-854)	1,385 (847-1,927)	1,356 (1,135-1,652)	1,852 (1,585-2,151)	6,013 (5,215-6,744)	7,643 (6,737-8,376)	7,952 (7,030-8,761)	6,501 (5,882-7,096)	7,054 (6,293-7,824)
Unintentional injuries	5,732 (4,421-6,639)	3,747 (2,939-4,379)	40,203 (33,653-46,926)	93,022 (78,389-106,267)	50,238 (45,800-54,738)	48,303 (44,804-51,483)	57,461 (53,282-60,030)	67,496 (62,858-70,396)	62,207 (58,025-64,912)	66,613 (61,560-69,227)	72,967 (66,057-76,039)
Falls	1,287 (797-1,827)	391 (274-480)	4,936 (3,453-6,126)	8,700 (5,755-11,186)	5,040 (4,109-5,807)	5,827 (4,879-6,612)	7,431 (6,466-8,126)	10,751 (9,367-11,693)	11,356 (9,729-12,321)	14,102 (11,804-15,112)	17,328 (14,410-18,627)
Drowning	380 (287-454)	138 (107-179)	4,506 (3,858-5,040)	47,343 (39,380-52,888)	26,841 (24,063-29,195)	24,726 (22,785-26,349)	23,877 (22,397-25,397)	20,388 (19,229-21,994)	14,879 (13,951-16,017)	14,186 (13,450-15,085)	13,712 (12,874-14,438)
Fire, heat, and hot substances	302 (210-352)	213 (139-258)	2,967 (2,079-3,477)	7,917 (4,997-9,972)	2,773 (2,108-3,238)	1,832 (1,422-2,092)	2,470 (1,931-2,763)	4,023 (3,181-4,507)	4,018 (3,235-4,476)	4,150 (3,460-4,674)	4,463 (3,730-4,957)
Poisonings	249 (118-413)	146 (74-220)	2,575 (1,202-4,401)	4,057 (2,376-6,093)	1,469 (1,019-1,731)	1,733 (1,185-2,005)	2,105 (1,541-2,548)	3,040 (2,325-3,699)	2,982 (2,267-3,545)	2,800 (2,115-3,345)	2,836 (2,089-3,244)
Exposure to mechanical forces	1,316 (845-1,594)	1,131 (673-1,516)	7,648 (5,450-9,085)	7,212 (4,917-9,220)	4,122 (3,566-4,840)	3,830 (3,459-4,505)	7,407 (6,237-8,034)	10,585 (8,720-11,445)	10,285 (8,491-11,044)	10,734 (8,630-11,498)	12,204 (9,285-13,092)
Unintentional firearm injuries	53 (33-69)	38 (23-54)	446 (270-629)	775 (395-1,193)	527 (405-679)	686 (580-821)	1,768 (1,474-2,082)	2,610 (1,910-3,082)	2,589 (1,911-3,033)	1,813 (1,350-2,111)	1,507 (1,111-1,719)
Unintentional suffocation	923 (484-1,193)	961 (512-1,338)	5,559 (3,385-6,797)	2,396 (1,492-3,704)	1,034 (790-1,650)	1,052 (876-1,597)	398 (324-557)	530 (417-753)	524 (421-734)	399 (311-550)	341 (266-451)
Other exposure to mechanical forces	340 (244-387)	131 (98-154)	1,643 (1,277-1,918)	4,042 (2,569-5,072)	2,561 (2,046-2,967)	2,092 (1,758-2,298)	5,240 (4,138-5,686)	7,445 (6,086-8,028)	7,172 (5,732-7,722)	8,522 (6,490-9,202)	10,357 (7,595-11,243)
Adverse effects of medical treatment	706 (476-849)	454 (313-561)	4,542 (2,761-6,001)	4,124 (1,867-6,579)	1,478 (1,048-1,902)	1,244 (941-1,488)	1,679 (1,232-1,940)	2,543 (1,840-2,947)	2,357 (1,707-2,699)	2,432 (1,730-2,800)	2,640 (1,954-3,005)
Animal contact	274 (235-315)	256 (211-311)	1,577 (1,293-1,888)	4,059 (3,058-5,476)	3,601 (2,944-4,333)	3,862 (3,222-4,575)	3,086 (2,650-3,524)	3,130 (2,694-3,540)	3,073 (2,674-3,513)	3,058 (2,684-3,474)	3,187 (2,807-3,598)
Venomous animal contact	239 (199-280)	222 (180-274)	1,351 (1,077-1,652)	3,363 (2,501-4,338)	3,086 (2,487-3,794)	3,468 (2,841-4,135)	2,569 (2,118-3,008)	2,615 (2,192-3,011)	2,571 (2,173-3,018)	2,669 (2,296-3,088)	2,777 (2,392-3,171)
Non-venomous animal contact	35 (27-44)	34 (25-44)	225 (153-317)	695 (355-1,365)	515 (364-691)	394 (304-492)	517 (401-634)	515 (394-637)	502 (361-624)	389 (286-488)	410 (305-511)
Foreign body	757 (474-922)	789 (528-964)	9,339 (6,439-12,673)	5,488 (3,324-9,256)	1,970 (1,541-2,479)	1,628 (1,348-1,937)	1,238 (1,017-1,451)	1,956 (1,609-2,316)	2,025 (1,668-2,421)	2,613 (2,168-3,032)	2,908 (2,400-3,546)
Pulmonary aspiration and foreign body in airway	723 (450-880)	768 (511-940)	8,954 (6,059-12,218)	4,853 (2,904-8,079)	1,606 (1,209-2,087)	1,333 (1,106-1,659)	1,063 (872-1,321)	1,692 (1,406-2,124)	1,753 (1,434-2,187)	2,068 (1,699-2,532)	2,324 (1,875-2,980)
Foreign body in other body part	34 (16-46)	21 (10-28)	384 (208-540)	635 (286-1,264)	365 (222-486)	295 (161-392)	175 (88-244)	263 (140-358)	273 (142-368)	545 (326-714)	584 (356-745)
Environmental heat and cold exposure	225 (98-286)	142 (57-183)	1,220 (549-1,562)	1,177 (532-1,592)	473 (277-591)	440 (249-530)	966 (585-1,151)	1,344 (818-1,627)	1,700 (1,092-2,129)	2,522 (1,602-3,210)	3,018 (1,981-4,044)
Other unintentional injuries	236 (207-269)	86 (72-102)	894 (741-1,060)	2,946 (2,105-3,629)	2,471 (2,187-2,768)	3,180 (2,859-3,493)	7,202 (6,479-7,822)	9,736 (8,876-10,517)	9,532 (8,869-10,268)	10,017 (8,810-10,677)	10,671 (8,834-11,432)
Self-harm and interpersonal violence	794 (593-956)	199 (153-246)	2,085 (1,602-2,586)	3,542 (2,720-4,383)	2,891 (2,190-3,457)	10,693 (8,823-11,631)	62,798 (54,357-66,710)	106,168 (92,622-112,502)	102,573 (89,194-109,553)	94,251 (82,163-101,109)	85,093 (74,321-92,224)

Cause	40 to 44	45 to 49	50 to 54	55 to 59	60 to 64	65 to 69	70 to 74	75 to 79	80 to 84	85 to 89	90 to 94	95 plus
Ischaemic stroke	9,920 (9,297-10,502)	19,836 (18,584-21,122)	37,539 (35,350-39,661)	54,346 (51,079-57,327)	94,353 (89,105-98,663)	154,766 (147,497-161,249)	194,443 (185,699-202,685)	232,121 (221,364-243,378)	184,211 (172,972-195,065)	107,922 (100,654-115,255)	47,309 (43,871-50,808)	13,021 (11,736-14,112)
Hemorrhagic stroke	57,350 (55,234-59,854)	76,566 (73,833-79,925)	118,421 (114,381-123,413)	141,850 (137,024-147,456)	145,791 (140,437-152,899)	194,995 (187,874-203,270)	222,062 (213,452-232,117)	188,154 (179,714-197,509)	142,381 (134,159-150,288)	74,806 (69,801-79,956)	28,875 (26,834-31,140)	6,977 (6,278-7,545)
Hypertensive heart disease	7,444 (5,776-9,033)	10,791 (8,402-13,405)	16,201 (12,670-19,485)	20,402 (16,313-24,662)	23,479 (18,832-29,230)	32,829 (26,499-40,341)	40,070 (31,970-48,652)	44,824 (35,488-53,695)	40,328 (32,098-49,977)	26,264 (20,094-31,158)	13,088 (9,617-14,942)	3,827 (2,654-4,370)
Cardiomyopathy and myocarditis	11,552 (9,215-13,521)	14,547 (11,829-16,952)	16,072 (13,389-18,821)	15,478 (13,145-18,078)	11,547 (9,727-13,382)	13,811 (11,711-15,986)	13,394 (10,217-14,649)	14,699 (10,594-16,129)	13,356 (8,516-15,034)	9,144 (5,889-10,319)	4,885 (3,008-5,561)	1,667 (981-1,917)
Myocarditis	1,158 (841-1,442)	827 (604-1,001)	861 (621-1,022)	972 (687-1,200)	774 (512-930)	978 (638-1,162)	1,282 (817-1,525)	1,476 (929-1,770)	1,546 (885-1,941)	1,278 (766-1,598)	981 (522-1,304)	416 (195-568)
Alcoholic cardiomyopathy	6,519 (4,839-8,130)	9,295 (7,119-11,426)	9,778 (7,646-12,085)	8,373 (6,638-10,287)	4,901 (3,841-5,960)	5,507 (4,414-7,084)	3,716 (2,614-4,458)	3,063 (1,997-3,572)	1,782 (938-2,151)	763 (391-928)	333 (171-407)	118 (57-142)
Other cardiomyopathy	3,875 (3,012-4,632)	4,425 (3,566-5,337)	5,433 (4,493-6,753)	6,133 (5,161-7,662)	5,873 (4,850-7,074)	7,325 (5,968-8,621)	8,396 (6,495-9,202)	10,161 (7,203-11,247)	10,027 (6,458-11,371)	7,103 (4,509-8,102)	3,570 (2,195-4,340)	1,132 (695-1,444)
Atrial fibrillation and flutter	167 (122-217)	311 (225-411)	536 (400-687)	899 (535-1,351)	1,602 (1,066-2,232)	3,258 (2,033-4,862)	6,063 (4,361-8,388)	9,036 (5,758-13,356)	15,986 (12,148-20,506)	14,850 (11,387-19,059)	7,109 (5,437-9,158)	1,755 (1,352-2,255)
Aortic aneurysm	1,930 (1,792-2,067)	2,720 (2,566-2,892)	3,904 (3,697-4,123)	5,571 (5,315-5,858)	7,294 (6,944-7,602)	10,482 (10,011-10,948)	12,842 (12,318-13,355)	14,660 (14,093-15,262)	12,618 (12,125-13,135)	7,929 (7,632-8,261)	3,508 (3,385-3,658)	801 (774-838)
Peripheral vascular disease	129 (85-161)	263 (169-320)	580 (374-729)	978 (610-1,201)	1,329 (858-1,640)	2,358 (1,524-2,928)	3,029 (2,142-4,011)	3,754 (2,668-5,048)	3,741 (2,736-5,162)	2,732 (2,024-3,812)	1,618 (1,181-2,342)	562 (426-841)
Endocarditis	1,746 (1,428-2,385)	1,780 (1,423-2,382)	2,079 (1,658-2,834)	2,181 (1,729-2,849)	2,399 (1,887-3,215)	2,966 (2,356-3,926)	3,245 (2,598-4,306)	3,634 (2,837-4,701)	3,244 (2,526-4,078)	2,128 (1,525-2,633)	1,033 (735-1,242)	306 (224-385)
Other cardiovascular and circulatory diseases	5,764 (4,942-7,861)	7,128 (5,954-9,590)	10,014 (8,556-12,950)	12,392 (10,688-16,229)	14,370 (12,074-18,416)	19,260 (16,238-24,866)	24,122 (20,827-31,690)	28,207 (24,883-35,660)	25,539 (22,769-32,093)	17,306 (15,870-21,157)	8,801 (8,216-10,559)	2,536 (2,386-2,963)
Chronic respiratory diseases	25,782 (24,684-26,950)	39,183 (37,421-40,741)	70,955 (67,871-74,257)	102,656 (98,167-106,585)	157,534 (150,643-163,910)	238,779 (227,346-247,720)	317,961 (304,073-329,154)	334,757 (320,779-345,643)	281,152 (269,288-291,123)	165,768 (158,581-172,233)	67,892 (65,019-70,464)	16,768 (16,009-17,462)
Chronic obstructive pulmonary disease	15,555 (13,516-17,763)	25,638 (22,282-28,744)	51,017 (45,042-56,269)	77,972 (69,873-84,671)	126,047 (114,763-135,194)	199,981 (185,299-212,496)	274,664 (258,199-288,815)	293,511 (277,899-307,355)	251,218 (239,087-262,581)	149,781 (142,384-156,536)	61,882 (59,285-64,550)	15,311 (14,620-16,024)
Pneumoconiosis	655 (582-765)	665 (582-795)	1,063 (944-1,256)	1,148 (1,006-1,430)	1,503 (1,318-1,854)	2,308 (2,060-2,784)	3,076 (2,808-3,527)	3,194 (2,954-3,581)	2,475 (2,302-2,729)	1,312 (1,215-1,437)	456 (423-505)	98 (91-109)
Silicosis	428 (375-514)	407 (354-505)	645 (566-798)	653 (568-814)	766 (663-936)	1,237 (1,076-1,512)	1,597 (1,421-1,872)	1,670 (1,491-1,943)	1,279 (1,132-1,484)	611 (540-709)	179 (143-208)	34 (25-39)
Asbestosis	45 (29-62)	45 (30-64)	71 (45-93)	96 (56-129)	141 (86-194)	242 (145-323)	364 (220-486)	459 (260-612)	420 (230-552)	248 (144-330)	92 (48-124)	18 (10-23)
Coal workers pneumoconiosis	56 (28-73)	69 (37-89)	114 (65-155)	146 (76-196)	218 (117-300)	364 (189-480)	511 (265-622)	479 (273-568)	418 (293-503)	242 (152-289)	83 (44-101)	16 (8-19)
Other pneumoconiosis	126 (102-164)	144 (110-206)	233 (181-328)	254 (185-375)	378 (259-573)	465 (333-713)	603 (452-906)	586 (448-845)	359 (280-520)	212 (165-320)	103 (82-158)	30 (25-43)
Asthma	7,647 (5,357-9,957)	10,259 (7,047-13,444)	15,293 (10,022-21,185)	18,662 (12,271-26,316)	23,962 (15,161-34,533)	28,341 (17,873-41,041)	30,179 (20,208-44,239)	27,426 (18,270-40,792)	18,460 (13,186-29,026)	9,344 (6,694-14,093)	3,299 (2,433-5,001)	796 (594-1,183)
Interstitial lung disease and pulmonary sarcoidosis	855 (540-1,300)	1,324 (831-1,930)	2,184 (1,359-3,159)	3,136 (1,961-4,452)	4,180 (2,579-5,908)	6,111 (3,777-8,185)	7,825 (4,852-10,011)	8,760 (5,532-10,774)	7,296 (4,501-8,934)	4,207 (2,514-5,116)	1,674 (1,020-2,004)	394 (252-472)
Other chronic respiratory diseases	1,069 (618-1,481)	1,297 (771-1,742)	1,398 (802-1,883)	1,737 (1,054-2,340)	1,841 (1,153-2,411)	2,038 (1,194-2,666)	2,217 (1,390-2,887)	1,866 (1,175-2,409)	1,702 (1,101-2,212)	1,123 (752-1,441)	581 (376-771)	169 (112-223)
Cirrhosis and other chronic liver diseases	67,414 (63,945-74,758)	81,809 (78,060-90,256)	93,906 (89,752-104,158)	88,205 (84,011-98,393)	76,225 (72,599-88,134)	71,475 (67,912-79,965)	59,243 (56,041-65,462)	45,314 (43,050-49,446)	27,941 (26,151-29,632)	13,422 (12,532-14,361)	4,782 (4,429-5,097)	1,252 (1,161-1,322)
Cirrhosis and other chronic liver diseases due to hepatitis B	23,578 (20,330-27,050)	25,198 (21,108-29,967)	28,136 (23,625-33,478)	26,774 (22,750-32,079)	24,239 (20,325-29,615)	23,205 (19,634-27,720)	19,752 (16,528-24,159)	15,023 (12,620-17,886)	8,738 (7,160-10,557)	3,860 (3,270-4,519)	1,203 (979-1,439)	279 (205-362)
Cirrhosis and other chronic liver diseases due to hepatitis C	18,235 (15,414-21,713)	23,170 (19,153-27,621)	26,606 (22,108-31,506)	25,074 (21,183-29,606)	21,443 (17,750-25,855)	20,188 (17,094-23,795)	16,845 (14,263-19,896)	12,529 (10,340-15,090)	7,768 (6,342-9,326)	3,646 (2,995-4,302)	1,317 (1,035-1,583)	343 (250-438)
Cirrhosis and other chronic liver diseases due to alcohol use	18,885 (16,343-21,936)	25,047 (21,126-29,437)	28,802 (24,418-34,123)	26,095 (21,813-30,797)	21,755 (17,580-26,507)	20,215 (16,973-23,495)	16,105 (13,502-19,027)	12,438 (10,473-14,743)	7,743 (6,479-9,068)	3,890 (3,400-4,410)	1,481 (1,268-1,703)	414 (336-496)
Cirrhosis and other chronic liver diseases due to other causes	6,716 (4,857-8,999)	8,394 (6,079-11,202)	10,361 (7,792-13,729)	10,263 (7,558-13,667)	8,788 (6,368-12,006)	7,866 (5,772-10,554)	6,542 (4,774-8,723)	5,325 (3,896-6,920)	3,691 (2,731-4,863)	2,026 (1,593-2,515)	781 (604-993)	215 (153-295)
Digestive diseases	25,585 (23,780-29,140)	30,130 (27,680-33,218)	34,645 (31,750-38,341)	38,722 (35,609-43,238)	41,324 (38,336-46,131)	51,274 (46,831-57,461)	56,918 (52,316-62,841)	56,981 (52,608-63,027)	45,948 (42,565-51,185)	28,985 (26,155-31,831)	13,648 (12,524-14,964)	3,780 (3,427-4,209)
Peptic ulcer disease	7,493 (6,516-8,936)	9,388 (8,106-10,938)	11,939 (10,526-13,674)	13,093 (11,512-14,697)	13,964 (12,271-15,795)	16,744 (14,613-18,786)	18,389 (16,026-20,388)	17,493 (15,600-19,278)	13,085 (11,648-14,432)	7,642 (6,810-8,446)	3,190 (2,862-3,545)	831 (745-932)
Gastritis and duodenitis	1,175 (1,000-1,466)	1,368 (1,170-1,625)	1,721 (1,492-2,020)	1,939 (1,666-2,250)	2,003 (1,707-2,289)	2,229 (1,906-2,550)	2,617 (2,212-2,954)	2,560 (2,174-2,903)	2,019 (1,724-2,289)	1,238 (1,064-1,390)	564 (486-635)	153 (132-172)
Appendicitis	1,570 (1,294-1,883)	1,330 (1,071-1,588)	1,423 (1,161-1,738)	1,453 (1,193-1,780)	1,533 (1,243-1,903)	1,551 (1,298-1,882)	1,696 (1,357-2,124)	1,474 (1,225-1,838)	1,103 (895-1,380)	648 (545-790)	271 (231-325)	72 (62-86)
Paralytic ileus and intestinal obstruction	4,537 (3,063-5,577)	5,267 (3,786-6,425)	5,768 (4,156-7,089)	7,078 (5,340-9,076)	7,764 (5,844-9,727)	10,039 (7,917-12,487)	10,413 (8,134-13,051)	10,165 (8,163-13,249)	8,728 (6,911-10,833)	5,893 (4,645-7,455)	2,826 (2,182-3,434)	782 (589-973)
Inguinal, femoral, and abdominal hernia	1,015 (688-1,291)	1,042 (768-1,338)	1,424 (1,022-1,843)	1,644 (1,199-2,180)	1,882 (1,366-2,486)	2,538 (1,835-3,239)	2,942 (2,075-3,624)	3,253 (2,355-3,943)	2,532 (1,908-3,316)	1,720 (1,344-2,170)	882 (704-1,083)	271 (215-361)
Inflammatory bowel disease	737 (580-914)	842 (638-1,012)	977 (750-1,130)	1,141 (858-1,289)	1,190 (920-1,316)	1,499 (1,156-1,679)	1,794 (1,363-1,971)	1,949 (1,466-2,145)	1,806 (1,263-1,994)	1,197 (814-1,330)	624 (413-703)	177 (116-201)
Vascular intestinal disorders	680 (558-857)	1,088 (898-1,362)	1,625 (1,383-2,047)	2,355 (2,057-3,038)	2,824 (2,451-3,600)	4,641 (4,072-6,135)	5,752 (4,865-7,282)	6,262 (5,169-7,846)	4,884 (3,831-6,005)	2,929 (2,168-3,408)	1,399 (1,087-1,615)	365 (273-427)
Gallbladder and biliary diseases	1,025 (900-1,376)	1,223 (1,079-1,562)	1,653 (1,439-2,106)	1,961 (1,737-2,461)	2,421 (2,119-3,077)	3,440 (3,093-4,515)	4,375 (3,892-5,390)	5,159 (4,525-6,303)	4,746 (4,205-5,782)	3,315 (2,923-4,017)	1,724 (1,548-2,039)	489 (430-591)
Pancreatitis	5,921 (4,936-6,979)	6,678 (5,450-7,840)	5,763 (4,806-6,869)	5,373 (4,375-6,316)	4,353 (3,510-5,258)	4,828 (3,776-5,874)	4,388 (3,422-5,234)	4,052 (3,126-4,941)	2,591 (2,047-3,139)	1,422 (1,093-1,682)	541 (407-655)	139 (111-183)
Other digestive diseases	1,431 (1,235-1,789)	1,905 (1,620-2,279)	2,351 (2,027-2,824)	2,683 (2,285-3,234)	3,390 (2,804-4,093)	3,766 (3,131-4,563)	4,554 (3,744-5,467)	4,615 (3,791-5,698)	4,455 (3,549-5,467)	2,981 (2,355-3,744)	1,628 (1,302-2,089)	501 (388-643)
Neurological disorders	8,139 (7,811-8,531)	9,432 (8,885-10,083)	12,142 (10,841-13,830)	15,871 (13,453-18,768)	22,068 (18,351-27,104)	40,218 (32,391-49,782)	75,033 (59,151-95,606)	128,548 (102,138-160,302)	157,981 (126,866-197,729)	135,430 (109,379-164,911)	80,913 (65,787-95,052)	22,029 (17,531-27,584)
Alzheimer's disease and other dementias	48 (20-96)	996 (577-1,534)	3,699 (2,468-5,323)	6,959 (4,644-9,884)	13,006 (9,406-17,980)	27,548 (20,122-36,803)	56,932 (41,459-76,847)	103,097 (76,910-134,282)	132,526 (101,256-172,850)	120,625 (93,413-151,094)	75,557 (60,784-90,161)	20,932 (16,308-26,572)
Parkinson's disease	213 (119-345)	338 (187-587)	629 (363-1,004)	1,123 (674-1,663)	2,304 (1,612-3,230)	5,366 (3,798-7,184)	11,589 (8,209-15,478)	19,858 (14,309-26,486)	21,523 (16,061-28,709)	12,906 (9,939-16,913)	4,699 (3,584-6,139)	937 (671-1,283)
Epilepsy	5,607 (5,358-5,872)	5,107 (4,847-5,370)	4,135 (3,973-4,297)	3,540 (3,385-3,685)	2,719 (2,538-2,902)	2,751 (2,558-2,937)	2,134 (1,990-2,274)	1,690 (1,585-1,785)	1,350 (1,239-1,453)	704 (646-757)	276 (257-292)	78 (73-83)
Multiple sclerosis	648 (579-851)	751 (656-937)	886 (751-1,051)	877 (714-1,041)	708 (559-838)	654 (529-775)	522 (405-611)	382 (284-432)	215 (157-236)	82 (57-92)	25 (18-27)	4 (3-5)
Motor neuron disease	629 (601-655)	942 (902-990)	1,239 (1,193-1,294)	1,661 (1,607-1,739)	1,760 (1,697-1,852)	2,176 (2,093-2,299)	2,106 (2,027-2,202)	1,782 (1,710-1,858)	1,084 (1,037-1,134)	426 (408-445)	96 (92-101)	16 (15-17)
Other neurological disorders	993 (938-1,034)	1,298 (1,213-1,356)	1,554 (1,452-1,627)	1,710 (1,596-1,791)	1,571 (1,443-1,652)	1,723 (1,589-1,811)	1,749 (1,623-1,837)	1,739 (1,625-1,822)	1,284 (1,202-1,348)	685 (646-717)	260 (248-272)	62 (59-64)
Mental and substance use disorders	28,623 (25,129-31,133)	31,090 (27,582-33,632)	28,269 (25,136-30,823)	22,250 (19,418-24,289)	14,025 (12,383-15,118)	12,345 (10,667-13,249)	7,831 (6,818-8,334)	5,821 (5,003-6,248)	3,088 (2,622-3,277)	1,553 (1,358-1,675)	598 (536-649)	171 (157-189)
Alcohol use disorders	18,315 (15,389-20,260)	22,370 (18,971-24,682)	21,095 (17,989-23,425)	17,129 (14,361-18,825)	10,905 (9,221-11,907)	9,132 (7,673-9,963)	5,262 (4,351-5,671)	3,550 (2,852-3,880)	1,564 (1,263-1,686)	695 (563-755)	236 (192-256)	67 (56-72)
Drug use disorders	10,272 (9,067-11,230)	8,687 (7,867-9,472)	7,175 (6,544-7,850)	5,120 (4,613-5,709)	3,120 (2,772-3,663)	3,213 (2,791-3,581)	2,569 (2,203-2,923)	2,271 (1,871-2,574)	1,524 (1,275-1,684)	857 (735-981)	362 (326-420)	103 (95-121)
Opioid use disorders	6,539 (4,899-7,432)	5,283 (3,908-5,963)	4,105 (3,049-4,675)	2,973 (2,327-3,431)	1,860 (1,520-2,231)	1,990 (1,645-2,310)	1,126 (882-1,356)	1,044 (781-1,258)	608 (443-711)	360 (259-454)	155 (118-193)	44 (35-54)
Cocaine use disorders	791 (552-1,099)	755 (505-1,051)	860 (549-1,181)	587 (399-776)	336 (215-441)	339 (217-447)	186 (118-242)	150 (95-194)	25 (15-32)	13 (8-18)	6 (4-7)	2 (1-2)
Amphetamine use disorders	448 (331-606)	401 (285-541)	376 (255-492)	267 (176-345)	154 (106-199)	152 (96-200)	61 (39-81)	50 (31-67)	12 (8-16)	7 (4-9)	3 (2-3)	1 (1-1)
Other drug use disorders	2,494 (2,032-3,256)	2,247 (1,933-3,129)	1,834 (1,594-2,487)	1,293 (1,082-1,657)	771 (637-1,008)	732 (547-902)	1,196 (970-1,444)	1,027 (826-1,235)	879 (714-1,040)	477 (408-572)	199 (176-245)	57 (50-71)
Eating disorders	35 (30-41)	33 (29-38)	0 (0-0)	0 (0-0)	-	-	-	-	-	-	-	-
Anorexia nervosa	31 (27-37)	24 (20-29)	0 (0-0)	0 (0-0)	-	-	-	-	-	-	-	-
Bulimia nervosa	4 (3-6)	9 (7-13)	0 (0-0)	0 (0-0)	-	-	-	-	-	-	-	-
Diabetes, urogenital, blood, and endocrine diseases	43,149 (41,137-45,402)	55,435 (52,769-58,023)	75,243 (71,916-78,070)	91,877 (88,526-94,891)	106,083 (102,342-110,442)	131,509 (126,430-136,781)	147,146 (140,960-153,344)	149,736 (144,182-155,847)	125,225 (120,015-130,832)	82,205 (78,561-85,991)	38,713 (37,119-40,413)	11,099 (10,634-11,546)
Diabetes mellitus	14,677 (14,168-15,299)	21,867 (21,181-22,560)	32,894 (31,920-33,889)	43,124 (41,838-44,376)	52,098 (50,467-53,661)	64,397 (62,181-66,624)	71,560 (68,891-74,048)	71,176 (68,337-73,832)	56,088 (53,458-58,569)	32,137 (30,383-33,747)	12,763 (12,139-13,343)	2,949 (2,802-3,082)
Acute glomerulonephritis	312 (277-354)	323 (291-360)	401 (360-449)	453 (411-494)	397 (357-446)	526 (479-577)	569 (514-635)	480 (435-528)	362 (328-403)	215 (193-240)	87 (77-98)	29 (23-38)
Chronic kidney disease	20,325 (18,466-22,075)	23,956 (21,618-25,978)	32,119 (28,977-34,859)	37,651 (34,543-40,535)	41,759 (38,630-45,849)	50,961 (46,469-56,377)	56,569 (52,619-61,931)	57,533 (53,523-62,743)	49,631 (46,598-54,164)	35,171 (33,182-38,252)	17,869 (16,844-19,251)	5,523 (5,174-5,853)
Chronic kidney disease due to diabetes mellitus	9,597 (7,450-11,664)	12,544 (10,090-14,958)	17,426 (13,571-20,682)	20,691 (16,549-23,961)	23,312 (18,981-27,275)	27,352 (21,617-32,164)	28,471 (22,748-33,530)	25,879 (20,386-31,172)	18,881 (14,460-23,143)	11,392 (8,530-14,783)	5,442 (3,959-7,010)	1,700 (1,171-2,231)
Chronic kidney disease due to hypertension	2,802 (2,155-3,641)	3,507 (2,709-4,544)	5,224 (3,939-6,891)	6,714 (5,121-8,661)	8,183 (6,445-10,271)	11,186 (8,699-14,218)	13,920 (10,978-17,535)	15,898 (12,449-20,119)	15,849 (12,779-19,312)	13,366 (10,866-16,410)	7,486 (6,105-8,974)	2,393 (1,905-2,901)
Chronic kidney disease due to glomerulonephritis	3,506 (2,284-4,921)	3,300 (2,176-4,667)	3,741 (2,505-5,309)	3,806 (2,590-5,293)	3,620 (2,383-5,180)	4,343 (2,857-6,295)	4,798 (3,209-6,934)	5,450 (3,729-7,805)	5,026 (3,554-6,806)	3,147 (2,199-4,386)	1,449 (1,021-1,962)	443 (268-708)
Chronic kidney disease due to other causes	4,419 (3,057-5,999)	4,605 (3,031-6,678)	5,729 (3,775-8,253)	6,440 (4,321-9,432)	6,644 (4,263-9,653)	8,080 (5,436-11,605)	9,381 (6,340-13,013)	10,307 (7,213-14,265)	9,875 (7,039-13,154)	7,267 (4,967-9,896)	3,492 (2,535-4,658)	987 (638-1,412)
Urinary diseases and male infertility	3,411 (3,246-3,577)	4,091 (3,877-4,301)	4,850 (4,620-5,083)	5,282 (5,049-5,498)	6,825 (6,446-7,138)	9,741 (9,251-10,159)	11,682 (11,098-12,190)	13,118 (12,496-13,668)	12,913 (12,252-13,499)	10,042 (9,545-10,509)	5,651 (5,412-5,887)	1,839 (1,764-1,914)
Interstitial nephritis and urinary tract infections	1,733 (1,407-1,982)	2,056 (1,661-2,351)	2,550 (2,088-2,892)	2,837 (2,363-3,200)	3,680 (3,003-4,215)	5,453 (4,544-6,236)	6,897 (5,800-7,686)	8,406 (7,344-9,182)	8,373 (7,373-9,064)	6,934 (6,181-7,477)	4,056 (3,663-4,343)	1,417 (1,303-1,506)
Urolithiasis	377 (297-533)	439 (342-630)	579 (467-798)	537 (442-686)	649 (526-894)	1,009 (822-1,380)	1,101 (920-1,499)	1,156 (974-1,567)	929 (773-1,264)	608 (507-827)	282 (232-381)	78 (64-103)
Other urinary diseases	1,300 (1,016-1,556)	1,597 (1,257-1,910)	1,721 (1,362-2,070)	1,908 (1,521-2,305)	2,496 (1,946-3,088)	3,279 (2,550-4,037)	3,684 (2,948-4,559)	3,555 (2,907-4,359)	3,610 (3,037-4,366)	2,499 (2,096-3,080)	1,312 (1,103-1,655)	343 (287-438)
Gynecological diseases	-	-	-	-	-	-	-	-	-	-	-	-
Uterine fibroids	-	-	-	-	-	-	-	-	-	-	-	-
Polycystic ovarian syndrome	-	-	-	-	-	-	-	-	-	-	-	-
Endometriosis	-	-	-	-	-	-	-	-	-	-	-	-
Genital prolapse	-	-	-	-	-	-	-	-	-	-	-	-
Other gynecological diseases	-	-	-	-	-	-	-	-	-	-	-	-
Hemoglobinopathies and hemolytic anaemias	1,842 (1,466-2,563)	2,181 (1,791-3,291)	1,621 (1,320-2,206)	1,610 (1,322-2,126)	1,561 (1,259-2,090)	1,969 (1,647-2,640)	2,524 (2,109-3,422)	3,128 (2,712-4,288)	2,335 (1,981-2,999)	1,962 (1,648-2,465)	972 (825-1,171)	306 (256-351)
Thalassemias	19 (16-25)	14 (13-20)	14 (12-20)	11 (9-16)	3 (3-5)	2 (1-2)	1 (1-1)	0 (0-0)	0 (0-0)	0 (0-0)	0 (0-0)	0 (0-0)
Sickle cell disorders	715 (575-1,085)	778 (577-1,276)	293 (230-451)	137 (108-203)	57 (47-81)	38 (33-51)	21 (18-26)	10 (8-12)	3 (3-4)	1 (1-1)	0 (0-0)	0 (0-0)
G6PD deficiency	778 (590-1,051)	999 (799-1,458)	792 (623-1,075)	740 (569-1,014)	556 (435-793)	507 (403-736)	438 (345-643)	330 (253-500)	125 (97-178)	62 (47-88)	15 (12-20)	3 (2-4)
Other hemoglobinopathies and hemolytic anaemias	330 (255-423)	389 (315-538)	521 (410-662)	723 (582-916)	945 (754-1,236)	1,423 (1,178-1,900)	2,064 (1,720-2,768)	2,788 (2,411-3,788)	2,206 (1,875-2,802)	1,899 (1,593-2,375)	957 (814-1,151)	303 (253-347)
Endocrine, metabolic, blood, and immune disorders	2,583 (1,982-2,852)	3,018 (2,308-3,317)	3,358 (2,611-3,662)	3,757 (2,904-4,118)	3,443 (2,743-3,856)	3,914 (3,015-4,603)	4,241 (3,528-5,058)	4,302 (3,496-5,107)	3,896 (3,037-4,593)	2,679 (2,113-3,116)	1,371 (1,110-1,587)	453 (362-527)
Musculoskeletal disorders	953 (798-1,108)	1,153 (987-1,338)	1,181 (1,007-1,330)	1,389 (1,207-1,606)	1,578 (1,373-1,791)	2,117 (1,838-2,383)	2,575 (2,203-2,831)	2,862 (2,438-3,139)	2,388 (2,022-2,594)	1,665 (1,425-1,808)	802 (699-878)	241 (220-261)
Rheumatoid arthritis	186 (138-234)	255 (192-311)	437 (325-512)	581 (438-654)	772 (601-869)	1,102 (839-1,232)	1,384 (1,061-1,531)	1,455 (1,152-1,644)	1,094 (870-1,218)	656 (501-767)	248 (188-298)	62 (49-74)
Other musculoskeletal disorders	767 (656-903)	898 (784-1,061)	744 (650-886)	808 (702-994)	806 (704-991)	1,015 (871-1,263)	1,191 (1,004-1,434)	1,407 (1,160-1,673)	1,294 (1,067-1,470)	1,010 (843-1,163)	554 (467-625)	179 (157-199)
Other non-communicable diseases	3,964 (3,113-4,361)	3,427 (2,573-3,979)	3,326 (2,357-3,754)	3,707 (2,467-4,246)	3,803 (2,529-4,424)	4,163 (2,650-4,893)	3,701 (1,994-4,631)	4,327 (2,404-5,685)	3,841 (2,080-5,086)	2,967 (1,653-4,005)	1,617 (905-2,114)	530 (287-713)
Congenital anomalies	2,757 (2,256-3,115)	1,760 (1,446-2,097)	1,536 (1,265-1,751)	1,407 (1,149-1,499)	1,284 (1,055-1,461)	1,059 (914-1,247)	-	-	-	-	-	-
Neural tube defects	49 (37-78)	47 (38-62)	35 (27-51)	29 (21-55)	22 (15-49)	18 (14-35)	-	-	-	-	-	-
Congenital heart anomalies	2,082 (1,705-2,404)	768 (633-933)	615 (508-734)	512 (438-610)	414 (354-515)	346 (302-449)	-	-	-	-	-	-
Orofacial clefts	-	-	-	-	-	-	-	-	-	-	-	-
Down's syndrome	176 (134-226)	351 (274-441)	343 (260-399)	369 (254-411)	279 (199-312)	121 (87-144)	-	-	-	-	-	-
Other chromosomal abnormalities	41 (15-51)	43 (19-51)	46 (24-55)	38 (19-46)	25 (14-31)	19 (12-25)	-	-	-	-	-	-
Congenital musculoskeletal and limb anomalies	25 (15-31)	30 (19-37)	29 (21-35)	18 (11-22)	20 (14-24)	19 (14-24)	-	-	-	-	-	-
Urogenital congenital anomalies	83 (56-110)	134 (90-170)	147 (104-183)	157 (110-192)	195 (140-249)	241 (175-301)	-	-	-	-	-	-
Digestive congenital anomalies	30 (18-37)	44 (33-52)	40 (27-46)	39 (27-45)	45 (32-55)	46 (31-54)	-	-	-	-	-	-
Other congenital anomalies	269 (203-341)	343 (269-416)	280 (222-338)	246 (193-284)	284 (225-345)	248 (198-308)	-	-	-	-	-	-
Skin and subcutaneous diseases	1,208 (586-1,498)	1,667 (879-2,205)	1,791 (899-2,164)	2,299 (1,145-2,827)	2,519 (1,312-3,165)	3,104 (1,579-3,882)	3,701 (1,994-4,631)	4,327 (2,404-5,685)	3,841 (2,080-5,086)	2,967 (1,653-4,005)	1,617 (905-2,114)	530 (287-713)
Cellulitis	235 (90-314)	320 (128-441)	443 (154-572)	532 (185-693)	466 (198-642)	507 (173-666)	556 (218-821)	643 (257-980)	560 (228-964)	420 (181-720)	230 (101-428)	72 (31-140)
Pyoderma	781 (362-984)	1,101 (551-1,453)	1,074 (524-1,410)	1,401 (662-1,848)	1,613 (752-2,042)	1,891 (922-2,426)	2,098 (1,011-2,626)	2,312 (1,183-2,960)	1,654 (866-2,143)	1,252 (639-1,622)	629 (329-840)	191 (93-247)
Decubitus ulcer	156 (83-229)	201 (114-318)	226 (121-339)	310 (168-464)	374 (206-556)	621 (336-926)	950 (528-1,349)	1,239 (685-1,792)	1,481 (775-2,040)	1,182 (605-1,628)	691 (369-927)	242 (122-331)
Other skin and subcutaneous diseases	36 (19-55)	45 (25-73)	48 (25-76)	57 (31-87)	65 (35-101)	86 (48-137)	96 (53-158)	133 (76-220)	146 (79-238)	114 (64-188)	67 (35-115)	26 (13-43)
Sudden infant death syndrome	-	-	-	-	-	-	-	-	-	-	-	-
Injuries	246,850 (234,022-254,926)	221,045 (209,005-229,613)	203,453 (190,535-211,081)	166,416 (155,690-172,213)	123,729 (114,560-128,424)	124,796 (114,055-129,579)	111,825 (99,749-116,582)	98,544 (86,390-103,045)	73,061 (63,530-76,983)	45,346 (39,134-48,118)	20,553 (17,417-21,787)	5,952 (5,081-6,309)
Transport injuries	94,684 (91,620-98,017)	78,160 (75,635-81,053)	71,997 (69,677-74,643)	56,101 (54,159-58,094)	41,122 (39,519-42,918)	38,253 (36,783-39,825)	32,092 (30,904-33,408)	24,971 (24,011-26,104)	15,624 (14,978-16,324)	7,771 (7,412-8,157)	2,729 (2,604-2,864)	669 (635-705)
Road injuries	88,187 (85,329-91,503)	72,292 (69,962-75,072)	66,446 (64,291-69,050)	51,652 (49,896-53,631)	38,270 (36,749-40,127)	35,536 (34,112-37,045)	29,948 (28,803-31,186)	23,207 (22,302-24,235)	14,603 (13,974-15,294)	7,251 (6,925-7,621)	2,506 (2,394-2,628)	595 (565-628)
Pedestrian road injuries	31,169 (28,293-34,622)	27,375 (25,023-30,199)	27,384 (24,854-30,065)	21,782 (19,678-23,969)	17,942 (16,360-19,811)	17,489 (16,147-19,162)	15,177 (13,977-16,566)	13,076 (12,114-14,270)	8,173 (7,573-8,935)	4,170 (3,864-4,554)	1,365 (1,255-1,500)	326 (300-362)
Cyclist road injuries	5,076 (4,405-5,679)	4,722 (4,154-5,342)	5,358 (4,712-5,976)	4,173 (3,665-4,610)	3,568 (3,148-3,994)	3,372 (2,998-3,791)	2,782 (2,474-3,091)	1,733 (1,536-1,916)	923 (834-1,046)	380 (337-434)	120 (106-142)	2 (2-2)
Motorcyclist road injuries	20,237 (17,415-21,996)	14,098 (12,286-15,584)	11,394 (9,709-12,589)	8,062 (7,165-8,924)	4,304 (3,748-4,886)	3,585 (2,936-3,993)	2,553 (2,135-2,830)	1,424 (1,103-1,586)	963 (770-1,080)	381 (309-433)	106 (87-122)	28 (23-32)
Motor vehicle road injuries	31,004 (26,896-36,354)	25,344 (21,984-29,193)	21,500 (18,466-25,470)	17,042 (14,651-19,856)	12,059 (10,440-14,196)	10,596 (9,136-12,476)	8,994 (7,609-10,568)	6,627 (5,704-7,669)	4,288 (3,710-4,980)	2,189 (1,895-2,555)	862 (748-979)	223 (189-251)
Other road injuries	701 (594-857)	753 (640-914)	810 (702-998)	593 (502-737)	396 (333-511)	494 (417-620)	441 (376-568)	346 (295-477)	256 (213-373)	131 (108-187)	52 (43-78)	16 (14-26)
Other transport injuries	6,497 (5,767-7,100)	5,868 (5,330-6,377)	5,550 (4,973-6,111)	4,449 (3,966-4,887)	2,852 (2,577-3,150)	2,718 (2,426-2,980)	2,145 (1,870-2,373)	1,764 (1,551-1,954)	1,021 (894-1,154)	521 (464-593)	223 (200-256)	74 (66-84)
Unintentional injuries	71,645 (63,932-74,868)	71,351 (64,554-75,050)	68,585 (61,305-72,363)	60,141 (53,968-63,292)	49,015 (42,940-51,765)	54,466 (47,005-57,581)	52,696 (44,276-56,012)	52,031 (43,676-55,380)	44,087 (36,496-47,319)	31,171 (25,559-33,561)	15,665 (12,814-16,787)	4,789 (3,937-5,122)
Falls	19,072 (15,733-20,434)	19,608 (16,136-20,957)	20,754 (16,936-22,087)	20,271 (16,680-21,555)	18,740 (15,020-20,146)	22,943 (18,346-24,750)	25,577 (19,682-27,879)	28,747 (22,068-31,266)	26,266 (19,979-28,612)	20,266 (15,293-22,090)	10,616 (8,085-11,467)	3,184 (2,418-3,435)
Drowning	12,659 (11,936-13,349)	11,342 (10,598-12,033)	10,588 (9,780-11,145)	8,295 (7,721-8,722)	6,439 (5,862-6,829)	6,317 (5,808-6,694)	5,659 (5,104-5,960)	4,203 (3,790-4,459)	2,973 (2,685-3,154)	1,540 (1,420-1,640)	502 (469-529)	118 (111-124)
Fire, heat, and hot substances	4,560 (3,843-5,033)	5,010 (4,140-5,557)	4,856 (4,002-5,372)	4,195 (3,431-4,655)	3,477 (2,826-3,814)	3,734 (3,053-4,087)	3,572 (2,978-3,957)	3,326 (2,779-3,642)	2,924 (2,459-3,186)	1,924 (1,667-2,117)	963 (841-1,066)	304 (267-327)
Poisonings	2,961 (2,249-3,335)	2,706 (1,966-3,073)	2,621 (1,895-2,960)	2,204 (1,597-2,493)	1,701 (1,182-1,951)	1,628 (1,130-1,838)	1,402 (1,004-1,602)	998 (687-1,124)	709 (492-807)	356 (262-405)	130 (103-158)	36 (31-49)
Exposure to mechanical forces	10,609 (7,909-11,357)	9,607 (7,576-10,233)	8,623 (6,861-9,198)	6,287 (5,036-6,705)	3,952 (3,163-4,298)	3,612 (2,852-3,907)	2,924 (2,321-3,149)	2,635 (2,148-2,848)	1,893 (1,520-2,064)	1,328 (1,083-1,449)	711 (561-781)	246 (196-273)
Unintentional firearm injuries	1,156 (889-1,321)	1,212 (934-1,379)	954 (719-1,099)	661 (513-763)	499 (383-588)	490 (373-575)	413 (313-481)	375 (297-436)	255 (204-285)	152 (120-175)	72 (60-83)	24 (21-29)
Unintentional suffocation	340 (271-482)	268 (209-350)	233 (186-325)	182 (144-234)	66 (45-80)	80 (56-107)	73 (51-100)	367 (230-444)	456 (277-579)	457 (264-553)	283 (163-337)	102 (57-122)
Other exposure to mechanical forces	9,113 (6,524-9,830)	8,127 (6,251-8,727)	7,436 (5,728-7,969)	5,444 (4,295-5,838)	3,387 (2,648-3,703)	3,041 (2,335-3,298)	2,439 (1,924-2,640)	1,893 (1,479-2,064)	1,182 (913-1,285)	720 (597-780)	356 (277-395)	120 (101-143)
Adverse effects of medical treatment	2,833 (2,092-3,214)	2,956 (2,155-3,377)	2,753 (2,072-3,098)	3,020 (2,336-3,500)	3,556 (2,607-4,124)	4,609 (3,414-5,323)	4,325 (3,358-5,151)	4,181 (3,210-4,905)	3,277 (2,536-4,006)	2,030 (1,594-2,494)	936 (740-1,160)	281 (224-370)
Animal contact	3,224 (2,858-3,568)	4,022 (3,552-4,517)	3,185 (2,823-3,527)	3,378 (3,016-3,758)	2,925 (2,595-3,269)	3,085 (2,737-3,453)	2,632 (2,313-2,917)	1,926 (1,657-2,149)	1,252 (1,066-1,407)	572 (481-655)	174 (148-198)	55 (47-65)
Venomous animal contact	2,784 (2,423-3,155)	3,439 (2,962-3,963)	2,614 (2,241-2,960)	2,819 (2,451-3,211)	2,458 (2,132-2,783)	2,615 (2,257-2,960)	2,222 (1,906-2,515)	1,527 (1,273-1,757)	1,041 (858-1,195)	456 (377-532)	127 (103-148)	35 (28-41)
Non-venomous animal contact	439 (311-551)	583 (413-756)	571 (450-701)	559 (430-712)	467 (370-579)	469 (369-588)	410 (325-510)	398 (303-509)	212 (164-265)	116 (90-145)	47 (37-59)	20 (16-26)
Foreign body	3,282 (2,701-3,933)	3,506 (2,848-4,277)	3,679 (3,042-4,463)	3,435 (2,740-4,169)	2,649 (2,128-3,150)	3,002 (2,368-3,606)	2,735 (2,114-3,078)	2,840 (2,089-3,225)	2,738 (1,915-3,177)	1,920 (1,295-2,265)	1,062 (689-1,268)	391 (242-471)
Pulmonary aspiration and foreign body in airway	2,722 (2,225-3,422)	3,239 (2,605-4,041)	3,421 (2,807-4,248)	3,220 (2,546-3,973)	2,437 (1,936-2,954)	2,836 (2,223-3,469)	2,575 (1,975-2,914)	2,641 (1,903-3,015)	2,585 (1,785-3,007)	1,821 (1,211-2,158)	1,015 (643-1,220)	375 (223-455)
Foreign body in other body part	559 (363-716)	267 (176-337)	258 (168-318)	215 (137-271)	212 (146-308)	166 (108-213)	160 (100-198)	198 (134-251)	152 (98-194)	99 (66-124)	46 (30-60)	16 (12-21)
Environmental heat and cold exposure	4,017 (2,514-5,531)	5,134 (3,290-7,987)	5,668 (3,518-9,079)	4,808 (3,042-7,774)	2,953 (1,893-4,444)	3,337 (2,117-4,858)	2,433 (1,475-3,335)	2,149 (1,277-2,837)	1,379 (900-1,880)	872 (598-1,241)	406 (279-599)	117 (77-180)
Other unintentional injuries	8,429 (6,778-9,009)	7,460 (6,029-7,983)	5,858 (4,712-6,250)	4,247 (3,529-4,544)	2,623 (2,208-2,816)	2,199 (1,838-2,357)	1,435 (1,302-1,533)	1,027 (923-1,101)	674 (595-730)	365 (319-402)	166 (143-182)	56 (46-63)
Self-harm and interpersonal violence	77,816 (67,765-85,390)	69,708 (61,206-77,107)	61,393 (53,137-67,337)	49,096 (41,466-53,755)	32,732 (27,314-35,390)	31,379 (25,553-33,975)	26,467 (21,024-28,795)	21,122 (16,728-22,866)	13,061 (10,207-14,075)	6,247 (4,849-6,704)	2,093 (1,654-2,248)	468 (386-512)

SR Table 4: Global deaths by cause and age group for males (with 95% uncertainty intervals), 2006

Cause	Early Neonatal	Late Neonatal	Post Neonatal	1 to 4	5 to 9	10 to 14	15 to 19	20 to 24	25 to 29	30 to 34	35 to 39	40 to 44	45 to 49
Self-harm	-	-	-	-	-	5,409<br>(4,381-5,988)	30,484<br>(27,058-33,207)	54,195<br>(47,487-58,854)	55,350<br>(49,555-60,832)	53,635<br>(48,681-58,552)	51,899<br>(44,301-56,682)	50,205<br>(44,404-55,989)	47,995<br>(41,646-52,855)
Self-harm by firearm	-	-	-	-	-	304<br>(221-441)	3,598<br>(2,756-5,075)	6,441<br>(4,913-8,845)	5,998<br>(4,406-8,003)	5,243<br>(3,888-7,096)	4,575<br>(3,553-6,120)	4,467<br>(3,593-6,103)	4,506<br>(3,477-6,328)
Self-harm by other specified means	-	-	-	-	-	5,104<br>(4,025-5,670)	26,886<br>(23,308-29,334)	47,754<br>(41,164-52,713)	49,352<br>(43,441-54,407)	48,392<br>(42,651-52,544)	47,324<br>(39,750-51,509)	45,738<br>(39,726-50,514)	43,489<br>(37,315-48,374)
Interpersonal violence	794<br>(593-956)	199<br>(153-246)	2,085<br>(1,602-2,586)	3,542<br>(2,720-4,383)	2,891<br>(2,190-3,457)	5,284<br>(3,979-6,192)	32,314<br>(24,836-35,606)	51,973<br>(40,758-57,709)	47,223<br>(36,315-53,000)	40,615<br>(30,788-47,663)	33,194<br>(24,604-38,652)	27,611<br>(20,275-33,735)	21,713<br>(15,182-27,656)
Assault by firearm	109<br>(65-166)	29<br>(17-43)	308<br>(182-461)	576<br>(361-865)	541<br>(332-779)	1,609<br>(905-1,983)	16,569<br>(10,214-19,007)	26,577<br>(16,935-30,183)	23,461<br>(14,734-26,814)	18,056<br>(10,901-20,906)	14,466<br>(8,907-17,285)	10,974<br>(6,407-14,022)	7,579<br>(4,360-10,064)
Assault by sharp object	73<br>(45-103)	19<br>(12-26)	168<br>(103-242)	445<br>(293-625)	410<br>(269-543)	844<br>(541-1,114)	8,346<br>(6,030-10,813)	13,496<br>(9,874-18,706)	12,870<br>(9,394-17,138)	11,711<br>(8,332-15,548)	9,575<br>(6,642-12,783)	8,209<br>(5,606-11,305)	6,624<br>(4,265-9,216)
Assault by other means	612<br>(438-754)	152<br>(110-191)	1,609<br>(1,209-2,002)	2,521<br>(1,872-3,093)	1,940<br>(1,372-2,300)	2,831<br>(2,067-3,415)	7,399<br>(5,618-9,873)	11,901<br>(9,021-15,189)	10,891<br>(7,900-14,441)	10,849<br>(7,817-14,684)	9,153<br>(6,612-12,351)	8,428<br>(5,896-11,305)	7,510<br>(4,956-10,011)
Forces of nature, conflict and terrorism, and executions and police conflict	193<br>(103-286)	114<br>(65-163)	434<br>(275-594)	3,327<br>(1,952-4,717)	2,492<br>(1,532-3,461)	2,658<br>(1,683-3,599)	6,767<br>(4,090-9,464)	11,656<br>(7,077-16,339)	8,379<br>(5,127-11,621)	6,002<br>(3,776-8,246)	4,212<br>(2,781-5,660)	2,706<br>(1,907-3,482)	1,825<br>(1,330-2,309)
Forces of nature, conflict and terrorism, and state actor violence	6<br>(4-8)	13<br>(9-17)	107<br>(72-141)	492<br>(331-650)	533<br>(362-704)	740<br>(496-977)	881<br>(603-1,161)	844<br>(575-1,113)	761<br>(517-1,005)	680<br>(458-895)	630<br>(418-827)	555<br>(370-724)	498<br>(334-649)
Conflict and terrorism	187<br>(98-279)	101<br>(52-150)	314<br>(163-467)	2,804<br>(1,453-4,177)	1,922<br>(993-2,867)	1,870<br>(966-2,791)	5,491<br>(2,855-8,156)	9,904<br>(5,345-14,514)	6,853<br>(3,647-10,083)	4,647<br>(2,455-6,849)	3,011<br>(1,627-4,405)	1,724<br>(996-2,447)	1,084<br>(657-1,506)
Executions and police conflict	-	-	13<br>(10-31)	30<br>(27-36)	37<br>(33-45)	49<br>(42-57)	395<br>(173-525)	909<br>(425-1,153)	765<br>(337-951)	675<br>(378-788)	571<br>(323-665)	426<br>(216-507)	243<br>(154-281)

Cause	50 to 54	55 to 59	60 to 64	65 to 69	70 to 74	75 to 79	80 to 84	85 to 89	90 to 94	95 plus
Self-harm	44,537<br>(38,385-49,390)	37,561<br>(31,323-41,803)	25,867<br>(21,049-28,164)	25,604<br>(20,149-27,913)	22,420<br>(17,233-24,175)	18,406<br>(13,923-19,921)	12,298<br>(9,363-13,262)	5,923<br>(4,577-6,373)	1,979<br>(1,534-2,116)	401<br>(316-429)
Self-harm by firearm	4,197<br>(3,321-5,837)	3,491<br>(2,693-4,739)	2,539<br>(2,008-3,474)	2,302<br>(1,761-3,111)	2,098<br>(1,558-2,850)	1,943<br>(1,479-2,532)	1,520<br>(1,165-1,983)	823<br>(596-1,038)	304<br>(221-403)	61<br>(46-81)
Self-harm by other specified means	40,340<br>(34,302-44,870)	34,070<br>(28,411-37,212)	23,328<br>(18,626-25,086)	23,302<br>(18,227-24,993)	20,322<br>(15,599-21,765)	16,463<br>(12,272-17,737)	10,778<br>(7,913-11,604)	5,100<br>(3,847-5,493)	1,675<br>(1,276-1,801)	340<br>(264-366)
Interpersonal violence	16,855<br>(11,748-21,538)	11,535<br>(7,967-14,772)	6,866<br>(4,901-8,815)	5,775<br>(3,993-7,465)	4,047<br>(2,851-5,268)	2,716<br>(1,975-3,566)	750<br>(558-1,060)	324<br>(240-477)	113<br>(81-170)	67<br>(47-101)
Assault by firearm	5,509<br>(3,108-7,599)	3,571<br>(2,208-4,945)	2,153<br>(1,306-2,932)	1,445<br>(834-2,081)	1,017<br>(581-1,503)	672<br>(380-978)	150<br>(79-230)	69<br>(34-101)	24<br>(12-37)	18<br>(9-23)
Assault by sharp object	4,993<br>(3,044-6,906)	3,228<br>(2,013-4,839)	2,060<br>(1,275-2,865)	1,617<br>(1,023-2,337)	1,068<br>(650-1,489)	645<br>(400-974)	203<br>(128-330)	81<br>(52-136)	34<br>(22-58)	16<br>(10-29)
Assault by other means	6,354<br>(4,063-8,481)	4,736<br>(3,087-6,148)	2,652<br>(1,814-3,618)	2,713<br>(1,862-3,597)	1,962<br>(1,362-2,602)	1,399<br>(973-1,869)	397<br>(285-591)	173<br>(130-275)	55<br>(40-91)	33<br>(24-53)
Forces of nature, conflict and terrorism, and executions and police conflict	1,479<br>(1,043-1,902)	1,079<br>(759-1,393)	860<br>(594-1,108)	699<br>(484-899)	569<br>(400-738)	421<br>(301-547)	302<br>(216-389)	156<br>(107-208)	67<br>(45-101)	26<br>(18-35)
Forces of nature, conflict and terrorism, and state actor violence	454<br>(303-595)	346<br>(232-455)	292<br>(194-386)	243<br>(162-321)	205<br>(137-272)	150<br>(101-198)	96<br>(64-127)	42<br>(28-55)	12<br>(8-16)	3<br>(2-4)
Conflict and terrorism	864<br>(492-1,234)	649<br>(385-910)	515<br>(296-732)	406<br>(232-578)	311<br>(178-443)	229<br>(133-324)	161<br>(94-227)	89<br>(52-126)	41<br>(24-58)	21<br>(13-29)
Executions and police conflict	162<br>(102-185)	84<br>(65-101)	53<br>(34-63)	51<br>(27-72)	53<br>(20-96)	42<br>(13-86)	45<br>(10-105)	26<br>(6-59)	15<br>(3-45)	3<br>(1-3)

SR Table 4: Global deaths by cause and age group for males (with 95% uncertainty intervals), 2010

Cause	Early Neonatal	Late Neonatal	Post Neonatal	1 to 4	5 to 9	10 to 14	15 to 19	20 to 24	25 to 29	30 to 34	35 to 39
All causes	1,233,699 (1,191,728-1,276,993)	333,464 (323,337-342,978)	1,043,177 (1,012,463-1,074,863)	946,647 (903,857-992,067)	293,187 (283,195-304,106)	248,130 (235,870-261,760)	396,845 (381,604-413,056)	573,579 (557,124-590,189)	652,773 (636,099-670,907)	747,891 (731,754-763,911)	896,914 (880,056-914,154)
Communicable, maternal, neonatal, and nutritional disorders	1,121,223 (1,077,628-1,164,794)	290,103 (279,436-300,560)	838,902 (805,656-873,937)	735,342 (692,833-782,715)	159,268 (148,933-170,061)	100,989 (93,729-109,082)	94,396 (87,298-104,242)	112,926 (105,765-123,014)	178,551 (167,042-192,836)	235,485 (223,807-249,349)	263,712 (250,935-278,945)
HIV/AIDS and tuberculosis	-	-	50,083 (46,976-53,332)	31,375 (28,916-34,139)	20,645 (19,708-21,674)	24,540 (23,224-25,974)	31,945 (29,547-34,402)	49,141 (45,752-53,519)	117,602 (107,648-128,154)	174,721 (164,147-186,340)	197,516 (186,744-208,608)
Tuberculosis	-	-	7,557 (6,232-9,142)	11,337 (9,297-13,686)	3,852 (3,302-4,455)	4,683 (4,129-5,321)	13,247 (12,053-14,609)	28,889 (26,650-31,457)	42,803 (39,916-46,221)	51,393 (48,018-54,887)	58,885 (54,915-62,677)
Drug-susceptible tuberculosis	-	-	7,043 (5,791-8,536)	10,557 (8,666-12,781)	3,568 (3,047-4,144)	4,314 (3,777-4,907)	12,175 (11,068-13,416)	26,431 (24,302-28,875)	39,012 (36,265-42,512)	46,645 (43,542-50,040)	53,233 (49,427-56,993)
Multidrug-resistant tuberculosis without extensive drug resistance	-	-	500 (380-651)	760 (567-997)	272 (218-337)	354 (285-439)	1,023 (827-1,262)	2,295 (1,894-2,767)	3,507 (2,926-4,150)	4,379 (3,712-5,153)	5,197 (4,396-6,126)
Extensively drug-resistant tuberculosis	-	-	15 (12-19)	21 (16-26)	12 (10-16)	16 (12-20)	50 (41-60)	163 (135-196)	285 (237-341)	369 (304-439)	456 (377-543)
HIV/AIDS	-	-	42,526 (39,867-45,394)	20,038 (18,799-21,308)	16,793 (16,025-17,608)	19,857 (18,651-21,160)	18,698 (16,730-20,721)	20,252 (17,600-23,828)	74,798 (65,516-85,033)	123,328 (112,884-133,754)	138,631 (128,312-148,737)
Drug-susceptible HIV/AIDS - Tuberculosis	-	-	11,884 (8,820-15,048)	5,999 (4,647-7,263)	4,802 (3,665-5,952)	5,458 (4,016-6,818)	5,445 (4,102-6,715)	5,382 (4,045-6,823)	17,188 (11,724-23,075)	25,072 (16,834-34,000)	27,659 (18,291-37,926)
Multidrug-resistant HIV/AIDS - Tuberculosis without extensive drug resistance	-	-	1,062 (673-1,561)	515 (338-756)	379 (246-541)	433 (273-630)	440 (277-640)	516 (332-747)	1,695 (1,055-2,544)	2,474 (1,482-3,739)	2,609 (1,569-3,925)
Extensively drug-resistant HIV/AIDS - Tuberculosis	-	-	17 (11-25)	7 (4-10)	5 (3-7)	6 (4-8)	6 (4-9)	18 (11-26)	77 (47-119)	139 (83-219)	134 (82-211)
HIV/AIDS resulting in other diseases	-	-	29,563 (25,481-33,950)	13,517 (11,908-15,378)	11,608 (10,312-13,045)	13,961 (12,223-15,804)	12,806 (10,754-15,108)	14,337 (11,932-17,656)	55,838 (46,420-67,132)	95,644 (82,756-109,600)	108,229 (93,385-122,867)
Diarrhoea, lower respiratory infections, and other common infectious diseases	106,916 (93,529-120,692)	91,425 (83,692-99,208)	537,363 (500,535-576,275)	383,817 (337,153-432,727)	91,221 (79,298-105,032)	52,165 (43,560-61,098)	44,278 (36,612-55,264)	43,581 (35,936-55,531)	43,190 (35,561-55,456)	43,030 (35,809-55,160)	47,749 (39,538-63,084)
Diarrhoeal diseases	13,357 (11,540-15,632)	23,773 (20,632-28,038)	189,786 (165,624-220,036)	140,517 (113,096-171,893)	21,361 (11,596-38,201)	10,855 (5,857-20,182)	12,451 (6,521-22,893)	14,149 (7,360-26,379)	14,936 (7,810-27,744)	14,599 (7,701-27,276)	16,489 (8,810-31,957)
Intestinal infectious diseases	-	-	585 (121-2,366)	13,541 (6,337-24,811)	24,041 (13,729-39,007)	16,181 (8,694-25,920)	10,314 (5,280-17,732)	6,359 (3,096-11,073)	4,576 (2,397-7,913)	3,393 (1,774-5,800)	2,700 (1,425-4,537)
Typhoid fever	-	-	105 (49-198)	12,013 (5,381-22,845)	21,024 (11,627-34,705)	13,662 (7,119-22,092)	8,917 (4,391-15,764)	5,322 (2,470-9,511)	3,521 (1,814-6,292)	2,521 (1,312-4,455)	2,008 (1,051-3,560)
Paratyphoid fever	-	-	15 (6-34)	1,219 (491-2,505)	2,979 (1,361-5,859)	2,504 (1,132-4,689)	1,382 (580-2,826)	1,018 (418-2,092)	990 (423-2,041)	851 (364-1,701)	671 (310-1,285)
Other intestinal infectious diseases	-	-	465 (31-2,190)	309 (22-1,400)	38 (3-180)	15 (1-75)	15 (1-70)	19 (1-98)	14 (1-61)	21 (2-102)	21 (2-96)
Lower respiratory infections	76,573 (67,367-87,164)	52,891 (47,249-59,030)	246,216 (225,240-267,613)	114,632 (97,400-133,734)	20,038 (15,069-23,761)	11,271 (8,773-13,126)	10,052 (7,918-12,028)	12,442 (9,892-14,947)	14,165 (11,255-16,878)	15,863 (13,031-18,354)	19,143 (15,578-22,982)
Upper respiratory infections	46 (26-75)	14 (8-25)	335 (203-541)	252 (137-451)	22 (12-37)	17 (10-27)	42 (28-63)	46 (31-67)	50 (35-72)	50 (33-70)	58 (40-83)
Otitis media	2 (1-3)	3 (2-6)	50 (25-125)	106 (47-213)	52 (29-91)	65 (37-121)	18 (11-33)	17 (10-31)	14 (9-24)	13 (8-22)	18 (11-29)
Meningitis	6,512 (4,119-9,048)	7,032 (5,541-8,958)	42,876 (34,877-53,393)	34,851 (27,706-44,833)	10,634 (6,270-16,194)	7,429 (4,632-11,451)	7,186 (4,621-11,513)	7,006 (4,381-11,030)	6,194 (3,904-10,064)	5,813 (3,599-9,560)	5,850 (3,753-9,413)
Pneumococcal meningitis	351 (205-536)	373 (247-543)	2,335 (1,636-3,248)	2,124 (1,355-3,235)	651 (368-1,059)	440 (255-772)	418 (243-736)	402 (219-710)	409 (238-711)	475 (285-797)	580 (334-1,023)
H influenzae type B meningitis	949 (589-1,396)	1,002 (737-1,375)	5,817 (4,347-7,878)	4,393 (3,119-6,102)	1,050 (588-1,672)	602 (346-985)	512 (311-875)	492 (300-847)	470 (283-802)	460 (271-780)	473 (293-775)
Meningococcal infection	3,269 (2,081-4,720)	3,524 (2,706-4,704)	21,264 (16,648-27,961)	18,577 (13,949-25,293)	5,953 (3,491-9,195)	4,082 (2,544-6,435)	3,976 (2,494-6,490)	3,809 (2,356-6,120)	3,200 (2,037-5,331)	2,769 (1,706-4,608)	2,699 (1,711-4,390)
Other meningitis	1,943 (1,186-2,632)	2,133 (1,643-2,774)	13,460 (10,441-17,173)	9,757 (7,318-12,639)	2,981 (1,740-4,813)	2,306 (1,382-3,573)	2,281 (1,415-3,663)	2,303 (1,428-3,687)	2,115 (1,323-3,373)	2,109 (1,293-3,557)	2,098 (1,296-3,417)
Encephalitis	413 (275-536)	589 (463-774)	6,974 (5,659-9,105)	7,414 (5,726-10,308)	3,058 (2,457-4,310)	2,589 (2,100-3,738)	2,082 (1,713-3,061)	2,013 (1,658-3,011)	1,903 (1,585-2,772)	2,028 (1,704-2,882)	2,129 (1,788-3,156)
Diphtheria	-	-	248 (129-459)	497 (222-1,041)	98 (50-184)	46 (27-75)	22 (14-35)	15 (10-23)	14 (9-21)	12 (8-18)	18 (12-26)
Whooping cough	-	-	21,132 (9,965-38,234)	19,093 (8,979-33,950)	1,974 (902-3,677)	427 (193-807)	192 (87-371)	132 (62-249)	76 (34-148)	59 (26-117)	40 (19-79)
Tetanus	9,974 (4,260-14,900)	6,990 (3,182-11,237)	2,868 (1,223-4,819)	1,329 (555-2,185)	821 (368-1,349)	582 (301-985)	898 (391-1,415)	847 (402-1,316)	913 (463-1,305)	919 (502-1,303)	1,090 (551-1,460)
Measles	-	-	25,169 (9,561-52,880)	50,213 (21,513-98,097)	8,532 (3,270-17,651)	2,504 (897-5,561)	928 (319-2,157)	434 (149-1,027)	203 (71-475)	124 (43-295)	79 (28-186)
Varicella and herpes zoster	41 (32-52)	133 (105-171)	1,123 (884-1,421)	1,372 (1,044-1,772)	590 (449-766)	201 (154-258)	94 (74-123)	119 (93-153)	146 (116-184)	157 (125-197)	134 (109-169)
Neglected tropical diseases and malaria	590 (307-906)	12,832 (8,819-18,739)	109,707 (79,931-149,323)	229,773 (175,481-293,996)	34,635 (25,422-44,724)	18,706 (13,702-24,225)	11,244 (8,633-14,251)	12,196 (9,397-15,293)	9,892 (7,640-12,350)	9,144 (6,925-11,443)	9,573 (7,060-12,074)
Malaria	-	12,325 (8,317-18,280)	103,441 (73,707-143,674)	216,509 (161,582-280,837)	27,386 (18,076-37,790)	14,211 (9,251-19,896)	6,847 (4,851-9,405)	7,434 (5,300-10,229)	6,176 (4,389-8,348)	5,802 (4,036-7,975)	5,952 (4,064-8,254)
Chagas disease	-	-	1 (1-1)	1 (0-1)	1 (1-1)	2 (2-3)	6 (5-9)	15 (12-21)	25 (21-32)	55 (47-67)	101 (88-116)
Leishmaniasis	-	-	1,281 (723-2,173)	1,816 (968-3,033)	872 (477-1,496)	592 (335-973)	779 (436-1,281)	956 (539-1,561)	818 (462-1,320)	736 (419-1,235)	849 (469-1,409)
Visceral leishmaniasis	-	-	1,281 (723-2,173)	1,816 (968-3,033)	872 (477-1,496)	592 (335-973)	779 (436-1,281)	956 (539-1,561)	818 (462-1,320)	736 (419-1,235)	849 (469-1,409)
African trypanosomiasis	-	-	-	86 (14-325)	234 (47-790)	282 (56-1,009)	358 (69-1,229)	383 (80-1,351)	323 (71-1,089)	277 (56-1,029)	242 (47-899)
Schistosomiasis	-	-	0 (0-0)	237 (133-412)	136 (85-225)	194 (123-294)	201 (138-279)	292 (209-392)	336 (237-458)	362 (265-484)	414 (305-563)
Cysticercosis	0 (0-0)	0 (0-0)	0 (0-0)	41 (27-62)	28 (20-39)	23 (18-30)	24 (19-31)	38 (30-48)	48 (39-60)	53 (43-65)	42 (35-51)
Cystic echinococcosis	-	-	-	119 (78-179)	54 (37-77)	36 (24-52)	18 (13-25)	25 (17-35)	36 (26-49)	37 (26-49)	44 (33-58)
Dengue	-	-	1,337 (378-2,103)	2,463 (886-3,723)	1,636 (692-2,595)	1,084 (372-1,596)	1,167 (256-1,888)	1,122 (202-1,748)	1,048 (172-1,631)	800 (134-1,261)	968 (129-1,526)
Yellow fever	-	2 (0-6)	39 (8-119)	370 (72-1,102)	742 (150-2,222)	650 (130-1,919)	608 (111-2,007)	521 (104-1,557)	396 (79-1,256)	300 (65-962)	231 (45-720)
Rabies	-	-	169 (87-236)	1,234 (491-2,005)	2,082 (697-3,611)	1,161 (438-1,722)	939 (467-1,423)	1,065 (490-1,523)	348 (138-446)	446 (192-571)	436 (188-560)
Intestinal nematode infections	-	-	196 (150-252)	2,075 (1,512-2,788)	336 (257-429)	73 (57-93)	27 (20-34)	20 (14-26)	16 (12-22)	20 (15-27)	19 (14-25)
Ascariasis	-	-	196 (150-252)	2,075 (1,512-2,788)	336 (257-429)	73 (57-93)	27 (20-34)	20 (14-26)	16 (12-22)	20 (15-27)	19 (14-25)
Ebola	0 (0-0)	0 (0-0)	0 (0-0)	0 (0-0)	0 (0-0)	0 (0-0)	0 (0-0)	0 (0-0)	0 (0-0)	0 (0-0)	0 (0-0)
Zika virus	0 (0-0)	0 (0-0)	0 (0-0)	0 (0-0)	0 (0-0)	0 (0-0)	0 (0-0)	0 (0-0)	0 (0-0)	0 (0-0)	0 (0-0)
Other neglected tropical diseases	590 (307-906)	506 (297-736)	3,242 (1,692-4,682)	4,824 (1,866-11,774)	1,129 (576-2,234)	399 (241-675)	269 (161-438)	326 (198-517)	321 (201-479)	256 (155-352)	276 (185-366)
Maternal disorders	-	-	-	-	-	-	-	-	-	-	-
Maternal haemorrhage	-	-	-	-	-	-	-	-	-	-	-
Maternal sepsis and other pregnancy related infections	-	-	-	-	-	-	-	-	-	-	-
Maternal hypertensive disorders	-	-	-	-	-	-	-	-	-	-	-
Maternal obstructed labour and uterine rupture	-	-	-	-	-	-	-	-	-	-	-
Maternal abortion, miscarriage, and ectopic pregnancy	-	-	-	-	-	-	-	-	-	-	-
Indirect maternal deaths	-	-	-	-	-	-	-	-	-	-	-
Late maternal deaths	-	-	-	-	-	-	-	-	-	-	-
Maternal deaths aggravated by HIV/AIDS	-	-	-	-	-	-	-	-	-	-	-
Other maternal disorders	-	-	-	-	-	-	-	-	-	-	-
Neonatal disorders	990,605 (946,650-1,034,801)	175,599 (165,461-187,070)	49,601 (43,202-56,926)	5,686 (4,797-6,609)	-	-	-	-	-	-	-
Neonatal preterm birth complications	378,677 (347,948-419,202)	49,435 (42,949-56,958)	17,569 (14,426-21,233)	1,417 (1,110-1,748)	-	-	-	-	-	-	-
Neonatal encephalopathy due to birth asphyxia and trauma	331,653 (291,045-368,225)	28,210 (23,364-33,556)	9,741 (7,557-12,242)	2,495 (2,002-2,977)	-	-	-	-	-	-	-
Neonatal sepsis and other neonatal infections	89,980 (60,736-119,465)	55,107 (43,470-67,450)	9,712 (7,142-12,770)	500 (216-906)	-	-	-	-	-	-	-
Hemolytic disease and other neonatal jaundice	27,100 (22,847-33,011)	12,060 (9,838-14,634)	2,479 (1,732-3,644)	137 (87-205)	-	-	-	-	-	-	-
Other neonatal disorders	163,195 (145,120-182,511)	30,787 (27,239-36,310)	10,100 (8,318-12,672)	1,137 (927-1,510)	-	-	-	-	-	-	-
Nutritional deficiencies	-	-	55,444 (48,480-64,669)	63,657 (49,127-82,362)	6,290 (5,009-8,158)	2,406 (1,947-3,180)	2,149 (1,778-3,120)	2,352 (1,897-3,315)	2,141 (1,732-3,097)	2,237 (1,862-3,163)	2,429 (2,009-3,497)
Protein-energy malnutrition	-	-	53,781 (46,826-62,773)	62,023 (47,552-80,625)	5,579 (4,404-7,235)	2,007 (1,574-2,718)	1,447 (1,160-2,112)	1,529 (1,216-2,074)	1,444 (1,152-2,092)	1,496 (1,228-2,054)	1,634 (1,324-2,411)
Iodine deficiency	-	-	143 (29-485)	115 (16-497)	44 (9-147)	12 (2-40)	36 (7-120)	43 (8-150)	48 (10-147)	45 (8-158)	41 (10-137)
Iron-deficiency anaemia	-	-	27 (12-49)	62 (32-99)	65 (36-95)	56 (33-75)	94 (64-124)	93 (71-120)	72 (58-94)	80 (61-100)	61 (51-80)
Other nutritional deficiencies	-	-	1,493 (1,000-2,031)	1,458 (881-2,092)	602 (436-815)	331 (254-433)	572 (427-881)	687 (477-1,163)	577 (404-954)	615 (420-1,012)	693 (485-1,110)
Other communicable, maternal, neonatal, and nutritional diseases	23,112 (13,859-35,108)	10,246 (6,794-14,978)	36,704 (24,667-53,393)	21,034 (14,498-29,785)	6,477 (4,888-8,342)	3,173 (2,700-3,675)	4,779 (4,217-5,330)	5,655 (5,130-6,161)	5,726 (5,227-6,237)	6,353 (5,785-6,920)	6,445 (5,908-6,989)
Sexually transmitted diseases excluding HIV	19,251 (10,721-30,614)	7,458 (4,126-11,722)	22,069 (12,496-35,035)	9,189 (5,068-14,733)	3,013 (1,653-4,873)	63 (46-80)	90 (64-118)	80 (54-102)	106 (72-139)	188 (129-241)	126 (84-161)
Syphilis	19,251 (10,721-30,614)	7,458 (4,126-11,722)	22,069 (12,496-35,035)	9,189 (5,068-14,733)	3,013 (1,653-4,873)	54 (39-68)	76 (54-100)	69 (47-88)	89 (60-116)	159 (109-204)	112 (75-143)
Chlamydial infection	-	-	-	-	-	0 (0-0)	0 (0-0)	0 (0-0)	0 (0-0)	0 (0-0)	0 (0-0)
Gonococcal infection	-	-	-	-	-	6 (5-8)	10 (7-14)	7 (5-9)	15 (10-19)	21 (14-27)	7 (5-9)
Other sexually transmitted diseases	-	-	-	-	-	3 (2-4)	4 (3-5)	4 (3-5)	3 (2-4)	8 (5-10)	7 (5-9)
Hepatitis	-	-	4,581 (3,977-5,359)	4,606 (3,707-5,519)	2,117 (1,709-2,566)	2,052 (1,711-2,428)	3,351 (2,935-3,826)	4,379 (3,984-4,816)	4,484 (4,053-4,950)	4,964 (4,491-5,483)	4,973 (4,539-5,462)
Acute hepatitis A	-	-	2,761 (1,838-3,688)	484 (268-752)	46 (27-74)	26 (15-41)	41 (23-65)	52 (27-87)	46 (21-83)	41 (18-74)	41 (18-77)
Hepatitis B	-	-	1,820 (1,008-2,786)	688 (393-1,070)	223 (116-380)	343 (172-556)	1,148 (746-1,605)	2,514 (1,842-3,250)	3,296 (2,641-3,968)	4,183 (3,588-4,791)	4,426 (3,879-4,942)
Hepatitis C	-	-	-	-	66 (39-101)	24 (15-35)	34 (21-50)	41 (22-68)	36 (17-65)	35 (17-63)	40 (18-75)
Acute hepatitis E	-	-	-	3,434 (2,678-4,229)	1,782 (1,431-2,175)	1,659 (1,337-2,021)	2,128 (1,595-2,621)	1,773 (1,118-2,408)	1,106 (613-1,705)	705 (365-1,181)	467 (211-819)
Other infectious diseases	3,861 (1,241-8,350)	2,789 (1,349-4,542)	10,055 (3,703-21,289)	7,240 (2,997-15,628)	1,346 (909-1,840)	1,057 (756-1,332)	1,338 (995-1,601)	1,196 (878-1,430)	1,135 (872-1,332)	1,201 (888-1,391)	1,346 (1,042-1,535)
Non-communicable diseases	104,937 (85,304-121,217)	38,852 (32,732-43,860)	156,078 (134,300-173,961)	91,531 (76,143-106,815)	47,694 (43,274-52,585)	51,132 (47,589-55,050)	92,133 (87,963-96,853)	135,002 (130,065-142,067)	172,626 (166,365-180,031)	245,544 (237,636-254,436)	370,746 (360,349-383,488)
Neoplasms	449 (382-478)	302 (267-322)	2,932 (2,741-3,205)	12,542 (11,502-14,240)	15,227 (13,896-16,598)	16,518 (15,331-17,568)	24,565 (23,141-25,818)	28,929 (27,565-30,155)	32,539 (31,106-33,831)	48,026 (46,203-49,630)	79,251 (76,871-81,691)
Lip and oral cavity cancer	-	-	-	-	-	-	242 (220-268)	505 (465-552)	923 (833-1,020)	1,626 (1,485-1,820)	2,891 (2,674-3,159)
Nasopharynx cancer	-	-	-	-	90 (80-100)	203 (179-228)	360 (331-391)	423 (390-459)	516 (473-567)	815 (757-884)	1,605 (1,486-1,723)
Other pharynx cancer	-	-	-	-	-	-	132 (116-149)	246 (221-274)	325 (289-365)	697 (636-764)	1,479 (1,359-1,619)
Oesophageal cancer	-	-	-	-	-	-	198 (181-216)	371 (348-397)	495 (460-533)	1,106 (1,039-1,190)	2,545 (2,419-2,689)
Stomach cancer	-	-	-	-	-	-	541 (510-576)	1,153 (1,091-1,233)	1,908 (1,817-2,031)	3,874 (3,697-4,087)	7,293 (7,009-7,631)
Colon and rectum cancer	-	-	-	-	-	-	745 (679-823)	1,664 (1,544-1,816)	2,299 (2,156-2,464)	3,788 (3,597-4,006)	6,014 (5,748-6,314)
Liver cancer	-	-	-	-	292 (254-337)	493 (437-564)	1,000 (909-1,111)	2,353 (2,111-2,667)	4,076 (3,662-4,652)	8,059 (7,480-8,758)	16,875 (15,939-17,923)
Liver cancer due to hepatitis B	-	-	-	-	64 (43-89)	168 (121-222)	434 (327-553)	1,266 (1,024-1,523)	2,429 (1,903-2,975)	4,863 (4,054-5,630)	9,948 (8,187-11,388)
Liver cancer due to hepatitis C	-	-	-	-	5 (4-7)	10 (7-14)	21 (16-29)	67 (50-89)	155 (106-224)	355 (266-459)	838 (633-1,101)
Liver cancer due to alcohol use	-	-	-	-	-	-	3 (2-4)	29 (18-42)	87 (53-135)	286 (204-394)	849 (589-1,175)
Liver cancer due to other causes	-	-	-	-	224 (189-264)	314 (258-375)	542 (433-663)	992 (811-1,186)	1,404 (1,099-1,844)	2,553 (2,014-3,226)	5,239 (4,218-6,690)
Gallbladder and biliary tract cancer	-	-	-	-	-	-	45 (38-52)	76 (60-92)	134 (99-161)	359 (266-430)	747 (551-910)
Pancreatic cancer	-	-	-	-	-	-	104 (99-110)	227 (215-240)	381 (364-403)	965 (922-1,012)	2,041 (1,971-2,116)
Larynx cancer	-	-	-	-	-	-	97 (89-107)	135 (127-143)	188 (177-199)	428 (397-463)	983 (915-1,058)
Tracheal, bronchus, and lung cancer	-	-	-	-	-	-	628 (590-674)	1,213 (1,132-1,326)	1,983 (1,869-2,113)	3,901 (3,714-4,141)	8,439 (8,069-8,888)
Malignant skin melanoma	-	-	-	-	-	-	124 (115-155)	236 (216-308)	359 (327-432)	601 (534-713)	958 (865-1,130)
Non-melanoma skin cancer	-	-	-	-	-	-	120 (113-127)	165 (156-175)	189 (178-201)	321 (303-341)	457 (435-481)
Non-melanoma skin cancer (squamous-cell carcinoma)	-	-	-	-	-	-	120 (113-127)	165 (156-175)	189 (178-201)	321 (303-341)	457 (435-481)
Breast cancer	-	-	-	-	-	-	37 (35-39)	38 (36-40)	48 (40-52)	84 (63-92)	170 (121-186)
Cervical cancer	-	-	-	-	-	-	-	-	-	-	-
Uterine cancer	-	-	-	-	-	-	-	-	-	-	-
Ovarian cancer	-	-	-	-	-	-	-	-	-	-	-
Prostate cancer	-	-	-	-	-	-	72 (58-82)	96 (75-109)	120 (95-137)	151 (117-170)	244 (184-267)
Testicular cancer	-	-	-	-	-	-	419 (384-465)	916 (850-1,001)	1,117 (1,038-1,212)	1,060 (996-1,136)	928 (876-992)
Kidney cancer	19 (16-21)	10 (9-11)	133 (123-147)	675 (606-752)	308 (279-337)	153 (138-164)	141 (129-152)	205 (187-219)	273 (251-288)	428 (396-450)	858 (797-897)
Bladder cancer	-	-	-	-	-	-	64 (59-70)	137 (126-149)	164 (151-180)	352 (327-384)	686 (643-735)
Brain and nervous system cancer	56 (39-65)	38 (29-44)	533 (413-634)	2,214 (1,869-2,655)	3,126 (2,656-3,617)	2,992 (2,488-3,440)	2,518 (2,117-2,864)	2,685 (2,227-3,054)	2,981 (2,547-3,415)	3,837 (3,275-4,295)	5,192 (4,449-5,928)
Thyroid cancer	-	-	-	-	-	66 (49-82)	113 (97-131)	175 (158-193)	212 (194-228)	229 (212-251)	318 (295-343)
Mesothelioma	-	-	-	-	-	-	58 (53-70)	72 (65-84)	114 (102-135)	137 (121-161)	252 (225-298)
Hodgkin lymphoma	6 (5-8)	1 (1-2)	35 (27-50)	255 (193-398)	782 (538-1,073)	908 (651-1,200)	962 (721-1,245)	924 (719-1,188)	987 (771-1,251)	1,000 (790-1,338)	1,056 (822-1,402)
Non-Hodgkin's lymphoma	28 (24-31)	21 (19-25)	252 (226-300)	1,497 (1,246-1,748)	2,441 (1,901-2,955)	2,313 (1,824-2,668)	2,329 (1,927-2,600)	2,655 (2,256-2,961)	2,460 (2,126-2,686)	2,926 (2,445-3,168)	3,663 (3,073-3,943)
Multiple myeloma	-	-	-	-	-	-	125 (95-145)	173 (128-199)	171 (134-199)	358 (289-435)	609 (502-741)
Leukaemia	105 (85-115)	92 (75-103)	1,043 (907-1,167)	4,511 (4,062-5,447)	5,578 (5,007-6,623)	5,879 (5,361-6,959)	6,871 (6,287-8,002)	6,402 (5,605-7,320)	5,152 (4,390-5,549)	4,999 (4,307-5,423)	5,772 (5,066-6,431)
Acute lymphoid leukaemia	29 (25-35)	25 (21-29)	333 (297-415)	1,783 (1,557-2,328)	2,567 (2,271-3,216)	2,545 (2,285-3,107)	2,371 (2,132-2,816)	1,833 (1,631-2,145)	1,268 (1,089-1,436)	1,132 (983-1,287)	1,146 (1,027-1,339)
Chronic lymphoid leukaemia	-	-	-	-	-	-	145 (129-183)	165 (146-204)	125 (105-146)	116 (99-135)	152 (136-182)
Acute myeloid leukaemia	19 (14-24)	19 (15-23)	212 (173-265)	1,056 (808-1,456)	1,015 (797-1,278)	1,045 (863-1,314)	1,366 (1,194-1,663)	1,324 (1,124-1,583)	1,177 (1,005-1,374)	1,217 (1,038-1,420)	1,445 (1,230-1,692)
Chronic myeloid leukaemia	-	-	-	-	-	-	212 (184-273)	293 (250-364)	487 (413-559)	518 (436-602)	584 (490-685)
Other leukaemia	57 (40-63)	49 (35-55)	498 (382-566)	1,671 (1,381-2,120)	1,997 (1,676-2,314)	2,289 (2,009-2,729)	2,777 (2,449-3,333)	2,788 (2,255-3,186)	2,095 (1,698-2,257)	2,016 (1,629-2,212)	2,445 (2,058-2,723)
Other neoplasms	235 (188-258)	139 (118-152)	936 (840-1,115)	3,390 (2,927-4,353)	2,611 (2,311-2,873)	3,511 (3,037-3,809)	6,518 (5,664-7,007)	5,682 (4,905-6,058)	4,966 (4,206-5,333)	5,926 (4,866-6,386)	7,176 (5,936-7,653)
Cardiovascular diseases	2,392 (2,165-2,658)	1,221 (1,131-1,325)	10,493 (9,815-11,391)	8,262 (7,556-8,981)	5,106 (4,691-5,586)	6,805 (6,374-7,259)	20,243 (19,246-21,546)	36,701 (35,181-38,808)	54,588 (52,305-57,366)	87,316 (84,256-91,027)	141,260 (136,443-146,671)
Rheumatic heart disease	-	-	-	1,637 (1,348-2,016)	1,173 (1,013-1,339)	2,123 (1,893-2,349)	4,035 (3,628-4,458)	4,328 (3,823-4,795)	4,288 (3,717-4,755)	4,750 (4,117-5,274)	5,576 (4,846-6,186)
Ischaemic heart disease	-	-	651 (555-776)	494 (409-597)	358 (309-420)	407 (363-454)	6,528 (6,110-7,107)	14,684 (13,958-15,648)	25,534 (24,277-27,065)	46,905 (44,903-49,313)	78,082 (74,921-81,523)
Cerebrovascular disease	1,094 (958-1,267)	535 (466-626)	3,785 (3,278-4,462)	2,579 (2,176-3,114)	1,773 (1,565-2,000)	2,129 (1,928-2,307)	4,548 (4,260-4,885)	9,921 (9,403-10,620)	13,437 (12,693-14,337)	21,507 (20,540-22,617)	37,952 (36,509-39,664)

Cause	40 to 44	45 to 49	50 to 54	55 to 59	60 to 64	65 to 69	70 to 74	75 to 79	80 to 84	85 to 89	90 to 94	95 plus
All causes	1,071,094 (1,054,574-1,088,316)	1,296,143 (1,275,312-1,317,161)	1,589,634 (1,565,875-1,615,070)	1,967,626 (1,941,198-1,996,359)	2,184,192 (2,158,169-2,213,322)	2,375,216 (2,348,434-2,403,699)	2,887,334 (2,855,422-2,922,149)	2,887,365 (2,861,648-2,917,656)	2,618,959 (2,599,932-2,639,444)	1,715,101 (1,706,550-1,724,190)	735,266 (732,428-738,246)	226,681 (225,888-227,499)
Communicable, maternal, neonatal, and nutritional disorders	260,091 (249,922-273,020)	249,219 (238,159-264,941)	229,708 (218,208-247,113)	219,099 (205,175-242,239)	219,734 (201,335-248,932)	230,292 (208,049-268,811)	271,509 (240,780-323,777)	264,662 (231,904-317,009)	253,146 (222,541-303,935)	179,477 (157,531-213,656)	82,260 (72,493-96,014)	28,843 (25,517-32,930)
HIV/AIDS and tuberculosis	191,248 (184,381-197,987)	169,626 (162,435-177,841)	139,728 (133,975-145,625)	113,380 (108,594-118,695)	91,744 (86,776-97,003)	81,434 (76,679-86,377)	80,256 (74,717-85,697)	60,773 (56,613-65,072)	44,221 (40,229-47,762)	22,696 (20,698-24,641)	7,166 (6,501-7,843)	1,770 (1,611-1,950)
Tuberculosis	64,719 (60,812-68,690)	72,758 (68,233-77,011)	79,754 (74,847-84,527)	81,702 (77,192-86,622)	73,767 (69,091-78,857)	70,601 (65,700-75,457)	73,624 (68,436-79,082)	57,083 (52,916-61,324)	43,112 (39,128-46,660)	22,264 (20,279-24,223)	7,056 (6,393-7,733)	1,748 (1,588-1,929)
Drug-susceptible tuberculosis	58,324 (54,720-62,043)	65,238 (60,793-69,243)	71,517 (67,141-76,235)	73,467 (69,037-78,264)	66,641 (62,131-71,700)	64,197 (59,741-68,664)	66,902 (61,870-71,892)	52,011 (48,148-55,950)	39,344 (35,736-42,716)	20,384 (18,573-22,191)	6,481 (5,849-7,108)	1,614 (1,466-1,781)
Multidrug-resistant tuberculosis without extensive drug resistance	5,868 (4,941-6,922)	6,851 (5,809-8,108)	7,513 (6,260-8,958)	7,585 (6,288-8,984)	6,628 (5,505-7,958)	6,015 (4,968-7,265)	6,297 (5,168-7,600)	4,759 (3,922-5,727)	3,532 (2,881-4,320)	1,762 (1,441-2,148)	538 (437-666)	125 (100-154)
Extensively drug-resistant tuberculosis	527 (437-627)	668 (563-800)	724 (603-870)	651 (542-779)	498 (410-599)	390 (321-477)	424 (350-517)	312 (255-382)	236 (190-294)	118 (94-147)	37 (29-47)	9 (7-11)
HIV/AIDS	126,529 (121,126-131,879)	96,869 (90,440-104,000)	59,974 (56,492-63,292)	31,678 (29,481-33,840)	17,977 (16,438-19,497)	10,832 (9,796-11,858)	6,632 (5,968-7,340)	3,690 (3,354-4,081)	1,109 (987-1,247)	433 (383-486)	110 (98-123)	22 (20-25)
Drug-susceptible HIV/AIDS - Tuberculosis	25,656 (17,103-34,840)	21,682 (14,624-28,856)	15,139 (10,616-19,236)	8,277 (6,239-10,297)	4,838 (3,757-5,970)	2,956 (2,330-3,612)	1,884 (1,493-2,258)	1,060 (843-1,268)	340 (273-404)	133 (107-157)	33 (27-39)	6 (5-7)
Multidrug-resistant HIV/AIDS - Tuberculosis without extensive drug resistance	2,364 (1,434-3,542)	2,007 (1,240-2,984)	1,429 (890-2,061)	799 (518-1,133)	457 (299-648)	272 (178-381)	171 (114-241)	95 (63-136)	30 (19-44)	12 (7-18)	3 (2-4)	1 (0-1)
Extensively drug-resistant HIV/AIDS - Tuberculosis	105 (64-160)	79 (48-121)	49 (31-74)	27 (17-41)	14 (9-21)	7 (5-11)	4 (3-7)	3 (2-4)	1 (1-1)	0 (0-0)	0 (0-0)	0 (0-0)
HIV/AIDS resulting in other diseases	98,405 (87,398-110,325)	73,101 (63,939-83,714)	43,357 (38,206-49,204)	22,575 (20,050-25,458)	12,669 (11,143-14,403)	7,597 (6,626-8,618)	4,573 (3,963-5,251)	2,533 (2,217-2,918)	738 (649-850)	288 (253-331)	74 (65-85)	16 (14-18)
Diarrhoea, lower respiratory infections, and other common infectious diseases	49,876 (41,191-64,555)	59,964 (49,948-77,633)	70,741 (58,925-91,074)	84,990 (69,975-111,864)	107,120 (86,739-140,360)	126,608 (101,417-168,059)	169,054 (133,964-226,732)	183,192 (148,637-240,095)	191,542 (159,428-246,805)	143,625 (119,725-179,106)	68,625 (58,580-83,178)	24,644 (21,376-28,963)
Diarrhoeal diseases	16,781 (8,532-32,532)	19,565 (10,453-38,152)	22,673 (11,764-44,525)	28,708 (15,085-55,743)	38,600 (20,554-75,587)	49,603 (26,257-94,121)	68,102 (36,674-125,751)	70,299 (37,579-128,294)	71,775 (40,669-127,953)	50,689 (29,639-87,546)	21,791 (13,287-36,627)	6,512 (4,127-10,423)
Intestinal infectious diseases	2,208 (1,186-3,638)	1,964 (1,055-3,291)	1,570 (819-2,716)	1,153 (552-2,086)	924 (456-1,664)	876 (487-1,432)	769 (452-1,225)	559 (327-893)	329 (190-533)	144 (85-228)	41 (24-65)	9 (5-16)
Typhoid fever	1,631 (850-2,880)	1,415 (753-2,393)	1,137 (579-1,992)	852 (398-1,590)	711 (337-1,320)	711 (391-1,191)	638 (364-1,022)	459 (263-751)	272 (156-443)	115 (66-184)	32 (18-51)	7 (4-11)
Paratyphoid fever	559 (259-1,087)	525 (239-1,064)	424 (183-871)	293 (119-611)	202 (85-425)	154 (70-301)	118 (56-225)	81 (39-147)	47 (22-86)	19 (9-36)	5 (2-10)	1 (0-2)
Other intestinal infectious diseases	18 (2-76)	24 (2-112)	9 (1-44)	9 (1-43)	11 (1-47)	11 (1-50)	13 (1-50)	20 (2-85)	11 (1-46)	11 (1-47)	4 (1-17)	2 (0-7)
Lower respiratory infections	22,441 (18,283-25,658)	29,285 (23,997-34,218)	37,723 (31,124-43,260)	46,261 (38,159-53,025)	59,538 (48,427-67,943)	67,952 (54,488-78,576)	92,555 (74,701-102,897)	105,673 (84,618-118,289)	113,462 (93,841-126,052)	89,358 (75,582-100,723)	45,545 (39,299-51,595)	17,767 (15,459-19,980)
Upper respiratory infections	59 (42-83)	70 (50-95)	70 (50-97)	76 (54-104)	75 (54-102)	63 (45-87)	56 (40-75)	54 (40-71)	37 (27-50)	24 (18-32)	9 (6-12)	2 (1-3)
Otitis media	20 (11-35)	53 (31-104)	60 (36-113)	26 (13-40)	25 (13-39)	27 (14-52)	26 (16-45)	26 (15-38)	28 (15-40)	18 (9-26)	9 (3-13)	3 (1-4)
Meningitis	4,953 (3,215-8,103)	5,489 (3,739-9,322)	5,252 (3,603-8,712)	5,077 (3,489-8,669)	4,690 (3,262-8,175)	4,669 (3,237-7,908)	3,882 (2,675-7,042)	3,150 (2,235-5,663)	2,782 (1,877-4,769)	1,508 (1,034-2,556)	509 (349-865)	122 (90-206)
Pneumococcal meningitis	560 (308-994)	663 (376-1,184)	633 (392-1,155)	618 (398-1,090)	612 (393-1,064)	598 (377-1,050)	495 (307-918)	395 (258-714)	331 (206-572)	169 (113-277)	57 (38-96)	15 (10-24)
H influenzae type B meningitis	398 (242-691)	446 (278-806)	425 (272-736)	408 (255-695)	392 (243-706)	374 (234-680)	299 (194-559)	240 (160-435)	217 (132-376)	115 (77-188)	37 (24-64)	8 (5-14)
Meningococcal infection	2,265 (1,433-3,854)	2,478 (1,651-4,342)	2,402 (1,561-4,070)	2,316 (1,570-4,028)	2,177 (1,473-4,025)	2,148 (1,423-3,861)	1,748 (1,185-3,247)	1,435 (967-2,631)	1,250 (802-2,155)	707 (476-1,252)	243 (164-431)	56 (38-100)
Other meningitis	1,731 (1,064-2,837)	1,901 (1,243-3,225)	1,791 (1,179-3,077)	1,735 (1,151-2,940)	1,509 (986-2,625)	1,549 (1,032-2,673)	1,340 (903-2,341)	1,081 (767-1,910)	984 (662-1,736)	517 (354-911)	171 (117-294)	43 (30-73)
Encephalitis	1,905 (1,576-2,929)	1,961 (1,579-3,049)	1,911 (1,536-3,083)	2,214 (1,778-3,413)	2,247 (1,802-3,486)	2,534 (2,011-3,870)	2,762 (2,239-4,066)	2,714 (2,261-3,804)	2,278 (1,847-3,124)	1,216 (973-1,645)	386 (306-546)	96 (77-139)
Diphtheria	18 (12-25)	20 (14-28)	22 (16-31)	17 (12-23)	-	-	-	-	-	-	-	-
Whooping cough	52 (24-102)	41 (18-81)	31 (14-61)	43 (19-85)	-	-	-	-	-	-	-	-
Tetanus	1,294 (640-1,725)	1,372 (665-1,808)	1,313 (628-1,768)	1,281 (624-1,673)	917 (467-1,268)	700 (358-959)	719 (327-954)	426 (182-545)	407 (193-540)	180 (86-241)	59 (29-80)	17 (8-22)
Measles	61 (21-145)	48 (17-113)	37 (13-87)	40 (14-95)	-	-	-	-	-	-	-	-
Varicella and herpes zoster	84 (68-104)	97 (77-120)	80 (63-100)	94 (75-119)	105 (83-131)	184 (148-232)	185 (148-232)	292 (235-361)	443 (349-572)	486 (380-619)	277 (214-359)	115 (87-148)
Neglected tropical diseases and malaria	8,917 (6,921-11,153)	8,485 (6,389-10,390)	7,821 (6,129-9,584)	8,211 (6,354-10,097)	8,479 (6,739-10,633)	8,693 (6,791-11,019)	7,279 (5,734-9,269)	5,653 (4,406-7,245)	3,053 (2,404-3,808)	2,182 (1,689-2,803)	462 (371-566)	164 (126-211)
Malaria	5,379 (3,832-7,490)	4,612 (3,253-6,425)	4,324 (3,058-6,041)	4,319 (3,080-6,087)	5,106 (3,549-7,281)	5,331 (3,590-7,908)	4,385 (2,951-6,495)	3,374 (2,226-5,051)	1,624 (1,142-2,340)	1,381 (930-2,041)	224 (156-315)	101 (67-147)
Chagas disease	152 (134-174)	226 (203-253)	319 (285-356)	404 (350-471)	550 (495-607)	563 (512-620)	619 (569-672)	390 (341-443)	304 (273-338)	187 (169-209)	71 (64-79)	15 (13-17)
Leishmaniasis	833 (449-1,351)	790 (440-1,286)	786 (448-1,293)	627 (348-1,057)	535 (300-896)	598 (339-982)	619 (348-1,026)	501 (284-842)	287 (159-477)	182 (100-297)	36 (17-60)	13 (5-23)
Visceral leishmaniasis	833 (449-1,351)	790 (440-1,286)	786 (448-1,293)	627 (348-1,057)	535 (300-896)	598 (339-982)	619 (348-1,026)	501 (284-842)	287 (159-477)	182 (100-297)	36 (17-60)	13 (5-23)
African trypanosomiasis	195 (34-658)	170 (29-665)	137 (24-527)	113 (21-461)	98 (16-396)	72 (12-282)	47 (8-215)	28 (5-103)	13 (2-53)	5 (1-20)	1 (0-5)	0 (0-1)
Schistosomiasis	449 (331-605)	478 (353-640)	541 (398-728)	632 (481-842)	636 (473-838)	618 (460-838)	546 (414-718)	406 (306-535)	215 (157-281)	90 (67-121)	25 (16-35)	4 (2-6)
Cysticercosis	41 (34-50)	37 (31-45)	43 (36-51)	46 (39-55)	28 (24-34)	25 (20-30)	15 (12-18)	12 (9-16)	6 (4-9)	2 (2-3)	2 (1-4)	0 (0-0)
Cystic echinococcosis	39 (30-51)	43 (32-55)	42 (33-53)	47 (37-60)	47 (38-59)	40 (31-50)	38 (30-47)	30 (23-37)	18 (14-22)	10 (8-12)	4 (2-7)	1 (0-1)
Dengue	797 (121-1,232)	886 (142-1,359)	811 (139-1,238)	997 (130-1,582)	736 (119-1,185)	827 (128-1,385)	416 (79-804)	331 (69-576)	298 (55-571)	182 (45-326)	58 (17-107)	15 (7-22)
Yellow fever	173 (34-532)	134 (25-397)	105 (20-337)	81 (17-240)	61 (12-202)	42 (8-134)	27 (5-82)	16 (3-50)	7 (1-21)	2 (0-7)	1 (0-2)	0 (0-0)
Rabies	566 (231-689)	796 (292-1,061)	411 (176-483)	594 (177-781)	373 (124-468)	272 (98-349)	238 (99-317)	315 (174-489)	118 (75-209)	51 (32-97)	8 (4-11)	3 (2-5)
Intestinal nematode infections	14 (10-18)	15 (11-20)	21 (16-27)	17 (13-23)	18 (14-23)	13 (9-17)	19 (14-24)	14 (11-19)	9 (7-12)	3 (2-4)	2 (1-3)	1 (0-1)
Ascariasis	14 (10-18)	15 (11-20)	21 (16-27)	17 (13-23)	18 (14-23)	13 (9-17)	19 (14-24)	14 (11-19)	9 (7-12)	3 (2-4)	2 (1-3)	1 (0-1)
Ebola	0 (0-0)	0 (0-0)	0 (0-0)	0 (0-0)	0 (0-0)	0 (0-0)	0 (0-0)	0 (0-0)	0 (0-0)	0 (0-0)	0 (0-0)	0 (0-0)
Zika virus	0 (0-0)	0 (0-0)	0 (0-0)	0 (0-0)	0 (0-0)	0 (0-0)	0 (0-0)	0 (0-0)	0 (0-0)	0 (0-0)	0 (0-0)	0 (0-0)
Other neglected tropical diseases	281 (198-375)	299 (190-391)	281 (206-364)	333 (250-427)	292 (221-380)	293 (218-381)	312 (231-412)	237 (171-313)	154 (111-209)	86 (62-115)	31 (24-42)	13 (10-22)
Maternal disorders	-	-	-	-	-	-	-	-	-	-	-	-
Maternal haemorrhage	-	-	-	-	-	-	-	-	-	-	-	-
Maternal sepsis and other pregnancy related infections	-	-	-	-	-	-	-	-	-	-	-	-
Maternal hypertensive disorders	-	-	-	-	-	-	-	-	-	-	-	-
Maternal obstructed labour and uterine rupture	-	-	-	-	-	-	-	-	-	-	-	-
Maternal abortion, miscarriage, and ectopic pregnancy	-	-	-	-	-	-	-	-	-	-	-	-
Indirect maternal deaths	-	-	-	-	-	-	-	-	-	-	-	-
Late maternal deaths	-	-	-	-	-	-	-	-	-	-	-	-
Maternal deaths aggravated by HIV/AIDS	-	-	-	-	-	-	-	-	-	-	-	-
Other maternal disorders	-	-	-	-	-	-	-	-	-	-	-	-
Neonatal disorders	-	-	-	-	-	-	-	-	-	-	-	-
Neonatal preterm birth complications	-	-	-	-	-	-	-	-	-	-	-	-
Neonatal encephalopathy due to birth asphyxia and trauma	-	-	-	-	-	-	-	-	-	-	-	-
Neonatal sepsis and other neonatal infections	-	-	-	-	-	-	-	-	-	-	-	-
Hemolytic disease and other neonatal jaundice	-	-	-	-	-	-	-	-	-	-	-	-
Other neonatal disorders	-	-	-	-	-	-	-	-	-	-	-	-
Nutritional deficiencies	2,648 (2,239-3,806)	2,925 (2,464-4,286)	3,547 (2,953-5,215)	4,437 (3,802-6,305)	5,105 (4,315-7,367)	6,679 (5,755-9,487)	8,402 (7,302-11,328)	9,375 (8,266-12,327)	9,947 (8,887-12,552)	8,612 (7,860-10,143)	5,176 (4,785-5,843)	2,032 (1,887-2,250)
Protein-energy malnutrition	1,866 (1,549-2,620)	1,841 (1,528-2,627)	2,405 (1,967-3,544)	3,019 (2,507-4,226)	3,620 (2,996-5,243)	4,725 (3,982-6,727)	6,262 (5,364-8,414)	7,206 (6,285-9,405)	7,996 (7,077-9,774)	7,027 (6,249-8,036)	4,349 (3,901-4,797)	1,733 (1,528-1,900)
Iodine deficiency	34 (9-105)	30 (8-85)	40 (12-111)	54 (17-153)	48 (15-137)	59 (20-168)	71 (24-171)	60 (20-140)	40 (13-100)	18 (5-50)	5 (1-17)	2 (0-9)
Iron-deficiency anaemia	58 (46-100)	58 (47-105)	48 (37-90)	61 (46-152)	50 (36-121)	67 (45-176)	89 (54-176)	80 (54-191)	75 (50-189)	48 (26-109)	29 (15-54)	16 (7-24)
Other nutritional deficiencies	690 (470-1,207)	996 (665-1,726)	1,054 (730-1,855)	1,303 (948-2,083)	1,387 (1,052-2,204)	1,827 (1,346-2,828)	1,980 (1,469-3,056)	2,030 (1,438-3,171)	1,837 (1,378-2,845)	1,518 (1,119-2,319)	793 (583-1,162)	281 (202-453)
Other communicable, maternal, neonatal, and nutritional diseases	7,403 (6,769-8,085)	8,219 (7,503-9,005)	7,870 (7,254-8,503)	8,081 (7,524-8,665)	7,285 (6,784-7,794)	6,878 (6,390-7,367)	6,517 (5,912-7,016)	5,668 (5,220-6,130)	4,384 (3,895-4,815)	2,362 (2,092-2,576)	831 (715-914)	233 (195-258)
Sexually transmitted diseases excluding HIV	91 (73-132)	84 (68-118)	140 (100-194)	205 (149-288)	67 (55-107)	75 (61-115)	83 (68-151)	81 (67-138)	56 (47-87)	39 (31-53)	20 (14-25)	11 (7-13)
Syphilis	83 (66-120)	76 (62-107)	131 (93-181)	190 (138-266)	63 (51-99)	71 (57-108)	77 (62-140)	75 (62-126)	51 (43-79)	35 (28-47)	17 (13-22)	8 (5-10)
Chlamydial infection	0 (0-0)	0 (0-0)	0 (0-0)	0 (0-0)	0 (0-0)	0 (0-0)	0 (0-0)	0 (0-0)	0 (0-0)	0 (0-0)	0 (0-0)	0 (0-0)
Gonococcal infection	5 (4-7)	4 (3-5)	4 (3-6)	7 (5-10)	3 (2-4)	2 (2-4)	5 (4-8)	4 (4-7)	3 (3-5)	2 (2-3)	2 (1-2)	1 (1-1)
Other sexually transmitted diseases	3 (3-5)	5 (4-6)	5 (4-7)	8 (6-11)	2 (2-3)	2 (2-3)	2 (2-4)	2 (2-4)	2 (2-3)	2 (2-3)	1 (1-1)	1 (1-2)
Hepatitis	6,165 (5,572-6,793)	6,676 (6,022-7,417)	6,196 (5,680-6,788)	6,198 (5,689-6,712)	5,100 (4,719-5,516)	4,655 (4,277-5,088)	4,163 (3,757-4,562)	3,533 (3,186-3,897)	2,482 (2,171-2,793)	1,138 (994-1,281)	308 (273-344)	64 (57-70)
Acute hepatitis A	52 (23-94)	64 (27-114)	67 (31-119)	71 (35-129)	62 (31-111)	56 (27-98)	55 (26-97)	48 (24-82)	34 (17-59)	15 (8-24)	4 (2-6)	1 (0-1)
Hepatitis B	5,615 (4,934-6,284)	6,058 (5,349-6,822)	5,602 (4,963-6,234)	5,578 (4,983-6,184)	4,533 (3,999-5,026)	4,073 (3,567-4,576)	3,556 (3,055-4,001)	2,931 (2,392-3,424)	1,828 (1,419-2,233)	687 (443-905)	150 (88-217)	25 (12-39)
Hepatitis C	63 (29-114)	92 (40-162)	116 (51-208)	151 (68-276)	151 (72-275)	127 (59-211)	107 (50-200)	92 (45-154)	64 (31-109)	31 (17-49)	9 (5-15)	2 (1-4)
Acute hepatitis E	436 (191-826)	463 (195-850)	411 (187-740)	397 (170-773)	355 (139-688)	399 (178-715)	446 (191-805)	462 (180-914)	556 (275-929)	405 (219-629)	146 (83-213)	36 (22-50)
Other infectious diseases	1,147 (931-1,262)	1,459 (1,170-1,610)	1,533 (1,250-1,691)	1,677 (1,368-1,837)	2,118 (1,758-2,329)	2,148 (1,819-2,359)	2,270 (1,806-2,567)	2,054 (1,743-2,280)	1,846 (1,455-2,106)	1,185 (936-1,337)	503 (396-576)	158 (124-181)
Non-communicable diseases	564,213 (550,673-579,076)	815,694 (797,769-833,860)	1,153,426 (1,131,169-1,176,111)	1,558,934 (1,529,783-1,586,113)	1,816,897 (1,783,965-1,846,956)	2,019,676 (1,980,306-2,051,774)	2,494,908 (2,441,505-2,536,722)	2,516,873 (2,465,261-2,557,738)	2,280,057 (2,231,409-2,314,956)	1,479,641 (1,447,253-1,505,151)	629,309 (616,136-640,043)	190,325 (186,374-194,026)
Neoplasms	137,209 (133,303-140,740)	215,510 (209,874-220,557)	330,631 (322,978-337,349)	484,091 (474,017-493,421)	564,004 (551,681-574,471)	594,652 (579,840-605,317)	657,357 (643,280-669,218)	584,416 (570,208-596,290)	435,446 (423,319-446,024)	237,011 (229,442-243,851)	82,426 (79,459-85,191)	21,335 (20,340-22,104)
Lip and oral cavity cancer	5,169 (4,793-5,592)	8,034 (7,602-8,486)	11,633 (11,112-12,260)	14,337 (13,765-14,932)	13,668 (13,107-14,269)	11,942 (11,394-12,445)	10,845 (10,366-11,282)	8,116 (7,751-8,454)	5,768 (5,461-6,045)	3,142 (2,970-3,291)	1,140 (1,077-1,196)	306 (289-321)
Nasopharynx cancer	3,020 (2,844-3,223)	4,213 (3,959-4,462)	5,398 (5,093-5,727)	6,435 (6,126-6,767)	5,631 (5,343-5,941)	4,786 (4,544-5,068)	4,053 (3,861-4,255)	2,684 (2,555-2,821)	1,736 (1,653-1,825)	754 (717-792)	249 (238-260)	72 (69-75)
Other pharynx cancer	3,279 (3,029-3,561)	5,847 (5,468-6,236)	9,193 (8,655-9,716)	11,359 (10,805-11,957)	11,026 (10,435-11,683)	9,389 (8,795-9,974)	8,105 (7,598-8,597)	5,389 (5,029-5,693)	3,653 (3,366-3,915)	1,591 (1,442-1,709)	444 (406-476)	83 (76-88)
Oesophageal cancer	6,499 (6,200-6,781)	13,029 (12,557-13,523)	22,697 (21,952-23,476)	36,475 (35,219-37,737)	41,631 (40,167-43,049)	42,393 (40,858-44,059)	42,448 (40,879-44,006)	34,265 (32,898-35,597)	22,533 (21,658-23,404)	9,972 (9,608-10,334)	2,814 (2,717-2,914)	588 (563-613)
Stomach cancer	14,186 (13,681-14,737)	23,480 (22,694-24,335)	36,348 (35,283-37,423)	56,188 (54,305-58,035)	66,794 (64,832-68,907)	71,047 (69,071-73,230)	81,219 (78,894-83,598)	70,340 (68,330-72,489)	49,293 (47,709-50,730)	23,869 (23,111-24,670)	7,868 (7,590-8,125)	2,097 (2,013-2,170)
Colon and rectum cancer	9,369 (9,051-9,725)	15,276 (14,820-15,768)	22,898 (22,305-23,456)	34,824 (33,935-35,770)	44,246 (43,080-45,365)	49,089 (47,863-50,380)	58,654 (56,996-60,365)	56,708 (55,093-58,405)	46,449 (45,018-48,019)	27,220 (26,272-28,108)	9,858 (9,518-10,194)	2,709 (2,611-2,806)
Liver cancer	29,401 (27,865-31,086)	40,052 (38,206-42,115)	52,606 (50,419-55,066)	69,150 (66,321-72,066)	67,071 (64,516-69,568)	62,037 (59,843-64,297)	60,178 (58,315-62,452)	47,696 (45,947-49,300)	30,017 (28,945-31,136)	12,985 (12,490-13,507)	3,548 (3,410-3,710)	742 (708-776)
Liver cancer due to hepatitis B	16,967 (13,784-19,338)	21,990 (17,641-25,023)	26,936 (21,557-31,092)	33,696 (26,836-38,944)	29,935 (22,559-34,919)	25,862 (20,050-30,013)	23,217 (17,624-27,415)	17,031 (13,283-20,238)	9,796 (7,230-12,023)	3,925 (3,081-4,760)	1,036 (799-1,278)	206 (138-265)
Liver cancer due to hepatitis C	1,792 (1,431-2,287)	3,480 (2,744-4,349)	6,349 (5,111-7,698)	9,922 (8,284-12,011)	11,868 (9,979-14,117)	11,674 (9,964-13,698)	12,536 (10,899-14,569)	11,523 (9,977-13,427)	8,393 (7,271-9,701)	4,135 (3,676-4,661)	1,237 (1,089-1,392)	288 (245-331)
Liver cancer due to alcohol use	1,757 (1,304-2,344)	2,870 (2,080-3,883)	4,410 (3,152-5,836)	5,691 (4,095-7,732)	6,565 (4,814-8,741)	7,372 (5,821-9,126)	7,935 (6,300-9,765)	5,829 (4,674-7,126)	3,519 (2,733-4,362)	1,500 (1,172-1,865)	352 (265-444)	65 (42-90)
Liver cancer due to other causes	8,884 (7,214-11,345)	11,713 (9,498-14,798)	14,910 (12,144-18,728)	19,840 (16,175-24,905)	18,704 (15,424-23,521)	17,128 (14,209-20,795)	16,490 (13,875-20,023)	13,313 (11,286-16,033)	8,309 (6,936-10,049)	3,425 (2,937-3,973)	923 (767-1,098)	183 (141-230)
Gallbladder and biliary tract cancer	1,372 (1,014-1,625)	2,184 (1,643-2,523)	3,679 (2,794-4,202)	5,381 (4,184-6,221)	6,701 (5,347-7,724)	7,494 (6,089-8,760)	8,967 (7,468-10,274)	8,838 (7,436-9,897)	7,121 (6,093-7,835)	4,061 (3,468-4,421)	1,456 (1,233-1,595)	401 (345-439)
Pancreatic cancer	4,156 (4,014-4,307)	7,618 (7,388-7,864)	12,763 (12,418-13,113)	19,649 (19,125-20,174)	24,218 (23,655-24,827)	25,955 (25,342-26,553)	28,437 (27,677-29,224)	24,252 (23,584-24,935)	17,608 (17,015-18,109)	9,363 (9,040-9,674)	3,074 (2,976-3,171)	738 (710-764)
Larynx cancer	2,478 (2,331-2,639)	5,102 (4,844-5,347)	9,079 (8,713-9,446)	12,397 (11,937-12,864)	13,325 (12,824-13,816)	12,578 (12,088-13,062)	11,531 (11,115-11,961)	8,428 (8,137-8,715)	5,789 (5,562-6,016)	2,687 (2,573-2,800)	794 (763-827)	170 (164-177)
Tracheal, bronchus, and lung cancer	20,223 (19,465-21,036)	40,685 (39,470-42,002)	74,487 (72,238-76,858)	120,407 (117,113-123,858)	149,709 (145,906-153,589)	161,584 (157,826-165,651)	177,623 (173,385-181,938)	151,434 (147,420-155,210)	102,744 (99,367-106,167)	47,128 (45,520-48,854)	12,978 (12,525-13,478)	2,643 (2,539-2,757)
Malignant skin melanoma	1,471 (1,304-1,704)	1,831 (1,647-2,091)	2,517 (2,318-2,949)	3,142 (2,899-3,498)	3,174 (2,886-3,434)	3,166 (2,797-3,426)	3,409 (2,949-3,615)	3,109 (2,547-3,292)	2,686 (2,128-2,835)	1,679 (1,278-1,783)	632 (475-669)	171 (127-183)
Non-melanoma skin cancer	746 (716-777)	1,002 (961-1,040)	1,545 (1,482-1,602)	2,183 (2,097-2,260)	3,348 (3,219-3,473)	3,663 (3,511-3,814)	4,276 (4,125-4,432)	4,135 (3,985-4,287)	3,831 (3,691-3,972)	2,696 (2,591-2,802)	1,312 (1,264-1,363)	524 (504-542)
Non-melanoma skin cancer (squamous-cell carcinoma)	746 (716-777)	1,002 (961-1,040)	1,545 (1,482-1,602)	2,183 (2,097-2,260)	3,348 (3,219-3,473)	3,663 (3,511-3,814)	4,276 (4,125-4,432)	4,135 (3,985-4,287)	3,831 (3,691-3,972)	2,696 (2,591-2,802)	1,312 (1,264-1,363)	524 (504-542)
Breast cancer	277 (191-304)	560 (379-613)	779 (521-843)	1,022 (682-1,106)	1,176 (766-1,270)	1,150 (790-1,237)	1,196 (837-1,285)	752 (578-800)	704 (531-753)	440 (335-472)	172 (136-186)	56 (44-60)
Cervical cancer	-	-	-	-	-	-	-	-	-	-	-	-
Uterine cancer	-	-	-	-	-	-	-	-	-	-	-	-
Ovarian cancer	-	-	-	-	-	-	-	-	-	-	-	-
Prostate cancer	564 (432-623)	1,690 (1,335-1,847)	4,811 (3,790-5,173)	11,490 (9,053-12,423)	23,690 (18,321-25,705)	35,729 (28,454-38,722)	53,134 (43,573-57,891)	62,290 (52,632-69,396)	57,631 (50,450-64,410)	42,916 (37,133-46,996)	19,708 (17,055-21,199)	5,766 (4,863-6,193)
Testicular cancer	680 (642-721)	569 (538-605)	472 (443-503)	362 (343-383)	352 (334-374)	333 (313-352)	379 (358-403)	299 (285-316)	308 (287-332)	165 (156-175)	65 (62-69)	22 (21-23)
Kidney cancer	1,656 (1,568-1,727)	3,284 (3,101-3,425)	5,627 (5,361-5,860)	8,311 (7,992-8,637)	9,246 (8,900-9,580)	9,103 (8,781-9,413)	10,107 (9,686-10,485)	9,553 (9,138-9,885)	7,570 (7,184-7,858)	4,358 (4,113-4,551)	1,444 (1,385-1,502)	356 (339-371)
Bladder cancer	1,354 (1,266-1,452)	2,617 (2,487-2,761)	4,659 (4,460-4,864)	7,631 (7,373-7,922)	11,269 (10,901-11,650)	13,700 (13,267-14,117)	18,680 (18,070-19,327)	19,822 (19,190-20,483)	19,014 (18,313-19,727)	12,714 (12,188-13,198)	4,967 (4,793-5,143)	1,423 (1,363-1,480)
Brain and nervous system cancer	6,924 (5,991-8,057)	8,237 (7,218-9,293)	10,182 (9,001-11,341)	12,574 (11,029-13,958)	12,434 (10,853-13,809)	11,191 (9,590-12,343)	10,152 (8,594-10,971)	7,632 (6,108-8,174)	4,527 (3,485-4,883)	1,907 (1,409-2,065)	490 (376-530)	103 (79-112)
Thyroid cancer	535 (475-572)	894 (799-956)	1,333 (1,200-1,409)	1,829 (1,673-1,920)	1,561 (1,477-1,684)	1,632 (1,542-1,750)	1,682 (1,594-1,824)	1,822 (1,670-1,898)	1,342 (1,266-1,407)	656 (625-690)	218 (208-229)	52 (50-55)
Mesothelioma	427 (381-487)	764 (696-867)	1,201 (1,101-1,323)	1,905 (1,758-2,107)	2,041 (1,882-2,236)	2,444 (2,263-2,598)	2,876 (2,657-3,075)	2,828 (2,635-3,007)	2,241 (2,103-2,380)	1,152 (1,076-1,226)	326 (303-347)	68 (65-73)
Hodgkin lymphoma	1,093 (826-1,442)	1,351 (1,062-1,725)	1,409 (1,185-1,849)	1,750 (1,446-2,244)	1,557 (1,291-1,984)	1,550 (1,284-1,941)	1,507 (1,277-1,904)	872 (735-1,044)	566 (472-667)	274 (231-312)	76 (66-89)	14 (13-17)
Non-Hodgkin's lymphoma	4,939 (4,185-5,304)	6,366 (5,336-6,812)	8,375 (7,086-8,907)	10,777 (9,155-11,600)	11,807 (10,612-12,913)	12,495 (10,924-13,611)	13,462 (11,856-14,586)	12,217 (10,744-13,385)	9,814 (8,152-10,453)	5,739 (4,698-6,060)	1,887 (1,592-2,019)	437 (374-475)
Multiple myeloma	1,163 (980-1,382)	1,645 (1,378-1,931)	2,798 (2,365-3,293)	4,216 (3,571-5,087)	5,063 (4,136-5,851)	5,546 (4,580-6,629)	6,347 (5,097-7,304)	5,914 (4,564-6,725)	4,797 (3,694-5,639)	2,897 (2,259-3,346)	904 (721-1,109)	195 (146-231)
Leukaemia	6,768 (5,994-7,381)	7,691 (6,870-8,345)	9,233 (8,262-10,185)	11,896 (10,421-12,991)	13,429 (12,012-14,194)	14,553 (13,129-15,361)	16,713 (15,343-17,872)	15,825 (14,783-16,526)	12,681 (12,070-13,230)	7,591 (7,233-7,945)	2,691 (2,590-2,821)	721 (689-762)
Acute lymphoid leukaemia	1,248 (1,110-1,427)	1,349 (1,177-1,494)	1,535 (1,339-1,703)	1,823 (1,576-2,032)	1,736 (1,477-1,892)	1,643 (1,395-1,813)	1,719 (1,497-1,940)	974 (862-1,087)	628 (573-699)	325 (294-364)	103 (94-113)	27 (24-29)
Chronic lymphoid leukaemia	237 (209-295)	388 (346-473)	701 (631-878)	1,193 (1,062-1,548)	1,737 (1,546-2,145)	2,140 (1,920-2,682)	2,859 (2,535-3,601)	2,771 (2,473-3,452)	2,694 (2,355-3,388)	1,846 (1,554-2,205)	714 (621-835)	206 (181-237)
Acute myeloid leukaemia	1,688 (1,461-1,949)	2,005 (1,725-2,307)	2,650 (2,353-3,004)	3,344 (2,858-3,795)	3,871 (3,272-4,293)	4,408 (3,736-4,867)	4,915 (4,043-5,434)	4,481 (3,611-4,874)	3,335 (2,699-3,689)	1,773 (1,404-1,986)	536 (457-637)	117 (104-149)
Chronic myeloid leukaemia	624 (540-729)	814 (694-923)	868 (746-993)	1,001 (861-1,146)	955 (832-1,067)	1,034 (918-1,143)	1,108 (1,001-1,231)	1,356 (1,234-1,499)	1,130 (1,039-1,257)	699 (645-776)	255 (235-278)	71 (64-80)
Other leukaemia	2,972 (2,451-3,253)	3,135 (2,609-3,456)	3,480 (2,878-3,805)	4,535 (3,682-4,873)	5,129 (4,326-5,543)	5,329 (4,515-5,714)	6,112 (5,230-6,636)	6,243 (5,657-6,767)	4,895 (4,516-5,356)	2,950 (2,731-3,228)	1,082 (992-1,159)	301 (277-326)
Other neoplasms	9,460 (7,759-10,025)	11,487 (9,638-12,130)	14,909 (12,476-15,704)	18,399 (15,483-19,359)	19,835 (16,870-20,817)	20,103 (17,461-21,093)	21,377 (18,813-22,327)	19,197 (17,127-19,977)	15,023 (13,330-15,683)	9,055 (8,307-9,506)	3,312 (3,100-3,465)	878 (828-920)
Cardiovascular diseases	227,806 (221,044-234,768)	343,034 (333,592-352,170)	498,325 (486,736-511,084)	669,659 (654,428-684,559)	783,665 (767,167-799,762)	886,056 (867,923-903,784)	1,150,728 (1,122,969-1,175,088)	1,175,958 (1,149,588-1,201,697)	1,107,793 (1,080,718-1,131,751)	719,911 (700,228-738,322)	310,037 (301,532-318,268)	99,192 (96,130-101,835)
Rheumatic heart disease	6,745 (5,838-7,540)	7,408 (6,485-8,214)	8,873 (7,905-9,744)	10,502 (9,510-11,460)	10,817 (9,753-11,784)	12,132 (11,005-13,256)	13,948 (12,712-15,008)	12,737 (11,848-13,572)	11,538 (10,787-12,256)	7,686 (7,250-8,102)	3,469 (3,312-3,639)	1,149 (1,105-1,198)
Ischaemic heart disease	127,008 (122,462-131,783)	198,149 (191,597-204,839)	288,235 (279,281-296,812)	379,863 (368,997-390,156)	432,606 (421,138-443,473)	470,966 (460,170-483,717)	607,153 (590,775-624,174)	615,670 (600,129-633,012)	593,946 (579,162-610,958)	398,619 (388,730-410,710)	180,215 (176,295-185,929)	59,783 (58,353-61,909)
Cerebrovascular disease	66,484 (64,282-69,045)	100,577 (97,143-104,219)	150,878 (145,975-156,151)	214,971 (208,122-221,521)	266,655 (259,027-275,576)	319,733 (310,084-329,451)	419,302 (403,279-434,135)	420,814 (402,250-439,228)	367,040 (345,021-386,772)	211,802 (197,380-226,906)	80,507 (74,298-87,348)	23,852 (21,499-25,992)

SR Table 4: Global deaths by cause and age group for males (with 95% uncertainty intervals), 2010

Cause	Early Neonatal	Late Neonatal	Post Neonatal	1 to 4	5 to 9	10 to 14	15 to 19	20 to 24	25 to 29	30 to 34	35 to 39
Ischaemic stroke	-	-	116 (101-139)	135 (115-162)	109 (96-123)	136 (122-153)	574 (520-634)	1,390 (1,259-1,525)	1,956 (1,760-2,168)	2,985 (2,699-3,272)	5,131 (4,657-5,603)
Hemorrhagic stroke	1,094 (958-1,267)	535 (466-626)	3,669 (3,168-4,329)	2,444 (2,056-2,960)	1,665 (1,466-1,884)	1,993 (1,805-2,165)	3,975 (3,726-4,258)	8,531 (8,070-9,140)	11,482 (10,877-12,199)	18,522 (17,670-19,509)	32,821 (31,493-34,353)
Hypertensive heart disease	-	-	193 (149-257)	74 (54-93)	73 (58-91)	108 (74-144)	717 (572-920)	1,316 (1,037-1,659)	1,846 (1,425-2,206)	2,839 (2,129-3,424)	4,651 (3,574-5,408)
Cardiomyopathy and myocarditis	485 (320-597)	260 (198-299)	2,056 (1,515-2,425)	1,313 (986-1,581)	669 (529-785)	801 (661-916)	2,004 (1,628-2,287)	2,834 (2,216-3,145)	4,511 (3,455-5,110)	5,659 (4,354-6,483)	7,783 (6,195-8,908)
Myocarditis	93 (49-142)	71 (48-93)	752 (510-936)	530 (350-701)	252 (183-309)	295 (216-341)	772 (552-974)	817 (617-1,021)	1,041 (756-1,335)	746 (566-925)	949 (705-1,180)
Alcoholic cardiomyopathy	-	-	-	-	-	-	126 (98-189)	425 (300-531)	1,240 (826-1,538)	2,486 (1,695-3,098)	3,810 (2,929-4,659)
Other cardiomyopathy	392 (265-485)	188 (144-222)	1,304 (957-1,592)	783 (574-1,012)	417 (319-504)	506 (410-592)	1,105 (869-1,287)	1,592 (1,227-1,813)	2,229 (1,629-2,573)	2,427 (1,842-2,802)	3,023 (2,276-3,461)
Atrial fibrillation and flutter	-	-	-	-	-	-	-	-	-	17 (12-23)	72 (53-95)
Aortic aneurysm	-	-	-	-	-	-	248 (225-274)	367 (341-401)	605 (559-661)	830 (773-898)	1,425 (1,329-1,547)
Peripheral vascular disease	-	-	-	-	-	-	-	-	-	-	-
Endocarditis	72 (45-96)	41 (27-55)	921 (682-1,203)	370 (272-479)	166 (123-209)	279 (215-358)	588 (444-794)	916 (694-1,251)	1,275 (1,006-1,722)	1,338 (1,043-1,789)	1,571 (1,261-2,146)
Other cardiovascular and circulatory diseases	741 (540-876)	385 (302-478)	2,887 (2,321-3,708)	1,796 (1,332-2,508)	894 (717-1,241)	957 (785-1,332)	1,575 (1,311-2,261)	2,336 (1,993-3,440)	3,093 (2,558-4,488)	3,470 (2,996-4,877)	4,148 (3,402-5,773)
Chronic respiratory diseases	0 (0-0)	0 (0-0)	0 (0-0)	6,619 (5,441-8,402)	2,230 (1,976-2,500)	2,507 (2,266-2,749)	4,499 (4,196-4,841)	6,716 (6,331-7,189)	8,694 (8,241-9,208)	10,784 (10,291-11,370)	16,011 (15,268-16,866)
Chronic obstructive pulmonary disease	-	-	0 (0-0)	1,170 (763-2,056)	379 (295-511)	326 (257-432)	1,798 (1,464-2,118)	2,767 (2,269-3,234)	3,701 (2,979-4,444)	5,293 (4,471-6,148)	8,284 (7,098-9,754)
Pneumoconiosis	-	-	-	-	-	-	19 (16-23)	35 (30-42)	69 (60-81)	127 (111-150)	356 (317-411)
Silicosis	-	-	-	-	-	-	2 (1-2)	6 (5-7)	23 (19-27)	62 (53-72)	223 (195-258)
Asbestosis	-	-	-	-	-	-	8 (7-11)	14 (12-17)	19 (16-24)	23 (17-30)	33 (24-46)
Coal workers pneumoconiosis	-	-	-	-	-	-	1 (0-1)	1 (1-1)	5 (3-6)	6 (3-8)	21 (12-27)
Other pneumoconiosis	-	-	-	-	-	-	8 (7-10)	14 (12-17)	23 (19-31)	36 (29-48)	78 (63-106)
Asthma	-	-	-	4,001 (2,924-5,445)	1,228 (982-1,490)	1,674 (1,410-1,956)	2,079 (1,662-2,454)	2,968 (2,327-3,550)	4,030 (3,095-4,735)	4,111 (3,077-4,977)	5,831 (4,305-7,134)
Interstitial lung disease and pulmonary sarcoidosis	-	-	-	127 (66-232)	55 (35-86)	41 (26-58)	116 (74-169)	189 (123-269)	275 (178-401)	375 (245-530)	574 (381-839)
Other chronic respiratory diseases	0 (0-0)	0 (0-0)	0 (0-0)	1,320 (596-2,256)	567 (372-790)	466 (283-662)	487 (314-660)	757 (506-1,002)	618 (362-857)	878 (524-1,229)	966 (561-1,327)
Cirrhosis and other chronic liver diseases	-	-	-	4,274 (3,292-7,206)	2,044 (1,706-3,037)	2,268 (1,964-3,220)	4,699 (4,279-5,736)	9,580 (8,730-12,156)	17,233 (15,833-19,598)	30,375 (28,426-34,130)	47,974 (45,246-53,581)
Cirrhosis and other chronic liver diseases due to hepatitis B	-	-	-	53 (33-92)	69 (46-106)	128 (90-187)	432 (299-604)	2,516 (1,897-3,433)	6,073 (4,726-7,846)	11,181 (9,495-13,443)	17,740 (14,912-20,918)
Cirrhosis and other chronic liver diseases due to hepatitis C	-	-	-	38 (25-65)	48 (32-77)	101 (68-151)	315 (209-468)	1,667 (1,206-2,259)	4,070 (3,032-5,261)	7,974 (6,687-9,500)	12,805 (10,361-15,759)
Cirrhosis and other chronic liver diseases due to alcohol use	-	-	-	-	-	-	86 (50-137)	758 (540-1,051)	2,148 (1,553-2,920)	6,068 (5,059-7,203)	12,119 (9,841-14,671)
Cirrhosis and other chronic liver diseases due to other causes	-	-	-	4,183 (3,227-7,015)	1,927 (1,606-2,859)	2,039 (1,768-2,907)	3,866 (3,459-4,670)	4,639 (3,744-5,881)	4,941 (3,780-6,341)	5,152 (3,887-6,640)	5,311 (3,800-7,122)
Digestive diseases	949 (765-1,154)	894 (690-1,082)	9,447 (7,213-12,124)	8,155 (6,174-11,097)	4,025 (3,468-4,698)	4,009 (3,513-4,580)	6,242 (5,614-7,169)	8,701 (7,927-9,935)	12,833 (11,696-14,271)	14,258 (13,099-16,444)	19,418 (18,032-22,780)
Peptic ulcer disease	-	-	-	790 (553-1,120)	450 (349-597)	617 (499-798)	1,172 (946-1,491)	2,043 (1,669-2,542)	2,578 (2,154-3,230)	3,422 (2,900-4,196)	4,591 (3,922-5,754)
Gastritis and duodenitis	-	-	-	333 (154-735)	89 (64-129)	80 (63-109)	197 (156-243)	276 (227-341)	442 (362-546)	737 (607-914)	869 (740-1,095)
Appendicitis	-	-	-	1,115 (671-1,564)	869 (692-1,109)	1,005 (814-1,278)	1,315 (1,090-1,634)	1,117 (939-1,366)	1,389 (1,196-1,747)	895 (765-1,113)	1,225 (1,047-1,512)
Paralytic ileus and intestinal obstruction	949 (765-1,154)	894 (690-1,082)	9,447 (7,213-12,124)	2,447 (1,625-3,236)	1,592 (1,188-1,957)	1,266 (947-1,563)	1,880 (1,368-2,351)	2,424 (1,806-3,079)	3,345 (2,499-4,249)	2,858 (2,210-3,696)	4,250 (3,027-5,347)
Inguinal, femoral, and abdominal hernia	-	-	-	605 (256-998)	201 (117-264)	234 (113-324)	311 (194-397)	499 (298-620)	568 (348-683)	488 (288-593)	561 (406-788)
Inflammatory bowel disease	-	-	-	680 (337-1,431)	137 (85-204)	126 (85-177)	170 (122-207)	276 (198-334)	417 (334-515)	467 (351-583)	527 (414-667)
Vascular intestinal disorders	-	-	-	325 (103-644)	52 (29-77)	32 (25-40)	65 (54-81)	131 (105-168)	201 (163-259)	195 (157-256)	382 (309-494)
Gallbladder and biliary diseases	-	-	-	185 (120-285)	83 (66-124)	157 (112-205)	193 (158-275)	313 (267-437)	392 (334-508)	494 (425-657)	750 (652-1,033)
Pancreatitis	-	-	-	96 (64-138)	73 (51-91)	105 (73-128)	378 (276-475)	1,143 (876-1,352)	2,771 (2,150-3,198)	3,719 (3,008-4,302)	5,050 (4,116-5,951)
Other digestive diseases	-	-	-	1,580 (755-2,691)	479 (309-699)	389 (292-544)	560 (449-719)	480 (401-603)	731 (611-934)	983 (820-1,233)	1,213 (1,030-1,521)
Neurological disorders	-	-	3,186 (2,899-3,641)	4,252 (3,586-5,077)	2,643 (2,377-2,944)	4,205 (3,840-4,581)	8,922 (8,274-9,547)	8,869 (8,251-9,451)	7,394 (6,945-7,884)	6,662 (6,313-7,038)	8,239 (7,806-8,702)
Alzheimer's disease and other dementias	-	-	-	-	-	-	-	-	-	-	-
Parkinson's disease	-	-	-	-	-	-	-	1 (1-2)	16 (7-32)	57 (30-105)	133 (71-233)
Epilepsy	-	-	1,837 (1,667-2,024)	3,297 (2,777-3,763)	2,173 (1,938-2,384)	3,264 (2,941-3,599)	6,815 (6,263-7,420)	7,094 (6,574-7,648)	6,262 (5,845-6,721)	5,486 (5,159-5,844)	6,512 (6,125-6,942)
Multiple sclerosis	-	-	-	-	-	-	-	55 (50-64)	113 (99-153)	239 (210-325)	389 (342-525)
Motor neuron disease	4 (3-4)	5 (5-6)	370 (303-428)	133 (98-157)	20 (18-22)	45 (42-49)	85 (80-91)	90 (84-96)	73 (68-78)	159 (150-169)	359 (337-379)
Other neurological disorders	-	-	979 (861-1,247)	823 (652-1,273)	450 (384-587)	895 (830-974)	2,021 (1,788-2,191)	1,628 (1,458-1,740)	930 (843-986)	721 (663-766)	846 (789-889)
Mental and substance use disorders	8 (4-14)	8 (6-12)	0 (0-0)	0 (0-0)	7 (5-8)	5 (4-6)	3,911 (3,371-4,314)	10,515 (9,281-11,689)	15,737 (13,933-17,096)	20,130 (17,647-21,643)	23,135 (20,212-24,873)
Alcohol use disorders	-	-	-	-	-	-	928 (677-1,032)	2,685 (2,058-3,055)	5,428 (4,345-6,051)	9,336 (7,494-10,441)	12,790 (10,229-14,117)
Drug use disorders	8 (4-14)	8 (6-12)	0 (0-0)	0 (0-0)	0 (0-0)	0 (0-0)	2,973 (2,556-3,474)	7,815 (6,935-8,905)	10,291 (8,930-11,407)	10,773 (9,463-11,759)	10,324 (9,102-11,368)
Opioid use disorders	8 (4-14)	8 (6-12)	0 (0-0)	0 (0-0)	0 (0-0)	0 (0-0)	2,206 (1,836-2,610)	5,604 (4,421-6,268)	7,257 (5,712-8,132)	6,979 (5,526-7,803)	6,653 (5,289-7,407)
Cocaine use disorders	0 (0-0)	0 (0-0)	0 (0-0)	0 (0-0)	0 (0-0)	0 (0-0)	113 (81-159)	314 (221-439)	447 (316-610)	779 (527-1,018)	740 (532-994)
Amphetamine use disorders	0 (0-0)	0 (0-0)	0 (0-0)	0 (0-0)	0 (0-0)	0 (0-0)	88 (63-130)	239 (173-335)	334 (248-469)	468 (342-612)	461 (335-615)
Other drug use disorders	0 (0-0)	0 (0-0)	0 (0-0)	0 (0-0)	0 (0-0)	0 (0-0)	566 (478-794)	1,657 (1,395-2,400)	2,254 (1,904-2,993)	2,546 (2,093-3,232)	2,470 (2,080-3,139)
Eating disorders	-	-	-	-	7 (5-8)	5 (4-6)	10 (8-12)	15 (13-18)	18 (15-22)	21 (18-26)	22 (18-26)
Anorexia nervosa	-	-	-	-	6 (5-7)	5 (4-5)	9 (8-11)	15 (13-17)	17 (14-21)	19 (16-23)	20 (17-25)
Bulimia nervosa	-	-	-	-	0 (0-1)	0 (0-1)	1 (0-1)	1 (1-1)	1 (1-1)	3 (2-4)	1 (1-2)
Diabetes, urogenital, blood, and endocrine diseases	2,203 (1,591-2,550)	1,543 (1,223-1,746)	14,840 (12,895-16,727)	14,090 (11,734-17,619)	6,904 (6,110-8,303)	7,658 (6,651-8,996)	12,459 (11,418-14,089)	18,961 (17,675-21,384)	19,025 (17,826-20,468)	23,603 (22,271-25,027)	30,910 (29,306-32,688)
Diabetes mellitus	34 (32-37)	47 (41-53)	429 (388-479)	366 (323-416)	490 (431-555)	644 (585-703)	1,331 (1,233-1,445)	2,620 (2,470-2,810)	3,609 (3,451-3,840)	5,792 (5,524-6,108)	8,630 (8,284-9,006)
Acute glomerulonephritis	-	-	50 (40-65)	73 (63-87)	91 (80-106)	100 (88-117)	128 (114-143)	173 (154-192)	151 (137-167)	183 (164-203)	265 (238-298)
Chronic kidney disease	-	-	2,679 (2,246-3,054)	2,861 (2,412-3,263)	2,127 (1,745-2,425)	2,371 (2,033-2,673)	5,687 (5,143-6,292)	9,414 (8,322-10,393)	10,020 (9,093-11,023)	11,898 (10,658-13,255)	15,685 (14,580-17,647)
Chronic kidney disease due to diabetes mellitus	-	-	73 (49-107)	88 (55-132)	80 (46-129)	119 (72-189)	426 (255-658)	1,342 (852-1,867)	2,182 (1,574-2,905)	3,663 (2,736-4,742)	6,249 (4,840-7,814)
Chronic kidney disease due to hypertension	-	-	18 (12-26)	28 (19-40)	36 (23-55)	66 (41-99)	251 (161-359)	632 (430-879)	867 (609-1,178)	1,250 (903-1,676)	1,910 (1,449-2,500)
Chronic kidney disease due to glomerulonephritis	-	-	1,049 (779-1,338)	1,140 (820-1,490)	837 (576-1,125)	981 (684-1,302)	2,387 (1,671-3,150)	3,851 (2,752-5,143)	3,732 (2,631-4,965)	3,566 (2,450-4,820)	3,562 (2,376-4,906)
Chronic kidney disease due to other causes	-	-	1,538 (1,232-1,859)	1,605 (1,234-1,974)	1,174 (884-1,513)	1,206 (879-1,555)	2,623 (1,892-3,353)	3,589 (2,505-4,770)	3,239 (2,223-4,423)	3,420 (2,358-4,746)	3,963 (2,790-5,275)
Urinary diseases and male infertility	188 (168-208)	263 (220-316)	3,095 (2,752-3,477)	1,704 (1,430-2,034)	708 (590-835)	601 (541-659)	1,355 (1,239-1,471)	1,912 (1,768-2,052)	1,911 (1,772-2,058)	2,232 (2,096-2,362)	2,671 (2,512-2,819)
Interstitial nephritis and urinary tract infections	101 (74-123)	154 (117-197)	2,157 (1,666-2,598)	867 (539-1,266)	339 (252-431)	322 (264-380)	723 (573-854)	1,049 (860-1,219)	985 (802-1,158)	1,096 (912-1,267)	1,354 (1,123-1,566)
Urolithiasis	-	-	-	-	89 (60-137)	70 (54-95)	145 (103-207)	161 (119-245)	138 (102-235)	148 (114-251)	202 (157-333)
Other urinary diseases	87 (67-108)	108 (77-144)	938 (630-1,367)	837 (455-1,245)	280 (193-370)	209 (153-261)	488 (357-632)	702 (531-869)	788 (618-952)	988 (789-1,158)	1,115 (875-1,315)
Gynecological diseases	-	-	-	-	-	-	-	-	-	-	-
Uterine fibroids	-	-	-	-	-	-	-	-	-	-	-
Polycystic ovarian syndrome	-	-	-	-	-	-	-	-	-	-	-
Endometriosis	-	-	-	-	-	-	-	-	-	-	-
Genital prolapse	-	-	-	-	-	-	-	-	-	-	-
Other gynecological diseases	-	-	-	-	-	-	-	-	-	-	-
Hemoglobinopathies and hemolytic anaemias	1,277 (771-1,594)	642 (413-776)	4,835 (3,285-6,059)	6,863 (4,797-10,039)	2,424 (1,893-3,512)	2,909 (2,115-3,909)	3,009 (2,272-4,201)	3,700 (2,966-5,563)	1,916 (1,479-2,662)	1,410 (1,079-1,925)	1,315 (1,023-1,780)
Thalassemias	241 (175-317)	175 (124-222)	972 (697-1,264)	1,357 (1,046-1,779)	637 (498-839)	257 (195-348)	155 (116-208)	97 (72-128)	41 (32-52)	23 (19-30)	20 (17-26)
Sickle cell disorders	937 (499-1,232)	438 (259-552)	3,610 (2,280-4,701)	5,106 (3,181-7,989)	1,571 (1,180-2,479)	2,359 (1,663-3,223)	2,389 (1,806-3,373)	2,768 (2,144-4,327)	1,194 (928-1,713)	744 (586-1,060)	606 (479-861)
G6PD deficiency	42 (27-58)	22 (15-31)	210 (139-297)	359 (250-505)	190 (141-252)	246 (177-325)	389 (288-518)	673 (512-920)	554 (403-731)	507 (370-669)	524 (391-690)
Other hemoglobinopathies and hemolytic anaemias	56 (39-75)	7 (5-10)	44 (30-60)	42 (31-58)	26 (20-33)	47 (36-59)	77 (59-100)	161 (126-224)	128 (96-166)	136 (104-175)	165 (127-208)
Endocrine, metabolic, blood, and immune disorders	703 (497-856)	591 (451-730)	3,753 (3,099-4,950)	2,223 (1,836-2,980)	1,064 (924-1,430)	1,033 (912-1,402)	948 (829-1,217)	1,143 (971-1,449)	1,419 (1,137-1,707)	2,088 (1,579-2,389)	2,344 (1,695-2,648)
Musculoskeletal disorders	-	-	-	-	299 (249-381)	404 (344-492)	460 (415-579)	587 (508-706)	576 (499-670)	610 (520-706)	780 (663-889)
Rheumatoid arthritis	-	-	-	-	57 (42-81)	66 (49-89)	54 (41-71)	65 (47-86)	56 (43-75)	58 (44-78)	89 (71-119)
Other musculoskeletal disorders	-	-	-	-	242 (200-317)	337 (290-422)	406 (362-514)	522 (448-628)	520 (450-609)	552 (464-632)	691 (587-787)
Other non-communicable diseases	98,933 (79,724-115,088)	34,878 (28,854-39,951)	115,179 (94,965-132,537)	33,337 (22,335-44,436)	9,209 (7,752-11,000)	6,755 (5,752-8,048)	6,134 (5,415-7,393)	5,444 (4,655-6,274)	4,007 (3,396-4,675)	3,780 (3,067-4,359)	3,768 (3,081-4,236)
Congenital anomalies	98,933 (79,724-115,088)	32,949 (27,429-38,208)	96,413 (77,306-113,440)	32,394 (21,325-43,468)	8,969 (7,514-10,756)	6,460 (5,385-7,761)	5,439 (4,776-6,622)	4,795 (4,126-5,654)	3,468 (2,924-4,235)	2,582 (2,196-3,069)	2,767 (2,312-3,300)
Neural tube defects	13,978 (7,835-24,513)	3,125 (1,662-5,910)	7,622 (3,274-15,106)	2,992 (532-7,554)	376 (182-796)	232 (136-465)	129 (89-247)	103 (72-212)	71 (51-136)	61 (44-113)	45 (34-70)
Congenital heart anomalies	32,256 (21,722-39,992)	15,056 (11,554-18,450)	49,789 (37,409-63,061)	16,068 (11,026-23,952)	5,621 (4,460-7,196)	4,302 (3,583-5,372)	3,689 (3,221-4,471)	3,402 (2,907-4,093)	2,574 (2,172-3,197)	1,812 (1,556-2,180)	2,054 (1,699-2,510)
Orofacial clefts	623 (322-1,151)	192 (97-366)	567 (247-1,203)	57 (17-175)	-	-	-	-	-	-	-
Down's syndrome	1,170 (642-2,441)	535 (339-1,047)	2,330 (1,474-5,016)	1,128 (566-3,558)	204 (149-338)	171 (127-249)	191 (152-285)	183 (144-246)	162 (130-219)	185 (145-244)	178 (139-238)
Other chromosomal abnormalities	2,749 (1,760-4,787)	1,084 (694-1,982)	2,344 (1,454-4,430)	537 (281-1,214)	118 (75-184)	85 (46-113)	108 (61-135)	102 (54-121)	73 (32-89)	54 (20-65)	54 (21-65)
Congenital musculoskeletal and limb anomalies	2,774 (1,703-5,541)	606 (402-1,119)	1,236 (792-2,558)	317 (180-873)	115 (84-189)	91 (69-133)	117 (86-155)	63 (44-80)	46 (36-60)	32 (22-41)	30 (22-38)
Urogenital congenital anomalies	3,199 (2,092-4,401)	815 (572-1,107)	1,535 (995-2,275)	297 (188-460)	132 (95-189)	128 (96-176)	75 (58-109)	79 (60-108)	65 (50-86)	65 (50-85)	71 (50-90)
Digestive congenital anomalies	9,884 (6,843-15,859)	3,817 (2,691-5,664)	8,663 (5,626-14,602)	1,698 (878-4,358)	206 (155-300)	105 (80-145)	58 (44-72)	40 (30-50)	37 (28-44)	28 (22-34)	31 (20-37)
Other congenital anomalies	32,300 (21,467-45,181)	7,720 (5,129-11,014)	22,328 (14,440-33,214)	9,300 (5,005-16,012)	2,196 (1,538-3,028)	1,346 (1,002-1,783)	1,072 (873-1,375)	823 (630-1,014)	441 (341-545)	346 (273-433)	305 (247-379)
Skin and subcutaneous diseases	-	-	1,617 (839-2,321)	943 (462-1,361)	240 (129-341)	294 (155-404)	695 (369-1,040)	649 (319-915)	538 (285-734)	1,198 (611-1,693)	1,001 (511-1,317)
Cellulitis	-	-	76 (29-148)	84 (30-128)	49 (21-70)	65 (32-94)	74 (42-117)	96 (48-144)	79 (34-110)	173 (61-231)	207 (80-275)
Pyoderma	-	-	1,495 (767-2,151)	743 (386-1,089)	139 (76-206)	171 (85-233)	484 (233-728)	439 (203-607)	362 (181-495)	866 (427-1,249)	626 (295-850)
Decubitus ulcer	-	-	-	55 (21-93)	26 (15-41)	29 (16-45)	81 (49-134)	66 (38-102)	63 (38-100)	118 (68-181)	133 (68-202)
Other skin and subcutaneous diseases	-	-	46 (23-78)	61 (24-104)	26 (15-42)	29 (17-47)	56 (33-98)	47 (25-77)	34 (20-54)	41 (24-71)	35 (20-56)
Sudden infant death syndrome	-	1,929 (1,362-2,399)	17,149 (13,219-21,150)	-	-	-	-	-	-	-	-
Injuries	7,539 (6,219-8,485)	4,509 (3,856-5,040)	48,197 (42,367-54,789)	119,774 (104,240-135,452)	86,225 (75,764-97,136)	96,008 (83,076-110,164)	210,316 (194,869-226,319)	325,652 (308,683-341,612)	301,596 (285,241-316,127)	266,862 (253,043-279,145)	262,455 (249,784-272,954)
Transport injuries	1,406 (1,103-1,836)	686 (570-862)	6,569 (5,502-7,937)	23,902 (20,599-27,816)	25,337 (23,253-27,614)	25,389 (23,614-27,237)	80,817 (76,881-86,279)	130,409 (125,382-138,231)	116,965 (112,691-123,165)	98,832 (95,474-103,679)	100,157 (96,988-104,432)
Road injuries	1,211 (940-1,609)	588 (487-749)	5,957 (4,994-7,261)	22,605 (19,383-26,348)	24,060 (22,067-26,261)	23,653 (22,078-25,554)	75,317 (71,566-80,383)	122,916 (118,140-130,529)	108,913 (104,876-115,028)	92,578 (89,520-97,185)	93,290 (90,123-97,391)
Pedestrian road injuries	480 (355-697)	230 (181-302)	2,322 (1,840-2,971)	12,037 (9,967-14,755)	12,935 (11,680-14,488)	11,230 (10,175-12,637)	19,540 (17,458-21,981)	31,706 (28,305-35,071)	28,726 (25,607-32,198)	28,144 (25,204-31,312)	31,870 (28,542-35,072)
Cyclist road injuries	-	-	-	770 (563-1,077)	1,422 (1,186-1,706)	2,004 (1,716-2,387)	3,633 (3,108-4,256)	3,896 (3,346-4,388)	3,679 (3,147-4,197)	3,737 (3,261-4,337)	4,058 (3,550-4,568)
Motorcyclist road injuries	109 (73-172)	74 (54-108)	690 (535-956)	1,480 (1,113-2,153)	1,501 (1,227-1,813)	2,515 (2,125-2,833)	22,209 (19,781-25,042)	37,156 (32,920-41,416)	31,434 (27,767-35,160)	23,729 (20,550-26,865)	22,738 (19,706-25,360)
Motor vehicle road injuries	596 (405-820)	273 (206-366)	2,799 (2,150-3,720)	8,045 (5,841-10,399)	7,896 (6,472-9,536)	7,551 (6,398-9,121)	29,308 (26,031-35,003)	49,154 (44,303-57,763)	44,198 (39,671-51,889)	36,348 (32,342-43,245)	33,950 (30,101-40,047)
Other road injuries	25 (15-43)	11 (8-18)	146 (108-233)	272 (193-413)	306 (251-403)	354 (291-472)	628 (535-799)	1,004 (854-1,266)	876 (736-1,093)	619 (526-744)	674 (566-803)
Other transport injuries	194 (127-276)	98 (70-128)	612 (464-790)	1,297 (803-1,798)	1,277 (1,067-1,551)	1,735 (1,483-2,024)	5,500 (4,860-6,280)	7,493 (6,763-8,252)	8,052 (7,197-8,943)	6,254 (5,701-6,908)	6,867 (6,250-7,614)
Unintentional injuries	5,172 (4,027-5,975)	3,307 (2,685-3,750)	37,291 (31,745-43,408)	80,605 (69,099-92,251)	45,707 (41,148-49,675)	43,809 (39,975-46,583)	49,486 (45,657-51,728)	65,800 (60,350-68,475)	60,343 (55,910-62,814)	60,908 (56,160-63,202)	68,231 (61,278-71,118)
Falls	1,192 (765-1,655)	338 (252-411)	4,604 (3,397-5,775)	7,827 (5,538-9,841)	4,824 (3,998-5,524)	5,626 (4,698-6,354)	6,515 (5,740-7,138)	10,871 (9,279-11,755)	11,265 (9,663-12,268)	13,151 (11,230-14,074)	16,653 (14,070-17,855)
Drowning	352 (263-425)	123 (96-157)	3,907 (3,393-4,384)	38,573 (32,703-43,097)	23,936 (21,354-26,016)	21,940 (19,918-23,362)	20,036 (18,628-21,442)	19,496 (18,209-20,869)	14,205 (13,357-15,251)	12,947 (12,202-13,843)	12,815 (11,973-13,487)
Fire, heat, and hot substances	280 (194-327)	196 (139-236)	2,757 (2,033-3,262)	7,128 (4,944-8,850)	2,566 (1,954-3,007)	1,689 (1,300-1,941)	2,139 (1,645-2,407)	3,759 (2,935-4,208)	3,815 (3,065-4,251)	3,723 (3,067-4,210)	4,058 (3,328-4,536)
Poisonings	232 (102-384)	140 (70-213)	2,496 (1,183-4,462)	3,749 (2,187-5,563)	1,333 (878-1,552)	1,494 (989-1,711)	1,741 (1,287-2,045)	2,851 (2,127-3,355)	2,785 (2,133-3,326)	2,457 (1,868-2,863)	2,571 (1,873-2,879)
Exposure to mechanical forces	1,117 (746-1,305)	948 (610-1,210)	6,658 (4,823-8,065)	6,660 (4,591-8,724)	3,849 (3,342-4,507)	3,509 (3,145-4,073)	6,406 (5,207-6,945)	10,396 (8,173-11,201)	9,923 (7,965-10,695)	9,396 (7,492-10,048)	11,011 (8,175-11,780)
Unintentional firearm injuries	49 (30-67)	36 (22-51)	458 (266-665)	740 (376-1,155)	466 (357-589)	604 (510-734)	1,656 (1,344-1,974)	2,457 (1,778-2,924)	2,558 (1,837-3,013)	1,811 (1,310-2,089)	1,472 (1,068-1,682)
Unintentional suffocation	765 (421-935)	796 (461-1,042)	4,677 (2,921-5,925)	2,245 (1,387-3,621)	1,004 (780-1,523)	1,028 (841-1,481)	373 (281-483)	557 (395-706)	556 (416-707)	396 (278-512)	358 (241-423)
Other exposure to mechanical forces	302 (212-347)	116 (90-134)	1,523 (1,179-1,810)	3,676 (2,439-4,664)	2,378 (1,835-2,736)	1,877 (1,521-2,073)	4,378 (3,369-4,755)	7,382 (5,704-8,002)	6,809 (5,320-7,374)	7,189 (5,447-7,779)	9,181 (6,616-9,928)
Adverse effects of medical treatment	652 (453-790)	412 (304-506)	4,427 (2,866-5,809)	3,979 (1,939-6,260)	1,412 (1,028-1,847)	1,191 (912-1,467)	1,611 (1,191-1,860)	2,574 (1,885-2,971)	2,468 (1,782-2,808)	2,504 (1,761-2,872)	2,728 (1,998-3,091)
Animal contact	233 (202-270)	215 (181-253)	1,373 (1,152-1,653)	3,620 (2,859-4,475)	3,191 (2,660-3,817)	3,495 (2,923-4,117)	2,802 (2,428-3,209)	2,942 (2,564-3,333)	2,980 (2,635-3,361)	2,919 (2,591-3,267)	3,083 (2,719-3,535)
Venomous animal contact	202 (170-240)	183 (151-220)	1,164 (949-1,436)	2,965 (2,304-3,658)	2,724 (2,243-3,314)	3,134 (2,588-3,736)	2,341 (1,979-2,734)	2,458 (2,088-2,847)	2,494 (2,140-2,875)	2,576 (2,240-2,918)	2,714 (2,359-3,124)
Non-venomous animal contact	31 (24-38)	31 (24-40)	209 (146-289)	655 (355-1,176)	468 (337-607)	361 (283-453)	461 (357-569)	484 (379-598)	486 (357-597)	343 (244-443)	369 (271-460)
Foreign body	700 (471-830)	734 (526-870)	9,147 (6,572-12,156)	5,379 (3,440-8,832)	1,915 (1,500-2,435)	1,588 (1,316-1,904)	1,164 (958-1,388)	1,933 (1,593-2,325)	2,003 (1,689-2,433)	2,564 (2,158-3,027)	2,865 (2,405-3,556)
Pulmonary aspiration and foreign body in airway	668 (451-795)	715 (512-849)	8,784 (6,153-11,738)	4,784 (2,988-7,955)	1,576 (1,186-2,075)	1,306 (1,097-1,641)	1,004 (841-1,267)	1,676 (1,415-2,142)	1,730 (1,476-2,216)	2,008 (1,689-2,556)	2,270 (1,871-3,011)
Foreign body in other body part	32 (15-44)	19 (10-26)	363 (199-502)	595 (280-1,154)	339 (212-451)	282 (157-389)	161 (81-237)	257 (136-362)	273 (142-387)	556 (333-742)	595 (360-780)
Environmental heat and cold exposure	201 (83-257)	120 (49-152)	1,109 (492-1,412)	1,064 (487-1,462)	438 (244-546)	408 (224-490)	838 (484-1,000)	1,233 (735-1,500)	1,534 (970-1,907)	2,224 (1,397-2,797)	2,671 (1,754-3,567)
Other unintentional injuries	214 (187-243)	79 (67-96)	814 (673-976)	2,625 (1,959-3,209)	2,244 (1,934-2,541)	2,870 (2,531-3,152)	6,233 (5,596-6,782)	9,747 (8,378-10,543)	9,365 (8,628-10,035)	9,022 (7,795-9,642)	9,776 (8,142-10,468)
Self-harm and interpersonal violence	721 (549-883)	181 (143-228)	1,904 (1,456-2,331)	3,191 (2,534-3,935)	2,633 (2,016-3,220)	10,053 (8,311-10,962)	58,253 (50,773-61,989)	104,496 (92,516-110,923)	103,001 (91,021-110,439)	91,752 (80,643-98,739)	82,403 (72,372-90,499)

Cause	40 to 44	45 to 49	50 to 54	55 to 59	60 to 64	65 to 69	70 to 74	75 to 79	80 to 84	85 to 89	90 to 94	95 plus
Ischaemic stroke	9,632 (8,927-10,289)	20,132 (18,716-21,381)	36,505 (34,163-38,652)	60,190 (56,881-63,509)	107,273 (101,776-111,987)	139,569 (133,171-145,637)	201,308 (192,245-209,785)	231,762 (221,037-242,977)	210,933 (197,683-222,874)	127,072 (118,085-136,511)	50,336 (46,420-54,603)	15,512 (13,994-16,922)
Hemorrhagic stroke	56,851 (54,800-59,227)	80,445 (77,456-83,776)	114,373 (110,051-119,048)	154,781 (149,040-161,010)	159,381 (153,568-166,287)	180,164 (173,000-188,194)	217,994 (208,717-228,041)	189,052 (179,359-198,815)	156,107 (146,604-165,291)	84,729 (78,571-91,071)	30,172 (27,855-32,778)	8,341 (7,507-9,107)
Hypertensive heart disease	7,645 (5,768-8,960)	11,549 (8,715-13,797)	17,054 (12,936-19,809)	23,787 (18,187-27,795)	27,755 (21,768-33,410)	33,159 (25,743-38,817)	44,419 (33,720-52,440)	50,658 (37,616-57,220)	50,431 (37,503-57,701)	34,341 (24,859-38,492)	15,706 (11,090-17,621)	5,031 (3,316-5,695)
Cardiomyopathy and myocarditis	10,037 (8,370-11,464)	12,752 (10,701-14,785)	15,593 (13,401-18,633)	16,441 (14,295-19,768)	14,631 (12,810-17,598)	12,367 (10,646-14,561)	14,260 (11,364-15,931)	14,867 (10,882-16,261)	15,249 (10,059-17,033)	11,434 (7,577-12,789)	5,216 (3,436-5,833)	1,904 (1,201-2,153)
Myocarditis	1,162 (853-1,492)	851 (649-1,071)	843 (619-1,031)	1,073 (779-1,374)	897 (635-1,109)	945 (645-1,128)	1,304 (882-1,511)	1,546 (1,047-1,800)	1,700 (1,087-2,026)	1,548 (1,015-1,854)	934 (588-1,140)	467 (252-605)
Alcoholic cardiomyopathy	5,072 (3,976-6,211)	7,489 (6,007-9,223)	9,132 (7,413-11,507)	8,682 (6,989-10,853)	6,853 (5,492-8,649)	4,292 (3,288-5,365)	4,345 (3,116-5,228)	3,045 (1,876-3,565)	2,385 (1,149-2,964)	969 (506-1,187)	353 (200-423)	139 (72-167)
Other cardiomyopathy	3,803 (2,928-4,399)	4,412 (3,553-5,094)	5,618 (4,633-6,942)	6,686 (5,735-8,500)	6,880 (5,830-8,606)	7,129 (6,077-8,596)	8,611 (7,011-9,557)	10,276 (7,626-11,239)	11,163 (7,499-12,467)	8,917 (5,750-10,090)	3,929 (2,528-4,535)	1,298 (846-1,590)
Atrial fibrillation and flutter	176 (129-228)	339 (245-450)	572 (428-733)	1,024 (610-1,532)	1,901 (1,261-2,639)	3,417 (2,141-5,119)	6,702 (4,815-9,239)	9,837 (6,272-14,568)	18,872 (14,355-24,086)	18,902 (14,538-24,254)	8,174 (6,259-10,554)	2,214 (1,710-2,839)
Aortic aneurysm	1,974 (1,876-2,088)	2,838 (2,713-2,986)	4,005 (3,823-4,199)	5,966 (5,740-6,227)	8,371 (8,020-8,716)	10,026 (9,580-10,428)	12,975 (12,455-13,531)	14,241 (13,699-14,767)	13,625 (13,131-14,164)	9,488 (9,130-9,891)	3,841 (3,717-3,992)	994 (961-1,039)
Peripheral vascular disease	119 (84-146)	244 (171-303)	581 (394-738)	1,064 (689-1,343)	1,698 (1,113-2,180)	2,103 (1,426-2,678)	3,486 (2,528-4,730)	3,767 (2,759-5,151)	4,439 (3,333-6,314)	3,483 (2,660-5,087)	1,780 (1,350-2,657)	703 (546-1,091)
Endocarditis	1,854 (1,511-2,565)	1,918 (1,557-2,598)	2,294 (1,833-3,144)	2,515 (1,986-3,301)	2,854 (2,251-3,859)	3,171 (2,505-4,245)	3,599 (2,849-4,751)	4,012 (3,119-5,253)	3,857 (3,011-4,885)	2,765 (1,956-3,413)	1,251 (879-1,493)	408 (288-504)
Other cardiovascular and circulatory diseases	5,767 (4,898-7,923)	7,260 (6,007-9,648)	10,240 (8,669-13,329)	13,526 (11,607-18,071)	16,376 (13,784-20,947)	18,981 (15,605-24,458)	24,884 (21,006-32,622)	29,355 (25,375-37,239)	28,797 (25,322-36,131)	21,392 (19,646-25,961)	9,877 (9,191-11,803)	3,154 (2,977-3,674)
Chronic respiratory diseases	23,991 (22,909-25,168)	38,363 (36,659-40,068)	66,916 (64,045-69,988)	108,008 (103,679-112,382)	162,607 (155,900-169,156)	219,050 (210,015-227,348)	305,652 (292,690-316,704)	328,500 (315,833-339,362)	303,689 (292,440-314,160)	186,914 (179,044-193,893)	72,333 (69,471-75,029)	20,088 (19,241-20,839)
Chronic obstructive pulmonary disease	14,226 (12,310-16,344)	24,877 (21,744-27,855)	47,312 (41,591-52,389)	81,517 (73,529-88,786)	130,271 (118,848-139,718)	182,195 (168,067-193,938)	262,821 (247,798-275,713)	287,271 (272,635-300,196)	270,819 (258,356-282,554)	168,316 (160,320-175,997)	65,566 (62,831-68,283)	18,344 (17,517-19,142)
Pneumoconiosis	602 (538-678)	710 (633-808)	941 (835-1,097)	1,266 (1,131-1,471)	1,559 (1,380-1,857)	1,944 (1,737-2,324)	2,735 (2,495-3,097)	3,069 (2,854-3,379)	2,675 (2,510-2,888)	1,572 (1,465-1,690)	496 (462-542)	122 (114-134)
Silicosis	399 (349-460)	460 (405-525)	570 (503-648)	756 (665-866)	812 (721-951)	1,009 (877-1,207)	1,352 (1,206-1,549)	1,569 (1,391-1,819)	1,389 (1,229-1,618)	750 (631-885)	189 (149-222)	44 (32-51)
Asbestosis	45 (30-60)	48 (33-67)	72 (47-94)	105 (62-140)	159 (100-216)	243 (154-314)	384 (241-485)	495 (273-609)	498 (264-598)	336 (184-403)	115 (58-141)	25 (13-31)
Coal workers pneumoconiosis	44 (24-54)	61 (35-78)	100 (54-139)	138 (71-185)	204 (110-272)	282 (166-373)	415 (220-510)	425 (241-503)	383 (272-473)	238 (164-304)	78 (44-100)	18 (10-23)
Other pneumoconiosis	115 (95-147)	141 (113-196)	200 (153-288)	267 (198-388)	383 (274-564)	410 (293-619)	584 (437-862)	581 (450-821)	405 (320-573)	248 (197-364)	114 (91-169)	35 (30-51)
Asthma	7,114 (5,048-9,153)	9,903 (6,905-12,708)	14,704 (9,813-20,281)	19,492 (12,954-26,556)	23,626 (15,263-33,591)	25,976 (16,630-38,408)	28,798 (19,506-41,840)	26,206 (17,861-38,592)	19,122 (13,674-29,150)	9,936 (7,208-14,545)	3,464 (2,560-5,240)	869 (652-1,264)
Interstitial lung disease and pulmonary sarcoidosis	913 (602-1,322)	1,454 (956-2,030)	2,440 (1,565-3,366)	3,707 (2,414-5,078)	5,054 (3,260-6,739)	6,785 (4,249-8,760)	8,864 (5,510-11,186)	9,935 (6,035-12,045)	9,077 (5,344-11,019)	5,723 (3,273-6,847)	2,116 (1,234-2,520)	547 (331-637)
Other chronic respiratory diseases	1,137 (671-1,543)	1,420 (848-1,914)	1,519 (873-2,004)	2,027 (1,210-2,689)	2,097 (1,331-2,698)	2,151 (1,273-2,806)	2,434 (1,544-3,136)	2,019 (1,252-2,573)	1,996 (1,270-2,544)	1,367 (917-1,742)	691 (444-915)	206 (137-271)
Cirrhosis and other chronic liver diseases	66,888 (63,518-73,743)	84,399 (80,509-95,022)	96,779 (92,911-107,852)	99,001 (94,767-111,770)	87,871 (83,865-101,810)	70,578 (67,224-79,281)	62,516 (59,376-69,975)	47,808 (45,347-52,183)	32,073 (30,095-34,188)	16,552 (15,371-17,518)	5,513 (5,068-5,878)	1,531 (1,411-1,619)
Cirrhosis and other chronic liver diseases due to hepatitis B	23,536 (20,316-27,222)	26,380 (22,145-31,710)	28,370 (23,711-34,080)	29,950 (25,384-36,176)	27,177 (22,793-33,198)	22,657 (19,034-27,199)	20,521 (17,224-25,188)	15,919 (13,405-19,223)	10,105 (8,347-12,246)	4,677 (3,963-5,513)	1,392 (1,133-1,680)	344 (254-443)
Cirrhosis and other chronic liver diseases due to hepatitis C	18,282 (15,358-21,830)	23,984 (19,756-28,385)	28,008 (23,200-33,471)	28,128 (23,826-33,177)	25,113 (20,809-30,212)	20,341 (17,253-23,929)	17,879 (15,105-21,059)	13,217 (10,861-15,820)	8,869 (7,218-10,660)	4,586 (3,784-5,377)	1,524 (1,199-1,823)	423 (310-539)
Cirrhosis and other chronic liver diseases due to alcohol use	18,462 (15,993-21,414)	25,322 (21,346-29,966)	29,877 (25,454-35,326)	29,189 (24,253-34,547)	25,274 (20,475-30,840)	19,779 (16,687-23,069)	17,203 (14,377-20,405)	13,038 (10,971-15,475)	8,862 (7,402-10,534)	4,826 (4,218-5,467)	1,690 (1,437-1,940)	503 (406-601)
Cirrhosis and other chronic liver diseases due to other causes	6,607 (4,778-8,824)	8,714 (6,315-11,645)	10,525 (7,883-13,990)	11,733 (8,644-15,895)	10,307 (7,434-14,176)	7,801 (5,686-10,624)	6,913 (5,063-9,178)	5,634 (4,133-7,432)	4,238 (3,095-5,578)	2,463 (1,956-3,056)	908 (704-1,164)	262 (186-358)
Digestive diseases	24,663 (22,985-28,484)	29,976 (27,952-33,916)	34,511 (32,150-39,195)	41,425 (38,873-47,495)	44,731 (42,103-51,481)	48,916 (45,621-56,095)	57,841 (53,842-66,789)	57,893 (53,908-66,793)	51,072 (47,843-58,900)	34,407 (31,535-38,871)	14,978 (13,963-16,861)	4,550 (4,200-5,184)
Peptic ulcer disease	6,556 (5,705-8,059)	8,604 (7,514-10,324)	10,878 (9,711-12,897)	13,011 (11,561-15,224)	13,930 (12,441-16,277)	14,586 (12,939-16,776)	17,144 (15,134-19,585)	16,606 (14,962-18,884)	13,508 (12,192-15,179)	8,248 (7,494-9,438)	3,228 (2,928-3,694)	904 (820-1,038)
Gastritis and duodenitis	1,124 (948-1,402)	1,368 (1,176-1,643)	1,614 (1,393-1,914)	2,034 (1,754-2,395)	2,070 (1,822-2,379)	2,082 (1,784-2,427)	2,540 (2,161-2,918)	2,597 (2,250-3,010)	2,155 (1,897-2,475)	1,308 (1,161-1,525)	565 (494-648)	175 (154-203)
Appendicitis	1,528 (1,297-1,885)	1,351 (1,124-1,661)	1,449 (1,224-1,811)	1,583 (1,343-1,960)	1,618 (1,389-1,983)	1,511 (1,276-1,829)	1,731 (1,453-2,165)	1,508 (1,289-1,858)	1,223 (1,020-1,481)	759 (646-907)	307 (267-360)	86 (75-104)
Paralytic ileus and intestinal obstruction	4,685 (3,314-5,921)	5,548 (4,186-7,061)	6,157 (4,569-7,903)	7,848 (6,144-10,392)	8,561 (6,667-11,196)	10,431 (8,361-13,344)	11,091 (8,855-14,552)	10,941 (8,915-14,538)	10,134 (8,081-12,760)	7,352 (5,819-9,333)	3,255 (2,530-3,998)	982 (739-1,229)
Inguinal, femoral, and abdominal hernia	994 (674-1,276)	1,036 (757-1,369)	1,433 (1,032-1,877)	1,744 (1,286-2,316)	1,948 (1,410-2,571)	2,473 (1,765-3,142)	2,969 (2,137-3,748)	3,286 (2,374-4,041)	2,755 (2,070-3,621)	2,020 (1,583-2,601)	969 (764-1,223)	317 (252-432)
Inflammatory bowel disease	739 (596-904)	859 (681-1,023)	1,006 (789-1,143)	1,264 (992-1,427)	1,365 (1,093-1,532)	1,488 (1,183-1,687)	1,871 (1,474-2,126)	2,014 (1,558-2,247)	1,984 (1,477-2,185)	1,408 (1,008-1,550)	656 (467-727)	212 (147-240)
Vascular intestinal disorders	680 (571-871)	1,116 (934-1,431)	1,705 (1,448-2,197)	2,642 (2,303-3,451)	3,402 (3,006-4,479)	4,386 (3,806-5,933)	6,327 (5,454-8,371)	6,427 (5,280-8,190)	5,715 (4,585-7,328)	3,696 (2,773-4,421)	1,565 (1,264-1,867)	448 (352-541)
Gallbladder and biliary diseases	1,027 (901-1,450)	1,289 (1,140-1,696)	1,712 (1,497-2,234)	2,185 (1,924-2,878)	2,805 (2,492-3,685)	3,432 (3,074-4,690)	4,620 (4,154-5,971)	5,437 (4,804-6,936)	5,587 (4,941-6,936)	4,237 (3,770-5,235)	1,999 (1,800-2,421)	638 (560-783)
Pancreatitis	5,914 (4,925-6,944)	6,839 (5,499-7,993)	6,118 (4,963-7,301)	6,142 (4,876-7,148)	5,242 (4,176-6,170)	4,730 (3,734-5,810)	4,802 (3,802-5,814)	4,306 (3,358-5,326)	3,072 (2,439-3,706)	1,806 (1,417-2,168)	622 (477-761)	175 (141-236)
Other digestive diseases	1,415 (1,217-1,735)	1,965 (1,661-2,313)	2,439 (2,044-2,868)	2,972 (2,512-3,521)	3,790 (3,079-4,545)	3,797 (3,076-4,569)	4,747 (3,823-5,745)	4,771 (3,825-5,842)	4,940 (3,891-6,152)	3,575 (2,781-4,508)	1,814 (1,456-2,372)	612 (474-793)
Neurological disorders	8,033 (7,648-8,429)	9,870 (9,238-10,575)	12,623 (11,231-14,353)	18,262 (15,454-21,628)	26,121 (21,818-32,042)	42,230 (34,043-52,318)	82,714 (64,943-105,298)	142,158 (112,344-177,985)	188,817 (151,157-235,875)	172,683 (139,610-210,397)	94,345 (76,869-110,919)	28,129 (22,312-35,137)
Alzheimer's disease and other dementias	50 (20-101)	1,092 (627-1,707)	3,869 (2,569-5,590)	8,150 (5,424-11,505)	15,354 (11,093-21,098)	28,905 (21,087-38,944)	62,682 (45,560-84,993)	114,407 (85,341-148,719)	158,633 (121,122-207,096)	153,632 (118,637-192,085)	88,073 (70,970-104,974)	26,719 (20,894-33,860)
Parkinson's disease	236 (133-387)	386 (212-670)	675 (394-1,077)	1,348 (811-1,995)	2,805 (1,967-3,943)	5,622 (3,989-7,562)	12,853 (9,029-17,109)	21,664 (15,486-28,963)	25,542 (18,948-34,444)	16,561 (12,670-21,767)	5,501 (4,162-7,188)	1,205 (860-1,652)
Epilepsy	5,422 (5,133-5,746)	5,177 (4,895-5,480)	4,114 (3,934-4,306)	3,933 (3,765-4,096)	3,011 (2,842-3,187)	2,766 (2,570-2,965)	2,258 (2,125-2,396)	1,793 (1,686-1,886)	1,562 (1,455-1,663)	893 (828-953)	318 (298-336)	99 (93-105)
Multiple sclerosis	629 (559-841)	746 (662-954)	907 (772-1,096)	960 (782-1,143)	851 (653-995)	691 (520-788)	577 (433-672)	403 (296-458)	246 (179-271)	105 (72-117)	29 (20-31)	5 (4-6)
Motor neuron disease	646 (614-677)	1,029 (982-1,078)	1,355 (1,296-1,416)	1,879 (1,812-1,958)	2,163 (2,086-2,259)	2,382 (2,289-2,492)	2,349 (2,258-2,453)	1,940 (1,853-2,035)	1,272 (1,220-1,336)	564 (539-593)	113 (108-119)	21 (20-22)
Other neurological disorders	1,049 (963-1,106)	1,440 (1,338-1,513)	1,701 (1,575-1,784)	1,992 (1,857-2,086)	1,937 (1,779-2,032)	1,864 (1,710-1,960)	1,994 (1,836-2,103)	1,952 (1,800-2,052)	1,561 (1,454-1,641)	927 (870-978)	310 (294-325)	80 (76-83)
Mental and substance use disorders	26,017 (22,412-27,775)	28,970 (25,241-31,055)	27,347 (23,968-29,553)	23,947 (20,975-25,788)	17,210 (15,182-18,555)	11,177 (9,494-11,902)	8,784 (7,668-9,377)	6,328 (5,389-6,746)	3,816 (3,219-4,068)	2,002 (1,751-2,158)	708 (629-771)	218 (200-245)
Alcohol use disorders	16,102 (12,882-17,603)	20,247 (16,679-22,188)	19,834 (16,581-21,870)	17,896 (15,158-19,576)	13,026 (11,070-14,144)	8,172 (6,844-8,878)	5,805 (4,865-6,301)	3,776 (3,003-4,175)	1,850 (1,491-2,001)	871 (709-947)	275 (221-301)	84 (71-91)
Drug use disorders	9,882 (8,755-11,009)	8,689 (7,997-9,478)	7,513 (6,913-8,211)	6,051 (5,497-6,835)	4,183 (3,757-4,871)	3,006 (2,538-3,365)	2,979 (2,498-3,362)	2,552 (2,054-2,920)	1,966 (1,614-2,156)	1,131 (963-1,276)	433 (385-505)	134 (123-159)
Opioid use disorders	6,269 (4,831-6,948)	5,239 (3,950-5,772)	4,244 (3,203-4,765)	3,488 (2,755-4,012)	2,511 (2,092-3,013)	1,876 (1,506-2,148)	1,369 (1,044-1,625)	1,235 (895-1,436)	832 (576-942)	486 (332-577)	188 (146-235)	58 (46-72)
Cocaine use disorders	723 (529-976)	731 (523-1,017)	892 (554-1,215)	690 (459-916)	441 (265-575)	304 (189-398)	202 (125-264)	157 (95-201)	30 (18-39)	16 (10-21)	7 (4-9)	2 (1-2)
Amphetamine use disorders	444 (338-591)	421 (310-585)	419 (287-585)	331 (220-430)	210 (142-274)	141 (89-182)	69 (45-88)	54 (32-69)	15 (10-19)	8 (6-11)	3 (2-4)	1 (1-1)
Other drug use disorders	2,446 (2,083-3,259)	2,299 (2,041-3,239)	1,959 (1,743-2,761)	1,542 (1,351-2,059)	1,021 (871-1,368)	685 (519-892)	1,339 (1,079-1,633)	1,107 (861-1,394)	1,088 (884-1,299)	620 (527-743)	235 (204-289)	73 (65-92)
Eating disorders	33 (29-38)	34 (30-39)	0 (0-0)	0 (0-0)	-	-	-	-	-	-	-	-
Anorexia nervosa	29 (25-34)	24 (21-28)	0 (0-0)	0 (0-0)	-	-	-	-	-	-	-	-
Bulimia nervosa	4 (3-6)	10 (8-12)	0 (0-0)	0 (0-0)	-	-	-	-	-	-	-	-
Diabetes, urogenital, blood, and endocrine diseases	44,500 (42,599-47,133)	60,596 (58,054-63,485)	81,501 (77,877-84,699)	108,689 (104,743-112,176)	124,381 (120,043-128,619)	140,406 (134,873-145,757)	162,517 (156,441-168,303)	166,010 (160,236-172,016)	150,129 (144,209-156,200)	104,363 (100,053-108,521)	46,139 (44,234-47,886)	14,310 (13,703-14,841)
Diabetes mellitus	15,199 (14,681-15,866)	23,894 (23,138-24,689)	35,745 (34,642-36,886)	50,831 (49,303-52,384)	60,652 (58,748-62,610)	68,600 (66,275-70,993)	78,600 (75,790-81,261)	78,693 (75,936-81,698)	67,093 (63,967-70,159)	40,541 (38,496-42,371)	15,247 (14,523-15,940)	3,793 (3,624-3,963)
Acute glomerulonephritis	313 (278-350)	367 (332-405)	366 (332-405)	515 (469-563)	461 (417-509)	505 (464-552)	599 (550-657)	560 (511-615)	441 (401-482)	252 (227-281)	98 (88-110)	35 (29-44)
Chronic kidney disease	21,092 (19,538-23,699)	26,373 (24,036-28,539)	34,859 (31,692-38,356)	45,074 (41,679-47,747)	49,276 (45,638-53,293)	54,929 (50,122-60,209)	63,013 (58,207-67,592)	64,545 (60,131-69,619)	59,896 (56,236-64,011)	45,021 (42,756-48,269)	21,319 (20,178-22,606)	7,160 (6,812-7,530)
Chronic kidney disease due to diabetes mellitus	9,998 (7,757-12,136)	13,975 (11,256-16,641)	19,041 (15,064-22,495)	24,974 (20,088-28,851)	27,621 (22,667-32,118)	29,675 (23,375-34,655)	31,812 (25,270-37,340)	29,253 (23,004-35,071)	23,122 (17,824-28,027)	14,767 (11,032-19,068)	6,566 (4,749-8,437)	2,199 (1,515-2,892)
Chronic kidney disease due to hypertension	2,949 (2,279-3,835)	3,962 (3,054-5,126)	5,662 (4,336-7,453)	8,175 (6,268-10,600)	9,682 (7,694-12,214)	12,194 (9,602-15,520)	15,567 (12,276-19,509)	18,091 (14,408-22,641)	19,065 (15,340-23,260)	16,757 (13,614-20,503)	8,902 (7,268-10,587)	3,092 (2,474-3,764)
Chronic kidney disease due to glomerulonephritis	3,564 (2,288-5,002)	3,456 (2,234-4,904)	3,961 (2,615-5,669)	4,358 (2,926-6,152)	4,256 (2,794-6,086)	4,490 (2,915-6,615)	5,287 (3,505-7,681)	5,821 (3,956-8,454)	5,816 (4,111-7,827)	4,000 (2,811-5,554)	1,661 (1,161-2,248)	554 (339-882)
Chronic kidney disease due to other causes	4,581 (3,148-6,189)	4,979 (3,278-7,181)	6,194 (4,052-8,868)	7,567 (5,081-10,992)	7,717 (5,020-11,149)	8,569 (5,785-12,396)	10,346 (7,042-14,302)	11,379 (7,876-15,679)	11,892 (8,480-15,800)	9,496 (6,563-12,791)	4,191 (3,031-5,609)	1,315 (847-1,864)
Urinary diseases and male infertility	3,472 (3,304-3,639)	4,351 (4,129-4,572)	5,137 (4,899-5,372)	6,058 (5,797-6,316)	7,876 (7,510-8,225)	9,921 (9,463-10,348)	12,826 (12,215-13,403)	14,086 (13,427-14,680)	15,341 (14,620-16,036)	12,561 (11,986-13,146)	6,664 (6,386-6,940)	2,344 (2,249-2,439)
Interstitial nephritis and urinary tract infections	1,782 (1,443-2,030)	2,207 (1,805-2,512)	2,768 (2,325-3,136)	3,315 (2,759-3,724)	4,403 (3,711-4,976)	5,602 (4,761-6,352)	7,775 (6,683-8,597)	9,162 (8,079-10,037)	10,107 (8,987-10,921)	8,873 (7,990-9,522)	4,855 (4,430-5,191)	1,834 (1,705-1,949)
Urolithiasis	329 (251-549)	410 (314-670)	506 (400-806)	530 (441-773)	661 (538-1,006)	871 (696-1,399)	1,063 (878-1,609)	1,106 (921-1,704)	1,041 (864-1,547)	696 (577-1,065)	301 (247-446)	89 (74-128)
Other urinary diseases	1,361 (1,043-1,600)	1,734 (1,359-2,047)	1,863 (1,472-2,210)	2,213 (1,798-2,632)	2,812 (2,242-3,327)	3,448 (2,665-4,150)	3,988 (3,213-4,788)	3,819 (3,104-4,585)	4,193 (3,574-5,058)	2,992 (2,508-3,648)	1,509 (1,259-1,848)	420 (349-528)
Gynecological diseases	-	-	-	-	-	-	-	-	-	-	-	-
Uterine fibroids	-	-	-	-	-	-	-	-	-	-	-	-
Polycystic ovarian syndrome	-	-	-	-	-	-	-	-	-	-	-	-
Endometriosis	-	-	-	-	-	-	-	-	-	-	-	-
Genital prolapse	-	-	-	-	-	-	-	-	-	-	-	-
Other gynecological diseases	-	-	-	-	-	-	-	-	-	-	-	-
Hemoglobinopathies and hemolytic anaemias	1,841 (1,458-2,574)	2,282 (1,889-3,441)	1,627 (1,321-2,218)	1,763 (1,416-2,328)	1,723 (1,387-2,316)	1,966 (1,615-2,649)	2,609 (2,167-3,551)	3,268 (2,816-4,511)	2,585 (2,208-3,312)	2,351 (1,987-2,971)	1,045 (879-1,274)	352 (299-418)
Thalassemias	18 (15-24)	14 (12-19)	12 (11-17)	9 (7-13)	4 (3-6)	2 (1-2)	1 (1-1)	0 (0-0)	0 (0-0)	0 (0-0)	0 (0-0)	0 (0-0)
Sickle cell disorders	756 (601-1,149)	849 (627-1,414)	328 (258-508)	162 (127-245)	79 (64-112)	46 (39-62)	23 (20-30)	10 (9-13)	4 (3-5)	1 (1-1)	0 (0-0)	0 (0-0)
G6PD deficiency	765 (580-1,036)	1,015 (794-1,481)	791 (622-1,075)	802 (611-1,096)	691 (541-976)	569 (451-817)	497 (391-722)	385 (296-588)	157 (121-226)	79 (60-114)	19 (15-27)	4 (3-6)
Other hemoglobinopathies and hemolytic anaemias	302 (235-391)	403 (317-556)	495 (385-625)	791 (622-1,006)	949 (752-1,229)	1,350 (1,096-1,796)	2,088 (1,731-2,776)	2,872 (2,464-3,911)	2,424 (2,072-3,073)	2,271 (1,921-2,853)	1,025 (863-1,245)	348 (295-413)
Endocrine, metabolic, blood, and immune disorders	2,583 (1,988-2,884)	3,330 (2,497-3,615)	3,767 (2,870-4,104)	4,447 (3,346-4,813)	4,394 (3,369-4,808)	4,485 (3,343-5,115)	4,870 (3,940-5,625)	4,858 (3,801-5,645)	4,774 (3,618-5,453)	3,639 (2,678-4,042)	1,765 (1,321-1,980)	625 (476-708)
Musculoskeletal disorders	952 (807-1,121)	1,216 (1,043-1,410)	1,178 (1,042-1,373)	1,540 (1,349-1,806)	1,816 (1,607-2,100)	2,133 (1,888-2,448)	2,688 (2,326-3,013)	3,031 (2,588-3,337)	2,730 (2,307-2,984)	2,051 (1,784-2,224)	904 (798-999)	303 (279-329)
Rheumatoid arthritis	167 (126-209)	249 (185-305)	391 (297-462)	619 (465-706)	846 (659-962)	1,057 (819-1,187)	1,390 (1,079-1,543)	1,510 (1,192-1,695)	1,232 (971-1,386)	765 (587-903)	275 (204-327)	77 (61-93)
Other musculoskeletal disorders	784 (675-924)	967 (837-1,157)	787 (685-960)	921 (801-1,130)	970 (843-1,209)	1,076 (951-1,364)	1,298 (1,110-1,592)	1,521 (1,259-1,835)	1,498 (1,241-1,705)	1,286 (1,083-1,475)	629 (542-713)	226 (195-251)
Other non-communicable diseases	4,154 (3,342-4,612)	3,760 (2,881-4,508)	3,616 (2,595-4,175)	4,312 (2,888-5,012)	4,491 (3,060-5,321)	4,477 (2,924-5,480)	4,112 (2,288-5,193)	4,772 (2,760-6,498)	4,493 (2,518-6,141)	3,747 (2,141-5,135)	1,925 (1,090-2,598)	669 (362-916)
Congenital anomalies	2,883 (2,388-3,266)	1,919 (1,587-2,242)	1,642 (1,337-1,841)	1,623 (1,313-1,721)	1,549 (1,272-1,729)	1,142 (976-1,343)	-	-	-	-	-	-
Neural tube defects	44 (33-76)	47 (39-64)	34 (27-52)	31 (23-60)	25 (18-51)	18 (14-35)	-	-	-	-	-	-
Congenital heart anomalies	2,188 (1,805-2,526)	815 (676-969)	628 (530-748)	579 (491-685)	487 (422-604)	360 (314-460)	-	-	-	-	-	-
Orofacial clefts	-	-	-	-	-	-	-	-	-	-	-	-
Down's syndrome	184 (141-229)	389 (303-479)	386 (283-425)	425 (287-469)	355 (234-396)	142 (94-163)	-	-	-	-	-	-
Other chromosomal abnormalities	44 (16-53)	48 (21-57)	53 (26-63)	44 (22-53)	31 (16-39)	21 (12-26)	-	-	-	-	-	-
Congenital musculoskeletal and limb anomalies	24 (16-30)	32 (21-41)	30 (22-37)	18 (12-23)	23 (16-28)	20 (15-25)	-	-	-	-	-	-
Urogenital congenital anomalies	89 (60-114)	150 (102-186)	161 (115-199)	187 (132-234)	231 (165-291)	262 (189-331)	-	-	-	-	-	-
Digestive congenital anomalies	30 (18-36)	47 (34-56)	41 (28-48)	45 (31-53)	53 (39-64)	48 (32-57)	-	-	-	-	-	-
Other congenital anomalies	280 (213-354)	390 (310-480)	310 (247-376)	295 (233-339)	344 (278-415)	271 (218-330)	-	-	-	-	-	-
Skin and subcutaneous diseases	1,271 (642-1,655)	1,841 (999-2,569)	1,973 (1,030-2,470)	2,689 (1,384-3,374)	2,943 (1,584-3,745)	3,335 (1,795-4,407)	4,112 (2,288-5,193)	4,772 (2,760-6,498)	4,493 (2,518-6,141)	3,747 (2,141-5,135)	1,925 (1,090-2,598)	669 (362-916)
Cellulitis	252 (110-354)	355 (156-518)	506 (188-646)	649 (242-851)	627 (267-873)	574 (214-778)	691 (282-1,033)	766 (311-1,180)	723 (304-1,243)	584 (250-1,009)	307 (138-584)	102 (45-205)
Pyoderma	819 (391-1,079)	1,215 (629-1,672)	1,177 (596-1,554)	1,613 (787-2,187)	1,812 (904-2,357)	2,026 (1,052-2,725)	2,313 (1,174-2,966)	2,553 (1,378-3,383)	1,968 (1,072-2,634)	1,625 (851-2,124)	781 (418-1,043)	248 (124-330)
Decubitus ulcer	161 (86-249)	220 (122-349)	237 (126-364)	359 (195-541)	426 (232-646)	642 (359-980)	1,000 (569-1,473)	1,307 (749-1,987)	1,628 (883-2,355)	1,387 (735-1,978)	760 (423-1,054)	286 (150-402)
Other skin and subcutaneous diseases	39 (23-61)	51 (29-85)	52 (28-87)	67 (37-106)	78 (44-125)	93 (54-152)	107 (60-181)	146 (85-246)	174 (96-287)	151 (87-255)	78 (42-138)	33 (17-57)
Sudden infant death syndrome	-	-	-	-	-	-	-	-	-	-	-	-
Injuries	246,789 (233,657-256,928)	231,230 (218,376-242,050)	206,500 (195,168-215,721)	189,593 (177,722-197,707)	147,561 (137,202-154,417)	125,248 (114,161-130,966)	120,917 (109,710-126,610)	105,830 (93,677-111,090)	85,755 (75,174-90,037)	55,982 (48,309-59,109)	23,697 (20,023-25,059)	7,513 (6,341-7,967)
Transport injuries	94,721 (91,653-98,644)	84,078 (81,349-87,160)	73,047 (70,668-75,751)	65,707 (63,453-68,232)	48,595 (46,947-50,489)	38,944 (37,533-40,499)	33,637 (32,395-34,922)	26,128 (25,187-27,246)	17,610 (16,904-18,316)	9,091 (8,699-9,524)	3,040 (2,900-3,189)	807 (767-845)
Road injuries	88,284 (85,365-91,825)	78,078 (75,587-80,928)	67,418 (65,126-69,945)	60,689 (58,579-63,009)	45,384 (43,850-47,170)	36,291 (35,016-37,727)	31,415 (30,273-32,618)	24,296 (23,399-25,330)	16,427 (15,762-17,120)	8,462 (8,106-8,875)	2,781 (2,654-2,912)	715 (680-749)
Pedestrian road injuries	32,125 (28,773-35,176)	30,366 (27,653-33,206)	27,301 (24,587-30,082)	25,907 (23,484-28,238)	21,627 (19,920-23,467)	17,884 (16,470-19,384)	16,000 (14,793-17,401)	13,855 (12,971-15,082)	9,298 (8,652-10,055)	4,867 (4,539-5,273)	1,513 (1,393-1,658)	391 (362-436)
Cyclist road injuries	4,963 (4,386-5,561)	4,971 (4,460-5,639)	5,159 (4,580-5,800)	4,705 (4,183-5,230)	4,170 (3,721-4,621)	3,430 (3,077-3,841)	2,926 (2,621-3,259)	1,855 (1,659-2,027)	1,064 (957-1,208)	464 (410-528)	136 (119-158)	2 (2-3)
Motorcyclist road injuries	20,595 (17,800-22,312)	16,145 (13,663-17,823)	12,302 (10,250-13,500)	10,300 (8,746-11,253)	5,506 (4,538-6,110)	3,983 (3,099-4,402)	2,847 (2,302-3,152)	1,551 (1,186-1,723)	1,157 (898-1,287)	476 (380-537)	131 (104-149)	36 (29-41)
Motor vehicle road injuries	29,905 (26,063-35,215)	25,815 (22,432-29,957)	21,841 (18,755-25,687)	19,119 (16,307-22,392)	13,643 (11,614-16,078)	10,506 (9,065-12,456)	9,187 (7,723-10,840)	6,681 (5,743-7,746)	4,624 (4,030-5,428)	2,504 (2,167-2,930)	942 (817-1,070)	267 (225-298)
Other road injuries	697 (596-845)	781 (667-971)	816 (706-1,048)	658 (563-841)	438 (373-556)	488 (422-638)	454 (393-597)	353 (305-482)	284 (245-403)	152 (129-219)	59 (49-90)	20 (17-31)
Other transport injuries	6,437 (5,825-7,279)	6,000 (5,518-6,831)	5,629 (5,142-6,495)	5,017 (4,564-5,682)	3,210 (2,956-3,714)	2,653 (2,430-2,988)	2,223 (2,007-2,461)	1,833 (1,624-2,039)	1,183 (1,061-1,336)	629 (560-711)	259 (234-300)	92 (82-104)
Unintentional injuries	68,692 (60,356-71,537)	70,688 (62,289-73,873)	66,149 (58,913-69,561)	65,720 (58,256-69,096)	56,388 (49,083-59,239)	52,941 (44,886-55,980)	56,199 (47,213-59,338)	55,693 (46,232-59,296)	51,637 (42,359-54,929)	38,613 (31,430-41,217)	18,090 (14,621-19,315)	6,043 (4,913-6,458)
Falls	19,282 (15,697-20,578)	20,709 (16,796-22,006)	20,927 (17,031-22,160)	23,298 (18,852-24,685)	21,970 (17,409-23,486)	23,402 (18,624-25,145)	27,781 (21,334-29,950)	31,497 (24,224-34,087)	31,176 (23,530-33,474)	25,456 (19,270-27,404)	12,454 (9,331-13,395)	4,086 (3,065-4,392)
Drowning	11,857 (11,088-12,492)	11,037 (10,372-11,653)	10,143 (9,478-10,666)	8,889 (8,138-9,339)	7,316 (6,634-7,672)	6,079 (5,422-6,423)	5,935 (5,330-6,241)	4,498 (4,012-4,745)	3,571 (3,199-3,750)	1,879 (1,734-1,981)	573 (536-602)	148 (139-155)
Fire, heat, and hot substances	3,850 (3,158-4,320)	4,334 (3,595-4,785)	4,400 (3,656-4,887)	4,159 (3,409-4,613)	3,886 (3,173-4,282)	3,214 (2,624-3,531)	3,646 (3,018-3,999)	3,251 (2,690-3,609)	3,189 (2,685-3,499)	2,193 (1,912-2,431)	1,000 (892-1,130)	349 (312-378)
Poisonings	2,721 (1,978-3,031)	2,576 (1,745-2,887)	2,430 (1,709-2,721)	2,335 (1,578-2,620)	1,861 (1,295-2,104)	1,490 (980-1,681)	1,406 (957-1,584)	995 (652-1,107)	768 (525-850)	389 (287-439)	136 (106-166)	41 (35-57)
Exposure to mechanical forces	10,374 (7,325-11,174)	9,927 (7,279-10,647)	8,276 (6,210-8,830)	7,070 (5,234-7,565)	4,617 (3,402-4,959)	3,556 (2,643-3,843)	3,063 (2,335-3,298)	2,754 (2,146-2,965)	2,134 (1,633-2,307)	1,549 (1,244-1,677)	797 (622-869)	300 (236-330)
Unintentional firearm injuries	1,077 (820-1,224)	1,155 (870-1,307)	894 (684-1,017)	677 (527-763)	535 (409-611)	453 (339-530)	414 (314-471)	368 (287-421)	273 (219-301)	170 (136-193)	77 (63-88)	27 (25-33)
Unintentional suffocation	367 (235-453)	305 (197-356)	256 (170-315)	243 (151-292)	110 (50-138)	115 (55-143)	116 (50-150)	403 (227-478)	512 (295-628)	521 (305-616)	333 (191-391)	128 (71-150)
Other exposure to mechanical forces	8,930 (5,996-9,689)	8,468 (5,948-9,149)	7,126 (5,176-7,663)	6,151 (4,457-6,608)	3,972 (2,843-4,304)	2,988 (2,170-3,240)	2,534 (1,914-2,735)	1,983 (1,499-2,139)	1,349 (999-1,447)	858 (685-917)	387 (303-436)	145 (120-171)
Adverse effects of medical treatment	2,886 (2,142-3,259)	3,086 (2,276-3,489)	2,871 (2,189-3,260)	3,388 (2,643-3,973)	3,981 (2,992-4,606)	4,692 (3,540-5,399)	4,655 (3,670-5,562)	4,450 (3,474-5,277)	3,769 (2,961-4,636)	2,532 (2,018-3,170)	1,077 (869-1,342)	346 (279-460)
Animal contact	3,130 (2,797-3,491)	3,962 (3,508-4,449)	3,089 (2,796-3,412)	3,593 (3,242-3,985)	2,980 (2,680-3,309)	3,011 (2,695-3,344)	2,662 (2,348-2,954)	1,993 (1,748-2,226)	1,385 (1,186-1,549)	648 (552-736)	198 (169-226)	64 (55-77)
Venomous animal contact	2,722 (2,375-3,069)	3,408 (2,921-3,881)	2,566 (2,269-2,875)	3,020 (2,628-3,412)	2,514 (2,211-2,815)	2,593 (2,273-2,922)	2,270 (1,951-2,554)	1,591 (1,353-1,829)	1,157 (970-1,319)	518 (429-605)	146 (121-171)	41 (33-48)
Non-venomous animal contact	408 (290-521)	554 (389-723)	523 (417-656)	572 (431-741)	466 (372-580)	418 (331-530)	392 (317-500)	402 (309-525)	229 (178-285)	130 (102-165)	52 (41-65)	23 (19-31)
Foreign body	3,035 (2,576-3,717)	3,178 (2,670-3,891)	3,530 (3,006-4,454)	3,539 (2,933-4,350)	3,210 (2,605-3,926)	2,736 (2,164-3,242)	2,933 (2,245-3,313)	2,978 (2,183-3,397)	3,186 (2,251-3,700)	2,430 (1,607-2,904)	1,211 (811-1,441)	498 (313-606)
Pulmonary aspiration and foreign body in airway	2,475 (2,068-3,211)	2,919 (2,439-3,665)	3,276 (2,754-4,207)	3,308 (2,714-4,133)	2,976 (2,380-3,667)	2,583 (2,018-3,118)	2,768 (2,074-3,152)	2,774 (1,986-3,181)	3,015 (2,098-3,532)	2,313 (1,495-2,781)	1,159 (758-1,387)	479 (292-584)
Foreign body in other body part	560 (365-729)	259 (173-330)	255 (167-318)	230 (152-293)	234 (171-351)	153 (98-197)	165 (108-205)	204 (142-256)	171 (115-224)	116 (81-151)	52 (34-67)	19 (14-25)
Environmental heat and cold exposure	3,140 (2,070-4,218)	4,018 (2,707-6,034)	4,827 (3,143-7,558)	4,619 (2,948-7,274)	3,495 (2,235-5,274)	2,659 (1,509-3,490)	2,594 (1,506-3,566)	2,207 (1,249-2,807)	1,611 (1,001-2,131)	1,063 (708-1,518)	442 (305-635)	141 (93-219)
Other unintentional injuries	8,417 (6,318-8,979)	7,859 (5,959-8,387)	5,655 (4,343-5,995)	4,832 (3,763-5,142)	3,071 (2,422-3,283)	2,104 (1,682-2,254)	1,524 (1,332-1,618)	1,071 (944-1,146)	848 (679-934)	473 (383-528)	203 (161-227)	71 (55-79)
Self-harm and interpersonal violence	73,226 (64,862-80,769)	67,562 (60,404-75,245)	59,657 (52,912-67,280)	51,838 (45,579-58,290)	37,499 (32,385-42,301)	28,996 (24,689-31,960)	27,373 (23,013-30,406)	21,322 (17,367-23,426)	14,757 (12,048-16,411)	7,436 (6,108-8,079)	2,287 (1,878-2,482)	576 (479-638)

SR Table 4: Global deaths by cause and age group for males (with 95% uncertainty intervals), 2010

Cause	Early Neonatal	Late Neonatal	Post Neonatal	1 to 4	5 to 9	10 to 14	15 to 19	20 to 24	25 to 29	30 to 34	35 to 39
Self-harm	-	-	-	-	-	4,952 (4,088-5,448)	27,305 (24,134-30,086)	53,107 (47,087-58,398)	55,266 (49,827-61,101)	51,617 (45,667-57,020)	50,018 (42,162-55,477)
Self-harm by firearm	-	-	-	-	-	274 (206-395)	3,247 (2,521-4,623)	6,214 (4,718-8,645)	6,066 (4,474-8,009)	5,293 (3,909-7,071)	4,528 (3,445-5,875)
Self-harm by other specified means	-	-	-	-	-	4,678 (3,749-5,177)	24,057 (20,894-26,389)	46,893 (40,958-51,477)	49,200 (43,839-54,157)	46,324 (40,712-51,036)	45,490 (38,469-50,412)
Interpersonal violence	721 (549-883)	181 (143-228)	1,904 (1,456-2,331)	3,191 (2,534-3,935)	2,633 (2,016-3,220)	5,101 (3,822-5,908)	30,948 (23,685-34,156)	51,389 (40,278-57,602)	47,735 (37,517-54,620)	40,135 (30,412-47,440)	32,385 (24,492-38,446)
Assault by firearm	103 (60-157)	27 (17-40)	293 (160-404)	556 (332-794)	502 (302-717)	1,654 (907-1,975)	16,920 (10,367-19,598)	26,923 (17,155-30,824)	24,424 (15,136-27,785)	19,172 (11,538-21,957)	14,948 (9,326-17,594)
Assault by sharp object	65 (42-91)	17 (11-24)	143 (90-208)	381 (270-547)	344 (245-486)	768 (506-1,057)	7,163 (5,271-9,884)	12,609 (9,475-18,199)	12,297 (9,223-16,867)	10,500 (7,556-14,702)	8,553 (6,169-11,879)
Assault by other means	552 (407-695)	137 (105-176)	1,468 (1,136-1,844)	2,254 (1,725-2,808)	1,787 (1,289-2,149)	2,679 (2,021-3,310)	6,865 (5,468-9,372)	11,856 (9,218-15,381)	11,014 (8,356-15,289)	10,463 (7,938-14,784)	8,885 (6,710-12,476)
Forces of nature, conflict and terrorism, and executions and police conflict	241 (136-354)	335 (110-574)	2,433 (618-4,434)	12,077 (3,743-21,178)	12,547 (3,347-22,690)	16,757 (4,144-30,696)	21,760 (7,169-37,424)	24,947 (10,428-40,174)	21,287 (8,159-35,085)	15,371 (5,917-25,459)	11,664 (4,438-19,501)
Forces of nature, conflict and terrorism, and state actor violence	124 (24-235)	272 (52-516)	2,225 (427-4,220)	10,250 (1,967-19,441)	11,283 (2,149-21,414)	15,460 (2,942-29,344)	17,706 (3,374-33,602)	17,405 (3,322-33,023)	15,836 (3,028-30,041)	11,474 (2,255-21,718)	9,011 (1,813-17,021)
Conflict and terrorism	117 (92-140)	63 (50-75)	196 (155-235)	1,796 (1,419-2,152)	1,228 (971-1,470)	1,248 (984-1,497)	3,703 (2,912-4,465)	6,779 (5,353-8,173)	4,781 (3,744-5,805)	3,382 (2,604-4,141)	2,249 (1,728-2,760)
Executions and police conflict	-	-	11 (9-27)	30 (27-36)	36 (32-43)	48 (39-57)	351 (140-476)	763 (379-993)	671 (286-859)	515 (269-645)	404 (246-538)

Cause	40 to 44	45 to 49	50 to 54	55 to 59	60 to 64	65 to 69	70 to 74	75 to 79	80 to 84	85 to 89	90 to 94	95 plus
Self-harm	47,496 (42,240-53,146)	46,838 (41,517-52,505)	43,369 (37,788-48,390)	39,751 (33,461-44,979)	29,704 (25,278-33,276)	23,729 (19,092-26,246)	23,077 (18,468-25,325)	18,594 (14,889-20,511)	13,898 (11,074-15,309)	7,051 (5,718-7,636)	2,159 (1,749-2,335)	497 (396-534)
Self-harm by firearm	4,197 (3,259-5,685)	4,445 (3,421-6,041)	4,422 (3,456-5,971)	3,862 (2,896-5,036)	3,043 (2,308-4,068)	2,414 (1,784-3,190)	2,213 (1,617-3,011)	1,902 (1,434-2,535)	1,621 (1,236-2,198)	982 (702-1,267)	340 (259-467)	76 (55-98)
Self-harm by other specified means	43,298 (38,122-48,410)	42,393 (36,634-47,836)	38,947 (33,592-44,089)	35,889 (30,441-40,213)	26,660 (22,557-29,689)	21,315 (17,339-23,122)	20,864 (16,631-22,663)	16,692 (13,088-18,012)	12,277 (9,763-13,284)	6,069 (4,884-6,551)	1,818 (1,441-1,959)	421 (336-453)
Interpersonal violence	25,730 (19,285-31,585)	20,723 (15,384-26,680)	16,288 (11,897-21,800)	12,087 (8,819-16,131)	7,795 (5,684-10,580)	5,268 (3,939-6,963)	4,296 (3,115-5,825)	2,728 (2,078-3,653)	859 (655-1,239)	385 (293-590)	128 (95-195)	79 (56-124)
Assault by firearm	11,058 (6,537-13,740)	7,866 (4,579-10,369)	5,816 (3,396-8,066)	3,986 (2,511-5,477)	2,450 (1,513-3,394)	1,515 (882-2,184)	1,112 (635-1,625)	719 (418-1,040)	171 (91-263)	80 (41-119)	29 (14-43)	21 (10-29)
Assault by sharp object	7,011 (4,994-9,905)	5,911 (4,107-8,451)	4,536 (3,001-6,507)	3,225 (2,103-5,100)	2,269 (1,511-3,409)	1,418 (959-2,126)	1,101 (712-1,569)	613 (402-947)	225 (146-382)	95 (61-167)	37 (24-64)	18 (11-35)
Assault by other means	7,661 (5,721-10,924)	6,946 (5,058-10,147)	5,936 (4,089-8,674)	4,876 (3,435-6,799)	3,076 (2,167-4,504)	2,334 (1,748-3,315)	2,083 (1,526-2,898)	1,396 (1,038-1,901)	462 (351-713)	210 (161-347)	62 (46-105)	39 (30-66)
Forces of nature, conflict and terrorism, and executions and police conflict	10,150 (3,358-17,525)	8,903 (2,753-15,726)	7,647 (2,228-13,677)	6,328 (1,813-11,348)	5,080 (1,463-9,112)	4,367 (1,183-7,914)	3,708 (1,013-6,733)	2,686 (745-4,869)	1,751 (500-3,125)	842 (258-1,486)	280 (101-480)	88 (39-139)
Forces of nature, conflict and terrorism, and state actor violence	8,341 (1,666-15,765)	7,681 (1,534-14,517)	6,785 (1,361-12,819)	5,629 (1,119-10,643)	4,542 (914-8,579)	3,963 (776-7,505)	3,389 (672-6,412)	2,442 (488-4,616)	1,551 (318-2,927)	728 (148-1,373)	222 (44-419)	58 (12-110)
Conflict and terrorism	1,488 (1,142-1,829)	1,008 (770-1,241)	730 (548-908)	616 (458-767)	481 (354-604)	354 (266-439)	263 (199-325)	200 (151-248)	154 (113-195)	89 (64-113)	45 (32-59)	28 (19-37)
Executions and police conflict	321 (185-409)	214 (146-262)	132 (85-158)	83 (67-105)	56 (38-71)	49 (23-78)	56 (19-107)	45 (12-97)	46 (11-99)	26 (6-55)	13 (3-37)	2 (1-2)

SR Table 4: Global deaths by cause and age group for males (with 95% uncertainty intervals), 2016

Cause	Early Neonatal	Late Neonatal	Post Neonatal	1 to 4	5 to 9	10 to 14	15 to 19	20 to 24	25 to 29	30 to 34	35 to 39
All causes	979,923 (929,543-1,032,635)	249,013 (238,398-259,855)	778,208 (743,346-813,029)	700,659 (664,512-738,443)	234,159 (228,476-240,215)	205,228 (201,907-208,660)	360,039 (351,756-368,626)	528,756 (515,301-542,831)	634,441 (618,926-651,039)	720,653 (702,473-740,649)	814,994 (793,992-837,731)
Communicable, maternal, neonatal, and nutritional disorders	887,261 (838,442-937,779)	214,245 (204,021-224,338)	606,049 (573,128-640,978)	530,688 (495,124-570,426)	125,501 (115,257-134,639)	84,854 (78,010-91,903)	90,527 (83,070-99,680)	102,008 (94,319-110,922)	147,251 (138,081-159,067)	190,051 (180,127-202,355)	209,020 (198,805-221,542)
HIV/AIDS and tuberculosis	-	-	27,798 (25,114-30,931)	16,689 (14,949-18,923)	13,524 (12,831-14,252)	19,061 (18,113-20,107)	34,065 (31,838-36,217)	42,696 (39,774-46,171)	89,127 (81,943-97,641)	130,305 (122,163-139,240)	144,424 (136,737-153,017)
Tuberculosis	-	-	5,439 (4,380-6,866)	7,610 (6,054-9,662)	2,672 (2,293-3,112)	3,535 (3,073-4,064)	10,552 (9,456-11,787)	23,751 (21,455-26,027)	36,042 (33,217-39,188)	44,315 (41,035-47,667)	50,229 (46,684-54,199)
Drug-susceptible tuberculosis	-	-	5,082 (4,088-6,469)	7,096 (5,663-9,034)	2,481 (2,129-2,890)	3,271 (2,848-3,781)	9,751 (8,745-10,918)	21,876 (19,732-24,060)	32,949 (30,337-36,038)	40,367 (37,198-43,435)	45,609 (42,190-49,054)
Multidrug-resistant tuberculosis without extensive drug resistance	-	-	342 (245-474)	493 (350-700)	180 (140-225)	247 (192-314)	748 (586-934)	1,710 (1,376-2,114)	2,756 (2,244-3,323)	3,498 (2,878-4,186)	4,086 (3,370-4,898)
Extensively drug-resistant tuberculosis	-	-	15 (11-19)	21 (16-27)	12 (9-16)	16 (12-21)	53 (42-66)	166 (134-206)	337 (266-416)	449 (354-564)	535 (417-673)
HIV/AIDS	-	-	22,360 (20,195-24,908)	9,079 (8,343-9,909)	10,851 (10,274-11,458)	15,526 (14,722-16,422)	23,513 (21,631-25,483)	18,945 (17,053-21,364)	53,084 (46,354-61,060)	85,991 (78,845-94,277)	94,195 (87,208-101,932)
Drug-susceptible HIV/AIDS - Tuberculosis	-	-	5,021 (3,352-6,530)	2,406 (1,690-3,022)	2,841 (2,035-3,595)	3,850 (2,704-4,938)	5,783 (4,085-7,556)	4,885 (3,645-6,151)	11,617 (8,181-15,549)	17,062 (11,708-23,080)	18,036 (12,346-24,936)
Multidrug-resistant HIV/AIDS - Tuberculosis without extensive drug resistance	-	-	481 (262-781)	219 (123-347)	234 (141-368)	312 (187-495)	472 (279-722)	422 (262-622)	1,059 (644-1,596)	1,601 (982-2,427)	1,636 (1,005-2,479)
Extensively drug-resistant HIV/AIDS - Tuberculosis	-	-	14 (8-23)	6 (4-10)	6 (3-9)	7 (4-11)	12 (7-18)	20 (12-30)	74 (43-116)	150 (89-236)	155 (93-243)
HIV/AIDS resulting in other diseases	-	-	16,844 (14,101-19,876)	6,448 (5,552-7,642)	7,771 (6,861-8,826)	11,356 (10,007-12,843)	17,246 (14,749-19,926)	13,618 (11,596-16,447)	40,335 (33,534-48,573)	67,178 (58,206-77,422)	74,368 (64,587-84,440)
Diarrhoea, lower respiratory infections, and other common infectious diseases	72,519 (62,502-83,201)	58,011 (51,687-64,987)	370,428 (339,043-406,857)	261,996 (227,263-299,405)	74,080 (62,599-85,927)	45,540 (38,250-54,172)	40,636 (33,174-50,823)	41,163 (33,646-51,545)	41,962 (34,383-53,422)	43,154 (35,754-54,334)	47,164 (39,278-61,299)
Diarrhoeal diseases	7,354 (6,144-8,796)	13,517 (11,215-16,032)	123,723 (104,266-148,035)	94,700 (75,142-116,760)	17,530 (9,450-31,416)	9,182 (4,855-17,040)	10,876 (5,746-20,075)	12,503 (6,313-22,708)	13,422 (6,816-24,776)	13,591 (7,090-24,888)	15,268 (7,801-29,027)
Intestinal infectious diseases	-	-	435 (99-1,501)	12,090 (5,959-22,193)	20,649 (11,722-33,545)	14,335 (7,726-22,899)	9,620 (4,934-16,444)	6,175 (2,940-10,832)	4,479 (2,329-7,795)	3,438 (1,817-5,980)	2,695 (1,420-4,576)
Typhoid fever	-	-	96 (46-183)	10,725 (5,119-20,318)	17,844 (9,812-29,030)	11,930 (6,170-19,154)	8,251 (4,103-14,574)	5,146 (2,329-9,196)	3,496 (1,803-6,186)	2,571 (1,329-4,575)	2,022 (1,060-3,519)
Paratyphoid fever	-	-	15 (6-32)	1,160 (476-2,474)	2,775 (1,270-5,399)	2,395 (1,094-4,476)	1,356 (561-2,750)	1,013 (412-2,061)	970 (411-2,009)	848 (366-1,684)	653 (299-1,254)
Other intestinal infectious diseases	-	-	324 (22-1,408)	206 (15-942)	30 (2-140)	11 (1-52)	12 (1-58)	16 (1-75)	13 (1-57)	20 (2-94)	20 (2-95)
Lower respiratory infections	53,913 (46,572-62,676)	34,532 (29,756-40,323)	175,555 (157,176-198,171)	81,759 (68,619-97,613)	17,213 (12,815-20,934)	10,230 (8,030-12,164)	9,370 (7,432-11,565)	11,983 (9,579-14,662)	14,320 (11,450-17,521)	16,492 (13,464-19,432)	19,450 (15,741-23,511)
Upper respiratory infections	32 (19-55)	9 (5-17)	249 (153-421)	190 (95-339)	16 (9-26)	13 (8-21)	35 (24-52)	40 (27-60)	46 (31-66)	46 (32-66)	50 (35-74)
Otitis media	1 (0-3)	2 (1-3)	35 (18-80)	78 (34-164)	39 (22-75)	48 (27-100)	13 (8-24)	12 (7-24)	11 (7-20)	11 (6-19)	16 (9-26)
Meningitis	5,441 (3,457-7,946)	5,431 (4,149-7,509)	36,352 (27,902-48,592)	28,538 (21,261-40,270)	10,286 (5,963-17,020)	7,584 (4,594-12,493)	7,535 (4,711-12,711)	7,526 (4,623-12,362)	6,812 (4,393-11,258)	6,522 (4,142-10,752)	6,534 (4,201-10,603)
Pneumococcal meningitis	310 (178-481)	310 (200-491)	2,132 (1,398-3,231)	1,878 (1,165-2,965)	688 (375-1,232)	483 (272-880)	465 (262-848)	454 (245-839)	471 (270-825)	545 (317-958)	667 (373-1,232)
H influenzae type B meningitis	870 (544-1,343)	863 (610-1,276)	5,515 (3,957-7,891)	4,030 (2,695-6,122)	1,144 (625-1,964)	675 (385-1,184)	584 (349-1,045)	569 (338-1,036)	547 (329-950)	539 (318-908)	555 (336-938)
Meningococcal infection	2,306 (1,433-3,415)	2,274 (1,689-3,143)	15,437 (11,613-21,519)	12,799 (9,102-18,293)	4,992 (2,868-8,365)	3,654 (2,197-6,059)	3,649 (2,305-6,133)	3,570 (2,201-5,832)	3,129 (1,984-5,410)	2,772 (1,736-4,842)	2,698 (1,725-4,518)
Other meningitis	1,956 (1,241-2,805)	1,984 (1,481-2,802)	13,268 (9,905-18,445)	9,831 (7,093-14,096)	3,462 (1,892-5,560)	2,772 (1,641-4,389)	2,837 (1,699-4,559)	2,933 (1,772-4,795)	2,666 (1,661-4,427)	2,667 (1,608-4,551)	2,614 (1,645-4,195)
Encephalitis	350 (227-480)	494 (337-709)	6,002 (4,469-8,517)	6,585 (4,898-9,838)	2,746 (2,003-4,139)	2,355 (1,780-3,589)	1,946 (1,512-2,939)	1,936 (1,493-2,978)	1,954 (1,539-2,925)	2,128 (1,685-3,165)	2,188 (1,742-3,306)
Diphtheria	-	-	142 (70-284)	267 (118-587)	56 (29-112)	28 (17-46)	14 (9-22)	10 (7-16)	10 (6-14)	9 (6-13)	12 (8-18)
Whooping cough	-	-	16,097 (8,437-27,608)	14,852 (7,752-25,590)	1,476 (744-2,669)	320 (164-580)	147 (75-263)	103 (54-186)	58 (29-107)	46 (23-84)	31 (16-58)
Tetanus	5,395 (2,367-8,627)	3,922 (1,736-6,531)	1,564 (692-2,714)	806 (317-1,563)	516 (210-927)	426 (211-767)	684 (266-1,230)	620 (274-1,055)	652 (334-991)	685 (373-1,008)	773 (392-1,086)
Measles	-	-	9,315 (3,418-20,646)	20,915 (7,811-44,096)	3,051 (1,087-6,873)	846 (299-1,932)	316 (113-732)	151 (53-347)	68 (25-158)	42 (15-99)	27 (10-61)
Varicella and herpes zoster	32 (24-41)	104 (80-135)	959 (744-1,259)	1,216 (924-1,586)	503 (385-663)	172 (134-227)	81 (62-106)	104 (80-133)	130 (102-165)	143 (113-185)	120 (95-152)
Neglected tropical diseases and malaria	580 (276-863)	9,109 (5,718-13,953)	88,513 (59,912-126,302)	179,432 (133,766-234,527)	27,745 (20,198-37,245)	15,615 (11,322-20,808)	9,939 (7,323-13,045)	10,935 (8,276-14,307)	8,840 (6,599-11,586)	8,424 (6,183-11,034)	9,063 (6,510-12,097)
Malaria	-	8,618 (5,292-13,336)	82,715 (54,503-121,129)	168,144 (120,417-223,964)	21,910 (14,542-31,305)	11,957 (7,938-17,042)	6,302 (4,379-8,794)	6,909 (4,844-9,676)	5,613 (3,971-7,863)	5,598 (3,926-7,841)	5,965 (4,111-8,457)
Chagas disease	-	-	1 (1-1)	0 (0-1)	1 (1-1)	2 (2-2)	5 (4-8)	12 (9-16)	17 (14-22)	43 (36-52)	88 (76-103)
Leishmaniasis	-	-	697 (379-1,162)	883 (481-1,487)	358 (197-626)	245 (131-412)	338 (181-553)	464 (252-760)	377 (211-613)	331 (181-545)	391 (213-657)
Visceral leishmaniasis	-	-	697 (379-1,162)	883 (481-1,487)	358 (197-626)	245 (131-412)	338 (181-553)	464 (252-760)	377 (211-613)	331 (181-545)	391 (213-657)
African trypanosomiasis	-	-	-	38 (6-178)	90 (15-360)	118 (21-447)	139 (24-491)	153 (28-577)	129 (24-450)	112 (18-467)	96 (17-346)
Schistosomiasis	-	-	0 (0-0)	154 (89-271)	99 (56-172)	153 (97-234)	168 (116-247)	244 (168-346)	288 (200-386)	314 (223-434)	357 (256-490)
Cysticercosis	0 (0-0)	0 (0-0)	0 (0-0)	31 (20-47)	22 (16-31)	20 (15-26)	23 (18-30)	36 (28-45)	47 (39-59)	52 (41-64)	41 (34-51)
Cystic echinococcosis	-	-	-	73 (47-109)	34 (23-49)	25 (17-37)	14 (9-20)	19 (13-27)	29 (21-39)	30 (22-40)	36 (27-47)
Dengue	-	-	1,193 (335-1,909)	2,391 (762-3,954)	1,613 (466-2,957)	1,195 (352-2,018)	1,401 (282-2,400)	1,466 (241-2,449)	1,333 (207-2,198)	1,040 (172-1,743)	1,254 (163-2,096)
Yellow fever	-	2 (0-6)	40 (8-130)	380 (68-1,240)	653 (126-1,962)	589 (120-1,702)	563 (102-1,653)	485 (92-1,425)	372 (74-1,103)	287 (56-872)	222 (43-699)
Rabies	-	-	90 (42-132)	948 (338-1,867)	1,494 (467-2,981)	811 (300-1,332)	671 (306-1,025)	779 (339-1,131)	262 (103-351)	316 (130-407)	299 (135-407)
Intestinal nematode infections	-	-	148 (110-196)	1,482 (1,018-2,032)	253 (193-332)	59 (45-76)	23 (17-30)	17 (12-23)	14 (10-19)	18 (13-24)	17 (12-22)
Ascariasis	-	-	148 (110-196)	1,482 (1,018-2,032)	253 (193-332)	59 (45-76)	23 (17-30)	17 (12-23)	14 (10-19)	18 (13-24)	17 (12-22)
Ebola	0 (0-0)	0 (0-0)	0 (0-0)	0 (0-0)	0 (0-0)	0 (0-0)	0 (0-0)	0 (0-0)	0 (0-0)	0 (0-0)	0 (0-0)
Zika virus	0 (0-0)	0 (0-0)	0 (0-0)	0 (0-2)	0 (0-2)	0 (0-3)	1 (0-3)	1 (0-5)	1 (0-5)	1 (0-4)	1 (0-5)
Other neglected tropical diseases	580 (276-863)	489 (285-734)	3,628 (1,947-5,461)	4,908 (2,056-10,010)	1,216 (586-2,307)	441 (267-742)	290 (161-497)	352 (212-570)	358 (220-561)	284 (170-421)	296 (197-411)
Maternal disorders	-	-	-	-	-	-	-	-	-	-	-
Maternal haemorrhage	-	-	-	-	-	-	-	-	-	-	-
Maternal sepsis and other pregnancy related infections	-	-	-	-	-	-	-	-	-	-	-
Maternal hypertensive disorders	-	-	-	-	-	-	-	-	-	-	-
Maternal obstructed labour and uterine rupture	-	-	-	-	-	-	-	-	-	-	-
Maternal abortion, miscarriage, and ectopic pregnancy	-	-	-	-	-	-	-	-	-	-	-
Indirect maternal deaths	-	-	-	-	-	-	-	-	-	-	-
Late maternal deaths	-	-	-	-	-	-	-	-	-	-	-
Maternal deaths aggravated by HIV/AIDS	-	-	-	-	-	-	-	-	-	-	-
Other maternal disorders	-	-	-	-	-	-	-	-	-	-	-
Neonatal disorders	794,883 (750,011-842,376)	138,809 (128,480-148,474)	43,585 (35,556-51,399)	5,316 (3,932-6,533)	-	-	-	-	-	-	-
Neonatal preterm birth complications	299,945 (271,331-332,405)	37,914 (31,016-43,524)	15,121 (11,371-18,784)	1,320 (837-1,790)	-	-	-	-	-	-	-
Neonatal encephalopathy due to birth asphyxia and trauma	273,124 (234,508-312,987)	22,682 (18,070-27,737)	8,490 (6,211-10,952)	2,337 (1,673-2,968)	-	-	-	-	-	-	-
Neonatal sepsis and other neonatal infections	79,674 (58,643-110,693)	48,077 (39,403-59,092)	9,303 (6,636-12,771)	515 (218-823)	-	-	-	-	-	-	-
Hemolytic disease and other neonatal jaundice	18,651 (15,285-23,172)	7,167 (5,743-8,925)	1,723 (1,161-2,647)	98 (59-147)	-	-	-	-	-	-	-
Other neonatal disorders	123,488 (107,345-140,288)	22,969 (19,141-26,555)	8,947 (6,876-11,145)	1,046 (798-1,318)	-	-	-	-	-	-	-
Nutritional deficiencies	-	-	42,705 (34,412-57,568)	49,606 (36,857-68,047)	5,045 (3,787-7,320)	2,115 (1,632-3,229)	1,930 (1,519-2,948)	2,227 (1,742-3,329)	2,070 (1,626-3,150)	2,209 (1,793-3,252)	2,381 (1,898-3,657)
Protein-energy malnutrition	-	-	41,353 (33,188-55,665)	48,237 (35,671-66,384)	4,497 (3,293-6,640)	1,785 (1,335-2,781)	1,294 (997-2,017)	1,436 (1,106-2,191)	1,384 (1,064-2,149)	1,465 (1,170-2,171)	1,594 (1,241-2,512)
Iodine deficiency	-	-	128 (22-427)	100 (12-401)	34 (7-116)	10 (2-28)	37 (7-118)	43 (7-163)	50 (10-173)	46 (9-160)	41 (10-126)
Iron-deficiency anaemia	-	-	27 (12-47)	63 (35-103)	59 (36-82)	52 (32-69)	97 (66-130)	98 (75-129)	79 (63-105)	84 (64-106)	64 (53-83)
Other nutritional deficiencies	-	-	1,196 (770-1,879)	1,205 (778-1,664)	454 (312-680)	268 (200-368)	502 (358-797)	650 (437-1,109)	556 (374-936)	614 (396-1,055)	683 (452-1,112)
Other communicable, maternal, neonatal, and nutritional diseases	19,280 (11,524-28,399)	8,316 (5,538-11,980)	33,020 (21,387-47,788)	17,650 (11,646-25,857)	5,107 (3,865-6,688)	2,523 (2,165-2,953)	3,956 (3,487-4,430)	4,986 (4,495-5,486)	5,252 (4,764-5,783)	5,958 (5,437-6,540)	5,989 (5,491-6,525)
Sexually transmitted diseases excluding HIV	15,758 (8,810-24,868)	5,967 (3,291-9,514)	19,897 (10,939-31,453)	8,128 (4,372-12,968)	2,563 (1,410-4,112)	62 (44-80)	90 (60-118)	82 (51-103)	96 (63-119)	198 (133-264)	112 (75-147)
Syphilis	15,758 (8,810-24,868)	5,967 (3,291-9,514)	19,897 (10,939-31,453)	8,128 (4,372-12,968)	2,563 (1,410-4,112)	53 (37-68)	76 (51-100)	71 (44-89)	80 (53-100)	168 (113-224)	99 (67-131)
Chlamydial infection	-	-	-	-	-	0 (0-0)	0 (0-0)	0 (0-0)	0 (0-0)	0 (0-0)	0 (0-0)
Gonococcal infection	-	-	-	-	-	6 (4-8)	10 (7-14)	7 (5-9)	13 (9-16)	22 (15-29)	7 (4-9)
Other sexually transmitted diseases	-	-	-	-	-	3 (2-4)	4 (2-5)	4 (3-5)	3 (2-3)	8 (5-11)	6 (4-8)
Hepatitis	-	-	2,687 (2,286-3,137)	2,576 (2,178-3,090)	1,363 (1,097-1,703)	1,505 (1,257-1,790)	2,655 (2,297-3,059)	3,783 (3,421-4,171)	4,008 (3,587-4,456)	4,576 (4,135-5,067)	4,536 (4,131-5,003)
Acute hepatitis A	-	-	1,591 (1,078-2,122)	286 (152-455)	31 (18-51)	20 (11-31)	33 (18-53)	45 (24-76)	39 (18-71)	36 (15-71)	34 (15-61)
Hepatitis B	-	-	1,097 (639-1,644)	411 (233-636)	153 (79-249)	272 (146-437)	1,012 (696-1,429)	2,335 (1,702-2,947)	3,100 (2,510-3,677)	3,945 (3,375-4,507)	4,070 (3,577-4,539)
Hepatitis C	-	-	-	-	49 (29-78)	20 (12-30)	30 (18-46)	39 (20-65)	34 (16-60)	33 (15-60)	36 (16-65)
Acute hepatitis E	-	-	-	1,879 (1,529-2,298)	1,130 (899-1,437)	1,193 (947-1,468)	1,580 (1,171-2,014)	1,364 (840-1,950)	835 (420-1,330)	562 (264-958)	396 (185-690)
Other infectious diseases	3,522 (1,053-7,829)	2,348 (1,173-3,868)	10,435 (3,434-22,066)	6,946 (2,620-14,117)	1,181 (781-1,620)	956 (678-1,245)	1,211 (889-1,503)	1,121 (821-1,371)	1,148 (860-1,423)	1,184 (900-1,411)	1,340 (1,026-1,584)
Non-communicable diseases	86,530 (73,600-98,758)	31,341 (27,867-35,672)	132,653 (117,886-148,604)	78,471 (66,797-90,994)	43,093 (39,249-47,623)	47,190 (43,925-50,694)	86,115 (81,925-91,369)	128,174 (122,861-135,784)	181,878 (174,485-189,832)	259,345 (249,483-271,048)	360,793 (347,020-376,677)
Neoplasms	402 (340-437)	272 (241-299)	2,781 (2,493-3,038)	12,300 (11,040-13,637)	14,890 (13,069-16,422)	15,985 (14,590-17,200)	23,383 (22,003-24,880)	27,941 (26,623-29,317)	36,443 (34,691-38,126)	52,335 (50,210-54,420)	74,462 (71,702-77,370)
Lip and oral cavity cancer	-	-	-	-	-	-	239 (212-267)	528 (472-587)	1,003 (875-1,142)	1,793 (1,585-2,041)	3,107 (2,771-3,477)
Nasopharynx cancer	-	-	-	-	80 (70-91)	183 (159-209)	326 (301-356)	390 (360-427)	587 (524-663)	875 (784-977)	1,353 (1,221-1,522)
Other pharynx cancer	-	-	-	-	-	-	131 (109-154)	259 (215-297)	362 (297-417)	791 (671-899)	1,663 (1,421-1,866)
Oesophageal cancer	-	-	-	-	-	-	191 (170-217)	364 (334-398)	550 (508-599)	1,202 (1,110-1,315)	2,470 (2,306-2,654)
Stomach cancer	-	-	-	-	-	-	446 (417-479)	981 (925-1,051)	1,931 (1,822-2,060)	3,783 (3,596-4,032)	5,958 (5,672-6,335)
Colon and rectum cancer	-	-	-	-	-	-	725 (644-812)	1,681 (1,530-1,848)	2,588 (2,382-2,803)	4,226 (3,916-4,525)	6,038 (5,648-6,431)
Liver cancer	-	-	-	-	297 (253-340)	498 (432-569)	954 (845-1,085)	2,301 (2,014-2,673)	4,880 (4,404-5,478)	8,727 (7,960-9,594)	14,072 (12,943-15,232)
Liver cancer due to hepatitis B	-	-	-	-	65 (43-89)	169 (121-222)	406 (301-518)	1,237 (984-1,539)	2,930 (2,284-3,532)	5,297 (4,405-6,230)	8,239 (6,720-9,595)
Liver cancer due to hepatitis C	-	-	-	-	5 (4-7)	10 (7-14)	21 (15-30)	68 (50-93)	182 (123-268)	396 (290-520)	768 (564-1,060)
Liver cancer due to alcohol use	-	-	-	-	-	-	3 (2-5)	31 (20-44)	97 (60-149)	322 (233-437)	867 (613-1,197)
Liver cancer due to other causes	-	-	-	-	227 (190-267)	318 (261-381)	523 (422-629)	966 (791-1,162)	1,671 (1,299-2,193)	2,712 (2,142-3,456)	4,198 (3,342-5,408)
Gallbladder and biliary tract cancer	-	-	-	-	-	-	43 (37-48)	72 (57-85)	142 (103-170)	382 (275-456)	736 (536-884)
Pancreatic cancer	-	-	-	-	-	-	102 (96-109)	228 (215-243)	443 (421-468)	1,112 (1,054-1,176)	2,043 (1,947-2,148)
Larynx cancer	-	-	-	-	-	-	88 (80-98)	126 (118-135)	197 (184-212)	453 (414-497)	969 (890-1,055)
Tracheal, bronchus, and lung cancer	-	-	-	-	-	-	558 (513-600)	1,105 (1,012-1,209)	2,166 (1,996-2,335)	4,076 (3,790-4,336)	7,122 (6,720-7,576)
Malignant skin melanoma	-	-	-	-	-	-	121 (109-156)	233 (209-315)	395 (354-487)	666 (578-799)	987 (859-1,184)
Non-melanoma skin cancer	-	-	-	-	-	-	112 (105-119)	155 (147-164)	204 (192-216)	345 (326-365)	424 (403-446)
Non-melanoma skin cancer (squamous-cell carcinoma)	-	-	-	-	-	-	112 (105-119)	155 (147-164)	204 (192-216)	345 (326-365)	424 (403-446)
Breast cancer	-	-	-	-	-	-	36 (34-39)	40 (36-43)	56 (45-62)	95 (72-109)	178 (133-200)
Cervical cancer	-	-	-	-	-	-	-	-	-	-	-
Uterine cancer	-	-	-	-	-	-	-	-	-	-	-
Ovarian cancer	-	-	-	-	-	-	-	-	-	-	-
Prostate cancer	-	-	-	-	-	-	69 (56-79)	95 (74-108)	134 (104-152)	170 (131-190)	264 (199-292)
Testicular cancer	-	-	-	-	-	-	389 (354-432)	873 (805-957)	1,104 (1,018-1,221)	1,075 (997-1,162)	918 (856-991)
Kidney cancer	18 (15-20)	9 (8-11)	132 (115-149)	713 (581-862)	318 (274-366)	160 (144-174)	141 (127-154)	207 (190-223)	326 (303-348)	494 (455-527)	879 (826-935)
Bladder cancer	-	-	-	-	-	-	63 (57-70)	138 (123-154)	183 (165-205)	402 (363-444)	731 (664-812)
Brain and nervous system cancer	50 (35-59)	35 (27-41)	527 (395-642)	2,205 (1,852-2,551)	3,154 (2,708-3,726)	2,959 (2,487-3,474)	2,393 (2,007-2,782)	2,622 (2,149-3,001)	3,433 (2,891-3,931)	4,307 (3,638-4,900)	5,233 (4,427-6,015)
Thyroid cancer	-	-	-	-	-	65 (49-80)	109 (93-126)	174 (156-194)	235 (212-258)	250 (226-276)	324 (293-359)
Mesothelioma	-	-	-	-	-	-	57 (52-69)	71 (64-79)	128 (115-144)	154 (134-172)	265 (233-303)
Hodgkin lymphoma	5 (3-6)	1 (1-1)	27 (22-38)	207 (157-320)	632 (442-928)	789 (568-1,069)	869 (649-1,135)	829 (642-1,072)	964 (752-1,221)	1,002 (791-1,339)	1,033 (800-1,376)
Non-Hodgkin's lymphoma	25 (22-28)	19 (16-23)	244 (206-299)	1,577 (1,161-1,950)	2,599 (1,964-3,207)	2,497 (1,957-2,940)	2,358 (1,939-2,635)	2,716 (2,254-3,031)	2,805 (2,353-3,082)	3,346 (2,781-3,665)	3,905 (3,226-4,260)
Multiple myeloma	-	-	-	-	-	-	117 (88-138)	177 (126-207)	215 (155-245)	420 (326-513)	657 (524-816)
Leukaemia	90 (69-102)	80 (61-92)	925 (751-1,045)	4,167 (3,535-4,712)	5,222 (4,384-5,913)	5,388 (4,819-6,143)	6,167 (5,639-7,259)	5,801 (5,161-6,708)	5,801 (5,000-6,380)	5,413 (4,662-5,887)	5,421 (4,822-6,093)
Acute lymphoid leukaemia	26 (22-30)	22 (18-26)	310 (260-367)	1,728 (1,436-1,998)	2,513 (2,133-2,907)	2,485 (2,208-2,867)	2,260 (1,995-2,664)	1,804 (1,632-2,115)	1,467 (1,275-1,684)	1,296 (1,118-1,479)	1,163 (1,035-1,360)
Chronic lymphoid leukaemia	-	-	-	-	-	-	124 (110-154)	151 (133-184)	155 (128-182)	132 (107-151)	139 (118-161)
Acute myeloid leukaemia	17 (13-21)	18 (14-21)	201 (160-244)	1,052 (799-1,305)	1,025 (806-1,275)	1,037 (862-1,251)	1,371 (1,202-1,701)	1,354 (1,168-1,618)	1,360 (1,152-1,603)	1,404 (1,205-1,654)	1,579 (1,345-1,852)
Chronic myeloid leukaemia	-	-	-	-	-	-	189 (160-243)	263 (225-327)	505 (422-582)	529 (446-625)	561 (468-670)
Other leukaemia	47 (32-55)	40 (27-48)	414 (298-476)	1,388 (1,076-1,596)	1,684 (1,337-1,924)	1,866 (1,582-2,125)	2,223 (1,976-2,726)	2,228 (1,857-2,602)	2,314 (1,868-2,530)	2,051 (1,670-2,292)	1,979 (1,701-2,218)
Other neoplasms	214 (178-235)	128 (115-142)	923 (829-1,041)	3,431 (3,032-3,987)	2,588 (2,276-2,869)	3,448 (3,017-3,799)	6,578 (5,747-7,100)	5,774 (4,954-6,213)	5,609 (4,742-6,065)	6,776 (5,570-7,349)	7,713 (6,355-8,274)
Cardiovascular diseases	1,801 (1,612-2,017)	923 (829-1,046)	7,850 (7,167-8,810)	6,312 (5,663-6,997)	4,196 (3,785-4,674)	5,808 (5,370-6,314)	18,224 (17,200-19,486)	33,710 (32,004-35,828)	56,936 (53,585-59,659)	90,764 (86,235-95,640)	136,041 (129,401-142,941)
Rheumatic heart disease	-	-	-	1,101 (927-1,320)	864 (744-1,000)	1,674 (1,494-1,874)	3,280 (2,955-3,664)	3,616 (3,248-4,043)	3,843 (3,485-4,265)	4,356 (3,941-4,822)	4,884 (4,425-5,396)
Ischaemic heart disease	-	-	411 (339-520)	375 (316-448)	296 (244-357)	338 (293-391)	5,966 (5,522-6,514)	13,666 (12,908-14,671)	27,120 (25,337-28,762)	49,520 (46,635-52,414)	77,338 (73,282-81,291)
Cerebrovascular disease	800 (688-959)	381 (326-470)	2,640 (2,299-3,145)	1,867 (1,606-2,175)	1,390 (1,222-1,587)	1,766 (1,610-1,964)	3,957 (3,675-4,282)	8,941 (8,390-9,533)	14,076 (13,056-14,989)	21,755 (20,475-22,934)	33,752 (31,932-35,569)

Cause	40 to 44	45 to 49	50 to 54	55 to 59	60 to 64	65 to 69	70 to 74	75 to 79	80 to 84	85 to 89	90 to 94	95 plus
All causes	989,346 (966,954-1,016,336)	1,273,353 (1,245,764-1,306,391)	1,638,752 (1,600,016-1,684,910)	2,022,292 (1,974,890-2,079,173)	2,597,722 (2,544,951-2,658,608)	2,812,500 (2,761,827-2,869,409)	2,870,449 (2,828,386-2,913,460)	3,116,275 (3,061,978-3,172,648)	2,906,359 (2,874,341-2,938,294)	2,209,059 (2,187,547-2,229,764)	1,064,752 (1,058,778-1,071,010)	288,323 (286,831-289,879)
Communicable, maternal, neonatal, and nutritional disorders	209,225 (199,992-221,825)	205,853 (195,846-220,304)	200,733 (189,550-218,643)	200,678 (186,532-222,482)	223,408 (202,279-254,003)	229,624 (206,429-265,745)	254,699 (225,944-300,256)	261,054 (227,532-308,324)	267,217 (232,031-318,442)	207,216 (181,060-243,410)	105,420 (92,713-121,651)	35,147 (30,761-40,248)
HIV/AIDS and tuberculosis	141,963 (136,424-147,376)	129,037 (123,028-135,590)	112,602 (106,801-117,818)	95,225 (90,025-100,117)	84,484 (79,607-89,694)	72,334 (68,094-77,164)	67,719 (63,036-72,758)	53,842 (49,891-57,814)	42,017 (38,530-45,683)	22,862 (21,052-24,781)	7,685 (7,059-8,360)	1,934 (1,765-2,132)
Tuberculosis	55,191 (51,522-58,888)	61,730 (57,468-65,770)	68,605 (63,913-73,208)	72,012 (67,223-76,561)	71,054 (66,334-75,859)	64,261 (60,055-68,787)	63,269 (58,626-68,234)	51,447 (47,580-55,364)	41,191 (37,673-44,870)	22,512 (20,696-24,438)	7,588 (6,960-8,261)	1,913 (1,745-2,110)
Drug-susceptible tuberculosis	49,983 (46,518-53,697)	55,827 (51,742-59,740)	61,893 (57,509-66,077)	64,996 (60,742-68,988)	64,240 (60,074-68,843)	58,475 (54,382-63,038)	57,768 (53,399-62,495)	46,977 (43,507-50,854)	37,637 (34,239-40,866)	20,619 (18,903-22,384)	6,977 (6,385-7,626)	1,769 (1,610-1,951)
Multidrug-resistant tuberculosis without extensive drug resistance	4,586 (3,753-5,449)	5,199 (4,336-6,159)	5,864 (4,800-7,012)	6,175 (5,085-7,325)	6,044 (4,974-7,243)	5,191 (4,229-6,241)	4,997 (4,028-6,009)	4,032 (3,296-4,846)	3,211 (2,623-3,902)	1,699 (1,375-2,070)	547 (437-669)	130 (102-162)
Extensively drug-resistant tuberculosis	622 (490-785)	704 (559-869)	848 (671-1,064)	842 (670-1,049)	770 (614-949)	595 (476-734)	504 (405-623)	438 (353-542)	344 (270-438)	193 (149-249)	64 (49-84)	15 (11-20)
HIV/AIDS	86,772 (82,914-90,902)	67,307 (62,761-72,494)	43,997 (41,067-47,113)	23,213 (21,231-25,491)	13,430 (12,245-14,988)	8,073 (7,362-9,026)	4,449 (3,995-5,052)	2,396 (2,158-2,716)	826 (739-931)	351 (312-396)	97 (86-110)	21 (19-23)
Drug-susceptible HIV/AIDS - Tuberculosis	16,653 (11,410-22,874)	13,362 (9,135-17,998)	9,518 (6,522-12,468)	5,049 (3,582-6,558)	2,828 (2,056-3,641)	1,581 (1,152-2,048)	917 (669-1,175)	476 (351-614)	171 (128-216)	74 (56-94)	21 (16-27)	4 (3-5)
Multidrug-resistant HIV/AIDS - Tuberculosis without extensive drug resistance	1,463 (896-2,210)	1,158 (706-1,763)	828 (505-1,246)	446 (276-663)	252 (158-376)	138 (87-205)	78 (49-115)	41 (26-61)	14 (9-21)	6 (4-9)	2 (1-3)	0 (0-0)
Extensively drug-resistant HIV/AIDS - Tuberculosis	126 (75-198)	89 (54-143)	59 (35-92)	31 (19-49)	18 (11-29)	10 (6-17)	5 (3-8)	3 (2-5)	1 (1-2)	0 (0-1)	0 (0-0)	0 (0-0)
HIV/AIDS resulting in other diseases	68,530 (61,564-75,988)	52,698 (46,139-59,663)	33,592 (29,431-38,043)	17,686 (15,414-20,421)	10,332 (9,013-11,982)	6,344 (5,559-7,328)	3,449 (2,983-4,104)	1,876 (1,634-2,203)	640 (557-746)	270 (235-317)	74 (65-86)	17 (15-19)
Diarrhoea, lower respiratory infections, and other common infectious diseases	49,311 (41,268-62,771)	57,890 (48,248-74,126)	69,169 (57,917-88,181)	84,766 (70,466-109,473)	116,468 (93,806-149,074)	134,084 (109,846-173,641)	164,812 (131,931-214,941)	185,981 (152,217-238,214)	205,384 (169,904-260,137)	168,501 (141,239-206,531)	89,170 (76,596-106,040)	30,180 (25,854-35,478)
Diarrhoeal diseases	15,632 (7,854-28,875)	17,686 (9,176-33,541)	20,153 (10,385-38,437)	25,696 (13,419-48,929)	37,669 (20,048-73,117)	45,806 (23,971-85,321)	60,094 (31,577-107,851)	64,787 (33,783-118,222)	70,225 (38,823-125,254)	51,282 (29,028-88,548)	23,569 (14,182-39,330)	7,101 (4,341-11,555)
Intestinal infectious diseases	2,184 (1,174-3,616)	1,940 (1,042-3,256)	1,574 (809-2,700)	1,172 (557-2,115)	1,066 (529-1,912)	952 (539-1,568)	806 (477-1,289)	620 (358-986)	386 (225-617)	167 (97-267)	48 (28-76)	11 (6-18)
Typhoid fever	1,622 (842-2,824)	1,401 (743-2,409)	1,141 (578-2,006)	869 (401-1,623)	820 (394-1,515)	771 (423-1,294)	667 (375-1,068)	509 (290-822)	317 (180-513)	133 (77-212)	38 (21-62)	8 (4-13)
Paratyphoid fever	543 (250-1,048)	518 (226-1,036)	424 (181-853)	295 (120-619)	236 (97-502)	170 (77-337)	127 (59-240)	94 (45-174)	57 (27-104)	23 (11-43)	6 (3-12)	1 (1-2)
Other intestinal infectious diseases	19 (1-100)	21 (2-90)	8 (1-37)	8 (1-37)	10 (1-44)	10 (1-45)	11 (1-49)	17 (2-84)	11 (1-50)	10 (1-41)	4 (1-18)	2 (0-7)
Lower respiratory infections	22,890 (18,548-26,677)	28,931 (23,604-34,465)	38,387 (31,353-44,295)	48,629 (40,016-56,726)	68,381 (54,819-78,362)	78,103 (63,124-90,905)	95,899 (77,540-107,442)	113,215 (90,532-128,168)	127,754 (104,253-144,011)	112,748 (94,542-128,613)	63,905 (55,351-73,001)	22,617 (19,440-25,658)
Upper respiratory infections	52 (37-73)	63 (45-89)	64 (47-87)	69 (50-93)	78 (56-104)	64 (47-85)	49 (36-66)	52 (37-70)	37 (27-50)	27 (20-36)	11 (8-14)	2 (2-3)
Otitis media	17 (9-33)	42 (23-86)	48 (28-92)	23 (11-38)	25 (13-43)	25 (13-45)	24 (14-39)	25 (14-38)	28 (14-43)	22 (9-36)	12 (4-18)	3 (1-5)
Meningitis	5,503 (3,472-9,245)	6,062 (4,074-10,223)	5,857 (3,922-10,064)	5,692 (3,851-9,879)	5,649 (3,952-10,160)	5,480 (3,771-9,803)	4,328 (2,986-8,102)	3,600 (2,561-6,559)	3,353 (2,277-5,834)	1,970 (1,347-3,391)	689 (481-1,183)	164 (122-289)
Pneumococcal meningitis	643 (348-1,205)	746 (401-1,380)	725 (432-1,355)	712 (451-1,292)	747 (463-1,349)	714 (445-1,302)	563 (338-1,069)	452 (282-836)	386 (238-675)	211 (141-355)	77 (52-128)	19 (12-33)
H influenzae type B meningitis	466 (287-819)	512 (312-918)	488 (303-904)	467 (288-807)	481 (294-911)	444 (272-835)	338 (219-627)	279 (180-514)	263 (163-461)	151 (103-254)	51 (33-88)	11 (7-20)
Meningococcal infection	2,281 (1,445-3,897)	2,502 (1,663-4,434)	2,440 (1,585-4,217)	2,384 (1,620-4,247)	2,396 (1,620-4,620)	2,275 (1,540-4,309)	1,789 (1,198-3,453)	1,548 (1,051-2,948)	1,380 (907-2,479)	855 (582-1,552)	305 (208-555)	73 (50-136)
Other meningitis	2,114 (1,284-3,589)	2,302 (1,473-4,087)	2,204 (1,433-3,713)	2,129 (1,418-3,663)	2,024 (1,315-3,613)	2,048 (1,368-3,555)	1,638 (1,094-2,870)	1,321 (914-2,374)	1,324 (885-2,354)	753 (519-1,359)	256 (176-443)	61 (42-104)
Encephalitis	1,971 (1,558-3,182)	2,019 (1,548-3,292)	2,019 (1,543-3,378)	2,404 (1,854-3,762)	2,794 (2,127-4,541)	2,963 (2,247-4,733)	2,972 (2,345-4,572)	3,102 (2,532-4,488)	2,860 (2,327-3,983)	1,628 (1,318-2,201)	569 (461-767)	142 (116-194)
Diphtheria	12 (9-17)	14 (10-20)	17 (12-22)	13 (9-17)	-	-	-	-	-	-	-	-
Whooping cough	40 (21-74)	31 (16-58)	24 (12-44)	33 (17-62)	-	-	-	-	-	-	-	-
Tetanus	914 (464-1,253)	1,000 (504-1,357)	941 (456-1,272)	934 (458-1,246)	701 (353-948)	517 (260-733)	472 (214-634)	306 (127-411)	309 (138-429)	156 (64-221)	56 (23-85)	15 (7-24)
Measles	21 (7-47)	16 (6-36)	12 (4-27)	13 (5-30)	-	-	-	-	-	-	-	-
Varicella and herpes zoster	75 (60-94)	88 (70-110)	73 (59-91)	87 (70-109)	106 (82-133)	175 (139-220)	169 (133-211)	275 (220-343)	431 (335-549)	500 (397-662)	311 (243-411)	125 (97-163)
Neglected tropical diseases and malaria	8,267 (6,206-10,766)	7,817 (5,651-9,913)	7,282 (5,522-9,251)	7,838 (5,846-9,891)	8,618 (6,694-11,094)	8,524 (6,570-10,919)	6,884 (5,368-8,984)	5,424 (4,181-7,129)	3,159 (2,424-3,973)	2,326 (1,789-3,035)	534 (416-666)	196 (154-250)
Malaria	5,303 (3,695-7,430)	4,469 (3,109-6,318)	4,246 (2,958-5,906)	4,300 (3,060-6,015)	5,193 (3,624-7,344)	5,109 (3,412-7,544)	4,273 (2,836-6,315)	3,328 (2,177-4,991)	1,631 (1,124-2,326)	1,388 (949-2,018)	230 (160-330)	111 (74-161)
Chagas disease	131 (113-152)	190 (167-221)	288 (246-335)	378 (316-449)	571 (507-648)	605 (544-676)	640 (571-719)	404 (341-477)	321 (287-359)	211 (187-238)	91 (80-103)	20 (17-22)
Leishmaniasis	389 (200-640)	354 (191-583)	347 (191-560)	296 (164-497)	327 (181-551)	448 (250-737)	360 (201-591)	324 (181-533)	201 (107-330)	143 (75-235)	19 (8-36)	9 (3-18)
Visceral leishmaniasis	389 (200-640)	354 (191-583)	347 (191-560)	296 (164-497)	327 (181-551)	448 (250-737)	360 (201-591)	324 (181-533)	201 (107-330)	143 (75-235)	19 (8-36)	9 (3-18)
African trypanosomiasis	75 (14-283)	70 (11-298)	56 (9-209)	44 (7-177)	35 (5-154)	28 (4-123)	19 (3-91)	13 (2-61)	6 (1-28)	2 (0-8)	0 (0-2)	0 (0-0)
Schistosomiasis	379 (275-519)	395 (287-547)	438 (314-603)	494 (357-670)	514 (381-697)	506 (365-687)	426 (312-578)	318 (233-431)	174 (125-238)	82 (59-113)	23 (15-32)	4 (2-6)
Cysticercosis	39 (32-47)	37 (30-44)	43 (36-51)	40 (34-47)	30 (25-36)	25 (21-30)	15 (12-18)	9 (7-11)	5 (4-7)	3 (2-3)	1 (1-1)	0 (0-0)
Cystic echinococcosis	31 (23-40)	34 (26-45)	34 (27-43)	37 (28-47)	37 (29-47)	34 (27-42)	26 (20-33)	22 (18-28)	15 (12-19)	10 (8-13)	3 (2-4)	1 (0-1)
Dengue	1,055 (150-1,797)	1,185 (192-1,949)	1,106 (183-1,728)	1,372 (190-2,146)	1,187 (201-2,008)	1,180 (196-1,950)	572 (131-1,070)	471 (117-787)	486 (107-930)	310 (85-540)	104 (34-188)	28 (14-43)
Yellow fever	170 (32-519)	125 (23-376)	98 (19-305)	75 (14-233)	56 (10-184)	38 (7-121)	25 (5-76)	14 (2-45)	6 (1-20)	2 (0-7)	1 (0-2)	0 (0-0)
Rabies	379 (160-467)	623 (229-854)	298 (125-348)	426 (129-590)	315 (105-402)	209 (69-271)	170 (68-233)	243 (119-353)	127 (57-186)	67 (26-98)	16 (4-25)	5 (2-7)
Intestinal nematode infections	12 (9-16)	14 (10-17)	19 (15-25)	16 (12-21)	18 (14-24)	12 (9-16)	17 (13-22)	14 (10-17)	9 (7-12)	3 (2-5)	2 (2-3)	1 (0-1)
Ascariasis	12 (9-16)	14 (10-17)	19 (15-25)	16 (12-21)	18 (14-24)	12 (9-16)	17 (13-22)	14 (10-17)	9 (7-12)	3 (2-5)	2 (2-3)	1 (0-1)
Ebola	0 (0-0)	0 (0-0)	0 (0-0)	1 (1-1)	0 (0-0)	0 (0-0)	0 (0-0)	0 (0-0)	0 (0-0)	0 (0-0)	0 (0-0)	0 (0-0)
Zika virus	1 (0-4)	1 (0-5)	1 (0-4)	1 (0-4)	0 (0-2)	0 (0-2)	0 (0-1)	0 (0-1)	0 (0-1)	0 (0-0)	0 (0-0)	0 (0-0)
Other neglected tropical diseases	304 (208-415)	321 (205-458)	308 (229-412)	358 (272-484)	334 (252-443)	329 (248-439)	340 (249-457)	263 (191-366)	177 (128-242)	105 (75-142)	42 (32-55)	17 (13-28)
Maternal disorders	-	-	-	-	-	-	-	-	-	-	-	-
Maternal haemorrhage	-	-	-	-	-	-	-	-	-	-	-	-
Maternal sepsis and other pregnancy related infections	-	-	-	-	-	-	-	-	-	-	-	-
Maternal hypertensive disorders	-	-	-	-	-	-	-	-	-	-	-	-
Maternal obstructed labour and uterine rupture	-	-	-	-	-	-	-	-	-	-	-	-
Maternal abortion, miscarriage, and ectopic pregnancy	-	-	-	-	-	-	-	-	-	-	-	-
Indirect maternal deaths	-	-	-	-	-	-	-	-	-	-	-	-
Late maternal deaths	-	-	-	-	-	-	-	-	-	-	-	-
Maternal deaths aggravated by HIV/AIDS	-	-	-	-	-	-	-	-	-	-	-	-
Other maternal disorders	-	-	-	-	-	-	-	-	-	-	-	-
Neonatal disorders	-	-	-	-	-	-	-	-	-	-	-	-
Neonatal preterm birth complications	-	-	-	-	-	-	-	-	-	-	-	-
Neonatal encephalopathy due to birth asphyxia and trauma	-	-	-	-	-	-	-	-	-	-	-	-
Neonatal sepsis and other neonatal infections	-	-	-	-	-	-	-	-	-	-	-	-
Hemolytic disease and other neonatal jaundice	-	-	-	-	-	-	-	-	-	-	-	-
Other neonatal disorders	-	-	-	-	-	-	-	-	-	-	-	-
Nutritional deficiencies	2,571 (2,088-3,844)	2,879 (2,354-4,453)	3,537 (2,879-5,398)	4,393 (3,643-6,465)	5,296 (4,451-7,788)	6,859 (5,794-9,892)	8,340 (7,129-11,696)	9,591 (8,297-13,023)	10,811 (9,560-13,885)	10,109 (9,130-11,912)	6,671 (6,164-7,441)	2,482 (2,310-2,734)
Protein-energy malnutrition	1,809 (1,455-2,731)	1,800 (1,451-2,779)	2,395 (1,919-3,662)	2,975 (2,423-4,443)	3,684 (3,025-5,478)	4,809 (4,017-6,978)	6,211 (5,223-8,829)	7,331 (6,237-9,989)	8,642 (7,565-10,943)	8,205 (7,248-9,500)	5,600 (4,992-6,155)	2,116 (1,879-2,313)
Iodine deficiency	32 (9-100)	32 (8-106)	43 (12-139)	56 (16-165)	52 (18-123)	65 (22-159)	73 (25-188)	64 (21-156)	46 (15-105)	24 (7-69)	7 (1-24)	3 (0-10)
Iron-deficiency anaemia	59 (48-92)	61 (50-97)	51 (41-86)	64 (49-132)	58 (45-127)	72 (50-161)	88 (55-165)	88 (61-170)	79 (53-172)	56 (31-105)	38 (19-64)	17 (8-27)
Other nutritional deficiencies	671 (443-1,143)	985 (649-1,685)	1,048 (686-1,801)	1,299 (904-2,095)	1,502 (1,089-2,299)	1,914 (1,388-2,899)	1,968 (1,418-3,011)	2,108 (1,447-3,208)	2,044 (1,513-3,020)	1,825 (1,346-2,588)	1,025 (753-1,474)	346 (247-531)
Other communicable, maternal, neonatal, and nutritional diseases	7,113 (6,501-7,812)	8,230 (7,475-9,044)	8,143 (7,517-8,888)	8,457 (7,824-9,147)	8,542 (7,959-9,126)	7,822 (7,238-8,434)	6,944 (6,206-7,548)	6,216 (5,672-6,733)	5,846 (4,658-6,646)	3,417 (2,698-3,854)	1,361 (1,015-1,577)	355 (260-412)
Sexually transmitted diseases excluding HIV	90 (71-120)	84 (66-112)	142 (101-195)	210 (150-299)	66 (53-95)	69 (56-101)	72 (58-126)	75 (62-112)	53 (43-82)	41 (31-52)	22 (16-28)	12 (8-15)
Syphilis	82 (64-109)	76 (59-101)	133 (94-182)	194 (138-277)	61 (50-89)	65 (53-95)	66 (53-116)	69 (57-102)	48 (39-74)	36 (28-46)	19 (14-24)	9 (6-11)
Chlamydial infection	0 (0-0)	0 (0-0)	0 (0-0)	0 (0-0)	0 (0-0)	0 (0-0)	0 (0-0)	0 (0-0)	0 (0-0)	0 (0-0)	0 (0-0)	0 (0-0)
Gonococcal infection	5 (4-7)	4 (3-5)	5 (3-6)	7 (5-11)	3 (2-4)	2 (2-3)	4 (3-7)	4 (3-6)	3 (2-4)	3 (2-3)	2 (1-2)	1 (1-1)
Other sexually transmitted diseases	3 (3-4)	5 (4-6)	5 (4-7)	8 (6-12)	2 (2-3)	2 (2-3)	2 (1-3)	2 (2-3)	2 (1-3)	2 (2-3)	1 (1-2)	2 (1-2)
Hepatitis	5,866 (5,277-6,497)	6,698 (6,007-7,458)	6,392 (5,805-7,047)	6,466 (5,900-7,114)	6,069 (5,597-6,595)	5,279 (4,820-5,786)	4,310 (3,915-4,746)	3,820 (3,431-4,225)	3,060 (2,693-3,446)	1,565 (1,381-1,774)	470 (418-527)	95 (85-106)
Acute hepatitis A	45 (19-84)	58 (28-105)	65 (29-117)	72 (36-122)	70 (34-123)	61 (30-109)	53 (28-91)	50 (24-87)	41 (19-76)	20 (10-34)	6 (3-11)	1 (1-2)
Hepatitis B	5,354 (4,639-6,049)	6,101 (5,378-6,920)	5,800 (5,122-6,471)	5,832 (5,094-6,565)	5,431 (4,772-6,047)	4,659 (4,114-5,285)	3,710 (3,152-4,200)	3,203 (2,615-3,679)	2,269 (1,748-2,757)	956 (656-1,246)	231 (131-342)	38 (17-60)
Hepatitis C	60 (27-108)	92 (43-167)	120 (55-214)	157 (80-288)	172 (81-311)	138 (65-241)	107 (50-191)	95 (46-164)	77 (40-134)	41 (23-66)	13 (8-22)	3 (1-5)
Acute hepatitis E	408 (175-767)	447 (210-813)	407 (183-740)	405 (175-811)	395 (153-781)	421 (187-776)	440 (198-821)	472 (182-917)	672 (341-1,135)	548 (301-857)	220 (123-315)	54 (32-76)
Other infectious diseases	1,156 (924-1,331)	1,448 (1,170-1,650)	1,609 (1,300-1,828)	1,781 (1,443-2,017)	2,408 (1,957-2,727)	2,474 (2,041-2,767)	2,563 (1,874-2,977)	2,321 (1,827-2,670)	2,734 (1,573-3,409)	1,812 (1,103-2,196)	869 (536-1,073)	248 (156-302)
Non-communicable diseases	550,506 (533,775-570,684)	840,816 (818,358-867,974)	1,227,943 (1,194,894-1,266,916)	1,630,252 (1,588,683-1,683,170)	2,198,832 (2,147,487-2,258,819)	2,434,691 (2,378,608-2,492,437)	2,493,890 (2,437,086-2,544,500)	2,740,109 (2,673,948-2,806,520)	2,541,854 (2,486,799-2,591,371)	1,929,758 (1,893,102-1,965,890)	924,473 (907,122-939,594)	243,446 (238,325-248,209)
Neoplasms	130,489 (125,797-134,643)	227,877 (220,489-234,494)	358,622 (347,208-369,501)	496,429 (482,162-510,173)	688,319 (669,877-706,363)	739,187 (719,396-758,367)	677,210 (660,274-693,229)	638,146 (619,570-655,509)	492,301 (477,065-507,692)	309,766 (299,509-319,685)	125,209 (120,588-129,740)	27,446 (26,258-28,638)
Lip and oral cavity cancer	5,496 (5,003-6,071)	8,713 (8,046-9,427)	12,929 (12,093-13,823)	16,132 (15,244-17,113)	17,436 (16,358-18,553)	15,201 (14,343-16,109)	11,876 (11,185-12,555)	9,554 (9,019-10,098)	7,084 (6,662-7,464)	4,310 (4,048-4,548)	1,762 (1,648-1,854)	423 (396-447)
Nasopharynx cancer	2,688 (2,430-3,026)	4,540 (4,112-5,024)	5,944 (5,421-6,518)	6,465 (5,997-6,989)	7,121 (6,571-7,699)	5,972 (5,534-6,416)	4,199 (3,902-4,522)	2,966 (2,746-3,192)	2,050 (1,891-2,214)	1,006 (935-1,080)	389 (365-416)	93 (87-98)
Other pharynx cancer	3,617 (3,144-4,005)	6,406 (5,673-7,029)	10,342 (9,273-11,221)	13,326 (12,070-14,382)	14,435 (12,979-15,577)	11,996 (10,771-13,020)	9,126 (8,207-9,955)	6,534 (5,887-7,085)	4,733 (4,213-5,269)	2,217 (1,994-2,445)	685 (615-754)	119 (107-130)
Oesophageal cancer	5,966 (5,631-6,352)	13,111 (12,479-13,848)	23,632 (22,547-24,915)	34,688 (33,082-36,514)	50,411 (47,887-52,885)	52,264 (49,637-55,119)	42,411 (40,378-44,655)	36,161 (34,403-38,101)	25,065 (23,913-26,405)	13,111 (12,593-13,657)	4,059 (3,904-4,219)	753 (720-786)
Stomach cancer	11,736 (11,185-12,383)	22,308 (21,429-23,338)	35,962 (34,496-37,503)	50,737 (48,683-53,035)	77,268 (73,964-80,762)	83,279 (79,794-86,913)	74,780 (72,106-77,619)	70,874 (68,055-73,949)	52,078 (50,023-54,137)	30,509 (29,204-31,749)	11,029 (10,588-11,473)	2,549 (2,450-2,654)
Colon and rectum cancer	9,442 (8,898-9,961)	16,547 (15,620-17,435)	25,404 (24,101-26,655)	36,995 (35,155-38,713)	54,119 (51,384-56,835)	61,886 (58,780-65,351)	60,071 (56,941-63,262)	63,157 (59,911-66,857)	52,221 (49,671-55,028)	35,852 (33,955-37,608)	15,366 (14,583-16,114)	3,458 (3,287-3,627)
Liver cancer	27,284 (25,271-29,306)	45,939 (42,674-49,114)	61,496 (57,700-65,296)	71,619 (67,891-75,807)	90,072 (85,344-95,104)	82,284 (77,726-87,161)	65,618 (62,121-68,675)	53,640 (50,869-56,185)	36,011 (34,198-37,811)	18,141 (17,237-19,039)	5,391 (5,129-5,643)	977 (931-1,026)
Liver cancer due to hepatitis B	15,690 (12,787-18,119)	25,468 (20,529-29,411)	31,808 (25,519-37,147)	34,441 (26,738-40,333)	41,463 (31,531-48,502)	34,896 (26,992-41,105)	25,286 (19,210-29,868)	19,381 (15,011-23,266)	12,037 (8,853-14,930)	5,719 (4,507-6,938)	1,563 (1,210-1,944)	286 (191-371)
Liver cancer due to hepatitis C	1,756 (1,378-2,262)	3,895 (3,035-4,907)	7,227 (5,765-8,802)	10,643 (8,845-12,849)	14,491 (12,079-17,490)	14,819 (12,614-17,534)	13,767 (11,859-16,109)	12,668 (10,914-14,890)	9,913 (8,537-11,486)	5,666 (5,043-6,385)	1,913 (1,673-2,172)	370 (314-430)
Liver cancer due to alcohol use	1,819 (1,358-2,451)	3,134 (2,238-4,280)	4,950 (3,463-6,598)	6,402 (4,579-8,748)	8,339 (6,112-11,123)	9,426 (7,507-11,777)	8,504 (6,725-10,422)	6,623 (5,376-8,074)	4,090 (3,146-5,081)	1,970 (1,557-2,423)	562 (422-712)	79 (52-111)
Liver cancer due to other causes	8,020 (6,504-10,220)	13,442 (10,858-16,889)	17,512 (14,067-22,249)	20,133 (16,354-25,204)	25,779 (21,228-32,476)	23,143 (19,204-28,548)	18,060 (15,150-22,003)	14,968 (12,520-18,037)	9,970 (8,266-12,146)	4,786 (4,073-5,615)	1,353 (1,121-1,622)	242 (187-305)
Gallbladder and biliary tract cancer	1,363 (997-1,584)	2,297 (1,691-2,653)	4,016 (3,034-4,544)	5,686 (4,327-6,579)	7,983 (6,218-9,119)	8,963 (7,184-10,223)	9,308 (7,705-10,670)	9,618 (7,960-10,757)	8,078 (6,829-8,952)	5,387 (4,544-5,900)	2,180 (1,834-2,386)	499 (424-544)
Pancreatic cancer	4,251 (4,035-4,452)	8,205 (7,870-8,556)	14,302 (13,739-14,904)	21,548 (20,704-22,423)	29,987 (28,889-31,083)	33,439 (32,057-34,629)	30,970 (29,726-32,104)	27,891 (26,557-29,102)	20,587 (19,474-21,519)	12,524 (11,927-13,031)	4,787 (4,607-4,961)	966 (930-1,000)
Larynx cancer	2,364 (2,198-2,551)	5,104 (4,838-5,442)	9,406 (8,942-9,954)	13,032 (12,424-13,750)	16,033 (15,299-16,849)	15,208 (14,482-15,968)	11,507 (11,057-11,993)	9,226 (8,850-9,663)	6,604 (6,280-6,929)	3,477 (3,317-3,633)	1,179 (1,124-1,237)	223 (213-234)
Tracheal, bronchus, and lung cancer	16,822 (16,004-17,763)	40,142 (38,136-42,136)	76,394 (72,689-79,957)	118,967 (113,502-124,568)	175,457 (167,599-182,805)	196,646 (188,726-204,377)	179,083 (172,368-185,550)	159,601 (153,097-165,987)	114,262 (108,759-120,377)	61,562 (58,509-64,841)	19,233 (18,240-20,459)	3,405 (3,227-3,629)
Malignant skin melanoma	1,527 (1,301-1,798)	1,911 (1,660-2,291)	2,801 (2,478-3,350)	3,513 (3,151-4,022)	3,771 (3,337-4,202)	4,129 (3,636-4,570)	3,829 (3,274-4,113)	3,660 (3,044-3,963)	3,098 (2,444-3,350)	2,203 (1,695-2,395)	1,021 (783-1,105)	234 (182-251)
Non-melanoma skin cancer	718 (681-756)	1,028 (973-1,084)	1,642 (1,553-1,735)	2,306 (2,178-2,448)	4,084 (3,872-4,304)	4,564 (4,333-4,823)	4,434 (4,211-4,674)	4,581 (4,348-4,844)	4,322 (4,110-4,561)	3,494 (3,318-3,676)	2,018 (1,931-2,112)	683 (654-708)
Non-melanoma skin cancer (squamous-cell carcinoma)	718 (681-756)	1,028 (973-1,084)	1,642 (1,553-1,735)	2,306 (2,178-2,448)	4,084 (3,872-4,304)	4,564 (4,333-4,823)	4,434 (4,211-4,674)	4,581 (4,348-4,844)	4,322 (4,110-4,561)	3,494 (3,318-3,676)	2,018 (1,931-2,112)	683 (654-708)
Breast cancer	283 (207-314)	624 (424-694)	892 (595-987)	1,114 (760-1,228)	1,527 (951-1,685)	1,485 (989-1,643)	1,302 (920-1,451)	841 (653-920)	834 (626-910)	590 (443-644)	277 (216-304)	74 (59-81)
Cervical cancer	-	-	-	-	-	-	-	-	-	-	-	-
Uterine cancer	-	-	-	-	-	-	-	-	-	-	-	-
Ovarian cancer	-	-	-	-	-	-	-	-	-	-	-	-
Prostate cancer	619 (465-692)	1,848 (1,419-2,047)	5,407 (4,162-5,942)	12,841 (10,069-14,184)	28,529 (22,000-31,462)	44,124 (34,995-48,426)	57,606 (46,345-62,863)	71,133 (58,916-78,798)	65,817 (56,569-73,724)	54,756 (47,276-61,089)	30,051 (25,933-32,330)	7,453 (6,351-8,135)
Testicular cancer	654 (613-701)	557 (522-596)	482 (448-518)	375 (350-402)	416 (390-447)	394 (370-420)	374 (352-398)	325 (305-346)	348 (322-377)	229 (213-247)	107 (99-117)	31 (28-33)
Kidney cancer	1,718 (1,604-1,841)	3,478 (3,261-3,695)	6,208 (5,788-6,610)	9,164 (8,614-9,749)	11,217 (10,606-11,968)	11,869 (11,225-12,480)	10,706 (10,162-11,249)	11,052 (10,397-11,639)	8,650 (8,053-9,130)	5,786 (5,417-6,105)	2,348 (2,212-2,478)	456 (433-479)
Bladder cancer	1,393 (1,275-1,522)	2,756 (2,559-2,954)	5,039 (4,782-5,318)	8,201 (7,801-8,669)	13,446 (12,797-14,106)	16,999 (16,163-17,813)	18,994 (18,202-19,770)	21,859 (20,866-22,933)	21,457 (20,160-22,628)	16,550 (15,730-17,439)	7,855 (7,517-8,172)	1,819 (1,737-1,895)
Brain and nervous system cancer	7,122 (6,122-8,357)	9,108 (7,884-10,484)	11,694 (10,323-13,471)	13,837 (12,074-15,781)	15,971 (13,874-17,724)	14,649 (12,540-16,140)	11,183 (9,483-12,089)	8,849 (7,307-9,556)	5,435 (4,250-5,906)	2,607 (1,951-2,879)	782 (597-866)	142 (109-159)
Thyroid cancer	557 (499-609)	986 (872-1,068)	1,514 (1,367-1,625)	2,009 (1,842-2,134)	1,945 (1,806-2,133)	2,005 (1,867-2,180)	1,805 (1,675-1,977)	2,091 (1,878-2,226)	1,601 (1,462-1,697)	893 (836-948)	331 (312-349)	69 (65-73)
Mesothelioma	456 (400-502)	864 (777-946)	1,385 (1,246-1,492)	2,126 (1,907-2,302)	2,315 (2,088-2,591)	3,011 (2,710-3,323)	3,220 (2,892-3,471)	3,254 (2,922-3,510)	2,625 (2,377-2,809)	1,505 (1,362-1,616)	537 (490-573)	92 (85-98)
Hodgkin lymphoma	1,043 (779-1,374)	1,311 (1,016-1,693)	1,403 (1,166-1,845)	1,756 (1,460-2,267)	1,740 (1,445-2,233)	1,741 (1,467-2,168)	1,451 (1,223-1,844)	897 (757-1,074)	601 (505-716)	326 (275-372)	106 (94-124)	18 (16-21)
Non-Hodgkin's lymphoma	5,313 (4,430-5,740)	7,263 (5,838-7,837)	9,839 (8,132-10,518)	12,149 (10,061-13,040)	15,017 (13,028-16,287)	16,100 (13,789-17,453)	15,115 (13,052-16,512)	13,954 (12,233-15,364)	11,352 (9,485-12,193)	7,404 (6,113-7,867)	2,925 (2,478-3,104)	578 (499-626)
Multiple myeloma	1,265 (1,056-1,509)	1,828 (1,507-2,150)	3,227 (2,682-3,874)	4,799 (3,974-5,826)	6,293 (5,106-7,304)	7,139 (5,875-8,637)	7,150 (5,691-8,307)	6,773 (5,261-7,752)	5,528 (4,281-6,508)	3,732 (2,964-4,358)	1,461 (1,154-1,789)	255 (196-309)
Leukaemia	6,551 (5,876-7,203)	8,223 (7,448-9,086)	10,217 (9,188-11,395)	12,634 (11,204-13,982)	16,325 (14,586-17,604)	18,116 (16,410-19,418)	17,582 (16,018-18,860)	17,471 (16,139-18,392)	14,174 (13,325-14,957)	9,567 (9,114-10,097)	4,191 (4,013-4,432)	918 (874-994)
Acute lymphoid leukaemia	1,308 (1,142-1,480)	1,534 (1,345-1,730)	1,785 (1,554-2,002)	2,063 (1,790-2,318)	2,263 (1,955-2,497)	2,152 (1,859-2,398)	1,873 (1,645-2,130)	1,144 (1,019-1,295)	752 (680-851)	434 (395-494)	153 (141-172)	37 (34-41)
Chronic lymphoid leukaemia	237 (208-285)	422 (378-513)	769 (684-941)	1,292 (1,149-1,684)	2,066 (1,844-2,577)	2,676 (2,332-3,329)	2,952 (2,628-3,715)	3,101 (2,730-3,849)	2,958 (2,559-3,643)	2,308 (1,971-2,863)	1,137 (997-1,350)	256 (228-302)
Acute myeloid leukaemia	1,843 (1,594-2,162)	2,212 (1,923-2,588)	3,026 (2,668-3,487)	3,797 (3,283-4,359)	4,666 (3,999-5,246)	5,588 (4,757-6,194)	5,450 (4,506-6,048)	5,104 (4,140-5,603)	3,832 (3,104-4,247)	2,293 (1,838-2,578)	857 (747-1,038)	155 (138-197)
Chronic myeloid leukaemia	592 (507-701)	801 (690-934)	872 (753-1,008)	1,009 (869-1,177)	1,058 (928-1,215)	1,152 (1,024-1,290)	1,040 (930-1,174)	1,384 (1,230-1,559)	1,170 (1,060-1,320)	804 (734-907)	378 (341-425)	79 (72-91)
Other leukaemia	2,572 (2,166-2,870)	3,254 (2,716-3,674)	3,764 (3,142-4,163)	4,473 (3,679-4,848)	6,272 (5,366-6,904)	6,548 (5,617-7,081)	6,266 (5,383-6,905)	6,737 (5,995-7,261)	5,462 (4,974-5,998)	3,727 (3,421-4,099)	1,665 (1,543-1,806)	392 (365-432)
Other neoplasms	10,240 (8,360-10,955)	12,779 (10,576-13,538)	17,043 (14,158-18,036)	20,411 (17,143-21,511)	25,400 (21,097-26,826)	25,725 (22,007-27,091)	23,509 (20,371-24,721)	22,184 (19,252-23,262)	17,688 (15,425-18,629)	12,030 (10,700-12,687)	5,139 (4,738-5,439)	1,159 (1,080-1,223)
Cardiovascular diseases	220,354 (211,930-229,521)	349,560 (336,994-362,616)	524,160 (506,633-543,996)	699,622 (674,735-726,298)	943,698 (914,053-976,133)	1,057,283 (1,025,607-1,088,719)	1,107,686 (1,076,281-1,135,841)	1,277,817 (1,237,763-1,318,790)	1,209,481 (1,174,734-1,241,559)	941,988 (914,182-966,211)	450,779 (437,881-463,069)	124,804 (120,687-128,290)
Rheumatic heart disease	5,903 (5,342-6,495)	6,769 (6,162-7,407)	8,194 (7,590-8,985)	9,620 (8,934-10,467)	11,634 (10,880-12,534)	12,816 (12,004-13,761)	12,998 (12,192-13,910)	12,655 (11,974-13,459)	12,120 (11,467-12,865)	9,138 (8,692-9,649)	4,681 (4,475-4,935)	1,383 (1,323-1,460)
Ischaemic heart disease	126,283 (121,056-132,083)	204,005 (195,891-212,209)	305,560 (293,795-317,592)	407,008 (390,753-425,117)	524,080 (504,625-544,945)	566,486 (546,720-588,608)	589,265 (569,339-608,633)	687,377 (660,408-717,406)	655,900 (636,084-679,215)	528,896 (514,379-548,145)	264,650 (258,163-273,927)	75,730 (73,939-78,346)
Cerebrovascular disease	59,927 (57,267-62,511)	102,205 (97,862-106,512)	157,610 (151,621-164,266)	213,571 (205,347-222,097)	317,427 (305,142-329,499)	373,452 (358,939-387,883)	390,316 (373,691-406,366)	434,327 (411,139-457,020)	384,364 (357,230-408,982)	267,488 (246,483-287,784)	110,670 (101,038-121,003)	29,089 (26,021-31,748)

SR Table 4: Global deaths by cause and age group for males (with 95% uncertainty intervals), 2016

Cause	Early Neonatal	Late Neonatal	Post Neonatal	1 to 4	5 to 9	10 to 14	15 to 19	20 to 24	25 to 29	30 to 34	35 to 39	40 to 44	45 to 49	50 to 54	55 to 59	60 to 64	65 to 69	70 to 74	75 to 79	80 to 84	85 to 89	90 to 94	95 plus
Ischaemic stroke	-	-	76 (64-93)	93 (80-111)	84 (71-98)	108 (94-123)	499 (450-557)	1,274 (1,128-1,408)	2,037 (1,814-2,285)	3,088 (2,749-3,432)	4,741 (4,226-5,271)	8,819 (8,026-9,590)	20,421 (18,866-21,990)	38,492 (35,497-41,213)	60,910 (56,485-65,304)	129,168 (121,657-136,758)	165,624 (155,893-174,654)	186,330 (176,090-195,980)	245,822 (231,572-261,033)	222,994 (206,617-238,172)	163,654 (150,747-177,366)	70,089 (63,957-76,328)	19,142 (17,148-20,933)
Hemorrhagic stroke	800 (688-959)	381 (326-470)	2,564 (2,227-3,058)	1,774 (1,520-2,081)	1,305 (1,149-1,495)	1,658 (1,510-1,845)	3,457 (3,214-3,735)	7,666 (7,191-8,208)	12,038 (11,161-12,802)	18,667 (17,525-19,689)	29,011 (27,410-30,586)	51,108 (48,756-53,316)	81,784 (78,100-85,616)	119,118 (113,944-124,538)	152,662 (146,052-159,565)	188,260 (179,902-196,492)	207,828 (198,396-217,704)	203,986 (193,576-214,300)	188,505 (177,903-199,668)	161,370 (149,854-172,080)	103,834 (95,273-111,891)	40,580 (37,031-44,168)	9,948 (8,902-10,909)
Hypertensive heart disease	-	-	125 (97-176)	61 (47-75)	67 (54-84)	104 (70-138)	674 (538-852)	1,258 (979-1,561)	1,956 (1,478-2,307)	3,050 (2,280-3,610)	4,728 (3,575-5,457)	7,728 (5,716-8,914)	12,198 (9,035-14,378)	18,627 (14,041-21,484)	25,868 (19,549-30,416)	35,553 (27,100-41,151)	41,896 (31,717-47,799)	46,537 (33,939-53,269)	58,146 (41,922-64,985)	60,812 (42,762-67,787)	48,134 (33,284-53,515)	23,983 (16,033-26,801)	6,565 (4,396-7,344)
Cardiomyopathy and myocarditis	385 (264-491)	205 (167-246)	1,562 (1,226-1,811)	1,070 (865-1,288)	610 (501-721)	730 (616-838)	1,924 (1,587-2,218)	2,633 (2,075-2,959)	4,722 (3,568-5,574)	5,970 (4,536-7,345)	7,812 (5,929-9,913)	10,178 (8,007-12,650)	11,270 (9,044-13,906)	15,174 (12,194-19,307)	17,534 (14,478-22,360)	17,805 (15,256-21,972)	16,275 (14,071-19,677)	14,288 (11,589-16,392)	16,809 (12,615-18,744)	16,834 (11,553-18,845)	15,091 (9,958-17,094)	8,351 (5,558-9,412)	2,384 (1,570-2,653)
Myocarditis	70 (39-117)	54 (38-70)	527 (396-640)	400 (308-527)	214 (165-270)	253 (194-303)	728 (515-938)	778 (578-964)	1,168 (827-1,445)	805 (607-985)	923 (667-1,159)	1,163 (834-1,538)	915 (693-1,171)	919 (685-1,141)	1,099 (820-1,498)	1,153 (818-1,446)	1,242 (850-1,480)	1,367 (938-1,596)	1,715 (1,185-2,018)	1,908 (1,272-2,260)	2,046 (1,380-2,474)	1,457 (943-1,820)	523 (320-650)
Alcoholic cardiomyopathy	-	-	-	-	-	-	107 (83-162)	305 (215-419)	1,138 (729-1,630)	2,423 (1,614-3,487)	3,641 (2,420-5,361)	4,895 (3,350-6,931)	5,854 (4,224-8,079)	8,169 (5,806-11,503)	8,959 (6,605-12,499)	8,188 (6,319-10,973)	5,883 (4,400-7,829)	3,660 (2,421-4,474)	3,715 (2,285-4,675)	2,267 (1,212-2,887)	1,369 (690-1,807)	517 (313-627)	172 (95-206)
Other cardiomyopathy	315 (219-400)	151 (123-183)	1,035 (797-1,217)	670 (531-830)	396 (313-477)	477 (396-562)	1,089 (864-1,278)	1,551 (1,197-1,755)	2,417 (1,765-2,856)	2,742 (2,022-3,321)	3,248 (2,389-3,953)	4,120 (3,117-4,893)	4,501 (3,597-5,291)	6,087 (4,954-7,430)	7,476 (6,335-9,512)	8,464 (7,162-10,788)	9,149 (7,822-11,172)	9,260 (7,674-10,627)	11,379 (8,621-12,621)	12,659 (8,640-14,252)	11,675 (7,583-13,254)	6,377 (4,091-7,348)	1,690 (1,121-1,976)
Atrial fibrillation and flutter	-	-	-	-	-	-	-	-	-	19 (14-26)	73 (53-97)	182 (134-240)	370 (270-492)	652 (489-842)	1,138 (687-1,701)	2,343 (1,557-3,265)	4,325 (2,708-6,481)	7,442 (5,380-10,225)	11,510 (7,314-16,959)	22,422 (17,068-28,778)	25,452 (19,544-32,489)	12,702 (9,723-16,392)	2,913 (2,241-3,699)
Aortic aneurysm	-	-	-	-	-	-	239 (220-262)	350 (326-380)	649 (601-698)	897 (839-961)	1,448 (1,350-1,554)	1,990 (1,887-2,105)	2,886 (2,743-3,044)	4,202 (3,979-4,495)	6,298 (5,964-6,694)	9,666 (9,164-10,299)	12,122 (11,442-12,932)	13,268 (12,610-14,066)	15,514 (14,709-16,487)	15,089 (14,372-16,009)	12,103 (11,550-12,737)	5,724 (5,500-6,018)	1,262 (1,215-1,326)
Peripheral vascular disease	-	-	-	-	-	-	-	-	-	-	-	121 (88-149)	236 (170-296)	597 (406-764)	1,158 (754-1,467)	2,064 (1,394-2,712)	2,761 (1,897-3,590)	3,379 (2,482-4,659)	4,566 (3,367-6,257)	4,915 (3,741-6,961)	4,684 (3,596-7,017)	2,852 (2,192-4,237)	891 (709-1,409)
Endocarditis	66 (43-86)	39 (25-53)	841 (641-1,136)	360 (275-482)	168 (128-222)	294 (230-388)	617 (464-856)	958 (727-1,302)	1,362 (1,060-1,840)	1,512 (1,182-2,006)	1,734 (1,396-2,351)	2,052 (1,645-2,829)	2,114 (1,693-2,892)	2,636 (2,119-3,618)	2,941 (2,308-3,876)	3,626 (2,868-4,949)	4,044 (3,206-5,402)	4,115 (3,261-5,398)	4,698 (3,637-6,196)	4,617 (3,652-5,908)	3,690 (2,653-4,610)	1,954 (1,360-2,328)	555 (391-688)
Other cardiovascular and circulatory diseases	550 (402-672)	299 (238-381)	2,271 (1,822-2,938)	1,478 (1,122-1,996)	801 (654-1,103)	903 (753-1,238)	1,568 (1,287-2,227)	2,289 (1,917-3,320)	3,208 (2,634-4,607)	3,685 (3,124-5,136)	4,272 (3,517-5,846)	5,988 (5,007-8,178)	7,508 (6,154-10,236)	10,908 (9,197-14,234)	14,486 (12,235-19,467)	19,498 (16,346-25,164)	23,105 (18,931-29,721)	26,078 (21,805-33,632)	32,216 (27,441-40,379)	32,408 (28,227-40,245)	27,313 (25,007-33,474)	15,212 (14,152-17,936)	4,033 (3,767-4,722)
Chronic respiratory diseases	0 (0-0)	0 (0-0)	0 (0-0)	4,794 (3,893-6,040)	1,745 (1,544-1,971)	2,083 (1,880-2,302)	3,926 (3,643-4,260)	6,069 (5,681-6,489)	8,254 (7,781-8,803)	10,397 (9,873-11,032)	14,908 (14,085-15,785)	22,198 (20,998-23,494)	37,392 (35,502-39,421)	66,694 (63,506-69,903)	107,899 (103,178-113,074)	187,059 (178,721-195,682)	244,132 (233,552-254,010)	295,435 (282,620-308,106)	335,406 (323,260-347,436)	327,134 (313,726-339,017)	230,314 (220,961-238,961)	98,872 (94,981-102,333)	25,169 (24,078-26,121)
Chronic obstructive pulmonary disease	-	-	0 (0-0)	811 (562-1,269)	280 (224-363)	256 (201-329)	1,553 (1,257-1,830)	2,438 (1,986-2,881)	3,549 (2,938-4,172)	5,069 (4,341-5,797)	7,593 (6,484-8,784)	12,975 (11,222-14,778)	24,224 (21,442-27,039)	47,116 (41,931-51,846)	80,764 (73,512-87,938)	150,225 (138,096-161,337)	204,131 (190,511-217,006)	252,721 (238,553-267,065)	292,723 (278,997-307,220)	290,910 (277,581-304,844)	207,623 (198,486-217,181)	89,597 (86,013-93,359)	22,930 (21,951-23,963)
Pneumoconiosis	-	-	-	-	-	-	15 (12-18)	28 (23-34)	72 (63-83)	110 (97-127)	221 (191-265)	429 (367-503)	766 (676-876)	1,027 (924-1,162)	1,213 (1,072-1,417)	1,891 (1,685-2,187)	2,211 (1,979-2,576)	2,519 (2,294-2,837)	2,980 (2,766-3,243)	2,764 (2,572-2,993)	1,886 (1,754-2,049)	720 (669-788)	147 (137-158)
Silicosis	-	-	-	-	-	-	1 (1-2)	4 (3-5)	22 (17-27)	49 (40-59)	121 (103-142)	266 (227-313)	509 (435-585)	647 (555-742)	715 (615-828)	1,036 (895-1,194)	1,181 (1,013-1,402)	1,180 (1,037-1,374)	1,430 (1,247-1,679)	1,398 (1,206-1,652)	897 (747-1,066)	286 (218-345)	46 (36-55)
Asbestosis	-	-	-	-	-	-	7 (6-8)	12 (10-15)	20 (17-25)	23 (19-30)	32 (24-44)	44 (31-60)	51 (36-68)	77 (53-99)	107 (68-142)	181 (114-246)	276 (188-351)	405 (269-515)	518 (305-643)	525 (295-656)	405 (235-501)	175 (94-223)	33 (18-41)
Coal workers pneumoconiosis	-	-	-	-	-	-	0 (0-0)	1 (0-1)	5 (3-6)	5 (3-6)	14 (7-19)	31 (16-42)	61 (31-79)	100 (49-133)	125 (58-169)	221 (115-288)	291 (177-379)	363 (199-458)	419 (224-513)	392 (278-477)	272 (183-348)	103 (64-141)	22 (11-28)
Other pneumoconiosis	-	-	-	-	-	-	6 (5-8)	11 (9-14)	25 (21-30)	33 (28-42)	55 (42-77)	87 (67-120)	145 (116-206)	203 (163-288)	267 (200-389)	453 (342-668)	463 (355-666)	572 (442-794)	613 (491-859)	448 (359-633)	311 (256-442)	155 (128-228)	45 (39-63)
Asthma	-	-	-	2,754 (2,011-3,689)	907 (731-1,109)	1,347 (1,147-1,564)	1,758 (1,423-2,087)	2,626 (2,094-3,153)	3,667 (2,896-4,338)	3,821 (2,915-4,554)	5,428 (4,090-6,655)	6,552 (4,773-8,173)	9,211 (6,616-11,650)	13,981 (9,777-18,877)	19,223 (13,339-25,711)	25,875 (17,566-36,371)	26,475 (18,247-36,803)	27,110 (18,883-38,373)	25,839 (18,413-36,767)	20,195 (14,602-29,097)	11,326 (8,482-15,532)	4,232 (3,253-5,959)	1,040 (798-1,444)
Interstitial lung disease and pulmonary sarcoidosis	-	-	-	111 (69-184)	50 (35-71)	39 (27-51)	116 (78-156)	196 (132-260)	308 (212-413)	428 (293-563)	632 (430-867)	1,013 (705-1,370)	1,625 (1,115-2,128)	2,862 (1,945-3,701)	4,405 (2,988-5,644)	6,454 (4,426-8,195)	8,706 (5,689-10,680)	10,402 (6,730-12,707)	11,575 (7,262-13,949)	10,941 (6,443-13,173)	7,721 (4,469-9,128)	3,333 (1,927-3,912)	769 (469-894)
Other chronic respiratory diseases	0 (0-0)	0 (0-0)	0 (0-0)	1,118 (596-1,780)	508 (366-682)	441 (291-598)	484 (325-655)	782 (537-1,041)	658 (395-894)	968 (601-1,330)	1,033 (617-1,407)	1,229 (751-1,659)	1,566 (933-2,097)	1,709 (1,016-2,226)	2,293 (1,404-3,084)	2,614 (1,645-3,448)	2,608 (1,573-3,396)	2,683 (1,703-3,455)	2,289 (1,412-2,970)	2,324 (1,475-2,957)	1,759 (1,177-2,235)	990 (628-1,321)	283 (190-372)
Cirrhosis and other chronic liver diseases	-	-	-	3,581 (2,951-5,747)	1,797 (1,502-2,658)	2,000 (1,718-2,857)	4,424 (3,841-5,367)	9,082 (8,129-11,493)	17,254 (15,512-20,061)	31,558 (28,760-36,362)	47,619 (43,682-53,840)	66,280 (61,683-74,708)	85,912 (80,256-99,763)	102,947 (96,188-117,506)	106,203 (99,597-120,420)	106,088 (98,825-123,493)	85,495 (79,403-99,001)	65,108 (60,777-72,445)	52,713 (48,957-58,203)	37,094 (34,219-40,717)	21,500 (19,791-23,069)	8,085 (7,399-8,701)	2,064 (1,869-2,224)
Cirrhosis and other chronic liver diseases due to hepatitis B	-	-	-	47 (31-78)	63 (43-95)	116 (81-168)	410 (279-568)	2,361 (1,706-3,221)	6,094 (4,694-7,923)	11,582 (9,716-14,040)	17,360 (14,445-20,889)	23,104 (19,884-27,436)	27,409 (22,540-33,486)	30,753 (25,282-38,048)	31,841 (26,616-39,273)	33,587 (27,926-42,256)	27,504 (22,899-33,920)	21,253 (17,620-25,970)	17,560 (14,548-20,997)	11,873 (9,679-14,402)	6,313 (5,310-7,416)	2,061 (1,677-2,535)	480 (352-623)
Cirrhosis and other chronic liver diseases due to hepatitis C	-	-	-	32 (20-53)	43 (27-69)	90 (60-134)	301 (193-439)	1,605 (1,161-2,156)	4,122 (3,053-5,394)	8,372 (6,902-10,196)	12,998 (10,739-16,072)	18,408 (15,327-22,005)	24,337 (20,093-29,027)	29,714 (24,589-35,979)	30,534 (25,739-36,438)	29,616 (24,500-36,028)	24,616 (20,707-30,334)	18,869 (15,838-22,392)	14,326 (11,665-17,499)	10,087 (8,141-12,238)	5,780 (4,720-6,818)	2,186 (1,702-2,635)	552 (401-704)
Cirrhosis and other chronic liver diseases due to alcohol use	-	-	-	-	-	-	81 (46-131)	718 (510-994)	2,124 (1,512-2,884)	6,299 (5,187-7,670)	12,222 (9,826-15,001)	18,444 (15,741-21,704)	25,291 (21,171-30,156)	31,286 (26,294-37,254)	31,439 (25,882-37,695)	30,135 (24,518-36,722)	23,739 (20,105-28,011)	17,807 (14,806-21,051)	14,621 (12,306-17,479)	10,259 (8,489-12,372)	6,206 (5,337-7,126)	2,543 (2,132-2,946)	671 (539-809)
Cirrhosis and other chronic liver diseases due to other causes	-	-	-	3,503 (2,892-5,628)	1,691 (1,415-2,514)	1,794 (1,536-2,561)	3,631 (3,130-4,426)	4,398 (3,457-5,650)	4,914 (3,724-6,382)	5,304 (3,998-6,861)	5,039 (3,580-6,845)	6,324 (4,506-8,496)	8,875 (6,376-12,214)	11,194 (8,213-14,887)	12,389 (9,085-16,899)	12,749 (9,125-17,741)	9,635 (7,003-13,099)	7,179 (5,219-9,740)	6,226 (4,540-8,274)	4,876 (3,582-6,515)	3,201 (2,583-3,984)	1,295 (1,002-1,675)	360 (257-491)
Digestive diseases	798 (633-987)	699 (535-867)	7,751 (5,733-9,772)	6,816 (5,467-8,935)	3,370 (2,887-3,943)	3,614 (3,176-4,155)	5,787 (5,153-6,574)	8,345 (7,580-9,571)	12,753 (11,629-14,502)	14,656 (13,420-16,927)	19,231 (17,593-22,493)	24,544 (22,795-28,452)	29,951 (27,605-34,130)	35,635 (32,869-41,364)	43,191 (40,315-50,787)	52,133 (48,801-61,096)	56,648 (52,725-65,195)	58,236 (53,855-68,359)	62,635 (57,983-73,667)	56,534 (52,963-66,242)	43,152 (40,022-50,032)	21,592 (20,142-24,494)	5,714 (5,333-6,546)
Peptic ulcer disease	-	-	-	527 (370-767)	312 (240-404)	471 (379-599)	965 (790-1,225)	1,765 (1,467-2,167)	2,281 (1,914-2,765)	3,132 (2,679-3,881)	3,945 (3,398-4,897)	5,734 (5,008-7,019)	7,835 (6,845-9,331)	10,266 (9,175-12,380)	12,303 (11,020-14,633)	15,144 (13,669-17,915)	15,576 (13,961-18,014)	15,666 (13,977-18,180)	16,513 (14,847-18,816)	13,929 (12,636-15,762)	9,614 (8,737-11,029)	4,143 (3,770-4,730)	1,048 (959-1,221)
Gastritis and duodenitis	-	-	-	287 (155-583)	75 (55-105)	72 (58-92)	190 (146-240)	269 (214-326)	459 (376-563)	785 (652-966)	868 (730-1,069)	1,133 (955-1,361)	1,453 (1,234-1,725)	1,739 (1,480-2,037)	2,042 (1,745-2,394)	2,499 (2,191-2,880)	2,515 (2,155-2,878)	2,651 (2,243-3,000)	2,813 (2,423-3,216)	2,458 (2,152-2,790)	1,680 (1,500-1,956)	743 (666-875)	212 (191-253)
Appendicitis	-	-	-	839 (569-1,185)	658 (518-839)	862 (707-1,093)	1,182 (964-1,457)	1,055 (890-1,274)	1,310 (1,116-1,649)	899 (766-1,122)	1,193 (1,017-1,474)	1,487 (1,269-1,816)	1,344 (1,129-1,713)	1,485 (1,278-1,855)	1,670 (1,452-2,115)	1,861 (1,614-2,308)	1,676 (1,439-2,062)	1,756 (1,475-2,231)	1,620 (1,379-2,003)	1,364 (1,152-1,655)	928 (798-1,123)	415 (367-494)	111 (98-132)
Paralytic ileus and intestinal obstruction	798 (633-987)	699 (535-867)	7,751 (5,733-9,772)	2,382 (1,610-3,247)	1,469 (1,126-1,834)	1,253 (960-1,573)	1,828 (1,357-2,252)	2,500 (1,912-3,247)	3,537 (2,754-4,597)	3,143 (2,485-4,192)	4,619 (3,447-5,870)	5,043 (3,669-6,541)	5,972 (4,515-7,856)	6,825 (5,173-9,148)	8,694 (7,004-11,853)	10,303 (8,240-13,790)	12,214 (10,009-15,894)	12,042 (9,820-16,093)	12,354 (10,110-16,734)	11,766 (9,496-15,096)	9,562 (7,596-12,351)	4,890 (3,771-6,050)	1,267 (965-1,596)
Inguinal, femoral, and abdominal hernia	-	-	-	452 (202-731)	150 (86-205)	201 (93-280)	272 (162-350)	480 (275-610)	545 (327-665)	459 (273-558)	514 (376-741)	987 (647-1,256)	1,011 (710-1,328)	1,448 (1,008-1,876)	1,791 (1,304-2,404)	2,170 (1,563-2,864)	2,662 (1,909-3,388)	2,955 (2,037-3,642)	3,458 (2,453-4,204)	2,994 (2,205-4,022)	2,373 (1,843-3,123)	1,314 (1,052-1,697)	388 (306-533)
Inflammatory bowel disease	-	-	-	577 (312-1,046)	120 (79-156)	118 (82-153)	167 (121-201)	270 (198-319)	439 (366-522)	510 (387-617)	544 (434-662)	779 (639-948)	900 (722-1,048)	1,092 (880-1,246)	1,367 (1,100-1,584)	1,634 (1,327-1,872)	1,794 (1,429-2,117)	1,960 (1,561-2,287)	2,196 (1,734-2,590)	2,162 (1,682-2,462)	1,711 (1,307-1,982)	959 (715-1,088)	252 (190-288)
Vascular intestinal disorders	-	-	-	268 (110-546)	46 (29-68)	29 (23-36)	61 (51-76)	131 (106-167)	211 (171-270)	209 (168-272)	397 (323-513)	703 (587-893)	1,143 (967-1,450)	1,822 (1,541-2,382)	2,910 (2,484-3,846)	4,157 (3,631-5,457)	5,518 (4,712-7,416)	6,345 (5,340-8,428)	7,429 (6,175-9,703)	6,402 (5,090-8,189)	4,840 (3,755-5,946)	2,411 (1,929-2,966)	577 (472-716)
Gallbladder and biliary diseases	-	-	-	150 (101-217)	69 (53-101)	156 (107-200)	187 (147-257)	313 (265-427)	413 (349-532)	532 (457-688)	775 (663-1,034)	1,066 (926-1,467)	1,357 (1,187-1,795)	1,881 (1,628-2,465)	2,412 (2,122-3,090)	3,423 (3,041-4,513)	4,242 (3,756-5,824)	4,858 (4,331-6,411)	6,061 (5,271-7,834)	6,351 (5,575-8,111)	5,602 (4,931-7,099)	3,084 (2,748-3,811)	834 (734-1,027)
Pancreatitis	-	-	-	77 (52-102)	60 (41-80)	91 (64-116)	359 (259-451)	1,082 (849-1,290)	2,816 (2,219-3,302)	3,948 (3,140-4,689)	5,150 (4,099-6,210)	6,164 (4,990-7,292)	6,917 (5,513-8,107)	6,461 (5,127-7,742)	6,788 (5,358-7,960)	6,486 (5,149-7,709)	5,905 (4,702-7,350)	4,942 (3,981-6,018)	4,925 (3,907-6,111)	3,547 (2,817-4,331)	2,379 (1,888-2,895)	945 (734-1,167)	225 (181-303)
Other digestive diseases	-	-	-	1,258 (717-2,042)	412 (295-601)	361 (289-494)	575 (455-747)	480 (398-604)	741 (609-922)	1,038 (882-1,276)	1,226 (1,048-1,525)	1,448 (1,235-1,787)	2,019 (1,720-2,384)	2,615 (2,168-3,068)	3,214 (2,635-3,809)	4,455 (3,602-5,348)	4,546 (3,652-5,424)	5,062 (4,076-6,084)	5,267 (4,213-6,482)	5,561 (4,393-6,908)	4,463 (3,466-5,659)	2,688 (2,129-3,547)	798 (624-1,041)
Neurological disorders	-	-	2,693 (2,474-2,967)	3,748 (3,300-4,294)	2,377 (2,076-2,692)	3,844 (3,462-4,285)	8,558 (7,756-9,498)	8,625 (7,963-9,424)	7,891 (7,379-8,516)	7,067 (6,626-7,659)	8,367 (7,858-8,962)	8,207 (7,757-8,717)	10,416 (9,693-11,218)	14,095 (12,434-16,182)	20,037 (16,875-23,697)	33,180 (27,435-40,759)	54,164 (43,525-67,235)	92,116 (72,186-117,205)	165,219 (130,656-207,219)	224,772 (179,976-281,737)	234,715 (188,171-285,808)	144,953 (118,212-171,135)	37,316 (29,630-46,781)
Alzheimer's disease and other dementias	-	-	-	-	-	-	-	-	-	-	-	51 (20-103)	1,199 (682-1,877)	4,489 (2,977-6,418)	8,912 (5,884-12,454)	19,884 (14,257-27,531)	37,168 (27,090-49,704)	69,681 (50,597-94,130)	132,694 (99,011-172,243)	189,066 (143,283-247,667)	209,105 (162,136-261,602)	135,098 (109,193-161,395)	35,418 (27,728-45,061)
Parkinson's disease	-	-	-	-	-	-	-	1 (1-3)	17 (7-35)	65 (34-119)	144 (77-251)	263 (148-425)	458 (252-787)	824 (476-1,331)	1,535 (913-2,257)	3,717 (2,570-5,230)	7,529 (5,356-10,231)	14,508 (10,271-19,244)	25,662 (18,354-34,307)	30,373 (22,359-41,132)	22,425 (16,963-29,779)	8,632 (6,464-11,429)	1,631 (1,167-2,244)
Epilepsy	-	-	1,431 (1,257-1,677)	2,802 (2,454-3,302)	1,893 (1,628-2,209)	2,884 (2,527-3,301)	6,474 (5,825-7,309)	6,818 (6,219-7,560)	6,588 (6,086-7,191)	5,731 (5,325-6,295)	6,553 (6,067-7,138)	5,464 (5,091-5,929)	5,316 (4,979-5,752)	4,329 (4,077-4,664)	4,154 (3,929-4,429)	3,608 (3,393-3,883)	3,185 (2,944-3,462)	2,356 (2,193-2,548)	1,955 (1,825-2,110)	1,779 (1,650-1,913)	1,128 (1,052-1,215)	491 (462-525)	125 (119-132)
Multiple sclerosis	-	-	-	-	-	-	-	53 (47-60)	119 (103-159)	259 (226-352)	399 (351-534)	640 (566-849)	750 (657-963)	971 (844-1,190)	1,046 (864-1,247)	998 (781-1,176)	871 (649-984)	646 (470-732)	465 (338-529)	279 (205-311)	136 (93-151)	45 (32-50)	7 (5-8)
Motor neuron disease	3 (3-4)	5 (4-5)	315 (262-364)	116 (88-138)	17 (16-19)	42 (38-45)	78 (72-84)	87 (81-93)	89 (83-95)	181 (167-193)	373 (343-399)	670 (620-707)	1,103 (1,028-1,163)	1,516 (1,426-1,588)	2,088 (1,968-2,183)	2,531 (2,390-2,642)	2,982 (2,822-3,135)	2,637 (2,479-2,794)	2,143 (1,996-2,286)	1,416 (1,320-1,507)	706 (658-750)	179 (167-191)	28 (26-30)
Other neurological disorders	-	-	947 (823-1,128)	830 (658-1,188)	467 (395-588)	918 (836-995)	2,007 (1,736-2,206)	1,666 (1,469-1,802)	1,077 (979-1,154)	832 (760-889)	898 (825-958)	1,119 (1,002-1,199)	1,590 (1,441-1,695)	1,966 (1,771-2,097)	2,302 (2,095-2,449)	2,441 (2,201-2,592)	2,429 (2,178-2,591)	2,287 (2,033-2,454)	2,300 (2,050-2,461)	1,859 (1,654-1,995)	1,216 (1,098-1,305)	508 (470-540)	106 (100-111)
Mental and substance use disorders	5 (3-8)	7 (5-10)	0 (0-0)	0 (0-0)	6 (5-7)	4 (4-5)	3,656 (3,124-4,307)	9,876 (8,416-11,414)	16,926 (14,658-19,330)	22,177 (18,781-25,297)	23,520 (20,022-26,585)	26,442 (22,173-29,654)	28,340 (23,575-31,784)	28,569 (24,713-32,231)	25,983 (21,940-29,364)	21,713 (18,447-24,183)	14,740 (12,109-16,239)	9,105 (7,644-9,850)	7,737 (6,325-8,452)	4,669 (3,781-5,074)	2,872 (2,369-3,151)	1,135 (981-1,238)	298 (271-340)
Alcohol use disorders	-	-	-	-	-	-	817 (582-925)	2,399 (1,838-2,817)	5,680 (4,415-6,574)	9,955 (7,749-11,664)	12,744 (9,998-14,828)	16,248 (12,553-18,886)	19,656 (15,442-22,390)	20,423 (16,581-23,613)	19,178 (15,649-22,097)	15,964 (12,960-17,870)	10,352 (8,280-11,517)	5,844 (4,721-6,368)	4,452 (3,494-4,898)	2,180 (1,733-2,389)	1,156 (915-1,266)	417 (332-460)	111 (93-122)
Drug use disorders	5 (3-8)	7 (5-10)	0 (0-0)	0 (0-0)	0 (0-0)	0 (0-0)	2,830 (2,371-3,457)	7,463 (6,287-8,983)	11,227 (9,664-13,309)	12,199 (10,307-14,263)	10,755 (9,128-12,323)	10,160 (8,753-11,851)	8,647 (7,719-9,828)	8,146 (7,119-9,444)	6,805 (5,944-8,038)	5,749 (4,762-7,036)	4,388 (3,513-5,136)	3,261 (2,493-3,800)	3,285 (2,516-3,840)	2,489 (1,885-2,839)	1,716 (1,386-1,968)	718 (608-837)	187 (164-227)
Opioid use disorders	5 (3-8)	7 (5-10)	0 (0-0)	0 (0-0)	0 (0-0)	0 (0-0)	2,156 (1,723-2,698)	5,427 (4,306-6,482)	7,996 (6,433-9,497)	8,016 (6,329-9,379)	7,081 (5,530-8,133)	6,579 (5,177-7,696)	5,249 (4,087-6,068)	4,681 (3,562-5,370)	3,990 (3,120-4,744)	3,576 (2,753-4,421)	2,843 (2,199-3,431)	1,585 (1,079-1,856)	1,695 (1,124-2,044)	1,135 (743-1,314)	798 (515-947)	320 (232-380)	84 (66-105)
Cocaine use disorders	0 (0-0)	0 (0-0)	0 (0-0)	0 (0-0)	0 (0-0)	0 (0-0)	94 (64-127)	268 (183-383)	455 (323-610)	828 (571-1,136)	713 (489-948)	688 (468-916)	685 (487-918)	897 (580-1,202)	729 (468-965)	542 (321-725)	390 (232-513)	192 (112-247)	176 (102-229)	34 (20-44)	22 (13-28)	10 (6-12)	3 (2-3)
Amphetamine use disorders	0 (0-0)	0 (0-0)	0 (0-0)	0 (0-0)	0 (0-0)	0 (0-0)	74 (55-107)	218 (160-319)	358 (252-490)	521 (372-719)	467 (331-629)	444 (339-589)	414 (300-567)	445 (312-604)	367 (241-488)	269 (174-361)	187 (112-244)	68 (42-86)	63 (38-82)	18 (11-23)	12 (8-15)	5 (4-6)	1 (1-2)
Other drug use disorders	0 (0-0)	0 (0-0)	0 (0-0)	0 (0-0)	0 (0-0)	0 (0-0)	506 (408-690)	1,550 (1,264-2,313)	2,417 (1,985-3,372)	2,833 (2,285-3,738)	2,493 (2,054-3,217)	2,449 (2,044-3,278)	2,299 (1,980-3,215)	2,124 (1,818-2,957)	1,720 (1,450-2,353)	1,362 (1,078-1,814)	968 (717-1,316)	1,416 (1,081-1,790)	1,351 (1,053-1,728)	1,302 (1,021-1,647)	884 (723-1,129)	384 (320-484)	99 (83-129)
Eating disorders	-	-	-	-	6 (5-7)	4 (4-5)	9 (7-11)	14 (12-17)	20 (16-23)	23 (19-28)	22 (18-26)	33 (28-38)	37 (32-41)	0 (0-0)	0 (0-0)	-	-	-	-	-	-	-	-
Anorexia nervosa	-	-	-	-	5 (4-6)	4 (3-5)	8 (7-10)	13 (11-16)	18 (15-22)	20 (16-24)	20 (16-24)	29 (25-33)	26 (22-29)	0 (0-0)	0 (0-0)	-	-	-	-	-	-	-	-
Bulimia nervosa	-	-	-	-	0 (0-1)	0 (0-1)	0 (0-1)	1 (1-1)	1 (1-2)	3 (2-4)	1 (1-2)	4 (3-6)	11 (9-14)	0 (0-0)	0 (0-0)	-	-	-	-	-	-	-	-
Diabetes, urogenital, blood, and endocrine diseases	1,863 (1,357-2,181)	1,231 (1,017-1,440)	12,795 (11,169-14,785)	11,793 (10,077-15,387)	6,066 (5,357-7,605)	7,177 (6,252-8,548)	11,906 (10,935-13,601)	18,653 (17,390-21,236)	20,352 (19,129-22,171)	25,500 (24,167-27,476)	31,903 (30,228-34,074)	46,576 (44,572-49,924)	65,841 (63,086-69,828)	91,774 (87,776-95,743)	124,193 (119,677-128,831)	158,639 (153,207-164,259)	174,621 (168,288-181,120)	181,215 (174,382-188,069)	191,308 (184,246-198,969)	181,143 (173,798-188,259)	137,690 (132,042-143,337)	69,451 (66,664-71,960)	19,304 (18,484-20,010)
Diabetes mellitus	28 (26-30)	37 (32-43)	345 (304-400)	303 (265-347)	431 (374-496)	609 (553-677)	1,293 (1,193-1,424)	2,586 (2,422-2,781)	3,977 (3,784-4,223)	6,384 (6,082-6,754)	9,223 (8,799-9,693)	16,429 (15,768-17,244)	26,460 (25,543-27,536)	40,938 (39,626-42,415)	59,285 (57,253-61,442)	78,273 (75,588-80,891)	85,754 (82,692-88,942)	88,743 (85,506-92,135)	91,743 (88,067-95,613)	82,894 (78,928-86,644)	53,822 (51,331-56,254)	22,885 (21,823-23,921)	5,189 (4,933-5,415)
Acute glomerulonephritis	-	-	54 (36-92)	67 (55-85)	77 (64-96)	89 (74-111)	96 (82-114)	135 (117-156)	184 (159-214)	200 (173-235)	206 (177-241)	273 (229-324)	418 (372-476)	425 (376-485)	511 (458-570)	618 (544-700)	668 (598-751)	631 (558-710)	656 (577-741)	554 (492-623)	390 (342-446)	152 (134-172)	54 (45-66)
Chronic kidney disease	-	-	2,130 (1,862-2,500)	2,332 (1,981-2,736)	1,809 (1,536-2,105)	2,066 (1,819-2,424)	5,282 (4,795-5,930)	9,166 (8,285-10,160)	10,565 (9,717-11,872)	12,583 (11,451-14,222)	15,771 (14,577-17,945)	21,565 (20,075-24,423)	28,418 (26,132-31,211)	38,836 (35,283-42,845)	50,644 (46,807-53,568)	62,273 (57,753-67,159)	67,850 (62,051-73,561)	69,512 (64,296-74,224)	73,308 (67,910-77,833)	70,786 (66,571-74,616)	59,180 (56,009-62,605)	32,078 (30,360-33,612)	9,587 (9,093-10,011)
Chronic kidney disease due to diabetes mellitus	-	-	59 (39-86)	72 (45-109)	68 (39-112)	100 (61-160)	387 (233-603)	1,254 (800-1,748)	2,318 (1,674-3,058)	3,804 (2,832-4,959)	5,973 (4,502-7,627)	10,030 (7,641-12,328)	15,107 (12,070-18,056)	21,258 (16,886-25,169)	28,101 (22,541-32,579)	34,985 (28,786-40,465)	36,648 (28,924-42,973)	35,171 (27,894-41,592)	33,305 (25,906-40,120)	27,258 (20,864-33,290)	19,428 (14,529-25,105)	9,779 (7,034-12,479)	2,942 (2,005-3,876)
Chronic kidney disease due to hypertension	-	-	14 (9-21)	22 (15-33)	30 (19-46)	56 (35-85)	229 (147-332)	606 (404-850)	898 (631-1,237)	1,304 (942-1,762)	1,877 (1,414-2,438)	2,941 (2,263-3,843)	4,301 (3,295-5,615)	6,355 (4,863-8,403)	9,075 (6,959-11,801)	12,490 (9,852-15,786)	15,231 (11,951-19,166)	17,125 (13,466-21,519)	20,334 (16,075-25,385)	22,371 (17,928-27,089)	21,716 (17,451-26,599)	13,049 (10,559-15,654)	4,090 (3,227-5,014)
Chronic kidney disease due to glomerulonephritis	-	-	849 (625-1,101)	936 (680-1,240)	714 (490-961)	855 (596-1,146)	2,222 (1,546-2,936)	3,766 (2,676-5,022)	3,917 (2,736-5,228)	3,791 (2,592-5,169)	3,716 (2,451-5,110)	3,726 (2,347-5,245)	3,630 (2,314-5,207)	4,310 (2,796-6,228)	4,837 (3,226-6,909)	5,201 (3,366-7,513)	5,478 (3,548-8,128)	5,703 (3,711-8,372)	6,612 (4,407-9,644)	6,736 (4,619-9,264)	5,145 (3,541-7,246)	2,578 (1,807-3,507)	728 (433-1,188)
Chronic kidney disease due to other causes	-	-	1,209 (963-1,490)	1,302 (1,011-1,651)	998 (749-1,302)	1,054 (775-1,362)	2,443 (1,772-3,154)	3,540 (2,480-4,697)	3,433 (2,332-4,694)	3,684 (2,541-5,072)	4,205 (2,976-5,594)	4,869 (3,323-6,632)	5,381 (3,506-7,888)	6,912 (4,492-9,945)	8,631 (5,784-12,594)	9,598 (6,259-13,964)	10,493 (7,021-15,245)	11,513 (7,813-15,813)	13,057 (9,009-18,114)	14,421 (10,179-19,310)	12,892 (8,886-17,300)	6,672 (4,869-8,860)	1,827 (1,186-2,613)
Urinary diseases and male infertility	146 (131-164)	188 (154-228)	2,562 (2,213-2,954)	1,382 (1,166-1,683)	584 (489-702)	545 (492-604)	1,364 (1,237-1,494)	1,974 (1,828-2,143)	2,019 (1,870-2,188)	2,427 (2,279-2,596)	2,853 (2,676-3,033)	3,668 (3,469-3,885)	4,656 (4,392-4,917)	5,630 (5,339-5,915)	6,835 (6,499-7,166)	10,052 (9,570-10,611)	12,215 (11,619-12,845)	13,867 (13,206-14,558)	16,303 (15,573-17,125)	18,231 (17,383-19,205)	16,456 (15,728-17,258)	10,006 (9,586-10,426)	3,177 (3,038-3,318)
Interstitial nephritis and urinary tract infections	87 (69-102)	121 (96-154)	1,938 (1,584-2,322)	748 (513-1,053)	304 (237-385)	307 (258-357)	770 (624-909)	1,143 (964-1,324)	1,112 (920-1,277)	1,265 (1,079-1,461)	1,516 (1,281-1,751)	1,985 (1,624-2,249)	2,490 (2,046-2,824)	3,216 (2,704-3,612)	3,995 (3,361-4,484)	5,955 (5,065-6,653)	7,392 (6,370-8,330)	8,832 (7,630-9,677)	11,077 (9,888-12,093)	12,319 (11,035-13,334)	11,843 (10,783-12,712)	7,527 (6,949-8,056)	2,519 (2,333-2,680)
Urolithiasis	-	-	-	-	78 (53-122)	68 (52-96)	149 (106-218)	166 (122-257)	146 (109-249)	158 (122-265)	200 (153-346)	325 (246-566)	427 (326-725)	538 (421-880)	541 (437-850)	840 (672-1,356)	1,078 (859-1,783)	1,067 (864-1,782)	1,262 (1,019-2,036)	1,233 (1,006-1,940)	968 (798-1,506)	448 (367-667)	123 (101-181)
Other urinary diseases	59 (48-73)	66 (50-92)	623 (458-890)	634 (365-948)	202 (147-267)	169 (133-209)	445 (324-576)	665 (498-824)	760 (597-929)	1,004 (817-1,167)	1,138 (905-1,341)	1,358 (1,056-1,609)	1,739 (1,381-2,065)	1,877 (1,476-2,259)	2,300 (1,881-2,768)	3,258 (2,577-3,955)	3,745 (2,908-4,515)	3,969 (3,215-4,868)	3,964 (3,162-4,803)	4,680 (3,881-5,642)	3,645 (3,021-4,439)	2,031 (1,667-2,511)	535 (430-673)
Gynecological diseases	-	-	-	-	-	-	-	-	-	-	-	-	-	-	-	-	-	-	-	-	-	-	-
Uterine fibroids	-	-	-	-	-	-	-	-	-	-	-	-	-	-	-	-	-	-	-	-	-	-	-
Polycystic ovarian syndrome	-	-	-	-	-	-	-	-	-	-	-	-	-	-	-	-	-	-	-	-	-	-	-
Endometriosis	-	-	-	-	-	-	-	-	-	-	-	-	-	-	-	-	-	-	-	-	-	-	-
Genital prolapse	-	-	-	-	-	-	-	-	-	-	-	-	-	-	-	-	-	-	-	-	-	-	-
Other gynecological diseases	-	-	-	-	-	-	-	-	-	-	-	-	-	-	-	-	-	-	-	-	-	-	-
Hemoglobinopathies and hemolytic anaemias	1,093 (661-1,382)	491 (324-612)	4,315 (2,955-5,553)	5,712 (4,389-8,959)	2,142 (1,705-3,325)	2,856 (2,143-3,941)	2,944 (2,251-4,138)	3,642 (2,945-5,479)	2,031 (1,552-2,833)	1,520 (1,155-2,055)	1,343 (1,057-1,830)	1,867 (1,486-2,618)	2,391 (1,968-3,562)	1,735 (1,400-2,350)	1,846 (1,473-2,439)	2,051 (1,629-2,733)	2,307 (1,872-3,072)	2,718 (2,235-3,663)	3,573 (3,056-4,947)	2,911 (2,472-3,795)	2,958 (2,480-3,763)	1,537 (1,291-1,864)	422 (358-512)
Thalassemias	189 (137-260)	120 (89-164)	710 (523-1,024)	985 (741-1,350)	511 (391-695)	189 (150-263)	120 (92-165)	83 (62-111)	44 (33-56)	24 (19-30)	16 (13-21)	18 (15-25)	17 (14-23)	14 (12-20)	9 (8-14)	5 (4-6)	2 (2-3)	1 (1-1)	0 (0-0)	0 (0-0)	0 (0-0)	0 (0-0)	0 (0-0)
Sickle cell disorders	848 (469-1,105)	349 (216-447)	3,403 (2,221-4,388)	4,446 (3,130-7,326)	1,460 (1,121-2,406)	2,426 (1,801-3,371)	2,426 (1,867-3,461)	2,817 (2,191-4,341)	1,310 (1,022-1,905)	854 (673-1,208)	710 (555-1,005)	870 (699-1,316)	981 (731-1,628)	408 (319-626)	223 (176-338)	114 (92-165)	71 (59-96)	35 (30-46)	15 (13-19)	5 (4-6)	2 (1-2)	0 (0-0)	0 (0-0)
G6PD deficiency	36 (23-51)	16 (11-23)	174 (112-261)	260 (181-384)	152 (114-210)	211 (154-286)	340 (251-459)	620 (477-846)	588 (421-762)	563 (405-734)	518 (388-690)	801 (606-1,080)	1,121 (870-1,596)	926 (714-1,221)	952 (724-1,279)	913 (707-1,275)	726 (577-1,038)	639 (504-925)	536 (412-815)	207 (159-300)	111 (86-160)	28 (22-40)	4 (3-6)
Other hemoglobinopathies and hemolytic anaemias	21 (14-30)	5 (4-8)	28 (18-43)	22 (15-33)	19 (15-26)	30 (23-38)	59 (46-78)	122 (94-171)	90 (65-115)	80 (59-101)	99 (75-128)	178 (139-233)	272 (209-370)	387 (296-483)	661 (518-828)	1,020 (791-1,308)	1,508 (1,197-1,969)	2,042 (1,678-2,710)	3,022 (2,550-4,101)	2,699 (2,290-3,472)	2,845 (2,384-3,584)	1,509 (1,270-1,824)	417 (354-506)
Endocrine, metabolic, blood, and immune disorders	596 (450-762)	516 (408-672)	3,389 (2,859-4,683)	1,997 (1,674-2,661)	1,023 (877-1,408)	1,013 (877-1,365)	926 (805-1,186)	1,150 (981-1,449)	1,576 (1,276-1,920)	2,385 (1,815-2,751)	2,506 (1,866-2,762)	2,774 (2,117-3,064)	3,497 (2,616-3,820)	4,210 (3,280-4,606)	5,071 (3,811-5,552)	5,371 (4,129-5,851)	5,825 (4,333-6,529)	5,745 (4,537-6,457)	5,724 (4,356-6,402)	5,767 (4,226-6,295)	4,884 (3,552-5,312)	2,793 (2,023-3,044)	876 (670-979)
Musculoskeletal disorders	-	-	-	-	273 (236-356)	376 (324-461)	433 (356-543)	563 (489-672)	628 (544-739)	664 (565-765)	791 (680-907)	958 (830-1,118)	1,293 (1,117-1,520)	1,282 (1,156-1,507)	1,630 (1,453-1,924)	2,211 (1,951-2,580)	2,671 (2,346-3,081)	2,925 (2,530-3,283)	3,368 (2,915-3,732)	3,155 (2,698-3,434)	2,627 (2,286-2,850)	1,384 (1,221-1,510)	387 (356-423)
Rheumatoid arthritis	-	-	-	-	51 (39-68)	58 (43-77)	46 (35-62)	53 (39-70)	55 (41-74)	55 (41-75)	74 (58-100)	139 (104-177)	243 (176-293)	394 (294-463)	589 (443-683)	990 (772-1,128)	1,265 (938-1,426)	1,441 (1,109-1,636)	1,606 (1,264-1,803)	1,384 (1,085-1,558)	969 (748-1,138)	385 (282-472)	98 (77-120)
Other musculoskeletal disorders	-	-	-	-	223 (190-302)	318 (279-395)	387 (343-488)	510 (438-621)	573 (496-677)	609 (518-705)	718 (610-824)	819 (703-970)	1,050 (910-1,270)	888 (782-1,096)	1,041 (917-1,300)	1,221 (1,071-1,533)	1,406 (1,236-1,771)	1,484 (1,292-1,839)	1,762 (1,474-2,113)	1,771 (1,477-2,018)	1,658 (1,413-1,896)	999 (859-1,109)	289 (255-323)
Other non-communicable diseases	81,657 (68,815-93,697)	28,205 (24,829-32,333)	98,784 (84,135-113,626)	29,128 (20,997-37,559)	8,373 (7,260-10,311)	6,298 (5,511-7,575)	5,819 (5,181-7,227)	5,312 (4,429-6,331)	4,441 (3,722-5,312)	4,228 (3,445-5,094)	3,951 (3,239-4,541)	4,459 (3,600-5,078)	4,235 (3,239-5,193)	4,165 (3,098-4,924)	5,065 (3,430-6,003)	5,793 (3,968-6,927)	5,750 (3,777-7,196)	4,853 (2,717-6,314)	5,761 (3,402-7,904)	5,571 (3,220-7,746)	5,134 (2,942-7,022)	3,013 (1,716-4,067)	945 (540-1,304)
Congenital anomalies	81,657 (68,815-93,697)	26,607 (23,412-30,886)	81,528 (68,881-96,365)	28,042 (19,974-36,286)	8,127 (7,030-10,040)	5,996 (5,139-7,271)	5,016 (4,442-6,298)	4,591 (3,867-5,552)	3,840 (3,233-4,653)	2,797 (2,361-3,346)	2,816 (2,392-3,432)	3,004 (2,513-3,511)	2,051 (1,665-2,444)	1,829 (1,494-2,110)	1,812 (1,441-1,951)	1,914 (1,571-2,168)	1,429 (1,203-1,674)	-	-	-	-	-	-
Neural tube defects	11,188 (6,386-20,029)	2,318 (1,303-4,286)	6,379 (3,044-12,407)	2,329 (505-5,813)	316 (160-681)	209 (122-435)	112 (74-241)	92 (63-198)	72 (52-134)	64 (47-116)	44 (33-70)	43 (32-77)	49 (40-65)	36 (29-54)	33 (24-63)	29 (22-56)	20 (16-37)	-	-	-	-	-	-
Congenital heart anomalies	25,755 (18,749-31,533)	12,013 (10,068-14,633)	40,559 (32,122-50,025)	13,611 (9,769-18,923)	4,953 (4,125-6,579)	3,890 (3,311-4,839)	3,313 (2,819-4,160)	3,182 (2,747-3,999)	2,823 (2,363-3,555)	1,922 (1,665-2,385)	2,060 (1,762-2,588)	2,258 (1,915-2,658)	830 (685-1,016)	663 (558-823)	603 (516-729)	577 (498-738)	434 (382-556)	-	-	-	-	-	-
Orofacial clefts	447 (231-901)	138 (68-289)	477 (193-1,067)	46 (14-132)	-	-	-	-	-	-	-	-	-	-	-	-	-	-	-	-	-	-	-
Down's syndrome	929 (622-1,518)	435 (326-681)	1,922 (1,409-3,389)	946 (569-2,435)	196 (152-269)	173 (133-238)	196 (158-290)	195 (152-259)	191 (156-251)	222 (176-289)	203 (157-269)	208 (157-262)	431 (345-541)	452 (327-513)	504 (339-566)	439 (289-492)	197 (132-224)	-	-	-	-	-	-
Other chromosomal abnormalities	2,624 (1,875-4,391)	1,041 (750-1,803)	2,358 (1,594-3,861)	547 (322-1,076)	128 (85-194)	93 (51-123)	115 (65-146)	110 (57-136)	91 (41-118)	66 (24-86)	59 (22-77)	48 (18-61)	52 (23-62)	62 (31-77)	53 (27-68)	39 (19-51)	30 (16-37)	-	-	-	-	-	-
Congenital musculoskeletal and limb anomalies	2,293 (1,541-4,123)	491 (359-799)	1,036 (735-1,850)	275 (175-646)	106 (81-164)	84 (67-120)	103 (78-137)	56 (39-70)	49 (38-62)	34 (23-43)	29 (21-37)	24 (16-29)	35 (24-45)	34 (25-41)	20 (13-26)	27 (20-34)	25 (19-32)	-	-	-	-	-	-
Urogenital congenital anomalies	2,777 (1,898-3,699)	708 (525-989)	1,442 (977-2,117)	282 (187-411)	127 (94-179)	120 (90-169)	71 (56-100)	80 (61-109)	72 (54-93)	71 (54-92)	72 (49-90)	93 (62-118)	167 (109-210)	181 (131-229)	212 (149-265)	297 (212-382)	323 (226-409)	-	-	-	-	-	-
Digestive congenital anomalies	7,843 (5,648-11,990)	2,978 (2,223-4,354)	7,387 (5,078-11,592)	1,452 (855-3,294)	183 (144-263)	97 (75-125)	54 (40-67)	38 (28-46)	41 (30-49)	30 (23-36)	32 (20-39)	31 (18-38)	49 (35-60)	45 (31-53)	48 (32-57)	65 (48-80)	59 (39-70)	-	-	-	-	-	-
Other congenital anomalies	27,800 (20,174-36,981)	6,483 (4,798-8,831)	19,968 (13,945-28,796)	8,554 (5,089-13,622)	2,119 (1,596-2,794)	1,330 (1,025-1,704)	1,053 (854-1,361)	838 (628-1,032)	501 (385-630)	389 (304-489)	318 (252-395)	299 (224-366)	437 (359-551)	356 (293-440)	340 (264-401)	440 (348-536)	341 (273-419)	-	-	-	-	-	-
Skin and subcutaneous diseases	-	-	1,790 (957-2,790)	1,086 (573-1,650)	246 (136-365)	302 (163-438)	802 (434-1,261)	721 (362-1,059)	601 (324-868)	1,430 (743-2,060)	1,135 (585-1,517)	1,455 (741-1,929)	2,184 (1,172-3,096)	2,336 (1,251-3,037)	3,254 (1,716-4,227)	3,879 (2,109-5,043)	4,321 (2,355-5,843)	4,853 (2,717-6,314)	5,761 (3,402-7,904)	5,571 (3,220-7,746)	5,134 (2,942-7,022)	3,013 (1,716-4,067)	945 (540-1,304)
Cellulitis	-	-	90 (39-195)	101 (43-159)	54 (25-80)	74 (37-118)	90 (49-147)	112 (58-187)	92 (44-141)	222 (85-308)	257 (108-357)	314 (140-445)	430 (197-657)	627 (250-826)	852 (340-1,161)	896 (392-1,259)	856 (334-1,184)	907 (384-1,457)	1,061 (429-1,617)	988 (411-1,693)	878 (373-1,512)	530 (237-1,031)	164 (71-327)
Pyoderma	-	-	1,657 (890-2,498)	848 (448-1,334)	137 (75-208)	167 (83-239)	560 (275-865)	489 (224-701)	404 (206-580)	1,030 (516-1,520)	708 (339-953)	938 (456-1,283)	1,452 (746-2,049)	1,386 (719-1,928)	1,925 (966-2,595)	2,348 (1,189-3,109)	2,571 (1,382-3,475)	2,749 (1,455-3,665)	3,122 (1,713-4,317)	2,524 (1,383-3,482)	2,302 (1,196-2,957)	1,253 (659-1,633)	365 (185-482)
Decubitus ulcer	-	-	-	68 (29-117)	27 (16-44)	30 (17-48)	86 (51-148)	67 (38-107)	65 (39-103)	128 (72-199)	132 (70-202)	159 (83-240)	243 (127-384)	262 (140-405)	399 (211-611)	536 (288-812)	774 (440-1,196)	1,073 (600-1,613)	1,407 (798-2,166)	1,843 (1,023-2,767)	1,739 (935-2,535)	1,096 (605-1,520)	371 (199-519)
Other skin and subcutaneous diseases	-	-	43 (23-76)	69 (32-115)	28 (17-46)	32 (17-51)	67 (39-123)	53 (29-90)	40 (23-64)	51 (30-88)	39 (22-61)	43 (25-69)	59 (33-97)	61 (34-101)	77 (42-124)	98 (55-161)	119 (72-197)	123 (69-213)	170 (101-291)	216 (120-363)	214 (118-347)	129 (68-214)	44 (23-74)
Sudden infant death syndrome	-	1,598 (1,111-2,035)	15,466 (11,256-19,105)	-	-	-	-	-	-	-	-	-	-	-	-	-	-	-	-	-	-	-	-
Injuries	6,132 (5,082-7,013)	3,427 (3,033-3,772)	39,505 (34,951-44,377)	91,499 (82,003-102,526)	65,565 (60,161-71,491)	73,183 (68,737-77,938)	183,398 (175,250-191,928)	298,575 (286,035-310,644)	305,313 (293,014-318,091)	271,257 (259,796-283,578)	245,181 (233,390-256,394)	229,615 (217,991-240,833)	226,684 (213,986-237,682)	210,076 (197,801-222,578)	191,363 (180,220-201,805)	175,482 (164,077-183,363)	148,185 (135,516-154,600)	121,860 (110,711-127,488)	115,112 (102,434-121,149)	97,288 (85,489-102,581)	72,085 (62,126-76,332)	34,859 (29,095-37,145)	9,730 (8,227-10,379)
Transport injuries	1,081 (822-1,502)	517 (422-668)	5,423 (4,460-6,734)	19,124 (16,326-22,553)	21,259 (19,119-23,758)	22,203 (20,591-24,308)	71,479 (67,585-76,629)	116,722 (111,623-124,193)	121,206 (115,936-128,508)	100,918 (96,550-106,450)	91,524 (87,498-96,513)	87,211 (83,842-91,483)	86,379 (82,973-90,263)	78,158 (75,143-81,305)	68,037 (65,436-70,792)	61,114 (58,741-63,604)	47,612 (45,844-49,395)	35,045 (33,555-36,541)	28,458 (27,256-29,722)	20,086 (19,218-20,980)	11,642 (11,104-12,199)	4,310 (4,104-4,502)	1,055 (1,001-1,106)
Road injuries	932 (703-1,297)	443 (357-574)	4,898 (4,000-6,124)	18,030 (15,385-21,286)	20,150 (18,112-22,539)	20,595 (19,069-22,461)	66,403 (62,731-71,616)	109,694 (104,724-116,970)	112,779 (107,730-120,037)	94,328 (90,342-99,537)	84,768 (80,919-89,535)	80,944 (77,856-84,967)	80,239 (77,072-83,613)	72,175 (69,361-75,048)	62,628 (60,155-65,142)	57,188 (54,856-59,631)	44,428 (42,662-46,095)	32,729 (31,346-34,115)	26,392 (25,248-27,568)	18,696 (17,929-19,539)	10,823 (10,331-11,328)	3,949 (3,762-4,117)	932 (882-978)
Pedestrian road injuries	363 (260-524)	170 (134-223)	1,881 (1,472-2,470)	9,294 (7,848-11,252)	10,330 (9,188-11,992)	9,465 (8,572-10,774)	16,362 (14,522-18,373)	26,641 (23,346-29,403)	30,483 (26,841-34,143)	27,907 (24,838-30,813)	26,522 (23,491-29,153)	27,529 (24,529-30,183)	30,986 (28,084-33,876)	28,879 (26,110-31,753)	25,446 (22,947-28,035)	27,191 (24,736-29,431)	21,837 (19,979-23,552)	16,364 (15,010-17,880)	14,889 (13,883-16,198)	10,536 (9,762-11,399)	6,266 (5,838-6,809)	2,137 (1,950-2,353)	513 (472-567)
Cyclist road injuries	-	-	-	632 (471-851)	1,219 (1,003-1,536)	1,734 (1,472-2,124)	3,268 (2,745-3,950)	3,569 (3,076-4,110)	4,048 (3,507-4,737)	3,911 (3,411-4,681)	3,741 (3,250-4,348)	4,678 (4,113-5,286)	5,291 (4,696-6,062)	5,641 (5,017-6,451)	4,728 (4,246-5,361)	5,331 (4,800-5,935)	4,279 (3,826-4,775)	3,111 (2,774-3,504)	2,113 (1,883-2,364)	1,256 (1,122-1,431)	634 (565-716)	202 (179-237)	3 (3-4)
Motorcyclist road injuries	84 (56-136)	54 (40-81)	553 (431-770)	1,165 (880-1,599)	1,250 (1,024-1,553)	2,142 (1,823-2,464)	19,616 (17,438-22,199)	33,445 (29,569-37,628)	32,247 (28,298-36,121)	24,046 (20,897-27,002)	20,819 (18,083-23,396)	18,822 (16,360-20,566)	16,866 (14,227-18,520)	13,594 (11,065-14,764)	11,158 (9,232-12,150)	7,347 (5,726-8,059)	5,072 (3,816-5,582)	3,132 (2,463-3,471)	1,747 (1,318-1,955)	1,431 (1,084-1,609)	649 (497-734)	191 (150-217)	50 (41-58)
Motor vehicle road injuries	468 (319-691)	210 (163-289)	2,353 (1,860-3,109)	6,729 (5,195-8,567)	7,105 (6,060-8,564)	6,950 (6,063-8,395)	26,604 (23,983-31,435)	45,137 (41,112-53,131)	45,135 (40,667-52,777)	37,859 (33,878-44,697)	33,081 (29,460-38,693)	29,278 (25,968-34,563)	26,318 (22,931-30,860)	23,208 (20,059-27,313)	20,614 (17,715-24,200)	16,807 (14,049-19,760)	12,686 (10,726-14,940)	9,661 (8,233-11,479)	7,265 (6,251-8,533)	5,148 (4,440-6,068)	3,087 (2,647-3,583)	1,339 (1,160-1,525)	340 (287-384)
Other road injuries	18 (11-29)	8 (6-13)	111 (81-178)	210 (154-319)	246 (198-333)	304 (247-399)	553 (467-702)	903 (756-1,131)	867 (729-1,082)	605 (505-722)	605 (495-729)	637 (533-782)	777 (662-956)	853 (742-1,101)	682 (583-886)	513 (434-642)	554 (479-705)	461 (397-598)	378 (330-512)	325 (281-440)	188 (161-266)	79 (67-119)	26 (22-40)
Other transport injuries	149 (101-211)	75 (57-99)	525 (405-697)	1,094 (722-1,432)	1,109 (941-1,361)	1,608 (1,382-1,922)	5,076 (4,388-5,894)	7,028 (6,259-8,008)	8,427 (7,531-9,582)	6,590 (5,931-7,455)	6,756 (6,102-7,646)	6,267 (5,649-7,190)	6,140 (5,581-7,221)	5,983 (5,376-6,988)	5,408 (4,858-6,321)	3,926 (3,557-4,698)	3,184 (2,884-3,679)	2,316 (2,071-2,627)	2,066 (1,820-2,333)	1,390 (1,243-1,622)	820 (733-940)	362 (326-429)	123 (109-143)
Unintentional injuries	4,103 (3,226-4,764)	2,560 (2,187-2,863)	31,781 (27,022-36,357)	63,246 (55,779-72,320)	37,169 (33,709-40,649)	36,707 (33,659-39,341)	42,528 (39,355-44,874)	57,555 (52,786-60,364)	60,681 (55,944-63,602)	60,645 (56,078-63,782)	61,747 (56,362-65,012)	63,418 (56,451-67,228)	69,606 (61,327-73,716)	67,441 (59,155-72,576)	67,006 (59,142-71,951)	68,092 (58,568-72,753)	63,668 (54,205-67,929)	58,386 (48,544-62,090)	62,318 (51,327-66,858)	60,120 (49,048-64,219)	50,161 (40,562-53,835)	26,921 (21,500-28,975)	7,869 (6,369-8,471)
Falls	969 (614-1,403)	245 (188-311)	3,906 (2,824-5,017)	6,843 (5,071-8,300)	4,226 (3,557-4,904)	5,237 (4,415-5,958)	5,751 (5,116-6,422)	9,729 (8,407-10,777)	12,014 (10,178-13,233)	13,667 (11,702-15,097)	15,407 (13,170-16,885)	18,440 (15,295-20,107)	21,799 (17,678-23,515)	22,732 (18,301-24,521)	24,850 (20,050-26,788)	27,915 (21,557-30,288)	29,014 (22,833-31,429)	30,140 (23,264-32,752)	36,192 (27,269-39,685)	37,125 (27,663-40,292)	33,517 (25,075-36,473)	18,833 (13,908-20,461)	5,407 (4,027-5,886)
Drowning	280 (211-343)	96 (74-129)	2,843 (2,460-3,356)	27,012 (23,418-30,885)	18,125 (16,164-20,123)	17,236 (15,758-18,707)	17,145 (15,696-18,382)	17,158 (15,977-18,408)	14,325 (13,351-15,310)	12,992 (12,102-13,949)	11,981 (11,117-12,839)	11,253 (10,401-11,989)	10,803 (9,963-11,504)	10,327 (9,567-10,999)	9,051 (8,404-9,664)	8,754 (7,861-9,256)	7,325 (6,412-7,737)	5,962 (5,286-6,261)	4,815 (4,249-5,077)	4,111 (3,634-4,342)	2,513 (2,312-2,650)	799 (745-838)	189 (176-199)
Fire, heat, and hot substances	233 (168-277)	162 (125-198)	2,365 (1,868-2,881)	5,867 (4,470-7,097)	2,264 (1,781-2,673)	1,540 (1,202-1,803)	1,872 (1,440-2,152)	3,359 (2,569-3,833)	3,685 (2,919-4,231)	3,708 (3,063-4,346)	3,856 (3,134-4,465)	3,609 (2,951-4,156)	3,894 (3,161-4,456)	4,154 (3,371-4,781)	4,085 (3,326-4,725)	4,308 (3,523-4,887)	3,686 (3,045-4,163)	3,455 (2,842-3,869)	3,431 (2,845-3,880)	3,368 (2,864-3,742)	2,666 (2,346-2,995)	1,391 (1,240-1,572)	419 (378-465)
Poisonings	189 (79-334)	103 (58-150)	2,337 (1,064-4,257)	3,157 (1,839-4,467)	1,070 (718-1,288)	1,167 (771-1,358)	1,376 (1,009-1,652)	2,298 (1,697-2,711)	2,641 (1,957-3,148)	2,338 (1,722-2,732)	2,211 (1,636-2,605)	2,426 (1,714-2,820)	2,436 (1,537-2,929)	2,393 (1,572-2,787)	2,213 (1,425-2,587)	2,146 (1,363-2,483)	1,697 (1,038-1,973)	1,316 (799-1,537)	1,029 (625-1,221)	802 (513-947)	482 (333-575)	177 (131-219)	49 (40-70)
Exposure to mechanical forces	823 (562-997)	703 (500-892)	5,285 (3,873-6,428)	5,607 (4,198-7,402)	3,391 (2,946-3,964)	3,085 (2,721-3,575)	5,374 (4,346-5,870)	8,784 (6,863-9,590)	9,927 (7,728-10,728)	9,033 (7,153-9,736)	9,083 (7,112-9,775)	8,659 (6,312-9,372)	9,760 (6,898-10,570)	8,441 (6,131-9,146)	6,841 (4,980-7,382)	5,559 (3,808-6,037)	4,236 (2,980-4,588)	3,109 (2,267-3,361)	2,979 (2,233-3,227)	2,345 (1,717-2,555)	1,883 (1,472-2,035)	1,108 (859-1,215)	396 (306-439)
Unintentional firearm injuries	41 (23-57)	30 (18-43)	432 (250-626)	649 (369-1,010)	424 (315-536)	541 (444-659)	1,521 (1,180-1,812)	2,264 (1,600-2,721)	2,459 (1,719-2,869)	1,815 (1,271-2,089)	1,454 (1,025-1,689)	1,049 (775-1,208)	1,111 (813-1,274)	868 (655-993)	671 (519-758)	576 (441-654)	488 (369-566)	387 (288-439)	373 (290-419)	285 (222-313)	200 (158-222)	100 (81-112)	32 (28-38)
Unintentional suffocation	559 (339-721)	588 (384-775)	3,618 (2,277-4,647)	2,002 (1,282-3,278)	972 (750-1,458)	989 (794-1,441)	333 (242-424)	502 (346-620)	610 (413-748)	441 (294-552)	355 (227-432)	376 (213-466)	331 (190-396)	291 (167-354)	270 (144-344)	186 (62-260)	191 (67-265)	166 (51-242)	479 (247-590)	564 (313-676)	626 (367-727)	458 (263-535)	185 (102-222)
Other exposure to mechanical forces	223 (158-256)	85 (69-99)	1,235 (995-1,466)	2,955 (2,183-3,713)	1,995 (1,569-2,288)	1,555 (1,281-1,733)	3,521 (2,724-3,849)	6,018 (4,640-6,558)	6,858 (5,235-7,465)	6,777 (5,190-7,341)	7,274 (5,628-7,936)	7,234 (5,084-7,852)	8,318 (5,695-9,079)	7,282 (5,162-7,943)	5,900 (4,233-6,375)	4,797 (3,257-5,230)	3,557 (2,459-3,865)	2,556 (1,855-2,784)	2,127 (1,551-2,312)	1,497 (1,057-1,616)	1,057 (809-1,140)	550 (416-613)	178 (148-207)
Adverse effects of medical treatment	524 (392-634)	326 (266-406)	3,932 (2,891-5,217)	3,525 (1,990-5,441)	1,305 (1,013-1,660)	1,128 (894-1,392)	1,565 (1,177-1,820)	2,538 (1,891-2,950)	2,559 (1,907-2,919)	2,675 (1,919-3,055)	2,843 (2,102-3,220)	3,016 (2,236-3,437)	3,237 (2,434-3,692)	3,083 (2,410-3,537)	3,726 (2,966-4,461)	4,829 (3,745-5,640)	5,501 (4,286-6,447)	4,969 (3,968-5,981)	4,988 (4,002-6,041)	4,394 (3,560-5,393)	3,199 (2,609-4,018)	1,608 (1,333-2,067)	442 (368-586)
Animal contact	172 (142-210)	155 (125-195)	1,109 (892-1,402)	3,244 (2,688-3,922)	2,758 (2,259-3,288)	3,091 (2,576-3,689)	2,530 (2,156-2,953)	2,691 (2,356-3,144)	2,862 (2,469-3,337)	2,901 (2,529-3,329)	3,046 (2,624-3,594)	3,088 (2,727-3,538)	3,974 (3,503-4,617)	3,119 (2,787-3,540)	3,685 (3,249-4,261)	3,437 (3,069-3,919)	3,309 (2,922-3,792)	2,746 (2,414-3,122)	2,153 (1,870-2,496)	1,578 (1,354-1,859)	780 (668-931)	253 (217-307)	82 (70-101)
Venomous animal contact	146 (113-183)	129 (101-164)	917 (694-1,181)	2,634 (1,990-3,218)	2,337 (1,847-2,853)	2,754 (2,247-3,339)	2,098 (1,737-2,511)	2,241 (1,888-2,689)	2,365 (1,971-2,834)	2,548 (2,178-2,981)	2,690 (2,312-3,215)	2,690 (2,320-3,141)	3,410 (2,912-4,045)	2,600 (2,268-3,001)	3,110 (2,656-3,656)	2,907 (2,520-3,381)	2,843 (2,440-3,302)	2,363 (2,025-2,755)	1,722 (1,418-2,051)	1,329 (1,125-1,608)	623 (512-765)	186 (152-229)	53 (43-66)
Non-venomous animal contact	26 (20-33)	26 (20-35)	192 (133-264)	609 (333-968)	421 (305-530)	337 (264-417)	433 (320-540)	450 (343-560)	497 (360-631)	353 (248-456)	356 (254-456)	399 (272-526)	563 (391-769)	520 (418-681)	575 (421-761)	530 (420-686)	467 (377-602)	383 (313-502)	431 (338-568)	249 (195-318)	156 (122-205)	68 (54-91)	29 (23-39)
Foreign body	602 (430-705)	630 (490-750)	8,509 (6,485-10,816)	5,060 (3,517-7,665)	1,875 (1,512-2,444)	1,577 (1,325-1,903)	1,113 (910-1,334)	1,885 (1,555-2,293)	2,046 (1,744-2,509)	2,700 (2,281-3,269)	2,937 (2,453-3,692)	3,117 (2,636-3,838)	2,990 (2,543-3,825)	3,507 (2,933-4,609)	3,700 (3,047-4,684)	3,758 (3,076-4,673)	3,427 (2,703-4,112)	2,982 (2,305-3,456)	3,276 (2,428-3,805)	3,578 (2,552-4,236)	3,111 (2,093-3,719)	1,810 (1,180-2,210)	618 (403-747)
Pulmonary aspiration and foreign body in airway	575 (411-676)	614 (477-734)	8,181 (6,222-10,464)	4,520 (3,129-6,955)	1,555 (1,245-2,102)	1,306 (1,101-1,670)	963 (805-1,226)	1,642 (1,380-2,109)	1,771 (1,510-2,301)	2,113 (1,803-2,700)	2,316 (1,926-3,084)	2,532 (2,133-3,304)	2,736 (2,313-3,599)	3,245 (2,708-4,309)	3,457 (2,830-4,452)	3,473 (2,773-4,366)	3,246 (2,525-3,941)	2,819 (2,155-3,289)	3,052 (2,198-3,573)	3,384 (2,387-4,035)	2,962 (1,950-3,554)	1,738 (1,118-2,134)	595 (377-721)
Foreign body in other body part	27 (13-38)	16 (8-22)	328 (176-453)	540 (269-958)	321 (204-429)	271 (151-370)	149 (74-222)	242 (126-344)	274 (145-382)	587 (349-773)	621 (368-808)	585 (369-759)	255 (164-328)	262 (176-331)	243 (160-313)	285 (213-438)	181 (123-236)	163 (108-202)	223 (154-285)	194 (130-257)	149 (107-195)	73 (51-93)	24 (18-33)
Environmental heat and cold exposure	144 (59-189)	78 (33-105)	825 (369-1,085)	868 (405-1,176)	383 (210-473)	364 (192-442)	740 (408-902)	1,057 (597-1,304)	1,468 (895-1,832)	2,127 (1,297-2,790)	2,413 (1,533-3,364)	2,845 (1,812-4,082)	3,215 (2,140-4,964)	4,138 (2,635-6,895)	4,326 (2,766-7,080)	3,767 (2,421-6,190)	3,046 (1,764-4,295)	2,252 (1,204-3,016)	2,333 (1,301-2,994)	1,738 (1,034-2,299)	1,304 (849-1,852)	625 (427-930)	167 (113-256)
Other unintentional injuries	167 (146-197)	63 (52-77)	671 (561-842)	2,063 (1,649-2,521)	1,770 (1,543-2,033)	2,283 (2,053-2,523)	5,062 (4,508-5,615)	8,058 (7,137-8,730)	9,155 (8,314-9,850)	8,503 (7,653-9,134)	7,970 (7,135-8,621)	6,965 (5,656-7,490)	7,500 (5,855-8,065)	5,546 (4,288-5,956)	4,528 (3,840-4,910)	3,618 (2,862-3,909)	2,427 (1,981-2,617)	1,454 (1,228-1,558)	1,123 (991-1,212)	1,080 (738-1,257)	707 (481-818)	317 (217-364)	101 (70-115)
Self-harm and interpersonal violence	583 (459-750)	146 (114-198)	1,623 (1,200-2,080)	2,777 (2,102-3,681)	2,335 (1,793-2,838)	9,198 (7,714-10,295)	54,540 (47,463-59,395)	99,634 (87,720-107,367)	106,148 (93,607-115,357)	97,297 (85,763-106,070)	83,597 (73,402-92,689)	73,729 (65,282-81,592)	67,140 (59,654-75,608)	61,912 (55,156-70,510)	54,246 (48,065-61,860)	44,384 (39,507-50,815)	35,709 (31,497-40,288)	27,514 (23,596-30,959)	23,658 (19,946-26,491)	16,612 (13,881-18,637)	9,985 (8,339-11,122)	3,492 (2,816-3,848)	747 (640-851)

SR Table 4: Global deaths by cause and age group for males (with 95% uncertainty intervals), 2016

Cause	Early Neonatal	Late Neonatal	Post Neonatal	1 to 4	5 to 9	10 to 14	15 to 19	20 to 24	25 to 29	30 to 34	35 to 39	40 to 44	45 to 49	50 to 54	55 to 59	60 to 64	65 to 69	70 to 74	75 to 79	80 to 84	85 to 89	90 to 94	95 plus
Self-harm	-	-	-	-	-	4,533 (3,759-5,226)	25,002 (22,016-28,007)	50,088 (44,511-55,363)	57,703 (51,608-64,771)	54,874 (47,900-60,578)	49,900 (42,609-55,900)	47,374 (41,752-53,739)	46,515 (40,804-52,243)	44,884 (39,328-51,620)	41,246 (35,534-47,190)	35,295 (30,261-39,672)	29,275 (24,383-33,101)	23,192 (19,363-26,089)	20,561 (16,831-22,931)	15,650 (12,786-17,448)	9,477 (7,752-10,475)	3,309 (2,651-3,613)	646 (532-711)
Self-harm by firearm	-	-	-	-	-	253 (194-355)	2,931 (2,297-4,096)	5,992 (4,578-8,038)	6,080 (4,530-7,766)	5,507 (4,114-7,099)	4,638 (3,528-5,852)	4,212 (3,227-5,515)	4,231 (3,171-5,694)	4,557 (3,522-5,944)	4,244 (3,110-5,468)	3,513 (2,574-4,534)	3,045 (2,217-3,985)	2,537 (1,851-3,366)	2,147 (1,611-2,781)	1,777 (1,296-2,334)	1,199 (849-1,526)	526 (373-683)	102 (75-132)
Self-harm by other specified means	-	-	-	-	-	4,280 (3,540-4,966)	22,071 (19,323-24,789)	44,097 (38,486-49,450)	51,624 (45,870-57,652)	49,367 (42,991-55,261)	45,262 (38,245-51,083)	43,161 (37,824-48,943)	42,284 (36,791-48,148)	40,326 (35,192-46,661)	37,003 (31,906-42,628)	31,782 (27,365-35,689)	26,230 (21,678-29,558)	20,655 (17,170-22,912)	18,414 (15,045-20,458)	13,873 (11,443-15,262)	8,277 (6,834-9,249)	2,783 (2,211-3,035)	544 (440-599)
Interpersonal violence	583 (459-750)	146 (114-198)	1,623 (1,200-2,080)	2,777 (2,102-3,681)	2,335 (1,793-2,838)	4,666 (3,612-5,438)	29,539 (22,583-33,380)	49,546 (38,684-55,503)	48,445 (38,427-55,609)	42,423 (32,428-51,258)	33,697 (25,940-40,393)	26,355 (19,826-32,580)	20,625 (15,620-26,381)	17,028 (12,578-22,768)	13,000 (9,615-17,758)	9,288 (6,932-12,975)	6,434 (4,821-8,831)	4,322 (3,298-5,945)	3,097 (2,349-4,290)	963 (752-1,401)	508 (396-817)	183 (138-291)	101 (75-162)
Assault by firearm	84 (49-130)	23 (13-33)	242 (125-336)	487 (269-703)	438 (266-619)	1,477 (800-1,795)	16,652 (10,297-19,684)	26,400 (16,955-30,941)	24,289 (15,345-27,966)	19,987 (12,204-23,188)	15,851 (9,933-18,755)	11,494 (6,993-14,090)	8,129 (4,786-10,583)	6,383 (3,719-8,602)	4,499 (2,802-6,209)	2,941 (1,819-4,073)	1,847 (1,068-2,698)	1,241 (730-1,831)	823 (471-1,177)	203 (112-311)	97 (51-147)	39 (19-61)	27 (14-38)
Assault by sharp object	53 (34-76)	14 (9-21)	115 (71-165)	329 (225-485)	284 (201-428)	672 (453-925)	6,221 (4,557-9,236)	11,423 (8,681-16,682)	12,493 (9,596-17,806)	10,998 (7,987-15,639)	8,544 (6,154-11,901)	6,957 (4,945-9,847)	5,709 (3,932-8,070)	4,624 (3,099-6,594)	3,379 (2,207-5,399)	2,700 (1,832-4,099)	1,727 (1,168-2,638)	1,099 (725-1,634)	687 (444-1,117)	245 (160-428)	124 (78-224)	53 (34-97)	23 (14-45)
Assault by other means	445 (342-590)	110 (83-155)	1,266 (933-1,646)	1,960 (1,447-2,636)	1,613 (1,176-2,002)	2,517 (1,918-3,143)	6,666 (5,303-9,052)	11,722 (9,209-15,414)	11,663 (9,058-16,004)	11,437 (8,827-16,297)	9,303 (7,032-13,332)	7,903 (5,930-11,339)	6,787 (5,169-9,894)	6,021 (4,451-9,040)	5,121 (3,701-7,319)	3,648 (2,624-5,475)	2,859 (2,141-4,145)	1,982 (1,513-2,812)	1,587 (1,189-2,234)	515 (407-817)	287 (232-499)	91 (68-158)	51 (40-86)
Forces of nature, conflict and terrorism, and executions and police conflict	365 (249-487)	203 (140-270)	677 (481-886)	6,353 (4,376-8,474)	4,802 (3,314-6,397)	5,074 (3,486-6,765)	14,850 (10,044-19,919)	24,664 (17,114-32,705)	17,277 (12,103-22,846)	12,398 (8,574-16,434)	8,314 (5,748-11,059)	5,258 (3,665-7,003)	3,559 (2,488-4,705)	2,565 (1,752-3,416)	2,074 (1,418-2,748)	1,692 (1,177-2,248)	1,197 (842-1,567)	915 (653-1,210)	678 (474-905)	470 (329-621)	296 (193-404)	136 (85-193)	58 (36-81)
Forces of nature, conflict and terrorism, and state actor violence	2 (1-3)	5 (3-7)	44 (28-60)	209 (133-289)	231 (148-320)	319 (203-442)	379 (239-527)	393 (241-556)	391 (235-559)	341 (205-487)	293 (175-419)	282 (161-417)	281 (156-422)	255 (142-381)	216 (119-324)	202 (114-301)	145 (81-218)	129 (72-192)	101 (57-150)	76 (41-114)	39 (21-59)	11 (6-16)	3 (2-5)
Conflict and terrorism	362 (247-484)	198 (135-265)	624 (425-834)	6,112 (4,176-8,180)	4,534 (3,071-6,102)	4,706 (3,148-6,373)	14,169 (9,427-19,226)	23,652 (16,106-31,768)	16,247 (11,080-21,728)	11,514 (7,734-15,516)	7,632 (5,079-10,332)	4,674 (3,081-6,379)	3,058 (1,986-4,196)	2,163 (1,382-2,993)	1,760 (1,119-2,432)	1,415 (912-1,949)	990 (648-1,352)	727 (473-996)	528 (335-732)	343 (213-479)	224 (129-322)	106 (60-153)	53 (30-76)
Executions and police conflict	-	-	10 (8-19)	31 (28-36)	37 (32-43)	49 (39-62)	302 (113-432)	619 (288-848)	640 (289-847)	542 (244-749)	388 (175-612)	302 (146-445)	220 (137-283)	147 (82-189)	97 (68-132)	75 (44-100)	61 (27-97)	59 (18-114)	49 (13-101)	51 (11-106)	33 (8-71)	20 (4-50)	2 (1-3)

SR Table 5: Global deaths by cause and age group for both sexes (with 95% uncertainty intervals), 1990

Cause	Early Neonatal	Late Neonatal	Post Neonatal	1 to 4	5 to 9	10 to 14	15 to 19	20 to 24	25 to 29	30 to 34	35 to 39
All causes	3,003,025 (2,941,548-3,059,741)	1,177,761 (1,153,960-1,202,683)	3,471,656 (3,400,689-3,535,864)	3,397,430 (3,317,942-3,472,873)	784,744 (777,308-792,168)	503,707 (498,353-509,502)	715,874 (707,570-723,886)	937,107 (926,108-948,177)	936,432 (925,079-948,463)	984,517 (973,174-996,257)	1,104,440 (1,091,320-1,118,690)
Communicable, maternal, neonatal, and nutritional disorders	2,756,899 (2,688,806-2,833,640)	1,052,708 (1,017,707-1,091,908)	2,902,871 (2,798,246-3,012,678)	2,689,314 (2,566,712-2,841,580)	451,867 (424,700-484,073)	217,012 (202,476-234,076)	222,434 (209,735-236,938)	291,173 (279,414-305,204)	312,020 (299,679-326,585)	316,491 (304,222-330,750)	313,366 (300,767-328,984)
HIV/AIDS and tuberculosis	-	-	45,313 (39,775-50,332)	60,531 (45,908-72,819)	19,243 (15,923-22,752)	18,200 (15,916-20,429)	43,957 (40,620-47,814)	89,694 (84,432-96,238)	130,585 (121,789-140,870)	148,406 (139,582-159,624)	153,091 (144,233-166,136)
Tuberculosis	-	-	27,235 (22,331-31,630)	48,085 (33,740-59,928)	17,872 (14,528-21,365)	17,742 (15,472-19,996)	39,637 (36,312-43,518)	68,631 (63,950-74,567)	87,346 (81,712-95,914)	100,598 (94,784-110,827)	110,706 (103,375-123,697)
Drug-susceptible tuberculosis	-	-	27,091 (22,172-31,466)	47,847 (33,558-59,664)	17,767 (14,439-21,237)	17,624 (15,377-19,872)	39,392 (36,086-43,290)	68,169 (63,464-74,105)	86,814 (81,207-95,472)	99,966 (94,187-110,100)	109,947 (102,698-122,892)
Multidrug-resistant tuberculosis without extensive drug resistance	-	-	144 (109-182)	238 (171-315)	105 (82-135)	118 (92-149)	245 (193-304)	462 (368-573)	532 (425-666)	632 (502-794)	759 (606-951)
Extensively drug-resistant tuberculosis	-	-	0 (0-0)	0 (0-0)	0 (0-0)	0 (0-0)	0 (0-0)	0 (0-0)	0 (0-0)	0 (0-0)	0 (0-0)
HIV/AIDS	-	-	18,078 (16,224-20,433)	12,445 (10,936-14,551)	1,371 (1,159-1,668)	459 (399-543)	4,320 (3,765-5,036)	21,063 (18,038-24,881)	43,239 (36,547-50,485)	47,808 (41,805-54,371)	42,385 (37,177-48,555)
Drug-susceptible HIV/AIDS - Tuberculosis	-	-	6,585 (5,301-7,700)	4,681 (3,795-5,556)	454 (355-566)	113 (81-145)	1,558 (1,304-1,873)	6,804 (5,172-8,531)	11,783 (8,523-15,465)	12,370 (9,022-16,065)	11,035 (8,127-14,067)
Multidrug-resistant HIV/AIDS - Tuberculosis without extensive drug resistance	-	-	28 (17-45)	19 (12-30)	2 (1-4)	1 (0-1)	6 (4-10)	30 (18-47)	64 (39-101)	83 (50-134)	77 (47-124)
Extensively drug-resistant HIV/AIDS - Tuberculosis	-	-	0 (0-0)	0 (0-0)	0 (0-0)	0 (0-0)	0 (0-0)	0 (0-0)	0 (0-0)	0 (0-0)	0 (0-0)
HIV/AIDS resulting in other diseases	-	-	11,464 (10,102-13,535)	7,745 (6,694-9,512)	914 (768-1,157)	345 (300-413)	2,756 (2,339-3,397)	14,229 (11,607-18,052)	31,392 (25,453-38,770)	35,356 (29,817-42,090)	31,273 (26,441-37,833)
Diarrhoea, lower respiratory infections, and other common infectious diseases	416,565 (356,571-470,768)	508,352 (429,553-558,102)	2,273,388 (2,160,648-2,420,415)	1,927,785 (1,731,841-2,198,377)	327,091 (282,372-379,285)	148,054 (126,986-171,949)	102,901 (86,242-122,712)	85,775 (71,939-104,933)	77,178 (64,521-96,048)	74,693 (62,418-92,303)	77,515 (65,057-99,206)
Diarrhoeal diseases	63,427 (55,797-73,135)	129,156 (109,767-146,630)	800,974 (710,260-880,224)	561,349 (450,179-667,825)	63,826 (35,363-105,793)	30,868 (17,579-52,747)	30,678 (17,848-50,557)	31,718 (19,351-52,611)	30,083 (18,633-49,841)	29,736 (18,470-49,986)	31,853 (19,591-55,208)
Intestinal infectious diseases	-	-	2,719 (554-9,421)	35,312 (16,548-67,335)	69,468 (37,997-115,548)	40,741 (21,497-64,290)	21,470 (10,936-37,024)	10,135 (5,077-17,616)	7,630 (3,956-13,067)	5,997 (3,172-10,123)	4,646 (2,513-7,636)
Typhoid fever	-	-	184 (84-362)	30,519 (13,261-61,064)	61,473 (32,286-103,510)	35,066 (18,090-55,907)	18,920 (9,480-33,301)	8,709 (4,256-15,470)	6,236 (3,178-10,715)	4,700 (2,445-7,952)	3,578 (1,895-6,106)
Paratyphoid fever	-	-	26 (10-57)	2,864 (1,132-6,265)	7,802 (3,448-15,759)	5,613 (2,552-10,838)	2,492 (1,068-4,974)	1,370 (589-2,824)	1,347 (584-2,746)	1,229 (556-2,445)	997 (473-1,912)
Other intestinal infectious diseases	-	-	2,509 (355-9,200)	1,929 (219-7,673)	193 (29-743)	62 (9-218)	58 (7-243)	56 (9-216)	47 (6-180)	68 (9-264)	71 (12-245)
Lower respiratory infections	240,501 (215,345-272,886)	256,946 (229,563-286,057)	979,447 (879,377-1,072,494)	558,061 (452,623-656,211)	54,674 (44,362-64,754)	24,008 (20,005-27,049)	18,578 (15,768-21,085)	19,619 (16,534-22,038)	19,834 (17,078-22,433)	20,597 (17,812-23,152)	23,537 (20,199-26,433)
Upper respiratory infections	131 (92-185)	64 (39-102)	1,207 (878-1,637)	1,011 (677-1,511)	75 (50-109)	47 (33-68)	101 (76-133)	97 (73-130)	96 (72-126)	93 (70-122)	106 (80-139)
Otitis media	15 (11-20)	69 (48-100)	639 (448-967)	887 (466-1,610)	489 (311-812)	399 (277-576)	197 (139-287)	121 (82-169)	124 (83-195)	115 (78-190)	113 (78-175)
Meningitis	10,961 (7,779-14,377)	19,981 (15,494-23,641)	111,722 (85,348-132,241)	107,315 (72,387-134,443)	24,671 (17,709-31,310)	15,125 (11,371-18,519)	12,542 (9,469-15,705)	10,476 (7,812-13,032)	8,815 (6,689-11,228)	8,016 (5,844-10,405)	7,769 (5,782-10,097)
Pneumococcal meningitis	542 (358-768)	984 (725-1,313)	6,041 (4,415-7,815)	5,797 (3,737-8,362)	1,347 (902-1,923)	821 (546-1,170)	669 (447-938)	541 (351-773)	537 (360-770)	622 (408-874)	724 (477-1,047)
H influenzae type B meningitis	1,428 (968-1,937)	2,530 (1,867-3,198)	13,362 (9,818-16,711)	11,723 (7,754-15,841)	2,155 (1,447-2,938)	1,107 (754-1,476)	809 (558-1,113)	665 (462-914)	604 (420-849)	580 (384-808)	577 (413-780)
Meningococcal infection	5,152 (3,602-6,876)	9,361 (7,152-11,464)	52,088 (39,717-63,005)	53,951 (37,616-69,079)	12,918 (9,141-16,720)	7,747 (5,536-9,751)	6,461 (4,731-8,195)	5,308 (3,864-6,834)	4,280 (3,059-5,578)	3,636 (2,583-4,836)	3,419 (2,460-4,479)
Other meningitis	3,839 (2,662-5,010)	7,106 (5,276-8,691)	40,232 (29,389-49,386)	35,844 (22,938-46,890)	8,251 (5,859-11,123)	5,451 (3,890-7,066)	4,603 (3,389-5,802)	3,962 (2,894-5,058)	3,394 (2,492-4,334)	3,178 (2,273-4,168)	3,048 (2,194-3,956)
Encephalitis	992 (538-1,343)	1,953 (954-2,978)	17,836 (9,389-26,166)	23,744 (11,145-41,244)	8,733 (5,309-12,327)	5,666 (3,742-7,776)	3,771 (2,688-4,940)	3,440 (2,524-4,521)	2,868 (2,073-3,577)	2,931 (2,205-3,723)	2,786 (2,127-3,555)
Diphtheria	-	-	2,544 (1,709-3,529)	5,679 (3,537-8,801)	849 (535-1,231)	355 (242-491)	162 (116-228)	100 (69-141)	88 (61-122)	78 (54-108)	110 (75-150)
Whooping cough	-	-	79,726 (35,619-154,405)	79,357 (35,320-154,442)	8,523 (3,709-17,305)	1,657 (703-3,452)	679 (279-1,445)	489 (201-1,053)	287 (112-634)	210 (82-475)	155 (59-347)
Tetanus	100,395 (52,231-152,301)	99,686 (50,961-135,552)	30,783 (16,940-48,286)	12,499 (6,092-20,800)	5,510 (2,581-8,687)	4,768 (2,199-8,566)	5,721 (2,745-9,739)	5,034 (2,401-8,750)	4,949 (2,576-8,115)	5,302 (3,015-8,718)	5,376 (2,993-8,637)
Measles	-	-	242,203 (92,881-495,140)	538,147 (227,765-1,007,167)	88,331 (36,321-175,462)	23,812 (8,687-51,831)	8,733 (2,982-20,323)	4,221 (1,427-10,027)	2,018 (676-4,872)	1,207 (406-2,900)	729 (244-1,743)
Varicella and herpes zoster	144 (119-171)	498 (408-602)	3,588 (2,948-4,372)	4,422 (3,362-5,629)	1,943 (1,534-2,423)	606 (504-722)	268 (225-318)	325 (275-384)	385 (328-450)	411 (351-480)	336 (285-392)
Neglected tropical diseases and malaria	1,022 (627-1,335)	21,529 (15,713-28,133)	196,525 (149,238-250,887)	421,504 (317,586-530,458)	68,531 (48,195-87,465)	34,953 (25,417-43,123)	19,463 (15,457-23,983)	19,756 (15,823-24,122)	14,674 (11,833-18,021)	14,063 (11,552-16,984)	15,443 (12,511-18,712)
Malaria	-	20,581 (14,779-26,928)	183,315 (137,230-236,920)	382,266 (282,547-490,115)	46,618 (30,962-63,599)	22,135 (15,357-29,169)	8,845 (6,505-11,417)	8,702 (6,416-11,152)	6,482 (4,785-8,219)	5,876 (4,340-7,533)	6,574 (4,848-8,604)
Chagas disease	-	-	8 (7-9)	5 (4-5)	5 (4-5)	11 (10-12)	29 (24-34)	68 (56-78)	139 (116-156)	267 (233-301)	408 (362-458)
Leishmaniasis	-	-	3,665 (1,931-6,687)	5,791 (2,816-10,772)	3,064 (1,557-5,708)	1,942 (1,014-3,402)	2,486 (1,322-4,228)	2,771 (1,530-4,822)	2,240 (1,227-3,763)	2,078 (1,140-3,537)	2,504 (1,351-4,308)
Visceral leishmaniasis	-	-	3,665 (1,931-6,687)	5,791 (2,816-10,772)	3,064 (1,557-5,708)	1,942 (1,014-3,402)	2,486 (1,322-4,228)	2,771 (1,530-4,822)	2,240 (1,227-3,763)	2,078 (1,140-3,537)	2,504 (1,351-4,308)
African trypanosomiasis	-	-	-	676 (154-2,000)	1,407 (434-3,238)	1,565 (545-3,330)	1,945 (680-3,867)	2,012 (731-4,070)	1,833 (613-3,847)	1,587 (548-3,378)	1,452 (444-3,255)
Schistosomiasis	-	-	0 (0-0)	902 (574-1,353)	375 (253-548)	468 (339-626)	411 (303-548)	529 (406-679)	601 (472-767)	662 (518-835)	814 (631-1,062)
Cysticercosis	0 (0-0)	0 (0-0)	0 (0-0)	172 (111-251)	102 (76-137)	73 (58-90)	64 (50-81)	92 (75-114)	111 (91-132)	122 (101-145)	104 (86-124)
Cystic echinococcosis	-	-	-	556 (360-847)	196 (131-281)	107 (72-151)	51 (35-70)	65 (46-90)	89 (66-119)	91 (67-120)	108 (81-141)
Dengue	-	-	1,166 (346-2,477)	2,218 (680-4,468)	1,111 (236-2,237)	652 (146-1,296)	520 (110-1,032)	486 (98-975)	369 (77-731)	281 (55-574)	270 (56-542)
Yellow fever	-	7 (1-23)	125 (25-367)	1,093 (209-3,432)	2,020 (406-6,113)	1,731 (385-5,203)	1,555 (309-4,652)	1,319 (280-3,943)	996 (205-3,021)	760 (159-2,277)	605 (127-1,826)
Rabies	-	-	1,273 (736-2,516)	6,253 (3,313-10,416)	10,129 (5,143-15,168)	5,221 (2,483-7,281)	2,975 (1,821-4,157)	3,177 (1,744-4,538)	1,320 (873-1,957)	1,875 (1,087-2,641)	2,100 (1,171-2,905)
Intestinal nematode infections	-	-	923 (728-1,145)	10,370 (7,685-13,469)	1,550 (1,212-1,948)	290 (233-359)	90 (73-111)	59 (48-72)	44 (35-55)	56 (45-68)	53 (42-64)
Ascariasis	-	-	923 (728-1,145)	10,370 (7,685-13,469)	1,550 (1,212-1,948)	290 (233-359)	90 (73-111)	59 (48-72)	44 (35-55)	56 (45-68)	53 (42-64)
Ebola	0 (0-0)	0 (0-0)	0 (0-0)	0 (0-0)	0 (0-0)	0 (0-0)	0 (0-0)	0 (0-0)	0 (0-0)	0 (0-0)	0 (0-0)
Zika virus	0 (0-0)	0 (0-0)	0 (0-0)	0 (0-0)	0 (0-0)	0 (0-0)	0 (0-0)	0 (0-0)	0 (0-0)	0 (0-0)	0 (0-0)
Other neglected tropical diseases	1,022 (627-1,335)	941 (643-1,334)	6,050 (3,914-8,563)	11,203 (5,406-20,890)	1,952 (1,171-3,160)	758 (491-1,099)	492 (298-672)	475 (294-610)	450 (288-569)	410 (233-512)	453 (251-547)
Maternal disorders	-	-	-	-	-	1,794 (1,504-2,098)	40,639 (36,516-44,207)	78,643 (72,401-84,330)	73,366 (67,588-78,150)	63,143 (58,440-67,241)	51,051 (46,906-54,780)
Maternal haemorrhage	-	-	-	-	-	372 (253-536)	11,747 (7,886-15,627)	27,212 (20,647-33,850)	26,349 (19,674-33,383)	22,528 (16,946-28,246)	17,950 (13,306-22,975)
Maternal sepsis and other pregnancy related infections	-	-	-	-	-	212 (139-302)	5,111 (3,338-7,624)	7,834 (4,822-11,874)	6,939 (4,654-9,953)	5,836 (3,802-8,286)	4,823 (3,210-7,038)
Maternal hypertensive disorders	-	-	-	-	-	293 (181-418)	5,207 (3,316-7,287)	9,073 (6,128-12,799)	8,019 (5,231-11,590)	6,749 (4,298-10,060)	5,556 (3,708-8,032)
Maternal obstructed labour and uterine rupture	-	-	-	-	-	60 (31-105)	1,177 (600-2,001)	2,681 (1,597-4,169)	2,805 (1,636-4,417)	2,803 (1,594-4,303)	2,633 (1,581-4,297)
Maternal abortion, miscarriage, and ectopic pregnancy	-	-	-	-	-	162 (98-242)	3,102 (1,935-4,669)	5,861 (3,689-8,930)	5,343 (3,505-8,130)	5,085 (3,298-7,326)	4,679 (2,885-6,718)
Indirect maternal deaths	-	-	-	-	-	251 (158-368)	4,995 (3,082-7,443)	9,792 (6,397-14,250)	9,840 (6,382-14,171)	8,463 (5,182-12,485)	6,056 (3,900-8,703)
Late maternal deaths	-	-	-	-	-	42 (21-76)	775 (446-1,273)	1,405 (775-2,391)	1,344 (745-2,310)	1,156 (660-1,912)	905 (490-1,514)
Maternal deaths aggravated by HIV/AIDS	-	-	-	-	-	0 (0-0)	38 (21-57)	129 (74-185)	164 (97-231)	142 (78-205)	100 (58-146)
Other maternal disorders	-	-	-	-	-	403 (282-574)	8,485 (5,697-12,391)	14,656 (9,621-21,018)	12,564 (8,515-17,441)	10,381 (6,767-14,733)	8,350 (5,577-12,370)
Neonatal disorders	2,282,583 (2,203,645-2,394,891)	496,910 (446,983-599,363)	135,043 (113,408-175,474)	12,747 (9,388-19,391)	-	-	-	-	-	-	-
Neonatal preterm birth complications	1,007,753 (944,070-1,081,685)	172,832 (149,744-230,072)	57,843 (48,343-78,429)	3,721 (2,702-6,056)	-	-	-	-	-	-	-
Neonatal encephalopathy due to birth asphyxia and trauma	678,445 (611,924-758,003)	73,382 (64,250-89,753)	22,999 (18,913-28,983)	4,615 (3,413-6,954)	-	-	-	-	-	-	-
Neonatal sepsis and other neonatal infections	144,509 (101,635-182,158)	107,571 (80,389-147,490)	17,382 (12,040-26,342)	989 (323-1,830)	-	-	-	-	-	-	-
Hemolytic disease and other neonatal jaundice	72,968 (58,433-93,139)	53,813 (43,616-67,395)	9,940 (7,738-12,771)	637 (444-905)	-	-	-	-	-	-	-
Other neonatal disorders	378,908 (330,032-431,470)	89,312 (71,151-132,363)	26,879 (20,903-40,455)	2,785 (1,865-5,377)	-	-	-	-	-	-	-
Nutritional deficiencies	-	-	160,727 (136,621-183,743)	197,775 (148,572-248,751)	15,327 (12,475-18,069)	5,792 (5,028-6,589)	4,637 (4,151-5,269)	4,954 (4,474-5,600)	4,829 (4,356-5,455)	4,662 (4,215-5,293)	4,599 (4,172-5,305)
Protein-energy malnutrition	-	-	154,628 (131,009-176,911)	190,247 (143,768-238,991)	12,948 (10,379-15,359)	4,616 (3,949-5,363)	3,160 (2,786-3,655)	3,075 (2,734-3,527)	3,121 (2,767-3,566)	2,957 (2,628-3,395)	2,963 (2,657-3,438)
Iodine deficiency	-	-	555 (185-1,338)	464 (116-1,348)	151 (52-351)	34 (9-85)	66 (23-151)	72 (24-183)	74 (26-171)	77 (27-194)	70 (24-156)
Iron-deficiency anaemia	-	-	414 (68-769)	180 (94-296)	197 (80-299)	161 (81-250)	182 (101-259)	174 (106-244)	140 (92-182)	149 (101-184)	148 (98-191)
Other nutritional deficiencies	-	-	5,130 (3,624-6,451)	6,884 (2,940-10,543)	2,030 (1,482-2,696)	982 (793-1,242)	1,229 (1,033-1,477)	1,633 (1,373-1,966)	1,494 (1,257-1,794)	1,479 (1,236-1,776)	1,418 (1,183-1,715)
Other communicable, maternal, neonatal, and nutritional diseases	56,729 (34,836-86,157)	25,916 (15,810-38,183)	91,875 (63,583-130,720)	68,973 (47,865-97,058)	21,675 (15,947-29,532)	8,219 (7,182-9,363)	10,838 (9,891-11,848)	12,352 (11,465-13,292)	11,388 (10,533-12,230)	11,523 (10,718-12,384)	11,666 (10,921-12,447)
Sexually transmitted diseases excluding HIV	49,118 (27,577-79,135)	19,966 (11,230-32,065)	55,692 (30,500-91,352)	31,734 (16,973-52,810)	10,564 (5,742-17,637)	213 (129-300)	821 (506-1,048)	942 (598-1,160)	1,115 (709-1,389)	1,068 (679-1,371)	983 (636-1,237)
Syphilis	49,118 (27,577-79,135)	19,966 (11,230-32,065)	55,692 (30,500-91,352)	31,734 (16,973-52,810)	10,564 (5,742-17,637)	149 (89-217)	337 (202-462)	369 (221-485)	389 (229-551)	468 (251-683)	317 (181-482)
Chlamydial infection	-	-	-	-	-	14 (8-19)	82 (51-108)	165 (106-211)	144 (94-184)	72 (47-89)	117 (77-142)
Gonococcal infection	-	-	-	-	-	34 (21-46)	224 (139-287)	254 (162-319)	409 (265-511)	349 (228-425)	336 (221-407)
Other sexually transmitted diseases	-	-	-	-	-	17 (10-23)	178 (112-230)	155 (99-195)	172 (112-217)	180 (119-220)	213 (139-258)
Hepatitis	-	-	16,114 (14,038-18,230)	20,950 (16,105-25,499)	7,448 (6,074-8,887)	5,783 (4,962-6,705)	7,597 (6,864-8,457)	9,292 (8,568-10,126)	8,423 (7,780-9,209)	8,447 (7,853-9,170)	8,721 (8,109-9,385)
Acute hepatitis A	-	-	10,134 (7,498-12,823)	2,408 (1,529-3,536)	179 (118-258)	85 (55-125)	117 (76-175)	143 (81-226)	97 (50-160)	77 (42-128)	75 (45-118)
Hepatitis B	-	-	5,980 (3,794-8,564)	3,076 (1,753-4,753)	799 (441-1,278)	1,008 (576-1,550)	2,893 (2,055-3,909)	5,934 (4,595-7,322)	6,719 (5,731-7,663)	7,465 (6,718-8,158)	7,987 (7,333-8,695)
Hepatitis C	-	-	-	-	203 (130-302)	68 (44-98)	84 (54-119)	98 (55-156)	71 (38-113)	62 (32-105)	73 (39-122)
Acute hepatitis E	-	-	-	15,465 (11,489-19,312)	6,268 (5,077-7,553)	4,622 (3,823-5,475)	4,503 (3,506-5,457)	3,118 (1,927-4,399)	1,535 (843-2,323)	843 (472-1,353)	586 (323-958)
Other infectious diseases	7,612 (2,568-13,930)	5,950 (2,385-9,067)	20,069 (7,860-34,430)	16,289 (7,144-30,499)	3,662 (2,732-4,579)	2,223 (1,727-2,685)	2,419 (1,919-2,842)	2,117 (1,656-2,473)	1,850 (1,453-2,115)	2,009 (1,589-2,302)	1,962 (1,579-2,229)
Non-communicable diseases	225,541 (168,673-274,600)	110,143 (80,364-138,160)	424,959 (346,129-500,837)	308,040 (236,933-370,348)	122,804 (109,992-134,943)	113,758 (105,278-121,244)	177,982 (170,096-184,094)	228,192 (221,248-234,433)	263,114 (254,796-269,936)	355,584 (345,960-364,208)	507,679 (494,732-519,286)
Neoplasms	1,101 (937-1,186)	756 (677-817)	9,577 (8,360-10,950)	32,293 (26,476-41,041)	32,882 (29,608-36,341)	30,834 (28,482-33,074)	41,449 (38,756-43,397)	47,845 (45,685-49,873)	58,376 (56,006-60,499)	90,490 (87,252-93,502)	146,034 (141,037-150,802)
Lip and oral cavity cancer	-	-	-	-	-	-	321 (297-349)	579 (540-627)	906 (837-985)	1,570 (1,470-1,691)	2,583 (2,420-2,763)
Nasopharynx cancer	-	-	-	-	231 (197-267)	388 (337-431)	704 (640-755)	842 (781-909)	997 (912-1,088)	1,503 (1,382-1,620)	2,486 (2,303-2,670)
Other pharynx cancer	-	-	-	-	-	-	259 (224-302)	357 (312-417)	501 (443-580)	873 (775-1,005)	1,581 (1,432-1,803)
Oesophageal cancer	-	-	-	-	-	-	439 (407-481)	734 (693-785)	918 (871-976)	1,826 (1,739-1,938)	4,011 (3,810-4,226)
Stomach cancer	-	-	-	-	-	-	1,488 (1,409-1,585)	3,299 (3,108-3,510)	5,517 (5,172-5,838)	10,788 (10,272-11,282)	18,294 (17,462-19,137)
Colon and rectum cancer	-	-	-	-	-	-	1,040 (949-1,168)	2,254 (2,087-2,485)	3,355 (3,159-3,641)	5,921 (5,557-6,426)	8,881 (8,423-9,531)
Liver cancer	-	-	-	-	656 (571-774)	880 (787-988)	1,705 (1,550-1,881)	3,490 (3,168-3,883)	6,024 (5,439-6,634)	11,100 (10,093-12,175)	20,538 (18,854-22,255)
Liver cancer due to hepatitis B	-	-	-	-	206 (144-289)	382 (284-494)	879 (683-1,086)	2,087 (1,727-2,463)	3,834 (3,109-4,489)	7,099 (5,917-8,191)	12,771 (10,451-14,656)
Liver cancer due to hepatitis C	-	-	-	-	15 (12-21)	26 (19-34)	49 (37-64)	120 (91-155)	243 (166-343)	503 (374-656)	1,016 (764-1,349)
Liver cancer due to alcohol use	-	-	-	-	-	-	6 (3-11)	41 (25-61)	115 (64-184)	395 (272-571)	1,159 (745-1,684)
Liver cancer due to other causes	-	-	-	-	434 (356-534)	472 (379-572)	770 (600-968)	1,243 (995-1,537)	1,832 (1,414-2,432)	3,102 (2,465-3,980)	5,592 (4,479-7,227)
Gallbladder and biliary tract cancer	-	-	-	-	-	-	95 (86-112)	159 (145-186)	284 (257-333)	706 (642-832)	1,396 (1,272-1,640)
Pancreatic cancer	-	-	-	-	-	-	163 (154-174)	324 (308-345)	562 (538-590)	1,232 (1,187-1,285)	2,512 (2,434-2,598)
Larynx cancer	-	-	-	-	-	-	187 (174-202)	254 (239-273)	315 (294-339)	657 (616-706)	1,393 (1,322-1,470)
Tracheal, bronchus, and lung cancer	-	-	-	-	-	-	1,014 (932-1,113)	1,932 (1,792-2,106)	3,338 (3,121-3,543)	6,627 (6,221-7,044)	13,546 (12,883-14,231)
Malignant skin melanoma	-	-	-	-	-	-	222 (186-257)	399 (349-469)	650 (564-733)	1,073 (921-1,185)	1,566 (1,349-1,725)
Non-melanoma skin cancer	-	-	-	-	-	-	191 (175-206)	252 (231-268)	301 (281-317)	444 (417-465)	602 (569-631)
Non-melanoma skin cancer (squamous-cell carcinoma)	-	-	-	-	-	-	191 (175-206)	252 (231-268)	301 (281-317)	444 (417-465)	602 (569-631)
Breast cancer	-	-	-	-	-	-	289 (214-357)	835 (669-1,045)	2,656 (2,220-3,193)	7,840 (6,794-9,160)	16,236 (14,467-18,702)
Cervical cancer	-	-	-	-	-	-	271 (207-318)	943 (746-1,155)	2,580 (2,187-3,117)	6,186 (5,218-7,563)	11,201 (9,574-13,808)
Uterine cancer	-	-	-	-	-	-	118 (104-132)	285 (252-313)	437 (391-481)	726 (649-791)	1,364 (1,212-1,478)
Ovarian cancer	-	-	-	-	-	-	470 (398-526)	663 (570-739)	904 (802-982)	1,425 (1,275-1,545)	2,544 (2,275-2,754)
Prostate cancer	-	-	-	-	-	-	64 (50-75)	76 (59-89)	86 (69-102)	108 (86-125)	162 (128-185)
Testicular cancer	-	-	-	-	-	-	507 (465-558)	1,013 (944-1,105)	1,228 (1,150-1,329)	1,195 (1,119-1,279)	967 (907-1,037)
Kidney cancer	38 (33-41)	21 (18-24)	295 (249-365)	1,312 (1,011-1,763)	656 (579-754)	270 (248-295)	201 (191-211)	263 (250-277)	356 (339-371)	514 (493-536)	928 (881-975)
Bladder cancer	-	-	-	-	-	-	123 (112-135)	195 (180-210)	258 (242-276)	490 (456-528)	887 (826-957)
Brain and nervous system cancer	152 (96-186)	104 (65-128)	1,588 (922-2,063)	5,723 (3,930-7,418)	6,891 (5,439-7,711)	5,504 (4,467-6,086)	4,316 (3,491-4,788)	4,111 (3,372-4,535)	4,493 (3,726-4,943)	5,400 (4,544-5,880)	6,935 (5,810-7,575)
Thyroid cancer	-	-	-	-	-	154 (124-183)	306 (259-350)	474 (407-535)	549 (483-608)	625 (546-695)	768 (675-848)
Mesothelioma	-	-	-	-	-	-	95 (79-116)	119 (93-150)	174 (139-216)	194 (157-237)	330 (276-394)
Hodgkin lymphoma	25 (11-32)	8 (3-11)	199 (81-267)	867 (377-1,159)	1,893 (1,034-2,502)	1,823 (1,176-2,160)	2,071 (1,454-2,432)	2,133 (1,585-2,436)	2,151 (1,678-2,419)	2,111 (1,644-2,380)	2,075 (1,571-2,331)
Non-Hodgkin's lymphoma	61 (50-68)	47 (40-57)	674 (511-869)	2,719 (2,075-3,775)	3,142 (2,551-3,711)	2,754 (2,333-3,104)	2,915 (2,586-3,141)	3,173 (2,899-3,516)	3,123 (2,827-3,398)	3,544 (3,145-3,825)	4,208 (3,826-4,485)
Multiple myeloma	-	-	-	-	-	-	230 (185-259)	270 (216-299)	245 (207-273)	378 (321-429)	654 (568-738)
Leukaemia	310 (236-345)	267 (222-314)	4,510 (3,415-5,711)	14,950 (11,204-22,175)	14,577 (12,607-18,079)	13,553 (12,107-16,125)	13,958 (12,250-15,970)	11,678 (10,056-13,178)	9,256 (7,929-10,065)	8,616 (7,496-9,407)	9,409 (8,085-10,278)
Acute lymphoid leukaemia	67 (57-89)	57 (45-84)	925 (649-1,566)	4,210 (2,987-7,551)	5,153 (4,295-7,081)	4,352 (3,787-5,805)	3,352 (2,969-4,400)	2,228 (1,979-2,902)	1,650 (1,455-2,140)	1,317 (1,165-1,658)	1,324 (1,163-1,657)
Chronic lymphoid leukaemia	-	-	-	-	-	-	284 (229-437)	273 (219-416)	234 (194-332)	231 (189-332)	302 (243-413)
Acute myeloid leukaemia	40 (30-49)	37 (28-48)	482 (367-729)	1,971 (1,479-3,276)	1,759 (1,428-2,259)	1,835 (1,562-2,244)	1,954 (1,712-2,333)	1,653 (1,448-1,929)	1,528 (1,349-1,769)	1,542 (1,370-1,755)	1,743 (1,549-1,965)
Chronic myeloid leukaemia	-	-	-	-	-	-	560 (458-716)	681 (564-817)	964 (818-1,141)	1,086 (928-1,247)	1,188 (1,037-1,340)
Other leukaemia	204 (139-238)	174 (128-208)	3,103 (2,041-4,036)	8,770 (6,119-12,248)	7,665 (6,237-9,094)	7,367 (6,137-8,405)	7,808 (6,227-8,847)	6,844 (5,377-7,785)	4,881 (3,796-5,506)	4,440 (3,542-4,996)	4,852 (3,814-5,403)
Other neoplasms	515 (446-548)	309 (269-360)	2,311 (1,940-2,986)	6,720 (5,425-9,961)	4,836 (4,296-5,654)	5,508 (5,062-6,069)	7,688 (7,232-8,153)	6,739 (6,357-7,134)	6,212 (5,822-6,579)	6,818 (6,433-7,178)	7,978 (7,500-8,408)
Cardiovascular diseases	6,463 (6,008-6,980)	4,587 (4,040-5,133)	38,982 (35,139-43,532)	33,472 (28,161-38,853)	16,923 (14,949-18,792)	18,464 (17,083-19,781)	44,271 (42,134-46,666)	66,679 (64,157-70,016)	81,787 (78,582-86,175)	117,771 (113,322-124,595)	175,456 (169,525-183,849)
Rheumatic heart disease	-	-	-	8,836 (6,989-11,279)	4,804 (4,012-5,692)	6,886 (6,063-7,644)	11,541 (10,460-12,678)	12,659 (11,322-13,967)	12,454 (10,620-13,956)	13,654 (11,612-15,476)	16,380 (13,878-18,418)
Ischaemic heart disease	-	-	2,435 (2,002-3,119)	2,155 (1,634-3,078)	1,223 (1,025-1,524)	1,229 (1,089-1,429)	12,469 (11,656-13,507)	21,595 (20,515-23,010)	31,049 (29,592-32,758)	52,392 (50,164-55,269)	81,131 (77,918-84,920)
Cerebrovascular disease	3,181 (2,802-3,642)	2,181 (1,826-2,674)	16,662 (13,638-20,782)	10,668 (8,467-14,317)	5,860 (5,087-6,792)	5,620 (5,134-6,265)	11,351 (10,721-12,312)	19,967 (19,003-21,429)	23,449 (22,230-25,155)	33,678 (32,108-36,003)	53,165 (50,851-56,460)

Cause	40 to 44	45 to 49	50 to 54	55 to 59	60 to 64	65 to 69	70 to 74	75 to 79	80 to 84	85 to 89	90 to 94	95 plus
All causes	1,168,985 (1,155,625-1,182,562)	1,373,049 (1,358,564-1,388,168)	1,894,860 (1,874,217-1,915,884)	2,428,818 (2,404,987-2,452,890)	3,195,687 (3,166,443-3,226,383)	3,680,469 (3,651,245-3,710,819)	4,015,893 (3,983,692-4,048,248)	4,294,701 (4,264,701-4,325,969)	3,628,935 (3,607,102-3,650,711)	2,431,500 (2,419,919-2,442,970)	1,067,561 (1,063,700-1,071,292)	341,114 (339,967-342,267)
Communicable, maternal, neonatal, and nutritional disorders	280,509 (268,260-295,049)	281,149 (265,899-300,290)	309,170 (289,023-336,834)	346,990 (321,680-382,291)	405,853 (368,643-461,698)	443,543 (398,517-509,129)	503,401 (446,273-587,498)	464,354 (410,831-537,624)	394,291 (348,093-454,314)	259,916 (230,606-295,608)	107,381 (95,217-120,533)	35,665 (32,172-39,411)
HIV/AIDS and tuberculosis	143,204 (135,193-156,012)	145,976 (136,742-160,047)	159,987 (149,332-177,547)	170,037 (158,313-188,352)	174,438 (160,132-195,566)	166,436 (152,531-187,970)	163,752 (148,743-185,091)	122,736 (111,080-140,157)	80,649 (70,888-97,221)	39,231 (34,350-48,078)	11,465 (9,980-14,157)	2,631 (2,263-3,311)
Tuberculosis	109,931 (103,226-122,825)	122,623 (114,491-137,064)	145,527 (135,276-162,371)	159,612 (148,027-178,268)	167,151 (152,920-188,330)	161,429 (147,611-182,940)	161,059 (146,005-182,578)	121,294 (109,527-138,740)	80,409 (70,634-96,986)	39,137 (34,267-48,002)	11,439 (9,950-14,127)	2,623 (2,255-3,303)
Drug-susceptible tuberculosis	109,170 (102,454-121,964)	121,824 (113,672-136,213)	144,454 (134,167-161,279)	158,392 (146,947-176,943)	165,735 (151,537-186,613)	160,044 (146,343-181,261)	159,605 (144,772-181,329)	120,135 (108,540-137,467)	79,698 (69,978-96,084)	38,785 (33,925-47,637)	11,337 (9,860-14,009)	2,598 (2,234-3,278)
Multidrug-resistant tuberculosis without extensive drug resistance	762 (602-951)	799 (640-997)	1,074 (850-1,364)	1,220 (969-1,523)	1,416 (1,124-1,772)	1,386 (1,103-1,731)	1,453 (1,145-1,830)	1,159 (913-1,464)	711 (534-961)	352 (259-505)	102 (72-152)	24 (17-39)
Extensively drug-resistant tuberculosis	0 (0-0)	0 (0-0)	0 (0-0)	0 (0-0)	0 (0-0)	0 (0-0)	0 (0-0)	0 (0-0)	0 (0-0)	0 (0-0)	0 (0-0)	0 (0-0)
HIV/AIDS	33,272 (29,481-37,696)	23,354 (20,320-27,033)	14,459 (12,694-16,555)	10,425 (9,064-12,231)	7,287 (6,248-8,625)	5,006 (4,252-5,941)	2,693 (2,238-3,201)	1,443 (1,219-1,714)	240 (190-307)	94 (76-120)	26 (22-34)	8 (7-9)
Drug-susceptible HIV/AIDS - Tuberculosis	9,233 (6,893-11,717)	7,104 (5,299-8,894)	4,682 (3,674-5,713)	3,455 (2,715-4,255)	2,465 (1,918-3,042)	1,721 (1,342-2,132)	981 (779-1,215)	524 (422-633)	74 (56-96)	28 (21-37)	8 (6-10)	2 (1-2)
Multidrug-resistant HIV/AIDS - Tuberculosis without extensive drug resistance	60 (37-96)	44 (27-70)	30 (18-48)	21 (13-34)	15 (9-23)	10 (6-16)	5 (3-8)	3 (2-5)	1 (0-1)	0 (0-0)	0 (0-0)	0 (0-0)
Extensively drug-resistant HIV/AIDS - Tuberculosis	0 (0-0)	0 (0-0)	0 (0-0)	0 (0-0)	0 (0-0)	0 (0-0)	0 (0-0)	0 (0-0)	0 (0-0)	0 (0-0)	0 (0-0)	0 (0-0)
HIV/AIDS resulting in other diseases	23,979 (20,443-28,955)	16,206 (13,629-19,862)	9,747 (8,377-11,741)	6,948 (5,941-8,521)	4,807 (4,071-5,876)	3,276 (2,756-4,033)	1,707 (1,391-2,131)	916 (764-1,148)	165 (128-222)	66 (52-88)	19 (15-24)	6 (6-8)
Diarrhoea, lower respiratory infections, and other common infectious diseases	75,601 (63,766-94,923)	88,470 (72,663-112,731)	113,040 (92,317-148,383)	141,108 (112,828-184,919)	192,973 (153,693-259,179)	237,657 (189,008-317,559)	301,957 (239,786-397,884)	306,132 (248,498-392,025)	287,532 (238,055-357,460)	201,377 (171,142-242,673)	86,341 (74,147-100,840)	29,126 (25,620-33,237)
Diarrhoeal diseases	30,710 (19,040-51,477)	38,428 (23,175-64,251)	51,312 (30,915-89,720)	68,583 (40,937-116,672)	99,185 (58,645-168,996)	125,170 (74,765-211,389)	164,545 (100,284-271,105)	156,294 (95,066-253,712)	141,877 (90,900-218,310)	90,509 (60,537-134,341)	34,790 (23,440-49,946)	9,800 (6,902-13,648)
Intestinal infectious diseases	3,578 (1,947-5,923)	3,135 (1,706-5,211)	2,703 (1,401-4,600)	2,124 (1,032-3,847)	1,822 (894-3,299)	1,539 (865-2,499)	1,179 (686-1,874)	791 (463-1,262)	439 (254-696)	216 (126-336)	66 (38-107)	17 (9-29)
Typhoid fever	2,724 (1,435-4,689)	2,343 (1,242-3,976)	2,058 (1,040-3,582)	1,656 (783-3,009)	1,467 (701-2,657)	1,283 (701-2,140)	989 (562-1,578)	638 (360-1,026)	354 (200-568)	164 (93-259)	48 (27-77)	10 (6-17)
Paratyphoid fever	794 (374-1,558)	720 (327-1,385)	615 (271-1,203)	435 (181-895)	319 (134-657)	215 (99-420)	144 (69-273)	90 (44-166)	51 (25-93)	24 (11-44)	7 (3-13)	2 (1-3)
Other intestinal infectious diseases	60 (8-252)	72 (12-267)	29 (4-112)	33 (5-126)	37 (6-136)	41 (7-167)	46 (7-177)	62 (10-229)	34 (6-111)	28 (5-99)	11 (2-35)	5 (1-15)
Lower respiratory infections	24,959 (21,539-27,647)	29,150 (25,243-33,065)	39,755 (34,446-44,635)	51,102 (43,960-57,682)	74,070 (63,046-82,408)	91,916 (78,024-102,798)	118,235 (102,957-132,441)	134,878 (118,430-150,407)	134,632 (118,409-149,101)	104,998 (93,306-116,807)	49,452 (43,836-55,803)	18,680 (16,342-21,041)
Upper respiratory infections	97 (73-127)	112 (84-144)	124 (94-159)	139 (107-179)	157 (120-202)	136 (104-176)	114 (87-147)	108 (84-139)	65 (50-85)	42 (32-54)	15 (11-19)	3 (2-5)
Otitis media	170 (111-269)	219 (130-341)	241 (143-389)	258 (175-404)	192 (131-272)	282 (191-410)	236 (161-346)	164 (110-232)	122 (82-178)	60 (42-84)	24 (17-35)	7 (5-9)
Meningitis	6,498 (4,937-8,364)	7,321 (5,522-9,651)	7,687 (5,898-9,998)	8,001 (6,078-10,225)	7,927 (6,061-10,363)	8,059 (6,214-10,124)	7,602 (5,891-9,326)	5,311 (4,123-6,598)	4,207 (3,318-5,074)	2,116 (1,681-2,577)	699 (554-840)	192 (158-226)
Pneumococcal meningitis	711 (458-1,090)	855 (538-1,316)	873 (602-1,271)	938 (672-1,270)	978 (708-1,334)	985 (694-1,327)	930 (652-1,286)	690 (504-918)	517 (376-686)	254 (194-323)	88 (69-109)	26 (19-34)
H influenzae type B meningitis	485 (337-674)	553 (382-796)	574 (403-789)	597 (422-809)	594 (415-825)	577 (403-780)	533 (387-707)	383 (278-518)	302 (219-395)	151 (111-196)	49 (35-65)	13 (9-19)
Meningococcal infection	2,875 (2,092-3,848)	3,201 (2,332-4,279)	3,415 (2,544-4,501)	3,531 (2,598-4,567)	3,529 (2,641-4,685)	3,504 (2,583-4,595)	3,160 (2,302-4,131)	2,264 (1,660-2,916)	1,734 (1,288-2,166)	912 (686-1,159)	305 (229-382)	82 (61-104)
Other meningitis	2,426 (1,764-3,081)	2,712 (2,024-3,603)	2,826 (2,154-3,716)	2,936 (2,181-3,825)	2,825 (2,119-3,688)	2,993 (2,273-3,963)	2,978 (2,249-3,751)	1,974 (1,502-2,499)	1,653 (1,305-2,083)	799 (630-1,005)	258 (202-322)	72 (55-91)
Encephalitis	2,424 (1,828-3,054)	2,526 (1,943-3,317)	2,736 (2,128-3,537)	3,218 (2,494-4,096)	3,735 (2,860-4,723)	4,016 (2,977-4,939)	3,948 (2,794-4,880)	3,481 (2,415-4,214)	2,550 (1,753-3,122)	1,291 (910-1,536)	369 (265-434)	92 (69-107)
Diphtheria	106 (74-145)	119 (85-168)	141 (102-197)	106 (75-145)	-	-	-	-	-	-	-	-
Whooping cough	154 (59-349)	132 (50-295)	121 (46-272)	148 (58-322)	-	-	-	-	-	-	-	-
Tetanus	6,224 (3,287-10,532)	6,755 (3,710-11,416)	7,751 (4,098-13,979)	6,930 (3,859-11,804)	5,593 (3,027-10,303)	6,011 (3,269-12,651)	5,606 (2,854-10,677)	4,333 (2,152-8,361)	2,578 (1,222-4,971)	1,021 (501-1,812)	294 (140-555)	77 (37-136)
Measles	487 (164-1,171)	342 (116-813)	264 (88-629)	250 (84-593)	-	-	-	-	-	-	-	-
Varicella and herpes zoster	196 (164-232)	232 (198-272)	203 (172-239)	249 (209-293)	292 (247-347)	527 (450-626)	493 (408-585)	773 (659-903)	1,062 (888-1,297)	1,125 (934-1,352)	631 (512-781)	258 (201-335)
Neglected tropical diseases and malaria	14,741 (11,955-17,882)	14,956 (12,195-18,069)	15,286 (12,760-18,353)	14,986 (12,559-17,904)	16,043 (13,412-19,187)	14,923 (12,472-17,957)	12,717 (10,569-15,673)	9,557 (7,903-11,676)	4,759 (3,902-5,723)	2,825 (2,255-3,496)	577 (452-706)	251 (178-372)
Malaria	6,085 (4,590-7,905)	5,712 (4,222-7,448)	5,972 (4,519-7,763)	6,183 (4,695-7,976)	8,234 (6,234-10,763)	7,331 (5,496-9,699)	6,111 (4,543-8,185)	4,603 (3,401-6,178)	2,366 (1,735-3,124)	1,594 (1,158-2,149)	271 (180-374)	158 (94-278)
Chagas disease	559 (500-617)	703 (637-777)	903 (817-1,002)	992 (895-1,105)	1,199 (1,107-1,297)	1,134 (1,051-1,222)	1,058 (993-1,122)	732 (673-795)	480 (444-518)	216 (201-234)	69 (64-76)	18 (17-20)
Leishmaniasis	2,501 (1,327-4,336)	2,423 (1,315-4,101)	2,454 (1,334-4,194)	1,842 (953-3,277)	1,508 (800-2,628)	1,604 (863-2,778)	1,766 (939-3,133)	1,419 (746-2,483)	738 (385-1,256)	456 (234-778)	92 (44-157)	32 (13-55)
Visceral leishmaniasis	2,501 (1,327-4,336)	2,423 (1,315-4,101)	2,454 (1,334-4,194)	1,842 (953-3,277)	1,508 (800-2,628)	1,604 (863-2,778)	1,766 (939-3,133)	1,419 (746-2,483)	738 (385-1,256)	456 (234-778)	92 (44-157)	32 (13-55)
African trypanosomiasis	1,296 (373-2,983)	1,170 (341-2,864)	1,024 (288-2,572)	958 (274-2,525)	770 (231-2,090)	580 (167-1,520)	416 (107-1,282)	250 (58-783)	116 (24-416)	41 (9-147)	10 (2-32)	2 (0-6)
Schistosomiasis	883 (676-1,159)	970 (765-1,250)	1,173 (914-1,525)	1,395 (1,092-1,799)	1,452 (1,153-1,873)	1,502 (1,171-1,897)	1,314 (1,028-1,685)	888 (696-1,110)	406 (320-513)	171 (136-215)	42 (30-56)	7 (4-10)
Cysticercosis	94 (79-112)	90 (76-109)	106 (90-125)	103 (89-121)	86 (72-101)	73 (62-86)	43 (36-52)	26 (22-32)	14 (11-17)	6 (5-8)	2 (1-2)	1 (0-1)
Cystic echinococcosis	93 (70-120)	104 (78-136)	119 (94-151)	128 (100-163)	133 (107-167)	116 (92-146)	93 (74-117)	86 (69-107)	47 (37-59)	24 (19-29)	7 (5-9)	2 (1-2)
Dengue	222 (44-451)	255 (55-506)	271 (59-566)	300 (70-614)	280 (61-582)	264 (68-523)	108 (31-186)	85 (27-144)	63 (21-113)	38 (16-66)	13 (5-21)	3 (1-4)
Yellow fever	487 (100-1,435)	402 (79-1,261)	327 (67-1,011)	265 (55-818)	202 (40-608)	146 (31-447)	92 (19-295)	49 (10-154)	18 (3-55)	6 (1-20)	2 (0-5)	0 (0-1)
Rabies	2,049 (1,357-3,376)	2,632 (1,589-4,155)	2,343 (1,607-4,891)	2,180 (1,153-3,381)	1,549 (917-2,122)	1,552 (828-2,468)	1,119 (759-1,952)	968 (641-1,809)	253 (150-470)	142 (72-304)	22 (10-50)	9 (4-21)
Intestinal nematode infections	35 (28-42)	39 (31-48)	61 (50-75)	55 (45-68)	65 (52-80)	47 (37-57)	69 (57-84)	51 (42-64)	29 (23-37)	10 (8-13)	6 (5-8)	2 (1-3)
Ascariasis	35 (28-42)	39 (31-48)	61 (50-75)	55 (45-68)	65 (52-80)	47 (37-57)	69 (57-84)	51 (42-64)	29 (23-37)	10 (8-13)	6 (5-8)	2 (1-3)
Ebola	0 (0-0)	0 (0-0)	0 (0-0)	0 (0-0)	0 (0-0)	0 (0-0)	0 (0-0)	0 (0-0)	0 (0-0)	0 (0-0)	0 (0-0)	0 (0-0)
Zika virus	0 (0-0)	0 (0-0)	0 (0-0)	0 (0-0)	0 (0-0)	0 (0-0)	0 (0-0)	0 (0-0)	0 (0-0)	0 (0-0)	0 (0-0)	0 (0-0)
Other neglected tropical diseases	438 (254-543)	455 (263-562)	530 (316-647)	585 (336-716)	565 (349-715)	575 (371-723)	528 (329-677)	400 (258-513)	230 (154-319)	119 (80-166)	42 (27-59)	18 (12-25)
Maternal disorders	30,664 (28,025-33,420)	15,791 (13,943-17,866)	2,578 (2,115-3,144)	-	-	-	-	-	-	-	-	-
Maternal haemorrhage	9,679 (6,732-13,100)	3,933 (2,719-5,435)	462 (268-735)	-	-	-	-	-	-	-	-	-
Maternal sepsis and other pregnancy related infections	3,062 (1,970-4,538)	1,708 (1,106-2,593)	286 (178-450)	-	-	-	-	-	-	-	-	-
Maternal hypertensive disorders	3,316 (1,991-5,134)	1,558 (957-2,373)	234 (121-392)	-	-	-	-	-	-	-	-	-
Maternal obstructed labour and uterine rupture	1,738 (950-2,970)	942 (492-1,560)	145 (62-277)	-	-	-	-	-	-	-	-	-
Maternal abortion, miscarriage, and ectopic pregnancy	3,239 (2,147-4,695)	2,106 (1,374-3,075)	321 (203-479)	-	-	-	-	-	-	-	-	-
Indirect maternal deaths	3,668 (2,222-5,630)	2,239 (1,310-3,320)	490 (261-768)	-	-	-	-	-	-	-	-	-
Late maternal deaths	565 (276-958)	305 (141-504)	53 (22-110)	-	-	-	-	-	-	-	-	-
Maternal deaths aggravated by HIV/AIDS	53 (29-79)	24 (13-37)	4 (2-6)	-	-	-	-	-	-	-	-	-
Other maternal disorders	5,344 (3,329-7,914)	2,976 (1,938-4,293)	584 (396-828)	-	-	-	-	-	-	-	-	-
Neonatal disorders	-	-	-	-	-	-	-	-	-	-	-	-
Neonatal preterm birth complications	-	-	-	-	-	-	-	-	-	-	-	-
Neonatal encephalopathy due to birth asphyxia and trauma	-	-	-	-	-	-	-	-	-	-	-	-
Neonatal sepsis and other neonatal infections	-	-	-	-	-	-	-	-	-	-	-	-
Hemolytic disease and other neonatal jaundice	-	-	-	-	-	-	-	-	-	-	-	-
Other neonatal disorders	-	-	-	-	-	-	-	-	-	-	-	-
Nutritional deficiencies	5,087 (4,615-5,833)	4,787 (4,309-5,692)	6,285 (5,622-7,296)	7,951 (7,119-9,141)	9,605 (8,621-11,028)	12,085 (10,796-13,953)	13,915 (12,541-15,955)	16,688 (15,134-18,618)	15,668 (14,351-17,202)	13,495 (12,494-14,656)	7,920 (7,365-8,530)	3,331 (3,120-3,566)
Protein-energy malnutrition	3,494 (3,155-3,999)	2,866 (2,578-3,370)	3,928 (3,488-4,601)	5,461 (4,868-6,228)	6,788 (6,103-7,805)	8,443 (7,608-9,808)	10,494 (9,528-11,898)	13,110 (12,069-14,452)	12,549 (11,578-13,727)	11,043 (10,209-12,075)	6,650 (6,112-7,240)	2,824 (2,521-3,058)
Iodine deficiency	54 (21-139)	49 (17-118)	72 (25-171)	86 (34-188)	86 (36-188)	115 (49-251)	115 (51-219)	105 (47-208)	71 (30-149)	36 (12-85)	11 (2-36)	5 (1-17)
Iron-deficiency anaemia	141 (113-197)	125 (100-183)	135 (103-243)	142 (111-233)	162 (130-221)	193 (147-276)	300 (198-442)	287 (192-455)	227 (149-359)	129 (78-203)	70 (42-110)	50 (30-73)
Other nutritional deficiencies	1,399 (1,139-1,749)	1,747 (1,407-2,299)	2,150 (1,713-2,772)	2,261 (1,777-2,941)	2,568 (2,095-3,240)	3,333 (2,674-4,294)	3,007 (2,417-3,947)	3,186 (2,548-4,236)	2,820 (2,232-3,778)	2,287 (1,801-3,270)	1,189 (912-1,882)	452 (347-802)
Other communicable, maternal, neonatal, and nutritional diseases	11,211 (10,453-12,009)	11,168 (10,363-12,002)	11,994 (11,196-12,907)	12,908 (12,030-13,969)	12,793 (11,803-13,869)	12,442 (11,455-13,542)	11,059 (10,063-12,184)	9,240 (8,353-10,190)	5,684 (4,974-6,641)	2,988 (2,551-3,519)	1,079 (885-1,262)	327 (261-377)
Sexually transmitted diseases excluding HIV	934 (615-1,152)	845 (572-1,056)	924 (600-1,197)	1,228 (757-1,592)	887 (496-1,133)	756 (517-929)	633 (424-802)	700 (429-878)	394 (262-482)	232 (161-278)	100 (69-125)	37 (25-48)
Syphilis	301 (162-428)	260 (152-372)	376 (239-571)	556 (354-830)	376 (217-484)	347 (226-441)	357 (223-484)	368 (195-498)	192 (114-252)	102 (63-129)	40 (25-55)	15 (9-21)
Chlamydial infection	128 (85-155)	110 (74-135)	101 (61-128)	129 (74-166)	102 (52-135)	49 (30-61)	43 (28-52)	54 (34-67)	46 (31-55)	29 (21-34)	13 (9-16)	4 (3-5)
Gonococcal infection	332 (224-402)	302 (205-371)	299 (183-376)	354 (202-450)	253 (131-332)	273 (169-340)	172 (114-210)	216 (136-265)	120 (83-145)	77 (57-92)	36 (26-44)	13 (9-15)
Other sexually transmitted diseases	173 (116-209)	173 (118-212)	148 (92-185)	189 (111-243)	156 (81-203)	87 (57-108)	61 (41-75)	63 (40-77)	36 (25-43)	24 (18-28)	11 (8-13)	5 (4-7)
Hepatitis	8,623 (7,967-9,400)	8,456 (7,790-9,192)	8,725 (8,116-9,434)	9,123 (8,505-9,804)	8,519 (7,945-9,197)	7,950 (7,386-8,601)	6,856 (6,341-7,489)	5,308 (4,885-5,788)	2,802 (2,546-3,087)	1,222 (1,111-1,353)	325 (296-359)	68 (62-75)
Acute hepatitis A	77 (42-127)	80 (44-137)	91 (51-149)	104 (60-168)	110 (64-176)	106 (63-163)	100 (59-150)	87 (52-136)	49 (29-73)	21 (14-30)	5 (4-8)	1 (1-2)
Hepatitis B	7,954 (7,170-8,743)	7,792 (7,013-8,546)	7,996 (7,292-8,762)	8,288 (7,550-9,049)	7,639 (6,862-8,427)	7,031 (6,296-7,742)	5,938 (5,273-6,557)	4,458 (3,842-5,105)	2,105 (1,701-2,478)	763 (560-955)	164 (102-226)	28 (15-42)
Hepatitis C	88 (46-144)	112 (57-196)	162 (87-282)	233 (128-381)	249 (133-422)	212 (116-339)	170 (98-282)	134 (75-220)	72 (40-116)	33 (20-51)	10 (6-15)	2 (1-4)
Acute hepatitis E	504 (239-940)	472 (232-852)	476 (245-809)	499 (234-896)	521 (228-987)	601 (301-1,034)	648 (327-1,099)	629 (283-1,101)	576 (320-902)	405 (240-601)	145 (92-208)	37 (23-51)
Other infectious diseases	1,655 (1,345-1,887)	1,868 (1,516-2,160)	2,345 (1,884-2,746)	2,556 (2,092-2,966)	3,387 (2,727-4,116)	3,736 (2,990-4,567)	3,570 (2,787-4,339)	3,232 (2,582-3,911)	2,489 (1,915-3,292)	1,535 (1,168-1,994)	654 (488-825)	221 (161-268)
Non-communicable diseases	666,140 (651,958-678,881)	900,273 (882,343-916,314)	1,391,670 (1,363,065-1,416,164)	1,902,336 (1,867,600-1,931,969)	2,620,153 (2,568,145-2,663,500)	3,084,581 (3,021,834-3,132,811)	3,375,880 (3,295,293-3,435,089)	3,700,780 (3,628,748-3,758,980)	3,134,929 (3,078,509-3,184,971)	2,106,027 (2,070,825-2,137,790)	932,354 (919,370-945,378)	296,787 (292,953-300,914)
Neoplasms	207,003 (200,520-212,685)	288,038 (279,923-296,087)	445,790 (435,242-457,307)	595,499 (582,614-609,670)	760,033 (745,827-776,902)	799,882 (785,775-815,459)	722,542 (708,694-739,443)	652,187 (639,214-668,463)	421,999 (411,725-434,665)	220,861 (215,177-228,412)	76,035 (73,678-78,742)	18,541 (17,864-19,257)
Lip and oral cavity cancer	4,510 (4,255-4,831)	6,851 (6,538-7,282)	10,317 (9,791-11,021)	12,339 (11,839-13,049)	14,032 (13,405-14,788)	12,172 (11,642-12,812)	9,266 (8,837-9,760)	7,715 (7,394-8,055)	5,300 (5,057-5,551)	2,984 (2,842-3,138)	1,153 (1,089-1,216)	315 (297-330)
Nasopharynx cancer	3,364 (3,089-3,671)	4,354 (4,016-4,742)	5,947 (5,477-6,473)	6,957 (6,474-7,521)	6,433 (5,979-6,943)	5,277 (4,951-5,603)	4,032 (3,792-4,269)	2,485 (2,352-2,614)	1,329 (1,247-1,416)	534 (501-569)	166 (156-175)	38 (36-40)
Other pharynx cancer	3,066 (2,812-3,456)	5,141 (4,752-5,753)	8,107 (7,528-9,015)	9,509 (8,868-10,514)	10,368 (9,622-11,532)	8,678 (8,006-9,613)	6,432 (5,875-7,204)	4,062 (3,716-4,498)	2,350 (2,100-2,584)	1,006 (908-1,100)	290 (265-314)	59 (55-63)
Oesophageal cancer	8,345 (7,914-8,802)	15,276 (14,600-16,032)	26,947 (25,725-28,127)	40,690 (38,862-42,596)	52,247 (49,742-54,825)	54,117 (51,645-56,903)	50,186 (47,486-53,017)	35,952 (34,313-37,857)	19,450 (18,556-20,548)	9,023 (8,646-9,477)	2,647 (2,533-2,779)	539 (514-564)
Stomach cancer	25,671 (24,402-26,810)	36,199 (34,712-37,634)	59,193 (56,964-61,540)	83,570 (80,868-86,307)	111,150 (107,262-115,228)	116,684 (112,964-120,627)	111,275 (107,356-115,593)	96,978 (93,840-100,264)	60,174 (58,190-62,427)	30,239 (29,229-31,395)	9,687 (9,356-10,088)	2,219 (2,127-2,306)
Colon and rectum cancer	12,060 (11,573-12,809)	18,265 (17,542-19,403)	29,255 (28,190-30,744)	41,893 (40,463-44,036)	58,819 (57,114-61,114)	68,617 (66,755-70,988)	67,228 (65,458-69,520)	73,897 (71,910-76,001)	55,543 (53,916-57,179)	33,144 (32,046-34,216)	12,753 (12,300-13,206)	3,306 (3,173-3,434)
Liver cancer	26,954 (24,266-29,050)	34,488 (31,319-37,333)	47,226 (43,783-50,668)	58,708 (54,443-62,165)	65,107 (61,259-68,841)	62,021 (57,840-65,701)	53,057 (49,744-56,215)	39,094 (36,901-41,254)	21,337 (20,222-22,819)	9,363 (8,947-9,937)	2,608 (2,491-2,773)	513 (487-544)
Liver cancer due to hepatitis B	16,059 (13,066-18,540)	19,296 (15,534-22,354)	24,422 (19,383-28,666)	27,667 (21,480-32,968)	27,530 (20,620-32,681)	23,976 (17,938-28,365)	19,600 (14,304-23,496)	12,828 (9,565-15,706)	6,119 (4,295-7,800)	2,487 (1,878-3,120)	647 (475-835)	123 (79-168)
Liver cancer due to hepatitis C	1,803 (1,405-2,328)	3,226 (2,489-4,091)	5,983 (4,712-7,285)	9,580 (7,958-11,520)	12,555 (10,527-14,928)	12,492 (10,653-14,565)	11,183 (9,672-13,023)	9,998 (8,614-11,617)	6,719 (5,806-7,817)	3,376 (2,966-3,836)	1,055 (917-1,189)	224 (189-264)
Liver cancer due to alcohol use	2,184 (1,523-3,021)	3,325 (2,206-4,664)	5,535 (3,789-7,474)	8,006 (5,431-11,148)	10,679 (7,835-14,190)	12,555 (9,789-15,553)	11,224 (8,757-14,147)	8,572 (6,680-10,691)	4,516 (3,386-5,728)	1,867 (1,375-2,368)	469 (327-621)	82 (49-118)
Liver cancer due to other causes	6,908 (5,512-8,996)	8,641 (6,927-11,136)	11,287 (9,122-14,430)	13,455 (10,794-17,126)	14,342 (11,782-18,034)	12,998 (10,747-15,918)	11,051 (9,129-13,648)	7,697 (6,423-9,209)	3,983 (3,276-4,779)	1,634 (1,374-1,915)	437 (362-524)	84 (64-107)
Gallbladder and biliary tract cancer	2,432 (2,222-2,781)	3,902 (3,600-4,429)	6,761 (6,313-7,509)	9,638 (9,054-10,780)	13,644 (12,907-15,069)	15,712 (14,928-17,248)	15,356 (14,599-16,925)	16,456 (15,841-17,638)	11,954 (11,517-12,676)	6,346 (6,080-6,707)	2,176 (2,089-2,287)	510 (486-537)
Pancreatic cancer	4,470 (4,305-4,619)	7,175 (6,992-7,357)	13,097 (12,617-13,519)	19,630 (19,179-20,088)	28,188 (27,437-28,873)	32,527 (31,873-33,166)	30,873 (30,213-31,521)	30,306 (29,571-30,994)	20,462 (19,849-21,046)	10,979 (10,636-11,327)	3,794 (3,653-3,925)	890 (853-925)
Larynx cancer	2,950 (2,800-3,096)	5,248 (5,013-5,505)	9,631 (9,141-10,089)	12,443 (11,918-12,997)	15,022 (14,401-15,657)	13,841 (13,278-14,408)	10,444 (9,978-10,879)	7,603 (7,293-7,929)	4,332 (4,125-4,549)	1,833 (1,751-1,923)	512 (490-535)	98 (94-102)
Tracheal, bronchus, and lung cancer	24,596 (23,644-25,633)	42,106 (40,666-43,984)	79,760 (77,148-83,009)	119,988 (116,236-124,293)	168,163 (163,394-174,274)	179,006 (174,500-183,859)	153,156 (149,432-157,437)	128,461 (125,432-131,643)	69,835 (67,620-71,933)	30,165 (29,232-31,123)	8,236 (7,933-8,522)	1,671 (1,600-1,733)
Malignant skin melanoma	2,004 (1,731-2,175)	2,101 (1,820-2,335)	2,857 (2,522-3,262)	3,237 (2,897-3,723)	3,778 (3,435-4,420)	3,879 (3,508-4,658)	3,349 (3,072-4,118)	3,410 (3,136-4,269)	2,582 (2,341-3,382)	1,485 (1,320-1,880)	588 (524-772)	176 (154-231)
Non-melanoma skin cancer	788 (749-823)	1,001 (952-1,041)	1,510 (1,437-1,594)	2,057 (1,973-2,148)	3,244 (3,100-3,400)	3,629 (3,478-3,792)	3,628 (3,490-3,774)	3,692 (3,545-3,840)	3,228 (3,092-3,362)	2,337 (2,235-2,439)	1,171 (1,116-1,231)	476 (453-501)
Non-melanoma skin cancer (squamous-cell carcinoma)	788 (749-823)	1,001 (952-1,041)	1,510 (1,437-1,594)	2,057 (1,973-2,148)	3,244 (3,100-3,400)	3,629 (3,478-3,792)	3,628 (3,490-3,774)	3,692 (3,545-3,840)	3,228 (3,092-3,362)	2,337 (2,235-2,439)	1,171 (1,116-1,231)	476 (453-501)
Breast cancer	23,646 (21,430-26,875)	29,833 (27,096-33,477)	38,172 (35,323-42,110)	40,942 (38,183-45,491)	40,815 (38,588-44,560)	38,729 (36,947-41,966)	30,706 (29,351-33,534)	28,991 (27,748-31,522)	21,166 (20,240-22,601)	13,212 (12,613-14,037)	5,416 (5,159-5,730)	1,687 (1,608-1,783)
Cervical cancer	15,939 (13,288-20,339)	19,714 (16,525-24,808)	23,693 (20,010-30,256)	25,319 (21,667-32,297)	24,649 (21,502-30,966)	21,581 (18,991-26,849)	17,046 (15,198-21,001)	12,861 (11,877-14,679)	8,252 (7,633-9,723)	4,010 (3,735-4,631)	1,366 (1,284-1,527)	370 (349-407)
Uterine cancer	1,997 (1,817-2,152)	3,252 (2,936-3,498)	5,234 (4,818-5,613)	6,810 (6,232-7,303)	9,072 (8,501-9,628)	9,619 (9,055-10,165)	7,894 (7,493-8,326)	7,054 (6,717-7,409)	4,689 (4,464-4,907)	2,462 (2,353-2,575)	895 (853-942)	235 (222-247)
Ovarian cancer	4,258 (3,862-4,479)	6,540 (6,073-6,869)	10,454 (9,831-10,944)	11,762 (11,160-12,221)	14,004 (13,271-14,628)	13,959 (13,365-14,529)	11,374 (10,956-11,800)	10,107 (9,708-10,528)	6,585 (6,304-6,891)	3,392 (3,246-3,538)	1,177 (1,127-1,228)	274 (260-287)
Prostate cancer	345 (274-398)	966 (771-1,121)	2,902 (2,346-3,247)	6,979 (5,606-7,890)	15,985 (12,900-17,987)	25,670 (21,240-28,320)	33,131 (28,258-36,169)	41,654 (36,780-46,447)	33,472 (28,712-36,805)	20,331 (18,047-22,431)	7,909 (7,137-8,946)	1,850 (1,690-2,204)
Testicular cancer	609 (570-657)	462 (429-507)	428 (392-481)	339 (314-369)	389 (356-433)	347 (321-379)	316 (289-350)	267 (247-293)	197 (181-216)	91 (85-99)	31 (29-34)	8 (7-9)
Kidney cancer	1,605 (1,504-1,682)	2,650 (2,512-2,759)	4,796 (4,524-5,031)	6,997 (6,708-7,292)	9,286 (8,879-9,656)	9,893 (9,565-10,206)	8,539 (8,272-8,803)	8,628 (8,332-8,893)	5,796 (5,605-5,984)	3,017 (2,907-3,125)	1,021 (979-1,066)	226 (215-237)
Bladder cancer	1,494 (1,405-1,603)	2,426 (2,293-2,593)	4,699 (4,423-5,000)	7,579 (7,233-8,002)	12,783 (12,095-13,367)	16,123 (15,534-16,771)	17,389 (16,844-18,129)	19,914 (19,289-20,542)	15,576 (15,088-16,045)	9,169 (8,878-9,457)	3,446 (3,331-3,562)	864 (828-895)
Brain and nervous system cancer	7,677 (6,390-8,388)	8,400 (7,071-9,106)	11,363 (9,502-12,237)	13,478 (11,718-14,382)	14,919 (13,197-15,863)	13,828 (12,509-14,733)	10,229 (9,314-11,007)	7,680 (7,125-8,430)	3,828 (3,501-4,240)	1,549 (1,415-1,709)	406 (368-443)	82 (75-90)
Thyroid cancer	1,096 (979-1,206)	1,503 (1,358-1,650)	2,373 (2,148-2,588)	3,038 (2,790-3,277)	3,343 (3,123-3,571)	3,521 (3,299-3,764)	3,173 (2,969-3,395)	3,017 (2,875-3,210)	2,051 (1,949-2,210)	1,061 (1,010-1,154)	347 (330-372)	72 (69-77)
Mesothelioma	499 (429-597)	635 (559-739)	1,095 (975-1,227)	1,728 (1,548-1,926)	2,105 (1,882-2,459)	2,470 (2,228-2,940)	2,299 (2,083-2,691)	2,509 (2,277-2,868)	1,588 (1,452-1,805)	697 (642-773)	203 (189-220)	42 (39-45)
Hodgkin lymphoma	1,921 (1,427-2,204)	1,982 (1,474-2,289)	2,359 (1,785-2,719)	2,833 (2,090-3,265)	3,096 (2,286-3,575)	3,050 (2,277-3,481)	2,460 (1,883-2,786)	1,692 (1,363-1,906)	973 (793-1,091)	418 (342-469)	108 (92-121)	20 (17-23)
Non-Hodgkin's lymphoma	5,031 (4,515-5,320)	5,775 (5,245-6,136)	8,036 (7,373-8,584)	10,287 (9,573-11,227)	13,578 (12,827-14,633)	15,298 (14,492-16,702)	14,347 (13,639-16,000)	13,412 (12,809-15,071)	9,322 (8,882-10,593)	4,945 (4,689-5,612)	1,633 (1,546-1,866)	373 (351-424)
Multiple myeloma	1,188 (1,006-1,307)	1,719 (1,483-1,892)	3,138 (2,748-3,507)	4,633 (4,114-5,278)	6,615 (5,765-7,583)	7,905 (7,044-9,222)	7,237 (6,631-8,555)	7,338 (6,759-9,082)	5,264 (4,887-6,799)	2,802 (2,582-3,662)	891 (820-1,175)	175 (158-223)
Leukaemia	9,091 (7,929-9,699)	9,488 (8,438-10,292)	11,880 (10,826-12,790)	14,523 (13,370-15,737)	17,796 (16,903-19,328)	19,310 (18,497-20,795)	17,852 (17,180-19,432)	17,780 (17,206-18,974)	12,472 (12,048-13,292)	7,027 (6,771-7,460)	2,628 (2,532-2,753)	697 (666-727)
Acute lymphoid leukaemia	1,230 (1,080-1,463)	1,234 (1,086-1,483)	1,497 (1,313-1,740)	1,805 (1,597-2,142)	1,906 (1,701-2,188)	1,771 (1,606-2,052)	1,512 (1,376-1,757)	1,028 (943-1,169)	625 (577-712)	311 (290-348)	104 (97-115)	26 (24-29)
Chronic lymphoid leukaemia	365 (311-464)	434 (384-547)	860 (770-1,026)	1,358 (1,231-1,648)	2,342 (2,134-2,717)	2,922 (2,709-3,363)	2,878 (2,660-3,375)	3,355 (3,085-3,830)	2,689 (2,479-3,094)	1,743 (1,625-1,999)	688 (648-770)	198 (186-213)
Acute myeloid leukaemia	1,834 (1,636-2,075)	2,038 (1,874-2,351)	2,594 (2,396-2,938)	3,131 (2,888-3,557)	3,907 (3,631-4,460)	4,382 (4,102-5,013)	4,017 (3,785-4,669)	3,885 (3,627-4,575)	2,487 (2,318-2,984)	1,212 (1,126-1,442)	402 (375-474)	92 (85-107)
Chronic myeloid leukaemia	1,299 (1,139-1,462)	1,406 (1,247-1,567)	1,717 (1,527-1,900)	1,980 (1,789-2,170)	2,238 (2,012-2,422)	2,387 (2,152-2,579)	2,078 (1,892-2,237)	2,391 (2,195-2,547)	1,780 (1,651-1,900)	984 (899-1,055)	387 (352-416)	107 (96-115)
Other leukaemia	4,364 (3,542-4,789)	4,376 (3,605-4,780)	5,212 (4,461-5,660)	6,249 (5,459-6,837)	7,403 (6,740-8,132)	7,847 (7,185-8,623)	7,367 (6,834-8,184)	7,121 (6,625-7,781)	4,891 (4,549-5,289)	2,778 (2,619-3,015)	1,046 (984-1,127)	274 (258-294)
Other neoplasms	9,395 (8,848-9,911)	10,587 (9,935-11,138)	14,562 (13,574-15,370)	17,593 (16,617-18,556)	21,404 (20,126-22,895)	22,418 (21,240-23,704)	20,266 (19,210-21,559)	19,172 (18,179-20,490)	12,893 (12,233-13,798)	7,241 (6,884-7,746)	2,775 (2,634-2,945)	756 (715-798)
Cardiovascular diseases	243,643 (236,335-254,267)	344,132 (334,047-358,398)	559,568 (544,899-579,477)	790,897 (770,985-817,079)	1,143,630 (1,117,282-1,175,871)	1,414,991 (1,380,824-1,458,668)	1,617,638 (1,576,088-1,671,307)	1,927,256 (1,881,957-1,985,633)	1,754,487 (1,709,279-1,812,690)	1,210,661 (1,179,469-1,251,140)	539,342 (523,167-558,182)	183,252 (176,675-189,041)
Rheumatic heart disease	18,534 (15,682-20,761)	19,766 (16,711-22,248)	26,950 (23,061-29,958)	31,153 (27,128-34,750)	36,229 (31,671-39,902)	38,776 (33,862-43,257)	40,417 (35,374-45,878)	36,047 (31,856-40,964)	26,137 (23,435-29,762)	15,423 (13,916-17,565)	6,191 (5,659-6,926)	1,785 (1,648-1,968)
Ischaemic heart disease	115,928 (111,895-121,161)	169,406 (164,244-175,990)	271,955 (263,567-282,877)	382,528 (371,847-395,465)	556,299 (541,538-574,333)	680,407 (662,902-702,682)	754,280 (732,366-783,852)	961,641 (938,264-993,767)	919,589 (899,794-951,210)	666,716 (652,696-690,126)	317,371 (309,876-328,916)	115,759 (113,016-119,375)
Cerebrovascular disease	77,159 (73,985-81,884)	115,153 (110,688-121,912)	197,581 (190,082-208,848)	289,758 (278,962-306,009)	429,846 (415,206-453,672)	541,550 (521,243-576,265)	637,394 (608,785-684,582)	704,309 (668,344-754,698)	590,421 (551,480-634,632)	362,872 (336,153-395,648)	138,135 (125,794-151,654)	41,764 (37,338-45,786)

SR Table 5: Global deaths by cause and age group for both sexes (with 95% uncertainty intervals), 1990

Cause	Early Neonatal	Late Neonatal	Post Neonatal	1 to 4	5 to 9	10 to 14	15 to 19	20 to 24	25 to 29	30 to 34	35 to 39	40 to 44	45 to 49	50 to 54	55 to 59	60 to 64	65 to 69	70 to 74	75 to 79	80 to 84	85 to 89	90 to 94	95 plus
Ischaemic stroke	-	-	779 (633-971)	627 (499-830)	454 (391-535)	409 (365-462)	1,548 (1,426-1,670)	3,007 (2,773-3,215)	3,614 (3,335-3,867)	4,782 (4,419-5,134)	7,401 (6,898-7,843)	11,524 (10,663-12,185)	22,173 (20,551-23,557)	44,869 (41,533-47,414)	74,533 (69,918-78,389)	167,801 (159,540-175,374)	229,234 (218,316-239,869)	281,808 (268,370-296,681)	392,632 (372,428-415,111)	358,065 (336,253-380,525)	234,252 (217,669-253,226)	92,059 (83,859-100,598)	29,042 (25,995-31,776)
Hemorrhagic stroke	3,181 (2,802-3,642)	2,181 (1,826-2,674)	15,883 (13,019-19,815)	10,041 (7,959-13,499)	5,406 (4,683-6,272)	5,211 (4,761-5,801)	9,803 (9,240-10,661)	16,961 (16,078-18,250)	19,834 (18,828-21,439)	28,896 (27,548-31,104)	45,763 (43,675-48,889)	65,635 (62,996-70,220)	92,980 (88,942-99,431)	152,712 (146,354-163,664)	215,225 (205,697-230,091)	262,045 (250,514-281,520)	312,317 (297,064-339,581)	355,586 (336,723-389,183)	311,677 (292,390-343,002)	232,356 (215,314-256,134)	128,620 (118,233-143,889)	46,076 (41,940-51,726)	12,723 (11,327-14,121)
Hypertensive heart disease	-	-	702 (446-878)	395 (243-512)	263 (170-329)	258 (162-324)	1,784 (1,263-2,158)	2,731 (1,996-3,226)	3,264 (2,498-3,821)	4,766 (3,688-5,557)	7,404 (5,838-8,658)	10,554 (8,389-12,202)	15,809 (12,173-18,376)	26,112 (20,652-30,187)	38,780 (30,584-44,732)	53,170 (41,454-60,821)	69,422 (53,349-79,375)	85,776 (64,305-98,200)	95,987 (75,536-108,397)	81,650 (65,240-92,863)	55,882 (44,084-65,080)	25,904 (20,921-29,687)	8,080 (6,189-9,825)
Cardiomyopathy and myocarditis	986 (722-1,166)	736 (551-910)	6,017 (4,567-8,054)	3,968 (2,628-5,535)	1,538 (1,248-1,973)	1,525 (1,325-1,934)	2,561 (2,245-3,266)	3,131 (2,786-3,829)	4,143 (3,669-4,905)	5,239 (4,561-5,960)	7,293 (6,243-8,480)	9,078 (7,712-10,555)	9,184 (7,669-10,545)	14,815 (12,391-18,134)	16,432 (13,828-18,778)	18,951 (15,739-21,352)	18,258 (14,831-19,885)	17,355 (13,022-19,039)	24,188 (16,281-27,418)	27,093 (15,625-32,112)	24,297 (12,710-29,336)	11,751 (6,087-14,309)	3,935 (2,046-4,755)
Myocarditis	224 (128-310)	218 (117-325)	2,490 (1,321-3,944)	1,792 (946-3,075)	690 (519-1,001)	606 (502-854)	1,055 (862-1,428)	1,164 (959-1,597)	1,171 (953-1,616)	912 (742-1,251)	1,078 (882-1,418)	1,136 (910-1,548)	826 (671-1,043)	976 (788-1,254)	1,262 (970-1,563)	1,160 (872-1,437)	1,336 (1,014-1,574)	1,574 (1,187-1,830)	2,343 (1,553-2,800)	2,822 (1,729-3,490)	2,942 (1,704-3,787)	1,713 (908-2,248)	690 (350-934)
Alcoholic cardiomyopathy	-	-	-	-	-	-	202 (167-280)	382 (312-473)	956 (747-1,200)	1,974 (1,522-2,507)	3,292 (2,532-4,277)	4,345 (3,315-5,648)	4,508 (3,450-5,750)	8,155 (6,169-11,013)	7,576 (5,987-9,649)	8,168 (6,543-10,559)	5,373 (4,572-6,604)	3,531 (2,863-4,290)	3,646 (2,990-4,343)	2,674 (1,907-3,247)	1,649 (1,053-2,078)	735 (442-935)	283 (171-360)
Other cardiomyopathy	761 (552-962)	518 (342-702)	3,527 (2,355-5,128)	2,176 (1,352-3,575)	848 (667-1,156)	920 (782-1,150)	1,305 (1,145-1,654)	1,585 (1,407-1,959)	2,016 (1,756-2,400)	2,353 (2,020-2,773)	2,923 (2,471-3,492)	3,597 (3,000-4,202)	3,849 (3,178-4,386)	5,684 (4,750-6,585)	7,593 (6,122-8,393)	9,623 (7,626-10,558)	11,549 (8,706-12,755)	12,250 (8,695-13,733)	18,199 (11,294-21,268)	21,597 (11,410-26,412)	19,706 (9,262-24,580)	9,303 (4,361-11,732)	2,961 (1,431-3,715)
Atrial fibrillation and flutter	-	-	-	-	-	-	-	-	-	26 (18-35)	102 (74-135)	215 (157-284)	373 (268-498)	675 (495-887)	1,215 (705-1,842)	2,567 (1,657-3,646)	4,987 (3,070-7,604)	8,741 (6,148-12,290)	14,886 (9,370-22,526)	27,662 (20,561-35,772)	28,465 (21,361-37,201)	13,291 (10,050-17,352)	3,659 (2,823-4,722)
Aortic aneurysm	-	-	-	-	-	-	352 (306-410)	473 (422-544)	680 (589-792)	895 (787-1,033)	1,369 (1,222-1,573)	1,701 (1,552-1,903)	2,297 (2,118-2,559)	3,570 (3,345-3,899)	5,738 (5,408-6,206)	9,049 (8,601-9,547)	12,997 (12,464-13,650)	15,237 (14,681-15,983)	18,289 (17,661-19,057)	15,522 (14,975-16,161)	9,805 (9,472-10,176)	4,006 (3,868-4,129)	1,051 (1,012-1,082)
Peripheral vascular disease	-	-	-	-	-	-	-	-	-	-	-	128 (85-159)	204 (134-249)	486 (305-626)	867 (566-1,081)	1,731 (1,185-2,215)	2,305 (1,695-2,995)	2,826 (2,032-3,701)	4,529 (3,313-6,461)	5,462 (3,890-8,262)	4,480 (3,061-7,334)	2,499 (1,594-4,132)	1,033 (625-1,674)
Endocarditis	153 (100-197)	103 (64-135)	2,047 (1,391-2,665)	892 (581-1,409)	393 (295-490)	462 (382-549)	879 (736-1,065)	1,216 (1,036-1,499)	1,559 (1,362-1,952)	1,533 (1,304-1,838)	1,783 (1,495-2,076)	2,043 (1,693-2,395)	1,819 (1,522-2,160)	2,356 (1,941-2,829)	2,761 (2,254-3,292)	3,681 (3,037-4,408)	4,357 (3,483-5,217)	4,653 (3,714-5,470)	5,695 (4,406-6,917)	5,434 (4,049-6,815)	3,942 (2,862-4,908)	1,990 (1,413-2,524)	672 (464-855)
Other cardiovascular and circulatory diseases	2,142 (1,775-2,458)	1,567 (1,197-1,840)	11,119 (8,808-13,681)	6,558 (4,850-9,005)	2,841 (2,144-3,417)	2,484 (1,888-2,865)	3,335 (2,566-3,892)	4,906 (3,837-5,671)	5,189 (4,220-6,178)	5,587 (4,650-6,553)	6,830 (5,393-7,846)	8,302 (6,700-9,552)	10,122 (8,123-11,482)	15,066 (12,024-16,822)	21,663 (16,962-24,122)	32,107 (24,944-35,596)	41,933 (32,585-46,526)	50,958 (37,335-56,833)	61,684 (48,296-67,577)	55,517 (46,204-60,594)	38,780 (33,227-42,058)	18,203 (16,277-19,695)	5,513 (5,080-6,034)
Chronic respiratory diseases	0 (0-0)	0 (0-0)	0 (0-0)	37,895 (30,914-44,272)	9,100 (7,780-10,322)	7,886 (7,065-8,696)	12,710 (11,641-13,583)	16,216 (15,111-17,369)	18,831 (17,617-20,027)	23,096 (21,482-24,375)	32,882 (30,329-34,589)	42,399 (38,886-44,345)	66,944 (59,925-70,218)	123,004 (109,125-128,972)	200,178 (177,443-210,412)	332,140 (295,428-347,724)	439,845 (392,121-460,860)	574,260 (511,530-602,471)	565,608 (505,994-593,016)	421,947 (372,954-444,836)	249,575 (221,210-262,998)	90,900 (79,823-96,268)	24,104 (21,608-25,455)
Chronic obstructive pulmonary disease	-	-	0 (0-0)	9,341 (6,228-16,693)	2,214 (1,501-3,133)	1,596 (1,138-2,072)	5,806 (4,707-6,697)	7,787 (6,585-8,859)	9,123 (7,602-10,402)	12,335 (10,403-13,938)	18,114 (15,193-20,645)	25,427 (21,653-28,897)	43,214 (36,105-48,751)	88,084 (75,504-97,582)	152,280 (131,297-166,828)	268,659 (234,408-290,069)	369,266 (323,630-395,741)	502,310 (441,132-535,215)	498,125 (440,601-526,652)	375,833 (329,730-399,222)	225,068 (198,145-238,796)	82,650 (72,494-87,796)	22,074 (19,576-23,338)
Pneumoconiosis	-	-	-	-	-	-	75 (57-110)	114 (82-185)	200 (136-337)	345 (249-553)	649 (479-1,078)	629 (494-1,090)	727 (576-1,232)	1,120 (911-1,785)	1,582 (1,315-2,419)	2,505 (2,105-3,767)	3,227 (2,779-4,462)	3,503 (3,028-4,727)	3,279 (2,911-4,066)	1,998 (1,805-2,335)	926 (839-1,086)	268 (242-316)	62 (57-72)
Silicosis	-	-	-	-	-	-	11 (7-17)	29 (17-46)	89 (53-155)	204 (138-346)	449 (316-753)	408 (303-781)	446 (327-834)	662 (505-1,191)	916 (720-1,591)	1,390 (1,114-2,281)	1,838 (1,501-2,849)	1,886 (1,540-2,696)	1,726 (1,467-2,220)	986 (839-1,224)	414 (352-507)	100 (83-120)	18 (16-22)
Asbestosis	-	-	-	-	-	-	26 (20-37)	33 (25-46)	32 (25-44)	37 (29-49)	41 (32-54)	43 (33-57)	42 (30-59)	69 (50-92)	103 (64-139)	157 (99-238)	207 (130-384)	244 (161-444)	261 (177-538)	186 (130-356)	94 (70-181)	28 (21-51)	6 (4-9)
Coal workers pneumoconiosis	-	-	-	-	-	-	6 (3-10)	7 (4-12)	22 (11-38)	25 (14-41)	46 (23-76)	67 (32-107)	97 (44-158)	159 (81-233)	247 (140-344)	442 (236-595)	685 (331-901)	801 (413-1,062)	762 (388-937)	518 (290-623)	240 (132-289)	62 (34-75)	12 (7-14)
Other pneumoconiosis	-	-	-	-	-	-	31 (23-53)	45 (30-86)	57 (33-114)	79 (49-148)	114 (68-215)	111 (71-202)	141 (89-247)	230 (149-392)	316 (211-535)	517 (341-865)	497 (325-845)	572 (381-966)	530 (371-830)	309 (228-479)	178 (135-278)	78 (61-119)	27 (23-36)
Asthma	-	-	-	24,406 (15,087-31,040)	5,536 (4,167-6,710)	5,389 (4,532-6,238)	6,086 (4,960-7,111)	7,346 (5,733-8,551)	8,518 (6,718-9,935)	9,103 (6,992-10,704)	12,456 (9,469-14,614)	14,353 (10,849-17,103)	20,532 (15,218-24,847)	30,174 (22,089-38,175)	41,162 (29,940-53,204)	54,003 (38,605-72,847)	58,653 (41,821-79,166)	58,950 (42,710-81,094)	54,353 (40,740-74,813)	36,288 (27,602-50,439)	18,915 (14,665-26,848)	6,225 (4,947-8,612)	1,501 (1,229-2,028)
Interstitial lung disease and pulmonary sarcoidosis	-	-	-	604 (193-1,600)	214 (106-468)	118 (71-215)	193 (111-368)	264 (157-490)	363 (226-635)	504 (313-861)	752 (478-1,301)	972 (619-1,680)	1,396 (871-2,412)	2,313 (1,424-4,006)	3,447 (2,149-5,830)	4,994 (3,285-8,201)	6,597 (4,570-10,425)	7,299 (5,176-11,058)	7,718 (5,818-11,032)	5,892 (4,551-8,419)	3,307 (2,600-4,733)	1,161 (926-1,646)	303 (247-442)
Other chronic respiratory diseases	0 (0-0)	0 (0-0)	0 (0-0)	3,544 (1,484-6,087)	1,136 (681-1,643)	784 (482-1,110)	550 (363-771)	703 (480-1,011)	628 (398-897)	809 (509-1,170)	910 (551-1,254)	1,018 (647-1,356)	1,075 (646-1,472)	1,313 (805-1,711)	1,707 (1,064-2,240)	1,978 (1,256-2,562)	2,102 (1,284-2,710)	2,199 (1,373-2,729)	2,133 (1,266-2,625)	1,936 (1,075-2,450)	1,358 (753-1,708)	596 (333-748)	164 (96-202)
Cirrhosis and other chronic liver diseases	-	-	-	16,035 (12,087-19,498)	5,335 (4,447-6,223)	5,152 (4,558-5,785)	7,962 (7,295-8,628)	13,236 (12,203-14,693)	18,808 (17,373-20,031)	31,061 (28,899-33,592)	46,638 (43,701-49,492)	58,269 (55,076-61,332)	69,524 (65,931-72,925)	86,993 (83,017-92,230)	94,810 (89,955-99,776)	100,688 (95,951-106,698)	90,727 (86,559-94,613)	73,908 (70,267-77,335)	59,258 (56,392-61,837)	38,259 (36,497-40,263)	20,441 (19,377-21,424)	7,163 (6,749-7,544)	2,055 (1,930-2,163)
Cirrhosis and other chronic liver diseases due to hepatitis B	-	-	-	123 (77-185)	104 (73-149)	185 (133-247)	496 (338-693)	2,817 (2,093-3,757)	6,079 (4,735-7,551)	10,875 (9,184-12,664)	16,479 (13,789-19,140)	19,419 (16,743-22,252)	21,362 (17,932-24,962)	25,687 (21,652-29,857)	28,362 (24,217-33,235)	30,235 (25,528-35,911)	27,281 (22,849-32,207)	23,204 (19,475-27,420)	17,713 (14,832-20,553)	10,137 (8,291-12,117)	4,733 (4,013-5,546)	1,412 (1,130-1,720)	349 (251-459)
Cirrhosis and other chronic liver diseases due to hepatitis C	-	-	-	97 (63-142)	81 (55-116)	145 (101-198)	345 (229-492)	1,723 (1,246-2,277)	3,801 (2,870-4,869)	7,417 (6,241-8,827)	11,705 (9,466-14,289)	15,211 (12,820-17,914)	18,417 (15,074-22,086)	22,867 (18,666-27,403)	24,496 (20,385-28,823)	25,736 (20,990-31,688)	23,340 (19,625-27,424)	18,603 (15,721-21,911)	14,230 (11,607-17,201)	9,555 (7,717-11,644)	5,303 (4,410-6,248)	1,881 (1,472-2,300)	516 (359-665)
Cirrhosis and other chronic liver diseases due to alcohol use	-	-	-	-	-	-	97 (55-152)	811 (572-1,097)	2,001 (1,459-2,666)	5,805 (4,864-6,878)	11,517 (9,527-13,853)	16,180 (14,024-18,714)	20,473 (17,275-23,905)	25,707 (21,703-29,905)	26,758 (22,281-31,372)	27,990 (22,788-33,574)	24,991 (21,125-29,149)	19,113 (16,104-22,593)	16,284 (13,785-19,079)	10,680 (8,869-12,524)	5,729 (5,002-6,474)	2,068 (1,754-2,376)	617 (499-740)
Cirrhosis and other chronic liver diseases due to other causes	-	-	-	15,815 (11,950-19,207)	5,150 (4,309-5,999)	4,822 (4,262-5,405)	7,024 (6,413-7,704)	7,886 (6,673-9,090)	6,928 (5,458-8,431)	6,964 (5,281-8,717)	7,965 (5,039-9,099)	7,459 (5,514-9,781)	9,272 (6,829-12,130)	12,732 (9,706-16,404)	15,195 (11,459-19,665)	16,726 (12,529-21,813)	15,114 (11,349-19,328)	12,990 (9,783-16,724)	11,032 (8,531-13,706)	7,887 (6,149-9,824)	4,676 (3,910-5,578)	1,802 (1,457-2,188)	573 (433-731)
Digestive diseases	2,776 (1,930-3,263)	3,547 (2,424-4,518)	28,413 (21,359-36,048)	33,497 (22,316-46,974)	11,880 (9,730-13,981)	9,528 (8,304-10,632)	13,496 (11,671-14,617)	16,496 (14,497-17,668)	20,626 (17,981-22,168)	22,319 (19,660-23,857)	27,515 (24,404-29,652)	31,519 (26,707-34,207)	37,888 (31,767-41,126)	48,675 (41,952-53,260)	62,514 (53,437-67,762)	77,676 (66,791-84,283)	87,221 (75,968-95,481)	91,033 (80,296-99,186)	96,519 (86,285-105,064)	79,230 (71,997-86,723)	52,982 (48,429-57,759)	23,467 (21,643-25,517)	7,005 (6,493-7,542)
Peptic ulcer disease	-	-	-	6,799 (4,252-9,785)	3,193 (2,428-4,092)	3,137 (2,656-3,706)	4,736 (4,125-5,335)	6,686 (5,873-7,380)	7,542 (6,633-8,302)	8,684 (7,578-9,498)	10,561 (9,319-11,679)	12,812 (11,063-13,997)	16,000 (13,561-17,472)	21,843 (18,677-23,873)	26,687 (22,834-28,834)	32,851 (28,623-35,777)	35,948 (31,428-39,224)	36,783 (32,174-40,391)	35,782 (31,448-39,226)	26,480 (23,486-29,161)	16,632 (14,993-18,189)	6,709 (6,129-7,269)	1,845 (1,695-1,986)
Gastritis and duodenitis	-	-	-	1,073 (406-1,914)	350 (218-534)	226 (166-301)	404 (321-492)	557 (457-659)	794 (670-936)	1,062 (905-1,229)	1,260 (1,085-1,479)	1,407 (1,202-1,609)	1,812 (1,525-2,073)	2,294 (1,909-2,634)	2,901 (2,416-3,336)	3,421 (2,877-3,970)	3,654 (3,081-4,311)	3,864 (3,257-4,582)	3,874 (3,273-4,764)	3,127 (2,605-3,997)	2,096 (1,737-2,691)	940 (775-1,206)	285 (236-357)
Appendicitis	-	-	-	5,634 (2,797-8,702)	2,562 (1,869-3,168)	2,083 (1,652-2,417)	2,806 (2,202-3,287)	2,212 (1,740-2,589)	2,425 (1,892-2,851)	1,598 (1,291-1,861)	1,794 (1,404-2,138)	1,678 (1,315-2,082)	1,787 (1,365-2,209)	1,907 (1,447-2,363)	2,807 (2,147-3,468)	2,824 (2,129-3,648)	2,648 (2,057-3,465)	2,462 (1,931-3,219)	2,272 (1,801-2,929)	1,582 (1,234-2,116)	951 (750-1,271)	385 (305-517)	105 (84-136)
Paralytic ileus and intestinal obstruction	2,776 (1,930-3,263)	3,547 (2,424-4,518)	28,413 (21,359-36,048)	5,464 (2,925-7,602)	2,575 (1,594-3,226)	1,873 (1,253-2,233)	2,704 (1,820-3,245)	2,759 (1,901-3,307)	3,656 (2,408-4,337)	3,091 (2,119-3,610)	4,169 (2,807-4,935)	4,734 (3,059-5,668)	5,732 (3,680-6,862)	6,764 (4,725-7,936)	9,352 (6,623-10,920)	10,418 (7,592-12,118)	12,385 (9,411-14,265)	12,080 (9,512-13,755)	12,714 (10,503-14,429)	11,160 (9,292-12,649)	8,157 (6,756-9,386)	3,984 (3,257-4,579)	1,230 (997-1,440)
Inguinal, femoral, and abdominal hernia	-	-	-	1,544 (608-2,603)	443 (273-588)	350 (199-464)	404 (246-525)	498 (291-667)	598 (358-769)	605 (316-794)	678 (436-844)	1,005 (652-1,251)	1,058 (722-1,331)	1,722 (1,217-2,181)	2,395 (1,624-2,973)	3,085 (2,052-3,899)	3,717 (2,481-4,734)	4,033 (2,615-5,181)	4,690 (3,068-5,968)	4,179 (2,854-5,200)	2,814 (1,953-3,455)	1,286 (878-1,592)	384 (269-471)
Inflammatory bowel disease	-	-	-	2,308 (724-6,496)	342 (180-654)	229 (143-375)	301 (196-448)	488 (314-716)	633 (442-894)	666 (466-924)	715 (518-948)	796 (598-1,000)	916 (668-1,159)	1,093 (849-1,298)	1,414 (1,102-1,697)	1,711 (1,367-1,979)	1,976 (1,590-2,258)	2,323 (1,868-2,657)	2,728 (2,186-3,001)	2,592 (2,079-2,831)	1,856 (1,458-2,027)	894 (692-982)	294 (232-324)
Vascular intestinal disorders	-	-	-	634 (209-1,148)	87 (54-121)	54 (42-64)	88 (72-101)	183 (148-211)	260 (211-295)	300 (240-340)	469 (387-530)	693 (584-772)	1,052 (896-1,171)	1,738 (1,495-1,957)	2,686 (2,356-3,081)	4,429 (3,867-4,921)	6,173 (5,548-6,874)	7,340 (6,648-8,059)	9,849 (8,936-10,849)	8,645 (7,786-9,409)	5,656 (5,108-6,142)	2,342 (2,140-2,536)	631 (571-687)
Gallbladder and biliary diseases	-	-	-	1,127 (476-1,728)	364 (259-465)	302 (236-359)	397 (301-445)	618 (480-681)	787 (630-876)	1,043 (825-1,151)	1,457 (1,135-1,606)	1,598 (1,261-1,812)	2,001 (1,540-2,262)	3,077 (2,344-3,525)	4,184 (3,183-4,754)	6,085 (4,740-7,088)	7,693 (6,009-8,832)	8,389 (6,842-9,632)	10,173 (8,480-11,588)	9,084 (7,785-10,429)	6,454 (5,701-7,283)	3,040 (2,703-3,409)	945 (855-1,047)
Pancreatitis	-	-	-	309 (150-484)	224 (148-306)	221 (164-287)	608 (457-769)	1,313 (1,041-1,634)	2,479 (2,047-2,954)	3,514 (2,937-4,147)	4,394 (3,707-5,241)	4,637 (3,873-5,564)	4,903 (4,039-5,950)	5,194 (4,284-6,434)	5,598 (4,581-6,888)	5,927 (4,901-7,279)	5,884 (4,879-7,063)	5,176 (4,300-6,098)	5,228 (4,471-6,135)	3,809 (3,266-4,556)	2,243 (1,927-2,668)	852 (728-985)	241 (207-278)
Other digestive diseases	-	-	-	8,605 (4,603-12,703)	1,739 (1,233-2,368)	1,052 (815-1,378)	1,048 (876-1,245)	1,181 (1,009-1,404)	1,451 (1,235-1,729)	1,755 (1,517-2,086)	2,018 (1,732-2,415)	2,159 (1,834-2,588)	2,626 (2,208-3,149)	3,423 (2,899-4,064)	4,490 (3,788-5,310)	6,304 (5,377-7,559)	7,142 (6,131-8,468)	8,585 (7,379-10,209)	9,209 (7,965-10,791)	8,572 (7,547-10,047)	6,123 (5,407-7,067)	3,035 (2,720-3,458)	1,043 (922-1,168)
Neurological disorders	-	-	9,565 (7,336-11,801)	12,271 (7,550-16,873)	6,929 (5,529-8,352)	8,929 (7,643-10,149)	14,210 (12,706-15,345)	13,773 (12,365-14,687)	11,283 (10,173-11,986)	10,607 (9,572-11,290)	10,822 (9,897-11,450)	9,281 (8,627-9,739)	10,039 (9,253-10,907)	14,297 (12,627-16,471)	19,844 (16,525-23,752)	32,890 (27,167-40,533)	56,629 (44,921-70,589)	99,766 (77,466-128,146)	192,552 (150,985-244,294)	251,807 (199,252-317,678)	240,108 (190,679-295,130)	145,888 (118,645-172,791)	46,108 (36,699-57,187)
Alzheimer's disease and other dementias	-	-	-	-	-	-	-	-	-	-	-	71 (30-139)	1,368 (812-2,073)	4,852 (3,266-6,931)	9,482 (6,267-13,358)	20,565 (14,840-28,221)	41,516 (30,511-55,686)	81,244 (58,887-109,812)	165,952 (123,278-215,315)	225,435 (171,678-291,549)	223,672 (174,734-278,889)	140,235 (113,292-167,483)	44,815 (35,404-56,033)
Parkinson's disease	-	-	-	-	-	-	-	1 (1-2)	16 (7-31)	56 (29-99)	116 (60-201)	172 (95-289)	252 (139-439)	478 (272-782)	931 (558-1,394)	2,323 (1,589-3,295)	5,140 (3,594-6,902)	10,152 (7,052-13,693)	19,621 (14,053-26,180)	21,690 (16,098-29,202)	13,938 (10,516-18,535)	4,854 (3,650-6,386)	1,086 (780-1,470)
Epilepsy	-	-	6,201 (4,583-7,790)	9,679 (5,815-13,272)	5,876 (4,638-7,142)	7,449 (6,282-8,566)	11,777 (10,304-12,837)	11,906 (10,524-12,807)	9,888 (8,801-10,520)	8,965 (7,965-9,566)	8,509 (7,594-9,050)	6,267 (5,692-6,606)	5,066 (4,626-5,352)	4,674 (4,239-4,930)	4,280 (3,859-4,527)	4,018 (3,553-4,286)	3,616 (3,175-3,935)	3,144 (2,741-3,446)	2,093 (1,898-2,252)	1,491 (1,351-1,647)	891 (805-997)	294 (269-325)	78 (72-87)
Multiple sclerosis	-	-	-	-	-	-	-	123 (99-176)	277 (226-373)	530 (425-644)	884 (722-1,064)	1,153 (955-1,310)	1,213 (1,045-1,431)	1,425 (1,275-1,743)	1,418 (1,274-1,841)	1,389 (1,257-1,783)	1,241 (1,125-1,590)	845 (763-1,073)	669 (606-853)	384 (337-481)	157 (126-177)	42 (34-49)	8 (6-9)
Motor neuron disease	9 (7-10)	12 (10-13)	1,147 (763-1,418)	433 (236-597)	51 (43-61)	77 (66-85)	113 (102-122)	93 (86-101)	86 (80-94)	165 (155-180)	318 (301-344)	522 (500-569)	799 (766-884)	1,218 (1,165-1,348)	1,762 (1,697-1,909)	2,350 (2,266-2,504)	2,756 (2,661-2,915)	2,239 (2,170-2,361)	1,939 (1,862-2,034)	1,058 (1,009-1,114)	405 (387-428)	82 (79-86)	14 (14-15)
Other neurological disorders	-	-	2,216 (1,579-3,171)	2,159 (1,282-3,496)	1,002 (786-1,323)	1,403 (1,193-1,677)	2,320 (2,146-2,511)	1,649 (1,557-1,757)	1,015 (962-1,077)	892 (850-938)	995 (946-1,038)	1,096 (1,018-1,149)	1,341 (1,256-1,405)	1,649 (1,512-1,753)	1,971 (1,834-2,088)	2,246 (2,110-2,370)	2,359 (2,235-2,472)	2,143 (2,035-2,237)	2,278 (2,162-2,378)	1,749 (1,659-1,837)	1,045 (988-1,102)	380 (358-405)	106 (100-113)
Mental and substance use disorders	18 (10-29)	29 (23-38)	0 (0-0)	0 (0-0)	16 (11-21)	14 (10-17)	7,085 (4,760-8,097)	12,776 (8,810-14,410)	16,166 (12,448-17,655)	20,180 (16,340-22,054)	22,704 (18,944-24,758)	21,397 (18,160-23,590)	18,888 (16,181-20,973)	21,805 (18,358-24,748)	17,855 (15,121-19,983)	15,668 (13,166-17,662)	10,412 (8,893-11,123)	6,640 (5,464-7,099)	5,512 (4,740-5,873)	2,865 (2,565-3,045)	1,539 (1,372-1,632)	579 (509-619)	173 (155-185)
Alcohol use disorders	-	-	-	-	-	-	1,277 (1,077-1,520)	2,696 (2,252-3,178)	5,093 (4,256-5,823)	8,925 (7,269-10,181)	12,157 (10,234-13,761)	13,392 (11,264-15,381)	13,721 (11,872-15,791)	15,841 (13,261-18,530)	13,130 (11,206-14,999)	10,996 (9,419-12,656)	6,841 (6,083-7,441)	3,585 (3,193-4,008)	2,804 (2,502-3,129)	1,263 (1,160-1,363)	592 (543-643)	194 (177-210)	58 (53-63)
Drug use disorders	18 (10-29)	29 (23-38)	0 (0-0)	0 (0-0)	0 (0-0)	0 (0-0)	5,770 (3,331-6,731)	10,025 (5,921-11,638)	11,018 (7,457-12,364)	11,202 (8,129-12,651)	10,499 (7,950-11,735)	7,952 (6,107-8,888)	5,120 (3,812-5,755)	5,965 (4,695-6,708)	4,725 (3,445-5,405)	4,671 (3,324-5,325)	3,571 (2,444-4,025)	3,055 (2,014-3,463)	2,707 (2,056-2,999)	1,602 (1,333-1,737)	947 (794-1,023)	385 (321-417)	115 (98-125)
Opioid use disorders	18 (10-29)	29 (23-38)	0 (0-0)	0 (0-0)	0 (0-0)	0 (0-0)	3,813 (2,293-4,554)	6,838 (4,062-8,133)	7,699 (5,143-8,856)	7,487 (5,365-8,718)	7,029 (5,150-8,083)	5,390 (4,035-6,242)	3,256 (2,443-3,779)	3,931 (2,923-4,560)	2,968 (2,082-3,522)	2,885 (1,927-3,417)	2,089 (1,366-2,504)	1,266 (815-1,545)	1,157 (814-1,371)	611 (451-718)	372 (282-427)	155 (113-175)	48 (35-54)
Cocaine use disorders	0 (0-0)	0 (0-0)	0 (0-0)	0 (0-0)	0 (0-0)	0 (0-0)	247 (123-321)	425 (232-537)	463 (283-577)	695 (459-849)	629 (414-770)	449 (301-549)	332 (213-416)	413 (271-521)	359 (216-455)	330 (196-424)	268 (159-335)	138 (74-184)	101 (60-129)	15 (10-19)	8 (5-11)	4 (2-4)	1 (1-1)
Amphetamine use disorders	0 (0-0)	0 (0-0)	0 (0-0)	0 (0-0)	0 (0-0)	0 (0-0)	208 (94-375)	343 (167-574)	337 (188-483)	345 (207-441)	311 (191-395)	222 (147-278)	163 (100-210)	166 (99-207)	153 (89-199)	142 (81-184)	116 (65-149)	49 (27-65)	37 (22-48)	7 (4-9)	4 (3-5)	1 (1-2)	0 (0-1)
Other drug use disorders	0 (0-0)	0 (0-0)	0 (0-0)	0 (0-0)	0 (0-0)	0 (0-0)	1,502 (722-1,827)	2,419 (1,285-2,899)	2,519 (1,505-2,933)	2,675 (1,735-3,056)	2,529 (1,644-2,887)	1,891 (1,220-2,190)	1,370 (857-1,587)	1,454 (1,008-1,692)	1,246 (844-1,475)	1,315 (880-1,548)	1,099 (742-1,274)	1,601 (1,002-1,852)	1,412 (1,031-1,586)	969 (818-1,077)	563 (473-623)	225 (190-248)	66 (56-73)
Eating disorders	-	-	-	-	16 (11-21)	14 (10-17)	38 (30-47)	55 (45-67)	55 (46-67)	53 (45-62)	49 (42-58)	53 (46-61)	47 (41-55)	0 (0-0)	0 (0-0)	-	-	-	-	-	-	-	-
Anorexia nervosa	-	-	-	-	15 (11-20)	13 (10-16)	37 (29-46)	54 (44-66)	54 (44-66)	50 (43-59)	47 (40-55)	48 (42-56)	40 (34-47)	0 (0-0)	0 (0-0)	-	-	-	-	-	-	-	-
Bulimia nervosa	-	-	-	-	1 (0-2)	1 (0-1)	1 (1-2)	1 (1-2)	1 (1-2)	3 (2-4)	2 (2-3)	5 (4-7)	8 (6-10)	0 (0-0)	0 (0-0)	-	-	-	-	-	-	-	-
Diabetes, urogenital, blood, and endocrine diseases	4,987 (3,823-5,659)	4,515 (3,451-5,240)	37,763 (32,343-42,107)	47,231 (33,257-57,556)	17,609 (15,114-20,152)	17,857 (15,437-19,823)	24,699 (22,388-26,468)	30,721 (28,848-32,894)	29,033 (27,435-30,502)	33,350 (31,762-35,055)	39,050 (37,579-40,923)	46,543 (44,896-48,826)	59,200 (57,084-62,015)	84,790 (82,109-89,095)	113,192 (109,631-118,516)	149,070 (144,501-156,219)	174,469 (168,986-182,867)	180,256 (174,019-189,028)	190,219 (183,633-198,823)	153,918 (148,691-161,250)	101,847 (98,281-106,176)	44,798 (43,241-46,737)	13,966 (13,381-14,530)
Diabetes mellitus	86 (81-92)	179 (158-202)	1,438 (1,292-1,638)	1,418 (1,175-1,681)	1,207 (1,039-1,382)	1,604 (1,457-1,750)	2,747 (2,595-2,912)	4,331 (4,135-4,580)	4,853 (4,677-5,060)	6,927 (6,697-7,221)	9,702 (9,410-10,038)	14,558 (14,117-15,058)	21,534 (20,800-22,314)	35,046 (33,922-36,265)	52,146 (50,517-53,966)	74,445 (72,045-77,275)	88,726 (85,776-92,084)	89,595 (86,156-93,485)	92,672 (88,886-96,576)	70,601 (67,439-74,197)	40,238 (38,521-42,191)	15,268 (14,586-16,024)	3,981 (3,787-4,181)
Acute glomerulonephritis	-	-	343 (279-420)	561 (434-714)	453 (379-548)	443 (362-550)	516 (435-615)	662 (564-798)	599 (503-715)	614 (537-706)	777 (674-901)	659 (569-760)	716 (638-806)	834 (735-936)	1,059 (955-1,181)	1,174 (1,045-1,322)	1,239 (1,108-1,384)	1,306 (1,154-1,478)	1,120 (992-1,272)	683 (606-782)	384 (342-438)	130 (116-148)	45 (39-54)
Chronic kidney disease	-	-	7,022 (5,802-7,987)	10,111 (8,187-11,833)	5,201 (4,421-5,857)	5,188 (4,604-5,736)	8,710 (8,142-9,626)	13,399 (12,305-14,824)	13,977 (13,054-15,232)	16,235 (15,175-17,751)	19,372 (17,985-21,163)	21,433 (20,118-23,577)	25,574 (23,940-27,965)	35,380 (33,527-38,934)	45,055 (42,826-49,347)	54,648 (52,060-59,811)	61,767 (58,922-68,539)	64,357 (61,404-70,960)	68,459 (65,557-74,739)	56,984 (54,797-61,955)	40,957 (39,345-43,993)	19,147 (18,338-20,333)	6,227 (5,936-6,563)
Chronic kidney disease due to diabetes mellitus	-	-	188 (123-272)	278 (172-418)	192 (107-306)	265 (161-424)	752 (454-1,170)	2,194 (1,382-3,042)	3,332 (2,432-4,361)	5,166 (3,880-6,474)	7,532 (5,779-9,305)	9,284 (7,163-11,333)	12,170 (9,595-14,803)	17,439 (13,550-21,252)	23,003 (18,256-27,292)	28,308 (23,202-33,460)	30,808 (24,164-37,109)	30,064 (23,701-36,115)	28,545 (22,209-34,817)	19,890 (14,894-24,828)	12,017 (8,761-15,842)	5,182 (3,638-6,854)	1,674 (1,099-2,274)
Chronic kidney disease due to hypertension	-	-	52 (34-75)	105 (69-153)	90 (58-136)	151 (97-226)	389 (255-552)	870 (596-1,197)	1,130 (780-1,539)	1,573 (1,156-2,089)	2,222 (1,675-2,882)	2,876 (2,208-3,680)	3,922 (3,013-5,040)	6,020 (4,601-7,911)	8,673 (6,661-11,265)	11,351 (9,004-14,404)	14,298 (11,169-18,110)	16,845 (13,106-21,214)	19,354 (15,397-24,181)	18,007 (14,514-22,096)	15,552 (12,523-18,866)	8,202 (6,656-9,765)	2,792 (2,224-3,399)
Chronic kidney disease due to glomerulonephritis	-	-	2,817 (2,048-3,574)	4,170 (2,984-5,500)	2,129 (1,473-2,842)	2,270 (1,622-2,946)	3,864 (2,787-4,954)	5,559 (4,073-7,164)	5,169 (3,668-6,726)	4,889 (3,419-6,608)	4,645 (3,149-6,312)	4,232 (2,752-5,752)	4,013 (2,590-5,587)	4,887 (3,260-6,926)	5,110 (3,503-7,118)	5,636 (3,640-8,006)	6,166 (4,080-8,877)	6,376 (4,251-9,118)	7,852 (5,286-11,186)	7,252 (4,989-9,832)	4,607 (3,204-6,508)	1,883 (1,284-2,536)	592 (357-943)
Chronic kidney disease due to other causes	-	-	3,965 (3,081-4,881)	5,557 (4,203-6,999)	2,791 (2,115-3,506)	2,502 (1,803-3,173)	3,705 (2,640-4,822)	4,775 (3,319-6,328)	4,346 (3,027-5,860)	4,608 (3,210-6,163)	4,974 (3,552-6,621)	5,041 (3,461-6,774)	5,470 (3,638-7,796)	7,034 (4,605-9,972)	8,268 (5,604-11,879)	9,353 (6,046-13,490)	10,496 (7,099-14,783)	11,073 (7,462-15,311)	12,707 (8,768-17,671)	11,834 (8,296-15,868)	8,781 (6,008-11,967)	3,879 (2,766-5,244)	1,168 (730-1,693)
Urinary diseases and male infertility	438 (392-503)	795 (617-1,033)	6,448 (5,640-7,470)	4,792 (3,847-6,021)	1,540 (1,271-1,863)	1,145 (1,013-1,320)	2,129 (1,961-2,286)	2,667 (2,503-2,900)	2,494 (2,347-2,670)	2,892 (2,748-3,077)	3,186 (3,029-3,354)	3,572 (3,393-3,748)	4,283 (4,068-4,519)	6,107 (5,823-6,472)	7,408 (7,092-7,830)	10,481 (10,022-11,015)	12,682 (12,118-13,255)	13,720 (13,072-14,361)	16,003 (15,354-16,709)	15,020 (14,454-15,583)	11,706 (11,271-12,136)	6,377 (6,153-6,619)	2,456 (2,358-2,552)
Interstitial nephritis and urinary tract infections	171 (122-223)	354 (218-507)	3,864 (2,938-4,698)	2,437 (1,505-3,429)	639 (456-815)	509 (388-615)	1,160 (950-1,391)	1,444 (1,156-1,675)	1,257 (1,028-1,437)	1,453 (1,232-1,641)	1,655 (1,415-1,866)	1,869 (1,607-2,108)	2,181 (1,823-2,465)	3,464 (2,992-3,836)	4,119 (3,492-4,559)	5,964 (5,067-6,580)	7,423 (6,496-8,122)	8,355 (7,298-9,138)	10,511 (9,441-11,296)	10,291 (9,416-10,987)	8,529 (7,883-9,115)	4,828 (4,477-5,174)	1,991 (1,848-2,103)
Urolithiasis	-	-	-	-	255 (123-358)	180 (96-234)	269 (153-345)	307 (172-394)	279 (148-366)	309 (168-398)	383 (237-482)	468 (286-598)	584 (352-734)	823 (511-1,015)	900 (582-1,092)	1,331 (904-1,617)	1,677 (1,123-2,035)	1,697 (1,076-2,027)	1,788 (1,222-2,072)	1,340 (906-1,566)	841 (545-990)	381 (243-456)	108 (73-132)
Other urinary diseases	267 (212-316)	441 (305-607)	2,584 (1,847-3,401)	2,356 (1,521-3,301)	646 (445-880)	456 (331-609)	701 (519-958)	915 (673-1,256)	958 (718-1,266)	1,131 (868-1,423)	1,147 (874-1,485)	1,235 (918-1,608)	1,518 (1,102-2,005)	1,819 (1,310-2,497)	2,389 (1,796-3,174)	3,186 (2,370-4,386)	3,582 (2,692-4,896)	3,668 (2,817-5,013)	3,704 (3,002-4,999)	3,389 (2,829-4,330)	2,336 (1,952-3,023)	1,168 (971-1,541)	357 (290-479)
Gynecological diseases	-	-	-	-	-	-	247 (179-292)	327 (236-386)	353 (273-404)	449 (350-512)	540 (432-619)	536 (433-620)	737 (597-842)	621 (501-715)	450 (364-522)	266 (222-314)	392 (322-463)	224 (184-266)	180 (156-217)	323 (256-396)	211 (174-256)	84 (70-103)	28 (24-34)
Uterine fibroids	-	-	-	-	-	-	17 (8-38)	23 (12-43)	38 (17-65)	76 (29-138)	166 (63-252)	205 (99-298)	347 (148-487)	337 (139-471)	217 (79-330)	102 (43-173)	138 (61-237)	58 (28-101)	44 (29-69)	82 (29-165)	44 (18-87)	17 (8-32)	5 (3-9)
Polycystic ovarian syndrome	-	-	-	-	-	-	3 (0-11)	29 (6-84)	70 (19-147)	61 (13-154)	74 (17-164)	69 (17-145)	112 (29-238)	177 (55-340)	-	-	-	-	-	-	-	-	-
Endometriosis	-	-	-	-	-	-	5 (1-12)	3 (1-7)	3 (1-6)	6 (2-12)	4 (2-7)	6 (3-10)	9 (4-17)	7 (3-14)	-	-	-	-	-	-	-	-	-
Genital prolapse	-	-	-	-	-	-	17 (4-50)	15 (4-33)	25 (9-49)	26 (5-46)	9 (3-18)	9 (3-16)	10 (3-19)	17 (4-42)	101 (16-188)	85 (21-149)	129 (36-215)	91 (35-141)	60 (36-85)	139 (48-234)	86 (35-138)	33 (16-51)	10 (6-16)
Other gynecological diseases	-	-	-	-	-	-	205 (138-254)	256 (166-324)	216 (118-300)	282 (160-383)	287 (162-436)	247 (139-390)	260 (122-492)	83 (33-194)	132 (63-239)	79 (42-141)	126 (69-207)	75 (46-122)	76 (55-101)	103 (60-185)	81 (54-135)	34 (23-53)	13 (9-18)
Hemoglobinopathies and hemolytic anaemias	2,538 (1,826-3,093)	1,852 (1,239-2,246)	12,022 (8,361-14,845)	23,450 (11,100-31,981)	6,681 (4,745-8,491)	7,495 (5,561-8,965)	8,473 (6,251-9,936)	7,248 (5,740-8,967)	4,436 (3,308-5,331)	3,565 (2,746-4,277)	2,807 (2,256-3,357)	3,224 (2,697-4,056)	3,492 (2,875-4,699)	3,279 (2,722-4,124)	3,074 (2,617-3,798)	3,265 (2,819-4,009)	3,977 (3,443-4,941)	5,311 (4,558-6,620)	5,577 (4,822-6,582)	4,844 (3,867-5,518)	4,524 (3,553-5,247)	1,980 (1,508-2,301)	613 (453-711)
Thalassemias	799 (599-1,032)	625 (469-841)	3,915 (2,426-5,146)	6,474 (3,498-9,166)	2,509 (1,762-3,355)	1,216 (985-1,588)	774 (602-997)	396 (298-486)	193 (148-227)	102 (82-121)	65 (54-75)	49 (41-57)	42 (34-47)	30 (24-35)	20 (16-24)	8 (6-10)	4 (3-5)	1 (1-2)	1 (1-1)	0 (0-0)	0 (0-0)	0 (0-0)	0 (0-0)
Sickle cell disorders	1,506 (941-2,003)	1,142 (737-1,453)	7,554 (5,272-9,513)	15,982 (7,017-22,860)	3,613 (2,424-4,647)	5,458 (3,788-6,852)	6,344 (4,486-7,529)	4,999 (3,833-6,227)	2,633 (1,845-3,210)	1,893 (1,362-2,309)	1,170 (900-1,401)	1,319 (1,074-1,682)	1,233 (993-1,664)	681 (535-895)	295 (245-386)	111 (94-141)	71 (61-88)	34 (27-41)	18 (13-21)	6 (4-8)	2 (1-3)	0 (0-1)	0 (0-0)
G6PD deficiency	56 (39-75)	43 (27-59)	321 (220-435)	687 (408-985)	340 (227-467)	472 (356-590)	771 (600-957)	973 (766-1,270)	869 (673-1,085)	822 (646-1,004)	703 (574-855)	781 (641-994)	1,034 (847-1,421)	794 (637-1,000)	666 (535-861)	490 (385-636)	367 (292-490)	289 (230-389)	211 (169-282)	79 (63-101)	45 (36-57)	13 (10-16)	2 (1-3)
Other hemoglobinopathies and hemolytic anaemias	177 (133-218)	43 (30-54)	232 (164-304)	307 (194-414)	218 (167-280)	349 (282-424)	584 (468-725)	879 (705-1,133)	742 (591-921)	749 (590-940)	868 (695-1,068)	1,074 (919-1,347)	1,183 (994-1,557)	1,774 (1,483-2,236)	2,093 (1,784-2,601)	2,656 (2,280-3,217)	3,535 (3,040-4,339)	4,987 (4,261-6,218)	5,348 (4,609-6,280)	4,758 (3,793-5,418)	4,477 (3,506-5,201)	1,966 (1,497-2,286)	611 (451-709)
Endocrine, metabolic, blood, and immune disorders	1,925 (1,287-2,189)	1,689 (1,177-2,039)	10,490 (8,104-12,426)	6,899 (4,959-8,707)	2,526 (2,161-2,998)	1,982 (1,725-2,281)	1,876 (1,580-2,080)	2,089 (1,738-2,312)	2,322 (1,983-2,589)	2,666 (2,322-3,018)	2,666 (2,359-3,048)	2,562 (2,251-2,918)	2,862 (2,495-3,223)	3,523 (2,970-3,846)	3,999 (3,467-4,427)	4,791 (4,229-5,433)	5,685 (4,894-6,398)	5,743 (4,933-6,468)	6,207 (5,206-6,968)	5,463 (4,531-6,209)	3,829 (3,198-4,222)	1,813 (1,461-2,034)	616 (520-711)
Musculoskeletal disorders	-	-	-	-	843 (576-1,116)	1,231 (952-1,590)	1,970 (1,471-2,510)	2,319 (1,754-2,938)	2,134 (1,749-2,722)	2,020 (1,670-2,510)	2,194 (1,820-2,669)	2,147 (1,814-2,496)	2,222 (1,901-2,566)	2,563 (2,147-2,959)	3,198 (2,721-3,639)	4,260 (3,737-4,790)	5,198 (4,565-5,743)	5,625 (4,914-6,149)	6,371 (5,663-6,977)	5,320 (4,732-5,879)	3,745 (3,321-4,100)	1,697 (1,470-1,862)	575 (512-637)
Rheumatoid arthritis	-	-	-	-	206 (107-363)	204 (126-330)	204 (134-336)	252 (169-423)	230 (170-356)	256 (189-379)	350 (257-498)	466 (356-587)	669 (515-810)	1,083 (829-1,258)	1,646 (1,280-1,883)	2,442 (1,956-2,748)	3,164 (2,565-3,504)	3,568 (2,904-3,934)	3,930 (3,168-4,286)	2,968 (2,354-3,257)	1,778 (1,372-1,975)	659 (499-741)	175 (129-200)
Other musculoskeletal disorders	-	-	-	-	637 (451-893)	1,027 (790-1,371)	1,765 (1,288-2,296)	2,067 (1,575-2,658)	1,905 (1,572-2,410)	1,763 (1,458-2,191)	1,845 (1,542-2,251)	1,681 (1,420-1,991)	1,553 (1,338-1,816)	1,480 (1,287-1,744)	1,553 (1,362-1,815)	1,818 (1,605-2,211)	2,034 (1,817-2,452)	2,058 (1,800-2,467)	2,441 (2,160-2,983)	2,351 (2,081-2,856)	1,967 (1,746-2,339)	1,038 (907-1,203)	400 (354-467)
Other non-communicable diseases	210,188 (155,479-259,373)	96,698 (68,724-124,174)	300,660 (228,062-373,647)	95,346 (60,039-136,231)	21,288 (16,578-26,875)	13,863 (11,113-16,729)	10,131 (8,239-11,905)	8,132 (6,692-9,558)	6,069 (5,066-7,072)	4,692 (3,877-5,362)	4,383 (3,591-5,001)	3,938 (3,207-4,545)	3,399 (2,683-3,941)	3,805 (2,969-4,481)	4,349 (3,363-5,269)	4,721 (3,610-5,746)	5,207 (3,894-6,570)	4,212 (2,699-5,760)	5,298 (3,572-7,447)	5,097 (3,365-7,246)	4,269 (2,672-5,815)	2,486 (1,473-3,430)	1,008 (544-1,427)
Congenital anomalies	210,188 (155,479-259,373)	91,492 (63,838-118,641)	254,282 (183,668-328,553)	93,544 (57,961-133,952)	20,773 (16,202-26,352)	13,357 (10,737-16,227)	9,313 (7,586-11,086)	7,414 (6,089-8,864)	5,440 (4,639-6,376)	3,673 (3,191-4,326)	3,427 (2,960-4,102)	2,860 (2,462-3,410)	1,925 (1,652-2,254)	1,839 (1,616-2,166)	1,766 (1,568-2,117)	1,751 (1,533-2,179)	1,455 (1,281-1,730)	-	-	-	-	-	-
Neural tube defects	42,206 (28,379-58,646)	15,244 (9,227-22,389)	33,146 (20,763-51,085)	9,334 (5,229-18,167)	1,936 (1,351-2,729)	1,158 (824-1,565)	644 (468-854)	443 (320-590)	245 (178-326)	184 (133-238)	139 (104-178)	107 (77-142)	86 (63-111)	80 (59-105)	77 (56-107)	62 (45-93)	58 (42-83)	-	-	-	-	-	-
Congenital heart anomalies	68,491 (44,501-90,097)	38,174 (25,491-49,977)	123,926 (95,691-160,457)	47,332 (33,343-64,980)	12,472 (9,466-16,226)	8,580 (6,792-10,435)	6,199 (5,004-7,493)	5,246 (4,347-6,357)	3,995 (3,359-4,758)	2,523 (2,188-3,033)	2,461 (2,120-2,936)	2,096 (1,789-2,528)	897 (776-1,057)	807 (706-960)	738 (664-895)	692 (618-864)	590 (523-728)	-	-	-	-	-	-
Orofacial clefts	2,159 (974-3,358)	896 (443-1,338)	1,820 (852-2,736)	226 (63-407)	-	-	-	-	-	-	-	-	-	-	-	-	-	-	-	-	-	-	-
Down's syndrome	2,243 (1,161-5,414)	1,370 (604-4,016)	5,648 (2,833-14,107)	2,572 (1,044-8,256)	381 (229-747)	269 (182-442)	241 (186-355)	217 (173-312)	214 (173-306)	220 (179-320)	200 (163-283)	180 (153-252)	329 (270-461)	323 (279-455)	308 (269-464)	253 (216-400)	109 (94-168)	-	-	-	-	-	-
Other chromosomal abnormalities	4,606 (2,463-8,299)	2,571 (1,301-5,071)	5,382 (2,702-12,219)	985 (433-2,960)	210 (123-394)	121 (78-182)	111 (75-158)	79 (57-109)	64 (44-82)	44 (31-55)	37 (24-44)	30 (19-37)	27 (18-33)	34 (23-46)	34 (22-43)	27 (18-35)	22 (16-29)	-	-	-	-	-	-
Congenital musculoskeletal and limb anomalies	5,270 (3,185-10,773)	1,545 (789-3,741)	3,079 (1,534-8,319)	971 (403-3,486)	293 (177-621)	230 (153-372)	213 (140-310)	109 (70-148)	80 (60-106)	59 (41-76)	47 (34-59)	36 (25-43)	39 (27-47)	42 (30-50)	39 (26-46)	45 (28-52)	51 (29-59)	-	-	-	-	-	-
Urogenital congenital anomalies	5,702 (3,881-7,423)	1,884 (1,061-2,589)	3,174 (1,690-4,731)	748 (354-1,282)	318 (204-461)	278 (185-380)	121 (88-168)	107 (79-142)	95 (70-125)	86 (64-111)	91 (65-116)	83 (60-106)	132 (89-172)	167 (115-215)	203 (147-252)	244 (178-309)	277 (212-351)	-	-	-	-	-	-
Digestive congenital anomalies	18,040 (13,297-27,661)	8,739 (5,602-15,690)	19,206 (11,298-41,388)	3,587 (1,721-11,661)	369 (258-661)	171 (136-248)	95 (77-127)	61 (49-80)	44 (37-55)	36 (29-44)	34 (27-40)	29 (22-33)	39 (30-47)	45 (34-52)	48 (37-55)	62 (49-76)	61 (47-71)	-	-	-	-	-	-
Other congenital anomalies	61,470 (36,768-101,175)	21,068 (10,014-39,722)	58,901 (27,119-107,602)	27,789 (9,310-59,157)	4,795 (2,713-8,093)	2,549 (1,653-3,938)	1,688 (1,172-2,439)	1,151 (781-1,722)	703 (492-1,023)	522 (378-740)	418 (309-580)	299 (230-404)	375 (277-514)	341 (250-459)	319 (234-431)	365 (276-485)	289 (222-376)	-	-	-	-	-	-
Skin and subcutaneous diseases	-	-	3,691 (1,690-5,141)	1,802 (817-2,677)	515 (245-745)	506 (263-676)	818 (435-1,050)	718 (387-904)	629 (354-796)	1,019 (553-1,296)	956 (545-1,188)	1,078 (656-1,386)	1,474 (902-1,902)	1,966 (1,220-2,558)	2,583 (1,644-3,461)	2,970 (1,902-3,886)	3,751 (2,447-5,068)	4,212 (2,699-5,760)	5,298 (3,572-7,447)	5,097 (3,365-7,246)	4,269 (2,672-5,815)	2,486 (1,473-3,430)	1,008 (544-1,427)
Cellulitis	-	-	155 (53-264)	146 (44-265)	69 (25-112)	68 (31-101)	77 (40-104)	78 (43-99)	56 (33-80)	99 (57-133)	114 (64-161)	125 (76-199)	161 (92-248)	265 (138-386)	342 (175-470)	392 (216-611)	428 (231-673)	458 (246-724)	564 (310-910)	521 (306-934)	399 (220-727)	233 (119-432)	95 (47-193)
Pyoderma	-	-	3,430 (1,559-4,894)	1,467 (656-2,279)	357 (160-535)	344 (161-481)	587 (290-778)	497 (241-645)	442 (228-568)	725 (357-947)	651 (336-842)	745 (409-949)	1,038 (590-1,324)	1,359 (777-1,795)	1,743 (1,018-2,373)	1,930 (1,150-2,545)	2,261 (1,395-3,178)	2,306 (1,384-3,101)	2,509 (1,684-3,560)	1,861 (1,232-2,819)	1,440 (921-2,123)	785 (489-1,233)	298 (178-459)
Decubitus ulcer	-	-	-	73 (31-123)	43 (27-61)	50 (32-71)	93 (59-137)	84 (53-115)	87 (56-117)	146 (93-208)	147 (94-213)	159 (104-230)	221 (147-333)	279 (178-428)	416 (271-641)	549 (366-822)	928 (600-1,351)	1,309 (868-1,871)	2,024 (1,254-2,905)	2,490 (1,524-3,443)	2,245 (1,238-2,955)	1,359 (722-1,845)	574 (275-777)
Other skin and subcutaneous diseases	-	-	106 (54-172)	116 (60-189)	46 (29-62)	44 (28-61)	61 (40-85)	59 (38-85)	43 (28-60)	49 (33-71)	44 (30-62)	49 (33-73)	54 (36-79)	62 (41-96)	81 (54-123)	99 (66-150)	135 (90-203)	138 (91-208)	201 (127-315)	225 (134-347)	185 (103-272)	110 (57-160)	42 (20-63)
Sudden infant death syndrome	-	5,206 (3,893-7,751)	42,687 (32,019-64,459)	-	-	-	-	-	-	-	-	-	-	-	-	-	-	-	-	-	-	-	-
Injuries	20,585 (16,514-23,391)	14,911 (11,676-18,457)	143,826 (121,449-168,305)	400,075 (319,858-457,569)	210,073 (190,779-225,875)	172,937 (162,746-183,126)	315,458 (305,594-325,044)	417,742 (406,803-426,918)	361,298 (351,069-369,122)	312,442 (301,753-320,408)	283,395 (272,996-291,412)	222,337 (214,603-228,971)	191,627 (183,907-197,873)	194,020 (185,492-201,677)	179,492 (171,519-186,063)	169,681 (161,019-176,574)	152,345 (141,823-159,313)	136,612 (125,930-143,516)	129,566 (118,731-136,597)	99,716 (90,256-106,401)	65,557 (57,469-70,530)	27,826 (24,006-30,206)	8,662 (7,408-9,375)
Transport injuries	3,423 (2,520-4,869)	2,547 (2,007-3,133)	20,880 (17,985-24,212)	76,322 (63,061-87,833)	63,685 (57,766-69,055)	47,202 (44,386-50,080)	106,841 (102,872-112,365)	140,134 (135,554-148,437)	118,832 (114,561-125,912)	99,612 (96,171-104,590)	91,298 (88,400-96,017)	71,020 (68,764-74,517)	59,407 (57,386-61,947)	58,372 (56,358-60,946)	53,893 (51,990-56,213)	48,051 (46,388-50,352)	40,477 (39,005-42,562)	34,272 (32,803-36,158)	28,302 (27,250-29,788)	17,102 (16,399-18,099)	8,006 (7,668-8,582)	2,542 (2,420-2,753)	668 (632-724)
Road injuries	2,864 (2,145-4,025)	2,214 (1,765-2,723)	19,134 (16,445-22,085)	72,146 (59,331-82,513)	61,025 (55,365-66,282)	44,497 (41,733-47,220)	100,362 (96,361-106,165)	132,421 (127,658-141,154)	111,306 (106,913-118,759)	93,251 (89,698-98,533)	85,028 (82,128-90,064)	65,886 (63,693-69,516)	54,919 (53,009-57,564)	53,892 (51,927-56,665)	50,094 (48,200-52,402)	44,821 (43,216-47,094)	37,893 (36,480-40,013)	32,256 (30,893-34,055)	26,560 (25,563-27,985)	16,094 (15,437-17,056)	7,500 (7,190-7,997)	2,351 (2,241-2,530)	603 (569-651)
Pedestrian road injuries	1,245 (881-1,982)	955 (693-1,319)	8,772 (6,935-11,134)	42,973 (34,635-52,484)	36,485 (32,285-41,543)	22,673 (20,622-26,200)	28,963 (26,531-33,980)	36,406 (33,183-42,932)	30,865 (27,688-36,473)	29,550 (26,743-34,663)	29,616 (27,019-34,496)	23,464 (21,414-27,195)	21,924 (20,039-25,300)	22,842 (20,871-26,134)	22,298 (20,511-25,424)	22,487 (20,912-25,303)	19,636 (18,229-22,063)	17,506 (16,336-19,674)	15,622 (14,706-17,227)	9,492 (8,926-10,389)	4,430 (4,145-4,852)	1,286 (1,195-1,429)	320 (295-357)
Cyclist road injuries	-	-	-	2,022 (1,161-2,668)	2,771 (2,217-3,238)	3,176 (2,726-3,572)	4,295 (3,751-4,771)	3,919 (3,361-4,388)	3,372 (2,923-3,751)	3,443 (2,955-3,820)	3,334 (2,804-3,744)	3,136 (2,687-3,494)	3,097 (2,657-3,445)	3,403 (2,953-3,777)	3,183 (2,754-3,563)	3,018 (2,580-3,358)	2,644 (2,314-2,897)	2,171 (1,892-2,415)	1,411 (1,236-1,549)	730 (642-813)	281 (243-324)	72 (61-91)	1 (1-1)
Motorcyclist road injuries	289 (204-444)	271 (177-446)	2,510 (1,977-3,344)	5,382 (3,744-7,754)	3,934 (3,278-4,943)	4,823 (4,263-5,546)	24,894 (22,706-29,034)	33,539 (30,161-39,154)	26,332 (23,164-31,170)	19,505 (17,188-22,607)	16,685 (14,462-19,656)	12,136 (10,537-13,997)	8,871 (7,913-10,286)	7,629 (6,724-8,910)	6,499 (5,689-7,777)	3,896 (3,418-4,798)	2,973 (2,616-3,543)	2,130 (1,885-2,570)	1,180 (1,047-1,368)	602 (529-738)	214 (186-273)	58 (49-80)	17 (14-23)
Motor vehicle road injuries	1,273 (747-1,997)	958 (651-1,303)	7,511 (5,371-9,848)	21,121 (13,695-27,662)	17,235 (13,292-20,787)	13,295 (10,572-15,665)	41,353 (35,316-46,742)	57,393 (48,375-64,933)	49,768 (42,370-56,622)	40,010 (33,725-45,712)	34,684 (29,258-39,439)	26,552 (22,722-30,058)	20,358 (17,286-23,073)	19,291 (16,542-22,210)	17,474 (15,021-19,763)	14,997 (13,053-16,976)	12,207 (10,591-13,741)	10,091 (8,869-11,349)	7,971 (7,102-8,951)	5,039 (4,560-5,713)	2,462 (2,241-2,752)	891 (799-983)	251 (220-274)
Other road injuries	56 (31-106)	30 (19-52)	340 (222-565)	647 (385-1,070)	599 (432-808)	530 (388-719)	859 (622-1,196)	1,164 (836-1,535)	970 (701-1,291)	742 (536-998)	709 (508-916)	598 (443-755)	668 (488-862)	727 (529-979)	641 (465-864)	423 (306-582)	433 (312-585)	358 (255-489)	376 (264-547)	231 (162-343)	114 (81-167)	44 (32-73)	14 (10-23)
Other transport injuries	560 (298-973)	333 (179-522)	1,747 (1,253-2,599)	4,176 (2,886-6,620)	2,660 (2,025-3,367)	2,705 (2,200-3,221)	6,479 (5,185-7,349)	7,713 (6,096-8,689)	7,525 (6,119-8,437)	6,361 (5,161-7,124)	6,270 (5,114-7,038)	5,134 (4,208-5,795)	4,488 (3,632-5,060)	4,481 (3,634-5,142)	3,799 (3,122-4,316)	3,229 (2,668-3,682)	2,584 (2,146-2,934)	2,017 (1,690-2,283)	1,742 (1,478-1,997)	1,008 (876-1,178)	505 (438-596)	191 (167-231)	65 (57-75)
Unintentional injuries	13,560 (10,255-15,721)	10,959 (8,064-14,299)	113,368 (92,792-136,714)	295,418 (227,668-348,486)	126,390 (112,058-137,827)	92,044 (85,217-98,016)	88,467 (82,845-92,542)	97,077 (90,886-101,460)	83,828 (78,443-88,401)	80,351 (74,780-84,034)	78,805 (73,472-82,822)	66,740 (61,961-70,687)	64,191 (59,545-67,978)	69,521 (64,056-74,806)	68,783 (63,617-73,091)	72,046 (65,916-77,192)	70,632 (62,901-76,082)	68,119 (59,898-73,917)	73,056 (64,364-79,130)	65,357 (56,903-70,918)	49,462 (42,017-54,030)	23,005 (19,353-25,198)	7,499 (6,304-8,181)
Falls	2,773 (1,614-3,817)	1,229 (747-1,658)	14,113 (9,119-18,603)	26,010 (16,165-35,273)	10,740 (8,603-12,982)	8,840 (7,363-10,202)	10,192 (8,990-11,346)	13,473 (11,905-15,091)	12,995 (11,509-14,428)	14,056 (12,521-15,437)	15,597 (13,951-17,274)	14,496 (13,087-16,431)	15,094 (13,415-17,059)	17,585 (15,960-19,587)	20,701 (18,580-23,169)	24,574 (21,859-27,252)	28,780 (24,261-32,186)	31,611 (26,738-35,485)	39,404 (33,440-43,782)	38,407 (32,312-42,528)	31,649 (26,798-35,092)	15,209 (12,432-16,708)	4,832 (4,014-5,323)
Drowning	765 (606-863)	461 (340-538)	14,741 (11,496-16,816)	166,283 (125,982-192,571)	72,196 (61,510-79,755)	48,783 (44,771-52,095)	36,769 (33,847-39,223)	30,033 (28,036-32,386)	20,938 (19,544-22,371)	18,134 (16,945-19,281)	16,016 (14,919-17,031)	12,328 (11,429-13,085)	10,340 (9,618-11,011)	10,908 (10,067-11,545)	9,474 (8,769-10,028)	9,022 (8,213-9,498)	7,809 (7,003-8,297)	6,872 (6,122-7,337)	5,511 (4,886-5,932)	3,649 (3,243-3,955)	1,827 (1,644-2,004)	517 (463-563)	118 (106-129)
Fire, heat, and hot substances	571 (426-646)	638 (376-788)	9,690 (5,977-11,486)	27,040 (14,284-34,952)	8,075 (5,888-9,396)	5,093 (3,882-5,728)	7,645 (5,719-8,639)	10,030 (7,613-11,242)	8,757 (6,925-9,753)	8,335 (6,748-9,155)	7,495 (6,217-8,182)	5,799 (4,935-6,362)	5,613 (4,826-6,130)	6,048 (5,284-6,586)	5,725 (4,969-6,276)	6,185 (5,430-6,788)	6,180 (5,284-6,870)	6,149 (5,223-6,800)	6,623 (5,634-7,285)	6,079 (4,991-6,772)	4,286 (3,483-4,752)	1,923 (1,518-2,127)	679 (528-749)
Poisonings	795 (336-1,406)	499 (271-755)	8,048 (3,614-13,015)	15,987 (8,653-23,733)	5,049 (3,710-6,360)	4,985 (3,806-6,462)	4,906 (3,751-6,523)	6,504 (4,865-8,891)	5,591 (4,199-7,564)	4,671 (3,626-6,238)	4,520 (3,435-5,873)	3,999 (3,129-5,125)	3,359 (2,580-4,290)	3,553 (2,843-4,611)	3,266 (2,590-4,277)	3,126 (2,447-3,910)	2,629 (2,072-3,384)	2,403 (1,909-3,070)	1,717 (1,397-2,196)	1,189 (984-1,567)	625 (528-800)	235 (196-299)	78 (62-96)
Exposure to mechanical forces	3,143 (2,283-3,909)	3,358 (2,147-5,298)	22,527 (17,915-29,548)	16,980 (10,572-22,977)	8,732 (7,342-10,722)	6,409 (5,861-7,675)	9,277 (8,633-10,835)	12,488 (11,526-14,619)	11,827 (10,901-13,625)	10,784 (10,030-12,295)	10,910 (10,011-12,747)	8,362 (7,568-9,723)	7,960 (7,235-8,980)	7,553 (6,760-8,598)	6,630 (6,025-7,424)	5,165 (4,625-5,789)	4,166 (3,735-4,663)	3,292 (2,954-3,641)	2,971 (2,707-3,264)	2,257 (2,028-2,466)	1,604 (1,413-1,758)	773 (672-853)	278 (245-312)
Unintentional firearm injuries	147 (87-175)	90 (59-115)	1,041 (708-1,322)	1,666 (1,016-2,326)	1,239 (1,008-1,454)	1,359 (1,148-1,553)	2,479 (2,076-2,906)	3,392 (2,510-3,983)	3,149 (2,344-3,711)	2,493 (1,875-2,840)	2,054 (1,463-2,349)	1,501 (1,115-1,735)	1,458 (1,076-1,694)	1,315 (869-1,601)	952 (729-1,099)	774 (643-917)	668 (551-783)	570 (461-659)	617 (521-711)	468 (394-537)	333 (264-384)	166 (133-192)	60 (48-70)
Unintentional suffocation	2,379 (1,616-3,101)	2,979 (1,811-4,849)	17,546 (13,601-24,519)	5,701 (3,564-9,369)	2,124 (1,702-3,398)	1,688 (1,419-2,549)	503 (429-718)	616 (525-894)	604 (514-847)	429 (360-603)	383 (326-512)	314 (262-429)	286 (245-376)	317 (265-427)	325 (273-447)	137 (101-166)	151 (109-189)	156 (115-201)	324 (262-458)	314 (259-481)	250 (203-380)	129 (106-203)	49 (41-84)
Other exposure to mechanical forces	616 (508-690)	288 (210-348)	3,940 (2,985-4,798)	9,612 (5,722-12,864)	5,369 (4,422-6,371)	3,362 (3,021-3,937)	6,295 (5,642-7,903)	8,481 (7,648-11,216)	8,074 (7,266-10,061)	7,862 (7,177-9,754)	8,473 (7,718-10,568)	6,548 (5,900-8,029)	6,215 (5,672-7,261)	5,922 (5,351-6,876)	5,353 (4,807-6,101)	4,254 (3,778-4,835)	3,347 (2,925-3,761)	2,566 (2,281-2,897)	2,030 (1,798-2,254)	1,474 (1,263-1,632)	1,021 (860-1,126)	479 (395-537)	168 (137-187)
Adverse effects of medical treatment	1,977 (1,260-2,316)	1,573 (846-2,049)	12,565 (6,288-16,481)	10,254 (3,834-15,775)	3,419 (2,148-4,304)	2,391 (1,746-2,738)	2,678 (2,034-3,049)	4,262 (3,229-4,801)	3,994 (3,064-4,470)	3,678 (2,803-4,116)	3,868 (3,008-4,341)	3,702 (2,871-4,186)	4,043 (3,129-4,554)	3,944 (3,044-4,478)	4,761 (3,714-5,422)	6,465 (4,998-7,441)	7,115 (5,409-8,196)	6,521 (5,082-7,505)	6,574 (5,149-7,469)	5,524 (4,108-6,362)	3,935 (2,859-4,548)	1,840 (1,277-2,160)	614 (420-723)
Animal contact	530 (379-628)	565 (335-716)	4,014 (2,593-4,930)	9,612 (5,720-13,927)	8,103 (5,339-10,218)	6,865 (4,637-8,450)	5,358 (3,661-6,492)	5,220 (3,671-6,166)	4,933 (3,549-5,850)	5,128 (3,480-6,190)	4,602 (3,354-5,527)	4,645 (3,232-5,535)	5,240 (3,846-6,204)	5,050 (3,439-6,035)	4,797 (3,509-5,650)	4,639 (3,222-5,522)	4,375 (3,004-5,262)	3,736 (2,473-4,582)	2,527 (1,749-3,052)	1,454 (1,004-1,765)	645 (462-786)	195 (145-232)	59 (46-68)
Venomous animal contact	416 (285-508)	453 (253-595)	3,145 (1,945-3,963)	7,742 (4,369-10,864)	6,803 (4,278-8,676)	6,091 (3,993-7,635)	4,498 (2,880-5,616)	4,214 (2,768-5,116)	4,039 (2,735-4,953)	4,421 (2,808-5,470)	3,879 (2,666-4,799)	3,987 (2,616-4,886)	4,460 (3,117-5,456)	4,230 (2,663-5,214)	3,988 (2,807-4,815)	3,917 (2,598-4,806)	3,734 (2,439-4,608)	3,185 (1,996-4,012)	2,055 (1,346-2,574)	1,192 (793-1,487)	506 (339-631)	142 (97-175)	38 (28-46)
Non-venomous animal contact	114 (84-151)	112 (76-171)	869 (600-1,408)	1,870 (942-4,462)	1,300 (961-2,019)	774 (575-1,079)	860 (639-1,119)	1,006 (765-1,327)	894 (673-1,177)	707 (557-933)	723 (554-942)	658 (496-852)	780 (596-1,025)	821 (630-1,049)	809 (622-1,042)	722 (563-896)	641 (494-788)	551 (426-689)	472 (358-576)	262 (196-330)	140 (105-174)	53 (40-65)	21 (16-25)
Foreign body	1,802 (1,105-2,189)	1,937 (1,092-2,775)	21,083 (12,649-30,323)	12,528 (6,257-22,209)	3,842 (2,622-5,024)	2,618 (1,952-3,037)	1,944 (1,512-2,211)	2,659 (2,087-3,020)	2,726 (2,180-3,092)	3,262 (2,617-3,734)	3,583 (2,866-4,109)	3,557 (2,830-4,090)	3,097 (2,499-3,537)	4,104 (3,305-4,939)	3,974 (3,247-4,651)	4,213 (3,485-4,858)	3,445 (2,832-3,858)	3,060 (2,469-3,394)	3,520 (2,793-3,925)	3,359 (2,589-3,782)	2,460 (1,825-2,826)	1,187 (846-1,402)	449 (319-531)
Pulmonary aspiration and foreign body in airway	1,617 (1,015-1,961)	1,787 (1,030-2,579)	18,105 (11,296-26,541)	9,517 (5,063-17,861)	2,876 (1,942-3,864)	2,014 (1,514-2,387)	1,364 (1,129-1,548)	1,913 (1,564-2,218)	2,049 (1,663-2,371)	2,455 (1,979-2,848)	2,712 (2,137-3,228)	2,833 (2,279-3,342)	2,710 (2,205-3,159)	3,660 (2,944-4,458)	3,545 (2,911-4,301)	3,799 (3,113-4,530)	3,132 (2,602-3,582)	2,711 (2,252-3,030)	3,141 (2,535-3,497)	3,035 (2,385-3,429)	2,213 (1,671-2,543)	1,076 (789-1,275)	411 (294-487)
Foreign body in other body part	186 (59-244)	149 (37-207)	2,978 (712-4,178)	3,011 (835-5,065)	967 (537-1,252)	604 (338-759)	580 (302-772)	746 (402-975)	676 (370-873)	807 (497-1,034)	871 (523-1,087)	724 (435-914)	387 (217-484)	444 (265-555)	429 (256-527)	413 (252-511)	313 (165-390)	349 (166-441)	379 (184-478)	324 (152-412)	246 (105-318)	111 (48-141)	38 (17-49)
Environmental heat and cold exposure	599 (295-738)	429 (174-582)	3,856 (1,677-4,862)	2,581 (1,295-3,441)	1,093 (695-1,323)	754 (494-873)	1,483 (914-1,770)	1,965 (1,161-2,410)	2,210 (1,350-2,881)	3,174 (1,897-4,213)	3,842 (2,484-5,144)	4,104 (2,660-5,875)	4,034 (2,621-5,772)	6,040 (4,051-9,880)	5,343 (3,620-8,120)	5,438 (3,792-8,464)	3,679 (2,507-4,967)	3,004 (1,988-3,716)	2,960 (1,952-3,719)	2,511 (1,614-3,077)	1,864 (1,143-2,269)	862 (493-1,062)	294 (151-355)
Other unintentional injuries	604 (535-704)	272 (219-318)	2,731 (2,144-3,363)	8,145 (5,213-10,633)	5,140 (4,325-6,040)	5,305 (4,699-6,017)	8,215 (7,401-9,606)	10,442 (9,358-12,592)	9,856 (8,835-11,640)	9,129 (8,305-10,567)	8,372 (7,595-9,571)	5,748 (5,275-6,553)	5,410 (4,925-6,112)	4,737 (4,305-5,344)	4,113 (3,722-4,679)	3,220 (2,919-3,610)	2,455 (2,180-2,823)	1,472 (1,335-1,730)	1,248 (1,123-1,447)	929 (818-1,098)	566 (497-680)	262 (227-314)	97 (82-117)
Self-harm and interpersonal violence	2,424 (1,825-2,826)	668 (495-806)	6,516 (4,835-7,923)	10,822 (8,145-14,393)	7,451 (5,908-8,973)	21,824 (19,465-24,194)	102,491 (95,185-108,998)	156,848 (143,941-164,425)	141,314 (128,605-148,793)	119,502 (108,150-126,640)	103,507 (93,661-110,143)	78,141 (71,618-85,673)	63,126 (57,459-68,850)	61,961 (56,758-68,288)	53,145 (48,448-57,821)	46,393 (41,612-49,744)	38,562 (34,218-40,941)	32,300 (28,316-34,211)	26,879 (23,405-28,430)	16,424 (14,124-17,410)	7,682 (6,493-8,165)	2,152 (1,840-2,287)	442 (392-469)

SR Table 5: Global deaths by cause and age group for both sexes (with 95% uncertainty intervals), 1990

Cause	Early Neonatal	Late Neonatal	Post Neonatal	1 to 4	5 to 9	10 to 14	15 to 19	20 to 24	25 to 29
Self-harm	-	-	-	-	-	13,094 (11,199-14,922)	69,647 (63,306-75,311)	105,355 (96,092-111,506)	92,631 (85,233-98,164)
Self-harm by firearm	-	-	-	-	-	639 (439-841)	5,442 (4,063-7,139)	8,548 (6,423-11,193)	7,889 (6,021-10,113)
Self-harm by other specified means	-	-	-	-	-	12,455 (10,676-14,204)	64,206 (58,050-69,621)	96,807 (87,194-102,459)	84,742 (76,810-89,826)
Interpersonal violence	2,424 (1,825-2,826)	668 (495-806)	6,516 (4,835-7,923)	10,822 (8,145-14,393)	7,451 (5,908-8,973)	8,730 (7,079-10,254)	32,844 (26,586-37,250)	51,493 (41,924-58,054)	48,683 (38,205-55,695)
Assault by firearm	170 (120-245)	58 (39-78)	696 (499-1,019)	1,042 (745-1,808)	982 (753-1,435)	2,087 (1,544-2,788)	13,586 (9,900-16,479)	21,715 (15,747-26,520)	20,586 (14,560-25,140)
Assault by sharp object	170 (126-246)	71 (41-94)	579 (375-750)	1,362 (925-1,928)	1,253 (877-1,527)	1,615 (1,183-1,944)	8,814 (6,581-10,856)	14,440 (10,737-18,035)	14,077 (10,257-17,643)
Assault by other means	2,085 (1,521-2,458)	538 (395-655)	5,241 (3,762-6,451)	8,418 (6,169-11,237)	5,216 (3,891-6,416)	5,028 (3,903-6,062)	10,443 (7,904-12,623)	15,338 (11,345-18,188)	14,020 (10,181-16,799)
Forces of nature, conflict and terrorism, and executions and police conflict	1,177 (1,019-1,332)	737 (618-860)	3,061 (2,321-3,813)	17,514 (13,569-21,478)	12,547 (8,813-16,382)	11,867 (8,004-15,796)	17,659 (13,967-21,321)	23,683 (19,832-27,626)	17,325 (14,168-20,358)
Forces of nature, conflict and terrorism, and state actor violence	47 (17-77)	130 (47-213)	1,079 (390-1,763)	5,477 (1,929-8,977)	5,568 (1,935-9,174)	5,856 (2,096-9,573)	5,105 (1,925-8,252)	4,354 (1,701-6,973)	3,880 (1,524-6,213)
Conflict and terrorism	1,130 (974-1,279)	607 (523-687)	1,916 (1,650-2,171)	11,924 (10,233-13,552)	6,860 (5,879-7,788)	5,897 (5,040-6,708)	11,829 (10,109-13,456)	16,588 (14,132-18,944)	11,056 (9,384-12,632)
Executions and police conflict	-	-	65 (51-94)	113 (72-181)	119 (93-143)	115 (90-143)	725 (265-979)	2,742 (790-3,737)	2,389 (637-3,271)

Cause	30 to 34	35 to 39	40 to 44	45 to 49	50 to 54	55 to 59	60 to 64	65 to 69	70 to 74
Self-harm	77,301 (71,420-82,712)	69,947 (63,225-75,888)	54,353 (50,657-60,671)	46,226 (42,580-50,481)	47,582 (43,747-52,792)	42,536 (38,253-46,776)	38,012 (33,809-40,999)	32,699 (28,474-34,839)	28,120 (24,195-29,959)
Self-harm by firearm	6,981 (5,443-8,957)	5,812 (4,676-7,611)	4,783 (4,018-6,681)	3,961 (3,335-5,584)	3,727 (3,211-5,313)	3,316 (2,817-4,522)	3,010 (2,400-3,993)	2,741 (2,112-3,411)	2,300 (1,706-2,642)
Self-harm by other specified means	70,321 (64,383-75,425)	64,136 (57,205-68,983)	49,570 (45,596-55,212)	42,266 (38,525-46,007)	43,856 (39,865-48,315)	39,220 (34,845-42,828)	35,001 (30,634-37,650)	29,958 (25,866-31,936)	25,820 (22,205-27,526)
Interpersonal violence	42,200 (32,768-49,027)	33,560 (25,551-39,118)	23,788 (17,924-28,905)	16,899 (13,006-20,937)	14,379 (11,155-18,649)	10,609 (8,060-13,569)	8,381 (6,583-10,599)	5,863 (4,677-7,200)	4,180 (3,400-5,079)
Assault by firearm	16,431 (11,087-20,683)	12,881 (8,676-16,126)	8,617 (5,707-11,409)	5,703 (3,635-7,691)	4,298 (2,746-6,118)	3,018 (2,008-4,321)	2,149 (1,428-2,941)	1,426 (927-1,971)	974 (675-1,353)
Assault by sharp object	12,378 (8,801-16,137)	9,678 (6,742-12,629)	6,815 (4,912-9,025)	4,834 (3,403-6,408)	3,957 (2,778-5,449)	2,838 (1,900-4,064)	2,365 (1,675-3,249)	1,515 (1,085-2,033)	1,090 (794-1,388)
Assault by other means	13,391 (9,632-16,245)	11,000 (7,903-13,274)	8,357 (6,122-10,495)	6,362 (4,815-8,247)	6,124 (4,612-8,102)	4,753 (3,633-6,142)	3,867 (3,053-5,172)	2,922 (2,352-3,682)	2,115 (1,693-2,638)
Forces of nature, conflict and terrorism, and executions and police conflict	12,977 (10,462-15,452)	9,785 (7,730-11,911)	6,435 (5,025-7,863)	4,904 (3,769-6,046)	4,165 (3,054-5,273)	3,671 (2,591-4,774)	3,191 (2,158-4,251)	2,674 (1,800-3,567)	1,922 (1,307-2,537)
Forces of nature, conflict and terrorism, and state actor violence	3,301 (1,287-5,300)	2,871 (1,124-4,607)	2,145 (865-3,428)	1,759 (723-2,798)	1,790 (723-2,863)	1,719 (668-2,766)	1,642 (628-2,647)	1,382 (520-2,233)	1,007 (409-1,612)
Conflict and terrorism	7,952 (6,741-9,085)	5,479 (4,663-6,250)	3,708 (3,158-4,223)	2,677 (2,282-3,042)	2,060 (1,754-2,342)	1,776 (1,513-2,020)	1,411 (1,198-1,610)	1,195 (1,016-1,363)	841 (715-959)
Executions and police conflict	1,724 (535-2,251)	1,434 (426-1,940)	582 (236-781)	468 (166-614)	316 (112-400)	175 (92-220)	138 (58-175)	96 (40-123)	74 (31-101)

Cause	75 to 79	80 to 84	85 to 89	90 to 94	95 plus
Self-harm	23,606 (20,181-25,096)	15,347 (13,046-16,312)	7,208 (6,033-7,687)	2,002 (1,689-2,136)	376 (328-401)
Self-harm by firearm	2,092 (1,476-2,356)	1,462 (1,008-1,663)	712 (507-799)	240 (181-276)	56 (46-65)
Self-harm by other specified means	21,514 (18,402-22,908)	13,885 (11,841-14,782)	6,496 (5,489-6,941)	1,762 (1,490-1,881)	321 (277-343)
Interpersonal violence	3,273 (2,663-4,042)	1,077 (880-1,354)	475 (384-588)	150 (128-188)	66 (53-84)
Assault by firearm	669 (479-945)	142 (85-205)	61 (36-85)	18 (12-26)	11 (6-15)
Assault by sharp object	814 (598-1,065)	279 (222-389)	111 (86-153)	41 (33-58)	16 (12-22)
Assault by other means	1,791 (1,456-2,226)	656 (534-841)	303 (238-386)	91 (72-114)	39 (30-51)
Forces of nature, conflict and terrorism, and executions and police conflict	1,330 (929-1,729)	833 (595-1,065)	407 (280-536)	128 (88-168)	52 (40-65)
Forces of nature, conflict and terrorism, and state actor violence	683 (295-1,075)	407 (182-634)	211 (90-335)	66 (28-105)	18 (7-28)
Conflict and terrorism	612 (520-700)	380 (322-435)	164 (137-190)	45 (37-52)	29 (24-34)
Executions and police conflict	34 (20-44)	46 (19-66)	32 (9-53)	16 (7-26)	5 (2-9)

SR Table 5: Global deaths by cause and age group for both sexes (with 95% uncertainty intervals), 1995

Cause	Early Neonatal	Late Neonatal	Post Neonatal	1 to 4	5 to 9	10 to 14	15 to 19	20 to 24	25 to 29	30 to 34	35 to 39
All causes	2,775,373 (2,718,155-2,836,381)	990,722 (969,624-1,012,586)	3,090,166 (3,016,087-3,161,682)	3,017,594 (2,934,023-3,097,419)	767,703 (760,188-774,695)	495,414 (491,209-499,646)	698,616 (690,366-706,489)	983,575 (971,224-997,362)	1,097,120 (1,079,250-1,116,716)	1,183,050 (1,165,570-1,201,595)	1,290,428 (1,270,697-1,310,969)
Communicable, maternal, neonatal, and nutritional disorders	2,544,700 (2,478,572-2,616,302)	884,189 (856,865-913,865)	2,590,368 (2,495,824-2,687,642)	2,425,525 (2,313,393-2,550,391)	438,640 (414,331-466,692)	216,857 (202,103-233,384)	220,267 (207,391-235,177)	315,548 (301,622-331,913)	387,712 (369,166-409,046)	416,087 (398,860-435,476)	403,496 (386,827-423,891)
HIV/AIDS and tuberculosis	-	-	78,827 (72,924-84,969)	76,962 (65,045-87,332)	23,634 (20,593-26,815)	18,312 (16,282-20,366)	44,639 (41,699-48,000)	115,954 (108,002-124,965)	203,157 (186,548-220,485)	242,524 (228,345-259,045)	236,291 (222,181-253,560)
Tuberculosis	-	-	23,652 (19,655-27,779)	40,575 (29,849-49,963)	15,599 (12,802-18,577)	16,378 (14,353-18,371)	37,104 (34,170-40,561)	65,874 (61,486-70,590)	86,353 (81,210-93,293)	100,731 (95,372-109,356)	110,905 (104,061-122,291)
Drug-susceptible tuberculosis	-	-	22,817 (18,920-26,817)	39,231 (28,817-48,170)	14,951 (12,231-17,781)	15,704 (13,695-17,660)	35,567 (32,760-38,911)	62,910 (58,783-67,670)	82,440 (77,340-89,485)	96,149 (90,716-104,324)	105,783 (99,323-116,776)
Multidrug-resistant tuberculosis without extensive drug resistance	-	-	828 (615-1,067)	1,332 (921-1,783)	641 (496-823)	667 (527-855)	1,517 (1,211-1,902)	2,908 (2,334-3,629)	3,834 (3,107-4,812)	4,475 (3,627-5,528)	4,974 (4,073-6,145)
Extensively drug-resistant tuberculosis	-	-	7 (6-10)	12 (9-16)	7 (5-9)	7 (5-9)	20 (15-25)	57 (41-75)	79 (57-104)	108 (80-145)	148 (108-201)
HIV/AIDS	-	-	55,175 (51,261-59,687)	36,386 (33,160-40,249)	8,034 (7,204-9,122)	1,935 (1,641-2,341)	7,535 (6,534-8,870)	50,079 (43,540-57,957)	116,805 (102,122-132,939)	141,793 (128,089-156,648)	125,386 (112,983-139,546)
Drug-susceptible HIV/AIDS - Tuberculosis	-	-	18,792 (14,444-22,120)	12,938 (10,201-15,269)	2,676 (2,113-3,141)	581 (441-732)	2,392 (1,864-2,963)	14,159 (10,267-18,227)	27,276 (18,994-36,895)	30,698 (21,275-41,893)	28,254 (19,490-37,777)
Multidrug-resistant HIV/AIDS - Tuberculosis without extensive drug resistance	-	-	581 (367-881)	378 (242-570)	74 (45-115)	16 (10-25)	95 (62-139)	501 (323-731)	931 (598-1,402)	1,071 (684-1,623)	990 (642-1,498)
Extensively drug-resistant HIV/AIDS - Tuberculosis	-	-	3 (2-4)	2 (1-2)	1 (1-2)	0 (0-0)	1 (1-1)	4 (2-5)	9 (6-15)	18 (10-30)	20 (11-33)
HIV/AIDS resulting in other diseases	-	-	35,799 (31,910-41,351)	23,070 (20,301-27,260)	5,283 (4,614-6,294)	1,337 (1,132-1,676)	5,047 (4,174-6,287)	35,417 (29,199-43,642)	88,589 (73,354-106,024)	110,006 (94,874-126,847)	96,122 (82,151-112,240)
Diarrhoea, lower respiratory infections, and other common infectious diseases	348,702 (296,808-391,565)	389,953 (338,675-424,072)	1,918,287 (1,813,451-2,041,485)	1,627,610 (1,451,071-1,861,286)	305,738 (265,199-353,694)	144,526 (124,515-167,399)	101,851 (85,926-121,924)	87,364 (74,102-107,891)	79,778 (67,660-99,256)	78,127 (65,867-96,398)	82,156 (69,492-104,344)
Diarrhoeal diseases	50,717 (44,917-57,312)	100,840 (88,607-112,945)	653,008 (574,035-718,546)	465,696 (374,329-552,145)	60,072 (33,584-98,247)	30,540 (17,708-51,763)	31,061 (18,288-51,347)	32,194 (19,812-53,833)	30,476 (18,975-50,578)	30,431 (18,820-51,143)	32,719 (20,215-57,181)
Intestinal infectious diseases	-	-	2,362 (475-7,933)	34,643 (16,223-64,980)	67,023 (36,700-109,381)	39,880 (21,400-63,051)	21,164 (10,684-36,788)	10,546 (5,180-18,376)	7,851 (4,100-13,386)	6,224 (3,322-10,544)	4,890 (2,665-8,077)
Typhoid fever	-	-	188 (86-370)	30,067 (12,818-58,491)	59,083 (31,626-98,377)	33,959 (17,477-54,053)	18,508 (9,215-32,749)	8,983 (4,391-16,081)	6,329 (3,202-10,970)	4,792 (2,457-8,248)	3,699 (1,959-6,349)
Paratyphoid fever	-	-	27 (11-59)	2,910 (1,124-6,358)	7,766 (3,505-15,468)	5,857 (2,684-11,068)	2,602 (1,087-5,209)	1,507 (640-3,012)	1,474 (634-2,924)	1,362 (597-2,724)	1,116 (529-2,090)
Other intestinal infectious diseases	-	-	2,147 (309-7,598)	1,665 (185-6,308)	173 (22-646)	65 (9-257)	54 (7-201)	57 (8-222)	48 (7-198)	70 (10-245)	76 (11-311)
Lower respiratory infections	215,027 (192,148-242,242)	206,934 (187,342-226,714)	840,707 (753,504-922,825)	462,462 (375,960-543,292)	53,641 (44,028-63,171)	24,382 (20,439-27,433)	18,876 (16,145-21,502)	20,822 (17,782-23,640)	21,971 (19,026-25,296)	23,220 (20,277-26,332)	27,259 (23,729-30,485)
Upper respiratory infections	119 (83-171)	53 (33-83)	1,059 (755-1,477)	883 (586-1,310)	71 (46-103)	45 (30-63)	93 (69-121)	97 (72-127)	105 (79-137)	102 (76-131)	109 (84-139)
Otitis media	11 (8-15)	52 (34-86)	428 (259-720)	733 (364-1,348)	444 (273-772)	364 (243-560)	160 (111-240)	103 (70-148)	113 (76-187)	94 (63-153)	95 (62-142)
Meningitis	11,016 (7,917-14,291)	17,756 (14,085-21,127)	102,587 (79,452-120,686)	96,485 (67,232-120,710)	24,484 (17,826-30,883)	15,685 (11,835-19,452)	13,217 (10,142-16,838)	11,322 (8,531-14,298)	9,714 (7,332-12,561)	8,882 (6,573-11,791)	8,586 (6,383-11,306)
Pneumococcal meningitis	536 (355-768)	850 (627-1,142)	5,316 (3,887-6,917)	5,150 (3,345-7,449)	1,326 (883-1,894)	837 (555-1,192)	696 (466-1,003)	582 (378-835)	585 (393-840)	674 (442-961)	785 (512-1,144)
H influenzae type B meningitis	1,452 (989-1,943)	2,254 (1,715-2,793)	12,302 (9,158-15,310)	10,587 (7,127-14,315)	2,149 (1,460-2,896)	1,150 (791-1,543)	853 (583-1,175)	722 (500-1,001)	667 (462-951)	646 (427-909)	639 (461-865)
Meningococcal infection	5,211 (3,680-6,961)	8,379 (6,704-10,199)	48,223 (37,817-58,620)	48,666 (34,387-62,130)	12,886 (9,175-16,523)	8,103 (5,862-10,228)	6,882 (5,120-8,982)	5,775 (4,244-7,456)	4,735 (3,374-6,186)	4,062 (2,903-5,485)	3,797 (2,766-5,028)
Other meningitis	3,817 (2,705-5,038)	6,273 (4,757-7,710)	36,746 (27,417-45,036)	32,082 (21,043-42,070)	8,123 (5,824-10,670)	5,596 (4,104-7,233)	4,786 (3,590-6,176)	4,242 (3,129-5,541)	3,726 (2,732-4,772)	3,500 (2,519-4,606)	3,364 (2,443-4,378)
Encephalitis	955 (541-1,270)	1,771 (978-2,504)	17,136 (9,774-23,855)	22,168 (11,703-35,603)	9,012 (5,847-12,377)	6,058 (4,245-8,183)	4,009 (2,957-5,092)	3,725 (2,796-4,867)	3,155 (2,307-3,913)	3,246 (2,489-4,090)	3,084 (2,407-3,873)
Diphtheria	-	-	1,911 (1,196-2,820)	4,224 (2,499-6,833)	675 (424-991)	292 (195-419)	138 (94-198)	84 (56-123)	74 (50-107)	66 (45-96)	94 (65-130)
Whooping cough	-	-	70,348 (32,772-136,497)	69,442 (32,297-132,604)	7,788 (3,353-16,322)	1,528 (664-3,188)	599 (261-1,237)	437 (187-892)	264 (109-555)	195 (80-411)	139 (56-297)
Tetanus	70,726 (33,649-104,551)	62,112 (30,626-86,808)	19,744 (10,371-32,487)	8,656 (4,363-13,826)	4,133 (1,939-6,441)	3,851 (1,803-6,726)	4,610 (2,296-7,521)	4,034 (1,994-6,716)	3,930 (2,158-6,079)	4,216 (2,500-6,556)	4,224 (2,470-6,347)
Measles	-	-	205,783 (79,264-426,681)	458,227 (194,560-872,431)	76,550 (30,553-151,158)	21,313 (7,604-46,562)	7,671 (2,639-17,996)	3,690 (1,239-8,740)	1,748 (594-4,197)	1,043 (351-2,488)	631 (211-1,501)
Varicella and herpes zoster	129 (107-154)	435 (362-516)	3,214 (2,652-3,870)	3,991 (3,075-5,029)	1,846 (1,468-2,295)	588 (482-707)	251 (209-301)	308 (261-362)	377 (319-446)	408 (348-481)	327 (276-383)
Neglected tropical diseases and malaria	1,085 (668-1,418)	23,871 (17,914-30,593)	215,025 (164,504-269,609)	453,360 (343,012-561,396)	71,754 (51,743-89,951)	37,837 (28,759-46,380)	20,013 (15,781-24,073)	20,464 (16,334-24,579)	15,422 (12,543-19,146)	14,890 (12,052-18,351)	16,010 (12,870-19,620)
Malaria	-	22,956 (17,058-29,654)	203,780 (154,836-259,397)	419,986 (312,144-526,707)	52,033 (36,114-68,764)	25,981 (18,719-33,549)	10,440 (7,783-13,281)	10,389 (7,751-13,300)	7,785 (5,917-9,850)	7,140 (5,309-9,109)	8,059 (5,916-10,645)
Chagas disease	-	-	5 (5-6)	3 (3-4)	4 (3-4)	9 (8-9)	23 (20-26)	53 (45-60)	100 (87-114)	197 (172-223)	316 (282-350)
Leishmaniasis	-	-	1,992 (1,035-3,618)	3,032 (1,453-5,500)	1,578 (810-2,919)	1,039 (541-1,820)	1,308 (694-2,300)	1,515 (819-2,698)	1,299 (701-2,214)	1,177 (631-2,032)	1,356 (727-2,375)
Visceral leishmaniasis	-	-	1,992 (1,035-3,618)	3,032 (1,453-5,500)	1,578 (810-2,919)	1,039 (541-1,820)	1,308 (694-2,300)	1,515 (819-2,698)	1,299 (701-2,214)	1,177 (631-2,032)	1,356 (727-2,375)
African trypanosomiasis	-	-	-	736 (171-2,119)	1,907 (577-4,439)	2,087 (706-4,296)	2,508 (876-5,033)	2,628 (846-5,711)	2,338 (788-5,102)	2,151 (699-4,899)	1,867 (575-4,568)
Schistosomiasis	-	-	0 (0-0)	768 (496-1,139)	345 (235-492)	449 (324-603)	401 (300-525)	529 (406-681)	620 (476-802)	678 (525-866)	810 (636-1,054)
Cysticercosis	0 (0-0)	0 (0-0)	0 (0-0)	158 (100-232)	96 (71-130)	74 (58-92)	63 (50-80)	86 (69-108)	115 (94-139)	133 (111-159)	106 (87-128)
Cystic echinococcosis	-	-	-	480 (313-725)	180 (121-259)	103 (70-149)	49 (35-67)	64 (46-88)	90 (66-120)	90 (66-118)	114 (88-148)
Dengue	-	-	1,329 (441-2,539)	2,328 (893-4,112)	1,350 (434-2,396)	764 (228-1,413)	616 (151-1,145)	571 (125-1,079)	445 (103-859)	343 (77-697)	339 (78-629)
Yellow fever	-	5 (1-15)	83 (17-259)	749 (150-2,284)	1,449 (298-4,373)	1,352 (289-3,967)	1,240 (247-3,633)	1,034 (212-3,061)	772 (163-2,243)	601 (125-1,825)	481 (93-1,470)
Rabies	-	-	1,062 (665-1,958)	5,186 (2,920-8,066)	9,478 (4,619-14,577)	4,932 (2,226-6,862)	2,775 (1,663-3,852)	3,002 (1,617-4,126)	1,271 (811-1,853)	1,844 (1,079-2,704)	1,988 (1,124-2,748)
Intestinal nematode infections	-	-	827 (643-1,056)	9,116 (6,888-11,748)	1,432 (1,108-1,802)	274 (219-339)	86 (70-105)	59 (47-73)	47 (38-59)	59 (47-73)	53 (43-65)
Ascariasis	-	-	827 (643-1,056)	9,116 (6,888-11,748)	1,432 (1,108-1,802)	274 (219-339)	86 (70-105)	59 (47-73)	47 (38-59)	59 (47-73)	53 (43-65)
Ebola	0 (0-0)	0 (0-0)	9 (6-11)	12 (9-15)	12 (9-15)	6 (4-8)	15 (11-18)	35 (26-45)	58 (43-74)	48 (36-61)	78 (58-99)
Zika virus	0 (0-0)	0 (0-0)	0 (0-0)	0 (0-0)	0 (0-0)	0 (0-0)	0 (0-0)	0 (0-0)	0 (0-0)	0 (0-0)	0 (0-0)
Other neglected tropical diseases	1,085 (668-1,418)	910 (609-1,262)	5,938 (3,962-8,539)	10,806 (5,014-21,226)	1,889 (1,151-3,035)	768 (507-1,096)	488 (307-689)	499 (314-665)	482 (311-612)	428 (257-536)	443 (270-534)
Maternal disorders	-	-	-	-	-	1,668 (1,395-1,976)	38,146 (34,248-41,472)	73,897 (68,065-79,185)	71,693 (66,133-76,330)	62,539 (57,694-66,554)	51,026 (47,007-54,706)
Maternal haemorrhage	-	-	-	-	-	350 (236-498)	10,870 (7,365-14,521)	25,270 (19,289-31,558)	26,103 (19,254-33,230)	22,557 (16,759-28,160)	17,989 (13,235-23,276)
Maternal sepsis and other pregnancy related infections	-	-	-	-	-	191 (121-276)	4,711 (2,965-7,138)	7,384 (4,561-11,167)	6,603 (4,405-9,449)	5,596 (3,566-8,050)	4,577 (2,993-6,704)
Maternal hypertensive disorders	-	-	-	-	-	293 (178-427)	5,184 (3,237-7,300)	8,907 (6,015-12,527)	8,024 (5,113-11,703)	6,913 (4,337-10,313)	5,817 (3,847-8,358)
Maternal obstructed labour and uterine rupture	-	-	-	-	-	56 (29-102)	1,076 (539-1,840)	2,212 (1,260-3,516)	2,461 (1,415-3,943)	2,507 (1,408-3,895)	2,451 (1,450-4,112)
Maternal abortion, miscarriage, and ectopic pregnancy	-	-	-	-	-	126 (75-200)	2,787 (1,697-4,279)	5,412 (3,420-8,151)	5,140 (3,354-7,803)	4,829 (3,098-7,118)	4,381 (2,642-6,486)
Indirect maternal deaths	-	-	-	-	-	244 (153-363)	4,925 (3,062-7,253)	9,640 (6,335-14,041)	9,876 (6,349-14,306)	8,581 (5,209-12,611)	6,384 (4,090-9,185)
Late maternal deaths	-	-	-	-	-	40 (17-77)	723 (366-1,301)	1,286 (615-2,406)	1,245 (614-2,349)	1,098 (570-1,946)	891 (440-1,571)
Maternal deaths aggravated by HIV/AIDS	-	-	-	-	-	0 (0-0)	73 (41-105)	291 (172-396)	397 (243-533)	363 (219-497)	273 (165-378)
Other maternal disorders	-	-	-	-	-	369 (248-548)	7,796 (5,197-11,374)	13,495 (8,754-19,396)	11,845 (7,934-16,710)	10,096 (6,496-14,401)	8,264 (5,452-12,389)
Neonatal disorders	2,136,632 (2,061,472-2,238,358)	444,808 (410,109-510,297)	119,360 (102,136-149,166)	11,971 (9,352-16,855)	-	-	-	-	-	-	-
Neonatal preterm birth complications	906,218 (852,780-983,825)	146,998 (129,691-185,311)	48,480 (40,861-64,157)	3,371 (2,562-5,017)	-	-	-	-	-	-	-
Neonatal encephalopathy due to birth asphyxia and trauma	648,581 (597,394-716,343)	66,771 (59,217-78,600)	20,990 (17,353-26,000)	4,468 (3,464-6,178)	-	-	-	-	-	-	-
Neonatal sepsis and other neonatal infections	149,874 (106,157-190,446)	105,681 (81,232-136,319)	16,973 (11,985-24,285)	1,001 (346-1,851)	-	-	-	-	-	-	-
Hemolytic disease and other neonatal jaundice	66,121 (54,284-80,733)	42,974 (35,825-51,761)	8,312 (6,612-10,351)	520 (376-721)	-	-	-	-	-	-	-
Other neonatal disorders	365,839 (321,779-412,799)	82,384 (68,742-111,930)	24,605 (19,891-34,717)	2,612 (1,845-4,628)	-	-	-	-	-	-	-
Nutritional deficiencies	-	-	169,392 (136,105-202,203)	190,834 (145,964-238,674)	16,020 (12,962-19,200)	6,430 (5,387-7,514)	5,009 (4,283-5,892)	5,790 (4,833-6,831)	5,949 (4,911-7,101)	5,724 (4,778-6,757)	5,982 (4,743-7,318)
Protein-energy malnutrition	-	-	164,249 (132,260-196,658)	184,192 (140,172-232,199)	13,807 (11,005-16,701)	5,271 (4,311-6,329)	3,519 (2,918-4,212)	3,845 (3,008-4,755)	4,164 (3,197-5,189)	3,914 (3,068-4,816)	4,242 (3,054-5,411)
Iodine deficiency	-	-	456 (149-1,126)	387 (81-1,139)	135 (48-339)	33 (10-81)	64 (22-154)	73 (22-183)	79 (27-187)	82 (29-201)	76 (27-201)
Iron-deficiency anaemia	-	-	230 (57-341)	139 (82-226)	184 (85-264)	154 (82-226)	178 (104-247)	177 (110-238)	155 (98-194)	163 (107-197)	162 (101-210)
Other nutritional deficiencies	-	-	4,457 (3,163-5,550)	6,115 (2,815-9,072)	1,893 (1,420-2,462)	972 (784-1,209)	1,247 (1,040-1,487)	1,694 (1,442-2,039)	1,551 (1,307-1,860)	1,564 (1,318-1,911)	1,503 (1,265-1,828)
Other communicable, maternal, neonatal, and nutritional diseases	58,280 (35,484-89,573)	25,558 (15,826-37,081)	89,476 (60,741-125,476)	64,789 (44,654-92,287)	21,496 (15,820-29,465)	8,082 (7,016-9,166)	10,610 (9,729-11,487)	12,080 (11,297-12,904)	11,713 (10,988-12,553)	12,284 (11,507-13,109)	12,031 (11,354-12,790)
Sexually transmitted diseases excluding HIV	51,460 (29,083-82,911)	20,305 (11,523-32,520)	57,516 (31,757-92,597)	32,438 (16,898-54,049)	10,874 (5,884-18,156)	216 (140-301)	795 (526-1,005)	925 (624-1,130)	1,163 (780-1,463)	1,131 (750-1,411)	1,004 (704-1,235)
Syphilis	51,460 (29,083-82,911)	20,305 (11,523-32,520)	57,516 (31,757-92,597)	32,438 (16,898-54,049)	10,874 (5,884-18,156)	151 (97-220)	328 (209-440)	357 (232-463)	403 (245-568)	507 (287-710)	329 (204-484)
Chlamydial infection	-	-	-	-	-	14 (8-19)	79 (54-101)	164 (111-207)	151 (104-190)	74 (51-91)	118 (84-142)
Gonococcal infection	-	-	-	-	-	34 (23-46)	216 (145-271)	251 (169-315)	429 (295-530)	363 (254-439)	341 (242-407)
Other sexually transmitted diseases	-	-	-	-	-	17 (11-23)	171 (116-216)	153 (103-192)	180 (124-225)	187 (132-227)	216 (153-257)
Hepatitis	-	-	14,330 (12,564-16,178)	18,054 (13,998-21,824)	7,214 (5,875-8,549)	5,764 (4,961-6,585)	7,532 (6,829-8,266)	9,173 (8,505-9,897)	8,779 (8,153-9,497)	9,182 (8,589-9,890)	9,089 (8,519-9,739)
Acute hepatitis A	-	-	9,024 (6,894-11,129)	2,012 (1,234-2,914)	169 (113-236)	86 (57-120)	118 (77-166)	146 (83-217)	110 (60-186)	91 (50-147)	87 (48-138)
Hepatitis B	-	-	5,306 (3,594-7,329)	2,474 (1,436-3,919)	700 (385-1,092)	919 (502-1,418)	2,683 (1,921-3,658)	5,661 (4,385-7,006)	6,852 (5,781-7,838)	7,993 (7,181-8,787)	8,228 (7,483-8,949)
Hepatitis C	-	-	-	-	198 (130-286)	68 (44-99)	85 (56-122)	100 (57-156)	79 (42-131)	72 (39-114)	83 (43-139)
Acute hepatitis E	-	-	-	13,568 (10,246-16,759)	6,147 (4,949-7,348)	4,691 (3,913-5,508)	4,646 (3,735-5,476)	3,266 (2,111-4,443)	1,738 (1,019-2,595)	1,026 (584-1,586)	690 (374-1,154)
Other infectious diseases	6,820 (2,460-12,356)	5,253 (2,296-7,886)	17,631 (7,224-28,834)	14,297 (6,769-27,529)	3,407 (2,619-4,257)	2,103 (1,689-2,528)	2,283 (1,896-2,664)	1,982 (1,628-2,295)	1,770 (1,466-1,981)	1,970 (1,626-2,223)	1,938 (1,649-2,185)
Non-communicable diseases	212,267 (161,870-254,689)	94,309 (72,726-115,384)	376,631 (313,496-436,499)	266,159 (208,538-314,886)	124,614 (112,693-136,184)	115,711 (108,018-122,766)	174,816 (167,390-180,832)	239,149 (232,485-245,365)	303,254 (294,885-311,673)	410,382 (401,569-419,675)	566,847 (554,887-580,241)
Neoplasms	978 (859-1,036)	648 (590-694)	8,156 (7,331-9,167)	28,949 (24,830-34,981)	35,202 (32,094-38,487)	32,091 (29,843-34,162)	40,041 (38,004-41,613)	49,968 (48,299-51,860)	67,939 (65,897-70,353)	104,053 (100,860-107,274)	157,036 (152,491-161,851)
Lip and oral cavity cancer	-	-	-	-	-	-	338 (312-368)	637 (592-695)	1,041 (961-1,145)	1,835 (1,706-1,986)	2,961 (2,777-3,164)
Nasopharynx cancer	-	-	-	-	241 (210-272)	379 (337-418)	631 (580-676)	832 (764-895)	1,204 (1,107-1,321)	1,757 (1,627-1,901)	2,475 (2,331-2,624)
Other pharynx cancer	-	-	-	-	-	-	275 (241-312)	388 (344-442)	549 (495-614)	981 (891-1,097)	1,773 (1,632-1,966)
Oesophageal cancer	-	-	-	-	-	-	380 (355-410)	752 (715-798)	1,105 (1,046-1,179)	2,121 (2,017-2,243)	4,167 (3,993-4,375)
Stomach cancer	-	-	-	-	-	-	1,271 (1,209-1,347)	3,172 (3,028-3,340)	6,226 (5,965-6,542)	11,682 (11,255-12,166)	18,001 (17,340-18,704)
Colon and rectum cancer	-	-	-	-	-	-	1,027 (940-1,127)	2,383 (2,205-2,586)	3,921 (3,621-4,237)	6,900 (6,431-7,391)	9,765 (9,213-10,392)
Liver cancer	-	-	-	-	763 (686-870)	920 (849-1,006)	1,567 (1,453-1,703)	3,662 (3,350-4,038)	7,640 (7,070-8,314)	13,946 (12,945-15,048)	21,832 (20,294-23,369)
Liver cancer due to hepatitis B	-	-	-	-	247 (175-329)	395 (300-496)	787 (620-962)	2,193 (1,850-2,565)	4,915 (4,030-5,724)	8,968 (7,476-10,283)	13,575 (11,192-15,518)
Liver cancer due to hepatitis C	-	-	-	-	17 (13-22)	27 (21-35)	48 (36-61)	129 (99-165)	298 (207-418)	612 (460-793)	1,115 (837-1,462)
Liver cancer due to alcohol use	-	-	-	-	-	-	6 (3-11)	48 (29-72)	151 (84-245)	515 (350-753)	1,371 (896-2,047)
Liver cancer due to other causes	-	-	-	-	499 (416-601)	498 (404-590)	725 (575-896)	1,293 (1,051-1,568)	2,276 (1,772-2,996)	3,851 (3,079-4,933)	5,771 (4,637-7,411)
Gallbladder and biliary tract cancer	-	-	-	-	-	-	90 (83-106)	161 (147-191)	319 (287-379)	787 (714-928)	1,522 (1,380-1,784)
Pancreatic cancer	-	-	-	-	-	-	159 (152-171)	341 (322-365)	643 (610-689)	1,396 (1,335-1,474)	2,739 (2,630-2,870)
Larynx cancer	-	-	-	-	-	-	169 (157-182)	253 (240-271)	354 (332-378)	719 (675-767)	1,495 (1,414-1,577)
Tracheal, bronchus, and lung cancer	-	-	-	-	-	-	962 (891-1,041)	2,012 (1,873-2,202)	4,016 (3,718-4,350)	7,847 (7,368-8,383)	14,576 (13,886-15,395)
Malignant skin melanoma	-	-	-	-	-	-	228 (200-259)	433 (384-505)	700 (612-783)	1,171 (1,016-1,296)	1,755 (1,549-1,964)
Non-melanoma skin cancer	-	-	-	-	-	-	176 (164-187)	257 (238-277)	352 (326-382)	501 (471-532)	639 (599-675)
Non-melanoma skin cancer (squamous-cell carcinoma)	-	-	-	-	-	-	176 (164-187)	257 (238-277)	352 (326-382)	501 (471-532)	639 (599-675)
Breast cancer	-	-	-	-	-	-	324 (237-384)	951 (755-1,136)	3,130 (2,550-3,657)	9,006 (7,782-10,250)	18,304 (16,385-20,658)
Cervical cancer	-	-	-	-	-	-	243 (185-279)	950 (747-1,119)	2,791 (2,405-3,266)	6,626 (5,710-7,801)	11,970 (10,332-14,043)
Uterine cancer	-	-	-	-	-	-	99 (90-109)	289 (253-317)	543 (477-598)	843 (750-920)	1,451 (1,283-1,568)
Ovarian cancer	-	-	-	-	-	-	485 (428-546)	717 (623-816)	1,031 (904-1,163)	1,631 (1,482-1,819)	2,859 (2,609-3,187)
Prostate cancer	-	-	-	-	-	-	65 (53-76)	83 (66-97)	100 (80-119)	127 (101-149)	190 (151-218)
Testicular cancer	-	-	-	-	-	-	501 (463-549)	1,038 (968-1,135)	1,274 (1,193-1,386)	1,273 (1,200-1,364)	1,043 (984-1,109)
Kidney cancer	34 (30-36)	18 (16-20)	248 (213-300)	1,152 (923-1,476)	651 (588-733)	274 (254-299)	199 (188-213)	266 (252-287)	380 (361-409)	563 (540-597)	1,039 (996-1,095)
Bladder cancer	-	-	-	-	-	-	116 (108-125)	199 (186-214)	296 (275-316)	554 (517-594)	964 (902-1,031)
Brain and nervous system cancer	138 (88-164)	92 (61-111)	1,407 (849-1,796)	5,275 (3,782-6,567)	7,732 (6,015-8,612)	5,921 (4,912-6,452)	4,252 (3,517-4,621)	4,455 (3,658-4,846)	5,414 (4,469-5,854)	6,449 (5,432-6,915)	7,790 (6,572-8,344)
Thyroid cancer	-	-	-	-	-	159 (129-186)	324 (265-378)	506 (428-567)	618 (547-679)	703 (620-775)	853 (754-939)
Mesothelioma	-	-	-	-	-	-	98 (82-117)	129 (102-157)	207 (163-248)	227 (184-268)	373 (314-435)
Hodgkin lymphoma	19 (9-23)	6 (3-8)	147 (66-188)	691 (337-888)	1,793 (1,007-2,378)	1,815 (1,191-2,164)	2,003 (1,455-2,332)	2,107 (1,604-2,376)	2,202 (1,676-2,451)	2,174 (1,691-2,415)	2,113 (1,656-2,332)
Non-Hodgkin's lymphoma	59 (48-64)	44 (38-51)	625 (505-770)	2,609 (2,084-3,412)	3,457 (2,828-4,040)	3,058 (2,606-3,411)	3,090 (2,731-3,333)	3,518 (3,222-3,898)	3,639 (3,377-3,992)	4,175 (3,755-4,493)	4,855 (4,415-5,191)
Multiple myeloma	-	-	-	-	-	-	223 (185-253)	304 (240-338)	313 (256-348)	459 (388-524)	766 (654-868)
Leukaemia	261 (214-288)	217 (186-251)	3,659 (2,933-4,445)	12,938 (10,491-17,702)	15,373 (13,610-18,680)	13,648 (12,250-15,793)	12,756 (11,532-14,368)	11,904 (10,489-13,501)	10,869 (9,495-12,040)	9,853 (8,799-10,973)	9,926 (8,886-10,980)
Acute lymphoid leukaemia	61 (53-81)	51 (41-73)	846 (626-1,339)	3,956 (2,970-6,453)	5,408 (4,569-7,316)	4,654 (4,110-6,026)	3,410 (3,057-4,329)	2,394 (2,158-3,056)	1,894 (1,676-2,554)	1,516 (1,356-1,964)	1,474 (1,331-1,861)
Chronic lymphoid leukaemia	-	-	-	-	-	-	255 (216-354)	281 (225-425)	280 (226-429)	270 (220-399)	325 (266-444)
Acute myeloid leukaemia	38 (29-46)	34 (27-43)	461 (360-655)	1,939 (1,487-2,966)	1,925 (1,562-2,473)	2,035 (1,764-2,431)	2,094 (1,853-2,475)	1,852 (1,633-2,174)	1,742 (1,541-2,044)	1,780 (1,603-2,061)	2,012 (1,814-2,295)
Chronic myeloid leukaemia	-	-	-	-	-	-	545 (450-659)	692 (579-825)	1,025 (870-1,207)	1,146 (986-1,322)	1,254 (1,095-1,412)
Other leukaemia	162 (118-185)	132 (102-154)	2,352 (1,656-2,941)	7,043 (5,377-9,336)	8,041 (6,792-9,384)	6,959 (5,811-7,789)	6,452 (5,295-7,125)	6,684 (5,443-7,449)	5,928 (4,745-6,613)	5,141 (4,227-5,703)	4,861 (4,011-5,306)
Other neoplasms	469 (417-505)	272 (245-311)	2,069 (1,764-2,632)	6,283 (5,197-8,787)	5,194 (4,677-5,957)	5,917 (5,478-6,518)	7,994 (7,574-8,465)	7,268 (6,905-7,643)	7,063 (6,740-7,455)	7,744 (7,395-8,157)	8,841 (8,463-9,276)
Cardiovascular diseases	5,614 (5,223-6,020)	3,618 (3,288-3,960)	32,495 (29,518-35,532)	27,469 (23,576-31,180)	16,443 (14,846-18,049)	17,654 (16,409-18,892)	41,675 (39,673-43,943)	67,710 (64,994-71,236)	93,557 (89,957-98,033)	135,331 (130,442-141,550)	198,148 (191,269-205,836)
Rheumatic heart disease	-	-	-	6,895 (5,426-8,555)	4,467 (3,808-5,207)	6,541 (5,789-7,284)	10,700 (9,685-11,766)	11,773 (10,560-12,988)	13,017 (11,514-14,533)	14,273 (12,501-16,065)	16,255 (14,314-18,112)
Ischaemic heart disease	-	-	2,047 (1,719-2,526)	1,705 (1,341-2,343)	1,163 (1,002-1,390)	1,174 (1,050-1,333)	12,107 (11,353-13,035)	22,719 (21,685-24,159)	36,081 (34,584-38,161)	61,141 (58,447-64,133)	94,400 (90,440-98,971)
Cerebrovascular disease	2,676 (2,380-3,000)	1,647 (1,427-1,943)	13,335 (11,377-16,133)	8,569 (6,992-11,039)	5,488 (4,880-6,257)	5,113 (4,716-5,610)	9,817 (9,280-10,623)	19,919 (18,942-21,205)	27,146 (25,798-29,021)	38,552 (36,820-40,875)	57,997 (55,580-61,188)

Cause	40 to 44	45 to 49	50 to 54	55 to 59	60 to 64	65 to 69	70 to 74	75 to 79	80 to 84	85 to 89	90 to 94	95 plus
All causes	1,515,838 (1,494,839-1,536,710)	1,675,868 (1,654,297-1,697,593)	1,933,480 (1,912,074-1,953,864)	2,648,322 (2,617,139-2,677,090)	3,399,630 (3,365,879-3,429,952)	4,177,284 (4,137,528-4,213,096)	4,613,349 (4,574,718-4,648,377)	4,366,860 (4,333,264-4,398,406)	4,149,779 (4,122,521-4,176,761)	2,941,456 (2,927,163-2,955,908)	1,371,834 (1,366,785-1,376,925)	434,987 (433,504-436,557)
Communicable, maternal, neonatal, and nutritional disorders	374,079 (359,274-393,270)	341,346 (324,901-363,178)	332,810 (313,647-359,856)	369,221 (343,710-407,210)	428,765 (391,368-489,520)	472,092 (425,978-543,585)	532,498 (472,829-621,815)	486,538 (432,718-565,411)	436,572 (388,080-502,329)	303,871 (270,536-345,685)	135,797 (121,084-152,261)	47,256 (42,649-52,386)
HIV/AIDS and tuberculosis	221,318 (210,159-234,524)	196,196 (185,762-209,205)	181,398 (171,528-195,064)	182,714 (171,921-196,965)	182,079 (169,764-198,543)	172,162 (159,530-190,306)	163,424 (149,827-183,348)	121,828 (111,524-136,500)	83,429 (74,385-96,517)	41,813 (37,178-48,936)	12,743 (11,338-15,135)	3,002 (2,621-3,595)
Tuberculosis	120,223 (113,275-131,423)	122,346 (115,022-134,024)	135,689 (126,365-149,169)	152,983 (142,228-167,735)	161,348 (149,078-178,014)	158,002 (145,510-176,023)	155,611 (141,947-175,408)	117,645 (107,527-132,316)	82,492 (73,439-95,690)	41,451 (36,790-48,589)	12,643 (11,243-15,032)	2,978 (2,596-3,571)
Drug-susceptible tuberculosis	114,321 (107,676-125,226)	116,537 (109,360-127,701)	129,535 (120,494-142,507)	145,688 (135,510-159,874)	153,341 (141,442-169,503)	150,054 (138,237-168,278)	147,921 (134,078-166,885)	111,609 (101,656-126,372)	78,274 (69,524-90,443)	39,407 (34,987-46,396)	12,044 (10,664-14,385)	2,841 (2,485-3,428)
Multidrug-resistant tuberculosis without extensive drug resistance	5,722 (4,663-7,004)	5,638 (4,578-6,946)	6,015 (4,862-7,467)	7,107 (5,744-8,692)	7,857 (6,363-9,722)	7,809 (6,279-9,685)	7,592 (6,078-9,633)	5,966 (4,741-7,514)	4,167 (3,248-5,438)	2,019 (1,562-2,686)	592 (449-806)	136 (101-193)
Extensively drug-resistant tuberculosis	180 (130-243)	170 (127-229)	139 (104-183)	189 (141-250)	150 (117-191)	139 (107-177)	98 (78-125)	70 (55-88)	51 (40-66)	25 (20-32)	7 (6-10)	2 (1-2)
HIV/AIDS	101,096 (92,666-110,968)	73,850 (66,369-81,979)	45,709 (41,804-50,191)	29,730 (26,833-33,153)	20,731 (18,575-23,205)	14,160 (12,574-16,093)	7,814 (6,855-8,927)	4,184 (3,648-4,750)	937 (790-1,184)	362 (307-459)	100 (85-128)	25 (22-30)
Drug-susceptible HIV/AIDS - Tuberculosis	24,539 (17,133-32,121)	19,690 (13,796-25,357)	13,803 (10,057-16,982)	9,309 (7,126-11,311)	6,706 (5,176-8,150)	4,657 (3,638-5,684)	2,674 (2,135-3,232)	1,451 (1,156-1,740)	308 (245-383)	115 (91-145)	31 (24-39)	6 (5-8)
Multidrug-resistant HIV/AIDS - Tuberculosis without extensive drug resistance	854 (555-1,280)	675 (434-1,006)	475 (300-711)	332 (214-496)	233 (151-343)	157 (102-231)	87 (56-130)	49 (31-74)	14 (9-21)	5 (3-8)	1 (1-2)	0 (0-0)
Extensively drug-resistant HIV/AIDS - Tuberculosis	13 (8-22)	8 (5-12)	4 (2-6)	3 (2-5)	2 (1-3)	1 (1-1)	0 (0-1)	0 (0-0)	0 (0-0)	0 (0-0)	0 (0-0)	0 (0-0)
HIV/AIDS resulting in other diseases	75,690 (64,921-87,455)	53,477 (45,825-63,730)	31,427 (27,427-37,234)	20,087 (17,464-23,840)	13,790 (11,947-16,321)	9,345 (8,072-11,215)	5,052 (4,269-6,126)	2,684 (2,282-3,224)	616 (505-838)	242 (200-335)	68 (56-91)	18 (16-22)
Diarrhoea, lower respiratory infections, and other common infectious diseases	86,058 (73,580-107,941)	95,401 (80,521-120,517)	113,062 (93,616-148,072)	147,290 (120,125-192,789)	203,419 (163,355-271,725)	254,848 (205,420-339,546)	325,482 (263,046-428,855)	323,556 (266,224-414,816)	320,383 (268,859-395,311)	236,594 (202,210-283,211)	109,199 (93,959-127,280)	38,239 (33,650-43,278)
Diarrhoeal diseases	33,431 (20,689-56,601)	38,843 (23,501-64,806)	49,746 (29,857-87,392)	68,487 (40,778-117,759)	101,553 (60,955-174,210)	129,628 (76,728-221,480)	170,255 (104,086-282,411)	163,209 (100,644-264,533)	151,787 (99,283-234,197)	100,688 (67,705-149,782)	40,539 (27,355-58,691)	11,496 (8,143-16,109)
Intestinal infectious diseases	4,025 (2,236-6,665)	3,210 (1,728-5,309)	2,649 (1,368-4,562)	2,140 (1,030-3,884)	1,872 (930-3,364)	1,628 (913-2,641)	1,259 (733-2,024)	849 (497-1,322)	479 (279-760)	235 (137-374)	73 (41-119)	19 (10-36)
Typhoid fever	2,992 (1,585-5,081)	2,345 (1,210-3,919)	1,973 (1,005-3,444)	1,630 (766-2,976)	1,475 (712-2,705)	1,336 (728-2,225)	1,042 (589-1,657)	683 (385-1,095)	381 (212-609)	175 (99-276)	52 (29-84)	11 (6-18)
Paratyphoid fever	969 (456-1,886)	792 (361-1,592)	646 (281-1,292)	480 (201-981)	360 (151-729)	248 (115-485)	167 (80-318)	105 (51-193)	60 (29-110)	28 (13-51)	8 (4-16)	2 (1-4)
Other intestinal infectious diseases	63 (8-213)	74 (10-292)	30 (5-116)	30 (5-116)	37 (5-139)	43 (7-177)	49 (8-197)	61 (10-233)	38 (7-146)	33 (5-121)	13 (2-41)	6 (1-22)
Lower respiratory infections	31,513 (27,651-35,441)	35,932 (31,714-40,388)	43,196 (37,504-48,030)	58,322 (50,767-65,686)	82,465 (70,173-91,649)	104,661 (89,075-114,884)	135,838 (118,461-150,129)	145,478 (127,010-162,344)	156,905 (137,245-172,886)	129,452 (115,708-143,866)	66,255 (58,896-74,589)	25,987 (22,729-29,130)
Upper respiratory infections	115 (87-152)	122 (93-157)	119 (92-154)	139 (106-182)	158 (120-201)	143 (110-183)	117 (90-152)	110 (84-141)	73 (56-92)	48 (37-61)	18 (14-24)	4 (3-6)
Otitis media	153 (103-246)	210 (115-347)	214 (117-359)	206 (141-316)	167 (114-242)	252 (167-366)	199 (139-283)	138 (98-191)	108 (75-150)	55 (39-75)	24 (16-33)	7 (5-9)
Meningitis	7,723 (5,886-10,066)	8,261 (6,330-10,927)	8,062 (6,201-10,620)	8,675 (6,796-11,109)	8,595 (6,663-11,311)	8,887 (6,936-11,315)	8,497 (6,681-10,603)	5,760 (4,517-7,326)	4,854 (3,870-5,977)	2,502 (2,001-3,096)	857 (686-1,047)	239 (198-280)
Pneumococcal meningitis	816 (522-1,255)	952 (601-1,461)	914 (627-1,337)	995 (701-1,366)	1,036 (750-1,442)	1,058 (742-1,444)	1,035 (724-1,439)	714 (513-966)	575 (409-767)	294 (225-374)	107 (84-132)	31 (23-42)
H influenzae type B meningitis	573 (396-804)	621 (436-897)	600 (416-851)	644 (458-869)	644 (451-906)	637 (446-873)	597 (435-795)	411 (297-570)	348 (251-449)	178 (132-232)	59 (43-78)	16 (11-23)
Meningococcal infection	3,457 (2,535-4,695)	3,626 (2,709-4,903)	3,582 (2,685-4,781)	3,854 (2,895-5,013)	3,852 (2,905-5,153)	3,909 (2,922-5,164)	3,533 (2,653-4,636)	2,512 (1,839-3,274)	2,042 (1,520-2,567)	1,093 (827-1,370)	378 (287-477)	103 (77-129)
Other meningitis	2,876 (2,101-3,691)	3,062 (2,313-4,072)	2,966 (2,275-3,967)	3,182 (2,383-4,111)	3,062 (2,286-4,055)	3,283 (2,529-4,337)	3,332 (2,564-4,228)	2,123 (1,625-2,721)	1,889 (1,493-2,406)	937 (746-1,193)	313 (248-387)	89 (69-113)
Encephalitis	2,991 (2,297-3,754)	2,866 (2,294-3,685)	2,762 (2,186-3,546)	3,509 (2,790-4,421)	4,065 (3,182-5,184)	4,509 (3,499-5,549)	4,478 (3,310-5,452)	3,888 (2,779-4,609)	2,987 (2,125-3,607)	1,589 (1,153-1,867)	482 (356-558)	122 (93-140)
Diphtheria	95 (66-134)	104 (72-146)	116 (82-161)	92 (65-129)	-	-	-	-	-	-	-	-
Whooping cough	152 (60-327)	120 (48-254)	105 (42-228)	131 (54-280)	-	-	-	-	-	-	-	-
Tetanus	5,218 (2,917-8,226)	5,216 (3,114-8,436)	5,685 (3,258-9,800)	5,146 (2,963-8,435)	4,261 (2,445-7,353)	4,624 (2,777-8,796)	4,353 (2,377-7,713)	3,366 (1,822-5,916)	2,104 (1,090-3,469)	836 (432-1,255)	247 (131-370)	67 (36-105)
Measles	436 (148-1,047)	290 (100-693)	220 (75-522)	209 (71-495)	-	-	-	-	-	-	-	-
Varicella and herpes zoster	207 (176-245)	227 (192-268)	189 (159-224)	234 (196-275)	282 (236-334)	515 (437-605)	486 (412-576)	758 (645-889)	1,086 (909-1,288)	1,188 (987-1,463)	704 (574-905)	299 (232-405)
Neglected tropical diseases and malaria	15,487 (12,757-19,021)	15,081 (12,248-18,471)	15,168 (12,617-18,913)	15,295 (12,802-18,700)	17,107 (14,333-20,881)	16,038 (13,426-19,698)	13,538 (11,372-16,652)	10,232 (8,532-12,536)	5,337 (4,483-6,439)	3,228 (2,665-3,972)	677 (534-825)	300 (209-470)
Malaria	7,480 (5,686-9,637)	6,684 (5,045-8,713)	6,973 (5,294-9,075)	7,203 (5,480-9,303)	9,831 (7,459-13,018)	8,934 (6,677-12,184)	7,577 (5,610-10,343)	5,744 (4,226-7,727)	3,054 (2,287-4,086)	2,051 (1,510-2,763)	361 (235-483)	210 (124-376)
Chagas disease	461 (413-511)	615 (555-676)	772 (702-847)	922 (830-1,015)	1,085 (1,007-1,173)	1,133 (1,053-1,217)	1,055 (986-1,124)	708 (654-775)	511 (477-551)	271 (251-291)	85 (78-93)	20 (19-22)
Leishmaniasis	1,394 (731-2,481)	1,370 (713-2,375)	1,386 (751-2,383)	1,125 (582-2,011)	910 (475-1,603)	963 (504-1,697)	1,051 (555-1,839)	828 (443-1,455)	465 (239-805)	285 (146-496)	63 (29-110)	20 (6-37)
Visceral leishmaniasis	1,394 (731-2,481)	1,370 (713-2,375)	1,386 (751-2,383)	1,125 (582-2,011)	910 (475-1,603)	963 (504-1,697)	1,051 (555-1,839)	828 (443-1,455)	465 (239-805)	285 (146-496)	63 (29-110)	20 (6-37)
African trypanosomiasis	1,652 (493-4,108)	1,552 (386-4,093)	1,421 (398-3,861)	1,231 (317-3,664)	989 (247-2,796)	791 (219-2,364)	540 (136-1,775)	319 (70-951)	145 (35-444)	52 (11-180)	13 (3-43)	3 (0-10)
Schistosomiasis	908 (718-1,147)	987 (778-1,235)	1,154 (912-1,463)	1,352 (1,053-1,709)	1,455 (1,139-1,819)	1,488 (1,159-1,881)	1,313 (1,045-1,681)	930 (741-1,164)	448 (358-562)	188 (145-237)	46 (34-61)	8 (5-11)
Cysticercosis	104 (87-125)	97 (81-116)	111 (94-132)	109 (93-129)	87 (74-103)	77 (65-91)	48 (40-57)	29 (24-36)	16 (12-19)	7 (6-9)	2 (2-3)	1 (1-1)
Cystic echinococcosis	97 (73-126)	106 (79-138)	102 (78-129)	129 (100-162)	126 (99-159)	119 (94-149)	100 (79-125)	76 (61-95)	57 (45-70)	30 (24-37)	8 (6-11)	2 (1-3)
Dengue	305 (64-599)	308 (69-586)	313 (72-623)	363 (89-700)	346 (82-662)	330 (86-629)	136 (40-227)	107 (36-176)	83 (28-143)	54 (22-89)	20 (8-33)	4 (2-7)
Yellow fever	383 (78-1,215)	303 (62-965)	254 (49-793)	200 (39-616)	156 (30-482)	109 (23-322)	71 (14-225)	40 (8-122)	16 (3-51)	6 (1-18)	1 (0-4)	0 (0-1)
Rabies	2,115 (1,363-3,135)	2,490 (1,496-3,800)	2,078 (1,427-4,415)	2,019 (1,079-3,134)	1,464 (860-1,961)	1,458 (772-2,303)	1,045 (715-1,802)	979 (656-1,742)	262 (159-458)	144 (76-302)	22 (10-52)	10 (4-22)
Intestinal nematode infections	39 (31-48)	41 (32-50)	60 (48-73)	55 (44-67)	66 (53-81)	48 (39-60)	71 (58-86)	54 (44-66)	33 (25-40)	12 (9-15)	7 (5-10)	2 (2-3)
Ascariasis	39 (31-48)	41 (32-50)	60 (48-73)	55 (44-67)	66 (53-81)	48 (39-60)	71 (58-86)	54 (44-66)	33 (25-40)	12 (9-15)	7 (5-10)	2 (2-3)
Ebola	60 (44-76)	62 (46-79)	45 (33-57)	36 (26-45)	32 (24-41)	12 (9-15)	5 (3-6)	0 (0-0)	0 (0-0)	0 (0-0)	0 (0-0)	0 (0-0)
Zika virus	0 (0-0)	0 (0-0)	0 (0-0)	0 (0-0)	0 (0-0)	0 (0-0)	0 (0-0)	0 (0-0)	0 (0-0)	0 (0-0)	0 (0-0)	0 (0-0)
Other neglected tropical diseases	490 (294-595)	466 (287-570)	501 (320-613)	552 (344-680)	560 (362-703)	576 (378-731)	528 (343-671)	418 (279-542)	248 (176-341)	129 (93-184)	46 (33-65)	20 (14-29)
Maternal disorders	31,844 (29,093-34,451)	16,032 (14,215-18,009)	2,772 (2,270-3,411)	-	-	-	-	-	-	-	-	-
Maternal haemorrhage	10,176 (7,121-13,686)	4,051 (2,767-5,585)	521 (310-825)	-	-	-	-	-	-	-	-	-
Maternal sepsis and other pregnancy related infections	3,102 (1,967-4,711)	1,665 (1,048-2,573)	284 (170-455)	-	-	-	-	-	-	-	-	-
Maternal hypertensive disorders	3,562 (2,166-5,535)	1,669 (1,040-2,553)	274 (141-463)	-	-	-	-	-	-	-	-	-
Maternal obstructed labour and uterine rupture	1,754 (971-2,966)	1,003 (540-1,632)	166 (71-324)	-	-	-	-	-	-	-	-	-
Maternal abortion, miscarriage, and ectopic pregnancy	3,299 (2,229-4,715)	2,034 (1,332-2,971)	307 (191-472)	-	-	-	-	-	-	-	-	-
Indirect maternal deaths	3,954 (2,402-6,036)	2,415 (1,416-3,553)	567 (316-893)	-	-	-	-	-	-	-	-	-
Late maternal deaths	556 (214-988)	306 (124-541)	59 (22-125)	-	-	-	-	-	-	-	-	-
Maternal deaths aggravated by HIV/AIDS	148 (86-209)	71 (41-102)	10 (6-16)	-	-	-	-	-	-	-	-	-
Other maternal disorders	5,292 (3,259-7,893)	2,818 (1,767-4,145)	584 (362-855)	-	-	-	-	-	-	-	-	-
Neonatal disorders	-	-	-	-	-	-	-	-	-	-	-	-
Neonatal preterm birth complications	-	-	-	-	-	-	-	-	-	-	-	-
Neonatal encephalopathy due to birth asphyxia and trauma	-	-	-	-	-	-	-	-	-	-	-	-
Neonatal sepsis and other neonatal infections	-	-	-	-	-	-	-	-	-	-	-	-
Hemolytic disease and other neonatal jaundice	-	-	-	-	-	-	-	-	-	-	-	-
Other neonatal disorders	-	-	-	-	-	-	-	-	-	-	-	-
Nutritional deficiencies	6,273 (5,393-7,377)	6,465 (5,078-8,096)	8,358 (6,439-10,518)	10,529 (8,225-12,950)	12,367 (9,967-14,975)	15,156 (12,569-18,022)	17,670 (14,695-21,089)	20,871 (17,636-24,474)	20,787 (17,886-23,975)	18,692 (16,072-21,443)	11,860 (10,204-13,721)	5,312 (4,511-6,211)
Protein-energy malnutrition	4,490 (3,703-5,392)	4,413 (3,128-5,794)	5,942 (4,211-7,868)	7,926 (5,823-10,147)	9,387 (7,175-11,744)	11,239 (9,013-13,790)	13,963 (11,365-16,846)	17,071 (14,234-20,222)	17,300 (14,717-20,230)	15,824 (13,404-18,378)	10,299 (8,706-12,122)	4,669 (3,867-5,566)
Iodine deficiency	64 (23-145)	57 (19-154)	75 (25-190)	94 (36-231)	91 (36-214)	126 (50-253)	131 (60-276)	114 (52-240)	83 (36-162)	42 (15-96)	14 (3-40)	7 (1-24)
Iron-deficiency anaemia	160 (134-205)	133 (111-181)	131 (105-231)	147 (120-226)	170 (139-217)	213 (169-280)	321 (230-451)	310 (222-467)	260 (187-379)	159 (89-241)	86 (49-127)	63 (39-87)
Other nutritional deficiencies	1,560 (1,292-1,936)	1,863 (1,503-2,480)	2,210 (1,780-2,902)	2,362 (1,909-3,042)	2,719 (2,244-3,414)	3,578 (2,919-4,520)	3,255 (2,657-4,268)	3,376 (2,714-4,484)	3,144 (2,554-4,191)	2,667 (2,140-3,799)	1,461 (1,150-2,359)	574 (451-1,051)
Other communicable, maternal, neonatal, and nutritional diseases	13,099 (12,395-13,855)	12,171 (11,454-12,954)	12,052 (11,346-12,858)	13,393 (12,613-14,321)	13,794 (12,888-14,818)	13,888 (12,901-14,968)	12,383 (11,342-13,431)	10,049 (9,183-10,957)	6,635 (5,917-7,605)	3,544 (3,126-4,090)	1,318 (1,135-1,518)	402 (339-455)
Sexually transmitted diseases excluding HIV	976 (689-1,154)	883 (636-1,069)	865 (615-1,095)	1,229 (845-1,545)	828 (520-1,014)	761 (562-910)	651 (459-811)	681 (425-867)	406 (278-493)	249 (180-299)	112 (82-134)	43 (29-53)
Syphilis	309 (190-401)	282 (171-374)	363 (250-527)	588 (391-825)	370 (228-471)	370 (258-456)	374 (242-495)	368 (192-512)	206 (120-272)	112 (68-146)	45 (29-57)	17 (10-22)
Chlamydial infection	135 (96-162)	113 (82-135)	92 (61-114)	122 (79-153)	91 (55-116)	46 (32-57)	42 (31-51)	50 (35-61)	45 (33-53)	30 (23-36)	14 (11-17)	5 (4-6)
Gonococcal infection	349 (249-417)	310 (228-371)	274 (185-338)	337 (218-416)	227 (138-286)	261 (183-317)	173 (126-206)	203 (138-245)	120 (87-140)	81 (63-95)	40 (31-47)	15 (11-17)
Other sexually transmitted diseases	181 (130-216)	178 (131-212)	136 (92-167)	182 (119-222)	140 (85-176)	84 (61-102)	62 (45-73)	59 (39-72)	36 (26-42)	26 (19-30)	12 (9-14)	6 (4-7)
Hepatitis	10,365 (9,735-11,082)	9,385 (8,768-10,060)	8,990 (8,431-9,601)	9,667 (9,084-10,319)	9,637 (9,082-10,249)	9,335 (8,771-9,930)	8,007 (7,474-8,568)	6,203 (5,754-6,663)	3,671 (3,357-3,999)	1,658 (1,523-1,813)	469 (432-511)	100 (93-110)
Acute hepatitis A	109 (61-173)	107 (60-168)	109 (63-177)	123 (72-191)	141 (83-217)	145 (86-226)	138 (88-205)	115 (70-178)	74 (46-111)	32 (22-46)	9 (6-13)	2 (1-3)
Hepatitis B	9,475 (8,666-10,304)	8,567 (7,849-9,325)	8,151 (7,431-8,864)	8,702 (7,919-9,448)	8,560 (7,726-9,308)	8,151 (7,373-8,894)	6,834 (6,051-7,576)	5,143 (4,440-5,846)	2,712 (2,186-3,190)	1,010 (737-1,266)	230 (142-316)	40 (20-59)
Hepatitis C	116 (62-189)	139 (75-237)	185 (102-312)	266 (148-441)	306 (170-512)	276 (152-444)	221 (119-368)	169 (95-268)	103 (56-169)	49 (31-73)	15 (9-23)	3 (2-6)
Acute hepatitis E	666 (321-1,207)	572 (295-1,006)	544 (285-946)	575 (282-1,020)	630 (288-1,201)	763 (394-1,259)	814 (420-1,378)	776 (337-1,382)	783 (440-1,229)	566 (347-832)	215 (134-304)	55 (35-76)
Other infectious diseases	1,758 (1,508-1,998)	1,903 (1,634-2,165)	2,198 (1,867-2,583)	2,497 (2,122-2,844)	3,329 (2,784-4,000)	3,792 (3,149-4,551)	3,724 (3,049-4,488)	3,165 (2,576-3,799)	2,558 (2,046-3,348)	1,637 (1,303-2,107)	737 (579-919)	259 (200-309)
Non-communicable diseases	850,448 (833,936-865,390)	1,093,010 (1,072,475-1,111,499)	1,403,337 (1,378,065-1,423,817)	2,073,500 (2,034,188-2,104,874)	2,785,229 (2,727,321-2,826,104)	3,527,150 (3,454,490-3,576,340)	3,921,848 (3,836,636-3,983,609)	3,745,273 (3,664,031-3,800,904)	3,598,764 (3,530,644-3,650,586)	2,558,712 (2,518,899-2,594,208)	1,200,438 (1,183,755-1,215,698)	376,366 (371,254-381,325)
Neoplasms	260,193 (253,271-266,775)	349,491 (340,895-357,698)	444,586 (435,644-453,990)	639,403 (626,412-652,604)	800,802 (785,574-817,552)	919,901 (904,777-937,255)	873,257 (858,680-892,521)	672,407 (659,671-688,891)	501,757 (490,726-515,911)	281,281 (273,896-290,256)	103,428 (100,374-107,126)	25,250 (24,313-26,112)
Lip and oral cavity cancer	5,544 (5,239-5,884)	8,130 (7,763-8,564)	10,557 (10,053-11,129)	13,821 (13,189-14,480)	15,123 (14,501-15,810)	14,271 (13,691-14,879)	11,254 (10,758-11,743)	8,093 (7,747-8,428)	6,196 (5,929-6,472)	3,755 (3,585-3,921)	1,534 (1,465-1,603)	422 (402-441)
Nasopharynx cancer	4,307 (4,051-4,571)	4,997 (4,722-5,293)	5,792 (5,460-6,207)	7,033 (6,625-7,544)	6,865 (6,482-7,305)	5,860 (5,571-6,188)	4,464 (4,251-4,685)	2,681 (2,557-2,816)	1,617 (1,541-1,701)	669 (636-704)	226 (216-237)	52 (50-54)
Other pharynx cancer	3,753 (3,501-4,105)	6,010 (5,670-6,519)	8,262 (7,783-8,992)	10,623 (10,021-11,471)	11,253 (10,525-12,249)	10,155 (9,470-10,986)	7,780 (7,171-8,459)	4,422 (4,059-4,820)	2,798 (2,539-3,039)	1,274 (1,156-1,375)	388 (358-413)	79 (74-84)
Oesophageal cancer	10,662 (10,169-11,186)	18,521 (17,806-19,329)	27,117 (26,125-28,226)	42,614 (40,941-44,429)	57,021 (54,688-59,555)	62,225 (59,831-64,986)	56,379 (53,907-58,984)	41,503 (39,587-43,710)	24,717 (23,614-26,064)	11,441 (10,984-12,021)	3,534 (3,396-3,720)	721 (689-755)
Stomach cancer	31,055 (30,001-32,078)	41,311 (39,971-42,690)	52,823 (51,264-54,504)	82,504 (79,870-85,181)	110,611 (107,377-113,990)	126,745 (123,334-130,615)	120,221 (117,059-123,950)	93,232 (90,655-96,366)	66,539 (64,433-68,994)	35,174 (34,025-36,526)	12,152 (11,754-12,618)	2,813 (2,698-2,923)
Colon and rectum cancer	15,112 (14,420-15,908)	22,377 (21,431-23,444)	29,616 (28,580-30,858)	46,259 (44,617-48,035)	62,064 (60,295-64,052)	79,887 (77,603-82,209)	84,714 (82,512-87,159)	72,333 (70,462-74,276)	64,002 (62,196-65,803)	41,357 (39,963-42,846)	17,124 (16,571-17,752)	4,485 (4,306-4,645)
Liver cancer	37,086 (34,503-39,455)	43,934 (40,670-46,667)	50,207 (46,727-53,329)	64,500 (60,005-67,658)	76,139 (71,641-79,647)	76,918 (72,458-80,495)	65,249 (62,054-68,267)	46,271 (43,829-48,569)	28,215 (26,906-29,961)	12,839 (12,288-13,481)	3,790 (3,627-3,976)	757 (720-798)
Liver cancer due to hepatitis B	22,236 (18,202-25,400)	24,618 (20,075-28,489)	26,002 (20,644-30,252)	30,637 (23,618-36,019)	32,539 (24,175-38,601)	29,584 (21,967-35,052)	23,032 (17,060-27,613)	15,766 (11,697-19,311)	8,375 (5,945-10,646)	3,450 (2,601-4,308)	948 (696-1,223)	180 (115-246)
Liver cancer due to hepatitis C	2,359 (1,843-3,031)	4,048 (3,124-5,104)	6,359 (5,046-7,737)	10,195 (8,448-12,242)	14,527 (12,247-17,253)	15,950 (13,648-18,481)	14,750 (12,784-17,056)	11,437 (9,909-13,232)	8,620 (7,443-10,020)	4,595 (4,079-5,177)	1,532 (1,336-1,718)	338 (287-396)
Liver cancer due to alcohol use	3,114 (2,128-4,384)	4,531 (3,018-6,355)	6,123 (4,164-8,292)	9,096 (6,315-12,314)	12,461 (9,068-16,398)	15,429 (12,208-18,907)	14,400 (11,406-17,751)	9,712 (7,563-12,029)	5,852 (4,361-7,380)	2,558 (1,911-3,242)	686 (487-902)	120 (70-175)
Liver cancer due to other causes	9,378 (7,544-12,221)	10,737 (8,683-13,675)	11,723 (9,430-14,835)	14,572 (11,776-18,218)	16,612 (13,623-20,818)	15,955 (13,236-19,170)	13,066 (10,811-15,885)	9,356 (7,793-11,241)	5,368 (4,404-6,514)	2,236 (1,889-2,617)	625 (520-750)	119 (91-153)
Gallbladder and biliary tract cancer	2,920 (2,667-3,363)	4,537 (4,194-5,105)	6,754 (6,287-7,564)	10,167 (9,557-11,369)	13,977 (13,240-15,530)	17,392 (16,543-19,046)	18,248 (17,446-19,969)	15,414 (14,767-16,710)	13,180 (12,655-14,040)	7,742 (7,439-8,178)	2,878 (2,761-3,028)	697 (666-734)
Pancreatic cancer	5,509 (5,328-5,740)	8,886 (8,640-9,224)	13,191 (12,857-13,610)	21,646 (21,080-22,311)	29,648 (28,962-30,477)	37,347 (36,483-38,341)	38,306 (37,446-39,363)	30,558 (29,802-31,411)	23,932 (23,228-24,685)	14,022 (13,584-14,503)	5,190 (5,012-5,379)	1,229 (1,183-1,271)
Larynx cancer	3,651 (3,469-3,822)	6,341 (6,046-6,650)	9,126 (8,712-9,552)	13,619 (12,989-14,240)	15,575 (14,916-16,189)	16,214 (15,509-16,872)	12,415 (11,909-12,857)	7,728 (7,414-8,018)	5,021 (4,801-5,253)	2,257 (2,161-2,353)	662 (634-690)	126 (121-130)
Tracheal, bronchus, and lung cancer	31,969 (30,725-33,221)	52,368 (50,751-54,196)	76,769 (74,688-79,177)	127,486 (123,555-131,862)	172,743 (168,203-177,521)	209,018 (203,847-214,147)	190,004 (185,947-194,279)	136,855 (133,590-140,258)	87,192 (84,716-89,542)	40,381 (39,076-41,712)	11,797 (11,414-12,168)	2,399 (2,301-2,486)
Malignant skin melanoma	2,427 (2,152-2,660)	2,732 (2,385-2,988)	2,994 (2,665-3,332)	3,778 (3,400-4,234)	3,979 (3,633-4,530)	4,590 (4,173-5,406)	4,498 (4,013-5,341)	3,515 (3,161-4,140)	3,147 (2,789-3,867)	1,961 (1,724-2,407)	823 (711-1,040)	244 (207-314)
Non-melanoma skin cancer	979 (935-1,026)	1,212 (1,153-1,264)	1,504 (1,447-1,554)	2,211 (2,134-2,285)	3,413 (3,305-3,522)	4,105 (3,963-4,250)	4,260 (4,134-4,379)	3,718 (3,606-3,832)	3,693 (3,578-3,809)	2,854 (2,764-2,954)	1,514 (1,461-1,570)	609 (582-632)
Non-melanoma skin cancer (squamous-cell carcinoma)	979 (935-1,026)	1,212 (1,153-1,264)	1,504 (1,447-1,554)	2,211 (2,134-2,285)	3,413 (3,305-3,522)	4,105 (3,963-4,250)	4,260 (4,134-4,379)	3,718 (3,606-3,832)	3,693 (3,578-3,809)	2,854 (2,764-2,954)	1,514 (1,461-1,570)	609 (582-632)
Breast cancer	29,318 (26,727-32,588)	37,129 (33,976-40,834)	40,732 (37,864-44,619)	46,821 (43,801-51,228)	43,381 (40,978-47,584)	43,278 (41,169-47,026)	39,514 (37,686-42,618)	29,032 (27,755-31,982)	24,732 (23,643-26,467)	16,801 (16,114-17,837)	7,390 (7,064-7,803)	2,345 (2,240-2,463)
Cervical cancer	18,627 (16,097-22,242)	22,162 (19,096-26,803)	23,751 (20,250-29,034)	26,403 (22,769-32,106)	25,248 (22,109-30,304)	22,988 (20,459-27,319)	19,519 (17,581-22,885)	12,421 (11,442-13,959)	9,287 (8,670-10,536)	4,791 (4,494-5,366)	1,717 (1,623-1,867)	461 (435-496)
Uterine cancer	2,541 (2,259-2,745)	3,929 (3,546-4,217)	5,321 (4,811-5,711)	7,402 (6,761-7,925)	9,499 (8,789-10,148)	10,670 (9,962-11,322)	9,578 (9,049-10,118)	6,923 (6,575-7,290)	5,377 (5,152-5,633)	3,002 (2,876-3,133)	1,158 (1,106-1,215)	301 (284-317)
Ovarian cancer	5,162 (4,799-5,654)	8,026 (7,566-8,691)	10,513 (9,930-11,360)	13,073 (12,420-13,857)	14,311 (13,724-15,162)	15,309 (14,638-16,115)	14,600 (13,991-15,294)	9,998 (9,611-10,508)	7,812 (7,500-8,185)	4,387 (4,207-4,591)	1,632 (1,560-1,709)	384 (365-403)
Prostate cancer	438 (351-509)	1,204 (978-1,371)	3,148 (2,549-3,516)	8,019 (6,492-8,974)	17,668 (14,215-19,825)	30,267 (25,421-33,202)	41,148 (35,477-44,726)	43,205 (37,405-47,469)	39,948 (33,501-43,718)	26,826 (22,613-28,880)	11,187 (9,699-12,233)	2,571 (2,242-2,848)
Testicular cancer	742 (700-787)	535 (503-572)	410 (377-449)	361 (335-388)	404 (376-434)	414 (385-444)	361 (338-385)	266 (250-283)	224 (210-240)	110 (104-117)	39 (36-41)	10 (9-11)
Kidney cancer	1,965 (1,888-2,063)	3,366 (3,249-3,507)	4,901 (4,740-5,064)	7,896 (7,624-8,197)	9,821 (9,547-10,130)	11,872 (11,536-12,243)	11,550 (11,254-11,847)	8,764 (8,538-8,997)	7,174 (6,949-7,414)	4,116 (3,956-4,276)	1,465 (1,410-1,526)	321 (307-334)
Bladder cancer	1,835 (1,738-1,939)	2,967 (2,833-3,125)	4,655 (4,467-4,876)	8,183 (7,863-8,518)	13,105 (12,690-13,544)	18,746 (18,140-19,382)	21,429 (20,823-22,114)	19,533 (19,007-20,057)	18,082 (17,555-18,618)	11,656 (11,298-12,066)	4,679 (4,524-4,836)	1,159 (1,113-1,200)
Brain and nervous system cancer	9,981 (8,470-10,675)	10,762 (9,196-11,472)	11,737 (10,114-12,440)	15,225 (13,402-16,058)	16,377 (14,308-17,229)	16,488 (14,715-17,310)	13,073 (11,838-13,814)	8,562 (7,693-9,110)	4,977 (4,471-5,288)	2,135 (1,875-2,281)	583 (507-622)	115 (103-122)
Thyroid cancer	1,330 (1,191-1,455)	1,739 (1,577-1,896)	2,457 (2,227-2,667)	3,303 (3,022-3,554)	3,602 (3,353-3,861)	4,029 (3,772-4,293)	3,824 (3,595-4,075)	3,080 (2,927-3,286)	2,354 (2,252-2,543)	1,309 (1,253-1,418)	461 (439-494)	100 (95-106)
Mesothelioma	616 (533-716)	769 (690-873)	1,131 (1,029-1,253)	1,878 (1,710-2,054)	2,176 (1,986-2,410)	2,680 (2,460-3,018)	2,848 (2,620-3,215)	2,404 (2,214-2,641)	1,789 (1,658-1,976)	849 (791-936)	259 (243-284)	52 (49-56)
Hodgkin lymphoma	2,187 (1,635-2,443)	2,182 (1,662-2,461)	2,185 (1,673-2,462)	2,795 (2,148-3,153)	3,027 (2,253-3,413)	3,178 (2,447-3,549)	2,658 (2,115-2,923)	1,587 (1,264-1,763)	1,056 (859-1,157)	482 (403-526)	130 (113-142)	25 (21-27)
Non-Hodgkin's lymphoma	6,300 (5,633-6,779)	7,186 (6,510-7,658)	8,796 (7,935-9,372)	11,652 (10,701-12,586)	15,015 (14,197-16,153)	18,024 (16,956-19,457)	18,674 (17,846-20,104)	14,663 (14,070-15,867)	11,939 (11,303-12,936)	6,852 (6,543-7,321)	2,395 (2,287-2,596)	535 (507-587)
Multiple myeloma	1,503 (1,279-1,666)	2,156 (1,850-2,367)	3,386 (2,945-3,770)	5,232 (4,595-5,851)	7,190 (6,205-8,037)	9,173 (8,062-10,310)	9,419 (8,342-10,793)	7,696 (6,760-8,908)	6,447 (5,698-7,782)	3,758 (3,224-4,576)	1,276 (1,133-1,587)	247 (215-305)
Leukaemia	11,240 (10,015-12,126)	11,349 (10,324-12,378)	11,939 (11,004-12,957)	15,665 (14,581-16,924)	18,732 (17,906-20,605)	22,066 (21,217-24,084)	22,302 (21,537-24,138)	17,905 (17,334-19,255)	14,621 (14,139-15,672)	8,950 (8,612-9,544)	3,578 (3,446-3,767)	945 (904-994)
Acute lymphoid leukaemia	1,525 (1,378-1,829)	1,498 (1,352-1,826)	1,527 (1,373-1,768)	2,013 (1,822-2,337)	2,044 (1,870-2,405)	2,088 (1,923-2,457)	1,865 (1,733-2,185)	1,052 (971-1,243)	722 (674-841)	380 (356-429)	137 (128-152)	35 (33-39)
Chronic lymphoid leukaemia	455 (389-587)	539 (480-683)	853 (769-1,010)	1,534 (1,385-1,838)	2,424 (2,224-2,813)	3,420 (3,162-3,927)	3,764 (3,477-4,360)	3,296 (3,025-3,696)	3,210 (2,927-3,628)	2,297 (2,093-2,574)	978 (915-1,070)	280 (262-300)
Acute myeloid leukaemia	2,281 (2,089-2,586)	2,573 (2,387-2,935)	2,785 (2,601-3,157)	3,627 (3,394-4,145)	4,297 (4,024-4,944)	5,287 (4,974-6,033)	5,443 (5,141-6,258)	4,204 (3,956-4,856)	3,106 (2,915-3,669)	1,622 (1,516-1,915)	567 (528-668)	128 (120-150)
Chronic myeloid leukaemia	1,472 (1,299-1,649)	1,611 (1,451-1,790)	1,676 (1,506-1,850)	2,085 (1,895-2,283)	2,236 (2,045-2,425)	2,613 (2,364-2,808)	2,582 (2,381-2,755)	2,217 (2,056-2,353)	1,983 (1,851-2,117)	1,219 (1,137-1,303)	511 (473-545)	137 (124-145)
Other leukaemia	5,506 (4,592-6,008)	5,128 (4,309-5,578)	5,097 (4,407-5,574)	6,407 (5,639-6,993)	7,732 (7,089-8,664)	8,658 (8,011-9,549)	8,647 (8,082-9,560)	7,136 (6,705-7,934)	5,601 (5,293-6,183)	3,433 (3,251-3,767)	1,386 (1,310-1,503)	365 (345-396)
Other neoplasms	11,437 (10,981-11,941)	12,674 (12,262-13,254)	14,813 (14,290-15,817)	19,233 (18,591-20,557)	22,835 (22,057-24,724)	25,991 (25,143-27,842)	24,968 (24,246-26,761)	20,043 (19,385-21,621)	15,686 (15,158-16,754)	9,531 (9,171-10,168)	3,868 (3,713-4,085)	1,048 (1,002-1,110)
Cardiovascular diseases	314,866 (306,016-325,076)	423,517 (411,400-436,675)	564,445 (550,984-580,705)	876,462 (854,909-899,310)	1,212,643 (1,184,606-1,243,310)	1,632,735 (1,594,448-1,677,165)	1,883,607 (1,837,302-1,937,555)	1,874,043 (1,828,582-1,929,778)	1,953,951 (1,904,090-2,016,388)	1,437,794 (1,400,829-1,483,949)	678,683 (659,426-700,588)	225,267 (216,792-232,326)
Rheumatic heart disease	20,949 (18,644-23,195)	20,951 (18,534-23,069)	23,614 (20,654-26,137)	30,122 (26,885-33,142)	34,813 (31,236-38,183)	39,156 (34,950-43,057)	40,405 (36,250-44,863)	35,170 (31,785-39,571)	28,508 (26,215-31,954)	17,661 (16,333-19,638)	7,673 (7,149-8,424)	2,278 (2,141-2,464)
Ischaemic heart disease	151,999 (145,896-158,164)	212,161 (204,477-219,987)	280,146 (271,432-289,246)	437,901 (424,213-451,061)	588,063 (572,120-604,121)	792,500 (771,265-813,508)	900,984 (877,178-928,487)	900,910 (876,862-929,684)	994,277 (973,008-1,025,001)	777,566 (761,446-803,657)	391,265 (382,367-404,081)	137,857 (134,543-142,351)
Cerebrovascular disease	99,548 (96,006-104,306)	139,475 (134,724-145,499)	197,978 (191,278-207,051)	312,809 (302,132-327,274)	462,411 (447,279-485,876)	630,010 (607,370-663,434)	730,458 (699,621-774,903)	711,321 (675,496-762,656)	682,746 (640,685-734,021)	442,002 (411,857-478,807)	179,219 (163,940-195,210)	53,286 (47,753-58,422)

SR Table 5: Global deaths by cause and age group for both sexes (with 95% uncertainty intervals), 1995

Cause	Early Neonatal	Late Neonatal	Post Neonatal	1 to 4	5 to 9	10 to 14	15 to 19	20 to 24	25 to 29	30 to 34	35 to 39	40 to 44	45 to 49	50 to 54	55 to 59	60 to 64	65 to 69	70 to 74	75 to 79	80 to 84	85 to 89	90 to 94	95 plus
Ischaemic stroke	-	-	608 (509-742)	472 (388-594)	407 (360-468)	365 (327-410)	1,411 (1,305-1,528)	2,999 (2,782-3,218)	4,039 (3,779-4,302)	5,397 (5,072-5,733)	8,055 (7,584-8,496)	14,956 (14,159-15,682)	27,262 (25,870-28,620)	44,023 (41,672-46,035)	83,813 (79,423-87,592)	178,243 (170,686-185,090)	274,990 (263,467-285,469)	333,624 (319,572-348,843)	380,633 (361,191-401,846)	404,452 (381,153-429,792)	283,203 (264,022-304,633)	118,864 (108,756-128,993)	36,776 (33,085-40,144)
Hemorrhagic stroke	2,676 (2,380-3,000)	1,647 (1,427-1,943)	12,727 (10,853-15,388)	8,097 (6,600-10,451)	5,080 (4,514-5,787)	4,748 (4,376-5,210)	8,406 (7,948-9,120)	16,919 (16,057-18,050)	23,106 (21,901-24,768)	33,155 (31,607-35,282)	49,942 (47,711-52,846)	84,591 (81,536-88,904)	112,213 (108,017-117,875)	153,956 (148,332-162,582)	228,997 (220,177-242,604)	284,168 (272,557-302,768)	355,020 (339,768-380,713)	396,834 (378,080-428,467)	330,688 (312,222-362,406)	278,294 (259,890-305,266)	158,799 (146,671-176,538)	60,355 (55,105-67,410)	16,510 (14,692-18,319)
Hypertensive heart disease	-	-	587 (400-721)	310 (203-389)	260 (174-316)	251 (170-312)	1,706 (1,287-1,995)	2,730 (2,085-3,172)	3,658 (2,810-4,216)	5,315 (4,180-6,104)	7,922 (6,351-9,078)	12,843 (10,382-14,549)	17,954 (14,081-20,411)	25,574 (20,418-28,981)	40,068 (32,727-45,045)	55,275 (43,747-61,743)	74,970 (59,649-84,443)	91,810 (72,938-102,280)	97,800 (77,660-108,718)	92,726 (75,206-105,879)	66,406 (53,440-78,759)	32,800 (26,642-37,730)	10,596 (8,020-12,666)
Cardiomyopathy and myocarditis	947 (681-1,112)	633 (484-757)	5,375 (4,254-6,784)	3,514 (2,517-4,629)	1,660 (1,386-2,086)	1,601 (1,429-1,963)	2,733 (2,471-3,347)	3,614 (3,256-4,260)	5,017 (4,385-5,891)	6,632 (5,734-7,542)	10,080 (8,463-11,602)	13,889 (11,427-16,140)	14,778 (12,350-17,126)	13,977 (11,778-15,818)	20,944 (18,028-23,809)	19,639 (16,816-21,896)	22,152 (18,774-23,941)	22,406 (16,839-24,522)	21,773 (15,613-24,099)	27,925 (17,377-32,271)	27,690 (15,535-32,911)	14,423 (7,904-17,269)	5,077 (2,738-6,043)
Myocarditis	211 (118-293)	180 (102-255)	2,164 (1,231-3,285)	1,547 (887-2,481)	770 (581-1,075)	613 (517-829)	1,070 (918-1,386)	1,285 (1,113-1,687)	1,477 (1,282-1,976)	1,123 (982-1,478)	1,261 (1,094-1,568)	1,497 (1,257-1,922)	1,052 (887-1,278)	986 (820-1,186)	1,378 (1,094-1,645)	1,280 (980-1,484)	1,553 (1,189-1,766)	1,961 (1,468-2,242)	2,206 (1,597-2,529)	3,104 (2,000-3,801)	3,550 (2,116-4,532)	2,160 (1,214-2,792)	906 (472-1,179)
Alcoholic cardiomyopathy	-	-	-	-	-	-	238 (191-337)	528 (389-663)	1,206 (880-1,468)	2,737 (2,052-3,358)	5,229 (3,991-6,434)	7,652 (5,901-9,378)	8,579 (6,796-10,362)	7,040 (5,740-8,535)	10,979 (9,152-13,246)	8,215 (6,956-10,149)	7,491 (6,471-9,600)	4,781 (3,972-5,747)	3,106 (2,544-3,592)	2,891 (2,091-3,434)	2,007 (1,265-2,477)	956 (565-1,203)	324 (201-401)
Other cardiomyopathy	736 (528-925)	453 (314-588)	3,211 (2,209-4,488)	1,966 (1,299-3,029)	890 (726-1,169)	988 (854-1,201)	1,425 (1,257-1,728)	1,800 (1,570-2,148)	2,333 (1,992-2,737)	2,771 (2,367-3,277)	3,590 (2,937-4,297)	4,740 (3,922-5,693)	5,147 (4,242-6,008)	5,951 (4,939-6,696)	8,587 (6,988-9,674)	10,144 (7,981-11,030)	13,108 (9,987-14,377)	15,663 (10,975-17,606)	16,461 (11,000-18,651)	21,931 (12,654-26,223)	22,133 (11,329-27,169)	11,307 (5,745-14,147)	3,847 (1,948-4,841)
Atrial fibrillation and flutter	-	-	-	-	-	-	-	-	-	30 (21-40)	112 (82-150)	264 (193-346)	454 (327-600)	712 (527-932)	1,344 (784-2,016)	2,781 (1,803-3,923)	5,737 (3,538-8,627)	10,746 (7,605-14,985)	14,976 (9,489-22,560)	31,841 (23,826-41,093)	35,122 (26,513-45,643)	17,413 (13,308-22,602)	4,781 (3,699-6,168)
Aortic aneurysm	-	-	-	-	-	-	384 (340-439)	539 (486-612)	807 (707-927)	1,063 (946-1,215)	1,590 (1,432-1,795)	2,157 (1,977-2,386)	2,847 (2,649-3,116)	3,836 (3,600-4,162)	6,313 (5,988-6,801)	9,590 (9,140-10,160)	14,431 (13,883-15,120)	18,580 (17,988-19,443)	18,631 (18,039-19,443)	18,147 (17,586-18,898)	12,335 (11,951-12,879)	5,336 (5,179-5,549)	1,396 (1,353-1,445)
Peripheral vascular disease	-	-	-	-	-	-	-	-	-	-	-	172 (111-210)	289 (178-363)	495 (317-608)	1,093 (690-1,358)	1,841 (1,244-2,294)	3,023 (2,110-3,766)	3,920 (2,762-5,080)	4,468 (3,160-6,041)	6,677 (4,644-10,072)	6,070 (4,061-9,618)	3,633 (2,253-5,829)	1,474 (876-2,374)
Endocarditis	151 (96-192)	96 (63-127)	2,014 (1,390-2,551)	893 (600-1,340)	423 (323-515)	508 (420-599)	967 (814-1,182)	1,354 (1,147-1,683)	1,777 (1,551-2,209)	1,824 (1,518-2,165)	2,193 (1,765-2,503)	2,682 (2,141-3,088)	2,321 (1,879-2,650)	2,501 (2,056-2,983)	3,198 (2,587-3,711)	4,068 (3,337-4,784)	5,095 (4,104-6,001)	5,735 (4,553-6,661)	6,028 (4,723-7,160)	6,478 (4,831-7,813)	5,051 (3,673-6,026)	2,733 (1,921-3,343)	935 (635-1,157)
Other cardiovascular and circulatory diseases	1,841 (1,520-2,127)	1,242 (991-1,447)	9,137 (7,463-11,389)	5,583 (4,269-7,474)	2,982 (2,209-3,501)	2,466 (1,914-2,843)	3,263 (2,635-3,867)	5,063 (4,074-6,017)	6,055 (4,872-7,164)	6,502 (5,380-7,536)	7,600 (6,192-8,820)	10,363 (8,255-11,762)	12,288 (9,943-13,716)	15,613 (12,606-17,617)	22,670 (17,966-25,280)	34,162 (26,520-37,756)	45,661 (35,524-50,000)	58,563 (44,342-63,763)	62,967 (47,465-68,602)	64,625 (52,578-69,705)	47,892 (41,013-51,121)	24,187 (21,564-25,646)	7,588 (7,119-8,192)
Chronic respiratory diseases	0 (0-0)	0 (0-0)	0 (0-0)	31,263 (25,344-36,950)	8,475 (7,292-9,604)	7,628 (6,902-8,424)	11,912 (11,087-12,895)	16,144 (15,261-17,314)	20,681 (19,543-22,048)	25,372 (23,836-26,912)	33,930 (31,746-35,630)	50,533 (46,569-52,713)	73,150 (65,551-76,382)	117,284 (104,209-122,932)	201,579 (179,736-210,956)	345,356 (307,457-361,856)	475,791 (426,724-496,978)	612,651 (547,725-639,916)	612,986 (547,839-639,880)	514,826 (453,149-538,933)	310,214 (275,140-325,404)	120,247 (105,398-126,713)	31,666 (28,268-33,265)
Chronic obstructive pulmonary disease	-	-	0 (0-0)	7,271 (4,938-12,531)	2,046 (1,426-2,849)	1,408 (1,062-1,824)	4,998 (4,221-5,839)	7,641 (6,545-8,677)	10,505 (9,044-11,813)	13,890 (11,902-15,568)	18,325 (15,843-20,811)	30,971 (26,845-34,804)	47,886 (40,691-53,543)	83,336 (71,198-92,551)	152,240 (131,743-166,452)	279,761 (244,234-302,828)	399,314 (352,982-426,573)	533,622 (471,907-564,626)	543,106 (479,896-571,805)	463,517 (407,703-488,524)	281,465 (247,790-297,080)	109,909 (96,286-116,124)	29,083 (25,837-30,603)
Pneumoconiosis	-	-	-	-	-	-	56 (45-73)	103 (82-149)	250 (178-396)	419 (309-652)	585 (478-896)	837 (681-1,313)	827 (677-1,329)	984 (809-1,493)	1,459 (1,231-2,157)	2,432 (2,074-3,633)	3,312 (2,903-4,562)	3,837 (3,384-5,041)	3,218 (2,869-4,119)	2,331 (2,130-2,726)	1,167 (1,068-1,350)	355 (323-414)	82 (75-93)
Silicosis	-	-	-	-	-	-	7 (5-10)	22 (16-32)	107 (67-175)	246 (174-393)	387 (306-596)	551 (432-963)	504 (392-888)	561 (439-983)	791 (631-1,323)	1,329 (1,092-2,124)	1,878 (1,561-2,801)	2,055 (1,741-2,815)	1,685 (1,447-2,199)	1,164 (1,008-1,444)	525 (451-632)	133 (113-158)	25 (22-29)
Asbestosis	-	-	-	-	-	-	22 (18-29)	34 (27-44)	40 (31-54)	44 (35-58)	45 (35-59)	56 (42-74)	53 (38-70)	71 (50-93)	118 (72-153)	173 (106-250)	247 (150-419)	312 (207-554)	291 (195-537)	243 (166-435)	136 (97-248)	44 (31-75)	9 (7-14)
Coal workers pneumoconiosis	-	-	-	-	-	-	4 (2-5)	6 (3-9)	28 (15-48)	29 (17-45)	42 (24-61)	80 (45-119)	102 (55-151)	136 (72-199)	212 (125-295)	379 (214-519)	594 (316-775)	804 (415-1,016)	665 (359-824)	540 (324-645)	277 (159-328)	78 (42-92)	16 (9-18)
Other pneumoconiosis	-	-	-	-	-	-	23 (18-33)	41 (30-68)	75 (44-143)	100 (64-179)	112 (79-188)	149 (105-235)	168 (117-273)	217 (148-357)	338 (239-533)	551 (381-877)	593 (421-926)	666 (475-1,058)	577 (409-892)	383 (291-575)	229 (180-339)	101 (81-146)	32 (27-43)
Asthma	-	-	-	20,189 (13,276-25,635)	5,045 (3,939-6,081)	5,274 (4,470-6,086)	6,047 (4,986-7,047)	7,325 (5,834-8,494)	8,798 (7,134-10,166)	9,552 (7,504-11,055)	13,064 (10,142-14,969)	16,210 (12,451-18,896)	21,444 (16,418-25,425)	29,076 (21,546-36,313)	42,041 (31,036-52,819)	55,362 (40,108-72,624)	62,807 (45,515-82,527)	63,216 (46,483-84,594)	55,631 (42,011-75,678)	39,261 (29,950-53,455)	21,355 (16,808-29,113)	7,489 (6,030-10,096)	1,834 (1,521-2,420)
Interstitial lung disease and pulmonary sarcoidosis	-	-	-	537 (188-1,311)	225 (118-465)	124 (77-213)	214 (125-388)	303 (184-528)	419 (261-706)	591 (376-967)	901 (580-1,479)	1,254 (809-2,039)	1,739 (1,121-2,789)	2,556 (1,591-4,190)	3,968 (2,537-6,354)	5,705 (3,764-8,942)	7,996 (5,655-11,866)	9,448 (6,885-13,381)	8,871 (6,843-11,891)	7,536 (6,114-9,940)	4,612 (3,804-6,060)	1,738 (1,461-2,276)	457 (391-633)
Other chronic respiratory diseases	0 (0-0)	0 (0-0)	0 (0-0)	3,267 (1,469-5,365)	1,160 (728-1,617)	821 (522-1,132)	597 (394-821)	772 (529-1,092)	710 (443-999)	920 (572-1,301)	1,056 (633-1,427)	1,261 (793-1,659)	1,255 (752-1,655)	1,331 (814-1,713)	1,872 (1,153-2,421)	2,097 (1,315-2,694)	2,362 (1,465-2,989)	2,526 (1,604-3,105)	2,160 (1,352-2,638)	2,181 (1,257-2,735)	1,616 (914-2,033)	756 (431-945)	210 (125-257)
Cirrhosis and other chronic liver diseases	-	-	-	13,771 (10,564-16,874)	5,221 (4,440-6,109)	5,330 (4,759-6,011)	8,302 (7,724-9,035)	14,168 (13,183-15,939)	21,618 (20,043-23,508)	36,160 (33,912-39,301)	53,396 (50,346-57,279)	75,026 (71,253-78,665)	83,945 (80,160-88,292)	91,345 (87,643-97,582)	105,330 (100,768-111,171)	110,462 (105,739-118,120)	104,375 (99,934-109,260)	87,637 (83,552-91,871)	63,048 (60,053-65,755)	44,367 (42,477-46,288)	24,450 (23,280-25,543)	8,943 (8,525-9,338)	2,616 (2,478-2,738)
Cirrhosis and other chronic liver diseases due to hepatitis B	-	-	-	105 (68-157)	104 (74-147)	192 (139-262)	524 (359-735)	3,023 (2,290-3,968)	7,199 (5,643-8,985)	12,983 (11,098-15,070)	18,749 (15,799-21,748)	25,365 (22,094-28,652)	25,522 (21,431-29,734)	26,699 (22,515-31,241)	31,092 (26,496-36,418)	33,806 (28,657-39,909)	31,585 (26,673-37,172)	26,601 (22,274-31,663)	19,752 (16,559-22,721)	12,308 (10,188-14,635)	5,818 (4,953-6,739)	1,797 (1,449-2,159)	451 (327-593)
Cirrhosis and other chronic liver diseases due to hepatitis C	-	-	-	84 (54-125)	78 (54-113)	151 (104-213)	374 (249-537)	1,851 (1,333-2,468)	4,145 (3,105-5,324)	8,378 (7,075-10,062)	13,390 (10,858-16,341)	19,065 (16,048-22,488)	22,148 (18,145-26,570)	24,180 (19,839-29,010)	27,121 (22,584-32,029)	27,978 (22,867-34,210)	26,783 (22,545-31,587)	22,447 (18,982-26,342)	14,984 (12,206-18,001)	10,765 (8,732-13,100)	6,109 (5,103-7,173)	2,236 (1,768-2,732)	633 (445-821)
Cirrhosis and other chronic liver diseases due to alcohol use	-	-	-	-	-	-	106 (61-165)	891 (636-1,232)	2,238 (1,630-3,037)	6,627 (5,573-7,885)	13,398 (11,069-16,192)	20,721 (17,999-23,771)	24,816 (20,962-28,988)	27,109 (22,934-31,615)	30,218 (25,076-35,628)	30,305 (24,647-36,394)	28,697 (24,121-33,275)	23,403 (19,622-27,594)	16,330 (13,899-18,964)	11,963 (9,952-13,938)	6,866 (5,991-7,737)	2,652 (2,254-3,049)	806 (649-969)
Cirrhosis and other chronic liver diseases due to other causes	-	-	-	13,582 (10,434-16,617)	5,039 (4,287-5,889)	4,986 (4,443-5,591)	7,299 (6,727-8,012)	8,402 (7,108-9,757)	8,036 (6,381-9,744)	8,172 (6,282-10,215)	7,860 (5,788-10,230)	9,874 (7,372-12,906)	11,459 (8,490-14,934)	13,356 (10,179-17,192)	16,900 (12,855-21,728)	18,373 (13,778-23,977)	17,310 (13,010-22,070)	15,186 (11,519-19,314)	11,983 (9,278-14,808)	9,331 (7,275-11,718)	5,658 (4,744-6,745)	2,257 (1,840-2,744)	726 (544-933)
Digestive diseases	2,378 (1,774-2,805)	2,715 (1,999-3,300)	23,707 (17,930-29,873)	26,184 (17,570-35,004)	11,257 (9,370-13,098)	9,487 (8,390-10,559)	13,583 (11,919-14,682)	17,024 (15,240-18,181)	22,199 (19,699-23,541)	24,396 (21,856-25,852)	29,909 (27,186-31,869)	37,140 (32,395-39,329)	41,965 (36,591-44,644)	47,998 (41,938-51,235)	65,593 (57,604-70,070)	80,006 (70,671-85,992)	95,461 (84,295-102,720)	101,618 (91,293-108,464)	96,564 (87,311-103,428)	88,618 (81,392-94,880)	62,992 (58,065-67,431)	29,771 (27,674-31,725)	9,008 (8,411-9,559)
Peptic ulcer disease	-	-	-	5,254 (3,490-7,413)	2,778 (2,178-3,551)	2,906 (2,447-3,496)	4,338 (3,801-4,958)	6,385 (5,650-7,056)	7,553 (6,754-8,288)	8,916 (7,889-9,698)	10,690 (9,520-11,704)	14,431 (12,708-15,614)	16,581 (14,368-17,904)	20,422 (17,760-21,998)	26,318 (22,988-28,209)	32,872 (28,925-35,408)	37,426 (32,916-40,444)	38,384 (34,146-41,484)	35,463 (31,410-38,373)	28,773 (25,865-31,097)	18,896 (17,138-20,347)	8,101 (7,453-8,650)	2,271 (2,098-2,433)
Gastritis and duodenitis	-	-	-	891 (377-1,562)	348 (226-522)	232 (175-307)	407 (329-497)	580 (482-685)	890 (741-1,057)	1,207 (1,029-1,404)	1,359 (1,165-1,603)	1,692 (1,484-1,926)	2,007 (1,713-2,291)	2,338 (1,988-2,654)	3,044 (2,570-3,476)	3,746 (3,198-4,280)	4,045 (3,457-4,680)	4,178 (3,594-4,780)	4,297 (3,715-5,037)	3,769 (3,226-4,542)	2,533 (2,170-3,072)	1,188 (1,017-1,431)	363 (310-431)
Appendicitis	-	-	-	4,649 (2,298-7,007)	2,390 (1,792-2,934)	2,133 (1,714-2,496)	3,013 (2,443-3,530)	2,405 (1,934-2,786)	2,683 (2,124-3,078)	1,785 (1,455-2,052)	1,993 (1,608-2,342)	1,972 (1,609-2,364)	1,965 (1,543-2,333)	1,931 (1,532-2,336)	3,052 (2,411-3,676)	3,028 (2,369-3,815)	2,973 (2,370-3,778)	2,769 (2,260-3,477)	2,347 (1,961-2,967)	1,761 (1,453-2,276)	1,112 (915-1,408)	476 (392-600)	133 (110-165)
Paralytic ileus and intestinal obstruction	2,378 (1,774-2,805)	2,715 (1,999-3,300)	23,707 (17,930-29,873)	5,155 (2,964-7,074)	2,707 (1,736-3,318)	2,085 (1,441-2,467)	3,017 (2,082-3,583)	3,147 (2,221-3,707)	4,146 (2,779-4,822)	3,586 (2,505-4,133)	4,817 (3,299-5,607)	5,600 (3,715-6,561)	6,473 (4,400-7,578)	7,281 (5,136-8,451)	10,609 (7,633-12,219)	11,802 (8,644-13,451)	14,466 (11,134-16,480)	14,507 (11,728-16,342)	13,531 (11,442-15,417)	13,022 (11,051-14,950)	10,147 (8,423-11,669)	5,244 (4,266-6,004)	1,646 (1,314-1,907)
Inguinal, femoral, and abdominal hernia	-	-	-	1,258 (523-2,074)	427 (268-552)	364 (209-480)	439 (267-567)	555 (323-735)	652 (386-815)	676 (348-875)	768 (489-943)	1,172 (754-1,427)	1,185 (806-1,454)	1,735 (1,234-2,148)	2,576 (1,799-3,145)	3,255 (2,174-4,049)	4,160 (2,842-5,160)	4,679 (3,144-5,808)	4,555 (3,028-5,678)	4,526 (3,180-5,537)	3,245 (2,310-3,891)	1,587 (1,121-1,929)	469 (341-573)
Inflammatory bowel disease	-	-	-	1,802 (685-4,726)	364 (198-659)	237 (155-371)	312 (212-454)	526 (347-777)	718 (502-1,035)	764 (540-1,071)	819 (608-1,091)	989 (751-1,255)	1,119 (821-1,413)	1,147 (900-1,385)	1,621 (1,274-1,955)	1,880 (1,498-2,206)	2,320 (1,843-2,660)	2,844 (2,315-3,242)	2,889 (2,276-3,206)	3,074 (2,412-3,329)	2,348 (1,814-2,564)	1,214 (921-1,332)	407 (315-452)
Vascular intestinal disorders	-	-	-	541 (192-956)	85 (53-118)	56 (44-66)	96 (77-113)	202 (162-235)	286 (230-328)	336 (264-387)	545 (443-632)	884 (740-1,006)	1,324 (1,127-1,502)	1,770 (1,535-1,989)	3,131 (2,784-3,671)	4,666 (4,147-5,264)	7,435 (6,751-8,478)	9,383 (8,592-10,465)	9,580 (8,626-10,436)	9,984 (9,075-10,821)	7,185 (6,526-7,783)	3,195 (2,921-3,460)	863 (781-946)
Gallbladder and biliary diseases	-	-	-	779 (413-1,144)	340 (259-433)	306 (251-370)	370 (308-425)	616 (520-688)	835 (698-924)	1,104 (921-1,215)	1,501 (1,281-1,646)	1,823 (1,554-1,986)	2,201 (1,802-2,409)	2,889 (2,382-3,202)	4,191 (3,447-4,627)	5,978 (4,906-6,759)	8,139 (6,725-9,134)	9,182 (7,886-10,170)	9,459 (8,120-10,539)	9,678 (8,545-10,801)	7,334 (6,641-8,034)	3,710 (3,326-4,046)	1,171 (1,067-1,272)
Pancreatitis	-	-	-	249 (136-380)	208 (146-284)	225 (171-293)	624 (499-786)	1,469 (1,229-1,829)	2,868 (2,456-3,459)	4,138 (3,627-4,955)	5,336 (4,717-6,423)	6,122 (5,401-7,458)	6,259 (5,497-7,676)	5,181 (4,509-6,379)	6,575 (5,658-8,075)	6,323 (5,437-7,821)	7,004 (6,004-8,546)	6,457 (5,528-7,629)	5,231 (4,491-6,169)	4,432 (3,842-5,299)	2,842 (2,411-3,309)	1,153 (980-1,307)	328 (275-372)
Other digestive diseases	-	-	-	5,605 (2,842-8,234)	1,610 (1,205-2,162)	943 (741-1,235)	967 (819-1,167)	1,138 (974-1,367)	1,570 (1,326-1,885)	1,883 (1,641-2,254)	2,081 (1,795-2,530)	2,456 (2,165-2,898)	2,851 (2,466-3,335)	3,305 (2,879-3,861)	4,475 (3,903-5,173)	6,455 (5,635-7,472)	7,493 (6,578-8,671)	9,234 (8,170-10,614)	9,210 (8,135-10,505)	9,599 (8,555-10,837)	7,352 (6,620-8,177)	3,903 (3,557-4,283)	1,358 (1,234-1,490)
Neurological disorders	-	-	8,879 (7,049-10,732)	11,177 (7,451-14,842)	7,193 (5,999-8,505)	9,322 (8,142-10,465)	14,447 (13,139-15,502)	14,523 (13,208-15,420)	12,834 (11,739-13,614)	12,164 (11,134-12,925)	12,088 (11,270-12,758)	11,565 (10,873-12,118)	12,257 (11,372-13,271)	15,034 (13,301-17,254)	21,932 (18,398-26,177)	36,054 (29,690-44,601)	65,135 (51,852-81,420)	120,288 (94,004-153,270)	198,369 (155,898-250,307)	293,155 (232,874-370,188)	298,252 (238,447-365,035)	191,598 (158,212-226,491)	60,715 (48,664-75,691)
Alzheimer's disease and other dementias	-	-	-	-	-	-	-	-	-	-	-	85 (36-165)	1,645 (974-2,492)	5,084 (3,430-7,223)	10,371 (6,870-14,578)	22,704 (16,416-31,209)	47,694 (35,017-64,121)	96,667 (70,510-129,910)	170,914 (127,142-221,489)	261,802 (201,319-338,366)	277,424 (216,784-343,723)	184,088 (150,228-219,240)	59,024 (47,020-74,220)
Parkinson's disease	-	-	-	-	-	-	-	1 (1-3)	19 (8-37)	66 (35-119)	136 (72-238)	225 (127-377)	323 (180-559)	521 (303-836)	1,083 (647-1,618)	2,564 (1,757-3,640)	6,179 (4,364-8,218)	13,108 (9,102-17,533)	20,132 (14,571-26,707)	25,727 (19,339-34,137)	17,620 (13,398-23,203)	6,421 (4,831-8,425)	1,403 (1,002-1,899)
Epilepsy	-	-	5,779 (4,498-7,044)	8,746 (5,829-11,588)	6,070 (5,028-7,175)	7,713 (6,662-8,729)	11,943 (10,642-12,966)	12,498 (11,190-13,338)	11,239 (10,167-11,995)	10,268 (9,221-10,955)	9,414 (8,650-9,952)	7,726 (7,138-8,136)	5,980 (5,506-6,277)	4,838 (4,393-5,085)	4,698 (4,263-4,940)	4,441 (3,921-4,714)	4,130 (3,629-4,449)	3,727 (3,248-4,034)	2,280 (2,058-2,436)	1,816 (1,656-1,972)	1,151 (1,041-1,258)	404 (373-437)	109 (101-119)
Multiple sclerosis	-	-	-	-	-	-	-	137 (108-193)	310 (249-420)	586 (482-727)	1,006 (830-1,220)	1,442 (1,204-1,672)	1,569 (1,329-1,786)	1,476 (1,319-1,735)	1,584 (1,437-1,998)	1,418 (1,299-1,756)	1,356 (1,225-1,661)	1,103 (972-1,326)	673 (588-806)	462 (382-536)	207 (154-229)	57 (45-64)	11 (8-12)
Motor neuron disease	8 (6-9)	11 (9-12)	982 (655-1,201)	384 (216-515)	54 (43-63)	78 (69-87)	108 (100-116)	102 (92-111)	102 (92-112)	198 (183-216)	385 (363-415)	689 (662-740)	1,040 (1,002-1,127)	1,355 (1,304-1,468)	1,979 (1,916-2,121)	2,524 (2,449-2,654)	3,109 (3,019-3,230)	2,960 (2,874-3,079)	2,047 (1,983-2,116)	1,316 (1,262-1,364)	550 (526-571)	121 (116-127)	21 (20-21)
Other neurological disorders	-	-	2,118 (1,554-2,965)	2,047 (1,273-3,184)	1,069 (848-1,400)	1,530 (1,301-1,823)	2,396 (2,183-2,629)	1,785 (1,670-1,916)	1,164 (1,089-1,244)	1,046 (984-1,107)	1,147 (1,099-1,196)	1,398 (1,349-1,452)	1,700 (1,642-1,771)	1,760 (1,660-1,844)	2,216 (2,098-2,311)	2,402 (2,292-2,502)	2,668 (2,564-2,769)	2,723 (2,622-2,817)	2,324 (2,216-2,426)	2,032 (1,939-2,125)	1,301 (1,242-1,363)	507 (482-534)	146 (138-154)
Mental and substance use disorders	17 (9-27)	25 (20-31)	0 (0-0)	0 (0-0)	17 (12-21)	14 (11-18)	7,119 (5,308-7,893)	14,840 (10,850-16,535)	20,269 (15,566-22,404)	25,673 (21,406-28,134)	30,050 (26,436-33,117)	33,397 (29,913-36,784)	29,607 (26,657-32,625)	23,015 (20,807-25,092)	25,546 (22,766-28,742)	18,552 (16,367-20,196)	14,232 (12,653-15,152)	8,389 (7,231-8,851)	5,994 (5,188-6,379)	3,573 (3,229-3,782)	2,004 (1,804-2,116)	799 (709-845)	239 (215-251)
Alcohol use disorders	-	-	-	-	-	-	1,302 (1,147-1,558)	3,226 (2,828-3,859)	6,184 (5,471-7,108)	11,542 (10,239-13,213)	17,303 (15,399-19,745)	21,548 (19,224-24,465)	21,649 (19,527-24,328)	17,332 (15,654-19,423)	19,403 (17,354-22,289)	13,452 (12,176-14,973)	9,624 (8,789-10,473)	4,834 (4,404-5,279)	3,009 (2,665-3,407)	1,510 (1,388-1,653)	749 (695-817)	254 (236-276)	77 (72-82)
Drug use disorders	17 (9-27)	25 (20-31)	0 (0-0)	0 (0-0)	0 (0-0)	0 (0-0)	5,778 (3,909-6,483)	11,550 (7,338-13,098)	14,017 (9,214-15,820)	14,065 (10,076-15,730)	12,688 (9,931-13,965)	11,781 (9,247-13,015)	7,894 (6,234-8,715)	5,684 (4,429-6,295)	6,144 (4,811-6,890)	5,101 (3,693-5,685)	4,608 (3,446-5,058)	3,555 (2,585-3,897)	2,985 (2,265-3,264)	2,063 (1,740-2,227)	1,255 (1,064-1,346)	545 (461-584)	162 (139-173)
Opioid use disorders	17 (9-27)	25 (20-31)	0 (0-0)	0 (0-0)	0 (0-0)	0 (0-0)	4,043 (2,719-4,617)	8,120 (5,124-9,442)	9,812 (6,164-11,328)	9,479 (6,336-10,878)	8,689 (6,317-9,869)	8,061 (5,847-9,120)	5,230 (3,940-5,948)	3,600 (2,601-4,101)	4,052 (2,928-4,717)	3,161 (2,109-3,647)	2,829 (1,979-3,240)	1,509 (1,031-1,769)	1,240 (879-1,470)	774 (591-919)	479 (364-557)	213 (154-241)	62 (47-71)
Cocaine use disorders	0 (0-0)	0 (0-0)	0 (0-0)	0 (0-0)	0 (0-0)	0 (0-0)	207 (116-257)	450 (260-557)	580 (362-712)	863 (572-1,040)	741 (537-937)	673 (476-838)	471 (327-576)	408 (278-496)	405 (262-515)	346 (219-437)	312 (201-388)	147 (87-191)	113 (70-143)	19 (13-25)	11 (8-14)	5 (3-6)	1 (1-2)
Amphetamine use disorders	0 (0-0)	0 (0-0)	0 (0-0)	0 (0-0)	0 (0-0)	0 (0-0)	172 (91-281)	369 (191-635)	454 (247-740)	440 (263-577)	357 (237-468)	323 (219-420)	225 (154-297)	173 (110-216)	178 (112-234)	156 (93-201)	137 (85-175)	53 (33-68)	43 (27-57)	9 (6-12)	5 (4-6)	2 (2-3)	1 (1-1)
Other drug use disorders	0 (0-0)	0 (0-0)	0 (0-0)	0 (0-0)	0 (0-0)	0 (0-0)	1,356 (792-1,592)	2,612 (1,550-3,037)	3,170 (1,906-3,643)	3,283 (2,233-3,732)	2,900 (2,119-3,228)	2,724 (1,952-3,035)	1,969 (1,446-2,205)	1,503 (1,083-1,675)	1,509 (1,137-1,713)	1,438 (1,012-1,633)	1,330 (989-1,494)	1,846 (1,287-2,054)	1,588 (1,143-1,769)	1,260 (1,061-1,386)	760 (641-829)	325 (277-353)	97 (84-106)
Eating disorders	-	-	-	-	17 (12-21)	14 (11-18)	38 (31-46)	63 (51-75)	68 (56-81)	66 (57-76)	59 (51-68)	69 (61-78)	63 (56-71)	0 (0-0)	0 (0-0)	-	-	-	-	-	-	-	-
Anorexia nervosa	-	-	-	-	16 (11-20)	14 (10-17)	38 (30-45)	62 (50-74)	67 (55-79)	63 (54-72)	56 (49-65)	62 (55-70)	53 (47-61)	0 (0-0)	0 (0-0)	-	-	-	-	-	-	-	-
Bulimia nervosa	-	-	-	-	1 (1-2)	1 (0-1)	1 (1-2)	1 (1-2)	2 (1-3)	3 (3-5)	3 (2-4)	7 (6-9)	10 (8-13)	0 (0-0)	0 (0-0)	-	-	-	-	-	-	-	-
Diabetes, urogenital, blood, and endocrine diseases	4,833 (3,664-5,474)	3,918 (3,003-4,449)	33,496 (28,968-37,041)	41,737 (29,416-50,769)	17,522 (15,249-20,099)	18,435 (15,955-20,436)	25,823 (23,243-27,745)	33,465 (31,428-35,905)	34,269 (32,272-35,984)	39,272 (37,419-41,174)	44,791 (42,959-46,814)	59,932 (57,777-62,710)	72,100 (69,606-75,171)	92,724 (89,643-96,877)	129,193 (125,244-134,697)	171,442 (166,055-178,963)	207,605 (201,250-217,167)	222,470 (215,205-232,154)	209,178 (201,543-217,708)	186,021 (179,546-194,472)	131,337 (126,721-136,931)	61,097 (58,837-63,439)	19,329 (18,544-20,056)
Diabetes mellitus	72 (67-77)	134 (120-149)	1,223 (1,112-1,363)	1,212 (1,025-1,410)	1,227 (1,084-1,371)	1,727 (1,581-1,880)	2,972 (2,815-3,138)	4,920 (4,703-5,186)	6,080 (5,830-6,358)	8,640 (8,317-9,037)	11,885 (11,528-12,296)	19,558 (18,923-20,193)	27,530 (26,629-28,358)	40,703 (39,513-42,018)	62,148 (60,251-64,120)	88,955 (86,184-91,961)	109,131 (105,610-113,227)	115,469 (111,547-119,907)	105,172 (101,042-109,488)	88,427 (84,877-92,378)	54,801 (52,482-57,372)	22,043 (21,116-23,074)	5,815 (5,534-6,090)
Acute glomerulonephritis	-	-	233 (197-280)	377 (310-453)	403 (351-477)	341 (293-406)	360 (320-409)	592 (522-676)	717 (630-812)	703 (632-787)	728 (651-816)	868 (775-967)	845 (766-935)	708 (637-785)	1,012 (925-1,104)	1,173 (1,063-1,296)	1,298 (1,192-1,433)	1,269 (1,148-1,419)	1,166 (1,049-1,310)	815 (735-908)	456 (413-509)	151 (137-168)	52 (46-61)
Chronic kidney disease	-	-	5,899 (5,035-6,596)	8,438 (6,902-9,728)	5,064 (4,382-5,629)	5,178 (4,660-5,743)	8,776 (8,209-9,652)	14,303 (13,230-15,624)	16,380 (15,277-17,678)	18,831 (17,563-20,358)	21,549 (19,983-23,305)	27,095 (25,538-29,402)	29,994 (28,344-32,500)	36,993 (35,117-40,362)	49,071 (46,768-53,372)	60,793 (58,131-65,957)	70,813 (67,701-77,625)	75,694 (72,535-82,628)	73,776 (70,530-80,334)	66,997 (64,366-72,275)	50,767 (48,768-53,939)	25,205 (24,128-26,684)	8,419 (8,024-8,838)
Chronic kidney disease due to diabetes mellitus	-	-	157 (104-227)	231 (145-345)	187 (106-297)	249 (152-401)	677 (412-1,045)	2,269 (1,437-3,131)	4,201 (3,081-5,438)	6,166 (4,694-7,755)	8,264 (6,346-10,235)	11,895 (9,214-14,380)	14,286 (11,434-17,362)	18,233 (14,083-22,243)	24,793 (19,585-29,199)	31,517 (25,908-37,182)	35,300 (27,704-42,198)	35,245 (27,796-42,047)	31,102 (24,162-37,846)	23,485 (17,536-29,309)	14,924 (10,961-19,524)	6,915 (4,908-9,110)	2,331 (1,559-3,130)
Chronic kidney disease due to hypertension	-	-	42 (29-61)	86 (58-124)	87 (57-131)	147 (95-218)	382 (248-545)	922 (627-1,261)	1,354 (936-1,853)	1,861 (1,378-2,464)	2,464 (1,856-3,176)	3,663 (2,796-4,664)	4,528 (3,496-5,846)	6,300 (4,823-8,209)	9,261 (7,186-11,934)	12,714 (10,152-16,115)	16,245 (12,734-20,652)	19,299 (15,059-24,341)	21,592 (17,166-26,836)	21,709 (17,532-26,453)	19,454 (15,728-23,533)	10,799 (8,871-12,775)	3,758 (3,009-4,552)
Chronic kidney disease due to glomerulonephritis	-	-	2,381 (1,753-3,005)	3,491 (2,529-4,559)	2,074 (1,459-2,746)	2,246 (1,605-2,931)	3,887 (2,825-4,990)	5,973 (4,377-7,746)	5,898 (4,149-7,734)	5,579 (3,906-7,457)	5,270 (3,620-7,150)	5,339 (3,500-7,275)	4,877 (3,198-6,748)	5,075 (3,357-7,164)	5,894 (4,048-8,080)	6,229 (4,041-8,862)	7,238 (4,849-10,317)	7,829 (5,293-11,130)	7,893 (5,299-11,413)	8,309 (5,848-11,200)	5,695 (4,055-7,937)	2,466 (1,708-3,289)	779 (470-1,243)
Chronic kidney disease due to other causes	-	-	3,319 (2,649-4,037)	4,631 (3,571-5,835)	2,715 (2,053-3,400)	2,536 (1,839-3,213)	3,830 (2,796-4,979)	5,140 (3,562-6,760)	4,926 (3,438-6,694)	5,225 (3,642-7,020)	5,551 (3,941-7,319)	6,198 (4,268-8,331)	6,304 (4,217-8,926)	7,385 (4,840-10,504)	9,123 (6,237-13,192)	10,334 (6,733-14,852)	12,030 (8,209-16,974)	13,321 (9,021-18,269)	13,190 (9,071-18,404)	13,494 (9,528-17,954)	10,694 (7,384-14,484)	5,025 (3,605-6,702)	1,551 (975-2,231)
Urinary diseases and male infertility	412 (372-462)	666 (534-828)	5,947 (5,290-6,729)	4,357 (3,577-5,310)	1,578 (1,336-1,874)	1,240 (1,105-1,395)	2,405 (2,222-2,639)	3,088 (2,904-3,304)	2,941 (2,787-3,123)	3,489 (3,315-3,683)	3,898 (3,692-4,109)	4,732 (4,512-4,977)	5,382 (5,122-5,674)	6,361 (6,049-6,746)	8,779 (8,360-9,251)	11,455 (10,927-12,001)	15,113 (14,436-15,787)	16,651 (15,905-17,334)	16,710 (15,965-17,415)	17,618 (16,968-18,291)	14,768 (14,226-15,277)	8,565 (8,245-8,880)	3,308 (3,167-3,430)
Interstitial nephritis and urinary tract infections	176 (131-221)	323 (220-437)	3,707 (2,932-4,419)	2,318 (1,532-3,121)	689 (515-849)	577 (464-677)	1,387 (1,149-1,613)	1,774 (1,473-2,008)	1,556 (1,318-1,736)	1,855 (1,615-2,057)	2,177 (1,894-2,413)	2,658 (2,328-2,938)	2,996 (2,590-3,308)	3,693 (3,172-4,077)	5,242 (4,511-5,747)	6,663 (5,691-7,287)	9,287 (8,142-10,046)	10,660 (9,514-11,506)	11,178 (10,069-11,983)	12,419 (11,421-13,193)	11,169 (10,428-11,824)	6,736 (6,272-7,127)	2,769 (2,581-2,917)
Urolithiasis	-	-	-	-	260 (145-351)	194 (115-247)	292 (180-371)	341 (202-423)	337 (185-422)	371 (216-457)	434 (282-523)	612 (398-744)	707 (461-851)	818 (528-974)	1,001 (679-1,175)	1,361 (935-1,610)	1,885 (1,323-2,214)	1,874 (1,281-2,168)	1,687 (1,205-1,959)	1,468 (1,076-1,695)	966 (672-1,155)	467 (321-573)	135 (95-176)
Other urinary diseases	235 (192-273)	344 (244-462)	2,239 (1,611-2,909)	2,038 (1,343-2,790)	628 (449-844)	469 (346-619)	726 (556-1,011)	973 (744-1,346)	1,048 (817-1,348)	1,263 (999-1,592)	1,287 (1,012-1,653)	1,462 (1,127-1,873)	1,680 (1,274-2,194)	1,851 (1,390-2,528)	2,536 (1,964-3,346)	3,431 (2,625-4,756)	3,941 (3,066-5,333)	4,117 (3,270-5,550)	3,845 (3,126-5,073)	3,731 (3,154-4,765)	2,633 (2,224-3,429)	1,362 (1,153-1,798)	403 (340-545)
Gynecological diseases	-	-	-	-	-	-	244 (188-280)	331 (251-384)	366 (293-416)	470 (383-529)	581 (488-670)	587 (490-672)	790 (674-905)	639 (529-728)	474 (391-547)	276 (231-320)	419 (352-492)	238 (200-282)	165 (143-207)	348 (277-425)	239 (196-290)	100 (83-122)	34 (29-41)
Uterine fibroids	-	-	-	-	-	-	16 (8-34)	22 (12-41)	37 (18-63)	75 (30-135)	169 (66-255)	220 (109-310)	367 (166-509)	346 (150-472)	226 (86-335)	105 (45-172)	147 (67-243)	60 (29-104)	41 (25-62)	88 (31-178)	49 (19-101)	20 (10-37)	6 (3-11)
Polycystic ovarian syndrome	-	-	-	-	-	-	3 (0-10)	28 (6-85)	71 (18-150)	60 (13-155)	76 (18-169)	73 (18-148)	112 (27-234)	177 (53-337)	-	-	-	-	-	-	-	-	-
Endometriosis	-	-	-	-	-	-	5 (1-10)	3 (1-6)	3 (1-7)	6 (2-12)	5 (2-7)	7 (4-12)	11 (5-19)	8 (3-14)	-	-	-	-	-	-	-	-	-
Genital prolapse	-	-	-	-	-	-	15 (4-48)	15 (3-34)	24 (8-45)	27 (4-48)	9 (3-18)	9 (3-16)	10 (3-18)	16 (4-39)	102 (16-189)	85 (22-147)	133 (37-222)	94 (39-148)	52 (30-77)	146 (48-245)	93 (38-155)	37 (17-58)	11 (6-17)
Other gynecological diseases	-	-	-	-	-	-	205 (144-247)	263 (177-329)	230 (129-313)	302 (179-407)	323 (191-469)	277 (167-418)	291 (144-512)	91 (37-204)	146 (74-255)	85 (46-150)	139 (75-227)	85 (54-133)	72 (53-97)	115 (66-203)	97 (64-157)	42 (30-66)	16 (12-22)
Hemoglobinopathies and hemolytic anaemias	2,526 (1,773-3,044)	1,621 (1,124-1,913)	11,062 (8,151-13,411)	21,434 (10,935-28,857)	6,651 (4,864-8,419)	7,906 (5,832-9,473)	9,198 (6,719-10,731)	7,989 (6,234-9,894)	5,087 (3,754-6,068)	4,024 (3,101-4,806)	3,071 (2,487-3,629)	3,833 (3,239-4,830)	3,909 (3,331-5,195)	3,385 (2,841-4,241)	3,189 (2,736-3,975)	3,460 (3,005-4,296)	4,330 (3,767-5,455)	6,024 (5,200-7,531)	5,691 (4,991-6,937)	5,381 (4,447-6,234)	5,492 (4,342-6,372)	2,585 (1,984-2,970)	831 (610-955)
Thalassemias	683 (518-858)	506 (351-658)	3,280 (2,201-4,249)	5,266 (3,322-7,143)	2,461 (1,853-3,309)	1,089 (887-1,418)	631 (504-783)	427 (313-516)	264 (191-316)	122 (98-145)	69 (57-84)	54 (46-68)	45 (39-56)	32 (28-40)	20 (17-27)	9 (8-13)	5 (4-7)	2 (2-3)	1 (1-1)	0 (0-0)	0 (0-0)	0 (0-0)	0 (0-0)
Sickle cell disorders	1,611 (999-2,102)	1,028 (681-1,274)	7,308 (5,135-9,189)	15,333 (6,887-21,287)	3,685 (2,592-4,737)	6,050 (4,194-7,523)	7,287 (5,135-8,669)	5,724 (4,336-7,114)	3,004 (2,093-3,667)	2,144 (1,548-2,599)	1,359 (1,047-1,623)	1,619 (1,322-2,053)	1,425 (1,176-1,913)	830 (657-1,082)	361 (297-472)	144 (121-187)	93 (80-116)	53 (43-64)	20 (16-24)	9 (6-10)	3 (2-3)	1 (0-1)	0 (0-0)
G6PD deficiency	57 (39-75)	38 (25-50)	303 (217-410)	646 (408-915)	346 (264-455)	482 (363-599)	763 (602-947)	1,046 (828-1,335)	1,105 (856-1,370)	982 (766-1,188)	855 (698-1,029)	1,047 (855-1,328)	1,205 (1,000-1,632)	939 (762-1,178)	780 (632-1,011)	650 (519-838)	502 (401-664)	429 (345-578)	266 (213-361)	108 (86-138)	59 (46-74)	14 (11-18)	3 (2-4)
Other hemoglobinopathies and hemolytic anaemias	175 (131-214)	50 (36-61)	171 (125-217)	190 (129-248)	158 (126-204)	285 (233-342)	516 (407-637)	793 (639-1,020)	714 (560-888)	775 (617-973)	787 (637-960)	1,113 (949-1,400)	1,235 (1,037-1,605)	1,585 (1,344-2,003)	2,027 (1,742-2,533)	2,657 (2,282-3,254)	3,730 (3,235-4,659)	5,541 (4,803-6,934)	5,404 (4,753-6,556)	5,264 (4,357-6,094)	5,431 (4,289-6,288)	2,570 (1,970-2,952)	829 (608-952)
Endocrine, metabolic, blood, and immune disorders	1,823 (1,212-2,064)	1,497 (1,038-1,762)	9,132 (7,318-10,671)	5,918 (4,585-7,376)	2,600 (2,267-3,099)	2,044 (1,837-2,380)	1,869 (1,630-2,112)	2,242 (1,861-2,455)	2,699 (2,250-2,969)	3,115 (2,683-3,474)	3,080 (2,699-3,464)	3,260 (2,807-3,625)	3,648 (3,149-4,059)	3,935 (3,334-4,302)	4,520 (3,884-5,003)	5,330 (4,650-5,943)	6,500 (5,682-7,327)	7,125 (6,222-8,075)	6,497 (5,678-7,412)	6,434 (5,540-7,350)	4,814 (4,205-5,391)	2,449 (2,059-2,806)	870 (747-991)
Musculoskeletal disorders	-	-	-	-	878 (632-1,150)	1,261 (1,020-1,593)	1,832 (1,449-2,270)	2,490 (1,905-3,063)	2,661 (2,071-3,361)	2,422 (1,957-2,943)	2,468 (2,060-2,894)	2,773 (2,304-3,186)	2,785 (2,400-3,151)	2,715 (2,306-3,064)	3,467 (2,970-3,837)	4,568 (4,000-5,091)	5,815 (5,095-6,381)	6,744 (5,938-7,267)	6,705 (5,868-7,251)	6,318 (5,540-6,939)	4,878 (4,161-5,284)	2,446 (1,980-2,633)	862 (712-938)
Rheumatoid arthritis	-	-	-	-	210 (118-363)	189 (130-294)	165 (121-256)	234 (160-412)	253 (177-432)	277 (203-431)	339 (257-489)	552 (425-726)	756 (583-911)	1,055 (814-1,207)	1,667 (1,308-1,884)	2,525 (2,041-2,814)	3,372 (2,758-3,721)	4,027 (3,368-4,443)	3,922 (3,253-4,340)	3,262 (2,719-3,734)	2,045 (1,671-2,444)	799 (633-985)	217 (173-276)
Other musculoskeletal disorders	-	-	-	-	668 (491-905)	1,072 (863-1,369)	1,667 (1,317-2,069)	2,256 (1,732-2,814)	2,408 (1,882-3,061)	2,145 (1,746-2,632)	2,129 (1,783-2,497)	2,220 (1,851-2,584)	2,028 (1,748-2,346)	1,660 (1,435-1,907)	1,800 (1,597-2,062)	2,044 (1,798-2,383)	2,443 (2,174-2,879)	2,717 (2,289-3,155)	2,783 (2,376-3,175)	3,056 (2,553-3,434)	2,833 (2,312-3,136)	1,647 (1,283-1,799)	645 (508-700)
Other non-communicable diseases	198,439 (149,303-240,897)	83,374 (62,537-104,012)	269,898 (212,769-326,592)	85,608 (57,298-118,226)	22,405 (18,117-27,095)	14,489 (12,127-17,054)	10,082 (8,526-11,558)	8,817 (7,483-10,140)	7,225 (6,228-8,311)	5,539 (4,634-6,261)	5,030 (4,181-5,665)	5,023 (4,156-5,669)	4,193 (3,365-4,805)	4,191 (3,261-4,883)	4,994 (3,821-5,992)	5,344 (4,020-6,396)	6,101 (4,490-7,623)	5,188 (3,334-6,963)	5,979 (3,994-8,254)	6,179 (4,057-8,650)	5,510 (3,391-7,414)	3,425 (1,959-4,640)	1,414 (743-1,951)
Congenital anomalies	198,439 (149,303-240,897)	78,783 (58,487-99,126)	228,143 (171,865-284,797)	83,670 (54,962-116,183)	21,833 (17,715-26,626)	13,912 (11,673-16,402)	9,128 (7,825-10,627)	7,981 (6,878-9,422)	6,479 (5,644-7,592)	4,313 (3,856-5,008)	3,882 (3,380-4,574)	3,636 (3,168-4,225)	2,390 (2,087-2,762)	1,943 (1,726-2,211)	1,981 (1,781-2,314)	1,886 (1,682-2,274)	1,635 (1,448-1,896)	-	-	-	-	-	-
Neural tube defects	36,677 (25,297-50,944)	11,370 (7,336-17,090)	25,281 (16,098-39,173)	7,471 (4,009-14,638)	1,792 (1,317-2,489)	1,019 (780-1,354)	521 (409-673)	418 (323-535)	290 (224-365)	207 (160-260)	144 (113-177)	135 (105-168)	100 (79-122)	78 (61-97)	75 (57-102)	60 (45-86)	59 (45-83)	-	-	-	-	-	-
Congenital heart anomalies	64,289 (44,132-81,973)	33,632 (23,847-42,572)	113,563 (91,192-143,280)	42,797 (32,598-56,861)	13,371 (10,785-16,830)	9,003 (7,390-10,657)	6,009 (5,062-7,200)	5,629 (4,862-6,730)	4,763 (4,171-5,680)	2,951 (2,626-3,489)	2,770 (2,445-3,245)	2,647 (2,300-3,134)	1,131 (985-1,304)	825 (713-964)	818 (737-966)	716 (644-869)	645 (574-775)	-	-	-	-	-	-
Orofacial clefts	1,945 (897-2,854)	746 (373-1,125)	1,562 (746-2,273)	201 (56-372)	-	-	-	-	-	-	-	-	-	-	-	-	-	-	-	-	-	-	-
Down's syndrome	2,171 (1,206-4,949)	1,184 (623-2,985)	5,248 (2,876-12,481)	2,337 (1,089-7,024)	392 (256-701)	305 (217-482)	270 (215-405)	248 (203-354)	254 (208-358)	270 (223-377)	244 (204-344)	243 (211-331)	420 (352-558)	372 (327-493)	374 (334-507)	297 (261-426)	131 (116-189)	-	-	-	-	-	-
Other chromosomal abnormalities	4,719 (2,682-8,410)	2,436 (1,361-4,562)	5,192 (2,846-10,912)	959 (473-2,681)	233 (152-394)	143 (100-208)	127 (91-179)	90 (68-124)	78 (57-97)	54 (39-64)	45 (30-53)	39 (26-47)	35 (24-43)	38 (27-51)	38 (27-48)	28 (20-36)	24 (18-33)	-	-	-	-	-	-
Congenital musculoskeletal and limb anomalies	5,025 (3,113-10,026)	1,344 (770-3,008)	2,753 (1,499-6,879)	844 (403-2,746)	305 (193-584)	243 (163-373)	208 (142-297)	119 (75-167)	96 (65-135)	71 (46-95)	53 (37-66)	45 (30-54)	49 (32-58)	45 (31-53)	42 (27-49)	48 (28-55)	58 (30-67)	-	-	-	-	-	-
Urogenital congenital anomalies	5,387 (3,693-7,001)	1,683 (1,010-2,236)	2,907 (1,702-4,063)	670 (356-1,066)	321 (220-444)	290 (205-384)	124 (93-173)	118 (91-156)	113 (85-144)	103 (78-129)	103 (76-129)	108 (80-135)	154 (107-197)	175 (126-223)	220 (164-267)	269 (205-338)	315 (247-396)	-	-	-	-	-	-
Digestive congenital anomalies	17,087 (12,651-26,347)	7,579 (5,204-12,724)	17,428 (11,049-35,413)	3,216 (1,738-9,539)	386 (287-642)	183 (149-256)	99 (83-130)	67 (58-87)	51 (43-64)	42 (36-52)	40 (31-46)	37 (28-42)	50 (39-58)	47 (36-53)	54 (42-61)	67 (54-79)	69 (55-79)	-	-	-	-	-	-
Other congenital anomalies	61,138 (37,522-99,593)	18,808 (9,926-33,742)	54,209 (27,193-96,182)	25,175 (9,646-51,714)	5,033 (3,110-8,048)	2,727 (1,889-4,047)	1,770 (1,276-2,479)	1,291 (912-1,885)	834 (601-1,191)	615 (459-851)	484 (372-652)	381 (302-502)	451 (346-591)	363 (278-479)	360 (274-477)	402 (314-524)	333 (264-421)	-	-	-	-	-	-
Skin and subcutaneous diseases	-	-	3,862 (1,825-5,357)	1,938 (881-2,863)	571 (272-807)	577 (295-761)	954 (506-1,220)	836 (445-1,040)	746 (411-942)	1,227 (670-1,535)	1,148 (654-1,418)	1,387 (823-1,740)	1,803 (1,094-2,280)	2,248 (1,375-2,891)	3,013 (1,883-3,991)	3,457 (2,163-4,445)	4,466 (2,880-6,007)	5,188 (3,334-6,963)	5,979 (3,994-8,254)	6,179 (4,057-8,650)	5,510 (3,391-7,414)	3,425 (1,959-4,640)	1,414 (743-1,951)
Cellulitis	-	-	172 (59-291)	162 (50-300)	78 (30-124)	81 (37-118)	92 (47-125)	98 (52-124)	71 (40-96)	133 (72-169)	156 (84-215)	186 (108-273)	234 (134-343)	334 (168-452)	453 (227-587)	488 (265-694)	554 (288-826)	623 (336-948)	673 (364-1,022)	678 (390-1,203)	553 (307-996)	335 (170-620)	135 (66-267)
Pyoderma	-	-	3,584 (1,672-5,083)	1,578 (705-2,415)	396 (178-580)	392 (184-538)	689 (343-901)	577 (279-739)	527 (268-680)	872 (433-1,120)	776 (401-992)	946 (512-1,199)	1,248 (703-1,575)	1,541 (885-2,035)	2,022 (1,164-2,701)	2,260 (1,321-2,918)	2,714 (1,651-3,739)	2,839 (1,720-3,806)	2,953 (1,926-4,104)	2,319 (1,517-3,415)	1,904 (1,188-2,735)	1,092 (647-1,646)	421 (241-617)
Decubitus ulcer	-	-	-	79 (34-130)	48 (29-65)	54 (35-76)	102 (63-144)	93 (57-125)	99 (62-135)	166 (98-228)	165 (103-236)	195 (123-281)	255 (166-379)	305 (194-456)	450 (288-685)	599 (393-886)	1,043 (664-1,503)	1,558 (1,032-2,206)	2,144 (1,349-3,109)	2,922 (1,804-4,054)	2,822 (1,549-3,685)	1,849 (953-2,437)	802 (378-1,067)
Other skin and subcutaneous diseases	-	-	106 (56-176)	118 (62-187)	50 (31-69)	50 (31-70)	71 (46-98)	68 (43-95)	50 (32-69)	57 (38-82)	50 (35-72)	61 (41-89)	66 (44-97)	68 (45-106)	89 (59-134)	110 (73-161)	155 (103-231)	167 (111-256)	209 (135-331)	260 (160-396)	232 (130-348)	149 (78-215)	57 (27-83)
Sudden infant death syndrome	-	4,592 (3,472-6,407)	37,893 (28,840-55,105)	-	-	-	-	-	-	-	-	-	-	-	-	-	-	-	-	-	-	-	-
Injuries	18,407 (14,516-21,048)	12,224 (9,561-14,561)	123,168 (104,415-142,981)	325,910 (267,396-371,781)	204,450 (187,410-218,367)	162,846 (153,745-171,113)	303,533 (293,494-311,898)	428,878 (417,586-438,156)	406,155 (393,980-415,162)	356,581 (344,038-366,034)	320,085 (306,129-329,599)	291,310 (278,076-301,127)	241,513 (230,067-250,042)	197,333 (186,939-204,087)	205,601 (194,917-212,675)	185,636 (174,928-192,295)	178,041 (166,157-185,192)	159,004 (147,167-165,840)	135,049 (124,169-141,860)	114,443 (104,059-120,569)	78,873 (70,311-83,761)	35,600 (31,123-38,048)	11,366 (9,712-12,172)
Transport injuries	3,449 (2,565-4,777)	2,076 (1,702-2,491)	18,243 (15,962-20,909)	63,659 (53,431-72,694)	63,868 (58,501-69,223)	47,142 (44,332-49,985)	106,252 (102,209-111,709)	148,940 (143,849-157,788)	139,264 (134,336-147,233)	117,407 (113,515-122,834)	104,550 (101,162-108,845)	94,505 (91,418-97,860)	74,160 (71,730-76,789)	60,874 (58,933-63,135)	60,125 (58,021-62,239)	53,408 (51,579-55,545)	47,293 (45,593-49,284)	40,544 (39,041-42,367)	30,319 (29,110-31,675)	20,202 (19,414-21,292)	10,037 (9,637-10,565)	3,338 (3,197-3,535)	883 (838-942)
Road injuries	2,937 (2,219-4,039)	1,820 (1,515-2,165)	16,691 (14,564-19,107)	60,058 (50,359-68,172)	61,055 (55,934-66,260)	44,283 (41,667-47,129)	99,389 (95,543-105,165)	140,564 (135,463-149,602)	130,562 (125,771-138,864)	110,054 (106,271-115,569)	97,424 (94,215-101,689)	87,808 (84,877-91,216)	68,572 (66,427-71,260)	56,203 (54,341-58,350)	55,751 (53,797-57,838)	49,795 (48,059-51,880)	44,154 (42,600-46,098)	38,119 (36,713-39,933)	28,424 (27,291-29,705)	18,987 (18,237-20,003)	9,395 (9,027-9,907)	3,082 (2,948-3,259)	796 (754-850)
Pedestrian road injuries	1,261 (897-1,996)	762 (577-994)	7,474 (6,071-9,236)	35,087 (28,930-42,040)	36,842 (33,169-41,162)	22,232 (20,444-24,962)	27,617 (25,266-31,653)	39,075 (35,637-44,914)	38,537 (35,151-44,563)	36,294 (33,359-41,812)	34,081 (31,295-38,500)	32,396 (29,748-36,344)	27,865 (25,784-31,069)	23,741 (21,771-26,282)	24,832 (22,912-27,436)	25,095 (23,510-27,687)	22,921 (21,462-25,169)	20,432 (19,196-22,309)	16,742 (15,852-18,198)	11,220 (10,625-12,116)	5,513 (5,220-5,961)	1,666 (1,570-1,834)	417 (387-472)
Cyclist road injuries	-	-	-	1,775 (1,103-2,279)	2,912 (2,385-3,368)	3,289 (2,866-3,662)	4,328 (3,807-4,753)	4,451 (3,827-4,937)	4,424 (3,852-4,937)	4,465 (3,828-4,942)	4,033 (3,414-4,514)	4,545 (3,865-5,028)	4,131 (3,563-4,572)	3,805 (3,350-4,211)	3,690 (3,257-4,102)	3,527 (3,063-3,870)	3,203 (2,820-3,545)	2,655 (2,335-2,938)	1,499 (1,321-1,639)	858 (763-950)	362 (319-422)	101 (87-125)	1 (1-2)
Motorcyclist road injuries	304 (208-455)	217 (145-343)	2,163 (1,731-2,818)	4,426 (3,193-6,243)	3,710 (3,173-4,417)	4,437 (3,937-5,077)	24,089 (21,991-27,616)	36,150 (32,710-40,844)	32,674 (29,191-37,447)	23,946 (21,479-27,360)	19,219 (17,300-22,006)	16,525 (14,816-18,452)	10,854 (9,770-12,229)	8,062 (7,235-9,230)	7,105 (6,380-8,314)	4,397 (3,949-5,272)	3,453 (3,100-4,031)	2,466 (2,214-2,909)	1,310 (1,170-1,504)	771 (696-909)	287 (254-351)	78 (68-103)	22 (19-29)
Motor vehicle road injuries	1,311 (849-1,984)	814 (597-1,050)	6,731 (5,066-8,628)	18,195 (12,972-23,378)	16,998 (13,606-20,427)	13,804 (11,547-16,244)	42,491 (36,896-48,232)	59,671 (51,186-68,236)	53,855 (46,451-62,379)	44,518 (38,548-51,508)	39,299 (34,076-44,801)	33,603 (29,193-38,383)	24,951 (21,581-28,142)	19,865 (17,296-22,775)	19,417 (17,044-22,015)	16,320 (14,352-18,396)	14,075 (12,325-15,811)	12,141 (10,718-13,543)	8,472 (7,616-9,384)	5,870 (5,354-6,520)	3,093 (2,814-3,407)	1,181 (1,059-1,280)	337 (291-363)
Other road injuries	61 (34-112)	27 (18-43)	322 (222-513)	575 (367-906)	593 (465-792)	521 (415-715)	864 (689-1,180)	1,217 (967-1,569)	1,072 (838-1,389)	832 (656-1,065)	791 (617-994)	738 (582-919)	772 (609-966)	730 (571-944)	707 (553-934)	455 (349-579)	502 (389-646)	424 (328-544)	401 (300-569)	269 (200-388)	140 (105-197)	56 (42-87)	18 (14-29)
Other transport injuries	512 (273-808)	256 (151-385)	1,553 (1,150-2,230)	3,602 (2,626-5,384)	2,814 (2,224-3,420)	2,859 (2,403-3,314)	6,864 (5,691-7,625)	8,376 (6,896-9,254)	8,702 (7,186-9,648)	7,353 (6,134-8,082)	7,126 (6,032-7,853)	6,697 (5,672-7,367)	5,588 (4,715-6,161)	4,670 (3,880-5,224)	4,375 (3,721-4,859)	3,613 (3,027-4,012)	3,139 (2,679-3,520)	2,425 (2,086-2,707)	1,895 (1,624-2,168)	1,215 (1,073-1,395)	642 (564-741)	256 (228-300)	87 (79-100)
Unintentional injuries	12,184 (9,191-14,010)	9,252 (6,901-11,442)	97,911 (80,138-116,735)	245,392 (196,418-286,255)	127,634 (114,972-138,037)	88,224 (81,726-93,761)	83,280 (77,617-87,051)	99,124 (93,207-103,298)	93,587 (88,036-97,273)	90,770 (84,962-94,143)	88,878 (83,369-92,622)	87,102 (81,382-90,949)	79,700 (74,514-83,369)	68,681 (63,354-72,196)	78,764 (72,952-82,899)	77,649 (70,958-82,124)	81,698 (73,373-86,994)	78,701 (69,424-84,226)	75,880 (66,443-81,667)	73,989 (64,866-79,469)	58,800 (50,639-63,216)	29,202 (25,004-31,454)	9,805 (8,191-10,561)
Falls	2,632 (1,581-3,671)	1,027 (671-1,317)	12,374 (8,350-15,799)	21,704 (14,399-29,003)	11,046 (9,015-12,928)	8,924 (7,552-10,066)	9,817 (8,746-10,769)	14,146 (12,810-15,835)	15,391 (13,715-16,950)	16,419 (14,727-17,877)	17,732 (15,984-19,531)	19,164 (17,385-21,601)	18,510 (16,726-20,786)	17,965 (16,194-19,362)	23,019 (20,585-25,058)	26,916 (23,716-29,272)	32,835 (27,760-36,091)	36,891 (31,300-40,690)	41,081 (34,913-45,204)	43,234 (36,819-47,195)	37,537 (32,455-40,728)	19,276 (16,241-20,892)	6,324 (5,256-6,960)
Drowning	682 (542-767)	367 (276-428)	12,190 (9,614-13,881)	135,010 (105,341-155,648)	74,053 (64,207-80,586)	45,366 (41,257-48,395)	33,117 (30,733-35,148)	30,711 (28,585-32,679)	24,005 (22,316-25,481)	21,264 (19,752-22,593)	18,701 (17,511-19,789)	16,661 (15,551-17,627)	13,300 (12,300-14,007)	11,072 (10,045-11,669)	11,156 (10,381-11,789)	10,089 (9,197-10,587)	9,184 (8,306-9,721)	7,902 (7,094-8,334)	6,155 (5,392-6,576)	4,591 (4,074-4,894)	2,368 (2,124-2,534)	709 (634-760)	160 (143-171)
Fire, heat, and hot substances	529 (393-591)	546 (345-650)	8,232 (5,327-9,701)	22,862 (13,087-29,080)	7,777 (5,822-8,917)	5,050 (3,934-5,641)	7,611 (5,809-8,520)	10,256 (7,895-11,382)	9,375 (7,501-10,278)	9,201 (7,649-9,954)	8,639 (7,257-9,272)	7,744 (6,678-8,397)	7,317 (6,335-7,902)	6,256 (5,343-6,699)	7,109 (6,177-7,717)	6,817 (5,959-7,363)	7,285 (6,318-7,950)	6,993 (6,069-7,617)	6,591 (5,586-7,177)	6,714 (5,529-7,347)	4,903 (3,964-5,403)	2,350 (1,838-2,556)	848 (633-925)
Poisonings	562 (272-973)	367 (220-517)	6,231 (3,113-10,197)	12,278 (7,106-17,970)	4,789 (3,734-5,978)	4,411 (3,619-5,620)	4,071 (3,283-5,153)	5,912 (4,782-7,829)	5,766 (4,651-7,683)	4,723 (3,926-6,248)	4,439 (3,626-5,523)	4,616 (3,860-5,820)	3,744 (3,076-4,559)	3,191 (2,658-3,924)	3,280 (2,815-4,140)	3,063 (2,512-3,813)	2,763 (2,305-3,377)	2,429 (2,033-2,972)	1,674 (1,415-2,087)	1,284 (1,113-1,676)	685 (604-868)	263 (234-333)	84 (74-107)
Exposure to mechanical forces	2,788 (1,916-3,385)	2,823 (1,710-4,145)	18,784 (14,985-22,843)	14,486 (9,459-19,024)	8,448 (7,207-10,269)	6,189 (5,676-7,409)	8,754 (8,151-9,878)	12,499 (11,640-14,251)	12,959 (12,132-14,956)	11,951 (11,187-13,662)	11,792 (10,732-13,329)	10,576 (9,588-12,114)	9,426 (8,543-10,321)	7,319 (6,539-7,885)	7,148 (6,352-7,755)	5,373 (4,797-5,840)	4,717 (4,200-5,133)	3,726 (3,313-4,039)	3,094 (2,780-3,344)	2,594 (2,318-2,789)	1,918 (1,688-2,070)	969 (845-1,050)	354 (309-385)
Unintentional firearm injuries	121 (75-141)	72 (52-90)	833 (608-1,058)	1,352 (873-1,924)	1,134 (929-1,331)	1,231 (1,082-1,437)	2,350 (2,079-2,823)	3,367 (2,504-3,890)	3,281 (2,421-3,730)	2,621 (1,971-2,914)	2,114 (1,529-2,354)	1,757 (1,287-1,976)	1,611 (1,217-1,821)	1,247 (822-1,496)	981 (767-1,107)	776 (649-889)	738 (608-842)	625 (511-706)	623 (512-701)	535 (441-593)	398 (312-441)	207 (165-232)	76 (61-87)
Unintentional suffocation	2,086 (1,313-2,650)	2,510 (1,455-3,787)	14,464 (11,041-18,545)	4,827 (3,164-7,421)	2,042 (1,659-3,210)	1,684 (1,406-2,562)	504 (431-723)	659 (563-969)	679 (589-980)	497 (425-706)	431 (359-585)	423 (356-582)	365 (312-477)	304 (262-410)	363 (314-482)	151 (107-177)	169 (115-205)	172 (118-210)	373 (286-494)	401 (306-569)	330 (245-461)	174 (133-254)	65 (48-96)
Other exposure to mechanical forces	580 (463-649)	241 (183-282)	3,486 (2,735-4,167)	8,307 (5,176-10,794)	5,272 (4,411-6,187)	3,273 (2,923-3,688)	5,900 (5,301-6,870)	8,473 (7,803-10,562)	8,999 (8,250-11,291)	8,833 (8,157-10,874)	9,248 (8,516-10,935)	8,397 (7,644-10,196)	7,450 (6,766-8,493)	5,768 (5,209-6,599)	5,804 (5,100-6,361)	4,446 (3,919-4,891)	3,809 (3,332-4,173)	2,929 (2,571-3,210)	2,097 (1,857-2,298)	1,658 (1,441-1,806)	1,190 (1,021-1,297)	589 (493-646)	213 (175-234)
Adverse effects of medical treatment	1,764 (1,162-2,074)	1,327 (778-1,668)	11,269 (5,952-14,677)	9,370 (3,662-14,380)	3,402 (2,217-4,201)	2,428 (1,826-2,766)	2,776 (2,126-3,136)	4,538 (3,496-5,050)	4,425 (3,385-4,885)	4,120 (3,179-4,555)	4,307 (3,398-4,758)	4,474 (3,523-4,981)	4,637 (3,687-5,140)	3,977 (3,174-4,434)	5,155 (4,129-5,749)	6,848 (5,449-7,737)	7,879 (6,167-8,941)	7,577 (6,054-8,588)	6,703 (5,319-7,581)	6,146 (4,684-6,987)	4,687 (3,496-5,340)	2,357 (1,682-2,716)	815 (570-944)
Animal contact	493 (361-584)	500 (324-595)	3,778 (2,535-4,611)	9,104 (5,725-12,497)	8,254 (5,672-10,332)	7,138 (4,855-8,822)	5,425 (3,779-6,471)	5,303 (3,847-6,194)	5,189 (3,811-6,050)	5,485 (3,752-6,485)	4,852 (3,550-5,749)	5,532 (3,954-6,502)	5,633 (4,176-6,567)	5,078 (3,445-6,005)	5,081 (3,764-5,906)	5,058 (3,558-5,991)	4,882 (3,404-5,788)	4,145 (2,751-4,996)	2,818 (1,943-3,360)	1,688 (1,199-2,020)	771 (558-912)	242 (182-282)	74 (59-84)
Venomous animal contact	391 (277-478)	405 (253-496)	3,013 (1,901-3,783)	7,347 (4,414-10,030)	6,845 (4,414-8,693)	6,358 (4,163-7,998)	4,600 (2,990-5,621)	4,288 (2,896-5,133)	4,155 (2,868-4,970)	4,655 (2,996-5,651)	4,110 (2,845-5,000)	4,707 (3,187-5,700)	4,742 (3,322-5,686)	4,271 (2,711-5,203)	4,216 (2,975-5,045)	4,267 (2,840-5,188)	4,153 (2,741-5,049)	3,535 (2,242-4,371)	2,304 (1,518-2,814)	1,374 (942-1,679)	598 (404-728)	176 (122-209)	48 (35-56)
Non-venomous animal contact	101 (75-139)	95 (67-144)	765 (539-1,273)	1,757 (911-4,011)	1,408 (1,067-2,111)	780 (582-1,080)	825 (634-1,078)	1,014 (796-1,326)	1,033 (830-1,318)	830 (673-1,078)	742 (606-957)	826 (669-1,052)	892 (706-1,156)	807 (634-1,043)	865 (681-1,112)	791 (636-988)	729 (576-887)	610 (472-760)	514 (389-633)	314 (239-384)	172 (132-209)	67 (52-81)	26 (21-32)
Foreign body	1,718 (1,051-2,078)	1,744 (1,018-2,349)	19,680 (12,290-27,551)	11,435 (6,148-19,476)	3,881 (2,734-4,879)	2,690 (2,050-3,087)	1,988 (1,594-2,260)	2,868 (2,268-3,259)	2,940 (2,320-3,275)	3,616 (2,860-4,096)	4,172 (3,296-4,801)	4,570 (3,637-5,186)	4,186 (3,381-4,799)	3,729 (3,017-4,276)	4,866 (3,980-5,618)	4,304 (3,566-4,855)	4,281 (3,511-4,770)	3,789 (3,042-4,171)	3,617 (2,821-4,033)	4,011 (2,997-4,550)	3,206 (2,262-3,738)	1,684 (1,156-1,999)	663 (452-795)
Pulmonary aspiration and foreign body in airway	1,571 (973-1,899)	1,629 (971-2,203)	17,377 (10,971-24,681)	8,962 (5,060-15,799)	2,928 (2,078-3,789)	2,098 (1,603-2,476)	1,428 (1,182-1,666)	2,102 (1,681-2,482)	2,199 (1,757-2,617)	2,727 (2,170-3,207)	3,233 (2,487-3,924)	3,681 (2,892-4,377)	3,722 (3,018-4,375)	3,322 (2,670-3,862)	4,400 (3,581-5,197)	3,877 (3,199-4,475)	3,914 (3,229-4,497)	3,404 (2,783-3,748)	3,209 (2,553-3,571)	3,620 (2,754-4,103)	2,903 (2,074-3,388)	1,542 (1,066-1,837)	613 (414-735)
Foreign body in other body part	147 (51-191)	115 (32-154)	2,303 (627-3,151)	2,473 (759-4,008)	953 (512-1,220)	592 (342-748)	560 (312-740)	767 (429-995)	741 (406-937)	889 (561-1,116)	940 (615-1,142)	888 (557-1,091)	464 (277-571)	407 (257-497)	465 (302-562)	427 (274-522)	367 (205-446)	385 (194-471)	409 (190-513)	391 (179-485)	303 (131-379)	142 (62-178)	50 (23-62)
Environmental heat and cold exposure	499 (242-611)	343 (141-448)	3,110 (1,362-3,912)	2,280 (1,164-3,028)	1,091 (681-1,316)	744 (479-854)	1,493 (921-1,752)	2,129 (1,282-2,539)	2,589 (1,585-3,138)	3,812 (2,316-4,830)	4,973 (3,300-6,687)	6,319 (4,104-8,742)	6,512 (4,276-9,470)	5,465 (3,786-7,937)	7,373 (5,150-11,148)	5,740 (4,006-8,822)	4,990 (3,504-7,108)	3,541 (2,375-4,533)	2,918 (1,877-3,511)	2,718 (1,793-3,324)	2,066 (1,325-2,542)	1,030 (609-1,261)	360 (188-432)
Other unintentional injuries	517 (462-582)	210 (174-243)	2,262 (1,835-2,723)	6,862 (4,660-8,677)	4,893 (4,211-5,784)	5,284 (4,719-5,926)	8,229 (7,484-9,179)	10,763 (9,810-12,648)	10,949 (10,052-12,971)	10,178 (9,380-11,372)	9,270 (8,523-10,347)	7,448 (6,904-8,246)	6,435 (5,924-7,031)	4,629 (4,261-5,083)	4,576 (4,193-5,040)	3,442 (3,146-3,777)	2,883 (2,591-3,197)	1,709 (1,567-1,906)	1,230 (1,114-1,386)	1,008 (894-1,149)	660 (570-754)	322 (270-369)	122 (98-141)
Self-harm and interpersonal violence	2,280 (1,651-2,594)	602 (448-698)	5,867 (4,283-6,799)	9,907 (7,636-12,013)	8,053 (6,201-9,190)	22,521 (20,132-24,422)	105,249 (96,426-111,096)	167,095 (150,609-175,048)	162,282 (145,616-170,366)	139,964 (124,698-147,580)	120,470 (106,198-128,669)	105,204 (93,147-112,554)	84,257 (74,650-90,648)	65,216 (57,602-69,511)	64,290 (55,794-68,745)	52,309 (45,226-55,514)	47,068 (40,871-49,770)	38,258 (33,442-40,273)	27,801 (24,409-29,288)	19,417 (16,887-20,409)	9,533 (8,171-10,060)	2,889 (2,496-3,048)	597 (533-631)

SR Table 5: Global deaths by cause and age group for both sexes (with 95% uncertainty intervals), 1995

Cause	Early Neonatal	Late Neonatal	Post Neonatal	1 to 4	5 to 9	10 to 14	15 to 19	20 to 24
Self-harm	-	-	-	-	-	13,161<br>(11,555-14,633)	67,705<br>(62,365-72,915)	107,776<br>(98,029-114,159)
Self-harm by firearm	-	-	-	-	-	668<br>(483-867)	5,715<br>(4,341-7,316)	8,702<br>(6,560-11,401)
Self-harm by other specified means	-	-	-	-	-	12,493<br>(10,931-13,872)	61,991<br>(56,951-66,872)	99,075<br>(90,046-105,180)
Interpersonal violence	2,280<br>(1,651-2,594)	602<br>(448-698)	5,867<br>(4,283-6,799)	9,907<br>(7,636-12,013)	8,053<br>(6,201-9,190)	9,360<br>(7,335-10,463)	37,543<br>(29,608-41,177)	59,319<br>(46,593-65,293)
Assault by firearm	173<br>(119-233)	55<br>(37-72)	661<br>(458-865)	1,031<br>(744-1,523)	1,010<br>(735-1,314)	2,363<br>(1,572-2,861)	16,886<br>(11,506-19,060)	25,520<br>(17,381-29,556)
Assault by sharp object	164<br>(120-219)	67<br>(38-88)	527<br>(324-658)	1,226<br>(854-1,551)	1,452<br>(963-1,753)	1,686<br>(1,192-1,976)	9,556<br>(6,853-11,658)	16,741<br>(12,017-20,587)
Assault by other means	1,943<br>(1,352-2,238)	480<br>(352-562)	4,679<br>(3,337-5,452)	7,650<br>(5,708-9,445)	5,591<br>(4,151-6,563)	5,310<br>(4,076-6,130)	11,102<br>(8,125-13,019)	17,058<br>(12,166-19,916)
Forces of nature, conflict and terrorism, and executions and police conflict	494<br>(376-613)	293<br>(221-365)	1,147<br>(867-1,430)	6,952<br>(5,187-8,730)	4,894<br>(3,660-6,186)	4,960<br>(3,664-6,306)	8,752<br>(6,644-10,936)	13,719<br>(10,586-16,882)
Forces of nature, conflict and terrorism, and state actor violence	12<br>(8-16)	32<br>(21-43)	266<br>(175-353)	1,319<br>(865-1,759)	1,390<br>(920-1,867)	1,640<br>(1,080-2,202)	1,636<br>(1,074-2,237)	1,642<br>(1,032-2,310)
Conflict and terrorism	482<br>(368-597)	261<br>(199-323)	834<br>(636-1,034)	5,534<br>(4,216-6,908)	3,380<br>(2,579-4,215)	3,209<br>(2,439-4,014)	6,610<br>(5,052-8,234)	9,739<br>(7,486-12,081)
Executions and police conflict	-	-	47<br>(38-70)	98<br>(64-174)	124<br>(92-146)	110<br>(90-133)	507<br>(231-629)	2,338<br>(687-2,967)

Cause	25 to 29	30 to 34	35 to 39	40 to 44	45 to 49	50 to 54	55 to 59	60 to 64
Self-harm	105,700<br>(97,437-111,614)	88,942<br>(81,455-94,160)	78,260<br>(70,580-83,244)	71,420<br>(64,686-77,021)	60,269<br>(54,182-65,011)	49,213<br>(44,038-52,101)	49,868<br>(43,923-53,144)	42,298<br>(37,085-44,844)
Self-harm by firearm	7,820<br>(5,940-9,827)	7,192<br>(5,534-9,170)	6,310<br>(5,054-8,047)	5,607<br>(4,625-7,430)	4,829<br>(3,963-6,740)	3,921<br>(3,358-5,375)	3,526<br>(2,957-4,782)	2,978<br>(2,390-3,971)
Self-harm by other specified means	97,880<br>(89,068-103,546)	81,750<br>(74,643-87,124)	71,950<br>(64,477-76,700)	65,812<br>(59,028-70,951)	55,440<br>(49,394-59,875)	45,293<br>(40,155-47,859)	46,343<br>(40,458-49,246)	39,320<br>(33,984-41,842)
Interpersonal violence	56,582<br>(43,426-62,649)	51,022<br>(37,989-57,298)	42,210<br>(30,999-47,998)	33,785<br>(24,037-38,746)	23,988<br>(16,950-28,067)	16,003<br>(11,424-19,013)	14,422<br>(9,883-17,074)	10,011<br>(7,203-11,605)
Assault by firearm	23,339<br>(15,974-27,613)	19,334<br>(12,664-23,725)	15,641<br>(10,197-19,112)	11,077<br>(7,111-14,281)	7,093<br>(4,376-9,347)	4,827<br>(3,004-6,688)	3,488<br>(2,287-4,831)	2,388<br>(1,562-3,186)
Assault by sharp object	17,133<br>(12,003-20,934)	15,772<br>(10,665-19,563)	12,927<br>(8,594-16,430)	10,727<br>(7,128-13,531)	7,499<br>(4,828-9,722)	4,635<br>(3,038-6,018)	4,210<br>(2,545-5,719)	2,990<br>(1,931-3,928)
Assault by other means	16,110<br>(11,495-18,784)	15,916<br>(10,835-18,880)	13,642<br>(9,012-16,426)	11,981<br>(7,880-14,656)	9,396<br>(6,199-11,485)	6,540<br>(4,475-7,893)	6,725<br>(4,388-8,038)	4,633<br>(3,167-5,516)
Forces of nature, conflict and terrorism, and executions and police conflict	11,022<br>(8,251-13,394)	8,440<br>(6,414-10,321)	6,187<br>(4,735-7,628)	4,499<br>(3,382-5,684)	3,395<br>(2,483-4,349)	2,563<br>(1,842-3,356)	2,421<br>(1,679-3,213)	2,271<br>(1,533-3,034)
Forces of nature, conflict and terrorism, and state actor violence	1,539<br>(963-2,182)	1,335<br>(844-1,887)	1,190<br>(742-1,684)	1,160<br>(674-1,692)	1,083<br>(588-1,636)	984<br>(526-1,490)	956<br>(513-1,442)	967<br>(503-1,483)
Conflict and terrorism	6,758<br>(5,203-8,368)	5,139<br>(3,938-6,409)	3,701<br>(2,838-4,612)	2,572<br>(1,983-3,188)	1,819<br>(1,416-2,243)	1,299<br>(1,016-1,587)	1,297<br>(997-1,611)	1,165<br>(896-1,444)
Executions and police conflict	2,724<br>(698-3,536)	1,966<br>(592-2,462)	1,296<br>(394-1,664)	768<br>(289-955)	494<br>(182-616)	280<br>(103-340)	169<br>(91-204)	138<br>(57-171)

Cause	65 to 69	70 to 74	75 to 79	80 to 84	85 to 89	90 to 94	95 plus
Self-harm	39,138<br>(33,635-41,579)	32,809<br>(28,334-34,614)	24,201<br>(20,859-25,538)	18,071<br>(15,675-19,049)	8,910<br>(7,574-9,411)	2,688<br>(2,288-2,842)	515<br>(450-544)
Self-harm by firearm	2,892<br>(2,240-3,697)	2,638<br>(1,981-3,130)	2,044<br>(1,528-2,360)	1,637<br>(1,143-1,849)	878<br>(615-994)	314<br>(231-350)	74<br>(60-83)
Self-harm by other specified means	36,246<br>(31,150-38,492)	30,171<br>(25,759-31,878)	22,157<br>(19,230-23,387)	16,434<br>(14,200-17,357)	8,031<br>(6,852-8,486)	2,374<br>(2,041-2,507)	441<br>(385-467)
Interpersonal violence	7,930<br>(5,908-9,451)	5,449<br>(4,184-6,493)	3,600<br>(2,824-4,229)	1,346<br>(1,027-1,615)	623<br>(479-754)	201<br>(167-246)	83<br>(66-102)
Assault by firearm	1,659<br>(1,073-2,251)	1,176<br>(806-1,617)	746<br>(525-999)	164<br>(99-228)	77<br>(45-102)	24<br>(15-34)	14<br>(8-19)
Assault by sharp object	2,175<br>(1,425-2,905)	1,472<br>(1,013-1,861)	910<br>(634-1,121)	362<br>(271-477)	151<br>(113-197)	56<br>(45-78)	20<br>(16-29)
Assault by other means	4,096<br>(2,964-4,855)	2,802<br>(2,094-3,304)	1,944<br>(1,507-2,248)	821<br>(626-983)	396<br>(293-484)	121<br>(92-148)	48<br>(36-60)
Forces of nature, conflict and terrorism, and executions and police conflict	1,982<br>(1,325-2,680)	1,500<br>(984-2,069)	1,050<br>(660-1,476)	835<br>(504-1,194)	503<br>(318-713)	170<br>(108-237)	80<br>(57-105)
Forces of nature, conflict and terrorism, and state actor violence	855<br>(435-1,319)	734<br>(364-1,138)	575<br>(272-908)	461<br>(203-746)	244<br>(105-395)	82<br>(34-134)	20<br>(9-34)
Conflict and terrorism	1,027<br>(783-1,283)	692<br>(534-855)	439<br>(339-545)	326<br>(244-411)	225<br>(171-281)	70<br>(55-85)	54<br>(42-67)
Executions and police conflict	99<br>(42-125)	74<br>(32-104)	35<br>(19-45)	48<br>(20-67)	34<br>(9-54)	19<br>(8-30)	5<br>(2-9)

SR Table 5: Global deaths by cause and age group for both sexes (with 95% uncertainty intervals), 2000

Cause	Early Neonatal	Late Neonatal	Post Neonatal	1 to 4	5 to 9	10 to 14	15 to 19	20 to 24	25 to 29	30 to 34	35 to 39
All causes	2,566,844 (2,512,460-2,623,714)	842,526 (825,373-860,420)	2,707,190 (2,637,577-2,771,990)	2,629,659 (2,542,244-2,710,350)	684,139 (677,822-691,529)	502,688 (498,139-507,566)	719,569 (710,360-728,698)	975,398 (960,385-989,758)	1,157,349 (1,133,833-1,181,772)	1,301,379 (1,279,222-1,323,009)	1,435,595 (1,411,112-1,461,157)
Communicable, maternal, neonatal, and nutritional disorders	2,346,039 (2,284,540-2,413,953)	748,648 (726,844-770,920)	2,262,364 (2,176,871-2,346,692)	2,132,685 (2,025,971-2,243,057)	402,400 (381,219-425,488)	214,691 (201,519-229,615)	222,068 (209,615-237,400)	325,697 (310,488-343,550)	456,828 (433,061-484,627)	511,786 (489,522-535,850)	506,628 (483,676-531,914)
HIV/AIDS and tuberculosis	-	-	114,918 (108,838-121,283)	90,799 (81,527-99,804)	35,106 (32,322-38,239)	26,343 (23,981-28,613)	48,620 (45,424-51,935)	132,977 (123,140-144,370)	278,534 (258,150-302,689)	342,016 (321,863-363,374)	339,711 (318,429-361,341)
Tuberculosis	-	-	21,101 (17,749-24,687)	34,486 (26,234-42,039)	13,090 (10,911-15,518)	15,135 (13,447-16,835)	37,340 (34,409-40,337)	64,828 (60,679-69,411)	85,367 (79,977-91,612)	99,475 (93,991-106,293)	110,499 (104,162-118,299)
Drug-susceptible tuberculosis	-	-	19,570 (16,459-22,885)	31,899 (24,199-39,141)	11,976 (10,039-14,162)	13,800 (12,177-15,388)	34,116 (31,488-36,990)	59,156 (55,244-63,639)	77,827 (72,786-83,507)	90,546 (85,345-96,864)	100,389 (94,394-108,015)
Multidrug-resistant tuberculosis without extensive drug resistance	-	-	1,513 (1,140-2,034)	2,559 (1,786-3,619)	1,096 (836-1,420)	1,313 (1,034-1,649)	3,167 (2,561-3,908)	5,525 (4,564-6,653)	7,329 (6,127-8,797)	8,694 (7,212-10,357)	9,812 (8,227-11,745)
Extensively drug-resistant tuberculosis	-	-	18 (14-23)	28 (21-36)	18 (14-23)	22 (18-28)	57 (46-70)	147 (119-180)	211 (171-256)	236 (191-286)	298 (245-364)
HIV/AIDS	-	-	93,817 (88,999-98,910)	56,313 (52,657-60,016)	22,017 (20,299-24,010)	11,208 (10,066-12,669)	11,281 (9,730-13,240)	68,148 (58,933-79,512)	193,167 (172,079-216,983)	242,541 (222,999-262,732)	229,213 (207,694-250,749)
Drug-susceptible HIV/AIDS - Tuberculosis	-	-	31,083 (23,871-37,122)	19,608 (15,627-23,063)	7,342 (5,846-8,642)	3,696 (3,056-4,363)	3,607 (2,806-4,435)	19,722 (13,965-25,566)	45,681 (30,056-62,640)	54,572 (35,094-75,122)	52,930 (34,567-72,526)
Multidrug-resistant HIV/AIDS - Tuberculosis without extensive drug resistance	-	-	2,280 (1,437-3,406)	1,411 (869-2,177)	488 (309-735)	245 (152-378)	304 (200-444)	1,559 (982-2,302)	3,456 (2,116-5,296)	4,186 (2,526-6,422)	4,050 (2,444-6,149)
Extensively drug-resistant HIV/AIDS - Tuberculosis	-	-	12 (8-17)	6 (4-10)	3 (2-4)	2 (1-3)	3 (2-4)	14 (9-21)	41 (25-63)	64 (39-101)	66 (40-105)
HIV/AIDS resulting in other diseases	-	-	60,443 (53,581-69,299)	35,288 (31,210-40,690)	14,185 (12,528-16,476)	7,265 (6,332-8,674)	7,367 (6,168-9,135)	46,853 (38,120-58,930)	143,989 (118,211-173,890)	183,718 (157,140-213,448)	172,166 (144,870-201,930)
Diarrhoea, lower respiratory infections, and other common infectious diseases	293,178 (250,678-330,864)	300,269 (269,115-324,910)	1,577,875 (1,485,407-1,674,643)	1,327,298 (1,179,696-1,517,124)	262,031 (229,455-303,371)	134,058 (116,152-155,248)	101,794 (86,417-121,435)	87,093 (73,841-107,413)	79,940 (68,129-99,354)	78,252 (66,000-97,446)	83,531 (71,216-105,266)
Diarrhoeal diseases	40,350 (35,700-45,338)	78,024 (69,976-87,230)	520,487 (456,809-579,232)	385,678 (307,631-460,425)	53,326 (31,248-87,673)	28,598 (17,279-47,556)	31,357 (19,156-52,146)	32,214 (19,747-54,718)	30,379 (18,995-50,889)	30,068 (18,950-50,289)	32,540 (20,403-56,957)
Intestinal infectious diseases	-	-	1,935 (391-6,045)	31,523 (14,956-58,158)	58,959 (32,785-97,412)	37,045 (19,630-58,549)	21,130 (10,744-36,617)	10,862 (5,370-18,831)	7,928 (4,116-13,613)	6,264 (3,358-10,670)	5,038 (2,724-8,356)
Typhoid fever	-	-	190 (88-367)	27,372 (11,942-53,650)	51,787 (27,972-85,841)	31,381 (15,991-50,202)	18,355 (9,131-32,353)	9,165 (4,411-16,376)	6,297 (3,207-10,950)	4,751 (2,460-8,206)	3,779 (1,999-6,485)
Paratyphoid fever	-	-	28 (11-61)	2,726 (1,096-5,808)	7,029 (3,247-14,100)	5,608 (2,603-10,814)	2,726 (1,155-5,341)	1,645 (693-3,332)	1,589 (683-3,222)	1,451 (644-2,878)	1,189 (560-2,253)
Other intestinal infectious diseases	-	-	1,717 (218-5,878)	1,425 (175-5,086)	144 (19-544)	56 (7-216)	50 (7-184)	52 (8-192)	42 (6-164)	62 (9-233)	70 (9-263)
Lower respiratory infections	187,747 (166,576-209,891)	163,600 (148,202-179,838)	700,526 (628,008-766,040)	370,718 (303,151-431,839)	46,806 (38,791-53,994)	24,186 (20,193-27,421)	19,578 (16,923-22,542)	21,182 (18,174-24,141)	22,824 (19,854-26,643)	24,274 (21,228-27,483)	29,030 (25,131-32,675)
Upper respiratory infections	109 (74-157)	42 (25-64)	905 (645-1,287)	756 (482-1,198)	59 (39-87)	43 (30-61)	93 (68-124)	89 (66-119)	98 (74-130)	100 (75-130)	109 (83-143)
Otitis media	7 (5-10)	34 (20-59)	275 (151-487)	496 (260-948)	302 (175-556)	267 (171-420)	115 (73-176)	76 (46-124)	81 (51-140)	57 (34-102)	68 (41-104)
Meningitis	11,024 (7,944-14,371)	15,870 (12,677-19,116)	92,522 (73,554-109,451)	84,911 (61,906-104,255)	22,384 (16,609-28,221)	15,663 (12,088-19,810)	14,124 (10,864-18,275)	11,826 (8,970-15,090)	10,139 (7,668-13,396)	9,282 (6,821-12,609)	9,030 (6,690-12,082)
Pneumococcal meningitis	527 (345-783)	733 (536-1,025)	4,528 (3,259-5,883)	4,467 (2,986-6,400)	1,202 (816-1,727)	828 (552-1,194)	736 (483-1,081)	602 (387-894)	602 (391-889)	697 (454-1,010)	823 (531-1,216)
H influenzae type B meningitis	1,449 (998-2,015)	1,994 (1,509-2,494)	11,011 (8,327-13,744)	9,310 (6,482-12,533)	1,970 (1,341-2,674)	1,150 (802-1,581)	912 (625-1,278)	756 (532-1,077)	700 (493-1,013)	678 (447-970)	679 (489-931)
Meningococcal infection	5,284 (3,723-6,987)	7,635 (6,196-9,478)	44,473 (35,563-53,845)	43,423 (31,445-54,474)	11,953 (8,819-15,630)	8,183 (5,988-10,605)	7,426 (5,564-9,824)	6,115 (4,501-8,076)	4,988 (3,577-6,715)	4,269 (3,057-5,934)	4,018 (2,899-5,451)
Other meningitis	3,764 (2,642-4,961)	5,508 (4,185-6,767)	32,511 (24,684-40,254)	27,712 (19,018-35,976)	7,260 (5,293-9,506)	5,503 (4,076-7,033)	5,049 (3,819-6,535)	4,352 (3,220-5,660)	3,849 (2,840-5,025)	3,638 (2,616-4,889)	3,510 (2,530-4,626)
Encephalitis	877 (539-1,118)	1,557 (996-1,990)	16,197 (10,236-21,192)	19,904 (12,098-29,461)	8,278 (5,863-10,916)	6,153 (4,614-8,101)	4,371 (3,344-5,510)	3,922 (3,103-5,072)	3,341 (2,561-4,150)	3,434 (2,756-4,416)	3,332 (2,694-4,242)
Diphtheria	-	-	1,610 (1,039-2,511)	3,613 (2,053-6,036)	571 (359-877)	260 (165-398)	128 (87-183)	77 (51-115)	67 (44-95)	59 (39-86)	84 (58-120)
Whooping cough	-	-	67,265 (30,887-126,252)	65,564 (29,938-122,002)	7,217 (3,265-14,527)	1,502 (671-3,058)	602 (260-1,268)	423 (185-875)	255 (107-537)	191 (79-408)	137 (57-292)
Tetanus	52,950 (24,098-80,719)	40,768 (21,099-59,820)	14,133 (6,863-23,029)	6,090 (2,943-10,115)	2,910 (1,386-4,821)	2,885 (1,353-4,851)	3,784 (1,815-6,100)	3,170 (1,560-5,181)	3,074 (1,721-4,856)	3,304 (1,989-5,223)	3,344 (1,909-5,022)
Measles	-	-	159,175 (60,864-338,369)	354,537 (146,875-679,556)	59,600 (23,875-119,403)	16,906 (6,155-36,813)	6,269 (2,182-14,375)	2,967 (1,013-6,916)	1,402 (483-3,294)	829 (283-1,949)	503 (173-1,186)
Varicella and herpes zoster	113 (92-138)	373 (309-445)	2,845 (2,361-3,391)	3,508 (2,730-4,377)	1,617 (1,286-1,993)	549 (459-665)	242 (203-291)	285 (241-339)	351 (298-410)	390 (335-458)	317 (270-370)
Neglected tropical diseases and malaria	1,120 (699-1,473)	26,960 (20,677-34,570)	235,816 (186,016-294,579)	482,172 (377,093-592,526)	71,854 (54,593-89,637)	39,150 (30,383-47,871)	21,289 (17,366-25,726)	21,437 (17,326-26,022)	16,026 (13,149-19,636)	15,207 (12,278-18,525)	16,302 (13,107-19,805)
Malaria	-	26,087 (19,684-33,639)	225,800 (175,601-283,551)	453,996 (351,193-561,430)	55,886 (40,404-72,517)	29,144 (21,269-37,402)	12,829 (9,742-16,407)	12,845 (9,715-16,503)	9,546 (7,336-12,328)	8,589 (6,581-11,250)	9,517 (7,103-12,354)
Chagas disease	-	-	3 (3-4)	2 (2-2)	3 (2-3)	6 (5-7)	17 (15-21)	40 (35-48)	71 (63-82)	138 (122-157)	233 (207-257)
Leishmaniasis	-	-	1,397 (743-2,536)	1,954 (948-3,525)	996 (522-1,833)	669 (347-1,189)	862 (453-1,555)	1,015 (544-1,813)	850 (451-1,442)	761 (398-1,320)	886 (460-1,591)
Visceral leishmaniasis	-	-	1,397 (743-2,536)	1,954 (948-3,525)	996 (522-1,833)	669 (347-1,189)	862 (453-1,555)	1,015 (544-1,813)	850 (451-1,442)	761 (398-1,320)	886 (460-1,591)
African trypanosomiasis	-	-	-	652 (166-2,032)	1,604 (513-3,814)	1,816 (657-3,843)	2,238 (729-4,745)	2,300 (818-4,742)	2,003 (667-4,481)	1,859 (572-4,211)	1,673 (523-3,922)
Schistosomiasis	-	-	0 (0-0)	631 (413-962)	297 (202-425)	414 (300-558)	387 (297-506)	507 (388-650)	589 (452-753)	650 (508-836)	768 (602-980)
Cysticercosis	0 (0-0)	0 (0-0)	0 (0-0)	139 (89-201)	84 (63-116)	67 (52-85)	62 (50-80)	85 (67-106)	113 (92-137)	126 (104-152)	103 (85-125)
Cystic echinococcosis	-	-	-	395 (258-600)	157 (106-225)	97 (67-137)	48 (33-64)	60 (42-82)	83 (61-114)	84 (61-110)	100 (76-132)
Dengue	-	-	1,298 (395-2,472)	2,194 (827-3,828)	1,283 (415-2,388)	763 (226-1,445)	678 (167-1,356)	615 (141-1,233)	485 (112-998)	377 (86-786)	390 (91-799)
Yellow fever	-	4 (1-12)	67 (14-213)	617 (117-1,836)	1,253 (257-3,767)	1,139 (242-3,393)	1,061 (214-3,066)	903 (190-2,660)	673 (131-2,024)	505 (104-1,577)	401 (81-1,250)
Rabies	-	-	816 (542-1,516)	4,059 (2,362-5,932)	7,319 (3,470-11,282)	4,011 (1,859-5,507)	2,512 (1,463-3,482)	2,502 (1,381-3,503)	1,078 (673-1,495)	1,613 (933-2,254)	1,708 (975-2,444)
Intestinal nematode infections	-	-	693 (540-870)	7,543 (5,667-9,735)	1,174 (913-1,449)	243 (196-302)	82 (66-100)	54 (43-66)	43 (34-53)	56 (45-69)	51 (40-62)
Ascariasis	-	-	693 (540-870)	7,543 (5,667-9,735)	1,174 (913-1,449)	243 (196-302)	82 (66-100)	54 (43-66)	43 (34-53)	56 (45-69)	51 (40-62)
Ebola	0 (0-0)	0 (0-0)	4 (3-5)	30 (22-38)	11 (8-14)	10 (7-13)	13 (9-16)	16 (12-20)	19 (14-24)	30 (22-38)	28 (21-36)
Zika virus	0 (0-0)	0 (0-0)	0 (0-0)	0 (0-0)	0 (0-0)	0 (0-0)	0 (0-0)	0 (0-0)	0 (0-0)	0 (0-0)	0 (0-0)
Other neglected tropical diseases	1,120 (699-1,473)	869 (588-1,163)	5,738 (3,889-8,040)	9,960 (4,835-19,202)	1,787 (1,079-2,860)	769 (513-1,110)	499 (309-711)	496 (330-673)	473 (326-611)	419 (268-539)	445 (289-542)
Maternal disorders	-	-	-	-	-	1,521 (1,281-1,791)	34,773 (31,200-38,086)	67,347 (61,023-72,331)	65,674 (60,228-70,061)	58,467 (53,912-62,663)	49,140 (45,376-52,745)
Maternal haemorrhage	-	-	-	-	-	313 (209-446)	9,648 (6,562-12,999)	21,746 (16,338-27,577)	22,219 (16,154-28,571)	20,039 (14,885-25,275)	16,922 (12,434-21,860)
Maternal sepsis and other pregnancy related infections	-	-	-	-	-	160 (101-230)	4,085 (2,567-6,119)	6,478 (3,937-9,876)	5,872 (3,880-8,431)	5,038 (3,220-7,295)	4,200 (2,774-6,127)
Maternal hypertensive disorders	-	-	-	-	-	285 (172-414)	5,034 (3,180-7,056)	8,683 (5,940-12,105)	7,856 (5,162-11,370)	6,843 (4,287-10,237)	5,913 (3,962-8,493)
Maternal obstructed labour and uterine rupture	-	-	-	-	-	56 (28-100)	1,064 (534-1,825)	2,166 (1,247-3,445)	2,415 (1,374-3,859)	2,485 (1,414-3,838)	2,417 (1,411-3,995)
Maternal abortion, miscarriage, and ectopic pregnancy	-	-	-	-	-	113 (67-186)	2,668 (1,610-4,065)	5,088 (3,188-7,698)	4,946 (3,287-7,393)	4,596 (3,020-6,649)	4,166 (2,586-5,966)
Indirect maternal deaths	-	-	-	-	-	237 (152-343)	4,870 (2,990-7,274)	9,701 (6,482-14,128)	10,154 (6,569-14,418)	8,812 (5,344-12,757)	6,445 (4,193-9,272)
Late maternal deaths	-	-	-	-	-	36 (16-73)	654 (329-1,192)	1,188 (575-2,224)	1,149 (562-2,155)	1,032 (545-1,865)	871 (428-1,526)
Maternal deaths aggravated by HIV/AIDS	-	-	-	-	-	1 (0-1)	81 (46-117)	354 (215-483)	559 (359-729)	524 (327-696)	407 (255-553)
Other maternal disorders	-	-	-	-	-	319 (213-468)	6,670 (4,464-9,893)	11,943 (7,866-17,115)	10,505 (7,146-14,545)	9,096 (5,957-12,967)	7,798 (5,120-11,476)
Neonatal disorders	2,000,633 (1,933,892-2,089,106)	399,518 (374,393-441,477)	105,917 (92,794-127,138)	11,253 (9,147-14,342)	-	-	-	-	-	-	-
Neonatal preterm birth complications	810,630 (758,756-897,810)	123,526 (111,206-150,788)	40,148 (34,352-51,519)	3,046 (2,433-4,065)	-	-	-	-	-	-	-
Neonatal encephalopathy due to birth asphyxia and trauma	624,595 (577,461-675,529)	60,981 (54,817-70,155)	19,846 (16,584-23,862)	4,451 (3,664-5,556)	-	-	-	-	-	-	-
Neonatal sepsis and other neonatal infections	153,794 (111,465-195,729)	103,723 (81,459-128,743)	16,926 (12,878-22,786)	906 (342-1,620)	-	-	-	-	-	-	-
Hemolytic disease and other neonatal jaundice	60,133 (50,901-72,607)	34,704 (29,208-41,214)	6,948 (5,389-8,828)	419 (313-576)	-	-	-	-	-	-	-
Other neonatal disorders	351,482 (312,497-393,751)	76,584 (65,902-95,304)	22,049 (17,939-29,415)	2,431 (1,840-3,749)	-	-	-	-	-	-	-
Nutritional deficiencies	-	-	149,771 (121,383-177,710)	165,598 (128,179-204,805)	14,645 (11,862-17,486)	6,148 (5,145-7,248)	5,074 (4,317-6,047)	5,366 (4,636-6,272)	5,802 (4,801-6,969)	5,965 (4,791-7,279)	5,859 (4,684-7,189)
Protein-energy malnutrition	-	-	145,319 (117,120-172,906)	159,929 (124,085-198,633)	12,694 (10,136-15,435)	5,025 (4,108-6,067)	3,477 (2,811-4,271)	3,291 (2,676-3,997)	3,932 (3,004-4,941)	4,088 (3,011-5,289)	4,042 (2,947-5,187)
Iodine deficiency	-	-	397 (117-981)	326 (75-1,033)	118 (35-287)	33 (9-90)	68 (23-168)	76 (22-190)	82 (25-198)	85 (29-199)	80 (28-177)
Iron-deficiency anaemia	-	-	100 (51-171)	111 (69-191)	153 (79-220)	135 (85-196)	169 (109-240)	161 (116-216)	131 (104-165)	133 (109-162)	124 (100-160)
Other nutritional deficiencies	-	-	3,955 (2,828-4,966)	5,231 (2,539-7,781)	1,680 (1,323-2,135)	955 (797-1,162)	1,360 (1,146-1,648)	1,837 (1,558-2,232)	1,657 (1,402-2,024)	1,659 (1,406-1,994)	1,614 (1,356-1,995)
Other communicable, maternal, neonatal, and nutritional diseases	51,108 (31,020-78,310)	21,902 (13,801-31,535)	78,067 (53,993-110,167)	55,565 (38,768-76,349)	18,764 (13,821-25,690)	7,472 (6,521-8,377)	10,517 (9,650-11,356)	11,477 (10,691-12,192)	10,852 (10,159-11,558)	11,879 (11,168-12,618)	12,084 (11,466-12,830)
Sexually transmitted diseases excluding HIV	44,967 (25,340-72,179)	17,076 (9,619-27,107)	50,531 (27,385-80,986)	28,393 (15,234-46,764)	9,669 (5,346-15,888)	193 (140-254)	726 (531-891)	838 (611-990)	1,061 (773-1,270)	984 (719-1,168)	946 (715-1,132)
Syphilis	44,967 (25,340-72,179)	17,076 (9,619-27,107)	50,531 (27,385-80,986)	28,393 (15,234-46,764)	9,669 (5,346-15,888)	132 (96-180)	282 (206-362)	301 (220-362)	329 (233-431)	392 (272-506)	284 (195-399)
Chlamydial infection	-	-	-	-	-	13 (9-17)	77 (56-96)	157 (113-190)	148 (107-181)	72 (53-86)	117 (88-136)
Gonococcal infection	-	-	-	-	-	32 (23-41)	204 (149-252)	236 (170-284)	409 (296-494)	342 (255-403)	334 (254-390)
Other sexually transmitted diseases	-	-	-	-	-	15 (11-20)	164 (119-203)	144 (104-173)	174 (126-212)	178 (132-209)	211 (160-245)
Hepatitis	-	-	12,037 (10,531-13,467)	14,545 (11,549-17,421)	6,063 (5,037-7,196)	5,236 (4,540-5,905)	7,460 (6,773-8,140)	8,689 (8,073-9,306)	8,048 (7,492-8,670)	8,926 (8,369-9,560)	9,177 (8,610-9,802)
Acute hepatitis A	-	-	7,475 (5,499-9,493)	1,589 (1,024-2,342)	141 (92-202)	78 (51-110)	115 (76-158)	132 (80-198)	97 (55-150)	88 (48-143)	90 (53-144)
Hepatitis B	-	-	4,562 (2,795-6,489)	2,041 (1,177-3,153)	586 (327-946)	852 (487-1,294)	2,656 (1,918-3,551)	5,358 (4,153-6,575)	6,265 (5,346-7,176)	7,759 (6,982-8,522)	8,302 (7,618-9,050)
Hepatitis C	-	-	-	-	171 (113-249)	62 (40-91)	83 (52-119)	93 (53-146)	72 (37-117)	69 (37-116)	86 (45-147)
Acute hepatitis E	-	-	-	10,915 (8,385-13,399)	5,165 (4,230-6,165)	4,244 (3,544-4,959)	4,607 (3,737-5,454)	3,105 (2,041-4,259)	1,614 (975-2,377)	1,008 (555-1,564)	700 (383-1,154)
Other infectious diseases	6,141 (2,421-10,842)	4,825 (2,270-7,204)	15,500 (7,043-27,026)	12,627 (6,352-21,878)	3,032 (2,326-3,719)	2,043 (1,638-2,414)	2,332 (1,926-2,661)	1,950 (1,623-2,242)	1,743 (1,454-1,948)	1,969 (1,630-2,211)	1,961 (1,703-2,202)
Non-communicable diseases	203,801 (159,181-243,756)	83,361 (67,763-99,355)	338,445 (285,208-387,026)	225,969 (179,692-265,721)	108,919 (98,979-118,806)	118,680 (111,956-125,503)	181,707 (174,079-188,119)	231,214 (224,292-238,226)	299,696 (291,315-308,289)	424,797 (415,247-435,070)	594,697 (581,927-608,726)
Neoplasms	885 (796-932)	568 (532-606)	6,828 (6,299-7,616)	25,497 (22,401-29,860)	31,286 (28,654-33,815)	34,789 (32,426-36,821)	42,603 (40,166-44,302)	47,561 (45,819-49,170)	67,056 (64,729-69,150)	110,500 (107,168-113,627)	167,388 (162,651-171,948)
Lip and oral cavity cancer	-	-	-	-	-	-	377 (347-413)	683 (635-744)	1,148 (1,061-1,250)	2,074 (1,930-2,248)	3,396 (3,164-3,655)
Nasopharynx cancer	-	-	-	-	185 (163-209)	368 (329-402)	616 (567-658)	702 (639-758)	1,046 (960-1,138)	1,815 (1,685-1,945)	2,597 (2,439-2,753)
Other pharynx cancer	-	-	-	-	-	-	303 (271-340)	429 (385-477)	597 (543-653)	1,071 (983-1,171)	1,956 (1,804-2,123)
Oesophageal cancer	-	-	-	-	-	-	383 (361-409)	684 (650-720)	1,074 (1,018-1,134)	2,220 (2,119-2,337)	4,377 (4,198-4,572)
Stomach cancer	-	-	-	-	-	-	1,270 (1,205-1,339)	2,707 (2,582-2,835)	5,519 (5,287-5,772)	11,305 (10,870-11,742)	17,576 (16,996-18,190)
Colon and rectum cancer	-	-	-	-	-	-	1,096 (1,008-1,191)	2,286 (2,121-2,458)	3,839 (3,588-4,087)	7,230 (6,816-7,630)	10,403 (9,937-10,945)
Liver cancer	-	-	-	-	634 (575-720)	1,018 (945-1,119)	1,628 (1,509-1,781)	3,208 (2,940-3,555)	7,449 (6,882-8,089)	15,845 (14,792-16,870)	24,533 (23,091-26,009)
Liver cancer due to hepatitis B	-	-	-	-	194 (138-259)	433 (330-541)	801 (622-985)	1,875 (1,564-2,200)	4,743 (3,931-5,526)	10,167 (8,596-11,560)	15,236 (12,731-17,245)
Liver cancer due to hepatitis C	-	-	-	-	14 (11-19)	28 (22-37)	50 (37-64)	122 (95-157)	305 (210-434)	694 (522-910)	1,271 (956-1,661)
Liver cancer due to alcohol use	-	-	-	-	-	-	6 (3-11)	45 (28-66)	148 (85-236)	537 (372-778)	1,437 (947-2,088)
Liver cancer due to other causes	-	-	-	-	426 (361-505)	557 (448-669)	769 (615-952)	1,165 (953-1,393)	2,253 (1,748-2,989)	4,446 (3,540-5,642)	6,589 (5,277-8,576)
Gallbladder and biliary tract cancer	-	-	-	-	-	-	92 (86-107)	154 (141-178)	321 (290-370)	843 (768-983)	1,674 (1,518-1,908)
Pancreatic cancer	-	-	-	-	-	-	174 (166-186)	329 (313-351)	651 (618-693)	1,494 (1,436-1,569)	2,977 (2,881-3,094)
Larynx cancer	-	-	-	-	-	-	170 (158-182)	233 (220-248)	329 (307-352)	716 (671-768)	1,452 (1,372-1,531)
Tracheal, bronchus, and lung cancer	-	-	-	-	-	-	1,001 (929-1,073)	1,821 (1,699-1,971)	3,849 (3,596-4,094)	8,367 (7,888-8,855)	15,248 (14,545-15,918)
Malignant skin melanoma	-	-	-	-	-	-	236 (212-270)	420 (387-493)	678 (607-775)	1,127 (995-1,267)	1,748 (1,563-1,986)
Non-melanoma skin cancer	-	-	-	-	-	-	190 (179-201)	242 (224-260)	342 (318-368)	531 (502-561)	686 (649-721)
Non-melanoma skin cancer (squamous-cell carcinoma)	-	-	-	-	-	-	190 (179-201)	242 (224-260)	342 (318-368)	531 (502-561)	686 (649-721)
Breast cancer	-	-	-	-	-	-	369 (279-423)	1,003 (825-1,167)	3,250 (2,678-3,692)	9,461 (8,318-10,601)	19,287 (17,476-21,435)
Cervical cancer	-	-	-	-	-	-	249 (184-277)	929 (697-1,072)	2,831 (2,364-3,158)	6,827 (5,779-7,717)	12,417 (10,736-13,987)
Uterine cancer	-	-	-	-	-	-	100 (90-111)	243 (215-266)	480 (429-526)	852 (771-920)	1,530 (1,374-1,647)
Ovarian cancer	-	-	-	-	-	-	513 (462-570)	687 (628-775)	1,059 (959-1,182)	1,703 (1,567-1,878)	3,029 (2,802-3,336)
Prostate cancer	-	-	-	-	-	-	70 (57-81)	84 (66-98)	107 (84-126)	139 (107-161)	212 (166-241)
Testicular cancer	-	-	-	-	-	-	492 (456-543)	984 (918-1,075)	1,202 (1,124-1,297)	1,205 (1,138-1,289)	1,024 (965-1,089)
Kidney cancer	33 (30-36)	17 (15-18)	237 (208-282)	1,100 (917-1,345)	612 (558-676)	302 (283-326)	216 (205-230)	271 (257-291)	398 (378-423)	603 (580-634)	1,119 (1,075-1,170)
Bladder cancer	-	-	-	-	-	-	118 (110-127)	187 (175-200)	271 (253-288)	561 (529-598)	1,006 (947-1,069)
Brain and nervous system cancer	123 (83-145)	80 (57-94)	1,201 (811-1,494)	4,618 (3,582-5,533)	6,911 (5,585-7,607)	6,593 (5,419-7,168)	4,576 (3,828-4,960)	4,298 (3,598-4,639)	5,465 (4,581-5,875)	7,034 (5,918-7,541)	8,638 (7,269-9,245)
Thyroid cancer	-	-	-	-	-	153 (127-176)	319 (267-365)	480 (419-537)	585 (525-641)	693 (621-759)	858 (769-934)
Mesothelioma	-	-	-	-	-	-	106 (91-126)	129 (105-153)	213 (173-249)	247 (208-286)	406 (351-465)
Hodgkin lymphoma	13 (8-15)	4 (2-4)	95 (54-115)	513 (301-675)	1,471 (900-1,902)	1,714 (1,160-2,042)	1,938 (1,438-2,259)	1,910 (1,487-2,193)	2,005 (1,581-2,299)	1,992 (1,578-2,264)	1,993 (1,564-2,239)
Non-Hodgkin's lymphoma	55 (46-60)	40 (36-46)	563 (490-676)	2,457 (2,029-3,035)	3,428 (2,805-3,940)	3,396 (2,936-3,770)	3,373 (2,984-3,629)	3,597 (3,246-3,913)	3,784 (3,506-4,088)	4,470 (4,076-4,758)	5,266 (4,843-5,596)
Multiple myeloma	-	-	-	-	-	-	246 (207-276)	299 (240-334)	327 (267-358)	516 (429-582)	866 (743-968)
Leukaemia	220 (189-243)	178 (158-208)	2,886 (2,455-3,499)	10,917 (9,248-14,141)	13,152 (11,791-15,671)	14,654 (13,403-16,553)	13,313 (11,784-14,511)	10,857 (9,600-11,854)	10,541 (9,098-11,308)	10,690 (9,296-11,473)	10,949 (9,484-11,723)
Acute lymphoid leukaemia	57 (49-73)	46 (38-63)	755 (597-1,103)	3,661 (2,926-5,466)	5,000 (4,306-6,528)	4,946 (4,376-6,379)	3,713 (3,356-4,579)	2,439 (2,226-2,945)	2,010 (1,814-2,517)	1,710 (1,551-2,140)	1,702 (1,559-2,066)
Chronic lymphoid leukaemia	-	-	-	-	-	-	265 (228-351)	253 (212-357)	278 (235-390)	305 (260-421)	360 (306-478)
Acute myeloid leukaemia	35 (27-43)	32 (26-40)	425 (346-581)	1,855 (1,455-2,655)	1,822 (1,491-2,271)	2,201 (1,893-2,611)	2,315 (2,042-2,701)	1,957 (1,722-2,229)	1,870 (1,649-2,156)	1,962 (1,739-2,234)	2,296 (2,047-2,572)
Chronic myeloid leukaemia	-	-	-	-	-	-	542 (442-651)	628 (521-737)	968 (822-1,149)	1,087 (937-1,255)	1,221 (1,061-1,383)
Other leukaemia	128 (100-145)	100 (84-115)	1,706 (1,302-2,070)	5,401 (4,237-6,766)	6,330 (5,329-7,214)	7,507 (6,350-8,204)	6,479 (5,128-7,148)	5,580 (4,460-6,178)	5,415 (4,253-6,002)	5,627 (4,476-6,170)	5,369 (4,249-5,837)
Other neoplasms	441 (393-476)	250 (229-279)	1,846 (1,616-2,318)	5,890 (4,958-7,998)	4,893 (4,438-5,568)	6,592 (6,120-7,198)	9,068 (8,509-9,575)	7,704 (7,292-8,107)	7,696 (7,252-8,096)	8,870 (8,243-9,305)	10,165 (9,623-10,609)
Cardiovascular diseases	5,019 (4,635-5,384)	2,864 (2,673-3,084)	26,903 (24,731-29,096)	21,759 (19,142-24,189)	13,445 (12,252-14,566)	17,320 (16,230-18,417)	41,973 (39,959-44,155)	63,816 (61,313-66,897)	90,851 (87,494-94,971)	138,290 (133,941-144,189)	205,186 (198,870-213,008)
Rheumatic heart disease	-	-	-	5,403 (4,359-6,587)	3,642 (3,137-4,192)	6,177 (5,499-6,851)	10,613 (9,534-11,710)	10,802 (9,668-11,934)	11,673 (10,327-13,085)	13,381 (11,728-14,955)	15,370 (13,501-17,104)
Ischaemic heart disease	-	-	1,696 (1,442-2,010)	1,277 (1,038-1,684)	945 (825-1,117)	1,142 (1,039-1,280)	12,535 (11,801-13,425)	22,358 (21,288-23,732)	36,312 (34,764-38,347)	63,713 (61,195-66,616)	98,479 (94,798-102,730)
Cerebrovascular disease	2,306 (2,050-2,594)	1,245 (1,095-1,441)	10,284 (8,832-12,212)	6,616 (5,519-8,372)	4,443 (3,989-5,011)	5,033 (4,672-5,467)	9,386 (8,927-10,024)	17,824 (16,983-18,873)	25,275 (24,125-26,684)	39,644 (37,985-41,694)	61,302 (58,989-64,079)

Cause	40 to 44	45 to 49	50 to 54	55 to 59	60 to 64	65 to 69	70 to 74	75 to 79	80 to 84	85 to 89	90 to 94	95 plus
All causes	1,634,206 (1,612,940-1,656,778)	1,977,351 (1,951,638-2,003,192)	2,206,754 (2,181,544-2,232,938)	2,544,869 (2,518,266-2,570,659)	3,531,775 (3,495,564-3,568,853)	4,295,892 (4,255,196-4,335,686)	5,079,539 (5,034,323-5,125,615)	4,974,477 (4,936,889-5,010,556)	4,154,566 (4,128,502-4,178,324)	3,319,504 (3,302,378-3,334,769)	1,670,102 (1,664,072-1,675,675)	553,566 (551,690-555,285)
Communicable, maternal, neonatal, and nutritional disorders	463,961 (446,786-484,115)	429,130 (409,865-454,379)	382,038 (363,668-409,841)	377,677 (354,267-414,322)	440,507 (404,724-497,866)	492,693 (447,123-567,816)	561,140 (499,410-654,615)	518,858 (462,836-604,382)	460,140 (409,457-532,820)	335,463 (300,345-382,109)	160,241 (143,748-180,499)	59,715 (53,811-66,311)
HIV/AIDS and tuberculosis	309,515 (295,744-324,402)	274,035 (261,311-288,673)	224,024 (214,457-235,764)	194,691 (184,730-206,164)	187,938 (177,386-200,116)	179,011 (167,268-191,945)	168,656 (155,511-183,788)	124,664 (114,754-136,043)	85,100 (76,375-95,904)	43,559 (39,364-49,381)	13,738 (12,429-15,655)	3,357 (2,994-3,841)
Tuberculosis	119,625 (112,935-126,951)	131,039 (123,518-140,180)	134,677 (126,167-144,610)	142,725 (133,101-153,397)	152,748 (141,859-163,721)	154,475 (142,874-167,209)	154,251 (141,458-169,865)	117,040 (107,016-128,771)	83,179 (74,286-94,047)	42,826 (38,606-48,659)	13,542 (12,231-15,459)	3,315 (2,953-3,800)
Drug-susceptible tuberculosis	108,232 (102,149-115,279)	118,369 (111,215-127,037)	122,061 (113,647-131,325)	129,751 (120,547-140,153)	138,382 (128,465-149,114)	140,362 (129,645-152,273)	140,359 (128,434-154,473)	106,799 (97,657-117,915)	75,987 (67,843-85,927)	39,232 (35,305-44,317)	12,474 (11,253-14,188)	3,066 (2,735-3,520)
Multidrug-resistant tuberculosis without extensive drug resistance	11,023 (9,186-13,182)	12,263 (10,211-14,763)	12,253 (10,173-14,790)	12,671 (10,448-15,365)	13,997 (11,471-17,066)	13,800 (11,204-16,936)	13,596 (11,039-16,742)	10,033 (8,035-12,447)	7,047 (5,592-8,907)	3,515 (2,787-4,471)	1,043 (817-1,338)	243 (188-319)
Extensively drug-resistant tuberculosis	370 (303-450)	407 (330-501)	363 (296-448)	303 (247-368)	369 (300-451)	313 (255-386)	296 (238-367)	208 (167-260)	145 (114-184)	79 (62-101)	25 (19-32)	6 (5-8)
HIV/AIDS	189,890 (177,038-202,325)	142,995 (131,756-154,934)	89,348 (83,836-95,231)	51,966 (48,154-55,913)	35,190 (32,316-38,478)	24,536 (22,493-26,802)	14,405 (13,065-15,897)	7,624 (6,894-8,356)	1,920 (1,696-2,181)	733 (646-835)	196 (174-223)	41 (38-46)
Drug-susceptible HIV/AIDS - Tuberculosis	47,242 (31,343-62,468)	39,264 (27,070-50,244)	27,270 (20,079-33,236)	16,788 (13,125-20,198)	11,590 (9,226-13,901)	8,236 (6,603-9,778)	4,916 (3,955-5,799)	2,637 (2,162-3,094)	660 (540-787)	248 (202-296)	65 (53-77)	12 (10-14)
Multidrug-resistant HIV/AIDS - Tuberculosis without extensive drug resistance	3,586 (2,190-5,411)	2,992 (1,864-4,458)	2,055 (1,305-3,056)	1,234 (789-1,843)	840 (539-1,247)	582 (376-862)	341 (219-509)	182 (116-267)	48 (31-71)	18 (12-27)	5 (3-7)	1 (1-1)
Extensively drug-resistant HIV/AIDS - Tuberculosis	50 (30-78)	34 (21-52)	18 (12-27)	10 (6-15)	7 (5-11)	4 (2-6)	2 (1-3)	1 (1-2)	0 (0-1)	0 (0-0)	0 (0-0)	0 (0-0)
HIV/AIDS resulting in other diseases	139,013 (119,048-160,829)	100,705 (86,859-118,168)	60,004 (52,864-69,651)	33,933 (29,906-39,507)	22,753 (19,975-26,585)	15,714 (13,773-18,285)	9,145 (8,005-10,681)	4,804 (4,200-5,543)	1,212 (1,044-1,420)	467 (402-548)	127 (110-147)	29 (25-32)
Diarrhoea, lower respiratory infections, and other common infectious diseases	87,618 (75,118-109,871)	103,854 (88,100-130,930)	118,723 (99,751-153,044)	143,716 (118,320-188,347)	207,645 (169,886-275,940)	265,307 (215,275-352,791)	345,849 (281,288-453,485)	349,752 (291,165-447,361)	340,008 (285,871-421,185)	263,652 (227,741-314,115)	130,870 (114,136-152,151)	49,316 (43,554-55,858)
Diarrhoeal diseases	33,200 (20,598-56,356)	40,841 (24,853-68,353)	48,727 (29,466-85,364)	65,177 (39,770-111,776)	99,514 (60,482-172,922)	131,608 (79,611-226,422)	175,813 (108,399-293,603)	170,277 (108,585-277,533)	162,180 (107,201-251,062)	108,639 (75,102-162,295)	45,421 (31,279-66,193)	13,512 (9,674-19,065)
Intestinal infectious diseases	4,162 (2,257-6,812)	3,580 (1,968-5,859)	2,672 (1,388-4,595)	2,076 (987-3,748)	1,877 (916-3,352)	1,718 (952-2,812)	1,365 (789-2,149)	943 (545-1,482)	526 (304-815)	249 (148-390)	79 (46-130)	22 (11-41)
Typhoid fever	3,074 (1,601-5,295)	2,572 (1,326-4,370)	1,954 (992-3,488)	1,555 (719-2,863)	1,459 (699-2,642)	1,401 (754-2,333)	1,126 (639-1,788)	754 (427-1,221)	422 (237-676)	186 (107-295)	56 (31-89)	12 (7-20)
Paratyphoid fever	1,029 (478-1,996)	935 (426-1,851)	690 (301-1,385)	493 (204-1,007)	384 (161-782)	277 (127-533)	190 (92-356)	123 (60-223)	69 (33-128)	31 (15-56)	9 (4-17)	2 (1-4)
Other intestinal infectious diseases	59 (8-222)	73 (10-273)	27 (4-97)	28 (4-105)	35 (5-124)	40 (6-150)	49 (7-195)	66 (11-237)	35 (6-125)	32 (6-105)	14 (3-46)	7 (1-28)
Lower respiratory infections	33,976 (29,970-37,989)	41,545 (36,842-46,823)	50,400 (44,472-55,833)	59,615 (51,463-66,854)	89,449 (75,866-99,206)	113,455 (95,695-124,174)	150,552 (130,456-166,810)	164,389 (143,970-182,786)	165,795 (144,644-183,632)	148,221 (131,274-164,864)	82,830 (73,647-93,567)	34,942 (30,501-39,186)
Upper respiratory infections	110 (85-141)	133 (102-172)	125 (95-164)	129 (101-166)	156 (121-203)	144 (113-183)	121 (94-153)	114 (87-145)	72 (56-93)	52 (40-67)	21 (16-28)	5 (4-7)
Otitis media	94 (58-155)	184 (92-334)	172 (85-286)	135 (84-217)	127 (81-199)	198 (126-318)	150 (100-218)	110 (77-152)	82 (55-117)	44 (30-59)	20 (12-28)	6 (4-8)
Meningitis	8,024 (6,127-10,694)	9,117 (6,987-12,388)	8,549 (6,700-11,475)	8,587 (6,761-11,359)	8,731 (6,842-11,852)	9,199 (7,275-12,304)	8,852 (7,016-11,506)	6,052 (4,897-7,997)	5,154 (4,139-6,565)	2,702 (2,190-3,520)	947 (761-1,200)	268 (224-328)
Pneumococcal meningitis	847 (532-1,323)	1,036 (652-1,593)	968 (663-1,434)	993 (689-1,395)	1,045 (755-1,514)	1,083 (756-1,537)	1,054 (732-1,503)	739 (540-1,017)	575 (399-807)	303 (230-394)	113 (88-142)	34 (24-46)
H influenzae type B meningitis	601 (417-860)	689 (487-1,017)	639 (450-915)	642 (457-885)	661 (462-952)	665 (466-947)	626 (460-863)	434 (321-619)	369 (266-489)	194 (143-262)	66 (48-89)	18 (12-26)
Meningococcal infection	3,589 (2,654-5,006)	4,016 (2,982-5,633)	3,796 (2,862-5,248)	3,802 (2,874-5,081)	3,916 (2,994-5,450)	4,032 (3,040-5,582)	3,672 (2,818-5,051)	2,605 (1,988-3,636)	2,180 (1,667-2,876)	1,196 (917-1,565)	424 (324-553)	117 (90-149)
Other meningitis	2,987 (2,191-3,886)	3,375 (2,574-4,592)	3,146 (2,455-4,316)	3,151 (2,373-4,213)	3,109 (2,333-4,210)	3,420 (2,656-4,590)	3,500 (2,728-4,495)	2,274 (1,772-3,064)	2,030 (1,596-2,644)	1,010 (811-1,323)	344 (272-433)	99 (76-127)
Encephalitis	3,214 (2,623-4,109)	3,398 (2,839-4,479)	3,026 (2,565-3,968)	3,446 (2,914-4,555)	4,287 (3,613-5,757)	4,788 (4,001-6,215)	4,987 (3,980-6,383)	4,358 (3,413-5,203)	3,374 (2,585-4,045)	1,820 (1,436-2,192)	574 (460-698)	153 (125-186)
Diphtheria	84 (59-119)	97 (66-137)	105 (73-152)	80 (54-112)	-	-	-	-	-	-	-	-
Whooping cough	145 (59-309)	124 (49-268)	102 (42-217)	122 (51-258)	-	-	-	-	-	-	-	-
Tetanus	4,061 (2,280-6,174)	4,361 (2,582-6,707)	4,490 (2,559-7,376)	3,972 (2,227-6,311)	3,240 (1,840-5,546)	3,700 (2,234-6,795)	3,534 (1,907-6,073)	2,760 (1,466-4,656)	1,744 (905-2,850)	689 (341-1,004)	207 (111-300)	57 (30-85)
Measles	348 (119-822)	241 (85-564)	173 (59-404)	163 (56-379)	-	-	-	-	-	-	-	-
Varicella and herpes zoster	198 (167-236)	233 (200-278)	181 (155-215)	215 (184-254)	263 (221-313)	496 (422-582)	474 (399-557)	749 (631-889)	1,080 (921-1,287)	1,236 (1,010-1,494)	772 (603-969)	351 (253-469)
Neglected tropical diseases and malaria	15,618 (12,762-19,074)	15,274 (12,571-18,337)	14,939 (12,346-18,214)	15,142 (12,484-18,529)	17,625 (14,452-21,714)	17,167 (14,003-21,311)	14,424 (11,824-17,819)	11,084 (9,119-13,746)	5,866 (4,820-7,181)	3,800 (3,030-4,780)	783 (620-947)	355 (245-572)
Malaria	8,876 (6,777-11,394)	7,903 (5,876-10,355)	7,959 (5,925-10,563)	8,207 (6,175-10,864)	11,205 (8,435-15,001)	10,615 (7,769-14,335)	9,005 (6,581-12,129)	6,875 (5,068-9,401)	3,667 (2,657-4,921)	2,600 (1,877-3,529)	437 (289-596)	260 (150-474)
Chagas disease	351 (319-389)	505 (463-551)	664 (610-720)	784 (705-871)	992 (918-1,072)	1,012 (939-1,085)	1,059 (987-1,133)	738 (677-803)	540 (500-581)	323 (302-345)	122 (113-131)	27 (25-29)
Leishmaniasis	905 (466-1,585)	871 (451-1,492)	878 (463-1,526)	725 (382-1,297)	661 (339-1,184)	800 (412-1,382)	752 (398-1,352)	625 (325-1,088)	367 (196-625)	243 (120-427)	43 (17-74)	17 (5-32)
Visceral leishmaniasis	905 (466-1,585)	871 (451-1,492)	878 (463-1,526)	725 (382-1,297)	661 (339-1,184)	800 (412-1,382)	752 (398-1,352)	625 (325-1,088)	367 (196-625)	243 (120-427)	43 (17-74)	17 (5-32)
African trypanosomiasis	1,428 (425-3,537)	1,276 (404-3,396)	1,160 (310-3,223)	1,041 (284-3,081)	835 (215-2,603)	692 (152-2,108)	448 (114-1,401)	268 (61-914)	121 (28-402)	45 (10-154)	10 (2-37)	2 (0-7)
Schistosomiasis	843 (662-1,075)	958 (751-1,208)	1,124 (884-1,423)	1,295 (1,023-1,614)	1,357 (1,094-1,736)	1,454 (1,152-1,825)	1,263 (1,003-1,560)	913 (732-1,132)	462 (366-577)	201 (159-251)	51 (38-66)	8 (5-12)
Cysticercosis	104 (88-125)	97 (81-116)	113 (96-134)	105 (90-125)	83 (70-99)	80 (68-95)	49 (41-59)	31 (25-37)	16 (13-21)	8 (6-9)	2 (2-3)	1 (1-1)
Cystic echinococcosis	91 (69-118)	104 (78-134)	102 (79-129)	105 (81-136)	117 (93-148)	116 (92-146)	95 (75-118)	81 (65-100)	45 (35-55)	32 (25-39)	13 (9-19)	2 (2-3)
Dengue	340 (77-731)	372 (82-787)	338 (84-722)	395 (101-833)	393 (92-800)	379 (104-758)	161 (48-289)	130 (44-233)	101 (33-191)	66 (25-120)	25 (10-46)	6 (3-10)
Yellow fever	318 (63-987)	252 (52-796)	205 (39-625)	167 (34-538)	126 (27-393)	93 (19-291)	58 (12-185)	33 (7-104)	14 (3-42)	5 (1-15)	1 (0-4)	0 (0-1)
Rabies	1,849 (1,189-2,730)	2,379 (1,340-3,419)	1,828 (1,203-3,569)	1,731 (896-2,592)	1,256 (707-1,658)	1,309 (665-1,926)	930 (631-1,559)	913 (639-1,559)	244 (155-412)	126 (74-241)	19 (10-37)	8 (4-17)
Intestinal nematode infections	37 (29-45)	43 (34-52)	58 (47-72)	53 (42-63)	61 (49-76)	48 (39-59)	69 (56-85)	53 (43-65)	32 (26-40)	12 (9-15)	8 (6-10)	3 (2-4)
Ascariasis	37 (29-45)	43 (34-52)	58 (47-72)	53 (42-63)	61 (49-76)	48 (39-59)	69 (56-85)	53 (43-65)	32 (26-40)	12 (9-15)	8 (6-10)	3 (2-4)
Ebola	28 (21-36)	30 (22-38)	14 (10-17)	18 (14-23)	11 (8-14)	8 (6-10)	4 (3-5)	1 (1-1)	0 (0-0)	0 (0-0)	0 (0-0)	0 (0-0)
Zika virus	0 (0-0)	0 (0-0)	0 (0-0)	0 (0-0)	0 (0-0)	0 (0-0)	0 (0-0)	0 (0-0)	0 (0-0)	0 (0-0)	0 (0-0)	0 (0-0)
Other neglected tropical diseases	448 (300-551)	483 (315-591)	496 (332-613)	516 (340-651)	525 (352-661)	562 (395-721)	530 (362-686)	424 (301-559)	257 (190-365)	141 (102-206)	50 (36-73)	22 (16-34)
Maternal disorders	31,239 (28,711-33,953)	15,916 (14,285-17,668)	2,895 (2,388-3,521)	-	-	-	-	-	-	-	-	-
Maternal haemorrhage	9,868 (6,915-13,254)	4,009 (2,795-5,489)	549 (331-869)	-	-	-	-	-	-	-	-	-
Maternal sepsis and other pregnancy related infections	2,814 (1,762-4,320)	1,565 (979-2,420)	279 (162-461)	-	-	-	-	-	-	-	-	-
Maternal hypertensive disorders	3,703 (2,274-5,635)	1,712 (1,073-2,576)	294 (153-494)	-	-	-	-	-	-	-	-	-
Maternal obstructed labour and uterine rupture	1,777 (999-3,000)	1,006 (533-1,658)	179 (79-342)	-	-	-	-	-	-	-	-	-
Maternal abortion, miscarriage, and ectopic pregnancy	3,111 (2,087-4,458)	2,005 (1,346-2,848)	310 (200-462)	-	-	-	-	-	-	-	-	-
Indirect maternal deaths	4,077 (2,456-6,088)	2,493 (1,481-3,690)	621 (351-987)	-	-	-	-	-	-	-	-	-
Late maternal deaths	561 (214-1,003)	302 (117-547)	64 (23-134)	-	-	-	-	-	-	-	-	-
Maternal deaths aggravated by HIV/AIDS	225 (135-308)	103 (61-145)	15 (8-23)	-	-	-	-	-	-	-	-	-
Other maternal disorders	5,102 (3,130-7,611)	2,720 (1,746-3,976)	584 (365-859)	-	-	-	-	-	-	-	-	-
Neonatal disorders	-	-	-	-	-	-	-	-	-	-	-	-
Neonatal preterm birth complications	-	-	-	-	-	-	-	-	-	-	-	-
Neonatal encephalopathy due to birth asphyxia and trauma	-	-	-	-	-	-	-	-	-	-	-	-
Neonatal sepsis and other neonatal infections	-	-	-	-	-	-	-	-	-	-	-	-
Hemolytic disease and other neonatal jaundice	-	-	-	-	-	-	-	-	-	-	-	-
Other neonatal disorders	-	-	-	-	-	-	-	-	-	-	-	-
Nutritional deficiencies	7,040 (5,576-8,652)	6,281 (5,201-7,734)	8,398 (6,527-10,564)	10,700 (8,338-13,462)	13,326 (10,186-16,700)	16,407 (13,411-19,943)	18,742 (15,469-22,580)	22,211 (18,650-26,181)	21,794 (18,554-25,412)	20,293 (17,417-23,542)	13,260 (11,406-15,260)	6,195 (5,220-7,273)
Protein-energy malnutrition	5,193 (3,805-6,635)	4,051 (3,122-5,156)	5,865 (4,192-7,703)	8,046 (5,798-10,402)	10,218 (7,250-13,254)	12,175 (9,397-15,212)	14,719 (11,689-18,063)	18,007 (14,824-21,548)	18,036 (15,098-21,346)	17,126 (14,454-20,179)	11,478 (9,665-13,523)	5,441 (4,502-6,504)
Iodine deficiency	68 (25-166)	64 (20-154)	84 (29-221)	97 (37-238)	97 (38-218)	142 (60-328)	149 (68-307)	130 (61-256)	87 (36-180)	48 (17-112)	17 (4-55)	8 (1-28)
Iron-deficiency anaemia	116 (95-197)	105 (87-187)	106 (82-233)	109 (83-222)	112 (87-202)	147 (107-273)	237 (158-422)	238 (158-494)	204 (142-376)	135 (87-216)	77 (45-116)	64 (42-91)
Other nutritional deficiencies	1,663 (1,396-2,094)	2,060 (1,696-2,704)	2,342 (1,893-3,071)	2,448 (1,983-3,216)	2,898 (2,438-3,658)	3,943 (3,252-5,001)	3,637 (3,012-4,802)	3,836 (3,123-5,082)	3,468 (2,846-4,589)	2,983 (2,417-4,069)	1,688 (1,358-2,678)	682 (537-1,225)
Other communicable, maternal, neonatal, and nutritional diseases	12,931 (12,268-13,683)	13,770 (12,984-14,647)	13,060 (12,411-13,739)	13,427 (12,642-14,225)	13,973 (13,118-14,856)	14,802 (13,842-15,755)	13,468 (12,472-14,392)	11,147 (10,301-11,933)	7,373 (6,695-8,175)	4,158 (3,753-4,593)	1,591 (1,418-1,735)	492 (426-537)
Sexually transmitted diseases excluding HIV	878 (680-1,017)	825 (646-962)	778 (587-928)	1,015 (755-1,247)	688 (502-808)	655 (529-764)	562 (456-676)	583 (446-679)	368 (275-427)	252 (194-287)	123 (97-140)	48 (36-55)
Syphilis	226 (171-283)	210 (162-263)	306 (232-418)	437 (325-613)	279 (210-333)	282 (224-342)	292 (229-368)	272 (196-335)	165 (114-201)	101 (73-121)	45 (32-53)	17 (11-20)
Chlamydial infection	134 (101-157)	117 (90-137)	87 (62-104)	112 (77-134)	82 (56-99)	45 (31-53)	42 (31-50)	52 (38-60)	46 (34-54)	34 (26-39)	17 (14-20)	6 (5-7)
Gonococcal infection	341 (258-399)	319 (244-371)	257 (185-304)	304 (209-361)	203 (138-244)	249 (178-295)	168 (129-196)	202 (152-234)	121 (89-140)	89 (70-101)	47 (37-54)	18 (14-20)
Other sexually transmitted diseases	177 (133-206)	180 (139-210)	127 (93-150)	162 (112-192)	124 (85-149)	79 (58-93)	59 (46-69)	57 (43-66)	35 (26-41)	27 (21-31)	14 (11-16)	7 (5-8)
Hepatitis	10,281 (9,682-10,891)	10,867 (10,192-11,602)	9,960 (9,401-10,528)	9,973 (9,367-10,584)	9,908 (9,365-10,498)	10,261 (9,618-10,907)	8,927 (8,350-9,473)	7,018 (6,541-7,478)	4,234 (3,895-4,561)	2,042 (1,886-2,202)	591 (549-634)	133 (123-143)
Acute hepatitis A	117 (66-185)	149 (84-232)	145 (86-228)	143 (86-225)	155 (94-240)	165 (97-246)	167 (105-252)	147 (89-219)	88 (58-128)	43 (28-60)	12 (8-18)	2 (2-4)
Hepatitis B	9,379 (8,606-10,137)	9,880 (9,034-10,713)	8,996 (8,243-9,710)	8,924 (8,089-9,668)	8,752 (7,872-9,514)	8,926 (8,012-9,771)	7,537 (6,646-8,292)	5,735 (4,933-6,445)	3,096 (2,490-3,612)	1,225 (868-1,533)	286 (176-392)	52 (26-78)
Hepatitis C	123 (64-201)	172 (95-287)	218 (116-369)	291 (163-487)	329 (181-547)	313 (174-487)	266 (152-440)	211 (118-329)	123 (70-193)	64 (40-96)	20 (13-31)	5 (3-8)
Acute hepatitis E	662 (321-1,220)	667 (342-1,164)	601 (328-1,045)	614 (286-1,069)	673 (320-1,245)	857 (430-1,456)	956 (500-1,595)	926 (422-1,651)	926 (541-1,438)	710 (424-1,035)	273 (170-386)	74 (48-101)
Other infectious diseases	1,772 (1,537-2,004)	2,078 (1,790-2,307)	2,322 (1,995-2,640)	2,440 (2,103-2,717)	3,376 (2,909-3,908)	3,885 (3,294-4,476)	3,980 (3,367-4,580)	3,546 (3,009-4,054)	2,771 (2,301-3,428)	1,865 (1,562-2,233)	876 (720-1,005)	312 (250-352)
Non-communicable diseases	870,546 (853,830-887,487)	1,265,310 (1,242,027-1,286,616)	1,594,756 (1,566,830-1,619,609)	1,974,313 (1,939,334-2,003,614)	2,893,517 (2,831,803-2,937,878)	3,616,061 (3,542,344-3,668,794)	4,339,092 (4,245,093-4,406,986)	4,299,649 (4,211,080-4,360,706)	3,576,028 (3,503,413-3,628,258)	2,893,626 (2,846,310-2,932,891)	1,466,077 (1,444,968-1,484,193)	479,102 (472,572-485,520)
Neoplasms	261,930 (254,982-268,315)	406,749 (397,829-414,869)	517,389 (507,427-527,263)	617,906 (606,444-629,947)	834,431 (820,174-849,010)	953,648 (938,708-969,272)	988,888 (973,691-1,005,320)	819,690 (803,976-834,650)	524,858 (512,760-536,298)	338,679 (329,847-347,601)	134,539 (130,469-138,633)	34,450 (33,198-35,527)
Lip and oral cavity cancer	6,175 (5,828-6,557)	9,570 (9,139-10,000)	12,208 (11,688-12,774)	13,771 (13,179-14,400)	16,403 (15,688-17,121)	15,104 (14,505-15,754)	13,011 (12,433-13,531)	9,808 (9,382-10,168)	6,670 (6,320-6,987)	4,453 (4,249-4,656)	1,967 (1,877-2,064)	564 (539-589)
Nasopharynx cancer	4,050 (3,845-4,271)	5,876 (5,592-6,167)	6,529 (6,188-6,889)	6,893 (6,525-7,269)	6,967 (6,634-7,352)	6,383 (6,086-6,693)	5,004 (4,790-5,223)	3,055 (2,925-3,186)	1,789 (1,707-1,880)	838 (802-878)	297 (285-312)	73 (69-76)
Other pharynx cancer	4,075 (3,803-4,385)	7,099 (6,704-7,562)	9,480 (8,978-10,102)	10,665 (10,088-11,368)	12,363 (11,604-13,120)	10,952 (10,267-11,728)	9,056 (8,415-9,685)	5,462 (5,089-5,841)	3,176 (2,877-3,411)	1,558 (1,416-1,670)	502 (465-531)	105 (98-111)
Oesophageal cancer	10,002 (9,632-10,411)	21,544 (20,743-22,394)	31,274 (30,152-32,422)	41,758 (40,363-43,230)	57,832 (55,643-60,067)	66,790 (64,343-69,227)	63,698 (61,228-66,319)	46,990 (45,117-49,040)	28,187 (27,085-29,406)	14,328 (13,786-14,887)	4,457 (4,294-4,650)	948 (909-986)
Stomach cancer	28,150 (27,240-28,986)	45,645 (44,275-47,049)	57,685 (56,000-59,575)	72,484 (70,264-74,639)	107,329 (104,256-110,477)	126,487 (123,386-129,756)	131,531 (128,120-135,044)	103,608 (100,836-106,625)	65,660 (63,670-67,703)	39,903 (38,584-41,351)	14,835 (14,337-15,365)	3,668 (3,520-3,794)
Colon and rectum cancer	15,487 (14,884-16,158)	25,813 (24,956-26,804)	34,633 (33,670-35,843)	44,616 (43,398-46,100)	65,315 (63,779-67,202)	80,793 (78,682-82,968)	94,969 (92,718-97,559)	89,699 (87,539-92,035)	63,004 (61,237-64,622)	48,023 (46,508-49,561)	21,885 (21,140-22,685)	6,063 (5,818-6,274)
Liver cancer	35,772 (33,670-37,694)	55,314 (52,159-58,272)	61,208 (57,009-64,055)	67,182 (63,498-70,274)	80,923 (76,956-84,236)	88,249 (83,826-91,792)	80,008 (76,196-83,162)	57,632 (54,592-59,667)	33,586 (32,083-35,123)	17,167 (16,396-17,842)	5,320 (5,083-5,539)	1,131 (1,068-1,186)
Liver cancer due to hepatitis B	21,320 (17,621-24,120)	31,289 (25,527-35,778)	31,887 (25,409-36,958)	32,352 (24,940-37,784)	35,366 (26,350-41,937)	34,960 (26,420-41,156)	28,749 (21,301-34,218)	19,180 (14,214-23,465)	10,759 (7,678-13,565)	5,001 (3,810-6,186)	1,399 (1,044-1,783)	282 (183-381)
Liver cancer due to hepatitis C	2,448 (1,919-3,142)	4,895 (3,799-6,164)	7,688 (6,100-9,350)	10,555 (8,740-12,752)	14,970 (12,469-17,912)	17,998 (15,413-20,908)	18,445 (16,006-21,261)	15,270 (13,291-17,627)	9,994 (8,666-11,543)	6,000 (5,370-6,683)	2,157 (1,896-2,412)	509 (439-588)
Liver cancer due to alcohol use	2,940 (2,067-4,091)	5,194 (3,495-7,318)	7,078 (4,746-9,719)	8,726 (5,902-12,068)	12,435 (9,025-16,689)	16,382 (12,580-20,591)	16,231 (12,547-20,235)	11,413 (8,817-14,229)	6,051 (4,450-7,794)	3,024 (2,242-3,838)	869 (610-1,156)	160 (93-237)
Liver cancer due to other causes	9,064 (7,375-11,699)	13,935 (11,336-17,648)	14,555 (11,768-18,410)	15,548 (12,602-19,584)	18,152 (14,981-22,862)	18,909 (15,574-22,905)	16,583 (13,821-20,146)	11,769 (9,924-14,061)	6,781 (5,614-8,262)	3,143 (2,663-3,666)	894 (733-1,077)	179 (138-228)
Gallbladder and biliary tract cancer	3,184 (2,899-3,570)	5,283 (4,875-5,876)	7,638 (7,141-8,379)	9,868 (9,206-10,957)	14,363 (13,497-15,819)	17,286 (16,355-18,998)	19,437 (18,558-21,269)	18,119 (17,316-19,270)	12,621 (12,076-13,425)	8,849 (8,458-9,298)	3,691 (3,525-3,869)	969 (922-1,014)
Pancreatic cancer	5,833 (5,649-6,039)	10,511 (10,244-10,795)	16,072 (15,723-16,471)	21,701 (21,217-22,268)	32,376 (31,652-33,147)	39,292 (38,442-40,317)	44,010 (43,105-45,110)	38,741 (37,789-39,759)	24,872 (24,193-25,536)	17,000 (16,479-17,594)	6,843 (6,601-7,105)	1,700 (1,632-1,757)
Larynx cancer	3,459 (3,305-3,618)	6,776 (6,465-7,068)	9,893 (9,462-10,308)	11,773 (11,293-12,253)	15,595 (14,946-16,227)	15,761 (15,110-16,374)	13,714 (13,196-14,215)	8,914 (8,582-9,226)	5,138 (4,906-5,356)	2,571 (2,469-2,677)	807 (777-839)	158 (152-164)
Tracheal, bronchus, and lung cancer	31,587 (30,323-32,925)	60,604 (58,736-62,504)	89,602 (87,285-92,350)	117,774 (114,379-121,428)	176,694 (172,176-181,704)	212,362 (207,034-217,741)	219,140 (214,048-224,691)	171,466 (167,583-175,302)	97,231 (94,633-99,862)	51,891 (50,310-53,518)	16,283 (15,722-16,815)	3,490 (3,341-3,617)
Malignant skin melanoma	2,492 (2,240-2,792)	3,022 (2,674-3,349)	3,633 (3,239-4,065)	3,706 (3,338-4,124)	4,400 (3,969-4,941)	4,632 (4,201-5,256)	5,108 (4,537-5,883)	4,636 (4,062-5,273)	3,246 (2,862-3,784)	2,387 (2,050-2,780)	1,100 (935-1,324)	337 (279-424)
Non-melanoma skin cancer	1,010 (962-1,056)	1,457 (1,400-1,514)	1,829 (1,767-1,882)	2,216 (2,152-2,277)	3,750 (3,637-3,868)	4,506 (4,369-4,628)	5,073 (4,934-5,203)	4,651 (4,516-4,771)	3,928 (3,796-4,054)	3,420 (3,304-3,531)	1,972 (1,903-2,036)	827 (791-859)
Non-melanoma skin cancer (squamous-cell carcinoma)	1,010 (962-1,056)	1,457 (1,400-1,514)	1,829 (1,767-1,882)	2,216 (2,152-2,277)	3,750 (3,637-3,868)	4,506 (4,369-4,628)	5,073 (4,934-5,203)	4,651 (4,516-4,771)	3,928 (3,796-4,054)	3,420 (3,304-3,531)	1,972 (1,903-2,036)	827 (791-859)
Breast cancer	30,598 (27,963-33,541)	42,004 (38,892-45,982)	47,672 (44,722-51,277)	47,110 (44,189-51,173)	47,445 (45,070-52,050)	44,050 (41,928-47,296)	42,258 (40,512-45,350)	36,262 (34,831-39,086)	24,061 (23,100-25,521)	19,308 (18,545-20,272)	9,456 (9,056-9,922)	3,164 (3,031-3,303)
Cervical cancer	19,215 (16,644-21,819)	24,904 (21,756-28,819)	26,243 (22,403-30,394)	26,043 (22,403-30,632)	26,003 (22,929-30,081)	23,136 (20,241-27,020)	20,555 (18,519-23,370)	14,144 (12,971-15,418)	9,307 (8,513-10,434)	5,402 (5,052-5,903)	2,074 (1,953-2,225)	563 (532-600)
Uterine cancer	2,640 (2,388-2,843)	4,651 (4,264-4,958)	6,253 (5,746-6,671)	7,283 (6,680-7,772)	10,176 (9,507-10,836)	10,891 (10,198-11,508)	10,314 (9,755-10,872)	8,347 (7,943-8,759)	5,391 (5,173-5,613)	3,474 (3,336-3,626)	1,454 (1,387-1,522)	391 (370-411)
Ovarian cancer	5,442 (5,050-5,929)	9,189 (8,682-9,852)	12,378 (11,735-13,224)	12,700 (12,120-13,524)	15,475 (14,834-16,296)	15,271 (14,625-16,067)	15,785 (15,166-16,526)	12,865 (12,395-13,492)	7,831 (7,542-8,162)	5,235 (5,033-5,455)	2,131 (2,039-2,236)	527 (502-551)
Prostate cancer	487 (383-557)	1,428 (1,155-1,610)	3,754 (3,024-4,138)	8,280 (6,566-9,136)	19,406 (15,464-21,476)	31,988 (26,101-34,860)	46,267 (39,083-50,340)	52,311 (45,042-57,632)	40,227 (34,181-44,030)	31,563 (26,349-34,055)	14,402 (12,121-15,575)	3,424 (2,890-3,749)
Testicular cancer	742 (701-787)	588 (553-626)	428 (401-464)	305 (286-325)	379 (355-408)	381 (358-405)	381 (357-406)	278 (263-294)	217 (202-233)	123 (116-130)	47 (44-50)	13 (12-13)
Kidney cancer	2,126 (2,049-2,219)	3,960 (3,818-4,118)	6,090 (5,900-6,309)	7,776 (7,573-8,004)	10,802 (10,461-11,183)	12,228 (11,921-12,577)	13,489 (13,131-13,872)	11,768 (11,447-12,109)	7,419 (7,187-7,628)	5,162 (4,971-5,350)	2,016 (1,932-2,097)	461 (442-479)
Bladder cancer	1,906 (1,810-2,009)	3,361 (3,213-3,524)	5,382 (5,178-5,623)	7,637 (7,368-7,926)	13,411 (13,008-13,850)	18,200 (17,696-18,758)	23,633 (22,970-24,386)	23,610 (22,964-24,239)	17,850 (17,367-18,321)	13,619 (13,216-14,049)	6,003 (5,801-6,209)	1,566 (1,504-1,620)
Brain and nervous system cancer	10,563 (9,014-11,236)	13,010 (11,216-13,826)	14,361 (12,406-15,196)	15,328 (13,415-16,146)	17,844 (15,710-18,779)	17,944 (15,859-18,837)	15,390 (13,630-16,151)	11,124 (9,528-11,752)	5,768 (4,794-6,161)	2,836 (2,253-3,048)	820 (658-879)	168 (137-181)
Thyroid cancer	1,340 (1,214-1,463)	1,927 (1,768-2,081)	2,624 (2,418-2,830)	3,217 (2,969-3,463)	3,697 (3,466-3,939)	4,175 (3,927-4,440)	4,203 (3,982-4,477)	3,688 (3,523-3,913)	2,409 (2,303-2,594)	1,517 (1,452-1,630)	585 (559-620)	137 (130-144)
Mesothelioma	662 (582-753)	909 (831-1,031)	1,342 (1,240-1,466)	1,926 (1,784-2,101)	2,357 (2,194-2,540)	2,793 (2,614-2,975)	3,132 (2,944-3,387)	3,028 (2,855-3,256)	1,815 (1,705-1,963)	1,019 (960-1,123)	338 (320-368)	71 (67-75)
Hodgkin lymphoma	2,051 (1,565-2,275)	2,271 (1,757-2,553)	2,238 (1,773-2,513)	2,439 (1,910-2,777)	2,803 (2,198-3,148)	2,971 (2,335-3,283)	2,672 (2,183-2,951)	1,675 (1,406-1,857)	979 (803-1,076)	510 (435-553)	148 (132-160)	30 (26-32)
Non-Hodgkin's lymphoma	6,803 (6,121-7,215)	8,419 (7,764-8,906)	10,432 (9,560-11,045)	12,221 (11,158-13,066)	16,403 (15,365-17,443)	19,314 (17,997-20,646)	21,118 (20,018-22,318)	18,803 (17,574-19,892)	12,886 (11,914-13,628)	8,603 (7,936-9,031)	3,250 (3,070-3,433)	752 (710-807)
Multiple myeloma	1,665 (1,424-1,823)	2,565 (2,250-2,785)	4,078 (3,580-4,499)	5,440 (4,766-6,058)	7,855 (6,777-8,685)	9,732 (8,490-10,823)	10,653 (9,330-11,763)	9,976 (8,541-11,218)	6,692 (5,750-7,685)	4,596 (3,723-5,356)	1,703 (1,447-2,028)	347 (285-407)
Leukaemia	11,538 (10,158-12,199)	13,427 (12,039-14,289)	14,137 (12,848-14,951)	15,604 (14,259-16,489)	20,204 (19,015-21,585)	23,273 (21,924-24,933)	25,401 (24,516-27,105)	22,846 (22,139-24,186)	15,202 (14,727-16,154)	10,794 (10,412-11,351)	4,652 (4,472-4,885)	1,264 (1,210-1,329)
Acute lymphoid leukaemia	1,701 (1,543-1,990)	1,860 (1,697-2,209)	1,885 (1,692-2,140)	2,092 (1,893-2,396)	2,328 (2,132-2,661)	2,291 (2,104-2,692)	2,248 (2,088-2,578)	1,325 (1,230-1,524)	779 (729-919)	453 (425-514)	176 (165-194)	48 (45-53)
Chronic lymphoid leukaemia	477 (417-599)	622 (566-771)	1,000 (914-1,174)	1,428 (1,305-1,732)	2,614 (2,402-3,015)	3,386 (3,155-3,967)	4,215 (3,905-4,900)	4,265 (3,962-4,855)	3,194 (2,910-3,543)	2,743 (2,512-3,058)	1,296 (1,211-1,407)	377 (352-405)
Acute myeloid leukaemia	2,567 (2,303-2,855)	3,096 (2,844-3,461)	3,491 (3,244-3,862)	3,879 (3,558-4,315)	4,986 (4,583-5,534)	5,918 (5,482-6,531)	6,620 (6,187-7,393)	5,897 (5,534-6,589)	3,553 (3,345-4,007)	2,178 (2,043-2,467)	800 (749-920)	185 (172-212)
Chronic myeloid leukaemia	1,429 (1,257-1,592)	1,672 (1,505-1,853)	1,762 (1,575-1,933)	1,827 (1,644-1,991)	2,161 (1,947-2,331)	2,376 (2,151-2,548)	2,574 (2,368-2,761)	2,575 (2,404-2,726)	1,725 (1,623-1,834)	1,296 (1,212-1,379)	590 (549-631)	162 (147-172)
Other leukaemia	5,364 (4,416-5,798)	6,178 (5,148-6,654)	5,999 (5,071-6,444)	6,377 (5,449-6,854)	8,115 (7,282-8,795)	9,303 (8,319-9,995)	9,744 (9,003-10,494)	8,784 (8,186-9,544)	5,950 (5,647-6,471)	4,124 (3,931-4,489)	1,790 (1,695-1,938)	492 (465-534)
Other neoplasms	12,877 (12,182-13,377)	15,623 (15,012-16,165)	18,292 (17,629-18,980)	20,189 (19,511-20,894)	25,861 (24,876-26,991)	28,702 (27,883-29,855)	29,878 (29,046-31,030)	26,182 (25,388-27,357)	17,696 (17,107-18,395)	12,529 (12,057-13,053)	5,500 (5,291-5,727)	1,539 (1,475-1,599)
Cardiovascular diseases	322,422 (313,348-332,029)	489,442 (477,116-502,749)	638,871 (624,098-654,328)	815,286 (796,511-833,897)	1,269,355 (1,239,233-1,299,277)	1,647,942 (1,609,217-1,687,931)	2,091,958 (2,042,146-2,138,928)	2,149,448 (2,098,550-2,200,294)	1,851,529 (1,802,605-1,903,994)	1,573,297 (1,533,183-1,619,650)	806,364 (782,846-830,907)	276,962 (266,698-285,059)
Rheumatic heart disease	18,770 (16,535-20,775)	21,336 (19,017-23,416)	23,452 (20,718-25,727)	25,990 (23,090-28,504)	33,159 (29,814-36,088)	38,375 (34,366-41,906)	40,790 (36,864-44,490)	35,782 (32,688-38,974)	28,190 (25,933-31,008)	19,887 (18,541-21,608)	9,301 (8,765-10,025)	2,963 (2,806-3,156)
Ischaemic heart disease	158,778 (153,381-164,523)	246,667 (238,955-255,027)	321,186 (312,136-330,762)	408,683 (397,706-419,294)	628,441 (612,541-644,671)	788,394 (767,391-810,176)	1,009,093 (982,560-1,036,424)	1,058,950 (1,032,068-1,085,321)	913,352 (892,440-941,697)	829,294 (811,009-852,929)	456,354 (446,148-470,763)	164,850 (160,959-170,124)
Cerebrovascular disease	101,370 (98,143-105,056)	163,866 (158,854-169,621)	223,226 (216,803-231,496)	293,523 (284,571-304,260)	480,887 (465,768-498,423)	653,254 (631,374-679,587)	823,123 (793,947-856,142)	805,416 (767,376-846,987)	668,547 (628,963-710,975)	501,399 (468,078-538,019)	218,468 (200,739-237,544)	67,944 (60,889-74,223)

SR Table 5: Global deaths by cause and age group for both sexes (with 95% uncertainty intervals), 2000

Cause	Early Neonatal	Late Neonatal	Post Neonatal	1 to 4	5 to 9	10 to 14	15 to 19	20 to 24	25 to 29	30 to 34	35 to 39
Ischaemic stroke	-	-	459 (385-556)	346 (288-428)	316 (281-362)	353 (317-390)	1,330 (1,232-1,433)	2,726 (2,517-2,932)	3,781 (3,555-4,019)	5,406 (5,103-5,730)	8,240 (7,782-8,647)
Hemorrhagic stroke	2,306 (2,050-2,594)	1,245 (1,095-1,441)	9,825 (8,433-11,670)	6,270 (5,225-7,939)	4,127 (3,709-4,654)	4,680 (4,345-5,083)	8,057 (7,644-8,618)	15,097 (14,382-16,015)	21,495 (20,488-22,730)	34,238 (32,783-36,044)	53,062 (50,985-55,630)
Hypertensive heart disease	-	-	467 (333-575)	225 (151-278)	196 (148-231)	245 (176-301)	1,654 (1,293-1,960)	2,437 (1,957-2,864)	3,380 (2,708-3,945)	5,251 (4,225-6,047)	8,035 (6,652-9,220)
Cardiomyopathy and myocarditis	941 (653-1,113)	566 (430-662)	5,151 (3,893-6,042)	3,144 (2,443-3,831)	1,566 (1,306-1,777)	1,821 (1,644-2,099)	3,159 (2,745-3,560)	3,955 (3,315-4,402)	5,734 (4,757-6,409)	6,680 (5,706-7,388)	10,065 (8,619-11,333)
Myocarditis	204 (116-282)	154 (97-205)	2,020 (1,265-2,787)	1,364 (881-1,996)	718 (551-861)	752 (628-913)	1,305 (1,053-1,523)	1,361 (1,102-1,577)	1,688 (1,368-1,986)	1,349 (1,117-1,594)	1,513 (1,193-1,751)
Alcoholic cardiomyopathy	-	-	-	-	-	-	254 (213-351)	612 (429-757)	1,416 (1,009-1,725)	2,429 (1,860-2,882)	4,781 (3,827-5,794)
Other cardiomyopathy	737 (505-925)	413 (295-519)	3,131 (2,070-4,239)	1,781 (1,201-2,564)	849 (689-1,067)	1,069 (929-1,269)	1,599 (1,384-1,846)	1,981 (1,634-2,267)	2,631 (2,149-3,031)	2,901 (2,429-3,278)	3,771 (3,117-4,235)
Atrial fibrillation and flutter	-	-	-	-	-	-	-	-	-	32 (23-44)	124 (91-165)
Aortic aneurysm	-	-	-	-	-	-	402 (362-454)	535 (486-595)	816 (725-917)	1,127 (1,017-1,262)	1,744 (1,588-1,938)
Peripheral vascular disease	-	-	-	-	-	-	-	-	-	-	-
Endocarditis	140 (93-176)	86 (59-110)	1,877 (1,370-2,385)	822 (590-1,186)	403 (316-481)	539 (448-633)	1,047 (882-1,276)	1,435 (1,222-1,774)	1,934 (1,686-2,418)	1,926 (1,635-2,365)	2,279 (1,958-2,746)
Other cardiovascular and circulatory diseases	1,632 (1,349-1,886)	967 (809-1,150)	7,428 (6,308-9,253)	4,272 (3,356-5,769)	2,248 (1,830-2,747)	2,362 (1,951-2,844)	3,177 (2,756-3,939)	4,470 (3,917-5,770)	5,726 (5,017-7,269)	6,537 (5,766-8,021)	7,789 (6,679-9,384)
Chronic respiratory diseases	0 (0-0)	0 (0-0)	0 (0-0)	23,516 (19,437-28,043)	6,735 (5,901-7,643)	6,831 (6,214-7,631)	11,432 (10,625-12,581)	14,773 (14,034-16,007)	19,241 (18,251-20,598)	24,541 (23,261-26,102)	33,605 (31,691-35,606)
Chronic obstructive pulmonary disease	-	-	0 (0-0)	5,086 (3,586-8,384)	1,442 (1,105-1,990)	1,191 (958-1,529)	4,588 (3,896-5,335)	6,500 (5,673-7,489)	9,276 (8,120-10,519)	13,079 (11,600-14,694)	17,648 (15,613-20,056)
Pneumoconiosis	-	-	-	-	-	-	51 (43-60)	77 (66-95)	191 (156-259)	416 (327-573)	592 (502-800)
Silicosis	-	-	-	-	-	-	5 (4-7)	14 (11-18)	74 (55-101)	239 (178-329)	381 (316-519)
Asbestosis	-	-	-	-	-	-	22 (18-27)	29 (24-36)	38 (32-48)	47 (38-62)	50 (39-65)
Coal workers pneumoconiosis	-	-	-	-	-	-	3 (1-4)	4 (2-5)	20 (12-29)	27 (17-39)	41 (23-56)
Other pneumoconiosis	-	-	-	-	-	-	21 (17-26)	31 (25-40)	59 (45-93)	103 (70-175)	121 (92-182)
Asthma	-	-	-	15,215 (10,537-19,380)	4,018 (3,191-4,915)	4,687 (4,015-5,435)	5,900 (4,933-6,969)	7,026 (5,682-8,211)	8,544 (6,988-9,877)	9,412 (7,570-10,857)	13,203 (10,497-15,279)
Interstitial lung disease and pulmonary sarcoidosis	-	-	-	443 (175-983)	196 (106-365)	126 (81-198)	242 (142-410)	339 (206-555)	469 (292-746)	657 (418-1,026)	1,031 (661-1,603)
Other chronic respiratory diseases	0 (0-0)	0 (0-0)	0 (0-0)	2,772 (1,316-4,329)	1,079 (718-1,461)	827 (541-1,119)	652 (430-878)	831 (570-1,140)	762 (477-1,047)	978 (607-1,387)	1,130 (685-1,536)
Cirrhosis and other chronic liver diseases	-	-	-	11,282 (8,800-14,050)	4,681 (4,019-5,535)	5,305 (4,769-6,046)	8,846 (8,216-9,821)	14,684 (13,634-16,937)	22,206 (20,638-24,793)	38,026 (35,991-41,715)	57,098 (54,097-62,643)
Cirrhosis and other chronic liver diseases due to hepatitis B	-	-	-	87 (55-137)	97 (68-135)	197 (144-263)	560 (389-778)	3,100 (2,332-4,171)	7,268 (5,689-9,158)	13,781 (11,817-16,045)	20,352 (17,136-23,489)
Cirrhosis and other chronic liver diseases due to hepatitis C	-	-	-	69 (44-103)	73 (50-105)	153 (106-219)	409 (274-588)	2,012 (1,459-2,663)	4,384 (3,280-5,663)	8,759 (7,332-10,494)	14,284 (11,474-17,470)
Cirrhosis and other chronic liver diseases due to alcohol use	-	-	-	-	-	-	116 (66-182)	968 (693-1,340)	2,391 (1,736-3,247)	6,923 (5,841-8,299)	14,162 (11,642-17,276)
Cirrhosis and other chronic liver diseases due to other causes	-	-	-	11,126 (8,697-13,846)	4,512 (3,880-5,315)	4,954 (4,457-5,662)	7,761 (7,158-8,534)	8,604 (7,258-10,054)	8,163 (6,490-9,946)	8,562 (6,619-10,663)	8,301 (6,087-10,823)
Digestive diseases	2,098 (1,631-2,467)	2,135 (1,657-2,534)	20,707 (16,373-25,880)	20,509 (13,710-27,752)	9,419 (7,999-10,960)	9,090 (8,177-10,110)	13,978 (12,502-15,164)	16,829 (15,320-18,004)	21,900 (19,953-23,198)	24,284 (22,497-25,971)	30,388 (28,134-32,571)
Peptic ulcer disease	-	-	-	3,410 (2,345-4,792)	1,748 (1,376-2,255)	2,127 (1,802-2,579)	3,331 (2,893-3,894)	5,061 (4,488-5,693)	6,037 (5,379-6,673)	7,569 (6,789-8,453)	9,349 (8,486-10,331)
Gastritis and duodenitis	-	-	-	685 (305-1,244)	274 (191-411)	220 (170-296)	398 (330-492)	550 (462-670)	853 (713-1,036)	1,261 (1,085-1,503)	1,404 (1,210-1,679)
Appendicitis	-	-	-	3,915 (1,865-6,026)	2,218 (1,700-2,759)	2,279 (1,873-2,695)	3,601 (2,999-4,225)	2,803 (2,244-3,244)	3,061 (2,451-3,514)	2,028 (1,669-2,359)	2,325 (1,885-2,729)
Paralytic ileus and intestinal obstruction	2,098 (1,631-2,467)	2,135 (1,657-2,534)	20,707 (16,373-25,880)	4,925 (3,051-6,844)	2,729 (1,873-3,294)	2,362 (1,701-2,748)	3,692 (2,607-4,297)	3,832 (2,771-4,427)	4,773 (3,335-5,429)	4,197 (3,048-4,828)	5,592 (3,953-6,385)
Inguinal, femoral, and abdominal hernia	-	-	-	1,047 (469-1,688)	407 (267-530)	376 (224-485)	503 (320-627)	632 (383-811)	709 (436-861)	712 (394-894)	839 (569-1,014)
Inflammatory bowel disease	-	-	-	1,428 (668-3,727)	325 (195-568)	261 (178-392)	350 (243-512)	559 (381-826)	774 (559-1,095)	855 (613-1,182)	924 (692-1,209)
Vascular intestinal disorders	-	-	-	477 (170-897)	80 (51-112)	58 (46-68)	106 (84-128)	223 (176-266)	312 (253-367)	360 (283-427)	602 (491-704)
Gallbladder and biliary diseases	-	-	-	640 (362-973)	303 (231-417)	344 (285-418)	397 (347-484)	630 (565-751)	847 (744-989)	1,156 (1,009-1,352)	1,592 (1,415-1,832)
Pancreatitis	-	-	-	210 (121-320)	189 (136-270)	245 (190-326)	684 (556-880)	1,585 (1,356-1,989)	3,133 (2,723-3,769)	4,377 (3,823-5,267)	5,749 (5,042-6,932)
Other digestive diseases	-	-	-	3,772 (1,542-6,162)	1,144 (792-1,630)	817 (628-1,099)	916 (769-1,132)	953 (802-1,195)	1,403 (1,166-1,736)	1,769 (1,523-2,170)	2,012 (1,736-2,483)
Neurological disorders	-	-	8,103 (6,697-9,578)	9,825 (7,011-12,620)	6,358 (5,450-7,367)	9,395 (8,378-10,414)	15,040 (13,855-16,122)	14,021 (12,923-14,864)	12,596 (11,607-13,294)	12,506 (11,597-13,191)	12,916 (12,194-13,582)
Alzheimer's disease and other dementias	-	-	-	-	-	-	-	-	-	-	-
Parkinson's disease	-	-	-	-	-	-	-	2 (1-3)	21 (9-41)	78 (41-142)	167 (88-289)
Epilepsy	-	-	5,265 (4,211-6,298)	7,646 (5,463-9,795)	5,339 (4,503-6,212)	7,670 (6,744-8,542)	12,363 (11,243-13,314)	11,985 (10,908-12,782)	10,932 (9,976-11,578)	10,495 (9,574-11,103)	10,025 (9,316-10,590)
Multiple sclerosis	-	-	-	-	-	-	-	139 (114-191)	309 (258-402)	564 (493-693)	988 (871-1,209)
Motor neuron disease	8 (6-9)	10 (9-11)	813 (569-990)	333 (203-431)	47 (39-55)	84 (74-94)	110 (102-117)	97 (90-105)	103 (95-114)	211 (196-230)	436 (413-471)
Other neurological disorders	-	-	2,025 (1,552-2,713)	1,846 (1,208-2,765)	972 (787-1,244)	1,640 (1,414-1,923)	2,568 (2,346-2,812)	1,798 (1,698-1,920)	1,232 (1,159-1,306)	1,159 (1,100-1,217)	1,300 (1,249-1,352)
Mental and substance use disorders	18 (9-30)	24 (19-30)	0 (0-0)	0 (0-0)	18 (13-21)	18 (14-21)	7,249 (6,028-7,923)	14,462 (11,943-15,872)	20,214 (17,072-22,107)	25,306 (21,262-27,190)	29,931 (26,447-32,088)
Alcohol use disorders	-	-	-	-	-	-	1,301 (1,116-1,522)	3,239 (2,807-3,784)	6,425 (5,605-7,354)	10,696 (9,188-12,162)	16,123 (14,240-17,914)
Drug use disorders	18 (9-30)	24 (19-30)	0 (0-0)	0 (0-0)	0 (0-0)	0 (0-0)	5,904 (4,612-6,556)	11,156 (8,703-12,423)	13,712 (10,541-15,251)	14,532 (11,154-16,027)	13,733 (11,335-14,932)
Opioid use disorders	18 (9-30)	24 (19-30)	0 (0-0)	0 (0-0)	0 (0-0)	0 (0-0)	4,179 (3,204-4,777)	8,010 (5,810-9,154)	9,633 (6,885-10,985)	9,486 (6,624-10,795)	8,987 (6,521-10,080)
Cocaine use disorders	0 (0-0)	0 (0-0)	0 (0-0)	0 (0-0)	0 (0-0)	0 (0-0)	204 (131-259)	404 (265-518)	555 (381-689)	934 (634-1,127)	883 (659-1,111)
Amphetamine use disorders	0 (0-0)	0 (0-0)	0 (0-0)	0 (0-0)	0 (0-0)	0 (0-0)	166 (109-215)	311 (210-395)	418 (282-535)	496 (335-621)	445 (325-575)
Other drug use disorders	0 (0-0)	0 (0-0)	0 (0-0)	0 (0-0)	0 (0-0)	0 (0-0)	1,354 (956-1,548)	2,431 (1,818-2,853)	3,107 (2,314-3,502)	3,617 (2,760-4,048)	3,418 (2,782-3,850)
Eating disorders	-	-	-	-	18 (13-21)	18 (14-21)	45 (36-52)	67 (56-78)	76 (64-87)	78 (68-87)	75 (66-84)
Anorexia nervosa	-	-	-	-	17 (13-20)	17 (13-20)	43 (34-51)	64 (53-75)	73 (61-84)	72 (63-81)	70 (61-79)
Bulimia nervosa	-	-	-	-	1 (1-2)	1 (1-2)	2 (1-3)	3 (2-4)	3 (3-5)	5 (4-8)	5 (4-6)
Diabetes, urogenital, blood, and endocrine diseases	4,586 (3,477-5,191)	3,350 (2,636-3,760)	29,389 (25,916-32,476)	35,468 (26,198-42,698)	15,484 (13,623-17,867)	18,641 (16,467-20,539)	27,493 (25,071-29,776)	33,759 (31,802-36,494)	35,338 (33,402-37,112)	42,399 (40,455-44,515)	49,579 (47,719-51,767)
Diabetes mellitus	61 (57-65)	102 (92-113)	1,022 (934-1,131)	1,034 (882-1,179)	1,123 (996-1,249)	1,791 (1,641-1,938)	3,254 (3,068-3,441)	5,080 (4,843-5,358)	6,449 (6,184-6,739)	9,397 (9,048-9,804)	13,289 (12,826-13,773)
Acute glomerulonephritis	-	-	171 (143-204)	277 (232-328)	321 (281-377)	363 (316-418)	338 (305-379)	441 (393-498)	598 (531-676)	669 (602-745)	669 (606-738)
Chronic kidney disease	-	-	5,078 (4,452-5,606)	6,963 (5,871-7,960)	4,515 (3,937-5,009)	5,314 (4,826-5,835)	9,445 (8,843-10,356)	14,325 (13,255-15,598)	16,797 (15,630-18,086)	20,419 (18,943-21,954)	23,885 (22,067-25,493)
Chronic kidney disease due to diabetes mellitus	-	-	133 (89-194)	191 (119-286)	162 (92-259)	263 (159-414)	721 (433-1,110)	2,097 (1,332-2,914)	4,129 (2,990-5,353)	6,857 (5,253-8,588)	9,360 (7,257-11,512)
Chronic kidney disease due to hypertension	-	-	35 (24-50)	69 (46-100)	75 (48-112)	149 (97-220)	408 (264-580)	901 (623-1,231)	1,361 (936-1,857)	2,026 (1,494-2,682)	2,759 (2,081-3,564)
Chronic kidney disease due to glomerulonephritis	-	-	2,050 (1,531-2,576)	2,887 (2,061-3,750)	1,843 (1,310-2,435)	2,303 (1,641-2,997)	4,157 (3,000-5,317)	6,022 (4,384-7,767)	6,117 (4,312-8,030)	5,904 (4,107-7,973)	5,658 (3,843-7,714)
Chronic kidney disease due to other causes	-	-	2,859 (2,305-3,468)	3,817 (2,953-4,737)	2,435 (1,854-3,052)	2,599 (1,898-3,308)	4,159 (3,015-5,368)	5,306 (3,722-6,955)	5,191 (3,649-6,955)	5,633 (3,943-7,542)	6,108 (4,340-8,059)
Urinary diseases and male infertility	384 (349-424)	552 (463-667)	5,491 (4,928-6,106)	3,879 (3,220-4,560)	1,473 (1,261-1,713)	1,297 (1,173-1,435)	2,717 (2,521-2,937)	3,405 (3,205-3,610)	3,255 (3,075-3,433)	3,865 (3,685-4,042)	4,336 (4,126-4,552)
Interstitial nephritis and urinary tract infections	190 (139-236)	299 (215-390)	3,643 (2,958-4,295)	2,200 (1,519-2,873)	714 (545-867)	655 (530-752)	1,653 (1,377-1,893)	2,076 (1,721-2,324)	1,836 (1,536-2,041)	2,146 (1,844-2,357)	2,518 (2,188-2,762)
Urolithiasis	-	-	-	-	227 (144-304)	204 (135-253)	322 (220-401)	352 (234-432)	337 (212-416)	394 (259-471)	455 (323-538)
Other urinary diseases	194 (153-233)	252 (180-349)	1,847 (1,302-2,490)	1,679 (1,069-2,350)	532 (369-725)	437 (319-593)	742 (572-1,055)	976 (748-1,360)	1,081 (847-1,418)	1,324 (1,068-1,702)	1,363 (1,088-1,777)
Gynecological diseases	-	-	-	-	-	-	238 (192-269)	325 (260-373)	369 (312-416)	474 (391-533)	605 (517-690)
Uterine fibroids	-	-	-	-	-	-	15 (7-33)	21 (12-38)	37 (18-61)	75 (32-131)	172 (72-261)
Polycystic ovarian syndrome	-	-	-	-	-	-	3 (0-9)	26 (6-79)	68 (18-149)	55 (12-151)	71 (19-155)
Endometriosis	-	-	-	-	-	-	4 (1-10)	3 (1-7)	3 (1-7)	6 (2-11)	5 (2-8)
Genital prolapse	-	-	-	-	-	-	14 (4-45)	13 (3-29)	20 (7-40)	25 (4-45)	8 (3-16)
Other gynecological diseases	-	-	-	-	-	-	203 (143-241)	262 (180-318)	240 (141-316)	312 (190-417)	349 (216-491)
Hemoglobinopathies and hemolytic anaemias	2,473 (1,699-2,996)	1,380 (965-1,613)	9,902 (7,411-11,971)	18,325 (9,911-24,618)	5,785 (4,394-7,492)	7,797 (5,906-9,254)	9,571 (7,126-11,346)	8,022 (6,485-10,132)	5,068 (3,856-6,109)	4,139 (3,277-5,035)	3,228 (2,665-3,863)
Thalassemias	601 (462-742)	413 (297-516)	2,687 (1,951-3,432)	4,057 (2,842-5,347)	1,979 (1,600-2,589)	1,099 (930-1,409)	619 (494-767)	340 (261-412)	238 (174-286)	131 (106-159)	72 (61-88)
Sickle cell disorders	1,683 (1,035-2,175)	908 (608-1,109)	6,810 (4,861-8,492)	13,533 (6,368-18,770)	3,396 (2,417-4,545)	5,956 (4,327-7,311)	7,699 (5,596-9,215)	6,045 (4,771-7,711)	3,149 (2,284-3,861)	2,255 (1,691-2,768)	1,448 (1,146-1,767)
G6PD deficiency	62 (43-81)	36 (25-48)	285 (205-377)	580 (397-794)	309 (236-403)	508 (384-629)	827 (651-1,024)	1,056 (847-1,345)	1,114 (880-1,376)	1,084 (853-1,312)	931 (767-1,123)
Other hemoglobinopathies and hemolytic anaemias	127 (94-157)	23 (17-27)	120 (90-152)	156 (114-201)	101 (83-128)	234 (190-279)	426 (328-522)	581 (479-741)	566 (447-696)	668 (534-842)	777 (653-943)
Endocrine, metabolic, blood, and immune disorders	1,668 (1,169-1,882)	1,316 (936-1,533)	7,726 (6,473-9,260)	4,989 (4,034-6,308)	2,267 (2,023-2,823)	2,079 (1,895-2,528)	1,931 (1,771-2,300)	2,161 (1,904-2,503)	2,801 (2,389-3,178)	3,437 (2,982-3,953)	3,566 (3,159-4,086)
Musculoskeletal disorders	-	-	-	-	787 (604-997)	1,347 (1,131-1,670)	1,991 (1,625-2,383)	2,347 (1,936-2,779)	2,705 (2,160-3,328)	2,683 (2,227-3,125)	2,779 (2,349-3,235)
Rheumatoid arthritis	-	-	-	-	166 (105-276)	185 (135-280)	151 (117-235)	182 (136-298)	222 (167-355)	275 (212-421)	351 (279-498)
Other musculoskeletal disorders	-	-	-	-	621 (481-809)	1,163 (957-1,433)	1,840 (1,467-2,233)	2,165 (1,788-2,586)	2,483 (1,991-3,038)	2,408 (1,983-2,826)	2,428 (2,038-2,816)
Other non-communicable diseases	191,187 (147,860-230,880)	74,409 (59,914-90,052)	246,516 (197,786-292,417)	78,114 (53,239-105,151)	20,706 (17,226-24,709)	15,945 (13,716-18,145)	11,101 (9,596-12,476)	8,963 (7,677-10,051)	7,589 (6,614-8,549)	6,261 (5,270-6,949)	5,826 (4,856-6,432)
Congenital anomalies	191,187 (147,860-230,880)	70,351 (56,247-85,416)	208,523 (160,880-253,338)	76,079 (50,789-103,791)	20,127 (16,736-24,218)	15,292 (13,114-17,555)	9,975 (8,821-11,466)	8,013 (7,033-9,197)	6,726 (5,994-7,746)	4,808 (4,317-5,410)	4,425 (3,865-4,965)
Neural tube defects	30,125 (20,555-43,871)	8,167 (5,350-12,564)	18,152 (11,271-29,866)	6,165 (2,770-13,690)	1,131 (793-1,684)	811 (614-1,127)	389 (305-526)	277 (215-370)	208 (166-271)	169 (136-213)	124 (103-153)
Congenital heart anomalies	62,658 (44,197-77,399)	30,662 (23,927-36,934)	106,755 (87,810-127,221)	38,697 (29,944-50,924)	12,541 (10,456-15,280)	10,193 (8,690-11,714)	6,669 (5,849-7,761)	5,683 (4,982-6,541)	4,991 (4,464-5,803)	3,340 (2,977-3,829)	3,180 (2,814-3,619)
Orofacial clefts	1,772 (854-2,568)	632 (323-951)	1,301 (627-1,953)	171 (49-323)	-	-	-	-	-	-	-
Down's syndrome	2,185 (1,303-4,714)	1,070 (643-2,330)	4,805 (2,905-10,361)	2,157 (1,116-6,048)	398 (271-662)	333 (239-477)	312 (257-433)	283 (235-383)	289 (240-389)	319 (262-417)	298 (247-388)
Other chromosomal abnormalities	5,102 (3,136-8,668)	2,433 (1,511-4,190)	5,236 (3,199-10,038)	977 (538-2,289)	246 (175-383)	172 (120-229)	163 (118-214)	117 (81-140)	98 (65-116)	72 (44-82)	65 (38-75)
Congenital musculoskeletal and limb anomalies	4,923 (3,094-9,780)	1,226 (748-2,555)	2,491 (1,444-5,878)	729 (373-2,189)	270 (182-498)	260 (177-394)	221 (160-319)	112 (74-156)	93 (65-128)	75 (49-98)	56 (41-70)
Urogenital congenital anomalies	5,228 (3,593-6,867)	1,564 (1,004-2,016)	2,720 (1,672-3,639)	610 (355-898)	302 (215-412)	302 (222-396)	133 (102-180)	118 (95-155)	115 (89-148)	112 (87-138)	115 (86-140)
Digestive congenital anomalies	16,727 (12,525-26,049)	6,897 (4,998-10,916)	16,048 (10,698-30,580)	3,004 (1,682-8,276)	375 (288-577)	205 (170-276)	110 (92-144)	72 (62-91)	55 (46-67)	45 (38-54)	45 (35-51)
Other congenital anomalies	62,469 (40,068-99,826)	17,701 (10,019-30,187)	51,013 (27,327-87,657)	23,570 (9,742-47,512)	4,863 (3,098-7,469)	3,016 (2,169-4,235)	1,977 (1,482-2,673)	1,350 (977-1,927)	876 (648-1,225)	675 (519-908)	543 (432-709)
Skin and subcutaneous diseases	-	-	3,957 (1,945-5,434)	2,035 (946-2,921)	579 (280-800)	653 (329-850)	1,125 (597-1,430)	950 (509-1,168)	863 (476-1,065)	1,453 (768-1,819)	1,401 (774-1,714)
Cellulitis	-	-	199 (67-330)	186 (57-328)	87 (33-136)	100 (45-144)	117 (58-160)	133 (63-170)	106 (48-134)	189 (86-249)	234 (106-299)
Pyoderma	-	-	3,650 (1,779-5,070)	1,635 (769-2,441)	388 (182-555)	429 (203-583)	802 (398-1,044)	634 (316-800)	585 (303-740)	1,004 (505-1,294)	911 (478-1,156)
Decubitus ulcer	-	-	-	90 (37-140)	51 (30-67)	65 (39-87)	120 (72-164)	105 (65-138)	114 (69-151)	193 (112-258)	195 (119-269)
Other skin and subcutaneous diseases	-	-	108 (55-178)	123 (60-194)	53 (32-75)	59 (35-82)	86 (54-118)	78 (48-107)	58 (37-79)	68 (44-94)	61 (40-83)
Sudden infant death syndrome	-	4,059 (3,106-5,383)	34,036 (26,829-47,223)	-	-	-	-	-	-	-	-
Injuries	17,004 (13,624-19,027)	10,517 (8,394-12,101)	106,380 (91,554-123,189)	271,005 (226,551-307,996)	172,819 (158,876-184,232)	169,317 (160,289-177,117)	315,795 (305,456-324,215)	418,487 (406,183-428,382)	400,825 (388,347-410,267)	364,796 (351,345-373,153)	334,270 (320,093-342,866)
Transport injuries	3,158 (2,425-4,076)	1,675 (1,434-1,947)	15,565 (13,808-17,646)	52,264 (44,574-59,166)	54,636 (50,360-58,605)	48,549 (45,981-51,411)	111,278 (107,150-116,818)	149,304 (144,154-157,041)	143,441 (138,567-150,275)	128,586 (124,705-134,085)	116,599 (113,008-120,919)
Road injuries	2,713 (2,122-3,531)	1,476 (1,282-1,715)	14,206 (12,625-16,078)	49,298 (41,791-55,783)	52,217 (48,197-56,057)	45,568 (43,222-48,416)	103,596 (99,612-109,112)	140,614 (135,804-148,611)	134,195 (129,550-141,735)	120,748 (116,977-126,298)	108,757 (105,286-113,313)
Pedestrian road injuries	1,143 (827-1,738)	599 (475-777)	6,091 (4,993-7,521)	27,972 (23,207-33,181)	30,631 (27,730-33,957)	22,901 (21,176-25,469)	28,748 (26,515-32,273)	38,070 (34,801-43,080)	39,401 (35,917-44,497)	40,640 (37,669-45,615)	38,304 (35,335-42,651)
Cyclist road injuries	-	-	-	1,518 (1,025-1,913)	2,597 (2,197-3,000)	3,720 (3,299-4,145)	4,841 (4,297-5,382)	4,586 (4,051-5,067)	4,835 (4,276-5,313)	5,367 (4,750-5,913)	4,896 (4,299-5,408)
Motorcyclist road injuries	303 (212-441)	176 (127-255)	1,879 (1,497-2,399)	3,665 (2,703-5,028)	3,189 (2,729-3,757)	4,546 (4,070-5,082)	25,442 (23,128-28,830)	36,914 (33,471-41,212)	34,327 (30,931-38,591)	27,886 (24,583-31,125)	22,898 (20,302-25,506)
Motor vehicle road injuries	1,206 (817-1,691)	677 (525-842)	5,944 (4,660-7,312)	15,650 (11,691-19,657)	15,265 (12,733-18,049)	13,883 (12,126-16,166)	43,684 (38,951-49,802)	59,850 (52,741-68,481)	54,582 (48,472-63,042)	45,982 (40,820-53,520)	41,814 (37,056-47,847)
Other road injuries	61 (36-107)	23 (17-34)	293 (209-445)	495 (339-770)	534 (444-702)	518 (439-680)	881 (744-1,167)	1,194 (1,007-1,506)	1,050 (881-1,330)	872 (737-1,100)	844 (712-1,033)
Other transport injuries	445 (265-664)	200 (131-279)	1,359 (1,047-1,954)	2,965 (2,217-4,234)	2,420 (1,999-2,927)	2,982 (2,561-3,390)	7,681 (6,465-8,576)	8,690 (7,517-9,652)	9,247 (7,900-10,310)	7,838 (6,751-8,588)	7,842 (6,720-8,632)
Unintentional injuries	10,858 (8,252-12,411)	7,762 (5,874-9,224)	84,146 (69,777-100,479)	198,381 (161,256-232,011)	103,930 (93,653-112,825)	91,185 (83,799-96,523)	83,319 (77,186-87,072)	92,807 (86,525-96,624)	90,089 (84,629-93,653)	89,888 (84,339-92,999)	89,480 (83,901-92,987)
Falls	2,389 (1,522-3,209)	843 (587-1,064)	10,635 (7,512-13,534)	17,509 (12,249-22,942)	9,291 (7,837-10,828)	8,972 (7,878-10,121)	9,957 (9,042-11,086)	13,158 (11,980-14,797)	14,835 (13,374-16,504)	16,682 (15,137-18,473)	18,496 (16,856-20,375)
Drowning	605 (481-684)	295 (231-347)	10,130 (8,148-11,561)	106,472 (81,826-121,225)	58,320 (50,376-63,220)	48,963 (43,909-51,645)	32,365 (29,643-34,337)	27,583 (25,584-29,327)	22,288 (20,732-23,612)	20,362 (18,869-21,454)	18,305 (17,177-19,261)
Fire, heat, and hot substances	481 (363-541)	460 (298-539)	6,774 (4,597-7,868)	18,398 (10,937-23,087)	6,406 (4,881-7,363)	4,731 (3,683-5,249)	7,669 (5,725-8,703)	9,941 (7,505-11,056)	9,016 (7,113-9,926)	8,700 (7,142-9,462)	8,350 (6,970-9,018)
Poisonings	431 (232-753)	292 (184-428)	5,069 (2,708-8,449)	9,263 (5,812-13,422)	3,611 (2,845-4,322)	4,347 (3,636-5,262)	3,892 (3,203-4,695)	5,107 (4,158-6,330)	5,292 (4,315-6,461)	4,632 (3,868-5,766)	4,341 (3,480-5,114)
Exposure to mechanical forces	2,486 (1,611-3,067)	2,358 (1,415-3,211)	15,971 (12,139-18,771)	12,566 (8,573-15,997)	7,268 (6,267-8,624)	6,120 (5,604-7,300)	8,910 (8,219-9,695)	11,862 (10,947-12,813)	12,674 (11,799-13,852)	12,187 (11,380-13,460)	12,201 (11,110-13,257)
Unintentional firearm injuries	90 (65-106)	58 (43-73)	650 (477-849)	1,083 (678-1,560)	880 (733-1,062)	1,050 (925-1,238)	2,202 (1,944-2,652)	3,108 (2,357-3,624)	3,032 (2,328-3,533)	2,351 (1,856-2,668)	1,935 (1,477-2,166)
Unintentional suffocation	1,849 (1,067-2,399)	2,085 (1,166-2,904)	12,120 (8,483-14,566)	4,232 (2,941-6,138)	1,733 (1,398-2,725)	1,646 (1,387-2,481)	535 (467-751)	650 (553-936)	679 (592-956)	494 (411-681)	433 (366-578)
Other exposure to mechanical forces	547 (426-612)	215 (169-249)	3,201 (2,576-3,778)	7,251 (4,775-9,160)	4,655 (3,983-5,347)	3,424 (3,073-3,760)	6,173 (5,542-6,798)	8,104 (7,450-9,125)	8,964 (8,262-10,206)	9,343 (8,673-10,803)	9,833 (8,974-10,997)
Adverse effects of medical treatment	1,541 (1,063-1,793)	1,089 (719-1,316)	9,752 (5,626-12,588)	8,249 (3,485-12,535)	3,012 (2,054-3,713)	2,402 (1,857-2,747)	2,906 (2,259-3,271)	4,652 (3,609-5,180)	4,537 (3,522-5,021)	4,289 (3,303-4,729)	4,587 (3,634-5,086)
Animal contact	444 (334-523)	434 (314-511)	3,483 (2,341-4,251)	8,284 (5,460-11,165)	7,418 (5,090-9,171)	7,144 (4,922-8,671)	5,675 (3,963-6,752)	5,242 (3,735-6,113)	5,111 (3,745-5,965)	5,515 (3,834-6,496)	4,973 (3,679-5,796)
Venomous animal contact	362 (263-439)	357 (248-434)	2,870 (1,861-3,626)	6,787 (4,236-8,847)	6,321 (4,131-7,936)	6,389 (4,276-7,886)	4,880 (3,212-5,925)	4,411 (2,948-5,291)	4,238 (2,926-5,078)	4,763 (3,111-5,731)	4,285 (3,006-5,111)
Non-venomous animal contact	82 (60-117)	77 (55-117)	613 (419-1,067)	1,497 (745-3,227)	1,097 (806-1,664)	755 (573-1,045)	795 (615-1,054)	832 (650-1,122)	873 (688-1,162)	752 (609-992)	687 (560-887)
Foreign body	1,616 (995-1,962)	1,544 (968-1,970)	17,972 (11,737-24,605)	9,871 (5,824-16,113)	3,464 (2,583-4,291)	2,617 (2,093-2,979)	1,982 (1,632-2,238)	2,798 (2,300-3,181)	2,888 (2,362-3,271)	3,426 (2,837-3,832)	3,979 (3,285-4,551)
Pulmonary aspiration and foreign body in airway	1,521 (937-1,847)	1,482 (935-1,896)	16,755 (10,858-23,111)	8,319 (4,909-13,962)	2,727 (2,016-3,418)	2,075 (1,673-2,415)	1,454 (1,241-1,706)	2,106 (1,730-2,494)	2,217 (1,825-2,669)	2,577 (2,141-3,014)	3,066 (2,510-3,661)
Foreign body in other body part	95 (44-122)	62 (28-80)	1,217 (553-1,625)	1,552 (668-2,577)	737 (455-942)	542 (335-681)	529 (321-693)	691 (431-891)	671 (412-849)	849 (554-1,054)	913 (615-1,120)
Environmental heat and cold exposure	413 (201-511)	273 (117-353)	2,437 (1,161-3,063)	2,008 (1,020-2,700)	915 (573-1,112)	725 (456-838)	1,464 (893-1,714)	1,895 (1,162-2,256)	2,372 (1,524-2,875)	3,323 (2,085-4,046)	4,345 (2,927-5,706)
Other unintentional injuries	452 (405-507)	172 (148-198)	1,922 (1,593-2,252)	5,760 (4,089-7,072)	4,224 (3,662-4,790)	5,164 (4,698-5,692)	8,499 (7,749-9,339)	10,568 (9,770-11,668)	11,074 (10,300-12,411)	10,770 (10,038-11,783)	9,904 (9,179-10,748)
Self-harm and interpersonal violence	1,956 (1,480-2,204)	509 (396-579)	4,727 (3,703-5,402)	8,358 (6,616-9,808)	6,865 (5,290-7,820)	22,711 (19,799-24,432)	107,768 (98,348-113,890)	157,432 (141,735-165,373)	153,792 (138,274-161,145)	136,447 (121,589-143,000)	121,217 (106,895-127,831)

Cause	40 to 44	45 to 49	50 to 54	55 to 59	60 to 64	65 to 69	70 to 74	75 to 79	80 to 84	85 to 89	90 to 94	95 plus
Ischaemic stroke	14,768 (14,042-15,426)	31,016 (29,612-32,429)	48,769 (46,573-50,879)	72,741 (69,276-75,797)	185,958 (178,109-193,203)	271,247 (260,530-280,789)	377,615 (363,335-391,818)	437,739 (416,535-458,216)	375,632 (353,657-397,527)	310,030 (289,442-332,040)	142,672 (130,991-154,846)	46,431 (41,606-50,822)
Hemorrhagic stroke	86,602 (83,679-90,123)	132,850 (128,406-138,029)	174,457 (168,815-181,358)	220,783 (212,862-230,520)	294,929 (283,662-308,369)	382,007 (366,964-402,303)	445,508 (426,798-468,653)	367,677 (350,070-389,309)	292,916 (274,879-315,199)	191,370 (177,446-207,825)	75,796 (69,658-83,265)	21,513 (19,240-23,743)
Hypertensive heart disease	12,699 (10,552-14,559)	20,061 (16,065-22,808)	27,393 (22,263-30,988)	37,395 (31,096-42,413)	54,224 (44,850-61,256)	74,970 (61,603-84,739)	95,161 (78,527-107,715)	102,728 (85,359-115,565)	92,240 (76,011-104,751)	74,897 (60,232-87,595)	40,031 (31,901-44,979)	13,856 (10,121-16,013)
Cardiomyopathy and myocarditis	14,684 (12,446-16,781)	16,975 (14,529-19,329)	17,683 (15,534-20,101)	16,595 (14,484-18,524)	21,028 (18,630-23,923)	21,023 (17,986-22,427)	24,441 (19,248-26,316)	27,044 (19,542-29,858)	25,289 (16,882-28,461)	27,305 (17,145-31,550)	16,545 (9,861-19,389)	6,255 (3,624-7,313)
Myocarditis	1,722 (1,347-2,062)	1,337 (1,059-1,510)	1,238 (988-1,429)	1,411 (1,079-1,609)	1,446 (1,028-1,657)	1,799 (1,305-2,062)	2,359 (1,669-2,723)	2,969 (1,954-3,481)	3,299 (2,124-3,993)	4,481 (2,595-5,698)	3,290 (1,646-4,315)	1,503 (692-1,966)
Alcoholic cardiomyopathy	7,886 (6,313-9,561)	9,883 (7,986-11,946)	9,619 (7,957-11,594)	7,218 (6,083-8,591)	9,537 (8,222-12,135)	6,646 (5,817-8,216)	5,842 (4,918-7,273)	4,070 (3,168-4,762)	2,489 (1,792-2,877)	1,994 (1,274-2,400)	1,010 (639-1,216)	349 (228-418)
Other cardiomyopathy	5,076 (4,148-5,945)	5,755 (4,833-6,720)	6,826 (5,833-7,861)	7,966 (6,746-8,916)	10,045 (8,430-11,191)	12,578 (10,198-13,641)	16,241 (12,188-18,032)	20,005 (13,979-22,709)	19,501 (12,622-22,621)	20,830 (12,628-25,072)	12,245 (7,275-15,390)	4,403 (2,596-5,731)
Atrial fibrillation and flutter	283 (206-370)	538 (389-708)	840 (624-1,094)	1,382 (810-2,073)	3,010 (1,966-4,250)	6,076 (3,736-9,157)	12,106 (8,616-16,884)	18,245 (11,587-27,339)	31,900 (23,996-40,975)	39,998 (30,242-51,931)	21,367 (16,283-27,806)	6,126 (4,692-7,932)
Aortic aneurysm	2,380 (2,205-2,588)	3,417 (3,208-3,685)	4,605 (4,384-4,906)	6,460 (6,177-6,874)	10,109 (9,709-10,567)	14,478 (13,970-15,093)	19,676 (19,068-20,429)	22,086 (21,470-22,945)	18,394 (17,851-19,078)	14,580 (14,174-15,130)	6,853 (6,651-7,105)	1,875 (1,817-1,944)
Peripheral vascular disease	193 (126-235)	354 (222-439)	664 (416-816)	1,003 (653-1,203)	2,155 (1,434-2,638)	2,984 (2,054-3,567)	4,742 (3,333-5,880)	5,904 (4,194-7,825)	6,399 (4,366-9,114)	7,310 (4,755-11,293)	4,859 (2,950-7,721)	2,038 (1,201-3,316)
Endocarditis	2,808 (2,438-3,406)	2,661 (2,273-3,153)	2,875 (2,431-3,474)	3,149 (2,671-3,749)	4,394 (3,726-5,289)	5,441 (4,528-6,477)	6,455 (5,248-7,539)	7,341 (5,819-8,808)	6,829 (5,233-8,152)	5,968 (4,387-7,115)	3,546 (2,477-4,288)	1,295 (859-1,555)
Other cardiovascular and circulatory diseases	10,458 (8,983-12,438)	13,566 (11,682-15,688)	16,947 (14,700-19,826)	21,106 (17,952-24,848)	31,948 (27,204-36,512)	42,947 (36,089-48,968)	56,372 (47,438-62,578)	65,950 (56,772-72,312)	60,388 (52,545-65,858)	52,658 (47,943-56,743)	29,041 (27,274-31,689)	9,760 (9,277-10,639)
Chronic respiratory diseases	47,604 (44,762-49,713)	77,973 (71,878-81,360)	118,488 (107,762-123,277)	183,495 (165,188-191,546)	328,407 (296,581-343,336)	471,653 (429,226-492,485)	623,270 (572,873-648,773)	619,936 (571,114-644,065)	523,385 (474,324-544,954)	352,123 (320,816-367,564)	143,155 (130,203-149,402)	40,484 (37,446-42,178)
Chronic obstructive pulmonary disease	27,815 (24,768-31,292)	50,434 (44,265-56,140)	83,931 (73,591-92,491)	137,104 (119,833-150,096)	263,607 (233,768-284,686)	394,888 (354,326-419,701)	540,035 (491,299-568,628)	544,077 (496,828-570,090)	469,966 (426,114-491,901)	320,015 (291,456-335,168)	130,794 (119,045-136,779)	37,213 (34,317-38,810)
Pneumoconiosis	690 (592-897)	946 (803-1,315)	1,043 (891-1,428)	1,254 (1,069-1,721)	2,169 (1,879-2,920)	3,153 (2,784-4,092)	3,943 (3,564-4,826)	3,616 (3,303-4,324)	2,400 (2,211-2,760)	1,467 (1,353-1,664)	476 (440-538)	111 (104-124)
Silicosis	422 (353-574)	569 (466-877)	590 (488-888)	662 (547-989)	1,107 (923-1,639)	1,740 (1,485-2,420)	2,069 (1,794-2,622)	1,864 (1,645-2,230)	1,158 (1,013-1,407)	659 (576-780)	177 (155-205)	33 (30-39)
Asbestosis	60 (45-80)	66 (48-88)	85 (60-109)	118 (75-151)	190 (119-258)	276 (168-402)	398 (246-583)	428 (265-688)	325 (207-475)	219 (141-327)	76 (48-112)	17 (11-22)
Coal workers pneumoconiosis	68 (35-94)	110 (64-150)	128 (72-181)	174 (102-239)	318 (178-433)	520 (286-672)	700 (375-875)	641 (370-771)	479 (318-576)	303 (191-354)	93 (55-109)	21 (12-24)
Other pneumoconiosis	140 (106-189)	200 (150-298)	239 (177-352)	300 (215-449)	554 (400-811)	618 (454-909)	776 (591-1,151)	683 (521-992)	436 (343-614)	286 (231-410)	129 (107-184)	40 (35-53)
Asthma	16,284 (12,601-18,902)	22,979 (17,720-27,026)	28,874 (21,874-35,519)	38,869 (29,148-48,339)	53,787 (39,049-69,241)	61,793 (45,014-80,016)	64,654 (48,864-84,074)	57,553 (43,492-75,896)	39,301 (30,207-52,904)	22,366 (17,857-29,431)	8,324 (6,790-10,838)	2,158 (1,802-2,741)
Interstitial lung disease and pulmonary sarcoidosis	1,473 (950-2,270)	2,187 (1,405-3,328)	3,150 (2,025-4,753)	4,420 (2,812-6,724)	6,623 (4,224-9,858)	9,353 (6,215-13,374)	11,909 (8,185-16,022)	12,245 (8,851-15,517)	9,535 (6,974-11,419)	6,556 (4,817-7,704)	2,677 (2,030-3,141)	738 (570-890)
Other chronic respiratory diseases	1,342 (852-1,751)	1,427 (880-1,901)	1,481 (916-1,910)	1,848 (1,157-2,428)	2,221 (1,448-2,831)	2,466 (1,552-3,103)	2,728 (1,805-3,375)	2,446 (1,584-2,943)	2,183 (1,414-2,640)	1,717 (1,084-2,081)	884 (542-1,091)	263 (166-324)
Cirrhosis and other chronic liver diseases	79,246 (75,714-85,944)	98,299 (94,100-104,904)	103,596 (99,940-112,010)	103,386 (99,388-111,242)	115,797 (111,212-126,049)	108,814 (104,530-116,079)	96,654 (92,448-101,709)	72,360 (69,405-76,313)	45,645 (43,365-47,829)	26,559 (25,342-27,862)	10,388 (9,936-10,863)	3,179 (3,017-3,315)
Cirrhosis and other chronic liver diseases due to hepatitis B	26,348 (22,672-30,100)	30,014 (25,441-35,110)	29,916 (25,156-35,295)	30,683 (26,237-35,890)	35,211 (29,727-41,863)	33,696 (28,482-39,814)	29,490 (24,854-35,255)	21,744 (18,312-25,444)	13,059 (10,809-15,484)	6,566 (5,607-7,586)	2,135 (1,726-2,558)	564 (412-731)
Cirrhosis and other chronic liver diseases due to hepatitis C	20,547 (17,242-24,246)	25,809 (21,208-31,068)	27,802 (22,830-33,548)	26,923 (22,764-31,775)	29,372 (24,150-35,650)	27,826 (23,497-32,695)	24,865 (20,960-29,357)	17,807 (14,532-21,481)	10,983 (8,924-13,321)	6,402 (5,321-7,529)	2,542 (2,017-3,100)	748 (532-973)
Cirrhosis and other chronic liver diseases due to alcohol use	22,234 (19,212-25,623)	29,083 (24,559-33,913)	30,956 (26,300-36,118)	29,317 (24,495-34,595)	31,930 (26,014-38,360)	29,209 (24,527-33,898)	25,562 (21,299-30,139)	19,226 (16,257-22,408)	11,844 (9,921-13,791)	7,439 (6,501-8,375)	3,116 (2,637-3,580)	1,002 (800-1,206)
Cirrhosis and other chronic liver diseases due to other causes	10,118 (7,541-13,219)	13,394 (9,916-17,428)	15,176 (11,585-19,309)	16,463 (12,443-21,132)	19,284 (14,485-25,005)	18,084 (13,634-23,057)	16,738 (12,757-21,288)	13,583 (10,548-16,796)	9,760 (7,577-12,157)	6,152 (5,179-7,336)	2,595 (2,112-3,164)	865 (647-1,115)
Digestive diseases	37,447 (33,729-39,938)	45,732 (41,696-48,373)	50,551 (46,108-53,543)	61,729 (55,610-65,891)	80,427 (73,273-85,657)	96,563 (87,426-103,337)	108,793 (99,487-115,388)	108,112 (99,218-114,980)	89,103 (82,336-95,400)	70,591 (65,337-74,713)	35,903 (33,699-37,925)	11,440 (10,704-12,102)
Peptic ulcer disease	12,477 (11,132-13,690)	15,942 (14,110-17,288)	18,763 (16,765-20,205)	21,617 (19,279-23,236)	28,938 (26,064-31,006)	33,981 (30,512-36,576)	36,826 (32,790-39,657)	34,884 (31,556-37,586)	26,894 (24,281-28,974)	18,960 (17,318-20,279)	8,623 (8,004-9,187)	2,541 (2,360-2,755)
Gastritis and duodenitis	1,653 (1,449-1,947)	2,170 (1,904-2,493)	2,407 (2,120-2,709)	2,864 (2,506-3,231)	3,654 (3,233-4,120)	4,217 (3,690-4,780)	4,503 (3,988-5,090)	4,560 (4,030-5,187)	4,145 (3,621-4,829)	2,987 (2,628-3,492)	1,414 (1,245-1,654)	446 (391-526)
Appendicitis	2,310 (1,886-2,702)	2,422 (1,960-2,840)	2,245 (1,847-2,649)	3,363 (2,672-4,014)	3,466 (2,778-4,205)	3,444 (2,751-4,234)	3,290 (2,686-4,049)	2,872 (2,409-3,532)	2,011 (1,660-2,561)	1,338 (1,127-1,657)	598 (509-738)	172 (147-212)
Paralytic ileus and intestinal obstruction	6,527 (4,509-7,468)	7,666 (5,501-8,709)	8,521 (6,272-9,698)	11,862 (8,755-13,552)	13,847 (10,306-15,694)	17,116 (13,218-19,426)	17,582 (14,288-19,631)	17,258 (14,667-19,655)	14,475 (12,272-16,323)	12,349 (10,208-14,005)	6,811 (5,514-7,668)	2,237 (1,754-2,520)
Inguinal, femoral, and abdominal hernia	1,280 (872-1,531)	1,384 (989-1,671)	1,916 (1,435-2,342)	2,513 (1,846-3,089)	3,416 (2,420-4,165)	4,234 (3,066-5,114)	5,052 (3,663-6,089)	5,187 (3,766-6,338)	4,393 (3,230-5,269)	3,476 (2,635-4,209)	1,838 (1,369-2,255)	569 (432-715)
Inflammatory bowel disease	1,117 (869-1,405)	1,380 (1,030-1,717)	1,423 (1,111-1,690)	1,727 (1,354-2,080)	2,166 (1,708-2,532)	2,615 (2,022-3,022)	3,407 (2,683-3,892)	3,694 (2,842-4,134)	3,425 (2,549-3,729)	2,987 (2,189-3,294)	1,655 (1,188-1,850)	587 (420-664)
Vascular intestinal disorders	999 (844-1,132)	1,616 (1,389-1,852)	2,194 (1,938-2,530)	3,114 (2,763-3,652)	5,315 (4,766-6,090)	7,796 (7,073-8,945)	11,160 (10,217-12,597)	12,467 (11,300-13,787)	10,033 (9,135-10,947)	8,582 (7,753-9,318)	4,200 (3,860-4,590)	1,183 (1,081-1,310)
Gallbladder and biliary diseases	1,890 (1,719-2,143)	2,467 (2,178-2,721)	3,199 (2,816-3,591)	3,963 (3,487-4,401)	6,009 (5,307-6,673)	8,188 (7,164-9,105)	9,919 (8,856-10,981)	10,712 (9,632-11,869)	9,613 (8,617-10,800)	8,326 (7,619-9,074)	4,576 (4,197-4,992)	1,556 (1,430-1,703)
Pancreatitis	6,889 (6,103-8,292)	7,719 (6,760-9,245)	6,462 (5,653-7,856)	6,509 (5,502-7,906)	7,321 (6,270-8,980)	7,647 (6,426-9,377)	7,783 (6,678-9,219)	6,707 (5,822-7,916)	4,656 (3,991-5,523)	3,363 (2,871-3,878)	1,458 (1,268-1,673)	421 (364-500)
Other digestive diseases	2,305 (2,023-2,764)	2,966 (2,621-3,476)	3,423 (3,046-3,985)	4,197 (3,744-4,877)	6,295 (5,594-7,219)	7,324 (6,523-8,428)	9,273 (8,363-10,510)	9,770 (8,817-11,075)	9,458 (8,480-10,703)	8,224 (7,486-9,141)	4,731 (4,365-5,211)	1,727 (1,586-1,930)
Neurological disorders	12,265 (11,643-12,825)	14,654 (13,673-15,823)	18,132 (16,023-20,677)	23,036 (19,402-27,501)	40,101 (33,119-49,589)	72,621 (58,160-90,734)	141,494 (110,437-180,237)	246,435 (195,633-309,166)	311,735 (249,244-390,688)	354,178 (286,746-431,299)	245,381 (203,558-287,975)	82,153 (66,472-101,182)
Alzheimer's disease and other dementias	93 (41-179)	1,994 (1,180-3,009)	6,123 (4,141-8,642)	10,817 (7,219-15,290)	24,945 (18,097-34,438)	52,810 (39,049-70,825)	112,331 (82,246-150,510)	208,951 (157,921-269,458)	276,649 (213,039-355,789)	328,188 (258,178-406,887)	235,430 (194,143-277,625)	79,848 (64,197-99,048)
Parkinson's disease	276 (154-455)	459 (254-792)	738 (432-1,171)	1,314 (798-1,942)	3,357 (2,310-4,729)	7,614 (5,413-10,303)	17,159 (12,065-22,942)	27,937 (20,271-37,261)	28,959 (21,738-38,677)	22,060 (16,952-28,892)	8,526 (6,511-11,105)	1,920 (1,387-2,593)
Epilepsy	8,072 (7,500-8,458)	7,057 (6,567-7,353)	5,531 (5,117-5,768)	4,787 (4,347-5,022)	4,791 (4,260-5,063)	4,599 (4,066-4,893)	4,309 (3,761-4,610)	2,840 (2,590-2,999)	2,094 (1,909-2,266)	1,493 (1,367-1,608)	569 (529-607)	159 (148-170)
Multiple sclerosis	1,478 (1,297-1,721)	1,777 (1,548-1,973)	1,835 (1,593-2,081)	1,644 (1,451-1,921)	1,568 (1,409-1,884)	1,401 (1,236-1,645)	1,212 (1,044-1,398)	895 (730-1,020)	484 (374-525)	247 (179-272)	75 (56-82)	15 (11-16)
Motor neuron disease	776 (748-828)	1,271 (1,226-1,366)	1,692 (1,634-1,806)	2,130 (2,070-2,277)	2,772 (2,692-2,917)	3,360 (3,258-3,500)	3,470 (3,366-3,584)	2,924 (2,824-3,019)	1,546 (1,488-1,602)	745 (712-777)	176 (168-184)	30 (29-31)
Other neurological disorders	1,570 (1,517-1,628)	2,096 (2,029-2,175)	2,213 (2,102-2,300)	2,344 (2,229-2,436)	2,669 (2,540-2,782)	2,837 (2,721-2,950)	3,012 (2,902-3,121)	2,888 (2,769-3,011)	2,004 (1,913-2,094)	1,444 (1,376-1,513)	605 (576-636)	180 (171-190)
Mental and substance use disorders	34,695 (31,025-37,432)	35,063 (31,375-37,957)	28,339 (25,318-30,688)	20,910 (18,624-22,579)	21,120 (18,778-22,855)	14,576 (12,971-15,436)	10,163 (9,008-10,679)	7,227 (6,479-7,601)	3,989 (3,604-4,182)	2,595 (2,380-2,730)	1,103 (1,013-1,159)	347 (324-365)
Alcohol use disorders	21,623 (19,177-24,034)	24,606 (21,781-27,255)	20,831 (18,419-22,985)	15,675 (13,955-17,113)	15,524 (13,879-17,076)	9,854 (8,817-10,594)	5,994 (5,377-6,442)	3,847 (3,402-4,215)	1,672 (1,501-1,810)	930 (854-1,005)	341 (317-365)	106 (99-113)
Drug use disorders	12,988 (11,010-14,176)	10,374 (8,837-11,298)	7,509 (6,456-8,150)	5,234 (4,260-5,704)	5,596 (4,625-6,135)	4,722 (3,724-5,151)	4,170 (3,399-4,522)	3,380 (2,851-3,623)	2,317 (2,000-2,471)	1,665 (1,473-1,770)	762 (679-810)	241 (219-256)
Opioid use disorders	8,501 (6,288-9,609)	6,548 (5,013-7,361)	4,598 (3,481-5,150)	3,177 (2,372-3,599)	3,491 (2,677-3,964)	2,871 (2,127-3,266)	1,830 (1,344-2,112)	1,415 (1,069-1,630)	823 (633-960)	605 (461-692)	276 (198-309)	83 (63-94)
Cocaine use disorders	842 (643-1,090)	719 (527-908)	602 (438-734)	418 (277-513)	390 (267-497)	345 (232-439)	181 (118-235)	129 (83-163)	24 (17-31)	16 (11-20)	8 (5-9)	2 (1-3)
Amphetamine use disorders	402 (308-517)	338 (250-421)	246 (181-302)	184 (124-231)	172 (119-215)	151 (101-191)	63 (43-80)	49 (32-62)	11 (8-15)	8 (6-10)	4 (3-4)	1 (1-2)
Other drug use disorders	3,243 (2,664-3,820)	2,768 (2,260-3,198)	2,063 (1,689-2,336)	1,455 (1,153-1,628)	1,542 (1,230-1,770)	1,355 (1,043-1,518)	2,096 (1,654-2,302)	1,788 (1,472-1,951)	1,458 (1,253-1,603)	1,035 (917-1,133)	475 (424-521)	154 (140-171)
Eating disorders	84 (75-93)	84 (75-93)	0 (0-0)	0 (0-0)	-	-	-	-	-	-	-	-
Anorexia nervosa	74 (66-83)	70 (62-78)	0 (0-0)	0 (0-0)	-	-	-	-	-	-	-	-
Bulimia nervosa	9 (8-12)	14 (12-18)	0 (0-0)	0 (0-0)	-	-	-	-	-	-	-	-
Diabetes, urogenital, blood, and endocrine diseases	66,096 (63,796-68,965)	88,711 (85,772-92,266)	110,617 (107,115-114,992)	139,325 (135,115-144,092)	192,694 (186,953-199,839)	236,946 (229,717-246,011)	264,075 (255,654-274,328)	260,655 (251,740-269,854)	211,870 (204,177-221,072)	162,858 (157,146-169,288)	81,505 (78,517-84,462)	26,904 (25,791-27,868)
Diabetes mellitus	22,186 (21,509-22,866)	34,271 (33,219-35,277)	48,892 (47,457-50,350)	67,535 (65,499-69,527)	99,966 (96,857-103,129)	124,748 (120,744-128,827)	137,829 (133,269-142,603)	133,615 (128,605-138,213)	103,171 (98,803-107,907)	69,577 (66,827-72,568)	30,377 (29,172-31,704)	8,399 (7,999-8,780)
Acute glomerulonephritis	685 (621-756)	841 (772-923)	709 (651-775)	815 (751-883)	1,065 (974-1,167)	1,266 (1,162-1,390)	1,280 (1,170-1,399)	1,081 (986-1,194)	837 (758-924)	536 (486-593)	165 (150-182)	58 (51-66)
Chronic kidney disease	29,567 (27,522-31,810)	37,235 (35,168-39,841)	44,326 (42,210-47,961)	53,736 (51,315-57,550)	69,293 (66,414-74,181)	82,824 (79,239-89,417)	90,631 (86,913-97,382)	90,403 (86,897-96,735)	75,115 (72,214-80,746)	61,690 (59,324-64,962)	32,798 (31,421-34,367)	11,426 (10,900-11,924)
Chronic kidney disease due to diabetes mellitus	13,090 (10,104-15,749)	18,139 (14,560-21,774)	22,138 (17,178-26,651)	27,548 (21,943-32,223)	35,967 (29,677-42,097)	41,619 (32,807-49,293)	42,337 (33,473-50,262)	37,723 (29,327-45,816)	26,675 (20,017-32,978)	18,374 (13,628-23,894)	9,034 (6,471-11,821)	3,139 (2,091-4,195)
Chronic kidney disease due to hypertension	3,944 (3,035-5,065)	5,599 (4,334-7,194)	7,370 (5,629-9,536)	10,126 (7,855-12,995)	14,246 (11,289-17,879)	19,193 (15,070-24,281)	23,041 (18,132-29,107)	25,884 (20,723-32,130)	24,864 (20,244-30,115)	23,645 (19,311-28,603)	13,986 (11,605-16,477)	5,107 (4,100-6,187)
Chronic kidney disease due to glomerulonephritis	5,740 (3,795-7,819)	5,866 (3,901-8,079)	6,151 (4,177-8,624)	6,212 (4,281-8,634)	7,328 (4,896-10,265)	8,234 (5,580-11,689)	9,346 (6,433-13,125)	9,940 (6,930-13,879)	8,538 (6,085-11,453)	6,565 (4,718-8,956)	3,078 (2,221-4,006)	1,005 (629-1,569)
Chronic kidney disease due to other causes	6,794 (4,691-9,166)	7,631 (5,070-10,835)	8,666 (5,711-12,346)	9,851 (6,703-14,254)	11,752 (7,778-16,716)	13,778 (9,466-19,379)	15,907 (10,917-21,629)	16,857 (11,784-22,992)	15,037 (10,711-19,971)	13,106 (9,064-17,629)	6,700 (4,925-8,829)	2,175 (1,385-3,088)
Urinary diseases and male infertility	5,229 (5,002-5,446)	6,413 (6,142-6,697)	7,314 (6,987-7,664)	8,485 (8,109-8,882)	12,581 (12,034-13,124)	15,943 (15,217-16,662)	19,377 (18,490-20,136)	20,691 (19,811-21,444)	19,984 (19,214-20,748)	18,711 (18,023-19,323)	11,595 (11,152-11,991)	4,591 (4,404-4,759)
Interstitial nephritis and urinary tract infections	3,093 (2,660-3,368)	3,736 (3,189-4,071)	4,484 (3,876-4,900)	5,112 (4,330-5,593)	7,699 (6,557-8,343)	10,044 (8,725-10,886)	13,029 (11,536-13,978)	14,677 (13,190-15,588)	14,623 (13,366-15,479)	14,696 (13,671-15,454)	9,501 (8,862-9,993)	3,959 (3,719-4,161)
Urolithiasis	596 (438-705)	788 (577-926)	892 (637-1,021)	906 (650-1,050)	1,368 (992-1,592)	1,774 (1,333-2,046)	1,960 (1,489-2,278)	1,831 (1,460-2,139)	1,465 (1,191-1,728)	1,107 (870-1,348)	563 (432-713)	171 (136-225)
Other urinary diseases	1,540 (1,208-1,982)	1,889 (1,476-2,486)	1,938 (1,490-2,632)	2,467 (1,915-3,342)	3,514 (2,719-4,938)	4,125 (3,251-5,648)	4,388 (3,534-5,992)	4,184 (3,447-5,578)	3,896 (3,306-5,070)	2,908 (2,494-3,837)	1,532 (1,311-2,054)	461 (396-628)
Gynecological diseases	633 (540-715)	875 (765-982)	682 (578-771)	485 (413-555)	305 (262-346)	469 (393-537)	272 (230-316)	197 (173-236)	388 (313-465)	273 (223-324)	121 (103-145)	44 (37-51)
Uterine fibroids	230 (126-318)	416 (210-557)	381 (186-503)	233 (98-339)	121 (57-185)	171 (81-271)	74 (41-119)	53 (36-74)	102 (38-203)	56 (22-114)	24 (12-44)	8 (4-13)
Polycystic ovarian syndrome	69 (18-137)	105 (26-220)	168 (52-322)	-	-	-	-	-	-	-	-	-
Endometriosis	9 (4-14)	15 (7-25)	10 (5-17)	-	-	-	-	-	-	-	-	-
Genital prolapse	9 (4-16)	10 (4-18)	17 (5-38)	96 (17-173)	85 (25-151)	138 (41-230)	101 (42-158)	58 (35-82)	157 (51-260)	103 (41-170)	43 (20-67)	14 (8-20)
Other gynecological diseases	316 (199-453)	329 (172-553)	107 (48-223)	157 (82-268)	99 (56-166)	160 (88-256)	97 (61-152)	87 (65-115)	129 (73-228)	114 (77-180)	54 (38-81)	22 (16-30)
Hemoglobinopathies and hemolytic anaemias	4,036 (3,433-5,106)	4,507 (3,837-5,941)	3,684 (3,131-4,626)	3,213 (2,766-4,024)	3,545 (3,101-4,382)	4,552 (3,967-5,805)	6,548 (5,675-8,323)	6,435 (5,676-7,940)	5,268 (4,536-6,309)	6,047 (5,026-7,011)	3,111 (2,454-3,537)	1,083 (809-1,222)
Thalassemias	51 (44-65)	43 (38-57)	37 (32-51)	20 (17-29)	10 (9-15)	5 (4-8)	3 (2-4)	1 (1-2)	0 (0-0)	0 (0-0)	0 (0-0)	0 (0-0)
Sickle cell disorders	1,790 (1,464-2,291)	1,716 (1,417-2,316)	975 (779-1,271)	405 (337-536)	170 (140-227)	108 (92-141)	67 (57-87)	27 (22-34)	9 (7-11)	3 (2-4)	1 (1-1)	0 (0-0)
G6PD deficiency	1,160 (956-1,468)	1,441 (1,201-1,940)	1,136 (934-1,420)	905 (744-1,172)	792 (636-1,016)	665 (536-882)	637 (519-849)	374 (299-509)	146 (117-188)	86 (68-109)	24 (19-30)	4 (3-6)
Other hemoglobinopathies and hemolytic anaemias	1,036 (885-1,285)	1,307 (1,111-1,670)	1,536 (1,325-1,926)	1,883 (1,615-2,362)	2,573 (2,224-3,147)	3,774 (3,273-4,756)	5,842 (5,056-7,399)	6,034 (5,296-7,446)	5,114 (4,416-6,128)	5,957 (4,951-6,886)	3,086 (2,431-3,507)	1,078 (806-1,217)
Endocrine, metabolic, blood, and immune disorders	3,760 (3,246-4,188)	4,569 (3,946-5,041)	5,010 (4,227-5,391)	5,055 (4,367-5,556)	5,939 (5,263-6,648)	7,145 (6,343-8,107)	8,138 (7,184-9,261)	8,231 (7,187-9,366)	7,108 (6,147-8,156)	6,024 (5,054-6,730)	3,338 (2,748-3,761)	1,303 (1,050-1,437)
Musculoskeletal disorders	2,988 (2,545-3,421)	3,378 (2,938-3,833)	3,280 (2,802-3,744)	3,580 (3,095-3,960)	4,850 (4,214-5,449)	6,157 (5,343-6,810)	7,399 (6,420-7,948)	8,093 (7,058-8,726)	6,692 (5,725-7,287)	5,831 (4,917-6,282)	3,184 (2,501-3,415)	1,212 (958-1,311)
Rheumatoid arthritis	527 (434-685)	884 (717-1,085)	1,218 (980-1,415)	1,657 (1,341-1,899)	2,604 (2,161-2,946)	3,530 (2,968-4,037)	4,290 (3,658-4,783)	4,449 (3,809-5,000)	3,343 (2,848-3,867)	2,316 (1,950-2,889)	947 (777-1,215)	274 (228-372)
Other musculoskeletal disorders	2,461 (2,077-2,782)	2,494 (2,122-2,837)	2,063 (1,762-2,349)	1,922 (1,655-2,172)	2,246 (1,943-2,567)	2,627 (2,263-2,987)	3,109 (2,539-3,459)	3,644 (2,957-3,976)	3,349 (2,703-3,651)	3,514 (2,754-3,809)	2,238 (1,670-2,420)	939 (696-1,011)
Other non-communicable diseases	5,852 (4,836-6,469)	5,308 (4,159-5,949)	5,238 (4,006-5,943)	5,662 (4,224-6,612)	6,334 (4,619-7,345)	7,140 (5,034-8,726)	6,398 (3,936-8,323)	7,693 (4,940-10,293)	7,221 (4,646-9,567)	6,917 (4,161-9,037)	4,556 (2,555-6,022)	1,970 (1,010-2,636)
Congenital anomalies	4,129 (3,575-4,618)	2,949 (2,581-3,308)	2,413 (2,089-2,662)	2,158 (1,874-2,376)	2,151 (1,921-2,475)	1,784 (1,585-2,010)	-	-	-	-	-	-
Neural tube defects	108 (86-137)	96 (79-116)	73 (60-91)	60 (47-88)	47 (37-75)	48 (39-69)	-	-	-	-	-	-
Congenital heart anomalies	3,036 (2,614-3,417)	1,381 (1,195-1,561)	1,016 (870-1,144)	828 (738-953)	787 (715-930)	677 (607-798)	-	-	-	-	-	-
Orofacial clefts	-	-	-	-	-	-	-	-	-	-	-	-
Down's syndrome	300 (258-385)	557 (465-684)	526 (435-599)	500 (410-567)	403 (337-485)	177 (145-215)	-	-	-	-	-	-
Other chromosomal abnormalities	55 (33-63)	51 (32-59)	56 (35-66)	47 (30-55)	34 (24-41)	28 (21-38)	-	-	-	-	-	-
Congenital musculoskeletal and limb anomalies	46 (33-55)	55 (38-66)	49 (36-58)	38 (27-45)	41 (29-48)	42 (30-49)	-	-	-	-	-	-
Urogenital congenital anomalies	118 (91-143)	193 (140-241)	205 (154-251)	237 (177-282)	305 (230-368)	365 (290-442)	-	-	-	-	-	-
Digestive congenital anomalies	42 (30-48)	61 (48-70)	57 (44-64)	56 (44-63)	74 (62-88)	74 (59-82)	-	-	-	-	-	-
Other congenital anomalies	425 (345-543)	554 (446-697)	431 (348-542)	391 (309-503)	461 (375-574)	372 (308-456)	-	-	-	-	-	-
Skin and subcutaneous diseases	1,722 (999-2,108)	2,360 (1,373-2,898)	2,825 (1,665-3,521)	3,504 (2,132-4,444)	4,183 (2,481-5,170)	5,356 (3,303-6,944)	6,398 (3,936-8,323)	7,693 (4,940-10,293)	7,221 (4,646-9,567)	6,917 (4,161-9,037)	4,556 (2,555-6,022)	1,970 (1,010-2,636)
Cellulitis	298 (138-382)	392 (180-497)	544 (254-691)	604 (276-763)	728 (347-937)	755 (339-986)	906 (445-1,210)	986 (498-1,407)	881 (480-1,428)	776 (422-1,307)	488 (247-872)	200 (99-385)
Pyoderma	1,123 (610-1,391)	1,569 (869-1,962)	1,831 (1,053-2,365)	2,304 (1,332-3,058)	2,663 (1,530-3,341)	3,236 (1,911-4,355)	3,494 (2,056-4,501)	3,782 (2,434-5,145)	2,869 (1,798-4,025)	2,512 (1,473-3,414)	1,517 (851-2,150)	606 (327-856)
Decubitus ulcer	228 (140-317)	315 (196-451)	363 (220-525)	493 (305-719)	664 (417-954)	1,185 (724-1,615)	1,798 (1,137-2,453)	2,654 (1,591-3,648)	3,185 (1,913-4,243)	3,338 (1,806-4,227)	2,350 (1,204-3,012)	1,084 (509-1,397)
Other skin and subcutaneous diseases	73 (48-104)	83 (54-118)	86 (56-126)	103 (67-150)	128 (84-183)	180 (115-256)	200 (130-291)	270 (170-414)	286 (181-425)	291 (164-434)	200 (105-299)	80 (40-122)
Sudden infant death syndrome	-	-	-	-	-	-	-	-	-	-	-	-
Injuries	299,699 (285,791-308,961)	282,911 (269,032-292,440)	229,959 (216,542-237,796)	192,879 (181,213-198,913)	197,750 (184,357-205,223)	187,139 (172,802-194,474)	179,307 (164,762-186,844)	155,969 (142,380-163,144)	118,397 (107,280-125,043)	90,415 (81,045-95,446)	43,784 (38,571-46,504)	14,750 (12,688-15,705)
Transport injuries	102,052 (98,783-105,265)	92,705 (89,583-95,932)	72,815 (70,600-75,167)	60,854 (58,934-62,865)	58,223 (56,154-60,307)	52,477 (50,625-54,545)	47,125 (45,375-49,003)	36,071 (34,731-37,513)	22,087 (21,222-23,194)	12,256 (11,771-12,844)	4,346 (4,168-4,572)	1,205 (1,141-1,270)
Road injuries	94,825 (91,848-98,021)	85,848 (83,062-88,980)	67,154 (65,189-69,473)	56,366 (54,573-58,282)	54,130 (52,227-56,269)	48,939 (47,151-51,106)	44,152 (42,578-45,975)	33,735 (32,483-35,084)	20,708 (19,900-21,712)	11,454 (11,014-11,997)	4,006 (3,843-4,216)	1,081 (1,022-1,141)
Pedestrian road injuries	34,099 (31,569-37,749)	34,774 (32,189-38,391)	28,332 (26,241-31,209)	24,646 (22,760-26,939)	27,078 (25,470-29,560)	25,439 (23,977-27,755)	23,715 (22,423-25,555)	19,674 (18,655-21,137)	12,200 (11,543-13,089)	6,701 (6,355-7,211)	2,142 (2,021-2,360)	560 (520-636)
Cyclist road injuries	5,172 (4,607-5,692)	5,634 (4,960-6,181)	4,855 (4,362-5,291)	3,969 (3,556-4,356)	4,076 (3,625-4,467)	3,843 (3,424-4,247)	3,253 (2,883-3,606)	1,870 (1,676-2,036)	924 (829-1,025)	452 (399-527)	137 (119-168)	2 (2-2)
Motorcyclist road injuries	18,666 (16,828-20,433)	14,463 (13,045-15,942)	9,806 (8,942-10,921)	7,616 (6,886-8,708)	4,996 (4,530-5,800)	4,090 (3,687-4,594)	2,974 (2,693-3,382)	1,590 (1,419-1,791)	942 (848-1,078)	387 (343-450)	109 (96-133)	32 (29-40)
Motor vehicle road injuries	36,104 (32,130-40,775)	30,069 (26,639-33,807)	23,352 (20,904-26,596)	19,450 (17,356-21,949)	17,501 (15,617-19,658)	15,026 (13,395-17,048)	13,725 (12,317-15,280)	10,126 (9,158-11,161)	6,339 (5,755-6,961)	3,746 (3,372-4,071)	1,546 (1,376-1,651)	462 (396-496)
Other road injuries	784 (663-948)	908 (768-1,099)	810 (675-998)	686 (574-862)	479 (399-595)	542 (449-667)	485 (400-607)	476 (385-648)	302 (241-427)	168 (133-230)	72 (56-103)	25 (19-38)
Other transport injuries	7,227 (6,143-7,914)	6,857 (5,902-7,456)	5,661 (4,929-6,174)	4,488 (3,882-4,916)	4,093 (3,547-4,470)	3,538 (3,056-3,877)	2,972 (2,581-3,279)	2,336 (2,051-2,615)	1,380 (1,227-1,549)	802 (716-903)	340 (310-383)	124 (113-138)
Unintentional injuries	86,993 (80,812-90,790)	90,943 (84,300-95,332)	78,143 (71,752-82,400)	71,626 (64,964-75,283)	82,920 (74,421-87,699)	85,826 (75,464-91,091)	88,626 (77,314-94,376)	88,250 (76,598-94,024)	76,863 (66,767-82,675)	67,055 (58,416-71,456)	35,838 (31,047-38,323)	12,724 (10,745-13,635)
Falls	19,737 (17,835-21,784)	22,177 (19,723-24,437)	20,986 (18,567-22,425)	22,869 (19,908-24,660)	29,342 (25,170-31,639)	36,355 (30,205-39,526)	42,479 (35,273-46,208)	49,189 (41,362-53,318)	46,024 (38,432-50,055)	43,739 (37,506-47,022)	24,359 (20,318-26,108)	8,497 (6,905-9,139)
Drowning	16,419 (15,364-17,282)	15,086 (14,122-15,842)	12,560 (11,533-13,168)	10,148 (9,352-10,625)	10,896 (9,796-11,416)	9,687 (8,737-10,174)	9,107 (8,144-9,516)	7,142 (6,156-7,584)	5,165 (4,414-5,494)	2,954 (2,594-3,153)	912 (813-969)	214 (191-227)
Fire, heat, and hot substances	7,717 (6,605-8,337)	8,184 (6,885-8,885)	7,129 (5,870-7,746)	6,056 (5,062-6,523)	7,327 (6,214-7,957)	7,090 (6,041-7,660)	7,414 (6,537-7,999)	6,985 (6,032-7,562)	6,233 (5,337-6,807)	5,022 (4,219-5,493)	2,570 (2,067-2,773)	972 (746-1,047)
Poisonings	4,293 (3,514-4,974)	4,065 (3,306-4,769)	3,430 (2,892-3,976)	2,915 (2,397-3,404)	3,023 (2,421-3,578)	2,798 (2,232-3,178)	2,554 (2,110-2,922)	1,761 (1,428-2,017)	1,311 (1,082-1,573)	766 (656-913)	291 (261-370)	92 (83-121)
Exposure to mechanical forces	10,509 (9,462-11,401)	10,763 (9,771-11,552)	8,169 (7,317-8,778)	6,579 (5,942-7,034)	5,581 (4,936-6,012)	4,881 (4,279-5,272)	4,141 (3,591-4,447)	3,523 (3,080-3,772)	2,721 (2,382-2,932)	2,232 (1,923-2,395)	1,184 (1,003-1,281)	453 (386-490)
Unintentional firearm injuries	1,595 (1,240-1,787)	1,635 (1,264-1,862)	1,218 (866-1,428)	842 (665-954)	763 (637-873)	705 (573-815)	659 (543-740)	655 (556-734)	524 (431-580)	433 (337-476)	235 (189-259)	92 (75-104)
Unintentional suffocation	408 (345-558)	413 (359-538)	354 (309-472)	322 (283-421)	141 (106-164)	169 (123-204)	165 (123-201)	421 (294-509)	476 (326-595)	444 (291-543)	243 (159-301)	91 (57-112)
Other exposure to mechanical forces	8,506 (7,601-9,546)	8,715 (7,876-9,523)	6,597 (5,946-7,230)	5,415 (4,851-5,885)	4,677 (4,081-5,069)	4,007 (3,469-4,340)	3,317 (2,826-3,585)	2,446 (2,105-2,644)	1,720 (1,503-1,873)	1,355 (1,177-1,459)	706 (600-769)	270 (223-294)
Adverse effects of medical treatment	4,790 (3,758-5,328)	5,338 (4,289-5,920)	4,376 (3,581-4,813)	5,053 (4,106-5,593)	7,229 (5,853-8,101)	8,255 (6,578-9,309)	8,203 (6,759-9,308)	7,724 (6,376-8,795)	6,024 (4,917-6,944)	4,935 (4,076-5,852)	2,643 (2,110-3,254)	951 (761-1,208)
Animal contact	5,518 (3,949-6,401)	6,284 (4,718-7,263)	5,313 (3,648-6,218)	4,994 (3,731-5,721)	5,179 (3,658-6,038)	5,253 (3,621-6,190)	4,530 (3,051-5,380)	3,071 (2,119-3,600)	1,880 (1,339-2,211)	883 (646-1,021)	288 (219-330)	93 (75-104)
Venomous animal contact	4,824 (3,300-5,736)	5,343 (3,813-6,335)	4,484 (2,912-5,384)	4,203 (3,014-4,933)	4,421 (2,924-5,277)	4,527 (2,997-5,426)	3,883 (2,515-4,717)	2,536 (1,668-3,040)	1,548 (1,091-1,855)	684 (476-815)	206 (146-243)	59 (44-68)
Non-venomous animal contact	693 (547-911)	942 (741-1,224)	829 (654-1,088)	791 (620-1,048)	758 (609-944)	725 (579-904)	647 (497-821)	535 (412-662)	332 (248-405)	199 (151-240)	82 (64-97)	34 (27-39)
Foreign body	4,575 (3,777-5,177)	4,596 (3,823-5,203)	4,439 (3,637-5,081)	3,882 (3,257-4,366)	4,649 (3,868-5,316)	4,148 (3,426-4,574)	4,318 (3,496-4,744)	4,347 (3,371-4,870)	4,124 (3,114-4,725)	3,873 (2,686-4,577)	2,256 (1,487-2,710)	977 (619-1,193)
Pulmonary aspiration and foreign body in airway	3,721 (3,008-4,388)	4,111 (3,365-4,767)	4,009 (3,270-4,687)	3,499 (2,925-4,066)	4,234 (3,474-4,900)	3,817 (3,152-4,321)	3,942 (3,205-4,348)	3,963 (3,069-4,448)	3,767 (2,838-4,312)	3,572 (2,461-4,238)	2,111 (1,385-2,550)	921 (577-1,133)
Foreign body in other body part	854 (583-1,029)	485 (312-583)	431 (291-519)	382 (257-457)	415 (292-507)	331 (199-394)	377 (222-449)	384 (215-464)	357 (194-434)	301 (154-367)	146 (78-175)	55 (30-66)
Environmental heat and cold exposure	5,826 (3,819-8,003)	6,921 (4,550-10,162)	6,599 (4,560-9,944)	5,069 (3,460-7,147)	6,215 (4,257-9,371)	4,477 (3,092-6,142)	4,009 (2,736-5,262)	3,141 (2,042-3,779)	2,392 (1,629-2,862)	1,973 (1,434-2,533)	999 (708-1,362)	348 (234-485)
Other unintentional injuries	7,609 (7,116-8,198)	7,528 (7,017-8,143)	5,142 (4,756-5,540)	4,060 (3,738-4,394)	3,480 (3,210-3,768)	2,881 (2,616-3,131)	1,870 (1,734-2,050)	1,366 (1,246-1,503)	991 (886-1,108)	677 (610-751)	337 (295-372)	126 (108-140)
Self-harm and interpersonal violence	105,829 (93,206-112,726)	95,658 (83,437-101,533)	76,273 (66,888-81,070)	58,249 (50,875-61,479)	54,673 (46,848-57,878)	47,211 (40,863-49,654)	42,302 (36,445-44,558)	30,765 (26,970-32,400)	18,817 (16,510-19,836)	10,771 (9,300-11,346)	3,494 (3,039-3,685)	768 (697-807)

SR Table 5: Global deaths by cause and age group for both sexes (with 95% uncertainty intervals), 2000

Cause	Early Neonatal	Late Neonatal	Post Neonatal	1 to 4	5 to 9	10 to 14	15 to 19	20 to 24
Self-harm	-	-	-	-	-	13,146<br>(11,475-14,273)	67,453<br>(62,564-72,553)	96,081<br>(88,983-101,746)
Self-harm by firearm	-	-	-	-	-	583<br>(425-767)	5,396<br>(4,102-7,054)	8,196<br>(6,201-10,818)
Self-harm by other specified means	-	-	-	-	-	12,563<br>(10,961-13,689)	62,057<br>(57,085-66,882)	87,885<br>(80,777-93,489)
Interpersonal violence	1,956<br>(1,480-2,204)	509<br>(396-579)	4,727<br>(3,703-5,402)	8,358<br>(6,616-9,808)	6,865<br>(5,290-7,820)	9,564<br>(7,378-10,613)	40,316<br>(31,779-44,094)	61,351<br>(48,422-67,328)
Assault by firearm	165<br>(110-216)	51<br>(34-63)	569<br>(398-732)	961<br>(706-1,306)	929<br>(675-1,163)	2,194<br>(1,413-2,657)	18,561<br>(12,362-20,922)	28,232<br>(18,827-31,777)
Assault by sharp object	147<br>(108-189)	59<br>(35-75)	412<br>(269-509)	1,005<br>(711-1,231)	1,153<br>(755-1,360)	1,821<br>(1,216-2,087)	10,252<br>(7,491-12,624)	16,400<br>(12,127-20,824)
Assault by other means	1,645<br>(1,196-1,872)	399<br>(306-459)	3,746<br>(2,884-4,360)	6,391<br>(4,858-7,614)	4,783<br>(3,623-5,543)	5,550<br>(4,299-6,380)	11,503<br>(8,876-13,848)	16,720<br>(12,454-19,521)
Forces of nature, conflict and terrorism, and executions and police conflict	1,032<br>(898-1,153)	571<br>(495-639)	1,942<br>(1,669-2,187)	12,002<br>(10,384-13,506)	7,388<br>(6,313-8,372)	6,872<br>(5,856-7,839)	13,430<br>(11,648-15,104)	18,945<br>(16,524-21,154)
Forces of nature, conflict and terrorism, and state actor violence	6<br>(4-8)	17<br>(11-23)	141<br>(93-187)	676<br>(447-906)	701<br>(464-942)	870<br>(580-1,165)	850<br>(565-1,133)	761<br>(504-1,016)
Conflict and terrorism	1,025<br>(894-1,145)	553<br>(483-618)	1,768<br>(1,543-1,973)	11,247<br>(9,841-12,532)	6,596<br>(5,766-7,352)	5,895<br>(5,151-6,578)	12,147<br>(10,617-13,562)	16,818<br>(14,669-18,796)
Executions and police conflict	-	-	33<br>(27-57)	79<br>(55-131)	91<br>(78-105)	108<br>(92-126)	432<br>(216-521)	1,366<br>(584-1,705)

Cause	25 to 29	30 to 34	35 to 39	40 to 44	45 to 49	50 to 54	55 to 59	60 to 64
Self-harm	96,384<br>(89,468-102,317)	85,735<br>(79,140-90,534)	78,350<br>(70,395-82,812)	70,738<br>(63,610-76,544)	68,099<br>(60,684-72,295)	56,999<br>(51,060-60,623)	45,443<br>(39,570-47,801)	43,343<br>(37,608-45,710)
Self-harm by firearm	7,324<br>(5,486-9,280)	6,588<br>(5,012-8,395)	6,004<br>(4,810-7,681)	5,618<br>(4,648-7,465)	5,158<br>(4,194-7,059)	4,365<br>(3,680-5,969)	3,384<br>(2,811-4,460)	2,908<br>(2,334-3,818)
Self-harm by other specified means	89,060<br>(82,666-94,770)	79,148<br>(72,544-83,954)	72,346<br>(64,135-76,484)	65,120<br>(58,290-69,874)	62,942<br>(55,711-67,141)	52,634<br>(46,636-55,967)	42,059<br>(36,273-44,267)	40,435<br>(34,711-42,608)
Interpersonal violence	57,408<br>(43,985-63,319)	50,711<br>(38,194-56,242)	42,867<br>(31,688-47,925)	35,091<br>(24,993-39,724)	27,559<br>(19,475-31,724)	19,274<br>(13,387-22,237)	12,806<br>(9,060-14,656)	11,330<br>(7,890-12,974)
Assault by firearm	24,735<br>(16,491-28,065)	20,140<br>(12,597-23,428)	16,784<br>(10,581-19,656)	12,296<br>(7,596-15,342)	8,403<br>(5,105-10,604)	5,675<br>(3,428-7,471)	3,670<br>(2,397-4,864)	2,641<br>(1,692-3,329)
Assault by sharp object	16,837<br>(12,072-20,264)	15,257<br>(10,769-18,448)	12,811<br>(8,786-15,747)	10,894<br>(7,269-13,340)	8,662<br>(5,664-10,655)	5,676<br>(3,675-7,004)	3,573<br>(2,258-4,594)	3,413<br>(2,159-4,193)
Assault by other means	15,836<br>(11,818-18,773)	15,315<br>(11,024-18,314)	13,272<br>(9,464-15,677)	11,901<br>(8,218-14,337)	10,494<br>(7,036-12,457)	7,922<br>(5,240-9,587)	5,563<br>(3,849-6,451)	5,276<br>(3,479-6,239)
Forces of nature, conflict and terrorism, and executions and police conflict	13,502<br>(11,782-15,075)	9,875<br>(8,551-11,113)	6,973<br>(6,017-7,854)	4,825<br>(4,149-5,478)	3,605<br>(3,098-4,090)	2,729<br>(2,343-3,105)	2,150<br>(1,836-2,454)	1,933<br>(1,619-2,234)
Forces of nature, conflict and terrorism, and state actor violence	717<br>(478-955)	634<br>(422-840)	578<br>(391-766)	527<br>(356-695)	476<br>(324-627)	396<br>(269-520)	332<br>(224-438)	358<br>(246-472)
Conflict and terrorism	11,219<br>(9,792-12,544)	7,769<br>(6,777-8,717)	5,326<br>(4,639-6,009)	3,657<br>(3,172-4,149)	2,681<br>(2,330-3,044)	2,096<br>(1,826-2,370)	1,693<br>(1,483-1,908)	1,466<br>(1,257-1,679)
Executions and police conflict	1,566<br>(593-1,940)	1,472<br>(564-1,789)	1,069<br>(393-1,316)	641<br>(253-787)	447<br>(206-535)	237<br>(112-280)	125<br>(82-151)	109<br>(56-136)

Cause	65 to 69	70 to 74	75 to 79	80 to 84	85 to 89	90 to 94	95 plus
Self-harm	39,209<br>(33,437-41,237)	35,687<br>(30,521-37,678)	26,398<br>(22,889-27,813)	17,538<br>(15,393-18,517)	10,079<br>(8,633-10,629)	3,252<br>(2,795-3,438)	668<br>(590-704)
Self-harm by firearm	2,648<br>(2,090-3,502)	2,597<br>(2,005-3,242)	2,253<br>(1,739-2,681)	1,540<br>(1,175-1,837)	947<br>(693-1,077)	361<br>(275-419)	87<br>(73-101)
Self-harm by other specified means	36,562<br>(31,369-38,567)	33,090<br>(28,140-34,906)	24,145<br>(20,830-25,502)	15,997<br>(13,879-16,934)	9,132<br>(7,849-9,634)	2,890<br>(2,500-3,063)	581<br>(513-612)
Interpersonal violence	8,002<br>(5,868-9,122)	6,615<br>(4,880-7,639)	4,367<br>(3,285-5,023)	1,279<br>(1,012-1,503)	692<br>(537-823)	243<br>(204-297)	100<br>(81-126)
Assault by firearm	1,771<br>(1,148-2,310)	1,369<br>(914-1,819)	902<br>(614-1,168)	177<br>(109-242)	90<br>(54-113)	31<br>(20-41)	19<br>(11-24)
Assault by sharp object	2,179<br>(1,425-2,702)	1,818<br>(1,199-2,222)	1,120<br>(738-1,332)	350<br>(267-443)	174<br>(130-221)	70<br>(57-97)	26<br>(20-37)
Assault by other means	4,052<br>(2,948-4,710)	3,428<br>(2,416-3,915)	2,346<br>(1,751-2,685)	752<br>(584-909)	428<br>(327-518)	141<br>(111-175)	56<br>(45-72)
Forces of nature, conflict and terrorism, and executions and police conflict	1,624<br>(1,372-1,869)	1,255<br>(1,040-1,470)	883<br>(736-1,033)	630<br>(532-738)	333<br>(271-398)	106<br>(83-135)	53<br>(43-64)
Forces of nature, conflict and terrorism, and state actor violence	298<br>(203-390)	279<br>(192-367)	203<br>(142-267)	138<br>(95-181)	76<br>(52-100)	25<br>(17-33)	6<br>(4-8)
Conflict and terrorism	1,242<br>(1,073-1,417)	906<br>(763-1,050)	640<br>(548-736)	432<br>(371-494)	214<br>(176-252)	56<br>(46-67)	41<br>(32-49)
Executions and police conflict	84<br>(41-107)	70<br>(34-98)	39<br>(21-52)	60<br>(20-95)	43<br>(10-71)	24<br>(8-46)	6<br>(2-9)

SR Table 5: Global deaths by cause and age group for both sexes (with 95% uncertainty intervals), 2006

Cause	Early Neonatal	Late Neonatal	Post Neonatal	1 to 4	5 to 9	10 to 14	15 to 19	20 to 24	25 to 29	30 to 34	35 to 39	40 to 44	45 to 49	50 to 54	55 to 59	60 to 64	65 to 69	70 to 74	75 to 79	80 to 84	85 to 89	90 to 94	95 plus
All causes	2,331,952 (2,278,220-2,395,068)	710,680 (694,406-728,145)	2,268,871 (2,211,980-2,326,069)	2,151,131 (2,077,451-2,229,886)	565,421 (558,582-572,680)	437,431 (432,693-442,638)	700,674 (690,948-711,032)	943,477 (929,348-959,413)	1,103,872 (1,079,901-1,132,283)	1,304,815 (1,282,448-1,327,794)	1,512,943 (1,486,161-1,537,948)	1,750,237 (1,730,011-1,770,184)	2,039,584 (2,014,503-2,064,703)	2,565,081 (2,536,781-2,592,828)	2,930,511 (2,900,547-2,959,294)	3,260,812 (3,231,992-3,289,521)	4,303,318 (4,264,919-4,342,364)	5,062,615 (5,023,042-5,103,119)	5,433,045 (5,393,498-5,475,881)	4,974,169 (4,945,770-5,005,225)	3,506,912 (3,492,516-3,521,554)	1,946,768 (1,940,298-1,953,016)	724,930 (722,675-727,039)
Communicable, maternal, neonatal, and nutritional disorders	2,121,848 (2,059,693-2,187,297)	625,343 (606,972-647,076)	1,872,174 (1,805,236-1,941,407)	1,735,743 (1,649,396-1,826,234)	346,388 (329,855-363,276)	204,137 (192,808-216,616)	213,769 (202,183-228,660)	303,363 (288,564-321,968)	452,928 (426,540-484,227)	548,847 (524,942-574,218)	562,752 (535,477-589,152)	520,784 (504,772-539,948)	469,357 (449,810-494,502)	423,971 (406,087-451,784)	388,867 (367,170-424,783)	407,144 (377,175-462,443)	469,272 (425,971-540,732)	556,982 (497,382-652,178)	533,654 (475,694-623,722)	502,694 (448,354-583,905)	362,453 (322,714-416,534)	183,257 (163,083-207,491)	75,484 (67,548-83,840)
HIV/AIDS and tuberculosis	-	-	122,005 (115,866-128,086)	83,156 (77,375-88,963)	44,656 (42,287-47,036)	44,253 (41,566-47,308)	56,561 (52,475-60,665)	120,031 (110,248-131,880)	284,996 (259,965-312,409)	389,211 (366,066-412,067)	403,987 (379,431-428,645)	372,466 (359,736-384,533)	318,271 (304,910-333,111)	257,982 (249,232-267,146)	201,080 (192,449-209,346)	168,989 (160,136-177,138)	160,329 (151,183-168,645)	154,712 (144,109-164,266)	115,922 (107,568-123,841)	82,465 (75,089-89,377)	42,714 (38,917-46,515)	13,746 (12,589-14,923)	3,517 (3,201-3,870)
Tuberculosis	-	-	17,456 (14,740-20,614)	26,915 (21,966-31,806)	10,199 (8,812-11,587)	11,868 (10,631-13,130)	31,453 (29,092-34,008)	57,014 (53,535-60,810)	74,632 (70,500-79,259)	87,233 (82,704-92,107)	97,450 (92,090-102,432)	105,439 (100,331-110,748)	116,294 (109,942-122,632)	129,569 (122,461-136,496)	129,947 (122,429-137,011)	125,259 (117,270-132,888)	131,681 (123,036-139,704)	136,677 (126,297-145,988)	106,729 (98,204-114,550)	79,711 (72,287-86,583)	41,704 (37,856-45,477)	13,475 (12,320-14,660)	3,461 (3,144-3,813)
Drug-susceptible tuberculosis	-	-	16,226 (13,639-19,208)	24,922 (20,377-29,482)	9,366 (8,088-10,679)	10,847 (9,723-12,035)	28,694 (26,378-31,154)	51,915 (48,505-55,549)	67,812 (63,907-72,021)	79,086 (74,733-83,749)	88,163 (83,136-92,926)	94,857 (89,870-100,031)	104,341 (98,214-110,262)	116,490 (109,949-122,969)	117,464 (110,273-124,397)	113,850 (106,398-120,974)	119,654 (111,149-127,623)	124,506 (114,809-133,280)	97,421 (89,467-104,679)	72,915 (66,390-79,425)	38,260 (34,726-41,707)	12,399 (11,335-13,533)	3,200 (2,908-3,514)
Multidrug-resistant tuberculosis without extensive drug resistance	-	-	1,205 (942-1,527)	1,955 (1,464-2,549)	810 (635-1,029)	991 (789-1,215)	2,669 (2,187-3,230)	4,877 (4,043-5,821)	6,484 (5,458-7,666)	7,746 (6,524-9,123)	8,815 (7,516-10,422)	9,993 (8,503-11,793)	11,254 (9,535-13,370)	12,371 (10,466-14,636)	11,889 (9,952-14,239)	10,957 (9,048-13,288)	11,521 (9,511-13,960)	11,698 (9,636-14,250)	8,934 (7,305-10,937)	6,521 (5,253-8,070)	3,301 (2,664-4,106)	1,028 (828-1,285)	248 (197-309)
Extensively drug-resistant tuberculosis	-	-	25 (20-31)	38 (29-49)	23 (18-30)	30 (24-38)	90 (73-110)	221 (181-265)	335 (277-402)	402 (332-480)	472 (392-563)	589 (491-711)	699 (583-835)	709 (578-860)	595 (490-718)	453 (371-558)	507 (415-624)	473 (384-587)	373 (303-465)	275 (220-346)	144 (114-183)	49 (39-62)	13 (10-17)
HIV/AIDS	-	-	104,549 (99,167-110,000)	56,241 (53,151-59,373)	34,457 (32,720-36,356)	32,385 (29,962-35,047)	25,108 (22,061-28,364)	63,018 (54,123-74,831)	210,365 (185,313-237,729)	301,978 (279,706-325,213)	306,537 (282,543-330,506)	267,027 (254,596-278,967)	201,978 (188,264-216,056)	128,412 (122,625-134,214)	71,133 (66,890-74,857)	43,730 (40,715-46,795)	28,648 (26,663-30,846)	18,035 (16,651-19,469)	9,193 (8,441-9,976)	2,754 (2,466-3,072)	1,010 (904-1,117)	271 (243-300)	56 (51-61)
Drug-susceptible HIV/AIDS - Tuberculosis	-	-	32,078 (23,977-40,222)	18,229 (14,153-22,291)	10,584 (8,180-12,868)	9,591 (7,156-11,689)	7,664 (5,913-9,391)	17,249 (12,436-22,525)	45,594 (29,416-63,513)	61,521 (38,597-85,606)	60,944 (38,138-85,369)	57,037 (36,227-77,357)	46,596 (30,748-62,847)	34,661 (24,412-43,992)	20,979 (15,630-25,630)	13,600 (10,406-16,376)	9,090 (7,233-10,808)	5,913 (4,790-6,935)	3,022 (2,457-3,561)	947 (786-1,115)	342 (283-402)	90 (75-106)	16 (14-19)
Multidrug-resistant HIV/AIDS - Tuberculosis without extensive drug resistance	-	-	2,754 (1,773-4,031)	1,529 (1,002-2,228)	812 (540-1,178)	714 (454-1,069)	594 (380-906)	1,619 (1,033-2,359)	4,080 (2,431-6,233)	5,436 (3,184-8,221)	5,275 (3,094-8,017)	4,898 (2,897-7,399)	4,077 (2,475-6,100)	3,087 (1,937-4,488)	1,862 (1,201-2,684)	1,181 (780-1,691)	774 (517-1,108)	495 (335-701)	251 (172-355)	80 (52-123)	29 (19-44)	8 (5-12)	1 (1-2)
Extensively drug-resistant HIV/AIDS - Tuberculosis	-	-	27 (18-41)	13 (8-19)	7 (5-11)	6 (4-9)	7 (4-10)	30 (19-45)	96 (58-151)	148 (89-233)	139 (84-218)	114 (70-177)	85 (53-131)	56 (35-85)	31 (19-46)	16 (10-23)	10 (7-15)	6 (4-8)	3 (2-4)	1 (1-2)	0 (0-1)	0 (0-0)	0 (0-0)
HIV/AIDS resulting in other diseases	-	-	69,689 (60,637-79,746)	36,470 (31,944-41,698)	23,054 (20,295-26,075)	22,074 (19,165-25,555)	16,844 (14,159-20,279)	44,120 (35,578-55,517)	160,594 (132,435-193,537)	234,873 (203,177-270,486)	240,180 (206,571-276,833)	204,978 (180,383-232,170)	151,219 (131,004-172,547)	90,609 (79,735-103,575)	48,261 (42,979-55,330)	28,933 (25,501-33,443)	18,775 (16,532-21,316)	11,622 (10,320-13,278)	5,916 (5,181-6,701)	1,726 (1,501-1,993)	638 (558-732)	173 (152-198)	38 (34-42)
Diarrhoea, lower respiratory infections, and other common infectious diseases	222,092 (191,955-245,638)	217,352 (200,523-232,582)	1,227,114 (1,159,305-1,296,825)	951,305 (864,762-1,056,899)	198,863 (175,480-225,417)	109,013 (94,641-125,636)	90,651 (77,573-109,461)	83,093 (70,741-104,051)	76,292 (65,263-94,948)	75,313 (64,571-93,256)	80,334 (69,220-102,592)	84,995 (73,303-104,683)	102,218 (88,102-127,386)	126,698 (107,669-160,697)	150,142 (126,061-191,012)	197,357 (163,782-260,131)	263,859 (217,690-345,365)	357,684 (294,018-463,508)	374,970 (312,010-473,409)	385,074 (328,142-474,982)	292,187 (252,032-350,422)	154,146 (133,845-179,322)	64,879 (57,218-73,383)
Diarrhoeal diseases	30,422 (27,221-34,438)	58,005 (51,953-65,162)	432,668 (385,789-482,260)	330,669 (279,352-384,546)	46,720 (29,740-75,045)	24,840 (16,136-41,189)	28,367 (18,136-46,738)	30,532 (19,231-51,489)	28,840 (18,753-48,724)	28,389 (17,862-47,291)	30,536 (19,536-52,979)	31,109 (19,538-52,026)	38,316 (24,357-64,950)	48,985 (30,272-86,462)	62,441 (39,421-106,163)	90,672 (57,402-156,311)	124,912 (77,663-212,758)	176,554 (112,734-292,236)	176,270 (110,882-285,207)	176,051 (118,927-273,925)	121,709 (84,032-182,619)	51,796 (36,706-75,767)	16,055 (11,698-22,695)
Intestinal infectious diseases	-	-	1,692 (383-5,684)	28,510 (13,674-53,331)	49,850 (28,185-81,577)	32,205 (17,233-50,544)	19,728 (10,072-33,851)	11,284 (5,525-19,857)	7,951 (4,114-13,764)	6,088 (3,185-10,404)	4,929 (2,629-8,231)	4,161 (2,274-6,883)	3,674 (1,984-6,161)	2,962 (1,534-5,079)	2,078 (994-3,764)	1,784 (879-3,198)	1,771 (988-2,888)	1,515 (871-2,401)	1,104 (662-1,748)	630 (366-996)	283 (166-446)	86 (50-135)	23 (13-39)
Typhoid fever	-	-	192 (90-367)	24,910 (11,039-47,481)	43,601 (23,809-71,719)	27,135 (13,864-43,834)	17,067 (8,453-29,692)	9,459 (4,402-16,805)	6,237 (3,157-10,971)	4,555 (2,364-7,984)	3,686 (1,934-6,452)	3,083 (1,608-5,428)	2,652 (1,377-4,532)	2,151 (1,086-3,837)	1,539 (727-2,877)	1,374 (651-2,528)	1,433 (767-2,402)	1,248 (705-1,995)	891 (503-1,432)	510 (285-817)	217 (123-348)	62 (34-99)	14 (7-23)
Paratyphoid fever	-	-	28 (11-62)	2,513 (1,025-5,287)	6,133 (2,812-12,233)	5,028 (2,296-9,507)	2,620 (1,104-5,311)	1,783 (740-3,654)	1,678 (726-3,352)	1,484 (647-2,926)	1,190 (559-2,275)	1,032 (476-2,042)	968 (438-1,922)	788 (342-1,577)	514 (210-1,068)	383 (160-798)	304 (139-593)	226 (107-423)	154 (74-279)	87 (42-160)	36 (17-67)	10 (5-19)	2 (1-4)
Other intestinal infectious diseases	-	-	1,472 (172-5,482)	1,088 (111-4,681)	116 (15-465)	42 (6-160)	40 (5-153)	43 (6-149)	36 (4-131)	48 (7-195)	52 (7-193)	46 (7-172)	55 (8-221)	23 (3-90)	24 (4-104)	27 (4-101)	33 (4-130)	41 (7-150)	58 (10-201)	34 (5-119)	30 (5-99)	14 (3-48)	7 (1-23)
Lower respiratory infections	153,051 (132,551-169,562)	124,512 (112,895-135,723)	566,254 (518,958-616,248)	291,220 (251,645-332,375)	40,982 (33,545-46,303)	21,859 (17,901-24,363)	19,119 (16,410-21,977)	21,614 (18,355-24,380)	23,124 (19,962-26,481)	25,323 (21,964-28,255)	29,770 (25,376-33,228)	35,415 (30,575-39,564)	44,427 (39,059-49,446)	58,824 (51,366-64,678)	70,091 (60,582-78,079)	90,270 (75,624-99,184)	120,518 (99,836-131,221)	162,555 (137,764-178,559)	183,806 (156,313-202,347)	196,244 (168,879-216,439)	163,266 (142,373-182,371)	99,554 (88,363-112,415)	47,872 (41,363-53,865)
Upper respiratory infections	93 (63-132)	33 (20-51)	755 (534-1,046)	610 (390-950)	46 (31-68)	34 (22-50)	83 (62-112)	81 (60-107)	85 (62-115)	86 (64-113)	101 (76-133)	104 (80-134)	118 (91-152)	127 (98-164)	130 (101-166)	133 (103-173)	131 (101-170)	113 (88-143)	113 (90-143)	77 (61-97)	53 (42-66)	23 (18-29)	6 (4-8)
Otitis media	4 (3-7)	17 (10-29)	143 (83-266)	263 (150-443)	155 (94-301)	146 (95-236)	57 (38-92)	44 (29-72)	49 (31-80)	34 (22-57)	43 (27-69)	61 (37-101)	107 (61-187)	115 (66-189)	90 (57-147)	79 (53-122)	127 (82-212)	101 (69-152)	79 (59-109)	67 (47-94)	38 (26-51)	17 (11-23)	6 (4-8)
Meningitis	10,701 (7,471-13,960)	13,827 (11,111-17,390)	84,207 (69,767-100,359)	74,951 (59,763-92,229)	20,229 (15,148-26,229)	14,325 (11,089-18,903)	13,609 (10,502-18,202)	11,867 (9,048-15,696)	10,012 (7,563-13,666)	9,200 (6,862-13,020)	8,950 (6,739-12,383)	7,932 (6,052-10,953)	9,010 (7,124-12,771)	8,936 (7,110-12,525)	8,676 (6,934-12,055)	8,130 (6,504-11,795)	8,726 (7,114-12,522)	8,630 (7,075-12,094)	6,036 (5,012-8,680)	5,509 (4,529-7,510)	2,898 (2,397-4,061)	1,030 (855-1,403)	296 (256-383)
Pneumococcal meningitis	512 (328-740)	638 (457-884)	4,032 (2,944-5,270)	3,906 (2,638-5,527)	1,092 (735-1,578)	762 (501-1,143)	715 (466-1,087)	607 (387-932)	595 (382-918)	692 (445-1,035)	820 (525-1,272)	842 (514-1,347)	1,028 (627-1,656)	1,006 (674-1,568)	1,007 (702-1,492)	991 (697-1,518)	1,030 (711-1,578)	1,016 (689-1,558)	724 (516-1,074)	610 (426-888)	310 (229-434)	118 (91-159)	37 (27-52)
H influenzae type B meningitis	1,414 (946-1,966)	1,753 (1,327-2,253)	10,152 (7,846-12,949)	8,251 (6,117-10,789)	1,792 (1,258-2,477)	1,054 (739-1,466)	886 (613-1,283)	764 (539-1,126)	699 (485-1,030)	679 (460-1,002)	677 (481-968)	600 (414-895)	686 (484-1,046)	673 (484-1,021)	652 (465-928)	619 (439-963)	634 (447-967)	610 (449-900)	436 (322-668)	396 (292-552)	207 (155-297)	72 (53-104)	20 (13-30)
Meningococcal infection	5,019 (3,444-6,702)	6,490 (5,212-8,245)	39,435 (32,328-48,186)	37,347 (29,032-46,796)	10,656 (7,889-14,161)	7,395 (5,545-9,838)	7,091 (5,334-9,679)	6,097 (4,487-8,418)	4,900 (3,522-6,790)	4,185 (3,031-6,063)	3,943 (2,885-5,582)	3,509 (2,617-5,199)	3,909 (2,993-5,815)	3,929 (3,035-5,831)	3,774 (2,910-5,400)	3,587 (2,749-5,415)	3,782 (2,940-5,763)	3,539 (2,736-5,271)	2,572 (1,968-3,974)	2,287 (1,750-3,293)	1,273 (996-1,831)	461 (362-653)	127 (98-175)
Other meningitis	3,756 (2,600-4,968)	4,946 (3,806-6,517)	30,588 (23,958-38,161)	25,446 (19,394-32,219)	6,689 (4,877-8,845)	5,114 (3,827-6,571)	4,918 (3,743-6,547)	4,398 (3,283-5,900)	3,818 (2,840-5,037)	3,644 (2,656-5,104)	3,511 (2,546-4,783)	2,981 (2,213-4,049)	3,386 (2,635-4,753)	3,328 (2,602-4,806)	3,243 (2,510-4,593)	2,933 (2,215-4,235)	3,280 (2,550-4,730)	3,464 (2,694-4,766)	2,304 (1,814-3,262)	2,215 (1,757-3,030)	1,108 (892-1,532)	379 (304-498)	112 (88-147)
Encephalitis	795 (545-965)	1,326 (1,031-1,584)	14,595 (11,154-17,899)	16,913 (12,742-24,275)	6,675 (5,494-8,942)	5,291 (4,432-7,136)	4,118 (3,543-5,333)	3,861 (3,371-5,279)	3,223 (2,794-4,264)	3,358 (2,923-4,456)	3,314 (2,895-4,457)	3,225 (2,815-4,401)	3,414 (2,913-4,835)	3,438 (2,934-4,831)	3,728 (3,162-5,137)	4,186 (3,555-5,871)	5,108 (4,330-7,040)	5,620 (4,731-7,514)	5,145 (4,347-6,309)	4,289 (3,411-5,333)	2,312 (1,910-2,861)	744 (618-938)	205 (173-258)
Diphtheria	-	-	770 (492-1,200)	1,702 (927-2,931)	294 (176-472)	134 (85-206)	63 (43-91)	41 (26-60)	35 (24-52)	31 (21-44)	45 (31-62)	44 (31-61)	52 (37-72)	59 (42-80)	44 (31-60)	-	-	-	-	-	-	-	-
Whooping cough	-	-	53,238 (25,722-96,668)	52,853 (25,092-96,077)	5,700 (2,680-10,590)	1,151 (537-2,220)	455 (216-868)	328 (157-631)	188 (85-375)	140 (64-284)	101 (46-204)	108 (49-221)	92 (42-188)	81 (36-165)	93 (43-188)	-	-	-	-	-	-	-	-
Tetanus	26,935 (13,373-35,871)	19,335 (10,659-26,257)	7,297 (3,909-10,225)	3,286 (1,754-5,168)	1,698 (862-2,733)	1,619 (852-2,593)	2,246 (1,169-3,438)	1,918 (1,038-2,913)	1,876 (1,135-2,714)	1,976 (1,233-2,789)	2,050 (1,199-2,958)	2,506 (1,441-3,468)	2,696 (1,604-3,876)	2,919 (1,752-4,199)	2,506 (1,446-3,811)	1,882 (1,117-2,793)	2,146 (1,326-3,436)	2,174 (1,279-3,061)	1,737 (966-2,696)	1,175 (617-1,809)	460 (237-610)	140 (77-191)	39 (21-53)
Measles	-	-	63,102 (23,656-139,777)	147,337 (59,590-301,828)	25,170 (9,595-53,522)	6,959 (2,452-15,914)	2,602 (908-6,070)	1,280 (446-3,026)	615 (215-1,455)	368 (129-870)	225 (78-527)	157 (55-366)	111 (39-260)	83 (29-196)	74 (26-173)	-	-	-	-	-	-	-	-
Varicella and herpes zoster	91 (75-110)	298 (246-355)	2,393 (1,999-2,860)	2,992 (2,409-3,659)	1,343 (1,074-1,689)	450 (370-548)	203 (169-243)	244 (204-295)	294 (250-344)	320 (273-374)	271 (229-321)	174 (147-206)	201 (169-236)	170 (145-199)	192 (163-229)	221 (186-262)	421 (359-501)	421 (353-495)	680 (572-810)	1,033 (861-1,237)	1,169 (952-1,411)	756 (585-938)	377 (259-521)
Neglected tropical diseases and malaria	1,122 (718-1,513)	27,514 (20,802-35,208)	241,793 (193,863-296,912)	505,002 (417,745-603,745)	73,440 (58,292-89,596)	38,020 (30,448-46,104)	21,391 (17,640-25,633)	22,247 (18,124-26,793)	16,691 (13,594-19,909)	15,518 (12,782-18,777)	16,663 (13,607-19,988)	16,035 (13,352-19,183)	15,613 (13,001-18,495)	15,847 (13,169-18,879)	15,727 (13,167-18,674)	18,383 (15,000-22,402)	17,937 (14,738-21,795)	15,784 (12,834-19,232)	12,261 (10,028-14,985)	6,755 (5,498-8,185)	4,546 (3,629-5,628)	1,017 (803-1,229)	444 (312-630)
Malaria	-	26,693 (19,994-34,447)	230,276 (182,790-285,767)	477,900 (388,251-576,638)	59,853 (45,294-75,823)	29,724 (22,415-37,535)	13,622 (10,700-17,121)	14,333 (11,166-18,164)	10,574 (8,161-13,393)	9,627 (7,402-12,340)	10,294 (7,906-13,378)	9,676 (7,475-12,399)	8,774 (6,789-11,231)	9,044 (6,941-11,618)	9,099 (6,997-11,757)	12,504 (9,348-16,395)	11,859 (8,744-15,582)	10,460 (7,728-13,814)	7,934 (5,847-10,547)	4,308 (3,176-5,633)	3,176 (2,336-4,220)	569 (397-746)	318 (194-508)
Chagas disease	-	-	2 (2-3)	2 (1-2)	2 (2-2)	4 (4-5)	12 (10-14)	27 (24-33)	51 (45-58)	97 (87-110)	172 (156-191)	268 (244-295)	393 (360-432)	558 (512-610)	704 (638-778)	862 (800-925)	950 (885-1,018)	994 (937-1,057)	794 (728-868)	596 (550-653)	361 (335-392)	154 (143-167)	38 (35-42)
Leishmaniasis	-	-	2,633 (1,405-4,680)	4,159 (2,043-7,439)	2,079 (1,107-3,750)	1,401 (741-2,443)	1,834 (978-3,120)	1,984 (1,059-3,486)	1,596 (838-2,735)	1,449 (787-2,525)	1,766 (934-3,074)	1,708 (884-2,959)	1,626 (858-2,803)	1,629 (877-2,810)	1,191 (607-2,140)	933 (482-1,677)	959 (506-1,668)	1,123 (593-2,029)	892 (463-1,577)	449 (233-788)	276 (144-478)	58 (25-104)	19 (6-36)
Visceral leishmaniasis	-	-	2,633 (1,405-4,680)	4,159 (2,043-7,439)	2,079 (1,107-3,750)	1,401 (741-2,443)	1,834 (978-3,120)	1,984 (1,059-3,486)	1,596 (838-2,735)	1,449 (787-2,525)	1,766 (934-3,074)	1,708 (884-2,959)	1,626 (858-2,803)	1,629 (877-2,810)	1,191 (607-2,140)	933 (482-1,677)	959 (506-1,668)	1,123 (593-2,029)	892 (463-1,577)	449 (233-788)	276 (144-478)	58 (25-104)	19 (6-36)
African trypanosomiasis	-	-	-	314 (84-896)	764 (228-1,929)	874 (277-2,033)	1,064 (345-2,582)	1,101 (328-2,711)	966 (284-2,392)	836 (261-2,093)	697 (200-1,824)	599 (169-1,730)	546 (151-1,510)	455 (136-1,236)	424 (114-1,257)	361 (90-1,059)	265 (67-843)	181 (45-590)	117 (30-388)	57 (14-181)	20 (5-64)	5 (1-20)	1 (0-4)
Schistosomiasis	-	-	0 (0-0)	515 (343-771)	264 (183-380)	361 (263-508)	348 (260-451)	457 (349-593)	526 (406-676)	580 (456-741)	691 (548-875)	765 (620-956)	851 (669-1,056)	1,050 (838-1,313)	1,224 (965-1,522)	1,232 (984-1,549)	1,294 (1,012-1,616)	1,183 (941-1,485)	873 (702-1,105)	459 (364-579)	210 (166-260)	58 (44-75)	10 (6-14)
Cysticercosis	0 (0-0)	0 (0-0)	0 (0-0)	109 (71-164)	68 (50-93)	55 (42-69)	49 (39-64)	70 (56-88)	91 (74-111)	107 (87-129)	84 (68-102)	83 (69-100)	81 (67-98)	101 (86-119)	94 (80-111)	70 (58-84)	61 (51-73)	40 (33-49)	26 (21-32)	17 (13-22)	6 (5-8)	2 (2-3)	1 (1-1)
Cystic echinococcosis	-	-	-	302 (202-450)	124 (85-181)	76 (51-110)	37 (25-51)	48 (33-67)	69 (50-95)	70 (51-94)	86 (65-112)	79 (60-101)	89 (68-116)	90 (69-114)	96 (73-124)	93 (72-118)	96 (75-122)	85 (67-106)	69 (55-86)	50 (39-62)	25 (20-31)	9 (7-12)	2 (2-4)
Dengue	-	-	2,002 (625-2,987)	3,423 (1,259-4,807)	2,149 (782-3,150)	1,320 (425-1,963)	1,279 (317-1,906)	1,226 (265-1,826)	1,035 (216-1,550)	818 (155-1,208)	878 (172-1,278)	795 (154-1,154)	884 (182-1,267)	858 (185-1,261)	955 (199-1,394)	874 (169-1,300)	916 (218-1,321)	432 (102-687)	361 (94-567)	295 (74-480)	192 (56-308)	77 (24-130)	18 (7-27)
Yellow fever	-	3 (1-9)	54 (11-166)	548 (110-1,634)	1,112 (219-3,352)	971 (188-2,984)	862 (177-2,575)	733 (146-2,249)	561 (111-1,861)	420 (82-1,266)	319 (65-958)	249 (51-735)	201 (41-650)	160 (31-493)	129 (26-414)	97 (20-290)	68 (14-207)	45 (9-139)	26 (5-81)	11 (2-34)	4 (1-13)	1 (0-3)	0 (0-1)
Rabies	-	-	476 (341-905)	2,682 (1,457-3,833)	4,341 (2,056-6,574)	2,295 (1,139-3,164)	1,723 (979-2,382)	1,729 (962-2,460)	729 (446-995)	1,075 (612-1,447)	1,207 (668-1,717)	1,350 (814-1,932)	1,683 (953-2,298)	1,352 (838-2,178)	1,257 (591-1,674)	829 (461-1,032)	900 (431-1,246)	650 (424-1,052)	680 (489-1,133)	193 (131-311)	102 (62-176)	16 (10-25)	7 (4-12)
Intestinal nematode infections	-	-	540 (422-669)	5,781 (4,497-7,340)	890 (712-1,100)	185 (151-231)	65 (51-80)	44 (34-54)	34 (27-42)	44 (35-55)	42 (33-52)	31 (24-38)	34 (28-42)	51 (42-62)	43 (34-52)	49 (39-60)	38 (30-46)	57 (46-70)	44 (36-54)	28 (23-36)	11 (8-14)	7 (5-9)	3 (2-4)
Ascariasis	-	-	540 (422-669)	5,781 (4,497-7,340)	890 (712-1,100)	185 (151-231)	65 (51-80)	44 (34-54)	34 (27-42)	44 (35-55)	42 (33-52)	31 (24-38)	34 (28-42)	51 (42-62)	43 (34-52)	49 (39-60)	38 (30-46)	57 (46-70)	44 (36-54)	28 (23-36)	11 (8-14)	7 (5-9)	3 (2-4)
Ebola	0 (0-0)	0 (0-0)	0 (0-0)	0 (0-0)	0 (0-0)	0 (0-0)	0 (0-0)	0 (0-0)	0 (0-0)	0 (0-0)	0 (0-0)	0 (0-0)	0 (0-0)	0 (0-0)	0 (0-0)	0 (0-0)	0 (0-0)	0 (0-0)	0 (0-0)	0 (0-0)	0 (0-0)	0 (0-0)	0 (0-0)
Zika virus	0 (0-0)	0 (0-0)	0 (0-0)	0 (0-0)	0 (0-0)	0 (0-0)	0 (0-0)	0 (0-0)	0 (0-0)	0 (0-0)	0 (0-0)	0 (0-0)	0 (0-0)	0 (0-0)	0 (0-0)	0 (0-0)	0 (0-0)	0 (0-0)	0 (0-0)	0 (0-0)	0 (0-0)	0 (0-0)	0 (0-0)
Other neglected tropical diseases	1,122 (718-1,513)	818 (555-1,076)	5,809 (3,766-7,752)	9,267 (5,010-16,885)	1,794 (1,121-2,826)	754 (501-1,116)	496 (315-715)	495 (335-662)	460 (323-614)	395 (267-502)	427 (301-531)	431 (305-536)	449 (318-563)	496 (348-617)	512 (356-639)	481 (336-622)	530 (385-706)	534 (369-715)	445 (314-584)	291 (215-398)	164 (119-232)	61 (45-84)	27 (21-43)
Maternal disorders	-	-	-	-	-	1,382 (1,156-1,653)	30,804 (27,848-33,644)	62,124 (56,730-66,924)	60,169 (55,340-64,093)	53,288 (49,331-56,775)	45,793 (42,486-48,853)	29,570 (27,091-31,942)	15,437 (13,885-17,058)	3,210 (2,639-3,966)	-	-	-	-	-	-	-	-	-
Maternal haemorrhage	-	-	-	-	-	269 (176-390)	8,426 (6,097-11,056)	19,663 (15,422-24,294)	19,575 (14,948-24,302)	17,521 (13,516-21,778)	15,606 (11,849-19,724)	9,365 (6,724-12,307)	3,950 (2,754-5,284)	618 (358-986)	-	-	-	-	-	-	-	-	-
Maternal sepsis and other pregnancy related infections	-	-	-	-	-	132 (83-195)	3,343 (2,190-4,789)	5,661 (3,656-8,329)	5,224 (3,586-7,343)	4,418 (2,965-6,304)	3,680 (2,494-5,265)	2,485 (1,577-3,752)	1,441 (917-2,236)	284 (161-471)	-	-	-	-	-	-	-	-	-
Maternal hypertensive disorders	-	-	-	-	-	284 (175-411)	4,855 (3,208-6,789)	8,630 (6,070-11,686)	7,772 (5,266-10,937)	6,703 (4,442-9,753)	5,858 (3,936-8,269)	3,734 (2,364-5,600)	1,730 (1,099-2,562)	345 (182-574)	-	-	-	-	-	-	-	-	-
Maternal obstructed labour and uterine rupture	-	-	-	-	-	54 (27-95)	1,025 (499-1,744)	2,087 (1,226-3,228)	2,299 (1,329-3,698)	2,405 (1,390-3,724)	2,377 (1,437-3,876)	1,748 (978-2,951)	991 (532-1,653)	197 (83-372)	-	-	-	-	-	-	-	-	-
Maternal abortion, miscarriage, and ectopic pregnancy	-	-	-	-	-	113 (67-178)	2,525 (1,539-3,797)	4,876 (3,142-7,117)	4,522 (3,040-6,469)	4,205 (2,859-6,008)	3,824 (2,428-5,353)	2,902 (1,993-4,193)	1,926 (1,315-2,745)	347 (227-502)	-	-	-	-	-	-	-	-	-
Indirect maternal deaths	-	-	-	-	-	228 (146-338)	4,534 (2,901-6,760)	9,276 (6,314-12,924)	9,757 (6,531-13,977)	8,522 (5,270-12,147)	6,191 (4,078-8,708)	3,947 (2,436-5,935)	2,437 (1,451-3,611)	711 (421-1,126)	-	-	-	-	-	-	-	-	-
Late maternal deaths	-	-	-	-	-	32 (14-67)	559 (271-1,037)	1,061 (500-1,972)	1,050 (534-1,981)	918 (471-1,650)	790 (364-1,424)	517 (176-951)	291 (108-525)	72 (26-149)	-	-	-	-	-	-	-	-	-
Maternal deaths aggravated by HIV/AIDS	-	-	-	-	-	2 (1-3)	69 (40-98)	332 (207-442)	545 (353-717)	583 (389-756)	436 (279-584)	253 (155-347)	113 (67-158)	18 (10-27)	-	-	-	-	-	-	-	-	-
Other maternal disorders	-	-	-	-	-	268 (179-394)	5,469 (3,786-7,729)	10,538 (7,432-14,541)	9,425 (6,721-12,606)	8,013 (5,528-10,848)	7,030 (4,838-10,114)	4,618 (2,885-6,731)	2,558 (1,649-3,707)	620 (393-943)	-	-	-	-	-	-	-	-	-
Neonatal disorders	1,850,761 (1,786,416-1,926,171)	360,156 (341,648-384,881)	96,692 (86,577-111,062)	10,575 (9,262-12,367)	-	-	-	-	-	-	-	-	-	-	-	-	-	-	-	-	-	-	-
Neonatal preterm birth complications	714,668 (671,789-794,626)	105,031 (96,087-121,964)	33,916 (29,722-40,913)	2,658 (2,266-3,203)	-	-	-	-	-	-	-	-	-	-	-	-	-	-	-	-	-	-	-
Neonatal encephalopathy due to birth asphyxia and trauma	603,238 (554,110-651,487)	55,717 (49,704-62,835)	19,453 (16,267-23,320)	4,487 (3,941-5,120)	-	-	-	-	-	-	-	-	-	-	-	-	-	-	-	-	-	-	-
Neonatal sepsis and other neonatal infections	155,352 (117,945-201,556)	101,762 (83,556-122,766)	17,463 (13,414-22,384)	797 (365-1,348)	-	-	-	-	-	-	-	-	-	-	-	-	-	-	-	-	-	-	-
Hemolytic disease and other neonatal jaundice	52,650 (46,171-62,135)	27,712 (24,034-32,499)	5,432 (4,258-7,052)	287 (213-376)	-	-	-	-	-	-	-	-	-	-	-	-	-	-	-	-	-	-	-
Other neonatal disorders	324,854 (294,308-355,883)	69,934 (62,421-81,353)	20,429 (17,286-25,397)	2,347 (1,897-3,202)	-	-	-	-	-	-	-	-	-	-	-	-	-	-	-	-	-	-	-
Nutritional deficiencies	-	-	109,771 (98,229-122,012)	133,077 (110,543-158,772)	11,970 (10,169-14,027)	4,818 (4,208-5,668)	4,231 (3,778-5,169)	4,561 (4,065-5,462)	4,532 (4,057-5,445)	4,425 (4,005-5,330)	4,476 (4,008-5,526)	5,252 (4,757-6,361)	5,060 (4,538-6,409)	6,498 (5,831-7,993)	8,268 (7,443-10,064)	9,730 (8,708-11,953)	13,434 (12,019-16,118)	15,715 (14,194-18,616)	19,284 (17,713-22,071)	20,163 (18,726-22,598)	18,541 (17,385-20,105)	12,549 (11,818-13,305)	6,057 (5,718-6,395)
Protein-energy malnutrition	-	-	105,865 (94,430-117,949)	128,121 (107,144-152,417)	10,362 (8,756-12,343)	3,825 (3,283-4,618)	2,669 (2,320-3,363)	2,495 (2,176-3,077)	2,659 (2,320-3,289)	2,545 (2,225-3,152)	2,644 (2,314-3,430)	3,396 (3,044-4,164)	2,807 (2,476-3,626)	3,859 (3,378-4,869)	5,476 (4,860-6,631)	6,611 (5,887-8,260)	8,996 (8,029-10,902)	11,309 (10,261-13,349)	14,543 (13,392-16,563)	15,752 (14,566-17,334)	14,878 (13,760-15,957)	10,410 (9,553-11,075)	5,106 (4,523-5,455)
Iodine deficiency	-	-	357 (108-890)	306 (69-954)	96 (29-251)	28 (7-74)	66 (21-158)	76 (22-204)	84 (25-215)	84 (27-205)	81 (27-179)	65 (23-154)	61 (20-160)	85 (32-213)	104 (42-253)	98 (41-226)	143 (58-300)	157 (67-309)	143 (61-293)	105 (41-214)	52 (19-123)	20 (5-59)	10 (1-36)
Iron-deficiency anaemia	-	-	58 (35-118)	110 (68-211)	143 (73-232)	120 (77-179)	162 (113-213)	163 (125-209)	130 (103-177)	135 (110-162)	121 (99-164)	110 (91-193)	101 (84-184)	103 (79-231)	105 (78-222)	89 (66-185)	119 (84-266)	212 (142-425)	230 (151-527)	202 (139-403)	130 (71-224)	94 (43-138)	85 (48-117)
Other nutritional deficiencies	-	-	3,491 (2,541-4,345)	4,538 (2,289-6,629)	1,369 (1,097-1,674)	845 (701-1,015)	1,334 (1,137-1,650)	1,826 (1,539-2,253)	1,658 (1,404-2,032)	1,661 (1,408-2,050)	1,630 (1,372-2,031)	1,681 (1,402-2,164)	2,091 (1,692-2,800)	2,452 (1,987-3,216)	2,583 (2,137-3,405)	2,933 (2,445-3,779)	4,175 (3,538-5,306)	4,038 (3,331-5,309)	4,367 (3,564-5,696)	4,104 (3,454-5,226)	3,480 (2,897-4,491)	2,024 (1,656-2,900)	856 (690-1,455)
Other communicable, maternal, neonatal, and nutritional diseases	47,872 (28,937-73,703)	20,321 (13,259-29,762)	74,799 (51,807-105,132)	52,628 (38,086-71,437)	17,459 (12,861-23,778)	6,652 (5,871-7,455)	10,131 (9,279-10,948)	11,308 (10,533-12,031)	10,248 (9,578-10,952)	11,093 (10,365-11,830)	11,499 (10,818-12,157)	12,466 (11,705-13,234)	12,757 (11,963-13,534)	13,736 (13,029-14,475)	13,650 (12,855-14,304)	12,683 (11,878-13,363)	13,714 (12,798-14,478)	13,086 (12,078-13,850)	11,218 (10,323-11,963)	8,237 (7,472-8,950)	4,466 (4,028-4,799)	1,799 (1,596-1,925)	586 (512-632)
Sexually transmitted diseases excluding HIV	42,251 (23,624-68,677)	15,873 (8,879-25,443)	50,136 (27,909-80,320)	28,772 (15,721-46,586)	9,680 (5,370-15,789)	154 (123-187)	593 (485-709)	731 (584-845)	892 (710-1,021)	825 (669-953)	807 (647-935)	777 (652-873)	732 (631-844)	703 (551-809)	887 (697-1,027)	496 (410-593)	545 (446-622)	448 (389-543)	465 (405-546)	331 (288-373)	234 (200-260)	127 (109-139)	55 (46-60)
Syphilis	42,251 (23,624-68,677)	15,873 (8,879-25,443)	50,136 (27,909-80,320)	28,772 (15,721-46,586)	9,680 (5,370-15,789)	105 (84-130)	222 (186-263)	257 (208-299)	255 (208-294)	304 (242-372)	217 (166-260)	169 (144-219)	148 (125-194)	250 (201-321)	362 (285-467)	171 (147-232)	204 (176-253)	198 (172-274)	186 (162-248)	127 (112-159)	82 (70-96)	41 (33-47)	17 (13-19)
Chlamydial infection	-	-	-	-	-	11 (8-14)	64 (50-78)	138 (111-162)	131 (101-152)	65 (50-74)	105 (83-119)	126 (98-140)	112 (92-125)	84 (64-95)	102 (74-116)	66 (51-74)	41 (31-47)	40 (31-45)	47 (38-53)	48 (38-53)	35 (29-39)	19 (16-21)	8 (7-9)
Gonococcal infection	-	-	-	-	-	26 (20-32)	170 (134-205)	208 (168-242)	354 (279-411)	299 (236-341)	298 (237-339)	318 (257-353)	303 (249-338)	247 (188-279)	277 (202-312)	161 (125-181)	228 (169-260)	155 (122-175)	182 (149-203)	122 (99-135)	91 (76-100)	52 (44-57)	22 (19-24)
Other sexually transmitted diseases	-	-	-	-	-	12 (10-15)	137 (108-167)	127 (103-148)	153 (119-177)	157 (123-179)	187 (150-213)	164 (134-182)	169 (142-189)	121 (92-136)	146 (108-165)	99 (77-111)	71 (54-81)	55 (43-62)	50 (42-56)	34 (29-38)	27 (23-29)	15 (13-16)	8 (7-9)
Hepatitis	-	-	10,660 (9,507-11,921)	12,495 (10,068-14,709)	5,128 (4,324-6,001)	4,657 (4,043-5,279)	7,291 (6,601-8,010)	8,654 (8,015-9,242)	7,677 (7,111-8,285)	8,365 (7,770-9,028)	8,755 (8,160-9,342)	9,886 (9,221-10,628)	9,857 (9,187-10,581)	10,436 (9,832-11,111)	10,031 (9,468-10,615)	8,790 (8,254-9,312)	9,173 (8,542-9,785)	8,467 (7,868-9,020)	6,852 (6,322-7,347)	4,554 (4,109-4,970)	2,055 (1,865-2,222)	628 (575-676)	144 (134-155)
Acute hepatitis A	-	-	6,582 (4,756-8,430)	1,317 (806-1,950)	114 (75-163)	66 (43-91)	107 (67-158)	129 (79-198)	90 (49-139)	78 (42-133)	77 (43-124)	94 (51-157)	107 (62-170)	124 (69-202)	125 (72-202)	120 (68-188)	123 (74-187)	125 (75-192)	114 (66-175)	79 (50-118)	34 (22-49)	11 (7-15)	2 (1-3)
Hepatitis B	-	-	4,078 (2,426-5,948)	1,673 (987-2,631)	475 (266-775)	715 (409-1,090)	2,453 (1,736-3,344)	5,086 (3,866-6,296)	5,781 (4,836-6,657)	7,144 (6,301-7,935)	7,840 (7,104-8,517)	8,969 (8,190-9,736)	8,912 (8,064-9,716)	9,419 (8,596-10,229)	8,981 (8,174-9,678)	7,747 (6,961-8,437)	7,953 (7,148-8,767)	7,173 (6,298-7,974)	5,631 (4,777-6,340)	3,325 (2,692-3,886)	1,237 (882-1,542)	307 (194-414)	57 (31-85)
Hepatitis C	-	-	-	-	140 (91-208)	52 (34-77)	76 (49-110)	89 (50-141)	66 (35-105)	62 (33-103)	75 (40-127)	107 (57-167)	145 (77-236)	208 (108-345)	276 (155-464)	282 (152-467)	269 (150-431)	234 (123-380)	191 (108-307)	126 (72-204)	59 (36-90)	20 (12-30)	5 (3-8)
Acute hepatitis E	-	-	-	9,505 (7,395-11,473)	4,399 (3,618-5,184)	3,823 (3,209-4,441)	4,655 (3,809-5,459)	3,350 (2,234-4,504)	1,741 (1,068-2,522)	1,082 (616-1,746)	762 (416-1,222)	717 (348-1,207)	694 (348-1,178)	686 (371-1,123)	649 (301-1,181)	641 (289-1,200)	828 (420-1,406)	935 (474-1,574)	916 (420-1,593)	1,023 (558-1,563)	725 (441-1,035)	291 (184-408)	80 (50-107)
Other infectious diseases	5,621 (2,369-10,119)	4,448 (2,279-6,527)	14,003 (6,929-24,277)	11,362 (6,410-17,892)	2,651 (2,015-3,226)	1,841 (1,447-2,171)	2,246 (1,822-2,550)	1,923 (1,552-2,182)	1,679 (1,385-1,890)	1,903 (1,524-2,131)	1,938 (1,622-2,142)	1,803 (1,573-1,976)	2,168 (1,843-2,363)	2,597 (2,216-2,832)	2,732 (2,330-2,935)	3,397 (2,905-3,714)	3,996 (3,359-4,354)	4,172 (3,458-4,575)	3,902 (3,267-4,238)	3,353 (2,810-3,763)	2,177 (1,835-2,402)	1,043 (850-1,142)	387 (316-426)
Non-communicable diseases	196,123 (159,190-226,607)	76,862 (66,331-86,111)	305,603 (263,871-343,165)	195,262 (156,782-225,574)	90,137 (82,802-98,165)	100,820 (94,735-107,085)	178,217 (171,827-185,149)	226,840 (220,263-234,906)	279,011 (271,089-287,911)	407,290 (397,553-418,346)	607,016 (594,096-621,686)	910,928 (894,283-929,262)	1,280,436 (1,257,032-1,302,252)	1,865,155 (1,833,270-1,893,684)	2,306,928 (2,266,359-2,338,753)	2,665,913 (2,611,956-2,704,000)	3,635,229 (3,558,969-3,688,852)	4,313,769 (4,221,340-4,383,538)	4,719,122 (4,626,719-4,790,356)	4,322,243 (4,234,879-4,389,466)	3,039,998 (2,984,321-3,085,874)	1,708,467 (1,683,405-1,730,773)	628,898 (620,051-638,080)
Neoplasms	845 (759-886)	537 (506-566)	5,710 (5,357-6,298)	22,985 (20,798-26,281)	26,247 (24,329-28,111)	29,731 (27,892-31,421)	44,111 (41,824-45,981)	47,847 (45,960-49,369)	59,492 (57,243-61,409)	101,218 (97,982-103,896)	170,175 (166,062-174,676)	273,060 (267,194-279,623)	398,596 (391,048-405,459)	606,976 (596,508-616,493)	746,190 (734,554-758,406)	801,201 (785,987-812,626)	986,476 (969,865-999,700)	1,029,928 (1,010,606-1,045,308)	943,803 (924,967-960,795)	684,507 (666,246-699,287)	383,739 (372,343-394,240)	167,921 (162,279-173,208)	49,185 (47,226-50,820)
Lip and oral cavity cancer	-	-	-	-	-	-	407 (380-442)	767 (719-821)	1,254 (1,155-1,357)	2,223 (2,080-2,404)	3,832 (3,597-4,105)	6,844 (6,455-7,288)	10,432 (10,002-10,884)	14,785 (14,191-15,394)	16,776 (16,161-17,405)	16,789 (16,038-17,499)	16,953 (16,248-17,557)	14,530 (13,837-15,084)	11,887 (11,362-12,334)	8,781 (8,342-9,169)	5,306 (5,027-5,557)	2,477 (2,364-2,595)	801 (760-837)
Nasopharynx cancer	-	-	-	-	134 (120-149)	293 (265-321)	582 (542-620)	620 (577-665)	784 (736-838)	1,432 (1,349-1,525)	2,599 (2,430-2,775)	4,229 (3,996-4,484)	5,414 (5,150-5,666)	7,707 (7,342-8,065)	7,898 (7,555-8,247)	6,493 (6,171-6,813)	6,279 (5,993-6,563)	5,217 (5,012-5,449)	3,396 (3,270-3,528)	2,122 (2,035-2,212)	916 (879-953)	352 (339-367)	97 (93-101)
Other pharynx cancer	-	-	-	-	-	-	310 (279-342)	471 (430-513)	649 (592-702)	1,148 (1,064-1,230)	2,137 (1,992-2,289)	4,380 (4,081-4,684)	7,688 (7,303-8,075)	11,511 (10,944-12,025)	12,911 (12,338-13,468)	12,858 (12,104-13,501)	12,399 (11,717-13,035)	10,369 (9,661-10,892)	6,772 (6,328-7,126)	4,337 (3,948-4,657)	1,991 (1,811-2,132)	665 (618-704)	149 (140-157)
Oesophageal cancer	-	-	-	-	-	-	374 (351-401)	646 (615-682)	898 (847-955)	1,907 (1,823-2,001)	4,197 (4,018-4,390)	9,804 (9,395-10,198)	18,117 (17,511-18,712)	34,336 (33,288-35,394)	46,277 (44,960-47,637)	51,018 (49,446-52,656)	62,384 (60,550-64,357)	63,005 (60,987-65,126)	51,097 (49,482-52,777)	33,249 (32,146-34,331)	15,872 (15,375-16,416)	5,533 (5,338-5,730)	1,253 (1,202-1,299)
Stomach cancer	-	-	-	-	-	-	1,200 (1,142-1,266)	2,382 (2,279-2,507)	4,077 (3,923-4,262)	8,945 (8,619-9,285)	16,294 (15,764-16,840)	27,141 (26,362-27,923)	38,984 (37,976-40,080)	62,720 (61,136-64,320)	79,906 (77,969-81,960)	88,746 (86,706-91,212)	117,070 (114,174-119,879)	125,576 (122,696-128,654)	112,302 (109,319-115,073)	77,796 (75,700-79,870)	40,957 (39,638-42,214)	17,340 (16,733-17,990)	4,998 (4,786-5,172)
Colon and rectum cancer	-	-	-	-	-	-	1,172 (1,088-1,267)	2,453 (2,292-2,628)	3,631 (3,452-3,821)	6,946 (6,636-7,253)	10,969 (10,481-11,424)	16,716 (16,133-17,316)	26,907 (26,202-27,671)	41,557 (40,475-42,675)	55,720 (54,394-57,210)	63,257 (61,994-64,600)	85,642 (83,669-87,679)	97,195 (95,188-99,157)	102,507 (99,989-104,952)	82,964 (80,806-85,131)	52,386 (50,643-54,072)	26,187 (25,294-27,153)	8,515 (8,143-8,832)
Liver cancer	-	-	-	-	445 (407-502)	762 (707-837)	1,614 (1,504-1,753)	2,992 (2,747-3,323)	5,410 (5,001-5,971)	12,420 (11,743-13,277)	24,090 (22,953-25,488)	36,854 (35,247-38,680)	48,445 (46,223-50,366)	73,225 (70,128-76,299)	80,413 (76,626-83,515)	78,594 (74,672-81,259)	88,307 (84,130-91,222)	87,505 (83,292-90,544)	69,928 (67,059-72,445)	44,282 (42,150-45,872)	20,772 (19,731-21,606)	7,218 (6,889-7,542)	1,708 (1,618-1,790)
Liver cancer due to hepatitis B	-	-	-	-	118 (85-158)	297 (225-378)	773 (602-955)	1,701 (1,414-1,991)	3,364 (2,756-3,951)	7,817 (6,679-8,880)	14,767 (12,292-16,651)	21,710 (17,917-24,665)	26,729 (21,574-30,535)	37,987 (30,596-43,866)	38,218 (29,757-44,855)	34,101 (25,253-40,274)	35,119 (26,555-41,626)	31,947 (23,594-38,045)	23,349 (17,318-28,692)	13,571 (9,665-17,211)	6,075 (4,675-7,494)	1,923 (1,444-2,431)	404 (262-548)
Liver cancer due to hepatitis C	-	-	-	-	12 (9-15)	23 (18-31)	49 (37-64)	120 (91-156)	260 (176-376)	615 (460-794)	1,308 (990-1,706)	2,588 (2,042-3,317)	4,813 (3,723-6,068)	9,081 (7,235-11,027)	12,860 (10,672-15,555)	14,808 (12,430-17,785)	17,745 (15,131-20,867)	19,925 (17,235-23,063)	18,753 (16,360-21,603)	13,875 (12,044-15,891)	7,353 (6,572-8,117)	2,961 (2,623-3,304)	813 (702-925)
Liver cancer due to alcohol use	-	-	-	-	-	-	6 (4-10)	46 (30-67)	136 (79-210)	478 (338-684)	1,400 (921-2,019)	2,904 (2,077-3,971)	4,810 (3,292-6,732)	8,212 (5,558-11,256)	10,385 (7,079-14,461)	11,785 (8,396-15,793)	16,186 (12,519-20,301)	17,000 (13,181-21,373)	13,114 (10,281-16,409)	7,863 (5,868-9,977)	3,442 (2,605-4,354)	1,093 (792-1,416)	227 (137-329)
Liver cancer due to other causes	-	-	-	-	315 (269-369)	441 (365-520)	786 (625-954)	1,125 (912-1,350)	1,651 (1,284-2,170)	3,510 (2,793-4,429)	6,616 (5,334-8,517)	9,652 (7,837-12,358)	12,092 (9,863-15,257)	17,945 (14,525-22,528)	18,950 (15,396-23,917)	17,900 (14,762-22,239)	19,256 (16,028-23,422)	18,633 (15,670-22,519)	14,712 (12,391-17,543)	8,972 (7,409-10,786)	3,901 (3,321-4,541)	1,240 (1,023-1,484)	265 (204-337)
Gallbladder and biliary tract cancer	-	-	-	-	-	-	96 (89-107)	158 (141-174)	311 (279-348)	847 (753-950)	1,824 (1,632-2,033)	3,554 (3,177-3,900)	5,778 (5,170-6,266)	9,114 (8,171-9,797)	11,718 (10,719-12,670)	14,244 (12,992-15,158)	17,955 (16,518-19,206)	20,008 (18,523-21,430)	20,088 (18,831-21,124)	15,983 (15,057-16,734)	9,538 (8,957-9,972)	4,535 (4,295-4,756)	1,467 (1,388-1,535)
Pancreatic cancer	-	-	-	-	-	-	183 (175-193)	338 (324-355)	611 (588-641)	1,479 (1,427-1,545)	3,171 (3,077-3,290)	6,380 (6,200-6,573)	11,425 (11,148-11,757)	19,839 (19,424-20,316)	28,395 (27,805-29,035)	33,968 (33,363-34,645)	44,668 (43,795-45,615)	48,581 (47,614-49,598)	46,426 (45,318-47,561)	34,569 (33,507-35,517)	20,021 (19,325-20,699)	8,837 (8,533-9,165)	2,536 (2,425-2,632)
Larynx cancer	-	-	-	-	-	-	163 (153-175)	217 (206-231)	282 (264-303)	650 (607-699)	1,366 (1,288-1,446)	3,245 (3,090-3,397)	6,272 (6,008-6,545)	10,734 (10,310-11,134)	13,164 (12,680-13,624)	13,593 (13,071-14,143)	15,365 (14,815-15,927)	13,176 (12,729-13,595)	9,781 (9,440-10,088)	6,172 (5,928-6,396)	2,794 (2,682-2,898)	945 (911-980)	204 (196-212)
Tracheal, bronchus, and lung cancer	-	-	-	-	-	-	1,076 (1,013-1,143)	1,816 (1,707-1,947)	3,194 (3,025-3,369)	7,356 (7,014-7,716)	15,366 (14,701-16,112)	32,824 (31,500-34,184)	59,555 (58,000-61,410)	108,553 (105,804-111,428)	149,177 (145,743-153,096)	172,492 (169,147-175,946)	223,751 (219,261-228,210)	235,449 (230,419-240,413)	206,859 (201,554-211,341)	132,977 (129,214-136,670)	64,048 (61,993-66,102)	22,391 (21,595-23,181)	5,373 (5,153-5,563)
Malignant skin melanoma	-	-	-	-	-	-	243 (225-280)	428 (399-510)	658 (607-771)	1,086 (985-1,234)	1,703 (1,547-1,967)	2,536 (2,297-2,881)	3,191 (2,845-3,598)	4,169 (3,763-4,691)	4,814 (4,332-5,317)	4,547 (4,064-4,947)	5,323 (4,745-5,805)	5,400 (4,726-5,960)	5,508 (4,730-5,972)	4,624 (3,930-5,132)	2,838 (2,368-3,126)	1,415 (1,190-1,611)	495 (398-600)
Non-melanoma skin cancer	-	-	-	-	-	-	205 (194-216)	239 (225-253)	285 (268-301)	482 (455-507)	704 (668-743)	1,081 (1,030-1,130)	1,468 (1,414-1,519)	2,199 (2,127-2,261)	2,753 (2,680-2,824)	3,692 (3,565-3,821)	4,812 (4,665-4,973)	5,418 (5,251-5,579)	5,498 (5,323-5,657)	5,241 (5,061-5,416)	3,914 (3,778-4,053)	2,480 (2,390-2,566)	1,210 (1,156-1,258)
Non-melanoma skin cancer (squamous-cell carcinoma)	-	-	-	-	-	-	205 (194-216)	239 (225-253)	285 (268-301)	482 (455-507)	704 (668-743)	1,081 (1,030-1,130)	1,468 (1,414-1,519)	2,199 (2,127-2,261)	2,753 (2,680-2,824)	3,692 (3,565-3,821)	4,812 (4,665-4,973)	5,418 (5,251-5,579)	5,498 (5,323-5,657)	5,241 (5,061-5,416)	3,914 (3,778-4,053)	2,480 (2,390-2,566)	1,210 (1,156-1,258)
Breast cancer	-	-	-	-	-	-	392 (312-432)	1,058 (899-1,181)	3,161 (2,761-3,470)	9,167 (8,368-9,972)	19,759 (18,344-21,383)	31,921 (29,790-34,257)	43,692 (41,008-46,989)	54,849 (52,184-57,933)	57,354 (54,584-60,743)	47,394 (45,557-50,676)	48,028 (46,191-51,338)	43,068 (41,414-45,748)	38,988 (37,525-41,830)	31,313 (30,054-32,886)	20,626 (19,770-21,555)	11,210 (10,725-11,729)	4,393 (4,184-4,584)
Cervical cancer	-	-	-	-	-	-	238 (170-260)	897 (649-991)	2,624 (2,083-2,845)	6,535 (5,226-7,042)	12,504 (10,162-13,506)	19,521 (16,298-20,977)	25,157 (20,952-27,104)	29,581 (24,410-32,166)	29,300 (24,426-31,763)	24,979 (21,078-27,319)	23,348 (19,769-25,415)	20,402 (17,667-22,139)	14,892 (13,251-15,806)	11,164 (9,930-11,960)	5,760 (5,182-6,132)	2,371 (2,214-2,505)	732 (688-773)
Uterine cancer	-	-	-	-	-	-	94 (85-105)	208 (191-224)	344 (317-371)	721 (665-770)	1,520 (1,379-1,624)	2,628 (2,431-2,802)	4,421 (4,121-4,657)	7,026 (6,556-7,414)	8,571 (8,047-9,074)	9,654 (9,158-10,147)	11,395 (10,830-11,957)	10,275 (9,808-10,706)	9,012 (8,638-9,424)	6,760 (6,479-7,072)	3,697 (3,541-3,850)	1,691 (1,615-1,770)	531 (501-561)
Ovarian cancer	-	-	-	-	-	-	537 (494-581)	722 (670-786)	1,062 (987-1,135)	1,709 (1,599-1,841)	3,186 (2,974-3,445)	5,836 (5,506-6,230)	9,944 (9,424-10,505)	14,680 (13,949-15,461)	15,879 (15,167-16,723)	15,466 (14,838-16,035)	16,728 (16,113-17,407)	16,132 (15,577-16,703)	13,963 (13,427-14,503)	10,335 (9,885-10,768)	5,715 (5,457-5,950)	2,555 (2,441-2,670)	746 (710-781)
Prostate cancer	-	-	-	-	-	-	76 (61-87)	90 (71-103)	111 (88-129)	147 (114-166)	233 (179-259)	541 (423-607)	1,584 (1,272-1,754)	4,523 (3,634-4,927)	10,359 (8,211-11,207)	20,400 (15,783-22,134)	35,151 (28,573-37,993)	49,394 (41,089-53,518)	60,037 (51,380-66,355)	51,268 (45,080-56,463)	35,077 (29,868-37,695)	17,642 (15,006-19,004)	4,687 (3,887-5,081)
Testicular cancer	-	-	-	-	-	-	450 (413-495)	930 (864-1,015)	1,109 (1,037-1,200)	1,091 (1,031-1,165)	959 (909-1,020)	711 (674-752)	572 (542-605)	470 (442-498)	333 (315-353)	317 (299-338)	352 (331-374)	364 (343-388)	296 (280-311)	257 (240-277)	128 (121-134)	55 (52-58)	17 (16-18)
Kidney cancer	35 (31-36)	17 (16-18)	240 (221-269)	1,177 (1,050-1,329)	590 (553-628)	311 (294-326)	261 (247-276)	326 (311-342)	433 (417-452)	681 (652-706)	1,267 (1,218-1,319)	2,335 (2,249-2,417)	4,393 (4,241-4,558)	7,395 (7,149-7,653)	10,332 (10,009-10,655)	10,795 (10,487-11,084)	13,605 (13,197-13,996)	14,276 (13,847-14,654)	14,362 (13,913-14,792)	10,983 (10,592-11,318)	6,211 (5,971-6,434)	2,713 (2,606-2,822)	732 (701-762)
Bladder cancer	-	-	-	-	-	-	117 (110-125)	186 (175-197)	235 (222-249)	521 (493-551)	991 (937-1,046)	1,950 (1,854-2,045)	3,445 (3,313-3,589)	6,162 (5,931-6,400)	9,142 (8,845-9,469)	12,429 (12,091-12,774)	18,389 (17,888-18,935)	23,107 (22,502-23,719)	26,350 (25,655-27,087)	23,178 (22,422-23,833)	15,040 (14,547-15,489)	7,394 (7,140-7,658)	2,235 (2,147-2,322)
Brain and nervous system cancer	108 (80-123)	70 (54-78)	961 (769-1,105)	3,999 (3,453-4,634)	5,583 (4,925-6,113)	5,463 (4,842-5,903)	4,723 (4,114-5,128)	4,345 (3,783-4,635)	4,902 (4,318-5,231)	6,622 (5,825-7,024)	8,925 (7,810-9,589)	11,291 (10,052-12,183)	13,189 (11,934-14,043)	17,146 (15,452-18,350)	19,046 (17,312-20,332)	17,873 (15,912-19,071)	19,339 (17,161-20,453)	16,975 (14,657-17,932)	13,405 (11,132-14,190)	7,952 (6,255-8,544)	3,489 (2,642-3,768)	1,110 (832-1,202)	255 (190-277)
Thyroid cancer	-	-	-	-	-	135 (114-154)	311 (265-364)	479 (429-528)	557 (507-609)	665 (610-717)	867 (797-936)	1,389 (1,289-1,494)	2,019 (1,887-2,149)	3,033 (2,859-3,228)	3,718 (3,510-3,949)	3,651 (3,464-3,867)	4,413 (4,203-4,650)	4,473 (4,274-4,725)	4,290 (4,122-4,500)	3,125 (3,000-3,291)	1,721 (1,650-1,821)	733 (698-770)	209 (198-219)
Mesothelioma	-	-	-	-	-	-	110 (97-126)	132 (114-152)	198 (172-225)	242 (213-273)	423 (374-475)	704 (638-783)	972 (902-1,086)	1,538 (1,433-1,663)	2,297 (2,159-2,481)	2,382 (2,233-2,549)	3,109 (2,941-3,271)	3,414 (3,239-3,586)	3,505 (3,333-3,695)	2,611 (2,477-2,748)	1,246 (1,184-1,313)	449 (425-473)	107 (101-113)
Hodgkin lymphoma	8 (6-10)	2 (2-3)	57 (42-72)	376 (257-528)	1,113 (772-1,435)	1,383 (998-1,686)	1,675 (1,301-2,001)	1,685 (1,354-2,014)	1,731 (1,430-2,064)	1,702 (1,419-2,055)	1,756 (1,431-2,092)	1,847 (1,468-2,146)	2,017 (1,676-2,375)	2,220 (1,881-2,674)	2,439 (2,064-2,923)	2,268 (1,887-2,722)	2,602 (2,186-3,054)	2,353 (2,030-2,769)	1,633 (1,448-1,885)	1,084 (947-1,207)	506 (451-552)	162 (149-175)	36 (33-40)
Non-Hodgkin's lymphoma	50 (44-54)	37 (34-42)	496 (452-571)	2,511 (2,172-2,831)	3,626 (2,965-4,176)	3,426 (2,902-3,874)	3,576 (3,173-3,847)	3,812 (3,441-4,129)	3,751 (3,461-4,011)	4,526 (4,051-4,794)	5,624 (5,116-5,945)	7,472 (6,758-7,851)	9,074 (8,353-9,563)	12,433 (11,473-13,126)	14,803 (13,771-15,942)	16,938 (15,880-18,061)	20,570 (19,274-21,930)	22,200 (21,020-23,524)	20,986 (19,642-22,269)	16,917 (15,420-17,745)	10,071 (9,044-10,524)	4,186 (3,862-4,408)	1,097 (1,027-1,160)
Multiple myeloma	-	-	-	-	-	-	260 (226-301)	302 (262-337)	299 (262-338)	525 (449-597)	935 (820-1,068)	1,835 (1,619-2,046)	2,799 (2,490-3,059)	4,904 (4,379-5,530)	6,742 (6,027-7,637)	8,166 (7,116-9,143)	10,532 (9,187-11,763)	11,217 (9,720-12,523)	11,208 (9,628-12,516)	8,940 (7,575-9,901)	5,198 (4,211-5,873)	2,107 (1,776-2,469)	494 (401-575)
Leukaemia	199 (168-217)	159 (141-178)	2,246 (2,002-2,585)	9,054 (7,914-11,171)	10,123 (9,210-11,608)	11,421 (10,457-12,832)	13,296 (11,922-14,741)	10,360 (9,091-11,117)	8,690 (7,651-9,242)	9,545 (8,303-10,179)	11,142 (9,696-11,949)	12,186 (10,874-12,887)	13,427 (12,062-14,191)	16,587 (14,810-17,423)	18,981 (17,118-19,790)	19,923 (18,299-20,799)	24,856 (23,001-25,925)	26,719 (25,123-27,926)	26,398 (25,121-27,472)	20,361 (19,638-21,153)	12,211 (11,768-12,694)	5,671 (5,452-5,913)	1,743 (1,669-1,824)
Acute lymphoid leukaemia	54 (48-68)	43 (38-55)	670 (583-896)	3,402 (2,892-4,586)	4,424 (3,955-5,454)	4,458 (4,033-5,505)	3,896 (3,533-4,685)	2,612 (2,391-3,028)	1,976 (1,792-2,282)	1,756 (1,580-2,093)	1,890 (1,740-2,242)	1,944 (1,767-2,253)	2,078 (1,867-2,403)	2,366 (2,106-2,664)	2,670 (2,393-2,976)	2,431 (2,144-2,697)	2,600 (2,331-2,957)	2,507 (2,286-2,832)	1,640 (1,513-1,844)	1,044 (975-1,204)	544 (508-616)	223 (210-247)	69 (64-75)
Chronic lymphoid leukaemia	-	-	-	-	-	-	276 (246-351)	248 (222-317)	228 (205-282)	275 (242-355)	379 (332-493)	510 (459-632)	652 (597-784)	1,156 (1,070-1,356)	1,751 (1,603-2,130)	2,470 (2,254-2,795)	3,630 (3,357-4,229)	4,223 (3,893-4,981)	4,824 (4,449-5,528)	4,313 (3,948-4,825)	2,984 (2,721-3,309)	1,551 (1,440-1,679)	521 (482-557)
Acute myeloid leukaemia	35 (27-40)	31 (26-37)	393 (336-503)	1,799 (1,478-2,418)	1,667 (1,390-2,008)	2,011 (1,747-2,349)	2,456 (2,189-2,851)	2,121 (1,866-2,389)	1,927 (1,712-2,177)	2,072 (1,837-2,312)	2,506 (2,237-2,772)	2,883 (2,579-3,185)	3,518 (3,210-3,857)	4,301 (3,924-4,704)	5,084 (4,603-5,521)	5,459 (4,873-5,859)	7,025 (6,311-7,528)	7,613 (6,827-8,182)	7,469 (6,753-8,000)	5,300 (4,802-5,724)	2,807 (2,540-3,047)	1,099 (1,031-1,219)	283 (265-319)
Chronic myeloid leukaemia	-	-	-	-	-	-	473 (392-567)	548 (458-659)	847 (717-987)	934 (802-1,073)	1,053 (910-1,194)	1,261 (1,102-1,425)	1,493 (1,327-1,677)	1,706 (1,501-1,894)	1,813 (1,626-2,012)	1,713 (1,517-1,879)	2,123 (1,901-2,281)	2,189 (1,995-2,349)	2,421 (2,252-2,577)	1,985 (1,861-2,128)	1,210 (1,134-1,288)	608 (570-648)	192 (176-205)
Other leukaemia	111 (83-125)	85 (69-96)	1,184 (945-1,388)	3,852 (3,170-4,640)	4,031 (3,381-4,500)	4,951 (4,214-5,428)	6,195 (5,031-6,732)	4,830 (3,880-5,229)	3,713 (3,019-4,004)	4,509 (3,589-4,890)	5,313 (4,223-5,762)	5,588 (4,675-5,978)	5,686 (4,744-6,133)	7,058 (5,904-7,548)	7,664 (6,412-8,203)	7,849 (6,813-8,316)	9,479 (8,304-9,985)	10,187 (9,128-10,759)	10,043 (9,219-10,733)	7,719 (7,221-8,201)	4,665 (4,418-4,981)	2,189 (2,073-2,334)	678 (641-726)
Other neoplasms	446 (394-482)	252 (233-276)	1,709 (1,550-2,074)	5,869 (5,091-7,711)	4,632 (4,258-5,054)	6,538 (6,005-7,000)	10,369 (9,422-10,940)	8,789 (8,020-9,208)	8,239 (7,444-8,679)	9,896 (8,742-10,423)	11,829 (10,557-12,372)	15,304 (13,690-15,922)	18,216 (16,605-18,869)	23,982 (21,971-24,802)	26,972 (25,272-27,865)	28,277 (26,459-29,308)	33,151 (31,378-34,199)	34,128 (32,393-35,205)	32,430 (31,085-33,378)	25,162 (23,905-26,046)	15,690 (14,942-16,266)	7,499 (7,159-7,794)	2,367 (2,260-2,467)
Cardiovascular diseases	4,267 (3,943-4,606)	2,326 (2,168-2,496)	21,312 (19,862-22,655)	17,405 (15,908-18,798)	10,205 (9,439-11,001)	13,873 (13,048-14,827)	40,176 (38,340-42,137)	61,220 (59,133-64,049)	84,666 (81,924-87,863)	135,001 (131,250-139,832)	212,073 (206,047-218,291)	340,092 (331,580-348,238)	501,051 (489,904-512,508)	752,240 (735,693-766,963)	954,191 (935,100-973,012)	1,133,698 (1,110,584-1,156,102)	1,661,889 (1,625,110-1,694,532)	2,041,639 (1,993,498-2,083,016)	2,358,458 (2,303,044-2,405,462)	2,235,747 (2,179,833-2,288,120)	1,581,019 (1,537,624-1,622,981)	903,581 (876,625-930,952)	351,425 (338,456-362,106)
Rheumatic heart disease	-	-	-	3,963 (3,337-4,682)	2,650 (2,305-3,007)	4,910 (4,436-5,451)	9,204 (8,288-10,097)	9,490 (8,513-10,460)	9,610 (8,485-10,676)	11,139 (9,914-12,377)	13,662 (12,194-15,029)	16,834 (14,954-18,460)	18,000 (16,130-19,684)	22,850 (20,501-24,669)	24,878 (22,509-26,771)	26,293 (23,626-28,436)	33,103 (29,858-35,604)	36,734 (33,427-39,526)	33,604 (30,871-35,859)	28,727 (26,718-30,633)	19,706 (18,522-20,920)	10,460 (9,972-11,037)	3,796 (3,635-3,973)
Ischaemic heart disease	-	-	1,395 (1,211-1,595)	999 (833-1,236)	749 (661-857)	901 (819-997)	12,724 (12,048-13,457)	22,585 (21,626-23,695)	35,720 (34,215-37,475)	65,350 (63,024-68,087)	105,135 (101,568-109,251)	171,732 (166,052-177,532)	264,867 (257,114-273,303)	391,781 (380,963-401,804)	479,598 (466,227-510,687)	584,478 (570,541-597,809)	847,060 (826,889-866,088)	1,021,107 (994,290-1,045,584)	1,215,523 (1,187,102-1,242,793)	1,159,774 (1,135,733-1,187,025)	849,219 (828,202-866,676)	512,396 (501,350-526,747)	205,981 (201,119-212,762)
Cerebrovascular disease	[illegible]	[illegible]	[illegible]	[illegible]	[illegible]	[illegible]	[illegible]	[illegible]	[illegible]	[illegible]	[illegible]	[illegible]	[illegible]	[illegible]	[illegible]	[illegible]	[illegible]	[illegible]	[illegible]	[illegible]	[illegible]	[illegible]	[illegible]

SR Table 5: Global deaths by cause and age group for both sexes (with 95% uncertainty intervals), 2006

Cause	Early Neonatal	Late Neonatal	Post Neonatal	1 to 4	5 to 9	10 to 14	15 to 19	20 to 24	25 to 29	30 to 34	35 to 39	40 to 44	45 to 49	50 to 54	55 to 59	60 to 64	65 to 69	70 to 74	75 to 79	80 to 84	85 to 89	90 to 94	95 plus
Ischaemic stroke	-	-	356 (307-417)	281 (242-338)	234 (212-261)	273 (249-298)	1,305 (1,227-1,395)	2,663 (2,468-2,845)	3,517 (3,294-3,738)	5,268 (4,970-5,569)	8,791 (8,305-9,272)	15,910 (15,226-16,574)	31,276 (29,786-32,734)	58,484 (56,055-60,901)	87,526 (83,723-91,265)	161,228 (154,409-167,268)	275,092 (264,590-284,978)	364,928 (350,471-378,581)	483,706 (458,968-506,314)	448,121 (419,373-472,911)	297,831 (275,467-319,423)	156,081 (143,336-169,862)	58,977 (52,451-64,702)
Hemorrhagic stroke	1,926 (1,706-2,180)	992 (882-1,141)	7,430 (6,552-8,614)	5,056 (4,262-6,281)	3,123 (2,861-3,455)	3,736 (3,491-3,989)	7,543 (7,191-7,946)	13,664 (13,087-14,411)	18,088 (17,270-19,061)	30,922 (29,824-32,305)	53,874 (51,966-56,155)	90,584 (87,724-93,571)	126,666 (122,942-130,816)	197,391 (191,737-203,406)	242,378 (235,298-250,169)	249,734 (241,425-259,403)	346,175 (333,621-359,324)	412,157 (395,994-428,830)	369,132 (350,127-387,715)	315,816 (295,045-334,388)	188,402 (174,552-201,791)	84,052 (77,247-91,308)	26,922 (23,864-29,464)
Hypertensive heart disease	-	-	365 (266-475)	172 (119-209)	141 (117-165)	200 (156-243)	1,540 (1,240-1,879)	2,309 (1,890-2,789)	3,100 (2,550-3,663)	4,955 (4,061-5,738)	8,020 (6,635-9,079)	13,038 (10,873-14,994)	20,083 (16,409-22,904)	30,900 (25,554-35,015)	40,995 (34,384-46,620)	47,744 (40,362-55,193)	70,807 (59,570-82,255)	91,012 (76,356-104,991)	107,117 (90,267-122,639)	105,587 (86,304-119,867)	79,279 (63,046-91,222)	47,916 (36,148-52,595)	19,334 (12,841-21,608)
Cardiomyopathy and myocarditis	860 (602-1,006)	496 (383-564)	4,393 (3,309-5,052)	2,753 (2,176-3,241)	1,271 (1,071-1,413)	1,541 (1,344-1,683)	3,432 (2,811-3,742)	4,355 (3,419-4,790)	6,591 (5,113-7,359)	8,176 (6,471-9,151)	10,898 (8,768-12,230)	15,763 (12,682-17,764)	19,588 (16,313-22,297)	22,257 (19,148-25,094)	22,655 (20,046-25,613)	17,377 (15,223-19,299)	21,827 (19,507-24,347)	23,441 (19,099-24,982)	29,150 (22,165-31,679)	31,924 (22,025-35,674)	26,080 (18,217-29,021)	17,223 (11,326-19,552)	8,046 (5,067-9,268)
Myocarditis	175 (105-237)	130 (88-163)	1,592 (1,151-2,042)	1,126 (816-1,543)	509 (413-579)	576 (466-648)	1,456 (1,063-1,692)	1,414 (1,044-1,657)	1,625 (1,222-1,946)	1,355 (1,048-1,548)	1,699 (1,254-1,943)	1,972 (1,489-2,283)	1,403 (1,070-1,581)	1,562 (1,191-1,751)	1,775 (1,334-1,997)	1,392 (959-1,601)	1,840 (1,286-2,108)	2,528 (1,716-2,925)	3,315 (2,183-3,851)	4,386 (2,673-5,277)	4,548 (2,777-5,455)	4,043 (2,143-5,132)	2,371 (1,082-3,079)
Alcoholic cardiomyopathy	-	-	-	-	-	-	246 (210-324)	689 (469-830)	1,885 (1,186-2,314)	3,477 (2,400-4,182)	5,076 (3,890-6,113)	8,285 (6,419-9,884)	11,827 (9,440-14,067)	12,667 (10,273-15,000)	11,291 (9,434-13,297)	6,516 (5,408-7,707)	7,701 (6,703-9,687)	5,378 (4,316-6,433)	5,124 (3,988-5,850)	3,659 (2,390-4,358)	1,979 (1,200-2,396)	1,116 (594-1,437)	464 (252-585)
Other cardiomyopathy	685 (482-833)	367 (276-439)	2,801 (1,973-3,547)	1,628 (1,161-2,172)	763 (629-890)	965 (837-1,093)	1,730 (1,437-1,960)	2,252 (1,754-2,534)	3,080 (2,340-3,501)	3,345 (2,680-3,749)	4,124 (3,297-4,572)	5,505 (4,504-6,231)	6,358 (5,411-7,300)	8,028 (6,914-9,495)	9,589 (8,389-11,293)	9,469 (8,358-10,742)	12,286 (10,611-13,512)	15,535 (12,415-16,895)	20,712 (15,313-22,900)	23,879 (16,299-27,121)	19,553 (13,611-22,267)	12,064 (8,370-14,830)	5,210 (3,563-6,831)
Atrial fibrillation and flutter	-	-	-	-	-	-	-	-	-	33 (23-44)	134 (98-176)	316 (232-410)	584 (423-766)	1,014 (761-1,309)	1,703 (1,019-2,531)	3,116 (2,068-4,363)	6,636 (4,124-9,899)	13,105 (9,370-18,112)	21,144 (13,545-31,400)	41,702 (31,853-53,194)	44,176 (33,949-56,798)	25,579 (19,803-32,779)	8,340 (6,502-10,601)
Aortic aneurysm	-	-	-	-	-	-	398 (365-441)	532 (491-586)	804 (732-885)	1,130 (1,038-1,242)	1,852 (1,712-2,024)	2,599 (2,437-2,777)	3,768 (3,578-4,010)	5,466 (5,214-5,762)	7,800 (7,487-8,201)	9,688 (9,330-10,055)	14,431 (13,934-14,981)	18,530 (17,891-19,146)	22,648 (21,973-23,350)	21,984 (21,305-22,622)	15,639 (15,190-16,119)	8,250 (8,027-8,530)	2,680 (2,595-2,793)
Peripheral vascular disease	-	-	-	-	-	-	-	-	-	-	-	201 (142-240)	378 (261-456)	789 (550-973)	1,344 (910-1,612)	1,883 (1,343-2,358)	3,412 (2,408-4,197)	4,628 (3,418-5,954)	6,796 (5,014-9,218)	8,550 (6,208-12,748)	7,572 (5,191-11,756)	5,814 (3,735-9,776)	2,826 (1,740-4,937)
Endocarditis	126 (90-155)	78 (57-97)	1,721 (1,331-2,237)	778 (595-1,109)	384 (318-449)	535 (456-633)	1,084 (919-1,317)	1,531 (1,297-1,899)	2,064 (1,769-2,546)	2,055 (1,742-2,531)	2,424 (2,079-2,977)	3,038 (2,661-3,780)	2,990 (2,569-3,636)	3,483 (2,979-4,276)	3,845 (3,272-4,627)	4,608 (3,907-5,514)	5,947 (5,001-7,151)	7,053 (5,858-8,308)	8,460 (6,850-10,121)	8,849 (6,828-10,634)	6,927 (5,098-8,190)	4,409 (3,083-5,322)	1,871 (1,225-2,243)
Other cardiovascular and circulatory diseases	1,355 (1,126-1,571)	760 (653-905)	5,651 (4,910-7,015)	3,402 (2,718-4,568)	1,652 (1,409-2,186)	1,778 (1,554-2,306)	2,945 (2,607-3,889)	4,091 (3,606-5,549)	5,172 (4,586-6,969)	5,974 (5,347-7,843)	7,283 (6,550-9,393)	10,078 (9,133-12,724)	12,875 (11,788-15,775)	17,825 (16,286-22,203)	22,308 (20,439-27,821)	27,550 (25,191-34,523)	37,399 (34,251-47,257)	48,943 (44,682-60,444)	61,177 (57,062-73,635)	64,714 (60,624-76,348)	50,684 (47,762-58,891)	31,401 (29,868-35,610)	12,653 (11,986-14,130)
Chronic respiratory diseases	0 (0-0)	0 (0-0)	0 (0-0)	15,731 (13,203-20,026)	4,762 (4,238-5,723)	5,128 (4,625-6,043)	9,247 (8,601-10,757)	12,380 (11,748-14,080)	15,304 (14,520-17,024)	19,799 (18,919-21,664)	29,102 (27,776-31,763)	42,284 (40,502-45,454)	66,333 (63,202-70,893)	116,180 (110,633-122,729)	172,976 (163,528-179,631)	263,670 (248,919-275,049)	401,455 (379,641-416,499)	542,720 (515,916-568,155)	578,447 (551,312-610,181)	518,427 (495,563-549,126)	338,780 (323,927-356,127)	155,331 (149,087-163,209)	47,890 (45,619-50,761)
Chronic obstructive pulmonary disease	-	-	0 (0-0)	3,036 (2,327-4,485)	848 (689-1,115)	752 (634-951)	3,664 (3,198-4,264)	5,146 (4,551-5,934)	6,735 (5,969-7,717)	9,939 (9,021-11,244)	15,010 (13,561-16,991)	24,373 (22,249-27,396)	41,552 (38,009-46,099)	81,847 (75,028-89,393)	128,991 (119,279-139,211)	208,242 (192,861-222,679)	332,148 (312,250-353,041)	465,530 (442,160-489,475)	503,005 (479,013-529,906)	460,400 (440,329-487,370)	304,936 (291,785-322,662)	141,471 (135,790-149,615)	43,838 (41,848-46,603)
Pneumoconiosis	-	-	-	-	-	-	43 (38-51)	59 (52-70)	103 (92-121)	240 (214-276)	533 (470-643)	711 (636-826)	726 (644-862)	1,153 (1,031-1,344)	1,275 (1,131-1,557)	1,660 (1,471-2,020)	2,531 (2,272-3,033)	3,401 (3,132-3,856)	3,558 (3,310-3,983)	2,821 (2,643-3,085)	1,536 (1,430-1,671)	568 (531-620)	143 (134-155)
Silicosis	-	-	-	-	-	-	4 (3-5)	9 (7-11)	32 (27-38)	120 (103-142)	332 (285-404)	440 (386-523)	422 (368-520)	666 (586-820)	685 (597-851)	808 (700-986)	1,294 (1,126-1,574)	1,700 (1,524-2,009)	1,773 (1,589-2,072)	1,368 (1,215-1,599)	667 (594-775)	207 (178-240)	44 (39-52)
Asbestosis	-	-	-	-	-	-	19 (16-23)	24 (21-30)	29 (25-36)	39 (32-50)	50 (39-64)	64 (47-82)	66 (49-84)	100 (72-125)	132 (90-166)	180 (121-234)	285 (181-366)	425 (266-550)	534 (319-682)	496 (286-617)	300 (184-370)	119 (68-143)	28 (16-32)
Coal workers pneumoconiosis	-	-	-	-	-	-	2 (1-2)	2 (1-3)	8 (5-10)	14 (8-17)	33 (20-42)	61 (32-78)	73 (42-93)	122 (74-163)	159 (89-209)	235 (133-316)	394 (221-511)	552 (310-666)	528 (327-619)	454 (337-538)	269 (180-317)	97 (60-117)	23 (16-27)
Other pneumoconiosis	-	-	-	-	-	-	18 (16-22)	24 (20-29)	35 (30-46)	66 (57-91)	118 (99-160)	146 (122-187)	165 (130-227)	265 (212-365)	299 (229-424)	438 (318-630)	558 (421-814)	723 (564-1,023)	723 (575-993)	503 (413-696)	300 (246-413)	146 (123-202)	48 (42-62)
Asthma	-	-	-	10,176 (7,344-13,440)	2,817 (2,348-3,540)	3,494 (2,991-4,258)	4,654 (3,965-5,823)	5,942 (4,974-7,162)	7,223 (5,956-8,677)	7,959 (6,526-9,285)	11,361 (9,196-13,507)	14,234 (11,362-16,900)	20,117 (15,697-23,943)	27,641 (21,170-33,750)	35,227 (26,496-43,116)	44,308 (32,348-56,961)	53,708 (39,507-69,005)	57,255 (43,567-74,547)	53,755 (41,957-69,394)	38,907 (30,748-50,105)	21,712 (17,685-27,989)	8,431 (7,067-10,507)	2,393 (2,068-2,916)
Interstitial lung disease and pulmonary sarcoidosis	-	-	-	322 (158-625)	146 (92-241)	107 (74-151)	237 (156-354)	342 (226-507)	473 (316-694)	662 (447-952)	1,038 (700-1,491)	1,564 (1,076-2,203)	2,397 (1,649-3,328)	3,781 (2,565-5,232)	5,347 (3,614-7,385)	7,191 (4,738-9,798)	10,452 (7,093-13,973)	13,637 (9,253-17,297)	15,423 (10,427-18,563)	13,731 (9,310-15,978)	8,808 (5,767-10,097)	3,907 (2,607-4,423)	1,198 (814-1,335)
Other chronic respiratory diseases	0 (0-0)	0 (0-0)	0 (0-0)	2,198 (1,166-3,273)	950 (665-1,236)	775 (524-1,000)	649 (438-841)	891 (619-1,155)	770 (490-1,012)	999 (640-1,350)	1,160 (720-1,530)	1,404 (905-1,794)	1,541 (969-1,971)	1,757 (1,101-2,258)	2,136 (1,367-2,774)	2,270 (1,520-2,858)	2,617 (1,653-3,282)	2,898 (1,951-3,579)	2,706 (1,825-3,260)	2,569 (1,750-3,099)	1,788 (1,257-2,160)	954 (649-1,158)	319 (212-393)
Cirrhosis and other chronic liver diseases	-	-	-	8,625 (6,995-11,382)	3,846 (3,331-4,977)	4,517 (4,072-5,368)	8,607 (8,014-9,735)	15,160 (14,151-17,827)	23,229 (21,658-25,707)	39,556 (37,474-43,838)	60,852 (57,854-67,089)	86,149 (82,313-93,555)	106,294 (102,257-114,521)	126,815 (122,188-137,090)	125,061 (120,681-135,570)	112,498 (108,103-124,022)	112,603 (108,455-120,569)	100,770 (96,521-107,576)	81,571 (78,444-85,610)	55,780 (53,172-58,329)	29,509 (28,071-30,814)	12,234 (11,671-12,766)	4,140 (3,934-4,317)
Cirrhosis and other chronic liver diseases due to hepatitis B	-	-	-	70 (45-112)	86 (60-121)	176 (127-242)	573 (398-797)	3,224 (2,404-4,324)	7,314 (5,670-9,262)	13,834 (11,785-16,229)	21,711 (18,265-25,366)	28,718 (24,733-32,774)	31,030 (25,933-36,905)	35,888 (30,077-42,439)	35,533 (30,426-42,345)	33,407 (28,103-40,448)	33,807 (28,568-40,057)	30,720 (25,869-36,959)	24,272 (20,295-28,794)	15,316 (12,536-18,276)	7,341 (6,277-8,503)	2,582 (2,115-3,084)	746 (547-957)
Cirrhosis and other chronic liver diseases due to hepatitis C	-	-	-	53 (34-81)	65 (44-96)	140 (97-207)	418 (279-609)	2,182 (1,598-2,916)	4,902 (3,665-6,348)	9,474 (7,944-11,302)	15,266 (12,187-18,682)	22,470 (18,872-26,547)	28,847 (23,702-34,705)	34,097 (28,098-40,572)	33,327 (27,940-39,030)	29,593 (24,357-36,042)	29,546 (25,093-34,920)	26,530 (22,408-31,180)	20,731 (16,961-25,017)	14,103 (11,485-17,034)	7,229 (6,051-8,479)	3,019 (2,416-3,646)	996 (718-1,288)
Cirrhosis and other chronic liver diseases due to alcohol use	-	-	-	-	-	-	116 (66-184)	1,026 (730-1,428)	2,702 (1,952-3,658)	7,588 (6,369-9,022)	15,142 (12,548-18,311)	24,046 (20,935-27,711)	32,280 (27,316-37,816)	38,213 (32,598-44,699)	36,104 (30,191-42,413)	31,064 (25,222-37,382)	30,816 (25,926-35,719)	26,294 (22,067-30,983)	21,499 (18,154-25,168)	14,744 (12,281-17,301)	8,087 (7,095-9,117)	3,576 (3,022-4,099)	1,288 (1,027-1,554)
Cirrhosis and other chronic liver diseases due to other causes	-	-	-	8,502 (6,904-11,216)	3,694 (3,206-4,779)	4,202 (3,784-4,982)	7,500 (6,911-8,384)	8,728 (7,384-10,313)	8,311 (6,679-10,124)	8,660 (6,817-10,767)	8,733 (6,481-11,309)	10,915 (8,156-14,164)	14,137 (10,593-18,236)	18,616 (14,320-23,686)	20,097 (15,400-25,649)	18,434 (13,797-24,033)	18,433 (13,964-23,558)	17,226 (13,090-21,996)	15,070 (11,673-18,578)	11,618 (9,045-14,548)	6,852 (5,755-8,148)	3,057 (2,485-3,726)	1,111 (829-1,443)
Digestive diseases	1,894 (1,486-2,250)	1,735 (1,378-2,012)	18,404 (14,930-22,504)	17,748 (13,084-24,323)	7,884 (6,936-9,153)	7,974 (7,228-8,842)	13,126 (11,885-14,142)	16,437 (15,023-17,634)	20,971 (19,487-22,392)	23,172 (21,691-25,186)	29,695 (27,985-32,718)	37,294 (34,587-40,937)	45,813 (42,703-48,490)	55,396 (52,057-59,586)	66,565 (61,909-71,236)	74,281 (68,948-78,986)	96,170 (87,861-101,827)	109,719 (101,063-116,031)	117,138 (108,287-123,665)	107,215 (99,137-113,016)	76,546 (70,476-80,506)	42,085 (39,462-44,175)	15,199 (14,194-15,987)
Peptic ulcer disease	-	-	-	2,305 (1,701-3,236)	1,113 (899-1,482)	1,479 (1,248-1,836)	2,464 (2,135-2,962)	3,996 (3,495-4,685)	4,618 (4,105-5,298)	5,972 (5,326-6,918)	7,753 (7,000-9,034)	10,599 (9,656-12,303)	13,416 (12,077-15,035)	17,853 (16,372-19,783)	19,998 (18,249-22,113)	22,925 (20,922-24,898)	28,979 (26,303-31,805)	32,824 (29,368-35,120)	33,154 (30,243-35,239)	27,832 (25,064-29,682)	18,191 (16,700-19,472)	8,734 (8,174-9,429)	2,865 (2,678-3,142)
Gastritis and duodenitis	-	-	-	558 (286-1,051)	194 (146-281)	171 (139-225)	350 (294-425)	501 (432-605)	723 (618-862)	1,151 (989-1,409)	1,328 (1,147-1,626)	1,648 (1,440-2,004)	1,988 (1,770-2,340)	2,567 (2,290-2,921)	2,947 (2,612-3,314)	3,261 (2,903-3,629)	4,012 (3,560-4,480)	4,667 (4,179-5,185)	4,903 (4,397-5,445)	4,549 (4,033-5,082)	3,153 (2,789-3,529)	1,645 (1,473-1,903)	543 (487-667)
Appendicitis	-	-	-	3,404 (1,815-5,379)	1,907 (1,567-2,374)	2,105 (1,782-2,524)	3,492 (2,957-4,149)	2,877 (2,380-3,376)	3,126 (2,591-3,665)	2,023 (1,711-2,365)	2,409 (2,008-2,787)	2,485 (2,081-2,872)	2,623 (2,172-3,038)	2,611 (2,202-3,014)	3,658 (2,992-4,311)	3,518 (2,854-4,141)	3,707 (3,001-4,371)	3,580 (2,958-4,215)	3,294 (2,765-3,890)	2,487 (2,069-2,972)	1,601 (1,358-1,883)	742 (640-871)	230 (197-278)
Paralytic ileus and intestinal obstruction	1,894 (1,486-2,250)	1,735 (1,378-2,012)	18,404 (14,930-22,504)	4,987 (3,228-6,631)	2,674 (1,986-3,165)	2,394 (1,778-2,758)	3,928 (2,864-4,473)	4,325 (3,245-4,883)	5,202 (3,829-5,880)	4,610 (3,469-5,260)	6,102 (4,566-6,898)	7,217 (5,243-8,189)	8,587 (6,501-9,578)	10,173 (7,869-11,454)	13,653 (10,456-15,245)	14,938 (11,512-16,734)	19,419 (15,053-21,725)	20,316 (16,524-22,704)	20,890 (17,580-23,907)	19,368 (16,183-21,793)	14,739 (12,212-16,577)	8,557 (6,890-9,503)	3,152 (2,431-3,510)
Inguinal, femoral, and abdominal hernia	-	-	-	870 (437-1,332)	337 (232-421)	337 (205-440)	454 (314-562)	627 (407-773)	677 (453-810)	640 (411-761)	767 (579-998)	1,241 (906-1,548)	1,379 (1,057-1,729)	2,070 (1,645-2,590)	2,611 (2,089-3,308)	3,139 (2,390-3,880)	4,163 (3,235-5,057)	4,903 (3,772-5,740)	5,392 (4,152-6,397)	5,119 (3,976-6,163)	3,682 (2,822-4,445)	2,071 (1,596-2,598)	723 (551-924)
Inflammatory bowel disease	-	-	-	1,339 (670-3,108)	271 (176-432)	233 (166-331)	363 (260-510)	588 (424-835)	778 (600-1,072)	882 (664-1,201)	968 (757-1,258)	1,206 (993-1,498)	1,515 (1,194-1,844)	1,739 (1,410-2,029)	2,125 (1,718-2,488)	2,290 (1,822-2,593)	2,920 (2,295-3,313)	3,816 (2,979-4,288)	4,365 (3,252-4,840)	4,636 (3,275-5,089)	3,565 (2,438-3,954)	2,219 (1,446-2,522)	888 (577-1,033)
Vascular intestinal disorders	-	-	-	424 (161-767)	73 (48-101)	54 (44-64)	109 (88-132)	239 (193-289)	331 (268-401)	372 (290-451)	638 (512-763)	1,086 (918-1,286)	1,823 (1,563-2,127)	2,698 (2,373-3,164)	3,955 (3,515-4,750)	5,238 (4,736-6,068)	8,853 (8,034-10,293)	11,890 (10,868-13,631)	14,850 (13,430-16,713)	13,666 (12,424-15,095)	9,409 (8,500-10,239)	5,054 (4,663-5,506)	1,654 (1,508-1,845)
Gallbladder and biliary diseases	-	-	-	530 (340-753)	249 (194-356)	311 (261-389)	388 (345-504)	636 (574-812)	823 (751-998)	1,122 (1,013-1,375)	1,590 (1,442-1,942)	1,973 (1,805-2,397)	2,556 (2,348-2,962)	3,672 (3,377-4,341)	4,523 (4,160-5,256)	5,668 (5,174-6,622)	8,180 (7,464-9,562)	9,999 (9,167-11,586)	11,964 (10,923-13,554)	12,085 (11,091-13,462)	9,423 (8,765-10,450)	5,761 (5,333-6,379)	2,292 (2,117-2,545)
Pancreatitis	-	-	-	198 (133-282)	170 (128-226)	235 (181-284)	733 (595-884)	1,785 (1,472-2,082)	3,468 (2,885-3,938)	4,821 (4,156-5,528)	6,280 (5,399-7,242)	7,625 (6,583-8,899)	9,030 (7,801-10,294)	8,338 (7,215-9,595)	8,563 (7,321-9,858)	7,416 (6,217-8,691)	8,920 (7,373-10,450)	8,709 (7,270-9,997)	8,212 (6,968-9,346)	6,317 (5,309-7,279)	3,864 (3,205-4,362)	1,794 (1,537-2,046)	584 (500-689)
Other digestive diseases	-	-	-	3,135 (1,396-4,916)	897 (621-1,227)	655 (518-868)	844 (718-1,022)	862 (750-1,062)	1,225 (1,048-1,494)	1,578 (1,366-1,920)	1,860 (1,627-2,276)	2,214 (1,981-2,642)	2,896 (2,584-3,373)	3,676 (3,309-4,213)	4,533 (4,096-5,186)	5,889 (5,221-6,693)	7,017 (6,184-7,926)	9,015 (8,071-10,032)	10,114 (9,008-11,240)	11,155 (9,925-12,399)	8,921 (8,119-9,851)	5,508 (5,099-6,134)	2,269 (2,094-2,570)
Neurological disorders	-	-	7,265 (6,343-8,218)	8,740 (6,814-10,713)	5,225 (4,657-5,835)	7,893 (7,220-8,613)	14,885 (14,028-15,733)	13,952 (13,049-14,663)	11,804 (11,157-12,364)	11,888 (11,234-12,535)	13,268 (12,612-13,984)	13,168 (12,607-13,756)	15,818 (14,801-17,024)	22,249 (19,636-25,568)	29,315 (24,577-34,987)	43,582 (35,914-53,640)	82,309 (66,172-102,559)	161,967 (126,917-206,133)	298,157 (235,943-374,337)	414,097 (331,865-516,780)	411,150 (334,861-498,037)	308,481 (256,859-360,107)	116,250 (94,290-142,143)
Alzheimer's disease and other dementias	-	-	-	-	-	-	-	-	-	-	-	102 (44-198)	2,164 (1,288-3,271)	7,596 (5,122-10,827)	13,826 (9,164-19,547)	27,180 (19,813-37,619)	59,656 (44,166-79,603)	128,960 (94,527-173,139)	252,205 (189,845-326,000)	365,654 (282,979-468,128)	380,441 (301,695-469,268)	295,821 (244,757-346,710)	112,995 (91,071-139,176)
Parkinson's disease	-	-	-	-	-	-	-	2 (1-3)	23 (10-45)	85 (45-156)	196 (105-342)	338 (189-556)	546 (300-949)	1,024 (599-1,637)	1,834 (1,111-2,726)	3,763 (2,596-5,282)	8,998 (6,338-12,125)	19,713 (13,852-26,419)	34,703 (24,990-46,642)	39,936 (29,840-53,366)	26,049 (20,022-34,242)	10,865 (8,256-14,144)	2,716 (1,957-3,676)
Epilepsy	-	-	4,675 (3,967-5,323)	6,697 (5,310-8,066)	4,312 (3,801-4,805)	6,289 (5,718-6,881)	12,015 (11,265-12,773)	11,760 (10,907-12,445)	10,119 (9,467-10,647)	9,833 (9,160-10,372)	10,207 (9,623-10,743)	8,581 (8,079-9,006)	7,406 (7,006-7,733)	6,629 (6,211-6,894)	5,623 (5,168-5,848)	4,858 (4,371-5,158)	4,988 (4,451-5,315)	4,780 (4,222-5,103)	3,375 (3,099-3,552)	2,883 (2,673-3,077)	1,857 (1,704-1,986)	768 (720-816)	245 (230-260)
Multiple sclerosis	-	-	-	-	-	-	-	146 (125-192)	301 (263-386)	551 (490-698)	953 (862-1,198)	1,458 (1,339-1,777)	1,846 (1,687-2,131)	2,107 (1,877-2,409)	2,117 (1,800-2,382)	1,720 (1,406-1,907)	1,583 (1,323-1,791)	1,295 (1,026-1,450)	1,016 (787-1,102)	665 (480-728)	291 (199-323)	97 (67-107)	22 (15-24)
Motor neuron disease	8 (7-9)	11 (10-12)	686 (539-794)	298 (202-369)	40 (37-45)	68 (64-73)	121 (114-128)	102 (96-107)	100 (95-106)	216 (205-228)	474 (452-499)	894 (863-933)	1,456 (1,403-1,539)	2,107 (2,044-2,209)	2,832 (2,756-2,965)	3,140 (3,048-3,313)	3,863 (3,748-4,035)	3,918 (3,799-4,051)	3,541 (3,415-3,664)	2,280 (2,184-2,376)	947 (907-988)	242 (232-252)	49 (46-51)
Other neurological disorders	-	-	1,905 (1,540-2,394)	1,745 (1,202-2,526)	872 (732-1,083)	1,536 (1,342-1,741)	2,749 (2,545-3,002)	1,942 (1,834-2,074)	1,262 (1,197-1,342)	1,204 (1,152-1,262)	1,438 (1,384-1,493)	1,795 (1,732-1,850)	2,401 (2,321-2,490)	2,785 (2,665-2,881)	3,082 (2,948-3,184)	2,922 (2,749-3,045)	3,222 (3,054-3,340)	3,300 (3,141-3,430)	3,318 (3,162-3,450)	2,679 (2,538-2,806)	1,565 (1,490-1,637)	688 (656-724)	223 (212-234)
Mental and substance use disorders	14 (8-25)	23 (19-29)	0 (0-0)	0 (0-0)	17 (13-19)	16 (13-19)	6,687 (6,002-7,207)	14,008 (12,453-15,032)	19,707 (17,686-21,328)	25,410 (22,550-27,136)	29,543 (26,260-31,560)	35,104 (31,642-37,708)	38,470 (34,934-41,056)	35,471 (32,195-38,045)	28,336 (25,396-30,360)	17,887 (16,147-18,989)	16,708 (14,994-17,657)	10,788 (9,654-11,342)	8,912 (7,985-9,367)	5,356 (4,807-5,606)	3,046 (2,810-3,207)	1,409 (1,334-1,494)	498 (474-528)
Alcohol use disorders	-	-	-	-	-	-	1,330 (1,052-1,471)	3,291 (2,676-3,694)	6,697 (5,684-7,553)	11,457 (9,538-12,825)	15,781 (13,151-17,267)	21,187 (18,269-23,326)	26,167 (22,743-28,554)	25,235 (22,082-27,572)	20,820 (18,015-22,648)	13,115 (11,439-14,127)	11,583 (10,135-12,405)	6,497 (5,613-6,934)	4,799 (4,104-5,142)	2,290 (1,991-2,424)	1,138 (1,003-1,206)	463 (419-490)	159 (148-167)
Drug use disorders	14 (8-25)	23 (19-29)	0 (0-0)	0 (0-0)	0 (0-0)	0 (0-0)	5,309 (4,716-5,823)	10,647 (9,371-11,578)	12,936 (11,308-14,256)	13,872 (12,280-15,005)	13,677 (12,211-14,681)	13,820 (12,544-14,795)	12,207 (11,403-12,981)	10,235 (9,551-10,998)	7,516 (6,937-8,148)	4,772 (4,354-5,237)	5,125 (4,563-5,524)	4,291 (3,789-4,638)	4,113 (3,600-4,402)	3,066 (2,700-3,267)	1,908 (1,740-2,050)	946 (890-1,024)	339 (319-368)
Opioid use disorders	14 (8-25)	23 (19-29)	0 (0-0)	0 (0-0)	0 (0-0)	0 (0-0)	3,708 (3,201-4,189)	7,460 (6,141-8,265)	8,946 (6,977-10,140)	8,811 (6,853-9,866)	8,525 (6,721-9,438)	8,502 (6,564-9,446)	7,168 (5,395-7,934)	5,736 (4,437-6,342)	4,286 (3,483-4,808)	2,769 (2,329-3,118)	3,082 (2,591-3,436)	1,829 (1,472-2,064)	1,773 (1,412-2,019)	1,094 (833-1,227)	690 (546-785)	334 (266-384)	111 (87-127)
Cocaine use disorders	0 (0-0)	0 (0-0)	0 (0-0)	0 (0-0)	0 (0-0)	0 (0-0)	202 (148-275)	425 (320-594)	558 (409-777)	978 (725-1,278)	983 (777-1,287)	1,035 (803-1,413)	1,012 (707-1,400)	1,046 (709-1,413)	717 (503-909)	416 (293-526)	426 (291-543)	207 (137-269)	169 (114-214)	32 (22-39)	20 (14-25)	10 (6-12)	4 (2-5)
Amphetamine use disorders	0 (0-0)	0 (0-0)	0 (0-0)	0 (0-0)	0 (0-0)	0 (0-0)	174 (133-235)	342 (265-451)	435 (326-559)	553 (414-707)	551 (419-720)	553 (435-718)	509 (392-671)	459 (350-582)	331 (245-416)	193 (141-240)	194 (138-242)	76 (53-96)	66 (46-82)	16 (12-20)	11 (8-13)	6 (4-7)	3 (1-4)
Other drug use disorders	0 (0-0)	0 (0-0)	0 (0-0)	0 (0-0)	0 (0-0)	0 (0-0)	1,226 (1,002-1,484)	2,420 (2,064-3,091)	2,998 (2,540-3,723)	3,530 (3,073-4,273)	3,618 (3,238-4,448)	3,729 (3,352-4,698)	3,518 (3,158-4,637)	2,994 (2,733-3,803)	2,182 (1,947-2,609)	1,394 (1,207-1,651)	1,423 (1,156-1,642)	2,179 (1,859-2,416)	2,105 (1,829-2,355)	1,924 (1,697-2,132)	1,188 (1,079-1,335)	596 (550-683)	222 (203-259)
Eating disorders	-	-	-	-	17 (13-19)	16 (13-19)	47 (38-55)	70 (59-81)	74 (63-85)	81 (72-90)	85 (74-94)	98 (89-109)	96 (87-108)	0 (0-0)	0 (0-0)	-	-	-	-	-	-	-	-
Anorexia nervosa	-	-	-	-	16 (13-18)	15 (12-18)	45 (36-52)	66 (54-76)	69 (58-80)	74 (65-82)	78 (69-87)	86 (78-96)	80 (72-90)	0 (0-0)	0 (0-0)	-	-	-	-	-	-	-	-
Bulimia nervosa	-	-	-	-	1 (1-1)	1 (1-1)	2 (2-3)	5 (4-6)	5 (4-6)	7 (6-9)	6 (5-8)	12 (10-15)	16 (14-20)	0 (0-0)	0 (0-0)	-	-	-	-	-	-	-	-
Diabetes, urogenital, blood, and endocrine diseases	4,191 (3,244-4,787)	2,812 (2,300-3,120)	26,095 (23,530-28,549)	30,638 (23,532-37,312)	13,215 (11,839-15,335)	16,431 (14,671-18,119)	27,236 (25,220-29,473)	34,069 (32,280-36,940)	34,129 (32,369-35,926)	42,231 (40,379-44,354)	52,918 (50,840-55,185)	73,869 (71,548-76,794)	98,421 (95,345-101,899)	139,282 (135,112-143,691)	172,607 (167,911-177,307)	207,032 (201,407-212,938)	262,926 (255,265-271,129)	300,934 (291,233-310,617)	314,351 (304,100-324,231)	283,078 (273,186-293,155)	201,272 (194,304-208,786)	107,907 (104,152-111,836)	39,974 (38,300-41,405)
Diabetes mellitus	53 (49-57)	81 (73-89)	823 (753-898)	924 (816-1,040)	999 (901-1,103)	1,641 (1,513-1,775)	3,282 (3,111-3,471)	5,248 (4,994-5,514)	6,628 (6,330-6,947)	9,891 (9,506-10,299)	14,603 (14,056-15,156)	25,620 (24,839-26,464)	39,444 (38,300-40,496)	62,548 (60,906-64,239)	84,169 (82,046-86,236)	108,223 (105,179-111,330)	138,462 (133,889-142,429)	156,994 (151,602-162,164)	161,035 (154,848-166,044)	138,405 (132,936-143,659)	85,807 (82,393-89,226)	39,494 (37,930-41,152)	12,185 (11,582-12,710)
Acute glomerulonephritis	-	-	117 (96-144)	184 (160-215)	186 (165-214)	214 (188-251)	301 (276-332)	307 (281-334)	295 (270-322)	410 (376-448)	522 (479-572)	545 (500-594)	548 (508-593)	653 (607-705)	744 (697-796)	703 (651-766)	905 (838-970)	1,025 (953-1,109)	885 (825-948)	720 (667-779)	460 (426-497)	192 (178-208)	71 (63-80)
Chronic kidney disease	-	-	4,500 (4,025-4,956)	5,958 (5,263-6,699)	3,832 (3,386-4,227)	4,687 (4,265-5,173)	9,810 (9,081-10,558)	14,638 (13,508-15,888)	15,880 (14,884-16,913)	19,644 (18,419-21,159)	25,252 (23,700-27,080)	32,583 (30,647-34,622)	40,379 (38,151-43,159)	55,891 (53,217-59,391)	66,381 (63,329-70,310)	74,887 (71,758-79,494)	92,535 (88,586-98,739)	105,102 (100,671-111,478)	110,088 (105,678-116,213)	100,332 (96,503-106,353)	76,919 (73,924-80,583)	44,439 (42,620-46,374)	17,447 (16,654-18,119)
Chronic kidney disease due to diabetes mellitus	-	-	116 (78-170)	166 (106-250)	131 (75-212)	212 (130-339)	743 (446-1,144)	1,992 (1,266-2,785)	3,340 (2,408-4,467)	6,111 (4,625-7,726)	9,978 (7,810-12,287)	14,819 (11,539-17,765)	20,106 (16,181-23,960)	28,959 (22,921-34,360)	35,062 (28,409-40,574)	40,104 (33,244-46,651)	47,616 (37,802-55,868)	50,552 (40,297-59,270)	47,200 (36,987-57,061)	36,125 (27,561-43,971)	23,794 (17,844-30,888)	12,861 (9,374-16,581)	5,033 (3,454-6,655)
Chronic kidney disease due to hypertension	-	-	31 (21-43)	59 (40-84)	63 (41-94)	129 (84-191)	436 (284-626)	969 (671-1,318)	1,357 (961-1,835)	2,026 (1,502-2,687)	3,060 (2,327-3,940)	4,512 (3,477-5,825)	6,018 (4,698-7,691)	9,314 (7,162-12,066)	12,140 (9,547-15,566)	14,953 (11,945-18,804)	20,716 (16,308-26,168)	26,573 (21,120-33,238)	31,225 (24,989-38,538)	32,560 (26,671-39,597)	29,753 (24,399-35,957)	18,895 (15,621-22,254)	7,674 (6,193-9,259)
Chronic kidney disease due to glomerulonephritis	-	-	1,819 (1,367-2,262)	2,463 (1,849-3,130)	1,544 (1,116-2,023)	1,992 (1,408-2,604)	4,247 (3,069-5,451)	6,174 (4,504-8,014)	6,058 (4,370-7,864)	5,933 (4,207-7,897)	5,884 (3,948-7,926)	6,053 (3,943-8,286)	6,219 (4,145-8,545)	7,376 (4,992-10,411)	7,680 (5,344-10,788)	7,603 (4,961-10,794)	9,252 (6,190-13,138)	10,402 (7,115-14,720)	11,997 (8,440-16,742)	11,883 (8,585-15,652)	7,839 (5,558-10,721)	4,041 (2,917-5,310)	1,526 (965-2,353)
Chronic kidney disease due to other causes	-	-	2,534 (2,045-3,017)	3,270 (2,615-3,991)	2,094 (1,611-2,620)	2,353 (1,750-2,965)	4,384 (3,187-5,584)	5,504 (3,875-7,215)	5,124 (3,614-6,855)	5,574 (3,950-7,511)	6,329 (4,556-8,344)	7,200 (4,941-9,645)	8,036 (5,389-11,465)	10,241 (6,795-14,420)	11,498 (7,830-16,591)	12,226 (7,997-17,433)	14,952 (10,253-21,098)	17,575 (12,012-24,079)	19,667 (13,757-26,912)	19,764 (14,295-26,237)	15,532 (10,772-20,955)	8,642 (6,331-11,346)	3,214 (2,053-4,518)
Urinary diseases and male infertility	325 (298-353)	432 (377-504)	4,866 (4,424-5,348)	3,325 (2,872-3,828)	1,297 (1,122-1,486)	1,144 (1,040-1,248)	2,729 (2,537-2,904)	3,562 (3,350-3,760)	3,330 (3,144-3,504)	3,933 (3,751-4,112)	4,593 (4,368-4,791)	5,711 (5,499-5,929)	6,976 (6,702-7,236)	8,794 (8,470-9,106)	10,169 (9,778-10,570)	12,506 (11,970-12,998)	17,685 (16,906-18,347)	21,318 (20,357-22,088)	25,202 (24,212-26,045)	26,932 (25,944-27,879)	23,408 (22,525-24,201)	15,421 (14,880-16,011)	6,708 (6,450-6,971)
Interstitial nephritis and urinary tract infections	176 (137-206)	246 (192-301)	3,278 (2,693-3,758)	1,906 (1,402-2,412)	661 (527-782)	604 (512-679)	1,681 (1,446-1,871)	2,200 (1,911-2,414)	1,913 (1,672-2,105)	2,238 (1,958-2,432)	2,731 (2,411-2,979)	3,452 (3,052-3,725)	4,209 (3,679-4,527)	5,505 (4,930-5,910)	6,373 (5,687-6,823)	7,707 (6,692-8,347)	11,408 (10,218-12,246)	14,526 (13,043-15,454)	18,162 (16,547-19,234)	20,220 (18,725-21,291)	18,681 (17,509-19,616)	12,848 (12,118-13,523)	5,900 (5,569-6,201)
Urolithiasis	-	-	-	-	174 (133-225)	163 (129-200)	293 (235-362)	328 (260-399)	281 (224-350)	323 (256-401)	426 (359-523)	563 (473-726)	705 (596-883)	970 (809-1,176)	996 (825-1,182)	1,134 (948-1,362)	1,708 (1,470-2,086)	1,869 (1,613-2,332)	2,045 (1,781-2,547)	1,803 (1,583-2,315)	1,245 (1,088-1,606)	680 (585-907)	231 (196-325)
Other urinary diseases	149 (119-181)	186 (142-240)	1,589 (1,174-2,099)	1,419 (964-1,905)	462 (344-592)	377 (288-477)	755 (600-957)	1,034 (838-1,313)	1,135 (938-1,394)	1,372 (1,157-1,635)	1,436 (1,194-1,745)	1,696 (1,389-2,018)	2,062 (1,690-2,461)	2,319 (1,908-2,832)	2,800 (2,331-3,489)	3,665 (2,993-4,648)	4,569 (3,777-5,615)	4,923 (4,104-6,141)	4,995 (4,226-6,145)	4,909 (4,256-5,981)	3,482 (2,986-4,339)	1,893 (1,627-2,380)	577 (487-748)
Gynecological diseases	-	-	-	-	-	-	211 (180-235)	303 (260-341)	359 (314-400)	463 (405-515)	616 (541-690)	667 (597-741)	932 (829-1,016)	767 (682-840)	540 (471-599)	328 (287-361)	519 (454-581)	331 (286-367)	251 (221-278)	491 (398-571)	345 (284-399)	156 (132-178)	58 (49-67)
Uterine fibroids	-	-	-	-	-	-	13 (7-25)	21 (13-35)	35 (19-57)	72 (37-115)	170 (87-245)	242 (153-321)	447 (270-573)	449 (263-558)	270 (148-361)	139 (81-188)	204 (117-284)	109 (72-147)	85 (65-104)	140 (67-230)	76 (35-134)	34 (18-54)	11 (7-17)
Polycystic ovarian syndrome	-	-	-	-	-	-	2 (0-6)	21 (5-64)	59 (17-131)	46 (12-124)	60 (19-128)	57 (18-108)	89 (26-179)	151 (53-288)	-	-	-	-	-	-	-	-	-
Endometriosis	-	-	-	-	-	-	3 (1-8)	3 (1-6)	3 (1-6)	7 (2-13)	4 (2-7)	8 (4-13)	16 (7-27)	11 (6-17)	-	-	-	-	-	-	-	-	-
Genital prolapse	-	-	-	-	-	-	10 (3-30)	8 (3-19)	15 (6-28)	19 (4-31)	7 (3-12)	8 (4-14)	9 (4-14)	15 (6-31)	80 (20-141)	70 (26-121)	122 (45-203)	97 (45-147)	56 (36-78)	170 (70-267)	116 (49-180)	50 (24-75)	17 (10-24)
Other gynecological diseases	-	-	-	-	-	-	183 (141-211)	250 (182-296)	247 (158-308)	319 (208-391)	376 (252-492)	351 (232-460)	372 (222-557)	141 (72-254)	190 (115-286)	119 (74-179)	192 (119-278)	125 (81-182)	110 (83-133)	182 (116-290)	154 (105-230)	73 (52-101)	31 (22-40)
Hemoglobinopathies and hemolytic anaemias	2,382 (1,601-2,932)	1,173 (823-1,387)	8,986 (6,931-10,886)	15,706 (9,439-21,074)	4,895 (3,811-6,429)	6,888 (5,390-8,172)	8,904 (6,928-10,807)	7,648 (6,352-9,860)	4,673 (3,712-5,749)	3,877 (3,197-4,778)	3,164 (2,654-3,831)	4,166 (3,556-5,249)	4,639 (4,009-6,082)	4,104 (3,506-5,114)	3,552 (3,078-4,431)	3,373 (2,971-4,181)	4,486 (3,930-5,665)	6,835 (6,003-8,630)	6,937 (6,175-8,635)	6,209 (5,435-7,561)	6,621 (5,640-7,842)	3,581 (2,975-4,232)	1,439 (1,102-1,642)
Thalassemias	504 (385-625)	327 (244-404)	2,070 (1,588-2,630)	3,003 (2,293-3,821)	1,360 (1,122-1,778)	725 (613-919)	588 (478-730)	263 (216-330)	143 (118-173)	99 (84-125)	76 (65-97)	55 (48-73)	41 (36-57)	35 (30-50)	26 (22-39)	11 (9-17)	6 (5-11)	3 (2-5)	1 (1-2)	0 (0-1)	0 (0-0)	0 (0-0)	0 (0-0)
Sickle cell disorders	1,724 (1,051-2,240)	799 (534-990)	6,564 (4,896-8,064)	12,072 (6,271-17,051)	3,205 (2,296-4,386)	5,615 (4,247-6,798)	7,178 (5,541-8,732)	5,910 (4,786-7,681)	3,144 (2,380-3,917)	2,263 (1,758-2,845)	1,481 (1,209-1,851)	1,893 (1,579-2,466)	1,914 (1,591-2,581)	1,153 (961-1,508)	509 (422-677)	210 (174-283)	133 (114-180)	72 (61-99)	31 (26-44)	12 (10-17)	4 (3-6)	1 (1-1)	0 (0-0)
G6PD deficiency	63 (43-84)	33 (23-43)	279 (202-373)	541 (381-727)	276 (212-353)	419 (322-520)	816 (658-1,014)	1,049 (855-1,343)	973 (790-1,200)	1,003 (819-1,226)	997 (822-1,201)	1,270 (1,050-1,594)	1,563 (1,310-2,070)	1,336 (1,105-1,664)	1,124 (927-1,438)	869 (698-1,125)	817 (665-1,092)	764 (624-1,022)	503 (401-699)	205 (164-270)	120 (95-154)	30 (24-39)	6 (4-8)
Other hemoglobinopathies and hemolytic anaemias	91 (67-114)	14 (10-17)	74 (57-94)	90 (68-114)	54 (45-67)	129 (104-151)	322 (254-391)	426 (355-541)	414 (340-503)	513 (425-640)	609 (509-733)	948 (805-1,180)	1,120 (954-1,413)	1,580 (1,340-1,939)	1,894 (1,630-2,329)	2,283 (1,991-2,803)	3,529 (3,065-4,458)	5,996 (5,245-7,597)	6,402 (5,682-7,968)	5,992 (5,250-7,288)	6,497 (5,536-7,688)	3,550 (2,948-4,194)	1,433 (1,095-1,634)
Endocrine, metabolic, blood, and immune disorders	1,432 (1,117-1,605)	1,127 (885-1,321)	6,803 (5,949-8,324)	4,542 (3,856-5,689)	2,007 (1,821-2,543)	1,858 (1,707-2,307)	1,999 (1,868-2,434)	2,363 (2,081-2,738)	2,965 (2,428-3,362)	4,013 (3,224-4,516)	4,168 (3,416-4,668)	4,577 (3,736-4,939)	5,504 (4,558-5,892)	6,526 (5,381-6,890)	7,051 (5,845-7,425)	7,012 (5,974-7,657)	8,335 (7,162-9,258)	9,329 (8,003-10,370)	9,953 (8,339-10,919)	9,990 (8,096-10,922)	7,712 (6,169-8,342)	4,624 (3,601-5,016)	2,066 (1,529-2,249)
Musculoskeletal disorders	-	-	-	-	661 (547-825)	1,162 (1,003-1,415)	2,068 (1,722-2,444)	2,284 (1,986-2,684)	2,325 (1,979-2,751)	2,537 (2,144-2,963)	2,902 (2,462-3,386)	3,213 (2,783-3,709)	3,544 (3,119-3,979)	3,881 (3,352-4,449)	4,361 (3,814-4,896)	4,809 (4,249-5,461)	6,252 (5,510-6,991)	7,585 (6,478-8,195)	8,776 (7,561-9,499)	8,337 (7,127-9,009)	6,511 (5,562-6,983)	3,851 (3,087-4,150)	1,666 (1,336-1,788)
Rheumatoid arthritis	-	-	-	-	121 (91-179)	142 (116-197)	140 (115-201)	148 (125-212)	157 (134-222)	218 (178-310)	331 (269-463)	524 (443-670)	822 (693-1,005)	1,352 (1,110-1,604)	1,880 (1,537-2,182)	2,378 (1,997-2,683)	3,400 (2,884-3,907)	4,257 (3,616-4,829)	4,690 (3,978-5,306)	3,949 (3,327-4,623)	2,556 (2,131-3,104)	1,144 (935-1,419)	364 (297-499)
Other musculoskeletal disorders	-	-	-	-	540 (445-683)	1,020 (876-1,230)	1,928 (1,585-2,270)	2,136 (1,850-2,498)	2,168 (1,819-2,528)	2,319 (1,950-2,679)	2,571 (2,170-2,977)	2,689 (2,291-3,044)	2,722 (2,369-3,100)	2,529 (2,153-2,923)	2,481 (2,153-2,809)	2,431 (2,113-2,849)	2,853 (2,477-3,271)	3,328 (2,784-3,738)	4,086 (3,363-4,498)	4,388 (3,542-4,760)	3,955 (3,189-4,266)	2,707 (2,088-2,921)	1,302 (990-1,403)
Other non-communicable diseases	184,904 (149,278-214,987)	69,419 (58,928-78,728)	226,817 (187,847-261,847)	73,390 (50,260-93,704)	18,076 (15,649-20,979)	14,094 (12,477-15,613)	12,075 (10,744-13,204)	9,484 (8,312-10,299)	7,384 (6,576-8,118)	6,478 (5,500-7,161)	6,488 (5,515-7,028)	6,694 (5,608-7,295)	6,095 (4,841-6,776)	6,666 (5,025-7,518)	7,326 (5,393-8,356)	7,254 (5,243-8,381)	8,439 (5,842-10,160)	7,719 (4,701-9,876)	9,509 (5,987-12,427)	9,697 (6,176-12,933)	8,428 (5,162-11,048)	5,667 (3,354-7,716)	2,670 (1,438-3,760)
Congenital anomalies	184,904 (149,278-214,987)	65,738 (55,702-74,811)	191,084 (153,462-224,082)	71,196 (47,866-91,687)	17,514 (15,133-20,414)	13,429 (12,030-15,056)	10,832 (9,757-12,190)	8,416 (7,425-9,321)	6,447 (5,840-7,249)	4,846 (4,318-5,420)	4,897 (4,352-5,458)	4,683 (4,099-5,135)	3,288 (2,894-3,658)	3,010 (2,601-3,278)	2,811 (2,357-2,979)	2,403 (2,079-2,604)	2,015 (1,780-2,220)	-	-	-	-	-	-
Neural tube defects	25,798 (17,708-38,072)	6,219 (4,168-9,562)	14,424 (8,766-24,189)	5,534 (2,287-11,778)	771 (502-1,273)	490 (356-734)	289 (228-417)	212 (164-316)	143 (114-204)	123 (100-167)	95 (79-120)	87 (72-116)	80 (68-98)	66 (56-84)	56 (45-84)	41 (33-68)	38 (31-58)	-	-	-	-	-	-
Congenital heart anomalies	58,676 (45,618-69,496)	28,757 (24,039-33,211)	96,896 (83,098-111,619)	35,028 (27,655-45,206)	10,721 (9,168-12,639)	8,959 (7,836-10,192)	7,332 (6,515-8,244)	5,956 (5,292-6,695)	4,733 (4,321-5,398)	3,357 (2,999-3,779)	3,546 (3,172-4,027)	3,442 (3,026-3,842)	1,469 (1,282-1,646)	1,226 (1,072-1,396)	1,032 (925-1,179)	805 (725-951)	719 (652-858)	-	-	-	-	-	-
Orofacial clefts	1,485 (840-2,305)	538 (303-872)	1,028 (565-1,721)	139 (48-287)	-	-	-	-	-	-	-	-	-	-	-	-	-	-	-	-	-	-	-
Down's syndrome	2,095 (1,426-3,831)	972 (700-1,679)	4,307 (2,997-7,882)	2,014 (1,188-5,028)	378 (279-558)	323 (249-425)	344 (294-456)	320 (270-411)	313 (267-405)	348 (295-445)	345 (289-433)	366 (309-439)	683 (570-795)	701 (563-761)	747 (544-813)	542 (403-591)	241 (183-264)	-	-	-	-	-	-
Other chromosomal abnormalities	5,694 (3,712-9,200)	2,599 (1,717-4,310)	5,600 (3,657-9,876)	1,076 (631-2,283)	261 (192-371)	182 (124-229)	200 (138-248)	151 (100-176)	120 (73-140)	88 (47-101)	82 (43-94)	74 (38-85)	70 (39-80)	76 (45-90)	68 (40-81)	43 (27-52)	37 (26-45)	-	-	-	-	-	-
Congenital musculoskeletal and limb anomalies	5,027 (3,174-10,300)	1,198 (778-2,410)	2,354 (1,459-5,368)	679 (364-2,071)	228 (166-401)	206 (161-301)	231 (174-321)	118 (81-154)	86 (66-111)	69 (48-88)	58 (43-71)	48 (35-57)	57 (42-68)	57 (43-68)	45 (33-55)	41 (31-48)	39 (32-47)	-	-	-	-	-	-
Urogenital congenital anomalies	5,364 (3,742-6,785)	1,575 (1,069-1,998)	2,706 (1,736-3,534)	595 (369-817)	278 (206-377)	284 (214-377)	147 (118-187)	131 (107-165)	113 (89-140)	115 (92-139)	127 (95-154)	135 (105-163)	221 (163-265)	266 (205-322)	293 (233-344)	339 (258-399)	417 (328-497)	-	-	-	-	-	-
Digestive congenital anomalies	16,402 (12,156-25,994)	6,584 (4,820-10,474)	14,705 (10,321-25,898)	2,942 (1,699-7,985)	344 (272-496)	189 (159-244)	120 (100-156)	79 (68-96)	57 (48-67)	48 (41-56)	49 (38-56)	47 (33-53)	67 (55-77)	72 (57-82)	72 (57-81)	77 (63-88)	84 (66-93)	-	-	-	-	-	-
Other congenital anomalies	64,364 (43,218-95,274)	17,297 (10,665-26,427)	49,065 (27,886-77,269)	23,189 (10,027-42,574)	4,532 (2,973-6,607)	2,796 (2,069-3,755)	2,169 (1,713-2,723)	1,449 (1,103-1,924)	880 (675-1,158)	699 (555-886)	596 (487-733)	484 (400-578)	640 (538-746)	546 (457-638)	499 (409-588)	517 (435-602)	440 (371-506)	-	-	-	-	-	-
Skin and subcutaneous diseases	-	-	4,238 (2,225-5,652)	2,194 (1,144-2,978)	562 (289-754)	665 (342-852)	1,243 (676-1,560)	1,068 (587-1,297)	937 (540-1,161)	1,632 (882-2,022)	1,591 (888-1,934)	2,011 (1,162-2,479)	2,807 (1,651-3,468)	3,656 (2,116-4,483)	4,515 (2,691-5,575)	4,851 (2,902-6,043)	6,424 (3,857-8,181)	7,719 (4,701-9,876)	9,509 (5,987-12,427)	9,697 (6,176-12,933)	8,428 (5,162-11,048)	5,667 (3,354-7,716)	2,670 (1,438-3,760)
Cellulitis	-	-	227 (81-364)	206 (70-352)	93 (37-138)	110 (52-153)	133 (70-173)	162 (80-203)	121 (59-153)	233 (108-298)	271 (129-338)	347 (172-452)	494 (241-618)	756 (350-951)	899 (408-1,126)	867 (424-1,112)	1,013 (461-1,298)	1,149 (572-1,533)	1,362 (678-1,870)	1,352 (716-2,159)	1,102 (593-1,804)	728 (364-1,257)	326 (161-621)
Pyoderma	-	-	3,909 (2,065-5,300)	1,759 (907-2,445)	362 (183-501)	426 (207-564)	872 (451-1,126)	697 (360-864)	632 (343-795)	1,120 (590-1,441)	1,035 (556-1,292)	1,313 (723-1,614)	1,865 (1,041-2,399)	2,340 (1,313-3,014)	2,880 (1,652-3,727)	3,111 (1,784-3,921)	3,912 (2,241-5,220)	4,325 (2,491-5,521)	4,841 (3,057-6,547)	4,000 (2,484-5,517)	3,300 (1,964-4,465)	2,027 (1,162-2,854)	868 (465-1,262)
Decubitus ulcer	-	-	-	105 (46-163)	51 (30-69)	65 (39-87)	134 (82-181)	118 (74-156)	117 (73-158)	201 (119-271)	215 (127-287)	264 (158-354)	350 (216-494)	450 (265-639)	603 (367-851)	729 (452-1,027)	1,297 (791-1,713)	2,013 (1,231-2,727)	2,985 (1,774-4,067)	3,963 (2,368-5,241)	3,696 (2,073-4,695)	2,668 (1,462-3,543)	1,361 (678-1,844)
Other skin and subcutaneous diseases	-	-	103 (57-163)	125 (64-197)	55 (33-82)	65 (38-92)	103 (64-147)	92 (57-125)	67 (43-93)	79 (51-111)	71 (47-98)	87 (56-121)	99 (64-140)	110 (69-156)	133 (85-191)	143 (95-204)	202 (130-289)	231 (151-338)	321 (208-486)	384 (248-578)	329 (200-526)	245 (139-407)	117 (62-206)
Sudden infant death syndrome	-	3,681 (2,802-4,580)	31,495 (25,618-42,192)	-	-	-	-	-	-	-	-	-	-	-	-	-	-	-	-	-	-	-	-
Injuries	13,981 (11,374-15,504)	8,474 (6,956-9,470)	91,094 (80,301-103,001)	220,125 (190,382-245,732)	128,896 (119,387-138,188)	132,474 (125,141-139,320)	308,688 (296,907-318,274)	413,274 (399,758-424,531)	371,933 (359,078-382,006)	348,679 (335,263-357,980)	343,175 (329,260-352,176)	318,525 (303,364-327,381)	289,791 (275,334-298,800)	275,955 (260,377-284,418)	234,716 (220,290-241,735)	187,755 (173,002-194,612)	198,817 (179,511-206,351)	191,864 (170,854-200,217)	180,269 (159,168-189,220)	149,232 (129,630-157,821)	104,460 (88,871-111,047)	55,044 (46,374-58,568)	20,548 (17,136-21,937)
Transport injuries	2,295 (1,866-2,816)	1,265 (1,113-1,457)	13,237 (11,890-14,948)	44,888 (39,224-50,074)	42,622 (39,714-45,854)	40,049 (37,777-42,685)	116,505 (112,442-122,483)	157,325 (152,353-165,289)	138,425 (133,851-145,029)	128,102 (124,371-133,458)	129,405 (125,482-134,368)	118,848 (115,324-122,386)	100,084 (97,266-103,337)	95,359 (92,568-98,215)	77,479 (75,158-79,892)	59,980 (57,936-62,318)	56,527 (54,689-58,486)	50,796 (49,033-52,607)	40,647 (39,183-42,233)	26,795 (25,802-27,939)	13,900 (13,347-14,497)	5,389 (5,174-5,619)	1,607 (1,522-1,683)
Road injuries	1,982 (1,634-2,442)	1,122 (989-1,308)	12,085 (10,856-13,611)	42,404 (37,248-47,168)	40,692 (37,918-43,862)	37,457 (35,418-39,999)	108,774 (104,814-114,536)	148,462 (143,854-156,636)	129,393 (125,152-135,818)	120,575 (117,162-125,920)	121,360 (117,580-126,471)	111,238 (107,881-114,815)	93,032 (90,352-96,057)	88,588 (86,102-91,433)	72,119 (70,027-74,407)	56,077 (54,185-58,200)	52,758 (51,040-54,675)	47,671 (46,060-49,397)	37,960 (36,612-39,475)	25,081 (24,134-26,159)	12,968 (12,454-13,527)	4,963 (4,770-5,171)	1,443 (1,363-1,513)
Pedestrian road injuries	813 (630-1,107)	446 (373-547)	4,901 (4,184-5,772)	23,094 (19,860-26,850)	22,126 (20,219-24,301)	17,843 (16,596-19,748)	30,983 (28,502-33,849)	39,071 (35,584-42,622)	35,245 (32,169-38,745)	39,061 (36,383-42,690)	43,461 (40,136-47,182)	40,753 (37,946-44,300)	36,247 (33,888-39,187)	37,375 (34,748-40,134)	31,614 (29,266-33,867)	27,466 (25,875-29,666)	27,139 (25,663-28,942)	25,526 (24,248-27,058)	22,102 (20,998-23,531)	14,658 (13,935-15,482)	7,546 (7,188-8,022)	2,682 (2,536-2,902)	747 (697-843)
Cyclist road injuries	-	-	-	1,347 (982-1,723)	2,089 (1,798-2,440)	3,016 (2,657-3,499)	5,481 (4,881-6,216)	4,933 (4,395-5,587)	4,592 (4,058-5,161)	5,353 (4,801-6,026)	5,736 (5,076-6,345)	6,479 (5,773-7,089)	6,156 (5,522-6,855)	6,842 (6,142-7,467)	5,519 (5,017-5,991)	4,585 (4,093-5,057)	4,426 (3,989-4,885)	3,771 (3,388-4,178)	2,321 (2,089-2,537)	1,206 (1,074-1,325)	544 (481-639)	185 (163-223)	3 (2-3)
Motorcyclist road injuries	230 (171-316)	129 (104-171)	1,622 (1,341-1,970)	3,180 (2,420-4,202)	2,564 (2,225-2,957)	3,766 (3,382-4,184)	28,116 (25,323-31,166)	40,519 (36,206-45,038)	34,010 (30,493-38,039)	28,537 (25,037-31,800)	27,189 (23,730-29,566)	23,653 (20,677-25,455)	16,806 (14,807-18,308)	14,225 (12,566-15,411)	10,369 (9,435-11,244)	5,830 (5,198-6,464)	4,846 (4,187-5,309)	3,608 (3,146-3,946)	2,025 (1,660-2,222)	1,308 (1,103-1,440)	542 (469-600)	166 (148-185)	52 (48-59)
Motor vehicle road injuries	895 (656-1,162)	528 (433-645)	5,306 (4,398-6,396)	14,334 (11,412-17,468)	13,425 (11,608-15,470)	12,336 (11,007-14,112)	43,266 (39,468-49,615)	62,677 (56,974-71,496)	54,498 (49,600-62,739)	46,756 (42,287-53,996)	44,050 (39,524-50,581)	39,458 (35,397-45,394)	32,818 (29,317-36,959)	29,119 (25,899-32,947)	23,795 (21,514-26,777)	17,716 (15,972-19,852)	15,767 (14,096-17,715)	14,238 (12,886-15,886)	10,975 (9,922-12,132)	7,533 (6,877-8,327)	4,133 (3,746-4,458)	1,840 (1,631-1,964)	606 (519-650)
Other road injuries	45 (29-72)	18 (14-27)	256 (192-389)	448 (330-672)	488 (412-629)	495 (424-619)	927 (816-1,188)	1,262 (1,103-1,585)	1,048 (911-1,281)	868 (767-1,058)	923 (809-1,099)	895 (801-1,102)	1,006 (888-1,225)	1,028 (911-1,255)	822 (725-1,028)	481 (419-594)	580 (507-714)	527 (459-660)	537 (464-715)	376 (321-517)	203 (169-275)	90 (73-122)	35 (27-51)
Other transport injuries	312 (214-434)	143 (104-186)	1,151 (935-1,531)	2,484 (1,937-3,361)	1,930 (1,664-2,272)	2,592 (2,292-2,919)	7,731 (6,825-8,526)	8,863 (7,944-9,625)	9,032 (8,081-9,876)	7,527 (6,827-8,198)	8,045 (7,271-8,874)	7,610 (6,876-8,296)	7,052 (6,499-7,583)	6,771 (6,141-7,344)	5,360 (4,908-5,799)	3,903 (3,565-4,218)	3,769 (3,373-4,092)	3,126 (2,794-3,386)	2,687 (2,415-2,944)	1,714 (1,552-1,875)	932 (843-1,033)	425 (393-472)	164 (153-182)
Unintentional injuries	9,511 (7,276-10,762)	6,452 (5,065-7,406)	72,897 (62,519-85,034)	160,813 (136,008-184,635)	76,029 (68,460-82,707)	69,592 (62,997-73,783)	80,674 (73,351-84,453)	89,928 (82,153-93,868)	82,346 (75,524-85,957)	86,674 (79,076-90,112)	92,796 (83,000-96,607)	91,744 (81,809-95,721)	92,962 (82,871-97,241)	92,590 (81,604-97,058)	86,120 (75,965-90,170)	77,532 (66,316-82,053)	92,641 (77,897-98,289)	97,453 (80,246-104,162)	103,908 (85,486-110,908)	99,278 (81,047-106,646)	78,954 (64,315-84,725)	45,425 (37,156-48,729)	17,863 (14,483-19,174)
Falls	2,214 (1,450-2,863)	722 (511-867)	9,679 (7,006-11,688)	15,729 (11,348-19,926)	7,796 (6,676-8,796)	7,911 (6,832-8,773)	10,345 (9,001-11,113)	13,521 (11,924-14,414)	13,879 (12,034-14,862)	16,941 (14,619-17,923)	20,858 (17,521-22,253)	22,696 (19,093-24,100)	24,181 (20,305-25,652)	26,959 (22,507-28,485)	28,865 (23,839-30,653)	30,428 (24,224-32,538)	41,211 (32,260-44,375)	50,151 (38,359-54,482)	60,346 (46,782-65,084)	62,296 (47,395-67,368)	53,982 (41,732-58,207)	32,134 (24,632-34,586)	12,366 (9,430-13,266)
Drowning	547 (427-626)	246 (197-293)	8,448 (6,959-9,464)	80,306 (64,261-90,420)	37,962 (33,094-41,430)	33,526 (29,925-35,560)	30,425 (27,796-31,991)	24,795 (23,301-26,275)	18,611 (17,344-19,651)	17,738 (16,467-18,603)	17,242 (15,871-17,975)	15,930 (14,556-16,639)	14,491 (13,371-15,193)	14,002 (12,692-14,652)	11,602 (10,486-12,113)	9,597 (8,442-10,061)	9,985 (8,856-10,485)	9,492 (8,319-9,924)	8,186 (6,943-8,656)	6,183 (5,228-6,563)	3,352 (2,877-3,563)	1,171 (1,010-1,235)	295 (257-313)
Fire, heat, and hot substances	438 (321-499)	396 (267-463)	5,837 (4,265-6,652)	15,545 (10,066-18,918)	5,190 (4,011-5,881)	3,828 (2,998-4,264)	6,951 (5,015-7,928)	9,536 (6,962-10,722)	8,449 (6,430-9,367)	8,139 (6,554-8,903)	7,652 (6,281-8,276)	7,103 (6,031-7,655)	7,874 (6,505-8,549)	7,622 (6,213-8,289)	6,811 (5,638-7,340)	5,930 (5,001-6,386)	7,063 (5,922-7,598)	7,058 (6,087-7,588)	7,297 (6,259-7,810)	6,990 (5,986-7,476)	4,913 (4,211-5,255)	2,728 (2,259-2,918)	1,121 (879-1,196)
Poisonings	360 (202-533)	242 (161-328)	4,434 (2,481-6,696)	7,436 (5,031-10,181)	2,496 (1,941-2,832)	3,109 (2,478-3,459)	3,662 (2,931-4,121)	4,676 (3,836-5,374)	4,264 (3,445-4,917)	4,020 (3,213-4,530)	4,160 (3,229-4,512)	4,228 (3,352-4,613)	3,732 (2,903-4,114)	3,839 (3,006-4,201)	3,321 (2,567-3,628)	2,567 (1,962-2,839)	2,678 (2,030-2,923)	2,482 (1,930-2,724)	1,879 (1,442-2,055)	1,469 (1,144-1,605)	792 (642-868)	334 (296-394)	112 (102-154)
Exposure to mechanical forces	2,080 (1,308-2,472)	1,861 (1,146-2,319)	12,623 (9,387-14,695)	11,092 (7,940-13,776)	6,212 (5,511-7,063)	5,273 (4,852-5,968)	9,236 (7,786-9,827)	12,157 (10,049-13,047)	12,095 (9,969-12,919)	12,442 (10,010-13,252)	13,981 (10,708-14,906)	12,473 (9,577-13,327)	11,359 (9,090-12,057)	10,219 (8,275-10,841)	8,148 (6,684-8,636)	5,427 (4,379-5,888)	5,189 (4,184-5,603)	4,404 (3,549-4,744)	3,997 (3,271-4,300)	3,323 (2,723-3,588)	2,464 (2,052-2,657)	1,418 (1,169-1,529)	586 (496-630)
Unintentional firearm injuries	73 (53-90)	52 (36-68)	594 (414-780)	1,005 (613-1,442)	744 (616-899)	865 (752-1,015)	2,041 (1,738-2,373)	2,900 (2,187-3,373)	2,930 (2,251-3,393)	2,184 (1,714-2,483)	1,758 (1,362-1,973)	1,430 (1,170-1,595)	1,496 (1,202-1,659)	1,170 (921-1,316)	874 (729-980)	659 (535-752)	683 (557-772)	625 (511-702)	672 (573-743)	571 (476-618)	427 (338-464)	265 (207-287)	118 (92-130)
Unintentional suffocation	1,505 (839-1,874)	1,622 (922-2,064)	9,070 (6,055-10,851)	3,647 (2,444-5,232)	1,530 (1,239-2,276)	1,467 (1,257-2,088)	549 (475-709)	680 (563-902)	665 (563-878)	500 (396-654)	444 (362-558)	427 (345-564)	419 (350-519)	423 (356-526)	397 (343-493)	123 (93-143)	154 (114-184)	152 (121-187)	431 (286-509)	532 (348-660)	521 (324-608)	319 (196-372)	116 (70-135)
Other exposure to mechanical forces	502 (374-565)	187 (151-215)	2,959 (2,440-3,411)	6,440 (4,612-7,886)	3,938 (3,316-4,440)	2,941 (2,492-3,195)	6,645 (5,331-7,156)	8,577 (6,990-9,237)	8,500 (6,798-9,112)	9,758 (7,452-10,490)	11,779 (8,674-12,660)	10,616 (7,794-11,435)	9,444 (7,323-10,128)	8,626 (6,782-9,217)	6,878 (5,444-7,342)	4,645 (3,643-5,053)	4,353 (3,408-4,724)	3,627 (2,824-3,922)	2,894 (2,277-3,118)	2,220 (1,771-2,407)	1,516 (1,260-1,636)	834 (698-901)	352 (292-382)
Adverse effects of medical treatment	1,321 (946-1,538)	910 (661-1,070)	8,562 (5,364-10,841)	7,312 (3,425-10,832)	2,597 (1,886-3,160)	2,139 (1,730-2,453)	2,838 (2,256-3,179)	4,702 (3,726-5,233)	4,492 (3,578-4,937)	4,264 (3,355-4,657)	4,710 (3,726-5,171)	5,052 (3,993-5,584)	5,723 (4,608-6,276)	5,070 (4,193-5,558)	5,788 (4,733-6,381)	7,243 (5,830-8,078)	8,913 (7,064-9,889)	8,821 (7,297-9,891)	8,706 (7,253-9,778)	7,521 (6,197-8,722)	5,356 (4,545-6,422)	2,972 (2,540-3,813)	1,204 (1,037-1,660)
Animal contact	380 (291-443)	364 (282-433)	3,209 (2,165-3,873)	7,687 (5,580-9,780)	6,408 (4,597-7,696)	6,449 (4,551-7,683)	5,348 (3,800-6,227)	5,114 (3,722-5,875)	4,846 (3,597-5,573)	5,192 (3,664-6,051)	4,801 (3,581-5,491)	5,426 (3,981-6,196)	6,086 (4,620-6,927)	5,842 (4,135-6,708)	5,210 (3,984-5,861)	4,983 (3,526-5,785)	5,394 (3,746-6,243)	4,884 (3,311-5,713)	3,407 (2,380-3,939)	2,178 (1,604-2,514)	1,023 (763-1,174)	354 (273-399)	120 (98-133)
Venomous animal contact	313 (230-379)	299 (223-364)	2,690 (1,694-3,350)	6,337 (4,349-8,003)	5,546 (3,779-6,802)	5,831 (4,030-7,074)	4,629 (3,101-5,536)	4,382 (3,031-5,179)	4,161 (2,952-4,924)	4,650 (3,143-5,523)	4,239 (3,042-4,944)	4,824 (3,420-5,643)	5,274 (3,871-6,150)	5,001 (3,365-5,870)	4,406 (3,248-5,089)	4,355 (2,969-5,140)	4,748 (3,177-5,573)	4,262 (2,766-5,072)	2,839 (1,885-3,372)	1,813 (1,295-2,118)	809 (583-948)	254 (183-293)	75 (58-85)
Non-venomous animal contact	67 (49-98)	65 (48-98)	519 (341-906)	1,350 (674-2,786)	862 (605-1,308)	618 (467-854)	719 (559-967)	732 (565-1,008)	685 (516-950)	543 (423-766)	563 (442-755)	602 (471-823)	812 (633-1,083)	841 (658-1,114)	804 (622-1,099)	628 (493-787)	646 (510-823)	621 (469-812)	568 (443-709)	365 (271-440)	214 (159-258)	100 (77-119)	45 (36-52)
Foreign body	1,433 (966-1,705)	1,349 (953-1,618)	16,558 (11,752-21,801)	9,039 (5,857-14,189)	3,102 (2,497-3,847)	2,394 (2,022-2,762)	1,923 (1,636-2,166)	2,774 (2,365-3,162)	2,828 (2,377-3,271)	3,401 (2,910-3,855)	3,736 (3,162-4,396)	4,348 (3,732-5,036)	4,648 (3,992-5,461)	4,960 (4,253-5,751)	4,886 (4,151-5,623)	3,927 (3,298-4,449)	4,495 (3,671-5,002)	4,463 (3,552-4,926)	5,043 (3,855-5,709)	5,422 (3,983-6,327)	4,462 (3,075-5,351)	2,917 (1,922-3,548)	1,525 (931-1,893)
Pulmonary aspiration and foreign body in airway	1,370 (911-1,628)	1,312 (919-1,571)	15,881 (11,084-21,073)	7,982 (5,107-12,630)	2,525 (2,007-3,171)	1,954 (1,657-2,322)	1,452 (1,246-1,695)	2,132 (1,821-2,567)	2,227 (1,877-2,671)	2,614 (2,206-3,080)	2,893 (2,437-3,558)	3,542 (2,967-4,228)	4,209 (3,547-5,020)	4,513 (3,815-5,346)	4,482 (3,781-5,210)	3,593 (2,974-4,156)	4,208 (3,428-4,728)	4,149 (3,291-4,600)	4,689 (3,556-5,327)	5,089 (3,672-5,959)	4,205 (2,835-5,068)	2,771 (1,796-3,389)	1,464 (878-1,826)
Foreign body in other body part	64 (39-83)	38 (24-48)	677 (439-989)	1,057 (586-1,816)	577 (390-769)	440 (290-543)	471 (314-602)	642 (436-809)	601 (414-737)	787 (547-972)	843 (601-1,030)	806 (599-978)	439 (328-528)	447 (337-552)	404 (312-502)	334 (253-437)	287 (204-342)	314 (227-364)	354 (249-410)	333 (233-387)	257 (172-302)	146 (96-168)	62 (41-72)
Environmental heat and cold exposure	331 (155-413)	212 (92-271)	1,932 (925-2,449)	1,759 (890-2,365)	765 (474-918)	588 (367-692)	1,286 (781-1,495)	1,637 (1,022-1,917)	2,015 (1,307-2,420)	3,026 (1,909-3,719)	3,632 (2,495-4,678)	4,895 (3,210-6,495)	6,379 (4,223-9,133)	7,059 (4,768-10,431)	6,222 (4,245-9,046)	4,086 (2,701-5,452)	4,570 (3,081-6,139)	3,679 (2,381-4,433)	3,449 (2,166-4,143)	2,671 (1,836-3,253)	1,853 (1,364-2,334)	1,023 (754-1,440)	390 (282-594)
Other unintentional injuries	406 (364-450)	148 (130-171)	1,616 (1,388-1,848)	4,909 (3,683-5,839)	3,501 (3,132-3,880)	4,377 (3,932-4,741)	8,661 (7,627-9,331)	11,016 (10,072-11,825)	10,866 (10,149-11,627)	11,512 (10,108-12,192)	12,023 (10,019-12,804)	9,595 (7,714-10,214)	8,490 (6,782-9,036)	7,018 (5,571-7,429)	5,268 (4,343-5,590)	3,345 (2,872-3,569)	3,144 (2,613-3,368)	2,017 (1,780-2,151)	1,597 (1,388-1,706)	1,225 (1,044-1,319)	757 (651-818)	374 (323-406)	144 (126-155)
Self-harm and interpersonal violence	1,609 (1,300-1,848)	430 (351-496)	3,751 (3,190-4,431)	6,887 (5,798-8,082)	5,203 (4,305-5,978)	17,953 (16,007-19,214)	102,243 (93,687-107,576)	152,228 (139,017-159,188)	141,262 (127,560-149,121)	126,541 (114,522-133,730)	115,630 (104,823-122,568)	104,348 (94,485-112,028)	94,152 (85,037-101,424)	85,752 (77,254-91,995)	69,402 (61,917-74,011)	48,811 (43,038-51,339)	48,384 (42,852-50,951)	42,567 (36,740-44,849)	34,913 (30,199-36,785)	22,561 (19,598-23,798)	11,283 (9,939-11,909)	4,111 (3,680-4,330)	1,017 (940-1,078)

SR Table 5: Global deaths by cause and age group for both sexes (with 95% uncertainty intervals), 2006

Cause	Early Neonatal	Late Neonatal	Post Neonatal	1 to 4	5 to 9	10 to 14	15 to 19	20 to 24	25 to 29	30 to 34
Self-harm	-	-	-	-	-	9,957 (8,845-10,800)	63,110 (58,965-67,506)	91,070 (85,116-96,425)	85,900 (80,373-91,750)	78,070 (73,501-83,321)
Self-harm by firearm	-	-	-	-	-	467 (350-621)	4,738 (3,649-6,430)	7,836 (6,063-10,487)	7,053 (5,286-9,169)	6,090 (4,602-7,984)
Self-harm by other specified means	-	-	-	-	-	9,490 (8,434-10,302)	58,372 (54,289-62,515)	83,234 (76,873-88,288)	78,847 (72,756-84,320)	71,980 (66,734-76,793)
Interpersonal violence	1,609 (1,300-1,848)	430 (351-496)	3,751 (3,190-4,431)	6,887 (5,798-8,082)	5,203 (4,305-5,978)	7,996 (6,460-8,965)	39,134 (31,348-42,608)	61,159 (49,400-66,593)	55,362 (44,143-61,930)	48,471 (38,238-55,246)
Assault by firearm	152 (105-208)	47 (33-59)	496 (351-650)	932 (691-1,213)	876 (643-1,093)	2,124 (1,371-2,504)	18,405 (12,082-20,931)	28,862 (19,141-32,570)	25,546 (16,969-28,934)	19,816 (12,530-22,688)
Assault by sharp object	127 (96-169)	52 (34-63)	305 (217-392)	784 (599-982)	688 (538-852)	1,320 (991-1,566)	9,846 (7,392-12,354)	15,875 (12,113-20,869)	15,136 (11,633-19,597)	14,105 (10,747-17,709)
Assault by other means	1,330 (1,055-1,540)	331 (273-389)	2,949 (2,498-3,557)	5,171 (4,233-6,157)	3,639 (2,936-4,295)	4,552 (3,688-5,347)	10,883 (8,916-13,548)	16,422 (12,978-20,007)	14,680 (11,544-18,466)	14,549 (11,329-18,418)
Forces of nature, conflict and terrorism, and executions and police conflict	567 (300-840)	328 (182-477)	1,210 (729-1,695)	7,537 (4,396-10,747)	5,041 (3,068-7,030)	4,880 (3,069-6,674)	9,266 (5,591-12,991)	13,791 (8,355-19,268)	9,900 (6,100-13,730)	7,363 (4,629-10,155)
Forces of nature, conflict and terrorism, and state actor violence	9 (6-12)	25 (16-32)	202 (135-266)	977 (655-1,289)	990 (671-1,308)	1,228 (825-1,624)	1,281 (877-1,689)	1,191 (811-1,572)	1,086 (737-1,433)	990 (667-1,304)
Conflict and terrorism	558 (292-829)	304 (159-451)	981 (511-1,459)	6,487 (3,372-9,650)	3,980 (2,062-5,929)	3,560 (1,844-5,306)	7,547 (3,925-11,212)	11,652 (6,268-17,099)	8,011 (4,252-11,799)	5,660 (2,981-8,350)
Executions and police conflict	-	-	27 (21-45)	74 (54-129)	72 (64-84)	93 (80-110)	438 (209-566)	948 (455-1,191)	803 (364-989)	712 (405-823)

Cause	35 to 39	40 to 44	45 to 49	50 to 54	55 to 59	60 to 64	65 to 69	70 to 74	75 to 79	80 to 84	85 to 89	90 to 94	95 plus
Self-harm	75,597 (68,170-80,603)	70,682 (64,995-76,614)	67,167 (60,911-72,269)	64,526 (58,326-69,578)	54,447 (48,446-58,526)	39,512 (34,900-41,677)	40,091 (34,721-42,632)	36,204 (30,951-38,115)	30,038 (25,575-31,659)	21,034 (18,058-22,179)	10,627 (9,234-11,245)	3,841 (3,403-4,036)	894 (805-942)
Self-harm by firearm	5,419 (4,310-7,011)	5,299 (4,359-6,984)	5,325 (4,275-7,152)	4,891 (3,995-6,541)	4,058 (3,283-5,324)	2,956 (2,372-3,839)	2,665 (2,115-3,482)	2,417 (1,913-3,115)	2,259 (1,776-2,809)	1,779 (1,406-2,217)	995 (770-1,219)	406 (326-508)	107 (92-128)
Self-harm by other specified means	70,178 (62,916-75,194)	65,384 (59,780-70,374)	61,841 (55,573-66,495)	59,635 (53,622-64,393)	50,390 (44,618-53,514)	36,556 (31,746-38,504)	37,426 (32,432-39,389)	33,787 (28,825-35,607)	27,779 (23,433-29,290)	19,255 (16,407-20,368)	9,632 (8,271-10,165)	3,435 (3,038-3,612)	787 (708-830)
Interpersonal violence	40,033 (31,497-45,364)	33,665 (25,706-39,741)	26,985 (20,487-32,411)	21,226 (15,946-25,841)	14,955 (11,059-17,832)	9,300 (7,199-11,015)	8,292 (6,390-9,871)	6,363 (4,939-7,426)	4,875 (3,840-5,778)	1,527 (1,288-1,949)	656 (563-839)	270 (233-349)	123 (101-168)
Assault by firearm	16,048 (10,451-18,641)	12,331 (7,796-15,272)	8,702 (5,388-11,123)	6,414 (4,004-8,475)	4,215 (2,866-5,510)	2,638 (1,778-3,387)	1,855 (1,250-2,453)	1,418 (970-1,837)	1,015 (711-1,326)	207 (136-287)	100 (64-133)	36 (24-50)	23 (14-30)
Assault by sharp object	11,691 (8,469-14,774)	10,207 (7,513-13,210)	8,275 (5,827-10,773)	6,277 (4,364-8,213)	4,182 (2,795-5,671)	2,731 (1,907-3,465)	2,219 (1,526-2,876)	1,715 (1,213-2,090)	1,225 (866-1,515)	423 (336-559)	170 (135-233)	82 (67-114)	32 (25-49)
Assault by other means	12,295 (9,554-15,744)	11,128 (8,474-14,017)	10,008 (7,367-12,593)	8,535 (6,097-10,600)	6,558 (4,772-8,045)	3,931 (2,972-4,757)	4,219 (3,223-5,120)	3,230 (2,455-3,817)	2,635 (2,034-3,101)	897 (742-1,162)	386 (325-509)	152 (129-200)	68 (58-96)
Forces of nature, conflict and terrorism, and executions and police conflict	5,344 (3,498-7,175)	3,586 (2,491-4,630)	2,593 (1,864-3,298)	2,254 (1,577-2,922)	1,714 (1,208-2,206)	1,432 (1,000-1,845)	1,265 (876-1,633)	1,048 (749-1,355)	801 (578-1,034)	598 (432-764)	322 (227-419)	120 (87-160)	61 (43-79)
Forces of nature, conflict and terrorism, and state actor violence	926 (614-1,215)	842 (562-1,098)	761 (512-991)	712 (478-930)	585 (394-768)	511 (342-673)	459 (310-602)	411 (276-543)	325 (218-427)	225 (152-295)	111 (74-146)	38 (26-50)	10 (7-13)
Conflict and terrorism	3,809 (2,045-5,583)	2,280 (1,305-3,253)	1,555 (932-2,169)	1,351 (766-1,936)	1,015 (604-1,420)	842 (485-1,193)	730 (418-1,039)	554 (317-789)	415 (241-588)	297 (172-423)	162 (94-231)	55 (32-77)	45 (28-61)
Executions and police conflict	610 (347-705)	464 (238-544)	277 (174-318)	191 (121-217)	114 (84-136)	79 (48-95)	75 (39-100)	83 (32-129)	61 (23-105)	75 (22-135)	49 (11-85)	27 (8-57)	6 (3-9)

SR Table 5: Global deaths by cause and age group for both sexes (with 95% uncertainty intervals), 2010

Cause	Early Neonatal	Late Neonatal	Post Neonatal	1 to 4	5 to 9	10 to 14	15 to 19	20 to 24	25 to 29	30 to 34	35 to 39	40 to 44	45 to 49	50 to 54	55 to 59	60 to 64	65 to 69	70 to 74	75 to 79	80 to 84	85 to 89	90 to 94	95 plus
All causes	2,141,045 (2,074,798-2,208,352)	625,439 (607,191-644,266)	2,002,554 (1,941,497-2,066,502)	1,861,921 (1,781,422-1,947,490)	529,438 (511,263-549,217)	435,375 (414,433-458,714)	663,202 (641,175-686,993)	917,496 (894,321-942,768)	1,065,485 (1,038,576-1,097,482)	1,210,601 (1,185,696-1,238,052)	1,423,972 (1,397,798-1,450,428)	1,681,553 (1,659,466-1,703,105)	2,037,013 (2,010,474-2,063,007)	2,529,143 (2,497,378-2,558,488)	3,165,652 (3,130,036-3,198,898)	3,631,906 (3,596,304-3,666,148)	4,085,960 (4,048,846-4,123,187)	5,184,367 (5,136,500-5,231,343)	5,485,418 (5,442,257-5,527,141)	5,493,549 (5,456,156-5,528,698)	4,172,927 (4,153,051-4,191,457)	2,090,675 (2,083,419-2,097,507)	869,340 (866,445-871,907)
Communicable, maternal, neonatal, and nutritional disorders	1,940,492 (1,869,581-2,010,162)	543,917 (525,090-562,709)	1,622,994 (1,558,691-1,690,573)	1,468,872 (1,387,491-1,561,799)	306,295 (290,790-321,894)	194,611 (183,427-206,404)	207,281 (196,088-222,090)	269,601 (256,384-286,650)	396,803 (373,250-425,309)	480,753 (458,846-505,908)	504,361 (482,109-528,222)	465,105 (451,489-482,112)	424,100 (406,780-446,296)	389,907 (372,501-414,874)	378,684 (355,842-413,813)	394,807 (365,344-444,749)	435,283 (393,962-501,115)	537,825 (481,200-625,544)	530,717 (471,106-618,254)	533,023 (475,070-618,833)	402,813 (357,957-459,911)	199,914 (178,426-226,306)	89,542 (79,872-99,426)
HIV/AIDS and tuberculosis	-	-	97,276 (91,633-103,517)	62,788 (58,445-67,393)	41,398 (39,471-43,428)	49,382 (46,810-52,084)	65,570 (61,238-69,978)	101,359 (93,504-111,300)	236,617 (215,007-261,094)	330,324 (308,954-351,553)	353,162 (332,817-373,282)	323,916 (313,682-333,230)	277,584 (265,417-291,234)	227,162 (218,847-234,443)	183,965 (176,182-191,461)	150,131 (142,617-156,961)	136,039 (128,675-143,626)	136,629 (127,622-145,116)	105,832 (98,502-112,698)	80,290 (73,525-86,029)	42,666 (38,984-45,812)	13,846 (12,716-14,971)	3,620 (3,305-3,982)
Tuberculosis	-	-	15,267 (12,927-18,465)	21,627 (18,243-25,180)	8,247 (7,190-9,424)	9,946 (8,909-11,082)	26,064 (24,119-28,236)	48,841 (45,632-51,953)	66,534 (62,931-70,532)	75,820 (71,908-79,967)	86,149 (81,399-90,814)	91,360 (86,703-95,700)	103,803 (98,470-109,301)	117,329 (111,643-123,111)	124,723 (118,503-131,489)	115,524 (108,750-121,972)	115,074 (107,728-122,400)	123,974 (115,160-132,253)	99,176 (91,627-106,144)	78,191 (71,376-83,979)	41,861 (38,177-45,018)	13,634 (12,511-14,764)	3,573 (3,258-3,934)
Drug-susceptible tuberculosis	-	-	14,232 (12,038-17,256)	20,094 (16,949-23,496)	7,606 (6,598-8,702)	9,126 (8,111-10,219)	23,852 (21,993-25,901)	44,565 (41,629-47,614)	60,600 (57,055-64,500)	68,835 (65,122-72,694)	78,038 (73,543-82,545)	82,534 (78,261-86,843)	93,550 (88,214-98,588)	105,805 (100,300-111,470)	112,755 (106,621-119,198)	104,863 (98,381-111,333)	104,993 (98,133-111,983)	112,984 (104,949-120,706)	90,617 (83,952-97,282)	71,462 (65,200-76,851)	38,375 (35,049-41,362)	12,537 (11,485-13,593)	3,299 (3,006-3,620)
Multidrug-resistant tuberculosis without extensive drug resistance	-	-	1,006 (789-1,263)	1,493 (1,153-1,898)	615 (484-764)	786 (635-963)	2,111 (1,742-2,539)	4,006 (3,313-4,756)	5,515 (4,634-6,479)	6,470 (5,477-7,565)	7,492 (6,417-8,773)	8,132 (6,885-9,514)	9,407 (7,999-11,079)	10,602 (8,882-12,518)	11,113 (9,285-13,201)	9,976 (8,329-11,970)	9,507 (7,911-11,418)	10,341 (8,588-12,406)	8,055 (6,643-9,714)	6,316 (5,174-7,673)	3,263 (2,671-3,969)	1,022 (828-1,257)	253 (203-318)
Extensively drug-resistant tuberculosis	-	-	29 (23-36)	41 (32-51)	25 (20-32)	34 (27-43)	101 (84-123)	269 (224-320)	420 (352-500)	515 (428-611)	620 (521-738)	694 (586-827)	847 (718-1,008)	922 (772-1,101)	856 (713-1,022)	686 (570-826)	573 (469-697)	649 (538-799)	504 (409-621)	413 (332-516)	224 (178-284)	75 (58-96)	21 (16-28)
HIV/AIDS	-	-	82,009 (76,920-87,416)	41,161 (38,564-43,942)	33,151 (31,673-34,775)	39,436 (37,125-41,957)	39,507 (35,624-43,574)	52,518 (44,726-62,216)	170,083 (148,023-194,526)	254,504 (233,309-275,838)	267,012 (246,322-287,679)	232,557 (222,825-241,311)	173,781 (162,217-186,245)	109,833 (104,502-115,255)	59,242 (55,484-62,904)	34,607 (32,148-37,200)	20,966 (19,366-22,680)	12,655 (11,594-13,764)	6,657 (6,078-7,275)	2,099 (1,874-2,344)	805 (719-898)	212 (190-235)	48 (44-52)
Drug-susceptible HIV/AIDS - Tuberculosis	-	-	22,964 (16,965-29,006)	12,272 (9,429-14,948)	9,448 (7,180-11,705)	10,694 (7,738-13,438)	11,234 (8,214-13,998)	13,661 (9,909-17,785)	36,194 (23,686-49,813)	47,124 (30,288-65,733)	48,528 (31,178-68,164)	42,839 (28,044-58,469)	36,807 (24,574-49,975)	27,199 (18,668-35,737)	16,013 (11,892-19,719)	9,764 (7,482-11,826)	6,036 (4,772-7,267)	3,792 (3,013-4,501)	2,008 (1,608-2,397)	682 (562-804)	257 (213-305)	66 (55-78)	12 (10-15)
Multidrug-resistant HIV/AIDS - Tuberculosis without extensive drug resistance	-	-	2,052 (1,303-2,996)	1,060 (688-1,564)	747 (482-1,069)	850 (530-1,245)	913 (567-1,348)	1,253 (791-1,857)	3,305 (1,959-5,063)	4,296 (2,481-6,583)	4,289 (2,504-6,600)	3,760 (2,245-5,681)	3,264 (1,979-4,904)	2,464 (1,503-3,593)	1,485 (944-2,094)	889 (575-1,256)	537 (344-755)	334 (220-467)	177 (117-251)	58 (37-88)	22 (14-34)	6 (4-9)	1 (1-2)
Extensively drug-resistant HIV/AIDS - Tuberculosis	-	-	31 (20-47)	14 (9-20)	10 (7-15)	11 (7-16)	13 (8-19)	35 (22-52)	122 (73-190)	197 (117-309)	186 (113-289)	145 (90-223)	110 (68-170)	73 (45-110)	42 (27-64)	22 (14-33)	12 (8-17)	7 (4-10)	4 (3-5)	1 (1-2)	1 (0-1)	0 (0-0)	0 (0-0)
HIV/AIDS resulting in other diseases	-	-	56,962 (49,191-65,411)	27,815 (24,449-31,818)	22,946 (20,430-25,795)	27,881 (24,431-31,671)	27,346 (23,039-32,428)	37,569 (30,466-46,917)	130,463 (107,272-158,224)	202,888 (175,781-233,009)	214,010 (186,268-242,694)	185,813 (167,243-206,282)	133,600 (115,718-152,146)	80,098 (70,316-90,947)	41,702 (37,194-47,647)	23,932 (21,189-27,215)	14,382 (12,670-16,188)	8,523 (7,509-9,658)	4,468 (3,932-5,093)	1,358 (1,195-1,564)	525 (464-603)	140 (124-160)	34 (31-38)
Diarrhoea, lower respiratory infections, and other common infectious diseases	180,597 (155,085-200,630)	171,679 (158,917-183,659)	1,039,818 (979,272-1,102,093)	770,399 (697,532-851,219)	172,705 (153,493-194,801)	99,118 (86,128-115,224)	82,416 (70,343-100,015)	78,665 (67,109-97,996)	74,585 (64,447-92,029)	72,470 (62,175-89,085)	78,042 (67,576-97,991)	81,016 (69,724-99,039)	99,048 (85,219-122,608)	124,377 (106,849-155,932)	156,077 (132,266-197,230)	203,565 (171,910-260,499)	254,875 (210,795-328,687)	356,739 (296,643-456,451)	381,242 (316,591-478,622)	414,277 (352,935-508,476)	328,905 (283,808-390,458)	168,918 (146,642-196,171)	77,586 (68,073-87,509)
Diarrhoeal diseases	22,936 (20,448-25,972)	44,375 (39,892-49,582)	361,200 (324,853-405,334)	273,236 (233,322-319,244)	41,418 (26,828-66,056)	22,245 (14,491-36,447)	25,152 (16,016-40,995)	27,801 (17,358-46,789)	27,177 (17,716-46,403)	26,696 (16,862-44,064)	28,878 (18,620-49,587)	29,057 (18,528-47,523)	35,797 (22,410-60,105)	45,576 (28,730-78,264)	61,221 (38,847-104,149)	85,450 (54,452-144,568)	113,868 (72,492-192,390)	165,740 (106,801-272,976)	170,319 (107,273-275,566)	180,023 (121,706-281,159)	129,456 (89,455-193,760)	55,852 (39,378-81,910)	17,783 (13,010-25,061)
Intestinal infectious diseases	-	-	1,528 (347-4,971)	25,748 (12,385-46,673)	43,966 (25,207-71,557)	29,411 (15,775-46,262)	18,355 (9,431-31,434)	11,169 (5,399-19,558)	8,087 (4,160-13,960)	6,073 (3,201-10,370)	4,927 (2,657-8,242)	4,165 (2,267-6,913)	3,766 (2,013-6,343)	3,030 (1,558-5,226)	2,254 (1,078-4,123)	1,837 (893-3,298)	1,778 (1,001-2,899)	1,594 (930-2,554)	1,211 (703-1,906)	722 (416-1,168)	328 (195-520)	97 (59-153)	26 (14-47)
Typhoid fever	-	-	197 (93-367)	22,594 (10,045-42,677)	38,422 (20,939-63,522)	24,822 (12,846-39,686)	15,841 (7,775-28,037)	9,320 (4,302-16,634)	6,291 (3,205-11,079)	4,497 (2,328-7,921)	3,651 (1,907-6,348)	3,063 (1,593-5,286)	2,698 (1,405-4,564)	2,185 (1,096-3,805)	1,655 (769-3,092)	1,404 (668-2,600)	1,430 (771-2,408)	1,310 (737-2,091)	978 (553-1,573)	584 (330-952)	250 (143-400)	71 (39-114)	15 (8-25)
Paratyphoid fever	-	-	29 (12-63)	2,311 (944-4,767)	5,444 (2,476-10,762)	4,551 (2,039-8,600)	2,480 (1,053-5,002)	1,811 (744-3,744)	1,767 (753-3,666)	1,534 (655-3,077)	1,231 (570-2,339)	1,062 (490-2,082)	1,017 (465-2,054)	824 (358-1,691)	577 (234-1,210)	406 (169-854)	316 (144-626)	248 (116-474)	176 (84-318)	103 (49-188)	43 (20-79)	12 (6-22)	2 (1-5)
Other intestinal infectious diseases	-	-	1,302 (160-4,758)	844 (109-3,296)	101 (13-492)	38 (5-152)	34 (4-138)	38 (5-155)	29 (4-103)	43 (6-167)	46 (7-175)	39 (7-129)	51 (7-171)	22 (3-75)	23 (3-93)	27 (4-98)	32 (4-145)	37 (6-136)	57 (9-224)	35 (6-116)	35 (6-135)	14 (3-42)	8 (2-29)
Lower respiratory infections	129,834 (110,593-146,105)	101,085 (91,190-110,159)	482,024 (438,116-523,969)	236,621 (204,366-268,315)	38,229 (31,001-43,534)	20,975 (17,126-23,684)	17,900 (15,121-20,423)	21,261 (17,790-23,792)	23,423 (19,873-26,431)	24,838 (21,193-27,317)	29,632 (24,949-32,859)	34,226 (28,945-38,076)	44,207 (38,085-49,906)	60,356 (52,353-66,013)	76,644 (65,426-84,905)	101,368 (85,119-110,031)	123,262 (100,588-134,147)	172,581 (143,499-188,535)	195,812 (163,297-215,655)	220,403 (186,429-244,292)	191,423 (165,005-215,399)	110,015 (96,271-124,617)	58,734 (50,393-66,356)
Upper respiratory infections	79 (52-116)	27 (17-43)	658 (445-961)	498 (318-801)	39 (25-60)	30 (20-44)	70 (51-97)	72 (53-95)	77 (57-103)	76 (57-101)	89 (66-117)	93 (70-122)	111 (86-142)	115 (89-149)	128 (98-165)	131 (102-169)	115 (90-146)	104 (82-133)	107 (83-133)	78 (62-98)	56 (43-71)	23 (18-29)	7 (5-9)
Otitis media	3 (2-5)	11 (7-19)	101 (60-184)	182 (106-307)	97 (59-160)	103 (65-172)	36 (24-61)	31 (21-50)	38 (25-67)	25 (17-42)	31 (21-49)	43 (27-77)	70 (45-122)	77 (50-131)	67 (45-106)	56 (39-82)	84 (54-135)	76 (53-121)	64 (48-90)	60 (43-86)	38 (26-49)	17 (11-23)	6 (4-8)
Meningitis	10,312 (7,155-13,598)	12,701 (10,118-16,908)	80,934 (66,425-102,788)	68,141 (54,129-88,697)	18,913 (14,195-25,050)	13,806 (10,578-18,444)	13,003 (10,016-17,818)	11,747 (8,914-15,883)	10,176 (7,644-14,116)	9,262 (6,905-13,207)	9,104 (6,783-12,766)	7,942 (6,044-11,255)	9,154 (7,229-13,254)	9,130 (7,245-13,007)	9,270 (7,365-13,181)	8,598 (6,950-12,742)	8,811 (7,202-13,109)	8,843 (7,313-12,693)	6,336 (5,319-9,271)	6,150 (5,054-8,524)	3,292 (2,740-4,638)	1,165 (965-1,608)	343 (296-447)
Pneumococcal meningitis	500 (317-721)	597 (423-855)	3,943 (2,844-5,353)	3,650 (2,401-5,330)	1,060 (716-1,559)	754 (496-1,173)	695 (445-1,066)	617 (383-966)	617 (389-978)	710 (450-1,079)	848 (535-1,363)	849 (504-1,391)	1,046 (628-1,699)	1,034 (685-1,637)	1,072 (736-1,623)	1,054 (735-1,614)	1,051 (717-1,625)	1,038 (698-1,617)	749 (526-1,147)	670 (463-978)	352 (258-499)	130 (99-183)	43 (31-61)
H influenzae type B meningitis	1,365 (912-1,864)	1,619 (1,232-2,205)	9,879 (7,504-13,078)	7,596 (5,523-10,288)	1,705 (1,180-2,396)	1,027 (721-1,479)	856 (592-1,266)	767 (532-1,164)	716 (499-1,071)	688 (463-1,048)	694 (488-1,033)	603 (421-923)	699 (483-1,118)	688 (489-1,053)	694 (490-1,041)	659 (468-1,042)	646 (453-1,004)	625 (456-940)	455 (331-708)	442 (324-628)	236 (177-341)	81 (59-118)	23 (15-35)
Meningococcal infection	4,713 (3,211-6,365)	5,810 (4,584-7,693)	36,934 (29,733-47,311)	33,104 (25,424-44,018)	9,873 (7,241-13,544)	7,077 (5,305-9,689)	6,724 (5,033-9,538)	6,012 (4,373-8,430)	4,952 (3,513-7,194)	4,188 (3,026-6,172)	3,981 (2,961-5,825)	3,489 (2,587-5,271)	3,945 (3,021-5,957)	3,989 (3,070-5,929)	4,027 (3,056-6,004)	3,760 (2,867-5,829)	3,785 (2,900-5,821)	3,609 (2,785-5,461)	2,704 (2,077-4,218)	2,563 (1,979-3,709)	1,448 (1,144-2,076)	527 (412-753)	148 (114-204)
Other meningitis	3,735 (2,566-4,942)	4,677 (3,515-6,642)	30,179 (23,454-39,374)	23,791 (17,965-32,061)	6,276 (4,607-8,493)	4,947 (3,703-6,506)	4,727 (3,565-6,347)	4,351 (3,268-5,958)	3,890 (2,911-5,183)	3,676 (2,663-5,316)	3,582 (2,616-5,045)	3,002 (2,215-4,208)	3,464 (2,680-4,971)	3,419 (2,662-4,944)	3,478 (2,680-4,969)	3,126 (2,359-4,563)	3,329 (2,559-4,780)	3,571 (2,783-4,932)	2,428 (1,906-3,434)	2,475 (1,970-3,462)	1,257 (1,003-1,791)	427 (339-581)	130 (101-173)
Encephalitis	716 (538-855)	1,179 (988-1,461)	13,405 (11,520-16,691)	14,922 (11,980-20,621)	5,874 (4,740-8,124)	4,737 (3,849-6,621)	3,696 (3,126-5,008)	3,703 (3,114-5,164)	3,223 (2,741-4,453)	3,298 (2,814-4,535)	3,281 (2,772-4,612)	3,192 (2,690-4,567)	3,457 (2,854-5,104)	3,507 (2,871-5,171)	4,156 (3,361-6,002)	4,485 (3,658-6,603)	5,014 (4,095-7,285)	5,812 (4,805-8,149)	5,415 (4,629-7,088)	4,871 (4,064-6,412)	2,746 (2,320-3,529)	884 (733-1,156)	253 (215-328)
Diphtheria	-	-	479 (302-760)	976 (555-1,692)	171 (104-276)	81 (54-117)	37 (26-53)	24 (17-34)	22 (16-31)	19 (13-27)	28 (20-39)	28 (21-39)	34 (25-45)	39 (29-52)	30 (23-40)	-	-	-	-	-	-	-	-
Whooping cough	-	-	44,961 (21,405-80,039)	43,896 (20,650-76,742)	4,520 (2,096-8,513)	928 (420-1,791)	363 (167-699)	264 (121-498)	155 (68-302)	113 (50-221)	82 (37-158)	87 (39-170)	74 (33-146)	65 (29-131)	78 (35-153)	-	-	-	-	-	-	-	-
Tetanus	16,639 (8,782-22,297)	12,044 (6,801-16,395)	4,783 (2,702-6,978)	2,239 (1,241-3,597)	1,165 (601-1,900)	1,214 (644-1,985)	1,655 (837-2,542)	1,394 (732-2,043)	1,436 (860-2,030)	1,480 (912-2,055)	1,560 (913-2,150)	1,901 (1,112-2,555)	2,103 (1,222-2,870)	2,259 (1,289-3,097)	1,980 (1,142-2,876)	1,432 (838-2,030)	1,562 (987-2,423)	1,599 (916-2,152)	1,343 (736-2,045)	964 (487-1,510)	378 (192-505)	119 (62-163)	33 (17-44)
Measles	-	-	47,562 (18,019-103,179)	101,248 (42,591-202,067)	17,162 (6,782-35,508)	5,200 (1,894-11,611)	1,971 (687-4,601)	981 (337-2,324)	499 (174-1,188)	300 (104-723)	184 (64-437)	125 (44-300)	88 (31-211)	66 (23-158)	63 (22-151)	-	-	-	-	-	-	-	-
Varicella and herpes zoster	78 (64-94)	257 (213-305)	2,182 (1,820-2,613)	2,692 (2,178-3,285)	1,150 (940-1,392)	390 (319-468)	177 (149-216)	219 (184-264)	270 (227-321)	289 (246-342)	245 (207-287)	157 (133-183)	186 (158-218)	156 (132-184)	186 (156-221)	209 (177-246)	382 (322-453)	389 (325-461)	636 (540-753)	1,006 (832-1,208)	1,187 (965-1,432)	747 (585-925)	400 (272-567)
Neglected tropical diseases and malaria	1,121 (710-1,471)	22,491 (16,951-29,311)	218,571 (173,637-271,435)	455,219 (370,884-543,341)	66,008 (52,487-79,452)	34,096 (26,775-41,538)	19,208 (15,498-23,346)	20,106 (16,502-23,987)	15,900 (13,021-18,974)	14,590 (11,957-17,555)	15,814 (12,879-18,806)	15,077 (12,525-18,180)	14,915 (12,086-17,756)	15,148 (12,611-17,942)	15,821 (13,140-18,782)	18,043 (14,931-21,924)	17,633 (14,600-21,441)	15,435 (12,618-18,936)	12,249 (10,119-14,914)	7,226 (6,007-8,674)	4,851 (3,948-5,956)	1,184 (933-1,436)	499 (368-774)
Malaria	-	21,670 (16,113-28,545)	206,444 (161,591-259,779)	429,338 (344,752-519,426)	53,869 (40,809-67,514)	26,538 (19,984-34,125)	12,292 (9,627-15,700)	12,899 (10,057-16,570)	10,021 (7,827-12,779)	9,137 (7,070-11,769)	9,848 (7,555-12,659)	9,171 (7,142-11,728)	8,383 (6,527-10,807)	8,673 (6,681-11,247)	8,965 (6,972-11,768)	11,862 (9,065-15,649)	11,303 (8,502-14,999)	10,024 (7,533-13,376)	7,793 (5,791-10,498)	4,339 (3,271-5,718)	3,136 (2,337-4,213)	597 (434-778)	335 (215-611)
Chagas disease	-	-	2 (2-2)	1 (1-2)	2 (1-2)	4 (4-4)	10 (8-13)	22 (19-27)	40 (35-47)	83 (74-94)	147 (133-164)	229 (209-251)	346 (320-377)	502 (464-547)	666 (603-743)	856 (787-925)	915 (851-984)	1,020 (953-1,093)	791 (723-861)	660 (611-719)	417 (382-464)	180 (167-195)	45 (41-49)
Leishmaniasis	-	-	2,457 (1,299-4,422)	3,518 (1,728-6,131)	1,628 (853-2,926)	1,121 (598-1,935)	1,467 (788-2,486)	1,715 (930-2,995)	1,453 (771-2,465)	1,303 (701-2,220)	1,529 (811-2,689)	1,521 (798-2,641)	1,459 (776-2,521)	1,473 (799-2,498)	1,177 (621-2,086)	1,009 (541-1,780)	1,149 (616-1,984)	1,172 (629-2,112)	952 (499-1,643)	544 (289-941)	351 (179-586)	67 (35-114)	24 (9-45)
Visceral leishmaniasis	-	-	2,457 (1,299-4,422)	3,518 (1,728-6,131)	1,628 (853-2,926)	1,121 (598-1,935)	1,467 (788-2,486)	1,715 (930-2,995)	1,453 (771-2,465)	1,303 (701-2,220)	1,529 (811-2,689)	1,521 (798-2,641)	1,459 (776-2,521)	1,473 (799-2,498)	1,177 (621-2,086)	1,009 (541-1,780)	1,149 (616-1,984)	1,172 (629-2,112)	952 (499-1,643)	544 (289-941)	351 (179-586)	67 (35-114)	24 (9-45)
African trypanosomiasis	-	-	-	167 (42-510)	460 (127-1,276)	550 (168-1,407)	647 (171-1,714)	643 (179-1,847)	556 (152-1,528)	480 (129-1,351)	420 (113-1,277)	351 (92-934)	329 (80-1,043)	279 (74-883)	248 (59-804)	212 (49-746)	161 (39-519)	111 (27-336)	68 (16-217)	33 (8-105)	12 (3-45)	3 (1-12)	1 (0-3)
Schistosomiasis	-	-	0 (0-0)	463 (298-710)	245 (172-347)	342 (243-459)	323 (243-418)	433 (335-555)	504 (386-643)	557 (438-706)	658 (522-818)	731 (581-927)	818 (649-1,020)	983 (778-1,228)	1,193 (960-1,471)	1,217 (967-1,520)	1,243 (995-1,553)	1,124 (898-1,396)	866 (692-1,063)	479 (379-599)	217 (175-269)	64 (49-82)	11 (7-16)
Cysticercosis	0 (0-0)	0 (0-0)	0 (0-0)	95 (62-140)	60 (44-81)	48 (37-61)	43 (34-56)	63 (50-79)	81 (66-101)	91 (74-112)	78 (64-95)	73 (60-88)	71 (58-86)	86 (73-103)	90 (76-107)	63 (52-76)	56 (47-68)	36 (30-44)	25 (20-32)	14 (11-18)	6 (5-8)	3 (2-6)	1 (1-1)
Cystic echinococcosis	-	-	-	231 (155-345)	98 (68-141)	63 (43-92)	30 (21-42)	40 (28-56)	60 (44-81)	62 (45-81)	73 (54-96)	65 (49-85)	74 (55-97)	79 (61-99)	90 (69-114)	88 (69-111)	81 (62-102)	75 (60-94)	62 (49-78)	45 (35-56)	30 (24-38)	10 (7-14)	3 (2-4)
Dengue	-	-	2,552 (925-3,731)	4,570 (1,924-6,377)	2,980 (1,317-4,298)	1,932 (726-2,795)	1,836 (530-2,711)	1,802 (419-2,693)	1,634 (359-2,350)	1,295 (283-1,906)	1,419 (293-2,081)	1,285 (276-1,930)	1,476 (315-2,178)	1,467 (312-2,166)	1,722 (353-2,528)	1,498 (304-2,240)	1,480 (348-2,288)	786 (191-1,322)	673 (170-1,100)	604 (145-993)	398 (119-668)	167 (51-295)	39 (16-65)
Yellow fever	-	3 (1-8)	56 (11-175)	519 (108-1,549)	1,036 (210-3,065)	912 (184-2,731)	816 (157-2,575)	681 (136-1,993)	523 (107-1,591)	399 (83-1,239)	310 (63-949)	236 (48-733)	188 (36-561)	153 (30-486)	120 (24-362)	89 (18-287)	64 (13-197)	42 (8-124)	24 (5-78)	11 (2-32)	4 (1-12)	1 (0-3)	0 (0-0)
Rabies	-	-	303 (221-652)	2,073 (1,106-3,260)	3,083 (1,402-5,106)	1,667 (849-2,409)	1,210 (695-1,719)	1,271 (657-1,785)	528 (322-757)	754 (409-1,070)	880 (491-1,272)	967 (576-1,392)	1,294 (727-1,794)	936 (615-1,641)	988 (445-1,340)	617 (340-769)	630 (300-905)	461 (309-753)	502 (356-835)	162 (110-248)	88 (53-140)	16 (9-25)	8 (4-12)
Intestinal nematode infections	-	-	470 (372-591)	4,877 (3,730-6,314)	729 (587-889)	155 (125-190)	53 (42-64)	36 (28-45)	29 (23-37)	37 (30-46)	35 (28-44)	26 (21-33)	30 (24-37)	45 (37-54)	39 (31-48)	43 (35-53)	32 (25-40)	49 (40-60)	40 (32-49)	26 (21-32)	10 (8-13)	7 (5-9)	2 (2-3)
Ascariasis	-	-	470 (372-591)	4,877 (3,730-6,314)	729 (587-889)	155 (125-190)	53 (42-64)	36 (28-45)	29 (23-37)	37 (30-46)	35 (28-44)	26 (21-33)	30 (24-37)	45 (37-54)	39 (31-48)	43 (35-53)	32 (25-40)	49 (40-60)	40 (32-49)	26 (21-32)	10 (8-13)	7 (5-9)	2 (2-3)
Ebola	0 (0-0)	0 (0-0)	0 (0-0)	0 (0-0)	0 (0-0)	0 (0-0)	0 (0-0)	0 (0-0)	0 (0-0)	0 (0-0)	0 (0-0)	0 (0-0)	0 (0-0)	0 (0-0)	0 (0-0)	0 (0-0)	0 (0-0)	0 (0-0)	0 (0-0)	0 (0-0)	0 (0-0)	0 (0-0)	0 (0-0)
Zika virus	0 (0-0)	0 (0-0)	0 (0-0)	0 (0-0)	0 (0-0)	0 (0-0)	0 (0-0)	0 (0-0)	0 (0-0)	0 (0-0)	0 (0-0)	0 (0-0)	0 (0-0)	0 (0-0)	0 (0-0)	0 (0-0)	0 (0-0)	0 (0-0)	0 (0-0)	0 (0-0)	0 (0-0)	0 (0-0)	0 (0-0)
Other neglected tropical diseases	1,121 (710-1,471)	818 (554-1,113)	6,287 (3,977-8,250)	9,366 (5,292-17,199)	1,819 (1,063-3,039)	765 (493-1,143)	482 (302-722)	501 (336-703)	471 (329-642)	391 (265-502)	416 (299-514)	422 (302-530)	448 (320-548)	470 (333-584)	525 (378-649)	488 (355-641)	519 (377-671)	535 (382-703)	452 (324-593)	310 (229-419)	181 (133-258)	67 (50-94)	32 (24-50)
Maternal disorders	-	-	-	-	-	1,336 (1,098-1,611)	27,035 (24,175-29,741)	54,455 (49,858-59,071)	55,251 (50,786-59,282)	48,609 (44,960-52,139)	42,205 (39,024-45,392)	28,104 (25,815-30,419)	14,915 (13,318-16,642)	3,448 (2,765-4,333)	-	-	-	-	-	-	-	-	-
Maternal haemorrhage	-	-	-	-	-	251 (164-373)	7,505 (5,518-9,773)	18,134 (14,674-21,918)	18,852 (14,837-23,195)	16,654 (12,865-20,425)	14,782 (11,259-18,596)	9,128 (6,550-12,006)	3,897 (2,804-5,303)	685 (390-1,097)	-	-	-	-	-	-	-	-	-
Maternal sepsis and other pregnancy related infections	-	-	-	-	-	127 (80-187)	2,694 (1,815-3,911)	4,416 (2,927-6,366)	4,524 (3,164-6,239)	3,877 (2,637-5,480)	3,205 (2,182-4,621)	2,250 (1,429-3,473)	1,366 (874-2,153)	285 (159-483)	-	-	-	-	-	-	-	-	-
Maternal hypertensive disorders	-	-	-	-	-	280 (170-406)	4,361 (2,897-5,964)	7,651 (5,449-10,118)	7,113 (4,862-9,853)	6,055 (4,038-8,640)	5,324 (3,645-7,487)	3,516 (2,183-5,235)	1,672 (1,060-2,430)	379 (196-658)	-	-	-	-	-	-	-	-	-
Maternal obstructed labour and uterine rupture	-	-	-	-	-	51 (26-89)	910 (450-1,538)	1,809 (1,043-2,840)	2,051 (1,181-3,279)	2,146 (1,233-3,257)	2,137 (1,280-3,507)	1,618 (897-2,647)	909 (492-1,525)	206 (86-387)	-	-	-	-	-	-	-	-	-
Maternal abortion, miscarriage, and ectopic pregnancy	-	-	-	-	-	123 (72-197)	2,303 (1,385-3,425)	4,167 (2,713-6,115)	4,112 (2,822-5,926)	3,744 (2,509-5,422)	3,472 (2,179-4,915)	2,759 (1,881-3,979)	1,901 (1,313-2,668)	366 (241-527)	-	-	-	-	-	-	-	-	-
Indirect maternal deaths	-	-	-	-	-	226 (143-331)	4,043 (2,658-5,925)	8,077 (5,700-11,110)	8,819 (5,986-12,167)	7,642 (4,758-10,884)	5,658 (3,757-8,002)	3,744 (2,309-5,626)	2,352 (1,443-3,499)	788 (445-1,222)	-	-	-	-	-	-	-	-	-
Late maternal deaths	-	-	-	-	-	30 (13-62)	493 (235-919)	930 (439-1,728)	973 (488-1,815)	859 (449-1,578)	738 (340-1,342)	495 (167-918)	279 (106-501)	77 (28-166)	-	-	-	-	-	-	-	-	-
Maternal deaths aggravated by HIV/AIDS	-	-	-	-	-	3 (2-4)	65 (37-92)	280 (171-381)	482 (311-632)	508 (322-667)	431 (272-576)	267 (164-367)	134 (83-185)	22 (13-33)	-	-	-	-	-	-	-	-	-
Other maternal disorders	-	-	-	-	-	246 (163-356)	4,661 (3,329-6,477)	8,989 (6,674-11,964)	8,326 (6,166-11,023)	7,124 (5,079-9,669)	6,458 (4,484-9,232)	4,327 (2,731-6,345)	2,405 (1,539-3,535)	639 (400-991)	-	-	-	-	-	-	-	-	-
Neonatal disorders	1,719,110 (1,651,610-1,786,624)	332,733 (316,816-349,743)	93,351 (84,184-103,280)	10,393 (9,183-11,673)	-	-	-	-	-	-	-	-	-	-	-	-	-	-	-	-	-	-	-
Neonatal preterm birth complications	659,962 (618,699-721,949)	95,380 (86,245-106,423)	32,164 (28,362-37,115)	2,608 (2,180-3,038)	-	-	-	-	-	-	-	-	-	-	-	-	-	-	-	-	-	-	-
Neonatal encephalopathy due to birth asphyxia and trauma	565,029 (516,271-610,824)	51,545 (45,313-58,391)	18,909 (15,317-23,086)	4,458 (3,855-5,056)	-	-	-	-	-	-	-	-	-	-	-	-	-	-	-	-	-	-	-
Neonatal sepsis and other neonatal infections	151,954 (118,179-202,814)	99,755 (84,151-121,110)	17,662 (13,803-21,944)	839 (426-1,292)	-	-	-	-	-	-	-	-	-	-	-	-	-	-	-	-	-	-	-
Hemolytic disease and other neonatal jaundice	46,924 (41,180-55,092)	23,039 (19,862-26,853)	4,760 (3,589-6,390)	246 (180-342)	-	-	-	-	-	-	-	-	-	-	-	-	-	-	-	-	-	-	-
Other neonatal disorders	295,241 (269,854-323,855)	63,014 (56,946-71,539)	19,855 (16,989-23,553)	2,242 (1,890-2,881)	-	-	-	-	-	-	-	-	-	-	-	-	-	-	-	-	-	-	-
Nutritional deficiencies	-	-	107,342 (96,998-120,297)	124,083 (102,995-149,885)	11,325 (9,615-13,442)	4,762 (4,182-5,599)	4,053 (3,602-4,967)	4,489 (3,956-5,456)	4,508 (4,036-5,480)	4,407 (3,973-5,288)	4,498 (4,042-5,606)	5,291 (4,799-6,454)	5,200 (4,655-6,541)	6,721 (6,002-8,345)	8,769 (7,971-10,626)	10,266 (9,272-12,579)	13,725 (12,439-16,603)	16,280 (14,807-19,159)	20,275 (18,686-23,293)	22,218 (20,735-24,881)	21,215 (19,906-22,886)	13,930 (13,200-14,740)	7,150 (6,770-7,530)
Protein-energy malnutrition	-	-	103,854 (93,708-116,407)	119,544 (98,427-144,511)	9,921 (8,382-11,948)	3,855 (3,331-4,634)	2,602 (2,259-3,280)	2,486 (2,125-3,090)	2,650 (2,301-3,289)	2,538 (2,214-3,132)	2,660 (2,308-3,464)	3,432 (3,068-4,246)	2,913 (2,549-3,733)	4,007 (3,505-5,101)	5,746 (5,142-6,899)	6,934 (6,190-8,569)	9,220 (8,254-11,228)	11,678 (10,638-13,771)	15,250 (14,074-17,470)	17,327 (16,135-19,037)	17,057 (15,782-18,184)	11,594 (10,806-12,278)	6,038 (5,330-6,448)
Iodine deficiency	-	-	341 (99-887)	308 (60-1,056)	92 (29-234)	26 (7-73)	65 (18-163)	78 (24-205)	86 (25-212)	84 (24-215)	81 (25-209)	67 (24-165)	62 (22-160)	90 (33-229)	112 (45-246)	108 (41-251)	148 (61-336)	164 (70-337)	153 (64-317)	114 (48-244)	62 (20-140)	20 (5-60)	12 (2-45)
Iron-deficiency anaemia	-	-	60 (37-115)	125 (77-238)	136 (74-225)	115 (72-171)	158 (112-205)	169 (134-211)	139 (109-180)	142 (114-172)	127 (104-168)	114 (96-189)	108 (91-186)	107 (84-222)	115 (89-230)	96 (72-185)	116 (83-245)	207 (143-390)	234 (157-476)	215 (149-406)	157 (72-254)	103 (47-146)	97 (48-138)
Other nutritional deficiencies	-	-	3,087 (2,334-3,856)	4,107 (2,314-6,085)	1,176 (946-1,457)	766 (632-921)	1,228 (1,029-1,531)	1,755 (1,477-2,190)	1,634 (1,394-2,031)	1,643 (1,386-2,027)	1,629 (1,360-2,042)	1,678 (1,394-2,183)	2,117 (1,728-2,806)	2,516 (2,059-3,319)	2,796 (2,288-3,599)	3,128 (2,618-3,974)	4,240 (3,594-5,414)	4,230 (3,511-5,403)	4,638 (3,831-6,047)	4,562 (3,819-5,806)	3,940 (3,302-4,969)	2,213 (1,824-3,006)	1,004 (815-1,721)
Other communicable, maternal, neonatal, and nutritional diseases	39,665 (24,081-60,170)	17,014 (11,403-24,627)	66,636 (45,662-93,741)	45,990 (33,180-62,865)	14,858 (11,076-20,324)	5,917 (5,209-6,615)	9,000 (8,232-9,756)	10,528 (9,756-11,189)	9,941 (9,275-10,581)	10,352 (9,643-11,014)	10,641 (9,917-11,270)	11,700 (10,998-12,470)	12,438 (11,594-13,250)	13,053 (12,287-13,780)	14,052 (13,264-14,774)	12,803 (11,999-13,501)	13,011 (12,060-13,756)	12,743 (11,789-13,526)	11,119 (10,228-11,761)	9,012 (8,159-9,673)	5,176 (4,630-5,521)	2,036 (1,754-2,183)	687 (588-737)
Sexually transmitted diseases excluding HIV	34,405 (19,483-54,347)	12,949 (7,159-20,326)	43,850 (24,542-70,236)	25,078 (13,717-40,613)	8,278 (4,591-13,387)	144 (115-174)	527 (434-635)	651 (547-764)	840 (689-952)	760 (627-870)	735 (606-855)	709 (611-792)	697 (622-801)	671 (536-756)	879 (673-993)	481 (402-558)	492 (403-554)	422 (367-504)	451 (395-527)	336 (292-374)	252 (219-276)	132 (114-144)	62 (53-67)
Syphilis	34,405 (19,483-54,347)	12,949 (7,159-20,326)	43,850 (24,542-70,236)	25,078 (13,717-40,613)	8,278 (4,591-13,387)	98 (78-118)	199 (164-231)	229 (189-262)	239 (194-275)	277 (222-325)	186 (141-218)	150 (129-187)	136 (120-171)	231 (186-281)	347 (273-421)	162 (140-210)	177 (154-209)	179 (158-245)	172 (155-222)	124 (110-153)	83 (71-97)	41 (33-46)	18 (14-20)
Chlamydial infection	-	-	-	-	-	10 (8-13)	57 (45-70)	123 (102-147)	123 (99-141)	60 (48-68)	98 (81-114)	116 (94-129)	108 (91-120)	82 (61-93)	104 (74-118)	65 (52-74)	38 (29-44)	39 (31-44)	47 (38-53)	50 (40-55)	39 (33-43)	20 (17-22)	9 (8-10)
Gonococcal infection	-	-	-	-	-	24 (19-30)	150 (121-184)	185 (155-220)	334 (271-381)	277 (226-316)	277 (228-321)	292 (242-326)	291 (247-324)	240 (180-272)	280 (204-316)	158 (127-179)	211 (158-239)	151 (120-169)	181 (149-204)	127 (102-140)	101 (85-110)	55 (47-60)	26 (22-28)
Other sexually transmitted diseases	-	-	-	-	-	12 (9-14)	121 (98-149)	113 (94-134)	144 (117-165)	145 (117-165)	174 (143-200)	151 (125-168)	162 (139-182)	117 (90-133)	148 (109-166)	97 (78-109)	66 (50-74)	53 (42-59)	50 (42-56)	36 (29-39)	29 (25-32)	16 (14-17)	9 (8-10)
Hepatitis	-	-	8,972 (8,007-9,993)	9,860 (8,197-11,412)	4,187 (3,538-4,903)	4,064 (3,552-4,619)	6,441 (5,829-7,091)	8,051 (7,437-8,613)	7,429 (6,904-8,007)	7,813 (7,279-8,389)	8,046 (7,450-8,608)	9,252 (8,603-9,980)	9,554 (8,826-10,312)	9,750 (9,116-10,425)	10,252 (9,609-10,875)	8,668 (8,153-9,211)	8,521 (7,891-9,115)	8,053 (7,490-8,616)	6,650 (6,131-7,128)	4,873 (4,390-5,301)	2,288 (2,068-2,486)	689 (628-741)	159 (146-171)
Acute hepatitis A	-	-	5,537 (4,095-6,993)	1,063 (668-1,564)	94 (59-136)	57 (36-80)	93 (59-142)	118 (68-184)	87 (47-142)	72 (38-119)	70 (37-113)	85 (46-143)	98 (51-159)	111 (62-184)	121 (69-195)	113 (65-182)	110 (62-175)	115 (66-185)	102 (60-159)	76 (46-117)	35 (22-53)	10 (6-15)	2 (1-4)
Hepatitis B	-	-	3,434 (2,206-4,874)	1,325 (772-2,041)	390 (216-632)	624 (343-986)	2,152 (1,517-2,958)	4,691 (3,637-5,819)	5,530 (4,657-6,429)	6,609 (5,856-7,392)	7,145 (6,438-7,875)	8,347 (7,493-9,128)	8,601 (7,751-9,404)	8,746 (7,896-9,496)	9,150 (8,244-9,917)	7,623 (6,872-8,294)	7,343 (6,550-8,080)	6,756 (5,903-7,564)	5,430 (4,607-6,136)	3,503 (2,775-4,156)	1,335 (919-1,681)	326 (200-458)	61 (31-94)
Hepatitis C	-	-	-	-	125 (78-186)	47 (30-70)	68 (43-99)	84 (50-132)	65 (36-107)	59 (33-96)	71 (36-123)	100 (54-165)	140 (72-237)	199 (107-327)	282 (154-451)	279 (152-468)	255 (140-403)	228 (120-379)	185 (105-309)	135 (75-218)	66 (41-101)	21 (13-32)	5 (3-9)
Acute hepatitis E	-	-	-	7,472 (5,978-8,938)	3,579 (2,989-4,220)	3,336 (2,827-3,888)	4,128 (3,342-4,880)	3,159 (2,080-4,172)	1,746 (1,037-2,538)	1,073 (613-1,670)	760 (393-1,269)	720 (375-1,273)	715 (368-1,205)	694 (369-1,153)	699 (339-1,284)	653 (301-1,203)	813 (427-1,342)	954 (478-1,650)	932 (408-1,638)	1,158 (650-1,774)	853 (522-1,256)	332 (209-467)	91 (58-123)
Other infectious diseases	5,259 (2,274-9,842)	4,065 (2,181-6,078)	13,815 (6,972-25,036)	11,051 (6,144-19,734)	2,393 (1,768-2,988)	1,709 (1,310-2,024)	2,032 (1,614-2,334)	1,826 (1,457-2,077)	1,672 (1,341-1,881)	1,779 (1,404-1,995)	1,861 (1,513-2,054)	1,740 (1,497-1,884)	2,187 (1,840-2,368)	2,632 (2,230-2,844)	2,921 (2,449-3,134)	3,653 (3,107-3,914)	3,999 (3,375-4,304)	4,268 (3,502-4,646)	4,018 (3,343-4,367)	3,803 (3,080-4,152)	2,636 (2,104-2,879)	1,215 (944-1,333)	465 (366-508)
Non-communicable diseases	188,103 (157,797-212,271)	73,531 (65,285-80,705)	290,900 (254,743-321,916)	182,004 (148,646-209,877)	85,353 (78,701-93,354)	95,103 (89,337-101,366)	160,238 (153,967-166,837)	223,683 (217,229-232,191)	280,107 (272,294-289,207)	389,702 (380,841-400,368)	585,235 (572,311-599,775)	897,197 (880,255-914,640)	1,309,932 (1,286,511-1,331,634)	1,859,882 (1,828,007-1,887,147)	2,521,588 (2,478,837-2,555,646)	3,015,766 (2,960,369-3,056,042)	3,451,883 (3,381,783-3,503,374)	4,442,299 (4,346,661-4,516,247)	4,763,503 (4,669,731-4,834,488)	4,791,188 (4,700,144-4,861,712)	3,643,654 (3,583,232-3,696,197)	1,829,163 (1,801,397-1,854,653)	754,337 (742,877-765,958)
Neoplasms	838 (741-882)	544 (504-575)	5,546 (5,239-5,991)	22,535 (20,814-25,169)	25,860 (23,875-27,763)	28,358 (26,685-30,000)	40,041 (37,971-41,616)	49,306 (47,149-50,854)	60,954 (58,384-62,725)	94,890 (91,940-97,416)	160,349 (156,221-164,059)	271,254 (265,611-276,665)	410,513 (402,491-417,820)	597,227 (586,974-606,533)	820,705 (806,843-833,325)	932,325 (915,510-945,294)	966,303 (948,706-979,556)	1,074,266 (1,054,169-1,089,752)	977,543 (954,981-995,519)	775,216 (753,179-793,543)	474,051 (458,658-487,949)	185,062 (178,509-191,076)	61,601 (59,140-63,618)
Lip and oral cavity cancer	-	-	-	-	-	-	395 (369-426)	798 (746-852)	1,335 (1,242-1,440)	2,293 (2,142-2,489)	4,019 (3,780-4,307)	7,205 (6,760-7,676)	11,188 (10,667-11,709)	15,832 (15,181-16,503)	19,452 (18,783-20,142)	19,700 (18,892-20,453)	17,425 (16,724-18,015)	16,116 (15,445-16,696)	13,003 (12,477-13,470)	10,440 (9,933-10,865)	6,706 (6,381-7,006)	2,905 (2,765-3,051)	1,015 (964-1,064)
Nasopharynx cancer	-	-	-	-	127 (114-139)	272 (245-299)	511 (479-546)	621 (584-657)	776 (729-832)	1,201 (1,137-1,274)	2,258 (2,134-2,384)	4,164 (3,971-4,384)	5,783 (5,515-6,063)	7,365 (7,050-7,710)	8,835 (8,493-9,204)	7,532 (7,231-7,852)	6,276 (6,013-6,557)	5,464 (5,254-5,693)	3,707 (3,564-3,854)	2,451 (2,352-2,552)	1,124 (1,079-1,170)	395 (380-413)	122 (117-128)
Other pharynx cancer	-	-	-	-	-	-	294 (264-323)	477 (433-523)	680 (618-748)	1,201 (1,109-1,296)	2,244 (2,089-2,416)	4,558 (4,252-4,872)	8,085 (7,663-8,506)	12,361 (11,771-12,964)	14,935 (14,291-15,609)	14,953 (14,213-15,712)	12,788 (12,094-13,490)	11,510 (10,867-12,091)	7,496 (7,041-7,857)	5,272 (4,891-5,600)	2,536 (2,344-2,699)	804 (751-849)	189 (179-198)
Oesophageal cancer	-	-	-	-	-	-	313 (293-336)	631 (599-666)	873 (824-932)	1,704 (1,625-1,805)	3,719 (3,564-3,878)	8,883 (8,547-9,236)	17,203 (16,670-17,777)	29,490 (28,667-30,280)	47,049 (45,627-48,389)	54,682 (53,122-56,181)	57,226 (55,529-59,046)	59,175 (57,493-60,883)	50,445 (48,784-51,937)	35,456 (34,283-36,657)	17,640 (17,071-18,209)	5,842 (5,641-6,059)	1,490 (1,432-1,544)
Stomach cancer	-	-	-	-	-	-	973 (923-1,031)	2,272 (2,174-2,397)	3,869 (3,723-4,058)	7,587 (7,343-7,899)	13,861 (13,423-14,364)	24,848 (24,187-25,564)	37,485 (36,528-38,465)	55,138 (53,846-56,458)	82,020 (79,913-84,262)	97,221 (94,779-99,711)	103,920 (101,471-106,520)	120,954 (118,068-124,005)	110,204 (107,275-112,962)	84,060 (81,448-86,384)	47,008 (45,459-48,522)	17,985 (17,324-18,644)	6,047 (5,797-6,266)
Colon and rectum cancer	-	-	-	-	-	-	1,068 (993-1,148)	2,502 (2,362-2,667)	3,727 (3,557-3,908)	6,627 (6,385-6,901)	10,666 (10,323-11,031)	17,103 (16,655-17,559)	28,440 (27,808-29,119)	42,574 (41,683-43,589)	61,732 (60,438-63,074)	75,564 (74,090-77,231)	83,073 (81,382-84,863)	102,981 (100,465-105,330)	104,755 (102,024-107,515)	92,995 (90,034-95,804)	64,751 (62,314-67,054)	28,086 (27,024-29,164)	10,469 (9,990-10,863)
Liver cancer	-	-	-	-	444 (404-500)	713 (654-791)	1,388 (1,289-1,524)	3,123 (2,874-3,460)	5,371 (4,928-5,968)	10,319 (9,713-11,050)	20,921 (19,949-22,047)	36,783 (35,159-38,606)	50,845 (48,849-53,021)	68,215 (65,396-71,015)	91,313 (87,494-95,059)	91,986 (88,350-95,050)	88,545 (85,353-91,588)	89,787 (85,912-92,908)	75,391 (72,461-77,972)	51,594 (49,202-53,738)	25,653 (24,467-26,770)	8,136 (7,753-8,542)	2,189 (2,077-2,305)
Liver cancer due to hepatitis B	-	-	-	-	119 (83-158)	270 (202-347)	640 (497-793)	1,769 (1,475-2,073)	3,322 (2,684-3,980)	6,424 (5,471-7,363)	12,644 (10,525-14,411)	21,493 (17,557-24,469)	27,779 (22,229-31,901)	34,343 (27,354-40,133)	43,202 (33,938-50,733)	39,372 (29,146-46,650)	34,930 (26,197-41,198)	32,543 (24,264-38,915)	25,232 (18,881-30,935)	15,657 (11,245-19,978)	7,115 (5,449-8,840)	2,133 (1,589-2,734)	502 (323-684)
Liver cancer due to hepatitis C	-	-	-	-	12 (9-15)	23 (17-30)	44 (33-59)	125 (95-162)	270 (181-395)	572 (424-742)	1,233 (925-1,635)	2,652 (2,087-3,393)	5,125 (3,982-6,416)	9,204 (7,309-11,210)	14,209 (11,776-17,203)	17,425 (14,616-20,949)	18,016 (15,300-21,213)	20,309 (17,559-23,607)	20,045 (17,461-23,211)	16,199 (14,063-18,560)	9,402 (8,397-10,424)	3,366 (2,996-3,767)	1,062 (917-1,204)
Liver cancer due to alcohol use	-	-	-	-	-	-	6 (3-9)	49 (31-69)	142 (84-221)	461 (324-641)	1,357 (893-1,955)	3,038 (2,162-4,173)	5,232 (3,575-7,352)	8,352 (5,849-11,225)	12,225 (8,438-16,868)	14,378 (10,449-19,211)	16,173 (12,540-20,036)	17,804 (14,010-22,052)	14,106 (10,982-17,583)	9,181 (6,916-11,639)	4,379 (3,360-5,482)	1,225 (891-1,581)	286 (176-411)
Liver cancer due to other causes	-	-	-	-	314 (268-365)	419 (346-493)	699 (562-843)	1,181 (965-1,415)	1,637 (1,269-2,154)	2,863 (2,261-3,619)	5,686 (4,570-7,266)	9,600 (7,807-12,324)	12,708 (10,293-16,077)	16,316 (13,212-20,534)	21,677 (17,638-27,417)	20,811 (17,132-26,050)	19,426 (16,079-23,501)	19,131 (16,058-23,302)	16,007 (13,524-19,253)	10,557 (8,772-12,711)	4,757 (4,054-5,532)	1,412 (1,166-1,685)	339 (259-429)
Gallbladder and biliary tract cancer	-	-	-	-	-	-	88 (82-97)	160 (143-177)	320 (283-353)	838 (739-930)	1,827 (1,615-2,015)	3,676 (3,235-4,004)	6,063 (5,346-6,533)	9,401 (8,345-10,096)	12,837 (11,483-13,834)	16,067 (14,624-17,148)	17,542 (15,990-18,818)	20,469 (18,849-21,976)	20,317 (18,865-21,544)	17,402 (16,247-18,296)	11,566 (10,832-12,152)	4,875 (4,602-5,136)	1,863 (1,762-1,958)
Pancreatic cancer	-	-	-	-	-	-	170 (163-178)	358 (345-372)	652 (631-679)	1,483 (1,435-1,538)	3,191 (3,104-3,289)	6,627 (6,453-6,821)	12,210 (11,937-12,493)	20,891 (20,478-21,335)	32,236 (31,597-32,898)	41,745 (40,998-42,570)	46,189 (45,351-47,093)	53,773 (52,580-54,970)	49,625 (48,340-50,852)	39,998 (38,656-41,174)	25,750 (24,786-26,662)	9,919 (9,554-10,281)	3,261 (3,103-3,394)
Larynx cancer	-	-	-	-	-	-	143 (134-154)	211 (200-224)	278 (260-299)	616 (573-663)	1,282 (1,209-1,364)	3,037 (2,887-3,204)	6,033 (5,772-6,303)	10,405 (10,022-10,784)	14,107 (13,635-14,582)	15,199 (14,677-15,702)	14,401 (13,933-14,886)	13,559 (13,129-13,997)	9,996 (9,689-10,299)	7,075 (6,812-7,329)	3,385 (3,256-3,513)	1,054 (1,016-1,094)	247 (236-256)
Tracheal, bronchus, and lung cancer	-	-	-	-	-	-	963 (914-1,019)	1,874 (1,777-1,992)	3,186 (3,029-3,336)	6,552 (6,209-6,915)	13,505 (13,018-14,072)	31,183 (30,126-32,195)	59,660 (58,074-61,279)	105,582 (102,916-108,418)	165,535 (161,585-169,529)	205,291 (200,976-209,423)	222,465 (218,051-227,151)	247,503 (242,015-252,692)	217,996 (212,507-223,324)	156,777 (151,943-161,710)	80,707 (77,806-83,696)	25,921 (24,825-26,946)	7,046 (6,718-7,339)
Malignant skin melanoma	-	-	-	-	-	-	223 (208-263)	435 (407-531)	682 (624-798)	1,077 (976-1,228)	1,699 (1,531-1,965)	2,531 (2,274-2,843)	3,275 (2,928-3,699)	4,398 (3,946-4,945)	5,321 (4,760-5,885)	5,626 (4,998-6,109)	5,454 (4,822-5,895)	6,159 (5,285-6,706)	5,802 (4,906-6,232)	5,394 (4,472-5,762)	3,751 (3,050-4,023)	1,615 (1,342-1,785)	633 (500-753)
Non-melanoma skin cancer	-	-	-	-	-	-	184 (175-193)	247 (237-259)	289 (277-302)	449 (428-470)	674 (646-705)	1,104 (1,067-1,145)	1,545 (1,494-1,593)	2,229 (2,160-2,293)	3,135 (3,040-3,229)	4,490 (4,349-4,634)	4,893 (4,711-5,075)	5,901 (5,715-6,089)	5,821 (5,625-6,013)	6,006 (5,802-6,209)	4,898 (4,725-5,072)	2,767 (2,663-2,874)	1,518 (1,449-1,579)
Non-melanoma skin cancer (squamous-cell carcinoma)	-	-	-	-	-	-	184 (175-193)	247 (237-259)	289 (277-302)	449 (428-470)	674 (646-705)	1,104 (1,067-1,145)	1,545 (1,494-1,593)	2,229 (2,160-2,293)	3,135 (3,040-3,229)	4,490 (4,349-4,634)	4,893 (4,711-5,075)	5,901 (5,715-6,089)	5,821 (5,625-6,013)	6,006 (5,802-6,209)	4,898 (4,725-5,072)	2,767 (2,663-2,874)	1,518 (1,449-1,579)
Breast cancer	-	-	-	-	-	-	378 (305-418)	1,087 (953-1,198)	3,300 (2,938-3,566)	8,963 (8,397-9,580)	19,265 (18,257-21,011)	32,038 (30,409-34,607)	45,064 (42,860-48,738)	56,041 (53,663-58,784)	62,284 (59,507-65,733)	55,261 (53,209-58,650)	47,395 (45,696-50,644)	46,304 (44,627-48,742)	39,302 (37,815-41,694)	33,773 (32,313-35,419)	25,658 (24,527-26,943)	11,872 (11,347-12,501)	5,394 (5,129-5,650)
Cervical cancer	-	-	-	-	-	-	201 (145-218)	851 (614-925)	2,605 (2,024-2,799)	6,151 (4,948-6,606)	11,940 (9,516-12,781)	19,147 (15,552-20,410)	25,518 (21,209-27,048)	29,564 (24,005-31,452)	31,304 (25,871-33,585)	27,146 (22,800-29,105)	22,447 (18,752-24,033)	20,666 (17,909-22,267)	14,666 (12,803-15,514)	11,798 (10,394-12,522)	6,686 (6,043-7,096)	2,507 (2,312-2,649)	864 (810-911)
Uterine cancer	-	-	-	-	-	-	79 (72-87)	208 (193-225)	345 (324-369)	642 (602-681)	1,387 (1,300-1,477)	2,613 (2,459-2,765)	4,534 (4,313-4,744)	6,896 (6,553-7,241)	9,255 (8,822-9,719)	11,225 (10,719-11,732)	10,964 (10,498-11,456)	11,020 (10,589-11,459)	9,077 (8,734-9,451)	7,400 (7,081-7,751)	4,565 (4,353-4,786)	1,826 (1,744-1,920)	656 (621-692)
Ovarian cancer	-	-	-	-	-	-	509 (467-553)	766 (715-818)	1,140 (1,060-1,206)	1,747 (1,644-1,842)	3,279 (3,069-3,456)	6,083 (5,763-6,387)	10,568 (10,019-11,033)	15,543 (14,762-16,188)	17,620 (16,766-18,234)	18,278 (17,562-18,937)	16,422 (15,857-16,949)	17,469 (16,786-18,112)	14,032 (13,472-14,554)	11,101 (10,624-11,602)	7,038 (6,672-7,377)	2,689 (2,568-2,821)	910 (865-951)
Prostate cancer	-	-	-	-	-	-	72 (58-82)	96 (75-109)	120 (95-137)	151 (117-170)	244 (184-267)	564 (432-623)	1,690 (1,335-1,847)	4,811 (3,790-5,173)	11,490 (9,053-12,423)	23,690 (18,321-25,705)	35,729 (28,454-38,722)	53,134 (43,573-57,891)	62,290 (52,632-69,396)	57,631 (50,450-64,410)	42,916 (37,133-46,996)	19,708 (17,055-21,199)	5,766 (4,863-6,193)
Testicular cancer	-	-	-	-	-	-	419 (384-465)	916 (850-1,001)	1,117 (1,038-1,212)	1,060 (996-1,136)	928 (876-992)	680 (642-721)	569 (538-605)	472 (443-503)	362 (343-383)	352 (334-374)	333 (313-352)	379 (358-403)	299 (285-316)	308 (287-332)	165 (156-175)	65 (62-69)	22 (21-23)
Kidney cancer	34 (31-36)	17 (16-18)	242 (228-262)	1,219 (1,120-1,318)	609 (572-650)	320 (302-338)	256 (242-268)	365 (350-381)	480 (460-498)	693 (662-718)	1,303 (1,247-1,348)	2,391 (2,304-2,468)	4,589 (4,407-4,745)	7,768 (7,493-8,012)	11,488 (11,104-11,831)	13,348 (12,959-13,719)	13,409 (13,043-13,756)	15,632 (15,112-16,111)	15,119 (14,605-15,552)	12,851 (12,328-13,303)	8,249 (7,861-8,569)	3,051 (2,922-3,170)	955 (909-994)
Bladder cancer	-	-	-	-	-	-	108 (101-116)	193 (182-206)	244 (229-261)	519 (490-551)	982 (934-1,035)	1,950 (1,856-2,049)	3,540 (3,409-3,691)	6,296 (6,066-6,532)	9,930 (9,628-10,248)	14,400 (14,020-14,786)	17,613 (17,148-18,067)	23,997 (23,366-24,701)	26,453 (25,744-27,230)	26,215 (25,346-27,106)	18,994 (18,288-19,665)	8,152 (7,861-8,441)	2,827 (2,709-2,930)
Brain and nervous system cancer	104 (80-117)	69 (54-78)	943 (770-1,077)	3,982 (3,500-4,567)	5,590 (4,936-6,267)	5,212 (4,630-5,742)	4,234 (3,744-4,665)	4,599 (4,044-5,007)	5,103 (4,552-5,509)	6,362 (5,726-6,891)	8,756 (7,825-9,595)	11,649 (10,540-12,901)	14,110 (12,878-15,314)	17,561 (16,062-19,273)	21,684 (19,839-23,792)	21,651 (19,530-23,188)	19,845 (17,618-21,116)	18,640 (16,023-19,627)	14,789 (12,094-15,726)	9,459 (7,312-10,173)	4,529 (3,311-4,922)	1,292 (965-1,400)	331 (241-363)
[truncated: 2,822,183 more chars]
