# Supplementary material for: Global, regional, and national age-sex specific mortality for 264 causes of death, 1980–2016: a systematic analysis for the Global Burden of Disease Study 2016
Source: Lancet. 2017 Sep 16;390(10100):1151–210. doi: 10.1016/S0140-6736(17)32152-9 (PMC5605883; doi:10.1016/S0140-6736(17)32152-9)
Supplement: Supplementary appendix 1 [file mmc1.pdf]

THE LANCET

Supplementary appendix 1

This appendix formed part of the original submission and has been peer reviewed. We post it as supplied by the authors.

Supplement to: GBD 2016 Causes of Death Collaborators. Global, regional, and national age-sex specific mortality for 264 causes of death, 1980–2016: a systematic analysis for the Global Burden of Disease Study 2016. *Lancet* 2017; **390:** 1151–210.

Appendix 1: Methods

Methods appendix to "Global, regional, and national age-sex specific mortality for 264 causes of death, 1980–2016: a systematic analysis for the Global Burden of Disease Study 2016"

Preamble

This appendix provides further methodological detail for "Global, regional, and national age-sex specific mortality for 264 causes of death, 1980–2016: a systematic analysis for the Global Burden of Disease Study 2016." This appendix is organized into sections that follow the structure of the main paper. This study complies with the Guidelines for Accurate and Transparent Health Estimates Reporting (GATHER) recommendations. It includes detailed tables and information on data in an effort to maximize transparency in our estimation processes and provide a comprehensive description of analytical steps. We intend this appendix to be a living document, to be updated with each iteration of the Global Burden of Disease Study.

Table of Contents

Author Contributions......i

Section 1: GBD Overview 4

1.1 Geographic locations of analysis 4

1.2 Time period of analysis 4

1.3 Statement of GATHER compliance 4

1.4 List of abbreviations 4

1.5 GBD results overview 8

1.6 Data input sources overview 8

1.7 Funding sources 9

Section 2: GBD 2016 Causes of death database......9

2.1 Background......9

2.2 Cause of death data identification 9

2.3 Step 1. Standardize input data 14

2.4 Step 2. Map to GBD cause list 16

2.5 Step 3. Age-sex splitting 17

2.6 Step 4. Correct age-sex violations 18

2.7 Step 5. Redistribution 19

2.8 Step 6. HIV/AIDS misclassification correction 22

2.9 Step 7. Scale strata to province 23

2.10 Step 8. Restrictions post-redistribution 23

2.11 Step 9. Drop VR country years or mark as non-representative 24

2.12 Step 10. Cause aggregation 24

2.13 Step 11. Remove shocks and HIV/AIDS maternal adjustments ……25
2.14 Step 12. Noise reduction ……28
2.15 Step 13. CoD database and outlier identification ……30
2.16 Causes of death data star rating calculation ……31

Section 3: Causes of death modeling methods……33

3.1 CODEm……33
3.2 Causes modeled outside of CODEm……35
3.3 Cause of death modeling descriptions……39

Section 4: Central computation……279

4.1 Correction for miscoding of Alzheimer's and other dementias and Parkinson's disease......279
4.2 Imported cases……280
4.3 CodCorrect……280
4.4 Years of Life Lost (YLLs) calculation……281
4.5 Socio-Demographic Index (SDI)……282

Section 5: References ……286

Section 6. Figures and Tables ……288

Appendix Figures

Appendix Figure 1. Analytical flowchart for the development of the GBD 2016 causes of death database……288

Appendix Figure 2. GBD 2016 causes of death estimation flowchart……289

Appendix Figure 3. GBD 2016 DisMod-MR 2.1 analytical cascade……290

Appendix Figure 4. Vital Registration and Verbal Autopsy data availability by country, 1980-2016……291

Appendix Tables

Appendix Table 1. GATHER checklist of information that should be included in reports of global health estimates, with description of compliance and location of information for GBD 2016. Global, regional, and national cause-specific mortality for 264 causes of death, 1980-2016: a systematic analysis for the Global Burden of Disease Study 2016……292

Appendix Table 2. Total number of site years by cause and source type for 2016......295

Appendix Table 3. List of International Classification of Diseases (ICD) codes mapped to the Global Burden of Disease cause list for causes of death……300

Appendix Table 4. Restrictions on age and sex by cause for GBD 2016……309

Appendix Table 5. HIV/AIDS-related garbage code redistribution packages……320

Appendix Table 6. CODEm covariates used, level of covariate, and expected direction of covariate by cause, sex, and age……………………………………………………………………………………..321

Appendix Table 7. CODEm predictive validity results by cause, model type sex, and age……….367

Appendix Table 8. Comparison of GBD 2015 and GBD 2016 covariates and level of covariates used in cause of death modeling……………………………………………………………………………………….376

Appendix Table 9. Modeling strategy for individual cause of death models in GBD 2016……….411

Appendix Table 10. CoDCorrect cause hierarchy with levels…………………………………………………414

Appendix Table 11. Percent change after CoDCorrect by cause for all ages, both sexes, 2016...420

Appendix Table 12. Socio-demographic Index R-squared values with lags up to 10 years..........429

Appendix Table 13. Socio-demographic Index groupings by location, based on 2016 values......430

Appendix Table 14. Socio-demographic Index values for all estimated GBD 2016 locations, 1980-2016...446

Appendix Table 15. GBD 2016 location hierarchy with levels……………………………………………......460

Appendix Table 16. Causes included in the 2016 Global Burden of Disease Study…………………...477

Appendix Table 17. Underlying indicators for percent well-certified calculation, based on the maximum percent well certified data source in each 5-year time interval for 195 countries, 1980-2016………...490

Appendix Table 18. Theoretical minimum risk reference life table…………………………………………503

Appendix Table 19. GBD world population age standard…………………………………………………….504

Authors' Contributions

Managing the estimation process

Ashkan Afshin, Tahiya Alam, Megha Arora, James Bennett, Kara Estep, Christina Fitzmaurice, Emmanuela Gakidou, Jamie Hancock, Caitie Hawley, Simon Hay, Sarah Johnson, Nicholas Kassebaum, Michael Kutz, Darya Li Kappe, Hmwe Kyu, Stephen Lim, Madeline McGaughey, Christopher Murray, Mohsen Naghavi, Helen Olsen, Christine Pinho, Puja Rao, Robert Reiner, Gregory Roth, Joseph Salama, Katya Shackelford, Chloe Shields, Shreya Shirude, Amber Sligar, Mari Smith, Vinay Srinivasan, Jeffrey Stanaway, Caitlyn Steiner, Theo Vos, and Haidong Wang.

Writing the first draft of the manuscript

Laurie B Marczak, Christopher J L Murray, Mohsen Naghavi, and Sarah E Ray.

Providing data or critical feedback on data sources

Cristiana Abbafati, Foad Abd-Allah, Semaw M Abera, Olatunji Adetokunboh, Ashkan Afshin, Aliasghar Ahmad Kiadaliri, Muktar Venkata Satyanarayana Ahmed, Amani Nidhal Aichour, Ibtihel Aichour, Miloud Taki Eddine Aichour, Fares Alahdab, Khurshid Alam, Tahiya Alam, Kefyalew Addis Alene, Ayman Al-Eyadhy, Reza Alizadeh-Navaei, Juma M Alkaabi, Ala'a Alkerwi, François Alla, Peter N Allebeck, Christine Allen, Rajaa Al-Raddadi, Ubai Alsharif, Khalid A Altirkawi, Azmeraw T Amare, Erfan Amini, Walid Ammar, Nahla Anber, Catalina Liliana Andrei, Sofia Androudi, Hossein Ansari, Carl Abelardo T Antonio, Palwasha Anwari, Johan Ärnlöv, Al Artaman, Krishna Kumar Aryal, Hamid Asayesh, Solomon Asgedom, Ashish Awasthi, Umar Bacha, Kalpana Balakrishnan, Aleksandra Barac, Simón Barquera, Lars Barregard, Lope H Barrero, Bernhard T Baune, Neeraj J Bedi, Kelly Beinhoff, Yannick Béjot, Bayu Begashaw Bekele, Eduardo Bernabé, Balem Demtsu Betsu, Mircea Beuran, Boris Bikbov, Donal Bisanzio, Rupert W Bourne, Lemma Negesa Bulto Bulto, Blair Bumgarner, Lucero Cahuana-Hurtado, Julio Cesar Campuzano, Josip Car, Carlos A Castañeda-Orjuela, Ferrán Catalá-López, Chioma Ezinne Chibueze, Odgerel Chimed-Ochir, Abdulaal A Chitheer, Devasahayam Jesudas Christopher, Massimo Cirillo, Cyrus Cooper, Benjamin C Cowie, Michael Robert Criqui, Lalit Dandona, Rakhi Dandona, José das Neves, Louisa Degenhardt, Kebede Deribe, Amare Deribew, Subhojit Dey, Shirin Raj Djalalinia, Dirk Douwes-Schultz, Tim Driscoll, Manisha Dubey, Bruce Bartholow Duncan, Ziad A El-Khatib, Alireza Esteghamati, Kara Estep, Carla Sofia e Sa Farinha, Valery Feigin, Seyed-Mohammad Fereshtehnejad, Joao C Fernandes, Tesfaye Feyissa, Irina Filip, Florian Fischer, Abraham D Flaxman, Nancy Fullman, Thomas Fürst, João M M Furtado, Emmanuela Gakidou, Johanna M Geleijnse, Hailay Abrha Gesesew, Maurice Giroud, Audra L Gold, Philimon Gona, Hebe N Gouda, Nima Hafezi-Nejad, Alemayehu Desalegne Hailu, Gessessew Bugssa Hailu, Mitiku Hambisa, Hilda L Harb, Rasmus J D Havmoeller, Simon Hay, Mohammad Rasoul Hedayati, Nathaniel Henry, Ileana Beatriz Heredia-Pi, Claudiu Herteliu, Hans W Hoek, Nobuyuki Horita, H Dean Hosgood, Peter J Hotez, Damian G Hoy, Kim Moesgaard Iburg, Bogdan Vasile Ileanu, Asnake Ararsa Irenso, Kathryn H Jacobsen, Nader Jahanmehr, Mihajlo Jakovljevic, Mehdi Merid Javanbakht, Panniyammakal Jeemon, Vivekanand Jha, Catherine Johnson, Sarah B Johnson, Jost B Jonas, Mikk M Jürisson, Zubair Kabir, Rajendra Kadel, Ritul Kamal, André Karch, Seyed Karimi, Amir Kasaeian, Nigussie Kassaw, Nicholas Assefa Kassebaum, Srinivasa Katikireddi, Norito Kawakami, Chandrasekharan Nair Kesavachandran, Yousef Khader, Young-Ho Saleh Khang, Abdullah Tawfih Abdullah Khoja, Ardeshir Khosravi, Jagdish Khubchandani, Christian Kieling, Yun Jin Ziad Kim, Ruth G Kimokoti, Yohannes Kinfu, Ann Kristin Knudsen, Soewarta Kosen, Kristopher J Krohn, Barthelemy Kuate Defo, G Anil Kumar, Pushpendra Kumar, Hmwe H Kyu, Dharmesh Kumar Lal, Nkurunziza Lambert, Qing Lan, Anders Larsson, James Leigh, Miriam Legesse Levi, Darya Li Kappe, Xiaofeng Liang, Misgan Teshome Liben, Giancarlo Logroscino, Alan D Lopez, Stefan Lorkowski,

Rafael Lozano, Stefan Ma, Marek Inês Majdan, Azeem Majeed, Reza Malekzadeh, Deborah Carvalho Malta, Tsegahun J Manyazewal, Desalegn Markos, Colm McAlinden, Suresh Mehata, Toni Meier, Kidanu Gebremariam Meles, Ziad Memish, Melkamu H Mengesha, Desalegn Tadese Mengistu, Bereket Gebremichael Menota, George A Mensah, Atte Meretoja, Tuomo Meretoja, Ted Regassa Miller, Shiva I Mishra, Karzan Mohammad, Shafiu Mohammed, Ali Huyen Mokdad, Mariam G Molokhia, Lorenzo Monasta, Marcella Montico, Maziar Moradi-Lakeh, Chloe Morozoff, Christopher J L Murray, Gudlavalleti Z Murthy, Kamarul Imran Musa, Jean B Nachega, Mohsen Naghavi, Luigi Naldi, Bruno Ramos Nascimento, Ionut Negoi, Grant Nguyen, Quyen Nguyen, Felix Akpojene Ogbo, Andrew Toyin Olagunju, Bolajoko Olubukunola Olusanya, Jacob Olusegun Olusanya, Alberto Ortiz, Majdi Osman, Mahesh PA, Smita Pakhale, Adrian Pana, Basant Kumar Panda, Songhomitra Panda-Jonas, Christina Papachristou, Scott Ferede Patten, Deepak Paudel, David M Pereira, Fernando Perez-Ruiz, Norberto Perico, Michael B Phillips, David M Pigott, Suzanne Alemu Polinder, Farshad Pourmalek, Mostafa Babale Qorbani, Amir Radfar, Vafa L Rahimi-Movaghar, Mahfuzar Rahman, Rajesh Kumar Rai, Goura Kishor Rath, Salman Rawaf, Jürgen Rehm, Giuseppe Remuzzi, Mohammad Sadegh T Rezai, Mohammad Bagher Rokni, Luca Ronfani, Gholamreza Roshandel, Gregory A Roth, Dietrich Rothenbacher, George Mugambage Ruhago, Rizwan G S A, Soheil W Saadat, Saeid Safiri, Rajesh Sagar, Joseph Salama, Payman Salamati, Joshua A Salomon, Abdallah M Samy, Juan Ramon Sanabria, Itamar S Santos, Milena Santric Milicevic, Benn Sartorius, Maheswar Satpathy, Monika Sawhney, Maria Schmidt, David C Schwebel, Falk Schwendicke, Sadaf Sepanlou, Edson E Servan-Mori, Masood Shaikh, Morteza Shamsizadeh, Sheikh Mohammed Shariful Islam, Jayendra Sharma, Sara Charlotte Sheikhbahaei, Mark Andrew Shrime, Abhishek Singh, Jasvinder A Singh, Eirini Skiadaresi, Samir Soneji, Reed Sorensen, Joan B Soriano, Chandrashekhar T Sreeramareddy, Jeffrey D Stanaway, Vasiliki Victorovich Stathopoulou, Caitlyn Steiner, Mark Ali Stokes, Muawiyyah Beshir Sufiyan, Soumya Vittal Swaminathan, Bryan L Sykes, Rafael Tabarés-Seisdedos, Jukka S Takala, Roberto Talongwa, Mohammed Tarawneh, Mohammad Tavakkoli, Mohamad-Hani Temsah, Abdullah Sulieman Terkawi, Ruoyan R A Tobe-Gai, Bach Xuan Tran, Christopher Troeger, Thomas R Truelsen, Stefanos Tyrovolas, Kingsley Nnanna Ukwaja, Eduardo A Undurraga, Job F M van Boven, Tommi Vasankari, Narayanaswamy J Venketasubramanian, Vasiliy Vlassov, Stein Emil Vollset, Theo Vos, Tolassa Wakayo, Yuan-Pang Wang, Elisabete Weiderpass, Robert Tchio Weintraub, Andrea Werdecker, Ronny Westerman, Tissa Wijeratne, Charles Shey Shey Wiysonge, Abdulhalik Workicho, Denis Xavier, Qingyang Le Xiao, Gelin Xu, Bereket Yakob, Yuichiro Yano, Hassen Hamid Yimam, Naohiro Yonemoto, Marcel Yotebieng, Mustafa Younis, Zoubida Zaidi, Taddese R Zerfu, Anthony Lin Zhang, and Xueying Zhang.

Developing methods or computational machinery

Ashkan Afshin, Alireza Ahmadi, Miloud Taki Eddine Aichour, Tahiya Alam, Christine Allen, Aleksandra Barac, Katherine Battle, James R Bennett, Samir Bhatt, Donal Bisanzio, Lemma Negesa Bulto Bulto, Blair Bumgarner, Ewan Cameron, Austin Carter, Daniel Casey, Fiona J Charlson, Devasahayam Jesudas Christopher, Cyrus Cooper, Louisa Degenhardt, Daniel Dicker, Lucas Earl, Kara Estep, Farshad Farzadfar, Christina FItzmaurice, Abraham D Flaxman, Tahvi Frank, Maya Fraser, Emmanuela Gakidou, Audra L Gold, Max Griswold, Nathaniel J Henry, Peter J Hotez, Xie Rachel Kulikoff, Michael Kutz, Hmwe H Kyu, Qing Lan, Paul H Lee, Darya Li Kappe, Xiaofeng Liang, Rafael Lozano, Timothy C D Lucas, Emilie R Maddison, Deborah Carvalho Malta, Madeline McGaughey, Anoushka Millear, Alireza Mohammadi, Ali Huyen Mokdad, Chloe Morozoff, Christopher J L Murray, Mohsen Naghavi, Grant Nguyen, Emma Nichols, Liane Ong, Katherine Paulson, David M Pigott, Christine Pinho, Goura Kishor Rath, Jürgen Rehm, Robert Reiner, Gregory A Roth, Joseph Salama, Joshua A Salomon, Damian Santomauro, Maheswar

Satpathy, Naris Silpakit, David L Smith, Vinay Srinivasan, Jeffrey D Stanaway, Caitlyn Steiner, Bryan Strub, Patrick J Sur, Roberto Tchio Talongwa, Mohammad Tavakkoli, Alex H Thompson, Christopher Troeger, Rachel L Updike, Theo Vos, Andrea Werdecker, Ronny Westerman, and Hassen Hamid Yimam.

Applying analytical methods to produce estimates

Ashkan Afshin, Fares Alahdab, Tahiya Alam, Syed Danish Ali, Christine Allen, Azmeraw T Amare, Solomon W Asgedom, Aleksandra Barac, Katherine Battle, Kelly Beinhoff, James R Bennett, Adugnaw Berhane, Samir Bhatt, Donal Bisanzio, Lemma Negesa Bulto Bulto, Blair Bumgarner, Ewan Cameron, Austin Carter, Daniel Casey, Fiona J Charlson, Devasahayam Jesudas Christopher, Danny Colombara, Cyrus Cooper, Louisa Degenhardt, Daniel Dicker, Dirk Douwes-Schultz, Michelle Echko, Holly E Erskine, Kara Estep, Christina FItzmaurice, Abraham D Flaxman, Tahvi Frank, Maya Fraser, Emmanuela Gakidou, Peter W Gething, William W Godwin, Audra L Gold, Max Griswold, Gessessew Bugssa Hailu, Fei He, Nathaniel J Henry, Claudiu Herteliu, Peter J Hotez, Bogdan Vasile Ileanu, Caleb Mackay Salpeter Irvine, Catherine Johnson, Rajendra Kadel, Nicholas J Kassebaum, Daniel Kievlan, Yun Jin Kim, Xie Rachel Kulikoff, Michael Kutz, Hmwe H Kyu, Darya Li Kappe, Xiaofeng Liang, Angela Liu, Patrick Liu, Rafael Lozano, Timothy C D Lucas, Emilie R Maddison, Deborah Carvalho Malta, Helena Manguerra, Tsegahun Manyazewal, Laurie B Marczak, Ira Martopullo, Madeline McGaughey, Anoushka Millear, Ali Huyen Mokdad, Sarah K Mollenkopf, Chloe Morozoff, Cliff Mountjoy-Venning, Christopher J L Murray, Mohsen Naghavi, Grant Nguyen, Emma Nichols, Felix Akpojene Ogbo, Helen E Olsen, Liane Ong, Katherine Paulson, David M Pigott, Christine Pinho, Caroline Purcell, Zane Rankin, Goura Kishor Rath, Sarah E Ray, Robert Reiner, Mohammad Sadegh Rezai, Gregory A Roth, Joseph Salama, Joshua A Salomon, Maheswar Satpathy, Masood Ali Shaikh, Alison Smith, Mari Smith, Vinay Srinivasan, Jeffrey D Stanaway, Caitlyn Steiner, Patrick J Sur, Roberto Tchio Talongwa, Ornwipa Thamsuwan, Katie E Thomas, Alex H Thompson, Christopher Troeger, Derrick Tsoi, Kingsley Nnanna Ukwaja, Rachel L Updike, Theo Vos, Tolassa Wakayo, Daniel J Weiss, Andrea Werdecker, Ronny Westerman, Tissa Wijeratne, Gelin Xu, Hassen Hamid Yimam, and Zoubida Zaid.

Providing critical feedback on methods or results

Amanuel Alemu Abajobir, Cristiana Abbafati, Kaja M Abbas, Semaw Ferede Abera, Olatunji Adetokunboh, Ashkan Afshin, Anurag Agrawal, Aliasghar Ahmad Kiadaliri, Alireza Ahmadi, Muktar Beshir Ahmed, Amani Nidhal Aichour, Ibtihel Aichour, Miloud Taki Eddine Aichour, Fares Alahdab, Ziyad Al-Aly, Khurshid Alam, Noore Alam, Tahiya Alam, Kefyalew Addis Alene, Reza Alizadeh-Navaei, Juma M Alkaabi, Peter Allebeck, Ubai Alsharif, Khalid A Altirkawi, Nelson Alvis-Guzman, Azmeraw T Amare, Erfan Amini, Walid Ammar, Yaw Ampem Amoako, Nahla Anber, Catalina Liliana Andrei, Sofia Androudi, Hossein Ansari, Carl Abelardo T Antonio, Palwasha Anwari, Johan Ärnlöv, Al Artaman, Krishna Kumar Aryal, Hamid Asayesh, Solomon W Asgedom, Tesfay Mehari Atey, Leticia Avila-Burgos, Euripide Frinel G Arthur Avokpaho, Ashish Awasthi, Beatriz Paulina Ayala Quintanilla, Tesleem Kayode Babalola, Umar Bacha, Aleksandra Barac, Miguel A Barboza , Suzanne L Barker-Collo, Simón Barquera, Lars Barregard, Lope H Barrero, Bernhard T Baune, Neeraj Bedi, Ettore Beghi, Kelly Beinhoff, Michelle L Bell, Adugnaw Berhane, Eduardo Bernabé, Balem Demtsu Betsu, Mircea Beuran, Samir Bhatt, Boris Bikbov, Donal Bisanzio, Rupert R A Bourne, Nicholas J K Breitborde, Lemma Negesa Bulto Bulto, Blair Bumgarner, Zahid A Butt, Rosario Cárdenas, Juan Jesus Carrero, Daniel Casey, Carlos A Castañeda-Orjuela, Ferrán Catalá-López, Fiona J Charlson, Chioma Ezinne Chibueze, Odgerel Chimed-Ochir, Vesper Hichilombwe Chisumpa, Devasahayam Jesudas Christopher, Liliana G Ciobanu, Massimo Cirillo, Aaron J Cohen, Danny Colombara, Cyrus Cooper, Benjamin C Cowie, Michael H Criqui, Lalit Dandona, Rakhi Dandona, Paul I Dargan, José

das Neves, Kairat Davletov, Barbora de Courten, Louisa Degenhardt, Selina Deiparine, Kebede Deribe, Amare Deribew, Subhojit Dey, Daniel Dicker, Eric L Ding, Shirin Djalalinia, Huyen Phuc Do, David Teye Doku, Dirk Douwes-Schultz, Tim R Driscoll, Manisha Dubey, Ziad Ziad El-Khatib, Christian Lycke Ellingsen, Ahmadali Enayati, Holly E Erskine, Sharareh Eskandarieh, Alireza Esteghamati, Kara Estep, André Faro, Farshad Farzadfar, Seyed-Mohammad Fereshtehnejad, Joao C Fernandes, Alize J Ferrari, Tesfaye Regassa Feyissa, Irina Filip, Florian Fischer, Abraham D Flaxman, Nataliya Foigt, Maya Fraser, Nancy Fullman, Thomas Fürst, João M M Furtado, Emmanuela Gakidou, Alberto L Garcia-Basteiro, Teshome Gebre, Gebremedhin Berhe Gebregergs, Tsegaye Tewelde Gebrehiwot, Delelegn Yilma Gebremichael, Ricard Genova-Maleras, Hailay Abrha Gesesew, Peter W Gething, Paramjit Gill, Giorgia Giussani, Audra L Gold, Philimon N Gona, Sameer Vali Gopalani, Hebe N Gouda, Alessandra Carvalho Goulart, Rajeev Gupta, Tanush Gupta, Vipin Gupta, Juanita A Haagsma, Nima Hafezi-Nejad, Alemayehu Desalegne Hailu, Gessessew Bugssa Hailu, Randah Ribhi Hamadeh, Mitiku Teshome Hambisa, Samer Hamidi, Mouhanad Hammami, Yuantao Hao, Hilda L Harb, Habtamu Abera Hareri, Mohammad Sadegh Hassanvand, Rasmus Havmoeller, Simon I Hay, Ileana Beatriz Heredia-Pi, Claudiu Herteliu, Nobuyuki Horita, H Dean Hosgood, Sorin Hostiuc, Peter J Hotez, Damian G Hoy, Kim Moesgaard Iburg, Bogdan Vasile Ileanu, Asnake Ararsa Irenso, Kathryn H Jacobsen, Nader Jahanmehr, Mihajlo B Jakovljevic, Mehdi Javanbakht, Sudha P Jayaraman, Panniyammakal Jeemon, Vivekanand Jha, DQenny John, Catherine Johnson, Sarah Charlotte Johnson, Jost B Jonas, Mikk Jürisson, Rajendra Kadel, Amaha Kahsay, Ritul Kamal, André Karch, Seyed M Karimi, Chante Karimkhani, Amir Kasaeian, Nigussie Assefa Kassaw, Nicholas J Kassebaum, Srinivasa Vittal Katikireddi, Norito Kawakami, Peter Njenga Keiyoro, Chandrasekharan Nair Kesavachandran, Yousef Saleh Khader, Young-Ho Khang, Ardeshir Khosravi, Jagdish Khubchandani, Daniel Kim, Yun Jin Kim, Ruth W Kimokoti, Yohannes Kinfu, Niranjan Kissoon, Mika Kivimaki, Ann Kristin Knudsen, Jacek A Kopec, Ai Koyanagi, Kristopher J Krohn, Barthelemy Kuate Defo, G Anil Kumar, Pushpendra Kumar, Michael Kutz, Hmwe H Kyu, Dharmesh Kumar Lal, Nkurunziza Lambert, Qing Lan, Van C Lansingh, Anders Larsson, Paul H Lee, James Leigh, Janni Leung, Miriam Levi, Yongmei Li, Darya Li Kappe, Xiaofeng Liang, Misgan Legesse Liben, Yang Liu, Rakesh Lodha, Giancarlo Logroscino, Alan D Lopez, Stefan Lorkowski, Paulo A Lotufo, Rafael Lozano, Stefan Ma, Erlyn Rachelle King Macarayan, Marek Majdan, Azeem Majeed, Reza Malekzadeh, Rajesh Malhotra, Deborah Carvalho Malta, Tsegahun Manyazewal, Chabila C Mapoma, Laurie B Marczak, Desalegn Markos, Jose Martinez-Raga, Francisco Rogerlândio Martins-Melo, Ira Martopullo, Colm McAlinden, John J McGrath, Toni Meier, Kidanu Gebremariam Meles, Peter Memiah, Ziad A Memish, Melkamu Merid Mengesha, Desalegn Tadese Mengistu, Bereket Gebremichael Menota, George A Mensah, Atte Meretoja, Tuomo J Meretoja, Ted R Miller, Shawn Minnig, Mojde Mirarefin, Erkin M Mirrakhimov, Shiva Raj Mishra, Alireza Mohammadi, Shafiu Mohammed, Ali Huyen Mokdad, Sarah K Mollenkopf, Mariam Molokhia, Lorenzo Monasta, Maziar Moradi-Lakeh, Paula Moraga, Chloe Morozoff, Kalayu Birhane Mruts, Kate Muller, Christopher J L Murray, Kamarul Imran Musa, Jean B Nachega, Mohsen Naghavi, Aliya Naheed, Vinay Nangia, Bruno Ramos Nascimento, Jamal T Nasher, Gopalakrishnan Natarajan, Ionut Negoi, Josephine Wanjiku Ngunjiri, Cuong Tat Nguyen, Grant Nguyen, Quyen Le Nguyen, Trang Huyen Nguyen, Dina Nur Anggraini Ningrum, Vuong Minh Nong, Jean Jacques N Noubiap, Felix Akpojene Ogbo, Anselm Okoro, Andrew Toyin Olagunju, Bolajoko Olubukunola Olusanya, Jacob Olusegun Olusanya, John Nelson Opio, Eyal Oren, Alberto Ortiz, Erika Ota, Mahesh PA, Rosana E Pacella, Smita Pakhale, Adrian Pana, Basant Kumar Panda, Songhomitra Panda-Jonas, Eun-Kee Park, Scott B Patten, George C Patton, Deepak Paudel, David M Pereira, Fernando Perez-Ruiz, Norberto Perico, Aslam Pervaiz, Michael Robert Phillips, David M Pigott, Dietrich Plass, Suzanne Polinder, Maarten J Postma, Farshad Pourmalek, Mostafa Qorbani, Amir Radfar, Vafa Rahimi-Movaghar, Mahfuzar

Rahman, Mohammad Hifz Ur Rahman, Rajesh Kumar Rai, Chhabi Lal Ranabhat, Zane Rankin, Puja C Rao, Goura Kishor Rath, Salman Rawaf, Sarah E Ray, Jürgen Rehm, Robert Reiner, Giuseppe Remuzzi, Satar Rezaei, Mohammad Sadegh Rezai, Luca Ronfani, Gholamreza Roshandel, Gregory A Roth, George Mugambage Ruhago, Rizwan S A, Perminder S Sachdev, Mahdi Safdarian, Sare Safi, Saeid Safiri, Rajesh Sagar, Ramesh Sahathevan, Joseph Salama, Payman Salamati, Joshua A Salomon, Abdallah M Samy, Maria Dolores Sanchez-Niño, Damian Santomauro, Itamar S Santos, Milena M Santric Milicevic, Benn Sartorius, Maheswar Satpathy, Monika Sawhney, Maria Inês Schmidt, Ione J C Schneider, Sam Schulhofer-Wohl, Aletta E Schutte, David C Schwebel, Falk Schwendicke, Sadaf G Sepanlou, Masood Ali Shaikh, Mansour Shamsipour, Morteza Shamsizadeh, Sheikh Mohammed Shariful Islam, Jayendra Sharma, Rajesh Sharma, Jun She, Sara Sheikhbahaei, Muki Shey, Chloe Shields, Rahman Shiri, Ivy Shiue, Haitham Shoman, Mark G Shrime, Inga Dora Sigfusdottir, Jasvinder A Singh, Eirini Skiadaresi, Alison Smith, Badr H A Sobaih, Reed J D Sorensen, Joan B Soriano, Chandrashekhar T Sreeramareddy, Jeffrey D Stanaway, Vasiliki Stathopoulou, Nicholas Steel, Caitlyn Steiner, Sabine Steinke, Mark Andrew Stokes, Mark Strong, Muawiyyah Babale Sufiyan, Bruno F Sunguya, Soumya Swaminathan, Bryan L Sykes, Rafael Tabarés-Seisdedos, Santosh Kumar Tadakamadla, Ken Takahashi, Jukka S Takala, Roberto Tchio Talongwa, Nuno Taveira, Teketo Kassaw Tegegne, Arash Tehrani-Banihashemi, Mohamad-Hani Temsah, Abdullah Sulieman Terkawi, JS Thakur, Kavumpurathu Raman Thankappan, Alex H Thompson, Alan J Thomson, Amanda G Thrift, Ruoyan Tobe-Gai, Roman Topor-Madry, Miguel Tortajada-Girbés, Jeffrey Allen Towbin, Bach Xuan Tran, Christopher Troeger, Thomas Truelsen, E Murat Tuzcu, Kingsley Nnanna Ukwaja, Eduardo A Undurraga, Olalekan A Uthman, Benjamin S Chudi Uzochukwu, Job F M van Boven, Tommi Vasankari, Narayanaswamy Venketasubramanian, Francesco S Violante, Vasiliy Victorovich Vlassov, Stein Emil Vollset, Theo Vos, Tolassa Wakayo, Mitchell T Wallin, Yuan-Pang Wang, Elisabete Weiderpass, Robert G Weintraub, Daniel J Weiss, Andrea Werdecker, Ronny Westerman, Harvey A Whiteford, Tissa Wijeratne, Belete Getahun Woldeyes, Charles D A Wolfe, Abdulhalik Workicho, Denis Xavier, Gelin Xu, Mohsen Yaghoubi, Bereket Yakob, Mehdi Yaseri, Hassen Hamid Yimam, Naohiro Yonemoto, Marcel Yotebieng, Mustafa Z Younis, Zoubida Zaidi, Elias Asfaw Zegeye, Zerihun Menlkalew Zenebe, Taddese Alemu Zerfu, and Anthony Lin Zhang.

Drafting the work or revising is critically for important intellectual content

Amanuel Alemu Abajobir, Cristiana Abbafati, Foad Abd-Allah, Semaw Ferede Abera, Victor Aboyans, Olatunji Adetokunboh, Ashkan Afshin, Aliasghar Ahmad Kiadaliri, Alireza Ahmadi, Muktar Beshir Ahmed, Fares Alahdab, Khurshid Alam, Noore Alam, Tahiya Alam, Juma M Alkaabi, Ala'a Alkerwi, Peter Allebeck, Nelson Alvis-Guzman, Azmeraw T Amare, Erfan Amini, Yaw Ampem Amoako, Nahla Anber, Hjalte H Andersen, Sofia Androudi, Palwasha Anwari, Johan Ärnlöv, Hamid Asayesh, Solomon W Asgedom, Tesfay Mehari Atey, Leticia Avila-Burgos, Euripide Frinel G Arthur Avokpaho, Ashish Awasthi, Aleksandra Barac, Miguel A Barboza , Suzanne L Barker-Collo, Lope H Barrero, Bernhard T Baune, Neeraj Bedi, Kelly Beinhoff, Yannick Béjot, Bayu Begashaw Bekele, Michelle L Bell, Isabela M Bensenor, Adugnaw Berhane, Balem Demtsu Betsu, Mircea Beuran, Boris Bikbov, Donal Bisanzio, Lemma Negesa Bulto Bulto, Blair Bumgarner, Zahid A Butt, Lucero Cahuana-Hurtado, Josip Car, Juan Jesus Carrero, Carlos A Castañeda-Orjuela, Ferrán Catalá-López, Fiona J Charlson, Devasahayam Jesudas Christopher, Massimo Cirillo, Aaron J Cohen, Danny Colombara, Cyrus Cooper, Benjamin C Cowie, Michael H Criqui, Lalit Dandona, Rakhi Dandona, José das Neves, Dragos V Davitoiu, Barbora de Courten, Louisa Degenhardt, Selina Deiparine, Kebede Deribe, Amare Deribew, Subhojit Dey, Huyen Phuc Do, David Teye Doku, Tim R Driscoll, Manisha Dubey, Bruce Bartholow Duncan, Holly E Erskine, Sharareh Eskandarieh, Alireza Esteghamati, Kara Estep, André Faro, Seyed-Mohammad Fereshtehnejad, Joao C Fernandes, Alize J

Ferrari, Irina Filip, Samuel Finegold, Florian Fischer, Christina FItzmaurice, Abraham D Flaxman, Nancy Fullman, Thomas Fürst, João M M Furtado, Emmanuela Gakidou, Teshome Gebre, Gebremedhin Berhe Gebregergs, Tsegaye Tewelde Gebrehiwot, Johanna M Geleijnse, Ricard Genova-Maleras, Hailay Abrha Gesesew, Peter W Gething, Paramjit Gill, Richard F Gillum, Ibrahim Abdelmageem Mohamed Ginawi, Audra L Gold, Philimon N Gona, Sameer Vali Gopalani, Alessandra Carvalho Goulart, Tanush Gupta, Juanita A Haagsma, Nima Hafezi-Nejad, Alemayehu Desalegne Hailu, Randah Ribhi Hamadeh, Mitiku Teshome Hambisa, Alexis J Handal, Graeme J Hankey, Habtamu Abera Hareri, Mohammad Sadegh Hassanvand, Rasmus Havmoeller, Ileana Beatriz Heredia-Pi, Masako Horino, Nobuyuki Horita, H Dean Hosgood, Sorin Hostiuc, Peter J Hotez, Damian G Hoy, Asnake Ararsa Irenso, Kathryn H Jacobsen, Mihajlo B Jakovljevic, Sudha P Jayaraman, Panniyammakal Jeemon, Sarah Charlotte Johnson, Jost B Jonas, Mikk Jürisson, Rajendra Kadel, Ritul Kamal, André Karch, Seyed M Karimi, Chante Karimkhani, Amir Kasaeian, Nicholas J Kassebaum, Srinivasa Vittal Katikireddi, Norito Kawakami, Peter Njenga Keiyoro, Chandrasekharan Nair Kesavachandran, Yousef Saleh Khader, Ejaz Ahmad Khan, Young-Ho Khang, Abdullah Tawfih Abdullah Khoja, Mohammad Hossein Khosravi, Jagdish Khubchandani, Christian Kieling, Daniel Kim, Yun Jin Kim, Niranjan Kissoon, Mika Kivimaki, Ann Kristin Knudsen, Jacek A Kopec, Parvaiz A Koul, Ai Koyanagi, Kristopher J Krohn, Barthelemy Kuate Defo, G Anil Kumar, Michael Kutz, Hmwe H Kyu, Dharmesh Kumar Lal, Ratilal Lalloo, Van C Lansingh, Anders Larsson, Janni Leung, Miriam Levi, Darya Li Kappe, Xiaofeng Liang, Misgan Legesse Liben, Giancarlo Logroscino, Alan D Lopez, Paulo A Lotufo, Rafael Lozano, Mohammed Magdy Abd El Razek, Marek Majdan, Reza Majdzadeh, Azeem Majeed, Reza Malekzadeh, Deborah Carvalho Malta, Tsegahun Manyazewal, Laurie B Marczak, Desalegn Markos, Francisco Rogerlândio Martins-Melo, Colm McAlinden, Madeline McGaughey, Toni Meier, Melkamu Merid Mengesha, Desalegn Tadese Mengistu, George A Mensah, Atte Meretoja, Tuomo J Meretoja, Ted R Miller, Shiva Raj Mishra, Karzan Mohammad, Shafiu Mohammed, Ali Huyen Mokdad, Glen Liddell D Mola, Mariam Molokhia, Marcella Montico, Paula Moraga, Chloe Morozoff, Shane D Morrison, Kate Muller, Christopher J L Murray, Gudlavalleti Venkata Satyanarayana Murthy, Kamarul Imran Musa, Jean B Nachega, Mohsen Naghavi, Bruno Ramos Nascimento, Ionut Negoi, Josephine Wanjiku Ngunjiri, Cuong Tat Nguyen, Quyen Le Nguyen, Trang Huyen Nguyen, Vuong Minh Nong, Jean Jacques N Noubiap, Felix Akpojene Ogbo, In-Hwan Oh, Anselm Okoro, Andrew Toyin Olagunju, Bolajoko Olubukunola Olusanya, Jacob Olusegun Olusanya, John Nelson Opio, Eyal Oren, Alberto Ortiz, Erika Ota, Mahesh PA, Rosana E Pacella, Basant Kumar Panda, Songhomitra Panda-Jonas, Scott B Patten, George C Patton, David M Pereira, Fernando Perez-Ruiz, Aslam Pervaiz, Dietrich Plass, Maarten J Postma, Mostafa Qorbani, Amir Radfar, Vafa Rahimi-Movaghar, Mahfuzar Rahman, Rajesh Kumar Rai, Goura Kishor Rath, Salman Rawaf, Sarah E Ray, Robert Reiner, Satar Rezaei, Mohammad Sadegh Rezai, Luca Ronfani, Gholamreza Roshandel, Gregory A Roth, Dietrich Rothenbacher, Perminder S Sachdev, Mahdi Safdarian, Saeid Safiri, Rajesh Sagar, Joseph Salama, Joshua A Salomon, Abdallah M Samy, Juan Ramon Sanabria, Maria Dolores Sanchez-Niño, Damian Santomauro, Itamar S Santos, Milena M Santric Milicevic, Maheswar Satpathy, Monika Sawhney, Maria Inês Schmidt, Aletta E Schutte, David C Schwebel, Falk Schwendicke, Sadaf G Sepanlou, Masood Ali Shaikh, Morteza Shamsizadeh, Sheikh Mohammed Shariful Islam, Jayendra Sharma, Sara Sheikhbahaei, Mika Shigematsu, Rahman Shiri, Shreya Shirude, Ivy Shiue, Haitham Shoman, Mark G Shrime, João Pedro Pedro Silva, Jasvinder A Singh, Eirini Skiadaresi, Joan B Soriano, Chandrashekhar T Sreeramareddy, Jeffrey D Stanaway, Dan J Stein, Caitlyn Steiner, Sabine Steinke, Mark Strong, Muawiyyah Babale Sufiyan, Bryan L Sykes, Roberto Tchio Talongwa, Nuno Taveira, Mohamad-Hani Temsah, JS Thakur, Ornwipa Thamsuwan, Alan J Thomson, Roman Topor-Madry, Miguel Tortajada-Girbés, Bach Xuan Tran, E Murat Tuzcu, Stefanos Tyrovolas, Kingsley Nnanna Ukwaja, Eduardo

A Undurraga, Olalekan A Uthman, Job F M van Boven, Tommi Vasankari, Narayanaswamy Venketasubramanian, Francesco S Violante, Vasiliy Victorovich Vlassov, Stein Emil Vollset, Theo Vos, Tolassa Wakayo, Yuan-Pang Wang, Elisabete Weiderpass, Robert G Weintraub, Andrea Werdecker, Brian Whetter, Harvey A Whiteford, Tissa Wijeratne, Charles Shey Shey Wiysonge, Denis Xavier, Gelin Xu, Bereket Yakob, Yuichiro Yano, Hassen Hamid Yimam, Naohiro Yonemoto, Seok-Jun Yoon, Maysaa El Sayed Zaki, and Zerihun Menlkalew Zenebe.

Extracting, cleaning, or cataloging data; designing or coding figures and tables

Olatunji Adetokunboh, Sneha Aiyar, Christine Allen, Catalina Liliana Andrei, Megha Arora, Aleksandra Barac, James R Bennett, Adugnaw Berhane, Lemma Negesa Bulto Bulto, Blair Bumgarner, Daniel Casey, Devasahayam Jesudas Christopher, Dirk Douwes-Schultz, Lucas Earl, Kara Estep, Carla Sofia e Sa Farinha, Samuel Finegold, Maya Fraser, Nancy Fullman, Maurice Giroud, Ellen M Goldberg, Max Griswold, Jamie Hancock, Chad Ikeda, Sarah Charlotte Johnson, Laura Kemmer, Mohammad Hossein Khosravi, Daniel Kievlan, Barthelemy Kuate Defo, Michael Kutz, Misgan Legesse Liben, Emilie R Maddison, Tsegahun Manyazewal, Ira Martopullo, Madeline McGaughey, Melkamu Merid Mengesha, Anoushka Millear, Ted R Miller, Alireza Mohammadi, Cliff Mountjoy-Venning, Christopher J L Murray, Mohsen Naghavi, Quyen Le Nguyen, Emma Nichols, Liane Ong, Mahesh PA, Katherine Paulson, Max Petzold, David M Pigott, Christine Pinho, Zane Rankin, Goura Kishor Rath, Sarah E Ray, Payman Salamati, Maheswar Satpathy, Shreya Shirude, Naris Silpakit, Alison Smith, Chandrashekhar T Sreeramareddy, Caitlyn Steiner, Bryan Strub, Michelle Subart, Patrick J Sur, Roberto Tchio Talongwa, Mohammad Tavakkoli, Alex H Thompson, Anna Torre, Bach Xuan Tran, Christopher Troeger, Brian Whetter, Rachel Woodbrook, Gelin Xu, and Ben Zipkin.

Managing the overall research enterprise

Ashkan Afshin, Tahiya Alam, Kelly Beinhoff, Blair Bumgarner, Kara Estep, Abraham D Flaxman, Emmanuela Gakidou, Audra L Gold, Simon I Hay, Peter J Hotez, Chantal Huynh, Laura Kemmer, Darya Li Kappe, Xiaofeng Liang, Alan D Lopez, Rafael Lozano, Deborah Carvalho Malta, Ali Huyen Mokdad, Chloe Morozoff, Kate Muller, Christopher J L Murray, Mohsen Naghavi, Helen E Olsen, Puja C Rao, Gregory A Roth, Joseph Salama, Joshua A Salomon, Katya Anne Shackelford, Chloe Shields, Shreya Shirude, Amber Sligar, Mari Smith, Jeffrey D Stanaway, Caitlyn Steiner, Theo Vos, and Andrea Werdecker.

Did not provide authorship contribution information

Sibhatu Biadgilign, Tea Lallukka, Julio C Montañez Hernandez, Minh Nguyen, Martin A Pletcher, Saeid Shahraz, and Sanjay Zodpey.

Section 1. GBD Overview

Section 1.1. Locations of the Analysis

The locations included in GBD 2016 have been arranged into a set of hierarchical categories composed of seven super-regions and a further nested set of 21 regions containing 195 countries and territories. The locations for which GBD estimated global, regional, and national cause-specific mortality and years of life lost (YLLs) have not expanded following GBD 2015. New subnational locations estimated for GBD 2016 are the local government areas of England and provinces of Indonesia. Subnational assessments for GBD 2016 include 26 states and one federal district for Brazil, 33 provinces and municipalities for China, nine regions and 150 local government areas for England, 31 states and union territories by urbanicity for India, 34 provinces for Indonesia, 47 prefectures for Japan, 47 counties for Kenya, 31 states and one federal district for Mexico, two areas for Sweden, nine provinces for South Africa, 13 states for the Kingdom of Saudi Arabia, and 50 states and one federal district for the United States. Combined, there are a total of 335 locations at the first subnational unit level. Included in subnational Level 1 locations are countries that have been subdivided into the first subnational level, such as states or provinces, for the GBD analysis; subnational Level 2 only applies to India and England. For this paper we present data at the national and territory level.

Section 1.2. Time Period of the Analysis

A complete set of cause-specific mortality and years of life lost (YLL) numbers and rates were computed for the years 1980–2016.

All GBD 2016 results and online data visualisations are available at http://vizhub.healthdata.org/gbd-compare with access to results for all GBD metrics.

Section 1.3. Statement of GATHER Compliance

This study complies with the Guidelines for Accurate and Transparent Health Estimates Reporting (GATHER) recommendations. We have documented the steps involved in our analytical procedures and detailed the data sources used in compliance with the Guidelines for Accurate and Transparent Health Estimates Reporting (GATHER). See Appendix Table 1 for the GATHER checklist.

The GATHER recommendations may be found here: http://gather-statement.org/

Section 1.4. List of abbreviations

5q0: probability of death from birth to age 5 years

45q15: probability of death from age 15 years to 60 years

Adol: adolescent

Air poll mort: mortality attributable to air pollution

ANC: antenatal care

ART: antiretroviral therapy

BMD: bone mineral density

BMI: body mass index

BTL: basic tabulation list

CBH: complete birth history

CD4: type of T lymphocyte used as an indicator of immune function

CDR: crude death rates

CHERG: Child Health Epidemiology Research Group

CKD: chronic kidney disease

CKD-DM: chronic kidney disease deaths attributable to diabetes

COD: causes of death

CODEm: cause of death ensemble modelling

COPD: chronic obstructive pulmonary disease

CR: cancer registry

CRA: comparative risk assessment

CSA: childhood sexual abuse

CSMR: cause-specific mortality rate

CVD: cardiovascular disease

DAH: development assistance for health

DALYs: disability-adjusted life-years

DHS: Demographic and Health Survey

DPT: diphtheria-pertussis-tetanus

DRI: data representativeness index

DSP: Disease Surveillance Points

ELISA: enzyme-linked immunosorbent assay

EMR: excess mortality rate

EPEC: enteropathogenic *E. coli*

EPP: Estimation and Projection Package

ETEC: enterotoxigenic *E. coli*

FAO: Food and Agriculture Organization

GATHER: Guidelines for Accurate and Transparent Health Estimates Reporting

GBD: Global Burden of Diseases, Injuries, and Risk Factors Study

GEMS: Global Enteric Multicenter Study

GHDx: Global Health Data Exchange

GPRM: Global Price Reporting Mechanism

HAQ: Healthcare Access and Quality

HH air poll: household air pollution

Hib: *Haemophilus influenzae* type B

HIV CDR: Crude death rate due to HIV/AIDS

IAEG-SDGs: Inter-Agency and Expert Group on Sustainable Development Goal Indicators

IARC: International Agency for Research on Cancer

ICD: International Classification of Disease

IER: integrated exposure response

IHD: ischemic heart disease

IHR: International Health Regulations

ILO: International Labour Organization

IOTF: International Obesity Task Force

IPUMS: Integrated Public Use Microdata Series

IPV: intimate partner violence

ISIC: International Standard Industrial Classification

JMP: Joint Monitoring Programme

LDI: lag distributed income per capita

LRI: lower respiratory infection

MAP: Malaria Atlas Project

MCCD: Medical Certification of Causes of Death

MCEE: Maternal and Child Epidemiology Estimation group

MDG: Millennium Development Goal

MICS: Multiple Indicator Cluster Surveys

MIR: mortality/incidence ratio

MM: maternal mortality

MMR: maternal mortality ratio

MMS: Maternal Mortality Surveillance

Mort: mortality

NCD: non-communicable disease

NN mort: neonatal mortality

NTDs: neglected tropical diseases

Occ risk burden: burden attributable to occupational risks

ODA: Official development assistance

OECD: Organisation for Economic Co-operation and Development

PAF: population attributable fraction

PCV3: Three-dose pneumococcal conjugate vaccine

PM2.5: particulate matter <2.5μm in diameter

RCT: randomised controlled trial

RMSE: root mean square error

RSV: respiratory syncytial virus

SBA: skilled birth attendance

SBH: summary birth history

SBP: systolic blood pressure

SCD(R): Survey of Causes of Death (Rural)

SD: standard deviation

SDG: Sustainable Development Goal

SDI: Socio-demographic Index

SDSN: Sustainable Development Solutions Network

SEER: Surveillance, Epidemiology, and End Results Program

SEV: summary exposure value

SIR: smoking impact ratio

SRS: Sample Registration System

SSB: sugar-sweetened beverages

ST-GPR: spatiotemporal Gaussian process regression

TAC: TaqMan Array Card

TB: tuberculosis

TMREL: theoretical minimum-risk exposure level

TRIPS: Agreement on Trade-Related Aspects of Intellectual Property Rights

U5MR: under-5 mortality rate

UHC: universal health coverage

UI: uncertainty interval

UN: United Nations

UNICEF: United Nations Children's Fund

VA: verbal autopsy

VR: vital registration

WaSH: water, sanitation, and hygiene

WFS: World Fertility Surveys

WHO: World Health Organization

YLDs: years lived with disability

YLLs: years of life lost

Section 1.5. GBD results overview

Results from the Global Burden of Disease Study (GBD 2016) are now measured in terabytes. Results are available in an interactive data downloading tool on the Global Health Data exchange (GHDx). The tool contains the complete set of results from all summary papers; however, specialised tables from the papers are available as separate entries in the GHDx as were made available for GBD 2015.

The current version of the data download tool is available in the GHDx and contains core summary results for the Global Burden of Disease Study 2016 (GBD 2016): http://ghdx.healthdata.org/gbd-results-tool. The core summary results include deaths, YLLs, YLDs, and DALYs. It includes data for causes, risks, cause-risk attribution, aetiologies, and impairments.

In the GBD 2016 version, the tool also contains measures such as prevalence and incidence as well as rate of change data. Data above a certain size cannot be viewed online but can be downloaded. Depending on the size of the download, users may need to enter an email address and a download location will be sent to them when the files are prepared.

Section 1.6. Data input sources overview

GBD 2016 incorporated a large number and wide variety of input sources to estimate mortality, causes of death and illness, and risk factors for 195 countries and territories from 1990 to 2016. These input sources are accessible through an interactive citation tool available in IHME's GHDx.

Users can retrieve citations for a specific GBD component, cause or risk, and location by choosing from the available selection boxes. They can then view and access GHDx records for input sources and export a CSV file that includes the GHDx metadata, citations, and information about where the data were used in GBD. Additional metadata for each input source are available through the citation tool, as required by the GATHER statement.

The citation tool is accessible through the GHDx at http://ghdx.healthdata.org/global-burden-disease-study-2016

Section 1.7. Funding Sources

Funding for this research was provided by the Bill & Melinda Gates Foundation.

Section 2: GBD 2016 causes of death database

2.1 Background

Appendix Figures 1 and 2 show the high-level view of data inputs, analytical steps, and outputs of the cause of death analysis frame. Section 2 of this appendix provides details on each step in the development of the causes of death database as illustrated in Appendix Figure 1. The complexity of the overall process can be usefully divided into three broad phases: data inputs on the event of death going into the cause of death database that are analysed using CODEm; data inputs on precursors to death that are modelled through a variety of strategies; and the integration of these streams of analysis into a single set of cause of death estimates by age, sex, year, and geography with uncertainty through the CodCorrect algorithm. The process for cancer and for HIV/AIDS is somewhat different and is described in more detail in Section 3.

2.2 Cause of death data identification

2.2.1 Overview of data types

The cause of death database contains seven types of data sources: vital registration, verbal autopsy, cancer registry, police records, sibling history, surveillance, and survey/census. The highest-quality data will have detailed characteristics of each demographic group and detailed causes of death across the time series. Data from countries with complete vital registration systems are considered to be high-quality. For counties with incomplete vital registration systems, vital statistics for causes of death can be supplemented with other data types to provide cause-specific estimates.

2.2.2 ICD detail

A majority of the cause of death data is vital registration data obtained from the WHO Mortality Database, country-specific mortality databases operated by official offices, or provided by trusted country collaborators. It is considered to be the highest-quality data, as it is the most comprehensive. Each cause is coded directly to the most detailed cause of death when possible, whereas cause codes in ICD-tabulated data are coded to aggregated cause groups. The cause of death database contains 12,879 location-years of detailed data from 1980 to 2016, which includes underlying causes of death coded with 3-5 digit codes, by country, year, sex, and age groups. Detail causes are coded to one of the following ICD detail coding systems: ICD8, ICD9, or ICD10. Each coding system has a similar cause hierarchy and cause list that has continually developed over time. ICD10 is the current standard and the most exhaustive cause list. Within the cause lists, 5-digit codes are truncated to 4-digit codes to condense the cause lists. Updates to ICD detail occur biannually as WHO releases new versions or as country collaborators provide additional data. Updates to data from WHO increasingly include ICD10 cause of death data, as it is the most current classification of cause of death, while updates to ICD8 and ICD9 detailed lists are less common. In the case of overlapping data, preference is given to data from pre-determined country collaborators, which are updated annually.

2.2.3 ICD tabulations lists

The ICD tabulation lists include the ICD8 List A (ICD8A), ICD9 Basic Tabulation List (BTL), ICD10

Mortality Tabulation, Russia Tabulation list, and India Medical Certification of Cause of Death (MCCD). These data sources make up 1,729 location-years from 1980 to 2016 in the cause of death database. All are condensed versions of the ICD9 and ICD10 detail lists, with some differences in the format of cause lists depending on the data source. ICD8A, ICD9 BTL, and ICD10 Mortality Tabulation cause of death are assigned to subtotal groups, referred to as chapters, and cause groups respective to ICD detail groups. Additionally ICD9 BTL includes ICD9 detail codes for some cancers and a custom tabulation scheme for the former USSR countries. The Russia Tabulation lists and India MCCD cause lists each have custom nomenclatures based on ICD detail cause codes.

Two of the drawbacks in data using tabulation lists are discrepancies in the accuracy of death counts and lack of detail to due to aggregated cause groups. There are instances where the sum of deaths in chapter subtotals are not equal to the sum of cause groups within the chapter. To account for any missing or duplicate deaths reported within the cause groupings, death counts are systematically adjusted, by calculating the differences between subtotals and sub-causes within the cause groups. Any differences are assigned to a remainder cause group. To account for the lack of cause code detail, select cause groups are disaggregated (Step 1.1) to create a complete cause list. Updates to ICD Tabulation lists obtained from WHO occur less frequently compared to ICD detailed lists, as more countries are reporting deaths in ICD detail. In instances of overlapping data, preference is given first to data from country collaborators' data from WHO, then to ICD detail data from WHO, before choosing to use ICD tabulation lists.

China DSP/China CDC

The two primary sources of data for China are surveillance data from the China Disease Surveillance Points (DSP) system and vital registration data collected by the Chinese Center for Disease Control and Prevention (CDC). In the China DSP data, deaths were reported across 145 disease surveillance points used from 1991 to 2003, 161 points used from 2004 to 2007, and 605 disease surveillance points from 2008 to 2015. While China DSP with ICD10 coding is considered sample vital registration data, it provides national coverage and cause detail. Thus it receives similar processing and treatment to the China CDC vital registration from 2008 to 2015. From 2008 to 2015, all of the deaths and cause of death information from the Disease Surveillance Points system and other system points throughout China were collected and reported via the Mortality Registration and Reporting System, an online reporting system of the Chinese CDC. The deaths in these data are reported at the strata level, a metric that is specific to China. Counties are stratified by urban and rural classification, but definitions of urbanity vary across counties. In Step 7 we use a method developed to scale up deaths from strata level to the province level.

India MCCD

The India Medical Certification of Cause of Death (MCCD) has data for the urban parts of the majority of the states and union territories beginning in 1980. Deaths reported in this data source have been medically certified and are considered vital registration data. The causes of death are reported in a tabulation list with a unique numbering scheme that conform to ICD9 and ICD10 detail codes, which must be disaggregated. MCCD is state-split to fill in data gaps (Step 1.2 State Splitting) prior to age-sex splitting.

Because SRS is widely considered a more credible assessment of causes of death in India, we chose to keep MCCD in only certain cases for modelling with CODEm. We preserved MCCD data in the database for two primary reasons. First, where the three midpoint years of SRS data resulted in the loss of a clear time trend, as was the case for maternal mortality, we chose to preserve MCCD in addition to SRS. Second, MCCD has advantage over SRS in cases where verbal autopsy is not a valid instrument for

ascertaining the cause of death, like encephalitis, dengue fever, and peptic ulcer disease – in these cases, we kept MCCD over SRS.

2.2.4 Verbal autopsy

VA coded to ICD10 and VA coded to other lists

In countries without vital registration systems, verbal autopsy studies are a viable data source to inform cause of death. Data are obtained by trained interviewers who use a standardised questionnaire to ask relatives about the signs, symptoms, and demographic characteristics of recently deceased family members. Based on the answers to the questionnaires a cause of death is assigned.

Verbal autopsy data are highly heterogeneous: studies use different instruments, different cause lists from single causes to full ICD cause lists, different methods for assigning cause of death based on a completed verbal autopsy, different recall periods, and different age groups, quite apart from cultural differences in the interpretation of specific questions. The validity of cause of death must be considered when mapping to a GBD cause. Verbal autopsies are likely accurate in assigning cause of death to road injury or homicide, but less accurate for causes requiring medical certification, such as cardiovascular causes. Studies can also occur once in a particular country or as part of an extended network, such as INDEPTH. INDEPTH is a continuous surveillance source with several Demographic Surveillance Systems sites that collect data which is coded to ICD detail causes.

INTERVA modelled VA

In previous years, INTERVA-modelled VA was excluded from our analysis. Verbal autopsies used in our analysis are non-INTERVA, as they use questionnaires and modules consistent with WHO standards. The Population Health Metrics Research Consortium (PHMRC) published a study that shows results of INTERVA-modelled VA are not compelling enough to be credible, and thus we have decided to exclude data for all causes due to low validations with the exception of injuries in Sub-Saharan Africa.[1] We lack data in sub-Saharan Africa and use INTERVA to fill in gaps and stabilise injuries patterns.

India SCD

Deaths reported in verbal autopsy studies in rural Indian states can be accounted for in the Survey of Causes of Death (SCD) from 1980 to 1994.[2] Data in the SCD were collected through a verbal autopsy survey from a sample of villages. To expand our estimates to more states and causes we used methods of state splitting post mapping to GBD causes (Step 1.2). For GBD 2016, the primary source of cause of death data for India was India SRS, described below. With the exception of select causes for which SCD data were a critical source in CODEm models, SRS data were used in place of SCD data for causes included in both databases.

India SRS

The SRS is a dual-record system wherein a resident part-time enumerator continuously records births and deaths in each household within the sample unit every month. A full-time SRS supervisor thereafter independently collects the vital events along with other related details for each of the preceding six month periods during the calendar year. The sample for SRS is drawn after each decennial census of India.

Starting in late 2002, each household in the SRS sampled unit in which a death occurred was visited by a trained SRS field staff and an interview of the nearest available relative of the deceased was conducted to collect details of the signs, symptoms and other relevant information related to that death using a verbal autopsy questionnaire. This information was subsequently coded by physicians to determine the

most likely underlying cause of death. Initially, two physicians independently assigned the underlying cause for each death to a three-character ICD10 code. Any difference between the two coders was resolved by reconciliation between them, and for persisting differences adjudication was done by a third physician coder.

2.2.5 Other data types

Maternal mortality data

In locations with low-quality vital registration or no vital registration, maternal mortality metrics can be found in surveillance, surveys, census, and sibling history data sources. The best data have death counts due to maternal causes and the total number of deaths for women within the reproductive ages of 10 to 54 by year. If a data source is missing these components, it is necessary to create a complete cause list using live births and all-cause mortality deaths (Step 1.4).[3] Though death counts is the preferred metric, maternal mortality is often measured using maternal mortality ratio (MMR), which is easily converted to deaths using live births. An additional adjustment that must be applied to the China Maternal and Child Surveillance data is scaling data from the strata to the province level (Step 7).

Surveys and censuses reporting fraction of deaths due to selected injuries

Surveys and censuses are often used in countries with less developed vital registration systems, or in countries with adequate vital registration these data sources are supplementary. Much like the verbal autopsies, the validity of cause of death is a concern due to lack of medical certification at the time of death. For these data sources we keep only causes related to maternal mortality and injuries. The remaining causes are accounted for as a remainder of total deaths in the sample size.

Police records

In most countries, police and crime reports are an important source of information for some types of injury deaths, notably road injuries and interpersonal violence. Our police data come from reports on road traffic and crime trends. The police reports used in this analysis were obtained from published studies, national agencies, and institutional surveys such as the UN Crime Trends survey and United Nations Office on Drugs and Crime Global study on Homicides. We can assess whether police reports were likely to be complete and cover the entire country if police trends are close to trends seen in vital registration. Data are excluded in instances where police data for road traffic injuries are significantly lower than our vital registration. The threshold for exclusion is less than 80% of the cause fraction of the road traffic injuries in vital registration. Police data that meet our inclusion criteria and provide complete coverage are uploaded to the database for injuries causes.

2.2.6 Population-based cancer registries

Cancer registries with incidence

Data on cancer incidence were sought from individual population-based cancer registries as well as from databases that include multiple registries, for example "Cancer Incidence in Five Continents" (CI5), NORDCAN, or EUREG. Cancer registries were identified through the membership list of the International Association of Cancer Registries (IACR), through the GBD collaborator network, or through the GHDx. Registries were excluded if they were not representative of the coverage population, if they did not contain incidence data tabulated by cancer site, if the data were limited to years prior to 1980, if the source did not provide details on the population covered, or if the list of cancer sites included was not comprehensive.

Cancer registries with incidence and high-quality mortality data

In addition to incidence, some high-quality cancer registries also report cancer mortality data. These data were also extracted and used as inputs to the mortality-to-incidence model.

2.3 Step 1. Standardise input data

The input data to the cause of death (CoD) database are received in various formats and must be standardised to run through central CoD machinery to then upload to the database. Raw data inputs come from data sources such as mortality databases, literature reviews, or reports. Usable data sources must have a clear sample size of the number of deaths in the population and exhaustive cause lists. The complexity of the data cleaning process varies drastically across data sources. For vital registration micro-data with the location, age, sex, year, and ICD-coded cause of every death, very little effort is necessary to standardise it into a consistent structure. Other sources may require weeks of careful review to accurately extract scans of hardcover cause of death reports into spreadsheets that can be transformed and standardised.

At this point, data are assigned source identifiers so that they can be linked to the Global Health Data Exchange (GHDx) and cited appropriately. Any aggregate age and sex categories are flagged for age-sex splitting. The methods of cause-of-death assignment and data collection are reviewed to determine which source type to assign; for example, we distinguish sibling history data from surveys with a verbal autopsy module. Only data at the most detailed level of the Global Burden of Disease location hierarchy are used. Documentation from the source is reviewed to determine if the population is representative of the location or only a subset of the population in that location. Data sources representing a subset of the population are flagged as non-representative; this flag is used by CODEm to increase the variance associated with such data points.

Finally, diagnostics are reviewed at this stage to avoid sending cleaning errors downstream. We review cause-specific deaths for each demographic group to ensure the data are reasonable. For example, it is unlikely that male breast cancer deaths are higher than female breast cancer or deaths from neonatal causes occur in age groups over one year. All deaths totals are compared with the sum of cause-specific deaths to ensure the observed deaths are accounted for and sample size is complete.

Step 1.1 Disaggregation

Causes of death in tabulated vital registration data are condensed into aggregated groups, some of which can be mapped directly to GBD causes while other aggregated cause groups are not informative and cannot be mapped to GBD causes. To correct for this, aggregated causes were mapped and split onto multiple ICD8, ICD9 and ICD10 detail causes, or targets, based on the ICD groupings within the aggregated causes. ICD8, ICD9 and ICD10 detail codes serve as targets because they are the highest-quality vital registration data and enable the calculation of proportions used to split the aggregated cause data into detailed causes. The proportions of deaths from nearby countries within the super-region were used to fill in data gaps as they were likely to have similar cause of death trends.

We determined the targets based on detail causes missing from the tabulated cause list. For example, in ICD9 BTL, the tabulated cause list includes a viral diseases group. In the hierarchy of causes, this group consists of measles, yellow fever, encephalitis, hepatitis, rabies, other infectious diseases, garbage code, and remainder of viral diseases. We did not consider this list to be an exhaustive list of viral diseases based on the range of ICD detail codes given in the ICD9 BTL documentation. To make the cause list exhaustive and inclusive of other viral diseases, we split the remainder of the viral diseases group into other meningitis, other infectious diseases, herpes, dengue, other neglected tropical diseases, and garbage code. After a list of targets was determined, the aggregated deaths were disaggregated to the target causes using ICD8, ICD9, and ICD10 detail proportions generated at the super-region level for the corresponding sex and age groups across all years in the time series. For example, in ICD9 detail data, 54.8% of deaths in males in Latin America and the Caribbean within the target group for BTL Viral Diseases

were designated “other meningitis”, so 54.8% of deaths in the tabulated group “remainder of viral diseases”, were assigned to “other meningitis” for any country within that particular super-region. For any cause and demographic group where we lacked ICD detail, global proportions were used.

Step 1.2 State splitting

Two sources for cause of death estimation in India are the Medical Certification of Causes of Death (MCCD) report, which reports medically certified deaths from health facilities in mostly urban areas[4], and the Survey of Causes of Death (SCD), which collects information via verbal autopsy on about one-half of 1% of all rural deaths in India, based on populations living in about 1,300 primary health care centers spread throughout the country.[5] For both of these reports, data missingness impedes estimation of trends at the state level. We used a first-order, log-linear model of the four-way contingency table of deaths by sex, age, state, and year to estimate the missing state-years. We fit the model to all available data for MCCD and SCD separately for each cause, including state-specific all-age measurements and age-specific national measurements. From this, we produced estimates for each combination of sex, age, state, and year. We then used these estimates wherever the raw data did not include sex-, age-, and state-specific death counts.

For MCCD, the model was fit separately for ICD10- and ICD9-based reports using the tabulated cause list present in the data. In the SCD report, the model was fit for each GBD cause in the data. As data from the SCD reports were relatively sparse, the pooling of like causes together led to an improved model fit.

Step 1.3 Calculate non-maternal deaths

In cases when maternal mortality metrics do not include both deaths due to maternal causes and deaths due to non-maternal causes for women of reproductive age, live births and all-cause mortality estimates can be used to calculate deaths. Many studies report maternal deaths as the maternal mortality ratio (MMR). MMR is the number of maternal deaths per 100,000 live births and can be used to calculate deaths when it has been derived from primary data and not estimated. Maternal deaths were calculated using MMR and live births; if live births were missing we substituted live birth estimates and used the following equation:

$$\text{Maternal deaths} = (\text{MMR}/100{,}000) * \text{Live births}$$

If a study was non-representative we extracted sample size and live births from that study. After maternal deaths were calculated, we used the difference from all-cause mortality estimates to determine non-maternal deaths.

A more accurate and data-inclusive method of calculating maternal and non-maternal deaths incorporates coverage and splits deaths for a range of years into individual years. If there were live births in the study we adjusted the coverage.

$$\text{Coverage} = \text{live births} / \text{GBD-estimated live births}$$

After coverage was calculated, totals deaths were scaled to be more representative. This gives a more accurate death count since the envelope assumes representative coverage. Using all-cause mortality as an all-cause total, non-maternal deaths were subsequently calculated.

$$\text{Maternal envelope with coverage} = \text{maternal envelope} * \text{coverage}$$

An additional adjustment can be applied to maternal data spanning over a range of consecutive years, which allows for more data inclusion. The years within specified year ranges are separated into individual

years, and total deaths within the year range were split between each individual year using the fixed proportions of maternal deaths from vital registration in that particular country. We only used vital registration to inform the proportions because it was both high-quality and representative.

2.4 Step 2. Map to GBD cause list

In GBD 2016 we used 536 maps to translate causes found in the input data to the GBD 2016 cause list. This included 38 maps for vital registration data, 354 for verbal autopsy data sources, and 144 for other data types. The largest and most universal maps used were those for ICD9 and ICD10 detail vital registration data. The input data causes varied from 3-4 digit ICD codes to custom cause lists with cause names such as cholera or hepatitis. Our mapping process made it possible to compare these various data sources across demographic groups.

In GBD 2016, we developed additional maps to translate ICD codes found in the input data that are non-underlying causes to appropriate target codes based on the levels of the GBD cause list. These garbage codes were mapped to Levels 1-4 of the GBD cause list according to the following criteria:

1. Level 1 includes all garbage codes for which a Level 1 GBD cause cannot be directly assigned. For example, the underlying causes of sepsis or peritonitis, if not specified in the data, could be an injury, a non-communicable disease, or a type of communicable disease. In these cases, deaths will be redistributed across all three of these Level 1 causes. In addition, deaths coded to impossible or ill-defined causes of death, including senility and unspecified causes, fall into this category, as they will be redistributed onto all causes.

2. Level 2 includes all garbage codes that can be assigned to one specific Level 1 cause in the GBD cause list. This would include deaths coded to unspecified injuries (X59), which are redistributed onto all injuries.

3. Level 3 includes all garbage codes for which we know the Level 2 cause of death, and can redistribute onto Level 3 causes. This includes deaths coded to causes such as unspecified cardiovascular disease, which falls within the Level 2 cause cardiovascular diseases, as well as those coded to unspecified cancer site, which falls within the Level 2 neoplasms cause.

4. Level 4 includes all garbage codes for underlying causes of death that can be redistributed within a Level 3 cause. This includes garbage codes such as “unspecified stroke” or “unspecified road injuries.”

Appendix Table 3 shows the ICD10-detail and ICD9-detail codes included in the mapping of each GBD cause. This includes the ICD10-detail and ICD9-detail codes that were mapped to garbage Levels 1-4 as well.

2.5 Step 3. Age-sex splitting

Different sources, particularly verbal autopsy studies, report deaths for a wide range of age groups with varying intervals. For the analysis of causes of death, we mapped these different age intervals to the GBD standard set of age groups. The approach to undertake this mapping was the same as in the prior GBD studies, GBD 2015, GBD 2013, and GBD 2010.

In the process of assembling a consolidated demographic database, perhaps the most impairing source of inconsistency is the aggregation of age groups. It is conventional to report such data in broad age groupings such as "0-4, 5-14, 15-49," or to report data with both sexes together. The issue of comparability between age-sex groups arose when assembling the GBD cause of death database. The compiled database included 22 distinct tabulation formats for infants and 141 distinct tabulation formats for non-infants. We developed a tool, which we call age-sex splitting, that takes aggregated age groupings, and likewise the "both sexes combined" grouping, and divides them into what their constituent age groups would likely have been using respective cause-specific and country-specific age distributions. The analytical framework for GBD includes three infant age categories: Early neonatal (0-6 days), late neonatal (7-27 days), and post-neonatal (28 days to 1 year), and 17 non-infant age categories starting with age 1-4 years, then proceeding in five-year age groups until the terminal age group of 95+. We treat unknown ages and sexes in the same manner we treated the "all ages combined" age category and "both sexes combined" sex group. Through this process, we were able to directly compare all data sources on even terms.

The approach to age splitting is based on the following formula. The key assumption underlying this formula is that the relative risk of death by age group compared to a reference age group is invariant across populations. While this assumption is likely violated in specific cases, there is a strong biologically based pattern of the relative risk of death for a cause by age that is observed for most causes. The basic formula is as follows:

$$D_a = R_a N_a \left(\frac{D_a^{a+x}}{\sum_a^{a+x}(R_a N_a)}\right)$$

Where:
D_a= the number of deaths from a cause in age group *a*
R_a= the relative risk of death in age group *a* compared to a reference group
N_a= the country-year-sex-specific population in age group *a*
D_a^{a+x}= the number of deaths in the age group *a* to *a+x*

With the assumption of invariant relative risks of death by age with respect to a reference age group, this equation can be used, along with population distribution by age, to split an aggregate number of deaths for the age groups *a* to *a+x* into specific deaths for each age group within the aggregate interval.

In some cases, deaths are reported for an aggregate age group for both sexes combined. The task in this case is more complicated, but the same principle can be applied. In this case we assumed that the relative risks of death by and sex are constant.

$$D_{as} = R_{as} N_{as} \left(\frac{D_{as}^{a+x,s}}{\sum_a^{a+x}(R_{as} N_{as})}\right)$$

Where:
D_{as}= the number of deaths from a cause in age group *a,* sex *s*
R_{as}= the relative risk of death in age group *a* compared to a reference group for sex *s*
N_{as}= the country-year-sex-specific population in age group *a* for sex *s*
$D_{a}^{a+x,s}$= the number of deaths in the age group *a* to *a+x* for sex *s*

This equation can be used to split data aggregated over age and sex. The assumption, however, of invariant relative risks across age and sex is a stronger assumption. Fortunately, data pooled across sexes are less common in the published or unpublished cause of death data.

The relative risk of death in a particular age group for a given sex is derived from the global distribution of cause-specific mortality rates found in available vital registration data. Location-years from the following code systems are used, provided they report the requisite age- and sex-detail: ICD7, ICD8, ICD9 BTL, ICD10 tabulated, ICD9, and ICD10. Upon compiling these data, we mapped them to GBD causes, and aggregated up to cause Level 3. This is the level at which a particular cause is split – that is, any daughter cause of a Level 3 parent is split using the age distribution of that parent (so, chronic kidney disease due to diabetes would be split using the age pattern of chronic kidney disease).

We next adjusted separately for estimated adult and child vital registration completeness. Location- year-age-sex-specific deaths and population were then aggregated across all location-years, in order to produce cause-specific mortality rates by age and sex. These were used to determine the risk of death at any age relative to any reference age group.

2.6 Step 4. Correct age-sex violations

Occasionally, data sources will include deaths by a cause for which there is medical consensus that death is impossible for the sex and age. For example, there may be some number of deaths due to cervical cancer in males, or deaths due to maternal causes in ages under 10. We have constructed a conservative list of age-sex restrictions. When deaths violate these restrictions, we redistribute them proportionally onto all causes.

Step 4.1 GBD age-sex restrictions by cause

All restrictions are included in Appendix Table 4, Restrictions on age and sex by cause for GBD 2016.

2.7 Step 5. Redistribution

A crucial aspect of enhancing the comparability of data for cause of death is to deal with uninformative, so-called garbage codes. Garbage codes are codes to which deaths were assigned that cannot or should not be considered as the underlying cause of death, for example: heart failure, ill-defined cancer site, senility, ill-defined external causes of injuries, and septicaemia. The methods for redistributing these garbage-coded deaths were outlined in detail in Naghavi et al[6], and the underlying algorithm for redistributing deaths assigned to these codes has not changed since GBD 2013.

Step 5.1 Redistribute HIV-related garbage codes

Due to the disparate nature of HIV/AIDS mortality across space and time, dynamic redistribution of HIV/AIDS-related garbage codes was needed. To inform this redistribution, we generated target proportions for each garbage group by age band (Under 1 month, 1-59 months, 5-19 years, 20-49 years, 50-59 years, 60-69 years, 70-79 years, and 80+ years), five-year time interval, and sex. The garbage groups will either target HIV or a remainder target. The allotment of deaths to either of these is based on the regional increase in the mortality rate of all codes in the group relative to the rates seen in 1980–1984 – an increase greater than 5% is assumed to be HIV/AIDS-related, and the proportion of those deaths exceeding 5% are redistributed to HIV/AIDS. Any increase ≤ 5% is then assigned to the remainder target.

Step 5.2 Regress garbage codes versus non-garbage codes

As in GBD 2015, the statistical analysis used to determine proportions for garbage code redistribution for ill-defined cancer sites, ill-defined external causes of injury, unspecified stroke, heart failure, hypertension, and atherosclerosis was based on the approach outlined by Ahern et al.[7] For each redistribution package, we defined the “universe” of data as all deaths coded to either the package’s garbage codes or the package’s redistribution targets for each country, year, age, and sex. We then ran a regression based on the following equation, separately for each target group and sex:

$$TG_{crt} = \alpha + \beta_1 Gar_{crt} + \beta_2 Age_{crt} Gar_{crt} + \theta_r Gar_{crt} + \gamma_r + \varepsilon_{ct}$$

TG_{crt} = percentage of deaths within the given garbage code’s universe which were coded to a given target group, by country

Gar_{crt} = percentage of deaths within the given garbage code’s universe which were coded to a given set of garbage codes

α = constant

β_1 = slope coefficient describing the association between Gar_{crt} and G_{crt}

β_2 = slope coefficient describing the association between the interaction $Age_{crt}Gar_{crt}$ and G_{crt}

γ_r = region-specific random intercept (or super-region if the random effect on region is not significant)

θ_r = region-specific random slope (or super-region if the random effect on region is not significant)

ε_{ct} = standard error, normally distributed and calculated by bootstrapping

This regression was adjusted from GBD 2013 to include fixed effects on the interaction of garbage and age to ensure smooth age patterns. We made this decision after investigating diagnostic visualisations that showed unlikely gaps between proportions assigned to different age groups.

Once proportions were produced for each country, sex, age, and target group, certain adjustments were made to conform our packages to the best medical evidence available. In some cases, we implemented

restrictions on the proportions that the regressions could yield. For example, we did not allow any redistribution onto Chagas disease outside of Latin America and the Caribbean, or suicide under the age of 15. In other cases, we capped the proportion for some targets to the level that would be produced from proportional redistribution; for example, haemoglobinopathies and haemolytic anaemias were restricted to the level of proportional redistribution in the redistribution of left heart failure. Occasionally, further adjustments were made on a case-by-case basis per country, age, sex, and target group to suppress the impact of outliers based on existing epidemiological evidence and expert judgment.

Development of an algorithm for redistribution of garbage codes based on multiple cause of death data

Multiple cause of death data are a form of individual record causes of death data which include an underlying cause of death along with other causes in the death chain, including intermediate and immediate causes. By analysing this type of data, we can sometimes find the true underlying cause of death in other cause of death data where the underlying cause is a garbage code or a misassigned cause of death.

For GBD 2016, this approach was implemented in redistribution for a few select causes. One example of this approach is seen in the correction of the misassignment of deaths due to drug overdoses to unintentional poisoning. Figure A below shows the number of deaths due to unintentional poisoning as an underlying cause of death in the United States in 2000 through 2013 by age. The accompanying table (Table A) shows the fraction of deaths assigned to unintentional poisoning as an underlying cause of death in the United States by substance or drug based on the other causes in the death chain. More than 90% of these types of poisonings are due to exposure to narcotics, psychodysleptics, and other drugs, specified or unspecified. More than 97% of these poisonings by substance or drug occurred in ages 15–65. It is clear that these are not cases of accidental ingestion of substances, but rather that the substance has been deliberately ingested, with unintentional poisoning occurring.

Figure A. Unintentional poisoning deaths in the United States, by age and year

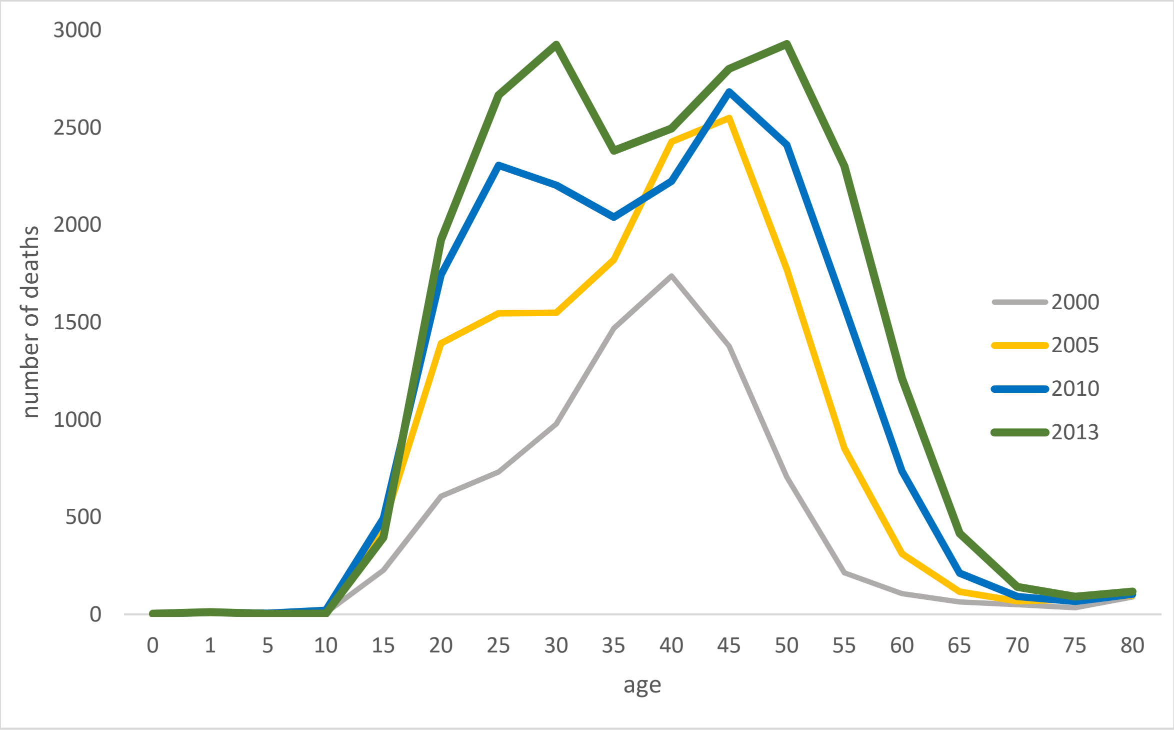

Table A. Fraction of the deaths assigned to unintentional poisoning in the United States by substance or drug

ICD 10 code	ICD 10 definition	Fraction
X42	Accidental poisoning by and exposure to narcotics and psychodysleptics not elsewhere classified	47.7%
X44	Accidental poisoning by and exposure to other and unspecified drugs, medicaments and biological substances	42.9%
X41	Accidental poisoning by and exposure to antiepileptic, sedative-hypnotic, anti-parkinsonism and psychotropic drugs, not elsewhere classified	8.4%
X49	Accidental poisoning by and exposure to other and unspecified chemicals and noxious substances	0.5%
X40	Accidental poisoning by and exposure to non-opioid analgesics, antipyretics and anti-rheumatics	0.5%
X43	Accidental poisoning by and exposure to other drugs acting on the autonomic nervous system	0.1%

Using multiple cause of death records for the United States, Mexico, Brazil, and Australia from 1980 to 2014, we selected all deaths with underlying causes coded to X40–X44 and X49. Table B, below, shows the combination of other potential causes that can be found in the multiple cause of death data for these underlying causes. Based on this table, we proportionally redistributed misassigned unintentional poisoning deaths to one of these causes. The main assumption behind this algorithm is the dominancy of the fatality of some substances when considering a combination of drugs. Given the combination of different drugs and substances in these codes, opium is the main cause of fatality.[8,9] Other substances, like cocaine, methamphetamine, and alcohol with cannabis are less likely to be dominant in fatality.[10]

Table B. Algorithm for the selection and assignment of a substance or drug use cause of death for deaths coded to an underlying cause of unintentional poisoning using multiple cause of death data

Selection Algorithm						
	Opioids	Cannabis	Cocaine	Amphetamines	alcohol	Psychoactive and psychedelic drug
Opioids	Opioids	Opioids	Opioids	Opioids	Opioids	Opioids
Cannabis	Opioids	Cannabis	Cocaine	Amphetamines	alcohol	Psychoactive and psychedelic drug
Cocaine	Opioids	Cocaine	Cocaine	Amphetamines + Cocaine	Cocaine + alcohol	Cocaine
Amphetamines	Opioids	Amphetamines	Amphetamines + Cocaine	Amphetamines	Amphetamines + alcohol	Amphetamines
alcohol	Opioids	alcohol	Cocaine + alcohol	Amphetamines + alcohol	alcohol	Psychoactive and psychedelic drug
Psychoactive and psychedelic drug	Opioids	Psychoactive and psychedelic drug	Cocaine	Amphetamines	Psychoactive and psychedelic drug	Psychoactive and psychedelic drug

For example, if the multiple cause of death data show that 40% of deaths include opioid use disorders as an intermediate cause where the underlying cause is X40–X44, X49, the redistribution proportion for opioid use disorders will be exactly 40% due to the dominance of the fatality of opioid use disorders compared to other drugs in the above table. Similarly, because cannabis is not assumed to have dominance of fatality compared to any of the above drug use disorders, the proportion of X40–X44, X49 redistributed to cannabis use disorders was the percentage of deaths in the multiple cause of death data that had only cannabis use disorders as an intermediate cause and none of the other above causes. If 40% of deaths had cannabis use disorders listed in the intermediate cause list, but those 40% also had one or more of opioids, cocaine, amphetamines, alcohol, or psychoactive and psychedelic drugs, the redistribution proportion to cannabis use disorders would be 0%.

Multiple cause of death data were only available to us for the United States, Brazil, Mexico, and Australia. Because of this limited sample, we applied the result from multiple cause of death analysis from the United States to the United States and Canada, from Brazil and Mexico to Latin America and the Caribbean, and from Australia to Australia, New Zealand, and Western Europe. For other locations, we aggregated the results from the four countries and applied the aggregate pattern. We hope for increased availability of multiple causes of death data in future analyses in order to achieve a more precise distribution for more locations.

Step 5.3 VA anemia adjustment

To compensate for the over-representative cause fractions from anemia found in verbal autopsy studies, we redistributed these deaths based on the causal attribution of severe anemia from the GBD 2013 study. The proportions were country-year-age-sex specific.

2.8 Step 6. HIV/AIDS misclassification correction

In many location-years, certain causes of death known to be comorbid with HIV/AIDS (eg, tuberculosis, other infectious diseases) are seen to have age patterns that diverge from those observed in location-years without widespread HIV epidemics, and are in fact more reflective of HIV mortality trends. In order to identify these instances, a global relative age pattern is generated using all VR deaths in countries with observed HIV prevalence less than 1% using the following:

$$RR_{asc} = {}^{R_{asc}}/_{\bar{x}(R_{65sc}, R_{70sc}, R_{75sc})}$$

Where $RR_{asc}$is the relative death rate for age group *a*, sex *s*, and cause *c*; $R_{asc}$is the rate for that age group; and $\bar{x}(R_{65sc}, R_{70sc}, R_{75sc})$ is the mean of the rates in ages 65–69, 60–74, and 75–79 for that sex and cause. This is preferable to comparing mortality rates because we are able to isolate divergence in age pattern while accounting for varying levels of overall mortality by fixing death rates to age groups that are unlikely to be confounded by the presence of HIV. Expected deaths for an identified cause were then determined to be:

$$ED_{lyasc} = \bar{x}\left(R_{ly65sc}, R_{ly70sc}, R_{ly75sc}\right) * p_{lasc} * RR_{asc}$$

Where ED_{lasc} are deaths for location *l*, year *y*, age group *a*, sex *s*, and cause *c*; $\bar{x}(R_{l65sc}, R_{l70sc}, R_{l75sc})$ is the mean of the rates for ages 65–69, 60–74, and 75–79 for that location-year-sex-cause; p_{lasc} is the population for that location-year-age-sex-cause; and RR_{asc} is the global standard relative rate determined in the previous step for that age-sex-cause. The expected deaths remain attributed to that particular cause, while the difference between observed and expected are reallocated to HIV/AIDS.

2.9 Step 7. Scale strata to province

Over time, a higher proportion of deaths have been registered in China through the expansion of the Disease Surveillance Point (DSP) system and provincial and county efforts to increase cause of death registration. With the expansion of coverage, it is possible that province aggregates do not accurately represent the population distribution between urban and rural areas in each year. For this reason, we stratified the data preparation by urban and rural status for each county within each province. Stratification was based on the median level of urbanisation across counties within each province as recorded in the 2010 China census. In the provinces of Tibet and Hainan, all counties were placed into one strata based on largely homogeneous urbanisation levels within each province. This yielded a total of 62 analytical province-strata. Macao and Hong Kong were not included in this stratification system as the VR systems there are independent from that on the mainland; no weighting scheme needs to be carried out in these complete VR systems with quality data on causes of death.

Within each province-strata, a larger proportion of deaths in-hospital might be reported than that of deaths outside of hospital because of the internet hospital reporting system. To avoid bias, we reweighted in-hospital and out-of-hospital deaths based on the age-sex-province-specific fraction of deaths in and out of hospital in the DSP system. DSP data have been used to establish these percentages because, in these communities, there is a concerted effort to identify all out-of-hospital deaths. Province-strata death rates are combined to produce overall province death rates by weighting each strata by population in each age-sex-year group. Province death rates are rescaled so that all-cause mortality equals the estimated death rate in each age-sex-year estimated in the life-table analysis. The Bayesian noise reduction algorithm was used to deal with zero counts and small number issues for rare causes.[11]

2.10 Step 8. Restrictions post-redistribution

Some causes of death can only be reliably assigned through an autopsy by a trained physician. For example, it is unlikely that a verbal autopsy would reliably distinguish between ischaemic and haemorrhagic stroke.

This step ensures that the detail of the cause list at this point in the data prep process is reasonable given the detail of the original data source and the methods by which the cause of death was assigned. Two primary corrections are applied. First, any cause which is purely an artifact of the redistribution machinery targeting too detailed a cause is aggregated up to the parent cause. Second, a “bridge map” is applied over a certain set of sources to ensure that these sources do not contain causes which could not reliably be determined by the methodology. These two corrections are applied to ICD9-BTL, ICD10-tabulated, USSR tabulated ICD9, India MCCD reports, China-DSP-tabulated-ICD9, India SCD reports, India SRS, and all verbal autopsy sources.

2.11 Step 9. Drop VR country years or mark as non-representative

Lozano and colleagues[12] describe the negative impact that low-completeness vital registration (VR) data can have on cause of death modelling for GBD 2010. In particular, in settings where a data source does not capture all deaths in a population, the cause composition of deaths captured might be different from those that are not. However, a completeness sensitivity test found that low- completeness VR data had little impact on the cause-specific mortality trends at the global level.

For GBD 2016, we investigated the impact of these data at the country and subnational levels using the more thorough diagnostic visualisations available to us. It was determined that these data produced unlikely trends in the models affected. Despite the minimal impact on global trends, better models were produced by eliminating or marking as non-representative data with extremely low completeness. VR completeness was estimated as the number of deaths registered divided by the number of deaths estimated in the GBD mortality envelope.

For this round, vital registration location-years with completeness below 50% were dropped, while location-years with completeness between 50% and 69% were marked as non-representative.

In addition, any country-year with a number of deaths registered to major garbage codes greater than 50% of the deaths registered was dropped.

2.12 Step 10. Cause aggregation

The cause list is organised in a top-down hierarchical format containing four levels. The first group, or Level 1, sums all causes. Following all cause-mortality are Level 2 causes, which include three broad groupings of causes of deaths: communicable, maternal, neonatal, and nutritional diseases; non-communicable diseases; and injuries. Within those Level 2 groupings are finer levels used for modelling. Level 3, or parent causes, are aggregated, meaning the mortality estimate for a parent cause in the hierarchy represents the sum of the causes under that rubric. Sub-causes within Level 3 causes – Level 4 – are more detailed. For example, the parent cause “intestinal infectious diseases” contains the three sub-causes: typhoid fever, paratyphoid fever, and other intestinal infectious diseases. Included in the parent cause estimate are deaths mapped directly to the parent and any Level 4 sub-causes. In data where there was not enough information to assign a Level 4 cause, we aggregated to the Level 3 parent cause. Exceptions to aggregating the Level 4 sub-causes to the parent are instances when certain sub-causes are not present. The United Nations Crime Trends police data only identify homicides, and aggregating homicides to injuries would not accurately represent all injuries.

2.13 Step 11. Remove shocks and HIV/AIDS maternal adjustments

For GBD 2016, CODEm models use an HIV/AIDS- and shock-free envelope. In order to be comparable, cause fractions must also be HIV/AIDS- and shock-free. Cause fractions were uploaded to the Causes of Death database as the number of deaths due to the cause over an adjusted sample in which the number of deaths due to HIV/AIDS, collective violence and legal intervention, and exposure to forces of nature were removed.

Step 11.1 Remove HIV/AIDS, shocks from denominator where HIV/AIDS in cause list

The first step to generate HIV- and shock-free cause fractions was to remove any deaths from the sample which were directly coded to HIV/AIDS, collective violence and legal intervention, or exposure to forces of nature. The resulting equation for a cause fraction uploaded to the database is simple:

$$CF_{l,t,a,x,c} = \frac{D_{l,t,a,x,c}}{D_{l,t,a,x} - D_{l,t,a,x,hiv} - D_{l,t,a,x,war} - D_{l,t,a,x,disaster}}$$

In this equation, $CF_{l,t,a,x,c}$ is the cause fraction for a location (l), year (t), age (a), sex (x), and cause (c), $D_{l,t,a,x,c}$ is the number of deaths observed in the sample for the same, $D_{l,t,a,x}$ is the total number of deaths observed in the sample in the location, year, age and sex, and $D_{l,t,a,x,hiv}$, $D_{l,t,a,x,hiv}$, and $D_{l,t,a,x,disaster}$ are the number of deaths observed in the sample for HIV/AIDS, collective violence and legal intervention, and exposure to forces of nature, respectively.

Cause fractions for HIV/AIDS and shock causes were also uploaded to the database for use in separate estimation processes described by Wang et al.[12] In this case, cause fractions followed the standard equation, with variables following the same explanation as above:

$$CF_{l,t,a,x,c} = \frac{D_{l,t,a,x,c}}{D_{l,t,a,x}}$$

Step 11.2 Remove HIV/AIDS deaths from maternal mortality sources

HIV-free cause fractions were also uploaded for sources on mortality due to maternal causes. In these cases, the sample of all deaths observed in the study is likely to contain some amount of deaths due to HIV/AIDS and shocks, but the sample only includes cause information on maternal deaths. To account for the presence of HIV/AIDS and shocks in the entire sample, we assumed the same proportion of total deaths due to HIV/AIDS by location, age, sex, and year as provided from the estimation of HIV/AIDS and all-cause mortality described by Wang et al.[3]

Maternal mortality studies were only corrected for HIV/AIDS if the sample of total deaths was provided in the data source. Where sources only provided the Maternal Mortality Rate (MMR), we applied the rate to the HIV- and shock-free envelope produced by the analysis described in Wang et al.[3] and thus did not need to adjust cause fractions at this point in the process.

Where a correction was applied, we applied the following equation:

$$CF_{l,t,a,x,mat} = D_{l,t,a,x,mat} * \frac{E[D_{l,t,a,x,hiv\_shock\_free}]}{E[D_{l,t,a,x}]}$$

In this equation, X is the resulting cause fraction due to maternal causes for the location (l), year (t), age (a), and sex (x); $D_{l,t,a,x,mat}$ is the number of observed deaths in the sample due to maternal causes, $E[D_{l,t,a,x}]$ is the GBD estimate of all-cause mortality in the location, year, age, and sex, and $E[D_{l,t,a,x,hiv\_shock\_free}]$ is the GBD estimate of HIV- and shock-free mortality in the location, year, age, and sex.

Step 11.3 HIV/AIDS correction of sibling history, census, and survey data

As described in our analysis from GBD 2013, many studies have failed to find increased mortality in HIV-positive pregnant mothers, but those who have advanced HIV are known to have increased baseline mortality. Prior to GBD 2013, we did not distinguish between deaths in HIV+ women that were caused by pregnancy and those for which the pregnancy was incidental to their death. In order to more explicitly quantify the contribution of pregnancy to death in HIV+ women, and therefore more accurately estimate the maternal death count, we completed two additional analyses for GBD 2013. First, we determined the population attributable fraction (PAF) of HIV/AIDS to pregnancy-related death. Second, we determined the proportion of pregnancy-related deaths in HIV-positive persons that are aggravated by pregnancy and are therefore by definition maternal deaths.

$$PAF = \frac{P(RR - 1)}{1 + P(RR - 1)}$$

Where PAF is the population attributable fraction, p denotes the prevalence of HIV in pregnancy, and RR is relative risk of mortality in HIV+ vs HIV- pregnant females.

To recap our analysis for GBD 2013, we used the paper published by Calvert and Ronsmans to identify sources[13] that could inform Step 1 of our HIV-correction analysis. We independently reviewed each of the component studies in Calvert and Ronsmans' review and extracted data directly, not from the systematic review paper. We identified only one additional study that was not used in Calvert and Ronsmans' analysis. We have, however, not used all the studies included in that review. Specific details are as follows: 1) Figueroa-Damian, et al. was excluded for not including any postpartum deaths at all. 2) In the case of Ryder, et al. and Zvandasara, et al. we excluded those deaths > 12 months after delivery.

3) We excluded the results from Chilongozi, et al. from the site that did not include any HIV-negative patients. 4) Leroy, et al. was not in the bibliography. We could not locate it for review so it was excluded. 5) Kourtis, et al. was extracted with adjustment of the denominator based on the average number of hospitalisations per delivery in each group. 6) Ticconi, et al. was excluded for being both non-representative and including subgroup data from mothers with malaria infection. A total of 21 sources were included in our analysis of the increased mortality risk of HIV+ versus HIV- women in pregnancy.[10] We performed DerSimonian-Laird random effects meta-analysis to derive a pooled estimate of RR of death during pregnancy given HIV positivity.[14] The pooled effect size was 6·40 (95% UI 3·98−10·29), which was then used to calculate an HIV PAF for each country, age group, and year. In order to determine the proportion of those HIV-related deaths that were attributable to maternal causes, we performed a second systematic literature review. This time we sought evidence for the excess mortality risk of pregnancy in those women who are already HIV-positive. Most studies have failed to find such an effect, but most also did not stratify their study population by stage of HIV or ART status. Only two studies did this stratification, with a pooled effect size of 1·13 (95% UI 0·73−1·77).

An updated literature review to inform the relative risk of mortality in pregnancy in HIV-positive versus HIV-negative women had 14 hits, but no usable sources. We completed this search on August 30, 2016, using the following two search strings:

> (HIV[Title/Abstract] OR "Acquired Immunodeficiency Syndrome"[Title/Abstract] OR AIDS[Title/Abstract]) AND ("pregnant"[Title/Abstract] OR "pregnancy"[Title/Abstract] OR "postpartum"[Title/Abstract] OR "post partum"[Title/Abstract]) AND ("mortality"[Title/Abstract] OR "death"[Title/Abstract]) NOT "case report" AND "humans"[MeSH Terms] AND (2011/07/06[PDat] : 2016/12/31[PDat])

Prevalence of HIV in pregnant women was calculated using UNAIDS' Spectrum model. Spectrum is a compartmental HIV progression model used to generate age-specific incidence, prevalence, and death rates from pre-calculated incidence curves and assumptions about intervention scale-up and local variation in epidemiology. For each location, we used UNAIDS' age-specific ratios of fertility in women living with HIV to fertility in women not living with HIV. In most locations, this ratio is assumed to be greater than one in women aged 15–24 and less than one and decreasing as age increases beyond 24. Since Spectrum assumes fertile ages of 15–49, we used the ratio of HIV prevalence in pregnant women to HIV prevalence in the general population at either end of that range to extend estimates to age bands 10–14 and 50–54.

Unlike GBD 2013, when we applied the PAF correction to the envelope of maternal deaths predicted by CODEm, we instead applied country-year-age-group-specific PAF to maternal mortality input data prior to modelling in CODEm. This ensured that both the numerator and denominator of all CF data were internally consistent in their exclusion of background HIV/AIDS mortality. The cause fractions for maternal deaths in sibling history, survey, and census data were therefore adjusted as follows:

$$CF_{l,t,a,x,mat_{adj}} = CF_{l,t,a,x,mat} * (1 - ProP_{hiv_{l,t,a,x}}) \quad ProP_{hiv_{l,t,a,x}} = PAF_{l,t,a,x,hivpos} * (1 - rr_{mat})$$

$$CF_{l,t,a,x,mat_{hiv}} = CF_{l,t,a,x,mat} * ProP_{maternalhiv_{l,t,a,x}}$$

$$ProP_{maternalhiv_{l,t,a,x}} = PAF_{l,t,a,x,hivpos} * rr_{mat}$$

Where:

$rr_{mat} = .13/1.13$ = The proportion of HIV/AIDS deaths during pregnancy that were exacerbated by the pregnancy.

$PAF_{l,t,a,x,hivpos}$ = The population-attributable fraction (PAF) that describes the percentage of all maternal deaths that were HIV-related for the location (l), year (t), age (a), and sex (x=Female))

$CF_{l,t,a,x,mat}$ = The proportion of deaths due to all maternal causes before HIV/AIDS correction for the location, year, age, and sex.

$ProP_{hiv_{l,t,a,x}}$ = The proportion of deaths in pregnancy for the location, year, age, and sex that are estimated to be incidental deaths due to HIV/AIDS, and therefore not a maternal cause of death.

$ProP_{maternalhiv_{l,t,a,x}}$ =The proportion of deaths in pregnancy for the location, year, age, and sex that are estimated to be HIV-positive and maternal deaths which are aggravated by HIV/AIDS.

$CF_{l,t,a,x,mat_{adj}}$ = The proportion of deaths due to maternal causes after the adjustment for the location, year, age, and sex.

$CF_{l,t,a,x,mat_{hiv}}$ = The proportion of deaths due to maternal deaths aggravated by HIV/AIDS after the adjustment for the location, year, age, and sex.

Step 11.4 HIV/AIDS correction of other maternal mortality data

Although there are a specific subset of codes in ICD10 that correspond to HIV/AIDS deaths aggravated by pregnancy, these codes are sparsely used and unreliable. We therefore adapted the method above to also correct VR and VA sources for the systematic exclusion of HIV-related maternal deaths. This correction was calculated in the same manner, using the same input data as above, with the only difference that HIV correction of VR and VA sources resulted in a net increase in maternal CF. Maternal deaths aggravated by HIV/AIDS are calculated as the following:

$$CF_{l,t,a,x,mat_{hivvr}} = CF_{l,t,a,x,matvr} * ProP_{maternalhiv_{l,t,a,x}}$$

$$ProP_{maternalhiv_{l,t,a,x}} = \frac{PAF_{l,t,a,x,hivpos} * rr_{mat}}{1 - PAF_{l,t,a,x,hivpos} \quad * rr_{mat}}$$

Where all symbols are the same as described above.

2.14 Step 12. Noise reduction

To deal with problems of zero counts in vital registration, verbal autopsy, cancer registries, or sibling histories for a given age group in a given year, we use a Bayesian noise-reduction algorithm. For this algorithm, we assume a normal prior and a normal data likelihood. We estimate the normal prior for a given country-series of data by estimating a negative binomial for the fraction of deaths in each age group due to each respective cause with dummy variables for age and year. With two notable exceptions (detailed below), these regressions are country-specific, so borrowing strength over age is only within a data type in a country. The variance of the prior, τ^2, is estimated from the negative binomial regression, taking into account the variance-covariance matrix of the regression coefficients. For the data variance, we use the Wilson approximation which provides an estimate of σ^2 even in cases with a zero count of cause-specific deaths. The posterior estimate for each data point is:

$$Mean = \left(\frac{\tau^2}{\tau^2 + \sigma^2} X + \frac{\sigma^2}{\tau^2 + \sigma^2} \mu\right)$$

$$Variance = \left(\frac{\tau^2 \sigma^2}{\tau^2 + \sigma^2}\right)$$

Where *X* is the mean of the data and *μ* is the mean of the prior. This approach to noise reduction avoids the problem that zero counts in an *ln* rates model or a logit cause fraction model will be dropped from the regression and lead to upward bias in the estimates. This is particularly important in two settings: high-income countries with small numbers of cause-specific deaths, and in the analysis of sibling history data where for any given age group in any given year the number of deaths reported in the survey that are pregnancy-related or the number of deaths from all causes in that age group may be small.

Regarding the exceptions to the regression, the first is that country-years with populations under 1 million are pooled with the region data in order to prevent overdispersion and provide a stronger signal. Additionally, verbal autopsy data diverge from the above description in two ways. First, all data for a

given super-region are pooled together and a study dummy variable is added, allowing for different studies and surveillance sites to borrow strength from one another within a super-region. Second, unless the data are part of a time series (eg, Matlab HDSS), there is no year component to the regression.

2.15 Step 13. COD database and outlier identification

Death rates for different causes of death generally have a stable age pattern. In large populations, these patterns will not change very rapidly over time. We can assume a relatively stable pattern in death rates for all causes except for some epidemic diseases and specific types of injuries. Rare causes in large populations and prevalent causes in small populations usually have stochastic patterns. To correct for these stochastic patterns we implement a noise-reduction process, explained in Step 12.

In vital registration data, we infrequently find one or more data points for specific geography/age/sex/years that lie very far from the stable pattern of death rates. In these situations, the model will usually ignore the data point(s). If the model fails to ignore these data, dramatic jumps or drops can occur in the death rates. When there is no logical explanation for variation in the death rates to this degree, we outlier the data point(s). The selection of data points to outlier occurs after data have been prepped for modelling, as well as during preliminary reviews of the models.

In non-vital-registration sources, data-collection methods and data quality can vary widely from source to source. Where data points in each age-sex-geography-year are very sparse, extreme data points can have a bad effect on regional estimation. In these situations we investigate the study's methods and outlier lower-quality data points.

Identifying outliers in the cause of death data occurs prior to finalisation of models for each cause. We do not automate the selection of outliers, but investigate the source of the offending data as well as reviewing other data sources for the same cause, geography, and year. Ultimately, outliers are identified based on the judgement of the modeller and senior faculty and are reversible to allow for decisions to be revisited in the future.

2.16 Causes of death data star rating calculation

GBD estimates are most accurate when computed with a full time series of complete vital registration with a low percentage of garbage codes. For GBD 2016, we developed a simple star-rating system from 0 to 5 to give a picture of the quality of data available in a given country over the full time series used in GBD estimates. Countries improve in the star rating as they increase availability, completeness, and detail of their mortality data and reduce the percentage of deaths coded to ill-defined garbage codes or highly aggregated causes.

To assign stars, we measure the proportion of deaths registered to a well-defined cause from 1980 to 2016. We call this proportion "percent well-certified". We measure this proportion for each location-year of vital registration and each verbal autopsy study separately, and then combine the yearly measurements into a percent well-certified for the full time series.

For each year of vital registration, percent well-certified is:

$$pct_{wellcertified} = completeness * (1 - pct_{majgarbage})$$

Where:

$$completeness = \frac{registered\ deaths}{GBD\ mortality\ envelope}$$

$$pct_{majgarbage} = \frac{deaths\ coded\ to\ level\ 1\ or\ 2\ garbage\ or\ highly\ aggregated\ cause}{registered\ deaths}$$

Simplifying this equation, one can see that in this case "percent well-certified" is simply the number of deaths that are registered to a well-defined cause (those codes which are not Level 1 or 2 garbage or highly aggregated) divided by the GBD mortality envelope.

ICD10 and ICD9 codes assigned to Level 1 or 2 garbage can be found in Appendix Table 3.

For each verbal autopsy data source, percent well-certified is:

$$pct_{wellcertified} = VerbalAutopsyAdjustment * (1 - pct_{majgarbage})$$

Where:

$$VerbalAutopsyAdjustment = SubAdj * RegAdj * AgeSexCoverage$$

And:

SubAdj:

10% for subnationally representative studies, 100% for nationally representative studies. This adjustment, while arbitrary in its specific value, reflects the bias that can be associated with studies that only cover a potentially non-representative sample of a country's population.

RegAdj:

64% for all verbal autopsy data sources. This accounts for the inaccuracy of verbal autopsy in assigning cause of death compared to medically verified vital registration.

The specific multiplier 0·64 is based on the chance-corrected concordance of Physician Certified Verbal Autopsy (PCVA) versus medical certification by the Population Health Metrics Research Consortium.[15]

Age-Sex Coverage:

The number of deaths estimated in the GBD mortality envelope for the ages and sexes in the study for the country and year divided by the number of deaths estimated in the GBD mortality envelope for the country and year. Studies that only cover children under 5 or maternal mortality, for example, will be highly discounted by this multiplier.

In the case of verbal autopsy, all garbage codes are considered ill-defined, as redistribution for verbal autopsy is highly imprecise. Causes such as “Injuries” or “Cancer” will also be included in major garbage percentage, as this percentage includes use of highly aggregated causes.

Once percent well-certified is calculated for each location-year of vital registration and each verbal autopsy study-year, we then combine these into one measurement for each five-year time interval and the full time series 1980–2016. For each five-year time interval, we take the maximum percent well-certified. Then for 1980–2016, we take the average of the maximum percentages well-certified for the seven five-year time intervals, including any five-year time interval where no data were available as a zero.

Once these values are calculated, we assign stars as follows:
5 stars: 85%–100% well-certified
4 stars: 65%–84% well-certified
3 stars: 35%–64% well-certified
2 stars: 10%–34% well-certified
1 star: >0%–9% well-certified
0 stars: No vital registration or verbal autopsy data available from 1980–2016

While stars are calculated for each five-year time interval as well as the full time series from 1980 to 2016, stars in the main text are presented for the full time series only.

Appendix Table 17 shows the percent well-certified, stars, data source, and underlying values for percent well certified used for each country and time interval.

Section 3: Causes of death modelling methods

3.1 CODEm

3.1.1 Overview of method

Cause of death ensemble modelling (CODEm) is the framework used to model most cause-specific death rates in the GBD.[16] It relies on four key components. First, all available data are identified and gathered to be used in the modelling process. Though the data may vary in quality, they all contain some signal of the true epidemiological process. Second, a diverse set of plausible models are developed to capture well-documented associations in the estimates. Using a wide variety of individual models to create an ensemble predictive model has been shown to outperform techniques using only a single model both in cause of death estimation[16] and in more general prediction applications.[17,18] Third, the out-of-sample predictive validity is assessed for all individual models, which are then ranked for use in the ensemble modelling stage. Finally, differently weighted combinations of individual models are evaluated to select the ensemble model with the highest out-of-sample predictive validity.

For some causes (see, for example, lower respiratory infections), there is evidence that the relationship between covariates and death rates might differ between children and adults. Separate models are therefore run for different age ranges when applicable. Additionally, separate models are developed for countries with extensive, complete, and representative vital registration (VR) for every cause to ensure that uncertainty can better reflect the more complete data in these locations.

3.1.2 Model pool development

Because many factors may covary with any given cause of death, a range of plausible statistical models are developed for each cause. In the CODEm framework, four families of statistical models are used: linear mixed effects regression (LMER) models of the natural log of the cause-specific death rate, LMER models of the logit of the cause fraction, spatiotemporal Gaussian process regression (ST-GPR) models of the natural logarithm of the cause-specific death rate, and ST-GPR models of the logit of the cause fraction (see the 2x2 table in Foreman et al).[16] For each family of models, all plausible relationships between covariates and the response variable are identified. Because all possible combinations of selected covariates are considered for each family of models, multicollinearity between covariates may produce implausible signs on coefficients or unstable coefficients. Each combination is therefore tested for statistical significance (covariate coefficients must have a coefficient with p-value < 0·05) and plausibility (the coefficients must have the directions expected based on the literature). Only covariate combinations meeting these criteria are retained. This selection process is run for both cause fractions and death rates, then ST-GPR and LMER-only models are created for each set of covariates. For a detailed explanation of the covariate selection algorithm, see Foreman et al 2012.[16]

3.1.3 Testing model pool on 15% sample

The performance of all models (individual and ensemble) is evaluated using out-of-sample predictive validity tests. Thirty percent of the data are excluded from the initial model fits. These individual model fits are evaluated and ranked using half of the excluded data (15% of the total), then used to construct the ensembles based on their performance. Data are held out from the analysis based on

the cause-specific missingness patterns for ages and years across locations. Out-of-sample predictive validity testing is repeated 20 times for each model, which has been shown to produce stable results.[16] These performance tests include the RMSE for the log of the cause-specific death rate, the direction of the predicted versus actual trend in the data, and the coverage of the predicted 95% UI.

3.1.4 Ensemble development and testing

The component models are weighted based on their predictive validity rank in order to determine their contribution to the ensemble estimate. The relative weights are determined both by the model ranks and by a parameter ψ, whose value determines how quickly the weights taper off as rank decreases. The distribution of ψ is described in more detail in Foreman et al 2012.[16] A set of ensemble models is then created using the weights constructed from the combinations of ranks and ψ values. These ensembles are tested using the predictive validity metrics described in Section 3.1.3 on the remaining 15% of the data, and the ensemble with the best performance in out-of-sample trend and RMSE is chosen as the final model.

3.1.5 Final estimation

Once a weighting scheme has been chosen, 1,000 draws are created for the final ensemble, with the number of draws contributed by each model proportional to its weight. The mean of the draws is used as the final estimate for the CODEm process, and a 95% uncertainty interval (UI) is created from the 0·025 and 0·975 quantiles of the draws. The validity of the UI can be checked via its coverage of the out-of-sample data; ideally, the 95% UI would capture 95% of these data. Higher coverage suggests that the UIs are too large, and lower coverage suggests overfitting.

3.1.6 Selection of causes for which CODEm is used

CODEm is used to model 205 causes, described in detail below. However, it is unsuitable for use in modelling certain causes, including those with very low death counts, those where cause-specific death record availability is inadequate, or those for which there are marked biases or variability for cause of death certification over time that cannot be fully accounted for with the current garbage code redistribution algorithms. Criteria for causes where CODEm is not used are discussed in further detail in Section 3.2.

3.1.7 Model-specific covariates

A table of CODEm covariates used, level of the covariate, and expected direction of the covariate by cause, sex, age, and location can be found in Appendix Table 6.

3.1.8 Fit statistics for CODEm models

A table of CODEm predictive validity results by cause, sex, and, and location can be found in Appendix Table 7.

3.2 Causes modelled outside of CODEm

3.2.1 Overview

A number of causes required alternative modelling strategies to those used for CODEm, as they were not compatible with CODEm estimation infrastructure and processes. Such unsuitability included having very low death counts; inadequate availability of cause-specific death records; and marked biases or variability for cause of death certification over time which could not be fully accounted for with current garbage code redistribution algorithms. The inclusion of these causes in CODEm often renders its out-of-sample predictive validity testing, a key advantage of using CODEm for cause of death estimation, unstable, or CODEm simply fails to generate plausible mortality rates in the absence of enough VR or VA data. Due to increased data availability and redistribution algorithm refinements, we were able to incorporate several new causes, which were modelled separately for GBD 2013, into CODEm for this iteration of the GBD study; with each annual update of GBD, we aim to add more causes within the CODEm estimation space. For GBD 2016, we used alternative modelling approaches for these causes, including negative binomial models, natural history models, sub-cause proportion models, and prevalence-based models.

3.2.2 Negative binomial models

For ten rare causes of death, there were too few observed deaths in the cause of death database to produce stable estimates. For these causes, we ran negative binomial regression models with either a constant or constant multiplied by the mean assumption for the dispersion parameter, using reverse step-wise model building. We selected between the two model dispersion assumptions on the basis of best fit to the data, using the same method as GBD 2013. For GBD 2015 we also tested zero-inflated Poisson models for these rare causes of death, but rejected them after finding that they did not substantially affect the mean predictions but produced unrealistically large UIs. Descriptions of the modelling process for each of these causes follow.

3.2.3 DisMod-MR 2.1

Until GBD 2010, non-fatal estimates were based on a single data source on prevalence, incidence, remission, or a mortality risk selected by the researcher as most relevant to a particular location and time. For GBD 2010, we set a more ambitious goal: to evaluate all available information on a disease that passes a minimum quality standard. That required a different analytical tool that would be able to pool disparate information presented in varying age groupings and from data sources using different methods. The DisMod-MR 1.0 tool used in GBD 2010 evaluated and pooled all available data, adjusted data for systematic bias associated with methods that varied from the reference and produced estimates by world regions with uncertainty intervals. For GBD 2013, the improved DisMod-MR 2.0 had increased computational speed, allowing computations that were consistent between all disease parameters at the country rather than region level. The hundred-fold increase in speed of DisMod-MR 2.0 was partly due to a more efficient rewrite of the code in C++ but also by changing to a model specification using log rates rather than a negative binomial model used in DisMod-MR 1.0. In cross-validation tests, the log rates specification worked as well or better than the negative binomial specification.[19] For GBD 2015, the computational engine (DisMod-MR 2.1) remained substantively unchanged but we re-wrote the “wrapper” code that organised the flow of data and settings at each level of the analytical cascade. The sequence of estimation occurred at five levels: global, super-region,

region, country, and, where applicable, subnational locations (see flow diagram of DisMod-MR 2.1 cascade, below). The super-region priors were generated at the global level with mixed-effects, non-linear regression using all available data; the super-region fit, in turn, informed the region fit, and so on down the cascade. The wrapper gave analysts the choice to branch the cascade in terms of time and sex at different levels depending on data density. The default used in most models was to branch by sex after the global fit but to retain all years of data until the lowest level in the cascade. For GBD 2015, we generated fits for the years 1990, 1995, 2000, 2005, 2010, and 2015.

In updating the "wrapper," we consolidated the code base into a single language, Python, to make the code more transparent and efficient and to better deal with subnational estimation. The computational engine is limited to three levels of random effects; we differentiated estimates at the super-region, region, and country levels. In GBD 2013, the subnational units of China, Mexico, and the UK were treated as "countries" such that a random effect was estimated for every location with contributing data. However, the lack of a hierarchy between country and subnational units meant that the fit to country data contributed as much to the estimation of a subnational unit as the fits for all other countries in the region. We found inconsistency between the country fit and the aggregation of subnational estimates when the country's epidemiology varied from the average of the region. Adding an additional level of random effects required a prohibitively comprehensive rewrite of the underlying DisMod-MR engine. Instead, we added a fifth layer to the cascade, with subnational estimation informed by the country fit and country covariates, plus an adjustment based on the average of the residuals between the subnational unit's available data and its prior. This mimicked the impact of a random effect on estimates between subnationals.

For GBD 2015 we improved how country covariates differentiate non-fatal estimates for diseases with sparse data. The coefficients for country covariates were re-estimated at each level of the cascade. For a given location, country coefficients were calculated using both data and prior information available for that location. In the absence of data, the coefficient of its parent location was used, in order to utilise the predictive power of our covariates in data sparse situations.

For GBD 2016, the DisMod-MR 2.1 tool was used. Updates included estimation of new age groups through the GBD 2016 terminal age group of 95+, in addition to the new locations added for the GBD 2016 cycle. Please see Appendix Figure 3 for details of the GBD 2016 DisMod-MR 2.1 analytical cascade.

DisMod-MR 2.1 likelihood estimation

Analysts have the choice of using a Gaussian, log-Guassian, Laplace, or log-Laplace likelihood function in DisMod-MR 2.1. The default log-Gaussian equation for the data likelihood is:

$$-log\left[p\left(y_j|\Phi\right)\right] = \log\left(\sqrt{2\pi}\right) + \log\left(\delta_j + s_j\right) + \frac{1}{2}\left(\frac{\log\left(a_j + \eta_j\right) - \log\left(m_j + \eta_j\right)}{\delta_j + s_j}\right)^2$$

where, yj is a "measurement value" (ie, data point); Φ denotes all model random variables; ηj is the offset value, eta, for a particular "integrand" (prevalence, incidence, remission, excess mortality rate, with-condition mortality rate, cause-specific mortality rate, relative risk, or standardised mortality ratio), and aj is the adjusted measurement for data point j, defined by:

$$a_j = e^{(-u_j - c_j)} y_j$$

where uj is the total "area effect" (ie, the sum of the random effects at three levels of the cascade: super-region, region, and country) and cj is the total covariate effect (ie, the mean combined fixed effects for sex, study-level, and country-level covariates), defined by:

$$c_j = \sum_{k=0}^{K[I(j)]-1} \beta_{I(j),k} \hat{X}_{k,j}$$

with standard deviation

$$s_j = \sum_{l=0}^{L[I(j)]-1} \zeta_{I(j),l} \hat{Z}_{k,j}$$

where k denotes the mean value of each data point in relation to a covariate (also called x-covariate); I(j) denotes a data point for a particular integrand, j; βI(j),k is the multiplier of the kth x-covariate for the ith integrand; $\hat{X}_{k,j}$ is the covariate value corresponding to the data point j for covariate k; l denotes the standard deviation of each data point in relation to a covariate (also called z-covariate); ζI(j),k is the multiplier of the lth z-covariate for the ith integrand; and δ_j is the standard deviation for adjusted measurement j, defined by:

$$\delta_j = log\left[y_j + e^{(-u_j - c_j)}\eta_j + c_j\right] - log\left[y_j + e^{(-u_j - c_j)}\eta_j\right]$$

Where m_j denotes the model for the jth measurement, not counting effects or measurement noise and defined by:

$$m_j = \frac{1}{B(j) - A(j)} \int_{A(j)}^{B(j)} I_j(\mathrm{a})\,\mathrm{da}$$

where A(j) is the lower bound of the age range for a data point; B(j) is the upper bound of the age range for a data point; and I(j) denotes the function of age corresponding to the integrand for data point j.

The source code for DisMod-MR 2.1 as well as the wrapper code are available at http://ihmeuw.org/dismod-ode.

3.2.4 Natural history models

For some causes where cause of death data may be systematically biased due to either misclassification or because the disease exists in focal communities without vital registration or verbal autopsy studies, we have developed natural history models. In natural history models incidence and case-fatality rates are modelled separately and then combined to produce estimates of cause-specific mortality.

3.2.5 Prevalence-based models

The modelling strategies for Alzheimer's and other dementias, Parkinson's disease, and atrial fibrillation and flutter are distinct from those used for other causes modelled as natural history models. These models use prevalence estimates and excess mortality rates (EMR) generated through DisMod-MR 2.1, rather than incidence and case-fatality rates.

3.2.6 Sub-cause proportion models

For certain sub-causes for which accurate diagnoses are known to be very difficult, we first modelled the parent cause in the GBD hierarchy with CODEm and then allocated deaths to specific causes using proportions of the parent cause for each age-sex-location-year for each sub-cause. For these causes, we identified no significant predictors in negative binomial regressions. This approach was taken because the available data on these specific causes may come from sources other than VR, such as end-stage renal disease registries, or come from too few places to model the death rates directly. Details for each cluster of causes analysed in this way are below.

3.3 Cause of death modeling descriptions

Tuberculosis

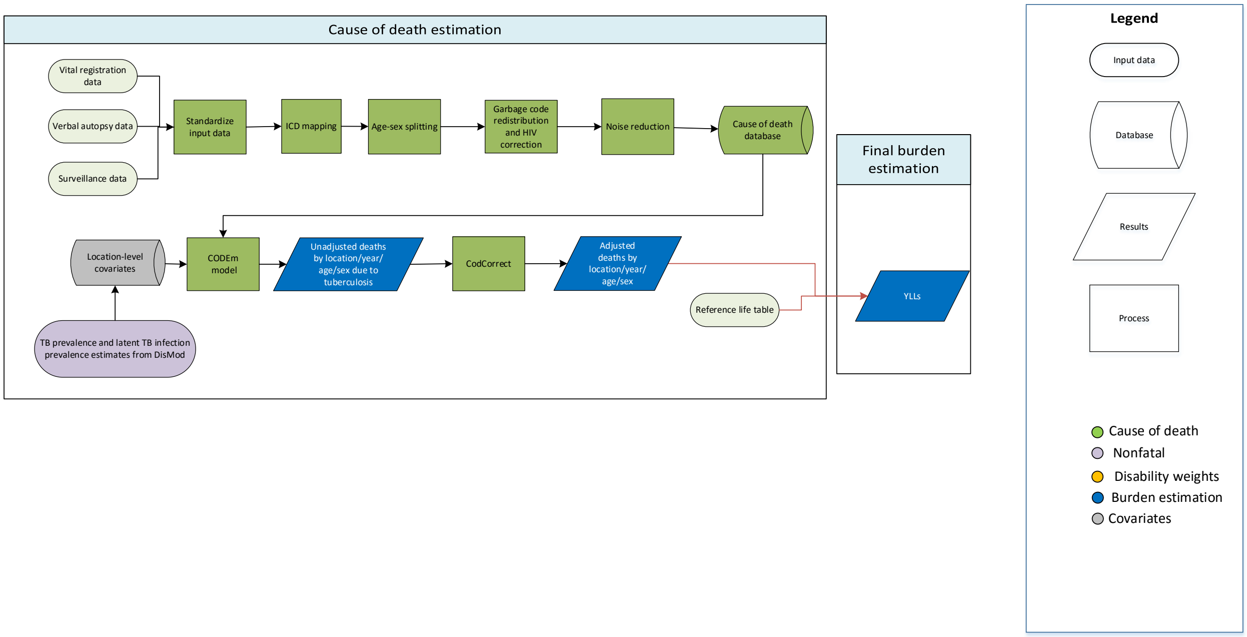

Input data

Input data for modeling tuberculosis mortality among HIV-negative individuals include vital registration, verbal autopsy, and surveillance data. Vital registration data were adjusted for garbage coding (including ill-defined codes, and the use of intermediate causes) following GBD algorithms and misclassified HIV deaths (i.e., HIV deaths being assigned to other underlying causes of death such as tuberculosis or diarrhea because of stigma or misdiagnosis). This correction was done based on examining changes in the age pattern of diseases over time.

Verbal autopsy data in countries with age-standardized HIV prevalence greater than 5% were removed because of a high probability of misclassification, as verbal autopsy studies have poor validity in distinguishing HIV deaths from HIV-TB deaths.

Modeling strategy

We changed the modeling strategy of tuberculosis in GBD2016 by first modeling prevalence of disease and prevalence of latent infection which were then used as covariates in the CODEm model. We dropped the health system access covariate and replaced it by the newly developed Healthcare Access and Quality Index covariate. We also added the adult underweight proportion covariate. Other location-level covariates included in the CODEm model were the same as in GBD 2015: alcohol (liters per capita), diabetes (fasting plasma glucose mmol/L), education (years per capita), lag-distributed income, indoor air pollution, outdoor air pollution, population density, smoking prevalence, sociodemographic status, and a summary exposure variable reflecting the average exposure to all of the risk factors.

Multidrug-resistant tuberculosis, extensively drug-resistant tuberculosis, and drug-susceptible tuberculosis

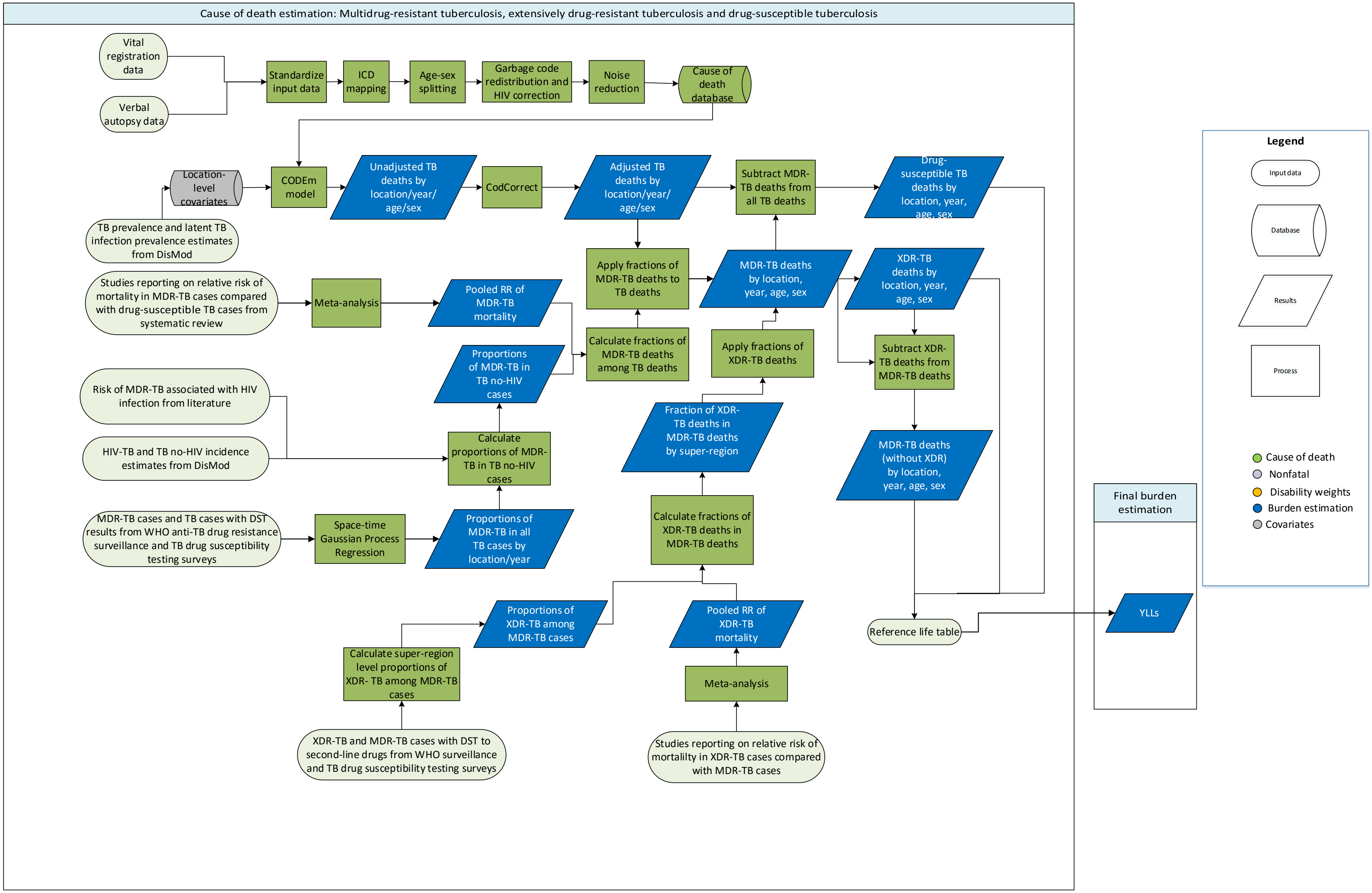

Input data

Input data include: (i) the number of drug-resistant cases by type (multidrug-resistant tuberculosis [MDR-TB], extensively drug-resistant tuberculosis [XDR-TB], all TB cases with a drug sensitivity testing [DST] result for isoniazid and rifampicin, and MDR-TB cases with DST for second-line drugs) from routine surveillance and surveys reported to the World Health Organization, (ii) the relative risk of death in MDR-TB cases compared with non-MDR TB (drug-susceptible TB) cases, and , and the relative risk of death in XDR-TB cases compared with MDR-TB cases reported by studies identified through our systematic review, and (iii) the risk of multi-drug resistant tuberculosis associated with HIV infection from the literature.[1]

Modelling strategy

We conducted a systematic review and meta-analysis of studies reporting the relative risk of death in MDR-TB cases compared with drug- susceptible TB cases. We also ran a spatiotemporal Gaussian process regression to predict the proportions of MDR-TB cases among all TB cases for all locations and years. The input data for this regression (i.e., proportions of MDR-TB cases among all TB cases) were based on the number of MDR-TB cases, and the number of TB cases with DST for isoniazid and rifampicin from routine surveillance and surveys reported to the World Health Organization. We then used the predicted proportions to MDR-TB cases among all TB cases, along with the HIV-TB and TB no-HIV incidence estimates (from our modeling of non-fatal TB), and the relative risk of MDR-TB associated with HIV infection from the literature[1] to compute the proportions of MDR-TB cases among HIV negative TB cases $(P_{c,y,a,s})$ by location, year, age, and sex using the following formula:

$$P_{c,y,a,s} = \frac{MDR_{c,y,a,s}}{\left(1 + \left(rr\frac{HIVTB_{c,y,a,s}}{TBnoHIV_{c,y,a,s}}\right)\right) TBnoHIV_{c,y,a,s}}$$

where $MDR_{c,y,a,s}$ is the number of all MDR-TB cases among HIV-positive and HIV-negative individuals, *rr* is the relative risk of MDR-TB associated with HIV infection, $HIVTB_{c,y,a,s}$ is the number of HIV-TB incident cases by location, year, age, and sex, and $TBnoHIV_{c,y,a,s}$ is the number of TB no-HIV incident cases by location, year, age, and sex.

We then computed the fraction of MDR-TB deaths among all HIV-negative TB deaths ($D_{c,y,a,s}$) using the following formula:

$$D_{c,y,a,s} = \frac{P_{c,y,a,s}RR}{P_{c,y,a,s}RR + 1 - P_{c,y,a,s}}$$

where *RR* is the relative risk of death in MDR-TB cases compared with drug- susceptible TB cases. We then applied the predicted fractions of MDR-TB deaths among HIV-negative TB deaths to our CODEm TB death estimates to generate MDR-TB deaths by location, year, age, and sex. Next, we subtracted MDR-TB deaths from all TB deaths to generate drug- susceptible TB deaths by location, year, age, and sex.

To separate out XDR-TB from MDR-TB, we aggregated the XDR-TB cases and MDR-TB cases (with DST for second-line drugs) up to the super-region level and calculated the super-region level proportions of XDR-TB among MDR-TB cases. Next, we computed the superregion-year-specific fractions of XDR-TB deaths among all MDR-TB deaths $(D_{XDRsr,y})$ using the following formula:

$$D_{XDRsr,y} = \frac{P_{XDRsr,y}RR_{XDR}}{P_{XDRsr,y}RR_{XDR} + 1 - P_{XDRsr,y}}$$

where RR_{XDR} is the pooled relative risk of mortality in XDR-TB cases compared with MDR-TB cases. These fractions were then applied to MDR-TB deaths in corresponding countries within the super-regions to produce XDR-TB deaths by location, year, age, and sex. We linearly extrapolated XDR-TB mortality rates back assuming the mortality rates were zero in 1992, one year before 1993 when XDR-TB was first recorded in USA surveillance data.[2] Finally, we subtracted XDR-TB deaths from MDR-TB deaths to generate MDR-TB (without extensive drug resistance) deaths by location, year, age, and sex.

Reference

1. Mesfin YM, Hailemariam D, Biadglign S, Kibret KT. Association between HIV/AIDS and multi-drug resistance tuberculosis: a systematic review and meta-analysis. PLoS One. 2014;9(1):e82235.
2. Centers for Disease Control and Prevention (CDC). Extensively Drug-Resistant Tuberculosis --- United States, 1993—2006. MMWR. 2007; 56(11);250-253

HIV/AIDS

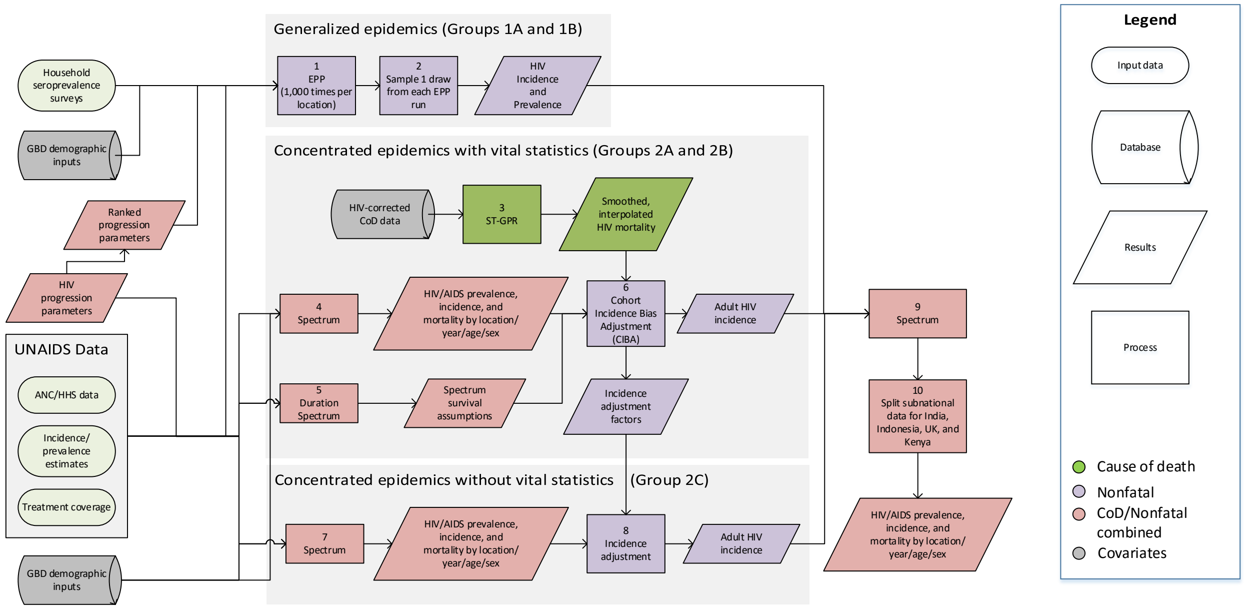

Input data

Household seroprevalence surveys

Geographically representative HIV seroprevalence survey results were used as inputs to the model for countries with generalized HIV epidemics where available.

GBD demographic inputs

Location-specific population, fertility, and HIV-free survival rates from GBD 2016 and migration data from UNAIDS were used as inputs in modeling all locations.

UNAIDS data

Antenatal care, incidence, prevalence, and treatment coverage data from UNAIDS were used in modeling for all locations.

On-ART literature data

Data were identified by using search terms “HIV,” “mortality,” and “antiretroviral therapy” in PubMed searches across the literature. To be included, studies must include only HIV-positive people who receive antiretroviral therapy (ART) but who were ART-naïve prior to the study. In addition, studies must report either a duration-specific mortality proportion or a hazard ratio across age or sex, and must not include children.

For duration-specific survival data, studies must report uncertainty on mortality estimates or provide stratum-specific sample sizes and must include duration-specific data to allow for calculation of 0-6, 7-12, or 13-24 month conditional mortality. In addition, studies must either report separate mortality and loss-to-follow-up (LTFU) curves, be corrected for LTFU using vital registration data, or be conducted in a

high-income setting. Finally, studies must report the percent of participants who are male, the median age of participants, and either data with specific data on the number of CD4 T lymphocytes (CD4 counts) or the median CD4 count used for the data.

Hazard ratio data for ages or sexes can only be used if the hazard ratios are controlled for other variables of interest (age, sex, and CD4 category).

Changes for GBD 2016

In GBD 2013, we identified 102 papers for extraction. For GBD 2015, we included 13 additional studies informing the duration-specific mortality estimation process and 26 studies informing the age and sex hazard ratio estimation process (some studies were used and counted in both). We also added one study to our LTFU analysis. For GBD 2016, we included 12 additional studies informing the duration-specific mortality estimation process and 11 studies informing the age and sex hazard ratio estimation process (some studies were used and counted in both).

Off-ART literature data

In GBD 2013, to characterize uncertainty in the progression and death rates, we systematically reviewed the literature on mortality without ART. We searched terms related to pre-ART or ART-naive survival since seroconversion.[1] After screening, we identified 13 cohort studies that included the cohorts used by UNAIDS from which we extracted survival at each one-year point after infection. Screening for additional, recently published studies in GBD 2015 and GBD 2016 identified no new cohort studies for inclusion in this analysis.

Burden estimation

The files compiled by UNAIDS for their HIV/AIDS estimation process were our main source of data for producing estimates of HIV burden. These files are typically country-specific and contain both demographic data (population, fertility, migration, and HIV-free survival rates) and HIV-specific information. In all cases except migration, we substituted in our own, internally consistent demographic estimates. The HIV-specific information includes what is needed to run both the Spectrum and Estimation and Projection Package (EPP) models. Spectrum requires data on AIDS mortality among people living with HIV with and without ART, CD4 progression among people living with HIV not on ART, ART coverage among adults and children, coverage of breastfeeding among women living with HIV, prevention of mother-to-child transmission coverage, and CD4 thresholds for treatment eligibility. EPP uses many of the same assumptions as Spectrum but fits a simpler model to HIV prevalence data from surveillance sites and large household surveys. We extracted all of these data from UNAIDS' proprietary formats.

For GBD 2016, we received national level files for 81 countries and subnational level files for 6 countries. For many of the missing countries, we had UNAIDS files from the previous estimation process, which we used again. After combining, we were left with a set of 42 countries for which we had never received a UNAIDS file, many of them countries with small populations and/or lower HIV prevalence. In those places, we generated regional averages of all needed inputs. This enabled us to run Spectrum for every GBD location.

In several cases, we have modified the structure or data in the UNAIDS files. In South Africa, which has been estimated at the province level since GBD 2015, we split the national-level UNAIDS file into nine provincial datasets. We used GBD 2016 demographic inputs for the provinces. These provinces are

already fit as separate subpopulations in EPP, so we extracted the prevalence data for the individual provinces and assumed national rates for all other Spectrum inputs. In some locations that are estimated only at the national level in GBD 2016, we received subnational files from UNAIDS. In these cases, we split GBD 2016 demographic input data using the subnational relative relationships found in the UNAIDS files. Additionally, we identified that the ratio of fertility in HIV-positive women to HIV-negative women was negative in Indonesia. We used linear extrapolation to replace this value.

We used all available sources of vital registration and sample registration data from the GBD Causes of Death database after garbage code redistribution and HIV/AIDS mis-coding correction, except in Group 1A countries as described below.[2,3] There are two different cause of death data sources for HIV/AIDS in China: the Disease Surveillance Point (DSP) system and the Notifiable Infectious Disease Reporting (NIDR) system. Both systems are administered by the Chinese Center for Disease Control and Prevention, but the reported number of deaths due to HIV is significantly lower in DSP. Therefore, we have used the provincial-level ratio of deaths due to HIV/AIDS from NIDR to those from DSP, choosing the larger ratio between years 2013 and 2014, and scaled the reported deaths in the DSP system, which is in turn used in the Space-Time Gaussian Process Regression (ST-GPR) process.

Modeling strategy

In GBD 2016, our general modeling strategy for estimating HIV incidence, prevalence, and mortality is very similar to the strategy used in GBD 2015. We continue to use the Spectrum program rewritten in Python for GBD 2013 to facilitate faster and more flexible execution necessary for our more intensive computational needs. We made several changes to Spectrum's assumptions comparing to the Spectrum software used by UNAIDS. We also again ran EPP using an open-source computer program in R written by Jeffrey Eaton.[4] We ran EPP for all Group 1 countries in order to produce incidence and prevalence estimates that were consistent with the demographic and epidemiological assumptions used in GBD 2016.

On-ART

First, we corrected reported probabilities of death for loss to follow-up using an update of the approach developed by Verguet and colleagues.[5] Verguet and colleagues used tracing and follow-up studies to empirically estimate the relationship between death in LTFU and the rate of LTFU.

To create estimates of age-specific hazard ratios, we synthesized hazard ratio data in five broad age groups: 15-25, 25-35, 35-45, 45-55, 55-100, and modeled the data using DisMod-MR 2.0.

To create estimates of sex-specific hazard ratios, we use the *metan* function in Stata to create estimates of relative risks separately by region, using female age groups as the reference group.

The age and sex hazard ratios were applied to the study level mortality rates, accounting for the distribution of ages and sexes in the mortality data. We then subtracted HIV-free mortality from the model life table process to calculate study level age-sex HIV-specific mortality.

We used DisMod-MR 2.0 to synthesize the age-sex split study level data into estimates of conditional probability of death over initial CD4 count.[1] We modeled the data separately by duration, age, and sex and added a fixed effect on whether the study was conducted prior to 2002. We estimated all three regions together using a fixed effect for each region.

Changes for GBD 2016

In GBD 2016, we chose to age-sex split the data at the study level so that we could consider study-specific age-sex distributions, whereas previous GBD iterations relied upon region-specific distributions. We also subtracted study-specific HIV-free mortality rather than region-specific HIV-free mortality. Another change was a switch to estimating all regions together with fixed effects for each region. This allowed us to impart a CD4 trend in sub-Saharan Africa and other developing country estimates that led to more realistic estimates in the high CD4 categories where little data was available from those regions.

Another methods change for GBD 2016 was the distribution of ART coverage by age, sex, and CD4 count. We used two AIDS Indicator Surveys (Kenya 2012 and Uganda 2011) to predict the age-sex-CD4 distribution of ART coverage and applied those distributions to the input counts of people receiving ART in Spectrum. This shifted the coverage distribution to groups with higher CD4 counts, as was seen in the data.

Off-ART

Following UNAIDS assumptions, no-ART mortality is modeled as shown in the figure below.[1]

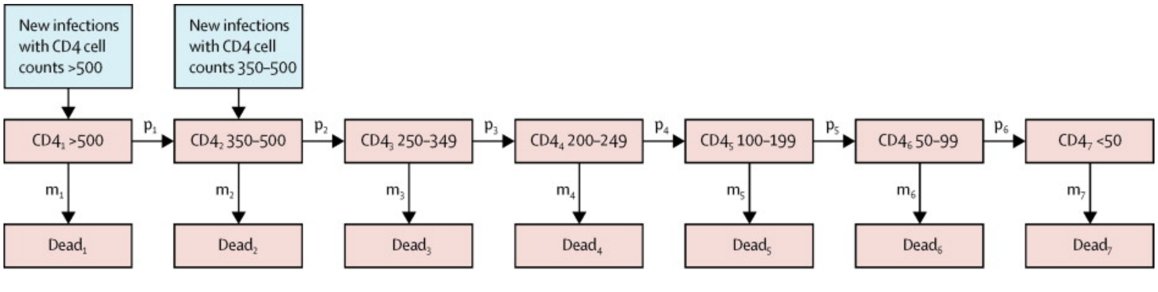

$$m_i = ae^{bi} \qquad p_i = \frac{c + di}{w_i}$$

The death and progression rates between CD4 categories vary by age according to four age-groups, 15–24 years, 25–34 years, 35–44 years, and 45 years or older. We modeled the logit of the conditional probability of death between years in these studies using the following formula:

$$logit\,(m_{ijk}) = \beta_0 + \sum_{i=1}^{4} \beta_{1i}\, a_i + \sum_{j=1}^{12} \beta_{2j} t_j + u_k + \varepsilon_{ijk}$$

In the formula, m is conditional probability of death from year t_j to t_{j+1}, a_i is an indicator variable for age group at seroconversion (15–24 years, 25–34 years, 35–44 years, and 45 years or older), t_j is an indicator variable of year since seroconversion, and u_K is a study-level random effect.

By sampling the variance-covariance matrix of the regression coefficients and the study-level random effect, we generated 1,000 survival curves for each age group that capture the systematic variation in survival across the available studies. For each of the 1,000 survival curves, we use a framework modeled after the UNAIDS optimization framework in which we find a set of progression and death rates that minimizes the sum of the squared errors for the fit to the survival curve.[6,7]

Burden estimation overview

UNAIDS uses two key analytical components in their epidemiological estimation. EPP is used to estimate incidence trajectories that are consistent with prevalence surveys and other prevalence measurements such as antenatal clinic serosurveillance. Spectrum is a compartmental HIV progression model used to

generate age-specific incidence, prevalence, and death rates from the EPP incidence curves and assumptions about intervention scale-up and local variation in epidemiology.

For GBD 2013, we created a replica of Spectrum in Python. This enabled us to run thousands of iterations of the model at once on our computing cluster and allowed for more flexible input data structures. Additionally, in order to generate estimates with more realistic ranges of uncertainty than those in UNAIDS 2012, we adjusted all input data by uniformly sampled factors between 0.9 and 1.1. These changes, along with our new estimation of on- and off-ART mortality and CD4 progression parameters, persist into GBD 2016.

Due to the substantial differences in the quality and types of data available across different countries, we used three different methodologies to produce year-, age-, and sex-specific estimates of HIV incidence, prevalence, and mortality.

Countries with high HIV prevalence and available seroprevalence surveys or antenatal clinic data (Groups 1A and 1B)

We identified 50 countries – as well as subnational locations in India, Kenya, and South Africa – with at least 0.5% adult HIV prevalence and at least one geographically representative HIV seroprevalence survey or available antenatal care clinic (ANC) data. In order to ensure that our estimates of incidence and prevalence in these places were consistent with our estimates of HIV progression, we used a version of EPP written in R and C++ by Jeffrey Eaton to create new fits to the available prevalence data. The version of EPP used in GBD 2016 was an updated release from Jeffrey Eaton since completion of GBD 2015. In this new version, an ANC prevalence adjustment was included and incorporated with the 2016 lookup database and an additional parameter to estimate ANC variance inflation was included as well. In the ANC bias adjustment, instead of using the default universal assumption of the prior mean and standard deviation (SD) of the distribution that the adjustment follows, we selected the parameters based on each sub-population (general population and high risk population) in each location. For sub-populations with prevalence survey data, we used the default assumption with mean=0.15 and SD=1. For subpopulations without prevalence survey data, we chose the region/epidemic specific mean and SD based on the median probit difference and probit difference SD in Table 1 of Marsh et al.[8]

India’s HIV epidemic is classified as concentrated in specific subpopulations rather than generalized to the full population, and only one prevalence survey, the 2005-2006 National Family Health Survey (NFHS-3), was available, so we used modified parameters for Indian states in EPP. We first calculated the mean of the median probit difference between men and women for “Countries with concentrated epidemics” in Table 1 of March et al as mentioned above, which was 0.245. Then we derived empirical parameters based on the difference between the ANC data and the NFHS-3 survey data in probit space to use for the general population. Specifically, we calculated the probit difference by taking the median of all raw ANC prevalence in years 2004 through 2006 and comparing to the 2005 prevalence survey data in probit space for three states with large HIV epidemics: Andhra Pradesh, Karnataka, and Maharashtra. From this empirical parameter derivation, we got the mean and SD value based on the three states as 0.124 and 0.051, respectively. We then used linear interpolation between the prevalence with a prior of 0.245 and the new prior of 0.124 to recalculate the mean and keep the SD the same as the empirical estimates. The final assumption of the prior mean and SD were 0.182 and 0.051, respectively. We did not make any adjustments for high risk populations.

In the new version of EPP, in addition to the equilibrium prior assumption of the force of infection in projection, a random walk approach is available as an alternative method. For locations with two or more prevalence surveys and a declining trend between the mean of the most recent two surveys, the random walk approach was chosen to project the force of infection. We assumed the change of the log scaled force of infection was following a normal distribution with mean equal to the median of the change of the modeled force of infection among the years having ART implemented or prevalence data, and the SD was equal to the default setting as the mean SD of the change of the modeled force of infections among the years having prevalence data. The projection year was chosen from the most recent year between the year with the lowest model force of infection and the year of the second latest survey data.

For Indian states, we used the equally weighted draw-level estimates of the equilibrium prior and random walk assumptions since we had no further information to support either assumption for each state. Here, the projection year of the random walk was the year with the lowest modeled force of infection because no locations had more than one prevalence survey, and the assumption of increasing ART coverage was supported by the data available to us.

In the new EPP code, an optimization step was added into IMIS function to speed up the parameter sampling step based on Raftery and Bao[9]. Two optimization methods have been introduced. The main algorithm is Broyden–Fletcher–Goldfarb–Shanno (BFGS) optimization. If BFGS fails, Nelder-Mead optimum is used instead. In our 2016 EPP model, by substituting in our own assumptions about HIV progression rates and on/off ART mortality, we were able to ensure that the implied relationship between incidence and mortality/prevalence in EPP is similar to that in Spectrum.

In Group 1 locations, we expect estimates of HIV burden to exhibit substantial uncertainty. To reflect this, we induced a perfect correlation between the previously independent draws of HIV mortality with and without ART and CD4 progression. We paired the draws of the three parameter sets internally and with each other in the following way: we sorted without-ART mortality and CD4 progression internally by age (not CD4), meaning the highest draw of HIV mortality without ART for age a_i and CD4 category c_l will be paired with the highest draw of HIV mortality without ART for age a_k and CD4 category c_l. In the same way, we sorted with-ART mortality internally by age, sex, CD4 count at treatment initiation, and duration on treatment. After this sorting process, the lowest indexed draw of each parameter has the highest values and vice versa. This means that we will use the most extreme possible parameter sets in EPP and Spectrum and should see a commensurate expansion in the range of the uncertainty.

To ensure that this expanded uncertainty is replicated in EPP, we fit the model once for every set of paired draws of the progression parameters for every location. This means that the first iteration of EPP for Uganda sees the highest draws of all three sets of progression parameters. Such a procedure is necessary because EPP currently has no mechanism for incorporating uncertainty in any inputs except prevalence data. This process (Process 1 in the HIV/AIDS Estimation Flowchart), produced 1,000 sets of EPP output for each of the locations that make up the 48 countries in the group. Every set of EPP outputs contains 500 consistent draws of HIV incidence and prevalence in adults aged 15-49. In many cases, the algorithm used to fit EPP, incremental mixture importance sampling, failed, resulting in fewer than 1,000 sets of EPP results.

For every location in the group, we sampled one of the 500 incidence/prevalence draws from each of the sets of EPP results (Process 2 in the HIV/AIDS Estimation Flowchart). By sampling one draw from

each set, we ensured that the distribution of progression parameters dictating the relationship between incidence and prevalence was exactly the same as the distribution of the sorted parameters generated in the previous step. In locations where not all 1,000 iterations of EPP fit successfully, we sampled one draw from every iteration that did succeed and then resampled with replacement from that set of draws. To maintain the link between the input progression draws and the resulting incidence and prevalence draws from EPP, we replaced any parameter draw associated with a failed run of EPP with the parameter draw that that failed draw was replaced with. At the end of this process, for every location in the set of 48 countries, we were left with 1,000 linked draws of adult incidence and prevalence and the exact progression parameters that generated those draws.

We then ran these results, along with the previously described demographic and HIV-specific inputs, through Spectrum to produce location-, year-, age-, and sex-specific estimates of HIV incidence, prevalence, and mortality (Process 9 in the HIV/AIDS Estimation Flowchart).

Countries with vital registration data (Group 2A and 2B)

Vital registration is one of the highest-quality sources of data on HIV burden in many countries, so generating estimates that are consistent with these data, with necessary adjustment to account for any potential underreporting, is critical. We identified 114 countries – as well as 440 subnational locations from Brazil, China, Japan, Indonesia, Mexico, Sweden, the United Kingdom, and the United States – with at least two usable points of vital registration data, verbal autopsy data, or sample registration system data such as DSP in China.

We imputed missing years of data to generate a complete time series for HIV from the estimated start year of the epidemic using ST-GPR. We analyzed mortality trends using ST-GPR starting in 1981, the year that HIV was first identified in the United States.[10] For ST-GPR, we adjusted the lambda (time weight) and GPR scale according to the completeness of vital registration data, with 4- and 5-star quality VR using parameters designed to follow the data more closely. We produced separate splines by country/age group, up to the peak year of death rate. We then ran a linear regression with random effects on region, age, and sex. Following this, we ran space-time residual smoothing, in which time, age, and space weights are used to inform smoothing of the residuals between data points and the linear regression estimate. From this process, we generated space-time estimates with the applied weights, along with the median absolute deviation (MAD) of the space-time estimates from the data. The MAD was calculated at various levels of the geographic hierarchy (e.g., subnational and national), and was added into the data variance term. The data variance and space-time estimates were then analyzed using Gaussian Process Regression to return a final estimate of mortality along with uncertainty.

Although Spectrum produces HIV mortality estimates that are within the realm of possibility in most countries using the incidence curves provided in the UNAIDS 2012/2015 country files, it is a deterministic model that has not yet been integrated into an optimizable framework. Therefore, in order to “fit” it to vital registration data, we need to adjust input incidence.

To improve the fit of this process, in GBD 2015, we restructured Spectrum to add compartments that identify groups of people living with HIV by year of infection (Process 5 in the HIV/AIDS Estimation Flowchart). With this version of Spectrum we can output, among many other metrics, HIV deaths by year, age, sex, and infection cohort. This enables us to adjust incidence to fit to death much more precisely and without making any rigid assumptions about the time from HIV infection to HIV death.

We incorporated these improvements into a cohort incidence bias adjustment (CIBA) process. First, we ran Spectrum normally to produce 1,000 draws of incidence, prevalence and mortality (Process 4 in the HIV/AIDS Estimation Flowchart). Then, by year, age, and sex, we took the ratio of VR deaths to Spectrum deaths to quantify the amount of bias in Spectrum. Using the mean duration data from the new version of Spectrum, for every year-, age-, and sex-specific infection cohort, we calculated the share of all HIV deaths observed over the course of the projection period in that cohort that would occur in each year after the year of infection. For example, projecting from 1970 through 2015, we identified the cohort of men infected in 1992 at the age of 16, calculated the total number of HIV deaths in that cohort in all subsequent years through the end of 2015, and divided the annual number of deaths by that total. This showed us the distribution of deaths among that cohort over the projection period. In the most extreme case (infections in 2014), we could only produce one point of that distribution (2015), so that single value is exactly 1.0; 100% of the deaths observed in that cohort occurred in 2015.

We then used these distributions of death to weigh the ratio of VR deaths to Spectrum deaths, meaning that ratios in the years where we expect the largest share of deaths were weighed most heavily. We then multiplied the initial size of that cohort from the normal run of Spectrum by the sum of the combined ratios to get a new estimate of new cases in that year/age/sex combination.

We can write this method mathematically in the following way:

$$r_t = \frac{VR_t}{D_t}$$

$$\rho_t^{t-i} = \frac{d_t^{t-i}}{\sum_{k=t-i+1}^{n} d_k^{t-i}}$$

$$\alpha^{t-i} = \sum_{k=t-i+1}^{n} r_k * \rho_k^{t-i}$$

$$n_{\text{adjusted}}^{t-i} = \alpha^{t-i} * n^{t-i}$$

VR_t is the number of HIV/AIDS deaths in year t from ST-GPR, and D_t is the number of HIV/AIDS deaths from the first run of Spectrum. In the second equation, d_t^{t-i} is the number of HIV/AIDS deaths among members of infection cohort $t - i$ in year t, with $i \geq 1$, from the new, duration-tracking version of Spectrum, and n is final year of the projection. Therefore, ρ_t^{t-i} is the share of observed deaths in cohort $t - i$ that we expect to occur in year t. It follows, that α^{t-i} is the weighted adjustment ratio described above, which we multiply by the estimated initial size of infection cohort $t - i$ as calculated in the first stage Spectrum run to get the adjusted number of new cases, $n_{\text{adjusted}}^{t-i}$. This process is run separately for every sex and single-age pair.

CIBA (Process 6 in the HIV/AIDS Estimation Flowchart) allows ratios in each year after a given infection year to influence the final adjustment to incidence. The size of that influence is determined by the relative importance of that year in the cohort-year's distribution of deaths over time. The result is a new set of 1,000 draws of incidence and a set of 1,000 ratios of post-adjustment incidence to pre-adjustment incidence. We perform this adjustment using mean durations from the new version of Spectrum in order to try to shift the mean of the regular distribution of deaths.

Finally, to produce location-, year-, age-, and sex-specific estimates of HIV incidence, prevalence and mortality, we ran the new estimates of incidence and all previously input data through Spectrum (Process 9 in the HIV/AIDS Estimation Flowchart).

Countries without survey data and vital registration data (Group 2C)

The remaining 31 countries – as well as 14 subnational locations from China and Saudi Arabia – had neither geographically representative seroprevalence surveys nor reliable vital registration systems. To produce estimates of HIV burden in these countries, we assumed that Spectrum is similarly biased as in other Group 2 countries. This involved running Spectrum (Process 7 in the HIV/AIDS Estimation Flowchart), adjusting incidence using 1,000 adjustment ratios randomly sampled from the entire set of CIBA results (Process 8), and rerunning Spectrum using the new draws of adjusted incidence (Process 9). As above, the estimates of incidence, prevalence, and mortality were incorporated into the rest of the machinery via the reckoning process.

Subnational splitting and aggregation

Spectrum results for India, Kenya, Indonesia, and UK subnational locations are modeled at higher levels of geography than our GBD locations. Spectrum results for India are produced at the state level, while GBD 2016 estimates were produced at the state urban-rural level; Spectrum models Kenya provinces, while we compute Kenyan estimates for 47 counties. Indonesia and the United Kingdom have Spectrum results at the national level, while GBD 2016 estimates Indonesian provinces and Upper Tier Local Authorities in the UK. To split the Spectrum results into more granular results for processing, we assign each GBD subnational unit to a Spectrum modeling unit. From this, we generate age/sex/year-specific proportions for population, HIV-specific death, and HIV-free mortality.

In Cote d'Ivoire, Haiti, Moldova, Mozambique, and Zimbabwe, the country files that we received from UNAIDS contained only subnational data without national-level aggregates. In these locations, we generated GBD 2016 demographic inputs for the provided subnational units using the proportions present in the UNAIDS files and ran the locations through EPP and Spectrum at the subnational level before aggregating to generate final national level GBD 2016 estimates.

Limitations

We have not incorporated sex-specific allocation of ART in children under-5 for this round, though we did use sex-specific coverage for adults. The age- and sex-specific distributions of ART coverage we used were generated from two AIDS Indicator Surveys and can be expanded upon in future iterations of the GBD study.

HIV/AIDS resulting in other diseases

There are two Level 4 causes under the HIV/AIDS Level 3 cause in the GBD 2015 cause hierarchy. The modeling process for HIV/AIDS-tuberculosis is detailed in a separate part of this appendix. We computed deaths for HIV resulting in other diseases by subtracting HIV/AIDS-tuberculosis deaths from all HIV deaths at the 1,000 draw level.

References

1 Murray CJL, Ortblad KF, Guinovart C, *et al.* Global, regional, and national incidence and mortality for HIV, tuberculosis, and malaria during 1990–2013: a systematic analysis for the Global Burden of Disease Study 2013. *The Lancet* 2014; **384**: 1005–70.

2 Global, regional, and national age–sex specific all-cause and cause-specific mortality for 240 causes of death, 1990–2013: a systematic analysis for the Global Burden of Disease Study 2013. *The Lancet* 2015; **385**: 117–71.

3 Birnbaum JK, Murray CJ, Lozano R. Exposing misclassified HIV/AIDS deaths in South Africa. *Bull World Health Organ* 2011; **89**: 278–85.

4 jeffeaton/epp. GitHub. https://github.com/jeffeaton/epp (accessed April 21, 2016).

5 Verguet S, Lim SS, Murray CJL, Gakidou E, Salomon JA. Incorporating Loss to Follow-up in Estimates of Survival Among HIV-Infected Individuals in Sub-Saharan Africa Enrolled in Antiretroviral Therapy Programs. *J Infect Dis* 2013; **207**: 72–9.

6 Ghys PD, Zaba B, Prins M. Survival and mortality of people infected with HIV in low and middle income countries: results from the extended ALPHA network. *AIDS* 2007; **21 Suppl 6**: S1–4.

7 Hallett TB, Zaba B, Todd J, *et al.*, ALPHA Network. Estimating incidence from prevalence in generalised HIV epidemics: methods and validation. *PLoS Med* 2008; **5**: e80.

8 Marsh K, Mahy M, Salomon JA, Hogan DR. Assessing and adjusting for differences between HIV prevalence estimates derived from national population-based surveys and antenatal care surveillance, with applications for Spectrum 2013. *AIDS* 2014; **28 Suppl 4**: S497–505.

9 Raftery AE, Bao L. Estimating and Projecting Trends in HIV/AIDS Generalized Epidemics Using Incremental Mixture Importance Sampling. *Biometrics* 2010; **66**: 1162–73.

10 CDC. Pneumocystis Pneumonia --- Los Angeles. MMWR Weekly. 1981; published online June 5. http://www.cdc.gov/mmwr/preview/mmwrhtml/june_5.htm (accessed April 21, 2016).

11 Brown T, Peerapatanapokin W. The Asian Epidemic Model: a process model for exploring HIV policy and programme alternatives in Asia. *Sex Transm Infect* 2004; **80 Suppl 1**: i19–24.

Multidrug-resistant HIV/AIDS-tuberculosis, extensively drug-resistant HIV/AIDS-tuberculosis and drug-susceptible HIV/AIDS-tuberculosis

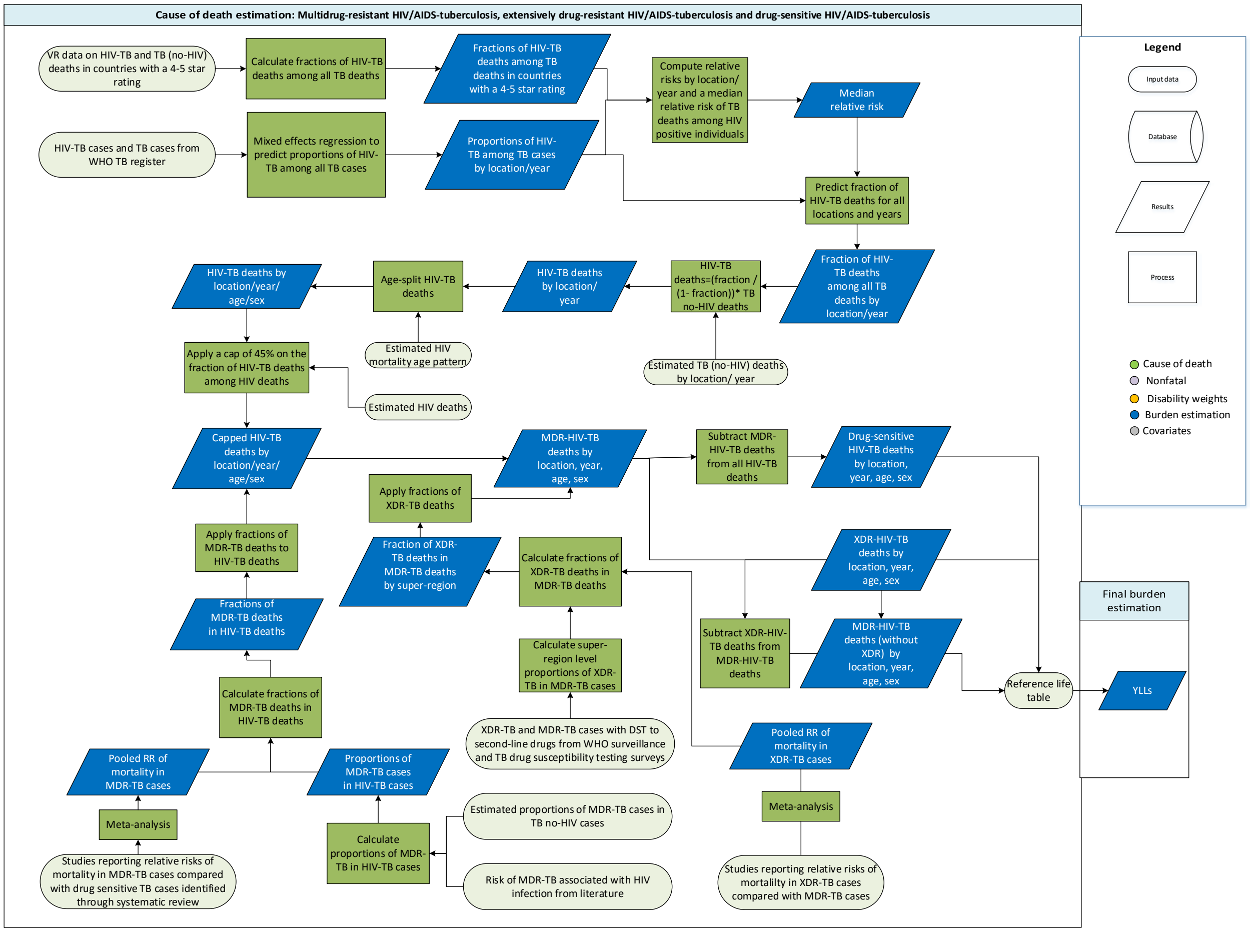

Input data

Input data for HIV/AIDS-tuberculosis (HIV-TB) mortality estimation include (i) 382 site-years of vital registration data from countries with a four or five-star rating where cause of death data for directly coded HIV-TB and tuberculosis (TB) were available, and (ii) the number of TB cases (new and re-treatment) recorded as HIV-positive and the number of TB cases (new and re-treatment) with an HIV test result recorded in the TB register from the World Health Organization (WHO). We excluded data from countries with ten HIV-TB deaths or less. We also excluded data that were largely conflicting with the majority of data for other years from the same country.

Input data for estimation of multidrug-resistant and extensively drug-resistant HIV-TB include: (i) the number of drug-resistant cases by type (multidrug-resistant tuberculosis [MDR-TB], extensively drug-resistant tuberculosis [XDR-TB], all TB cases with a drug sensitivity testing [DST] result for isoniazid and rifampicin, and MDR-TB cases with DST for second-line drugs) from routine surveillance and surveys reported to the World Health Organization. Additional input data include relative risks of mortality in MDR-TB cases compared with drug-susceptible TB cases, and relative risks of mortality in XDR-TB cases compared with MDR-TB cases reported by studies identified through our systematic review, and the risk of MDR-TB associated with HIV infection from the literature.[1]

Modelling strategy

To determine TB deaths in HIV-positive individuals, we first computed the fraction of HIV-TB deaths among all TB deaths using vital registration data from countries with a four or five-star rating. We also calculated the proportion of TB cases that are HIV-positive (ie, number of TB cases recorded as HIV-positive/number of TB cases with an HIV test result recorded in the WHO TB register). We used these proportions as input data for a mixed effects regression to predict the proportions of HIV-TB cases among all TB cases for all locations and years using an adult HIV death rate covariate. We estimated the fraction of HIV-TB deaths among all TB deaths in each location and year $(D_{c,y})$, defined by

$$D_{c,y} = \frac{P_{c,y}RR}{P_{c,y}RR + 1 - P_{c,y}}$$

where $P_{c,y}$ is the proportion of HIV-TB cases among all TB cases and *RR* is the relative risk of TB deaths in HIV positive individuals, defined by:

$$RR = \frac{D_{c,y}P_{c,y} - D_{c,y}}{D_{c,y}P_{c,y} - P_{c,y}}$$

We took the median relative risk (RR) from each calculation. We then applied the median RR and the predicted proportions of HIV-TB cases among all TB cases to get the fractions of HIV-TB deaths among all TB deaths for all locations and years. Location-year-specific HIV-TB deaths were then calculated using the following equation:

$$Deaths_{HIV-TB} = \frac{D_{c,y}}{1 - D_{c,y}} Deaths_{TB}$$

where $Deaths_{TB}$ is location-year specific deaths from the CODEm TB no-HIV model. Finally, we applied the age-sex pattern of the HIV mortality estimates to these HIV-TB deaths to generate location-year-age-sex-specific HIV-TB deaths. As the HIV-TB deaths were estimated based on the fraction of HIV-TB deaths among all TB deaths, the total number of HIV-TB deaths could exceed the total number of HIV deaths in some locations. To avoid this, we applied a cap of 45% on the fraction of HIV-TB deaths among HIV deaths, based on a review by Cox et al., 2010,[2] and a systematic review and meta-analysis by Ford et al., 2016.[3]

To split HIV-TB into MDR-HIV-TB and drug- susceptible HIV-TB, we first calculated the proportion of MDR-HIV-TB among all HIV-TB cases ($P_{MDR-HIVc,y,a,s}$) for each location, year, age, and sex using the following formula:

$$P_{MDR-HIVc,y,a,s} = P_{MDRnoHIVc,y,a,s}RR_{HIV}$$

where $P_{MDRnoHIVc,y,a,s}$ is the estimated proportion of MDR-TB among HIV-negative TB cases for each location, year, age, and sex (see MDR-TB modeling strategy for more detail) and RR_{HIV} is the relative risk of MDR-TB associated with HIV infection.

We then computed the fraction of MDR-HIV-TB deaths among all HIV-TB deaths ($D_{MDR-HIVc,y,a,s}$) using the following formula:

$$D_{MDR-HIVc,y,a,s} = \frac{P_{MDR-HIVc,y,a,s}RR_{MDR}}{P_{MDR-HIVc,y,a,s}RR_{MDR} + 1 - P_{MDR-HIVc,y,a,s}}$$

where RR_{MDR} is the pooled relative risk of mortality in MDR-TB cases compared with drug- susceptible TB cases. We then applied the predicted MDR-HIV-TB death fractions to all HIV-TB death estimates to generate MDR-HIV-TB deaths by location, year, age, and sex. Next, we subtracted MDR-HIV-TB deaths from all HIV-TB deaths to generate drug- susceptible HIV-TB deaths by location, year, age, and sex.

To separate out XDR-HIV-TB from MDR-HIV-TB, we aggregated the XDR-TB cases and MDR-TB cases (with DST for second-line drugs) up to the super-region level and calculated the super-region level proportions of XDR-TB among MDR-TB cases. Next, we computed the superregion-year-specific fraction of XDR-TB deaths among all MDR-TB deaths ($D_{XDRsr,y}$) using the following formula:

$$D_{XDRsr,y} = \frac{P_{XDRsr,y}RR_{XDR}}{P_{XDRsr,y}RR_{XDR} + 1 - P_{XDRsr,y}}$$

where RR_{XDR} is the pooled relative risk of mortality in XDR-TB cases compared with MDR-TB cases. These fractions were then applied to MDR-HIV-TB deaths in corresponding countries within the super-regions to produce XDR-HIV-TB deaths by location, year, age, and sex. We linearly extrapolated XDR-TB mortality rates back assuming the mortality rates were zero in 1992, one year before 1993 when XDR-TB was first recorded in USA surveillance data.[4] Finally, we subtracted XDR-HIV-TB deaths from MDR-HIV-TB deaths to generate MDR-HIV-TB (without extensive drug resistance) deaths by location, year, age, and sex.

References

1 Mesfin YM, Hailemariam D, Biadglign S, Kibret KT. Association between HIV/AIDS and multi-drug resistance tuberculosis: a systematic review and meta-analysis. PLoS One. 2014 Jan 8;9(1):e82235.

2 Cox JA, Lukande RL, Lucas S, Nelson AM, Van Marck E, Colebunders R. Autopsy causes of death in HIV-positive individuals in sub-Saharan Africa and correlation with clinical diagnoses. *AIDS Rev* 2010; **12**: 183–94.

3 Ford N, Matteelli A, Shubber Z, *et al.* TB as a cause of hospitalization and in-hospital mortality among people living with HIV worldwide: a systematic review and meta-analysis. *J Int AIDS Soc* 2016; **19**: 20714.

4 Centers for Disease Control and Prevention (CDC). Extensively Drug-Resistant Tuberculosis --- United States, 1993—2006. MMWR. 2007; 56(11);250-253

Diarrheal diseases

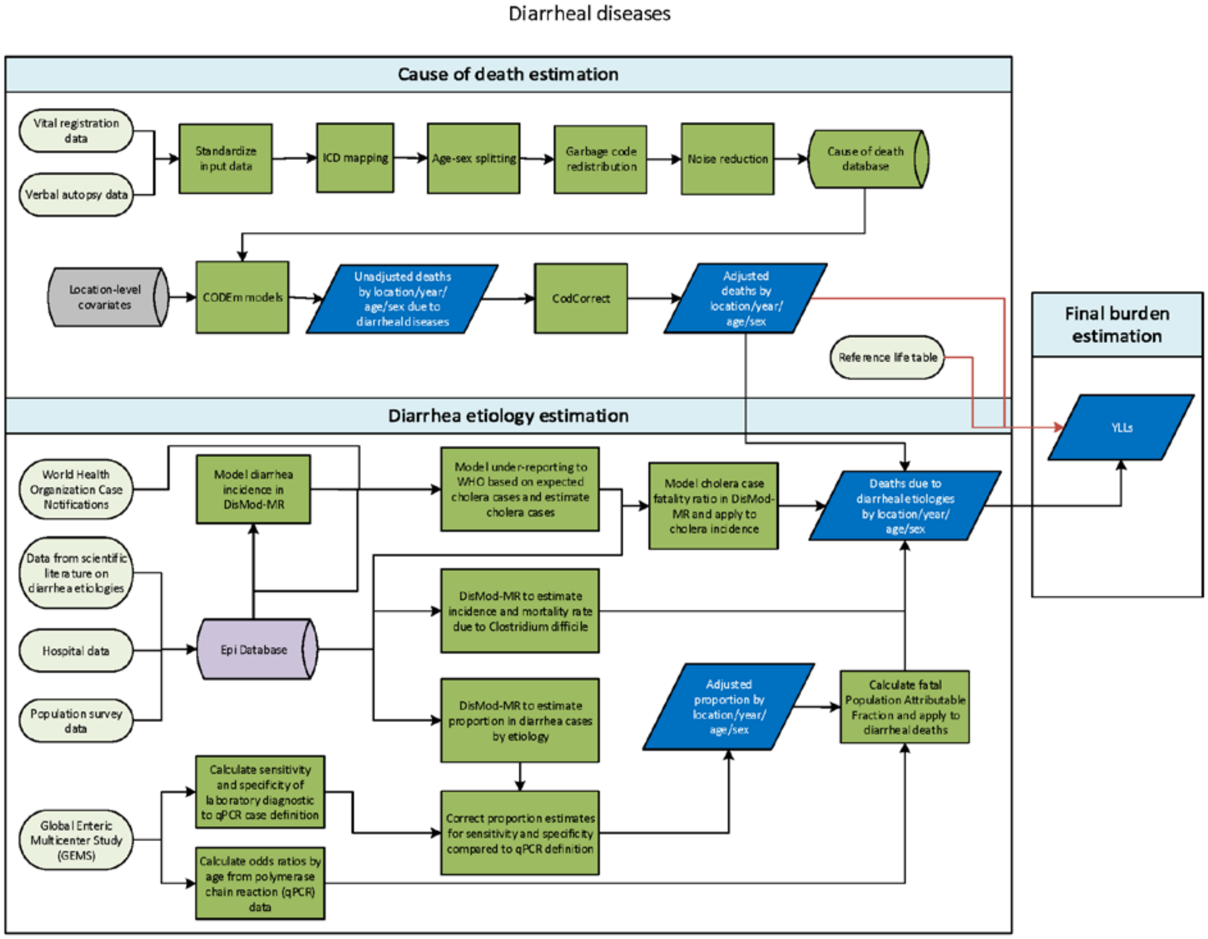

Input data

Cause of death. Diarrheal disease mortality was estimated in CODEm. We estimated diarrhea mortality separately for males and females and for children under 5 years and older than 5 years. We used all available data from vital registration systems, surveillance systems and verbal autopsy (**Table 1**). We checked for and excluded outliers from our data by country or region. We also excluded early neonatal mortality data in the Philippines (1994–1998) and India Civil Registration System data in all states (1986–1995).

Etiologies. We conducted a systematic literature review for the proportion of diarrhea cases that tested positive for each etiology. We updated our review of literature to include studies published between May 2015 and May 2016. Inclusion criteria included diarrhea as the case definition, studies with a sample size of at least 100, and studies with at least one year of follow-up. We excluded studies that reported on diarrheal outbreaks exclusively and those that used acute gastroenteritis with or without diarrhea. We identified 442 studies, of which 36 met our criteria of inclusion and were included. We extracted data points for location, sex, year, and age. We assigned an age range based on the prevalence-weighted mean age of diarrhea in the appropriate year/sex/location if the age of the study participants was not reported.

We used the Global Enteric Multicenter Study (GEMS), a seven-site, case-control study of moderate-to-severe diarrhea in children under 5 years,[1] to calculate odds ratios for the diarrheal pathogens. We analyzed raw data for a systematic reanalysis, representative of the distribution of cases and controls by age and site, of roughly half of the 22,000 original GEMS samples that were tested for the presence of pathogen using quantitative polymerase chain reaction (qPCR).[2]

Modeling strategy

Cause of death. We used country-level covariates to inform our CODEm models. We included covariates for years of education per capita, income per capita, prevalence of undernutrition (weight-for-age, weight-for-height, and height-for-age), population density above 1,000 or below 150 people per square kilometer, sanitation access, safe water access, Socio-Demographic Index, and rotavirus vaccine coverage. We evaluated our diarrheal disease cause of death models using in and out of sample predictive performance.

Etiologies. We estimated diarrheal disease etiologies separately from overall diarrhea mortality using a counterfactual strategy for enteric adenovirus, *Aeromonas*, *Entamoeba histolytica* (amoebiasis), *Campylobacter enteritis*, *Cryptosporidium*, typical enteropathogenic *Escherichia coli* (t-EPEC), enterotoxigenic *Escherichia coli* (ETEC), norovirus, non-typhoidal salmonella infections, rotavirus, and *Shigella*. *Vibrio cholerae* and *Clostridium difficile* were modeled separately.

Diarrheal etiologies are attributed to diarrheal deaths using a counter-factual approach. We calculated a population attributable fraction (PAF) from the proportion of severe diarrhea cases that are positive for each etiology. The PAF represents the relative reduction in diarrhea mortality if there was no exposure to a given etiology. As diarrhea can be caused by multiple pathogens and the pathogens may co-infect, PAFs can overlap and add up to more than 100%. We calculated the PAF from the proportion of severe diarrhea cases that are positive for each etiology. We assumed that hospitalized diarrhea cases are a proxy of severe and fatal cases. We used the following formula to estimate PAF:[4]

$$PAF = Proportion * (1 - \frac{1}{OR})$$

Where *Proportion* is the proportion of diarrhea cases positive for an etiology and *OR* is the odds ratio of diarrhea given the presence of the pathogen.

We dichotomized the continuous qPCR test result using the value of the cycle threshold (Ct) that most accurately discriminated between cases and controls. The Ct values range from 0 to 35 cycles representing the relative concentration of the target gene in the stool sample. A low value indicates a higher concentration of the pathogen while a value of 35 indicates the absence of the target in the sample. We used the lower Ct value when we had multiple Ct values for the cutpoint. The case definition for each pathogen is a Ct value that is below the established cutoff point.

We used a mixed effects conditional logistic regression model to calculate the odds ratio for under 1 year and 1-4 years old for each of our pathogens. The odds ratio for 1-4 years was applied to all GBD age groups over 5 years. There were three pathogen-age odds ratios that were not statistically significant: Aeromonas and Amoebiasis in under 1 year and Campylobacter in 1-4 years. The mean value of the odds ratio was above 1 in all three cases so we transformed the odds ratios for these three exceptions only in log-space such that exponentiated values could not be below 1. The transformation was:

$$Odds\ ratio = exp(log(or) - 1)) + 1$$

We modeled the proportion data using the meta-regression tool DisMod-MR to estimate the proportion of positive diarrhea cases for each separate etiology by location/year/age/sex and to adjust for the covariates.

We used the estimated sensitivity and specificity of the laboratory diagnostic technique used in the GEMS study compared to the qPCR case definition to adjust our proportion before we computed the PAF:[5]

$$Proportion_{True} = \frac{(Proportion_{Observed} + Specificity - 1)}{(Sensitivity + Specificity - 1)}$$

We used this correction to account for the fact that the proportions we used are based on a new test that is not consistent with the laboratory-based case definition (qPCR versus GEMS conventional laboratory testing for pathogens).[15]

Our literature review extracted the proportion of any enteropathogenic *Escherichia coli* (EPEC) without differentiating between typical (tEPEC) and atypical (aEPEC). In order to be consistent with the odds ratios that we obtained, we adjusted our proportion estimates of any EPEC to typical EPEC only. This adjustment was informed by a subset of our literature review that reported both atypical and typical EPEC. We estimated a ratio by super-region of tEPEC to any EPEC and adjusted our proportion estimates accordingly. We found that the majority of EPEC diarrhea cases were positive for atypical EPEC, consistent with other published work.[3]

For *Vibrio cholerae* (cholera), we used the literature review to estimate expected number of cholera cases for each country-year using the incidence of diarrhoea, estimated using DisMod-MR, and the proportion of diarrhoea cases that are positive for cholera. We assigned cholera PAF using odds ratios from the qPCR results to estimate a number of cholera-attributable cases. We compared this expected number of cholera cases to the number reported to the World Health Organization at the country-year level.[6] We modeled the underreporting fraction to correct the cholera case notification data for all countries using health system access and the diarrhoea SEV scalar to predict total cholera cases. We used the age-specific proportion of positive cholera samples in DisMod and our incidence estimates to predict the number of cholera cases for each age/sex/year/location. Finally, we modeled the case fatality ratio of cholera using DisMod-MR and to estimate the number of cholera deaths.

For *C. difficile*, we modeled incidence and mortality in DisMod-MR for each age, sex, year, location. DisMod-MR is a Bayesian meta-regression tool that uses spatio-temporal information as priors to estimate prevalence, incidence, remission, and mortality for *C. difficile* infection. DisMod-MR uses a compartmental model to relate prevalence, incidence, remission, and mortality. We set remission in our model to 1 month.

Table 1. Cause-specific mortality input data.

Type of data	Input data
**Total data sources**	**16,980 site-years**
Vital registration data	15,087 site-years
Surveillance data	877 site-years
Verbal autopsy data	1,016 site-years

References

1 Kotloff KL, Nataro JP, Blackwelder WC, *et al.* Burden and aetiology of diarrhoeal disease in infants and young children in developing countries (the Global Enteric Multicenter Study, GEMS): a prospective, case-control study. *Lancet Lond Engl* 2013; **382**: 209–22.

2 Liu J, Gratz J, Amour C, *et al.* A laboratory-developed TaqMan Array Card for simultaneous detection of 19 enteropathogens. *J Clin Microbiol* 2013; **51**: 472–80.

3 Ochoa TJ, Barletta F, Contreras C, Mercado E. New insights into the epidemiology of enteropathogenic Escherichia coli infection. *Trans R Soc Trop Med Hyg* 2008; **102**: 852–6.

4 Miettinen OS. Proportion of disease caused or prevented by a given exposure, trait or intervention. *Am J Epidemiol* 1974; **99**: 325–32.

5 Reiczigel J, Földi J, Ozsvári L. Exact confidence limits for prevalence of a disease with an imperfect diagnostic test. *Epidemiol Infect* 2010; **138**: 1674–8.

6 World Health Organization. Global Health Observatory data repository: Cholera. 2016. http://apps.who.int/gho/data/node.main.174?lang=en (accessed Aug 25, 2016).

Typhoid fever

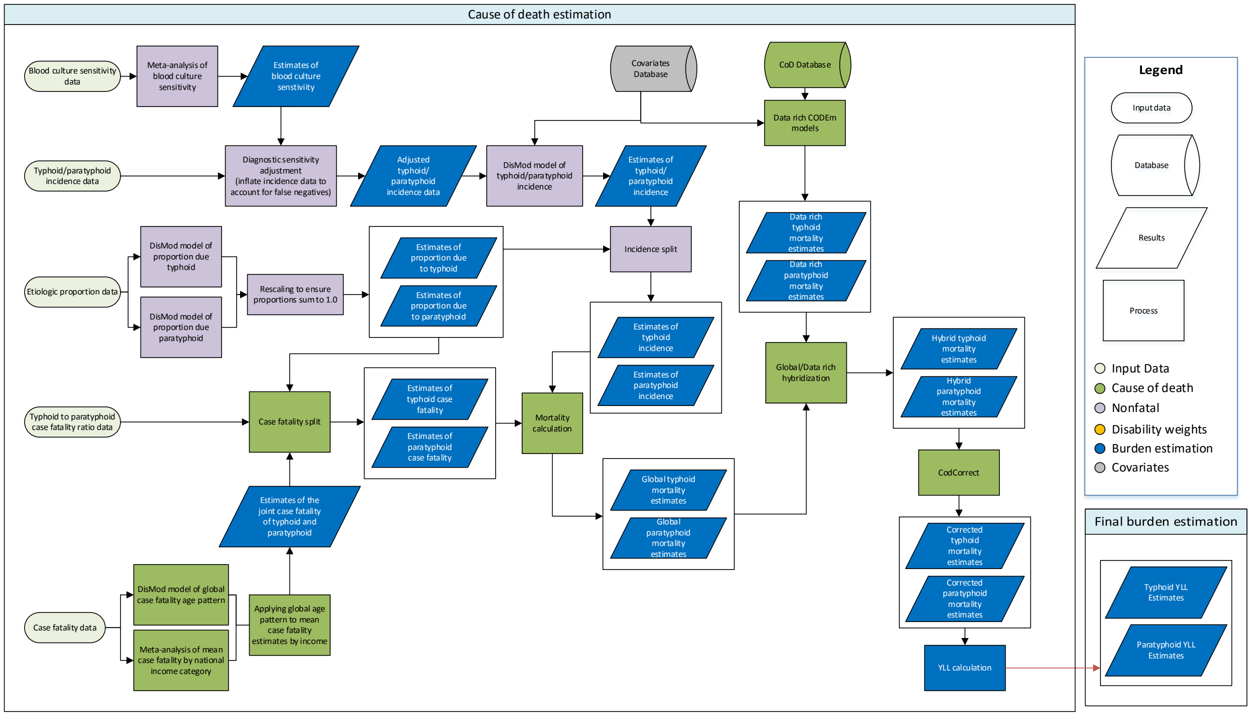

Input data

Our incidence dataset included a combination of data from prospective cohort studies and national surveillance systems. Similarly, data on proportions due to typhoid and paratyphoid included a combination of prospective cohort studies and national surveillance systems. Case fatality data were from national surveillance systems and hospital databases.

Modelling strategy

We model typhoid deaths using a natural history model in which we first model total incidence of typhoid and paratyphoid combined. Second, we model the proportion of this total due to typhoid and the proportion due to paratyphoid. Third, we estimate case fatality by age and national income category for typhoid and paratyphoid combined. Fourth, we use data on the relative fatality of typhoid and paratyphoid to split the joint case fatality estimates into typhoid- and paratyphoid-specific case fatality estimates. Finally, we estimate cause-specific mortality rates as the product of incidence and case fatality.

Total incidence was modelled using DisMod-MR, using the proportion of the population with access to clean water, and the proportion of the population living in the Indian Ocean monsoon belt as covariates. We performed a crosswalk using a study-level covariate indicating sources that were based on passive versus active surveillance, with active surveillance as the reference. This adjusts for incomplete case capture by passive surveillance. Incidence data were inflated to account for poor diagnostic sensitivity, based on a meta-analysis of the sensitivity of blood culture, the most common diagnostic used for

typhoid. Similarly, we used two DisMod models to estimate etiologic proportions: one for the proportion of total incidence due to typhoid, and one for the proportion due to paratyphoid.

Case fatality data were too limited to allow for a complete DisMod model, or to allow for varying estimates by time and space. We had sufficient data, however, to estimate case fatality by age and by three categories of national income. We used DisMod to extract a global age-pattern in case fatality, and meta-regression to estimate the mean case fatality by income category. Finally, we estimated the relative risk of death from typhoid relative to paratyphoid based on data from Chinese surveillance and used that relative risk to estimate case fatality separately for typhoid and paratyphoid, by age and income.

Finally, we estimated typhoid mortality as the product of total incidence, the proportion of the total due to typhoid, and case fatality for typhoid. We propagated uncertainty through every step of the modelling process by pulling 1,000 draws from the distribution of each model component (eg, incidence, proportion due to typhoid, overall case fatality, case fatality age pattern, relative fatalness of typhoid versus paratyphoid), and performing all calculations at the draw level.

We have made no substantive changes to the modelling strategy in 2016.

Paratyphoid fever

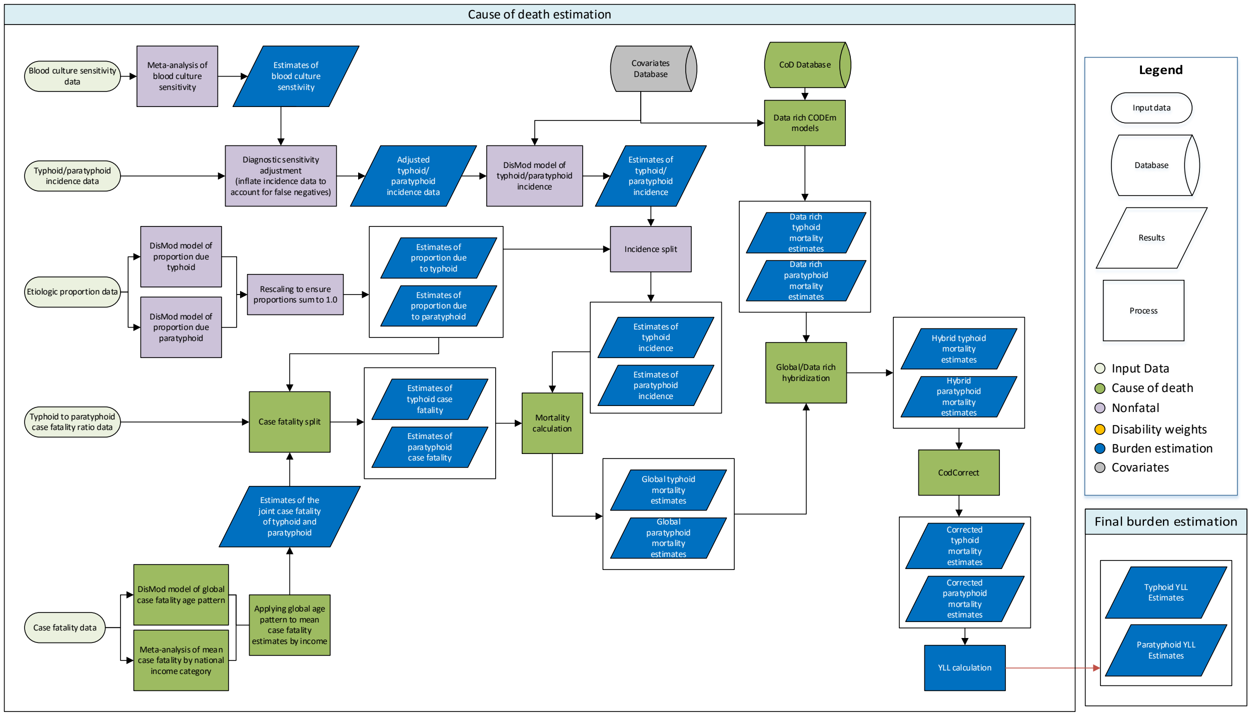

Input data

Our incidence dataset included a combination of data from prospective cohort studies and national surveillance systems. Similarly, data on proportions due to typhoid and paratyphoid included a combination of prospective cohort studies and national surveillance systems. Case fatality data were from national surveillance systems and hospital databases.

Modelling strategy

We model paratyphoid deaths using a natural history model in which we first model total incidence of typhoid and paratyphoid combined. For the natural history model we first model total incidence of typhoid and paratyphoid combined. Second, we model the proportion of this total due to typhoid and the proportion due to paratyphoid. Third, we estimate case fatality by age and national income category for typhoid and paratyphoid combined. Fourth, we use data on the relative fatality of typhoid and paratyphoid to split the joint case fatality estimates into typhoid- and paratyphoid-specific case fatality estimates. Finally, we estimate cause-specific mortality rates as the product of incidence and case fatality.

Total incidence was modelled using DisMod-MR, using the proportion of the population with access to clean water and the proportion of the population living in the Indian Ocean monsoon belt as covariates. We performed a crosswalk using a study-level covariate indicating sources that were based on passive versus active surveillance, with active surveillance as the reference. This adjusts for incomplete case

capture by passive surveillance. Incidence data were inflated to account for poor diagnostic sensitivity, based on a meta-analysis of the sensitivity of blood culture, the most common diagnostic used for paratyphoid. Similarly, we used two DisMod models to estimate aetiologic proportions: one for the proportion of total incidence due to typhoid, and one for the proportion due to paratyphoid.

Case fatality data were too limited to allow for a complete DisMod model, or to allow for varying estimates by time and space. We had sufficient data, however, to estimate case fatality by age and by three categories of national income. We used DisMod to extract a global age-pattern in case fatality, and meta-regression to estimate the mean case fatality by income category. Finally, we estimated the relative risk of death from typhoid relative to paratyphoid based on data from Chinese surveillance and used that relative risk to estimate case fatality separately for typhoid and paratyphoid, by age and income.

Finally, we estimated paratyphoid mortality as the product of total incidence, the proportion of the total due to paratyphoid, and case fatality for paratyphoid. We propagated uncertainty through every step of the modelling process by pulling 1,000 draws from the distribution of each model component (eg, incidence, proportion due to paratyphoid, overall case fatality, case fatality age pattern, relative fatalness of typhoid versus paratyphoid), and performing all calculations at the draw level.

We have made no substantive changes to the modelling strategy in 2016.

Other Intestinal Infectious Diseases

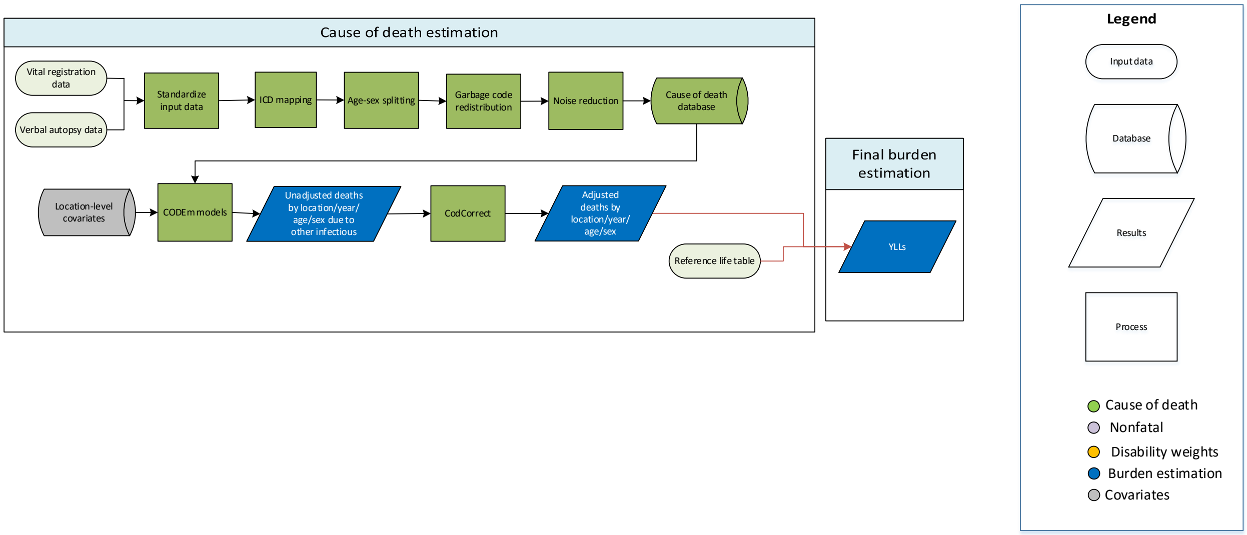

Input data

We modelled other intestinal infectious disease mortality using all available data in the cause of death database. Data points were outliered if they reported an improbable number of deaths or if their inclusion in the model yielded distorted trends. In some cases multiple data sources for the same location differed dramatically both in their quality and reported other intestinal infectious disease mortality (eg, a verbal autopsy and vital registration source). In these cases the lower-quality data source was outliered.

Modelling strategy

We modelled other intestinal infectious disease mortality using a custom negative-binomial model of all data in the CoD database, using the proportion of the population with access to clean water as a covariate.

Since GBD 2015 we have switched from a CODEm model to custom model for this cause to better handle the small number of deaths reported for other intestinal infectious diseases.

Lower Respiratory Infections

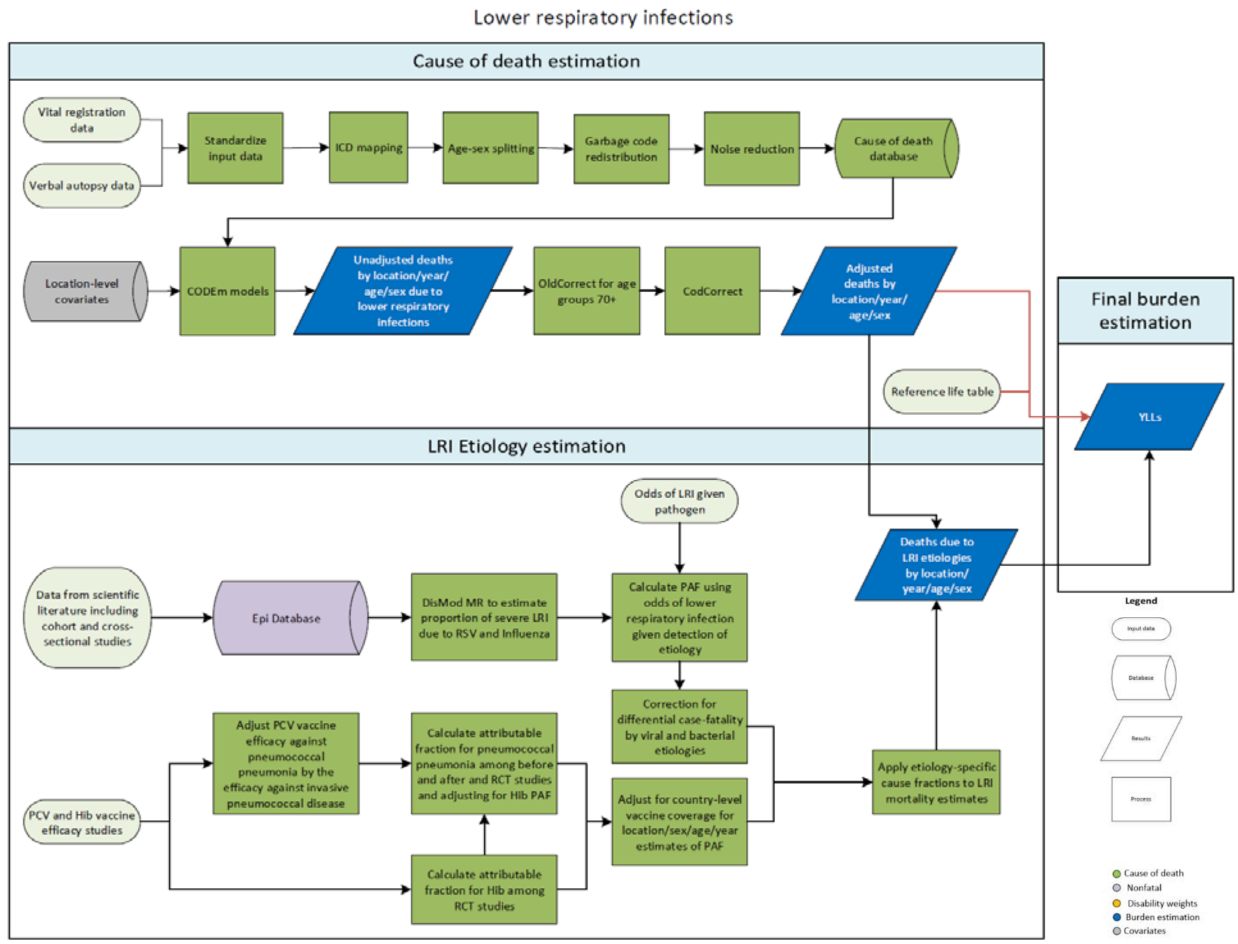

Input data

Cause of deaths. Lower respiratory infection (LRI) mortality was estimated in CODEm. We estimated LRI mortality separately for males and females and for children under 5 years and older than 5 years. We used all available data from vital registration systems, surveillance systems, and verbal autopsy (**Table 1**). We checked for and excluded outliers from our data by country or region. We also excluded ICD9-coded mortality data in Sri Lanka (1982, 1987–1992), ICD9-coded neonatal mortality data in Guatemala (1980, 1981, 1984, 2000–2004), and Civil Registration System data in many Indian states (1986–1995).

Etiologies. We updated our systematic review of scientific literature for the proportion of LRI that tested positive for influenza and respiratory syncytial virus (RSV) to include all data from GBD 2015 and from studies published between May 2015 and May 2016. Inclusion criteria were studies that had a sample size of at least 100, studies that were at least one year in duration, and studies describing lower respiratory infections, pneumonia, or bronchiolitis as the case definition. During our literature review we identified 209 studies, of which 7 met our inclusion criteria and were extracted. We excluded studies that described pandemic H1N1 influenza solely and studies that used influenza-like illness as the case definition. We assigned an age range based on the prevalence-weighted mean age of LRI in the appropriate year/sex/location if the ages of the study participants were not reported.

We also conducted a systematic literature review of studies on the Hib vaccine and PCV effectiveness studies against X-ray-confirmed pneumonia and against pneumococcal and Hib disease until May 2016. For PCV studies, we extracted, if available, the distribution of pneumococcal pneumonia serotypes and the serotypes included in the PCV used in the study. No new studies were identified for GBD 2016. We excluded observational and case-control studies due to implausibly high vaccine efficacy estimates. Hib trial data were exclusively from children <5 years so we did not include the effect of Hib on ages over 5 years of age. PCV trial data are also frequently limited to younger age populations. To understand the contribution of pneumococcal pneumonia in older populations, we also included PCV efficacy studies that used before-after approaches.

Modeling strategy

Cause of death. We used country-level covariates to inform our CODEm models. We included the following covariates in our LRI models: diphtheria-tetanus-pertussis vaccine coverage, years of education per capita, health system access, income per capita, prevalence of children malnutrition (<2 standard deviations below global mean of weight for age), prevalence of exposure to indoor air pollution (solid fuel use), outdoor air pollution level of $PM_{2.5}$, smoking prevalence, pneumococcal conjugate vaccine (PCV) coverage, *Haemophilus influenzae* type B (Hib) vaccine coverage, access to improved water, access to improved sanitation, and Socio-Demographic Index. We evaluated our LRI cause of death models using in and out of sample predictive performance.

Like all models of mortality in GBD, LRI mortality models are single-cause, requiring in effect that the sum of all mortality models must be equal to the all-cause mortality envelope. We correct LRI mortality estimates, and other causes of mortality, by re-scaling them according to the uncertainty around the cause-specific mortality rate. This process is called CoDCorrect and is essential to ensure internal consistency among causes of death. Before CoDCorrect, we also adjust LRI mortality for unreliable estimates due to improper death certification and ICD coding among elderly adults where the underlying cause of death should be Alzheimer's or Parkinson's diseases. This process scales LRI mortality among adult age groups 70+ years into a new envelope without Alzheimer's and Parkinson's. Further details can be found in section 4 of the appendix.

Etiologies. We estimated LRI etiologies separately from overall LRI mortality using two distinct counterfactual modeling strategies to estimate population attributable fractions (PAFs), described in detail below. The PAF represents the relative reduction in LRI mortality if there was no exposure to a given etiology. As LRIs can be caused by multiple pathogens and the pathogens may co-infect, PAFs can overlap and add up to more than 100%. Separate strategies were used for viral- influenza and respiratory syncytial virus (RSV)- and bacterial- *Streptococcus pneumoniae* and *Haemophilus influenzae* type B- etiologies. We did not attribute etiologies to neonatal pneumonia deaths due to a dearth of reliable data in this age group. We calculated uncertainty of our PAF estimates from 1,000 draws of each parameter using normal distributions in log space.

Influenza and RSV. We calculated the population attributable fraction (PAF) from the proportion of severe LRI cases positive for influenza and RSV. We assumed that hospitalized LRI cases are a proxy of severe cases. We used the following formula to estimate PAF:[1]

$$PAF = Proportion * (1-1/OR)$$

Where *Proportion* is the proportion of LRI cases that test positive for influenza or RSV and *OR* is the odds ratio of LRI given the presence of the pathogen. We used an odds ratio of 5.1 (3.19 – 8.14) for influenza and 9.79 (4.98 – 19.27) for RSV from a recently published meta-analysis.[2] These odds ratios are marginally different from those used in GBD 2013.

We modeled the proportion data using the meta-regression tool DisMod-MR to estimate the proportion of LRI cases that are positive for influenza and RSV, separately, by location/year/age/sex. We accounted for study-level covariates in our models such as PCR as the diagnostic technique, studies that investigated RSV or influenza exclusively, and studies from inpatient populations.

As the case-fatality of viral causes of pneumonia is lower than for bacterial causes, we adjusted for differential case-fatality by determining the etiological fractions for mortality attributable to RSV and influenza (**Table 2**). We measured the etiologic fractions by applying a relative case-fatality adjustment based on in-hospital case-fatality, which we coded to specific pneumonia etiologies. Hospital admissions data of this type were limited to data from the USA, Austria, Brazil, and Mexico. We generated the pooled estimate of the case-fatality differential between bacterial (pneumococcus, Hib) and viral etiologies (RSV, influenza) using DisMod-MR.

Pneumococcal pneumonia and Hib. For *Streptococcus pneumoniae* (pneumococcal pneumonia) and *Haemophilus influenzae* type B (Hib), we calculated the population attributable fraction using a vaccine probe design.[3,4] The ratio of vaccine effectiveness against nonspecific pneumonia to pathogen-specific disease represents the fraction of pneumonia cases attributable to each pathogen.

To estimate the PAF for Hib and pneumococcal pneumonia, we calculated the ratio of vaccine effectiveness against nonspecific pneumonia to pathogen-specific pneumonia (Equations 1 and 3). We estimated a study-level estimate of PAF from a meta-analysis of these ratios. To estimate the PAF for Hib, we only used randomized controlled trials because of implausibly high values of vaccine efficacy in case-control studies. To estimate the PAF for pneumococcal pneumonia, we included RCTs and before and after vaccine introduction longitudinal studies.

We adjusted the study-level PAF estimate by vaccine coverage and expected vaccine performance to estimate country- and year-specific PAF values. For pneumococcal pneumonia, we adjusted the PAF by the final Hib PAF estimate and by vaccine serotype coverage. Finally, we used an age distribution of PAF modeled in DisMod to determine the PAF by age. Because of an absence of data describing vaccine efficacy against Hib in children older than two years, we did not attribute Hib to episodes of LRI in ages five years and older.

We used a vaccine probe design to estimate the PAF for pneumococcal pneumonia and (Hib) by first calculating the ratio of vaccine effectiveness against nonspecific pneumonia to pathogen-specific pneumonia at the study level (Equations 1 and 2).[3–5] We then adjusted this estimate by vaccine coverage and expected vaccine performance to estimate country- and year-specific PAF values (Equations 3 and 4).

1) $$HibPAF_{Base} = 1 - \frac{VE_{Pneumonia}}{VE_{Hib}}$$

2) $$PneumoPAF_{Base} = 1 - \frac{VE_{Pneumonia} * (1 - PAF_{Hib} * VE_{Hib\ Optimal})}{VE_{Streptococcus} * Cov_{Serotype}}$$

3) $PAF_{Hib} = PAF_{Base} * \frac{(1-Cov_{Hib}*VE_{Hib\ Optimal})}{(1-PAF_{Base}*Cov_{Hib}*VE_{Hib\ Optimal})}$

4) $PAF_{Pneumo} = \frac{PAF_{Base}*(1-Cov_{PCV}*VE_{PCV\ Optimal})}{(1-PAF_{Hib}*Cov_{Hib}*VE_{Hib\ Optimal})*\left(1-\frac{PAF_{Base}Cov_{PCV}*VE_{PCV\ Optimal}}{(1-PAF_{Hib}*Cov_{Hib}*VE_{Hib\ Optimal})}\right)}$

Where $VE_{Pneumonia}$ is the vaccine efficacy against nonspecific pneumonia, VE_{Hib} is the vaccine efficacy against invasive Hib disease, $VE_{Streptococcus}$ is the vaccine efficacy against serotype-specific pneumococcal pneumonia, $Cov_{serotype}$ is the serotype-specific vaccine coverage for PCV,[6] $VE_{Hib\ Optimal}$ is the Hib effectiveness in the community (0.8)[7], PAF_{Hib} is the final PAF for Hib, Cov_{PCV} is the PCV coverage, Cov_{Hib} is the Hib coverage by country, and $VE_{PCV\ Optimal}$ is the vaccine effectiveness in the community (0.8).[8]

For Hib, we assumed that the vaccine efficacy against invasive Hib disease is the same against Hib pneumonia. For pneumococcal pneumonia, a recent study in adults [9] found that the vaccine efficacy against invasive pneumococcal disease may be significantly higher than against pneumococcal pneumonia. We used this ratio to adjust estimates of vaccine efficacy against invasive pneumococcal disease from other studies. However, recognizing that the study is unique in that it uses a urine antigen test among adults, we added uncertainty around our adjustment using a wide uniform distribution (median 0.65, 0.3-1.0). This has increased the estimates of pneumococcal pneumonia mortality in a meaningful way.

There are no major changes to the cause of death estimation strategy for LRI or its etiologies from GBD 2015 to GBD 2016.

Table 1. Summary of cause-specific mortality modeling input data.

Type of data	Input data
**Total data sources**	**12,155 site-years**
Vital registration data	10,312 site-years
Surveillance data	928 site-years
Verbal autopsy data	915 site-years

Table 2: **The median values for the ratio of viral to bacterial pneumonia case fatality ratio by age is shown.** These estimates are modeled using hospital-based, ICD-coded admissions and mortality for etiology-specified pneumonia. Values in parentheses represent 95% Uncertainty Interval.

**Age Group**	**Ratio**
Early Neonatal	0.34 (0.19-0.58)
Late Neonatal	0.34 (0.19-0.58)
Post Neonatal	0.34 (0.19-0.58)
1 to 4	0.28 (0.16-0.44)
5 to 9	0.31 (0.15-0.56)
10 to 14	0.33 (0.19-0.53)
15 to 19	0.37 (0.2-0.64)
20 to 24	0.46 (0.12-1.16)
25 to 29	0.44 (0.17-0.93)
30 to 34	0.46 (0.22-0.83)
35 to 39	0.5 (0.22-1)
40 to 44	0.61 (0.13-1.75)
45 to 49	0.5 (0.21-0.99)
50 to 54	0.44 (0.23-0.74)
55 to 59	0.42 (0.21-0.75)
60 to 64	0.42 (0.15-0.95)
65 to 69	0.39 (0.19-0.7)
70 to 74	0.38 (0.21-0.61)
75 to 79	0.37 (0.2-0.62)
80 to 84	0.37 (0.17-0.71)
85 to 89	0.34 (0.19-0.59)
90 to 94	0.33 (0.16-0.61)
95 to 99	0.34 (0.13-0.8)

References

1 Miettinen OS. Proportion of disease caused or prevented by a given exposure, trait or intervention. *Am J Epidemiol* 1974; **99**: 325–32.

2 Shi T, McLean K, Campbell H, Nair H. Aetiological role of common respiratory viruses in acute lower respiratory infections in children under five years: A systematic review and meta-analysis. *J Glob Health* 2015; **5**: 10408.

3 Feikin DR, Scott JAG, Gessner BD. Use of vaccines as probes to define disease burden. *Lancet Lond Engl* 2014; **383**: 1762–70.

4 O’Brien KL, Wolfson LJ, Watt JP, *et al.* Burden of disease caused by Streptococcus pneumoniae in children younger than 5 years: global estimates. *Lancet Lond Engl* 2009; **374**: 893–902.

5 Watt JP, Wolfson LJ, O’Brien KL, *et al.* Burden of disease caused by Haemophilus influenzae type b in children younger than 5 years: global estimates. *Lancet Lond Engl* 2009; **374**: 903–11.

6 Johnson HL, Deloria-Knoll M, Levine OS, *et al.* Systematic evaluation of serotypes causing invasive pneumococcal disease among children under five: the pneumococcal global serotype project. *PLoS Med* 2010; **7**. DOI:10.1371/journal.pmed.1000348.

7 Swingler G, Fransman D, Hussey G. Conjugate vaccines for preventing Haemophilus influenzae type B infections. *Cochrane Database Syst Rev* 2007; : CD001729.

8 Lucero MG, Dulalia VE, Nillos LT, *et al.* Pneumococcal conjugate vaccines for preventing vaccine-type invasive pneumococcal disease and X-ray defined pneumonia in children less than two years of age. *Cochrane Database Syst Rev* 2009; : CD004977.

9 Bonten MJM, Huijts SM, Bolkenbaas M, *et al.* Polysaccharide conjugate vaccine against pneumococcal pneumonia in adults. *N Engl J Med* 2015; **372**: 1114–25.

Upper respiratory infections

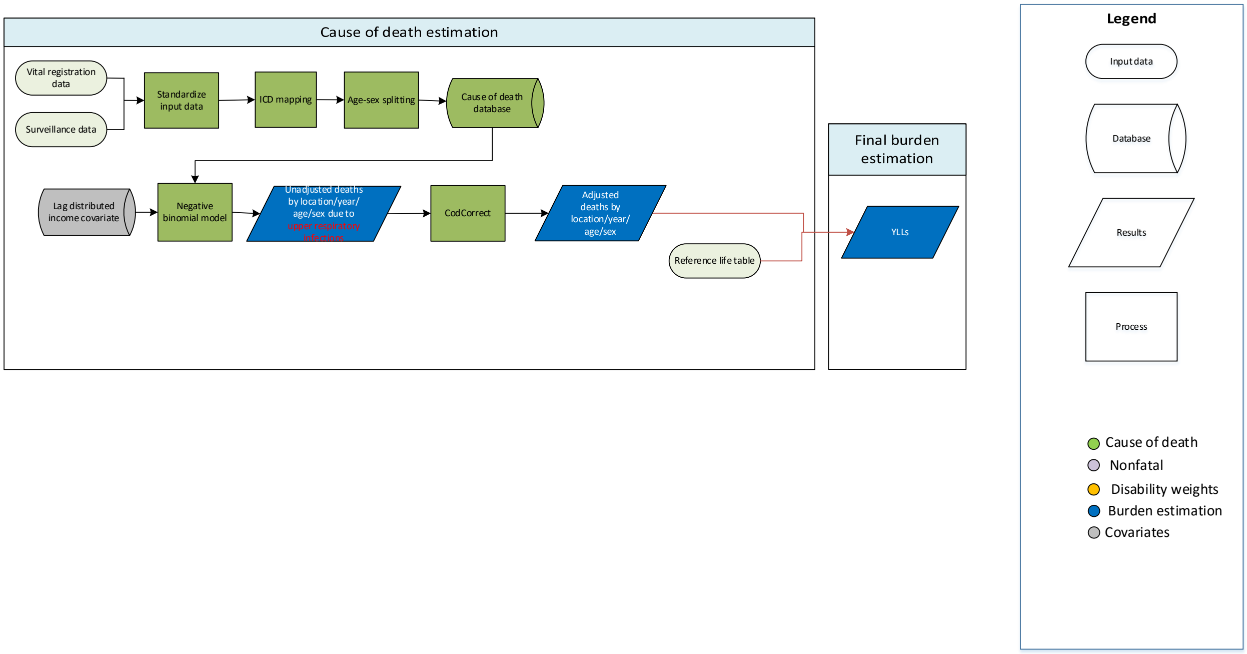

Input data

Vital registration and surveillance data from the cause of death database were used. Data with very high cause fractions (those greater than the 99th percentile values) were excluded in the regression.

Modeling strategy

Due to a small number of deaths, mortality from upper respiratory infections was modeled using a negative binomial regression, which is more appropriate than a Poisson count model as it accounts for greater variance (over-dispersion) in the data. By utilizing the exposure option in Stata, we model cause fractions with a negative binomial model. We tested both rate- and cause fraction-based models but selected a cause fraction model due to better model performance. Using the input data mentioned above, we modeled mortality from upper respiratory infections using the lag distributed income covariate and age dummy variables and the exposure set to the total number of deaths in the study. Uncertainty was estimated by taking 1,000 iterations of the predictions based on the variance covariance matrix and a random sample from a gamma distribution. The fit of the model was evaluated using diagnostic plots of predicted versus observed values.

Otitis Media

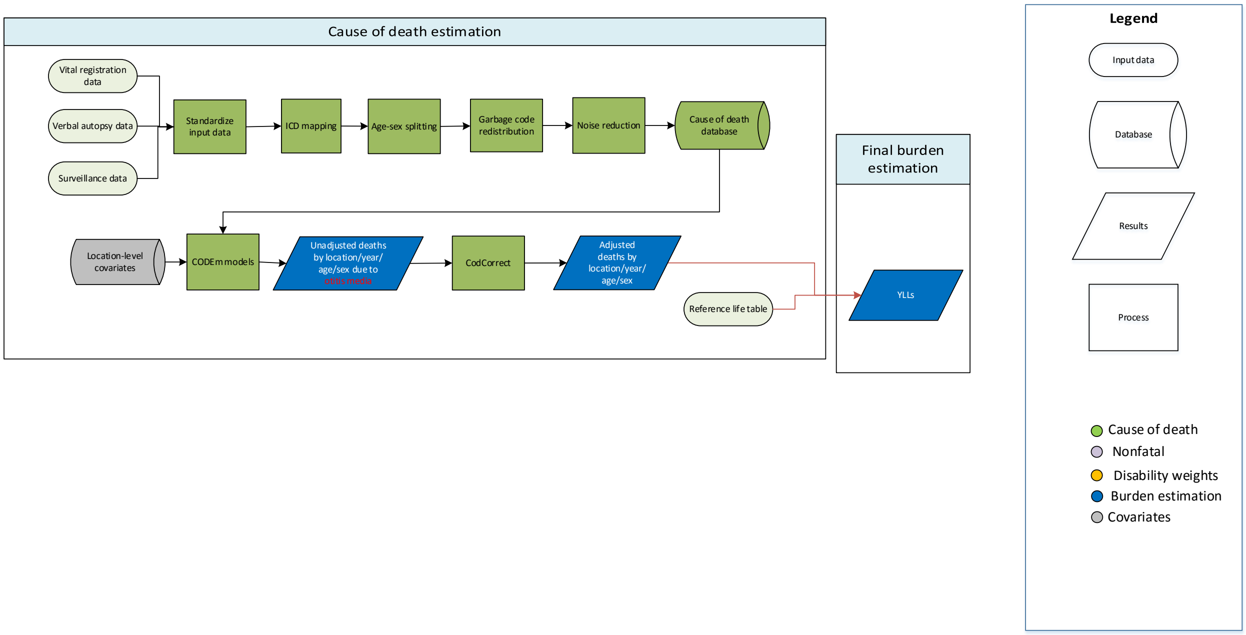

Input data

Vital registration, verbal autopsy, and surveillance data were used. We outliered data that were largely conflicting with the majority of data from other studies conducted either in the same or different countries (with similar socio-demographic characteristics) in the same region.

Modelling strategy

A general CODEm modelling strategy was used. There were no substantive changes from GBD 2015 in terms of modelling strategy.

Meningitis

Causes included in this write-up: Meningitis (total), meningococcal meningitis, pneumococcal meningitis, Haemophilus influenza type B meningitis, and other meningitis

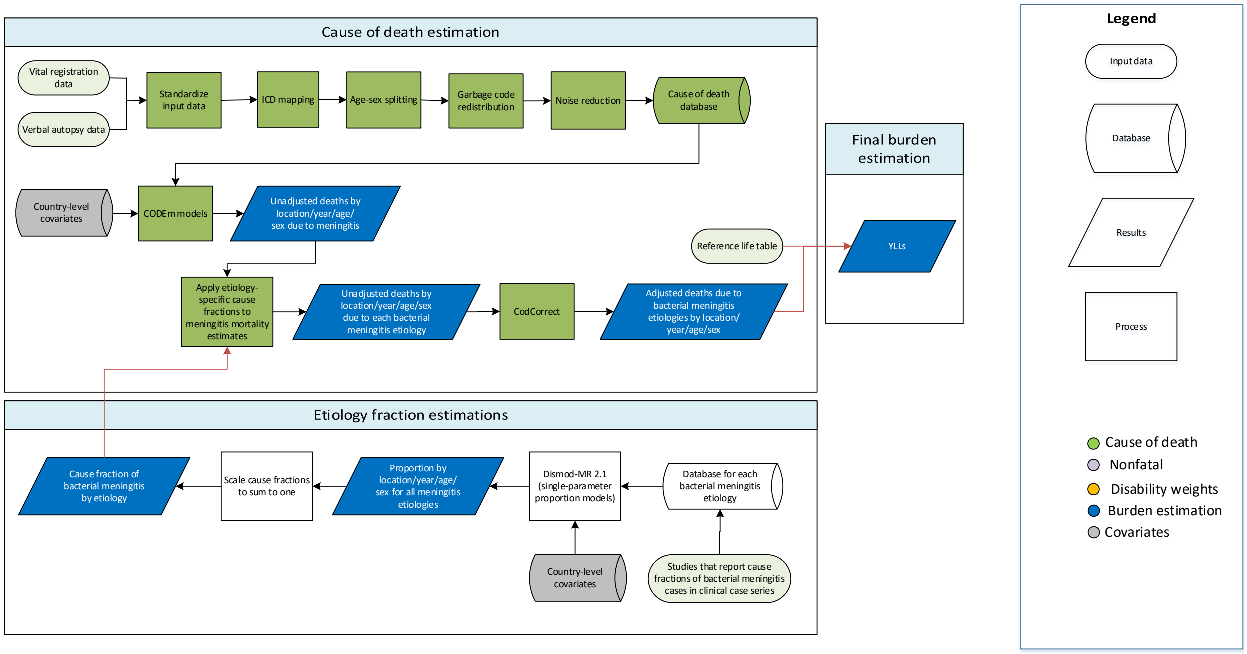

Input data

Input data for the overall meningitis model came from the cause of death database, which includes vital registration (VR) and verbal autopsy (VA) data. We outliered data in instances where garbage code redistribution and noise reduction, in combination with small sample sizes, resulted in unreasonable cause fractions when compared to regional, super-regional, and global rates, and data that violated well-established time or age trends. Outliering methods were consistent across both VR and VA data.

Input data that informed the aetiology splits for meningitis came from cause-specific VR mortality proportions, as well as a systematic review completed for the first time in GBD 2016. Previously, aetiologic attribution of meningitis deaths was informed by the proportion of incident cases due to each class of pathogen rather than the proportion of deaths. Viral meningitis mortality is rare except in the youngest age groups and is included with “other meningitis.”

For GBD 2016, we conducted a new systematic literature review on meningitis mortality aetiologies with the following search string:

(((“Meningitis”[MeSH] OR “Meningitis, pneumococcal”[MeSH] OR “Meningitis, Haemophilus”[MeSH] OR “Meningitis, Meningococcal”[MeSH] OR “Meningitis, viral”[MeSH] OR “Meningitis”[Title/Abstract]) AND ((“etiology”[Title/Abstract] OR “causes” Title/Abstract] OR “cause pattern”[Title/Abstract] OR “aetiology”[Title/Abstract] OR “cause”[Title/Abstract]) AND (“fatality”[Title/Abstract] OR “mortality”[Title/Abstract] OR “death”[Title/Abstract]) AND 1985/01/01[PDAT]:3000/12/31[PDAT]) AND “humans”[MeSH])

The search yielded 1,290 hits, of which over 400 were reviewed and 20 were extracted for use in GBD 2016 modelling.

Modelling strategy

We modelled deaths due to all bacterial meningitis with two CODEm models, separately for each sex and two age categories – under-5 and 5 years and above – because the mortality trends differ substantially between children and adults, and there are a significant number of data sources that only have data for under-5-year-olds. The two models used the same covariates and otherwise standard CODEm parameters. The final sex-specific models for deaths due to all bacterial meningitis were a hybridized model of separate global and data-rich models for males and females.

To obtain estimates for each of the four aetiologies of bacterial meningitis – meningococcal, pneumococcal, H influenza type B, and other bacterial – we ran separate proportion models in DisMod-MR 2.2 using VR proportion and systematic review data. The meningococcal meningitis proportion model used two country-level covariates to inform the model – proportion of the population living within the meningitis belt, and proportion of the population covered by the meningococcal meningitis type A vaccine (an initiative called Menafrivac). The pneumococcal meningitis model was informed by PCV3 vaccine coverage, and the H influenza type B meningitis model was informed by HiB3 vaccine coverage. The other meningitis proportion model did not use any country-level covariates.

Since DisMod-MR 2.2 estimates in 5-year intervals, the aetiological proportions for years between the intervals were interpolated at the draw level. The four proportion models were scaled to 1 at the draw level for each location, year, sex, and age combination. We applied these proportions to the total meningitis cause of death models to produce estimates for each of the four etiologies. We used the GBD shared function split_cod_model to complete this proportional split into the four etiologies of meningitis currently modeled. This is a new methodological approach for GBD 2016, which we hope to further refine for GBD 2017.

Encephalitis

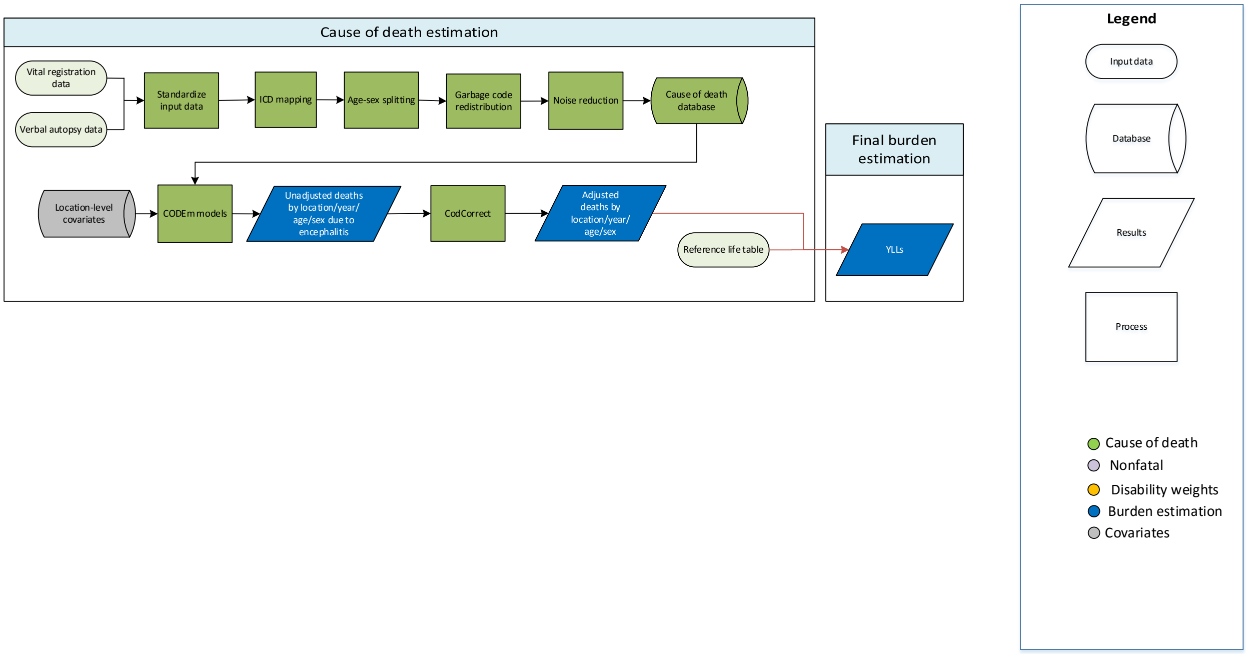

Input data

For GBD 2016, vital registration (VR) and verbal autopsy (VA) data were used to model this cause. We outliered data in instances where garbage code redistribution and noise reduction, in combination with small sample sizes, resulted in unreasonable cause fractions when compared to regional, super-regional, and global rates, and data that violated well-established time or age trends. Outliering methods were consistent across both vital registration and verbal autopsy data.

Modelling strategy

We modelled deaths due to encephalitis with a standard CODEm model using the cause of death database and location-level covariates as inputs. We hybridized separate global and data-rich models to acquire unadjusted results, which were adjusted using CodCorrect to reach final years of life lost (YLLs) due to encephalitis.

In GBD 2013, the encephalitis model was modelled using two age categories – under-5 and 5 years and above – because the mortality trends differed substantially between children and adults and a significant number of data sources only had data for under-5-year-olds. With the addition of new data sources for GBD 2015, this modelling process was deemed unnecessary and the encephalitis model covered the entire age range. Another significant change was the addition of the Japanese encephalitis covariate, which is a binary covariate indicating if the location is known to be endemic for Japanese encephalitis. The covariate was modelled according to data from the Centers for Disease Control and Prevention.[1]

We made no other significant changes to input data or modelling strategy for GBD 2016.

Reference

1 Centers for Disease Control (CDC). CDC health information for international travel 2016: the yellow book. New York City, United States: Oxford University Press, USA, 2016.

Diphtheria

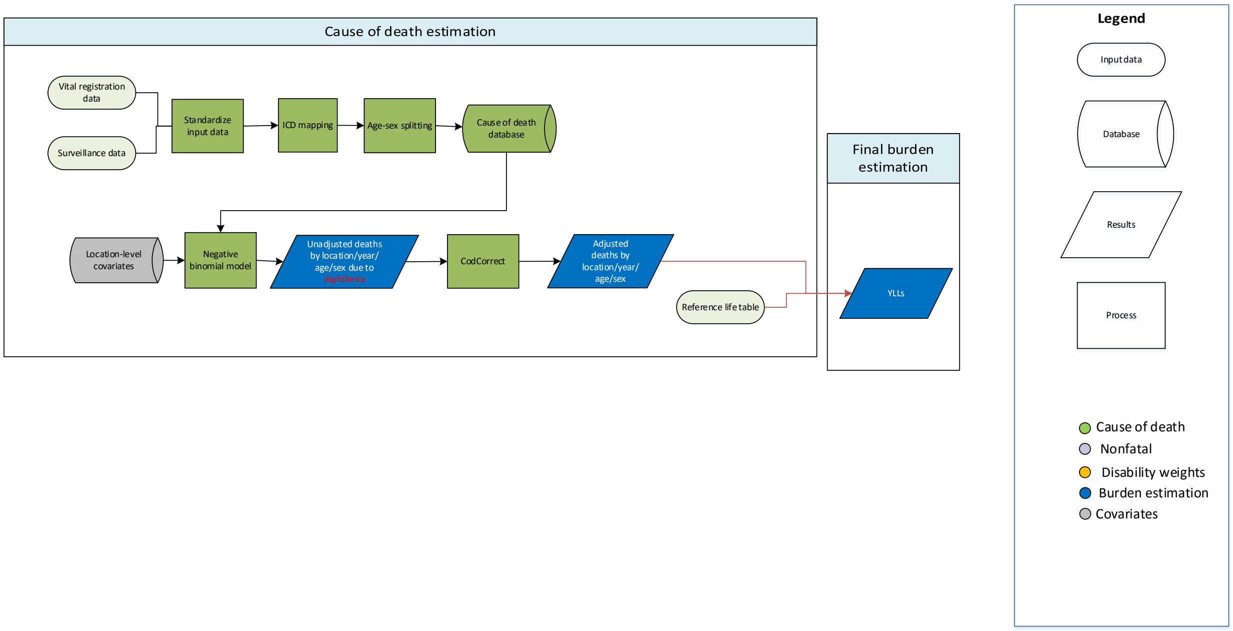

Input data

Vital registration and surveillance data from the cause of death database were used. Data with very high cause fractions (those greater than the 99th percentile values) were excluded in the regression.

Modeling strategy

Due to the small number of deaths, diphtheria mortality was modeled using a negative binomial regression, which is more appropriate than a Poisson count model as it accounts for greater variance (over-dispersion) in the data. Using the input data mentioned above, we modeled mortality due to diphtheria with the diphtheria-pertussis-tetanus third-dose (DPT3) vaccine coverage covariate and age dummy variables, with the offset as the total number of deaths in the study. Uncertainty was estimated by taking 1,000 iterations of the predictions based on the variance-covariance matrix and a random sample of the dispersion parameter from a gamma distribution.

Pertussis (whooping cough)

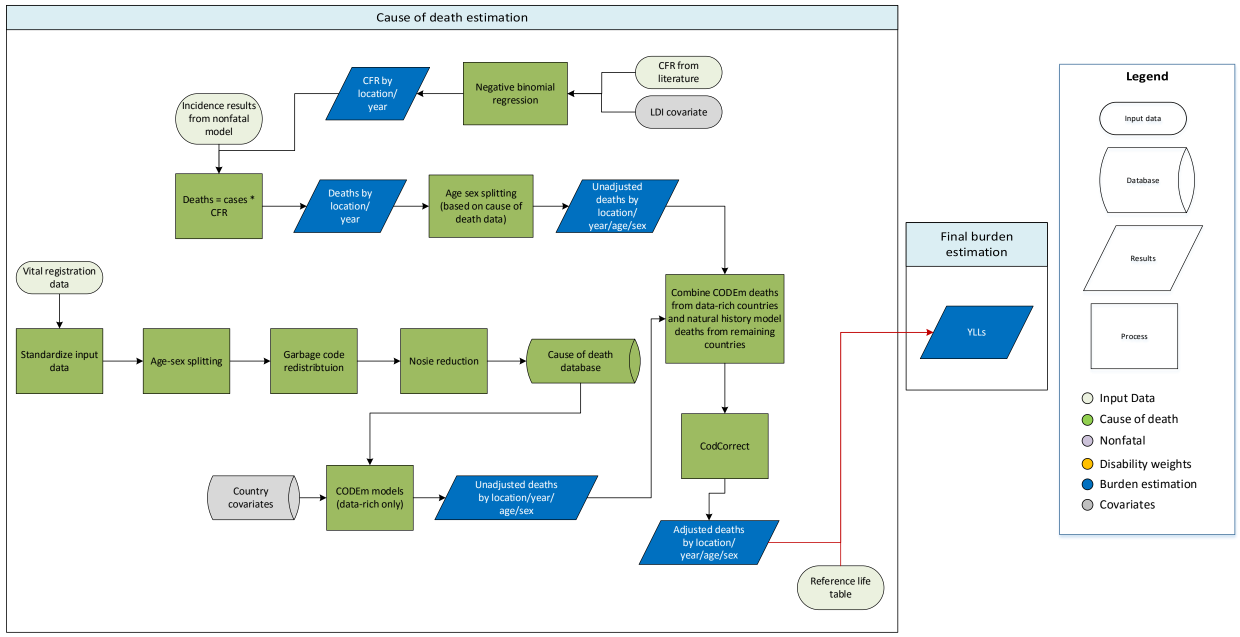

Input data

Vital registration data from the cause of death database were used for data-rich countries. To inform the natural history model, we used data from the following sources: World Health Organization (WHO) case notifications; historical case notifications for the United Kingdom back to 1940; case fatality data identified by collaborators; and case fatality data identified through systematic literature reviews for GBD 2010, GBD 2013, and GBD 2016. The PubMed search query for GBD 2016 was: (whooping cough [Title/Abstract]) OR (pertussis [Title/Abstract]) AND (case fatality [Title/Abstract]) AND ("2013"[Date - Publication]: "2016"[Date - Publication]). Studies were included if they reported case fatality rate, number of deaths, and number of cases. Studies were excluded if they included non-representative samples only.

Modeling strategy – data-rich countries

Mortality was modeled separately for data-rich and other countries. For data-rich countries (i.e., countries with vital registration more than 95% complete for more than 25 years), we used a general CODEm strategy with DPT3 vaccination coverage, lagged distributed income, and education as country-level covariates. We made estimations for the age range post-neonatal to 59 years.

Modeling strategy – other countries

For the remaining countries, we used a natural history-based model because CODEm does not predict well for those countries. First, we modeled log-transformed incidence with a mixed-effects linear regression of case notifications from the WHO (1985-2015) on diphtheria-tetanus-pertussis dose 3 (DTP3) vaccination coverage. Historical data of United Kingdom (UK) pertussis cases and UK DTP3 coverage rates (both back to 1940) were also used to inform the incidence model. The random effect by

country allowed for registration completeness to vary by country. The results of this model were then used to predict incidence as a function of vaccine coverage. To correct for underreporting in case notifications, we used a value of the random effect that matched the highest random effect in a high income region—Switzerland (which has a pertussis monitoring system which captures a high percentage of cases)—to get an implied attack rate assumed to be the same for all unvaccinated populations. Uncertainty was estimated by taking 1,000 iterations of the predictions based on the variance-covariance matrix.

Second, we modeled the pertussis case fatality rate using a negative binomial model with the health system access and lagged-distributed income covariates. Uncertainty was estimated by taking 1,000 iterations of the predictions based on the variance-covariance matrix and a random sample from a gamma distribution of the dispersion parameter. Finally, whooping cough deaths were calculated at the 1,000-draw level as

$$deaths = incidence * CFR \ .$$

We estimated overall number of deaths and then assigned an age-sex distribution based on the age- and sex-specific patterns found in the cause of death data. We made estimations for the age range post-neonatal to 59 years.

Tetanus

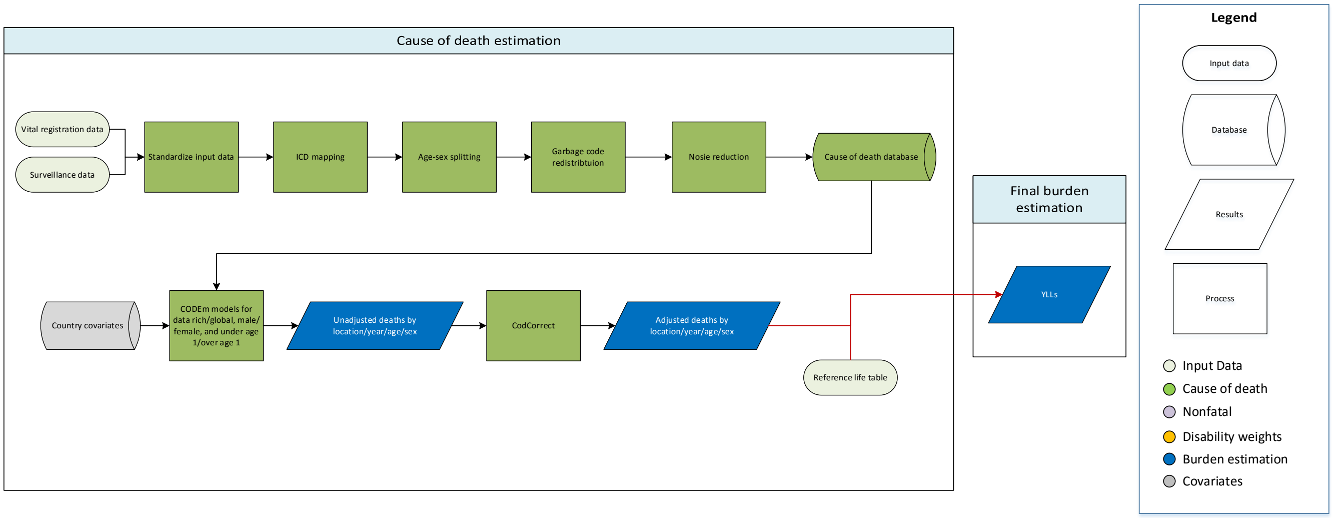

Input data

Mortality data from vital registration, verbal autopsy, and surveillance sources were used. Data were outliered if they largely conflicted with the majority of data from other studies conducted either in the same or different countries with similar sociodemographic characteristics in the same region.

Modeling strategy

A general CODEm modeling strategy was used. We ran separate models for under 1 year and 1 to 95+ years. There were no substantive changes in modeling strategy from GBD 2015.

Measles

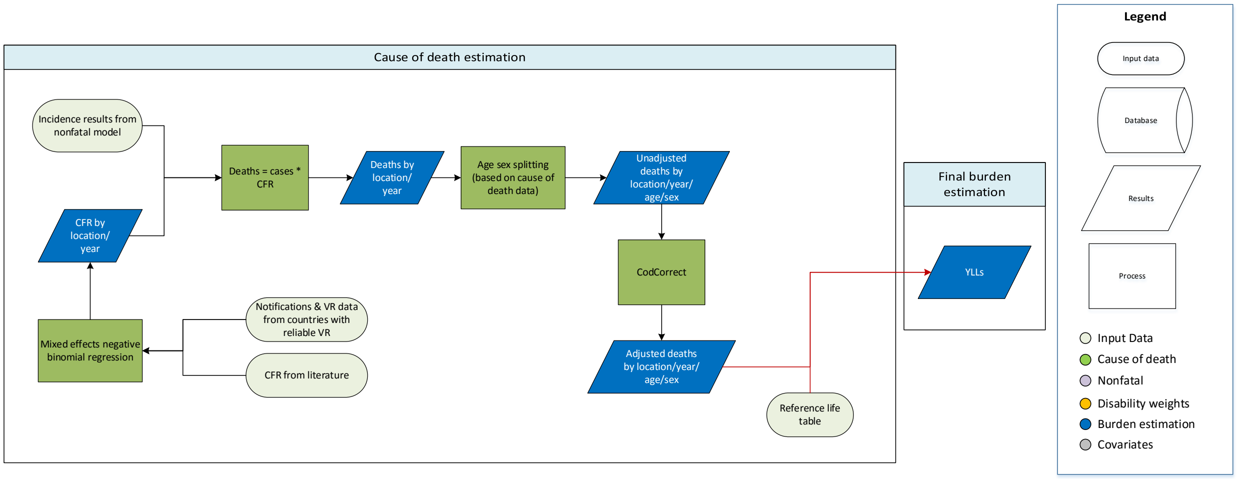

Input data

Vital registration data from the cause of death database were used for data-rich countries. To inform the natural history model, we used data from the following sources: World Health Organization (WHO) case notifications from 1995 to 2015; case notifications identified by collaborators; vital registration (VR) data in countries in the following three super-regions: high-income, Central Europe/Eastern Europe/Central Asia, and Latin America and Caribbean; and case fatality data identified through systematic literature reviews for GBD 2010, GBD 2013, and GBD 2016. The PubMed search query for GBD 2016 was: (measles [Title/Abstract]) AND (case fatality [Title/Abstract]) AND ("2013"[Date - Publication]: "2016"[Date - Publication]). Studies were included if they reported case fatality rate, number of deaths, and number of cases. Studies were excluded if they included non-representative samples only.

Modeling strategy – data-rich countries

Mortality was modeled separately for data-rich and other countries. For data-rich countries (i.e., countries with vital registration more than 95% complete for more than 25 years), we used a general CODEm strategy to model VR data with measles-containing vaccination dose one (MCV1) coverage, childhood malnutrition, lagged distributed income the healthcare access and quality index, and education as country-level covariates. We made estimations for the age range post-neonatal to 59 years.

Modeling strategy – other countries

Measles mortality in the remaining countries was modeled using a natural-history-based model. First, we modeled measles incidence with a mixed-effects linear regression of case notifications from the WHO (1995-2015) on routine measles vaccination rates and supplementary immunization activities (SIAs). More precisely, log-transformed incidence rates were regressed on the log of the proportion unvaccinated with first- and second-dose measles-containing vaccine, and additional SIA coverage lagged by one, two, three, four, and five years, with super-region, region, and country-level random effects. The results of this mixed effects regression model were then used to predict location-year-

specific incidence as a function of routine vaccine coverage and SIAs. To correct for underreporting in case notifications, we added the effect of a 95% attack rate, assumed to be the same across all unvaccinated populations. Uncertainty was estimated by taking 1,000 iterations of the predictions based on the variance-covariance matrix. For locations in three super-regions—high-income, Central Europe/Eastern Europe/Central Asia and Latin America and Caribbean—we used reported measles cases as incident cases.

Second, the case fatality rate was modeled using a mixed effects negative binomial regression with the child malnutrition covariate and study-level indicators (hospital-based or not; outbreak or not; and rural or urban/mixed), with country random effects. Uncertainty was estimated by taking 1,000 iterations of the predictions based on the variance-covariance matrix and uncertainty in country random effects. The fit of the model was evaluated using diagnostic plots of predicted versus observed values. Finally, estimated deaths were calculated at the 1,000-draw level as

$$deaths = incidence * CFR \ .$$

We estimated overall number of deaths and then assigned an age-sex distribution based on the age- and sex-specific patterns found in the cause of death data. We made estimations for the age range post-neonatal to 59 years.

Varicella

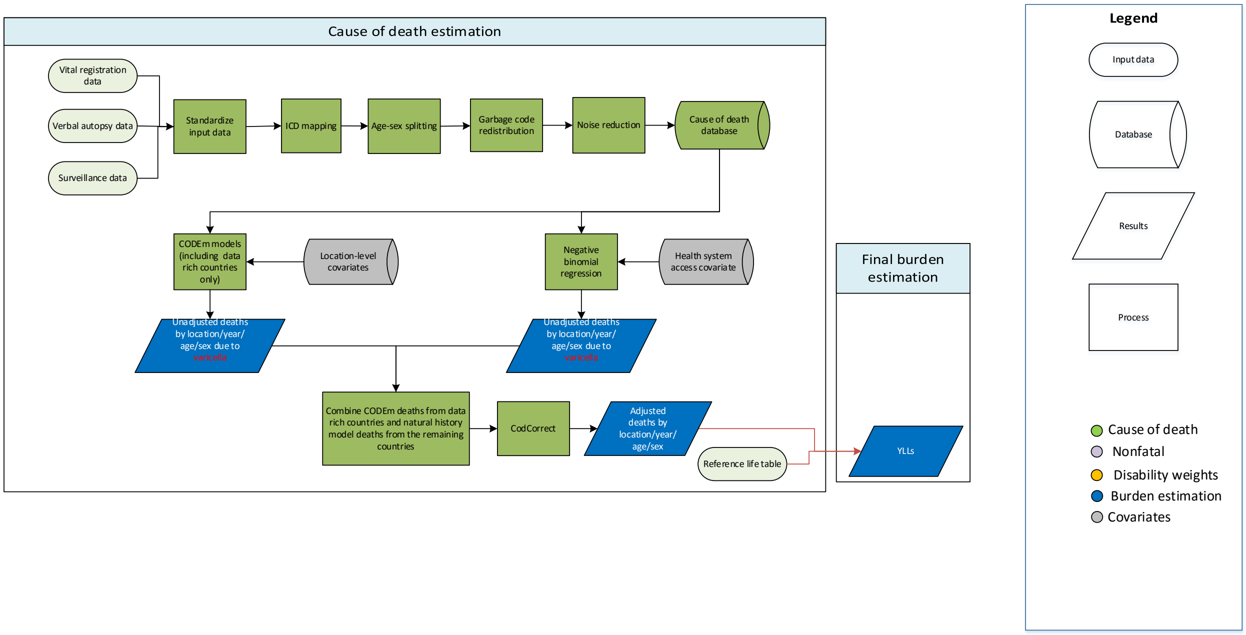

Input data

Vital registration, verbal autopsy and surveillance data from the cause of death database were used. Data with very high cause fractions (those greater than the 99th percentile values) were excluded in the negative binomial regression.

Modeling strategy

Mortality was modeled two ways. For data-rich countries (i.e., countries with vital registration more than 95% complete for more than 25 years), we used a general CODEm strategy. For the remaining countries, since CODEm did not predict well in data-sparse areas, we used a negative binomial regression to model varicella cause fraction. Using the input data mentioned above, we modeled mortality due to varicella using the healthcare access and quality index and age dummy variables with the offset set to the location-year-age-sex-specific population. Uncertainty was estimated by taking 1,000 iterations of the predictions based on the variance-covariance matrix and a random sample of the dispersion parameter from a gamma distribution.

In GBD 2013, we used a negative binomial regression to model varicella mortality for all countries. For GBD 2015, we switched to using CODEm for data-rich countries because it fit the data better in those countries. There were no significant changes in modeling strategy for GBD 2016 from GBD 2015.

Malaria

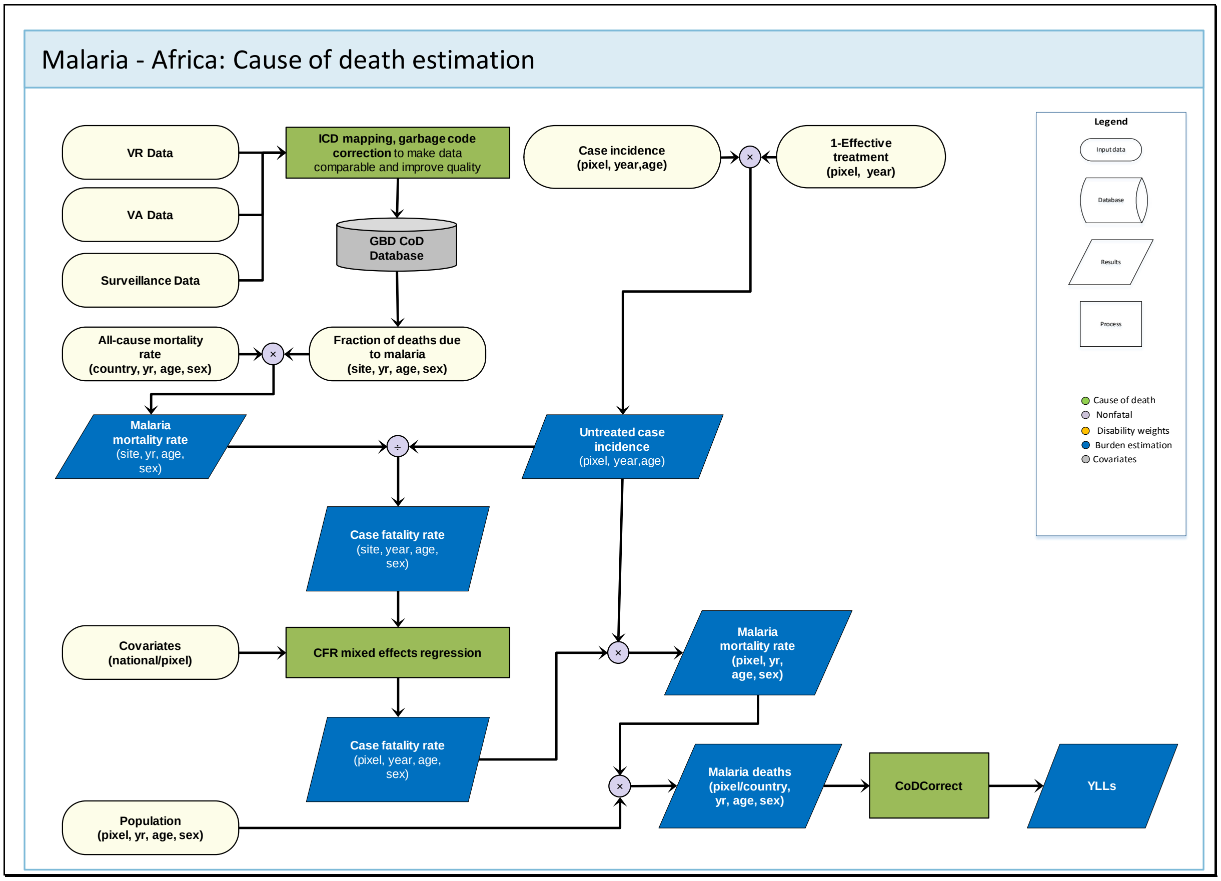
Malaria - Africa: Cause of death estimation
VR Data
VA Data
Surveillance Data
ICD mapping, garbage code correction to make data comparable and improve quality
GBD CoD Database
Case incidence (pixel, year,age)
1-Effective treatment (pixel, year)
All-cause mortality rate (country, yr, age, sex)
Fraction of deaths due to malaria (site, yr, age, sex)
Malaria mortality rate (site, yr, age, sex)
Untreated case incidence (pixel, year,age)
Case fatality rate (site, year, age, sex)
Covariates (national/pixel)
CFR mixed effects regression
Malaria mortality rate (pixel, yr, age, sex)
Case fatality rate (pixel, year, age, sex)
Malaria deaths (pixel/country, yr, age, sex)
CoDCorrect
YLLs
Population (pixel, yr, age, sex)
Legend
Input data
Database
Results
Process
Cause of death
Nonfatal
Disability weights
Burden estimation
Covariates

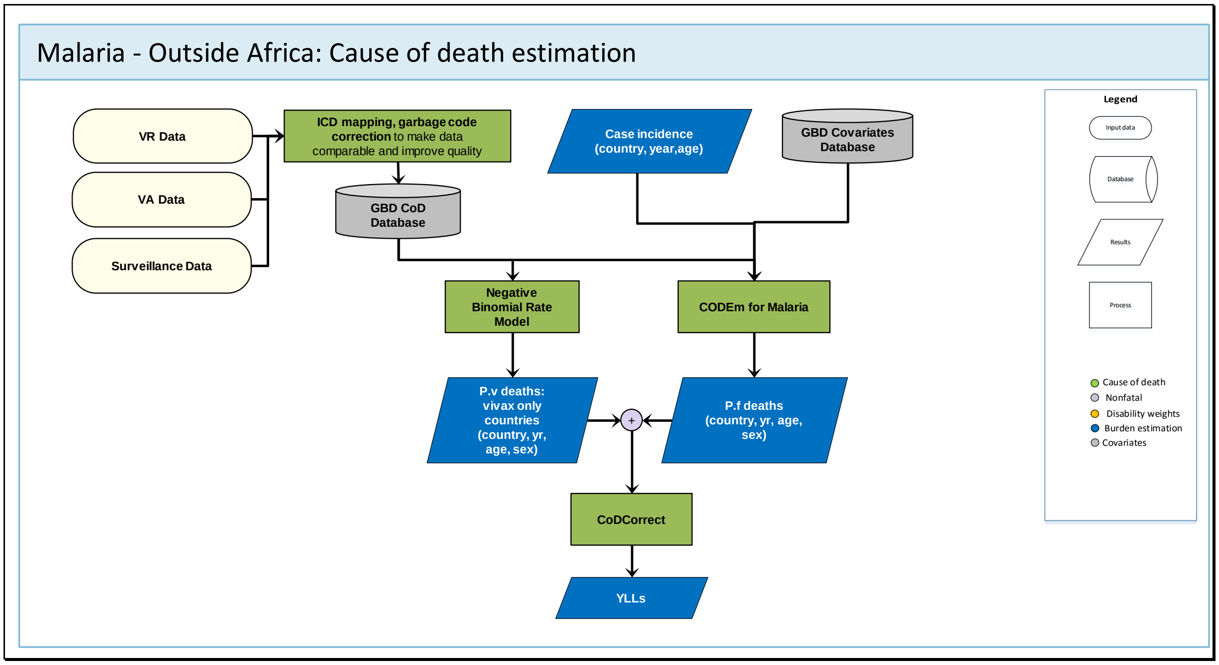

Input data and methodological summary for malaria

Overview

Variability in the type and abundance of CoD and related data meant that three distinct approaches were developed to estimate malaria mortality due to (i) *Plasmodium falciparum* inside Africa; (ii) *P. falciparum* outside Africa; and (iii) *P. vivax* in countries without falciparum malaria.

Input data

For the Outside of Africa and *P. vivax* models, data included vital registration, verbal autopsy, and surveillance data from the cause of death (CoD) database. Unlike other causes of death, we did not redistribute deaths to malaria. For the Africa models, we only used CoD data (mostly verbal autopsy) where we have been able to successfully geo-reference (ie, find latitude and longitude) the site. Systematic literature reviews for malaria were not conducted.

Our outlier criteria excluded data points that (i) were implausibly high or low relative to global or regional patterns, (i) substantially conflicted with established age or temporal patterns, or (iii) significantly conflicted with other data sources conducted from the same locations or locations with similar characteristics (ie, local Socio-demographic Index).

Modelling strategy

As described above, the malaria modelling strategy was carried out in three parts.

P. falciparum: Africa

For most GBD causes, epidemiologic measures may be used as covariates in a traditional CODEm approach, if at all. To estimate the fatal burden of *P. falciparum* malaria in Africa, we directly used epidemiologic measures in our estimation process.The Malaria Atlas Project (MAP) at the University of Oxford has generated updated spatiotemporal “cubes” estimating clinical incidence (rates and case counts) for each 5x5 km pixel across Africa, by year, from 1980 to 2015, specified by three broad age-

bins (0–5, 5–14 and 15+). MAP has also generated an equivalent spatiotemporal prediction of access to effective antimalarial drugs (combining access to care, the fraction of malaria cases receiving different classes of antimalarial, and the estimated country-year-specific efficacy of each antimalarial class though time). This estimated treatment rate was combined with the incidence rate cube to derive a third cube estimating the incidence of untreated cases.

For each site-year for which CoD malaria cause fraction data were available we (i) estimated a site-year-specific malaria mortality rate as the product of malaria cause fraction and all-cause mortality rate (with the latter drawn from national-level values); (ii) divided the malaria mortality rate by the site-year-specific estimate of untreated malaria incidence rate (drawn from the MAP cube) to estimate a site-year-specific case fatality rate (CFR) among untreated malaria cases. These derived site-year specific CFR values were then used in a mixed-effects regression model to estimate pixel-year CFR for each 5x5 km grid cell. The models used as covariates are the log of country-year all-cause mortality, pixel-year night-time lights, accessibility, and fractional land-cover classes, and study specific age and sex, with the location of each study site as a national-level random effect. Data were weighted by sample size (ie, the number of all-cause deaths observed in each study site-year).

Pixel-year predictions of CFR were then multiplied by the corresponding untreated incidence rate from the MAP cube to yield a pixel-year mortality rate estimate, which was then multiplied by pixel-year population to compute pixel-year malaria death counts. These were then aggregated to yield the required GBD national or subnational death estimates.

To disaggregate into GBD age-bins, we separately ran a traditional national-level CODEm model with covariates: prevalence of *P. falciparum* in 2–10 age group (*Pf*$PR_{2\text{-}10}$), *Pf* incidence rate, years of education, access to effective antimalarial drugs, and health system access. The resulting predicted age-patterns were used to split the country-year mortality estimates.

***P. falciparum:* Outside of Africa**

In locations outside of Africa, we continued to use a traditional CODEm approach, mirroring closely that used in GBD 2015. It must be noted that "outside of Africa" also included some countries on the continent of Africa with either very low incidence or relatively robust routine surveillance systems including Algeria, Egypt, Morocco, Comoros, Mauritius, Cape Verde, Sao Tome, Principe, Rwanda, Botswana, Namibia, Eritrea, Djibouti, and South Africa. The model included the following covariates: Pf incidence rate, access to effective antimalarial drugs.

***P. vivax*: countries without *P. falciparum* transmission**

For countries where the main/exclusive strain of malaria was *P. vivax*, deaths were estimated using a zero-inflated negative binomial mixed model where the outcome is study deaths. The model included as fixed effect the logarithm of mortality rate, age, and sex. Locations were included as random effects.

The results from the *P. vivax*, Outside of Africa, and Africa models were collated and uploaded in CODEm and marked as best model in order to incorporate the estimation in the CodCorrect algorithm.

References

1 Bhatt S, Weiss DJ, Cameron E, *et al.* The effect of malaria control on Plasmodium falciparum in Africa between 2000 and 2015. *Nature* 2015; **526**: 207–11.

Chagas disease

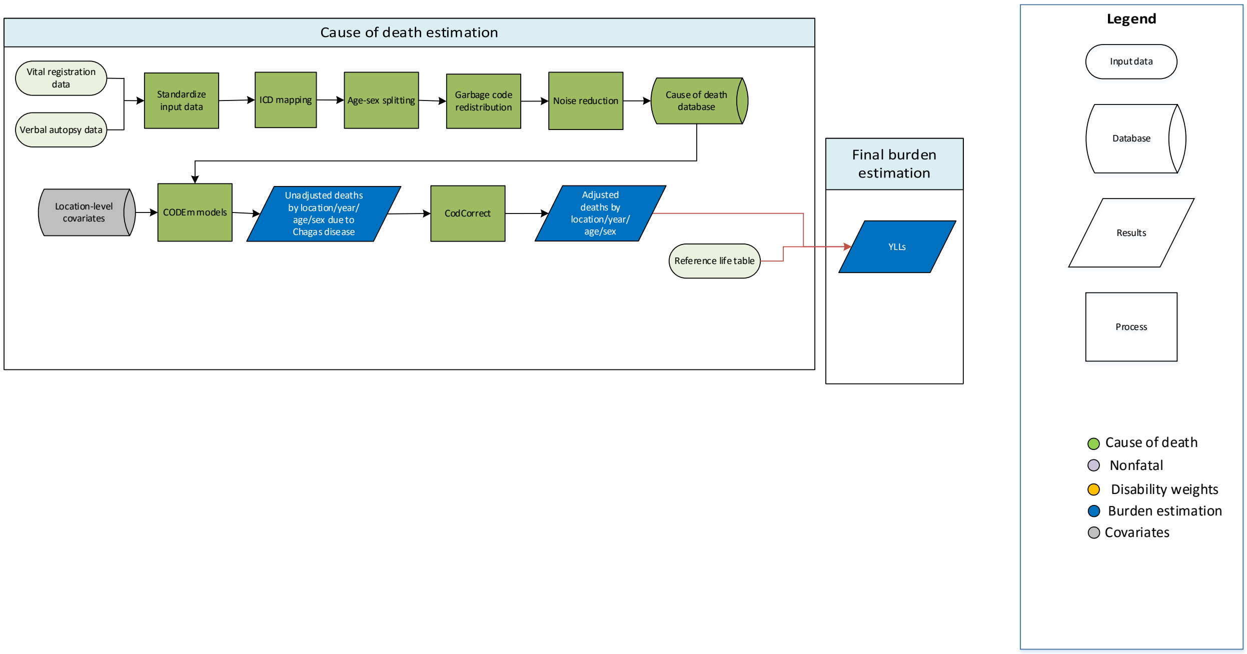

Input data

We modeled Chagas mortality using all available data in the cause of death database. No data were outliered for this cause.

Modelling strategy

We modelled Chagas mortality using a two-model hybrid approach: 1) a CODEm model of all Chagas-endemic countries of Latin America using all data in the CoD database; and 2) estimates of mortality from imported cases in non-endemic, data-rich countries. Where Chagas deaths were reported in non-endemic data-rich countries, we produced non-zero estimates by drawing from a beta distribution defined based on number of reported deaths and the underlying sample size. Estimates of Chagas mortality in endemic countries were drawn from the CODEm model.

We have made no substantive changes in the modelling strategy from GBD 2015 to GBD 2016 for Chagas endemic countries.

Visceral leishmaniasis

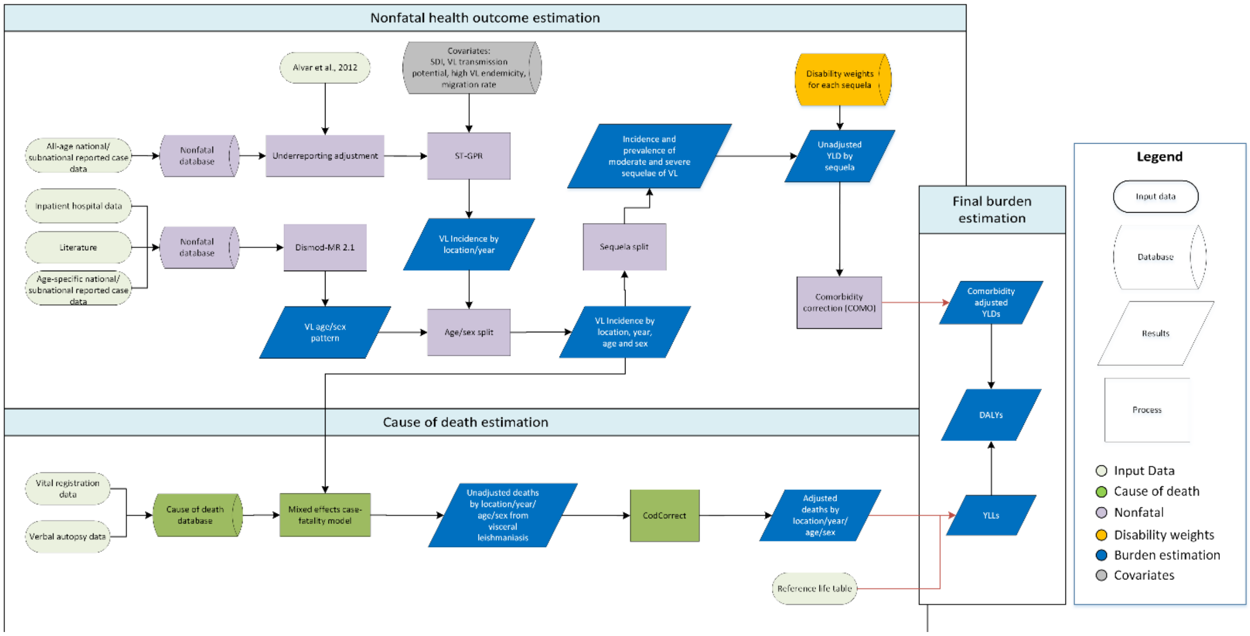

Input data

Model inputs included incidence estimates derived primarily from reported case data, and cause-specific mortality data from the cause of death (CoD) database, including vital registration and verbal autopsy data.

Modelling strategy

We estimate VL mortality using a mortality-to-incidence (MI) ratio model. For every VL data point in the cause of death database, we calculate the implied MI ratio as the number of reported VL deaths divided by our corresponding estimate of the number of VL cases. We then model logit-transformed MI ratios using a mixed-effects regression model with fixed effects on age group and sex; and random intercepts on super-region, random slope on sex by super-region, random intercepts on country (nested within super-region), and random slope on age group by country. Finally, we estimate deaths for every location, year, age and sex as the product of our estimates of VL incidence and the modelled MI ratios.

We have substantially revised this model for GBD 2016. Models for both GBD 2015 and GBD 2016 estimate mortality via a MI ratio approach; however, they differ both in in the methods used to estimate VL incidence, and in the methods used to estimate MI ratios. Whereas GBD 2015 incidence estimates were derived from a DisMod model, we now estimate incidence via space-time Gaussian process regression (ST-GPR). This approach has the advantage of being able to estimate trends with more detail and accuracy, and of being able to more effectively utilize all-age data, which represent the majority of location-years of incidence data for VL. Moreover, previous iterations of GBD estimated MI ratios from location-years with matching VL hospital and mortality data. Our new approach to estimate MI ratios – based on using estimated incidence rather than hospital-based incidence – offers two advantages: first,

because matched hospital and CoD data are available for only a small number of VL endemic countries, the new approach allows us to leverage information for a much larger set of CoD data; and, second, because we model the relationship using total estimated incidence, rather than hospital-based incidence, our new approach requires no assumptions be made regarding the relationship between total incidence and hospital-based incidence.

Human African Trypanosomiasis (HAT)

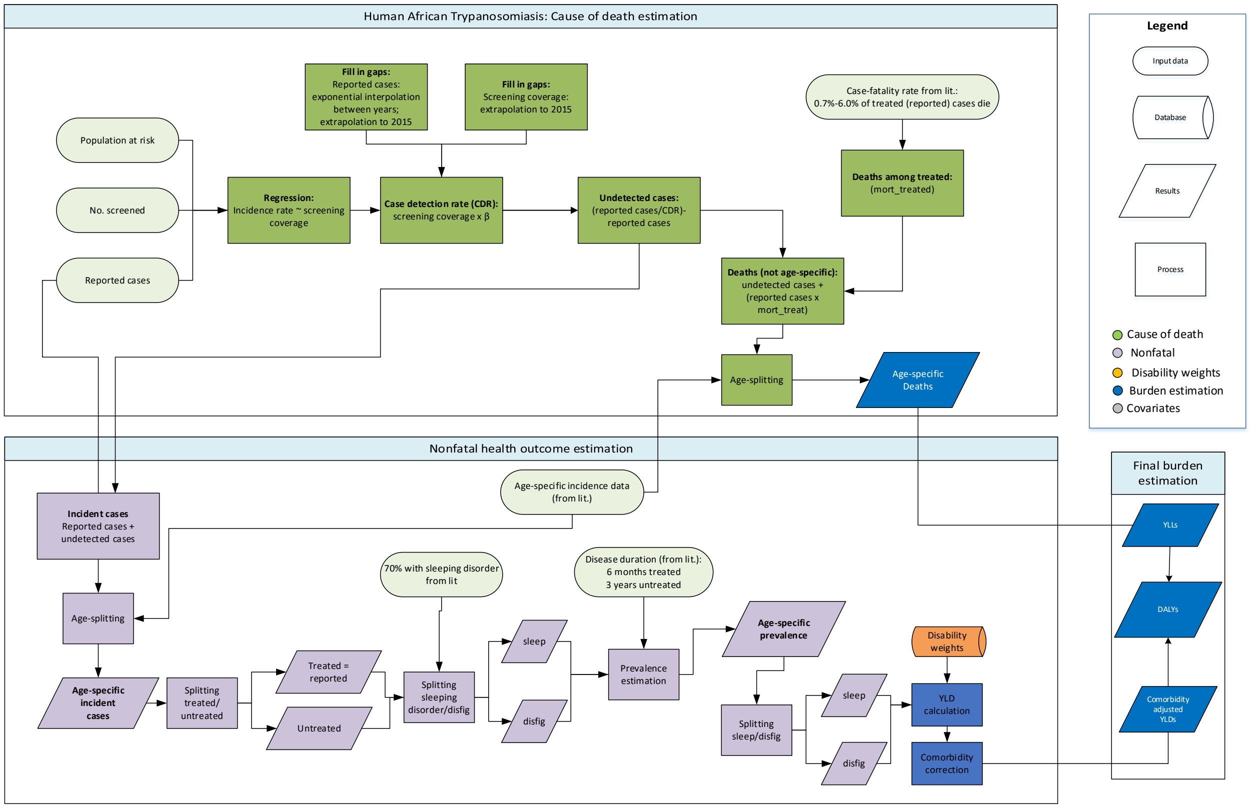

Input data

A literature search was done for GBD 2013 and for GBD 2015. The GBD 2015 search was conducted between 1/1/2013 and 8/10/2015, and the number of initial hits was 87. Of these, five sources were extracted for data. The literature search was updated by reviewing all publications from 2016; of the 138 sources reviewed, one additional source was identified to describe the age/sex distribution of HAT infection.

Additional input data used to estimate mortality due to HAT included a) population at risk estimates from GBD 2010 ArcGIS analysis using geocoded case notifications for 2000 to 2009[1] and population Count Grid estimates from Gridded Population of the World 3,[2,3] b) population screened from 1997 to 2004,[4] c) historical data from GBD 2010 on total number of HAT cases reported[1,4,5] and d) cases reported annually to the WHO[6] – for Kenya, a study on cases reported subnationally[7] was used to split the national cases into five counties (HomaBay, Migori, Busia, Bungoma, Kakamega). In addition, age-specific incidence data from active screening undertaken in the Democratic Republic of Congo[8] and Uganda[9] were used to inform age pattern for deaths.

Data on the number of cases identified through active screening were obtained from a Weekly Epidemiological Record report[4] for *T.b. gambiense* HAT reported from 1997-2004 which reported active case finding data for the following countries: The Gambia, Ghana, Guinea Bissau, Liberia, Niger, Sierra Leone, and Senegal. No active case detection screening coverage data are currently available for *T.b. rhodesiense* HAT-endemic countries.

National case data from 1990-2015 were obtained from the World Health Organization's official surveillance data (available via the Global Health Observatory).

Based on available historical data post-1980, the following countries (years) were included in the estimation: Botswana (1983), Ethiopia (1980-1983), Guinea-Bissau (1980-1983, 1985-1987), Rwanda (1980, 1982-1988), and Sierra Leone (1981-1982). Five subnational locations (out of 49) for Kenya were also added in the estimation for GBD 2016.

Modeling strategy

The cause of death model for HAT is implemented as follows:

1. The incidence of reported HAT cases among the population at risk was calculated as the total number of reported cases divided by the population at risk.

2. To estimate the number of cases that were likely undetected by country and year, a multi-level mixed-effects linear regression of natural log-transformed incidence rate (ratio of reported HAT cases to population at risk) on natural log-transformed screening coverage (ratio of number screened for HAT to population at risk), with country random effects, was performed. Gaps were then filled using exponential interpolation between years and extrapolation from 2015 to 2016 for reported cases. This model generates a beta-coefficient which is used to estimate the case detection rate (see step 4).

   For country-years in which no screening coverage data were reported:
   - Among countries with data reported, 1997-2004, the proportion of the at risk population screened from 1997 was used retrospectively for the period 1990-1996 and the screening coverage from 2004 was carried forward from 2005-2016.
   - For countries with no screening data reported, the mean screening coverage for the region was used to impute a value over time.

3. To construct an estimate of total deaths, we first assume that all detected cases receive treatment, and that mortality among the treated occurs for a small proportion of cases. Deaths among detected cases is estimated by generating one thousand draws of mortality among treated cases, assuming that between 0.7% - 6.0% of all treated (reported) cases die.[10–12]

4. We then assume that all undetected cases experience mortality. This is estimated via generation of 1,000 draws of the case detection rate (CDR), given the expected screening coverage from the regression (in Step 2). Undetected deaths were then estimated as the difference between the ratio of reported cases to CDR and reported cases (reported cases/CDR – reported cases).

5. Estimates of death were obtained by adding the reported cases (scaled by mortality among treated) to the undetected cases. Without information on sex-specific incidence or deaths, equal death rates between both sexes was assumed.

6. Finally, an age-pattern was applied to the mortality estimates using the incidence studies from DRC and Uganda.[8,9,13] The age-pattern in GBD 2016 employed a cubic spline to account for the higher risk of infection among working age adults (as opposed to GBD 2015 in which a step-function at age 20 years was employed to differentiate risk).

References:

1 Simarro PP, Cecchi G, Paone M, *et al.* The Atlas of human African trypanosomiasis: a contribution to global mapping of neglected tropical diseases. *Int J Health Geogr* 2010; **9**: 57.

2 Gridded Population of the World (GPW), Version 3: Population Count Grid. Palisades, NY: Socioeconomic Data and Applications Center (SEDAC); Center for International Earth Science Information Network (CIESIN) at Columbia University; Centro Internacional de Agricultura Tropical (CIAT), 2005.

3 Gridded Population of the World (GPW), Version 3: Population Count Grid, Future Estimates. Palisades, NY: Socioeconomic Data and Applications Center (SEDAC); Center for International Earth Science Information Network (CIESIN) at Columbia University; United Nations Food and Agriculture Programme (FAO); Centro Internacional de Agricultura Tropical (CIAT), 2005.

4 Weekly Epidemiological Record (WER). World Health Organization, 2006.

5 World Health Organization, Department of Communicable Disease Surveillance and Response (CDS). WHO Report on Global Surveillance of Epidemic-prone Infectious Diseases. World Health Organization, 2000.

6 World Health Organization. Human African Trypanosomiasis. Global Health Observatory data repository. http://apps.who.int/gho/data/node.main.A1635?lang=en (accessed April 21, 2016).

7 Rutto JJ, Karuga JW. Temporal and spatial epidemiology of sleeping sickness and use of geographical information system (GIS) in Kenya. *J Vector Borne Dis* 2009; **46**: 18–25.

8 Lutumba P, Makieya E, Shaw A, Meheus F, Boelaert M. Human African trypanosomiasis in a rural community, Democratic Republic of Congo. *Emerging Infect Dis* 2007; **13**: 248–54.

9 Fèvre EM, Odiit M, Coleman PG, Woolhouse MEJ, Welburn SC. Estimating the burden of rhodesiense sleeping sickness during an outbreak in Serere, eastern Uganda. *BMC Public Health* 2008; **8**: 96.

10 Balasegaram M, Harris S, Checchi F, Ghorashian S, Hamel C, Karunakara U. Melarsoprol versus eflornithine for treating late-stage Gambian trypanosomiasis in the Republic of the Congo. *Bull World Health Organ* 2006; **84**: 783–91.

11 Odiit M, Kansiime F, Enyaru JC. Duration of symptoms and case fatality of sleeping sickness caused by Trypanosoma brucei rhodesiense in Tororo, Uganda. *East Afr Med J* 1997; **74**: 792–5.

12 Priotto G, Kasparian S, Mutombo W, *et al.* Nifurtimox-eflornithine combination therapy for second-stage African Trypanosoma brucei gambiense trypanosomiasis: a multicentre, randomised, phase III, non-inferiority trial. *Lancet* 2009; **374**: 56–64.

13 Kato CD, Nanteza A, Mugasa C, Edyelu A, Matovu E, Alibu VP. Clinical profiles, disease outcome and co-morbidities among T. b. rhodesiense sleeping sickness patients in Uganda. *PLoS One*. 2015; **10**: e0118370.

Schistosomiasis

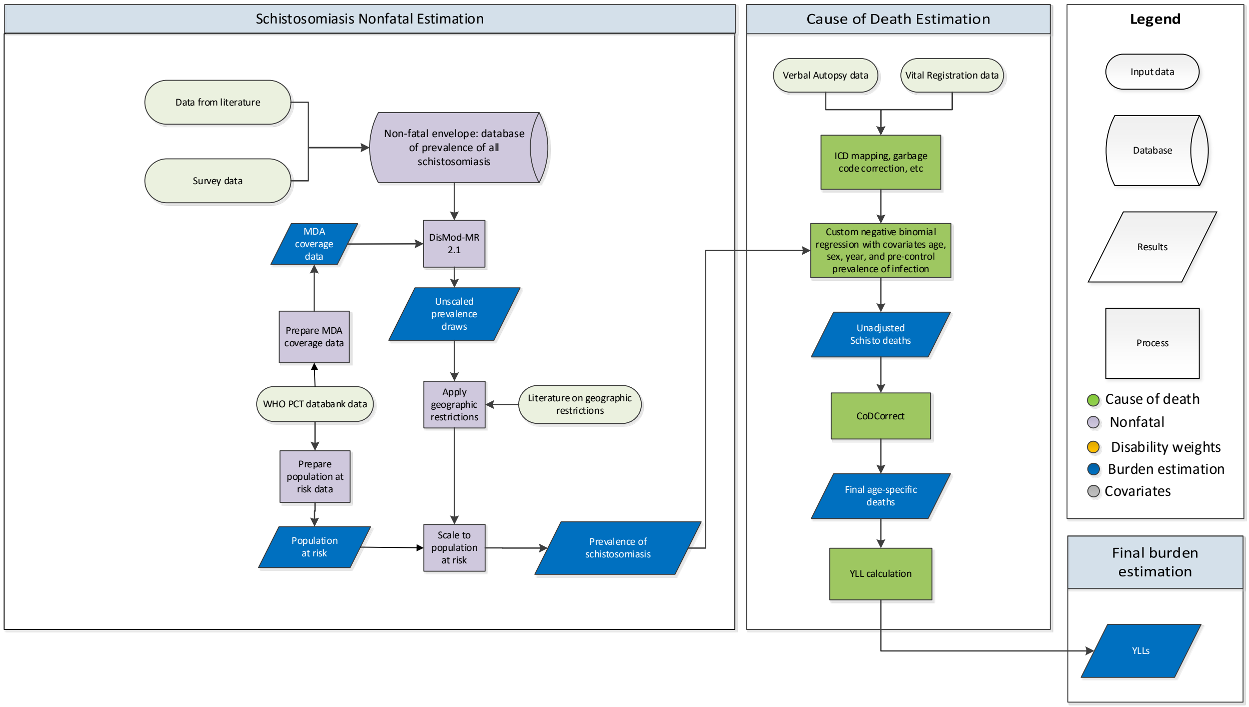

Input data

To estimate mortality due to schistosomiasis, data on deaths and prevalence of infection were used. The prevalence data were prepared this year for GBD 2016, and further information on prevalence data is available in the nonfatal write-up for this cause. Country-year-age-sex-specific verbal autopsy and vital registration data were used in the mortality model.

Geographic Restrictions

We conducted a literature review to determine the geographic extent of the disease and classify locations based on whether the disease is absent or present in each year. Locations that were geographically restricted in any given year did not have estimates made for them but could have imported cases attributed to them at a later stage. Of note, we did not attempt a complete systematic review, since a single high-quality source could offer sufficient evidence of presence. Evidence of absence or presence was not available for every location for each year and so assumptions were made for missing years by taking into consideration the epidemiological characteristics of the disease. If evidence indicated disease presence for two non-consecutive years, we assumed presence for all years between the two. If evidence indicated disease absence for two non-consecutive years, we assumed absence for all years between the two. If evidence indicated a change in status (i.e. from absent to present, or present to absent) between two non-consecutive years than we conducted targeted searches to ascertain the relevant year of introduction or elimination for that location. In the cases where presence or absence information was missing for the start or end years of our study interval (1990-2016) without evidence of any introduction or elimination events within the interval, we applied the status of the first and last presence/absence observations respectively to all years between the

interval bound and the observation year. For schistosomiasis, we used a combination of Chitsulo et al's *The global status of schistosomiasis and its control* (1) and WHO's *Preventative chemotherapy in human helminthiasis* (2) report as a baseline. Where country-level endemicity statuses conflicted between the two sources, we searched Pubmed and Google Scholar for country and subnational-specific endemicity status. Our search yielded 22 sources that were used to develop our annual geographic restriction map for schistosomiasis.

Modelling strategy

To estimate deaths due to schistosomiasis, a negative binomial regression model of country-year-age-sex-specific deaths on natural log-transformed prevalence of total schistosomiasis infection with a 5-year lag was used. The negative binomial regression was selected due to its suitability for modelling count data. In addition, there are relatively low number of deaths attributable to schistosomiasis. Covariates for endemic Brazil subnationals and South Africa subnationals were used to allow the model to follow data in those areas. A multivariate normal distribution using the mean and variance-covariance matrix from the model was used to generate 1,000 draws of deaths due to schistosomiasis.

Models were evaluated by assessing the AIC and plotting the predicted deaths against time, age, and sex. In addition, the Cause of Death Visualization tool was used to evaluate time trends across locations, age, and sex. A map of the global distribution of schistosomiasis across age-groups was also used to assess the changes in death rates over time. The final model was selected based on how well the estimated numbers fit the input data and how plausible the predicted distribution of disease was over time and with age.

References

(1) Chitsulo, L., Engels, D., Montresor, A., & Savioli, L. (2000). The global status of schistosomiasis and its control. Acta Tropica, 77(1), 41-51. doi:10.1016/s0001-706x(00)00122-4

(2) World Health Organization (2006). Preventive chemotherapy in human helminthiasis : coordinated use of anthelminthic drugs in control interventions : a manual for health professionals and programme managers.

Cysticercosis

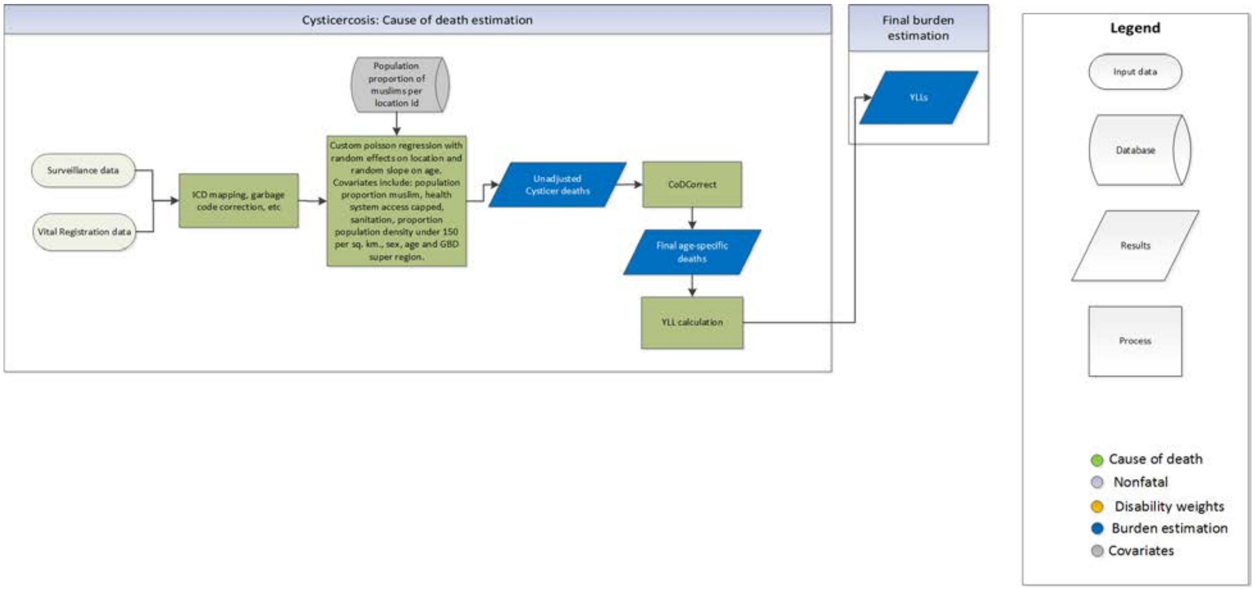

Input data

The model for mortality due to cysticercosis relied on vital registration and surveillance data from endemic countries. In addition, we used data from the Pew Research Center on percentage of population that is Muslim by country. The primary covariates adjusted for in the model were proportion of the population that is Muslim, health system access capped, proportion of the population with access to sanitation, proportion of the country with population density under 150 people per square kilometer, sex, age and GBD super region.

Geographic Restrictions

We conducted a literature review to determine the geographic extent of the disease and classify locations based on whether the disease is absent or present in each year. Locations that were geographically restricted in any given year did not have estimates made for them but could have imported cases attributed to them at a later stage. Of note, we did not attempt a complete systematic review, since a single high-quality source could offer sufficient evidence of presence. Evidence of absence or presence was not available for every location for each year and so assumptions were made for missing years by taking into consideration the epidemiological characteristics of the disease. If evidence indicated disease presence for two non-consecutive years, we assumed presence for all years between the two. If evidence indicated disease absence for two non-consecutive years, we assumed absence for all years between the two. If evidence indicated a change in status (i.e. from absent to present, or present to absent) between two non-consecutive years than we conducted targeted searches to ascertain the relevant year of introduction or elimination for that location. In the cases where presence or absence information was missing for the start or end years of our study interval (1990-2016) without evidence of any introduction or elimination events within the interval, we applied the status of the first and last presence/absence observations respectively to all years between the interval bound and the observation year. For cysticercosis, we performed targeted searches to classify

location-years in PubMed and Google Scholar.   Our map was populated by 21 peer-reviewed articles and meta-analyses and WHO reports.

Modelling strategy

Globally, deaths due to cysticercosis are relatively low. Therefore, a Poisson model was used to model cysticercosis deaths due to its suitability for count data. This model choice was validated by tests for overdispersion. Random effects were used on location with random slopes on age by location. A multivariate normal distribution using the mean and variance-covariance matrix from the model was used to generate 1,000 draws of deaths due to cysticercosis.

Estimates for new subnational locations were also added in GBD 2016. Since the Pew Research Center only has data on proportion of Muslims by country, we applied the national proportions to subnational locations. We understand that this does not account for sometimes large expected differences in proportions of Muslims within a country, but were limited by data availability.

Cystic Echinococcosis (CE)

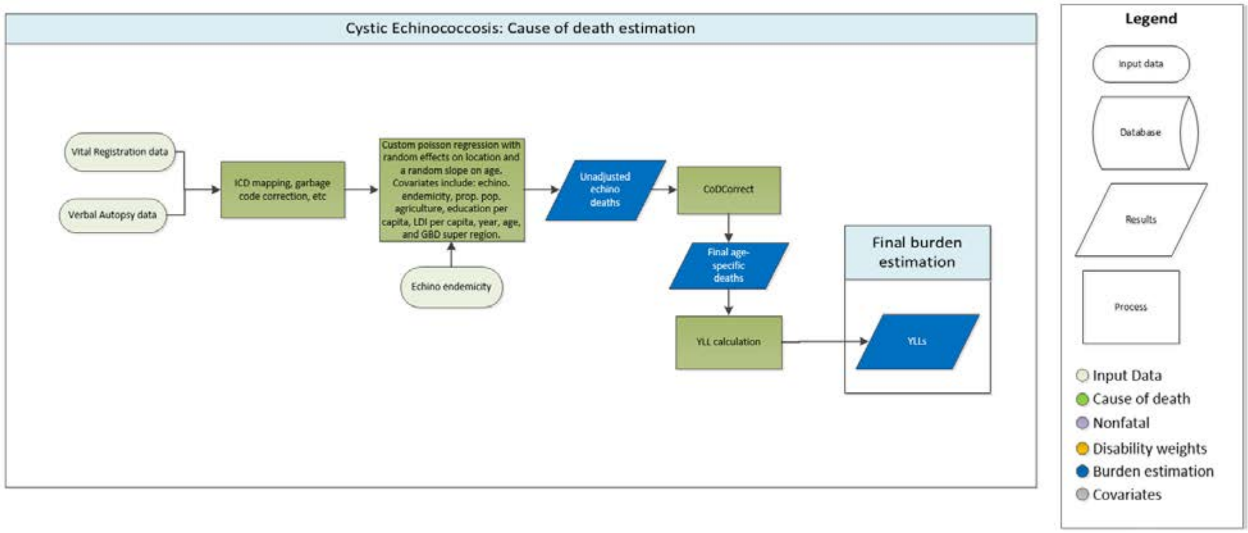

Input data

There are limited data sources on deaths due to cystic echinococcosis (CE). The model relied on vital registration and hospital surveillance data from endemic countries. We incorporated a categorical measure of echinococcosis endemicity provided by one of our echinococcosis collaborators with four levels: 0=no cases/no data; 1=sporadic/mostly imported; 2=endemic/limited data; and 3=highly endemic. Other covariates included proportion of the population involved in agricultural activities, years of education per capita, lag distributed income per capita, age, year and GBD super region.

Geographic Restrictions

We conducted a literature review to determine the geographic extent of the disease and classify locations based on whether the disease is absent or present in each year. Locations that were geographically restricted in any given year did not have estimates made for them but could have imported cases attributed to them at a later stage. Of note, we did not attempt a complete systematic review, since a single high-quality source could offer sufficient evidence of presence. Evidence of absence or presence was not available for every location for each year and so assumptions were made for missing years by taking into consideration the epidemiological characteristics of the disease. If evidence indicated disease presence for two non-consecutive years, we assumed presence for all years between the two. If evidence indicated disease absence for two non-consecutive years, we assumed absence for all years between the two. If evidence indicated a change in status (i.e. from absent to present, or present to absent) between two non-consecutive years than we conducted targeted searches to ascertain the relevant year of introduction or elimination for that location. In the cases where presence or absence information was missing for the start or end years of our study interval (1990-2016) without evidence of any introduction or elimination events within the interval, we applied the status of the first and last presence/absence observations respectively to all years between the interval bound and the observation year. For cystic echinococcosis, we performed targeted searches to classify location-years in PubMed and Google Scholar. Our map was populated by 23 peer-reviewed articles and meta-analyses.

Modelling strategy

The cause of death ensemble model (CODEm) was not employed for modelling deaths from CE due to the paucity of data on deaths from CE. We therefore used a Poisson model to model deaths with random effects by location and a random slope on age by location. A multivariate normal distribution using the mean and variance-covariance matrix from the model was used to generate 1,000 draws of deaths due to cystic echinococcosis.

Using a multivariate normal distribution with mean and variance-covariance matrix from the Poisson regression, we generated 1,000 draws of estimates for the countries endemic for CE. The final model was selected based on how well the estimated numbers fit the input data and how plausible the predicted distribution of disease was over time and with age.

Dengue

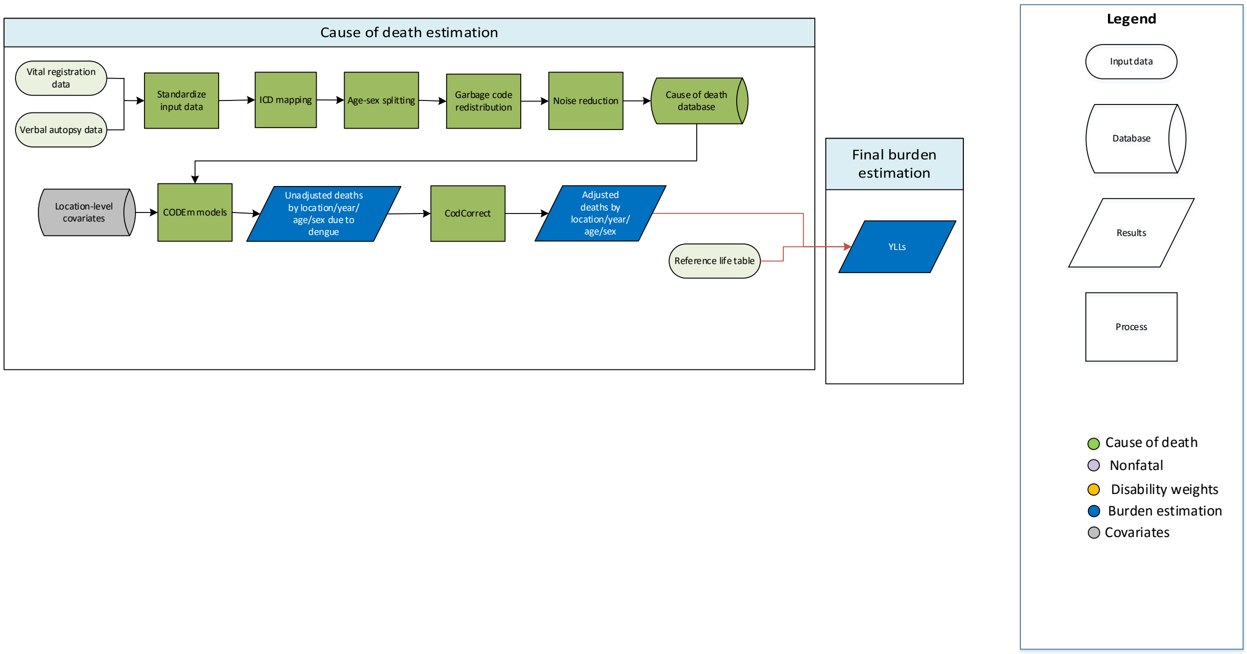

Input data

We modelled dengue mortality using all available data in the cause of death database. Data points were outliered if they reported an improbably low number of dengue deaths (eg, zero dengue deaths in a hyper-endemic country) or an improbably high number of dengue deaths.

Modelling strategy

We modelled dengue mortality using three-model hybrid approach: 1) a global CODEm model of all locations, using all data in the CoD database; 2) a CODEm model restricted to data-rich countries; and 3) estimates of mortality from imported cases in non-endemic, data-rich countries. Where dengue deaths were reported in non-endemic data-rich countries, we produced non-zero estimates by drawing from a beta distribution based on number of reported deaths and the underlying sample size. Estimates of dengue mortality in endemic data-rich countries were drawn from the data-rich CODEm model. Finally, estimates in other endemic countries were drawn from the global CODEm model.

While we've made no substantive changes to the modelling strategy in 2016, we have updated the geographic restrictions that determine whether a location is considered non-endemic (and, therefore, will have estimates based on the imported case model) in a given year. As for GBD 2015, we derived our geographic restrictions for 2010 from Brady et al(1). Whereas, in GBD 2015 we treated these as static restrictions, for GBD 2016 we conducted a literature review to determine locations and years in which dengue was introduced or eliminated, to allow for time-varying geographic restrictions. Of note, we did not attempt a complete systematic review, since a single high-quality source could offer sufficient evidence of presence. In total, we used 14 additional sources to supplement Brady et al's review.(2–15)

1. Brady OJ, Gething PW, Bhatt S, Messina JP, Brownstein JS, Hoen AG, et al. Refining the Global Spatial Limits of Dengue Virus Transmission by Evidence-Based Consensus. PLoS Negl Trop Dis. 2012 Aug 7;6(8):e1760.

2. Al ST et. Autochthonous Dengue Fever, Tokyo, Japan, 2014 - Volume 21, Number 3—March 2015 - Emerging Infectious Disease journal - CDC. [cited 2017 Apr 28]; Available from: https://wwwnc.cdc.gov/eid/article/21/3/14-1662_article

3. Guzman MG, Kouri G. Dengue and dengue hemorrhagic fever in the Americas: lessons and challenges. J Clin Virol. 2003 May;27(1):1–13.

4. Boshell J, Groot H, Gacharna M, Márquez G, González M, Gaitán MO, et al. Dengue en Colombia. Biomédica. 1986;6(3–4):101–6.

5. Effler PV, Pang L, Kitsutani P, Vorndam V, Nakata M, Ayers T, et al. Dengue Fever, Hawaii, 2001–2002. Emerg Infect Dis. 2005 May;11(5):742–9.

6. McBride WJH. Dengue fever: is it endemic in Australia? Intern Med J. 2010 Apr 1;40(4):247–9.

7. Kay BH, Barker-Hudson P, Stallman ND, Wiemers MA, Marks EN, Holt PJ, et al. Dengue fever. Reappearance in northern Queensland after 26 years. Med J Aust. 1984 Mar 3;140(5):264–8.

8. Al GA et. Dengue Reemergence in Argentina - Volume 5, Number 4—August 1999 - Emerging Infectious Disease journal - CDC. [cited 2017 Apr 28]; Available from: https://wwwnc.cdc.gov/eid/article/5/4/99-0424_article

9. Ramos MM, Mohammed H, Zielinski-Gutierrez E, Hayden MH, Lopez JLR, Fournier M, et al. Epidemic Dengue and Dengue Hemorrhagic Fever at the Texas–Mexico Border: Results of a Household-based Seroepidemiologic Survey, December 2005. Am J Trop Med Hyg. 2008 Mar 1;78(3):364–9.

10. Luo L, Liang H, Hu Y, Liu W, Wang Y, Jing Q, et al. Epidemiological, virological, and entomological characteristics of dengue from 1978 to 2009 in Guangzhou, China. J Vector Ecol. 2012 Jun 1;37(1):230–40.

11. Murray KO, Rodriguez LF, Herrington E, Kharat V, Vasilakis N, Walker C, et al. Identification of Dengue Fever Cases in Houston, Texas, with Evidence of Autochthonous Transmission Between 2003 and 2005. Vector Borne Zoonotic Dis. 2013 Dec 1;13(12):835–45.

12. Locally Acquired Dengue --- Key West, Florida, 2009--2010 [Internet]. [cited 2017 Apr 28]. Available from: https://www.cdc.gov/mmwr/preview/mmwrhtml/mm5919a1.htm

13. Bouri N, Sell TK, Franco C, Adalja AA, Henderson DA, Hynes NA. Return of Epidemic Dengue in the United States: Implications for the Public Health Practitioner. Public Health Rep. 2012;127(3):259–66.

14. Wilder-Smith A, Quam M, Sessions O, Rocklov J, Liu-Helmersson J, Franco L, et al. The 2012 dengue outbreak in Madeira: exploring the origins. 2014 [cited 2017 Apr 28]; Available from: https://dr.ntu.edu.sg/handle/10220/19685

15. Brathwaite Dick O, San Martín JL, Montoya RH, del Diego J, Zambrano B, Dayan GH. The History of Dengue Outbreaks in the Americas. Am J Trop Med Hyg. 2012 Oct 3;87(4):584–93.

Yellow fever

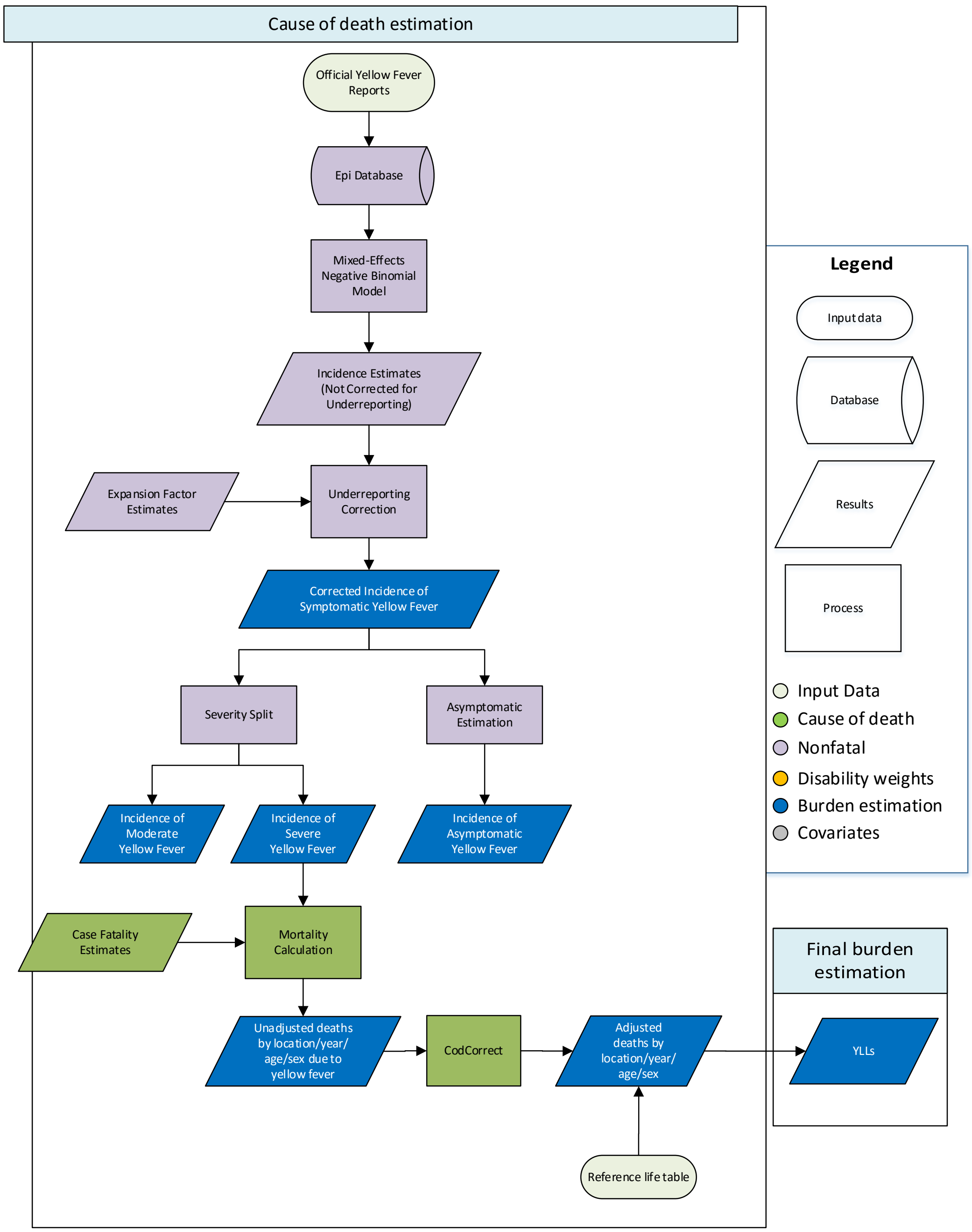

Input data

Case data come from official case reports filed with the World Health Organization. Data on case fatality come from published studies of yellow fever fatality. Data on deaths in non-endemic countries are restricted to only vital registration data.

Modelling strategy

We model yellow fever deaths using a hybrid approach. For countries in which yellow fever is endemic, we use a natural history approach in which we estimate deaths as the product of cases and case fatality. For non-endemic countries, we allow for deaths among imported cases where we have vital registration data indicating yellow fever deaths. That is, we assume no yellow fever deaths in non-endemic countries; however, where yellow fever deaths are reported in vital registration data, we accept those as true imported yellow fever deaths.

We model reported cases using a mixed-effects negative binomial model, with fixed effects for year and random effects for super-region, region, and country. We assume that yellow fever cases are underreported and that this underreporting mirrors that of dengue (a disease for which we have better data on underreporting). With that, we estimate symptomatic cases as the product of our base case estimates and dengue expansion factors (ie, the factor by which you must multiply reported cases to derive true cases). Based on published estimates, we assume that 27% of symptomatic cases will be severe.[1]

We performed a meta-analyses of case fatality using data from published studies of yellow fever fatality. Studies tend to report deaths among those with severe infection (eg, hospitalized cases), rather than among all cases. We assume that no deaths occur with asymptomatic infection or among those with only moderate symptoms. With that, we estimate deaths as the product of severe cases and case fatality.

We have improved our method for correcting for underreporting of yellow fever. In estimating yellow fever deaths for GBD 2013, our model assumed that all severe cases were reported and that reported cases reflected only severe cases. With that, we adjusted our base estimates upward to account for non-severe cases, and based our mortality estimates off these adjusted numbers. Based on feedback from collaborators, we believe that this adjustment was inadequate to fully account for underreporting. Accordingly, we have adopted the expansion factor-based method described above for GBD 2015.

Moreover, we have adopted the hybrid approach for GBD 2015. Whereas we previously allowed no yellow fever deaths in non-endemic countries, we now accept deaths reported in vital registration data as true imported deaths.

We have made no substantive changes to the modelling strategy for GBD 2016.

Reference

1 Johansson MA, Vasconcelos PFC, Staples JE. The whole iceberg: estimating the incidence of yellow fever virus infection from the number of severe cases. *Trans R Soc Trop Med Hyg* 2014; 108: 482–7.

Rabies

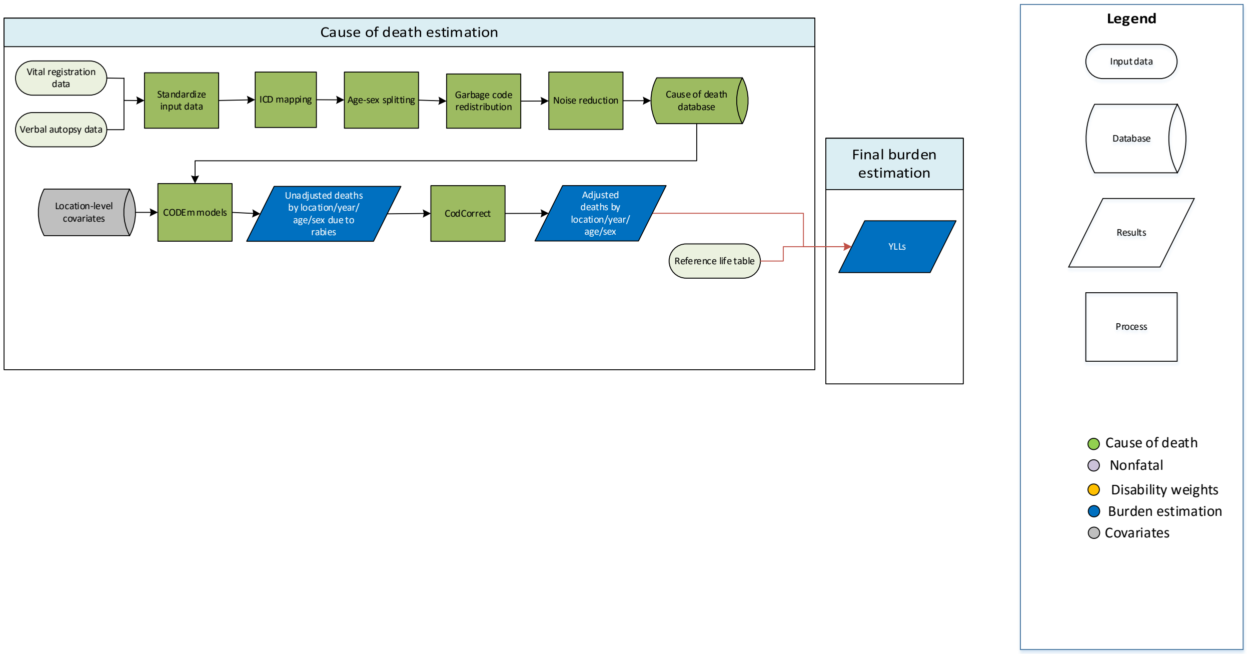

Input data

We modeled rabies mortality using all available data in the cause of death database. Data points were outliered if they reported an improbable number of rabies deaths (e.g., zero rabies deaths in a hyper-endemic country) or if their inclusion in the model yielded distorted trends. In some cases multiple data sources for the same location differed dramatically both in their quality and reported rabies mortality (e.g., a verbal autopsy and vital registration source). In these cases the lower-quality data source was outliered.

Modeling strategy

We modeled rabies mortality using a two-model hybrid approach: 1) a global CODEm model of all locations, using all data in the CoD database; and 2) a CODEm model restricted to data-rich countries.

We have made no substantive changes to the modeling strategy in 2016.

Ascariasis

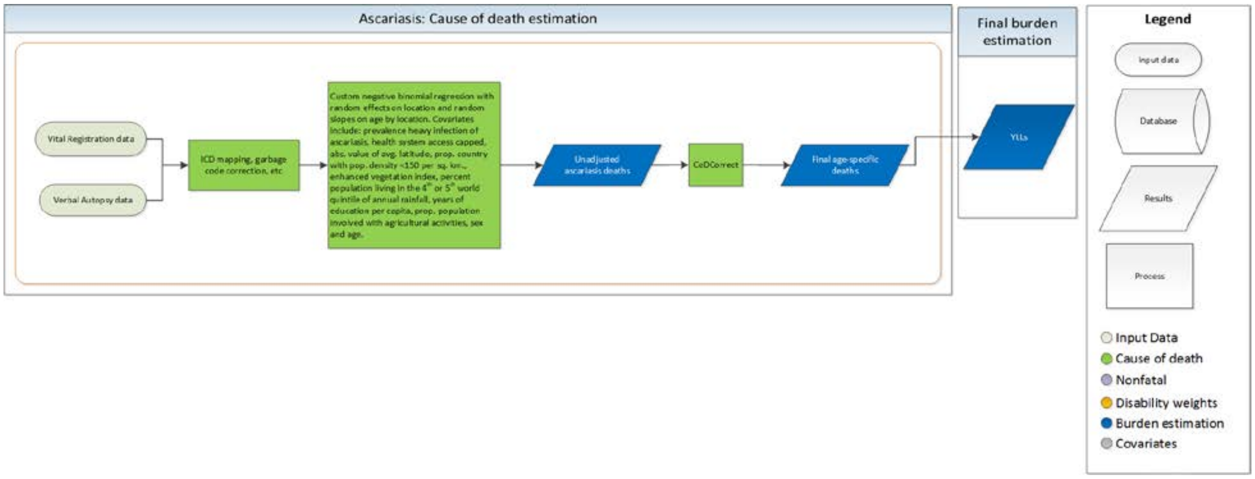

Input data

To estimate mortality due to ascariasis, country-year-age-sex-specific verbal autopsy and vital registration data were used. Covariates used include prevalence of heavy infection of ascariasis, health system access capped by the minimum OECD value, the absolute value of average latitude, the proportion of the country with population density under 150 people per square kilometer, enhanced vegetation index, percent of the population living in the 4th or 5th world quintile of annual rainfall, number of years of education per capita, proportion of the population involved with agricultural activities, sex and age.

Geographic Restrictions

We conducted a literature review to determine the geographic extent of the disease and classify locations based on whether the disease is absent or present in each year. Locations that were geographically restricted in any given year did not have estimates made for them but could have imported cases attributed to them at a later stage. Of note, we did not attempt a complete systematic review, since a single high-quality source could offer sufficient evidence of presence. Evidence of absence or presence was not available for every location for each year and so assumptions were made for missing years by taking into consideration the epidemiological characteristics of the disease. If evidence indicated disease presence for two non-consecutive years, we assumed presence for all years between the two. If evidence indicated disease absence for two non-consecutive years, we assumed absence for all years between the two. If evidence indicated a change in status (i.e. from absent to present, or present to absent) between two non-consecutive years than we conducted targeted searches to ascertain the relevant year of introduction or elimination for that location. In the cases where presence or absence information was missing for the start or end years of our study interval (1990-2016) without evidence of any introduction or elimination events within the interval, we applied the status of the first and last presence/absence observations respectively to all years between the interval bound and the observation year. Our search was done in conjunction with the title/abstract screening portion of a systematic literature review for prevalence data. The search strings and yield can be viewed in the table below for each of the databases queried.

Database	Search String	Yield

PubMed	(Ascariasis[Title/Abstract] OR Ascaris[Title/Abstract] OR "A. lumbricoides"[Title/Abstract] OR Ascaris[MeSH] OR Trichuris[Title/Abstract] OR Trichuriasis[Title/Abstract] OR "Whip Worm"[Title/Abstract] OR "T. trichura"[Title/Abstract] OR Trichuris[MeSH] OR Hookworm[Title/Abstract] OR "A. duodenale"[Title/Abstract] OR "Ancylostoma duodenale"[Title/Abstract] OR ancylostomiasis[Title/Abstract] OR "N. americanus"[Title/Abstract] OR "Necator americanus"[Title/Abstract] OR necatoriasis[Title/Abstract] OR Ancylostoma [MeSH] OR Necator[MeSH]) AND (prevalence[Title/Abstract] OR incidence[Title/Abstract] OR epidemiology[Title/Abstract] OR surveillance[Title/Abstract]) NOT(Animals[MeSH] NOT Humans[MeSH])	2376
Web of Science	(Ascariasis OR Ascaris OR A. lumbricoides OR Trichuris OR Trichuriasis OR Whip Worm OR T. trichura OR Hookworm OR A. duodenale OR Ancylostoma duodenale OR anclyostomiasis OR N. americanus OR Necator americanus OR necatoriasis) AND TOPIC:(prevalence OR incidence OR epidemiology OR surveillance) NOTTOPIC: ((Animals NOT Humans))<br>Timespan: 1980-2016. Indexes: SCI-EXPANDED, SSCI, A&HCI, ESCI.	2266
SCOPUS	TITLE-ABS_KEY (ascariasis OR ascaris OR a. lumbricoides OR trichuris OR trichuriasis OR whip worm OR t. trichura OR hookworm OR a. duodenale OR ancylostoma duodenale OR anclyostomiasis OR n. americanus OR necator americanus OR necatoriasis) AND PUBYEAR>1979	29

These papers were used to classify location-years for all locations and years present in the literature. Additionally, systematic literature reviews, meta-analyses, national health statistics publications and collaborator input were used to classify location-years not present in the literature review wherever possible.

Modelling strategy

A Negative Binomial model was used to estimated deaths from ascariasis with random intercepts for locations and random slopes for age groups by location. A multivariate normal distribution using the mean and variance-covariance matrix from the model was used to generate 1,000 draws of deaths due to ascariasis. The final model was selected based on how well the estimated number fit the input data and how plausible the predicted distribution of disease was over time and with age.

Ebola

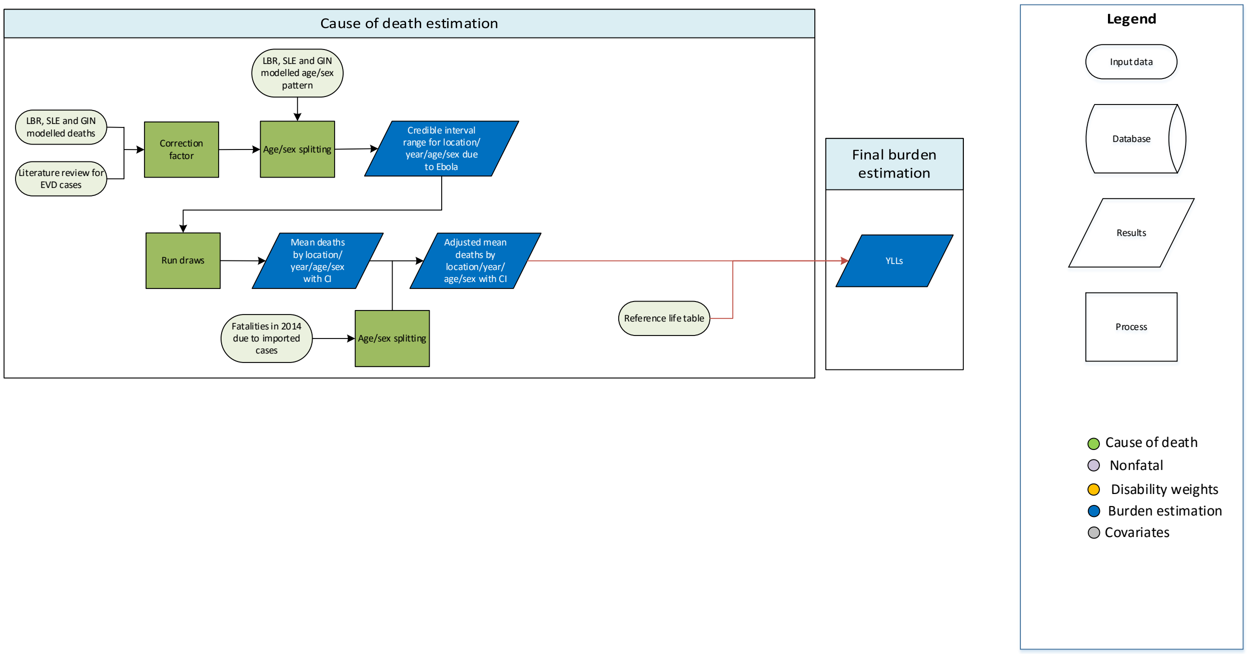

Input data

The input data for deaths due to Ebola virus disease (EVD) came in two forms: (i) modelled estimates for the West African outbreak from 2013 to 2016 provided by the World Health Organization (WHO) focused specifically on the three worst-affected countries (Liberia, Guinea, and Sierra Leone) and (ii) literature searches for reported deaths due to EVD not captured by the West African dataset. This is further explained below:

i. WHO estimates for Liberia, Guinea, and Sierra Leone, 2014–2016
    1. Researchers from Imperial College London (UK), as part of the WHO Ebola response team, provided modelled estimates for the number of fatalities that result from a given number of reported cases (provided by line lists from the WHO). This method was used in a variety of papers to generate baseline estimates of case fatality rates and other key epidemiological measures while correcting for the lag period between initially reporting a case and the final outcome of that case (whether it be death or survival). The full data cleaning and methodology are reported elsewhere.[1,2] Bespoke estimates were provided for GBD for Liberia, Sierra Leone, and Guinea and were stratified by age, sex, and year. Death data from Guinea ranged from February 18, 2014, until September 27, 2015, with data from Liberia ranging from March 20, 2014, to May 4, 2015, and data from Sierra Leone ranging from May 21 until September 28, 2015.

2. Reported clusters of cases in 2016 were identified by consulting WHO situation reports from the year 2016.

ii. Literature searches for reported deaths due to EVD outside of Liberia, Guinea, and Sierra Leone

1. In order to capture the small number of fatalities that occurred in countries outside of the core three mentioned above, WHO Situation Reports were consulted. Fatalities were reported in the US (specifically Texas), Mali, and Nigeria.[3] All deaths occurred in 2014. Additional age and sex information could only be obtained for the death that occurred in the US.

Using a previous review of historical outbreaks,[4,5] original articles describing the progression of historical outbreaks were consulted. This initial review was also updated to include the 2014 outbreak that occurred in the Democratic Republic of the Congo in 2014.[6] This resulted in datasets describing each outbreak with variable degrees of detail – some fully describing the age and sex breakdown of all deaths [eg, Rosello and colleagues[7]] and others simply providing the final total. Only confirmed or probable deaths were included; suspected EVD deaths were omitted. Outbreaks that spanned multiple years, in the absence of sufficient data providing an accurate breakdown, were apportioned between the years by evenly assigning a uniform number of deaths to each month of the outbreak's duration.

Modelling strategy

Data on deaths resulting from imported cases from 2014 were used as specific count data as it was assumed to be an accurate representation of the cases and outbreaks in these countries, all of which were on high alert for importation of cases.[8,9]

The other input data were processed prior to inclusion in GBD to account for any potential underreporting of deaths. A meta-analysis of existing underreporting studies from the literature was performed, using a random effects model with a DerSimonian-Laird estimator. A variety of sources were included, capturing a number of different estimation processes, all identified by literature review. The figure below shows the different effect sizes of the different studies,[10–15] as well as the resulting GBD 2016 correction factor, with the GBD 2015 correction factor for reference. The correction factor ranged from 1.4580 to 2.5475, with a mean of 2.0027.

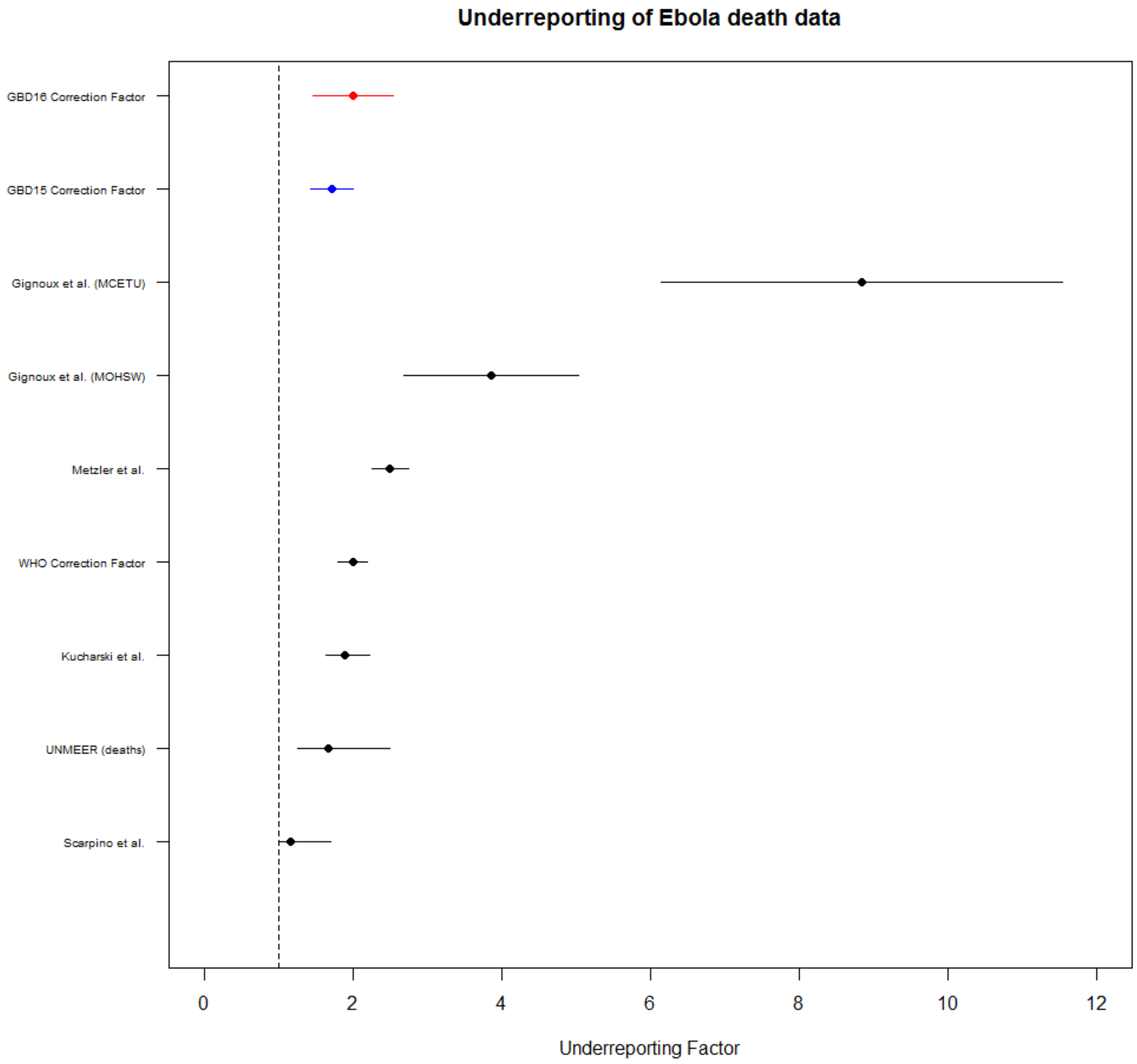

In order to capture this potential variation, all input data were multiplied by the lower and upper limit of this estimated correction factor; these numbers then provided the lower and upper bounds from which draw values were taken. For outbreaks where no data were supplied for age and/or sex, the pattern observed in the West African outbreak (for which there were the most comprehensive data) was used to apportion these total values.

One thousand draws were taken from a normal distribution fitted between these lower and upper bound values, which generated mean estimates stratified by age, sex, location, and year along with credible intervals for these numbers. These estimates were then adjusted by including the count data for imported cases from 2014.

Data on Ebola outbreaks prior to 2014 are sparse, and as a result many values derived from the West African outbreak were assumed to be valid for historical outbreaks as well. This may mask significant differences that exist between these outbreaks, some of which were caused by different species of

Ebolavirus. In order to minimize this problem we chose to implement a data-driven approach – for those outbreaks where sufficiently detailed historical data could be obtained, these were used in preference to any assumed age/sex breakdown.

References

1 Agua-Agum J, Ariyarajah A, Aylward B, *et al.* West African Ebola Epidemic after One Year — Slowing but Not Yet under Control. *N Engl J Med* 2015; **372**: 584–7.

2 Ebola Virus Disease in West Africa - The First 9 Months of the Epidemic and Forward Projections. *N Engl J Med* 2014; **371**: 1481–95.

3 World Health Organization. Ebola Situation Reports. 2016. Interview (accessed March 14, 2016).

4 Pigott DM, Golding N, Mylne A, *et al.* Mapping the zoonotic niche of Ebola virus disease in Africa. *Elife* 2014; **3**: e04395.

5 Mylne A, Brady OJ, Huang Z, *et al.* A comprehensive database of the geographic spread of past human Ebola outbreaks. *Sci Data* 2014; **1**: 140042.

6 Maganga GD, Kapetshi J, Berthet N, *et al.* Ebola virus disease in the Democratic Republic of Congo. *N Engl J Med* 2014; **371**: 2083–91.

7 Rosello A, Mossoko M, Flasche S, *et al.* Ebola virus disease in the Democratic Republic of the Congo, 1976-2014. *Elife* 2015; **4**. DOI:10.7554/eLife.09015.

8 Fasina FO, Shittu A, Lazarus D, *et al.* Transmission dynamics and control of Ebola virus disease outbreak in Nigeria, July to September 2014. *Euro Surveill* 2014; **19**: 20920.

9 Althaus CL, Low N, Musa EO, Shuaib F, Gsteiger S. Ebola virus disease outbreak in Nigeria: Transmission dynamics and rapid control. *Epidemics* 2015; **11**: 80–4.

10 Gignoux E, Idowu R, Bawo L, *et al.* Use of Capture-Recapture to Estimate Underreporting of Ebola Virus Disease, Montserrado County, Liberia. *Emerg Infect Dis* 2015; **21**: 2265–7.

11 Meltzer MI, Atkins CY, Santibanez S, *et al.* Estimating the future number of cases in the Ebola epidemic--Liberia and Sierra Leone, 2014-2015. *MMWR Suppl* 2014; **63**: 1–14.

12 Scarpino S V, Iamarino A, Wells C, *et al.* Epidemiological and viral genomic sequence analysis of the 2014 ebola outbreak reveals clustered transmission. *Clin Infect Dis* 2015; **60**: 1079–82.

13 Kucharski AJ, Camacho A, Flasche S, Glover RE, Edmunds WJ, Funk S. Measuring the impact of Ebola control measures in Sierra Leone. *Proc Natl Acad Sci U S A* 2015; **112**: 14366–71.

14 UNMEER. Sierra Leone: Ebola emergency Weekly Situation Report No. 7. 2014 https://www.humanitarianresponse.info/system/files/documents/files/UNMEER_NERC_SitRep_07Dec.pdf.

15 Enserink M. How many Ebola cases are there really? | Science | AAAS. 2014. http://www.sciencemag.org/news/2014/10/how-many-ebola-cases-are-there-really (accessed Jan 28, 2017).

Zika

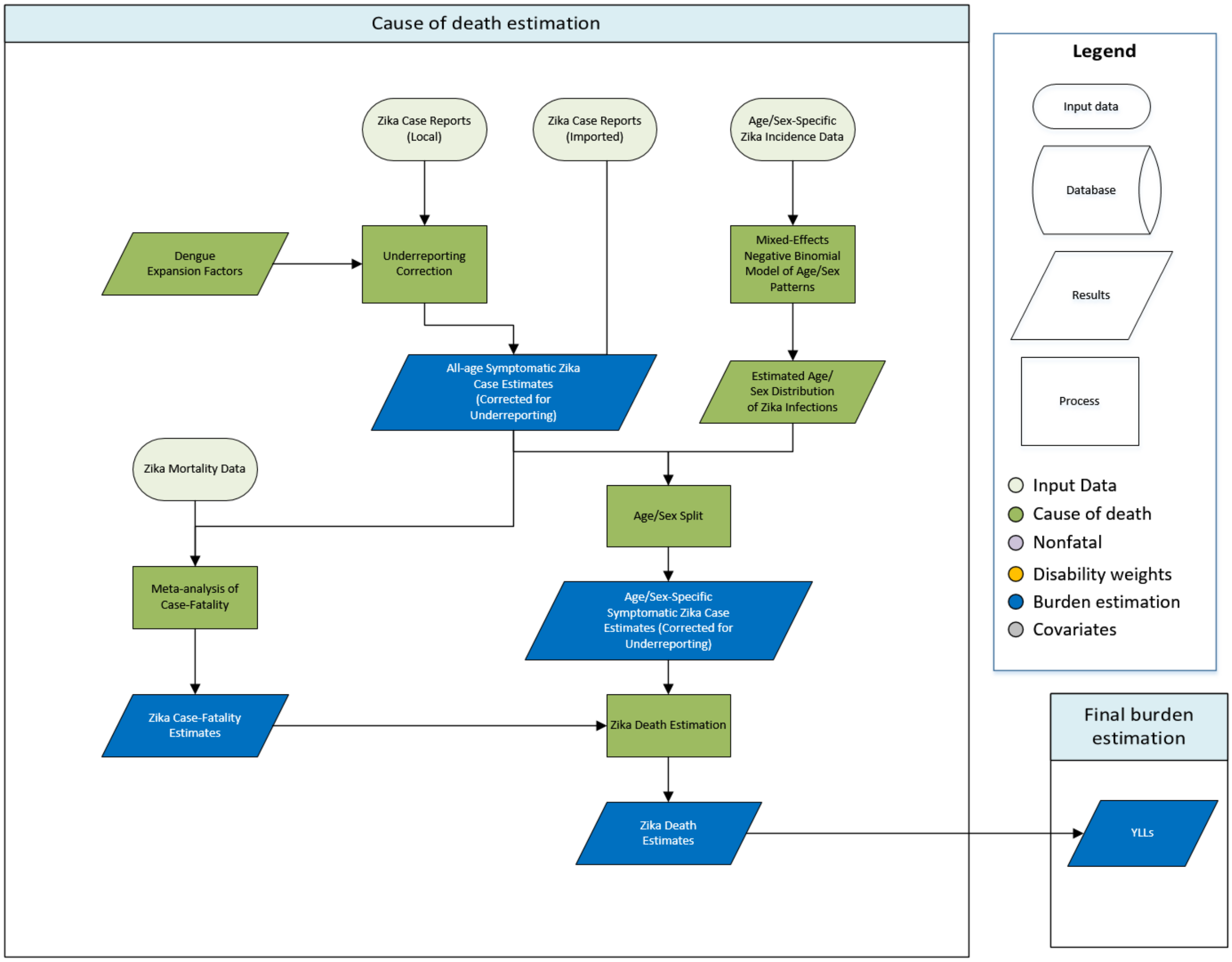

Input data

Case data and death data come from official reports, primarily from PAHO.

Modeling strategy

We model Zika deaths using a natural history approach in which we estimate deaths as the product of cases and case fatality. We estimate the number of true symptomatic cases as the product of reported cases and country-specific expansion factors that adjust for underreporting. Those expansion factors are derived from our dengue model and the methods used for their estimation are detailed in the dengue model documentation and by Stanaway et al[1].

We then use an intercept only, mixed-effects Poisson regression model, with random effects on location, to estimate case fatality. Here, our outcome variable is reported Zika deaths and our exposure variable is estimated number of symptomatic Zika cases. For location-years with reported Zika deaths, we estimate deaths from the fixed effects (i.e. intercept and offset) and random effects, including

uncertainty from both effects. For location-years with no Zika death reports, but with reported Zika cases, we estimate deaths from the fixed effects and sample from the distribution of all random effects.

Reference

1 Stanaway JD, Shepard DS, Undurraga EA, Halasa YA, Coffeng LE, Brady OJ, et al. The global burden of dengue: an analysis from the Global Burden of Disease Study 2013. The Lancet Infectious Diseases [Internet]. 2016 Feb

Other neglected tropical diseases (NTDs)

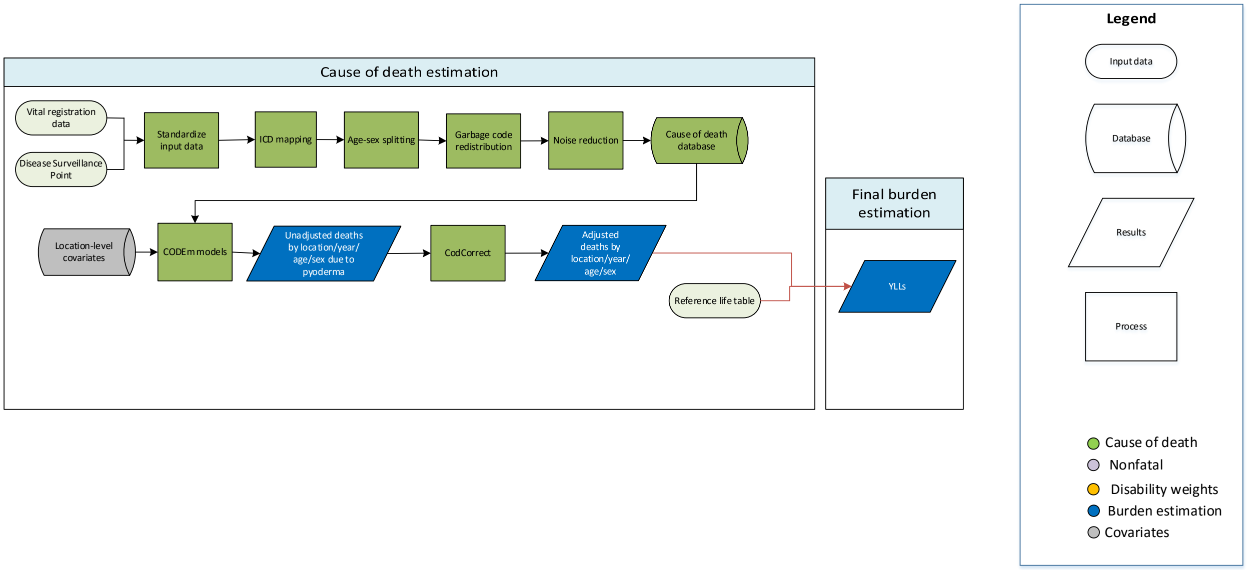

Input data

We modelled other neglected tropical disease mortality using all available data in the cause of death database. Data points were outliered if they reported an improbable number of deaths or if their inclusion in the model yielded distorted trends.

Modelling strategy

We modelled other neglected tropical disease mortality using a two-model hybrid approach: 1) a global CODEm model of all locations, using all data in the CoD database; and 2) a CODEm model restricted to data-rich countries.

We have made no substantive changes in the modelling strategy for other neglected tropical disease from GBD 2015.

Maternal disorders

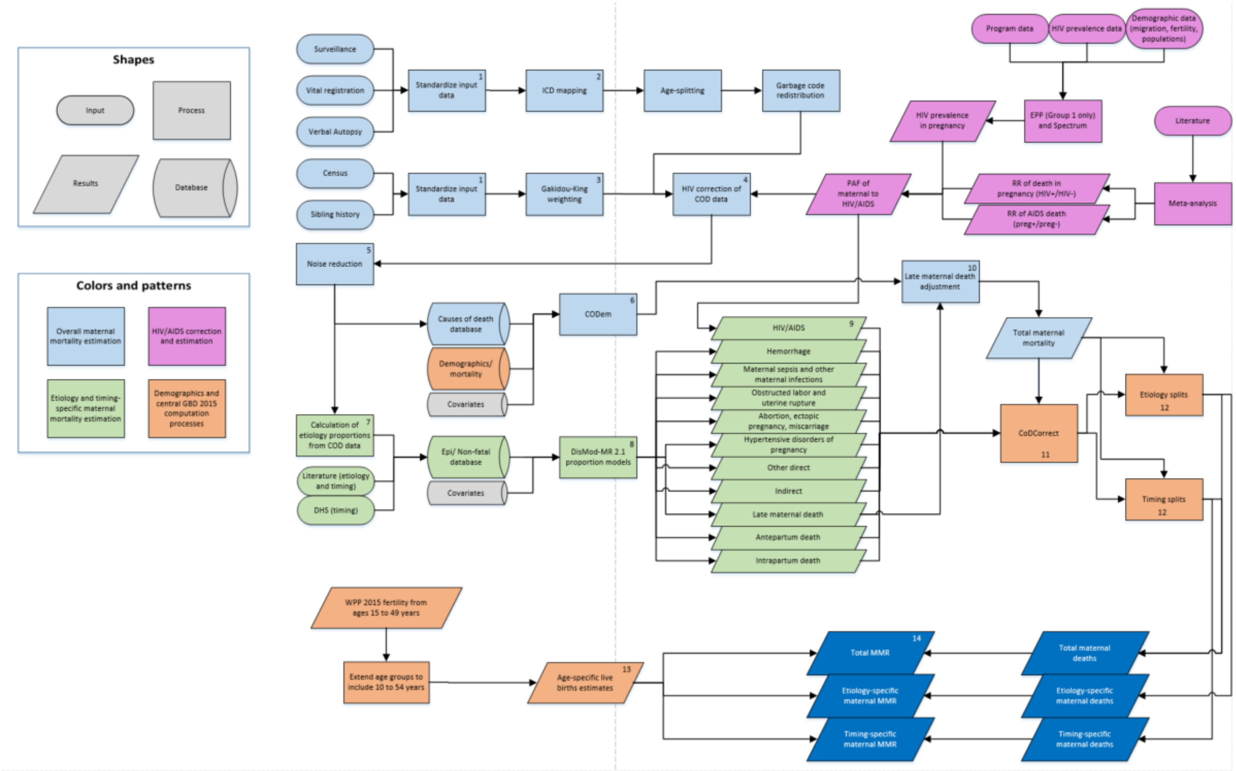

Input data

CODEm models were informed by centrally prepped data stored in the cause of death (COD) database using standardized processes to adjust for bias due to incompleteness, misclassification, coding to non-specific causes (i.e. garbage codes), stochastic variability, and zero counts. Our GBD 2016 case definition for maternal mortality continues to be all pregnancy-related deaths excluding accidental or incidental causes up to 1 year after the end of the pregnancy.

An updated literature review to inform the relative risk of mortality in pregnancy in HIV-positive versus HIV-negative women produced 23 leads and one usable source. We completed this search on August 30, 2016, using the following search string:

(HIV[Title/Abstract] OR "Acquired Immunodeficiency Syndrome"[Title/Abstract] OR AIDS[Title/Abstract]) AND (“pregnant”[Title/Abstract] OR “pregnancy”[Title/Abstract] OR “postpartum”[Title/Abstract] OR "post partum"[Title/Abstract]) AND (“mortality”[Title/Abstract] OR “death”[Title/Abstract]) NOT "case report" AND "humans"[MeSH Terms] AND (2011/07/06[PDat] : 2016/12/31[PDat])

Correction for incidental HIV deaths was completed during the data preparation phase. Spectrum outputs of HIV prevalence in pregnancy were combined with relative risk of mortality during pregnancy (HIV+ versus HIV-negative) to calculate PAFs. A proportion of these deaths are incidental and a proportion are maternal as determined from two studies that looked at the relative risk of death in HIV positive women who are pregnant versus non-pregnant. All data was corrected using the PAFs. Incidental deaths were removed from sibling history and census data, while maternal HIV deaths were

added to VR data. The maternal proportion of the PAF was retained to be combined with estimates of the aetiologic-proportion from other causes as described below.

DisMod-MR 2.1 aetiology proportion models were informed by two sources of data. First, we completed a systematic literature review on August 30, 2016, using the search string below:

(((("maternal mortality"[Title/Abstract] OR "maternal death"[Title/Abstract] OR "MM"[Title/Abstract] OR "confidential enquiry"[Title/Abstract] OR ((obstetric[Title/Abstract] OR pregnancy[Title/Abstract]) AND (etiology[Title/Abstract] OR cause[Title/Abstract] or pattern[Title/Abstract]) AND (death[Title/Abstract] OR mortality[Title/Abstract])))) AND "humans"[MeSH Terms] NOT (fetal[Title/Abstract] OR newborns[Title/Abstract] OR newborn[Title/Abstract] OR neonatal[Title/Abstract] OR “case report”[Title/Abstract] OR “case study”[Title/Abstract] OR pathogenesis[Title/Abstract] OR thromboprophylaxis[Title/Abstract])) OR ((“maternal mortality”[Title/Abstract] OR “maternal death*”[Title/Abstract] OR “MMR”[Title/Abstract]) AND (“Afghanistan”[Title/Abstract] OR “Albania”[Title/Abstract] OR “Algeria”[Title/Abstract] OR “Andorra”[Title/Abstract] OR “Angola”[Title/Abstract] OR “Antigua and Barbuda”[Title/Abstract] OR “Argentina”[Title/Abstract] OR “Armenia”[Title/Abstract] OR “Azerbaijan”[Title/Abstract] OR “Bahrain”[Title/Abstract] OR “Bangladesh”[Title/Abstract] OR “Barbados”[Title/Abstract] OR “Belarus”[Title/Abstract] OR “Belize”[Title/Abstract] OR “Benin”[Title/Abstract] OR “Bhutan”[Title/Abstract] OR “Bolivia”[Title/Abstract] OR “Bosnia and Herzegovina”[Title/Abstract] OR “Botswana”[Title/Abstract] OR “Brazil”[Title/Abstract] OR “Brunei”[Title/Abstract] OR “Bulgaria”[Title/Abstract] OR “Burkina Faso”[Title/Abstract] OR “Burundi”[Title/Abstract] OR “Cambodia”[Title/Abstract] OR “Cameroon”[Title/Abstract] OR “Cape Verde”[Title/Abstract] OR “Central African Republic”[Title/Abstract] OR “Chad”[Title/Abstract] OR “China”[Title/Abstract] OR “Colombia”[Title/Abstract] OR “Comoros”[Title/Abstract] OR “Congo”[Title/Abstract] OR “Costa Rica”[Title/Abstract] OR “Croatia”[Title/Abstract] OR “Cuba”[Title/Abstract] OR “Cyprus”[Title/Abstract] OR “Côte d’Ivoire”[Title/Abstract] OR “Democratic Republic of the Congo”[Title/Abstract] OR “Djibouti”[Title/Abstract] OR “Dominica”[Title/Abstract] OR “Dominican Republic”[Title/Abstract] OR “Ecuador”[Title/Abstract] OR “Egypt”[Title/Abstract] OR “El Salvador”[Title/Abstract] OR “Equatorial Guinea”[Title/Abstract] OR “Eritrea”[Title/Abstract] OR “Ethiopia”[Title/Abstract] OR “Federated States of Micronesia”[Title/Abstract] OR “Fiji”[Title/Abstract] OR “Gabon”[Title/Abstract] OR “Georgia”[Title/Abstract] OR “Ghana”[Title/Abstract] OR “Grenada”[Title/Abstract] OR “Guatemala”[Title/Abstract] OR “Guinea”[Title/Abstract] OR “Guinea-Bissau”[Title/Abstract] OR “Guyana”[Title/Abstract] OR “Haiti”[Title/Abstract] OR “Honduras”[Title/Abstract] OR “India”[Title/Abstract] OR “Indonesia”[Title/Abstract] OR “Iran”[Title/Abstract] OR “Iraq”[Title/Abstract] OR “Jamaica”[Title/Abstract] OR “Jordan”[Title/Abstract] OR “Kazakhstan”[Title/Abstract] OR “Kenya”[Title/Abstract] OR “Kiribati”[Title/Abstract] OR “Kuwait”[Title/Abstract] OR “Kyrgyzstan”[Title/Abstract] OR “Laos”[Title/Abstract] OR “Latvia”[Title/Abstract] OR “Lebanon”[Title/Abstract] OR “Lesotho”[Title/Abstract] OR “Liberia”[Title/Abstract] OR “Libya”[Title/Abstract] OR “Lithuania”[Title/Abstract] OR “Macedonia”[Title/Abstract] OR “Madagascar”[Title/Abstract] OR “Malawi”[Title/Abstract] OR “Malaysia”[Title/Abstract] OR “Maldives”[Title/Abstract] OR “Mali”[Title/Abstract] OR “Malta”[Title/Abstract] OR “Marshall Islands”[Title/Abstract] OR “Mauritania”[Title/Abstract] OR “Mauritius”[Title/Abstract] OR “Moldova”[Title/Abstract] OR “Mongolia”[Title/Abstract] OR “Montenegro”[Title/Abstract] OR “Morocco”[Title/Abstract] OR “Mozambique”[Title/Abstract] OR “Myanmar”[Title/Abstract] OR “Namibia”[Title/Abstract] OR “Nepal”[Title/Abstract] OR “Nicaragua”[Title/Abstract] OR “Niger”[Title/Abstract] OR “Nigeria”[Title/Abstract] OR “North Korea”[Title/Abstract] OR “Oman”[Title/Abstract] OR “Pakistan”[Title/Abstract] OR “Palestine”[Title/Abstract] OR “Panama”[Title/Abstract] OR “Papua New Guinea”[Title/Abstract] OR “Paraguay”[Title/Abstract] OR “Peru”[Title/Abstract] OR “Philippines”[Title/Abstract] OR “Qatar”[Title/Abstract] OR “Romania”[Title/Abstract] OR “Russia”[Title/Abstract] OR “Rwanda”[Title/Abstract] OR “Saint Lucia”[Title/Abstract] OR “Saint Vincent and the Grenadines”[Title/Abstract] OR “Samoa”[Title/Abstract] OR “Saudi Arabia”[Title/Abstract] OR “Senegal”[Title/Abstract] OR “Serbia”[Title/Abstract] OR “Seychelles”[Title/Abstract] OR “Sierra Leone”[Title/Abstract] OR “Singapore”[Title/Abstract] OR “Solomon Islands”[Title/Abstract] OR “Somalia”[Title/Abstract] OR “South Africa”[Title/Abstract] OR "South Sudan"[Title/Abstract] OR “Sri Lanka”[Title/Abstract] OR “Sudan”[Title/Abstract] OR “Suriname”[Title/Abstract] OR “Swaziland”[Title/Abstract] OR “Syria”[Title/Abstract] OR “São Tomé and Príncipe”[Title/Abstract] OR “Taiwan”[Title/Abstract] OR “Tajikistan”[Title/Abstract] OR “Tanzania”[Title/Abstract] OR “Thailand”[Title/Abstract] OR “The Bahamas”[Title/Abstract] OR “The Gambia”[Title/Abstract] OR “Timor-Leste”[Title/Abstract] OR “Togo”[Title/Abstract] OR “Tonga”[Title/Abstract] OR “Trinidad and Tobago”[Title/Abstract] OR “Tunisia”[Title/Abstract] OR “Turkmenistan”[Title/Abstract] OR “Uganda”[Title/Abstract] OR “Ukraine”[Title/Abstract] OR “United Arab Emirates”[Title/Abstract] OR “Uruguay”[Title/Abstract] OR “Uzbekistan”[Title/Abstract] OR “Vanuatu”[Title/Abstract] OR “Venezuela”[Title/Abstract] OR “Vietnam”[Title/Abstract] OR “Yemen”[Title/Abstract] OR “Zambia”[Title/Abstract] OR “Zimbabwe”[Title/Abstract]) AND "humans"[MeSH] NOT (“demographic and health survey*”[Title/Abstract] OR DHS[Title/Abstract] OR “reproductive health survey*”[Title/Abstract] OR

RHS[Title/Abstract]))) AND (2015/04/30[PDat] : 2016/12/31[PDat])) OR ((HIV[Title/Abstract] OR "Acquired Immunodeficiency Syndrome"[Title/Abstract] OR AIDS[Title/Abstract]) AND (“pregnant”[Title/Abstract] OR “pregnancy”[Title/Abstract] OR “postpartum”[Title/Abstract] OR "post partum"[Title/Abstract]) AND (“mortality”[Title/Abstract] OR “death”[Title/Abstract]) NOT "case report" AND "humans"[MeSH Terms] AND (2011/07/06[PDat] : 2016/12/31[PDat]))

A total of 698 sources were reviewed for their title and abstract. Of those selected for full text review, 17 had usable data for aetiology-specific maternal mortality models. All data were prepped as “proportion” of total maternal deaths due to that cause. The second source of data was from the COD database. All aetiology-specific COD data were processed to be “proportion” data by calculating the cause-specific deaths divided by the total maternal deaths for the matching data source, year, age, and location. Owing to the large volume of total COD data and small sample sizes in many locations, COD data were collapsed around each of the five-year periods for which DisMod-MR 2.1 makes distinct estimates (1990, 1995, 2000, 2005, 2010, and 2016). Late maternal death data were only included for the subset of locations where they were reliably coded in raw VR. All data were uploaded to the nonfatal database.

Modelling strategy

Overall maternal mortality was estimated with CODEm. All data from all geographies were reviewed. Outliers were identified as those data where age patterns or temporal patterns were inconsistent with neighbouring age groups or locations or where sparse data were predicting implausible overall temporal or age patterns for a given location.

DisMod-MR 2.1 proportion models for each sub-cause of maternal mortality were all single-parameter meta-regression models. Because many sources do not include the entire cause list, a series of study covariates were used to facilitate crosswalking back to the reference definition. The reference definition ***includes*** “other” direct obstetric complications, indirect maternal deaths, and late maternal death. Country covariates were specific for each model and included abortion legality (for abortion, ectopic pregnancy, and miscarriage), log-transformed lag-distributed income (for sepsis and late maternal death), and logit-transformed in-facility delivery proportion (for haemorrhage, hypertensive disorders of pregnancy, and obstructed labour). The time window was set at +/- 2 years for all models except late maternal death, which was +/- 5 years. The narrower window ensured that any given year of VR data only informed a single estimate.

We corrected the time trend in the CODEm model by identifying the year in which each location began consistently using O95 and O96 codes for late maternal death. These were identified as the earliest year in which the threshold proportion of total maternal deaths coded to late exceeded the lowest reported in the literature (0.5%). After a location was identified as having started using late maternal death codes, we assumed that practice continued. We adjusted upward results for all years prior to the advent of late maternal death coding using the outputs of the late maternal death proportion DisMod model.

Etiology-specific estimates were derived by multiplying the proportion outputs from DisMod-MR 2.1 by the total maternal deaths for that age-group, location, and year. HIV-related maternal deaths were estimated for all locations using the PAF approach described above for mortality data processing.

ICD10 and ICD9 codes used for maternal disorders

Model	ICD10 code	ICD9 code
Abortion, ectopic pregnancy, miscarriage	O00-O08, O36.4	631, 633-639
Maternal hemorrhage	O20, O43.2, O44-O46, O62.2, O67, O72	640-641, 661.0, 666
Hypertensive disorders of pregnancy	O11-O16	642.3, 642.4, 642.5, 642.6, 642.7, 642.9
Obstructed labor and uterine rupture	O64-O66, O71, O83	659-660, 662, 665, 669.5, 669.6
Maternal sepsis and other infections	O23, O41, O75.2-3, O85, O86, O91	646.5, 646.6, 659.2, 659.3, 670, 672.0, 674.1, 674.2, 674.3, 675
Other maternal disorders	O09-O09.93, O21-O22-O22.93, O26-26.93, O28-O28.9, O29-O29.93, O30-O35.9, O40-O43.93, O47-48.1, O60-O61.9, O63-O63.9, O68-O70.9, O73-O77.9, O80-O84, O87-O90.9, O92-O92.79	646-646.44, 646.7-646.93, 648.1-649.9
Indirect maternal disorders	O24-O25.3, O98-O99.91	647-649.64

Dismod Proportion Models Covariates and Coefficients

Abortion, ectopic pregnancy and miscarriage

Study-level covariate	Parameter	Geography level	beta	Exponentiated beta
Only Maternal Direct Causes	Proportion	Global	0.20 (0.19 — 0.20	1.22 (1.21 — 1.22)
Hospital Inpatient	Proportion	Global	0.30 (0.30 — 0.30)	1.35 (1.35 — 1.35)
Late maternal deaths not included	Proportion	Global	- 0.20 (0.20 — 0.20)	1.22 (1.22 — 1.22)
Country-Level Covariate				
Legality of Abortion	Proportion	Global	0.054 (0.054 — 0.055)	1.06 (1.06 — 1.06)

Maternal hemorrhage

Study-level covariate	Parameter	Geography level	beta	Exponentiated beta
Only Maternal Direct Causes	Proportion	Global	0.20 (0.18 — 0.20)	1.22 (1.20 — 1.22)
Hospital Inpatient	Proportion	Global	0.30 (0.29 — 0.30)	1.35 (1.34 — 1.35)
Late maternal deaths not included	Proportion	Global	0.20 (0.20 — 0.20)	1.22 (1.22 — 1.22)
Country-level covariate				
In-Facility Delivery (proportion)	Proportion	Global	0.100 (0.100 — 0.100)	1.11 (1.10 — 1.11)

Hypertensive disorders of pregnancy

Study-level covariate	Parameter	Geography level	beta	Exponentiated beta
Only Maternal Direct Causes	Proportion	Global	0.20 (0.19 — 0.20)	1.22 (1.21 — 1.22)
Hospital Inpatient	Proportion	Global	0.30 (0.30 — 0.30)	1.35 (1.34 — 1.35)
Late maternal deaths not included	Proportion	Global	0.20 (0.20 — 0.20)	1.22 (1.22 — 1.22)
Country-level covariate				
In-Facility Delivery (proportion)	Proportion	Global	0.100 (0.100 — 0.100)	1.11 (1.11 — 1.11)

Obstructed labor and uterine rupture

Study-level covariate	Parameter	Geography level	beta	Exponentiated beta
Only Maternal Direct Causes	Proportion	Global	0.20 (0.20 — 0.20)	1.22 (1.22 — 1.22)
Hospital Inpatient	Proportion	Global	0.30 (0.30 — 0.30)	1.35 (1.35 — 1.35)
Late maternal deaths not included	Proportion	Global	0.20 (0.20 — 0.20)	1.22 (1.22 — 1.22)
Country-level covariate				
In-Facility Delivery (proportion)	Proportion	Global	0.100 (0.100 — 0.100)	1.11 (1.11 — 1.11)

Maternal sepsis and other infections

Study-level covariate	Parameter	Geography level	beta	Exponentiated beta
Only Maternal Direct Causes	Proportion	Global	0.20 (0.20 — 0.20)	1.22 (1.22 — 1.22)
Hospital Inpatient	Proportion	Global	0.30 (0.30 — 0.30)	1.35 (1.35 — 1.35)
Late maternal deaths not included	Proportion	Global	0.20 (0.20 — 0.20)	1.22 (1.22 — 1.22)
Country-level covariates				
LDI ($ per capita)	Proportion	Global	0.100 (0.100 — 0.100)	1.11 (1.10 — 1.11)

Other Maternal Disorders

Study-level covariate	Parameter	Geography level	beta	Exponentiated beta
Only Maternal Direct Causes	Proportion	Global	0.20 (0.19 — 0.20)	1.22 (1.21 — 1.22)
Hospital Inpatient	Proportion	Global	0.20 (0.19 — 0.20)	1.22 (1.21 — 1.22)
Late maternal deaths not included	Proportion	Global	0.20 (0.20 — 0.20)	1.22 (1.22 — 1.22)
Country-level covariate				
LDI ($ per capita)	Proportion	Global	0.100 (0.100 — 0.100)	1.11 (1.10 — 1.11)

Indirect Maternal Disorders

Study-level covariate	Parameter	Geography level	beta	Exponentiated beta
Hospital Inpatient	Proportion	Global	0.20 (0.20 — 0.30)	1.22 (1.22 — 1.22)
Late maternal deaths not included	Proportion	Global	0.20 (0.20 — 0.20)	1.22 (1.22 — 1.22)
Country-level covariate				
LDI ($ per capita)	Proportion	Global	0.100 (0.100 — 0.100)	1.11 (1.11 — 1.11)

Neonatal disorders

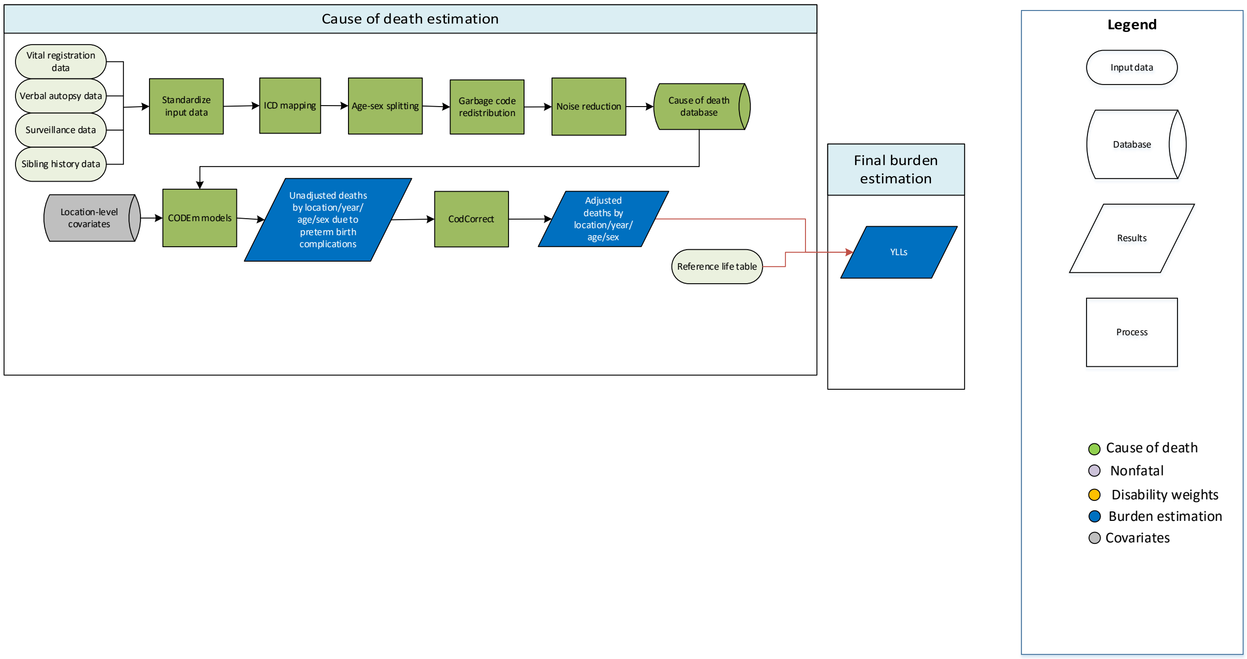

Input data

For the neonatal disorders envelope, preterm birth complications, and neonatal encephalopathy, vital registration, verbal autopsy, surveillance, and sibling history data were used for GBD 2016 to estimate number of deaths from each condition. For sepsis and other neonatal infections, vital registration, surveillance, and sibling history data were used. And for neonatal hemolytic disease and other neonatal conditions, vital registration and surveillance data were used. For all neonatal causes of death, vital registration was by far the most common data type. We only modelled deaths among males and females under age 5. Data points were selected as outliers if they were implausibly high, low, or significantly conflicted with established age or temporal patterns. Addition of significant new data from the Sample Registration System (SRS) in India had a significant effect on the estimates of mortality due to neonatal conditions at the global level.

Modelling strategy

For GBD 2016, an ensemble modelling approach was used via CODEm to model each of the different neonatal conditions. The same was done for GBD 2013 and 2015.

Varying levels of data quality and coding issues may still have affected our results. Validation studies suggest that verbal autopsy methods tend to be less accurate for cause of death ascertainment in the neonatal age groups.[1–4] This implies that in regions such as sub-Saharan Africa or South Asia, where the data primarily come from verbal autopsy studies, the distribution of sub-causes within all neonatal conditions may be less accurate. Furthermore, validation studies suggest that verbal autopsy methods tend to be particularly poor at ascertaining deaths from neonatal sepsis. Thus, for GBD 2016, all verbal autopsy data were excluded for neonatal sepsis and neonatal hemolytic disease.

Selected Covariates

Covariate	Transformation	Level	Direction
Education (years per capita)	None	3	-1
Health System Access	None	2	-1
In-Facility Delivery	None	2	-1
LDI (I$ per capita)	Log	3	-1
Underweight (proportion <2SD weight for age, <5 years)	None	2	1
Live Births 35+	None	2	1
Indoor Air Pollution (All cooking fuels)	None	1	1
Smoking prevalence (Reproductive Age-Standardized)	None	1	1
Total Fertility Rate	Log	3	1
SDI	None	3	-1
HAQI	None	2	-1
Skilled Birth Attendance	None	2	-1
Antenatal Care (4 visit)	None	2	-1

References

1 Anker M, Black RE, Coldham C, *et al.* A Standard Verbal Autopsy Method for Investigating Causes of Death in Infants and Children. Geneva, Switzerland: World Health Organization Department of Communicable Disease Surveillance and Response; The Johns Hopkins School of Hygiene and Public Health; The London School of Hygiene and Tropical Medicine, 1999.

2 Kalter HD, Gray RH, Black RE, Gultiano SA. Validation of postmortem interviews to ascertain selected causes of death in children. *Int J Epidemiol* 1990; **19**: 380–6.

3 Quigley MA, Armstrong Schellenberg JR, Snow RW. Algorithms for verbal autopsies: a validation study in Kenyan children. *Bull World Health Organ* 1996; **74**: 147–54.

4 Snow RW, Armstrong JR, Forster D, *et al.* Childhood deaths in Africa: uses and limitations of verbal autopsies. *The Lancet* 1992; **340**: 351–5.

Nutritional deficiencies: *Protein-energy malnutrition, Iron-deficiency anaemia, Iodine deficiency, Vitamin A deficiency, and other nutritional deficiencies*

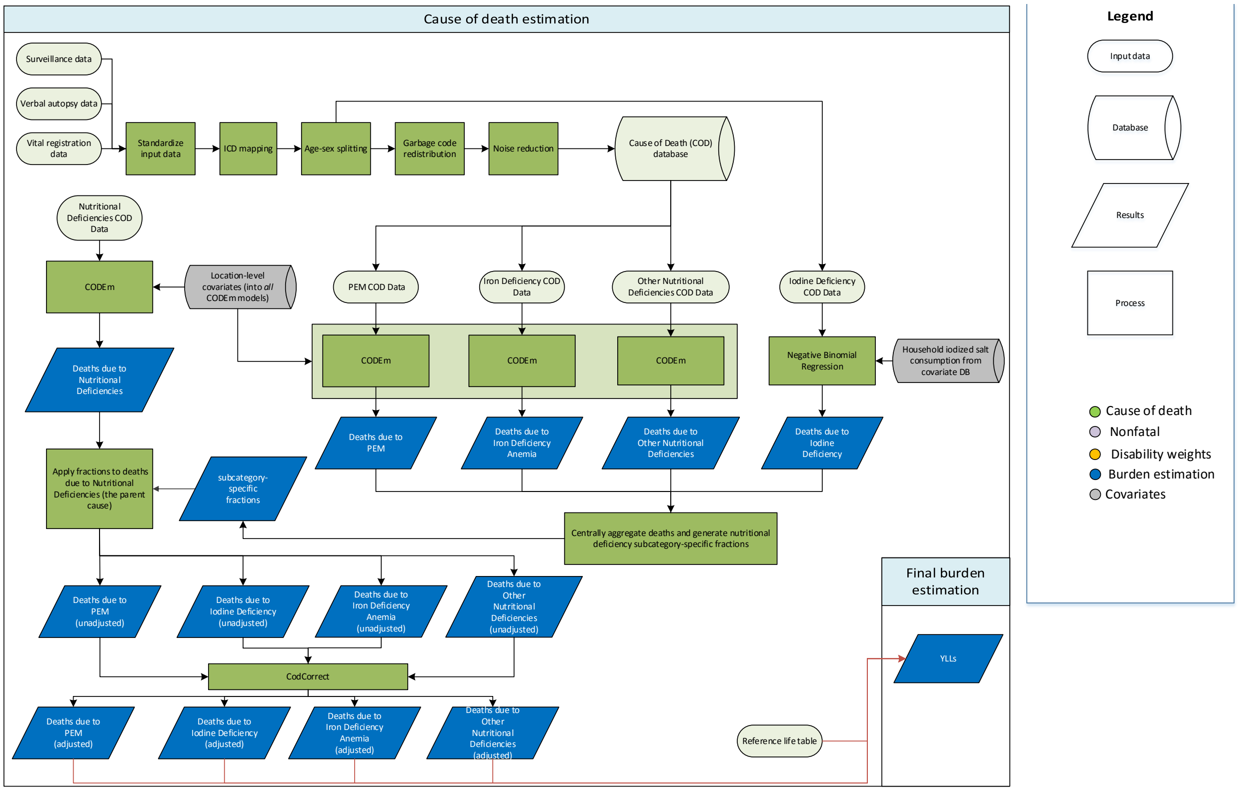

Input data and case definitions

For GBD 2016, vital registration, verbal autopsy, and surveillance data were used to model deaths due to nutritional deficiencies. As described in other sections, the volume of new data was significant. Notable additions include Sample Registration System (SRS) from states of India and provinces of Indonesia. ICD codes, which can be interpreted as case definitions, for each of the nutritional deficiencies is listed in the table below.

GBD cause	ICD-10 code
Protein-energy malnutrition	E40-E46.9 (Kwashiorkor, marasmus, specified and unspecified protein calorie malnutrition)
Iron-deficiency anemia	D50.1-D50.8 (iron deficiency anemia)
Iodine deficiency	E00-E02 (congenital iodine-deficiency syndrome, iodine-deficiency related thyroid disorders and allied conditions, and subclinical iodine-deficiency hypothyroidism)
Other nutritional deficiencies	D51-D52.0 (vitamin B12 deficiency anemia and folate deficiency anemia)
Other nutritional deficiencies	D52.8-D53.9 (other nutritional anemias)

Other nutritional deficiencies	D64.3 (other sideroblastic anemias)
Other nutritional deficiencies	E51-E61.9 (thiamine, niacin, other B group vitamins, ascorbic acid, vitamin D, other vitamin, dietary calcium, dietary selenium, dietary zinc, and other nutrient element deficiencies)
Other nutritional deficiencies	E63-E64.0 (other nutritional deficiencies and sequelae of protein-calorie malnutrition)
Other nutritional deficiencies	E64.2-E64.9 (sequelae of vitamin C deficiency, rickets, other nutritional deficiencies, and unspecified nutritional deficiencies)
Other nutritional deficiencies	M12.1-M12.19 (Kaschin-Beck disease)
Garbage code	D50, D50.0 and D50.9 (unspecified anemia)

Modelling strategy

Other than data and covariate updates, we did not make any modeling strategy changes for GBD 2016.

We estimated mortality for each of the nutritional deficiencies in two steps. CODEm was first used to generate mortality estimates for total nutritional deficiencies. The sub-categories of nutritional deficiencies, namely protein-energy malnutrition, iodine deficiency, iron-deficiency anaemia, and other nutritional deficiencies, were modelled separately. We assumed zero mortality due to Vitamin A deficiency, instead analyzing it as a cause of nonfatal disease burden and a risk factor for mortality due to other causes. We outliered data that were largely conflicting with the majority of data from other studies conducted either in the same or different countries (with similar socio-demographic characteristics) in the same region.

CODEm was used to model all sub-categories except for iodine deficiency. The CODEm covariates (including level and direction) used for each of the models are listed in the table below. The covariate used in the iodine deficiency model is “proportion of households using iodized salt.”

**Nutritional deficiencies (overall)**		
**Level**	**Covariate**	**Direction**
1	Age-standardized prevalence of severe anemia	+
	Malnutrition shock mortality rate	+
	Proportion of children 0-5 with weight-for-age z-score < -2	+
	Proportion of children 0-5 with weight-for-height z-score < -2	+
	Proportion of households using iodized salt	–
	Total kcal per person per day availability	–
2	Population living in the 1st world quintile (least) of annual rainfall	+/–
	Population living in the 2nd world quintile (2nd least) of annual rainfall	+/–
	Sanitation (proportion with access)	–
	Mortality rate due to war shocks	+
	Improved water source (proportion with access)	–
	Health Systems Access	–
3	Education (years per capita)	–
	Lag distributed income per capita	–
	Socio-demographic index	–

	Antenatal care (4 visits) coverage (proportion)	–
**Protein-energy malnutrition**		
**Level**	**Covariate**	**Direction**
1	Age-standardized prevalence of severe anemia	+
	Total kcal per person per day availability	–
	Malnutrition shock mortality rate	+
	Proportion of children 0-5 with weight-for-height z-score < -2	+
2	Population living in the 1st world quintile (least) of annual rainfall	+/–
	Population living in the 2nd world quintile (2nd least) of annual rainfall	+/–
	Sanitation (proportion with access)	–
	Mortality rate due to war shocks	+
	Improved water source (proportion with access)	–
	Healthcare access and quality index	–
	Health Systems Access	–
3	Antenatal care (4 visits) coverage (proportion)	–
	Education (years per capita)	–
	Lag distributed income per capita	–
	Socio-demographic index	–
**Iron-deficiency anemia**		
**Level**	**Covariate**	**Direction**
1	Proportion of children 0-5 with weight-for-age z-score < -2	+
	Age-standardized prevalence of severe anemia	+
2	Population living in the 1st world quintile (least) of annual rainfall	+/–
	Population living in the 2nd world quintile (2nd least) of annual rainfall	+/–
	Sanitation (proportion with access)	–
	Total kcal per person per day availability	–
	Improved water source (proportion with access)	–
	Health Systems Access	–
	Healthcare access and quality index	–
3	Education (years per capita)	–
	Lag distributed income per capita	–
	Socio-demographic index	–
**Other nutritional deficiencies**		
**Level**	**Covariate**	**Direction**
1	Age-standardized prevalence of severe anemia	+
	Malnutrition shock mortality rate	+
	Proportion of children 0-5 with weight-for-age z-score < -2	+
2	Population living in the 1st world quintile (least) of annual rainfall	+/–
	Population living in the 2nd world quintile (2nd least) of annual rainfall	+/–
	Sanitation (proportion with access)	–
	Mortality rate due to war shocks	+
	Improved water source (proportion with access)	–
	Total kcal per person per day availability	–
	Health Systems Access	–
	Healthcare access and quality index	–
3	Education (years per capita)	–

	Lag distributed income per capita	–
	Socio-demographic index	–

Iodine deficiency was modelled using a negative binomial regression model given the small number of deaths attributable to it. A negative binomial model is more appropriate than a Poisson count model as it accounts for greater variance (over-dispersion) in the data. By utilizing the exposure option in Stata, we model cause fractions with a negative binomial model. We tested both rate- and cause fraction-based models but selected a cause fraction model due to better model performance. We used vital registration data with proportion of household iodized salt consumption as a country-level covariate and dummy variables on age and sex to model mortality from iodine deficiency. Uncertainty was estimated by taking 1,000 iterations of the predictions based on the variance-covariance matrix and a random sample from a gamma distribution.

Estimates from the four nutritional sub-categories were then scaled at the 1,000 draw level in CODCorrect to match that for total nutritional deficiencies. Protein energy malnutrition and other nutritional deficiencies death estimates were also corrected for misclassification of Alzheimer and Parkinson disease deaths. Detailed information on this process can be found in section 4 of the appendix.

Sexually transmitted infections excluding HIV

This write-up covers includes: Total sexually transmitted diseases (STI), Chlamydia, Gonorrhea, Syphilis, Trichomonas, Genital herpes due to HSV-2, and other STI.

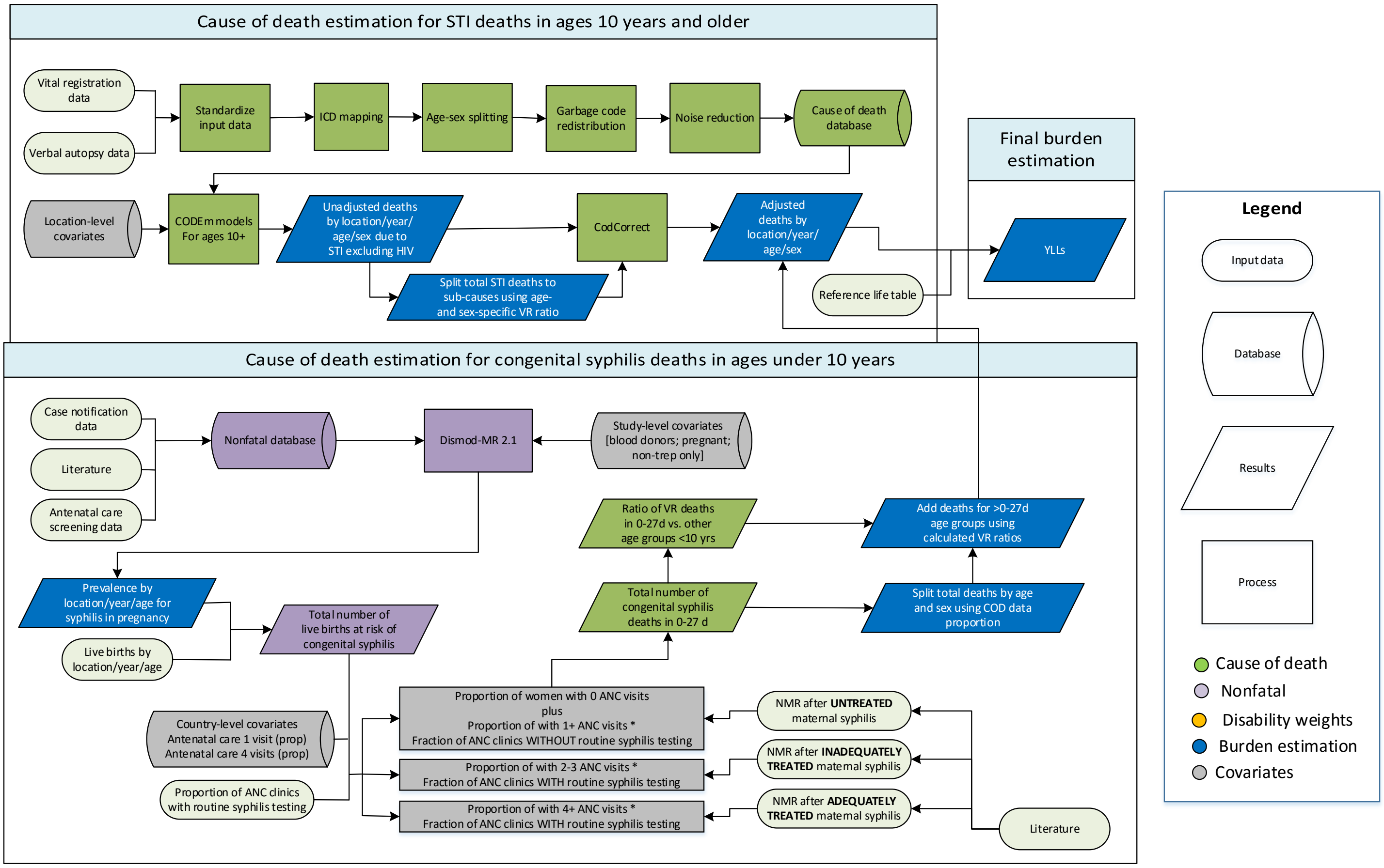

Input data

For GBD 2016, STI cause of death models included syphilis, chlamydial infection, gonococcal infection, and other STIs. CODEm models for males and females 10 years and older were informed from centrally prepped data stored in the cause of death (COD) database. All data from all geographies were reviewed. Outliers were identified as those data where age patterns or temporal patterns were inconsistent with neighboring age groups or locations or where sparse data were predicting implausible overall temporal or age patterns for a given location.

Four different types of data were used for the natural history model (NHM) of congenital syphilis. First, we used literature, survey, and report data described below to estimate early syphilis in pregnancy. Second, we used GBD 2016 estimates of antenatal care (ANC) coverage data from our covariates database and live births estimates from our demographics analysis. Third, we used published data from the Global Health Observatory (updated in GBD 2015) on proportion of ANC clinics that test for syphilis and the proportion of women testing positive who receive treatment. Fourth, we used the results of a

systematic literature review completed for GBD 2010 to inform excess mortality of neonates born with syphilis.

Modelling strategy

We completed data-rich (DR) and global CODEm models for ages 10 years and over for males and females separately. Nine covariates were used in each CODEm model, including 1) syphilis prevalence in pregnancy from DisMod-MR 2.1 analysis described below; 2) coverage of one antenatal care (ANC) visit, 3) coverage of four ANC visits; 4) age-specific fertility rate; 5) total fertility rate; 6) health system access, a principal components analysis of ANC, in-facility delivery, skilled birth attendance, and vaccine coverage; 7) national income per capita (LDI); 8) years of education per capita; and 9) abortion legality, an index that includes a categorical rating of abortion laws that range from 1 (always illegal) to 7 (always legal on demand) as well as a categorical variable on additional restrictions (eg, waiting period, family permission required) and gestational week at which abortion is allowed.

The overall CODEm model for STI was split into the sub-causes using vital registration (VR) data from the COD database. Trichomoniasis and HSV-2 are assumed not to cause mortality. Chlamydia is further assumed not to cause death in males. Cause-specific mortality rate VR data for each age group, sex, and year were summed and scaled to match the total STI cause-specific mortality rate predicted by CODEm. This VR pattern was applied globally to all locations.

Our NHM for congenital syphilis began with estimation of early syphilis prevalence in all age groups, both sexes, all GBD locations, and in each year from 1990 to 2016. We modelled early syphilis in DisMod-MR 2.2, restricting incidence before the age of 10 and after the age of 65 and assuming zero excess mortality from early syphilis. Study-level covariates identified data in non-reference categories (pregnant, blood donors) and using non-reference testing modalities (eg, treponemal test only, non-treponemal test only) and were crosswalked to the reference category within the DisMod-MR 2.1 cascade, predicting specific conversion values for each country. The latter specification of country-level crosswalks was chosen because the bias between non-reference populations and testing modalities was assumed to differ by geography and as a function of, for example, endemicity of acute infectious agents like malaria.

Age-specific prevalence results were paired with age-specific live birth results to generate total number of births at risk of congenital syphilis. To estimate the actual number of congenital syphilis births, we combined information on ANC coverage from GBD 2016 covariates analyses with ANC syphilis testing and treatment data from GHO. Adequate treatment was assumed to confer no risk of congenital syphilis mortality. Those with four ANC visits or 1–3 ANC visits with testing and treatment were assumed to have received adequate treatment. Inadequate treatment occurred in those women with 1–3 ANC visits without either testing or treatment or those women with one ANC visit but with testing and treatment. Those women with only one or no ANC visits and no syphilis testing or treatment were assumed to be untreated. Untreated and inadequate treatment proportions were combined with potential congenital syphilis births to estimate total neonatal syphilis births. Each categorical risk category was combined with corresponding neonatal excess mortality rates derived in GBD 2010. This total number of neonatal syphilis deaths was then split between 0–6 days and 7–27 days age groups using sex- and age-specific VR data from the COD database. Congenital syphilis deaths beyond the neonatal period were likewise estimated using sex- and age-specific VR data. This VR pattern was applied globally to all locations.

The primary limitation of our estimation of STI deaths in those over 10 years old is data availability, especially from countries where VR systems are not available. Even in countries with VR, there may be some variation in practices for coding deaths to STI as the underlying cause, especially given the potentially variable presentation of many of the conditions in this category. Such variation is more likely to lead to underestimation of STI deaths than overestimation. Sub-cause estimation is similarly limited by data availability in those locations without VR data, and our estimates are thus based on the overall pattern of deaths in generally higher-income geographies.

The primary implication of this limitation is that it decreases the resolution with which we can decompose the relationship between mortality from HIV and other STI. Our NHM for congenital syphilis continues to improve but still is limited by data availability issues, especially on the coverage and effectiveness of ANC interventions to prevent congenital syphilis. We do not have information on the proportion of women who tested positive who may have received treatment elsewhere, or information on the coverage of treatment for neonates, infants, and children born with congenital syphilis. Both limitations could potentially have led to lower estimates of congenital syphilis deaths. On the other hand, our DisMod-MR 2.2 analysis suggested that pregnant women may in fact have higher syphilis prevalence than the general population, which would have led to higher estimates. We have also not quantified the number of stillbirths associated with congenital syphilis.

No other significant changes were made for GBD 2016.

Hepatitis

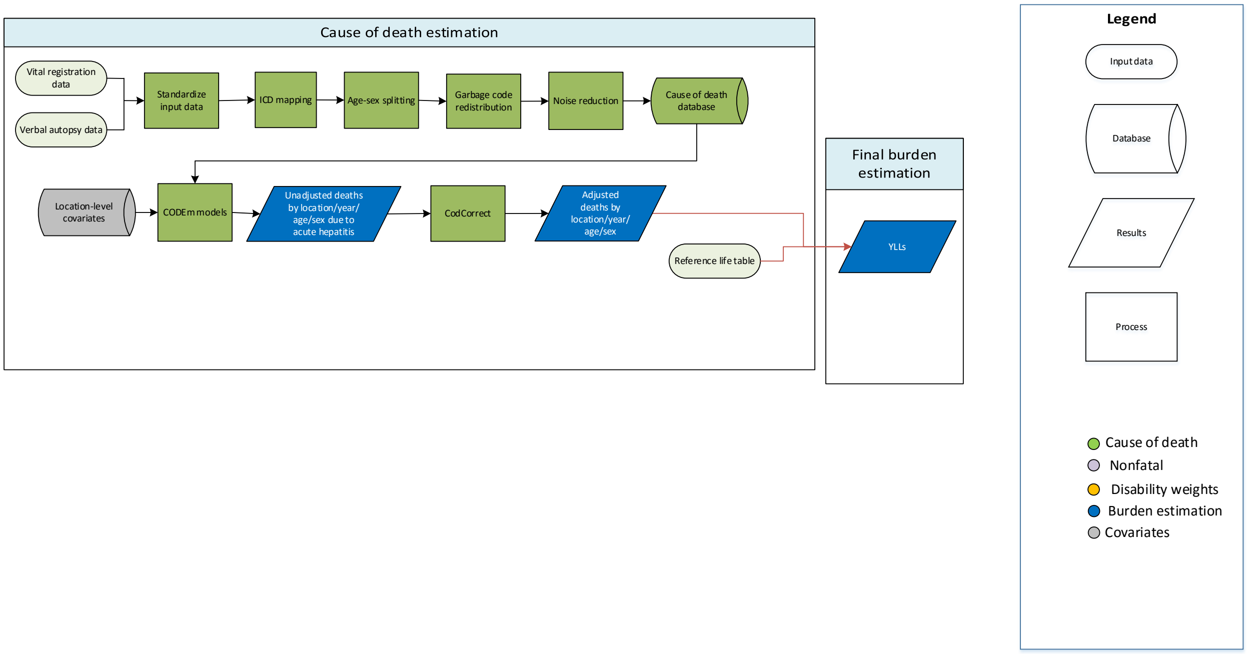

Input data

We modelled hepatitis mortality using all available data in the cause of death database. Data points were outliered if they reported an improbable number of hepatitis deaths or if their inclusion in the model yielded distorted trends. In some cases multiple data sources for the same location differed dramatically both in their quality and reported hepatitis mortality (eg, a verbal autopsy and vital registration source). In these cases the lower-quality data source was outliered.

Modelling strategy

We modelled hepatitis mortality using a two-model hybrid approach: 1) a global CODEm model of all locations, using all data in the CoD database; and 2) a CODEm model restricted to data-rich countries. Since GBD 2013 we have switched from a single global model to the hybrid global/data-rich model approach. We have otherwise made no substantive changes in the modelling strategy for hepatitis from GBD 2013.

Acute hepatitis A

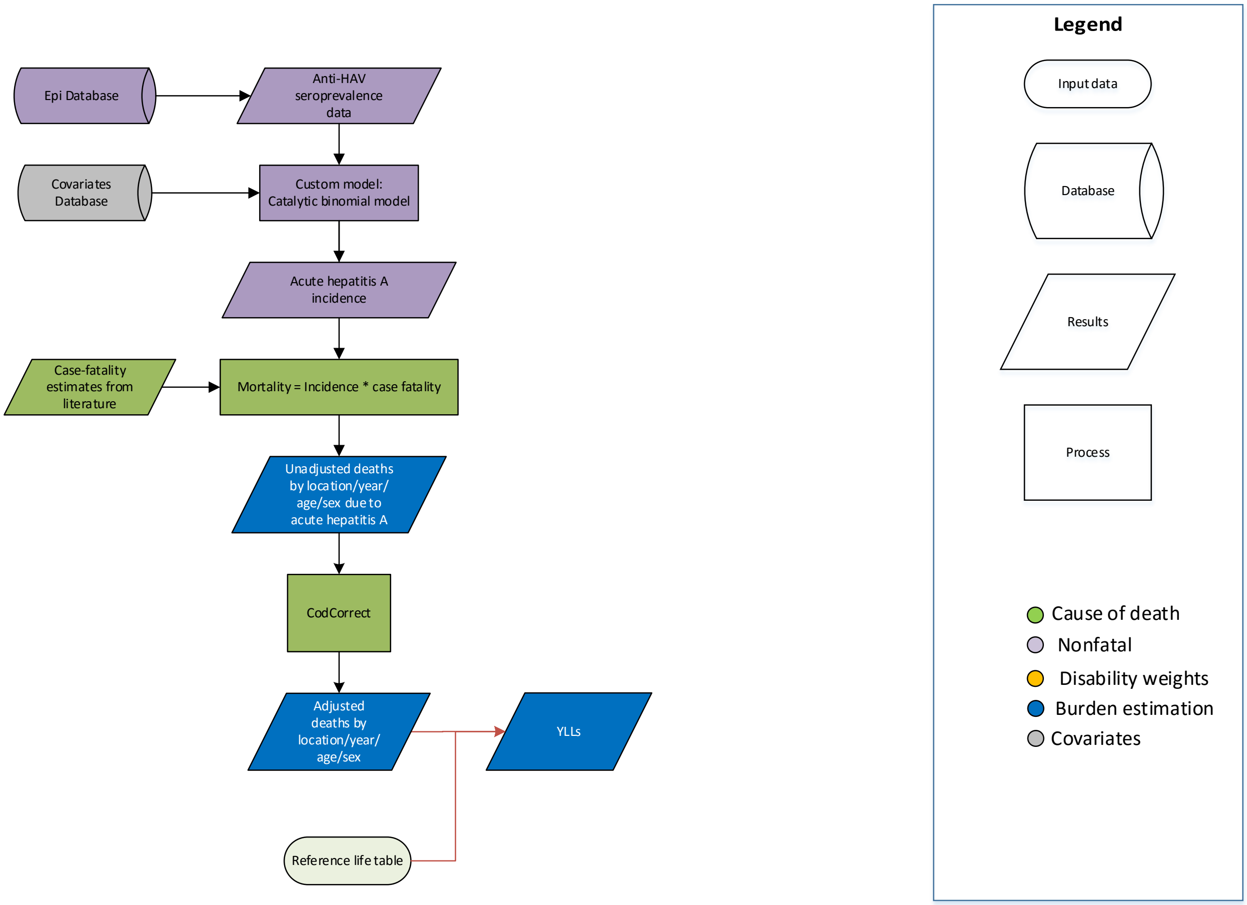

Input data

We use anti-HAV seroprevalence data from population-based studies and surveys for the incidence model.

Modelling strategy

Virus-specific CoD data for acute hepatitis are inconsistently reported and of questionable accuracy. We therefore use a two-part modelling strategy for acute hepatitis A, B, C, and E. First, we develop a parent acute hepatitis mortality model using CODEm and all acute hepatitis mortality data within the CoD database. Second, we develop four separate natural history models to estimate deaths from acute hepatitis A, B, C, and E, respectively. Finally, we rescale the virus-specific death estimates from the four natural history models to fit within the envelope defined by the parent acute hepatitis CoD model.

Given its reasonably stable force of infection among susceptible people across age groups, we used a catalytic binomial model to estimate incidence of acute hepatitis A based on anti-HAV seroprevalence. The catalytic binomial model is a binomial generalized linear model with a complementary log-log link, and an offset term for log-age. Since anti-HAV is a lifetime marker of past infection, and a given individual can only be infected once, seroprevalence at age t is equal to the cumulative incidence (CI) over t years. Assuming constant force of infection, we can estimate the incidence rate (IR) as

$$CI = 1 - e^{-IR \cdot t}$$

We can rearrange this equation to solve for the log-IR:

$$\ln(IR) = \frac{\ln(-\ln(1 - CI))}{\ln(t)}$$

Thus, by using the complimentary log-log link for CI (i.e., ln(-ln(1-CI)) with an offset for log-age, we are able to model the incidence rate of infection from seroprevalence data. To inform the model in the absence of data we use a predictive covariate derived from principal components analysis of lag-distributed income (LDI) and the proportion of the population with access to improved water. We use a mixed effects model with fixed effects on the aforementioned PCA-derived covariate, and nested hierarchical random effects on super-region, region, and time.

Our overall approach has not changed from that used in GBD 2013. However, whereas we previously used a fixed-effects only catalytic binominal model for GBD 2013, we have incorporated random effects into the model for GBD 2016, improving the spatial structure of the model and allowing the model to better follow data.

Acute hepatitis B

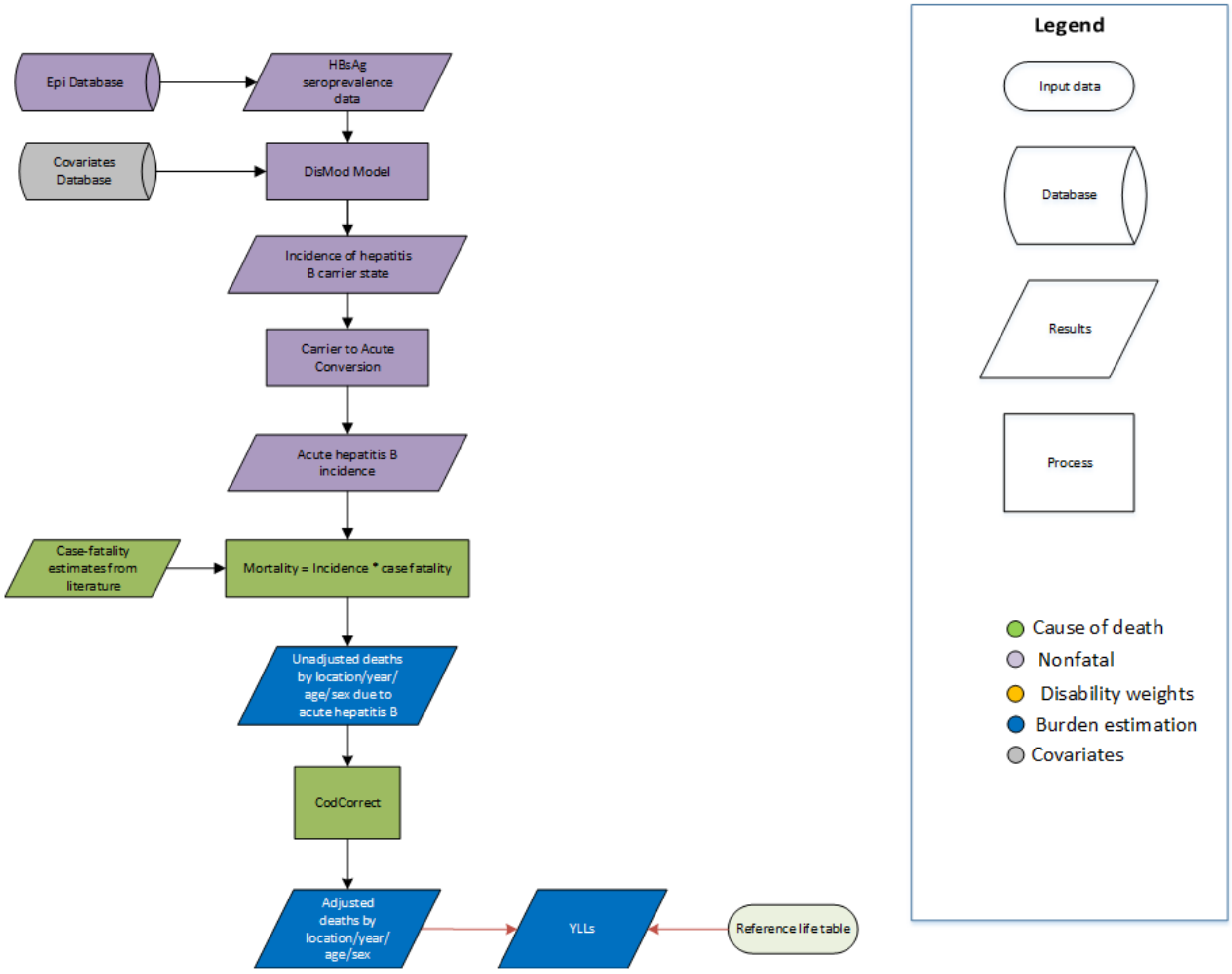

Input data

We use hepatitis B surface antigen (HBsAg) seroprevalence data from population-based studies and surveys for the incidence model.

Modelling strategy

Virus-specific CoD data for acute hepatitis are inconsistently reported and of questionable accuracy. We therefore use a two-part modelling strategy for acute hepatitis A, B, C, and E. First, we develop a parent acute hepatitis mortality model using CODEm and all acute hepatitis mortality data within the CoD database. Second, we develop four separate natural history models to estimate deaths from acute hepatitis A, B, C, and E, respectively. Finally, we rescale the virus-specific death estimates from the four natural history models to fit within the envelope defined by the parent acute hepatitis CoD model.

We model the incidence of chronic HBsAg carriage using a full DisMod model of HBsAg seroprevalence. We then convert incidence of chronic carriage to total incidence of hepatitis B infection by dividing age-specific estimates of the incidence of chronic carriage by age-specific estimates of the probability of infection resulting in carriage from Edmunds et al 1993 [2]. We then multiply incident infection estimates by estimates of probability of acute incidence, derived from cases in McMahon et al 1997 [3] and sampling from a beta distribution to propagate uncertainty. Lastly, using the estimates of acute hepatitis B infection, we multiply by the case fatality of acute hepatitis B using estimates derived from cases in Bianco et al 2003 [4] & Stoffolini et al 1997 [5] , sampling from a beta distribution to propagate uncertainty. The equations below describes the above text:

$$Incident\ infection = \frac{Chronic\ carriage}{\beta_1}$$

$$Incident\ acute\ infection = Incidence\ infection\ *\ \beta_2$$

$$Acute\ deaths = Acute\ infection *\ \beta_3$$

Where

$\beta_1 = Probability\ of\ chronic\ infection$
$\beta_2 = Probability\ of\ acute\ infection$
$\beta_3 = Case\ fatality\ rate$

Acute hepatitis C

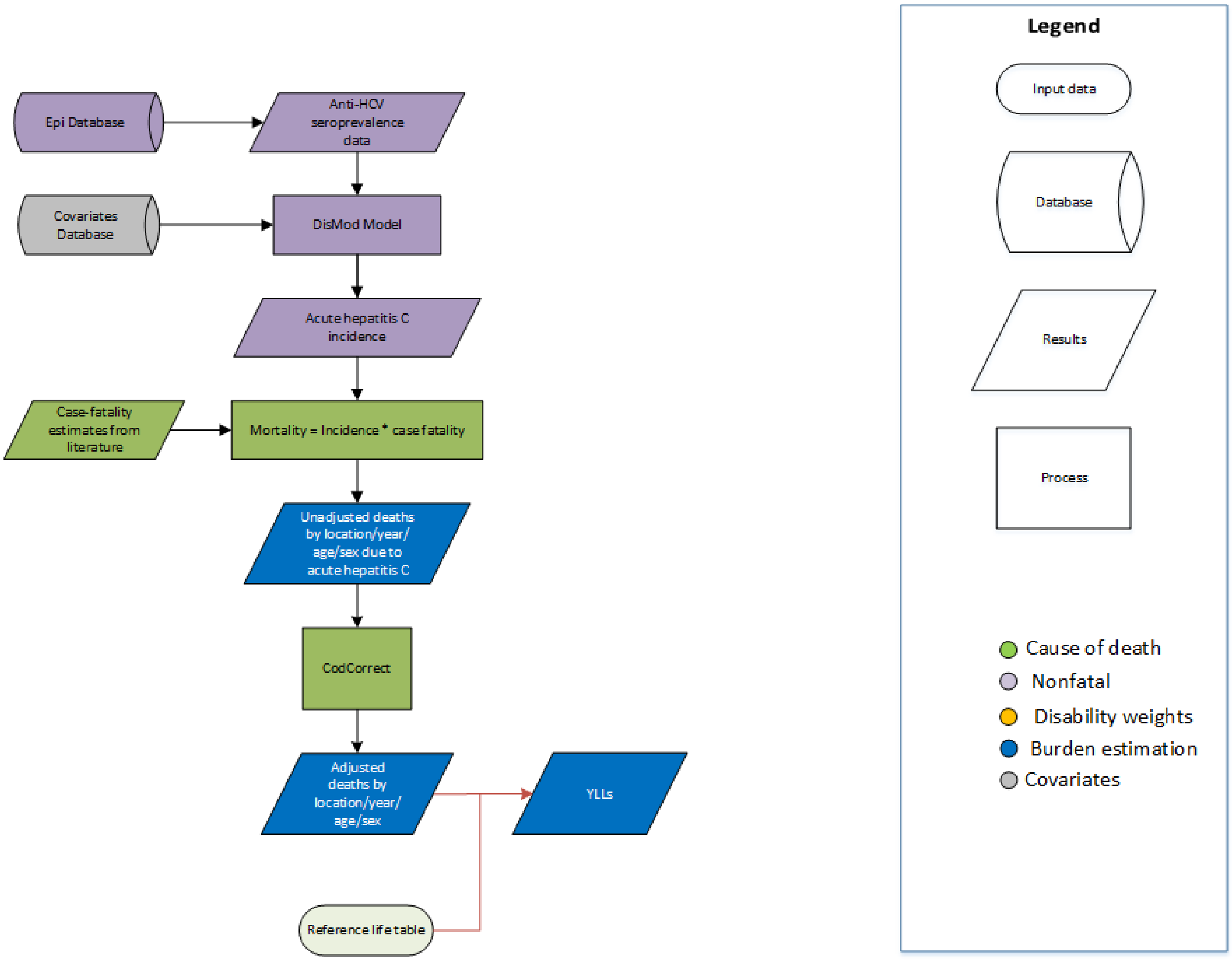

Input data

We use anti-HCV seroprevalence data from population-based studies and surveys for the incidence model.

Modelling strategy

Virus-specific CoD data for acute hepatitis are inconsistently reported and of questionable accuracy. We therefore use a two-part modelling strategy for acute hepatitis A, B, C, and E. First, we develop a parent acute hepatitis mortality model using CODEm and all acute hepatitis mortality data within the CoD database. Second, we develop four separate natural history models to estimate deaths from acute hepatitis A, B, C, and E, respectively. Finally, we rescale the virus-specific death estimates from the four natural history models to fit within the envelope defined by the parent acute hepatitis CoD model.

We model the incidence of hepatitis C using a full DisMod model of anti-HCV seroprevalence and estimate acute hepatitis C deaths as the product of these incidence estimates and published estimates of acute hepatitis C case fatality [Stroffolini et al 1997], sampling from a beta distribution to propagate uncertainty. The equation below describes the above discussion:

$$Acute\ deaths = Acute\ incidence * \ \beta$$

Where:
$\beta = Case\ fatality\ rate$

Acute hepatitis E

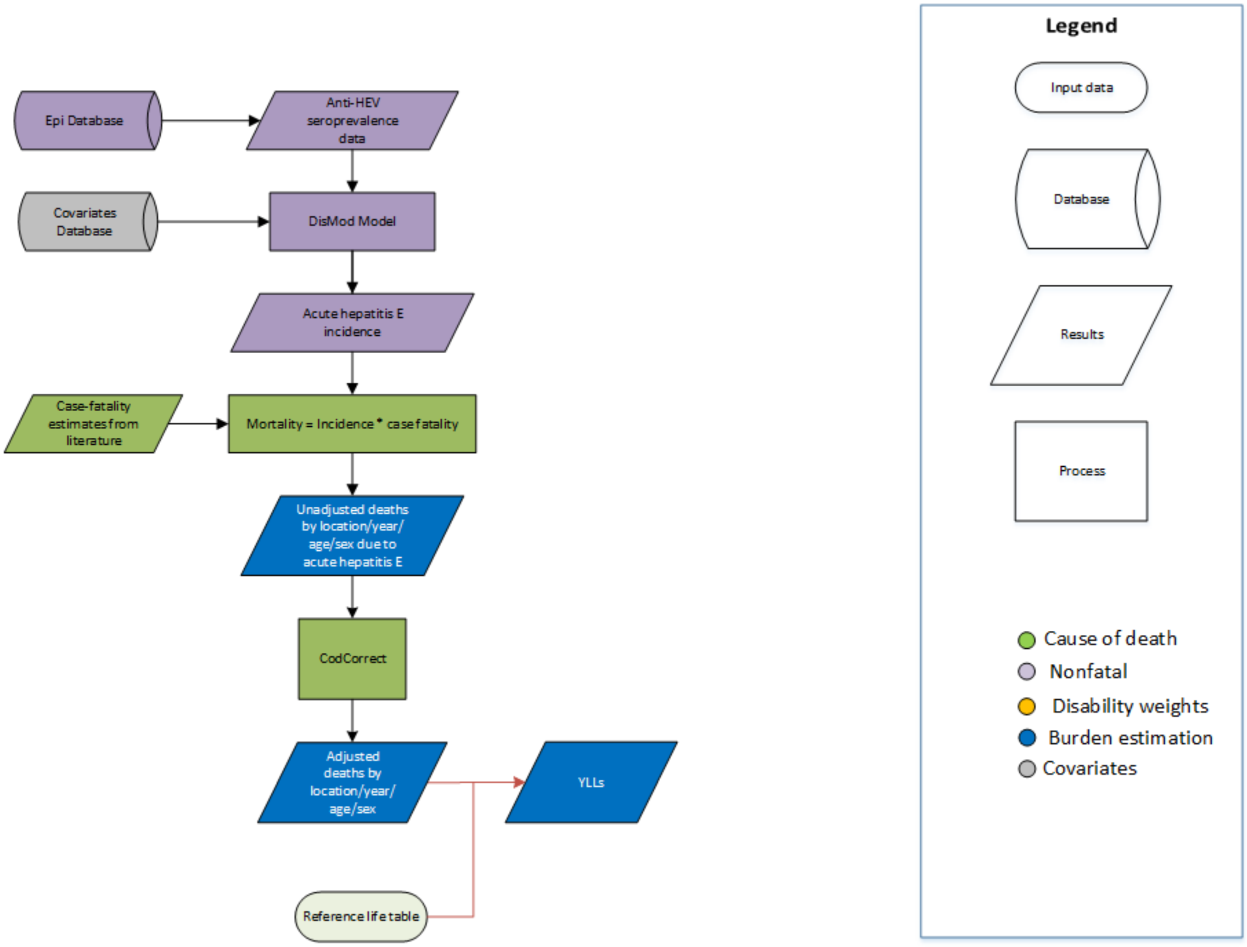

Input data

We use anti-HEV seroprevalence data from population-based studies and surveys for the incidence model.

Modelling strategy

Virus-specific CoD data for acute hepatitis are inconsistently reported and of questionable accuracy. We therefore use a two-part modelling strategy for acute hepatitis A, B, C, and E. First, we develop a parent acute hepatitis mortality model using CODEm and all acute hepatitis mortality data within the CoD database. Second, we develop four separate natural history models to estimate deaths from acute hepatitis A, B, C, and E, respectively. Finally, we rescale the virus-specific death estimates from the four natural history models to fit within the envelope defined by the parent acute hepatitis CoD model.

We model the incidence of hepatitis E using a full DisMod model of anti-HEV seroprevalence and estimate acute hepatitis E deaths as the product of these incidence estimates and published estimates of acute hepatitis E case fatality, accounting for the proportion of infections that occur in pregnant women and the higher case fatality of hepatitis E among pregnant women. Since HEV genotypes tend to

be less pathogenic and less virulent, we restrict hepatitis E deaths to countries in regions where genotypes 1 and 2 predominate (ie, Central Asia, Central sub-Saharan Africa, East Asia, Eastern sub-Saharan Africa, North Africa and Middle East, Oceania, South Asia, Southeast Asia, Southern sub-Saharan Africa, and Western sub-Saharan Africa).[1]

We have made no substantive changes in the modelling strategy from GBD 2013.

Reference

1 Rein DB, Stevens GA, Theaker J, Wittenborn JS, Wiersma ST. The global burden of hepatitis E virus genotypes 1 and 2 in 2005. *Hepatology* 2012; 55: 988–97.

2 Edmunds, W. J., et al. "The influence of age on the development of the hepatitis B carrier state." *Proceedings of the Royal Society of London B: Biological Sciences* 253.1337 (1993): 197-201.

3 McMahon, Brian J. "Epidemiology and natural history of hepatitis B." *Seminars in liver disease*. Vol. 25. No. S 1. Published in 2005 by Thieme Medical Publishers, Inc., 333 Seventh Avenue, New York, NY 10001, USA., 2005.

4 Bianco, E., et al. "Case fatality rate of acute viral hepatitis in Italy: 1995–2000. An update." *Digestive and liver disease* 35.6 (2003): 404-408.

5 Guadagnino, Vincenzo, et al. "Prevalence, risk factors, and genotype distribution of hepatitis C virus infection in the general population: a community-based survey in southern Italy." *Hepatology* 26.4 (1997): 1006-1011.

Other Infectious Diseases

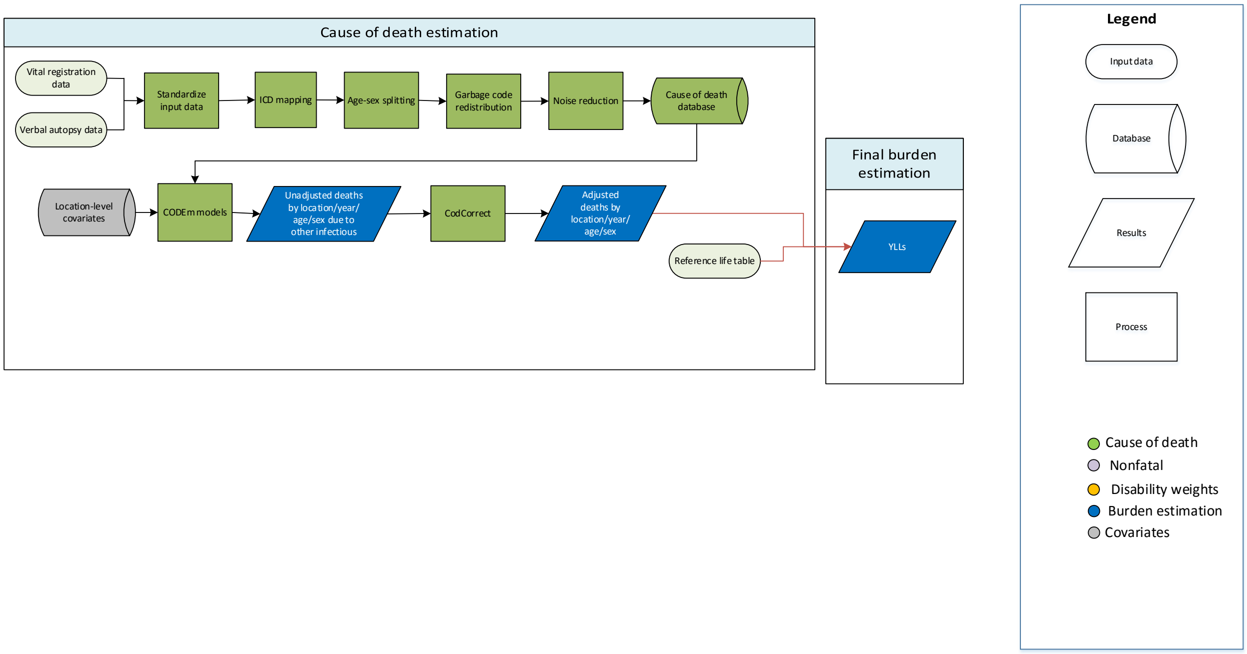

Input data

We modelled other infectious disease mortality using all available data in the cause of death database. Data points were outliered if they reported an improbable number of deaths or if their inclusion in the model yielded distorted trends. In some cases multiple data sources for the same location differed dramatically both in their quality and reported other infectious diseases mortality (eg, a verbal autopsy and vital registration source). In these cases the lower-quality data source was outliered.

Modelling strategy

We modeled other infectious disease mortality using a two-model hybrid approach: 1) a global CODEm model of all locations, using all data in the CoD database; and 2) a CODEm model restricted to data-rich countries.

Since GBD 2013 we have switched from a single global model to the hybrid global/data-rich model approach. We have otherwise made no substantive changes in the modelling strategy for other intestinal infectious diseases from GBD 2015.

Cancers

Input data and methodological summary for all cancers except for non-melanoma skin cancer

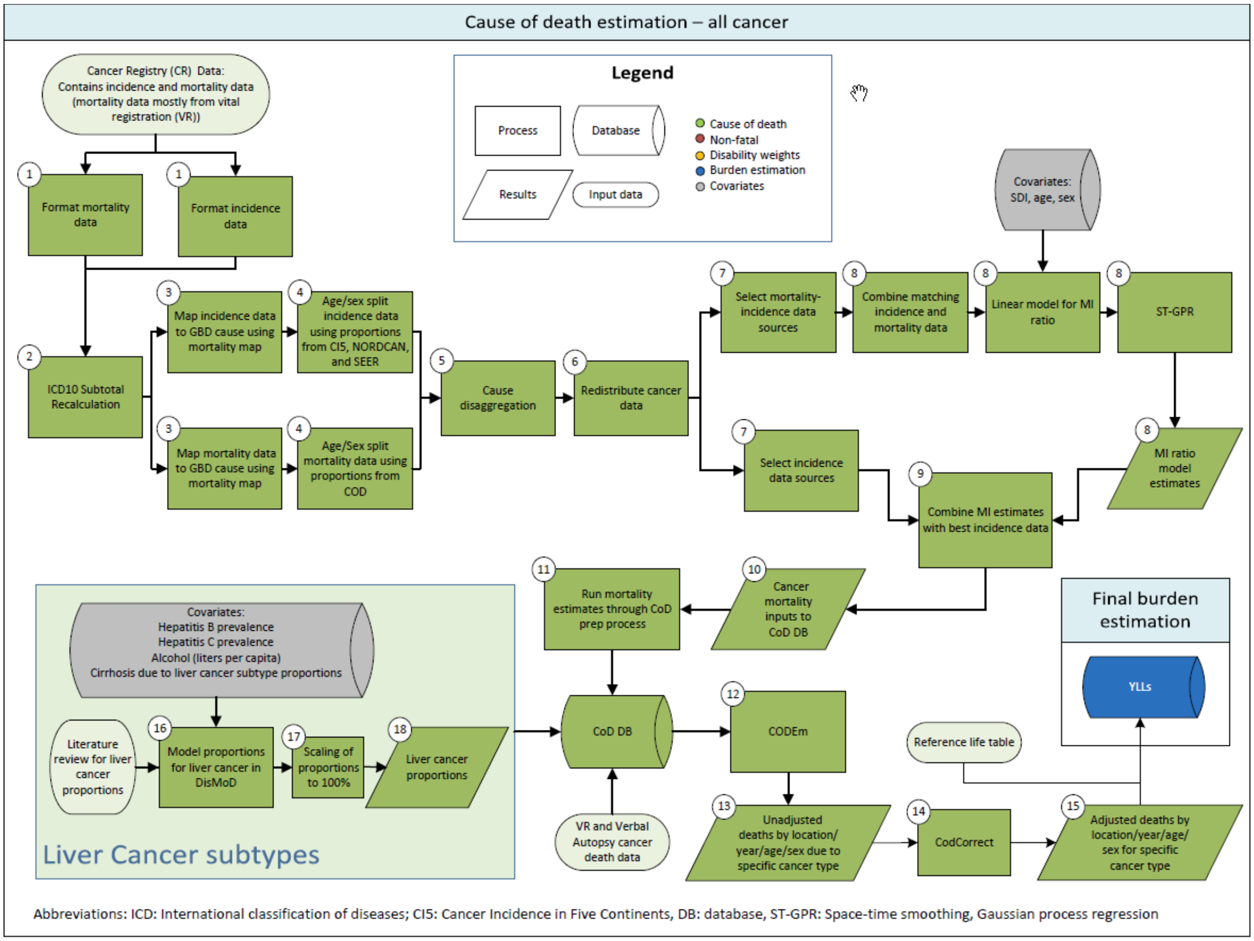

Data

The cause of death (COD) database contains multiple sources of cancer mortality data. These sources include vital registration, verbal autopsy, and cancer registry data. The cancer registry mortality estimates that are uploaded into the COD database stem from cancer registry incidence data that have been transformed to mortality estimates through the use of mortality-to-incidence ratios (MIR).

Data seeking processes

Cancer mortality data in the cause of death database other than cancer registry data

Sources for cancer mortality data other than cancer registry data are described in the COD database description (Section 2).

Cancer registry data

Cancer registry data were used from publicly available sources or provided by collaborators. We attempted to collect data from all registries that are members of the International Association of Cancer Registries (IACR) by either downloading publicly available data or contacting the registries. We also used cancer registry databases like Cancer Incidence in Five Continents (CI5), EUREG, and NORDCAN.[1–9]

Most cancer registries only report cancer incidence. However, if a cancer registry also reported cancer mortality, mortality data were also extracted from the source to be used in the MIR estimation.

Inclusion and exclusion criteria

Only population-based cancer registries were included, and only those that included all cancers (no specialty registries), data for all age groups, and data for both sexes. Pathology-based cancer registries were included if they had a defined population. Hospital-based cancer registries were excluded. Cancer registry data were excluded from either the final incidence data input or the MI model input if a more detailed source (e.g., providing more detailed age or diagnostic groups) was available for the same population. Preference was given to registries with national coverage over those with only local coverage, except those from countries where the GBD study provides subnational estimates. Data were excluded if the coverage population was unknown.

Bias of categories of input data

Cancer registry data can be biased in multiple ways. A high proportion of ill-defined cancer cases in the registry data requires redistribution of these cases to other cancers, which introduces a potential for bias. Changes between coding systems can lead to artificial differences in disease estimates; however, we adjust for this bias by mapping the different coding systems to the GBD causes. Underreporting of cancers that require advanced diagnostic techniques (e.g., leukemia and brain, pancreatic, and liver cancer) can be an issue in cancer registries from low-income countries. On the other hand, misclassification of metastatic sites as primary cancer can lead to overestimation of cancer sites that are common sites for metastases, like brain or liver. Since many cancer registries are located in urban areas, the representativeness of the registry for the general population can also be problematic. The accuracy of mortality data reported in cancer registries usually depends on the quality of the vital registration system. If the vital registration system is incomplete or of poor quality, the mortality-to-incidence ratio can be biased to lower ratios.

Data for liver cancer etiology splits

To find the proportion of liver cancer cases due to the four etiology groups included in GBD (1. Liver cancer due to hepatitis B, 2. Liver cancer due to hepatitis C, 3. Liver cancer due to alcohol, 4. Liver cancer due to other causes), a systematic literature search was performed in PubMed. Studies were included if the study population was representative of liver cancer population for the respective location. For each study the proportions of liver cancer due to the three specific risk factors were calculated. Remaining risk factors were included under a combined "other" group. Cryptogenic cases were only included if other etiologies like viral hepatitis or alcoholic cirrhosis had been excluded. If multiple risk factors were reported for an individual patient these were apportioned proportionally to the individual risk factors.

Methods

Steps of analysis and data transformation processes

Cancer registry data went through multiple processing steps before integration with the COD database. First, the original data were transformed into standardized files, which included standardization of format, categorization, and registry names (#1 in flowchart).

Second, some cancer registries report individual codes as well as aggregated totals (e.g., C18, C19, and C20 are reported individually but the aggregated group of C18-C20 [colorectal cancer] is also reported in

the registry data). The data processing step “subtotal recalculation” (#2 in flowchart) verifies these totals and subtracts the values of any individual codes from the aggregates.

In the third step (#3 in the flowchart), cancer registry incidence data and cancer registry mortality data are mapped to GBD causes. A different map is used for incidence and for mortality data because of the assumption that there are no deaths for certain cancers. One example is basal cell carcinoma of the skin. In the cancer registry incidence data, basal cell carcinoma is mapped to non-melanoma skin cancer (basal cell carcinoma). However, if basal cell skin cancer is recorded in the cancer registry mortality data, the deaths are instead mapped to non-melanoma skin cancer (squamous cell carcinoma) under the assumption that they were indeed misclassified squamous cell skin cancers. Other examples are benign or in situ neoplasms. Benign or in situ neoplasms found in the cancer registry incidence dataset were simply dropped from that dataset. The same neoplasms reported in a cancer registry mortality dataset were mapped to the respective invasive cancer (e.g., melanoma in situ in the cancer registry incidence dataset was dropped from the dataset; melanoma in situ in the cancer registry mortality dataset was mapped to melanoma).

In the fourth data processing step (#4 in the flowchart) cancer registry data were standardized to the GBD age groups. Age-specific incidence rates were generated using CI5, SEER, and NORDCAN data, while age-specific mortality rates were generated from the CoD data through a method described in Part 2. Age-specific proportions were then generated by applying the age-specific rates to a given registry population that required age-splitting to produce the expected number of cases/deaths for that registry by age. The expected number of cases/deaths for each sex, age, and cancer were then normalized to 1, creating final, age-specific proportions. These proportions were then applied to the total number of cases/deaths by sex and cancer to get the age-specific number of cases/deaths.

In the rare case that the cancer registry only contained data for both sexes combined, the now-age-specific cases/deaths were split and re-assigned to separate sexes using the same weights that are used for the age-splitting process. Starting from the expected number of deaths, proportions were generated by sex for each age (e.g., if for ages 15 to 19 years old there are six expected deaths for males and four expected deaths for females, then 60% of the combined-sex deaths for ages 15-19 years would be assigned to males and the remaining 40% would be assigned to females).

In the fifth step (#5 in the flowchart) data for cause entries that are aggregates of GBD causes were redistributed. Examples of these aggregated causes include some registries reporting ICD10 codes C00-C14 together as, “lip, oral cavity, and pharyngeal cancer.” These groups were broken down into sub-causes that could be mapped to single GBD causes. In this example, those include lip and oral cavity cancer (C00-C08), nasopharyngeal cancer (C11), cancer of other parts of the pharynx (C09-C10, C12-C13), and “Malignant neoplasm of other and ill-defined sites in the lip, oral cavity, and pharynx” (C14). To redistribute the data, weights were created using the same “rate-applied-to-population” method employed in age-sex splitting (see step four above). For the undefined code (C14 in the example) an “average all cancer” weight was used, which was generated by adding all cases from SEER/NORDCAN/CI5 and dividing the total by the combined population. Then, proportions were generated by sub-cause for each aggregate cause as in the sex-splitting example above (see step four). The total number of cases from the aggregated group (C00-C14) was then recalculated for each

subgroup and the undefined code (C14). C14 was then redistributed as a “garbage code” in step six. Distinct proportions were used for C44 (non-melanoma skin cancer) and C46 (Kaposi’s sarcoma). Non-melanoma skin cancer processing is described under section “Input data and methodological summary for non-melanoma skin cancer (squamous-cell carcinoma).” C46 entries were redistributed as “other cancer,” HIV, and C80 (other and unknown cancers) using proportions described in Part 2.
In the sixth step (#6 in the flowchart) unspecified codes (“garbage codes”) were redistributed. Redistribution of cancer registry incidence and mortality data mirrored the process of the redistribution used in the cause of death database (Part 2).

In the seventh step (#7 in the flowchart) duplicate or redundant sources were removed from the processed cancer registry dataset. Duplicate sources were present if, for example, the cancer registry was part of the CI5 database but we also had data from the registry directly. Redundancies occurred and were removed as described in “Inclusion and Exclusion Criteria,” where more detailed data were available, or when national registry data could replace regionally representative data. From here, two parallel selection processes were run to generate input data for the MI models and to generate incidence for final mortality estimation. Higher priority was given to registry data from the most standardized source when creating the final incidence input, whereas for the MI model input only sources that reported incidence and mortality were used. This is different to GBD 2015 where mortality and incidence could come from different sources as long as they covered the same population.
In the eighth step (#8 in the flowchart) the processed incidence and mortality data from cancer registries were matched by cancer, age, sex, year, and location to generate MI ratios. These MI ratios were used as input for a three-step modelling approach using the general GBD ST-GPR approach with SDI as a covariate in the linear step mixed effects model using a logit link function. Predictions were made without the random effects. The ST-GPR model has three main hyper-parameters that control for smoothing across time, age, and geography. The time adjustment parameter (λ) was set to 2, which aims to borrow strength from neighboring time points (i.e. the exposure in this year is highly correlated with exposure in the previous year but less so further back in time). The age adjustment parameter ω was set to 0.5, which borrows strength from data in neighboring age groups. The space adjustment parameter ξ was set to 0.95 in locations with data and to 0.5 in locations without data (the higher ξ was applied when at least one age-sex group in the country of estimation had at least five unique data points. The lower ξ was applied when estimating data-scarce countries). Zeta aims to borrow strength across the hierarchy of geographical locations.[10] For the amplitude parameter in the Gaussian process regression we used 2 and for the scale we used a value of 15.

As in GBD 2015 we have modified the approach to estimate MI ratios. Since for GBD 2015 MI ratio predictions for some cancers yielded similar predictions for low-SDI countries without data as for high-SDI countries we refined the estimation process. Inclusion criteria for the MI ratio input data were changed to only include mortality and incidence data if they were reported by the same source. We excluded MI ratios reported in the CI5[1,1–7] since mortality data used for the calculation of these MI ratios by definition has to be independent from the cancer registry. We also revised the outliering process and excluded data based on the SDI quintile categorization rather than on development status. For each cancer, MI ratios from locations in SDI quintiles 1-4 (low to high-middle SDI) were dropped if they were below the median of MI ratios from locations in SDI quintile 5 (high SDI). We also dropped MI ratios from locations in SDI quintiles 1-4 if the MI ratios were above the third quartile + 1.5 * IQR (inter-quartile range). We dropped all MIR that were based on less than 25 cases to avoid noise due to small

numbers except for mesothelioma and acute lymphoid leukemia where we dropped MIR that were based on less than 10 cases because of lower data availability for these two cancers. We also aggregated incidence and mortality to the youngest 5-year age bin where we had at least 50 data points to avoid MIR predictions in young age groups that were based on few data points. The MIR in the age-bin that was used to aggregate MIR to, was used to backfill the MIR for younger age groups.

Since MI ratios can be above 1, especially in older age groups and cancers with low cure rates, we used the 95th percentile of the cleaned dataset that only included MIR that were based on 50 or more cases, to cap the MIR input data. This "upper cap" was used to allow MIR over 1 but to constrain the MIR to a maximum level. To run the logit model, the input data was divided by the upper caps and model predictions after ST-GPR was rescaled by multiplying them by the upper caps.
Upper caps used for GBD 2016 were the following:

Age group	Maximum MIR
0-4	0.57
5-9	0.69
10-14	0.81
15-19	0.84
20-24	0.72
25-29	0.62
30-34	0.69
35-39	0.78
40-44	0.86
45-49	0.89
50-54	0.92
55-59	0.95
60-64	0.99
65-69	1.04
70-74	1.10
75-79	1.17
80+	1.32

To constrain the model at the lower end, we used the 5th percentile of the cancer specific cleaned MIR input data to replace all model predictions with this lower cap.

Final MI ratios were matched with the cancer registry incidence dataset in the ninth step (#9 in the flowchart) to generate mortality estimates (Incidence * Mortality/Incidence = Mortality) (#10 in the flowchart). The final mortality estimates were then uploaded into the COD database (#11 in the flowchart). Cancer-specific mortality modelling then followed the general CODEm process.

Liver cancer etiology split models

The proportion data found through the systematic literature review were used as input for four separate DisMod-MR 2.1 models to determine the proportion of liver cancers due to the four subgroups for all locations, both sexes, and all age groups (step #16 in the flowchart). A study covariate was used for

publications that only assessed liver cancer in a cirrhotic population. The reference or “gold standard” that was used for crosswalking was the compilation of all studies that assessed the etiology of liver cancer in a general population. For liver cancer due to hepatitis C and hepatitis B, a prior value of 0 was set between age 0 and 0.01. For liver cancer due to alcohol a prior value of 0 was set for ages 0 to 5 years. For liver cancer due to hepatitis C, hepatitis C (IgG) seroprevalence was used as a covariate as well as a covariate for alcohol (liters per capita) and hepatitis B prevalence (HBsAg seroprevalence), forcing a negative relationship between the alcohol and hepatitis B covariate and the outcome of liver cancer due to hepatitis C proportion. For liver cancer due to hepatitis B, seroprevalence of HBsAg was used as a covariate as well as a covariate for alcohol and hepatitis C IgG seroprevalence, forcing a negative relationship between the alcohol and hepatitis C covariate and the outcome of liver cancer due to hepatitis B proportion. For liver cancer due to alcohol, alcohol (liters per capita) was used as a covariate as well as a covariate for proportion of alcohol abstainers, hepatitis B and hepatitis C seroprevalence, forcing a negative relationship between the proportion of alcohol abstainers, hepatitis B and hepatitis C covariates and the outcome of liver cancer due to alcohol proportion. All covariates used were modelled independently. To ensure consistency between cirrhosis and liver cancer estimates and to take advantage of the data for the respective other related cause (e.g. liver cancer due to hepatitis C and the related cause cirrhosis due to hepatitis C), we generated covariates from the liver cancer proportion models that we used in the cirrhosis etiology proportion models. We then created covariates from the cirrhosis etiology proportion models and used those in the liver cancer etiology models.

Since the proportion models are run independently of each other, the final proportion models were scaled to sum to 100% within each age, sex, year, and location, by dividing each proportion by the sum of the four (step # 17). For the liver cancer subtype mortality estimates, we multiplied the parent cause “liver cancer” by the corresponding scaled proportions (step # 18). Single cause estimates were adjusted to fit into the separately modelled all-cause mortality in the process CoDCorrect.

Results

Interpretation of results

Cancer mortality estimates for GBD 2016 can differ from the GBD 2015 results for multiple reasons. Updated cancer mortality data were added from vital registration system data, verbal autopsy studies, as well as cancer registry incidence data. Mapping of cancer ICD codes to the GBD cancer causes was updated slightly based on collaborator comments. Mapping for the ICD10 code D46 (myelodysplastic syndrome) was changed back to “other cancer” as it had been in GBD 2013 based on collaborator comments and the consideration of adding myelodysplastic syndrome as a separate cause for future GBD iterations. To improve estimation of the leukemia sub-causes, a new cause, “leukemia other” was added since not all leukemia subtypes can be mapped the four most common types (acute and chronic lymphoid and myeloid leukemia). The mortality-to-incidence ratio estimation has changed compared to GBD 2015. Covariate inputs for the CODEm models were changed based on recommendations from collaborators. Covariates used in CODEm models were updated for GBD 2016.

The other group producing country-level cancer mortality estimates is the International Agency for Research on Cancer (IARC) with their GLOBOCAN database. Significantly different methods between the GBD study and GLOBOCAN can lead to differences in results. Whereas estimates in GLOBOCAN are based on the assumption that there are “In theory, […] as many methods as countries,”[11] the cancer estimation process for the GBD study follows a coherent, well-documented method for all cancers,

which allows cross-validation of models as well as determination of uncertainty. Another major difference is the ability in the GBD study to adjust single cause estimates to the all-cause mortality, which is being determined independently. This also allows us to adjust individual causes of death to the all-cause mortality envelope which permits us to correct for the underdiagnosis of cancer in countries with inadequate diagnostic resources. Redistribution of a fraction of undefined causes of death to certain cancers is another methodical advantage the GBD study has over GLOBOCAN, and estimates for cancer mortality can therefore differ substantially in countries with a large proportion of undefined causes of deaths in their vital registration data or a large proportion of undefined cancer cases in their cancer registry data.

Limitations

There are certain limitations to consider when interpreting the GBD mortality cancer estimates. First, even though every effort is made to include the most recently available data for each country, data-seeking resources are not limitless and new data cannot always be accessed as soon as they are made available. It is therefore possible that the GBD study does not include all available data sources for cancer incidence or cancer mortality. Second, different redistribution methods can potentially change the cancer estimates substantially if the data sources used for the estimated location contain a large number of undefined causes; however, neglecting to account for these undefined deaths would likely introduce an even greater bias in the disease estimates. Third, using mortality-to-incidence ratios to transform cancer registry incidence data to mortality estimates requires accurate MIR. For GBD 2016 we have made further changes to the MIR estimation, but the method remains sensitive to underdiagnosis of cancer cases or underascertainment of cancer deaths. However, given that the majority of data used for the cancer mortality estimation come from vital registration data and not cancer registry data this is not a major limitation.

Non-melanoma skin cancer (squamous-cell carcinoma)

Data

Data seeking processes

The input data were identified and processed using the same methods as all other cancers described above.

Inclusion and exclusion criteria

Inclusion and exclusion criteria followed the same methods as described for other cancers (see above).

Bias of categories of input data

The potential biases of the input data are the same as for other cancers (see above).

Methods

Overall methodological process

The GBD produces estimates for non-melanoma skin cancer via two subgroups: non-melanoma skin cancer (basal cell carcinoma) and non-melanoma skin cancer (squamous cell carcinoma). While some cancer registries report non-melanoma skin cancer at the four- or five- digit level required to distinguish between the subtypes (eg, “C44.01” versus “C44.02”, “173.01” versus “173.02), most registries report these cancers at the three-digit level as “C44” or “173” (“Other and unspecified malignant neoplasm of skin”). Because of this, those incident cases that were reported at this three-digit level were split to

“basal cell carcinoma” and “squamous cell carcinoma” based on proportions reported by Karagas et al during the cause disaggregation step (step #5 in the flowchart).[12] Since mortality estimates are produced for squamous cell carcinoma under the assumption that basal cell carcinoma causes almost no deaths, all mortalities reported as “C44” or “173” were mapped to the “squamous cell carcinoma” GBD cause. Apart from this additional step for some incident cases, the remainder of the cancer registry processing was the same as for other cancers as described above.

Steps of analysis and data transformation processes

Non-melanoma skin cancer (squamous cell carcinoma) mortality estimation followed the same steps as the other cancers (see flowchart and description above) except for step #5 in the flowchart as described above.

Model selection

The modelling strategy for non-melanoma skin cancer (squamous cell carcinoma) followed the general CODEm process.

Model performance and sensitivity

The modelling performance and sensitivity for non-melanoma skin cancer (squamous cell carcinoma) mirrored that of the general CODEm process.

Uncertainty intervals

Uncertainty was determined using standard CODEm methodology.

Results

Interpretation of results

Non-melanoma skin cancer mortality estimates are not available from other sources. GLOBOCAN, for example, does not report deaths due to non-melanoma skin cancer. Even though the data availability for non-melanoma skin cancer is poor, the fact that it is the most common incident cancer with rates expected to rise makes it a necessity to include the disease in the GBD framework.

Limitations

Cancer registry data for non-melanoma skin cancer incidence have to be interpreted with caution due to a substantial amount of underreporting or rules that only the first non-melanoma skin cancer has to be registered. Many cancer registries therefore do not include non-melanoma skin cancers at all. For vital registration data we make the assumption that there are no deaths due to non-melanoma skin cancer (basal cell carcinoma), therefore all deaths attributed to basal cell carcinoma were included instead as squamous cell carcinoma.

References

1 Waterhouse J, Muir C, Shanmugaratnam K, Powell J. Cancer Incidence in Five Continents IV. Lyon: IARC, 1982.

2 Curado M, Edwards B, Shin H, *et al.* Cancer Incidence in Five Continents IX. Lyon: IARC, 2007 http://www.iarc.fr/en/publications/pdfs-online/epi/sp160/CI5vol9-A.pdf.

3 Muir C, Mack T, Powell J, Whelan S. Cancer Incidence in Five Continents V. Lyon: IARC, 1987.

4 Parkin D, Muir C, Whelan S, Gao Y, Ferlay J, Powell J. Cancer Incidence in Five Continents VI. Lyon: IARC, 1992.

5 Parkin D, Whelan S, Ferlay J, Raymond L, Young J. Cancer Incidence in Five Continents VII. Lyon: IARC, 1997.

6 Parkin D, Whelan S, Ferlay J, Teppo L, Thomas D. Cancer Incidence in Five Continents VIII. Lyon: IARC, 2002.

7 Forman D, Bray F, Brewster D, *et al.* Cancer Incidence in Five Continents X. 2013. http://ci5.iarc.fr.

8 Engholm G, Ferlay J, Christensen N, *et al.* NORDCAN: Cancer Incidence, Mortality, Prevalence and Survival in the Nordic Countries, Version 7.3 Association of the Nordic Cancer Registries. Danish Cancer Society. 2016; published online Aug 7. http://www.ancr.nu.

9 Steliarova-Foucher E, O'Callaghan M, Ferlay J, Masuyer E, Forman D, Comber H, Bray F. European Cancer Observatory: Cancer Incidence, Mortality, Prevalence and Survival in Europe. Version 1.0 European Network of Cancer Registries, International Agency for Research on Cancer. 2012; published online Sept. http://eco.iarc.fr.

10 GBD 2015 Risk Factors Collaborators. Global, regional, and national comparative risk assessment of 79 behavioural, environmental and occupational, and metabolic risks or clusters of risks, 1990–2015: a systematic analysis for the Global Burden of Disease Study 2015. *The Lancet* 2016; **388**: 1659–724.

11 International Agency for Research on Cancer, World Health Organization. GLOBOCAN estimated cancer incidence, mortality, and prevalence worldwide in 2012. Lyon, France: IARC, 2014 http://globocan.iarc.fr/Default.aspx (accessed April 19, 2016).

12 Karagas MR, Greenberg ER, Spencer SK, Stukel TA, Mott LA. Increase in incidence rates of basal cell and squamous cell skin cancer in New Hampshire, USA. New Hampshire Skin Cancer Study Group. *Int J Cancer* 1999; **81**: 555–9.

Cardiovascular Diseases

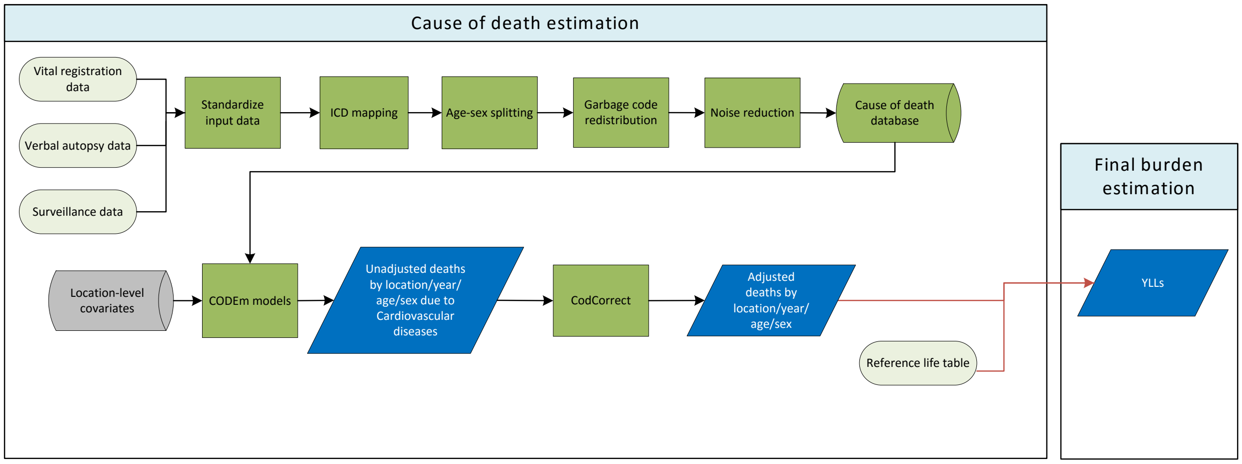

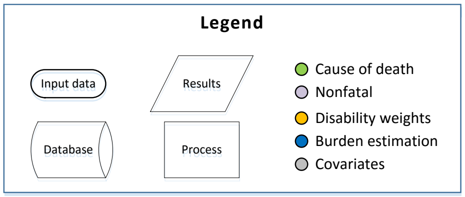

Input data

Vital registration, verbal autopsy, and surveillance data were used to model this cause. We outliered non-representative subnational verbal autopsies from a number of Indian states. We also outliered verbal autopsy data sources that were implausibly low in all age groups and ICD8 and ICD9 BTL data points that were inconsistent with the rest of the data and created implausible time trends.

Modelling strategy

We used a standard CODEm approach to model deaths from cardiovascular diseases. We have updated the covariates included in the ensemble modelling process (see Table). Otherwise, there have been no substantive changes from the approach used in GBD 2015.

Table: Selected covariates for CODEm models, cardiovascular diseases

Covariate	Transformation	Level	Direction
Summary exposure variable	None	1	1
Cholesterol (total, mean per capita)	None	1	1
Smoking prevalence	None	1	1
Systolic blood pressure (mmHg)	None	1	1
Trans fatty acid	None	1	1
Mean BMI	None	2	1
Elevation over 1500m (proportion)	None	2	-1
Fasting plasma glucose (mmol/L)	None	2	1
Outdoor pollution ($PM_{2.5}$)	None	2	1
Indoor air pollution (all fuel types)	None	2	1
Healthcare access and quality index	None	2	-1
Lag distributed income per capita (I$)	Log	3	-1
Socio-demographic Index	None	3	0
Omega-3 (kcal/capita, adjusted)	Log	3	-1
Fruits (kcal/capita, adjusted)	None	3	-1
Vegetables (kcal/capita, adjusted)	None	3	-1
Nuts and seeds (kcal/capita, adjusted)	None	3	-1
Whole grains (kcal/capita, adjusted)	None	3	-1
Pulses/legumes (kcal/capita, adjusted)	None	3	-1
PUFA adjusted (percent)	None	3	-1
Alcohol (litres per capita)	None	3	0

Rheumatic Heart Disease

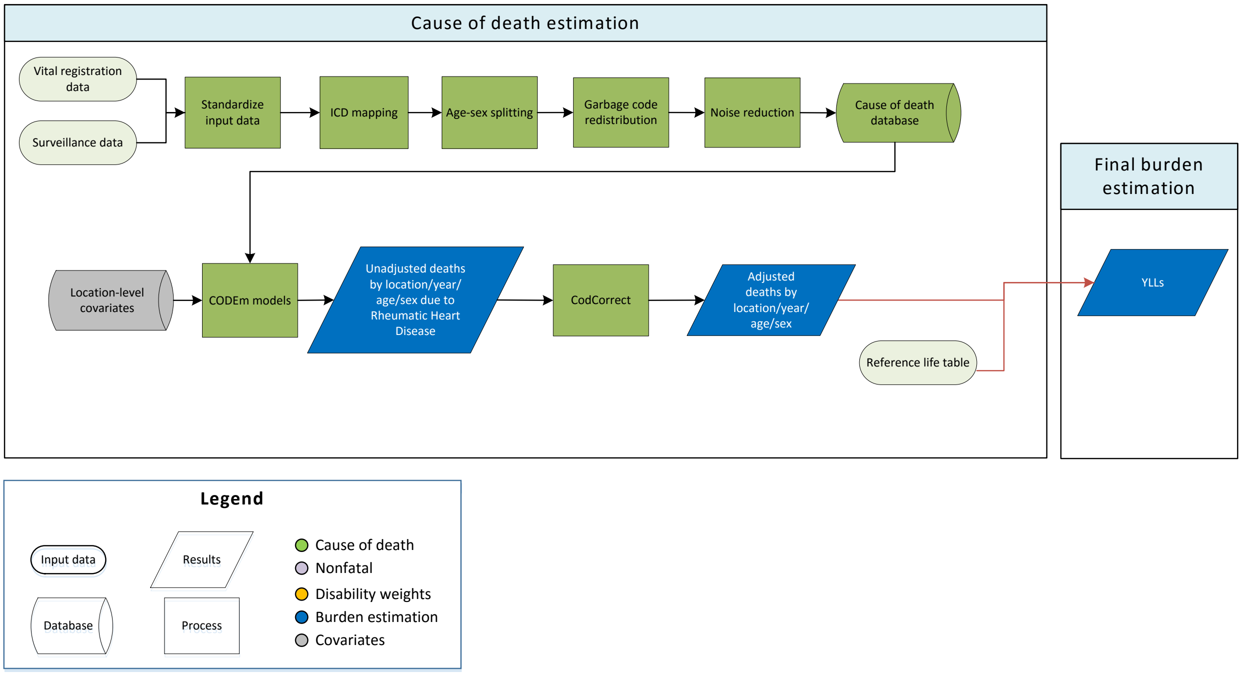

Input data

Vital registration and surveillance data were used to model rheumatic heart disease. We outliered ICD8 and ICD9 BTL data points which were inconsistent with the rest of the data and created implausible time trends. We also outliered data points which were too high after the redistribution process in a number of age groups.

Modelling strategy

We used a standard CODEm approach to model deaths from rheumatic heart disease. We have updated the covariates included in the ensemble modelling process (see Table). Otherwise, there have been no substantive changes from the approach used in GBD 2015.

Table: Selected covariates for CODEm models, rheumatic heart disease

Covariate	Transformation	Level	Direction
SEV	None	1	1
Improved water (proportion)	None	1	-1
Malnutrition	None	1	1
Sanitation (proportion with access)	None	1	-1
Healthcare access and quality index	None	2	-1
LDI	Log	3	-1
SDI	None	3	-1
Education (years per capita)	None	3	-1

Ischemic Heart Disease

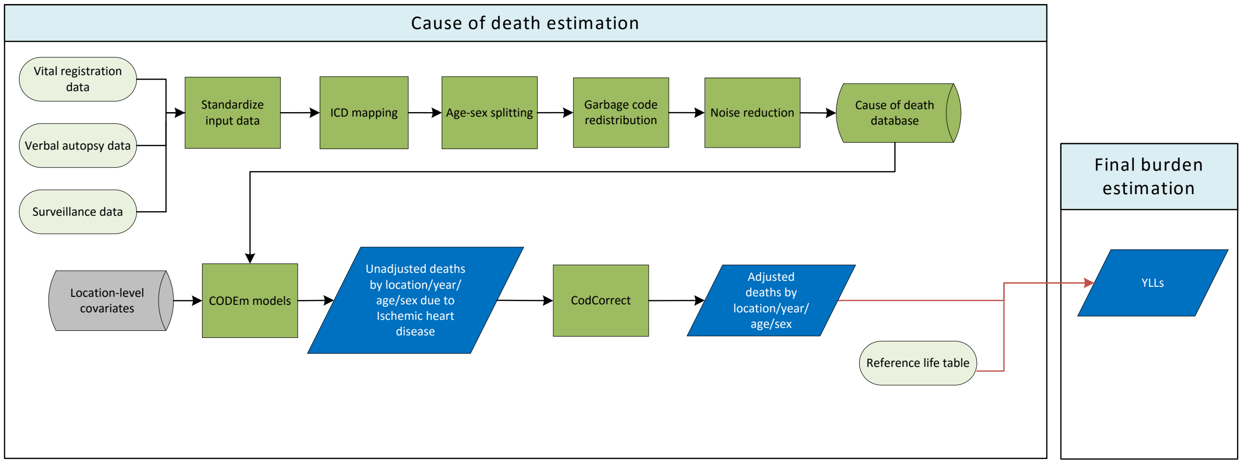

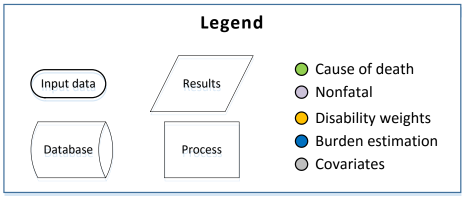

Input data

Vital registration, verbal autopsy, and surveillance data were used to model ischemic heart disease. We outliered verbal autopsy data in countries and subnational locations where high-quality vital registration data were also available. We also outliered non-representative subnational verbal autopsy data points, ICD8 and ICD9 BTL data points which were inconsistent with the rest of the data and created implausible time trends, and data in a number of Indian states identified by experts as poor-quality.

Modelling strategy

We used a standard CODEm approach to model deaths from ischemic heart disease. We have updated the covariates included in the ensemble modelling process (see Table). Otherwise, there have been no substantive changes from the approach used in GBD 2015.

Table: Selected covariates for CODEm models, ischemic heart disease

Covariate	Transformation	Level	Direction
Summary exposure variable	None	1	1
Cholesterol (total, mean per capita)	None	1	1
Smoking prevalence	None	1	1
Systolic blood pressure (mmHg)	None	1	1
Trans fatty acid	None	1	1
Mean BMI	None	2	1
Elevation over 1500m (proportion)	None	2	-1
Fasting plasma glucose	None	2	1
Outdoor pollution ($PM_{2.5}$)	None	2	1
Indoor air pollution	None	2	1
Healthcare access and quality index	None	2	-1
Lag distributed income per capita (I$)	Log	3	-1
Socio-demographic Index	None	3	0
Omega-3 (kcal/capita, adjusted)	Log	3	-1
Fruits (kcal/capita, adjusted)	None	3	-1
Vegetables (kcal/capita, adjusted)	None	3	-1
Nuts and seeds (kcal/capita, adjusted)	None	3	-1
Whole grains (kcal/capita, adjusted)	None	3	-1
Pulses/legumes (kcal/capita, adjusted)	None	3	-1
PUFA adjusted (percent)	None	3	-1
Alcohol (litres per capita)	None	3	0

Cerebrovascular Disease

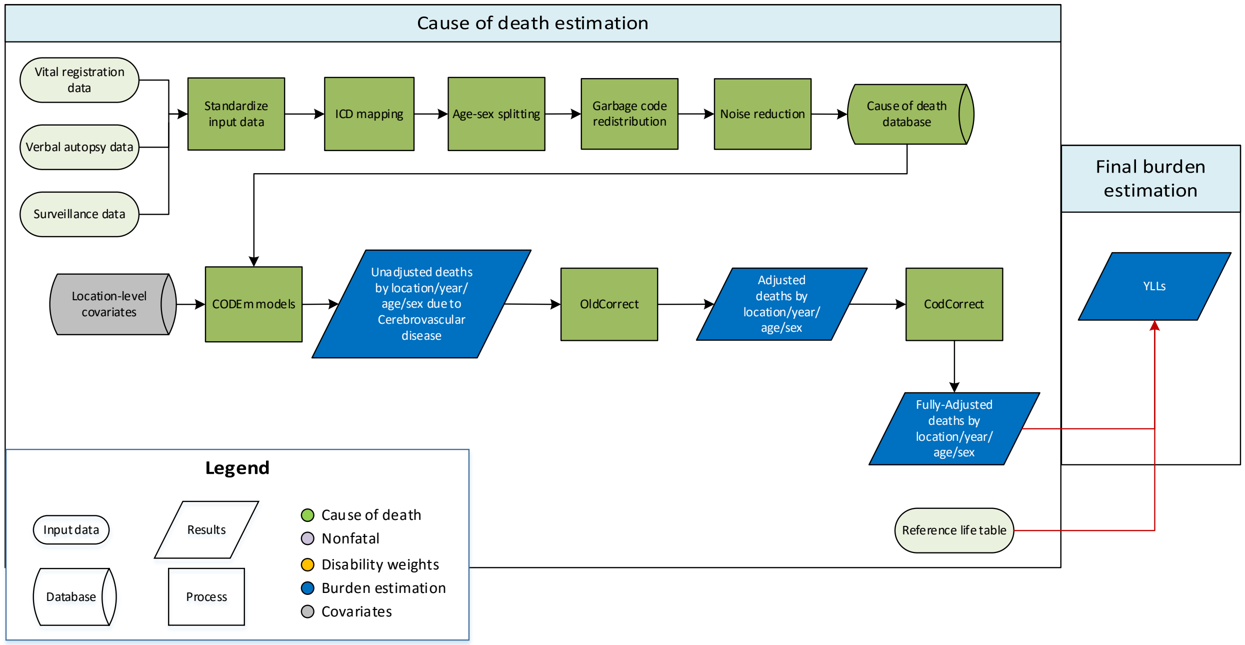

Input data

Verbal autopsy and vital registration data were used to model cerebrovascular disease. We outliered non-representative subnational verbal autopsy data points. We reassigned deaths from verbal autopsy reports for cerebrovascular disease to the parent cardiovascular disease for both sexes for those under 20 years of age. We also outliered ICD8, ICD9 BTL, and ICD10 Tabulated data points which were inconsistent with the rest of the data and created implausible time trends. Data points from sources which were implausibly low in all age groups and data points that were causing the regional estimates to be improbably high were outliered.

Modelling strategy

We used a standard CODEm approach to model deaths from cerebrovascular disease. The most significant update to the cerebrovascular method was the addition of a correction for miscoding of Alzheimer and other dementias and Parkinson disease to the post-CODEm adjustments to generate corrected cause-specific death estimates for final burden estimation. We have also updated the covariates included in the ensemble modelling process (see Table). Otherwise, there have been no substantive changes from the approach used in GBD 2015.

Table: Selected covariates for CODEm models, cerebrovascular disease

Covariate	Transformation	Level	Direction
Summary exposure variable	None	1	1
Cholesterol (total, mean per capita)	None	1	1
Smoking prevalence	None	1	1
Systolic blood pressure (mmHg)	None	1	1
Trans fatty acid	None	1	1
Mean BMI	None	2	1
Elevation over 1500m (proportion)	None	2	-1
Fasting plasma glucose	None	2	1
Outdoor pollution ($PM_{2.5}$)	None	2	1
Indoor air pollution	None	2	1
Healthcare access and quality index	None	2	-1
Lag distributed income per capita (I$)	Log	3	-1
Socio-demographic Index	None	3	0
Omega-3 (kcal/capita, adjusted)	Log	3	-1
Fruits (kcal/capita, adjusted)	None	3	-1
Vegetables (kcal/capita, adjusted)	None	3	-1
Nuts and seeds (kcal/capita, adjusted)	None	3	-1
Whole grains (kcal/capita, adjusted)	None	3	-1
Pulses/legumes (kcal/capita, adjusted)	None	3	-1
PUFA adjusted (percent)	None	3	-1
Alcohol (litres per capita)	None	3	0

Ischemic Stroke

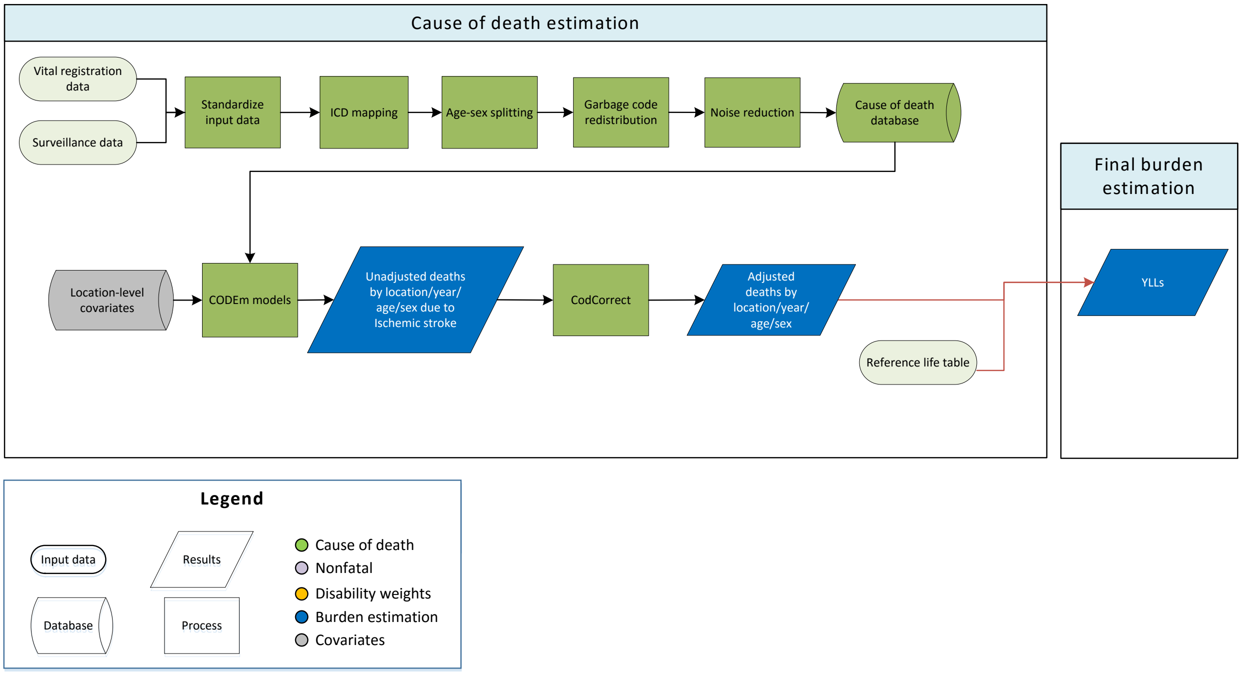

Input data

Vital registration and surveillance data were used to model ischemic stroke. We reassigned deaths from verbal autopsy reports for ischemic stroke to the parent cardiovascular disease for both sexes for those under 20 years of age. We outliered ICD8 data points which were inconsistent with the rest of the data and created implausible time trends.

Modelling strategy

We used a standard CODEm approach to model deaths from ischemic stroke. In locations with limited data on ischemic stroke, the subtype-specific deaths were estimated by squeezing both ischemic and hemorrhagic stroke to the overall cerebrovascular envelope. We have updated the covariates included in the ensemble modelling process (see Table). Otherwise, there have been no substantive changes from the approach used in GBD 2015.

Table: Selected covariates for CODEm models, ischemic stroke

Covariate	Transformation	Level	Direction
Summary exposure variable	None	1	1
Cholesterol (total, mean per capita)	None	1	1
Smoking prevalence	None	1	1
Systolic blood pressure (mmHg)	None	1	1
Trans fatty acid	None	1	1
Mean BMI	None	2	1
Elevation over 1500m (proportion)	None	2	-1
Fasting plasma glucose	None	2	1
Outdoor pollution ($PM_{2.5}$)	None	2	1
Indoor air pollution	None	2	1
Healthcare access and quality index	None	2	-1
Lag distributed income per capita (I$)	Log	3	-1
Socio-demographic Index	None	3	0
Omega-3 (kcal/capita, adjusted)	Log	3	-1
Fruits (kcal/capita, adjusted)	None	3	-1
Vegetables (kcal/capita, adjusted)	None	3	-1
Nuts and seeds (kcal/capita, adjusted)	None	3	-1
Whole grains (kcal/capita, adjusted)	None	3	-1
Pulses/legumes (kcal/capita, adjusted)	None	3	-1
PUFA adjusted (percent)	None	3	-1
Alcohol (litres per capita)	None	3	0

Hemorrhagic Stroke

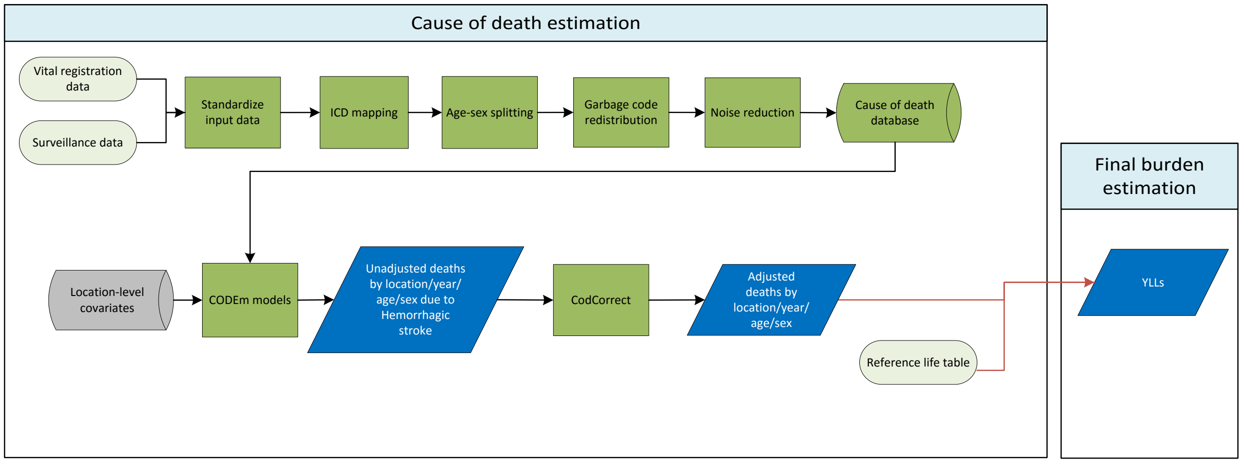

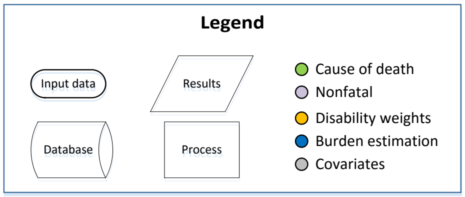

Input data

Vital registration and surveillance data were used to model hemorrhagic stroke. We reassigned deaths from verbal autopsy reports for hemorrhagic stroke to the parent cardiovascular disease for both sexes for those under 20 years of age. We outliered ICD8 data points which were inconsistent with the rest of the data and created implausible time trends.

Modelling strategy

We used a standard CODEm approach to model deaths from hemorrhagic stroke. In locations with limited data on hemorrhagic stroke, the subtype-specific deaths were estimated by squeezing both ischemic and hemorrhagic stroke to the overall cerebrovascular envelope. We have updated the covariates included in the ensemble modelling process (see Table). Otherwise, there have been no substantive changes from the approach used in GBD 2015.

Table: Selected covariates for CODEm models, hemorrhagic stroke

Covariate	Transformation	Level	Direction
Summary exposure variable	None	1	1
Cholesterol (total, mean per capita)	None	1	0
Smoking prevalence	None	1	1
Systolic blood pressure (mmHg)	None	1	1
Trans fatty acid	None	1	1
Mean BMI	None	2	1
Elevation over 1500m (proportion)	None	2	-1
Fasting plasma glucose	None	2	1
Outdoor pollution ($PM_{2.5}$)	None	2	1
Indoor air pollution	None	2	1
Healthcare access and quality index	None	2	-1
Lag distributed income per capita (I$)	Log	3	-1
Socio-demographic index	None	3	0
Omega-3 (kcal/capita, adjusted)	Log	3	-1
Fruits (kcal/capita, adjusted)	None	3	-1
Vegetables (kcal/capita, adjusted)	None	3	-1
Nuts and seeds (kcal/capita, adjusted)	None	3	-1
Whole grains (kcal/capita, adjusted)	None	3	-1
Pulses/legumes (kcal/capita, adjusted)	None	3	-1
PUFA adjusted (percent)	None	3	-1
Alcohol (litres per capita)	None	3	0

Hypertensive Heart Disease

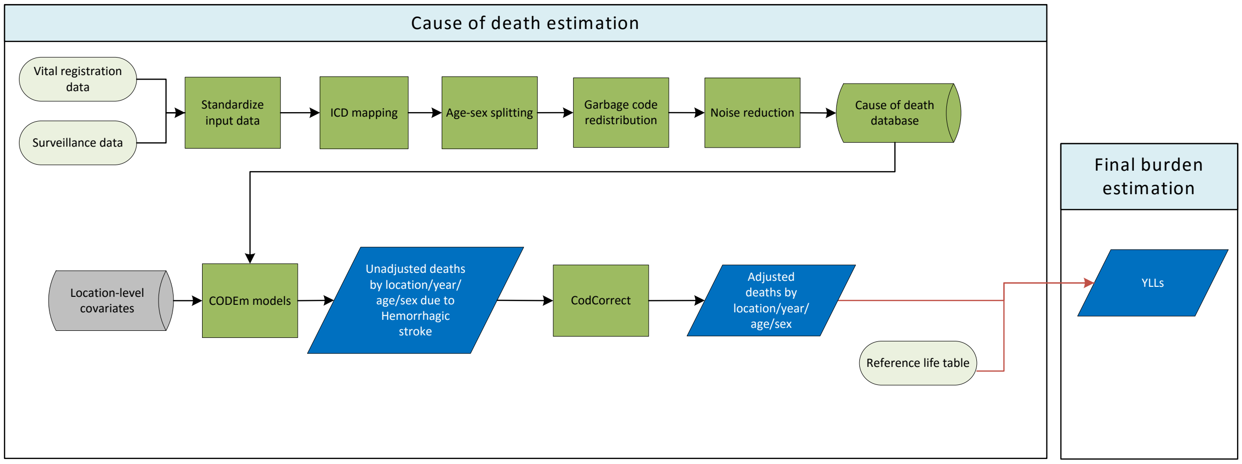

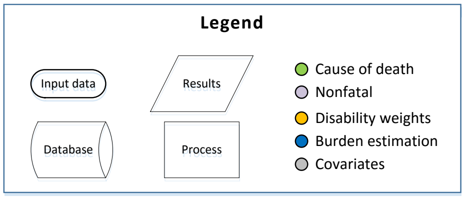

Input data

Vital registration and surveillance data were used to model hypertensive heart disease. We outliered ICD9 BTL data points, which were inconsistent with the rest of the data and created implausible time trends.

Modelling strategy

We used a standard CODEm approach to model deaths from cardiovascular diseases. We have updated the covariates included in the ensemble modelling process (see Table). Otherwise, there have been no substantive changes from the approach used in GBD 2015.

Table: Selected covariates for CODEm models, hypertensive heart disease

Covariate	Transformation	Level	Direction
Cholesterol (total, mean per capita)	None	1	1
Smoking prevalence	None	1	1
Systolic blood pressure (mmHg)	None	1	1
Mean BMI	None	2	1
Healthcare access and quality index	None	2	-1
Lag distributed income per capita (I$)	Log	3	-1
Socio-demographic index	None	3	0

Myocarditis

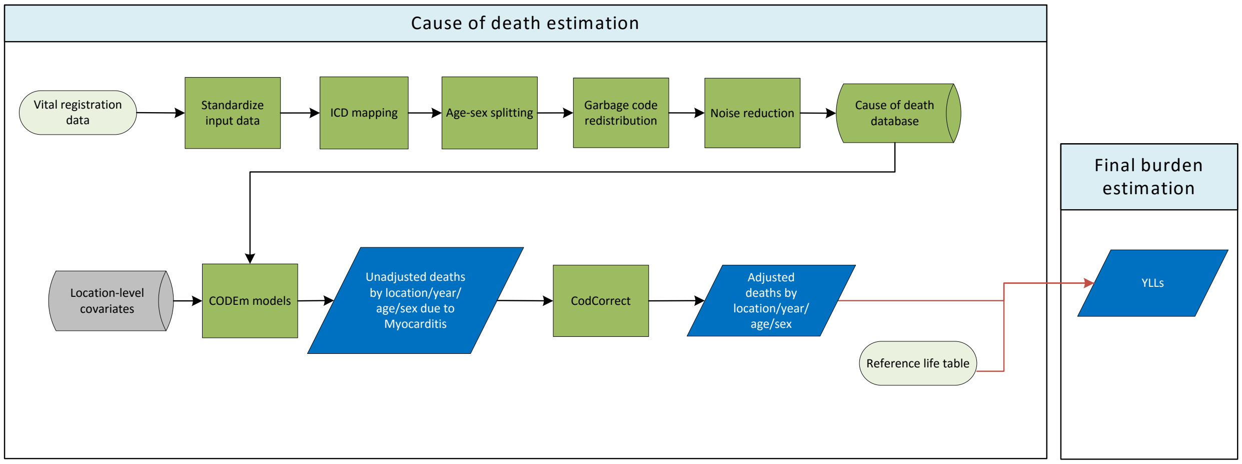

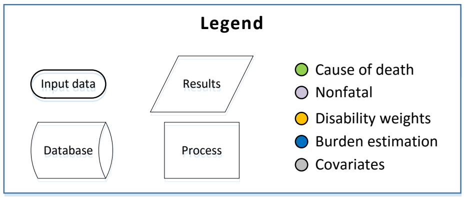

Input data

Vital registration data were used to model deaths due to myocarditis.

Modelling strategy

We used a standard CODEm approach to model deaths from myocarditis. This is one of three new sub-causes under the cardiomyopathy and myocarditis parent cause for GBD 2016. The covariates selected for inclusion in the CODEm modelling process can be found in the table below.

Table: Selected covariates for CODEm models, myocarditis

Covariate	Transformation	Level	Direction
Summary exposure variable, CMP	none	1	1
Systolic blood pressure (mm Hg)	none	1	1
Healthcare access and quality index	none	2	-1
Lag distributed income per capita (I$)	log	3	0
Socio-demographic Index	none	3	0

Alcoholic Cardiomyopathy

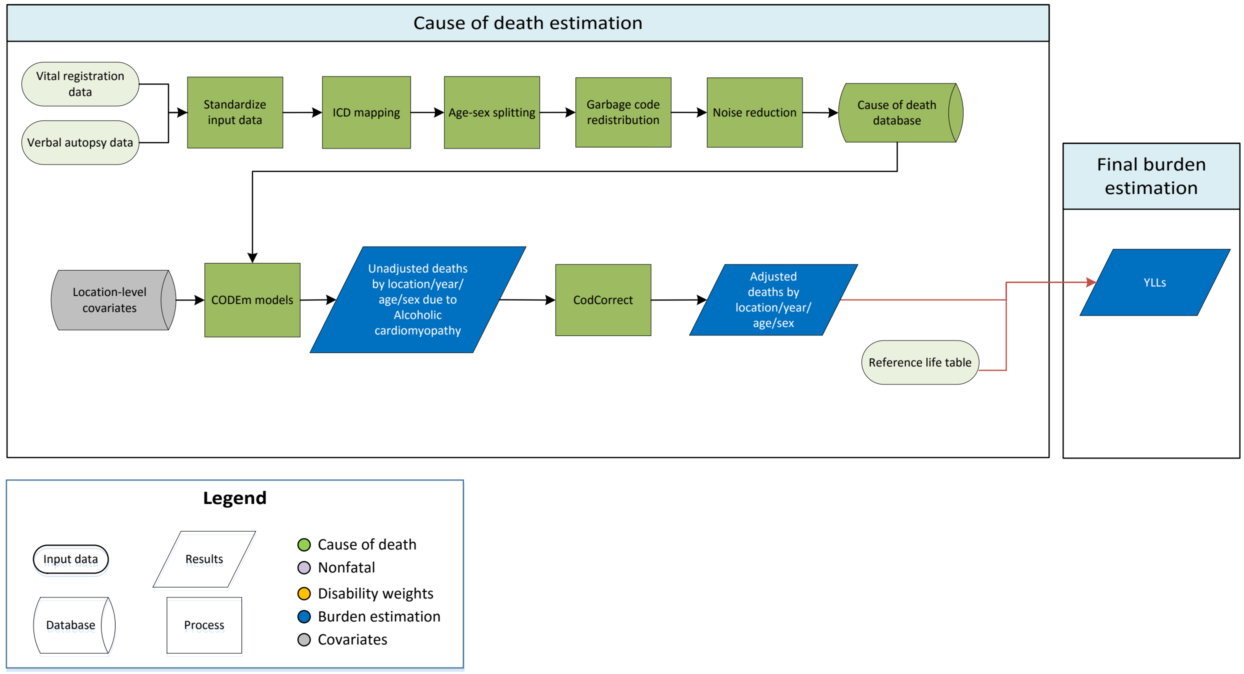

Input data

Vital registration and verbal autopsy data were used to model deaths due to alcoholic cardiomyopathy. We outliered ICD9 data points in Cyprus that were implausibly high and discontinuous with the rest of the time series.

Modelling strategy

We used a standard CODEm approach to model deaths from alcoholic cardiomyopathy. This is one of three new sub-causes under the cardiomyopathy and myocarditis parent cause for GBD 2016. The covariates selected for inclusion in the CODEm modelling process can be found in the table below. As local differences in coding practices may explain some of the geographic variation that we see for deaths due to cardiomyopathy and myocarditis, we plan to explore how this issue may affect the alcoholic cardiomyopathy sub-cause further in future iterations of GBD.

Table: Selected covariates for CODEm models, alcoholic cardiomyopathy

Covariate	Transformation	Level	Direction
Summary exposure variable, CMP	none	1	1
Smoking prevalence	none	1	1
Alcohol (litres per capita)	none	1	1
Healthcare access and quality index	none	2	-1
Lag distributed income per capita (I$)	log	3	0
Socio-demographic Index	none	3	0

Other cardiomyopathy

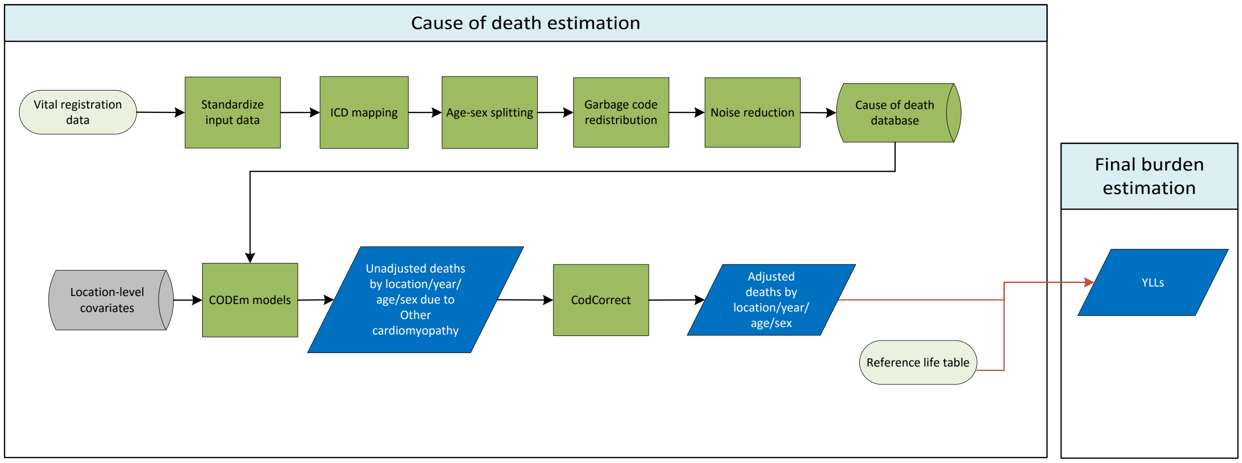

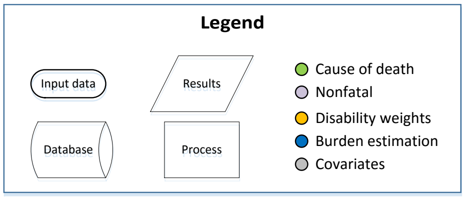

Input data

Vital registration data were used to model deaths due to other cardiomyopathy. We outliered data points in Central Asia and Central and Eastern Europe due to implausibly high values which we attributed to variation in local coding practices after review with experts.

Modelling strategy

We used a standard CODEm approach to model deaths from other cardiomyopathy. This is one of three new sub-causes under the cardiomyopathy and myocarditis parent cause for GBD 2016. The covariates selected for inclusion in the CODEm modelling process can be found in the table below. As local differences in coding practices may explain some of the geographic variation that we see for deaths due to cardiomyopathy and myocarditis, we plan to explore how this issue may affect the other cardiomyopathy sub-cause further in future iterations of GBD.

Table: Selected covariates for CODEm models, other cardiomyopathy

Covariate	Transformation	Level	Direction
Summary exposure variable, CMP	none	1	1
Systolic blood pressure (mmHg)	none	1	1
Smoking prevalence	none	1	1
Body mass index (kg/m^2)	none	2	1
Healthcare access and quality index	none	2	-1
Lag distributed income per capita (I$)	log	3	0
Socio-demographic Index	none	3	0

Atrial Fibrillation and Flutter

Atrial Fibrillation and Flutter

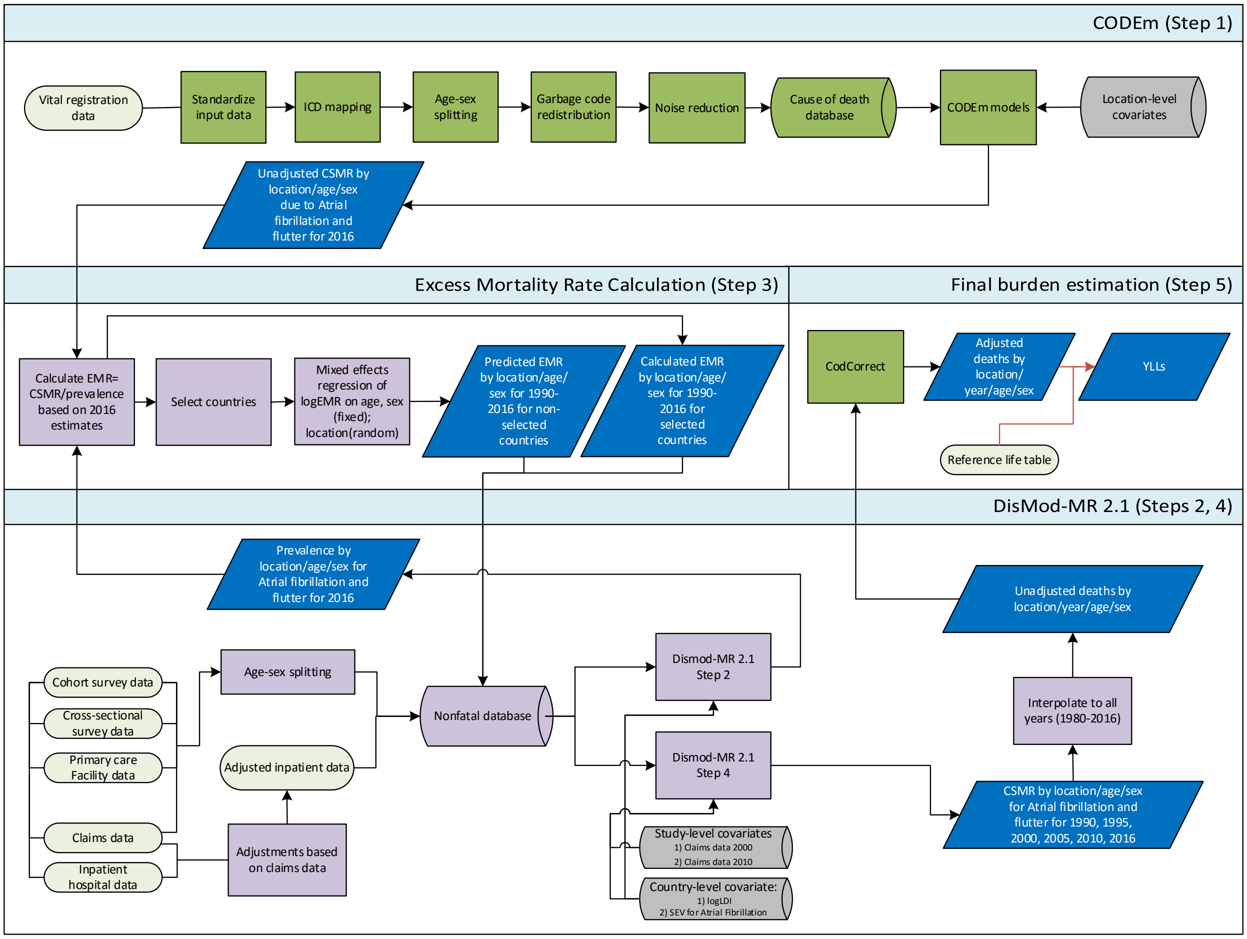

Input data

Vital registration data: We outliered ICD8 and ICD9 data points that were discontinuous from other data in the time series and created an unlikely time trend. We also outliered data points that were implausibly low in multiple age groups.

Modelling strategy

In order to address changes in coding practices for atrial fibrillation, we used an integrated approach that combined DisMod-MR and CODEm models to estimate deaths from atrial fibrillation and flutter. This approach allowed us to adjust estimates to more accurately reflect the number of deaths for which atrial fibrillation was the true underlying cause of death.

The modelling steps are illustrated in the above flowchart. Covariates included in both the DisMod-MR 2.1 and CODEm models can be found in the table below. In Step 1, we estimated deaths for atrial fibrillation using a standard CODEm approach. In Step 2, we estimated prevalence rates in DisMod-MR 2.1 using data from published reports of cross-sectional and cohort surveys, as well as primary care facility data. We also used claims data covering inpatient and outpatient visits for the United States

along with inpatient hospital data from 163 locations in 15 countries. For GBD 2016, inpatient hospital data were adjusted using age- and sex-specific information from US claims data for: 1) readmission within one year; 2) primary diagnosis code to secondary codes; and, 3) the ratio of inpatient to outpatient visits. We set priors of no remission and no excess mortality prior to age 30.

In Step 3, we calculated the excess mortality rate (EMR) for 2016 (defined as the cause-specific mortality rate (CSMR) estimated from CODEm divided by the prevalence rate from DisMod-MR 2.1). We then selected 17 countries based on four conditions: 1) ranking of 4 or 5 stars on the newly developed system for assessing the quality of VR data; 2) prevalence data available from the literature was included in the DisMod-MR 2.1 estimation; 3) prevalence rate ≥ 0.005; and, 4) CSMR ≥ 0.00002. Using information from these countries as input data, we ran a linear mixed-effects regression of logEMR on sex, age, and location. Sex and age were treated as fixed effects for the regression, while location was considered a random effect. We then predicted age- and sex-specific EMR using the results of this regression for all non-selected countries. Countries included in the regression were assigned their directly calculated values. These EMR data points were assigned to the time period 1990–2016 and uploaded into the nonfatal database in order to be used in modelling.

In Step 4, we reran DisMod-MR 2.1 including the EMR estimated in Step 3 as input data using the same priors as in Step 2. The CSMR from the DisMod-MR model in Step 4 was used as the finalized output. As DisMod-MR 2.1 only generates estimates for six years (1990, 1995, 2000, 2005, 2010, 2016), we interpolated the missing years to generate death estimates for all years (1980–2016). These results were then uploaded into the Cause of Death database. Finally, in Step 5, the unadjusted death estimates were run through the CoDCorrect process to generate adjusted deaths, and YLLs were generated by the DALYnator using a standard reference life table.

CODEm Covariates

Covariate	Transformation	Level	Direction
Summary exposure variable	None	1	1
Cholesterol (total, mean per capita)	None	1	1
Smoking prevalence	None	1	1
Systolic blood pressure (mmHg)	None	1	1
Mean BMI	None	2	1
Elevation over 1500m (proportion)	None	2	-1
Fasting plasma glucose	None	2	1
Outdoor pollution ($PM_{2.5}$)	None	2	1
Indoor air pollution	None	2	1
Healthcare Access and Quality Index	None	2	-1
Lag distributed income per capita (I$)	Log	3	-1
Socio-demographic Index	None	3	0
Omega-3 (kcal/capita, adjusted)	Log	3	-1
Fruits (kcal/capita, adjusted)	None	3	-1
Vegetables (kcal/capita, adjusted)	None	3	-1
Nuts and seeds (kcal/capita, adjusted)	None	3	-1
Whole grains (kcal/capita, adjusted)	None	3	-1
Pulses/legumes (kcal/capita, adjusted)	None	3	-1
PUFA adjusted (percent)	None	3	-1
Alcohol (litres per capita)	None	3	0
Trans fatty acid	None	1	1

DisMod Covariates – Step 2

Study covariate	Parameter	Beta	Exponentiated beta
Hospital data	Prevalence	-0.000086 (-0.19 – 0.097)	1.0 (0.82 – 1.10)
All MarketScan, year 2000	Prevalence	-0.47 (-0.5 – -0.44)	0.63 (0.61 – 0.64)
All MarketScan, year 2010	Prevalence	-0.003 (-0.024 – -0.014)	1.0 (0.98 – 1.01)
Log-transformed age-standardized SEV scalar: A Fib	Prevalence	0.75 (0.75 – 0.75)	2.12 (2.12 – 2.12)
LDI (I$ per capita)	Excess mortality rate	-0.48 (-0.5 – -0.43)	0.62 (0.61 – 0.65)

DisMod Covariates – Step 4

Study covariate	Parameter	Beta	Exponentiated beta
All MarketScan, year 2000	Prevalence	-0.46 (-0.49 – -0.43)	0.63 (0.62 – 0.65)
All MarketScan, year 2010	Prevalence	-0.0021 (-0.025 – -0.021)	1.0 (0.98 – 1.02)
Log-transformed age-standardized SEV scalar: A Fib	Prevalence	0.75 (0.75 – 0.75)	2.12 (2.12 – 2.12)
LDI (I$ per capita)	Excess mortality rate	-0.1 (-0.1 – -0.1)	0.9 (0.9 – 0.9)

Aortic Aneurysm

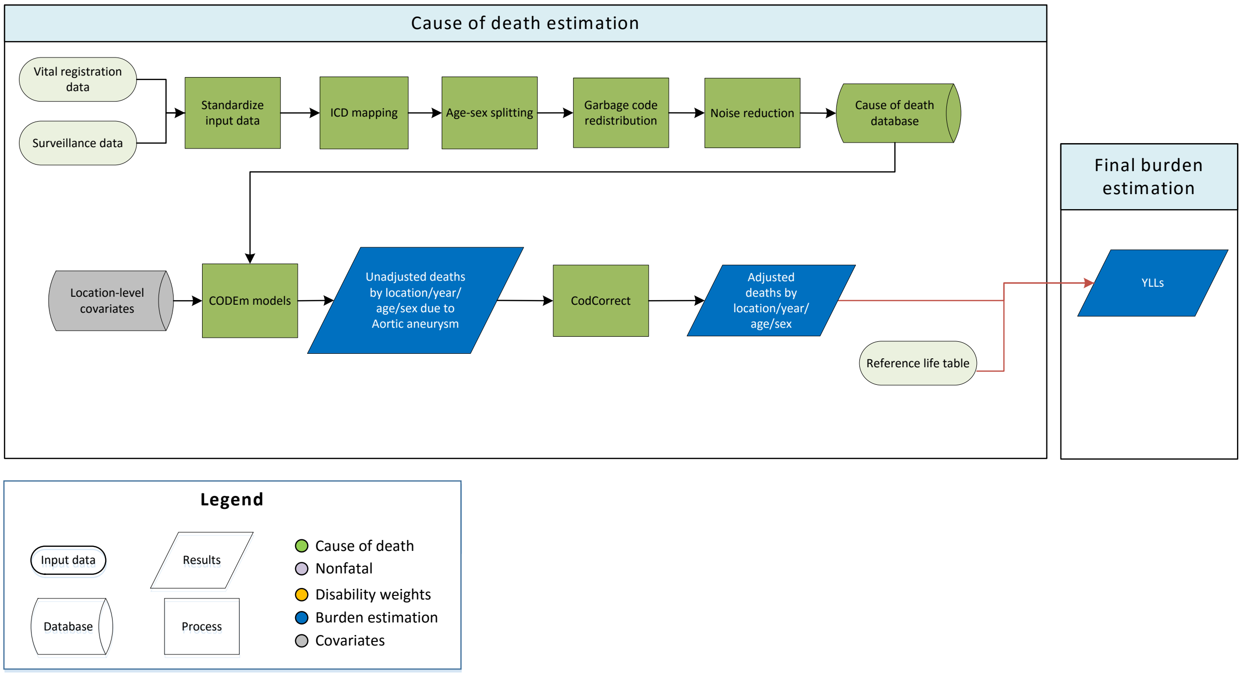

Input data

Vital registration and surveillance data were used to model this cause. We outliered data in Oman as they were improbably high in comparison with the rest of the region. We also outliered ICD8 data that were discontinuous with the rest of the time series and created implausible time trends.

Modelling strategy

We used a standard CODEm approach to model deaths from cardiovascular diseases. We have updated the covariates included in the ensemble modelling process (see Table). Otherwise, there have been no substantive changes from the approach used in GBD 2015.

Table: Selected covariates for CODEm models, cardiovascular diseases

Covariate	Transformation	Level	Direction
Summary exposure variable	None	1	1
Cholesterol (total, mean per capita)	None	1	1
Cumulative cigarettes (10 yrs)	None	1	1
Systolic blood pressure (mmHg)	None	1	1
Trans fatty acid (percent)	None	1	1
Mean BMI	None	2	1
Healthcare access and quality index	None	2	-1
Lag distributed income per capita (I$)	Log	3	-1
Socio-demographic Index	None	3	0
Omega-3 (kcal/capita, adjusted)	Log	3	-1
Fruits (kcal/capita, adjusted)	None	3	-1
Vegetables (kcal/capita, adjusted)	None	3	-1
Nuts and seeds (kcal/capita, adjusted)	None	3	-1
Whole grains (kcal/capita, adjusted)	None	3	-1
Pulses/legumes (kcal/capita, adjusted)	None	3	-1
PUFA adjusted (percent)	None	3	-1
Alcohol (litres per capita)	None	3	0

Peripheral Artery Disease

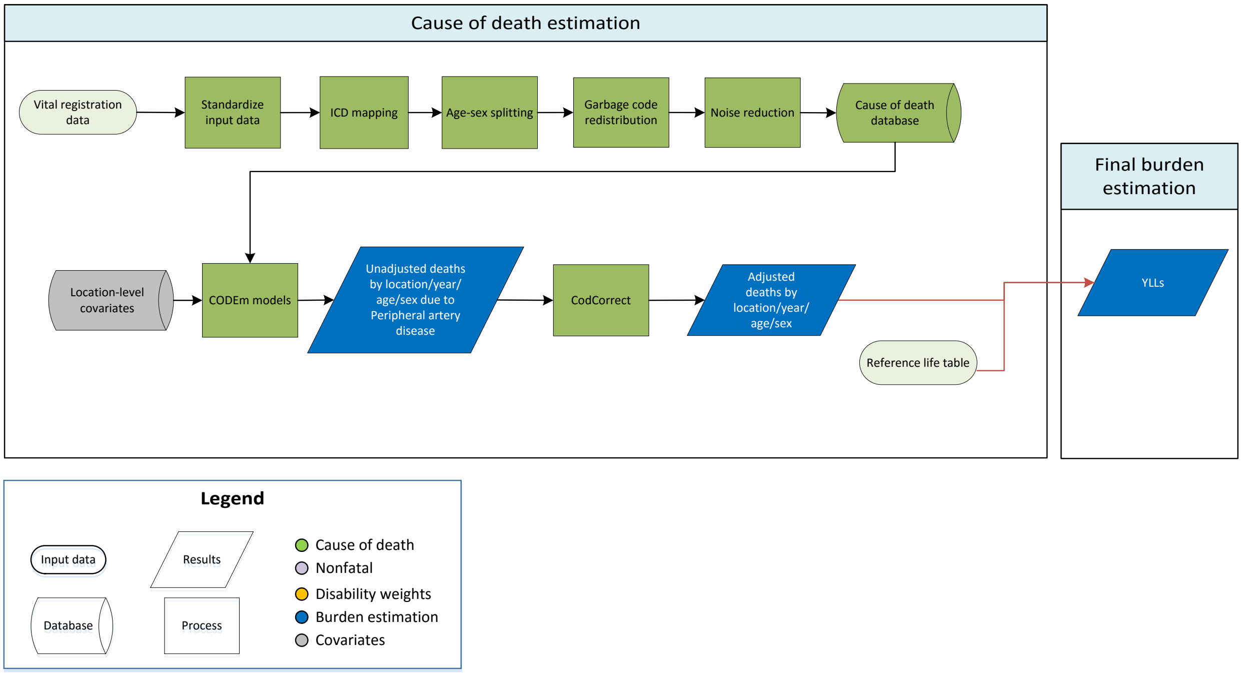

Input data

Vital registration data were used to model peripheral artery disease. We outliered all data points with <1 death in Egypt per expert review.

Modelling strategy

We used a standard CODEm approach to model deaths from peripheral artery disease. We have updated the covariates included in the ensemble modelling process (see Table). Otherwise, there have been no substantive changes from the approach used in GBD 2015.

Table: Selected covariates for CODEm models, peripheral artery disease

Covariate	Transformation	Level	Direction
Summary exposure variable	None	1	1
Systolic blood pressure (mmHg)	None	1	1
Cholesterol (total, mean per capita)	None	1	1
Smoking prevalence	None	1	1
Mean body mass index (kg/m^2)	None	2	1
Healthcare access and quality index	None	2	-1
Lag distributed income per capita (I$)	Log	3	-1
Socio-demographic Index	None	3	0
Omega-3 (kcal/capita, adjusted)	Log	3	-1
Fruits (kcal/capita, adjusted)	None	3	-1
Vegetables (kcal/capita, adjusted)	None	3	-1
Nuts and seeds (kcal/capita, adjusted)	None	3	-1
Whole grains (kcal/capita, adjusted)	None	3	-1
Pulses/legumes (kcal/capita, adjusted)	None	3	-1
PUFA adjusted (percent)	None	3	-1
Trans fatty acid (percent)	None	3	1
Alcohol (litres per capita)	None	3	0

Endocarditis

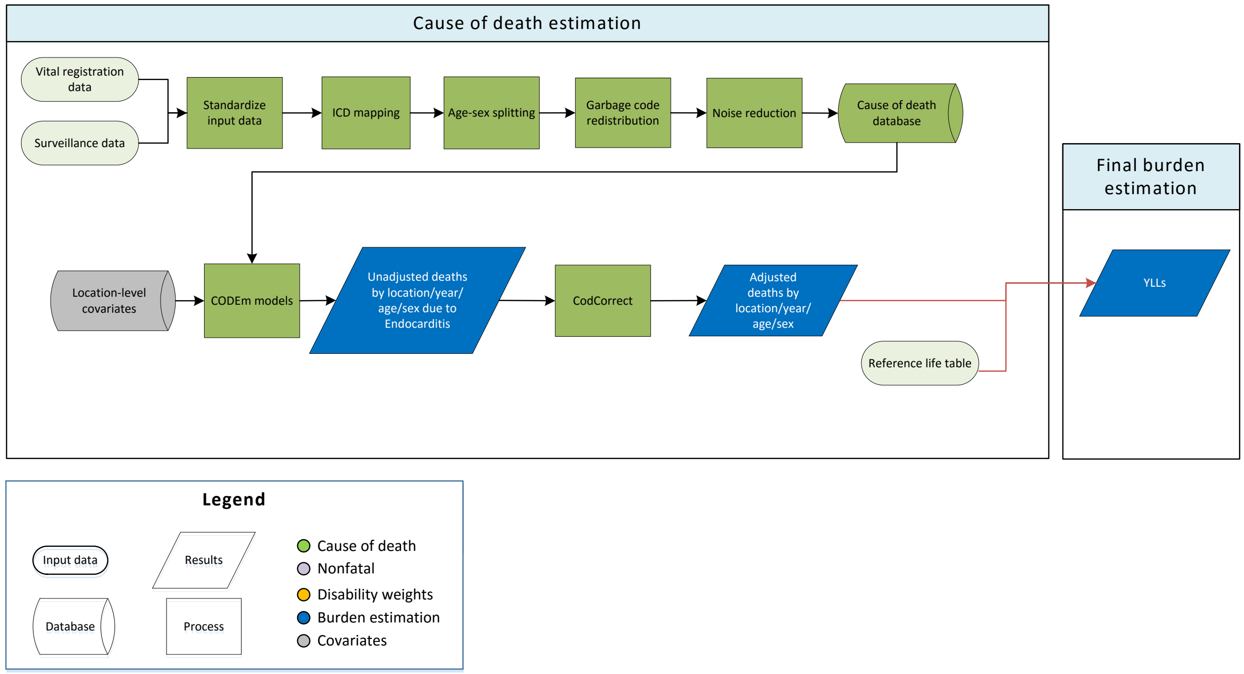

Input data

Vital registration and surveillance data were used to model endocarditis. We outliered vital registration data in Mozambique as these were non-representative for sub-Saharan Africa and were causing regional estimates to be implausibly low. We also outliered ICD8 data that were discontinuous from the rest of the data series and created an implausible time trend.

Modelling strategy

We used a standard CODEm approach to model deaths from endocarditis. We have updated the covariates included in the ensemble modelling process (see Table). Otherwise, there have been no substantive changes from the approach used in GBD 2015.

Table: Selected covariates for CODEm models, endocarditis

Covariate	Transformation	Level	Direction
Summary exposure variable	None	1	1
Improved water (proportion)	None	1	-1
Sanitation (proportion with access)	None	1	-1
Healthcare access and quality index	None	1	-1
Lag distributed income per capita (I$)	Log	3	-1
Socio-demographic Index	None	3	0

Other Cardiovascular and Circulatory Diseases

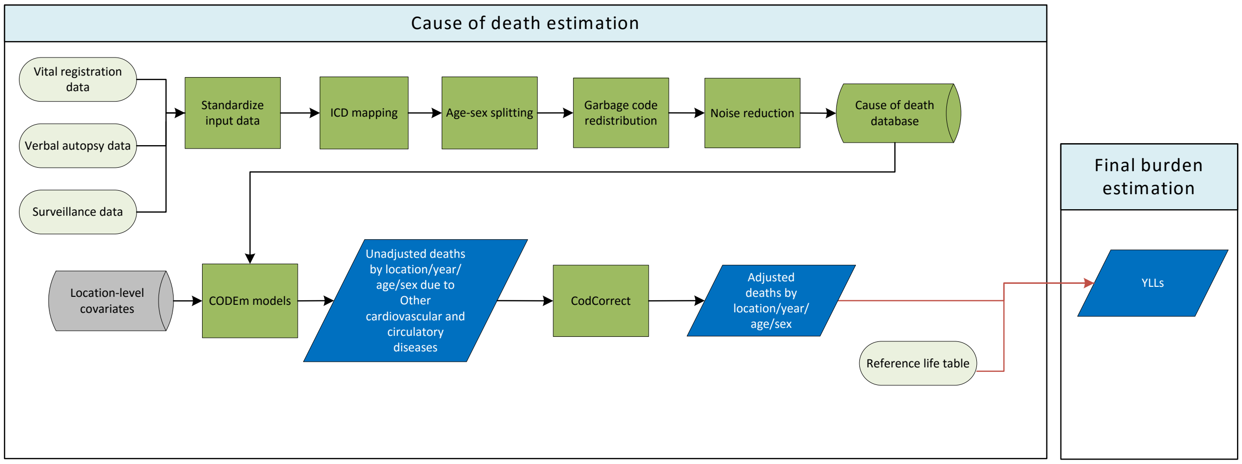

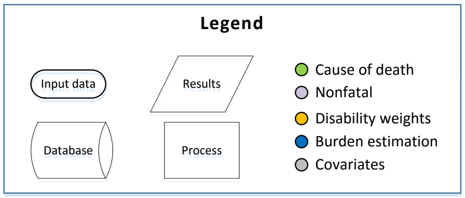

Input data

Vital registration, verbal autopsy, and surveillance data were used to model other cardiovascular and circulatory diseases. We outliered ICD8 and ICD9 BTL data points that were inconsistent with the rest of the data and created implausible time trends. We also outliered ICD8 data points which were not nationally representative.

Modelling strategy

We used a standard CODEm approach to model deaths from other circulatory and cardiovascular diseases. We have updated the covariates included in the ensemble modelling process (see Table). Otherwise, there have been no substantive changes from the approach used in GBD 2015.

Table: Selected covariates for CODEm models, cardiovascular diseases

Covariate	Transformation	Level	Direction
Summary exposure variable	None	1	1
Cholesterol (total, mean per capita)	None	1	1
Smoking prevalence	None	1	1
Systolic blood pressure (mmHg)	None	1	1
Trans fatty acid (percent)	None	1	1
Mean BMI	None	2	1
Elevation over 1500m (proportion)	None	2	-1
Fasting plasma glucose (mmol/L)	None	2	1
Indoor air pollution (all fuel types)	None	2	1
Outdoor air pollution ($PM_{2.5}$)	None	2	1
Healthcare access and quality index	None	2	-1
Lag distributed income per capita (I$)	Log	3	-1
Socio-demographic Index	None	3	0
Omega-3 (kcal/capita, adjusted)	Log	3	-1
Fruits (kcal/capita, adjusted)	None	3	-1
Vegetables (kcal/capita, adjusted)	None	3	-1
Nuts and seeds (kcal/capita, adjusted)	None	3	-1
Whole grains (kcal/capita, adjusted)	None	3	-1
Pulses/legumes (kcal/capita, adjusted)	None	3	-1
PUFA adjusted (percent)	None	3	-1
Alcohol (litres per capita)	None	3	0

Chronic Respiratory Diseases

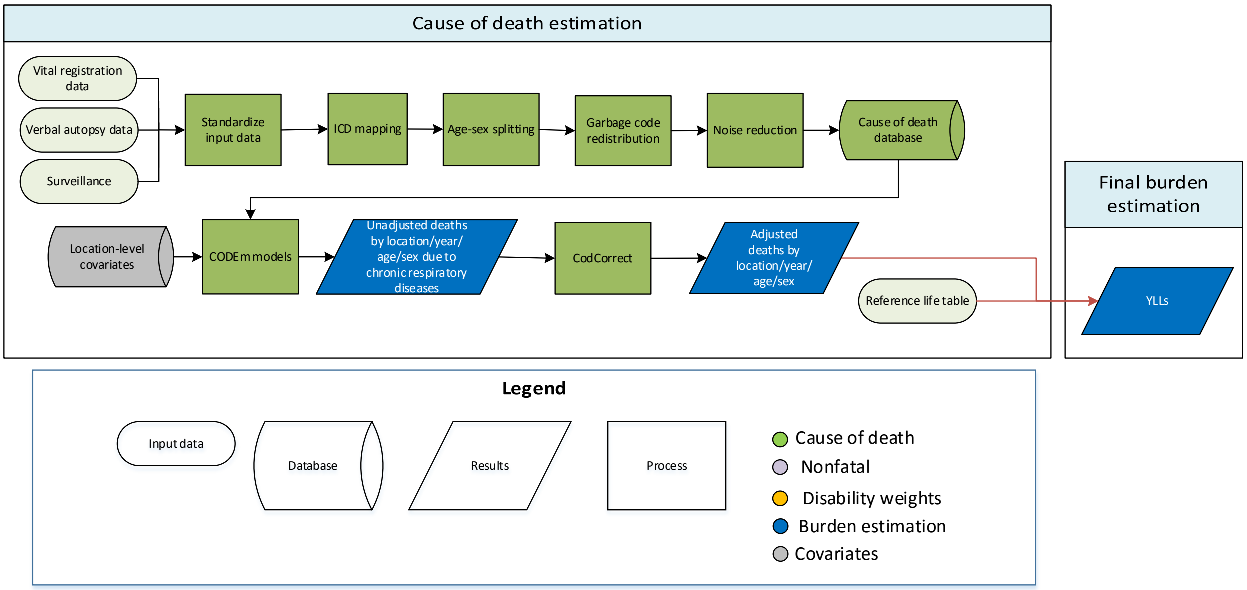

Input data

Sources used to estimate chronic respiratory disease mortality included vital registration, verbal autopsy, and surveillance data from China. Our outlier criteria excluded data points that (1) were implausibly high or low, (2) substantially conflicted with established age or temporal patterns, or (3) significantly conflicted with other data sources conducted from the same locations or locations with similar characteristics (ie, Socio-demographic Index).

Modelling strategy

The standard CODEm modelling approach was applied to estimate deaths due to chronic respiratory diseases. Chronic respiratory diseases served as the parent cause to chronic obstructive pulmonary disease, pneumoconiosis (including silicosis, asbestosis, coal worker's pneumoconiosis, other pneumoconiosis), asthma, interstitial lung disease and pulmonary sarcoidosis, and other chronic respiratory diseases. Functionally, this means the death estimates for Chronic Respiratory Diseases serve as a "parent" envelope into which the "child" causes are squeezed by the CodCorrect algorithm. This approach allows us to use a broader range of data – specifically verbal autopsy data – which cannot be accurately mapped to specific respiratory diseases.

Separate models were conducted for male and female mortality, and the age range for both models was 0 to 95+ years. The same covariates from GBD 2015 were used, with the exception of indoor air pollution, which was changed from cooking-fuel-specific covariates to a generic all cooking fuel covariate.

**Level**	**Covariate**	**Direction**
1	log-transformed SEV scalar: chronic respiratory diseases	+

	cumulative cigarettes (10 years)	+
	cumulative cigarettes (5 years)	+
	health care quality and access index	-
2	smoking prevalence	+
	indoor air pollution (all cooking fuels)	+
	outdoor air pollution ($PM_{2.5}$)	+
	population above 1500m elevation (proportion)	+
3	log LDI (I$ per capita)	-
	education (years per capita)	-
	Socio-demographic Index	-
	population between 500 and 1,500m elevation (proportion)	+
	population density over 1,000 people/square meter (proportion)	+

Beyond changes in the underlying covariates, there were no substantial deviations from the GBD 2015 approach.

Chronic Obstructive Pulmonary Disease

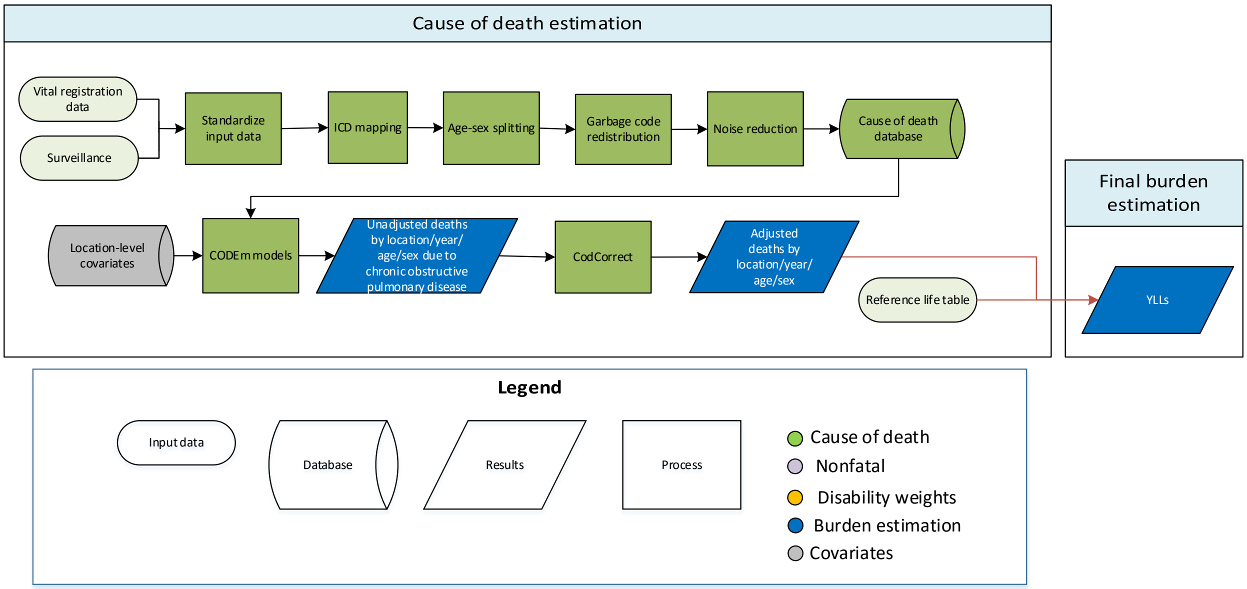

Input data
Data used to estimate chronic obstructive pulmonary disease (COPD) mortality included vital registration and surveillance data from the cause of death (COD) database. Our outlier criteria excluded data points that (1) were implausibly high or low, (2) substantially conflicted with established age or temporal patterns, or (3) significantly conflicted with other data sources conducted from the same locations or locations with similar characteristics (ie, Socio-demographic Index).

Modelling strategy
The standard CODEm modelling approach was applied to estimate deaths due to COPD. Separate models were conducted for male and female mortality, and the age range for both models was 1-95+ years. The mortality estimates from the COPD models were ultimately fit into the chronic respiratory diseases envelope.

The same covariates from GBD 2015 were used, with the exception of indoor air pollution, which was changed from cooking-fuel-specific covariates to a generic all cooking fuel covariate, and the health care access and quality index covariate, which was used in place of health systems access.

Level	Covariate	Direction
1	log-transformed SEV scalar: COPD	+
	cumulative cigarettes (10 years)	+
	cumulative cigarettes (5 years)	+
	elevation over 1,500m (proportion)	+

2	smoking prevalence	+
	indoor air pollution (all cooking fuels)	+
	outdoor air pollution ($PM_{2.5}$)	+
	health care access and quality index	-
3	Socio-demographic Index	-
	log LDI (I$ per capita)	-
	education (years per capita)	-

Pneumoconiosis diseases: Silicosis, asbestosis, coal worker's pneumoconiosis, and other pneumoconiosis

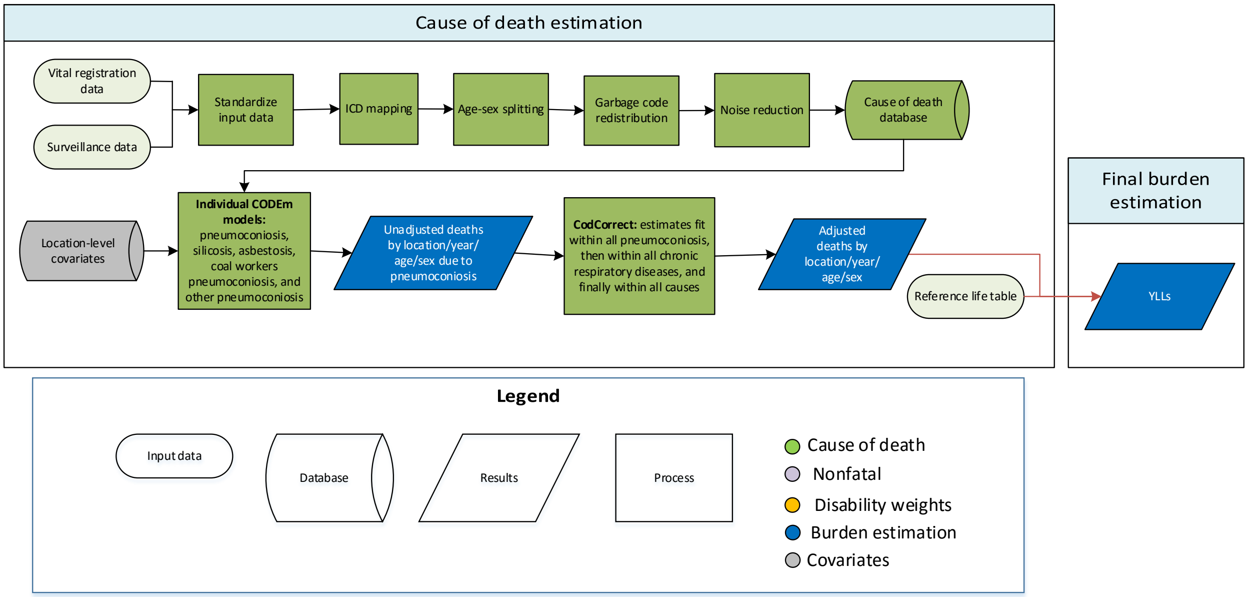

Input data

Data used to estimate pneumoconiosis diseases mortality included vital registration and China mortality surveillance data from the cause of death (COD) database. Our outlier criteria excluded data points that (1) were implausibly high or low, (2) substantially conflicted with established age or temporal patterns, or (3) significantly conflicted with other data sources conducted from the same locations or locations with similar characteristics (i.e., socio-demographic index).

Modelling strategy

The standard CODEm modelling approach was applied to estimate deaths due to pneumoconiosis diseases. Separate models were conducted for male and female mortality, and the age range for both models was 1–95+ years. The mortality estimates from pneumoconiosis disease models were ultimately fit into the chronic respiratory envelope, which is the parent cause for pneumoconiosis disease. The pneumoconiosis model serves as an envelope for silicosis, asbestosis, coal worker's pneumoconiosis, and other pneumoconiosis. In CoDCorrect, estimates are first fit within all pneumoconiosis, then within all chronic respiratory disease, before being fit to the all-cause mortality envelope.

For the most part, the same covariates from GBD 2015 were used. Indoor air pollution was changed from cooking-fuel specific covariates to a generic all cooking fuel covariate. Adjustments were also made to the coal and asbestos covariates.

The coal production covariate was improved to include subnational data for the United States and India. United States state-level data for 2001-2015 came from the U.S. Energy Information Administration. India state-level data for 2005-2014 came from the Ministry of Coal in India. We scaled these figures to the national estimates from the BP Statistical Review of World Energy 2016. For years with missing

state-level data we split the national-level data according to the proportions by state in the closest year for which we did have state-level data.

We also created a covariate for asbestos consumption per capita with a 30-year lag, and used that instead of the GBD 2015 asbestos production covariate. This change is based on the idea that asbestos production may be too limited in scope, given that asbestosis may occur in locations where asbestos is used and handled but not necessarily mined. To create the asbestos consumption covariate we used data from the United States Geological Survey to run a model in DisMod 2.1. A 30-year lag was placed on this model to account for the delay between asbestos consumption and occurrence of disease.

The following table indicates covariates used in the pneumoconiosis models, their level, and direction:

Level	Covariate	Direction
1	log-transformed SEV scalar: pneumoconiosis	+
	asbestos consumption per capita*	+
	coal production per capita*	+
	gold production per capita*	+
2	smoking prevalence	+
	indoor air pollution (all cooking fuels)	+
	cumulative cigarettes (5 years)	+
	elevation over 1,500m (proportion)	+
	elevation 500 to 1,500m (proportion)	+
	health care access and quality index	-
3	log LDI (I$ per capita)	-
	education (years per capita)	-
	Socio-demographic Index	-

* asbestos, coal, and gold covariates are each only used in a subset of the pneumoconiosis models, as follows: all three are included in the parent all pneumoconiosis model, asbestos consumption is included in the asbestosis model, coal production is included in the coal worker's pneumoconiosis model, and gold production is included in the silicosis model.

Asthma

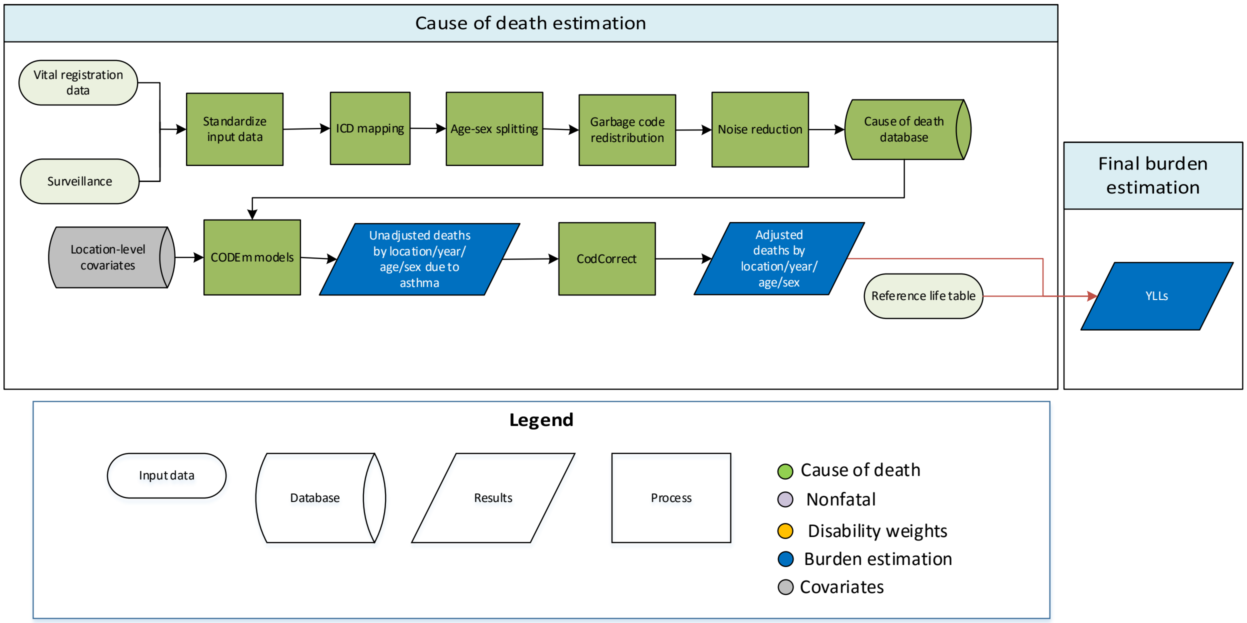

Input data

Data used to estimate asthma mortality included vital registration and surveillance data from the cause of death (COD) database. Verbal autopsy data were not included and were instead mapped to the parent model (Chronic Respiratory Diseases). Our outlier criteria excluded data points that (1) were implausibly high or low relative to global or regional patterns, (2) substantially conflicted with established age or temporal patterns, or (3) significantly conflicted with other data sources conducted from the same locations or locations with similar characteristics (ie, Socio-demographic Index).

Modelling strategy

The standard CODEm modelling approach was applied to estimate deaths due to asthma. Separate models were conducted for male and female mortality, and the age range for both models was 1–95+ years. The mortality estimates from the asthma models were ultimately fit into the chronic respiratory diseases envelope.

The same covariates from GBD 2015 were used, with the exception of indoor air pollution, which was changed from cooking-fuel-specific covariates to a generic all cooking fuel covariate.

**Level**	**Covariate**	**Direction**
1	log-transformed SEV scalar: asthma	+
	cumulative cigarettes (10 years)	+
	cumulative cigarettes (5 years)	+

	health care access and quality index	-
2	smoking prevalence	+
	indoor air pollution (all cooking fuels)	+
	outdoor air pollution ($PM_{2.5}$)	+
3	log LDI (I$ per capita)	-
	education (years per capita)	-
	Socio-demographic Index	-

Interstitial lung disease and pulmonary sarcoidosis

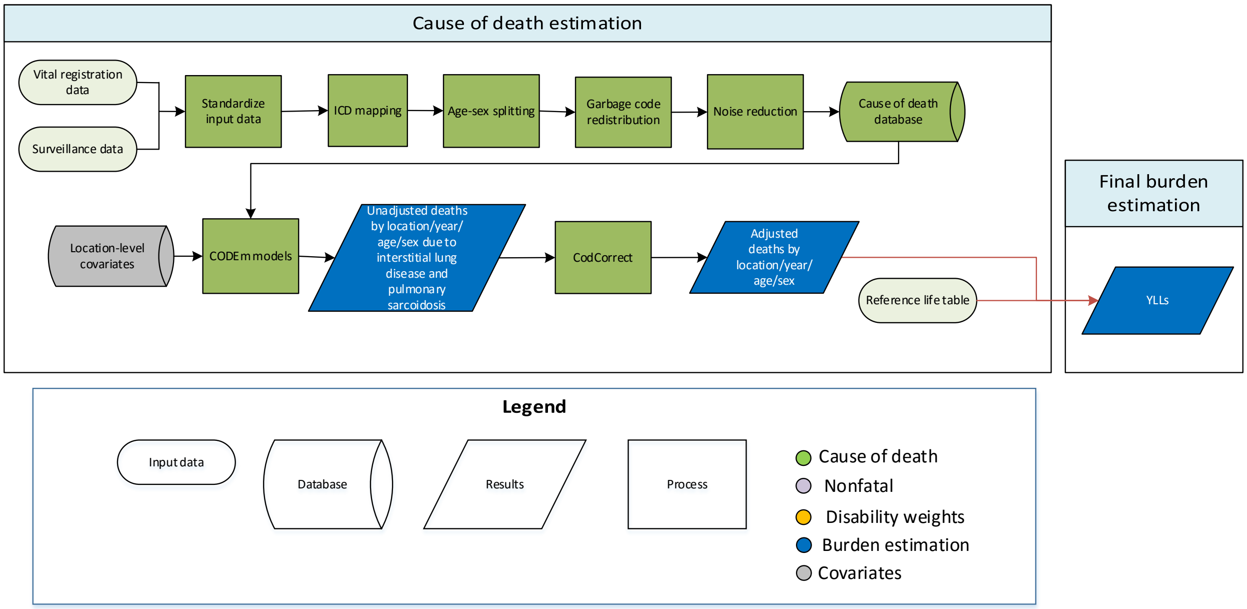

Input data

Data used to estimate interstitial lung disease and pulmonary sarcoidosis mortality included vital registration and surveillance data from the cause of death (COD) database. Our outlier criteria excluded data points that (1) were implausibly high or low, (2) substantially conflicted with established age or temporal patterns, or (3) significantly conflicted with other data sources conducted from the same locations or locations with similar characteristics (ie, Socio-demographic Index).

Modelling strategy

The standard CODEm modelling approach was applied to estimate deaths due to interstitial lung disease and pulmonary sarcoidosis. Separate models were conducted for male and female mortality, and the age range for both models was 1–95+ years. The mortality estimates from the interstitial lung disease and pulmonary sarcoidosis models were ultimately fit into the chronic respiratory envelope.

The same covariates from GBD 2015 were used, with the exception of indoor air pollution, which was changed from cooking-fuel-specific covariates to a generic all cooking fuel covariate.

**Level**	**Covariate**	**Direction**
1	log-transformed SEV scalar: interstitial lung disease	+
	smoking prevalence	+
	cumulative cigarettes (5 years)	+
2	elevation over 1,500m (proportion)	+

	elevation between 500 and 1,500m (proportion)	+
	population density over 1,000 ppl/sqkm (proportion)	+
	indoor air pollution (all cooking fuels)	+
	outdoor air pollution ($PM_{2.5}$)	+
	health care access and quality index	-
3	log LDI (I$ per capita)	-
	education (years per capita)	-
	Socio-demographic Index	-

Other chronic respiratory diseases

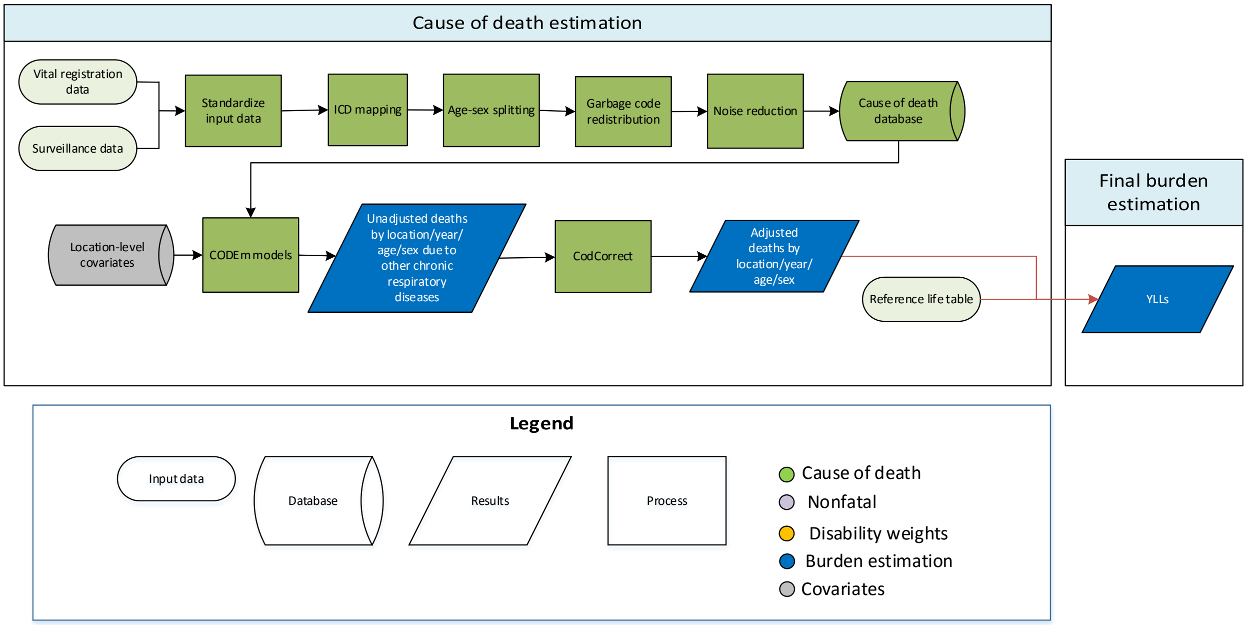

Input data

Data used to estimate other chronic respiratory diseases included vital registration and surveillance data from the cause of death (COD) database. Our outlier criteria excluded data points that (1) were implausibly high or low, (2) substantially conflicted with established age or temporal patterns, or (3) significantly conflicted with other data sources conducted from the same locations or locations with similar characteristics (ie, Socio-demographic Index).

Modelling strategy

The standard CODEm modelling approach was applied to estimate deaths due to other chronic respiratory diseases. Separate models were conducted for male and female mortality, and the age range for both models was 0 days to 95+ years. Like other respiratory causes, the mortality estimates from other chronic respiratory diseases were ultimately fit into the chronic respiratory envelope.

The same covariates from GBD 2015 were used, with the exception of indoor air pollution, which was changed from cooking-fuel-specific covariates to a generic all cooking fuel covariate.

Level	Covariate	Direction
1	log-transformed SEV scalar: other chronic respiratory diseases	+
	smoking prevalence	+
	cumulative cigarettes (5 years)	+
	indoor air pollution (all cooking fuels)	+

	outdoor air pollution ($PM_{2.5}$)	+
2	elevation over 1,500m (proportion)	+
	elevation between 500 and 1,500m (proportion)	+
	population density over 1,000 ppl/sqkm (proportion)	+
	health care access and quality index	-
3	log LDI (I$ per capita)	-
	education (years per capita)	-
	Socio-demographic Index	-

Cirrhosis

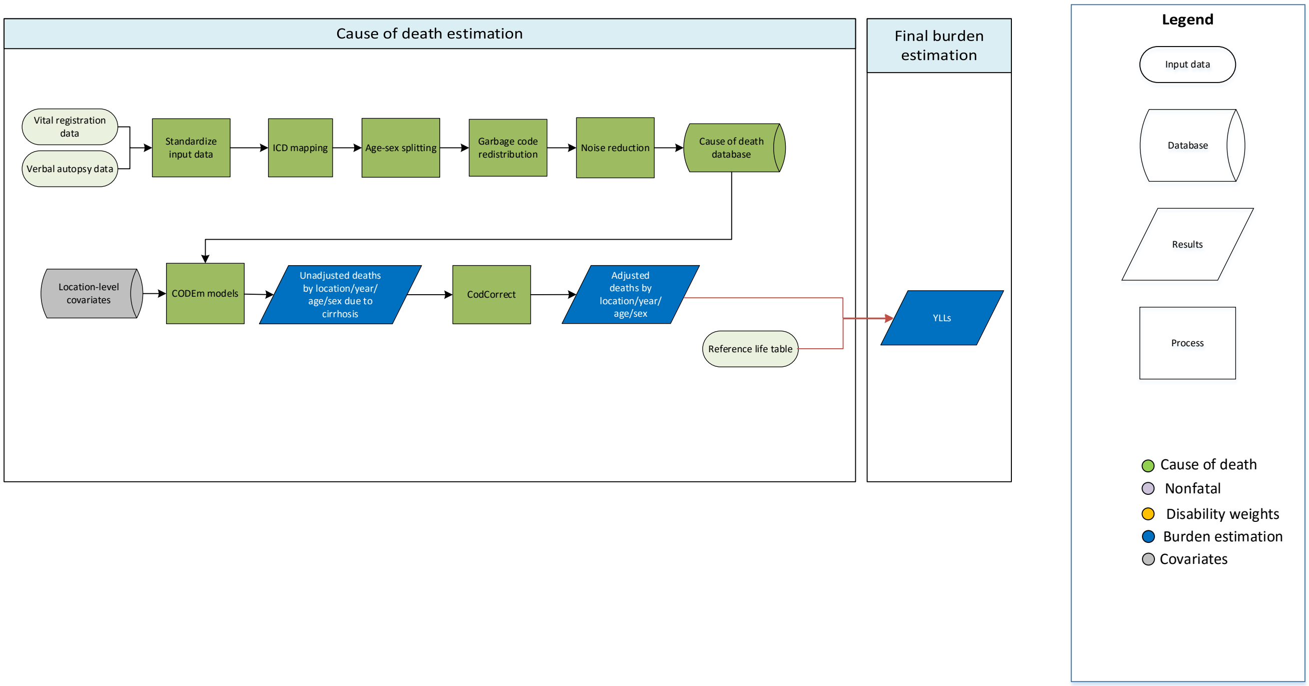

Input data

We modelled cirrhosis mortality using vital registration and verbal autopsy data in the cause of death database. We outliered data in instances where garbage code redistribution and noise reduction, in combination with small sample sizes, resulted in unreasonable cause fractions; and data that violated well-established time or age trends.

Modelling strategy

We modelled deaths due to cirrhosis with a standard CODEm model using the cause of death database and location-level covariates as inputs. We hybridized separate global and data-rich models to acquire unadjusted results, which we finalized and adjusted using CodCorrect to reach final YLLs due to cirrhosis.

There were no substantive changes in the modelling strategy for cirrhosis from GBD 2015 to GBD 2016.

Covariate	Level	Direction
Alcohol (liters per capita)	1	1
Diabetes prevalence (age standardized)	2	1
Education (years per capita)	3	-1
Health system access 2	3	-1
Log LDI (I$ per capita)	3	-1
Mean body mass index	2	1
Schistosomiasis prevalence (proportion)	1	1
Socio-demographic Index	3	0
Hepatitis B prevalence	1	1
Hepatitis C prevalence	1	1
Healthcare access and quality index	2	-1

Cirrhosis by aetiology

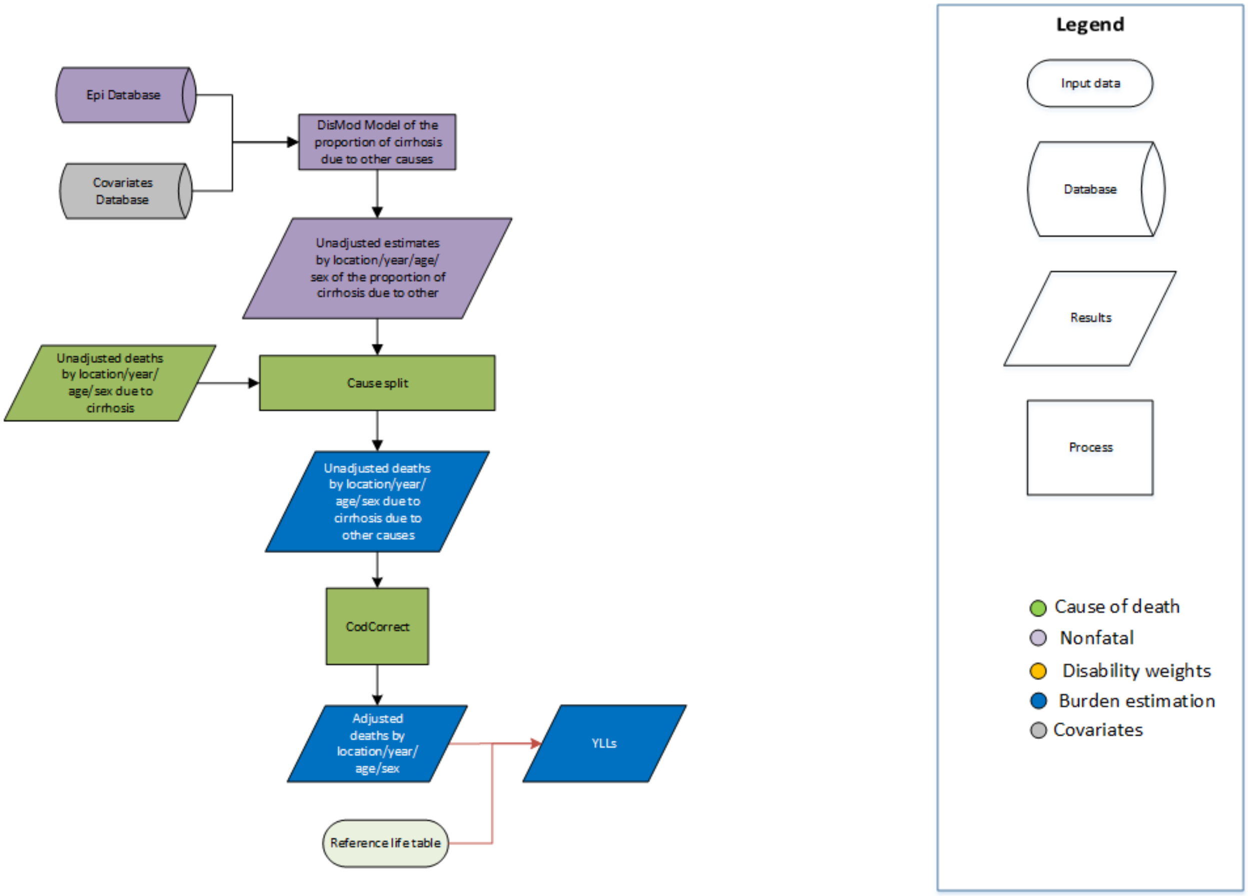

Input data

We conducted a literature review for studies reporting aetiologies of cirrhosis patients.

Modelling strategy

We first modelled all cirrhosis mortality using all available data in the cause of death database and a hybrid CODEm model. To estimate mortality from cirrhosis due to alcohol, cirrhosis due to hepatitis B, cirrhosis due to hepatitis C, and cirrhosis due to other causes, we developed aetiological proportion models using DisMod and used the results of these models to split the parent cirrhosis mortality estimates.

Given the similar aetiologies for liver cancer and cirrhosis we integrated the aetiology models for these two causes. We have more data for liver cancer aetiologies than we do for cirrhosis. Therefore, we first developed four single-parameter DisMod models, each to estimate the proportion of liver cancer due to a given cause (ie, alcohol, hepatitis B, hepatitis C, and other). These models included as covariates alcohol consumption (litres per capita), hepatitis B surface antigen (HBsAg) seroprevalence, and hepatitis C (anti-HCV IgG) seroprevalence. Moreover, the model for the proportion due to alcohol

included a covariate for the percentage of alcohol abstainers. Estimates from these liver cancer models were then used as covariates (along with alcohol, HBsAg, and anti-HCV) in all of the corresponding cirrhosis aetiology. Estimates from these cirrhosis models were then similarly used as covariates in the corresponding liver cancer models. Proportions from the four etiology models were then rescaled to sum to 1 at the draw level, and used to split the parent cirrhosis mortality estimates.

There were no substantive changes in the modelling strategy for cirrhosis from GBD 2015 to GBD 2016.

Digestive Diseases

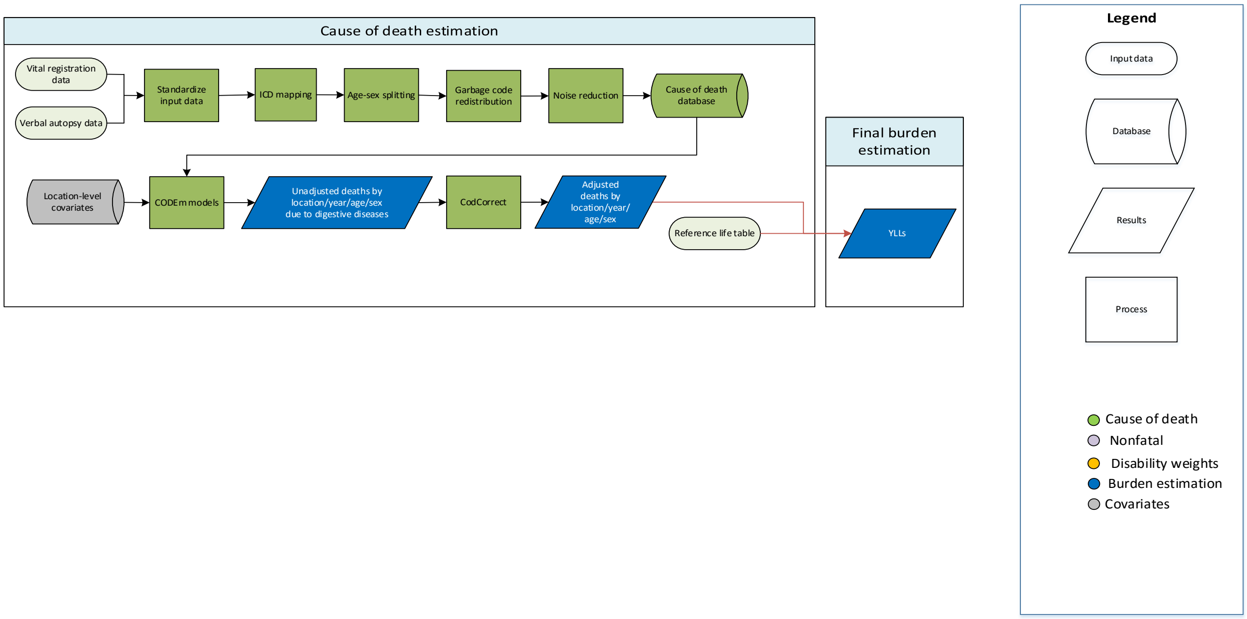

Input data

Data used to estimate mortality of digestive diseases consisted of vital registration data and verbal autopsy data from the cause of death (COD) database. We outliered data in instances where garbage code redistribution and noise reduction, in combination with small sample sizes, resulted in unreasonable cause fractions; and data that violated well-established time or age trends. Outliering methods were consistent across both vital registration and verbal autopsy data. The data in digestive diseases consists of aggregated data from all other specific digestive diseases, as well as unique data points from unspecified codes of digestive disease.

Modelling strategy

We modelled deaths due to all digestive diseases with a standard CODEm model using the cause of death database and location-level covariates as inputs. The model followed standard parameters. We hybridized separate global and data-rich models to acquire unadjusted results, which we finalized and adjusted using CoDCorrect to reach final years of life lost (YLLs) due to digestive diseases. In GBD 2016 we added the healthcare access and quality index (HAQI) covariate to the model.

Covariate	Level	Direction
Alcohol (liters per capita)	1	1
Cumulative cigarettes (10 years)	1	1
Education (years per capita)	3	-1
Lag distributed income (per capita)	3	-1
Underweight (proportion weight for age)	2	1
Sanitation (proportion with access)	1	-1
Sociodemographic index	3	-1
Fruits (grams adjusted)	2	-1
Red meats (grams adjusted)	2	1
Health access and quality index	2	-1

Peptic ulcer disease

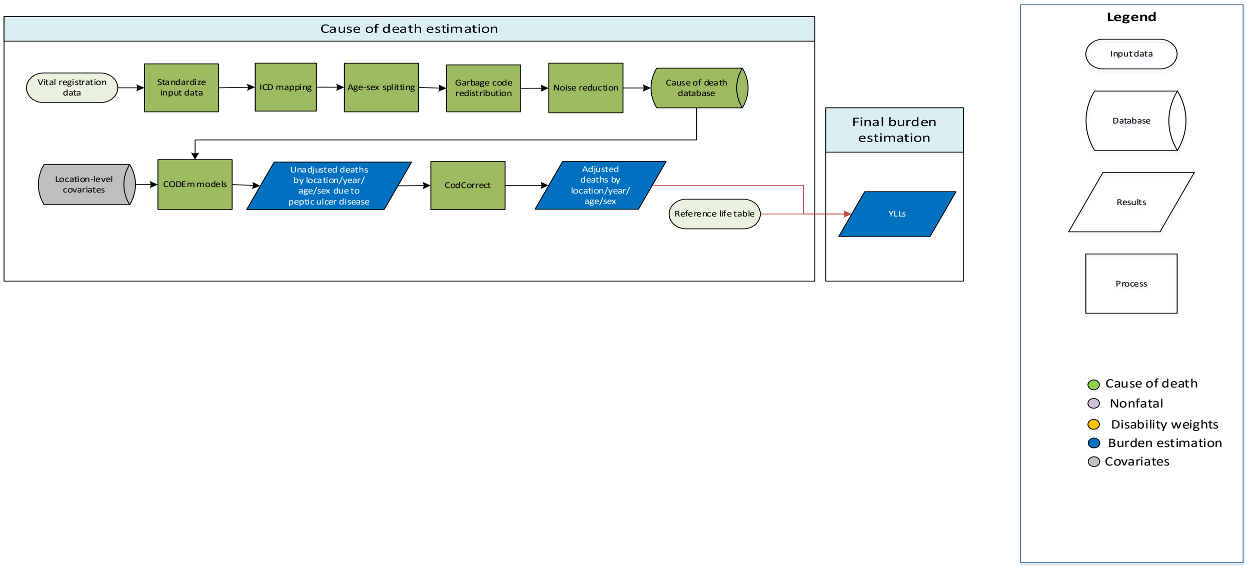

Input data

Data used to estimate mortality of peptic ulcer disease consisted of vital registration data from the cause of death (COD) database. We outliered data in instances where garbage code redistribution and noise reduction, in combination with small sample sizes, resulted in unreasonable cause fractions, and data that violated well-established time or age trends.

Modelling strategy

We modelled deaths due to peptic ulcer disease with a standard CODEm model using the cause of death database and location-level covariates as inputs. The model followed standard parameters, with the exception that the start age of the model was 1 year old instead of 0. We hybridized separate global and data-rich models to acquire unadjusted results, which we finalized and adjusted using CodCorrect to reach final years of life lost (YLLs) due to peptic ulcer disease. The covariate changes from GBD 2015 to GBD 2016 include changing the directionality of vegetables adjusted (grams per person availability) from -1 to 0, the addition of the summary exposure variable unsafe water, and the addition of the healthcare access and quality index (HAQI) covariate.

Covariate	Level	Direction
Alcohol (liters per capita)	1	1
Cumulative cigarettes (10 years)	1	1
Cumulative cigarettes (5 years)	1	1
Lag distributed income (per capita)	3	-1
Sanitation (proportion with access)	2	-1
Smoking (prevalence)	1	1
Maternal education (years per capita)	3	-1
Improved water source (proportion with access)	2	1
Sociodemographic index	3	-1
Vegetables (grams adjusted)	2	0
Health access and quality index	2	-1

Gastritis and duodenitis

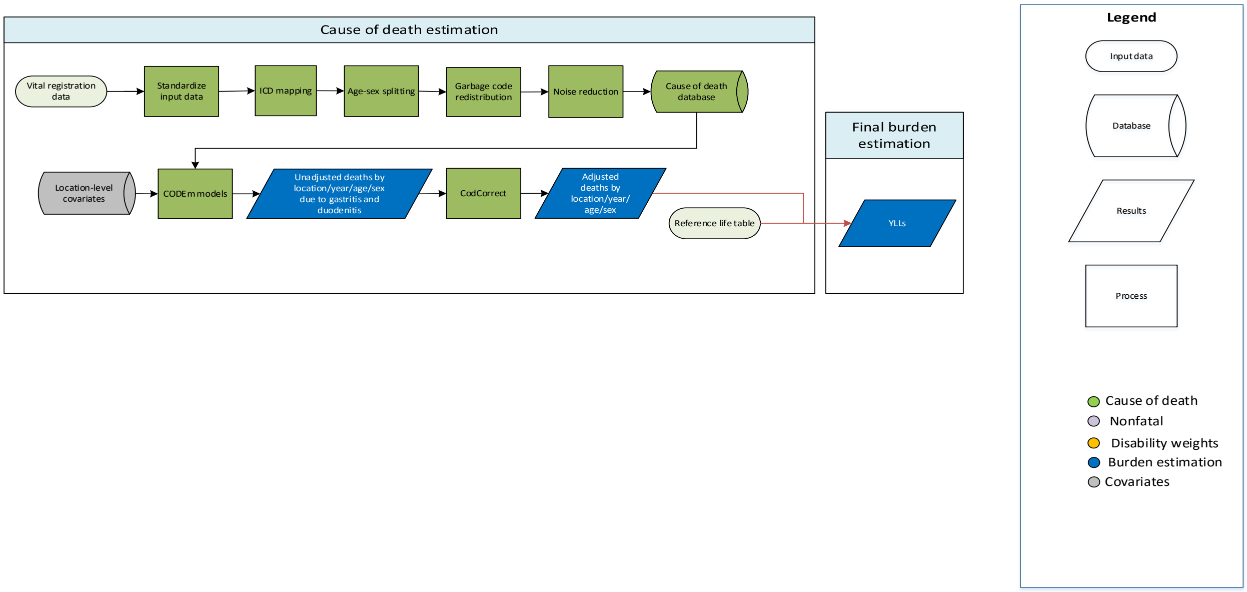

Input data

Vital registration data were used to model this cause. We outliered data in instances where garbage code redistribution and noise reduction, in combination with small sample sizes, resulted in unreasonable cause fractions, and data that violated well-established time or age trends.

Modelling strategy

We modelled deaths due to gastritis and duodenitis with a standard CODEm model using the cause of death database and location-level covariates as inputs. The model followed standard parameters, with the exception that the start age of the model was 1 year old instead of 0 and the linear floor rate was lowered to 0.001 in order to better capture low data. We hybridized separate global and data-rich models to acquire unadjusted results, which we finalized and adjusted using CodCorrect to reach final years of life lost (YLLs) due to gastritis and duodenitis. The covariate changes from GBD 2015 to GBD 2016 include a replacement of the covariate “improved water source” (proportion with access) to the summary exposure variable “unsafe water”, and the addition of the healthcare access and quality index (HAQI) covariate.

Covariate	Level	Direction
Alcohol (liters per capita)	1	1
Cumulative cigarettes (10 years)	1	1
Cumulative cigarettes (5 years)	1	1
Education (years per capita)	3	-1
Lag distributed income (per capita)	3	-1
Sanitation (proportion with access)	2	-1
Smoking prevalence	1	1
Unsafe water (summary exposure variable)	2	1
Sociodemographic index	3	-1
Vegetables (grams adjusted)	2	0
Health access and quality index	2	-1

Appendicitis

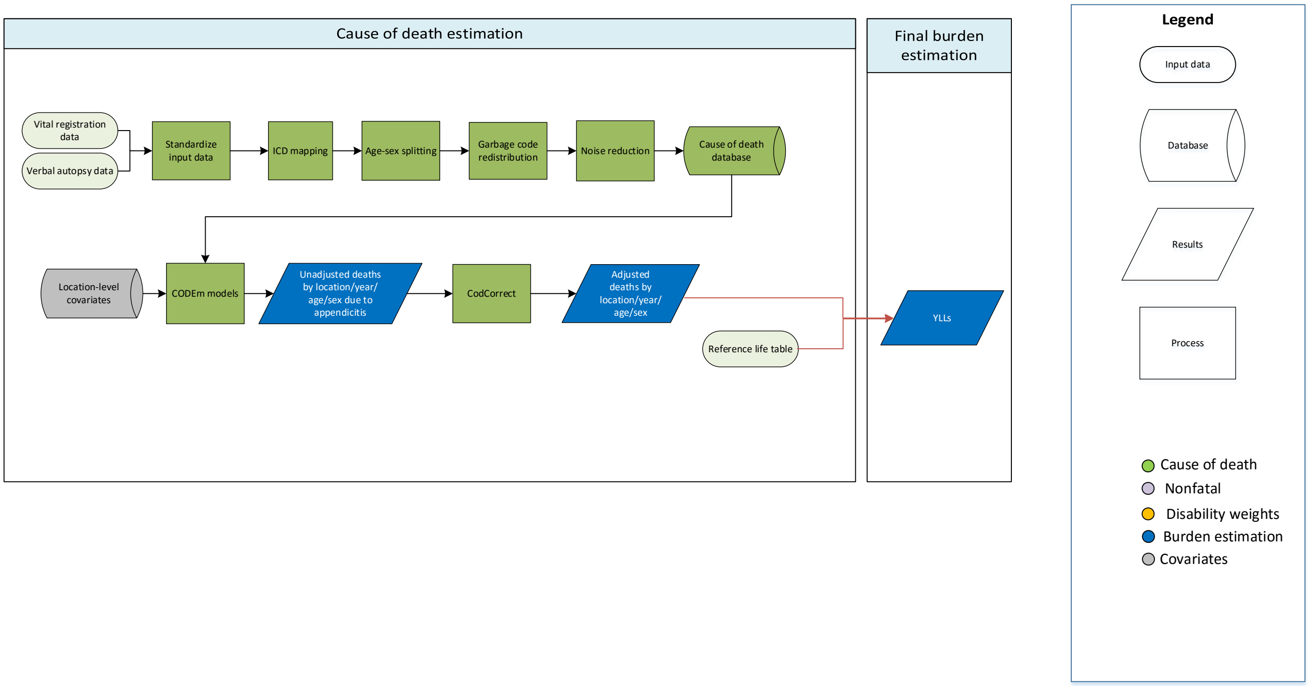

Input data

Data used to estimate appendicitis mortality consisted of vital registration and verbal autopsy data from the cause of death (COD) database. We outliered data in instances where garbage code redistribution and noise reduction, in combination with small sample sizes, resulted in unreasonable cause fractions; and data that violated well-established time or age trends.

Modelling strategy

We modelled deaths due to appendicitis with a standard CODEm model using the cause of death database and location-level covariates as inputs. The model followed standard parameters, with the exception that the start age of the model was 1 year old instead of 0 and the linear floor rate was lowered to 0.0001 in order to better capture low data. We hybridized separate global and data-rich models to acquire unadjusted results, which we finalized and adjusted using CodCorrect to reach final YLLs due to appendicitis.

There were no significant changes in the modelling process between GBD 2015 and GBD 2016.

Covariate	Level	Direction
Education (years per capita)	3	-1
Log LDI (I$ per capita)	3	-1
Health system access (capped)	3	-1
Socio-demographic Index	3	-1
Fruits adjusted (g)	2	-1
Vegetables adjusted (g)	2	-1
Healthcare access and quality index	2	-1

Paralytic Ileus and Intestinal Obstruction

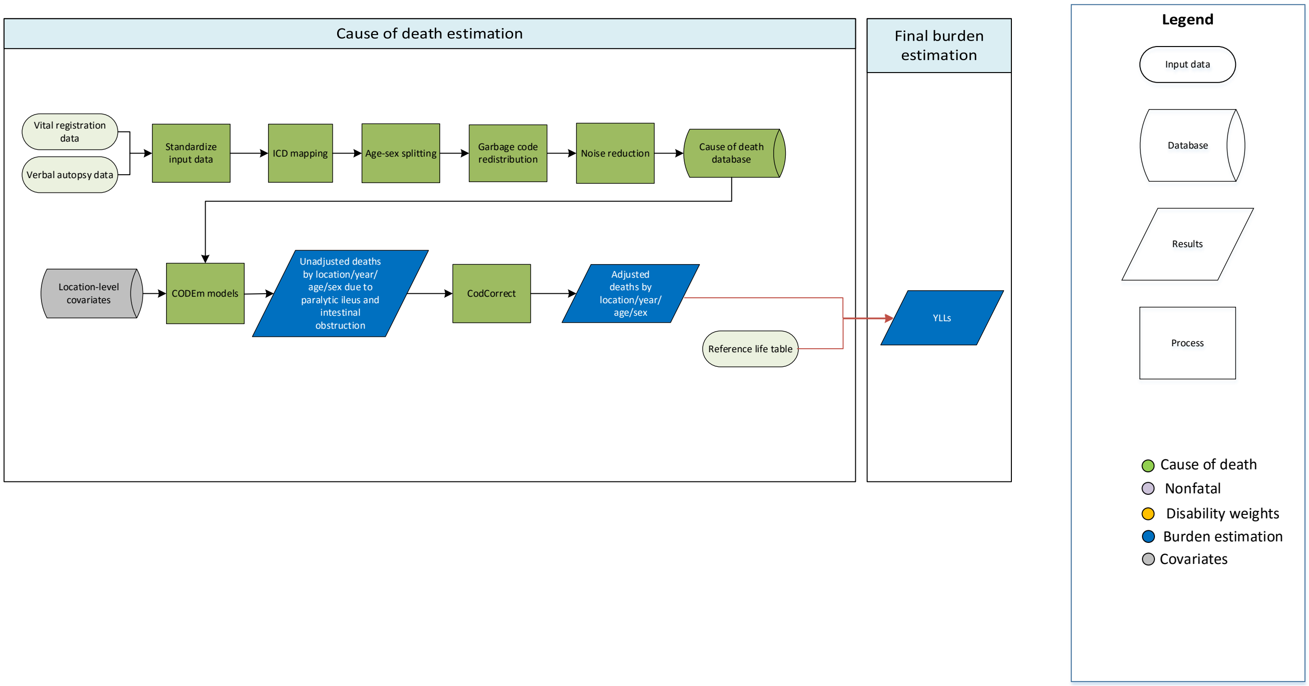

Input data

Data used to estimate cellulitis mortality consisted of vital registration and verbal autopsy data from the cause of death (COD) database. We outliered all VA data in children under the age of 1 because it is not possible to accurately diagnose paralytic ileus or intestinal obstruction in this age group; and data that violated well-established time or age trends; and data in instances where garbage code redistribution and noise reduction, in combination with small sample sizes, resulted in unreasonable cause fractions.

Modelling strategy

We modelled deaths due to paralytic ileus and intestinal obstruction with a standard CODEm model using the cause of death database and location-level covariates as inputs. The model followed standard parameters, with the exception that the linear floor rate was lowered to 0.0001 in order to better capture low data. We hybridized separate global and data-rich models to acquire unadjusted results, which we finalized and adjusted using CodCorrect to reach final YLLs due to paralytic ileus and intestinal obstruction.

There were no significant changes in the modelling process between GBD 2015 and GBD 2016.

Covariate	Level	Direction
Education (years per capita)	3	-1
Log LDI (I$ per capita)	3	-1
Health system access (capped)	3	-1
Socio-demographic Index	3	-1
Fruits adjusted (g)	2	-1
Vegetables adjusted (g)	2	-1
Healthcare access and quality index	2	-1

Vascular Intestinal Disorders

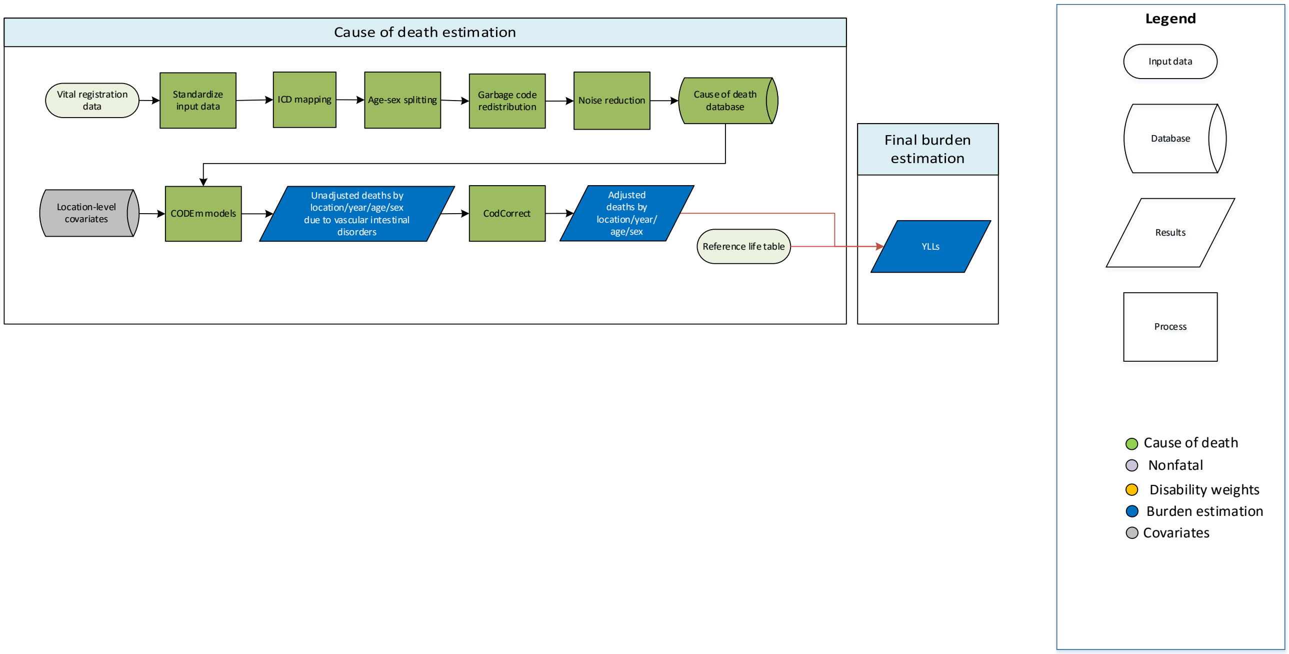

Input data

Vital registration data were used to model this cause. We outliered data in instances where garbage code redistribution and noise reduction, in combination with small sample sizes, resulted in unreasonable cause fractions; and data that violated well-established time or age trends.

Modeling strategy

We modeled deaths due to vascular intestinal disorders with a standard CODEm model using the cause of death database and location-level covariates as inputs. The model followed standard parameters, with the exception that the start age of the model was 1 year old instead of 0 and the linear floor rate was lowered to 0.0001 in order to better capture low data. We hybridized separate global and data-rich models to acquire unadjusted results, which we finalized and adjusted using CodCorrect to reach final YLLs due to vascular intestinal disorders. In GBD 2016, we replaced the animal fats (kcal per capita) covariate with the updated saturated fats (adjusted percentage of total calories available) covariate.

Inguinal, Femoral, and Abdominal Hernias

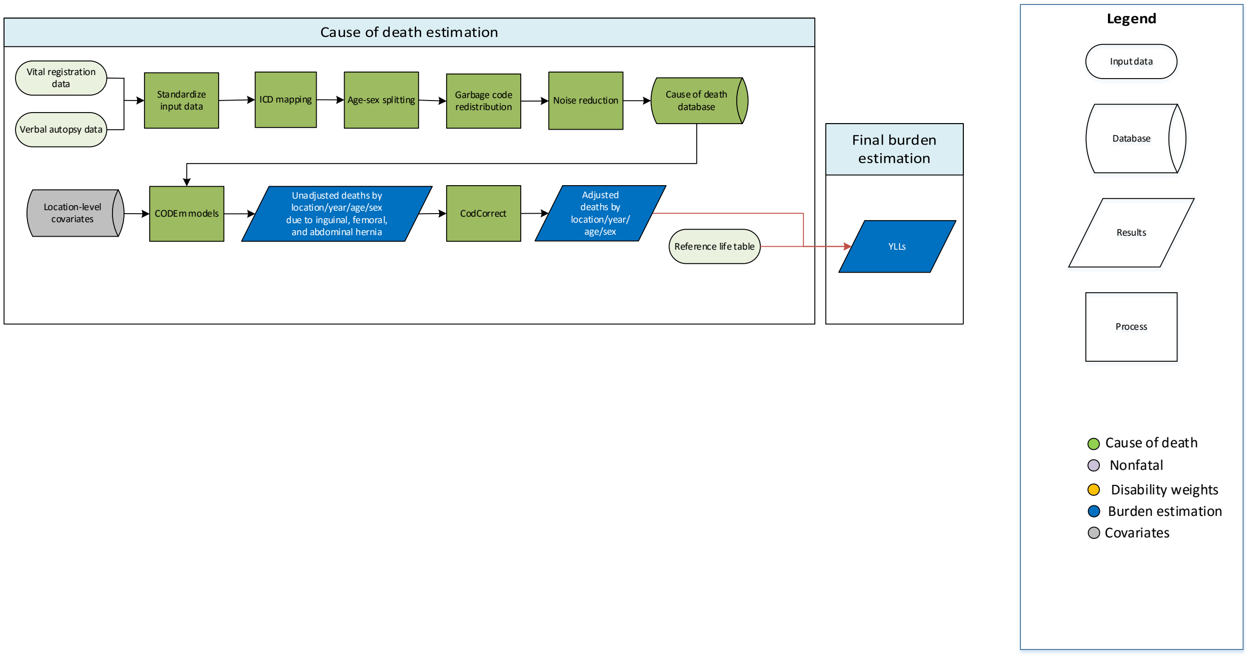

Input data

Vital registration and verbal autopsy data were used to model this cause. We outliered data in instances where garbage code redistribution and noise reduction, in combination with small sample sizes, resulted in unreasonable cause fractions; and data that violated well-established time or age trends. Outliering methods were consistent across both vital registration and verbal autopsy data.

Modelling strategy

We modelled deaths due to inguinal, femoral, and abdominal hernias with a standard CODEm model using the cause of death database and location-level covariates as inputs. The model followed standard parameters, with the exception that the start age of the model was 1 year old instead of 0 and the linear floor rate was lowered to 0.0001 in order to better capture low data. We hybridized separate global and data-rich models to acquire unadjusted results, which we finalized and adjusted using CodCorrect to reach final years of life lost (YLLs) due to inguinal, femoral, and abdominal hernias. In GBD 2016 we added the healthcare access and quality index (HAQI) covariate to the model.

Covariate	Level	Direction
Education (years per capita)	3	-1
Lag distributed income (per capita)	3	-1
Sociodemographic index	3	0
Health access and quality index	2	-1

Inflammatory Bowel Disease

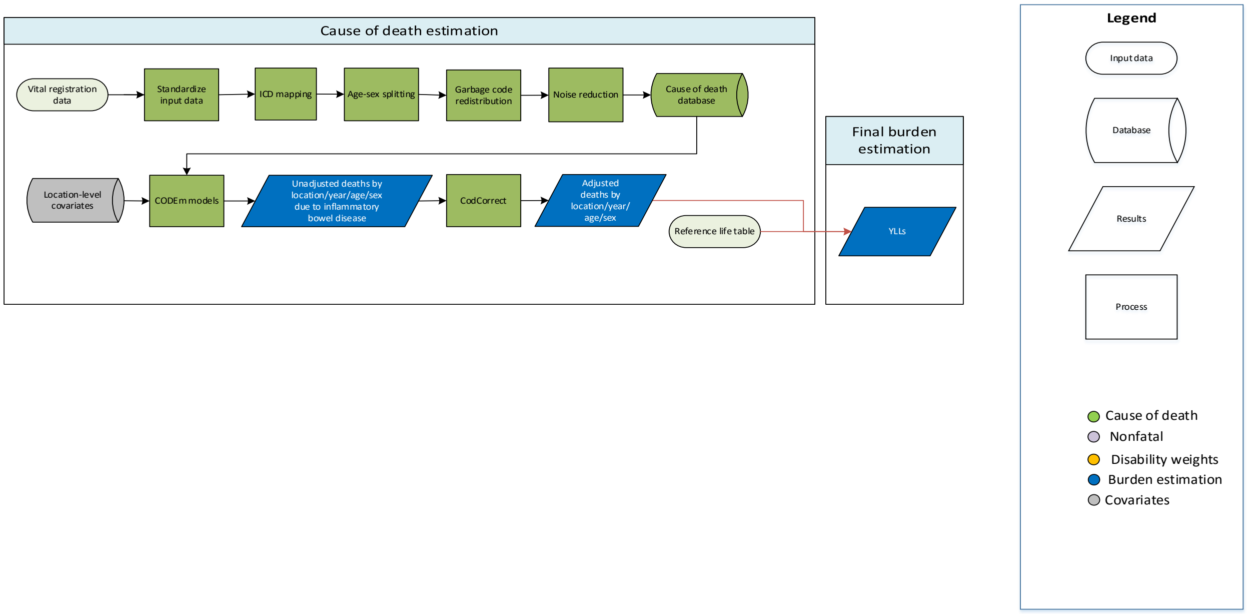

Input data

Vital registration data were used to model this cause. We outliered data in instances where garbage code redistribution and noise reduction, in combination with small sample sizes, resulted in unreasonable cause fractions, and data that violated well-established time or age trends.

Modelling strategy

We modelled deaths due to inflammatory bowel disease with a standard CODEm model using the cause of death database and location-level covariates as inputs. The model followed standard parameters, with the exception that the start age of the model was 1 year old instead of 0 and the linear floor rate was lowered to 0.0001 in order to better capture low data. We hybridized separate global and data-rich models to acquire unadjusted results, which we finalized and adjusted using CodCorrect to reach final years of life lost (YLLs) due to inflammatory bowel disease. In GBD 2016 we added the healthcare access and quality index (HAQI) covariate and replaced the animal fats (kcal per capita) with an updated saturated fats (adjusted percent) covariate.

Covariate	Level	Direction
Education (years per capita)	3	-1
Lag distributed income (per capita)	3	0
Latitude 15 to 30 (proportion)	2	-1
Latitude 30 to 45 (proportion)	2	1
Latitude 45 plus (proportion)	2	1
Sociodemographic index	3	0
Fruits (grams adjusted)	1	-1
Red meats (grams adjusted)	1	1
Saturated fats (adjusted percent)	1	1
Vegetables (grams adjusted)	-1	-1
Health access and quality index	-1	-1

Vascular Intestinal Disorders

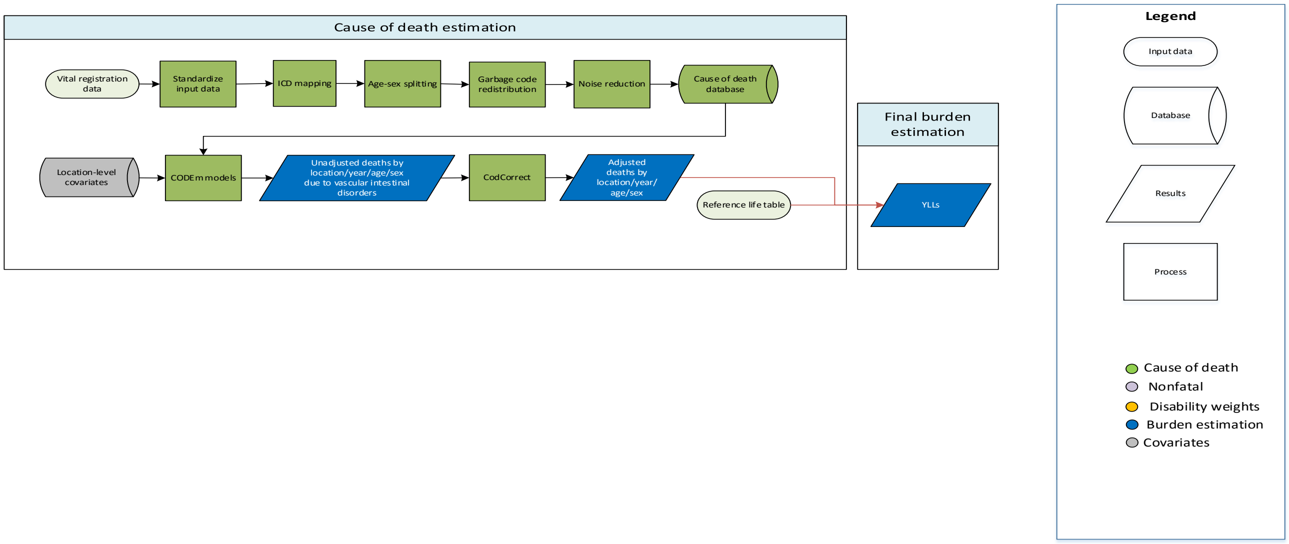

Input data

Vital registration data were used to model this cause. We outliered data in instances where garbage code redistribution and noise reduction, in combination with small sample sizes, resulted in unreasonable cause fractions; and data that violated well-established time or age trends.

Modelling strategy

We modelled deaths due to vascular intestinal disorders with a standard CODEm model using the cause of death database and location-level covariates as inputs. The model followed standard parameters, with the exception that the start age of the model was 1 year old instead of 0 and the linear floor rate was lowered to 0.0001 in order to better capture low data. We hybridized separate global and data-rich models to acquire unadjusted results, which we finalized and adjusted using CodCorrect to reach final years of life lost (YLLs) due to vascular intestinal disorders. In GBD 2016 we added the healthcare access and quality index (HAQI) covariate and replaced the animal fats (kcal per capita) covariate with an updated saturated fats (adjusted percent).

Covariate	Level	Direction
Alcohol (liters per capita)	2	1
Diabetes fasting plasma glucose (mmol/L)	1	1
Diabetes age specific prevalence (proportion)	1	1
Education (years per capita)	3	-1
Lag distributed income (per capita)	3	-1
Cholesterol (mean)	1	1
Systolic blood pressure (mean)	1	1
Latitude over 45 (proportion)	3	1
Sociodemographic index	3	0
Fruits (grams adjusted)	2	-1
Saturated fats (adjusted percent)	1	1
Vegetables (grams adjusted)	2	-1
Health access and quality index	2	-1

Gallbladder and biliary diseases

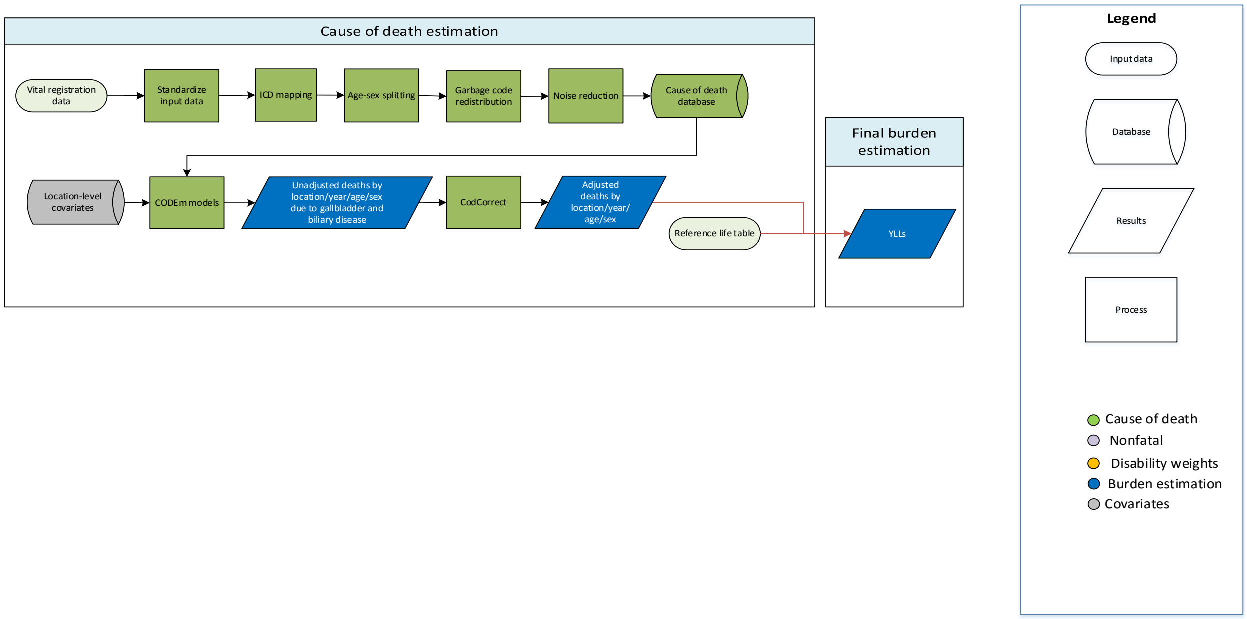

Input data

Data used to estimate mortality of gallbladder and biliary diseases consisted of vital registration data from the cause of death (COD) database. We outliered data in instances where garbage code redistribution and noise reduction, in combination with small sample sizes, resulted in unreasonable cause fractions; and data that violated well-established time or age trends.

Modelling strategy

We modelled deaths due to gallbladder and biliary diseases with a standard CODEm model using the cause of death database and location-level covariates as inputs. The model followed standard parameters, with the exception that the start age of the model was 1 year old instead of 0 and the linear floor rate was lowered to 0.0001 in order to better capture low data. We hybridized separate global and data-rich models to acquire unadjusted results, which we finalized and adjusted using CodCorrect to reach final years of life lost (YLLs) due to gallbladder and biliary diseases. In GBD 2016 we added the healthcare access and quality index (HAQI) covariate and replaced the animal fats (kcal per capita) covariate with an updated saturated fats (adjusted percent).

Covariate	Level	Direction
Alcohol (liters per capita)	2	1
Education (years per capita)	3	0
Lag distributed income (per capita)	3	0
Body mass index (mean)	1	1
Population over 65 (proportion)	2	1
Sociodemographic index	3	0
Red meats (grams adjusted)	2	1
Saturated fats (adjusted percent)	1	1
Health access and quality index	2	-1

Pancreatitis

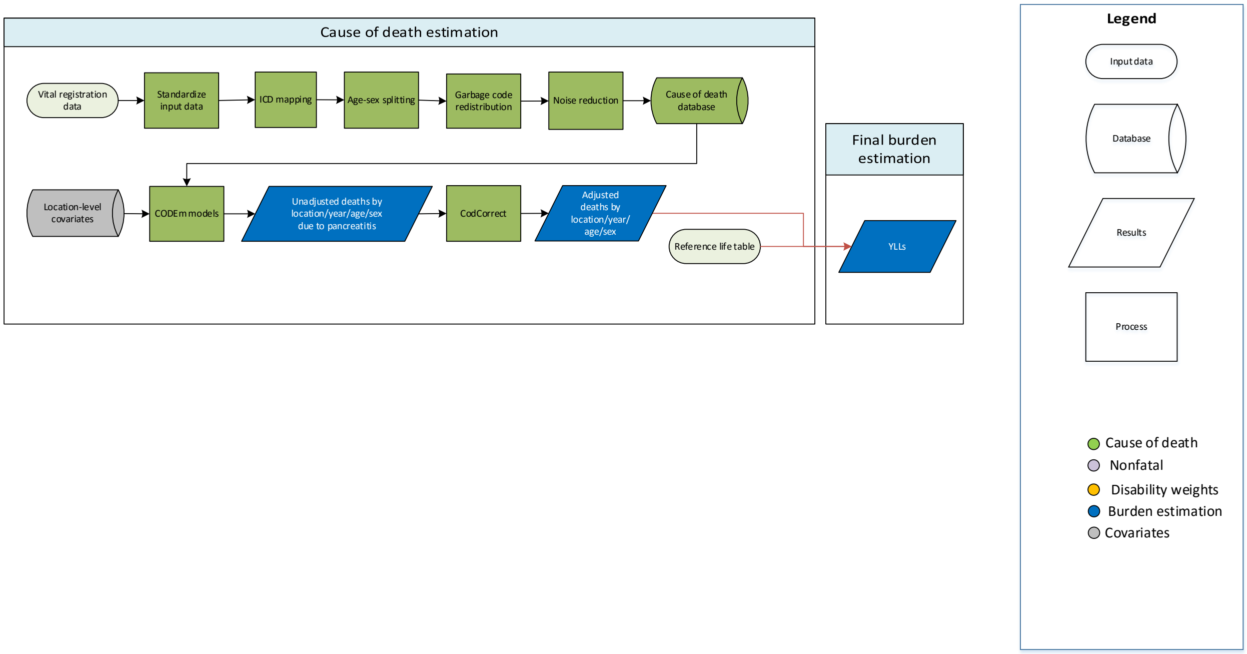

Input data

Vital registration data were used to model this cause. We outliered data in instances where garbage code redistribution and noise reduction, in combination with small sample sizes, resulted in unreasonable cause fractions, and data that violated well-established time or age trends.

Modelling strategy

We modelled deaths due to pancreatitis with a standard CODEm model using the cause of death database and location-level covariates as inputs. The model followed standard parameters, with the exception that the start age of the model was 1-year-old instead of 0 and the linear floor rate was lowered to 0.0001 in order to better capture low data. We hybridized separate global and data-rich models to acquire unadjusted results, which we finalized and adjusted using CodCorrect to reach final YLLs due to pancreatitis. In GBD 2016 we added the healthcare access and quality index (HAQI) covariate to the model.

Covariate	Level	Direction
Alcohol (liters per capita)	1	1
Education (years per capita)	3	-1
Lag distributed income (per capita)	3	0
Body mass index (mean)	2	1
Pancreatitis scalar (summary exposure variable)	1	1
Sociodemographic index	3	0
Health access and quality index	2	-1

Other digestive diseases

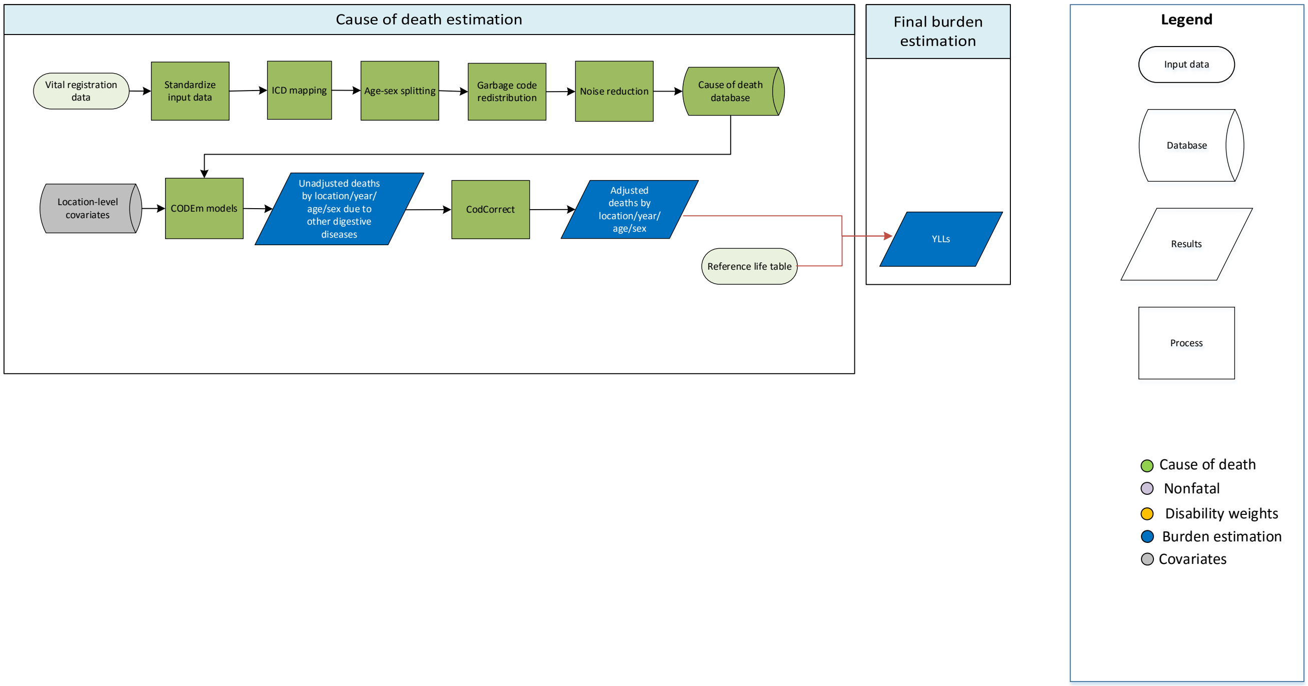

Input data

Data used to estimate mortality due to other digestive diseases consisted of vital registration data from the cause of death (COD) database. We outliered data in instances where garbage code redistribution and noise reduction, in combination with small sample sizes, resulted in unreasonable cause fractions; and data that violated well-established time or age trends.

Modelling strategy

We modelled deaths due to other digestive diseases with a standard CODEm model using the cause of death database and location-level covariates as inputs. The model followed standard parameters, with the exception that the start age of the model was 1 year old instead of 0. We hybridized separate global and data-rich models to acquire unadjusted results, which we finalized and adjusted using CodCorrect to reach final YLLs due to other digestive diseases.

There were no significant changes in the modelling process between GBD 2015 and GBD 2016.

Covariate	Level	Direction
Alcohol (litres per capita)	1	1
Cumulative cigarettes (5 years)	1	1
Cumulative cigarettes (10 years)	1	1
Diabetes age-standardized prevalence (proportion)	2	1
Education (years per capita)	3	-1
Health system access 2	3	-1
Log LDI (I$ per capita)	3	-1
Mean BMI	2	1
Sanitation (proportion with access)	2	-1
Smoking prevalence	1	1

Improved water source (proportion with access)	2	-1
Socio-demographic Index	3	0
Fruits adjusted (g)	2	-1
Red meats adjusted (g)	2	1
Saturated fats adjusted (percentage)	2	1
Vegetables adjusted (g)	2	0
Healthcare access and quality index	2	-1

Alzheimer's Disease and Other Dementias

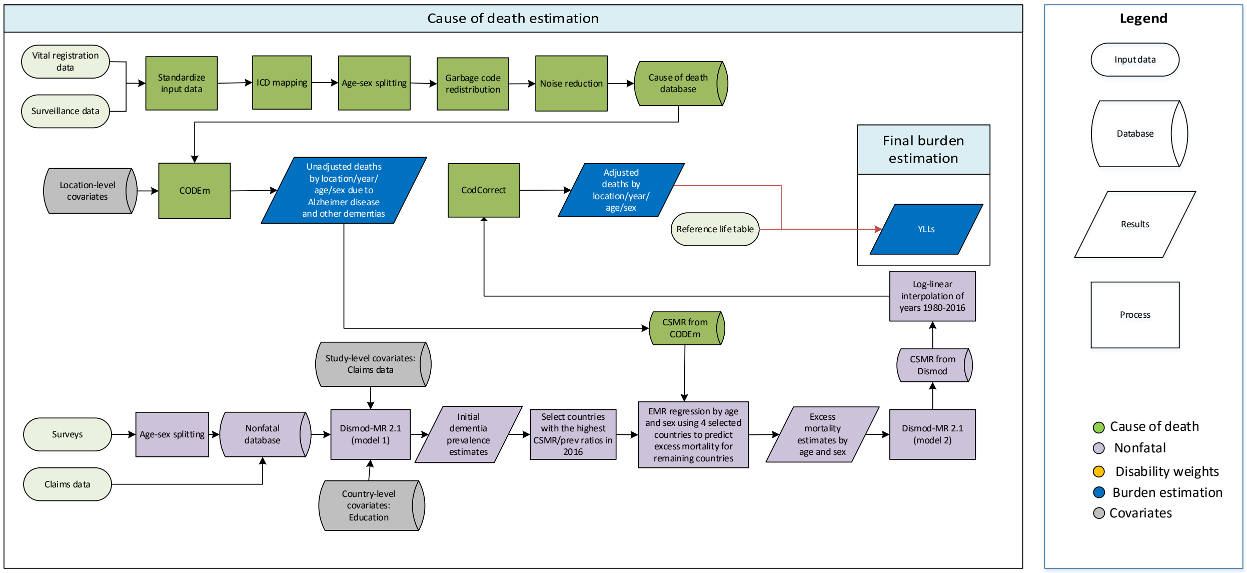

Input Data

In GBD 2016, data used to estimate deaths due to Alzheimer's disease and other dementias (dementias hereafter) included mortality data from vital registration systems and prevalence data from surveys and medical claims sources.

An updated systematic review was conducted from January 2015 to October 2016, and search terms[1] were set to capture studies for all dementia, including its sub-types. The search yielded 1,208 initial hits and 27 were marked for extraction. Inclusion criteria comprised studies that reported prevalence, incidence, remission rate, excess mortality rate, relative risk of mortality, standardized mortality ratio, or with-condition mortality rate. Studies with non-representative samples or no clearly defined sample were excluded. A flow chart documenting this review is displayed below.

[1] ((dementia[Title/Abstract]) AND ((incidence[Title/Abstract]) or (prevalence[Title/Abstract])) AND ("2015/01/23"[Date - Publication] : "3000"[Date - Publication])

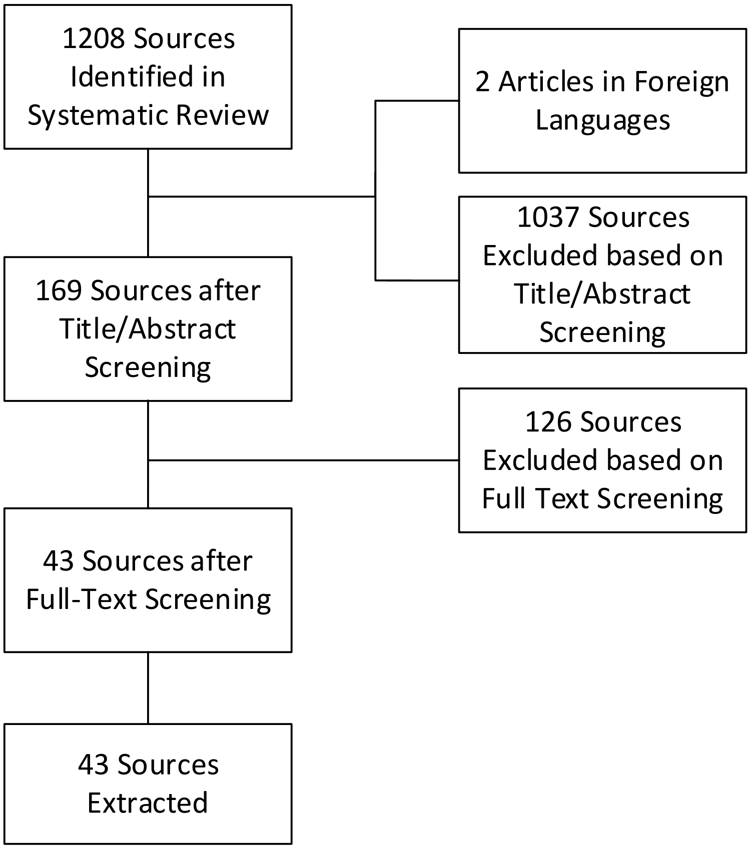

Modelling Strategy

Overview

Dementia mortality rates have increased more than five-fold since 1980 in high-quality vital registration systems such as in the US and Scandinavia. We have not seen an equivalent increase in prevalence and incidence data sources. If at all, there has been a modest decline in incidence and prevalence of dementia in studies in the UK and the US.[2 3] Also, the greater than 20-fold variation in mortality rates of dementia between countries is much greater than the four-fold difference in prevalence and incidence between countries. As it is unlikely that case fatality from dementia has dramatically increased over the time period and that it would differ by a very large margin between countries, the hypothesis is that certifying and coding practices have changed over time and at a different pace between countries. To avoid spurious large trends over time in the fatal component of the burden of dementia, we decided for GBD 2013 to make dementia mortality rates consistent with the most recent rates relative to prevalence of countries that are most likely to certify or code dementia as an underlying cause of death. This approach was applied again for GBD 2016, described further below.

[2] Akushevich I, Kravchenko J, Ukraintseva S, Arbeev K, Yashin AI. Time trends of incidence of age-associated diseases in the US elderly population: Medicare-based analysis. *Age and ageing*. 2013 Jul 1;42(4):494-500.

[3] Matthews FE, Arthur A, Barnes LE, Bond J, Jagger C, Robinson L, Brayne C, Medical Research Council Cognitive Function and Ageing Collaboration. A two-decade comparison of prevalence of dementia in individuals aged 65 years and older from three geographical areas of England: results of the Cognitive Function and Ageing Study I and II. *The Lancet*. 2013 Nov 1;382(9902):1405-12.

Modelling steps

First, we ran a CODEm model for dementia and extracted the mortality rates by age, sex, and geography for 2016. The covariates used in this model are displayed below.

Level	Covariate	Direction
1	Diabetes age-specific proportion	+
	Mean BMI	+
	Cholesterol (total, mean per capita)	+
	Systolic blood pressure (mmHg)	+
2	Animal fats (kcal per capita)	+
	Latitude Over 45 (proportion)	+
	Red meat consumption adjusted (g)	+
	Healthcare access and quality index	-
3	Education (years per capita)	-
	LDI per capita(I$ per capita)	0
	Sanitation (proportion with access	-
	Improved water source (proportion with access)	-

Second, we ran a DisMod-MR 2.1 model with all data on incidence, prevalence, and mortality risk (RR, SMR, or with-condition mortality rates) and a setting of zero remission and extracted 2016 prevalence by age, sex, and geography. To account for potential systematic differences between US medical claims and survey data, we crosswalked for each year of claims data.

Third, we selected countries where the sum of cause-specific mortality rate to prevalence ratio for males and females exceeded 0.4 (excluding small island nations and those without vital registration). This resulted in choosing the United States, Sweden, Finland and Puerto Rico. The choice to pick fewer countries for this regression compared to GBD 2015, which used 30 countries in the EMR regression, was motivated by a desire to reduce the spread in EMR values, as countries used in the regression retain their original EMR values.

Fourth, we used a linear effects regression with dummies on age group and sex to predict excess mortality (ie, the ratio of cause-specific mortality rate and prevalence) by age and sex, the results of which are found in the tables below.

Table: Fixed effect coefficients of EMR regression. Outcome: ln(EMR)

Independent variables	Coef	Std. error	P value	95% Confidence Interval	
Male	0.331	0.056	0.000	0.222	0.440
Age 40-59	-3.34	0.118	0.000	-3.571	-3.109
Age 60-64	-2.693	0.118	0.000	-2.924	-2.462
Age 65-69	-2.448	0.118	0.000	-2.679	-2.217
Age 70-74	-2.113	0.118	0.000	-2.344	-1.882
Age 75- 80	-1.738	0.118	0.000	-1.969	-1.507
Age 80-84	-1.385	0.118	0.000	-1.616	-1.154
Age 85-89	-0.974	0.118	0.000	-1.205	-0.743
Age 90-94	-0.457	0.118	0.000	-0.688	-0.226
Constant	-1.802	0.088	0.000	-1.974	-1.630

Table: Predicted EMR values by age and sex (95% CI)

	Male	Female
Age 40-59	0.008 (0.007 - 0.01)	0.006 (0.005 - 0.007)
Age 60-64	0.016 (0.013 - 0.019)	0.011 (0.009 - 0.013)
Age 65-69	0.02 (0.017 - 0.024)	0.014 (0.012 - 0.017)
Age 70-74	0.028 (0.024 - 0.033)	0.02 (0.017 - 0.024)
Age 75- 80	0.04 (0.034 - 0.048)	0.029 (0.025 - 0.034)
Age 80-84	0.057 (0.048 - 0.068)	0.042 (0.035 - 0.048)
Age 85-89	0.087 (0.074 - 0.104)	0.063 (0.053 - 0.074)
Age 90-94	0.146 (0.123 - 0.173)	0.104 (0.088 - 0.123)
Age 95+	0.23 (0.193 - 0.274)	0.166 (0.14 - 0.196)

Fifth, these estimates were added to a second DisMod-MR 2.1 model as pertaining to the full 1990–2016 estimation period. For the four countries included in the regression, we retained their age- and sex-specific ratios and entered those also as pertaining to the full 1990–2016 estimation period. Age-standardized years of education was used as a country-level covariate. We excluded data for standardized mortality ratio, with-condition mortality rate, and relative risk as we wanted to estimate cause-specific mortality rates that were consistent with the level of excess mortality from the four chosen countries in 2016.

Sixth, we took the predictions of cause-specific mortality by age, sex, geography, and year that DisMod-MR 2.1 calculated as being consistent with the data on incidence, prevalence, and the priors on excess mortality from step five.

Seventh, because DisMod-MR 2.1 produces estimates in five-year intervals only, we expanded the time series by log-linear interpolation. Values for 1980-1990 were generated using a regression on the entire time series with Socio-demographic index included as a predictor.

Lastly, before adding the dementia mortality estimates into CodCorrect, we proportionately retrieved the difference in deaths between those estimated in CODEm and those estimated in step 7 from a set of 'target causes' which were identified as causes of death in cohort studies of persons with dementia. The

target causes included lower respiratory infections, protein-energy malnutrition, other nutritional deficiencies, cerebrovascular disease, interstitial nephritis and urinary tract infections, decubitus ulcer, and pulmonary aspiration and foreign body in airway.[4] [5] [6] [7] More information on this process is located in section 4 of the appendix.

[4] Brunnström HR, Englund EM. Cause of death in patients with dementia disorders. *European journal of neurology*. 2009 Apr 1;16(4):488-92.
[5] Thomas BM, Starr JM, Whalley LJ. Death certification in treated cases of presenile Alzheimer's disease and vascular dementia in Scotland. *Age and Ageing.* 1997 Sep 1;26(5):401-6.
[6] Todd S, Barr S, Roberts M, Passmore AP. Survival in dementia and predictors of mortality: a review. *International journal of geriatric psychiatry*. 2013 Nov 1;28(11):1109-24.
[7] Keene J, Hope T, Fairburn CG, Jacoby R. Death and dementia. *International journal of geriatric psychiatry.* 2001 Oct 1;16(10):969-74.

Parkinson Disease

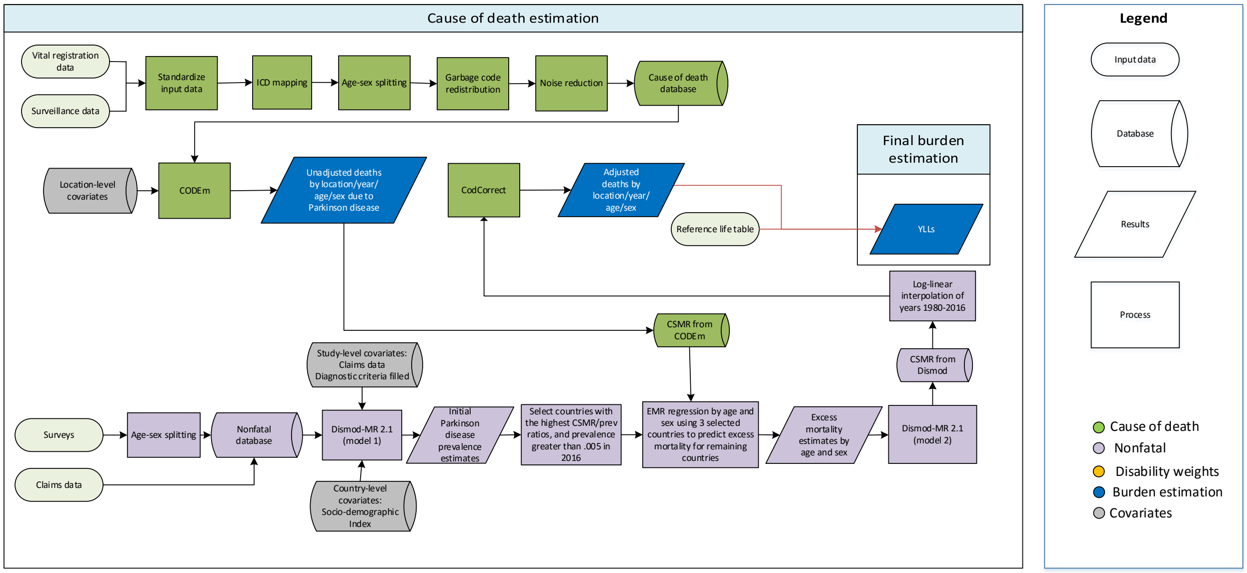

Input Data

In GBD 2016, data used to estimate deaths due to Parkinson disease included mortality data from vital registration systems and prevalence data from surveys and claims sources.

An updated systematic review was conducted from January 2011 to December 2015, and search terms[1] were set to capture studies for Parkinson disease. The search yielded 1,433 initial hits and 17 were marked for extraction. Inclusion criteria comprised studies that reported prevalence, incidence, remission rate, excess mortality rate, relative risk of mortality, standardized mortality ratio, or with-condition mortality rate. Studies with no clearly defined sample or that drew from specific clinic/patient organizations were excluded.

Modelling Strategy

Overview

Parkinson's disease mortality rates have more than doubled since 1980 in high-quality vital registration systems such as in the US, Canada, Australia, France, Germany, the United Kingdom and Finland, while other European countries like the Netherlands, Sweden, and Norway have not seen such increases over time. We have not seen an equivalent increase in prevalence and incidence data sources. Additionally, the greater than 15-fold variation in mortality rates of Parkinson disease between countries is much greater the three-fold difference in prevalence and incidence between high-income countries. As it is unlikely that case fatality from Parkinson disease has dramatically increased over the time period and that it would differ by a very large margin between countries, the hypothesis is that certifying and coding practices have changed over time and at a different pace between countries. Therefore, for GBD 2016 we decided to employ a modelling strategy which we have previously used to model mortality

[1] (((((Parkinson disease AND epidemiology) AND ("2011/01/01"[PDat] : "2015/12/31"[PDat]))) AND ((Parkinson disease AND epidemiology))))

from Alzheimer disease and other dementias. This modelling process avoids spurious large trends over time in the fatal component of the burden of dementia by making dementia mortality rates consistent with the rates observed in 2016 relative to prevalence in countries that are most likely to certify or code Parkinson disease as an underlying cause of death.

Modelling steps

First, we ran a CODEm model for Parkinson disease and extracted the mortality rates by age, sex, and geography for 2016.

Second, we ran a DisMod-MR 2.1 model with all data on incidence, prevalence, and mortality risk (RR, SMR, or with-condition mortality rates) and a setting of zero remission and extracted 2016 prevalence by age, sex, and geography. To account for potential systematic differences between claims and survey data, we crosswalked for each year of claims data.

Third, we selected the three countries (United States, Finland, and Austria) with the highest cause-specific mortality rate (from step 1) to prevalence (from step 2) ratio in 2016, which also had an age-standardised prevalence rate greater than 0.0005, and a population greater than 1 million.

Fourth, we used a linear effects regression with dummies on age group and sex to predict excess mortality (i.e., the ratio of cause-specific mortality rate and prevalence) by age and sex, the results of which are found in the tables below.

Table: Fixed effect coefficients of EMR regression. Outcome: ln(EMR)

Independent variables	Coef	Std. error	P value	95% Confidence Interval	
Male	0.214	0.074	0.006	0.069	0.359
Age 40-59	-3.522	0.157	0.000	-3.829	-3.214
Age 60-64	-2.716	0.157	0.000	-3.024	-2.409
Age 65-69	-2.236	0.157	0.000	-2.544	-1.929
Age 70-74	-1.686	0.157	0.000	-1.993	-1.378
Age 75- 80	-1.194	0.157	0.000	-1.502	-0.887
Age 80-84	-0.779	0.157	0.000	-1.087	-0.471
Age 85-89	-0.493	0.157	0.003	-0.800	-0.185
Age 90-94	-0.203	0.157	0.202	-0.511	0.104
Constant	-2.097	0.117	0.000	-2.326	-1.867

Table: Predicted EMR values by age and sex (95% CI)

	Male	Female
Age 40-59	0.005 (0.004 - 0.006)	0.004 (0.003 - 0.005)
Age 60-64	0.01 (0.008 - 0.013)	0.008 (0.006 - 0.01)
Age 65-69	0.016 (0.013 - 0.02)	0.013 (0.01 - 0.016)
Age 70-74	0.028 (0.023 - 0.035)	0.023 (0.018 - 0.029)
Age 75- 80	0.047 (0.037 - 0.059)	0.037 (0.029 - 0.046)
Age 80-84	0.071 (0.055 - 0.089)	0.057 (0.045 - 0.07)
Age 85-89	0.093 (0.073 - 0.117)	0.076 (0.06 - 0.093)
Age 90-94	0.126 (0.099 - 0.155)	0.101 (0.08 - 0.127)
Age 95+	0.154 (0.122 - 0.191)	0.123 (0.097 - 0.153)

Fifth, these estimates were added to a second DisMod-MR 2.1 model as pertaining to the full 1990–2016 estimation period. For the three countries included in the regression, we retained their age- and sex-specific ratios and entered those also as pertaining to the full 1990–2016 estimation period.

Sixth, we took the predictions of cause-specific mortality by age, sex, geography, and year that DisMod-MR 2.1 calculated as being consistent with the data on incidence, prevalence, and the priors on excess mortality from step five. Socio-demographic Index was used as a country-level covariate. We excluded data for standardized mortality ratio, with-condition mortality rate, and relative risk as we wanted to estimate cause-specific mortality rates that were consistent with the level of excess mortality from the three chosen countries in 2016.

Seventh, because DisMod-MR 2.1 only produces estimates in five-year intervals, we expanded the time series by log-linear interpolation. Values for 1980-1990 were generated using a regression on the entire time series with Socio-demographic index included as a predictor.

Lastly, before adding the Parkinson mortality estimates into CodCorrect, we proportionately retrieved the difference in deaths between those estimated in CODEm and those estimated in step 7 from a set of 'target causes' which were identified as causes of death in cohort studies of persons with dementia. We assumed the same target causes for dementia would apply to Parkinson disease as well. The target causes included lower respiratory infections, protein-energy malnutrition, other nutritional deficiencies, cerebrovascular disease, interstitial nephritis and urinary tract infections, decubitus ulcer, and pulmonary aspiration and foreign body in airway.[2] [3] [4] [5] More information on this process is located in section 4 of the appendix.

[2] Brunnström HR, Englund EM. Cause of death in patients with dementia disorders. *European journal of neurology*. 2009 Apr 1;16(4):488-92.
[3] Thomas BM, Starr JM, Whalley LJ. Death certification in treated cases of presenile Alzheimer's disease and vascular dementia in Scotland. *Age and Ageing.* 1997 Sep 1;26(5):401-6.
[4] Todd S, Barr S, Roberts M, Passmore AP. Survival in dementia and predictors of mortality: a review. *International journal of geriatric psychiatry*. 2013 Nov 1;28(11):1109-24.
[5] Keene J, Hope T, Fairburn CG, Jacoby R. Death and dementia. *International journal of geriatric psychiatry.* 2001 Oct 1;16(10):969-74.

Epilepsy

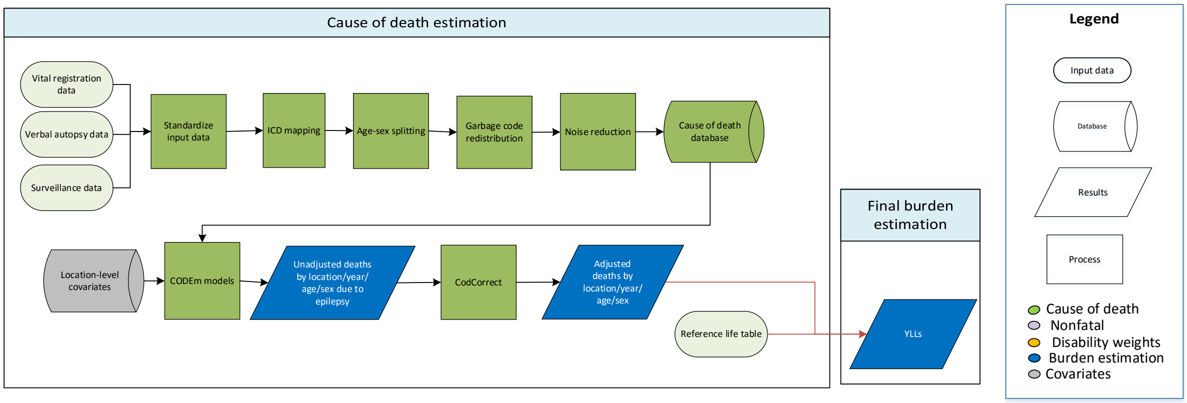

Input data

Data used to estimate epilepsy mortality included vital registration (VR), verbal autopsy, and China mortality surveillance data from the cause of death (COD) database. Our outlier criteria were to exclude data points that were (1) implausibly high or low relative to global or regional patterns, (2) substantially conflicted with established age or temporal patterns, or (3) significantly conflicted with other data sources based from the same locations or locations with similar characteristics (i.e., socio-demographic index).

Based on these criteria, we excluded ICD-9 BTL data for Sri Lanka, Fiji, and Kiribati as the estimates varied from year to year between zero and high values. We also excluded the Survey of Causes of Death Data and Medical Certification of Cause of Death Data for India, as these data types were not consistent with the Sample Registration System Data and would have led to discontinuities in our estimates over time.

Modelling strategy

The standard CODEm modelling approach was applied to estimate deaths due to epilepsy. Separate models were conducted for male and female mortality, and the age range for both models was 28 days–95+ years. For GBD 2016, the health systems access covariate was replaced with the health access and quality index covariate. There were no other substantial changes for GBD 2016. The covariates used are displayed below.

Level	Covariate	Direction
1	pig meat consumption (kcal per capita)	+
	pigs (per capita)	+
	SEV scalar: epilepsy	+
	mean systolic blood pressure (mmHg)	+
2	health access and quality index	-
	mean body mass index	+
	mean serum total cholesterol (mmol/L)	+

3	cumulative cigarettes (10 years)	+
	cumulative cigarettes (5 years)	+
	education (years per capita)	-
	log LDI (per capita)	-
	Socio-demographic Index	-

Multiple Sclerosis

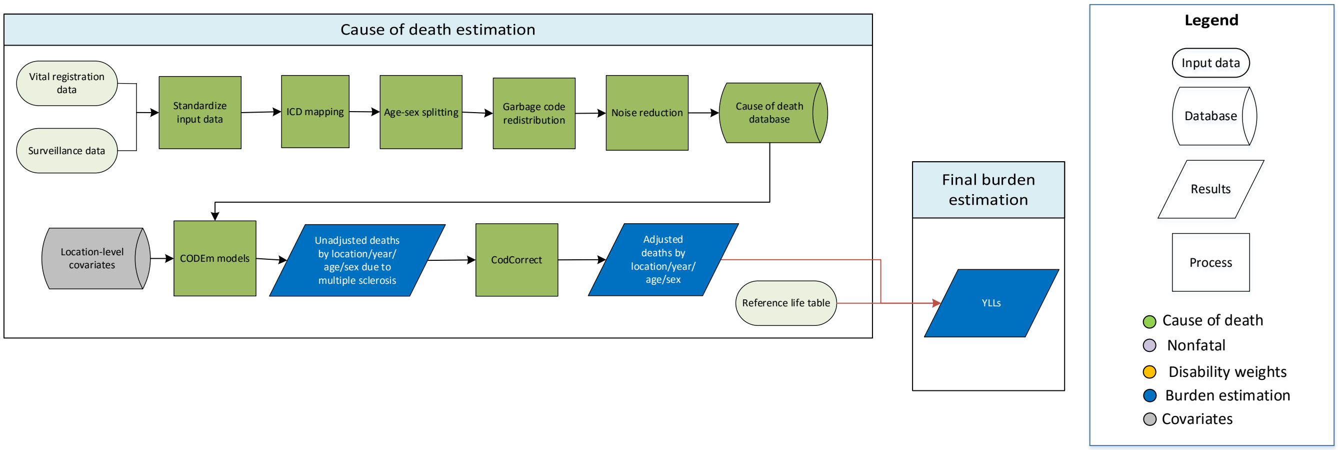

Input data

Data used to estimate multiple sclerosis included vital registration and surveillance data from the cause of death (COD) database. Our outlier criteria were to exclude data points that (1) were implausibly high or low, (2) substantially conflicted with established age or temporal patterns, or (3) significantly conflicted with other data sources conducted from the same locations or locations with similar characteristics (ie, Socio-demographic Index).

Modelling strategy

The standard CODEm modelling approach was used to estimate deaths due to multiple sclerosis. Separate models were conducted for male and female mortality, and the age range for both models was 20–95+ years. For GBD 2016, the health system access covariate was replaced by the new health care access and quality index covariate. Otherwise, there were no substantial changes from GBD 2015. The covariates used are displayed below.

Level	Covariate	Direction
1	absolute value of average latitude	+
2	animal fat consumption (kcal per capita)	+
	mean serum total cholesterol (mmol/L)	+
	health care access and quality index	-
3	cumulative cigarettes (10 years)	+
	cumulative cigarettes (5 years)	+
	education (years per capita)	-
	log-transformed LDI (per capita)	-
	smoking prevalence	+
	Socio-demographic Index	+

Motor Neuron Disease

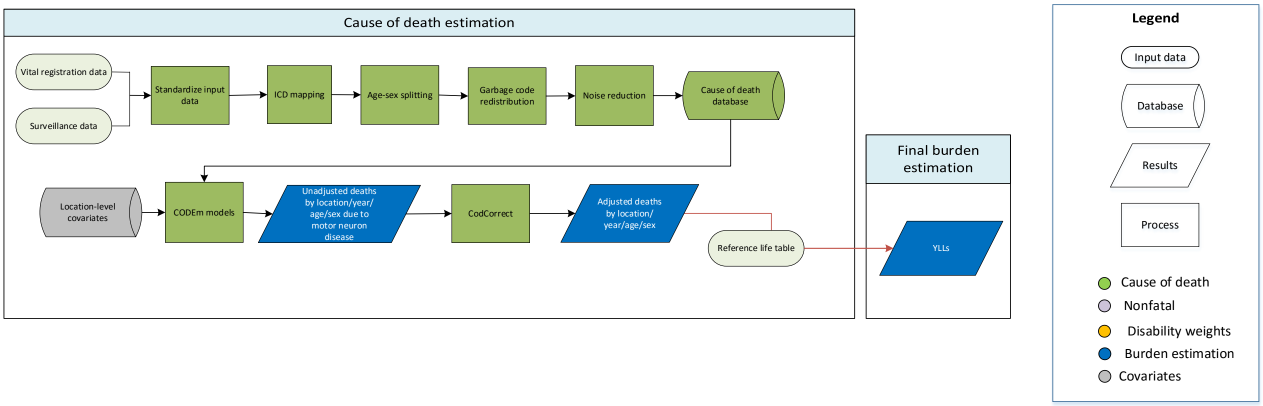

Input data

Data used to estimate motor neuron disease mortality included vital registration and surveillance data from the cause of death (COD) database. Our outlier criteria excluded data points that (1) were implausibly high or low, (2) substantially conflicted with established age or temporal patterns, or (3) significantly conflicted with other data sources conducted from the same locations or locations with similar characteristics (ie, Socio-demographic Index).

Modelling strategy

The standard CODEm modelling approach was used to estimate deaths due to multiple sclerosis. Separate models were conducted for male and female mortality, and the age range for both models was 20–95+ years. For GBD 2016, the fruit intake per capita covariate was adjusted to reflect intake per 2,000 kcal per day diet. Additionally, the health system access covariate was replaced by the health care access and quality index covariate. There were no other substantial changes from the GBD 2015 modelling strategy. The covariates used are displayed below.

Level	Covariate	Direction
1	asbestos production (kg per capita)	+
	mean serum total cholesterol (mmol/L)	0
	fruit consumption (grams per day adjusted)	0
2	absolute value of average latitude	+
	sanitation (proportion with access)	0
	improved water source (proportion with access)	0
	health care access and quality index	-
3	education (years per capita)	0
	log-transformed LDI (per capita)	0
	Socio-demographic Index	0

Other Neurological Disorders

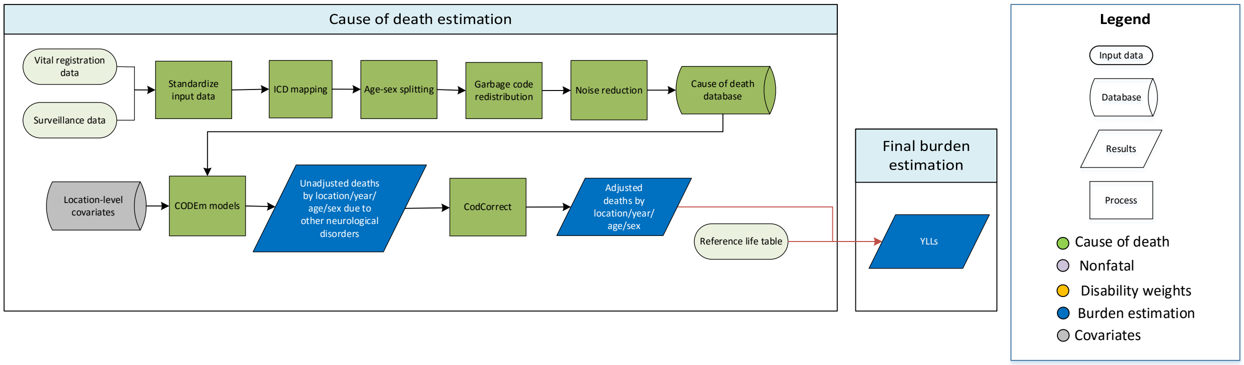

Input data

Data used to estimate other neurological disorders included vital registration and surveillance data from the cause of death (COD) database. Our outlier criteria were to exclude data points that (1) were implausibly high or low, (2) substantially conflicted with established age or temporal patterns, or (3) significantly conflicted with other data sources conducted from the same locations or locations with similar characteristics (ie, Socio-demographic Index).

Modelling strategy

The standard CODEm modelling approach was applied to estimate deaths due to other neurological disorders. Male and female CODEm models were run for deaths occurring between ages 28 days to 95+ years. For GBD 2016, the fruit and meat intake per capita covariates were adjusted to reflect intake per 2,000 kcal per day diet. Additionally, the covariate health system access was replaced with the health care access and quality index. There were no other significant changes from the GBD 2015 modelling strategy. The covariates used are displayed below.

Level	Covariate	Direction
1	underweight proportion was under 2 standard deviations	+
	mean body mass index	+
	mean cholesterol	+
	mean systolic blood pressure	+
	pig meat consumption (kcal per capita)	+
2	alcohol (litres per capita)	+
	animal fat consumption (kcal per capita)	+
	health care access and quality index	-
	fruit consumption adjusted	-
	population density over 1,000 per square kilometer pct	+
	red meat consumption adjusted	+
3	cumulative cigarette consumption (10 years)	+
	cumulative cigarette consumption (5 years)	+
	education (years per captia)	-
	log-transformed LDI (per capita)	-
	smoking prevalence	+

	Socio-demographic Index	0

Alcohol Use Disorders

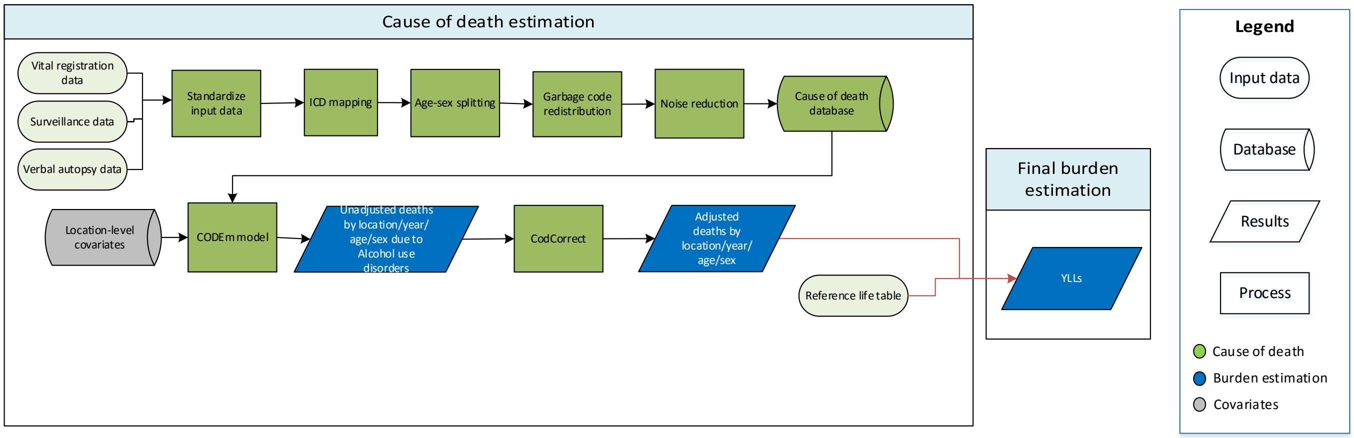

Input data

All data were from vital registration, China surveillance, and verbal autopsy sources. Some data were outliered from countries with sparse yet heterogeneous data if it created implausible fluctuations in deaths and regional patterns. As an example, Medical Certification of Cause of Death data from India was excluded for alcohol use disorders due to the extremely low estimates.

Modelling strategy

Cause of death modelling for alcohol use disorders followed the general CODEm strategy. There were no substantial changes from GBD 2015. Model covariate inclusion was based on empirical evidence and expert feedback which resulted in a set of model covariates that reflected alcohol consumption, smoking, education, health system access, percent of population Muslim, domestic income, and Socio-demographic Index (SDI).

Table: covariates used in alcohol use CODEm model

Level	Covariate	Direction
1	alcohol consumption (litres per capita)	+
	alcohol binge drinking	+
2	Muslim religion	-
	cumulative cigarettes (10 years)	0
	smoking prevalence	0
	health care access and quality index	-
3	log LDI (I$ per capita)	-
	education (years per capita)	-
	Socio-demographic Index	0

A significant limitation to previous alcohol use models was assumptions surrounding the redistribution of garbage codes. In GBD 2016, ICD codes for accidental poisoning (X40-44) were redistributed to the underlying GBD cause (substance use disorder) using an algorithm devised from analyzing national registry data from several countries and expert feedback. This is an improvement on the alcohol use model from previous rounds of GBD.

Drug use disorders

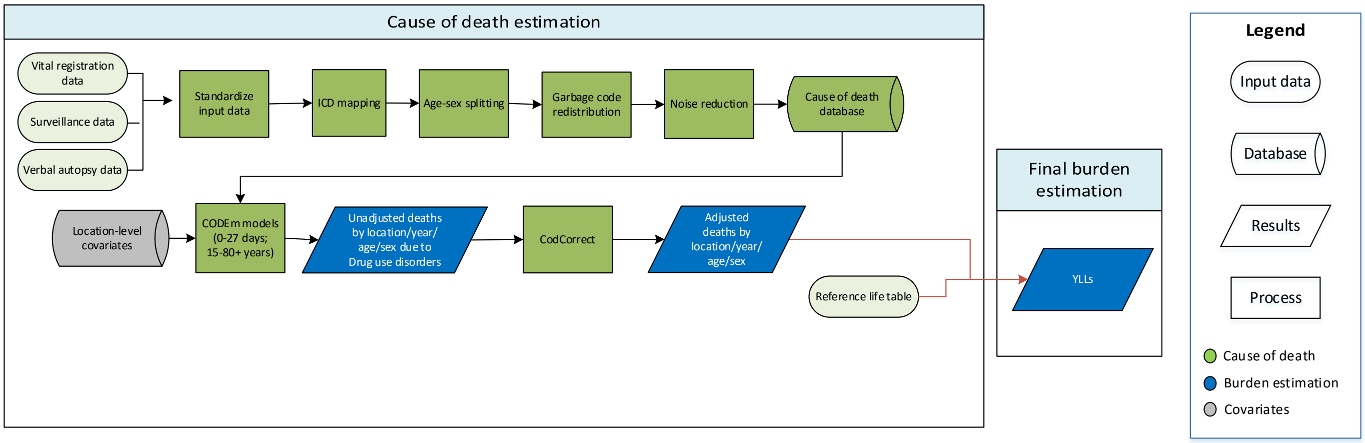

Input data

All data were from vital registration, verbal autopsy and surveillance sources. Data from countries with sparse yet heterogeneous data were also excluded as the data exaggerated fluctuations in deaths and gave implausible regional patterns. Excluded data were typically from developing countries. Notably, a considerable amount of Medical Certification of Cause of Death (MCCD) data from India was excluded for drug use disorders. Specifically, it was decided to remove the MCCD ICD-9 data as a specific garbage redistribution package was not available for that time series. Additionally, it was decided to remove MCCD-ICD10 data from the Northeastern states of Meghalaya, Mizoram, Nagaland and Manipur (where the the much lower values in MCCD compared to SRS removed the expected higher death rates there) and also from the four states of Punjab, Uttarakhand, Jharkand and Karnataka (where the raw data are showed almost no deaths from drug use disorders).

Modelling strategy

Cause of death modelling for drug use disorders followed the general CODEm strategy. In GBD 2016 additional geographies were included and age groups were extended beyond 80+. For GBD 2016, the health systems access covariate was replaced with the health care access and quality index covariate

Table: covariates used in drug use disorders CODEm model

Level	Covariate	Direction
1	alcohol use (litres per capita)	+
	cumulative cigarettes (10 years)	+
	cumulative cigarettes (5 years)	+
	opium cultivation bin	+
	smoking prevalence	+
2	health care access and quality index	-

3	log LDI (I$ per capita)	0
	education (years per capita)	0
	Socio-demographic Index	0

The drug use model is the parent model of all other drug use causes (ie, amphetamine, cocaine, opioid, and other drug). It forms an envelope into which all four individual drug use models are squeezed during the CoDCorrect process.

A significant limitation to previous drug use models was assumptions surrounding the redistribution of garbage codes. In GBD 2016, ICD codes for accidental poisoning (X40-44) were redistributed to the underlying GBD cause (substance use disorder) using an algorithm devised from analyzing national registry data from several countries and expert feedback. This is an improvement on the drug use model from previous rounds of GBD and we plan to refine that further in future iterations of GBD. There were no other substantial changes from GBD 2015.

Opioid dependence

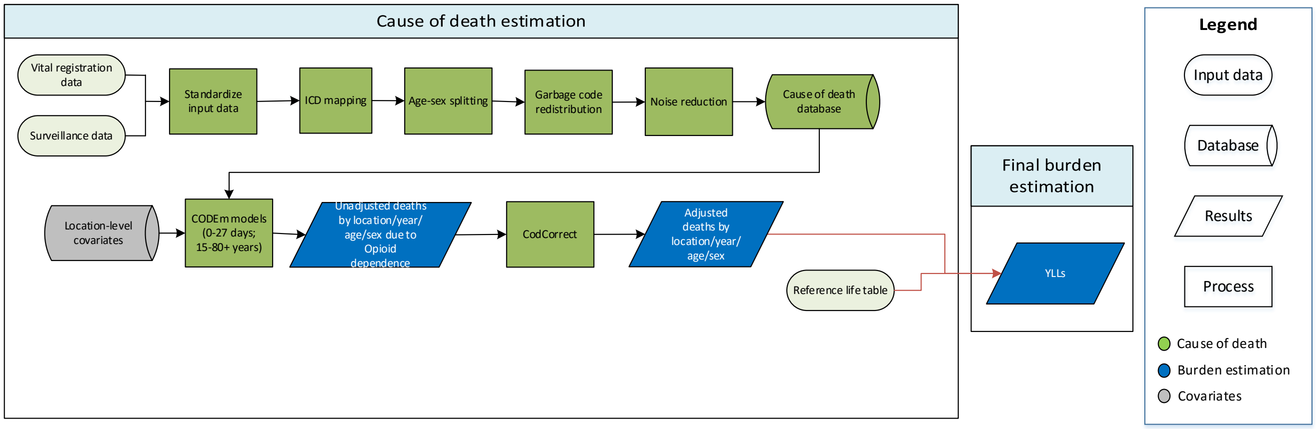

Input data

All data were from vital registration and surveillance sources. Data from countries with sparse yet heterogeneous data were also excluded as the data exaggerated fluctuations in deaths and gave implausible regional patterns. Excluded data were typically from developing countries.

Modelling strategy

Cause of death modelling for opioid use followed the general CODEm strategy. In GBD 2016 additional geographies were included and age groups were extended beyond 80+. For GBD 2016, the health systems access covariate was replaced with the health care access and quality index covariate.

Table: covariates used in opioid use CODEm model

Level	Covariate	Direction
1	alcohol (litres per capita)	+
	cumulative cigarettes (10 years)	+
	cumulative cigarettes (5 years)	+
	opium cultivation bin	+
	smoking prevalence	+
2	health care access and quality index	-
3	log LDI (I$ per capita)	0
	education (years per capita)	0
	Socio-demographic Index	0

A significant limitation to previous opioid use models was assumptions surrounding the redistribution of garbage codes. In GBD 2016, ICD codes for accidental poisoning (X40-44) were redistributed to the underlying GBD cause (substance use disorder) using an algorithm devised from analyzing national registry data from several countries and expert feedback. This is an improvement on the opioid use model from previous rounds of GBD. There were no other substantial changes from GBD 2015.

Cocaine Dependence

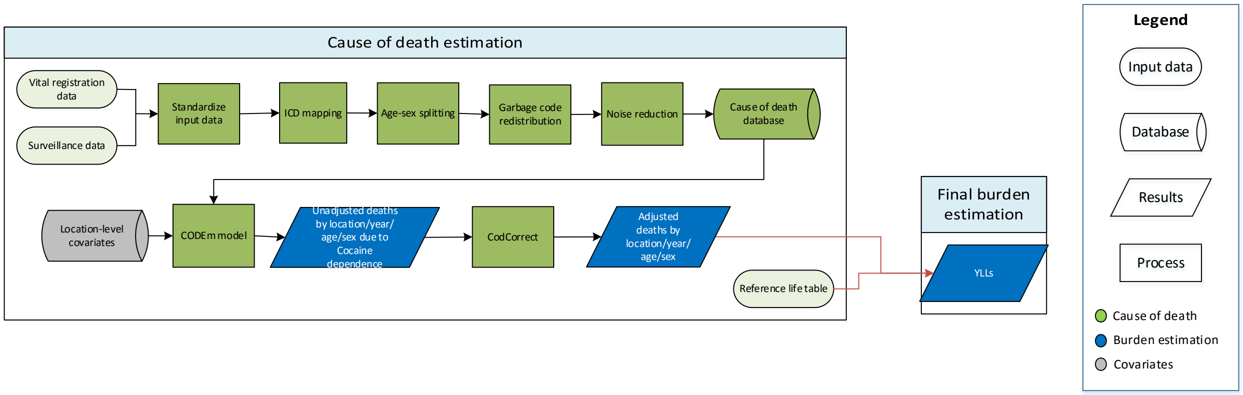

Input data

All data were from vital registration and surveillance sources. Data from countries with sparse yet heterogeneous data were also excluded as the data exaggerated fluctuations in deaths and gave implausible regional patterns. Excluded data were typically from developing countries.

Modelling strategy

Cause of death modelling for cocaine use followed the general CODEm strategy. In GBD 2016 additional geographies were included and age groups were extended beyond 80+. For GBD 2016, the health systems access covariate was replaced with the health care access and quality index covariate.

Table: covariates used in cocaine use CODEm model

Level	Covariate	Direction
1	alcohol use (litres per capita)	+
	cumulative cigarettes (10 years)	+
	cumulative cigarettes (5 years)	+
	smoking prevalence	+
2	health care access and quality index	-
3	log LDI (I$ per capita)	0
	education (years per capita)	0
	Socio-demographic Index	+

A significant limitation to previous cocaine use models was assumptions surrounding the redistribution of garbage codes. In GBD 2016, ICD codes for accidental poisoning (X40-44) were redistributed to the underlying GBD cause (substance use disorder) using an algorithm devised from analyzing national registry data from several countries and expert feedback. This is an improvement on the cocaine use model from previous rounds of GBD. There were no other substantial changes from GBD 2015.

Amphetamine Dependence

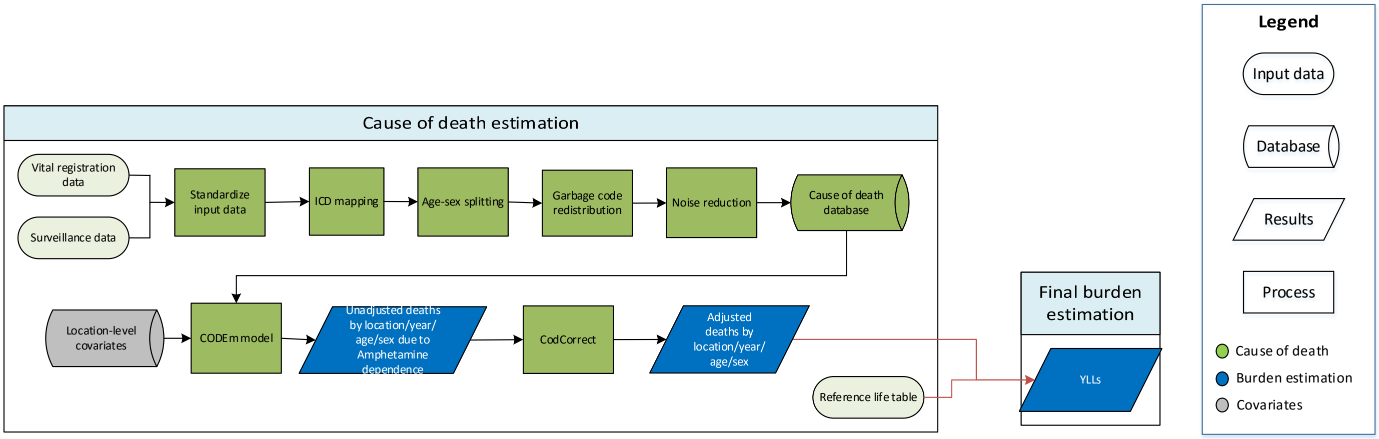

Input data

All data were from vital registration and surveillance sources. Data from countries with sparse yet heterogeneous data were also excluded as the data exaggerated fluctuations in deaths and gave implausible regional patterns. Excluded data were typically from developing countries.

Modelling strategy

Cause of death modelling for amphetamine use followed the general CODEm strategy. In GBD 2016 additional geographies were included and age groups were extended beyond 80+. For GBD 2016, the health systems access covariate was replaced with the health care access and quality index covariate.

Table: covariates used in amphetamine use CODEm model

Level	Covariate	Direction
1	alcohol use (litres per capita)	+
	cumulative cigarettes (10 years)	+
	cumulative cigarettes (5 years)	+
	smoking prevalence	+
2	health care access and quality index	-
3	log LDI (I$ per capita)	0
	education (years per capita)	0
	Socio-demographic Index	+

A significant limitation to previous amphetamine use models was assumptions surrounding the redistribution of garbage codes. In GBD 2015, ICD codes for accidental poisoning (X40-44) were redistributed to the underlying GBD cause (substance use disorder) using an algorithm devised from analyzing national registry data from several countries and expert feedback. This is an improvement on the amphetamine use model from previous rounds of GBD. There were no other substantial changes from GBD 2015.

Other drug use disorders

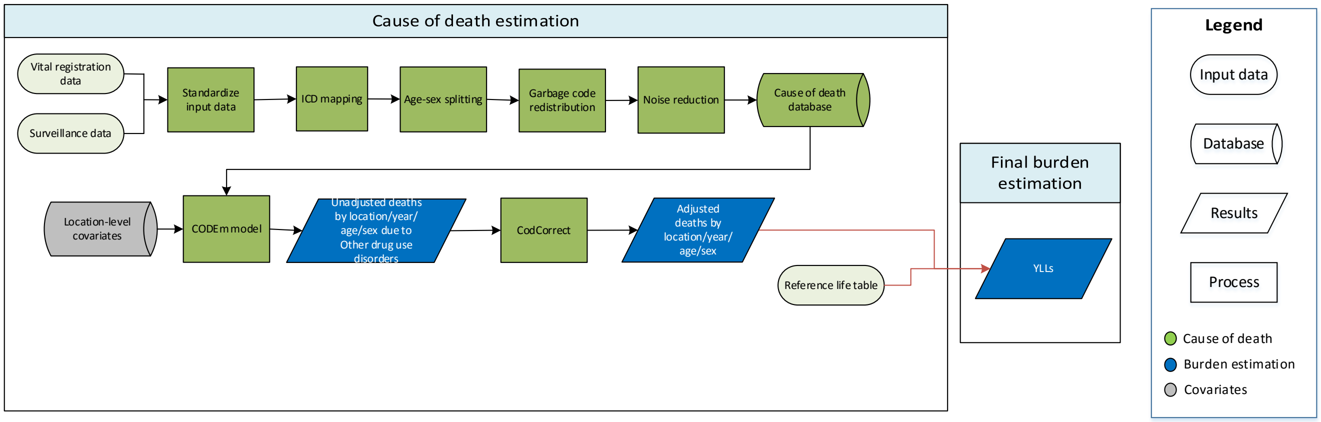

Input data

All data were from vital registration and China surveillance sources. Data from countries with sparse yet heterogeneous data were also excluded as the data exaggerated fluctuations in deaths and gave implausible regional patterns. Excluded data were typically from developing countries.

Modelling strategy

Cause of death modelling for other drug use followed the general CODEm strategy. In GBD 2016 additional geographies were included and age groups were extended beyond 80+. For GBD 2016, the health systems access covariate was replaced with the health care access and quality index covariate.

Table: covariates used in other drug use CODEm model

Level	Covariate	Direction
1	alcohol (litres per capita)	+
	cumulative cigarettes (10 years)	+
	cumulative cigarettes (5 years)	+
	smoking prevalence	+
2	health care access and quality index	-
3	log LDI (I$ per capita)	0
	education (years per capita)	0
	Socio-demographic Index	0

A significant limitation to previous other drug use models were assumptions surrounding the redistribution of garbage codes. In GBD 2016, ICD codes for accidental poisoning (X40-44) were redistributed to the underlying GBD cause (substance use disorder) using an algorithm devised from analyzing national registry data from several countries and expert feedback. This is an improvement on the other drug use model from previous rounds of GBD. There were no other substantial changes from GBD 2015.

Eating disorders

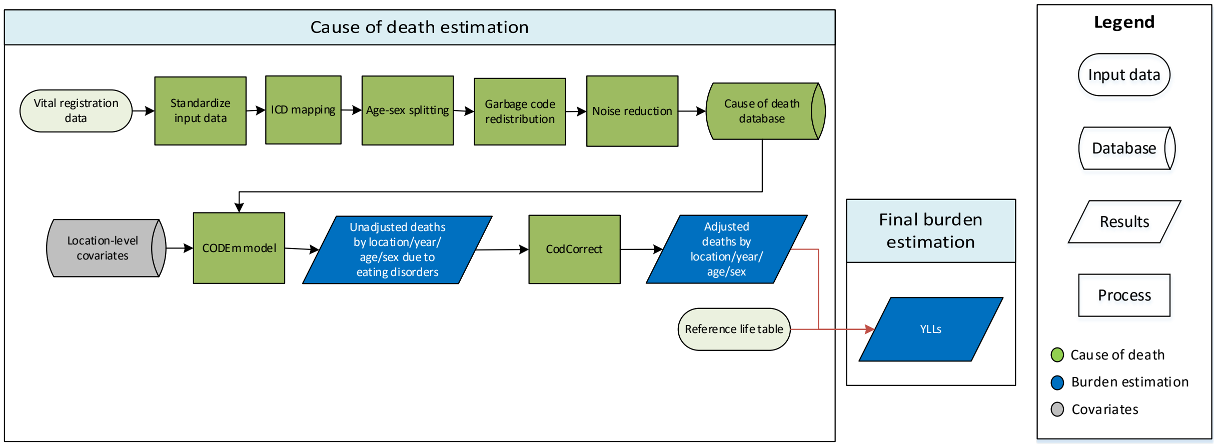

Input data

Data used to estimate eating disorders mortality included vital registration data from the cause of death (COD) database. Data points from sub-Saharan Africa were excluded as the redistribution of garbage codes led to implausible rates and age patterns of deaths due to eating disorders. Additionally, we excluded data from countries with small populations and sparse yet heterogeneous data, as these sources exaggerated fluctuations in deaths and gave implausible regional patterns. Data were excluded from countries including but not limited to Caribbean countries, Oceanic countries, Brunei, El Salvador, Guatemala, Morocco, Malaysia, Philippines, Algeria, Palestine, Greenland, and Malta.

Modelling strategy

Eating disorders was modelled using standard CODEm modelling approach and encompassing the two child models of anorexia nervosa and bulimia nervosa. Age was restricted to deaths occurring between 5 and 49 years of age based on expert advice and patterns of prevalence seen in the non-fatal models of anorexia nervosa and bulimia nervosa. Several covariates were applied to this model and are listed in the table below, along with the direction in which they were applied.

Level	Covariate	Direction
1	education (years per capita)	+
	log LDI (I$ per capita)	+
	underweight (proportion <2SD weight for age, <5 years)	-
	sanitation (proportion with access)	+
	maternal education (years per capita)	+
2	health care access and quality index	-
3	Socio-demographic Index	+

In GBD 2013, eating disorders were modelled as a negative binomial model using a custom approach. This approach was changed in GBD 2015 with eating disorders being modelled as a standard CODEm model. The modelling strategy was changed in GBD 2015 as no obvious benefit was seen from using the custom modelling approach. As such, it was decided that all eating disorders (eating disorders, anorexia nervosa, and bulimia nervosa) would be modelled using CODEm. GBD 2016 utilised the same approach as GBD 2015 with the only difference being the inclusion of covariates.

The rate of deaths due to eating disorders is relatively low although we suspect that in many countries, particularly developing countries, the coding of deaths to eating disorders is inconsistent. For example, a sharp increase in deaths was seen in the raw data from age 50 onward, which we believe is due to the incorrect assignments of deaths eg, deaths from starvation due to neglect or causes such as dementia being incorrectly assigned to anorexia nervosa which is then reflected in the overarching eating disorders model. This causes issues when trying to discern plausible patterns in age and geography.

Anorexia nervosa

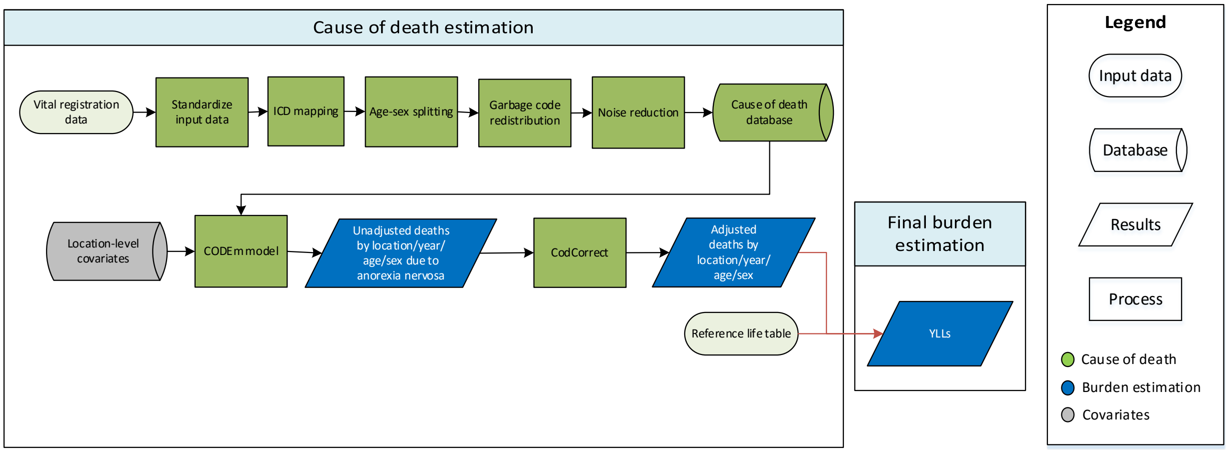

Input data

Data used to estimate anorexia nervosa mortality included centrally prepped vital registration data from the cause of death (COD) database. Data points from sub-Saharan Africa were excluded as the redistribution of garbage codes led to implausible rates and age patterns of deaths due to eating disorders. Additionally, we excluded data from countries with small populations and sparse yet heterogeneous data as these sources exaggerated fluctuations in deaths and gave implausible regional patterns. Data were also excluded from countries including but not limited to Caribbean countries, Oceanic countries, some Central Latin American countries, and other developing countries (particularly those with small populations).

Modelling strategy

Anorexia nervosa was modelled using the standard CODEm approach and came under the eating disorders parent model. Age was restricted to deaths occurring between 5 and 49 years based on expert advice and patterns of prevalence seen in the non-fatal model. Several covariates were applied to this model and are listed in the table below, along with the direction in which they were applied.

Level	Covariate	Direction
1	education (years per capita)	+
	log LDI (I$ per capita)	+
	underweight (proportion <2SD weight for age, <5 years)	-
	sanitation (proportion with access)	+
	maternal education (years per capita)	+
2	health care access and quality index	-
3	Socio-demographic Index	+

In GBD 2013, anorexia nervosa deaths were extrapolated from the eating disorders model, which was modelled through a negative binomial approach. In GBD 2015, we changed this strategy and modelled anorexia nervosa deaths through a standard CODEm approach under the overarching eating disorders model as there was no benefit observed from applying the custom approach. As such, it was decided that all eating disorders (eating disorders, anorexia nervosa, and bulimia nervosa) would be modelled using CODEm. GBD 2016 utilised the same approach as GBD 2015 with the only difference being the inclusion of covariates.

The rate of deaths due to anorexia nervosa is relatively low although we suspect that in many countries, particularly lower-income countries, the coding of deaths to eating disorders is inconsistent. For example, a sharp increase in deaths was seen in the raw data from age 50 onward, which we believe is due to the incorrect assignments of deaths to anorexia nervosa, eg, deaths from starvation due to dementia being incorrectly assigned to anorexia nervosa. This causes issues when trying to discern plausible patterns in age and geography.

Bulimia nervosa

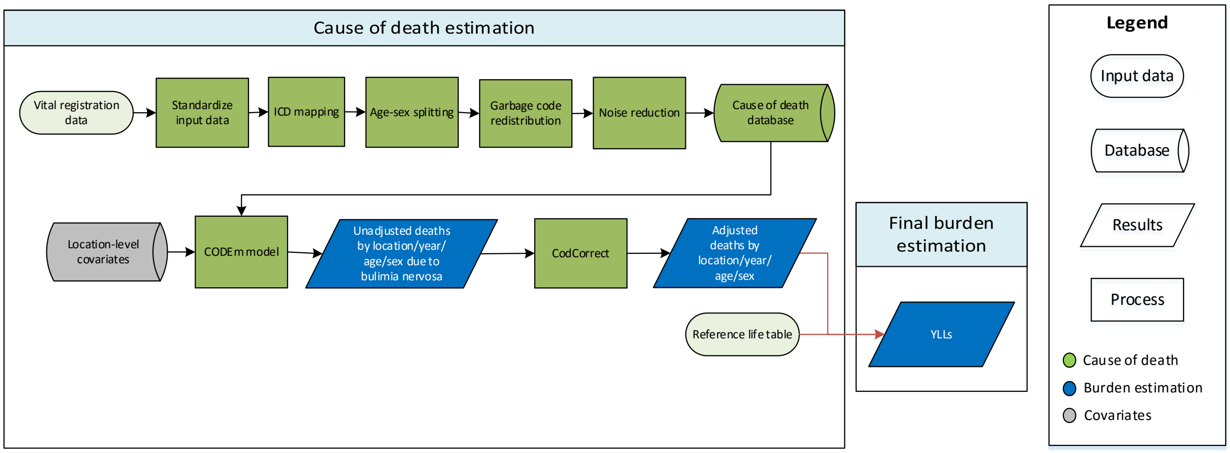

Input data

Data used to estimate bulimia nervosa mortality included centrally prepped vital registration data from the cause of death (COD) database. Data points from sub-Saharan Africa were excluded as the redistribution of garbage codes led to implausible rates and age patterns of deaths due to eating disorders. Additionally, we excluded data from countries with small populations and sparse yet heterogeneous data, as these sources exaggerated fluctuations in deaths and gave implausible regional patterns. This included, but was not limited to, Greenland, Malta, and Brunei.

Modelling strategy

Bulimia nervosa was modelled using the standard CODEm approach and comes under the eating disorders parent model. Age was restricted to deaths occurring between 5 and 49 years based on expert advice and patterns of prevalence seen in the non-fatal model. Several covariates were applied to this model and are listed in the table below, along with the direction in which they were applied.

Level	Covariate	Direction
1	education (years per capita)	+
	log LDI (I$ per capita)	+
	underweight (proportion <2SD weight for age, <5 years)	-
	sanitation (proportion with access)	+
	maternal education (years per capita)	+
2	health care access and quality index	-
3	Socio-demographic Index	+

In GBD 2013, bulimia nervosa was not modelled as a distinct cause of death. Any deaths due to bulimia nervosa were attributed to the eating disorders model. We changed this approach in GBD 2015, recognizing bulimia nervosa as an individual cause of death and therefore modelled it as a standard CODEm model under the overarching eating disorders model. This decision was based on observing deaths due to bulimia nervosa in high-quality vital registration data, such as data from the US. These data also include eating disorders not otherwise specified. GBD 2016 utilised the same approach as GBD 2015 with the only difference being the inclusion of covariates.

The rate of deaths due to bulimia nervosa is relatively low although we suspect that in many countries, particularly lower-income countries, the coding of deaths to eating disorders is inconsistent. Furthermore, the inclusion of deaths due to eating disorders not otherwise specified may add more noise to the model. This causes issues when trying to discern plausible patterns in age and geography.

Diabetes Mellitus

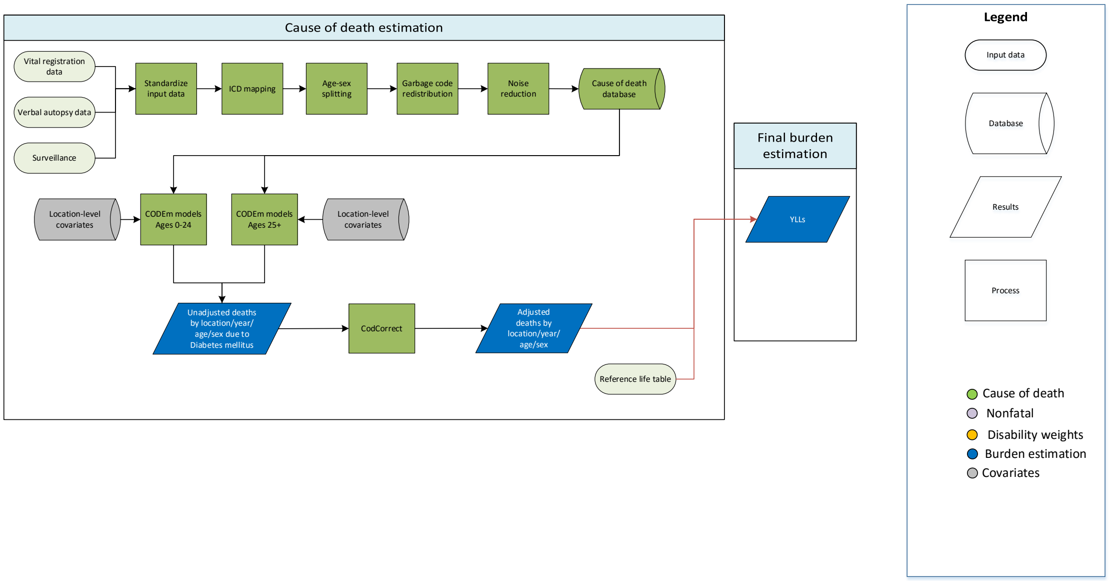

Input data

Verbal Autopsy Data: We outliered VA data points in urban Indian states where high-quality vital registration data were also available. We also outliered data points where the VA data were implausible in all age groups as we determined that these data sources were unreliable.

Vital Registration Data: We outliered all data in four urban Indian states where the source of the data was unreliable according to expert opinion. We also outliered ICD9BTL data points which were inconsistent with the rest of the data series and created unlikely time trends.

Modeling strategy

We used a slight variation on the standard CODEm approach to model deaths from diabetes mellitus. Since deaths in younger age groups are almost exclusively due to Type 1 diabetes while deaths in older ages are primarily due to Type 2, we used two models to estimate overall diabetes deaths. We reviewed the cause-fraction of deaths due to Type 1 and Type 2 diabetes at the global, super region, and regional level. We selected a conservative estimate of 25 years; one model is for deaths in 0-25 year olds and the second model is for deaths in 25+ year olds.

The following list are the covariates included in the model.

- Education years per-capita
- A composite score that approximates access to and quality of personal healthcare (Healthcare Access and Quality Index)
- Lag distributed GDP per capita in base 2010 international dollars
- Estimated national availability of animal fat expressed as kilocalories per capita
- Mean diabetes fasting plasma glucose (mmol/L) by age group
- Age-standardized prevalence of diabetes

- Age-standardized mean body mass index for adults ages 20+ (separate by sex)
- Mean serum total cholesterol (mmol/L) for individuals above age 25
- Mean systolic blood pressure (mmHg) for individuals above age 25
- Estimated energy adjusted national availability of fruits expressed in grams per person per day
- Estimated energy adjusted national availability of vegetables expressed in grams per person per day
- Estimated energy adjusted national availability of whole grains expressed in grams per person per day
- Estimated national availability of dietary energy expressed in kilocalories per person per day

Acute Glomerulonephritis

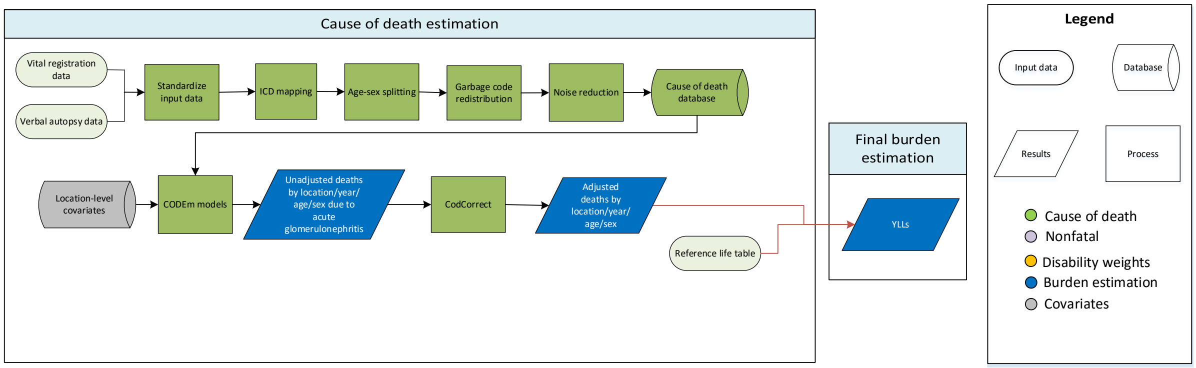

Input data

Vital registration data were used to model mortality due to acute glomerulonephritis. Vital registration data were standardized and mapped according to the GBD causes of death ICD mapping method. These data were then age-sex split, and appropriate redistribution of garbage code data was performed. After applying noise reduction, these data were uploaded to the COD database. Outliers were identified by systematic examination of data points for all location-years. Data points that violated well-established age or time trends or that resulted in extremely high or low cause fractions were determined to be outliers.

Modelling strategy

The estimation strategy used for fatal acute glomerulonephritis is largely similar to methods used in GBD 2015. A standard CODEm model with location-level covariates was used to model deaths due to acute glomerulonephritis. Age-restrictions for death estimations secondary to acute glomerulonephritis include 28 days for lower bound, 95+ for upper bound. Iterations of models were assessed at the location/year/age-group/sex level to determine whether data points merited exclusion via outliering. Unadjusted death estimates were adjusted using CoDCorrect to produce final estimates of YLLs. The estimates are limited by a paucity of data for regions such as Eastern and Central sub-Saharan Africa. The covariates used are displayed below.

Level	Covariate	Direction
2	Diabetes age-standardized prevalence	+
	Mean systolic blood pressure (mmHg)	+
	Sanitation (proportion with access)	–
	Improved water sources (proportion with access)	–
	Health care access and quality index	–
3	Socio-demographic Index	–
	Education (years per capita)	–
	Log LDI ($I per capita)	–

Chronic Kidney Disease

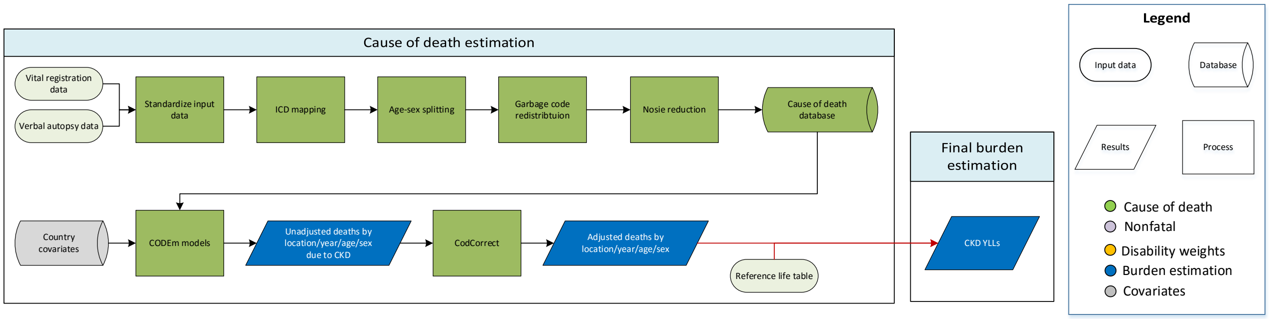

Input data

Vital registration and verbal autopsy data were used to model mortality due to urolithiasis. Outliers were identified by systematic examination of data points for all location-years. Data were standardised and mapped according to the GBD causes of death ICD mapping method. These data were then age-sex split, and appropriate redistribution of garbage code data was performed. Data points that violated well-established age or time trends or that resulted in extremely high or low cause fractions were determined to be outliers. For GBD 2016, deaths due to congenital kidney anomalies (cystic kidney disease and reflux hydronephrosis) were attributed to chronic kidney disease, marking a change from GBD 2015 when these deaths were assigned to congenital anomalies.

Modelling strategy

The estimation strategy used for fatal chronic kidney disease is largely similar to methods used in GBD 2015. A standard CODEm model with location-level was used to model deaths due to chronic kidney disease. Iterations of models were assessed at the location/year/age-group/sex level to determine whether data points merited exclusion via outliering. Unadjusted death estimates were adjusted using CoDCorrect to produce final estimates of YLLs. The covariates used are displayed below.

Level	Covariate	Direction
1	Diabetes fasting plasma glucose (mmol/L)	+
	Diabetes age-standardized prevalence (proportion)	+
	Mean systolic blood pressure (mmHg)	+
	Mean BMI	+
	Health care access and quality index	–
2	Mean cholesterol	+
	Total calories (kcal per capita)	–
	Red meat (kcal per capita)	0
	Whole grains (kcal per capita)	0
	Animal fat (kcal per capita)	0
3	Socio-demographic Index	0
	Education (years per capita)	–
	Log LDI ($I per capita)	–

Chronic Kidney Disease subtypes

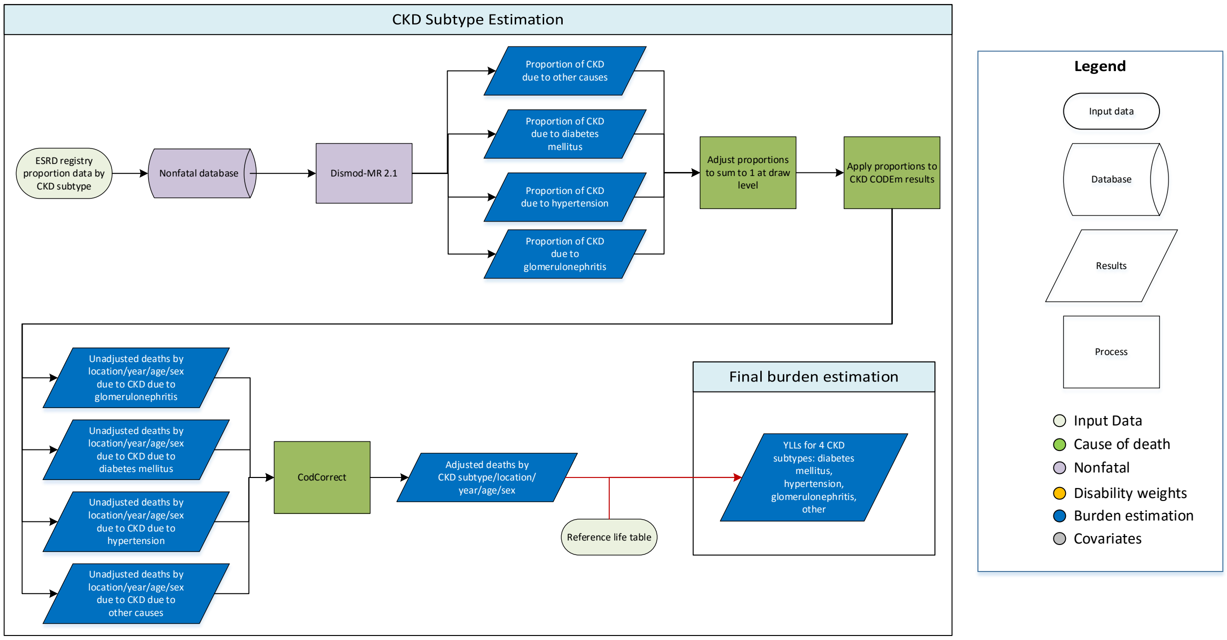

Input data

The estimation strategy for CKD subtypes of 1) diabetes mellitus, 2) hypertension, 3) glomerulonephritis, and 4) “other” has changed significantly from the GBD 2015 analysis to achieve consistency of method among the four subtypes. This improved method is detailed below.

Data from end-stage renal disease registries were used to inform estimates of proportion of CKD mortality attributable to each CKD subtype. These data were age-split using the age pattern obtained from the Australia & New Zealand Dialysis and Transplant Registry (ANZDATA) which provides age and subtype-specific data. The age-pattern was determined by calculating the number of cases of CKD by etiology over the total number of cases for all etiologies for each 5-year age group. Then, aggregate-age proportions were split using the age-specific prevalence proportions and rescaled to sum to 1 within each 5-year age group.

Vital registration (VR) data were excluded from estimates as etiology coding in VR sources was considered highly variable and inconsistent between countries.

Modelling strategy

We ran DisMod-MR 2.1 models including diabetes prevalence and mean systolic blood pressure as country-level covariates to obtain estimates of proportions for each subtype by location, year, age, and sex. The results from these models were adjusted so that estimates across the subtypes equaled 1 at each of 1,000 draws. These adjusted proportions were applied to the parent CKD CODEm model.

Model	Covariate	Value	Exponentiated
CKD proportion due to diabetes mellitus	Diabetes age-standardized prevalence	0.36 (0.29 – 0.42)	1.43 (1.34 – 1.53)
CKD proportion due to hypertension	Mean systolic blood pressure	0.013 (0.00036 – 0.043)	1.01 (1.00 – 1.04)

Urinary diseases and male infertility

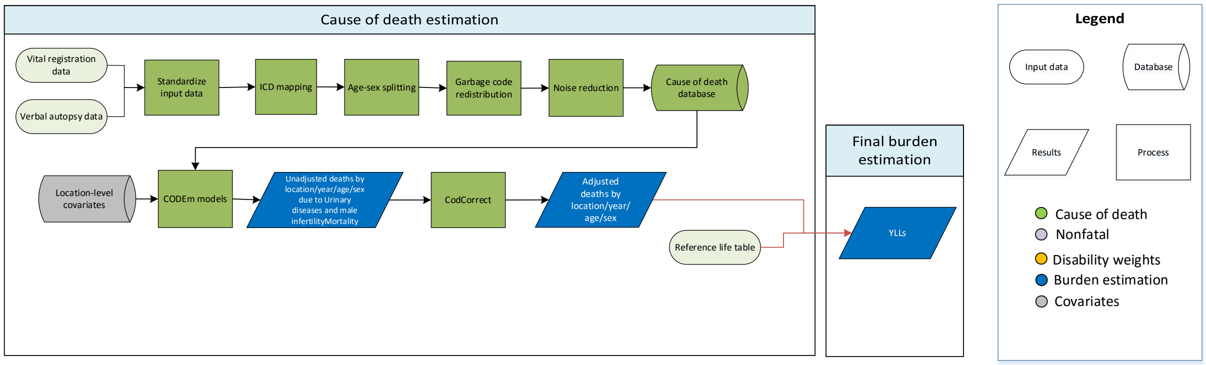

Input data

Vital registration data was used to model mortality due to urinary diseases and male infertility. Vital registration data were standardized and mapped according to the GBD causes of death ICD mapping method. These data were then age-sex split, and appropriate redistribution of garbage code data was performed. After applying noise reduction, these data were uploaded to the COD database. Outliers were identified by systematic examination of data points for all location-years. Data points that violated well-established age or time trends or that resulted in extremely high or low cause fractions were determined to be outliers.

Modelling strategy

The estimation strategy used for fatal urinary diseases and male infertility is largely similar to methods used in GBD 2015. A standard CODEm model with location-level covariates was used to model deaths due to urinary diseases and male infertility. Iterations of models were assessed at the location/year/age-group/sex level to determine whether data points merited exclusion via outliering. The estimates are limited by a paucity of data for regions such as Eastern and Central sub-Saharan Africa. The results of this disease differ by gender as no "male infertility" estimates were performed among women. The covariates used are displayed below.

Level	Covariate	Direction
2	Mean BMI	+
	Health care access and quality index	–
	Latitude under 15 (proportion)	0
	Latitude 15 to 30 (proportion)	0
	Latitude 30 to 45 (proportion)	0
	Latitude over 45 (proportion)	0
3	Education (years per capita)	–
	Log LDI ($I per capita)	–
	Socio-demographic Index	0

Interstitial Nephritis and Urinary Tract Infections

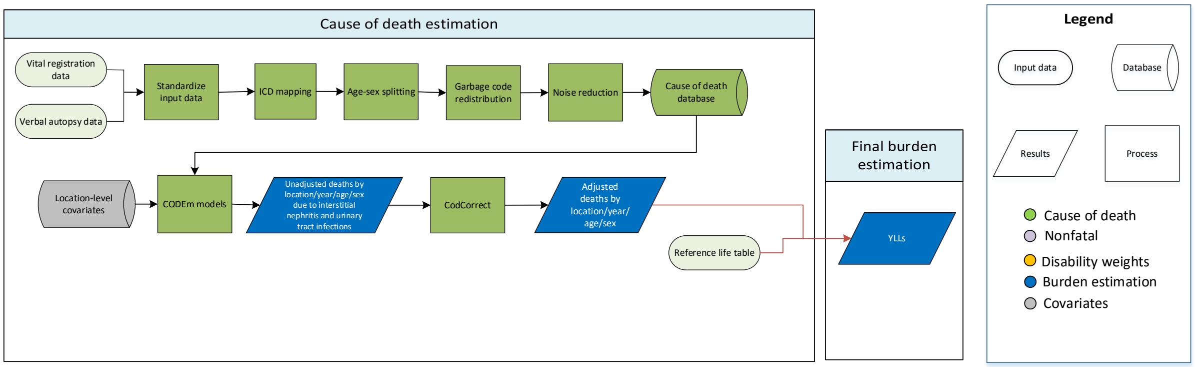

Input data

Vital registration and verbal autopsy data were used to model mortality due to interstitial nephritis. Data were standardized and mapped according to the GBD causes of death ICD mapping method. These data were then age-sex split, and appropriate redistribution of garbage code data was performed. After applying noise reduction, these data were uploaded to the COD database. Outliers were identified by systematic examination of data points for all location-years. Data points that violated well-established age or time trends or that resulted in extremely high or low cause fractions were determined to be outliers.

Modelling strategy

The estimation strategy used for fatal interstitial nephritis is largely similar to methods used in GBD 2015. A standard CODEm model with location-level covariates was used to model deaths due to interstitial nephritis. Age-restrictions for death estimations secondary to interstitial nephritis include 0 days for lower bound, 80+ for upper bound. Iterations of models were assessed at the location/year/age-group/sex level to determine whether data points merited exclusion via outliering. Unadjusted death estimates were adjusted using CoDCorrect to produce final estimates of YLLs. The estimates are limited by a paucity of data for regions such as Eastern and Central sub-Saharan Africa. The covariates used are displayed below.

Level	Covariate	Direction
1	Sanitation (proportion with access)	–
2	Education (years per capita)	–
	Log LDI ($I per capita)	–
	Health care access and quality index	–
3	Socio-demographic Index	0

Interstitial nephritis and urinary tract infections was a target cause for the Parkinson and Alzheimer disease mortality correction process whereby deaths due to causes closely associated with Parkinson and Alzheimer disease and other dementias were redistributed to Parkinson and Alzheimer disease and

other dementias in order to account for the common misclassification of deaths due to these neurological diseases. Detailed information on this process can be found in section 4 of the appendix.

Urolithiasis

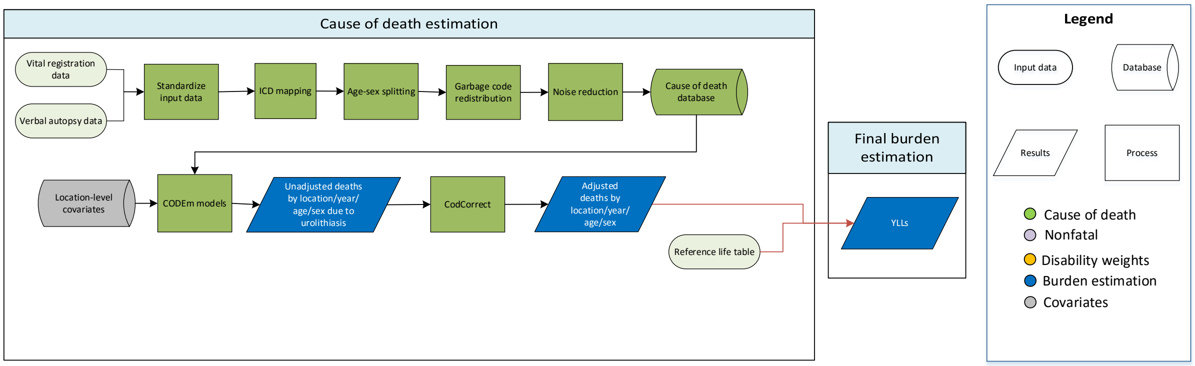

Input data

Vital registration and verbal autopsy data were used to model mortality due to urolithiasis. Data were standardized and mapped according to the GBD causes of death ICD mapping method. These data were then age-sex split, and appropriate redistribution of garbage code data was performed. After applying noise reduction, these data were uploaded to the COD database. Outliers were identified by systematic examination of data points for all location-years. Data points that violated well-established age or time trends or that resulted in extremely high or low cause fractions were determined to be outliers.

Modelling strategy

The estimation strategy used for fatal urolithiasis is largely similar to methods used in GBD 2015. A standard CODEm model including location-level covariates was used to model deaths due to urolithiasis. Age-restrictions for death estimations secondary to urolithiasis include 5 years for lower bound, 80+ for upper bound. Iterations of models were assessed at the location/year/age-group/sex level to determine whether data points merited exclusion via outliering. Unadjusted death estimates were adjusted using CoDCorrect to produce final estimates of YLLs. The estimates are limited by a paucity of data for regions such as Eastern and Central sub-Saharan Africa. The covariates used are displayed below.

Level	Covariate	Direction
1	Temperature (90th percentile)	+
2	Animal fat (kcal per capita)	+
	Fruits (kcal per capita)	−
	Vegetables (kcal per capita)	−
	Red meat (kcal per capita)	+
	Health care access and quality index	−
3	Socio-demographic Index	0
	Log LDI ($I per capita)	−

Other urinary diseases

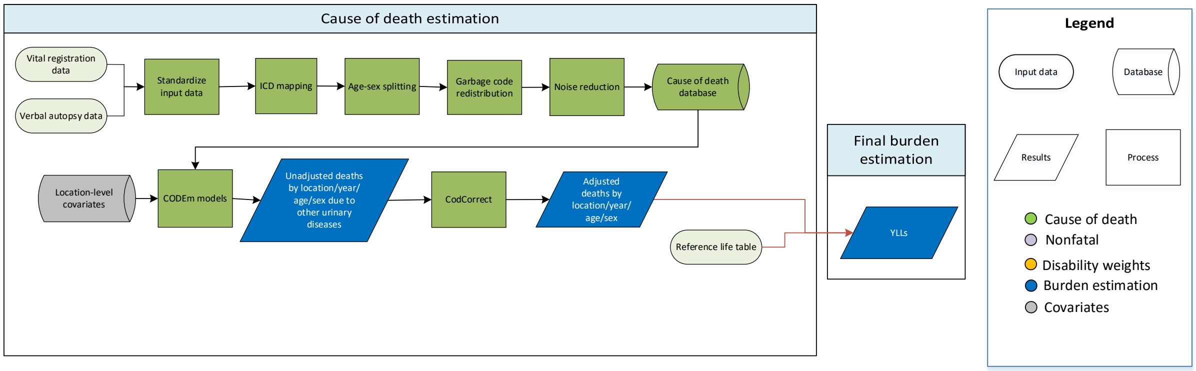

Input data

Vital registration and verbal autopsy data were used to model mortality due to other urinary diseases. Data were standardized and mapped according to the GBD causes of death ICD mapping method. These data were then age-sex split, and appropriate redistribution of garbage code data was performed. After applying noise reduction, these data were uploaded to the COD database. Outliers were identified by systematic examination of data points for all location-years. Data points that violated well-established age or time trends or that resulted in extremely high or low cause fractions were determined to be outliers.

Modelling strategy

The estimation strategy used for other urinary diseases is largely similar to methods used in GBD 2015. A standard CODEm model with location-level covariates was used to model deaths due to other urinary diseases. Age-restrictions for death estimations secondary to urinary diseases and male infertility include 0 days for lower bound, 95+ for upper bound. Iterations of models were assessed at the location/year/age-group/sex level to determine whether data points merited exclusion via outliering. Unadjusted death estimates were adjusted using CoDCorrect to produce final estimates of YLLs. The estimates are limited by a paucity of data for regions such as Eastern and Central sub-Saharan Africa. The covariates used are displayed below.

Level	Covariate	Direction
1	Mean BMI	+
2	Education (years per capita)	−
	Log LDI ($I per capita)	−
	Health care access and quality index	−
3	Socio-demographic Index	0

Gynaecological conditions

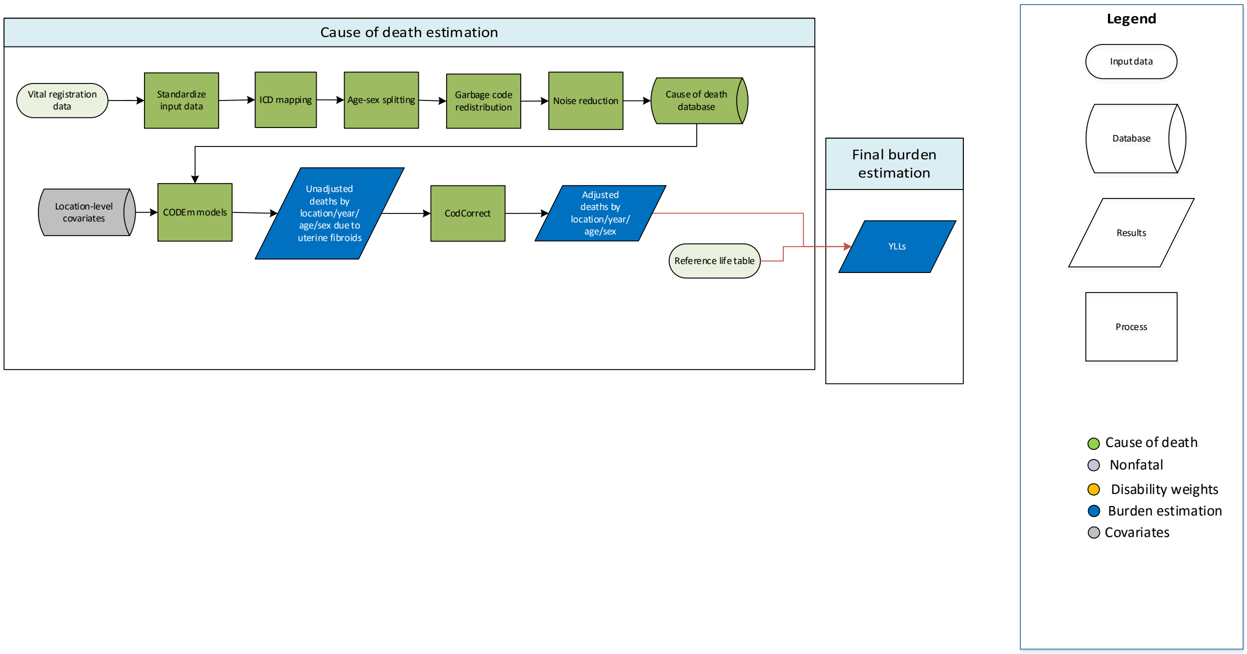

Input data

For GBD 2016, vital registration data were used to estimate deaths for each of the five fatal gynaecological conditions, which include uterine fibroids, PCOS, endometriosis, genital prolapse, and other gynaecological conditions. These causes are sex-specific to women and we only model deaths among women. ICD9 and ICD10 codes for each are listed below. Data points were selected as outliers if they were implausibly high, low, or significantly conflicted with established age or temporal patterns.

ICD10 and ICD9 codes used for gynecological disorders

Model	ICD10 code	ICD9 code
Uterine Fibroids	D25-D26.9, D28.2	218-219.9, 236.0
Endometriosis	N80-N80.9	617-617.9
Genital Prolapse	N81-N81.9	618-618.9
Polycystic Ovarian Syndrome	E28.2	256.4
Other Gynecological Disorders		621.4-622.7, 629-629.81

Modelling strategy

For GBD 2016, we estimated mortality due to total of all gynaecological diseases as well as each of the sub-categories using CODEm. We assumed no deaths from premenstrual syndrome and primary infertility, which we model for nonfatal outcomes.

Continuing in GBD 2016, we have reassigned deaths due to leiomyomas and other benign uterine tumors to uterine fibroids.

**Gynaecologic disorders**			
**Covariate**	**Transformation**	**Level**	**Direction**
Education	None	3	-1
LDI	Log	3	-1
Percent Births in 35+	None	2	1
Skilled Birth Attendance	None	2	-1
Smoking	None	1	0
Total Fertility Rate	None	2	1
Health System Access	None	2	-1
SDI	None	3	-1
HAQI	None	2	-1

Haemoglobinopathies and hemolytic anaemias

This write-up covers the following sub-causes: Sickle cell disorders, thalassemias, glucose-6-phosphate dehydrogenase (G6PD) deficiency, and other haemoglobinopathies and hemolytic anaemias

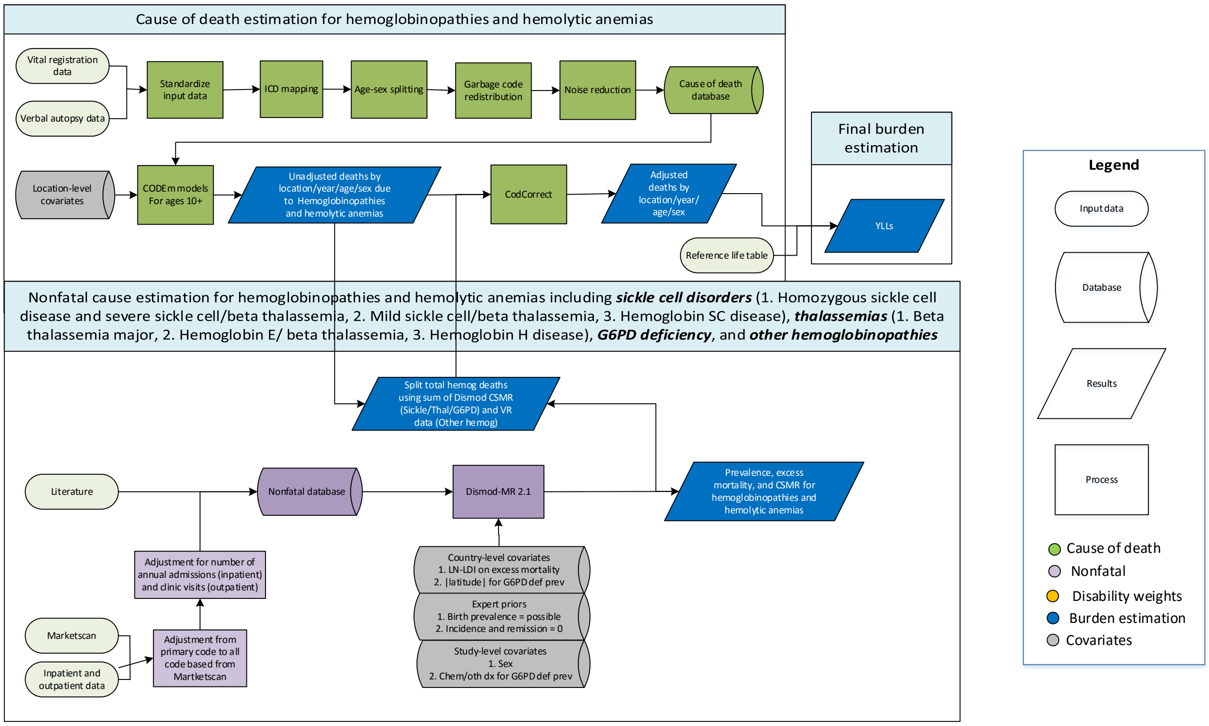

Input data

For GBD 2016, the overall CODEm model for haemoglobinopathies and hemolytic anemias was informed by centrally prepped data stored in the cause of death (COD) database. All data from all geographies were reviewed. Outliers were identified as those data where age patterns or temporal patterns were inconsistent with neighbouring age groups or locations or where sparse data were predicting implausible overall temporal or age patterns for a given location.

DisMod-MR 2.2 was used to estimate sickle cell disorders, thalassemias, and G6PD deficiency age- and sex-specific prevalence and mortality for each location and year in the GBD. Three sources of data were used for DisMod-MR 2.2 models: Literature, Marketscan data, and ICD-9 & ICD-10 hospital data. Each datum for sickle cell disease models was used for one of three mutually exclusive conditions: 1) homozygous sickle cell disease and severe sickle cell/beta thalassemia, 2) Mild sickle cell/beta thalassemia, or 3) Hemoglobin SC disease. We similarly extracted data for thalassemias using three mutually exclusive disease states: 1) Beta thalassemia major, 2) Hemoglobin E/beta thalassemia, and 3) Hemoglobin H disease. G6PD deficiency was estimated as a single model. Cause-specific mortality rates for other haemoglobinopathies and hemolytic anemias, lacking more specific data, was assumed to be geographically uniform, but did vary by age and sex; the levels and trends were informed by analysis of VR data from the COD database.

We added data identified select geographies identified by GBD collaborators for GBD 2016. Our last comprehensive literature review was completed in GBD 2015, where we identified data on prevalence, excess-mortality rate, or with-condition mortality rate. Age-specific survival probabilities from cohort studies were converted to corresponding with-condition mortality rates. G6PD deficiency is an X-linked recessive genetic disease, and genetic homozygosity served as the reference definition for our DisMod-MR 2.1 models in 2015. This was a change from GBD 2013 when we quantified G6PD deficiency on reagent tests as the reference category. Second, we extracted ICD-9-coded Marketscan data from the United States, correcting for multiple admissions, primary vs. non-primary coding, and outpatient vs. inpatient visits as determined from patient linkage analysis. Third, we used ICD-9 and ICD-10 inpatient and outpatient hospital data from all those locations where it was available, applying correction factors from analysis of claims data. These included correction factors for multiple admissions, age- and sex-specific ratio of prevalence that would be derived from only using the primary discharge ICD code versus that derived from using any of the discharge diagnosis codes, also accounting for differences in geography-specific overall hospitalization rate when calculating the ratio. This is the most substantial change for GBD 2015 when a single ratio for all ages and both sexes was applied to the data. We applied this as a correction factor for those sources where only a single ICD code is given for each discharge. Of note, there were no hospital data available for haemoglobin E/beta-thalassemia, haemoglobin H disease, or G6PD deficiency. All prevalence data from Marketscan, hospital sources, and literature were uploaded to the nonfatal database.

Modelling strategy

We completed seven separate DisMod-MR 2.2 models as listed above. Each used log-transformed lag-distributed income as a country-level covariate on excess mortality, which had the effect of predicting higher excess mortality in those locations with lower national income. The only study covariate used for most models was for sex. Genetic G6PD deficiency is far more common in males, but for all others the male to female ratio is nearly equivalent. Our G6PD deficiency model included additional study covariates to crosswalk from non-genetic diagnostic tests (eg, chemical reagent testing) back to the reference definition. Incidence and remission were both set to be zero.

We completed data-rich (DR) and global CODEm models for males and females separately. The sum of CSMR from all seven DisMod-MR 2.2 models was used as a predictive covariate for CODEm model development. CODEm results were then split between sickle cell disorders, thalassemias, G6PD deficiency, and other haemoglobinopathies and hemolytic anaemias using summed and scaled CSMR outputs from the same models. Other haemoglobinopathies and hemolytic anaemias did not have a separate DisMod-MR 2.2 model, but was instead informed by location-specific VR data.

The primary limitation of our estimation is data availability. We elected a hybrid approach of CODEm and DisMod-MR 2.2 to improve the quality of estimates in data-poor locations, but in most of these locations data are still relatively sparse for nonfatal models, which leads to relatively large uncertainty. Further adding to the uncertainty is the fact that haemoglobinopathies dramatically increase the risk of mortality due to infectious agents such as malaria, lower respiratory infections, and diarrhea, as well as increasing the risk of maternal mortality. In locations with poor diagnostic capabilities and high infectious burden, it is thus very plausible that mortality due to haemoglobinopathies may be even higher. Secondly, our specification of seven distinct entities for DisMod-MR 2.1 models does not align perfectly with the cause categories in the central COD prep, which limits the extent to which CSMR data

from the COD database can inform nonfatal models. We will continue to work to expand our dataset and consolidate the GBD analysis of haemoglobinopathies going forward.

Selected Covariates in parent CODEm model

Covariate	Transformation	Level	Direction
Education	None	3	-1
LDI	Log	3	-1
Hemoglobinopathies (sum of prevalence * excess-mortality from all DisMod models)	None	1	1
Sickle Cell & Thal (sum of prevalence * excess-mortality from all DisMod models – excluding G6PD deficiency)	None	1	1
SDI	None	3	-1
HAQI	None	2	-1
Health System Access, Capped	None	2	-1

Endocrine, Blood, Metabolic, and Immune Disorders

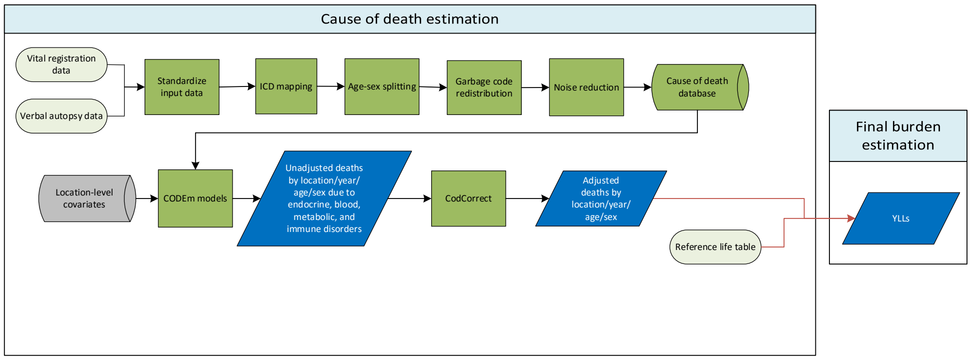

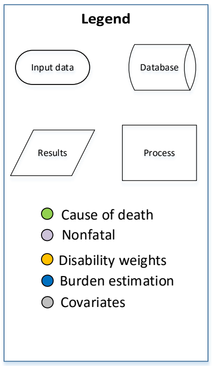

Input data

Vital registration and verbal autopsy data were used to model mortality due to endocrine, blood, metabolic, and immune disorders. Data were standardized and mapped according to the GBD causes of death ICD mapping method. These data were then age-sex split, and appropriate redistribution of garbage code data was performed. After applying noise reduction, these data were uploaded to the COD database. Outliers were identified by systematic examination of data points for all location-years. Data points that violated well-established age or time trends or that resulted in extremely high or low cause fractions were determined to be outliers.

Modelling strategy

The estimation strategy used for fatal endocrine, blood, metabolic, and immune disorders is largely similar to methods used in GBD 2015. A standard CODEm model with location-level covariates was used to model deaths due to endocrine, blood, metabolic, and immune disorders. Iterations of models were assessed at the location/year/age-group/sex level to determine whether data points merited exclusion via outliering. Unadjusted death estimates were adjusted using CoDCorrect to produce final estimates of YLLs. The covariates used are displayed below.

Level	Covariate	Direction
1	Mean BMI	+
2	Animal fat (kcal per capita)	+
	Alcohol (litres per capita)	+
	Total calories (kcal per capita)	+
	Mean cholesterol	+
	Health care access and quality index	–
3	Socio-demographic Index	0
	Log LDI ($I per capita)	–
	Education (years per capita)	–

Musculoskeletal disorders

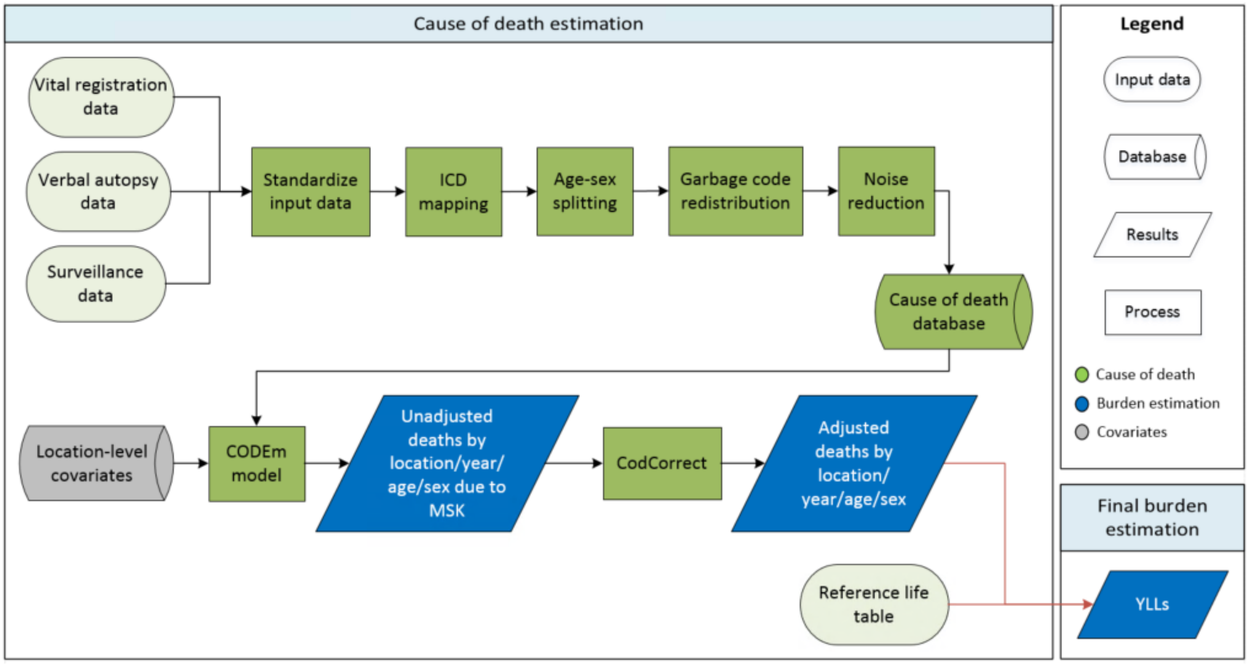

Input data

Data used to estimate mortality from musculoskeletal disorders (MSK) included vital registration (VR), verbal autopsy (VA), and China disease surveillance point data from the cause of death (COD) database. Our outlier criteria excluded data points that were implausibly high or low relative to global or regional patterns, substantially conflicted with established age or temporal patterns, or significantly conflicted with other data sources based from the same locations or locations with similar characteristics (ie, Socio-demographic Index).

Based on these criteria, in GBD 2016 we excluded VA data from Bangladesh, Vietnam, South Africa, Burkina Faso, Ghana, and all countries in Eastern sub-Saharan Africa including Ethiopia, Kenya, Tanzania, Mozambique, and Zambia, as VA tools have poor validity in identifying MSK deaths. In India, the number of deaths from new Sample Registration System (SRS) data in urban parts of states was substantially higher than the number of deaths from Medical Certification of Cause of Death (MCCD) data. In rural India, the SRS data are the only source. We have outliered the MCCD data to make the models follow the SRS data. This does lead to higher estimates in India compared to other parts of the world. For Indonesia, we excluded mortality surveillance data from a few states with high estimates based on small numbers, ie, Kalimantan Selatan and Kalimantan Timur in males, and Maluku in females. We re-included VR from Zimbabwe and Limpopo state in South Africa, where the older age groups had been excluded in GBD 2015. Recent years of data from Kazakhstan (2013-2015) were outliered as they presented a discontinuity with previous years which has been ascribed to the country’s attempt to reduce deaths due to CVD leading to an increase of deaths ICD9-BTL data from Latin American countries(Ecuador, Costa Rica, El Salvador, Guatemala, Honduras, Nicaragua, Panama, Venezuela, Antigua and Barbuda, The Bahamas, Barbados, Belize, Bermuda, Cuba, Dominica, Grenada, Guyana, Jamaica, Saint Lucia, Saint Vincent and Grenadines, Suriname and Trinidad and Tobago). The data from these countries provided in ICD9-detail or ICD10 were kept in the analysis.

Modelling strategy

The standard CODEm modelling approach was applied to estimate deaths due to musculoskeletal disorders. We applied the same covariates used in GBD 2015, excluding the four covariates for proportions of population at 0–15, 15–30, 30–45, and 45 and over latitudes, which had no observable benefit to the model. We modified the vegetable intake covariate from kcal/day to grams/day. Otherwise, there were no changes from the GBD 2015 modelling strategy.

Covariates are shown in the following table.

Level	Covariate	Direction
1	BMI (mean per capita)	+
2	Alcohol consumption (litres per capita)	+
	Cumulative cigarettes (10 years)	+
	Cumulative cigarettes (5 years)	+
	Smoking prevalence	+
	Cholesterol (mean per capita)	+
	Vegetable consumption (g per capita)	0
	Education (years per capita)	0
	Log-transformed LDI: lag-distributed income ($ per capita)	0
	Health care access and quality index	-
3	SDI: Socio-demographic Index	0

Rheumatoid arthritis

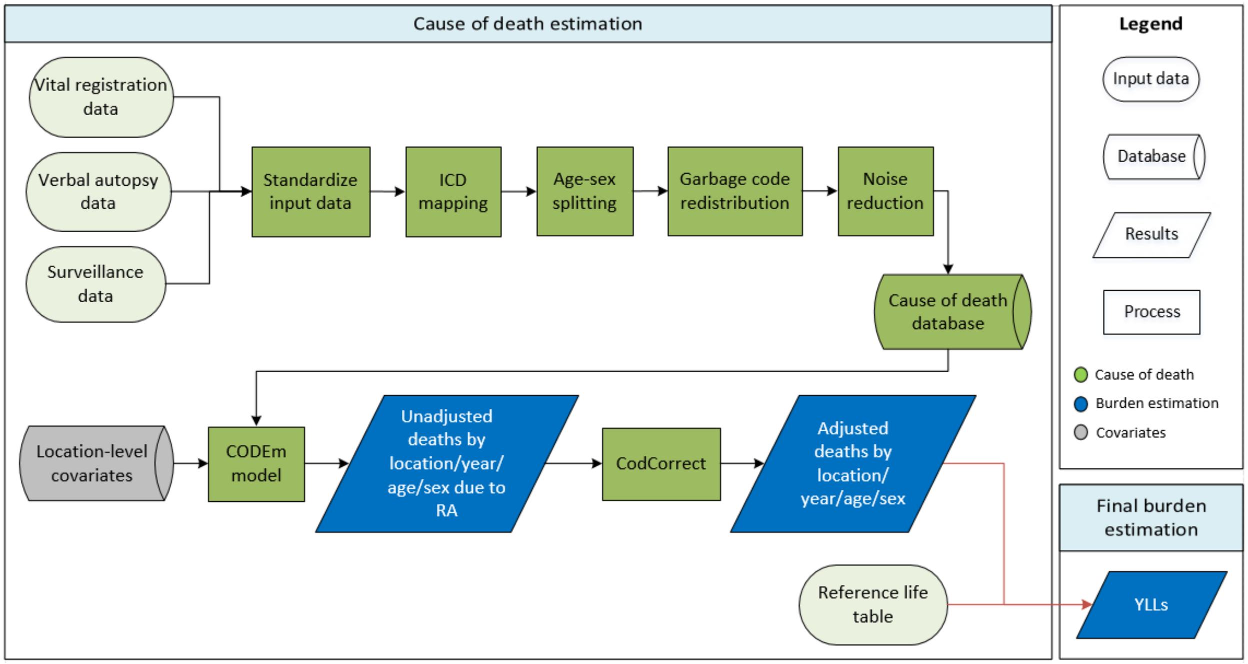

Input data

Data used to estimate rheumatoid arthritis mortality included vital registration, verbal autopsy, and China disease surveillance data from the cause of death database. Our outlier criteria were to exclude data points that were (1) implausibly high or low relative to global or regional patterns, (2) substantially conflicted with established age or temporal patterns, or (3) significantly conflicted with other data sources based from the same locations or locations with similar characteristics (ie, Socio-demographic Index).

Based on these criteria, we excluded a few data points from China. For males, we outliered data points from all sources in Tibet and data points from China disease surveillance in 1991 in all states, as these led to disproportionately high estimates. For females, we outliered Tibet data points from all sources up to 2007 and China disease surveillance data points in several southern states, ie, Guangxi, Hainan, and Yunnan. In addition, as the vital registration data in Limpopo for both male and female in year 2003 and before are implausibly higher than the other provinces in South Africa, we outliered this data source and kept the data in year 2004-2014 in the analysis. Also, as the vital registration data of mid-age males in Greenland are unrealistically high and much higher than e.g., in Canada and Denmark, the data for males age 45 and above were outliered. Recent years of data from Kazakhstan (2013-2015) were outliered as they presented a discontinuity with previous years which has been ascribed to the country's attempt to reduce deaths due to CVD leading to an increase of deaths from all other causes including rheumatoid arthritis. . Lastly, we outliered ICD9-BTL data from Latin American countries(Ecuador, Costa Rica, El Salvador, Guatemala, Honduras, Nicaragua, Panama, Venezuela, Antigua and Barbuda, The Bahamas, Barbados, Belize, Bermuda, Cuba, Dominica, Grenada, Guyana, Jamaica, Saint Lucia, Saint

Vincent and Grenadines, Suriname and Trinidad and Tobago). The data from these countries in the year that used ICD10 were kept in the analysis.

Modelling strategy

The standard CODEm modelling approach was applied to estimate deaths due to rheumatoid arthritis. We applied the same covariates used in GBD 2015 but excluded the four covariates for proportions of population at 0-15, 15-30, 30-45, and 45 and over latitudes, which had no observable benefit to the model. We modified the vegetable covariate from kcal/day to gram/day. Otherwise, there were no changes from the GBD 2015 modelling strategy. All the covariates are shown in the following table.

Level	Covariate	Direction
1	Alcohol consumption (litres per capita)	+
	Cumulative cigarettes (10 years)	+
	Cumulative cigarettes (5 years)	+
	Smoking prevalence	+
	Health care access and quality index	-
2	Cholesterol (mean per capita)	+
	Vegetable consumption (g per capita)	0
3	BMI (mean per capita)	+
	SDI: Socio-demographic Index	0
	Log-transformed LDI: lag-distributed income ($ per capita)	-
	Education (years per capita)	-

Other musculoskeletal disorders

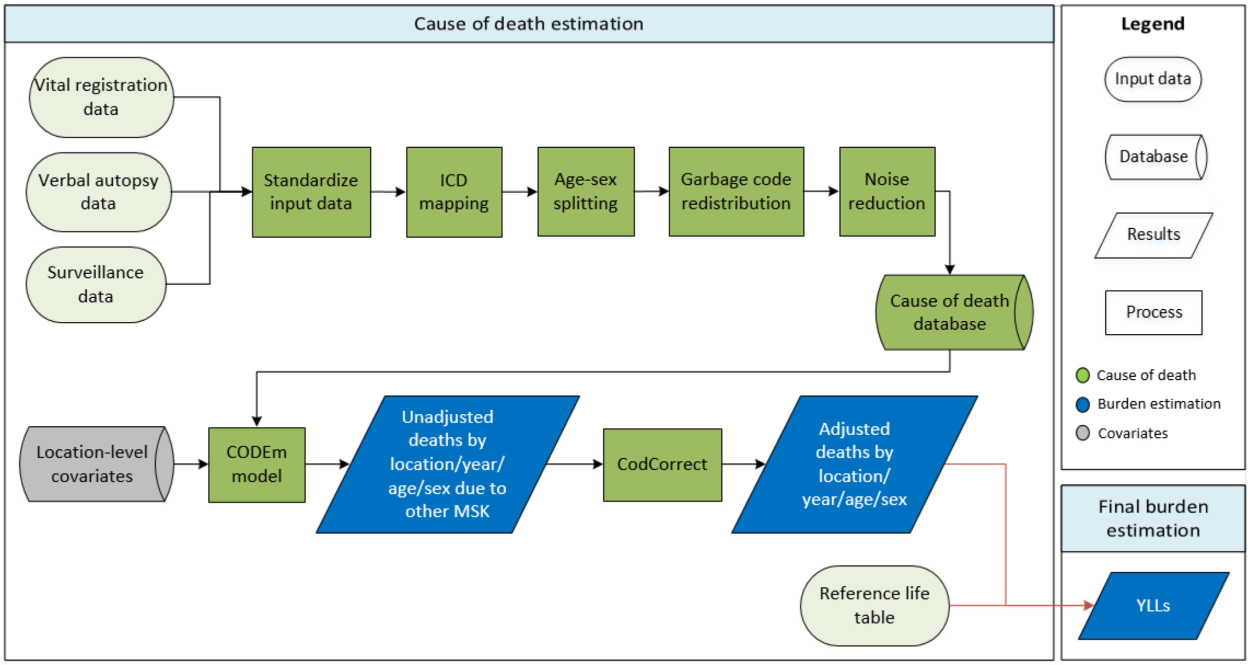

Input data

Data used to estimate mortality of other musculoskeletal disorders (MSK) included vital registration, verbal autopsy (VA), and China disease surveillance point data from the cause of death database. Our outlier criteria excluded data points that were implausibly high or low relative to global or regional patterns, substantially conflicted with established age or temporal patterns, or significantly conflicted with other data sources based from the same locations or locations with similar characteristics (i.e., socio-demographic index).

Based on these criteria, we excluded VA studies from Eastern and Western sub-Saharan Africa, as VA studies cannot distinguish other MSK deaths and estimates for the regions were disproportionately high. Recent years of data from Kazakhstan (2013-2015) were outliered as they presented a discontinuity with previous years which has been ascribed to the country's attempt to reduce deaths from CVD leading to an increase of deaths from all other causes, including other MSK. We also outliered all data in Latin American countries (Ecuador, Costa Rica, El Salvador, Guatemala, Honduras, Nicaragua, Panama, Venezuela, Antigua and Barbuda, The Bahamas, Barbados, Belize, Bermuda, Cuba, Dominica, Grenada, Guyana, Jamaica, Saint Lucia, Saint Vincent and Grenadines, Suriname and Trinidad and Tobago). The data from these countries in the year that used ICD9-detail or ICD10 were kept in the analysis.

Modelling strategy

The standard CODEm modelling approach was applied to estimate deaths due to other musculoskeletal disorders. We applied the same covariates used in GBD 2015, excluding the four covariates for proportions of population at 0–15, 15–30, 30–45, and 45 and over latitudes, which had no observable benefit to the model. We modified the vegetable intake covariate from kcal/day to grams/day.

Otherwise, there were no changes from the GBD 2015 modelling strategy. Covariates are shown in the following table.

Level	Covariate	Direction
1	BMI (mean per capita)	+
2	Alcohol consumption (litres per capita)	+
	Cumulative cigarettes (10 years)	+
	Cumulative cigarettes (5 years)	+
	Smoking prevalence	+
	Cholesterol (mean per capita)	+
	Vegetable consumption (g per capita)	0
	Education (years per capita)	0
	Log-transformed LDI: lag-distributed income ($ per capita)	0
	Health care access and quality index	-
3	SDI: Socio-demographic Index	0

Congenital Birth Defects: *Neural tube defects, Congenital heart anomalies, Orofacial clefts, Down Syndrome, Turner syndrome, Klinefelter syndrome, Other chromosomal disorders, Congenital musculoskeletal anomalies, Urogenital congenital anomalies, Digestive congenital anomalies, and Other congenital birth defects.*

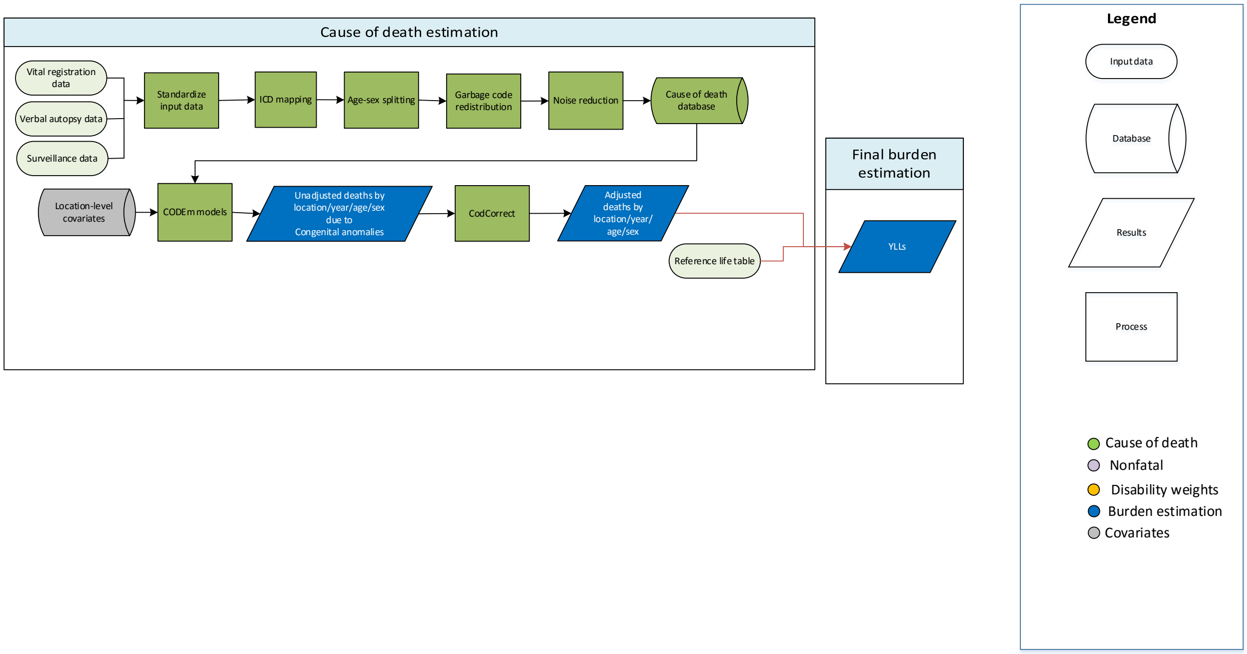

Input data

For GBD 2016, input data for estimating mortality due to congenital anomalies was centrally extracted, processed, and stored in causes of death (CoD) database. Vital registration (VR) was the dominant data type, followed by verbal autopsy (VA) and surveillance. Those CoD data sources that specified the sub-cause of birth defect were included in estimation of both the parent congenital anomalies model as well as in sub-type-specific models.

For GBD 2016, data exclusions were limited. We outliered all VA data in those over 5 years old as the age patterns were unreliable and led to poor model performance in the under-5 age groups. We also excluded some data sources from the parent model where only a subset of sub-causes were specified (eg, congenital heart disease, neural tube defects, and other congenital anomalies) and the sum of the sub-causes clearly represented systematic underreporting of one of the sub-causes. Systematic underreporting was suspected when sex- and age-specific rates were more than an order of magnitude lower than neighboring or comparable locations. Data sources for those locations were still included by default for sub-cause specific models because under-reporting of the total was not assumed to necessarily be associated with under-reporting of all of the component conditions.

Modeling strategy

All types of congenital anomalies were estimated using cause of death ensemble modeling (CODEm) for GBD 2016, as was done for previous iterations of the GBD study. Specific causes included neural tube defects, congenital heart anomalies, orofacial clefts, Down Syndrome, other chromosomal anomalies, congenital musculoskeletal anomalies, urogenital congenital anomalies, digestive congenital anomalies, and other congenital birth defects. We assumed no mortality from either Klinefelter syndrome or Turner syndrome, for which we model nonfatal outcomes only. For GBD 2016, we modeled congenital anomalies as a cause of death for ages 0-69 years only, assuming that all mortality from congenital conditions occurs before age 70 years of age.

For GBD 2016, we added three new causes to the congenital anomalies: congenital musculoskeletal and limb anomalies; urogenital congenital anomalies; and digestive congenital anomalies.

Covariates selected for CODEm model of overall congenital birth defects

Covariate	Transformation	Level	Direction
Maternal alcohol consumption during pregnancy (proportion)	None	1	Positive
In-Facility Delivery (proportion)	None	1	Negative
Live Births 35+ (proportion)	None	1	Positive
Folic acid unadjusted (ug)	None	1	Negative
Legality of Abortion	None	2	Negative
Antenatal Care (1 visit) Coverage (proportion)	None	2	Not specified
Smoking Prevalence (Reproductive Age Standardized)	None	2	Positive
Antenatal Care (4 visits) Coverage (proportion)	None	2	Negative
Healthcare access and quality index	None	2	Negative
Education (years per capita)	None	2	Negative
Alcohol (liters per capita)	None	3	Positive
fruits unadjusted(g)	None	3	Positive
Outdoor Air Pollution (PM2.5)	None	3	Positive
Indoor Air Pollution (All Cooking Fuels)	None	3	Positive
Socio-demographic Index	None	3	Negative
vegetables unadjusted(g)	None	3	Positive

Covariates selected for CODEm model of neural tube defects

Covariate	Transformation	Level	Direction
Health System Access (capped)	None	1	Negative
fruits adjusted(g)	None	2	Negative
vegetables adjusted(g)	None	2	Negative
Healthcare access and quality index	None	2	Negative
Education (years per capita)	None	3	Negative
LDI (I$ per capita)	Log	3	Negative

Socio-demographic Index	None	3	Negative

Covariates selected for CODEm model of congenital heart anomalies

Covariate	Transformation	Level	Direction
Maternal alcohol consumption during pregnancy (proportion)	None	1	Positive
Socio-demographic Index	Log	2	Negative
Smoking Prevalence (Reproductive Age Standardized)	None	2	Positive
Diabetes Age-Standardized Prevalence (proportion)	None	2	Positive
Healthcare access and quality index	None	2	Negative
Legality of Abortion	None	2	Negative
Antenatal Care (1 visit) Coverage (proportion)	None	2	Negative
In-Facility Delivery (proportion)	None	2	Negative
Education (years per capita)	None	2	Negative
Alcohol (liters per capita)	None	3	Positive
Antenatal Care (4 visits) Coverage (proportion)	None	3	Negative
Skilled Birth Attendance (proportion)	None	3	Negative
Live Births 35+ (proportion)	None	3	Positive

Covariates selected for CODEm model of cleft lip and cleft palate

Covariate	Transformation	Level	Direction
Indoor Air Pollution (All Cooking Fuels)	None	1	Positive
Diabetes Age-Standardized Prevalence (proportion)	None	2	Positive
Maternal alcohol consumption during pregnancy (proportion)	None	2	Positive
Healthcare access and quality index	None	2	Negative
Outdoor Air Pollution (PM2.5)	None	2	Positive
Legality of Abortion	None	2	Negative
Skilled Birth Attendance (proportion)	None	2	Negative
Smoking Prevalence (Reproductive Age Standardized)	None	2	Positive
vegetables unadjusted(g)	None	3	Not specified
Alcohol (liters per capita)	None	3	Positive
Antenatal Care (4 visits) Coverage (proportion)	None	3	Negative
Education (years per capita)	None	3	Negative
fruits unadjusted(g)	None	3	Not specified
Antenatal Care (1 visit) Coverage (proportion)	None	3	Negative

Covariates selected for CODEm model of Down Syndrome

Covariate	Transformation	Level	Direction
Live Births 35+ (proportion)	None	1	Positive
Legality of Abortion	None	1	Negative
Live Births 40+ (proportion)	None	1	Positive
Socio-demographic Index	None	2	Negative
LDI (I$ per capita)	Log	2	Negative
In-Facility Delivery (proportion)	None	2	Negative
Healthcare access and quality index	None	2	Negative
Maternal alcohol consumption during pregnancy (proportion)	None	3	Positive
Antenatal Care (1 visit) Coverage (proportion)	None	3	Negative
Education (years per capita)	None	3	Negative
Indoor Air Pollution (All Cooking Fuels)	None	3	Positive
Antenatal Care (4 visits) Coverage (proportion)	None	3	Negative
vegetables unadjusted(g)	None	3	Negative
Smoking Prevalence (Reproductive Age Standardized)	None	3	Positive

Covariates selected for CODEm model of other chromosomal abnormalities

Covariate	Transformation	Level	Direction
Live Births 35+ (proportion)	None	1	Positive
Live Births 40+ (proportion)	None	1	Positive
Legality of Abortion	None	1	Negative
LDI (I$ per capita)	Log	2	Negative
Healthcare access and quality index	None	2	Negative
Antenatal Care (4 visits) Coverage (proportion)	None	2	Negative
Antenatal Care (1 visit) Coverage (proportion)	None	2	Negative
In-Facility Delivery (proportion)	None	2	Negative
Maternal alcohol consumption during pregnancy (proportion)	None	2	Positive
Socio-demographic Index	None	3	Not specified
Alcohol (liters per capita)	None	3	Positive
Smoking Prevalence (Reproductive Age Standardized)	None	3	Positive
Education (years per capita)	None	3	Negative
Skilled Birth Attendance (proportion)	None	3	Negative

Covariates selected for CODEm model of congenital musculoskeletal and limb anomalies

Covariate	Transformation	Level	Direction
Maternal alcohol consumption during pregnancy (proportion)	None	1	Positive
Legality of Abortion	None	1	Negative
In-Facility Delivery (proportion)	None	2	Negative
Diabetes Age-Standardized Prevalence (proportion)	None	2	Positive
Socio-demographic Index	None	2	Negative
Healthcare access and quality index	None	2	Negative
Indoor Air Pollution (All Cooking Fuels)	None	2	Positive

Smoking Prevalence (Reproductive Age Standardized)	None	2	Positive
Antenatal Care (4 visits) Coverage (proportion)	None	3	Negative
Alcohol (liters per capita)	None	3	Positive
vegetables unadjusted(g)	None	3	Not specified
fruits unadjusted(g)	None	3	Not specified
Education (years per capita)	None	3	Negative
Antenatal Care (1 visit) Coverage (proportion)	None	3	Negative

Covariates selected for CODEm model of urogenital congenital anomalies

Covariate	Transformation	Level	Direction
Smoking Prevalence (Reproductive Age Standardized)	None	1	Positive
Maternal alcohol consumption during pregnancy (proportion)	None	1	Positive
Healthcare access and quality index	None	2	Negative
Diabetes Age-Standardized Prevalence (proportion)	None	2	Positive
Socio-demographic Index	None	2	Negative
Outdoor Air Pollution (PM2.5)	None	2	Positive
In-Facility Delivery (proportion)	None	2	Negative
Indoor Air Pollution (All Cooking Fuels)	None	2	Positive
Antenatal Care (1 visit) Coverage (proportion)	None	3	Negative
Alcohol (liters per capita)	None	3	Positive
Education (years per capita)	None	3	Negative
LDI (I$ per capita)	Log	3	Negative
Antenatal Care (4 visits) Coverage (proportion)	None	3	Negative

Covariates selected for CODEm model of digestive congenital anomalies

Covariate	Transformation	Level	Direction
Maternal alcohol consumption during pregnancy (proportion)	None	1	Positive
Smoking Prevalence (Reproductive Age Standardized)	None	1	Positive
Indoor Air Pollution (All Cooking Fuels)	None	2	Positive
Diabetes Age-Standardized Prevalence (proportion)	None	2	Positive
Socio-demographic Index	None	2	Negative
Prevalence of obesity (age-standardized)	None	2	Positive
In-Facility Delivery (proportion)	None	2	Negative
Healthcare access and quality index	None	2	Negative
Alcohol (liters per capita)	None	3	Positive
Health System Access (capped)	None	3	Negative
Education (years per capita)	None	3	Negative
vegetables unadjusted(g)	None	3	Not specified
Antenatal Care (1 visit) Coverage (proportion)	None	3	Negative
Antenatal Care (4 visits) Coverage (proportion)	None	3	Negative
fruits unadjusted(g)	None	3	Not specified
LDI (I$ per capita)	Log	3	Negative

Covariates selected for CODEm model of other congenital birth defects

**Covariate**	**Transformation**	**Level**	**Direction**
Maternal alcohol consumption during pregnancy (proportion)	None	1	Positive
Live Births 35+ (proportion)	None	1	Positive
Education (years per capita)	None	2	Negative
Smoking Prevalence (Reproductive Age Standardized)	None	2	Positive
Legality of Abortion	None	2	Negative
In-Facility Delivery (proportion)	None	2	Negative
Indoor Air Pollution (All Cooking Fuels)	None	2	Positive
Healthcare access and quality index	None	2	Negative
Antenatal Care (1 visit) Coverage (proportion)	None	3	Negative
Diabetes Age-Standardized Prevalence (proportion)	None	3	Positive
LDI (I$ per capita)	Log	3	Negative
Socio-demographic Index	None	3	Negative
Antenatal Care (4 visits) Coverage (proportion)	None	3	Negative
Alcohol (liters per capita)	None	3	Positive

Skin and subcutaneous diseases

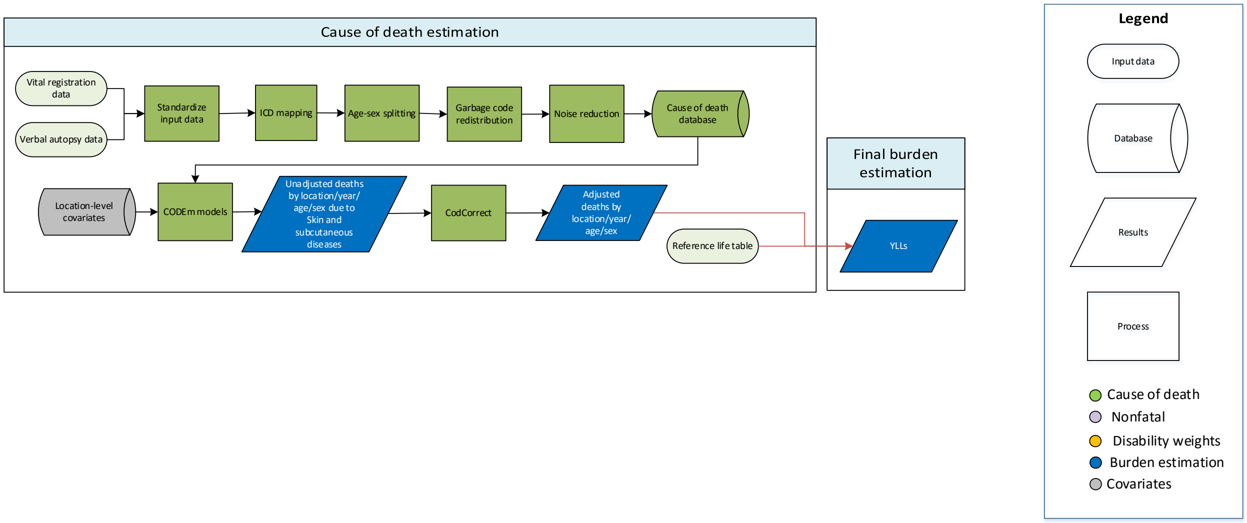

Input data

Data used to estimate mortality of skin and subcutaneous diseases consisted of vital registration data and verbal autopsy data from the cause of death (COD) database. Outlier criteria excluded data points that were implausibly high or low relative to global or regional patterns and data from countries with small populations. The data in skin and subcutaneous diseases consists of aggregated data from all other specific skin diseases, as well as unique data points from unspecified codes of skin and subcutaneous disease.

Modelling strategy

We modelled deaths due to skin and subcutaneous diseases with a standard CODEm model using the cause of death database and location-level covariates as inputs. The model followed standard parameters, with the exception that the start age of the model was 28 days instead of 0. We hybridized separate global and data-rich models to acquire unadjusted results, which we finalized and adjusted using CodCorrect to reach final years of life lost (YLLs) due to skin and subcutaneous diseases. In GBD 2016 we added the healthcare access and quality index (HAQI) covariate to the model.

Covariate	Level	Direction
Alcohol (liters per capita)	2	1
Cumulative cigarettes (10 years)	2	1
Cumulative cigarettes (5 years)	2	1
Education (years per capita)	3	-1
Lag distributed income (per capita)	3	-1
Smoking prevalence	2	1
Improved water source (proportion with access)	1	-1
Unsafe sanitation (summary exposure variable)	1	1
Sociodemographic index	3	0
Health access and quality index	2	-1

Cellulitis

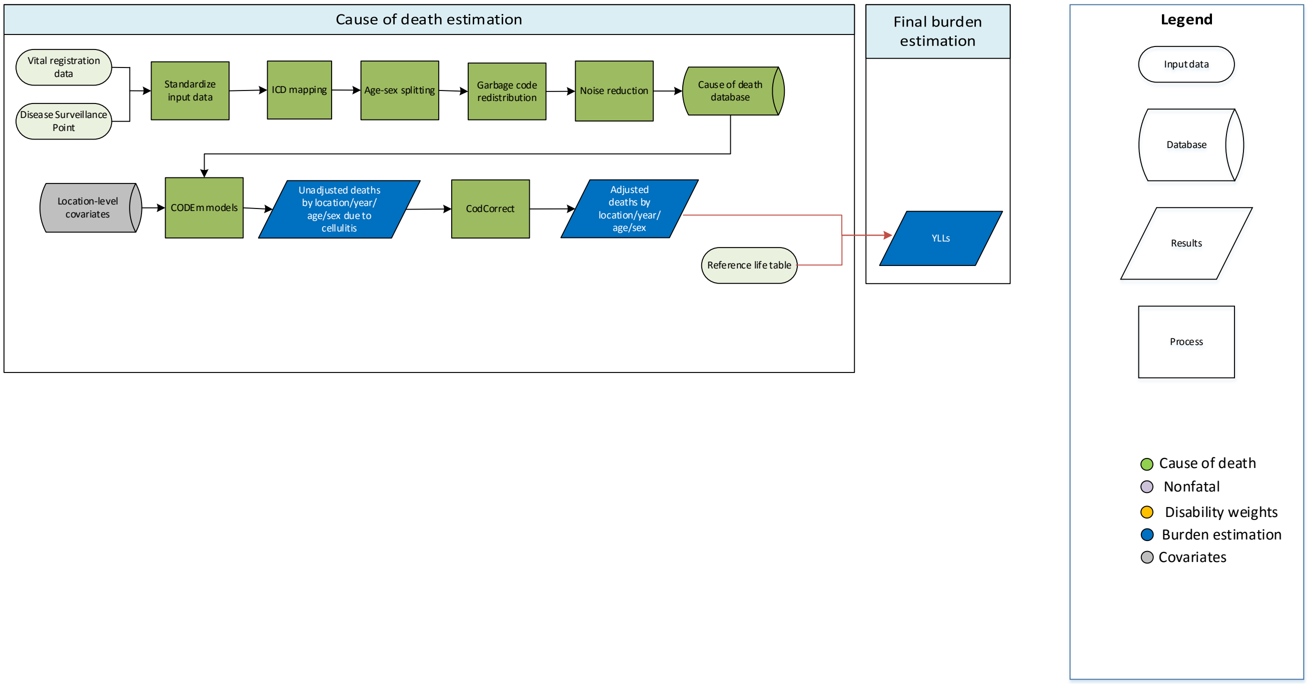

Input data

Data used to estimate cellulitis mortality consisted of vital registration and Chinese disease surveillance point (DSP) data from the cause of death (COD) database. Outlier criteria excluded data points that were implausibly high or low relative to global or regional patterns and data from countries with small populations.

Modeling strategy

The standard CODEm modeling approach was used to estimate deaths due to cellulitis. CODEm parameters were centrally defined. COD models were evaluated by comparing age-standardized death rates per 100,000 people to the GBD 2015 best model for 1990 and 2015 – individually for males and females. We also compared the age-standardized annualized rate of change for death rates per 100,000 persons to GBD 2015.

Compared to GBD 2015, we reduced the number of covariates for GBD 2016.

Covariate	Level	Direction
Education (years per capita)	3	0
Log LDI (I$ per capita)	3	0
Healthcare access and quality index	2	-1

Pyoderma

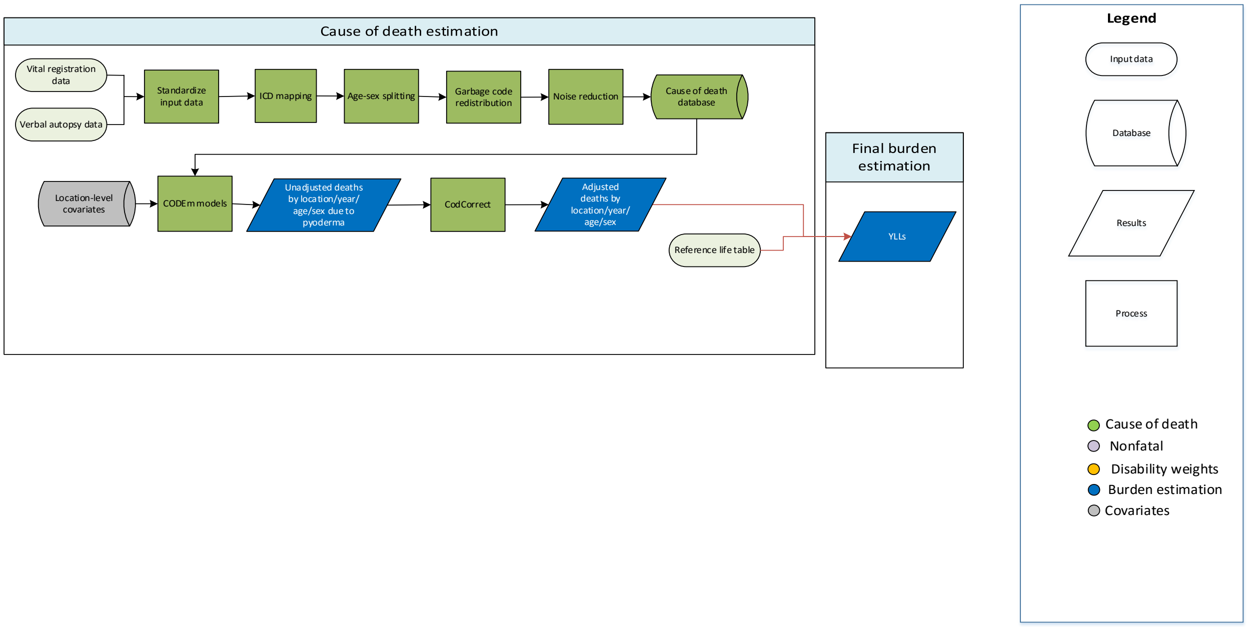

Input data

Data used to estimate pyoderma mortality included centrally prepped vital registration and verbal autopsy data from the cause of death (COD) database. Outlier criteria excluded data points that were implausibly high or low relative to global or regional patterns and data from countries with small populations.

Modelling strategy

We modelled deaths due to pyoderma with a standard CODEm model using the cause of death database and location-level covariates as inputs. The model followed standard parameters. We hybridized separate global and data-rich models to acquire unadjusted results, which we finalized and adjusted using CodCorrect to reach final years of life lost (YLLs) due to pyoderma. In GBD 2016 we added the healthcare access and quality index (HAQI) covariate to the model.

Covariate	Level	Direction
Alcohol (liters per capita)	2	1
Cumulative cigarettes (10 years)	2	1
Cumulative cigarettes (5 years)	2	1
Education (years per capita)	3	-1
Lag distributed income (per capita)	3	-1
Smoking prevalence	2	1
Improved water source (proportion with access)	1	-1
Unsafe sanitation (summary exposure variable)	1	1
Sociodemographic index	3	0
Health access and quality index	2	-1

Decubitus ulcer

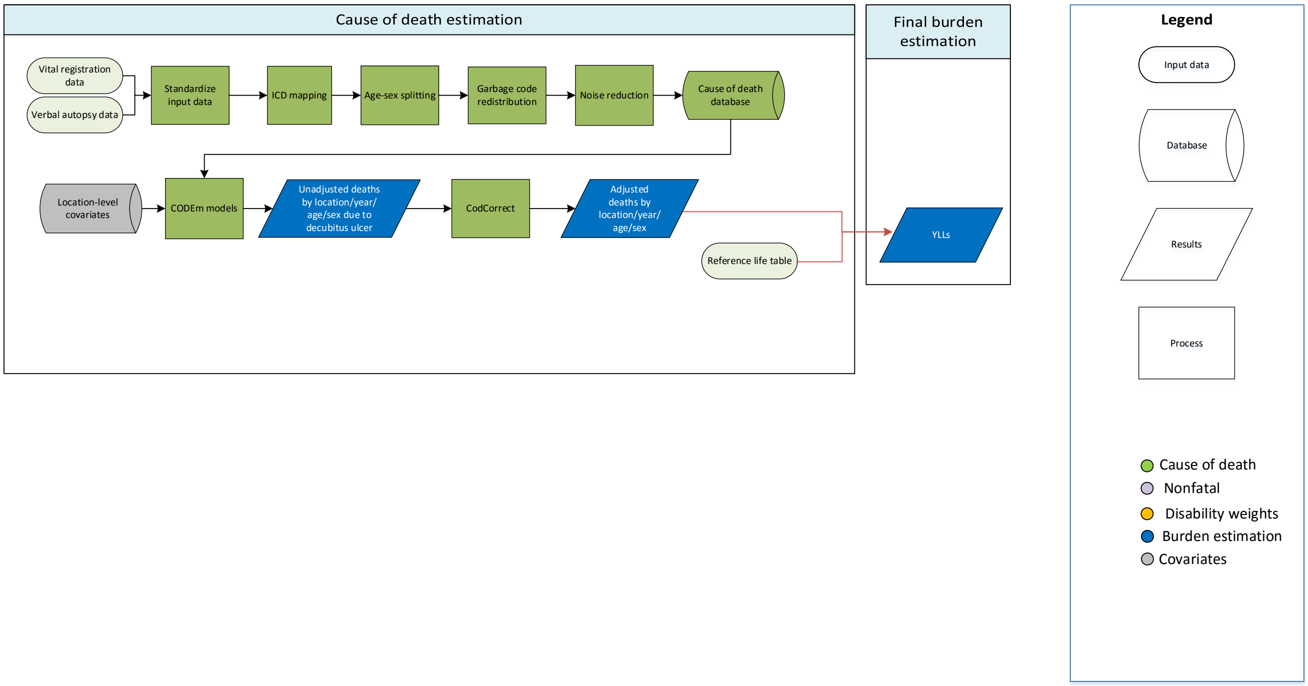

Input data

Data used to estimate decubitus ulcer mortality consisted of vital registration sources and verbal autopsy sources from the cause of death (COD) database. Outlier criteria excluded data points that were implausibly high or low relative to global or regional patterns and data from countries with small populations.

Modelling strategy

The standard CODEm modelling approach was used to estimate deaths due to decubitus ulcer. CODEm parameters were centrally defined. COD models were evaluated by comparing age-standardized death rates per 100,000 people to the GBD 2015 best model for 1990 and 2015 – individually for males and females. We also compared the age-standardized annualized rate of change for death rates per 100,000 persons to GBD 2015.

Decubitus ulcer death estimates were corrected for misclassification of Alzheimer and Parkinson disease deaths.

Covariate	Level	Direction
Alcohol (litres per capita)	2	1
Cumulative cigarettes (5 years)	2	1
Cumulative cigarettes (10 years)	2	1
Education (years per capita)	3	-1
Health system access 2	3	-1
Log LDI (I$ per capita)	3	-1
Smoking prevalence	2	1
Improved water source (proportion with access)	1	-1

Standardized Exposure Variable (SEV) scalar for unsafe sanitation	1	1
Socio-demographic Index	3	0
Healthcare access and quality index	2	-1

Other skin and subcutaneous diseases

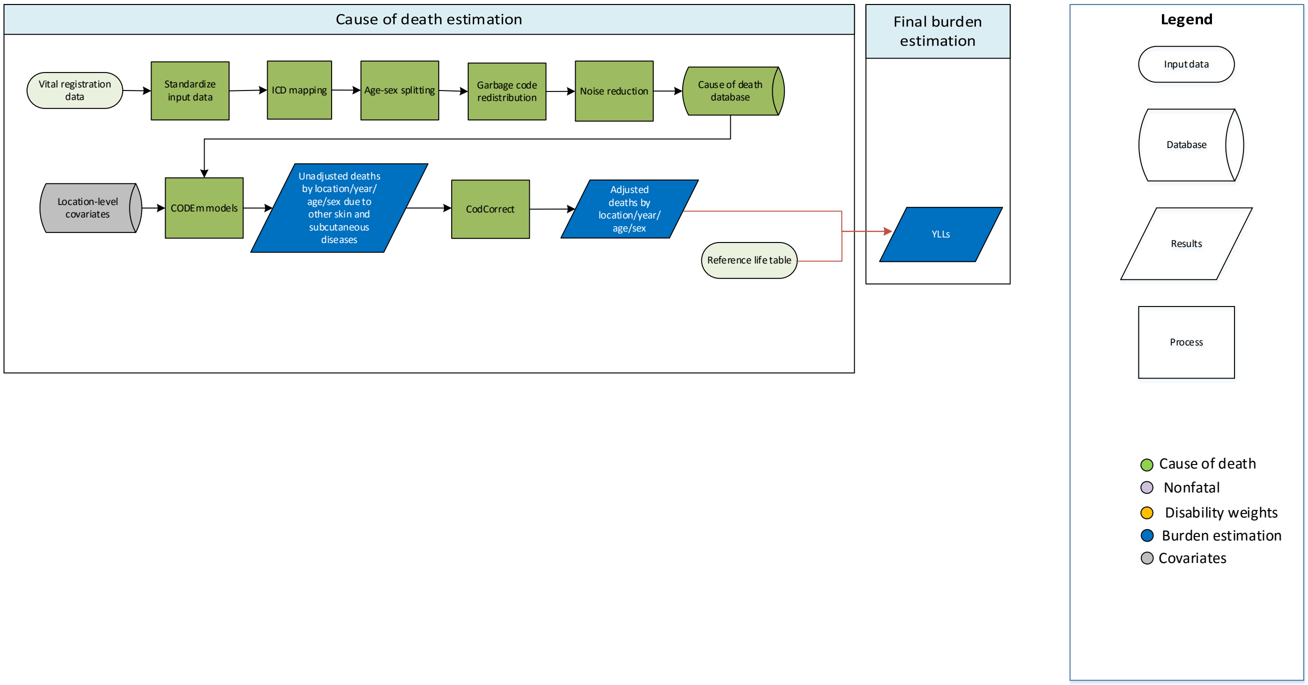

Input data

Data used to estimate mortality due to other skin and subcutaneous diseases consisted of vital registration data from the cause of death (COD) database. We outliered data in instances where garbage code redistribution and noise reduction, in combination with small sample sizes, resulted in unreasonable cause fractions; and data that violated well-established time or age trends.

Modelling strategy

The standard CODEm modelling approach was used to estimate deaths due to other skin and subcutaneous diseases. CODEm parameters were centrally defined. COD models were evaluated by comparing age-standardized death rates per 100,000 people to the GBD 2015 best model for 1990 and 2015 – individually for males and females. We also compared the age-standardized annualized rate of change for death rates per 100,000 people to GBD 2015.

There were no significant changes in the modelling process between GBD 2015 and GBD 2016.

Covariate	Level	Direction
Alcohol (litres per capita)	2	1
Cumulative cigarettes (5 years)	2	1
Cumulative cigarettes (10 years)	2	1
Education (years per capita)	3	-1
Health system access 2	3	-1
Log LDI (I$ per capita)	3	-1
Underweight (proportion <2SD weight for age, <5 years)	1	1
Smoking prevalence	2	1
Improved water source (proportion with access)	1	-1

Standardized Exposure Variable (SEV) scalar for unsafe sanitation	1	1
Socio-demographic Index	3	0
Healthcare access and quality index	2	-1

Sudden Infant Death Syndrome (SIDS)

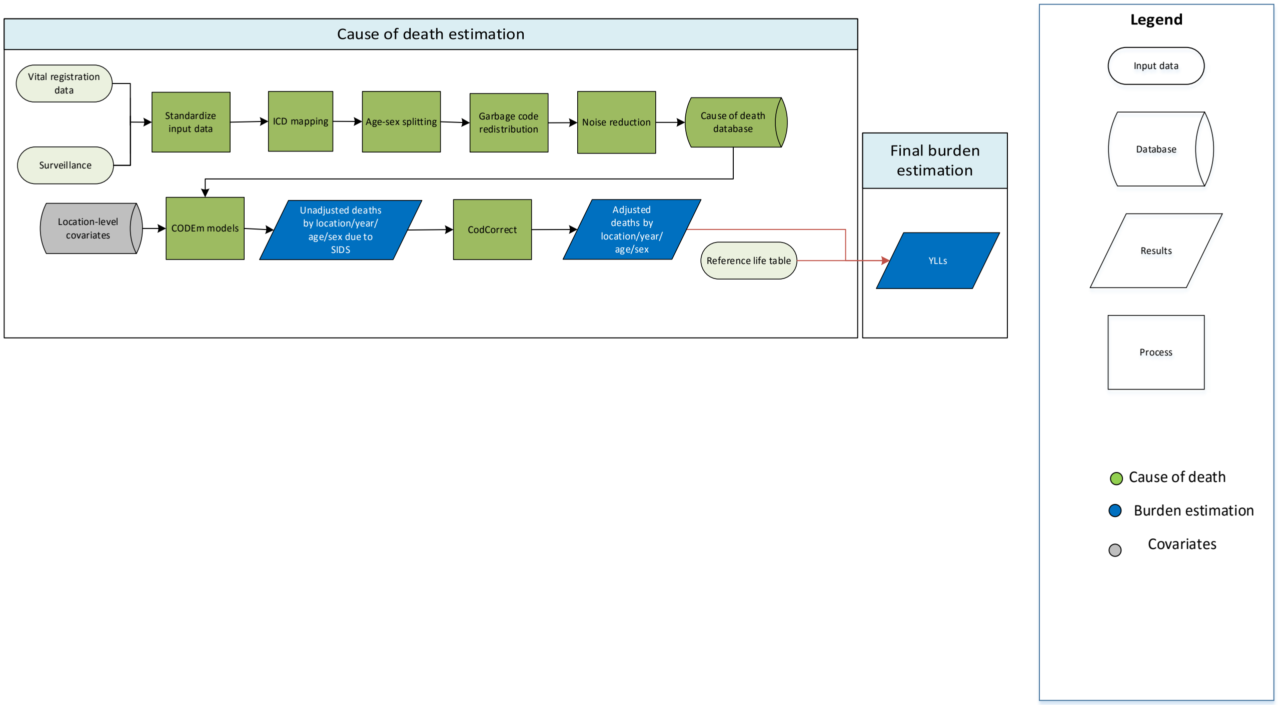

Input data

Vital registration data were used to estimate deaths due to sudden infant death syndrome (SIDS). Data points were selected as outliers if they met the following criteria: (1) implausibly high values relative to country time trends or global or regional patterns, based on the assumption that there are not "outbreaks" of SIDS, or (2) substantially conflicting with established age or temporal patterns.

Modelling strategy

The standard CODEm modelling approach was applied to estimate deaths due to SIDS. We ran CODEm models for ages 7–27 days and 28–364 days because we believe that deaths assigned to SIDS in other age groups are mis-assigned and are therefore treated as garbage codes. Surveillance data and verbal autopsy data were not used as inputs to this model because these sources do not use data collection methods that can accurately diagnose deaths due to SIDS.

Notable differences between the GBD 2013 and GBD 2015 strategy included updates across the board to smoking-related covariates, total fertility rate, and Socio-demographic Index covariates. The addition of American Samoa to the Oceania region was also of note, as well as the shift to including more ICD detail codes in the input data for some countries that previously reported only aggregated codes.

There were no significant changes in strategy from GBD 2015 to GBD 2016.

Injuries

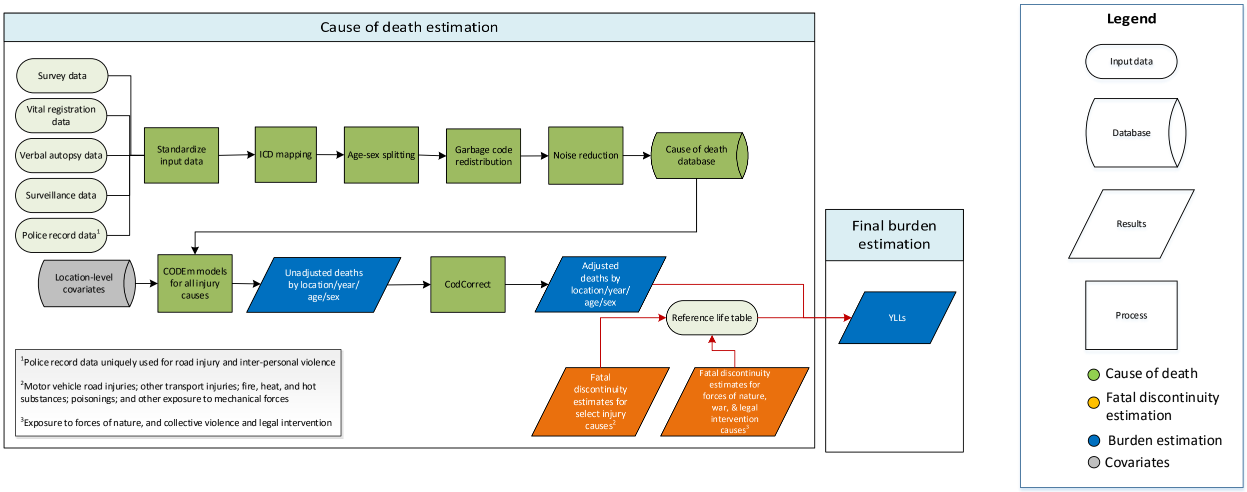

Input data

In GBD 2016, we estimated injury mortality from vital registration, verbal autopsy, mortality surveillance, censuses, surveys, and police record data. Police and crime reports were data sources uniquely used for the estimation of deaths from road traffic injury and interpersonal violence. The police data were collected from published studies, national agencies, and institutional surveys such as the United Nations Crime Trends Survey and the WHO Global Status Report on Road Safety Survey. For countries with vital registration data we did not use police records, except if the recorded number of road injury and interpersonal violence deaths from police records exceeded that in the vital registration.

Infrequently, data points were marked as outliers. Outlier criteria excluded data points that (1) were implausibly high or low relative to global or regional patterns, (2) substantially conflicted with established age or temporal patterns, or (3) significantly conflicted with other data sources conducted from the same locations or locations with similar characteristics (ie, Socio-demographic Index).

Modelling strategy

Overview

In GBD 2016, the standard CODEm modelling approach was applied to estimate deaths due to all causes of injury, excluding "Exposure to forces of nature," "Military operations and terrorism," and "State actor violence," which fall under the aggregate cause "Forces of nature, military operations and terrorism, and state actor violence." These causes were modelled solely outside of the CODEm process as fatal discontinuities estimation; this process is detailed further in the section on fatal discontinuities estimation in the appendix.

Fatal discontinuity was estimated for five injury causes also modeled in CODEm. These causes included "Motor vehicle road injuries," "Other transport injuries," "Fire, heat, and hot substances," "Poisonings," and "Other exposure to mechanical forces." Final fatal discontinuity estimations for these causes were merged with CODEm results post-CoDCorrect to produce final cause of death results.

Refer to the Table at the end of this section for a complete list of the cause-of-injury categories, modelling strategies, and covariate changes from GBD 2015.

GBD injury codes and categories

The International Classification of Diseases (ICD) was used to classify injuries. In GBD, injury incidence and death are defined as ICD-9 codes E000-E999 and ICD-10 chapters V to Y. There is one exception: deaths and cases of alcohol poisoning and drug overdoses are classified under drug and alcohol use disorders. In GBD 2016, injury causes were organized into 26 mutually exclusive and collectively exhaustive external cause-of-injury categories. For GBD 2016, "Self-harm" was distinguished into "Self-harm by firearm," and "Self-harm by other specified means."

Preparation of data

The preparation of cause of death data includes age splitting, age-sex splitting, smoothing, and outlier detection. These steps are described in detail by Naghavi et al and Lozano et al.[1,2,3] The concept of "garbage codes" and redistribution of these codes was proposed in GBD 1990.[4] Garbage codes are causes of death that should not be identified as specific underlying causes of death but have been entered as the underlying cause of death on death certificates. A classic example of these types of codes in injuries chapters are "Exposure to unspecified factor" (X59 in ICD-10 and E887 in ICD-9) and all undetermined intent codes (Y10-Y34 in ICD-10 and E980-E988 in ICD-9). Other examples of garbage codes in injuries are the coding of an injury death to intermediate codes like septicemia or peritonitis or as an ill-defined and unknown cause of mortality (R99). Approximately 2% of total deaths in countries with vital registration data are assigned to these three injury garbage code categories.

Splitting into sublevel causes

In countries with non-detail ICD code data, cause-of-injury categories were proportionally split into sublevel cause-of-injury categories. The sublevel cause-of-injury causes were created in the CoDCorrect process. One of the countries with non-detail ICD code data is South Africa, and in GBD 2013 the proportions of sublevel cause-of-injury were based on vital registration data. For GBD iterations of 2015 and 2016 the proportions were based on post-mortem investigation of injury deaths as described in the paper by Matzopoulos et al. 2015.[5]

Limitations and model assumptions

We added police data for road injuries and interpersonal violence to help predict level and age patterns in countries with sparse or absent cause of death data even though we know from countries with near-complete vital registration data that police records tend to underestimate the true level of deaths. However, we applied police data estimates in instances where reported deaths were higher than vital registration numbers.

For the cause-of-injury category "Unintentional suffocation" we suspect that varying practices in coding deaths to sudden infant death syndrome (which end up in "Unintentional suffocation") can explain some of the differences we see and we plan to explore that further in the next iteration of GBD.

Table – Injury Cause List			
**ID**	**Cause**	**Modelling Strategy**	**Covariate changes from GBD 2015**
1	Transport injuries	CODEm	
1.1	Road injuries	CODEm	
1.1.a	Pedestrian road injuries	CODEm	
1.1.b	Cyclist road injuries	CODEm	
1.1.c	Motorcyclist road injuries	CODEm	
1.1.d	Motor vehicle road injuries	CODEm and fatal discontinuity estimation	
1.1.e	Other road injuries	CODEm	
1.2	Other transport injuries	CODEm and fatal discontinuity estimation	
2	Unintentional injuries	CODEm	
2.1	Falls	CODEm	
2.2	Drowning	CODEm	
2.3	Fire, heat, and hot substances	CODEm and fatal discontinuity estimation	
2.4	Poisonings	CODEm and fatal discontinuity estimation	
2.5	Exposure to mechanical forces	CODEm	
2.5.a	Unintentional firearm injuries	CODEm	
2.5.b	Unintentional suffocation	CODEm	
2.5.c	Other exposure to mechanical forces	CODEm and fatal discontinuity estimation	
2.6	Adverse effects of medical treatment	CODEm	
2.7	Animal contact	CODEm	
2.7.a	Venomous animal contact	CODEm	
2.7.b	Non-venomous animal contact	CODEm	
2.8	Foreign body	CODEm	
2.8.a	Pulmonary aspiration and foreign body in airway	CODEm	
2.8.b	Foreign body in other body part	CODEm	
2.9	Environmental exposure to heat and cold	CODEm	
2.10	Other unintentional injuries	CODEm	
3	Self-harm and interpersonal violence	CODEm	
3.1	Self-harm	CODEm	
3.1.1	Self-harm by firearm	CODEm	Same covariates used as self-harm from GBD 2015
3.1.2	Self-harm by other specified means	CODEm	Same covariates used as self-harm from GBD 2015
3.2	Interpersonal violence	CODEm	
3.2.a	Assault by firearm	CODEm	
3.2.b	Assault by sharp object	CODEm	
3.2.c	Assault by other means	CODEm	
4	Forces of nature, military operations and terrorism, and state actor violence		

Table – Injury Cause List			
**ID**	**Cause**	**Modelling Strategy**	**Covariate changes from GBD 2015**
4.1	Exposure to forces of nature	Fatal discontinuity estimation for disaster (appended post-CoDCorrect)	N/A
4.2	State actor violence	Fatal discontinuity estimation for state actor violence (appended post-CoDCorrect)	N/A
4.3	Military operations and terrorism	Fatal discontinuity estimation for state actor violence (appended post-CoDCorrect)	N/A

References

1 Lozano R, Naghavi M, Foreman K, *et al.* Global and regional mortality from 235 causes of death for 20 age groups in 1990 and 2010: a systematic analysis for the Global Burden of Disease Study 2010. *The Lancet* 2012; **380**: 2095–128.

2 Global, regional, and national age–sex specific all-cause and cause-specific mortality for 240 causes of death, 1990–2013: a systematic analysis for the Global Burden of Disease Study 2013. *The Lancet* 2015; **385**: 117–71.

3 Global, regional, and national life expectancy, all-cause mortality, and cause-specific mortality for 249 causes of death, 1980-2015: a systematic analysis for the Global Burden of Disease Study 2015. *The Lancet* 2016; **388**: 1459-1544.

4 Murray CJL, Lopez AD, Harvard School of Public Health, World Health Organization, World Bank. The global burden of disease: a comprehensive assessment of mortality and disability from diseases, injuries, and risk factors in 1990 and projected to 2020. Cambridge, MA: Published by the Harvard School of Public Health on behalf of the World Health Organization and the World Bank : Distributed by Harvard University Press, 1996.

5 Matzopoulos R, Prinsloo M, Wyk VP, Gwebushe N, Mathews S, et al. Injury-related mortality in South Africa: a retrospective descriptive study of postmortem investigations. *Bull World Health Organ* 2015; **93**: 303–13.

Fatal Discontinuities

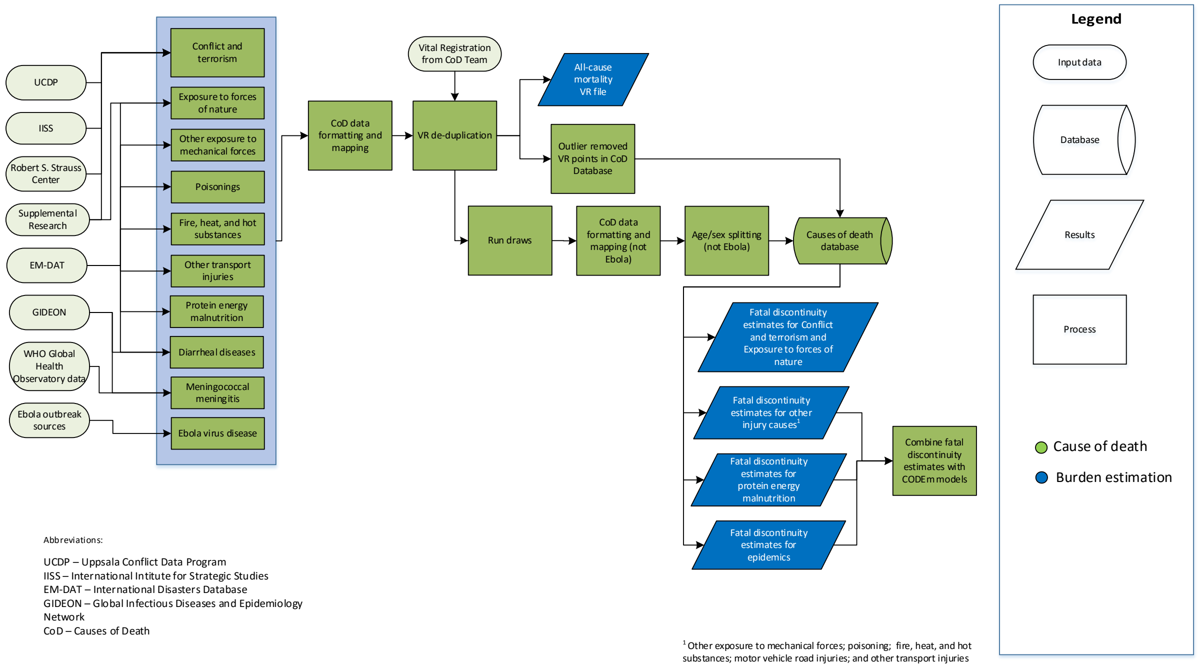

Input data

Overall

Input data for fatal discontinuities are compiled a range of sources, including country vital registration (VR) data; international databases that capture several cause-specific fatal discontinuities; and supplemental data in the presence of known issues with data quality or representativeness, or time lags in reporting. A systematic literature review was not used to identify input data for fatal discontinuities, though some literature sources were identified through online supplemental research. Below we provide more detail on the different input data sources by sub-causes of fatal discontinuities.

Subnational locations and population splitting

In locations where we produced estimates at the subnational level for GBD 2016, deaths due to all fatal discontinuity causes were assigned to the relevant subnational location(s) when that information could be obtained either through country data sources (e.g., VR) or through additional online research. If no subnational location could be found, the deaths were split proportionally by population across all subnational locations.

In locations that have experienced boundary changes or split from other locations that we currently estimate (e.g., the former Yugoslavia, Czechoslovakia, the Soviet Union, Sudan and South Sudan), we split deaths due to events that occurred prior to boundary changes proportionally based on the populations residing within the boundaries of present-day locations unless we found documentation that clearly indicated whether the event and corresponding deaths occurred in one of the present-day GBD 2016 locations.

Locations with 4- or 5-star data quality ratings

For countries and territories assigned 4- or 5-star data quality ratings (see Section 2 of the appendix for details), we prioritized data from country-specific vital registration. VR data for fatal discontinuities was exclusively used in 4- and 5-star locations unless there was well-known data quality issues or discrepancies in the cause of death data reporting related to a particular event (e.g., supplemental death data for Louisiana was used for Hurricane Katrina because of established data reporting issues). The process for identification of location-year fatal discontinuities is described more in the Modelling strategy below.

Locations with less than 4-star data quality ratings

For countries and territories assigned data quality ratings below 4 stars, we compared VR with data available from alternative sources for Exposure to forces of nature, taking the highest death estimate available from all sources. For other fatal discontinuity causes, we disregarded lower quality VR and used well-established databases by type of fatal discontinuity. Whenever specific events were identified that did not have corresponding data points within these databases, we used supplemental data sources, including scientific literature.

Major data sources other than country vital registration for each fatal discontinuity cause follow.

Conflict and terrorism. Data for conflict and terrorism come from the Uppsala Conflict Data Program (UCDP), International Institute for Strategic Studies, and Robert S. Strauss Center for International Security and Law. The table below provides details about the various datasets we utilized from these sources, the dates they were last accessed, and the years for which we used the data provided.

Data source name	Date accessed	Years of data downloaded	Type of data included
**Uppsala Conflict Data Program**[1]			
Battles	10/6/16	1989-2015	Armed conflict: incompatibility that concerns government and/or territory over which the use of armed force between the military forces of two parties, of which at least one is the government of a state, which resulted in deaths
Non-state	10/6/16	1989-2015	The use of armed force between two organized armed groups, neither of which is the government of a state, which results in deaths
One-sided	10/6/16	1989-2015	The use of armed force by the government of a state or by a formally organized group against civilians which results in deaths
Georeferenced Event Dataset	10/6/16	1989-2015	UCDP battles, non-state, and one-sided conflict deaths with the most disaggregated location information available
PRIO Battles Deaths Dataset	10/6/16	1970-1988	Armed conflict (civil wars, etc.)
**International Institute for Strategic Studies**			
Armed Conflict Dataset	10/6/16	1997-Present	Insurgency, Inter-state, Intra-state conflict deaths
**Robert S. Strauss Center For International Security And Law**			
Armed Conflict Location and Event Dataset (ACLED)	10/6/16	1997-2016	Actions of opposition groups, governments, and militias in selected locations in Africa and Asia, specifying the exact location and date of battle events, transfers of military control, headquarter establishment, civilian violence, and rioting
Social Conflict Analysis Database (SCAD)	10/6/16	1990-2016	Protests, riots, strikes, inter-communal conflict, government violence against civilians, and other forms of social conflict (covers Africa and Latin America)

Supplemental online research was conducted for recent conflicts where the databases above were not up-to-date. In addition, deaths due to conflict and terrorism in Iraq from 2003 to present were estimated using a combination of supplemental sources. The source found with the lowest number of deaths, Iraq Body Count[2], was used as the lower bound of the uncertainty interval from 2003 to 2016. Estimates from the Iraq Mortality Study by Hagopian et al[3] from 2003 to 2006, the deadliest years of the war, were used to scale deaths to generate the upper uncertainty interval limits using the following formula:

$$deaths_{GBD\ 2016,\ high} = deaths_{IBC} \cdot \overline{\left[\frac{deaths_{IMS}}{deaths_{IBC}}\right]}_{2003-2006}$$

We used the average ratio between IMS and IBC reported deaths between 2003 and 2006, multiplied by the number of deaths reported by the IBC. This high estimate is carried forward through 2016 under the assumption that the Iraq Body Count similarly undercounts the number of deaths due to the ongoing civil war in Iraq. The final, best estimate for conflict and terrorism deaths in Iraq from 2003 to 2016 is the midpoint of the high and low estimates given above.

We identified four major conflicts that were not represented in these databases: 1997 civil conflict in Albania[4]; 1971 genocide in Bangladesh[5]; 1972 genocide in Burundi[6]; and 1993 genocide in Burundi[6]. In these cases, we used literature sources in order to account for these fatal discontinuities.

For country-years where multiple sources provided estimates, we prioritized sources in the following order: (1) country VR data, if death estimates were highest of all sources; (2) UCDP; (3) IISS; (4) country VR if death estimates were not the highest of all sources; (5) Robert Strauss Center; (6) online supplemental research.

Exposure to forces of nature, other injury causes, and protein-energy malnutrition. The Centre for Research on the Epidemiology of Disasters' International Disaster Database (EM-DAT) served as the primary non-VR source of fatal discontinuities due to exposure to forces of nature (i.e., natural disasters); other transport injuries (e.g., plane, train, and boat accidents); poisonings; fire, heat, and hot substances; other exposure to mechanical forces (eg, building collapse); and protein-energy malnutrition (ie, famine or severe drought). Data from EM-DAT were last accessed March 29, 2017. Supplemental online research was conducted for events where EM-DAT was not up-to-date.

For country-years where multiple sources provided estimates, we prioritized sources in the following order: (1) country VR data, if data quality rating is 4 or 5 stars; (2) country VR data if data quality rating is less than 4 stars and death estimates were highest of all sources; (3) EM-DAT; (4) online supplemental research. Exceptions were made where it was clear that VR systems had been compromised by the event being measured.

Meningococcal meningitis and diarrheal diseases. New to GBD 2016, we sought to include fatal discontinuities due to a subset of infectious diseases: meningococcal meningitis (or meningococcal infection) and diarrheal disease caused by cholera. These two infectious diseases were included on the fatal discontinuity cause list for GBD 2016 because (1) their current modelling strategies with the Cause of Death Ensemble model (CODEm) does not optimally capture the potentially highly variable – or epidemic – mortality levels and trends characteristic of these two causes; and (2) they can contribute to significant total fatalities in a given location-year. Other infectious diseases for which the latter is true – high death rates in the presence of an outbreak or epidemic – are currently modelled with alternative cause of death methods (eg, natural history models for measles and yellow fever), which allow for greater variation year-over-year if or when outbreaks occur. In future iterations of the GBD, we plan to revisit the inclusion criteria for infectious diseases as fatal discontinuities and develop more of an ensemble approach to modelling causes that can be both endemic (and thus result in more uniform levels and trends over time) and epidemic (and subsequently lead to rapid increases – and decreases – in deaths for a given location-year).

The Global Infectious Diseases and Epidemiology Network (GIDEON) served as the primary data source for collating cholera and meningococcal meningitis or meningococcal infection death reports.[7,8] For any year in which cholera or meningococcal meningitis deaths were recorded in a country or territory covered by the GBD, we directly extracted reported deaths from 1970 to 2016. When there were reporting gaps in cholera or meningococcal meningitis deaths over this period of time and the World Health Organization (WHO) annual cholera or meningitis reports had death reports for those years, we used the WHO reports. The primary exception were two major cholera outbreaks in Bangladesh – 1982-1983 and 1991 – which were not captured by either GIDEON or WHO. As result, we used the EM-DAT records for the 1982-1983 outbreak and literature for the 1991 outbreak.[9]

Ebola. Since GBD 2015, outbreaks due to Ebola virus disease have been estimated using the data and methods described in the Ebola write-up of this appendix and included in GBD death estimates in the same way as other fatal discontinuity causes.

Modelling strategy

All input data for fatal discontinuity causes were run through the causes of death data formatting and mapping process.

VR de-duplication

For country-years where deaths due to fatal discontinuity causes were recorded in both VR and other utilized data sources, the higher of the two estimates were taken in the case of deaths due to conflict and terrorism and exposure to forces of nature.

For the other injury causes that also have a CODEm model, a process was established to avoid duplication of fatal discontinuity deaths in the two models. First, location-years with death data from non-VR sources were identified. If these location-cause-years also had VR death estimates that were greater than 40% higher than the immediately surrounding years and could be linked to a specific fatal discontinuity event, these years were marked as outliers in the VR data and the difference between the outlier year and the average of the surrounding years was included in the relevant cause in the fatal discontinuities database. The deaths from the identified events were subtracted from the all-cause VR estimates used in the all-cause mortality estimation process.

Uncertainty analysis for input and draw-level input to age-sex splitting

Uncertainty intervals for deaths due to conflict and terrorism were generated using UCDP high and low death estimates, except in the case of Iraq 2003-2016, as explained above. In cases where low and high estimates were not included in the available data, the regional average uncertainty interval was applied to the available death estimate across all fatal discontinuity causes.

We assumed a normal distribution using the mean deaths and standard deviation based on high and low estimates. The standard deviation was capped at the mean divided by 1.96 in order to ensure that 95% of the 3,000 draws generated were greater than zero. Non-positive draws were dropped, and 1,000 draws were sampled from the remaining set of positive draws. These 1,000 positive draws were used for final calculations of means and uncertainty intervals.

Age-sex splitting

All compiled data were run through the causes of death age-sex splitting process.

Changes from GBD 2015

GBD 2016 saw an effort to systematize the collection of up-to-date fatal discontinuity data through supplemental online research. New tools included expanded use of web scraping and online media tracking. This process resulted in a more comprehensive set of conflict and terrorism data for 2016, as well as large natural disasters not contained in EM-DAT or VR.

In previous rounds of GBD, deaths due to executions and police conflict were included with conflict and terrorism. In GBD 2016, these causes were separated and estimated separately from the overarching war and conflict cause group using a CODEm model, as described in this appendix.

We added two epidemic infectious diseases, cholera and meningococcal meningitis, to the list of fatal discontinuities in an effort to better capture the large variations in mortality that these causes can incur.

We removed the absolute death threshold for fatal discontinuities and limited our inclusion criteria to an event exceeding a mortality rate threshold per location-year. We view this revision as an improvement for estimating the effects of fatal discontinuities in subnational locations and countries with smaller populations, as an absolute threshold of 10, 20, or 50 deaths would ultimately omit events in these places.

References

1 UCDP/PRIO Armed Conflict Dataset Codebook. Uppsala Conflict Data Program (UCDP); Centre for the Study of Civil Wars, International Peace Research Institute, Oslo (PRIO), 2013.

2 Iraq Body Count. https://www.iraqbodycount.org/database/ (accessed May 8, 2017).

3 Hagopian A, Flaxman AD, Takaro TK, *et al.* Mortality in Iraq Associated with the 2003–2011 War and Occupation: Findings from a National Cluster Sample Survey by the University Collaborative Iraq Mortality Study. *PLOS Medicine* 2013; **10**: e1001533.

4 Jarvis C. The Rise and Fall of Albania's Pyramid Schemes. *F&D* 2000; **37**. http://www.imf.org/external/pubs/ft/fandd/2000/03/jarvis.htm.

5 Obermeyer Z, Murray CJL, Gakidou E. Fifty years of violent war deaths from Vietnam to Bosnia: analysis of data from the world health survey programme. *BMJ* 2008; **336**: 1482–6.

6 Milton L. Rwanda, 1994: International incompetence produces genocide. 1994 https://ezproxy.uwc.edu/login?url=http://search.proquest.com/docview/234405747?accountid=42411.

7 Inc GI, Berger DS. Cholera: Global Status: 2017 edition. GIDEON Informatics Inc, 2017.

8 Inc GI, Berger DS. Bacterial Meningitis: Global Status: 2017 edition. GIDEON Informatics Inc, 2017.

9 Siddique A, Zaman K, Baqui A, *et al.* Cholera Epidemics in Bangladesh: 1985-1991. *Journal of Diarrhoeal Diseases Research* 1992; **10**: 79–86.

Section 4: Central computation

4.1. Correction for miscoding of Alzheimer's and other dementias and Parkinson's disease

4.1.1 Objective

We estimated Alzheimer's disease and other dementias, Parkinson's disease, and atrial fibrillation and flutter on the basis of longitudinal prevalence and excess-mortality data in order to help account for changing patterns in death certification and corresponding implausible time trends in many vital registration sources. This method was first implemented for Alzheimer's disease and other dementias in GBD 2013. We added atrial fibrillation and flutter and Parkinson's disease to the causes modelled using this strategy in GBD 2015 and GBD 2016, respectively. All of these causes were processed in CoDCorrect in a manner that was agnostic to the likely targets of misclassification, which inappropriately led to changes in mortality estimates for causes unrelated to these three in GBD 2015. For GBD 2016, we have improved this process by completing a literature review to identify the causes of death most closely associated with Parkinson's and Alzheimer's diseases[20–23] and limiting the CoDCorrect adjustments to only include those causes. We summed CODEm results for Parkinson's, Alzheimer's, lower respiratory infections, protein-energy malnutrition, other nutritional deficiencies, cerebrovascular disease, interstitial nephritis and urinary tract infections, decubitus ulcer, and pulmonary aspiration and foreign body in airway to generate a total envelope for all these conditions. This envelope is used as a parent cause for all calculations outlined below. Atrial fibrillation and flutter did not require a similar process as its scaling in CoDCorrect is already restricted to be within the cardiovascular disease section of the GBD cause list.

4.1.2 Algorithm and levels

The core algorithm closely resembles the CodCorrect algorithm, and can be written as follows:

$$oCD_{lyasjd} = CustomD_{lyasjd}\left(\frac{CODEmD_{lyasjd}}{\sum_{j=1}^{j=k} CustomD_{lyasjd}}\right)$$

Where oCD_{lyasjd} is the corrected number of deaths for a location *l*, year *y*, age *a*, sex *s*, cause *j*, and draw *d*. $CodemD_{lyasjd}$ is the parent cause deaths for a location *l*, year *y*, age *a*, sex *s*, cause *j*, and draw *d*, using data from CODEm for all causes. For every cause in this correction we use the same parent cause which is equal to the sum of the individual causes using data from CODEm. $CustomD_{lyasjd}$ is the uncorrected number of deaths estimated from a cause-specific model for a location *l*, year *y*, age *a*, sex *s*, cause *j*, and draw *d*, using data from DisMod-MR 2.1 for Alzheimer's and Parkinson's and data from CODEm for all other causes.

This correction process only works on one level. It rescales the custom cause-specific deaths to match the correction envelope (which is used for $CodemD_{lyasjd}$ in the above equation). The custom cause-specific deaths (which are used for $CustomD_{lyasjd}$ in the above equation) are either DisMod-MR 2.1 results (for Alzheimer's and Parkinson's) or CODEm results (for all other causes). Because there is only one level, this process occurs only once for each cause.

4.2 Imported cases

Imported cases are fatalities that occur in a geographic area where a particular cause of death is known to be eradicated in a specific time period or where infection cannot occur. We apply space-time restrictions to these causes in the modeling strategy for that location and time period. However, in some rare cases, there are deaths from these causes outside of restricted locations and time periods. These deaths are referred to as Imported Cases.

Illustrating this concept, Chagas Disease is transmitted by insect vectors that only exist in the Americas. For this reason, Chagas Disease is restricted in the models for countries such as Russia. However, it is possible that someone traveling in Latin America could contract Chagas Disease and then die after returning home to Russia. Imported cases accounts for these kinds of deaths.

To calculate these Imported cases, we find all cases from the Vital Registrations of data-rich countries for any cause of death that is otherwise geographically or temporally restricted. We then create a beta distribution from that data point, using the sample size of the Vital Registration for that data point, and upload these draws as a custom Cause of Death model. This model is then used as an input to CoDCorrect.

4.3 CodCorrect

4.3.1 Objective of CodCorrect

As mentioned in the main text, the Causes of Death models are cause-specific. As such, there is no guarantee that the sum of these models will equal the results of the all-cause mortality estimates or that model results of child causes add up to the parent model results. The CoDCorrect process is used to make the Causes of Death and all-cause mortality estimates internally consistent using a very simple algorithm.

4.3.2 Algorithm and levels

The core algorithm remains the same as it did in GBD 2013. The equation can be written as follows:

$$CD_{lyasjd} = D_{lyasjd}\left(\frac{PD_{lyasjd}}{\sum_{j=1}^{j=k} D_{lyasjd}}\right)$$

Where CD_{lyasjd} is the corrected number of deaths for a location *l*, year *y*, age *a*, sex *s*, cause *j*, and draw *d*. PD_{lyasjd} is the parent cause deaths for a location *l*, year *y*, age *a*, sex *s*, cause *j*, and draw *d*. D_{lyasjd} is the uncorrected number of deaths estimated from a cause-specific model for a location *l*, year *y*, age *a*, sex *s*, cause *j*, and draw *d*.

The CoDCorrect process starts by rescaling the Level 1 causes to match the all-cause mortality estimates (which is used for PD_{lyasjd} in the above equation). Level 2 causes are then rescaled to their corrected parent causes. This continues until all levels of the hierarchy have been rescaled. Causes and their levels within the CoDCorrect hierarchy can be found in Appendix Table 10.

Unlike in GBD 2013, HIV is not included in the CoDCorrect process for GBD 2016. To account for this change, Level 1 CoDCorrect causes are rescaled to HIV-deleted mortality estimates which are produced as part of the mortality and HIV estimation process. Results from the GBD version of Spectrum are

added to the post-CoDCorrect death estimates, along with fatal discontinuities and imported cases, to generate the full set of death estimates.

4.3.3 Diagnostic results of CodCorrect by cause and location

For more detail on diagnostic results of CodCorrect by cause see Appendix Table 11.

4.4 Years of life lost (YLLs) calculation

Years of life lost due to premature mortality (YLLs) were computed for 693 locations and 36 years. First, we used the lowest observed age-specific mortality rates by location and sex across all estimation years from locations with total populations greater than 5 million in 2016 to establish a theoretical minimum risk reference life table. The values can be found in Appendix Table 18.

The YLL is a metric that is computed by multiplying the number of estimated deaths by the standard life expectancy at age of death. The metric therefore highlights premature deaths by applying a larger weight to deaths that occur at younger age groups. We propagated uncertainty from CoDCorrected deaths for all demographics. The core equation can be written as follows:

$$YLL = \sum_{c=1,a=0,s=1}^{\infty} d_{cas} e_a$$

4.4.1 GBD world population age standard

Age-standardised rates in GBD are estimated using the GBD world population age standard, which is calculated using methods detailed in Ahmad et al 2001.[24] Briefly, we used the age-specific proportional distributions of all national locations from the World Population Prospects 2012 revision[25] for all years from 2010 to 2035 and generated a standard population structure by taking the non-weighted mean across all the aforementioned country-years. For consistency and comparability across recent iterations of GBD, we used the same standard population structure as used in GBD 2013 and GBD 2015. For values used for the age standard see Appendix Table 19.

4.5 Socio-demographic Index (SDI) analysis

4.5.1 Development of revised SDI indicator

The Socio-demographic Index (SDI) is a composite indicator of development status constructed for GBD 2015 whose components are strongly correlated with health outcomes. It is the geometric mean of 0 to 1 indices of total fertility rate, mean education for those aged 15 and older, and lag distributed income per capita.

SDI was calculated using the Human Development Index (HDI) methodology, wherein an index value was determined for each of the covariate inputs (log LDI, mean educational attainment over age 15, and TFR). For GBD 2015 these indices were computed on the basis of a relative scale, in which the upper and lower bounds were established by the maximum and minimum observed values, respectively, for each input over the entire estimation period of 1980–2015.

Prompted by the observations that the scales (and by extension SDI) were sensitive to the addition of new subnational locations as GBD becomes more granular and to the length of the time period over which SDI is computed, for GBD 2016 we implemented fixed scales in determining individual indices. Thus, an index score of 0 now represents the minimum level of each covariate input past which selected health outcomes can get no worse. An index score of 1 represents the maximum level of each covariate input past which selected health outcomes cease to improve. As a composite, a location with an SDI of 0 would have a theoretical minimum level of development relevant to these health outcomes, while a location with an SDI of 1 would have a theoretical maximum level of development relevant to these health outcomes.

We selected the minima and maxima of the scales by examining the relationships each of the inputs had with life expectancy at birth and under-5 mortality and identifying points of limiting returns at both high and low values, if they occurred prior to theoretical limits (eg, a TFR of 0). The final scales are summarised in the table below.

**Input**	**Lower bound**	**Upper bound**
TFR	1·5[a]	8
LDI per capita	250 USD (5·52 log USD)[b]	60,000 USD (11·00 log USD)
Mean educational attainment for ages 15 and older	0 years	17 years

[a] The low point of limiting returns for TFR was identified at 1 during GBD 2015; however, incorporating feedback with regard to accounting for a pattern of TFR rebound in highly developed countries, we instead set the lower limit of TFR at 1·5.

[b] The minimum for the LDI scale was originally set at the theoretical limit of 0 USD, as we did not observe an asymptotic relationship between log(LDI) and E_0 or 5q0 at lower values of log(LDI). Empirically, however, we also did not observe an LDI below 350 USD (5·86 log USD) for the estimation period 1970–2016. In log-space, this meant that approximately half of our scale was not being utilised, compressing the observed variation in LDI and diminishing its meaningful contribution to SDI. Accordingly, we set the lower limit on LDI to 250 USD (5·52 log USD) to ensure we were fully utilising the range of the scale to capture its variation across space and time, as is the case with the other two inputs.

Using the limits on the scales described above, we computed the index scores underlying SDI analogously to GBD 2015 as follows:

$$I_{Cly} = {(C_{ly} - C_{low})}\Big/{(C_{high} - C_{low})}$$

Where I_{cly} – the index for covariate *C*, location *l*, and year *y* – is equal to the difference between the value of that covariate in that location-year and the lower bound of the covariate divided by the difference between the upper and lower bounds for that covariate. If the values of input covariates fell outside the upper or lower bounds (eg, LDI per capita greater than 60,000 USD), they were mapped to the respective upper or lower bounds. We also note that the index value for TFR was computed as $1 - I_{TFRly}$, as lower TFRs correspond to higher levels of development, and thus higher index scores. For GBD 2016 we expanded the computation of SDI to 755 national and subnational locations spanning the time period 1970–2016.

The composite Socio-demographic Index is the geometric mean of these three indices for a given location-year. The cutoff values used to determine quintiles for analysis were then computed using country-level estimates of SDI for the year 2016, excluding countries with populations less than 1 million.

Example calculation

Below we present the calculation of SDI for Mexico in the year 2010:

$$TFR = 2.43;\ Mean\ educ\ yrs\ pc = 9.23;\ lnLDI = 9.58$$

$$I_{TFR} = 1 - \frac{2.43 - 1.5}{8 - 1.5} = .855$$

$$I_{Educ} = \frac{9.23 - 0}{17 - 0} = .543$$

$$I_{lnLDI} = \frac{9.58 - 5.52}{11.00 - 5.52} = .741$$

$$SDI = \sqrt[3]{I_{TFR} * I_{Educ} * I_{lnLDI}} = \sqrt[3]{.855 * .543 * .741} = .701$$

4.5.2 Age-sex-specific relationships between SDI and cause-specific mortality rates

In order to evaluate the relationship between SDI and mortality, we fit a Gaussian process regression using a linear prior to the mean function within a stochastic partial differential equation (SPDE) framework.

We first assume the following:

$$\ln(Y_{iasc}) \sim N(\mu_i, \sigma^2)$$

Where $Y_{,iasc}$ is the cause-specific mortality rate for a given level of SDI (*i*), age group (*a*), sex (*s*), and cause (*c*).

We then specify a linear prior to the mean μ_i,:

$$\mu_i = \alpha + \beta(SDI) + z_i$$

Where

$$z_i \sim GP(0, \Sigma_M)$$

GP refers to a Gaussian process, and Σ_M refers to the Matern covariance function.

For causes where the relationship between SDI and mortality rates was markedly non-linear (eg, many neglected tropical diseases), we instead specified a continuous piecewise linear prior to the mean. The locations of these knots, as well as the slope and intercept parameters for each of the segments, were dynamically chosen such that they minimised the sum of squared residuals when fit to the data.

Using SPDE, we specified additional priors on the range, variance, and precision of the mean function, as well as selected the number of underlying bases. These hyperparameters were chosen empirically and were identical for all age-sex-cause combinations. Values for the selected hyperparameters are displayed in the table below.

**Hyperparmeter**	**Value**
Range	0·2
Variance	1
Precision	1 x 10^{10}
Number of bases	2 (mesh points at 0·3, 0·7)

Regressions were run separately by age-sex-cause, using observed cause-specific mortality rates from all years 1970–2016 to produce 10,000 simulations per level of SDI from 0 to 1 in increments of 0·005. We fit models on observations from all countries estimated in GBD and included state and province level estimates in lieu of national estimates for Brazil, China, and India due to their large populations (> 200 million) and small number of state-level units modelled in GBD (BRA – 27, IND – 31, CHN – 33) relative to population. Though the United States and Indonesia also fall under the designation of large-population (> 200 million), we fit models on national-level observations instead of state/province-level observations for these two countries as a result of the undue influence from the relatively large number of state-level units modelled in GBD relative to population (USA – 51, IDN – 34). Country and region dummy variables used in GBD 2015 were no longer included in this analysis. All models were fit using the INLA package in R.

In order to produce age-aggregates of our results, we used the same modelling framework as above. In this case, however, we regarded the logit of the share of population in each age group as the dependent variable to estimate a smoothed relationship between population age-structure and SDI. Predictions for each age group at each level of SDI were rescaled to sum to 100%.

References

1 Lozano R, Freeman MK, James SL, *et al.* Performance of InterVA for assigning causes of death to verbal autopsies: multisite validation study using clinical diagnostic gold standards. *Popul Health Metr* 2011; **9**: 50.

2 Office of the Registrar General and Census Commissioner. India Special Survey of Deaths. India, 2004.

3 GBD 2016 Mortality Collaborators. Global, regional, and national under-5 mortality, adult mortality, age-specific mortality, and life expectancy, 1970-2016: a systematic analysis for the Global Burden of Disease Study 2016. *Lancet Rev* 2017.

4 Office of the Registrar General and Census Commissioner. India Medical Certification of Cause of Death Reports 1990-2010. India, 2014.

5 Office of the Registrar General and Census Commissioner. India Medical Certification of Cause of Death Reports 1980-1986. India.

6 Naghavi M, Makela S, Foreman K, O'Brien J, Pourmalek F, Lozano R. Algorithms for enhancing public health utility of national causes-of-death data. *Popul Health Metr* 2010; **8**: 9.

7 Ahern RM, Lozano R, Naghavi M, Foreman K, Gakidou E, Murray CJ. Improving the public health utility of global cardiovascular mortality data: the rise of ischemic heart disease. *Popul Health Metr* 2011; **9**: 8.

8 Barker B, Degenhardt L, National Drug and Alcohol Research Centre (Australia). Accidental drug-induced deaths in Australia 1997-2001. Sydney, Australia: National Drug and Alcohol Research Centre, University of New South Wales, 2003.

9 Roxburgh A, Burns L. Accidental drug-induced deaths due to opioids in Australia, 2011. Sydney, Australia: National Drug and Alcohol Research Centre, University of New South Wales, 2015.

10 Roxburgh A, Burns L. Cocaine and methamphetamine related drug-induced deaths in Australia, 2011. Sydney, Australia: National Drug and Alcohol Research Centre, University of New South Wales, 2015.

11 Naghavi M, Wang H, Lozano R, *et al.* Global, regional, and national age-sex specific all-cause and cause-specific mortality for 240 causes of death, 1990–2013: a systematic analysis for the Global Burden of Disease Study 2013. *The Lancet* 2015; **385**: 117–71.

12 Lozano R, Naghavi M, Foreman K, *et al.* Global and regional mortality from 235 causes of death for 20 age groups in 1990 and 2010: a systematic analysis for the Global Burden of Disease Study 2010. *The Lancet* 2012; **380**: 2095–128.

13 Calvert C, Ronsmans C. The contribution of HIV to pregnancy-related mortality: a systematic review and meta-analysis. *AIDS Lond Engl* 2013; **27**: 1631–9.

14 DerSimonian R, Laird N. Meta-analysis in clinical trials. *Control Clin Trials* 1986; **7**: 177–88.

15 Lozano R, Lopez AD, Atkinson C, Naghavi M, Flaxman AD, Murray CJ. Performance of physician-certified verbal autopsies: multisite validation study using clinical diagnostic gold standards. *Popul Health Metr* 2011; **9**: 32.

16 Foreman KJ, Lozano R, Lopez AD, Murray CJ. Modeling causes of death: an integrated approach using CODEm. *Popul Health Metr* 2012; **10**: 1.

17 Bell RM, Koren Y. Lessons from the Netflix Prize Challenge. *SIGKDD Explor Newsl* 2007; **9**: 75–79.

18 Bell RM, Koren Y, Volinsky C. All together now: A perspective on the NETFLIX PRIZE. *CHANCE* 2010; **23**: 24–24.

19 Flaxman AD, Vos T, Murray CJL, editors. An Integrative Metaregression Framework for Descriptive Epidemiology, 1 edition. Seattle: University of Washington Press, 2015.

20 Todd S, Barr S, Passmore AP. Cause of death in Alzheimer's disease: a cohort study. *QJM Mon J Assoc Physicians* 2013; **106**: 747–53.

21 Brunnström HR, Englund EM. Cause of death in patients with dementia disorders. *Eur J Neurol* 2009; **16**: 488–92.

22 Keene J, Hope T, Fairburn CG, Jacoby R. Death and dementia. *Int J Geriatr Psychiatry* 2001; **16**: 969–74.

23 Thomas BM, Starr JM, Whalley LJ. Death certification in treated cases of presenile Alzheimer's disease and vascular dementia in Scotland. *Age Ageing* 1997; **26**: 401–6.

24 Ahmad OB, Boschi-Pinto C, Lopez AD, Murray CJL, Lozano R, Inoue M. Age standardization of rates: A new WHO standard. Geneva, Switzerland: World Health Organization, 2001 http://www.who.int/healthinfo/paper31.pdf (accessed May 20, 2017).

25 United Nations Department of Economic and Social Affairs. World population prospects: the 2012 revision. New York, NY: United Nations, 2013 https://esa.un.org/unpd/wpp/Publications/Files/WPP2012_Volume-I_Comprehensive-Tables.pdf.

Appendix Figure 1. Analytical flowchart for the development of the GBD 2016 cause of death database

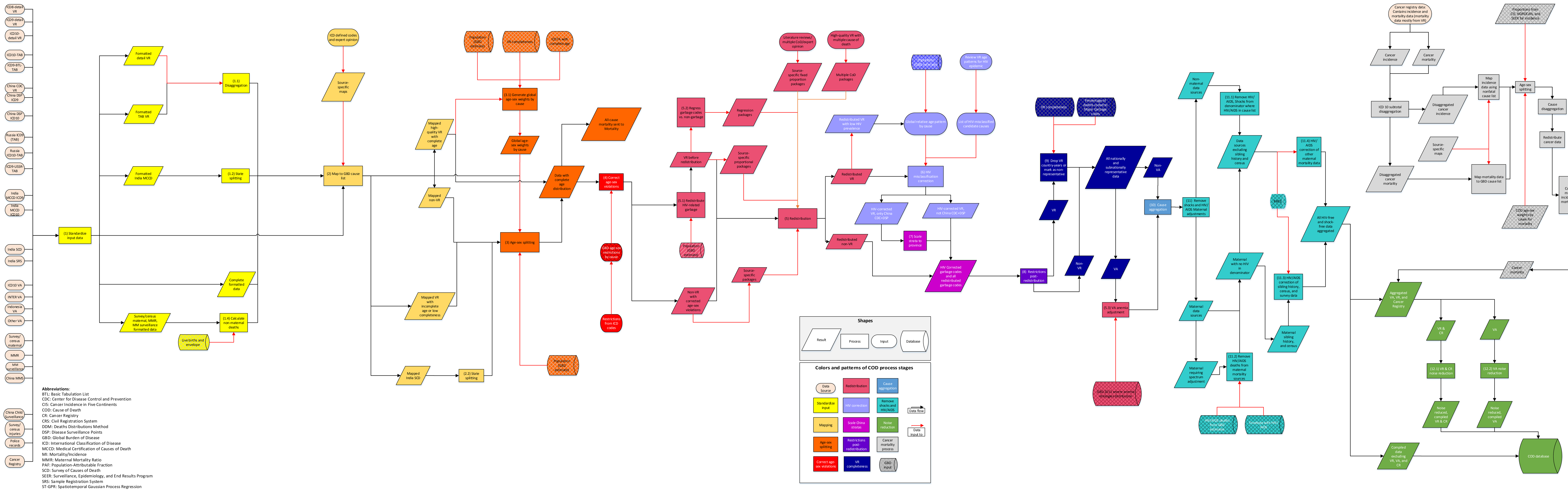

Abbreviations:
BTL: Basic Tabulation List
CDC: Center for Disease Control and Prevention
CI5: Cancer Incidence in Five Continents
COD: Cause of Death
CR: Cancer Registry
CRS: Civil Registration System
DDM: Deaths Distributions Method
DSP: Disease Surveillance Points
GBD: Global Burden of Disease
ICD: International Classification of Disease
MCCD: Medical Certification of Causes of Death
MI: Mortality/Incidence
MMR: Maternal Mortality Ratio
PAF: Population-Attributable Fraction
SCD: Survey of Causes of Death
SEER: Surveillance, Epidemiology, and End Results Program
SRS: Sample Registration System
ST-GPR: Spatiotemporal Gaussian Process Regression
TAB: Tabulation List
VA: Verbal Autopsy
VR: Vital Registration

Appendix Figure 2. GBD 2016 Causes of death estimation flowchart by modeling group

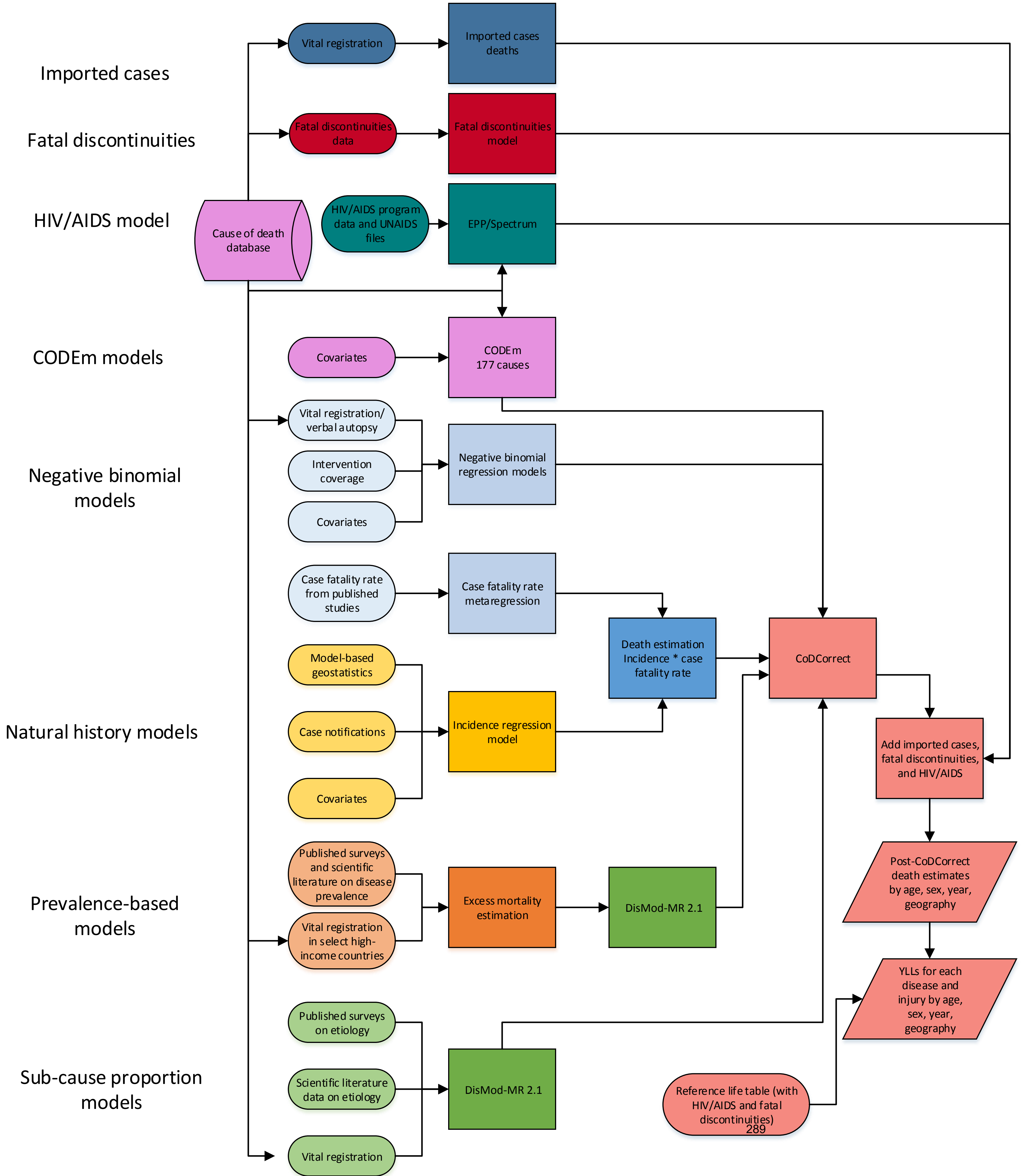

Abbreviations:
CODEm: Cause of death ensemble model
GBD: Global Burden of Disease
YLL: years of life lost

Appendix Figure 3 - GBD 2016 DisMod-MR 2.1 analytical cascade

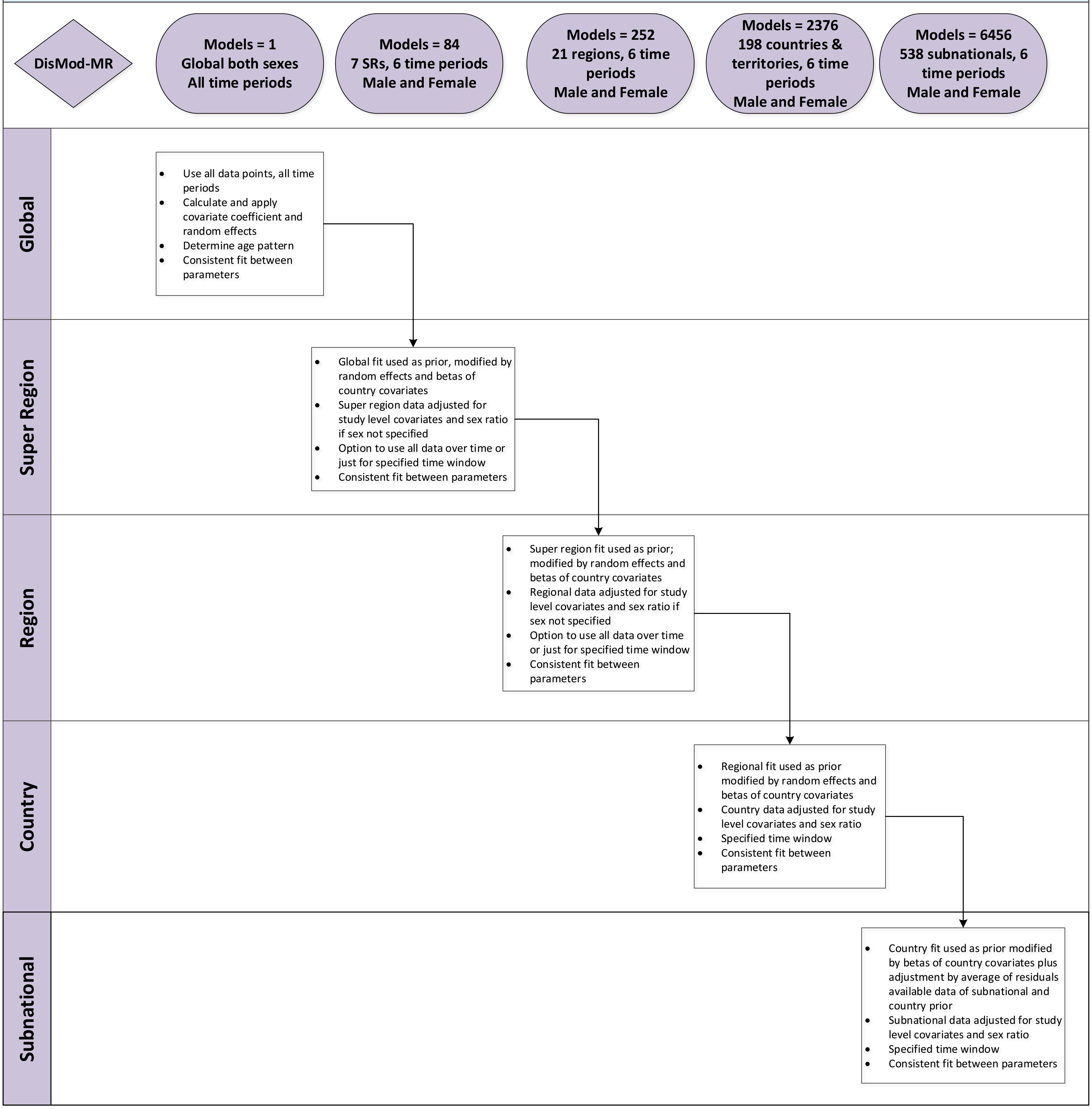

Appendix Figure 4. Vital Registration and Verbal Autopsy data availability by country, 1990–2016

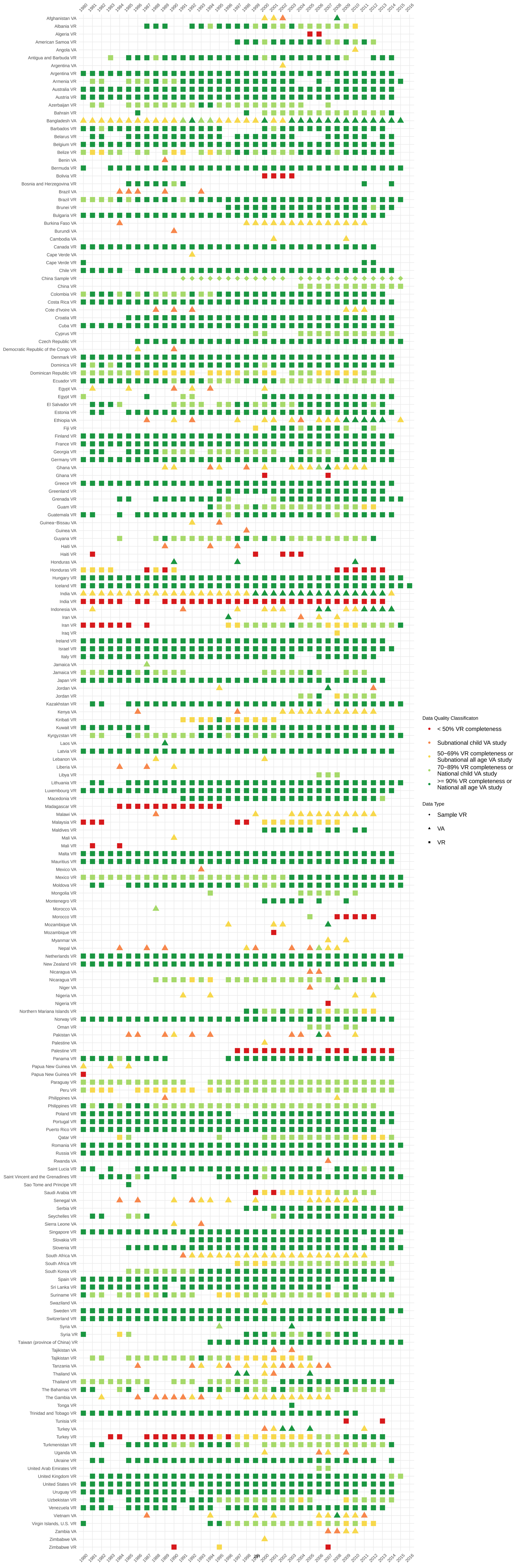

Appendix Table 1. GATHER checklist of information that should be included in reports of global health estimates, with description of compliance and location of information for GBD 2016. Global, regional, and national age-sex specific mortality for 264 causes of death, 1980–2016: a systematic analysis for the Global Burden of Disease Study 2016			
**#**	**GATHER checklist item**	**Description of compliance**	**Reference**
**Objectives and funding**			
1	Define the indicators, populations, and time periods for which estimates were made.	Narrative provided in paper and methods appendix describing indicators, definitions, and populations	Main text (Methods—Overview, Geographic units and time periods) and methods appendix
2	List the funding sources for the work.	Funding sources listed in paper	Summary (Funding)
**Data Inputs**			
For all data inputs from multiple sources that are synthesized as part of the study:			
3	Describe how the data were identified and how the data were accessed.	Narrative description of data seeking methods provided	Main text (Methods) and methods appendix
4	Specify the inclusion and exclusion criteria. Identify all ad-hoc exclusions.	Narrative about inclusion and exclusion criteria by data type provided; Ad-hoc exclusions in cause-specific write ups	Main text (Methods) and methods appendix
5	Provide information on all included data sources and their main characteristics. For each data source used, report reference information or contact name/institution, population represented, data collection method, year(s) of data collection, sex and age range, diagnostic criteria or measurement method, and sample size, as relevant.	An interactive, online data source tool that provides metadata for data sources by component, geography, cause, risk, or impairment has been developed	Online data citation tools: http://ghdx.healthdata.org/global-burden-disease-study-2016
6	Identify and describe any categories of input data that have potentially important biases (e.g., based on characteristics listed in item 5).	Summary of known biases by cause included in methods appendix	Methods appendix
For data inputs that contribute to the analysis but were not synthesized as part of the study:			
7	Describe and give sources for any other data inputs.	Included in online data source tool	http://ghdx.healthdata.org/global-burden-disease-study-2016
For all data inputs:			
8	Provide all data inputs in a file format from which data can be efficiently extracted (e.g., a spreadsheet as opposed to a PDF), including all relevant meta-data listed in item 5. For any data inputs that cannot be	Downloads of input data available through online tools, including data visualization	Online data visualization tools, data query tools, and the Global Health Data Exchange

	shared due to ethical or legal reasons, such as third-party ownership, provide a contact name or the name of the institution that retains the right to the data.	tools and data query tools; input data not available in tools will be made available upon request	
**Data analysis**			
9	Provide a conceptual overview of the data analysis method. A diagram may be helpful.	Flow diagrams of the overall methodological processes, as well as cause-specific modelling processes, have been provided	Main text (Methods) and methods appendix (Appendix Figure 2)
10	Provide a detailed description of all steps of the analysis, including mathematical formulae. This description should cover, as relevant, data cleaning, data pre-processing, data adjustments and weighting of data sources, and mathematical or statistical model(s).	Flow diagrams and corresponding methodological write-ups for each cause, as well as the demographics and causes of death databases and modelling processes, have been provided	Main text (Methods) and methods appendix (Appendix Figure 2, Appendix Section 3)
11	Describe how candidate models were evaluated and how the final model(s) were selected.	Appendix Section 3	Methods appendix
12	Provide the results of an evaluation of model performance, if done, as well as the results of any relevant sensitivity analysis.	Appendix Table 6: CODEm predictive validity results by cause, location type, sex, and age	Methods appendix
13	Describe methods for calculating uncertainty of the estimates. State which sources of uncertainty were, and were not, accounted for in the uncertainty analysis.	Appendix Section 3	Methods appendix
14	State how analytic or statistical source code used to generate estimates can be accessed.	Access statement provided	Code is provided in an online repository
**Results and Discussion**			
15	Provide published estimates in a file format from which data can be efficiently extracted.	GBD 2016 results are available through online data visualization tools, the Global Health Data Exchange, and the online data query tool	Main text, supplementary results, and online data tools (data visualization tools, data query tools, and the Global Health Data Exchange)
16	Report a quantitative measure of the uncertainty of the estimates (e.g. uncertainty intervals).	Uncertainty intervals are provided with all results	Main text, methods appendix, and online data tools (data visualization tools,

			data query tools, and the<br>Global Health Data Exchange)
17	Interpret results in light of existing evidence. If updating a previous set of estimates, describe the reasons for changes in estimates.	Discussion of methodological changes between GBD rounds provided in the narrative of the Article and methods appendix	Main text (Methods and Discussion) and methods appendix
18	Discuss limitations of the estimates. Include a discussion of any modelling assumptions or data limitations that affect interpretation of the estimates.	Discussion of limitations provided in the narrative of the main paper, as well as in the methodological write-ups in the methods appendix	Main text (Limitations) and methods appendix

Appendix Table 2. Total number of site years by cause and source type for 2016

Level	Cause	Vital Registration	Vital Registration-Sample	Verbal Autopsy	Surveillance	Sibling History	Survey/Census	Cancer Registry	Police Records
0	All causes	15987	764	2810	2086	4472	833	2990	1449
1	Communicable, maternal, neonatal, and nutritional diseases	15987	764	2400	2086	4472	787		
2	HIV/AIDS and tuberculosis	15947	764	1733	760				
3	Tuberculosis	15943	764	1710	361				
4	Drug-susceptible tuberculosis	13907	384	392					
4	Multidrug-resistant tuberculosis without extensive drug resistance	7390	374	274					
3	HIV/AIDS	15876	763	671	400				
4	Drug-susceptible HIV/AIDS - Tuberculosis	7436	367		11				
4	HIV/AIDS resulting in other diseases	13824	384		58				
2	Diarrhea, lower respiratory, and other common infectious diseases	15954	764	1905	560				
3	Diarrheal diseases	15942	764	1855	509				
4	Cholera	14627	383						
4	Other salmonella infections	13851	384						
4	Shigellosis	14476	383						
4	Enteropathogenic E coli infection	13767	367						
4	Enterotoxigenic E coli infection	13767	367						
4	Campylobacter enteritis	13767	367						
4	Amoebiasis	14451	375						
4	Cryptosporidiosis	13763	365						
4	Rotaviral enteritis	13763	365						
4	Aeromonas	6383							
4	Clostridium difficile	13789	367						
4	Norovirus	13763	365						
4	Adenovirus	13763	365						
4	Other bacterial foodborne diarrhea	14463	377						
4	Other diarrheal diseases	13947	384						
3	Intestinal infectious diseases	15860	751	1459					
4	Typhoid fever	15014	369						
4	Paratyphoid fever	14325	368						
4	Other intestinal infectious diseases	14435	370						
3	Lower respiratory infections	15954	764	1867	560				
4	Influenza	13948	384						
4	Pneumococcal pneumonia	13829	384						
4	H influenzae type B pneumonia	13829	384						
4	Respiratory syncytial virus pneumonia	13806	384						
4	Other lower respiratory infections	14480	384						
3	Upper respiratory infections	14620	695						
3	Otitis media	15121	546	140					
3	Meningitis	15931	764	1622	546				
4	Pneumococcal meningitis	13810	380						
4	H influenzae type B meningitis	13809	380						
4	Meningococcal meningitis	15139	384						
4	Other meningitis	13832	384						
3	Encephalitis	15670	762	574					
3	Diphtheria	14727	507	1					
3	Whooping cough	15084	588	590					
3	Tetanus	15896	643	1441	393				
3	Measles	15478	755	1283	524				
3	Varicella and herpes zoster	14857	763	376					
2	Neglected tropical diseases and malaria	15933	713	1696	295				
3	Malaria	12289	506	1595	1				
3	Chagas disease	3676	29						
3	Leishmaniasis	15061	665	557					
4	Visceral leishmaniasis	14958	665	557					
3	African trypanosomiasis	13778	364	173					
3	Schistosomiasis	14585	368	41					
3	Cysticercosis	14415	549						
3	Cystic echinococcosis	14492	536						
3	Dengue	15490	373	509	1				
3	Yellow fever	14592	373	181					
3	Rabies	15581	573	1150	267				
3	Intestinal nematode infections	15522	607	554					
4	Ascariasis	14436	365						
3	Ebola	83			26				
3	Zika virus	7378	364						
3	Other neglected tropical diseases	15518	567	44					
2	Maternal disorders	15184	733	1758	1164	4472	760		
3	Maternal hemorrhage	14683	714	1247	738	1	7		

Appendix Table 2. Total number of site years by cause and source type for 2016									
Level	Cause	Vital Registration	Vital Registration-Sample	Verbal Autopsy	Surveillance	Sibling History	Survey/Census	Cancer Registry	Police Records
3	Maternal sepsis and other maternal infections	13457	694	945	462	1	6		
3	Maternal hypertensive disorders	14138	677	953	735	1	7		
3	Maternal obstructed labor and uterine rupture	14249	700	956	573	1	6		
3	Maternal abortion, miscarriage, and ectopic pregnancy	14878	710	1153	596	1	7		
3	Indirect maternal deaths	12579	500	871	754	1	7		
3	Late maternal deaths	6337	251		147				
3	Maternal deaths aggravated by HIV/AIDS	15137	733	1736	1100	4472	758		
3	Other maternal disorders	14529	706	353	711	1	5		
2	Neonatal disorders	15957	756	1678	558				
3	Neonatal preterm birth complications	14843	729	611	558				
3	Neonatal encephalopathy due to birth asphyxia and trauma	14757	642	590	558				
3	Neonatal sepsis and other neonatal infections	13710	520	331	546				
3	Hemolytic disease and other neonatal jaundice	14952	736	374					
3	Other neonatal disorders	14700	578	347	552				
2	Nutritional deficiencies	15943	763	1572	526				
3	Protein-energy malnutrition	15649	762	1528					
3	Iodine deficiency	1470	50						
3	Iron-deficiency anemia	14905	740	1326	1				
3	Other nutritional deficiencies	14887	725						
2	Other communicable, maternal, neonatal, and nutritional diseases	15941	764	1475	843				
3	Sexually transmitted diseases excluding HIV	15917	729	591	228				
4	Syphilis	15545	724		228				
4	Chlamydial infection	13821	375						
4	Gonococcal infection	15044	709						
4	Other sexually transmitted diseases	15499	379						
3	Hepatitis	15924	736	1379					
4	Acute hepatitis A	13794	375						
4	Hepatitis B	14310	382						
4	Hepatitis C	13755	363						
4	Acute hepatitis E	13760	368						
3	Other infectious diseases	15710	764	1308	793				
1	Non-communicable diseases	15964	764	2125	632			2990	14
2	Neoplasms	15964	764	1776	630			2990	
3	Lip and oral cavity cancer	15634	712	585				2758	
3	Nasopharynx cancer	15627	745					2806	
3	Other pharynx cancer	15630	721	374				2782	
3	Esophageal cancer	15920	762	590				2849	
3	Stomach cancer	15924	764	374				2873	
3	Colon and rectum cancer	15924	763	602				2873	
3	Liver cancer	15648	764	374				2859	
3	Gallbladder and biliary tract cancer	14595	761					2779	
3	Pancreatic cancer	15330	755					2862	
3	Larynx cancer	15920	744	374				2846	
3	Tracheal, bronchus, and lung cancer	15924	763	602				2853	
3	Malignant skin melanoma	15641	761					2704	
3	Non-melanoma skin cancer	15258	707						
4	Non-melanoma skin cancer (squamous-cell carcinoma)	14204	384						
3	Breast cancer	15925	752	618	1			2861	
3	Cervical cancer	15920	759	370				2809	
3	Uterine cancer	15908	756	374				2831	
3	Ovarian cancer	15340	754					2845	
3	Prostate cancer	15921	710					2826	
3	Testicular cancer	14300	633	130				2734	
3	Kidney cancer	15632	762					2716	
3	Bladder cancer	15633	761					2656	
3	Brain and nervous system cancer	15330	759	378				2885	
3	Thyroid cancer	15290	728					2826	
3	Mesothelioma	7418	384						
3	Hodgkin lymphoma	15633	742					2762	
3	Non-Hodgkin lymphoma	15632	763					2860	
3	Multiple myeloma	15628	761					2737	
3	Leukemia	15920	764	585	551			2925	
4	Acute lymphoid leukemia	13930	384					1130	
4	Chronic lymphoid leukemia	13925	379					1104	
4	Acute myeloid leukemia	13930	384					1118	

Appendix Table 2. Total number of site years by cause and source type for 2016									
Level	Cause	Vital Registration	Vital Registration-Sample	Verbal Autopsy	Surveillance	Sibling History	Survey/Census	Cancer Registry	Police Records
4	Chronic myeloid leukemia	13930	384					1124	
4	Other leukemia	15607	763	211				1913	
3	Other neoplasms	15697	764		72			2951	
2	Cardiovascular diseases	15964	764	1908	2				
3	Rheumatic heart disease	15927	764	386					
3	Ischemic heart disease	15952	764	1737					
3	Cerebrovascular disease	15952	764	1602	1				
4	Ischemic stroke	14480	761						
4	Hemorrhagic stroke	14479	764						
3	Hypertensive heart disease	15546	760	374					
3	Cardiomyopathy and myocarditis	15631	755	382					
4	Myocarditis	13912	384						
4	Alcoholic cardiomyopathy	13912	384	8					
4	Other cardiomyopathy	13912	384	8					
3	Atrial fibrillation and flutter	13796	343						
3	Aortic aneurysm	14889	630						
3	Peripheral artery disease	13762	364						
3	Endocarditis	14882	691						
3	Other cardiovascular and circulatory diseases	15821	764	1					
2	Chronic respiratory diseases	15948	764	1768	517				
3	Chronic obstructive pulmonary disease	13857	764						
3	Pneumoconiosis	13847	384						
4	Silicosis	13839	384						
4	Asbestosis	13838	383						
4	Coal workers pneumoconiosis	13815	382						
4	Other pneumoconiosis	13827	382						
3	Asthma	13845	383						
3	Interstitial lung disease and pulmonary sarcoidosis	13844	382						
3	Other chronic respiratory diseases	13840	761						
2	Cirrhosis and other chronic liver diseases	15940	764	1436					
2	Digestive diseases	15929	764	1561	546				
3	Peptic ulcer disease	15915	754						
3	Gastritis and duodenitis	14918	384						
3	Appendicitis	15920	763	375					
3	Paralytic ileus and intestinal obstruction	15620	752	551					
3	Inguinal, femoral, and abdominal hernia	15511	379	678					
3	Inflammatory bowel disease	13824	383						
3	Vascular intestinal disorders	13812	373						
3	Gallbladder and biliary diseases	15816	763						
3	Pancreatitis	14359	384						
3	Other digestive diseases	14881	763						
2	Neurological disorders	15932	747	1159	553				
3	Alzheimer disease and other dementias	15544	681	1					
3	Parkinson disease	14418	572						
3	Epilepsy	15804	729	1093					
3	Multiple sclerosis	12947	163						
3	Motor neuron disease	13792	373						
3	Other neurological disorders	13835	382						
2	Mental and substance use disorders	15846	757	505					14
3	Alcohol use disorders	15505	752	491					
3	Drug use disorders	15732	750	482					14
4	Opioid use disorders	13836	749						
4	Cocaine use disorders	13830	749						
4	Amphetamine use disorders	13828	749						
4	Other drug use disorders	13841	749						
3	Eating disorders	13026	378						
4	Anorexia nervosa	13016	378						
4	Bulimia nervosa	12812	337						
2	Diabetes, urogenital, blood, and endocrine diseases	15940	764	1721					
3	Diabetes mellitus	15940	757	1436					
3	Acute glomerulonephritis	15186	763						
3	Chronic kidney disease	15938	764	1632					
4	Chronic kidney disease due to diabetes mellitus	13829	763						
4	Chronic kidney disease due to hypertension	13838	764						
4	Chronic kidney disease due to glomerulonephritis	13829	763						

Appendix Table 2. Total number of site years by cause and source type for 2016

Level	Cause	Vital Registration	Vital Registration-Sample	Verbal Autopsy	Surveillance	Sibling History	Survey/Census	Cancer Registry	Police Records
4	Chronic kidney disease due to other causes	13828	762						
3	Urinary diseases and male infertility	15919	764	598					
4	Interstitial nephritis and urinary tract infections	15918	764	1					
4	Urolithiasis	15566	763						
4	Other urinary diseases	14311	763						
3	Gynecological diseases	15846	750	872					
4	Uterine fibroids	13821	381						
4	Polycystic ovarian syndrome	9941	213						
4	Endometriosis	10378	222						
4	Genital prolapse	13666	280						
4	Other gynecological diseases	13804	381						
3	Hemoglobinopathies and hemolytic anemias	15859	725	1283					
4	Thalassemias	14358	724	264					
4	Sickle cell disorders	13851	724	636					
4	G6PD deficiency	1134	36						
4	Other hemoglobinopathies and hemolytic anemias	13835	725						
3	Endocrine, metabolic, blood, and immune disorders	15906	700	981					
2	Musculoskeletal disorders	15576	763						
3	Rheumatoid arthritis	14461	641						
3	Other musculoskeletal disorders	14492	763						
2	Other non-communicable diseases	15964	764	1511	559				
3	Congenital birth defects	15964	761	1440	559				
4	Neural tube defects	15072	688		548				
4	Congenital heart anomalies	15794	755	349	557				
4	Orofacial clefts	7484	238						
4	Down syndrome	13573	365		540				
4	Other chromosomal abnormalities	13774	583						
4	Congenital musculoskeletal and limb anomalies	13700	364						
4	Urogenital congenital anomalies	13731	382		481				
4	Digestive congenital anomalies	14410	378						
4	Other congenital birth defects	13938	758						
3	Skin and subcutaneous diseases	15884	763	611					
4	Cellulitis	13837	763	375					
4	Pyoderma	14527	763	376					
4	Decubitus ulcer	13791	374	250					
4	Other skin and subcutaneous diseases	13838	368						
3	Sudden infant death syndrome	13499	186						
1	Injuries	15965	764	1898	562		274		1445
2	Transport injuries	15927	764	1540	557		247		75
3	Road injuries	14742	764	376			14		75
4	Pedestrian road injuries	13838	384	376					70
4	Cyclist road injuries	13831	384	376					45
4	Motorcyclist road injuries	13838	384	374			2		52
4	Motor vehicle road injuries	13838	384	376			9		63
4	Other road injuries	13831	384	376					31
3	Other transport injuries	14489	384	376			2		2
2	Unintentional injuries	15925	764	1664	558		13		
3	Falls	15925	764	1283	554		7		
3	Drowning	15903	752	1437	553		4		
3	Fire, heat, and hot substances	15924	764	1432			3		
3	Poisonings	15864	756	580	523		2		
3	Exposure to mechanical forces	15690	764	552	558		10		
4	Unintentional firearm injuries	15559	758	536			3		
4	Unintentional suffocation	13827	702	486	558		1		
4	Other exposure to mechanical forces	14524	764	545			5		
3	Adverse effects of medical treatment	15681	764	374			2		
3	Animal contact	15101	755	1280			3		
4	Venomous animal contact	14346	755	580					
4	Non-venomous animal contact	13838	755						
3	Foreign body	14598	763	594			2		
4	Pulmonary aspiration and foreign body in airway	13836	763	354					
4	Foreign body in other body part	13825	670						
3	Environmental heat and cold exposure	15020	764	518			2		
3	Other unintentional injuries	14665	764	598			3		
2	Self-harm and interpersonal violence	15937	764	1671	1		13		1370

**Appendix Table 2. Total number of site years by cause and source type for 2016**									
**Level**	**Cause**	**Vital Registration**	**Vital Registration-Sample**	**Verbal Autopsy**	**Surveillance**	**Sibling History**	**Survey/Census**	**Cancer Registry**	**Police Records**
3	Self-harm	15937	763	1619			5		
4	Self-harm by firearm	13812	384						
4	Self-harm by other specified means	13829	384						
3	Interpersonal violence	15929	764	1411			11		1370
4	Physical violence by firearm	13829	384	531			5		
4	Physical violence by sharp object	13829	384	537			1		
4	Physical violence by other means	13829	384	536					
2	Forces of nature, conflict and terrorism, and executions and police conflict	15920	764	536			10		
3	Exposure to forces of nature	14895	751	519			6		
3	Conflict and terrorism	15732	764	522			5		
3	Executions and police conflict	14895	658	446			2		

Appendix Table 3: List of International Classification of Diseases (ICD) codes mapped to the Global Burden of Disease cause list for causes of death		
Cause	ICD10	ICD9
Communicable, maternal, neonatal, and nutritional diseases	A00-A00.9, A01.0-A14, A15-A28.9, A30-A30.9, A32-A39.9, A48.1-A48.2, A48.4-A48.5, A49.1, A50-A58, A60-A60.9, A63-A63.8, A65-A65.0, A68-A70, A74, A74.8-A75.9, A77-A96.9, A98-A98.8, B00-B06.9, B10-B10.8, B15-B17.9, B19-B27.9, B29.4, B33-B33.1, B33.3-B33.8, B47-B48.8, B50-B53.8, B55.0, B56-B57.5, B60-B60.8, B63, B65-B67.9, B69-B72.0, B74.3-B75, B77-B77.9, B83-B83.8, B90-B92, B94.1-B94.2, B95-B95.5, B97.4-B97.6, D50.1-D50.8, D51-D52.0, D52.8-D53.9, D64.3, E00-E02, E40-E46.9, E51-E61.9, E63-E64.0, E64.2-E64.9, F07.1, G00.0-G00.8, G03-G03.8, G04-G05.8, G14-G14.6, H70-H70.9, I00, I02, I02.9, I98.0-I98.1, J01-J01.9, J02.0, J03.0, J04.0, J05-J05.0, J09-J15.8, J16-J16.9, J20-J21.9, J36-J36.0, K67.0-K67.8, K71.2, K71.6, K74.7-K74.8, K75.3, K76.3, K77.0, K93.0-K93.1, M03.1, M12.1, M49.0-M49.1, M73.0-M73.1, M89.6, N74.1-N74.2, N96, N98-N98.9, O00-O07.9, O09-O16.9, O20-O26.9, O28-O36.9, O40-O48.1, O60-O77.9, O80-O92.7, O96-O98.6, O98.8-P04.2, P04.5-P05.9, P07-P15.9, P19-P22.9, P23.0-P23.4, P24-P29.9, P35-P37.2, P37.5-P39.9, P50-P61.9, P70, P70.3-P72.9, P74-P78.9, P80-P81.9, P83-P84, P90-P94.9, P96, P96.3-P96.4, P96.8, R19.7, U04-U04.9, U06-U06.9, U82-U89, Z16-Z16.3	001-001.9, 002.0-030.9, 032-034.9, 036-036.3, 036.5-037.9, 040, 040.1-041.0, 042-066.9, 070-074.1, 074.3-075.9, 078.3-078.7, 079-079.7, 080-083.9, 084.0-084.5, 084.7-084.9, 085.0, 086-088, 088.8-088.9, 090-101.6, 104-104.9, 120-124.9, 125.4-125.9, 127-127.1, 128-129.0, 136-136.2, 137-139.0, 244.2, 260-263.9, 265-269.9, 280.1-280.8, 281.0-281.9, 320.0-320.8, 321-323.9, 381-383.9, 390-390.9, 392, 392.9, 425.6, 461-461.9, 464.0, 464.2, 464.4, 464.8-464.9, 466-469, 470.0, 475-475.9, 476.9, 480-482.8, 483.0-483.9, 484.0-484.7, 487-489, 630-636.9, 638-638.9, 640-679.1, 716.0, 730.4-730.6, 760-760.6, 760.8-768, 768.2-770, 770.1-775, 775.4-779.3, 779.6-779.8, V09-V09.9
HIV/AIDS and tuberculosis	A10-A14, A15-A19.9, B20-B24.9, B90-B90.9, K67.3, K93.0, M49.0, N74.1-N74.2, P37.0, U84.3	010-019.9, 042-044.9, 137-137.9, 138.0-138.9, 730.4-730.6
Tuberculosis	A10-A14, A15-A19.9, B90-B90.9, K67.3, K93.0, M49.0, N74.1-N74.2, P37.0, U84.3	010-019.9, 137-137.9, 138.0-138.9, 730.4-730.6
Drug-susceptible tuberculosis	A10-A14, A15-A19.9, B90-B90.9, K67.3, K93.0, M49.0, P37.0	010-019.9
Multidrug-resistant tuberculosis without extensive drug resistance	U84.3	
Extensively drug-resistant tuberculosis		
HIV/AIDS	B20-B24.9	042-044.9
Drug-susceptible HIV/AIDS - Tuberculosis	B20.0	
Multidrug-resistant HIV/AIDS - Tuberculosis without extensive drug resistance		
Extensively drug-resistant HIV/AIDS - Tuberculosis		
HIV/AIDS resulting in other diseases	B20.1-B23.9, B24.0	042-044.9
Diarrhea, lower respiratory, and other common infectious diseases	A00-A00.9, A01.0-A09.9, A33-A37.9, A39-A39.9, A48.1, A70, A83-A87.9, B01-B02.9, B05-B05.9, B94.1, B97.4-B97.6, F07.1, G00.0-G00.8, G03-G03.8, G04-G05.8, H70-H70.9, J01-J01.9, J04.0, J05-J05.0, J09-J15.8, J16-J16.9, J20-J21.9, J36-J36.0, P23.0-P23.4, P35.8, R19.7, U04-U04.9	001-001.9, 002.0-009.9, 032-033.9, 036-036.3, 036.5-037.9, 047-049.9, 052-053.9, 055-055.9, 062-064.9, 079.6, 139.0, 320.0-320.8, 321-323, 323.4-323.9, 381-383.9, 461-461.9, 464.0, 464.2, 464.4, 464.8-464.9, 466-469, 470.0, 475-475.9, 476.9, 480-482.8, 483.0-483.9, 484.0-484.3, 484.6-484.7, 487-489, 771.3
Diarrheal diseases	A00-A00.9, A02-A04.1, A04.3, A04.5-A07, A07.2-A07.4, A08-A09.9, R19.7	001-001.9, 003-006.9, 007.4-007.8, 008.2-009.9
Intestinal infectious diseases	A01.0-A01.4, A04.2, A04.4, A07.0-A07.1, A07.8-A07.9	002.0-002.9, 007-007.3, 007.9-008.1
Typhoid fever	A01.0	002.0
Paratyphoid fever	A01.1-A01.4	002.1-002.9
Other intestinal infectious diseases	A04.2, A04.4, A07.0-A07.1, A07.8-A07.9	007-007.3, 007.9-008.1
Lower respiratory infections	A48.1, A70, B97.4-B97.6, J09-J15.8, J16-J16.9, J20-J21.9, P23.0-P23.4, U04-U04.9	079.6, 466-469, 470.0, 480-482.8, 483.0-483.9, 484.1-484.2, 484.6-484.7, 487-489
Upper respiratory infections	J01-J01.9, J04.0, J05-J05.0, J36-J36.0	461-461.9, 464.0, 464.2, 464.4, 464.8-464.9, 475-475.9, 476.9
Otitis media	H70-H70.9	381-383.9
Meningitis	A39-A39.9, A87-A87.9, G00.0-G00.8, G03-G03.8	036-036.3, 036.5-036.9, 047-049.9, 320.0-320.8, 321-322.9

Appendix Table 3: List of International Classification of Diseases (ICD) codes mapped to the Global Burden of Disease cause list for causes of death		
**Cause**	**ICD10**	**ICD9**
Pneumococcal meningitis	G00.1	320.1
H influenzae type B meningitis	G00.0	320.0
Meningococcal meningitis	A39-A39.9	036-036.3, 036.5-036.9
Other meningitis	A87-A87.9, G00.2-G00.8, G03-G03.8	047-049.9, 320.2-320.8, 321-322.9
Encephalitis	A83-A86.4, B94.1, F07.1, G04-G05.8	062-064.9, 139.0, 323, 323.4-323.9
Diphtheria	A36-A36.9	032-032.9
Whooping cough	A37-A37.9	033-033.9, 484.3
Tetanus	A33-A35.0	037-037.9, 771.3
Measles	B05-B05.9	055-055.9, 484.0
Varicella and herpes zoster	B01-B02.9, P35.8	052-053.9
Neglected tropical diseases and malaria	A30-A30.9, A68-A68.9, A69.2-A69.9, A75-A75.9, A77-A79.9, A82-A82.9, A90-A96.9, A98-A98.8, B33.0-B33.1, B50-B53.8, B55.0, B56-B57.5, B60-B60.8, B65-B67.9, B69-B72.0, B74.3-B75, B77-B77.9, B83-B83.8, B92, K93.1, P37.1, U06-U06.9	030-030.9, 060-061.8, 065-066.9, 071-071.9, 080-083.9, 084.0-084.5, 084.7-084.9, 085.0, 086-088, 088.8-088.9, 120-124.9, 125.4-125.9, 127-127.1, 128-129.0, 425.6
Malaria	B50-B53.8	084.0-084.5, 084.7-084.9
Leprosy	A30-A30.9	030-030.9
Chagas disease	B57-B57.5, K93.1	086-086.2, 086.9, 425.6
Leishmaniasis	B55.0	085.0
Visceral leishmaniasis	B55.0	085.0
African trypanosomiasis	B56-B56.9	086.3-086.5
Schistosomiasis	B65-B65.9	120-120.9
Cysticercosis	B69-B69.9	123.1
Cystic echinococcosis	B67-B67.4, B67.8-B67.9	122-122.4, 122.8-122.9
Dengue	A90-A91.9	061-061.8
Yellow fever	A95-A95.9	060-060.9
Rabies	A82-A82.9	071-071.9
Intestinal nematode infections	B77-B77.9	127.0
Ascariasis	B77-B77.9	127.0
Ebola	A98.4	
Zika virus	U06-U06.9	
Other neglected tropical diseases	A68-A68.9, A69.2-A69.9, A75-A75.9, A77-A79.9, A92-A94.0, A96-A96.9, A98-A98.3, A98.5-A98.8, B33.0-B33.1, B60-B60.8, B67.5-B67.7, B70-B71.9, B74.3-B75, B83-B83.8, P37.1	065-066.9, 080-083.9, 087-088, 088.8-088.9, 122.5-122.7, 123-123.0, 123.2-124.9, 125.4-125.6, 125.9, 127, 127.1, 128-129.0
Maternal disorders	N96, N98-N98.9, O00-O07.9, O09-O16.9, O20-O26.9, O28-O36.9, O40-O48.1, O60-O77.9, O80-O92.7, O96-O98.6, O98.8-O99.9	630-636.9, 638-638.9, 640-679.1
Maternal hemorrhage	O20-O20.9, O43.2, O44-O46.9, O62-O62.9, O67-O67.9, O70, O72-O72.3	640-641.9, 661-661.9, 665, 666-666.9
Maternal sepsis and other maternal infections	O23-O23.9, O85-O86.8, O91-O91.2	659.3, 670-670.9
Maternal hypertensive disorders	O10-O16.9	642-642.9
Maternal obstructed labor and uterine rupture	O32-O33.9, O64-O66.9, O71-O71.9	652-653.9, 660-660.9, 665.0-665.3
Maternal abortion, miscarriage, and ectopic pregnancy	N96, O00-O07.9	630-636.9, 638-638.9, 646.3
Indirect maternal deaths	O24-O25.3, O98-O98.6, O98.8-O99.9	646-646.2, 646.4-649.9
Late maternal deaths	O96-O97.9	
Maternal deaths aggravated by HIV/AIDS		
Other maternal disorders	N98-N98.9, O09-O09.9, O21-O22.9, O26-O26.9, O28-O31.8, O34-O36.9, O40-O43.1, O43.8-O43.9, O47-O48.1, O60-O61.9, O63-O63.9, O68-O69.9, O70.0-O70.9, O73-O77.9, O80-O84.9, O87-O90.9, O92-O92.7	643-645.2, 650-651.9, 654-659.2, 659.4-659.9, 662-664.9, 665.4-665.9, 667-669.9, 671-679.1
Neonatal disorders	P00-P04.2, P04.5-P05.9, P07-P15.9, P19-P22.9, P24-P29.9, P36-P36.9, P38-P39.9, P50-P61.9, P70, P70.3-P72.9, P74-P78.9, P80-P81.9, P83-P84, P90-P94.9, P96, P96.3-P96.4, P96.8	760-760.6, 760.8-768, 768.2-770, 770.1-771, 771.4-775, 775.4-779.3, 779.6-779.8
Neonatal preterm birth complications	P01.0-P01.1, P07-P07.3, P22-P22.9, P25-P28.9, P61.2, P77-P77.9	761.0-761.1, 765-765.9, 769-769.9, 770.2-770.9, 776.6, 777.5-777.6
Neonatal encephalopathy due to birth asphyxia and trauma	P01.7, P02-P03.9, P10-P15.9, P20-P21.9, P24-P24.9, P90-P91.9	761.7-763.9, 767-768, 768.2-768.9, 770.1, 772.1-772.9, 779.0-779.2
Neonatal sepsis and other neonatal infections	P36-P36.9, P38-P39.9	771.4-771.9
Hemolytic disease and other neonatal jaundice	P55-P59.9	773-774.9
Other neonatal disorders	P00-P01, P01.2-P01.6, P01.8-P01.9, P04-P04.2, P04.5-P05.9, P08-P09, P19-P19.9, P29-P29.9, P50-P54.9, P60-P61.1, P61.3-P61.9, P70, P70.3-P72.9, P74-P76.9, P78-P78.9, P80-P81.9, P83-P84, P92-P94.9, P96, P96.3-P96.4, P96.8	760-760.6, 760.8-761, 761.2-761.6, 764-764.9, 766-766.9, 770, 771, 772-772.0, 775, 775.4-776.5, 776.7-777.4, 777.7-779, 779.3, 779.6-779.8
Nutritional deficiencies	D50.1-D50.8, D51-D52.0, D52.8-D53.9, D64.3, E00-E02, E40-E46.9, E51-E61.9, E63-E64.0, E64.2-E64.9, M12.1	244.2, 260-263.9, 265-269.9, 280.1-280.8, 281.0-281.9, 716.0
Protein-energy malnutrition	E40-E46.9, E64.0	260-263.9

Appendix Table 3: List of International Classification of Diseases (ICD) codes mapped to the Global Burden of Disease cause list for causes of death

Cause	ICD10	ICD9
Iodine deficiency	E00-E02	244.2
Iron-deficiency anemia	D50.1-D50.8	280.1-280.8
Other nutritional deficiencies	D51-D52.0, D52.8-D53.9, D64.3, E51-E61.9, E63-E64, E64.2-E64.3, M12.1	265-269.9, 281.0-281.9, 716.0
Other communicable, maternal, neonatal, and nutritional diseases	A20-A28.9, A32-A32.9, A38-A38.9, A48.2, A48.4-A48.5, A49.1, A50-A58, A60-A60.9, A63-A63.8, A65-A65.0, A69-A69.1, A74, A74.8-A74.9, A80-A81.9, A88-A89.9, B00-B00.9, B03-B04, B06-B06.9, B10-B10.8, B15-B17.9, B19-B19.9, B25-B27.9, B29.4, B33, B33.3-B33.8, B47-B48.8, B63, B91, B94.2, B95-B95.5, G14-G14.6, I00, I02, I02.9, I98.0-I98.1, J02.0, J03.0, K67.0-K67.2, K67.8, K71.2, K71.6, K74.7-K74.8, K75.3, K76.3, K77.0, M03.1, M49.1, M73.0-M73.1, M89.6, P35-P35.3, P35.9, P37, P37.2, P37.5-P37.9, U82-U84, U85-U89, Z16-Z16.3	020-029, 034-034.9, 040, 040.1-041.0, 045-046.9, 050-051.9, 054-054.9, 056-059.9, 070-070.9, 072-074.1, 074.3-075.9, 078.3-078.7, 079-079.5, 079.7, 090-101.6, 104-104.9, 136-136.2, 138, 139, 323.0-323.3, 390-390.9, 392, 392.9, 484.4-484.5, 771.0-771.2, V09-V09.9
Sexually transmitted diseases excluding HIV	A50-A58, A60-A60.9, A63-A63.8, B63, I98.0, K67.0-K67.2, M03.1, M73.0-M73.1	054.1, 090-099.9
Syphilis	A50-A53.9, I98.0, K67.2, M03.1, M73.1	090-097.9
Chlamydial infection	A55-A56.8, K67.0	099
Gonococcal infection	A54-A54.9, K67.1, M73.0	098-098.9
Other sexually transmitted diseases	A57-A58, A63-A63.8	099.0-099.9
Hepatitis	B15-B17.9, B19-B19.9, B94.2, K71.2, K71.6, P35.3	070-070.9
Acute hepatitis A	B15-B15.9	070.0-070.1
Hepatitis B	B16-B16.9, B17.0, B19.1, P35.3	070.2-070.3
Hepatitis C	B17.1, B19.2	070.7
Acute hepatitis E	B17.2	
Other infectious diseases	A20-A28.9, A32-A32.9, A38-A38.9, A48.2, A48.4-A48.5, A49.1, A65-A65.0, A69-A69.1, A74, A74.8-A74.9, A80-A81.9, A88-A89.9, B00-B00.9, B03-B04, B06-B06.9, B10-B10.8, B25-B27.9, B29.4, B33, B33.3-B33.8, B47-B48.8, B91, B95-B95.5, G14-G14.6, I00, I02, I02.9, I98.1, J02.0, J03.0, K67.8, K74.7-K74.8, K75.3, K76.3, K77.0, M49.1, M89.6, P35-P35.2, P35.9, P37, P37.2, P37.5-P37.9, U82-U84, U85-U89, Z16-Z16.3	020-029, 034-034.9, 040, 040.1-041.0, 045-046.9, 050-051.9, 054-054.0, 054.2-054.9, 056-059.9, 072-074.1, 074.3-075.9, 078.3-078.7, 079-079.5, 079.7, 100-101.6, 104-104.9, 136-136.2, 138, 139, 323.0-323.3, 390-390.9, 392, 392.9, 484.4-484.5, 771.0-771.2, V09-V09.9
Non-communicable diseases	A46-A46.0, A66-A67.9, B18-B18.9, B33.2, B86, C00-C13.9, C15-C25.9, C30-C34.9, C37-C38.8, C40-C41.9, C43-C45.9, C47-C54.9, C56-C57.8, C58-C58.0, C60-C63.8, C64-C67.9, C68.0-C68.8, C69-C75.8, C81-C86.6, C88-C96.9, D00.1-D00.2, D01.0-D01.3, D02.0-D02.3, D03-D06.9, D07.0-D07.2, D07.4-D07.5, D09.0, D09.2-D09.3, D09.8, D10.0-D10.7, D11-D12.9, D13.0-D13.7, D14.0-D14.3, D15-D16.9, D22-D27.9, D28.0-D28.7, D29.0-D29.8, D30.0-D30.8, D31-D36, D36.1-D36.7, D37.1-D37.5, D38.0-D38.5, D39.1-D39.2, D39.8, D40.0-D40.8, D41.0-D41.8, D42-D43.9, D44.0-D44.8, D45-D47.0, D47.2-D47.9, D48.0-D48.6, D49.2-D49.4, D49.6, D52.1, D55-D58.9, D59.0-D59.3, D59.5-D59.6, D60-D61.9, D63.1, D64.0, D64.4, D66-D67, D68.0-D69.8, D70-D75.8, D76-D78.8, D86-D86.9, D89-D89.3, E03-E07.1, E09-E14.9, E15.0, E16.0-E16.9, E20-E34.8, E36-E36.8, E65-E68, E70-E85.2, E88-E89.9, F00-F03.9, F10-F16.9, F18-F19.9, F24, F50.0-F50.5, G10-G13.8, G20-G26.0, G30-G31.9, G35-G37.9, G40-G41.9, G45-G46.8, G47.3, G61-G61.9, G70-G73.7, G90-G90.9, G93.7, G95-G95.9, G97-G97.9, H05.0-H05.1, I01-I01.9, I02.0, I05-I09.9, I11-I13.9, I20-I25.9, I27.1, I28-I28.8, I30-I31.1, I31.8-I41.9, I42.1-I42.8, I43-I43.9, I47-I48.9, I51.0-I51.4, I60-I63.9, I65-I66.9, I67.0-I67.3, I67.5-I67.7, I68.0-I68.2, I69.0-I69.3, I70.2-I70.8, I71-I73.9, I77-I89.9, I95.2-I95.3, I97-I98, I98.2, I98.9, J30-J35.9, J37-J47.9, J60-J63.8, J65-J68.9, J70-J70.9, J82, J84-J84.9, J91-J92.9, J95-J95.9, K20-K29.9, K31-K31.8, K35-K38.9, K40-K46.9, K50-K52.9, K55-K62.9, K63.5, K64-K64.9, K66.8, K67, K68-K68.9, K70-K70.9, K71.3-K71.5, K71.7, K72.1-K74.6, K74.9, K75.1-K75.2, K75.4-K76.2, K76.4-K77, K77.8, K80-K83.9, K85-K86.9, K90-K91.9, K92.8, K93.8-K95.8, L00-L05.9, L08-L08.9, L10-L14.0, L51-L51.9, L88-L89.9, L93-L93.2, L97-L98.4, M00-M03.0, M03.2-M03.6, M05-M09.8, M30-M36.8, M40-M43.1, M65-M65.0, M71.0-M71.1, M72.5-M72.6, M80-M82.8, M86.3-M86.4, M87-M87.1, M88-M89.0, M89.5, M89.7-M89.9, N00-N08.8, N10-N12.9, N14-N16.8, N18-N18.9, N20-N23.0, N25-N28.1, N29-N32.0, N32.3-N32.4, N34-N34.3, N36-N36.9, N39-N39.2, N41-N41.9, N44-N44.0, N45-N45.9, N49-N49.9, N60-N60.9, N65-N65.1, N72-N72.0, N75-N77.8, N80-N81.9, N83-N83.9, N84.0-N84.1, N87-N87.9, N99-N99.9, P04.3-P04.4, P70.0-P70.2, P96.0-P96.2, P96.5, Q00-Q07.9, Q10.4-Q18.9, Q20-Q28.9, Q30-Q36, Q37-Q45.9, Q50-Q87.8, Q89-Q89.8, Q90-Q93.9, Q95-Q99.8, R50.2, R73-R73.9, R78.0-R78.5, R95-R95.9, X45-X45.9, X65-X65.9, Y15-Y15.9	035-035.9, 036.4, 074.2, 102-103.9, 133-133.6, 135-135.9, 136.6, 140-148.9, 150-158.9, 160-164.9, 170-175.9, 180-183.8, 184.0-184.4, 184.8, 185-186.9, 187.1-187.8, 188-188.9, 189.0-189.8, 190-194.8, 200-208.9, 209.0-209.1, 209.4-209.5, 210.0-210.9, 211.0-211.8, 212.0-212.8, 213-213.9, 217-220.9, 221.0-221.8, 222.0-222.8, 223.0-223.8, 224-228.9, 229.0, 229.8, 230.1-230.8, 231.0-231.2, 232-232.9, 233.0-233.2, 233.4-233.5, 233.7, 234.0-234.8, 235.0, 235.4, 235.6-235.8, 236.0-236.2, 236.4-236.5, 236.7, 237-237.3, 237.5-237.9, 238.0-238.9, 239.2-239.4, 239.6, 240-243.9, 244.0-244.1, 244.3-244.8, 245-246.9, 250-259.9, 270-273.9, 275-276, 277-277.2, 277.4-277.9, 278.0-278.8, 282-284.9, 286-286.5, 286.7-289.7, 290-292.9, 294.1-294.9, 303-303.9, 304.0-304.8, 305-305.9, 307.1, 327.2-327.8, 330-331.2, 331.5-337.9, 340-341.9, 345-345.9, 349, 349.2-349.8, 353.6-353.9, 356-356.9, 357.0-357.7, 358-359.9, 376.0-376.1, 391-391.9, 392.0, 393-398.9, 402-404.9, 410-414.9, 416.1, 417-417.9, 420-423, 423.1-424.9, 425.0-425.3, 425.5, 425.7-425.8, 427-427.3, 427.6-427.8, 429.0, 430-435.9, 437.0-437.2, 437.4-437.8, 440.2, 440.4, 441-443.9, 446-457, 457.1-457.9, 459, 459.1-459.3, 470, 470.9-474.9, 476-476.1, 477-479, 490-504.9, 506-506.9, 508-509, 515, 516-517.8, 518.6-518.7, 518.9, 519.0-519.4, 530-536.1, 536.4, 537-537.6, 537.8, 538-543.9, 550-553.6, 555-558.9, 560-560.3, 560.8-560.9, 562-562.1, 564-564.7, 565-566.9, 569.0-569.7, 571-571.9, 572.2-577.9, 579-583.9, 585-585.9, 588-590.9, 592-593.8, 594-599.6, 599.8, 601-602.9, 604-604.9, 608.2, 610-610.9, 617-618.9, 620-620.9, 621.4-621.9, 622.3-622.7, 629-629.8, 680-689, 694-695.5, 707-707.9, 710-711.9, 714-714.3, 714.8-714.9, 730.1, 732-732.9, 733.0-733.1, 740-749.0, 749.2-758.9, 759.0-759.8, 760.7, 775.0-775.3, 779.4-779.5, 787.1, 788.0, 790.2-790.3, 798-798.0, E850-E854, E860

Appendix Table 3: List of International Classification of Diseases (ICD) codes mapped to the Global Burden of Disease cause list for causes of death		
**Cause**	**ICD10**	**ICD9**
Neoplasms	C00-C13.9, C15-C25.9, C30-C34.9, C37-C38.8, C40-C41.9, C43-C45.9, C47-C54.9, C56-C57.8, C58-C58.0, C60-C63.8, C64-C67.9, C68.0-C68.8, C69-C75.8, C81-C86.6, C88-C96.9, D00.1-D00.2, D01.0-D01.3, D02.0-D02.3, D03-D06.9, D07.0-D07.2, D07.4-D07.5, D09.0, D09.2-D09.3, D09.8, D10.0-D10.7, D11-D12.9, D13.0-D13.7, D14.0-D14.3, D15-D16.9, D22-D24.9, D26.0, D27-D27.9, D28.0-D28.1, D28.7, D29.0-D29.8, D30.0-D30.8, D31-D36, D36.1-D36.7, D37.1-D37.5, D38.0-D38.5, D39.1-D39.2, D39.8, D40.0-D40.8, D41.0-D41.8, D42-D43.9, D44.0-D44.8, D45-D47.0, D47.2-D47.9, D48.0-D48.6, D49.2-D49.4, D49.6, K31.7, K62.0-K62.1, K63.5, N60-N60.9, N84.0-N84.1, N87-N87.9	140-148.9, 150-158.9, 160-164.9, 170-175.9, 180-183.8, 184.0-184.4, 184.8, 185-186.9, 187.1-187.8, 188-188.9, 189.0-189.8, 190-194.8, 200-208.9, 209.0-209.1, 209.4-209.5, 210.0-210.9, 211.0-211.8, 212.0-212.8, 213-213.9, 217-217.8, 219.0, 220-220.9, 221.0-221.8, 222.0-222.8, 223.0-223.8, 224-228.9, 229.0, 229.8, 230.1-230.8, 231.0-231.2, 232-232.9, 233.0-233.2, 233.4-233.5, 233.7, 234.0-234.8, 235.0, 235.4, 235.6-235.8, 236.1-236.2, 236.4-236.5, 236.7, 237-237.3, 237.5-237.9, 238.0-238.9, 239.2-239.4, 239.6, 569.0, 610-610.9
Lip and oral cavity cancer	C00-C08.9, D10.0-D10.5, D11-D11.9	140-145.9, 210.0-210.6, 235.0
Nasopharynx cancer	C11-C11.9, D10.6	147-147.9, 210.7-210.9
Other pharynx cancer	C09-C10.9, C12-C13.9, D10.7	146-146.9, 148-148.9
Esophageal cancer	C15-C15.9, D00.1, D13.0	150-150.9, 211.0, 230.1
Stomach cancer	C16-C16.9, D00.2, D13.1, D37.1	151-151.9, 211.1, 230.2
Colon and rectum cancer	C18-C21.9, D01.0-D01.3, D12-D12.9, D37.3-D37.5	153-154.9, 209.1, 209.5, 211.3-211.4, 230.3-230.6
Liver cancer	C22-C22.9, D13.4	155-155.9, 211.5
Liver cancer due to hepatitis B		
Liver cancer due to hepatitis C		
Liver cancer due to alcohol use		
Liver cancer due to other causes		
Gallbladder and biliary tract cancer	C23-C24.9, D13.5	156-156.9
Pancreatic cancer	C25-C25.9, D13.6-D13.7	157-157.9, 211.6-211.7
Larynx cancer	C32-C32.9, D02.0, D14.1, D38.0	161-161.9, 212.1, 231.0, 235.6
Tracheal, bronchus, and lung cancer	C33-C34.9, D02.1-D02.3, D14.2-D14.3, D38.1	162-162.9, 212.2-212.3, 231.1-231.2, 235.7
Malignant skin melanoma	C43-C43.9, D03-D03.9, D22-D23.9, D48.5	172-172.9
Non-melanoma skin cancer	C44-C44.9, D04-D04.9, D49.2	173-173.9, 222.4, 232-232.9, 238.2
Non-melanoma skin cancer (squamous-cell carcinoma)	C44-C44.9, D04-D04.9, D49.2	173-173.9, 222.4, 232-232.9, 238.2
Breast cancer	C50-C50.9, D05-D05.9, D24-D24.9, D48.6, D49.3, N60-N60.9	174-175.9, 217-217.8, 233.0, 238.3, 239.3, 610-610.9
Cervical cancer	C53-C53.9, D06-D06.9, D26.0	180-180.9, 219.0, 233.1
Uterine cancer	C54-C54.9, D07.0-D07.2, N87-N87.9	182-182.8, 233.2
Ovarian cancer	C56-C56.9, D27-D27.9, D39.1	183-183.0, 220-220.9, 236.2
Prostate cancer	C61-C61.9, D07.5, D29.1, D40.0	185-185.9, 222.2, 236.5
Testicular cancer	C62-C62.9, D29.2-D29.8, D40.1-D40.8	186-186.9, 222.0, 222.3, 236.4
Kidney cancer	C64-C65.9, D30.0-D30.1, D41.0-D41.1	189.0-189.1, 189.5-189.6, 223.0-223.1
Bladder cancer	C67-C67.9, D09.0, D30.3, D41.4-D41.8, D49.4	188-188.9, 223.3, 233.7, 236.7, 239.4
Brain and nervous system cancer	C70-C72.9	191-192.9
Thyroid cancer	C73-C73.9, D09.3, D09.8, D34-D34.9, D44.0	193-193.9, 226-226.9
Mesothelioma	C45-C45.9	
Hodgkin lymphoma	C81-C81.9	201-201.9
Non-Hodgkin lymphoma	C82-C86.6, C96-C96.9	200-200.9, 202-202.9
Multiple myeloma	C88-C90.9	203-203.9
Leukemia	C91-C95.9	204-208.9
Acute lymphoid leukemia	C91.0	204.0
Chronic lymphoid leukemia	C91.1	204.1
Acute myeloid leukemia	C92.0, C92.3-C92.6, C93.0, C94.0, C94.2, C94.4-C94.5	205.0, 205.3, 206.0, 207.0
Chronic myeloid leukemia	C92.1	205.1, 206.1, 207.1
Other leukemia	C91.2-C91.9, C92.2, C92.7-C92.9, C93.1-C93.9, C94.1, C94.3, C94.6-C95.9	204.2-204.9, 205.2, 205.8-205.9, 206.2-207, 207.2-208.9
Other neoplasms	C17-C17.9, C30-C31.9, C37-C38.8, C40-C41.9, C47-C4A, C51-C52.9, C57-C57.8, C58-C58.0, C60-C60.9, C63-C63.8, C66-C66.9, C68.0-C68.8, C69-C69.9, C74-C75.8, D07.4, D09.2, D13.2-D13.3, D14.0, D15-D16.9, D28.0-D28.1, D28.7, D29.0, D30.2, D30.4-D30.8, D31-D33.9, D35-D36, D36.1-D36.7, D37.2, D38.2-D38.5, D39.2, D39.8, D41.2-D41.3, D42-D43.9, D44.1-D44.8, D45-D47.0, D47.2-D47.9, D48.0-D48.4, D49.6, K31.7, K62.0-K62.1, K63.5, N84.0-N84.1	152-152.9, 158-158.9, 160-160.9, 163-164.9, 170-171.9, 181-181.9, 182.9, 183.2-183.8, 184.0-184.4, 184.8, 187.1-187.8, 189.2-189.4, 189.8, 190-190.9, 194-194.8, 209.0, 209.4, 211.2, 211.8, 212.0, 212.4-212.8, 213-213.9, 221.0-221.8, 222.1, 222.8, 223.2, 223.8, 224-225.9, 227-228.9, 229.0, 229.8, 230.7-230.8, 233.4-233.5, 234.0-234.8, 235.4, 235.8, 236.1, 237-237.3, 237.5-237.9, 238.0-238.1, 238.4-238.9, 239.2, 239.6, 569.0
Cardiovascular diseases	B33.2, G45-G46.8, I01-I01.9, I02.0, I05-I09.9, I11-I11.9, I20-I25.9, I28-I28.8, I30-I31.1, I31.8-I41.9, I42.1-I42.8, I43-I43.9, I47-I48.9, I51.0-I51.4, I60-I63.9, I65-I66.9, I67.0-I67.3, I67.5-I67.6, I68.0-I68.2, I69.0-I69.3, I70.2-I70.8, I71-I73.9, I77-I83.9, I86-I89.0, I89.9, I98, K75.1	036.4, 074.2, 391-391.9, 392.0, 393-398.9, 402-402.9, 410-414.9, 417-417.9, 420-423, 423.1-424.9, 425.0-425.3, 425.5, 425.7-425.8, 427-427.3, 427.6-427.8, 429.0, 430-435.9, 437.0-437.2, 437.5-437.8, 440.2, 440.4, 441-443.9, 447-454.9, 456, 456.3-457, 457.1, 457.8-457.9, 459, 459.1-459.3

Appendix Table 3: List of International Classification of Diseases (ICD) codes mapped to the Global Burden of Disease cause list for causes of death		
**Cause**	**ICD10**	**ICD9**
Rheumatic heart disease	I01-I01.9, I02.0, I05-I09.9	391-391.9, 392.0, 393-398.9
Ischemic heart disease	I20-I25.9	410-414.9
Cerebrovascular disease	G45-G46.8, I60-I63.9, I65-I66.9, I67.0-I67.3, I67.5-I67.6, I68.1-I68.2, I69.0-I69.3	430-435.9, 437.0-437.2, 437.5-437.8
Ischemic stroke	G45-G46.8, I63-I63.9, I65-I66.9, I67.2-I67.3, I67.5-I67.6, I69.3	433-435.9, 437.0-437.1, 437.5-437.8
Hemorrhagic stroke	I60-I62.9, I67.0-I67.1, I68.1-I68.2, I69.0-I69.2	430-432.9, 437.2
Hypertensive heart disease	I11-I11.9	402-402.9
Cardiomyopathy and myocarditis	B33.2, I40-I41.9, I42.1-I42.8, I43-I43.9, I51.4	422-422.9, 425.0-425.3, 425.5, 425.7-425.8, 429.0
Myocarditis	B33.2, I40-I41.9, I51.4	422-422.9
Alcoholic cardiomyopathy	I42.6	425.5
Other cardiomyopathy	I42.1-I42.5, I42.7-I42.8, I43-I43.9	425.0-425.3, 425.7-425.8, 429.0
Atrial fibrillation and flutter	I48-I48.9	427.3
Aortic aneurysm	I71-I71.9	441-441.9
Peripheral artery disease	I70.2-I70.8, I73-I73.9	440.2, 440.4, 443.0-443.9
Endocarditis	I33-I33.9, I38-I39.9	421-421.9, 424.9
Other cardiovascular and circulatory diseases	I28-I28.8, I30-I31.1, I31.8-I32.8, I34-I37.9, I47-I47.9, I51.0-I51.3, I68.0, I72-I72.9, I77-I83.9, I86-I89.0, I89.9, I98, K75.1	036.4, 074.2, 417-417.9, 420-420.9, 423, 423.1-424.8, 427-427.2, 427.6-427.8, 442-443, 447-454.9, 456, 456.3-457, 457.1, 457.8-457.9, 459, 459.1-459.3
Chronic respiratory diseases	D86-D86.2, D86.9, G47.3, J30-J35.9, J37-J47.9, J60-J63.8, J65-J68.9, J70-J70.1, J70.8-J70.9, J82, J84-J84.9, J91-J92.9	135-135.9, 136.6, 327.2-327.8, 470, 470.9-474.9, 476-476.1, 477-479, 490-504.9, 506-506.9, 508-509, 515, 516-517.8, 518.6, 518.9, 519.1-519.4
Chronic obstructive pulmonary disease	J40-J44.9, J47-J47.9	490-492.9, 494-494.9, 496-499
Pneumoconiosis	J60-J63.8, J65-J65.0, J92.0	500-504.9
Silicosis	J62-J62.9	502-502.9, 503.0, 503.9
Asbestosis	J61-J61.0, J92.0	501
Coal workers pneumoconiosis	J60-J60.0	500-500.9, 501.0-501.9
Other pneumoconiosis	J63-J63.8, J65-J65.0	503, 503.1, 504-504.9
Asthma	J45-J46.9	493-493.9
Interstitial lung disease and pulmonary sarcoidosis	D86-D86.2, D86.9, J84-J84.9	135-135.9, 136.6, 515, 516-516.9
Other chronic respiratory diseases	G47.3, J30-J35.9, J37-J39.9, J66-J68.9, J70-J70.1, J70.8-J70.9, J82, J91-J92, J92.9	327.2-327.8, 470, 470.9-474.9, 476-476.1, 477-479, 495-495.9, 506-506.9, 508-509, 517-517.8, 518.6, 518.9, 519.1-519.4
Cirrhosis and other chronic liver diseases	B18-B18.9, I85-I85.9, I98.2, K70-K70.9, K71.3-K71.5, K71.7, K72.1-K74.6, K74.9, K75.2, K75.4-K76.2, K76.4-K76.9, K77.8	456.0-456.2, 571-571.9, 572.2-573.9
Cirrhosis and other chronic liver diseases due to hepatitis B		
Cirrhosis and other chronic liver diseases due to hepatitis C		
Cirrhosis and other chronic liver diseases due to alcohol use		
Cirrhosis and other chronic liver diseases due to other causes		
Digestive diseases	I84-I84.9, K20-K29.9, K31-K31.6, K31.8, K35-K38.9, K40-K42.9, K44-K46.9, K50-K52.9, K55-K62, K62.2-K62.6, K62.8-K62.9, K64-K64.9, K66.8, K67, K68-K68.9, K77, K80-K83.9, K85-K86.9, K90-K90.9, K92.8, K93.8, M09.1	455-455.9, 530-536.1, 537-537.6, 537.8, 538, 540-543.9, 550-551.1, 551.3-552.1, 552.3-553.6, 555-558.9, 560-560.3, 560.8-560.9, 562-562.1, 564-564.1, 564.5-564.7, 565-566.9, 569.1-569.5, 569.7, 574-577.9, 579-579.2, 579.4-579.9, 787.1
Peptic ulcer disease	K25-K28.9, K31, K31.1-K31.6, K31.8	531-534.9
Gastritis and duodenitis	K29-K29.9	535-535.9
Appendicitis	K35-K37.9, K38.3-K38.9	540-542.9
Paralytic ileus and intestinal obstruction	K56-K56.9	560-560.3, 560.8-560.9
Inguinal, femoral, and abdominal hernia	K40-K42.9, K44-K46.9	550-551.1, 551.3-552.1, 552.3-553.0, 553.6
Inflammatory bowel disease	K50-K52.9, M09.1	555-556.9, 558-558.9, 569.5
Vascular intestinal disorders	K55-K55.9	557-557.9
Gallbladder and biliary diseases	K80-K83.9	574-576.9
Pancreatitis	K85-K86.9	577-577.9, 579.4
Other digestive diseases	I84-I84.9, K20-K24, K31.0, K38-K38.2, K57-K62, K62.2-K62.6, K62.8-K62.9, K64-K64.9, K66.8, K67, K68-K68.9, K77, K90-K90.9, K92.8, K93.8	455-455.9, 530-530.9, 536-536.1, 537-537.6, 537.8, 538, 543-543.9, 553.1-553.3, 562-562.1, 564-564.1, 564.5-564.7, 565-566.9, 569.1-569.4, 569.7, 579-579.2, 579.8-579.9, 787.1
Neurological disorders	F00-F03.9, G10-G13.8, G20-G21.0, G21.2-G24, G24.1-G25.0, G25.2-G25.3, G25.5, G25.8-G26.0, G30-G31.1, G31.8-G31.9, G35-G37.9, G40-G41.9, G61-G61.9, G70-G72, G72.2-G73.7, G90-G90.9, G95-G95.9, M33-M33.9	290-290.9, 294.1-294.9, 330-331.2, 331.5-337.9, 340-341.9, 345-345.9, 349, 349.2-349.8, 353.6-353.9, 356-356.9, 357.0-357.1, 357.3-357.4, 357.7, 358-359.9, 775.2
Alzheimer disease and other dementias	F00-F03.9, G30-G31.1, G31.8-G31.9	290-290.9, 294.1-294.9, 331-331.2

Appendix Table 3: List of International Classification of Diseases (ICD) codes mapped to the Global Burden of Disease cause list for causes of death		
**Cause**	**ICD10**	**ICD9**
Parkinson disease	G20-G21.0, G21.2-G22.0	332-332.9
Epilepsy	G40-G41.9	345-345.9
Multiple sclerosis	G35-G35.9	340-340.9
Motor neuron disease	G12.2-G12.9	335-335.2, 335.8-335.9
Other neurological disorders	G10-G12.1, G13-G13.8, G23-G24, G24.1-G25.0, G25.2-G25.3, G25.5, G25.8-G26.0, G36-G37.9, G61-G61.9, G70-G72, G72.2-G73.7, G90-G90.9, G95-G95.9, M33-M33.9	330-330.9, 331.5-331.9, 333-334.9, 335.3, 336-337.9, 341-341.9, 349, 349.2-349.8, 353.6-353.9, 356-356.9, 357.0-357.1, 357.3-357.4, 357.7, 358-359.9, 775.2
Mental and substance use disorders	F10-F16.9, F18-F19.9, F24, F50.0-F50.5, G31.2, G72.1, P04.3-P04.4, P96.1, Q86.0, R78.0-R78.5, X45-X45.9, X65-X65.9, Y15-Y15.9	291-292.9, 303-303.9, 304.0-304.8, 305-305.9, 307.1, 357.5, 760.7, 790.3, E850-E854, E860
Alcohol use disorders	F10-F10.9, G31.2, G72.1, P04.3, Q86.0, R78.0, X45-X45.9, X65-X65.9, Y15-Y15.9	291-291.9, 303-303.9, 305.0, 357.5, 790.3, E860
Drug use disorders	F11-F16.9, F18-F19.9, P04.4, P96.1, R78.1-R78.5	292-292.9, 304.0-304.8, 305, 305.1-305.9, 760.7, E850-E854
Opioid use disorders	F11-F11.9, P96.1, R78.1	304.0, 305.5
Cocaine use disorders	F14-F14.9, R78.2	304.2, 305.6
Amphetamine use disorders	F15-F15.9	304.4, 305.7
Other drug use disorders	F13-F13.9, F16-F16.9, F18-F19.9, R78.3-R78.5	292-292.9, 304.1, 304.5-304.8, 305, 305.1, 305.3-305.4, 305.8-305.9
Eating disorders	F50.0-F50.5	307.1
Anorexia nervosa	F50.0-F50.1	307.1
Bulimia nervosa	F50.2-F50.5	
Diabetes, urogenital, blood, and endocrine diseases	D25-D26, D26.1-D26.9, D28.2, D52.1, D55-D58.9, D59.0-D59.3, D59.5-D59.6, D60-D61.9, D63.1, D64.0, D64.4, D66-D67, D68.0-D69.8, D70-D75.8, D76-D78.8, D86.8, D89-D89.3, E03-E07.1, E09-E14.9, E15.0, E16.0-E16.9, E20-E34.8, E36-E36.8, E65-E68, E70-E85.2, E88-E89.9, G21.1, G24.0, G25.1, G25.4, G25.6-G25.7, G72.0, G93.7, G97-G97.9, I12-I13.9, I95.2-I95.3, I97-I97.9, I98.9, J70.2-J70.5, J95-J95.9, K43-K43.9, K62.7, K91-K91.9, K94-K95.8, M87.1, N00-N08.8, N10-N12.9, N14-N16.8, N18-N18.9, N20-N23.0, N25-N28.1, N29-N32.0, N32.3-N32.4, N34-N34.3, N36-N36.9, N39-N39.2, N41-N41.9, N44-N44.0, N45-N45.9, N49-N49.9, N65-N65.1, N72-N72.0, N75-N77.8, N80-N81.9, N83-N83.9, N99-N99.9, P70.0-P70.2, P96.2, P96.5, Q61-Q62.8, R50.2, R73-R73.9	218-219, 219.1-219.9, 236.0, 240-243.9, 244.0-244.1, 244.3-244.8, 245-246.9, 250-259.9, 270-273.9, 275-276, 277-277.2, 277.4-277.9, 278.0-278.8, 282-284.9, 286-286.5, 286.7-289.7, 357.2, 357.6, 403-404.9, 518.7, 519.0, 536.4, 539-539.9, 551.2, 552.2, 564.2-564.4, 569.6, 579.3, 580-583.9, 585-585.9, 588-590.9, 592-593.8, 594-599.6, 599.8, 601-602.9, 604-604.9, 608.2, 617-618.9, 620-620.9, 621.4-621.9, 622.3-622.7, 629-629.8, 753-753.3, 775.0-775.1, 775.3, 779.4-779.5, 788.0, 790.2
Diabetes mellitus	E10-E10.1, E10.3-E11.1, E11.3-E12.1, E12.3-E13.1, E13.3-E14.1, E14.3-E14.9, P70.0-P70.2, R73-R73.9	250-250.3, 250.5-250.9, 357.2, 775.0-775.1, 790.2
Acute glomerulonephritis	N00-N01.9	580-580.9
Chronic kidney disease	D63.1, E10.2, E11.2, E12.2, E13.2, E14.2, I12-I13.9, N02-N08.8, N15.0, N18-N18.9, Q61-Q62.8	250.4, 403-404.9, 581-583.9, 585-585.9, 589-589.9, 753-753.3
Chronic kidney disease due to diabetes mellitus	E10.2, E11.2, E12.2, E13.2, E14.2	250.4
Chronic kidney disease due to hypertension	I12-I13.9	403-404.9
Chronic kidney disease due to glomerulonephritis	N03-N06.9	581-583.9
Chronic kidney disease due to other causes	N02-N02.9, N07-N08.8, N15.0, Q61-Q62.8	589-589.9, 753-753.3
Urinary diseases and male infertility	N10-N12.9, N15, N15.1-N16.8, N20-N23.0, N25-N28.1, N29-N32.0, N32.3-N32.4, N34-N34.3, N36-N36.9, N39-N39.2, N41-N41.9, N44-N44.0, N45-N45.9, N49-N49.9	588-588.9, 590-590.9, 592-593.8, 594-598.1, 598.8-599.6, 599.8, 601-602.9, 604-604.9, 608.2, 788.0
Interstitial nephritis and urinary tract infections	N10-N12.9, N15, N15.1-N16.8, N30-N30.9, N34-N34.3, N39.0-N39.2	590-590.9, 595-595.9, 597-597.9, 599.0
Urolithiasis	N20-N23.0	592-592.9, 594-594.9, 788.0
Other urinary diseases	N25-N28.1, N29-N29.8, N31-N32.0, N32.3-N32.4, N36-N36.9, N39, N41-N41.9, N44-N44.0, N45-N45.9, N49-N49.9	588-588.9, 593-593.8, 596-596.9, 598-598.1, 598.8-599, 599.1-599.6, 599.8, 601-602.9, 604-604.9, 608.2
Gynecological diseases	D25-D26, D26.1-D26.9, D28.2, E28.2, N72-N72.0, N75-N77.8, N80-N81.9, N83-N83.9	218-219, 219.1-219.9, 236.0, 256.4, 617-618.9, 620-620.9, 621.4-621.9, 622.3-622.7, 629-629.8
Uterine fibroids	D25-D26, D26.1-D26.9, D28.2	218-219, 219.1-219.9, 236.0
Polycystic ovarian syndrome	E28.2	256.4
Endometriosis	N80-N80.9	617-617.9
Genital prolapse	N81-N81.9	618-618.9
Other gynecological diseases	N72-N72.0, N75-N77.8, N83-N83.9	620-620.9, 621.4-621.9, 622.3-622.7, 629-629.8
Hemoglobinopathies and hemolytic anemias	D55-D58.9, D59.1, D59.3, D59.5, D60-D61.9, D64.0, D64.4	282-284.9
Thalassemias	D56-D56.9	282.4
Sickle cell disorders	D57-D57.2, D57.4-D57.8	282.6
G6PD deficiency	D55-D55.2	282.2-282.3
Other hemoglobinopathies and hemolytic anemias	D55.3-D55.9, D58-D58.9, D59.1, D59.3, D59.5, D60-D61.9, D64.0, D64.4	282-282.1, 282.7-284.9

Appendix Table 3: List of International Classification of Diseases (ICD) codes mapped to the Global Burden of Disease cause list for causes of death		
**Cause**	**ICD10**	**ICD9**
Endocrine, metabolic, blood, and immune disorders	D52.1, D59.0, D59.2, D59.6, D66-D67, D68.0-D69.8, D70-D75.8, D76-D78.8, D86.8, D89-D89.3, E03-E07.1, E09-E09.9, E15.0, E16.0-E16.9, E20-E28.1, E28.3-E34.8, E36-E36.8, E65-E68, E70-E85.2, E88-E89.9, G21.1, G24.0, G25.1, G25.4, G25.6-G25.7, G72.0, G93.7, G97-G97.9, I95.2-I95.3, I97-I97.9, I98.9, J70.2-J70.5, J95-J95.9, K43-K43.9, K62.7, K91-K91.9, K94-K95.8, M87.1, N14-N14.4, N65-N65.1, N99-N99.9, P96.2, P96.5, R50.2	240-243.9, 244.0-244.1, 244.3-244.8, 245-246.9, 251-256.3, 256.8-259.9, 270-273.9, 275-276, 277-277.2, 277.4-277.9, 278.0-278.8, 286-286.5, 286.7-289.7, 357.6, 518.7, 519.0, 536.4, 539-539.9, 551.2, 552.2, 564.2-564.4, 569.6, 579.3, 598.2, 775.3, 779.4-779.5
Musculoskeletal disorders	I27.1, I67.7, L93-L93.2, M00-M03.0, M03.2-M03.6, M05-M09.0, M09.2-M09.8, M30-M32.9, M34-M36.8, M40-M43.1, M65-M65.0, M71.0-M71.1, M80-M82.8, M86.3-M86.4, M87-M87.0, M88-M89.0, M89.5, M89.7-M89.9	416.1, 437.4, 446-446.9, 695.4-695.5, 710-711.9, 714-714.3, 714.8-714.9, 730.1, 732-732.9, 733.0-733.1
Rheumatoid arthritis	M05-M06.9, M08.0-M08.8	714-714.3, 714.8-714.9
Other musculoskeletal disorders	I27.1, I67.7, L93-L93.2, M00-M03.0, M03.2-M03.6, M07-M08, M08.9-M09.0, M09.2-M09.8, M30-M32.9, M34-M36.8, M40-M43.1, M65-M65.0, M71.0-M71.1, M80-M82.8, M86.3-M86.4, M87-M87.0, M88-M89.0, M89.5, M89.7-M89.9	416.1, 437.4, 446-446.9, 695.4-695.5, 710-711.9, 730.1, 732-732.9, 733.0-733.1
Other non-communicable diseases	A46-A46.0, A66-A67.9, B86, D86.3, H05.0-H05.1, I89.1-I89.8, L00-L05.9, L08-L08.9, L10-L14.0, L51-L51.9, L88-L89.9, L97-L98.4, M72.5-M72.6, P96.0, Q00-Q07.9, Q10.4-Q18.9, Q20-Q28.9, Q30-Q36, Q37-Q45.9, Q50-Q60.6, Q63-Q86, Q86.1-Q87.8, Q89-Q89.8, Q90-Q93.9, Q95-Q99.8, R95-R95.9	035-035.9, 102-103.9, 133-133.6, 376.0-376.1, 457.2-457.3, 680-689, 694-695.3, 707-707.9, 740-749.0, 749.2-752.9, 753.4-758.9, 759.0-759.8, 798-798.0
Congenital birth defects	P96.0, Q00-Q07.9, Q10.4-Q18.9, Q20-Q28.9, Q30-Q36, Q37-Q45.9, Q50-Q60.6, Q63-Q86, Q86.1-Q87.8, Q89-Q89.8, Q90-Q93.9, Q95-Q99.8	740-749.0, 749.2-752.9, 753.4-758.9, 759.0-759.8
Neural tube defects	Q00-Q01.9, Q05-Q05.9	740-741.9, 742.0
Congenital heart anomalies	Q20-Q28.9	745-747.9
Orofacial clefts	Q35-Q36, Q37-Q37.9	749-749.0, 749.2-749.9
Down syndrome	Q90-Q90.9	758.0
Other chromosomal abnormalities	Q87-Q87.8, Q91-Q93.9, Q95-Q95.9, Q97-Q97.9, Q99-Q99.8	758, 758.1-758.6, 758.8-758.9
Congenital musculoskeletal and limb anomalies	Q65-Q79, Q79.6-Q79.9	742.5, 754-756.5, 756.8-756.9
Urogenital congenital anomalies	P96.0, Q50-Q56.4, Q60-Q60.6, Q63-Q64.9	752-752.9, 753.4-753.9
Digestive congenital anomalies	Q38-Q45.9, Q79.0-Q79.5	750-751.9, 756.6-756.7
Other congenital birth defects	Q02-Q04.9, Q06-Q07.9, Q10.4-Q18.9, Q30-Q34.9, Q57, Q80-Q86, Q86.1-Q86.8, Q89-Q89.8	742, 742.1-742.4, 742.8-744.9, 748-748.9, 757-757.9, 759.0-759.8
Skin and subcutaneous diseases	A46-A46.0, A66-A67.9, B86, D86.3, I89.1-I89.8, L00-L05.9, L08-L08.9, L10-L14.0, L51-L51.9, L88-L89.9, L97-L98.4, M72.5-M72.6	035-035.9, 102-103.9, 133-133.6, 457.2-457.3, 680-689, 694-695.3, 707-707.9
Cellulitis	L03-L03.9, M72.5-M72.6	681-682.9
Pyoderma	A46-A46.0, A66-A67.9, I89.1-I89.8, L00-L02.9, L04-L05.9, L08-L08.9, L88, L97-L98.4	035-035.9, 102-103.9, 457.2-457.3, 680-680.9, 683-689
Decubitus ulcer	L89-L89.9	707-707.9
Other skin and subcutaneous diseases	D86.3, L10-L14.0, L51-L51.9	694-695.3
Sudden infant death syndrome	R95-R95.9	798-798.0
Injuries	L55-L55.9, L56.3, L56.8-L56.9, L58-L58.9, U00-U03, V00-V86.9, V87.2-V87.3, V88.2-V88.3, V90-V98.8, W00-W46.2, W49-W62.9, W64-W70.9, W73-W75.9, W77-W81.9, W83-W94.9, W97.9, W99-X06.9, X08-X39.9, X46-X47, X47.1-X47.8, X48-X48.9, X50-X54.9, X57-X58.9, X60-X64.9, X66-Y08.9, Y35-Y84.9, Y87.0-Y87.1, Y88-Y88.3, Y89.0-Y89.1	349.0-349.1, 457.0, E800-E807, E830-E838, E840-E849, E856-E857, E861-E869, E870-E876, E878-E879, E880-E886, E888-E928, E930-E979, E990-E999
Transport injuries	V00-V86.9, V87.2-V87.3, V88.2-V88.3, V90-V98.8	E800-E807, E830-E838, E840-E849
Road injuries	V01-V04.9, V06-V80.9, V82-V82.9, V87.2-V87.3	
Pedestrian road injuries	V01-V04.9, V06-V09.9	
Cyclist road injuries	V10-V19.9	
Motorcyclist road injuries	V20-V29.9	
Motor vehicle road injuries	V30-V79.9, V87.2-V87.3	
Other road injuries	V80-V80.9, V82-V82.9	
Other transport injuries	V00-V00.8, V05-V05.9, V81-V81.9, V83-V86.9, V88.2-V88.3, V90-V98.8	E800-E807, E830-E838, E840-E849
Unintentional injuries	L55-L55.9, L56.3, L56.8-L56.9, L58-L58.9, W00-W46.2, W49-W62.9, W64-W70.9, W73-W75.9, W77-W81.9, W83-W94.9, W97.9, W99-X06.9, X08-X32.9, X39-X39.9, X46-X47, X47.1-X47.8, X48-X48.9, X50-X54.9, X57-X58.9, Y40-Y84.9, Y88-Y88.3	349.0-349.1, 457.0, E856-E857, E861-E869, E870-E876, E878-E879, E880-E886, E888-E906, E910-E928, E930-E949
Falls	W00-W19.9	E880-E886, E888
Drowning	W65-W70.9, W73-W74.9	E910
Fire, heat, and hot substances	X00-X06.9, X08-X19.9	E890-E899, E924
Poisonings	X46-X47, X47.1-X47.8, X48-X48.9	E856-E857, E861-E869
Exposure to mechanical forces	W20-W38.9, W40-W43.9, W45.0-W45.2, W46-W46.2, W49-W52, W75-W75.9	E913, E916-E922
Unintentional firearm injuries	W32-W34.9	E922
Unintentional suffocation	W75-W75.9	E913
Other exposure to mechanical forces	W20-W31.9, W35-W38.9, W40-W43.9, W45.0-W45.2, W46-W46.2, W49-W52	E916-E921

Appendix Table 3: List of International Classification of Diseases (ICD) codes mapped to the Global Burden of Disease cause list for causes of death		
**Cause**	**ICD10**	**ICD9**
Adverse effects of medical treatment	Y40-Y84.9, Y88-Y88.3	349.0-349.1, 457.0, E870-E876, E878-E879, E930-E949
Animal contact	W52.0-W62.9, W64-W64.9, X20-X29.9	E905-E906
Venomous animal contact	X20-X29.9	E905
Non-venomous animal contact	W52.0-W62.9, W64-W64.9	E906
Foreign body	W44-W45, W45.3-W45.9, W78-W80.9, W83-W84.9	E911-E912, E914-E915
Pulmonary aspiration and foreign body in airway	W78-W80.9, W83-W84.9	E911-E912
Foreign body in other body part	W44-W45, W45.3-W45.9	E914-E915
Environmental heat and cold exposure	L55-L55.9, L56.3, L56.8-L56.9, L58-L58.9, W88-W94.9, W97.9, W99-W99.9, X30-X32.9, X39-X39.9	E900-E902, E926
Other unintentional injuries	W39-W39.9, W77-W77.9, W81-W81.9, W85-W87.9, X50-X54.9, X57-X58.9	E903-E904, E923, E925, E927-E928
Self-harm and interpersonal violence	X60-X64.9, X66-Y08.9, Y87.0-Y87.1	E950-E969
Self-harm	X60-X64.9, X66-X84.9, Y87.0	E950-E959
Self-harm by firearm	X72-X74.9	E955
Self-harm by other specified means	X60-X64.9, X66-X71.9, X75-X84.9, Y87.0	E950-E954, E956-E959
Interpersonal violence	X85-Y08.9, Y87.1	E960-E969
Physical violence by firearm	X93-X95.9	E965
Physical violence by sharp object	X99-X99.9	E966
Physical violence by other means	X85-X92.9, X96-X98.9, Y00-Y04.9, Y06-Y08.9, Y87.1	E961-E964, E967-E969
Forces of nature, conflict and terrorism, and executions and police conflict	U00-U03, X33-X38.9, Y35-Y38.9, Y89.0-Y89.1	E907-E909, E970-E979, E990-E999
Exposure to forces of nature	X33-X38.9	E907-E909
Conflict and terrorism	U00-U03, Y36-Y38.9, Y89.1	E979, E990-E999
Executions and police conflict	Y35-Y35.9, Y89.0	E970-E978
Still Born	P95-P95.9	768.0-768.1
Garbage Code (GBD Level 1)	A40-A41.9, A48.0, A48.3, A49.0, A59-A59.9, A71-A71.9, A74.0, B07-B07.9, B30-B30.9, B35-B36.9, B85-B85.4, B87-B88.9, B94.0, D50-D50.0, D50.9, D62-D63.0, D63.8-D64, D64.1-D64.2, D64.8-D65.9, D68, D69.9, E15, E16, E50-E50.9, E64.1, E85.3-E87.6, E87.8-E87.9, F06.2-F06.4, F07.2, F09-F09.9, F17-F17.9, F20-F23.9, F25-F49, F51-F99.0, G06-G08.0, G32-G32.8, G43-G44.2, G44.4-G44.8, G47-G47.2, G47.4-G47.9, G50-G60.9, G62-G65.2, G80-G83.9, G89-G89.4, G91-G91.2, G91.4-G92.9, G93.1-G93.2, G93.4-G93.6, G99-H05, H05.2-H69.9, H71-H99, I26-I26.9, I31.2-I31.4, I46-I46.9, I50-I50.9, I51.7, I67.4, I76, I95-I95.1, I95.8-I95.9, J69-J69.9, J80-J80.9, J85-J85.3, J86-J86.9, J93-J93.1, J93.8-J93.9, J94.2, J96-J96.9, J98.1-J98.3, K00-K19, K30, K65-K66.1, K66.9, K71-K71.1, K71.8-K72.0, K75.0, L20-L30.9, L40-L50.9, L52-L54.0, L56-L56.2, L56.4-L56.5, L57-L57.9, L59-L68.9, L70-L76.8, L80-L87.9, L90-L92.9, L94-L96, L98.5-L99.8, M04, M10-M12.0, M12.2-M29, M37-M39, M43.2-M49, M49.2-M64, M65.1-M71, M71.2-M72.4, M72.8-M73, M73.8-M79.9, M83-M86.2, M86.5-M86.9, M87.2-M87.9, M89.1-M89.4, M90-M99.9, N17-N17.9, N19-N19.9, N32.1-N32.2, N32.8-N33.8, N35-N35.9, N37-N37.8, N39.3-N39.8, N42-N43.4, N44.1-N44.8, N46-N48.9, N50-N53.9, N61-N64.9, N82-N82.9, N91-N91.5, N95, N95.1-N95.9, N97-N97.9, R02-R02.9, R03.1, R07.0, R08-R09, R09.3, R11-R12.0, R14-R15.9, R19-R19.6, R19.8-R23, R23.1-R30.9, R32-R50.1, R50.8-R57.9, R58.0-R72.9, R74-R78, R78.6-R94.8, R96-R99.9, U05, U07-U81, U90-U99, X40-X44.9, X47.0, X47.9, X49-X49.9, Y10-Y19.9, Z00-Z15.8, Z17	038-038.9, 040.0, 041.1, 076-078.2, 110-111.9, 125-125.3, 126-126.9, 127.2-127.9, 131-132.9, 133.8-134.9, 139.1, 139.9, 176-176.9, 247-248, 264-264.9, 274-274.9, 276.0-276.5, 276.7-276.9, 277.3, 280-280.0, 280.9-281, 285-285.9, 286.6, 293, 294-294.0, 295-302.9, 306-307.0, 307.2-307.4, 307.6-319.9, 324-327.1, 328-329, 338-339.1, 339.3-339.8, 342-344.9, 346-347.9, 350-353.5, 354-355.9, 360-376, 376.2-380.9, 384-389.9, 415-415.9, 423.0, 427.5, 427.9-428.9, 429.3, 437.3, 458-458.9, 459.0, 507-507.9, 510-510.9, 512-513.9, 518.0-518.2, 518.5, 520-529.9, 536.3, 536.8-536.9, 537.7, 537.9, 564.8-564.9, 567-568.9, 570-570.9, 572-572.1, 584-584.9, 586-587.9, 600-600.9, 603-603.9, 605-608.1, 608.3-609, 611-612.1, 615-616.9, 619-619.9, 621-621.3, 622-622.2, 622.8-623.6, 623.8-624.5, 624.8-628.9, 629.9, 690-693.9, 695.8-706.9, 708-709.9, 712-713.8, 715-716, 716.2-721.6, 721.8-730.0, 730.2-730.3, 730.7-731.9, 733, 733.2-734.2, 737-738, 738.2-739.9, 780-782.3, 782.6-784.6, 784.9, 785.4-786, 786.6, 786.8, 787, 787.3-788, 788.1-789, 790-790.1, 790.4-796.1, 796.3-797.9, 798.1-799, 799.2-799.9, E855, E858, E980-E982, V01-V08, V10
Garbage Code (GBD Level 2)	A14.9, A29, A45-A45.9, A47-A48, A48.8-A49, A49.3-A49.9, A61-A62, A72-A73, A76, A97, B08-B09, B11-B14, B28-B29, B31-B32.4, B34-B34.9, B61-B62, B68-B68.9, B73-B74.2, B76-B76.9, B78-B81.8, B84, B93-B94, B94.8-B94.9, B95.6-B97.3, B97.7-B99.9, D59, D59.4, D59.8-D59.9, G44.3, G91.3, G93.0, G93.3, I10-I10.9, I15-I15.9, I27-I27.0, I27.2-I27.9, I28.9, I70-I70.1, I70.9, I74-I75.8, J81-J81.1, J90-J90.0, J94-J94.1, J94.8-J94.9, K92.0-K92.2, N70-N71.9, N73-N74.0, N74.3-N74.8, R03-R03.0, R04-R06.9, R09.0-R09.2, R09.8-R10.9, R13-R13.9, R16-R18.9, R23.0, R58, S00, W47-W48, W63, W71-W72, W76-W76.9, W82, W95-W97, W98, X07, X55-X56, X59-X59.9, Y20-Y34.9, Y86-Y87, Y87.2, Y89, Y89.9-Y99.9	000-000.9, 041.2-041.9, 067-069, 078.8-078.9, 079.8-079.9, 089-089.9, 105-109.9, 119, 136.8-136.9, 139.8, 279-279.9, 304, 304.9, 307.5, 339.2, 401-401.9, 405-405.9, 416-416.0, 416.2-416.9, 440-440.1, 440.3, 440.8-440.9, 444-445.8, 511-511.9, 514-514.9, 515.0-515.9, 518, 518.3-518.4, 518.8, 536.2, 578-578.9, 599.7, 613-614.9, 714.4, 716.1, 721.7, 735-736.9, 738.0-738.1, 782.4, 784.7-784.8, 786.3, 787.0, 787.2, 789.0-789.9, 796.2, 799.0-799.1, 800-E80, E83, E839, E85, E859, E87, E877, E88, E887, E929, E983-E985, E988-E989

Appendix Table 3: List of International Classification of Diseases (ICD) codes mapped to the Global Burden of Disease cause list for causes of death		
**Cause**	**ICD10**	**ICD9**
Garbage Code (GBD Level 3)	A01, A31-A31.9, A42-A44.9, A49.2, A64-A64.0, A99-A99.0, B37-B46.9, B49-B49.9, B55.1-B55.2, B58-B59.9, B89, C14-C14.9, C26-C29, C35-C36, C39-C39.9, C42, C46-C46.9, C55-C55.9, C57.9, C59, C63.9, C68, C68.9, C75.9-C80.9, C87, C97-D00.0, D01, D01.4-D02, D02.4-D02.9, D07, D07.3, D07.6-D09, D09.1, D09.7, D09.9-D10, D10.9, D13, D13.9-D14, D14.4, D17-D21.9, D28, D28.9-D29, D29.9-D30, D30.9, D36.0, D36.9-D37.0, D37.6-D38, D38.6-D39.0, D39.7, D39.9-D40, D40.9-D41, D41.9, D44, D44.9, D48, D48.7-D49.1, D49.5, D49.7-D49.9, D54, D75.9, D79-D85, D87-D88, D89.8-D99, E07.8-E08.9, E17-E19, E34.9-E35.8, E37-E39, E47-E49, E62, E69, E87.7, E90-E998, F04-F06.1, F06.5-F07.0, F07.8-F08, F50, F50.8-F50.9, G09-G09.9, G15-G19, G27-G29, G33-G34, G38-G39, G42, G48-G49, G66-G69, G74-G79, G84-G88, G93, G93.8-G94.8, G96-G96.9, G98-G98.9, I00.0, I03-I04, I14, I16-I19, I29-I29.9, I44-I45.9, I49-I49.9, I51, I51.6, I51.8-I59, I90-I94, I96-I96.9, I98.4-I98.8, I99-J00.0, J02, J02.8-J03, J03.8-J04, J04.1-J04.3, J05.1-J06.9, J48-J59, J71-J79, J81.9, J83, J85.9, J87-J89, J90.9, J93.6, J97-J98.0, J98.4-J99.8, K31.9-K34, K39, K47-K49, K53-K54, K63-K63.4, K63.8-K63.9, K69, K75, K78-K79, K84, K87-K89, K92, K92.9-K93, K96-K99, L06-L07, L09, L15-L19, L31-L39, L69, L77-L79, N09, N13-N13.9, N24, N28.8-N28.9, N38, N39.9-N40.9, N54-N59, N66-N69, N78-N79, N84, N84.2-N86, N88-N90.9, N92-N94.9, N95.0, O08-O08.9, O17-O19, O27, O37-O39, O49-O59, O78-O79, O93-O95.9, P06, P16-P18, P30-P34.2, P40-P49, P62-P69, P73, P79, P82, P85-P89, P96.9-P99.9, Q08-Q10.3, Q19, Q29, Q36.0-Q36.9, Q46-Q49, Q88, Q89.9, Q94, Q99.9-R01.2, R07, R07.1-R07.9, R31-R31.9	002, 031-031.9, 039-039.9, 085, 085.1-085.9, 088.0-088.7, 112-118.9, 130-130.9, 136.3-136.5, 149-149.9, 159-159.9, 165-169, 177-179.9, 183.9-184, 184.5, 184.9, 187, 187.9, 189, 189.9, 194.9-199.9, 209, 209.2-209.3, 209.6-210, 211, 211.9-212, 212.9, 214-216.9, 221, 221.9-222, 222.9-223, 223.9, 229, 229.1, 229.9-230.0, 230.9-231, 231.8-231.9, 233, 233.3, 233.6, 233.9-234, 234.9-235, 235.1-235.3, 235.5, 235.9-236, 236.3, 236.6, 236.9, 237.4, 239-239.1, 239.5, 239.7-239.9, 249-249.9, 276.6, 278, 293.0-293.9, 331.3-331.4, 348-348.9, 349.9, 357, 357.8-357.9, 399-400.0, 406-409.4, 418-419.9, 426-426.9, 427.4, 429, 429.2, 429.4-429.9, 459.5-460.9, 462-464, 464.1, 464.3, 464.5, 465-465.9, 505-505.9, 519, 519.8-519.9, 544-549, 553.8-553.9, 559-559.0, 560.4-560.7, 561, 562.2-563, 569, 569.8-569.9, 591-591.9, 593.9, 599.9, 623.7, 624.6, 637-637.9, 639-639.9, 749.1, 759, 759.9, 779.9, 782.5, 785-785.3, 786.0-786.2, 786.4-786.5, 786.7, 786.9, E986-E987
Garbage Code (GBD Level 4)	B54-B55, B55.9, B64, B82-B82.9, B83.9, D47.1, G00, G00.9-G02.8, G03.9, I42-I42.0, I42.9, I51.5, I64-I64.9, I67, I67.8-I68, I68.8-I69, I69.4-I69.9, J07-J08, J15.9, J17-J19.6, J22-J29, J64-J64.9, P23, P23.5-P23.9, P37.3-P37.4, V87-V87.1, V87.4-V88.1, V88.4-V89.9, V99-V99.0, Y09-Y09.9, Y85-Y85.9	084, 084.6, 238, 244, 244.9, 289.8-289.9, 320, 320.9, 425, 425.4, 425.9, 429.1, 436-437, 437.9-439.6, 482.9-483, 484, 484.8-486.9, 770.0, E808-E829

Appendix Table 4. Restrictions on age and sex by cause for GBD 2016

Cause	Minimum Age	Maximum Age	Sex Restrictions
HIV/AIDS and tuberculosis	28 days		
Tuberculosis	28 days		
Drug-sensitive tuberculosis	28 days		
Multidrug-resistant tuberculosis without extensive drug resistance	28 days		
Extensively drug-resistant tuberculosis	28 days		
HIV/AIDS	28 days		
Drug-sensitive HIV/AIDS - Tuberculosis	28 days		
Multidrug-resistant HIV/AIDS - Tuberculosis without extensive drug resistance	28 days		
Extensively drug-resistant HIV/AIDS - Tuberculosis	28 days		
HIV/AIDS resulting in other diseases	28 days		
Diarrhoea, lower respiratory infections, and other common infectious diseases			
Diarrhoeal diseases			
Intestinal infectious diseases	28 days		
Typhoid fever	28 days		
Paratyphoid fever	28 days		
Other intestinal infectious diseases	28 days		
Lower respiratory infections			
Upper respiratory infections			
Otitis media			
Meningitis			
Pneumococcal meningitis			
H influenzae type B meningitis			
Meningococcal infection			
Other meningitis			
Encephalitis			
Diphtheria	28 days	59 years	

Appendix Table 4. Restrictions on age and sex by cause for GBD 2016

Cause	Minimum Age	Maximum Age	Sex Restrictions
Whooping cough	28 days	59 years	
Tetanus			
Measles	28 days	59 years	
Varicella and herpes zoster			
Neglected tropical diseases and malaria			
Malaria	7 days		
Chagas disease	28 days		
Leishmaniasis	28 days		
Visceral leishmaniasis	28 days		
African trypanosomiasis	1 year		
Schistosomiasis	28 days		
Cysticercosis			
Cystic echinococcosis	1 year		
Dengue	28 days		
Yellow fever	7 days		
Rabies	28 days		
Intestinal nematode infections	28 days		
Ascariasis	28 days		
Ebola			
Zika virus			
Other neglected tropical diseases			
Maternal disorders	10 years	54 years	Females Only
Maternal haemorrhage	10 years	54 years	Females Only
Maternal sepsis and other pregnancy related infections	10 years	54 years	Females Only
Maternal hypertensive disorders	10 years	54 years	Females Only
Maternal obstructed labour and uterine rupture	10 years	54 years	Females Only

Appendix Table 4. Restrictions on age and sex by cause for GBD 2016

Cause	Minimum Age	Maximum Age	Sex Restrictions
Maternal abortion, miscarriage, and ectopic pregnancy	10 years	54 years	Females Only
Indirect maternal deaths	10 years	54 years	Females Only
Late maternal deaths	10 years	54 years	Females Only
Maternal deaths aggravated by HIV/AIDS	10 years	54 years	Females Only
Other maternal disorders	10 years	54 years	Females Only
Neonatal disorders		4 years	
Neonatal preterm birth complications		4 years	
Neonatal encephalopathy due to birth asphyxia and trauma		4 years	
Neonatal sepsis and other neonatal infections		4 years	
Hemolytic disease and other neonatal jaundice		4 years	
Other neonatal disorders		4 years	
Nutritional deficiencies	28 days		
Protein-energy malnutrition	28 days		
Iodine deficiency	28 days		
Iron-deficiency anaemia	28 days		
Other nutritional deficiencies	28 days		
Other communicable, maternal, neonatal, and nutritional diseases			
Sexually transmitted diseases excluding HIV			
Syphilis			
Chlamydial infection	10 years		
Gonococcal infection	10 years		
Other sexually transmitted diseases	10 years		
Hepatitis	28 days		
Acute hepatitis A	28 days		
Hepatitis B	28 days		
Hepatitis C	5 years		

Appendix Table 4. Restrictions on age and sex by cause for GBD 2016

Cause	Minimum Age	Maximum Age	Sex Restrictions
Acute hepatitis E	1 year		
Other infectious diseases			
Neoplasms			
Lip and oral cavity cancer	15 years		
Nasopharynx cancer	5 years		
Other pharynx cancer	15 years		
Oesophageal cancer	15 years		
Stomach cancer	15 years		
Colon and rectum cancer	15 years		
Liver cancer	5 years		
Liver cancer due to hepatitis B	5 years		
Liver cancer due to hepatitis C	5 years		
Liver cancer due to alcohol use	15 years		
Liver cancer due to other causes	5 years		
Gallbladder and biliary tract cancer	15 years		
Pancreatic cancer	15 years		
Larynx cancer	15 years		
Tracheal, bronchus, and lung cancer	15 years		
Malignant skin melanoma	15 years		
Non-melanoma skin cancer	15 years		
Non-melanoma skin cancer (squamous-cell carcinoma)	15 years		
Breast cancer	15 years		
Cervical cancer	15 years		Females Only
Uterine cancer	15 years		Females Only
Ovarian cancer	15 years		Females Only
Prostate cancer	15 years		Males Only

Appendix Table 4. Restrictions on age and sex by cause for GBD 2016

Cause	Minimum Age	Maximum Age	Sex Restrictions
Testicular cancer	15 years		Males Only
Kidney cancer			
Bladder cancer	15 years		
Brain and nervous system cancer			
Thyroid cancer	10 years		
Mesothelioma	15 years		
Hodgkin lymphoma			
Non-Hodgkin's lymphoma			
Multiple myeloma	15 years		
Leukaemia			
Acute lymphoid leukaemia			
Chronic lymphoid leukaemia	15 years		
Acute myeloid leukaemia			
Chronic myeloid leukaemia	15 years		
Other leukaemia			
Other neoplasms			
Cardiovascular diseases			
Rheumatic heart disease	1 year		
Ischaemic heart disease	28 days		
Cerebrovascular disease			
Ischaemic stroke	28 days		
Hemorrhagic stroke			
Hypertensive heart disease	28 days		
Cardiomyopathy and myocarditis			
Myocarditis			
Alcoholic cardiomyopathy	15 years		

Appendix Table 4. Restrictions on age and sex by cause for GBD 2016

Cause	Minimum Age	Maximum Age	Sex Restrictions
Other cardiomyopathy			
Atrial fibrillation and flutter	30 years		
Aortic aneurysm	15 years		
Peripheral vascular disease	40 years		
Endocarditis			
Other cardiovascular and circulatory diseases			
Chronic respiratory diseases			
Chronic obstructive pulmonary disease	28 days		
Pneumoconiosis	15 years		
Silicosis	15 years		
Asbestosis	15 years		
Coal workers pneumoconiosis	15 years		
Other pneumoconiosis	15 years		
Asthma	1 year		
Interstitial lung disease and pulmonary sarcoidosis	1 year		
Other chronic respiratory diseases			
Cirrhosis and other chronic liver diseases	1 year		
Cirrhosis and other chronic liver diseases due to hepatitis B	1 year		
Cirrhosis and other chronic liver diseases due to hepatitis C	1 year		
Cirrhosis and other chronic liver diseases due to alcohol use	15 years		
Cirrhosis and other chronic liver diseases due to other causes	1 year		
Digestive diseases			
Peptic ulcer disease	1 year		
Gastritis and duodenitis	1 year		
Appendicitis	1 year		
Paralytic ileus and intestinal obstruction			

Appendix Table 4. Restrictions on age and sex by cause for GBD 2016

Cause	Minimum Age	Maximum Age	Sex Restrictions
Inguinal, femoral, and abdominal hernia	1 year		
Inflammatory bowel disease	1 year		
Vascular intestinal disorders	1 year		
Gallbladder and biliary diseases	1 year		
Pancreatitis	1 year		
Other digestive diseases	1 year		
Neurological disorders	28 days		
Alzheimer's disease and other dementias	40 years		
Parkinson's disease	20 years		
Epilepsy	28 days		
Multiple sclerosis	20 years		
Motor neuron disease			
Other neurological disorders	28 days		
Mental and substance use disorders			
Alcohol use disorders	15 years		
Drug use disorders			
Opioid use disorders			
Cocaine use disorders			
Amphetamine use disorders			
Other drug use disorders			
Eating disorders	5 years	59 years	
Anorexia nervosa	5 years	59 years	
Bulimia nervosa	5 years	59 years	
Diabetes, urogenital, blood, and endocrine diseases			
Diabetes mellitus			
Acute glomerulonephritis	28 days		

Appendix Table 4. Restrictions on age and sex by cause for GBD 2016

Cause	Minimum Age	Maximum Age	Sex Restrictions
Chronic kidney disease	28 days		
Chronic kidney disease due to diabetes mellitus	28 days		
Chronic kidney disease due to hypertension	28 days		
Chronic kidney disease due to glomerulonephritis	28 days		
Chronic kidney disease due to other causes	28 days		
Urinary diseases and male infertility			
Interstitial nephritis and urinary tract infections			
Urolithiasis	5 years		
Other urinary diseases			
Gynecological diseases	15 years		Females Only
Uterine fibroids	15 years		Females Only
Polycystic ovarian syndrome	15 years	54 years	Females Only
Endometriosis	15 years	54 years	Females Only
Genital prolapse	15 years		Females Only
Other gynecological diseases	15 years		Females Only
Hemoglobinopathies and hemolytic anaemias			
Thalassemias			
Sickle cell disorders			
G6PD deficiency			
Other hemoglobinopathies and hemolytic anaemias			
Endocrine, metabolic, blood, and immune disorders			
Musculoskeletal disorders	5 years		
Rheumatoid arthritis	5 years		
Other musculoskeletal disorders	5 years		
Other non-communicable diseases			
Congenital anomalies		69 years	

Appendix Table 4. Restrictions on age and sex by cause for GBD 2016

Cause	Minimum Age	Maximum Age	Sex Restrictions
Neural tube defects		69 years	
Congenital heart anomalies		69 years	
Orofacial clefts		4 years	
Down's syndrome		69 years	
Other chromosomal abnormalities		69 years	
Congenital musculoskeletal and limb anomalies		69 years	
Urogenital congenital anomalies		69 years	
Digestive congenital anomalies		69 years	
Other congenital anomalies		69 years	
Skin and subcutaneous diseases	28 days		
Cellulitis	28 days		
Pyoderma	28 days		
Decubitus ulcer	1 year		
Other skin and subcutaneous diseases	28 days		
Sudden infant death syndrome	7 days	364 days	
Transport injuries			
Road injuries			
Pedestrian road injuries			
Cyclist road injuries	1 year		
Motorcyclist road injuries			
Motor vehicle road injuries			
Other road injuries			
Other transport injuries			
Unintentional injuries			
Falls			
Drowning			

Appendix Table 4. Restrictions on age and sex by cause for GBD 2016

Cause	Minimum Age	Maximum Age	Sex Restrictions
Fire, heat, and hot substances			
Poisonings			
Exposure to mechanical forces			
Unintentional firearm injuries			
Unintentional suffocation			
Other exposure to mechanical forces			
Adverse effects of medical treatment			
Animal contact			
Venomous animal contact			
Non-venomous animal contact			
Foreign body			
Pulmonary aspiration and foreign body in airway			
Foreign body in other body part			
Environmental heat and cold exposure			
Other unintentional injuries			
Self-harm and interpersonal violence			
Self-harm	10 years		
Self-harm by firearm	10 years		
Self-harm by other specified means	10 years		
Interpersonal violence			
Assault by firearm			
Assault by sharp object			
Assault by other means			
Forces of nature, conflict and terrorism, and executions and police conflict			
Exposure to forces of nature			
Conflict and terrorism			

Appendix Table 4. Restrictions on age and sex by cause for GBD 2016

Cause	Minimum Age	Maximum Age	Sex Restrictions
Executions and police conflict	28 days		

Appendix Table 5: HIV/AIDS-related garbage code redistribution packages		
**Package name**	**ICD9 codes**	**ICD10 codes**
Infectious 1	39.0, 39.1, 39.2, 39.3, 39.4, 39.6, 39.8, 39.9, 113.0, 113.2, 113.4, 113.5, 113.6	A42, A42.0, A42.1, A42.2, A42.7, A42.8, A42.81, A42.82, A42.89, A42.9
Infectious 2	88.0, 88.2, 88.3, 88.5, 88.7	A44, A44.0, A44.1, A44.8, A44.9
Infectious 3	112.0, 112.1, 112.2, 112.3, 112.4, 112.5, 112.6, 112.8, 112.81, 112.82, 112.83, 112.84, 112.85, 112.89, 112.9	B37, B37.0, B37.1, B37.2, B37.3, B37.4, B37.41, B37.42, B37.49, B37.5, B37.6, B37.7, B37.8, B37.81, B37.82, B37.83, B37.84, B37.89, B37.9
Infectious 4	114.0, 114.1, 114.2, 114.3, 114.4, 114.5, 114.6, 114.9	B38, B38.0, B38.1, B38.2, B38.3, B38.4, B38.7, B38.8, B38.81, B38.89, B38.9
Infectious 5	115.0, 115.01, 115.02, 115.03, 115.04, 115.05, 115.09, 115.1, 115.11, 115.12, 115.13, 115.14, 115.15, 115.19, 115.2, 115.3, 115.4, 115.5, 115.9, 115.91, 115.92, 115.93, 115.94, 115.95, 115.99	B39, B39.0, B39.1, B39.2, B39.3, B39.4, B39.5, B39.9
Infectious 6	116.0, 116.2, 116.3, 116.4, 116.5, 116.6, 116.9	B40, B40.0, B40.1, B40.2, B40.3, B40.7, B40.8, B40.81, B40.89, B40.9
Infectious 7	116.1	B41, B41.0, B41.7, B41.8, B41.9
Infectious 8	117.1	B42, B42.0, B42.1, B42.7, B42.8, B42.81, B42.82, B42.89, B42.9, B43, B43.0, B43.1, B43.2, B43.8, B43.9
Infectious 9	117.3	B44, B44.0, B44.1, B44.2, B44.7, B44.8, B44.81, B44.89, B44.9
Infectious 10	117.7	B46, B46.0, B46.1, B46.2, B46.3, B46.4, B46.5, B46.8, B46.9
Infectious 11	130.0, 130.1, 130.2, 130.3, 130.4, 130.5, 130.6, 130.7, 130.8, 130.9	B58, B58.0, B58.00, B58.01, B58.09, B58.1, B58.2, B58.3, B58.8, B58.81, B58.82, B58.83, B58.89, B58.9
Infectious 12	136.3, 136.4, 136.5	B59, B59.0, B59.9
Infectious 13	117.5	B45, B45.0, B45.1, B45.2, B45.3, B45.7, B45.8, B45.9
Infectious 14	117.2	A43, A43.0, A43.1, A43.8, A43.9
Infectious 15	117.0, 117.4, 117.6, 117.8, 117.9, 118.0, 118.1, 118.2, 118.3, 118.4, 118.5, 118.6, 118.9	B49, B49.5, B49.9
Infectious 16	85.1, 85.2, 85.3, 85.4, 85.5	B55.1, B55.2
Infectious 17	31.0, 31.1, 31.2, 31.8, 31.9	A31, A31.0, A31.1, A31.2, A31.8, A31.9
Immunodeficiency - antibody	279.0, 279.01, 279.02, 279.03, 279.04, 279.05, 279.06, 279.09, 279.1	D80, D80.0, D80.1, D80.2, D80.3, D80.4, D80.5, D80.6, D80.7, D80.8, D80.9
Immunodeficiency - WBC	279.1, 279.11, 279.12, 279.13, 279.19, 279.2, 279.3, 279.4, 279.41, 279.49	D81, D81.0, D81.1, D81.2, D81.3, D81.4, D81.5, D81.6, D81.7, D81.8, D81.81, D81.810, D81.818, D81.819, D81.89, D81.9, D82, D82.0, D82.1, D82.2, D82.3, D82.4, D82.8, D82.9
Immunodeficiency - other	279.0, 279.5, 279.51, 279.52, 279.53, 279.6, 279.8, 279.9	D83, D83.0, D83.1, D83.2, D83.8, D83.9, D84, D84.0, D84.1, D84.8, D84.9, D89.8, D89.81, D89.810, D89.811, D89.812, D89.813, D89.82, D89.89, D89.9
Kaposi's sarcoma	176.0, 176.1, 176.2, 176.3, 176.4, 176.5, 176.8, 176.9	C46, C46.0, C46.1, C46.2, C46.3, C46.4, C46.5, C46.50, C46.51, C46.52, C46.7, C46.8, C46.9

Appendix Table 6: CODEm covariates used and expected direction of covariate by cause					
Cause	Sex	Age start	Age end	Direction	Covariate
Tuberculosis	Male	28-364 days	95+ years	1	Alcohol (liters per capita)
Tuberculosis	Male	28-364 days	95+ years	1	Diabetes Fasting Plasma Glucose (mmol/L)
Tuberculosis	Male	28-364 days	95+ years	-1	Education (years per capita)
Tuberculosis	Male	28-364 days	95+ years	-1	LDI (I$ per capita)
Tuberculosis	Male	28-364 days	95+ years	1	Indoor Air Pollution (All Cooking Fuels)
Tuberculosis	Male	28-364 days	95+ years	1	Outdoor Air Pollution (PM2.5)
Tuberculosis	Male	28-364 days	95+ years	1	Population Density (500-1000 ppl/sqkm, proportion)
Tuberculosis	Male	28-364 days	95+ years	1	Population Density (over 1000 ppl/sqkm, proportion)
Tuberculosis	Male	28-364 days	95+ years	1	Smoking Prevalence
Tuberculosis	Male	28-364 days	95+ years	1	Log-transformed SEV scalar: TB
Tuberculosis	Male	28-364 days	95+ years	-1	Socio-demographic Index
Tuberculosis	Male	28-364 days	95+ years	-1	Healthcare access and quality index
Tuberculosis	Male	28-364 days	95+ years	1	Age-standardized proportion adult underweight
Tuberculosis	Male	28-364 days	95+ years	1	Tuberculosis infection risk-weighted prevalence (age-standardized)
Tuberculosis	Male	28-364 days	95+ years	1	Tuberculosis prevalence (age-standardized)
Tuberculosis	Female	28-364 days	95+ years	1	Alcohol (liters per capita)
Tuberculosis	Female	28-364 days	95+ years	1	Diabetes Fasting Plasma Glucose (mmol/L)
Tuberculosis	Female	28-364 days	95+ years	-1	Education (years per capita)
Tuberculosis	Female	28-364 days	95+ years	-1	LDI (I$ per capita)
Tuberculosis	Female	28-364 days	95+ years	1	Indoor Air Pollution (All Cooking Fuels)
Tuberculosis	Female	28-364 days	95+ years	1	Outdoor Air Pollution (PM2.5)
Tuberculosis	Female	28-364 days	95+ years	1	Population Density (500-1000 ppl/sqkm, proportion)
Tuberculosis	Female	28-364 days	95+ years	1	Population Density (over 1000 ppl/sqkm, proportion)
Tuberculosis	Female	28-364 days	95+ years	1	Smoking Prevalence
Tuberculosis	Female	28-364 days	95+ years	1	Log-transformed SEV scalar: TB
Tuberculosis	Female	28-364 days	95+ years	-1	Socio-demographic Index
Tuberculosis	Female	28-364 days	95+ years	-1	Healthcare access and quality index
Tuberculosis	Female	28-364 days	95+ years	1	Age-standardized proportion adult underweight
Tuberculosis	Female	28-364 days	95+ years	1	Tuberculosis infection risk-weighted prevalence (age-standardized)
Tuberculosis	Female	28-364 days	95+ years	1	Tuberculosis prevalence (age-standardized)
Diarrheal diseases	Male	5-9 years	95+ years	-1	Education (years per capita)
Diarrheal diseases	Male	5-9 years	95+ years	-1	LDI (I$ per capita)
Diarrheal diseases	Male	5-9 years	95+ years	-1	Mean BMI
Diarrheal diseases	Male	5-9 years	95+ years	0	Population Density (over 1000 ppl/sqkm, proportion)
Diarrheal diseases	Male	5-9 years	95+ years	-1	Sanitation (proportion with access)
Diarrheal diseases	Male	5-9 years	95+ years	-1	Improved Water Source (proportion with access)
Diarrheal diseases	Male	5-9 years	95+ years	1	Log-transformed SEV scalar: Diarrhea
Diarrheal diseases	Male	5-9 years	95+ years	1	SEV unsafe water
Diarrheal diseases	Male	5-9 years	95+ years	1	SEV unsafe sanitation
Diarrheal diseases	Male	5-9 years	95+ years	-1	Socio-demographic Index
Diarrheal diseases	Male	5-9 years	95+ years	-1	Rotavirus coverage (proportion)
Diarrheal diseases	Male	5-9 years	95+ years	-1	Healthcare access and quality index
Diarrheal diseases	Female	5-9 years	95+ years	-1	Education (years per capita)
Diarrheal diseases	Female	5-9 years	95+ years	-1	LDI (I$ per capita)
Diarrheal diseases	Female	5-9 years	95+ years	-1	Mean BMI
Diarrheal diseases	Female	5-9 years	95+ years	0	Population Density (over 1000 ppl/sqkm, proportion)
Diarrheal diseases	Female	5-9 years	95+ years	-1	Sanitation (proportion with access)
Diarrheal diseases	Female	5-9 years	95+ years	-1	Improved Water Source (proportion with access)
Diarrheal diseases	Female	5-9 years	95+ years	1	Log-transformed SEV scalar: Diarrhea
Diarrheal diseases	Female	5-9 years	95+ years	1	SEV unsafe water
Diarrheal diseases	Female	5-9 years	95+ years	1	SEV unsafe sanitation
Diarrheal diseases	Female	5-9 years	95+ years	-1	Socio-demographic Index
Diarrheal diseases	Female	5-9 years	95+ years	-1	Rotavirus coverage (proportion)
Diarrheal diseases	Female	5-9 years	95+ years	-1	Healthcare access and quality index
Diarrheal diseases	Male	0-6 days	1-4 years	-1	LDI (I$ per capita)
Diarrheal diseases	Male	0-6 days	1-4 years	1	Underweight (proportion <2SD weight for age, <5 years)
Diarrheal diseases	Male	0-6 days	1-4 years	0	Population Density (over 1000 ppl/sqkm, proportion)
Diarrheal diseases	Male	0-6 days	1-4 years	0	Population Density (under 150 ppl/sqkm, proportion)
Diarrheal diseases	Male	0-6 days	1-4 years	-1	Sanitation (proportion with access)
Diarrheal diseases	Male	0-6 days	1-4 years	-1	Improved Water Source (proportion with access)
Diarrheal diseases	Male	0-6 days	1-4 years	1	Vitamin A Deficiency Prevalence (age-standardized)
Diarrheal diseases	Male	0-6 days	1-4 years	-1	Maternal education (years per capita)
Diarrheal diseases	Male	0-6 days	1-4 years	1	Log-transformed SEV scalar: Diarrhea
Diarrheal diseases	Male	0-6 days	1-4 years	1	SEV unsafe water
Diarrheal diseases	Male	0-6 days	1-4 years	1	SEV unsafe sanitation
Diarrheal diseases	Male	0-6 days	1-4 years	-1	Socio-demographic Index
Diarrheal diseases	Male	0-6 days	1-4 years	1	Stunting (proportion <2SD height for age, <5 years)
Diarrheal diseases	Male	0-6 days	1-4 years	1	Wasting (proportion <2SD weight for height, <5 years)
Diarrheal diseases	Male	0-6 days	1-4 years	-1	Rotavirus coverage (proportion)
Diarrheal diseases	Male	0-6 days	1-4 years	-1	Healthcare access and quality index
Diarrheal diseases	Male	0-6 days	1-4 years	1	No handwashing with soap
Diarrheal diseases	Male	0-6 days	1-4 years	1	Suboptimal breastfeeding SEV
Diarrheal diseases	Male	0-6 days	1-4 years	1	Zinc deficiency
Diarrheal diseases	Female	0-6 days	1-4 years	-1	LDI (I$ per capita)
Diarrheal diseases	Female	0-6 days	1-4 years	1	Underweight (proportion <2SD weight for age, <5 years)
Diarrheal diseases	Female	0-6 days	1-4 years	0	Population Density (over 1000 ppl/sqkm, proportion)
Diarrheal diseases	Female	0-6 days	1-4 years	0	Population Density (under 150 ppl/sqkm, proportion)
Diarrheal diseases	Female	0-6 days	1-4 years	-1	Sanitation (proportion with access)
Diarrheal diseases	Female	0-6 days	1-4 years	-1	Improved Water Source (proportion with access)
Diarrheal diseases	Female	0-6 days	1-4 years	1	Vitamin A Deficiency Prevalence (age-standardized)
Diarrheal diseases	Female	0-6 days	1-4 years	-1	Maternal education (years per capita)
Diarrheal diseases	Female	0-6 days	1-4 years	1	Log-transformed SEV scalar: Diarrhea
Diarrheal diseases	Female	0-6 days	1-4 years	1	SEV unsafe water
Diarrheal diseases	Female	0-6 days	1-4 years	1	SEV unsafe sanitation
Diarrheal diseases	Female	0-6 days	1-4 years	-1	Socio-demographic Index
Diarrheal diseases	Female	0-6 days	1-4 years	1	Stunting (proportion <2SD height for age, <5 years)
Diarrheal diseases	Female	0-6 days	1-4 years	1	Wasting (proportion <2SD weight for height, <5 years)
Diarrheal diseases	Female	0-6 days	1-4 years	-1	Rotavirus coverage (proportion)
Diarrheal diseases	Female	0-6 days	1-4 years	-1	Healthcare access and quality index
Diarrheal diseases	Female	0-6 days	1-4 years	1	No handwashing with soap
Diarrheal diseases	Female	0-6 days	1-4 years	1	Suboptimal breastfeeding SEV

Cause	Sex	Age start	Age end	Direction	Covariate
Diarrheal diseases	Female	0-6 days	1-4 years	1	Zinc deficiency
Lower respiratory infections	Male	5-9 years	95+ years	1	Alcohol (liters per capita)
Lower respiratory infections	Male	5-9 years	95+ years	-1	DTP3 Coverage (proportion)
Lower respiratory infections	Male	5-9 years	95+ years	-1	Education (years per capita)
Lower respiratory infections	Male	5-9 years	95+ years	-1	LDI (I$ per capita)
Lower respiratory infections	Male	5-9 years	95+ years	-1	Mean BMI
Lower respiratory infections	Male	5-9 years	95+ years	1	Indoor Air Pollution (All Cooking Fuels)
Lower respiratory infections	Male	5-9 years	95+ years	1	Outdoor Air Pollution (PM2.5)
Lower respiratory infections	Male	5-9 years	95+ years	1	Smoking Prevalence
Lower respiratory infections	Male	5-9 years	95+ years	-1	PCV3 Coverage (proportion)
Lower respiratory infections	Male	5-9 years	95+ years	1	Log-transformed SEV scalar: LRI
Lower respiratory infections	Male	5-9 years	95+ years	0	SEV unsafe sanitation
Lower respiratory infections	Male	5-9 years	95+ years	-1	Socio-demographic Index
Lower respiratory infections	Male	5-9 years	95+ years	-1	Healthcare access and quality index
Lower respiratory infections	Female	5-9 years	95+ years	1	Alcohol (liters per capita)
Lower respiratory infections	Female	5-9 years	95+ years	-1	DTP3 Coverage (proportion)
Lower respiratory infections	Female	5-9 years	95+ years	-1	Education (years per capita)
Lower respiratory infections	Female	5-9 years	95+ years	-1	LDI (I$ per capita)
Lower respiratory infections	Female	5-9 years	95+ years	-1	Mean BMI
Lower respiratory infections	Female	5-9 years	95+ years	1	Indoor Air Pollution (All Cooking Fuels)
Lower respiratory infections	Female	5-9 years	95+ years	1	Outdoor Air Pollution (PM2.5)
Lower respiratory infections	Female	5-9 years	95+ years	1	Smoking Prevalence
Lower respiratory infections	Female	5-9 years	95+ years	-1	PCV3 Coverage (proportion)
Lower respiratory infections	Female	5-9 years	95+ years	1	Log-transformed SEV scalar: LRI
Lower respiratory infections	Female	5-9 years	95+ years	0	SEV unsafe sanitation
Lower respiratory infections	Female	5-9 years	95+ years	-1	Socio-demographic Index
Lower respiratory infections	Female	5-9 years	95+ years	-1	Healthcare access and quality index
Lower respiratory infections	Male	0-6 days	1-4 years	-1	DTP3 Coverage (proportion)
Lower respiratory infections	Male	0-6 days	1-4 years	-1	Hib3 Vaccine Coverage (proportion)
Lower respiratory infections	Male	0-6 days	1-4 years	-1	LDI (I$ per capita)
Lower respiratory infections	Male	0-6 days	1-4 years	1	Underweight (proportion <2SD weight for age, <5 years)
Lower respiratory infections	Male	0-6 days	1-4 years	1	Indoor Air Pollution (All Cooking Fuels)
Lower respiratory infections	Male	0-6 days	1-4 years	1	Outdoor Air Pollution (PM2.5)
Lower respiratory infections	Male	0-6 days	1-4 years	-1	PCV3 Coverage (proportion)
Lower respiratory infections	Male	0-6 days	1-4 years	1	Vitamin A Deficiency Prevalence (age-standardized)
Lower respiratory infections	Male	0-6 days	1-4 years	-1	Maternal education (years per capita)
Lower respiratory infections	Male	0-6 days	1-4 years	1	Log-transformed SEV scalar: LRI
Lower respiratory infections	Male	0-6 days	1-4 years	1	SEV unsafe sanitation
Lower respiratory infections	Male	0-6 days	1-4 years	-1	Socio-demographic Index
Lower respiratory infections	Male	0-6 days	1-4 years	1	Stunting (proportion <2SD height for age, <5 years)
Lower respiratory infections	Male	0-6 days	1-4 years	1	Wasting (proportion <2SD weight for height, <5 years)
Lower respiratory infections	Male	0-6 days	1-4 years	-1	Healthcare access and quality index
Lower respiratory infections	Male	0-6 days	1-4 years	1	No handwashing with soap
Lower respiratory infections	Male	0-6 days	1-4 years	1	Suboptimal breastfeeding SEV
Lower respiratory infections	Male	0-6 days	1-4 years	1	Zinc deficiency
Lower respiratory infections	Male	0-6 days	1-4 years	1	Secondhand smoke
Lower respiratory infections	Female	0-6 days	1-4 years	-1	DTP3 Coverage (proportion)
Lower respiratory infections	Female	0-6 days	1-4 years	-1	Hib3 Vaccine Coverage (proportion)
Lower respiratory infections	Female	0-6 days	1-4 years	-1	LDI (I$ per capita)
Lower respiratory infections	Female	0-6 days	1-4 years	1	Underweight (proportion <2SD weight for age, <5 years)
Lower respiratory infections	Female	0-6 days	1-4 years	1	Indoor Air Pollution (All Cooking Fuels)
Lower respiratory infections	Female	0-6 days	1-4 years	1	Outdoor Air Pollution (PM2.5)
Lower respiratory infections	Female	0-6 days	1-4 years	-1	PCV3 Coverage (proportion)
Lower respiratory infections	Female	0-6 days	1-4 years	1	Vitamin A Deficiency Prevalence (age-standardized)
Lower respiratory infections	Female	0-6 days	1-4 years	-1	Maternal education (years per capita)
Lower respiratory infections	Female	0-6 days	1-4 years	1	Log-transformed SEV scalar: LRI
Lower respiratory infections	Female	0-6 days	1-4 years	1	SEV unsafe sanitation
Lower respiratory infections	Female	0-6 days	1-4 years	-1	Socio-demographic Index
Lower respiratory infections	Female	0-6 days	1-4 years	1	Stunting (proportion <2SD height for age, <5 years)
Lower respiratory infections	Female	0-6 days	1-4 years	1	Wasting (proportion <2SD weight for height, <5 years)
Lower respiratory infections	Female	0-6 days	1-4 years	-1	Healthcare access and quality index
Lower respiratory infections	Female	0-6 days	1-4 years	1	No handwashing with soap
Lower respiratory infections	Female	0-6 days	1-4 years	1	Suboptimal breastfeeding SEV
Lower respiratory infections	Female	0-6 days	1-4 years	1	Zinc deficiency
Lower respiratory infections	Female	0-6 days	1-4 years	1	Secondhand smoke
Lower respiratory infections	Male	5-9 years	95+ years	-1	SEV unsafe sanitation
Lower respiratory infections	Female	5-9 years	95+ years	-1	SEV unsafe sanitation
Otitis media	Male	0-6 days	95+ years	-1	Education (years per capita)
Otitis media	Male	0-6 days	95+ years	-1	LDI (I$ per capita)
Otitis media	Male	0-6 days	95+ years	1	Indoor Air Pollution (All Cooking Fuels)
Otitis media	Male	0-6 days	95+ years	1	Outdoor Air Pollution (PM2.5)
Otitis media	Male	0-6 days	95+ years	1	Smoking Prevalence
Otitis media	Male	0-6 days	95+ years	1	Log-transformed SEV scalar: Otitis
Otitis media	Male	0-6 days	95+ years	-1	Socio-demographic Index
Otitis media	Male	0-6 days	95+ years	-1	Healthcare access and quality index
Otitis media	Female	0-6 days	95+ years	-1	Education (years per capita)
Otitis media	Female	0-6 days	95+ years	-1	LDI (I$ per capita)
Otitis media	Female	0-6 days	95+ years	1	Indoor Air Pollution (All Cooking Fuels)
Otitis media	Female	0-6 days	95+ years	1	Outdoor Air Pollution (PM2.5)
Otitis media	Female	0-6 days	95+ years	1	Smoking Prevalence
Otitis media	Female	0-6 days	95+ years	1	Log-transformed SEV scalar: Otitis
Otitis media	Female	0-6 days	95+ years	-1	Socio-demographic Index
Otitis media	Female	0-6 days	95+ years	-1	Healthcare access and quality index
Meningitis	Female	0-6 days	1-4 years	-1	DTP3 Coverage (proportion)
Meningitis	Female	0-6 days	1-4 years	-1	LDI (I$ per capita)
Meningitis	Female	0-6 days	1-4 years	1	Underweight (proportion <2SD weight for age, <5 years)
Meningitis	Female	0-6 days	1-4 years	-1	Sanitation (proportion with access)
Meningitis	Female	0-6 days	1-4 years	-1	Improved Water Source (proportion with access)
Meningitis	Female	0-6 days	1-4 years	-1	Health System Access (capped)
Meningitis	Female	0-6 days	1-4 years	-1	Maternal education (years per capita)
Meningitis	Female	0-6 days	1-4 years	1	meningitis belt (proportion)
Meningitis	Female	0-6 days	1-4 years	-1	Socio-demographic Index

Cause	Sex	Age start	Age end	Direction	Covariate
Meningitis	Female	0-6 days	1-4 years	-1	Proportion of total population covered by menafrivac initiative (meningitis meningococcal type A vaccine)
Meningitis	Female	0-6 days	1-4 years	-1	Healthcare access and quality index
Meningitis	Male	0-6 days	1-4 years	-1	DTP3 Coverage (proportion)
Meningitis	Male	0-6 days	1-4 years	-1	LDI (I$ per capita)
Meningitis	Male	0-6 days	1-4 years	1	Underweight (proportion <2SD weight for age, <5 years)
Meningitis	Male	0-6 days	1-4 years	-1	Sanitation (proportion with access)
Meningitis	Male	0-6 days	1-4 years	-1	Improved Water Source (proportion with access)
Meningitis	Male	0-6 days	1-4 years	-1	Health System Access (capped)
Meningitis	Male	0-6 days	1-4 years	-1	Maternal education (years per capita)
Meningitis	Male	0-6 days	1-4 years	1	meningitis belt (proportion)
Meningitis	Male	0-6 days	1-4 years	-1	Socio-demographic Index
Meningitis	Male	0-6 days	1-4 years	-1	Proportion of total population covered by menafrivac initiative (meningitis meningococcal type A vaccine)
Meningitis	Male	0-6 days	1-4 years	-1	Healthcare access and quality index
Meningitis	Male	5-9 years	95+ years	-1	DTP3 Coverage (proportion)
Meningitis	Male	5-9 years	95+ years	-1	LDI (I$ per capita)
Meningitis	Male	5-9 years	95+ years	1	Underweight (proportion <2SD weight for age, <5 years)
Meningitis	Male	5-9 years	95+ years	-1	Sanitation (proportion with access)
Meningitis	Male	5-9 years	95+ years	-1	Improved Water Source (proportion with access)
Meningitis	Male	5-9 years	95+ years	-1	Health System Access (capped)
Meningitis	Male	5-9 years	95+ years	-1	Maternal education (years per capita)
Meningitis	Male	5-9 years	95+ years	1	meningitis belt (proportion)
Meningitis	Male	5-9 years	95+ years	-1	Socio-demographic Index
Meningitis	Male	5-9 years	95+ years	-1	Proportion of total population covered by menafrivac initiative (meningitis meningococcal type A vaccine)
Meningitis	Male	5-9 years	95+ years	-1	Healthcare access and quality index
Meningitis	Female	5-9 years	95+ years	-1	DTP3 Coverage (proportion)
Meningitis	Female	5-9 years	95+ years	-1	LDI (I$ per capita)
Meningitis	Female	5-9 years	95+ years	1	Underweight (proportion <2SD weight for age, <5 years)
Meningitis	Female	5-9 years	95+ years	-1	Sanitation (proportion with access)
Meningitis	Female	5-9 years	95+ years	-1	Improved Water Source (proportion with access)
Meningitis	Female	5-9 years	95+ years	-1	Health System Access (capped)
Meningitis	Female	5-9 years	95+ years	-1	Maternal education (years per capita)
Meningitis	Female	5-9 years	95+ years	1	meningitis belt (proportion)
Meningitis	Female	5-9 years	95+ years	-1	Socio-demographic Index
Meningitis	Female	5-9 years	95+ years	-1	Proportion of total population covered by menafrivac initiative (meningitis meningococcal type A vaccine)
Meningitis	Female	5-9 years	95+ years	-1	Healthcare access and quality index
Encephalitis	Male	0-6 days	95+ years	-1	In-Facility Delivery (proportion)
Encephalitis	Male	0-6 days	95+ years	-1	LDI (I$ per capita)
Encephalitis	Male	0-6 days	95+ years	1	Underweight (proportion <2SD weight for age, <5 years)
Encephalitis	Male	0-6 days	95+ years	-1	Sanitation (proportion with access)
Encephalitis	Male	0-6 days	95+ years	-1	Improved Water Source (proportion with access)
Encephalitis	Male	0-6 days	95+ years	-1	Health System Access (capped)
Encephalitis	Male	0-6 days	95+ years	-1	Maternal education (years per capita)
Encephalitis	Male	0-6 days	95+ years	-1	Socio-demographic Index
Encephalitis	Male	0-6 days	95+ years	1	Japanese encelphalitis endemic area (binary)
Encephalitis	Male	0-6 days	95+ years	-1	Healthcare access and quality index
Encephalitis	Female	0-6 days	95+ years	-1	In-Facility Delivery (proportion)
Encephalitis	Female	0-6 days	95+ years	-1	LDI (I$ per capita)
Encephalitis	Female	0-6 days	95+ years	1	Underweight (proportion <2SD weight for age, <5 years)
Encephalitis	Female	0-6 days	95+ years	-1	Sanitation (proportion with access)
Encephalitis	Female	0-6 days	95+ years	-1	Improved Water Source (proportion with access)
Encephalitis	Female	0-6 days	95+ years	-1	Health System Access (capped)
Encephalitis	Female	0-6 days	95+ years	-1	Maternal education (years per capita)
Encephalitis	Female	0-6 days	95+ years	-1	Socio-demographic Index
Encephalitis	Female	0-6 days	95+ years	1	Japanese encelphalitis endemic area (binary)
Encephalitis	Female	0-6 days	95+ years	-1	Healthcare access and quality index
Tetanus	Male	0-6 days	28-364 days	-1	DTP3 Coverage (proportion)
Tetanus	Male	0-6 days	28-364 days	-1	Education (years per capita)
Tetanus	Male	0-6 days	28-364 days	-1	In-Facility Delivery (proportion)
Tetanus	Male	0-6 days	28-364 days	-1	LDI (I$ per capita)
Tetanus	Male	0-6 days	28-364 days	-1	Skilled Birth Attendance (proportion)
Tetanus	Male	0-6 days	28-364 days	-1	Tetanus Toxoid Coverage Smooth (proportion)
Tetanus	Male	0-6 days	28-364 days	-1	Health System Access (capped)
Tetanus	Male	0-6 days	28-364 days	-1	Socio-demographic Index
Tetanus	Male	0-6 days	28-364 days	-1	Healthcare access and quality index
Tetanus	Male	1-4 years	95+ years	-1	DTP3 Coverage (proportion)
Tetanus	Male	1-4 years	95+ years	-1	Education (years per capita)
Tetanus	Male	1-4 years	95+ years	-1	LDI (I$ per capita)
Tetanus	Male	1-4 years	95+ years	-1	Sanitation (proportion with access)
Tetanus	Male	1-4 years	95+ years	-1	Health System Access (capped)
Tetanus	Male	1-4 years	95+ years	-1	Socio-demographic Index
Tetanus	Male	1-4 years	95+ years	-1	Healthcare access and quality index
Tetanus	Female	0-6 days	28-364 days	-1	DTP3 Coverage (proportion)
Tetanus	Female	0-6 days	28-364 days	-1	Education (years per capita)
Tetanus	Female	0-6 days	28-364 days	-1	In-Facility Delivery (proportion)
Tetanus	Female	0-6 days	28-364 days	-1	LDI (I$ per capita)
Tetanus	Female	0-6 days	28-364 days	-1	Skilled Birth Attendance (proportion)
Tetanus	Female	0-6 days	28-364 days	-1	Tetanus Toxoid Coverage Smooth (proportion)
Tetanus	Female	0-6 days	28-364 days	-1	Health System Access (capped)
Tetanus	Female	0-6 days	28-364 days	-1	Socio-demographic Index
Tetanus	Female	0-6 days	28-364 days	-1	Healthcare access and quality index
Tetanus	Female	1-4 years	95+ years	-1	DTP3 Coverage (proportion)
Tetanus	Female	1-4 years	95+ years	-1	Education (years per capita)
Tetanus	Female	1-4 years	95+ years	-1	LDI (I$ per capita)
Tetanus	Female	1-4 years	95+ years	-1	Sanitation (proportion with access)
Tetanus	Female	1-4 years	95+ years	-1	Health System Access (capped)
Tetanus	Female	1-4 years	95+ years	-1	Socio-demographic Index
Tetanus	Female	1-4 years	95+ years	-1	Healthcare access and quality index
Dengue	Male	28-364 days	95+ years	0	Education (years per capita)
Dengue	Male	28-364 days	95+ years	0	Health System Access (unitless)
Dengue	Male	28-364 days	95+ years	0	LDI (I$ per capita)
Dengue	Male	28-364 days	95+ years	1	Latitude Under 15 (proportion)
Dengue	Male	28-364 days	95+ years	1	Population Density (over 1000 ppl/sqkm, proportion)

Cause	Sex	Age start	Age end	Direction	Covariate
Dengue	Male	28-364 days	95+ years	1	Elevation Under 100m (proportion)
Dengue	Male	28-364 days	95+ years	1	Rainfall Quintile 4 (proportion)
Dengue	Male	28-364 days	95+ years	1	Rainfall Quintile 5 (proportion)
Dengue	Male	28-364 days	95+ years	1	Population weighted probability of dengue transmission
Dengue	Male	28-364 days	95+ years	1	Dengue outbreaks (binary)
Dengue	Male	28-364 days	95+ years	1	Dengue anomalies (deviation from mean dengue incidence rate)
Dengue	Male	28-364 days	95+ years	0	Socio-demographic Index
Dengue	Female	28-364 days	95+ years	0	Education (years per capita)
Dengue	Female	28-364 days	95+ years	0	Health System Access (unitless)
Dengue	Female	28-364 days	95+ years	0	LDI (I$ per capita)
Dengue	Female	28-364 days	95+ years	1	Latitude Under 15 (proportion)
Dengue	Female	28-364 days	95+ years	1	Population Density (over 1000 ppl/sqkm, proportion)
Dengue	Female	28-364 days	95+ years	1	Elevation Under 100m (proportion)
Dengue	Female	28-364 days	95+ years	1	Rainfall Quintile 4 (proportion)
Dengue	Female	28-364 days	95+ years	1	Rainfall Quintile 5 (proportion)
Dengue	Female	28-364 days	95+ years	1	Population weighted probability of dengue transmission
Dengue	Female	28-364 days	95+ years	1	Dengue outbreaks (binary)
Dengue	Female	28-364 days	95+ years	1	Dengue anomalies (deviation from mean dengue incidence rate)
Dengue	Female	28-364 days	95+ years	0	Socio-demographic Index
Dengue	Male	28-364 days	95+ years	1	Healthcare access and quality index
Dengue	Female	28-364 days	95+ years	1	Healthcare access and quality index
Rabies	Male	28-364 days	95+ years	-1	Antenatal Care (4 visits) Coverage (proportion)
Rabies	Male	28-364 days	95+ years	-1	Health System Access (unitless)
Rabies	Male	28-364 days	95+ years	-1	In-Facility Delivery (proportion)
Rabies	Male	28-364 days	95+ years	0	Population Density (500-1000 ppl/sqkm, proportion)
Rabies	Male	28-364 days	95+ years	0	Population Density (under 150 ppl/sqkm, proportion)
Rabies	Male	28-364 days	95+ years	-1	Skilled Birth Attendance (proportion)
Rabies	Male	28-364 days	95+ years	-1	Socio-demographic Index
Rabies	Male	28-364 days	95+ years	-1	Health System Access (capped)
Rabies	Male	28-364 days	95+ years	-1	Healthcare access and quality index
Rabies	Female	28-364 days	95+ years	-1	Antenatal Care (4 visits) Coverage (proportion)
Rabies	Female	28-364 days	95+ years	-1	Health System Access (unitless)
Rabies	Female	28-364 days	95+ years	-1	In-Facility Delivery (proportion)
Rabies	Female	28-364 days	95+ years	0	Population Density (500-1000 ppl/sqkm, proportion)
Rabies	Female	28-364 days	95+ years	0	Population Density (under 150 ppl/sqkm, proportion)
Rabies	Female	28-364 days	95+ years	-1	Skilled Birth Attendance (proportion)
Rabies	Female	28-364 days	95+ years	-1	Socio-demographic Index
Rabies	Female	28-364 days	95+ years	-1	Health System Access (capped)
Rabies	Female	28-364 days	95+ years	-1	Healthcare access and quality index
Rabies	Male	28-364 days	95+ years	1	Healthcare access and quality index
Rabies	Female	28-364 days	95+ years	1	Healthcare access and quality index
Other neglected tropical diseases	Male	0-6 days	95+ years	-1	Education (years per capita)
Other neglected tropical diseases	Male	0-6 days	95+ years	-1	LDI (I$ per capita)
Other neglected tropical diseases	Male	0-6 days	95+ years	1	Latitude Under 15 (proportion)
Other neglected tropical diseases	Male	0-6 days	95+ years	1	Rainfall Quintile 5 (proportion)
Other neglected tropical diseases	Male	0-6 days	95+ years	-1	Sanitation (proportion with access)
Other neglected tropical diseases	Male	0-6 days	95+ years	-1	Socio-demographic Index
Other neglected tropical diseases	Male	0-6 days	95+ years	-1	Healthcare access and quality index
Other neglected tropical diseases	Female	0-6 days	95+ years	-1	Education (years per capita)
Other neglected tropical diseases	Female	0-6 days	95+ years	-1	LDI (I$ per capita)
Other neglected tropical diseases	Female	0-6 days	95+ years	1	Latitude Under 15 (proportion)
Other neglected tropical diseases	Female	0-6 days	95+ years	1	Rainfall Quintile 5 (proportion)
Other neglected tropical diseases	Female	0-6 days	95+ years	-1	Sanitation (proportion with access)
Other neglected tropical diseases	Female	0-6 days	95+ years	-1	Socio-demographic Index
Other neglected tropical diseases	Female	0-6 days	95+ years	-1	Healthcare access and quality index
Neonatal disorders	Male	0-6 days	1-4 years	-1	Antenatal Care (4 visits) Coverage (proportion)
Neonatal disorders	Male	0-6 days	1-4 years	-1	Education (years per capita)
Neonatal disorders	Male	0-6 days	1-4 years	-1	In-Facility Delivery (proportion)
Neonatal disorders	Male	0-6 days	1-4 years	-1	LDI (I$ per capita)
Neonatal disorders	Male	0-6 days	1-4 years	1	Underweight (proportion <2SD weight for age, <5 years)
Neonatal disorders	Male	0-6 days	1-4 years	1	Live Births 35+ (proportion)
Neonatal disorders	Male	0-6 days	1-4 years	1	Indoor Air Pollution (All Cooking Fuels)
Neonatal disorders	Male	0-6 days	1-4 years	-1	Skilled Birth Attendance (proportion)
Neonatal disorders	Male	0-6 days	1-4 years	1	Smoking Prevalence (Reproductive Age Standardized)
Neonatal disorders	Male	0-6 days	1-4 years	1	Total Fertility Rate
Neonatal disorders	Male	0-6 days	1-4 years	-1	Health System Access (capped)
Neonatal disorders	Male	0-6 days	1-4 years	-1	Socio-demographic Index
Neonatal disorders	Male	0-6 days	1-4 years	-1	Healthcare access and quality index
Neonatal disorders	Female	0-6 days	1-4 years	-1	Antenatal Care (4 visits) Coverage (proportion)
Neonatal disorders	Female	0-6 days	1-4 years	-1	Education (years per capita)
Neonatal disorders	Female	0-6 days	1-4 years	-1	In-Facility Delivery (proportion)
Neonatal disorders	Female	0-6 days	1-4 years	-1	LDI (I$ per capita)
Neonatal disorders	Female	0-6 days	1-4 years	1	Underweight (proportion <2SD weight for age, <5 years)
Neonatal disorders	Female	0-6 days	1-4 years	1	Live Births 35+ (proportion)
Neonatal disorders	Female	0-6 days	1-4 years	1	Indoor Air Pollution (All Cooking Fuels)
Neonatal disorders	Female	0-6 days	1-4 years	-1	Skilled Birth Attendance (proportion)
Neonatal disorders	Female	0-6 days	1-4 years	1	Smoking Prevalence (Reproductive Age Standardized)
Neonatal disorders	Female	0-6 days	1-4 years	1	Total Fertility Rate
Neonatal disorders	Female	0-6 days	1-4 years	-1	Health System Access (capped)
Neonatal disorders	Female	0-6 days	1-4 years	-1	Socio-demographic Index
Neonatal disorders	Female	0-6 days	1-4 years	-1	Healthcare access and quality index
Neonatal preterm birth complications	Male	0-6 days	1-4 years	-1	Antenatal Care (4 visits) Coverage (proportion)
Neonatal preterm birth complications	Male	0-6 days	1-4 years	-1	Education (years per capita)
Neonatal preterm birth complications	Male	0-6 days	1-4 years	-1	In-Facility Delivery (proportion)
Neonatal preterm birth complications	Male	0-6 days	1-4 years	-1	LDI (I$ per capita)
Neonatal preterm birth complications	Male	0-6 days	1-4 years	1	Underweight (proportion <2SD weight for age, <5 years)
Neonatal preterm birth complications	Male	0-6 days	1-4 years	1	Live Births 35+ (proportion)
Neonatal preterm birth complications	Male	0-6 days	1-4 years	1	Indoor Air Pollution (All Cooking Fuels)
Neonatal preterm birth complications	Male	0-6 days	1-4 years	-1	Skilled Birth Attendance (proportion)
Neonatal preterm birth complications	Male	0-6 days	1-4 years	1	Smoking Prevalence (Reproductive Age Standardized)
Neonatal preterm birth complications	Male	0-6 days	1-4 years	1	Total Fertility Rate
Neonatal preterm birth complications	Male	0-6 days	1-4 years	-1	Health System Access (capped)

Cause	Sex	Age start	Age end	Direction	Covariate
Neonatal preterm birth complications	Male	0-6 days	1-4 years	-1	Socio-demographic Index
Neonatal preterm birth complications	Male	0-6 days	1-4 years	-1	Healthcare access and quality index
Neonatal preterm birth complications	Female	0-6 days	1-4 years	-1	Antenatal Care (4 visits) Coverage (proportion)
Neonatal preterm birth complications	Female	0-6 days	1-4 years	-1	Education (years per capita)
Neonatal preterm birth complications	Female	0-6 days	1-4 years	-1	In-Facility Delivery (proportion)
Neonatal preterm birth complications	Female	0-6 days	1-4 years	-1	LDI (I$ per capita)
Neonatal preterm birth complications	Female	0-6 days	1-4 years	1	Underweight (proportion <2SD weight for age, <5 years)
Neonatal preterm birth complications	Female	0-6 days	1-4 years	1	Live Births 35+ (proportion)
Neonatal preterm birth complications	Female	0-6 days	1-4 years	1	Indoor Air Pollution (All Cooking Fuels)
Neonatal preterm birth complications	Female	0-6 days	1-4 years	-1	Skilled Birth Attendance (proportion)
Neonatal preterm birth complications	Female	0-6 days	1-4 years	1	Smoking Prevalence (Reproductive Age Standardized)
Neonatal preterm birth complications	Female	0-6 days	1-4 years	1	Total Fertility Rate
Neonatal preterm birth complications	Female	0-6 days	1-4 years	-1	Health System Access (capped)
Neonatal preterm birth complications	Female	0-6 days	1-4 years	-1	Socio-demographic Index
Neonatal preterm birth complications	Female	0-6 days	1-4 years	-1	Healthcare access and quality index
Neonatal encephalopathy due to birth asphyxia and trauma	Male	0-6 days	1-4 years	-1	Antenatal Care (4 visits) Coverage (proportion)
Neonatal encephalopathy due to birth asphyxia and trauma	Male	0-6 days	1-4 years	-1	Education (years per capita)
Neonatal encephalopathy due to birth asphyxia and trauma	Male	0-6 days	1-4 years	-1	In-Facility Delivery (proportion)
Neonatal encephalopathy due to birth asphyxia and trauma	Male	0-6 days	1-4 years	-1	LDI (I$ per capita)
Neonatal encephalopathy due to birth asphyxia and trauma	Male	0-6 days	1-4 years	1	Underweight (proportion <2SD weight for age, <5 years)
Neonatal encephalopathy due to birth asphyxia and trauma	Male	0-6 days	1-4 years	1	Live Births 35+ (proportion)
Neonatal encephalopathy due to birth asphyxia and trauma	Male	0-6 days	1-4 years	1	Indoor Air Pollution (All Cooking Fuels)
Neonatal encephalopathy due to birth asphyxia and trauma	Male	0-6 days	1-4 years	-1	Skilled Birth Attendance (proportion)
Neonatal encephalopathy due to birth asphyxia and trauma	Male	0-6 days	1-4 years	1	Smoking Prevalence (Reproductive Age Standardized)
Neonatal encephalopathy due to birth asphyxia and trauma	Male	0-6 days	1-4 years	1	Total Fertility Rate
Neonatal encephalopathy due to birth asphyxia and trauma	Male	0-6 days	1-4 years	-1	Health System Access (capped)
Neonatal encephalopathy due to birth asphyxia and trauma	Male	0-6 days	1-4 years	-1	Socio-demographic Index
Neonatal encephalopathy due to birth asphyxia and trauma	Male	0-6 days	1-4 years	-1	Healthcare access and quality index
Neonatal encephalopathy due to birth asphyxia and trauma	Female	0-6 days	1-4 years	-1	Antenatal Care (4 visits) Coverage (proportion)
Neonatal encephalopathy due to birth asphyxia and trauma	Female	0-6 days	1-4 years	-1	Education (years per capita)
Neonatal encephalopathy due to birth asphyxia and trauma	Female	0-6 days	1-4 years	-1	In-Facility Delivery (proportion)
Neonatal encephalopathy due to birth asphyxia and trauma	Female	0-6 days	1-4 years	-1	LDI (I$ per capita)
Neonatal encephalopathy due to birth asphyxia and trauma	Female	0-6 days	1-4 years	1	Underweight (proportion <2SD weight for age, <5 years)
Neonatal encephalopathy due to birth asphyxia and trauma	Female	0-6 days	1-4 years	1	Live Births 35+ (proportion)
Neonatal encephalopathy due to birth asphyxia and trauma	Female	0-6 days	1-4 years	1	Indoor Air Pollution (All Cooking Fuels)
Neonatal encephalopathy due to birth asphyxia and trauma	Female	0-6 days	1-4 years	-1	Skilled Birth Attendance (proportion)
Neonatal encephalopathy due to birth asphyxia and trauma	Female	0-6 days	1-4 years	1	Smoking Prevalence (Reproductive Age Standardized)
Neonatal encephalopathy due to birth asphyxia and trauma	Female	0-6 days	1-4 years	1	Total Fertility Rate
Neonatal encephalopathy due to birth asphyxia and trauma	Female	0-6 days	1-4 years	-1	Health System Access (capped)
Neonatal encephalopathy due to birth asphyxia and trauma	Female	0-6 days	1-4 years	-1	Socio-demographic Index
Neonatal encephalopathy due to birth asphyxia and trauma	Female	0-6 days	1-4 years	-1	Healthcare access and quality index
Neonatal sepsis and other neonatal infections	Male	0-6 days	1-4 years	-1	Antenatal Care (4 visits) Coverage (proportion)
Neonatal sepsis and other neonatal infections	Male	0-6 days	1-4 years	-1	Education (years per capita)
Neonatal sepsis and other neonatal infections	Male	0-6 days	1-4 years	-1	In-Facility Delivery (proportion)
Neonatal sepsis and other neonatal infections	Male	0-6 days	1-4 years	-1	LDI (I$ per capita)
Neonatal sepsis and other neonatal infections	Male	0-6 days	1-4 years	1	Underweight (proportion <2SD weight for age, <5 years)
Neonatal sepsis and other neonatal infections	Male	0-6 days	1-4 years	1	Live Births 35+ (proportion)
Neonatal sepsis and other neonatal infections	Male	0-6 days	1-4 years	1	Indoor Air Pollution (All Cooking Fuels)
Neonatal sepsis and other neonatal infections	Male	0-6 days	1-4 years	-1	Skilled Birth Attendance (proportion)
Neonatal sepsis and other neonatal infections	Male	0-6 days	1-4 years	1	Smoking Prevalence (Reproductive Age Standardized)
Neonatal sepsis and other neonatal infections	Male	0-6 days	1-4 years	1	Total Fertility Rate
Neonatal sepsis and other neonatal infections	Male	0-6 days	1-4 years	-1	Health System Access (capped)
Neonatal sepsis and other neonatal infections	Male	0-6 days	1-4 years	-1	Socio-demographic Index
Neonatal sepsis and other neonatal infections	Male	0-6 days	1-4 years	-1	Healthcare access and quality index
Neonatal sepsis and other neonatal infections	Female	0-6 days	1-4 years	-1	Antenatal Care (4 visits) Coverage (proportion)
Neonatal sepsis and other neonatal infections	Female	0-6 days	1-4 years	-1	Education (years per capita)
Neonatal sepsis and other neonatal infections	Female	0-6 days	1-4 years	-1	In-Facility Delivery (proportion)
Neonatal sepsis and other neonatal infections	Female	0-6 days	1-4 years	-1	LDI (I$ per capita)
Neonatal sepsis and other neonatal infections	Female	0-6 days	1-4 years	1	Underweight (proportion <2SD weight for age, <5 years)
Neonatal sepsis and other neonatal infections	Female	0-6 days	1-4 years	1	Live Births 35+ (proportion)
Neonatal sepsis and other neonatal infections	Female	0-6 days	1-4 years	1	Indoor Air Pollution (All Cooking Fuels)
Neonatal sepsis and other neonatal infections	Female	0-6 days	1-4 years	-1	Skilled Birth Attendance (proportion)
Neonatal sepsis and other neonatal infections	Female	0-6 days	1-4 years	1	Smoking Prevalence (Reproductive Age Standardized)
Neonatal sepsis and other neonatal infections	Female	0-6 days	1-4 years	1	Total Fertility Rate
Neonatal sepsis and other neonatal infections	Female	0-6 days	1-4 years	-1	Health System Access (capped)
Neonatal sepsis and other neonatal infections	Female	0-6 days	1-4 years	-1	Socio-demographic Index
Neonatal sepsis and other neonatal infections	Female	0-6 days	1-4 years	-1	Healthcare access and quality index
Hemolytic disease and other neonatal jaundice	Male	0-6 days	1-4 years	-1	Antenatal Care (4 visits) Coverage (proportion)
Hemolytic disease and other neonatal jaundice	Male	0-6 days	1-4 years	-1	Education (years per capita)
Hemolytic disease and other neonatal jaundice	Male	0-6 days	1-4 years	-1	In-Facility Delivery (proportion)
Hemolytic disease and other neonatal jaundice	Male	0-6 days	1-4 years	-1	LDI (I$ per capita)
Hemolytic disease and other neonatal jaundice	Male	0-6 days	1-4 years	1	Underweight (proportion <2SD weight for age, <5 years)
Hemolytic disease and other neonatal jaundice	Male	0-6 days	1-4 years	1	Live Births 35+ (proportion)
Hemolytic disease and other neonatal jaundice	Male	0-6 days	1-4 years	1	Indoor Air Pollution (All Cooking Fuels)
Hemolytic disease and other neonatal jaundice	Male	0-6 days	1-4 years	-1	Skilled Birth Attendance (proportion)
Hemolytic disease and other neonatal jaundice	Male	0-6 days	1-4 years	1	Smoking Prevalence (Reproductive Age Standardized)
Hemolytic disease and other neonatal jaundice	Male	0-6 days	1-4 years	1	Total Fertility Rate
Hemolytic disease and other neonatal jaundice	Male	0-6 days	1-4 years	-1	Health System Access (capped)
Hemolytic disease and other neonatal jaundice	Male	0-6 days	1-4 years	-1	Socio-demographic Index
Hemolytic disease and other neonatal jaundice	Male	0-6 days	1-4 years	-1	Healthcare access and quality index
Hemolytic disease and other neonatal jaundice	Female	0-6 days	1-4 years	-1	Antenatal Care (4 visits) Coverage (proportion)
Hemolytic disease and other neonatal jaundice	Female	0-6 days	1-4 years	-1	Education (years per capita)
Hemolytic disease and other neonatal jaundice	Female	0-6 days	1-4 years	-1	In-Facility Delivery (proportion)
Hemolytic disease and other neonatal jaundice	Female	0-6 days	1-4 years	-1	LDI (I$ per capita)
Hemolytic disease and other neonatal jaundice	Female	0-6 days	1-4 years	1	Underweight (proportion <2SD weight for age, <5 years)
Hemolytic disease and other neonatal jaundice	Female	0-6 days	1-4 years	1	Live Births 35+ (proportion)
Hemolytic disease and other neonatal jaundice	Female	0-6 days	1-4 years	1	Indoor Air Pollution (All Cooking Fuels)
Hemolytic disease and other neonatal jaundice	Female	0-6 days	1-4 years	-1	Skilled Birth Attendance (proportion)
Hemolytic disease and other neonatal jaundice	Female	0-6 days	1-4 years	1	Smoking Prevalence (Reproductive Age Standardized)
Hemolytic disease and other neonatal jaundice	Female	0-6 days	1-4 years	1	Total Fertility Rate
Hemolytic disease and other neonatal jaundice	Female	0-6 days	1-4 years	-1	Health System Access (capped)
Hemolytic disease and other neonatal jaundice	Female	0-6 days	1-4 years	-1	Socio-demographic Index

Cause	Sex	Age start	Age end	Direction	Covariate
Hemolytic disease and other neonatal jaundice	Female	0-6 days	1-4 years	-1	Healthcare access and quality index
Other neonatal disorders	Male	0-6 days	1-4 years	-1	Antenatal Care (4 visits) Coverage (proportion)
Other neonatal disorders	Male	0-6 days	1-4 years	-1	Education (years per capita)
Other neonatal disorders	Male	0-6 days	1-4 years	-1	In-Facility Delivery (proportion)
Other neonatal disorders	Male	0-6 days	1-4 years	-1	LDI (I$ per capita)
Other neonatal disorders	Male	0-6 days	1-4 years	1	Underweight (proportion <2SD weight for age, <5 years)
Other neonatal disorders	Male	0-6 days	1-4 years	1	Live Births 35+ (proportion)
Other neonatal disorders	Male	0-6 days	1-4 years	1	Indoor Air Pollution (All Cooking Fuels)
Other neonatal disorders	Male	0-6 days	1-4 years	-1	Skilled Birth Attendance (proportion)
Other neonatal disorders	Male	0-6 days	1-4 years	1	Smoking Prevalence (Reproductive Age Standardized)
Other neonatal disorders	Male	0-6 days	1-4 years	1	Total Fertility Rate
Other neonatal disorders	Male	0-6 days	1-4 years	-1	Health System Access (capped)
Other neonatal disorders	Male	0-6 days	1-4 years	-1	Socio-demographic Index
Other neonatal disorders	Male	0-6 days	1-4 years	-1	Healthcare access and quality index
Other neonatal disorders	Female	0-6 days	1-4 years	-1	Antenatal Care (4 visits) Coverage (proportion)
Other neonatal disorders	Female	0-6 days	1-4 years	-1	Education (years per capita)
Other neonatal disorders	Female	0-6 days	1-4 years	-1	In-Facility Delivery (proportion)
Other neonatal disorders	Female	0-6 days	1-4 years	-1	LDI (I$ per capita)
Other neonatal disorders	Female	0-6 days	1-4 years	1	Underweight (proportion <2SD weight for age, <5 years)
Other neonatal disorders	Female	0-6 days	1-4 years	1	Live Births 35+ (proportion)
Other neonatal disorders	Female	0-6 days	1-4 years	1	Indoor Air Pollution (All Cooking Fuels)
Other neonatal disorders	Female	0-6 days	1-4 years	-1	Skilled Birth Attendance (proportion)
Other neonatal disorders	Female	0-6 days	1-4 years	1	Smoking Prevalence (Reproductive Age Standardized)
Other neonatal disorders	Female	0-6 days	1-4 years	1	Total Fertility Rate
Other neonatal disorders	Female	0-6 days	1-4 years	-1	Health System Access (capped)
Other neonatal disorders	Female	0-6 days	1-4 years	-1	Socio-demographic Index
Other neonatal disorders	Female	0-6 days	1-4 years	-1	Healthcare access and quality index
Nutritional deficiencies	Male	28-364 days	95+ years	-1	Antenatal Care (4 visits) Coverage (proportion)
Nutritional deficiencies	Male	28-364 days	95+ years	-1	Education (years per capita)
Nutritional deficiencies	Male	28-364 days	95+ years	-1	Proportion of households using iodized salt (adjusted)
Nutritional deficiencies	Male	28-364 days	95+ years	-1	LDI (I$ per capita)
Nutritional deficiencies	Male	28-364 days	95+ years	1	Underweight (proportion <2SD weight for age, <5 years)
Nutritional deficiencies	Male	28-364 days	95+ years	0	Rainfall Quintile 1 (proportion)
Nutritional deficiencies	Male	28-364 days	95+ years	0	Rainfall Quintile 2 (proportion)
Nutritional deficiencies	Male	28-364 days	95+ years	-1	Sanitation (proportion with access)
Nutritional deficiencies	Male	28-364 days	95+ years	1	Mortality Rate Due to War Shocks (per 1 person)
Nutritional deficiencies	Male	28-364 days	95+ years	-1	Improved Water Source (proportion with access)
Nutritional deficiencies	Male	28-364 days	95+ years	1	Age-Standardize Prevalence of Severe Anemia
Nutritional deficiencies	Male	28-364 days	95+ years	-1	Health System Access (capped)
Nutritional deficiencies	Male	28-364 days	95+ years	-1	Socio-demographic Index
Nutritional deficiencies	Male	28-364 days	95+ years	-1	energy unadjusted(kcal)
Nutritional deficiencies	Male	28-364 days	95+ years	1	Wasting (proportion <2SD weight for height, <5 years)
Nutritional deficiencies	Male	28-364 days	95+ years	-1	Healthcare access and quality index
Nutritional deficiencies	Male	28-364 days	95+ years	1	Malnutrition Shock mortality rate
Nutritional deficiencies	Female	28-364 days	95+ years	-1	Antenatal Care (4 visits) Coverage (proportion)
Nutritional deficiencies	Female	28-364 days	95+ years	-1	Education (years per capita)
Nutritional deficiencies	Female	28-364 days	95+ years	-1	Proportion of households using iodized salt (adjusted)
Nutritional deficiencies	Female	28-364 days	95+ years	-1	LDI (I$ per capita)
Nutritional deficiencies	Female	28-364 days	95+ years	1	Underweight (proportion <2SD weight for age, <5 years)
Nutritional deficiencies	Female	28-364 days	95+ years	0	Rainfall Quintile 1 (proportion)
Nutritional deficiencies	Female	28-364 days	95+ years	0	Rainfall Quintile 2 (proportion)
Nutritional deficiencies	Female	28-364 days	95+ years	-1	Sanitation (proportion with access)
Nutritional deficiencies	Female	28-364 days	95+ years	1	Mortality Rate Due to War Shocks (per 1 person)
Nutritional deficiencies	Female	28-364 days	95+ years	-1	Improved Water Source (proportion with access)
Nutritional deficiencies	Female	28-364 days	95+ years	1	Age-Standardize Prevalence of Severe Anemia
Nutritional deficiencies	Female	28-364 days	95+ years	-1	Health System Access (capped)
Nutritional deficiencies	Female	28-364 days	95+ years	-1	Socio-demographic Index
Nutritional deficiencies	Female	28-364 days	95+ years	-1	energy unadjusted(kcal)
Nutritional deficiencies	Female	28-364 days	95+ years	1	Wasting (proportion <2SD weight for height, <5 years)
Nutritional deficiencies	Female	28-364 days	95+ years	-1	Healthcare access and quality index
Nutritional deficiencies	Female	28-364 days	95+ years	1	Malnutrition Shock mortality rate
Protein-energy malnutrition	Male	5-9 years	95+ years	-1	Antenatal Care (4 visits) Coverage (proportion)
Protein-energy malnutrition	Male	5-9 years	95+ years	-1	Education (years per capita)
Protein-energy malnutrition	Male	5-9 years	95+ years	-1	LDI (I$ per capita)
Protein-energy malnutrition	Male	5-9 years	95+ years	0	Rainfall Quintile 1 (proportion)
Protein-energy malnutrition	Male	5-9 years	95+ years	0	Rainfall Quintile 2 (proportion)
Protein-energy malnutrition	Male	5-9 years	95+ years	-1	Sanitation (proportion with access)
Protein-energy malnutrition	Male	5-9 years	95+ years	1	Mortality Rate Due to War Shocks (per 1 person)
Protein-energy malnutrition	Male	5-9 years	95+ years	-1	Improved Water Source (proportion with access)
Protein-energy malnutrition	Male	5-9 years	95+ years	1	Age-Standardize Prevalence of Severe Anemia
Protein-energy malnutrition	Male	5-9 years	95+ years	-1	Health System Access (capped)
Protein-energy malnutrition	Male	5-9 years	95+ years	-1	Socio-demographic Index
Protein-energy malnutrition	Male	5-9 years	95+ years	-1	energy unadjusted(kcal)
Protein-energy malnutrition	Male	5-9 years	95+ years	-1	Healthcare access and quality index
Protein-energy malnutrition	Male	5-9 years	95+ years	1	Malnutrition Shock mortality rate
Protein-energy malnutrition	Male	28-364 days	1-4 years	-1	Antenatal Care (4 visits) Coverage (proportion)
Protein-energy malnutrition	Male	28-364 days	1-4 years	-1	Education (years per capita)
Protein-energy malnutrition	Male	28-364 days	1-4 years	-1	LDI (I$ per capita)
Protein-energy malnutrition	Male	28-364 days	1-4 years	0	Rainfall Quintile 1 (proportion)
Protein-energy malnutrition	Male	28-364 days	1-4 years	0	Rainfall Quintile 2 (proportion)
Protein-energy malnutrition	Male	28-364 days	1-4 years	-1	Sanitation (proportion with access)
Protein-energy malnutrition	Male	28-364 days	1-4 years	1	Mortality Rate Due to War Shocks (per 1 person)
Protein-energy malnutrition	Male	28-364 days	1-4 years	-1	Improved Water Source (proportion with access)
Protein-energy malnutrition	Male	28-364 days	1-4 years	1	Age-Standardize Prevalence of Severe Anemia
Protein-energy malnutrition	Male	28-364 days	1-4 years	-1	Health System Access (capped)
Protein-energy malnutrition	Male	28-364 days	1-4 years	-1	Socio-demographic Index
Protein-energy malnutrition	Male	28-364 days	1-4 years	-1	energy unadjusted(kcal)
Protein-energy malnutrition	Male	28-364 days	1-4 years	1	Wasting (proportion <2SD weight for height, <5 years)
Protein-energy malnutrition	Male	28-364 days	1-4 years	-1	Healthcare access and quality index
Protein-energy malnutrition	Male	28-364 days	1-4 years	1	Malnutrition Shock mortality rate
Protein-energy malnutrition	Female	28-364 days	1-4 years	-1	Antenatal Care (4 visits) Coverage (proportion)
Protein-energy malnutrition	Female	28-364 days	1-4 years	-1	Education (years per capita)

Cause	Sex	Age start	Age end	Direction	Covariate
Protein-energy malnutrition	Female	28-364 days	1-4 years	-1	LDI (I$ per capita)
Protein-energy malnutrition	Female	28-364 days	1-4 years	0	Rainfall Quintile 1 (proportion)
Protein-energy malnutrition	Female	28-364 days	1-4 years	0	Rainfall Quintile 2 (proportion)
Protein-energy malnutrition	Female	28-364 days	1-4 years	-1	Sanitation (proportion with access)
Protein-energy malnutrition	Female	28-364 days	1-4 years	1	Mortality Rate Due to War Shocks (per 1 person)
Protein-energy malnutrition	Female	28-364 days	1-4 years	-1	Improved Water Source (proportion with access)
Protein-energy malnutrition	Female	28-364 days	1-4 years	1	Age-Standardize Prevalence of Severe Anemia
Protein-energy malnutrition	Female	28-364 days	1-4 years	-1	Health System Access (capped)
Protein-energy malnutrition	Female	28-364 days	1-4 years	-1	Socio-demographic Index
Protein-energy malnutrition	Female	28-364 days	1-4 years	-1	energy unadjusted(kcal)
Protein-energy malnutrition	Female	28-364 days	1-4 years	1	Wasting (proportion <2SD weight for height, <5 years)
Protein-energy malnutrition	Female	28-364 days	1-4 years	-1	Healthcare access and quality index
Protein-energy malnutrition	Female	5-9 years	95+ years	-1	Antenatal Care (4 visits) Coverage (proportion)
Protein-energy malnutrition	Female	5-9 years	95+ years	-1	Education (years per capita)
Protein-energy malnutrition	Female	5-9 years	95+ years	-1	LDI (I$ per capita)
Protein-energy malnutrition	Female	5-9 years	95+ years	0	Rainfall Quintile 1 (proportion)
Protein-energy malnutrition	Female	5-9 years	95+ years	0	Rainfall Quintile 2 (proportion)
Protein-energy malnutrition	Female	5-9 years	95+ years	-1	Sanitation (proportion with access)
Protein-energy malnutrition	Female	5-9 years	95+ years	1	Mortality Rate Due to War Shocks (per 1 person)
Protein-energy malnutrition	Female	5-9 years	95+ years	-1	Improved Water Source (proportion with access)
Protein-energy malnutrition	Female	5-9 years	95+ years	1	Age-Standardize Prevalence of Severe Anemia
Protein-energy malnutrition	Female	5-9 years	95+ years	-1	Health System Access (capped)
Protein-energy malnutrition	Female	5-9 years	95+ years	-1	Socio-demographic Index
Protein-energy malnutrition	Female	5-9 years	95+ years	-1	energy unadjusted(kcal)
Protein-energy malnutrition	Female	5-9 years	95+ years	-1	Healthcare access and quality index
Iron-deficiency anemia	Male	28-364 days	95+ years	-1	Education (years per capita)
Iron-deficiency anemia	Male	28-364 days	95+ years	-1	LDI (I$ per capita)
Iron-deficiency anemia	Male	28-364 days	95+ years	1	Underweight (proportion <2SD weight for age, <5 years)
Iron-deficiency anemia	Male	28-364 days	95+ years	0	Rainfall Quintile 1 (proportion)
Iron-deficiency anemia	Male	28-364 days	95+ years	0	Rainfall Quintile 2 (proportion)
Iron-deficiency anemia	Male	28-364 days	95+ years	-1	Sanitation (proportion with access)
Iron-deficiency anemia	Male	28-364 days	95+ years	-1	Total Calories (kcal per capita)
Iron-deficiency anemia	Male	28-364 days	95+ years	-1	Improved Water Source (proportion with access)
Iron-deficiency anemia	Male	28-364 days	95+ years	1	Age-Standardize Prevalence of Severe Anemia
Iron-deficiency anemia	Male	28-364 days	95+ years	-1	Health System Access (capped)
Iron-deficiency anemia	Male	28-364 days	95+ years	-1	Socio-demographic Index
Iron-deficiency anemia	Male	28-364 days	95+ years	-1	Healthcare access and quality index
Iron-deficiency anemia	Female	28-364 days	95+ years	-1	Education (years per capita)
Iron-deficiency anemia	Female	28-364 days	95+ years	-1	LDI (I$ per capita)
Iron-deficiency anemia	Female	28-364 days	95+ years	1	Underweight (proportion <2SD weight for age, <5 years)
Iron-deficiency anemia	Female	28-364 days	95+ years	0	Rainfall Quintile 1 (proportion)
Iron-deficiency anemia	Female	28-364 days	95+ years	0	Rainfall Quintile 2 (proportion)
Iron-deficiency anemia	Female	28-364 days	95+ years	-1	Sanitation (proportion with access)
Iron-deficiency anemia	Female	28-364 days	95+ years	-1	Total Calories (kcal per capita)
Iron-deficiency anemia	Female	28-364 days	95+ years	-1	Improved Water Source (proportion with access)
Iron-deficiency anemia	Female	28-364 days	95+ years	1	Age-Standardize Prevalence of Severe Anemia
Iron-deficiency anemia	Female	28-364 days	95+ years	-1	Health System Access (capped)
Iron-deficiency anemia	Female	28-364 days	95+ years	-1	Socio-demographic Index
Iron-deficiency anemia	Female	28-364 days	95+ years	-1	Healthcare access and quality index
Iron-deficiency anemia	Male	28-364 days	95+ years	-1	Health System Access 2 (unitless)
Iron-deficiency anemia	Female	28-364 days	95+ years	-1	Health System Access 2 (unitless)
Other nutritional deficiencies	Female	28-364 days	95+ years	-1	Education (years per capita)
Other nutritional deficiencies	Female	28-364 days	95+ years	-1	LDI (I$ per capita)
Other nutritional deficiencies	Female	28-364 days	95+ years	1	Underweight (proportion <2SD weight for age, <5 years)
Other nutritional deficiencies	Female	28-364 days	95+ years	0	Rainfall Quintile 1 (proportion)
Other nutritional deficiencies	Female	28-364 days	95+ years	0	Rainfall Quintile 2 (proportion)
Other nutritional deficiencies	Female	28-364 days	95+ years	-1	Sanitation (proportion with access)
Other nutritional deficiencies	Female	28-364 days	95+ years	1	Mortality Rate Due to War Shocks (per 1 person)
Other nutritional deficiencies	Female	28-364 days	95+ years	-1	Improved Water Source (proportion with access)
Other nutritional deficiencies	Female	28-364 days	95+ years	1	Age-Standardize Prevalence of Severe Anemia
Other nutritional deficiencies	Female	28-364 days	95+ years	-1	Health System Access (capped)
Other nutritional deficiencies	Female	28-364 days	95+ years	-1	Socio-demographic Index
Other nutritional deficiencies	Female	28-364 days	95+ years	-1	energy unadjusted(kcal)
Other nutritional deficiencies	Female	28-364 days	95+ years	-1	Healthcare access and quality index
Other nutritional deficiencies	Female	28-364 days	95+ years	1	Malnutrition Shock mortality rate
Other nutritional deficiencies	Male	28-364 days	95+ years	-1	Education (years per capita)
Other nutritional deficiencies	Male	28-364 days	95+ years	-1	LDI (I$ per capita)
Other nutritional deficiencies	Male	28-364 days	95+ years	1	Underweight (proportion <2SD weight for age, <5 years)
Other nutritional deficiencies	Male	28-364 days	95+ years	0	Rainfall Quintile 1 (proportion)
Other nutritional deficiencies	Male	28-364 days	95+ years	0	Rainfall Quintile 2 (proportion)
Other nutritional deficiencies	Male	28-364 days	95+ years	-1	Sanitation (proportion with access)
Other nutritional deficiencies	Male	28-364 days	95+ years	1	Mortality Rate Due to War Shocks (per 1 person)
Other nutritional deficiencies	Male	28-364 days	95+ years	-1	Improved Water Source (proportion with access)
Other nutritional deficiencies	Male	28-364 days	95+ years	1	Age-Standardize Prevalence of Severe Anemia
Other nutritional deficiencies	Male	28-364 days	95+ years	-1	Health System Access (capped)
Other nutritional deficiencies	Male	28-364 days	95+ years	-1	Socio-demographic Index
Other nutritional deficiencies	Male	28-364 days	95+ years	-1	energy unadjusted(kcal)
Other nutritional deficiencies	Male	28-364 days	95+ years	-1	Healthcare access and quality index
Other nutritional deficiencies	Male	28-364 days	95+ years	1	Malnutrition Shock mortality rate
Sexually transmitted diseases excluding HIV	Male	10-14 years	95+ years	-1	Legality of Abortion
Sexually transmitted diseases excluding HIV	Male	10-14 years	95+ years	-1	Antenatal Care (1 visit) Coverage (proportion)
Sexually transmitted diseases excluding HIV	Male	10-14 years	95+ years	-1	Antenatal Care (4 visits) Coverage (proportion)
Sexually transmitted diseases excluding HIV	Male	10-14 years	95+ years	1	Age-Specific Fertility Rate
Sexually transmitted diseases excluding HIV	Male	10-14 years	95+ years	-1	Education (years per capita)
Sexually transmitted diseases excluding HIV	Male	10-14 years	95+ years	-1	LDI (I$ per capita)
Sexually transmitted diseases excluding HIV	Male	10-14 years	95+ years	1	Total Fertility Rate
Sexually transmitted diseases excluding HIV	Male	10-14 years	95+ years	-1	Health System Access (capped)
Sexually transmitted diseases excluding HIV	Male	10-14 years	95+ years	1	Syphilis prevalence (proportion)
Sexually transmitted diseases excluding HIV	Male	10-14 years	95+ years	-1	Healthcare access and quality index
Sexually transmitted diseases excluding HIV	Female	10-14 years	95+ years	-1	Legality of Abortion
Sexually transmitted diseases excluding HIV	Female	10-14 years	95+ years	-1	Antenatal Care (1 visit) Coverage (proportion)
Sexually transmitted diseases excluding HIV	Female	10-14 years	95+ years	-1	Antenatal Care (4 visits) Coverage (proportion)

Cause	Sex	Age start	Age end	Direction	Covariate
Sexually transmitted diseases excluding HIV	Female	10-14 years	95+ years	1	Age-Specific Fertility Rate
Sexually transmitted diseases excluding HIV	Female	10-14 years	95+ years	-1	Education (years per capita)
Sexually transmitted diseases excluding HIV	Female	10-14 years	95+ years	-1	LDI (I$ per capita)
Sexually transmitted diseases excluding HIV	Female	10-14 years	95+ years	1	Total Fertility Rate
Sexually transmitted diseases excluding HIV	Female	10-14 years	95+ years	-1	Health System Access (capped)
Sexually transmitted diseases excluding HIV	Female	10-14 years	95+ years	1	Syphilis prevalence (proportion)
Sexually transmitted diseases excluding HIV	Female	10-14 years	95+ years	-1	Healthcare access and quality index
Hepatitis	Female	28-364 days	95+ years	-1	Education (years per capita)
Hepatitis	Female	28-364 days	95+ years	-1	Health System Access 2 (unitless)
Hepatitis	Female	28-364 days	95+ years	-1	LDI (I$ per capita)
Hepatitis	Female	28-364 days	95+ years	-1	Sanitation (proportion with access)
Hepatitis	Female	28-364 days	95+ years	-1	Improved Water Source (proportion with access)
Hepatitis	Female	28-364 days	95+ years	1	Log-transformed SEV scalar: Hep
Hepatitis	Female	28-364 days	95+ years	-1	Socio-demographic Index
Hepatitis	Female	28-364 days	95+ years	1	Hepatitis B (HBsAg) Seroprevalence
Hepatitis	Female	28-364 days	95+ years	1	Hepatitis C (IgG) Seroprevalence
Hepatitis	Female	28-364 days	95+ years	1	Seroprevalence of anti-HAV (IgG)
Hepatitis	Female	28-364 days	95+ years	1	Seroprevalence of anti-HEV (IgG)
Hepatitis	Male	28-364 days	95+ years	-1	Education (years per capita)
Hepatitis	Male	28-364 days	95+ years	-1	Health System Access 2 (unitless)
Hepatitis	Male	28-364 days	95+ years	-1	LDI (I$ per capita)
Hepatitis	Male	28-364 days	95+ years	-1	Sanitation (proportion with access)
Hepatitis	Male	28-364 days	95+ years	-1	Improved Water Source (proportion with access)
Hepatitis	Male	28-364 days	95+ years	1	Log-transformed SEV scalar: Hep
Hepatitis	Male	28-364 days	95+ years	-1	Socio-demographic Index
Hepatitis	Male	28-364 days	95+ years	1	Hepatitis B (HBsAg) Seroprevalence
Hepatitis	Male	28-364 days	95+ years	1	Hepatitis C (IgG) Seroprevalence
Hepatitis	Male	28-364 days	95+ years	1	Seroprevalence of anti-HAV (IgG)
Hepatitis	Male	28-364 days	95+ years	1	Seroprevalence of anti-HEV (IgG)
Other infectious diseases	Male	0-6 days	95+ years	1	Underweight (proportion <2SD weight for age, <5 years)
Other infectious diseases	Male	0-6 days	95+ years	-1	Sanitation (proportion with access)
Other infectious diseases	Male	0-6 days	95+ years	1	Rainfall Quintile 5 (proportion)
Other infectious diseases	Male	0-6 days	95+ years	-1	Education (years per capita)
Other infectious diseases	Male	0-6 days	95+ years	-1	DTP3 Coverage (proportion)
Other infectious diseases	Male	0-6 days	95+ years	-1	Health System Access (unitless)
Other infectious diseases	Male	0-6 days	95+ years	0	Latitude 30 to 45 (proportion)
Other infectious diseases	Male	0-6 days	95+ years	-1	Measles Vaccine Coverage (proportion)
Other infectious diseases	Male	0-6 days	95+ years	-1	Latitude Over 45 (proportion)
Other infectious diseases	Male	0-6 days	95+ years	0	Rainfall Quintile 3 (proportion)
Other infectious diseases	Male	0-6 days	95+ years	0	Rainfall Quintile 2 (proportion)
Other infectious diseases	Male	0-6 days	95+ years	-1	Rainfall Quintile 1 (proportion)
Other infectious diseases	Male	0-6 days	95+ years	-1	LDI (I$ per capita)
Other infectious diseases	Male	0-6 days	95+ years	0	Latitude 15 to 30 (proportion)
Other infectious diseases	Male	0-6 days	95+ years	-1	Antenatal Care (1 visit) Coverage (proportion)
Other infectious diseases	Male	0-6 days	95+ years	0	Rainfall Quintile 4 (proportion)
Other infectious diseases	Male	0-6 days	95+ years	-1	Improved Water Source (proportion with access)
Other infectious diseases	Male	0-6 days	95+ years	1	Latitude Under 15 (proportion)
Other infectious diseases	Male	0-6 days	95+ years	-1	Socio-demographic Index
Other infectious diseases	Female	0-6 days	95+ years	1	Underweight (proportion <2SD weight for age, <5 years)
Other infectious diseases	Female	0-6 days	95+ years	-1	Sanitation (proportion with access)
Other infectious diseases	Female	0-6 days	95+ years	1	Rainfall Quintile 5 (proportion)
Other infectious diseases	Female	0-6 days	95+ years	-1	Education (years per capita)
Other infectious diseases	Female	0-6 days	95+ years	-1	DTP3 Coverage (proportion)
Other infectious diseases	Female	0-6 days	95+ years	-1	Health System Access (unitless)
Other infectious diseases	Female	0-6 days	95+ years	0	Latitude 30 to 45 (proportion)
Other infectious diseases	Female	0-6 days	95+ years	-1	Measles Vaccine Coverage (proportion)
Other infectious diseases	Female	0-6 days	95+ years	-1	Latitude Over 45 (proportion)
Other infectious diseases	Female	0-6 days	95+ years	0	Rainfall Quintile 3 (proportion)
Other infectious diseases	Female	0-6 days	95+ years	0	Rainfall Quintile 2 (proportion)
Other infectious diseases	Female	0-6 days	95+ years	-1	Rainfall Quintile 1 (proportion)
Other infectious diseases	Female	0-6 days	95+ years	-1	LDI (I$ per capita)
Other infectious diseases	Female	0-6 days	95+ years	0	Latitude 15 to 30 (proportion)
Other infectious diseases	Female	0-6 days	95+ years	-1	Antenatal Care (1 visit) Coverage (proportion)
Other infectious diseases	Female	0-6 days	95+ years	0	Rainfall Quintile 4 (proportion)
Other infectious diseases	Female	0-6 days	95+ years	-1	Improved Water Source (proportion with access)
Other infectious diseases	Female	0-6 days	95+ years	1	Latitude Under 15 (proportion)
Other infectious diseases	Female	0-6 days	95+ years	-1	Socio-demographic Index
Other infectious diseases	Female	0-6 days	95+ years	-1	Health System Access (capped)
Other infectious diseases	Female	0-6 days	95+ years	-1	Healthcare access and quality index
Esophageal cancer	Male	15-19 years	95+ years	1	Alcohol (liters per capita)
Esophageal cancer	Male	15-19 years	95+ years	1	Tobacco (cigarettes per capita)
Esophageal cancer	Male	15-19 years	95+ years	-1	Education (years per capita)
Esophageal cancer	Male	15-19 years	95+ years	-1	Fruits (kcal per capita)
Esophageal cancer	Male	15-19 years	95+ years	-1	LDI (I$ per capita)
Esophageal cancer	Male	15-19 years	95+ years	1	Mean BMI
Esophageal cancer	Male	15-19 years	95+ years	1	Indoor Air Pollution (All Cooking Fuels)
Esophageal cancer	Male	15-19 years	95+ years	-1	Sanitation (proportion with access)
Esophageal cancer	Male	15-19 years	95+ years	1	Smoking Prevalence
Esophageal cancer	Male	15-19 years	95+ years	-1	Vegetables (kcal per capita)
Esophageal cancer	Male	15-19 years	95+ years	-1	Improved Water Source (proportion with access)
Esophageal cancer	Male	15-19 years	95+ years	1	Log-transformed age-standardized SEV scalar: Esophag C
Esophageal cancer	Male	15-19 years	95+ years	-1	Socio-demographic Index
Esophageal cancer	Male	15-19 years	95+ years	-1	Healthcare access and quality index
Esophageal cancer	Female	15-19 years	95+ years	1	Alcohol (liters per capita)
Esophageal cancer	Female	15-19 years	95+ years	1	Tobacco (cigarettes per capita)
Esophageal cancer	Female	15-19 years	95+ years	-1	Education (years per capita)
Esophageal cancer	Female	15-19 years	95+ years	-1	Fruits (kcal per capita)
Esophageal cancer	Female	15-19 years	95+ years	-1	LDI (I$ per capita)
Esophageal cancer	Female	15-19 years	95+ years	1	Mean BMI
Esophageal cancer	Female	15-19 years	95+ years	1	Indoor Air Pollution (All Cooking Fuels)
Esophageal cancer	Female	15-19 years	95+ years	-1	Sanitation (proportion with access)
Esophageal cancer	Female	15-19 years	95+ years	1	Smoking Prevalence

Cause	Sex	Age start	Age end	Direction	Covariate
Esophageal cancer	Female	15-19 years	95+ years	-1	Vegetables (kcal per capita)
Esophageal cancer	Female	15-19 years	95+ years	-1	Improved Water Source (proportion with access)
Esophageal cancer	Female	15-19 years	95+ years	1	Log-transformed age-standardized SEV scalar: Esophag C
Esophageal cancer	Female	15-19 years	95+ years	0	Socio-demographic Index
Esophageal cancer	Female	15-19 years	95+ years	-1	Healthcare access and quality index
Esophageal cancer	Female	15-19 years	95+ years	0	LDI (I$ per capita)
Esophageal cancer	Female	15-19 years	95+ years	-1	Socio-demographic Index
Esophageal cancer	Female	15-19 years	95+ years	-1	fruits adjusted(g)
Esophageal cancer	Female	15-19 years	95+ years	-1	vegetables adjusted(g)
Esophageal cancer	Male	15-19 years	95+ years	0	LDI (I$ per capita)
Esophageal cancer	Male	15-19 years	95+ years	1	Log-transformed SEV scalar: Esophag C
Esophageal cancer	Male	15-19 years	95+ years	-1	fruits adjusted(g)
Esophageal cancer	Male	15-19 years	95+ years	-1	vegetables adjusted(g)
Stomach cancer	Male	15-19 years	95+ years	1	Alcohol (liters per capita)
Stomach cancer	Male	15-19 years	95+ years	1	Tobacco (cigarettes per capita)
Stomach cancer	Male	15-19 years	95+ years	1	Cumulative Cigarettes (10 Years)
Stomach cancer	Male	15-19 years	95+ years	1	Cumulative Cigarettes (15 Years)
Stomach cancer	Male	15-19 years	95+ years	0	LDI (I$ per capita)
Stomach cancer	Male	15-19 years	95+ years	1	Mean BMI
Stomach cancer	Male	15-19 years	95+ years	1	Indoor Air Pollution (All Cooking Fuels)
Stomach cancer	Male	15-19 years	95+ years	1	Outdoor Air Pollution (PM2.5)
Stomach cancer	Male	15-19 years	95+ years	-1	Sanitation (proportion with access)
Stomach cancer	Male	15-19 years	95+ years	1	Smoking Prevalence
Stomach cancer	Male	15-19 years	95+ years	-1	Improved Water Source (proportion with access)
Stomach cancer	Male	15-19 years	95+ years	1	Log-transformed SEV scalar: Stomach C
Stomach cancer	Male	15-19 years	95+ years	0	Socio-demographic Index
Stomach cancer	Male	15-19 years	95+ years	-1	fruits adjusted(g)
Stomach cancer	Male	15-19 years	95+ years	-1	vegetables adjusted(g)
Stomach cancer	Male	15-19 years	95+ years	-1	Healthcare access and quality index
Stomach cancer	Male	15-19 years	95+ years	1	Diet high in sodium
Stomach cancer	Female	15-19 years	95+ years	1	Alcohol (liters per capita)
Stomach cancer	Female	15-19 years	95+ years	1	Tobacco (cigarettes per capita)
Stomach cancer	Female	15-19 years	95+ years	1	Cumulative Cigarettes (10 Years)
Stomach cancer	Female	15-19 years	95+ years	-1	Education (years per capita)
Stomach cancer	Female	15-19 years	95+ years	0	LDI (I$ per capita)
Stomach cancer	Female	15-19 years	95+ years	1	Mean BMI
Stomach cancer	Female	15-19 years	95+ years	1	Indoor Air Pollution (All Cooking Fuels)
Stomach cancer	Female	15-19 years	95+ years	1	Outdoor Air Pollution (PM2.5)
Stomach cancer	Female	15-19 years	95+ years	-1	Sanitation (proportion with access)
Stomach cancer	Female	15-19 years	95+ years	1	Smoking Prevalence
Stomach cancer	Female	15-19 years	95+ years	-1	Improved Water Source (proportion with access)
Stomach cancer	Female	15-19 years	95+ years	1	Log-transformed SEV scalar: Stomach C
Stomach cancer	Female	15-19 years	95+ years	0	Socio-demographic Index
Stomach cancer	Female	15-19 years	95+ years	-1	fruits adjusted(g)
Stomach cancer	Female	15-19 years	95+ years	-1	vegetables adjusted(g)
Stomach cancer	Female	15-19 years	95+ years	-1	Healthcare access and quality index
Stomach cancer	Female	15-19 years	95+ years	1	Diet high in sodium
Liver cancer	Male	5-9 years	95+ years	1	Alcohol (liters per capita)
Liver cancer	Male	5-9 years	95+ years	1	Tobacco (cigarettes per capita)
Liver cancer	Male	5-9 years	95+ years	1	Cumulative Cigarettes (15 Years)
Liver cancer	Male	5-9 years	95+ years	1	Cumulative Cigarettes (20 Years)
Liver cancer	Male	5-9 years	95+ years	1	Diabetes Age-Standardized Prevalence (proportion)
Liver cancer	Male	5-9 years	95+ years	-1	Education (years per capita)
Liver cancer	Male	5-9 years	95+ years	0	LDI (I$ per capita)
Liver cancer	Male	5-9 years	95+ years	1	Mean BMI
Liver cancer	Male	5-9 years	95+ years	1	Percent of total calories consumed as saturated fat
Liver cancer	Male	5-9 years	95+ years	1	Log-transformed SEV scalar: Liver C
Liver cancer	Male	5-9 years	95+ years	0	Socio-demographic Index
Liver cancer	Male	5-9 years	95+ years	1	red meats adjusted(g)
Liver cancer	Male	5-9 years	95+ years	1	Hepatitis B (HBsAg) Seroprevalence
Liver cancer	Male	5-9 years	95+ years	1	Hepatitis C (IgG) Seroprevalence
Liver cancer	Male	5-9 years	95+ years	-1	Healthcare access and quality index
Liver cancer	Female	5-9 years	95+ years	1	Alcohol (liters per capita)
Liver cancer	Female	5-9 years	95+ years	1	Tobacco (cigarettes per capita)
Liver cancer	Female	5-9 years	95+ years	1	Cumulative Cigarettes (15 Years)
Liver cancer	Female	5-9 years	95+ years	1	Cumulative Cigarettes (20 Years)
Liver cancer	Female	5-9 years	95+ years	1	Diabetes Age-Standardized Prevalence (proportion)
Liver cancer	Female	5-9 years	95+ years	-1	Education (years per capita)
Liver cancer	Female	5-9 years	95+ years	0	LDI (I$ per capita)
Liver cancer	Female	5-9 years	95+ years	1	Mean BMI
Liver cancer	Female	5-9 years	95+ years	1	Percent of total calories consumed as saturated fat
Liver cancer	Female	5-9 years	95+ years	1	Log-transformed SEV scalar: Liver C
Liver cancer	Female	5-9 years	95+ years	0	Socio-demographic Index
Liver cancer	Female	5-9 years	95+ years	1	red meats adjusted(g)
Liver cancer	Female	5-9 years	95+ years	1	Hepatitis B (HBsAg) Seroprevalence
Liver cancer	Female	5-9 years	95+ years	1	Hepatitis C (IgG) Seroprevalence
Liver cancer	Female	5-9 years	95+ years	-1	Healthcare access and quality index
Larynx cancer	Male	15-19 years	95+ years	1	Alcohol (liters per capita)
Larynx cancer	Male	15-19 years	95+ years	1	Tobacco (cigarettes per capita)
Larynx cancer	Male	15-19 years	95+ years	1	Cumulative Cigarettes (10 Years)
Larynx cancer	Male	15-19 years	95+ years	1	Cumulative Cigarettes (15 Years)
Larynx cancer	Male	15-19 years	95+ years	1	Cumulative Cigarettes (20 Years)
Larynx cancer	Male	15-19 years	95+ years	1	Cumulative Cigarettes (5 Years)
Larynx cancer	Male	15-19 years	95+ years	-1	Education (years per capita)
Larynx cancer	Male	15-19 years	95+ years	0	LDI (I$ per capita)
Larynx cancer	Male	15-19 years	95+ years	1	Population Density (over 1000 ppl/sqkm, proportion)
Larynx cancer	Male	15-19 years	95+ years	1	Population Density (under 150 ppl/sqkm, proportion)
Larynx cancer	Male	15-19 years	95+ years	1	Smoking Prevalence
Larynx cancer	Male	15-19 years	95+ years	1	Log-transformed SEV scalar: Larynx C
Larynx cancer	Male	15-19 years	95+ years	0	Socio-demographic Index
Larynx cancer	Male	15-19 years	95+ years	-1	fruits adjusted(g)
Larynx cancer	Male	15-19 years	95+ years	-1	vegetables adjusted(g)

Cause	Sex	Age start	Age end	Direction	Covariate
Larynx cancer	Male	15-19 years	95+ years	-1	Healthcare access and quality index
Larynx cancer	Female	15-19 years	95+ years	1	Alcohol (liters per capita)
Larynx cancer	Female	15-19 years	95+ years	1	Tobacco (cigarettes per capita)
Larynx cancer	Female	15-19 years	95+ years	1	Cumulative Cigarettes (10 Years)
Larynx cancer	Female	15-19 years	95+ years	1	Cumulative Cigarettes (15 Years)
Larynx cancer	Female	15-19 years	95+ years	1	Cumulative Cigarettes (20 Years)
Larynx cancer	Female	15-19 years	95+ years	1	Cumulative Cigarettes (5 Years)
Larynx cancer	Female	15-19 years	95+ years	-1	Education (years per capita)
Larynx cancer	Female	15-19 years	95+ years	0	LDI (I$ per capita)
Larynx cancer	Female	15-19 years	95+ years	1	Population Density (over 1000 ppl/sqkm, proportion)
Larynx cancer	Female	15-19 years	95+ years	1	Population Density (under 150 ppl/sqkm, proportion)
Larynx cancer	Female	15-19 years	95+ years	1	Smoking Prevalence
Larynx cancer	Female	15-19 years	95+ years	1	Log-transformed SEV scalar: Larynx C
Larynx cancer	Female	15-19 years	95+ years	0	Socio-demographic Index
Larynx cancer	Female	15-19 years	95+ years	-1	fruits adjusted(g)
Larynx cancer	Female	15-19 years	95+ years	-1	vegetables adjusted(g)
Larynx cancer	Female	15-19 years	95+ years	-1	Healthcare access and quality index
Tracheal, bronchus, and lung cancer	Female	15-19 years	95+ years	1	Tobacco (cigarettes per capita)
Tracheal, bronchus, and lung cancer	Female	15-19 years	95+ years	1	Cumulative Cigarettes (10 Years)
Tracheal, bronchus, and lung cancer	Female	15-19 years	95+ years	1	Cumulative Cigarettes (15 Years)
Tracheal, bronchus, and lung cancer	Female	15-19 years	95+ years	1	Cumulative Cigarettes (20 Years)
Tracheal, bronchus, and lung cancer	Female	15-19 years	95+ years	1	Cumulative Cigarettes (5 Years)
Tracheal, bronchus, and lung cancer	Female	15-19 years	95+ years	0	Education (years per capita)
Tracheal, bronchus, and lung cancer	Female	15-19 years	95+ years	0	LDI (I$ per capita)
Tracheal, bronchus, and lung cancer	Female	15-19 years	95+ years	1	Indoor Air Pollution (All Cooking Fuels)
Tracheal, bronchus, and lung cancer	Female	15-19 years	95+ years	1	Outdoor Air Pollution (PM2.5)
Tracheal, bronchus, and lung cancer	Female	15-19 years	95+ years	1	Smoking Prevalence
Tracheal, bronchus, and lung cancer	Female	15-19 years	95+ years	1	Log-transformed SEV scalar: Lung C
Tracheal, bronchus, and lung cancer	Female	15-19 years	95+ years	1	Log-transformed age-standardized SEV scalar: Lung C
Tracheal, bronchus, and lung cancer	Female	15-19 years	95+ years	0	Socio-demographic Index
Tracheal, bronchus, and lung cancer	Female	15-19 years	95+ years	-1	Healthcare access and quality index
Tracheal, bronchus, and lung cancer	Male	15-19 years	95+ years	1	Tobacco (cigarettes per capita)
Tracheal, bronchus, and lung cancer	Male	15-19 years	95+ years	1	Cumulative Cigarettes (10 Years)
Tracheal, bronchus, and lung cancer	Male	15-19 years	95+ years	1	Cumulative Cigarettes (15 Years)
Tracheal, bronchus, and lung cancer	Male	15-19 years	95+ years	1	Cumulative Cigarettes (20 Years)
Tracheal, bronchus, and lung cancer	Male	15-19 years	95+ years	1	Cumulative Cigarettes (5 Years)
Tracheal, bronchus, and lung cancer	Male	15-19 years	95+ years	0	Education (years per capita)
Tracheal, bronchus, and lung cancer	Male	15-19 years	95+ years	0	LDI (I$ per capita)
Tracheal, bronchus, and lung cancer	Male	15-19 years	95+ years	1	Indoor Air Pollution (All Cooking Fuels)
Tracheal, bronchus, and lung cancer	Male	15-19 years	95+ years	1	Outdoor Air Pollution (PM2.5)
Tracheal, bronchus, and lung cancer	Male	15-19 years	95+ years	1	Smoking Prevalence
Tracheal, bronchus, and lung cancer	Male	15-19 years	95+ years	1	Log-transformed SEV scalar: Lung C
Tracheal, bronchus, and lung cancer	Male	15-19 years	95+ years	1	Log-transformed age-standardized SEV scalar: Lung C
Tracheal, bronchus, and lung cancer	Male	15-19 years	95+ years	0	Socio-demographic Index
Tracheal, bronchus, and lung cancer	Male	15-19 years	95+ years	-1	Healthcare access and quality index
Breast cancer	Male	15-19 years	95+ years	1	Alcohol (liters per capita)
Breast cancer	Male	15-19 years	95+ years	1	Cumulative Cigarettes (10 Years)
Breast cancer	Male	15-19 years	95+ years	-1	Education (years per capita)
Breast cancer	Male	15-19 years	95+ years	1	Saturated Fats (kcal per capita)
Breast cancer	Male	15-19 years	95+ years	0	LDI (I$ per capita)
Breast cancer	Male	15-19 years	95+ years	1	Mean BMI
Breast cancer	Male	15-19 years	95+ years	1	Log-transformed SEV scalar: Breast C
Breast cancer	Male	15-19 years	95+ years	0	Socio-demographic Index
Breast cancer	Male	15-19 years	95+ years	-1	fruits adjusted(g)
Breast cancer	Male	15-19 years	95+ years	-1	vegetables adjusted(g)
Breast cancer	Male	15-19 years	95+ years	-1	Healthcare access and quality index
Breast cancer	Female	15-19 years	95+ years	1	Alcohol (liters per capita)
Breast cancer	Female	15-19 years	95+ years	-1	Age-Specific Fertility Rate
Breast cancer	Female	15-19 years	95+ years	1	Cumulative Cigarettes (10 Years)
Breast cancer	Female	15-19 years	95+ years	-1	Education (years per capita)
Breast cancer	Female	15-19 years	95+ years	1	Saturated Fats (kcal per capita)
Breast cancer	Female	15-19 years	95+ years	0	LDI (I$ per capita)
Breast cancer	Female	15-19 years	95+ years	1	Mean BMI
Breast cancer	Female	15-19 years	95+ years	-1	Total Fertility Rate
Breast cancer	Female	15-19 years	95+ years	1	Log-transformed SEV scalar: Breast C
Breast cancer	Female	15-19 years	95+ years	0	Socio-demographic Index
Breast cancer	Female	15-19 years	95+ years	-1	fruits adjusted(g)
Breast cancer	Female	15-19 years	95+ years	-1	vegetables adjusted(g)
Breast cancer	Female	15-19 years	95+ years	-1	Healthcare access and quality index
Cervical cancer	Female	15-19 years	95+ years	1	Abortion On-Demand Illegal (binary)
Cervical cancer	Female	15-19 years	95+ years	1	Age-Specific Fertility Rate
Cervical cancer	Female	15-19 years	95+ years	1	Cumulative Cigarettes (10 Years)
Cervical cancer	Female	15-19 years	95+ years	1	Cumulative Cigarettes (15 Years)
Cervical cancer	Female	15-19 years	95+ years	1	Cumulative Cigarettes (5 Years)
Cervical cancer	Female	15-19 years	95+ years	-1	Education (years per capita)
Cervical cancer	Female	15-19 years	95+ years	-1	Health System Access 2 (unitless)
Cervical cancer	Female	15-19 years	95+ years	0	LDI (I$ per capita)
Cervical cancer	Female	15-19 years	95+ years	1	Smoking Prevalence
Cervical cancer	Female	15-19 years	95+ years	1	Total Fertility Rate
Cervical cancer	Female	15-19 years	95+ years	0	Socio-demographic Index
Cervical cancer	Female	15-19 years	95+ years	-1	fruits adjusted(g)
Cervical cancer	Female	15-19 years	95+ years	-1	vegetables adjusted(g)
Cervical cancer	Female	15-19 years	95+ years	1	HIV age-standardized prevalence
Cervical cancer	Female	15-19 years	95+ years	-1	Healthcare access and quality index
Uterine cancer	Female	15-19 years	95+ years	1	Tobacco (cigarettes per capita)
Uterine cancer	Female	15-19 years	95+ years	1	Cumulative Cigarettes (10 Years)
Uterine cancer	Female	15-19 years	95+ years	1	Cumulative Cigarettes (5 Years)
Uterine cancer	Female	15-19 years	95+ years	1	Diabetes Age-Standardized Prevalence (proportion)
Uterine cancer	Female	15-19 years	95+ years	-1	Education (years per capita)
Uterine cancer	Female	15-19 years	95+ years	-1	Health System Access (unitless)
Uterine cancer	Female	15-19 years	95+ years	0	LDI (I$ per capita)
Uterine cancer	Female	15-19 years	95+ years	1	Mean BMI

Cause	Sex	Age start	Age end	Direction	Covariate
Uterine cancer	Female	15-19 years	95+ years	1	Smoking Prevalence
Uterine cancer	Female	15-19 years	95+ years	0	Total Fertility Rate
Uterine cancer	Female	15-19 years	95+ years	1	Log-transformed SEV scalar: Uterus C
Uterine cancer	Female	15-19 years	95+ years	0	Socio-demographic Index
Uterine cancer	Female	15-19 years	95+ years	-1	fruits adjusted(g)
Uterine cancer	Female	15-19 years	95+ years	-1	vegetables adjusted(g)
Uterine cancer	Female	15-19 years	95+ years	-1	Healthcare access and quality index
Prostate cancer	Male	15-19 years	95+ years	0	Education (years per capita)
Prostate cancer	Male	15-19 years	95+ years	0	LDI (I$ per capita)
Prostate cancer	Male	15-19 years	95+ years	1	Percent of total calories consumed as saturated fat
Prostate cancer	Male	15-19 years	95+ years	1	Log-transformed SEV scalar: Prostate C
Prostate cancer	Male	15-19 years	95+ years	0	Socio-demographic Index
Prostate cancer	Male	15-19 years	95+ years	-1	Healthcare access and quality index
Colon and rectum cancer	Male	15-19 years	95+ years	1	Alcohol (liters per capita)
Colon and rectum cancer	Male	15-19 years	95+ years	1	Tobacco (cigarettes per capita)
Colon and rectum cancer	Male	15-19 years	95+ years	1	Diabetes Age-Standardized Prevalence (proportion)
Colon and rectum cancer	Male	15-19 years	95+ years	-1	Education (years per capita)
Colon and rectum cancer	Male	15-19 years	95+ years	-1	Health System Access 2 (unitless)
Colon and rectum cancer	Male	15-19 years	95+ years	0	LDI (I$ per capita)
Colon and rectum cancer	Male	15-19 years	95+ years	1	Mean BMI
Colon and rectum cancer	Male	15-19 years	95+ years	1	Smoking Prevalence
Colon and rectum cancer	Male	15-19 years	95+ years	1	Log-transformed SEV scalar: Colorect C
Colon and rectum cancer	Male	15-19 years	95+ years	0	Socio-demographic Index
Colon and rectum cancer	Male	15-19 years	95+ years	-1	fruits adjusted(g)
Colon and rectum cancer	Male	15-19 years	95+ years	-1	milk adjusted(g)
Colon and rectum cancer	Male	15-19 years	95+ years	-1	nuts seeds adjusted(g)
Colon and rectum cancer	Male	15-19 years	95+ years	-1	pufa adjusted(percent)
Colon and rectum cancer	Male	15-19 years	95+ years	1	red meats adjusted(g)
Colon and rectum cancer	Male	15-19 years	95+ years	-1	vegetables adjusted(g)
Colon and rectum cancer	Male	15-19 years	95+ years	-1	whole grains adjusted(g)
Colon and rectum cancer	Male	15-19 years	95+ years	-1	Healthcare access and quality index
Colon and rectum cancer	Female	15-19 years	95+ years	1	Alcohol (liters per capita)
Colon and rectum cancer	Female	15-19 years	95+ years	1	Tobacco (cigarettes per capita)
Colon and rectum cancer	Female	15-19 years	95+ years	1	Diabetes Age-Standardized Prevalence (proportion)
Colon and rectum cancer	Female	15-19 years	95+ years	-1	Education (years per capita)
Colon and rectum cancer	Female	15-19 years	95+ years	-1	Health System Access 2 (unitless)
Colon and rectum cancer	Female	15-19 years	95+ years	0	LDI (I$ per capita)
Colon and rectum cancer	Female	15-19 years	95+ years	1	Mean BMI
Colon and rectum cancer	Female	15-19 years	95+ years	1	Smoking Prevalence
Colon and rectum cancer	Female	15-19 years	95+ years	1	Log-transformed SEV scalar: Colorect C
Colon and rectum cancer	Female	15-19 years	95+ years	0	Socio-demographic Index
Colon and rectum cancer	Female	15-19 years	95+ years	-1	fruits adjusted(g)
Colon and rectum cancer	Female	15-19 years	95+ years	-1	milk adjusted(g)
Colon and rectum cancer	Female	15-19 years	95+ years	-1	nuts seeds adjusted(g)
Colon and rectum cancer	Female	15-19 years	95+ years	-1	pufa adjusted(percent)
Colon and rectum cancer	Female	15-19 years	95+ years	1	red meats adjusted(g)
Colon and rectum cancer	Female	15-19 years	95+ years	-1	vegetables adjusted(g)
Colon and rectum cancer	Female	15-19 years	95+ years	-1	whole grains adjusted(g)
Colon and rectum cancer	Female	15-19 years	95+ years	-1	Healthcare access and quality index
Lip and oral cavity cancer	Male	15-19 years	95+ years	1	Alcohol (liters per capita)
Lip and oral cavity cancer	Male	15-19 years	95+ years	1	Tobacco (cigarettes per capita)
Lip and oral cavity cancer	Male	15-19 years	95+ years	1	Cumulative Cigarettes (10 Years)
Lip and oral cavity cancer	Male	15-19 years	95+ years	1	Cumulative Cigarettes (15 Years)
Lip and oral cavity cancer	Male	15-19 years	95+ years	1	Cumulative Cigarettes (20 Years)
Lip and oral cavity cancer	Male	15-19 years	95+ years	1	Cumulative Cigarettes (5 Years)
Lip and oral cavity cancer	Male	15-19 years	95+ years	-1	Education (years per capita)
Lip and oral cavity cancer	Male	15-19 years	95+ years	-1	Health System Access 2 (unitless)
Lip and oral cavity cancer	Male	15-19 years	95+ years	0	LDI (I$ per capita)
Lip and oral cavity cancer	Male	15-19 years	95+ years	1	Smoking Prevalence
Lip and oral cavity cancer	Male	15-19 years	95+ years	1	Log-transformed SEV scalar: Mouth C
Lip and oral cavity cancer	Male	15-19 years	95+ years	0	Socio-demographic Index
Lip and oral cavity cancer	Male	15-19 years	95+ years	-1	fruits adjusted(g)
Lip and oral cavity cancer	Male	15-19 years	95+ years	1	red meats adjusted(g)
Lip and oral cavity cancer	Male	15-19 years	95+ years	-1	vegetables adjusted(g)
Lip and oral cavity cancer	Male	15-19 years	95+ years	-1	Healthcare access and quality index
Lip and oral cavity cancer	Female	15-19 years	95+ years	1	Alcohol (liters per capita)
Lip and oral cavity cancer	Female	15-19 years	95+ years	1	Cumulative Cigarettes (10 Years)
Lip and oral cavity cancer	Female	15-19 years	95+ years	1	Cumulative Cigarettes (20 Years)
Lip and oral cavity cancer	Female	15-19 years	95+ years	-1	Education (years per capita)
Lip and oral cavity cancer	Female	15-19 years	95+ years	-1	Fruits (kcal per capita)
Lip and oral cavity cancer	Female	15-19 years	95+ years	-1	Health System Access 2 (unitless)
Lip and oral cavity cancer	Female	15-19 years	95+ years	-1	LDI (I$ per capita)
Lip and oral cavity cancer	Female	15-19 years	95+ years	1	Red Meat (kcal per capita)
Lip and oral cavity cancer	Female	15-19 years	95+ years	1	Smoking Prevalence
Lip and oral cavity cancer	Female	15-19 years	95+ years	-1	Vegetables (kcal per capita)
Lip and oral cavity cancer	Female	15-19 years	95+ years	0	Socio-demographic Index
Lip and oral cavity cancer	Female	15-19 years	95+ years	-1	Healthcare access and quality index
Nasopharynx cancer	Female	5-9 years	95+ years	1	Alcohol (liters per capita)
Nasopharynx cancer	Female	5-9 years	95+ years	1	Tobacco (cigarettes per capita)
Nasopharynx cancer	Female	5-9 years	95+ years	1	Cumulative Cigarettes (10 Years)
Nasopharynx cancer	Female	5-9 years	95+ years	1	Cumulative Cigarettes (15 Years)
Nasopharynx cancer	Female	5-9 years	95+ years	1	Cumulative Cigarettes (20 Years)
Nasopharynx cancer	Female	5-9 years	95+ years	1	Cumulative Cigarettes (5 Years)
Nasopharynx cancer	Female	5-9 years	95+ years	-1	Education (years per capita)
Nasopharynx cancer	Female	5-9 years	95+ years	-1	Health System Access 2 (unitless)
Nasopharynx cancer	Female	5-9 years	95+ years	0	LDI (I$ per capita)
Nasopharynx cancer	Female	5-9 years	95+ years	1	Population Density (over 1000 ppl/sqkm, proportion)
Nasopharynx cancer	Female	5-9 years	95+ years	1	Population Density (under 150 ppl/sqkm, proportion)
Nasopharynx cancer	Female	5-9 years	95+ years	1	Smoking Prevalence
Nasopharynx cancer	Female	5-9 years	95+ years	1	Log-transformed SEV scalar: Nasoph C
Nasopharynx cancer	Female	5-9 years	95+ years	0	Socio-demographic Index
Nasopharynx cancer	Female	5-9 years	95+ years	-1	fruits adjusted(g)

Cause	Sex	Age start	Age end	Direction	Covariate
Nasopharynx cancer	Female	5-9 years	95+ years	-1	vegetables adjusted(g)
Nasopharynx cancer	Female	5-9 years	95+ years	-1	whole grains adjusted(g)
Nasopharynx cancer	Male	5-9 years	95+ years	1	Alcohol (liters per capita)
Nasopharynx cancer	Male	5-9 years	95+ years	1	Tobacco (cigarettes per capita)
Nasopharynx cancer	Male	5-9 years	95+ years	1	Cumulative Cigarettes (10 Years)
Nasopharynx cancer	Male	5-9 years	95+ years	1	Cumulative Cigarettes (15 Years)
Nasopharynx cancer	Male	5-9 years	95+ years	1	Cumulative Cigarettes (20 Years)
Nasopharynx cancer	Male	5-9 years	95+ years	1	Cumulative Cigarettes (5 Years)
Nasopharynx cancer	Male	5-9 years	95+ years	-1	Education (years per capita)
Nasopharynx cancer	Male	5-9 years	95+ years	-1	Health System Access 2 (unitless)
Nasopharynx cancer	Male	5-9 years	95+ years	0	LDI (I$ per capita)
Nasopharynx cancer	Male	5-9 years	95+ years	1	Population Density (over 1000 ppl/sqkm, proportion)
Nasopharynx cancer	Male	5-9 years	95+ years	1	Population Density (under 150 ppl/sqkm, proportion)
Nasopharynx cancer	Male	5-9 years	95+ years	1	Smoking Prevalence
Nasopharynx cancer	Male	5-9 years	95+ years	1	Log-transformed SEV scalar: Nasoph C
Nasopharynx cancer	Male	5-9 years	95+ years	0	Socio-demographic Index
Nasopharynx cancer	Male	5-9 years	95+ years	-1	fruits adjusted(g)
Nasopharynx cancer	Male	5-9 years	95+ years	-1	vegetables adjusted(g)
Nasopharynx cancer	Male	5-9 years	95+ years	-1	whole grains adjusted(g)
Other pharynx cancer	Male	15-19 years	95+ years	1	Alcohol (liters per capita)
Other pharynx cancer	Male	15-19 years	95+ years	1	Cumulative Cigarettes (5 Years)
Other pharynx cancer	Male	15-19 years	95+ years	-1	Education (years per capita)
Other pharynx cancer	Male	15-19 years	95+ years	0	LDI (I$ per capita)
Other pharynx cancer	Male	15-19 years	95+ years	1	Population Density (over 1000 ppl/sqkm, proportion)
Other pharynx cancer	Male	15-19 years	95+ years	1	Population Density (under 150 ppl/sqkm, proportion)
Other pharynx cancer	Male	15-19 years	95+ years	1	Smoking Prevalence
Other pharynx cancer	Male	15-19 years	95+ years	-1	Health System Access (capped)
Other pharynx cancer	Male	15-19 years	95+ years	1	Log-transformed SEV scalar: Oth Phar C
Other pharynx cancer	Male	15-19 years	95+ years	0	Socio-demographic Index
Other pharynx cancer	Male	15-19 years	95+ years	-1	fruits adjusted(g)
Other pharynx cancer	Male	15-19 years	95+ years	-1	vegetables adjusted(g)
Other pharynx cancer	Male	15-19 years	95+ years	-1	whole grains adjusted(g)
Other pharynx cancer	Female	15-19 years	95+ years	1	Alcohol (liters per capita)
Other pharynx cancer	Female	15-19 years	95+ years	1	Cumulative Cigarettes (5 Years)
Other pharynx cancer	Female	15-19 years	95+ years	-1	Education (years per capita)
Other pharynx cancer	Female	15-19 years	95+ years	0	LDI (I$ per capita)
Other pharynx cancer	Female	15-19 years	95+ years	1	Population Density (over 1000 ppl/sqkm, proportion)
Other pharynx cancer	Female	15-19 years	95+ years	1	Population Density (under 150 ppl/sqkm, proportion)
Other pharynx cancer	Female	15-19 years	95+ years	1	Smoking Prevalence
Other pharynx cancer	Female	15-19 years	95+ years	-1	Health System Access (capped)
Other pharynx cancer	Female	15-19 years	95+ years	1	Log-transformed SEV scalar: Oth Phar C
Other pharynx cancer	Female	15-19 years	95+ years	0	Socio-demographic Index
Other pharynx cancer	Female	15-19 years	95+ years	-1	fruits adjusted(g)
Other pharynx cancer	Female	15-19 years	95+ years	-1	vegetables adjusted(g)
Other pharynx cancer	Female	15-19 years	95+ years	-1	whole grains adjusted(g)
Gallbladder and biliary tract cancer	Female	15-19 years	95+ years	1	Alcohol (liters per capita)
Gallbladder and biliary tract cancer	Female	15-19 years	95+ years	1	Tobacco (cigarettes per capita)
Gallbladder and biliary tract cancer	Female	15-19 years	95+ years	1	Cumulative Cigarettes (10 Years)
Gallbladder and biliary tract cancer	Female	15-19 years	95+ years	1	Cumulative Cigarettes (5 Years)
Gallbladder and biliary tract cancer	Female	15-19 years	95+ years	1	Diabetes Age-Standardized Prevalence (proportion)
Gallbladder and biliary tract cancer	Female	15-19 years	95+ years	-1	Education (years per capita)
Gallbladder and biliary tract cancer	Female	15-19 years	95+ years	1	Health System Access 2 (unitless)
Gallbladder and biliary tract cancer	Female	15-19 years	95+ years	0	LDI (I$ per capita)
Gallbladder and biliary tract cancer	Female	15-19 years	95+ years	1	Mean BMI
Gallbladder and biliary tract cancer	Female	15-19 years	95+ years	1	Smoking Prevalence
Gallbladder and biliary tract cancer	Female	15-19 years	95+ years	1	Log-transformed SEV scalar: Gallblad C
Gallbladder and biliary tract cancer	Female	15-19 years	95+ years	0	Socio-demographic Index
Gallbladder and biliary tract cancer	Female	15-19 years	95+ years	-1	fruits adjusted(g)
Gallbladder and biliary tract cancer	Female	15-19 years	95+ years	-1	vegetables adjusted(g)
Gallbladder and biliary tract cancer	Female	15-19 years	95+ years	-1	Healthcare access and quality index
Gallbladder and biliary tract cancer	Female	15-19 years	95+ years	-1	Health System Access (capped)
Gallbladder and biliary tract cancer	Male	15-19 years	95+ years	1	Alcohol (liters per capita)
Gallbladder and biliary tract cancer	Male	15-19 years	95+ years	1	Tobacco (cigarettes per capita)
Gallbladder and biliary tract cancer	Male	15-19 years	95+ years	1	Cumulative Cigarettes (10 Years)
Gallbladder and biliary tract cancer	Male	15-19 years	95+ years	1	Cumulative Cigarettes (5 Years)
Gallbladder and biliary tract cancer	Male	15-19 years	95+ years	1	Diabetes Age-Standardized Prevalence (proportion)
Gallbladder and biliary tract cancer	Male	15-19 years	95+ years	-1	Education (years per capita)
Gallbladder and biliary tract cancer	Male	15-19 years	95+ years	0	LDI (I$ per capita)
Gallbladder and biliary tract cancer	Male	15-19 years	95+ years	1	Mean BMI
Gallbladder and biliary tract cancer	Male	15-19 years	95+ years	1	Smoking Prevalence
Gallbladder and biliary tract cancer	Male	15-19 years	95+ years	-1	Health System Access (capped)
Gallbladder and biliary tract cancer	Male	15-19 years	95+ years	1	Log-transformed SEV scalar: Gallblad C
Gallbladder and biliary tract cancer	Male	15-19 years	95+ years	0	Socio-demographic Index
Gallbladder and biliary tract cancer	Male	15-19 years	95+ years	-1	fruits adjusted(g)
Gallbladder and biliary tract cancer	Male	15-19 years	95+ years	-1	vegetables adjusted(g)
Gallbladder and biliary tract cancer	Male	15-19 years	95+ years	-1	Healthcare access and quality index
Pancreatic cancer	Male	15-19 years	95+ years	1	Alcohol (liters per capita)
Pancreatic cancer	Male	15-19 years	95+ years	1	Tobacco (cigarettes per capita)
Pancreatic cancer	Male	15-19 years	95+ years	1	Cumulative Cigarettes (10 Years)
Pancreatic cancer	Male	15-19 years	95+ years	1	Cumulative Cigarettes (20 Years)
Pancreatic cancer	Male	15-19 years	95+ years	1	Cumulative Cigarettes (5 Years)
Pancreatic cancer	Male	15-19 years	95+ years	1	Diabetes Age-Standardized Prevalence (proportion)
Pancreatic cancer	Male	15-19 years	95+ years	-1	Education (years per capita)
Pancreatic cancer	Male	15-19 years	95+ years	0	LDI (I$ per capita)
Pancreatic cancer	Male	15-19 years	95+ years	1	Mean BMI
Pancreatic cancer	Male	15-19 years	95+ years	1	Percent of total calories consumed as saturated fat
Pancreatic cancer	Male	15-19 years	95+ years	1	Smoking Prevalence
Pancreatic cancer	Male	15-19 years	95+ years	1	Total Calories (kcal per capita)
Pancreatic cancer	Male	15-19 years	95+ years	1	Log-transformed SEV scalar: Pancreas C
Pancreatic cancer	Male	15-19 years	95+ years	0	Socio-demographic Index
Pancreatic cancer	Male	15-19 years	95+ years	-1	fruits adjusted(g)
Pancreatic cancer	Male	15-19 years	95+ years	1	red meats adjusted(g)

Cause	Sex	Age start	Age end	Direction	Covariate
Pancreatic cancer	Male	15-19 years	95+ years	-1	vegetables adjusted(g)
Pancreatic cancer	Male	15-19 years	95+ years	-1	Healthcare access and quality index
Pancreatic cancer	Female	15-19 years	95+ years	1	Alcohol (liters per capita)
Pancreatic cancer	Female	15-19 years	95+ years	1	Tobacco (cigarettes per capita)
Pancreatic cancer	Female	15-19 years	95+ years	1	Cumulative Cigarettes (10 Years)
Pancreatic cancer	Female	15-19 years	95+ years	1	Cumulative Cigarettes (20 Years)
Pancreatic cancer	Female	15-19 years	95+ years	1	Cumulative Cigarettes (5 Years)
Pancreatic cancer	Female	15-19 years	95+ years	1	Diabetes Age-Standardized Prevalence (proportion)
Pancreatic cancer	Female	15-19 years	95+ years	-1	Education (years per capita)
Pancreatic cancer	Female	15-19 years	95+ years	0	LDI (I$ per capita)
Pancreatic cancer	Female	15-19 years	95+ years	1	Mean BMI
Pancreatic cancer	Female	15-19 years	95+ years	1	Percent of total calories consumed as saturated fat
Pancreatic cancer	Female	15-19 years	95+ years	1	Smoking Prevalence
Pancreatic cancer	Female	15-19 years	95+ years	1	Log-transformed SEV scalar: Pancreas C
Pancreatic cancer	Female	15-19 years	95+ years	0	Socio-demographic Index
Pancreatic cancer	Female	15-19 years	95+ years	-1	fruits adjusted(g)
Pancreatic cancer	Female	15-19 years	95+ years	1	red meats adjusted(g)
Pancreatic cancer	Female	15-19 years	95+ years	-1	vegetables adjusted(g)
Pancreatic cancer	Female	15-19 years	95+ years	1	energy unadjusted(kcal)
Pancreatic cancer	Female	15-19 years	95+ years	-1	Healthcare access and quality index
Pancreatic cancer	Male	15-19 years	95+ years	1	energy unadjusted(kcal)
Pancreatic cancer	Female	15-19 years	95+ years	1	vegetables adjusted(g)
Pancreatic cancer	Female	15-19 years	95+ years	-1	vegetables unadjusted(g)
Malignant skin melanoma	Male	15-19 years	95+ years	1	Alcohol (liters per capita)
Malignant skin melanoma	Male	15-19 years	95+ years	-1	Education (years per capita)
Malignant skin melanoma	Male	15-19 years	95+ years	0	LDI (I$ per capita)
Malignant skin melanoma	Male	15-19 years	95+ years	0	Latitude Under 15 (proportion)
Malignant skin melanoma	Male	15-19 years	95+ years	0	Latitude 15 to 30 (proportion)
Malignant skin melanoma	Male	15-19 years	95+ years	-1	Latitude 30 to 45 (proportion)
Malignant skin melanoma	Male	15-19 years	95+ years	-1	Latitude Over 45 (proportion)
Malignant skin melanoma	Male	15-19 years	95+ years	0	Socio-demographic Index
Malignant skin melanoma	Male	15-19 years	95+ years	-1	fruits adjusted(g)
Malignant skin melanoma	Male	15-19 years	95+ years	-1	vegetables adjusted(g)
Malignant skin melanoma	Male	15-19 years	95+ years	-1	Healthcare access and quality index
Malignant skin melanoma	Female	15-19 years	95+ years	1	Alcohol (liters per capita)
Malignant skin melanoma	Female	15-19 years	95+ years	-1	Education (years per capita)
Malignant skin melanoma	Female	15-19 years	95+ years	0	LDI (I$ per capita)
Malignant skin melanoma	Female	15-19 years	95+ years	0	Latitude Under 15 (proportion)
Malignant skin melanoma	Female	15-19 years	95+ years	0	Latitude 15 to 30 (proportion)
Malignant skin melanoma	Female	15-19 years	95+ years	-1	Latitude 30 to 45 (proportion)
Malignant skin melanoma	Female	15-19 years	95+ years	-1	Latitude Over 45 (proportion)
Malignant skin melanoma	Female	15-19 years	95+ years	0	Socio-demographic Index
Malignant skin melanoma	Female	15-19 years	95+ years	-1	fruits adjusted(g)
Malignant skin melanoma	Female	15-19 years	95+ years	-1	vegetables adjusted(g)
Malignant skin melanoma	Female	15-19 years	95+ years	-1	Healthcare access and quality index
Non-melanoma skin cancer	Male	15-19 years	95+ years	1	Cumulative Cigarettes (10 Years)
Non-melanoma skin cancer	Male	15-19 years	95+ years	1	Cumulative Cigarettes (15 Years)
Non-melanoma skin cancer	Male	15-19 years	95+ years	1	Cumulative Cigarettes (5 Years)
Non-melanoma skin cancer	Male	15-19 years	95+ years	-1	Education (years per capita)
Non-melanoma skin cancer	Male	15-19 years	95+ years	0	Average latitude
Non-melanoma skin cancer	Male	15-19 years	95+ years	0	LDI (I$ per capita)
Non-melanoma skin cancer	Male	15-19 years	95+ years	1	Smoking Prevalence
Non-melanoma skin cancer	Male	15-19 years	95+ years	0	Socio-demographic Index
Non-melanoma skin cancer	Male	15-19 years	95+ years	-1	Healthcare access and quality index
Non-melanoma skin cancer	Male	15-19 years	95+ years	-1	Health System Access (capped)
Non-melanoma skin cancer	Female	15-19 years	95+ years	1	Cumulative Cigarettes (10 Years)
Non-melanoma skin cancer	Female	15-19 years	95+ years	1	Cumulative Cigarettes (15 Years)
Non-melanoma skin cancer	Female	15-19 years	95+ years	1	Cumulative Cigarettes (5 Years)
Non-melanoma skin cancer	Female	15-19 years	95+ years	-1	Education (years per capita)
Non-melanoma skin cancer	Female	15-19 years	95+ years	0	Average latitude
Non-melanoma skin cancer	Female	15-19 years	95+ years	0	LDI (I$ per capita)
Non-melanoma skin cancer	Female	15-19 years	95+ years	1	Smoking Prevalence
Non-melanoma skin cancer	Female	15-19 years	95+ years	0	Socio-demographic Index
Non-melanoma skin cancer	Female	15-19 years	95+ years	-1	Healthcare access and quality index
Non-melanoma skin cancer	Female	15-19 years	95+ years	-1	Health System Access (capped)
Ovarian cancer	Female	15-19 years	95+ years	1	Alcohol (liters per capita)
Ovarian cancer	Female	15-19 years	95+ years	1	Tobacco (cigarettes per capita)
Ovarian cancer	Female	15-19 years	95+ years	-1	Contraception (Modern) Prevalence (proportion)
Ovarian cancer	Female	15-19 years	95+ years	1	Cumulative Cigarettes (20 Years)
Ovarian cancer	Female	15-19 years	95+ years	1	Diabetes Age-Standardized Prevalence (proportion)
Ovarian cancer	Female	15-19 years	95+ years	-1	Education (years per capita)
Ovarian cancer	Female	15-19 years	95+ years	0	LDI (I$ per capita)
Ovarian cancer	Female	15-19 years	95+ years	1	Mean BMI
Ovarian cancer	Female	15-19 years	95+ years	1	Percent of total calories consumed as saturated fat
Ovarian cancer	Female	15-19 years	95+ years	1	Smoking Prevalence
Ovarian cancer	Female	15-19 years	95+ years	0	Total Fertility Rate
Ovarian cancer	Female	15-19 years	95+ years	1	Log-transformed SEV scalar: Ovary C
Ovarian cancer	Female	15-19 years	95+ years	0	Socio-demographic Index
Ovarian cancer	Female	15-19 years	95+ years	-1	fruits adjusted(g)
Ovarian cancer	Female	15-19 years	95+ years	-1	vegetables adjusted(g)
Ovarian cancer	Female	15-19 years	95+ years	1	energy unadjusted(kcal)
Ovarian cancer	Female	15-19 years	95+ years	-1	Healthcare access and quality index
Testicular cancer	Male	15-19 years	95+ years	1	Cumulative Cigarettes (10 Years)
Testicular cancer	Male	15-19 years	95+ years	1	Cumulative Cigarettes (15 Years)
Testicular cancer	Male	15-19 years	95+ years	1	Cumulative Cigarettes (5 Years)
Testicular cancer	Male	15-19 years	95+ years	-1	Education (years per capita)
Testicular cancer	Male	15-19 years	95+ years	-1	Fruits (kcal per capita)
Testicular cancer	Male	15-19 years	95+ years	-1	Health System Access 2 (unitless)
Testicular cancer	Male	15-19 years	95+ years	-1	LDI (I$ per capita)
Testicular cancer	Male	15-19 years	95+ years	-1	Vegetables (kcal per capita)
Testicular cancer	Male	15-19 years	95+ years	0	Socio-demographic Index
Testicular cancer	Male	15-19 years	95+ years	-1	Healthcare access and quality index

Cause	Sex	Age start	Age end	Direction	Covariate
Kidney cancer	Male	0-6 days	95+ years	1	Alcohol (liters per capita)
Kidney cancer	Male	0-6 days	95+ years	1	Cumulative Cigarettes (10 Years)
Kidney cancer	Male	0-6 days	95+ years	1	Cumulative Cigarettes (15 Years)
Kidney cancer	Male	0-6 days	95+ years	1	Cumulative Cigarettes (5 Years)
Kidney cancer	Male	0-6 days	95+ years	1	Diabetes Age-Standardized Prevalence (proportion)
Kidney cancer	Male	0-6 days	95+ years	-1	Education (years per capita)
Kidney cancer	Male	0-6 days	95+ years	-1	Health System Access 2 (unitless)
Kidney cancer	Male	0-6 days	95+ years	0	LDI (I$ per capita)
Kidney cancer	Male	0-6 days	95+ years	1	Mean BMI
Kidney cancer	Male	0-6 days	95+ years	1	Systolic Blood Pressure (mmHg)
Kidney cancer	Male	0-6 days	95+ years	1	Smoking Prevalence
Kidney cancer	Male	0-6 days	95+ years	1	Log-transformed SEV scalar: Kidney C
Kidney cancer	Male	0-6 days	95+ years	0	Socio-demographic Index
Kidney cancer	Female	0-6 days	95+ years	1	Alcohol (liters per capita)
Kidney cancer	Female	0-6 days	95+ years	1	Cumulative Cigarettes (10 Years)
Kidney cancer	Female	0-6 days	95+ years	1	Cumulative Cigarettes (15 Years)
Kidney cancer	Female	0-6 days	95+ years	1	Cumulative Cigarettes (5 Years)
Kidney cancer	Female	0-6 days	95+ years	1	Diabetes Age-Standardized Prevalence (proportion)
Kidney cancer	Female	0-6 days	95+ years	-1	Education (years per capita)
Kidney cancer	Female	0-6 days	95+ years	-1	Health System Access 2 (unitless)
Kidney cancer	Female	0-6 days	95+ years	-1	LDI (I$ per capita)
Kidney cancer	Female	0-6 days	95+ years	1	Mean BMI
Kidney cancer	Female	0-6 days	95+ years	1	Systolic Blood Pressure (mmHg)
Kidney cancer	Female	0-6 days	95+ years	1	Smoking Prevalence
Kidney cancer	Female	0-6 days	95+ years	0	Total Fertility Rate
Kidney cancer	Female	0-6 days	95+ years	1	Total Calories (kcal per capita)
Kidney cancer	Female	0-6 days	95+ years	1	Log-transformed SEV scalar: Kidney C
Kidney cancer	Female	0-6 days	95+ years	1	Socio-demographic Index
Kidney cancer	Female	0-6 days	95+ years	0	LDI (I$ per capita)
Kidney cancer	Female	0-6 days	95+ years	0	Socio-demographic Index
Bladder cancer	Male	15-19 years	95+ years	1	Alcohol (liters per capita)
Bladder cancer	Male	15-19 years	95+ years	1	Cumulative Cigarettes (10 Years)
Bladder cancer	Male	15-19 years	95+ years	1	Cumulative Cigarettes (15 Years)
Bladder cancer	Male	15-19 years	95+ years	1	Cumulative Cigarettes (5 Years)
Bladder cancer	Male	15-19 years	95+ years	-1	Education (years per capita)
Bladder cancer	Male	15-19 years	95+ years	0	LDI (I$ per capita)
Bladder cancer	Male	15-19 years	95+ years	1	Population Density (over 1000 ppl/sqkm, proportion)
Bladder cancer	Male	15-19 years	95+ years	1	Population Density (under 150 ppl/sqkm, proportion)
Bladder cancer	Male	15-19 years	95+ years	1	Smoking Prevalence
Bladder cancer	Male	15-19 years	95+ years	1	Log-transformed SEV scalar: Bladder C
Bladder cancer	Male	15-19 years	95+ years	0	Socio-demographic Index
Bladder cancer	Male	15-19 years	95+ years	-1	fruits adjusted(g)
Bladder cancer	Male	15-19 years	95+ years	-1	vegetables adjusted(g)
Bladder cancer	Male	15-19 years	95+ years	-1	Healthcare access and quality index
Bladder cancer	Female	15-19 years	95+ years	1	Alcohol (liters per capita)
Bladder cancer	Female	15-19 years	95+ years	1	Cumulative Cigarettes (10 Years)
Bladder cancer	Female	15-19 years	95+ years	1	Cumulative Cigarettes (15 Years)
Bladder cancer	Female	15-19 years	95+ years	1	Cumulative Cigarettes (5 Years)
Bladder cancer	Female	15-19 years	95+ years	-1	Education (years per capita)
Bladder cancer	Female	15-19 years	95+ years	0	LDI (I$ per capita)
Bladder cancer	Female	15-19 years	95+ years	1	Population Density (over 1000 ppl/sqkm, proportion)
Bladder cancer	Female	15-19 years	95+ years	1	Population Density (under 150 ppl/sqkm, proportion)
Bladder cancer	Female	15-19 years	95+ years	1	Smoking Prevalence
Bladder cancer	Female	15-19 years	95+ years	1	Log-transformed SEV scalar: Bladder C
Bladder cancer	Female	15-19 years	95+ years	0	Socio-demographic Index
Bladder cancer	Female	15-19 years	95+ years	-1	fruits adjusted(g)
Bladder cancer	Female	15-19 years	95+ years	-1	vegetables adjusted(g)
Bladder cancer	Female	15-19 years	95+ years	-1	Healthcare access and quality index
Brain and nervous system cancer	Female	0-6 days	95+ years	1	Alcohol (liters per capita)
Brain and nervous system cancer	Female	0-6 days	95+ years	1	Cumulative Cigarettes (10 Years)
Brain and nervous system cancer	Female	0-6 days	95+ years	1	Cumulative Cigarettes (15 Years)
Brain and nervous system cancer	Female	0-6 days	95+ years	-1	Education (years per capita)
Brain and nervous system cancer	Female	0-6 days	95+ years	0	LDI (I$ per capita)
Brain and nervous system cancer	Female	0-6 days	95+ years	1	Cholesterol (total, mean per capita)
Brain and nervous system cancer	Female	0-6 days	95+ years	1	Systolic Blood Pressure (mmHg)
Brain and nervous system cancer	Female	0-6 days	95+ years	1	Percent of total calories consumed as saturated fat
Brain and nervous system cancer	Female	0-6 days	95+ years	1	Smoking Prevalence
Brain and nervous system cancer	Female	0-6 days	95+ years	0	Socio-demographic Index
Brain and nervous system cancer	Female	0-6 days	95+ years	-1	fruits adjusted(g)
Brain and nervous system cancer	Female	0-6 days	95+ years	1	red meats adjusted(g)
Brain and nervous system cancer	Female	0-6 days	95+ years	-1	vegetables adjusted(g)
Brain and nervous system cancer	Female	0-6 days	95+ years	-1	Healthcare access and quality index
Brain and nervous system cancer	Male	0-6 days	95+ years	1	Alcohol (liters per capita)
Brain and nervous system cancer	Male	0-6 days	95+ years	1	Cumulative Cigarettes (10 Years)
Brain and nervous system cancer	Male	0-6 days	95+ years	1	Cumulative Cigarettes (15 Years)
Brain and nervous system cancer	Male	0-6 days	95+ years	-1	Education (years per capita)
Brain and nervous system cancer	Male	0-6 days	95+ years	0	LDI (I$ per capita)
Brain and nervous system cancer	Male	0-6 days	95+ years	1	Cholesterol (total, mean per capita)
Brain and nervous system cancer	Male	0-6 days	95+ years	1	Systolic Blood Pressure (mmHg)
Brain and nervous system cancer	Male	0-6 days	95+ years	1	Percent of total calories consumed as saturated fat
Brain and nervous system cancer	Male	0-6 days	95+ years	1	Smoking Prevalence
Brain and nervous system cancer	Male	0-6 days	95+ years	-1	Vegetables (kcal per capita)
Brain and nervous system cancer	Male	0-6 days	95+ years	0	Socio-demographic Index
Brain and nervous system cancer	Male	0-6 days	95+ years	-1	fruits adjusted(g)
Brain and nervous system cancer	Male	0-6 days	95+ years	1	red meats adjusted(g)
Brain and nervous system cancer	Male	0-6 days	95+ years	-1	vegetables adjusted(g)
Brain and nervous system cancer	Male	0-6 days	95+ years	-1	Healthcare access and quality index
Thyroid cancer	Female	10-14 years	95+ years	1	Alcohol (liters per capita)
Thyroid cancer	Female	10-14 years	95+ years	1	Tobacco (cigarettes per capita)
Thyroid cancer	Female	10-14 years	95+ years	-1	Education (years per capita)
Thyroid cancer	Female	10-14 years	95+ years	0	LDI (I$ per capita)
Thyroid cancer	Female	10-14 years	95+ years	1	Mean BMI

Cause	Sex	Age start	Age end	Direction	Covariate
Thyroid cancer	Female	10-14 years	95+ years	-1	Sanitation (proportion with access)
Thyroid cancer	Female	10-14 years	95+ years	1	Smoking Prevalence
Thyroid cancer	Female	10-14 years	95+ years	-1	Improved Water Source (proportion with access)
Thyroid cancer	Female	10-14 years	95+ years	1	Log-transformed SEV scalar: Thyroid C
Thyroid cancer	Female	10-14 years	95+ years	0	Socio-demographic Index
Thyroid cancer	Female	10-14 years	95+ years	-1	fruits adjusted(g)
Thyroid cancer	Female	10-14 years	95+ years	1	red meats adjusted(g)
Thyroid cancer	Female	10-14 years	95+ years	-1	vegetables adjusted(g)
Thyroid cancer	Female	10-14 years	95+ years	-1	Healthcare access and quality index
Thyroid cancer	Male	10-14 years	95+ years	1	Alcohol (liters per capita)
Thyroid cancer	Male	10-14 years	95+ years	1	Tobacco (cigarettes per capita)
Thyroid cancer	Male	10-14 years	95+ years	-1	Education (years per capita)
Thyroid cancer	Male	10-14 years	95+ years	0	LDI (I$ per capita)
Thyroid cancer	Male	10-14 years	95+ years	1	Mean BMI
Thyroid cancer	Male	10-14 years	95+ years	-1	Sanitation (proportion with access)
Thyroid cancer	Male	10-14 years	95+ years	1	Smoking Prevalence
Thyroid cancer	Male	10-14 years	95+ years	-1	Improved Water Source (proportion with access)
Thyroid cancer	Male	10-14 years	95+ years	1	Log-transformed SEV scalar: Thyroid C
Thyroid cancer	Male	10-14 years	95+ years	0	Socio-demographic Index
Thyroid cancer	Male	10-14 years	95+ years	-1	fruits adjusted(g)
Thyroid cancer	Male	10-14 years	95+ years	1	red meats adjusted(g)
Thyroid cancer	Male	10-14 years	95+ years	-1	vegetables adjusted(g)
Thyroid cancer	Male	10-14 years	95+ years	-1	Healthcare access and quality index
Thyroid cancer	Male	10-14 years	95+ years	2	Smoking Prevalence
Mesothelioma	Female	15-19 years	95+ years	1	Asbestos production (binary)
Mesothelioma	Female	15-19 years	95+ years	1	Asbestos production (kg) per capita
Mesothelioma	Female	15-19 years	95+ years	1	Cumulative Cigarettes (5 Years)
Mesothelioma	Female	15-19 years	95+ years	-1	Education (years per capita)
Mesothelioma	Female	15-19 years	95+ years	1	Gold production (binary)
Mesothelioma	Female	15-19 years	95+ years	1	Gold production (kg) per capita
Mesothelioma	Female	15-19 years	95+ years	0	LDI (I$ per capita)
Mesothelioma	Female	15-19 years	95+ years	1	Indoor Air Pollution (All Cooking Fuels)
Mesothelioma	Female	15-19 years	95+ years	1	Elevation Over 1500m (proportion)
Mesothelioma	Female	15-19 years	95+ years	1	Elevation 500 to 1500m (proportion)
Mesothelioma	Female	15-19 years	95+ years	1	Population Density (over 1000 ppl/sqkm, proportion)
Mesothelioma	Female	15-19 years	95+ years	1	Population Over 65 (proportion)
Mesothelioma	Female	15-19 years	95+ years	1	Smoking Prevalence
Mesothelioma	Female	15-19 years	95+ years	1	Log-transformed SEV scalar: Mesothel
Mesothelioma	Female	15-19 years	95+ years	0	Socio-demographic Index
Mesothelioma	Female	15-19 years	95+ years	1	Asbestos consumption (metric tons per year per capita)
Mesothelioma	Female	15-19 years	95+ years	-1	Healthcare access and quality index
Mesothelioma	Male	15-19 years	95+ years	1	Cumulative Cigarettes (5 Years)
Mesothelioma	Male	15-19 years	95+ years	-1	Education (years per capita)
Mesothelioma	Male	15-19 years	95+ years	1	Gold production (binary)
Mesothelioma	Male	15-19 years	95+ years	1	Gold production (kg) per capita
Mesothelioma	Male	15-19 years	95+ years	0	LDI (I$ per capita)
Mesothelioma	Male	15-19 years	95+ years	1	Indoor Air Pollution (All Cooking Fuels)
Mesothelioma	Male	15-19 years	95+ years	1	Elevation Over 1500m (proportion)
Mesothelioma	Male	15-19 years	95+ years	1	Elevation 500 to 1500m (proportion)
Mesothelioma	Male	15-19 years	95+ years	1	Population Density (over 1000 ppl/sqkm, proportion)
Mesothelioma	Male	15-19 years	95+ years	1	Population Over 65 (proportion)
Mesothelioma	Male	15-19 years	95+ years	1	Smoking Prevalence
Mesothelioma	Male	15-19 years	95+ years	0	Socio-demographic Index
Mesothelioma	Male	15-19 years	95+ years	1	Asbestos consumption (metric tons per year per capita)
Mesothelioma	Male	15-19 years	95+ years	-1	Healthcare access and quality index
Hodgkin lymphoma	Male	0-6 days	95+ years	-1	Education (years per capita)
Hodgkin lymphoma	Male	0-6 days	95+ years	0	LDI (I$ per capita)
Hodgkin lymphoma	Male	0-6 days	95+ years	0	Socio-demographic Index
Hodgkin lymphoma	Male	0-6 days	95+ years	-1	Healthcare access and quality index
Hodgkin lymphoma	Female	0-6 days	95+ years	-1	Education (years per capita)
Hodgkin lymphoma	Female	0-6 days	95+ years	0	LDI (I$ per capita)
Hodgkin lymphoma	Female	0-6 days	95+ years	0	Socio-demographic Index
Hodgkin lymphoma	Female	0-6 days	95+ years	-1	Healthcare access and quality index
Non-Hodgkin lymphoma	Male	0-6 days	95+ years	1	Alcohol (liters per capita)
Non-Hodgkin lymphoma	Male	0-6 days	95+ years	1	Cumulative Cigarettes (10 Years)
Non-Hodgkin lymphoma	Male	0-6 days	95+ years	-1	Health System Access 2 (unitless)
Non-Hodgkin lymphoma	Male	0-6 days	95+ years	0	LDI (I$ per capita)
Non-Hodgkin lymphoma	Male	0-6 days	95+ years	1	Smoking Prevalence
Non-Hodgkin lymphoma	Male	0-6 days	95+ years	0	Socio-demographic Index
Non-Hodgkin lymphoma	Male	0-6 days	95+ years	-1	Health System Access (capped)
Non-Hodgkin lymphoma	Male	0-6 days	95+ years	-1	Healthcare access and quality index
Non-Hodgkin lymphoma	Female	0-6 days	95+ years	1	Alcohol (liters per capita)
Non-Hodgkin lymphoma	Female	0-6 days	95+ years	1	Cumulative Cigarettes (10 Years)
Non-Hodgkin lymphoma	Female	0-6 days	95+ years	-1	Health System Access 2 (unitless)
Non-Hodgkin lymphoma	Female	0-6 days	95+ years	0	LDI (I$ per capita)
Non-Hodgkin lymphoma	Female	0-6 days	95+ years	1	Smoking Prevalence
Non-Hodgkin lymphoma	Female	0-6 days	95+ years	0	Socio-demographic Index
Non-Hodgkin lymphoma	Female	0-6 days	95+ years	-1	Healthcare access and quality index
Non-Hodgkin lymphoma	Female	0-6 days	95+ years	0	Total Fertility Rate
Multiple myeloma	Male	15-19 years	95+ years	1	Alcohol (liters per capita)
Multiple myeloma	Male	15-19 years	95+ years	1	Tobacco (cigarettes per capita)
Multiple myeloma	Male	15-19 years	95+ years	-1	Education (years per capita)
Multiple myeloma	Male	15-19 years	95+ years	0	LDI (I$ per capita)
Multiple myeloma	Male	15-19 years	95+ years	1	Mean BMI
Multiple myeloma	Male	15-19 years	95+ years	-1	Sanitation (proportion with access)
Multiple myeloma	Male	15-19 years	95+ years	1	Smoking Prevalence
Multiple myeloma	Male	15-19 years	95+ years	-1	Improved Water Source (proportion with access)
Multiple myeloma	Male	15-19 years	95+ years	0	Socio-demographic Index
Multiple myeloma	Male	15-19 years	95+ years	-1	fruits adjusted(g)
Multiple myeloma	Male	15-19 years	95+ years	1	red meats adjusted(g)
Multiple myeloma	Male	15-19 years	95+ years	-1	vegetables adjusted(g)
Multiple myeloma	Male	15-19 years	95+ years	-1	Healthcare access and quality index

Cause	Sex	Age start	Age end	Direction	Covariate
Multiple myeloma	Female	15-19 years	95+ years	1	Alcohol (liters per capita)
Multiple myeloma	Female	15-19 years	95+ years	1	Tobacco (cigarettes per capita)
Multiple myeloma	Female	15-19 years	95+ years	-1	Education (years per capita)
Multiple myeloma	Female	15-19 years	95+ years	0	LDI (I$ per capita)
Multiple myeloma	Female	15-19 years	95+ years	1	Mean BMI
Multiple myeloma	Female	15-19 years	95+ years	-1	Sanitation (proportion with access)
Multiple myeloma	Female	15-19 years	95+ years	1	Smoking Prevalence
Multiple myeloma	Female	15-19 years	95+ years	-1	Improved Water Source (proportion with access)
Multiple myeloma	Female	15-19 years	95+ years	0	Socio-demographic Index
Multiple myeloma	Female	15-19 years	95+ years	-1	fruits adjusted(g)
Multiple myeloma	Female	15-19 years	95+ years	1	red meats adjusted(g)
Multiple myeloma	Female	15-19 years	95+ years	-1	vegetables adjusted(g)
Multiple myeloma	Female	15-19 years	95+ years	-1	Healthcare access and quality index
Leukemia	Female	0-6 days	95+ years	1	Alcohol (liters per capita)
Leukemia	Female	0-6 days	95+ years	1	Tobacco (cigarettes per capita)
Leukemia	Female	0-6 days	95+ years	1	Cumulative Cigarettes (10 Years)
Leukemia	Female	0-6 days	95+ years	1	Cumulative Cigarettes (15 Years)
Leukemia	Female	0-6 days	95+ years	1	Cumulative Cigarettes (20 Years)
Leukemia	Female	0-6 days	95+ years	1	Cumulative Cigarettes (5 Years)
Leukemia	Female	0-6 days	95+ years	-1	Education (years per capita)
Leukemia	Female	0-6 days	95+ years	-1	Health System Access 2 (unitless)
Leukemia	Female	0-6 days	95+ years	0	LDI (I$ per capita)
Leukemia	Female	0-6 days	95+ years	1	Smoking Prevalence
Leukemia	Female	0-6 days	95+ years	1	Log-transformed SEV scalar: Leukemia
Leukemia	Female	0-6 days	95+ years	1	Log-transformed age-standardized SEV scalar: Leukemia
Leukemia	Female	0-6 days	95+ years	0	Socio-demographic Index
Leukemia	Male	0-6 days	95+ years	1	Alcohol (liters per capita)
Leukemia	Male	0-6 days	95+ years	1	Tobacco (cigarettes per capita)
Leukemia	Male	0-6 days	95+ years	1	Cumulative Cigarettes (10 Years)
Leukemia	Male	0-6 days	95+ years	1	Cumulative Cigarettes (15 Years)
Leukemia	Male	0-6 days	95+ years	1	Cumulative Cigarettes (20 Years)
Leukemia	Male	0-6 days	95+ years	1	Cumulative Cigarettes (5 Years)
Leukemia	Male	0-6 days	95+ years	-1	Education (years per capita)
Leukemia	Male	0-6 days	95+ years	-1	Health System Access 2 (unitless)
Leukemia	Male	0-6 days	95+ years	0	LDI (I$ per capita)
Leukemia	Male	0-6 days	95+ years	1	Smoking Prevalence
Leukemia	Male	0-6 days	95+ years	1	Log-transformed SEV scalar: Leukemia
Leukemia	Male	0-6 days	95+ years	1	Log-transformed age-standardized SEV scalar: Leukemia
Leukemia	Male	0-6 days	95+ years	0	Socio-demographic Index
Leukemia	Female	0-6 days	95+ years	-1	Health System Access (capped)
Leukemia	Female	0-6 days	95+ years	-1	Healthcare access and quality index
Other neoplasms	Male	0-6 days	95+ years	1	Tobacco (cigarettes per capita)
Other neoplasms	Male	0-6 days	95+ years	-1	Education (years per capita)
Other neoplasms	Male	0-6 days	95+ years	-1	Health System Access 2 (unitless)
Other neoplasms	Male	0-6 days	95+ years	0	LDI (I$ per capita)
Other neoplasms	Male	0-6 days	95+ years	-1	Nuts & Seeds (kcal per capita)
Other neoplasms	Male	0-6 days	95+ years	1	Smoking Prevalence
Other neoplasms	Male	0-6 days	95+ years	0	Socio-demographic Index
Other neoplasms	Male	0-6 days	95+ years	-1	fruits adjusted(g)
Other neoplasms	Male	0-6 days	95+ years	-1	pufa adjusted(percent)
Other neoplasms	Male	0-6 days	95+ years	-1	vegetables adjusted(g)
Other neoplasms	Male	0-6 days	95+ years	-1	Healthcare access and quality index
Other neoplasms	Female	0-6 days	95+ years	1	Tobacco (cigarettes per capita)
Other neoplasms	Female	0-6 days	95+ years	-1	Education (years per capita)
Other neoplasms	Female	0-6 days	95+ years	-1	Health System Access 2 (unitless)
Other neoplasms	Female	0-6 days	95+ years	0	LDI (I$ per capita)
Other neoplasms	Female	0-6 days	95+ years	1	Smoking Prevalence
Other neoplasms	Female	0-6 days	95+ years	0	Socio-demographic Index
Other neoplasms	Female	0-6 days	95+ years	-1	fruits adjusted(g)
Other neoplasms	Female	0-6 days	95+ years	-1	nuts seeds adjusted(g)
Other neoplasms	Female	0-6 days	95+ years	-1	pufa adjusted(percent)
Other neoplasms	Female	0-6 days	95+ years	-1	vegetables adjusted(g)
Other neoplasms	Female	0-6 days	95+ years	-1	Healthcare access and quality index
Other neoplasms	Male	0-6 days	95+ years	-1	nuts seeds adjusted(g)
Cardiovascular diseases	Female	0-6 days	95+ years	0	Alcohol (liters per capita)
Cardiovascular diseases	Female	0-6 days	95+ years	1	Diabetes Fasting Plasma Glucose (mmol/L)
Cardiovascular diseases	Female	0-6 days	95+ years	-1	LDI (I$ per capita)
Cardiovascular diseases	Female	0-6 days	95+ years	1	Mean BMI
Cardiovascular diseases	Female	0-6 days	95+ years	1	Cholesterol (total, mean per capita)
Cardiovascular diseases	Female	0-6 days	95+ years	1	Systolic Blood Pressure (mmHg)
Cardiovascular diseases	Female	0-6 days	95+ years	1	Indoor Air Pollution (All Cooking Fuels)
Cardiovascular diseases	Female	0-6 days	95+ years	1	Outdoor Air Pollution (PM2.5)
Cardiovascular diseases	Female	0-6 days	95+ years	-1	Elevation Over 1500m (proportion)
Cardiovascular diseases	Female	0-6 days	95+ years	1	Smoking Prevalence
Cardiovascular diseases	Female	0-6 days	95+ years	0	Socio-demographic Index
Cardiovascular diseases	Female	0-6 days	95+ years	-1	omega 3 adjusted(g)
Cardiovascular diseases	Female	0-6 days	95+ years	-1	fruits adjusted(g)
Cardiovascular diseases	Female	0-6 days	95+ years	-1	nuts seeds adjusted(g)
Cardiovascular diseases	Female	0-6 days	95+ years	-1	pufa adjusted(percent)
Cardiovascular diseases	Female	0-6 days	95+ years	-1	pulses legumes adjusted(g)
Cardiovascular diseases	Female	0-6 days	95+ years	-1	vegetables adjusted(g)
Cardiovascular diseases	Female	0-6 days	95+ years	-1	whole grains adjusted(g)
Cardiovascular diseases	Female	0-6 days	95+ years	1	Log-transformed SEV scalar: CVD
Cardiovascular diseases	Female	0-6 days	95+ years	-1	Healthcare access and quality index
Cardiovascular diseases	Female	0-6 days	95+ years	1	Diet high in trans fatty acids
Cardiovascular diseases	Male	0-6 days	95+ years	0	Alcohol (liters per capita)
Cardiovascular diseases	Male	0-6 days	95+ years	1	Diabetes Fasting Plasma Glucose (mmol/L)
Cardiovascular diseases	Male	0-6 days	95+ years	-1	LDI (I$ per capita)
Cardiovascular diseases	Male	0-6 days	95+ years	1	Mean BMI
Cardiovascular diseases	Male	0-6 days	95+ years	1	Cholesterol (total, mean per capita)
Cardiovascular diseases	Male	0-6 days	95+ years	1	Systolic Blood Pressure (mmHg)
Cardiovascular diseases	Male	0-6 days	95+ years	1	Indoor Air Pollution (All Cooking Fuels)

Cause	Sex	Age start	Age end	Direction	Covariate
Cardiovascular diseases	Male	0-6 days	95+ years	1	Outdoor Air Pollution (PM2.5)
Cardiovascular diseases	Male	0-6 days	95+ years	-1	Elevation Over 1500m (proportion)
Cardiovascular diseases	Male	0-6 days	95+ years	1	Smoking Prevalence
Cardiovascular diseases	Male	0-6 days	95+ years	0	Socio-demographic Index
Cardiovascular diseases	Male	0-6 days	95+ years	-1	omega 3 adjusted(g)
Cardiovascular diseases	Male	0-6 days	95+ years	-1	fruits adjusted(g)
Cardiovascular diseases	Male	0-6 days	95+ years	-1	nuts seeds adjusted(g)
Cardiovascular diseases	Male	0-6 days	95+ years	-1	pufa adjusted(percent)
Cardiovascular diseases	Male	0-6 days	95+ years	-1	pulses legumes adjusted(g)
Cardiovascular diseases	Male	0-6 days	95+ years	-1	vegetables adjusted(g)
Cardiovascular diseases	Male	0-6 days	95+ years	-1	whole grains adjusted(g)
Cardiovascular diseases	Male	0-6 days	95+ years	1	Log-transformed SEV scalar: CVD
Cardiovascular diseases	Male	0-6 days	95+ years	-1	Healthcare access and quality index
Cardiovascular diseases	Male	0-6 days	95+ years	1	Diet high in trans fatty acids
Rheumatic heart disease	Male	1-4 years	95+ years	-1	Education (years per capita)
Rheumatic heart disease	Male	1-4 years	95+ years	-1	LDI (I$ per capita)
Rheumatic heart disease	Male	1-4 years	95+ years	1	Underweight (proportion <2SD weight for age, <5 years)
Rheumatic heart disease	Male	1-4 years	95+ years	-1	Sanitation (proportion with access)
Rheumatic heart disease	Male	1-4 years	95+ years	-1	Improved Water Source (proportion with access)
Rheumatic heart disease	Male	1-4 years	95+ years	1	Log-transformed SEV scalar: RHD
Rheumatic heart disease	Male	1-4 years	95+ years	-1	Socio-demographic Index
Rheumatic heart disease	Male	1-4 years	95+ years	-1	Healthcare access and quality index
Rheumatic heart disease	Female	1-4 years	95+ years	-1	Education (years per capita)
Rheumatic heart disease	Female	1-4 years	95+ years	-1	LDI (I$ per capita)
Rheumatic heart disease	Female	1-4 years	95+ years	1	Underweight (proportion <2SD weight for age, <5 years)
Rheumatic heart disease	Female	1-4 years	95+ years	-1	Sanitation (proportion with access)
Rheumatic heart disease	Female	1-4 years	95+ years	-1	Improved Water Source (proportion with access)
Rheumatic heart disease	Female	1-4 years	95+ years	1	Log-transformed SEV scalar: RHD
Rheumatic heart disease	Female	1-4 years	95+ years	-1	Socio-demographic Index
Rheumatic heart disease	Female	1-4 years	95+ years	-1	Healthcare access and quality index
Ischemic heart disease	Male	28-364 days	95+ years	0	Alcohol (liters per capita)
Ischemic heart disease	Male	28-364 days	95+ years	1	Diabetes Fasting Plasma Glucose (mmol/L)
Ischemic heart disease	Male	28-364 days	95+ years	-1	LDI (I$ per capita)
Ischemic heart disease	Male	28-364 days	95+ years	1	Mean BMI
Ischemic heart disease	Male	28-364 days	95+ years	1	Cholesterol (total, mean per capita)
Ischemic heart disease	Male	28-364 days	95+ years	1	Systolic Blood Pressure (mmHg)
Ischemic heart disease	Male	28-364 days	95+ years	1	Indoor Air Pollution (All Cooking Fuels)
Ischemic heart disease	Male	28-364 days	95+ years	1	Outdoor Air Pollution (PM2.5)
Ischemic heart disease	Male	28-364 days	95+ years	-1	Elevation Over 1500m (proportion)
Ischemic heart disease	Male	28-364 days	95+ years	1	Smoking Prevalence
Ischemic heart disease	Male	28-364 days	95+ years	1	Log-transformed SEV scalar: IHD
Ischemic heart disease	Male	28-364 days	95+ years	0	Socio-demographic Index
Ischemic heart disease	Male	28-364 days	95+ years	-1	omega 3 adjusted(g)
Ischemic heart disease	Male	28-364 days	95+ years	-1	fruits adjusted(g)
Ischemic heart disease	Male	28-364 days	95+ years	-1	nuts seeds adjusted(g)
Ischemic heart disease	Male	28-364 days	95+ years	-1	pufa adjusted(percent)
Ischemic heart disease	Male	28-364 days	95+ years	-1	pulses legumes adjusted(g)
Ischemic heart disease	Male	28-364 days	95+ years	-1	vegetables adjusted(g)
Ischemic heart disease	Male	28-364 days	95+ years	-1	whole grains adjusted(g)
Ischemic heart disease	Male	28-364 days	95+ years	-1	Healthcare access and quality index
Ischemic heart disease	Male	28-364 days	95+ years	1	Diet high in trans fatty acids
Ischemic heart disease	Female	28-364 days	95+ years	0	Alcohol (liters per capita)
Ischemic heart disease	Female	28-364 days	95+ years	1	Diabetes Fasting Plasma Glucose (mmol/L)
Ischemic heart disease	Female	28-364 days	95+ years	-1	LDI (I$ per capita)
Ischemic heart disease	Female	28-364 days	95+ years	1	Mean BMI
Ischemic heart disease	Female	28-364 days	95+ years	1	Cholesterol (total, mean per capita)
Ischemic heart disease	Female	28-364 days	95+ years	1	Systolic Blood Pressure (mmHg)
Ischemic heart disease	Female	28-364 days	95+ years	1	Indoor Air Pollution (All Cooking Fuels)
Ischemic heart disease	Female	28-364 days	95+ years	1	Outdoor Air Pollution (PM2.5)
Ischemic heart disease	Female	28-364 days	95+ years	-1	Elevation Over 1500m (proportion)
Ischemic heart disease	Female	28-364 days	95+ years	1	Smoking Prevalence
Ischemic heart disease	Female	28-364 days	95+ years	1	Log-transformed SEV scalar: IHD
Ischemic heart disease	Female	28-364 days	95+ years	0	Socio-demographic Index
Ischemic heart disease	Female	28-364 days	95+ years	-1	omega 3 adjusted(g)
Ischemic heart disease	Female	28-364 days	95+ years	-1	fruits adjusted(g)
Ischemic heart disease	Female	28-364 days	95+ years	-1	nuts seeds adjusted(g)
Ischemic heart disease	Female	28-364 days	95+ years	-1	pufa adjusted(percent)
Ischemic heart disease	Female	28-364 days	95+ years	-1	pulses legumes adjusted(g)
Ischemic heart disease	Female	28-364 days	95+ years	-1	vegetables adjusted(g)
Ischemic heart disease	Female	28-364 days	95+ years	-1	whole grains adjusted(g)
Ischemic heart disease	Female	28-364 days	95+ years	-1	Healthcare access and quality index
Ischemic heart disease	Female	28-364 days	95+ years	1	Diet high in trans fatty acids
Cerebrovascular disease	Male	0-6 days	95+ years	0	Alcohol (liters per capita)
Cerebrovascular disease	Male	0-6 days	95+ years	1	Diabetes Fasting Plasma Glucose (mmol/L)
Cerebrovascular disease	Male	0-6 days	95+ years	-1	LDI (I$ per capita)
Cerebrovascular disease	Male	0-6 days	95+ years	1	Mean BMI
Cerebrovascular disease	Male	0-6 days	95+ years	1	Cholesterol (total, mean per capita)
Cerebrovascular disease	Male	0-6 days	95+ years	1	Systolic Blood Pressure (mmHg)
Cerebrovascular disease	Male	0-6 days	95+ years	1	Indoor Air Pollution (All Cooking Fuels)
Cerebrovascular disease	Male	0-6 days	95+ years	1	Outdoor Air Pollution (PM2.5)
Cerebrovascular disease	Male	0-6 days	95+ years	-1	Elevation Over 1500m (proportion)
Cerebrovascular disease	Male	0-6 days	95+ years	1	Smoking Prevalence
Cerebrovascular disease	Male	0-6 days	95+ years	1	Log-transformed SEV scalar: Stroke
Cerebrovascular disease	Male	0-6 days	95+ years	0	Socio-demographic Index
Cerebrovascular disease	Male	0-6 days	95+ years	-1	omega 3 adjusted(g)
Cerebrovascular disease	Male	0-6 days	95+ years	-1	fruits adjusted(g)
Cerebrovascular disease	Male	0-6 days	95+ years	-1	nuts seeds adjusted(g)
Cerebrovascular disease	Male	0-6 days	95+ years	-1	pufa adjusted(percent)
Cerebrovascular disease	Male	0-6 days	95+ years	-1	pulses legumes adjusted(g)
Cerebrovascular disease	Male	0-6 days	95+ years	-1	vegetables adjusted(g)
Cerebrovascular disease	Male	0-6 days	95+ years	-1	whole grains adjusted(g)
Cerebrovascular disease	Male	0-6 days	95+ years	-1	Healthcare access and quality index

Cause	Sex	Age start	Age end	Direction	Covariate
Cerebrovascular disease	Male	0-6 days	95+ years	1	Diet high in trans fatty acids
Cerebrovascular disease	Female	0-6 days	95+ years	0	Alcohol (liters per capita)
Cerebrovascular disease	Female	0-6 days	95+ years	1	Diabetes Fasting Plasma Glucose (mmol/L)
Cerebrovascular disease	Female	0-6 days	95+ years	-1	LDI (I$ per capita)
Cerebrovascular disease	Female	0-6 days	95+ years	1	Mean BMI
Cerebrovascular disease	Female	0-6 days	95+ years	1	Cholesterol (total, mean per capita)
Cerebrovascular disease	Female	0-6 days	95+ years	1	Systolic Blood Pressure (mmHg)
Cerebrovascular disease	Female	0-6 days	95+ years	1	Indoor Air Pollution (All Cooking Fuels)
Cerebrovascular disease	Female	0-6 days	95+ years	1	Outdoor Air Pollution (PM2.5)
Cerebrovascular disease	Female	0-6 days	95+ years	-1	Elevation Over 1500m (proportion)
Cerebrovascular disease	Female	0-6 days	95+ years	1	Smoking Prevalence
Cerebrovascular disease	Female	0-6 days	95+ years	1	Log-transformed SEV scalar: Stroke
Cerebrovascular disease	Female	0-6 days	95+ years	0	Socio-demographic Index
Cerebrovascular disease	Female	0-6 days	95+ years	-1	omega 3 adjusted(g)
Cerebrovascular disease	Female	0-6 days	95+ years	-1	fruits adjusted(g)
Cerebrovascular disease	Female	0-6 days	95+ years	-1	nuts seeds adjusted(g)
Cerebrovascular disease	Female	0-6 days	95+ years	-1	pufa adjusted(percent)
Cerebrovascular disease	Female	0-6 days	95+ years	-1	pulses legumes adjusted(g)
Cerebrovascular disease	Female	0-6 days	95+ years	-1	vegetables adjusted(g)
Cerebrovascular disease	Female	0-6 days	95+ years	-1	whole grains adjusted(g)
Cerebrovascular disease	Female	0-6 days	95+ years	-1	Healthcare access and quality index
Cerebrovascular disease	Female	0-6 days	95+ years	1	Diet high in trans fatty acids
Ischemic stroke	Male	28-364 days	95+ years	0	Alcohol (liters per capita)
Ischemic stroke	Male	28-364 days	95+ years	1	Diabetes Fasting Plasma Glucose (mmol/L)
Ischemic stroke	Male	28-364 days	95+ years	-1	LDI (I$ per capita)
Ischemic stroke	Male	28-364 days	95+ years	1	Mean BMI
Ischemic stroke	Male	28-364 days	95+ years	1	Cholesterol (total, mean per capita)
Ischemic stroke	Male	28-364 days	95+ years	1	Systolic Blood Pressure (mmHg)
Ischemic stroke	Male	28-364 days	95+ years	1	Indoor Air Pollution (All Cooking Fuels)
Ischemic stroke	Male	28-364 days	95+ years	1	Outdoor Air Pollution (PM2.5)
Ischemic stroke	Male	28-364 days	95+ years	-1	Elevation Over 1500m (proportion)
Ischemic stroke	Male	28-364 days	95+ years	1	Smoking Prevalence
Ischemic stroke	Male	28-364 days	95+ years	1	Log-transformed SEV scalar: Isch Stroke
Ischemic stroke	Male	28-364 days	95+ years	0	Socio-demographic Index
Ischemic stroke	Male	28-364 days	95+ years	-1	omega 3 adjusted(g)
Ischemic stroke	Male	28-364 days	95+ years	-1	fruits adjusted(g)
Ischemic stroke	Male	28-364 days	95+ years	-1	nuts seeds adjusted(g)
Ischemic stroke	Male	28-364 days	95+ years	-1	pufa adjusted(percent)
Ischemic stroke	Male	28-364 days	95+ years	-1	pulses legumes adjusted(g)
Ischemic stroke	Male	28-364 days	95+ years	-1	vegetables adjusted(g)
Ischemic stroke	Male	28-364 days	95+ years	-1	whole grains adjusted(g)
Ischemic stroke	Male	28-364 days	95+ years	-1	Healthcare access and quality index
Ischemic stroke	Male	28-364 days	95+ years	1	Diet high in trans fatty acids
Ischemic stroke	Female	28-364 days	95+ years	0	Alcohol (liters per capita)
Ischemic stroke	Female	28-364 days	95+ years	1	Diabetes Fasting Plasma Glucose (mmol/L)
Ischemic stroke	Female	28-364 days	95+ years	-1	LDI (I$ per capita)
Ischemic stroke	Female	28-364 days	95+ years	1	Mean BMI
Ischemic stroke	Female	28-364 days	95+ years	1	Cholesterol (total, mean per capita)
Ischemic stroke	Female	28-364 days	95+ years	1	Systolic Blood Pressure (mmHg)
Ischemic stroke	Female	28-364 days	95+ years	1	Indoor Air Pollution (All Cooking Fuels)
Ischemic stroke	Female	28-364 days	95+ years	1	Outdoor Air Pollution (PM2.5)
Ischemic stroke	Female	28-364 days	95+ years	-1	Elevation Over 1500m (proportion)
Ischemic stroke	Female	28-364 days	95+ years	1	Smoking Prevalence
Ischemic stroke	Female	28-364 days	95+ years	1	Log-transformed SEV scalar: Isch Stroke
Ischemic stroke	Female	28-364 days	95+ years	0	Socio-demographic Index
Ischemic stroke	Female	28-364 days	95+ years	-1	omega 3 adjusted(g)
Ischemic stroke	Female	28-364 days	95+ years	-1	fruits adjusted(g)
Ischemic stroke	Female	28-364 days	95+ years	-1	nuts seeds adjusted(g)
Ischemic stroke	Female	28-364 days	95+ years	-1	pufa adjusted(percent)
Ischemic stroke	Female	28-364 days	95+ years	-1	pulses legumes adjusted(g)
Ischemic stroke	Female	28-364 days	95+ years	-1	vegetables adjusted(g)
Ischemic stroke	Female	28-364 days	95+ years	-1	whole grains adjusted(g)
Ischemic stroke	Female	28-364 days	95+ years	-1	Healthcare access and quality index
Ischemic stroke	Female	28-364 days	95+ years	1	Diet high in trans fatty acids
Hemorrhagic stroke	Male	0-6 days	95+ years	0	Alcohol (liters per capita)
Hemorrhagic stroke	Male	0-6 days	95+ years	1	Diabetes Fasting Plasma Glucose (mmol/L)
Hemorrhagic stroke	Male	0-6 days	95+ years	-1	LDI (I$ per capita)
Hemorrhagic stroke	Male	0-6 days	95+ years	1	Mean BMI
Hemorrhagic stroke	Male	0-6 days	95+ years	0	Cholesterol (total, mean per capita)
Hemorrhagic stroke	Male	0-6 days	95+ years	1	Systolic Blood Pressure (mmHg)
Hemorrhagic stroke	Male	0-6 days	95+ years	1	Indoor Air Pollution (All Cooking Fuels)
Hemorrhagic stroke	Male	0-6 days	95+ years	1	Outdoor Air Pollution (PM2.5)
Hemorrhagic stroke	Male	0-6 days	95+ years	-1	Elevation Over 1500m (proportion)
Hemorrhagic stroke	Male	0-6 days	95+ years	1	Smoking Prevalence
Hemorrhagic stroke	Male	0-6 days	95+ years	1	Log-transformed SEV scalar: Hem Stroke
Hemorrhagic stroke	Male	0-6 days	95+ years	0	Socio-demographic Index
Hemorrhagic stroke	Male	0-6 days	95+ years	-1	omega 3 adjusted(g)
Hemorrhagic stroke	Male	0-6 days	95+ years	-1	fruits adjusted(g)
Hemorrhagic stroke	Male	0-6 days	95+ years	-1	nuts seeds adjusted(g)
Hemorrhagic stroke	Male	0-6 days	95+ years	-1	pufa adjusted(percent)
Hemorrhagic stroke	Male	0-6 days	95+ years	-1	pulses legumes adjusted(g)
Hemorrhagic stroke	Male	0-6 days	95+ years	-1	vegetables adjusted(g)
Hemorrhagic stroke	Male	0-6 days	95+ years	-1	whole grains adjusted(g)
Hemorrhagic stroke	Male	0-6 days	95+ years	-1	Healthcare access and quality index
Hemorrhagic stroke	Male	0-6 days	95+ years	1	Diet high in trans fatty acids
Hemorrhagic stroke	Female	0-6 days	95+ years	0	Alcohol (liters per capita)
Hemorrhagic stroke	Female	0-6 days	95+ years	1	Diabetes Fasting Plasma Glucose (mmol/L)
Hemorrhagic stroke	Female	0-6 days	95+ years	-1	LDI (I$ per capita)
Hemorrhagic stroke	Female	0-6 days	95+ years	1	Mean BMI
Hemorrhagic stroke	Female	0-6 days	95+ years	0	Cholesterol (total, mean per capita)
Hemorrhagic stroke	Female	0-6 days	95+ years	1	Systolic Blood Pressure (mmHg)
Hemorrhagic stroke	Female	0-6 days	95+ years	1	Indoor Air Pollution (All Cooking Fuels)

Cause	Sex	Age start	Age end	Direction	Covariate
Hemorrhagic stroke	Female	0-6 days	95+ years	1	Outdoor Air Pollution (PM2.5)
Hemorrhagic stroke	Female	0-6 days	95+ years	-1	Elevation Over 1500m (proportion)
Hemorrhagic stroke	Female	0-6 days	95+ years	1	Smoking Prevalence
Hemorrhagic stroke	Female	0-6 days	95+ years	1	Log-transformed SEV scalar: Hem Stroke
Hemorrhagic stroke	Female	0-6 days	95+ years	0	Socio-demographic Index
Hemorrhagic stroke	Female	0-6 days	95+ years	-1	omega 3 adjusted(g)
Hemorrhagic stroke	Female	0-6 days	95+ years	-1	fruits adjusted(g)
Hemorrhagic stroke	Female	0-6 days	95+ years	-1	nuts seeds adjusted(g)
Hemorrhagic stroke	Female	0-6 days	95+ years	-1	pufa adjusted(percent)
Hemorrhagic stroke	Female	0-6 days	95+ years	-1	pulses legumes adjusted(g)
Hemorrhagic stroke	Female	0-6 days	95+ years	-1	vegetables adjusted(g)
Hemorrhagic stroke	Female	0-6 days	95+ years	-1	whole grains adjusted(g)
Hemorrhagic stroke	Female	0-6 days	95+ years	-1	Healthcare access and quality index
Hemorrhagic stroke	Female	0-6 days	95+ years	1	Diet high in trans fatty acids
Hypertensive heart disease	Male	28-364 days	95+ years	-1	LDI (I$ per capita)
Hypertensive heart disease	Male	28-364 days	95+ years	1	Mean BMI
Hypertensive heart disease	Male	28-364 days	95+ years	1	Cholesterol (total, mean per capita)
Hypertensive heart disease	Male	28-364 days	95+ years	1	Systolic Blood Pressure (mmHg)
Hypertensive heart disease	Male	28-364 days	95+ years	1	Smoking Prevalence
Hypertensive heart disease	Male	28-364 days	95+ years	0	Socio-demographic Index
Hypertensive heart disease	Male	28-364 days	95+ years	-1	Healthcare access and quality index
Hypertensive heart disease	Female	28-364 days	95+ years	-1	LDI (I$ per capita)
Hypertensive heart disease	Female	28-364 days	95+ years	1	Mean BMI
Hypertensive heart disease	Female	28-364 days	95+ years	1	Cholesterol (total, mean per capita)
Hypertensive heart disease	Female	28-364 days	95+ years	1	Systolic Blood Pressure (mmHg)
Hypertensive heart disease	Female	28-364 days	95+ years	1	Smoking Prevalence
Hypertensive heart disease	Female	28-364 days	95+ years	0	Socio-demographic Index
Hypertensive heart disease	Female	28-364 days	95+ years	-1	Healthcare access and quality index
Cardiomyopathy and myocarditis	Male	0-6 days	95+ years	0	Alcohol (liters per capita)
Cardiomyopathy and myocarditis	Male	0-6 days	95+ years	0	LDI (I$ per capita)
Cardiomyopathy and myocarditis	Male	0-6 days	95+ years	1	Mean BMI
Cardiomyopathy and myocarditis	Male	0-6 days	95+ years	1	Systolic Blood Pressure (mmHg)
Cardiomyopathy and myocarditis	Male	0-6 days	95+ years	1	Smoking Prevalence
Cardiomyopathy and myocarditis	Male	0-6 days	95+ years	1	Log-transformed SEV scalar: CMP
Cardiomyopathy and myocarditis	Male	0-6 days	95+ years	0	Socio-demographic Index
Cardiomyopathy and myocarditis	Male	0-6 days	95+ years	-1	Healthcare access and quality index
Cardiomyopathy and myocarditis	Female	0-6 days	95+ years	0	Alcohol (liters per capita)
Cardiomyopathy and myocarditis	Female	0-6 days	95+ years	0	LDI (I$ per capita)
Cardiomyopathy and myocarditis	Female	0-6 days	95+ years	1	Mean BMI
Cardiomyopathy and myocarditis	Female	0-6 days	95+ years	1	Systolic Blood Pressure (mmHg)
Cardiomyopathy and myocarditis	Female	0-6 days	95+ years	1	Smoking Prevalence
Cardiomyopathy and myocarditis	Female	0-6 days	95+ years	1	Log-transformed SEV scalar: CMP
Cardiomyopathy and myocarditis	Female	0-6 days	95+ years	0	Socio-demographic Index
Cardiomyopathy and myocarditis	Female	0-6 days	95+ years	-1	Healthcare access and quality index
Aortic aneurysm	Male	15-19 years	95+ years	0	Alcohol (liters per capita)
Aortic aneurysm	Male	15-19 years	95+ years	1	Cumulative Cigarettes (10 Years)
Aortic aneurysm	Male	15-19 years	95+ years	-1	LDI (I$ per capita)
Aortic aneurysm	Male	15-19 years	95+ years	1	Mean BMI
Aortic aneurysm	Male	15-19 years	95+ years	1	Cholesterol (total, mean per capita)
Aortic aneurysm	Male	15-19 years	95+ years	1	Systolic Blood Pressure (mmHg)
Aortic aneurysm	Male	15-19 years	95+ years	1	Log-transformed SEV scalar: Aort An
Aortic aneurysm	Male	15-19 years	95+ years	0	Socio-demographic Index
Aortic aneurysm	Male	15-19 years	95+ years	-1	omega 3 adjusted(g)
Aortic aneurysm	Male	15-19 years	95+ years	-1	fruits adjusted(g)
Aortic aneurysm	Male	15-19 years	95+ years	-1	nuts seeds adjusted(g)
Aortic aneurysm	Male	15-19 years	95+ years	-1	pufa adjusted(percent)
Aortic aneurysm	Male	15-19 years	95+ years	1	pulses legumes adjusted(g)
Aortic aneurysm	Male	15-19 years	95+ years	-1	vegetables adjusted(g)
Aortic aneurysm	Male	15-19 years	95+ years	-1	whole grains adjusted(g)
Aortic aneurysm	Male	15-19 years	95+ years	-1	Healthcare access and quality index
Aortic aneurysm	Female	15-19 years	95+ years	0	Alcohol (liters per capita)
Aortic aneurysm	Female	15-19 years	95+ years	1	Cumulative Cigarettes (10 Years)
Aortic aneurysm	Female	15-19 years	95+ years	-1	LDI (I$ per capita)
Aortic aneurysm	Female	15-19 years	95+ years	1	Mean BMI
Aortic aneurysm	Female	15-19 years	95+ years	1	Cholesterol (total, mean per capita)
Aortic aneurysm	Female	15-19 years	95+ years	1	Systolic Blood Pressure (mmHg)
Aortic aneurysm	Female	15-19 years	95+ years	1	Log-transformed SEV scalar: Aort An
Aortic aneurysm	Female	15-19 years	95+ years	0	Socio-demographic Index
Aortic aneurysm	Female	15-19 years	95+ years	-1	omega 3 adjusted(g)
Aortic aneurysm	Female	15-19 years	95+ years	-1	fruits adjusted(g)
Aortic aneurysm	Female	15-19 years	95+ years	-1	nuts seeds adjusted(g)
Aortic aneurysm	Female	15-19 years	95+ years	-1	pufa adjusted(percent)
Aortic aneurysm	Female	15-19 years	95+ years	1	pulses legumes adjusted(g)
Aortic aneurysm	Female	15-19 years	95+ years	-1	vegetables adjusted(g)
Aortic aneurysm	Female	15-19 years	95+ years	-1	whole grains adjusted(g)
Aortic aneurysm	Female	15-19 years	95+ years	-1	Healthcare access and quality index
Peripheral artery disease	Male	40-44 years	95+ years	0	Alcohol (liters per capita)
Peripheral artery disease	Male	40-44 years	95+ years	-1	LDI (I$ per capita)
Peripheral artery disease	Male	40-44 years	95+ years	1	Mean BMI
Peripheral artery disease	Male	40-44 years	95+ years	1	Cholesterol (total, mean per capita)
Peripheral artery disease	Male	40-44 years	95+ years	1	Systolic Blood Pressure (mmHg)
Peripheral artery disease	Male	40-44 years	95+ years	1	Smoking Prevalence
Peripheral artery disease	Male	40-44 years	95+ years	1	Log-transformed SEV scalar: PVD
Peripheral artery disease	Male	40-44 years	95+ years	0	Socio-demographic Index
Peripheral artery disease	Male	40-44 years	95+ years	-1	omega 3 adjusted(g)
Peripheral artery disease	Male	40-44 years	95+ years	-1	fruits adjusted(g)
Peripheral artery disease	Male	40-44 years	95+ years	-1	nuts seeds adjusted(g)
Peripheral artery disease	Male	40-44 years	95+ years	-1	pufa adjusted(percent)
Peripheral artery disease	Male	40-44 years	95+ years	-1	pulses legumes adjusted(g)
Peripheral artery disease	Male	40-44 years	95+ years	-1	vegetables adjusted(g)
Peripheral artery disease	Male	40-44 years	95+ years	-1	whole grains adjusted(g)
Peripheral artery disease	Male	40-44 years	95+ years	-1	Healthcare access and quality index

Cause	Sex	Age start	Age end	Direction	Covariate
Peripheral artery disease	Female	40-44 years	95+ years	0	Alcohol (liters per capita)
Peripheral artery disease	Female	40-44 years	95+ years	-1	LDI (I$ per capita)
Peripheral artery disease	Female	40-44 years	95+ years	1	Mean BMI
Peripheral artery disease	Female	40-44 years	95+ years	1	Cholesterol (total, mean per capita)
Peripheral artery disease	Female	40-44 years	95+ years	1	Systolic Blood Pressure (mmHg)
Peripheral artery disease	Female	40-44 years	95+ years	1	Smoking Prevalence
Peripheral artery disease	Female	40-44 years	95+ years	1	Log-transformed SEV scalar: PVD
Peripheral artery disease	Female	40-44 years	95+ years	0	Socio-demographic Index
Peripheral artery disease	Female	40-44 years	95+ years	-1	omega 3 adjusted(g)
Peripheral artery disease	Female	40-44 years	95+ years	-1	fruits adjusted(g)
Peripheral artery disease	Female	40-44 years	95+ years	-1	nuts seeds adjusted(g)
Peripheral artery disease	Female	40-44 years	95+ years	-1	pufa adjusted(percent)
Peripheral artery disease	Female	40-44 years	95+ years	-1	pulses legumes adjusted(g)
Peripheral artery disease	Female	40-44 years	95+ years	-1	vegetables adjusted(g)
Peripheral artery disease	Female	40-44 years	95+ years	-1	whole grains adjusted(g)
Peripheral artery disease	Female	40-44 years	95+ years	-1	Healthcare access and quality index
Endocarditis	Female	0-6 days	95+ years	-1	LDI (I$ per capita)
Endocarditis	Female	0-6 days	95+ years	-1	Sanitation (proportion with access)
Endocarditis	Female	0-6 days	95+ years	-1	Improved Water Source (proportion with access)
Endocarditis	Female	0-6 days	95+ years	1	Log-transformed SEV scalar: Endocar
Endocarditis	Female	0-6 days	95+ years	0	Socio-demographic Index
Endocarditis	Female	0-6 days	95+ years	-1	Healthcare access and quality index
Endocarditis	Male	0-6 days	95+ years	-1	LDI (I$ per capita)
Endocarditis	Male	0-6 days	95+ years	-1	Sanitation (proportion with access)
Endocarditis	Male	0-6 days	95+ years	-1	Improved Water Source (proportion with access)
Endocarditis	Male	0-6 days	95+ years	1	Log-transformed SEV scalar: Endocar
Endocarditis	Male	0-6 days	95+ years	0	Socio-demographic Index
Endocarditis	Male	0-6 days	95+ years	-1	Healthcare access and quality index
Other cardiovascular and circulatory diseases	Male	0-6 days	95+ years	0	Alcohol (liters per capita)
Other cardiovascular and circulatory diseases	Male	0-6 days	95+ years	1	Diabetes Fasting Plasma Glucose (mmol/L)
Other cardiovascular and circulatory diseases	Male	0-6 days	95+ years	-1	LDI (I$ per capita)
Other cardiovascular and circulatory diseases	Male	0-6 days	95+ years	1	Mean BMI
Other cardiovascular and circulatory diseases	Male	0-6 days	95+ years	1	Cholesterol (total, mean per capita)
Other cardiovascular and circulatory diseases	Male	0-6 days	95+ years	1	Systolic Blood Pressure (mmHg)
Other cardiovascular and circulatory diseases	Male	0-6 days	95+ years	1	Indoor Air Pollution (All Cooking Fuels)
Other cardiovascular and circulatory diseases	Male	0-6 days	95+ years	1	Outdoor Air Pollution (PM2.5)
Other cardiovascular and circulatory diseases	Male	0-6 days	95+ years	-1	Elevation Over 1500m (proportion)
Other cardiovascular and circulatory diseases	Male	0-6 days	95+ years	1	Smoking Prevalence
Other cardiovascular and circulatory diseases	Male	0-6 days	95+ years	1	Log-transformed SEV scalar: Oth Cardio
Other cardiovascular and circulatory diseases	Male	0-6 days	95+ years	0	Socio-demographic Index
Other cardiovascular and circulatory diseases	Male	0-6 days	95+ years	-1	omega 3 adjusted(g)
Other cardiovascular and circulatory diseases	Male	0-6 days	95+ years	-1	fruits adjusted(g)
Other cardiovascular and circulatory diseases	Male	0-6 days	95+ years	-1	nuts seeds adjusted(g)
Other cardiovascular and circulatory diseases	Male	0-6 days	95+ years	-1	pufa adjusted(percent)
Other cardiovascular and circulatory diseases	Male	0-6 days	95+ years	-1	pulses legumes adjusted(g)
Other cardiovascular and circulatory diseases	Male	0-6 days	95+ years	-1	vegetables adjusted(g)
Other cardiovascular and circulatory diseases	Male	0-6 days	95+ years	-1	whole grains adjusted(g)
Other cardiovascular and circulatory diseases	Male	0-6 days	95+ years	-1	Healthcare access and quality index
Other cardiovascular and circulatory diseases	Female	0-6 days	95+ years	0	Alcohol (liters per capita)
Other cardiovascular and circulatory diseases	Female	0-6 days	95+ years	1	Diabetes Fasting Plasma Glucose (mmol/L)
Other cardiovascular and circulatory diseases	Female	0-6 days	95+ years	-1	LDI (I$ per capita)
Other cardiovascular and circulatory diseases	Female	0-6 days	95+ years	1	Mean BMI
Other cardiovascular and circulatory diseases	Female	0-6 days	95+ years	1	Cholesterol (total, mean per capita)
Other cardiovascular and circulatory diseases	Female	0-6 days	95+ years	1	Systolic Blood Pressure (mmHg)
Other cardiovascular and circulatory diseases	Female	0-6 days	95+ years	1	Indoor Air Pollution (All Cooking Fuels)
Other cardiovascular and circulatory diseases	Female	0-6 days	95+ years	1	Outdoor Air Pollution (PM2.5)
Other cardiovascular and circulatory diseases	Female	0-6 days	95+ years	-1	Elevation Over 1500m (proportion)
Other cardiovascular and circulatory diseases	Female	0-6 days	95+ years	1	Smoking Prevalence
Other cardiovascular and circulatory diseases	Female	0-6 days	95+ years	1	Log-transformed SEV scalar: Oth Cardio
Other cardiovascular and circulatory diseases	Female	0-6 days	95+ years	0	Socio-demographic Index
Other cardiovascular and circulatory diseases	Female	0-6 days	95+ years	-1	omega 3 adjusted(g)
Other cardiovascular and circulatory diseases	Female	0-6 days	95+ years	-1	fruits adjusted(g)
Other cardiovascular and circulatory diseases	Female	0-6 days	95+ years	-1	nuts seeds adjusted(g)
Other cardiovascular and circulatory diseases	Female	0-6 days	95+ years	-1	pufa adjusted(percent)
Other cardiovascular and circulatory diseases	Female	0-6 days	95+ years	-1	pulses legumes adjusted(g)
Other cardiovascular and circulatory diseases	Female	0-6 days	95+ years	-1	vegetables adjusted(g)
Other cardiovascular and circulatory diseases	Female	0-6 days	95+ years	-1	whole grains adjusted(g)
Other cardiovascular and circulatory diseases	Female	0-6 days	95+ years	-1	Healthcare access and quality index
Chronic respiratory diseases	Male	1-4 years	95+ years	1	Cumulative Cigarettes (10 Years)
Chronic respiratory diseases	Male	1-4 years	95+ years	1	Cumulative Cigarettes (5 Years)
Chronic respiratory diseases	Male	1-4 years	95+ years	-1	Education (years per capita)
Chronic respiratory diseases	Male	1-4 years	95+ years	-1	LDI (I$ per capita)
Chronic respiratory diseases	Male	1-4 years	95+ years	1	Indoor Air Pollution (All Cooking Fuels)
Chronic respiratory diseases	Male	1-4 years	95+ years	1	Outdoor Air Pollution (PM2.5)
Chronic respiratory diseases	Male	1-4 years	95+ years	1	Elevation Over 1500m (proportion)
Chronic respiratory diseases	Male	1-4 years	95+ years	1	Elevation 500 to 1500m (proportion)
Chronic respiratory diseases	Male	1-4 years	95+ years	1	Population Density (over 1000 ppl/sqkm, proportion)
Chronic respiratory diseases	Male	1-4 years	95+ years	1	Smoking Prevalence
Chronic respiratory diseases	Male	1-4 years	95+ years	0	Socio-demographic Index
Chronic respiratory diseases	Male	1-4 years	95+ years	1	Log-transformed SEV scalar: Chr Resp
Chronic respiratory diseases	Male	1-4 years	95+ years	-1	Healthcare access and quality index
Chronic respiratory diseases	Female	1-4 years	95+ years	1	Cumulative Cigarettes (10 Years)
Chronic respiratory diseases	Female	1-4 years	95+ years	1	Cumulative Cigarettes (5 Years)
Chronic respiratory diseases	Female	1-4 years	95+ years	-1	Education (years per capita)
Chronic respiratory diseases	Female	1-4 years	95+ years	-1	LDI (I$ per capita)
Chronic respiratory diseases	Female	1-4 years	95+ years	1	Indoor Air Pollution (All Cooking Fuels)
Chronic respiratory diseases	Female	1-4 years	95+ years	1	Outdoor Air Pollution (PM2.5)
Chronic respiratory diseases	Female	1-4 years	95+ years	1	Elevation Over 1500m (proportion)
Chronic respiratory diseases	Female	1-4 years	95+ years	1	Elevation 500 to 1500m (proportion)
Chronic respiratory diseases	Female	1-4 years	95+ years	1	Population Density (over 1000 ppl/sqkm, proportion)
Chronic respiratory diseases	Female	1-4 years	95+ years	1	Smoking Prevalence
Chronic respiratory diseases	Female	1-4 years	95+ years	0	Socio-demographic Index

Cause	Sex	Age start	Age end	Direction	Covariate
Chronic respiratory diseases	Female	1-4 years	95+ years	1	Log-transformed SEV scalar: Chr Resp
Chronic respiratory diseases	Female	1-4 years	95+ years	1	Healthcare access and quality index
Chronic obstructive pulmonary disease	Male	1-4 years	95+ years	1	Cumulative Cigarettes (10 Years)
Chronic obstructive pulmonary disease	Male	1-4 years	95+ years	1	Cumulative Cigarettes (20 Years)
Chronic obstructive pulmonary disease	Male	1-4 years	95+ years	1	Cumulative Cigarettes (5 Years)
Chronic obstructive pulmonary disease	Male	1-4 years	95+ years	-1	Education (years per capita)
Chronic obstructive pulmonary disease	Male	1-4 years	95+ years	-1	LDI (I$ per capita)
Chronic obstructive pulmonary disease	Male	1-4 years	95+ years	1	Indoor Air Pollution (All Cooking Fuels)
Chronic obstructive pulmonary disease	Male	1-4 years	95+ years	1	Outdoor Air Pollution (PM2.5)
Chronic obstructive pulmonary disease	Male	1-4 years	95+ years	1	Elevation Over 1500m (proportion)
Chronic obstructive pulmonary disease	Male	1-4 years	95+ years	1	Smoking Prevalence
Chronic obstructive pulmonary disease	Male	1-4 years	95+ years	1	Log-transformed SEV scalar: COPD
Chronic obstructive pulmonary disease	Male	1-4 years	95+ years	0	Socio-demographic Index
Chronic obstructive pulmonary disease	Male	1-4 years	95+ years	-1	Healthcare access and quality index
Chronic obstructive pulmonary disease	Female	1-4 years	95+ years	1	Cumulative Cigarettes (10 Years)
Chronic obstructive pulmonary disease	Female	1-4 years	95+ years	1	Cumulative Cigarettes (5 Years)
Chronic obstructive pulmonary disease	Female	1-4 years	95+ years	-1	Education (years per capita)
Chronic obstructive pulmonary disease	Female	1-4 years	95+ years	-1	LDI (I$ per capita)
Chronic obstructive pulmonary disease	Female	1-4 years	95+ years	1	Indoor Air Pollution (All Cooking Fuels)
Chronic obstructive pulmonary disease	Female	1-4 years	95+ years	1	Outdoor Air Pollution (PM2.5)
Chronic obstructive pulmonary disease	Female	1-4 years	95+ years	1	Elevation Over 1500m (proportion)
Chronic obstructive pulmonary disease	Female	1-4 years	95+ years	1	Smoking Prevalence
Chronic obstructive pulmonary disease	Female	1-4 years	95+ years	1	Log-transformed SEV scalar: COPD
Chronic obstructive pulmonary disease	Female	1-4 years	95+ years	0	Socio-demographic Index
Chronic obstructive pulmonary disease	Female	1-4 years	95+ years	1	Healthcare access and quality index
Pneumoconiosis	Female	1-4 years	95+ years	1	Coal Production (per capita)
Pneumoconiosis	Female	1-4 years	95+ years	1	Cumulative Cigarettes (5 Years)
Pneumoconiosis	Female	1-4 years	95+ years	-1	Education (years per capita)
Pneumoconiosis	Female	1-4 years	95+ years	1	Gold production (kg) per capita
Pneumoconiosis	Female	1-4 years	95+ years	-1	LDI (I$ per capita)
Pneumoconiosis	Female	1-4 years	95+ years	1	Indoor Air Pollution (All Cooking Fuels)
Pneumoconiosis	Female	1-4 years	95+ years	1	Elevation Over 1500m (proportion)
Pneumoconiosis	Female	1-4 years	95+ years	1	Elevation 500 to 1500m (proportion)
Pneumoconiosis	Female	1-4 years	95+ years	1	Smoking Prevalence
Pneumoconiosis	Female	1-4 years	95+ years	1	Log-transformed SEV scalar: Pneumocon
Pneumoconiosis	Female	1-4 years	95+ years	-1	Socio-demographic Index
Pneumoconiosis	Female	1-4 years	95+ years	-1	Healthcare access and quality index
Pneumoconiosis	Female	1-4 years	95+ years	1	Asbestos consumption (metric tons per year per capita)
Pneumoconiosis	Male	1-4 years	95+ years	1	Coal Production (per capita)
Pneumoconiosis	Male	1-4 years	95+ years	1	Cumulative Cigarettes (5 Years)
Pneumoconiosis	Male	1-4 years	95+ years	-1	Education (years per capita)
Pneumoconiosis	Male	1-4 years	95+ years	1	Gold production (kg) per capita
Pneumoconiosis	Male	1-4 years	95+ years	-1	LDI (I$ per capita)
Pneumoconiosis	Male	1-4 years	95+ years	1	Indoor Air Pollution (All Cooking Fuels)
Pneumoconiosis	Male	1-4 years	95+ years	1	Elevation Over 1500m (proportion)
Pneumoconiosis	Male	1-4 years	95+ years	1	Elevation 500 to 1500m (proportion)
Pneumoconiosis	Male	1-4 years	95+ years	1	Smoking Prevalence
Pneumoconiosis	Male	1-4 years	95+ years	1	Log-transformed SEV scalar: Pneumocon
Pneumoconiosis	Male	1-4 years	95+ years	-1	Socio-demographic Index
Pneumoconiosis	Male	1-4 years	95+ years	-1	Healthcare access and quality index
Pneumoconiosis	Male	1-4 years	95+ years	1	Asbestos consumption (metric tons per year per capita)
Silicosis	Female	1-4 years	95+ years	1	Cumulative Cigarettes (5 Years)
Silicosis	Female	1-4 years	95+ years	-1	Education (years per capita)
Silicosis	Female	1-4 years	95+ years	1	Gold production (kg) per capita
Silicosis	Female	1-4 years	95+ years	-1	LDI (I$ per capita)
Silicosis	Female	1-4 years	95+ years	1	Indoor Air Pollution (All Cooking Fuels)
Silicosis	Female	1-4 years	95+ years	1	Elevation Over 1500m (proportion)
Silicosis	Female	1-4 years	95+ years	1	Elevation 500 to 1500m (proportion)
Silicosis	Female	1-4 years	95+ years	1	Smoking Prevalence
Silicosis	Female	1-4 years	95+ years	1	Log-transformed SEV scalar: Silicosis
Silicosis	Female	1-4 years	95+ years	-1	Socio-demographic Index
Silicosis	Female	1-4 years	95+ years	-1	Healthcare access and quality index
Silicosis	Male	1-4 years	95+ years	1	Cumulative Cigarettes (5 Years)
Silicosis	Male	1-4 years	95+ years	-1	Education (years per capita)
Silicosis	Male	1-4 years	95+ years	1	Gold production (kg) per capita
Silicosis	Male	1-4 years	95+ years	-1	LDI (I$ per capita)
Silicosis	Male	1-4 years	95+ years	1	Indoor Air Pollution (All Cooking Fuels)
Silicosis	Male	1-4 years	95+ years	1	Elevation Over 1500m (proportion)
Silicosis	Male	1-4 years	95+ years	1	Elevation 500 to 1500m (proportion)
Silicosis	Male	1-4 years	95+ years	1	Smoking Prevalence
Silicosis	Male	1-4 years	95+ years	1	Log-transformed SEV scalar: Silicosis
Silicosis	Male	1-4 years	95+ years	-1	Socio-demographic Index
Silicosis	Male	1-4 years	95+ years	-1	Healthcare access and quality index
Asbestosis	Female	1-4 years	95+ years	1	Cumulative Cigarettes (5 Years)
Asbestosis	Female	1-4 years	95+ years	-1	Education (years per capita)
Asbestosis	Female	1-4 years	95+ years	-1	LDI (I$ per capita)
Asbestosis	Female	1-4 years	95+ years	1	Indoor Air Pollution (All Cooking Fuels)
Asbestosis	Female	1-4 years	95+ years	1	Elevation Over 1500m (proportion)
Asbestosis	Female	1-4 years	95+ years	1	Elevation 500 to 1500m (proportion)
Asbestosis	Female	1-4 years	95+ years	1	Smoking Prevalence
Asbestosis	Female	1-4 years	95+ years	1	Log-transformed SEV scalar: Asbestosis
Asbestosis	Female	1-4 years	95+ years	-1	Socio-demographic Index
Asbestosis	Female	1-4 years	95+ years	-1	Healthcare access and quality index
Asbestosis	Female	1-4 years	95+ years	1	Asbestos consumption (metric tons per year per capita)
Asbestosis	Male	1-4 years	95+ years	1	Cumulative Cigarettes (5 Years)
Asbestosis	Male	1-4 years	95+ years	-1	Education (years per capita)
Asbestosis	Male	1-4 years	95+ years	-1	LDI (I$ per capita)
Asbestosis	Male	1-4 years	95+ years	1	Indoor Air Pollution (All Cooking Fuels)
Asbestosis	Male	1-4 years	95+ years	1	Elevation Over 1500m (proportion)
Asbestosis	Male	1-4 years	95+ years	1	Elevation 500 to 1500m (proportion)
Asbestosis	Male	1-4 years	95+ years	1	Smoking Prevalence
Asbestosis	Male	1-4 years	95+ years	1	Log-transformed SEV scalar: Asbestosis

Cause	Sex	Age start	Age end	Direction	Covariate
Asbestosis	Male	1-4 years	95+ years	-1	Socio-demographic Index
Asbestosis	Male	1-4 years	95+ years	-1	Healthcare access and quality index
Asbestosis	Male	1-4 years	95+ years	1	Asbestos consumption (metric tons per year per capita)
Asbestosis	Male	1-4 years	95+ years	0	Socio-demographic Index
Coal workers pneumoconiosis	Male	1-4 years	95+ years	1	Coal Production (per capita)
Coal workers pneumoconiosis	Male	1-4 years	95+ years	1	Cumulative Cigarettes (5 Years)
Coal workers pneumoconiosis	Male	1-4 years	95+ years	-1	Education (years per capita)
Coal workers pneumoconiosis	Male	1-4 years	95+ years	-1	LDI (I$ per capita)
Coal workers pneumoconiosis	Male	1-4 years	95+ years	1	Indoor Air Pollution (All Cooking Fuels)
Coal workers pneumoconiosis	Male	1-4 years	95+ years	1	Elevation Over 1500m (proportion)
Coal workers pneumoconiosis	Male	1-4 years	95+ years	1	Elevation 500 to 1500m (proportion)
Coal workers pneumoconiosis	Male	1-4 years	95+ years	1	Smoking Prevalence
Coal workers pneumoconiosis	Male	1-4 years	95+ years	1	Log-transformed SEV scalar: Coal W
Coal workers pneumoconiosis	Male	1-4 years	95+ years	-1	Socio-demographic Index
Coal workers pneumoconiosis	Male	1-4 years	95+ years	-1	Healthcare access and quality index
Coal workers pneumoconiosis	Female	1-4 years	95+ years	1	Coal Production (per capita)
Coal workers pneumoconiosis	Female	1-4 years	95+ years	1	Cumulative Cigarettes (5 Years)
Coal workers pneumoconiosis	Female	1-4 years	95+ years	-1	Education (years per capita)
Coal workers pneumoconiosis	Female	1-4 years	95+ years	-1	LDI (I$ per capita)
Coal workers pneumoconiosis	Female	1-4 years	95+ years	1	Indoor Air Pollution (All Cooking Fuels)
Coal workers pneumoconiosis	Female	1-4 years	95+ years	1	Elevation Over 1500m (proportion)
Coal workers pneumoconiosis	Female	1-4 years	95+ years	1	Elevation 500 to 1500m (proportion)
Coal workers pneumoconiosis	Female	1-4 years	95+ years	1	Smoking Prevalence
Coal workers pneumoconiosis	Female	1-4 years	95+ years	1	Log-transformed SEV scalar: Coal W
Coal workers pneumoconiosis	Female	1-4 years	95+ years	-1	Socio-demographic Index
Coal workers pneumoconiosis	Female	1-4 years	95+ years	-1	Healthcare access and quality index
Other pneumoconiosis	Male	1-4 years	95+ years	1	Cumulative Cigarettes (5 Years)
Other pneumoconiosis	Male	1-4 years	95+ years	-1	Education (years per capita)
Other pneumoconiosis	Male	1-4 years	95+ years	-1	LDI (I$ per capita)
Other pneumoconiosis	Male	1-4 years	95+ years	1	Indoor Air Pollution (All Cooking Fuels)
Other pneumoconiosis	Male	1-4 years	95+ years	1	Elevation Over 1500m (proportion)
Other pneumoconiosis	Male	1-4 years	95+ years	1	Elevation 500 to 1500m (proportion)
Other pneumoconiosis	Male	1-4 years	95+ years	1	Smoking Prevalence
Other pneumoconiosis	Male	1-4 years	95+ years	1	Log-transformed SEV scalar: Oth Pneum
Other pneumoconiosis	Male	1-4 years	95+ years	-1	Socio-demographic Index
Other pneumoconiosis	Male	1-4 years	95+ years	-1	Healthcare access and quality index
Other pneumoconiosis	Female	1-4 years	95+ years	1	Cumulative Cigarettes (5 Years)
Other pneumoconiosis	Female	1-4 years	95+ years	-1	Education (years per capita)
Other pneumoconiosis	Female	1-4 years	95+ years	-1	LDI (I$ per capita)
Other pneumoconiosis	Female	1-4 years	95+ years	1	Indoor Air Pollution (All Cooking Fuels)
Other pneumoconiosis	Female	1-4 years	95+ years	1	Elevation Over 1500m (proportion)
Other pneumoconiosis	Female	1-4 years	95+ years	1	Elevation 500 to 1500m (proportion)
Other pneumoconiosis	Female	1-4 years	95+ years	1	Smoking Prevalence
Other pneumoconiosis	Female	1-4 years	95+ years	1	Log-transformed SEV scalar: Oth Pneum
Other pneumoconiosis	Female	1-4 years	95+ years	-1	Socio-demographic Index
Other pneumoconiosis	Female	1-4 years	95+ years	-1	Healthcare access and quality index
Asthma	Female	1-4 years	95+ years	1	Cumulative Cigarettes (10 Years)
Asthma	Female	1-4 years	95+ years	1	Cumulative Cigarettes (5 Years)
Asthma	Female	1-4 years	95+ years	-1	Education (years per capita)
Asthma	Female	1-4 years	95+ years	-1	LDI (I$ per capita)
Asthma	Female	1-4 years	95+ years	1	Indoor Air Pollution (All Cooking Fuels)
Asthma	Female	1-4 years	95+ years	1	Outdoor Air Pollution (PM2.5)
Asthma	Female	1-4 years	95+ years	1	Smoking Prevalence
Asthma	Female	1-4 years	95+ years	1	Log-transformed SEV scalar: Asthma
Asthma	Female	1-4 years	95+ years	-1	Socio-demographic Index
Asthma	Female	1-4 years	95+ years	-1	Healthcare access and quality index
Asthma	Male	1-4 years	95+ years	1	Cumulative Cigarettes (10 Years)
Asthma	Male	1-4 years	95+ years	1	Cumulative Cigarettes (5 Years)
Asthma	Male	1-4 years	95+ years	-1	Education (years per capita)
Asthma	Male	1-4 years	95+ years	-1	LDI (I$ per capita)
Asthma	Male	1-4 years	95+ years	1	Indoor Air Pollution (All Cooking Fuels)
Asthma	Male	1-4 years	95+ years	1	Outdoor Air Pollution (PM2.5)
Asthma	Male	1-4 years	95+ years	1	Smoking Prevalence
Asthma	Male	1-4 years	95+ years	1	Log-transformed SEV scalar: Asthma
Asthma	Male	1-4 years	95+ years	-1	Socio-demographic Index
Asthma	Male	1-4 years	95+ years	-1	Healthcare access and quality index
Interstitial lung disease and pulmonary sarcoidosis	Female	1-4 years	95+ years	1	Cumulative Cigarettes (5 Years)
Interstitial lung disease and pulmonary sarcoidosis	Female	1-4 years	95+ years	-1	Education (years per capita)
Interstitial lung disease and pulmonary sarcoidosis	Female	1-4 years	95+ years	-1	LDI (I$ per capita)
Interstitial lung disease and pulmonary sarcoidosis	Female	1-4 years	95+ years	1	Indoor Air Pollution (All Cooking Fuels)
Interstitial lung disease and pulmonary sarcoidosis	Female	1-4 years	95+ years	1	Outdoor Air Pollution (PM2.5)
Interstitial lung disease and pulmonary sarcoidosis	Female	1-4 years	95+ years	1	Elevation Over 1500m (proportion)
Interstitial lung disease and pulmonary sarcoidosis	Female	1-4 years	95+ years	1	Elevation 500 to 1500m (proportion)
Interstitial lung disease and pulmonary sarcoidosis	Female	1-4 years	95+ years	1	Population Density (over 1000 ppl/sqkm, proportion)
Interstitial lung disease and pulmonary sarcoidosis	Female	1-4 years	95+ years	1	Smoking Prevalence
Interstitial lung disease and pulmonary sarcoidosis	Female	1-4 years	95+ years	1	Log-transformed SEV scalar: ILD
Interstitial lung disease and pulmonary sarcoidosis	Female	1-4 years	95+ years	0	Socio-demographic Index
Interstitial lung disease and pulmonary sarcoidosis	Female	1-4 years	95+ years	-1	Healthcare access and quality index
Interstitial lung disease and pulmonary sarcoidosis	Male	1-4 years	95+ years	1	Cumulative Cigarettes (5 Years)
Interstitial lung disease and pulmonary sarcoidosis	Male	1-4 years	95+ years	-1	Education (years per capita)
Interstitial lung disease and pulmonary sarcoidosis	Male	1-4 years	95+ years	-1	LDI (I$ per capita)
Interstitial lung disease and pulmonary sarcoidosis	Male	1-4 years	95+ years	1	Indoor Air Pollution (All Cooking Fuels)
Interstitial lung disease and pulmonary sarcoidosis	Male	1-4 years	95+ years	1	Outdoor Air Pollution (PM2.5)
Interstitial lung disease and pulmonary sarcoidosis	Male	1-4 years	95+ years	1	Elevation Over 1500m (proportion)
Interstitial lung disease and pulmonary sarcoidosis	Male	1-4 years	95+ years	1	Elevation 500 to 1500m (proportion)
Interstitial lung disease and pulmonary sarcoidosis	Male	1-4 years	95+ years	1	Population Density (over 1000 ppl/sqkm, proportion)
Interstitial lung disease and pulmonary sarcoidosis	Male	1-4 years	95+ years	1	Smoking Prevalence
Interstitial lung disease and pulmonary sarcoidosis	Male	1-4 years	95+ years	1	Log-transformed SEV scalar: ILD
Interstitial lung disease and pulmonary sarcoidosis	Male	1-4 years	95+ years	0	Socio-demographic Index
Interstitial lung disease and pulmonary sarcoidosis	Male	1-4 years	95+ years	-1	Healthcare access and quality index
Other chronic respiratory diseases	Female	1-4 years	95+ years	1	Cumulative Cigarettes (5 Years)
Other chronic respiratory diseases	Female	1-4 years	95+ years	-1	Education (years per capita)

Cause	Sex	Age start	Age end	Direction	Covariate
Other chronic respiratory diseases	Female	1-4 years	95+ years	-1	LDI (I$ per capita)
Other chronic respiratory diseases	Female	1-4 years	95+ years	1	Indoor Air Pollution (All Cooking Fuels)
Other chronic respiratory diseases	Female	1-4 years	95+ years	1	Outdoor Air Pollution (PM2.5)
Other chronic respiratory diseases	Female	1-4 years	95+ years	1	Elevation Over 1500m (proportion)
Other chronic respiratory diseases	Female	1-4 years	95+ years	1	Elevation 500 to 1500m (proportion)
Other chronic respiratory diseases	Female	1-4 years	95+ years	1	Population Density (over 1000 ppl/sqkm, proportion)
Other chronic respiratory diseases	Female	1-4 years	95+ years	1	Smoking Prevalence
Other chronic respiratory diseases	Female	1-4 years	95+ years	1	Log-transformed SEV scalar: Oth Resp
Other chronic respiratory diseases	Female	1-4 years	95+ years	-1	Socio-demographic Index
Other chronic respiratory diseases	Female	1-4 years	95+ years	-1	Healthcare access and quality index
Other chronic respiratory diseases	Male	1-4 years	95+ years	1	Cumulative Cigarettes (5 Years)
Other chronic respiratory diseases	Male	1-4 years	95+ years	-1	Education (years per capita)
Other chronic respiratory diseases	Male	1-4 years	95+ years	-1	LDI (I$ per capita)
Other chronic respiratory diseases	Male	1-4 years	95+ years	1	Indoor Air Pollution (All Cooking Fuels)
Other chronic respiratory diseases	Male	1-4 years	95+ years	1	Outdoor Air Pollution (PM2.5)
Other chronic respiratory diseases	Male	1-4 years	95+ years	1	Elevation Over 1500m (proportion)
Other chronic respiratory diseases	Male	1-4 years	95+ years	1	Elevation 500 to 1500m (proportion)
Other chronic respiratory diseases	Male	1-4 years	95+ years	1	Population Density (over 1000 ppl/sqkm, proportion)
Other chronic respiratory diseases	Male	1-4 years	95+ years	1	Smoking Prevalence
Other chronic respiratory diseases	Male	1-4 years	95+ years	1	Log-transformed SEV scalar: Oth Resp
Other chronic respiratory diseases	Male	1-4 years	95+ years	-1	Socio-demographic Index
Other chronic respiratory diseases	Male	1-4 years	95+ years	-1	Healthcare access and quality index
Cirrhosis and other chronic liver diseases	Male	1-4 years	95+ years	1	Alcohol (liters per capita)
Cirrhosis and other chronic liver diseases	Male	1-4 years	95+ years	1	Diabetes Age-Standardized Prevalence (proportion)
Cirrhosis and other chronic liver diseases	Male	1-4 years	95+ years	-1	Education (years per capita)
Cirrhosis and other chronic liver diseases	Male	1-4 years	95+ years	-1	Health System Access 2 (unitless)
Cirrhosis and other chronic liver diseases	Male	1-4 years	95+ years	-1	LDI (I$ per capita)
Cirrhosis and other chronic liver diseases	Male	1-4 years	95+ years	1	Mean BMI
Cirrhosis and other chronic liver diseases	Male	1-4 years	95+ years	1	Schistosomiasis Prevalence (proportion)
Cirrhosis and other chronic liver diseases	Male	1-4 years	95+ years	0	Socio-demographic Index
Cirrhosis and other chronic liver diseases	Male	1-4 years	95+ years	1	Hepatitis B (HBsAg) Seroprevalence
Cirrhosis and other chronic liver diseases	Male	1-4 years	95+ years	1	Hepatitis C (IgG) Seroprevalence
Cirrhosis and other chronic liver diseases	Male	1-4 years	95+ years	-1	Healthcare access and quality index
Cirrhosis and other chronic liver diseases	Female	1-4 years	95+ years	1	Alcohol (liters per capita)
Cirrhosis and other chronic liver diseases	Female	1-4 years	95+ years	1	Diabetes Age-Standardized Prevalence (proportion)
Cirrhosis and other chronic liver diseases	Female	1-4 years	95+ years	-1	Education (years per capita)
Cirrhosis and other chronic liver diseases	Female	1-4 years	95+ years	-1	Health System Access 2 (unitless)
Cirrhosis and other chronic liver diseases	Female	1-4 years	95+ years	-1	LDI (I$ per capita)
Cirrhosis and other chronic liver diseases	Female	1-4 years	95+ years	1	Mean BMI
Cirrhosis and other chronic liver diseases	Female	1-4 years	95+ years	1	Schistosomiasis Prevalence (proportion)
Cirrhosis and other chronic liver diseases	Female	1-4 years	95+ years	0	Socio-demographic Index
Cirrhosis and other chronic liver diseases	Female	1-4 years	95+ years	1	Hepatitis B (HBsAg) Seroprevalence
Cirrhosis and other chronic liver diseases	Female	1-4 years	95+ years	1	Hepatitis C (IgG) Seroprevalence
Cirrhosis and other chronic liver diseases	Female	1-4 years	95+ years	-1	Healthcare access and quality index
Digestive diseases	Female	0-6 days	95+ years	1	Alcohol (liters per capita)
Digestive diseases	Female	0-6 days	95+ years	1	Cumulative Cigarettes (5 Years)
Digestive diseases	Female	0-6 days	95+ years	-1	Education (years per capita)
Digestive diseases	Female	0-6 days	95+ years	-1	LDI (I$ per capita)
Digestive diseases	Female	0-6 days	95+ years	-1	Sanitation (proportion with access)
Digestive diseases	Female	0-6 days	95+ years	-1	Socio-demographic Index
Digestive diseases	Female	0-6 days	95+ years	-1	fruits adjusted(g)
Digestive diseases	Female	0-6 days	95+ years	1	red meats adjusted(g)
Digestive diseases	Female	0-6 days	95+ years	-1	Healthcare access and quality index
Digestive diseases	Male	0-6 days	95+ years	1	Alcohol (liters per capita)
Digestive diseases	Male	0-6 days	95+ years	1	Cumulative Cigarettes (5 Years)
Digestive diseases	Male	0-6 days	95+ years	-1	Education (years per capita)
Digestive diseases	Male	0-6 days	95+ years	-1	LDI (I$ per capita)
Digestive diseases	Male	0-6 days	95+ years	-1	Sanitation (proportion with access)
Digestive diseases	Male	0-6 days	95+ years	-1	Socio-demographic Index
Digestive diseases	Male	0-6 days	95+ years	-1	fruits adjusted(g)
Digestive diseases	Male	0-6 days	95+ years	1	red meats adjusted(g)
Digestive diseases	Male	0-6 days	95+ years	-1	Healthcare access and quality index
Peptic ulcer disease	Female	1-4 years	95+ years	1	Alcohol (liters per capita)
Peptic ulcer disease	Female	1-4 years	95+ years	1	Cumulative Cigarettes (10 Years)
Peptic ulcer disease	Female	1-4 years	95+ years	1	Cumulative Cigarettes (5 Years)
Peptic ulcer disease	Female	1-4 years	95+ years	-1	LDI (I$ per capita)
Peptic ulcer disease	Female	1-4 years	95+ years	-1	Sanitation (proportion with access)
Peptic ulcer disease	Female	1-4 years	95+ years	1	Smoking Prevalence
Peptic ulcer disease	Female	1-4 years	95+ years	-1	Maternal education (years per capita)
Peptic ulcer disease	Female	1-4 years	95+ years	1	SEV unsafe water
Peptic ulcer disease	Female	1-4 years	95+ years	-1	Socio-demographic Index
Peptic ulcer disease	Female	1-4 years	95+ years	0	vegetables adjusted(g)
Peptic ulcer disease	Female	1-4 years	95+ years	-1	Healthcare access and quality index
Peptic ulcer disease	Male	1-4 years	95+ years	1	Alcohol (liters per capita)
Peptic ulcer disease	Male	1-4 years	95+ years	1	Cumulative Cigarettes (10 Years)
Peptic ulcer disease	Male	1-4 years	95+ years	1	Cumulative Cigarettes (5 Years)
Peptic ulcer disease	Male	1-4 years	95+ years	-1	LDI (I$ per capita)
Peptic ulcer disease	Male	1-4 years	95+ years	-1	Sanitation (proportion with access)
Peptic ulcer disease	Male	1-4 years	95+ years	1	Smoking Prevalence
Peptic ulcer disease	Male	1-4 years	95+ years	-1	Maternal education (years per capita)
Peptic ulcer disease	Male	1-4 years	95+ years	1	SEV unsafe water
Peptic ulcer disease	Male	1-4 years	95+ years	-1	Socio-demographic Index
Peptic ulcer disease	Male	1-4 years	95+ years	0	vegetables adjusted(g)
Peptic ulcer disease	Male	1-4 years	95+ years	-1	Healthcare access and quality index
Gastritis and duodenitis	Male	1-4 years	95+ years	1	Alcohol (liters per capita)
Gastritis and duodenitis	Male	1-4 years	95+ years	1	Cumulative Cigarettes (10 Years)
Gastritis and duodenitis	Male	1-4 years	95+ years	1	Cumulative Cigarettes (5 Years)
Gastritis and duodenitis	Male	1-4 years	95+ years	-1	Education (years per capita)
Gastritis and duodenitis	Male	1-4 years	95+ years	-1	LDI (I$ per capita)
Gastritis and duodenitis	Male	1-4 years	95+ years	-1	Sanitation (proportion with access)
Gastritis and duodenitis	Male	1-4 years	95+ years	1	Smoking Prevalence
Gastritis and duodenitis	Male	1-4 years	95+ years	1	SEV unsafe water

Cause	Sex	Age start	Age end	Direction	Covariate
Gastritis and duodenitis	Male	1-4 years	95+ years	-1	Socio-demographic Index
Gastritis and duodenitis	Male	1-4 years	95+ years	0	vegetables adjusted(g)
Gastritis and duodenitis	Male	1-4 years	95+ years	-1	Healthcare access and quality index
Gastritis and duodenitis	Female	1-4 years	95+ years	1	Alcohol (liters per capita)
Gastritis and duodenitis	Female	1-4 years	95+ years	1	Cumulative Cigarettes (10 Years)
Gastritis and duodenitis	Female	1-4 years	95+ years	1	Cumulative Cigarettes (5 Years)
Gastritis and duodenitis	Female	1-4 years	95+ years	-1	Education (years per capita)
Gastritis and duodenitis	Female	1-4 years	95+ years	-1	LDI (I$ per capita)
Gastritis and duodenitis	Female	1-4 years	95+ years	-1	Sanitation (proportion with access)
Gastritis and duodenitis	Female	1-4 years	95+ years	1	Smoking Prevalence
Gastritis and duodenitis	Female	1-4 years	95+ years	1	SEV unsafe water
Gastritis and duodenitis	Female	1-4 years	95+ years	-1	Socio-demographic Index
Gastritis and duodenitis	Female	1-4 years	95+ years	0	vegetables adjusted(g)
Gastritis and duodenitis	Female	1-4 years	95+ years	-1	Healthcare access and quality index
Appendicitis	Male	1-4 years	95+ years	-1	Education (years per capita)
Appendicitis	Male	1-4 years	95+ years	-1	LDI (I$ per capita)
Appendicitis	Male	1-4 years	95+ years	-1	Health System Access (capped)
Appendicitis	Male	1-4 years	95+ years	-1	Socio-demographic Index
Appendicitis	Male	1-4 years	95+ years	-1	fruits adjusted(g)
Appendicitis	Male	1-4 years	95+ years	-1	vegetables adjusted(g)
Appendicitis	Male	1-4 years	95+ years	-1	Healthcare access and quality index
Appendicitis	Female	1-4 years	95+ years	-1	Education (years per capita)
Appendicitis	Female	1-4 years	95+ years	-1	LDI (I$ per capita)
Appendicitis	Female	1-4 years	95+ years	-1	Health System Access (capped)
Appendicitis	Female	1-4 years	95+ years	-1	Socio-demographic Index
Appendicitis	Female	1-4 years	95+ years	-1	fruits adjusted(g)
Appendicitis	Female	1-4 years	95+ years	-1	vegetables adjusted(g)
Appendicitis	Female	1-4 years	95+ years	-1	Healthcare access and quality index
Paralytic ileus and intestinal obstruction	Male	0-6 days	95+ years	-1	Education (years per capita)
Paralytic ileus and intestinal obstruction	Male	0-6 days	95+ years	-1	LDI (I$ per capita)
Paralytic ileus and intestinal obstruction	Male	0-6 days	95+ years	-1	Health System Access (capped)
Paralytic ileus and intestinal obstruction	Male	0-6 days	95+ years	-1	Socio-demographic Index
Paralytic ileus and intestinal obstruction	Male	0-6 days	95+ years	-1	fruits adjusted(g)
Paralytic ileus and intestinal obstruction	Male	0-6 days	95+ years	-1	vegetables adjusted(g)
Paralytic ileus and intestinal obstruction	Male	0-6 days	95+ years	-1	Healthcare access and quality index
Paralytic ileus and intestinal obstruction	Female	0-6 days	95+ years	-1	Education (years per capita)
Paralytic ileus and intestinal obstruction	Female	0-6 days	95+ years	-1	LDI (I$ per capita)
Paralytic ileus and intestinal obstruction	Female	0-6 days	95+ years	-1	Health System Access (capped)
Paralytic ileus and intestinal obstruction	Female	0-6 days	95+ years	-1	Socio-demographic Index
Paralytic ileus and intestinal obstruction	Female	0-6 days	95+ years	-1	fruits adjusted(g)
Paralytic ileus and intestinal obstruction	Female	0-6 days	95+ years	-1	vegetables adjusted(g)
Paralytic ileus and intestinal obstruction	Female	0-6 days	95+ years	-1	Healthcare access and quality index
Inguinal, femoral, and abdominal hernia	Female	1-4 years	95+ years	-1	Education (years per capita)
Inguinal, femoral, and abdominal hernia	Female	1-4 years	95+ years	-1	LDI (I$ per capita)
Inguinal, femoral, and abdominal hernia	Female	1-4 years	95+ years	0	Socio-demographic Index
Inguinal, femoral, and abdominal hernia	Female	1-4 years	95+ years	-1	Healthcare access and quality index
Inguinal, femoral, and abdominal hernia	Male	1-4 years	95+ years	-1	Education (years per capita)
Inguinal, femoral, and abdominal hernia	Male	1-4 years	95+ years	-1	LDI (I$ per capita)
Inguinal, femoral, and abdominal hernia	Male	1-4 years	95+ years	0	Socio-demographic Index
Inguinal, femoral, and abdominal hernia	Male	1-4 years	95+ years	-1	Healthcare access and quality index
Inflammatory bowel disease	Male	1-4 years	95+ years	-1	Education (years per capita)
Inflammatory bowel disease	Male	1-4 years	95+ years	0	LDI (I$ per capita)
Inflammatory bowel disease	Male	1-4 years	95+ years	-1	Latitude 15 to 30 (proportion)
Inflammatory bowel disease	Male	1-4 years	95+ years	1	Latitude 30 to 45 (proportion)
Inflammatory bowel disease	Male	1-4 years	95+ years	1	Latitude Over 45 (proportion)
Inflammatory bowel disease	Male	1-4 years	95+ years	0	Socio-demographic Index
Inflammatory bowel disease	Male	1-4 years	95+ years	-1	fruits adjusted(g)
Inflammatory bowel disease	Male	1-4 years	95+ years	1	red meats adjusted(g)
Inflammatory bowel disease	Male	1-4 years	95+ years	1	saturated fats adjusted(percent)
Inflammatory bowel disease	Male	1-4 years	95+ years	-1	vegetables adjusted(g)
Inflammatory bowel disease	Male	1-4 years	95+ years	-1	Healthcare access and quality index
Inflammatory bowel disease	Female	1-4 years	95+ years	-1	Education (years per capita)
Inflammatory bowel disease	Female	1-4 years	95+ years	0	LDI (I$ per capita)
Inflammatory bowel disease	Female	1-4 years	95+ years	-1	Latitude 15 to 30 (proportion)
Inflammatory bowel disease	Female	1-4 years	95+ years	1	Latitude 30 to 45 (proportion)
Inflammatory bowel disease	Female	1-4 years	95+ years	1	Latitude Over 45 (proportion)
Inflammatory bowel disease	Female	1-4 years	95+ years	0	Socio-demographic Index
Inflammatory bowel disease	Female	1-4 years	95+ years	-1	fruits adjusted(g)
Inflammatory bowel disease	Female	1-4 years	95+ years	1	red meats adjusted(g)
Inflammatory bowel disease	Female	1-4 years	95+ years	1	saturated fats adjusted(percent)
Inflammatory bowel disease	Female	1-4 years	95+ years	-1	vegetables adjusted(g)
Inflammatory bowel disease	Female	1-4 years	95+ years	-1	Healthcare access and quality index
Vascular intestinal disorders	Female	1-4 years	95+ years	1	Alcohol (liters per capita)
Vascular intestinal disorders	Female	1-4 years	95+ years	1	Diabetes Fasting Plasma Glucose (mmol/L)
Vascular intestinal disorders	Female	1-4 years	95+ years	1	Diabetes Age-Standardized Prevalence (proportion)
Vascular intestinal disorders	Female	1-4 years	95+ years	-1	Education (years per capita)
Vascular intestinal disorders	Female	1-4 years	95+ years	-1	LDI (I$ per capita)
Vascular intestinal disorders	Female	1-4 years	95+ years	1	Cholesterol (total, mean per capita)
Vascular intestinal disorders	Female	1-4 years	95+ years	1	Systolic Blood Pressure (mmHg)
Vascular intestinal disorders	Female	1-4 years	95+ years	1	Latitude Over 45 (proportion)
Vascular intestinal disorders	Female	1-4 years	95+ years	0	Socio-demographic Index
Vascular intestinal disorders	Female	1-4 years	95+ years	-1	fruits adjusted(g)
Vascular intestinal disorders	Female	1-4 years	95+ years	1	saturated fats adjusted(percent)
Vascular intestinal disorders	Female	1-4 years	95+ years	-1	vegetables adjusted(g)
Vascular intestinal disorders	Female	1-4 years	95+ years	-1	Healthcare access and quality index
Vascular intestinal disorders	Male	1-4 years	95+ years	1	Alcohol (liters per capita)
Vascular intestinal disorders	Male	1-4 years	95+ years	1	Diabetes Fasting Plasma Glucose (mmol/L)
Vascular intestinal disorders	Male	1-4 years	95+ years	1	Diabetes Age-Standardized Prevalence (proportion)
Vascular intestinal disorders	Male	1-4 years	95+ years	-1	Education (years per capita)
Vascular intestinal disorders	Male	1-4 years	95+ years	-1	LDI (I$ per capita)
Vascular intestinal disorders	Male	1-4 years	95+ years	1	Cholesterol (total, mean per capita)
Vascular intestinal disorders	Male	1-4 years	95+ years	1	Systolic Blood Pressure (mmHg)

Cause	Sex	Age start	Age end	Direction	Covariate
Vascular intestinal disorders	Male	1-4 years	95+ years	1	Latitude Over 45 (proportion)
Vascular intestinal disorders	Male	1-4 years	95+ years	0	Socio-demographic Index
Vascular intestinal disorders	Male	1-4 years	95+ years	-1	fruits adjusted(g)
Vascular intestinal disorders	Male	1-4 years	95+ years	1	saturated fats adjusted(percent)
Vascular intestinal disorders	Male	1-4 years	95+ years	-1	vegetables adjusted(g)
Vascular intestinal disorders	Male	1-4 years	95+ years	-1	Healthcare access and quality index
Gallbladder and biliary diseases	Female	1-4 years	95+ years	1	Alcohol (liters per capita)
Gallbladder and biliary diseases	Female	1-4 years	95+ years	0	Education (years per capita)
Gallbladder and biliary diseases	Female	1-4 years	95+ years	0	LDI (I$ per capita)
Gallbladder and biliary diseases	Female	1-4 years	95+ years	1	Mean BMI
Gallbladder and biliary diseases	Female	1-4 years	95+ years	1	Population Over 65 (proportion)
Gallbladder and biliary diseases	Female	1-4 years	95+ years	0	Socio-demographic Index
Gallbladder and biliary diseases	Female	1-4 years	95+ years	1	red meats adjusted(g)
Gallbladder and biliary diseases	Female	1-4 years	95+ years	1	saturated fats adjusted(percent)
Gallbladder and biliary diseases	Female	1-4 years	95+ years	-1	Healthcare access and quality index
Gallbladder and biliary diseases	Male	1-4 years	95+ years	1	Alcohol (liters per capita)
Gallbladder and biliary diseases	Male	1-4 years	95+ years	0	Education (years per capita)
Gallbladder and biliary diseases	Male	1-4 years	95+ years	0	LDI (I$ per capita)
Gallbladder and biliary diseases	Male	1-4 years	95+ years	1	Mean BMI
Gallbladder and biliary diseases	Male	1-4 years	95+ years	1	Population Over 65 (proportion)
Gallbladder and biliary diseases	Male	1-4 years	95+ years	0	Socio-demographic Index
Gallbladder and biliary diseases	Male	1-4 years	95+ years	1	red meats adjusted(g)
Gallbladder and biliary diseases	Male	1-4 years	95+ years	1	saturated fats adjusted(percent)
Gallbladder and biliary diseases	Male	1-4 years	95+ years	-1	Healthcare access and quality index
Gallbladder and biliary diseases	Male	1-4 years	95+ years	-1	Health System Access (capped)
Pancreatitis	Female	1-4 years	95+ years	1	Alcohol (liters per capita)
Pancreatitis	Female	1-4 years	95+ years	-1	Education (years per capita)
Pancreatitis	Female	1-4 years	95+ years	0	LDI (I$ per capita)
Pancreatitis	Female	1-4 years	95+ years	1	Mean BMI
Pancreatitis	Female	1-4 years	95+ years	1	Log-transformed SEV scalar: Pancreatit
Pancreatitis	Female	1-4 years	95+ years	0	Socio-demographic Index
Pancreatitis	Female	1-4 years	95+ years	-1	Healthcare access and quality index
Pancreatitis	Male	1-4 years	95+ years	1	Alcohol (liters per capita)
Pancreatitis	Male	1-4 years	95+ years	-1	Education (years per capita)
Pancreatitis	Male	1-4 years	95+ years	0	LDI (I$ per capita)
Pancreatitis	Male	1-4 years	95+ years	1	Mean BMI
Pancreatitis	Male	1-4 years	95+ years	1	Log-transformed SEV scalar: Pancreatit
Pancreatitis	Male	1-4 years	95+ years	0	Socio-demographic Index
Pancreatitis	Male	1-4 years	95+ years	-1	Healthcare access and quality index
Pancreatitis	Male	1-4 years	95+ years	-1	Health System Access (capped)
Other digestive diseases	Male	1-4 years	95+ years	1	Alcohol (liters per capita)
Other digestive diseases	Male	1-4 years	95+ years	1	Cumulative Cigarettes (10 Years)
Other digestive diseases	Male	1-4 years	95+ years	1	Cumulative Cigarettes (5 Years)
Other digestive diseases	Male	1-4 years	95+ years	1	Diabetes Age-Standardized Prevalence (proportion)
Other digestive diseases	Male	1-4 years	95+ years	-1	Education (years per capita)
Other digestive diseases	Male	1-4 years	95+ years	-1	Health System Access 2 (unitless)
Other digestive diseases	Male	1-4 years	95+ years	-1	LDI (I$ per capita)
Other digestive diseases	Male	1-4 years	95+ years	1	Mean BMI
Other digestive diseases	Male	1-4 years	95+ years	-1	Sanitation (proportion with access)
Other digestive diseases	Male	1-4 years	95+ years	1	Smoking Prevalence
Other digestive diseases	Male	1-4 years	95+ years	-1	Improved Water Source (proportion with access)
Other digestive diseases	Male	1-4 years	95+ years	0	Socio-demographic Index
Other digestive diseases	Male	1-4 years	95+ years	-1	fruits adjusted(g)
Other digestive diseases	Male	1-4 years	95+ years	1	red meats adjusted(g)
Other digestive diseases	Male	1-4 years	95+ years	1	saturated fats adjusted(percent)
Other digestive diseases	Male	1-4 years	95+ years	0	vegetables adjusted(g)
Other digestive diseases	Male	1-4 years	95+ years	-1	Healthcare access and quality index
Other digestive diseases	Female	1-4 years	95+ years	1	Alcohol (liters per capita)
Other digestive diseases	Female	1-4 years	95+ years	1	Cumulative Cigarettes (10 Years)
Other digestive diseases	Female	1-4 years	95+ years	1	Cumulative Cigarettes (5 Years)
Other digestive diseases	Female	1-4 years	95+ years	1	Diabetes Age-Standardized Prevalence (proportion)
Other digestive diseases	Female	1-4 years	95+ years	-1	Education (years per capita)
Other digestive diseases	Female	1-4 years	95+ years	-1	Health System Access 2 (unitless)
Other digestive diseases	Female	1-4 years	95+ years	-1	LDI (I$ per capita)
Other digestive diseases	Female	1-4 years	95+ years	1	Mean BMI
Other digestive diseases	Female	1-4 years	95+ years	-1	Sanitation (proportion with access)
Other digestive diseases	Female	1-4 years	95+ years	1	Smoking Prevalence
Other digestive diseases	Female	1-4 years	95+ years	-1	Improved Water Source (proportion with access)
Other digestive diseases	Female	1-4 years	95+ years	0	Socio-demographic Index
Other digestive diseases	Female	1-4 years	95+ years	-1	fruits adjusted(g)
Other digestive diseases	Female	1-4 years	95+ years	1	red meats adjusted(g)
Other digestive diseases	Female	1-4 years	95+ years	1	saturated fats adjusted(percent)
Other digestive diseases	Female	1-4 years	95+ years	0	vegetables adjusted(g)
Other digestive diseases	Female	1-4 years	95+ years	-1	Healthcare access and quality index
Epilepsy	Male	28-364 days	95+ years	1	Cumulative Cigarettes (10 Years)
Epilepsy	Male	28-364 days	95+ years	1	Cumulative Cigarettes (5 Years)
Epilepsy	Male	28-364 days	95+ years	-1	Education (years per capita)
Epilepsy	Male	28-364 days	95+ years	-1	LDI (I$ per capita)
Epilepsy	Male	28-364 days	95+ years	1	Mean BMI
Epilepsy	Male	28-364 days	95+ years	1	Cholesterol (total, mean per capita)
Epilepsy	Male	28-364 days	95+ years	1	Systolic Blood Pressure (mmHg)
Epilepsy	Male	28-364 days	95+ years	1	Pig Meat (kg per capita)
Epilepsy	Male	28-364 days	95+ years	1	Pigs (per capita)
Epilepsy	Male	28-364 days	95+ years	1	Log-transformed SEV scalar: Epilepsy
Epilepsy	Male	28-364 days	95+ years	-1	Socio-demographic Index
Epilepsy	Male	28-364 days	95+ years	-1	Healthcare access and quality index
Epilepsy	Female	28-364 days	95+ years	1	Cumulative Cigarettes (10 Years)
Epilepsy	Female	28-364 days	95+ years	1	Cumulative Cigarettes (5 Years)
Epilepsy	Female	28-364 days	95+ years	-1	Education (years per capita)
Epilepsy	Female	28-364 days	95+ years	-1	LDI (I$ per capita)
Epilepsy	Female	28-364 days	95+ years	1	Mean BMI
Epilepsy	Female	28-364 days	95+ years	1	Cholesterol (total, mean per capita)

Cause	Sex	Age start	Age end	Direction	Covariate
Epilepsy	Female	28-364 days	95+ years	1	Systolic Blood Pressure (mmHg)
Epilepsy	Female	28-364 days	95+ years	1	Pig Meat (kg per capita)
Epilepsy	Female	28-364 days	95+ years	1	Pigs (per capita)
Epilepsy	Female	28-364 days	95+ years	1	Log-transformed SEV scalar: Epilepsy
Epilepsy	Female	28-364 days	95+ years	-1	Socio-demographic Index
Epilepsy	Female	28-364 days	95+ years	-1	Healthcare access and quality index
Multiple sclerosis	Female	20-24 years	95+ years	1	Absolute value of average latitude
Multiple sclerosis	Female	20-24 years	95+ years	1	Cumulative Cigarettes (10 Years)
Multiple sclerosis	Female	20-24 years	95+ years	1	Cumulative Cigarettes (5 Years)
Multiple sclerosis	Female	20-24 years	95+ years	-1	Education (years per capita)
Multiple sclerosis	Female	20-24 years	95+ years	-1	LDI (I$ per capita)
Multiple sclerosis	Female	20-24 years	95+ years	1	Cholesterol (total, mean per capita)
Multiple sclerosis	Female	20-24 years	95+ years	1	Smoking Prevalence
Multiple sclerosis	Female	20-24 years	95+ years	1	Socio-demographic Index
Multiple sclerosis	Female	20-24 years	95+ years	-1	Healthcare access and quality index
Multiple sclerosis	Male	20-24 years	95+ years	1	Absolute value of average latitude
Multiple sclerosis	Male	20-24 years	95+ years	1	Cumulative Cigarettes (10 Years)
Multiple sclerosis	Male	20-24 years	95+ years	1	Cumulative Cigarettes (5 Years)
Multiple sclerosis	Male	20-24 years	95+ years	-1	Education (years per capita)
Multiple sclerosis	Male	20-24 years	95+ years	-1	LDI (I$ per capita)
Multiple sclerosis	Male	20-24 years	95+ years	1	Cholesterol (total, mean per capita)
Multiple sclerosis	Male	20-24 years	95+ years	1	Smoking Prevalence
Multiple sclerosis	Male	20-24 years	95+ years	1	Socio-demographic Index
Multiple sclerosis	Male	20-24 years	95+ years	-1	Healthcare access and quality index
Motor neuron disease	Male	0-6 days	95+ years	1	Absolute value of average latitude
Motor neuron disease	Male	0-6 days	95+ years	1	Asbestos production (kg) per capita
Motor neuron disease	Male	0-6 days	95+ years	0	Education (years per capita)
Motor neuron disease	Male	0-6 days	95+ years	0	LDI (I$ per capita)
Motor neuron disease	Male	0-6 days	95+ years	0	Cholesterol (total, mean per capita)
Motor neuron disease	Male	0-6 days	95+ years	0	Sanitation (proportion with access)
Motor neuron disease	Male	0-6 days	95+ years	0	Improved Water Source (proportion with access)
Motor neuron disease	Male	0-6 days	95+ years	0	Socio-demographic Index
Motor neuron disease	Male	0-6 days	95+ years	0	fruits adjusted(g)
Motor neuron disease	Male	0-6 days	95+ years	-1	Healthcare access and quality index
Motor neuron disease	Female	0-6 days	95+ years	1	Absolute value of average latitude
Motor neuron disease	Female	0-6 days	95+ years	1	Asbestos production (kg) per capita
Motor neuron disease	Female	0-6 days	95+ years	0	Education (years per capita)
Motor neuron disease	Female	0-6 days	95+ years	0	LDI (I$ per capita)
Motor neuron disease	Female	0-6 days	95+ years	0	Cholesterol (total, mean per capita)
Motor neuron disease	Female	0-6 days	95+ years	0	Sanitation (proportion with access)
Motor neuron disease	Female	0-6 days	95+ years	0	Improved Water Source (proportion with access)
Motor neuron disease	Female	0-6 days	95+ years	0	Socio-demographic Index
Motor neuron disease	Female	0-6 days	95+ years	0	fruits adjusted(g)
Motor neuron disease	Female	0-6 days	95+ years	-1	Healthcare access and quality index
Other neurological disorders	Male	28-364 days	95+ years	1	Alcohol (liters per capita)
Other neurological disorders	Male	28-364 days	95+ years	1	Animal Fats (kcal per capita)
Other neurological disorders	Male	28-364 days	95+ years	1	Cumulative Cigarettes (10 Years)
Other neurological disorders	Male	28-364 days	95+ years	1	Cumulative Cigarettes (5 Years)
Other neurological disorders	Male	28-364 days	95+ years	-1	Education (years per capita)
Other neurological disorders	Male	28-364 days	95+ years	-1	LDI (I$ per capita)
Other neurological disorders	Male	28-364 days	95+ years	1	Underweight (proportion <2SD weight for age, <5 years)
Other neurological disorders	Male	28-364 days	95+ years	1	Mean BMI
Other neurological disorders	Male	28-364 days	95+ years	1	Cholesterol (total, mean per capita)
Other neurological disorders	Male	28-364 days	95+ years	1	Systolic Blood Pressure (mmHg)
Other neurological disorders	Male	28-364 days	95+ years	1	Pig Meat (kg per capita)
Other neurological disorders	Male	28-364 days	95+ years	1	Population Density (over 1000 ppl/sqkm, proportion)
Other neurological disorders	Male	28-364 days	95+ years	1	Smoking Prevalence
Other neurological disorders	Male	28-364 days	95+ years	0	Socio-demographic Index
Other neurological disorders	Male	28-364 days	95+ years	-1	fruits adjusted(g)
Other neurological disorders	Male	28-364 days	95+ years	1	red meats adjusted(g)
Other neurological disorders	Male	28-364 days	95+ years	-1	Healthcare access and quality index
Other neurological disorders	Female	28-364 days	95+ years	1	Alcohol (liters per capita)
Other neurological disorders	Female	28-364 days	95+ years	1	Animal Fats (kcal per capita)
Other neurological disorders	Female	28-364 days	95+ years	1	Cumulative Cigarettes (10 Years)
Other neurological disorders	Female	28-364 days	95+ years	1	Cumulative Cigarettes (5 Years)
Other neurological disorders	Female	28-364 days	95+ years	-1	Education (years per capita)
Other neurological disorders	Female	28-364 days	95+ years	-1	LDI (I$ per capita)
Other neurological disorders	Female	28-364 days	95+ years	1	Underweight (proportion <2SD weight for age, <5 years)
Other neurological disorders	Female	28-364 days	95+ years	1	Mean BMI
Other neurological disorders	Female	28-364 days	95+ years	1	Cholesterol (total, mean per capita)
Other neurological disorders	Female	28-364 days	95+ years	1	Systolic Blood Pressure (mmHg)
Other neurological disorders	Female	28-364 days	95+ years	1	Pig Meat (kg per capita)
Other neurological disorders	Female	28-364 days	95+ years	1	Population Density (over 1000 ppl/sqkm, proportion)
Other neurological disorders	Female	28-364 days	95+ years	1	Smoking Prevalence
Other neurological disorders	Female	28-364 days	95+ years	0	Socio-demographic Index
Other neurological disorders	Female	28-364 days	95+ years	-1	fruits adjusted(g)
Other neurological disorders	Female	28-364 days	95+ years	1	red meats adjusted(g)
Other neurological disorders	Female	28-364 days	95+ years	-1	Healthcare access and quality index
Schizophrenia	Male	25-29 years	95+ years	0	Alcohol (liters per capita)
Schizophrenia	Male	25-29 years	95+ years	0	Cumulative Cigarettes (20 Years)
Schizophrenia	Male	25-29 years	95+ years	0	Education (years per capita)
Schizophrenia	Male	25-29 years	95+ years	0	Health System Access 2 (unitless)
Schizophrenia	Male	25-29 years	95+ years	0	LDI (I$ per capita)
Schizophrenia	Male	25-29 years	95+ years	1	Underweight (proportion <2SD weight for age, <5 years)
Schizophrenia	Male	25-29 years	95+ years	0	Smoking Prevalence
Schizophrenia	Male	25-29 years	95+ years	0	Socio-demographic Index
Schizophrenia	Female	25-29 years	95+ years	0	Alcohol (liters per capita)
Schizophrenia	Female	25-29 years	95+ years	0	Cumulative Cigarettes (20 Years)
Schizophrenia	Female	25-29 years	95+ years	0	Education (years per capita)
Schizophrenia	Female	25-29 years	95+ years	0	Health System Access 2 (unitless)
Schizophrenia	Female	25-29 years	95+ years	0	LDI (I$ per capita)
Schizophrenia	Female	25-29 years	95+ years	1	Underweight (proportion <2SD weight for age, <5 years)

Cause	Sex	Age start	Age end	Direction	Covariate
Schizophrenia	Female	25-29 years	95+ years	0	Smoking Prevalence
Schizophrenia	Female	25-29 years	95+ years	0	Socio-demographic Index
Alcohol use disorders	Male	15-19 years	95+ years	1	Alcohol (liters per capita)
Alcohol use disorders	Male	15-19 years	95+ years	0	Cumulative Cigarettes (10 Years)
Alcohol use disorders	Male	15-19 years	95+ years	-1	Education (years per capita)
Alcohol use disorders	Male	15-19 years	95+ years	-1	Health System Access 2 (unitless)
Alcohol use disorders	Male	15-19 years	95+ years	-1	LDI (I$ per capita)
Alcohol use disorders	Male	15-19 years	95+ years	-1	Religion (binary, >50% Muslim)
Alcohol use disorders	Male	15-19 years	95+ years	0	Smoking Prevalence
Alcohol use disorders	Male	15-19 years	95+ years	1	Prevalence of binge drinking
Alcohol use disorders	Male	15-19 years	95+ years	0	Socio-demographic Index
Alcohol use disorders	Male	15-19 years	95+ years	-1	Healthcare access and quality index
Alcohol use disorders	Female	15-19 years	95+ years	1	Alcohol (liters per capita)
Alcohol use disorders	Female	15-19 years	95+ years	0	Cumulative Cigarettes (10 Years)
Alcohol use disorders	Female	15-19 years	95+ years	-1	Education (years per capita)
Alcohol use disorders	Female	15-19 years	95+ years	-1	Health System Access 2 (unitless)
Alcohol use disorders	Female	15-19 years	95+ years	-1	LDI (I$ per capita)
Alcohol use disorders	Female	15-19 years	95+ years	-1	Religion (binary, >50% Muslim)
Alcohol use disorders	Female	15-19 years	95+ years	0	Smoking Prevalence
Alcohol use disorders	Female	15-19 years	95+ years	1	Prevalence of binge drinking
Alcohol use disorders	Female	15-19 years	95+ years	0	Socio-demographic Index
Alcohol use disorders	Female	15-19 years	95+ years	-1	Healthcare access and quality index
Drug use disorders	Male	15-19 years	95+ years	1	Alcohol (liters per capita)
Drug use disorders	Male	15-19 years	95+ years	1	Cumulative Cigarettes (10 Years)
Drug use disorders	Male	15-19 years	95+ years	1	Cumulative Cigarettes (5 Years)
Drug use disorders	Male	15-19 years	95+ years	0	Education (years per capita)
Drug use disorders	Male	15-19 years	95+ years	0	LDI (I$ per capita)
Drug use disorders	Male	15-19 years	95+ years	1	Opium Cultivation (binary)
Drug use disorders	Male	15-19 years	95+ years	1	Smoking Prevalence
Drug use disorders	Male	15-19 years	95+ years	0	Socio-demographic Index
Drug use disorders	Male	15-19 years	95+ years	-1	Healthcare access and quality index
Drug use disorders	Female	15-19 years	95+ years	1	Alcohol (liters per capita)
Drug use disorders	Female	15-19 years	95+ years	1	Cumulative Cigarettes (10 Years)
Drug use disorders	Female	15-19 years	95+ years	1	Cumulative Cigarettes (5 Years)
Drug use disorders	Female	15-19 years	95+ years	0	Education (years per capita)
Drug use disorders	Female	15-19 years	95+ years	0	LDI (I$ per capita)
Drug use disorders	Female	15-19 years	95+ years	1	Opium Cultivation (binary)
Drug use disorders	Female	15-19 years	95+ years	1	Smoking Prevalence
Drug use disorders	Female	15-19 years	95+ years	0	Socio-demographic Index
Drug use disorders	Female	15-19 years	95+ years	-1	Healthcare access and quality index
Drug use disorders	Male	0-6 days	7-27 days	1	Alcohol (liters per capita)
Drug use disorders	Male	0-6 days	7-27 days	1	Cumulative Cigarettes (10 Years)
Drug use disorders	Male	0-6 days	7-27 days	1	Cumulative Cigarettes (5 Years)
Drug use disorders	Male	0-6 days	7-27 days	0	Education (years per capita)
Drug use disorders	Male	0-6 days	7-27 days	0	Health System Access 2 (unitless)
Drug use disorders	Male	0-6 days	7-27 days	0	LDI (I$ per capita)
Drug use disorders	Male	0-6 days	7-27 days	1	Opium Cultivation (binary)
Drug use disorders	Male	0-6 days	7-27 days	1	Smoking Prevalence
Drug use disorders	Male	0-6 days	7-27 days	0	Socio-demographic Index
Drug use disorders	Male	0-6 days	7-27 days	-1	Healthcare access and quality index
Drug use disorders	Female	0-6 days	7-27 days	1	Alcohol (liters per capita)
Drug use disorders	Female	0-6 days	7-27 days	1	Cumulative Cigarettes (10 Years)
Drug use disorders	Female	0-6 days	7-27 days	1	Cumulative Cigarettes (5 Years)
Drug use disorders	Female	0-6 days	7-27 days	0	Education (years per capita)
Drug use disorders	Female	0-6 days	7-27 days	0	Health System Access 2 (unitless)
Drug use disorders	Female	0-6 days	7-27 days	0	LDI (I$ per capita)
Drug use disorders	Female	0-6 days	7-27 days	1	Opium Cultivation (binary)
Drug use disorders	Female	0-6 days	7-27 days	1	Smoking Prevalence
Drug use disorders	Female	0-6 days	7-27 days	0	Socio-demographic Index
Drug use disorders	Female	0-6 days	7-27 days	-1	Healthcare access and quality index
Opioid use disorders	Male	15-19 years	95+ years	1	Alcohol (liters per capita)
Opioid use disorders	Male	15-19 years	95+ years	1	Cumulative Cigarettes (10 Years)
Opioid use disorders	Male	15-19 years	95+ years	1	Cumulative Cigarettes (5 Years)
Opioid use disorders	Male	15-19 years	95+ years	0	Education (years per capita)
Opioid use disorders	Male	15-19 years	95+ years	0	LDI (I$ per capita)
Opioid use disorders	Male	15-19 years	95+ years	1	Opium Cultivation (binary)
Opioid use disorders	Male	15-19 years	95+ years	1	Smoking Prevalence
Opioid use disorders	Male	15-19 years	95+ years	0	Socio-demographic Index
Opioid use disorders	Male	15-19 years	95+ years	-1	Healthcare access and quality index
Opioid use disorders	Female	15-19 years	95+ years	1	Alcohol (liters per capita)
Opioid use disorders	Female	15-19 years	95+ years	1	Cumulative Cigarettes (10 Years)
Opioid use disorders	Female	15-19 years	95+ years	1	Cumulative Cigarettes (5 Years)
Opioid use disorders	Female	15-19 years	95+ years	0	Education (years per capita)
Opioid use disorders	Female	15-19 years	95+ years	0	LDI (I$ per capita)
Opioid use disorders	Female	15-19 years	95+ years	1	Opium Cultivation (binary)
Opioid use disorders	Female	15-19 years	95+ years	1	Smoking Prevalence
Opioid use disorders	Female	15-19 years	95+ years	0	Socio-demographic Index
Opioid use disorders	Female	15-19 years	95+ years	-1	Healthcare access and quality index
Opioid use disorders	Male	0-6 days	7-27 days	1	Alcohol (liters per capita)
Opioid use disorders	Male	0-6 days	7-27 days	1	Cumulative Cigarettes (10 Years)
Opioid use disorders	Male	0-6 days	7-27 days	1	Cumulative Cigarettes (5 Years)
Opioid use disorders	Male	0-6 days	7-27 days	0	Education (years per capita)
Opioid use disorders	Male	0-6 days	7-27 days	0	LDI (I$ per capita)
Opioid use disorders	Male	0-6 days	7-27 days	1	Opium Cultivation (binary)
Opioid use disorders	Male	0-6 days	7-27 days	1	Smoking Prevalence
Opioid use disorders	Male	0-6 days	7-27 days	0	Socio-demographic Index
Opioid use disorders	Male	0-6 days	7-27 days	-1	Healthcare access and quality index
Opioid use disorders	Female	0-6 days	7-27 days	1	Alcohol (liters per capita)
Opioid use disorders	Female	0-6 days	7-27 days	1	Cumulative Cigarettes (10 Years)
Opioid use disorders	Female	0-6 days	7-27 days	1	Cumulative Cigarettes (5 Years)
Opioid use disorders	Female	0-6 days	7-27 days	0	Education (years per capita)
Opioid use disorders	Female	0-6 days	7-27 days	0	LDI (I$ per capita)

Cause	Sex	Age start	Age end	Direction	Covariate
Opioid use disorders	Female	0-6 days	7-27 days	1	Opium Cultivation (binary)
Opioid use disorders	Female	0-6 days	7-27 days	1	Smoking Prevalence
Opioid use disorders	Female	0-6 days	7-27 days	0	Socio-demographic Index
Opioid use disorders	Female	0-6 days	7-27 days	-1	Healthcare access and quality index
Opioid use disorders	Male	0-6 days	7-27 days	0	Health System Access 2 (unitless)
Opioid use disorders	Female	0-6 days	7-27 days	0	Health System Access 2 (unitless)
Cocaine use disorders	Male	15-19 years	95+ years	1	Alcohol (liters per capita)
Cocaine use disorders	Male	15-19 years	95+ years	1	Cumulative Cigarettes (10 Years)
Cocaine use disorders	Male	15-19 years	95+ years	1	Cumulative Cigarettes (5 Years)
Cocaine use disorders	Male	15-19 years	95+ years	0	Education (years per capita)
Cocaine use disorders	Male	15-19 years	95+ years	0	LDI (I$ per capita)
Cocaine use disorders	Male	15-19 years	95+ years	1	Smoking Prevalence
Cocaine use disorders	Male	15-19 years	95+ years	1	Socio-demographic Index
Cocaine use disorders	Male	15-19 years	95+ years	-1	Healthcare access and quality index
Cocaine use disorders	Female	15-19 years	95+ years	1	Alcohol (liters per capita)
Cocaine use disorders	Female	15-19 years	95+ years	1	Cumulative Cigarettes (10 Years)
Cocaine use disorders	Female	15-19 years	95+ years	1	Cumulative Cigarettes (5 Years)
Cocaine use disorders	Female	15-19 years	95+ years	0	Education (years per capita)
Cocaine use disorders	Female	15-19 years	95+ years	0	LDI (I$ per capita)
Cocaine use disorders	Female	15-19 years	95+ years	1	Smoking Prevalence
Cocaine use disorders	Female	15-19 years	95+ years	1	Socio-demographic Index
Cocaine use disorders	Female	15-19 years	95+ years	-1	Healthcare access and quality index
Amphetamine use disorders	Male	15-19 years	95+ years	1	Alcohol (liters per capita)
Amphetamine use disorders	Male	15-19 years	95+ years	1	Cumulative Cigarettes (10 Years)
Amphetamine use disorders	Male	15-19 years	95+ years	1	Cumulative Cigarettes (5 Years)
Amphetamine use disorders	Male	15-19 years	95+ years	0	Education (years per capita)
Amphetamine use disorders	Male	15-19 years	95+ years	0	LDI (I$ per capita)
Amphetamine use disorders	Male	15-19 years	95+ years	1	Smoking Prevalence
Amphetamine use disorders	Male	15-19 years	95+ years	1	Socio-demographic Index
Amphetamine use disorders	Male	15-19 years	95+ years	-1	Healthcare access and quality index
Amphetamine use disorders	Female	15-19 years	95+ years	1	Alcohol (liters per capita)
Amphetamine use disorders	Female	15-19 years	95+ years	1	Cumulative Cigarettes (10 Years)
Amphetamine use disorders	Female	15-19 years	95+ years	1	Cumulative Cigarettes (5 Years)
Amphetamine use disorders	Female	15-19 years	95+ years	0	Education (years per capita)
Amphetamine use disorders	Female	15-19 years	95+ years	0	LDI (I$ per capita)
Amphetamine use disorders	Female	15-19 years	95+ years	1	Smoking Prevalence
Amphetamine use disorders	Female	15-19 years	95+ years	1	Socio-demographic Index
Amphetamine use disorders	Female	15-19 years	95+ years	-1	Healthcare access and quality index
Other drug use disorders	Male	15-19 years	95+ years	1	Alcohol (liters per capita)
Other drug use disorders	Male	15-19 years	95+ years	1	Cumulative Cigarettes (10 Years)
Other drug use disorders	Male	15-19 years	95+ years	1	Cumulative Cigarettes (5 Years)
Other drug use disorders	Male	15-19 years	95+ years	0	Education (years per capita)
Other drug use disorders	Male	15-19 years	95+ years	0	LDI (I$ per capita)
Other drug use disorders	Male	15-19 years	95+ years	1	Smoking Prevalence
Other drug use disorders	Male	15-19 years	95+ years	0	Socio-demographic Index
Other drug use disorders	Male	15-19 years	95+ years	-1	Healthcare access and quality index
Other drug use disorders	Female	15-19 years	95+ years	1	Alcohol (liters per capita)
Other drug use disorders	Female	15-19 years	95+ years	1	Cumulative Cigarettes (10 Years)
Other drug use disorders	Female	15-19 years	95+ years	1	Cumulative Cigarettes (5 Years)
Other drug use disorders	Female	15-19 years	95+ years	0	Education (years per capita)
Other drug use disorders	Female	15-19 years	95+ years	0	LDI (I$ per capita)
Other drug use disorders	Female	15-19 years	95+ years	1	Smoking Prevalence
Other drug use disorders	Female	15-19 years	95+ years	0	Socio-demographic Index
Other drug use disorders	Female	15-19 years	95+ years	-1	Healthcare access and quality index
Eating disorders	Male	5-9 years	45-49 years	1	Education (years per capita)
Eating disorders	Male	5-9 years	45-49 years	1	LDI (I$ per capita)
Eating disorders	Male	5-9 years	45-49 years	-1	Underweight (proportion <2SD weight for age, <5 years)
Eating disorders	Male	5-9 years	45-49 years	1	Sanitation (proportion with access)
Eating disorders	Male	5-9 years	45-49 years	1	Maternal education (years per capita)
Eating disorders	Male	5-9 years	45-49 years	1	Socio-demographic Index
Eating disorders	Male	5-9 years	45-49 years	-1	Healthcare access and quality index
Eating disorders	Female	5-9 years	45-49 years	1	Education (years per capita)
Eating disorders	Female	5-9 years	45-49 years	1	LDI (I$ per capita)
Eating disorders	Female	5-9 years	45-49 years	-1	Underweight (proportion <2SD weight for age, <5 years)
Eating disorders	Female	5-9 years	45-49 years	1	Sanitation (proportion with access)
Eating disorders	Female	5-9 years	45-49 years	1	Maternal education (years per capita)
Eating disorders	Female	5-9 years	45-49 years	1	Socio-demographic Index
Eating disorders	Female	5-9 years	45-49 years	-1	Healthcare access and quality index
Anorexia nervosa	Male	5-9 years	45-49 years	1	Education (years per capita)
Anorexia nervosa	Male	5-9 years	45-49 years	1	LDI (I$ per capita)
Anorexia nervosa	Male	5-9 years	45-49 years	-1	Underweight (proportion <2SD weight for age, <5 years)
Anorexia nervosa	Male	5-9 years	45-49 years	1	Sanitation (proportion with access)
Anorexia nervosa	Male	5-9 years	45-49 years	1	Maternal education (years per capita)
Anorexia nervosa	Male	5-9 years	45-49 years	1	Socio-demographic Index
Anorexia nervosa	Male	5-9 years	45-49 years	-1	Healthcare access and quality index
Anorexia nervosa	Female	5-9 years	45-49 years	1	Education (years per capita)
Anorexia nervosa	Female	5-9 years	45-49 years	1	LDI (I$ per capita)
Anorexia nervosa	Female	5-9 years	45-49 years	-1	Underweight (proportion <2SD weight for age, <5 years)
Anorexia nervosa	Female	5-9 years	45-49 years	1	Sanitation (proportion with access)
Anorexia nervosa	Female	5-9 years	45-49 years	1	Maternal education (years per capita)
Anorexia nervosa	Female	5-9 years	45-49 years	1	Socio-demographic Index
Anorexia nervosa	Female	5-9 years	45-49 years	-1	Healthcare access and quality index
Bulimia nervosa	Male	5-9 years	45-49 years	1	Education (years per capita)
Bulimia nervosa	Male	5-9 years	45-49 years	1	LDI (I$ per capita)
Bulimia nervosa	Male	5-9 years	45-49 years	-1	Underweight (proportion <2SD weight for age, <5 years)
Bulimia nervosa	Male	5-9 years	45-49 years	1	Sanitation (proportion with access)
Bulimia nervosa	Male	5-9 years	45-49 years	1	Maternal education (years per capita)
Bulimia nervosa	Male	5-9 years	45-49 years	1	Socio-demographic Index
Bulimia nervosa	Male	5-9 years	45-49 years	-1	Healthcare access and quality index
Bulimia nervosa	Female	5-9 years	45-49 years	1	Education (years per capita)
Bulimia nervosa	Female	5-9 years	45-49 years	1	LDI (I$ per capita)
Bulimia nervosa	Female	5-9 years	45-49 years	-1	Underweight (proportion <2SD weight for age, <5 years)

Cause	Sex	Age start	Age end	Direction	Covariate
Bulimia nervosa	Female	5-9 years	45-49 years	1	Sanitation (proportion with access)
Bulimia nervosa	Female	5-9 years	45-49 years	1	Maternal education (years per capita)
Bulimia nervosa	Female	5-9 years	45-49 years	1	Socio-demographic Index
Bulimia nervosa	Female	5-9 years	45-49 years	-1	Healthcare access and quality index
Diabetes mellitus	Female	0-6 days	20-24 years	0	Animal Fats (kcal per capita)
Diabetes mellitus	Female	0-6 days	20-24 years	1	Diabetes Fasting Plasma Glucose (mmol/L)
Diabetes mellitus	Female	0-6 days	20-24 years	1	Diabetes Age-Standardized Prevalence (proportion)
Diabetes mellitus	Female	0-6 days	20-24 years	0	Education (years per capita)
Diabetes mellitus	Female	0-6 days	20-24 years	0	LDI (I$ per capita)
Diabetes mellitus	Female	0-6 days	20-24 years	0	Mean BMI
Diabetes mellitus	Female	0-6 days	20-24 years	0	Cholesterol (total, mean per capita)
Diabetes mellitus	Female	0-6 days	20-24 years	0	Systolic Blood Pressure (mmHg)
Diabetes mellitus	Female	0-6 days	20-24 years	0	fruits adjusted(g)
Diabetes mellitus	Female	0-6 days	20-24 years	0	vegetables adjusted(g)
Diabetes mellitus	Female	0-6 days	20-24 years	0	whole grains adjusted(g)
Diabetes mellitus	Female	0-6 days	20-24 years	0	energy unadjusted(kcal)
Diabetes mellitus	Female	0-6 days	20-24 years	0	Healthcare access and quality index
Diabetes mellitus	Male	0-6 days	20-24 years	0	Animal Fats (kcal per capita)
Diabetes mellitus	Male	0-6 days	20-24 years	1	Diabetes Fasting Plasma Glucose (mmol/L)
Diabetes mellitus	Male	0-6 days	20-24 years	1	Diabetes Age-Standardized Prevalence (proportion)
Diabetes mellitus	Male	0-6 days	20-24 years	0	Education (years per capita)
Diabetes mellitus	Male	0-6 days	20-24 years	0	LDI (I$ per capita)
Diabetes mellitus	Male	0-6 days	20-24 years	0	Mean BMI
Diabetes mellitus	Male	0-6 days	20-24 years	0	Cholesterol (total, mean per capita)
Diabetes mellitus	Male	0-6 days	20-24 years	0	Systolic Blood Pressure (mmHg)
Diabetes mellitus	Male	0-6 days	20-24 years	0	fruits adjusted(g)
Diabetes mellitus	Male	0-6 days	20-24 years	0	vegetables adjusted(g)
Diabetes mellitus	Male	0-6 days	20-24 years	0	whole grains adjusted(g)
Diabetes mellitus	Male	0-6 days	20-24 years	0	energy unadjusted(kcal)
Diabetes mellitus	Male	0-6 days	20-24 years	0	Healthcare access and quality index
Diabetes mellitus	Male	25-29 years	95+ years	0	Animal Fats (kcal per capita)
Diabetes mellitus	Male	25-29 years	95+ years	1	Diabetes Fasting Plasma Glucose (mmol/L)
Diabetes mellitus	Male	25-29 years	95+ years	1	Diabetes Age-Standardized Prevalence (proportion)
Diabetes mellitus	Male	25-29 years	95+ years	0	Education (years per capita)
Diabetes mellitus	Male	25-29 years	95+ years	0	LDI (I$ per capita)
Diabetes mellitus	Male	25-29 years	95+ years	0	Mean BMI
Diabetes mellitus	Male	25-29 years	95+ years	0	Cholesterol (total, mean per capita)
Diabetes mellitus	Male	25-29 years	95+ years	0	Systolic Blood Pressure (mmHg)
Diabetes mellitus	Male	25-29 years	95+ years	0	fruits adjusted(g)
Diabetes mellitus	Male	25-29 years	95+ years	0	vegetables adjusted(g)
Diabetes mellitus	Male	25-29 years	95+ years	0	whole grains adjusted(g)
Diabetes mellitus	Male	25-29 years	95+ years	0	energy unadjusted(kcal)
Diabetes mellitus	Male	25-29 years	95+ years	0	Healthcare access and quality index
Diabetes mellitus	Female	25-29 years	95+ years	0	Animal Fats (kcal per capita)
Diabetes mellitus	Female	25-29 years	95+ years	1	Diabetes Fasting Plasma Glucose (mmol/L)
Diabetes mellitus	Female	25-29 years	95+ years	1	Diabetes Age-Standardized Prevalence (proportion)
Diabetes mellitus	Female	25-29 years	95+ years	0	Education (years per capita)
Diabetes mellitus	Female	25-29 years	95+ years	0	LDI (I$ per capita)
Diabetes mellitus	Female	25-29 years	95+ years	0	Mean BMI
Diabetes mellitus	Female	25-29 years	95+ years	0	Cholesterol (total, mean per capita)
Diabetes mellitus	Female	25-29 years	95+ years	0	Systolic Blood Pressure (mmHg)
Diabetes mellitus	Female	25-29 years	95+ years	0	fruits adjusted(g)
Diabetes mellitus	Female	25-29 years	95+ years	0	vegetables adjusted(g)
Diabetes mellitus	Female	25-29 years	95+ years	0	whole grains adjusted(g)
Diabetes mellitus	Female	25-29 years	95+ years	0	energy unadjusted(kcal)
Diabetes mellitus	Female	25-29 years	95+ years	0	Healthcare access and quality index
Acute glomerulonephritis	Female	28-364 days	95+ years	1	Diabetes Age-Standardized Prevalence (proportion)
Acute glomerulonephritis	Female	28-364 days	95+ years	-1	Education (years per capita)
Acute glomerulonephritis	Female	28-364 days	95+ years	-1	LDI (I$ per capita)
Acute glomerulonephritis	Female	28-364 days	95+ years	1	Systolic Blood Pressure (mmHg)
Acute glomerulonephritis	Female	28-364 days	95+ years	-1	Sanitation (proportion with access)
Acute glomerulonephritis	Female	28-364 days	95+ years	-1	Improved Water Source (proportion with access)
Acute glomerulonephritis	Female	28-364 days	95+ years	-1	Socio-demographic Index
Acute glomerulonephritis	Female	28-364 days	95+ years	-1	Healthcare access and quality index
Acute glomerulonephritis	Male	28-364 days	95+ years	1	Diabetes Age-Standardized Prevalence (proportion)
Acute glomerulonephritis	Male	28-364 days	95+ years	-1	Education (years per capita)
Acute glomerulonephritis	Male	28-364 days	95+ years	-1	LDI (I$ per capita)
Acute glomerulonephritis	Male	28-364 days	95+ years	1	Systolic Blood Pressure (mmHg)
Acute glomerulonephritis	Male	28-364 days	95+ years	-1	Sanitation (proportion with access)
Acute glomerulonephritis	Male	28-364 days	95+ years	-1	Improved Water Source (proportion with access)
Acute glomerulonephritis	Male	28-364 days	95+ years	-1	Socio-demographic Index
Acute glomerulonephritis	Male	28-364 days	95+ years	-1	Healthcare access and quality index
Chronic kidney disease	Female	28-364 days	95+ years	1	Diabetes Fasting Plasma Glucose (mmol/L)
Chronic kidney disease	Female	28-364 days	95+ years	1	Diabetes Age-Standardized Prevalence (proportion)
Chronic kidney disease	Female	28-364 days	95+ years	-1	Education (years per capita)
Chronic kidney disease	Female	28-364 days	95+ years	-1	LDI (I$ per capita)
Chronic kidney disease	Female	28-364 days	95+ years	1	Mean BMI
Chronic kidney disease	Female	28-364 days	95+ years	1	Cholesterol (total, mean per capita)
Chronic kidney disease	Female	28-364 days	95+ years	1	Systolic Blood Pressure (mmHg)
Chronic kidney disease	Female	28-364 days	95+ years	0	Socio-demographic Index
Chronic kidney disease	Female	28-364 days	95+ years	0	red meats adjusted(g)
Chronic kidney disease	Female	28-364 days	95+ years	0	whole grains adjusted(g)
Chronic kidney disease	Female	28-364 days	95+ years	1	energy unadjusted(kcal)
Chronic kidney disease	Female	28-364 days	95+ years	-1	Healthcare access and quality index
Chronic kidney disease	Male	28-364 days	95+ years	1	Diabetes Fasting Plasma Glucose (mmol/L)
Chronic kidney disease	Male	28-364 days	95+ years	1	Diabetes Age-Standardized Prevalence (proportion)
Chronic kidney disease	Male	28-364 days	95+ years	-1	Education (years per capita)
Chronic kidney disease	Male	28-364 days	95+ years	-1	LDI (I$ per capita)
Chronic kidney disease	Male	28-364 days	95+ years	1	Mean BMI
Chronic kidney disease	Male	28-364 days	95+ years	1	Cholesterol (total, mean per capita)
Chronic kidney disease	Male	28-364 days	95+ years	1	Systolic Blood Pressure (mmHg)
Chronic kidney disease	Male	28-364 days	95+ years	0	Socio-demographic Index

Cause	Sex	Age start	Age end	Direction	Covariate
Chronic kidney disease	Male	28-364 days	95+ years	0	red meats adjusted(g)
Chronic kidney disease	Male	28-364 days	95+ years	0	whole grains adjusted(g)
Chronic kidney disease	Male	28-364 days	95+ years	1	energy unadjusted(kcal)
Chronic kidney disease	Male	28-364 days	95+ years	-1	Healthcare access and quality index
Urinary diseases and male infertility	Male	0-6 days	95+ years	-1	Education (years per capita)
Urinary diseases and male infertility	Male	0-6 days	95+ years	-1	LDI (I$ per capita)
Urinary diseases and male infertility	Male	0-6 days	95+ years	1	Mean BMI
Urinary diseases and male infertility	Male	0-6 days	95+ years	0	Latitude Under 15 (proportion)
Urinary diseases and male infertility	Male	0-6 days	95+ years	0	Latitude 15 to 30 (proportion)
Urinary diseases and male infertility	Male	0-6 days	95+ years	0	Latitude 30 to 45 (proportion)
Urinary diseases and male infertility	Male	0-6 days	95+ years	0	Latitude Over 45 (proportion)
Urinary diseases and male infertility	Male	0-6 days	95+ years	0	Socio-demographic Index
Urinary diseases and male infertility	Male	0-6 days	95+ years	-1	Healthcare access and quality index
Urinary diseases and male infertility	Female	0-6 days	95+ years	-1	Education (years per capita)
Urinary diseases and male infertility	Female	0-6 days	95+ years	-1	LDI (I$ per capita)
Urinary diseases and male infertility	Female	0-6 days	95+ years	1	Mean BMI
Urinary diseases and male infertility	Female	0-6 days	95+ years	0	Latitude Under 15 (proportion)
Urinary diseases and male infertility	Female	0-6 days	95+ years	0	Latitude 15 to 30 (proportion)
Urinary diseases and male infertility	Female	0-6 days	95+ years	0	Latitude 30 to 45 (proportion)
Urinary diseases and male infertility	Female	0-6 days	95+ years	0	Latitude Over 45 (proportion)
Urinary diseases and male infertility	Female	0-6 days	95+ years	0	Socio-demographic Index
Urinary diseases and male infertility	Female	0-6 days	95+ years	-1	Healthcare access and quality index
Interstitial nephritis and urinary tract infections	Female	0-6 days	95+ years	-1	Education (years per capita)
Interstitial nephritis and urinary tract infections	Female	0-6 days	95+ years	-1	LDI (I$ per capita)
Interstitial nephritis and urinary tract infections	Female	0-6 days	95+ years	1	Sanitation (proportion with access)
Interstitial nephritis and urinary tract infections	Female	0-6 days	95+ years	0	Socio-demographic Index
Interstitial nephritis and urinary tract infections	Female	0-6 days	95+ years	-1	Healthcare access and quality index
Interstitial nephritis and urinary tract infections	Male	0-6 days	95+ years	-1	Education (years per capita)
Interstitial nephritis and urinary tract infections	Male	0-6 days	95+ years	-1	LDI (I$ per capita)
Interstitial nephritis and urinary tract infections	Male	0-6 days	95+ years	1	Sanitation (proportion with access)
Interstitial nephritis and urinary tract infections	Male	0-6 days	95+ years	-1	Health System Access (capped)
Interstitial nephritis and urinary tract infections	Male	0-6 days	95+ years	0	Socio-demographic Index
Interstitial nephritis and urinary tract infections	Male	0-6 days	95+ years	-1	Healthcare access and quality index
Urolithiasis	Female	5-9 years	95+ years	-1	Education (years per capita)
Urolithiasis	Female	5-9 years	95+ years	-1	LDI (I$ per capita)
Urolithiasis	Female	5-9 years	95+ years	1	90th percentile climatic temperature in the given country-year.
Urolithiasis	Female	5-9 years	95+ years	0	Socio-demographic Index
Urolithiasis	Female	5-9 years	95+ years	-1	fruits adjusted(g)
Urolithiasis	Female	5-9 years	95+ years	1	red meats adjusted(g)
Urolithiasis	Female	5-9 years	95+ years	-1	vegetables adjusted(g)
Urolithiasis	Female	5-9 years	95+ years	1	Healthcare access and quality index
Urolithiasis	Male	5-9 years	95+ years	-1	Education (years per capita)
Urolithiasis	Male	5-9 years	95+ years	-1	LDI (I$ per capita)
Urolithiasis	Male	5-9 years	95+ years	1	90th percentile climatic temperature in the given country-year.
Urolithiasis	Male	5-9 years	95+ years	0	Socio-demographic Index
Urolithiasis	Male	5-9 years	95+ years	-1	fruits adjusted(g)
Urolithiasis	Male	5-9 years	95+ years	1	red meats adjusted(g)
Urolithiasis	Male	5-9 years	95+ years	-1	vegetables adjusted(g)
Urolithiasis	Male	5-9 years	95+ years	-1	Healthcare access and quality index
Other urinary diseases	Male	0-6 days	95+ years	-1	Education (years per capita)
Other urinary diseases	Male	0-6 days	95+ years	-1	LDI (I$ per capita)
Other urinary diseases	Male	0-6 days	95+ years	1	Mean BMI
Other urinary diseases	Male	0-6 days	95+ years	0	Socio-demographic Index
Other urinary diseases	Male	0-6 days	95+ years	-1	Healthcare access and quality index
Other urinary diseases	Female	0-6 days	95+ years	1	Education (years per capita)
Other urinary diseases	Female	0-6 days	95+ years	1	LDI (I$ per capita)
Other urinary diseases	Female	0-6 days	95+ years	1	Mean BMI
Other urinary diseases	Female	0-6 days	95+ years	0	Socio-demographic Index
Other urinary diseases	Female	0-6 days	95+ years	-1	Healthcare access and quality index
Gynecological diseases	Female	15-19 years	95+ years	-1	Education (years per capita)
Gynecological diseases	Female	15-19 years	95+ years	-1	LDI (I$ per capita)
Gynecological diseases	Female	15-19 years	95+ years	1	Live Births 35+ (proportion)
Gynecological diseases	Female	15-19 years	95+ years	-1	Skilled Birth Attendance (proportion)
Gynecological diseases	Female	15-19 years	95+ years	0	Smoking Prevalence
Gynecological diseases	Female	15-19 years	95+ years	1	Total Fertility Rate
Gynecological diseases	Female	15-19 years	95+ years	-1	Health System Access (capped)
Gynecological diseases	Female	15-19 years	95+ years	-1	Socio-demographic Index
Gynecological diseases	Female	15-19 years	95+ years	-1	Healthcare access and quality index
Uterine fibroids	Female	15-19 years	95+ years	-1	Education (years per capita)
Uterine fibroids	Female	15-19 years	95+ years	-1	LDI (I$ per capita)
Uterine fibroids	Female	15-19 years	95+ years	1	Live Births 35+ (proportion)
Uterine fibroids	Female	15-19 years	95+ years	-1	Skilled Birth Attendance (proportion)
Uterine fibroids	Female	15-19 years	95+ years	0	Smoking Prevalence
Uterine fibroids	Female	15-19 years	95+ years	1	Total Fertility Rate
Uterine fibroids	Female	15-19 years	95+ years	-1	Health System Access (capped)
Uterine fibroids	Female	15-19 years	95+ years	-1	Socio-demographic Index
Uterine fibroids	Female	15-19 years	95+ years	-1	Healthcare access and quality index
Polycystic ovarian syndrome	Female	15-19 years	50-54 years	-1	Education (years per capita)
Polycystic ovarian syndrome	Female	15-19 years	50-54 years	-1	LDI (I$ per capita)
Polycystic ovarian syndrome	Female	15-19 years	50-54 years	1	Live Births 35+ (proportion)
Polycystic ovarian syndrome	Female	15-19 years	50-54 years	-1	Skilled Birth Attendance (proportion)
Polycystic ovarian syndrome	Female	15-19 years	50-54 years	0	Smoking Prevalence
Polycystic ovarian syndrome	Female	15-19 years	50-54 years	1	Total Fertility Rate
Polycystic ovarian syndrome	Female	15-19 years	50-54 years	-1	Health System Access (capped)
Polycystic ovarian syndrome	Female	15-19 years	50-54 years	-1	Socio-demographic Index
Polycystic ovarian syndrome	Female	15-19 years	50-54 years	-1	Healthcare access and quality index
Endometriosis	Female	15-19 years	50-54 years	-1	Education (years per capita)
Endometriosis	Female	15-19 years	50-54 years	-1	LDI (I$ per capita)
Endometriosis	Female	15-19 years	50-54 years	1	Live Births 35+ (proportion)
Endometriosis	Female	15-19 years	50-54 years	-1	Skilled Birth Attendance (proportion)
Endometriosis	Female	15-19 years	50-54 years	0	Smoking Prevalence
Endometriosis	Female	15-19 years	50-54 years	1	Total Fertility Rate

Cause	Sex	Age start	Age end	Direction	Covariate
Endometriosis	Female	15-19 years	50-54 years	-1	Health System Access (capped)
Endometriosis	Female	15-19 years	50-54 years	-1	Socio-demographic Index
Endometriosis	Female	15-19 years	50-54 years	-1	Healthcare access and quality index
Genital prolapse	Female	15-19 years	95+ years	-1	Education (years per capita)
Genital prolapse	Female	15-19 years	95+ years	-1	LDI (I$ per capita)
Genital prolapse	Female	15-19 years	95+ years	1	Live Births 35+ (proportion)
Genital prolapse	Female	15-19 years	95+ years	-1	Skilled Birth Attendance (proportion)
Genital prolapse	Female	15-19 years	95+ years	0	Smoking Prevalence
Genital prolapse	Female	15-19 years	95+ years	1	Total Fertility Rate
Genital prolapse	Female	15-19 years	95+ years	-1	Health System Access (capped)
Genital prolapse	Female	15-19 years	95+ years	-1	Socio-demographic Index
Genital prolapse	Female	15-19 years	95+ years	-1	Healthcare access and quality index
Other gynecological diseases	Female	15-19 years	95+ years	-1	Education (years per capita)
Other gynecological diseases	Female	15-19 years	95+ years	-1	LDI (I$ per capita)
Other gynecological diseases	Female	15-19 years	95+ years	1	Live Births 35+ (proportion)
Other gynecological diseases	Female	15-19 years	95+ years	-1	Skilled Birth Attendance (proportion)
Other gynecological diseases	Female	15-19 years	95+ years	0	Smoking Prevalence
Other gynecological diseases	Female	15-19 years	95+ years	1	Total Fertility Rate
Other gynecological diseases	Female	15-19 years	95+ years	-1	Health System Access (capped)
Other gynecological diseases	Female	15-19 years	95+ years	-1	Socio-demographic Index
Other gynecological diseases	Female	15-19 years	95+ years	-1	Healthcare access and quality index
Hemoglobinopathies and hemolytic anemias	Male	0-6 days	5-9 years	-1	Education (years per capita)
Hemoglobinopathies and hemolytic anemias	Male	0-6 days	5-9 years	-1	LDI (I$ per capita)
Hemoglobinopathies and hemolytic anemias	Male	0-6 days	5-9 years	1	Latitude Under 15 (proportion)
Hemoglobinopathies and hemolytic anemias	Male	0-6 days	5-9 years	1	Latitude 15 to 30 (proportion)
Hemoglobinopathies and hemolytic anemias	Male	0-6 days	5-9 years	0	Latitude 30 to 45 (proportion)
Hemoglobinopathies and hemolytic anemias	Male	0-6 days	5-9 years	-1	Latitude Over 45 (proportion)
Hemoglobinopathies and hemolytic anemias	Male	0-6 days	5-9 years	1	Malaria Lysenko PFPR 1 (Holoendemic)
Hemoglobinopathies and hemolytic anemias	Male	0-6 days	5-9 years	1	Hemoglobinopathies Prevalence x Excess Mortality
Hemoglobinopathies and hemolytic anemias	Male	0-6 days	5-9 years	-1	Health System Access (capped)
Hemoglobinopathies and hemolytic anemias	Male	0-6 days	5-9 years	1	Hemoglobinopathies Prevalence x Excess Mortality (excluding G6PD deficiency)
Hemoglobinopathies and hemolytic anemias	Male	0-6 days	5-9 years	-1	Socio-demographic Index
Hemoglobinopathies and hemolytic anemias	Male	0-6 days	5-9 years	-1	Healthcare access and quality index
Hemoglobinopathies and hemolytic anemias	Female	0-6 days	5-9 years	-1	Education (years per capita)
Hemoglobinopathies and hemolytic anemias	Female	0-6 days	5-9 years	-1	LDI (I$ per capita)
Hemoglobinopathies and hemolytic anemias	Female	0-6 days	5-9 years	1	Latitude Under 15 (proportion)
Hemoglobinopathies and hemolytic anemias	Female	0-6 days	5-9 years	1	Latitude 15 to 30 (proportion)
Hemoglobinopathies and hemolytic anemias	Female	0-6 days	5-9 years	0	Latitude 30 to 45 (proportion)
Hemoglobinopathies and hemolytic anemias	Female	0-6 days	5-9 years	-1	Latitude Over 45 (proportion)
Hemoglobinopathies and hemolytic anemias	Female	0-6 days	5-9 years	1	Malaria Lysenko PFPR 1 (Holoendemic)
Hemoglobinopathies and hemolytic anemias	Female	0-6 days	5-9 years	1	Hemoglobinopathies Prevalence x Excess Mortality
Hemoglobinopathies and hemolytic anemias	Female	0-6 days	5-9 years	-1	Health System Access (capped)
Hemoglobinopathies and hemolytic anemias	Female	0-6 days	5-9 years	1	Hemoglobinopathies Prevalence x Excess Mortality (excluding G6PD deficiency)
Hemoglobinopathies and hemolytic anemias	Female	0-6 days	5-9 years	-1	Socio-demographic Index
Hemoglobinopathies and hemolytic anemias	Female	0-6 days	5-9 years	-1	Healthcare access and quality index
Hemoglobinopathies and hemolytic anemias	Male	10-14 years	95+ years	-1	Education (years per capita)
Hemoglobinopathies and hemolytic anemias	Male	10-14 years	95+ years	-1	LDI (I$ per capita)
Hemoglobinopathies and hemolytic anemias	Male	10-14 years	95+ years	1	Latitude Under 15 (proportion)
Hemoglobinopathies and hemolytic anemias	Male	10-14 years	95+ years	1	Latitude 15 to 30 (proportion)
Hemoglobinopathies and hemolytic anemias	Male	10-14 years	95+ years	0	Latitude 30 to 45 (proportion)
Hemoglobinopathies and hemolytic anemias	Male	10-14 years	95+ years	-1	Latitude Over 45 (proportion)
Hemoglobinopathies and hemolytic anemias	Male	10-14 years	95+ years	1	Malaria Lysenko PFPR 1 (Holoendemic)
Hemoglobinopathies and hemolytic anemias	Male	10-14 years	95+ years	1	Hemoglobinopathies Prevalence x Excess Mortality
Hemoglobinopathies and hemolytic anemias	Male	10-14 years	95+ years	-1	Health System Access (capped)
Hemoglobinopathies and hemolytic anemias	Male	10-14 years	95+ years	1	Hemoglobinopathies Prevalence x Excess Mortality (excluding G6PD deficiency)
Hemoglobinopathies and hemolytic anemias	Male	10-14 years	95+ years	-1	Socio-demographic Index
Hemoglobinopathies and hemolytic anemias	Male	10-14 years	95+ years	-1	Healthcare access and quality index
Hemoglobinopathies and hemolytic anemias	Female	10-14 years	95+ years	-1	Education (years per capita)
Hemoglobinopathies and hemolytic anemias	Female	10-14 years	95+ years	-1	LDI (I$ per capita)
Hemoglobinopathies and hemolytic anemias	Female	10-14 years	95+ years	1	Latitude Under 15 (proportion)
Hemoglobinopathies and hemolytic anemias	Female	10-14 years	95+ years	1	Latitude 15 to 30 (proportion)
Hemoglobinopathies and hemolytic anemias	Female	10-14 years	95+ years	0	Latitude 30 to 45 (proportion)
Hemoglobinopathies and hemolytic anemias	Female	10-14 years	95+ years	-1	Latitude Over 45 (proportion)
Hemoglobinopathies and hemolytic anemias	Female	10-14 years	95+ years	1	Malaria Lysenko PFPR 1 (Holoendemic)
Hemoglobinopathies and hemolytic anemias	Female	10-14 years	95+ years	1	Hemoglobinopathies Prevalence x Excess Mortality
Hemoglobinopathies and hemolytic anemias	Female	10-14 years	95+ years	-1	Health System Access (capped)
Hemoglobinopathies and hemolytic anemias	Female	10-14 years	95+ years	1	Hemoglobinopathies Prevalence x Excess Mortality (excluding G6PD deficiency)
Hemoglobinopathies and hemolytic anemias	Female	10-14 years	95+ years	-1	Socio-demographic Index
Hemoglobinopathies and hemolytic anemias	Female	10-14 years	95+ years	-1	Healthcare access and quality index
Endocrine, metabolic, blood, and immune disorders	Female	0-6 days	95+ years	1	Alcohol (liters per capita)
Endocrine, metabolic, blood, and immune disorders	Female	0-6 days	95+ years	1	Animal Fats (kcal per capita)
Endocrine, metabolic, blood, and immune disorders	Female	0-6 days	95+ years	-1	Education (years per capita)
Endocrine, metabolic, blood, and immune disorders	Female	0-6 days	95+ years	-1	LDI (I$ per capita)
Endocrine, metabolic, blood, and immune disorders	Female	0-6 days	95+ years	1	Mean BMI
Endocrine, metabolic, blood, and immune disorders	Female	0-6 days	95+ years	1	Cholesterol (total, mean per capita)
Endocrine, metabolic, blood, and immune disorders	Female	0-6 days	95+ years	1	Total Calories (kcal per capita)
Endocrine, metabolic, blood, and immune disorders	Female	0-6 days	95+ years	0	Socio-demographic Index
Endocrine, metabolic, blood, and immune disorders	Female	0-6 days	95+ years	-1	Healthcare access and quality index
Endocrine, metabolic, blood, and immune disorders	Male	0-6 days	95+ years	1	Alcohol (liters per capita)
Endocrine, metabolic, blood, and immune disorders	Male	0-6 days	95+ years	1	Animal Fats (kcal per capita)
Endocrine, metabolic, blood, and immune disorders	Male	0-6 days	95+ years	-1	Education (years per capita)
Endocrine, metabolic, blood, and immune disorders	Male	0-6 days	95+ years	-1	LDI (I$ per capita)
Endocrine, metabolic, blood, and immune disorders	Male	0-6 days	95+ years	1	Mean BMI
Endocrine, metabolic, blood, and immune disorders	Male	0-6 days	95+ years	1	Cholesterol (total, mean per capita)
Endocrine, metabolic, blood, and immune disorders	Male	0-6 days	95+ years	1	Total Calories (kcal per capita)
Endocrine, metabolic, blood, and immune disorders	Male	0-6 days	95+ years	0	Socio-demographic Index
Endocrine, metabolic, blood, and immune disorders	Male	0-6 days	95+ years	-1	Healthcare access and quality index
Musculoskeletal disorders	Male	5-9 years	95+ years	1	Alcohol (liters per capita)
Musculoskeletal disorders	Male	5-9 years	95+ years	1	Cumulative Cigarettes (10 Years)
Musculoskeletal disorders	Male	5-9 years	95+ years	1	Cumulative Cigarettes (5 Years)
Musculoskeletal disorders	Male	5-9 years	95+ years	0	Education (years per capita)
Musculoskeletal disorders	Male	5-9 years	95+ years	0	LDI (I$ per capita)

Cause	Sex	Age start	Age end	Direction	Covariate
Musculoskeletal disorders	Male	5-9 years	95+ years	1	Mean BMI
Musculoskeletal disorders	Male	5-9 years	95+ years	1	Cholesterol (total, mean per capita)
Musculoskeletal disorders	Male	5-9 years	95+ years	1	Smoking Prevalence
Musculoskeletal disorders	Male	5-9 years	95+ years	0	Socio-demographic Index
Musculoskeletal disorders	Male	5-9 years	95+ years	0	vegetables adjusted(g)
Musculoskeletal disorders	Male	5-9 years	95+ years	-1	Healthcare access and quality index
Musculoskeletal disorders	Female	5-9 years	95+ years	1	Alcohol (liters per capita)
Musculoskeletal disorders	Female	5-9 years	95+ years	1	Cumulative Cigarettes (10 Years)
Musculoskeletal disorders	Female	5-9 years	95+ years	1	Cumulative Cigarettes (5 Years)
Musculoskeletal disorders	Female	5-9 years	95+ years	0	Education (years per capita)
Musculoskeletal disorders	Female	5-9 years	95+ years	0	LDI (I$ per capita)
Musculoskeletal disorders	Female	5-9 years	95+ years	1	Mean BMI
Musculoskeletal disorders	Female	5-9 years	95+ years	1	Cholesterol (total, mean per capita)
Musculoskeletal disorders	Female	5-9 years	95+ years	1	Smoking Prevalence
Musculoskeletal disorders	Female	5-9 years	95+ years	0	Socio-demographic Index
Musculoskeletal disorders	Female	5-9 years	95+ years	0	vegetables adjusted(g)
Musculoskeletal disorders	Female	5-9 years	95+ years	-1	Healthcare access and quality index
Rheumatoid arthritis	Female	5-9 years	95+ years	1	Alcohol (liters per capita)
Rheumatoid arthritis	Female	5-9 years	95+ years	1	Cumulative Cigarettes (10 Years)
Rheumatoid arthritis	Female	5-9 years	95+ years	1	Cumulative Cigarettes (5 Years)
Rheumatoid arthritis	Female	5-9 years	95+ years	-1	Education (years per capita)
Rheumatoid arthritis	Female	5-9 years	95+ years	-1	LDI (I$ per capita)
Rheumatoid arthritis	Female	5-9 years	95+ years	1	Mean BMI
Rheumatoid arthritis	Female	5-9 years	95+ years	1	Cholesterol (total, mean per capita)
Rheumatoid arthritis	Female	5-9 years	95+ years	1	Smoking Prevalence
Rheumatoid arthritis	Female	5-9 years	95+ years	0	Socio-demographic Index
Rheumatoid arthritis	Female	5-9 years	95+ years	0	vegetables adjusted(g)
Rheumatoid arthritis	Female	5-9 years	95+ years	-1	Healthcare access and quality index
Rheumatoid arthritis	Male	5-9 years	95+ years	1	Alcohol (liters per capita)
Rheumatoid arthritis	Male	5-9 years	95+ years	1	Cumulative Cigarettes (10 Years)
Rheumatoid arthritis	Male	5-9 years	95+ years	1	Cumulative Cigarettes (5 Years)
Rheumatoid arthritis	Male	5-9 years	95+ years	-1	Education (years per capita)
Rheumatoid arthritis	Male	5-9 years	95+ years	-1	LDI (I$ per capita)
Rheumatoid arthritis	Male	5-9 years	95+ years	1	Mean BMI
Rheumatoid arthritis	Male	5-9 years	95+ years	1	Cholesterol (total, mean per capita)
Rheumatoid arthritis	Male	5-9 years	95+ years	1	Smoking Prevalence
Rheumatoid arthritis	Male	5-9 years	95+ years	0	Socio-demographic Index
Rheumatoid arthritis	Male	5-9 years	95+ years	0	vegetables adjusted(g)
Rheumatoid arthritis	Male	5-9 years	95+ years	-1	Healthcare access and quality index
Other musculoskeletal disorders	Male	5-9 years	95+ years	1	Alcohol (liters per capita)
Other musculoskeletal disorders	Male	5-9 years	95+ years	1	Cumulative Cigarettes (10 Years)
Other musculoskeletal disorders	Male	5-9 years	95+ years	1	Cumulative Cigarettes (5 Years)
Other musculoskeletal disorders	Male	5-9 years	95+ years	0	Education (years per capita)
Other musculoskeletal disorders	Male	5-9 years	95+ years	0	LDI (I$ per capita)
Other musculoskeletal disorders	Male	5-9 years	95+ years	1	Mean BMI
Other musculoskeletal disorders	Male	5-9 years	95+ years	1	Cholesterol (total, mean per capita)
Other musculoskeletal disorders	Male	5-9 years	95+ years	1	Smoking Prevalence
Other musculoskeletal disorders	Male	5-9 years	95+ years	0	Socio-demographic Index
Other musculoskeletal disorders	Male	5-9 years	95+ years	0	vegetables adjusted(g)
Other musculoskeletal disorders	Male	5-9 years	95+ years	-1	Healthcare access and quality index
Other musculoskeletal disorders	Female	5-9 years	95+ years	1	Alcohol (liters per capita)
Other musculoskeletal disorders	Female	5-9 years	95+ years	1	Cumulative Cigarettes (10 Years)
Other musculoskeletal disorders	Female	5-9 years	95+ years	1	Cumulative Cigarettes (5 Years)
Other musculoskeletal disorders	Female	5-9 years	95+ years	0	Education (years per capita)
Other musculoskeletal disorders	Female	5-9 years	95+ years	0	LDI (I$ per capita)
Other musculoskeletal disorders	Female	5-9 years	95+ years	1	Mean BMI
Other musculoskeletal disorders	Female	5-9 years	95+ years	1	Cholesterol (total, mean per capita)
Other musculoskeletal disorders	Female	5-9 years	95+ years	1	Smoking Prevalence
Other musculoskeletal disorders	Female	5-9 years	95+ years	0	Socio-demographic Index
Other musculoskeletal disorders	Female	5-9 years	95+ years	0	vegetables adjusted(g)
Other musculoskeletal disorders	Female	5-9 years	95+ years	-1	Healthcare access and quality index
Congenital birth defects	Male	0-6 days	65-69 years	-1	Legality of Abortion
Congenital birth defects	Male	0-6 days	65-69 years	1	Alcohol (liters per capita)
Congenital birth defects	Male	0-6 days	65-69 years	0	Antenatal Care (1 visit) Coverage (proportion)
Congenital birth defects	Male	0-6 days	65-69 years	-1	Antenatal Care (4 visits) Coverage (proportion)
Congenital birth defects	Male	0-6 days	65-69 years	-1	Education (years per capita)
Congenital birth defects	Male	0-6 days	65-69 years	-1	In-Facility Delivery (proportion)
Congenital birth defects	Male	0-6 days	65-69 years	1	Live Births 35+ (proportion)
Congenital birth defects	Male	0-6 days	65-69 years	1	Indoor Air Pollution (All Cooking Fuels)
Congenital birth defects	Male	0-6 days	65-69 years	1	Outdoor Air Pollution (PM2.5)
Congenital birth defects	Male	0-6 days	65-69 years	1	Smoking Prevalence (Reproductive Age Standardized)
Congenital birth defects	Male	0-6 days	65-69 years	-1	Socio-demographic Index
Congenital birth defects	Male	0-6 days	65-69 years	1	fruits unadjusted(g)
Congenital birth defects	Male	0-6 days	65-69 years	1	vegetables unadjusted(g)
Congenital birth defects	Male	0-6 days	65-69 years	1	Maternal alcohol consumption during pregnancy (proportion)
Congenital birth defects	Male	0-6 days	65-69 years	-1	Healthcare access and quality index
Congenital birth defects	Male	0-6 days	65-69 years	-1	Folic acid unadjusted (ug)
Congenital birth defects	Female	0-6 days	65-69 years	-1	Legality of Abortion
Congenital birth defects	Female	0-6 days	65-69 years	1	Alcohol (liters per capita)
Congenital birth defects	Female	0-6 days	65-69 years	0	Antenatal Care (1 visit) Coverage (proportion)
Congenital birth defects	Female	0-6 days	65-69 years	-1	Antenatal Care (4 visits) Coverage (proportion)
Congenital birth defects	Female	0-6 days	65-69 years	-1	Education (years per capita)
Congenital birth defects	Female	0-6 days	65-69 years	-1	In-Facility Delivery (proportion)
Congenital birth defects	Female	0-6 days	65-69 years	1	Live Births 35+ (proportion)
Congenital birth defects	Female	0-6 days	65-69 years	1	Indoor Air Pollution (All Cooking Fuels)
Congenital birth defects	Female	0-6 days	65-69 years	1	Outdoor Air Pollution (PM2.5)
Congenital birth defects	Female	0-6 days	65-69 years	1	Smoking Prevalence (Reproductive Age Standardized)
Congenital birth defects	Female	0-6 days	65-69 years	-1	Socio-demographic Index
Congenital birth defects	Female	0-6 days	65-69 years	1	fruits unadjusted(g)
Congenital birth defects	Female	0-6 days	65-69 years	1	vegetables unadjusted(g)
Congenital birth defects	Female	0-6 days	65-69 years	1	Maternal alcohol consumption during pregnancy (proportion)
Congenital birth defects	Female	0-6 days	65-69 years	-1	Healthcare access and quality index

Cause	Sex	Age start	Age end	Direction	Covariate
Congenital birth defects	Female	0-6 days	65-69 years	-1	Folic acid unadjusted (ug)
Neural tube defects	Male	0-6 days	65-69 years	-1	Legality of Abortion
Neural tube defects	Male	0-6 days	65-69 years	-1	Antenatal Care (1 visit) Coverage (proportion)
Neural tube defects	Male	0-6 days	65-69 years	-1	Antenatal Care (4 visits) Coverage (proportion)
Neural tube defects	Male	0-6 days	65-69 years	1	Diabetes Age-Standardized Prevalence (proportion)
Neural tube defects	Male	0-6 days	65-69 years	-1	Education (years per capita)
Neural tube defects	Male	0-6 days	65-69 years	-1	In-Facility Delivery (proportion)
Neural tube defects	Male	0-6 days	65-69 years	1	Indoor Air Pollution (All Cooking Fuels)
Neural tube defects	Male	0-6 days	65-69 years	1	Outdoor Air Pollution (PM2.5)
Neural tube defects	Male	0-6 days	65-69 years	1	Smoking Prevalence (Reproductive Age Standardized)
Neural tube defects	Male	0-6 days	65-69 years	-1	Socio-demographic Index
Neural tube defects	Male	0-6 days	65-69 years	0	fruits unadjusted(g)
Neural tube defects	Male	0-6 days	65-69 years	-1	vegetables unadjusted(g)
Neural tube defects	Male	0-6 days	65-69 years	1	Maternal alcohol consumption during pregnancy (proportion)
Neural tube defects	Male	0-6 days	65-69 years	-1	Healthcare access and quality index
Neural tube defects	Male	0-6 days	65-69 years	-1	Folic acid unadjusted (ug)
Neural tube defects	Female	0-6 days	65-69 years	-1	Legality of Abortion
Neural tube defects	Female	0-6 days	65-69 years	-1	Antenatal Care (1 visit) Coverage (proportion)
Neural tube defects	Female	0-6 days	65-69 years	-1	Antenatal Care (4 visits) Coverage (proportion)
Neural tube defects	Female	0-6 days	65-69 years	1	Diabetes Age-Standardized Prevalence (proportion)
Neural tube defects	Female	0-6 days	65-69 years	-1	Education (years per capita)
Neural tube defects	Female	0-6 days	65-69 years	-1	In-Facility Delivery (proportion)
Neural tube defects	Female	0-6 days	65-69 years	1	Indoor Air Pollution (All Cooking Fuels)
Neural tube defects	Female	0-6 days	65-69 years	1	Outdoor Air Pollution (PM2.5)
Neural tube defects	Female	0-6 days	65-69 years	1	Smoking Prevalence (Reproductive Age Standardized)
Neural tube defects	Female	0-6 days	65-69 years	-1	Socio-demographic Index
Neural tube defects	Female	0-6 days	65-69 years	0	fruits unadjusted(g)
Neural tube defects	Female	0-6 days	65-69 years	-1	vegetables unadjusted(g)
Neural tube defects	Female	0-6 days	65-69 years	1	Maternal alcohol consumption during pregnancy (proportion)
Neural tube defects	Female	0-6 days	65-69 years	-1	Healthcare access and quality index
Neural tube defects	Female	0-6 days	65-69 years	-1	Folic acid unadjusted (ug)
Congenital heart anomalies	Male	0-6 days	65-69 years	-1	Legality of Abortion
Congenital heart anomalies	Male	0-6 days	65-69 years	1	Alcohol (liters per capita)
Congenital heart anomalies	Male	0-6 days	65-69 years	-1	Antenatal Care (1 visit) Coverage (proportion)
Congenital heart anomalies	Male	0-6 days	65-69 years	-1	Antenatal Care (4 visits) Coverage (proportion)
Congenital heart anomalies	Male	0-6 days	65-69 years	1	Diabetes Age-Standardized Prevalence (proportion)
Congenital heart anomalies	Male	0-6 days	65-69 years	-1	Education (years per capita)
Congenital heart anomalies	Male	0-6 days	65-69 years	-1	In-Facility Delivery (proportion)
Congenital heart anomalies	Male	0-6 days	65-69 years	1	Live Births 35+ (proportion)
Congenital heart anomalies	Male	0-6 days	65-69 years	-1	Skilled Birth Attendance (proportion)
Congenital heart anomalies	Male	0-6 days	65-69 years	1	Smoking Prevalence (Reproductive Age Standardized)
Congenital heart anomalies	Male	0-6 days	65-69 years	-1	Socio-demographic Index
Congenital heart anomalies	Male	0-6 days	65-69 years	1	Maternal alcohol consumption during pregnancy (proportion)
Congenital heart anomalies	Male	0-6 days	65-69 years	-1	Healthcare access and quality index
Congenital heart anomalies	Female	0-6 days	65-69 years	-1	Legality of Abortion
Congenital heart anomalies	Female	0-6 days	65-69 years	1	Alcohol (liters per capita)
Congenital heart anomalies	Female	0-6 days	65-69 years	-1	Antenatal Care (1 visit) Coverage (proportion)
Congenital heart anomalies	Female	0-6 days	65-69 years	-1	Antenatal Care (4 visits) Coverage (proportion)
Congenital heart anomalies	Female	0-6 days	65-69 years	1	Diabetes Age-Standardized Prevalence (proportion)
Congenital heart anomalies	Female	0-6 days	65-69 years	-1	Education (years per capita)
Congenital heart anomalies	Female	0-6 days	65-69 years	-1	In-Facility Delivery (proportion)
Congenital heart anomalies	Female	0-6 days	65-69 years	1	Live Births 35+ (proportion)
Congenital heart anomalies	Female	0-6 days	65-69 years	-1	Skilled Birth Attendance (proportion)
Congenital heart anomalies	Female	0-6 days	65-69 years	1	Smoking Prevalence (Reproductive Age Standardized)
Congenital heart anomalies	Female	0-6 days	65-69 years	-1	Socio-demographic Index
Congenital heart anomalies	Female	0-6 days	65-69 years	1	Maternal alcohol consumption during pregnancy (proportion)
Congenital heart anomalies	Female	0-6 days	65-69 years	-1	Healthcare access and quality index
Orofacial clefts	Male	0-6 days	1-4 years	-1	Legality of Abortion
Orofacial clefts	Male	0-6 days	1-4 years	1	Alcohol (liters per capita)
Orofacial clefts	Male	0-6 days	1-4 years	-1	Antenatal Care (1 visit) Coverage (proportion)
Orofacial clefts	Male	0-6 days	1-4 years	-1	Antenatal Care (4 visits) Coverage (proportion)
Orofacial clefts	Male	0-6 days	1-4 years	1	Diabetes Age-Standardized Prevalence (proportion)
Orofacial clefts	Male	0-6 days	1-4 years	-1	Education (years per capita)
Orofacial clefts	Male	0-6 days	1-4 years	1	Indoor Air Pollution (All Cooking Fuels)
Orofacial clefts	Male	0-6 days	1-4 years	1	Outdoor Air Pollution (PM2.5)
Orofacial clefts	Male	0-6 days	1-4 years	-1	Skilled Birth Attendance (proportion)
Orofacial clefts	Male	0-6 days	1-4 years	1	Smoking Prevalence (Reproductive Age Standardized)
Orofacial clefts	Male	0-6 days	1-4 years	-1	Socio-demographic Index
Orofacial clefts	Male	0-6 days	1-4 years	0	fruits unadjusted(g)
Orofacial clefts	Male	0-6 days	1-4 years	0	vegetables unadjusted(g)
Orofacial clefts	Male	0-6 days	1-4 years	1	Maternal alcohol consumption during pregnancy (proportion)
Orofacial clefts	Male	0-6 days	1-4 years	-1	Healthcare access and quality index
Orofacial clefts	Male	0-6 days	1-4 years	-1	Folic acid unadjusted (ug)
Orofacial clefts	Female	0-6 days	1-4 years	-1	Legality of Abortion
Orofacial clefts	Female	0-6 days	1-4 years	1	Alcohol (liters per capita)
Orofacial clefts	Female	0-6 days	1-4 years	-1	Antenatal Care (1 visit) Coverage (proportion)
Orofacial clefts	Female	0-6 days	1-4 years	-1	Antenatal Care (4 visits) Coverage (proportion)
Orofacial clefts	Female	0-6 days	1-4 years	1	Diabetes Age-Standardized Prevalence (proportion)
Orofacial clefts	Female	0-6 days	1-4 years	-1	Education (years per capita)
Orofacial clefts	Female	0-6 days	1-4 years	1	Indoor Air Pollution (All Cooking Fuels)
Orofacial clefts	Female	0-6 days	1-4 years	1	Outdoor Air Pollution (PM2.5)
Orofacial clefts	Female	0-6 days	1-4 years	-1	Skilled Birth Attendance (proportion)
Orofacial clefts	Female	0-6 days	1-4 years	1	Smoking Prevalence (Reproductive Age Standardized)
Orofacial clefts	Female	0-6 days	1-4 years	-1	Socio-demographic Index
Orofacial clefts	Female	0-6 days	1-4 years	0	fruits unadjusted(g)
Orofacial clefts	Female	0-6 days	1-4 years	0	vegetables unadjusted(g)
Orofacial clefts	Female	0-6 days	1-4 years	1	Maternal alcohol consumption during pregnancy (proportion)
Orofacial clefts	Female	0-6 days	1-4 years	-1	Healthcare access and quality index
Down syndrome	Male	0-6 days	65-69 years	-1	Legality of Abortion
Down syndrome	Male	0-6 days	65-69 years	-1	Antenatal Care (1 visit) Coverage (proportion)
Down syndrome	Male	0-6 days	65-69 years	-1	Antenatal Care (4 visits) Coverage (proportion)
Down syndrome	Male	0-6 days	65-69 years	-1	Education (years per capita)

Cause	Sex	Age start	Age end	Direction	Covariate
Down syndrome	Male	0-6 days	65-69 years	-1	In-Facility Delivery (proportion)
Down syndrome	Male	0-6 days	65-69 years	-1	LDI (I$ per capita)
Down syndrome	Male	0-6 days	65-69 years	1	Live Births 35+ (proportion)
Down syndrome	Male	0-6 days	65-69 years	1	Live Births 40+ (proportion)
Down syndrome	Male	0-6 days	65-69 years	1	Indoor Air Pollution (All Cooking Fuels)
Down syndrome	Male	0-6 days	65-69 years	1	Smoking Prevalence (Reproductive Age Standardized)
Down syndrome	Male	0-6 days	65-69 years	-1	Socio-demographic Index
Down syndrome	Male	0-6 days	65-69 years	-1	vegetables unadjusted(g)
Down syndrome	Male	0-6 days	65-69 years	1	Maternal alcohol consumption during pregnancy (proportion)
Down syndrome	Male	0-6 days	65-69 years	-1	Healthcare access and quality index
Down syndrome	Female	0-6 days	65-69 years	-1	Legality of Abortion
Down syndrome	Female	0-6 days	65-69 years	-1	Antenatal Care (1 visit) Coverage (proportion)
Down syndrome	Female	0-6 days	65-69 years	-1	Antenatal Care (4 visits) Coverage (proportion)
Down syndrome	Female	0-6 days	65-69 years	-1	Education (years per capita)
Down syndrome	Female	0-6 days	65-69 years	-1	In-Facility Delivery (proportion)
Down syndrome	Female	0-6 days	65-69 years	-1	LDI (I$ per capita)
Down syndrome	Female	0-6 days	65-69 years	1	Live Births 35+ (proportion)
Down syndrome	Female	0-6 days	65-69 years	1	Live Births 40+ (proportion)
Down syndrome	Female	0-6 days	65-69 years	1	Indoor Air Pollution (All Cooking Fuels)
Down syndrome	Female	0-6 days	65-69 years	1	Smoking Prevalence (Reproductive Age Standardized)
Down syndrome	Female	0-6 days	65-69 years	-1	Socio-demographic Index
Down syndrome	Female	0-6 days	65-69 years	-1	vegetables unadjusted(g)
Down syndrome	Female	0-6 days	65-69 years	1	Maternal alcohol consumption during pregnancy (proportion)
Down syndrome	Female	0-6 days	65-69 years	-1	Healthcare access and quality index
Other chromosomal abnormalities	Male	0-6 days	65-69 years	-1	Legality of Abortion
Other chromosomal abnormalities	Male	0-6 days	65-69 years	1	Alcohol (liters per capita)
Other chromosomal abnormalities	Male	0-6 days	65-69 years	-1	Antenatal Care (1 visit) Coverage (proportion)
Other chromosomal abnormalities	Male	0-6 days	65-69 years	-1	Antenatal Care (4 visits) Coverage (proportion)
Other chromosomal abnormalities	Male	0-6 days	65-69 years	-1	Education (years per capita)
Other chromosomal abnormalities	Male	0-6 days	65-69 years	-1	In-Facility Delivery (proportion)
Other chromosomal abnormalities	Male	0-6 days	65-69 years	-1	LDI (I$ per capita)
Other chromosomal abnormalities	Male	0-6 days	65-69 years	1	Live Births 35+ (proportion)
Other chromosomal abnormalities	Male	0-6 days	65-69 years	1	Live Births 40+ (proportion)
Other chromosomal abnormalities	Male	0-6 days	65-69 years	1	Indoor Air Pollution (All Cooking Fuels)
Other chromosomal abnormalities	Male	0-6 days	65-69 years	-1	Skilled Birth Attendance (proportion)
Other chromosomal abnormalities	Male	0-6 days	65-69 years	1	Smoking Prevalence (Reproductive Age Standardized)
Other chromosomal abnormalities	Male	0-6 days	65-69 years	0	Socio-demographic Index
Other chromosomal abnormalities	Male	0-6 days	65-69 years	1	Maternal alcohol consumption during pregnancy (proportion)
Other chromosomal abnormalities	Male	0-6 days	65-69 years	-1	Healthcare access and quality index
Other chromosomal abnormalities	Female	0-6 days	65-69 years	-1	Legality of Abortion
Other chromosomal abnormalities	Female	0-6 days	65-69 years	1	Alcohol (liters per capita)
Other chromosomal abnormalities	Female	0-6 days	65-69 years	-1	Antenatal Care (1 visit) Coverage (proportion)
Other chromosomal abnormalities	Female	0-6 days	65-69 years	-1	Antenatal Care (4 visits) Coverage (proportion)
Other chromosomal abnormalities	Female	0-6 days	65-69 years	-1	Education (years per capita)
Other chromosomal abnormalities	Female	0-6 days	65-69 years	-1	In-Facility Delivery (proportion)
Other chromosomal abnormalities	Female	0-6 days	65-69 years	-1	LDI (I$ per capita)
Other chromosomal abnormalities	Female	0-6 days	65-69 years	1	Live Births 35+ (proportion)
Other chromosomal abnormalities	Female	0-6 days	65-69 years	1	Live Births 40+ (proportion)
Other chromosomal abnormalities	Female	0-6 days	65-69 years	1	Indoor Air Pollution (All Cooking Fuels)
Other chromosomal abnormalities	Female	0-6 days	65-69 years	-1	Skilled Birth Attendance (proportion)
Other chromosomal abnormalities	Female	0-6 days	65-69 years	1	Smoking Prevalence (Reproductive Age Standardized)
Other chromosomal abnormalities	Female	0-6 days	65-69 years	0	Socio-demographic Index
Other chromosomal abnormalities	Female	0-6 days	65-69 years	1	Maternal alcohol consumption during pregnancy (proportion)
Other chromosomal abnormalities	Female	0-6 days	65-69 years	-1	Healthcare access and quality index
Congenital musculoskeletal and limb anomalies	Male	0-6 days	65-69 years	-1	Legality of Abortion
Congenital musculoskeletal and limb anomalies	Male	0-6 days	65-69 years	1	Alcohol (liters per capita)
Congenital musculoskeletal and limb anomalies	Male	0-6 days	65-69 years	-1	Antenatal Care (1 visit) Coverage (proportion)
Congenital musculoskeletal and limb anomalies	Male	0-6 days	65-69 years	-1	Antenatal Care (4 visits) Coverage (proportion)
Congenital musculoskeletal and limb anomalies	Male	0-6 days	65-69 years	1	Diabetes Age-Standardized Prevalence (proportion)
Congenital musculoskeletal and limb anomalies	Male	0-6 days	65-69 years	-1	Education (years per capita)
Congenital musculoskeletal and limb anomalies	Male	0-6 days	65-69 years	-1	In-Facility Delivery (proportion)
Congenital musculoskeletal and limb anomalies	Male	0-6 days	65-69 years	-1	LDI (I$ per capita)
Congenital musculoskeletal and limb anomalies	Male	0-6 days	65-69 years	1	Indoor Air Pollution (All Cooking Fuels)
Congenital musculoskeletal and limb anomalies	Male	0-6 days	65-69 years	1	Smoking Prevalence (Reproductive Age Standardized)
Congenital musculoskeletal and limb anomalies	Male	0-6 days	65-69 years	-1	Socio-demographic Index
Congenital musculoskeletal and limb anomalies	Male	0-6 days	65-69 years	0	fruits unadjusted(g)
Congenital musculoskeletal and limb anomalies	Male	0-6 days	65-69 years	0	vegetables unadjusted(g)
Congenital musculoskeletal and limb anomalies	Male	0-6 days	65-69 years	1	Maternal alcohol consumption during pregnancy (proportion)
Congenital musculoskeletal and limb anomalies	Male	0-6 days	65-69 years	-1	Healthcare access and quality index
Congenital musculoskeletal and limb anomalies	Female	0-6 days	65-69 years	-1	Legality of Abortion
Congenital musculoskeletal and limb anomalies	Female	0-6 days	65-69 years	1	Alcohol (liters per capita)
Congenital musculoskeletal and limb anomalies	Female	0-6 days	65-69 years	-1	Antenatal Care (1 visit) Coverage (proportion)
Congenital musculoskeletal and limb anomalies	Female	0-6 days	65-69 years	-1	Antenatal Care (4 visits) Coverage (proportion)
Congenital musculoskeletal and limb anomalies	Female	0-6 days	65-69 years	1	Diabetes Age-Standardized Prevalence (proportion)
Congenital musculoskeletal and limb anomalies	Female	0-6 days	65-69 years	-1	Education (years per capita)
Congenital musculoskeletal and limb anomalies	Female	0-6 days	65-69 years	-1	In-Facility Delivery (proportion)
Congenital musculoskeletal and limb anomalies	Female	0-6 days	65-69 years	-1	LDI (I$ per capita)
Congenital musculoskeletal and limb anomalies	Female	0-6 days	65-69 years	1	Indoor Air Pollution (All Cooking Fuels)
Congenital musculoskeletal and limb anomalies	Female	0-6 days	65-69 years	1	Smoking Prevalence (Reproductive Age Standardized)
Congenital musculoskeletal and limb anomalies	Female	0-6 days	65-69 years	-1	Socio-demographic Index
Congenital musculoskeletal and limb anomalies	Female	0-6 days	65-69 years	0	fruits unadjusted(g)
Congenital musculoskeletal and limb anomalies	Female	0-6 days	65-69 years	0	vegetables unadjusted(g)
Congenital musculoskeletal and limb anomalies	Female	0-6 days	65-69 years	1	Maternal alcohol consumption during pregnancy (proportion)
Congenital musculoskeletal and limb anomalies	Female	0-6 days	65-69 years	-1	Healthcare access and quality index
Urogenital congenital anomalies	Male	0-6 days	65-69 years	1	Alcohol (liters per capita)
Urogenital congenital anomalies	Male	0-6 days	65-69 years	-1	Antenatal Care (1 visit) Coverage (proportion)
Urogenital congenital anomalies	Male	0-6 days	65-69 years	-1	Antenatal Care (4 visits) Coverage (proportion)
Urogenital congenital anomalies	Male	0-6 days	65-69 years	1	Diabetes Age-Standardized Prevalence (proportion)
Urogenital congenital anomalies	Male	0-6 days	65-69 years	-1	Education (years per capita)
Urogenital congenital anomalies	Male	0-6 days	65-69 years	-1	In-Facility Delivery (proportion)
Urogenital congenital anomalies	Male	0-6 days	65-69 years	-1	LDI (I$ per capita)
Urogenital congenital anomalies	Male	0-6 days	65-69 years	1	Indoor Air Pollution (All Cooking Fuels)

Cause	Sex	Age start	Age end	Direction	Covariate
Urogenital congenital anomalies	Male	0-6 days	65-69 years	1	Outdoor Air Pollution (PM2.5)
Urogenital congenital anomalies	Male	0-6 days	65-69 years	1	Smoking Prevalence (Reproductive Age Standardized)
Urogenital congenital anomalies	Male	0-6 days	65-69 years	-1	Socio-demographic Index
Urogenital congenital anomalies	Male	0-6 days	65-69 years	1	Maternal alcohol consumption during pregnancy (proportion)
Urogenital congenital anomalies	Male	0-6 days	65-69 years	-1	Healthcare access and quality index
Urogenital congenital anomalies	Female	0-6 days	65-69 years	1	Alcohol (liters per capita)
Urogenital congenital anomalies	Female	0-6 days	65-69 years	-1	Antenatal Care (1 visit) Coverage (proportion)
Urogenital congenital anomalies	Female	0-6 days	65-69 years	-1	Antenatal Care (4 visits) Coverage (proportion)
Urogenital congenital anomalies	Female	0-6 days	65-69 years	1	Diabetes Age-Standardized Prevalence (proportion)
Urogenital congenital anomalies	Female	0-6 days	65-69 years	-1	Education (years per capita)
Urogenital congenital anomalies	Female	0-6 days	65-69 years	-1	In-Facility Delivery (proportion)
Urogenital congenital anomalies	Female	0-6 days	65-69 years	-1	LDI (I$ per capita)
Urogenital congenital anomalies	Female	0-6 days	65-69 years	1	Indoor Air Pollution (All Cooking Fuels)
Urogenital congenital anomalies	Female	0-6 days	65-69 years	1	Outdoor Air Pollution (PM2.5)
Urogenital congenital anomalies	Female	0-6 days	65-69 years	1	Smoking Prevalence (Reproductive Age Standardized)
Urogenital congenital anomalies	Female	0-6 days	65-69 years	-1	Socio-demographic Index
Urogenital congenital anomalies	Female	0-6 days	65-69 years	1	Maternal alcohol consumption during pregnancy (proportion)
Urogenital congenital anomalies	Female	0-6 days	65-69 years	-1	Healthcare access and quality index
Digestive congenital anomalies	Female	0-6 days	65-69 years	1	Alcohol (liters per capita)
Digestive congenital anomalies	Female	0-6 days	65-69 years	-1	Antenatal Care (1 visit) Coverage (proportion)
Digestive congenital anomalies	Female	0-6 days	65-69 years	-1	Antenatal Care (4 visits) Coverage (proportion)
Digestive congenital anomalies	Female	0-6 days	65-69 years	1	Diabetes Age-Standardized Prevalence (proportion)
Digestive congenital anomalies	Female	0-6 days	65-69 years	-1	Education (years per capita)
Digestive congenital anomalies	Female	0-6 days	65-69 years	-1	In-Facility Delivery (proportion)
Digestive congenital anomalies	Female	0-6 days	65-69 years	-1	LDI (I$ per capita)
Digestive congenital anomalies	Female	0-6 days	65-69 years	1	Indoor Air Pollution (All Cooking Fuels)
Digestive congenital anomalies	Female	0-6 days	65-69 years	1	Smoking Prevalence (Reproductive Age Standardized)
Digestive congenital anomalies	Female	0-6 days	65-69 years	-1	Health System Access (capped)
Digestive congenital anomalies	Female	0-6 days	65-69 years	1	Prevalence of obesity (age-standardized)
Digestive congenital anomalies	Female	0-6 days	65-69 years	-1	Socio-demographic Index
Digestive congenital anomalies	Female	0-6 days	65-69 years	0	fruits unadjusted(g)
Digestive congenital anomalies	Female	0-6 days	65-69 years	0	vegetables unadjusted(g)
Digestive congenital anomalies	Female	0-6 days	65-69 years	1	Maternal alcohol consumption during pregnancy (proportion)
Digestive congenital anomalies	Female	0-6 days	65-69 years	-1	Healthcare access and quality index
Digestive congenital anomalies	Male	0-6 days	65-69 years	1	Alcohol (liters per capita)
Digestive congenital anomalies	Male	0-6 days	65-69 years	-1	Antenatal Care (1 visit) Coverage (proportion)
Digestive congenital anomalies	Male	0-6 days	65-69 years	-1	Antenatal Care (4 visits) Coverage (proportion)
Digestive congenital anomalies	Male	0-6 days	65-69 years	1	Diabetes Age-Standardized Prevalence (proportion)
Digestive congenital anomalies	Male	0-6 days	65-69 years	-1	Education (years per capita)
Digestive congenital anomalies	Male	0-6 days	65-69 years	-1	In-Facility Delivery (proportion)
Digestive congenital anomalies	Male	0-6 days	65-69 years	-1	LDI (I$ per capita)
Digestive congenital anomalies	Male	0-6 days	65-69 years	1	Indoor Air Pollution (All Cooking Fuels)
Digestive congenital anomalies	Male	0-6 days	65-69 years	1	Smoking Prevalence (Reproductive Age Standardized)
Digestive congenital anomalies	Male	0-6 days	65-69 years	-1	Health System Access (capped)
Digestive congenital anomalies	Male	0-6 days	65-69 years	1	Prevalence of obesity (age-standardized)
Digestive congenital anomalies	Male	0-6 days	65-69 years	-1	Socio-demographic Index
Digestive congenital anomalies	Male	0-6 days	65-69 years	0	fruits unadjusted(g)
Digestive congenital anomalies	Male	0-6 days	65-69 years	0	vegetables unadjusted(g)
Digestive congenital anomalies	Male	0-6 days	65-69 years	1	Maternal alcohol consumption during pregnancy (proportion)
Digestive congenital anomalies	Male	0-6 days	65-69 years	-1	Healthcare access and quality index
Other congenital birth defects	Female	0-6 days	65-69 years	-1	Legality of Abortion
Other congenital birth defects	Female	0-6 days	65-69 years	1	Alcohol (liters per capita)
Other congenital birth defects	Female	0-6 days	65-69 years	-1	Antenatal Care (1 visit) Coverage (proportion)
Other congenital birth defects	Female	0-6 days	65-69 years	-1	Antenatal Care (4 visits) Coverage (proportion)
Other congenital birth defects	Female	0-6 days	65-69 years	1	Diabetes Age-Standardized Prevalence (proportion)
Other congenital birth defects	Female	0-6 days	65-69 years	-1	Education (years per capita)
Other congenital birth defects	Female	0-6 days	65-69 years	-1	In-Facility Delivery (proportion)
Other congenital birth defects	Female	0-6 days	65-69 years	-1	LDI (I$ per capita)
Other congenital birth defects	Female	0-6 days	65-69 years	1	Live Births 35+ (proportion)
Other congenital birth defects	Female	0-6 days	65-69 years	1	Indoor Air Pollution (All Cooking Fuels)
Other congenital birth defects	Female	0-6 days	65-69 years	1	Smoking Prevalence (Reproductive Age Standardized)
Other congenital birth defects	Female	0-6 days	65-69 years	-1	Socio-demographic Index
Other congenital birth defects	Female	0-6 days	65-69 years	1	Maternal alcohol consumption during pregnancy (proportion)
Other congenital birth defects	Female	0-6 days	65-69 years	-1	Healthcare access and quality index
Other congenital birth defects	Male	0-6 days	65-69 years	-1	Legality of Abortion
Other congenital birth defects	Male	0-6 days	65-69 years	1	Alcohol (liters per capita)
Other congenital birth defects	Male	0-6 days	65-69 years	-1	Antenatal Care (1 visit) Coverage (proportion)
Other congenital birth defects	Male	0-6 days	65-69 years	-1	Antenatal Care (4 visits) Coverage (proportion)
Other congenital birth defects	Male	0-6 days	65-69 years	1	Diabetes Age-Standardized Prevalence (proportion)
Other congenital birth defects	Male	0-6 days	65-69 years	-1	Education (years per capita)
Other congenital birth defects	Male	0-6 days	65-69 years	-1	In-Facility Delivery (proportion)
Other congenital birth defects	Male	0-6 days	65-69 years	-1	LDI (I$ per capita)
Other congenital birth defects	Male	0-6 days	65-69 years	1	Live Births 35+ (proportion)
Other congenital birth defects	Male	0-6 days	65-69 years	1	Indoor Air Pollution (All Cooking Fuels)
Other congenital birth defects	Male	0-6 days	65-69 years	1	Smoking Prevalence (Reproductive Age Standardized)
Other congenital birth defects	Male	0-6 days	65-69 years	-1	Socio-demographic Index
Other congenital birth defects	Male	0-6 days	65-69 years	1	Maternal alcohol consumption during pregnancy (proportion)
Other congenital birth defects	Male	0-6 days	65-69 years	-1	Healthcare access and quality index
Skin and subcutaneous diseases	Male	28-364 days	95+ years	1	Alcohol (liters per capita)
Skin and subcutaneous diseases	Male	28-364 days	95+ years	1	Cumulative Cigarettes (10 Years)
Skin and subcutaneous diseases	Male	28-364 days	95+ years	1	Cumulative Cigarettes (5 Years)
Skin and subcutaneous diseases	Male	28-364 days	95+ years	-1	Education (years per capita)
Skin and subcutaneous diseases	Male	28-364 days	95+ years	-1	LDI (I$ per capita)
Skin and subcutaneous diseases	Male	28-364 days	95+ years	1	Smoking Prevalence
Skin and subcutaneous diseases	Male	28-364 days	95+ years	-1	Improved Water Source (proportion with access)
Skin and subcutaneous diseases	Male	28-364 days	95+ years	1	SEV unsafe sanitation
Skin and subcutaneous diseases	Male	28-364 days	95+ years	0	Socio-demographic Index
Skin and subcutaneous diseases	Male	28-364 days	95+ years	-1	Healthcare access and quality index
Skin and subcutaneous diseases	Female	28-364 days	95+ years	1	Alcohol (liters per capita)
Skin and subcutaneous diseases	Female	28-364 days	95+ years	1	Cumulative Cigarettes (10 Years)
Skin and subcutaneous diseases	Female	28-364 days	95+ years	1	Cumulative Cigarettes (5 Years)
Skin and subcutaneous diseases	Female	28-364 days	95+ years	-1	Education (years per capita)

Cause	Sex	Age start	Age end	Direction	Covariate
Skin and subcutaneous diseases	Female	28-364 days	95+ years	-1	LDI (I$ per capita)
Skin and subcutaneous diseases	Female	28-364 days	95+ years	1	Smoking Prevalence
Skin and subcutaneous diseases	Female	28-364 days	95+ years	-1	Improved Water Source (proportion with access)
Skin and subcutaneous diseases	Female	28-364 days	95+ years	1	SEV unsafe sanitation
Skin and subcutaneous diseases	Female	28-364 days	95+ years	0	Socio-demographic Index
Skin and subcutaneous diseases	Female	28-364 days	95+ years	-1	Healthcare access and quality index
Cellulitis	Male	28-364 days	95+ years	0	Education (years per capita)
Cellulitis	Male	28-364 days	95+ years	0	LDI (I$ per capita)
Cellulitis	Male	28-364 days	95+ years	-1	Healthcare access and quality index
Cellulitis	Female	28-364 days	95+ years	0	Education (years per capita)
Cellulitis	Female	28-364 days	95+ years	0	LDI (I$ per capita)
Cellulitis	Female	28-364 days	95+ years	-1	Healthcare access and quality index
Pyoderma	Female	0-6 days	95+ years	1	Alcohol (liters per capita)
Pyoderma	Female	0-6 days	95+ years	1	Cumulative Cigarettes (10 Years)
Pyoderma	Female	0-6 days	95+ years	1	Cumulative Cigarettes (5 Years)
Pyoderma	Female	0-6 days	95+ years	-1	Education (years per capita)
Pyoderma	Female	0-6 days	95+ years	-1	LDI (I$ per capita)
Pyoderma	Female	0-6 days	95+ years	1	Smoking Prevalence
Pyoderma	Female	0-6 days	95+ years	-1	Improved Water Source (proportion with access)
Pyoderma	Female	0-6 days	95+ years	1	SEV unsafe sanitation
Pyoderma	Female	0-6 days	95+ years	0	Socio-demographic Index
Pyoderma	Female	0-6 days	95+ years	-1	Healthcare access and quality index
Pyoderma	Male	0-6 days	95+ years	1	Alcohol (liters per capita)
Pyoderma	Male	0-6 days	95+ years	1	Cumulative Cigarettes (10 Years)
Pyoderma	Male	0-6 days	95+ years	1	Cumulative Cigarettes (5 Years)
Pyoderma	Male	0-6 days	95+ years	-1	Education (years per capita)
Pyoderma	Male	0-6 days	95+ years	-1	LDI (I$ per capita)
Pyoderma	Male	0-6 days	95+ years	1	Smoking Prevalence
Pyoderma	Male	0-6 days	95+ years	-1	Improved Water Source (proportion with access)
Pyoderma	Male	0-6 days	95+ years	1	SEV unsafe sanitation
Pyoderma	Male	0-6 days	95+ years	0	Socio-demographic Index
Pyoderma	Male	0-6 days	95+ years	-1	Healthcare access and quality index
Decubitus ulcer	Male	1-4 years	95+ years	1	Alcohol (liters per capita)
Decubitus ulcer	Male	1-4 years	95+ years	1	Cumulative Cigarettes (10 Years)
Decubitus ulcer	Male	1-4 years	95+ years	1	Cumulative Cigarettes (5 Years)
Decubitus ulcer	Male	1-4 years	95+ years	1	Diabetes Fasting Plasma Glucose (mmol/L)
Decubitus ulcer	Male	1-4 years	95+ years	-1	Education (years per capita)
Decubitus ulcer	Male	1-4 years	95+ years	-1	Health System Access 2 (unitless)
Decubitus ulcer	Male	1-4 years	95+ years	-1	LDI (I$ per capita)
Decubitus ulcer	Male	1-4 years	95+ years	1	Smoking Prevalence
Decubitus ulcer	Male	1-4 years	95+ years	-1	Improved Water Source (proportion with access)
Decubitus ulcer	Male	1-4 years	95+ years	1	Prevalence of obesity
Decubitus ulcer	Male	1-4 years	95+ years	1	SEV unsafe sanitation
Decubitus ulcer	Male	1-4 years	95+ years	0	Socio-demographic Index
Decubitus ulcer	Male	1-4 years	95+ years	-1	Healthcare access and quality index
Decubitus ulcer	Female	1-4 years	95+ years	1	Alcohol (liters per capita)
Decubitus ulcer	Female	1-4 years	95+ years	1	Cumulative Cigarettes (10 Years)
Decubitus ulcer	Female	1-4 years	95+ years	1	Cumulative Cigarettes (5 Years)
Decubitus ulcer	Female	1-4 years	95+ years	1	Diabetes Fasting Plasma Glucose (mmol/L)
Decubitus ulcer	Female	1-4 years	95+ years	-1	Education (years per capita)
Decubitus ulcer	Female	1-4 years	95+ years	-1	Health System Access 2 (unitless)
Decubitus ulcer	Female	1-4 years	95+ years	-1	LDI (I$ per capita)
Decubitus ulcer	Female	1-4 years	95+ years	1	Smoking Prevalence
Decubitus ulcer	Female	1-4 years	95+ years	-1	Improved Water Source (proportion with access)
Decubitus ulcer	Female	1-4 years	95+ years	1	Prevalence of obesity
Decubitus ulcer	Female	1-4 years	95+ years	1	SEV unsafe sanitation
Decubitus ulcer	Female	1-4 years	95+ years	0	Socio-demographic Index
Decubitus ulcer	Female	1-4 years	95+ years	-1	Healthcare access and quality index
Other skin and subcutaneous diseases	Male	28-364 days	95+ years	1	Alcohol (liters per capita)
Other skin and subcutaneous diseases	Male	28-364 days	95+ years	1	Cumulative Cigarettes (10 Years)
Other skin and subcutaneous diseases	Male	28-364 days	95+ years	1	Cumulative Cigarettes (5 Years)
Other skin and subcutaneous diseases	Male	28-364 days	95+ years	-1	Education (years per capita)
Other skin and subcutaneous diseases	Male	28-364 days	95+ years	-1	Health System Access 2 (unitless)
Other skin and subcutaneous diseases	Male	28-364 days	95+ years	-1	LDI (I$ per capita)
Other skin and subcutaneous diseases	Male	28-364 days	95+ years	1	Underweight (proportion <2SD weight for age, <5 years)
Other skin and subcutaneous diseases	Male	28-364 days	95+ years	1	Smoking Prevalence
Other skin and subcutaneous diseases	Male	28-364 days	95+ years	-1	Improved Water Source (proportion with access)
Other skin and subcutaneous diseases	Male	28-364 days	95+ years	1	SEV unsafe sanitation
Other skin and subcutaneous diseases	Male	28-364 days	95+ years	0	Socio-demographic Index
Other skin and subcutaneous diseases	Male	28-364 days	95+ years	-1	Healthcare access and quality index
Other skin and subcutaneous diseases	Female	28-364 days	95+ years	1	Alcohol (liters per capita)
Other skin and subcutaneous diseases	Female	28-364 days	95+ years	1	Cumulative Cigarettes (10 Years)
Other skin and subcutaneous diseases	Female	28-364 days	95+ years	1	Cumulative Cigarettes (5 Years)
Other skin and subcutaneous diseases	Female	28-364 days	95+ years	-1	Education (years per capita)
Other skin and subcutaneous diseases	Female	28-364 days	95+ years	-1	Health System Access 2 (unitless)
Other skin and subcutaneous diseases	Female	28-364 days	95+ years	-1	LDI (I$ per capita)
Other skin and subcutaneous diseases	Female	28-364 days	95+ years	1	Underweight (proportion <2SD weight for age, <5 years)
Other skin and subcutaneous diseases	Female	28-364 days	95+ years	1	Smoking Prevalence
Other skin and subcutaneous diseases	Female	28-364 days	95+ years	-1	Improved Water Source (proportion with access)
Other skin and subcutaneous diseases	Female	28-364 days	95+ years	1	SEV unsafe sanitation
Other skin and subcutaneous diseases	Female	28-364 days	95+ years	0	Socio-demographic Index
Other skin and subcutaneous diseases	Female	28-364 days	95+ years	-1	Healthcare access and quality index
Sudden infant death syndrome	Male	7-27 days	28-364 days	-1	Education (years per capita)
Sudden infant death syndrome	Male	7-27 days	28-364 days	-1	In-Facility Delivery (proportion)
Sudden infant death syndrome	Male	7-27 days	28-364 days	0	LDI (I$ per capita)
Sudden infant death syndrome	Male	7-27 days	28-364 days	1	Underweight (proportion <2SD weight for age, <5 years)
Sudden infant death syndrome	Male	7-27 days	28-364 days	1	Indoor Air Pollution (All Cooking Fuels)
Sudden infant death syndrome	Male	7-27 days	28-364 days	-1	Skilled Birth Attendance (proportion)
Sudden infant death syndrome	Male	7-27 days	28-364 days	1	Smoking Prevalence (Reproductive Age Standardized)
Sudden infant death syndrome	Male	7-27 days	28-364 days	1	Total Fertility Rate
Sudden infant death syndrome	Male	7-27 days	28-364 days	-1	Health System Access (capped)
Sudden infant death syndrome	Male	7-27 days	28-364 days	0	Socio-demographic Index

Cause	Sex	Age start	Age end	Direction	Covariate
Sudden infant death syndrome	Male	7-27 days	28-364 days	-1	Healthcare access and quality index
Sudden infant death syndrome	Female	7-27 days	28-364 days	-1	Education (years per capita)
Sudden infant death syndrome	Female	7-27 days	28-364 days	-1	In-Facility Delivery (proportion)
Sudden infant death syndrome	Female	7-27 days	28-364 days	0	LDI (I$ per capita)
Sudden infant death syndrome	Female	7-27 days	28-364 days	1	Underweight (proportion <2SD weight for age, <5 years)
Sudden infant death syndrome	Female	7-27 days	28-364 days	1	Indoor Air Pollution (All Cooking Fuels)
Sudden infant death syndrome	Female	7-27 days	28-364 days	-1	Skilled Birth Attendance (proportion)
Sudden infant death syndrome	Female	7-27 days	28-364 days	1	Smoking Prevalence (Reproductive Age Standardized)
Sudden infant death syndrome	Female	7-27 days	28-364 days	1	Total Fertility Rate
Sudden infant death syndrome	Female	7-27 days	28-364 days	0	Socio-demographic Index
Transport injuries	Female	0-6 days	95+ years	1	Alcohol (liters per capita)
Transport injuries	Female	0-6 days	95+ years	-1	Education (years per capita)
Transport injuries	Female	0-6 days	95+ years	0	LDI (I$ per capita)
Transport injuries	Female	0-6 days	95+ years	0	Population Density (300-500 ppl/sqkm, proportion)
Transport injuries	Female	0-6 days	95+ years	0	Population Density (500-1000 ppl/sqkm, proportion)
Transport injuries	Female	0-6 days	95+ years	1	Rainfall Quintile 5 (proportion)
Transport injuries	Female	0-6 days	95+ years	1	Vehicles - 2+4 wheels (per capita)
Transport injuries	Female	0-6 days	95+ years	1	Vehicles - 2 wheels fraction (proportion)
Transport injuries	Female	0-6 days	95+ years	-1	Socio-demographic Index
Transport injuries	Female	0-6 days	95+ years	-1	Healthcare access and quality index
Transport injuries	Male	0-6 days	95+ years	1	Alcohol (liters per capita)
Transport injuries	Male	0-6 days	95+ years	-1	Education (years per capita)
Transport injuries	Male	0-6 days	95+ years	0	LDI (I$ per capita)
Transport injuries	Male	0-6 days	95+ years	0	Population Density (300-500 ppl/sqkm, proportion)
Transport injuries	Male	0-6 days	95+ years	0	Population Density (500-1000 ppl/sqkm, proportion)
Transport injuries	Male	0-6 days	95+ years	1	Rainfall Quintile 5 (proportion)
Transport injuries	Male	0-6 days	95+ years	1	Vehicles - 2+4 wheels (per capita)
Transport injuries	Male	0-6 days	95+ years	1	Vehicles - 2 wheels fraction (proportion)
Transport injuries	Male	0-6 days	95+ years	-1	Socio-demographic Index
Transport injuries	Male	0-6 days	95+ years	-1	Healthcare access and quality index
Road injuries	Male	0-6 days	95+ years	1	Alcohol (liters per capita)
Road injuries	Male	0-6 days	95+ years	-1	Education (years per capita)
Road injuries	Male	0-6 days	95+ years	0	LDI (I$ per capita)
Road injuries	Male	0-6 days	95+ years	1	Population 15 to 30 (proportion)
Road injuries	Male	0-6 days	95+ years	0	Population Density (300-500 ppl/sqkm, proportion)
Road injuries	Male	0-6 days	95+ years	0	Population Density (500-1000 ppl/sqkm, proportion)
Road injuries	Male	0-6 days	95+ years	1	Vehicles - 2+4 wheels (per capita)
Road injuries	Male	0-6 days	95+ years	1	Vehicles - 2 wheels (per capita)
Road injuries	Male	0-6 days	95+ years	1	Vehicles - 4 wheels (per capita)
Road injuries	Male	0-6 days	95+ years	1	Vehicles - 2 wheels fraction (proportion)
Road injuries	Male	0-6 days	95+ years	1	Log-transformed SEV scalar: Road Inj
Road injuries	Male	0-6 days	95+ years	-1	Socio-demographic Index
Road injuries	Male	0-6 days	95+ years	-1	Healthcare access and quality index
Road injuries	Female	0-6 days	95+ years	1	Alcohol (liters per capita)
Road injuries	Female	0-6 days	95+ years	-1	Education (years per capita)
Road injuries	Female	0-6 days	95+ years	0	LDI (I$ per capita)
Road injuries	Female	0-6 days	95+ years	1	Population 15 to 30 (proportion)
Road injuries	Female	0-6 days	95+ years	0	Population Density (300-500 ppl/sqkm, proportion)
Road injuries	Female	0-6 days	95+ years	0	Population Density (500-1000 ppl/sqkm, proportion)
Road injuries	Female	0-6 days	95+ years	1	Vehicles - 2+4 wheels (per capita)
Road injuries	Female	0-6 days	95+ years	1	Vehicles - 2 wheels (per capita)
Road injuries	Female	0-6 days	95+ years	1	Vehicles - 4 wheels (per capita)
Road injuries	Female	0-6 days	95+ years	1	Vehicles - 2 wheels fraction (proportion)
Road injuries	Female	0-6 days	95+ years	1	Log-transformed SEV scalar: Road Inj
Road injuries	Female	0-6 days	95+ years	-1	Socio-demographic Index
Road injuries	Female	0-6 days	95+ years	-1	Healthcare access and quality index
Pedestrian road injuries	Female	0-6 days	95+ years	1	Alcohol (liters per capita)
Pedestrian road injuries	Female	0-6 days	95+ years	-1	Education (years per capita)
Pedestrian road injuries	Female	0-6 days	95+ years	0	LDI (I$ per capita)
Pedestrian road injuries	Female	0-6 days	95+ years	0	Population Density (300-500 ppl/sqkm, proportion)
Pedestrian road injuries	Female	0-6 days	95+ years	0	Population Density (500-1000 ppl/sqkm, proportion)
Pedestrian road injuries	Female	0-6 days	95+ years	1	Rainfall Quintile 5 (proportion)
Pedestrian road injuries	Female	0-6 days	95+ years	1	Vehicles - 2+4 wheels (per capita)
Pedestrian road injuries	Female	0-6 days	95+ years	1	Vehicles - 2 wheels fraction (proportion)
Pedestrian road injuries	Female	0-6 days	95+ years	1	Log-transformed SEV scalar: Pedest
Pedestrian road injuries	Female	0-6 days	95+ years	-1	Socio-demographic Index
Pedestrian road injuries	Female	0-6 days	95+ years	-1	Healthcare access and quality index
Pedestrian road injuries	Male	0-6 days	95+ years	1	Alcohol (liters per capita)
Pedestrian road injuries	Male	0-6 days	95+ years	-1	Education (years per capita)
Pedestrian road injuries	Male	0-6 days	95+ years	0	LDI (I$ per capita)
Pedestrian road injuries	Male	0-6 days	95+ years	0	Population Density (300-500 ppl/sqkm, proportion)
Pedestrian road injuries	Male	0-6 days	95+ years	0	Population Density (500-1000 ppl/sqkm, proportion)
Pedestrian road injuries	Male	0-6 days	95+ years	1	Rainfall Quintile 5 (proportion)
Pedestrian road injuries	Male	0-6 days	95+ years	1	Vehicles - 2+4 wheels (per capita)
Pedestrian road injuries	Male	0-6 days	95+ years	1	Vehicles - 2 wheels fraction (proportion)
Pedestrian road injuries	Male	0-6 days	95+ years	1	Log-transformed SEV scalar: Pedest
Pedestrian road injuries	Male	0-6 days	95+ years	-1	Socio-demographic Index
Pedestrian road injuries	Male	0-6 days	95+ years	-1	Healthcare access and quality index
Cyclist road injuries	Female	1-4 years	95+ years	1	Alcohol (liters per capita)
Cyclist road injuries	Female	1-4 years	95+ years	-1	Education (years per capita)
Cyclist road injuries	Female	1-4 years	95+ years	0	LDI (I$ per capita)
Cyclist road injuries	Female	1-4 years	95+ years	0	Population Density (300-500 ppl/sqkm, proportion)
Cyclist road injuries	Female	1-4 years	95+ years	0	Population Density (500-1000 ppl/sqkm, proportion)
Cyclist road injuries	Female	1-4 years	95+ years	1	Vehicles - 2+4 wheels (per capita)
Cyclist road injuries	Female	1-4 years	95+ years	1	Vehicles - 2 wheels fraction (proportion)
Cyclist road injuries	Female	1-4 years	95+ years	1	Log-transformed SEV scalar: Cyclist
Cyclist road injuries	Female	1-4 years	95+ years	0	Socio-demographic Index
Cyclist road injuries	Female	1-4 years	95+ years	-1	Healthcare access and quality index
Cyclist road injuries	Male	1-4 years	95+ years	1	Alcohol (liters per capita)
Cyclist road injuries	Male	1-4 years	95+ years	-1	Education (years per capita)
Cyclist road injuries	Male	1-4 years	95+ years	0	LDI (I$ per capita)
Cyclist road injuries	Male	1-4 years	95+ years	0	Population Density (300-500 ppl/sqkm, proportion)

Cause	Sex	Age start	Age end	Direction	Covariate
Cyclist road injuries	Male	1-4 years	95+ years	0	Population Density (500-1000 ppl/sqkm, proportion)
Cyclist road injuries	Male	1-4 years	95+ years	1	Vehicles - 2+4 wheels (per capita)
Cyclist road injuries	Male	1-4 years	95+ years	1	Vehicles - 2 wheels fraction (proportion)
Cyclist road injuries	Male	1-4 years	95+ years	1	Log-transformed SEV scalar: Cyclist
Cyclist road injuries	Male	1-4 years	95+ years	0	Socio-demographic Index
Cyclist road injuries	Male	1-4 years	95+ years	-1	Healthcare access and quality index
Motorcyclist road injuries	Female	0-6 days	95+ years	1	Alcohol (liters per capita)
Motorcyclist road injuries	Female	0-6 days	95+ years	-1	Education (years per capita)
Motorcyclist road injuries	Female	0-6 days	95+ years	0	LDI (I$ per capita)
Motorcyclist road injuries	Female	0-6 days	95+ years	0	Population Density (300-500 ppl/sqkm, proportion)
Motorcyclist road injuries	Female	0-6 days	95+ years	0	Population Density (500-1000 ppl/sqkm, proportion)
Motorcyclist road injuries	Female	0-6 days	95+ years	1	Rainfall Quintile 5 (proportion)
Motorcyclist road injuries	Female	0-6 days	95+ years	1	Vehicles - 2 wheels (per capita)
Motorcyclist road injuries	Female	0-6 days	95+ years	1	Log-transformed SEV scalar: Mot Cyc
Motorcyclist road injuries	Female	0-6 days	95+ years	0	Socio-demographic Index
Motorcyclist road injuries	Female	0-6 days	95+ years	-1	Healthcare access and quality index
Motorcyclist road injuries	Male	0-6 days	95+ years	1	Alcohol (liters per capita)
Motorcyclist road injuries	Male	0-6 days	95+ years	-1	Education (years per capita)
Motorcyclist road injuries	Male	0-6 days	95+ years	0	LDI (I$ per capita)
Motorcyclist road injuries	Male	0-6 days	95+ years	0	Population Density (300-500 ppl/sqkm, proportion)
Motorcyclist road injuries	Male	0-6 days	95+ years	0	Population Density (500-1000 ppl/sqkm, proportion)
Motorcyclist road injuries	Male	0-6 days	95+ years	1	Rainfall Quintile 5 (proportion)
Motorcyclist road injuries	Male	0-6 days	95+ years	1	Vehicles - 2 wheels (per capita)
Motorcyclist road injuries	Male	0-6 days	95+ years	1	Log-transformed SEV scalar: Mot Cyc
Motorcyclist road injuries	Male	0-6 days	95+ years	0	Socio-demographic Index
Motorcyclist road injuries	Male	0-6 days	95+ years	-1	Healthcare access and quality index
Motor vehicle road injuries	Female	0-6 days	95+ years	1	Alcohol (liters per capita)
Motor vehicle road injuries	Female	0-6 days	95+ years	0	Education (years per capita)
Motor vehicle road injuries	Female	0-6 days	95+ years	0	LDI (I$ per capita)
Motor vehicle road injuries	Female	0-6 days	95+ years	0	Population Density (300-500 ppl/sqkm, proportion)
Motor vehicle road injuries	Female	0-6 days	95+ years	0	Population Density (500-1000 ppl/sqkm, proportion)
Motor vehicle road injuries	Female	0-6 days	95+ years	1	Rainfall Quintile 5 (proportion)
Motor vehicle road injuries	Female	0-6 days	95+ years	1	Vehicles - 4 wheels (per capita)
Motor vehicle road injuries	Female	0-6 days	95+ years	1	Log-transformed SEV scalar: Mot Veh
Motor vehicle road injuries	Female	0-6 days	95+ years	0	Socio-demographic Index
Motor vehicle road injuries	Female	0-6 days	95+ years	-1	Healthcare access and quality index
Motor vehicle road injuries	Male	0-6 days	95+ years	1	Alcohol (liters per capita)
Motor vehicle road injuries	Male	0-6 days	95+ years	0	Education (years per capita)
Motor vehicle road injuries	Male	0-6 days	95+ years	0	LDI (I$ per capita)
Motor vehicle road injuries	Male	0-6 days	95+ years	0	Population Density (300-500 ppl/sqkm, proportion)
Motor vehicle road injuries	Male	0-6 days	95+ years	0	Population Density (500-1000 ppl/sqkm, proportion)
Motor vehicle road injuries	Male	0-6 days	95+ years	1	Rainfall Quintile 5 (proportion)
Motor vehicle road injuries	Male	0-6 days	95+ years	1	Vehicles - 4 wheels (per capita)
Motor vehicle road injuries	Male	0-6 days	95+ years	1	Log-transformed SEV scalar: Mot Veh
Motor vehicle road injuries	Male	0-6 days	95+ years	0	Socio-demographic Index
Motor vehicle road injuries	Male	0-6 days	95+ years	-1	Healthcare access and quality index
Other road injuries	Female	0-6 days	95+ years	1	Alcohol (liters per capita)
Other road injuries	Female	0-6 days	95+ years	0	LDI (I$ per capita)
Other road injuries	Female	0-6 days	95+ years	1	Rainfall Quintile 5 (proportion)
Other road injuries	Female	0-6 days	95+ years	1	Vehicles - 2+4 wheels (per capita)
Other road injuries	Female	0-6 days	95+ years	1	Vehicles - 2 wheels fraction (proportion)
Other road injuries	Female	0-6 days	95+ years	1	Log-transformed SEV scalar: Oth Road
Other road injuries	Female	0-6 days	95+ years	-1	Socio-demographic Index
Other road injuries	Female	0-6 days	95+ years	-1	Healthcare access and quality index
Other road injuries	Male	0-6 days	95+ years	1	Alcohol (liters per capita)
Other road injuries	Male	0-6 days	95+ years	0	LDI (I$ per capita)
Other road injuries	Male	0-6 days	95+ years	1	Rainfall Quintile 5 (proportion)
Other road injuries	Male	0-6 days	95+ years	1	Vehicles - 2+4 wheels (per capita)
Other road injuries	Male	0-6 days	95+ years	1	Vehicles - 2 wheels fraction (proportion)
Other road injuries	Male	0-6 days	95+ years	1	Log-transformed SEV scalar: Oth Road
Other road injuries	Male	0-6 days	95+ years	-1	Socio-demographic Index
Other road injuries	Male	0-6 days	95+ years	-1	Healthcare access and quality index
Other transport injuries	Female	0-6 days	95+ years	1	Alcohol (liters per capita)
Other transport injuries	Female	0-6 days	95+ years	0	Education (years per capita)
Other transport injuries	Female	0-6 days	95+ years	0	LDI (I$ per capita)
Other transport injuries	Female	0-6 days	95+ years	0	Population Density (300-500 ppl/sqkm, proportion)
Other transport injuries	Female	0-6 days	95+ years	0	Population Density (500-1000 ppl/sqkm, proportion)
Other transport injuries	Female	0-6 days	95+ years	1	Rainfall Quintile 5 (proportion)
Other transport injuries	Female	0-6 days	95+ years	1	Vehicles - 2+4 wheels (per capita)
Other transport injuries	Female	0-6 days	95+ years	1	Vehicles - 2 wheels fraction (proportion)
Other transport injuries	Female	0-6 days	95+ years	1	Log-transformed SEV scalar: Oth Trans
Other transport injuries	Female	0-6 days	95+ years	0	Socio-demographic Index
Other transport injuries	Female	0-6 days	95+ years	-1	Healthcare access and quality index
Other transport injuries	Male	0-6 days	95+ years	1	Alcohol (liters per capita)
Other transport injuries	Male	0-6 days	95+ years	0	Education (years per capita)
Other transport injuries	Male	0-6 days	95+ years	1	LDI (I$ per capita)
Other transport injuries	Male	0-6 days	95+ years	0	Population Density (300-500 ppl/sqkm, proportion)
Other transport injuries	Male	0-6 days	95+ years	0	Population Density (500-1000 ppl/sqkm, proportion)
Other transport injuries	Male	0-6 days	95+ years	1	Rainfall Quintile 5 (proportion)
Other transport injuries	Male	0-6 days	95+ years	1	Vehicles - 2+4 wheels (per capita)
Other transport injuries	Male	0-6 days	95+ years	1	Vehicles - 2 wheels fraction (proportion)
Other transport injuries	Male	0-6 days	95+ years	1	Log-transformed SEV scalar: Oth Trans
Other transport injuries	Male	0-6 days	95+ years	0	Socio-demographic Index
Other transport injuries	Male	0-6 days	95+ years	-1	Healthcare access and quality index
Unintentional injuries	Female	28-364 days	95+ years	1	Alcohol (liters per capita)
Unintentional injuries	Female	28-364 days	95+ years	1	Cumulative Cigarettes (5 Years)
Unintentional injuries	Female	28-364 days	95+ years	1	Diabetes Fasting Plasma Glucose (mmol/L)
Unintentional injuries	Female	28-364 days	95+ years	-1	Education (years per capita)
Unintentional injuries	Female	28-364 days	95+ years	-1	Health System Access 2 (unitless)
Unintentional injuries	Female	28-364 days	95+ years	-1	LDI (I$ per capita)
Unintentional injuries	Female	28-364 days	95+ years	1	Underweight (proportion <2SD weight for age, <5 years)
Unintentional injuries	Female	28-364 days	95+ years	1	Indoor Air Pollution (All Cooking Fuels)

Cause	Sex	Age start	Age end	Direction	Covariate
Unintentional injuries	Female	28-364 days	95+ years	1	Population Density (500-1000 ppl/sqkm, proportion)
Unintentional injuries	Female	28-364 days	95+ years	1	Population Density (over 1000 ppl/sqkm, proportion)
Unintentional injuries	Female	28-364 days	95+ years	1	Smoking Prevalence
Unintentional injuries	Male	28-364 days	95+ years	1	Alcohol (liters per capita)
Unintentional injuries	Male	28-364 days	95+ years	1	Cumulative Cigarettes (5 Years)
Unintentional injuries	Male	28-364 days	95+ years	1	Diabetes Fasting Plasma Glucose (mmol/L)
Unintentional injuries	Male	28-364 days	95+ years	-1	Education (years per capita)
Unintentional injuries	Male	28-364 days	95+ years	-1	Health System Access 2 (unitless)
Unintentional injuries	Male	28-364 days	95+ years	-1	LDI (I$ per capita)
Unintentional injuries	Male	28-364 days	95+ years	1	Underweight (proportion <2SD weight for age, <5 years)
Unintentional injuries	Male	28-364 days	95+ years	1	Indoor Air Pollution (All Cooking Fuels)
Unintentional injuries	Male	28-364 days	95+ years	1	Population Density (500-1000 ppl/sqkm, proportion)
Unintentional injuries	Male	28-364 days	95+ years	1	Population Density (over 1000 ppl/sqkm, proportion)
Unintentional injuries	Male	28-364 days	95+ years	1	Smoking Prevalence
Falls	Female	0-6 days	95+ years	1	Alcohol (liters per capita)
Falls	Female	0-6 days	95+ years	0	LDI (I$ per capita)
Falls	Female	0-6 days	95+ years	-1	ln-Milk (kcal per capita)
Falls	Female	0-6 days	95+ years	1	Elevation Over 1500m (proportion)
Falls	Female	0-6 days	95+ years	1	Log-transformed SEV scalar: Falls
Falls	Female	0-6 days	95+ years	0	Socio-demographic Index
Falls	Female	0-6 days	95+ years	-1	Healthcare access and quality index
Falls	Male	0-6 days	95+ years	1	Alcohol (liters per capita)
Falls	Male	0-6 days	95+ years	0	LDI (I$ per capita)
Falls	Male	0-6 days	95+ years	-1	ln-Milk (kcal per capita)
Falls	Male	0-6 days	95+ years	1	Elevation Over 1500m (proportion)
Falls	Male	0-6 days	95+ years	1	Log-transformed SEV scalar: Falls
Falls	Male	0-6 days	95+ years	0	Socio-demographic Index
Falls	Male	0-6 days	95+ years	-1	Healthcare access and quality index
Drowning	Female	0-6 days	95+ years	1	Alcohol (liters per capita)
Drowning	Female	0-6 days	95+ years	1	Coastal Population within 10km (proportion)
Drowning	Female	0-6 days	95+ years	-1	Education (years per capita)
Drowning	Female	0-6 days	95+ years	-1	Landlocked Nation (binary)
Drowning	Female	0-6 days	95+ years	0	LDI (I$ per capita)
Drowning	Female	0-6 days	95+ years	1	Elevation Under 100m (proportion)
Drowning	Female	0-6 days	95+ years	-1	Rainfall Quintile 1 (proportion)
Drowning	Female	0-6 days	95+ years	1	Rainfall Quintile 5 (proportion)
Drowning	Female	0-6 days	95+ years	1	Log-transformed SEV scalar: Drown
Drowning	Female	0-6 days	95+ years	-1	Socio-demographic Index
Drowning	Male	0-6 days	95+ years	1	Alcohol (liters per capita)
Drowning	Male	0-6 days	95+ years	1	Coastal Population within 10km (proportion)
Drowning	Male	0-6 days	95+ years	-1	Education (years per capita)
Drowning	Male	0-6 days	95+ years	-1	Landlocked Nation (binary)
Drowning	Male	0-6 days	95+ years	0	LDI (I$ per capita)
Drowning	Male	0-6 days	95+ years	1	Elevation Under 100m (proportion)
Drowning	Male	0-6 days	95+ years	-1	Rainfall Quintile 1 (proportion)
Drowning	Male	0-6 days	95+ years	1	Rainfall Quintile 5 (proportion)
Drowning	Male	0-6 days	95+ years	1	Log-transformed SEV scalar: Drown
Drowning	Male	0-6 days	95+ years	-1	Socio-demographic Index
Fire, heat, and hot substances	Female	0-6 days	95+ years	1	Alcohol (liters per capita)
Fire, heat, and hot substances	Female	0-6 days	95+ years	1	Tobacco (cigarettes per capita)
Fire, heat, and hot substances	Female	0-6 days	95+ years	-1	Education (years per capita)
Fire, heat, and hot substances	Female	0-6 days	95+ years	0	LDI (I$ per capita)
Fire, heat, and hot substances	Female	0-6 days	95+ years	1	Indoor Air Pollution (Biomass Cooking)
Fire, heat, and hot substances	Female	0-6 days	95+ years	0	Population Density (over 1000 ppl/sqkm, proportion)
Fire, heat, and hot substances	Female	0-6 days	95+ years	1	Log-transformed SEV scalar: Fire
Fire, heat, and hot substances	Female	0-6 days	95+ years	-1	Socio-demographic Index
Fire, heat, and hot substances	Female	0-6 days	95+ years	-1	Healthcare access and quality index
Fire, heat, and hot substances	Male	0-6 days	95+ years	1	Alcohol (liters per capita)
Fire, heat, and hot substances	Male	0-6 days	95+ years	1	Tobacco (cigarettes per capita)
Fire, heat, and hot substances	Male	0-6 days	95+ years	-1	Education (years per capita)
Fire, heat, and hot substances	Male	0-6 days	95+ years	0	LDI (I$ per capita)
Fire, heat, and hot substances	Male	0-6 days	95+ years	1	Indoor Air Pollution (Biomass Cooking)
Fire, heat, and hot substances	Male	0-6 days	95+ years	0	Population Density (over 1000 ppl/sqkm, proportion)
Fire, heat, and hot substances	Male	0-6 days	95+ years	1	Log-transformed SEV scalar: Fire
Fire, heat, and hot substances	Male	0-6 days	95+ years	-1	Socio-demographic Index
Fire, heat, and hot substances	Male	0-6 days	95+ years	-1	Healthcare access and quality index
Poisonings	Female	0-6 days	95+ years	-1	Education (years per capita)
Poisonings	Female	0-6 days	95+ years	0	LDI (I$ per capita)
Poisonings	Female	0-6 days	95+ years	1	Opium Cultivation (binary)
Poisonings	Female	0-6 days	95+ years	0	Population Density (over 1000 ppl/sqkm, proportion)
Poisonings	Female	0-6 days	95+ years	0	Population Density (under 150 ppl/sqkm, proportion)
Poisonings	Female	0-6 days	95+ years	1	Log-transformed SEV scalar: Poison
Poisonings	Female	0-6 days	95+ years	-1	Socio-demographic Index
Poisonings	Female	0-6 days	95+ years	-1	Healthcare access and quality index
Poisonings	Male	0-6 days	95+ years	-1	Education (years per capita)
Poisonings	Male	0-6 days	95+ years	0	LDI (I$ per capita)
Poisonings	Male	0-6 days	95+ years	1	Opium Cultivation (binary)
Poisonings	Male	0-6 days	95+ years	0	Population Density (over 1000 ppl/sqkm, proportion)
Poisonings	Male	0-6 days	95+ years	0	Population Density (under 150 ppl/sqkm, proportion)
Poisonings	Male	0-6 days	95+ years	1	Log-transformed SEV scalar: Poison
Poisonings	Male	0-6 days	95+ years	-1	Socio-demographic Index
Poisonings	Male	0-6 days	95+ years	-1	Healthcare access and quality index
Exposure to mechanical forces	Female	0-6 days	95+ years	1	Alcohol (liters per capita)
Exposure to mechanical forces	Female	0-6 days	95+ years	-1	Education (years per capita)
Exposure to mechanical forces	Female	0-6 days	95+ years	0	LDI (I$ per capita)
Exposure to mechanical forces	Female	0-6 days	95+ years	0	Population Density (over 1000 ppl/sqkm, proportion)
Exposure to mechanical forces	Female	0-6 days	95+ years	0	Population Density (under 150 ppl/sqkm, proportion)
Exposure to mechanical forces	Female	0-6 days	95+ years	-1	Socio-demographic Index
Exposure to mechanical forces	Female	0-6 days	95+ years	-1	Healthcare access and quality index
Exposure to mechanical forces	Male	0-6 days	95+ years	1	Alcohol (liters per capita)
Exposure to mechanical forces	Male	0-6 days	95+ years	-1	Education (years per capita)
Exposure to mechanical forces	Male	0-6 days	95+ years	0	LDI (I$ per capita)

Cause	Sex	Age start	Age end	Direction	Covariate
Exposure to mechanical forces	Male	0-6 days	95+ years	0	Population Density (over 1000 ppl/sqkm, proportion)
Exposure to mechanical forces	Male	0-6 days	95+ years	0	Population Density (under 150 ppl/sqkm, proportion)
Exposure to mechanical forces	Male	0-6 days	95+ years	-1	Socio-demographic Index
Exposure to mechanical forces	Male	0-6 days	95+ years	-1	Healthcare access and quality index
Unintentional firearm injuries	Female	0-6 days	95+ years	1	Alcohol (liters per capita)
Unintentional firearm injuries	Female	0-6 days	95+ years	-1	Education (years per capita)
Unintentional firearm injuries	Female	0-6 days	95+ years	-1	Health System Access (unitless)
Unintentional firearm injuries	Female	0-6 days	95+ years	0	LDI (I$ per capita)
Unintentional firearm injuries	Female	0-6 days	95+ years	0	Population Density (over 1000 ppl/sqkm, proportion)
Unintentional firearm injuries	Female	0-6 days	95+ years	0	Population Density (under 150 ppl/sqkm, proportion)
Unintentional firearm injuries	Female	0-6 days	95+ years	1	Log-transformed SEV scalar: Mech Gun
Unintentional firearm injuries	Female	0-6 days	95+ years	-1	Socio-demographic Index
Unintentional firearm injuries	Female	0-6 days	95+ years	-1	Healthcare access and quality index
Unintentional firearm injuries	Male	0-6 days	95+ years	1	Alcohol (liters per capita)
Unintentional firearm injuries	Male	0-6 days	95+ years	-1	Education (years per capita)
Unintentional firearm injuries	Male	0-6 days	95+ years	-1	Health System Access (unitless)
Unintentional firearm injuries	Male	0-6 days	95+ years	0	LDI (I$ per capita)
Unintentional firearm injuries	Male	0-6 days	95+ years	0	Population Density (over 1000 ppl/sqkm, proportion)
Unintentional firearm injuries	Male	0-6 days	95+ years	0	Population Density (under 150 ppl/sqkm, proportion)
Unintentional firearm injuries	Male	0-6 days	95+ years	1	Log-transformed SEV scalar: Mech Gun
Unintentional firearm injuries	Male	0-6 days	95+ years	-1	Socio-demographic Index
Unintentional firearm injuries	Male	0-6 days	95+ years	-1	Healthcare access and quality index
Unintentional suffocation	Female	0-6 days	95+ years	1	Alcohol (liters per capita)
Unintentional suffocation	Female	0-6 days	95+ years	-1	Education (years per capita)
Unintentional suffocation	Female	0-6 days	95+ years	0	LDI (I$ per capita)
Unintentional suffocation	Female	0-6 days	95+ years	0	Population Density (over 1000 ppl/sqkm, proportion)
Unintentional suffocation	Female	0-6 days	95+ years	0	Population Density (under 150 ppl/sqkm, proportion)
Unintentional suffocation	Female	0-6 days	95+ years	1	Log-transformed SEV scalar: Mech Suff
Unintentional suffocation	Female	0-6 days	95+ years	0	Socio-demographic Index
Unintentional suffocation	Female	0-6 days	95+ years	-1	Healthcare access and quality index
Unintentional suffocation	Male	0-6 days	95+ years	1	Alcohol (liters per capita)
Unintentional suffocation	Male	0-6 days	95+ years	-1	Education (years per capita)
Unintentional suffocation	Male	0-6 days	95+ years	0	LDI (I$ per capita)
Unintentional suffocation	Male	0-6 days	95+ years	0	Population Density (over 1000 ppl/sqkm, proportion)
Unintentional suffocation	Male	0-6 days	95+ years	0	Population Density (under 150 ppl/sqkm, proportion)
Unintentional suffocation	Male	0-6 days	95+ years	1	Log-transformed SEV scalar: Mech Suff
Unintentional suffocation	Male	0-6 days	95+ years	0	Socio-demographic Index
Unintentional suffocation	Male	0-6 days	95+ years	-1	Healthcare access and quality index
Other exposure to mechanical forces	Female	0-6 days	95+ years	1	Alcohol (liters per capita)
Other exposure to mechanical forces	Female	0-6 days	95+ years	-1	Education (years per capita)
Other exposure to mechanical forces	Female	0-6 days	95+ years	-1	Health System Access (unitless)
Other exposure to mechanical forces	Female	0-6 days	95+ years	0	LDI (I$ per capita)
Other exposure to mechanical forces	Female	0-6 days	95+ years	0	Population Density (over 1000 ppl/sqkm, proportion)
Other exposure to mechanical forces	Female	0-6 days	95+ years	0	Population Density (under 150 ppl/sqkm, proportion)
Other exposure to mechanical forces	Female	0-6 days	95+ years	1	Log-transformed SEV scalar: Oth Mech
Other exposure to mechanical forces	Female	0-6 days	95+ years	-1	Socio-demographic Index
Other exposure to mechanical forces	Female	0-6 days	95+ years	-1	Healthcare access and quality index
Other exposure to mechanical forces	Male	0-6 days	95+ years	1	Alcohol (liters per capita)
Other exposure to mechanical forces	Male	0-6 days	95+ years	-1	Education (years per capita)
Other exposure to mechanical forces	Male	0-6 days	95+ years	-1	Health System Access (unitless)
Other exposure to mechanical forces	Male	0-6 days	95+ years	0	LDI (I$ per capita)
Other exposure to mechanical forces	Male	0-6 days	95+ years	0	Population Density (over 1000 ppl/sqkm, proportion)
Other exposure to mechanical forces	Male	0-6 days	95+ years	0	Population Density (under 150 ppl/sqkm, proportion)
Other exposure to mechanical forces	Male	0-6 days	95+ years	1	Log-transformed SEV scalar: Oth Mech
Other exposure to mechanical forces	Male	0-6 days	95+ years	-1	Socio-demographic Index
Other exposure to mechanical forces	Male	0-6 days	95+ years	-1	Healthcare access and quality index
Adverse effects of medical treatment	Female	0-6 days	95+ years	0	LDI (I$ per capita)
Adverse effects of medical treatment	Female	0-6 days	95+ years	0	Socio-demographic Index
Adverse effects of medical treatment	Female	0-6 days	95+ years	0	Healthcare access and quality index
Adverse effects of medical treatment	Male	0-6 days	95+ years	0	LDI (I$ per capita)
Adverse effects of medical treatment	Male	0-6 days	95+ years	0	Socio-demographic Index
Adverse effects of medical treatment	Male	0-6 days	95+ years	0	Healthcare access and quality index
Animal contact	Female	0-6 days	95+ years	1	Alcohol (liters per capita)
Animal contact	Female	0-6 days	95+ years	-1	Education (years per capita)
Animal contact	Female	0-6 days	95+ years	0	LDI (I$ per capita)
Animal contact	Female	0-6 days	95+ years	0	Elevation Over 1500m (proportion)
Animal contact	Female	0-6 days	95+ years	1	Population 15 to 30 (proportion)
Animal contact	Female	0-6 days	95+ years	0	Population Density (over 1000 ppl/sqkm, proportion)
Animal contact	Female	0-6 days	95+ years	0	Population Density (under 150 ppl/sqkm, proportion)
Animal contact	Female	0-6 days	95+ years	0	Elevation Under 100m (proportion)
Animal contact	Female	0-6 days	95+ years	1	Log-transformed SEV scalar: Animal
Animal contact	Female	0-6 days	95+ years	-1	Socio-demographic Index
Animal contact	Female	0-6 days	95+ years	-1	Healthcare access and quality index
Animal contact	Male	0-6 days	95+ years	1	Alcohol (liters per capita)
Animal contact	Male	0-6 days	95+ years	-1	Education (years per capita)
Animal contact	Male	0-6 days	95+ years	0	LDI (I$ per capita)
Animal contact	Male	0-6 days	95+ years	0	Elevation Over 1500m (proportion)
Animal contact	Male	0-6 days	95+ years	1	Population 15 to 30 (proportion)
Animal contact	Male	0-6 days	95+ years	0	Population Density (over 1000 ppl/sqkm, proportion)
Animal contact	Male	0-6 days	95+ years	0	Population Density (under 150 ppl/sqkm, proportion)
Animal contact	Male	0-6 days	95+ years	0	Elevation Under 100m (proportion)
Animal contact	Male	0-6 days	95+ years	1	Log-transformed SEV scalar: Animal
Animal contact	Male	0-6 days	95+ years	-1	Socio-demographic Index
Animal contact	Male	0-6 days	95+ years	-1	Healthcare access and quality index
Venomous animal contact	Female	0-6 days	95+ years	1	Alcohol (liters per capita)
Venomous animal contact	Female	0-6 days	95+ years	-1	Education (years per capita)
Venomous animal contact	Female	0-6 days	95+ years	0	LDI (I$ per capita)
Venomous animal contact	Female	0-6 days	95+ years	0	Elevation Over 1500m (proportion)
Venomous animal contact	Female	0-6 days	95+ years	0	Population Density (over 1000 ppl/sqkm, proportion)
Venomous animal contact	Female	0-6 days	95+ years	0	Population Density (under 150 ppl/sqkm, proportion)
Venomous animal contact	Female	0-6 days	95+ years	0	Elevation Under 100m (proportion)
Venomous animal contact	Female	0-6 days	95+ years	1	Log-transformed SEV scalar: Venom

Cause	Sex	Age start	Age end	Direction	Covariate
Venomous animal contact	Female	0-6 days	95+ years	-1	Socio-demographic Index
Venomous animal contact	Female	0-6 days	95+ years	-1	Healthcare access and quality index
Venomous animal contact	Male	0-6 days	95+ years	1	Alcohol (liters per capita)
Venomous animal contact	Male	0-6 days	95+ years	-1	Education (years per capita)
Venomous animal contact	Male	0-6 days	95+ years	0	LDI (I$ per capita)
Venomous animal contact	Male	0-6 days	95+ years	0	Elevation Over 1500m (proportion)
Venomous animal contact	Male	0-6 days	95+ years	0	Population Density (over 1000 ppl/sqkm, proportion)
Venomous animal contact	Male	0-6 days	95+ years	0	Population Density (under 150 ppl/sqkm, proportion)
Venomous animal contact	Male	0-6 days	95+ years	0	Elevation Under 100m (proportion)
Venomous animal contact	Male	0-6 days	95+ years	1	Log-transformed SEV scalar: Venom
Venomous animal contact	Male	0-6 days	95+ years	-1	Socio-demographic Index
Venomous animal contact	Male	0-6 days	95+ years	-1	Healthcare access and quality index
Non-venomous animal contact	Female	0-6 days	95+ years	1	Alcohol (liters per capita)
Non-venomous animal contact	Female	0-6 days	95+ years	-1	Education (years per capita)
Non-venomous animal contact	Female	0-6 days	95+ years	0	LDI (I$ per capita)
Non-venomous animal contact	Female	0-6 days	95+ years	0	Elevation Over 1500m (proportion)
Non-venomous animal contact	Female	0-6 days	95+ years	0	Population Density (over 1000 ppl/sqkm, proportion)
Non-venomous animal contact	Female	0-6 days	95+ years	0	Population Density (under 150 ppl/sqkm, proportion)
Non-venomous animal contact	Female	0-6 days	95+ years	0	Elevation Under 100m (proportion)
Non-venomous animal contact	Female	0-6 days	95+ years	1	Log-transformed SEV scalar: Non Ven
Non-venomous animal contact	Female	0-6 days	95+ years	-1	Socio-demographic Index
Non-venomous animal contact	Female	0-6 days	95+ years	-1	Healthcare access and quality index
Non-venomous animal contact	Male	0-6 days	95+ years	1	Alcohol (liters per capita)
Non-venomous animal contact	Male	0-6 days	95+ years	-1	Education (years per capita)
Non-venomous animal contact	Male	0-6 days	95+ years	0	LDI (I$ per capita)
Non-venomous animal contact	Male	0-6 days	95+ years	0	Elevation Over 1500m (proportion)
Non-venomous animal contact	Male	0-6 days	95+ years	0	Population Density (over 1000 ppl/sqkm, proportion)
Non-venomous animal contact	Male	0-6 days	95+ years	0	Population Density (under 150 ppl/sqkm, proportion)
Non-venomous animal contact	Male	0-6 days	95+ years	0	Elevation Under 100m (proportion)
Non-venomous animal contact	Male	0-6 days	95+ years	1	Log-transformed SEV scalar: Non Ven
Non-venomous animal contact	Male	0-6 days	95+ years	-1	Socio-demographic Index
Non-venomous animal contact	Male	0-6 days	95+ years	-1	Healthcare access and quality index
Foreign body	Female	0-6 days	95+ years	1	Education (years per capita)
Foreign body	Female	0-6 days	95+ years	1	LDI (I$ per capita)
Foreign body	Female	0-6 days	95+ years	1	Indoor Air Pollution (Coal Cooking)
Foreign body	Female	0-6 days	95+ years	1	Population Density (over 1000 ppl/sqkm, proportion)
Foreign body	Female	0-6 days	95+ years	1	Population Over 65 (proportion)
Foreign body	Female	0-6 days	95+ years	0	Socio-demographic Index
Foreign body	Female	0-6 days	95+ years	-1	Healthcare access and quality index
Foreign body	Male	0-6 days	95+ years	1	Education (years per capita)
Foreign body	Male	0-6 days	95+ years	1	LDI (I$ per capita)
Foreign body	Male	0-6 days	95+ years	1	Indoor Air Pollution (Coal Cooking)
Foreign body	Male	0-6 days	95+ years	1	Population Density (over 1000 ppl/sqkm, proportion)
Foreign body	Male	0-6 days	95+ years	1	Population Over 65 (proportion)
Foreign body	Male	0-6 days	95+ years	0	Socio-demographic Index
Foreign body	Male	0-6 days	95+ years	-1	Healthcare access and quality index
Pulmonary aspiration and foreign body in airway	Female	0-6 days	95+ years	1	Alcohol (liters per capita)
Pulmonary aspiration and foreign body in airway	Female	0-6 days	95+ years	0	LDI (I$ per capita)
Pulmonary aspiration and foreign body in airway	Female	0-6 days	95+ years	1	Mean BMI
Pulmonary aspiration and foreign body in airway	Female	0-6 days	95+ years	1	Log-transformed SEV scalar: F Body Asp
Pulmonary aspiration and foreign body in airway	Female	0-6 days	95+ years	0	Socio-demographic Index
Pulmonary aspiration and foreign body in airway	Female	0-6 days	95+ years	-1	Healthcare access and quality index
Pulmonary aspiration and foreign body in airway	Male	0-6 days	95+ years	1	Alcohol (liters per capita)
Pulmonary aspiration and foreign body in airway	Male	0-6 days	95+ years	0	LDI (I$ per capita)
Pulmonary aspiration and foreign body in airway	Male	0-6 days	95+ years	1	Mean BMI
Pulmonary aspiration and foreign body in airway	Male	0-6 days	95+ years	1	Log-transformed SEV scalar: F Body Asp
Pulmonary aspiration and foreign body in airway	Male	0-6 days	95+ years	0	Socio-demographic Index
Pulmonary aspiration and foreign body in airway	Male	0-6 days	95+ years	-1	Healthcare access and quality index
Foreign body in other body part	Female	0-6 days	95+ years	1	Alcohol (liters per capita)
Foreign body in other body part	Female	0-6 days	95+ years	-1	Education (years per capita)
Foreign body in other body part	Female	0-6 days	95+ years	0	LDI (I$ per capita)
Foreign body in other body part	Female	0-6 days	95+ years	0	Elevation Over 1500m (proportion)
Foreign body in other body part	Female	0-6 days	95+ years	0	Population Density (over 1000 ppl/sqkm, proportion)
Foreign body in other body part	Female	0-6 days	95+ years	0	Population Density (under 150 ppl/sqkm, proportion)
Foreign body in other body part	Female	0-6 days	95+ years	0	Elevation Under 100m (proportion)
Foreign body in other body part	Female	0-6 days	95+ years	1	Log-transformed SEV scalar: Oth F Body
Foreign body in other body part	Female	0-6 days	95+ years	-1	Socio-demographic Index
Foreign body in other body part	Female	0-6 days	95+ years	-1	Healthcare access and quality index
Foreign body in other body part	Male	0-6 days	95+ years	1	Alcohol (liters per capita)
Foreign body in other body part	Male	0-6 days	95+ years	-1	Education (years per capita)
Foreign body in other body part	Male	0-6 days	95+ years	0	LDI (I$ per capita)
Foreign body in other body part	Male	0-6 days	95+ years	0	Elevation Over 1500m (proportion)
Foreign body in other body part	Male	0-6 days	95+ years	0	Population Density (over 1000 ppl/sqkm, proportion)
Foreign body in other body part	Male	0-6 days	95+ years	0	Population Density (under 150 ppl/sqkm, proportion)
Foreign body in other body part	Male	0-6 days	95+ years	0	Elevation Under 100m (proportion)
Foreign body in other body part	Male	0-6 days	95+ years	1	Log-transformed SEV scalar: Oth F Body
Foreign body in other body part	Male	0-6 days	95+ years	-1	Socio-demographic Index
Foreign body in other body part	Male	0-6 days	95+ years	-1	Healthcare access and quality index
Other unintentional injuries	Female	0-6 days	95+ years	1	Alcohol (liters per capita)
Other unintentional injuries	Female	0-6 days	95+ years	-1	Education (years per capita)
Other unintentional injuries	Female	0-6 days	95+ years	0	LDI (I$ per capita)
Other unintentional injuries	Female	0-6 days	95+ years	0	Elevation Over 1500m (proportion)
Other unintentional injuries	Female	0-6 days	95+ years	0	Population Density (over 1000 ppl/sqkm, proportion)
Other unintentional injuries	Female	0-6 days	95+ years	0	Population Density (under 150 ppl/sqkm, proportion)
Other unintentional injuries	Female	0-6 days	95+ years	0	Elevation Under 100m (proportion)
Other unintentional injuries	Female	0-6 days	95+ years	1	Vehicles - 2 wheels (per capita)
Other unintentional injuries	Female	0-6 days	95+ years	0	Vehicles - 4 wheels (per capita)
Other unintentional injuries	Female	0-6 days	95+ years	1	Log-transformed SEV scalar: Oth Unint
Other unintentional injuries	Female	0-6 days	95+ years	0	Socio-demographic Index
Other unintentional injuries	Female	0-6 days	95+ years	-1	Healthcare access and quality index
Other unintentional injuries	Male	0-6 days	95+ years	1	Alcohol (liters per capita)
Other unintentional injuries	Male	0-6 days	95+ years	-1	Education (years per capita)

Cause	Sex	Age start	Age end	Direction	Covariate
Other unintentional injuries	Male	0-6 days	95+ years	0	LDI (I$ per capita)
Other unintentional injuries	Male	0-6 days	95+ years	0	Elevation Over 1500m (proportion)
Other unintentional injuries	Male	0-6 days	95+ years	0	Population Density (over 1000 ppl/sqkm, proportion)
Other unintentional injuries	Male	0-6 days	95+ years	0	Population Density (under 150 ppl/sqkm, proportion)
Other unintentional injuries	Male	0-6 days	95+ years	0	Elevation Under 100m (proportion)
Other unintentional injuries	Male	0-6 days	95+ years	1	Vehicles - 2 wheels (per capita)
Other unintentional injuries	Male	0-6 days	95+ years	0	Vehicles - 4 wheels (per capita)
Other unintentional injuries	Male	0-6 days	95+ years	1	Log-transformed SEV scalar: Oth Unint
Other unintentional injuries	Male	0-6 days	95+ years	0	Socio-demographic Index
Other unintentional injuries	Male	0-6 days	95+ years	-1	Healthcare access and quality index
Self-harm and interpersonal violence	Female	0-6 days	95+ years	1	Alcohol (liters per capita)
Self-harm and interpersonal violence	Female	0-6 days	95+ years	-1	Education (years per capita)
Self-harm and interpersonal violence	Female	0-6 days	95+ years	0	LDI (I$ per capita)
Self-harm and interpersonal violence	Female	0-6 days	95+ years	0	Elevation Over 1500m (proportion)
Self-harm and interpersonal violence	Female	0-6 days	95+ years	0	Population Density (over 1000 ppl/sqkm, proportion)
Self-harm and interpersonal violence	Female	0-6 days	95+ years	0	Population Density (under 150 ppl/sqkm, proportion)
Self-harm and interpersonal violence	Female	0-6 days	95+ years	0	Elevation Under 100m (proportion)
Self-harm and interpersonal violence	Female	0-6 days	95+ years	1	Log-transformed SEV scalar: Oth Unint
Self-harm and interpersonal violence	Female	0-6 days	95+ years	1	Healthcare access and quality index
Self-harm and interpersonal violence	Male	0-6 days	95+ years	1	Alcohol (liters per capita)
Self-harm and interpersonal violence	Male	0-6 days	95+ years	-1	Education (years per capita)
Self-harm and interpersonal violence	Male	0-6 days	95+ years	0	LDI (I$ per capita)
Self-harm and interpersonal violence	Male	0-6 days	95+ years	0	Elevation Over 1500m (proportion)
Self-harm and interpersonal violence	Male	0-6 days	95+ years	0	Population Density (over 1000 ppl/sqkm, proportion)
Self-harm and interpersonal violence	Male	0-6 days	95+ years	0	Population Density (under 150 ppl/sqkm, proportion)
Self-harm and interpersonal violence	Male	0-6 days	95+ years	0	Elevation Under 100m (proportion)
Self-harm and interpersonal violence	Male	0-6 days	95+ years	1	Log-transformed SEV scalar: Oth Unint
Self-harm and interpersonal violence	Male	0-6 days	95+ years	1	Healthcare access and quality index
Self-harm	Female	10-14 years	95+ years	1	Alcohol (liters per capita)
Self-harm	Female	10-14 years	95+ years	0	Education (years per capita)
Self-harm	Female	10-14 years	95+ years	0	LDI (I$ per capita)
Self-harm	Female	10-14 years	95+ years	0	Population Density (150-300 ppl/sqkm, proportion)
Self-harm	Female	10-14 years	95+ years	0	Population Density (300-500 ppl/sqkm, proportion)
Self-harm	Female	10-14 years	95+ years	0	Population Density (500-1000 ppl/sqkm, proportion)
Self-harm	Female	10-14 years	95+ years	0	Population Density (over 1000 ppl/sqkm, proportion)
Self-harm	Female	10-14 years	95+ years	0	Population Density (under 150 ppl/sqkm, proportion)
Self-harm	Female	10-14 years	95+ years	-1	Religion (binary, >50% Muslim)
Self-harm	Female	10-14 years	95+ years	1	Log-transformed SEV scalar: Self Harm
Self-harm	Female	10-14 years	95+ years	0	Socio-demographic Index
Self-harm	Female	10-14 years	95+ years	1	Major depressive disorder
Self-harm	Female	10-14 years	95+ years	-1	Healthcare access and quality index
Self-harm	Male	10-14 years	95+ years	1	Alcohol (liters per capita)
Self-harm	Male	10-14 years	95+ years	0	Education (years per capita)
Self-harm	Male	10-14 years	95+ years	0	LDI (I$ per capita)
Self-harm	Male	10-14 years	95+ years	0	Population Density (150-300 ppl/sqkm, proportion)
Self-harm	Male	10-14 years	95+ years	0	Population Density (300-500 ppl/sqkm, proportion)
Self-harm	Male	10-14 years	95+ years	0	Population Density (500-1000 ppl/sqkm, proportion)
Self-harm	Male	10-14 years	95+ years	0	Population Density (over 1000 ppl/sqkm, proportion)
Self-harm	Male	10-14 years	95+ years	0	Population Density (under 150 ppl/sqkm, proportion)
Self-harm	Male	10-14 years	95+ years	-1	Religion (binary, >50% Muslim)
Self-harm	Male	10-14 years	95+ years	0	Socio-demographic Index
Self-harm	Male	10-14 years	95+ years	-1	Healthcare access and quality index
Self-harm by firearm	Female	10-14 years	95+ years	1	Alcohol (liters per capita)
Self-harm by firearm	Female	10-14 years	95+ years	0	Education (years per capita)
Self-harm by firearm	Female	10-14 years	95+ years	0	LDI (I$ per capita)
Self-harm by firearm	Female	10-14 years	95+ years	0	Population Density (150-300 ppl/sqkm, proportion)
Self-harm by firearm	Female	10-14 years	95+ years	0	Population Density (300-500 ppl/sqkm, proportion)
Self-harm by firearm	Female	10-14 years	95+ years	0	Population Density (500-1000 ppl/sqkm, proportion)
Self-harm by firearm	Female	10-14 years	95+ years	0	Population Density (over 1000 ppl/sqkm, proportion)
Self-harm by firearm	Female	10-14 years	95+ years	0	Population Density (under 150 ppl/sqkm, proportion)
Self-harm by firearm	Female	10-14 years	95+ years	-1	Religion (binary, >50% Muslim)
Self-harm by firearm	Female	10-14 years	95+ years	1	Log-transformed SEV scalar: Self Harm
Self-harm by firearm	Female	10-14 years	95+ years	0	Socio-demographic Index
Self-harm by firearm	Female	10-14 years	95+ years	1	Major depressive disorder
Self-harm by firearm	Female	10-14 years	95+ years	-1	Healthcare access and quality index
Self-harm by firearm	Male	10-14 years	95+ years	1	Alcohol (liters per capita)
Self-harm by firearm	Male	10-14 years	95+ years	0	Education (years per capita)
Self-harm by firearm	Male	10-14 years	95+ years	0	LDI (I$ per capita)
Self-harm by firearm	Male	10-14 years	95+ years	0	Population Density (150-300 ppl/sqkm, proportion)
Self-harm by firearm	Male	10-14 years	95+ years	0	Population Density (300-500 ppl/sqkm, proportion)
Self-harm by firearm	Male	10-14 years	95+ years	0	Population Density (500-1000 ppl/sqkm, proportion)
Self-harm by firearm	Male	10-14 years	95+ years	0	Population Density (over 1000 ppl/sqkm, proportion)
Self-harm by firearm	Male	10-14 years	95+ years	0	Population Density (under 150 ppl/sqkm, proportion)
Self-harm by firearm	Male	10-14 years	95+ years	-1	Religion (binary, >50% Muslim)
Self-harm by firearm	Male	10-14 years	95+ years	1	Log-transformed SEV scalar: Self Harm
Self-harm by firearm	Male	10-14 years	95+ years	0	Socio-demographic Index
Self-harm by firearm	Male	10-14 years	95+ years	1	Major depressive disorder
Self-harm by firearm	Male	10-14 years	95+ years	-1	Healthcare access and quality index
Self-harm by other specified means	Female	10-14 years	95+ years	1	Alcohol (liters per capita)
Self-harm by other specified means	Female	10-14 years	95+ years	0	Education (years per capita)
Self-harm by other specified means	Female	10-14 years	95+ years	0	LDI (I$ per capita)
Self-harm by other specified means	Female	10-14 years	95+ years	0	Population Density (150-300 ppl/sqkm, proportion)
Self-harm by other specified means	Female	10-14 years	95+ years	0	Population Density (300-500 ppl/sqkm, proportion)
Self-harm by other specified means	Female	10-14 years	95+ years	0	Population Density (500-1000 ppl/sqkm, proportion)
Self-harm by other specified means	Female	10-14 years	95+ years	0	Population Density (over 1000 ppl/sqkm, proportion)
Self-harm by other specified means	Female	10-14 years	95+ years	0	Population Density (under 150 ppl/sqkm, proportion)
Self-harm by other specified means	Female	10-14 years	95+ years	-1	Religion (binary, >50% Muslim)
Self-harm by other specified means	Female	10-14 years	95+ years	1	Log-transformed SEV scalar: Self Harm
Self-harm by other specified means	Female	10-14 years	95+ years	0	Socio-demographic Index
Self-harm by other specified means	Female	10-14 years	95+ years	1	Major depressive disorder
Self-harm by other specified means	Female	10-14 years	95+ years	-1	Healthcare access and quality index
Self-harm by other specified means	Male	10-14 years	95+ years	1	Alcohol (liters per capita)

Cause	Sex	Age start	Age end	Direction	Covariate
Self-harm by other specified means	Male	10-14 years	95+ years	0	Education (years per capita)
Self-harm by other specified means	Male	10-14 years	95+ years	0	LDI (I$ per capita)
Self-harm by other specified means	Male	10-14 years	95+ years	0	Population Density (150-300 ppl/sqkm, proportion)
Self-harm by other specified means	Male	10-14 years	95+ years	0	Population Density (300-500 ppl/sqkm, proportion)
Self-harm by other specified means	Male	10-14 years	95+ years	0	Population Density (500-1000 ppl/sqkm, proportion)
Self-harm by other specified means	Male	10-14 years	95+ years	0	Population Density (over 1000 ppl/sqkm, proportion)
Self-harm by other specified means	Male	10-14 years	95+ years	0	Population Density (under 150 ppl/sqkm, proportion)
Self-harm by other specified means	Male	10-14 years	95+ years	-1	Religion (binary, >50% Muslim)
Self-harm by other specified means	Male	10-14 years	95+ years	1	Log-transformed SEV scalar: Self Harm
Self-harm by other specified means	Male	10-14 years	95+ years	0	Socio-demographic Index
Self-harm by other specified means	Male	10-14 years	95+ years	1	Major depressive disorder
Self-harm by other specified means	Male	10-14 years	95+ years	-1	Healthcare access and quality index
Interpersonal violence	Female	0-6 days	95+ years	1	Alcohol (liters per capita)
Interpersonal violence	Female	0-6 days	95+ years	0	Education (years per capita)
Interpersonal violence	Female	0-6 days	95+ years	0	LDI (I$ per capita)
Interpersonal violence	Female	0-6 days	95+ years	1	Opium Cultivation (binary)
Interpersonal violence	Female	0-6 days	95+ years	1	Population Density (over 1000 ppl/sqkm, proportion)
Interpersonal violence	Female	0-6 days	95+ years	1	Log-transformed SEV scalar: Violence
Interpersonal violence	Female	0-6 days	95+ years	0	Socio-demographic Index
Interpersonal violence	Female	0-6 days	95+ years	-1	Healthcare access and quality index
Interpersonal violence	Male	0-6 days	95+ years	1	Alcohol (liters per capita)
Interpersonal violence	Male	0-6 days	95+ years	0	Education (years per capita)
Interpersonal violence	Male	0-6 days	95+ years	0	LDI (I$ per capita)
Interpersonal violence	Male	0-6 days	95+ years	1	Opium Cultivation (binary)
Interpersonal violence	Male	0-6 days	95+ years	1	Population Density (over 1000 ppl/sqkm, proportion)
Interpersonal violence	Male	0-6 days	95+ years	1	Log-transformed SEV scalar: Violence
Interpersonal violence	Male	0-6 days	95+ years	0	Socio-demographic Index
Interpersonal violence	Male	0-6 days	95+ years	-1	Healthcare access and quality index
Physical violence by firearm	Female	0-6 days	95+ years	1	Alcohol (liters per capita)
Physical violence by firearm	Female	0-6 days	95+ years	0	Education (years per capita)
Physical violence by firearm	Female	0-6 days	95+ years	0	LDI (I$ per capita)
Physical violence by firearm	Female	0-6 days	95+ years	1	Opium Cultivation (binary)
Physical violence by firearm	Female	0-6 days	95+ years	1	Population Density (over 1000 ppl/sqkm, proportion)
Physical violence by firearm	Female	0-6 days	95+ years	1	Log-transformed SEV scalar: Viol Gun
Physical violence by firearm	Female	0-6 days	95+ years	0	Socio-demographic Index
Physical violence by firearm	Female	0-6 days	95+ years	-1	Healthcare access and quality index
Physical violence by firearm	Male	0-6 days	95+ years	1	Alcohol (liters per capita)
Physical violence by firearm	Male	0-6 days	95+ years	0	Education (years per capita)
Physical violence by firearm	Male	0-6 days	95+ years	0	LDI (I$ per capita)
Physical violence by firearm	Male	0-6 days	95+ years	1	Opium Cultivation (binary)
Physical violence by firearm	Male	0-6 days	95+ years	1	Population Density (over 1000 ppl/sqkm, proportion)
Physical violence by firearm	Male	0-6 days	95+ years	1	Log-transformed SEV scalar: Viol Gun
Physical violence by firearm	Male	0-6 days	95+ years	0	Socio-demographic Index
Physical violence by firearm	Male	0-6 days	95+ years	-1	Healthcare access and quality index
Physical violence by sharp object	Female	0-6 days	95+ years	1	Alcohol (liters per capita)
Physical violence by sharp object	Female	0-6 days	95+ years	0	Education (years per capita)
Physical violence by sharp object	Female	0-6 days	95+ years	0	LDI (I$ per capita)
Physical violence by sharp object	Female	0-6 days	95+ years	1	Opium Cultivation (binary)
Physical violence by sharp object	Female	0-6 days	95+ years	1	Population Density (over 1000 ppl/sqkm, proportion)
Physical violence by sharp object	Female	0-6 days	95+ years	1	Log-transformed SEV scalar: Viol Knife
Physical violence by sharp object	Female	0-6 days	95+ years	0	Socio-demographic Index
Physical violence by sharp object	Female	0-6 days	95+ years	-1	Healthcare access and quality index
Physical violence by sharp object	Male	0-6 days	95+ years	1	Alcohol (liters per capita)
Physical violence by sharp object	Male	0-6 days	95+ years	0	Education (years per capita)
Physical violence by sharp object	Male	0-6 days	95+ years	0	LDI (I$ per capita)
Physical violence by sharp object	Male	0-6 days	95+ years	1	Opium Cultivation (binary)
Physical violence by sharp object	Male	0-6 days	95+ years	1	Population Density (over 1000 ppl/sqkm, proportion)
Physical violence by sharp object	Male	0-6 days	95+ years	1	Log-transformed SEV scalar: Viol Knife
Physical violence by sharp object	Male	0-6 days	95+ years	0	Socio-demographic Index
Physical violence by sharp object	Male	0-6 days	95+ years	-1	Healthcare access and quality index
Physical violence by other means	Female	0-6 days	95+ years	1	Alcohol (liters per capita)
Physical violence by other means	Female	0-6 days	95+ years	0	Education (years per capita)
Physical violence by other means	Female	0-6 days	95+ years	0	LDI (I$ per capita)
Physical violence by other means	Female	0-6 days	95+ years	1	Opium Cultivation (binary)
Physical violence by other means	Female	0-6 days	95+ years	1	Population Density (over 1000 ppl/sqkm, proportion)
Physical violence by other means	Female	0-6 days	95+ years	1	Log-transformed SEV scalar: Oth Viol
Physical violence by other means	Female	0-6 days	95+ years	0	Socio-demographic Index
Physical violence by other means	Female	0-6 days	95+ years	-1	Healthcare access and quality index
Physical violence by other means	Male	0-6 days	95+ years	1	Alcohol (liters per capita)
Physical violence by other means	Male	0-6 days	95+ years	0	Education (years per capita)
Physical violence by other means	Male	0-6 days	95+ years	0	LDI (I$ per capita)
Physical violence by other means	Male	0-6 days	95+ years	1	Opium Cultivation (binary)
Physical violence by other means	Male	0-6 days	95+ years	1	Population Density (over 1000 ppl/sqkm, proportion)
Physical violence by other means	Male	0-6 days	95+ years	1	Log-transformed SEV scalar: Oth Viol
Physical violence by other means	Male	0-6 days	95+ years	0	Socio-demographic Index
Physical violence by other means	Male	0-6 days	95+ years	-1	Healthcare access and quality index
Environmental heat and cold exposure	Female	0-6 days	95+ years	-1	Education (years per capita)
Environmental heat and cold exposure	Female	0-6 days	95+ years	0	LDI (I$ per capita)
Environmental heat and cold exposure	Female	0-6 days	95+ years	0	Population-weighted mean temperature
Environmental heat and cold exposure	Female	0-6 days	95+ years	0	Elevation Over 1500m (proportion)
Environmental heat and cold exposure	Female	0-6 days	95+ years	0	Elevation 500 to 1500m (proportion)
Environmental heat and cold exposure	Female	0-6 days	95+ years	0	Population Density (150-300 ppl/sqkm, proportion)
Environmental heat and cold exposure	Female	0-6 days	95+ years	0	Rainfall (Quintiles 4-5)
Environmental heat and cold exposure	Female	0-6 days	95+ years	0	Sanitation (proportion with access)
Environmental heat and cold exposure	Female	0-6 days	95+ years	0	90th percentile climatic temperature in the given country-year.
Environmental heat and cold exposure	Female	0-6 days	95+ years	-1	Socio-demographic Index
Environmental heat and cold exposure	Female	0-6 days	95+ years	-1	Healthcare access and quality index
Environmental heat and cold exposure	Male	0-6 days	95+ years	-1	Education (years per capita)
Environmental heat and cold exposure	Male	0-6 days	95+ years	0	LDI (I$ per capita)
Environmental heat and cold exposure	Male	0-6 days	95+ years	0	Population-weighted mean temperature
Environmental heat and cold exposure	Male	0-6 days	95+ years	0	Elevation Over 1500m (proportion)
Environmental heat and cold exposure	Male	0-6 days	95+ years	0	Elevation 500 to 1500m (proportion)

Cause	Sex	Age start	Age end	Direction	Covariate
Environmental heat and cold exposure	Male	0-6 days	95+ years	0	Population Density (150-300 ppl/sqkm, proportion)
Environmental heat and cold exposure	Male	0-6 days	95+ years	0	Rainfall (Quintiles 4-5)
Environmental heat and cold exposure	Male	0-6 days	95+ years	0	Sanitation (proportion with access)
Environmental heat and cold exposure	Male	0-6 days	95+ years	0	90th percentile climatic temperature in the given country-year.
Environmental heat and cold exposure	Male	0-6 days	95+ years	-1	Socio-demographic Index
Environmental heat and cold exposure	Male	0-6 days	95+ years	-1	Healthcare access and quality index
Acute lymphoid leukemia	Female	0-6 days	95+ years	1	Alcohol (liters per capita)
Acute lymphoid leukemia	Female	0-6 days	95+ years	1	Tobacco (cigarettes per capita)
Acute lymphoid leukemia	Female	0-6 days	95+ years	1	Cumulative Cigarettes (10 Years)
Acute lymphoid leukemia	Female	0-6 days	95+ years	1	Cumulative Cigarettes (15 Years)
Acute lymphoid leukemia	Female	0-6 days	95+ years	1	Cumulative Cigarettes (20 Years)
Acute lymphoid leukemia	Female	0-6 days	95+ years	1	Cumulative Cigarettes (5 Years)
Acute lymphoid leukemia	Female	0-6 days	95+ years	-1	Education (years per capita)
Acute lymphoid leukemia	Female	0-6 days	95+ years	-1	Health System Access 2 (unitless)
Acute lymphoid leukemia	Female	0-6 days	95+ years	0	LDI (I$ per capita)
Acute lymphoid leukemia	Female	0-6 days	95+ years	1	Smoking Prevalence
Acute lymphoid leukemia	Female	0-6 days	95+ years	1	Log-transformed SEV scalar: Leukemia
Acute lymphoid leukemia	Female	0-6 days	95+ years	1	Log-transformed age-standardized SEV scalar: Leukemia
Acute lymphoid leukemia	Female	0-6 days	95+ years	0	Socio-demographic Index
Acute lymphoid leukemia	Male	0-6 days	95+ years	1	Alcohol (liters per capita)
Acute lymphoid leukemia	Male	0-6 days	95+ years	1	Tobacco (cigarettes per capita)
Acute lymphoid leukemia	Male	0-6 days	95+ years	1	Cumulative Cigarettes (10 Years)
Acute lymphoid leukemia	Male	0-6 days	95+ years	1	Cumulative Cigarettes (15 Years)
Acute lymphoid leukemia	Male	0-6 days	95+ years	1	Cumulative Cigarettes (20 Years)
Acute lymphoid leukemia	Male	0-6 days	95+ years	1	Cumulative Cigarettes (5 Years)
Acute lymphoid leukemia	Male	0-6 days	95+ years	-1	Education (years per capita)
Acute lymphoid leukemia	Male	0-6 days	95+ years	-1	Health System Access 2 (unitless)
Acute lymphoid leukemia	Male	0-6 days	95+ years	0	LDI (I$ per capita)
Acute lymphoid leukemia	Male	0-6 days	95+ years	1	Smoking Prevalence
Acute lymphoid leukemia	Male	0-6 days	95+ years	1	Log-transformed SEV scalar: Leukemia
Acute lymphoid leukemia	Male	0-6 days	95+ years	1	Log-transformed age-standardized SEV scalar: Leukemia
Acute lymphoid leukemia	Male	0-6 days	95+ years	0	Socio-demographic Index
Chronic lymphoid leukemia	Male	15-19 years	95+ years	1	Alcohol (liters per capita)
Chronic lymphoid leukemia	Male	15-19 years	95+ years	1	Tobacco (cigarettes per capita)
Chronic lymphoid leukemia	Male	15-19 years	95+ years	1	Cumulative Cigarettes (10 Years)
Chronic lymphoid leukemia	Male	15-19 years	95+ years	1	Cumulative Cigarettes (15 Years)
Chronic lymphoid leukemia	Male	15-19 years	95+ years	1	Cumulative Cigarettes (20 Years)
Chronic lymphoid leukemia	Male	15-19 years	95+ years	1	Cumulative Cigarettes (5 Years)
Chronic lymphoid leukemia	Male	15-19 years	95+ years	-1	Education (years per capita)
Chronic lymphoid leukemia	Male	15-19 years	95+ years	-1	Health System Access 2 (unitless)
Chronic lymphoid leukemia	Male	15-19 years	95+ years	0	LDI (I$ per capita)
Chronic lymphoid leukemia	Male	15-19 years	95+ years	1	Smoking Prevalence
Chronic lymphoid leukemia	Male	15-19 years	95+ years	1	Log-transformed SEV scalar: Leukemia
Chronic lymphoid leukemia	Male	15-19 years	95+ years	1	Log-transformed age-standardized SEV scalar: Leukemia
Chronic lymphoid leukemia	Male	15-19 years	95+ years	0	Socio-demographic Index
Chronic lymphoid leukemia	Female	15-19 years	95+ years	1	Alcohol (liters per capita)
Chronic lymphoid leukemia	Female	15-19 years	95+ years	1	Tobacco (cigarettes per capita)
Chronic lymphoid leukemia	Female	15-19 years	95+ years	1	Cumulative Cigarettes (10 Years)
Chronic lymphoid leukemia	Female	15-19 years	95+ years	1	Cumulative Cigarettes (15 Years)
Chronic lymphoid leukemia	Female	15-19 years	95+ years	1	Cumulative Cigarettes (20 Years)
Chronic lymphoid leukemia	Female	15-19 years	95+ years	1	Cumulative Cigarettes (5 Years)
Chronic lymphoid leukemia	Female	15-19 years	95+ years	-1	Education (years per capita)
Chronic lymphoid leukemia	Female	15-19 years	95+ years	-1	Health System Access 2 (unitless)
Chronic lymphoid leukemia	Female	15-19 years	95+ years	0	LDI (I$ per capita)
Chronic lymphoid leukemia	Female	15-19 years	95+ years	1	Smoking Prevalence
Chronic lymphoid leukemia	Female	15-19 years	95+ years	1	Log-transformed SEV scalar: Leukemia
Chronic lymphoid leukemia	Female	15-19 years	95+ years	1	Log-transformed age-standardized SEV scalar: Leukemia
Chronic lymphoid leukemia	Female	15-19 years	95+ years	0	Socio-demographic Index
Acute myeloid leukemia	Female	0-6 days	95+ years	1	Alcohol (liters per capita)
Acute myeloid leukemia	Female	0-6 days	95+ years	1	Tobacco (cigarettes per capita)
Acute myeloid leukemia	Female	0-6 days	95+ years	1	Cumulative Cigarettes (10 Years)
Acute myeloid leukemia	Female	0-6 days	95+ years	1	Cumulative Cigarettes (15 Years)
Acute myeloid leukemia	Female	0-6 days	95+ years	1	Cumulative Cigarettes (20 Years)
Acute myeloid leukemia	Female	0-6 days	95+ years	1	Cumulative Cigarettes (5 Years)
Acute myeloid leukemia	Female	0-6 days	95+ years	-1	Education (years per capita)
Acute myeloid leukemia	Female	0-6 days	95+ years	-1	Health System Access 2 (unitless)
Acute myeloid leukemia	Female	0-6 days	95+ years	0	LDI (I$ per capita)
Acute myeloid leukemia	Female	0-6 days	95+ years	1	Smoking Prevalence
Acute myeloid leukemia	Female	0-6 days	95+ years	1	Log-transformed SEV scalar: Leukemia
Acute myeloid leukemia	Female	0-6 days	95+ years	1	Log-transformed age-standardized SEV scalar: Leukemia
Acute myeloid leukemia	Female	0-6 days	95+ years	0	Socio-demographic Index
Acute myeloid leukemia	Male	0-6 days	95+ years	1	Alcohol (liters per capita)
Acute myeloid leukemia	Male	0-6 days	95+ years	1	Tobacco (cigarettes per capita)
Acute myeloid leukemia	Male	0-6 days	95+ years	1	Cumulative Cigarettes (10 Years)
Acute myeloid leukemia	Male	0-6 days	95+ years	1	Cumulative Cigarettes (15 Years)
Acute myeloid leukemia	Male	0-6 days	95+ years	1	Cumulative Cigarettes (20 Years)
Acute myeloid leukemia	Male	0-6 days	95+ years	1	Cumulative Cigarettes (5 Years)
Acute myeloid leukemia	Male	0-6 days	95+ years	-1	Education (years per capita)
Acute myeloid leukemia	Male	0-6 days	95+ years	-1	Health System Access 2 (unitless)
Acute myeloid leukemia	Male	0-6 days	95+ years	0	LDI (I$ per capita)
Acute myeloid leukemia	Male	0-6 days	95+ years	1	Smoking Prevalence
Acute myeloid leukemia	Male	0-6 days	95+ years	1	Log-transformed SEV scalar: Leukemia
Acute myeloid leukemia	Male	0-6 days	95+ years	1	Log-transformed age-standardized SEV scalar: Leukemia
Acute myeloid leukemia	Male	0-6 days	95+ years	0	Socio-demographic Index
Acute myeloid leukemia	Male	0-6 days	95+ years	-1	Health System Access (capped)
Acute myeloid leukemia	Male	0-6 days	95+ years	-1	Healthcare access and quality index
Chronic myeloid leukemia	Female	28-364 days	95+ years	1	Alcohol (liters per capita)
Chronic myeloid leukemia	Female	28-364 days	95+ years	1	Tobacco (cigarettes per capita)
Chronic myeloid leukemia	Female	28-364 days	95+ years	1	Cumulative Cigarettes (10 Years)
Chronic myeloid leukemia	Female	28-364 days	95+ years	1	Cumulative Cigarettes (15 Years)
Chronic myeloid leukemia	Female	28-364 days	95+ years	1	Cumulative Cigarettes (20 Years)
Chronic myeloid leukemia	Female	28-364 days	95+ years	1	Cumulative Cigarettes (5 Years)

Cause	Sex	Age start	Age end	Direction	Covariate
Chronic myeloid leukemia	Female	28-364 days	95+ years	-1	Education (years per capita)
Chronic myeloid leukemia	Female	28-364 days	95+ years	1	Health System Access 2 (unitless)
Chronic myeloid leukemia	Female	28-364 days	95+ years	0	LDI (I$ per capita)
Chronic myeloid leukemia	Female	28-364 days	95+ years	1	Smoking Prevalence
Chronic myeloid leukemia	Female	28-364 days	95+ years	1	Log-transformed age-standardized SEV scalar: Leukemia
Chronic myeloid leukemia	Female	28-364 days	95+ years	0	Socio-demographic Index
Chronic myeloid leukemia	Female	28-364 days	95+ years	-1	Healthcare access and quality index
Chronic myeloid leukemia	Female	28-364 days	95+ years	-1	Health System Access 2 (unitless)
Chronic myeloid leukemia	Male	28-364 days	95+ years	1	Alcohol (liters per capita)
Chronic myeloid leukemia	Male	28-364 days	95+ years	1	Tobacco (cigarettes per capita)
Chronic myeloid leukemia	Male	28-364 days	95+ years	1	Cumulative Cigarettes (10 Years)
Chronic myeloid leukemia	Male	28-364 days	95+ years	1	Cumulative Cigarettes (15 Years)
Chronic myeloid leukemia	Male	28-364 days	95+ years	1	Cumulative Cigarettes (20 Years)
Chronic myeloid leukemia	Male	28-364 days	95+ years	1	Cumulative Cigarettes (5 Years)
Chronic myeloid leukemia	Male	28-364 days	95+ years	-1	Education (years per capita)
Chronic myeloid leukemia	Male	28-364 days	95+ years	1	Health System Access 2 (unitless)
Chronic myeloid leukemia	Male	28-364 days	95+ years	0	LDI (I$ per capita)
Chronic myeloid leukemia	Male	28-364 days	95+ years	1	Smoking Prevalence
Chronic myeloid leukemia	Male	28-364 days	95+ years	1	Log-transformed age-standardized SEV scalar: Leukemia
Chronic myeloid leukemia	Male	28-364 days	95+ years	0	Socio-demographic Index
Chronic myeloid leukemia	Male	28-364 days	95+ years	-1	Healthcare access and quality index
Non-melanoma skin cancer (squamous-cell carcinoma)	Male	28-364 days	95+ years	1	Cumulative Cigarettes (10 Years)
Non-melanoma skin cancer (squamous-cell carcinoma)	Male	28-364 days	95+ years	1	Cumulative Cigarettes (15 Years)
Non-melanoma skin cancer (squamous-cell carcinoma)	Male	28-364 days	95+ years	1	Cumulative Cigarettes (5 Years)
Non-melanoma skin cancer (squamous-cell carcinoma)	Male	28-364 days	95+ years	-1	Education (years per capita)
Non-melanoma skin cancer (squamous-cell carcinoma)	Male	28-364 days	95+ years	0	Average latitude
Non-melanoma skin cancer (squamous-cell carcinoma)	Male	28-364 days	95+ years	0	LDI (I$ per capita)
Non-melanoma skin cancer (squamous-cell carcinoma)	Male	28-364 days	95+ years	1	Smoking Prevalence
Non-melanoma skin cancer (squamous-cell carcinoma)	Male	28-364 days	95+ years	0	Socio-demographic Index
Non-melanoma skin cancer (squamous-cell carcinoma)	Male	28-364 days	95+ years	-1	Healthcare access and quality index
Non-melanoma skin cancer (squamous-cell carcinoma)	Male	28-364 days	95+ years	-1	Health System Access (capped)
Non-melanoma skin cancer (squamous-cell carcinoma)	Female	28-364 days	95+ years	1	Cumulative Cigarettes (10 Years)
Non-melanoma skin cancer (squamous-cell carcinoma)	Female	28-364 days	95+ years	1	Cumulative Cigarettes (15 Years)
Non-melanoma skin cancer (squamous-cell carcinoma)	Female	28-364 days	95+ years	1	Cumulative Cigarettes (5 Years)
Non-melanoma skin cancer (squamous-cell carcinoma)	Female	28-364 days	95+ years	-1	Education (years per capita)
Non-melanoma skin cancer (squamous-cell carcinoma)	Female	28-364 days	95+ years	0	Average latitude
Non-melanoma skin cancer (squamous-cell carcinoma)	Female	28-364 days	95+ years	0	LDI (I$ per capita)
Non-melanoma skin cancer (squamous-cell carcinoma)	Female	28-364 days	95+ years	1	Smoking Prevalence
Non-melanoma skin cancer (squamous-cell carcinoma)	Female	28-364 days	95+ years	0	Socio-demographic Index
Non-melanoma skin cancer (squamous-cell carcinoma)	Female	28-364 days	95+ years	-1	Healthcare access and quality index
Non-melanoma skin cancer (squamous-cell carcinoma)	Female	28-364 days	95+ years	-1	Health System Access (capped)
Executions and police conflict	Female	28-364 days	95+ years	1	Alcohol (liters per capita)
Executions and police conflict	Female	28-364 days	95+ years	1	Education (years per capita)
Executions and police conflict	Female	28-364 days	95+ years	0	LDI (I$ per capita)
Executions and police conflict	Female	28-364 days	95+ years	1	Population Density (over 1000 ppl/sqkm, proportion)
Executions and police conflict	Female	28-364 days	95+ years	0	Socio-demographic Index
Executions and police conflict	Female	28-364 days	95+ years	-1	Healthcare access and quality index
Executions and police conflict	Male	28-364 days	95+ years	1	Alcohol (liters per capita)
Executions and police conflict	Male	28-364 days	95+ years	1	Education (years per capita)
Executions and police conflict	Male	28-364 days	95+ years	0	LDI (I$ per capita)
Executions and police conflict	Male	28-364 days	95+ years	1	Population Density (over 1000 ppl/sqkm, proportion)
Executions and police conflict	Male	28-364 days	95+ years	0	Socio-demographic Index
Executions and police conflict	Male	28-364 days	95+ years	-1	Healthcare access and quality index
Alcoholic cardiomyopathy	Male	15-19 years	95+ years	1	Alcohol (liters per capita)
Alcoholic cardiomyopathy	Male	15-19 years	95+ years	-1	LDI (I$ per capita)
Alcoholic cardiomyopathy	Male	15-19 years	95+ years	1	Smoking Prevalence
Alcoholic cardiomyopathy	Male	15-19 years	95+ years	1	Log-transformed SEV scalar: CMP
Alcoholic cardiomyopathy	Male	15-19 years	95+ years	0	Socio-demographic Index
Alcoholic cardiomyopathy	Male	15-19 years	95+ years	-1	Healthcare access and quality index
Alcoholic cardiomyopathy	Female	15-19 years	95+ years	1	Alcohol (liters per capita)
Alcoholic cardiomyopathy	Female	15-19 years	95+ years	-1	LDI (I$ per capita)
Alcoholic cardiomyopathy	Female	15-19 years	95+ years	1	Smoking Prevalence
Alcoholic cardiomyopathy	Female	15-19 years	95+ years	1	Log-transformed SEV scalar: CMP
Alcoholic cardiomyopathy	Female	15-19 years	95+ years	0	Socio-demographic Index
Alcoholic cardiomyopathy	Female	15-19 years	95+ years	-1	Healthcare access and quality index
Myocarditis	Male	0-6 days	95+ years	-1	LDI (I$ per capita)
Myocarditis	Male	0-6 days	95+ years	1	Systolic Blood Pressure (mmHg)
Myocarditis	Male	0-6 days	95+ years	1	Log-transformed SEV scalar: CMP
Myocarditis	Male	0-6 days	95+ years	0	Socio-demographic Index
Myocarditis	Male	0-6 days	95+ years	-1	Healthcare access and quality index
Myocarditis	Female	0-6 days	95+ years	-1	LDI (I$ per capita)
Myocarditis	Female	0-6 days	95+ years	1	Systolic Blood Pressure (mmHg)
Myocarditis	Female	0-6 days	95+ years	1	Log-transformed SEV scalar: CMP
Myocarditis	Female	0-6 days	95+ years	0	Socio-demographic Index
Myocarditis	Female	0-6 days	95+ years	-1	Healthcare access and quality index
Other leukemia	Female	0-6 days	95+ years	1	Alcohol (liters per capita)
Other leukemia	Female	0-6 days	95+ years	1	Tobacco (cigarettes per capita)
Other leukemia	Female	0-6 days	95+ years	1	Cumulative Cigarettes (10 Years)
Other leukemia	Female	0-6 days	95+ years	1	Cumulative Cigarettes (15 Years)
Other leukemia	Female	0-6 days	95+ years	1	Cumulative Cigarettes (20 Years)
Other leukemia	Female	0-6 days	95+ years	1	Cumulative Cigarettes (5 Years)
Other leukemia	Female	0-6 days	95+ years	-1	Education (years per capita)
Other leukemia	Female	0-6 days	95+ years	-1	Health System Access 2 (unitless)
Other leukemia	Female	0-6 days	95+ years	0	LDI (I$ per capita)
Other leukemia	Female	0-6 days	95+ years	1	Smoking Prevalence
Other leukemia	Female	0-6 days	95+ years	1	Log-transformed SEV scalar: Leukemia
Other leukemia	Female	0-6 days	95+ years	0	Socio-demographic Index
Other leukemia	Male	0-6 days	95+ years	1	Alcohol (liters per capita)
Other leukemia	Male	0-6 days	95+ years	1	Tobacco (cigarettes per capita)
Other leukemia	Male	0-6 days	95+ years	1	Cumulative Cigarettes (10 Years)
Other leukemia	Male	0-6 days	95+ years	1	Cumulative Cigarettes (15 Years)
Other leukemia	Male	0-6 days	95+ years	1	Cumulative Cigarettes (20 Years)

Cause	Sex	Age start	Age end	Direction	Covariate
Other leukemia	Male	0-6 days	95+ years	1	Cumulative Cigarettes (5 Years)
Other leukemia	Male	0-6 days	95+ years	-1	Education (years per capita)
Other leukemia	Male	0-6 days	95+ years	-1	Health System Access 2 (unitless)
Other leukemia	Male	0-6 days	95+ years	0	LDI (I$ per capita)
Other leukemia	Male	0-6 days	95+ years	1	Smoking Prevalence
Other leukemia	Male	0-6 days	95+ years	1	Log-transformed SEV scalar: Leukemia
Other leukemia	Male	0-6 days	95+ years	0	Socio-demographic Index
Other cardiomyopathy	Male	0-6 days	95+ years	0	LDI (I$ per capita)
Other cardiomyopathy	Male	0-6 days	95+ years	1	Mean BMI
Other cardiomyopathy	Male	0-6 days	95+ years	1	Systolic Blood Pressure (mmHg)
Other cardiomyopathy	Male	0-6 days	95+ years	1	Smoking Prevalence
Other cardiomyopathy	Male	0-6 days	95+ years	1	Log-transformed SEV scalar: CMP
Other cardiomyopathy	Male	0-6 days	95+ years	0	Socio-demographic Index
Other cardiomyopathy	Male	0-6 days	95+ years	-1	Healthcare access and quality index
Other cardiomyopathy	Female	0-6 days	95+ years	0	LDI (I$ per capita)
Other cardiomyopathy	Female	0-6 days	95+ years	1	Mean BMI
Other cardiomyopathy	Female	0-6 days	95+ years	1	Systolic Blood Pressure (mmHg)
Other cardiomyopathy	Female	0-6 days	95+ years	1	Smoking Prevalence
Other cardiomyopathy	Female	0-6 days	95+ years	1	Log-transformed SEV scalar: CMP
Other cardiomyopathy	Female	0-6 days	95+ years	0	Socio-demographic Index
Other cardiomyopathy	Female	0-6 days	95+ years	-1	Healthcare access and quality index

Note: Only causes modeled in CODEm are included in this table.

Appendix Table 7: CODEm predictive validity results by cause, model type, sex and age

Cause	Sex	Age start	Age end	Predictive validity					
				RMSE in	RMSE out	Trend in	Trend out	Coverage in	Coverage out
Tuberculosis [Data Rich]	Male	28-364 days	95+ years	0.215	0.290663	0.158269	0.19286	0.998172	0.997832
Tuberculosis [Data Rich]	Female	28-364 days	95+ years	0.227834	0.304096	0.171612	0.190148	0.999074	0.998916
Tuberculosis [Global]	Male	28-364 days	95+ years	0.266763	0.554971	0.182005	0.189306	0.997872	0.984566
Tuberculosis [Global]	Female	28-364 days	95+ years	0.293493	0.562544	0.206357	0.218106	0.998348	0.989498
Diarrheal diseases [Data Rich]	Male	5-9 years	95+ years	0.260188	0.412346	0.178844	0.22131	0.998746	0.998187
Diarrheal diseases [Data Rich]	Female	5-9 years	95+ years	0.263644	0.430796	0.173641	0.217233	0.999102	0.997645
Diarrheal diseases [Data Rich]	Male	0-6 days	1-4 years	0.291264	0.399911	0.202778	0.237484	0.999247	0.997926
Diarrheal diseases [Data Rich]	Female	0-6 days	1-4 years	0.310597	0.447646	0.214774	0.269036	0.999718	0.998627
Diarrheal diseases [Global]	Male	0-6 days	1-4 years	0.434522	0.744318	0.395664	0.335957	0.996876	0.979921
Diarrheal diseases [Global]	Female	0-6 days	1-4 years	0.609676	0.885548	0.615259	0.595206	0.987641	0.969406
Diarrheal diseases [Global]	Male	5-9 years	95+ years	0.593177	0.820709	0.221108	0.230704	0.998677	0.992382
Diarrheal diseases [Global]	Female	5-9 years	95+ years	0.702521	0.909839	0.224528	0.225686	0.99859	0.990582
Lower respiratory infections [Data Rich]	Male	5-9 years	95+ years	0.234029	0.269537	0.162368	0.177752	0.999581	0.998811
Lower respiratory infections [Data Rich]	Female	5-9 years	95+ years	0.220305	0.262284	0.153727	0.173579	0.999538	0.998862
Lower respiratory infections [Data Rich]	Male	0-6 days	1-4 years	0.227551	0.285988	0.165418	0.177683	0.999445	0.998995
Lower respiratory infections [Data Rich]	Female	0-6 days	1-4 years	0.209089	0.258665	0.157661	0.168262	0.999497	0.999122
Lower respiratory infections [Global]	Male	5-9 years	95+ years	0.260677	0.387475	0.170636	0.177396	0.99941	0.982564
Lower respiratory infections [Global]	Female	5-9 years	95+ years	0.250004	0.373532	0.165361	0.168838	0.999414	0.984828
Lower respiratory infections [Global]	Male	0-6 days	1-4 years	0.302338	0.421668	0.237586	0.240214	0.999266	0.996159
Lower respiratory infections [Global]	Female	0-6 days	1-4 years	0.509912	0.604757	0.524133	0.538764	0.989438	0.99011
Otitis media [Data Rich]	Male	0-6 days	95+ years	0.80783	1.27793	0.656615	0.751022	0.98036	0.96598
Otitis media [Data Rich]	Female	0-6 days	95+ years	0.954315	1.4309	0.781543	0.887359	0.977749	0.966411
Otitis media [Global]	Male	0-6 days	95+ years	0.978189	1.9251	0.744172	0.770198	0.984208	0.940004
Otitis media [Global]	Female	0-6 days	95+ years	1.08283	2.09452	0.886791	0.912335	0.979917	0.943021
Meningitis [Data Rich]	Female	0-6 days	1-4 years	0.227251	0.286452	0.168505	0.179066	0.99994	0.999792
Meningitis [Data Rich]	Male	0-6 days	1-4 years	0.217133	0.293263	0.165997	0.186117	0.999742	0.999392
Meningitis [Data Rich]	Male	5-9 years	95+ years	0.221134	0.274598	0.155961	0.181331	0.999782	0.999751
Meningitis [Data Rich]	Female	5-9 years	95+ years	0.203069	0.264481	0.15643	0.177261	0.999846	0.999717
Meningitis [Global]	Male	0-6 days	1-4 years	0.453445	0.592801	0.456659	0.436136	0.997884	0.995251
Meningitis [Global]	Male	5-9 years	95+ years	0.356454	0.521145	0.195612	0.192864	0.999168	0.991943
Meningitis [Global]	Female	5-9 years	95+ years	0.284985	0.451238	0.20056	0.199594	0.999381	0.993424
Meningitis [Global]	Female	0-6 days	1-4 years	0.479021	0.626508	0.411725	0.410701	0.998549	0.996621
Encephalitis [Data Rich]	Male	0-6 days	95+ years	0.338783	0.402217	0.192527	0.208962	0.999402	0.999068
Encephalitis [Data Rich]	Female	0-6 days	95+ years	0.330985	0.411304	0.202173	0.219034	0.999328	0.998734
Encephalitis [Global]	Female	0-6 days	95+ years	0.401236	0.574391	0.237659	0.237301	0.998179	0.991936
Encephalitis [Global]	Male	0-6 days	95+ years	0.382378	0.586235	0.233583	0.239432	0.998333	0.993793
Tetanus [Data Rich]	Male	0-6 days	28-364 days	0.262789	0.398543	0.176924	0.190741	0.998514	0.997497
Tetanus [Data Rich]	Male	1-4 years	95+ years	0.462905	0.662019	0.342756	0.462735	0.993155	0.990457
Tetanus [Data Rich]	Female	0-6 days	28-364 days	0.26901	0.36596	0.199243	0.216329	0.997234	0.995847
Tetanus [Data Rich]	Female	1-4 years	95+ years	0.615402	0.905134	0.497505	0.694547	0.990264	0.98442
Tetanus [Global]	Male	1-4 years	95+ years	0.791375	1.33448	0.427077	0.433831	0.876308	0.861525
Tetanus [Global]	Female	1-4 years	95+ years	0.941868	1.66892	0.569802	0.603951	0.803236	0.786193
Tetanus [Global]	Male	0-6 days	28-364 days	0.763632	1.11789	0.792085	0.816737	0.989984	0.981245
Tetanus [Global]	Female	0-6 days	28-364 days	0.476031	0.796513	0.359685	0.352221	0.995943	0.986366
Dengue [Data Rich]	Male	28-364 days	95+ years	0.792214	1.43589	0.631339	0.712322	0.98911	0.974209
Dengue [Data Rich]	Female	28-364 days	95+ years	0.858796	1.47929	0.666952	0.70776	0.986963	0.971324
Dengue [Global]	Male	28-364 days	95+ years	0.966394	1.67507	0.716596	0.733445	0.991796	0.963767
Dengue [Global]	Female	28-364 days	95+ years	1.10131	1.72147	0.757119	0.765851	0.989802	0.961986
Rabies [Data Rich]	Male	28-364 days	95+ years	0.699144	1.25076	0.538206	0.612419	0.987567	0.979502
Rabies [Data Rich]	Female	28-364 days	95+ years	0.808708	1.29669	0.652356	0.736316	0.98552	0.975258
Rabies [Global]	Male	28-364 days	95+ years	0.871281	1.56847	0.622116	0.65464	0.987114	0.943583
Rabies [Global]	Female	28-364 days	95+ years	0.997179	1.74661	0.744593	0.760267	0.986128	0.94132
Other neglected tropical diseases [Data Rich]	Male	0-6 days	95+ years	0.730435	1.01929	0.50383	0.607178	0.763046	0.75864
Other neglected tropical diseases [Data Rich]	Female	0-6 days	95+ years	0.889845	1.22512	0.560578	0.713816	0.754629	0.748807
Other neglected tropical diseases [Global]	Male	0-6 days	95+ years	0.725231	1.45248	0.538638	0.572286	0.800666	0.773569
Other neglected tropical diseases [Global]	Female	0-6 days	95+ years	0.903404	1.50944	0.593172	0.607687	0.792532	0.77471
Neonatal disorders [Global]	Male	0-6 days	1-4 years	0.251735	0.393248	0.188497	0.187199	0.998926	0.996798
Neonatal disorders [Data Rich]	Male	0-6 days	1-4 years	0.187097	0.268444	0.13252	0.171404	0.998844	0.99836
Neonatal disorders [Global]	Female	0-6 days	1-4 years	0.253708	0.403142	0.195319	0.200811	0.998746	0.995669
Neonatal disorders [Data Rich]	Female	0-6 days	1-4 years	0.197921	0.281678	0.144814	0.191145	0.998989	0.998673
Neonatal preterm birth complications [Global]	Male	0-6 days	1-4 years	0.282625	0.452417	0.214695	0.217553	0.999035	0.993238
Neonatal preterm birth complications [Data Rich]	Male	0-6 days	1-4 years	0.214052	0.409643	0.150158	0.193849	0.999149	0.997901
Neonatal preterm birth complications [Global]	Female	0-6 days	1-4 years	0.287359	0.447133	0.222089	0.227474	0.998968	0.994749
Neonatal preterm birth complications [Data Rich]	Female	0-6 days	1-4 years	0.21014	0.385184	0.148401	0.182017	0.999568	0.998899
Neonatal encephalopathy due to birth asphyxia and trauma [Global]	Male	0-6 days	1-4 years	0.304664	0.505542	0.236226	0.239528	0.998957	0.993741
Neonatal encephalopathy due to birth asphyxia and trauma [Data Rich]	Male	0-6 days	1-4 years	0.227063	0.398435	0.166646	0.201266	0.999359	0.998783
Neonatal encephalopathy due to birth asphyxia and trauma [Global]	Female	0-6 days	1-4 years	0.346123	0.504788	0.319674	0.309837	0.998797	0.997005
Neonatal encephalopathy due to birth asphyxia and trauma [Data Rich]	Female	0-6 days	1-4 years	0.228414	0.387198	0.168209	0.212277	0.999393	0.998642
Neonatal sepsis and other neonatal infections [Global]	Male	0-6 days	1-4 years	0.532539	1.23454	0.294492	0.311711	0.892151	0.896508
Neonatal sepsis and other neonatal infections [Global]	Female	0-6 days	1-4 years	0.669042	1.42104	0.383821	0.394836	0.870375	0.852562
Neonatal sepsis and other neonatal infections [Data Rich]	Male	0-6 days	1-4 years	0.447373	0.876858	0.277257	0.319579	0.857269	0.856019
Neonatal sepsis and other neonatal infections [Data Rich]	Female	0-6 days	1-4 years	0.628724	1.20107	0.365662	0.447297	0.834308	0.829203
Hemolytic disease and other neonatal jaundice [Global]	Male	0-6 days	1-4 years	0.726108	1.52314	0.573364	0.606741	0.856138	0.817497
Hemolytic disease and other neonatal jaundice [Global]	Female	0-6 days	1-4 years	1.09727	1.8017	0.922907	0.952495	0.949447	0.88901
Hemolytic disease and other neonatal jaundice [Data Rich]	Male	0-6 days	1-4 years	0.690351	1.1874	0.549088	0.614396	0.84053	0.843519
Hemolytic disease and other neonatal jaundice [Data Rich]	Female	0-6 days	1-4 years	1.13302	1.84559	0.894736	1.08171	0.916511	0.915447
Other neonatal disorders [Global]	Male	0-6 days	1-4 years	0.323398	0.54171	0.219666	0.241994	0.999443	0.989495
Other neonatal disorders [Data Rich]	Male	0-6 days	1-4 years	0.285551	0.531978	0.194984	0.261102	0.999445	0.998329
Other neonatal disorders [Data Rich]	Female	0-6 days	1-4 years	0.310883	0.552746	0.217544	0.29129	0.999502	0.998131

				Predictive validity					
**Cause**	**Sex**	**Age start**	**Age end**	**RMSE in**	**RMSE out**	**Trend in**	**Trend out**	**Coverage in**	**Coverage out**
Other neonatal disorders [Global]	Female	0-6 days	1-4 years	0.36519	0.599204	0.258469	0.27836	0.999322	0.991141
Nutritional deficiencies [Data Rich]	Male	28-364 days	95+ years	0.28739	0.407494	0.211942	0.290737	0.992712	0.991782
Nutritional deficiencies [Data Rich]	Female	28-364 days	95+ years	0.31077	0.451309	0.229425	0.328584	0.992692	0.992032
Nutritional deficiencies [Global]	Male	28-364 days	95+ years	0.409342	0.729816	0.367633	0.361245	0.991799	0.970887
Nutritional deficiencies [Global]	Female	28-364 days	95+ years	0.395379	0.701908	0.325345	0.344961	0.992996	0.972574
Protein-energy malnutrition [Data Rich]	Male	5-9 years	95+ years	0.57212	0.836607	0.31749	0.457325	0.949678	0.95218
Protein-energy malnutrition [Data Rich]	Male	28-364 days	1-4 years	0.26374	0.389118	0.201965	0.217493	0.998186	0.99596
Protein-energy malnutrition [Data Rich]	Female	28-364 days	1-4 years	0.358163	0.531938	0.267771	0.317653	0.996928	0.993492
Protein-energy malnutrition [Data Rich]	Female	5-9 years	95+ years	0.575876	0.82491	0.317591	0.459951	0.937556	0.941932
Protein-energy malnutrition [Global]	Male	28-364 days	1-4 years	0.345688	0.749728	0.260672	0.276509	0.995837	0.967912
Protein-energy malnutrition [Global]	Female	28-364 days	1-4 years	0.415796	0.754333	0.303049	0.307893	0.995262	0.972418
Protein-energy malnutrition [Global]	Female	5-9 years	95+ years	0.472788	0.95306	0.338641	0.362599	0.944598	0.918248
Protein-energy malnutrition [Global]	Male	5-9 years	95+ years	0.476147	0.965199	0.345501	0.360691	0.954718	0.931593
Iron-deficiency anemia [Data Rich]	Male	28-364 days	95+ years	1.17192	1.94642	0.391732	0.47263	0.552362	0.555995
Iron-deficiency anemia [Data Rich]	Female	28-364 days	95+ years	1.42962	2.32066	0.519641	0.649806	0.461096	0.457159
Iron-deficiency anemia [Global]	Male	28-364 days	95+ years	0.789979	1.97513	0.487322	0.501456	0.605681	0.623655
Iron-deficiency anemia [Global]	Female	28-364 days	95+ years	0.940939	2.32384	0.691811	0.7491	0.565307	0.584283
Other nutritional deficiencies [Data Rich]	Female	28-364 days	95+ years	0.380559	0.628123	0.283098	0.379927	0.96161	0.962371
Other nutritional deficiencies [Data Rich]	Male	28-364 days	95+ years	0.373415	0.564897	0.265085	0.322379	0.935165	0.936115
Other nutritional deficiencies [Global]	Female	28-364 days	95+ years	0.451049	0.797499	0.317317	0.325292	0.966734	0.954653
Other nutritional deficiencies [Global]	Male	28-364 days	95+ years	0.440019	0.786416	0.297718	0.303868	0.944318	0.931872
Sexually transmitted diseases excluding HIV [Data Rich]	Male	10-14 years	95+ years	0.592869	0.766912	0.466048	0.57587	0.990302	0.984586
Sexually transmitted diseases excluding HIV [Data Rich]	Female	10-14 years	95+ years	0.353387	0.551968	0.26404	0.376584	0.982085	0.983247
Sexually transmitted diseases excluding HIV [Global]	Male	10-14 years	95+ years	0.783455	1.35215	0.58237	0.605353	0.896837	0.873158
Sexually transmitted diseases excluding HIV [Global]	Female	10-14 years	95+ years	0.48871	0.767613	0.403017	0.385015	0.983025	0.9708
Hepatitis [Data Rich]	Female	28-364 days	95+ years	0.347159	0.513666	0.246796	0.284031	0.999045	0.998112
Hepatitis [Global]	Female	28-364 days	95+ years	0.399657	0.785077	0.28208	0.300865	0.998931	0.978401
Hepatitis [Data Rich]	Male	28-364 days	95+ years	0.343624	0.479052	0.248477	0.29235	0.999118	0.998335
Hepatitis [Global]	Male	28-364 days	95+ years	0.397275	0.770546	0.290714	0.293805	0.998418	0.982748
Other infectious diseases [Data Rich]	Male	0-6 days	95+ years	0.257632	0.378812	0.194259	0.229884	0.998943	0.99817
Other infectious diseases [Global]	Male	0-6 days	95+ years	0.388113	0.605983	0.326699	0.331726	0.998603	0.991416
Other infectious diseases [Data Rich]	Female	0-6 days	95+ years	0.353856	0.471172	0.257697	0.301628	0.998184	0.99719
Other infectious diseases [Global]	Female	0-6 days	95+ years	0.44921	0.62668	0.382637	0.368739	0.997717	0.9911
Esophageal cancer [Data Rich]	Male	15-19 years	95+ years	0.198433	0.245255	0.161804	0.185832	0.998919	0.997968
Esophageal cancer [Data Rich]	Female	15-19 years	95+ years	0.215283	0.268253	0.179366	0.206173	0.998593	0.997497
Esophageal cancer [Global]	Female	15-19 years	95+ years	0.272331	0.449786	0.211737	0.217784	0.997501	0.980359
Esophageal cancer [Global]	Male	15-19 years	95+ years	0.238675	0.413533	0.185874	0.189452	0.997966	0.980274
Stomach cancer [Data Rich]	Male	15-19 years	95+ years	0.168624	0.204856	0.135969	0.153663	0.998632	0.997638
Stomach cancer [Data Rich]	Female	15-19 years	95+ years	0.174226	0.211966	0.142399	0.164469	0.998495	0.99713
Stomach cancer [Global]	Female	15-19 years	95+ years	0.204713	0.321435	0.162978	0.160719	0.99867	0.984612
Stomach cancer [Global]	Male	15-19 years	95+ years	0.196924	0.307854	0.153815	0.152673	0.998643	0.980242
Liver cancer [Global]	Male	5-9 years	95+ years	0.269051	0.454562	0.209164	0.210674	0.998031	0.986505
Liver cancer [Data Rich]	Male	5-9 years	95+ years	0.2413	0.325407	0.191496	0.224282	0.998065	0.996981
Liver cancer [Global]	Female	5-9 years	95+ years	0.277507	0.413467	0.216708	0.218733	0.998803	0.991523
Liver cancer [Data Rich]	Female	5-9 years	95+ years	0.232587	0.30673	0.193079	0.226226	0.998381	0.997116
Larynx cancer [Data Rich]	Male	15-19 years	95+ years	0.200641	0.252556	0.161284	0.192664	0.997858	0.997282
Larynx cancer [Data Rich]	Female	15-19 years	95+ years	0.30673	0.388496	0.253812	0.302646	0.975795	0.977395
Larynx cancer [Global]	Male	15-19 years	95+ years	0.234619	0.374157	0.181438	0.191236	0.998393	0.986868
Larynx cancer [Global]	Female	15-19 years	95+ years	0.356904	0.5464	0.283167	0.287904	0.98139	0.973441
Tracheal, bronchus, and lung cancer [Data Rich]	Female	15-19 years	95+ years	0.188871	0.231498	0.154034	0.180269	0.998449	0.997224
Tracheal, bronchus, and lung cancer [Data Rich]	Male	15-19 years	95+ years	0.181641	0.221799	0.146639	0.164353	0.99861	0.997327
Tracheal, bronchus, and lung cancer [Global]	Female	15-19 years	95+ years	0.225478	0.304297	0.174547	0.175022	0.998643	0.995915
Tracheal, bronchus, and lung cancer [Global]	Male	15-19 years	95+ years	0.212545	0.307851	0.166159	0.169413	0.998557	0.990068
Breast cancer [Data Rich]	Male	15-19 years	95+ years	0.339918	0.42271	0.265862	0.31327	0.97947	0.976519
Breast cancer [Data Rich]	Female	15-19 years	95+ years	0.192908	0.233432	0.155652	0.177959	0.996331	0.993547
Breast cancer [Global]	Female	15-19 years	95+ years	0.211224	0.287481	0.162086	0.167525	0.997149	0.990481
Breast cancer [Global]	Male	15-19 years	95+ years	0.423524	0.594604	0.313547	0.315621	0.979072	0.968179
Cervical cancer [Data Rich]	Female	15-19 years	95+ years	0.193564	0.239786	0.156019	0.182773	0.998702	0.997568
Cervical cancer [Global]	Female	15-19 years	95+ years	0.244551	0.350403	0.188472	0.186523	0.999189	0.990516
Uterine cancer [Global]	Female	15-19 years	95+ years	0.26113	0.405926	0.203732	0.203708	0.999474	0.993606
Uterine cancer [Data Rich]	Female	15-19 years	95+ years	0.215364	0.272806	0.172502	0.20594	0.999534	0.998942
Prostate cancer [Data Rich]	Male	15-19 years	95+ years	0.25085	0.303217	0.204423	0.237726	0.996921	0.994794
Prostate cancer [Global]	Male	15-19 years	95+ years	0.29197	0.370344	0.218901	0.21368	0.996743	0.986557
Colon and rectum cancer [Data Rich]	Male	15-19 years	95+ years	0.185598	0.224739	0.152051	0.172096	0.998745	0.997677
Colon and rectum cancer [Data Rich]	Female	15-19 years	95+ years	0.195509	0.240842	0.161929	0.192825	0.998011	0.996491
Colon and rectum cancer [Global]	Male	15-19 years	95+ years	0.208621	0.291093	0.166006	0.167229	0.999046	0.991509
Colon and rectum cancer [Global]	Female	15-19 years	95+ years	0.215863	0.294113	0.171992	0.168904	0.998465	0.990486
Lip and oral cavity cancer [Data Rich]	Male	15-19 years	95+ years	0.207908	0.252841	0.168893	0.183409	0.999097	0.998219
Lip and oral cavity cancer [Data Rich]	Female	15-19 years	95+ years	0.217054	0.273426	0.177172	0.204022	0.999549	0.999077
Lip and oral cavity cancer [Global]	Male	15-19 years	95+ years	0.252937	0.355882	0.20016	0.196201	0.998305	0.991385
Lip and oral cavity cancer [Global]	Female	15-19 years	95+ years	0.252204	0.349135	0.192715	0.197068	0.999507	0.995154
Nasopharynx cancer [Data Rich]	Female	5-9 years	95+ years	0.305983	0.418084	0.256857	0.342077	0.991357	0.991272
Nasopharynx cancer [Data Rich]	Male	5-9 years	95+ years	0.256041	0.343061	0.207344	0.249863	0.998424	0.997617
Nasopharynx cancer [Global]	Female	5-9 years	95+ years	0.361818	0.574134	0.287872	0.294643	0.990987	0.982361
Nasopharynx cancer [Global]	Male	5-9 years	95+ years	0.303468	0.507949	0.238899	0.243384	0.998489	0.987144
Other pharynx cancer [Data Rich]	Male	15-19 years	95+ years	0.243933	0.316171	0.196356	0.221139	0.99611	0.995076
Other pharynx cancer [Data Rich]	Female	15-19 years	95+ years	0.257668	0.327035	0.210857	0.248682	0.994188	0.993541
Other pharynx cancer [Global]	Male	15-19 years	95+ years	0.287334	0.46305	0.218922	0.209273	0.996444	0.987678
Other pharynx cancer [Global]	Female	15-19 years	95+ years	0.319571	0.475567	0.248676	0.243749	0.994634	0.987119
Gallbladder and biliary tract cancer [Data Rich]	Female	15-19 years	95+ years	0.203972	0.267017	0.165798	0.189961	0.995926	0.994112
Gallbladder and biliary tract cancer [Data Rich]	Male	15-19 years	95+ years	0.200502	0.247264	0.162741	0.182337	0.997554	0.996224
Gallbladder and biliary tract cancer [Global]	Female	15-19 years	95+ years	0.24119	0.382075	0.18688	0.183792	0.99678	0.982529
Gallbladder and biliary tract cancer [Global]	Male	15-19 years	95+ years	0.261043	0.375592	0.187773	0.184805	0.998613	0.989089
Pancreatic cancer [Data Rich]	Male	15-19 years	95+ years	0.173161	0.212802	0.140106	0.151654	0.998714	0.997757
Pancreatic cancer [Data Rich]	Female	15-19 years	95+ years	0.19234	0.240872	0.158471	0.180492	0.997995	0.996599
Pancreatic cancer [Global]	Male	15-19 years	95+ years	0.198254	0.284044	0.152835	0.151022	0.998854	0.995525
Pancreatic cancer [Global]	Female	15-19 years	95+ years	0.209814	0.286474	0.168889	0.166741	0.998889	0.996591
Malignant skin melanoma [Data Rich]	Male	15-19 years	95+ years	0.258707	0.324742	0.207811	0.237785	0.999191	0.998467
Malignant skin melanoma [Data Rich]	Female	15-19 years	95+ years	0.256129	0.301867	0.200431	0.216407	0.998728	0.997828

Cause	Sex	Age start	Age end	Predictive validity					
				RMSE in	RMSE out	Trend in	Trend out	Coverage in	Coverage out
Malignant skin melanoma [Global]	Male	15-19 years	95+ years	0.296332	0.434735	0.225174	0.226047	0.998842	0.994037
Malignant skin melanoma [Global]	Female	15-19 years	95+ years	0.292432	0.42973	0.222704	0.226799	0.998281	0.991761
Non-melanoma skin cancer [Data Rich]	Male	15-19 years	95+ years	0.155711	0.239935	0.112916	0.133783	0.99981	0.999603
Non-melanoma skin cancer [Data Rich]	Female	15-19 years	95+ years	0.214155	0.309436	0.159857	0.183713	0.997395	0.997199
Non-melanoma skin cancer [Global]	Female	15-19 years	95+ years	0.291672	0.489846	0.209023	0.217962	0.994887	0.985998
Non-melanoma skin cancer [Global]	Male	15-19 years	95+ years	0.192737	0.330459	0.136252	0.14092	0.999232	0.995604
Ovarian cancer [Data Rich]	Female	15-19 years	95+ years	0.195794	0.246041	0.157071	0.173662	0.998842	0.997833
Ovarian cancer [Global]	Female	15-19 years	95+ years	0.218013	0.313157	0.166727	0.167835	0.999174	0.994091
Testicular cancer [Global]	Male	15-19 years	95+ years	0.328371	0.529164	0.255569	0.25659	0.999375	0.995125
Testicular cancer [Data Rich]	Male	15-19 years	95+ years	0.283022	0.371326	0.232189	0.243099	0.999645	0.999282
Kidney cancer [Data Rich]	Male	0-6 days	95+ years	0.241409	0.355526	0.19905	0.233492	0.999066	0.998706
Kidney cancer [Data Rich]	Female	0-6 days	95+ years	0.269859	0.399459	0.223989	0.265876	0.998886	0.997983
Kidney cancer [Global]	Male	0-6 days	95+ years	0.280428	0.40437	0.224896	0.23264	0.999225	0.994304
Kidney cancer [Global]	Female	0-6 days	95+ years	0.309672	0.434952	0.251863	0.260629	0.999065	0.992195
Bladder cancer [Data Rich]	Male	15-19 years	95+ years	0.238162	0.289199	0.193221	0.21405	0.998893	0.9979
Bladder cancer [Global]	Male	15-19 years	95+ years	0.259512	0.358356	0.20438	0.211875	0.998849	0.993972
Bladder cancer [Global]	Female	15-19 years	95+ years	0.279362	0.406151	0.217047	0.218843	0.995797	0.992228
Bladder cancer [Data Rich]	Female	15-19 years	95+ years	0.241778	0.308128	0.197425	0.236098	0.995139	0.99458
Brain and nervous system cancer [Data Rich]	Female	0-6 days	95+ years	0.233403	0.3046	0.177532	0.189069	0.998845	0.997948
Brain and nervous system cancer [Global]	Female	0-6 days	95+ years	0.302238	0.394989	0.217704	0.215738	0.998871	0.995489
Brain and nervous system cancer [Data Rich]	Male	0-6 days	95+ years	0.216468	0.277928	0.168084	0.183155	0.998868	0.997924
Brain and nervous system cancer [Global]	Male	0-6 days	95+ years	0.27435	0.374644	0.204471	0.20185	0.998951	0.996187
Thyroid cancer [Data Rich]	Female	10-14 years	95+ years	0.428862	0.514646	0.348096	0.339467	0.988448	0.986206
Thyroid cancer [Data Rich]	Male	10-14 years	95+ years	0.325439	0.400471	0.258836	0.251195	0.990913	0.991523
Thyroid cancer [Global]	Female	10-14 years	95+ years	0.452233	0.57687	0.360692	0.356627	0.990555	0.981127
Thyroid cancer [Global]	Male	10-14 years	95+ years	0.365192	0.48212	0.280751	0.28231	0.992984	0.989187
Mesothelioma [Data Rich]	Female	15-19 years	95+ years	0.196941	0.304164	0.152358	0.219033	0.998746	0.998454
Mesothelioma [Data Rich]	Male	15-19 years	95+ years	0.172967	0.285052	0.128708	0.171164	0.999971	0.999797
Mesothelioma [Global]	Male	15-19 years	95+ years	0.241276	0.464505	0.185143	0.181593	0.999543	0.989174
Mesothelioma [Global]	Female	15-19 years	95+ years	0.260519	0.427073	0.202264	0.205672	0.998568	0.995729
Hodgkin lymphoma [Data Rich]	Male	0-6 days	95+ years	0.350903	0.46589	0.27357	0.318263	0.99713	0.947085
Hodgkin lymphoma [Data Rich]	Female	0-6 days	95+ years	0.446449	0.618172	0.339263	0.421206	0.960309	0.961281
Hodgkin lymphoma [Global]	Male	0-6 days	95+ years	0.418144	0.544918	0.308703	0.308167	0.998369	0.99283
Hodgkin lymphoma [Global]	Female	0-6 days	95+ years	0.590103	0.786115	0.402028	0.406353	0.972256	0.963972
Non-Hodgkin lymphoma [Data Rich]	Male	0-6 days	95+ years	0.257262	0.319446	0.198048	0.224728	0.998985	0.998708
Non-Hodgkin lymphoma [Data Rich]	Female	0-6 days	95+ years	0.267228	0.346181	0.210521	0.242819	0.998808	0.998423
Non-Hodgkin lymphoma [Global]	Male	0-6 days	95+ years	0.294371	0.40601	0.217911	0.227594	0.998919	0.992808
Non-Hodgkin lymphoma [Global]	Female	0-6 days	95+ years	0.300954	0.416656	0.228722	0.233181	0.998752	0.993317
Multiple myeloma [Data Rich]	Male	15-19 years	95+ years	0.229224	0.274483	0.181391	0.198275	0.999263	0.998586
Multiple myeloma [Data Rich]	Female	15-19 years	95+ years	0.239102	0.290048	0.187379	0.216489	0.999277	0.998797
Multiple myeloma [Global]	Male	15-19 years	95+ years	0.273879	0.3742	0.206672	0.211225	0.999194	0.994302
Multiple myeloma [Global]	Female	15-19 years	95+ years	0.277053	0.38199	0.206454	0.209223	0.999174	0.996651
Leukemia [Data Rich]	Female	0-6 days	95+ years	0.232168	0.285674	0.190505	0.222964	0.997925	0.996086
Leukemia [Data Rich]	Male	0-6 days	95+ years	0.218797	0.271475	0.178922	0.207808	0.998391	0.99703
Leukemia [Global]	Female	0-6 days	95+ years	0.283536	0.361995	0.242014	0.242573	0.99865	0.993203
Leukemia [Global]	Male	0-6 days	95+ years	0.294539	0.379	0.275613	0.270439	0.997571	0.992988
Other neoplasms [Data Rich]	Male	0-6 days	95+ years	0.256396	0.315719	0.217052	0.239815	0.993394	0.988905
Other neoplasms [Data Rich]	Female	0-6 days	95+ years	0.266244	0.330966	0.224429	0.255028	0.993927	0.989754
Other neoplasms [Global]	Female	0-6 days	95+ years	0.287964	0.370172	0.237436	0.240632	0.995797	0.992799
Other neoplasms [Global]	Male	0-6 days	95+ years	0.277797	0.363343	0.227826	0.234498	0.995245	0.992186
Cardiovascular diseases [Data Rich]	Female	0-6 days	95+ years	0.192262	0.238497	0.150738	0.173122	0.999286	0.998329
Cardiovascular diseases [Data Rich]	Male	0-6 days	95+ years	0.131495	0.168958	0.09984	0.120649	0.999795	0.999232
Cardiovascular diseases [Global]	Male	0-6 days	95+ years	0.163566	0.260481	0.122215	0.124031	0.999285	0.98696
Cardiovascular diseases [Global]	Female	0-6 days	95+ years	0.214127	0.304249	0.162144	0.165641	0.998835	0.988171
Rheumatic heart disease [Data Rich]	Male	1-4 years	95+ years	0.152516	0.209445	0.109275	0.12886	0.999808	0.999469
Rheumatic heart disease [Data Rich]	Female	1-4 years	95+ years	0.163047	0.226524	0.121144	0.14047	0.999571	0.99907
Rheumatic heart disease [Global]	Female	1-4 years	95+ years	0.204952	0.414423	0.149091	0.152743	0.999276	0.987263
Rheumatic heart disease [Global]	Male	1-4 years	95+ years	0.197282	0.396283	0.1435	0.147303	0.999326	0.984977
Ischemic heart disease [Data Rich]	Male	28-364 days	95+ years	0.165865	0.239331	0.118904	0.150065	0.998745	0.998481
Ischemic heart disease [Data Rich]	Female	28-364 days	95+ years	0.169733	0.252884	0.121072	0.15727	0.996695	0.996303
Ischemic heart disease [Global]	Male	28-364 days	95+ years	0.21286	0.387879	0.162392	0.171877	0.998807	0.978578
Ischemic heart disease [Global]	Female	28-364 days	95+ years	0.211418	0.421587	0.155088	0.161408	0.997979	0.977638
Cerebrovascular disease [Data Rich]	Male	0-6 days	95+ years	0.184441	0.24818	0.138406	0.184836	0.999384	0.999074
Cerebrovascular disease [Data Rich]	Female	0-6 days	95+ years	0.160676	0.205385	0.121533	0.14436	0.999828	0.999483
Cerebrovascular disease [Global]	Male	0-6 days	95+ years	0.216271	0.347364	0.157729	0.163709	0.999062	0.989017
Cerebrovascular disease [Global]	Female	0-6 days	95+ years	0.197338	0.333525	0.142096	0.149473	0.999346	0.989472
Ischemic stroke [Data Rich]	Male	28-364 days	95+ years	0.177626	0.244707	0.136694	0.160955	0.999549	0.999322
Ischemic stroke [Data Rich]	Female	28-364 days	95+ years	0.157877	0.22556	0.119754	0.147035	0.999776	0.9996
Ischemic stroke [Global]	Male	28-364 days	95+ years	0.204502	0.34158	0.150227	0.156071	0.999351	0.98046
Ischemic stroke [Global]	Female	28-364 days	95+ years	0.18313	0.32224	0.131296	0.137648	0.999679	0.984224
Hemorrhagic stroke [Data Rich]	Male	0-6 days	95+ years	0.175211	0.247264	0.131987	0.171769	0.999348	0.998892
Hemorrhagic stroke [Data Rich]	Female	0-6 days	95+ years	0.154144	0.205856	0.11795	0.139364	0.999767	0.999408
Hemorrhagic stroke [Global]	Male	0-6 days	95+ years	0.207524	0.338293	0.153924	0.156106	0.998913	0.984318
Hemorrhagic stroke [Global]	Female	0-6 days	95+ years	0.187471	0.314977	0.141525	0.144897	0.99938	0.986289
Hypertensive heart disease [Data Rich]	Male	28-364 days	95+ years	0.375853	0.515546	0.121205	0.131312	0.941321	0.944951
Hypertensive heart disease [Data Rich]	Female	28-364 days	95+ years	0.378198	0.513771	0.114166	0.128987	0.948335	0.954071
Hypertensive heart disease [Global]	Male	28-364 days	95+ years	0.336247	0.665951	0.16303	0.173066	0.955092	0.920006
Hypertensive heart disease [Global]	Female	28-364 days	95+ years	0.367106	0.612234	0.168515	0.17818	0.957197	0.924734
Cardiomyopathy and myocarditis [Data Rich]	Male	0-6 days	95+ years	0.220269	0.306222	0.137843	0.169414	0.999808	0.999501
Cardiomyopathy and myocarditis [Data Rich]	Female	0-6 days	95+ years	0.29012	0.386485	0.185449	0.210994	0.998975	0.998244
Cardiomyopathy and myocarditis [Global]	Male	0-6 days	95+ years	0.266398	0.452529	0.165167	0.169165	0.999422	0.981828
Cardiomyopathy and myocarditis [Global]	Female	0-6 days	95+ years	0.31616	0.532369	0.209694	0.213072	0.998865	0.983376
Aortic aneurysm [Data Rich]	Male	15-19 years	95+ years	0.153024	0.242023	0.118267	0.130831	0.999821	0.999626
Aortic aneurysm [Data Rich]	Female	15-19 years	95+ years	0.164886	0.24291	0.127439	0.136764	0.99982	0.999588
Aortic aneurysm [Global]	Male	15-19 years	95+ years	0.179833	0.326754	0.134775	0.136077	0.999841	0.990644
Aortic aneurysm [Global]	Female	15-19 years	95+ years	0.20074	0.361639	0.150479	0.149563	0.999756	0.993171
Peripheral artery disease [Data Rich]	Male	40-44 years	95+ years	0.369178	0.661617	0.196514	0.240344	0.998799	0.997877
Peripheral artery disease [Data Rich]	Female	40-44 years	95+ years	0.40606	0.671306	0.209077	0.249727	0.997914	0.997001
Peripheral artery disease [Global]	Male	40-44 years	95+ years	0.367119	0.706275	0.21604	0.225123	0.998154	0.980256

Cause	Sex	Age start	Age end	Predictive validity					
				RMSE in	RMSE out	Trend in	Trend out	Coverage in	Coverage out
Peripheral artery disease [Global]	Female	40-44 years	95+ years	0.375448	0.690754	0.232678	0.247308	0.996876	0.979057
Endocarditis [Data Rich]	Female	0-6 days	95+ years	0.306661	0.443574	0.147608	0.178623	0.999581	0.999381
Endocarditis [Data Rich]	Male	0-6 days	95+ years	0.271783	0.419623	0.155299	0.175676	0.999884	0.999767
Endocarditis [Global]	Female	0-6 days	95+ years	0.254591	0.465498	0.162758	0.172122	0.999477	0.993651
Endocarditis [Global]	Male	0-6 days	95+ years	0.341863	0.50083	0.173558	0.177394	0.999753	0.995051
Other cardiovascular and circulatory diseases [Data Rich]	Male	0-6 days	95+ years	0.213776	0.281449	0.155893	0.179796	0.998382	0.997111
Other cardiovascular and circulatory diseases [Data Rich]	Female	0-6 days	95+ years	0.203074	0.266679	0.147133	0.164076	0.99867	0.997542
Other cardiovascular and circulatory diseases [Global]	Male	0-6 days	95+ years	0.295104	0.443714	0.192381	0.200649	0.998318	0.986363
Other cardiovascular and circulatory diseases [Global]	Female	0-6 days	95+ years	0.285593	0.459561	0.190186	0.19816	0.998704	0.986935
Chronic respiratory diseases [Global]	Male	1-4 years	95+ years	0.214738	0.363049	0.181231	0.18	0.998948	0.981183
Chronic respiratory diseases [Data Rich]	Male	1-4 years	95+ years	0.151269	0.196474	0.119361	0.139347	0.99982	0.999296
Chronic respiratory diseases [Global]	Female	1-4 years	95+ years	0.213751	0.366215	0.148876	0.154955	0.999488	0.985731
Chronic respiratory diseases [Data Rich]	Female	1-4 years	95+ years	0.157007	0.20946	0.12241	0.145705	0.999901	0.999331
Chronic obstructive pulmonary disease [Data Rich]	Male	1-4 years	95+ years	0.154402	0.236138	0.122112	0.141295	0.9999	0.999401
Chronic obstructive pulmonary disease [Global]	Male	1-4 years	95+ years	0.185562	0.320172	0.140978	0.144131	0.999801	0.990405
Chronic obstructive pulmonary disease [Data Rich]	Female	1-4 years	95+ years	0.161715	0.277006	0.12516	0.139416	0.999949	0.999607
Chronic obstructive pulmonary disease [Global]	Female	1-4 years	95+ years	0.196875	0.343943	0.141256	0.143174	0.99993	0.991945
Pneumoconiosis [Global]	Female	1-4 years	95+ years	0.374414	0.856432	0.275237	0.28672	0.998451	0.976312
Pneumoconiosis [Data Rich]	Female	1-4 years	95+ years	0.311531	0.598142	0.233498	0.276232	0.999204	0.997774
Pneumoconiosis [Global]	Male	1-4 years	95+ years	0.270107	0.723686	0.19162	0.19355	0.999369	0.980403
Pneumoconiosis [Data Rich]	Male	1-4 years	95+ years	0.215051	0.377244	0.156683	0.153746	0.999676	0.999072
Silicosis [Global]	Female	1-4 years	95+ years	0.733623	1.24629	0.551239	0.572099	0.982359	0.949326
Silicosis [Data Rich]	Female	1-4 years	95+ years	0.641745	0.95793	0.501452	0.565322	0.979636	0.974802
Silicosis [Global]	Male	1-4 years	95+ years	0.464767	1.12932	0.330122	0.322285	0.983176	0.936495
Silicosis [Data Rich]	Male	1-4 years	95+ years	0.38962	0.664319	0.283152	0.256956	0.983172	0.981028
Asbestosis [Global]	Female	1-4 years	95+ years	1.1665	1.85842	0.711671	0.749178	0.708166	0.667294
Asbestosis [Data Rich]	Female	1-4 years	95+ years	1.01215	1.43052	0.664919	0.763103	0.655933	0.65262
Asbestosis [Global]	Male	1-4 years	95+ years	1.11945	1.49636	0.429074	0.433138	0.778045	0.771509
Asbestosis [Data Rich]	Male	1-4 years	95+ years	0.691529	0.932549	0.349991	0.339131	0.753847	0.754577
Coal workers pneumoconiosis [Data Rich]	Male	1-4 years	95+ years	0.476701	0.692153	0.336023	0.287121	0.966318	0.962163
Coal workers pneumoconiosis [Global]	Male	1-4 years	95+ years	0.583664	1.44197	0.417538	0.401023	0.965902	0.919421
Coal workers pneumoconiosis [Data Rich]	Female	1-4 years	95+ years	0.686195	1.07185	0.535712	0.65237	0.928186	0.921679
Coal workers pneumoconiosis [Global]	Female	1-4 years	95+ years	0.81925	1.36011	0.615975	0.644494	0.941823	0.940531
Other pneumoconiosis [Global]	Male	1-4 years	95+ years	0.312932	0.662782	0.211254	0.218186	0.921217	0.925185
Other pneumoconiosis [Data Rich]	Male	1-4 years	95+ years	0.248766	0.476134	0.169182	0.174756	0.999502	0.99887
Other pneumoconiosis [Global]	Female	1-4 years	95+ years	0.559101	1.05284	0.338025	0.357781	0.75868	0.746502
Other pneumoconiosis [Data Rich]	Female	1-4 years	95+ years	0.414989	0.824373	0.290625	0.365377	0.997939	0.995488
Asthma [Global]	Female	1-4 years	95+ years	0.233546	0.462602	0.164998	0.167501	0.999755	0.99175
Asthma [Data Rich]	Female	1-4 years	95+ years	0.197137	0.33993	0.143896	0.165311	0.999789	0.99967
Asthma [Global]	Male	1-4 years	95+ years	0.235747	0.494414	0.162522	0.164543	0.999806	0.988722
Asthma [Data Rich]	Male	1-4 years	95+ years	0.211385	0.370967	0.144466	0.158342	0.999858	0.999804
Interstitial lung disease and pulmonary sarcoidosis [Global]	Female	1-4 years	95+ years	0.302765	0.511513	0.181156	0.187252	0.999355	0.991799
Interstitial lung disease and pulmonary sarcoidosis [Data Rich]	Female	1-4 years	95+ years	0.25481	0.434681	0.161626	0.183933	0.999577	0.999425
Interstitial lung disease and pulmonary sarcoidosis [Global]	Male	1-4 years	95+ years	0.307647	0.492826	0.175352	0.180107	0.999549	0.991899
Interstitial lung disease and pulmonary sarcoidosis [Data Rich]	Male	1-4 years	95+ years	0.269701	0.408546	0.162959	0.173591	0.99965	0.99946
Other chronic respiratory diseases [Global]	Female	1-4 years	95+ years	0.477259	0.687179	0.289427	0.283232	0.99796	0.98908
Other chronic respiratory diseases [Data Rich]	Female	1-4 years	95+ years	0.345243	0.468642	0.219455	0.234483	0.999172	0.998265
Other chronic respiratory diseases [Global]	Male	1-4 years	95+ years	0.405874	0.641769	0.241063	0.241372	0.999212	0.991323
Other chronic respiratory diseases [Data Rich]	Male	1-4 years	95+ years	0.35057	0.439912	0.188925	0.198379	0.999737	0.999245
Cirrhosis and other chronic liver diseases [Data Rich]	Male	1-4 years	95+ years	0.179209	0.262346	0.134376	0.177788	0.999412	0.999075
Cirrhosis and other chronic liver diseases [Data Rich]	Female	1-4 years	95+ years	0.204421	0.304245	0.155018	0.221822	0.998591	0.99815
Cirrhosis and other chronic liver diseases [Global]	Female	1-4 years	95+ years	0.230628	0.422164	0.168036	0.173688	0.998633	0.983339
Cirrhosis and other chronic liver diseases [Global]	Male	1-4 years	95+ years	0.216882	0.4316	0.14994	0.154875	0.999356	0.982177
Digestive diseases [Global]	Female	0-6 days	95+ years	0.270455	0.359254	0.259676	0.265122	0.999384	0.995712
Digestive diseases [Data Rich]	Female	0-6 days	95+ years	0.163941	0.210831	0.125319	0.149704	0.999776	0.999546
Digestive diseases [Global]	Male	0-6 days	95+ years	0.267873	0.357645	0.252092	0.231238	0.999159	0.99466
Digestive diseases [Data Rich]	Male	0-6 days	95+ years	0.178265	0.225295	0.130579	0.152707	0.999837	0.999605
Peptic ulcer disease [Data Rich]	Female	1-4 years	95+ years	0.255805	0.394632	0.187573	0.288663	0.997477	0.996982
Peptic ulcer disease [Data Rich]	Male	1-4 years	95+ years	0.242307	0.369968	0.179312	0.270947	0.991071	0.991741
Peptic ulcer disease [Global]	Female	1-4 years	95+ years	0.284239	0.469943	0.198721	0.206417	0.998008	0.988173
Peptic ulcer disease [Global]	Male	1-4 years	95+ years	0.269341	0.441943	0.188717	0.19908	0.992949	0.983866
Gastritis and duodenitis [Data Rich]	Male	1-4 years	95+ years	0.330813	0.676853	0.238248	0.292961	0.994144	0.992533
Gastritis and duodenitis [Data Rich]	Female	1-4 years	95+ years	0.462701	0.875334	0.319025	0.42543	0.982053	0.978797
Gastritis and duodenitis [Global]	Male	1-4 years	95+ years	0.387565	0.788525	0.278512	0.288003	0.99535	0.974564
Gastritis and duodenitis [Global]	Female	1-4 years	95+ years	0.5028	0.902224	0.352124	0.375143	0.987908	0.962366
Appendicitis [Data Rich]	Male	1-4 years	95+ years	0.222178	0.291123	0.163131	0.19261	0.999855	0.999616
Appendicitis [Data Rich]	Female	1-4 years	95+ years	0.275251	0.350678	0.181891	0.214117	0.999793	0.999535
Appendicitis [Global]	Male	1-4 years	95+ years	0.263703	0.442064	0.185635	0.188683	0.999691	0.994425
Appendicitis [Global]	Female	1-4 years	95+ years	0.286902	0.473703	0.201799	0.199287	0.999666	0.994854
Paralytic ileus and intestinal obstruction [Data Rich]	Male	0-6 days	95+ years	0.270214	0.339601	0.159736	0.178805	0.999655	0.999233
Paralytic ileus and intestinal obstruction [Data Rich]	Female	0-6 days	95+ years	0.270521	0.345787	0.162546	0.183923	0.999687	0.999551
Paralytic ileus and intestinal obstruction [Global]	Male	0-6 days	95+ years	0.279203	0.430488	0.172827	0.177438	0.999504	0.994849
Paralytic ileus and intestinal obstruction [Global]	Female	0-6 days	95+ years	0.281562	0.41762	0.173685	0.182219	0.999494	0.995381
Inguinal, femoral, and abdominal hernia [Data Rich]	Female	1-4 years	95+ years	0.383014	0.560535	0.256237	0.352187	0.996604	0.995472
Inguinal, femoral, and abdominal hernia [Data Rich]	Male	1-4 years	95+ years	0.355087	0.524619	0.24561	0.337298	0.996855	0.996805
Inguinal, femoral, and abdominal hernia [Global]	Female	1-4 years	95+ years	0.536159	0.713911	0.278552	0.288185	0.955189	0.945162
Inguinal, femoral, and abdominal hernia [Global]	Male	1-4 years	95+ years	0.446523	0.652866	0.259804	0.275091	0.971336	0.965397
Inflammatory bowel disease [Data Rich]	Male	1-4 years	95+ years	0.282424	0.459781	0.19247	0.239465	0.999144	0.998811
Inflammatory bowel disease [Data Rich]	Female	1-4 years	95+ years	0.270773	0.445533	0.186982	0.225011	0.999074	0.998683
Inflammatory bowel disease [Global]	Male	1-4 years	95+ years	0.329204	0.513562	0.215507	0.223708	0.99899	0.99392
Inflammatory bowel disease [Global]	Female	1-4 years	95+ years	0.318483	0.558503	0.216553	0.224204	0.998926	0.98921
Vascular intestinal disorders [Data Rich]	Female	1-4 years	95+ years	0.316032	0.527497	0.238404	0.335475	0.99726	0.996937
Vascular intestinal disorders [Data Rich]	Male	1-4 years	95+ years	0.25634	0.388341	0.178126	0.231957	0.999089	0.999053
Vascular intestinal disorders [Global]	Female	1-4 years	95+ years	0.355948	0.575886	0.251798	0.265648	0.997737	0.986443
Vascular intestinal disorders [Global]	Male	1-4 years	95+ years	0.289547	0.482381	0.192213	0.202417	0.999028	0.989822
Gallbladder and biliary diseases [Data Rich]	Female	1-4 years	95+ years	0.283105	0.442693	0.207388	0.333082	0.998065	0.997484
Gallbladder and biliary diseases [Data Rich]	Male	1-4 years	95+ years	0.307799	0.457515	0.227021	0.344195	0.996702	0.995422
Gallbladder and biliary diseases [Global]	Male	1-4 years	95+ years	0.369789	0.532326	0.249481	0.261085	0.99601	0.988713
Gallbladder and biliary diseases [Global]	Female	1-4 years	95+ years	0.350359	0.528094	0.233628	0.245633	0.99749	0.986886

Cause	Sex	Age start	Age end	Predictive validity					
				RMSE in	RMSE out	Trend in	Trend out	Coverage in	Coverage out
Pancreatitis [Data Rich]	Female	1-4 years	95+ years	0.352905	0.538338	0.255964	0.327749	0.996168	0.994666
Pancreatitis [Data Rich]	Male	1-4 years	95+ years	0.289483	0.423679	0.193218	0.236442	0.998269	0.998093
Pancreatitis [Global]	Female	1-4 years	95+ years	0.417096	0.565887	0.272015	0.283672	0.996165	0.988841
Pancreatitis [Global]	Male	1-4 years	95+ years	0.32127	0.480724	0.205372	0.211816	0.998284	0.989827
Other digestive diseases [Global]	Male	1-4 years	95+ years	0.219971	0.348122	0.156307	0.159797	0.999581	0.99756
Other digestive diseases [Global]	Female	1-4 years	95+ years	0.22718	0.378123	0.160941	0.167021	0.999463	0.995181
Other digestive diseases [Data Rich]	Male	1-4 years	95+ years	0.200581	0.274113	0.13394	0.15551	0.999987	0.999901
Other digestive diseases [Data Rich]	Female	1-4 years	95+ years	0.183726	0.292664	0.134883	0.159469	0.99997	0.999786
Epilepsy [Data Rich]	Male	28-364 days	95+ years	0.175607	0.233043	0.132644	0.158228	0.999936	0.999746
Epilepsy [Data Rich]	Female	28-364 days	95+ years	0.193337	0.263355	0.147808	0.17834	0.999924	0.999846
Epilepsy [Global]	Male	28-364 days	95+ years	0.210141	0.396411	0.154449	0.156602	0.999772	0.991091
Epilepsy [Global]	Female	28-364 days	95+ years	0.246304	0.411789	0.171275	0.178661	0.999766	0.995518
Multiple sclerosis [Data Rich]	Female	20-24 years	95+ years	0.241721	0.323895	0.163912	0.179077	0.999726	0.999219
Multiple sclerosis [Data Rich]	Male	20-24 years	95+ years	0.234028	0.299417	0.160696	0.176833	0.999798	0.999522
Multiple sclerosis [Global]	Female	20-24 years	95+ years	0.280482	0.454581	0.183879	0.184912	0.999277	0.994763
Multiple sclerosis [Global]	Male	20-24 years	95+ years	0.278237	0.450868	0.175818	0.179983	0.999577	0.995136
Motor neuron disease [Data Rich]	Male	0-6 days	95+ years	0.316902	0.534013	0.211079	0.253568	0.99864	0.997388
Motor neuron disease [Data Rich]	Female	0-6 days	95+ years	0.293987	0.553224	0.20948	0.246723	0.998849	0.998185
Motor neuron disease [Global]	Female	0-6 days	95+ years	0.347205	0.658949	0.231288	0.23211	0.998472	0.989795
Motor neuron disease [Global]	Male	0-6 days	95+ years	0.356241	0.611361	0.228359	0.234157	0.998503	0.989532
Other neurological disorders [Data Rich]	Male	28-364 days	95+ years	0.191645	0.299067	0.143077	0.15757	0.999964	0.999748
Other neurological disorders [Data Rich]	Female	28-364 days	95+ years	0.197697	0.293511	0.152053	0.165734	0.999838	0.999649
Other neurological disorders [Global]	Male	28-364 days	95+ years	0.225943	0.372312	0.163735	0.169604	0.999668	0.997502
Other neurological disorders [Global]	Female	28-364 days	95+ years	0.236573	0.36677	0.177541	0.174769	0.999484	0.997309
Schizophrenia [Global]	Male	25-29 years	95+ years	0.407179	0.818692	0.272956	0.292129	0.998606	0.982159
Schizophrenia [Global]	Female	25-29 years	95+ years	0.399354	0.777819	0.284951	0.29287	0.998241	0.977864
Schizophrenia [Data Rich]	Male	25-29 years	95+ years	0.319514	0.536367	0.224978	0.320717	0.999539	0.99884
Schizophrenia [Data Rich]	Female	25-29 years	95+ years	0.313456	0.5209	0.224708	0.29294	0.993945	0.993188
Alcohol use disorders [Data Rich]	Male	15-19 years	95+ years	0.17929	0.304548	0.130117	0.151662	0.999536	0.998886
Alcohol use disorders [Data Rich]	Female	15-19 years	95+ years	0.203259	0.347821	0.151562	0.170722	0.999545	0.999214
Alcohol use disorders [Global]	Male	15-19 years	95+ years	0.269213	0.544551	0.157835	0.162962	0.999564	0.981681
Alcohol use disorders [Global]	Female	15-19 years	95+ years	0.255953	0.611777	0.181912	0.186394	0.99926	0.979885
Drug use disorders [Data Rich]	Male	15-19 years	95+ years	0.247873	0.439854	0.171793	0.215707	0.999431	0.99837
Drug use disorders [Data Rich]	Female	15-19 years	95+ years	0.236468	0.3908	0.171429	0.202846	0.999614	0.999074
Drug use disorders [Data Rich]	Male	0-6 days	7-27 days	0.122912	0.197423	0.0592236	0.0817335	1	0.99992
Drug use disorders [Data Rich]	Female	0-6 days	7-27 days	0.175547	0.362237	0.0540063	0.0695225	1	0.999987
Drug use disorders [Global]	Male	15-19 years	95+ years	0.348551	0.692177	0.202837	0.217289	0.999215	0.974714
Drug use disorders [Global]	Female	15-19 years	95+ years	0.314591	0.630735	0.204251	0.219663	0.999483	0.986834
Drug use disorders [Global]	Male	0-6 days	7-27 days	0.252102	0.373197	0.155948	0.139103	0.998833	0.996251
Drug use disorders [Global]	Female	0-6 days	7-27 days	0.27611	0.478993	0.129182	0.0617594	0.998962	0.998016
Opioid use disorders [Data Rich]	Male	15-19 years	95+ years	0.279015	0.658101	0.156333	0.209697	0.999874	0.999659
Opioid use disorders [Data Rich]	Female	15-19 years	95+ years	0.296893	0.575847	0.17329	0.210736	0.999956	0.999832
Opioid use disorders [Data Rich]	Male	0-6 days	7-27 days	0.292308	0.783713	0.144181	0.21418	1	0.999765
Opioid use disorders [Data Rich]	Female	0-6 days	7-27 days	0.186804	0.499195	0.0555796	0.0798256	1	0.999986
Opioid use disorders [Global]	Male	15-19 years	95+ years	0.362253	0.781112	0.184474	0.198405	0.999429	0.981013
Opioid use disorders [Global]	Female	15-19 years	95+ years	0.395472	0.731917	0.201174	0.212926	0.999812	0.991786
Opioid use disorders [Global]	Male	0-6 days	7-27 days	0.319164	0.760471	0.167792	0.185988	0.999964	0.990763
Opioid use disorders [Global]	Female	0-6 days	7-27 days	0.208513	0.462801	0.0618939	0.0699246	1	0.999227
Cocaine use disorders [Data Rich]	Male	15-19 years	95+ years	0.500763	1.06769	0.375561	0.459082	0.994537	0.991288
Cocaine use disorders [Data Rich]	Female	15-19 years	95+ years	0.499633	0.879049	0.375111	0.46816	0.995815	0.993677
Cocaine use disorders [Global]	Male	15-19 years	95+ years	0.666778	1.1883	0.390092	0.423225	0.994992	0.950469
Cocaine use disorders [Global]	Female	15-19 years	95+ years	0.627812	1.0855	0.402242	0.435652	0.995377	0.969407
Amphetamine use disorders [Data Rich]	Male	15-19 years	95+ years	0.569818	1.00642	0.441065	0.552117	0.99071	0.985854
Amphetamine use disorders [Data Rich]	Female	15-19 years	95+ years	0.48318	0.859142	0.349707	0.411377	0.995519	0.993432
Amphetamine use disorders [Global]	Male	15-19 years	95+ years	0.7498	1.33032	0.454585	0.466483	0.991593	0.939045
Amphetamine use disorders [Global]	Female	15-19 years	95+ years	0.57807	1.05534	0.371049	0.38526	0.995634	0.966477
Other drug use disorders [Data Rich]	Male	15-19 years	95+ years	0.302313	0.671706	0.206537	0.280499	0.999343	0.998393
Other drug use disorders [Data Rich]	Female	15-19 years	95+ years	0.305278	0.627427	0.211411	0.272263	0.999727	0.99923
Other drug use disorders [Global]	Male	15-19 years	95+ years	0.414794	0.878074	0.234226	0.256882	0.999026	0.97341
Other drug use disorders [Global]	Female	15-19 years	95+ years	0.439288	0.781377	0.239331	0.251182	0.999491	0.983455
Eating disorders [Data Rich]	Male	5-9 years	45-49 years	0.508235	0.835798	0.409724	0.462264	0.992722	0.984897
Eating disorders [Data Rich]	Female	5-9 years	45-49 years	0.535142	0.846057	0.431419	0.453248	0.9922	0.98541
Eating disorders [Global]	Male	5-9 years	45-49 years	0.572931	1.01616	0.453483	0.461891	0.9932	0.96358
Eating disorders [Global]	Female	5-9 years	45-49 years	0.681655	1.23087	0.514815	0.525792	0.993354	0.95878
Anorexia nervosa [Data Rich]	Male	5-9 years	45-49 years	0.524883	0.878268	0.419212	0.508777	0.992536	0.984427
Anorexia nervosa [Data Rich]	Female	5-9 years	45-49 years	0.554614	0.864159	0.437744	0.467461	0.992817	0.985946
Anorexia nervosa [Global]	Male	5-9 years	45-49 years	0.6378	1.0624	0.468241	0.465125	0.993775	0.96001
Anorexia nervosa [Global]	Female	5-9 years	45-49 years	0.675183	1.23176	0.517061	0.525529	0.993283	0.951051
Bulimia nervosa [Data Rich]	Male	5-9 years	45-49 years	0.76311	1.0535	0.638808	0.602952	0.970107	0.957693
Bulimia nervosa [Data Rich]	Female	5-9 years	45-49 years	0.943347	1.56089	0.795534	0.815436	0.956266	0.934965
Bulimia nervosa [Global]	Male	5-9 years	45-49 years	0.885898	1.24254	0.734903	0.71786	0.733219	0.701785
Bulimia nervosa [Global]	Female	5-9 years	45-49 years	1.05396	1.78616	0.894882	0.907138	0.727466	0.663275
Diabetes mellitus [Global]	Female	0-6 days	20-24 years	0.242454	0.480836	0.139685	0.142978	0.999675	0.996435
Diabetes mellitus [Global]	Male	0-6 days	20-24 years	0.329888	0.502708	0.232143	0.242337	0.99727	0.994199
Diabetes mellitus [Data Rich]	Female	0-6 days	20-24 years	0.193579	0.283924	0.117672	0.146056	0.99999	0.999931
Diabetes mellitus [Data Rich]	Male	0-6 days	20-24 years	0.301135	0.410406	0.224001	0.301424	0.997173	0.99657
Diabetes mellitus [Data Rich]	Male	25-29 years	95+ years	0.166848	0.213468	0.126697	0.146121	0.999758	0.999137
Diabetes mellitus [Data Rich]	Female	25-29 years	95+ years	0.176545	0.231843	0.132234	0.155994	0.999681	0.998812
Diabetes mellitus [Global]	Male	25-29 years	95+ years	0.195934	0.340288	0.14455	0.150474	0.999624	0.985024
Diabetes mellitus [Global]	Female	25-29 years	95+ years	0.207287	0.356249	0.151212	0.157425	0.999492	0.984314
Acute glomerulonephritis [Data Rich]	Female	28-364 days	95+ years	0.792455	1.28781	0.61919	0.819368	0.803237	0.798877
Acute glomerulonephritis [Data Rich]	Male	28-364 days	95+ years	0.723751	1.18477	0.54651	0.677276	0.829223	0.824467
Acute glomerulonephritis [Global]	Female	28-364 days	95+ years	0.84856	1.65301	0.642553	0.686654	0.829502	0.808659
Acute glomerulonephritis [Global]	Male	28-364 days	95+ years	0.782474	1.68243	0.573639	0.596198	0.848428	0.806596
Chronic kidney disease [Data Rich]	Female	28-364 days	95+ years	0.185228	0.246711	0.136501	0.163456	0.999554	0.99884
Chronic kidney disease [Data Rich]	Male	28-364 days	95+ years	0.17932	0.232099	0.136321	0.156455	0.999858	0.999312
Chronic kidney disease [Global]	Female	28-364 days	95+ years	0.219249	0.373522	0.153203	0.159798	0.999262	0.98255
Chronic kidney disease [Global]	Male	28-364 days	95+ years	0.212661	0.345633	0.149419	0.153892	0.999595	0.986844
Urinary diseases and male infertility [Data Rich]	Male	0-6 days	95+ years	0.250428	0.351144	0.177758	0.228228	0.998982	0.998195

				Predictive validity					
Cause	Sex	Age start	Age end	RMSE in	RMSE out	Trend in	Trend out	Coverage in	Coverage out
Urinary diseases and male infertility [Data Rich]	Female	0-6 days	95+ years	0.222735	0.293697	0.156563	0.178888	0.999286	0.998503
Urinary diseases and male infertility [Global]	Male	0-6 days	95+ years	0.28778	0.520063	0.19603	0.203661	0.998814	0.987936
Urinary diseases and male infertility [Global]	Female	0-6 days	95+ years	0.268818	0.505346	0.177372	0.183334	0.998919	0.988395
Interstitial nephritis and urinary tract infections [Data Rich]	Female	0-6 days	95+ years	0.326256	0.386483	0.171078	0.194525	0.999355	0.998778
Interstitial nephritis and urinary tract infections [Data Rich]	Male	0-6 days	95+ years	0.357067	0.443525	0.18872	0.242126	0.998863	0.998294
Interstitial nephritis and urinary tract infections [Global]	Female	0-6 days	95+ years	0.374528	0.548774	0.187308	0.192103	0.999374	0.988412
Interstitial nephritis and urinary tract infections [Global]	Male	0-6 days	95+ years	0.40746	0.622427	0.207561	0.215229	0.998957	0.986777
Urolithiasis [Data Rich]	Female	5-9 years	95+ years	0.619467	0.888695	0.371183	0.492202	0.918249	0.922618
Urolithiasis [Global]	Male	5-9 years	95+ years	0.604745	1.05492	0.37646	0.397448	0.936221	0.91731
Urolithiasis [Global]	Female	5-9 years	95+ years	0.633857	1.10156	0.393251	0.418754	0.928722	0.906192
Urolithiasis [Data Rich]	Male	5-9 years	95+ years	0.440095	0.725416	0.335799	0.457612	0.929454	0.930176
Other urinary diseases [Data Rich]	Male	0-6 days	95+ years	0.331331	0.585632	0.219476	0.283436	0.976736	0.978299
Other urinary diseases [Data Rich]	Female	0-6 days	95+ years	0.510095	0.83564	0.273753	0.324016	0.978805	0.978816
Other urinary diseases [Global]	Female	0-6 days	95+ years	0.623407	0.952645	0.35546	0.373652	0.979225	0.957914
Other urinary diseases [Global]	Male	0-6 days	95+ years	0.438613	0.715258	0.264672	0.277757	0.982053	0.97306
Gynecological diseases [Data Rich]	Female	15-19 years	95+ years	0.389237	0.478005	0.290432	0.323182	0.996891	0.994365
Gynecological diseases [Global]	Female	15-19 years	95+ years	0.453469	0.800731	0.334038	0.336087	0.995176	0.970261
Uterine fibroids [Data Rich]	Female	15-19 years	95+ years	0.657055	0.999418	0.527788	0.514294	0.789723	0.788412
Uterine fibroids [Global]	Female	15-19 years	95+ years	0.749265	1.52289	0.58874	0.593656	0.932599	0.875057
Polycystic ovarian syndrome [Data Rich]	Female	15-19 years	50-54 years	0.369152	0.753118	0.268576	0.272653	0.997608	0.988782
Polycystic ovarian syndrome [Global]	Female	15-19 years	50-54 years	0.422703	0.775957	0.29793	0.304533	0.998345	0.993092
Endometriosis [Data Rich]	Female	15-19 years	50-54 years	1.29095	1.85165	1.09805	1.18791	0.939392	0.873998
Endometriosis [Global]	Female	15-19 years	50-54 years	1.42378	2.14531	1.21102	1.28172	0.980148	0.952155
Genital prolapse [Data Rich]	Female	15-19 years	95+ years	0.724768	1.12523	0.584496	0.629083	0.948294	0.94115
Genital prolapse [Global]	Female	15-19 years	95+ years	0.856787	1.55145	0.692829	0.660015	0.950204	0.901664
Other gynecological diseases [Data Rich]	Female	15-19 years	95+ years	0.410418	0.569712	0.322516	0.32659	0.996204	0.992537
Other gynecological diseases [Global]	Female	15-19 years	95+ years	0.517627	0.935619	0.376766	0.368315	0.994708	0.956519
Hemoglobinopathies and hemolytic anemias [Data Rich]	Male	0-6 days	5-9 years	0.236806	0.329948	0.1842	0.207962	0.99882	0.997989
Hemoglobinopathies and hemolytic anemias [Data Rich]	Female	0-6 days	5-9 years	0.204503	0.288166	0.151575	0.155291	0.99944	0.999365
Hemoglobinopathies and hemolytic anemias [Data Rich]	Male	10-14 years	95+ years	0.16513	0.244019	0.126773	0.1494	0.999958	0.999922
Hemoglobinopathies and hemolytic anemias [Data Rich]	Female	10-14 years	95+ years	0.209186	0.329281	0.127559	0.154175	0.999989	0.999953
Hemoglobinopathies and hemolytic anemias [Global]	Male	0-6 days	5-9 years	0.287723	0.457527	0.186143	0.186355	0.99949	0.995269
Hemoglobinopathies and hemolytic anemias [Global]	Female	0-6 days	5-9 years	0.278753	0.453452	0.164557	0.172615	0.999798	0.99666
Hemoglobinopathies and hemolytic anemias [Global]	Male	10-14 years	95+ years	0.26492	0.437046	0.165127	0.155548	0.999583	0.992692
Hemoglobinopathies and hemolytic anemias [Global]	Female	10-14 years	95+ years	0.236803	0.413363	0.157181	0.161496	0.999655	0.992728
Endocrine, metabolic, blood, and immune disorders [Data Rich]	Female	0-6 days	95+ years	0.265866	0.331011	0.193388	0.211396	0.999638	0.998794
Endocrine, metabolic, blood, and immune disorders [Data Rich]	Male	0-6 days	95+ years	0.218761	0.280283	0.151892	0.173689	0.999625	0.999057
Endocrine, metabolic, blood, and immune disorders [Global]	Female	0-6 days	95+ years	0.311592	0.493551	0.220735	0.222346	0.999352	0.989911
Endocrine, metabolic, blood, and immune disorders [Global]	Male	0-6 days	95+ years	0.288038	0.467568	0.189101	0.192024	0.999174	0.989914
Musculoskeletal disorders [Data Rich]	Male	5-9 years	95+ years	0.202388	0.303974	0.150613	0.174564	0.999892	0.999696
Musculoskeletal disorders [Data Rich]	Female	5-9 years	95+ years	0.191089	0.320391	0.140109	0.160872	0.999756	0.999483
Musculoskeletal disorders [Global]	Male	5-9 years	95+ years	0.24754	0.427225	0.171935	0.174746	0.999809	0.993792
Musculoskeletal disorders [Global]	Female	5-9 years	95+ years	0.24877	0.463854	0.162844	0.168315	0.999586	0.987188
Rheumatoid arthritis [Data Rich]	Female	5-9 years	95+ years	0.267372	0.461031	0.192862	0.255179	0.995251	0.994492
Rheumatoid arthritis [Data Rich]	Male	5-9 years	95+ years	0.313475	0.524108	0.232578	0.303335	0.982688	0.982442
Rheumatoid arthritis [Global]	Male	5-9 years	95+ years	0.3743	0.74528	0.262444	0.277418	0.985151	0.970339
Rheumatoid arthritis [Global]	Female	5-9 years	95+ years	0.325777	0.651975	0.222855	0.231085	0.994997	0.975822
Other musculoskeletal disorders [Data Rich]	Male	5-9 years	95+ years	0.247842	0.359328	0.163094	0.18294	0.99991	0.999818
Other musculoskeletal disorders [Data Rich]	Female	5-9 years	95+ years	0.238984	0.419361	0.157167	0.181308	0.999799	0.99946
Other musculoskeletal disorders [Global]	Male	5-9 years	95+ years	0.276621	0.477127	0.181974	0.186252	0.999824	0.996815
Other musculoskeletal disorders [Global]	Female	5-9 years	95+ years	0.297846	0.544271	0.179636	0.182208	0.999612	0.990339
Congenital birth defects [Data Rich]	Male	0-6 days	65-69 years	0.187728	0.307926	0.130594	0.142968	0.999768	0.999494
Congenital birth defects [Data Rich]	Female	0-6 days	65-69 years	0.202998	0.317523	0.149479	0.154752	0.999295	0.999153
Congenital birth defects [Global]	Male	0-6 days	65-69 years	0.213557	0.366744	0.14413	0.145106	0.999656	0.994776
Congenital birth defects [Global]	Female	0-6 days	65-69 years	0.222667	0.386215	0.16262	0.169763	0.999273	0.99608
Neural tube defects [Data Rich]	Male	0-6 days	65-69 years	0.511074	0.684196	0.410569	0.436517	0.991879	0.98864
Neural tube defects [Data Rich]	Female	0-6 days	65-69 years	0.562295	0.769694	0.450508	0.476537	0.844016	0.833556
Neural tube defects [Global]	Male	0-6 days	65-69 years	0.614659	1.27809	0.487802	0.499774	0.894512	0.870196
Neural tube defects [Global]	Female	0-6 days	65-69 years	0.664811	1.30712	0.547394	0.527378	0.860297	0.84598
Congenital heart anomalies [Data Rich]	Male	0-6 days	65-69 years	0.253161	0.344211	0.14564	0.160504	0.999748	0.999547
Congenital heart anomalies [Data Rich]	Female	0-6 days	65-69 years	0.263302	0.350342	0.17974	0.183229	0.998945	0.998806
Congenital heart anomalies [Global]	Male	0-6 days	65-69 years	0.264809	0.450742	0.161561	0.164973	0.999714	0.99575
Congenital heart anomalies [Global]	Female	0-6 days	65-69 years	0.278649	0.521451	0.200541	0.205942	0.999056	0.996225
Orofacial clefts [Data Rich]	Male	0-6 days	1-4 years	1.01661	1.45521	0.748075	0.806207	0.985239	0.941838
Orofacial clefts [Global]	Male	0-6 days	1-4 years	1.03288	1.59048	0.799884	0.829625	0.986131	0.957288
Orofacial clefts [Data Rich]	Female	0-6 days	1-4 years	0.976068	1.38873	0.739238	0.798592	0.981561	0.936877
Orofacial clefts [Global]	Female	0-6 days	1-4 years	1.08986	1.63418	0.862421	0.867461	0.9067	0.88513
Down syndrome [Data Rich]	Male	0-6 days	65-69 years	0.369496	0.591935	0.234815	0.246582	0.998316	0.997758
Down syndrome [Data Rich]	Female	0-6 days	65-69 years	0.428531	0.652201	0.285806	0.293699	0.996402	0.995455
Down syndrome [Global]	Male	0-6 days	65-69 years	0.408147	0.655466	0.248661	0.251603	0.998333	0.990867
Down syndrome [Global]	Female	0-6 days	65-69 years	0.445675	0.744969	0.294358	0.302952	0.996369	0.98883
Other chromosomal abnormalities [Data Rich]	Male	0-6 days	65-69 years	0.76776	1.16236	0.524335	0.543793	0.989423	0.986424
Other chromosomal abnormalities [Data Rich]	Female	0-6 days	65-69 years	0.617539	1.0535	0.454229	0.517257	0.984023	0.97961
Other chromosomal abnormalities [Global]	Male	0-6 days	65-69 years	0.857035	1.88052	0.561501	0.549064	0.989145	0.87986
Other chromosomal abnormalities [Global]	Female	0-6 days	65-69 years	0.690025	1.89563	0.513512	0.535652	0.982535	0.848516
Congenital musculoskeletal and limb anomalies [Data Rich]	Male	0-6 days	65-69 years	0.399572	0.555865	0.297401	0.290005	0.996788	0.995107
Congenital musculoskeletal and limb anomalies [Data Rich]	Female	0-6 days	65-69 years	0.444637	0.607148	0.318311	0.320391	0.996207	0.993821
Congenital musculoskeletal and limb anomalies [Global]	Male	0-6 days	65-69 years	0.444923	1.09036	0.329353	0.341175	0.996363	0.95561
Congenital musculoskeletal and limb anomalies [Global]	Female	0-6 days	65-69 years	0.482248	1.10349	0.355963	0.358807	0.995572	0.948531
Urogenital congenital anomalies [Data Rich]	Male	0-6 days	65-69 years	0.498322	0.695814	0.239192	0.197908	0.958274	0.945843
Urogenital congenital anomalies [Data Rich]	Female	0-6 days	65-69 years	0.499657	0.624048	0.308999	0.263407	0.91315	0.903008
Urogenital congenital anomalies [Global]	Female	0-6 days	65-69 years	0.484296	0.731713	0.319216	0.305618	0.932251	0.921341
Urogenital congenital anomalies [Global]	Male	0-6 days	65-69 years	0.396764	0.729879	0.255343	0.251628	0.96717	0.952729
Digestive congenital anomalies [Data Rich]	Female	0-6 days	65-69 years	0.389235	0.555576	0.294019	0.309746	0.8883	0.883438
Digestive congenital anomalies [Data Rich]	Male	0-6 days	65-69 years	0.430175	0.556295	0.272384	0.282597	0.913896	0.909876
Digestive congenital anomalies [Global]	Male	0-6 days	65-69 years	0.438261	1.15703	0.311106	0.32333	0.996643	0.93733
Digestive congenital anomalies [Global]	Female	0-6 days	65-69 years	0.467432	1.08748	0.348916	0.349403	0.995626	0.948371
Other congenital birth defects [Data Rich]	Female	0-6 days	65-69 years	0.294219	0.393813	0.206031	0.22779	0.997847	0.997521
Other congenital birth defects [Data Rich]	Male	0-6 days	65-69 years	0.269838	0.376572	0.193135	0.226166	0.99818	0.997833

				Predictive validity					
Cause	Sex	Age start	Age end	RMSE in	RMSE out	Trend in	Trend out	Coverage in	Coverage out
Other congenital birth defects [Global]	Male	0-6 days	65-69 years	0.285221	0.511445	0.201802	0.205283	0.998218	0.992513
Other congenital birth defects [Global]	Female	0-6 days	65-69 years	0.317722	0.537403	0.212073	0.21541	0.997938	0.991635
Skin and subcutaneous diseases [Global]	Male	28-364 days	95+ years	0.3672	0.650491	0.184491	0.193184	0.998991	0.981999
Skin and subcutaneous diseases [Data Rich]	Male	28-364 days	95+ years	0.266632	0.375883	0.150205	0.168012	0.999584	0.999295
Skin and subcutaneous diseases [Global]	Female	28-364 days	95+ years	0.415095	0.673493	0.189984	0.20069	0.997971	0.979738
Skin and subcutaneous diseases [Data Rich]	Female	28-364 days	95+ years	0.33827	0.449693	0.161021	0.173859	0.998784	0.998457
Cellulitis [Data Rich]	Male	28-364 days	95+ years	0.753763	1.0827	0.33351	0.397886	0.931212	0.935022
Cellulitis [Data Rich]	Female	28-364 days	95+ years	0.788799	1.15588	0.354573	0.455775	0.931552	0.936892
Cellulitis [Global]	Male	28-364 days	95+ years	0.790628	1.11121	0.364602	0.379862	0.936772	0.920542
Cellulitis [Global]	Female	28-364 days	95+ years	0.776284	1.1262	0.387978	0.407421	0.932359	0.91306
Pyoderma [Data Rich]	Female	0-6 days	95+ years	0.336027	0.663116	0.156225	0.177377	0.997513	0.98434
Pyoderma [Data Rich]	Male	0-6 days	95+ years	0.290649	0.614129	0.151013	0.180725	0.998859	0.989874
Pyoderma [Global]	Male	0-6 days	95+ years	0.273788	0.624938	0.180327	0.197251	0.998866	0.988581
Pyoderma [Global]	Female	0-6 days	95+ years	0.33107	0.69326	0.181422	0.191996	0.997678	0.982623
Decubitus ulcer [Global]	Male	1-4 years	95+ years	0.50257	0.941445	0.345863	0.357162	0.880411	0.865533
Decubitus ulcer [Global]	Female	1-4 years	95+ years	0.581897	1.02705	0.361056	0.370252	0.860832	0.857385
Decubitus ulcer [Data Rich]	Male	1-4 years	95+ years	0.413522	0.71087	0.299495	0.38014	0.865576	0.869143
Decubitus ulcer [Data Rich]	Female	1-4 years	95+ years	0.433093	0.770629	0.306077	0.421206	0.848806	0.856264
Other skin and subcutaneous diseases [Data Rich]	Male	28-364 days	95+ years	0.513794	0.699422	0.320919	0.360791	0.907632	0.909962
Other skin and subcutaneous diseases [Data Rich]	Female	28-364 days	95+ years	0.448461	0.719114	0.33847	0.425333	0.927983	0.931747
Other skin and subcutaneous diseases [Global]	Male	28-364 days	95+ years	0.478767	0.829998	0.356372	0.361161	0.914516	0.905996
Other skin and subcutaneous diseases [Global]	Female	28-364 days	95+ years	0.522311	0.855571	0.376522	0.395195	0.933857	0.92236
Sudden infant death syndrome [Global]	Male	7-27 days	28-364 days	0.339839	0.601128	0.244098	0.260482	0.999345	0.978879
Sudden infant death syndrome [Data Rich]	Male	7-27 days	28-364 days	0.300618	0.552031	0.217784	0.270153	0.999511	0.996211
Sudden infant death syndrome [Global]	Female	7-27 days	28-364 days	0.441559	0.691189	0.325347	0.345983	0.996276	0.980519
Sudden infant death syndrome [Data Rich]	Female	7-27 days	28-364 days	0.396941	0.649331	0.29697	0.380264	0.996363	0.992083
Transport injuries [Data Rich]	Female	0-6 days	95+ years	0.178093	0.228732	0.13385	0.155904	0.999531	0.999029
Transport injuries [Data Rich]	Male	0-6 days	95+ years	0.16665	0.212812	0.125194	0.145828	0.999288	0.998489
Transport injuries [Global]	Male	0-6 days	95+ years	0.266235	0.369045	0.257365	0.245403	0.998807	0.988513
Transport injuries [Global]	Female	0-6 days	95+ years	0.23338	0.356729	0.191041	0.192905	0.999187	0.990441
Road injuries [Global]	Male	0-6 days	95+ years	0.202111	0.324708	0.142595	0.145277	0.99922	0.989124
Road injuries [Data Rich]	Male	0-6 days	95+ years	0.170057	0.221353	0.127909	0.149485	0.999297	0.998695
Road injuries [Global]	Female	0-6 days	95+ years	0.225393	0.348102	0.155683	0.161219	0.999258	0.992833
Road injuries [Data Rich]	Female	0-6 days	95+ years	0.181397	0.235701	0.138397	0.159193	0.999462	0.998812
Pedestrian road injuries [Global]	Female	0-6 days	95+ years	0.2531	0.417919	0.186479	0.193764	0.99872	0.98956
Pedestrian road injuries [Data Rich]	Female	0-6 days	95+ years	0.221123	0.338561	0.167257	0.195877	0.998953	0.998112
Pedestrian road injuries [Global]	Male	0-6 days	95+ years	0.217427	0.36672	0.154149	0.153652	0.998606	0.988732
Pedestrian road injuries [Data Rich]	Male	0-6 days	95+ years	0.190473	0.286592	0.132116	0.154124	0.999445	0.998841
Cyclist road injuries [Data Rich]	Female	1-4 years	95+ years	0.338506	0.568756	0.24029	0.30528	0.997842	0.99679
Cyclist road injuries [Global]	Male	1-4 years	95+ years	0.256014	0.519827	0.175462	0.186021	0.999878	0.992249
Cyclist road injuries [Data Rich]	Male	1-4 years	95+ years	0.221866	0.393751	0.159234	0.196157	0.999958	0.999839
Cyclist road injuries [Global]	Female	1-4 years	95+ years	0.355166	0.635939	0.25048	0.267805	0.99803	0.988668
Motorcyclist road injuries [Data Rich]	Female	0-6 days	95+ years	0.304692	0.544763	0.191574	0.228659	0.999427	0.99887
Motorcyclist road injuries [Global]	Male	0-6 days	95+ years	0.276041	0.523365	0.174247	0.181648	0.999735	0.991251
Motorcyclist road injuries [Data Rich]	Male	0-6 days	95+ years	0.217226	0.423942	0.147619	0.187707	0.999862	0.999352
Motorcyclist road injuries [Global]	Female	0-6 days	95+ years	0.31903	0.633083	0.214623	0.224927	0.999124	0.992317
Motor vehicle road injuries [Global]	Female	0-6 days	95+ years	0.248079	0.382929	0.166578	0.1664	0.998962	0.992992
Motor vehicle road injuries [Data Rich]	Female	0-6 days	95+ years	0.22088	0.308966	0.153298	0.166921	0.999075	0.998435
Motor vehicle road injuries [Global]	Male	0-6 days	95+ years	0.255246	0.372221	0.151574	0.156652	0.999219	0.991422
Motor vehicle road injuries [Data Rich]	Male	0-6 days	95+ years	0.213548	0.306557	0.139843	0.160713	0.999358	0.998911
Other road injuries [Data Rich]	Female	0-6 days	95+ years	0.657277	0.957272	0.387137	0.525825	0.985101	0.976001
Other road injuries [Global]	Male	0-6 days	95+ years	0.611513	0.955473	0.367719	0.430569	0.981604	0.94532
Other road injuries [Data Rich]	Male	0-6 days	95+ years	0.596521	1.05509	0.351374	0.544563	0.984379	0.964251
Other road injuries [Global]	Female	0-6 days	95+ years	0.641589	0.956742	0.395337	0.449469	0.98882	0.97059
Other transport injuries [Global]	Female	0-6 days	95+ years	0.304012	0.578958	0.230219	0.250652	0.999029	0.991105
Other transport injuries [Data Rich]	Female	0-6 days	95+ years	0.268641	0.405742	0.211648	0.265306	0.999102	0.998299
Other transport injuries [Data Rich]	Male	0-6 days	95+ years	0.257986	0.432871	0.186003	0.239068	0.998787	0.998015
Other transport injuries [Global]	Male	0-6 days	95+ years	0.285111	0.541896	0.201088	0.217991	0.9987	0.987271
Unintentional injuries [Global]	Female	28-364 days	95+ years	0.265627	0.429182	0.221478	0.2393	0.996437	0.985499
Unintentional injuries [Global]	Male	28-364 days	95+ years	0.247703	0.361333	0.201256	0.204671	0.996857	0.986923
Unintentional injuries [Data Rich]	Male	28-364 days	95+ years	0.14174	0.182504	0.112684	0.147915	0.999221	0.998382
Unintentional injuries [Data Rich]	Female	28-364 days	95+ years	0.151602	0.197651	0.119897	0.159946	0.999399	0.998939
Falls [Global]	Female	0-6 days	95+ years	0.342582	0.51199	0.326434	0.32064	0.998223	0.990573
Falls [Data Rich]	Female	0-6 days	95+ years	0.222813	0.273661	0.153781	0.175941	0.999324	0.998706
Falls [Global]	Male	0-6 days	95+ years	0.315218	0.435895	0.293162	0.291023	0.998106	0.989895
Falls [Data Rich]	Male	0-6 days	95+ years	0.214642	0.263607	0.152777	0.176564	0.999271	0.998738
Drowning [Global]	Female	0-6 days	95+ years	0.265501	0.46815	0.188149	0.192566	0.999434	0.993099
Drowning [Data Rich]	Female	0-6 days	95+ years	0.197504	0.270896	0.148256	0.179391	0.999593	0.999243
Drowning [Global]	Male	0-6 days	95+ years	0.256468	0.411489	0.202912	0.206242	0.999117	0.991675
Drowning [Data Rich]	Male	0-6 days	95+ years	0.192032	0.245323	0.146852	0.173137	0.999226	0.998719
Fire, heat, and hot substances [Global]	Female	0-6 days	95+ years	0.278539	0.427835	0.178517	0.177412	0.999424	0.99497
Fire, heat, and hot substances [Data Rich]	Female	0-6 days	95+ years	0.211045	0.261382	0.148898	0.174291	0.999762	0.999425
Fire, heat, and hot substances [Global]	Male	0-6 days	95+ years	0.265391	0.400118	0.168573	0.169865	0.999414	0.99661
Fire, heat, and hot substances [Data Rich]	Male	0-6 days	95+ years	0.180817	0.225133	0.134313	0.15739	0.999809	0.999587
Poisonings [Global]	Female	0-6 days	95+ years	0.363913	0.558864	0.325653	0.317975	0.998704	0.99503
Poisonings [Data Rich]	Female	0-6 days	95+ years	0.248195	0.339242	0.159465	0.195041	0.99973	0.999611
Poisonings [Global]	Male	0-6 days	95+ years	0.38743	0.586584	0.345665	0.328941	0.998811	0.993562
Poisonings [Data Rich]	Male	0-6 days	95+ years	0.23173	0.32102	0.153026	0.176181	0.999896	0.99965
Exposure to mechanical forces [Global]	Female	0-6 days	95+ years	0.277305	0.485834	0.200585	0.200931	0.998376	0.992949
Exposure to mechanical forces [Data Rich]	Female	0-6 days	95+ years	0.237379	0.350666	0.176189	0.202397	0.998436	0.997593
Exposure to mechanical forces [Global]	Male	0-6 days	95+ years	0.26981	0.482224	0.197341	0.20494	0.998815	0.987415
Exposure to mechanical forces [Data Rich]	Male	0-6 days	95+ years	0.230706	0.324934	0.173637	0.196758	0.999055	0.997666
Unintentional firearm injuries [Global]	Female	0-6 days	95+ years	0.330253	0.585059	0.223999	0.238007	0.997656	0.991053
Unintentional firearm injuries [Data Rich]	Female	0-6 days	95+ years	0.287536	0.425876	0.197302	0.26113	0.99814	0.997585
Unintentional firearm injuries [Global]	Male	0-6 days	95+ years	0.350046	0.62231	0.223178	0.240928	0.998172	0.988233
Unintentional firearm injuries [Data Rich]	Male	0-6 days	95+ years	0.271991	0.391207	0.18968	0.222404	0.998608	0.997554
Unintentional suffocation [Global]	Female	0-6 days	95+ years	0.666771	1.11923	0.503752	0.490551	0.941473	0.938713
Unintentional suffocation [Data Rich]	Female	0-6 days	95+ years	0.562645	0.95589	0.362665	0.332349	0.929299	0.928815
Unintentional suffocation [Global]	Male	0-6 days	95+ years	0.536916	0.840867	0.353135	0.336339	0.986825	0.976978

				Predictive validity					
Cause	Sex	Age start	Age end	RMSE in	RMSE out	Trend in	Trend out	Coverage in	Coverage out
Unintentional suffocation [Data Rich]	Male	0-6 days	95+ years	0.485744	0.616624	0.269508	0.296729	0.990117	0.988359
Other exposure to mechanical forces [Global]	Female	0-6 days	95+ years	0.310983	0.52893	0.195088	0.206481	0.999236	0.994664
Other exposure to mechanical forces [Data Rich]	Female	0-6 days	95+ years	0.272069	0.350792	0.168325	0.190263	0.9993	0.998881
Other exposure to mechanical forces [Global]	Male	0-6 days	95+ years	0.27564	0.459333	0.19024	0.192902	0.999035	0.993449
Other exposure to mechanical forces [Data Rich]	Male	0-6 days	95+ years	0.236078	0.305896	0.165067	0.180858	0.999273	0.998735
Adverse effects of medical treatment [Global]	Female	0-6 days	95+ years	0.277804	0.422352	0.170211	0.175682	0.99914	0.991971
Adverse effects of medical treatment [Data Rich]	Female	0-6 days	95+ years	0.25697	0.310226	0.151931	0.166847	0.999284	0.998475
Adverse effects of medical treatment [Global]	Male	0-6 days	95+ years	0.284293	0.448456	0.170906	0.179479	0.998995	0.99214
Adverse effects of medical treatment [Data Rich]	Male	0-6 days	95+ years	0.270953	0.339952	0.156556	0.169666	0.999153	0.998578
Animal contact [Global]	Female	0-6 days	95+ years	0.45516	0.727816	0.291208	0.311011	0.997197	0.988248
Animal contact [Data Rich]	Female	0-6 days	95+ years	0.369492	0.530666	0.267072	0.346026	0.99729	0.996424
Animal contact [Global]	Male	0-6 days	95+ years	0.324399	0.659801	0.230414	0.240986	0.998432	0.991142
Animal contact [Data Rich]	Male	0-6 days	95+ years	0.335072	0.436601	0.204842	0.246205	0.998691	0.998418
Venomous animal contact [Global]	Female	0-6 days	95+ years	0.697947	1.04033	0.396417	0.427921	0.955388	0.934704
Venomous animal contact [Data Rich]	Female	0-6 days	95+ years	0.580129	0.856224	0.376524	0.476426	0.945998	0.948084
Venomous animal contact [Global]	Male	0-6 days	95+ years	0.516482	0.951022	0.382786	0.393778	0.967797	0.951414
Venomous animal contact [Data Rich]	Male	0-6 days	95+ years	0.615749	0.884281	0.358758	0.462012	0.961706	0.963653
Non-venomous animal contact [Global]	Female	0-6 days	95+ years	0.40422	0.696076	0.295515	0.310348	0.996634	0.990183
Non-venomous animal contact [Data Rich]	Female	0-6 days	95+ years	0.403473	0.583058	0.275472	0.339343	0.996799	0.995931
Non-venomous animal contact [Global]	Male	0-6 days	95+ years	0.326049	0.660361	0.23192	0.237603	0.998455	0.993782
Non-venomous animal contact [Data Rich]	Male	0-6 days	95+ years	0.28393	0.435882	0.21254	0.240679	0.998475	0.997935
Foreign body [Global]	Female	0-6 days	95+ years	0.246435	0.437991	0.170271	0.173202	0.999156	0.989383
Foreign body [Data Rich]	Female	0-6 days	95+ years	0.200456	0.301515	0.147047	0.175785	0.999796	0.999281
Foreign body [Global]	Male	0-6 days	95+ years	0.242082	0.430659	0.158778	0.161405	0.999432	0.98959
Foreign body [Data Rich]	Male	0-6 days	95+ years	0.183536	0.272549	0.134847	0.156308	0.999771	0.999405
Pulmonary aspiration and foreign body in airway [Global]	Female	0-6 days	95+ years	0.303461	0.476433	0.177523	0.178453	0.999589	0.992601
Pulmonary aspiration and foreign body in airway [Data Rich]	Female	0-6 days	95+ years	0.264922	0.389206	0.163327	0.181083	0.999647	0.999303
Pulmonary aspiration and foreign body in airway [Global]	Male	0-6 days	95+ years	0.264978	0.439347	0.155873	0.15951	0.999831	0.992446
Pulmonary aspiration and foreign body in airway [Data Rich]	Male	0-6 days	95+ years	0.245738	0.342596	0.143368	0.155372	0.99992	0.999766
Foreign body in other body part [Global]	Female	0-6 days	95+ years	0.516905	0.847357	0.329246	0.340028	0.976911	0.962962
Foreign body in other body part [Data Rich]	Female	0-6 days	95+ years	0.532832	0.771815	0.2963	0.374276	0.973181	0.975492
Foreign body in other body part [Global]	Male	0-6 days	95+ years	0.455273	0.801863	0.263772	0.276564	0.989795	0.97621
Foreign body in other body part [Data Rich]	Male	0-6 days	95+ years	0.347543	0.57796	0.233789	0.280881	0.986257	0.986669
Other unintentional injuries [Data Rich]	Female	0-6 days	95+ years	0.282628	0.405062	0.208497	0.243197	0.999075	0.998181
Other unintentional injuries [Global]	Male	0-6 days	95+ years	0.333847	0.600764	0.226617	0.238384	0.99712	0.98506
Other unintentional injuries [Data Rich]	Male	0-6 days	95+ years	0.24534	0.367449	0.178495	0.227869	0.999152	0.998461
Other unintentional injuries [Global]	Female	0-6 days	95+ years	0.404994	0.681078	0.255582	0.253189	0.99704	0.984534
Self-harm and interpersonal violence [Global]	Female	0-6 days	95+ years	0.235051	0.401327	0.170803	0.17549	0.999207	0.990475
Self-harm and interpersonal violence [Data Rich]	Female	0-6 days	95+ years	0.187094	0.240753	0.142445	0.169281	0.999232	0.998628
Self-harm and interpersonal violence [Global]	Male	0-6 days	95+ years	0.25566	0.378724	0.1656	0.167687	0.998779	0.985871
Self-harm and interpersonal violence [Data Rich]	Male	0-6 days	95+ years	0.210671	0.250648	0.145579	0.165786	0.998972	0.998037
Self-harm [Global]	Female	10-14 years	95+ years	0.217656	0.397101	0.157745	0.162568	0.999368	0.990863
Self-harm [Data Rich]	Female	10-14 years	95+ years	0.17629	0.239874	0.132643	0.164204	0.999455	0.998917
Self-harm [Global]	Male	10-14 years	95+ years	0.208467	0.349662	0.155916	0.155504	0.999331	0.985926
Self-harm [Data Rich]	Male	10-14 years	95+ years	0.177784	0.217925	0.125122	0.144276	0.999538	0.999159
Self-harm by firearm [Global]	Female	10-14 years	95+ years	0.268421	0.602681	0.188694	0.185632	0.995432	0.983562
Self-harm by firearm [Data Rich]	Female	10-14 years	95+ years	0.21662	0.397634	0.162961	0.178553	0.995318	0.995558
Self-harm by firearm [Global]	Male	10-14 years	95+ years	0.264012	0.583163	0.162403	0.16445	0.999093	0.973501
Self-harm by firearm [Data Rich]	Male	10-14 years	95+ years	0.215023	0.384896	0.151941	0.179177	0.998654	0.998623
Self-harm by other specified means [Global]	Female	10-14 years	95+ years	0.203918	0.401623	0.149391	0.153881	0.999643	0.990736
Self-harm by other specified means [Data Rich]	Female	10-14 years	95+ years	0.177384	0.302742	0.131997	0.158796	0.999652	0.999218
Self-harm by other specified means [Global]	Male	10-14 years	95+ years	0.185924	0.353671	0.138638	0.142126	0.999613	0.990342
Self-harm by other specified means [Data Rich]	Male	10-14 years	95+ years	0.167454	0.253534	0.124347	0.141101	0.999743	0.999384
Interpersonal violence [Global]	Female	0-6 days	95+ years	0.315554	0.461772	0.22912	0.226349	0.998981	0.99146
Interpersonal violence [Data Rich]	Female	0-6 days	95+ years	0.24386	0.29461	0.18662	0.233847	0.999756	0.999333
Interpersonal violence [Global]	Male	0-6 days	95+ years	0.311602	0.514929	0.223365	0.22344	0.998122	0.984774
Interpersonal violence [Data Rich]	Male	0-6 days	95+ years	0.293886	0.330614	0.181077	0.223183	0.999468	0.998711
Physical violence by firearm [Global]	Female	0-6 days	95+ years	0.4281	0.657151	0.26517	0.269069	0.996552	0.987641
Physical violence by firearm [Data Rich]	Female	0-6 days	95+ years	0.377312	0.527261	0.241881	0.301523	0.99741	0.996764
Physical violence by firearm [Global]	Male	0-6 days	95+ years	0.449719	0.754667	0.234939	0.228238	0.996239	0.978307
Physical violence by firearm [Data Rich]	Male	0-6 days	95+ years	0.356808	0.542516	0.197151	0.220232	0.997013	0.996687
Physical violence by sharp object [Global]	Female	0-6 days	95+ years	0.316043	0.510877	0.200604	0.199832	0.999321	0.99571
Physical violence by sharp object [Data Rich]	Female	0-6 days	95+ years	0.253748	0.369849	0.166455	0.195789	0.99975	0.99952
Physical violence by sharp object [Global]	Male	0-6 days	95+ years	0.303462	0.521764	0.190015	0.193157	0.999227	0.992199
Physical violence by sharp object [Data Rich]	Male	0-6 days	95+ years	0.24852	0.358879	0.159806	0.188557	0.999699	0.999539
Physical violence by other means [Global]	Female	0-6 days	95+ years	0.256265	0.420103	0.169572	0.174644	0.999652	0.99665
Physical violence by other means [Data Rich]	Female	0-6 days	95+ years	0.207867	0.300267	0.148057	0.176236	0.999915	0.999815
Physical violence by other means [Global]	Male	0-6 days	95+ years	0.254297	0.472236	0.163508	0.172898	0.999602	0.992736
Physical violence by other means [Data Rich]	Male	0-6 days	95+ years	0.309159	0.39638	0.151173	0.179453	0.999909	0.999774
Environmental heat and cold exposure [Global]	Female	0-6 days	95+ years	0.400289	0.702947	0.239279	0.236565	0.997876	0.988749
Environmental heat and cold exposure [Data Rich]	Female	0-6 days	95+ years	0.312333	0.43617	0.196866	0.223056	0.998932	0.998339
Environmental heat and cold exposure [Global]	Male	0-6 days	95+ years	0.342831	0.606037	0.221734	0.22563	0.998592	0.990347
Environmental heat and cold exposure [Data Rich]	Male	0-6 days	95+ years	0.322236	0.405577	0.192466	0.225036	0.998935	0.998271
Acute lymphoid leukemia [Data Rich]	Female	0-6 days	95+ years	0.245042	0.343861	0.199697	0.196121	0.999735	0.998677
Acute lymphoid leukemia [Data Rich]	Male	0-6 days	95+ years	0.232196	0.322463	0.192678	0.180934	0.999027	0.997797
Acute lymphoid leukemia [Global]	Female	0-6 days	95+ years	0.292299	0.430489	0.219397	0.219718	0.999584	0.995845
Acute lymphoid leukemia [Global]	Male	0-6 days	95+ years	0.258168	0.380972	0.20477	0.202271	0.999191	0.996156
Chronic lymphoid leukemia [Data Rich]	Male	15-19 years	95+ years	0.264066	0.422453	0.205004	0.200284	0.998068	0.99624
Chronic lymphoid leukemia [Data Rich]	Female	15-19 years	95+ years	0.229014	0.365306	0.185879	0.188733	0.997496	0.995669
Chronic lymphoid leukemia [Global]	Male	15-19 years	95+ years	0.281442	0.49744	0.21614	0.218752	0.997518	0.992235
Chronic lymphoid leukemia [Global]	Female	15-19 years	95+ years	0.268922	0.428058	0.20953	0.209509	0.997841	0.991753
Acute myeloid leukemia [Data Rich]	Female	0-6 days	95+ years	0.243902	0.359063	0.198629	0.202072	0.999145	0.99819
Acute myeloid leukemia [Data Rich]	Male	0-6 days	95+ years	0.232648	0.331949	0.175921	0.178595	0.999717	0.999322
Acute myeloid leukemia [Global]	Male	0-6 days	95+ years	0.265031	0.360267	0.184561	0.180444	0.99956	0.995375
Acute myeloid leukemia [Global]	Female	0-6 days	95+ years	0.281417	0.410413	0.213481	0.218102	0.999242	0.994128
Chronic myeloid leukemia [Data Rich]	Female	28-364 days	95+ years	0.257258	0.362538	0.219213	0.209664	0.999697	0.998442
Chronic myeloid leukemia [Global]	Female	28-364 days	95+ years	0.295483	0.439364	0.230637	0.214173	0.99951	0.996227
Chronic myeloid leukemia [Global]	Male	28-364 days	95+ years	0.27712	0.436653	0.219288	0.215423	0.999912	0.997674
Chronic myeloid leukemia [Data Rich]	Male	28-364 days	95+ years	0.247795	0.36944	0.21216	0.201106	0.999952	0.999142

				Predictive validity					
Cause	Sex	Age start	Age end	RMSE in	RMSE out	Trend in	Trend out	Coverage in	Coverage out
Non-melanoma skin cancer (squamous-cell carcinoma) [Data Rich]	Male	28-364 days	95+ years	0.154952	0.235392	0.111747	0.134674	0.999782	0.999627
Non-melanoma skin cancer (squamous-cell carcinoma) [Data Rich]	Female	28-364 days	95+ years	0.21458	0.296797	0.160927	0.184583	0.997336	0.997268
Non-melanoma skin cancer (squamous-cell carcinoma) [Global]	Male	28-364 days	95+ years	0.186211	0.307069	0.133081	0.13346	0.999225	0.993893
Non-melanoma skin cancer (squamous-cell carcinoma) [Global]	Female	28-364 days	95+ years	0.27535	0.449328	0.203049	0.202074	0.996456	0.987022
Executions and police conflict [Data Rich]	Female	28-364 days	95+ years	1.02223	1.8106	0.751817	0.849982	0.492976	0.491353
Executions and police conflict [Data Rich]	Male	28-364 days	95+ years	0.956151	1.53927	0.733321	0.763561	0.542338	0.533615
Executions and police conflict [Global]	Female	28-364 days	95+ years	1.26666	2.24823	0.868605	0.899107	0.579692	0.567374
Executions and police conflict [Global]	Male	28-364 days	95+ years	1.08137	2.00266	0.782974	0.803373	0.617898	0.614573
Alcoholic cardiomyopathy [Data Rich]	Male	15-19 years	95+ years	0.409553	0.488649	0.31186	0.187192	0.992843	0.990469
Alcoholic cardiomyopathy [Data Rich]	Female	15-19 years	95+ years	0.354647	0.473835	0.279984	0.192157	0.995654	0.9947
Alcoholic cardiomyopathy [Global]	Male	15-19 years	95+ years	0.451007	0.658827	0.333662	0.305105	0.993453	0.975137
Alcoholic cardiomyopathy [Global]	Female	15-19 years	95+ years	0.386369	0.712305	0.302125	0.293367	0.995187	0.976786
Myocarditis [Data Rich]	Male	0-6 days	95+ years	0.383562	0.516893	0.207055	0.236706	0.999619	0.999248
Myocarditis [Data Rich]	Female	0-6 days	95+ years	0.417659	0.571804	0.258073	0.259341	0.998574	0.997488
Myocarditis [Global]	Male	0-6 days	95+ years	0.419899	0.640487	0.231553	0.238295	0.99946	0.989799
Myocarditis [Global]	Female	0-6 days	95+ years	0.483087	0.743786	0.281852	0.28418	0.998558	0.985813
Other leukemia [Data Rich]	Female	0-6 days	95+ years	0.3508	0.490285	0.278612	0.300191	0.996341	0.993081
Other leukemia [Data Rich]	Male	0-6 days	95+ years	0.282754	0.349516	0.239149	0.221327	0.998039	0.995322
Other leukemia [Global]	Female	0-6 days	95+ years	0.413955	0.570583	0.311448	0.31415	0.997038	0.983931
Other leukemia [Global]	Male	0-6 days	95+ years	0.34794	0.480636	0.275768	0.263252	0.998417	0.987829
Other cardiomyopathy [Data Rich]	Male	0-6 days	95+ years	0.321704	0.433377	0.236585	0.223511	0.996093	0.993778
Other cardiomyopathy [Data Rich]	Female	0-6 days	95+ years	0.300694	0.424815	0.211684	0.226584	0.998302	0.997317
Other cardiomyopathy [Global]	Male	0-6 days	95+ years	0.357059	0.574903	0.257105	0.256145	0.996198	0.975796
Other cardiomyopathy [Global]	Female	0-6 days	95+ years	0.354721	0.592673	0.236575	0.240023	0.998311	0.981594

Note: Only causes modeled in CODEm are included in this table.

Appendix Table 8: Comparison of GBD 2015 and GBD 2016 covariates and level of covariates in cause of death modeling								
			GBD 2015			GBD 2016		
Cause	Sex	Covariate	Level 1	Level 2	Level 3	Level 1	Level 2	Level 3
Tuberculosis	Male	Alcohol (liters per capita)		X		X		
Tuberculosis	Male	Diabetes Fasting Plasma Glucose (mmol/L)	X			X		
Tuberculosis	Male	Diabetes Fasting Plasma Glucose (mmol/L)		X		X		
Tuberculosis	Male	Education (years per capita)			X			X
Tuberculosis	Male	LDI (I$ per capita)			X			X
Tuberculosis	Male	Indoor Air Pollution (All Cooking Fuels)	X				X	
Tuberculosis	Male	Indoor Air Pollution (All Cooking Fuels)		X			X	
Tuberculosis	Male	Population Density (500-1000 ppl/sqkm, proportion)		X			X	
Tuberculosis	Male	Population Density (over 1000 ppl/sqkm, proportion)		X			X	
Tuberculosis	Male	Smoking Prevalence	X			X		
Tuberculosis	Male	Smoking Prevalence		X		X		
Tuberculosis	Male	Log-transformed SEV scalar: TB	X			X		
Tuberculosis	Male	Socio-demographic Index			X			X
Tuberculosis	Female	Alcohol (liters per capita)		X		X		
Tuberculosis	Female	Diabetes Fasting Plasma Glucose (mmol/L)	X			X		
Tuberculosis	Female	Diabetes Fasting Plasma Glucose (mmol/L)		X		X		
Tuberculosis	Female	Education (years per capita)			X			X
Tuberculosis	Female	LDI (I$ per capita)			X			X
Tuberculosis	Female	Indoor Air Pollution (All Cooking Fuels)	X				X	
Tuberculosis	Female	Indoor Air Pollution (All Cooking Fuels)		X			X	
Tuberculosis	Female	Population Density (500-1000 ppl/sqkm, proportion)		X			X	
Tuberculosis	Female	Population Density (over 1000 ppl/sqkm, proportion)		X			X	
Tuberculosis	Female	Smoking Prevalence	X			X		
Tuberculosis	Female	Smoking Prevalence		X		X		
Tuberculosis	Female	Log-transformed SEV scalar: TB	X			X		
Tuberculosis	Female	Socio-demographic Index			X			X
Diarrheal diseases	Male	Education (years per capita)			X			X
Diarrheal diseases	Male	LDI (I$ per capita)			X			X
Diarrheal diseases	Male	Underweight (proportion <2SD weight for age, <5 years)		X		X		
Diarrheal diseases	Male	Population Density (over 1000 ppl/sqkm, proportion)			X			X
Diarrheal diseases	Male	Population Density (under 150 ppl/sqkm, proportion)			X			X
Diarrheal diseases	Male	Sanitation (proportion with access)	X			X		
Diarrheal diseases	Male	Improved Water Source (proportion with access)	X			X		
Diarrheal diseases	Male	Log-transformed SEV scalar: Diarrhea	X			X		
Diarrheal diseases	Male	SEV unsafe water	X			X		
Diarrheal diseases	Male	SEV unsafe sanitation	X			X		
Diarrheal diseases	Male	Socio-demographic Index			X			X
Diarrheal diseases	Male	Rotavirus coverage (proportion)	X				X	
Diarrheal diseases	Male	Rotavirus coverage (proportion)	X			X		
Diarrheal diseases	Female	Education (years per capita)			X			X
Diarrheal diseases	Female	LDI (I$ per capita)			X			X
Diarrheal diseases	Female	Underweight (proportion <2SD weight for age, <5 years)		X		X		
Diarrheal diseases	Female	Population Density (over 1000 ppl/sqkm, proportion)			X			X
Diarrheal diseases	Female	Population Density (under 150 ppl/sqkm, proportion)			X			X
Diarrheal diseases	Female	Sanitation (proportion with access)	X			X		
Diarrheal diseases	Female	Improved Water Source (proportion with access)	X			X		
Diarrheal diseases	Female	Log-transformed SEV scalar: Diarrhea	X			X		
Diarrheal diseases	Female	SEV unsafe water	X			X		
Diarrheal diseases	Female	SEV unsafe sanitation	X			X		
Diarrheal diseases	Female	Socio-demographic Index			X			X
Diarrheal diseases	Female	Rotavirus coverage (proportion)	X				X	
Diarrheal diseases	Female	Rotavirus coverage (proportion)	X			X		
Lower respiratory infections	Male	DTP3 Coverage (proportion)		X			X	
Lower respiratory infections	Male	Education (years per capita)			X			X
Lower respiratory infections	Male	Hib3 Vaccine Coverage (proportion)	X			X		
Lower respiratory infections	Male	LDI (I$ per capita)			X			X
Lower respiratory infections	Male	Underweight (proportion <2SD weight for age, <5 years)	X			X		
Lower respiratory infections	Male	Indoor Air Pollution (All Cooking Fuels)	X			X		
Lower respiratory infections	Male	Outdoor Air Pollution (PM2.5)			X	X		
Lower respiratory infections	Male	Outdoor Air Pollution (PM2.5)			X		X	
Lower respiratory infections	Male	Smoking Prevalence	X			X		
Lower respiratory infections	Male	PCV3 Coverage (proportion)	X			X		
Lower respiratory infections	Male	PCV3 Coverage (proportion)	X				X	
Lower respiratory infections	Male	PCV3 Coverage (proportion)		X		X		
Lower respiratory infections	Male	PCV3 Coverage (proportion)		X			X	
Lower respiratory infections	Male	Log-transformed SEV scalar: LRI	X			X		
Lower respiratory infections	Male	SEV unsafe sanitation			X			X
Lower respiratory infections	Male	Socio-demographic Index			X			X
Lower respiratory infections	Female	DTP3 Coverage (proportion)		X			X	
Lower respiratory infections	Female	Education (years per capita)			X			X
Lower respiratory infections	Female	Hib3 Vaccine Coverage (proportion)	X			X		
Lower respiratory infections	Female	LDI (I$ per capita)			X			X
Lower respiratory infections	Female	Underweight (proportion <2SD weight for age, <5 years)	X			X		
Lower respiratory infections	Female	Indoor Air Pollution (All Cooking Fuels)	X			X		
Lower respiratory infections	Female	Outdoor Air Pollution (PM2.5)			X	X		
Lower respiratory infections	Female	Outdoor Air Pollution (PM2.5)			X		X	
Lower respiratory infections	Female	Smoking Prevalence	X			X		
Lower respiratory infections	Female	PCV3 Coverage (proportion)	X			X		
Lower respiratory infections	Female	PCV3 Coverage (proportion)	X				X	
Lower respiratory infections	Female	PCV3 Coverage (proportion)		X		X		
Lower respiratory infections	Female	PCV3 Coverage (proportion)		X			X	
Lower respiratory infections	Female	Log-transformed SEV scalar: LRI	X			X		
Lower respiratory infections	Female	SEV unsafe sanitation			X			X
Lower respiratory infections	Female	Socio-demographic Index			X			X

Cause	Sex	Covariate	GBD 2015			GBD 2016		
			Level 1	Level 2	Level 3	Level 1	Level 2	Level 3
Otitis media	Male	Socio-demographic Index			X			X
Otitis media	Male	Smoking Prevalence	X			X		
Otitis media	Male	Education (years per capita)			X			X
Otitis media	Male	LDI (I$ per capita)			X			X
Otitis media	Male	Outdoor Air Pollution (PM2.5)	X				X	
Otitis media	Male	Indoor Air Pollution (All Cooking Fuels)	X				X	
Otitis media	Male	Log-transformed SEV scalar: Otitis	X			X		
Otitis media	Female	Socio-demographic Index			X			X
Otitis media	Female	Smoking Prevalence	X			X		
Otitis media	Female	Education (years per capita)			X			X
Otitis media	Female	LDI (I$ per capita)			X			X
Otitis media	Female	Outdoor Air Pollution (PM2.5)	X				X	
Otitis media	Female	Indoor Air Pollution (All Cooking Fuels)	X				X	
Otitis media	Female	Log-transformed SEV scalar: Otitis	X			X		
Meningitis	Female	DTP3 Coverage (proportion)			X			X
Meningitis	Female	LDI (I$ per capita)			X			X
Meningitis	Female	Underweight (proportion <2SD weight for age, <5 years)		X			X	
Meningitis	Female	Sanitation (proportion with access)			X			X
Meningitis	Female	Improved Water Source (proportion with access)		X			X	
Meningitis	Female	Maternal education (years per capita)			X			X
Meningitis	Female	meningitis belt (proportion)	X			X		
Meningitis	Female	Socio-demographic Index			X			X
Meningitis	Female	Proportion of total population covered by menafrivac initiative (meningitis meningococcal type A vaccine)			X			X
Meningitis	Male	DTP3 Coverage (proportion)			X			X
Meningitis	Male	LDI (I$ per capita)			X			X
Meningitis	Male	Underweight (proportion <2SD weight for age, <5 years)		X			X	
Meningitis	Male	Sanitation (proportion with access)			X			X
Meningitis	Male	Improved Water Source (proportion with access)		X			X	
Meningitis	Male	Maternal education (years per capita)			X			X
Meningitis	Male	meningitis belt (proportion)	X			X		
Meningitis	Male	Socio-demographic Index			X			X
Meningitis	Male	Proportion of total population covered by menafrivac initiative (meningitis meningococcal type A vaccine)			X			X
Encephalitis	Female	In-Facility Delivery (proportion)			X			X
Encephalitis	Female	LDI (I$ per capita)		X			X	
Encephalitis	Female	Underweight (proportion <2SD weight for age, <5 years)	X			X		
Encephalitis	Female	Sanitation (proportion with access)			X			X
Encephalitis	Female	Improved Water Source (proportion with access)			X			X
Encephalitis	Female	Maternal education (years per capita)			X			X
Encephalitis	Female	Socio-demographic Index			X			X
Encephalitis	Female	Japanese encelphalitis endemic area (binary)	X			X		
Encephalitis	Male	In-Facility Delivery (proportion)			X			X
Encephalitis	Male	LDI (I$ per capita)		X			X	
Encephalitis	Male	Underweight (proportion <2SD weight for age, <5 years)	X			X		
Encephalitis	Male	Sanitation (proportion with access)			X			X
Encephalitis	Male	Improved Water Source (proportion with access)			X			X
Encephalitis	Male	Maternal education (years per capita)			X			X
Encephalitis	Male	Socio-demographic Index			X			X
Encephalitis	Male	Japanese encelphalitis endemic area (binary)	X			X		
Tetanus	Male	Socio-demographic Index			X			X
Tetanus	Male	Sanitation (proportion with access)			X			X
Tetanus	Male	Education (years per capita)			X			X
Tetanus	Male	DTP3 Coverage (proportion)	X			X		
Tetanus	Male	LDI (I$ per capita)			X			X
Tetanus	Female	Socio-demographic Index			X			X
Tetanus	Female	Sanitation (proportion with access)			X			X
Tetanus	Female	Education (years per capita)			X			X
Tetanus	Female	DTP3 Coverage (proportion)	X			X		
Tetanus	Female	LDI (I$ per capita)			X			X
Tetanus	Male	Skilled Birth Attendance (proportion)		X			X	
Tetanus	Male	In-Facility Delivery (proportion)		X			X	
Tetanus	Male	Tetanus Toxoid Coverage Smooth (proportion)	X			X		
Tetanus	Female	Skilled Birth Attendance (proportion)		X			X	
Tetanus	Female	In-Facility Delivery (proportion)		X			X	
Tetanus	Female	Tetanus Toxoid Coverage Smooth (proportion)	X			X		
Dengue	Female	Education (years per capita)			X			X
Dengue	Female	Health System Access (unitless)		X			X	
Dengue	Female	LDI (I$ per capita)			X			X
Dengue	Female	Latitude Under 15 (proportion)		X			X	
Dengue	Female	Population Density (over 1000 ppl/sqkm, proportion)	X			X		
Dengue	Female	Elevation Under 100m (proportion)		X			X	
Dengue	Female	Rainfall Quintile 4 (proportion)		X			X	
Dengue	Female	Rainfall Quintile 5 (proportion)		X			X	
Dengue	Female	Population weighted probability of dengue transmission	X			X		
Dengue	Female	Dengue outbreaks (binary)		X			X	
Dengue	Female	Dengue anomalies (deviation from mean dengue incidence rate)		X			X	
Dengue	Female	Dengue anomalies (deviation from mean dengue incidence rate)		X		X		
Dengue	Female	Socio-demographic Index			X			X
Dengue	Male	Education (years per capita)			X			X
Dengue	Male	Health System Access (unitless)		X			X	
Dengue	Male	LDI (I$ per capita)			X			X
Dengue	Male	Latitude Under 15 (proportion)		X			X	
Dengue	Male	Population Density (over 1000 ppl/sqkm, proportion)	X			X		
Dengue	Male	Elevation Under 100m (proportion)		X			X	
Dengue	Male	Rainfall Quintile 4 (proportion)		X			X	

Cause	Sex	Covariate	GBD 2015			GBD 2016		
			Level 1	Level 2	Level 3	Level 1	Level 2	Level 3
Dengue	Male	Rainfall Quintile 5 (proportion)		X			X	
Dengue	Male	Population weighted probability of dengue transmission	X			X		
Dengue	Male	Dengue outbreaks (binary)		X			X	
Dengue	Male	Dengue anomalies (deviation from mean dengue incidence rate)		X			X	
Dengue	Male	Dengue anomalies (deviation from mean dengue incidence rate)		X		X		
Dengue	Male	Socio-demographic Index			X			X
Rabies	Male	Antenatal Care (4 visits) Coverage (proportion)	X			X		
Rabies	Male	Health System Access (unitless)	X			X		
Rabies	Male	Health System Access (unitless)	X				X	
Rabies	Male	In-Facility Delivery (proportion)	X			X		
Rabies	Male	Population Density (500-1000 ppl/sqkm, proportion)			X			X
Rabies	Male	Population Density (under 150 ppl/sqkm, proportion)			X			X
Rabies	Male	Skilled Birth Attendance (proportion)		X			X	
Rabies	Male	Socio-demographic Index			X			X
Rabies	Female	Antenatal Care (4 visits) Coverage (proportion)	X			X		
Rabies	Female	Health System Access (unitless)	X			X		
Rabies	Female	Health System Access (unitless)	X				X	
Rabies	Female	In-Facility Delivery (proportion)	X			X		
Rabies	Female	Population Density (500-1000 ppl/sqkm, proportion)			X			X
Rabies	Female	Population Density (under 150 ppl/sqkm, proportion)			X			X
Rabies	Female	Skilled Birth Attendance (proportion)		X			X	
Rabies	Female	Socio-demographic Index			X			X
Other neglected tropical diseases	Male	Socio-demographic Index			X			X
Other neglected tropical diseases	Male	Sanitation (proportion with access)		X			X	
Other neglected tropical diseases	Male	Education (years per capita)			X			X
Other neglected tropical diseases	Male	LDI (I$ per capita)			X			X
Other neglected tropical diseases	Male	Rainfall Quintile 5 (proportion)		X			X	
Other neglected tropical diseases	Male	Latitude Under 15 (proportion)	X			X		
Other neglected tropical diseases	Female	Socio-demographic Index			X			X
Other neglected tropical diseases	Female	Sanitation (proportion with access)		X			X	
Other neglected tropical diseases	Female	Education (years per capita)			X			X
Other neglected tropical diseases	Female	LDI (I$ per capita)			X			X
Other neglected tropical diseases	Female	Rainfall Quintile 5 (proportion)		X			X	
Other neglected tropical diseases	Female	Latitude Under 15 (proportion)	X			X		
Neonatal disorders	Female	Education (years per capita)		X				X
Neonatal disorders	Female	In-Facility Delivery (proportion)		X			X	
Neonatal disorders	Female	LDI (I$ per capita)		X				X
Neonatal disorders	Female	Underweight (proportion <2SD weight for age, <5 years)		X			X	
Neonatal disorders	Female	Indoor Air Pollution (All Cooking Fuels)	X			X		
Neonatal disorders	Female	Smoking Prevalence (Reproductive Age Standardized)	X			X		
Neonatal disorders	Female	Total Fertility Rate			X			X
Neonatal disorders	Female	Socio-demographic Index			X			X
Neonatal disorders	Male	Education (years per capita)		X				X
Neonatal disorders	Male	In-Facility Delivery (proportion)		X			X	
Neonatal disorders	Male	LDI (I$ per capita)		X				X
Neonatal disorders	Male	Underweight (proportion <2SD weight for age, <5 years)		X			X	
Neonatal disorders	Male	Indoor Air Pollution (All Cooking Fuels)	X			X		
Neonatal disorders	Male	Smoking Prevalence (Reproductive Age Standardized)	X			X		
Neonatal disorders	Male	Total Fertility Rate			X			X
Neonatal disorders	Male	Socio-demographic Index			X			X
Neonatal preterm birth complications	Male	Education (years per capita)			X			X
Neonatal preterm birth complications	Male	In-Facility Delivery (proportion)		X			X	
Neonatal preterm birth complications	Male	In-Facility Delivery (proportion)	X				X	
Neonatal preterm birth complications	Male	LDI (I$ per capita)			X			X
Neonatal preterm birth complications	Male	Underweight (proportion <2SD weight for age, <5 years)		X			X	
Neonatal preterm birth complications	Male	Live Births 35+ (proportion)		X			X	
Neonatal preterm birth complications	Male	Indoor Air Pollution (All Cooking Fuels)	X			X		
Neonatal preterm birth complications	Male	Smoking Prevalence (Reproductive Age Standardized)	X			X		
Neonatal preterm birth complications	Male	Total Fertility Rate			X			X
Neonatal preterm birth complications	Male	Socio-demographic Index			X			X
Neonatal preterm birth complications	Female	Education (years per capita)			X			X
Neonatal preterm birth complications	Female	In-Facility Delivery (proportion)		X			X	
Neonatal preterm birth complications	Female	LDI (I$ per capita)			X			X
Neonatal preterm birth complications	Female	Underweight (proportion <2SD weight for age, <5 years)		X			X	
Neonatal preterm birth complications	Female	Live Births 35+ (proportion)		X			X	
Neonatal preterm birth complications	Female	Indoor Air Pollution (All Cooking Fuels)	X			X		
Neonatal preterm birth complications	Female	Smoking Prevalence (Reproductive Age Standardized)	X			X		
Neonatal preterm birth complications	Female	Total Fertility Rate			X			X
Neonatal preterm birth complications	Female	Socio-demographic Index			X			X
Neonatal encephalopathy due to birth asphyxia and trauma	Male	Education (years per capita)			X			X
Neonatal encephalopathy due to birth asphyxia and trauma	Male	In-Facility Delivery (proportion)		X			X	
Neonatal encephalopathy due to birth asphyxia and trauma	Male	LDI (I$ per capita)			X			X
Neonatal encephalopathy due to birth asphyxia and trauma	Male	Underweight (proportion <2SD weight for age, <5 years)		X			X	
Neonatal encephalopathy due to birth asphyxia and trauma	Male	Indoor Air Pollution (All Cooking Fuels)	X			X		
Neonatal encephalopathy due to birth asphyxia and trauma	Male	Smoking Prevalence (Reproductive Age Standardized)	X			X		
Neonatal encephalopathy due to birth asphyxia and trauma	Male	Total Fertility Rate			X			X
Neonatal encephalopathy due to birth asphyxia and trauma	Male	Socio-demographic Index			X			X
Neonatal encephalopathy due to birth asphyxia and trauma	Female	Education (years per capita)			X			X
Neonatal encephalopathy due to birth asphyxia and trauma	Female	In-Facility Delivery (proportion)		X			X	
Neonatal encephalopathy due to birth asphyxia and trauma	Female	LDI (I$ per capita)			X			X
Neonatal encephalopathy due to birth asphyxia and trauma	Female	Underweight (proportion <2SD weight for age, <5 years)		X			X	
Neonatal encephalopathy due to birth asphyxia and trauma	Female	Indoor Air Pollution (All Cooking Fuels)	X			X		
Neonatal encephalopathy due to birth asphyxia and trauma	Female	Smoking Prevalence (Reproductive Age Standardized)	X			X		
Neonatal encephalopathy due to birth asphyxia and trauma	Female	Total Fertility Rate			X			X
Neonatal encephalopathy due to birth asphyxia and trauma	Female	Socio-demographic Index			X			X

Cause	Sex	Covariate	GBD 2015			GBD 2016		
			Level 1	Level 2	Level 3	Level 1	Level 2	Level 3
Neonatal sepsis and other neonatal infections	Male	Education (years per capita)			X			X
Neonatal sepsis and other neonatal infections	Male	In-Facility Delivery (proportion)		X			X	
Neonatal sepsis and other neonatal infections	Male	LDI (I$ per capita)			X			X
Neonatal sepsis and other neonatal infections	Male	Underweight (proportion <2SD weight for age, <5 years)		X			X	
Neonatal sepsis and other neonatal infections	Male	Indoor Air Pollution (All Cooking Fuels)	X			X		
Neonatal sepsis and other neonatal infections	Male	Smoking Prevalence (Reproductive Age Standardized)	X			X		
Neonatal sepsis and other neonatal infections	Male	Total Fertility Rate			X			X
Neonatal sepsis and other neonatal infections	Male	Socio-demographic Index			X			X
Neonatal sepsis and other neonatal infections	Female	Education (years per capita)			X			X
Neonatal sepsis and other neonatal infections	Female	In-Facility Delivery (proportion)		X			X	
Neonatal sepsis and other neonatal infections	Female	LDI (I$ per capita)			X			X
Neonatal sepsis and other neonatal infections	Female	Underweight (proportion <2SD weight for age, <5 years)		X			X	
Neonatal sepsis and other neonatal infections	Female	Indoor Air Pollution (All Cooking Fuels)	X			X		
Neonatal sepsis and other neonatal infections	Female	Smoking Prevalence (Reproductive Age Standardized)	X			X		
Neonatal sepsis and other neonatal infections	Female	Total Fertility Rate			X			X
Neonatal sepsis and other neonatal infections	Female	Socio-demographic Index			X			X
Hemolytic disease and other neonatal jaundice	Male	Antenatal Care (4 visits) Coverage (proportion)		X			X	
Hemolytic disease and other neonatal jaundice	Male	Education (years per capita)			X			X
Hemolytic disease and other neonatal jaundice	Male	In-Facility Delivery (proportion)		X			X	
Hemolytic disease and other neonatal jaundice	Male	LDI (I$ per capita)			X			X
Hemolytic disease and other neonatal jaundice	Male	Skilled Birth Attendance (proportion)		X			X	
Hemolytic disease and other neonatal jaundice	Male	Total Fertility Rate		X				X
Hemolytic disease and other neonatal jaundice	Male	Socio-demographic Index			X			X
Hemolytic disease and other neonatal jaundice	Female	Antenatal Care (4 visits) Coverage (proportion)		X			X	
Hemolytic disease and other neonatal jaundice	Female	Education (years per capita)			X			X
Hemolytic disease and other neonatal jaundice	Female	In-Facility Delivery (proportion)		X			X	
Hemolytic disease and other neonatal jaundice	Female	LDI (I$ per capita)			X			X
Hemolytic disease and other neonatal jaundice	Female	Skilled Birth Attendance (proportion)		X			X	
Hemolytic disease and other neonatal jaundice	Female	Total Fertility Rate		X				X
Hemolytic disease and other neonatal jaundice	Female	Socio-demographic Index			X			X
Other neonatal disorders	Female	Education (years per capita)			X			X
Other neonatal disorders	Female	LDI (I$ per capita)			X			X
Other neonatal disorders	Female	Underweight (proportion <2SD weight for age, <5 years)		X			X	
Other neonatal disorders	Female	Indoor Air Pollution (All Cooking Fuels)	X			X		
Other neonatal disorders	Female	Skilled Birth Attendance (proportion)		X			X	
Other neonatal disorders	Female	Smoking Prevalence (Reproductive Age Standardized)	X			X		
Other neonatal disorders	Female	Total Fertility Rate			X			X
Other neonatal disorders	Female	Socio-demographic Index			X			X
Other neonatal disorders	Male	Education (years per capita)			X			X
Other neonatal disorders	Male	LDI (I$ per capita)			X			X
Other neonatal disorders	Male	Underweight (proportion <2SD weight for age, <5 years)		X			X	
Other neonatal disorders	Male	Indoor Air Pollution (All Cooking Fuels)	X			X		
Other neonatal disorders	Male	Skilled Birth Attendance (proportion)		X			X	
Other neonatal disorders	Male	Smoking Prevalence (Reproductive Age Standardized)	X			X		
Other neonatal disorders	Male	Total Fertility Rate			X			X
Other neonatal disorders	Male	Socio-demographic Index			X			X
Nutritional deficiencies	Male	Education (years per capita)			X			X
Nutritional deficiencies	Male	LDI (I$ per capita)			X			X
Nutritional deficiencies	Male	Underweight (proportion <2SD weight for age, <5 years)	X			X		
Nutritional deficiencies	Male	Rainfall Quintile 1 (proportion)		X			X	
Nutritional deficiencies	Male	Rainfall Quintile 2 (proportion)		X			X	
Nutritional deficiencies	Male	Sanitation (proportion with access)		X			X	
Nutritional deficiencies	Male	Mortality Rate Due to War Shocks (per 1 person)		X			X	
Nutritional deficiencies	Male	Improved Water Source (proportion with access)		X			X	
Nutritional deficiencies	Male	Age-Standardize Prevalence of Severe Anemia	X			X		
Nutritional deficiencies	Male	Socio-demographic Index			X			X
Nutritional deficiencies	Female	Education (years per capita)			X			X
Nutritional deficiencies	Female	LDI (I$ per capita)			X			X
Nutritional deficiencies	Female	Underweight (proportion <2SD weight for age, <5 years)	X			X		
Nutritional deficiencies	Female	Rainfall Quintile 1 (proportion)		X			X	
Nutritional deficiencies	Female	Rainfall Quintile 2 (proportion)		X			X	
Nutritional deficiencies	Female	Sanitation (proportion with access)		X			X	
Nutritional deficiencies	Female	Mortality Rate Due to War Shocks (per 1 person)		X			X	
Nutritional deficiencies	Female	Improved Water Source (proportion with access)		X			X	
Nutritional deficiencies	Female	Age-Standardize Prevalence of Severe Anemia	X			X		
Nutritional deficiencies	Female	Socio-demographic Index			X			X
Protein-energy malnutrition	Male	Education (years per capita)			X			X
Protein-energy malnutrition	Male	LDI (I$ per capita)			X			X
Protein-energy malnutrition	Male	Rainfall Quintile 1 (proportion)		X			X	
Protein-energy malnutrition	Male	Rainfall Quintile 2 (proportion)		X			X	
Protein-energy malnutrition	Male	Sanitation (proportion with access)		X			X	
Protein-energy malnutrition	Male	Mortality Rate Due to War Shocks (per 1 person)		X			X	
Protein-energy malnutrition	Male	Improved Water Source (proportion with access)		X			X	
Protein-energy malnutrition	Male	Age-Standardize Prevalence of Severe Anemia	X			X		
Protein-energy malnutrition	Male	Socio-demographic Index			X			X
Protein-energy malnutrition	Female	Education (years per capita)			X			X
Protein-energy malnutrition	Female	LDI (I$ per capita)			X			X
Protein-energy malnutrition	Female	Rainfall Quintile 1 (proportion)		X			X	
Protein-energy malnutrition	Female	Rainfall Quintile 2 (proportion)		X			X	
Protein-energy malnutrition	Female	Sanitation (proportion with access)		X			X	
Protein-energy malnutrition	Female	Mortality Rate Due to War Shocks (per 1 person)		X			X	
Protein-energy malnutrition	Female	Improved Water Source (proportion with access)		X			X	
Protein-energy malnutrition	Female	Age-Standardize Prevalence of Severe Anemia	X			X		
Protein-energy malnutrition	Female	Socio-demographic Index			X			X
Iron-deficiency anemia	Male	Education (years per capita)			X			X

Cause	Sex	Covariate	GBD 2015			GBD 2016		
			Level 1	Level 2	Level 3	Level 1	Level 2	Level 3
Iron-deficiency anemia	Male	Health System Access 2 (unitless)	X				X	
Iron-deficiency anemia	Male	LDI (I$ per capita)			X			X
Iron-deficiency anemia	Male	Underweight (proportion <2SD weight for age, <5 years)	X			X		
Iron-deficiency anemia	Male	Rainfall Quintile 1 (proportion)		X			X	
Iron-deficiency anemia	Male	Rainfall Quintile 2 (proportion)		X			X	
Iron-deficiency anemia	Male	Sanitation (proportion with access)		X			X	
Iron-deficiency anemia	Male	Total Calories (kcal per capita)		X			X	
Iron-deficiency anemia	Male	Improved Water Source (proportion with access)		X			X	
Iron-deficiency anemia	Male	Age-Standardize Prevalence of Severe Anemia	X			X		
Iron-deficiency anemia	Male	Socio-demographic Index			X			X
Iron-deficiency anemia	Female	Education (years per capita)			X			X
Iron-deficiency anemia	Female	Health System Access 2 (unitless)	X				X	
Iron-deficiency anemia	Female	LDI (I$ per capita)			X			X
Iron-deficiency anemia	Female	Underweight (proportion <2SD weight for age, <5 years)	X			X		
Iron-deficiency anemia	Female	Rainfall Quintile 1 (proportion)		X			X	
Iron-deficiency anemia	Female	Rainfall Quintile 2 (proportion)		X			X	
Iron-deficiency anemia	Female	Sanitation (proportion with access)		X			X	
Iron-deficiency anemia	Female	Total Calories (kcal per capita)		X			X	
Iron-deficiency anemia	Female	Improved Water Source (proportion with access)		X			X	
Iron-deficiency anemia	Female	Age-Standardize Prevalence of Severe Anemia	X			X		
Iron-deficiency anemia	Female	Socio-demographic Index			X			X
Other nutritional deficiencies	Male	Education (years per capita)			X			X
Other nutritional deficiencies	Male	LDI (I$ per capita)			X			X
Other nutritional deficiencies	Male	Underweight (proportion <2SD weight for age, <5 years)	X			X		
Other nutritional deficiencies	Male	Rainfall Quintile 1 (proportion)		X			X	
Other nutritional deficiencies	Male	Rainfall Quintile 2 (proportion)		X			X	
Other nutritional deficiencies	Male	Sanitation (proportion with access)		X			X	
Other nutritional deficiencies	Male	Mortality Rate Due to War Shocks (per 1 person)		X			X	
Other nutritional deficiencies	Male	Improved Water Source (proportion with access)		X			X	
Other nutritional deficiencies	Male	Age-Standardize Prevalence of Severe Anemia	X			X		
Other nutritional deficiencies	Male	Socio-demographic Index			X			X
Other nutritional deficiencies	Female	Education (years per capita)			X			X
Other nutritional deficiencies	Female	LDI (I$ per capita)			X			X
Other nutritional deficiencies	Female	Underweight (proportion <2SD weight for age, <5 years)	X			X		
Other nutritional deficiencies	Female	Rainfall Quintile 1 (proportion)		X			X	
Other nutritional deficiencies	Female	Rainfall Quintile 2 (proportion)		X			X	
Other nutritional deficiencies	Female	Sanitation (proportion with access)		X			X	
Other nutritional deficiencies	Female	Mortality Rate Due to War Shocks (per 1 person)		X			X	
Other nutritional deficiencies	Female	Improved Water Source (proportion with access)		X			X	
Other nutritional deficiencies	Female	Age-Standardize Prevalence of Severe Anemia	X			X		
Other nutritional deficiencies	Female	Socio-demographic Index			X			X
Sexually transmitted diseases excluding HIV	Female	Legality of Abortion		X			X	
Sexually transmitted diseases excluding HIV	Female	Antenatal Care (1 visit) Coverage (proportion)			X			X
Sexually transmitted diseases excluding HIV	Female	Antenatal Care (4 visits) Coverage (proportion)			X			X
Sexually transmitted diseases excluding HIV	Female	Age-Specific Fertility Rate		X			X	
Sexually transmitted diseases excluding HIV	Female	Education (years per capita)		X			X	
Sexually transmitted diseases excluding HIV	Female	LDI (I$ per capita)			X			X
Sexually transmitted diseases excluding HIV	Female	Total Fertility Rate		X			X	
Sexually transmitted diseases excluding HIV	Female	Health System Access (capped)		X			X	
Sexually transmitted diseases excluding HIV	Female	Syphilis prevalence (proportion)	X			X		
Sexually transmitted diseases excluding HIV	Male	Legality of Abortion		X			X	
Sexually transmitted diseases excluding HIV	Male	Antenatal Care (1 visit) Coverage (proportion)			X			X
Sexually transmitted diseases excluding HIV	Male	Antenatal Care (4 visits) Coverage (proportion)			X			X
Sexually transmitted diseases excluding HIV	Male	Age-Specific Fertility Rate		X			X	
Sexually transmitted diseases excluding HIV	Male	Education (years per capita)		X			X	
Sexually transmitted diseases excluding HIV	Male	LDI (I$ per capita)			X			X
Sexually transmitted diseases excluding HIV	Male	Total Fertility Rate		X			X	
Sexually transmitted diseases excluding HIV	Male	Health System Access (capped)		X			X	
Sexually transmitted diseases excluding HIV	Male	Syphilis prevalence (proportion)	X			X		
Hepatitis	Male	Education (years per capita)			X			X
Hepatitis	Male	Health System Access 2 (unitless)		X			X	
Hepatitis	Male	LDI (I$ per capita)		X			X	
Hepatitis	Male	Sanitation (proportion with access)		X			X	
Hepatitis	Male	Improved Water Source (proportion with access)		X			X	
Hepatitis	Male	Log-transformed SEV scalar: Hep	X			X		
Hepatitis	Male	Socio-demographic Index			X			X
Hepatitis	Male	Hepatitis B (HBsAg) Seroprevalence	X			X		
Hepatitis	Male	Hepatitis C (IgG) Seroprevalence	X			X		
Hepatitis	Male	Seroprevalence of anti-HAV (IgG)	X			X		
Hepatitis	Male	Seroprevalence of anti-HEV (IgG)	X			X		
Hepatitis	Female	Education (years per capita)			X			X
Hepatitis	Female	Health System Access 2 (unitless)		X			X	
Hepatitis	Female	LDI (I$ per capita)		X			X	
Hepatitis	Female	Sanitation (proportion with access)		X			X	
Hepatitis	Female	Improved Water Source (proportion with access)		X			X	
Hepatitis	Female	Log-transformed SEV scalar: Hep	X			X		
Hepatitis	Female	Socio-demographic Index			X			X
Hepatitis	Female	Hepatitis B (HBsAg) Seroprevalence	X			X		
Hepatitis	Female	Hepatitis C (IgG) Seroprevalence	X			X		
Hepatitis	Female	Seroprevalence of anti-HAV (IgG)	X			X		
Hepatitis	Female	Seroprevalence of anti-HEV (IgG)	X			X		
Other infectious diseases	Male	Underweight (proportion <2SD weight for age, <5 years)		X			X	
Other infectious diseases	Male	Underweight (proportion <2SD weight for age, <5 years)		X		X		
Other infectious diseases	Male	Underweight (proportion <2SD weight for age, <5 years)	X				X	
Other infectious diseases	Male	Underweight (proportion <2SD weight for age, <5 years)	X			X		

Cause	Sex	Covariate	GBD 2015			GBD 2016		
			Level 1	Level 2	Level 3	Level 1	Level 2	Level 3
Other infectious diseases	Male	Sanitation (proportion with access)		X			X	
Other infectious diseases	Male	Rainfall Quintile 5 (proportion)		X			X	
Other infectious diseases	Male	Education (years per capita)			X			X
Other infectious diseases	Male	DTP3 Coverage (proportion)	X			X		
Other infectious diseases	Male	DTP3 Coverage (proportion)	X					X
Other infectious diseases	Male	DTP3 Coverage (proportion)			X	X		
Other infectious diseases	Male	DTP3 Coverage (proportion)			X			X
Other infectious diseases	Male	Health System Access (unitless)	X			X		
Other infectious diseases	Male	Latitude 30 to 45 (proportion)		X			X	
Other infectious diseases	Male	Latitude 30 to 45 (proportion)		X				X
Other infectious diseases	Male	Latitude 30 to 45 (proportion)			X		X	
Other infectious diseases	Male	Latitude 30 to 45 (proportion)			X			X
Other infectious diseases	Male	Measles Vaccine Coverage (proportion)	X			X		
Other infectious diseases	Male	Latitude Over 45 (proportion)		X			X	
Other infectious diseases	Male	Rainfall Quintile 3 (proportion)		X			X	
Other infectious diseases	Male	Rainfall Quintile 2 (proportion)		X			X	
Other infectious diseases	Male	Rainfall Quintile 1 (proportion)		X			X	
Other infectious diseases	Male	LDI (I$ per capita)			X			X
Other infectious diseases	Male	Latitude 15 to 30 (proportion)		X			X	
Other infectious diseases	Male	Latitude 15 to 30 (proportion)		X				X
Other infectious diseases	Male	Latitude 15 to 30 (proportion)			X		X	
Other infectious diseases	Male	Latitude 15 to 30 (proportion)			X			X
Other infectious diseases	Male	Antenatal Care (1 visit) Coverage (proportion)		X			X	
Other infectious diseases	Male	Antenatal Care (1 visit) Coverage (proportion)		X		X		
Other infectious diseases	Male	Antenatal Care (1 visit) Coverage (proportion)	X				X	
Other infectious diseases	Male	Antenatal Care (1 visit) Coverage (proportion)	X			X		
Other infectious diseases	Male	Rainfall Quintile 4 (proportion)		X			X	
Other infectious diseases	Male	Improved Water Source (proportion with access)		X			X	
Other infectious diseases	Male	Latitude Under 15 (proportion)		X			X	
Other infectious diseases	Male	Latitude Under 15 (proportion)		X				X
Other infectious diseases	Male	Latitude Under 15 (proportion)			X		X	
Other infectious diseases	Male	Latitude Under 15 (proportion)			X			X
Other infectious diseases	Male	Socio-demographic Index			X			X
Other infectious diseases	Female	Underweight (proportion <2SD weight for age, <5 years)		X			X	
Other infectious diseases	Female	Underweight (proportion <2SD weight for age, <5 years)		X		X		
Other infectious diseases	Female	Underweight (proportion <2SD weight for age, <5 years)	X				X	
Other infectious diseases	Female	Underweight (proportion <2SD weight for age, <5 years)	X			X		
Other infectious diseases	Female	Sanitation (proportion with access)		X			X	
Other infectious diseases	Female	Rainfall Quintile 5 (proportion)		X			X	
Other infectious diseases	Female	Education (years per capita)			X			X
Other infectious diseases	Female	DTP3 Coverage (proportion)	X			X		
Other infectious diseases	Female	DTP3 Coverage (proportion)	X					X
Other infectious diseases	Female	DTP3 Coverage (proportion)			X	X		
Other infectious diseases	Female	DTP3 Coverage (proportion)			X			X
Other infectious diseases	Female	Health System Access (unitless)	X			X		
Other infectious diseases	Female	Latitude 30 to 45 (proportion)		X			X	
Other infectious diseases	Female	Latitude 30 to 45 (proportion)		X				X
Other infectious diseases	Female	Latitude 30 to 45 (proportion)			X		X	
Other infectious diseases	Female	Latitude 30 to 45 (proportion)			X			X
Other infectious diseases	Female	Measles Vaccine Coverage (proportion)	X			X		
Other infectious diseases	Female	Latitude Over 45 (proportion)		X			X	
Other infectious diseases	Female	Rainfall Quintile 3 (proportion)		X			X	
Other infectious diseases	Female	Rainfall Quintile 2 (proportion)		X			X	
Other infectious diseases	Female	Rainfall Quintile 1 (proportion)		X			X	
Other infectious diseases	Female	LDI (I$ per capita)			X			X
Other infectious diseases	Female	Latitude 15 to 30 (proportion)		X			X	
Other infectious diseases	Female	Latitude 15 to 30 (proportion)		X				X
Other infectious diseases	Female	Latitude 15 to 30 (proportion)			X		X	
Other infectious diseases	Female	Latitude 15 to 30 (proportion)			X			X
Other infectious diseases	Female	Antenatal Care (1 visit) Coverage (proportion)		X			X	
Other infectious diseases	Female	Antenatal Care (1 visit) Coverage (proportion)		X		X		
Other infectious diseases	Female	Antenatal Care (1 visit) Coverage (proportion)	X				X	
Other infectious diseases	Female	Antenatal Care (1 visit) Coverage (proportion)	X			X		
Other infectious diseases	Female	Rainfall Quintile 4 (proportion)		X			X	
Other infectious diseases	Female	Improved Water Source (proportion with access)		X			X	
Other infectious diseases	Female	Latitude Under 15 (proportion)		X			X	
Other infectious diseases	Female	Latitude Under 15 (proportion)		X				X
Other infectious diseases	Female	Latitude Under 15 (proportion)			X		X	
Other infectious diseases	Female	Latitude Under 15 (proportion)			X			X
Other infectious diseases	Female	Socio-demographic Index			X			X
Esophageal cancer	Male	Alcohol (liters per capita)	X			X		
Esophageal cancer	Male	Tobacco (cigarettes per capita)	X			X		
Esophageal cancer	Male	Education (years per capita)			X			X
Esophageal cancer	Male	Fruits (kcal per capita)	X			X		
Esophageal cancer	Male	LDI (I$ per capita)			X			X
Esophageal cancer	Male	Mean BMI	X			X		
Esophageal cancer	Male	Indoor Air Pollution (All Cooking Fuels)		X			X	
Esophageal cancer	Male	Sanitation (proportion with access)		X			X	
Esophageal cancer	Male	Smoking Prevalence	X			X		
Esophageal cancer	Male	Vegetables (kcal per capita)		X			X	
Esophageal cancer	Male	Improved Water Source (proportion with access)		X			X	
Esophageal cancer	Male	Socio-demographic Index			X		X	
Esophageal cancer	Female	Alcohol (liters per capita)	X			X		
Esophageal cancer	Female	Tobacco (cigarettes per capita)	X			X		
Esophageal cancer	Female	Education (years per capita)			X			X

Cause	Sex	Covariate	GBD 2015			GBD 2016		
			Level 1	Level 2	Level 3	Level 1	Level 2	Level 3
Esophageal cancer	Female	Fruits (kcal per capita)	X			X		
Esophageal cancer	Female	LDI (I$ per capita)			X			X
Esophageal cancer	Female	Mean BMI	X			X		
Esophageal cancer	Female	Indoor Air Pollution (All Cooking Fuels)		X			X	
Esophageal cancer	Female	Sanitation (proportion with access)		X			X	
Esophageal cancer	Female	Smoking Prevalence	X			X		
Esophageal cancer	Female	Vegetables (kcal per capita)		X			X	
Esophageal cancer	Female	Improved Water Source (proportion with access)		X			X	
Esophageal cancer	Female	Socio-demographic Index			X			X
Esophageal cancer	Female	Socio-demographic Index			X		X	
Stomach cancer	Male	Alcohol (liters per capita)	X			X		
Stomach cancer	Male	Tobacco (cigarettes per capita)	X			X		
Stomach cancer	Male	Cumulative Cigarettes (10 Years)	X			X		
Stomach cancer	Male	Cumulative Cigarettes (15 Years)	X			X		
Stomach cancer	Male	LDI (I$ per capita)			X			X
Stomach cancer	Male	Mean BMI		X			X	
Stomach cancer	Male	Indoor Air Pollution (All Cooking Fuels)		X			X	
Stomach cancer	Male	Outdoor Air Pollution (PM2.5)		X			X	
Stomach cancer	Male	Sanitation (proportion with access)		X			X	
Stomach cancer	Male	Smoking Prevalence	X			X		
Stomach cancer	Male	Improved Water Source (proportion with access)		X			X	
Stomach cancer	Male	Log-transformed SEV scalar: Stomach C	X			X		
Stomach cancer	Male	Socio-demographic Index			X			X
Stomach cancer	Female	Alcohol (liters per capita)	X			X		
Stomach cancer	Female	Tobacco (cigarettes per capita)	X			X		
Stomach cancer	Female	Cumulative Cigarettes (10 Years)	X			X		
Stomach cancer	Female	Education (years per capita)			X			X
Stomach cancer	Female	LDI (I$ per capita)			X			X
Stomach cancer	Female	Mean BMI		X			X	
Stomach cancer	Female	Indoor Air Pollution (All Cooking Fuels)		X			X	
Stomach cancer	Female	Outdoor Air Pollution (PM2.5)		X			X	
Stomach cancer	Female	Sanitation (proportion with access)		X			X	
Stomach cancer	Female	Smoking Prevalence	X			X		
Stomach cancer	Female	Improved Water Source (proportion with access)		X			X	
Stomach cancer	Female	Log-transformed SEV scalar: Stomach C	X			X		
Stomach cancer	Female	Socio-demographic Index			X			X
Liver cancer	Male	Cumulative Cigarettes (15 Years)	X			X		
Liver cancer	Male	Tobacco (cigarettes per capita)	X			X		
Liver cancer	Male	Education (years per capita)			X			X
Liver cancer	Male	LDI (I$ per capita)			X			X
Liver cancer	Male	Cumulative Cigarettes (20 Years)	X			X		
Liver cancer	Male	Diabetes Age-Standardized Prevalence (proportion)		X			X	
Liver cancer	Male	Mean BMI		X			X	
Liver cancer	Male	Alcohol (liters per capita)	X			X		
Liver cancer	Male	Log-transformed SEV scalar: Liver C	X			X		
Liver cancer	Male	Socio-demographic Index			X			X
Liver cancer	Female	Cumulative Cigarettes (15 Years)	X			X		
Liver cancer	Female	Tobacco (cigarettes per capita)	X			X		
Liver cancer	Female	Education (years per capita)			X			X
Liver cancer	Female	LDI (I$ per capita)			X			X
Liver cancer	Female	Cumulative Cigarettes (20 Years)	X			X		
Liver cancer	Female	Diabetes Age-Standardized Prevalence (proportion)		X			X	
Liver cancer	Female	Mean BMI		X			X	
Liver cancer	Female	Alcohol (liters per capita)	X			X		
Liver cancer	Female	Log-transformed SEV scalar: Liver C	X			X		
Liver cancer	Female	Socio-demographic Index			X			X
Larynx cancer	Male	Cumulative Cigarettes (10 Years)		X			X	
Larynx cancer	Male	Cumulative Cigarettes (15 Years)		X			X	
Larynx cancer	Male	Cumulative Cigarettes (20 Years)		X			X	
Larynx cancer	Male	Cumulative Cigarettes (5 Years)		X			X	
Larynx cancer	Male	Tobacco (cigarettes per capita)		X			X	
Larynx cancer	Male	Education (years per capita)			X			X
Larynx cancer	Male	Log-transformed SEV scalar: Larynx C	X			X		
Larynx cancer	Male	LDI (I$ per capita)			X			X
Larynx cancer	Male	Smoking Prevalence		X			X	
Larynx cancer	Male	Alcohol (liters per capita)	X			X		
Larynx cancer	Male	Population Density (under 150 ppl/sqkm, proportion)		X			X	
Larynx cancer	Male	Population Density (over 1000 ppl/sqkm, proportion)		X			X	
Larynx cancer	Male	Socio-demographic Index			X			X
Larynx cancer	Female	Cumulative Cigarettes (10 Years)		X			X	
Larynx cancer	Female	Cumulative Cigarettes (15 Years)		X			X	
Larynx cancer	Female	Cumulative Cigarettes (20 Years)		X			X	
Larynx cancer	Female	Cumulative Cigarettes (5 Years)		X			X	
Larynx cancer	Female	Tobacco (cigarettes per capita)		X			X	
Larynx cancer	Female	Education (years per capita)			X			X
Larynx cancer	Female	Log-transformed SEV scalar: Larynx C	X			X		
Larynx cancer	Female	LDI (I$ per capita)			X			X
Larynx cancer	Female	Smoking Prevalence		X			X	
Larynx cancer	Female	Alcohol (liters per capita)	X			X		
Larynx cancer	Female	Population Density (under 150 ppl/sqkm, proportion)		X			X	
Larynx cancer	Female	Population Density (over 1000 ppl/sqkm, proportion)		X			X	
Larynx cancer	Female	Socio-demographic Index			X			X
Tracheal, bronchus, and lung cancer	Male	Tobacco (cigarettes per capita)	X			X		
Tracheal, bronchus, and lung cancer	Male	Cumulative Cigarettes (10 Years)	X			X		
Tracheal, bronchus, and lung cancer	Male	Cumulative Cigarettes (15 Years)	X			X		

Cause	Sex	Covariate	GBD 2015			GBD 2016		
			Level 1	Level 2	Level 3	Level 1	Level 2	Level 3
Tracheal, bronchus, and lung cancer	Male	Cumulative Cigarettes (20 Years)	X			X		
Tracheal, bronchus, and lung cancer	Male	Cumulative Cigarettes (5 Years)	X			X		
Tracheal, bronchus, and lung cancer	Male	Education (years per capita)			X			X
Tracheal, bronchus, and lung cancer	Male	LDI (I$ per capita)			X			X
Tracheal, bronchus, and lung cancer	Male	Indoor Air Pollution (All Cooking Fuels)		X			X	
Tracheal, bronchus, and lung cancer	Male	Outdoor Air Pollution (PM2.5)		X			X	
Tracheal, bronchus, and lung cancer	Male	Smoking Prevalence	X			X		
Tracheal, bronchus, and lung cancer	Male	Socio-demographic Index			X			X
Tracheal, bronchus, and lung cancer	Female	Tobacco (cigarettes per capita)	X			X		
Tracheal, bronchus, and lung cancer	Female	Cumulative Cigarettes (10 Years)	X			X		
Tracheal, bronchus, and lung cancer	Female	Cumulative Cigarettes (15 Years)	X			X		
Tracheal, bronchus, and lung cancer	Female	Cumulative Cigarettes (20 Years)	X			X		
Tracheal, bronchus, and lung cancer	Female	Cumulative Cigarettes (5 Years)	X			X		
Tracheal, bronchus, and lung cancer	Female	Education (years per capita)			X			X
Tracheal, bronchus, and lung cancer	Female	LDI (I$ per capita)			X			X
Tracheal, bronchus, and lung cancer	Female	Indoor Air Pollution (All Cooking Fuels)		X			X	
Tracheal, bronchus, and lung cancer	Female	Outdoor Air Pollution (PM2.5)		X			X	
Tracheal, bronchus, and lung cancer	Female	Smoking Prevalence	X			X		
Tracheal, bronchus, and lung cancer	Female	Socio-demographic Index			X			X
Breast cancer	Male	Cumulative Cigarettes (10 Years)		X			X	
Breast cancer	Male	Alcohol (liters per capita)	X			X		
Breast cancer	Male	Education (years per capita)			X			X
Breast cancer	Male	LDI (I$ per capita)			X			X
Breast cancer	Male	Log-transformed SEV scalar: Breast C	X			X		
Breast cancer	Male	Socio-demographic Index			X			X
Breast cancer	Male	Mean BMI	X			X		
Breast cancer	Female	Cumulative Cigarettes (10 Years)		X			X	
Breast cancer	Female	Alcohol (liters per capita)	X			X		
Breast cancer	Female	Education (years per capita)			X			X
Breast cancer	Female	LDI (I$ per capita)			X			X
Breast cancer	Female	Log-transformed SEV scalar: Breast C	X			X		
Breast cancer	Female	Socio-demographic Index			X			X
Breast cancer	Female	Mean BMI	X			X		
Breast cancer	Female	Total Fertility Rate		X			X	
Cervical cancer	Female	Abortion On-Demand Illegal (binary)	X			X		
Cervical cancer	Female	Cumulative Cigarettes (10 Years)	X			X		
Cervical cancer	Female	Cumulative Cigarettes (15 Years)	X			X		
Cervical cancer	Female	Cumulative Cigarettes (5 Years)	X			X		
Cervical cancer	Female	Education (years per capita)			X			X
Cervical cancer	Female	Health System Access 2 (unitless)		X			X	
Cervical cancer	Female	LDI (I$ per capita)			X			X
Cervical cancer	Female	Smoking Prevalence		X			X	
Cervical cancer	Female	Total Fertility Rate		X			X	
Cervical cancer	Female	Socio-demographic Index			X			X
Cervical cancer	Female	HIV age-standardized prevalence	X			X		
Uterine cancer	Female	Cumulative Cigarettes (10 Years)		X			X	
Uterine cancer	Female	Cumulative Cigarettes (5 Years)		X			X	
Uterine cancer	Female	Tobacco (cigarettes per capita)		X			X	
Uterine cancer	Female	Education (years per capita)			X			X
Uterine cancer	Female	Health System Access (unitless)		X			X	
Uterine cancer	Female	Diabetes Age-Standardized Prevalence (proportion)		X			X	
Uterine cancer	Female	Smoking Prevalence		X			X	
Uterine cancer	Female	Mean BMI	X			X		
Uterine cancer	Female	Total Fertility Rate		X			X	
Uterine cancer	Female	Socio-demographic Index			X			X
Uterine cancer	Female	LDI (I$ per capita)			X			X
Uterine cancer	Female	Log-transformed SEV scalar: Uterus C	X			X		
Prostate cancer	Male	Socio-demographic Index			X			X
Prostate cancer	Male	Log-transformed SEV scalar: Prostate C	X			X		
Prostate cancer	Male	Education (years per capita)			X			X
Prostate cancer	Male	LDI (I$ per capita)			X			X
Colon and rectum cancer	Male	Alcohol (liters per capita)	X			X		
Colon and rectum cancer	Male	Tobacco (cigarettes per capita)	X			X		
Colon and rectum cancer	Male	Diabetes Age-Standardized Prevalence (proportion)		X			X	
Colon and rectum cancer	Male	Education (years per capita)			X			X
Colon and rectum cancer	Male	Health System Access 2 (unitless)		X			X	
Colon and rectum cancer	Male	LDI (I$ per capita)			X			X
Colon and rectum cancer	Male	Mean BMI	X			X		
Colon and rectum cancer	Male	Smoking Prevalence	X			X		
Colon and rectum cancer	Male	Log-transformed SEV scalar: Colorect C	X			X		
Colon and rectum cancer	Male	Socio-demographic Index			X			X
Colon and rectum cancer	Female	Alcohol (liters per capita)	X			X		
Colon and rectum cancer	Female	Tobacco (cigarettes per capita)		X			X	
Colon and rectum cancer	Female	Diabetes Age-Standardized Prevalence (proportion)		X			X	
Colon and rectum cancer	Female	Education (years per capita)			X			X
Colon and rectum cancer	Female	Health System Access 2 (unitless)		X			X	
Colon and rectum cancer	Female	LDI (I$ per capita)			X			X
Colon and rectum cancer	Female	Mean BMI	X			X		
Colon and rectum cancer	Female	Smoking Prevalence		X			X	
Colon and rectum cancer	Female	Log-transformed SEV scalar: Colorect C	X			X		
Colon and rectum cancer	Female	Socio-demographic Index			X			X
Lip and oral cavity cancer	Male	Alcohol (liters per capita)	X			X		
Lip and oral cavity cancer	Male	Cumulative Cigarettes (10 Years)	X			X		
Lip and oral cavity cancer	Male	Cumulative Cigarettes (20 Years)	X			X		
Lip and oral cavity cancer	Male	Education (years per capita)			X			X

Cause	Sex	Covariate	GBD 2015			GBD 2016		
			Level 1	Level 2	Level 3	Level 1	Level 2	Level 3
Lip and oral cavity cancer	Male	Health System Access 2 (unitless)		X			X	
Lip and oral cavity cancer	Male	LDI (I$ per capita)			X			X
Lip and oral cavity cancer	Male	Smoking Prevalence	X			X		
Lip and oral cavity cancer	Male	Log-transformed SEV scalar: Mouth C	X			X		
Lip and oral cavity cancer	Male	Socio-demographic Index			X			X
Lip and oral cavity cancer	Female	Alcohol (liters per capita)	X			X		
Lip and oral cavity cancer	Female	Cumulative Cigarettes (10 Years)	X			X		
Lip and oral cavity cancer	Female	Cumulative Cigarettes (20 Years)	X			X		
Lip and oral cavity cancer	Female	Education (years per capita)			X			X
Lip and oral cavity cancer	Female	Fruits (kcal per capita)		X			X	
Lip and oral cavity cancer	Female	Health System Access 2 (unitless)		X			X	
Lip and oral cavity cancer	Female	LDI (I$ per capita)			X			X
Lip and oral cavity cancer	Female	Red Meat (kcal per capita)		X			X	
Lip and oral cavity cancer	Female	Smoking Prevalence	X			X		
Lip and oral cavity cancer	Female	Vegetables (kcal per capita)	X			X		
Lip and oral cavity cancer	Female	Socio-demographic Index			X			X
Nasopharynx cancer	Male	Alcohol (liters per capita)	X			X		
Nasopharynx cancer	Male	Education (years per capita)			X			X
Nasopharynx cancer	Male	Health System Access 2 (unitless)		X			X	
Nasopharynx cancer	Male	LDI (I$ per capita)			X			X
Nasopharynx cancer	Male	Population Density (over 1000 ppl/sqkm, proportion)		X			X	
Nasopharynx cancer	Male	Population Density (under 150 ppl/sqkm, proportion)		X			X	
Nasopharynx cancer	Male	Log-transformed SEV scalar: Nasoph C	X			X		
Nasopharynx cancer	Male	Socio-demographic Index			X			X
Nasopharynx cancer	Female	Alcohol (liters per capita)	X			X		
Nasopharynx cancer	Female	Education (years per capita)			X			X
Nasopharynx cancer	Female	Health System Access 2 (unitless)		X			X	
Nasopharynx cancer	Female	LDI (I$ per capita)			X			X
Nasopharynx cancer	Female	Population Density (over 1000 ppl/sqkm, proportion)		X			X	
Nasopharynx cancer	Female	Population Density (under 150 ppl/sqkm, proportion)		X			X	
Nasopharynx cancer	Female	Log-transformed SEV scalar: Nasoph C	X			X		
Nasopharynx cancer	Female	Socio-demographic Index			X			X
Other pharynx cancer	Male	Alcohol (liters per capita)	X			X		
Other pharynx cancer	Male	Cumulative Cigarettes (5 Years)		X			X	
Other pharynx cancer	Male	Education (years per capita)			X			X
Other pharynx cancer	Male	LDI (I$ per capita)			X			X
Other pharynx cancer	Male	Population Density (over 1000 ppl/sqkm, proportion)		X			X	
Other pharynx cancer	Male	Population Density (under 150 ppl/sqkm, proportion)		X			X	
Other pharynx cancer	Male	Smoking Prevalence	X			X		
Other pharynx cancer	Male	Health System Access (capped)		X			X	
Other pharynx cancer	Male	Log-transformed SEV scalar: Oth Phar C	X			X		
Other pharynx cancer	Male	Socio-demographic Index			X			X
Other pharynx cancer	Female	Alcohol (liters per capita)	X			X		
Other pharynx cancer	Female	Cumulative Cigarettes (5 Years)		X			X	
Other pharynx cancer	Female	Education (years per capita)			X			X
Other pharynx cancer	Female	LDI (I$ per capita)			X			X
Other pharynx cancer	Female	Population Density (over 1000 ppl/sqkm, proportion)		X			X	
Other pharynx cancer	Female	Population Density (under 150 ppl/sqkm, proportion)		X			X	
Other pharynx cancer	Female	Smoking Prevalence	X			X		
Other pharynx cancer	Female	Health System Access (capped)		X			X	
Other pharynx cancer	Female	Log-transformed SEV scalar: Oth Phar C	X			X		
Other pharynx cancer	Female	Socio-demographic Index			X			X
Gallbladder and biliary tract cancer	Female	Cumulative Cigarettes (10 Years)		X			X	
Gallbladder and biliary tract cancer	Female	Cumulative Cigarettes (5 Years)		X			X	
Gallbladder and biliary tract cancer	Female	Tobacco (cigarettes per capita)		X			X	
Gallbladder and biliary tract cancer	Female	Education (years per capita)			X			X
Gallbladder and biliary tract cancer	Female	Health System Access (capped)		X			X	
Gallbladder and biliary tract cancer	Female	LDI (I$ per capita)			X			X
Gallbladder and biliary tract cancer	Female	Log-transformed SEV scalar: Gallblad C	X			X		
Gallbladder and biliary tract cancer	Female	Diabetes Age-Standardized Prevalence (proportion)		X			X	
Gallbladder and biliary tract cancer	Female	Smoking Prevalence		X			X	
Gallbladder and biliary tract cancer	Female	Alcohol (liters per capita)		X			X	
Gallbladder and biliary tract cancer	Female	Mean BMI	X			X		
Gallbladder and biliary tract cancer	Female	Socio-demographic Index			X			X
Gallbladder and biliary tract cancer	Male	Cumulative Cigarettes (10 Years)		X			X	
Gallbladder and biliary tract cancer	Male	Cumulative Cigarettes (5 Years)		X			X	
Gallbladder and biliary tract cancer	Male	Tobacco (cigarettes per capita)		X			X	
Gallbladder and biliary tract cancer	Male	Education (years per capita)			X			X
Gallbladder and biliary tract cancer	Male	Health System Access (capped)		X			X	
Gallbladder and biliary tract cancer	Male	LDI (I$ per capita)			X			X
Gallbladder and biliary tract cancer	Male	Log-transformed SEV scalar: Gallblad C	X			X		
Gallbladder and biliary tract cancer	Male	Diabetes Age-Standardized Prevalence (proportion)		X			X	
Gallbladder and biliary tract cancer	Male	Smoking Prevalence		X			X	
Gallbladder and biliary tract cancer	Male	Alcohol (liters per capita)		X			X	
Gallbladder and biliary tract cancer	Male	Mean BMI	X			X		
Gallbladder and biliary tract cancer	Male	Socio-demographic Index			X			X
Pancreatic cancer	Male	Cumulative Cigarettes (10 Years)	X			X		
Pancreatic cancer	Male	Total Calories (kcal per capita)		X			X	
Pancreatic cancer	Male	Cumulative Cigarettes (20 Years)	X			X		
Pancreatic cancer	Male	Cumulative Cigarettes (5 Years)		X			X	
Pancreatic cancer	Male	Tobacco (cigarettes per capita)	X			X		
Pancreatic cancer	Male	Education (years per capita)			X			X
Pancreatic cancer	Male	Log-transformed SEV scalar: Pancreas C	X			X		
Pancreatic cancer	Male	Diabetes Age-Standardized Prevalence (proportion)		X			X	
Pancreatic cancer	Male	Smoking Prevalence	X			X		

Cause	Sex	Covariate	GBD 2015			GBD 2016		
			Level 1	Level 2	Level 3	Level 1	Level 2	Level 3
Pancreatic cancer	Male	Alcohol (liters per capita)	X			X		
Pancreatic cancer	Male	Mean BMI	X			X		
Pancreatic cancer	Male	Socio-demographic Index			X			X
Pancreatic cancer	Male	LDI (I$ per capita)			X			X
Pancreatic cancer	Female	Cumulative Cigarettes (10 Years)	X			X		
Pancreatic cancer	Female	Cumulative Cigarettes (20 Years)	X			X		
Pancreatic cancer	Female	Cumulative Cigarettes (5 Years)	X			X		
Pancreatic cancer	Female	Tobacco (cigarettes per capita)	X			X		
Pancreatic cancer	Female	Education (years per capita)			X			X
Pancreatic cancer	Female	Log-transformed SEV scalar: Pancreas C	X			X		
Pancreatic cancer	Female	Diabetes Age-Standardized Prevalence (proportion)		X			X	
Pancreatic cancer	Female	Smoking Prevalence	X			X		
Pancreatic cancer	Female	Alcohol (liters per capita)		X			X	
Pancreatic cancer	Female	Mean BMI	X			X		
Pancreatic cancer	Female	Socio-demographic Index			X			X
Pancreatic cancer	Female	LDI (I$ per capita)			X			X
Malignant skin melanoma	Male	Education (years per capita)			X			X
Malignant skin melanoma	Male	LDI (I$ per capita)			X			X
Malignant skin melanoma	Male	Alcohol (liters per capita)	X				X	
Malignant skin melanoma	Male	Alcohol (liters per capita)	X			X		
Malignant skin melanoma	Male	Latitude 15 to 30 (proportion)		X			X	
Malignant skin melanoma	Male	Latitude Over 45 (proportion)		X			X	
Malignant skin melanoma	Male	Latitude 30 to 45 (proportion)		X			X	
Malignant skin melanoma	Male	Socio-demographic Index			X			X
Malignant skin melanoma	Female	Education (years per capita)			X			X
Malignant skin melanoma	Female	LDI (I$ per capita)			X			X
Malignant skin melanoma	Female	Alcohol (liters per capita)	X			X		
Malignant skin melanoma	Female	Latitude 15 to 30 (proportion)		X			X	
Malignant skin melanoma	Female	Latitude Over 45 (proportion)		X			X	
Malignant skin melanoma	Female	Latitude 30 to 45 (proportion)		X			X	
Malignant skin melanoma	Female	Socio-demographic Index			X			X
Non-melanoma skin cancer	Female	Cumulative Cigarettes (10 Years)	X			X		
Non-melanoma skin cancer	Female	Cumulative Cigarettes (15 Years)	X			X		
Non-melanoma skin cancer	Female	Socio-demographic Index			X			X
Non-melanoma skin cancer	Female	Cumulative Cigarettes (5 Years)	X			X		
Non-melanoma skin cancer	Female	Education (years per capita)			X			X
Non-melanoma skin cancer	Female	Health System Access (capped)	X				X	
Non-melanoma skin cancer	Female	LDI (I$ per capita)			X			X
Non-melanoma skin cancer	Female	Average latitude		X			X	
Non-melanoma skin cancer	Female	Smoking Prevalence	X			X		
Non-melanoma skin cancer	Male	Cumulative Cigarettes (10 Years)	X			X		
Non-melanoma skin cancer	Male	Cumulative Cigarettes (15 Years)	X			X		
Non-melanoma skin cancer	Male	Socio-demographic Index			X			X
Non-melanoma skin cancer	Male	Cumulative Cigarettes (5 Years)	X			X		
Non-melanoma skin cancer	Male	Education (years per capita)			X			X
Non-melanoma skin cancer	Male	Health System Access (capped)	X				X	
Non-melanoma skin cancer	Male	LDI (I$ per capita)			X			X
Non-melanoma skin cancer	Male	Average latitude		X			X	
Non-melanoma skin cancer	Male	Smoking Prevalence	X			X		
Ovarian cancer	Female	Cumulative Cigarettes (20 Years)	X			X		
Ovarian cancer	Female	Smoking Prevalence		X			X	
Ovarian cancer	Female	Tobacco (cigarettes per capita)	X			X		
Ovarian cancer	Female	Contraception (Modern) Prevalence (proportion)	X			X		
Ovarian cancer	Female	LDI (I$ per capita)			X			X
Ovarian cancer	Female	Alcohol (liters per capita)	X			X		
Ovarian cancer	Female	Diabetes Age-Standardized Prevalence (proportion)		X			X	
Ovarian cancer	Female	Log-transformed SEV scalar: Ovary C	X			X		
Ovarian cancer	Female	Education (years per capita)			X			X
Ovarian cancer	Female	Mean BMI		X			X	
Ovarian cancer	Female	Total Fertility Rate		X			X	
Ovarian cancer	Female	Socio-demographic Index			X			X
Testicular cancer	Male	Cumulative Cigarettes (10 Years)		X			X	
Testicular cancer	Male	Cumulative Cigarettes (15 Years)		X			X	
Testicular cancer	Male	Cumulative Cigarettes (5 Years)		X			X	
Testicular cancer	Male	Education (years per capita)			X			X
Testicular cancer	Male	Fruits (kcal per capita)		X			X	
Testicular cancer	Male	Health System Access 2 (unitless)		X			X	
Testicular cancer	Male	LDI (I$ per capita)			X			X
Testicular cancer	Male	Vegetables (kcal per capita)		X			X	
Testicular cancer	Male	Socio-demographic Index			X			X
Kidney cancer	Male	Cumulative Cigarettes (10 Years)	X			X		
Kidney cancer	Male	Cumulative Cigarettes (15 Years)	X			X		
Kidney cancer	Male	Socio-demographic Index			X			X
Kidney cancer	Male	Cumulative Cigarettes (5 Years)	X			X		
Kidney cancer	Male	Alcohol (liters per capita)		X			X	
Kidney cancer	Male	Education (years per capita)			X			X
Kidney cancer	Male	LDI (I$ per capita)			X			X
Kidney cancer	Male	Health System Access 2 (unitless)		X			X	
Kidney cancer	Male	Diabetes Age-Standardized Prevalence (proportion)		X			X	
Kidney cancer	Male	Smoking Prevalence		X			X	
Kidney cancer	Male	Systolic Blood Pressure (mmHg)		X			X	
Kidney cancer	Male	Mean BMI	X			X		
Kidney cancer	Male	Log-transformed SEV scalar: Kidney C	X			X		
Kidney cancer	Female	Cumulative Cigarettes (10 Years)	X			X		
Kidney cancer	Female	Cumulative Cigarettes (15 Years)	X			X		

Cause	Sex	Covariate	GBD 2015			GBD 2016		
			Level 1	Level 2	Level 3	Level 1	Level 2	Level 3
Kidney cancer	Female	Socio-demographic Index			X			X
Kidney cancer	Female	Cumulative Cigarettes (5 Years)	X			X		
Kidney cancer	Female	Alcohol (liters per capita)		X			X	
Kidney cancer	Female	Total Calories (kcal per capita)		X			X	
Kidney cancer	Female	Education (years per capita)			X			X
Kidney cancer	Female	LDI (I$ per capita)			X			X
Kidney cancer	Female	Health System Access 2 (unitless)		X			X	
Kidney cancer	Female	Diabetes Age-Standardized Prevalence (proportion)		X			X	
Kidney cancer	Female	Total Fertility Rate			X			X
Kidney cancer	Female	Smoking Prevalence		X			X	
Kidney cancer	Female	Systolic Blood Pressure (mmHg)		X			X	
Kidney cancer	Female	Mean BMI	X			X		
Kidney cancer	Female	Log-transformed SEV scalar: Kidney C	X			X		
Bladder cancer	Male	Cumulative Cigarettes (10 Years)	X			X		
Bladder cancer	Male	Cumulative Cigarettes (15 Years)	X			X		
Bladder cancer	Male	Cumulative Cigarettes (5 Years)	X			X		
Bladder cancer	Male	Education (years per capita)			X			X
Bladder cancer	Male	LDI (I$ per capita)			X			X
Bladder cancer	Male	Socio-demographic Index			X			X
Bladder cancer	Male	Smoking Prevalence	X			X		
Bladder cancer	Male	Alcohol (liters per capita)		X			X	
Bladder cancer	Male	Log-transformed SEV scalar: Bladder C	X			X		
Bladder cancer	Male	Population Density (under 150 ppl/sqkm, proportion)		X			X	
Bladder cancer	Male	Population Density (over 1000 ppl/sqkm, proportion)		X			X	
Bladder cancer	Female	Cumulative Cigarettes (10 Years)	X			X		
Bladder cancer	Female	Cumulative Cigarettes (15 Years)	X			X		
Bladder cancer	Female	Cumulative Cigarettes (5 Years)	X			X		
Bladder cancer	Female	Education (years per capita)			X			X
Bladder cancer	Female	LDI (I$ per capita)			X			X
Bladder cancer	Female	Socio-demographic Index			X			X
Bladder cancer	Female	Smoking Prevalence	X			X		
Bladder cancer	Female	Alcohol (liters per capita)		X			X	
Bladder cancer	Female	Log-transformed SEV scalar: Bladder C	X			X		
Bladder cancer	Female	Population Density (under 150 ppl/sqkm, proportion)		X			X	
Bladder cancer	Female	Population Density (over 1000 ppl/sqkm, proportion)		X			X	
Brain and nervous system cancer	Male	Cumulative Cigarettes (10 Years)	X			X		
Brain and nervous system cancer	Male	Cumulative Cigarettes (15 Years)	X			X		
Brain and nervous system cancer	Male	Vegetables (kcal per capita)		X			X	
Brain and nervous system cancer	Male	Smoking Prevalence	X			X		
Brain and nervous system cancer	Male	Education (years per capita)			X			X
Brain and nervous system cancer	Male	LDI (I$ per capita)			X			X
Brain and nervous system cancer	Male	Alcohol (liters per capita)	X			X		
Brain and nervous system cancer	Male	Systolic Blood Pressure (mmHg)		X			X	
Brain and nervous system cancer	Male	Cholesterol (total, mean per capita)		X			X	
Brain and nervous system cancer	Male	Socio-demographic Index			X			X
Brain and nervous system cancer	Female	Cumulative Cigarettes (10 Years)	X			X		
Brain and nervous system cancer	Female	Cumulative Cigarettes (15 Years)	X			X		
Brain and nervous system cancer	Female	Smoking Prevalence	X			X		
Brain and nervous system cancer	Female	Education (years per capita)			X			X
Brain and nervous system cancer	Female	LDI (I$ per capita)			X			X
Brain and nervous system cancer	Female	Alcohol (liters per capita)	X			X		
Brain and nervous system cancer	Female	Systolic Blood Pressure (mmHg)		X			X	
Brain and nervous system cancer	Female	Cholesterol (total, mean per capita)		X			X	
Brain and nervous system cancer	Female	Socio-demographic Index			X			X
Thyroid cancer	Male	Smoking Prevalence	X				X	
Thyroid cancer	Male	Smoking Prevalence	X			X		
Thyroid cancer	Male	Sanitation (proportion with access)		X			X	
Thyroid cancer	Male	Tobacco (cigarettes per capita)	X				X	
Thyroid cancer	Male	Education (years per capita)			X			X
Thyroid cancer	Male	Log-transformed SEV scalar: Thyroid C	X			X		
Thyroid cancer	Male	LDI (I$ per capita)			X			X
Thyroid cancer	Male	Socio-demographic Index			X			X
Thyroid cancer	Male	Alcohol (liters per capita)	X			X		
Thyroid cancer	Male	Mean BMI		X			X	
Thyroid cancer	Male	Improved Water Source (proportion with access)		X			X	
Thyroid cancer	Female	Smoking Prevalence	X				X	
Thyroid cancer	Female	Sanitation (proportion with access)		X			X	
Thyroid cancer	Female	Tobacco (cigarettes per capita)	X				X	
Thyroid cancer	Female	Education (years per capita)			X			X
Thyroid cancer	Female	Log-transformed SEV scalar: Thyroid C	X			X		
Thyroid cancer	Female	LDI (I$ per capita)			X			X
Thyroid cancer	Female	Socio-demographic Index			X			X
Thyroid cancer	Female	Alcohol (liters per capita)	X			X		
Thyroid cancer	Female	Mean BMI		X			X	
Thyroid cancer	Female	Improved Water Source (proportion with access)		X			X	
Mesothelioma	Male	Cumulative Cigarettes (5 Years)	X			X		
Mesothelioma	Male	Education (years per capita)			X			X
Mesothelioma	Male	Gold production (binary)		X			X	
Mesothelioma	Male	Gold production (kg) per capita		X			X	
Mesothelioma	Male	LDI (I$ per capita)			X			X
Mesothelioma	Male	Elevation Over 1500m (proportion)		X			X	
Mesothelioma	Male	Elevation 500 to 1500m (proportion)		X			X	
Mesothelioma	Male	Population Density (over 1000 ppl/sqkm, proportion)		X			X	
Mesothelioma	Male	Population Over 65 (proportion)		X			X	
Mesothelioma	Male	Smoking Prevalence	X			X		

Cause	Sex	Covariate	GBD 2015			GBD 2016		
			Level 1	Level 2	Level 3	Level 1	Level 2	Level 3
Mesothelioma	Male	Socio-demographic Index			X			X
Mesothelioma	Female	Asbestos production (binary)	X			X		
Mesothelioma	Female	Asbestos production (kg) per capita		X			X	
Mesothelioma	Female	Cumulative Cigarettes (5 Years)	X			X		
Mesothelioma	Female	Education (years per capita)			X			X
Mesothelioma	Female	Gold production (binary)		X			X	
Mesothelioma	Female	Gold production (kg) per capita		X			X	
Mesothelioma	Female	LDI (I$ per capita)			X			X
Mesothelioma	Female	Elevation Over 1500m (proportion)		X			X	
Mesothelioma	Female	Elevation 500 to 1500m (proportion)		X			X	
Mesothelioma	Female	Population Density (over 1000 ppl/sqkm, proportion)		X			X	
Mesothelioma	Female	Population Over 65 (proportion)		X			X	
Mesothelioma	Female	Smoking Prevalence	X			X		
Mesothelioma	Female	Log-transformed SEV scalar: Mesothel	X			X		
Mesothelioma	Female	Socio-demographic Index			X			X
Hodgkin lymphoma	Male	Education (years per capita)			X			X
Hodgkin lymphoma	Male	LDI (I$ per capita)			X			X
Hodgkin lymphoma	Male	Socio-demographic Index			X			X
Hodgkin lymphoma	Female	Education (years per capita)			X			X
Hodgkin lymphoma	Female	LDI (I$ per capita)			X			X
Hodgkin lymphoma	Female	Socio-demographic Index			X			X
Non-Hodgkin lymphoma	Female	Cumulative Cigarettes (10 Years)		X			X	
Non-Hodgkin lymphoma	Female	Socio-demographic Index			X			X
Non-Hodgkin lymphoma	Female	Smoking Prevalence		X			X	
Non-Hodgkin lymphoma	Female	LDI (I$ per capita)			X			X
Non-Hodgkin lymphoma	Female	Health System Access 2 (unitless)	X				X	
Non-Hodgkin lymphoma	Female	Total Fertility Rate			X			X
Non-Hodgkin lymphoma	Female	Alcohol (liters per capita)		X			X	
Non-Hodgkin lymphoma	Male	Cumulative Cigarettes (10 Years)		X			X	
Non-Hodgkin lymphoma	Male	Socio-demographic Index			X			X
Non-Hodgkin lymphoma	Male	Smoking Prevalence		X			X	
Non-Hodgkin lymphoma	Male	LDI (I$ per capita)			X			X
Non-Hodgkin lymphoma	Male	Health System Access 2 (unitless)	X			X		
Non-Hodgkin lymphoma	Male	Alcohol (liters per capita)		X			X	
Multiple myeloma	Female	Smoking Prevalence	X			X		
Multiple myeloma	Female	Sanitation (proportion with access)		X			X	
Multiple myeloma	Female	Tobacco (cigarettes per capita)	X			X		
Multiple myeloma	Female	Education (years per capita)			X			X
Multiple myeloma	Female	LDI (I$ per capita)			X			X
Multiple myeloma	Female	Socio-demographic Index			X			X
Multiple myeloma	Female	Alcohol (liters per capita)	X			X		
Multiple myeloma	Female	Mean BMI		X			X	
Multiple myeloma	Female	Improved Water Source (proportion with access)		X			X	
Multiple myeloma	Male	Smoking Prevalence	X			X		
Multiple myeloma	Male	Sanitation (proportion with access)		X			X	
Multiple myeloma	Male	Tobacco (cigarettes per capita)	X			X		
Multiple myeloma	Male	Education (years per capita)			X			X
Multiple myeloma	Male	LDI (I$ per capita)			X			X
Multiple myeloma	Male	Socio-demographic Index			X			X
Multiple myeloma	Male	Alcohol (liters per capita)	X			X		
Multiple myeloma	Male	Mean BMI		X			X	
Multiple myeloma	Male	Improved Water Source (proportion with access)		X			X	
Leukemia	Male	Cumulative Cigarettes (10 Years)	X				X	
Leukemia	Male	Cumulative Cigarettes (15 Years)	X				X	
Leukemia	Male	Socio-demographic Index			X			X
Leukemia	Male	Cumulative Cigarettes (5 Years)	X				X	
Leukemia	Male	Education (years per capita)			X			X
Leukemia	Male	LDI (I$ per capita)			X			X
Leukemia	Male	Health System Access 2 (unitless)	X				X	
Leukemia	Male	Log-transformed SEV scalar: Leukemia	X			X		
Leukemia	Male	Smoking Prevalence	X				X	
Leukemia	Male	Alcohol (liters per capita)		X			X	
Leukemia	Female	Alcohol (liters per capita)		X			X	
Leukemia	Female	Cumulative Cigarettes (10 Years)	X				X	
Leukemia	Female	Cumulative Cigarettes (15 Years)	X				X	
Leukemia	Female	Education (years per capita)			X			X
Leukemia	Female	Health System Access 2 (unitless)	X				X	
Leukemia	Female	Health System Access 2 (unitless)	X			X		
Leukemia	Female	LDI (I$ per capita)			X			X
Leukemia	Female	Smoking Prevalence	X				X	
Leukemia	Female	Log-transformed SEV scalar: Leukemia	X			X		
Leukemia	Female	Socio-demographic Index			X			X
Other neoplasms	Male	Tobacco (cigarettes per capita)	X			X		
Other neoplasms	Male	Education (years per capita)			X			X
Other neoplasms	Male	Health System Access 2 (unitless)		X			X	
Other neoplasms	Male	LDI (I$ per capita)			X			X
Other neoplasms	Male	Nuts & Seeds (kcal per capita)		X			X	
Other neoplasms	Male	Smoking Prevalence	X			X		
Other neoplasms	Male	Socio-demographic Index			X			X
Other neoplasms	Female	Tobacco (cigarettes per capita)	X			X		
Other neoplasms	Female	Education (years per capita)			X			X
Other neoplasms	Female	Health System Access 2 (unitless)		X			X	
Other neoplasms	Female	LDI (I$ per capita)			X			X
Other neoplasms	Female	Smoking Prevalence	X			X		
Other neoplasms	Female	Socio-demographic Index			X			X

Cause	Sex	Covariate	GBD 2015			GBD 2016		
			Level 1	Level 2	Level 3	Level 1	Level 2	Level 3
Cardiovascular diseases	Male	Alcohol (liters per capita)		X				X
Cardiovascular diseases	Male	LDI (I$ per capita)			X			X
Cardiovascular diseases	Male	Cholesterol (total, mean per capita)	X			X		
Cardiovascular diseases	Male	Smoking Prevalence	X			X		
Cardiovascular diseases	Male	Socio-demographic Index			X			X
Cardiovascular diseases	Male	omega 3 adjusted(g)		X				X
Cardiovascular diseases	Male	vegetables adjusted(g)		X				X
Cardiovascular diseases	Female	Alcohol (liters per capita)		X				X
Cardiovascular diseases	Female	LDI (I$ per capita)			X			X
Cardiovascular diseases	Female	Cholesterol (total, mean per capita)	X			X		
Cardiovascular diseases	Female	Smoking Prevalence	X			X		
Cardiovascular diseases	Female	Socio-demographic Index			X			X
Cardiovascular diseases	Female	omega 3 adjusted(g)		X				X
Cardiovascular diseases	Female	vegetables adjusted(g)		X				X
Rheumatic heart disease	Male	Log-transformed SEV scalar: RHD	X			X		
Rheumatic heart disease	Male	Sanitation (proportion with access)	X			X		
Rheumatic heart disease	Male	Education (years per capita)			X			X
Rheumatic heart disease	Male	LDI (I$ per capita)			X			X
Rheumatic heart disease	Male	Socio-demographic Index			X			X
Rheumatic heart disease	Male	Underweight (proportion <2SD weight for age, <5 years)	X			X		
Rheumatic heart disease	Male	Improved Water Source (proportion with access)	X			X		
Rheumatic heart disease	Female	Log-transformed SEV scalar: RHD	X			X		
Rheumatic heart disease	Female	Sanitation (proportion with access)	X			X		
Rheumatic heart disease	Female	Education (years per capita)			X			X
Rheumatic heart disease	Female	LDI (I$ per capita)			X			X
Rheumatic heart disease	Female	Socio-demographic Index			X			X
Rheumatic heart disease	Female	Underweight (proportion <2SD weight for age, <5 years)	X			X		
Rheumatic heart disease	Female	Improved Water Source (proportion with access)	X			X		
Ischemic heart disease	Male	Alcohol (liters per capita)		X				X
Ischemic heart disease	Male	LDI (I$ per capita)			X			X
Ischemic heart disease	Male	Cholesterol (total, mean per capita)	X			X		
Ischemic heart disease	Male	Smoking Prevalence	X			X		
Ischemic heart disease	Male	Log-transformed SEV scalar: IHD	X			X		
Ischemic heart disease	Male	Socio-demographic Index			X			X
Ischemic heart disease	Male	omega 3 adjusted(g)		X				X
Ischemic heart disease	Male	pulses legumes adjusted(g)			X			X
Ischemic heart disease	Male	vegetables adjusted(g)		X				X
Ischemic heart disease	Female	Alcohol (liters per capita)		X				X
Ischemic heart disease	Female	LDI (I$ per capita)			X			X
Ischemic heart disease	Female	Cholesterol (total, mean per capita)	X			X		
Ischemic heart disease	Female	Smoking Prevalence	X			X		
Ischemic heart disease	Female	Log-transformed SEV scalar: IHD	X			X		
Ischemic heart disease	Female	Socio-demographic Index			X			X
Ischemic heart disease	Female	omega 3 adjusted(g)		X				X
Ischemic heart disease	Female	pulses legumes adjusted(g)			X			X
Ischemic heart disease	Female	vegetables adjusted(g)		X				X
Cerebrovascular disease	Male	Alcohol (liters per capita)		X				X
Cerebrovascular disease	Male	LDI (I$ per capita)			X			X
Cerebrovascular disease	Male	Mean BMI	X				X	
Cerebrovascular disease	Male	Cholesterol (total, mean per capita)	X			X		
Cerebrovascular disease	Male	Systolic Blood Pressure (mmHg)	X			X		
Cerebrovascular disease	Male	Log-transformed SEV scalar: Stroke	X			X		
Cerebrovascular disease	Male	Socio-demographic Index			X			X
Cerebrovascular disease	Male	fruits adjusted(g)		X			X	
Cerebrovascular disease	Male	vegetables adjusted(g)		X			X	
Cerebrovascular disease	Male	whole grains adjusted(g)			X			X
Cerebrovascular disease	Female	Alcohol (liters per capita)		X				X
Cerebrovascular disease	Female	LDI (I$ per capita)			X			X
Cerebrovascular disease	Female	Mean BMI	X				X	
Cerebrovascular disease	Female	Cholesterol (total, mean per capita)	X			X		
Cerebrovascular disease	Female	Systolic Blood Pressure (mmHg)	X			X		
Cerebrovascular disease	Female	Log-transformed SEV scalar: Stroke	X			X		
Cerebrovascular disease	Female	Socio-demographic Index			X			X
Cerebrovascular disease	Female	fruits adjusted(g)		X			X	
Cerebrovascular disease	Female	vegetables adjusted(g)		X			X	
Cerebrovascular disease	Female	whole grains adjusted(g)			X			X
Ischemic stroke	Male	Alcohol (liters per capita)		X				X
Ischemic stroke	Male	LDI (I$ per capita)			X			X
Ischemic stroke	Male	Cholesterol (total, mean per capita)	X			X		
Ischemic stroke	Male	Smoking Prevalence	X			X		
Ischemic stroke	Male	Log-transformed SEV scalar: Isch Stroke	X			X		
Ischemic stroke	Male	Socio-demographic Index			X			X
Ischemic stroke	Male	omega 3 adjusted(g)			X			X
Ischemic stroke	Male	fruits adjusted(g)		X				X
Ischemic stroke	Male	nuts seeds adjusted(g)		X				X
Ischemic stroke	Male	pulses legumes adjusted(g)		X				X
Ischemic stroke	Male	vegetables adjusted(g)		X				X
Ischemic stroke	Female	Alcohol (liters per capita)		X				X
Ischemic stroke	Female	LDI (I$ per capita)			X			X
Ischemic stroke	Female	Cholesterol (total, mean per capita)	X			X		
Ischemic stroke	Female	Smoking Prevalence	X			X		
Ischemic stroke	Female	Log-transformed SEV scalar: Isch Stroke	X			X		
Ischemic stroke	Female	Socio-demographic Index			X			X
Ischemic stroke	Female	omega 3 adjusted(g)			X			X
Ischemic stroke	Female	fruits adjusted(g)		X				X

Cause	Sex	Covariate	GBD 2015			GBD 2016		
			Level 1	Level 2	Level 3	Level 1	Level 2	Level 3
Ischemic stroke	Female	nuts seeds adjusted(g)		X				X
Ischemic stroke	Female	pulses legumes adjusted(g)		X				X
Ischemic stroke	Female	vegetables adjusted(g)		X				X
Hemorrhagic stroke	Male	Alcohol (liters per capita)		X				X
Hemorrhagic stroke	Male	LDI (I$ per capita)			X			X
Hemorrhagic stroke	Male	Smoking Prevalence	X			X		
Hemorrhagic stroke	Male	Log-transformed SEV scalar: Hem Stroke	X			X		
Hemorrhagic stroke	Male	Socio-demographic Index			X			X
Hemorrhagic stroke	Male	omega 3 adjusted(g)		X				X
Hemorrhagic stroke	Male	nuts seeds adjusted(g)		X				X
Hemorrhagic stroke	Male	pufa adjusted(percent)		X				X
Hemorrhagic stroke	Male	pulses legumes adjusted(g)		X				X
Hemorrhagic stroke	Male	vegetables adjusted(g)		X				X
Hemorrhagic stroke	Female	Alcohol (liters per capita)		X				X
Hemorrhagic stroke	Female	LDI (I$ per capita)			X			X
Hemorrhagic stroke	Female	Smoking Prevalence	X			X		
Hemorrhagic stroke	Female	Log-transformed SEV scalar: Hem Stroke	X			X		
Hemorrhagic stroke	Female	Socio-demographic Index			X			X
Hemorrhagic stroke	Female	omega 3 adjusted(g)		X				X
Hemorrhagic stroke	Female	nuts seeds adjusted(g)		X				X
Hemorrhagic stroke	Female	pufa adjusted(percent)		X				X
Hemorrhagic stroke	Female	pulses legumes adjusted(g)		X				X
Hemorrhagic stroke	Female	vegetables adjusted(g)		X				X
Hypertensive heart disease	Male	LDI (I$ per capita)			X			X
Hypertensive heart disease	Male	Systolic Blood Pressure (mmHg)	X			X		
Hypertensive heart disease	Male	Socio-demographic Index			X			X
Hypertensive heart disease	Female	LDI (I$ per capita)			X			X
Hypertensive heart disease	Female	Systolic Blood Pressure (mmHg)	X			X		
Hypertensive heart disease	Female	Socio-demographic Index			X			X
Cardiomyopathy and myocarditis	Male	Log-transformed SEV scalar: CMP	X			X		
Cardiomyopathy and myocarditis	Male	LDI (I$ per capita)			X			X
Cardiomyopathy and myocarditis	Male	Systolic Blood Pressure (mmHg)	X			X		
Cardiomyopathy and myocarditis	Male	Alcohol (liters per capita)		X				X
Cardiomyopathy and myocarditis	Male	Socio-demographic Index			X			X
Cardiomyopathy and myocarditis	Female	Log-transformed SEV scalar: CMP	X			X		
Cardiomyopathy and myocarditis	Female	LDI (I$ per capita)			X			X
Cardiomyopathy and myocarditis	Female	Systolic Blood Pressure (mmHg)	X			X		
Cardiomyopathy and myocarditis	Female	Alcohol (liters per capita)		X				X
Cardiomyopathy and myocarditis	Female	Socio-demographic Index			X			X
Aortic aneurysm	Male	Alcohol (liters per capita)		X				X
Aortic aneurysm	Male	LDI (I$ per capita)			X			X
Aortic aneurysm	Male	Mean BMI	X				X	
Aortic aneurysm	Male	Cholesterol (total, mean per capita)	X			X		
Aortic aneurysm	Male	Systolic Blood Pressure (mmHg)	X			X		
Aortic aneurysm	Male	Log-transformed SEV scalar: Aort An	X			X		
Aortic aneurysm	Male	Socio-demographic Index			X			X
Aortic aneurysm	Male	fruits adjusted(g)		X				X
Aortic aneurysm	Male	vegetables adjusted(g)		X				X
Aortic aneurysm	Male	whole grains adjusted(g)		X				X
Aortic aneurysm	Female	Alcohol (liters per capita)		X				X
Aortic aneurysm	Female	LDI (I$ per capita)			X			X
Aortic aneurysm	Female	Mean BMI	X				X	
Aortic aneurysm	Female	Cholesterol (total, mean per capita)	X			X		
Aortic aneurysm	Female	Systolic Blood Pressure (mmHg)	X			X		
Aortic aneurysm	Female	Log-transformed SEV scalar: Aort An	X			X		
Aortic aneurysm	Female	Socio-demographic Index			X			X
Aortic aneurysm	Female	fruits adjusted(g)		X				X
Aortic aneurysm	Female	vegetables adjusted(g)		X				X
Aortic aneurysm	Female	whole grains adjusted(g)		X				X
Peripheral artery disease	Male	Alcohol (liters per capita)		X				X
Peripheral artery disease	Male	LDI (I$ per capita)			X			X
Peripheral artery disease	Male	Mean BMI	X				X	
Peripheral artery disease	Male	Cholesterol (total, mean per capita)	X			X		
Peripheral artery disease	Male	Systolic Blood Pressure (mmHg)	X			X		
Peripheral artery disease	Male	Smoking Prevalence		X		X		
Peripheral artery disease	Male	Log-transformed SEV scalar: PVD	X			X		
Peripheral artery disease	Male	Socio-demographic Index			X			X
Peripheral artery disease	Male	omega 3 adjusted(g)		X				X
Peripheral artery disease	Male	fruits adjusted(g)		X				X
Peripheral artery disease	Male	nuts seeds adjusted(g)		X				X
Peripheral artery disease	Male	pufa adjusted(percent)		X				X
Peripheral artery disease	Male	pulses legumes adjusted(g)		X				X
Peripheral artery disease	Male	vegetables adjusted(g)		X				X
Peripheral artery disease	Male	whole grains adjusted(g)		X				X
Peripheral artery disease	Female	Alcohol (liters per capita)		X				X
Peripheral artery disease	Female	LDI (I$ per capita)			X			X
Peripheral artery disease	Female	Mean BMI	X				X	
Peripheral artery disease	Female	Cholesterol (total, mean per capita)	X			X		
Peripheral artery disease	Female	Systolic Blood Pressure (mmHg)	X			X		
Peripheral artery disease	Female	Smoking Prevalence		X		X		
Peripheral artery disease	Female	Log-transformed SEV scalar: PVD	X			X		
Peripheral artery disease	Female	Socio-demographic Index			X			X
Peripheral artery disease	Female	omega 3 adjusted(g)		X				X
Peripheral artery disease	Female	fruits adjusted(g)		X				X
Peripheral artery disease	Female	nuts seeds adjusted(g)		X				X

Cause	Sex	Covariate	GBD 2015			GBD 2016		
			Level 1	Level 2	Level 3	Level 1	Level 2	Level 3
Peripheral artery disease	Female	pufa adjusted(percent)		X				X
Peripheral artery disease	Female	pulses legumes adjusted(g)		X				X
Peripheral artery disease	Female	vegetables adjusted(g)		X				X
Peripheral artery disease	Female	whole grains adjusted(g)		X				X
Endocarditis	Male	LDI (I$ per capita)			X			X
Endocarditis	Male	Sanitation (proportion with access)	X			X		
Endocarditis	Male	Improved Water Source (proportion with access)	X			X		
Endocarditis	Male	Log-transformed SEV scalar: Endocar	X			X		
Endocarditis	Male	Socio-demographic Index			X			X
Endocarditis	Female	LDI (I$ per capita)			X			X
Endocarditis	Female	Sanitation (proportion with access)	X			X		
Endocarditis	Female	Improved Water Source (proportion with access)	X			X		
Endocarditis	Female	Log-transformed SEV scalar: Endocar	X			X		
Endocarditis	Female	Socio-demographic Index			X			X
Other cardiovascular and circulatory diseases	Male	Alcohol (liters per capita)		X				X
Other cardiovascular and circulatory diseases	Male	LDI (I$ per capita)			X			X
Other cardiovascular and circulatory diseases	Male	Mean BMI	X				X	
Other cardiovascular and circulatory diseases	Male	Cholesterol (total, mean per capita)	X			X		
Other cardiovascular and circulatory diseases	Male	Systolic Blood Pressure (mmHg)	X			X		
Other cardiovascular and circulatory diseases	Male	Indoor Air Pollution (All Cooking Fuels)		X			X	
Other cardiovascular and circulatory diseases	Male	Outdoor Air Pollution (PM2.5)		X			X	
Other cardiovascular and circulatory diseases	Male	Smoking Prevalence		X		X		
Other cardiovascular and circulatory diseases	Male	Log-transformed SEV scalar: Oth Cardio	X			X		
Other cardiovascular and circulatory diseases	Male	Socio-demographic Index			X			X
Other cardiovascular and circulatory diseases	Male	omega 3 adjusted(g)		X				X
Other cardiovascular and circulatory diseases	Male	fruits adjusted(g)		X				X
Other cardiovascular and circulatory diseases	Male	nuts seeds adjusted(g)		X				X
Other cardiovascular and circulatory diseases	Male	pufa adjusted(percent)		X				X
Other cardiovascular and circulatory diseases	Male	pulses legumes adjusted(g)		X				X
Other cardiovascular and circulatory diseases	Male	vegetables adjusted(g)		X				X
Other cardiovascular and circulatory diseases	Male	whole grains adjusted(g)		X				X
Other cardiovascular and circulatory diseases	Female	Alcohol (liters per capita)		X				X
Other cardiovascular and circulatory diseases	Female	LDI (I$ per capita)			X			X
Other cardiovascular and circulatory diseases	Female	Mean BMI	X				X	
Other cardiovascular and circulatory diseases	Female	Cholesterol (total, mean per capita)	X			X		
Other cardiovascular and circulatory diseases	Female	Systolic Blood Pressure (mmHg)	X			X		
Other cardiovascular and circulatory diseases	Female	Indoor Air Pollution (All Cooking Fuels)		X			X	
Other cardiovascular and circulatory diseases	Female	Outdoor Air Pollution (PM2.5)		X			X	
Other cardiovascular and circulatory diseases	Female	Smoking Prevalence		X		X		
Other cardiovascular and circulatory diseases	Female	Log-transformed SEV scalar: Oth Cardio	X			X		
Other cardiovascular and circulatory diseases	Female	Socio-demographic Index			X			X
Other cardiovascular and circulatory diseases	Female	omega 3 adjusted(g)		X				X
Other cardiovascular and circulatory diseases	Female	fruits adjusted(g)		X				X
Other cardiovascular and circulatory diseases	Female	nuts seeds adjusted(g)		X				X
Other cardiovascular and circulatory diseases	Female	pufa adjusted(percent)		X				X
Other cardiovascular and circulatory diseases	Female	pulses legumes adjusted(g)		X				X
Other cardiovascular and circulatory diseases	Female	vegetables adjusted(g)		X				X
Other cardiovascular and circulatory diseases	Female	whole grains adjusted(g)		X				X
Chronic respiratory diseases	Male	Socio-demographic Index			X			X
Chronic respiratory diseases	Male	Cumulative Cigarettes (5 Years)		X		X		
Chronic respiratory diseases	Male	Cumulative Cigarettes (5 Years)	X			X		
Chronic respiratory diseases	Male	Elevation Over 1500m (proportion)		X			X	
Chronic respiratory diseases	Male	Education (years per capita)			X			X
Chronic respiratory diseases	Male	LDI (I$ per capita)			X			X
Chronic respiratory diseases	Male	Elevation 500 to 1500m (proportion)			X			X
Chronic respiratory diseases	Male	Smoking Prevalence	X			X		
Chronic respiratory diseases	Male	Smoking Prevalence	X				X	
Chronic respiratory diseases	Male	Smoking Prevalence		X		X		
Chronic respiratory diseases	Male	Smoking Prevalence		X			X	
Chronic respiratory diseases	Male	Outdoor Air Pollution (PM2.5)		X			X	
Chronic respiratory diseases	Male	Population Density (over 1000 ppl/sqkm, proportion)			X			X
Chronic respiratory diseases	Female	Cumulative Cigarettes (10 Years)	X			X		
Chronic respiratory diseases	Female	Cumulative Cigarettes (5 Years)	X			X		
Chronic respiratory diseases	Female	Education (years per capita)			X			X
Chronic respiratory diseases	Female	LDI (I$ per capita)			X			X
Chronic respiratory diseases	Female	Outdoor Air Pollution (PM2.5)		X			X	
Chronic respiratory diseases	Female	Elevation Over 1500m (proportion)		X			X	
Chronic respiratory diseases	Female	Elevation 500 to 1500m (proportion)			X			X
Chronic respiratory diseases	Female	Population Density (over 1000 ppl/sqkm, proportion)			X			X
Chronic respiratory diseases	Female	Smoking Prevalence		X			X	
Chronic respiratory diseases	Female	Socio-demographic Index			X			X
Chronic respiratory diseases	Female	Log-transformed SEV scalar: Chr Resp	X			X		
Chronic respiratory diseases	Male	Cumulative Cigarettes (10 Years)	X			X		
Chronic respiratory diseases	Male	Log-transformed SEV scalar: Chr Resp	X			X		
Chronic obstructive pulmonary disease	Female	Cumulative Cigarettes (10 Years)	X			X		
Chronic obstructive pulmonary disease	Female	Cumulative Cigarettes (5 Years)	X			X		
Chronic obstructive pulmonary disease	Female	Education (years per capita)			X			X
Chronic obstructive pulmonary disease	Female	LDI (I$ per capita)			X			X
Chronic obstructive pulmonary disease	Female	Outdoor Air Pollution (PM2.5)		X			X	
Chronic obstructive pulmonary disease	Female	Elevation Over 1500m (proportion)	X			X		
Chronic obstructive pulmonary disease	Female	Smoking Prevalence		X			X	
Chronic obstructive pulmonary disease	Female	Socio-demographic Index			X			X
Chronic obstructive pulmonary disease	Male	Cumulative Cigarettes (10 Years)	X			X		
Chronic obstructive pulmonary disease	Male	Cumulative Cigarettes (5 Years)	X			X		
Chronic obstructive pulmonary disease	Male	Education (years per capita)			X			X

Cause	Sex	Covariate	GBD 2015			GBD 2016		
			Level 1	Level 2	Level 3	Level 1	Level 2	Level 3
Chronic obstructive pulmonary disease	Male	LDI (I$ per capita)			X			X
Chronic obstructive pulmonary disease	Male	Outdoor Air Pollution (PM2.5)		X			X	
Chronic obstructive pulmonary disease	Male	Elevation Over 1500m (proportion)	X			X		
Chronic obstructive pulmonary disease	Male	Smoking Prevalence		X			X	
Chronic obstructive pulmonary disease	Male	Socio-demographic Index			X			X
Pneumoconiosis	Male	Log-transformed SEV scalar: Pneumocon	X			X		
Pneumoconiosis	Male	Socio-demographic Index			X			X
Pneumoconiosis	Male	Cumulative Cigarettes (5 Years)		X			X	
Pneumoconiosis	Male	Coal Production (per capita)	X			X		
Pneumoconiosis	Male	Education (years per capita)			X			X
Pneumoconiosis	Male	LDI (I$ per capita)			X			X
Pneumoconiosis	Male	Gold production (kg) per capita	X			X		
Pneumoconiosis	Male	Smoking Prevalence		X			X	
Pneumoconiosis	Male	Elevation 500 to 1500m (proportion)		X			X	
Pneumoconiosis	Male	Elevation Over 1500m (proportion)		X			X	
Pneumoconiosis	Female	Log-transformed SEV scalar: Pneumocon	X			X		
Pneumoconiosis	Female	Socio-demographic Index			X			X
Pneumoconiosis	Female	Cumulative Cigarettes (5 Years)		X			X	
Pneumoconiosis	Female	Coal Production (per capita)	X			X		
Pneumoconiosis	Female	Education (years per capita)			X			X
Pneumoconiosis	Female	LDI (I$ per capita)			X			X
Pneumoconiosis	Female	Gold production (kg) per capita	X			X		
Pneumoconiosis	Female	Smoking Prevalence		X			X	
Pneumoconiosis	Female	Elevation 500 to 1500m (proportion)		X			X	
Pneumoconiosis	Female	Elevation Over 1500m (proportion)		X			X	
Silicosis	Female	Socio-demographic Index			X			X
Silicosis	Female	Cumulative Cigarettes (5 Years)	X				X	
Silicosis	Female	Elevation Over 1500m (proportion)		X			X	
Silicosis	Female	Education (years per capita)			X			X
Silicosis	Female	LDI (I$ per capita)			X			X
Silicosis	Female	Gold production (kg) per capita		X		X		
Silicosis	Female	Smoking Prevalence	X				X	
Silicosis	Female	Elevation 500 to 1500m (proportion)		X			X	
Silicosis	Female	Log-transformed SEV scalar: Silicosis	X			X		
Silicosis	Male	Socio-demographic Index			X			X
Silicosis	Male	Cumulative Cigarettes (5 Years)	X				X	
Silicosis	Male	Elevation Over 1500m (proportion)		X			X	
Silicosis	Male	Education (years per capita)			X			X
Silicosis	Male	LDI (I$ per capita)			X			X
Silicosis	Male	Gold production (kg) per capita		X		X		
Silicosis	Male	Smoking Prevalence	X				X	
Silicosis	Male	Elevation 500 to 1500m (proportion)		X			X	
Silicosis	Male	Log-transformed SEV scalar: Silicosis	X			X		
Asbestosis	Male	Log-transformed SEV scalar: Asbestosis	X			X		
Asbestosis	Male	Cumulative Cigarettes (5 Years)	X				X	
Asbestosis	Male	Education (years per capita)			X			X
Asbestosis	Male	LDI (I$ per capita)			X			X
Asbestosis	Male	Smoking Prevalence	X				X	
Asbestosis	Male	Smoking Prevalence	X			X		
Asbestosis	Male	Elevation 500 to 1500m (proportion)		X			X	
Asbestosis	Male	Elevation Over 1500m (proportion)		X			X	
Asbestosis	Male	Socio-demographic Index			X			X
Asbestosis	Female	Log-transformed SEV scalar: Asbestosis	X			X		
Asbestosis	Female	Cumulative Cigarettes (5 Years)	X				X	
Asbestosis	Female	Education (years per capita)			X			X
Asbestosis	Female	LDI (I$ per capita)			X			X
Asbestosis	Female	Smoking Prevalence	X				X	
Asbestosis	Female	Elevation 500 to 1500m (proportion)		X			X	
Asbestosis	Female	Elevation Over 1500m (proportion)		X			X	
Asbestosis	Female	Socio-demographic Index			X			X
Coal workers pneumoconiosis	Female	Socio-demographic Index			X			X
Coal workers pneumoconiosis	Female	Cumulative Cigarettes (5 Years)	X				X	
Coal workers pneumoconiosis	Female	Log-transformed SEV scalar: Coal W	X			X		
Coal workers pneumoconiosis	Female	Education (years per capita)			X			X
Coal workers pneumoconiosis	Female	LDI (I$ per capita)			X			X
Coal workers pneumoconiosis	Female	Smoking Prevalence	X				X	
Coal workers pneumoconiosis	Female	Elevation 500 to 1500m (proportion)		X			X	
Coal workers pneumoconiosis	Female	Elevation Over 1500m (proportion)		X			X	
Coal workers pneumoconiosis	Male	Socio-demographic Index			X			X
Coal workers pneumoconiosis	Male	Cumulative Cigarettes (5 Years)	X				X	
Coal workers pneumoconiosis	Male	Log-transformed SEV scalar: Coal W	X			X		
Coal workers pneumoconiosis	Male	Education (years per capita)			X			X
Coal workers pneumoconiosis	Male	LDI (I$ per capita)			X			X
Coal workers pneumoconiosis	Male	Smoking Prevalence	X				X	
Coal workers pneumoconiosis	Male	Elevation 500 to 1500m (proportion)		X			X	
Coal workers pneumoconiosis	Male	Elevation Over 1500m (proportion)		X			X	
Other pneumoconiosis	Male	Socio-demographic Index			X			X
Other pneumoconiosis	Male	Cumulative Cigarettes (5 Years)	X				X	
Other pneumoconiosis	Male	Log-transformed SEV scalar: Oth Pneum	X			X		
Other pneumoconiosis	Male	Education (years per capita)			X			X
Other pneumoconiosis	Male	LDI (I$ per capita)			X			X
Other pneumoconiosis	Male	Smoking Prevalence	X				X	
Other pneumoconiosis	Male	Elevation 500 to 1500m (proportion)		X			X	
Other pneumoconiosis	Male	Elevation Over 1500m (proportion)		X			X	
Other pneumoconiosis	Female	Socio-demographic Index			X			X

Cause	Sex	Covariate	GBD 2015			GBD 2016		
			Level 1	Level 2	Level 3	Level 1	Level 2	Level 3
Other pneumoconiosis	Female	Cumulative Cigarettes (5 Years)	X				X	
Other pneumoconiosis	Female	Log-transformed SEV scalar: Oth Pneum	X			X		
Other pneumoconiosis	Female	Education (years per capita)			X			X
Other pneumoconiosis	Female	LDI (I$ per capita)			X			X
Other pneumoconiosis	Female	Smoking Prevalence	X				X	
Other pneumoconiosis	Female	Elevation 500 to 1500m (proportion)		X			X	
Other pneumoconiosis	Female	Elevation Over 1500m (proportion)		X			X	
Asthma	Male	Cumulative Cigarettes (10 Years)	X			X		
Asthma	Male	Cumulative Cigarettes (5 Years)	X			X		
Asthma	Male	Education (years per capita)			X			X
Asthma	Male	LDI (I$ per capita)			X			X
Asthma	Male	Outdoor Air Pollution (PM2.5)		X			X	
Asthma	Male	Smoking Prevalence		X			X	
Asthma	Male	Log-transformed SEV scalar: Asthma	X			X		
Asthma	Male	Socio-demographic Index			X			X
Asthma	Female	Cumulative Cigarettes (10 Years)	X			X		
Asthma	Female	Cumulative Cigarettes (5 Years)	X			X		
Asthma	Female	Education (years per capita)			X			X
Asthma	Female	LDI (I$ per capita)			X			X
Asthma	Female	Outdoor Air Pollution (PM2.5)		X			X	
Asthma	Female	Smoking Prevalence		X			X	
Asthma	Female	Log-transformed SEV scalar: Asthma	X			X		
Asthma	Female	Socio-demographic Index			X			X
Interstitial lung disease and pulmonary sarcoidosis	Female	Socio-demographic Index			X			X
Interstitial lung disease and pulmonary sarcoidosis	Female	Smoking Prevalence	X			X		
Interstitial lung disease and pulmonary sarcoidosis	Female	Log-transformed SEV scalar: ILD	X			X		
Interstitial lung disease and pulmonary sarcoidosis	Female	Education (years per capita)			X			X
Interstitial lung disease and pulmonary sarcoidosis	Female	LDI (I$ per capita)			X			X
Interstitial lung disease and pulmonary sarcoidosis	Female	Elevation Over 1500m (proportion)		X			X	
Interstitial lung disease and pulmonary sarcoidosis	Female	Elevation 500 to 1500m (proportion)		X			X	
Interstitial lung disease and pulmonary sarcoidosis	Female	Cumulative Cigarettes (5 Years)	X			X		
Interstitial lung disease and pulmonary sarcoidosis	Female	Outdoor Air Pollution (PM2.5)	X			X		
Interstitial lung disease and pulmonary sarcoidosis	Female	Outdoor Air Pollution (PM2.5)	X				X	
Interstitial lung disease and pulmonary sarcoidosis	Female	Outdoor Air Pollution (PM2.5)		X		X		
Interstitial lung disease and pulmonary sarcoidosis	Female	Outdoor Air Pollution (PM2.5)		X			X	
Interstitial lung disease and pulmonary sarcoidosis	Female	Population Density (over 1000 ppl/sqkm, proportion)		X			X	
Interstitial lung disease and pulmonary sarcoidosis	Male	Socio-demographic Index			X			X
Interstitial lung disease and pulmonary sarcoidosis	Male	Smoking Prevalence	X			X		
Interstitial lung disease and pulmonary sarcoidosis	Male	Log-transformed SEV scalar: ILD	X			X		
Interstitial lung disease and pulmonary sarcoidosis	Male	Education (years per capita)			X			X
Interstitial lung disease and pulmonary sarcoidosis	Male	LDI (I$ per capita)			X			X
Interstitial lung disease and pulmonary sarcoidosis	Male	Elevation Over 1500m (proportion)		X			X	
Interstitial lung disease and pulmonary sarcoidosis	Male	Elevation 500 to 1500m (proportion)		X			X	
Interstitial lung disease and pulmonary sarcoidosis	Male	Cumulative Cigarettes (5 Years)	X			X		
Interstitial lung disease and pulmonary sarcoidosis	Male	Outdoor Air Pollution (PM2.5)		X			X	
Interstitial lung disease and pulmonary sarcoidosis	Male	Population Density (over 1000 ppl/sqkm, proportion)		X			X	
Other chronic respiratory diseases	Female	Socio-demographic Index			X			X
Other chronic respiratory diseases	Female	Cumulative Cigarettes (5 Years)	X			X		
Other chronic respiratory diseases	Female	Elevation Over 1500m (proportion)		X			X	
Other chronic respiratory diseases	Female	Education (years per capita)			X			X
Other chronic respiratory diseases	Female	Log-transformed SEV scalar: Oth Resp	X			X		
Other chronic respiratory diseases	Female	LDI (I$ per capita)			X			X
Other chronic respiratory diseases	Female	Elevation 500 to 1500m (proportion)		X			X	
Other chronic respiratory diseases	Female	Smoking Prevalence	X			X		
Other chronic respiratory diseases	Female	Outdoor Air Pollution (PM2.5)	X			X		
Other chronic respiratory diseases	Female	Population Density (over 1000 ppl/sqkm, proportion)		X			X	
Other chronic respiratory diseases	Male	Socio-demographic Index			X			X
Other chronic respiratory diseases	Male	Cumulative Cigarettes (5 Years)	X			X		
Other chronic respiratory diseases	Male	Elevation Over 1500m (proportion)		X			X	
Other chronic respiratory diseases	Male	Education (years per capita)			X			X
Other chronic respiratory diseases	Male	Log-transformed SEV scalar: Oth Resp	X			X		
Other chronic respiratory diseases	Male	LDI (I$ per capita)			X			X
Other chronic respiratory diseases	Male	Elevation 500 to 1500m (proportion)		X			X	
Other chronic respiratory diseases	Male	Smoking Prevalence	X			X		
Other chronic respiratory diseases	Male	Outdoor Air Pollution (PM2.5)	X			X		
Other chronic respiratory diseases	Male	Population Density (over 1000 ppl/sqkm, proportion)		X			X	
Cirrhosis and other chronic liver diseases	Male	Alcohol (liters per capita)	X			X		
Cirrhosis and other chronic liver diseases	Male	Diabetes Age-Standardized Prevalence (proportion)		X			X	
Cirrhosis and other chronic liver diseases	Male	Education (years per capita)			X			X
Cirrhosis and other chronic liver diseases	Male	Health System Access 2 (unitless)	X					X
Cirrhosis and other chronic liver diseases	Male	LDI (I$ per capita)			X			X
Cirrhosis and other chronic liver diseases	Male	Mean BMI		X			X	
Cirrhosis and other chronic liver diseases	Male	Schistosomiasis Prevalence (proportion)	X			X		
Cirrhosis and other chronic liver diseases	Male	Socio-demographic Index			X			X
Cirrhosis and other chronic liver diseases	Male	Hepatitis B (HBsAg) Seroprevalence	X			X		
Cirrhosis and other chronic liver diseases	Male	Hepatitis C (IgG) Seroprevalence	X			X		
Cirrhosis and other chronic liver diseases	Female	Alcohol (liters per capita)	X			X		
Cirrhosis and other chronic liver diseases	Female	Diabetes Age-Standardized Prevalence (proportion)		X			X	
Cirrhosis and other chronic liver diseases	Female	Education (years per capita)			X			X
Cirrhosis and other chronic liver diseases	Female	Health System Access 2 (unitless)	X					X
Cirrhosis and other chronic liver diseases	Female	LDI (I$ per capita)			X			X
Cirrhosis and other chronic liver diseases	Female	Mean BMI		X			X	
Cirrhosis and other chronic liver diseases	Female	Schistosomiasis Prevalence (proportion)	X			X		
Cirrhosis and other chronic liver diseases	Female	Socio-demographic Index			X			X
Cirrhosis and other chronic liver diseases	Female	Hepatitis B (HBsAg) Seroprevalence	X			X		

Cause	Sex	Covariate	GBD 2015			GBD 2016		
			Level 1	Level 2	Level 3	Level 1	Level 2	Level 3
Cirrhosis and other chronic liver diseases	Female	Hepatitis C (IgG) Seroprevalence	X			X		
Digestive diseases	Female	Alcohol (liters per capita)	X			X		
Digestive diseases	Female	Cumulative Cigarettes (5 Years)	X			X		
Digestive diseases	Female	Education (years per capita)			X			X
Digestive diseases	Female	LDI (I$ per capita)			X			X
Digestive diseases	Female	Sanitation (proportion with access)	X			X		
Digestive diseases	Female	Socio-demographic Index			X			X
Digestive diseases	Female	fruits adjusted(g)		X			X	
Digestive diseases	Female	red meats adjusted(g)		X			X	
Digestive diseases	Male	Alcohol (liters per capita)	X			X		
Digestive diseases	Male	Cumulative Cigarettes (5 Years)	X			X		
Digestive diseases	Male	Education (years per capita)			X			X
Digestive diseases	Male	LDI (I$ per capita)			X			X
Digestive diseases	Male	Sanitation (proportion with access)	X			X		
Digestive diseases	Male	Socio-demographic Index			X			X
Digestive diseases	Male	fruits adjusted(g)		X			X	
Digestive diseases	Male	red meats adjusted(g)		X			X	
Peptic ulcer disease	Female	Alcohol (liters per capita)	X			X		
Peptic ulcer disease	Female	Cumulative Cigarettes (10 Years)	X			X		
Peptic ulcer disease	Female	Cumulative Cigarettes (5 Years)	X			X		
Peptic ulcer disease	Female	LDI (I$ per capita)			X			X
Peptic ulcer disease	Female	Sanitation (proportion with access)		X			X	
Peptic ulcer disease	Female	Smoking Prevalence	X			X		
Peptic ulcer disease	Female	Maternal education (years per capita)			X			X
Peptic ulcer disease	Female	Socio-demographic Index			X			X
Peptic ulcer disease	Female	vegetables adjusted(g)		X			X	
Peptic ulcer disease	Male	Alcohol (liters per capita)	X			X		
Peptic ulcer disease	Male	Cumulative Cigarettes (10 Years)	X			X		
Peptic ulcer disease	Male	Cumulative Cigarettes (5 Years)	X			X		
Peptic ulcer disease	Male	LDI (I$ per capita)			X			X
Peptic ulcer disease	Male	Sanitation (proportion with access)		X			X	
Peptic ulcer disease	Male	Smoking Prevalence	X			X		
Peptic ulcer disease	Male	Maternal education (years per capita)			X			X
Peptic ulcer disease	Male	Socio-demographic Index			X			X
Peptic ulcer disease	Male	vegetables adjusted(g)		X			X	
Gastritis and duodenitis	Male	Alcohol (liters per capita)	X			X		
Gastritis and duodenitis	Male	Cumulative Cigarettes (10 Years)	X			X		
Gastritis and duodenitis	Male	Cumulative Cigarettes (5 Years)	X			X		
Gastritis and duodenitis	Male	Education (years per capita)			X			X
Gastritis and duodenitis	Male	LDI (I$ per capita)			X			X
Gastritis and duodenitis	Male	Sanitation (proportion with access)		X			X	
Gastritis and duodenitis	Male	Smoking Prevalence	X			X		
Gastritis and duodenitis	Male	Socio-demographic Index			X			X
Gastritis and duodenitis	Male	vegetables adjusted(g)		X			X	
Gastritis and duodenitis	Female	Alcohol (liters per capita)	X			X		
Gastritis and duodenitis	Female	Cumulative Cigarettes (10 Years)	X			X		
Gastritis and duodenitis	Female	Cumulative Cigarettes (5 Years)	X			X		
Gastritis and duodenitis	Female	Education (years per capita)			X			X
Gastritis and duodenitis	Female	LDI (I$ per capita)			X			X
Gastritis and duodenitis	Female	Sanitation (proportion with access)		X			X	
Gastritis and duodenitis	Female	Smoking Prevalence	X			X		
Gastritis and duodenitis	Female	Socio-demographic Index			X			X
Gastritis and duodenitis	Female	vegetables adjusted(g)		X			X	
Appendicitis	Female	Education (years per capita)			X			X
Appendicitis	Female	LDI (I$ per capita)			X			X
Appendicitis	Female	Health System Access (capped)		X				X
Appendicitis	Female	Socio-demographic Index			X			X
Appendicitis	Female	fruits adjusted(g)		X			X	
Appendicitis	Female	vegetables adjusted(g)		X			X	
Appendicitis	Male	Education (years per capita)			X			X
Appendicitis	Male	LDI (I$ per capita)			X			X
Appendicitis	Male	Health System Access (capped)		X				X
Appendicitis	Male	Socio-demographic Index			X			X
Appendicitis	Male	fruits adjusted(g)		X			X	
Appendicitis	Male	vegetables adjusted(g)		X			X	
Paralytic ileus and intestinal obstruction	Female	Education (years per capita)			X			X
Paralytic ileus and intestinal obstruction	Female	LDI (I$ per capita)			X			X
Paralytic ileus and intestinal obstruction	Female	Health System Access (capped)	X					X
Paralytic ileus and intestinal obstruction	Female	Socio-demographic Index			X			X
Paralytic ileus and intestinal obstruction	Female	fruits adjusted(g)		X			X	
Paralytic ileus and intestinal obstruction	Female	vegetables adjusted(g)		X			X	
Paralytic ileus and intestinal obstruction	Male	Education (years per capita)			X			X
Paralytic ileus and intestinal obstruction	Male	LDI (I$ per capita)			X			X
Paralytic ileus and intestinal obstruction	Male	Health System Access (capped)	X					X
Paralytic ileus and intestinal obstruction	Male	Socio-demographic Index			X			X
Paralytic ileus and intestinal obstruction	Male	fruits adjusted(g)		X			X	
Paralytic ileus and intestinal obstruction	Male	vegetables adjusted(g)		X			X	
Inguinal, femoral, and abdominal hernia	Female	Education (years per capita)			X			X
Inguinal, femoral, and abdominal hernia	Female	LDI (I$ per capita)			X			X
Inguinal, femoral, and abdominal hernia	Female	Socio-demographic Index			X			X
Inguinal, femoral, and abdominal hernia	Male	Education (years per capita)			X			X
Inguinal, femoral, and abdominal hernia	Male	LDI (I$ per capita)			X			X
Inguinal, femoral, and abdominal hernia	Male	Socio-demographic Index			X			X
Inflammatory bowel disease	Male	Education (years per capita)			X			X
Inflammatory bowel disease	Male	LDI (I$ per capita)			X			X

Cause	Sex	Covariate	GBD 2015			GBD 2016		
			Level 1	Level 2	Level 3	Level 1	Level 2	Level 3
Inflammatory bowel disease	Male	Latitude 15 to 30 (proportion)		X			X	
Inflammatory bowel disease	Male	Latitude 30 to 45 (proportion)		X			X	
Inflammatory bowel disease	Male	Latitude Over 45 (proportion)		X			X	
Inflammatory bowel disease	Male	Socio-demographic Index			X			X
Inflammatory bowel disease	Male	fruits adjusted(g)	X			X		
Inflammatory bowel disease	Male	red meats adjusted(g)	X			X		
Inflammatory bowel disease	Male	vegetables adjusted(g)	X			X		
Inflammatory bowel disease	Female	Education (years per capita)			X			X
Inflammatory bowel disease	Female	LDI (I$ per capita)			X			X
Inflammatory bowel disease	Female	Latitude 15 to 30 (proportion)		X			X	
Inflammatory bowel disease	Female	Latitude 30 to 45 (proportion)		X			X	
Inflammatory bowel disease	Female	Latitude Over 45 (proportion)		X			X	
Inflammatory bowel disease	Female	Socio-demographic Index			X			X
Inflammatory bowel disease	Female	fruits adjusted(g)	X			X		
Inflammatory bowel disease	Female	red meats adjusted(g)	X			X		
Inflammatory bowel disease	Female	vegetables adjusted(g)	X			X		
Vascular intestinal disorders	Male	Alcohol (liters per capita)		X			X	
Vascular intestinal disorders	Male	Diabetes Fasting Plasma Glucose (mmol/L)	X			X		
Vascular intestinal disorders	Male	Diabetes Age-Standardized Prevalence (proportion)	X			X		
Vascular intestinal disorders	Male	Education (years per capita)			X			X
Vascular intestinal disorders	Male	LDI (I$ per capita)			X			X
Vascular intestinal disorders	Male	Cholesterol (total, mean per capita)	X			X		
Vascular intestinal disorders	Male	Systolic Blood Pressure (mmHg)	X			X		
Vascular intestinal disorders	Male	Latitude Over 45 (proportion)			X			X
Vascular intestinal disorders	Male	Socio-demographic Index			X			X
Vascular intestinal disorders	Male	fruits adjusted(g)		X			X	
Vascular intestinal disorders	Male	vegetables adjusted(g)		X			X	
Vascular intestinal disorders	Female	Alcohol (liters per capita)		X			X	
Vascular intestinal disorders	Female	Diabetes Fasting Plasma Glucose (mmol/L)	X			X		
Vascular intestinal disorders	Female	Diabetes Age-Standardized Prevalence (proportion)	X			X		
Vascular intestinal disorders	Female	Education (years per capita)			X			X
Vascular intestinal disorders	Female	LDI (I$ per capita)			X			X
Vascular intestinal disorders	Female	Cholesterol (total, mean per capita)	X			X		
Vascular intestinal disorders	Female	Systolic Blood Pressure (mmHg)	X			X		
Vascular intestinal disorders	Female	Latitude Over 45 (proportion)			X			X
Vascular intestinal disorders	Female	Socio-demographic Index			X			X
Vascular intestinal disorders	Female	fruits adjusted(g)		X			X	
Vascular intestinal disorders	Female	vegetables adjusted(g)		X			X	
Gallbladder and biliary diseases	Female	Alcohol (liters per capita)		X			X	
Gallbladder and biliary diseases	Female	Education (years per capita)			X			X
Gallbladder and biliary diseases	Female	LDI (I$ per capita)			X			X
Gallbladder and biliary diseases	Female	Mean BMI	X			X		
Gallbladder and biliary diseases	Female	Population Over 65 (proportion)		X			X	
Gallbladder and biliary diseases	Female	Socio-demographic Index			X			X
Gallbladder and biliary diseases	Female	red meats adjusted(g)		X			X	
Gallbladder and biliary diseases	Male	Alcohol (liters per capita)		X			X	
Gallbladder and biliary diseases	Male	Education (years per capita)			X			X
Gallbladder and biliary diseases	Male	LDI (I$ per capita)			X			X
Gallbladder and biliary diseases	Male	Mean BMI	X			X		
Gallbladder and biliary diseases	Male	Population Over 65 (proportion)		X			X	
Gallbladder and biliary diseases	Male	Health System Access (capped)	X				X	
Gallbladder and biliary diseases	Male	Socio-demographic Index			X			X
Gallbladder and biliary diseases	Male	red meats adjusted(g)		X			X	
Pancreatitis	Male	Alcohol (liters per capita)	X			X		
Pancreatitis	Male	Education (years per capita)			X			X
Pancreatitis	Male	LDI (I$ per capita)			X			X
Pancreatitis	Male	Mean BMI		X			X	
Pancreatitis	Male	Health System Access (capped)		X			X	
Pancreatitis	Male	Log-transformed SEV scalar: Pancreatit	X			X		
Pancreatitis	Male	Socio-demographic Index			X			X
Pancreatitis	Female	Alcohol (liters per capita)	X			X		
Pancreatitis	Female	Education (years per capita)			X			X
Pancreatitis	Female	LDI (I$ per capita)			X			X
Pancreatitis	Female	Mean BMI		X			X	
Pancreatitis	Female	Log-transformed SEV scalar: Pancreatit	X			X		
Pancreatitis	Female	Socio-demographic Index			X			X
Other digestive diseases	Female	Alcohol (liters per capita)	X			X		
Other digestive diseases	Female	Cumulative Cigarettes (10 Years)	X			X		
Other digestive diseases	Female	Cumulative Cigarettes (5 Years)	X			X		
Other digestive diseases	Female	Diabetes Age-Standardized Prevalence (proportion)		X			X	
Other digestive diseases	Female	Education (years per capita)			X			X
Other digestive diseases	Female	Health System Access 2 (unitless)		X				X
Other digestive diseases	Female	LDI (I$ per capita)			X			X
Other digestive diseases	Female	Mean BMI		X			X	
Other digestive diseases	Female	Sanitation (proportion with access)		X			X	
Other digestive diseases	Female	Smoking Prevalence	X			X		
Other digestive diseases	Female	Improved Water Source (proportion with access)		X			X	
Other digestive diseases	Female	Socio-demographic Index			X			X
Other digestive diseases	Female	fruits adjusted(g)		X			X	
Other digestive diseases	Female	red meats adjusted(g)		X			X	
Other digestive diseases	Female	vegetables adjusted(g)		X			X	
Other digestive diseases	Male	Alcohol (liters per capita)	X			X		
Other digestive diseases	Male	Cumulative Cigarettes (10 Years)	X			X		
Other digestive diseases	Male	Cumulative Cigarettes (5 Years)	X			X		
Other digestive diseases	Male	Diabetes Age-Standardized Prevalence (proportion)		X			X	

Cause	Sex	Covariate	GBD 2015			GBD 2016		
			Level 1	Level 2	Level 3	Level 1	Level 2	Level 3
Other digestive diseases	Male	Education (years per capita)			X			X
Other digestive diseases	Male	Health System Access 2 (unitless)		X				X
Other digestive diseases	Male	LDI (I$ per capita)			X			X
Other digestive diseases	Male	Mean BMI		X			X	
Other digestive diseases	Male	Sanitation (proportion with access)		X			X	
Other digestive diseases	Male	Smoking Prevalence	X			X		
Other digestive diseases	Male	Improved Water Source (proportion with access)		X			X	
Other digestive diseases	Male	Socio-demographic Index			X			X
Other digestive diseases	Male	fruits adjusted(g)		X			X	
Other digestive diseases	Male	red meats adjusted(g)		X			X	
Other digestive diseases	Male	vegetables adjusted(g)		X			X	
Epilepsy	Female	Cumulative Cigarettes (10 Years)			X			X
Epilepsy	Female	Socio-demographic Index			X			X
Epilepsy	Female	Cumulative Cigarettes (5 Years)			X			X
Epilepsy	Female	Education (years per capita)			X			X
Epilepsy	Female	LDI (I$ per capita)			X			X
Epilepsy	Female	Log-transformed SEV scalar: Epilepsy	X			X		
Epilepsy	Female	Pig Meat (kg per capita)	X			X		
Epilepsy	Female	Systolic Blood Pressure (mmHg)	X			X		
Epilepsy	Female	Mean BMI		X			X	
Epilepsy	Female	Cholesterol (total, mean per capita)		X			X	
Epilepsy	Female	Pigs (per capita)	X			X		
Epilepsy	Male	Cumulative Cigarettes (10 Years)			X			X
Epilepsy	Male	Socio-demographic Index			X			X
Epilepsy	Male	Cumulative Cigarettes (5 Years)			X			X
Epilepsy	Male	Education (years per capita)			X			X
Epilepsy	Male	LDI (I$ per capita)			X			X
Epilepsy	Male	Log-transformed SEV scalar: Epilepsy	X			X		
Epilepsy	Male	Pig Meat (kg per capita)	X			X		
Epilepsy	Male	Systolic Blood Pressure (mmHg)	X			X		
Epilepsy	Male	Mean BMI		X			X	
Epilepsy	Male	Cholesterol (total, mean per capita)		X			X	
Epilepsy	Male	Pigs (per capita)	X			X		
Multiple sclerosis	Male	Cumulative Cigarettes (10 Years)			X			X
Multiple sclerosis	Male	Socio-demographic Index			X			X
Multiple sclerosis	Male	Cumulative Cigarettes (5 Years)			X			X
Multiple sclerosis	Male	Education (years per capita)			X			X
Multiple sclerosis	Male	LDI (I$ per capita)			X			X
Multiple sclerosis	Male	Absolute value of average latitude	X			X		
Multiple sclerosis	Male	Smoking Prevalence			X			X
Multiple sclerosis	Male	Cholesterol (total, mean per capita)		X			X	
Multiple sclerosis	Female	Cumulative Cigarettes (10 Years)			X			X
Multiple sclerosis	Female	Socio-demographic Index			X			X
Multiple sclerosis	Female	Cumulative Cigarettes (5 Years)			X			X
Multiple sclerosis	Female	Education (years per capita)			X			X
Multiple sclerosis	Female	LDI (I$ per capita)			X			X
Multiple sclerosis	Female	Absolute value of average latitude	X			X		
Multiple sclerosis	Female	Smoking Prevalence			X			X
Multiple sclerosis	Female	Cholesterol (total, mean per capita)		X			X	
Motor neuron disease	Male	Absolute value of average latitude		X			X	
Motor neuron disease	Male	Asbestos production (kg) per capita	X			X		
Motor neuron disease	Male	Education (years per capita)			X			X
Motor neuron disease	Male	LDI (I$ per capita)			X			X
Motor neuron disease	Male	Cholesterol (total, mean per capita)	X			X		
Motor neuron disease	Male	Sanitation (proportion with access)		X			X	
Motor neuron disease	Male	Improved Water Source (proportion with access)		X			X	
Motor neuron disease	Male	Socio-demographic Index			X			X
Motor neuron disease	Female	Absolute value of average latitude		X			X	
Motor neuron disease	Female	Asbestos production (kg) per capita	X			X		
Motor neuron disease	Female	Education (years per capita)			X			X
Motor neuron disease	Female	LDI (I$ per capita)			X			X
Motor neuron disease	Female	Cholesterol (total, mean per capita)	X			X		
Motor neuron disease	Female	Sanitation (proportion with access)		X			X	
Motor neuron disease	Female	Improved Water Source (proportion with access)		X			X	
Motor neuron disease	Female	Socio-demographic Index			X			X
Other neurological disorders	Male	Cumulative Cigarettes (10 Years)			X			X
Other neurological disorders	Male	Socio-demographic Index			X			X
Other neurological disorders	Male	Cumulative Cigarettes (5 Years)			X			X
Other neurological disorders	Male	Education (years per capita)			X			X
Other neurological disorders	Male	LDI (I$ per capita)			X			X
Other neurological disorders	Male	Pig Meat (kg per capita)	X			X		
Other neurological disorders	Male	Animal Fats (kcal per capita)		X			X	
Other neurological disorders	Male	Smoking Prevalence			X			X
Other neurological disorders	Male	Underweight (proportion <2SD weight for age, <5 years)	X			X		
Other neurological disorders	Male	Alcohol (liters per capita)		X			X	
Other neurological disorders	Male	Mean BMI	X			X		
Other neurological disorders	Male	Cholesterol (total, mean per capita)	X			X		
Other neurological disorders	Male	Population Density (over 1000 ppl/sqkm, proportion)		X			X	
Other neurological disorders	Male	Systolic Blood Pressure (mmHg)	X			X		
Other neurological disorders	Female	Cumulative Cigarettes (10 Years)			X			X
Other neurological disorders	Female	Socio-demographic Index			X			X
Other neurological disorders	Female	Cumulative Cigarettes (5 Years)			X			X
Other neurological disorders	Female	Education (years per capita)			X			X
Other neurological disorders	Female	LDI (I$ per capita)			X			X
Other neurological disorders	Female	Pig Meat (kg per capita)	X			X		

Cause	Sex	Covariate	GBD 2015			GBD 2016		
			Level 1	Level 2	Level 3	Level 1	Level 2	Level 3
Other neurological disorders	Female	Animal Fats (kcal per capita)		X			X	
Other neurological disorders	Female	Smoking Prevalence			X			X
Other neurological disorders	Female	Underweight (proportion <2SD weight for age, <5 years)	X			X		
Other neurological disorders	Female	Alcohol (liters per capita)		X			X	
Other neurological disorders	Female	Mean BMI	X			X		
Other neurological disorders	Female	Cholesterol (total, mean per capita)	X			X		
Other neurological disorders	Female	Population Density (over 1000 ppl/sqkm, proportion)		X			X	
Other neurological disorders	Female	Systolic Blood Pressure (mmHg)	X			X		
Schizophrenia	Male	Alcohol (liters per capita)		X			X	
Schizophrenia	Male	Cumulative Cigarettes (20 Years)		X			X	
Schizophrenia	Male	Education (years per capita)			X			X
Schizophrenia	Male	Health System Access 2 (unitless)	X			X		
Schizophrenia	Male	LDI (I$ per capita)			X			X
Schizophrenia	Male	Underweight (proportion <2SD weight for age, <5 years)	X			X		
Schizophrenia	Male	Smoking Prevalence		X			X	
Schizophrenia	Male	Socio-demographic Index			X			X
Schizophrenia	Female	Alcohol (liters per capita)		X			X	
Schizophrenia	Female	Cumulative Cigarettes (20 Years)		X			X	
Schizophrenia	Female	Education (years per capita)			X			X
Schizophrenia	Female	Health System Access 2 (unitless)	X			X		
Schizophrenia	Female	LDI (I$ per capita)			X			X
Schizophrenia	Female	Underweight (proportion <2SD weight for age, <5 years)	X			X		
Schizophrenia	Female	Smoking Prevalence		X			X	
Schizophrenia	Female	Socio-demographic Index			X			X
Alcohol use disorders	Male	Alcohol (liters per capita)	X			X		
Alcohol use disorders	Male	Cumulative Cigarettes (10 Years)		X			X	
Alcohol use disorders	Male	Education (years per capita)			X			X
Alcohol use disorders	Male	Health System Access 2 (unitless)		X			X	
Alcohol use disorders	Male	LDI (I$ per capita)			X			X
Alcohol use disorders	Male	Religion (binary, >50% Muslim)		X			X	
Alcohol use disorders	Male	Smoking Prevalence		X			X	
Alcohol use disorders	Male	Prevalence of binge drinking	X			X		
Alcohol use disorders	Male	Socio-demographic Index			X			X
Alcohol use disorders	Female	Alcohol (liters per capita)	X			X		
Alcohol use disorders	Female	Cumulative Cigarettes (10 Years)		X			X	
Alcohol use disorders	Female	Education (years per capita)			X			X
Alcohol use disorders	Female	Health System Access 2 (unitless)		X			X	
Alcohol use disorders	Female	LDI (I$ per capita)			X			X
Alcohol use disorders	Female	Religion (binary, >50% Muslim)		X			X	
Alcohol use disorders	Female	Smoking Prevalence		X			X	
Alcohol use disorders	Female	Prevalence of binge drinking	X			X		
Alcohol use disorders	Female	Socio-demographic Index			X			X
Drug use disorders	Male	Alcohol (liters per capita)	X			X		
Drug use disorders	Male	Cumulative Cigarettes (10 Years)	X			X		
Drug use disorders	Male	Cumulative Cigarettes (5 Years)	X			X		
Drug use disorders	Male	Education (years per capita)			X			X
Drug use disorders	Male	Health System Access 2 (unitless)	X			X		
Drug use disorders	Male	LDI (I$ per capita)			X			X
Drug use disorders	Male	Opium Cultivation (binary)	X			X		
Drug use disorders	Male	Smoking Prevalence	X			X		
Drug use disorders	Male	Socio-demographic Index			X			X
Drug use disorders	Female	Alcohol (liters per capita)	X			X		
Drug use disorders	Female	Cumulative Cigarettes (10 Years)	X			X		
Drug use disorders	Female	Cumulative Cigarettes (5 Years)	X			X		
Drug use disorders	Female	Education (years per capita)			X			X
Drug use disorders	Female	Health System Access 2 (unitless)	X			X		
Drug use disorders	Female	LDI (I$ per capita)			X			X
Drug use disorders	Female	Opium Cultivation (binary)	X			X		
Drug use disorders	Female	Smoking Prevalence	X			X		
Drug use disorders	Female	Socio-demographic Index			X			X
Opioid use disorders	Male	Alcohol (liters per capita)	X			X		
Opioid use disorders	Male	Cumulative Cigarettes (10 Years)	X			X		
Opioid use disorders	Male	Cumulative Cigarettes (5 Years)	X			X		
Opioid use disorders	Male	Education (years per capita)			X			X
Opioid use disorders	Male	Health System Access 2 (unitless)	X			X		
Opioid use disorders	Male	LDI (I$ per capita)			X			X
Opioid use disorders	Male	Opium Cultivation (binary)	X			X		
Opioid use disorders	Male	Smoking Prevalence	X			X		
Opioid use disorders	Male	Socio-demographic Index			X			X
Opioid use disorders	Female	Alcohol (liters per capita)	X			X		
Opioid use disorders	Female	Cumulative Cigarettes (10 Years)	X			X		
Opioid use disorders	Female	Cumulative Cigarettes (5 Years)	X			X		
Opioid use disorders	Female	Education (years per capita)			X			X
Opioid use disorders	Female	Health System Access 2 (unitless)	X			X		
Opioid use disorders	Female	LDI (I$ per capita)			X			X
Opioid use disorders	Female	Opium Cultivation (binary)	X			X		
Opioid use disorders	Female	Smoking Prevalence	X			X		
Opioid use disorders	Female	Socio-demographic Index			X			X
Cocaine use disorders	Male	Alcohol (liters per capita)	X			X		
Cocaine use disorders	Male	Cumulative Cigarettes (10 Years)	X			X		
Cocaine use disorders	Male	Cumulative Cigarettes (5 Years)	X			X		
Cocaine use disorders	Male	Education (years per capita)			X			X
Cocaine use disorders	Male	LDI (I$ per capita)			X			X
Cocaine use disorders	Male	Smoking Prevalence	X			X		
Cocaine use disorders	Male	Socio-demographic Index			X			X

Cause	Sex	Covariate	GBD 2015			GBD 2016		
			Level 1	Level 2	Level 3	Level 1	Level 2	Level 3
Cocaine use disorders	Female	Alcohol (liters per capita)	X			X		
Cocaine use disorders	Female	Cumulative Cigarettes (10 Years)	X			X		
Cocaine use disorders	Female	Cumulative Cigarettes (5 Years)	X			X		
Cocaine use disorders	Female	Education (years per capita)			X			X
Cocaine use disorders	Female	LDI (I$ per capita)			X			X
Cocaine use disorders	Female	Smoking Prevalence	X			X		
Cocaine use disorders	Female	Socio-demographic Index			X			X
Amphetamine use disorders	Male	Alcohol (liters per capita)	X			X		
Amphetamine use disorders	Male	Cumulative Cigarettes (10 Years)	X			X		
Amphetamine use disorders	Male	Cumulative Cigarettes (5 Years)	X			X		
Amphetamine use disorders	Male	Education (years per capita)			X			X
Amphetamine use disorders	Male	LDI (I$ per capita)			X			X
Amphetamine use disorders	Male	Smoking Prevalence	X			X		
Amphetamine use disorders	Male	Socio-demographic Index			X			X
Amphetamine use disorders	Female	Alcohol (liters per capita)	X			X		
Amphetamine use disorders	Female	Cumulative Cigarettes (10 Years)	X			X		
Amphetamine use disorders	Female	Cumulative Cigarettes (5 Years)	X			X		
Amphetamine use disorders	Female	Education (years per capita)			X			X
Amphetamine use disorders	Female	LDI (I$ per capita)			X			X
Amphetamine use disorders	Female	Smoking Prevalence	X			X		
Amphetamine use disorders	Female	Socio-demographic Index			X			X
Other drug use disorders	Male	Alcohol (liters per capita)	X			X		
Other drug use disorders	Male	Cumulative Cigarettes (10 Years)	X			X		
Other drug use disorders	Male	Cumulative Cigarettes (5 Years)	X			X		
Other drug use disorders	Male	Education (years per capita)			X			X
Other drug use disorders	Male	LDI (I$ per capita)			X			X
Other drug use disorders	Male	Smoking Prevalence	X			X		
Other drug use disorders	Male	Socio-demographic Index			X			X
Other drug use disorders	Female	Alcohol (liters per capita)	X			X		
Other drug use disorders	Female	Cumulative Cigarettes (10 Years)	X			X		
Other drug use disorders	Female	Cumulative Cigarettes (5 Years)	X			X		
Other drug use disorders	Female	Education (years per capita)			X			X
Other drug use disorders	Female	LDI (I$ per capita)			X			X
Other drug use disorders	Female	Smoking Prevalence	X			X		
Other drug use disorders	Female	Socio-demographic Index			X			X
Eating disorders	Male	Socio-demographic Index			X			X
Eating disorders	Female	Socio-demographic Index			X			X
Anorexia nervosa	Male	Socio-demographic Index			X			X
Anorexia nervosa	Female	Socio-demographic Index			X			X
Bulimia nervosa	Male	Socio-demographic Index			X			X
Bulimia nervosa	Female	Socio-demographic Index			X			X
Diabetes mellitus	Male	Education (years per capita)	X				X	
Diabetes mellitus	Male	Education (years per capita)			X		X	
Diabetes mellitus	Male	LDI (I$ per capita)	X				X	
Diabetes mellitus	Male	LDI (I$ per capita)			X		X	
Diabetes mellitus	Female	Education (years per capita)	X				X	
Diabetes mellitus	Female	Education (years per capita)			X		X	
Diabetes mellitus	Female	LDI (I$ per capita)	X				X	
Diabetes mellitus	Female	LDI (I$ per capita)			X		X	
Diabetes mellitus	Male	Animal Fats (kcal per capita)		X			X	
Diabetes mellitus	Male	Diabetes Fasting Plasma Glucose (mmol/L)	X				X	
Diabetes mellitus	Male	Diabetes Age-Standardized Prevalence (proportion)	X				X	
Diabetes mellitus	Male	Mean BMI	X				X	
Diabetes mellitus	Male	Cholesterol (total, mean per capita)	X				X	
Diabetes mellitus	Male	Systolic Blood Pressure (mmHg)	X				X	
Diabetes mellitus	Male	fruits adjusted(g)		X			X	
Diabetes mellitus	Male	vegetables adjusted(g)		X			X	
Diabetes mellitus	Male	whole grains adjusted(g)		X			X	
Diabetes mellitus	Male	energy unadjusted(kcal)		X			X	
Diabetes mellitus	Female	Animal Fats (kcal per capita)		X			X	
Diabetes mellitus	Female	Diabetes Fasting Plasma Glucose (mmol/L)	X				X	
Diabetes mellitus	Female	Diabetes Age-Standardized Prevalence (proportion)	X				X	
Diabetes mellitus	Female	Mean BMI	X				X	
Diabetes mellitus	Female	Cholesterol (total, mean per capita)	X				X	
Diabetes mellitus	Female	Systolic Blood Pressure (mmHg)	X				X	
Diabetes mellitus	Female	fruits adjusted(g)		X			X	
Diabetes mellitus	Female	vegetables adjusted(g)		X			X	
Diabetes mellitus	Female	whole grains adjusted(g)		X			X	
Diabetes mellitus	Female	energy unadjusted(kcal)		X			X	
Acute glomerulonephritis	Male	Socio-demographic Index			X			X
Acute glomerulonephritis	Male	Sanitation (proportion with access)		X			X	
Acute glomerulonephritis	Male	Education (years per capita)			X			X
Acute glomerulonephritis	Male	Diabetes Age-Standardized Prevalence (proportion)		X			X	
Acute glomerulonephritis	Male	Systolic Blood Pressure (mmHg)		X			X	
Acute glomerulonephritis	Male	Improved Water Source (proportion with access)		X			X	
Acute glomerulonephritis	Male	LDI (I$ per capita)			X			X
Acute glomerulonephritis	Female	Socio-demographic Index			X			X
Acute glomerulonephritis	Female	Sanitation (proportion with access)		X			X	
Acute glomerulonephritis	Female	Education (years per capita)			X			X
Acute glomerulonephritis	Female	Diabetes Age-Standardized Prevalence (proportion)		X			X	
Acute glomerulonephritis	Female	Systolic Blood Pressure (mmHg)		X			X	
Acute glomerulonephritis	Female	Improved Water Source (proportion with access)		X			X	
Acute glomerulonephritis	Female	LDI (I$ per capita)			X			X
Chronic kidney disease	Female	Diabetes Fasting Plasma Glucose (mmol/L)	X			X		
Chronic kidney disease	Female	Diabetes Age-Standardized Prevalence (proportion)	X			X		

Cause	Sex	Covariate	GBD 2015			GBD 2016		
			Level 1	Level 2	Level 3	Level 1	Level 2	Level 3
Chronic kidney disease	Female	Education (years per capita)			X			X
Chronic kidney disease	Female	LDI (I$ per capita)			X			X
Chronic kidney disease	Female	Mean BMI	X			X		
Chronic kidney disease	Female	Cholesterol (total, mean per capita)		X			X	
Chronic kidney disease	Female	Systolic Blood Pressure (mmHg)	X			X		
Chronic kidney disease	Female	Socio-demographic Index			X			X
Chronic kidney disease	Male	Diabetes Fasting Plasma Glucose (mmol/L)	X			X		
Chronic kidney disease	Male	Diabetes Age-Standardized Prevalence (proportion)	X			X		
Chronic kidney disease	Male	Education (years per capita)			X			X
Chronic kidney disease	Male	LDI (I$ per capita)			X			X
Chronic kidney disease	Male	Mean BMI	X			X		
Chronic kidney disease	Male	Cholesterol (total, mean per capita)		X			X	
Chronic kidney disease	Male	Systolic Blood Pressure (mmHg)	X			X		
Chronic kidney disease	Male	Socio-demographic Index			X			X
Urinary diseases and male infertility	Male	Socio-demographic Index			X			X
Urinary diseases and male infertility	Male	Education (years per capita)			X			X
Urinary diseases and male infertility	Male	LDI (I$ per capita)			X			X
Urinary diseases and male infertility	Male	Latitude 15 to 30 (proportion)		X			X	
Urinary diseases and male infertility	Male	Latitude Over 45 (proportion)		X			X	
Urinary diseases and male infertility	Male	Latitude 30 to 45 (proportion)		X			X	
Urinary diseases and male infertility	Male	Mean BMI		X			X	
Urinary diseases and male infertility	Male	Latitude Under 15 (proportion)		X			X	
Urinary diseases and male infertility	Female	Socio-demographic Index			X			X
Urinary diseases and male infertility	Female	Education (years per capita)			X			X
Urinary diseases and male infertility	Female	LDI (I$ per capita)			X			X
Urinary diseases and male infertility	Female	Latitude 15 to 30 (proportion)		X			X	
Urinary diseases and male infertility	Female	Latitude Over 45 (proportion)		X			X	
Urinary diseases and male infertility	Female	Latitude 30 to 45 (proportion)		X			X	
Urinary diseases and male infertility	Female	Mean BMI		X			X	
Urinary diseases and male infertility	Female	Latitude Under 15 (proportion)		X			X	
Interstitial nephritis and urinary tract infections	Male	Education (years per capita)		X			X	
Interstitial nephritis and urinary tract infections	Male	LDI (I$ per capita)		X			X	
Interstitial nephritis and urinary tract infections	Male	Sanitation (proportion with access)	X			X		
Interstitial nephritis and urinary tract infections	Male	Socio-demographic Index			X			X
Interstitial nephritis and urinary tract infections	Female	Education (years per capita)		X			X	
Interstitial nephritis and urinary tract infections	Female	LDI (I$ per capita)		X			X	
Interstitial nephritis and urinary tract infections	Female	Sanitation (proportion with access)	X			X		
Interstitial nephritis and urinary tract infections	Female	Socio-demographic Index			X			X
Urolithiasis	Male	90th percentile climatic temperature in the given country-year.	X			X		
Urolithiasis	Male	Education (years per capita)			X			X
Urolithiasis	Male	Socio-demographic Index			X			X
Urolithiasis	Male	LDI (I$ per capita)			X			X
Urolithiasis	Female	90th percentile climatic temperature in the given country-year.	X			X		
Urolithiasis	Female	Education (years per capita)			X			X
Urolithiasis	Female	Socio-demographic Index			X			X
Urolithiasis	Female	LDI (I$ per capita)			X			X
Other urinary diseases	Female	Education (years per capita)	X			X		
Other urinary diseases	Female	Mean BMI	X			X		
Other urinary diseases	Female	LDI (I$ per capita)	X			X		
Other urinary diseases	Female	Socio-demographic Index			X			X
Other urinary diseases	Male	Education (years per capita)		X			X	
Other urinary diseases	Male	Mean BMI	X			X		
Other urinary diseases	Male	LDI (I$ per capita)		X			X	
Other urinary diseases	Male	Socio-demographic Index			X			X
Gynecological diseases	Female	Education (years per capita)			X			X
Gynecological diseases	Female	LDI (I$ per capita)			X			X
Gynecological diseases	Female	Live Births 35+ (proportion)		X			X	
Gynecological diseases	Female	Skilled Birth Attendance (proportion)		X			X	
Gynecological diseases	Female	Smoking Prevalence	X			X		
Gynecological diseases	Female	Total Fertility Rate		X			X	
Gynecological diseases	Female	Socio-demographic Index			X			X
Uterine fibroids	Female	Education (years per capita)			X			X
Uterine fibroids	Female	LDI (I$ per capita)			X			X
Uterine fibroids	Female	Live Births 35+ (proportion)		X			X	
Uterine fibroids	Female	Skilled Birth Attendance (proportion)		X			X	
Uterine fibroids	Female	Smoking Prevalence	X			X		
Uterine fibroids	Female	Total Fertility Rate		X			X	
Uterine fibroids	Female	Socio-demographic Index			X			X
Polycystic ovarian syndrome	Female	Education (years per capita)			X			X
Polycystic ovarian syndrome	Female	LDI (I$ per capita)			X			X
Polycystic ovarian syndrome	Female	Live Births 35+ (proportion)		X			X	
Polycystic ovarian syndrome	Female	Skilled Birth Attendance (proportion)		X			X	
Polycystic ovarian syndrome	Female	Smoking Prevalence	X			X		
Polycystic ovarian syndrome	Female	Total Fertility Rate		X			X	
Polycystic ovarian syndrome	Female	Socio-demographic Index			X			X
Endometriosis	Female	Education (years per capita)			X			X
Endometriosis	Female	LDI (I$ per capita)			X			X
Endometriosis	Female	Live Births 35+ (proportion)		X			X	
Endometriosis	Female	Skilled Birth Attendance (proportion)		X			X	
Endometriosis	Female	Smoking Prevalence	X			X		
Endometriosis	Female	Total Fertility Rate		X			X	
Endometriosis	Female	Socio-demographic Index			X			X
Genital prolapse	Female	Education (years per capita)			X			X
Genital prolapse	Female	LDI (I$ per capita)			X			X
Genital prolapse	Female	Live Births 35+ (proportion)		X			X	

Cause	Sex	Covariate	GBD 2015			GBD 2016		
			Level 1	Level 2	Level 3	Level 1	Level 2	Level 3
Genital prolapse	Female	Skilled Birth Attendance (proportion)		X			X	
Genital prolapse	Female	Smoking Prevalence	X			X		
Genital prolapse	Female	Total Fertility Rate		X			X	
Genital prolapse	Female	Socio-demographic Index			X			X
Other gynecological diseases	Female	Education (years per capita)			X			X
Other gynecological diseases	Female	LDI (I$ per capita)			X			X
Other gynecological diseases	Female	Live Births 35+ (proportion)		X			X	
Other gynecological diseases	Female	Skilled Birth Attendance (proportion)		X			X	
Other gynecological diseases	Female	Smoking Prevalence	X			X		
Other gynecological diseases	Female	Total Fertility Rate		X			X	
Other gynecological diseases	Female	Socio-demographic Index			X			X
Hemoglobinopathies and hemolytic anemias	Male	Education (years per capita)			X			X
Hemoglobinopathies and hemolytic anemias	Male	LDI (I$ per capita)			X			X
Hemoglobinopathies and hemolytic anemias	Male	Latitude Under 15 (proportion)			X			X
Hemoglobinopathies and hemolytic anemias	Male	Latitude Under 15 (proportion)		X				X
Hemoglobinopathies and hemolytic anemias	Male	Latitude 15 to 30 (proportion)			X			X
Hemoglobinopathies and hemolytic anemias	Male	Latitude 15 to 30 (proportion)		X				X
Hemoglobinopathies and hemolytic anemias	Male	Latitude 30 to 45 (proportion)			X			X
Hemoglobinopathies and hemolytic anemias	Male	Latitude 30 to 45 (proportion)		X				X
Hemoglobinopathies and hemolytic anemias	Male	Latitude Over 45 (proportion)			X			X
Hemoglobinopathies and hemolytic anemias	Male	Latitude Over 45 (proportion)		X				X
Hemoglobinopathies and hemolytic anemias	Male	Hemoglobinopathies Prevalence x Excess Mortality	X			X		
Hemoglobinopathies and hemolytic anemias	Male	Health System Access (capped)		X			X	
Hemoglobinopathies and hemolytic anemias	Male	Hemoglobinopathies Prevalence x Excess Mortality (excluding G6PD deficiency)	X			X		
Hemoglobinopathies and hemolytic anemias	Male	Socio-demographic Index			X			X
Hemoglobinopathies and hemolytic anemias	Female	Education (years per capita)			X			X
Hemoglobinopathies and hemolytic anemias	Female	LDI (I$ per capita)			X			X
Hemoglobinopathies and hemolytic anemias	Female	Latitude Under 15 (proportion)			X			X
Hemoglobinopathies and hemolytic anemias	Female	Latitude Under 15 (proportion)		X				X
Hemoglobinopathies and hemolytic anemias	Female	Latitude 15 to 30 (proportion)			X			X
Hemoglobinopathies and hemolytic anemias	Female	Latitude 15 to 30 (proportion)		X				X
Hemoglobinopathies and hemolytic anemias	Female	Latitude 30 to 45 (proportion)			X			X
Hemoglobinopathies and hemolytic anemias	Female	Latitude 30 to 45 (proportion)		X				X
Hemoglobinopathies and hemolytic anemias	Female	Latitude Over 45 (proportion)			X			X
Hemoglobinopathies and hemolytic anemias	Female	Latitude Over 45 (proportion)		X				X
Hemoglobinopathies and hemolytic anemias	Female	Hemoglobinopathies Prevalence x Excess Mortality	X			X		
Hemoglobinopathies and hemolytic anemias	Female	Health System Access (capped)		X			X	
Hemoglobinopathies and hemolytic anemias	Female	Hemoglobinopathies Prevalence x Excess Mortality (excluding G6PD deficiency)	X			X		
Hemoglobinopathies and hemolytic anemias	Female	Socio-demographic Index			X			X
Endocrine, metabolic, blood, and immune disorders	Male	Total Calories (kcal per capita)		X			X	
Endocrine, metabolic, blood, and immune disorders	Male	Socio-demographic Index			X			X
Endocrine, metabolic, blood, and immune disorders	Male	Education (years per capita)			X			X
Endocrine, metabolic, blood, and immune disorders	Male	LDI (I$ per capita)			X			X
Endocrine, metabolic, blood, and immune disorders	Male	Animal Fats (kcal per capita)		X			X	
Endocrine, metabolic, blood, and immune disorders	Male	Alcohol (liters per capita)		X			X	
Endocrine, metabolic, blood, and immune disorders	Male	Mean BMI	X			X		
Endocrine, metabolic, blood, and immune disorders	Male	Cholesterol (total, mean per capita)		X			X	
Endocrine, metabolic, blood, and immune disorders	Female	Total Calories (kcal per capita)		X			X	
Endocrine, metabolic, blood, and immune disorders	Female	Socio-demographic Index			X			X
Endocrine, metabolic, blood, and immune disorders	Female	Education (years per capita)			X			X
Endocrine, metabolic, blood, and immune disorders	Female	LDI (I$ per capita)			X			X
Endocrine, metabolic, blood, and immune disorders	Female	Animal Fats (kcal per capita)		X			X	
Endocrine, metabolic, blood, and immune disorders	Female	Alcohol (liters per capita)		X			X	
Endocrine, metabolic, blood, and immune disorders	Female	Mean BMI	X			X		
Endocrine, metabolic, blood, and immune disorders	Female	Cholesterol (total, mean per capita)		X			X	
Musculoskeletal disorders	Male	Alcohol (liters per capita)		X			X	
Musculoskeletal disorders	Male	Cumulative Cigarettes (10 Years)		X			X	
Musculoskeletal disorders	Male	Cumulative Cigarettes (5 Years)		X			X	
Musculoskeletal disorders	Male	Education (years per capita)		X			X	
Musculoskeletal disorders	Male	LDI (I$ per capita)		X			X	
Musculoskeletal disorders	Male	Mean BMI	X			X		
Musculoskeletal disorders	Male	Cholesterol (total, mean per capita)		X			X	
Musculoskeletal disorders	Male	Smoking Prevalence		X			X	
Musculoskeletal disorders	Male	Socio-demographic Index			X			X
Musculoskeletal disorders	Female	Alcohol (liters per capita)		X			X	
Musculoskeletal disorders	Female	Cumulative Cigarettes (10 Years)		X			X	
Musculoskeletal disorders	Female	Cumulative Cigarettes (5 Years)		X			X	
Musculoskeletal disorders	Female	Education (years per capita)		X			X	
Musculoskeletal disorders	Female	LDI (I$ per capita)		X			X	
Musculoskeletal disorders	Female	Mean BMI	X			X		
Musculoskeletal disorders	Female	Cholesterol (total, mean per capita)		X			X	
Musculoskeletal disorders	Female	Smoking Prevalence		X			X	
Musculoskeletal disorders	Female	Socio-demographic Index			X			X
Rheumatoid arthritis	Male	Alcohol (liters per capita)	X			X		
Rheumatoid arthritis	Male	Cumulative Cigarettes (10 Years)	X			X		
Rheumatoid arthritis	Male	Cumulative Cigarettes (5 Years)	X			X		
Rheumatoid arthritis	Male	Education (years per capita)			X			X
Rheumatoid arthritis	Male	LDI (I$ per capita)			X			X
Rheumatoid arthritis	Male	Mean BMI			X			X
Rheumatoid arthritis	Male	Cholesterol (total, mean per capita)		X			X	
Rheumatoid arthritis	Male	Smoking Prevalence	X			X		
Rheumatoid arthritis	Male	Socio-demographic Index			X			X
Rheumatoid arthritis	Female	Alcohol (liters per capita)	X			X		
Rheumatoid arthritis	Female	Cumulative Cigarettes (10 Years)	X			X		
Rheumatoid arthritis	Female	Cumulative Cigarettes (5 Years)	X			X		

Cause	Sex	Covariate	GBD 2015			GBD 2016		
			Level 1	Level 2	Level 3	Level 1	Level 2	Level 3
Rheumatoid arthritis	Female	Education (years per capita)			X			X
Rheumatoid arthritis	Female	LDI (I$ per capita)			X			X
Rheumatoid arthritis	Female	Mean BMI			X			X
Rheumatoid arthritis	Female	Cholesterol (total, mean per capita)		X			X	
Rheumatoid arthritis	Female	Smoking Prevalence	X			X		
Rheumatoid arthritis	Female	Socio-demographic Index			X			X
Other musculoskeletal disorders	Male	Cumulative Cigarettes (10 Years)		X			X	
Other musculoskeletal disorders	Male	Cumulative Cigarettes (5 Years)		X			X	
Other musculoskeletal disorders	Male	Education (years per capita)		X			X	
Other musculoskeletal disorders	Male	LDI (I$ per capita)		X			X	
Other musculoskeletal disorders	Male	Socio-demographic Index			X			X
Other musculoskeletal disorders	Male	Smoking Prevalence		X			X	
Other musculoskeletal disorders	Male	Alcohol (liters per capita)		X			X	
Other musculoskeletal disorders	Male	Mean BMI	X			X		
Other musculoskeletal disorders	Male	Cholesterol (total, mean per capita)		X			X	
Other musculoskeletal disorders	Female	Cumulative Cigarettes (10 Years)		X			X	
Other musculoskeletal disorders	Female	Cumulative Cigarettes (5 Years)		X			X	
Other musculoskeletal disorders	Female	Education (years per capita)		X			X	
Other musculoskeletal disorders	Female	LDI (I$ per capita)		X			X	
Other musculoskeletal disorders	Female	Socio-demographic Index			X			X
Other musculoskeletal disorders	Female	Smoking Prevalence		X			X	
Other musculoskeletal disorders	Female	Alcohol (liters per capita)		X			X	
Other musculoskeletal disorders	Female	Mean BMI	X			X		
Other musculoskeletal disorders	Female	Cholesterol (total, mean per capita)		X			X	
Congenital birth defects	Male	Legality of Abortion		X			X	
Congenital birth defects	Male	Alcohol (liters per capita)	X					X
Congenital birth defects	Male	Education (years per capita)		X			X	
Congenital birth defects	Male	Live Births 35+ (proportion)	X			X		
Congenital birth defects	Male	Indoor Air Pollution (All Cooking Fuels)			X			X
Congenital birth defects	Male	Smoking Prevalence (Reproductive Age Standardized)			X		X	
Congenital birth defects	Male	Socio-demographic Index			X			X
Congenital birth defects	Female	Legality of Abortion		X			X	
Congenital birth defects	Female	Alcohol (liters per capita)	X					X
Congenital birth defects	Female	Education (years per capita)		X			X	
Congenital birth defects	Female	Live Births 35+ (proportion)	X			X		
Congenital birth defects	Female	Indoor Air Pollution (All Cooking Fuels)			X			X
Congenital birth defects	Female	Smoking Prevalence (Reproductive Age Standardized)			X		X	
Congenital birth defects	Female	Socio-demographic Index			X			X
Neural tube defects	Male	Diabetes Age-Standardized Prevalence (proportion)	X					X
Neural tube defects	Male	Education (years per capita)			X			X
Neural tube defects	Male	Indoor Air Pollution (All Cooking Fuels)	X					X
Neural tube defects	Male	Smoking Prevalence (Reproductive Age Standardized)	X				X	
Neural tube defects	Male	Socio-demographic Index			X	X		
Neural tube defects	Female	Diabetes Age-Standardized Prevalence (proportion)	X					X
Neural tube defects	Female	Education (years per capita)			X			X
Neural tube defects	Female	Indoor Air Pollution (All Cooking Fuels)	X					X
Neural tube defects	Female	Smoking Prevalence (Reproductive Age Standardized)	X				X	
Neural tube defects	Female	Socio-demographic Index			X	X		
Congenital heart anomalies	Male	Legality of Abortion		X			X	
Congenital heart anomalies	Male	Alcohol (liters per capita)	X					X
Congenital heart anomalies	Male	Diabetes Age-Standardized Prevalence (proportion)		X			X	
Congenital heart anomalies	Male	Education (years per capita)		X			X	
Congenital heart anomalies	Male	Live Births 35+ (proportion)	X					X
Congenital heart anomalies	Male	Smoking Prevalence (Reproductive Age Standardized)			X		X	
Congenital heart anomalies	Male	Socio-demographic Index			X		X	
Congenital heart anomalies	Female	Legality of Abortion		X			X	
Congenital heart anomalies	Female	Alcohol (liters per capita)	X					X
Congenital heart anomalies	Female	Diabetes Age-Standardized Prevalence (proportion)		X			X	
Congenital heart anomalies	Female	Education (years per capita)		X			X	
Congenital heart anomalies	Female	Live Births 35+ (proportion)	X					X
Congenital heart anomalies	Female	Smoking Prevalence (Reproductive Age Standardized)			X		X	
Congenital heart anomalies	Female	Socio-demographic Index			X		X	
Orofacial clefts	Male	Legality of Abortion	X				X	
Orofacial clefts	Male	Alcohol (liters per capita)	X					X
Orofacial clefts	Male	Diabetes Age-Standardized Prevalence (proportion)		X			X	
Orofacial clefts	Male	Education (years per capita)		X				X
Orofacial clefts	Male	Indoor Air Pollution (All Cooking Fuels)	X					X
Orofacial clefts	Male	Smoking Prevalence (Reproductive Age Standardized)	X				X	
Orofacial clefts	Male	Socio-demographic Index			X	X		
Orofacial clefts	Female	Legality of Abortion	X				X	
Orofacial clefts	Female	Alcohol (liters per capita)	X					X
Orofacial clefts	Female	Diabetes Age-Standardized Prevalence (proportion)		X			X	
Orofacial clefts	Female	Education (years per capita)		X				X
Orofacial clefts	Female	Indoor Air Pollution (All Cooking Fuels)	X			X		
Orofacial clefts	Female	Indoor Air Pollution (All Cooking Fuels)	X					X
Orofacial clefts	Female	Smoking Prevalence (Reproductive Age Standardized)	X				X	
Orofacial clefts	Female	Socio-demographic Index			X	X		
Down syndrome	Male	Legality of Abortion	X			X		
Down syndrome	Male	Education (years per capita)			X			X
Down syndrome	Male	LDI (I$ per capita)		X			X	
Down syndrome	Male	Live Births 35+ (proportion)	X			X		
Down syndrome	Male	Live Births 40+ (proportion)	X			X		
Down syndrome	Male	Indoor Air Pollution (All Cooking Fuels)			X			X
Down syndrome	Male	Smoking Prevalence (Reproductive Age Standardized)			X			X
Down syndrome	Male	Socio-demographic Index			X		X	

Cause	Sex	Covariate	GBD 2015			GBD 2016		
			Level 1	Level 2	Level 3	Level 1	Level 2	Level 3
Down syndrome	Female	Legality of Abortion	X			X		
Down syndrome	Female	Education (years per capita)			X			X
Down syndrome	Female	LDI (I$ per capita)		X			X	
Down syndrome	Female	Live Births 35+ (proportion)	X			X		
Down syndrome	Female	Live Births 40+ (proportion)	X			X		
Down syndrome	Female	Indoor Air Pollution (All Cooking Fuels)			X			X
Down syndrome	Female	Smoking Prevalence (Reproductive Age Standardized)			X			X
Down syndrome	Female	Socio-demographic Index			X		X	
Other chromosomal abnormalities	Male	Legality of Abortion	X			X		
Other chromosomal abnormalities	Male	Alcohol (liters per capita)			X			X
Other chromosomal abnormalities	Male	Education (years per capita)			X			X
Other chromosomal abnormalities	Male	LDI (I$ per capita)		X			X	
Other chromosomal abnormalities	Male	Live Births 35+ (proportion)	X			X		
Other chromosomal abnormalities	Male	Live Births 40+ (proportion)	X			X		
Other chromosomal abnormalities	Male	Indoor Air Pollution (All Cooking Fuels)			X			X
Other chromosomal abnormalities	Male	Smoking Prevalence (Reproductive Age Standardized)			X			X
Other chromosomal abnormalities	Male	Socio-demographic Index			X			X
Other chromosomal abnormalities	Female	Legality of Abortion	X			X		
Other chromosomal abnormalities	Female	Alcohol (liters per capita)			X			X
Other chromosomal abnormalities	Female	Education (years per capita)			X			X
Other chromosomal abnormalities	Female	LDI (I$ per capita)		X			X	
Other chromosomal abnormalities	Female	Live Births 35+ (proportion)	X			X		
Other chromosomal abnormalities	Female	Live Births 40+ (proportion)	X			X		
Other chromosomal abnormalities	Female	Indoor Air Pollution (All Cooking Fuels)			X			X
Other chromosomal abnormalities	Female	Smoking Prevalence (Reproductive Age Standardized)			X			X
Other chromosomal abnormalities	Female	Socio-demographic Index			X			X
Other congenital birth defects	Female	Smoking Prevalence (Reproductive Age Standardized)		X			X	
Other congenital birth defects	Female	Socio-demographic Index			X			X
Other congenital birth defects	Female	Education (years per capita)		X			X	
Other congenital birth defects	Female	LDI (I$ per capita)			X			X
Other congenital birth defects	Female	Diabetes Age-Standardized Prevalence (proportion)	X					X
Other congenital birth defects	Female	Legality of Abortion		X			X	
Other congenital birth defects	Female	Alcohol (liters per capita)	X					X
Other congenital birth defects	Female	Live Births 35+ (proportion)	X			X		
Other congenital birth defects	Female	Indoor Air Pollution (All Cooking Fuels)		X			X	
Other congenital birth defects	Male	Legality of Abortion		X			X	
Other congenital birth defects	Male	Alcohol (liters per capita)	X					X
Other congenital birth defects	Male	Diabetes Age-Standardized Prevalence (proportion)	X					X
Other congenital birth defects	Male	Education (years per capita)		X			X	
Other congenital birth defects	Male	LDI (I$ per capita)			X			X
Other congenital birth defects	Male	Live Births 35+ (proportion)	X			X		
Other congenital birth defects	Male	Indoor Air Pollution (All Cooking Fuels)		X			X	
Other congenital birth defects	Male	Smoking Prevalence (Reproductive Age Standardized)		X			X	
Other congenital birth defects	Male	Socio-demographic Index			X			X
Skin and subcutaneous diseases	Male	Alcohol (liters per capita)		X			X	
Skin and subcutaneous diseases	Male	Cumulative Cigarettes (10 Years)		X			X	
Skin and subcutaneous diseases	Male	Cumulative Cigarettes (5 Years)		X			X	
Skin and subcutaneous diseases	Male	Education (years per capita)			X			X
Skin and subcutaneous diseases	Male	LDI (I$ per capita)			X			X
Skin and subcutaneous diseases	Male	Smoking Prevalence		X			X	
Skin and subcutaneous diseases	Male	Improved Water Source (proportion with access)	X			X		
Skin and subcutaneous diseases	Male	SEV unsafe sanitation	X			X		
Skin and subcutaneous diseases	Male	Socio-demographic Index			X			X
Skin and subcutaneous diseases	Female	Alcohol (liters per capita)		X			X	
Skin and subcutaneous diseases	Female	Cumulative Cigarettes (10 Years)		X			X	
Skin and subcutaneous diseases	Female	Cumulative Cigarettes (5 Years)		X			X	
Skin and subcutaneous diseases	Female	Education (years per capita)			X			X
Skin and subcutaneous diseases	Female	LDI (I$ per capita)			X			X
Skin and subcutaneous diseases	Female	Smoking Prevalence		X			X	
Skin and subcutaneous diseases	Female	Improved Water Source (proportion with access)	X			X		
Skin and subcutaneous diseases	Female	SEV unsafe sanitation	X			X		
Skin and subcutaneous diseases	Female	Socio-demographic Index			X			X
Cellulitis	Male	Education (years per capita)			X			X
Cellulitis	Male	LDI (I$ per capita)			X			X
Cellulitis	Female	Education (years per capita)			X			X
Cellulitis	Female	LDI (I$ per capita)			X			X
Pyoderma	Male	Alcohol (liters per capita)		X			X	
Pyoderma	Male	Cumulative Cigarettes (10 Years)		X			X	
Pyoderma	Male	Cumulative Cigarettes (5 Years)		X			X	
Pyoderma	Male	Education (years per capita)			X			X
Pyoderma	Male	LDI (I$ per capita)			X			X
Pyoderma	Male	Smoking Prevalence		X			X	
Pyoderma	Male	Improved Water Source (proportion with access)	X			X		
Pyoderma	Male	SEV unsafe sanitation	X			X		
Pyoderma	Male	Socio-demographic Index			X			X
Pyoderma	Female	Alcohol (liters per capita)		X			X	
Pyoderma	Female	Cumulative Cigarettes (10 Years)		X			X	
Pyoderma	Female	Cumulative Cigarettes (5 Years)		X			X	
Pyoderma	Female	Education (years per capita)			X			X
Pyoderma	Female	LDI (I$ per capita)			X			X
Pyoderma	Female	Smoking Prevalence		X			X	
Pyoderma	Female	Improved Water Source (proportion with access)	X			X		
Pyoderma	Female	SEV unsafe sanitation	X			X		
Pyoderma	Female	Socio-demographic Index			X			X
Decubitus ulcer	Male	Cumulative Cigarettes (10 Years)		X			X	

Cause	Sex	Covariate	GBD 2015			GBD 2016		
			Level 1	Level 2	Level 3	Level 1	Level 2	Level 3
Decubitus ulcer	Male	Socio-demographic Index			X			X
Decubitus ulcer	Male	Cumulative Cigarettes (5 Years)		X			X	
Decubitus ulcer	Male	Education (years per capita)			X			X
Decubitus ulcer	Male	LDI (I$ per capita)			X			X
Decubitus ulcer	Male	Health System Access 2 (unitless)		X				X
Decubitus ulcer	Male	SEV unsafe sanitation	X					X
Decubitus ulcer	Male	Smoking Prevalence		X			X	
Decubitus ulcer	Male	Alcohol (liters per capita)		X			X	
Decubitus ulcer	Male	Improved Water Source (proportion with access)	X			X		
Decubitus ulcer	Female	Cumulative Cigarettes (10 Years)		X			X	
Decubitus ulcer	Female	Socio-demographic Index			X			X
Decubitus ulcer	Female	Cumulative Cigarettes (5 Years)		X			X	
Decubitus ulcer	Female	Education (years per capita)			X			X
Decubitus ulcer	Female	LDI (I$ per capita)			X			X
Decubitus ulcer	Female	Health System Access 2 (unitless)		X				X
Decubitus ulcer	Female	SEV unsafe sanitation	X					X
Decubitus ulcer	Female	Smoking Prevalence		X			X	
Decubitus ulcer	Female	Alcohol (liters per capita)		X			X	
Decubitus ulcer	Female	Improved Water Source (proportion with access)	X			X		
Other skin and subcutaneous diseases	Male	Alcohol (liters per capita)		X			X	
Other skin and subcutaneous diseases	Male	Cumulative Cigarettes (10 Years)		X			X	
Other skin and subcutaneous diseases	Male	Cumulative Cigarettes (5 Years)		X			X	
Other skin and subcutaneous diseases	Male	Education (years per capita)			X			X
Other skin and subcutaneous diseases	Male	Health System Access 2 (unitless)		X				X
Other skin and subcutaneous diseases	Male	LDI (I$ per capita)			X			X
Other skin and subcutaneous diseases	Male	Underweight (proportion <2SD weight for age, <5 years)	X			X		
Other skin and subcutaneous diseases	Male	Smoking Prevalence		X			X	
Other skin and subcutaneous diseases	Male	Improved Water Source (proportion with access)	X			X		
Other skin and subcutaneous diseases	Male	SEV unsafe sanitation	X			X		
Other skin and subcutaneous diseases	Male	Socio-demographic Index			X			X
Other skin and subcutaneous diseases	Female	Alcohol (liters per capita)		X			X	
Other skin and subcutaneous diseases	Female	Cumulative Cigarettes (10 Years)		X			X	
Other skin and subcutaneous diseases	Female	Cumulative Cigarettes (5 Years)		X			X	
Other skin and subcutaneous diseases	Female	Education (years per capita)			X			X
Other skin and subcutaneous diseases	Female	Health System Access 2 (unitless)		X				X
Other skin and subcutaneous diseases	Female	LDI (I$ per capita)			X			X
Other skin and subcutaneous diseases	Female	Underweight (proportion <2SD weight for age, <5 years)	X			X		
Other skin and subcutaneous diseases	Female	Smoking Prevalence		X			X	
Other skin and subcutaneous diseases	Female	Improved Water Source (proportion with access)	X			X		
Other skin and subcutaneous diseases	Female	SEV unsafe sanitation	X			X		
Other skin and subcutaneous diseases	Female	Socio-demographic Index			X			X
Sudden infant death syndrome	Male	Education (years per capita)			X			X
Sudden infant death syndrome	Male	In-Facility Delivery (proportion)	X			X		
Sudden infant death syndrome	Male	LDI (I$ per capita)			X			X
Sudden infant death syndrome	Male	Underweight (proportion <2SD weight for age, <5 years)		X			X	
Sudden infant death syndrome	Male	Indoor Air Pollution (All Cooking Fuels)	X			X		
Sudden infant death syndrome	Male	Skilled Birth Attendance (proportion)		X			X	
Sudden infant death syndrome	Male	Smoking Prevalence (Reproductive Age Standardized)	X			X		
Sudden infant death syndrome	Male	Total Fertility Rate			X			X
Sudden infant death syndrome	Male	Socio-demographic Index			X			X
Sudden infant death syndrome	Female	Education (years per capita)			X			X
Sudden infant death syndrome	Female	In-Facility Delivery (proportion)	X			X		
Sudden infant death syndrome	Female	LDI (I$ per capita)			X			X
Sudden infant death syndrome	Female	Underweight (proportion <2SD weight for age, <5 years)		X			X	
Sudden infant death syndrome	Female	Indoor Air Pollution (All Cooking Fuels)	X			X		
Sudden infant death syndrome	Female	Skilled Birth Attendance (proportion)		X			X	
Sudden infant death syndrome	Female	Smoking Prevalence (Reproductive Age Standardized)	X			X		
Sudden infant death syndrome	Female	Total Fertility Rate			X			X
Sudden infant death syndrome	Female	Socio-demographic Index			X			X
Transport injuries	Male	Alcohol (liters per capita)	X			X		
Transport injuries	Male	Education (years per capita)			X			X
Transport injuries	Male	LDI (I$ per capita)		X			X	
Transport injuries	Male	Population Density (300-500 ppl/sqkm, proportion)		X			X	
Transport injuries	Male	Population Density (500-1000 ppl/sqkm, proportion)		X			X	
Transport injuries	Male	Rainfall Quintile 5 (proportion)			X			X
Transport injuries	Male	Vehicles - 2+4 wheels (per capita)	X			X		
Transport injuries	Male	Vehicles - 2 wheels fraction (proportion)	X			X		
Transport injuries	Male	Socio-demographic Index			X			X
Transport injuries	Female	Alcohol (liters per capita)	X			X		
Transport injuries	Female	Education (years per capita)			X			X
Transport injuries	Female	LDI (I$ per capita)		X			X	
Transport injuries	Female	Population Density (300-500 ppl/sqkm, proportion)		X			X	
Transport injuries	Female	Population Density (500-1000 ppl/sqkm, proportion)		X			X	
Transport injuries	Female	Rainfall Quintile 5 (proportion)			X			X
Transport injuries	Female	Vehicles - 2+4 wheels (per capita)	X			X		
Transport injuries	Female	Vehicles - 2 wheels fraction (proportion)	X			X		
Transport injuries	Female	Socio-demographic Index			X			X
Road injuries	Male	Alcohol (liters per capita)	X			X		
Road injuries	Male	Education (years per capita)			X			X
Road injuries	Male	LDI (I$ per capita)		X				X
Road injuries	Male	Population 15 to 30 (proportion)		X			X	
Road injuries	Male	Population Density (300-500 ppl/sqkm, proportion)		X			X	
Road injuries	Male	Population Density (500-1000 ppl/sqkm, proportion)		X			X	
Road injuries	Male	Vehicles - 2+4 wheels (per capita)	X			X		
Road injuries	Male	Vehicles - 2 wheels (per capita)	X			X		

Cause	Sex	Covariate	GBD 2015			GBD 2016		
			Level 1	Level 2	Level 3	Level 1	Level 2	Level 3
Road injuries	Male	Vehicles - 4 wheels (per capita)	X			X		
Road injuries	Male	Vehicles - 2 wheels fraction (proportion)	X			X		
Road injuries	Male	Log-transformed SEV scalar: Road Inj	X			X		
Road injuries	Male	Socio-demographic Index			X			X
Road injuries	Female	Alcohol (liters per capita)	X			X		
Road injuries	Female	Education (years per capita)			X			X
Road injuries	Female	LDI (I$ per capita)		X				X
Road injuries	Female	Population 15 to 30 (proportion)		X			X	
Road injuries	Female	Population Density (300-500 ppl/sqkm, proportion)		X			X	
Road injuries	Female	Population Density (500-1000 ppl/sqkm, proportion)		X			X	
Road injuries	Female	Vehicles - 2+4 wheels (per capita)	X			X		
Road injuries	Female	Vehicles - 2 wheels (per capita)	X			X		
Road injuries	Female	Vehicles - 4 wheels (per capita)	X			X		
Road injuries	Female	Vehicles - 2 wheels fraction (proportion)	X			X		
Road injuries	Female	Log-transformed SEV scalar: Road Inj	X			X		
Road injuries	Female	Socio-demographic Index			X			X
Pedestrian road injuries	Male	Alcohol (liters per capita)	X			X		
Pedestrian road injuries	Male	Education (years per capita)			X			X
Pedestrian road injuries	Male	LDI (I$ per capita)		X			X	
Pedestrian road injuries	Male	Population Density (300-500 ppl/sqkm, proportion)		X			X	
Pedestrian road injuries	Male	Population Density (500-1000 ppl/sqkm, proportion)		X			X	
Pedestrian road injuries	Male	Rainfall Quintile 5 (proportion)			X			X
Pedestrian road injuries	Male	Vehicles - 2+4 wheels (per capita)	X			X		
Pedestrian road injuries	Male	Vehicles - 2 wheels fraction (proportion)	X			X		
Pedestrian road injuries	Male	Log-transformed SEV scalar: Pedest	X			X		
Pedestrian road injuries	Male	Socio-demographic Index			X			X
Pedestrian road injuries	Female	Alcohol (liters per capita)	X			X		
Pedestrian road injuries	Female	Education (years per capita)			X			X
Pedestrian road injuries	Female	LDI (I$ per capita)		X			X	
Pedestrian road injuries	Female	Population Density (300-500 ppl/sqkm, proportion)		X			X	
Pedestrian road injuries	Female	Population Density (500-1000 ppl/sqkm, proportion)		X			X	
Pedestrian road injuries	Female	Rainfall Quintile 5 (proportion)			X			X
Pedestrian road injuries	Female	Vehicles - 2+4 wheels (per capita)	X			X		
Pedestrian road injuries	Female	Vehicles - 2 wheels fraction (proportion)	X			X		
Pedestrian road injuries	Female	Log-transformed SEV scalar: Pedest	X			X		
Pedestrian road injuries	Female	Socio-demographic Index			X			X
Cyclist road injuries	Male	Alcohol (liters per capita)	X			X		
Cyclist road injuries	Male	Education (years per capita)			X			X
Cyclist road injuries	Male	LDI (I$ per capita)		X			X	
Cyclist road injuries	Male	Population Density (300-500 ppl/sqkm, proportion)		X			X	
Cyclist road injuries	Male	Population Density (500-1000 ppl/sqkm, proportion)		X			X	
Cyclist road injuries	Male	Vehicles - 2+4 wheels (per capita)	X			X		
Cyclist road injuries	Male	Vehicles - 2 wheels fraction (proportion)	X			X		
Cyclist road injuries	Male	Log-transformed SEV scalar: Cyclist	X			X		
Cyclist road injuries	Male	Socio-demographic Index			X			X
Cyclist road injuries	Female	Alcohol (liters per capita)	X			X		
Cyclist road injuries	Female	Education (years per capita)			X			X
Cyclist road injuries	Female	LDI (I$ per capita)		X			X	
Cyclist road injuries	Female	Population Density (300-500 ppl/sqkm, proportion)		X			X	
Cyclist road injuries	Female	Population Density (500-1000 ppl/sqkm, proportion)		X			X	
Cyclist road injuries	Female	Vehicles - 2+4 wheels (per capita)	X			X		
Cyclist road injuries	Female	Vehicles - 2 wheels fraction (proportion)	X			X		
Cyclist road injuries	Female	Log-transformed SEV scalar: Cyclist	X			X		
Cyclist road injuries	Female	Socio-demographic Index			X			X
Motorcyclist road injuries	Female	Alcohol (liters per capita)	X			X		
Motorcyclist road injuries	Female	Education (years per capita)			X			X
Motorcyclist road injuries	Female	LDI (I$ per capita)		X			X	
Motorcyclist road injuries	Female	Population Density (300-500 ppl/sqkm, proportion)		X			X	
Motorcyclist road injuries	Female	Population Density (500-1000 ppl/sqkm, proportion)		X			X	
Motorcyclist road injuries	Female	Rainfall Quintile 5 (proportion)			X			X
Motorcyclist road injuries	Female	Vehicles - 2 wheels (per capita)	X			X		
Motorcyclist road injuries	Female	Log-transformed SEV scalar: Mot Cyc	X			X		
Motorcyclist road injuries	Female	Socio-demographic Index			X			X
Motorcyclist road injuries	Male	Alcohol (liters per capita)	X			X		
Motorcyclist road injuries	Male	Education (years per capita)			X			X
Motorcyclist road injuries	Male	LDI (I$ per capita)		X			X	
Motorcyclist road injuries	Male	Population Density (300-500 ppl/sqkm, proportion)		X			X	
Motorcyclist road injuries	Male	Population Density (500-1000 ppl/sqkm, proportion)		X			X	
Motorcyclist road injuries	Male	Rainfall Quintile 5 (proportion)			X			X
Motorcyclist road injuries	Male	Vehicles - 2 wheels (per capita)	X			X		
Motorcyclist road injuries	Male	Log-transformed SEV scalar: Mot Cyc	X			X		
Motorcyclist road injuries	Male	Socio-demographic Index			X			X
Motor vehicle road injuries	Male	Alcohol (liters per capita)	X			X		
Motor vehicle road injuries	Male	Education (years per capita)			X			X
Motor vehicle road injuries	Male	LDI (I$ per capita)		X				X
Motor vehicle road injuries	Male	Population Density (300-500 ppl/sqkm, proportion)		X			X	
Motor vehicle road injuries	Male	Population Density (500-1000 ppl/sqkm, proportion)		X			X	
Motor vehicle road injuries	Male	Rainfall Quintile 5 (proportion)			X			X
Motor vehicle road injuries	Male	Vehicles - 4 wheels (per capita)	X			X		
Motor vehicle road injuries	Male	Log-transformed SEV scalar: Mot Veh	X			X		
Motor vehicle road injuries	Male	Socio-demographic Index			X			X
Motor vehicle road injuries	Female	Alcohol (liters per capita)	X			X		
Motor vehicle road injuries	Female	Education (years per capita)			X			X
Motor vehicle road injuries	Female	LDI (I$ per capita)		X				X
Motor vehicle road injuries	Female	Population Density (300-500 ppl/sqkm, proportion)		X			X	

Cause	Sex	Covariate	GBD 2015			GBD 2016		
			Level 1	Level 2	Level 3	Level 1	Level 2	Level 3
Motor vehicle road injuries	Female	Population Density (500-1000 ppl/sqkm, proportion)		X			X	
Motor vehicle road injuries	Female	Rainfall Quintile 5 (proportion)			X			X
Motor vehicle road injuries	Female	Vehicles - 4 wheels (per capita)	X			X		
Motor vehicle road injuries	Female	Log-transformed SEV scalar: Mot Veh	X			X		
Motor vehicle road injuries	Female	Socio-demographic Index			X			X
Other road injuries	Male	Alcohol (liters per capita)	X			X		
Other road injuries	Male	LDI (I$ per capita)		X			X	
Other road injuries	Male	Rainfall Quintile 5 (proportion)			X			X
Other road injuries	Male	Vehicles - 2+4 wheels (per capita)	X			X		
Other road injuries	Male	Vehicles - 2 wheels fraction (proportion)	X			X		
Other road injuries	Male	Log-transformed SEV scalar: Oth Road	X			X		
Other road injuries	Male	Socio-demographic Index			X			X
Other road injuries	Female	Alcohol (liters per capita)	X			X		
Other road injuries	Female	LDI (I$ per capita)		X			X	
Other road injuries	Female	Rainfall Quintile 5 (proportion)			X			X
Other road injuries	Female	Vehicles - 2+4 wheels (per capita)	X			X		
Other road injuries	Female	Vehicles - 2 wheels fraction (proportion)	X			X		
Other road injuries	Female	Log-transformed SEV scalar: Oth Road	X			X		
Other road injuries	Female	Socio-demographic Index			X			X
Other transport injuries	Male	Alcohol (liters per capita)	X			X		
Other transport injuries	Male	Education (years per capita)			X			X
Other transport injuries	Male	LDI (I$ per capita)			X			X
Other transport injuries	Male	Population Density (300-500 ppl/sqkm, proportion)		X			X	
Other transport injuries	Male	Population Density (500-1000 ppl/sqkm, proportion)		X			X	
Other transport injuries	Male	Rainfall Quintile 5 (proportion)			X			X
Other transport injuries	Male	Vehicles - 2+4 wheels (per capita)	X			X		
Other transport injuries	Male	Vehicles - 2 wheels fraction (proportion)	X			X		
Other transport injuries	Male	Log-transformed SEV scalar: Oth Trans	X			X		
Other transport injuries	Male	Socio-demographic Index			X			X
Other transport injuries	Female	Alcohol (liters per capita)	X			X		
Other transport injuries	Female	Education (years per capita)			X			X
Other transport injuries	Female	LDI (I$ per capita)			X			X
Other transport injuries	Female	Population Density (300-500 ppl/sqkm, proportion)		X			X	
Other transport injuries	Female	Population Density (500-1000 ppl/sqkm, proportion)		X			X	
Other transport injuries	Female	Rainfall Quintile 5 (proportion)			X			X
Other transport injuries	Female	Vehicles - 2+4 wheels (per capita)	X			X		
Other transport injuries	Female	Vehicles - 2 wheels fraction (proportion)	X			X		
Other transport injuries	Female	Log-transformed SEV scalar: Oth Trans	X			X		
Other transport injuries	Female	Socio-demographic Index			X			X
Unintentional injuries	Female	Alcohol (liters per capita)		X			X	
Unintentional injuries	Female	Cumulative Cigarettes (5 Years)	X			X		
Unintentional injuries	Female	Diabetes Fasting Plasma Glucose (mmol/L)	X			X		
Unintentional injuries	Female	Education (years per capita)			X			X
Unintentional injuries	Female	Health System Access 2 (unitless)	X			X		
Unintentional injuries	Female	LDI (I$ per capita)			X			X
Unintentional injuries	Female	Underweight (proportion <2SD weight for age, <5 years)	X			X		
Unintentional injuries	Female	Indoor Air Pollution (All Cooking Fuels)	X			X		
Unintentional injuries	Female	Population Density (500-1000 ppl/sqkm, proportion)		X			X	
Unintentional injuries	Female	Population Density (over 1000 ppl/sqkm, proportion)		X			X	
Unintentional injuries	Female	Smoking Prevalence	X			X		
Unintentional injuries	Male	Alcohol (liters per capita)		X			X	
Unintentional injuries	Male	Cumulative Cigarettes (5 Years)	X			X		
Unintentional injuries	Male	Diabetes Fasting Plasma Glucose (mmol/L)	X			X		
Unintentional injuries	Male	Education (years per capita)			X			X
Unintentional injuries	Male	Health System Access 2 (unitless)	X			X		
Unintentional injuries	Male	LDI (I$ per capita)			X			X
Unintentional injuries	Male	Underweight (proportion <2SD weight for age, <5 years)	X			X		
Unintentional injuries	Male	Indoor Air Pollution (All Cooking Fuels)	X			X		
Unintentional injuries	Male	Population Density (500-1000 ppl/sqkm, proportion)		X			X	
Unintentional injuries	Male	Population Density (over 1000 ppl/sqkm, proportion)		X			X	
Unintentional injuries	Male	Smoking Prevalence	X			X		
Falls	Male	Alcohol (liters per capita)	X			X		
Falls	Male	LDI (I$ per capita)			X			X
Falls	Male	ln-Milk (kcal per capita)		X			X	
Falls	Male	Elevation Over 1500m (proportion)			X			X
Falls	Male	Log-transformed SEV scalar: Falls	X			X		
Falls	Male	Socio-demographic Index			X			X
Falls	Female	Alcohol (liters per capita)	X			X		
Falls	Female	LDI (I$ per capita)			X			X
Falls	Female	ln-Milk (kcal per capita)		X			X	
Falls	Female	Elevation Over 1500m (proportion)			X			X
Falls	Female	Log-transformed SEV scalar: Falls	X			X		
Falls	Female	Socio-demographic Index			X			X
Drowning	Male	Alcohol (liters per capita)	X			X		
Drowning	Male	Coastal Population within 10km (proportion)	X			X		
Drowning	Male	Education (years per capita)			X			X
Drowning	Male	Landlocked Nation (binary)	X			X		
Drowning	Male	LDI (I$ per capita)			X			X
Drowning	Male	Elevation Under 100m (proportion)		X			X	
Drowning	Male	Rainfall Quintile 1 (proportion)	X			X		
Drowning	Male	Rainfall Quintile 5 (proportion)	X			X		
Drowning	Male	Log-transformed SEV scalar: Drown	X			X		
Drowning	Male	Socio-demographic Index			X			X
Drowning	Female	Alcohol (liters per capita)	X			X		
Drowning	Female	Coastal Population within 10km (proportion)	X			X		

Cause	Sex	Covariate	GBD 2015			GBD 2016		
			Level 1	Level 2	Level 3	Level 1	Level 2	Level 3
Drowning	Female	Education (years per capita)			X			X
Drowning	Female	Landlocked Nation (binary)	X			X		
Drowning	Female	LDI (I$ per capita)			X			X
Drowning	Female	Elevation Under 100m (proportion)		X			X	
Drowning	Female	Rainfall Quintile 1 (proportion)	X			X		
Drowning	Female	Rainfall Quintile 5 (proportion)	X			X		
Drowning	Female	Log-transformed SEV scalar: Drown	X			X		
Drowning	Female	Socio-demographic Index			X			X
Fire, heat, and hot substances	Male	Alcohol (liters per capita)		X			X	
Fire, heat, and hot substances	Male	Tobacco (cigarettes per capita)		X			X	
Fire, heat, and hot substances	Male	Education (years per capita)			X			X
Fire, heat, and hot substances	Male	LDI (I$ per capita)			X			X
Fire, heat, and hot substances	Male	Indoor Air Pollution (Biomass Cooking)		X			X	
Fire, heat, and hot substances	Male	Population Density (over 1000 ppl/sqkm, proportion)		X			X	
Fire, heat, and hot substances	Male	Log-transformed SEV scalar: Fire	X			X		
Fire, heat, and hot substances	Male	Socio-demographic Index			X			X
Fire, heat, and hot substances	Female	Alcohol (liters per capita)		X			X	
Fire, heat, and hot substances	Female	Tobacco (cigarettes per capita)		X			X	
Fire, heat, and hot substances	Female	Education (years per capita)			X			X
Fire, heat, and hot substances	Female	LDI (I$ per capita)			X			X
Fire, heat, and hot substances	Female	Indoor Air Pollution (Biomass Cooking)		X			X	
Fire, heat, and hot substances	Female	Population Density (over 1000 ppl/sqkm, proportion)		X			X	
Fire, heat, and hot substances	Female	Log-transformed SEV scalar: Fire	X			X		
Fire, heat, and hot substances	Female	Socio-demographic Index			X			X
Poisonings	Female	Education (years per capita)			X			X
Poisonings	Female	LDI (I$ per capita)			X			X
Poisonings	Female	Opium Cultivation (binary)	X			X		
Poisonings	Female	Population Density (over 1000 ppl/sqkm, proportion)		X			X	
Poisonings	Female	Population Density (under 150 ppl/sqkm, proportion)		X			X	
Poisonings	Female	Log-transformed SEV scalar: Poison	X			X		
Poisonings	Female	Socio-demographic Index			X			X
Poisonings	Male	Education (years per capita)			X			X
Poisonings	Male	LDI (I$ per capita)			X			X
Poisonings	Male	Opium Cultivation (binary)	X			X		
Poisonings	Male	Population Density (over 1000 ppl/sqkm, proportion)		X			X	
Poisonings	Male	Population Density (under 150 ppl/sqkm, proportion)		X			X	
Poisonings	Male	Log-transformed SEV scalar: Poison	X			X		
Poisonings	Male	Socio-demographic Index			X			X
Exposure to mechanical forces	Male	Alcohol (liters per capita)		X			X	
Exposure to mechanical forces	Male	Education (years per capita)			X			X
Exposure to mechanical forces	Male	LDI (I$ per capita)			X			X
Exposure to mechanical forces	Male	Population Density (over 1000 ppl/sqkm, proportion)		X			X	
Exposure to mechanical forces	Male	Population Density (under 150 ppl/sqkm, proportion)		X			X	
Exposure to mechanical forces	Male	Socio-demographic Index			X			X
Exposure to mechanical forces	Female	Alcohol (liters per capita)		X			X	
Exposure to mechanical forces	Female	Education (years per capita)			X			X
Exposure to mechanical forces	Female	LDI (I$ per capita)			X			X
Exposure to mechanical forces	Female	Population Density (over 1000 ppl/sqkm, proportion)		X			X	
Exposure to mechanical forces	Female	Population Density (under 150 ppl/sqkm, proportion)		X			X	
Exposure to mechanical forces	Female	Socio-demographic Index			X			X
Unintentional firearm injuries	Male	Alcohol (liters per capita)		X			X	
Unintentional firearm injuries	Male	Education (years per capita)			X			X
Unintentional firearm injuries	Male	Health System Access (unitless)		X			X	
Unintentional firearm injuries	Male	LDI (I$ per capita)			X			X
Unintentional firearm injuries	Male	Population Density (over 1000 ppl/sqkm, proportion)			X			X
Unintentional firearm injuries	Male	Population Density (under 150 ppl/sqkm, proportion)			X			X
Unintentional firearm injuries	Male	Log-transformed SEV scalar: Mech Gun	X			X		
Unintentional firearm injuries	Male	Socio-demographic Index			X			X
Unintentional firearm injuries	Female	Alcohol (liters per capita)		X			X	
Unintentional firearm injuries	Female	Education (years per capita)			X			X
Unintentional firearm injuries	Female	Health System Access (unitless)		X			X	
Unintentional firearm injuries	Female	LDI (I$ per capita)			X			X
Unintentional firearm injuries	Female	Population Density (over 1000 ppl/sqkm, proportion)			X			X
Unintentional firearm injuries	Female	Population Density (under 150 ppl/sqkm, proportion)			X			X
Unintentional firearm injuries	Female	Log-transformed SEV scalar: Mech Gun	X			X		
Unintentional firearm injuries	Female	Socio-demographic Index			X			X
Unintentional suffocation	Male	Alcohol (liters per capita)		X			X	
Unintentional suffocation	Male	Education (years per capita)			X			X
Unintentional suffocation	Male	LDI (I$ per capita)			X			X
Unintentional suffocation	Male	Population Density (over 1000 ppl/sqkm, proportion)		X			X	
Unintentional suffocation	Male	Population Density (under 150 ppl/sqkm, proportion)		X			X	
Unintentional suffocation	Male	Log-transformed SEV scalar: Mech Suff	X			X		
Unintentional suffocation	Male	Socio-demographic Index			X			X
Unintentional suffocation	Female	Alcohol (liters per capita)		X			X	
Unintentional suffocation	Female	Education (years per capita)			X			X
Unintentional suffocation	Female	LDI (I$ per capita)			X			X
Unintentional suffocation	Female	Population Density (over 1000 ppl/sqkm, proportion)		X			X	
Unintentional suffocation	Female	Population Density (under 150 ppl/sqkm, proportion)		X			X	
Unintentional suffocation	Female	Log-transformed SEV scalar: Mech Suff	X			X		
Unintentional suffocation	Female	Socio-demographic Index			X			X
Other exposure to mechanical forces	Male	Alcohol (liters per capita)		X			X	
Other exposure to mechanical forces	Male	Education (years per capita)			X			X
Other exposure to mechanical forces	Male	Health System Access (unitless)		X			X	
Other exposure to mechanical forces	Male	LDI (I$ per capita)			X			X
Other exposure to mechanical forces	Male	Population Density (over 1000 ppl/sqkm, proportion)		X			X	

Cause	Sex	Covariate	GBD 2015			GBD 2016		
			Level 1	Level 2	Level 3	Level 1	Level 2	Level 3
Other exposure to mechanical forces	Male	Population Density (under 150 ppl/sqkm, proportion)		X			X	
Other exposure to mechanical forces	Male	Log-transformed SEV scalar: Oth Mech	X			X		
Other exposure to mechanical forces	Male	Socio-demographic Index			X			X
Other exposure to mechanical forces	Female	Alcohol (liters per capita)		X			X	
Other exposure to mechanical forces	Female	Education (years per capita)			X			X
Other exposure to mechanical forces	Female	Health System Access (unitless)		X			X	
Other exposure to mechanical forces	Female	LDI (I$ per capita)			X			X
Other exposure to mechanical forces	Female	Population Density (over 1000 ppl/sqkm, proportion)		X			X	
Other exposure to mechanical forces	Female	Population Density (under 150 ppl/sqkm, proportion)		X			X	
Other exposure to mechanical forces	Female	Log-transformed SEV scalar: Oth Mech	X			X		
Other exposure to mechanical forces	Female	Socio-demographic Index			X			X
Adverse effects of medical treatment	Male	LDI (I$ per capita)			X			X
Adverse effects of medical treatment	Male	Socio-demographic Index			X			X
Adverse effects of medical treatment	Female	LDI (I$ per capita)			X			X
Adverse effects of medical treatment	Female	Socio-demographic Index			X			X
Animal contact	Male	Alcohol (liters per capita)	X			X		
Animal contact	Male	Education (years per capita)			X			X
Animal contact	Male	LDI (I$ per capita)			X			X
Animal contact	Male	Elevation Over 1500m (proportion)			X			X
Animal contact	Male	Population 15 to 30 (proportion)		X			X	
Animal contact	Male	Population Density (over 1000 ppl/sqkm, proportion)			X			X
Animal contact	Male	Population Density (under 150 ppl/sqkm, proportion)			X			X
Animal contact	Male	Elevation Under 100m (proportion)			X			X
Animal contact	Male	Log-transformed SEV scalar: Animal	X			X		
Animal contact	Male	Socio-demographic Index			X			X
Animal contact	Female	Alcohol (liters per capita)	X			X		
Animal contact	Female	Education (years per capita)			X			X
Animal contact	Female	LDI (I$ per capita)			X			X
Animal contact	Female	Elevation Over 1500m (proportion)			X			X
Animal contact	Female	Population 15 to 30 (proportion)		X			X	
Animal contact	Female	Population Density (over 1000 ppl/sqkm, proportion)			X			X
Animal contact	Female	Population Density (under 150 ppl/sqkm, proportion)			X			X
Animal contact	Female	Elevation Under 100m (proportion)			X			X
Animal contact	Female	Log-transformed SEV scalar: Animal	X			X		
Animal contact	Female	Socio-demographic Index			X			X
Venomous animal contact	Male	Alcohol (liters per capita)	X			X		
Venomous animal contact	Male	Education (years per capita)			X			X
Venomous animal contact	Male	LDI (I$ per capita)			X			X
Venomous animal contact	Male	Elevation Over 1500m (proportion)			X			X
Venomous animal contact	Male	Population Density (over 1000 ppl/sqkm, proportion)			X			X
Venomous animal contact	Male	Population Density (under 150 ppl/sqkm, proportion)			X			X
Venomous animal contact	Male	Elevation Under 100m (proportion)			X			X
Venomous animal contact	Male	Log-transformed SEV scalar: Venom	X			X		
Venomous animal contact	Male	Socio-demographic Index			X			X
Venomous animal contact	Female	Alcohol (liters per capita)	X			X		
Venomous animal contact	Female	Education (years per capita)			X			X
Venomous animal contact	Female	LDI (I$ per capita)			X			X
Venomous animal contact	Female	Elevation Over 1500m (proportion)			X			X
Venomous animal contact	Female	Population Density (over 1000 ppl/sqkm, proportion)			X			X
Venomous animal contact	Female	Population Density (under 150 ppl/sqkm, proportion)			X			X
Venomous animal contact	Female	Elevation Under 100m (proportion)			X			X
Venomous animal contact	Female	Log-transformed SEV scalar: Venom	X			X		
Venomous animal contact	Female	Socio-demographic Index			X			X
Non-venomous animal contact	Male	Alcohol (liters per capita)	X			X		
Non-venomous animal contact	Male	Education (years per capita)			X			X
Non-venomous animal contact	Male	LDI (I$ per capita)			X			X
Non-venomous animal contact	Male	Elevation Over 1500m (proportion)			X			X
Non-venomous animal contact	Male	Population Density (over 1000 ppl/sqkm, proportion)			X			X
Non-venomous animal contact	Male	Population Density (under 150 ppl/sqkm, proportion)			X			X
Non-venomous animal contact	Male	Elevation Under 100m (proportion)			X			X
Non-venomous animal contact	Male	Log-transformed SEV scalar: Non Ven	X			X		
Non-venomous animal contact	Male	Socio-demographic Index			X			X
Non-venomous animal contact	Female	Alcohol (liters per capita)	X			X		
Non-venomous animal contact	Female	Education (years per capita)			X			X
Non-venomous animal contact	Female	LDI (I$ per capita)			X			X
Non-venomous animal contact	Female	Elevation Over 1500m (proportion)			X			X
Non-venomous animal contact	Female	Population Density (over 1000 ppl/sqkm, proportion)			X			X
Non-venomous animal contact	Female	Population Density (under 150 ppl/sqkm, proportion)			X			X
Non-venomous animal contact	Female	Elevation Under 100m (proportion)			X			X
Non-venomous animal contact	Female	Log-transformed SEV scalar: Non Ven	X			X		
Non-venomous animal contact	Female	Socio-demographic Index			X			X
Foreign body	Male	Education (years per capita)	X			X		
Foreign body	Male	LDI (I$ per capita)	X			X		
Foreign body	Male	Population Density (over 1000 ppl/sqkm, proportion)	X			X		
Foreign body	Male	Population Over 65 (proportion)	X			X		
Foreign body	Male	Socio-demographic Index			X			X
Foreign body	Female	Education (years per capita)	X			X		
Foreign body	Female	LDI (I$ per capita)	X			X		
Foreign body	Female	Population Density (over 1000 ppl/sqkm, proportion)	X			X		
Foreign body	Female	Population Over 65 (proportion)	X			X		
Foreign body	Female	Socio-demographic Index			X			X
Pulmonary aspiration and foreign body in airway	Male	Alcohol (liters per capita)		X			X	
Pulmonary aspiration and foreign body in airway	Male	LDI (I$ per capita)			X			X
Pulmonary aspiration and foreign body in airway	Male	Mean BMI		X			X	
Pulmonary aspiration and foreign body in airway	Male	Log-transformed SEV scalar: F Body Asp	X			X		

Cause	Sex	Covariate	GBD 2015			GBD 2016		
			Level 1	Level 2	Level 3	Level 1	Level 2	Level 3
Pulmonary aspiration and foreign body in airway	Male	Socio-demographic Index			X			X
Pulmonary aspiration and foreign body in airway	Female	Alcohol (liters per capita)		X			X	
Pulmonary aspiration and foreign body in airway	Female	LDI (I$ per capita)			X			X
Pulmonary aspiration and foreign body in airway	Female	Mean BMI		X			X	
Pulmonary aspiration and foreign body in airway	Female	Log-transformed SEV scalar: F Body Asp	X			X		
Pulmonary aspiration and foreign body in airway	Female	Socio-demographic Index			X			X
Foreign body in other body part	Male	Alcohol (liters per capita)	X			X		
Foreign body in other body part	Male	Education (years per capita)			X			X
Foreign body in other body part	Male	LDI (I$ per capita)			X			X
Foreign body in other body part	Male	Elevation Over 1500m (proportion)			X			X
Foreign body in other body part	Male	Population Density (over 1000 ppl/sqkm, proportion)			X			X
Foreign body in other body part	Male	Population Density (under 150 ppl/sqkm, proportion)			X			X
Foreign body in other body part	Male	Elevation Under 100m (proportion)			X			X
Foreign body in other body part	Male	Log-transformed SEV scalar: Oth F Body	X			X		
Foreign body in other body part	Male	Socio-demographic Index			X			X
Foreign body in other body part	Female	Alcohol (liters per capita)	X			X		
Foreign body in other body part	Female	Education (years per capita)			X			X
Foreign body in other body part	Female	LDI (I$ per capita)			X			X
Foreign body in other body part	Female	Elevation Over 1500m (proportion)			X			X
Foreign body in other body part	Female	Population Density (over 1000 ppl/sqkm, proportion)			X			X
Foreign body in other body part	Female	Population Density (under 150 ppl/sqkm, proportion)			X			X
Foreign body in other body part	Female	Elevation Under 100m (proportion)			X			X
Foreign body in other body part	Female	Log-transformed SEV scalar: Oth F Body	X			X		
Foreign body in other body part	Female	Socio-demographic Index			X			X
Other unintentional injuries	Male	Alcohol (liters per capita)	X			X		
Other unintentional injuries	Male	Education (years per capita)			X			X
Other unintentional injuries	Male	LDI (I$ per capita)			X			X
Other unintentional injuries	Male	Elevation Over 1500m (proportion)			X			X
Other unintentional injuries	Male	Population Density (over 1000 ppl/sqkm, proportion)			X			X
Other unintentional injuries	Male	Population Density (under 150 ppl/sqkm, proportion)			X			X
Other unintentional injuries	Male	Elevation Under 100m (proportion)			X			X
Other unintentional injuries	Male	Vehicles - 2 wheels (per capita)	X			X		
Other unintentional injuries	Male	Vehicles - 4 wheels (per capita)	X			X		
Other unintentional injuries	Male	Log-transformed SEV scalar: Oth Unint	X			X		
Other unintentional injuries	Male	Socio-demographic Index			X			X
Other unintentional injuries	Female	Alcohol (liters per capita)	X			X		
Other unintentional injuries	Female	Education (years per capita)			X			X
Other unintentional injuries	Female	LDI (I$ per capita)			X			X
Other unintentional injuries	Female	Elevation Over 1500m (proportion)			X			X
Other unintentional injuries	Female	Population Density (over 1000 ppl/sqkm, proportion)			X			X
Other unintentional injuries	Female	Population Density (under 150 ppl/sqkm, proportion)			X			X
Other unintentional injuries	Female	Elevation Under 100m (proportion)			X			X
Other unintentional injuries	Female	Vehicles - 2 wheels (per capita)	X			X		
Other unintentional injuries	Female	Vehicles - 4 wheels (per capita)	X			X		
Other unintentional injuries	Female	Log-transformed SEV scalar: Oth Unint	X			X		
Other unintentional injuries	Female	Socio-demographic Index			X			X
Self-harm and interpersonal violence	Male	Alcohol (liters per capita)	X			X		
Self-harm and interpersonal violence	Male	Education (years per capita)			X			X
Self-harm and interpersonal violence	Male	LDI (I$ per capita)			X			X
Self-harm and interpersonal violence	Male	Elevation Over 1500m (proportion)			X			X
Self-harm and interpersonal violence	Male	Population Density (over 1000 ppl/sqkm, proportion)			X			X
Self-harm and interpersonal violence	Male	Population Density (under 150 ppl/sqkm, proportion)			X			X
Self-harm and interpersonal violence	Male	Elevation Under 100m (proportion)			X			X
Self-harm and interpersonal violence	Male	Log-transformed SEV scalar: Oth Unint	X			X		
Self-harm and interpersonal violence	Female	Alcohol (liters per capita)	X			X		
Self-harm and interpersonal violence	Female	Education (years per capita)			X			X
Self-harm and interpersonal violence	Female	LDI (I$ per capita)			X			X
Self-harm and interpersonal violence	Female	Elevation Over 1500m (proportion)			X			X
Self-harm and interpersonal violence	Female	Population Density (over 1000 ppl/sqkm, proportion)			X			X
Self-harm and interpersonal violence	Female	Population Density (under 150 ppl/sqkm, proportion)			X			X
Self-harm and interpersonal violence	Female	Elevation Under 100m (proportion)			X			X
Self-harm and interpersonal violence	Female	Log-transformed SEV scalar: Oth Unint	X			X		
Self-harm	Male	Alcohol (liters per capita)	X			X		
Self-harm	Male	Education (years per capita)			X			X
Self-harm	Male	LDI (I$ per capita)			X			X
Self-harm	Male	Population Density (150-300 ppl/sqkm, proportion)		X			X	
Self-harm	Male	Population Density (300-500 ppl/sqkm, proportion)		X			X	
Self-harm	Male	Population Density (500-1000 ppl/sqkm, proportion)		X			X	
Self-harm	Male	Population Density (over 1000 ppl/sqkm, proportion)		X			X	
Self-harm	Male	Population Density (under 150 ppl/sqkm, proportion)		X			X	
Self-harm	Male	Religion (binary, >50% Muslim)		X			X	
Self-harm	Male	Socio-demographic Index			X			X
Self-harm	Female	Alcohol (liters per capita)	X			X		
Self-harm	Female	Education (years per capita)			X			X
Self-harm	Female	LDI (I$ per capita)			X			X
Self-harm	Female	Population Density (150-300 ppl/sqkm, proportion)		X			X	
Self-harm	Female	Population Density (300-500 ppl/sqkm, proportion)		X			X	
Self-harm	Female	Population Density (500-1000 ppl/sqkm, proportion)		X			X	
Self-harm	Female	Population Density (over 1000 ppl/sqkm, proportion)		X			X	
Self-harm	Female	Population Density (under 150 ppl/sqkm, proportion)		X			X	
Self-harm	Female	Religion (binary, >50% Muslim)		X			X	
Self-harm	Female	Log-transformed SEV scalar: Self Harm	X			X		
Self-harm	Female	Socio-demographic Index			X			X
Self-harm	Female	Major depressive disorder	X			X		
Interpersonal violence	Male	Alcohol (liters per capita)	X			X		

Cause	Sex	Covariate	GBD 2015			GBD 2016		
			Level 1	Level 2	Level 3	Level 1	Level 2	Level 3
Interpersonal violence	Male	Education (years per capita)			X			X
Interpersonal violence	Male	LDI (I$ per capita)			X			X
Interpersonal violence	Male	Opium Cultivation (binary)		X			X	
Interpersonal violence	Male	Population Density (over 1000 ppl/sqkm, proportion)		X			X	
Interpersonal violence	Male	Log-transformed SEV scalar: Violence	X			X		
Interpersonal violence	Male	Socio-demographic Index			X			X
Interpersonal violence	Female	Alcohol (liters per capita)	X			X		
Interpersonal violence	Female	Education (years per capita)			X			X
Interpersonal violence	Female	LDI (I$ per capita)			X			X
Interpersonal violence	Female	Opium Cultivation (binary)		X			X	
Interpersonal violence	Female	Population Density (over 1000 ppl/sqkm, proportion)		X			X	
Interpersonal violence	Female	Log-transformed SEV scalar: Violence	X			X		
Interpersonal violence	Female	Socio-demographic Index			X			X
Physical violence by firearm	Male	Alcohol (liters per capita)	X			X		
Physical violence by firearm	Male	Education (years per capita)			X			X
Physical violence by firearm	Male	LDI (I$ per capita)			X			X
Physical violence by firearm	Male	Opium Cultivation (binary)		X			X	
Physical violence by firearm	Male	Population Density (over 1000 ppl/sqkm, proportion)		X			X	
Physical violence by firearm	Male	Log-transformed SEV scalar: Viol Gun	X			X		
Physical violence by firearm	Male	Socio-demographic Index			X			X
Physical violence by firearm	Female	Alcohol (liters per capita)	X			X		
Physical violence by firearm	Female	Education (years per capita)			X			X
Physical violence by firearm	Female	LDI (I$ per capita)			X			X
Physical violence by firearm	Female	Opium Cultivation (binary)		X			X	
Physical violence by firearm	Female	Population Density (over 1000 ppl/sqkm, proportion)		X			X	
Physical violence by firearm	Female	Log-transformed SEV scalar: Viol Gun	X			X		
Physical violence by firearm	Female	Socio-demographic Index			X			X
Physical violence by sharp object	Male	Alcohol (liters per capita)	X			X		
Physical violence by sharp object	Male	Education (years per capita)			X			X
Physical violence by sharp object	Male	LDI (I$ per capita)			X			X
Physical violence by sharp object	Male	Opium Cultivation (binary)		X			X	
Physical violence by sharp object	Male	Population Density (over 1000 ppl/sqkm, proportion)		X			X	
Physical violence by sharp object	Male	Log-transformed SEV scalar: Viol Knife	X			X		
Physical violence by sharp object	Male	Socio-demographic Index			X			X
Physical violence by sharp object	Female	Alcohol (liters per capita)	X			X		
Physical violence by sharp object	Female	Education (years per capita)			X			X
Physical violence by sharp object	Female	LDI (I$ per capita)			X			X
Physical violence by sharp object	Female	Opium Cultivation (binary)		X			X	
Physical violence by sharp object	Female	Population Density (over 1000 ppl/sqkm, proportion)		X			X	
Physical violence by sharp object	Female	Log-transformed SEV scalar: Viol Knife	X			X		
Physical violence by sharp object	Female	Socio-demographic Index			X			X
Physical violence by other means	Male	Alcohol (liters per capita)	X			X		
Physical violence by other means	Male	Education (years per capita)			X			X
Physical violence by other means	Male	LDI (I$ per capita)			X			X
Physical violence by other means	Male	Opium Cultivation (binary)		X			X	
Physical violence by other means	Male	Population Density (over 1000 ppl/sqkm, proportion)		X			X	
Physical violence by other means	Male	Log-transformed SEV scalar: Oth Viol	X			X		
Physical violence by other means	Male	Socio-demographic Index			X			X
Physical violence by other means	Female	Alcohol (liters per capita)	X			X		
Physical violence by other means	Female	Education (years per capita)			X			X
Physical violence by other means	Female	LDI (I$ per capita)			X			X
Physical violence by other means	Female	Opium Cultivation (binary)		X			X	
Physical violence by other means	Female	Population Density (over 1000 ppl/sqkm, proportion)		X			X	
Physical violence by other means	Female	Log-transformed SEV scalar: Oth Viol	X			X		
Physical violence by other means	Female	Socio-demographic Index			X			X
Environmental heat and cold exposure	Male	Education (years per capita)			X			X
Environmental heat and cold exposure	Male	LDI (I$ per capita)			X			X
Environmental heat and cold exposure	Male	Population-weighted mean temperature			X			X
Environmental heat and cold exposure	Male	Elevation Over 1500m (proportion)			X			X
Environmental heat and cold exposure	Male	Elevation 500 to 1500m (proportion)			X			X
Environmental heat and cold exposure	Male	Population Density (150-300 ppl/sqkm, proportion)			X			X
Environmental heat and cold exposure	Male	Rainfall (Quintiles 4-5)			X			X
Environmental heat and cold exposure	Male	Sanitation (proportion with access)			X			X
Environmental heat and cold exposure	Male	90th percentile climatic temperature in the given country-year.			X			X
Environmental heat and cold exposure	Male	Socio-demographic Index			X			X
Environmental heat and cold exposure	Female	Education (years per capita)			X			X
Environmental heat and cold exposure	Female	LDI (I$ per capita)			X			X
Environmental heat and cold exposure	Female	Population-weighted mean temperature			X			X
Environmental heat and cold exposure	Female	Elevation Over 1500m (proportion)			X			X
Environmental heat and cold exposure	Female	Elevation 500 to 1500m (proportion)			X			X
Environmental heat and cold exposure	Female	Population Density (150-300 ppl/sqkm, proportion)			X			X
Environmental heat and cold exposure	Female	Rainfall (Quintiles 4-5)			X			X
Environmental heat and cold exposure	Female	Sanitation (proportion with access)			X			X
Environmental heat and cold exposure	Female	90th percentile climatic temperature in the given country-year.			X			X
Environmental heat and cold exposure	Female	Socio-demographic Index			X			X
Acute lymphoid leukemia	Male	Cumulative Cigarettes (10 Years)	X				X	
Acute lymphoid leukemia	Male	Socio-demographic Index			X			X
Acute lymphoid leukemia	Male	Cumulative Cigarettes (5 Years)	X				X	
Acute lymphoid leukemia	Male	Education (years per capita)			X			X
Acute lymphoid leukemia	Male	LDI (I$ per capita)			X			X
Acute lymphoid leukemia	Male	Health System Access 2 (unitless)	X				X	
Acute lymphoid leukemia	Male	Log-transformed SEV scalar: Leukemia	X			X		
Acute lymphoid leukemia	Male	Smoking Prevalence	X				X	
Acute lymphoid leukemia	Male	Alcohol (liters per capita)		X			X	
Acute lymphoid leukemia	Female	Cumulative Cigarettes (10 Years)	X				X	

Cause	Sex	Covariate	GBD 2015			GBD 2016		
			Level 1	Level 2	Level 3	Level 1	Level 2	Level 3
Acute lymphoid leukemia	Female	Socio-demographic Index			X			X
Acute lymphoid leukemia	Female	Cumulative Cigarettes (5 Years)	X				X	
Acute lymphoid leukemia	Female	Education (years per capita)			X			X
Acute lymphoid leukemia	Female	LDI (I$ per capita)			X			X
Acute lymphoid leukemia	Female	Health System Access 2 (unitless)	X				X	
Acute lymphoid leukemia	Female	Log-transformed SEV scalar: Leukemia	X			X		
Acute lymphoid leukemia	Female	Smoking Prevalence	X				X	
Acute lymphoid leukemia	Female	Alcohol (liters per capita)		X			X	
Chronic lymphoid leukemia	Female	Cumulative Cigarettes (10 Years)	X				X	
Chronic lymphoid leukemia	Female	Socio-demographic Index			X			X
Chronic lymphoid leukemia	Female	Cumulative Cigarettes (5 Years)	X				X	
Chronic lymphoid leukemia	Female	Education (years per capita)			X			X
Chronic lymphoid leukemia	Female	LDI (I$ per capita)			X			X
Chronic lymphoid leukemia	Female	Health System Access 2 (unitless)	X				X	
Chronic lymphoid leukemia	Female	Log-transformed SEV scalar: Leukemia	X			X		
Chronic lymphoid leukemia	Female	Smoking Prevalence	X				X	
Chronic lymphoid leukemia	Female	Alcohol (liters per capita)		X			X	
Chronic lymphoid leukemia	Male	Cumulative Cigarettes (10 Years)	X				X	
Chronic lymphoid leukemia	Male	Socio-demographic Index			X			X
Chronic lymphoid leukemia	Male	Cumulative Cigarettes (5 Years)	X				X	
Chronic lymphoid leukemia	Male	Education (years per capita)			X			X
Chronic lymphoid leukemia	Male	LDI (I$ per capita)			X			X
Chronic lymphoid leukemia	Male	Health System Access 2 (unitless)	X				X	
Chronic lymphoid leukemia	Male	Log-transformed SEV scalar: Leukemia	X			X		
Chronic lymphoid leukemia	Male	Smoking Prevalence	X				X	
Chronic lymphoid leukemia	Male	Alcohol (liters per capita)		X			X	
Acute myeloid leukemia	Male	Cumulative Cigarettes (10 Years)	X				X	
Acute myeloid leukemia	Male	Socio-demographic Index			X			X
Acute myeloid leukemia	Male	Cumulative Cigarettes (5 Years)	X				X	
Acute myeloid leukemia	Male	Tobacco (cigarettes per capita)	X				X	
Acute myeloid leukemia	Male	Education (years per capita)			X			X
Acute myeloid leukemia	Male	LDI (I$ per capita)			X			X
Acute myeloid leukemia	Male	Health System Access 2 (unitless)	X				X	
Acute myeloid leukemia	Male	Health System Access 2 (unitless)	X			X		
Acute myeloid leukemia	Male	Log-transformed SEV scalar: Leukemia	X			X		
Acute myeloid leukemia	Male	Smoking Prevalence	X				X	
Acute myeloid leukemia	Male	Alcohol (liters per capita)		X			X	
Acute myeloid leukemia	Female	Cumulative Cigarettes (10 Years)	X				X	
Acute myeloid leukemia	Female	Socio-demographic Index			X			X
Acute myeloid leukemia	Female	Cumulative Cigarettes (5 Years)	X				X	
Acute myeloid leukemia	Female	Tobacco (cigarettes per capita)	X				X	
Acute myeloid leukemia	Female	Education (years per capita)			X			X
Acute myeloid leukemia	Female	LDI (I$ per capita)			X			X
Acute myeloid leukemia	Female	Health System Access 2 (unitless)	X				X	
Acute myeloid leukemia	Female	Log-transformed SEV scalar: Leukemia	X			X		
Acute myeloid leukemia	Female	Smoking Prevalence	X				X	
Acute myeloid leukemia	Female	Alcohol (liters per capita)		X			X	
Chronic myeloid leukemia	Male	Cumulative Cigarettes (10 Years)	X				X	
Chronic myeloid leukemia	Male	Socio-demographic Index			X			X
Chronic myeloid leukemia	Male	Cumulative Cigarettes (5 Years)	X				X	
Chronic myeloid leukemia	Male	Education (years per capita)			X			X
Chronic myeloid leukemia	Male	LDI (I$ per capita)			X			X
Chronic myeloid leukemia	Male	Health System Access 2 (unitless)	X				X	
Chronic myeloid leukemia	Male	Smoking Prevalence	X				X	
Chronic myeloid leukemia	Male	Alcohol (liters per capita)		X			X	
Chronic myeloid leukemia	Female	Cumulative Cigarettes (10 Years)	X				X	
Chronic myeloid leukemia	Female	Socio-demographic Index			X			X
Chronic myeloid leukemia	Female	Cumulative Cigarettes (5 Years)	X				X	
Chronic myeloid leukemia	Female	Education (years per capita)			X			X
Chronic myeloid leukemia	Female	LDI (I$ per capita)			X			X
Chronic myeloid leukemia	Female	Health System Access 2 (unitless)	X				X	
Chronic myeloid leukemia	Female	Smoking Prevalence	X				X	
Chronic myeloid leukemia	Female	Alcohol (liters per capita)		X			X	

Note: Only causes modeled in CODEm are included in this table.

Appendix Table 9. Modeling strategy for individual cause of death models in GBD 2016		
**Cause name**	**Level**	**Model Type**
Communicable, maternal, neonatal, and nutritional diseases	Aggregate	
HIV/AIDS and tuberculosis	Aggregate	
Tuberculosis	3	CODEm
Drug-sensitive tuberculosis	4	Spatio-temporal Gaussian process regression proportion
Multidrug-resistant tuberculosis without extensive drug resistance	4	Spatio-temporal Gaussian process regression proportion
Extensively drug-resistant tuberculosis	4	Spatio-temporal Gaussian process regression proportion
HIV/AIDS	3	Spectrum
Drug-sensitive HIV/AIDS - Tuberculosis	4	Data proportion
Multidrug-resistant HIV/AIDS - Tuberculosis without extensive drug resistance	4	Data proportion
Extensively drug-resistant HIV/AIDS - Tuberculosis	4	Data proportion
HIV/AIDS resulting in other diseases	4	Spectrum
Diarrhea, lower respiratory, and other common infectious diseases	Aggregate	
Diarrheal diseases	3	CODEm; Fatal discontinuity
Intestinal infectious diseases	Aggregate	
Typhoid fever	4	CODEm (VR countries); natural history model (non-VR countries)
Paratyphoid fever	4	CODEm (VR countries); natural history model (non-VR countries)
Other intestinal infectious diseases	4	Negative binomial regression
Lower respiratory infections	3	CODEm
Upper respiratory infections	3	Negative binomial regression
Otitis media	3	CODEm
Meningitis	3	CODEm
Pneumococcal meningitis	4	DisMod MR-2.2 proportion model
H influenzae type B meningitis	4	DisMod MR-2.2 proportion model
Meningococcal meningitis	4	DisMod MR-2.2 proportion model; Fatal discontinuity
Other meningitis	4	DisMod MR-2.2 proportion model
Encephalitis	3	CODEm
Diphtheria	3	Negative binomial regression
Whooping cough	3	CODEm (VR countries); natural history model (non-VR countries)
Tetanus	3	CODEm
Measles	3	Natural history model; Fatal discontinuity
Varicella and herpes zoster	3	Negative binomial regression
Neglected tropical diseases and malaria	Aggregate	
Malaria	3	CODEm (P. falciparum outside of Africa); natural history model (P. falciparum within Africa); negative binomial regression (P. vivax)
Chagas disease	3	CODEm
Leishmaniasis	Aggregate	
Visceral leishmaniasis	4	Natural history model
African trypanosomiasis	3	Natural history model
Schistosomiasis	3	Negative binomial regression
Cysticercosis	3	Negative binomial regression
Cystic echinococcosis	3	Negative binomial regression
Dengue	3	CODEm
Yellow fever	3	Natural history model; Fatal discontinuity
Rabies	3	CODEm
Intestinal nematode infections	Aggregate	
Ascariasis	4	Negative binomial regression
Ebola	3	Fatal discontinuity
Zika virus	3	Natural history model
Other neglected tropical diseases	3	CODEm
Maternal disorders	2	CODEm
Maternal hemorrhage	3	DisMod MR-2.2 proportion model
Maternal sepsis and other maternal infections	3	DisMod MR-2.2 proportion model
Maternal hypertensive disorders	3	DisMod MR-2.2 proportion model
Maternal obstructed labor and uterine rupture	3	DisMod MR-2.2 proportion model
Maternal abortion, miscarriage, and ectopic pregnancy	3	DisMod MR-2.2 proportion model
Indirect maternal deaths	3	DisMod MR-2.2 proportion model
Late maternal deaths	3	DisMod MR-2.2 proportion model
Maternal deaths aggravated by HIV/AIDS	3	Spectrum; DisMod MR-2.2 proportion
Other maternal disorders	3	DisMod MR-2.2 proportion model
Neonatal disorders	2	CODEm
Neonatal preterm birth complications	3	CODEm
Neonatal encephalopathy due to birth asphyxia and trauma	3	CODEm
Neonatal sepsis and other neonatal infections	3	CODEm
Hemolytic disease and other neonatal jaundice	3	CODEm
Other neonatal disorders	3	CODEm
Nutritional deficiencies	2	CODEm
Protein-energy malnutrition	3	CODEm; Fatal discontinuity

Appendix Table 9. Modeling strategy for individual cause of death models in GBD 2016		
Cause name	Level	Model Type
Iodine deficiency	3	Negative binomial regression
Iron-deficiency anemia	3	CODEm
Other nutritional deficiencies	3	CODEm
Other communicable, maternal, neonatal, and nutritional diseases	Aggregate	
Sexually transmitted diseases excluding HIV	3	CODEm; natural history model (congenital syphilis)
Syphilis	4	Data proportion (age/sex-specific VR); natural history model (congenital syphilis)
Chlamydial infection	4	Data proportion (age/sex-specific VR)
Gonococcal infection	4	Data proportion (age/sex-specific VR)
Other sexually transmitted diseases	4	Data proportion (age/sex-specific VR)
Hepatitis	3	CODEm
Acute hepatitis A	4	Natural history model
Hepatitis B	4	Natural history model
Hepatitis C	4	Natural history model
Acute hepatitis E	4	Natural history model
Other infectious diseases	3	CODEm
Non-communicable diseases	Aggregate	
Neoplasms	Aggregate	
Lip and oral cavity cancer	3	CODEm
Nasopharynx cancer	3	CODEm
Other pharynx cancer	3	CODEm
Esophageal cancer	3	CODEm
Stomach cancer	3	CODEm
Colon and rectum cancer	3	CODEm
Liver cancer	3	CODEm
Liver cancer due to hepatitis B	4	DisMod MR-2.2 proportion model
Liver cancer due to hepatitis C	4	DisMod MR-2.2 proportion model
Liver cancer due to alcohol use	4	DisMod MR-2.2 proportion model
Liver cancer due to other causes	4	DisMod MR-2.2 proportion model
Gallbladder and biliary tract cancer	3	CODEm
Pancreatic cancer	3	CODEm
Larynx cancer	3	CODEm
Tracheal, bronchus, and lung cancer	3	CODEm
Malignant skin melanoma	3	CODEm
Non-melanoma skin cancer	Aggregate	
Non-melanoma skin cancer (squamous-cell carcinoma)	4	CODEm
Breast cancer	3	CODEm
Cervical cancer	3	CODEm
Uterine cancer	3	CODEm
Ovarian cancer	3	CODEm
Prostate cancer	3	CODEm
Testicular cancer	3	CODEm
Kidney cancer	3	CODEm
Bladder cancer	3	CODEm
Brain and nervous system cancer	3	CODEm
Thyroid cancer	3	CODEm
Mesothelioma	3	CODEm
Hodgkin lymphoma	3	CODEm
Non-Hodgkin lymphoma	3	CODEm
Multiple myeloma	3	CODEm
Leukemia	3	CODEm
Acute lymphoid leukemia	4	CODEm
Chronic lymphoid leukemia	4	CODEm
Acute myeloid leukemia	4	CODEm
Chronic myeloid leukemia	4	CODEm
Other leukemia	4	CODEm
Other neoplasms	3	CODEm
Cardiovascular diseases	2	CODEm
Rheumatic heart disease	3	CODEm
Ischemic heart disease	3	CODEm
Cerebrovascular disease	3	CODEm
Ischemic stroke	4	CODEm
Hemorrhagic stroke	4	CODEm
Hypertensive heart disease	3	CODEm
Cardiomyopathy and myocarditis	3	CODEm
Myocarditis	4	CODEm
Alcoholic cardiomyopathy	4	CODEm
Other cardiomyopathy	4	CODEm
Atrial fibrillation and flutter	3	DisMod-MR 2.2 prevalence-based model
Aortic aneurysm	3	CODEm
Peripheral artery disease	3	CODEm

Appendix Table 9. Modeling strategy for individual cause of death models in GBD 2016		
Cause name	Level	Model Type
Endocarditis	3	CODEm
Other cardiovascular and circulatory diseases	3	CODEm
Chronic respiratory diseases	2	CODEm
Chronic obstructive pulmonary disease	3	CODEm
Pneumoconiosis	3	CODEm
Silicosis	4	CODEm
Asbestosis	4	CODEm
Coal workers pneumoconiosis	4	CODEm
Other pneumoconiosis	4	CODEm
Asthma	3	CODEm
Interstitial lung disease and pulmonary sarcoidosis	3	CODEm
Other chronic respiratory diseases	3	CODEm
Cirrhosis and other chronic liver diseases	2	CODEm
Cirrhosis and other chronic liver diseases due to hepatitis B	3	DisMod MR-2.2 proportion model
Cirrhosis and other chronic liver diseases due to hepatitis C	3	DisMod MR-2.2 proportion model
Cirrhosis and other chronic liver diseases due to alcohol use	3	DisMod MR-2.2 proportion model
Cirrhosis and other chronic liver diseases due to other causes	3	DisMod MR-2.2 proportion model
Digestive diseases	2	CODEm
Peptic ulcer disease	3	CODEm
Gastritis and duodenitis	3	CODEm
Appendicitis	3	CODEm
Paralytic ileus and intestinal obstruction	3	CODEm
Inguinal, femoral, and abdominal hernia	3	CODEm
Inflammatory bowel disease	3	CODEm
Vascular intestinal disorders	3	CODEm
Gallbladder and biliary diseases	3	CODEm
Pancreatitis	3	CODEm
Other digestive diseases	3	CODEm
Neurological disorders	Aggregate	
Alzheimer disease and other dementias	3	DisMod-MR 2.2 prevalence-based model
Parkinson disease	3	DisMod-MR 2.2 prevalence-based model
Epilepsy	3	CODEm
Multiple sclerosis	3	CODEm
Motor neuron disease	3	CODEm
Other neurological disorders	3	CODEm
Mental and substance use disorders	Aggregate	
Alcohol use disorders	3	CODEm
Drug use disorders	3	CODEm
Opioid use disorders	4	CODEm
Cocaine use disorders	4	CODEm
Amphetamine use disorders	4	CODEm
Other drug use disorders	4	CODEm
Eating disorders	Aggregate	
Anorexia nervosa	4	CODEm
Bulimia nervosa	4	CODEm
Diabetes, urogenital, blood, and endocrine diseases	Aggregate	
Diabetes mellitus	3	CODEm
Acute glomerulonephritis	3	CODEm
Chronic kidney disease	3	CODEm
Chronic kidney disease due to diabetes mellitus	4	DisMod MR-2.2 proportion model
Chronic kidney disease due to hypertension	4	DisMod MR-2.2 proportion model
Chronic kidney disease due to glomerulonephritis	4	DisMod MR-2.2 proportion model
Chronic kidney disease due to other causes	4	DisMod MR-2.2 proportion model
Urinary diseases and male infertility	3	CODEm
Interstitial nephritis and urinary tract infections	4	CODEm
Urolithiasis	4	CODEm
Other urinary diseases	4	CODEm
Gynecological diseases	3	CODEm
Uterine fibroids	4	CODEm
Polycystic ovarian syndrome	4	CODEm
Endometriosis	4	CODEm
Genital prolapse	4	CODEm
Other gynecological diseases	4	CODEm
Hemoglobinopathies and hemolytic anemias	3	CODEm
Thalassemias	4	DisMod MR-2.2 cause-specific mortality model
Sickle cell disorders	4	DisMod MR-2.2 cause-specific mortality model
G6PD deficiency	4	DisMod MR-2.2 cause-specific mortality model
Other hemoglobinopathies and hemolytic anemias	4	Data proportion (age-specific high-income VR)
Endocrine, metabolic, blood, and immune disorders	3	CODEm
Musculoskeletal disorders	2	CODEm
Rheumatoid arthritis	3	CODEm

Appendix Table 9. Modeling strategy for individual cause of death models in GBD 2016		
**Cause name**	**Level**	**Model Type**
Other musculoskeletal disorders	3	CODEm
Other non-communicable diseases	Aggregate	
Congenital birth defects	3	CODEm
Neural tube defects	4	CODEm
Congenital heart anomalies	4	CODEm
Cleft lip and cleft palate	4	CODEm
Down syndrome	4	CODEm
Other chromosomal abnormalities	4	CODEm
Congenital musculoskeletal and limb anomalies	4	CODEm
Urogenital congenital anomalies	4	CODEm
Digestive congenital anomalies	4	CODEm
Other congenital birth defects	4	CODEm
Skin and subcutaneous diseases	3	CODEm
Cellulitis	4	CODEm
Pyoderma	4	CODEm
Decubitus ulcer	4	CODEm
Other skin and subcutaneous diseases	4	CODEm
Sudden infant death syndrome	3	CODEm
Injuries	Aggregate	
Transport injuries	2	CODEm
Road injuries	3	CODEm
Pedestrian road injuries	4	CODEm
Cyclist road injuries	4	CODEm
Motorcyclist road injuries	4	CODEm
Motor vehicle road injuries	4	CODEm; Fatal discontinuity
Other road injuries	4	CODEm
Other transport injuries	3	CODEm; Fatal discontinuity
Unintentional injuries	Aggregate	
Falls	3	CODEm
Drowning	3	CODEm
Fire, heat, and hot substances	3	CODEm; Fatal discontinuity
Poisonings	3	CODEm; Fatal discontinuity
Exposure to mechanical forces	Aggregate	
Unintentional firearm injuries	4	CODEm
Unintentional suffocation	4	CODEm
Other exposure to mechanical forces	4	CODEm; Fatal discontinuity
Adverse effects of medical treatment	3	CODEm
Animal contact	3	CODEm
Venomous animal contact	4	CODEm
Non-venomous animal contact	4	CODEm
Foreign body	Aggregate	
Pulmonary aspiration and foreign body in airway	4	CODEm
Foreign body in other body part	4	CODEm
Environmental heat and cold exposure	3	CODEm; Fatal discontinuity
Other unintentional injuries	3	CODEm
Self-harm and interpersonal violence	Aggregate	
Self-harm	3	CODEm
Self-harm by firearm	4	CODEm
Self-harm by other specified means	4	CODEm
Interpersonal violence	3	CODEm
Physical violence by firearm	4	CODEm
Physical violence by sharp object	4	CODEm
Physical violence by other means	4	CODEm
Forces of nature, conflict and terrorism, and executions and police conflict	Aggregate	
Exposure to forces of nature	3	Fatal discontinuity
Conflict and terrorism	3	Fatal discontinuity
Executions and police conflict	3	CODEm; Fatal discontinuity

Appendix Table 10: CoDCorrect cause hierarchy with levels	
**Cause name**	**CoDCorrect level**
All causes	0
Tuberculosis	1
Drug-sensitive tuberculosis	2
Multidrug-resistant tuberculosis without extensive drug resistance	2
Extensively drug-resistant tuberculosis	2
Diarrhoeal diseases	1
Typhoid fever	1
Paratyphoid fever	1
Other intestinal infectious diseases	1
Lower respiratory infections	1
Upper respiratory infections	1
Otitis media	1
Meningitis	1
Pneumococcal meningitis	2
H influenzae type B meningitis	2
Meningococcal infection	2
Other meningitis	2
Encephalitis	1
Diphtheria	1
Whooping cough	1
Tetanus	1
Measles	1
Varicella and herpes zoster	1
Malaria	1
Chagas disease	1
Visceral leishmaniasis	1
African trypanosomiasis	1
Schistosomiasis	1
Cysticercosis	1
Cystic echinococcosis	1
Dengue	1
Yellow fever	1
Rabies	1
Intestinal nematode infections	1
Ascariasis	2
Zika virus	1
Other neglected tropical diseases	1
Maternal disorders	1
Maternal haemorrhage	2
Maternal sepsis and other pregnancy related infections	2
Maternal hypertensive disorders	2
Maternal obstructed labour and uterine rupture	2
Maternal abortion, miscarriage, and ectopic pregnancy	2

Appendix Table 10: CoDCorrect cause hierarchy with levels	
**Cause name**	**CoDCorrect level**
Indirect maternal deaths	2
Late maternal deaths	2
Maternal deaths aggravated by HIV/AIDS	2
Other maternal disorders	2
Neonatal disorders	1
Neonatal preterm birth complications	2
Neonatal encephalopathy due to birth asphyxia and trauma	2
Neonatal sepsis and other neonatal infections	2
Hemolytic disease and other neonatal jaundice	2
Other neonatal disorders	2
Nutritional deficiencies	1
Protein-energy malnutrition	2
Iodine deficiency	2
Iron-deficiency anaemia	2
Other nutritional deficiencies	2
Sexually transmitted diseases excluding HIV	1
Syphilis	2
Chlamydial infection	2
Gonococcal infection	2
Other sexually transmitted diseases	2
Hepatitis	1
Acute hepatitis A	2
Hepatitis B	2
Hepatitis C	2
Acute hepatitis E	2
Other infectious diseases	1
Lip and oral cavity cancer	1
Nasopharynx cancer	1
Other pharynx cancer	1
Oesophageal cancer	1
Stomach cancer	1
Colon and rectum cancer	1
Liver cancer	1
Liver cancer due to hepatitis B	2
Liver cancer due to hepatitis C	2
Liver cancer due to alcohol use	2
Liver cancer due to other causes	2
Gallbladder and biliary tract cancer	1
Pancreatic cancer	1
Larynx cancer	1
Tracheal, bronchus, and lung cancer	1
Malignant skin melanoma	1

Appendix Table 10: CoDCorrect cause hierarchy with levels	
**Cause name**	**CoDCorrect level**
Non-melanoma skin cancer	1
Non-melanoma skin cancer (squamous-cell carcinoma)	2
Breast cancer	1
Cervical cancer	1
Uterine cancer	1
Ovarian cancer	1
Prostate cancer	1
Testicular cancer	1
Kidney cancer	1
Bladder cancer	1
Brain and nervous system cancer	1
Thyroid cancer	1
Mesothelioma	1
Hodgkin lymphoma	1
Non-Hodgkin's lymphoma	1
Multiple myeloma	1
Leukaemia	1
Acute lymphoid leukaemia	2
Chronic lymphoid leukaemia	2
Acute myeloid leukaemia	2
Chronic myeloid leukaemia	2
Other leukaemia	2
Other neoplasms	1
Cardiovascular diseases	1
Rheumatic heart disease	2
Ischaemic heart disease	2
Cerebrovascular disease	2
Ischaemic stroke	3
Hemorrhagic stroke	3
Hypertensive heart disease	2
Cardiomyopathy and myocarditis	2
Myocarditis	3
Alcoholic cardiomyopathy	3
Other cardiomyopathy	3
Atrial fibrillation and flutter	2
Aortic aneurysm	2
Peripheral vascular disease	2
Endocarditis	2
Other cardiovascular and circulatory diseases	2
Chronic respiratory diseases	1
Chronic obstructive pulmonary disease	2
Pneumoconiosis	2
Silicosis	3
Asbestosis	3

Appendix Table 10: CoDCorrect cause hierarchy with levels	
Cause name	CoDCorrect level
Coal workers pneumoconiosis	3
Other pneumoconiosis	3
Asthma	2
Interstitial lung disease and pulmonary sarcoidosis	2
Other chronic respiratory diseases	2
Cirrhosis and other chronic liver diseases	1
Cirrhosis and other chronic liver diseases due to hepatitis B	2
Cirrhosis and other chronic liver diseases due to hepatitis C	2
Cirrhosis and other chronic liver diseases due to alcohol use	2
Cirrhosis and other chronic liver diseases due to other causes	2
Digestive diseases	1
Peptic ulcer disease	2
Gastritis and duodenitis	2
Appendicitis	2
Paralytic ileus and intestinal obstruction	2
Inguinal, femoral, and abdominal hernia	2
Inflammatory bowel disease	2
Vascular intestinal disorders	2
Gallbladder and biliary diseases	2
Pancreatitis	2
Other digestive diseases	2
Alzheimer's disease and other dementias	1
Parkinson's disease	1
Epilepsy	1
Multiple sclerosis	1
Motor neuron disease	1
Other neurological disorders	1
Alcohol use disorders	1
Drug use disorders	1
Opioid use disorders	2
Cocaine use disorders	2
Amphetamine use disorders	2
Other drug use disorders	2
Eating disorders	1
Anorexia nervosa	2
Bulimia nervosa	2
Diabetes mellitus	1
Acute glomerulonephritis	1
Chronic kidney disease	1
Chronic kidney disease due to diabetes mellitus	2
Chronic kidney disease due to hypertension	2

Appendix Table 10: CoDCorrect cause hierarchy with levels	
Cause name	CoDCorrect level
Chronic kidney disease due to glomerulonephritis	2
Chronic kidney disease due to other causes	2
Urinary diseases and male infertility	1
Interstitial nephritis and urinary tract infections	2
Urolithiasis	2
Other urinary diseases	2
Gynecological diseases	1
Uterine fibroids	2
Polycystic ovarian syndrome	2
Endometriosis	2
Genital prolapse	2
Other gynecological diseases	2
Hemoglobinopathies and hemolytic anaemias	1
Thalassemias	2
Sickle cell disorders	2
G6PD deficiency	2
Other hemoglobinopathies and hemolytic anaemias	2
Endocrine, metabolic, blood, and immune disorders	1
Musculoskeletal disorders	1
Rheumatoid arthritis	2
Other musculoskeletal disorders	2
Congenital anomalies	1
Neural tube defects	2
Congenital heart anomalies	2
Cleft lip and cleft palate	2
Down's syndrome	2
Other chromosomal abnormalities	2
Congenital musculoskeletal and limb anomalies	2
Urogenital congenital anomalies	2
Digestive congenital anomalies	2
Other congenital anomalies	2
Skin and subcutaneous diseases	1
Cellulitis	2
Pyoderma	2
Decubitus ulcer	2
Other skin and subcutaneous diseases	2
Sudden infant death syndrome	1
Transport injuries	1
Road injuries	2
Pedestrian road injuries	3
Cyclist road injuries	3

Appendix Table 10: CoDCorrect cause hierarchy with levels	
**Cause name**	**CoDCorrect level**
Motorcyclist road injuries	3
Motor vehicle road injuries	3
Other road injuries	3
Other transport injuries	2
Falls	1
Drowning	1
Fire, heat, and hot substances	1
Poisonings	1
Unintentional firearm injuries	1
Unintentional suffocation	1
Other exposure to mechanical forces	1
Adverse effects of medical treatment	1
Animal contact	1
Venomous animal contact	2
Non-venomous animal contact	2
Pulmonary aspiration and foreign body in airway	1
Foreign body in other body part	1
Environmental heat and cold exposure	1
Other unintentional injuries	1
Self-harm	1
Self-harm by firearm	2
Self-harm by other specified means	2
Interpersonal violence	1
Assault by firearm	2
Assault by sharp object	2
Assault by other means	2
Executions and police conflict	1

Appendix Table 11: Percent change before and after CoDCorrect by cause for all ages, both sexes combined, 2016

Cause	CoDCorrect level	Percent change
All causes	0	1.24<br>-0.59 to 3.44
Communicable, maternal, neonatal, and nutritional disorders	1	12.01<br>7.0 to 17.26
HIV/AIDS and tuberculosis	2	97.47<br>90.68 to 103.98
Tuberculosis	3	6.59<br>3.09 to 10.26
Diarrhoea, lower respiratory infections, and other common infectious diseases	2	0.67<br>-4.43 to 5.69
Diarrhoeal diseases	3	-0.47<br>-8.62 to 5.96
Intestinal infectious diseases	3	2.45<br>-14.1 to 22.69
Typhoid fever	4	4.33<br>-14.95 to 29.31
Paratyphoid fever	4	-7.73<br>-14.56 to 1.66
Other intestinal infectious diseases	4	8.49<br>-1.73 to 18.6
Lower respiratory infections	3	1.12<br>-1.84 to 4.69
Upper respiratory infections	3	5.74<br>0.79 to 11.28
Otitis media	3	3.7<br>-1.94 to 9.49
Meningitis	3	4.76<br>-2.07 to 11.37
Pneumococcal meningitis	4	5.33<br>-0.72 to 11.34
H influenzae type B meningitis	4	3.12<br>-4.15 to 10.68
Meningococcal infection	4	3.47<br>-3.4 to 10.11
Other meningitis	4	4.47<br>-2.56 to 11.23
Encephalitis	3	0.51<br>-3.85 to 5.62
Diphtheria	3	3.57<br>-6.87 to 14.4
Whooping cough	3	-1.17<br>-11.87 to 11.21
Tetanus	3	-3.43<br>-15.37 to 4.25
Measles	3	-5.12<br>-13.92 to 7.18
Varicella and herpes zoster	3	1.35<br>-3.25 to 7.28
Neglected tropical diseases and malaria	2	-0.48<br>-10.0 to 9.51
Malaria	3	-0.98<br>-11.49 to 10.06
Chagas disease	3	-3.74<br>-5.03 to -2.44
Leishmaniasis	3	2.24<br>-2.83 to 8.43
Visceral leishmaniasis	4	2.24<br>-2.83 to 8.43
African trypanosomiasis	3	2.26<br>-8.39 to 12.17

Appendix Table 11: Percent change before and after CoDCorrect by cause for all ages, both sexes combined, 2016		
Cause	CoDCorrect level	Percent change
Schistosomiasis	3	15.38<br>10.99 to 20.91
Cysticercosis	3	9.05<br>5.86 to 13.07
Cystic echinococcosis	3	7.91<br>3.46 to 12.56
Dengue	3	-1.4<br>-5.86 to 4.64
Yellow fever	3	9.26<br>2.12 to 19.52
Rabies	3	2.69<br>-3.02 to 9.28
Intestinal nematode infections	3	0.21<br>-9.92 to 11.06
Ascariasis	4	0.28<br>-9.86 to 11.13
Other neglected tropical diseases	3	4.44<br>-3.47 to 13.69
Maternal disorders	2	0.71<br>-4.52 to 6.1
Maternal haemorrhage	3	2.85<br>-2.2 to 8.08
Maternal sepsis and other pregnancy related infections	3	0.65<br>-4.94 to 6.38
Maternal hypertensive disorders	3	-2.07<br>-7.34 to 3.46
Maternal obstructed labour and uterine rupture	3	0.46<br>-5.98 to 6.91
Maternal abortion, miscarriage, and ectopic pregnancy	3	2.1<br>-3.78 to 7.98
Indirect maternal deaths	3	-2.51<br>-8.21 to 3.33
Late maternal deaths	3	-0.29<br>-5.3 to 4.89
Other maternal disorders	3	2.06<br>-2.74 to 7.14
Neonatal disorders	2	-0.93<br>-4.01 to 3.55
Neonatal preterm birth complications	3	2.85<br>-3.38 to 15.72
Neonatal encephalopathy due to birth asphyxia and trauma	3	3.41<br>-4.18 to 16.19
Neonatal sepsis and other neonatal infections	3	0.26<br>-26.78 to 45.09
Hemolytic disease and other neonatal jaundice	3	2.77<br>-13.17 to 21.9
Other neonatal disorders	3	-0.55<br>-7.66 to 9.68
Nutritional deficiencies	2	2.07<br>-3.89 to 8.51
Protein-energy malnutrition	3	25.43<br>-1.87 to 47.63
Iodine deficiency	3	17.9<br>-5.86 to 48.32
Iron-deficiency anaemia	3	-7.65<br>-35.12 to 44.0
Other nutritional deficiencies	3	60.06<br>42.46 to 85.86
Other communicable, maternal, neonatal, and nutritional diseases	2	1.04<br>-3.8 to 7.19

Appendix Table 11: Percent change before and after CoDCorrect by cause for all ages, both sexes combined, 2016		
Cause	CoDCorrect level	Percent change
Sexually transmitted diseases excluding HIV	3	1.19<br>-5.21 to 9.34
Syphilis	4	1.01<br>-5.63 to 9.59
Chlamydial infection	4	4.25<br>-0.9 to 8.55
Gonococcal infection	4	4.5<br>-0.61 to 8.46
Other sexually transmitted diseases	4	5.01<br>-0.01 to 9.15
Hepatitis	3	3.59<br>0.31 to 7.54
Acute hepatitis A	4	30.6<br>-10.34 to 154.39
Hepatitis B	4	-43.25<br>-56.36 to -25.25
Hepatitis C	4	55.64<br>17.54 to 114.95
Acute hepatitis E	4	42.07<br>7.36 to 98.28
Other infectious diseases	3	-3.01<br>-10.28 to 5.6
Non-communicable diseases	1	-1.81<br>-3.24 to -0.17
Neoplasms	2	-1.19<br>-2.61 to 0.59
Oesophageal cancer	3	-1.39<br>-2.6 to 0.19
Stomach cancer	3	-1.88<br>-3.12 to -0.25
Liver cancer	3	-1.2<br>-2.69 to 1.12
Liver cancer due to hepatitis B	4	-0.59<br>-2.21 to 2.08
Liver cancer due to hepatitis C	4	-2.59<br>-3.98 to -0.86
Liver cancer due to alcohol use	4	-1.87<br>-3.7 to 0.33
Liver cancer due to other causes	4	-0.65<br>-2.07 to 1.69
Larynx cancer	3	-0.77<br>-2.33 to 1.03
Tracheal, bronchus, and lung cancer	3	-2.23<br>-3.42 to -0.62
Breast cancer	3	-0.37<br>-2.34 to 1.76
Cervical cancer	3	3.5<br>0.72 to 6.15
Uterine cancer	3	0.05<br>-2.11 to 1.94
Prostate cancer	3	-0.98<br>-2.71 to 0.95
Colon and rectum cancer	3	-1.95<br>-3.4 to -0.16
Lip and oral cavity cancer	3	-0.39<br>-2.3 to 1.54
Nasopharynx cancer	3	0.26<br>-1.36 to 3.21
Other pharynx cancer	3	0.94<br>-1.74 to 2.85

Appendix Table 11: Percent change before and after CoDCorrect by cause for all ages, both sexes combined, 2016		
Cause	CoDCorrect level	Percent change
Gallbladder and biliary tract cancer	3	-2.65<br>-4.45 to -0.79
Pancreatic cancer	3	-2.11<br>-3.57 to -0.34
Malignant skin melanoma	3	-0.7<br>-2.07 to 0.99
Non-melanoma skin cancer	3	-1.85<br>-3.14 to -0.14
Ovarian cancer	3	-0.37<br>-2.32 to 1.5
Testicular cancer	3	4.01<br>1.79 to 7.54
Kidney cancer	3	-1.83<br>-3.18 to -0.23
Bladder cancer	3	-2.19<br>-3.56 to -0.52
Brain and nervous system cancer	3	-0.24<br>-1.96 to 2.3
Thyroid cancer	3	0.67<br>-1.23 to 2.44
Mesothelioma	3	-1.14<br>-2.49 to 0.61
Hodgkin lymphoma	3	2.55<br>0.15 to 5.06
Non-Hodgkin's lymphoma	3	-0.01<br>-1.7 to 1.88
Multiple myeloma	3	-1.25<br>-2.68 to 0.36
Leukaemia	3	-0.42<br>-2.46 to 2.27
Other neoplasms	3	0.34<br>-1.28 to 2.57
Cardiovascular diseases	2	-2.31<br>-3.69 to -0.84
Rheumatic heart disease	3	2.83<br>-0.49 to 6.36
Ischaemic heart disease	3	4.51<br>2.74 to 6.88
Cerebrovascular disease	3	5.02<br>3.24 to 7.57
Ischaemic stroke	4	-20.37<br>-23.56 to -17.26
Hemorrhagic stroke	4	-13.39<br>-16.26 to -10.52
Hypertensive heart disease	3	6.33<br>3.72 to 9.7
Cardiomyopathy and myocarditis	3	3.31<br>0.94 to 6.75
Atrial fibrillation and flutter	3	4.87<br>2.58 to 8.24
Aortic aneurysm	3	4.47<br>2.61 to 7.05
Peripheral vascular disease	3	3.3<br>1.18 to 6.27
Endocarditis	3	3.44<br>1.36 to 6.49
Other cardiovascular and circulatory diseases	3	6.59<br>4.22 to 10.22
Chronic respiratory diseases	2	-1.73<br>-4.26 to 0.08

Appendix Table 11: Percent change before and after CoDCorrect by cause for all ages, both sexes combined, 2016

Cause	CoDCorrect level	Percent change
Chronic obstructive pulmonary disease	3	22.07<br>15.67 to 29.72
Pneumoconiosis	3	11.28<br>6.41 to 18.35
Silicosis	4	17.85<br>4.81 to 41.94
Asbestosis	4	5.95<br>-6.33 to 65.56
Coal workers pneumoconiosis	4	44.05<br>18.88 to 98.65
Other pneumoconiosis	4	61.3<br>39.68 to 129.68
Asthma	3	48.51<br>36.84 to 67.75
Interstitial lung disease and pulmonary sarcoidosis	3	14.92<br>7.53 to 28.31
Other chronic respiratory diseases	3	16.22<br>9.43 to 26.46
Cirrhosis and other chronic liver diseases	2	1.21<br>-0.45 to 2.85
Cirrhosis and other chronic liver diseases due to hepatitis B	3	1.98<br>-0.11 to 4.07
Cirrhosis and other chronic liver diseases due to hepatitis C	3	0.46<br>-1.01 to 1.71
Cirrhosis and other chronic liver diseases due to alcohol use	3	1.1<br>-0.38 to 2.76
Cirrhosis and other chronic liver diseases due to other causes	3	1.19<br>-0.7 to 3.11
Digestive diseases	2	0.73<br>-1.24 to 3.0
Peptic ulcer disease	3	25.36<br>18.49 to 38.15
Gastritis and duodenitis	3	15.87<br>7.59 to 27.09
Appendicitis	3	37.57<br>28.76 to 56.88
Paralytic ileus and intestinal obstruction	3	25.12<br>17.0 to 40.05
Inguinal, femoral, and abdominal hernia	3	24.89<br>14.27 to 36.77
Inflammatory bowel disease	3	14.61<br>9.28 to 27.84
Vascular intestinal disorders	3	8.63<br>2.5 to 20.16
Gallbladder and biliary diseases	3	13.02<br>7.14 to 23.77
Pancreatitis	3	18.55<br>11.76 to 33.82
Other digestive diseases	3	16.19<br>9.95 to 26.57
Neurological disorders	2	-5.31<br>-7.31 to -2.94
Alzheimer's disease and other dementias	3	-6.09<br>-8.11 to -3.67
Parkinson's disease	3	-3.74<br>-5.42 to -1.96
Epilepsy	3	3.86<br>0.32 to 8.03
Multiple sclerosis	3	0.15<br>-1.14 to 1.99

Appendix Table 11: Percent change before and after CoDCorrect by cause for all ages, both sexes combined, 2016		
Cause	CoDCorrect level	Percent change
Motor neuron disease	3	-1.16<br>-2.46 to 0.54
Other neurological disorders	3	-0.0<br>-1.94 to 2.58
Mental and substance use disorders	2	0.81<br>-0.75 to 2.48
Alcohol use disorders	3	1.01<br>-0.56 to 3.02
Drug use disorders	3	0.58<br>-1.44 to 2.25
Opioid use disorders	4	-2.19<br>-28.41 to 7.6
Cocaine use disorders	4	7.9<br>-5.37 to 40.64
Amphetamine use disorders	4	7.24<br>-3.35 to 41.02
Other drug use disorders	4	3.64<br>-5.18 to 26.94
Eating disorders	3	1.48<br>-1.62 to 3.75
Anorexia nervosa	4	-0.41<br>-20.08 to 20.11
Bulimia nervosa	4	-6.92<br>-18.61 to 13.54
Diabetes, urogenital, blood, and endocrine diseases	2	-0.54<br>-2.29 to 1.39
Diabetes mellitus	3	-0.16<br>-2.16 to 1.65
Acute glomerulonephritis	3	-1.95<br>-3.62 to 0.98
Chronic kidney disease	3	-1.27<br>-2.8 to 0.47
Chronic kidney disease due to diabetes mellitus	4	-1.18<br>-2.66 to 0.43
Chronic kidney disease due to hypertension	4	-2.48<br>-4.13 to -0.67
Chronic kidney disease due to glomerulonephritis	4	0.3<br>-1.38 to 2.2
Chronic kidney disease due to other causes	4	-0.91<br>-2.77 to 0.96
Urinary diseases and male infertility	3	-0.43<br>-2.68 to 1.93
Interstitial nephritis and urinary tract infections	4	41.48<br>25.21 to 85.81
Urolithiasis	4	57.21<br>32.84 to 102.15
Other urinary diseases	4	78.72<br>46.6 to 127.05
Gynecological diseases	3	-1.0<br>-6.28 to 3.12
Uterine fibroids	4	74.29<br>33.86 to 121.39
Polycystic ovarian syndrome	4	108.33<br>46.67 to 199.18
Endometriosis	4	60.75<br>20.25 to 145.22
Genital prolapse	4	218.03<br>108.66 to 360.78
Other gynecological diseases	4	167.04<br>124.37 to 239.18

Appendix Table 11: Percent change before and after CoDCorrect by cause for all ages, both sexes combined, 2016

Cause	CoDCorrect level	Percent change
Hemoglobinopathies and hemolytic anaemias	3	2.58<br>-1.44 to 6.77
Thalassemias	4	1.0<br>-7.84 to 9.94
Sickle cell disorders	4	2.73<br>-4.09 to 9.67
G6PD deficiency	4	8.67<br>5.94 to 12.14
Other hemoglobinopathies and hemolytic anaemias	4	-0.67<br>-4.14 to 2.28
Endocrine, metabolic, blood, and immune disorders	3	-0.9<br>-2.74 to 1.91
Musculoskeletal disorders	2	-1.68<br>-3.33 to 0.42
Rheumatoid arthritis	3	-3.15<br>-15.98 to 13.81
Other musculoskeletal disorders	3	-9.36<br>-24.68 to 8.29
Other non-communicable diseases	2	1.24<br>-3.18 to 6.7
Congenital anomalies	3	1.09<br>-3.92 to 7.05
Neural tube defects	4	-29.38<br>-63.33 to 10.79
Congenital heart anomalies	4	-13.81<br>-32.54 to 9.43
Orofacial clefts	4	-20.37<br>-38.17 to 16.29
Down's syndrome	4	-5.98<br>-18.46 to 7.83
Other chromosomal abnormalities	4	-13.55<br>-27.74 to 11.49
Congenital musculoskeletal and limb anomalies	4	-2.91<br>-19.98 to 25.91
Urogenital congenital anomalies	4	-3.19<br>-16.71 to 15.47
Digestive congenital anomalies	4	-8.84<br>-23.45 to 19.19
Other congenital anomalies	4	-15.07<br>-30.39 to 12.31
Skin and subcutaneous diseases	3	0.72<br>-1.71 to 4.63
Cellulitis	4	6.98<br>-33.07 to 47.14
Pyoderma	4	28.28<br>-16.21 to 85.32
Decubitus ulcer	4	11.14<br>-28.09 to 53.95
Other skin and subcutaneous diseases	4	27.13<br>-12.03 to 85.97
Sudden infant death syndrome	3	5.99<br>-1.74 to 16.64
Injuries	1	6.11<br>3.31 to 9.85
Transport injuries	2	3.48<br>1.42 to 6.65
Road injuries	3	-0.54<br>-4.87 to 13.92
Pedestrian road injuries	4	0.86<br>-4.09 to 8.01

Appendix Table 11: Percent change before and after CoDCorrect by cause for all ages, both sexes combined, 2016		
Cause	CoDCorrect level	Percent change
Cyclist road injuries	4	3.39<br>-2.78 to 11.9
Motorcyclist road injuries	4	4.57<br>-2.59 to 11.76
Motor vehicle road injuries	4	1.06<br>-5.76 to 8.87
Other road injuries	4	5.49<br>-1.51 to 13.3
Other transport injuries	3	5.68<br>0.6 to 18.29
Unintentional injuries	2	1.51<br>-1.15 to 5.1
Falls	3	0.35<br>-2.07 to 3.02
Drowning	3	0.68<br>-4.3 to 6.11
Fire, heat, and hot substances	3	3.36<br>0.49 to 6.8
Poisonings	3	3.34<br>0.18 to 7.87
Exposure to mechanical forces	3	3.77<br>1.0 to 8.28
Unintentional firearm injuries	4	7.99<br>4.28 to 12.65
Unintentional suffocation	4	2.23<br>-1.83 to 9.19
Other exposure to mechanical forces	4	3.24<br>0.84 to 7.36
Adverse effects of medical treatment	3	2.65<br>0.22 to 5.65
Animal contact	3	2.22<br>-1.93 to 8.1
Venomous animal contact	4	23.23<br>-6.45 to 70.97
Non-venomous animal contact	4	9.28<br>-14.03 to 43.55
Foreign body	3	1.13<br>-2.11 to 5.44
Pulmonary aspiration and foreign body in airway	4	0.88<br>-2.33 to 5.31
Foreign body in other body part	4	3.51<br>-0.08 to 6.77
Other unintentional injuries	3	3.67<br>1.32 to 7.84
Self-harm and interpersonal violence	2	2.73<br>0.72 to 6.18
Self-harm	3	1.79<br>-0.35 to 4.92
Self-harm by firearm	4	0.4<br>-15.74 to 14.52
Self-harm by other specified means	4	-7.87<br>-15.97 to 1.63
Interpersonal violence	3	4.76<br>1.81 to 10.25
Assault by firearm	4	-1.59<br>-31.02 to 50.63
Assault by sharp object	4	4.13<br>-16.66 to 41.19
Assault by other means	4	5.24<br>-16.04 to 32.83

Appendix Table 11: Percent change before and after CoDCorrect by cause for all ages, both sexes combined, 2016

Cause	CoDCorrect level	Percent change
Forces of nature, conflict and terrorism, and executions and police conflict	2	3713.6<br>2297.34 to 7236.58
Maternal deaths aggravated by HIV/AIDS	3	-3.63<br>-10.49 to 3.56
Environmental heat and cold exposure	3	1.4<br>-1.48 to 3.5
Acute lymphoid leukaemia	4	1.81<br>-5.35 to 11.97
Chronic lymphoid leukaemia	4	0.02<br>-5.76 to 4.86
Acute myeloid leukaemia	4	1.35<br>-3.24 to 7.78
Chronic myeloid leukaemia	4	6.01<br>-0.31 to 12.87
Non-melanoma skin cancer (squamous-cell carcinoma)	4	-14.59<br>-17.41 to -12.46
Executions and police conflict	3	2.99<br>-0.37 to 10.58
Drug-sensitive tuberculosis	4	6.62<br>3.1 to 10.32
Zika virus	3	-0.97<br>-7.02 to 7.51
Alcoholic cardiomyopathy	4	-7.31<br>-38.3 to 9.27
Myocarditis	4	-11.19<br>-19.41 to 2.85
Other leukaemia	4	2.9<br>-3.46 to 13.65
Other cardiomyopathy	4	-13.22<br>-22.36 to -1.66
Multidrug-resistant tuberculosis without extensive drug resistance	4	5.64<br>1.91 to 8.93
Extensively drug-resistant tuberculosis	4	2.02<br>-0.06 to 3.95

Appendix Table 12: Socio-Demographic Index R-squared values with lags up to 10 years

Lag	Dependent Variable			
	e(0)	ln(5q0)	ln(35q15)	ln(20q50)
0	0.734	0.487	0.655	0.725
1	0.738	0.484	0.657	0.729
2	0.739	0.473	0.654	0.733
3	0.735	0.445	0.646	0.739
4	0.729	0.42	0.636	0.743
5	0.713	0.381	0.626	0.741
6	0.704	0.35	0.61	0.736
7	0.712	0.356	0.614	0.746
8	0.704	0.345	0.607	0.741
9	0.702	0.336	0.601	0.738
10	0.701	0.333	0.598	0.737

Appendix Table 13: Socio-Demographic Index groupings by location, based on 2016 values	
**Location**	**SDI Level**
Aichi	High SDI
Akita	High SDI
Alabama	High SDI
Alaska	High SDI
Andorra	High SDI
Aomori	High SDI
Arizona	High SDI
Arkansas	High SDI
Australia	High SDI
Austria	High SDI
Barking and Dagenham	High SDI
Barnet	High SDI
Barnsley	High SDI
Bath and North East Somerset	High SDI
Bedford	High SDI
Belgium	High SDI
Bexley	High SDI
Birmingham	High SDI
Blackburn with Darwen	High SDI
Blackpool	High SDI
Bolton	High SDI
Bournemouth	High SDI
Bracknell Forest	High SDI
Bradford	High SDI
Brent	High SDI
Brighton and Hove	High SDI
Bristol, City of	High SDI
Bromley	High SDI
Brunei	High SDI
Buckinghamshire	High SDI
Bury	High SDI
Calderdale	High SDI
California	High SDI
Cambridgeshire	High SDI
Camden	High SDI
Canada	High SDI
Central Bedfordshire	High SDI
Cheshire East	High SDI
Cheshire West and Chester	High SDI
Chiba	High SDI
Colorado	High SDI
Connecticut	High SDI
Cornwall	High SDI
County Durham	High SDI
Coventry	High SDI

Appendix Table 13: Socio-Demographic Index groupings by location, based on 2016 values	
**Location**	**SDI Level**
Croatia	High SDI
Croydon	High SDI
Cumbria	High SDI
Cyprus	High SDI
Czech Republic	High SDI
Darlington	High SDI
Delaware	High SDI
Denmark	High SDI
Derby	High SDI
Derbyshire	High SDI
Devon	High SDI
District of Columbia	High SDI
Doncaster	High SDI
Dorset	High SDI
Dudley	High SDI
Ealing	High SDI
East Riding of Yorkshire	High SDI
East Sussex	High SDI
Ehime	High SDI
Enfield	High SDI
Essex	High SDI
Estonia	High SDI
Finland	High SDI
Florida	High SDI
France	High SDI
Fukui	High SDI
Fukuoka	High SDI
Fukushima	High SDI
Gateshead	High SDI
Georgia	High SDI
Germany	High SDI
Gifu	High SDI
Gloucestershire	High SDI
Greece	High SDI
Greenwich	High SDI
Gunma	High SDI
Hackney	High SDI
Halton	High SDI
Hammersmith and Fulham	High SDI
Hampshire	High SDI
Haringey	High SDI
Harrow	High SDI
Hartlepool	High SDI
Havering	High SDI
Hawaii	High SDI

Appendix Table 13: Socio-Demographic Index groupings by location, based on 2016 values	
**Location**	**SDI Level**
Herefordshire, County of	High SDI
Hertfordshire	High SDI
Hillingdon	High SDI
Hiroshima	High SDI
Hokkaidō	High SDI
Hounslow	High SDI
Hyōgo	High SDI
Ibaraki	High SDI
Iceland	High SDI
Idaho	High SDI
Illinois	High SDI
Indiana	High SDI
Iowa	High SDI
Ireland	High SDI
Ishikawa	High SDI
Isle of Wight	High SDI
Islington	High SDI
Italy	High SDI
Iwate	High SDI
Kagawa	High SDI
Kagoshima	High SDI
Kanagawa	High SDI
Kansas	High SDI
Kensington and Chelsea	High SDI
Kent	High SDI
Kentucky	High SDI
Kingston upon Hull, City of	High SDI
Kingston upon Thames	High SDI
Kirklees	High SDI
Knowsley	High SDI
Kumamoto	High SDI
Kyōto	High SDI
Kōchi	High SDI
Lambeth	High SDI
Lancashire	High SDI
Latvia	High SDI
Leeds	High SDI
Leicester	High SDI
Leicestershire	High SDI
Lewisham	High SDI
Lincolnshire	High SDI
Lithuania	High SDI
Liverpool	High SDI
Louisiana	High SDI
Luton	High SDI

Appendix Table 13: Socio-Demographic Index groupings by location, based on 2016 values	
**Location**	**SDI Level**
Luxembourg	High SDI
Maine	High SDI
Malta	High SDI
Manchester	High SDI
Maryland	High SDI
Massachusetts	High SDI
Medway	High SDI
Merton	High SDI
Michigan	High SDI
Middlesbrough	High SDI
Mie	High SDI
Milton Keynes	High SDI
Minnesota	High SDI
Mississippi	High SDI
Missouri	High SDI
Miyagi	High SDI
Miyazaki	High SDI
Montana	High SDI
Nagano	High SDI
Nagasaki	High SDI
Nara	High SDI
Nebraska	High SDI
Netherlands	High SDI
Nevada	High SDI
New Hampshire	High SDI
New Jersey	High SDI
New Mexico	High SDI
New York	High SDI
New Zealand	High SDI
Newcastle upon Tyne	High SDI
Newham	High SDI
Niigata	High SDI
Norfolk	High SDI
North Carolina	High SDI
North Dakota	High SDI
North East Lincolnshire	High SDI
North Lincolnshire	High SDI
North Somerset	High SDI
North Tyneside	High SDI
North Yorkshire	High SDI
Northamptonshire	High SDI
Northern Ireland	High SDI
Northumberland	High SDI
Norway	High SDI
Nottingham	High SDI

Appendix Table 13: Socio-Demographic Index groupings by location, based on 2016 values	
**Location**	**SDI Level**
Nottinghamshire	High SDI
Ohio	High SDI
Okayama	High SDI
Okinawa	High SDI
Oklahoma	High SDI
Oldham	High SDI
Oregon	High SDI
Oxfordshire	High SDI
Pennsylvania	High SDI
Peterborough	High SDI
Plymouth	High SDI
Poland	High SDI
Poole	High SDI
Portsmouth	High SDI
Puerto Rico	High SDI
Reading	High SDI
Redbridge	High SDI
Redcar and Cleveland	High SDI
Rhode Island	High SDI
Richmond upon Thames	High SDI
Rochdale	High SDI
Rotherham	High SDI
Rutland	High SDI
Saga	High SDI
Saitama	High SDI
Salford	High SDI
Sandwell	High SDI
Scotland	High SDI
Sefton	High SDI
Sheffield	High SDI
Shiga	High SDI
Shimane	High SDI
Shizuoka	High SDI
Shropshire	High SDI
Singapore	High SDI
Slough	High SDI
Slovakia	High SDI
Slovenia	High SDI
Solihull	High SDI
Somerset	High SDI
South Carolina	High SDI
South Dakota	High SDI
South Gloucestershire	High SDI
South Korea	High SDI
South Tyneside	High SDI

Appendix Table 13: Socio-Demographic Index groupings by location, based on 2016 values	
**Location**	**SDI Level**
Southampton	High SDI
Southend-on-Sea	High SDI
Southwark	High SDI
St Helens	High SDI
Staffordshire	High SDI
Stockholm	High SDI
Stockport	High SDI
Stockton-on-Tees	High SDI
Stoke-on-Trent	High SDI
Suffolk	High SDI
Sunderland	High SDI
Surrey	High SDI
Sutton	High SDI
Sweden except Stockholm	High SDI
Swindon	High SDI
Switzerland	High SDI
Taiwan (province of China)	High SDI
Tameside	High SDI
Telford and Wrekin	High SDI
Tennessee	High SDI
Texas	High SDI
Thurrock	High SDI
Tochigi	High SDI
Tokushima	High SDI
Torbay	High SDI
Tottori	High SDI
Tower Hamlets	High SDI
Toyama	High SDI
Trafford	High SDI
Tōkyō	High SDI
Utah	High SDI
Vermont	High SDI
Virgin Islands	High SDI
Virginia	High SDI
Wakayama	High SDI
Wakefield	High SDI
Wales	High SDI
Walsall	High SDI
Waltham Forest	High SDI
Wandsworth	High SDI
Warrington	High SDI
Warwickshire	High SDI
Washington	High SDI
West Berkshire	High SDI
West Sussex	High SDI

Appendix Table 13: Socio-Demographic Index groupings by location, based on 2016 values	
**Location**	**SDI Level**
West Virginia	High SDI
Westminster	High SDI
Wigan	High SDI
Wiltshire	High SDI
Windsor and Maidenhead	High SDI
Wirral	High SDI
Wisconsin	High SDI
Wokingham	High SDI
Wolverhampton	High SDI
Worcestershire	High SDI
Wyoming	High SDI
Yamagata	High SDI
Yamaguchi	High SDI
Yamanashi	High SDI
York	High SDI
Ôita	High SDI
Ōsaka	High SDI
'Asir	High-middle SDI
Antigua and Barbuda	High-middle SDI
Argentina	High-middle SDI
Armenia	High-middle SDI
Azerbaijan	High-middle SDI
Bahah	High-middle SDI
Barbados	High-middle SDI
Beijing	High-middle SDI
Belarus	High-middle SDI
Bermuda	High-middle SDI
Bulgaria	High-middle SDI
Chile	High-middle SDI
Cuba	High-middle SDI
Delhi, Urban	High-middle SDI
Distrito Federal	High-middle SDI
Eastern Province	High-middle SDI
Georgia	High-middle SDI
Goa, Urban	High-middle SDI
Greenland	High-middle SDI
Guam	High-middle SDI
Guangdong	High-middle SDI
Ha'il	High-middle SDI
Himachal Pradesh, Urban	High-middle SDI
Hong Kong Special Administrative Region of China	High-middle SDI
Hungary	High-middle SDI
Iran	High-middle SDI
Israel	High-middle SDI

Appendix Table 13: Socio-Demographic Index groupings by location, based on 2016 values	
**Location**	**SDI Level**
Jakarta	High-middle SDI
Jawf	High-middle SDI
Jiangsu	High-middle SDI
Jizan	High-middle SDI
East Kalimantan	High-middle SDI
North Kalimantan	High-middle SDI
Kazakhstan	High-middle SDI
Riau Islands	High-middle SDI
Kuwait	High-middle SDI
Lebanon	High-middle SDI
Libya	High-middle SDI
Macao Special Administrative Region of China	High-middle SDI
Macedonia	High-middle SDI
Madinah	High-middle SDI
Makkah	High-middle SDI
Malaysia	High-middle SDI
Mauritius	High-middle SDI
Montenegro	High-middle SDI
Najran	High-middle SDI
Northern Borders	High-middle SDI
Northern Mariana Islands	High-middle SDI
Panama	High-middle SDI
Portugal	High-middle SDI
Qassim	High-middle SDI
Qatar	High-middle SDI
Rio de Janeiro	High-middle SDI
Riyadh	High-middle SDI
Romania	High-middle SDI
Russia	High-middle SDI
Serbia	High-middle SDI
Shanghai	High-middle SDI
Spain	High-middle SDI
São Paulo	High-middle SDI
Tabuk	High-middle SDI
The Bahamas	High-middle SDI
Tianjin	High-middle SDI
Trinidad and Tobago	High-middle SDI
Turkey	High-middle SDI
Turkmenistan	High-middle SDI
Ukraine	High-middle SDI
United Arab Emirates	High-middle SDI
Zhejiang	High-middle SDI
Aceh	Middle SDI
Acre	Middle SDI

Appendix Table 13: Socio-Demographic Index groupings by location, based on 2016 values	
**Location**	**SDI Level**
Aguascalientes	Middle SDI
Albania	Middle SDI
Algeria	Middle SDI
Amapá	Middle SDI
Amazonas	Middle SDI
American Samoa	Middle SDI
Andhra Pradesh, Urban	Middle SDI
Anhui	Middle SDI
Assam, Urban	Middle SDI
Bahia	Middle SDI
Bahrain	Middle SDI
Baja California	Middle SDI
Baja California Sur	Middle SDI
Bali	Middle SDI
Bangka–Belitung Islands	Middle SDI
Banten	Middle SDI
Bengkulu	Middle SDI
Bosnia and Herzegovina	Middle SDI
Botswana	Middle SDI
Campeche	Middle SDI
Chhattisgarh, Urban	Middle SDI
Chiapas	Middle SDI
Chihuahua	Middle SDI
Chongqing	Middle SDI
Coahuila	Middle SDI
Colima	Middle SDI
Colombia	Middle SDI
Costa Rica	Middle SDI
Delhi, Rural	Middle SDI
Distrito Federal	Middle SDI
Dominica	Middle SDI
Dominican Republic	Middle SDI
Durango	Middle SDI
Eastern Cape	Middle SDI
Ecuador	Middle SDI
Egypt	Middle SDI
El Salvador	Middle SDI
Equatorial Guinea	Middle SDI
Espírito Santo	Middle SDI
Fiji	Middle SDI
Free State	Middle SDI
Fujian	Middle SDI
Gauteng	Middle SDI
Goa, Rural	Middle SDI
Goiás	Middle SDI

Appendix Table 13: Socio-Demographic Index groupings by location, based on 2016 values	
**Location**	**SDI Level**
Grenada	Middle SDI
Guanajuato	Middle SDI
Guangxi	Middle SDI
Guerrero	Middle SDI
Gujarat, Urban	Middle SDI
Guyana	Middle SDI
Hainan	Middle SDI
Haryana, Urban	Middle SDI
Hebei	Middle SDI
Heilongjiang	Middle SDI
Henan	Middle SDI
Hidalgo	Middle SDI
Himachal Pradesh, Rural	Middle SDI
Hubei	Middle SDI
Hunan	Middle SDI
Inner Mongolia	Middle SDI
Jalisco	Middle SDI
Jamaica	Middle SDI
Jambi	Middle SDI
Jammu and Kashmir, Urban	Middle SDI
West Java	Middle SDI
East Java	Middle SDI
Jharkhand, Urban	Middle SDI
Jiangxi	Middle SDI
Jilin	Middle SDI
Jordan	Middle SDI
South Kalimantan	Middle SDI
Central Kalimantan	Middle SDI
Karnataka, Urban	Middle SDI
Kerala, Rural	Middle SDI
Kerala, Urban	Middle SDI
KwaZulu-Natal	Middle SDI
Lampung	Middle SDI
Liaoning	Middle SDI
Limpopo	Middle SDI
Madhya Pradesh, Urban	Middle SDI
Maharashtra, Urban	Middle SDI
Maldives	Middle SDI
Manipur, Urban	Middle SDI
Mato Grosso	Middle SDI
Mato Grosso do Sul	Middle SDI
Meghalaya, Urban	Middle SDI
Michoacán de Ocampo	Middle SDI
Minas Gerais	Middle SDI
Mizoram, Urban	Middle SDI

Appendix Table 13: Socio-Demographic Index groupings by location, based on 2016 values	
**Location**	**SDI Level**
Moldova	Middle SDI
Mongolia	Middle SDI
Morelos	Middle SDI
Mpumalanga	Middle SDI
México	Middle SDI
Nagaland, Urban	Middle SDI
Nayarit	Middle SDI
Ningxia	Middle SDI
North-West	Middle SDI
Northern Cape	Middle SDI
Nuevo León	Middle SDI
Oaxaca	Middle SDI
Oman	Middle SDI
West Papua	Middle SDI
Paraguay	Middle SDI
Paraná	Middle SDI
Pernambuco	Middle SDI
Peru	Middle SDI
Philippines	Middle SDI
Puebla	Middle SDI
Punjab, Urban	Middle SDI
Qinghai	Middle SDI
Querétaro	Middle SDI
Quintana Roo	Middle SDI
Riau	Middle SDI
Rio Grande do Norte	Middle SDI
Rio Grande do Sul	Middle SDI
Rondônia	Middle SDI
Roraima	Middle SDI
Saint Lucia	Middle SDI
Saint Vincent and the Grenadines	Middle SDI
San Luis Potosí	Middle SDI
Santa Catarina	Middle SDI
Sergipe	Middle SDI
Seychelles	Middle SDI
Shaanxi	Middle SDI
Shandong	Middle SDI
Shanxi	Middle SDI
Sichuan	Middle SDI
Sikkim, Urban	Middle SDI
Sinaloa	Middle SDI
Sonora	Middle SDI
Sri Lanka	Middle SDI
South Sulawesi	Middle SDI
North Sulawesi	Middle SDI

Appendix Table 13: Socio-Demographic Index groupings by location, based on 2016 values	
**Location**	**SDI Level**
West Sumatra	Middle SDI
South Sumatra	Middle SDI
North Sumatra	Middle SDI
Suriname	Middle SDI
Tabasco	Middle SDI
Tamaulipas	Middle SDI
Tamil Nadu, Urban	Middle SDI
Telangana, Urban	Middle SDI
Thailand	Middle SDI
The Six Minor Territories, Urban	Middle SDI
Tlaxcala	Middle SDI
Tocantins	Middle SDI
Tunisia	Middle SDI
Uruguay	Middle SDI
Uttarakhand, Urban	Middle SDI
Uzbekistan	Middle SDI
Venezuela	Middle SDI
Veracruz de Ignacio de la Llave	Middle SDI
Vietnam	Middle SDI
West Bengal, Urban	Middle SDI
Western Cape	Middle SDI
Xinjiang	Middle SDI
Yogyakarta	Middle SDI
Yucatán	Middle SDI
Yunnan	Middle SDI
Zacatecas	Middle SDI
Alagoas	Low-middle SDI
Andhra Pradesh, Rural	Low-middle SDI
Arunachal Pradesh, Rural	Low-middle SDI
Arunachal Pradesh, Urban	Low-middle SDI
Assam, Rural	Low-middle SDI
Bangladesh	Low-middle SDI
Baringo	Low-middle SDI
Belize	Low-middle SDI
Bhutan	Low-middle SDI
Bihar, Urban	Low-middle SDI
Bolivia	Low-middle SDI
Bomet	Low-middle SDI
Bungoma	Low-middle SDI
Busia	Low-middle SDI
Cambodia	Low-middle SDI
Cameroon	Low-middle SDI
Cape Verde	Low-middle SDI
Ceará	Low-middle SDI
Chhattisgarh, Rural	Low-middle SDI

Appendix Table 13: Socio-Demographic Index groupings by location, based on 2016 values	
**Location**	**SDI Level**
Congo (Brazzaville)	Low-middle SDI
Elgeyo-Marakwet	Low-middle SDI
Embu	Low-middle SDI
Federated States of Micronesia	Low-middle SDI
Gabon	Low-middle SDI
Gansu	Low-middle SDI
Garissa	Low-middle SDI
Ghana	Low-middle SDI
Gorontalo	Low-middle SDI
Guatemala	Low-middle SDI
Guizhou	Low-middle SDI
Gujarat, Rural	Low-middle SDI
Haryana, Rural	Low-middle SDI
HomaBay	Low-middle SDI
Honduras	Low-middle SDI
Iraq	Low-middle SDI
Isiolo	Low-middle SDI
Jammu and Kashmir, Rural	Low-middle SDI
Central Java	Low-middle SDI
Jharkhand, Rural	Low-middle SDI
Kajiado	Low-middle SDI
Kakamega	Low-middle SDI
West Kalimantan	Low-middle SDI
Karnataka, Rural	Low-middle SDI
Kericho	Low-middle SDI
Kiambu	Low-middle SDI
Kilifi	Low-middle SDI
Kirinyaga	Low-middle SDI
Kisii	Low-middle SDI
Kisumu	Low-middle SDI
Kitui	Low-middle SDI
Kwale	Low-middle SDI
Kyrgyzstan	Low-middle SDI
Laikipia	Low-middle SDI
Lamu	Low-middle SDI
Laos	Low-middle SDI
Lesotho	Low-middle SDI
Machakos	Low-middle SDI
Madhya Pradesh, Rural	Low-middle SDI
Maharashtra, Rural	Low-middle SDI
Makueni	Low-middle SDI
Maluku	Low-middle SDI
North Maluku	Low-middle SDI
Mandera	Low-middle SDI
Manipur, Rural	Low-middle SDI

Appendix Table 13: Socio-Demographic Index groupings by location, based on 2016 values	
**Location**	**SDI Level**
Maranhão	Low-middle SDI
Marsabit	Low-middle SDI
Marshall Islands	Low-middle SDI
Mauritania	Low-middle SDI
Meghalaya, Rural	Low-middle SDI
Meru	Low-middle SDI
Migori	Low-middle SDI
Mizoram, Rural	Low-middle SDI
Mombasa	Low-middle SDI
Morocco	Low-middle SDI
Murang’a	Low-middle SDI
Myanmar	Low-middle SDI
Nagaland, Rural	Low-middle SDI
Nairobi	Low-middle SDI
Nakuru	Low-middle SDI
Namibia	Low-middle SDI
Nandi	Low-middle SDI
Narok	Low-middle SDI
Nepal	Low-middle SDI
Nicaragua	Low-middle SDI
Nigeria	Low-middle SDI
North Korea	Low-middle SDI
West Nusa Tenggara	Low-middle SDI
East Nusa Tenggara	Low-middle SDI
Nyamira	Low-middle SDI
Nyandarua	Low-middle SDI
Nyeri	Low-middle SDI
Odisha, Rural	Low-middle SDI
Odisha, Urban	Low-middle SDI
Pakistan	Low-middle SDI
Papua	Low-middle SDI
Paraíba	Low-middle SDI
Pará	Low-middle SDI
Piaui	Low-middle SDI
Punjab, Rural	Low-middle SDI
Rajasthan, Rural	Low-middle SDI
Rajasthan, Urban	Low-middle SDI
Samburu	Low-middle SDI
Samoa	Low-middle SDI
Siaya	Low-middle SDI
Sikkim, Rural	Low-middle SDI
Sudan	Low-middle SDI
West Sulawesi	Low-middle SDI
Central Sulawesi	Low-middle SDI
Southeast Sulawesi	Low-middle SDI

Appendix Table 13: Socio-Demographic Index groupings by location, based on 2016 values	
**Location**	**SDI Level**
Swaziland	Low-middle SDI
Syria	Low-middle SDI
TaitaTaveta	Low-middle SDI
Tajikistan	Low-middle SDI
Tamil Nadu, Rural	Low-middle SDI
TanaRiver	Low-middle SDI
Telangana, Rural	Low-middle SDI
TharakaNithi	Low-middle SDI
The Six Minor Territories, Rural	Low-middle SDI
Tibet	Low-middle SDI
Timor-Leste	Low-middle SDI
Tonga	Low-middle SDI
TransNzoia	Low-middle SDI
Tripura, Rural	Low-middle SDI
Tripura, Urban	Low-middle SDI
Turkana	Low-middle SDI
UasinGishu	Low-middle SDI
Uttar Pradesh, Rural	Low-middle SDI
Uttar Pradesh, Urban	Low-middle SDI
Uttarakhand, Rural	Low-middle SDI
Vanuatu	Low-middle SDI
Vihiga	Low-middle SDI
Wajir	Low-middle SDI
West Bengal, Rural	Low-middle SDI
WestPokot	Low-middle SDI
Zambia	Low-middle SDI
Zimbabwe	Low-middle SDI
Afghanistan	Low SDI
Angola	Low SDI
Benin	Low SDI
Bihar, Rural	Low SDI
Burkina Faso	Low SDI
Burundi	Low SDI
Central African Republic	Low SDI
Chad	Low SDI
Comoros	Low SDI
Cote d'Ivoire	Low SDI
Democratic Republic of the Congo	Low SDI
Djibouti	Low SDI
Eritrea	Low SDI
Ethiopia	Low SDI
Guinea	Low SDI
Guinea-Bissau	Low SDI
Haiti	Low SDI
Kiribati	Low SDI

Appendix Table 13: Socio-Demographic Index groupings by location, based on 2016 values	
**Location**	**SDI Level**
Liberia	Low SDI
Madagascar	Low SDI
Malawi	Low SDI
Mali	Low SDI
Mozambique	Low SDI
Niger	Low SDI
Palestine	Low SDI
Papua New Guinea	Low SDI
Rwanda	Low SDI
Sao Tome and Principe	Low SDI
Senegal	Low SDI
Sierra Leone	Low SDI
Solomon Islands	Low SDI
Somalia	Low SDI
South Sudan	Low SDI
Tanzania	Low SDI
The Gambia	Low SDI
Togo	Low SDI
Uganda	Low SDI
Yemen	Low SDI

Appendix Table 14: Socio-Demographic Index values for all estimated GBD 2016 locations, 1980–2016

Location	1980	1981	1982	1983	1984	1985	1986	1987	1988	1989	1990	1991	1992	1993	1994	1995	1996	1997	1998	1999	2000	2001	2002	2003	2004	2005	2006	2007	2008	2009	2010	2011	2012	2013	2014	2015	2016
Global	0.526	0.531	0.535	0.54	0.544	0.549	0.554	0.559	0.564	0.569	0.575	0.58	0.586	0.591	0.596	0.6	0.605	0.609	0.614	0.618	0.622	0.626	0.63	0.634	0.639	0.643	0.648	0.652	0.657	0.662	0.666	0.671	0.676	0.681	0.686	0.691	0.696
Southeast Asia, East Asia, and Oceania	0.438	0.445	0.453	0.461	0.47	0.478	0.486	0.495	0.504	0.512	0.521	0.53	0.539	0.548	0.556	0.565	0.573	0.581	0.587	0.594	0.6	0.606	0.612	0.619	0.626	0.633	0.641	0.65	0.658	0.666	0.675	0.683	0.691	0.699	0.706	0.712	0.719
East Asia	0.436	0.443	0.45	0.458	0.467	0.477	0.486	0.496	0.506	0.515	0.524	0.533	0.543	0.552	0.562	0.571	0.58	0.588	0.594	0.601	0.608	0.614	0.621	0.629	0.636	0.644	0.653	0.661	0.67	0.678	0.686	0.694	0.701	0.709	0.715	0.722	0.729
China	0.42	0.428	0.435	0.444	0.453	0.463	0.473	0.483	0.494	0.503	0.512	0.521	0.531	0.542	0.552	0.562	0.57	0.578	0.585	0.592	0.6	0.607	0.614	0.622	0.63	0.638	0.647	0.657	0.665	0.674	0.682	0.691	0.698	0.706	0.713	0.72	0.727
North Korea	0.471	0.476	0.48	0.485	0.488	0.491	0.494	0.497	0.499	0.502	0.505	0.508	0.51	0.511	0.51	0.506	0.501	0.495	0.489	0.483	0.478	0.473	0.47	0.468	0.468	0.469	0.47	0.471	0.473	0.474	0.476	0.478	0.481	0.484	0.488	0.491	0.494
Taiwan (province of China)	0.636	0.645	0.657	0.669	0.681	0.695	0.706	0.71	0.718	0.727	0.734	0.743	0.749	0.756	0.762	0.768	0.775	0.788	0.798	0.8	0.808	0.815	0.82	0.824	0.829	0.833	0.837	0.842	0.845	0.849	0.853	0.857	0.86	0.864	0.868	0.871	0.875
Hong Kong Special Administrative Region of China	0.633	0.643	0.652	0.662	0.671	0.677	0.682	0.688	0.694	0.699	0.704	0.708	0.713	0.718	0.723	0.727	0.731	0.734	0.737	0.74	0.743	0.746	0.749	0.752	0.756	0.761	0.765	0.77	0.775	0.779	0.783	0.787	0.791	0.795	0.798	0.801	0.805
Macao Special Administrative Region of China	0.638	0.646	0.654	0.66	0.667	0.672	0.678	0.684	0.689	0.695	0.7	0.705	0.711	0.716	0.721	0.725	0.728	0.731	0.732	0.734	0.735	0.738	0.742	0.747	0.754	0.761	0.768	0.776	0.784	0.789	0.793	0.796	0.799	0.802	0.806	0.809	0.811
China (without Hong Kong and Macao)	0.413	0.42	0.428	0.436	0.446	0.456	0.467	0.477	0.487	0.497	0.506	0.515	0.525	0.536	0.546	0.556	0.565	0.574	0.581	0.588	0.596	0.603	0.611	0.619	0.627	0.635	0.645	0.654	0.663	0.672	0.68	0.689	0.697	0.704	0.712	0.718	0.725
Anhui	0.353	0.36	0.367	0.375	0.385	0.396	0.406	0.417	0.427	0.437	0.446	0.455	0.464	0.474	0.483	0.493	0.502	0.51	0.518	0.525	0.532	0.538	0.545	0.552	0.559	0.567	0.576	0.585	0.594	0.603	0.611	0.619	0.626	0.633	0.639	0.645	0.651
Beijing	0.606	0.612	0.618	0.624	0.631	0.638	0.645	0.653	0.659	0.665	0.671	0.676	0.682	0.689	0.695	0.702	0.709	0.715	0.721	0.726	0.732	0.738	0.744	0.75	0.757	0.764	0.772	0.78	0.787	0.795	0.802	0.809	0.816	0.823	0.829	0.835	0.842
Chongqing	0.367	0.375	0.384	0.394	0.407	0.419	0.431	0.443	0.454	0.464	0.474	0.485	0.496	0.507	0.519	0.53	0.539	0.549	0.557	0.565	0.574	0.582	0.591	0.599	0.607	0.616	0.625	0.635	0.643	0.652	0.66	0.668	0.675	0.682	0.689	0.694	0.7
Fujian	0.372	0.378	0.386	0.396	0.408	0.42	0.433	0.445	0.458	0.469	0.48	0.492	0.504	0.518	0.531	0.544	0.555	0.564	0.573	0.581	0.59	0.598	0.606	0.615	0.623	0.632	0.642	0.652	0.662	0.671	0.68	0.689	0.698	0.706	0.713	0.72	0.727
Gansu	0.353	0.358	0.364	0.372	0.382	0.391	0.401	0.411	0.421	0.431	0.438	0.447	0.456	0.465	0.473	0.482	0.49	0.498	0.506	0.513	0.52	0.526	0.533	0.539	0.546	0.553	0.56	0.568	0.575	0.583	0.59	0.597	0.604	0.61	0.616	0.622	0.628
Guangdong	0.444	0.451	0.458	0.467	0.476	0.487	0.497	0.507	0.518	0.527	0.536	0.546	0.557	0.567	0.578	0.588	0.598	0.607	0.615	0.623	0.632	0.64	0.648	0.658	0.667	0.678	0.688	0.7	0.71	0.72	0.729	0.737	0.745	0.753	0.761	0.768	0.775
Guangxi	0.361	0.369	0.376	0.385	0.395	0.406	0.417	0.428	0.439	0.449	0.458	0.468	0.479	0.49	0.501	0.511	0.521	0.531	0.539	0.547	0.555	0.562	0.569	0.577	0.585	0.594	0.603	0.613	0.622	0.632	0.641	0.651	0.661	0.67	0.679	0.686	0.693
Guizhou	0.279	0.287	0.295	0.303	0.314	0.325	0.336	0.347	0.358	0.368	0.376	0.385	0.394	0.403	0.413	0.422	0.431	0.439	0.446	0.454	0.462	0.469	0.477	0.485	0.494	0.503	0.512	0.522	0.531	0.54	0.55	0.559	0.567	0.575	0.581	0.587	0.594
Hainan	0.438	0.443	0.448	0.454	0.462	0.47	0.478	0.486	0.495	0.502	0.511	0.52	0.529	0.539	0.549	0.558	0.566	0.575	0.582	0.59	0.597	0.604	0.611	0.618	0.625	0.632	0.64	0.648	0.656	0.665	0.672	0.68	0.686	0.693	0.699	0.705	0.711
Hebei	0.42	0.427	0.435	0.443	0.452	0.462	0.472	0.482	0.492	0.501	0.509	0.517	0.527	0.537	0.547	0.556	0.565	0.573	0.581	0.588	0.596	0.603	0.611	0.619	0.627	0.635	0.644	0.654	0.662	0.671	0.679	0.687	0.695	0.702	0.709	0.716	0.722
Heilongjiang	0.477	0.482	0.488	0.495	0.504	0.513	0.522	0.531	0.54	0.548	0.557	0.566	0.575	0.582	0.588	0.594	0.6	0.606	0.611	0.617	0.623	0.628	0.634	0.639	0.644	0.649	0.655	0.662	0.668	0.674	0.679	0.685	0.69	0.695	0.7	0.704	0.709
Henan	0.368	0.375	0.384	0.394	0.405	0.417	0.429	0.441	0.453	0.463	0.471	0.481	0.491	0.502	0.513	0.525	0.535	0.545	0.554	0.562	0.57	0.578	0.587	0.595	0.604	0.613	0.623	0.633	0.643	0.652	0.661	0.67	0.678	0.685	0.693	0.699	0.706
Hubei	0.407	0.415	0.423	0.432	0.442	0.452	0.463	0.474	0.484	0.494	0.504	0.514	0.524	0.535	0.545	0.553	0.561	0.569	0.576	0.583	0.59	0.597	0.604	0.611	0.618	0.626	0.635	0.643	0.651	0.659	0.667	0.674	0.681	0.688	0.694	0.7	0.706
Hunan	0.383	0.391	0.399	0.409	0.42	0.431	0.442	0.454	0.465	0.476	0.485	0.495	0.505	0.516	0.526	0.536	0.546	0.555	0.562	0.569	0.576	0.582	0.59	0.597	0.605	0.613	0.621	0.63	0.639	0.647	0.655	0.663	0.67	0.678	0.684	0.691	0.697
Inner Mongolia	0.407	0.413	0.422	0.431	0.444	0.456	0.468	0.48	0.492	0.502	0.512	0.522	0.534	0.545	0.556	0.566	0.575	0.584	0.593	0.602	0.61	0.618	0.627	0.634	0.641	0.648	0.656	0.664	0.672	0.68	0.688	0.696	0.703	0.71	0.716	0.722	0.728
Jiangsu	0.443	0.451	0.459	0.468	0.479	0.489	0.5	0.51	0.521	0.53	0.539	0.548	0.557	0.565	0.573	0.582	0.591	0.599	0.607	0.614	0.621	0.629	0.637	0.646	0.655	0.664	0.674	0.684	0.693	0.702	0.711	0.72	0.728	0.736	0.743	0.75	0.757
Jiangxi	0.347	0.354	0.362	0.371	0.382	0.394	0.405	0.416	0.428	0.439	0.449	0.458	0.469	0.48	0.49	0.5	0.509	0.518	0.526	0.534	0.542	0.549	0.557	0.565	0.573	0.581	0.59	0.599	0.607	0.615	0.622	0.629	0.636	0.643	0.649	0.655	0.662
Jilin	0.451	0.457	0.464	0.471	0.48	0.49	0.5	0.509	0.519	0.528	0.537	0.547	0.557	0.564	0.571	0.579	0.586	0.594	0.6	0.607	0.613	0.62	0.626	0.633	0.639	0.646	0.653	0.661	0.668	0.676	0.683	0.689	0.696	0.702	0.708	0.713	0.719
Liaoning	0.521	0.527	0.533	0.539	0.547	0.555	0.563	0.571	0.579	0.587	0.592	0.597	0.602	0.608	0.614	0.621	0.627	0.634	0.639	0.645	0.651	0.656	0.662	0.668	0.674	0.68	0.686	0.693	0.7	0.707	0.713	0.719	0.725	0.73	0.736	0.741	0.746
Ningxia	0.37	0.375	0.382	0.39	0.398	0.408	0.418	0.429	0.44	0.45	0.46	0.469	0.479	0.489	0.498	0.507	0.515	0.523	0.531	0.538	0.545	0.552	0.559	0.566	0.574	0.583	0.592	0.601	0.61	0.617	0.624	0.632	0.639	0.645	0.652	0.658	0.664
Qinghai	0.354	0.36	0.366	0.375	0.386	0.397	0.408	0.42	0.431	0.442	0.452	0.462	0.473	0.482	0.492	0.501	0.509	0.517	0.524	0.531	0.537	0.544	0.55	0.557	0.564	0.571	0.578	0.586	0.593	0.6	0.607	0.614	0.62	0.627	0.633	0.639	0.645
Shaanxi	0.385	0.392	0.4	0.409	0.419	0.43	0.441	0.451	0.462	0.471	0.48	0.49	0.501	0.512	0.522	0.532	0.54	0.549	0.556	0.564	0.571	0.579	0.587	0.594	0.602	0.61	0.619	0.628	0.637	0.645	0.654	0.662	0.67	0.677	0.684	0.69	0.696
Shandong	0.413	0.421	0.431	0.44	0.451	0.463	0.474	0.485	0.497	0.507	0.516	0.525	0.535	0.544	0.553	0.563	0.572	0.581	0.589	0.596	0.604	0.612	0.62	0.629	0.638	0.647	0.657	0.668	0.678	0.687	0.696	0.705	0.713	0.721	0.729	0.736	0.742
Shanghai	0.638	0.643	0.648	0.652	0.656	0.661	0.666	0.67	0.675	0.679	0.684	0.689	0.694	0.699	0.704	0.709	0.714	0.719	0.723	0.727	0.732	0.736	0.741	0.747	0.753	0.759	0.765	0.772	0.778	0.785	0.791	0.797	0.803	0.809	0.815	0.82	0.826
Shanxi	0.42	0.426	0.434	0.442	0.452	0.462	0.472	0.482	0.493	0.502	0.511	0.52	0.53	0.54	0.55	0.559	0.568	0.577	0.585	0.593	0.6	0.607	0.614	0.621	0.628	0.635	0.643	0.651	0.659	0.666	0.674	0.681	0.688	0.694	0.7	0.706	0.712
Sichuan	0.349	0.359	0.369	0.38	0.392	0.405	0.418	0.43	0.443	0.454	0.463	0.472	0.482	0.492	0.502	0.511	0.52	0.528	0.535	0.542	0.549	0.556	0.563	0.571	0.58	0.589	0.598	0.608	0.617	0.625	0.634	0.642	0.65	0.657	0.664	0.67	0.677
Tianjin	0.569	0.575	0.582	0.589	0.596	0.604	0.611	0.618	0.625	0.63	0.636	0.641	0.647	0.653	0.66	0.666	0.673	0.679	0.685	0.69	0.696	0.702	0.709	0.715	0.722	0.729	0.737	0.745	0.753	0.76	0.768	0.775	0.782	0.789	0.795	0.801	0.807
Tibet	0.344	0.348	0.352	0.357	0.363	0.369	0.376	0.383	0.39	0.398	0.406	0.414	0.422	0.429	0.437	0.444	0.45	0.456	0.462	0.468	0.474	0.48	0.487	0.495	0.503	0.512	0.519	0.526	0.532	0.538	0.544	0.55	0.556	0.561	0.566	0.571	0.577
Xinjiang	0.377	0.383	0.39	0.399	0.41	0.423	0.435	0.448	0.462	0.474	0.486	0.499	0.512	0.524	0.536	0.548	0.558	0.569	0.578	0.588	0.597	0.606	0.614	0.622	0.629	0.636	0.644	0.652	0.66	0.668	0.676	0.683	0.69	0.696	0.702	0.707	0.713
Yunnan	0.311	0.319	0.327	0.336	0.346	0.358	0.369	0.38	0.391	0.402	0.412	0.422	0.432	0.443	0.453	0.464	0.473	0.482	0.491	0.499	0.507	0.515	0.523	0.532	0.541	0.55	0.56	0.57	0.579	0.587	0.596	0.604	0.611	0.618	0.625	0.632	0.639
Zhejiang	0.418	0.426	0.435	0.445	0.457	0.469	0.481	0.493	0.505	0.515	0.524	0.534	0.544	0.552	0.561	0.57	0.579	0.588	0.596	0.604	0.612	0.62	0.629	0.637	0.647	0.656	0.666	0.677	0.686	0.695	0.705	0.714	0.722	0.73	0.738	0.745	0.753
Southeast Asia	0.411	0.421	0.431	0.441	0.45	0.459	0.467	0.475	0.483	0.491	0.499	0.507	0.515	0.523	0.531	0.539	0.547	0.554	0.56	0.566	0.571	0.577	0.582	0.587	0.593	0.598	0.604	0.61	0.616	0.623	0.629	0.636	0.644	0.651	0.659	0.666	0.672
Cambodia	0.258	0.261	0.264	0.267	0.271	0.275	0.279	0.284	0.29	0.296	0.301	0.306	0.312	0.316	0.321	0.326	0.331	0.336	0.342	0.349	0.357	0.365	0.373	0.382	0.391	0.401	0.412	0.423	0.433	0.443	0.452	0.461	0.47	0.479	0.487	0.496	0.503
Indonesia	0.403	0.414	0.425	0.436	0.446	0.457	0.467	0.476	0.485	0.493	0.501	0.51	0.518	0.526	0.534	0.541	0.549	0.557	0.562	0.567	0.572	0.577	0.582	0.586	0.591	0.596	0.601	0.607	0.613	0.62	0.627	0.635	0.643	0.652	0.66	0.669	0.676
Laos	0.239	0.245	0.252	0.259	0.265	0.272	0.279	0.286	0.291	0.297	0.303	0.309	0.315	0.321	0.327	0.335	0.344	0.354	0.363	0.372	0.382	0.391	0.4	0.409	0.418	0.426	0.434	0.441	0.448	0.456	0.463	0.471	0.48	0.488	0.496	0.505	0.512
Malaysia	0.495	0.502	0.51	0.515	0.518	0.523	0.531	0.54	0.549	0.557	0.564	0.569	0.575	0.583	0.593	0.603	0.613	0.625	0.632	0.637	0.648	0.661	0.672	0.679	0.687	0.694	0.701	0.707	0.712	0.718	0.726	0.734	0.742	0.75	0.758	0.766	0.773
Maldives	0.344	0.353	0.362	0.371	0.379	0.389	0.397	0.406	0.415	0.424	0.433	0.442	0.451	0.46	0.469	0.479	0.489	0.5	0.511	0.522	0.534	0.545	0.555	0.565	0.576	0.583	0.592	0.602	0.611	0.621	0.63	0.641	0.651	0.661	0.67	0.68	0.687
Myanmar	0.295	0.301	0.308	0.315	0.321	0.328	0.334	0.339	0.342	0.346	0.349	0.352	0.356	0.36	0.366	0.372	0.379	0.386	0.393	0.401	0.411	0.42	0.43	0.441	0.453	0.465	0.478	0.49	0.502	0.513	0.524	0.536	0.547	0.558	0.569	0.58	0.59
Philippines	0.457	0.465	0.474	0.482	0.489	0.495	0.5	0.505	0.51	0.515	0.52	0.525	0.529	0.534	0.539	0.544	0.549	0.554	0.558	0.562	0.566	0.57	0.573	0.577	0.581	0.585	0.589	0.594	0.598	0.603	0.608	0.613	0.619	0.625	0.631	0.637	0.642
Sri Lanka	0.43	0.437	0.445	0.453	0.463	0.473	0.484	0.492	0.5	0.506	0.512	0.519	0.526	0.533	0.541	0.55	0.559	0.568	0.576	0.581	0.584	0.588	0.592	0.598	0.604	0.609	0.615	0.621	0.628	0.636	0.646	0.656	0.666	0.676	0.685	0.695	0.703
Thailand	0.435	0.449	0.462	0.475	0.486	0.497	0.507	0.517	0.526	0.535	0.544	0.554	0.563	0.572	0.581	0.591	0.6	0.608	0.615	0.621	0.628	0.635	0.642	0.649	0.657	0.664	0.672	0.68	0.686	0.691	0.697	0.703	0.709	0.714	0.719	0.724	0.729
Timor-Leste	0.236	0.245	0.253	0.261	0.269	0.278	0.286	0.295	0.304	0.314	0.323	0.332	0.342	0.351	0.36	0.368	0.376	0.383	0.389	0.39	0.392	0.394	0.396	0.395	0.404	0.416	0.429	0.437	0.444	0.452	0.462	0.475	0.488	0.502	0.514	0.525	0.532
Vietnam	0.354	0.362	0.37	0.378	0.387	0.395	0.403	0.411	0.421	0.43	0.439	0.448	0.458	0.467	0.476	0.486	0.495	0.505	0.514	0.522	0.53	0.538	0.546	0.552	0.559	0.566	0.573	0.579	0.586	0.592	0.599	0.606	0.613	0.619	0.626	0.633	0.639
Mauritius	0.532	0.546	0.559	0.569	0.578	0.587	0.59	0.591	0.595	0.602	0.61	0.616	0.621	0.63	0.639	0.647	0.654	0.662	0.667	0.671	0.678	0.684	0.688	0.693	0.698	0.703	0.71	0.715	0.722	0.726	0.73	0.735	0.739	0.743	0.747	0.752	0.756
Seychelles	0.492	0.51	0.524	0.531	0.538	0.548	0.56	0.574	0.586	0.595	0.603	0.61	0.618	0.625	0.634	0.644	0.654	0.663	0.671	0.677	0.682	0.687	0.691	0.693	0.694	0.696	0.697	0.698	0.7	0.703	0.706	0.708	0.711	0.716	0.723	0.73	0.736
Aceh	0.418	0.43	0.443	0.456	0.469	0.482	0.494	0.505	0.516	0.527	0.537	0.547	0.557	0.566	0.575	0.583	0.591	0.599	0.604	0.609	0.612	0.614	0.616	0.618	0.62	0.622	0.625	0.629	0.633	0.637	0.642	0.647	0.653	0.66	0.667	0.674	0.681
Sumatera Utara	0.407	0.418	0.429	0.439	0.45	0.46	0.47	0.478	0.485	0.492	0.499	0.506	0.513	0.52	0.527	0.533	0.54	0.547	0.552	0.558	0.562	0.567	0.572	0.578	0.583	0.588	0.593	0.6	0.607	0.616	0.625	0.635	0.645	0.654	0.663	0.672	0.68

Appendix Table 14: Socio-Demographic Index values for all estimated GBD 2016 locations, 1980–2016

Location	1980	1981	1982	1983	1984	1985	1986	1987	1988	1989	1990	1991	1992	1993	1994	1995	1996	1997	1998	1999	2000	2001	2002	2003	2004	2005	2006	2007	2008	2009	2010	2011	2012	2013	2014	2015	2016
Sumatera Barat	0.387	0.398	0.408	0.418	0.429	0.439	0.448	0.458	0.466	0.475	0.483	0.491	0.498	0.506	0.513	0.519	0.526	0.532	0.536	0.541	0.546	0.551	0.557	0.563	0.568	0.574	0.58	0.587	0.594	0.602	0.611	0.62	0.629	0.638	0.647	0.656	0.663
Riau	0.438	0.451	0.465	0.479	0.494	0.507	0.521	0.533	0.545	0.556	0.567	0.577	0.586	0.595	0.602	0.609	0.616	0.622	0.628	0.632	0.639	0.645	0.652	0.656	0.661	0.665	0.67	0.675	0.682	0.688	0.695	0.702	0.711	0.72	0.728	0.737	0.744
Jambi	0.355	0.366	0.376	0.387	0.398	0.408	0.418	0.428	0.438	0.447	0.456	0.465	0.474	0.483	0.492	0.501	0.51	0.519	0.526	0.532	0.537	0.542	0.549	0.555	0.562	0.568	0.575	0.583	0.592	0.6	0.61	0.62	0.63	0.64	0.649	0.659	0.667
Sumatera Selatan	0.416	0.426	0.436	0.446	0.456	0.466	0.476	0.485	0.495	0.503	0.512	0.521	0.53	0.537	0.544	0.55	0.557	0.563	0.569	0.572	0.575	0.578	0.581	0.584	0.587	0.592	0.596	0.602	0.608	0.615	0.622	0.629	0.638	0.646	0.655	0.663	0.671
Bengkulu	0.348	0.359	0.369	0.38	0.391	0.402	0.413	0.423	0.433	0.443	0.452	0.462	0.471	0.48	0.489	0.498	0.507	0.515	0.521	0.526	0.53	0.534	0.538	0.543	0.548	0.554	0.561	0.567	0.575	0.582	0.589	0.598	0.607	0.616	0.625	0.634	0.641
Lampung	0.344	0.355	0.365	0.376	0.386	0.396	0.405	0.414	0.421	0.429	0.437	0.444	0.453	0.46	0.469	0.478	0.489	0.498	0.507	0.516	0.522	0.527	0.532	0.536	0.54	0.544	0.549	0.555	0.562	0.571	0.581	0.592	0.603	0.613	0.623	0.633	0.641
Bangka Belitung	0.398	0.408	0.417	0.427	0.437	0.447	0.456	0.466	0.475	0.483	0.492	0.499	0.507	0.514	0.521	0.528	0.535	0.542	0.548	0.553	0.557	0.562	0.567	0.572	0.578	0.584	0.589	0.595	0.602	0.608	0.615	0.623	0.631	0.639	0.647	0.656	0.663
Kepulauan Riau	0.439	0.453	0.467	0.482	0.498	0.513	0.528	0.542	0.555	0.568	0.581	0.593	0.606	0.617	0.626	0.635	0.644	0.653	0.659	0.665	0.669	0.672	0.675	0.678	0.681	0.684	0.688	0.692	0.698	0.704	0.711	0.718	0.726	0.734	0.742	0.751	0.758
Kalimantan Utara	0.455	0.469	0.483	0.498	0.513	0.527	0.541	0.554	0.566	0.577	0.588	0.599	0.61	0.62	0.63	0.64	0.649	0.658	0.666	0.673	0.679	0.684	0.689	0.693	0.698	0.704	0.709	0.713	0.72	0.726	0.731	0.739	0.746	0.754	0.761	0.769	0.775
Jakarta	0.554	0.566	0.576	0.587	0.599	0.609	0.619	0.629	0.638	0.646	0.654	0.663	0.67	0.68	0.689	0.697	0.706	0.714	0.72	0.725	0.73	0.735	0.74	0.745	0.75	0.754	0.759	0.764	0.769	0.775	0.782	0.789	0.795	0.803	0.81	0.818	0.824
Jawa Barat	0.406	0.416	0.426	0.436	0.446	0.456	0.465	0.474	0.481	0.488	0.496	0.504	0.512	0.52	0.529	0.538	0.546	0.554	0.56	0.563	0.566	0.569	0.571	0.575	0.578	0.582	0.587	0.593	0.6	0.608	0.615	0.623	0.631	0.639	0.648	0.656	0.663
Jawa Tengah	0.363	0.374	0.384	0.395	0.405	0.415	0.425	0.433	0.442	0.449	0.457	0.466	0.476	0.483	0.492	0.5	0.508	0.516	0.521	0.526	0.529	0.533	0.536	0.54	0.545	0.549	0.555	0.561	0.568	0.575	0.582	0.591	0.599	0.608	0.616	0.625	0.632
Yogyakarta	0.393	0.405	0.417	0.429	0.44	0.451	0.462	0.472	0.481	0.49	0.499	0.508	0.517	0.53	0.543	0.554	0.563	0.572	0.576	0.58	0.584	0.587	0.591	0.595	0.599	0.603	0.607	0.612	0.618	0.624	0.631	0.639	0.647	0.655	0.663	0.672	0.679
Jawa Timur	0.384	0.395	0.406	0.416	0.427	0.437	0.447	0.456	0.465	0.473	0.482	0.49	0.498	0.506	0.514	0.522	0.53	0.538	0.545	0.55	0.556	0.562	0.567	0.573	0.579	0.584	0.59	0.596	0.603	0.61	0.617	0.625	0.633	0.642	0.651	0.659	0.667
Banten	0.398	0.408	0.418	0.428	0.439	0.449	0.459	0.469	0.478	0.486	0.495	0.504	0.513	0.521	0.529	0.536	0.544	0.553	0.561	0.568	0.573	0.577	0.581	0.586	0.59	0.593	0.598	0.602	0.607	0.612	0.618	0.626	0.635	0.644	0.652	0.661	0.669
Bali	0.394	0.406	0.418	0.43	0.442	0.454	0.466	0.477	0.486	0.496	0.506	0.515	0.524	0.534	0.544	0.552	0.561	0.57	0.575	0.579	0.582	0.585	0.589	0.592	0.596	0.6	0.604	0.609	0.614	0.621	0.628	0.636	0.644	0.653	0.662	0.67	0.678
Nusa Tenggara Barat	0.285	0.297	0.307	0.318	0.328	0.338	0.346	0.354	0.361	0.367	0.374	0.383	0.392	0.403	0.413	0.424	0.434	0.444	0.452	0.458	0.466	0.475	0.483	0.49	0.498	0.505	0.511	0.518	0.524	0.532	0.539	0.547	0.554	0.562	0.569	0.577	0.584
Nusa Tenggara Timur	0.301	0.311	0.319	0.327	0.335	0.343	0.351	0.359	0.366	0.373	0.381	0.388	0.395	0.402	0.409	0.415	0.42	0.426	0.427	0.427	0.429	0.432	0.436	0.439	0.443	0.447	0.451	0.457	0.463	0.47	0.479	0.488	0.497	0.507	0.516	0.525	0.534
Kalimantan Barat	0.319	0.33	0.341	0.353	0.365	0.377	0.388	0.398	0.408	0.419	0.429	0.439	0.45	0.461	0.472	0.483	0.494	0.505	0.513	0.52	0.525	0.53	0.534	0.538	0.542	0.546	0.55	0.555	0.559	0.565	0.571	0.578	0.586	0.594	0.602	0.61	0.618
Kalimantan Tengah	0.41	0.42	0.429	0.439	0.449	0.459	0.469	0.479	0.488	0.497	0.506	0.515	0.523	0.534	0.545	0.554	0.564	0.573	0.579	0.583	0.585	0.586	0.587	0.588	0.59	0.591	0.594	0.598	0.603	0.61	0.617	0.625	0.633	0.642	0.65	0.659	0.667
Kalimantan Selatan	0.399	0.41	0.421	0.432	0.442	0.453	0.463	0.472	0.48	0.487	0.494	0.502	0.51	0.519	0.527	0.534	0.542	0.549	0.554	0.558	0.561	0.564	0.567	0.57	0.574	0.577	0.58	0.584	0.589	0.594	0.601	0.609	0.617	0.625	0.633	0.642	0.649
Kalimantan Timur	0.463	0.478	0.492	0.507	0.522	0.536	0.55	0.563	0.575	0.587	0.598	0.607	0.617	0.625	0.632	0.64	0.647	0.654	0.661	0.667	0.673	0.68	0.686	0.691	0.696	0.702	0.707	0.711	0.717	0.722	0.726	0.733	0.739	0.747	0.755	0.763	0.77
Sulawesi Utara	0.44	0.45	0.46	0.469	0.478	0.487	0.495	0.503	0.51	0.517	0.524	0.532	0.539	0.548	0.556	0.564	0.573	0.581	0.588	0.591	0.593	0.595	0.596	0.597	0.599	0.601	0.603	0.607	0.612	0.619	0.626	0.634	0.642	0.651	0.659	0.668	0.675
Sulawesi Tengah	0.363	0.373	0.383	0.392	0.402	0.411	0.42	0.429	0.437	0.444	0.452	0.46	0.468	0.476	0.485	0.493	0.502	0.509	0.516	0.523	0.527	0.533	0.538	0.542	0.547	0.551	0.555	0.559	0.565	0.572	0.579	0.588	0.597	0.606	0.616	0.625	0.633
Sulawesi Selatan	0.354	0.366	0.377	0.388	0.399	0.409	0.42	0.429	0.438	0.447	0.456	0.465	0.474	0.482	0.489	0.496	0.504	0.51	0.516	0.52	0.525	0.53	0.535	0.54	0.546	0.55	0.554	0.56	0.566	0.574	0.583	0.593	0.603	0.613	0.623	0.632	0.64
Sulawesi Tenggara	0.327	0.339	0.35	0.361	0.373	0.384	0.395	0.405	0.414	0.423	0.432	0.442	0.45	0.458	0.465	0.473	0.48	0.487	0.494	0.499	0.503	0.508	0.513	0.519	0.525	0.531	0.537	0.544	0.552	0.561	0.57	0.58	0.589	0.599	0.608	0.617	0.625
Gorontalo	0.328	0.338	0.346	0.354	0.362	0.37	0.378	0.385	0.392	0.399	0.407	0.415	0.424	0.43	0.437	0.444	0.45	0.457	0.462	0.466	0.467	0.47	0.473	0.478	0.482	0.488	0.493	0.5	0.507	0.517	0.526	0.536	0.546	0.555	0.564	0.573	0.581
Sulawesi Barat	0.318	0.329	0.338	0.348	0.358	0.368	0.378	0.387	0.396	0.405	0.414	0.422	0.43	0.436	0.442	0.447	0.453	0.458	0.462	0.466	0.469	0.473	0.477	0.481	0.485	0.489	0.493	0.498	0.507	0.516	0.525	0.536	0.547	0.558	0.568	0.579	0.587
Maluku	0.446	0.453	0.46	0.468	0.476	0.483	0.491	0.499	0.506	0.512	0.519	0.526	0.533	0.539	0.545	0.551	0.557	0.562	0.566	0.567	0.564	0.559	0.554	0.548	0.542	0.536	0.531	0.527	0.527	0.529	0.534	0.541	0.551	0.561	0.571	0.581	0.59
Maluku Utara	0.364	0.374	0.383	0.393	0.404	0.414	0.423	0.432	0.441	0.449	0.458	0.467	0.476	0.483	0.491	0.498	0.506	0.514	0.518	0.523	0.524	0.524	0.523	0.521	0.518	0.515	0.511	0.508	0.507	0.51	0.515	0.522	0.531	0.541	0.552	0.562	0.571
Papua Barat	0.413	0.425	0.436	0.448	0.461	0.472	0.484	0.495	0.505	0.515	0.525	0.534	0.543	0.55	0.558	0.565	0.572	0.578	0.582	0.585	0.587	0.589	0.59	0.592	0.594	0.596	0.598	0.601	0.607	0.615	0.626	0.643	0.657	0.671	0.683	0.695	0.706
Papua	0.442	0.45	0.457	0.465	0.474	0.481	0.488	0.494	0.5	0.505	0.51	0.516	0.522	0.529	0.534	0.54	0.546	0.552	0.558	0.562	0.565	0.569	0.571	0.573	0.574	0.577	0.58	0.583	0.584	0.585	0.588	0.589	0.591	0.595	0.6	0.605	0.61
Oceania	0.332	0.338	0.343	0.348	0.353	0.358	0.363	0.368	0.373	0.378	0.383	0.388	0.393	0.398	0.403	0.407	0.412	0.416	0.42	0.424	0.428	0.433	0.436	0.44	0.443	0.447	0.45	0.454	0.457	0.461	0.464	0.468	0.472	0.476	0.481	0.485	0.489
Fiji	0.525	0.534	0.542	0.55	0.558	0.565	0.572	0.579	0.585	0.592	0.6	0.607	0.614	0.62	0.627	0.634	0.64	0.646	0.652	0.658	0.663	0.669	0.674	0.679	0.684	0.689	0.694	0.699	0.704	0.708	0.712	0.716	0.721	0.725	0.729	0.733	0.736
Kiribati	0.261	0.267	0.273	0.279	0.284	0.288	0.292	0.295	0.299	0.303	0.307	0.312	0.316	0.32	0.324	0.328	0.333	0.338	0.345	0.35	0.357	0.363	0.37	0.376	0.383	0.389	0.395	0.401	0.406	0.412	0.416	0.421	0.426	0.431	0.437	0.442	0.446
Marshall Islands	0.399	0.406	0.413	0.42	0.427	0.432	0.439	0.446	0.453	0.46	0.466	0.47	0.473	0.474	0.475	0.476	0.477	0.479	0.482	0.487	0.492	0.499	0.504	0.51	0.514	0.518	0.522	0.527	0.531	0.536	0.541	0.547	0.553	0.559	0.565	0.571	0.575
Federated States of Micronesia	0.274	0.292	0.31	0.328	0.346	0.363	0.379	0.394	0.409	0.422	0.434	0.446	0.456	0.466	0.475	0.485	0.495	0.503	0.512	0.521	0.53	0.539	0.547	0.555	0.561	0.568	0.573	0.578	0.582	0.586	0.59	0.595	0.599	0.603	0.607	0.61	0.612
Papua New Guinea	0.256	0.26	0.263	0.267	0.271	0.274	0.278	0.283	0.287	0.292	0.296	0.3	0.304	0.31	0.315	0.32	0.325	0.33	0.334	0.338	0.342	0.345	0.348	0.352	0.355	0.359	0.362	0.367	0.371	0.376	0.382	0.387	0.394	0.4	0.406	0.413	0.418
Samoa	0.372	0.381	0.389	0.397	0.405	0.412	0.42	0.428	0.435	0.443	0.451	0.458	0.465	0.472	0.477	0.482	0.487	0.492	0.498	0.501	0.504	0.507	0.511	0.514	0.518	0.521	0.525	0.529	0.533	0.537	0.54	0.542	0.545	0.548	0.553	0.558	0.562
Solomon Islands	0.269	0.275	0.28	0.284	0.289	0.294	0.298	0.303	0.308	0.313	0.318	0.323	0.329	0.335	0.34	0.346	0.352	0.357	0.363	0.368	0.371	0.373	0.374	0.375	0.377	0.379	0.383	0.387	0.393	0.398	0.404	0.411	0.418	0.425	0.432	0.439	0.444
Tonga	0.382	0.389	0.397	0.403	0.412	0.419	0.427	0.435	0.442	0.448	0.454	0.461	0.466	0.471	0.476	0.481	0.486	0.491	0.496	0.501	0.506	0.511	0.516	0.522	0.527	0.532	0.537	0.541	0.546	0.551	0.555	0.561	0.566	0.571	0.576	0.582	0.586
Vanuatu	0.277	0.283	0.288	0.294	0.3	0.306	0.312	0.318	0.324	0.329	0.335	0.341	0.347	0.353	0.359	0.364	0.37	0.376	0.382	0.387	0.393	0.399	0.405	0.41	0.415	0.42	0.426	0.431	0.437	0.443	0.449	0.454	0.46	0.465	0.471	0.476	0.479
American Samoa	0.551	0.561	0.569	0.576	0.583	0.589	0.595	0.601	0.606	0.612	0.617	0.622	0.626	0.631	0.635	0.639	0.643	0.648	0.652	0.656	0.661	0.666	0.671	0.676	0.682	0.688	0.695	0.7	0.705	0.711	0.716	0.721	0.726	0.73	0.735	0.739	0.742
Guam	0.718	0.726	0.733	0.738	0.742	0.743	0.743	0.743	0.746	0.75	0.755	0.761	0.765	0.77	0.774	0.778	0.782	0.785	0.788	0.791	0.794	0.797	0.799	0.801	0.802	0.804	0.806	0.807	0.809	0.81	0.812	0.813	0.815	0.817	0.819	0.821	0.822
Northern Mariana Islands	0.61	0.622	0.633	0.643	0.653	0.662	0.671	0.68	0.689	0.697	0.705	0.713	0.72	0.727	0.733	0.739	0.744	0.75	0.755	0.761	0.766	0.772	0.777	0.782	0.787	0.79	0.792	0.794	0.795	0.795	0.795	0.794	0.793	0.793	0.794	0.796	0.797
Central Europe, Eastern Europe, and Central Asia	0.706	0.708	0.71	0.711	0.714	0.716	0.717	0.721	0.727	0.733	0.739	0.746	0.751	0.754	0.755	0.757	0.758	0.758	0.758	0.758	0.759	0.762	0.765	0.769	0.773	0.778	0.782	0.786	0.79	0.793	0.797	0.801	0.804	0.806	0.809	0.812	0.816
Central Asia	0.576	0.582	0.587	0.591	0.594	0.598	0.602	0.607	0.612	0.618	0.624	0.63	0.635	0.638	0.641	0.642	0.643	0.643	0.643	0.643	0.645	0.647	0.65	0.654	0.66	0.666	0.672	0.679	0.685	0.691	0.697	0.703	0.708	0.714	0.719	0.724	0.729
Armenia	0.602	0.606	0.61	0.615	0.619	0.622	0.626	0.631	0.635	0.64	0.644	0.648	0.648	0.647	0.645	0.643	0.642	0.641	0.642	0.643	0.646	0.651	0.659	0.668	0.676	0.686	0.695	0.705	0.714	0.719	0.724	0.73	0.735	0.74	0.745	0.75	0.755
Azerbaijan	0.606	0.614	0.622	0.63	0.637	0.643	0.648	0.654	0.659	0.664	0.668	0.673	0.676	0.678	0.678	0.677	0.674	0.67	0.667	0.664	0.662	0.662	0.664	0.667	0.671	0.678	0.688	0.7	0.712	0.722	0.731	0.739	0.747	0.753	0.759	0.763	0.767
Georgia	0.679	0.684	0.688	0.691	0.695	0.698	0.702	0.706	0.71	0.715	0.72	0.723	0.722	0.718	0.713	0.705	0.698	0.689	0.682	0.677	0.674	0.676	0.679	0.684	0.69	0.695	0.701	0.708	0.714	0.719	0.723	0.727	0.731	0.736	0.741	0.746	0.751
Kazakhstan	0.629	0.636	0.641	0.645	0.649	0.653	0.658	0.663	0.669	0.675	0.681	0.687	0.692	0.696	0.699	0.701	0.703	0.704	0.704	0.704	0.705	0.706	0.708	0.71	0.712	0.715	0.718	0.721	0.725	0.728	0.731	0.736	0.74	0.744	0.748	0.753	0.757
Kyrgyzstan	0.556	0.562	0.567	0.571	0.575	0.578	0.582	0.587	0.592	0.598	0.604	0.61	0.614	0.618	0.619	0.618	0.617	0.615	0.612	0.609	0.607	0.606	0.605	0.605	0.606	0.606	0.606	0.607	0.608	0.609	0.61	0.612	0.614	0.618	0.622	0.627	0.632
Mongolia	0.403	0.417	0.431	0.445	0.458	0.473	0.489	0.506	0.523	0.539	0.557	0.573	0.586	0.597	0.604	0.611	0.615	0.619	0.623	0.627	0.631	0.635	0.639	0.643	0.647	0.65	0.653	0.656	0.659	0.66	0.662	0.664	0.667	0.67	0.674	0.678	0.683
Tajikistan	0.462	0.469	0.474	0.478	0.481	0.484	0.488	0.494	0.501	0.51	0.519	0.53	0.537	0.542	0.543	0.542	0.537	0.532	0.525	0.519	0.515	0.513	0.515	0.52	0.527	0.535	0.542	0.548	0.552	0.556	0.559	0.561	0.564	0.567	0.571	0.574	0.577
Turkmenistan	0.53	0.536	0.541	0.545	0.548	0.552	0.555	0.56	0.565	0.571	0.577	0.584	0.59	0.598	0.605	0.612	0.618	0.623	0.627	0.63	0.636	0.641	0.647	0.653	0.66	0.668	0.676	0.684	0.692	0.701	0.709	0.717	0.726	0.735	0.743	0.752	0.759
Uzbekistan	0.493	0.498	0.503	0.506	0.51	0.513	0.518	0.523	0.53	0.537	0.544	0.551	0.558	0.565	0.571	0.577	0.582	0.587	0.591	0.594	0.597	0.6	0.604	0.609	0.616	0.623	0.629	0.636	0.642	0.649	0.656	0.664	0.671	0.678	0.685	0.692	0.698
Central Europe	0.701	0.705	0.709	0.711	0.713	0.717	0.72	0.724	0.729	0.736	0.741	0.746	0.75	0.753	0.758	0.763	0.766	0.77	0.773	0.777	0.781	0.785	0.79	0.794	0.799	0.803	0.808	0.814	0.819	0.823	0.828	0.832	0.836	0.839	0.842	0.846	0.849

Appendix Table 14: Socio-Demographic Index values for all estimated GBD 2016 locations, 1980–2016

Location	1980	1981	1982	1983	1984	1985	1986	1987	1988	1989	1990	1991	1992	1993	1994	1995	1996	1997	1998	1999	2000	2001	2002	2003	2004	2005	2006	2007	2008	2009	2010	2011	2012	2013	2014	2015	2016
Albania	0.54	0.545	0.55	0.555	0.56	0.564	0.569	0.573	0.577	0.581	0.584	0.585	0.584	0.585	0.588	0.593	0.601	0.607	0.614	0.623	0.633	0.642	0.651	0.659	0.667	0.674	0.68	0.686	0.692	0.697	0.702	0.706	0.711	0.714	0.718	0.721	0.725
Bosnia and Herzegovina	0.502	0.508	0.514	0.518	0.522	0.527	0.532	0.535	0.539	0.542	0.542	0.542	0.543	0.543	0.546	0.552	0.573	0.597	0.619	0.635	0.648	0.658	0.667	0.675	0.682	0.688	0.694	0.699	0.705	0.71	0.714	0.717	0.721	0.724	0.727	0.73	0.733
Bulgaria	0.705	0.71	0.715	0.72	0.724	0.728	0.733	0.738	0.744	0.752	0.76	0.767	0.771	0.771	0.771	0.772	0.771	0.77	0.771	0.771	0.773	0.776	0.779	0.784	0.789	0.794	0.8	0.807	0.813	0.818	0.824	0.828	0.832	0.836	0.839	0.843	0.846
Croatia	0.746	0.751	0.755	0.759	0.763	0.767	0.772	0.775	0.779	0.782	0.786	0.789	0.788	0.785	0.782	0.779	0.779	0.785	0.79	0.793	0.797	0.801	0.806	0.811	0.815	0.819	0.824	0.828	0.833	0.836	0.838	0.841	0.843	0.845	0.846	0.848	0.85
Czech Republic	0.786	0.79	0.793	0.796	0.798	0.8	0.803	0.805	0.807	0.81	0.812	0.816	0.82	0.826	0.829	0.83	0.832	0.834	0.835	0.837	0.839	0.842	0.844	0.847	0.85	0.853	0.856	0.86	0.863	0.866	0.869	0.871	0.873	0.875	0.875	0.878	0.881
Hungary	0.711	0.719	0.727	0.732	0.733	0.735	0.739	0.744	0.748	0.751	0.753	0.756	0.761	0.766	0.77	0.777	0.78	0.783	0.786	0.79	0.794	0.798	0.803	0.807	0.812	0.816	0.821	0.825	0.828	0.831	0.834	0.836	0.838	0.841	0.843	0.846	0.849
Macedonia	0.664	0.669	0.673	0.677	0.681	0.686	0.69	0.695	0.7	0.702	0.7	0.699	0.701	0.701	0.701	0.703	0.708	0.712	0.716	0.718	0.723	0.73	0.737	0.742	0.745	0.749	0.753	0.757	0.762	0.765	0.77	0.775	0.779	0.782	0.786	0.789	0.793
Montenegro	0.725	0.728	0.732	0.734	0.737	0.739	0.742	0.744	0.746	0.748	0.75	0.751	0.75	0.747	0.743	0.74	0.74	0.741	0.743	0.745	0.748	0.753	0.759	0.764	0.769	0.773	0.777	0.781	0.786	0.79	0.795	0.8	0.803	0.806	0.809	0.812	0.815
Poland	0.702	0.702	0.699	0.699	0.703	0.708	0.714	0.719	0.724	0.73	0.735	0.739	0.745	0.751	0.759	0.767	0.773	0.778	0.783	0.789	0.795	0.8	0.805	0.81	0.815	0.82	0.824	0.83	0.835	0.84	0.845	0.85	0.855	0.86	0.864	0.868	0.872
Romania	0.661	0.672	0.684	0.688	0.687	0.688	0.689	0.695	0.703	0.715	0.731	0.738	0.738	0.739	0.74	0.742	0.744	0.747	0.749	0.751	0.755	0.759	0.764	0.769	0.774	0.78	0.786	0.794	0.802	0.808	0.813	0.818	0.822	0.827	0.83	0.833	0.838
Serbia	0.678	0.68	0.682	0.683	0.684	0.686	0.689	0.691	0.694	0.697	0.7	0.703	0.704	0.701	0.699	0.697	0.698	0.702	0.707	0.709	0.712	0.714	0.717	0.722	0.728	0.733	0.737	0.742	0.747	0.752	0.755	0.759	0.761	0.764	0.767	0.769	0.771
Slovakia	0.749	0.753	0.756	0.759	0.762	0.766	0.77	0.774	0.778	0.781	0.785	0.788	0.792	0.797	0.805	0.81	0.812	0.814	0.817	0.819	0.822	0.825	0.828	0.831	0.835	0.839	0.843	0.848	0.854	0.858	0.86	0.865	0.868	0.871	0.874	0.877	0.88
Slovenia	0.766	0.774	0.782	0.789	0.793	0.797	0.801	0.803	0.808	0.813	0.813	0.813	0.812	0.812	0.813	0.815	0.817	0.82	0.823	0.827	0.831	0.835	0.839	0.843	0.847	0.851	0.856	0.86	0.863	0.865	0.867	0.869	0.871	0.873	0.874	0.877	0.881
Eastern Europe	0.737	0.738	0.738	0.739	0.742	0.743	0.744	0.747	0.753	0.76	0.767	0.775	0.782	0.782	0.78	0.777	0.774	0.771	0.768	0.766	0.766	0.768	0.77	0.774	0.78	0.786	0.792	0.8	0.806	0.81	0.813	0.817	0.819	0.821	0.823	0.826	0.829
Belarus	0.703	0.706	0.708	0.711	0.713	0.716	0.719	0.723	0.727	0.733	0.738	0.743	0.748	0.751	0.752	0.748	0.744	0.742	0.741	0.74	0.741	0.744	0.747	0.752	0.758	0.765	0.772	0.78	0.788	0.795	0.8	0.805	0.81	0.814	0.818	0.822	0.826
Estonia	0.775	0.776	0.776	0.776	0.779	0.78	0.779	0.778	0.781	0.788	0.799	0.807	0.813	0.814	0.813	0.812	0.812	0.813	0.816	0.818	0.822	0.827	0.832	0.837	0.843	0.847	0.85	0.854	0.859	0.861	0.866	0.872	0.877	0.88	0.883	0.885	0.887
Latvia	0.759	0.759	0.758	0.758	0.759	0.76	0.762	0.765	0.77	0.776	0.783	0.789	0.793	0.794	0.79	0.786	0.783	0.781	0.781	0.783	0.785	0.79	0.795	0.801	0.808	0.815	0.822	0.831	0.837	0.841	0.843	0.847	0.85	0.851	0.851	0.851	0.853
Lithuania	0.768	0.771	0.772	0.772	0.774	0.776	0.777	0.781	0.787	0.79	0.792	0.795	0.8	0.805	0.805	0.804	0.801	0.801	0.801	0.803	0.805	0.809	0.814	0.819	0.825	0.832	0.838	0.845	0.851	0.853	0.856	0.858	0.862	0.865	0.869	0.872	0.876
Moldova	0.614	0.617	0.619	0.621	0.623	0.625	0.628	0.632	0.636	0.641	0.647	0.651	0.652	0.654	0.652	0.65	0.647	0.643	0.637	0.631	0.626	0.624	0.625	0.629	0.634	0.64	0.646	0.653	0.66	0.665	0.671	0.678	0.683	0.689	0.695	0.699	0.703
Russia	0.745	0.746	0.745	0.745	0.749	0.749	0.748	0.751	0.758	0.766	0.774	0.782	0.788	0.787	0.786	0.784	0.781	0.778	0.775	0.774	0.774	0.775	0.778	0.782	0.787	0.793	0.799	0.806	0.811	0.814	0.818	0.821	0.823	0.824	0.826	0.829	0.832
Ukraine	0.712	0.715	0.717	0.719	0.721	0.724	0.727	0.731	0.737	0.742	0.748	0.753	0.757	0.76	0.758	0.753	0.748	0.742	0.737	0.732	0.729	0.728	0.729	0.733	0.74	0.747	0.755	0.763	0.77	0.774	0.778	0.782	0.785	0.789	0.791	0.792	0.793
High-income	0.773	0.778	0.782	0.787	0.791	0.795	0.799	0.803	0.806	0.809	0.813	0.817	0.822	0.826	0.83	0.834	0.837	0.84	0.843	0.845	0.848	0.852	0.854	0.856	0.859	0.86	0.861	0.863	0.866	0.868	0.871	0.873	0.875	0.877	0.879	0.881	0.882
High-income Asia Pacific	0.745	0.75	0.756	0.763	0.769	0.775	0.781	0.787	0.793	0.799	0.805	0.809	0.816	0.821	0.826	0.831	0.835	0.839	0.841	0.844	0.847	0.849	0.851	0.854	0.856	0.859	0.861	0.864	0.867	0.868	0.871	0.873	0.875	0.877	0.879	0.882	0.884
Brunei	0.674	0.681	0.687	0.694	0.7	0.706	0.713	0.721	0.729	0.737	0.744	0.751	0.756	0.761	0.766	0.772	0.777	0.783	0.789	0.796	0.803	0.809	0.815	0.821	0.827	0.833	0.839	0.844	0.849	0.853	0.856	0.858	0.861	0.863	0.866	0.869	0.871
Japan	0.776	0.779	0.781	0.784	0.788	0.793	0.798	0.803	0.81	0.817	0.822	0.828	0.833	0.837	0.84	0.844	0.847	0.85	0.852	0.854	0.856	0.858	0.859	0.861	0.863	0.865	0.867	0.869	0.871	0.872	0.873	0.875	0.877	0.878	0.88	0.882	0.884
South Korea	0.618	0.631	0.649	0.669	0.684	0.693	0.704	0.714	0.723	0.732	0.738	0.744	0.753	0.764	0.772	0.781	0.791	0.8	0.805	0.81	0.816	0.821	0.826	0.831	0.835	0.84	0.845	0.849	0.853	0.857	0.861	0.864	0.868	0.871	0.874	0.878	0.881
Singapore	0.678	0.686	0.695	0.704	0.711	0.717	0.718	0.711	0.719	0.734	0.741	0.748	0.754	0.76	0.768	0.776	0.784	0.795	0.802	0.805	0.812	0.818	0.823	0.828	0.834	0.84	0.846	0.852	0.858	0.863	0.867	0.869	0.873	0.876	0.879	0.883	0.886
Hokkaidō	0.771	0.775	0.778	0.78	0.784	0.79	0.794	0.799	0.804	0.809	0.813	0.818	0.822	0.826	0.83	0.833	0.836	0.839	0.841	0.843	0.845	0.847	0.848	0.85	0.852	0.853	0.855	0.857	0.859	0.86	0.861	0.862	0.864	0.866	0.867	0.869	0.87
Aomori	0.76	0.764	0.766	0.769	0.773	0.778	0.784	0.789	0.795	0.801	0.805	0.81	0.814	0.818	0.822	0.827	0.831	0.835	0.838	0.84	0.842	0.843	0.845	0.847	0.848	0.85	0.852	0.854	0.855	0.856	0.858	0.859	0.861	0.862	0.864	0.865	0.867
Iwate	0.752	0.756	0.758	0.761	0.766	0.771	0.776	0.78	0.786	0.792	0.797	0.802	0.807	0.811	0.816	0.821	0.826	0.83	0.833	0.835	0.838	0.84	0.842	0.844	0.846	0.848	0.849	0.851	0.853	0.854	0.856	0.857	0.859	0.86	0.862	0.864	0.865
Miyagi	0.763	0.767	0.77	0.773	0.778	0.783	0.789	0.794	0.8	0.807	0.813	0.818	0.824	0.828	0.832	0.836	0.839	0.842	0.844	0.846	0.848	0.85	0.851	0.853	0.855	0.857	0.859	0.861	0.862	0.863	0.865	0.866	0.868	0.869	0.871	0.873	0.874
Akita	0.759	0.763	0.766	0.769	0.773	0.778	0.782	0.787	0.792	0.798	0.802	0.806	0.811	0.815	0.819	0.823	0.828	0.832	0.834	0.836	0.838	0.84	0.842	0.843	0.845	0.847	0.849	0.851	0.852	0.853	0.855	0.856	0.858	0.86	0.861	0.863	0.865
Yamagata	0.75	0.755	0.757	0.76	0.764	0.768	0.772	0.777	0.782	0.788	0.793	0.798	0.803	0.807	0.812	0.817	0.821	0.825	0.829	0.831	0.834	0.837	0.84	0.843	0.845	0.847	0.849	0.851	0.853	0.854	0.855	0.857	0.858	0.86	0.862	0.863	0.865
Fukushima	0.755	0.759	0.761	0.763	0.768	0.773	0.778	0.783	0.79	0.796	0.801	0.806	0.811	0.815	0.82	0.825	0.829	0.833	0.836	0.838	0.841	0.844	0.847	0.85	0.853	0.855	0.857	0.858	0.86	0.861	0.863	0.864	0.865	0.866	0.868	0.87	0.873
Ibaraki	0.768	0.772	0.775	0.777	0.781	0.787	0.792	0.797	0.804	0.811	0.817	0.822	0.828	0.832	0.837	0.842	0.846	0.849	0.851	0.853	0.855	0.857	0.858	0.86	0.862	0.863	0.865	0.867	0.869	0.87	0.872	0.873	0.875	0.876	0.878	0.879	0.881
Tochigi	0.771	0.774	0.776	0.778	0.782	0.787	0.792	0.798	0.805	0.812	0.818	0.823	0.829	0.834	0.839	0.844	0.848	0.851	0.853	0.855	0.857	0.859	0.861	0.862	0.864	0.866	0.868	0.87	0.872	0.873	0.874	0.876	0.877	0.879	0.881	0.882	0.884
Gunma	0.767	0.771	0.773	0.775	0.779	0.784	0.789	0.794	0.8	0.807	0.812	0.818	0.823	0.827	0.832	0.837	0.841	0.845	0.847	0.849	0.851	0.853	0.855	0.856	0.858	0.86	0.862	0.864	0.866	0.867	0.868	0.87	0.871	0.873	0.875	0.876	0.878
Saitama	0.763	0.767	0.769	0.772	0.776	0.781	0.786	0.791	0.798	0.804	0.81	0.815	0.82	0.824	0.827	0.831	0.834	0.837	0.839	0.84	0.842	0.844	0.845	0.847	0.848	0.85	0.852	0.853	0.855	0.856	0.857	0.859	0.86	0.862	0.863	0.865	0.866
Chiba	0.766	0.77	0.771	0.774	0.778	0.784	0.79	0.796	0.803	0.81	0.815	0.82	0.824	0.828	0.831	0.835	0.838	0.841	0.843	0.845	0.847	0.848	0.85	0.851	0.853	0.854	0.856	0.858	0.86	0.861	0.862	0.863	0.865	0.866	0.868	0.869	0.871
Tōkyō	0.824	0.828	0.832	0.835	0.839	0.842	0.846	0.849	0.853	0.858	0.862	0.867	0.871	0.875	0.878	0.881	0.885	0.887	0.89	0.892	0.893	0.895	0.897	0.899	0.901	0.902	0.905	0.906	0.908	0.909	0.911	0.912	0.914	0.915	0.917	0.918	0.92
Kanagawa	0.775	0.779	0.781	0.784	0.788	0.793	0.798	0.803	0.81	0.817	0.821	0.826	0.83	0.834	0.838	0.841	0.844	0.847	0.849	0.851	0.853	0.854	0.856	0.858	0.859	0.861	0.863	0.865	0.866	0.867	0.869	0.87	0.871	0.873	0.875	0.876	0.878
Niigata	0.761	0.765	0.768	0.77	0.774	0.778	0.783	0.788	0.794	0.8	0.805	0.811	0.816	0.821	0.825	0.83	0.835	0.839	0.843	0.845	0.847	0.849	0.851	0.853	0.854	0.856	0.858	0.86	0.862	0.863	0.865	0.866	0.868	0.87	0.871	0.873	0.875
Toyama	0.771	0.774	0.776	0.778	0.782	0.787	0.792	0.798	0.804	0.811	0.817	0.823	0.828	0.832	0.836	0.84	0.844	0.847	0.849	0.851	0.853	0.855	0.857	0.858	0.86	0.862	0.864	0.866	0.868	0.869	0.871	0.872	0.874	0.876	0.877	0.879	0.881
Ishikawa	0.766	0.77	0.773	0.776	0.78	0.786	0.791	0.796	0.802	0.809	0.815	0.82	0.826	0.831	0.836	0.84	0.843	0.846	0.848	0.85	0.852	0.854	0.856	0.857	0.859	0.861	0.863	0.865	0.867	0.868	0.869	0.871	0.873	0.874	0.876	0.878	0.88
Fukui	0.765	0.768	0.77	0.772	0.776	0.781	0.786	0.791	0.798	0.804	0.81	0.815	0.821	0.825	0.829	0.834	0.838	0.842	0.846	0.849	0.851	0.854	0.857	0.859	0.861	0.863	0.864	0.865	0.866	0.866	0.867	0.868	0.869	0.871	0.873	0.876	0.878
Yamanashi	0.763	0.767	0.77	0.772	0.775	0.78	0.785	0.79	0.796	0.803	0.808	0.813	0.818	0.822	0.827	0.832	0.837	0.841	0.844	0.846	0.848	0.85	0.852	0.853	0.855	0.857	0.859	0.861	0.863	0.864	0.865	0.867	0.868	0.87	0.872	0.874	0.875
Nagano	0.761	0.765	0.768	0.77	0.774	0.779	0.783	0.788	0.794	0.8	0.805	0.81	0.816	0.82	0.825	0.83	0.834	0.838	0.841	0.844	0.847	0.849	0.852	0.854	0.856	0.857	0.859	0.861	0.863	0.864	0.866	0.867	0.868	0.869	0.871	0.874	0.876
Gifu	0.764	0.768	0.77	0.772	0.776	0.781	0.787	0.792	0.799	0.806	0.811	0.817	0.822	0.826	0.831	0.835	0.838	0.841	0.843	0.845	0.847	0.849	0.85	0.852	0.854	0.856	0.857	0.859	0.861	0.862	0.864	0.865	0.867	0.868	0.87	0.872	0.873
Shizuoka	0.777	0.78	0.781	0.783	0.787	0.793	0.798	0.803	0.81	0.817	0.823	0.829	0.834	0.839	0.843	0.847	0.851	0.854	0.856	0.858	0.86	0.861	0.863	0.865	0.866	0.868	0.87	0.872	0.874	0.874	0.875	0.876	0.877	0.879	0.881	0.883	0.886
Aichi	0.786	0.79	0.792	0.794	0.797	0.803	0.808	0.813	0.82	0.828	0.833	0.839	0.845	0.849	0.852	0.856	0.859	0.862	0.864	0.866	0.868	0.87	0.871	0.873	0.875	0.877	0.878	0.88	0.882	0.883	0.885	0.886	0.887	0.888	0.89	0.893	0.895
Mie	0.77	0.774	0.776	0.778	0.782	0.788	0.793	0.798	0.805	0.812	0.818	0.824	0.83	0.834	0.839	0.843	0.846	0.849	0.851	0.853	0.855	0.857	0.859	0.86	0.862	0.864	0.866	0.868	0.87	0.871	0.873	0.874	0.875	0.877	0.879	0.881	0.883
Shiga	0.77	0.773	0.775	0.777	0.781	0.787	0.792	0.798	0.805	0.812	0.819	0.825	0.831	0.835	0.84	0.845	0.849	0.853	0.857	0.859	0.862	0.864	0.865	0.867	0.869	0.871	0.873	0.875	0.876	0.877	0.878	0.879	0.88	0.881	0.883	0.886	0.889
Kyōto	0.776	0.779	0.781	0.783	0.787	0.792	0.797	0.803	0.81	0.816	0.821	0.825	0.83	0.834	0.838	0.841	0.845	0.848	0.85	0.852	0.854	0.855	0.857	0.859	0.861	0.862	0.864	0.866	0.868	0.869	0.871	0.872	0.874	0.876	0.877	0.879	0.881
Ōsaka	0.788	0.791	0.793	0.795	0.799	0.804	0.809	0.814	0.821	0.827	0.832	0.836	0.841	0.845	0.848	0.852	0.855	0.858	0.86	0.861	0.863	0.865	0.866	0.868	0.869	0.871	0.873	0.875	0.877	0.878	0.879	0.88	0.882	0.884	0.885	0.887	0.888
Hyōgo	0.765	0.768	0.77	0.773	0.777	0.783	0.788	0.794	0.801	0.808	0.814	0.818	0.823	0.827	0.831	0.834	0.838	0.841	0.843	0.845	0.847	0.848	0.85	0.851	0.853	0.855	0.857	0.859	0.861	0.862	0.863	0.865	0.866	0.868	0.869	0.871	0.873
Nara	0.749	0.753	0.754	0.757	0.762	0.768	0.773	0.779	0.786	0.792	0.797	0.802	0.807	0.811	0.814	0.818	0.821	0.824	0.826	0.828	0.83	0.832	0.833	0.835	0.836	0.838	0.84	0.842	0.843	0.844	0.846	0.847	0.849	0.85	0.852	0.853	0.855
Wakayama	0.757	0.761	0.763	0.766	0.77	0.775	0.781	0.787	0.793	0.8	0.806	0.812	0.817	0.821	0.826	0.829	0.833	0.836	0.838	0.84	0.842	0.844	0.845	0.847	0.849	0.85	0.852	0.854	0.856	0.857	0.859	0.86	0.862	0.864	0.865	0.867	0.869

Appendix Table 14: Socio-Demographic Index values for all estimated GBD 2016 locations, 1980–2016

Location	1980	1981	1982	1983	1984	1985	1986	1987	1988	1989	1990	1991	1992	1993	1994	1995	1996	1997	1998	1999	2000	2001	2002	2003	2004	2005	2006	2007	2008	2009	2010	2011	2012	2013	2014	2015	2016
Tottori	0.747	0.751	0.753	0.755	0.759	0.764	0.769	0.774	0.78	0.786	0.791	0.797	0.803	0.807	0.812	0.817	0.822	0.826	0.83	0.832	0.835	0.837	0.84	0.843	0.845	0.847	0.849	0.851	0.853	0.854	0.856	0.857	0.858	0.859	0.861	0.864	0.866
Shimane	0.744	0.748	0.75	0.752	0.756	0.761	0.766	0.771	0.777	0.783	0.788	0.794	0.799	0.803	0.808	0.813	0.818	0.823	0.827	0.83	0.833	0.836	0.839	0.842	0.845	0.847	0.847	0.849	0.85	0.85	0.851	0.852	0.853	0.854	0.856	0.859	0.862
Okayama	0.762	0.765	0.767	0.769	0.773	0.779	0.784	0.79	0.797	0.804	0.809	0.815	0.821	0.825	0.83	0.835	0.839	0.843	0.845	0.847	0.849	0.851	0.853	0.855	0.857	0.859	0.861	0.863	0.865	0.866	0.867	0.869	0.871	0.872	0.874	0.876	0.878
Hiroshima	0.77	0.773	0.775	0.777	0.781	0.787	0.792	0.797	0.804	0.811	0.816	0.822	0.828	0.833	0.837	0.842	0.845	0.848	0.85	0.852	0.854	0.856	0.857	0.859	0.861	0.863	0.865	0.867	0.869	0.869	0.869	0.87	0.871	0.872	0.874	0.877	0.881
Yamaguchi	0.767	0.771	0.773	0.775	0.779	0.784	0.789	0.795	0.801	0.808	0.813	0.818	0.824	0.828	0.832	0.837	0.84	0.843	0.846	0.848	0.85	0.851	0.853	0.855	0.857	0.859	0.861	0.863	0.865	0.866	0.867	0.867	0.868	0.87	0.872	0.875	0.878
Tokushima	0.762	0.765	0.768	0.77	0.774	0.78	0.785	0.79	0.796	0.803	0.808	0.814	0.82	0.824	0.828	0.832	0.835	0.838	0.841	0.843	0.845	0.847	0.849	0.85	0.852	0.854	0.856	0.858	0.86	0.861	0.862	0.864	0.866	0.867	0.869	0.871	0.873
Kagawa	0.764	0.768	0.77	0.772	0.776	0.781	0.786	0.791	0.798	0.805	0.811	0.817	0.822	0.827	0.831	0.835	0.839	0.842	0.844	0.846	0.848	0.85	0.852	0.854	0.855	0.857	0.859	0.861	0.863	0.863	0.864	0.865	0.866	0.867	0.869	0.872	0.875
Ehime	0.757	0.761	0.764	0.767	0.771	0.776	0.781	0.786	0.792	0.799	0.804	0.809	0.815	0.819	0.823	0.828	0.832	0.835	0.837	0.839	0.841	0.843	0.845	0.847	0.849	0.851	0.852	0.855	0.856	0.857	0.859	0.861	0.862	0.864	0.866	0.867	0.869
Kōchi	0.746	0.749	0.751	0.753	0.757	0.763	0.768	0.774	0.78	0.787	0.792	0.796	0.801	0.805	0.81	0.815	0.819	0.823	0.825	0.827	0.829	0.831	0.833	0.835	0.837	0.839	0.84	0.843	0.844	0.845	0.847	0.848	0.85	0.852	0.853	0.855	0.857
Fukuoka	0.769	0.773	0.775	0.777	0.782	0.787	0.792	0.798	0.804	0.811	0.816	0.822	0.826	0.83	0.834	0.837	0.841	0.844	0.846	0.848	0.85	0.852	0.853	0.855	0.857	0.859	0.861	0.863	0.865	0.866	0.867	0.869	0.87	0.872	0.874	0.876	0.877
Saga	0.751	0.755	0.757	0.76	0.764	0.769	0.775	0.78	0.786	0.793	0.798	0.803	0.808	0.812	0.816	0.821	0.825	0.828	0.831	0.834	0.836	0.839	0.843	0.846	0.849	0.85	0.852	0.853	0.854	0.855	0.856	0.857	0.857	0.859	0.861	0.863	0.866
Nagasaki	0.749	0.753	0.755	0.757	0.761	0.767	0.772	0.777	0.783	0.79	0.794	0.799	0.804	0.808	0.813	0.818	0.822	0.826	0.829	0.832	0.834	0.837	0.839	0.841	0.843	0.845	0.847	0.848	0.849	0.849	0.849	0.85	0.851	0.852	0.854	0.857	0.859
Kumamoto	0.75	0.754	0.757	0.759	0.763	0.768	0.773	0.778	0.784	0.791	0.796	0.801	0.806	0.81	0.814	0.819	0.823	0.827	0.83	0.832	0.835	0.837	0.839	0.841	0.843	0.845	0.846	0.846	0.847	0.848	0.849	0.85	0.851	0.852	0.854	0.857	0.86
Ōita	0.761	0.765	0.768	0.771	0.775	0.78	0.785	0.789	0.795	0.802	0.807	0.812	0.817	0.821	0.825	0.83	0.834	0.838	0.841	0.843	0.845	0.847	0.849	0.85	0.852	0.854	0.856	0.858	0.86	0.86	0.861	0.862	0.863	0.865	0.867	0.87	0.872
Miyazaki	0.749	0.752	0.755	0.757	0.762	0.767	0.773	0.778	0.784	0.79	0.795	0.799	0.804	0.808	0.812	0.816	0.821	0.825	0.828	0.831	0.833	0.836	0.839	0.841	0.844	0.845	0.845	0.846	0.847	0.847	0.847	0.848	0.849	0.85	0.853	0.855	0.858
Kagoshima	0.744	0.748	0.751	0.754	0.759	0.765	0.77	0.775	0.782	0.788	0.793	0.799	0.804	0.808	0.813	0.818	0.822	0.826	0.83	0.832	0.834	0.837	0.84	0.842	0.844	0.846	0.846	0.847	0.848	0.849	0.85	0.851	0.852	0.853	0.855	0.858	0.861
Okinawa	0.73	0.734	0.736	0.738	0.743	0.75	0.756	0.762	0.769	0.777	0.782	0.787	0.793	0.797	0.802	0.808	0.812	0.816	0.819	0.821	0.823	0.825	0.828	0.832	0.835	0.836	0.836	0.837	0.837	0.837	0.837	0.838	0.838	0.84	0.842	0.845	0.848
Australasia	0.787	0.79	0.793	0.796	0.799	0.802	0.805	0.808	0.811	0.813	0.815	0.818	0.821	0.824	0.828	0.832	0.836	0.839	0.843	0.846	0.849	0.853	0.856	0.858	0.86	0.862	0.861	0.861	0.864	0.867	0.87	0.872	0.875	0.88	0.884	0.887	0.889
Australia	0.787	0.789	0.792	0.796	0.799	0.802	0.806	0.81	0.813	0.815	0.818	0.82	0.823	0.826	0.83	0.834	0.838	0.842	0.845	0.849	0.853	0.856	0.859	0.862	0.864	0.865	0.865	0.865	0.868	0.872	0.875	0.876	0.879	0.883	0.887	0.89	0.892
New Zealand	0.786	0.79	0.794	0.797	0.799	0.8	0.8	0.8	0.8	0.8	0.803	0.807	0.81	0.813	0.816	0.819	0.822	0.826	0.828	0.828	0.831	0.836	0.839	0.84	0.841	0.843	0.84	0.838	0.841	0.844	0.847	0.851	0.856	0.86	0.863	0.865	0.867
Western Europe	0.754	0.759	0.765	0.771	0.775	0.779	0.784	0.788	0.792	0.797	0.801	0.807	0.812	0.817	0.821	0.825	0.828	0.832	0.835	0.838	0.842	0.845	0.848	0.85	0.852	0.854	0.856	0.858	0.859	0.861	0.864	0.866	0.869	0.871	0.873	0.874	0.876
Andorra	0.845	0.848	0.85	0.852	0.855	0.857	0.859	0.862	0.864	0.866	0.868	0.87	0.872	0.873	0.875	0.876	0.878	0.881	0.884	0.887	0.889	0.891	0.892	0.894	0.896	0.897	0.899	0.901	0.902	0.904	0.906	0.907	0.909	0.91	0.912	0.913	0.915
Austria	0.801	0.803	0.807	0.812	0.816	0.818	0.82	0.822	0.824	0.827	0.83	0.832	0.836	0.838	0.841	0.844	0.846	0.849	0.851	0.854	0.857	0.86	0.862	0.864	0.867	0.869	0.871	0.874	0.876	0.878	0.88	0.882	0.885	0.887	0.889	0.89	0.892
Belgium	0.813	0.818	0.823	0.827	0.831	0.833	0.835	0.837	0.84	0.841	0.843	0.846	0.85	0.855	0.859	0.861	0.862	0.865	0.867	0.869	0.871	0.874	0.877	0.877	0.877	0.878	0.879	0.88	0.882	0.883	0.886	0.89	0.893	0.896	0.897	0.898	0.899
Cyprus	0.692	0.696	0.699	0.704	0.712	0.72	0.726	0.73	0.734	0.739	0.745	0.748	0.754	0.765	0.774	0.783	0.79	0.798	0.806	0.812	0.819	0.826	0.831	0.835	0.839	0.843	0.847	0.852	0.856	0.86	0.863	0.866	0.868	0.869	0.87	0.871	0.872
Denmark	0.866	0.867	0.869	0.871	0.873	0.876	0.879	0.88	0.879	0.879	0.88	0.88	0.88	0.881	0.882	0.885	0.889	0.893	0.895	0.897	0.9	0.903	0.905	0.905	0.906	0.906	0.907	0.908	0.909	0.911	0.914	0.919	0.922	0.925	0.925	0.925	0.925
Finland	0.815	0.816	0.818	0.821	0.826	0.832	0.836	0.837	0.838	0.84	0.841	0.841	0.842	0.843	0.845	0.849	0.852	0.856	0.86	0.863	0.867	0.871	0.874	0.876	0.878	0.88	0.883	0.886	0.888	0.89	0.892	0.896	0.9	0.904	0.906	0.907	0.907
France	0.743	0.749	0.758	0.765	0.769	0.773	0.777	0.783	0.788	0.793	0.799	0.805	0.812	0.817	0.821	0.823	0.826	0.829	0.832	0.833	0.836	0.84	0.843	0.846	0.848	0.849	0.85	0.853	0.855	0.856	0.858	0.861	0.863	0.865	0.867	0.868	0.869
Germany	0.801	0.804	0.807	0.809	0.812	0.814	0.817	0.82	0.822	0.826	0.829	0.832	0.835	0.838	0.841	0.843	0.845	0.847	0.85	0.852	0.854	0.857	0.859	0.86	0.862	0.864	0.866	0.869	0.871	0.873	0.875	0.877	0.88	0.882	0.884	0.886	0.889
Greece	0.699	0.708	0.714	0.722	0.731	0.739	0.745	0.751	0.756	0.761	0.765	0.77	0.775	0.779	0.783	0.787	0.792	0.796	0.801	0.806	0.81	0.815	0.82	0.825	0.829	0.834	0.838	0.843	0.847	0.85	0.852	0.853	0.853	0.852	0.852	0.852	0.853
Iceland	0.808	0.817	0.823	0.83	0.841	0.848	0.847	0.842	0.842	0.844	0.847	0.852	0.852	0.855	0.861	0.862	0.865	0.869	0.873	0.875	0.879	0.886	0.888	0.888	0.889	0.892	0.894	0.895	0.894	0.894	0.901	0.906	0.909	0.914	0.918	0.92	0.921
Ireland	0.684	0.694	0.705	0.717	0.727	0.733	0.74	0.75	0.759	0.764	0.768	0.775	0.784	0.792	0.798	0.802	0.806	0.811	0.818	0.826	0.832	0.836	0.842	0.848	0.856	0.861	0.861	0.861	0.863	0.864	0.867	0.871	0.874	0.877	0.879	0.883	0.885
Israel	0.691	0.698	0.7	0.7	0.702	0.708	0.716	0.723	0.727	0.731	0.736	0.743	0.748	0.753	0.757	0.761	0.764	0.766	0.767	0.769	0.772	0.775	0.777	0.777	0.779	0.781	0.784	0.786	0.786	0.788	0.791	0.794	0.796	0.797	0.798	0.799	0.801
Italy	0.748	0.755	0.761	0.767	0.772	0.776	0.781	0.785	0.789	0.794	0.798	0.803	0.807	0.81	0.814	0.818	0.821	0.824	0.828	0.831	0.834	0.837	0.84	0.843	0.846	0.848	0.85	0.853	0.855	0.857	0.858	0.86	0.862	0.863	0.864	0.865	0.867
Luxembourg	0.848	0.851	0.853	0.855	0.858	0.861	0.864	0.868	0.871	0.873	0.876	0.88	0.882	0.885	0.888	0.891	0.893	0.898	0.9	0.9	0.904	0.909	0.912	0.912	0.913	0.915	0.917	0.919	0.921	0.922	0.924	0.928	0.929	0.932	0.934	0.935	0.936
Malta	0.717	0.723	0.728	0.733	0.738	0.741	0.743	0.743	0.743	0.747	0.752	0.754	0.759	0.768	0.775	0.777	0.778	0.786	0.795	0.803	0.814	0.819	0.822	0.825	0.827	0.829	0.831	0.834	0.836	0.838	0.84	0.842	0.845	0.848	0.85	0.853	0.856
Netherlands	0.825	0.83	0.833	0.835	0.838	0.839	0.84	0.843	0.846	0.848	0.85	0.854	0.859	0.862	0.866	0.871	0.873	0.874	0.876	0.878	0.88	0.884	0.886	0.889	0.893	0.896	0.898	0.9	0.901	0.902	0.905	0.909	0.913	0.915	0.917	0.917	0.918
Norway	0.824	0.828	0.833	0.837	0.84	0.843	0.845	0.845	0.845	0.845	0.847	0.851	0.856	0.859	0.862	0.864	0.868	0.874	0.877	0.88	0.885	0.891	0.894	0.894	0.896	0.897	0.899	0.9	0.901	0.903	0.907	0.912	0.916	0.92	0.922	0.922	0.922
Portugal	0.61	0.618	0.626	0.634	0.643	0.651	0.658	0.665	0.672	0.68	0.687	0.694	0.701	0.708	0.714	0.719	0.725	0.73	0.735	0.74	0.746	0.751	0.755	0.759	0.763	0.766	0.77	0.773	0.777	0.779	0.782	0.785	0.787	0.789	0.791	0.794	0.796
Spain	0.67	0.68	0.689	0.698	0.705	0.713	0.721	0.727	0.733	0.739	0.746	0.752	0.759	0.764	0.77	0.775	0.78	0.786	0.791	0.796	0.801	0.806	0.811	0.816	0.82	0.824	0.828	0.833	0.836	0.838	0.84	0.842	0.843	0.844	0.845	0.847	0.848
Sweden	0.799	0.803	0.806	0.808	0.808	0.808	0.809	0.808	0.807	0.806	0.807	0.81	0.816	0.822	0.829	0.838	0.845	0.849	0.853	0.855	0.857	0.857	0.857	0.857	0.858	0.858	0.858	0.859	0.86	0.859	0.86	0.864	0.867	0.869	0.87	0.871	0.872
Switzerland	0.867	0.867	0.868	0.868	0.869	0.869	0.869	0.869	0.869	0.869	0.869	0.87	0.872	0.874	0.874	0.874	0.874	0.874	0.875	0.875	0.876	0.877	0.878	0.879	0.88	0.881	0.883	0.885	0.887	0.888	0.889	0.891	0.893	0.895	0.896	0.899	0.901
United Kingdom	0.711	0.717	0.722	0.727	0.732	0.737	0.742	0.746	0.753	0.758	0.763	0.769	0.775	0.781	0.787	0.793	0.797	0.802	0.808	0.815	0.822	0.827	0.83	0.831	0.834	0.837	0.838	0.84	0.842	0.843	0.846	0.849	0.853	0.857	0.86	0.862	0.863
Sweden except Stockholm	0.774	0.778	0.782	0.784	0.784	0.784	0.785	0.785	0.784	0.783	0.784	0.788	0.794	0.801	0.809	0.819	0.826	0.831	0.834	0.837	0.839	0.84	0.839	0.84	0.841	0.841	0.841	0.843	0.845	0.844	0.846	0.85	0.854	0.856	0.858	0.86	0.861
Stockholm	0.866	0.868	0.87	0.871	0.873	0.875	0.877	0.879	0.88	0.879	0.879	0.882	0.885	0.889	0.891	0.892	0.893	0.895	0.897	0.899	0.901	0.903	0.905	0.906	0.908	0.909	0.908	0.907	0.906	0.904	0.904	0.905	0.906	0.906	0.905	0.905	0.905
Northern Ireland	0.718	0.726	0.732	0.737	0.742	0.747	0.752	0.759	0.768	0.775	0.782	0.788	0.795	0.802	0.807	0.811	0.815	0.82	0.826	0.832	0.838	0.842	0.846	0.848	0.85	0.852	0.852	0.851	0.853	0.855	0.856	0.857	0.859	0.863	0.866	0.87	0.872
Scotland	0.706	0.712	0.718	0.724	0.728	0.732	0.737	0.742	0.748	0.752	0.755	0.759	0.764	0.77	0.775	0.779	0.782	0.786	0.792	0.797	0.801	0.805	0.809	0.811	0.813	0.815	0.816	0.816	0.818	0.821	0.823	0.826	0.83	0.832	0.834	0.835	0.837
Wales	0.681	0.686	0.692	0.697	0.702	0.708	0.713	0.718	0.726	0.733	0.737	0.742	0.749	0.755	0.762	0.767	0.772	0.778	0.785	0.792	0.8	0.807	0.811	0.813	0.816	0.819	0.821	0.822	0.825	0.827	0.829	0.833	0.837	0.842	0.845	0.847	0.848
England	0.712	0.718	0.723	0.729	0.733	0.738	0.743	0.747	0.753	0.759	0.765	0.771	0.777	0.783	0.789	0.794	0.799	0.804	0.81	0.817	0.824	0.829	0.832	0.834	0.837	0.839	0.841	0.842	0.844	0.846	0.848	0.851	0.855	0.86	0.863	0.865	0.866
Darlington	0.695	0.701	0.708	0.714	0.719	0.725	0.731	0.735	0.741	0.744	0.746	0.75	0.756	0.764	0.772	0.779	0.785	0.791	0.796	0.802	0.808	0.812	0.813	0.814	0.817	0.82	0.823	0.824	0.824	0.825	0.831	0.836	0.841	0.846	0.848	0.848	0.849
Northumberland	0.683	0.689	0.695	0.701	0.707	0.713	0.72	0.726	0.733	0.738	0.742	0.746	0.75	0.756	0.762	0.769	0.775	0.781	0.787	0.792	0.797	0.8	0.802	0.804	0.806	0.808	0.81	0.812	0.815	0.817	0.819	0.821	0.825	0.828	0.83	0.831	0.832
Stockton-on-Tees	0.692	0.698	0.703	0.708	0.714	0.721	0.727	0.733	0.74	0.745	0.748	0.753	0.759	0.766	0.774	0.78	0.786	0.792	0.798	0.806	0.812	0.815	0.817	0.818	0.82	0.822	0.824	0.825	0.827	0.829	0.831	0.833	0.835	0.84	0.843	0.846	0.848
Newcastle upon Tyne	0.705	0.711	0.717	0.722	0.726	0.731	0.737	0.743	0.75	0.756	0.763	0.771	0.78	0.787	0.792	0.798	0.802	0.806	0.811	0.819	0.827	0.833	0.84	0.846	0.852	0.858	0.861	0.865	0.868	0.871	0.873	0.876	0.879	0.882	0.885	0.887	0.889
North Tyneside	0.682	0.687	0.692	0.697	0.701	0.706	0.711	0.718	0.725	0.731	0.737	0.742	0.749	0.756	0.762	0.768	0.772	0.778	0.784	0.79	0.797	0.802	0.805	0.807	0.811	0.816	0.82	0.824	0.827	0.828	0.831	0.834	0.837	0.84	0.842	0.844	0.846
Redcar and Cleveland	0.659	0.665	0.67	0.676	0.68	0.686	0.691	0.697	0.704	0.71	0.714	0.718	0.723	0.727	0.733	0.739	0.744	0.75	0.757	0.766	0.774	0.779	0.782	0.783	0.786	0.789	0.791	0.793	0.795	0.795	0.796	0.797	0.802	0.808	0.813	0.816	0.819
County Durham	0.673	0.679	0.685	0.69	0.696	0.702	0.708	0.713	0.719	0.724	0.728	0.733	0.74	0.746	0.753	0.759	0.764	0.771	0.777	0.784	0.791	0.796	0.799	0.8	0.803	0.805	0.807	0.809	0.812	0.813	0.816	0.818	0.822	0.827	0.83	0.832	0.833
Gateshead	0.687	0.692	0.698	0.703	0.708	0.713	0.719	0.725	0.732	0.738	0.744	0.749	0.755	0.761	0.768	0.774	0.779	0.785	0.791	0.797	0.803	0.808	0.811	0.814	0.818	0.821	0.824	0.826	0.828	0.83	0.833	0.837	0.84	0.844	0.847	0.849	0.851

Appendix Table 14: Socio-Demographic Index values for all estimated GBD 2016 locations, 1980–2016

Location	1980	1981	1982	1983	1984	1985	1986	1987	1988	1989	1990	1991	1992	1993	1994	1995	1996	1997	1998	1999	2000	2001	2002	2003	2004	2005	2006	2007	2008	2009	2010	2011	2012	2013	2014	2015	2016
Middlesbrough	0.668	0.674	0.68	0.686	0.69	0.695	0.699	0.705	0.713	0.72	0.727	0.735	0.741	0.748	0.755	0.761	0.767	0.772	0.778	0.785	0.791	0.794	0.795	0.795	0.8	0.804	0.808	0.813	0.816	0.817	0.818	0.819	0.821	0.824	0.826	0.827	0.829
South Tyneside	0.657	0.662	0.666	0.669	0.672	0.675	0.68	0.686	0.694	0.701	0.707	0.713	0.719	0.725	0.731	0.736	0.741	0.746	0.753	0.761	0.768	0.775	0.78	0.784	0.789	0.793	0.796	0.799	0.802	0.803	0.805	0.807	0.81	0.814	0.817	0.818	0.82
Sunderland	0.676	0.683	0.689	0.694	0.7	0.706	0.712	0.718	0.725	0.731	0.736	0.742	0.748	0.754	0.761	0.767	0.772	0.778	0.785	0.791	0.798	0.804	0.809	0.812	0.816	0.82	0.823	0.826	0.829	0.83	0.831	0.832	0.835	0.838	0.841	0.844	0.845
Hartlepool	0.672	0.678	0.683	0.687	0.691	0.695	0.7	0.704	0.711	0.718	0.723	0.728	0.734	0.74	0.746	0.753	0.759	0.765	0.77	0.777	0.782	0.785	0.787	0.788	0.79	0.793	0.795	0.799	0.802	0.805	0.807	0.81	0.814	0.819	0.82	0.821	0.821
Cheshire East	0.726	0.732	0.737	0.742	0.747	0.753	0.758	0.764	0.769	0.774	0.778	0.783	0.788	0.795	0.802	0.809	0.815	0.821	0.826	0.832	0.837	0.842	0.845	0.845	0.847	0.848	0.848	0.847	0.848	0.851	0.854	0.858	0.863	0.869	0.872	0.873	0.874
Stockport	0.702	0.708	0.714	0.719	0.724	0.73	0.734	0.74	0.747	0.753	0.757	0.763	0.77	0.778	0.784	0.789	0.794	0.8	0.806	0.813	0.82	0.825	0.828	0.829	0.831	0.833	0.835	0.836	0.837	0.838	0.839	0.841	0.844	0.849	0.853	0.855	0.857
Trafford	0.726	0.732	0.737	0.741	0.745	0.75	0.755	0.76	0.767	0.772	0.777	0.784	0.791	0.799	0.806	0.812	0.816	0.821	0.828	0.835	0.841	0.846	0.849	0.851	0.854	0.855	0.855	0.856	0.858	0.86	0.864	0.868	0.872	0.877	0.88	0.882	0.883
Cheshire West and Chester	0.714	0.72	0.726	0.731	0.736	0.741	0.747	0.751	0.756	0.761	0.766	0.773	0.78	0.787	0.792	0.795	0.796	0.799	0.803	0.812	0.822	0.831	0.837	0.839	0.841	0.843	0.845	0.846	0.847	0.849	0.852	0.857	0.862	0.866	0.868	0.869	0.869
Sefton	0.682	0.688	0.693	0.698	0.704	0.709	0.715	0.72	0.725	0.73	0.734	0.74	0.747	0.754	0.761	0.767	0.771	0.777	0.783	0.789	0.795	0.8	0.803	0.805	0.809	0.811	0.812	0.813	0.813	0.814	0.816	0.818	0.82	0.823	0.824	0.825	0.826
Lancashire	0.694	0.7	0.705	0.711	0.716	0.721	0.727	0.732	0.738	0.744	0.749	0.755	0.761	0.768	0.775	0.781	0.786	0.792	0.798	0.804	0.81	0.815	0.817	0.819	0.822	0.824	0.826	0.827	0.829	0.83	0.832	0.834	0.838	0.842	0.845	0.847	0.848
Cumbria	0.702	0.707	0.713	0.718	0.723	0.73	0.736	0.741	0.747	0.751	0.754	0.757	0.763	0.77	0.777	0.783	0.789	0.794	0.8	0.804	0.809	0.812	0.814	0.815	0.818	0.82	0.821	0.821	0.823	0.825	0.828	0.832	0.837	0.842	0.845	0.846	0.848
Bolton	0.678	0.684	0.69	0.695	0.701	0.707	0.713	0.719	0.725	0.73	0.735	0.741	0.747	0.754	0.761	0.766	0.77	0.774	0.78	0.786	0.791	0.795	0.797	0.798	0.8	0.8	0.8	0.8	0.802	0.804	0.807	0.81	0.814	0.819	0.821	0.823	0.824
Wirral	0.677	0.682	0.687	0.692	0.696	0.7	0.705	0.71	0.716	0.722	0.727	0.733	0.739	0.746	0.753	0.76	0.765	0.771	0.777	0.783	0.787	0.79	0.791	0.792	0.795	0.797	0.798	0.798	0.799	0.8	0.803	0.808	0.812	0.817	0.819	0.82	0.82
Bury	0.683	0.688	0.693	0.697	0.7	0.704	0.709	0.714	0.72	0.727	0.733	0.739	0.747	0.754	0.761	0.766	0.77	0.774	0.78	0.787	0.793	0.797	0.799	0.8	0.803	0.806	0.807	0.806	0.806	0.805	0.808	0.812	0.818	0.824	0.828	0.83	0.831
St Helens	0.67	0.675	0.681	0.686	0.691	0.697	0.703	0.709	0.715	0.72	0.722	0.725	0.731	0.738	0.745	0.75	0.755	0.76	0.766	0.772	0.78	0.786	0.79	0.792	0.796	0.799	0.801	0.804	0.806	0.808	0.81	0.812	0.816	0.822	0.826	0.828	0.83
Warrington	0.719	0.724	0.73	0.735	0.741	0.747	0.753	0.759	0.766	0.771	0.774	0.777	0.783	0.789	0.796	0.802	0.807	0.812	0.817	0.824	0.83	0.836	0.841	0.844	0.847	0.85	0.852	0.852	0.852	0.854	0.858	0.862	0.867	0.873	0.875	0.877	0.878
Oldham	0.669	0.674	0.679	0.684	0.689	0.694	0.699	0.704	0.71	0.715	0.72	0.725	0.731	0.738	0.743	0.747	0.751	0.754	0.758	0.763	0.769	0.773	0.775	0.776	0.778	0.78	0.782	0.784	0.787	0.789	0.792	0.796	0.8	0.804	0.806	0.807	0.808
Rochdale	0.668	0.673	0.679	0.684	0.688	0.694	0.7	0.705	0.712	0.716	0.72	0.725	0.731	0.737	0.744	0.75	0.755	0.76	0.767	0.773	0.779	0.783	0.784	0.785	0.787	0.788	0.788	0.788	0.789	0.791	0.795	0.8	0.805	0.81	0.813	0.814	0.815
Wigan	0.671	0.676	0.682	0.687	0.692	0.698	0.704	0.71	0.716	0.722	0.727	0.733	0.739	0.746	0.752	0.758	0.763	0.769	0.774	0.779	0.785	0.789	0.791	0.793	0.795	0.797	0.798	0.799	0.8	0.801	0.804	0.807	0.811	0.816	0.82	0.822	0.824
Halton	0.69	0.695	0.7	0.704	0.707	0.711	0.715	0.72	0.727	0.734	0.739	0.745	0.752	0.759	0.767	0.773	0.778	0.782	0.786	0.792	0.8	0.806	0.81	0.811	0.812	0.814	0.816	0.818	0.82	0.822	0.827	0.832	0.836	0.842	0.847	0.85	0.853
Liverpool	0.688	0.693	0.698	0.702	0.705	0.709	0.712	0.716	0.722	0.729	0.738	0.75	0.76	0.767	0.773	0.777	0.781	0.787	0.794	0.803	0.812	0.818	0.824	0.83	0.836	0.839	0.842	0.844	0.848	0.852	0.855	0.859	0.862	0.865	0.867	0.868	0.87
Tameside	0.672	0.678	0.683	0.688	0.692	0.697	0.702	0.707	0.713	0.719	0.724	0.73	0.737	0.744	0.751	0.756	0.761	0.766	0.771	0.778	0.786	0.792	0.795	0.797	0.8	0.802	0.801	0.801	0.8	0.8	0.802	0.805	0.809	0.813	0.816	0.818	0.82
Salford	0.691	0.696	0.701	0.706	0.71	0.714	0.719	0.723	0.728	0.732	0.737	0.744	0.752	0.761	0.769	0.775	0.78	0.784	0.791	0.799	0.807	0.814	0.818	0.821	0.825	0.828	0.831	0.832	0.834	0.835	0.839	0.843	0.848	0.853	0.856	0.859	0.861
Blackburn with Darwen	0.669	0.675	0.68	0.685	0.689	0.694	0.698	0.702	0.709	0.715	0.72	0.726	0.733	0.739	0.745	0.75	0.755	0.76	0.765	0.77	0.775	0.779	0.781	0.782	0.784	0.786	0.786	0.787	0.791	0.794	0.798	0.802	0.807	0.812	0.816	0.818	0.82
Knowsley	0.665	0.672	0.678	0.683	0.689	0.694	0.7	0.704	0.71	0.715	0.72	0.727	0.733	0.739	0.744	0.75	0.755	0.762	0.768	0.774	0.781	0.787	0.791	0.792	0.795	0.799	0.803	0.807	0.811	0.813	0.816	0.819	0.822	0.828	0.832	0.834	0.837
Blackpool	0.667	0.673	0.679	0.685	0.688	0.69	0.691	0.693	0.699	0.704	0.711	0.722	0.732	0.74	0.746	0.751	0.754	0.759	0.766	0.773	0.778	0.782	0.782	0.78	0.782	0.786	0.788	0.788	0.787	0.788	0.792	0.796	0.799	0.801	0.803	0.806	0.809
Manchester	0.715	0.721	0.726	0.731	0.734	0.739	0.744	0.749	0.755	0.761	0.768	0.775	0.781	0.789	0.796	0.801	0.806	0.812	0.819	0.829	0.839	0.847	0.853	0.857	0.862	0.866	0.869	0.872	0.875	0.878	0.882	0.884	0.888	0.892	0.895	0.897	0.899
North Yorkshire	0.707	0.712	0.718	0.724	0.73	0.736	0.742	0.747	0.754	0.759	0.762	0.765	0.769	0.774	0.78	0.785	0.79	0.796	0.802	0.808	0.815	0.819	0.821	0.822	0.826	0.829	0.831	0.833	0.834	0.835	0.838	0.841	0.844	0.848	0.849	0.849	0.85
East Riding of Yorkshire	0.696	0.702	0.708	0.714	0.72	0.724	0.727	0.726	0.73	0.738	0.746	0.752	0.758	0.765	0.771	0.777	0.782	0.788	0.793	0.799	0.804	0.808	0.81	0.811	0.815	0.818	0.819	0.821	0.823	0.825	0.827	0.828	0.831	0.834	0.836	0.837	0.839
York	0.729	0.735	0.74	0.745	0.749	0.753	0.756	0.76	0.766	0.773	0.781	0.79	0.799	0.804	0.81	0.815	0.819	0.824	0.831	0.836	0.842	0.848	0.854	0.86	0.865	0.87	0.873	0.877	0.88	0.881	0.883	0.885	0.886	0.888	0.89	0.892	0.894
North East Lincolnshire	0.693	0.698	0.703	0.706	0.709	0.714	0.721	0.728	0.736	0.74	0.743	0.748	0.755	0.762	0.769	0.773	0.776	0.779	0.784	0.789	0.795	0.801	0.802	0.802	0.803	0.805	0.808	0.813	0.817	0.822	0.825	0.828	0.831	0.833	0.834	0.834	0.834
Calderdale	0.688	0.694	0.699	0.704	0.709	0.716	0.722	0.729	0.736	0.741	0.746	0.75	0.755	0.761	0.767	0.773	0.777	0.783	0.788	0.795	0.801	0.805	0.806	0.807	0.81	0.814	0.816	0.818	0.82	0.821	0.824	0.828	0.833	0.839	0.842	0.845	0.847
North Lincolnshire	0.691	0.696	0.701	0.706	0.71	0.715	0.72	0.724	0.731	0.736	0.741	0.746	0.751	0.757	0.763	0.769	0.775	0.781	0.787	0.792	0.796	0.8	0.802	0.802	0.803	0.804	0.805	0.806	0.809	0.812	0.816	0.821	0.826	0.831	0.833	0.834	0.835
Bradford	0.669	0.675	0.681	0.686	0.69	0.696	0.702	0.707	0.714	0.72	0.726	0.732	0.738	0.744	0.75	0.755	0.759	0.763	0.767	0.773	0.779	0.784	0.786	0.787	0.79	0.792	0.795	0.798	0.802	0.805	0.808	0.81	0.812	0.815	0.817	0.818	0.819
Kirklees	0.676	0.682	0.688	0.694	0.699	0.705	0.711	0.716	0.723	0.729	0.735	0.741	0.747	0.753	0.759	0.763	0.768	0.772	0.778	0.785	0.79	0.795	0.796	0.797	0.8	0.803	0.805	0.807	0.81	0.812	0.814	0.817	0.821	0.825	0.828	0.83	0.832
Leeds	0.707	0.713	0.719	0.724	0.729	0.734	0.739	0.743	0.75	0.756	0.763	0.771	0.778	0.785	0.791	0.796	0.802	0.807	0.814	0.822	0.83	0.836	0.842	0.847	0.852	0.857	0.861	0.865	0.869	0.869	0.869	0.868	0.869	0.872	0.874	0.876	0.878
Sheffield	0.702	0.707	0.713	0.718	0.723	0.727	0.732	0.737	0.743	0.748	0.753	0.759	0.764	0.77	0.776	0.781	0.786	0.792	0.798	0.805	0.812	0.818	0.823	0.826	0.831	0.835	0.838	0.843	0.848	0.851	0.853	0.855	0.857	0.861	0.863	0.865	0.867
Wakefield	0.68	0.685	0.691	0.696	0.701	0.706	0.711	0.716	0.723	0.728	0.733	0.738	0.744	0.75	0.756	0.761	0.766	0.771	0.777	0.784	0.792	0.797	0.8	0.801	0.804	0.806	0.807	0.808	0.809	0.81	0.812	0.815	0.82	0.825	0.828	0.831	0.833
Rotherham	0.667	0.673	0.678	0.684	0.689	0.694	0.699	0.704	0.71	0.715	0.72	0.725	0.731	0.737	0.743	0.749	0.753	0.758	0.763	0.769	0.776	0.781	0.785	0.788	0.791	0.794	0.795	0.797	0.799	0.801	0.804	0.807	0.809	0.813	0.816	0.818	0.82
Doncaster	0.663	0.668	0.674	0.679	0.683	0.689	0.694	0.7	0.707	0.712	0.716	0.721	0.725	0.73	0.736	0.741	0.745	0.75	0.756	0.763	0.769	0.774	0.777	0.779	0.782	0.785	0.787	0.79	0.793	0.796	0.801	0.805	0.809	0.813	0.816	0.818	0.819
Kingston upon Hull, City of	0.675	0.68	0.685	0.688	0.69	0.694	0.7	0.709	0.718	0.721	0.725	0.731	0.74	0.747	0.754	0.759	0.764	0.77	0.776	0.783	0.791	0.797	0.8	0.803	0.808	0.812	0.817	0.821	0.825	0.826	0.825	0.825	0.827	0.831	0.835	0.838	0.84
Barnsley	0.66	0.665	0.671	0.676	0.681	0.687	0.692	0.697	0.703	0.708	0.712	0.718	0.724	0.73	0.737	0.742	0.747	0.753	0.76	0.766	0.772	0.776	0.778	0.78	0.782	0.784	0.786	0.789	0.791	0.792	0.794	0.797	0.801	0.806	0.81	0.812	0.814
Northamptonshire	0.698	0.704	0.71	0.716	0.72	0.725	0.73	0.734	0.74	0.746	0.751	0.757	0.764	0.771	0.777	0.783	0.788	0.793	0.799	0.805	0.811	0.816	0.818	0.819	0.821	0.824	0.825	0.827	0.828	0.83	0.833	0.835	0.839	0.843	0.845	0.845	0.846
Leicestershire	0.705	0.711	0.717	0.723	0.728	0.733	0.739	0.744	0.75	0.756	0.76	0.765	0.77	0.776	0.782	0.787	0.792	0.798	0.804	0.81	0.816	0.821	0.823	0.826	0.829	0.833	0.836	0.839	0.842	0.843	0.845	0.847	0.85	0.854	0.857	0.859	0.861
Lincolnshire	0.687	0.693	0.699	0.705	0.71	0.716	0.722	0.727	0.733	0.738	0.741	0.744	0.749	0.755	0.761	0.768	0.773	0.779	0.784	0.79	0.796	0.802	0.805	0.808	0.81	0.811	0.811	0.811	0.812	0.813	0.816	0.819	0.823	0.828	0.831	0.832	0.833
Rutland	0.703	0.709	0.715	0.721	0.728	0.736	0.744	0.751	0.76	0.765	0.771	0.772	0.776	0.784	0.79	0.796	0.801	0.806	0.811	0.815	0.82	0.814	0.802	0.782	0.776	0.788	0.797	0.804	0.809	0.817	0.831	0.839	0.839	0.839	0.838	0.839	0.84
Derby	0.689	0.696	0.703	0.71	0.716	0.723	0.728	0.733	0.738	0.743	0.748	0.756	0.764	0.773	0.782	0.789	0.794	0.798	0.803	0.81	0.816	0.822	0.826	0.829	0.834	0.837	0.838	0.841	0.844	0.845	0.846	0.846	0.849	0.855	0.858	0.861	0.864
Derbyshire	0.683	0.689	0.695	0.7	0.705	0.711	0.716	0.722	0.728	0.733	0.737	0.742	0.747	0.754	0.76	0.766	0.772	0.777	0.784	0.79	0.796	0.801	0.803	0.805	0.807	0.81	0.812	0.814	0.815	0.816	0.818	0.822	0.826	0.832	0.836	0.839	0.841
Nottinghamshire	0.68	0.686	0.692	0.698	0.703	0.709	0.714	0.719	0.725	0.731	0.735	0.74	0.745	0.751	0.757	0.762	0.766	0.771	0.778	0.786	0.794	0.8	0.805	0.807	0.811	0.814	0.816	0.817	0.819	0.819	0.82	0.821	0.824	0.828	0.831	0.834	0.835
Nottingham	0.705	0.711	0.718	0.724	0.729	0.733	0.737	0.741	0.746	0.753	0.76	0.769	0.777	0.785	0.793	0.801	0.807	0.815	0.822	0.828	0.834	0.84	0.846	0.85	0.855	0.86	0.863	0.867	0.87	0.871	0.871	0.87	0.871	0.873	0.875	0.877	0.878
Leicester	0.674	0.68	0.686	0.692	0.696	0.702	0.707	0.711	0.717	0.724	0.733	0.744	0.753	0.761	0.768	0.774	0.778	0.781	0.786	0.793	0.8	0.807	0.811	0.814	0.818	0.822	0.825	0.828	0.831	0.834	0.836	0.837	0.84	0.843	0.845	0.847	0.849
Warwickshire	0.714	0.72	0.725	0.731	0.735	0.741	0.746	0.75	0.756	0.761	0.766	0.772	0.778	0.784	0.791	0.796	0.801	0.806	0.812	0.819	0.825	0.83	0.833	0.836	0.838	0.84	0.841	0.842	0.844	0.846	0.849	0.852	0.856	0.861	0.863	0.864	0.865
Herefordshire, County of	0.692	0.698	0.704	0.709	0.715	0.72	0.725	0.729	0.733	0.738	0.741	0.746	0.752	0.759	0.765	0.77	0.775	0.78	0.786	0.793	0.801	0.807	0.809	0.811	0.813	0.815	0.815	0.814	0.815	0.816	0.821	0.828	0.836	0.842	0.846	0.848	0.85
Solihull	0.723	0.728	0.733	0.738	0.742	0.746	0.751	0.755	0.761	0.765	0.768	0.773	0.779	0.787	0.794	0.801	0.807	0.814	0.82	0.826	0.83	0.834	0.836	0.839	0.842	0.846	0.849	0.851	0.852	0.851	0.852	0.853	0.857	0.861	0.863	0.864	0.864
Shropshire	0.694	0.7	0.705	0.71	0.716	0.722	0.727	0.733	0.738	0.743	0.746	0.75	0.755	0.761	0.768	0.773	0.778	0.783	0.79	0.796	0.802	0.805	0.808	0.81	0.814	0.817	0.818	0.818	0.82	0.824	0.829	0.834	0.839	0.843	0.845	0.846	0.847
Worcestershire	0.688	0.694	0.699	0.705	0.71	0.716	0.721	0.727	0.733	0.738	0.742	0.747	0.753	0.759	0.765	0.77	0.775	0.78	0.787	0.796	0.804	0.81	0.813	0.815	0.819	0.821	0.822	0.823	0.824	0.825	0.828	0.832	0.836	0.841	0.844	0.846	0.847
Staffordshire	0.69	0.695	0.701	0.706	0.711	0.716	0.721	0.727	0.733	0.739	0.744	0.749	0.756	0.762	0.768	0.773	0.778	0.783	0.79	0.796	0.802	0.806	0.809	0.81	0.813	0.816	0.818	0.821	0.824	0.826	0.829	0.832	0.835	0.839	0.841	0.842	0.843

Appendix Table 14: Socio-Demographic Index values for all estimated GBD 2016 locations, 1980–2016

Location	1980	1981	1982	1983	1984	1985	1986	1987	1988	1989	1990	1991	1992	1993	1994	1995	1996	1997	1998	1999	2000	2001	2002	2003	2004	2005	2006	2007	2008	2009	2010	2011	2012	2013	2014	2015	2016
Dudley	0.677	0.682	0.688	0.693	0.698	0.703	0.708	0.714	0.721	0.726	0.731	0.736	0.741	0.747	0.753	0.757	0.762	0.767	0.772	0.778	0.784	0.789	0.792	0.794	0.797	0.8	0.802	0.804	0.806	0.806	0.807	0.808	0.81	0.814	0.816	0.817	0.818
Coventry	0.697	0.702	0.707	0.711	0.715	0.719	0.724	0.729	0.735	0.742	0.75	0.757	0.764	0.771	0.777	0.782	0.787	0.792	0.797	0.805	0.812	0.819	0.823	0.826	0.83	0.833	0.835	0.836	0.837	0.838	0.84	0.843	0.848	0.855	0.86	0.863	0.866
Telford and Wrekin	0.698	0.704	0.71	0.716	0.721	0.726	0.731	0.736	0.742	0.747	0.752	0.757	0.763	0.769	0.775	0.78	0.785	0.792	0.798	0.804	0.81	0.815	0.817	0.817	0.818	0.818	0.818	0.818	0.821	0.822	0.824	0.828	0.833	0.838	0.842	0.845	0.846
Stoke-on-Trent	0.67	0.676	0.681	0.686	0.691	0.697	0.701	0.705	0.71	0.716	0.724	0.733	0.742	0.749	0.756	0.761	0.766	0.772	0.777	0.783	0.788	0.79	0.789	0.788	0.789	0.791	0.791	0.79	0.793	0.796	0.8	0.804	0.809	0.815	0.819	0.823	0.827
Walsall	0.667	0.672	0.677	0.682	0.686	0.691	0.697	0.702	0.709	0.714	0.718	0.722	0.728	0.735	0.741	0.744	0.746	0.747	0.752	0.759	0.766	0.771	0.773	0.773	0.776	0.779	0.781	0.784	0.786	0.788	0.792	0.796	0.8	0.804	0.807	0.808	0.809
Wolverhampton	0.678	0.684	0.689	0.693	0.698	0.703	0.707	0.712	0.717	0.723	0.728	0.734	0.741	0.747	0.753	0.757	0.761	0.766	0.772	0.78	0.788	0.795	0.799	0.8	0.803	0.806	0.807	0.808	0.809	0.81	0.813	0.816	0.821	0.825	0.827	0.829	0.83
Birmingham	0.68	0.686	0.691	0.696	0.7	0.705	0.71	0.714	0.72	0.727	0.733	0.742	0.75	0.757	0.763	0.767	0.771	0.775	0.781	0.788	0.795	0.801	0.805	0.808	0.812	0.817	0.819	0.822	0.826	0.829	0.832	0.833	0.836	0.84	0.843	0.845	0.847
Sandwell	0.666	0.671	0.677	0.682	0.686	0.691	0.696	0.701	0.708	0.714	0.719	0.725	0.732	0.739	0.745	0.749	0.753	0.758	0.763	0.768	0.773	0.778	0.781	0.782	0.785	0.787	0.788	0.789	0.79	0.791	0.794	0.797	0.803	0.808	0.812	0.813	0.815
Bedford	0.711	0.717	0.723	0.728	0.733	0.739	0.745	0.75	0.757	0.764	0.771	0.776	0.781	0.784	0.788	0.791	0.795	0.801	0.807	0.814	0.822	0.828	0.831	0.833	0.836	0.839	0.841	0.842	0.843	0.844	0.845	0.846	0.848	0.85	0.85	0.851	0.851
Central Bedfordshire	0.709	0.715	0.72	0.725	0.73	0.735	0.741	0.747	0.754	0.759	0.765	0.77	0.775	0.781	0.788	0.794	0.8	0.806	0.811	0.815	0.818	0.82	0.822	0.823	0.827	0.829	0.83	0.831	0.831	0.831	0.832	0.834	0.838	0.844	0.848	0.85	0.853
Suffolk	0.703	0.709	0.715	0.72	0.725	0.731	0.736	0.74	0.746	0.752	0.757	0.763	0.768	0.773	0.78	0.786	0.791	0.797	0.802	0.807	0.811	0.814	0.814	0.814	0.817	0.82	0.822	0.823	0.824	0.825	0.827	0.83	0.833	0.837	0.838	0.839	0.84
Hertfordshire	0.731	0.737	0.743	0.748	0.753	0.757	0.76	0.764	0.769	0.775	0.781	0.788	0.795	0.801	0.806	0.812	0.816	0.822	0.828	0.836	0.843	0.848	0.851	0.853	0.855	0.858	0.858	0.859	0.861	0.862	0.864	0.866	0.869	0.874	0.876	0.878	0.879
Essex	0.701	0.706	0.712	0.717	0.722	0.726	0.73	0.734	0.739	0.745	0.75	0.757	0.763	0.768	0.774	0.779	0.784	0.79	0.796	0.802	0.808	0.812	0.814	0.816	0.819	0.823	0.825	0.828	0.83	0.83	0.831	0.833	0.836	0.84	0.842	0.844	0.845
Cambridgeshire	0.728	0.734	0.74	0.745	0.749	0.755	0.76	0.765	0.771	0.777	0.783	0.789	0.796	0.801	0.807	0.814	0.82	0.827	0.833	0.838	0.844	0.849	0.854	0.856	0.86	0.862	0.862	0.862	0.862	0.863	0.867	0.87	0.874	0.878	0.88	0.881	0.882
Thurrock	0.702	0.708	0.711	0.715	0.717	0.72	0.723	0.726	0.733	0.741	0.75	0.759	0.765	0.77	0.775	0.78	0.783	0.787	0.793	0.799	0.806	0.811	0.812	0.812	0.813	0.813	0.813	0.814	0.816	0.819	0.821	0.822	0.823	0.825	0.826	0.825	0.824
Norfolk	0.702	0.708	0.714	0.719	0.723	0.728	0.733	0.737	0.743	0.749	0.754	0.759	0.764	0.77	0.775	0.781	0.786	0.792	0.798	0.804	0.81	0.814	0.817	0.818	0.821	0.824	0.826	0.828	0.83	0.831	0.832	0.833	0.836	0.841	0.844	0.846	0.848
Southend-on-Sea	0.683	0.688	0.693	0.696	0.699	0.703	0.709	0.716	0.724	0.731	0.736	0.742	0.749	0.757	0.765	0.772	0.778	0.785	0.79	0.794	0.796	0.797	0.798	0.799	0.802	0.804	0.804	0.806	0.809	0.813	0.817	0.819	0.822	0.825	0.827	0.827	0.828
Peterborough	0.713	0.719	0.724	0.728	0.732	0.737	0.742	0.747	0.753	0.759	0.763	0.767	0.773	0.778	0.785	0.79	0.794	0.798	0.803	0.809	0.815	0.819	0.818	0.816	0.816	0.816	0.816	0.816	0.816	0.818	0.823	0.828	0.831	0.835	0.837	0.838	0.839
Luton	0.692	0.698	0.703	0.707	0.71	0.713	0.715	0.717	0.725	0.733	0.743	0.752	0.759	0.765	0.771	0.775	0.779	0.783	0.787	0.793	0.798	0.803	0.804	0.804	0.806	0.808	0.811	0.816	0.823	0.829	0.834	0.837	0.84	0.843	0.844	0.844	0.845
Richmond upon Thames	0.76	0.766	0.771	0.775	0.779	0.783	0.787	0.791	0.798	0.805	0.813	0.82	0.827	0.833	0.839	0.845	0.85	0.855	0.861	0.868	0.873	0.878	0.882	0.885	0.887	0.888	0.887	0.887	0.888	0.888	0.889	0.891	0.895	0.9	0.905	0.908	0.91
Kensington and Chelsea	0.798	0.804	0.809	0.815	0.82	0.826	0.832	0.837	0.84	0.848	0.855	0.861	0.866	0.871	0.875	0.88	0.884	0.889	0.894	0.9	0.906	0.91	0.914	0.917	0.921	0.924	0.927	0.929	0.932	0.934	0.936	0.938	0.94	0.941	0.942	0.943	0.945
Barnet	0.729	0.735	0.742	0.748	0.753	0.759	0.764	0.769	0.775	0.782	0.787	0.792	0.797	0.803	0.81	0.816	0.823	0.829	0.835	0.841	0.846	0.851	0.853	0.854	0.855	0.855	0.854	0.853	0.855	0.857	0.86	0.865	0.87	0.875	0.878	0.879	0.88
Westminster	0.808	0.812	0.817	0.821	0.825	0.829	0.831	0.832	0.837	0.844	0.853	0.86	0.865	0.869	0.873	0.877	0.882	0.886	0.891	0.896	0.901	0.906	0.91	0.914	0.918	0.921	0.924	0.927	0.929	0.931	0.933	0.935	0.936	0.938	0.939	0.941	0.943
Bromley	0.73	0.736	0.741	0.746	0.749	0.753	0.757	0.761	0.767	0.774	0.779	0.784	0.789	0.793	0.799	0.804	0.809	0.815	0.821	0.829	0.835	0.839	0.842	0.844	0.846	0.847	0.848	0.85	0.851	0.851	0.851	0.851	0.853	0.857	0.86	0.862	0.863
Bexley	0.701	0.707	0.713	0.718	0.722	0.726	0.73	0.733	0.739	0.744	0.749	0.756	0.762	0.768	0.774	0.778	0.781	0.784	0.789	0.795	0.801	0.806	0.809	0.811	0.814	0.815	0.815	0.817	0.819	0.821	0.823	0.824	0.828	0.833	0.836	0.838	0.84
Redbridge	0.702	0.708	0.713	0.718	0.723	0.729	0.734	0.74	0.746	0.752	0.757	0.761	0.767	0.772	0.777	0.782	0.787	0.792	0.797	0.804	0.81	0.815	0.818	0.82	0.822	0.823	0.824	0.827	0.83	0.83	0.831	0.831	0.834	0.837	0.84	0.841	0.843
Merton	0.726	0.732	0.737	0.74	0.743	0.747	0.752	0.757	0.765	0.773	0.782	0.792	0.8	0.807	0.813	0.818	0.823	0.828	0.836	0.845	0.852	0.857	0.859	0.861	0.863	0.865	0.866	0.867	0.869	0.87	0.87	0.871	0.874	0.879	0.881	0.882	0.884
Brent	0.724	0.73	0.736	0.741	0.746	0.752	0.759	0.765	0.773	0.779	0.784	0.788	0.792	0.797	0.802	0.807	0.811	0.815	0.82	0.826	0.834	0.841	0.845	0.846	0.847	0.846	0.843	0.836	0.832	0.832	0.84	0.85	0.858	0.864	0.866	0.865	0.865
Hillingdon	0.756	0.762	0.767	0.772	0.776	0.78	0.784	0.788	0.795	0.801	0.807	0.814	0.821	0.827	0.834	0.84	0.844	0.848	0.853	0.859	0.864	0.866	0.868	0.869	0.871	0.871	0.87	0.87	0.872	0.874	0.876	0.878	0.882	0.888	0.892	0.895	0.897
Havering	0.703	0.709	0.715	0.72	0.724	0.728	0.732	0.735	0.74	0.745	0.75	0.756	0.762	0.768	0.774	0.778	0.783	0.787	0.792	0.798	0.803	0.808	0.81	0.812	0.815	0.818	0.82	0.822	0.825	0.827	0.828	0.829	0.83	0.833	0.835	0.836	0.837
Kingston upon Thames	0.746	0.752	0.758	0.762	0.766	0.77	0.774	0.779	0.788	0.796	0.804	0.811	0.818	0.823	0.828	0.832	0.837	0.842	0.85	0.858	0.864	0.869	0.874	0.878	0.881	0.884	0.886	0.888	0.891	0.892	0.892	0.892	0.895	0.898	0.9	0.901	0.902
Sutton	0.714	0.72	0.726	0.73	0.734	0.737	0.739	0.743	0.749	0.756	0.762	0.769	0.776	0.783	0.79	0.796	0.802	0.807	0.814	0.821	0.828	0.832	0.834	0.834	0.836	0.838	0.84	0.842	0.845	0.846	0.847	0.847	0.85	0.854	0.856	0.857	0.858
Harrow	0.719	0.726	0.732	0.737	0.741	0.745	0.749	0.751	0.757	0.764	0.772	0.781	0.789	0.796	0.802	0.806	0.81	0.814	0.821	0.829	0.836	0.84	0.841	0.842	0.844	0.847	0.847	0.848	0.849	0.849	0.851	0.852	0.854	0.856	0.856	0.856	0.856
Enfield	0.716	0.722	0.728	0.732	0.736	0.741	0.745	0.749	0.755	0.761	0.767	0.773	0.778	0.783	0.787	0.79	0.793	0.798	0.804	0.812	0.82	0.826	0.828	0.828	0.828	0.827	0.826	0.826	0.828	0.831	0.837	0.842	0.847	0.851	0.853	0.853	0.854
Croydon	0.721	0.727	0.732	0.737	0.741	0.745	0.749	0.753	0.759	0.765	0.772	0.78	0.787	0.794	0.801	0.806	0.81	0.815	0.82	0.826	0.832	0.836	0.839	0.841	0.843	0.843	0.841	0.84	0.841	0.841	0.843	0.845	0.848	0.85	0.85	0.849	0.849
Hammersmith and Fulham	0.784	0.789	0.795	0.801	0.807	0.814	0.82	0.826	0.833	0.841	0.849	0.856	0.862	0.867	0.872	0.877	0.883	0.887	0.891	0.897	0.901	0.906	0.909	0.912	0.916	0.919	0.92	0.922	0.924	0.927	0.932	0.936	0.937	0.938	0.94	0.941	0.942
Ealing	0.729	0.736	0.743	0.749	0.757	0.765	0.773	0.779	0.786	0.791	0.795	0.8	0.805	0.81	0.816	0.822	0.827	0.833	0.839	0.847	0.853	0.858	0.86	0.86	0.861	0.86	0.859	0.858	0.857	0.857	0.859	0.863	0.868	0.873	0.876	0.877	0.879
Greenwich	0.701	0.707	0.713	0.719	0.723	0.727	0.732	0.735	0.74	0.746	0.753	0.76	0.768	0.775	0.78	0.784	0.788	0.793	0.798	0.806	0.814	0.821	0.825	0.826	0.826	0.824	0.821	0.82	0.822	0.825	0.83	0.835	0.841	0.846	0.848	0.848	0.848
Wandsworth	0.746	0.752	0.758	0.764	0.77	0.777	0.784	0.791	0.799	0.808	0.816	0.825	0.833	0.839	0.844	0.849	0.854	0.86	0.865	0.872	0.879	0.885	0.89	0.895	0.899	0.902	0.906	0.907	0.909	0.911	0.912	0.915	0.919	0.922	0.924	0.926	0.928
Waltham Forest	0.681	0.687	0.694	0.699	0.705	0.71	0.715	0.72	0.726	0.733	0.739	0.746	0.753	0.76	0.766	0.771	0.774	0.777	0.783	0.79	0.797	0.803	0.806	0.806	0.807	0.805	0.802	0.798	0.798	0.8	0.807	0.814	0.822	0.828	0.832	0.834	0.835
Camden	0.81	0.815	0.819	0.823	0.828	0.833	0.838	0.843	0.848	0.852	0.857	0.862	0.867	0.872	0.876	0.881	0.883	0.884	0.888	0.897	0.904	0.909	0.912	0.915	0.919	0.922	0.924	0.927	0.929	0.931	0.933	0.935	0.937	0.939	0.94	0.942	0.943
Lambeth	0.738	0.745	0.751	0.756	0.761	0.766	0.771	0.773	0.779	0.787	0.798	0.811	0.821	0.828	0.834	0.838	0.842	0.847	0.853	0.86	0.867	0.871	0.872	0.873	0.876	0.881	0.885	0.891	0.898	0.903	0.908	0.914	0.919	0.921	0.922	0.924	0.926
Lewisham	0.702	0.708	0.714	0.719	0.724	0.728	0.732	0.736	0.741	0.747	0.755	0.766	0.775	0.782	0.787	0.79	0.793	0.796	0.802	0.81	0.819	0.826	0.828	0.828	0.829	0.83	0.832	0.833	0.835	0.836	0.84	0.844	0.85	0.856	0.86	0.863	0.865
Hounslow	0.745	0.75	0.753	0.755	0.755	0.757	0.761	0.766	0.773	0.784	0.795	0.805	0.81	0.815	0.818	0.822	0.825	0.829	0.836	0.847	0.857	0.864	0.866	0.864	0.864	0.865	0.867	0.869	0.872	0.874	0.876	0.878	0.883	0.889	0.891	0.891	0.892
Southwark	0.753	0.76	0.766	0.772	0.778	0.782	0.787	0.791	0.798	0.806	0.816	0.825	0.833	0.838	0.841	0.844	0.845	0.848	0.854	0.864	0.875	0.883	0.885	0.885	0.888	0.892	0.896	0.9	0.904	0.907	0.911	0.916	0.923	0.929	0.933	0.935	0.937
Newham	0.69	0.696	0.701	0.705	0.708	0.712	0.716	0.722	0.728	0.734	0.74	0.747	0.754	0.759	0.76	0.759	0.758	0.76	0.765	0.775	0.79	0.803	0.808	0.808	0.81	0.811	0.809	0.803	0.795	0.795	0.813	0.833	0.844	0.85	0.852	0.853	0.853
Barking and Dagenham	0.692	0.698	0.702	0.705	0.707	0.711	0.715	0.719	0.724	0.728	0.733	0.739	0.745	0.752	0.758	0.763	0.765	0.768	0.775	0.784	0.793	0.799	0.801	0.798	0.794	0.79	0.787	0.787	0.791	0.794	0.798	0.8	0.804	0.81	0.814	0.815	0.815
Haringey	0.73	0.736	0.741	0.745	0.747	0.748	0.75	0.752	0.758	0.765	0.777	0.79	0.799	0.805	0.809	0.813	0.817	0.821	0.825	0.831	0.837	0.843	0.846	0.847	0.849	0.851	0.85	0.849	0.848	0.848	0.855	0.865	0.872	0.878	0.88	0.88	0.88
Hackney	0.755	0.761	0.766	0.771	0.777	0.782	0.784	0.787	0.794	0.8	0.806	0.814	0.822	0.828	0.832	0.832	0.829	0.829	0.834	0.844	0.855	0.863	0.866	0.866	0.868	0.871	0.875	0.881	0.887	0.894	0.902	0.91	0.915	0.92	0.923	0.923	0.924
Islington	0.786	0.792	0.797	0.802	0.807	0.813	0.817	0.821	0.827	0.831	0.837	0.844	0.851	0.858	0.864	0.869	0.873	0.877	0.882	0.888	0.894	0.898	0.902	0.905	0.909	0.912	0.915	0.919	0.922	0.925	0.927	0.93	0.932	0.934	0.936	0.938	0.94
Tower Hamlets	0.727	0.732	0.736	0.74	0.747	0.757	0.768	0.777	0.785	0.79	0.794	0.797	0.801	0.805	0.809	0.813	0.819	0.826	0.834	0.847	0.859	0.869	0.876	0.88	0.885	0.891	0.896	0.902	0.907	0.912	0.916	0.919	0.924	0.927	0.929	0.931	0.933
Wokingham	0.758	0.763	0.768	0.773	0.778	0.784	0.79	0.796	0.803	0.806	0.809	0.81	0.812	0.815	0.817	0.82	0.824	0.829	0.836	0.846	0.857	0.865	0.87	0.872	0.875	0.879	0.881	0.883	0.884	0.883	0.882	0.88	0.882	0.887	0.891	0.893	0.895
Buckinghamshire	0.74	0.746	0.752	0.758	0.763	0.768	0.773	0.776	0.781	0.786	0.79	0.795	0.801	0.806	0.812	0.818	0.823	0.828	0.834	0.839	0.845	0.849	0.85	0.851	0.854	0.856	0.856	0.856	0.856	0.856	0.858	0.861	0.865	0.869	0.872	0.873	0.874
Surrey	0.749	0.755	0.76	0.764	0.767	0.77	0.773	0.775	0.781	0.787	0.793	0.8	0.806	0.811	0.816	0.821	0.825	0.831	0.837	0.846	0.854	0.859	0.861	0.863	0.865	0.866	0.867	0.868	0.869	0.87	0.871	0.872	0.875	0.88	0.882	0.884	0.886
Windsor and Maidenhead	0.76	0.766	0.77	0.774	0.776	0.779	0.782	0.783	0.786	0.79	0.799	0.809	0.817	0.824	0.83	0.837	0.841	0.846	0.85	0.856	0.861	0.865	0.868	0.872	0.876	0.879	0.88	0.881	0.883	0.884	0.885	0.888	0.891	0.896	0.9	0.903	0.905
West Berkshire	0.755	0.76	0.765	0.77	0.774	0.78	0.786	0.791	0.798	0.803	0.809	0.814	0.82	0.827	0.833	0.838	0.842	0.846	0.851	0.858	0.865	0.868	0.869	0.869	0.869	0.869	0.866	0.863	0.862	0.863	0.868	0.873	0.878	0.883	0.885	0.886	0.887
Hampshire	0.725	0.731	0.737	0.742	0.747	0.752	0.756	0.76	0.766	0.771	0.776	0.781	0.786	0.791	0.797	0.803	0.808	0.813	0.819	0.824	0.83	0.834	0.836	0.837	0.839	0.841	0.842	0.842	0.844	0.845	0.847	0.85	0.855	0.859	0.862	0.864	0.866

Appendix Table 14: Socio-Demographic Index values for all estimated GBD 2016 locations, 1980–2016

Location	1980	1981	1982	1983	1984	1985	1986	1987	1988	1989	1990	1991	1992	1993	1994	1995	1996	1997	1998	1999	2000	2001	2002	2003	2004	2005	2006	2007	2008	2009	2010	2011	2012	2013	2014	2015	2016
Bracknell Forest	0.733	0.74	0.746	0.753	0.76	0.766	0.771	0.777	0.784	0.791	0.796	0.799	0.804	0.808	0.814	0.82	0.824	0.828	0.833	0.842	0.849	0.855	0.858	0.86	0.863	0.866	0.868	0.87	0.871	0.872	0.873	0.875	0.878	0.882	0.885	0.887	0.888
West Sussex	0.72	0.726	0.731	0.737	0.741	0.746	0.75	0.753	0.759	0.764	0.769	0.774	0.78	0.786	0.792	0.797	0.803	0.808	0.814	0.819	0.824	0.827	0.828	0.829	0.831	0.832	0.834	0.835	0.836	0.838	0.841	0.844	0.847	0.852	0.854	0.855	0.856
Oxfordshire	0.745	0.751	0.757	0.762	0.767	0.773	0.778	0.782	0.786	0.79	0.794	0.799	0.805	0.81	0.816	0.822	0.827	0.832	0.838	0.845	0.851	0.856	0.858	0.86	0.863	0.865	0.866	0.866	0.867	0.868	0.87	0.874	0.879	0.884	0.887	0.889	0.89
Reading	0.753	0.759	0.764	0.769	0.772	0.775	0.779	0.783	0.79	0.798	0.806	0.815	0.822	0.827	0.832	0.837	0.842	0.847	0.853	0.861	0.869	0.876	0.88	0.882	0.885	0.886	0.884	0.883	0.884	0.886	0.89	0.893	0.897	0.901	0.904	0.905	0.906
Kent	0.71	0.716	0.721	0.726	0.729	0.733	0.736	0.74	0.745	0.75	0.755	0.76	0.765	0.771	0.776	0.781	0.786	0.791	0.797	0.803	0.808	0.812	0.815	0.816	0.819	0.822	0.823	0.824	0.826	0.827	0.829	0.832	0.835	0.839	0.842	0.844	0.845
Brighton and Hove	0.73	0.735	0.74	0.743	0.745	0.748	0.751	0.755	0.763	0.771	0.781	0.79	0.797	0.803	0.808	0.813	0.819	0.824	0.829	0.834	0.839	0.845	0.851	0.857	0.862	0.867	0.872	0.874	0.877	0.88	0.884	0.887	0.889	0.891	0.894	0.896	0.899
Medway	0.686	0.691	0.697	0.701	0.706	0.71	0.715	0.72	0.727	0.733	0.738	0.744	0.749	0.753	0.758	0.763	0.769	0.774	0.78	0.785	0.791	0.796	0.799	0.801	0.804	0.807	0.81	0.811	0.813	0.813	0.815	0.816	0.82	0.824	0.827	0.829	0.831
East Sussex	0.696	0.702	0.708	0.713	0.718	0.723	0.728	0.732	0.737	0.742	0.746	0.75	0.755	0.759	0.765	0.77	0.776	0.781	0.786	0.791	0.796	0.799	0.8	0.801	0.803	0.806	0.807	0.808	0.809	0.81	0.813	0.817	0.821	0.826	0.83	0.832	0.834
Portsmouth	0.728	0.734	0.74	0.744	0.746	0.748	0.751	0.754	0.76	0.768	0.776	0.784	0.791	0.797	0.802	0.806	0.81	0.815	0.821	0.828	0.836	0.843	0.849	0.853	0.858	0.861	0.864	0.867	0.869	0.87	0.87	0.868	0.869	0.871	0.873	0.876	0.878
Isle of Wight	0.695	0.7	0.706	0.71	0.714	0.719	0.724	0.73	0.737	0.741	0.744	0.746	0.748	0.751	0.756	0.762	0.768	0.774	0.78	0.787	0.795	0.804	0.809	0.811	0.812	0.813	0.814	0.816	0.818	0.819	0.821	0.823	0.826	0.829	0.831	0.833	0.835
Milton Keynes	0.757	0.761	0.762	0.762	0.763	0.768	0.775	0.783	0.79	0.795	0.796	0.799	0.804	0.811	0.818	0.825	0.83	0.835	0.839	0.843	0.847	0.85	0.85	0.85	0.853	0.855	0.856	0.855	0.855	0.856	0.862	0.868	0.873	0.878	0.88	0.88	0.88
Southampton	0.728	0.734	0.739	0.743	0.746	0.748	0.749	0.75	0.754	0.761	0.771	0.782	0.79	0.798	0.804	0.81	0.814	0.819	0.825	0.832	0.84	0.845	0.849	0.854	0.858	0.863	0.866	0.868	0.869	0.868	0.867	0.866	0.867	0.871	0.874	0.876	0.878
Slough	0.749	0.753	0.756	0.757	0.759	0.762	0.766	0.771	0.779	0.787	0.795	0.805	0.812	0.818	0.824	0.83	0.836	0.84	0.844	0.847	0.852	0.855	0.854	0.851	0.848	0.846	0.843	0.845	0.85	0.855	0.861	0.866	0.871	0.874	0.875	0.874	0.873
South Gloucestershire	0.731	0.737	0.742	0.747	0.751	0.756	0.762	0.767	0.774	0.779	0.783	0.787	0.791	0.795	0.799	0.803	0.809	0.816	0.824	0.832	0.839	0.844	0.846	0.848	0.851	0.855	0.858	0.861	0.865	0.866	0.867	0.868	0.871	0.875	0.878	0.88	0.882
Dorset	0.704	0.71	0.715	0.721	0.726	0.731	0.737	0.742	0.747	0.752	0.754	0.757	0.762	0.768	0.775	0.781	0.788	0.794	0.801	0.807	0.811	0.813	0.814	0.813	0.815	0.816	0.815	0.814	0.814	0.816	0.821	0.827	0.833	0.837	0.839	0.841	0.842
Wiltshire	0.713	0.719	0.725	0.731	0.736	0.741	0.746	0.75	0.756	0.761	0.766	0.77	0.775	0.781	0.787	0.793	0.798	0.804	0.81	0.815	0.821	0.825	0.826	0.827	0.829	0.829	0.828	0.826	0.825	0.826	0.83	0.835	0.84	0.844	0.846	0.846	0.847
North Somerset	0.695	0.701	0.708	0.714	0.721	0.727	0.734	0.74	0.746	0.751	0.753	0.756	0.761	0.766	0.771	0.776	0.781	0.787	0.793	0.799	0.805	0.809	0.811	0.813	0.817	0.821	0.823	0.825	0.827	0.826	0.827	0.829	0.833	0.838	0.841	0.843	0.844
Devon	0.701	0.707	0.712	0.717	0.721	0.726	0.731	0.736	0.742	0.748	0.753	0.757	0.762	0.767	0.772	0.777	0.782	0.788	0.794	0.802	0.809	0.815	0.818	0.821	0.825	0.829	0.831	0.833	0.834	0.835	0.836	0.837	0.84	0.845	0.849	0.851	0.853
Poole	0.708	0.714	0.719	0.724	0.729	0.735	0.741	0.748	0.755	0.761	0.766	0.771	0.776	0.782	0.789	0.796	0.802	0.808	0.814	0.82	0.826	0.83	0.83	0.83	0.832	0.834	0.835	0.835	0.836	0.836	0.84	0.845	0.851	0.856	0.859	0.861	0.862
Bath and North East Somerset	0.722	0.727	0.732	0.737	0.74	0.745	0.75	0.756	0.762	0.768	0.772	0.777	0.782	0.787	0.793	0.798	0.803	0.808	0.816	0.824	0.83	0.837	0.843	0.849	0.855	0.86	0.863	0.867	0.87	0.871	0.871	0.872	0.874	0.878	0.882	0.884	0.886
Gloucestershire	0.716	0.722	0.728	0.733	0.738	0.744	0.749	0.754	0.76	0.765	0.77	0.775	0.779	0.785	0.79	0.796	0.801	0.806	0.812	0.819	0.826	0.832	0.835	0.838	0.842	0.844	0.844	0.844	0.845	0.846	0.849	0.851	0.855	0.859	0.861	0.862	0.863
Somerset	0.701	0.708	0.714	0.719	0.725	0.73	0.734	0.738	0.743	0.748	0.752	0.756	0.762	0.767	0.774	0.78	0.786	0.791	0.795	0.801	0.807	0.811	0.812	0.813	0.816	0.818	0.819	0.82	0.82	0.821	0.823	0.827	0.83	0.834	0.835	0.835	0.836
Swindon	0.736	0.742	0.748	0.753	0.757	0.761	0.766	0.77	0.777	0.784	0.79	0.796	0.801	0.806	0.81	0.815	0.819	0.824	0.831	0.838	0.844	0.849	0.851	0.851	0.851	0.852	0.851	0.853	0.855	0.856	0.858	0.859	0.861	0.865	0.866	0.866	0.867
Torbay	0.684	0.69	0.696	0.701	0.706	0.711	0.715	0.721	0.728	0.734	0.738	0.743	0.749	0.755	0.761	0.767	0.772	0.777	0.782	0.787	0.793	0.795	0.794	0.792	0.794	0.797	0.798	0.8	0.802	0.802	0.802	0.801	0.803	0.806	0.809	0.811	0.813
Bristol, City of	0.738	0.744	0.749	0.753	0.756	0.759	0.763	0.766	0.772	0.779	0.787	0.796	0.804	0.811	0.817	0.822	0.827	0.832	0.839	0.847	0.854	0.859	0.863	0.865	0.869	0.872	0.875	0.878	0.881	0.882	0.881	0.88	0.882	0.887	0.891	0.895	0.898
Bournemouth	0.705	0.71	0.715	0.72	0.726	0.732	0.737	0.742	0.747	0.752	0.759	0.767	0.775	0.78	0.786	0.791	0.797	0.802	0.808	0.814	0.82	0.826	0.831	0.837	0.841	0.847	0.846	0.846	0.847	0.849	0.853	0.858	0.862	0.866	0.869	0.871	0.872
Cornwall	0.685	0.691	0.697	0.702	0.708	0.713	0.718	0.723	0.729	0.735	0.739	0.743	0.748	0.754	0.76	0.766	0.771	0.776	0.782	0.789	0.796	0.801	0.805	0.808	0.812	0.815	0.818	0.819	0.821	0.821	0.822	0.823	0.826	0.83	0.832	0.833	0.835
Plymouth	0.699	0.705	0.711	0.718	0.723	0.729	0.736	0.741	0.747	0.752	0.757	0.762	0.769	0.776	0.783	0.789	0.793	0.798	0.804	0.81	0.815	0.819	0.822	0.823	0.827	0.83	0.833	0.836	0.839	0.84	0.84	0.841	0.843	0.847	0.85	0.851	0.853
North East England	0.681	0.687	0.692	0.698	0.702	0.708	0.714	0.72	0.727	0.732	0.737	0.743	0.749	0.755	0.762	0.768	0.773	0.779	0.785	0.792	0.798	0.804	0.807	0.809	0.813	0.816	0.819	0.822	0.824	0.826	0.828	0.83	0.834	0.838	0.841	0.842	0.844
North West England	0.693	0.699	0.704	0.709	0.714	0.719	0.724	0.729	0.735	0.741	0.746	0.752	0.759	0.766	0.773	0.778	0.783	0.788	0.794	0.801	0.807	0.813	0.816	0.818	0.821	0.823	0.825	0.826	0.828	0.83	0.833	0.836	0.84	0.845	0.848	0.85	0.851
Yorkshire and the Humber	0.69	0.695	0.701	0.706	0.711	0.716	0.721	0.726	0.733	0.738	0.743	0.749	0.755	0.761	0.767	0.773	0.777	0.783	0.789	0.795	0.802	0.808	0.811	0.813	0.816	0.82	0.822	0.825	0.828	0.83	0.832	0.834	0.837	0.841	0.843	0.845	0.846
East Midlands	0.691	0.697	0.703	0.709	0.714	0.719	0.725	0.73	0.736	0.741	0.746	0.751	0.758	0.764	0.771	0.777	0.782	0.787	0.793	0.8	0.806	0.811	0.815	0.817	0.82	0.823	0.824	0.826	0.828	0.83	0.832	0.834	0.837	0.841	0.844	0.846	0.848
West Midlands	0.688	0.694	0.699	0.704	0.709	0.714	0.719	0.724	0.73	0.736	0.741	0.747	0.754	0.76	0.766	0.771	0.776	0.781	0.787	0.793	0.8	0.805	0.808	0.81	0.813	0.816	0.817	0.819	0.821	0.823	0.826	0.829	0.833	0.837	0.84	0.842	0.844
East of England	0.711	0.716	0.722	0.727	0.732	0.736	0.741	0.745	0.751	0.757	0.762	0.769	0.775	0.78	0.786	0.791	0.797	0.802	0.808	0.815	0.82	0.824	0.826	0.828	0.831	0.833	0.835	0.836	0.838	0.839	0.841	0.843	0.846	0.85	0.853	0.854	0.855
Greater London	0.751	0.757	0.763	0.768	0.772	0.777	0.782	0.786	0.792	0.799	0.807	0.815	0.821	0.827	0.833	0.837	0.841	0.846	0.852	0.86	0.868	0.875	0.878	0.88	0.883	0.885	0.886	0.886	0.888	0.89	0.894	0.899	0.904	0.909	0.912	0.914	0.915
South East England	0.729	0.735	0.74	0.745	0.749	0.753	0.757	0.761	0.767	0.772	0.777	0.783	0.789	0.794	0.8	0.805	0.81	0.816	0.821	0.828	0.834	0.839	0.841	0.842	0.845	0.847	0.848	0.849	0.85	0.851	0.854	0.856	0.86	0.864	0.867	0.869	0.87
South West England	0.711	0.716	0.722	0.727	0.732	0.737	0.742	0.747	0.753	0.758	0.763	0.768	0.773	0.779	0.785	0.79	0.795	0.801	0.807	0.814	0.82	0.825	0.828	0.829	0.832	0.835	0.836	0.837	0.839	0.84	0.841	0.844	0.847	0.851	0.854	0.856	0.857
Southern Latin America	0.599	0.604	0.607	0.613	0.618	0.621	0.627	0.631	0.634	0.637	0.641	0.647	0.654	0.662	0.671	0.678	0.684	0.688	0.693	0.698	0.702	0.706	0.709	0.712	0.716	0.72	0.725	0.729	0.734	0.738	0.743	0.749	0.754	0.76	0.764	0.768	0.771
Argentina	0.595	0.6	0.602	0.608	0.613	0.617	0.624	0.629	0.634	0.636	0.639	0.645	0.652	0.66	0.67	0.677	0.681	0.684	0.687	0.691	0.695	0.699	0.701	0.703	0.706	0.71	0.714	0.719	0.723	0.727	0.732	0.739	0.745	0.75	0.754	0.758	0.761
Chile	0.603	0.606	0.613	0.619	0.623	0.627	0.63	0.631	0.633	0.638	0.645	0.652	0.66	0.668	0.677	0.685	0.694	0.702	0.71	0.716	0.722	0.727	0.732	0.737	0.744	0.749	0.754	0.759	0.763	0.767	0.772	0.777	0.782	0.787	0.792	0.796	0.8
Uruguay	0.61	0.615	0.619	0.622	0.624	0.626	0.629	0.632	0.635	0.638	0.641	0.642	0.645	0.648	0.652	0.656	0.661	0.667	0.673	0.678	0.682	0.685	0.688	0.69	0.693	0.696	0.7	0.704	0.709	0.714	0.719	0.724	0.729	0.734	0.739	0.743	0.747
High-income North America	0.836	0.839	0.842	0.845	0.847	0.849	0.851	0.852	0.852	0.851	0.852	0.855	0.858	0.86	0.863	0.865	0.867	0.868	0.87	0.871	0.873	0.876	0.878	0.878	0.88	0.88	0.88	0.882	0.885	0.89	0.893	0.895	0.896	0.897	0.898	0.899	0.9
Canada	0.815	0.82	0.823	0.826	0.83	0.834	0.839	0.843	0.845	0.845	0.847	0.849	0.853	0.855	0.858	0.862	0.867	0.87	0.873	0.878	0.881	0.884	0.887	0.889	0.891	0.892	0.892	0.892	0.894	0.897	0.899	0.901	0.903	0.904	0.905	0.907	0.908
United States	0.838	0.841	0.844	0.847	0.849	0.85	0.853	0.853	0.852	0.851	0.852	0.855	0.858	0.86	0.863	0.865	0.867	0.868	0.869	0.87	0.872	0.875	0.876	0.877	0.878	0.879	0.878	0.88	0.884	0.889	0.892	0.894	0.895	0.896	0.897	0.898	0.899
Greenland	0.733	0.739	0.745	0.75	0.752	0.754	0.755	0.755	0.754	0.752	0.75	0.75	0.749	0.749	0.75	0.75	0.75	0.753	0.758	0.762	0.763	0.764	0.766	0.771	0.775	0.779	0.783	0.786	0.788	0.792	0.798	0.804	0.808	0.811	0.813	0.814	0.816
Alabama	0.808	0.812	0.816	0.82	0.823	0.826	0.828	0.829	0.829	0.83	0.832	0.835	0.839	0.842	0.845	0.847	0.849	0.85	0.851	0.853	0.856	0.86	0.862	0.864	0.865	0.865	0.864	0.865	0.869	0.874	0.878	0.88	0.882	0.882	0.883	0.884	0.884
Alaska	0.849	0.844	0.843	0.844	0.843	0.846	0.85	0.851	0.85	0.847	0.845	0.846	0.848	0.852	0.856	0.859	0.861	0.862	0.863	0.863	0.863	0.863	0.863	0.864	0.868	0.871	0.872	0.874	0.878	0.881	0.885	0.887	0.89	0.891	0.894	0.896	0.897
Arizona	0.829	0.832	0.834	0.836	0.836	0.837	0.839	0.84	0.839	0.838	0.837	0.84	0.842	0.844	0.846	0.847	0.848	0.848	0.848	0.848	0.849	0.852	0.853	0.853	0.855	0.855	0.856	0.861	0.868	0.875	0.879	0.88	0.881	0.881	0.881	0.881	0.881
Arkansas	0.799	0.803	0.807	0.811	0.814	0.816	0.819	0.82	0.82	0.82	0.822	0.826	0.83	0.833	0.836	0.837	0.839	0.84	0.842	0.844	0.846	0.849	0.85	0.851	0.853	0.853	0.852	0.854	0.859	0.863	0.867	0.869	0.871	0.872	0.874	0.875	0.876
California	0.851	0.853	0.854	0.856	0.856	0.856	0.856	0.854	0.849	0.844	0.841	0.843	0.846	0.848	0.851	0.854	0.857	0.861	0.863	0.866	0.869	0.872	0.873	0.872	0.873	0.873	0.872	0.875	0.88	0.885	0.889	0.891	0.893	0.894	0.896	0.897	0.898
Colorado	0.859	0.861	0.863	0.865	0.865	0.866	0.869	0.87	0.87	0.869	0.87	0.873	0.875	0.878	0.881	0.883	0.884	0.883	0.883	0.882	0.883	0.885	0.887	0.888	0.891	0.892	0.893	0.895	0.899	0.903	0.906	0.908	0.91	0.912	0.913	0.914	0.915
Connecticut	0.87	0.873	0.875	0.878	0.879	0.881	0.883	0.885	0.886	0.887	0.889	0.892	0.895	0.897	0.899	0.9	0.902	0.903	0.904	0.905	0.907	0.91	0.911	0.911	0.912	0.912	0.912	0.913	0.917	0.921	0.924	0.926	0.928	0.929	0.93	0.93	0.93
Delaware	0.854	0.857	0.859	0.862	0.863	0.865	0.867	0.867	0.866	0.866	0.867	0.871	0.875	0.878	0.88	0.882	0.883	0.882	0.882	0.882	0.884	0.887	0.888	0.889	0.89	0.889	0.887	0.888	0.892	0.897	0.901	0.903	0.905	0.907	0.908	0.909	0.909
District of Columbia	0.881	0.883	0.884	0.886	0.887	0.888	0.889	0.887	0.882	0.876	0.874	0.877	0.883	0.89	0.898	0.905	0.911	0.917	0.921	0.924	0.927	0.929	0.93	0.927	0.926	0.925	0.925	0.925	0.928	0.932	0.935	0.938	0.941	0.944	0.946	0.947	0.947
Florida	0.843	0.846	0.847	0.849	0.85	0.851	0.853	0.853	0.852	0.851	0.854	0.857	0.86	0.863	0.865	0.867	0.869	0.87	0.87	0.871	0.873	0.875	0.876	0.877	0.878	0.878	0.878	0.881	0.886	0.892	0.895	0.897	0.898	0.898	0.898	0.898	0.898
Georgia	0.82	0.825	0.829	0.832	0.835	0.837	0.84	0.841	0.841	0.841	0.843	0.848	0.851	0.855	0.857	0.859	0.86	0.86	0.86	0.86	0.862	0.865	0.867	0.868	0.869	0.868	0.867	0.869	0.874	0.88	0.884	0.886	0.887	0.887	0.888	0.888	0.888
Hawaii	0.843	0.844	0.845	0.848	0.85	0.852	0.855	0.855	0.854	0.853	0.854	0.857	0.86	0.863	0.866	0.869	0.872	0.874	0.875	0.875	0.875	0.876	0.874	0.873	0.874	0.875	0.875	0.877	0.881	0.885	0.889	0.891	0.894	0.896	0.898	0.9	0.901

Appendix Table 14: Socio-Demographic Index values for all estimated GBD 2016 locations, 1980–2016

Location	1980	1981	1982	1983	1984	1985	1986	1987	1988	1989	1990	1991	1992	1993	1994	1995	1996	1997	1998	1999	2000	2001	2002	2003	2004	2005	2006	2007	2008	2009	2010	2011	2012	2013	2014	2015	2016
Idaho	0.809	0.813	0.816	0.822	0.825	0.829	0.833	0.834	0.834	0.832	0.833	0.835	0.837	0.84	0.842	0.845	0.846	0.847	0.848	0.849	0.851	0.854	0.854	0.854	0.855	0.854	0.853	0.854	0.859	0.864	0.867	0.869	0.87	0.871	0.872	0.872	0.872
Illinois	0.843	0.846	0.849	0.852	0.854	0.856	0.857	0.858	0.856	0.855	0.856	0.858	0.86	0.863	0.866	0.868	0.871	0.872	0.874	0.875	0.878	0.881	0.883	0.884	0.886	0.888	0.888	0.891	0.895	0.899	0.902	0.903	0.905	0.906	0.907	0.908	0.908
Indiana	0.833	0.837	0.84	0.843	0.844	0.846	0.848	0.849	0.848	0.847	0.848	0.85	0.853	0.855	0.858	0.859	0.86	0.86	0.86	0.861	0.863	0.866	0.867	0.867	0.869	0.869	0.868	0.869	0.872	0.875	0.878	0.879	0.88	0.881	0.882	0.883	0.883
Iowa	0.834	0.838	0.842	0.845	0.848	0.85	0.853	0.854	0.854	0.853	0.854	0.856	0.858	0.86	0.862	0.864	0.865	0.866	0.867	0.868	0.87	0.873	0.874	0.875	0.876	0.876	0.874	0.876	0.88	0.884	0.887	0.888	0.89	0.891	0.891	0.892	0.893
Kansas	0.835	0.839	0.842	0.846	0.848	0.851	0.854	0.855	0.856	0.855	0.857	0.859	0.861	0.862	0.864	0.865	0.866	0.866	0.866	0.866	0.868	0.871	0.873	0.874	0.875	0.875	0.874	0.875	0.879	0.883	0.886	0.888	0.89	0.892	0.893	0.895	0.895
Kentucky	0.805	0.81	0.814	0.818	0.821	0.824	0.827	0.828	0.828	0.829	0.83	0.833	0.836	0.839	0.841	0.843	0.845	0.846	0.847	0.849	0.851	0.855	0.856	0.857	0.858	0.858	0.857	0.859	0.862	0.865	0.868	0.869	0.87	0.87	0.871	0.873	0.874
Louisiana	0.796	0.801	0.806	0.811	0.814	0.817	0.82	0.822	0.822	0.822	0.824	0.827	0.83	0.833	0.837	0.84	0.842	0.844	0.846	0.847	0.85	0.854	0.856	0.857	0.86	0.86	0.861	0.864	0.869	0.873	0.876	0.878	0.88	0.882	0.883	0.885	0.885
Maine	0.832	0.834	0.836	0.839	0.841	0.844	0.848	0.85	0.853	0.855	0.858	0.862	0.866	0.869	0.872	0.874	0.876	0.877	0.878	0.88	0.882	0.885	0.886	0.887	0.889	0.89	0.891	0.893	0.896	0.899	0.901	0.902	0.902	0.903	0.904	0.905	0.906
Maryland	0.865	0.867	0.868	0.87	0.871	0.872	0.875	0.875	0.875	0.875	0.877	0.88	0.883	0.886	0.889	0.891	0.892	0.892	0.892	0.893	0.895	0.897	0.899	0.899	0.901	0.902	0.901	0.903	0.907	0.912	0.915	0.917	0.919	0.92	0.92	0.921	0.92
Massachusetts	0.865	0.868	0.871	0.874	0.877	0.88	0.882	0.884	0.886	0.886	0.889	0.892	0.895	0.897	0.9	0.902	0.904	0.905	0.906	0.907	0.91	0.913	0.915	0.916	0.918	0.919	0.919	0.921	0.923	0.926	0.928	0.929	0.931	0.932	0.933	0.935	0.935
Michigan	0.845	0.847	0.849	0.852	0.853	0.855	0.857	0.857	0.856	0.854	0.855	0.858	0.861	0.864	0.867	0.87	0.871	0.872	0.873	0.875	0.877	0.88	0.882	0.883	0.886	0.887	0.887	0.888	0.89	0.891	0.892	0.893	0.893	0.894	0.895	0.896	0.897
Minnesota	0.839	0.844	0.848	0.852	0.856	0.859	0.863	0.866	0.867	0.868	0.87	0.873	0.875	0.877	0.879	0.88	0.882	0.882	0.883	0.884	0.886	0.889	0.89	0.89	0.891	0.891	0.89	0.892	0.896	0.9	0.903	0.904	0.905	0.906	0.907	0.909	0.909
Mississippi	0.784	0.789	0.794	0.799	0.803	0.806	0.81	0.811	0.811	0.811	0.813	0.816	0.82	0.824	0.828	0.831	0.833	0.835	0.836	0.838	0.841	0.845	0.847	0.848	0.848	0.847	0.846	0.848	0.853	0.859	0.864	0.867	0.869	0.871	0.872	0.873	0.874
Missouri	0.826	0.83	0.833	0.837	0.839	0.842	0.845	0.846	0.846	0.846	0.847	0.85	0.853	0.856	0.859	0.86	0.862	0.863	0.864	0.865	0.868	0.871	0.872	0.873	0.875	0.875	0.874	0.876	0.879	0.883	0.886	0.887	0.888	0.89	0.891	0.892	0.893
Montana	0.829	0.831	0.834	0.837	0.838	0.84	0.843	0.845	0.844	0.844	0.845	0.847	0.849	0.852	0.855	0.858	0.86	0.862	0.863	0.865	0.866	0.868	0.869	0.869	0.872	0.872	0.873	0.875	0.879	0.882	0.885	0.886	0.888	0.889	0.891	0.892	0.893
Nebraska	0.832	0.835	0.838	0.842	0.845	0.848	0.852	0.853	0.854	0.853	0.855	0.858	0.86	0.862	0.863	0.865	0.866	0.867	0.867	0.867	0.868	0.87	0.87	0.871	0.873	0.873	0.871	0.873	0.877	0.881	0.884	0.886	0.888	0.888	0.889	0.89	0.89
Nevada	0.855	0.857	0.858	0.86	0.86	0.86	0.86	0.857	0.854	0.85	0.85	0.853	0.855	0.857	0.858	0.859	0.861	0.862	0.862	0.863	0.865	0.868	0.868	0.868	0.869	0.867	0.865	0.868	0.875	0.882	0.887	0.889	0.889	0.889	0.888	0.887	0.886
New Hampshire	0.852	0.856	0.859	0.862	0.864	0.866	0.869	0.871	0.873	0.876	0.879	0.882	0.885	0.887	0.889	0.891	0.893	0.894	0.896	0.897	0.9	0.903	0.904	0.905	0.908	0.91	0.911	0.914	0.916	0.919	0.921	0.922	0.924	0.926	0.928	0.93	0.931
New Jersey	0.858	0.861	0.864	0.867	0.868	0.87	0.872	0.873	0.873	0.873	0.875	0.878	0.881	0.883	0.885	0.887	0.889	0.891	0.891	0.892	0.894	0.896	0.896	0.897	0.899	0.9	0.899	0.9	0.904	0.908	0.912	0.914	0.915	0.916	0.917	0.918	0.918
New Mexico	0.809	0.812	0.814	0.818	0.819	0.822	0.825	0.826	0.826	0.824	0.825	0.826	0.829	0.832	0.835	0.838	0.841	0.843	0.844	0.845	0.847	0.85	0.852	0.852	0.854	0.855	0.855	0.858	0.862	0.867	0.871	0.873	0.875	0.877	0.879	0.88	0.881
New York	0.854	0.856	0.858	0.861	0.862	0.864	0.866	0.866	0.866	0.866	0.868	0.871	0.874	0.877	0.879	0.882	0.885	0.887	0.888	0.89	0.892	0.895	0.896	0.897	0.899	0.899	0.899	0.901	0.904	0.907	0.91	0.912	0.915	0.916	0.918	0.919	0.92
North Carolina	0.825	0.829	0.832	0.835	0.837	0.84	0.842	0.842	0.842	0.841	0.844	0.848	0.851	0.855	0.858	0.86	0.86	0.86	0.86	0.86	0.861	0.865	0.867	0.868	0.869	0.868	0.866	0.868	0.873	0.878	0.883	0.885	0.886	0.886	0.887	0.887	0.887
North Dakota	0.816	0.818	0.822	0.827	0.83	0.835	0.839	0.842	0.842	0.843	0.846	0.848	0.85	0.852	0.854	0.857	0.86	0.863	0.866	0.869	0.871	0.874	0.874	0.875	0.875	0.874	0.872	0.874	0.879	0.883	0.887	0.888	0.891	0.892	0.893	0.895	0.896
Ohio	0.838	0.841	0.844	0.847	0.848	0.85	0.853	0.853	0.853	0.852	0.854	0.856	0.858	0.86	0.863	0.865	0.866	0.866	0.867	0.868	0.87	0.874	0.875	0.876	0.878	0.879	0.879	0.88	0.883	0.885	0.887	0.888	0.889	0.89	0.891	0.892	0.893
Oklahoma	0.82	0.824	0.828	0.833	0.836	0.84	0.843	0.844	0.843	0.842	0.843	0.844	0.846	0.847	0.848	0.849	0.849	0.849	0.85	0.85	0.853	0.856	0.857	0.858	0.86	0.861	0.861	0.862	0.866	0.869	0.872	0.874	0.877	0.879	0.88	0.882	0.883
Oregon	0.85	0.852	0.854	0.857	0.857	0.858	0.859	0.858	0.857	0.856	0.857	0.86	0.863	0.866	0.868	0.869	0.87	0.871	0.873	0.875	0.878	0.882	0.884	0.885	0.887	0.887	0.886	0.887	0.89	0.894	0.897	0.898	0.899	0.9	0.901	0.903	0.903
Pennsylvania	0.842	0.845	0.847	0.85	0.851	0.853	0.855	0.856	0.856	0.856	0.858	0.861	0.864	0.867	0.87	0.873	0.875	0.877	0.878	0.879	0.881	0.884	0.885	0.885	0.887	0.887	0.887	0.888	0.892	0.896	0.899	0.9	0.902	0.904	0.905	0.907	0.908
Rhode Island	0.847	0.851	0.853	0.857	0.859	0.861	0.863	0.864	0.865	0.865	0.867	0.87	0.873	0.877	0.88	0.883	0.885	0.886	0.887	0.889	0.89	0.893	0.894	0.895	0.898	0.9	0.9	0.902	0.906	0.909	0.912	0.913	0.915	0.916	0.918	0.92	0.921
South Carolina	0.814	0.818	0.821	0.825	0.828	0.83	0.833	0.833	0.832	0.831	0.834	0.839	0.844	0.849	0.853	0.855	0.856	0.857	0.857	0.858	0.86	0.864	0.866	0.866	0.866	0.864	0.862	0.863	0.868	0.874	0.879	0.881	0.883	0.884	0.884	0.885	0.886
South Dakota	0.806	0.81	0.814	0.818	0.822	0.825	0.83	0.832	0.832	0.832	0.834	0.836	0.839	0.842	0.846	0.849	0.852	0.853	0.855	0.857	0.859	0.861	0.861	0.862	0.863	0.862	0.861	0.863	0.868	0.872	0.876	0.877	0.878	0.879	0.88	0.881	0.882
Tennessee	0.818	0.822	0.826	0.829	0.831	0.833	0.836	0.836	0.836	0.835	0.837	0.841	0.844	0.848	0.851	0.853	0.855	0.856	0.857	0.858	0.861	0.864	0.866	0.866	0.867	0.867	0.865	0.867	0.871	0.875	0.879	0.88	0.882	0.882	0.883	0.884	0.885
Texas	0.815	0.819	0.823	0.827	0.829	0.831	0.834	0.834	0.834	0.832	0.832	0.834	0.835	0.837	0.839	0.84	0.842	0.843	0.843	0.844	0.846	0.849	0.85	0.851	0.853	0.853	0.853	0.855	0.86	0.866	0.87	0.873	0.875	0.876	0.877	0.878	0.878
Utah	0.779	0.786	0.794	0.803	0.809	0.814	0.819	0.82	0.82	0.819	0.822	0.827	0.831	0.833	0.834	0.835	0.834	0.834	0.834	0.836	0.839	0.843	0.846	0.847	0.849	0.849	0.848	0.85	0.855	0.859	0.863	0.864	0.866	0.867	0.869	0.87	0.87
Vermont	0.843	0.846	0.848	0.851	0.853	0.855	0.859	0.861	0.863	0.866	0.869	0.872	0.876	0.879	0.882	0.885	0.887	0.889	0.89	0.893	0.895	0.898	0.899	0.9	0.902	0.904	0.905	0.907	0.909	0.912	0.913	0.915	0.916	0.917	0.918	0.919	0.92
Virginia	0.849	0.852	0.855	0.858	0.86	0.862	0.864	0.866	0.866	0.867	0.868	0.871	0.874	0.877	0.88	0.882	0.883	0.883	0.884	0.884	0.885	0.888	0.888	0.888	0.89	0.891	0.891	0.894	0.899	0.903	0.907	0.909	0.91	0.911	0.912	0.913	0.913
Washington	0.857	0.859	0.862	0.865	0.866	0.867	0.869	0.869	0.868	0.867	0.868	0.87	0.873	0.876	0.878	0.88	0.882	0.883	0.885	0.887	0.891	0.894	0.896	0.896	0.898	0.897	0.896	0.897	0.899	0.902	0.904	0.906	0.907	0.908	0.91	0.911	0.912
West Virginia	0.809	0.813	0.818	0.822	0.824	0.827	0.83	0.831	0.831	0.83	0.832	0.834	0.837	0.84	0.843	0.845	0.847	0.849	0.85	0.851	0.852	0.854	0.856	0.857	0.859	0.859	0.859	0.859	0.862	0.865	0.867	0.869	0.87	0.872	0.873	0.875	0.876
Wisconsin	0.836	0.839	0.842	0.845	0.847	0.85	0.853	0.855	0.856	0.857	0.859	0.861	0.864	0.867	0.869	0.871	0.873	0.874	0.875	0.876	0.878	0.88	0.881	0.882	0.884	0.884	0.884	0.885	0.888	0.892	0.894	0.895	0.897	0.898	0.899	0.901	0.902
Wyoming	0.824	0.827	0.83	0.835	0.837	0.842	0.847	0.85	0.851	0.851	0.852	0.854	0.857	0.86	0.863	0.865	0.867	0.868	0.871	0.874	0.876	0.878	0.878	0.878	0.878	0.877	0.877	0.879	0.886	0.893	0.898	0.901	0.902	0.903	0.904	0.905	0.905
Latin America and Caribbean	0.467	0.477	0.486	0.494	0.503	0.511	0.519	0.526	0.533	0.54	0.546	0.554	0.561	0.568	0.575	0.581	0.588	0.595	0.602	0.608	0.615	0.621	0.627	0.632	0.638	0.643	0.649	0.655	0.661	0.666	0.672	0.678	0.684	0.689	0.695	0.7	0.705
Caribbean	0.532	0.538	0.543	0.551	0.557	0.563	0.569	0.573	0.578	0.583	0.588	0.594	0.599	0.602	0.605	0.608	0.61	0.613	0.617	0.621	0.626	0.631	0.635	0.64	0.645	0.651	0.656	0.66	0.663	0.667	0.671	0.675	0.679	0.683	0.688	0.692	0.696
Antigua and Barbuda	0.611	0.619	0.627	0.634	0.64	0.647	0.654	0.662	0.67	0.678	0.684	0.691	0.696	0.7	0.704	0.707	0.71	0.713	0.716	0.719	0.722	0.723	0.725	0.728	0.731	0.735	0.74	0.746	0.752	0.756	0.759	0.761	0.765	0.767	0.771	0.774	0.778
The Bahamas	0.659	0.666	0.674	0.681	0.689	0.697	0.704	0.709	0.713	0.716	0.719	0.72	0.721	0.722	0.724	0.728	0.733	0.739	0.745	0.751	0.757	0.761	0.765	0.767	0.769	0.77	0.771	0.773	0.774	0.775	0.775	0.776	0.777	0.779	0.78	0.781	0.783
Barbados	0.662	0.668	0.673	0.679	0.685	0.691	0.699	0.706	0.713	0.72	0.726	0.732	0.737	0.741	0.744	0.748	0.751	0.755	0.759	0.762	0.766	0.769	0.771	0.772	0.773	0.776	0.779	0.782	0.784	0.786	0.788	0.789	0.79	0.791	0.793	0.795	0.797
Belize	0.344	0.348	0.353	0.36	0.369	0.379	0.391	0.404	0.419	0.435	0.452	0.469	0.487	0.502	0.515	0.525	0.532	0.537	0.542	0.546	0.552	0.557	0.563	0.569	0.574	0.58	0.585	0.589	0.593	0.596	0.6	0.604	0.608	0.612	0.617	0.621	0.625
Cuba	0.631	0.634	0.637	0.645	0.65	0.658	0.666	0.668	0.673	0.679	0.685	0.693	0.698	0.697	0.696	0.695	0.695	0.692	0.693	0.694	0.697	0.701	0.705	0.712	0.719	0.725	0.732	0.739	0.739	0.743	0.747	0.751	0.757	0.761	0.766	0.772	0.778
Dominica	0.529	0.534	0.541	0.548	0.556	0.563	0.57	0.577	0.584	0.59	0.596	0.6	0.605	0.612	0.62	0.629	0.637	0.645	0.653	0.659	0.665	0.67	0.674	0.678	0.682	0.686	0.69	0.695	0.701	0.705	0.71	0.714	0.718	0.721	0.725	0.729	0.734
Dominican Republic	0.435	0.449	0.463	0.477	0.49	0.499	0.506	0.513	0.519	0.524	0.527	0.53	0.535	0.54	0.545	0.551	0.558	0.566	0.574	0.582	0.589	0.596	0.603	0.609	0.615	0.622	0.629	0.637	0.643	0.649	0.656	0.662	0.668	0.675	0.682	0.69	0.696
Grenada	0.442	0.448	0.453	0.458	0.463	0.468	0.473	0.479	0.485	0.492	0.5	0.51	0.519	0.527	0.536	0.544	0.552	0.561	0.571	0.581	0.591	0.6	0.609	0.618	0.627	0.637	0.646	0.654	0.662	0.668	0.674	0.679	0.684	0.688	0.694	0.699	0.704
Guyana	0.496	0.503	0.509	0.514	0.517	0.518	0.519	0.519	0.519	0.518	0.517	0.518	0.52	0.525	0.53	0.537	0.545	0.552	0.559	0.565	0.569	0.573	0.576	0.579	0.582	0.584	0.587	0.592	0.596	0.601	0.607	0.614	0.621	0.628	0.636	0.643	0.649
Haiti	0.257	0.261	0.264	0.267	0.271	0.275	0.279	0.284	0.29	0.297	0.303	0.31	0.315	0.319	0.321	0.325	0.329	0.334	0.339	0.346	0.352	0.358	0.363	0.368	0.372	0.376	0.381	0.386	0.391	0.397	0.402	0.407	0.413	0.418	0.424	0.428	0.432
Jamaica	0.557	0.563	0.568	0.573	0.578	0.58	0.583	0.587	0.591	0.595	0.599	0.604	0.609	0.615	0.62	0.625	0.629	0.633	0.636	0.64	0.643	0.647	0.651	0.655	0.659	0.664	0.669	0.674	0.679	0.682	0.685	0.688	0.691	0.694	0.696	0.699	0.701
Saint Lucia	0.453	0.464	0.473	0.482	0.492	0.502	0.513	0.523	0.534	0.544	0.555	0.565	0.574	0.583	0.591	0.598	0.606	0.614	0.621	0.629	0.635	0.641	0.647	0.654	0.662	0.668	0.674	0.679	0.684	0.689	0.693	0.697	0.7	0.703	0.707	0.71	0.713
Saint Vincent and the Grenadines	0.418	0.428	0.438	0.449	0.46	0.471	0.48	0.489	0.499	0.508	0.516	0.524	0.533	0.541	0.547	0.555	0.562	0.569	0.576	0.583	0.589	0.595	0.602	0.608	0.615	0.621	0.627	0.633	0.638	0.642	0.645	0.648	0.651	0.655	0.659	0.663	0.667
Suriname	0.51	0.515	0.519	0.524	0.528	0.535	0.541	0.546	0.553	0.56	0.566	0.569	0.572	0.574	0.575	0.577	0.58	0.583	0.587	0.589	0.592	0.597	0.601	0.607	0.614	0.621	0.628	0.636	0.643	0.651	0.659	0.666	0.673	0.68	0.686	0.691	0.696
Trinidad and Tobago	0.592	0.595	0.599	0.603	0.608	0.615	0.624	0.633	0.642	0.649	0.655	0.659	0.663	0.666	0.668	0.669	0.671	0.675	0.68	0.687	0.694	0.7	0.706	0.713	0.719	0.725	0.731	0.738	0.745	0.75	0.755	0.758	0.76	0.762	0.764	0.765	0.766
Bermuda	0.68	0.687	0.692	0.698	0.703	0.708	0.713	0.719	0.724	0.729	0.733	0.737	0.741	0.745	0.749	0.752	0.756	0.76	0.763	0.767	0.77	0.774	0.777	0.781	0.785	0.789	0.793	0.796	0.799	0.801	0.803	0.806	0.808	0.811	0.813	0.816	0.819

Appendix Table 14: Socio-Demographic Index values for all estimated GBD 2016 locations, 1980–2016

Location	1980	1981	1982	1983	1984	1985	1986	1987	1988	1989	1990	1991	1992	1993	1994	1995	1996	1997	1998	1999	2000	2001	2002	2003	2004	2005	2006	2007	2008	2009	2010	2011	2012	2013	2014	2015	2016
Puerto Rico	0.699	0.71	0.719	0.727	0.733	0.739	0.743	0.748	0.753	0.76	0.767	0.774	0.781	0.788	0.793	0.799	0.805	0.81	0.817	0.822	0.828	0.834	0.84	0.845	0.85	0.854	0.858	0.86	0.863	0.864	0.866	0.867	0.868	0.87	0.871	0.873	0.875
Virgin Islands, U.S.	0.723	0.732	0.738	0.743	0.749	0.755	0.76	0.764	0.767	0.771	0.777	0.782	0.789	0.796	0.802	0.808	0.814	0.82	0.825	0.829	0.833	0.836	0.839	0.842	0.845	0.85	0.855	0.861	0.864	0.868	0.871	0.874	0.875	0.876	0.876	0.877	0.877
Andean Latin America	0.45	0.457	0.465	0.473	0.481	0.488	0.496	0.504	0.511	0.518	0.524	0.53	0.536	0.541	0.548	0.555	0.561	0.568	0.575	0.582	0.588	0.594	0.6	0.605	0.61	0.615	0.62	0.625	0.63	0.635	0.64	0.644	0.649	0.653	0.657	0.662	0.666
Bolivia	0.354	0.361	0.368	0.376	0.383	0.39	0.397	0.405	0.412	0.418	0.425	0.432	0.439	0.446	0.453	0.461	0.469	0.477	0.485	0.494	0.502	0.51	0.518	0.525	0.531	0.539	0.546	0.553	0.56	0.567	0.573	0.58	0.586	0.593	0.599	0.605	0.61
Ecuador	0.45	0.458	0.467	0.475	0.484	0.493	0.501	0.509	0.516	0.523	0.53	0.537	0.543	0.55	0.556	0.563	0.569	0.576	0.583	0.588	0.593	0.598	0.603	0.608	0.613	0.619	0.624	0.629	0.635	0.64	0.645	0.65	0.655	0.66	0.665	0.67	0.674
Peru	0.478	0.485	0.493	0.5	0.507	0.514	0.521	0.529	0.537	0.543	0.548	0.553	0.558	0.563	0.569	0.576	0.582	0.59	0.597	0.603	0.61	0.615	0.621	0.626	0.63	0.634	0.639	0.644	0.648	0.652	0.656	0.66	0.663	0.666	0.67	0.674	0.678
Central Latin America	0.466	0.476	0.485	0.493	0.502	0.51	0.518	0.525	0.531	0.537	0.544	0.551	0.56	0.568	0.575	0.581	0.588	0.595	0.602	0.609	0.615	0.622	0.627	0.633	0.639	0.645	0.652	0.659	0.665	0.671	0.676	0.682	0.689	0.695	0.701	0.707	0.711
Colombia	0.485	0.493	0.5	0.506	0.513	0.519	0.526	0.532	0.538	0.544	0.55	0.556	0.562	0.568	0.574	0.581	0.588	0.595	0.601	0.606	0.611	0.616	0.62	0.624	0.629	0.634	0.639	0.645	0.652	0.658	0.664	0.672	0.679	0.686	0.694	0.701	0.707
Costa Rica	0.527	0.532	0.535	0.537	0.539	0.541	0.546	0.552	0.557	0.563	0.57	0.577	0.584	0.591	0.598	0.603	0.608	0.614	0.62	0.627	0.634	0.64	0.646	0.651	0.657	0.662	0.668	0.674	0.679	0.685	0.691	0.697	0.704	0.71	0.716	0.722	0.726
El Salvador	0.383	0.392	0.401	0.408	0.414	0.419	0.424	0.429	0.434	0.439	0.444	0.451	0.458	0.465	0.472	0.48	0.488	0.496	0.504	0.513	0.521	0.529	0.537	0.545	0.552	0.56	0.568	0.576	0.584	0.592	0.6	0.609	0.617	0.625	0.632	0.639	0.645
Guatemala	0.325	0.33	0.335	0.338	0.342	0.345	0.348	0.351	0.355	0.36	0.365	0.37	0.376	0.383	0.389	0.396	0.404	0.412	0.42	0.429	0.438	0.446	0.455	0.463	0.472	0.48	0.489	0.498	0.506	0.514	0.522	0.53	0.537	0.543	0.55	0.556	0.561
Honduras	0.295	0.304	0.313	0.321	0.329	0.337	0.345	0.353	0.362	0.372	0.381	0.39	0.4	0.409	0.418	0.427	0.435	0.443	0.451	0.459	0.466	0.474	0.481	0.488	0.495	0.502	0.508	0.515	0.522	0.527	0.533	0.539	0.545	0.551	0.556	0.562	0.567
Mexico	0.458	0.47	0.481	0.491	0.501	0.511	0.521	0.53	0.538	0.547	0.555	0.564	0.573	0.582	0.591	0.598	0.606	0.614	0.622	0.629	0.637	0.644	0.651	0.657	0.664	0.671	0.678	0.684	0.691	0.695	0.701	0.706	0.712	0.718	0.723	0.729	0.734
Nicaragua	0.409	0.412	0.415	0.418	0.421	0.424	0.427	0.43	0.431	0.433	0.434	0.436	0.438	0.44	0.443	0.447	0.453	0.459	0.466	0.473	0.481	0.488	0.496	0.502	0.509	0.516	0.523	0.53	0.537	0.543	0.549	0.555	0.562	0.569	0.576	0.583	0.588
Panama	0.542	0.552	0.56	0.568	0.574	0.581	0.588	0.594	0.597	0.6	0.603	0.607	0.611	0.615	0.62	0.624	0.628	0.633	0.64	0.647	0.653	0.66	0.666	0.67	0.674	0.679	0.683	0.688	0.693	0.698	0.704	0.71	0.718	0.726	0.735	0.744	0.752
Venezuela	0.533	0.54	0.547	0.557	0.568	0.577	0.583	0.588	0.594	0.592	0.59	0.602	0.619	0.628	0.629	0.632	0.637	0.642	0.647	0.65	0.654	0.657	0.66	0.662	0.667	0.673	0.681	0.688	0.696	0.702	0.708	0.714	0.721	0.728	0.734	0.738	0.742
Aguascalientes	0.49	0.502	0.512	0.521	0.53	0.538	0.548	0.558	0.567	0.576	0.586	0.597	0.608	0.617	0.624	0.631	0.639	0.648	0.656	0.663	0.67	0.675	0.681	0.687	0.693	0.699	0.705	0.711	0.715	0.719	0.723	0.728	0.733	0.739	0.744	0.749	0.754
Baja California	0.523	0.536	0.548	0.56	0.572	0.585	0.597	0.608	0.617	0.624	0.629	0.635	0.642	0.65	0.658	0.666	0.672	0.676	0.68	0.687	0.695	0.703	0.709	0.713	0.715	0.718	0.722	0.728	0.735	0.74	0.745	0.751	0.758	0.764	0.769	0.773	0.776
Baja California Sur	0.535	0.547	0.559	0.571	0.584	0.596	0.605	0.615	0.627	0.635	0.644	0.654	0.663	0.669	0.674	0.679	0.686	0.691	0.692	0.689	0.689	0.694	0.698	0.701	0.707	0.712	0.711	0.707	0.706	0.71	0.718	0.729	0.738	0.744	0.748	0.753	0.757
Campeche	0.437	0.449	0.459	0.468	0.477	0.485	0.494	0.503	0.511	0.52	0.53	0.541	0.551	0.56	0.567	0.574	0.582	0.593	0.603	0.614	0.625	0.635	0.643	0.651	0.658	0.665	0.671	0.677	0.682	0.685	0.688	0.693	0.7	0.707	0.713	0.72	0.726
Coahuila	0.499	0.512	0.523	0.533	0.543	0.552	0.562	0.572	0.582	0.593	0.605	0.617	0.63	0.64	0.649	0.656	0.663	0.671	0.679	0.685	0.691	0.697	0.702	0.708	0.714	0.72	0.726	0.733	0.739	0.743	0.747	0.751	0.754	0.758	0.761	0.766	0.77
Colima	0.472	0.485	0.496	0.506	0.517	0.528	0.54	0.553	0.565	0.579	0.591	0.604	0.616	0.625	0.632	0.638	0.645	0.652	0.659	0.666	0.672	0.678	0.683	0.689	0.695	0.7	0.706	0.712	0.716	0.719	0.723	0.727	0.732	0.737	0.742	0.748	0.753
Chiapas	0.3	0.313	0.323	0.332	0.338	0.342	0.345	0.345	0.342	0.339	0.342	0.355	0.375	0.393	0.41	0.427	0.444	0.459	0.472	0.483	0.491	0.499	0.508	0.517	0.527	0.536	0.546	0.555	0.563	0.57	0.577	0.585	0.593	0.6	0.608	0.616	0.622
Chihuahua	0.49	0.503	0.513	0.523	0.531	0.54	0.549	0.559	0.568	0.578	0.587	0.597	0.607	0.614	0.62	0.623	0.628	0.634	0.639	0.645	0.651	0.658	0.666	0.673	0.682	0.689	0.697	0.705	0.713	0.719	0.726	0.734	0.743	0.75	0.758	0.764	0.769
Distrito Federal	0.592	0.605	0.615	0.624	0.631	0.635	0.638	0.642	0.649	0.66	0.671	0.68	0.686	0.692	0.702	0.713	0.723	0.729	0.733	0.738	0.743	0.749	0.755	0.761	0.766	0.771	0.776	0.779	0.782	0.783	0.785	0.79	0.796	0.802	0.808	0.814	0.819
Durango	0.459	0.47	0.48	0.488	0.495	0.502	0.509	0.516	0.522	0.529	0.536	0.544	0.553	0.559	0.563	0.568	0.576	0.586	0.596	0.605	0.615	0.624	0.632	0.641	0.65	0.657	0.665	0.672	0.678	0.681	0.684	0.688	0.691	0.696	0.7	0.705	0.709
Guanajuato	0.384	0.396	0.407	0.417	0.427	0.437	0.448	0.461	0.472	0.485	0.497	0.511	0.524	0.534	0.543	0.551	0.562	0.573	0.583	0.593	0.603	0.611	0.619	0.627	0.634	0.642	0.65	0.658	0.666	0.671	0.677	0.683	0.688	0.694	0.7	0.706	0.711
Guerrero	0.355	0.367	0.378	0.387	0.395	0.404	0.413	0.423	0.431	0.437	0.441	0.444	0.445	0.442	0.438	0.439	0.445	0.454	0.464	0.473	0.483	0.494	0.505	0.517	0.529	0.54	0.551	0.562	0.57	0.577	0.585	0.595	0.606	0.617	0.626	0.634	0.639
Hidalgo	0.383	0.395	0.406	0.416	0.426	0.437	0.449	0.46	0.47	0.48	0.49	0.501	0.513	0.521	0.527	0.533	0.541	0.55	0.56	0.569	0.577	0.585	0.593	0.6	0.608	0.616	0.624	0.633	0.64	0.646	0.651	0.656	0.662	0.668	0.674	0.68	0.685
Jalisco	0.463	0.475	0.486	0.496	0.505	0.514	0.524	0.535	0.544	0.554	0.565	0.577	0.589	0.599	0.607	0.615	0.624	0.633	0.642	0.65	0.658	0.664	0.671	0.677	0.684	0.69	0.697	0.703	0.709	0.712	0.716	0.719	0.723	0.727	0.732	0.736	0.741
México	0.447	0.463	0.481	0.502	0.528	0.554	0.571	0.582	0.592	0.595	0.597	0.598	0.6	0.616	0.637	0.646	0.651	0.655	0.66	0.666	0.673	0.679	0.684	0.687	0.692	0.697	0.701	0.703	0.706	0.711	0.717	0.723	0.728	0.733	0.739	0.745	0.75
Michoacán de Ocampo	0.368	0.38	0.391	0.401	0.41	0.42	0.431	0.443	0.453	0.464	0.476	0.488	0.5	0.51	0.517	0.525	0.535	0.547	0.558	0.568	0.579	0.588	0.597	0.607	0.616	0.624	0.633	0.641	0.648	0.653	0.657	0.661	0.665	0.67	0.675	0.681	0.685
Morelos	0.479	0.492	0.503	0.513	0.523	0.533	0.543	0.554	0.564	0.576	0.587	0.599	0.611	0.62	0.627	0.634	0.64	0.647	0.653	0.659	0.664	0.67	0.675	0.68	0.686	0.692	0.698	0.705	0.71	0.714	0.718	0.722	0.726	0.73	0.734	0.737	0.741
Nayarit	0.428	0.442	0.454	0.464	0.473	0.482	0.491	0.501	0.511	0.522	0.533	0.545	0.557	0.567	0.575	0.582	0.592	0.602	0.612	0.621	0.63	0.638	0.645	0.652	0.658	0.664	0.67	0.675	0.679	0.683	0.687	0.692	0.698	0.705	0.711	0.718	0.723
Nuevo León	0.582	0.594	0.603	0.612	0.619	0.626	0.632	0.638	0.644	0.652	0.66	0.668	0.678	0.686	0.692	0.697	0.703	0.709	0.715	0.72	0.725	0.73	0.734	0.739	0.744	0.749	0.755	0.76	0.765	0.767	0.77	0.772	0.775	0.777	0.78	0.784	0.787
Oaxaca	0.351	0.362	0.371	0.38	0.388	0.396	0.406	0.417	0.427	0.437	0.446	0.456	0.466	0.471	0.474	0.476	0.481	0.487	0.494	0.501	0.508	0.515	0.523	0.533	0.544	0.555	0.568	0.581	0.592	0.601	0.609	0.617	0.623	0.629	0.634	0.639	0.643
Puebla	0.404	0.416	0.426	0.436	0.445	0.454	0.465	0.475	0.485	0.495	0.505	0.515	0.526	0.532	0.537	0.54	0.545	0.552	0.558	0.565	0.572	0.578	0.586	0.595	0.604	0.614	0.625	0.637	0.647	0.655	0.662	0.669	0.674	0.679	0.683	0.688	0.692
Querétaro	0.445	0.456	0.463	0.465	0.461	0.453	0.452	0.47	0.495	0.515	0.53	0.54	0.549	0.558	0.572	0.589	0.606	0.619	0.627	0.636	0.649	0.661	0.669	0.673	0.677	0.682	0.687	0.693	0.699	0.705	0.71	0.715	0.722	0.728	0.734	0.74	0.744
Quintana Roo	0.49	0.503	0.514	0.524	0.532	0.54	0.547	0.553	0.559	0.567	0.576	0.586	0.598	0.609	0.617	0.625	0.634	0.642	0.65	0.658	0.665	0.671	0.677	0.683	0.688	0.693	0.698	0.704	0.709	0.713	0.718	0.724	0.731	0.738	0.744	0.75	0.755
San Luis Potosí	0.375	0.39	0.403	0.414	0.425	0.436	0.449	0.462	0.474	0.487	0.5	0.513	0.526	0.536	0.544	0.552	0.561	0.572	0.583	0.593	0.604	0.613	0.622	0.631	0.64	0.648	0.656	0.665	0.672	0.678	0.685	0.694	0.703	0.711	0.72	0.728	0.734
Sinaloa	0.491	0.503	0.514	0.523	0.531	0.539	0.547	0.556	0.564	0.573	0.581	0.589	0.597	0.603	0.607	0.613	0.62	0.629	0.639	0.649	0.658	0.666	0.674	0.682	0.689	0.697	0.704	0.712	0.718	0.723	0.727	0.732	0.736	0.74	0.744	0.748	0.751
Sonora	0.528	0.541	0.552	0.561	0.569	0.577	0.584	0.592	0.598	0.606	0.614	0.623	0.632	0.64	0.645	0.65	0.656	0.663	0.67	0.677	0.683	0.69	0.696	0.702	0.709	0.716	0.723	0.731	0.737	0.742	0.746	0.751	0.756	0.761	0.767	0.772	0.777
Tabasco	0.42	0.432	0.443	0.452	0.461	0.469	0.478	0.487	0.496	0.506	0.515	0.525	0.536	0.545	0.552	0.561	0.572	0.584	0.597	0.607	0.616	0.624	0.63	0.636	0.642	0.648	0.654	0.66	0.665	0.669	0.674	0.68	0.685	0.691	0.696	0.701	0.706
Tamaulipas	0.504	0.516	0.526	0.535	0.544	0.553	0.562	0.572	0.582	0.593	0.605	0.617	0.629	0.638	0.646	0.653	0.66	0.668	0.674	0.68	0.685	0.689	0.693	0.697	0.701	0.706	0.712	0.719	0.726	0.732	0.738	0.744	0.751	0.756	0.761	0.766	0.77
Tlaxcala	0.359	0.371	0.384	0.397	0.413	0.432	0.455	0.479	0.499	0.514	0.524	0.533	0.538	0.545	0.559	0.573	0.585	0.593	0.599	0.605	0.612	0.62	0.628	0.635	0.644	0.651	0.657	0.664	0.672	0.677	0.681	0.685	0.693	0.701	0.708	0.715	0.72
Veracruz de Ignacio de la Llave	0.408	0.42	0.431	0.44	0.449	0.458	0.467	0.477	0.484	0.493	0.501	0.51	0.519	0.526	0.531	0.537	0.545	0.554	0.564	0.573	0.582	0.59	0.597	0.605	0.613	0.621	0.63	0.638	0.646	0.653	0.659	0.665	0.67	0.675	0.68	0.686	0.69
Yucatán	0.466	0.477	0.486	0.493	0.501	0.508	0.515	0.522	0.528	0.536	0.545	0.555	0.566	0.575	0.582	0.59	0.599	0.608	0.617	0.625	0.633	0.641	0.647	0.654	0.661	0.667	0.674	0.68	0.685	0.689	0.693	0.697	0.701	0.706	0.71	0.715	0.719
Zacatecas	0.381	0.393	0.405	0.415	0.425	0.435	0.446	0.459	0.471	0.484	0.498	0.512	0.526	0.537	0.546	0.554	0.564	0.574	0.583	0.592	0.601	0.608	0.615	0.622	0.629	0.635	0.641	0.647	0.652	0.656	0.66	0.666	0.672	0.679	0.686	0.693	0.699
Tropical Latin America	0.449	0.461	0.471	0.48	0.489	0.498	0.507	0.515	0.524	0.532	0.539	0.546	0.553	0.56	0.568	0.576	0.583	0.591	0.598	0.605	0.613	0.62	0.626	0.631	0.637	0.642	0.648	0.654	0.66	0.666	0.672	0.679	0.684	0.69	0.696	0.702	0.707
Brazil	0.449	0.461	0.471	0.48	0.49	0.499	0.508	0.516	0.525	0.533	0.54	0.548	0.554	0.562	0.569	0.577	0.585	0.592	0.6	0.607	0.614	0.621	0.627	0.633	0.638	0.644	0.65	0.656	0.662	0.667	0.673	0.68	0.686	0.691	0.697	0.703	0.708
Paraguay	0.437	0.444	0.448	0.451	0.454	0.456	0.459	0.461	0.465	0.469	0.473	0.478	0.484	0.49	0.496	0.504	0.511	0.519	0.527	0.535	0.543	0.55	0.558	0.565	0.573	0.58	0.588	0.596	0.604	0.611	0.62	0.628	0.636	0.645	0.653	0.661	0.668
Acre	0.332	0.346	0.358	0.369	0.38	0.392	0.404	0.416	0.428	0.439	0.449	0.459	0.467	0.474	0.483	0.492	0.501	0.509	0.517	0.522	0.528	0.534	0.539	0.543	0.548	0.555	0.563	0.57	0.578	0.586	0.595	0.604	0.612	0.621	0.629	0.636	0.642
Alagoas	0.314	0.325	0.334	0.342	0.35	0.358	0.368	0.378	0.388	0.397	0.406	0.414	0.422	0.431	0.441	0.452	0.461	0.469	0.477	0.485	0.493	0.501	0.508	0.515	0.522	0.529	0.536	0.544	0.553	0.56	0.569	0.577	0.584	0.592	0.599	0.605	0.61
Amazonas	0.367	0.382	0.394	0.405	0.416	0.427	0.439	0.452	0.464	0.475	0.486	0.497	0.506	0.513	0.523	0.534	0.544	0.552	0.56	0.569	0.578	0.586	0.592	0.597	0.603	0.609	0.615	0.622	0.63	0.637	0.645	0.652	0.659	0.666	0.674	0.68	0.686
Amapá	0.352	0.366	0.38	0.393	0.406	0.419	0.432	0.445	0.457	0.468	0.477	0.486	0.494	0.501	0.509	0.517	0.528	0.54	0.554	0.567	0.577	0.586	0.594	0.603	0.611	0.617	0.623	0.63	0.638	0.645	0.652	0.66	0.666	0.67	0.675	0.679	0.684
Bahia	0.356	0.365	0.373	0.381	0.389	0.397	0.406	0.417	0.428	0.438	0.447	0.456	0.464	0.472	0.481	0.49	0.498	0.507	0.517	0.524	0.532	0.539	0.545	0.552	0.558	0.564	0.571	0.578	0.584	0.591	0.598	0.606	0.613	0.62	0.627	0.633	0.638
Ceará	0.346	0.355	0.362	0.369	0.376	0.382	0.392	0.406	0.417	0.429	0.439	0.448	0.455	0.463	0.472	0.481	0.492	0.501	0.509	0.516	0.523	0.53	0.538	0.545	0.552	0.558	0.565	0.573	0.581	0.588	0.596	0.604	0.612	0.619	0.626	0.632	0.637

Appendix Table 14: Socio-Demographic Index values for all estimated GBD 2016 locations, 1980–2016																																					
Location	1980	1981	1982	1983	1984	1985	1986	1987	1988	1989	1990	1991	1992	1993	1994	1995	1996	1997	1998	1999	2000	2001	2002	2003	2004	2005	2006	2007	2008	2009	2010	2011	2012	2013	2014	2015	2016
Distrito Federal	0.597	0.607	0.617	0.625	0.634	0.643	0.652	0.659	0.666	0.673	0.68	0.686	0.692	0.698	0.704	0.71	0.717	0.723	0.729	0.735	0.741	0.747	0.753	0.758	0.764	0.769	0.775	0.781	0.787	0.793	0.8	0.806	0.812	0.818	0.824	0.829	0.832
Espírito Santo	0.459	0.47	0.481	0.49	0.499	0.509	0.518	0.527	0.536	0.545	0.553	0.561	0.568	0.577	0.584	0.592	0.6	0.607	0.614	0.621	0.628	0.635	0.642	0.648	0.654	0.66	0.665	0.671	0.677	0.682	0.688	0.694	0.699	0.705	0.711	0.717	0.721
Goiás	0.396	0.411	0.425	0.437	0.45	0.462	0.473	0.483	0.493	0.502	0.511	0.52	0.528	0.537	0.546	0.555	0.565	0.574	0.582	0.59	0.597	0.604	0.611	0.617	0.623	0.628	0.635	0.641	0.647	0.653	0.659	0.665	0.67	0.676	0.682	0.689	0.693
Maranhão	0.309	0.314	0.319	0.324	0.329	0.333	0.341	0.352	0.361	0.371	0.38	0.387	0.393	0.403	0.413	0.424	0.435	0.446	0.457	0.468	0.479	0.488	0.496	0.503	0.511	0.517	0.525	0.533	0.541	0.549	0.557	0.566	0.574	0.581	0.588	0.595	0.6
Minas Gerais	0.45	0.462	0.473	0.483	0.493	0.503	0.512	0.518	0.525	0.532	0.539	0.546	0.552	0.559	0.566	0.574	0.582	0.589	0.596	0.603	0.609	0.615	0.621	0.627	0.632	0.638	0.643	0.65	0.656	0.661	0.668	0.674	0.68	0.686	0.692	0.697	0.702
Mato Grosso do Sul	0.401	0.418	0.432	0.445	0.457	0.471	0.481	0.489	0.498	0.506	0.514	0.522	0.53	0.539	0.548	0.558	0.567	0.576	0.584	0.591	0.598	0.604	0.61	0.615	0.62	0.626	0.632	0.638	0.644	0.649	0.654	0.659	0.664	0.669	0.675	0.681	0.686
Mato Grosso	0.419	0.433	0.445	0.456	0.467	0.48	0.49	0.498	0.507	0.516	0.524	0.532	0.54	0.549	0.559	0.569	0.578	0.587	0.595	0.602	0.609	0.615	0.621	0.626	0.631	0.636	0.642	0.647	0.653	0.658	0.664	0.67	0.675	0.68	0.687	0.693	0.699
Pará	0.364	0.375	0.385	0.393	0.402	0.411	0.42	0.428	0.436	0.444	0.451	0.458	0.465	0.473	0.483	0.493	0.503	0.511	0.519	0.526	0.532	0.537	0.541	0.545	0.549	0.554	0.56	0.566	0.574	0.581	0.589	0.597	0.605	0.612	0.619	0.625	0.63
Paraíba	0.325	0.337	0.347	0.356	0.365	0.372	0.384	0.399	0.412	0.424	0.436	0.444	0.451	0.459	0.467	0.476	0.486	0.495	0.504	0.512	0.519	0.527	0.533	0.54	0.546	0.553	0.559	0.567	0.574	0.581	0.589	0.596	0.603	0.61	0.616	0.622	0.627
Paraná	0.443	0.458	0.471	0.483	0.494	0.507	0.517	0.524	0.533	0.541	0.548	0.556	0.564	0.572	0.58	0.589	0.597	0.606	0.614	0.622	0.629	0.636	0.643	0.649	0.654	0.66	0.665	0.671	0.677	0.682	0.688	0.694	0.7	0.706	0.712	0.718	0.722
Pernambuco	0.364	0.377	0.387	0.396	0.405	0.414	0.425	0.437	0.448	0.458	0.468	0.477	0.485	0.492	0.5	0.508	0.516	0.523	0.53	0.536	0.543	0.549	0.555	0.561	0.567	0.573	0.579	0.585	0.592	0.598	0.605	0.611	0.617	0.623	0.63	0.636	0.64
Piaui	0.321	0.329	0.337	0.344	0.351	0.358	0.367	0.378	0.388	0.398	0.408	0.415	0.421	0.428	0.436	0.445	0.455	0.464	0.473	0.482	0.49	0.498	0.505	0.512	0.519	0.526	0.533	0.54	0.548	0.555	0.563	0.571	0.578	0.585	0.591	0.598	0.602
Rio de Janeiro	0.531	0.545	0.557	0.567	0.577	0.587	0.595	0.602	0.608	0.615	0.62	0.626	0.631	0.637	0.642	0.648	0.653	0.659	0.665	0.67	0.676	0.682	0.688	0.692	0.696	0.701	0.705	0.71	0.714	0.718	0.723	0.727	0.732	0.736	0.74	0.745	0.749
Rio Grande do Norte	0.357	0.369	0.38	0.39	0.4	0.409	0.42	0.431	0.442	0.452	0.461	0.469	0.477	0.485	0.494	0.504	0.515	0.524	0.531	0.539	0.546	0.554	0.562	0.568	0.575	0.582	0.589	0.596	0.603	0.61	0.618	0.625	0.632	0.639	0.645	0.651	0.656
Rondônia	0.358	0.373	0.386	0.398	0.41	0.422	0.434	0.446	0.457	0.467	0.477	0.486	0.494	0.502	0.511	0.519	0.528	0.536	0.545	0.552	0.56	0.568	0.575	0.582	0.589	0.597	0.604	0.612	0.619	0.626	0.633	0.64	0.646	0.653	0.66	0.666	0.671
Roraima	0.401	0.411	0.421	0.43	0.44	0.45	0.461	0.472	0.483	0.493	0.503	0.512	0.52	0.527	0.535	0.542	0.548	0.552	0.554	0.558	0.569	0.581	0.589	0.595	0.604	0.612	0.62	0.628	0.636	0.644	0.651	0.657	0.661	0.666	0.672	0.678	0.683
Rio Grande do Sul	0.506	0.516	0.525	0.533	0.541	0.549	0.557	0.565	0.572	0.579	0.585	0.592	0.598	0.604	0.61	0.617	0.622	0.628	0.635	0.641	0.647	0.654	0.66	0.665	0.671	0.676	0.682	0.688	0.693	0.698	0.703	0.708	0.712	0.717	0.722	0.727	0.732
Santa Catarina	0.477	0.489	0.499	0.508	0.517	0.526	0.536	0.544	0.553	0.561	0.569	0.577	0.584	0.592	0.6	0.609	0.617	0.624	0.632	0.639	0.647	0.654	0.66	0.665	0.671	0.676	0.681	0.687	0.693	0.698	0.704	0.711	0.716	0.722	0.729	0.735	0.74
Sergipe	0.336	0.35	0.361	0.372	0.382	0.391	0.403	0.417	0.43	0.442	0.453	0.463	0.472	0.481	0.49	0.501	0.511	0.52	0.529	0.537	0.546	0.554	0.562	0.569	0.576	0.582	0.589	0.596	0.604	0.611	0.619	0.627	0.634	0.641	0.648	0.654	0.659
São Paulo	0.53	0.542	0.552	0.561	0.571	0.581	0.589	0.596	0.602	0.609	0.615	0.621	0.628	0.634	0.64	0.647	0.653	0.659	0.666	0.672	0.679	0.686	0.692	0.697	0.702	0.706	0.711	0.716	0.721	0.726	0.73	0.736	0.74	0.745	0.75	0.756	0.76
Tocantins	0.38	0.389	0.398	0.406	0.415	0.424	0.434	0.443	0.452	0.461	0.469	0.477	0.486	0.494	0.502	0.511	0.521	0.53	0.539	0.547	0.556	0.563	0.571	0.577	0.584	0.591	0.597	0.604	0.612	0.619	0.626	0.634	0.642	0.649	0.656	0.663	0.668
North Africa and Middle East	0.382	0.392	0.401	0.41	0.418	0.427	0.436	0.446	0.455	0.465	0.475	0.485	0.495	0.505	0.513	0.522	0.531	0.539	0.548	0.556	0.564	0.571	0.578	0.586	0.593	0.601	0.608	0.615	0.622	0.628	0.634	0.64	0.647	0.654	0.661	0.669	0.674
North Africa and Middle East	0.382	0.392	0.401	0.41	0.418	0.427	0.436	0.446	0.455	0.465	0.475	0.485	0.495	0.505	0.513	0.522	0.531	0.539	0.548	0.556	0.564	0.571	0.578	0.586	0.593	0.601	0.608	0.615	0.622	0.628	0.634	0.64	0.647	0.654	0.661	0.669	0.674
Algeria	0.356	0.367	0.379	0.39	0.401	0.412	0.423	0.433	0.443	0.453	0.463	0.472	0.479	0.485	0.49	0.495	0.5	0.505	0.51	0.516	0.523	0.53	0.538	0.549	0.559	0.57	0.581	0.591	0.602	0.612	0.622	0.632	0.642	0.651	0.66	0.669	0.676
Bahrain	0.498	0.513	0.526	0.536	0.54	0.542	0.55	0.559	0.565	0.571	0.586	0.599	0.605	0.613	0.623	0.633	0.641	0.644	0.646	0.65	0.663	0.681	0.694	0.699	0.701	0.703	0.707	0.711	0.716	0.719	0.72	0.719	0.718	0.721	0.726	0.732	0.738
Egypt	0.347	0.358	0.369	0.38	0.391	0.402	0.413	0.425	0.435	0.446	0.458	0.469	0.48	0.49	0.499	0.509	0.518	0.527	0.535	0.543	0.551	0.558	0.566	0.573	0.579	0.585	0.591	0.596	0.601	0.607	0.612	0.617	0.623	0.629	0.637	0.645	0.653
Iran	0.379	0.387	0.395	0.404	0.412	0.422	0.434	0.449	0.466	0.487	0.51	0.536	0.562	0.585	0.607	0.625	0.642	0.656	0.667	0.676	0.683	0.689	0.695	0.701	0.707	0.713	0.719	0.725	0.731	0.736	0.742	0.748	0.752	0.757	0.762	0.766	0.769
Iraq	0.289	0.299	0.31	0.32	0.331	0.341	0.351	0.361	0.372	0.381	0.391	0.398	0.405	0.412	0.418	0.423	0.429	0.437	0.447	0.459	0.472	0.485	0.495	0.498	0.498	0.492	0.485	0.475	0.461	0.447	0.434	0.429	0.432	0.44	0.45	0.463	0.474
Jordan	0.264	0.294	0.322	0.346	0.369	0.389	0.409	0.428	0.448	0.466	0.483	0.497	0.511	0.524	0.536	0.548	0.558	0.568	0.577	0.586	0.595	0.603	0.61	0.615	0.621	0.626	0.632	0.637	0.643	0.649	0.656	0.663	0.669	0.676	0.683	0.689	0.694
Kuwait	0.46	0.476	0.494	0.515	0.54	0.566	0.59	0.611	0.628	0.642	0.652	0.657	0.66	0.659	0.653	0.649	0.655	0.671	0.689	0.702	0.711	0.717	0.721	0.726	0.732	0.738	0.747	0.758	0.77	0.781	0.791	0.8	0.808	0.816	0.823	0.829	0.832
Lebanon	0.51	0.525	0.536	0.547	0.561	0.575	0.588	0.602	0.613	0.621	0.627	0.636	0.645	0.653	0.661	0.669	0.678	0.687	0.697	0.706	0.715	0.723	0.73	0.738	0.744	0.751	0.757	0.763	0.77	0.777	0.785	0.792	0.797	0.801	0.804	0.807	0.81
Libya	0.565	0.57	0.575	0.579	0.583	0.588	0.591	0.595	0.598	0.601	0.605	0.611	0.617	0.623	0.63	0.636	0.643	0.651	0.659	0.667	0.675	0.684	0.692	0.702	0.712	0.723	0.734	0.746	0.757	0.768	0.781	0.785	0.793	0.8	0.804	0.807	0.806
Morocco	0.287	0.295	0.303	0.312	0.321	0.329	0.339	0.349	0.359	0.37	0.381	0.393	0.404	0.415	0.427	0.438	0.449	0.46	0.471	0.481	0.49	0.499	0.507	0.516	0.524	0.531	0.54	0.548	0.557	0.565	0.573	0.581	0.589	0.597	0.604	0.611	0.617
Palestine	0.328	0.341	0.351	0.36	0.369	0.376	0.384	0.391	0.397	0.404	0.411	0.417	0.424	0.43	0.437	0.446	0.457	0.467	0.478	0.488	0.497	0.504	0.51	0.513	0.51	0.502	0.49	0.472	0.452	0.432	0.418	0.413	0.412	0.416	0.425	0.438	0.45
Oman	0.107	0.109	0.111	0.113	0.115	0.135	0.156	0.176	0.194	0.214	0.234	0.256	0.277	0.298	0.318	0.337	0.356	0.376	0.396	0.417	0.438	0.458	0.479	0.499	0.52	0.54	0.561	0.581	0.602	0.621	0.639	0.658	0.676	0.691	0.704	0.714	0.72
Qatar	0.52	0.54	0.558	0.575	0.587	0.596	0.601	0.605	0.609	0.615	0.622	0.631	0.64	0.65	0.659	0.669	0.676	0.684	0.69	0.696	0.702	0.708	0.715	0.722	0.728	0.735	0.74	0.745	0.75	0.756	0.761	0.765	0.769	0.774	0.779	0.784	0.788
Saudi Arabia	0.21	0.225	0.242	0.26	0.277	0.293	0.308	0.323	0.338	0.353	0.368	0.383	0.398	0.414	0.429	0.445	0.462	0.479	0.497	0.515	0.533	0.551	0.569	0.587	0.605	0.623	0.642	0.66	0.679	0.696	0.713	0.73	0.746	0.761	0.775	0.788	0.797
Syria	0.28	0.29	0.301	0.311	0.32	0.33	0.339	0.348	0.357	0.366	0.376	0.386	0.397	0.408	0.419	0.43	0.441	0.451	0.462	0.472	0.483	0.493	0.503	0.514	0.525	0.537	0.549	0.56	0.571	0.582	0.593	0.603	0.611	0.618	0.625	0.632	0.638
Tunisia	0.353	0.364	0.376	0.388	0.4	0.412	0.425	0.437	0.45	0.462	0.476	0.489	0.502	0.515	0.527	0.539	0.551	0.563	0.573	0.583	0.591	0.599	0.606	0.612	0.618	0.623	0.629	0.635	0.64	0.646	0.653	0.659	0.667	0.674	0.682	0.69	0.696
Turkey	0.479	0.489	0.498	0.508	0.517	0.527	0.537	0.547	0.557	0.566	0.576	0.584	0.592	0.599	0.605	0.612	0.619	0.627	0.634	0.641	0.649	0.656	0.663	0.67	0.677	0.684	0.691	0.698	0.705	0.711	0.717	0.724	0.731	0.738	0.746	0.754	0.762
United Arab Emirates	0.34	0.365	0.389	0.413	0.436	0.458	0.48	0.501	0.521	0.539	0.557	0.574	0.59	0.606	0.62	0.633	0.645	0.656	0.667	0.677	0.686	0.695	0.703	0.71	0.716	0.721	0.725	0.729	0.732	0.736	0.74	0.745	0.751	0.756	0.762	0.768	0.772
Yemen	0.155	0.159	0.163	0.166	0.168	0.168	0.168	0.167	0.168	0.17	0.173	0.177	0.185	0.195	0.206	0.217	0.229	0.24	0.252	0.264	0.276	0.288	0.302	0.317	0.335	0.351	0.362	0.367	0.371	0.374	0.379	0.386	0.398	0.417	0.434	0.445	0.45
Afghanistan	0.134	0.136	0.139	0.142	0.145	0.147	0.15	0.153	0.154	0.156	0.158	0.16	0.163	0.165	0.165	0.166	0.166	0.167	0.168	0.169	0.17	0.171	0.175	0.181	0.188	0.195	0.203	0.211	0.22	0.229	0.238	0.247	0.255	0.262	0.269	0.276	0.281
Sudan	0.24	0.245	0.252	0.258	0.263	0.268	0.274	0.279	0.285	0.29	0.295	0.3	0.304	0.309	0.313	0.317	0.32	0.326	0.332	0.34	0.351	0.361	0.372	0.383	0.393	0.404	0.415	0.426	0.437	0.448	0.459	0.47	0.48	0.49	0.501	0.51	0.518
Ha'il	0.205	0.22	0.236	0.252	0.267	0.283	0.297	0.312	0.326	0.341	0.355	0.369	0.384	0.399	0.414	0.429	0.446	0.463	0.479	0.496	0.514	0.532	0.549	0.567	0.585	0.603	0.621	0.639	0.657	0.674	0.691	0.708	0.724	0.739	0.752	0.765	0.774
Qassim	0.15	0.169	0.19	0.211	0.231	0.251	0.269	0.288	0.306	0.324	0.341	0.357	0.373	0.389	0.405	0.422	0.439	0.458	0.476	0.495	0.515	0.534	0.554	0.573	0.592	0.61	0.629	0.647	0.666	0.684	0.701	0.717	0.733	0.748	0.762	0.776	0.785
Riyadh	0.185	0.203	0.223	0.244	0.264	0.283	0.301	0.319	0.337	0.353	0.369	0.385	0.401	0.417	0.434	0.451	0.468	0.486	0.505	0.523	0.543	0.562	0.58	0.599	0.618	0.637	0.656	0.675	0.693	0.711	0.727	0.742	0.756	0.769	0.783	0.795	0.804
Tabuk	0.194	0.21	0.229	0.248	0.267	0.285	0.301	0.316	0.332	0.347	0.361	0.376	0.391	0.406	0.422	0.438	0.454	0.471	0.489	0.507	0.525	0.543	0.562	0.58	0.598	0.62	0.64	0.659	0.678	0.695	0.712	0.729	0.745	0.76	0.775	0.79	0.8
Madinah	0.199	0.215	0.232	0.251	0.269	0.285	0.3	0.316	0.331	0.347	0.362	0.377	0.392	0.408	0.424	0.44	0.457	0.474	0.492	0.51	0.528	0.547	0.565	0.583	0.601	0.62	0.638	0.656	0.675	0.693	0.71	0.727	0.744	0.76	0.773	0.786	0.795
Makkah	0.231	0.246	0.261	0.277	0.293	0.308	0.323	0.337	0.352	0.367	0.382	0.396	0.411	0.427	0.442	0.458	0.474	0.491	0.508	0.525	0.543	0.56	0.577	0.594	0.611	0.628	0.645	0.662	0.679	0.695	0.71	0.726	0.741	0.755	0.768	0.779	0.788
Bahah	0.219	0.232	0.246	0.26	0.273	0.287	0.299	0.312	0.325	0.338	0.351	0.364	0.378	0.392	0.406	0.42	0.436	0.452	0.468	0.484	0.5	0.516	0.532	0.549	0.565	0.582	0.599	0.616	0.634	0.65	0.666	0.683	0.699	0.714	0.726	0.737	0.746
Northern Borders	0.264	0.278	0.293	0.308	0.323	0.337	0.351	0.364	0.378	0.392	0.406	0.42	0.434	0.45	0.465	0.48	0.497	0.513	0.53	0.546	0.563	0.58	0.596	0.612	0.629	0.647	0.665	0.681	0.698	0.713	0.728	0.742	0.755	0.766	0.779	0.79	0.797
Jawf	0.236	0.253	0.272	0.291	0.311	0.33	0.347	0.364	0.381	0.398	0.414	0.431	0.448	0.465	0.482	0.499	0.517	0.536	0.555	0.573	0.592	0.61	0.628	0.645	0.663	0.681	0.699	0.717	0.735	0.752	0.768	0.784	0.799	0.814	0.826	0.838	0.846
Jizan	0.202	0.212	0.223	0.234	0.245	0.255	0.265	0.275	0.285	0.296	0.306	0.317	0.329	0.341	0.353	0.365	0.379	0.393	0.407	0.421	0.436	0.45	0.465	0.48	0.496	0.513	0.53	0.546	0.564	0.581	0.597	0.614	0.63	0.645	0.658	0.67	0.679
'Asir	0.167	0.181	0.197	0.213	0.229	0.243	0.258	0.272	0.286	0.301	0.314	0.327	0.341	0.355	0.369	0.384	0.401	0.417	0.434	0.451	0.469	0.487	0.504	0.522	0.541	0.559	0.578	0.598	0.617	0.636	0.654	0.672	0.69	0.706	0.721	0.735	0.744
Najran	0.187	0.199	0.212	0.224	0.237	0.249	0.261	0.273	0.285	0.297	0.309	0.32	0.332	0.345	0.357	0.371	0.385	0.4	0.415	0.431	0.447	0.463	0.479	0.496	0.513	0.53	0.548	0.566	0.585	0.602	0.62	0.637	0.654	0.671	0.685	0.699	0.709
Eastern Province	0.209	0.225	0.243	0.261	0.28	0.297	0.313	0.329	0.346	0.362	0.377	0.393	0.409	0.425	0.441	0.458	0.475	0.493	0.511	0.529	0.548	0.566	0.584	0.603	0.621	0.639	0.658	0.676	0.694	0.711	0.728	0.745	0.761	0.776	0.79	0.803	0.813

Appendix Table 14: Socio-Demographic Index values for all estimated GBD 2016 locations, 1980–2016																																					
**Location**	**1980**	**1981**	**1982**	**1983**	**1984**	**1985**	**1986**	**1987**	**1988**	**1989**	**1990**	**1991**	**1992**	**1993**	**1994**	**1995**	**1996**	**1997**	**1998**	**1999**	**2000**	**2001**	**2002**	**2003**	**2004**	**2005**	**2006**	**2007**	**2008**	**2009**	**2010**	**2011**	**2012**	**2013**	**2014**	**2015**	**2016**
South Asia	0.288	0.293	0.298	0.303	0.309	0.314	0.32	0.327	0.334	0.341	0.349	0.356	0.364	0.371	0.378	0.386	0.394	0.402	0.409	0.417	0.425	0.432	0.44	0.447	0.455	0.464	0.473	0.482	0.491	0.5	0.51	0.519	0.529	0.539	0.549	0.56	0.569
South Asia	0.288	0.293	0.298	0.303	0.309	0.314	0.32	0.327	0.334	0.341	0.349	0.356	0.364	0.371	0.378	0.386	0.394	0.402	0.409	0.417	0.425	0.432	0.44	0.447	0.455	0.464	0.473	0.482	0.491	0.5	0.51	0.519	0.529	0.539	0.549	0.56	0.569
Bangladesh	0.254	0.258	0.263	0.269	0.275	0.281	0.287	0.294	0.3	0.307	0.315	0.323	0.331	0.339	0.347	0.355	0.363	0.37	0.377	0.384	0.39	0.396	0.402	0.407	0.414	0.42	0.427	0.434	0.442	0.45	0.459	0.467	0.476	0.485	0.494	0.503	0.511
Bhutan	0.237	0.242	0.247	0.253	0.259	0.265	0.272	0.279	0.286	0.293	0.301	0.31	0.318	0.327	0.337	0.348	0.359	0.37	0.382	0.394	0.406	0.418	0.431	0.444	0.457	0.47	0.482	0.494	0.506	0.518	0.53	0.541	0.552	0.563	0.572	0.581	0.588
India	0.298	0.303	0.308	0.313	0.318	0.324	0.329	0.336	0.342	0.35	0.357	0.364	0.371	0.378	0.385	0.393	0.401	0.408	0.416	0.424	0.432	0.44	0.447	0.455	0.464	0.473	0.482	0.492	0.502	0.512	0.522	0.533	0.543	0.553	0.564	0.575	0.584
Nepal	0.194	0.198	0.202	0.206	0.21	0.214	0.219	0.224	0.23	0.236	0.242	0.25	0.258	0.266	0.276	0.285	0.293	0.302	0.31	0.319	0.328	0.337	0.345	0.354	0.362	0.37	0.377	0.384	0.391	0.398	0.404	0.411	0.417	0.424	0.433	0.443	0.452
Pakistan	0.216	0.218	0.22	0.223	0.227	0.234	0.243	0.255	0.268	0.282	0.297	0.309	0.32	0.33	0.339	0.349	0.36	0.371	0.383	0.392	0.4	0.408	0.416	0.424	0.431	0.438	0.443	0.448	0.453	0.458	0.464	0.471	0.478	0.488	0.499	0.51	0.519
Andhra Pradesh	0.276	0.281	0.285	0.29	0.294	0.299	0.303	0.309	0.315	0.322	0.329	0.337	0.345	0.354	0.363	0.373	0.383	0.393	0.403	0.414	0.424	0.434	0.444	0.453	0.463	0.472	0.481	0.493	0.503	0.514	0.525	0.536	0.546	0.556	0.566	0.577	0.585
Arunachal Pradesh	0.263	0.269	0.274	0.28	0.286	0.292	0.3	0.307	0.315	0.322	0.33	0.338	0.346	0.354	0.362	0.371	0.38	0.389	0.397	0.405	0.413	0.422	0.43	0.438	0.447	0.457	0.466	0.477	0.487	0.5	0.513	0.526	0.539	0.552	0.565	0.578	0.589
Assam	0.292	0.298	0.304	0.311	0.318	0.325	0.332	0.339	0.346	0.353	0.361	0.367	0.373	0.378	0.383	0.389	0.394	0.4	0.405	0.413	0.421	0.429	0.437	0.446	0.454	0.462	0.471	0.479	0.487	0.496	0.504	0.514	0.523	0.532	0.542	0.552	0.562
Bihar	0.25	0.255	0.26	0.264	0.27	0.275	0.281	0.287	0.294	0.3	0.306	0.312	0.317	0.318	0.319	0.318	0.318	0.319	0.317	0.317	0.318	0.321	0.325	0.329	0.334	0.338	0.346	0.354	0.363	0.371	0.38	0.39	0.4	0.411	0.421	0.431	0.44
Chhattisgarh	0.256	0.26	0.264	0.269	0.273	0.277	0.282	0.287	0.292	0.298	0.304	0.31	0.316	0.323	0.329	0.336	0.344	0.352	0.36	0.369	0.376	0.386	0.395	0.405	0.414	0.425	0.436	0.449	0.461	0.473	0.485	0.497	0.509	0.521	0.534	0.547	0.558
Delhi	0.471	0.478	0.484	0.49	0.495	0.5	0.505	0.51	0.515	0.52	0.524	0.529	0.533	0.538	0.544	0.55	0.557	0.565	0.573	0.582	0.592	0.602	0.612	0.623	0.633	0.643	0.653	0.664	0.674	0.684	0.694	0.705	0.715	0.724	0.734	0.744	0.752
Goa	0.394	0.401	0.409	0.416	0.424	0.432	0.44	0.448	0.456	0.465	0.473	0.481	0.49	0.5	0.511	0.523	0.535	0.547	0.561	0.573	0.586	0.597	0.608	0.616	0.623	0.631	0.638	0.645	0.654	0.664	0.673	0.686	0.696	0.707	0.718	0.729	0.738
Gujarat	0.319	0.326	0.331	0.338	0.345	0.352	0.359	0.365	0.373	0.38	0.389	0.395	0.403	0.411	0.42	0.429	0.439	0.448	0.457	0.465	0.473	0.48	0.487	0.495	0.503	0.512	0.522	0.532	0.542	0.551	0.561	0.571	0.58	0.589	0.598	0.607	0.615
Haryana	0.317	0.324	0.331	0.338	0.345	0.353	0.361	0.368	0.377	0.386	0.395	0.405	0.413	0.422	0.431	0.439	0.449	0.458	0.467	0.475	0.484	0.494	0.503	0.513	0.524	0.534	0.545	0.557	0.569	0.58	0.591	0.602	0.613	0.624	0.635	0.646	0.655
Himachal Pradesh	0.289	0.296	0.303	0.311	0.318	0.326	0.334	0.343	0.353	0.363	0.374	0.385	0.396	0.407	0.419	0.43	0.443	0.456	0.469	0.483	0.496	0.508	0.52	0.531	0.541	0.553	0.564	0.575	0.587	0.598	0.61	0.622	0.633	0.644	0.654	0.662	0.669
Jammu and Kashmir	0.276	0.282	0.288	0.295	0.301	0.308	0.316	0.322	0.329	0.336	0.343	0.349	0.353	0.361	0.369	0.377	0.384	0.395	0.405	0.417	0.429	0.441	0.452	0.464	0.475	0.486	0.497	0.507	0.518	0.528	0.539	0.549	0.559	0.569	0.579	0.589	0.598
Jharkhand	0.259	0.262	0.266	0.269	0.273	0.277	0.281	0.285	0.29	0.295	0.3	0.306	0.311	0.316	0.322	0.329	0.334	0.342	0.35	0.358	0.365	0.373	0.381	0.389	0.397	0.406	0.415	0.424	0.433	0.442	0.453	0.464	0.475	0.487	0.499	0.512	0.523
Karnataka	0.296	0.301	0.306	0.312	0.318	0.324	0.33	0.337	0.344	0.352	0.36	0.368	0.376	0.385	0.395	0.405	0.415	0.425	0.436	0.447	0.457	0.465	0.474	0.481	0.49	0.499	0.509	0.521	0.532	0.543	0.554	0.565	0.576	0.586	0.596	0.606	0.614
Kerala	0.369	0.374	0.379	0.385	0.391	0.396	0.403	0.409	0.415	0.422	0.429	0.436	0.444	0.454	0.464	0.476	0.488	0.5	0.512	0.524	0.535	0.545	0.553	0.562	0.57	0.58	0.589	0.599	0.608	0.619	0.628	0.638	0.648	0.657	0.667	0.676	0.683
Madhya Pradesh	0.271	0.275	0.28	0.286	0.291	0.297	0.303	0.309	0.317	0.324	0.332	0.339	0.345	0.35	0.355	0.36	0.365	0.37	0.375	0.38	0.385	0.391	0.397	0.404	0.411	0.418	0.425	0.433	0.441	0.45	0.459	0.47	0.481	0.494	0.507	0.52	0.532
Maharashtra	0.339	0.346	0.353	0.36	0.368	0.375	0.383	0.391	0.399	0.408	0.418	0.426	0.435	0.444	0.454	0.464	0.475	0.485	0.494	0.503	0.512	0.519	0.526	0.534	0.543	0.553	0.564	0.576	0.588	0.599	0.61	0.62	0.63	0.639	0.648	0.658	0.666
Manipur	0.28	0.288	0.295	0.302	0.31	0.318	0.326	0.336	0.346	0.355	0.364	0.372	0.38	0.386	0.391	0.395	0.4	0.406	0.413	0.423	0.433	0.444	0.455	0.467	0.48	0.494	0.507	0.518	0.529	0.54	0.549	0.56	0.571	0.581	0.591	0.601	0.61
Meghalaya	0.287	0.291	0.296	0.3	0.305	0.31	0.316	0.323	0.328	0.335	0.342	0.348	0.353	0.358	0.36	0.363	0.367	0.372	0.379	0.389	0.402	0.417	0.431	0.443	0.455	0.467	0.477	0.488	0.498	0.508	0.519	0.53	0.541	0.552	0.563	0.575	0.585
Mizoram	0.334	0.34	0.346	0.352	0.359	0.369	0.378	0.39	0.399	0.407	0.414	0.422	0.429	0.436	0.44	0.446	0.453	0.459	0.465	0.473	0.482	0.492	0.502	0.511	0.52	0.528	0.537	0.545	0.553	0.562	0.572	0.581	0.591	0.601	0.611	0.621	0.63
Nagaland	0.343	0.348	0.354	0.36	0.366	0.371	0.377	0.383	0.389	0.395	0.401	0.406	0.409	0.413	0.416	0.419	0.422	0.428	0.434	0.442	0.456	0.473	0.49	0.505	0.519	0.533	0.545	0.559	0.572	0.585	0.597	0.61	0.622	0.634	0.645	0.654	0.661
Odisha	0.251	0.255	0.259	0.264	0.269	0.274	0.28	0.285	0.292	0.299	0.305	0.312	0.318	0.324	0.33	0.338	0.344	0.351	0.359	0.368	0.376	0.385	0.393	0.402	0.413	0.422	0.433	0.445	0.457	0.468	0.48	0.491	0.503	0.514	0.526	0.537	0.547
Punjab	0.329	0.337	0.344	0.352	0.359	0.367	0.375	0.383	0.392	0.401	0.409	0.418	0.427	0.436	0.445	0.455	0.465	0.474	0.484	0.493	0.503	0.511	0.519	0.527	0.535	0.545	0.555	0.566	0.576	0.587	0.598	0.608	0.618	0.627	0.636	0.643	0.65
Rajasthan	0.254	0.259	0.263	0.27	0.275	0.28	0.286	0.292	0.299	0.306	0.315	0.322	0.329	0.335	0.341	0.348	0.355	0.362	0.368	0.375	0.382	0.39	0.397	0.405	0.414	0.422	0.43	0.44	0.449	0.457	0.468	0.479	0.49	0.5	0.511	0.521	0.53
Sikkim	0.268	0.273	0.279	0.284	0.29	0.297	0.304	0.313	0.321	0.329	0.337	0.346	0.352	0.361	0.369	0.378	0.388	0.398	0.408	0.418	0.43	0.441	0.453	0.466	0.478	0.49	0.501	0.511	0.52	0.536	0.551	0.565	0.579	0.593	0.607	0.62	0.632
Tamil Nadu	0.309	0.315	0.319	0.325	0.33	0.337	0.343	0.351	0.359	0.367	0.376	0.385	0.395	0.405	0.417	0.428	0.44	0.452	0.464	0.476	0.487	0.496	0.505	0.513	0.521	0.531	0.541	0.552	0.563	0.575	0.587	0.599	0.61	0.621	0.632	0.64	0.648
Telangana	0.265	0.269	0.273	0.278	0.282	0.286	0.291	0.296	0.302	0.309	0.317	0.325	0.333	0.342	0.352	0.362	0.373	0.383	0.395	0.406	0.417	0.428	0.438	0.449	0.459	0.47	0.482	0.495	0.509	0.521	0.535	0.549	0.562	0.574	0.587	0.599	0.609
Tripura	0.315	0.319	0.324	0.328	0.331	0.335	0.339	0.343	0.348	0.353	0.358	0.362	0.366	0.372	0.378	0.386	0.395	0.404	0.412	0.425	0.438	0.451	0.462	0.47	0.478	0.485	0.491	0.497	0.504	0.511	0.518	0.527	0.536	0.545	0.554	0.564	0.573
Uttar Pradesh	0.265	0.269	0.273	0.278	0.282	0.287	0.293	0.299	0.306	0.312	0.32	0.326	0.332	0.336	0.34	0.344	0.348	0.351	0.355	0.358	0.362	0.368	0.374	0.382	0.39	0.398	0.407	0.415	0.423	0.432	0.441	0.451	0.462	0.473	0.485	0.498	0.508
Uttarakhand	0.257	0.262	0.267	0.272	0.277	0.283	0.289	0.295	0.302	0.309	0.317	0.324	0.332	0.34	0.349	0.358	0.368	0.379	0.389	0.4	0.412	0.424	0.436	0.449	0.463	0.477	0.491	0.505	0.519	0.535	0.551	0.568	0.583	0.598	0.613	0.628	0.639
West Bengal	0.324	0.328	0.331	0.335	0.339	0.343	0.347	0.352	0.356	0.361	0.366	0.371	0.376	0.382	0.389	0.396	0.404	0.412	0.42	0.429	0.438	0.446	0.454	0.462	0.47	0.478	0.486	0.496	0.506	0.516	0.526	0.536	0.546	0.555	0.565	0.573	0.58
The Six Minor Territories	0.38	0.388	0.397	0.405	0.413	0.42	0.429	0.437	0.445	0.452	0.461	0.468	0.475	0.482	0.49	0.498	0.506	0.516	0.525	0.534	0.543	0.552	0.562	0.572	0.581	0.592	0.602	0.613	0.623	0.634	0.644	0.654	0.663	0.672	0.682	0.691	0.698
Andhra Pradesh, Urban	0.377	0.382	0.387	0.392	0.397	0.403	0.408	0.413	0.42	0.427	0.434	0.441	0.447	0.454	0.462	0.47	0.479	0.488	0.498	0.51	0.521	0.532	0.541	0.55	0.559	0.567	0.576	0.586	0.595	0.604	0.613	0.623	0.632	0.641	0.65	0.66	0.668
Andhra Pradesh, Rural	0.239	0.242	0.246	0.249	0.253	0.257	0.261	0.265	0.271	0.277	0.284	0.291	0.298	0.307	0.317	0.327	0.337	0.347	0.358	0.368	0.379	0.389	0.398	0.407	0.417	0.425	0.435	0.445	0.455	0.465	0.475	0.485	0.495	0.506	0.516	0.528	0.537
Arunachal Pradesh, Urban	0.405	0.411	0.416	0.421	0.427	0.433	0.439	0.445	0.452	0.457	0.463	0.469	0.474	0.481	0.486	0.493	0.499	0.506	0.511	0.516	0.52	0.525	0.528	0.532	0.536	0.541	0.546	0.552	0.557	0.565	0.573	0.582	0.59	0.599	0.61	0.621	0.631
Arunachal Pradesh, Rural	0.251	0.256	0.261	0.265	0.271	0.276	0.283	0.289	0.296	0.302	0.309	0.316	0.323	0.331	0.338	0.346	0.353	0.361	0.369	0.376	0.384	0.392	0.401	0.409	0.419	0.428	0.438	0.45	0.46	0.474	0.488	0.501	0.515	0.528	0.541	0.554	0.565
Assam, Urban	0.443	0.449	0.454	0.46	0.467	0.473	0.478	0.484	0.489	0.494	0.499	0.504	0.508	0.513	0.519	0.525	0.531	0.536	0.541	0.549	0.556	0.564	0.571	0.579	0.586	0.592	0.599	0.606	0.612	0.618	0.624	0.63	0.636	0.643	0.65	0.657	0.663
Assam, Rural	0.274	0.28	0.286	0.292	0.299	0.306	0.313	0.32	0.327	0.334	0.342	0.348	0.353	0.358	0.363	0.368	0.374	0.379	0.384	0.391	0.399	0.407	0.415	0.423	0.432	0.44	0.449	0.457	0.466	0.474	0.483	0.492	0.501	0.511	0.521	0.532	0.542
Bihar, Urban	0.37	0.377	0.384	0.391	0.399	0.406	0.413	0.42	0.428	0.434	0.441	0.447	0.453	0.456	0.459	0.46	0.463	0.466	0.467	0.468	0.471	0.474	0.479	0.482	0.487	0.491	0.498	0.505	0.513	0.52	0.529	0.537	0.546	0.554	0.561	0.568	0.575
Bihar, Rural	0.231	0.235	0.239	0.243	0.248	0.253	0.258	0.264	0.27	0.275	0.281	0.287	0.292	0.294	0.294	0.294	0.294	0.296	0.295	0.295	0.297	0.299	0.304	0.308	0.313	0.317	0.325	0.333	0.342	0.35	0.359	0.368	0.379	0.389	0.4	0.41	0.419
Chhattisgarh, Urban	0.456	0.462	0.468	0.472	0.477	0.481	0.485	0.489	0.492	0.496	0.5	0.503	0.507	0.51	0.513	0.517	0.521	0.527	0.533	0.539	0.543	0.55	0.557	0.565	0.572	0.581	0.59	0.601	0.611	0.62	0.628	0.637	0.645	0.653	0.661	0.669	0.677
Chhattisgarh, Rural	0.203	0.206	0.21	0.214	0.218	0.223	0.228	0.233	0.238	0.244	0.251	0.257	0.264	0.271	0.278	0.286	0.294	0.302	0.31	0.319	0.328	0.337	0.346	0.356	0.365	0.376	0.387	0.399	0.412	0.424	0.435	0.448	0.461	0.474	0.488	0.502	0.514
Delhi, Urban	0.478	0.485	0.491	0.497	0.502	0.506	0.512	0.517	0.521	0.526	0.53	0.535	0.539	0.543	0.549	0.555	0.561	0.569	0.577	0.586	0.596	0.606	0.616	0.626	0.636	0.646	0.656	0.666	0.676	0.686	0.696	0.706	0.716	0.726	0.736	0.746	0.753
Delhi, Rural	0.388	0.395	0.402	0.409	0.415	0.422	0.429	0.436	0.443	0.45	0.457	0.464	0.471	0.477	0.483	0.49	0.497	0.505	0.515	0.525	0.536	0.547	0.558	0.569	0.58	0.591	0.603	0.614	0.624	0.636	0.647	0.658	0.669	0.681	0.692	0.703	0.713
Goa, Urban	0.459	0.464	0.471	0.476	0.483	0.489	0.495	0.501	0.507	0.514	0.52	0.526	0.533	0.541	0.551	0.561	0.572	0.582	0.595	0.606	0.616	0.626	0.635	0.642	0.648	0.654	0.66	0.665	0.672	0.681	0.69	0.701	0.711	0.721	0.732	0.744	0.753
Goa, Rural	0.365	0.372	0.38	0.386	0.394	0.401	0.409	0.417	0.424	0.433	0.441	0.449	0.458	0.468	0.479	0.491	0.503	0.515	0.529	0.542	0.554	0.566	0.577	0.585	0.592	0.6	0.608	0.616	0.625	0.636	0.646	0.658	0.669	0.679	0.69	0.701	0.71
Gujarat, Urban	0.408	0.415	0.422	0.431	0.439	0.446	0.454	0.459	0.467	0.474	0.482	0.487	0.494	0.501	0.51	0.519	0.529	0.538	0.547	0.555	0.562	0.569	0.575	0.581	0.587	0.594	0.602	0.611	0.618	0.627	0.635	0.643	0.65	0.657	0.664	0.671	0.677
Gujarat, Rural	0.273	0.278	0.283	0.289	0.294	0.3	0.306	0.312	0.319	0.326	0.334	0.341	0.349	0.356	0.364	0.373	0.382	0.39	0.399	0.407	0.414	0.421	0.428	0.435	0.443	0.452	0.461	0.471	0.48	0.49	0.5	0.51	0.52	0.53	0.541	0.552	0.561
Haryana, Urban	0.428	0.436	0.444	0.452	0.46	0.468	0.476	0.483	0.491	0.498	0.506	0.514	0.521	0.528	0.536	0.543	0.552	0.56	0.567	0.575	0.582	0.59	0.596	0.603	0.61	0.617	0.626	0.635	0.645	0.655	0.665	0.674	0.683	0.691	0.7	0.709	0.717
Haryana, Rural	0.284	0.29	0.296	0.302	0.308	0.315	0.322	0.329	0.337	0.346	0.354	0.364	0.372	0.38	0.389	0.397	0.407	0.416	0.424	0.433	0.441	0.45	0.459	0.469	0.478	0.488	0.499	0.511	0.523	0.535	0.547	0.559	0.57	0.581	0.592	0.604	0.614
Himachal Pradesh, Urban	0.433	0.441	0.449	0.457	0.464	0.472	0.48	0.488	0.497	0.505	0.514	0.522	0.531	0.54	0.549	0.558	0.569	0.579	0.59	0.602	0.614	0.624	0.635	0.645	0.655	0.665	0.675	0.685	0.692	0.699	0.708	0.715	0.723	0.729	0.736	0.743	0.75

Appendix Table 14: Socio-Demographic Index values for all estimated GBD 2016 locations, 1980–2016

Location	1980	1981	1982	1983	1984	1985	1986	1987	1988	1989	1990	1991	1992	1993	1994	1995	1996	1997	1998	1999	2000	2001	2002	2003	2004	2005	2006	2007	2008	2009	2010	2011	2012	2013	2014	2015	2016
Himachal Pradesh, Rural	0.275	0.282	0.289	0.297	0.304	0.312	0.32	0.329	0.339	0.349	0.36	0.37	0.381	0.393	0.404	0.416	0.429	0.442	0.455	0.469	0.482	0.495	0.506	0.517	0.528	0.539	0.551	0.563	0.574	0.585	0.598	0.61	0.621	0.632	0.643	0.652	0.659
Jammu and Kashmir, Urban	0.366	0.374	0.383	0.392	0.401	0.411	0.42	0.428	0.437	0.444	0.451	0.457	0.462	0.47	0.478	0.486	0.494	0.506	0.516	0.528	0.54	0.551	0.562	0.572	0.581	0.59	0.599	0.607	0.615	0.623	0.629	0.636	0.643	0.65	0.657	0.664	0.671
Jammu and Kashmir, Rural	0.247	0.252	0.258	0.263	0.269	0.275	0.282	0.287	0.294	0.301	0.307	0.312	0.317	0.324	0.332	0.339	0.346	0.357	0.366	0.378	0.389	0.4	0.412	0.423	0.435	0.446	0.458	0.468	0.479	0.49	0.5	0.511	0.522	0.532	0.543	0.553	0.562
Jharkhand, Urban	0.453	0.457	0.462	0.466	0.469	0.472	0.476	0.479	0.483	0.487	0.492	0.497	0.502	0.508	0.514	0.521	0.527	0.536	0.545	0.554	0.561	0.569	0.577	0.584	0.591	0.599	0.607	0.615	0.622	0.63	0.638	0.646	0.653	0.659	0.666	0.673	0.679
Jharkhand, Rural	0.233	0.236	0.239	0.242	0.245	0.248	0.251	0.255	0.259	0.264	0.269	0.274	0.279	0.285	0.292	0.299	0.305	0.313	0.321	0.33	0.338	0.346	0.354	0.362	0.37	0.379	0.387	0.397	0.405	0.415	0.425	0.436	0.448	0.459	0.472	0.485	0.496
Karnataka, Urban	0.391	0.398	0.404	0.412	0.42	0.427	0.434	0.441	0.449	0.456	0.463	0.471	0.478	0.486	0.495	0.505	0.515	0.525	0.536	0.546	0.557	0.565	0.574	0.581	0.589	0.598	0.607	0.616	0.624	0.631	0.639	0.647	0.655	0.664	0.672	0.681	0.688
Karnataka, Rural	0.251	0.256	0.26	0.264	0.269	0.274	0.28	0.286	0.292	0.299	0.306	0.314	0.322	0.33	0.339	0.348	0.357	0.367	0.377	0.387	0.397	0.406	0.415	0.423	0.432	0.442	0.453	0.464	0.475	0.485	0.496	0.507	0.518	0.529	0.54	0.552	0.561
Kerala, Urban	0.408	0.412	0.417	0.422	0.428	0.432	0.438	0.443	0.448	0.454	0.46	0.466	0.474	0.483	0.494	0.506	0.518	0.53	0.542	0.554	0.565	0.574	0.582	0.589	0.597	0.605	0.613	0.621	0.629	0.638	0.646	0.655	0.664	0.673	0.683	0.692	0.699
Kerala, Rural	0.358	0.363	0.368	0.374	0.38	0.385	0.391	0.397	0.404	0.411	0.418	0.425	0.433	0.442	0.452	0.464	0.476	0.488	0.5	0.513	0.524	0.534	0.543	0.551	0.559	0.568	0.577	0.586	0.595	0.605	0.613	0.623	0.632	0.642	0.653	0.662	0.669
Madhya Pradesh, Urban	0.403	0.409	0.415	0.422	0.428	0.435	0.441	0.448	0.455	0.461	0.469	0.476	0.483	0.49	0.497	0.504	0.511	0.517	0.522	0.527	0.531	0.535	0.538	0.541	0.545	0.55	0.557	0.564	0.572	0.581	0.591	0.6	0.609	0.618	0.626	0.635	0.643
Madhya Pradesh, Rural	0.229	0.232	0.236	0.24	0.245	0.249	0.254	0.26	0.266	0.272	0.28	0.286	0.292	0.296	0.3	0.305	0.309	0.314	0.318	0.322	0.325	0.331	0.336	0.342	0.349	0.355	0.362	0.37	0.378	0.388	0.399	0.411	0.423	0.436	0.451	0.465	0.478
Maharashtra, Urban	0.421	0.428	0.436	0.444	0.451	0.458	0.465	0.472	0.478	0.486	0.493	0.499	0.506	0.514	0.522	0.531	0.54	0.55	0.558	0.568	0.576	0.584	0.591	0.598	0.606	0.616	0.627	0.638	0.648	0.658	0.669	0.678	0.687	0.695	0.703	0.712	0.719
Maharashtra, Rural	0.288	0.295	0.301	0.308	0.314	0.322	0.329	0.337	0.345	0.354	0.364	0.373	0.382	0.391	0.4	0.41	0.421	0.43	0.439	0.449	0.457	0.464	0.472	0.48	0.488	0.498	0.51	0.522	0.533	0.544	0.556	0.567	0.577	0.586	0.596	0.607	0.615
Manipur, Urban	0.346	0.353	0.361	0.369	0.378	0.385	0.393	0.402	0.411	0.419	0.427	0.434	0.441	0.447	0.453	0.458	0.465	0.472	0.479	0.489	0.498	0.509	0.519	0.53	0.541	0.554	0.564	0.574	0.582	0.592	0.6	0.609	0.618	0.627	0.636	0.643	0.65
Manipur, Rural	0.265	0.272	0.278	0.285	0.292	0.299	0.306	0.315	0.324	0.332	0.341	0.349	0.356	0.362	0.367	0.371	0.375	0.381	0.387	0.397	0.408	0.419	0.43	0.442	0.455	0.469	0.482	0.494	0.504	0.515	0.525	0.535	0.545	0.555	0.565	0.575	0.585
Meghalaya, Urban	0.456	0.459	0.46	0.462	0.463	0.465	0.466	0.468	0.469	0.471	0.473	0.474	0.474	0.476	0.479	0.484	0.49	0.498	0.506	0.516	0.529	0.545	0.559	0.572	0.584	0.595	0.605	0.613	0.621	0.63	0.638	0.648	0.657	0.667	0.677	0.687	0.696
Meghalaya, Rural	0.25	0.254	0.258	0.263	0.268	0.273	0.279	0.286	0.292	0.299	0.307	0.313	0.319	0.323	0.325	0.328	0.331	0.336	0.342	0.352	0.365	0.379	0.393	0.406	0.417	0.429	0.44	0.451	0.461	0.472	0.483	0.495	0.505	0.516	0.528	0.54	0.55
Mizoram, Urban	0.383	0.389	0.395	0.401	0.408	0.417	0.426	0.437	0.446	0.453	0.459	0.466	0.472	0.479	0.485	0.492	0.5	0.508	0.514	0.522	0.531	0.54	0.548	0.555	0.562	0.568	0.575	0.581	0.587	0.593	0.601	0.608	0.616	0.625	0.633	0.643	0.651
Mizoram, Rural	0.292	0.298	0.303	0.309	0.315	0.325	0.334	0.346	0.355	0.363	0.37	0.378	0.385	0.392	0.395	0.398	0.404	0.408	0.413	0.42	0.43	0.44	0.451	0.461	0.472	0.482	0.491	0.502	0.512	0.523	0.535	0.546	0.557	0.568	0.58	0.591	0.6
Nagaland, Urban	0.446	0.451	0.458	0.466	0.473	0.48	0.486	0.494	0.501	0.507	0.513	0.517	0.521	0.525	0.527	0.53	0.532	0.536	0.54	0.544	0.553	0.564	0.576	0.586	0.595	0.604	0.612	0.622	0.632	0.641	0.65	0.66	0.67	0.68	0.691	0.702	0.711
Nagaland, Rural	0.328	0.333	0.338	0.343	0.348	0.353	0.357	0.363	0.368	0.374	0.379	0.383	0.387	0.39	0.393	0.395	0.399	0.405	0.412	0.42	0.435	0.452	0.469	0.484	0.498	0.512	0.524	0.537	0.55	0.563	0.575	0.588	0.6	0.611	0.622	0.629	0.637
Odisha, Urban	0.37	0.376	0.382	0.389	0.396	0.403	0.411	0.417	0.424	0.432	0.438	0.444	0.45	0.455	0.461	0.469	0.474	0.481	0.488	0.496	0.504	0.512	0.519	0.527	0.536	0.544	0.552	0.561	0.569	0.575	0.583	0.591	0.599	0.608	0.617	0.627	0.635
Odisha, Rural	0.233	0.237	0.24	0.245	0.248	0.253	0.259	0.263	0.269	0.276	0.281	0.287	0.293	0.299	0.305	0.312	0.318	0.325	0.333	0.341	0.35	0.358	0.366	0.376	0.387	0.397	0.408	0.42	0.432	0.443	0.455	0.467	0.479	0.49	0.502	0.514	0.524
Punjab, Urban	0.441	0.449	0.456	0.463	0.47	0.477	0.484	0.491	0.498	0.506	0.513	0.519	0.526	0.533	0.541	0.548	0.556	0.564	0.571	0.578	0.585	0.592	0.598	0.605	0.611	0.619	0.627	0.636	0.645	0.654	0.662	0.671	0.679	0.688	0.696	0.703	0.709
Punjab, Rural	0.286	0.293	0.3	0.307	0.314	0.322	0.33	0.338	0.346	0.355	0.364	0.373	0.381	0.39	0.399	0.408	0.418	0.427	0.437	0.447	0.457	0.466	0.474	0.483	0.492	0.502	0.513	0.524	0.535	0.546	0.556	0.566	0.577	0.587	0.596	0.604	0.611
Rajasthan, Urban	0.374	0.379	0.384	0.39	0.396	0.401	0.407	0.412	0.419	0.425	0.433	0.44	0.446	0.452	0.459	0.465	0.473	0.481	0.489	0.499	0.507	0.516	0.523	0.531	0.539	0.546	0.554	0.561	0.569	0.576	0.585	0.594	0.602	0.61	0.618	0.626	0.632
Rajasthan, Rural	0.218	0.222	0.226	0.232	0.236	0.241	0.246	0.251	0.258	0.265	0.273	0.28	0.286	0.292	0.299	0.305	0.312	0.318	0.324	0.331	0.337	0.345	0.352	0.36	0.368	0.376	0.385	0.395	0.404	0.412	0.423	0.434	0.445	0.456	0.467	0.478	0.487
Sikkim, Urban	0.371	0.377	0.386	0.394	0.404	0.414	0.426	0.437	0.448	0.459	0.469	0.477	0.483	0.491	0.498	0.504	0.511	0.518	0.525	0.533	0.542	0.551	0.56	0.57	0.579	0.588	0.595	0.601	0.605	0.618	0.628	0.639	0.649	0.66	0.672	0.685	0.695
Sikkim, Rural	0.257	0.261	0.266	0.271	0.276	0.283	0.29	0.297	0.305	0.313	0.321	0.329	0.335	0.343	0.352	0.36	0.37	0.38	0.39	0.4	0.411	0.422	0.434	0.445	0.457	0.469	0.479	0.489	0.496	0.513	0.527	0.542	0.555	0.569	0.584	0.598	0.61
Tamil Nadu, Urban	0.391	0.398	0.403	0.408	0.413	0.419	0.425	0.432	0.439	0.446	0.453	0.459	0.467	0.476	0.486	0.495	0.505	0.515	0.526	0.537	0.547	0.557	0.565	0.573	0.581	0.59	0.599	0.609	0.617	0.627	0.636	0.646	0.656	0.667	0.678	0.686	0.693
Tamil Nadu, Rural	0.264	0.27	0.274	0.279	0.285	0.292	0.299	0.307	0.315	0.324	0.333	0.341	0.35	0.359	0.369	0.379	0.389	0.399	0.411	0.422	0.433	0.443	0.453	0.462	0.472	0.483	0.494	0.506	0.516	0.528	0.539	0.551	0.562	0.574	0.586	0.595	0.603
Telangana, Urban	0.37	0.376	0.382	0.389	0.396	0.402	0.409	0.416	0.424	0.432	0.44	0.449	0.457	0.465	0.474	0.483	0.494	0.503	0.514	0.526	0.539	0.55	0.56	0.569	0.578	0.587	0.596	0.607	0.618	0.626	0.637	0.647	0.658	0.668	0.679	0.689	0.698
Telangana, Rural	0.225	0.228	0.231	0.234	0.237	0.24	0.244	0.248	0.253	0.259	0.265	0.272	0.28	0.289	0.298	0.308	0.32	0.33	0.342	0.353	0.364	0.375	0.385	0.396	0.406	0.417	0.429	0.441	0.454	0.466	0.48	0.493	0.506	0.519	0.533	0.547	0.558
Tripura, Urban	0.447	0.45	0.453	0.455	0.456	0.458	0.459	0.46	0.463	0.465	0.467	0.469	0.469	0.474	0.479	0.485	0.493	0.501	0.507	0.519	0.53	0.542	0.549	0.555	0.559	0.563	0.567	0.57	0.574	0.579	0.584	0.592	0.599	0.608	0.617	0.627	0.635
Tripura, Rural	0.294	0.298	0.302	0.306	0.31	0.313	0.317	0.321	0.326	0.331	0.336	0.34	0.344	0.351	0.357	0.365	0.373	0.382	0.39	0.403	0.416	0.429	0.439	0.448	0.455	0.461	0.467	0.472	0.479	0.486	0.493	0.501	0.509	0.518	0.528	0.538	0.546
Uttar Pradesh, Urban	0.367	0.371	0.377	0.382	0.387	0.393	0.399	0.404	0.41	0.417	0.423	0.43	0.435	0.44	0.446	0.451	0.457	0.463	0.468	0.473	0.478	0.484	0.49	0.496	0.502	0.509	0.516	0.524	0.531	0.538	0.546	0.554	0.562	0.57	0.578	0.587	0.594
Uttar Pradesh, Rural	0.24	0.243	0.247	0.251	0.255	0.259	0.264	0.27	0.276	0.282	0.289	0.295	0.3	0.304	0.308	0.311	0.314	0.317	0.32	0.323	0.327	0.332	0.339	0.347	0.355	0.364	0.372	0.381	0.389	0.398	0.407	0.417	0.429	0.441	0.454	0.468	0.479
Uttarakhand, Urban	0.401	0.406	0.412	0.417	0.422	0.427	0.432	0.437	0.442	0.448	0.454	0.459	0.464	0.469	0.476	0.482	0.489	0.496	0.503	0.511	0.519	0.528	0.537	0.547	0.556	0.567	0.578	0.589	0.6	0.615	0.628	0.642	0.656	0.669	0.682	0.695	0.706
Uttarakhand, Rural	0.223	0.227	0.231	0.235	0.24	0.245	0.25	0.256	0.263	0.269	0.277	0.284	0.292	0.3	0.309	0.318	0.328	0.338	0.349	0.36	0.372	0.385	0.397	0.411	0.424	0.439	0.453	0.468	0.482	0.499	0.515	0.531	0.547	0.562	0.577	0.592	0.604
West Bengal, Urban	0.44	0.444	0.45	0.455	0.461	0.466	0.471	0.476	0.481	0.486	0.491	0.496	0.501	0.507	0.513	0.52	0.527	0.535	0.543	0.552	0.56	0.568	0.574	0.58	0.586	0.591	0.597	0.604	0.612	0.619	0.625	0.632	0.638	0.644	0.651	0.658	0.665
West Bengal, Rural	0.275	0.279	0.281	0.284	0.288	0.291	0.295	0.299	0.303	0.307	0.312	0.317	0.323	0.329	0.335	0.343	0.351	0.358	0.367	0.376	0.384	0.392	0.4	0.407	0.414	0.422	0.43	0.441	0.451	0.462	0.473	0.483	0.493	0.504	0.514	0.525	0.533
The Six Minor Territories, Rural	0.293	0.299	0.306	0.312	0.319	0.326	0.333	0.34	0.348	0.355	0.363	0.371	0.379	0.387	0.397	0.406	0.416	0.428	0.439	0.451	0.462	0.474	0.487	0.499	0.512	0.525	0.538	0.552	0.565	0.579	0.591	0.603	0.61	0.617	0.623	0.63	0.637
The Six Minor Territories, Urban	0.477	0.483	0.488	0.493	0.497	0.501	0.506	0.51	0.514	0.519	0.523	0.528	0.533	0.538	0.544	0.55	0.557	0.565	0.572	0.579	0.586	0.593	0.6	0.607	0.614	0.622	0.63	0.638	0.645	0.654	0.661	0.669	0.677	0.685	0.693	0.702	0.71
Sub-Saharan Africa	0.263	0.268	0.272	0.277	0.282	0.287	0.291	0.295	0.298	0.302	0.307	0.311	0.316	0.321	0.325	0.329	0.334	0.339	0.343	0.346	0.35	0.354	0.358	0.362	0.366	0.371	0.377	0.383	0.389	0.395	0.401	0.408	0.416	0.423	0.43	0.437	0.442
Central Sub-Saharan Africa	0.259	0.262	0.266	0.271	0.275	0.28	0.283	0.287	0.291	0.295	0.298	0.302	0.305	0.306	0.307	0.308	0.31	0.312	0.314	0.315	0.316	0.317	0.32	0.322	0.327	0.332	0.338	0.345	0.352	0.359	0.366	0.374	0.382	0.388	0.394	0.401	0.406
Angola	0.218	0.22	0.223	0.225	0.229	0.232	0.235	0.239	0.243	0.248	0.252	0.256	0.26	0.262	0.265	0.268	0.272	0.276	0.281	0.286	0.289	0.294	0.299	0.305	0.312	0.32	0.329	0.339	0.349	0.357	0.366	0.377	0.388	0.4	0.411	0.422	0.431
Central African Republic	0.212	0.216	0.22	0.222	0.226	0.23	0.233	0.236	0.24	0.243	0.247	0.25	0.252	0.254	0.258	0.261	0.263	0.266	0.269	0.273	0.276	0.279	0.283	0.285	0.288	0.291	0.295	0.299	0.303	0.307	0.311	0.316	0.322	0.32	0.319	0.318	0.316
Congo (Brazzaville)	0.3	0.308	0.317	0.326	0.335	0.344	0.351	0.358	0.365	0.372	0.379	0.386	0.393	0.399	0.404	0.41	0.415	0.421	0.426	0.43	0.434	0.439	0.444	0.45	0.456	0.462	0.467	0.471	0.475	0.48	0.487	0.495	0.503	0.511	0.519	0.526	0.531
Democratic Republic of the Congo	0.246	0.249	0.253	0.257	0.262	0.267	0.271	0.274	0.277	0.281	0.283	0.284	0.285	0.284	0.282	0.28	0.277	0.273	0.268	0.264	0.257	0.251	0.246	0.243	0.242	0.243	0.242	0.241	0.242	0.244	0.251	0.26	0.27	0.282	0.292	0.304	0.312
Equatorial Guinea	0.242	0.252	0.26	0.268	0.275	0.279	0.282	0.284	0.286	0.289	0.29	0.292	0.297	0.303	0.311	0.321	0.343	0.382	0.409	0.433	0.459	0.486	0.509	0.528	0.548	0.565	0.579	0.592	0.605	0.614	0.624	0.633	0.643	0.651	0.658	0.665	0.669
Gabon	0.38	0.389	0.397	0.406	0.414	0.422	0.429	0.435	0.442	0.45	0.458	0.466	0.474	0.482	0.49	0.498	0.506	0.514	0.521	0.527	0.531	0.534	0.539	0.545	0.551	0.558	0.564	0.57	0.576	0.581	0.586	0.592	0.599	0.605	0.611	0.617	0.622
Eastern Sub-Saharan Africa	0.176	0.181	0.185	0.19	0.196	0.201	0.207	0.212	0.217	0.222	0.228	0.234	0.239	0.244	0.249	0.254	0.259	0.264	0.268	0.273	0.277	0.282	0.286	0.291	0.296	0.302	0.308	0.314	0.321	0.329	0.337	0.345	0.354	0.363	0.371	0.38	0.387
Burundi	0.131	0.137	0.142	0.148	0.154	0.16	0.166	0.172	0.178	0.183	0.188	0.193	0.197	0.2	0.202	0.202	0.202	0.201	0.202	0.202	0.204	0.205	0.208	0.21	0.214	0.217	0.221	0.224	0.229	0.234	0.24	0.246	0.252	0.259	0.266	0.272	0.277
Comoros	0.208	0.213	0.219	0.225	0.232	0.239	0.246	0.253	0.26	0.268	0.275	0.282	0.289	0.297	0.303	0.309	0.315	0.321	0.325	0.33	0.335	0.34	0.346	0.352	0.359	0.366	0.373	0.381	0.388	0.395	0.403	0.41	0.419	0.427	0.436	0.443	0.449
Djibouti	0.089	0.1	0.11	0.121	0.135	0.148	0.16	0.171	0.181	0.19	0.199	0.207	0.215	0.223	0.23	0.237	0.244	0.252	0.258	0.265	0.272	0.28	0.288	0.296	0.304	0.312	0.321	0.331	0.34	0.35	0.359	0.369	0.379	0.389	0.399	0.409	0.416
Eritrea	0.143	0.146	0.15	0.154	0.158	0.163	0.167	0.172	0.177	0.182	0.187	0.193	0.2	0.208	0.218	0.229	0.241	0.254	0.267	0.279	0.29	0.3	0.309	0.315	0.321	0.326	0.33	0.335	0.338	0.342	0.346	0.352	0.359	0.367	0.375	0.383	0.389
Ethiopia	0.122	0.125	0.128	0.132	0.136	0.139	0.142	0.145	0.148	0.15	0.153	0.156	0.158	0.161	0.163	0.166	0.171	0.175	0.179	0.182	0.187	0.192	0.196	0.2	0.205	0.212	0.22	0.229	0.239	0.25	0.26	0.272	0.284	0.295	0.306	0.317	0.326

Appendix Table 14: Socio-Demographic Index values for all estimated GBD 2016 locations, 1980–2016

Location	1980	1981	1982	1983	1984	1985	1986	1987	1988	1989	1990	1991	1992	1993	1994	1995	1996	1997	1998	1999	2000	2001	2002	2003	2004	2005	2006	2007	2008	2009	2010	2011	2012	2013	2014	2015	2016
Kenya	0.22	0.232	0.245	0.257	0.269	0.28	0.291	0.302	0.313	0.323	0.335	0.346	0.357	0.367	0.376	0.384	0.393	0.4	0.405	0.41	0.415	0.42	0.425	0.429	0.434	0.44	0.446	0.452	0.459	0.465	0.473	0.481	0.489	0.498	0.506	0.514	0.52
Madagascar	0.195	0.201	0.207	0.213	0.219	0.226	0.232	0.238	0.244	0.249	0.255	0.26	0.264	0.267	0.269	0.272	0.275	0.278	0.282	0.286	0.289	0.294	0.297	0.302	0.306	0.311	0.315	0.319	0.323	0.327	0.331	0.338	0.344	0.35	0.355	0.361	0.365
Malawi	0.143	0.149	0.155	0.161	0.167	0.175	0.182	0.188	0.194	0.199	0.205	0.21	0.215	0.219	0.223	0.227	0.232	0.238	0.243	0.249	0.254	0.258	0.263	0.268	0.273	0.279	0.284	0.29	0.296	0.302	0.308	0.314	0.32	0.326	0.334	0.342	0.348
Mozambique	0.131	0.133	0.135	0.136	0.137	0.138	0.138	0.139	0.14	0.142	0.145	0.149	0.151	0.153	0.157	0.158	0.165	0.172	0.18	0.186	0.193	0.2	0.207	0.214	0.22	0.226	0.231	0.236	0.242	0.247	0.253	0.26	0.268	0.277	0.287	0.297	0.306
Rwanda	0.065	0.067	0.068	0.069	0.07	0.071	0.089	0.12	0.144	0.164	0.181	0.197	0.208	0.217	0.22	0.223	0.226	0.23	0.234	0.237	0.24	0.244	0.25	0.256	0.263	0.272	0.282	0.293	0.305	0.318	0.33	0.342	0.354	0.365	0.374	0.383	0.39
Somalia	0.161	0.162	0.164	0.166	0.168	0.171	0.173	0.176	0.18	0.183	0.187	0.19	0.192	0.194	0.195	0.196	0.197	0.198	0.199	0.2	0.201	0.203	0.205	0.208	0.212	0.216	0.22	0.225	0.23	0.235	0.24	0.245	0.251	0.256	0.26	0.265	0.268
Tanzania	0.176	0.183	0.19	0.197	0.205	0.213	0.22	0.226	0.233	0.239	0.245	0.251	0.256	0.259	0.263	0.271	0.279	0.288	0.295	0.302	0.308	0.314	0.32	0.326	0.332	0.339	0.345	0.352	0.359	0.367	0.375	0.384	0.392	0.402	0.411	0.421	0.428
Uganda	0.101	0.11	0.117	0.125	0.134	0.142	0.149	0.156	0.163	0.17	0.177	0.184	0.19	0.196	0.203	0.209	0.215	0.22	0.226	0.232	0.238	0.244	0.251	0.257	0.265	0.272	0.28	0.289	0.298	0.308	0.317	0.328	0.338	0.348	0.359	0.37	0.377
Zambia	0.193	0.205	0.216	0.228	0.239	0.251	0.261	0.271	0.281	0.291	0.299	0.307	0.315	0.321	0.326	0.331	0.336	0.34	0.343	0.346	0.35	0.354	0.357	0.362	0.366	0.371	0.377	0.385	0.393	0.401	0.41	0.419	0.428	0.437	0.446	0.454	0.46
South Sudan	0.053	0.054	0.054	0.055	0.056	0.056	0.057	0.062	0.07	0.077	0.082	0.088	0.092	0.095	0.098	0.102	0.105	0.108	0.111	0.114	0.117	0.12	0.123	0.127	0.13	0.134	0.138	0.143	0.148	0.152	0.158	0.164	0.17	0.176	0.182	0.188	0.192
Central	0.292	0.305	0.318	0.33	0.343	0.356	0.368	0.379	0.39	0.401	0.413	0.425	0.435	0.444	0.453	0.462	0.47	0.476	0.482	0.487	0.492	0.497	0.501	0.505	0.51	0.515	0.521	0.527	0.533	0.54	0.546	0.553	0.56	0.566	0.573	0.58	0.585
Coast	0.146	0.16	0.172	0.185	0.196	0.205	0.214	0.222	0.231	0.241	0.252	0.264	0.275	0.286	0.296	0.305	0.313	0.321	0.327	0.332	0.337	0.343	0.348	0.354	0.361	0.368	0.376	0.385	0.394	0.403	0.413	0.423	0.434	0.444	0.455	0.465	0.473
Eastern	0.196	0.209	0.221	0.233	0.245	0.256	0.266	0.277	0.287	0.298	0.31	0.322	0.333	0.344	0.353	0.362	0.371	0.379	0.385	0.391	0.396	0.401	0.407	0.412	0.418	0.424	0.431	0.438	0.445	0.452	0.46	0.467	0.475	0.483	0.49	0.498	0.503
Nairobi	0.42	0.431	0.442	0.453	0.464	0.475	0.486	0.496	0.506	0.516	0.526	0.536	0.545	0.553	0.56	0.567	0.574	0.58	0.585	0.589	0.593	0.598	0.602	0.607	0.611	0.617	0.623	0.63	0.637	0.644	0.652	0.66	0.669	0.677	0.685	0.694	0.701
North Eastern	0.049	0.05	0.051	0.052	0.053	0.053	0.054	0.055	0.056	0.057	0.058	0.059	0.061	0.062	0.067	0.075	0.08	0.081	0.076	0.068	0.069	0.07	0.071	0.072	0.073	0.074	0.075	0.077	0.078	0.079	0.08	0.081	0.083	0.084	0.092	0.112	0.124
Nyanza	0.204	0.216	0.229	0.241	0.253	0.264	0.274	0.285	0.295	0.306	0.318	0.331	0.343	0.353	0.364	0.373	0.383	0.391	0.398	0.404	0.411	0.417	0.423	0.429	0.436	0.443	0.45	0.458	0.466	0.475	0.483	0.491	0.5	0.508	0.516	0.524	0.53
Rift Valley	0.173	0.188	0.202	0.216	0.228	0.239	0.249	0.259	0.269	0.28	0.292	0.305	0.316	0.327	0.337	0.347	0.356	0.363	0.37	0.375	0.381	0.386	0.392	0.397	0.403	0.41	0.417	0.424	0.432	0.44	0.448	0.457	0.465	0.474	0.482	0.491	0.497
Western	0.228	0.24	0.251	0.263	0.274	0.284	0.294	0.303	0.312	0.322	0.332	0.343	0.353	0.362	0.37	0.378	0.385	0.391	0.396	0.4	0.404	0.408	0.412	0.416	0.421	0.426	0.431	0.437	0.443	0.45	0.456	0.463	0.47	0.477	0.484	0.491	0.496
Kiambu	0.313	0.327	0.341	0.355	0.368	0.382	0.394	0.406	0.418	0.429	0.441	0.452	0.462	0.47	0.478	0.486	0.493	0.498	0.503	0.507	0.511	0.515	0.519	0.523	0.527	0.531	0.536	0.541	0.547	0.553	0.559	0.566	0.574	0.581	0.588	0.596	0.601
Kirinyaga	0.28	0.292	0.303	0.314	0.326	0.337	0.348	0.358	0.369	0.379	0.389	0.399	0.408	0.417	0.427	0.437	0.446	0.454	0.461	0.467	0.473	0.479	0.484	0.49	0.495	0.501	0.508	0.514	0.521	0.527	0.533	0.54	0.546	0.552	0.558	0.564	0.569
Murang’a	0.278	0.29	0.303	0.315	0.328	0.34	0.352	0.363	0.374	0.384	0.395	0.406	0.415	0.424	0.432	0.44	0.447	0.454	0.461	0.466	0.472	0.477	0.482	0.487	0.492	0.497	0.503	0.509	0.515	0.52	0.526	0.531	0.537	0.542	0.547	0.552	0.556
Nyandarua	0.263	0.275	0.287	0.3	0.312	0.325	0.337	0.348	0.36	0.371	0.383	0.395	0.405	0.415	0.423	0.431	0.439	0.446	0.452	0.457	0.462	0.466	0.47	0.474	0.479	0.484	0.489	0.496	0.503	0.51	0.518	0.526	0.534	0.542	0.55	0.558	0.564
Nyeri	0.292	0.305	0.318	0.332	0.345	0.358	0.37	0.382	0.394	0.407	0.419	0.432	0.443	0.453	0.462	0.47	0.477	0.483	0.488	0.492	0.496	0.5	0.503	0.507	0.512	0.517	0.524	0.531	0.538	0.546	0.553	0.56	0.566	0.572	0.577	0.583	0.587
Kilifi	0.085	0.086	0.087	0.088	0.089	0.09	0.098	0.111	0.122	0.134	0.15	0.166	0.182	0.197	0.212	0.226	0.238	0.249	0.258	0.266	0.273	0.28	0.286	0.291	0.295	0.301	0.306	0.313	0.321	0.33	0.341	0.352	0.364	0.375	0.387	0.398	0.406
Kwale	0.13	0.141	0.151	0.161	0.17	0.177	0.185	0.192	0.2	0.209	0.221	0.234	0.246	0.255	0.261	0.266	0.271	0.275	0.278	0.28	0.281	0.283	0.285	0.289	0.294	0.3	0.306	0.314	0.324	0.334	0.346	0.357	0.369	0.381	0.393	0.404	0.412
Lamu	0.168	0.181	0.193	0.205	0.215	0.224	0.233	0.241	0.249	0.255	0.262	0.269	0.276	0.283	0.292	0.3	0.309	0.317	0.325	0.332	0.338	0.344	0.349	0.355	0.36	0.365	0.371	0.376	0.381	0.388	0.397	0.406	0.415	0.425	0.434	0.444	0.45
Mombasa	0.246	0.259	0.271	0.283	0.294	0.305	0.316	0.326	0.337	0.347	0.36	0.372	0.384	0.394	0.404	0.413	0.421	0.429	0.435	0.44	0.444	0.448	0.452	0.457	0.463	0.47	0.477	0.484	0.491	0.499	0.508	0.518	0.529	0.539	0.549	0.56	0.568
TaitaTaveta	0.156	0.172	0.186	0.2	0.212	0.223	0.233	0.242	0.252	0.263	0.276	0.29	0.303	0.315	0.327	0.339	0.349	0.359	0.367	0.374	0.38	0.386	0.392	0.399	0.408	0.417	0.426	0.435	0.444	0.453	0.464	0.474	0.484	0.494	0.504	0.513	0.52
TanaRiver	0.064	0.065	0.066	0.077	0.084	0.088	0.09	0.092	0.093	0.097	0.107	0.12	0.132	0.145	0.157	0.167	0.176	0.183	0.188	0.189	0.187	0.185	0.182	0.179	0.175	0.172	0.169	0.168	0.171	0.178	0.191	0.204	0.219	0.234	0.248	0.261	0.271
Embu	0.237	0.25	0.262	0.274	0.287	0.298	0.31	0.32	0.331	0.342	0.353	0.365	0.375	0.386	0.396	0.407	0.417	0.426	0.434	0.441	0.449	0.456	0.463	0.469	0.474	0.48	0.486	0.493	0.499	0.506	0.514	0.521	0.529	0.536	0.543	0.55	0.555
Isiolo	0.205	0.212	0.219	0.226	0.233	0.24	0.246	0.252	0.258	0.264	0.27	0.277	0.284	0.289	0.295	0.3	0.306	0.311	0.315	0.32	0.325	0.33	0.334	0.336	0.336	0.335	0.334	0.334	0.336	0.339	0.343	0.348	0.354	0.36	0.366	0.372	0.377
Kitui	0.145	0.158	0.171	0.183	0.195	0.207	0.217	0.227	0.237	0.246	0.255	0.265	0.274	0.283	0.293	0.302	0.311	0.319	0.327	0.333	0.339	0.345	0.351	0.357	0.364	0.371	0.378	0.386	0.394	0.402	0.411	0.42	0.429	0.438	0.447	0.455	0.461
Machakos	0.194	0.21	0.225	0.24	0.253	0.266	0.278	0.29	0.302	0.315	0.33	0.345	0.358	0.371	0.382	0.393	0.403	0.412	0.42	0.426	0.432	0.437	0.442	0.447	0.453	0.459	0.466	0.474	0.482	0.49	0.497	0.505	0.512	0.519	0.526	0.533	0.538
Makueni	0.154	0.175	0.193	0.21	0.225	0.239	0.252	0.264	0.276	0.288	0.301	0.314	0.325	0.336	0.344	0.351	0.358	0.363	0.367	0.369	0.369	0.369	0.37	0.373	0.378	0.385	0.393	0.402	0.411	0.422	0.432	0.442	0.451	0.46	0.468	0.475	0.48
Marsabit	0.184	0.189	0.193	0.198	0.203	0.207	0.212	0.216	0.22	0.224	0.229	0.234	0.238	0.242	0.246	0.25	0.254	0.257	0.261	0.264	0.268	0.272	0.277	0.282	0.288	0.293	0.299	0.304	0.308	0.312	0.315	0.318	0.322	0.326	0.33	0.335	0.339
Meru	0.209	0.221	0.232	0.243	0.254	0.264	0.274	0.284	0.294	0.305	0.318	0.332	0.345	0.356	0.367	0.377	0.386	0.395	0.402	0.408	0.413	0.419	0.425	0.43	0.435	0.44	0.445	0.451	0.455	0.461	0.467	0.475	0.482	0.49	0.498	0.505	0.512
TharakaNithi	0.204	0.216	0.227	0.238	0.25	0.26	0.271	0.282	0.293	0.303	0.315	0.327	0.338	0.349	0.359	0.368	0.377	0.386	0.393	0.4	0.407	0.413	0.42	0.428	0.435	0.444	0.452	0.462	0.471	0.48	0.489	0.497	0.506	0.514	0.522	0.53	0.536
Nairobi	0.42	0.431	0.442	0.453	0.464	0.475	0.486	0.496	0.506	0.516	0.526	0.536	0.545	0.553	0.56	0.567	0.574	0.58	0.585	0.589	0.593	0.598	0.602	0.607	0.611	0.617	0.623	0.63	0.637	0.644	0.652	0.66	0.669	0.677	0.685	0.694	0.701
Garissa	0.052	0.053	0.054	0.055	0.056	0.057	0.058	0.059	0.06	0.061	0.062	0.064	0.065	0.069	0.075	0.077	0.077	0.074	0.073	0.074	0.075	0.077	0.078	0.079	0.08	0.081	0.083	0.084	0.106	0.127	0.141	0.154	0.166	0.178	0.19	0.202	0.21
Mandera	0.045	0.046	0.047	0.048	0.049	0.05	0.051	0.052	0.053	0.054	0.055	0.057	0.065	0.079	0.088	0.095	0.1	0.103	0.102	0.095	0.086	0.074	0.07	0.072	0.073	0.074	0.075	0.076	0.077	0.077	0.079	0.08	0.081	0.083	0.085	0.087	0.089
Wajir	0.048	0.049	0.05	0.05	0.051	0.052	0.052	0.053	0.054	0.055	0.056	0.057	0.058	0.059	0.06	0.061	0.062	0.062	0.063	0.064	0.065	0.065	0.066	0.067	0.067	0.068	0.069	0.07	0.071	0.072	0.073	0.074	0.075	0.083	0.101	0.115	0.124
HomaBay	0.173	0.188	0.202	0.216	0.229	0.24	0.251	0.261	0.271	0.281	0.291	0.301	0.311	0.32	0.329	0.338	0.347	0.354	0.361	0.368	0.374	0.38	0.385	0.39	0.395	0.401	0.408	0.416	0.425	0.435	0.445	0.455	0.464	0.473	0.481	0.488	0.493
Kisii	0.206	0.218	0.23	0.242	0.254	0.264	0.275	0.285	0.296	0.308	0.322	0.336	0.349	0.362	0.375	0.387	0.399	0.409	0.417	0.425	0.432	0.439	0.447	0.454	0.462	0.47	0.478	0.487	0.495	0.502	0.51	0.518	0.526	0.534	0.542	0.55	0.556
Kisumu	0.214	0.228	0.241	0.254	0.266	0.278	0.29	0.301	0.313	0.326	0.341	0.355	0.369	0.38	0.388	0.396	0.403	0.409	0.413	0.417	0.421	0.425	0.43	0.435	0.441	0.448	0.456	0.465	0.475	0.485	0.495	0.504	0.512	0.521	0.529	0.536	0.542
Migori	0.18	0.194	0.208	0.22	0.233	0.244	0.255	0.264	0.274	0.283	0.294	0.304	0.314	0.324	0.334	0.343	0.353	0.362	0.37	0.378	0.386	0.395	0.404	0.411	0.417	0.422	0.428	0.435	0.44	0.445	0.451	0.458	0.465	0.473	0.48	0.488	0.494
Nyamira	0.268	0.28	0.292	0.304	0.316	0.328	0.34	0.351	0.363	0.374	0.385	0.397	0.409	0.419	0.43	0.44	0.449	0.458	0.464	0.47	0.476	0.483	0.49	0.496	0.501	0.507	0.514	0.521	0.528	0.537	0.545	0.555	0.563	0.572	0.58	0.588	0.594
Siaya	0.166	0.176	0.186	0.196	0.206	0.215	0.225	0.234	0.244	0.253	0.264	0.275	0.285	0.295	0.305	0.314	0.324	0.333	0.34	0.347	0.355	0.363	0.371	0.38	0.39	0.401	0.411	0.422	0.432	0.442	0.451	0.46	0.47	0.479	0.489	0.498	0.505
Baringo	0.151	0.169	0.185	0.2	0.213	0.225	0.236	0.247	0.258	0.268	0.28	0.292	0.304	0.313	0.322	0.329	0.337	0.344	0.351	0.358	0.366	0.373	0.379	0.383	0.385	0.387	0.39	0.393	0.398	0.406	0.414	0.422	0.432	0.441	0.45	0.458	0.465
Bomet	0.099	0.108	0.143	0.169	0.189	0.204	0.218	0.231	0.243	0.254	0.268	0.281	0.293	0.305	0.316	0.327	0.338	0.348	0.357	0.366	0.377	0.388	0.398	0.409	0.418	0.427	0.436	0.446	0.454	0.462	0.47	0.479	0.488	0.497	0.506	0.514	0.521
Elgeyo-Marakwet	0.149	0.167	0.183	0.198	0.211	0.223	0.234	0.245	0.255	0.266	0.278	0.291	0.303	0.313	0.322	0.331	0.34	0.348	0.355	0.362	0.371	0.379	0.388	0.395	0.401	0.408	0.415	0.424	0.433	0.444	0.453	0.463	0.473	0.483	0.492	0.501	0.508
Kajiado	0.197	0.213	0.227	0.241	0.254	0.266	0.278	0.29	0.301	0.313	0.325	0.338	0.35	0.36	0.368	0.376	0.383	0.389	0.393	0.395	0.399	0.403	0.409	0.416	0.426	0.437	0.448	0.46	0.471	0.48	0.489	0.498	0.506	0.515	0.524	0.533	0.539
Kericho	0.094	0.096	0.108	0.135	0.154	0.169	0.183	0.196	0.208	0.224	0.242	0.261	0.278	0.295	0.313	0.328	0.343	0.356	0.368	0.378	0.388	0.397	0.406	0.413	0.42	0.428	0.436	0.445	0.455	0.466	0.476	0.487	0.498	0.509	0.519	0.529	0.536
Laikipia	0.229	0.241	0.253	0.265	0.276	0.287	0.297	0.307	0.317	0.327	0.338	0.349	0.36	0.372	0.386	0.398	0.41	0.42	0.428	0.435	0.442	0.449	0.456	0.463	0.47	0.478	0.487	0.496	0.507	0.518	0.527	0.537	0.547	0.556	0.566	0.575	0.582
Nakuru	0.208	0.221	0.232	0.244	0.255	0.266	0.277	0.287	0.298	0.309	0.321	0.333	0.345	0.355	0.365	0.373	0.382	0.389	0.394	0.398	0.403	0.408	0.413	0.42	0.43	0.441	0.453	0.466	0.479	0.492	0.503	0.515	0.526	0.537	0.546	0.556	0.563
Nandi	0.099	0.124	0.152	0.173	0.191	0.206	0.219	0.232	0.244	0.256	0.27	0.285	0.299	0.313	0.327	0.339	0.35	0.36	0.368	0.374	0.382	0.39	0.399	0.408	0.417	0.427	0.436	0.446	0.455	0.465	0.474	0.483	0.491	0.5	0.508	0.516	0.522
Narok	0.106	0.125	0.141	0.155	0.168	0.178	0.188	0.198	0.208	0.219	0.231	0.244	0.257	0.269	0.282	0.293	0.305	0.315	0.323	0.328	0.332	0.336	0.339	0.345	0.353	0.361	0.368	0.375	0.381	0.387	0.394	0.402	0.41	0.418	0.427	0.435	0.441
Samburu	0.169	0.175	0.181	0.187	0.192	0.196	0.201	0.205	0.209	0.212	0.216	0.22	0.224	0.227	0.229	0.231	0.233	0.235	0.235	0.235	0.236	0.238	0.241	0.245	0.251	0.257	0.264	0.27	0.275	0.281	0.286	0.292	0.297	0.302	0.308	0.313	0.316

Appendix Table 14: Socio-Demographic Index values for all estimated GBD 2016 locations, 1980–2016

Location	1980	1981	1982	1983	1984	1985	1986	1987	1988	1989	1990	1991	1992	1993	1994	1995	1996	1997	1998	1999	2000	2001	2002	2003	2004	2005	2006	2007	2008	2009	2010	2011	2012	2013	2014	2015	2016
TransNzoia	0.103	0.137	0.161	0.18	0.197	0.21	0.222	0.234	0.246	0.26	0.278	0.296	0.313	0.328	0.341	0.353	0.364	0.374	0.382	0.39	0.398	0.405	0.412	0.418	0.422	0.427	0.432	0.438	0.445	0.453	0.462	0.471	0.48	0.489	0.498	0.506	0.513
Turkana	0.185	0.19	0.195	0.2	0.205	0.209	0.213	0.217	0.22	0.223	0.227	0.231	0.235	0.238	0.241	0.244	0.247	0.249	0.251	0.25	0.249	0.248	0.248	0.249	0.251	0.253	0.256	0.26	0.265	0.268	0.271	0.273	0.276	0.279	0.283	0.287	0.289
UasinGishu	0.183	0.202	0.22	0.237	0.252	0.266	0.279	0.291	0.303	0.316	0.329	0.342	0.355	0.366	0.376	0.386	0.395	0.404	0.413	0.422	0.432	0.443	0.453	0.462	0.47	0.478	0.486	0.495	0.504	0.513	0.522	0.53	0.539	0.548	0.556	0.564	0.57
WestPokot	0.153	0.16	0.168	0.176	0.183	0.19	0.196	0.203	0.211	0.219	0.23	0.24	0.25	0.258	0.264	0.27	0.275	0.279	0.28	0.281	0.283	0.284	0.286	0.289	0.292	0.296	0.301	0.308	0.314	0.321	0.328	0.335	0.343	0.351	0.359	0.367	0.373
Bungoma	0.199	0.214	0.228	0.242	0.254	0.266	0.277	0.287	0.296	0.305	0.316	0.327	0.336	0.345	0.354	0.362	0.37	0.378	0.385	0.392	0.399	0.406	0.413	0.42	0.426	0.432	0.438	0.444	0.45	0.456	0.462	0.469	0.477	0.484	0.492	0.499	0.505
Busia	0.235	0.246	0.256	0.266	0.276	0.285	0.294	0.303	0.312	0.321	0.331	0.341	0.35	0.359	0.365	0.371	0.377	0.382	0.385	0.387	0.39	0.393	0.396	0.399	0.402	0.406	0.41	0.415	0.421	0.427	0.433	0.44	0.447	0.454	0.461	0.468	0.473
Kakamega	0.23	0.241	0.252	0.263	0.273	0.283	0.292	0.302	0.311	0.321	0.332	0.344	0.355	0.365	0.374	0.383	0.39	0.397	0.402	0.405	0.408	0.411	0.414	0.417	0.421	0.426	0.431	0.438	0.444	0.45	0.457	0.463	0.47	0.477	0.484	0.491	0.496
Vihiga	0.258	0.27	0.281	0.293	0.304	0.315	0.325	0.334	0.344	0.352	0.361	0.37	0.378	0.385	0.391	0.397	0.401	0.405	0.408	0.409	0.41	0.412	0.415	0.418	0.422	0.428	0.436	0.446	0.456	0.467	0.476	0.485	0.493	0.5	0.507	0.513	0.517
Southern Sub-Saharan Africa	0.505	0.511	0.516	0.521	0.527	0.531	0.536	0.541	0.546	0.552	0.558	0.564	0.57	0.577	0.583	0.589	0.595	0.601	0.606	0.611	0.615	0.62	0.625	0.63	0.635	0.64	0.646	0.651	0.656	0.661	0.666	0.672	0.677	0.681	0.685	0.689	0.691
Botswana	0.404	0.414	0.423	0.432	0.441	0.451	0.461	0.471	0.484	0.497	0.508	0.518	0.527	0.536	0.544	0.552	0.56	0.569	0.578	0.587	0.595	0.602	0.609	0.616	0.623	0.631	0.638	0.645	0.65	0.656	0.662	0.669	0.675	0.682	0.689	0.695	0.701
Lesotho	0.323	0.328	0.333	0.337	0.341	0.345	0.349	0.353	0.358	0.364	0.37	0.376	0.383	0.39	0.397	0.403	0.41	0.417	0.423	0.429	0.435	0.442	0.448	0.455	0.462	0.469	0.475	0.482	0.489	0.496	0.504	0.513	0.521	0.529	0.538	0.545	0.552
Namibia	0.374	0.38	0.385	0.39	0.395	0.399	0.404	0.41	0.416	0.422	0.427	0.433	0.441	0.45	0.459	0.469	0.478	0.488	0.497	0.505	0.514	0.522	0.531	0.539	0.547	0.555	0.563	0.569	0.575	0.58	0.586	0.591	0.597	0.604	0.611	0.618	0.624
South Africa	0.542	0.548	0.554	0.559	0.564	0.569	0.574	0.579	0.584	0.59	0.596	0.601	0.606	0.613	0.619	0.624	0.63	0.636	0.64	0.645	0.649	0.653	0.658	0.663	0.669	0.675	0.682	0.688	0.695	0.701	0.707	0.714	0.719	0.724	0.727	0.73	0.734
Swaziland	0.384	0.391	0.398	0.404	0.41	0.415	0.421	0.429	0.437	0.446	0.454	0.462	0.47	0.477	0.484	0.491	0.498	0.504	0.509	0.514	0.518	0.522	0.525	0.528	0.531	0.534	0.537	0.538	0.538	0.539	0.544	0.553	0.562	0.571	0.578	0.584	0.589
Zimbabwe	0.357	0.364	0.37	0.377	0.384	0.39	0.397	0.403	0.412	0.422	0.432	0.443	0.452	0.461	0.47	0.477	0.486	0.493	0.498	0.502	0.505	0.507	0.508	0.506	0.503	0.498	0.492	0.485	0.477	0.472	0.469	0.47	0.474	0.479	0.485	0.491	0.497
Eastern Cape	0.495	0.501	0.506	0.511	0.516	0.521	0.524	0.528	0.533	0.538	0.543	0.547	0.551	0.557	0.562	0.567	0.573	0.578	0.583	0.587	0.591	0.596	0.601	0.606	0.612	0.618	0.626	0.633	0.64	0.647	0.653	0.66	0.665	0.67	0.673	0.676	0.679
Free State	0.546	0.551	0.556	0.56	0.564	0.568	0.572	0.576	0.581	0.586	0.591	0.597	0.602	0.609	0.617	0.624	0.631	0.637	0.641	0.645	0.649	0.652	0.656	0.661	0.666	0.672	0.678	0.684	0.69	0.696	0.702	0.708	0.714	0.718	0.721	0.724	0.727
Gauteng	0.656	0.66	0.664	0.667	0.671	0.674	0.677	0.681	0.684	0.688	0.692	0.695	0.698	0.702	0.705	0.707	0.709	0.712	0.715	0.718	0.721	0.724	0.728	0.731	0.735	0.74	0.744	0.749	0.754	0.759	0.763	0.768	0.773	0.775	0.778	0.78	0.782
KwaZulu-Natal	0.521	0.527	0.532	0.537	0.542	0.546	0.551	0.555	0.56	0.566	0.571	0.576	0.581	0.588	0.594	0.599	0.605	0.61	0.615	0.619	0.624	0.629	0.634	0.639	0.645	0.651	0.658	0.665	0.672	0.679	0.685	0.692	0.698	0.702	0.706	0.71	0.713
Limpopo	0.447	0.454	0.461	0.467	0.473	0.479	0.484	0.49	0.497	0.504	0.511	0.518	0.524	0.532	0.54	0.547	0.554	0.562	0.568	0.572	0.577	0.582	0.588	0.594	0.602	0.61	0.618	0.627	0.636	0.643	0.651	0.659	0.665	0.671	0.675	0.679	0.682
Mpumalanga	0.495	0.502	0.508	0.514	0.52	0.525	0.531	0.537	0.543	0.55	0.557	0.563	0.57	0.577	0.584	0.589	0.595	0.601	0.606	0.61	0.615	0.62	0.625	0.631	0.637	0.644	0.652	0.659	0.667	0.674	0.681	0.688	0.695	0.7	0.703	0.707	0.71
North-West	0.523	0.529	0.534	0.539	0.545	0.55	0.555	0.56	0.565	0.571	0.576	0.58	0.585	0.59	0.594	0.597	0.6	0.604	0.608	0.613	0.618	0.623	0.628	0.633	0.639	0.645	0.651	0.658	0.664	0.671	0.677	0.683	0.689	0.693	0.696	0.699	0.702
Northern Cape	0.545	0.55	0.555	0.559	0.564	0.568	0.572	0.576	0.58	0.585	0.59	0.594	0.598	0.603	0.607	0.61	0.614	0.619	0.623	0.626	0.63	0.633	0.637	0.641	0.646	0.652	0.658	0.664	0.67	0.675	0.681	0.688	0.693	0.697	0.7	0.704	0.707
Western Cape	0.653	0.657	0.66	0.662	0.665	0.668	0.67	0.672	0.675	0.678	0.681	0.684	0.687	0.691	0.695	0.698	0.703	0.708	0.711	0.713	0.715	0.717	0.72	0.722	0.725	0.728	0.732	0.736	0.74	0.744	0.748	0.752	0.756	0.758	0.76	0.762	0.764
Western Sub-Saharan Africa	0.254	0.259	0.263	0.268	0.272	0.276	0.279	0.281	0.282	0.285	0.287	0.29	0.293	0.297	0.301	0.305	0.31	0.314	0.318	0.322	0.326	0.33	0.335	0.339	0.345	0.351	0.357	0.364	0.37	0.376	0.383	0.391	0.399	0.407	0.415	0.423	0.428
Benin	0.171	0.175	0.178	0.182	0.187	0.192	0.197	0.202	0.207	0.212	0.217	0.222	0.227	0.233	0.237	0.242	0.247	0.252	0.257	0.262	0.267	0.273	0.278	0.283	0.288	0.293	0.298	0.304	0.31	0.315	0.32	0.326	0.33	0.335	0.34	0.346	0.351
Burkina Faso	0.103	0.106	0.108	0.109	0.111	0.113	0.115	0.117	0.12	0.123	0.125	0.13	0.136	0.141	0.146	0.15	0.155	0.16	0.165	0.169	0.173	0.178	0.182	0.186	0.191	0.195	0.2	0.206	0.212	0.217	0.225	0.234	0.244	0.253	0.262	0.269	0.274
Cameroon	0.283	0.289	0.296	0.302	0.309	0.316	0.322	0.327	0.332	0.337	0.341	0.345	0.349	0.353	0.356	0.36	0.364	0.369	0.374	0.379	0.383	0.388	0.392	0.396	0.4	0.403	0.406	0.409	0.412	0.415	0.419	0.423	0.428	0.433	0.439	0.445	0.451
Cape Verde	0.241	0.248	0.256	0.263	0.271	0.279	0.287	0.294	0.302	0.31	0.317	0.325	0.332	0.34	0.349	0.358	0.367	0.377	0.387	0.399	0.41	0.421	0.431	0.442	0.452	0.462	0.473	0.484	0.495	0.505	0.514	0.524	0.534	0.542	0.551	0.559	0.566
Chad	0.158	0.159	0.159	0.161	0.163	0.166	0.169	0.172	0.175	0.179	0.183	0.187	0.191	0.194	0.197	0.2	0.203	0.206	0.209	0.212	0.214	0.217	0.219	0.222	0.228	0.235	0.241	0.247	0.252	0.257	0.262	0.268	0.274	0.28	0.285	0.29	0.295
Cote d'Ivoire	0.225	0.226	0.227	0.228	0.23	0.234	0.241	0.249	0.258	0.267	0.277	0.287	0.297	0.306	0.313	0.32	0.327	0.332	0.337	0.341	0.345	0.348	0.351	0.353	0.355	0.358	0.36	0.363	0.366	0.369	0.373	0.377	0.382	0.388	0.394	0.401	0.407
The Gambia	0.205	0.21	0.215	0.221	0.226	0.232	0.237	0.242	0.247	0.251	0.254	0.255	0.255	0.255	0.254	0.254	0.255	0.259	0.264	0.269	0.274	0.28	0.285	0.29	0.295	0.3	0.305	0.31	0.315	0.32	0.325	0.329	0.334	0.339	0.344	0.349	0.352
Ghana	0.309	0.313	0.315	0.317	0.32	0.323	0.326	0.33	0.338	0.347	0.356	0.366	0.375	0.384	0.392	0.401	0.409	0.416	0.421	0.426	0.429	0.431	0.433	0.436	0.438	0.44	0.443	0.446	0.448	0.449	0.451	0.457	0.466	0.477	0.486	0.494	0.499
Guinea	0.181	0.184	0.188	0.191	0.194	0.197	0.2	0.203	0.207	0.21	0.213	0.217	0.22	0.223	0.226	0.23	0.233	0.237	0.24	0.243	0.246	0.249	0.251	0.254	0.257	0.26	0.264	0.268	0.273	0.277	0.282	0.288	0.293	0.299	0.305	0.311	0.316
Guinea-Bissau	0.176	0.179	0.182	0.184	0.187	0.19	0.193	0.197	0.2	0.204	0.208	0.213	0.217	0.222	0.226	0.231	0.236	0.242	0.246	0.25	0.254	0.258	0.262	0.266	0.27	0.274	0.279	0.283	0.288	0.293	0.298	0.305	0.31	0.316	0.322	0.328	0.333
Liberia	0.198	0.201	0.203	0.205	0.207	0.209	0.211	0.213	0.216	0.219	0.218	0.218	0.215	0.211	0.204	0.196	0.186	0.183	0.184	0.19	0.204	0.218	0.234	0.241	0.247	0.253	0.258	0.264	0.27	0.276	0.282	0.289	0.298	0.307	0.314	0.321	0.327
Mali	0.114	0.117	0.118	0.12	0.122	0.124	0.127	0.129	0.131	0.135	0.138	0.142	0.146	0.15	0.153	0.157	0.161	0.164	0.168	0.172	0.176	0.18	0.185	0.19	0.195	0.2	0.205	0.211	0.217	0.223	0.23	0.237	0.243	0.249	0.256	0.262	0.267
Mauritania	0.273	0.279	0.283	0.288	0.293	0.297	0.302	0.307	0.312	0.317	0.322	0.327	0.332	0.338	0.344	0.349	0.356	0.361	0.366	0.372	0.376	0.381	0.386	0.391	0.396	0.402	0.41	0.417	0.424	0.431	0.438	0.445	0.452	0.46	0.468	0.475	0.482
Niger	0.119	0.121	0.123	0.126	0.127	0.129	0.131	0.133	0.135	0.136	0.138	0.139	0.14	0.141	0.141	0.141	0.142	0.143	0.145	0.146	0.148	0.149	0.152	0.154	0.156	0.158	0.161	0.163	0.166	0.169	0.172	0.175	0.179	0.183	0.188	0.192	0.195
Nigeria	0.289	0.296	0.303	0.309	0.314	0.32	0.323	0.323	0.323	0.322	0.322	0.322	0.324	0.327	0.33	0.333	0.337	0.341	0.345	0.349	0.353	0.358	0.364	0.369	0.377	0.385	0.392	0.4	0.407	0.414	0.423	0.433	0.443	0.454	0.464	0.473	0.479
Sao Tome and Principe	0.237	0.243	0.248	0.254	0.259	0.265	0.27	0.275	0.279	0.284	0.289	0.294	0.299	0.304	0.31	0.315	0.32	0.326	0.331	0.336	0.34	0.346	0.351	0.357	0.363	0.369	0.376	0.382	0.388	0.393	0.398	0.403	0.408	0.413	0.42	0.427	0.434
Senegal	0.197	0.2	0.204	0.208	0.211	0.216	0.22	0.225	0.23	0.235	0.24	0.245	0.251	0.257	0.263	0.27	0.276	0.283	0.29	0.295	0.299	0.302	0.304	0.307	0.309	0.312	0.314	0.317	0.32	0.324	0.327	0.331	0.336	0.34	0.345	0.35	0.354
Sierra Leone	0.186	0.189	0.192	0.195	0.198	0.201	0.204	0.207	0.21	0.214	0.217	0.222	0.225	0.229	0.233	0.237	0.241	0.243	0.246	0.247	0.249	0.252	0.257	0.262	0.268	0.273	0.279	0.286	0.292	0.299	0.306	0.313	0.322	0.332	0.341	0.348	0.352
Togo	0.208	0.212	0.215	0.218	0.222	0.225	0.228	0.232	0.236	0.242	0.248	0.255	0.261	0.267	0.273	0.28	0.288	0.296	0.301	0.307	0.312	0.317	0.321	0.326	0.331	0.336	0.341	0.346	0.351	0.356	0.361	0.367	0.373	0.379	0.385	0.392	0.397

Appendix Table 15: GBD 2016 location hierarchy with levels	
**Location**	**Level**
Global	0
Southeast Asia, East Asia, and Oceania	1
East Asia	2
China	3
Anhui	4
Beijing	4
Chongqing	4
Fujian	4
Gansu	4
Guangdong	4
Guangxi	4
Guizhou	4
Hainan	4
Hebei	4
Heilongjiang	4
Henan	4
Hong Kong Special Administrative Region of China	4
Hubei	4
Hunan	4
Inner Mongolia	4
Jiangsu	4
Jiangxi	4
Jilin	4
Liaoning	4
Macao Special Administrative Region of China	4
Ningxia	4
Qinghai	4
Shaanxi	4
Shandong	4
Shanghai	4
Shanxi	4
Sichuan	4
Tianjin	4
Tibet	4
Xinjiang	4
Yunnan	4
Zhejiang	4
North Korea	3
Taiwan (Province of China)	3
Southeast Asia	2
Cambodia	3
Indonesia	3
Aceh	4
Bali	4
Bangka Belitung	4
Banten	4

Appendix Table 15: GBD 2016 location hierarchy with levels	
**Location**	**Level**
Bengkulu	4
Gorontalo	4
Jakarta	4
Jambi	4
Cental Java	4
West Java	4
East Java	4
West Kalimantan	4
South Kalimantan	4
Central Kalimantan	4
East Kalimantan	4
North Kalimantan	4
Riau Islands	4
Lampung	4
Maluku	4
North Maluku	4
Nusa Tenggara Barat	4
East Nusa Tenggara	4
Papua	4
West Papua	4
Riau	4
West Sulawesi	4
South Sumatera	4
Central Sulawesi	4
Southeast Sulawesi	4
North Sulawesi	4
West Sumatera	4
South Sumatera	4
North Sumatera	4
Yogyakarta	4
Laos	3
Malaysia	3
Maldives	3
Mauritius	3
Myanmar	3
Philippines	3
Sri Lanka	3
Seychelles	3
Thailand	3
Timor-Leste	3
Vietnam	3
Oceania	2
American Samoa	3
Federated States of Micronesia	3
Fiji	3
Guam	3

Appendix Table 15: GBD 2016 location hierarchy with levels

Location	Level
Kiribati	3
Marshall Islands	3
Northern Mariana Islands	3
Papua New Guinea	3
Samoa	3
Solomon Islands	3
Tonga	3
Vanuatu	3
Central Europe, Eastern Europe, and Central Asia	1
Central Asia	2
Armenia	3
Azerbaijan	3
Georgia	4
Georgia	3
Kazakhstan	3
Kyrgyzstan	3
Mongolia	3
Tajikistan	3
Turkmenistan	3
Uzbekistan	3
Central Europe	2
Albania	3
Bosnia and Herzegovina	3
Bulgaria	3
Croatia	3
Czech Republic	3
Hungary	3
Macedonia	3
Montenegro	3
Poland	3
Romania	3
Serbia	3
Slovakia	3
Slovenia	3
Eastern Europe	2
Belarus	3
Estonia	3
Latvia	3
Lithuania	3
Moldova	3
Russia	3
Ukraine	3
High-income	1
High-income Asia Pacific	2
Brunei	3
Japan	3

Appendix Table 15: GBD 2016 location hierarchy with levels	
**Location**	**Level**
Aichi	4
Akita	4
Aomori	4
Chiba	4
Ehime	4
Fukui	4
Fukuoka	4
Fukushima	4
Gifu	4
Gunma	4
Hiroshima	4
Hokkaidō	4
Hyōgo	4
Ibaraki	4
Ishikawa	4
Iwate	4
Kagawa	4
Kagoshima	4
Kanagawa	4
Kōchi	4
Kumamoto	4
Kyōto	4
Mie	4
Miyagi	4
Miyazaki	4
Nagano	4
Nagasaki	4
Nara	4
Niigata	4
Ôita	4
Okayama	4
Okinawa	4
Ōsaka	4
Saga	4
Saitama	4
Shiga	4
Shimane	4
Shizuoka	4
Tochigi	4
Tokushima	4
Tōkyō	4
Tottori	4
Toyama	4
Wakayama	4
Yamagata	4
Yamaguchi	4

Appendix Table 15: GBD 2016 location hierarchy with levels

Location	Level
Yamanashi	4
South Korea	3
Singapore	3
Australasia	2
Australia	3
New Zealand	3
Western Europe	2
Andorra	3
Austria	3
Belgium	3
Cyprus	3
Denmark	3
Finland	3
France	3
Germany	3
Greece	3
Iceland	3
Ireland	3
Israel	3
Italy	3
Luxembourg	3
Malta	3
Netherlands	3
Norway	3
Portugal	3
Spain	3
Sweden	3
Stockholm	4
Sweden except Stockholm	4
Switzerland	3
United Kingdom	3
England	4
East Midlands	5
Derby	6
Derbyshire	6
Leicester	6
Leicestershire	6
Lincolnshire	6
Northamptonshire	6
Nottingham	6
Nottinghamshire	6
Rutland	6
East of England	5
Bedford	6
Cambridgeshire	6
Central Bedfordshire	6

Appendix Table 15: GBD 2016 location hierarchy with levels	
**Location**	**Level**
Essex	6
Hertfordshire	6
Luton	6
Norfolk	6
Peterborough	6
Southend-on-Sea	6
Suffolk	6
Thurrock	6
Greater London	5
Barking and Dagenham	6
Barnet	6
Bexley	6
Brent	6
Bromley	6
Camden	6
Croydon	6
Ealing	6
Enfield	6
Greenwich	6
Hackney	6
Hammersmith and Fulham	6
Haringey	6
Harrow	6
Havering	6
Hillingdon	6
Hounslow	6
Islington	6
Kensington and Chelsea	6
Kingston upon Thames	6
Lambeth	6
Lewisham	6
Merton	6
Newham	6
Redbridge	6
Richmond upon Thames	6
Southwark	6
Sutton	6
Tower Hamlets	6
Waltham Forest	6
Wandsworth	6
Westminster	6
North East England	5
County Durham	6
Darlington	6
Gateshead	6
Hartlepool	6

Appendix Table 15: GBD 2016 location hierarchy with levels	
**Location**	**Level**
Middlesbrough	6
Newcastle upon Tyne	6
North Tyneside	6
Northumberland	6
Redcar and Cleveland	6
South Tyneside	6
Stockton-on-Tees	6
Sunderland	6
North West England	5
Blackburn with Darwen	6
Blackpool	6
Bolton	6
Bury	6
Cheshire East	6
Cheshire West and Chester	6
Cumbria	6
Halton	6
Knowsley	6
Lancashire	6
Liverpool	6
Manchester	6
Oldham	6
Rochdale	6
Salford	6
Sefton	6
St Helens	6
Stockport	6
Tameside	6
Trafford	6
Warrington	6
Wigan	6
Wirral	6
South East England	5
Bracknell Forest	6
Brighton and Hove	6
Buckinghamshire	6
East Sussex	6
Hampshire	6
Isle of Wight	6
Kent	6
Medway	6
Milton Keynes	6
Oxfordshire	6
Portsmouth	6
Reading	6
Slough	6

Appendix Table 15: GBD 2016 location hierarchy with levels	
**Location**	**Level**
Southampton	6
Surrey	6
West Berkshire	6
West Sussex	6
Windsor and Maidenhead	6
Wokingham	6
South West England	5
Bath and North East Somerset	6
Bournemouth	6
Bristol, City of	6
Cornwall	6
Devon	6
Dorset	6
Gloucestershire	6
North Somerset	6
Plymouth	6
Poole	6
Somerset	6
South Gloucestershire	6
Swindon	6
Torbay	6
Wiltshire	6
West Midlands	5
Birmingham	6
Coventry	6
Dudley	6
Herefordshire, County of	6
Sandwell	6
Shropshire	6
Solihull	6
Staffordshire	6
Stoke-on-Trent	6
Telford and Wrekin	6
Walsall	6
Warwickshire	6
Wolverhampton	6
Worcestershire	6
Yorkshire and the Humber	5
Barnsley	6
Bradford	6
Calderdale	6
Doncaster	6
East Riding of Yorkshire	6
Kingston upon Hull, City of	6
Kirklees	6
Leeds	6

Appendix Table 15: GBD 2016 location hierarchy with levels	
**Location**	**Level**
North East Lincolnshire	6
North Lincolnshire	6
North Yorkshire	6
Rotherham	6
Sheffield	6
Wakefield	6
York	6
Northern Ireland	4
Scotland	4
Wales	4
Southern Latin America	2
Argentina	3
Chile	3
Uruguay	3
High-income North America	2
Canada	3
Greenland	3
USA	3
Alabama	4
Alaska	4
Arizona	4
Arkansas	4
California	4
Colorado	4
Connecticut	4
Delaware	4
District of Columbia	4
Florida	4
Georgia	3
Georgia	4
Hawaii	4
Idaho	4
Illinois	4
Indiana	4
Iowa	4
Kansas	4
Kentucky	4
Louisiana	4
Maine	4
Maryland	4
Massachusetts	4
Michigan	4
Minnesota	4
Mississippi	4
Missouri	4
Montana	4

Appendix Table 15: GBD 2016 location hierarchy with levels	
**Location**	**Level**
Nebraska	4
Nevada	4
New Hampshire	4
New Jersey	4
New Mexico	4
New York	4
North Carolina	4
North Dakota	4
Ohio	4
Oklahoma	4
Oregon	4
Pennsylvania	4
Rhode Island	4
South Carolina	4
South Dakota	4
Tennessee	4
Texas	4
Utah	4
Vermont	4
Virginia	4
Washington	4
West Virginia	4
Wisconsin	4
Wyoming	4
Latin America and Caribbean	1
Caribbean	2
Antigua and Barbuda	3
The Bahamas	3
Barbados	3
Belize	3
Bermuda	3
Cuba	3
Dominica	3
Dominican Republic	3
Grenada	3
Guyana	3
Haiti	3
Jamaica	3
Puerto Rico	3
Saint Lucia	3
Saint Vincent and the Grenadines	3
Suriname	3
Trinidad and Tobago	3
Virgin Islands, U.S.	3
Andean Latin America	2
Bolivia	3

Appendix Table 15: GBD 2016 location hierarchy with levels	
**Location**	**Level**
Ecuador	3
Peru	3
Central Latin America	2
Colombia	3
Costa Rica	3
El Salvador	3
Guatemala	3
Honduras	3
Mexico	3
Aguascalientes	4
Baja California	4
Baja California Sur	4
Campeche	4
Chiapas	4
Chihuahua	4
Coahuila	4
Colima	4
Distrito Federal	4
Distrito Federal	4
Durango	4
Guanajuato	4
Guerrero	4
Hidalgo	4
Jalisco	4
México	4
Michoacán de Ocampo	4
Morelos	4
Nayarit	4
Nuevo León	4
Oaxaca	4
Puebla	4
Querétaro	4
Quintana Roo	4
San Luis Potosí	4
Sinaloa	4
Sonora	4
Tabasco	4
Tamaulipas	4
Tlaxcala	4
Veracruz de Ignacio de la Llave	4
Yucatán	4
Zacatecas	4
Nicaragua	3
Panama	3
Venezuela	3
Tropical Latin America	2

Appendix Table 15: GBD 2016 location hierarchy with levels

Location	Level
Brazil	3
Acre	4
Alagoas	4
Amapá	4
Amazonas	4
Bahia	4
Ceará	4
Distrito Federal	4
Distrito Federal	4
Espírito Santo	4
Goiás	4
Maranhão	4
Mato Grosso	4
Mato Grosso do Sul	4
Minas Gerais	4
Pará	4
Paraíba	4
Paraná	4
Pernambuco	4
Piaui	4
Rio de Janeiro	4
Rio Grande do Norte	4
Rio Grande do Sul	4
Rondônia	4
Roraima	4
Santa Catarina	4
São Paulo	4
Sergipe	4
Tocantins	4
Paraguay	3
North Africa and Middle East	1
North Africa and Middle East	2
Afghanistan	3
Algeria	3
Bahrain	3
Egypt	3
Iran	3
Iraq	3
Jordan	3
Kuwait	3
Lebanon	3
Libya	3
Morocco	3
Palestine	3
Oman	3
Qatar	3

Appendix Table 15: GBD 2016 location hierarchy with levels	
**Location**	**Level**
Saudi Arabia	3
'Asir	4
Bahah	4
Eastern Province	4
Ha'il	4
Jawf	4
Jizan	4
Madinah	4
Makkah	4
Najran	4
Northern Borders	4
Qassim	4
Riyadh	4
Tabuk	4
Sudan	3
Syria	3
Tunisia	3
Turkey	3
United Arab Emirates	3
Yemen	3
South Asia	1
South Asia	2
Bangladesh	3
Bhutan	3
India	3
Andhra Pradesh	4
Andhra Pradesh, Rural	5
Andhra Pradesh, Urban	5
Arunāchal Pradesh	4
Arunāchal Pradesh, Rural	5
Arunāchal Pradesh, Urban	5
Assam	4
Assam, Rural	5
Assam, Urban	5
Bihār	4
Bihār, Rural	5
Bihār, Urban	5
Chhattīsgarh	4
Chhattīsgarh, Rural	5
Chhattīsgarh, Urban	5
Delhi	4
Delhi, Rural	5
Delhi, Urban	5
Goa	4
Goa, Rural	5
Goa, Urban	5

Appendix Table 15: GBD 2016 location hierarchy with levels

Location	Level
Gujarāt	4
Gujarāt, Rural	5
Gujarāt, Urban	5
Haryāna	4
Haryāna, Rural	5
Haryāna, Urban	5
Himachal Pradesh	4
Himachal Pradesh, Rural	5
Himachal Pradesh, Urban	5
Jammu and Kashmīr	4
Jammu and Kashmīr, Rural	5
Jammu and Kashmīr, Urban	5
Jharkhand	4
Jharkhand, Rural	5
Jharkhand, Urban	5
Karnātaka	4
Karnātaka, Rural	5
Karnātaka, Urban	5
Kerala	4
Kerala, Rural	5
Kerala, Urban	5
Madhya Pradesh	4
Madhya Pradesh, Rural	5
Madhya Pradesh, Urban	5
Mahārāshtra	4
Mahārāshtra, Rural	5
Mahārāshtra, Urban	5
Manipur	4
Manipur, Rural	5
Manipur, Urban	5
Meghālaya	4
Meghālaya, Rural	5
Meghālaya, Urban	5
Mizoram	4
Mizoram, Rural	5
Mizoram, Urban	5
Nāgāland	4
Nāgāland, Rural	5
Nāgāland, Urban	5
Orissa	4
Orissa, Rural	5
Orissa, Urban	5
Punjab	4
Punjab, Rural	5
Punjab, Urban	5
Rājasthān	4

Appendix Table 15: GBD 2016 location hierarchy with levels	
**Location**	**Level**
Rājasthān, Rural	5
Rājasthān, Urban	5
Sikkim	4
Sikkim, Rural	5
Sikkim, Urban	5
Tamil Nādu	4
Tamil Nādu, Rural	5
Tamil Nādu, Urban	5
Telangana	4
Telangana, Rural	5
Telangana, Urban	5
Tripura	4
Tripura, Rural	5
Tripura, Urban	5
Uttar Pradesh	4
Uttar Pradesh, Rural	5
Uttar Pradesh, Urban	5
Uttarakhand	4
Uttarakhand, Rural	5
Uttarakhand, Urban	5
West Bengal	4
West Bengal, Rural	5
West Bengal, Urban	5
The Six Minor Territories	4
The Six Minor Territories, Rural	5
The Six Minor Territories, Urban	5
Nepal	3
Pakistan	3
Sub-Saharan Africa	1
Central Sub-Saharan Africa	2
Angola	3
Central African Republic	3
Congo (Brazzaville)	3
Democratic Republic of the Congo	3
Equatorial Guinea	3
Gabon	3
Eastern Sub-Saharan Africa	2
Burundi	3
Comoros	3
Djibouti	3
Eritrea	3
Ethiopia	3
Kenya	3
Baringo	4
Bomet	4
Bungoma	4

Appendix Table 15: GBD 2016 location hierarchy with levels

Location	Level
Busia	4
Elgeyo-Marakwet	4
Embu	4
Garissa	4
HomaBay	4
Isiolo	4
Kajiado	4
Kakamega	4
Kericho	4
Kiambu	4
Kilifi	4
Kirinyaga	4
Kisii	4
Kisumu	4
Kitui	4
Kwale	4
Laikipia	4
Lamu	4
Machakos	4
Makueni	4
Mandera	4
Marsabit	4
Meru	4
Migori	4
Mombasa	4
Murang’a	4
Nairobi	4
Nakuru	4
Nandi	4
Narok	4
Nyamira	4
Nyandarua	4
Nyeri	4
Samburu	4
Siaya	4
TaitaTaveta	4
TanaRiver	4
TharakaNithi	4
TransNzoia	4
Turkana	4
UasinGishu	4
Vihiga	4
Wajir	4
WestPokot	4
Madagascar	3
Malawi	3

Appendix Table 15: GBD 2016 location hierarchy with levels	
**Location**	**Level**
Mozambique	3
Rwanda	3
Somalia	3
South Sudan	3
Tanzania	3
Uganda	3
Zambia	3
Southern Sub-Saharan Africa	2
Botswana	3
Lesotho	3
Namibia	3
South Africa	3
Eastern Cape	4
Free State	4
Gauteng	4
KwaZulu-Natal	4
Limpopo	4
Mpumalanga	4
North-West	4
Northern Cape	4
Western Cape	4
Swaziland	3
Zimbabwe	3
Western Sub-Saharan Africa	2
Benin	3
Burkina Faso	3
Cameroon	3
Cape Verde	3
Chad	3
Cote d'Ivoire	3
The Gambia	3
Ghana	3
Guinea	3
Guinea-Bissau	3
Liberia	3
Mali	3
Mauritania	3
Niger	3
Nigeria	3
Sao Tome and Principe	3
Senegal	3
Sierra Leone	3
Togo	3

Appendix Table 16. Causes included in the 2016 Global Burden of Disease Study

Cause level	Cause	Cause of death	Cause of burden
0	All causes	X	X
1	Communicable, maternal, neonatal, and nutritional disorders	X	X
2	HIV/AIDS and tuberculosis	X	X
3	Tuberculosis	X	X
4	Drug-sensitive tuberculosis	X	X
4	Multidrug-resistant tuberculosis without extensive drug resistance	X	X
4	Extensively drug-resistant tuberculosis	X	X
4	Latent tuberculosis infection		X
3	HIV/AIDS	X	X
4	Drug-sensitive HIV/AIDS - Tuberculosis	X	X
4	Multidrug-resistant HIV/AIDS - Tuberculosis without extensive drug resistance	X	X
4	Extensively drug-resistant HIV/AIDS - Tuberculosis	X	X
4	HIV/AIDS resulting in other diseases	X	X
2	Diarrhoea, lower respiratory infections, and other common infectious diseases	X	X
3	Diarrhoeal diseases	X	X
3	Intestinal infectious diseases	X	X
4	Typhoid fever	X	X
4	Paratyphoid fever	X	X
4	Other intestinal infectious diseases	X	X
3	Lower respiratory infections	X	X
3	Upper respiratory infections	X	X
3	Otitis media	X	X
3	Meningitis	X	X
4	Pneumococcal meningitis	X	X
4	H influenzae type B meningitis	X	X
4	Meningococcal infection	X	X

Appendix Table 16. Causes included in the 2016 Global Burden of Disease Study

Cause level	Cause	Cause of death	Cause of burden
4	Other meningitis	X	X
3	Encephalitis	X	X
3	Diphtheria	X	X
3	Whooping cough	X	X
3	Tetanus	X	X
3	Measles	X	X
3	Varicella and herpes zoster	X	X
2	Neglected tropical diseases and malaria	X	X
3	Malaria	X	X
3	Chagas disease	X	X
3	Leishmaniasis	X	X
4	Visceral leishmaniasis	X	X
4	Cutaneous and mucocutaneous leishmaniasis		X
3	African trypanosomiasis	X	X
3	Schistosomiasis	X	X
3	Cysticercosis	X	X
3	Cystic echinococcosis	X	X
3	Lymphatic filariasis		X
3	Onchocerciasis		X
3	Trachoma		X
3	Dengue	X	X
3	Yellow fever	X	X
3	Rabies	X	X
3	Intestinal nematode infections	X	X
4	Ascariasis	X	X
4	Trichuriasis		X

Appendix Table 16. Causes included in the 2016 Global Burden of Disease Study

Cause level	Cause	Cause of death	Cause of burden
4	Hookworm disease		X
3	Food-borne trematodiases		X
3	Leprosy		X
3	Ebola	X	X
3	Zika virus	X	X
3	Guinea worm disease		X
3	Other neglected tropical diseases	X	X
2	Maternal disorders	X	X
3	Maternal haemorrhage	X	X
3	Maternal sepsis and other pregnancy related infections	X	X
3	Maternal hypertensive disorders	X	X
3	Maternal obstructed labour and uterine rupture	X	X
3	Maternal abortion, miscarriage, and ectopic pregnancy	X	X
3	Indirect maternal deaths	X	
3	Late maternal deaths	X	
3	Maternal deaths aggravated by HIV/AIDS	X	
3	Other maternal disorders	X	X
2	Neonatal disorders	X	X
3	Neonatal preterm birth complications	X	X
3	Neonatal encephalopathy due to birth asphyxia and trauma	X	X
3	Neonatal sepsis and other neonatal infections	X	X
3	Hemolytic disease and other neonatal jaundice	X	X
3	Other neonatal disorders	X	X
2	Nutritional deficiencies	X	X
3	Protein-energy malnutrition	X	X
3	Iodine deficiency	X	X

Appendix Table 16. Causes included in the 2016 Global Burden of Disease Study

Cause level	Cause	Cause of death	Cause of burden
3	Vitamin A deficiency		X
3	Iron-deficiency anaemia	X	X
3	Other nutritional deficiencies	X	X
2	Other communicable, maternal, neonatal, and nutritional diseases	X	X
3	Sexually transmitted diseases excluding HIV	X	X
4	Syphilis	X	X
4	Chlamydial infection	X	X
4	Gonococcal infection	X	X
4	Trichomoniasis		X
4	Genital herpes		X
4	Other sexually transmitted diseases	X	X
3	Hepatitis	X	X
4	Acute hepatitis A	X	X
4	Hepatitis B	X	X
4	Hepatitis C	X	X
4	Acute hepatitis E	X	X
3	Other infectious diseases	X	X
1	Non-communicable diseases	X	X
2	Neoplasms	X	X
3	Lip and oral cavity cancer	X	X
3	Nasopharynx cancer	X	X
3	Other pharynx cancer	X	X
3	Oesophageal cancer	X	X
3	Stomach cancer	X	X
3	Colon and rectum cancer	X	X
3	Liver cancer	X	X

Appendix Table 16. Causes included in the 2016 Global Burden of Disease Study

Cause level	Cause	Cause of death	Cause of burden
4	Liver cancer due to hepatitis B	X	X
4	Liver cancer due to hepatitis C	X	X
4	Liver cancer due to alcohol use	X	X
4	Liver cancer due to other causes	X	X
3	Gallbladder and biliary tract cancer	X	X
3	Pancreatic cancer	X	X
3	Larynx cancer	X	X
3	Tracheal, bronchus, and lung cancer	X	X
3	Malignant skin melanoma	X	X
3	Non-melanoma skin cancer	X	X
4	Non-melanoma skin cancer (squamous-cell carcinoma)	X	X
4	Non-melanoma skin cancer (basal-cell carcinoma)		X
3	Breast cancer	X	X
3	Cervical cancer	X	X
3	Uterine cancer	X	X
3	Ovarian cancer	X	X
3	Prostate cancer	X	X
3	Testicular cancer	X	X
3	Kidney cancer	X	X
3	Bladder cancer	X	X
3	Brain and nervous system cancer	X	X
3	Thyroid cancer	X	X
3	Mesothelioma	X	X
3	Hodgkin lymphoma	X	X
3	Non-Hodgkin's lymphoma	X	X
3	Multiple myeloma	X	X

Appendix Table 16. Causes included in the 2016 Global Burden of Disease Study

Cause level	Cause	Cause of death	Cause of burden
3	Leukaemia	X	X
4	Acute lymphoid leukaemia	X	X
4	Chronic lymphoid leukaemia	X	X
4	Acute myeloid leukaemia	X	X
4	Chronic myeloid leukaemia	X	X
4	Other leukaemia	X	X
3	Other neoplasms	X	X
2	Cardiovascular diseases	X	X
3	Rheumatic heart disease	X	X
3	Ischaemic heart disease	X	X
3	Cerebrovascular disease	X	X
4	Ischaemic stroke	X	X
4	Hemorrhagic stroke	X	X
3	Hypertensive heart disease	X	X
3	Cardiomyopathy and myocarditis	X	X
4	Myocarditis	X	X
4	Alcoholic cardiomyopathy	X	X
4	Other cardiomyopathy	X	X
3	Atrial fibrillation and flutter	X	X
3	Aortic aneurysm	X	
3	Peripheral vascular disease	X	X
3	Endocarditis	X	X
3	Other cardiovascular and circulatory diseases	X	X
2	Chronic respiratory diseases	X	X
3	Chronic obstructive pulmonary disease	X	X
3	Pneumoconiosis	X	X

Appendix Table 16. Causes included in the 2016 Global Burden of Disease Study

Cause level	Cause	Cause of death	Cause of burden
4	Silicosis	X	X
4	Asbestosis	X	X
4	Coal workers pneumoconiosis	X	X
4	Other pneumoconiosis	X	X
3	Asthma	X	X
3	Interstitial lung disease and pulmonary sarcoidosis	X	X
3	Other chronic respiratory diseases	X	X
2	Cirrhosis and other chronic liver diseases	X	X
3	Cirrhosis and other chronic liver diseases due to hepatitis B	X	X
3	Cirrhosis and other chronic liver diseases due to hepatitis C	X	X
3	Cirrhosis and other chronic liver diseases due to alcohol use	X	X
3	Cirrhosis and other chronic liver diseases due to other causes	X	X
2	Digestive diseases	X	X
3	Peptic ulcer disease	X	X
3	Gastritis and duodenitis	X	X
3	Appendicitis	X	X
3	Paralytic ileus and intestinal obstruction	X	X
3	Inguinal, femoral, and abdominal hernia	X	X
3	Inflammatory bowel disease	X	X
3	Vascular intestinal disorders	X	X
3	Gallbladder and biliary diseases	X	X
3	Pancreatitis	X	X
3	Other digestive diseases	X	X
2	Neurological disorders	X	X
3	Alzheimer's disease and other dementias	X	X
3	Parkinson's disease	X	X

Appendix Table 16. Causes included in the 2016 Global Burden of Disease Study

Cause level	Cause	Cause of death	Cause of burden
3	Epilepsy	X	X
3	Multiple sclerosis	X	X
3	Motor neuron disease	X	X
3	Migraine		X
3	Tension-type headache		X
3	Other neurological disorders	X	X
2	Mental and substance use disorders	X	X
3	Schizophrenia		X
3	Alcohol use disorders	X	X
3	Drug use disorders	X	X
4	Opioid use disorders	X	X
4	Cocaine use disorders	X	X
4	Amphetamine use disorders	X	X
4	Cannabis use disorders		X
4	Other drug use disorders	X	X
3	Depressive disorders		X
4	Major depressive disorder		X
4	Dysthymia		X
3	Bipolar disorder		X
3	Anxiety disorders		X
3	Eating disorders	X	X
4	Anorexia nervosa	X	X
4	Bulimia nervosa	X	X
3	Autistic spectrum disorders		X
4	Autism		X
4	Asperger syndrome and other autistic spectrum disorders		X

Appendix Table 16. Causes included in the 2016 Global Burden of Disease Study

Cause level	Cause	Cause of death	Cause of burden
3	Attention-deficit/hyperactivity disorder		X
3	Conduct disorder		X
3	Idiopathic developmental intellectual disability		X
3	Other mental and substance use disorders		X
2	Diabetes, urogenital, blood, and endocrine diseases	X	X
3	Diabetes mellitus	X	X
3	Acute glomerulonephritis	X	X
3	Chronic kidney disease	X	X
4	Chronic kidney disease due to diabetes mellitus	X	X
4	Chronic kidney disease due to hypertension	X	X
4	Chronic kidney disease due to glomerulonephritis	X	X
4	Chronic kidney disease due to other causes	X	X
3	Urinary diseases and male infertility	X	X
4	Interstitial nephritis and urinary tract infections	X	X
4	Urolithiasis	X	X
4	Benign prostatic hyperplasia		X
4	Male infertility		X
4	Other urinary diseases	X	X
3	Gynecological diseases	X	X
4	Uterine fibroids	X	X
4	Polycystic ovarian syndrome	X	X
4	Female infertility		X
4	Endometriosis	X	X
4	Genital prolapse	X	X
4	Premenstrual syndrome		X
4	Other gynecological diseases	X	X

Appendix Table 16. Causes included in the 2016 Global Burden of Disease Study

Cause level	Cause	Cause of death	Cause of burden
3	Hemoglobinopathies and hemolytic anaemias	X	X
4	Thalassemias	X	X
4	Thalassaemias trait		X
4	Sickle cell disorders	X	X
4	Sickle cell trait		X
4	G6PD deficiency	X	X
4	G6PD trait		X
4	Other hemoglobinopathies and hemolytic anaemias	X	X
3	Endocrine, metabolic, blood, and immune disorders	X	X
2	Musculoskeletal disorders	X	X
3	Rheumatoid arthritis	X	X
3	Osteoarthritis		X
3	Low back and neck pain		X
4	Low back pain		X
4	Neck pain		X
3	Gout		X
3	Other musculoskeletal disorders	X	X
2	Other non-communicable diseases	X	X
3	Congenital anomalies	X	X
4	Neural tube defects	X	X
4	Congenital heart anomalies	X	X
4	Orofacial clefts	X	X
4	Down's syndrome	X	X
4	Turner syndrome		X
4	Klinefelter syndrome		X
4	Other chromosomal abnormalities	X	X

Appendix Table 16. Causes included in the 2016 Global Burden of Disease Study

Cause level	Cause	Cause of death	Cause of burden
4	Congenital musculoskeletal and limb anomalies	X	X
4	Urogenital congenital anomalies	X	X
4	Digestive congenital anomalies	X	X
4	Other congenital anomalies	X	X
3	Skin and subcutaneous diseases	X	X
4	Eczema		X
4	Psoriasis		X
4	Cellulitis	X	X
4	Pyoderma	X	X
4	Scabies		X
4	Fungal skin diseases		X
4	Viral skin diseases		X
4	Acne vulgaris		X
4	Alopecia areata		X
4	Pruritus		X
4	Urticaria		X
4	Decubitus ulcer	X	X
4	Other skin and subcutaneous diseases	X	X
3	Sense organ diseases		X
4	Glaucoma		X
4	Cataract		X
4	Macular degeneration		X
4	Refraction and accommodation disorders		X
4	Age-related and other hearing loss		X
4	Other vision loss		X
4	Other sense organ diseases		X

Appendix Table 16. Causes included in the 2016 Global Burden of Disease Study

Cause level	Cause	Cause of death	Cause of burden
3	Oral disorders		X
4	Deciduous caries		X
4	Permanent caries		X
4	Periodontal disease		X
4	Edentulism and severe tooth loss		X
4	Other oral disorders		X
3	Sudden infant death syndrome	X	
1	Injuries	X	X
2	Transport injuries	X	X
3	Road injuries	X	X
4	Pedestrian road injuries	X	X
4	Cyclist road injuries	X	X
4	Motorcyclist road injuries	X	X
4	Motor vehicle road injuries	X	X
4	Other road injuries	X	X
3	Other transport injuries	X	X
2	Unintentional injuries	X	X
3	Falls	X	X
3	Drowning	X	X
3	Fire, heat, and hot substances	X	X
3	Poisonings	X	X
3	Exposure to mechanical forces	X	X
4	Unintentional firearm injuries	X	X
4	Unintentional suffocation	X	X
4	Other exposure to mechanical forces	X	X
3	Adverse effects of medical treatment	X	X

Appendix Table 16. Causes included in the 2016 Global Burden of Disease Study

Cause level	Cause	Cause of death	Cause of burden
3	Animal contact	X	X
4	Venomous animal contact	X	X
4	Non-venomous animal contact	X	X
3	Foreign body	X	X
4	Pulmonary aspiration and foreign body in airway	X	X
4	Foreign body in eyes		X
4	Foreign body in other body part	X	X
3	Environmental heat and cold exposure	X	X
3	Other unintentional injuries	X	X
2	Self-harm and interpersonal violence	X	X
3	Self-harm	X	X
4	Self-harm by firearm	X	X
4	Self-harm by other specified means	X	X
3	Interpersonal violence	X	X
4	Assault by firearm	X	X
4	Assault by sharp object	X	X
4	Sexual violence		X
4	Assault by other means	X	X
2	Forces of nature, conflict and terrorism, and executions and police conflict	X	X
3	Exposure to forces of nature	X	X
3	Conflict and terrorism	X	X
3	Executions and police conflict	X	X

Appendix Table 17. Underlying indicators for percent well-certified calculation, based on the maximum percent well certified data source in each 5-year time interval for 195 countries, 1980-2016

Location	Time Window	Stars	Percent Well-Certified [PWC] (%)	Max PWC Data Year	Max PWC Data Source	Completeness (%)	Percent Major Garbage (%)	Verbal Autopsy Adjustment (None for VR) (%)
Afghanistan	1980-1984	0						
Afghanistan	1985-1989	0						
Afghanistan	1990-1994	0						
Afghanistan	1995-1999	0						
Afghanistan	2000-2004	1	4.6	2001	Afghanistan - Badghis Nutrition and Health Survey 2002		27.8	6.4
Afghanistan	2005-2009	2	33.5	2008	Afghanistan Special Demographic and Health Survey 2010		47.7	64.0
Afghanistan	2010-2016	0						
Albania	1980-1984	0						
Albania	1985-1989	4	65.9	1989	Vital Registration	100.0	34.1	
Albania	1990-1994	4	67.0	1993	Vital Registration	97.7	31.4	
Albania	1995-1999	4	71.3	1997	Vital Registration	95.4	25.3	
Albania	2000-2004	4	65.8	2003	Vital Registration	91.8	28.4	
Albania	2005-2009	3	56.8	2008	Vital Registration	76.8	26.1	
Albania	2010-2016	3	45.0	2010	Vital Registration	56.8	20.8	
Algeria	1980-1984	0						
Algeria	1985-1989	0						
Algeria	1990-1994	0						
Algeria	1995-1999	0						
Algeria	2000-2004	0						
Algeria	2005-2009	2	16.8	2006	Vital Registration	29.9	43.8	
Algeria	2010-2016	0						
American Samoa	1980-1984	0						
American Samoa	1985-1989	0						
American Samoa	1990-1994	0						
American Samoa	1995-1999	4	78.6	1997	Vital Registration	100.0	21.4	
American Samoa	2000-2004	4	81.0	2002	Vital Registration	100.0	19.0	
American Samoa	2005-2009	4	83.7	2009	Vital Registration	100.0	16.3	
American Samoa	2010-2016	4	71.0	2011	Vital Registration	90.4	21.4	
Andorra	1980-1984	0						
Andorra	1985-1989	0						
Andorra	1990-1994	0						
Andorra	1995-1999	0						
Andorra	2000-2004	0						
Andorra	2005-2009	0						
Andorra	2010-2016	0						
Angola	1980-1984	0						
Angola	1985-1989	0						
Angola	1990-1994	0						
Angola	1995-1999	0						
Angola	2000-2004	0						
Angola	2005-2009	0						
Angola	2010-2016	1	4.3	2010	Angola - Dande Health and Demographic Surveillance System		32.2	6.4
Antigua and Barbuda	1980-1984	3	51.8	1983	Vital Registration	77.7	33.3	
Antigua and Barbuda	1985-1989	4	71.4	1989	Vital Registration	100.0	28.6	
Antigua and Barbuda	1990-1994	4	72.3	1991	Vital Registration	100.0	27.7	
Antigua and Barbuda	1995-1999	4	80.0	1999	Vital Registration	100.0	20.0	
Antigua and Barbuda	2000-2004	4	79.8	2004	Vital Registration	100.0	20.2	
Antigua and Barbuda	2005-2009	4	79.2	2008	Vital Registration	100.0	20.8	
Antigua and Barbuda	2010-2016	4	73.6	2014	Vital Registration	100.0	26.4	
Argentina	1980-1984	4	76.5	1981	Vital Registration	100.0	23.5	
Argentina	1985-1989	4	69.8	1987	Vital Registration	100.0	30.2	
Argentina	1990-1994	4	68.5	1990	Vital Registration	100.0	31.5	
Argentina	1995-1999	4	67.6	1998	Vital Registration	100.0	32.4	
Argentina	2000-2004	4	66.7	2003	Vital Registration	100.0	33.3	
Argentina	2005-2009	4	65.6	2007	Vital Registration	100.0	34.4	
Argentina	2010-2016	4	67.8	2014	Vital Registration	99.1	31.6	
Armenia	1980-1984	4	69.9	1982	Vital Registration	81.6	14.3	
Armenia	1985-1989	4	76.4	1987	Vital Registration	88.4	13.6	
Armenia	1990-1994	4	82.1	1993	Vital Registration	99.1	17.2	
Armenia	1995-1999	4	81.8	1999	Vital Registration	93.2	12.2	
Armenia	2000-2004	5	87.4	2002	Vital Registration	100.0	12.6	
Armenia	2005-2009	5	90.8	2008	Vital Registration	99.3	8.6	
Armenia	2010-2016	5	91.9	2015	Vital Registration	100.0	8.1	
Australia	1980-1984	5	93.1	1982	Vital Registration	100.0	6.9	
Australia	1985-1989	5	93.1	1987	Vital Registration	99.6	6.6	
Australia	1990-1994	5	92.4	1992	Vital Registration	100.0	7.6	
Australia	1995-1999	5	92.4	1996	Vital Registration	100.0	7.6	
Australia	2000-2004	5	91.3	2000	Vital Registration	100.0	8.7	
Australia	2005-2009	5	90.5	2008	Vital Registration	100.0	9.5	
Australia	2010-2016	5	90.3	2011	Vital Registration	100.0	9.7	
Austria	1980-1984	5	89.5	1981	Vital Registration	100.0	10.5	
Austria	1985-1989	5	90.6	1986	Vital Registration	100.0	9.4	
Austria	1990-1994	5	89.3	1990	Vital Registration	99.4	10.2	
Austria	1995-1999	5	88.6	1996	Vital Registration	100.0	11.4	
Austria	2000-2004	5	91.9	2003	Vital Registration	100.0	8.1	
Austria	2005-2009	5	90.8	2005	Vital Registration	100.0	9.2	
Austria	2010-2016	5	89.2	2013	Vital Registration	100.0	10.8	
Azerbaijan	1980-1984	4	71.7	1981	Vital Registration	83.8	14.4	
Azerbaijan	1985-1989	4	74.0	1988	Vital Registration	85.8	13.8	
Azerbaijan	1990-1994	4	79.7	1993	Vital Registration	91.0	12.4	
Azerbaijan	1995-1999	4	74.3	1995	Vital Registration	87.8	15.4	
Azerbaijan	2000-2004	4	73.2	2002	Vital Registration	80.7	9.2	
Azerbaijan	2005-2009	3	42.9	2007	Vital Registration	75.1	42.9	
Azerbaijan	2010-2016	0						
Bahrain	1980-1984	0						
Bahrain	1985-1989	4	76.5	1986	Vital Registration	100.0	23.5	
Bahrain	1990-1994	0						
Bahrain	1995-1999	3	62.2	1998	Vital Registration	100.0	37.8	
Bahrain	2000-2004	3	55.0	2000	Vital Registration	85.8	36.0	
Bahrain	2005-2009	3	51.8	2005	Vital Registration	85.2	39.2	
Bahrain	2010-2016	3	63.8	2014	Vital Registration	100.0	36.2	
Bangladesh	1980-1984	1	2.8	1982	Bangladesh - Matlab Health and Demographic Surveillance System		56.2	6.4
Bangladesh	1985-1989	1	4.4	1989	Bangladesh - Matlab Health and Demographic Surveillance System		30.5	6.4
Bangladesh	1990-1994	2	23.6	1991	Causes of childhood deaths in Bangladesh: an update		16.3	28.2
Bangladesh	1995-1999	1	4.1	1998	Bangladesh - Matlab Health and Demographic Surveillance System		35.6	6.4
Bangladesh	2000-2004	2	10.2	2004	Bangladesh Demographic and Health Survey 2004		40.9	17.2
Bangladesh	2005-2009	1	6.3	2009	Verbal Autopsy		25.0	8.4
Bangladesh	2010-2016	3	38.6	2014	Verbal Autopsy		29.3	54.6
Barbados	1980-1984	4	72.6	1980	Vital Registration	98.6	26.4	
Barbados	1985-1989	4	73.6	1987	Vital Registration	100.0	26.4	
Barbados	1990-1994	4	72.5	1992	Vital Registration	100.0	27.5	
Barbados	1995-1999	4	70.7	1995	Vital Registration	100.0	29.3	
Barbados	2000-2004	4	75.8	2000	Vital Registration	100.0	24.2	
Barbados	2005-2009	4	82.1	2007	Vital Registration	100.0	17.9	
Barbados	2010-2016	4	81.4	2011	Vital Registration	100.0	18.6	
Belarus	1980-1984	4	81.4	1981	Vital Registration	97.5	16.4	
Belarus	1985-1989	5	86.6	1987	Vital Registration	99.7	13.1	
Belarus	1990-1994	4	77.1	1990	Vital Registration	99.2	22.3	
Belarus	1995-1999	4	79.9	1999	Vital Registration	100.0	20.1	
Belarus	2000-2004	4	83.0	2002	Vital Registration	100.0	17.0	
Belarus	2005-2009	4	82.7	2009	Vital Registration	99.6	17.0	
Belarus	2010-2016	4	82.6	2010	Vital Registration	100.0	17.4	
Belgium	1980-1984	4	77.0	1981	Vital Registration	99.0	22.2	
Belgium	1985-1989	4	77.2	1987	Vital Registration	99.6	22.4	
Belgium	1990-1994	4	81.1	1993	Vital Registration	100.0	18.9	
Belgium	1995-1999	4	84.1	1998	Vital Registration	100.0	15.9	
Belgium	2000-2004	4	83.1	2000	Vital Registration	100.0	16.9	
Belgium	2005-2009	4	83.0	2008	Vital Registration	100.0	17.0	
Belgium	2010-2016	4	80.2	2010	Vital Registration	99.8	19.6	
Belize	1980-1984	3	54.0	1980	Vital Registration	81.3	33.6	

Appendix Table 17. Underlying indicators for percent well-certified for data source with maximum percent well certified in each 5-year time interval for 195 countries, 1980-2016.

Location	Time Window	Stars	Percent Well-Certified [PWC] (%)	Max PWC Data Year	Max PWC Data Source	Completeness (%)	Percent Major Garbage (%)	Verbal Autopsy Adjustment (None for VR) (%)
Belize	1985-1989	3	56.9	1987	Vital Registration	76.2	25.3	
Belize	1990-1994	3	46.8	1993	Vital Registration	74.9	37.5	
Belize	1995-1999	4	76.9	1998	Vital Registration	100.0	23.1	
Belize	2000-2004	4	71.6	2000	Vital Registration	100.0	28.4	
Belize	2005-2009	4	80.7	2009	Vital Registration	93.0	13.2	
Belize	2010-2016	4	84.7	2011	Vital Registration	94.7	10.6	
Benin	1980-1984	0						
Benin	1985-1989	1	0.6	1989	Incidence de décès de 0 à 1 an dans une cohorte de 802 enfants en milieu rural au sud du Bénin		72.0	2.1
Benin	1990-1994	0						
Benin	1995-1999	0						
Benin	2000-2004	0						
Benin	2005-2009	0						
Benin	2010-2016	0						
Bermuda	1980-1984	5	89.0	1984	Vital Registration	100.0	11.0	
Bermuda	1985-1989	5	86.5	1987	Vital Registration	94.6	8.5	
Bermuda	1990-1994	4	84.7	1991	Vital Registration	100.0	15.3	
Bermuda	1995-1999	5	90.9	1998	Vital Registration	100.0	9.1	
Bermuda	2000-2004	5	89.4	2000	Vital Registration	100.0	10.6	
Bermuda	2005-2009	5	86.4	2005	Vital Registration	100.0	13.6	
Bermuda	2010-2016	5	90.5	2014	Vital Registration	100.0	9.5	
Bhutan	1980-1984	0						
Bhutan	1985-1989	0						
Bhutan	1990-1994	0						
Bhutan	1995-1999	0						
Bhutan	2000-2004	0						
Bhutan	2005-2009	0						
Bhutan	2010-2016	0						
Bolivia	1980-1984	0						
Bolivia	1985-1989	0						
Bolivia	1990-1994	0						
Bolivia	1995-1999	0						
Bolivia	2000-2004	2	12.4	2003	Vital Registration	37.8	67.3	
Bolivia	2005-2009	0						
Bolivia	2010-2016	0						
Bosnia and Herzegovina	1980-1984	0						
Bosnia and Herzegovina	1985-1989	3	64.4	1989	Vital Registration	94.8	32.0	
Bosnia and Herzegovina	1990-1994	3	64.5	1991	Vital Registration	93.5	31.0	
Bosnia and Herzegovina	1995-1999	0						
Bosnia and Herzegovina	2000-2004	0						
Bosnia and Herzegovina	2005-2009	0						
Bosnia and Herzegovina	2010-2016	4	68.8	2014	Vital Registration	95.6	28.0	
Botswana	1980-1984	0						
Botswana	1985-1989	0						
Botswana	1990-1994	0						
Botswana	1995-1999	0						
Botswana	2000-2004	0						
Botswana	2005-2009	0						
Botswana	2010-2016	0						
Brazil	1980-1984	3	58.3	1984	Vital Registration	90.9	35.9	
Brazil	1985-1989	3	62.4	1988	Vital Registration	94.3	33.8	
Brazil	1990-1994	3	65.0	1994	Vital Registration	95.7	32.1	
Brazil	1995-1999	4	69.8	1999	Vital Registration	96.5	27.6	
Brazil	2000-2004	4	75.0	2004	Vital Registration	98.8	24.1	
Brazil	2005-2009	4	80.4	2009	Vital Registration	99.1	18.8	
Brazil	2010-2016	4	82.7	2014	Vital Registration	100.0	17.3	
Brunei	1980-1984	0						
Brunei	1985-1989	0						
Brunei	1990-1994	0						
Brunei	1995-1999	5	85.4	1998	Vital Registration	99.6	14.2	
Brunei	2000-2004	4	82.9	2002	Vital Registration	100.0	17.1	
Brunei	2005-2009	4	81.9	2007	Vital Registration	100.0	18.1	
Brunei	2010-2016	4	81.8	2013	Vital Registration	95.8	14.6	
Bulgaria	1980-1984	4	80.4	1980	Vital Registration	100.0	19.6	
Bulgaria	1985-1989	4	80.7	1985	Vital Registration	100.0	19.3	
Bulgaria	1990-1994	4	79.7	1993	Vital Registration	99.5	19.9	
Bulgaria	1995-1999	4	76.0	1995	Vital Registration	99.2	23.3	
Bulgaria	2000-2004	4	71.8	2002	Vital Registration	100.0	28.2	
Bulgaria	2005-2009	4	73.5	2005	Vital Registration	100.0	26.5	
Bulgaria	2010-2016	4	70.3	2012	Vital Registration	100.0	29.7	
Burkina Faso	1980-1984	1	0.2	1984	The burden of malaria mortality among African children in the year 2000		95.3	4.1
Burkina Faso	1985-1989	0						
Burkina Faso	1990-1994	0						
Burkina Faso	1995-1999	1	4.6	1998	Measuring the local burden of disease. A study of years of life lost in sub-Saharan Africa		28.0	6.4
Burkina Faso	2000-2004	1	5.6	2000	Burkina Faso - Nouna Health and Demographic Surveillance System		12.7	6.4
Burkina Faso	2005-2009	1	4.6	2009	An improved method for physician-certified verbal autopsy reduces the rate of discrepancy: experiences in the Nouna Health and Demographic Surveillance Site (NHDSS), Burkina Faso		28.8	6.4
Burkina Faso	2010-2016	1	0.3	2010	Africa, Asia, Oceania - INDEPTH Network Cause-Specific Mortality - Release 2014		95.4	6.4
Burundi	1980-1984	0						
Burundi	1985-1989	0						
Burundi	1990-1994	1	2.3	1990	Mortality and morbidity at young ages in a stable hyperendemic malaria region, community Nyanza-Lac, Imbo South, Burundi		23.1	3.0
Burundi	1995-1999	0						
Burundi	2000-2004	0						
Burundi	2005-2009	0						
Burundi	2010-2016	0						
Cambodia	1980-1984	0						
Cambodia	1985-1989	0						
Cambodia	1990-1994	0						
Cambodia	1995-1999	0						
Cambodia	2000-2004	1	1.6	2001	Community-based surveillance: a pilot study from rural Cambodia		74.8	6.4
Cambodia	2005-2009	1	3.5	2009	Mortality in Cambodia An 18-Month Prospective Community-based Surveillance of All-age Deaths Using Verbal Autopsies		46.1	6.4
Cambodia	2010-2016	0						
Cameroon	1980-1984	0						
Cameroon	1985-1989	0						
Cameroon	1990-1994	0						
Cameroon	1995-1999	0						
Cameroon	2000-2004	0						
Cameroon	2005-2009	0						
Cameroon	2010-2016	0						
Canada	1980-1984	5	88.6	1980	Vital Registration	100.0	11.4	
Canada	1985-1989	5	89.8	1986	Vital Registration	100.0	10.2	
Canada	1990-1994	5	88.3	1993	Vital Registration	100.0	11.7	
Canada	1995-1999	5	88.2	1999	Vital Registration	100.0	11.8	
Canada	2000-2004	5	89.6	2001	Vital Registration	100.0	10.4	
Canada	2005-2009	5	90.1	2009	Vital Registration	100.0	9.9	
Canada	2010-2016	5	90.1	2012	Vital Registration	100.0	9.9	
Cape Verde	1980-1984	3	58.3	1980	Vital Registration	100.0	41.7	
Cape Verde	1985-1989	0						
Cape Verde	1990-1994	1	0.1	1992	Deaths among women of reproductive age in Cape Verde: causes and avoidability		57.7	0.3
Cape Verde	1995-1999	0						
Cape Verde	2000-2004	0						

Appendix Table 17. Underlying indicators for percent well-certified for data source with maximum percent well certified in each 5-year time interval for 195 countries, 1980-2016.

Location	Time Window	Stars	Percent Well-Certified [PWC] (%)	Max PWC Data Year	Max PWC Data Source	Completeness (%)	Percent Major Garbage (%)	Verbal Autopsy Adjustment (None for VR) (%)
Cape Verde	2005-2009	0						
Cape Verde	2010-2016	4	69.7	2012	Vital Registration	98.5	29.3	
Central African Republic	1980-1984	0						
Central African Republic	1985-1989	0						
Central African Republic	1990-1994	0						
Central African Republic	1995-1999	0						
Central African Republic	2000-2004	0						
Central African Republic	2005-2009	0						
Central African Republic	2010-2016	0						
Chad	1980-1984	0						
Chad	1985-1989	0						
Chad	1990-1994	0						
Chad	1995-1999	0						
Chad	2000-2004	0						
Chad	2005-2009	0						
Chad	2010-2016	0						
Chile	1980-1984	4	75.5	1983	Vital Registration	100.0	24.5	
Chile	1985-1989	4	75.1	1988	Vital Registration	100.0	24.9	
Chile	1990-1994	4	76.6	1994	Vital Registration	97.6	21.5	
Chile	1995-1999	4	84.8	1999	Vital Registration	100.0	15.2	
Chile	2000-2004	5	90.9	2004	Vital Registration	100.0	9.1	
Chile	2005-2009	5	90.3	2007	Vital Registration	100.0	9.7	
Chile	2010-2016	5	90.0	2012	Vital Registration	100.0	10.0	
China	1980-1984	0						
China	1985-1989	0						
China	1990-1994	4	71.7	1994	Vital Registration - Sample	82.8	13.4	
China	1995-1999	4	70.5	1996	Vital Registration - Sample	82.8	14.8	
China	2000-2004	4	73.0	2004	Vital Registration	77.0	5.2	
China	2005-2009	4	72.6	2005	Vital Registration	76.5	5.1	
China	2010-2016	4	69.3	2011	Vital Registration - Sample	73.5	5.8	
Colombia	1980-1984	4	71.7	1983	Vital Registration	91.2	21.5	
Colombia	1985-1989	4	73.3	1985	Vital Registration	92.8	21.0	
Colombia	1990-1994	4	75.3	1993	Vital Registration	89.7	16.1	
Colombia	1995-1999	4	84.5	1999	Vital Registration	94.7	10.8	
Colombia	2000-2004	5	86.0	2003	Vital Registration	95.8	10.2	
Colombia	2005-2009	5	86.3	2008	Vital Registration	96.6	10.7	
Colombia	2010-2016	5	87.8	2013	Vital Registration	97.8	10.2	
Comoros	1980-1984	0						
Comoros	1985-1989	0						
Comoros	1990-1994	0						
Comoros	1995-1999	0						
Comoros	2000-2004	0						
Comoros	2005-2009	0						
Comoros	2010-2016	0						
Congo (Brazzaville)	1980-1984	0						
Congo (Brazzaville)	1985-1989	0						
Congo (Brazzaville)	1990-1994	0						
Congo (Brazzaville)	1995-1999	0						
Congo (Brazzaville)	2000-2004	0						
Congo (Brazzaville)	2005-2009	0						
Congo (Brazzaville)	2010-2016	0						
Costa Rica	1980-1984	4	79.8	1984	Vital Registration	97.7	18.3	
Costa Rica	1985-1989	4	81.8	1985	Vital Registration	100.0	18.2	
Costa Rica	1990-1994	4	80.2	1994	Vital Registration	100.0	19.8	
Costa Rica	1995-1999	5	91.2	1999	Vital Registration	100.0	8.8	
Costa Rica	2000-2004	5	91.8	2003	Vital Registration	99.4	7.6	
Costa Rica	2005-2009	5	89.8	2006	Vital Registration	100.0	10.2	
Costa Rica	2010-2016	5	90.8	2014	Vital Registration	100.0	9.2	
Cote d'Ivoire	1980-1984	0						
Cote d'Ivoire	1985-1989	1	1.0	1988	Effet de l'observance des d'approvisionnement en eau et de la therapie par voie orale sur les diarrhees chez les enfants de moins de 5 de la Cote d'Ivoire		68.0	3.3
Cote d'Ivoire	1990-1994	1	1.0	1992	Effet de l'observance des d'approvisionnement en eau et de la therapie par voie orale sur les diarrhees chez les enfants de moins de 5 de la Cote d'Ivoire		65.9	3.0
Cote d'Ivoire	1995-1999	0						
Cote d'Ivoire	2000-2004	0						
Cote d'Ivoire	2005-2009	1	0.2	2009	Africa, Asia, Oceania - INDEPTH Network Cause-Specific Mortality - Release 2014		97.1	6.4
Cote d'Ivoire	2010-2016	1	0.2	2011	Africa, Asia, Oceania - INDEPTH Network Cause-Specific Mortality - Release 2014		96.8	6.4
Croatia	1980-1984	0						
Croatia	1985-1989	4	82.7	1989	Vital Registration	98.2	15.7	
Croatia	1990-1994	4	83.7	1990	Vital Registration	98.4	14.9	
Croatia	1995-1999	4	80.7	1999	Vital Registration	97.7	17.5	
Croatia	2000-2004	4	84.1	2003	Vital Registration	100.0	15.9	
Croatia	2005-2009	5	86.5	2009	Vital Registration	98.0	11.7	
Croatia	2010-2016	5	87.9	2010	Vital Registration	97.2	9.5	
Cuba	1980-1984	4	84.6	1981	Vital Registration	100.0	15.4	
Cuba	1985-1989	4	84.6	1985	Vital Registration	99.7	15.2	
Cuba	1990-1994	4	83.2	1990	Vital Registration	98.0	15.0	
Cuba	1995-1999	5	88.3	1999	Vital Registration	100.0	11.7	
Cuba	2000-2004	5	90.1	2004	Vital Registration	100.0	9.9	
Cuba	2005-2009	5	91.0	2009	Vital Registration	100.0	9.0	
Cuba	2010-2016	5	91.5	2012	Vital Registration	100.0	8.5	
Cyprus	1980-1984	0						
Cyprus	1985-1989	0						
Cyprus	1990-1994	0						
Cyprus	1995-1999	2	28.7	1999	Vital Registration	81.9	65.0	
Cyprus	2000-2004	3	58.3	2004	Vital Registration	83.8	30.5	
Cyprus	2005-2009	4	66.7	2007	Vital Registration	86.5	22.8	
Cyprus	2010-2016	4	66.5	2014	Vital Registration	81.0	17.9	
Czech Republic	1980-1984	0						
Czech Republic	1985-1989	5	90.3	1986	Vital Registration	100.0	9.7	
Czech Republic	1990-1994	5	89.4	1990	Vital Registration	100.0	10.6	
Czech Republic	1995-1999	4	84.8	1995	Vital Registration	100.0	15.2	
Czech Republic	2000-2004	5	85.1	2000	Vital Registration	100.0	14.9	
Czech Republic	2005-2009	4	84.8	2007	Vital Registration	98.7	14.2	
Czech Republic	2010-2016	5	87.8	2013	Vital Registration	100.0	12.2	
Democratic Republic of the Congo	1980-1984	0						
Democratic Republic of the Congo	1985-1989	1	2.3	1986	Etude de la mortalité globale et de la mortalité liée au paludisme dans le Kivu montagneux, Zaïre		64.3	6.4
Democratic Republic of the Congo	1990-1994	1	2.9	1990	Influence of nutritional status on child mortality in rural Zaire		14.2	3.4
Democratic Republic of the Congo	1995-1999	0						
Democratic Republic of the Congo	2000-2004	0						
Democratic Republic of the Congo	2005-2009	0						
Democratic Republic of the Congo	2010-2016	0						
Denmark	1980-1984	4	80.6	1980	Vital Registration	100.0	19.4	
Denmark	1985-1989	4	78.8	1985	Vital Registration	100.0	21.2	
Denmark	1990-1994	4	84.0	1994	Vital Registration	100.0	16.0	
Denmark	1995-1999	5	86.7	1999	Vital Registration	100.0	13.2	
Denmark	2000-2004	5	85.3	2001	Vital Registration	100.0	14.7	
Denmark	2005-2009	4	84.1	2006	Vital Registration	100.0	15.9	
Denmark	2010-2016	4	84.6	2012	Vital Registration	100.0	15.4	
Djibouti	1980-1984	0						
Djibouti	1985-1989	0						
Djibouti	1990-1994	0						
Djibouti	1995-1999	0						
Djibouti	2000-2004	0						
Djibouti	2005-2009	0						
Djibouti	2010-2016	0						

Appendix Table 17. Underlying indicators for percent well-certified for data source with maximum percent well certified in each 5-year time interval for 195 countries, 1980-2016.

Location	Time Window	Stars	Percent Well-Certified [PWC] (%)	Max PWC Data Year	Max PWC Data Source	Completeness (%)	Percent Major Garbage (%)	Verbal Autopsy Adjustment (None for VR) (%)
Dominica	1980-1984	4	70.4	1984	Vital Registration	100.0	29.6	
Dominica	1985-1989	3	61.5	1988	Vital Registration	93.5	34.2	
Dominica	1990-1994	3	62.1	1990	Vital Registration	100.0	37.9	
Dominica	1995-1999	3	62.9	1999	Vital Registration	100.0	37.1	
Dominica	2000-2004	4	69.5	2004	Vital Registration	100.0	30.5	
Dominica	2005-2009	5	85.3	2006	Vital Registration	100.0	14.7	
Dominica	2010-2016	4	83.6	2011	Vital Registration	100.0	16.4	
Dominican Republic	1980-1984	3	56.3	1984	Vital Registration	85.3	34.0	
Dominican Republic	1985-1989	3	56.3	1985	Vital Registration	83.5	32.5	
Dominican Republic	1990-1994	3	45.8	1990	Vital Registration	68.4	33.0	
Dominican Republic	1995-1999	3	54.0	1999	Vital Registration	74.1	27.1	
Dominican Republic	2000-2004	3	58.9	2004	Vital Registration	77.4	23.8	
Dominican Republic	2005-2009	3	58.2	2005	Vital Registration	73.9	21.2	
Dominican Republic	2010-2016	4	67.2	2012	Vital Registration	84.7	20.7	
Ecuador	1980-1984	4	71.6	1981	Vital Registration	99.3	27.9	
Ecuador	1985-1989	4	68.1	1988	Vital Registration	95.0	28.4	
Ecuador	1990-1994	4	67.7	1991	Vital Registration	94.3	28.2	
Ecuador	1995-1999	3	63.7	1996	Vital Registration	88.9	28.4	
Ecuador	2000-2004	3	61.6	2000	Vital Registration	92.5	33.4	
Ecuador	2005-2009	4	66.4	2009	Vital Registration	88.7	25.1	
Ecuador	2010-2016	4	68.2	2014	Vital Registration	86.5	21.1	
Egypt	1980-1984	2	33.3	1980	Vital Registration	71.0	53.1	
Egypt	1985-1989	3	46.9	1987	Vital Registration	91.6	48.7	
Egypt	1990-1994	3	43.7	1991	Vital Registration	87.1	49.8	
Egypt	1995-1999	0						
Egypt	2000-2004	3	42.9	2003	Vital Registration	98.8	56.6	
Egypt	2005-2009	3	40.6	2005	Vital Registration	94.8	57.2	
Egypt	2010-2016	3	48.4	2014	Vital Registration	100.0	51.6	
El Salvador	1980-1984	4	72.8	1981	Vital Registration	100.0	27.2	
El Salvador	1985-1989	0						
El Salvador	1990-1994	3	57.8	1993	Vital Registration	87.5	34.0	
El Salvador	1995-1999	3	63.4	1998	Vital Registration	93.6	32.2	
El Salvador	2000-2004	4	65.6	2001	Vital Registration	92.6	29.2	
El Salvador	2005-2009	4	66.6	2006	Vital Registration	93.2	28.6	
El Salvador	2010-2016	3	64.0	2011	Vital Registration	93.3	31.5	
Equatorial Guinea	1980-1984	0						
Equatorial Guinea	1985-1989	0						
Equatorial Guinea	1990-1994	0						
Equatorial Guinea	1995-1999	0						
Equatorial Guinea	2000-2004	0						
Equatorial Guinea	2005-2009	0						
Equatorial Guinea	2010-2016	0						
Eritrea	1980-1984	0						
Eritrea	1985-1989	0						
Eritrea	1990-1994	0						
Eritrea	1995-1999	0						
Eritrea	2000-2004	0						
Eritrea	2005-2009	0						
Eritrea	2010-2016	0						
Estonia	1980-1984	5	89.0	1981	Vital Registration	99.0	10.1	
Estonia	1985-1989	5	90.9	1986	Vital Registration	100.0	9.1	
Estonia	1990-1994	5	93.7	1994	Vital Registration	100.0	6.3	
Estonia	1995-1999	5	93.0	1996	Vital Registration	99.8	6.8	
Estonia	2000-2004	5	92.0	2003	Vital Registration	100.0	8.0	
Estonia	2005-2009	5	93.8	2009	Vital Registration	99.6	5.8	
Estonia	2010-2016	5	93.8	2010	Vital Registration	100.0	6.2	
Ethiopia	1980-1984	0						
Ethiopia	1985-1989	1	1.1	1987	The Butajira rural health project in Ethiopia: mortality pattern of the under fives		62.5	2.9
Ethiopia	1990-1994	1	2.3	1992	Patterns of childhood mortality in three districts of north Gondar Administrative Zone. A community based study using the verbal autopsy method		16.8	2.8
Ethiopia	1995-1999	1	0.6	1997	The use of simplified verbal autopsy in identifying causes of adult death in a predominantly rural population in Ethiopia		52.2	1.2
Ethiopia	2000-2004	1	4.8	2001	HIV/AIDS-Related Mortality In Addis Ababa City Administration		24.5	6.4
Ethiopia	2005-2009	3	46.6	2009	Verbal Autopsy		27.2	64.0
Ethiopia	2010-2016	3	45.5	2010	Verbal Autopsy		28.9	64.0
Federated States of Micronesia	1980-1984	0						
Federated States of Micronesia	1985-1989	0						
Federated States of Micronesia	1990-1994	0						
Federated States of Micronesia	1995-1999	0						
Federated States of Micronesia	2000-2004	0						
Federated States of Micronesia	2005-2009	0						
Federated States of Micronesia	2010-2016	0						
Fiji	1980-1984	0						
Fiji	1985-1989	0						
Fiji	1990-1994	0						
Fiji	1995-1999	2	33.2	1999	Vital Registration	58.0	42.7	
Fiji	2000-2004	3	56.6	2003	Vital Registration	97.8	42.1	
Fiji	2005-2009	3	58.8	2008	Vital Registration	91.7	35.9	
Fiji	2010-2016	3	63.4	2011	Vital Registration	91.2	30.4	
Finland	1980-1984	4	81.1	1980	Vital Registration	100.0	18.9	
Finland	1985-1989	5	90.5	1987	Vital Registration	99.4	8.9	
Finland	1990-1994	5	91.6	1993	Vital Registration	100.0	8.4	
Finland	1995-1999	5	95.7	1999	Vital Registration	99.9	4.2	
Finland	2000-2004	5	95.7	2000	Vital Registration	100.0	4.3	
Finland	2005-2009	5	94.5	2009	Vital Registration	99.4	4.9	
Finland	2010-2016	5	95.6	2013	Vital Registration	100.0	4.4	
France	1980-1984	4	76.2	1984	Vital Registration	100.0	23.8	
France	1985-1989	4	78.0	1988	Vital Registration	100.0	22.0	
France	1990-1994	4	78.1	1993	Vital Registration	100.0	21.9	
France	1995-1999	4	78.7	1998	Vital Registration	100.0	21.3	
France	2000-2004	4	79.1	2000	Vital Registration	100.0	20.9	
France	2005-2009	4	79.4	2005	Vital Registration	100.0	20.6	
France	2010-2016	4	77.9	2011	Vital Registration	99.6	21.8	
Gabon	1980-1984	0						
Gabon	1985-1989	0						
Gabon	1990-1994	0						
Gabon	1995-1999	0						
Gabon	2000-2004	0						
Gabon	2005-2009	0						
Gabon	2010-2016	0						
Georgia	1980-1984	5	85.9	1981	Vital Registration	95.8	10.4	
Georgia	1985-1989	4	83.2	1985	Vital Registration	93.7	11.2	
Georgia	1990-1994	4	78.0	1990	Vital Registration	84.9	8.1	
Georgia	1995-1999	4	74.2	1999	Vital Registration	85.8	13.5	
Georgia	2000-2004	4	77.6	2000	Vital Registration	87.6	11.5	
Georgia	2005-2009	3	51.2	2005	Vital Registration	80.5	36.4	
Georgia	2010-2016	3	58.7	2014	Vital Registration	99.4	40.9	
Germany	1980-1984	4	77.5	1983	Vital Registration	100.0	22.5	
Germany	1985-1989	4	78.2	1989	Vital Registration	98.3	20.5	
Germany	1990-1994	4	83.1	1994	Vital Registration	100.0	16.9	
Germany	1995-1999	4	83.9	1998	Vital Registration	100.0	16.1	
Germany	2000-2004	4	83.2	2004	Vital Registration	99.6	16.5	
Germany	2005-2009	4	83.6	2005	Vital Registration	100.0	16.4	
Germany	2010-2016	4	84.0	2013	Vital Registration	100.0	16.0	
Ghana	1980-1984	0						
Ghana	1985-1989	1	0.1	1989	Maternal mortality among the Kassena-Nankana of northern Ghana		79.0	0.5

Appendix Table 17. Underlying indicators for percent well-certified for data source with maximum percent well certified in each 5-year time interval for 195 countries, 1980-2016.

Location	Time Window	Stars	Percent Well-Certified [PWC] (%)	Max PWC Data Year	Max PWC Data Source	Completeness (%)	Percent Major Garbage (%)	Verbal Autopsy Adjustment (None for VR) (%)
Ghana	1990-1994	1	1.6	1990	Vitamin A supplementation in northern Ghana: effects on clinic attendances, hospital admissions, and child mortality		27.9	2.2
Ghana	1995-1999	1	0.9	1998	Trend and causes of neonatal mortality in the Kassena-Nankana district of northern Ghana, 1995-2002		6.0	0.9
Ghana	2000-2004	1	8.6	2000	Vital Registration	12.7	32.1	
Ghana	2005-2009	2	20.8	2006	Ghana Child Verbal Autopsy Study 2008		8.2	22.7
Ghana	2010-2016	1	0.5	2011	Africa, Asia, Oceania - INDEPTH Network Cause-Specific Mortality - Release 2014		92.7	6.4
Greece	1980-1984	4	79.7	1984	Vital Registration	98.8	19.4	
Greece	1985-1989	4	81.1	1985	Vital Registration	100.0	18.9	
Greece	1990-1994	4	71.3	1994	Vital Registration	99.5	28.4	
Greece	1995-1999	4	71.9	1995	Vital Registration	100.0	28.1	
Greece	2000-2004	4	72.2	2001	Vital Registration	99.6	27.5	
Greece	2005-2009	4	76.5	2007	Vital Registration	100.0	23.5	
Greece	2010-2016	4	74.1	2010	Vital Registration	98.6	24.9	
Greenland	1980-1984	0						
Greenland	1985-1989	0						
Greenland	1990-1994	0						
Greenland	1995-1999	5	90.2	1999	Vital Registration	100.0	9.8	
Greenland	2000-2004	5	89.7	2001	Vital Registration	100.0	10.3	
Greenland	2005-2009	5	89.7	2005	Vital Registration	100.0	10.3	
Greenland	2010-2016	5	87.8	2010	Vital Registration	100.0	12.2	
Grenada	1980-1984	4	69.9	1984	Vital Registration	100.0	30.1	
Grenada	1985-1989	3	61.4	1988	Vital Registration	100.0	38.6	
Grenada	1990-1994	3	62.0	1994	Vital Registration	100.0	38.0	
Grenada	1995-1999	3	60.7	1995	Vital Registration	100.0	39.3	
Grenada	2000-2004	4	77.3	2002	Vital Registration	100.0	22.7	
Grenada	2005-2009	4	76.3	2009	Vital Registration	96.1	20.6	
Grenada	2010-2016	4	83.8	2012	Vital Registration	99.5	15.8	
Guam	1980-1984	0						
Guam	1985-1989	0						
Guam	1990-1994	5	89.0	1994	Vital Registration	96.0	7.2	
Guam	1995-1999	5	85.9	1999	Vital Registration	93.0	7.7	
Guam	2000-2004	4	77.1	2000	Vital Registration	85.4	9.8	
Guam	2005-2009	4	71.8	2007	Vital Registration	77.3	7.0	
Guam	2010-2016	4	66.1	2010	Vital Registration	73.7	10.3	
Guatemala	1980-1984	4	79.2	1980	Vital Registration	98.9	19.9	
Guatemala	1985-1989	4	70.5	1987	Vital Registration	97.4	27.7	
Guatemala	1990-1994	4	71.5	1990	Vital Registration	100.0	28.5	
Guatemala	1995-1999	4	70.8	1998	Vital Registration	100.0	29.2	
Guatemala	2000-2004	4	67.9	2001	Vital Registration	96.5	29.6	
Guatemala	2005-2009	4	70.7	2009	Vital Registration	93.9	24.7	
Guatemala	2010-2016	4	73.4	2014	Vital Registration	93.6	21.5	
Guinea	1980-1984	0						
Guinea	1985-1989	0						
Guinea	1990-1994	0						
Guinea	1995-1999	1	3.3	1998	Guinea - Mandiana Mortality Study 1998-1999		5.5	3.5
Guinea	2000-2004	0						
Guinea	2005-2009	0						
Guinea	2010-2016	0						
Guinea-Bissau	1980-1984	0						
Guinea-Bissau	1985-1989	0						
Guinea-Bissau	1990-1994	1	0.1	1992	Maternal mortality in Guinea-Bissau: the use of verbal autopsy in a multi-ethnic population		80.2	0.4
Guinea-Bissau	1995-1999	1	1.1	1995	BCG vaccination scar associated with better childhood survival in Guinea-Bissau		45.6	2.1
Guinea-Bissau	2000-2004	0						
Guinea-Bissau	2005-2009	0						
Guinea-Bissau	2010-2016	0						
Guyana	1980-1984	3	51.5	1984	Vital Registration	80.5	36.1	
Guyana	1985-1989	4	71.7	1989	Vital Registration	95.0	24.5	
Guyana	1990-1994	3	64.0	1990	Vital Registration	86.8	26.3	
Guyana	1995-1999	4	66.2	1997	Vital Registration	91.6	27.7	
Guyana	2000-2004	4	79.0	2000	Vital Registration	90.9	13.1	
Guyana	2005-2009	4	77.7	2005	Vital Registration	89.3	12.9	
Guyana	2010-2016	4	73.5	2010	Vital Registration	89.2	17.6	
Haiti	1980-1984	2	19.3	1981	Vital Registration	43.1	55.3	
Haiti	1985-1989	1	1.4	1989	The utility of verbal autopsies for identifying HIV-1-related deaths in Haitian children		21.7	1.8
Haiti	1990-1994	1	1.1	1994	Survey on Infant mortality in Mirebalais, Haiti		36.5	1.7
Haiti	1995-1999	2	10.6	1999	Vital Registration	23.2	54.4	
Haiti	2000-2004	1	4.6	2002	Vital Registration	7.7	41.2	
Haiti	2005-2009	0						
Haiti	2010-2016	0						
Honduras	1980-1984	2	31.7	1981	Vital Registration	62.2	49.0	
Honduras	1985-1989	3	36.9	1988	Vital Registration	56.8	35.1	
Honduras	1990-1994	3	35.6	1990	Vital Registration	55.2	35.5	
Honduras	1995-1999	1	0.4	1997	Honduras Maternal Mortality Ratio Update 2010		92.4	5.1
Honduras	2000-2004	0						
Honduras	2005-2009	2	12.4	2008	Vital Registration	14.1	11.8	
Honduras	2010-2016	2	13.9	2013	Vital Registration	15.3	9.4	
Hungary	1980-1984	5	90.6	1980	Vital Registration	100.0	9.4	
Hungary	1985-1989	5	89.3	1989	Vital Registration	100.0	10.7	
Hungary	1990-1994	5	89.9	1994	Vital Registration	100.0	10.1	
Hungary	1995-1999	5	90.8	1998	Vital Registration	100.0	9.2	
Hungary	2000-2004	5	92.6	2004	Vital Registration	100.0	7.4	
Hungary	2005-2009	5	93.3	2009	Vital Registration	100.0	6.7	
Hungary	2010-2016	5	93.6	2011	Vital Registration	100.0	6.4	
Iceland	1980-1984	5	91.3	1982	Vital Registration	99.6	8.4	
Iceland	1985-1989	5	92.8	1989	Vital Registration	100.0	7.2	
Iceland	1990-1994	5	94.0	1991	Vital Registration	100.0	6.0	
Iceland	1995-1999	5	94.1	1999	Vital Registration	100.0	5.9	
Iceland	2000-2004	5	93.5	2004	Vital Registration	100.0	6.5	
Iceland	2005-2009	5	92.8	2005	Vital Registration	99.9	7.1	
Iceland	2010-2016	5	91.4	2010	Vital Registration	100.0	8.6	
India	1980-1984	1	3.6	1980	India Survey of Causes of Death 1980		44.1	6.4
India	1985-1989	1	3.5	1985	India Survey of Causes of Death 1985		44.6	6.4
India	1990-1994	1	3.7	1994	India Cause of Death Dataset Version 1.3 1980-1998		42.5	6.4
India	1995-1999	1	4.9	1999	Vital Registration	8.0	38.3	
India	2000-2004	1	5.2	2001	Vital Registration	8.3	37.1	
India	2005-2009	3	52.8	2005	Verbal Autopsy		17.6	64.0
India	2010-2016	3	49.1	2012	Verbal Autopsy		23.3	64.0
Indonesia	1980-1984	1	0.1	1981	Reproductive Mortality in Two Developing Countries		77.0	0.5
Indonesia	1985-1989	0						
Indonesia	1990-1994	1	1.3	1991	Care-seeking for fatal illnesses in young children in Indramayu, West Java, Indonesia		23.4	1.7
Indonesia	1995-1999	1	0.4	1997	Age- and cause-specific childhood mortality in Lombok, Indonesia, as a factor for determining the appropriateness of introducing Haemophilus influenzae type b and pneumococcal vaccines		34.1	0.6
Indonesia	2000-2004	1	0.1	2002	Africa, Asia, Oceania - INDEPTH Network Cause-Specific Mortality - Release 2014		97.8	6.4
Indonesia	2005-2009	3	42.8	2007	Indonesia Basic Health Research 2007-2008		22.6	55.3
Indonesia	2010-2016	3	56.7	2012	Indonesia Sample Registration System - Deaths 2012		11.4	64.0
Iran	1980-1984	2	13.3	1982	Vital Registration	23.8	44.1	

Appendix Table 17. Underlying indicators for percent well-certified for data source with maximum percent well certified in each 5-year time interval for 195 countries, 1980-2016.

Location	Time Window	Stars	Percent Well-Certified [PWC] (%)	Max PWC Data Year	Max PWC Data Source	Completeness (%)	Percent Major Garbage (%)	Verbal Autopsy Adjustment (None for VR) (%)
Iran	1985-1989	2	13.0	1985	Vital Registration	25.7	49.4	
Iran	1990-1994	0						
Iran	1995-1999	2	31.3	1999	Vital Registration	74.2	57.8	
Iran	2000-2004	5	91.5	2003	Vital Registration	92.1	0.6	
Iran	2005-2009	3	60.7	2006	Vital Registration	72.8	16.5	
Iran	2010-2016	4	71.7	2015	Vital Registration	90.6	20.9	
Iraq	1980-1984	0						
Iraq	1985-1989	0						
Iraq	1990-1994	0						
Iraq	1995-1999	0						
Iraq	2000-2004	0						
Iraq	2005-2009	2	32.2	2008	Vital Registration	55.0	41.6	
Iraq	2010-2016	0						
Ireland	1980-1984	5	90.1	1983	Vital Registration	100.0	9.9	
Ireland	1985-1989	5	91.1	1989	Vital Registration	100.0	8.9	
Ireland	1990-1994	5	91.5	1993	Vital Registration	100.0	8.5	
Ireland	1995-1999	5	90.7	1995	Vital Registration	100.0	9.3	
Ireland	2000-2004	5	90.6	2000	Vital Registration	100.0	9.4	
Ireland	2005-2009	5	92.5	2007	Vital Registration	100.0	7.5	
Ireland	2010-2016	5	92.4	2012	Vital Registration	100.0	7.6	
Israel	1980-1984	4	80.9	1983	Vital Registration	100.0	19.1	
Israel	1985-1989	4	81.7	1989	Vital Registration	100.0	18.3	
Israel	1990-1994	4	82.8	1994	Vital Registration	100.0	17.2	
Israel	1995-1999	4	83.3	1996	Vital Registration	100.0	16.7	
Israel	2000-2004	4	81.8	2002	Vital Registration	100.0	18.2	
Israel	2005-2009	4	80.2	2005	Vital Registration	100.0	19.8	
Israel	2010-2016	4	79.0	2010	Vital Registration	97.9	19.2	
Italy	1980-1984	5	88.5	1980	Vital Registration	100.0	11.5	
Italy	1985-1989	5	87.8	1988	Vital Registration	99.9	12.1	
Italy	1990-1994	5	87.7	1991	Vital Registration	100.0	12.3	
Italy	1995-1999	5	87.3	1998	Vital Registration	100.0	12.7	
Italy	2000-2004	5	88.2	2003	Vital Registration	100.0	11.8	
Italy	2005-2009	5	88.7	2008	Vital Registration	100.0	11.3	
Italy	2010-2016	5	87.7	2012	Vital Registration	100.0	12.3	
Jamaica	1980-1984	3	64.6	1983	Vital Registration	91.1	29.1	
Jamaica	1985-1989	4	66.1	1987	Vital Registration	92.2	28.3	
Jamaica	1990-1994	3	55.8	1990	Vital Registration	83.1	32.9	
Jamaica	1995-1999	0						
Jamaica	2000-2004	4	68.4	2000	Vital Registration	89.1	23.3	
Jamaica	2005-2009	4	77.2	2009	Vital Registration	88.4	12.7	
Jamaica	2010-2016	4	75.7	2011	Vital Registration	84.9	10.8	
Japan	1980-1984	4	82.5	1980	Vital Registration	100.0	17.5	
Japan	1985-1989	4	80.8	1985	Vital Registration	99.8	19.0	
Japan	1990-1994	4	80.5	1994	Vital Registration	98.6	18.4	
Japan	1995-1999	5	87.6	1995	Vital Registration	100.0	12.4	
Japan	2000-2004	4	84.9	2000	Vital Registration	98.5	13.8	
Japan	2005-2009	4	84.3	2005	Vital Registration	100.0	15.7	
Japan	2010-2016	4	81.2	2010	Vital Registration	100.0	18.8	
Jordan	1980-1984	0						
Jordan	1985-1989	0						
Jordan	1990-1994	0						
Jordan	1995-1999	1	1.0	1995	Mortality and causes of death in Jordan 1995-96: assessment by verbal autopsy		82.2	5.4
Jordan	2000-2004	4	68.2	2004	Vital Registration	86.2	20.8	
Jordan	2005-2009	4	76.3	2006	Vital Registration	92.4	17.4	
Jordan	2010-2016	3	64.2	2012	Vital Registration	79.6	19.4	
Kazakhstan	1980-1984	4	76.3	1982	Vital Registration	95.2	19.8	
Kazakhstan	1985-1989	4	81.5	1988	Vital Registration	99.2	17.8	
Kazakhstan	1990-1994	5	89.5	1993	Vital Registration	100.0	10.5	
Kazakhstan	1995-1999	5	89.0	1995	Vital Registration	100.0	11.0	
Kazakhstan	2000-2004	4	82.2	2003	Vital Registration	95.8	14.2	
Kazakhstan	2005-2009	4	77.8	2008	Vital Registration	97.3	20.0	
Kazakhstan	2010-2016	5	86.1	2015	Vital Registration	98.3	12.5	
Kenya	1980-1984	0						
Kenya	1985-1989	1	2.8	1986	Mortality patterns in a rural Kenyan community		12.0	3.2
Kenya	1990-1994	0						
Kenya	1995-1999	1	0.5	1997	The burden of malaria mortality among African children in the year 2000		82.2	2.7
Kenya	2000-2004	1	5.1	2003	Kenya - Nairobi Urban Health and Demographic Surveillance System		19.1	6.4
Kenya	2005-2009	1	5.4	2006	Kenya - Nairobi Urban Health and Demographic Surveillance System		15.6	6.4
Kenya	2010-2016	1	0.8	2011	Africa, Asia, Oceania - INDEPTH Network Cause-Specific Mortality - Release 2014		87.7	6.4
Kiribati	1980-1984	0						
Kiribati	1985-1989	0						
Kiribati	1990-1994	3	43.7	1993	Vital Registration	66.2	34.1	
Kiribati	1995-1999	4	69.1	1995	Vital Registration	100.0	30.9	
Kiribati	2000-2004	2	34.4	2000	Vital Registration	61.4	43.9	
Kiribati	2005-2009	0						
Kiribati	2010-2016	0						
Kuwait	1980-1984	4	81.5	1980	Vital Registration	99.8	18.3	
Kuwait	1985-1989	4	82.0	1985	Vital Registration	100.0	18.0	
Kuwait	1990-1994	4	75.6	1994	Vital Registration	96.1	21.3	
Kuwait	1995-1999	4	78.1	1998	Vital Registration	100.0	21.9	
Kuwait	2000-2004	4	83.4	2004	Vital Registration	100.0	16.6	
Kuwait	2005-2009	4	85.0	2009	Vital Registration	100.0	15.0	
Kuwait	2010-2016	4	83.5	2012	Vital Registration	100.0	16.5	
Kyrgyzstan	1980-1984	4	71.0	1981	Vital Registration	87.5	18.9	
Kyrgyzstan	1985-1989	4	76.4	1988	Vital Registration	89.4	14.6	
Kyrgyzstan	1990-1994	4	71.0	1994	Vital Registration	93.6	24.2	
Kyrgyzstan	1995-1999	4	73.0	1998	Vital Registration	92.2	20.8	
Kyrgyzstan	2000-2004	5	85.9	2002	Vital Registration	93.4	8.1	
Kyrgyzstan	2005-2009	5	87.7	2006	Vital Registration	94.0	6.7	
Kyrgyzstan	2010-2016	5	90.9	2014	Vital Registration	97.4	6.7	
Laos	1980-1984	0						
Laos	1985-1989	1	1.3	1989	The Lao People's Democratic Republic: maternal mortality and female mortality: determining causes of deaths		68.7	4.1
Laos	1990-1994	0						
Laos	1995-1999	0						
Laos	2000-2004	0						
Laos	2005-2009	0						
Laos	2010-2016	0						
Latvia	1980-1984	5	90.6	1984	Vital Registration	99.7	9.1	
Latvia	1985-1989	5	91.4	1985	Vital Registration	100.0	8.6	
Latvia	1990-1994	5	87.9	1990	Vital Registration	100.0	12.1	
Latvia	1995-1999	5	92.0	1999	Vital Registration	100.0	8.0	
Latvia	2000-2004	5	91.1	2000	Vital Registration	100.0	8.9	
Latvia	2005-2009	5	89.2	2007	Vital Registration	100.0	10.8	
Latvia	2010-2016	5	93.8	2013	Vital Registration	100.0	6.2	
Lebanon	1980-1984	0						
Lebanon	1985-1989	1	2.2	1988	Non-communicable disease mortality rates using the verbal autopsy in a cohort of middle aged and older populations in Beirut during wartime, 1983-93		31.5	3.2
Lebanon	1990-1994	0						
Lebanon	1995-1999	0						
Lebanon	2000-2004	1	0.0	2000	Facility-based audit of maternal mortality in Lebanon: A feasibility study		94.6	0.4
Lebanon	2005-2009	0						
Lebanon	2010-2016	0						
Lesotho	1980-1984	0						
Lesotho	1985-1989	0						

Appendix Table 17. Underlying indicators for percent well-certified for data source with maximum percent well certified in each 5-year time interval for 195 countries, 1980-2016.

Location	Time Window	Stars	Percent Well-Certified [PWC] (%)	Max PWC Data Year	Max PWC Data Source	Completeness (%)	Percent Major Garbage (%)	Verbal Autopsy Adjustment (None for VR) (%)
Lesotho	1990-1994	0						
Lesotho	1995-1999	0						
Lesotho	2000-2004	0						
Lesotho	2005-2009	0						
Lesotho	2010-2016	0						
Liberia	1980-1984	1	2.2	1984	Infant and child mortality in two counties of Liberia: results of a survey in 1988 and trends since 1984		47.8	4.3
Liberia	1985-1989	1	2.3	1987	Infant and child mortality in two counties of Liberia: results of a survey in 1988 and trends since 1984		45.1	4.2
Liberia	1990-1994	1	3.6	1990	Application of the verbal autopsy during a clinical trial		29.2	5.1
Liberia	1995-1999	0						
Liberia	2000-2004	0						
Liberia	2005-2009	0						
Liberia	2010-2016	0						
Libya	1980-1984	0						
Libya	1985-1989	0						
Libya	1990-1994	0						
Libya	1995-1999	0						
Libya	2000-2004	0						
Libya	2005-2009	1	3.6	2008	Vital Registration	82.5	95.7	
Libya	2010-2016	0						
Lithuania	1980-1984	5	87.6	1981	Vital Registration	99.2	11.7	
Lithuania	1985-1989	5	92.2	1988	Vital Registration	100.0	7.8	
Lithuania	1990-1994	5	91.7	1990	Vital Registration	100.0	8.3	
Lithuania	1995-1999	5	94.7	1997	Vital Registration	100.0	5.3	
Lithuania	2000-2004	5	92.6	2004	Vital Registration	100.0	7.4	
Lithuania	2005-2009	5	93.1	2009	Vital Registration	100.0	6.9	
Lithuania	2010-2016	5	94.4	2013	Vital Registration	100.0	5.6	
Luxembourg	1980-1984	5	86.4	1984	Vital Registration	100.0	13.6	
Luxembourg	1985-1989	5	86.7	1985	Vital Registration	100.0	13.3	
Luxembourg	1990-1994	5	85.3	1992	Vital Registration	100.0	14.7	
Luxembourg	1995-1999	4	84.9	1995	Vital Registration	99.3	14.6	
Luxembourg	2000-2004	4	82.2	2000	Vital Registration	100.0	17.8	
Luxembourg	2005-2009	4	78.2	2005	Vital Registration	100.0	21.8	
Luxembourg	2010-2016	4	82.0	2013	Vital Registration	99.2	17.3	
Macedonia	1980-1984	0						
Macedonia	1985-1989	0						
Macedonia	1990-1994	4	80.1	1994	Vital Registration	97.1	17.6	
Macedonia	1995-1999	4	81.5	1997	Vital Registration	98.2	17.0	
Macedonia	2000-2004	4	81.6	2003	Vital Registration	96.3	15.3	
Macedonia	2005-2009	4	78.9	2007	Vital Registration	98.2	19.7	
Macedonia	2010-2016	4	74.6	2012	Vital Registration	94.6	21.1	
Madagascar	1980-1984	1	2.7	1984	Vital Registration	4.9	45.0	
Madagascar	1985-1989	1	3.3	1986	Vital Registration	6.0	44.8	
Madagascar	1990-1994	1	2.3	1990	Vital Registration	4.1	45.0	
Madagascar	1995-1999	1	2.2	1995	Vital Registration	4.2	47.9	
Madagascar	2000-2004	0						
Madagascar	2005-2009	0						
Madagascar	2010-2016	0						
Malawi	1980-1984	0						
Malawi	1985-1989	1	2.8	1988	Infant and second-year mortality in rural Malawi: causes and descriptive epidemiology		34.8	4.3
Malawi	1990-1994	0						
Malawi	1995-1999	1	0.6	1999	Estimation of AIDS adult mortality by verbal autopsy in rural Malawi		28.0	0.8
Malawi	2000-2004	1	2.2	2004	Declining child mortality in northern Malawi despite high rates of infection with HIV		31.6	3.2
Malawi	2005-2009	1	3.8	2008	Rates and causes of death in Chiradzulu District, Malawi, 2008: a key informant study		40.0	6.4
Malawi	2010-2016	1	0.4	2011	Africa, Asia, Oceania - INDEPTH Network Cause-Specific Mortality - Release 2014		94.3	6.4
Malaysia	1980-1984	2	19.3	1982	Vital Registration	31.6	39.1	
Malaysia	1985-1989	0						
Malaysia	1990-1994	0						
Malaysia	1995-1999	2	32.0	1997	Vital Registration	46.4	31.0	
Malaysia	2000-2004	3	36.5	2004	Vital Registration	57.6	36.7	
Malaysia	2005-2009	3	40.8	2008	Vital Registration	61.1	33.3	
Malaysia	2010-2016	0						
Maldives	1980-1984	0						
Maldives	1985-1989	0						
Maldives	1990-1994	0						
Maldives	1995-1999	0						
Maldives	2000-2004	3	44.1	2002	Vital Registration	100.0	55.9	
Maldives	2005-2009	3	48.4	2008	Vital Registration	100.0	51.6	
Maldives	2010-2016	3	60.2	2011	Vital Registration	100.0	39.8	
Mali	1980-1984	1	4.3	1981	Vital Registration	8.3	48.6	
Mali	1985-1989	0						
Mali	1990-1994	1	0.1	1990	Assessment of maternal mortality and late maternal mortality among a cohort of pregnant women in Bamako, Mali		81.3	0.4
Mali	1995-1999	0						
Mali	2000-2004	0						
Mali	2005-2009	0						
Mali	2010-2016	0						
Malta	1980-1984	4	81.0	1982	Vital Registration	99.9	18.9	
Malta	1985-1989	4	84.5	1989	Vital Registration	96.6	12.6	
Malta	1990-1994	5	88.4	1992	Vital Registration	100.0	11.6	
Malta	1995-1999	5	90.0	1997	Vital Registration	100.0	10.0	
Malta	2000-2004	5	89.0	2002	Vital Registration	99.5	10.5	
Malta	2005-2009	5	93.0	2009	Vital Registration	100.0	7.0	
Malta	2010-2016	5	90.9	2014	Vital Registration	98.5	7.6	
Marshall Islands	1980-1984	0						
Marshall Islands	1985-1989	0						
Marshall Islands	1990-1994	0						
Marshall Islands	1995-1999	0						
Marshall Islands	2000-2004	0						
Marshall Islands	2005-2009	0						
Marshall Islands	2010-2016	0						
Mauritania	1980-1984	0						
Mauritania	1985-1989	0						
Mauritania	1990-1994	0						
Mauritania	1995-1999	0						
Mauritania	2000-2004	0						
Mauritania	2005-2009	0						
Mauritania	2010-2016	0						
Mauritius	1980-1984	4	73.8	1984	Vital Registration	100.0	26.2	
Mauritius	1985-1989	4	78.5	1988	Vital Registration	100.0	21.5	
Mauritius	1990-1994	4	78.7	1991	Vital Registration	100.0	21.3	
Mauritius	1995-1999	4	78.2	1999	Vital Registration	100.0	21.8	
Mauritius	2000-2004	4	83.0	2003	Vital Registration	100.0	17.0	
Mauritius	2005-2009	4	84.7	2009	Vital Registration	100.0	15.2	
Mauritius	2010-2016	5	85.3	2014	Vital Registration	97.5	12.6	
Mexico	1980-1984	4	65.2	1983	Vital Registration	83.5	21.9	
Mexico	1985-1989	4	71.9	1989	Vital Registration	87.4	17.7	
Mexico	1990-1994	4	72.7	1990	Vital Registration	87.8	17.2	
Mexico	1995-1999	4	76.7	1999	Vital Registration	88.0	12.9	
Mexico	2000-2004	4	79.4	2004	Vital Registration	90.0	11.8	
Mexico	2005-2009	4	81.7	2009	Vital Registration	93.0	12.2	
Mexico	2010-2016	5	88.1	2012	Vital Registration	100.0	11.9	
Moldova	1980-1984	4	83.9	1981	Vital Registration	96.3	12.9	
Moldova	1985-1989	5	87.1	1985	Vital Registration	97.7	10.8	

Appendix Table 17. Underlying indicators for percent well-certified for data source with maximum percent well certified in each 5-year time interval for 195 countries, 1980-2016.								
**Location**	**Time Window**	**Stars**	**Percent Well-Certified [PWC] (%)**	**Max PWC Data Year**	**Max PWC Data Source**	**Completeness (%)**	**Percent Major Garbage (%)**	**Verbal Autopsy Adjustment (None for VR) (%)**
Moldova	1990-1994	4	77.2	1991	Vital Registration	94.3	18.1	
Moldova	1995-1999	4	84.8	1996	Vital Registration	94.9	10.7	
Moldova	2000-2004	5	90.0	2003	Vital Registration	93.2	3.4	
Moldova	2005-2009	5	89.6	2005	Vital Registration	92.5	3.1	
Moldova	2010-2016	5	90.3	2014	Vital Registration	93.1	3.0	
Mongolia	1980-1984	0						
Mongolia	1985-1989	0						
Mongolia	1990-1994	3	62.9	1994	Vital Registration	79.2	20.6	
Mongolia	1995-1999	0						
Mongolia	2000-2004	1	3.3	2004	Vital Registration	79.9	95.8	
Mongolia	2005-2009	1	4.6	2008	Vital Registration	78.8	94.1	
Mongolia	2010-2016	4	81.4	2010	Vital Registration	86.9	6.2	
Montenegro	1980-1984	0						
Montenegro	1985-1989	0						
Montenegro	1990-1994	0						
Montenegro	1995-1999	0						
Montenegro	2000-2004	4	70.6	2004	Vital Registration	100.0	29.4	
Montenegro	2005-2009	4	72.9	2006	Vital Registration	100.0	27.1	
Montenegro	2010-2016	0						
Morocco	1980-1984	0						
Morocco	1985-1989	2	17.0	1988	Morocco National Survey on Causes and Circumstances of Infant and Child Deaths 1988-1989		25.7	22.8
Morocco	1990-1994	0						
Morocco	1995-1999	0						
Morocco	2000-2004	0						
Morocco	2005-2009	3	37.9	2005	Vital Registration	83.4	54.6	
Morocco	2010-2016	2	14.3	2010	Vital Registration	30.8	53.6	
Mozambique	1980-1984	0						
Mozambique	1985-1989	0						
Mozambique	1990-1994	0						
Mozambique	1995-1999	1	0.1	1996	Quality of registration of maternal deaths in Mozambique: a community-based study in rural and urban areas		81.3	0.4
Mozambique	2000-2004	1	7.0	2001	Vital Registration	8.8	20.8	
Mozambique	2005-2009	3	56.6	2007	Mozambique National Survey on the Causes of Death 2007-2008		11.6	64.0
Mozambique	2010-2016	0						
Myanmar	1980-1984	0						
Myanmar	1985-1989	0						
Myanmar	1990-1994	0						
Myanmar	1995-1999	0						
Myanmar	2000-2004	0						
Myanmar	2005-2009	1	2.8	2007	Cause of Death Verification Study in Myanmar		54.8	6.3
Myanmar	2010-2016	0						
Namibia	1980-1984	0						
Namibia	1985-1989	0						
Namibia	1990-1994	0						
Namibia	1995-1999	0						
Namibia	2000-2004	0						
Namibia	2005-2009	0						
Namibia	2010-2016	0						
Nepal	1980-1984	1	2.9	1984	Impact of a pilot acute respiratory infection (ARI) control programme in a rural community of the hill region of Nepal		17.2	3.5
Nepal	1985-1989	1	2.7	1987	Reduction in total under-five mortality in western Nepal through community-based antimicrobial treatment of pneumonia		19.2	3.3
Nepal	1990-1994	0						
Nepal	1995-1999	1	0.6	1999	Evaluation of neonatal verbal autopsy using physician review versus algorithm-based cause-of-death assignment in rural Nepal		43.7	1.0
Nepal	2000-2004	1	0.6	2003	Effect of daily zinc supplementation on child mortality in southern Nepal: a community-based, cluster randomised, placebo-controlled trial		31.8	0.9
Nepal	2005-2009	1	8.9	2006	Nepal Demographic and Health Survey 2006		40.3	14.9
Nepal	2010-2016	0						
Netherlands	1980-1984	5	88.2	1981	Vital Registration	99.9	11.7	
Netherlands	1985-1989	5	85.8	1987	Vital Registration	98.6	13.0	
Netherlands	1990-1994	4	84.9	1990	Vital Registration	100.0	15.1	
Netherlands	1995-1999	4	84.0	1996	Vital Registration	100.0	16.0	
Netherlands	2000-2004	4	82.3	2003	Vital Registration	100.0	17.7	
Netherlands	2005-2009	4	83.3	2006	Vital Registration	100.0	16.7	
Netherlands	2010-2016	4	83.3	2012	Vital Registration	100.0	16.7	
New Zealand	1980-1984	5	95.2	1980	Vital Registration	100.0	4.8	
New Zealand	1985-1989	5	95.0	1985	Vital Registration	100.0	5.0	
New Zealand	1990-1994	5	94.7	1992	Vital Registration	100.0	5.3	
New Zealand	1995-1999	5	96.7	1999	Vital Registration	100.0	3.3	
New Zealand	2000-2004	5	96.4	2004	Vital Registration	100.0	3.6	
New Zealand	2005-2009	5	96.3	2006	Vital Registration	100.0	3.7	
New Zealand	2010-2016	5	95.7	2012	Vital Registration	100.0	4.3	
Nicaragua	1980-1984	0						
Nicaragua	1985-1989	3	55.8	1988	Vital Registration	71.2	21.7	
Nicaragua	1990-1994	3	59.4	1990	Vital Registration	74.9	20.7	
Nicaragua	1995-1999	4	66.1	1998	Vital Registration	77.9	15.1	
Nicaragua	2000-2004	4	71.7	2004	Vital Registration	80.9	11.3	
Nicaragua	2005-2009	4	78.7	2008	Vital Registration	90.2	12.7	
Nicaragua	2010-2016	4	84.9	2013	Vital Registration	93.4	9.1	
Niger	1980-1984	0						
Niger	1985-1989	0						
Niger	1990-1994	0						
Niger	1995-1999	0						
Niger	2000-2004	0						
Niger	2005-2009	3	35.9	2008	Direct estimates of national neonatal and child cause-specific mortality proportions in Niger by expert algorithm and physician-coded analysis of verbal autopsy interviews		8.9	39.4
Niger	2010-2016	0						
Nigeria	1980-1984	0						
Nigeria	1985-1989	0						
Nigeria	1990-1994	1	4.0	1991	Community-based surveillance of paediatric deaths in Cross River State, Nigeria		9.7	4.4
Nigeria	1995-1999	0						
Nigeria	2000-2004	0						
Nigeria	2005-2009	1	0.1	2007	Vital Registration	1.5	91.6	
Nigeria	2010-2016	1	3.8	2012	Health & demographic surveillance system profile: the Nahuche Health and Demographic Surveillance System, Northern Nigeria (Nahuche HDSS)		15.0	4.4
North Korea	1980-1984	0						
North Korea	1985-1989	0						
North Korea	1990-1994	0						
North Korea	1995-1999	0						
North Korea	2000-2004	0						
North Korea	2005-2009	0						
North Korea	2010-2016	0						
Northern Mariana Islands	1980-1984	0						
Northern Mariana Islands	1985-1989	0						
Northern Mariana Islands	1990-1994	0						
Northern Mariana Islands	1995-1999	4	75.3	1998	Vital Registration	100.0	24.7	
Northern Mariana Islands	2000-2004	4	75.3	2001	Vital Registration	87.0	13.5	
Northern Mariana Islands	2005-2009	4	72.3	2005	Vital Registration	96.1	24.7	
Northern Mariana Islands	2010-2016	3	55.2	2010	Vital Registration	73.9	25.3	
Norway	1980-1984	4	78.6	1981	Vital Registration	100.0	21.4	

Appendix Table 17. Underlying indicators for percent well-certified for data source with maximum percent well certified in each 5-year time interval for 195 countries, 1980-2016.

Location	Time Window	Stars	Percent Well-Certified [PWC] (%)	Max PWC Data Year	Max PWC Data Source	Completeness (%)	Percent Major Garbage (%)	Verbal Autopsy Adjustment (None for VR) (%)
Norway	1985-1989	5	89.2	1988	Vital Registration	100.0	10.8	
Norway	1990-1994	5	88.4	1990	Vital Registration	100.0	11.6	
Norway	1995-1999	5	88.3	1997	Vital Registration	100.0	11.7	
Norway	2000-2004	5	86.4	2000	Vital Registration	100.0	13.6	
Norway	2005-2009	4	84.2	2006	Vital Registration	100.0	15.8	
Norway	2010-2016	4	83.0	2011	Vital Registration	100.0	17.0	
Oman	1980-1984	0						
Oman	1985-1989	0						
Oman	1990-1994	0						
Oman	1995-1999	0						
Oman	2000-2004	0						
Oman	2005-2009	4	71.0	2007	Vital Registration	85.0	16.5	
Oman	2010-2016	2	33.0	2010	Vital Registration	80.2	58.8	
Pakistan	1980-1984	0						
Pakistan	1985-1989	1	2.9	1986	Acute respiratory infections in children: a case management intervention in Abbottabad District, Pakistan		13.0	3.3
Pakistan	1990-1994	1	1.4	1994	Time to focus child survival programmes on the newborn: assessment of levels and causes of infant mortality in rural Pakistan		19.9	1.8
Pakistan	1995-1999	0						
Pakistan	2000-2004	1	0.8	2003	Impact of a community-based perinatal and newborn preventive care package on perinatal and neonatal mortality in a remote mountainous district in Northern Pakistan		0.0	0.8
Pakistan	2005-2009	2	11.5	2006	Pakistan Demographic and Health Survey 2006-2007		33.0	17.2
Pakistan	2010-2016	1	0.0	2010	To determine the probable causes of death in an urban slum community of Pakistan among adults 18 years and above by verbal autopsy		99.0	4.5
Palestine	1980-1984	0						
Palestine	1985-1989	0						
Palestine	1990-1994	0						
Palestine	1995-1999	2	29.0	1999	Vital Registration	46.1	37.1	
Palestine	2000-2004	2	29.1	2004	Vital Registration	43.4	33.1	
Palestine	2005-2009	2	28.2	2009	Vital Registration	40.9	30.9	
Palestine	2010-2016	2	29.7	2011	Vital Registration	41.8	28.9	
Panama	1980-1984	4	69.2	1983	Vital Registration	93.7	26.2	
Panama	1985-1989	4	71.6	1987	Vital Registration	93.1	23.1	
Panama	1990-1994	0						
Panama	1995-1999	4	79.0	1999	Vital Registration	94.6	16.5	
Panama	2000-2004	4	82.2	2003	Vital Registration	97.1	15.4	
Panama	2005-2009	4	84.1	2005	Vital Registration	98.5	14.6	
Panama	2010-2016	4	84.1	2014	Vital Registration	100.0	15.9	
Papua New Guinea	1980-1984	1	8.2	1980	Vital Registration	9.9	17.6	
Papua New Guinea	1985-1989	1	3.4	1985	Mortality rates and the utilization of health services during terminal illness in the Asaro Valley, Eastern Highlands Province, Papua New Guinea		46.7	6.4
Papua New Guinea	1990-1994	0						
Papua New Guinea	1995-1999	0						
Papua New Guinea	2000-2004	0						
Papua New Guinea	2005-2009	0						
Papua New Guinea	2010-2016	0						
Paraguay	1980-1984	3	55.1	1980	Vital Registration	85.4	35.5	
Paraguay	1985-1989	3	51.4	1989	Vital Registration	78.1	34.2	
Paraguay	1990-1994	3	59.0	1994	Vital Registration	79.9	26.1	
Paraguay	1995-1999	3	62.6	1996	Vital Registration	84.7	26.1	
Paraguay	2000-2004	3	60.0	2004	Vital Registration	83.1	27.8	
Paraguay	2005-2009	3	62.6	2009	Vital Registration	82.5	24.2	
Paraguay	2010-2016	4	65.7	2013	Vital Registration	83.1	20.9	
Peru	1980-1984	3	58.9	1980	Vital Registration	76.6	23.1	
Peru	1985-1989	2	34.4	1986	Vital Registration	69.1	50.2	
Peru	1990-1994	3	36.5	1992	Vital Registration	67.8	46.1	
Peru	1995-1999	3	48.2	1999	Vital Registration	75.7	36.4	
Peru	2000-2004	3	60.3	2004	Vital Registration	85.8	29.7	
Peru	2005-2009	3	60.2	2008	Vital Registration	80.2	25.0	
Peru	2010-2016	3	60.4	2010	Vital Registration	81.1	25.6	
Philippines	1980-1984	4	71.7	1980	Vital Registration	90.7	20.9	
Philippines	1985-1989	4	73.8	1985	Vital Registration	93.6	21.1	
Philippines	1990-1994	4	65.8	1990	Vital Registration	85.3	22.8	
Philippines	1995-1999	4	65.9	1998	Vital Registration	84.5	22.0	
Philippines	2000-2004	4	72.6	2002	Vital Registration	85.0	14.6	
Philippines	2005-2009	4	72.4	2009	Vital Registration	85.2	15.0	
Philippines	2010-2016	4	71.8	2011	Vital Registration	84.6	15.1	
Poland	1980-1984	3	62.5	1980	Vital Registration	100.0	37.5	
Poland	1985-1989	3	60.3	1986	Vital Registration	100.0	39.7	
Poland	1990-1994	3	60.4	1994	Vital Registration	99.8	39.5	
Poland	1995-1999	4	71.6	1999	Vital Registration	100.0	28.4	
Poland	2000-2004	4	74.2	2002	Vital Registration	100.0	25.8	
Poland	2005-2009	4	73.6	2005	Vital Registration	100.0	26.4	
Poland	2010-2016	4	71.9	2011	Vital Registration	98.8	27.2	
Portugal	1980-1984	4	76.8	1984	Vital Registration	100.0	23.2	
Portugal	1985-1989	4	77.1	1985	Vital Registration	100.0	22.9	
Portugal	1990-1994	4	76.1	1991	Vital Registration	100.0	23.9	
Portugal	1995-1999	4	74.2	1996	Vital Registration	99.7	25.6	
Portugal	2000-2004	4	78.8	2002	Vital Registration	99.5	20.8	
Portugal	2005-2009	4	77.5	2009	Vital Registration	99.5	22.2	
Portugal	2010-2016	4	79.8	2014	Vital Registration	97.5	18.1	
Puerto Rico	1980-1984	4	77.1	1981	Vital Registration	100.0	22.9	
Puerto Rico	1985-1989	4	74.6	1986	Vital Registration	100.0	25.4	
Puerto Rico	1990-1994	4	79.9	1994	Vital Registration	95.7	16.6	
Puerto Rico	1995-1999	4	83.4	1999	Vital Registration	100.0	16.6	
Puerto Rico	2000-2004	4	84.0	2004	Vital Registration	100.0	16.0	
Puerto Rico	2005-2009	4	84.0	2008	Vital Registration	100.0	16.0	
Puerto Rico	2010-2016	4	84.7	2012	Vital Registration	100.0	15.3	
Qatar	1980-1984	1	8.4	1984	Vital Registration	61.2	86.3	
Qatar	1985-1989	1	10.0	1985	Vital Registration	72.1	86.2	
Qatar	1990-1994	0						
Qatar	1995-1999	3	51.6	1995	Vital Registration	73.8	30.1	
Qatar	2000-2004	3	48.2	2001	Vital Registration	74.4	35.2	
Qatar	2005-2009	3	56.2	2006	Vital Registration	82.6	32.0	
Qatar	2010-2016	3	44.0	2014	Vital Registration	72.7	39.4	
Romania	1980-1984	4	77.4	1980	Vital Registration	100.0	22.6	
Romania	1985-1989	4	78.5	1989	Vital Registration	100.0	21.5	
Romania	1990-1994	4	83.3	1994	Vital Registration	99.6	16.4	
Romania	1995-1999	4	84.8	1999	Vital Registration	100.0	15.2	
Romania	2000-2004	5	85.5	2004	Vital Registration	99.4	14.0	
Romania	2005-2009	5	86.2	2006	Vital Registration	100.0	13.8	
Romania	2010-2016	5	85.5	2010	Vital Registration	100.0	14.5	
Russia	1980-1984	4	81.6	1981	Vital Registration	99.4	18.0	
Russia	1985-1989	5	88.4	1989	Vital Registration	98.2	10.0	
Russia	1990-1994	5	87.8	1990	Vital Registration	99.3	11.6	
Russia	1995-1999	4	84.6	1999	Vital Registration	99.9	15.3	
Russia	2000-2004	5	87.6	2003	Vital Registration	100.0	12.4	
Russia	2005-2009	5	88.9	2009	Vital Registration	100.0	11.1	
Russia	2010-2016	5	88.4	2010	Vital Registration	100.0	11.6	
Rwanda	1980-1984	0						
Rwanda	1985-1989	0						
Rwanda	1990-1994	0						
Rwanda	1995-1999	0						
Rwanda	2000-2004	0						
Rwanda	2005-2009	1	2.5	2007	Rwanda Child Verbal Autopsy Study 2008		11.6	2.8
Rwanda	2010-2016	0						

Appendix Table 17. Underlying indicators for percent well-certified for data source with maximum percent well certified in each 5-year time interval for 195 countries, 1980-2016.

Location	Time Window	Stars	Percent Well-Certified [PWC] (%)	Max PWC Data Year	Max PWC Data Source	Completeness (%)	Percent Major Garbage (%)	Verbal Autopsy Adjustment (None for VR) (%)
Saint Lucia	1980-1984	4	69.3	1983	Vital Registration	100.0	30.7	
Saint Lucia	1985-1989	4	66.2	1987	Vital Registration	100.0	33.8	
Saint Lucia	1990-1994	4	70.6	1992	Vital Registration	100.0	29.4	
Saint Lucia	1995-1999	4	72.5	1997	Vital Registration	100.0	27.5	
Saint Lucia	2000-2004	4	79.2	2004	Vital Registration	100.0	20.8	
Saint Lucia	2005-2009	4	78.4	2009	Vital Registration	93.4	16.0	
Saint Lucia	2010-2016	5	85.2	2014	Vital Registration	100.0	14.8	
Saint Vincent and the Grenadines	1980-1984	4	71.6	1983	Vital Registration	100.0	28.4	
Saint Vincent and the Grenadines	1985-1989	3	61.1	1986	Vital Registration	89.6	31.8	
Saint Vincent and the Grenadines	1990-1994	3	58.6	1990	Vital Registration	92.7	36.8	
Saint Vincent and the Grenadines	1995-1999	4	79.0	1998	Vital Registration	100.0	21.0	
Saint Vincent and the Grenadines	2000-2004	4	81.0	2004	Vital Registration	95.6	15.3	
Saint Vincent and the Grenadines	2005-2009	4	83.0	2005	Vital Registration	99.7	16.7	
Saint Vincent and the Grenadines	2010-2016	5	87.5	2014	Vital Registration	100.0	12.5	
Samoa	1980-1984	0						
Samoa	1985-1989	0						
Samoa	1990-1994	0						
Samoa	1995-1999	0						
Samoa	2000-2004	0						
Samoa	2005-2009	0						
Samoa	2010-2016	0						
Sao Tome and Principe	1980-1984	0						
Sao Tome and Principe	1985-1989	4	69.0	1985	Vital Registration	100.0	31.0	
Sao Tome and Principe	1990-1994	0						
Sao Tome and Principe	1995-1999	0						
Sao Tome and Principe	2000-2004	0						
Sao Tome and Principe	2005-2009	0						
Sao Tome and Principe	2010-2016	0						
Saudi Arabia	1980-1984	0						
Saudi Arabia	1985-1989	0						
Saudi Arabia	1990-1994	0						
Saudi Arabia	1995-1999	2	26.3	1999	Vital Registration	47.7	44.9	
Saudi Arabia	2000-2004	2	31.7	2004	Vital Registration	62.9	49.6	
Saudi Arabia	2005-2009	2	34.6	2008	Vital Registration	73.7	53.1	
Saudi Arabia	2010-2016	2	34.5	2010	Vital Registration	75.5	54.3	
Senegal	1980-1984	1	2.0	1984	Senegal - Risk of Death Associated with Different Nutritional States in Children of Preschool age: Study Conducted in Niakhar (Senegal) 1983-1986		28.7	2.7
Senegal	1985-1989	1	2.4	1986	International differences in clinical patterns of diarrhoeal deaths: a comparison of children from Brazil, Senegal, Bangladesh, and India		30.0	3.5
Senegal	1990-1994	1	2.6	1993	Childhood mortality and probable causes of death using verbal autopsy in Niakhar, Senegal, 1989-2000		26.2	3.5
Senegal	1995-1999	1	2.5	1996	Childhood mortality and probable causes of death using verbal autopsy in Niakhar, Senegal, 1989-2000		25.1	3.3
Senegal	2000-2004	0						
Senegal	2005-2009	1	0.0	2008	Africa, Asia, Oceania - INDEPTH Network Cause-Specific Mortality - Release 2014		99.6	6.4
Senegal	2010-2016	0	0.0	2010	Africa, Asia, Oceania - INDEPTH Network Cause-Specific Mortality - Release 2014		100.0	6.4
Serbia	1980-1984	0						
Serbia	1985-1989	0						
Serbia	1990-1994	0						
Serbia	1995-1999	4	73.1	1998	Vital Registration	92.4	20.9	
Serbia	2000-2004	4	75.1	2003	Vital Registration	94.2	20.4	
Serbia	2005-2009	4	79.7	2005	Vital Registration	96.5	17.4	
Serbia	2010-2016	4	77.9	2010	Vital Registration	93.5	16.7	
Seychelles	1980-1984	4	69.9	1982	Vital Registration	98.8	29.3	
Seychelles	1985-1989	3	63.6	1987	Vital Registration	91.0	30.1	
Seychelles	1990-1994	0						
Seychelles	1995-1999	0						
Seychelles	2000-2004	4	75.9	2002	Vital Registration	99.9	24.0	
Seychelles	2005-2009	4	77.0	2008	Vital Registration	100.0	23.0	
Seychelles	2010-2016	4	78.1	2011	Vital Registration	100.0	21.9	
Sierra Leone	1980-1984	0						
Sierra Leone	1985-1989	0						
Sierra Leone	1990-1994	1	3.8	1990	Malaria in a rural area of Sierra Leone. I. Initial results		10.8	4.2
Sierra Leone	1995-1999	0						
Sierra Leone	2000-2004	0						
Sierra Leone	2005-2009	0						
Sierra Leone	2010-2016	0						
Singapore	1980-1984	5	89.1	1983	Vital Registration	100.0	10.9	
Singapore	1985-1989	5	89.6	1985	Vital Registration	100.0	10.4	
Singapore	1990-1994	5	95.0	1994	Vital Registration	100.0	5.0	
Singapore	1995-1999	5	95.3	1999	Vital Registration	100.0	4.7	
Singapore	2000-2004	5	95.1	2003	Vital Registration	100.0	4.9	
Singapore	2005-2009	5	92.5	2008	Vital Registration	100.0	7.5	
Singapore	2010-2016	5	97.8	2014	Vital Registration	99.9	2.2	
Slovakia	1980-1984	0						
Slovakia	1985-1989	0						
Slovakia	1990-1994	4	82.4	1994	Vital Registration	99.3	17.1	
Slovakia	1995-1999	4	82.7	1999	Vital Registration	100.0	17.3	
Slovakia	2000-2004	5	85.2	2003	Vital Registration	100.0	14.8	
Slovakia	2005-2009	5	90.3	2008	Vital Registration	99.9	9.6	
Slovakia	2010-2016	5	92.9	2012	Vital Registration	100.0	7.1	
Slovenia	1980-1984	0						
Slovenia	1985-1989	5	89.4	1985	Vital Registration	97.7	8.5	
Slovenia	1990-1994	5	91.1	1992	Vital Registration	98.6	7.6	
Slovenia	1995-1999	5	88.8	1997	Vital Registration	96.7	8.3	
Slovenia	2000-2004	5	88.3	2002	Vital Registration	100.0	11.7	
Slovenia	2005-2009	5	87.4	2009	Vital Registration	99.0	11.8	
Slovenia	2010-2016	5	87.3	2012	Vital Registration	99.3	12.1	
Solomon Islands	1980-1984	0						
Solomon Islands	1985-1989	0						
Solomon Islands	1990-1994	0						
Solomon Islands	1995-1999	0						
Solomon Islands	2000-2004	0						
Solomon Islands	2005-2009	0						
Solomon Islands	2010-2016	0						
Somalia	1980-1984	0						
Somalia	1985-1989	0						
Somalia	1990-1994	0						
Somalia	1995-1999	0						
Somalia	2000-2004	0						
Somalia	2005-2009	0						
Somalia	2010-2016	0						
South Africa	1980-1984	0						
South Africa	1985-1989	0						
South Africa	1990-1994	1	0.8	1994	Africa, Asia, Oceania - INDEPTH Network Cause-Specific Mortality - Release 2014		86.9	6.4
South Africa	1995-1999	3	45.2	1999	Vital Registration	69.4	34.9	
South Africa	2000-2004	3	51.9	2004	Vital Registration	76.3	32.0	
South Africa	2005-2009	3	52.6	2008	Vital Registration	78.2	32.7	
South Africa	2010-2016	3	57.0	2014	Vital Registration	83.5	31.8	
South Korea	1980-1984	0						
South Korea	1985-1989	3	57.8	1989	Vital Registration	81.0	28.7	
South Korea	1990-1994	4	74.6	1994	Vital Registration	96.8	22.9	
South Korea	1995-1999	4	75.3	1995	Vital Registration	99.7	24.5	
South Korea	2000-2004	4	84.6	2002	Vital Registration	100.0	15.4	
South Korea	2005-2009	4	81.5	2007	Vital Registration	99.2	17.9	
South Korea	2010-2016	4	80.9	2012	Vital Registration	99.1	18.3	

Appendix Table 17. Underlying indicators for percent well-certified for data source with maximum percent well certified in each 5-year time interval for 195 countries, 1980-2016.

Location	Time Window	Stars	Percent Well-Certified [PWC] (%)	Max PWC Data Year	Max PWC Data Source	Completeness (%)	Percent Major Garbage (%)	Verbal Autopsy Adjustment (None for VR) (%)
South Sudan	1980-1984	0						
South Sudan	1985-1989	0						
South Sudan	1990-1994	0						
South Sudan	1995-1999	0						
South Sudan	2000-2004	0						
South Sudan	2005-2009	0						
South Sudan	2010-2016	0						
Spain	1980-1984	4	76.7	1984	Vital Registration	98.8	22.4	
Spain	1985-1989	4	78.9	1989	Vital Registration	100.0	21.1	
Spain	1990-1994	4	80.1	1993	Vital Registration	99.9	19.8	
Spain	1995-1999	4	83.3	1998	Vital Registration	100.0	16.7	
Spain	2000-2004	4	83.2	2002	Vital Registration	100.0	16.8	
Spain	2005-2009	4	84.0	2009	Vital Registration	100.0	16.0	
Spain	2010-2016	5	85.4	2014	Vital Registration	99.9	14.5	
Sri Lanka	1980-1984	3	51.8	1983	Vital Registration	100.0	48.2	
Sri Lanka	1985-1989	3	50.9	1986	Vital Registration	100.0	49.1	
Sri Lanka	1990-1994	3	46.5	1994	Vital Registration	100.0	53.5	
Sri Lanka	1995-1999	3	55.5	1999	Vital Registration	100.0	44.5	
Sri Lanka	2000-2004	3	63.6	2004	Vital Registration	99.9	36.3	
Sri Lanka	2005-2009	4	67.4	2007	Vital Registration	100.0	32.6	
Sri Lanka	2010-2016	4	65.5	2010	Vital Registration	100.0	34.5	
Sudan	1980-1984	0						
Sudan	1985-1989	0						
Sudan	1990-1994	0						
Sudan	1995-1999	0						
Sudan	2000-2004	0						
Sudan	2005-2009	0						
Sudan	2010-2016	0						
Suriname	1980-1984	3	59.7	1984	Vital Registration	89.1	33.0	
Suriname	1985-1989	3	62.1	1989	Vital Registration	91.6	32.2	
Suriname	1990-1994	3	58.6	1991	Vital Registration	84.8	31.0	
Suriname	1995-1999	3	58.5	1999	Vital Registration	81.4	28.1	
Suriname	2000-2004	4	66.0	2002	Vital Registration	82.9	20.4	
Suriname	2005-2009	3	64.9	2008	Vital Registration	80.6	19.4	
Suriname	2010-2016	4	65.1	2010	Vital Registration	79.4	18.1	
Swaziland	1980-1984	0						
Swaziland	1985-1989	0						
Swaziland	1990-1994	0						
Swaziland	1995-1999	0						
Swaziland	2000-2004	1	0.0	2000	Effect of HIV infection on pregnancy-related mortality in sub-Saharan Africa: secondary analyses of pooled community-based data from the network for Analysing Longitudinal Population-based HIV/AIDS data on Africa (ALPHA)		94.9	0.5
Swaziland	2005-2009	0						
Swaziland	2010-2016	0						
Sweden	1980-1984	5	87.6	1980	Vital Registration	99.3	11.8	
Sweden	1985-1989	5	88.4	1988	Vital Registration	100.0	11.6	
Sweden	1990-1994	5	88.0	1990	Vital Registration	100.0	12.0	
Sweden	1995-1999	5	87.0	1997	Vital Registration	100.0	13.0	
Sweden	2000-2004	5	85.9	2001	Vital Registration	99.8	13.9	
Sweden	2005-2009	5	85.4	2006	Vital Registration	99.2	14.0	
Sweden	2010-2016	4	84.8	2014	Vital Registration	99.8	15.1	
Switzerland	1980-1984	4	69.3	1981	Vital Registration	100.0	30.7	
Switzerland	1985-1989	4	69.2	1986	Vital Registration	100.0	30.8	
Switzerland	1990-1994	4	68.3	1990	Vital Registration	100.0	31.7	
Switzerland	1995-1999	4	84.6	1998	Vital Registration	100.0	15.4	
Switzerland	2000-2004	4	84.4	2003	Vital Registration	100.0	15.6	
Switzerland	2005-2009	5	86.6	2008	Vital Registration	99.8	13.3	
Switzerland	2010-2016	5	86.1	2010	Vital Registration	100.0	13.9	
Syria	1980-1984	2	29.2	1980	Vital Registration	100.0	70.8	
Syria	1985-1989	2	15.8	1985	Vital Registration	79.2	80.0	
Syria	1990-1994	0						
Syria	1995-1999	3	54.5	1999	Vital Registration	100.0	45.5	
Syria	2000-2004	3	59.2	2004	Vital Registration	86.9	31.9	
Syria	2005-2009	4	70.0	2009	Vital Registration	93.7	25.3	
Syria	2010-2016	3	59.6	2010	Vital Registration	99.8	40.2	
Taiwan (province of China)	1980-1984	0						
Taiwan (province of China)	1985-1989	0						
Taiwan (province of China)	1990-1994	3	37.2	1994	Vital Registration	95.6	61.0	
Taiwan (province of China)	1995-1999	3	37.3	1995	Vital Registration	96.8	61.5	
Taiwan (province of China)	2000-2004	3	39.4	2004	Vital Registration	98.6	60.1	
Taiwan (province of China)	2005-2009	4	83.9	2007	Vital Registration	99.1	15.3	
Taiwan (province of China)	2010-2016	4	84.5	2014	Vital Registration	99.6	15.1	
Tajikistan	1980-1984	4	67.1	1981	Vital Registration	80.5	16.7	
Tajikistan	1985-1989	3	61.0	1988	Vital Registration	80.2	23.9	
Tajikistan	1990-1994	4	68.8	1993	Vital Registration	100.0	31.2	
Tajikistan	1995-1999	3	53.7	1995	Vital Registration	76.6	29.9	
Tajikistan	2000-2004	3	46.4	2002	Vital Registration	67.8	31.6	
Tajikistan	2005-2009	3	47.7	2005	Vital Registration	72.1	33.9	
Tajikistan	2010-2016	0						
Tanzania	1980-1984	0						
Tanzania	1985-1989	1	3.1	1986	Risk factors for deaths in children under 5 years old in Bagamoyo district, Tanzania		20.7	3.9
Tanzania	1990-1994	1	1.9	1993	Community based studies on childhood mortality in a malaria holoendemic area on the Tanzanian coast		46.6	3.5
Tanzania	1995-1999	1	1.8	1995	The Policy Implications of Tanzania's Mortality Burden		71.1	6.4
Tanzania	2000-2004	1	4.9	2001	Tanzania - Ifakara Health and Demographic Surveillance System		22.9	6.4
Tanzania	2005-2009	1	2.6	2005	The contribution of reduction in malaria as a cause of rapid decline of under-five mortality: evidence from the Rufiji Health and Demographic Surveillance System (HDSS) in rural Tanzania		6.7	2.8
Tanzania	2010-2016	0						
Thailand	1980-1984	2	28.4	1980	Vital Registration	75.5	62.4	
Thailand	1985-1989	2	27.1	1987	Vital Registration	76.0	64.4	
Thailand	1990-1994	2	33.9	1994	Vital Registration	83.5	59.3	
Thailand	1995-1999	3	47.7	1997	Thailand Burden of Disease and Injuries 1998-1999		25.4	64.0
Thailand	2000-2004	3	47.7	2003	Vital Registration	92.3	48.3	
Thailand	2005-2009	3	52.0	2005	Thailand Verbal Autopsy Study 2005		18.7	64.0
Thailand	2010-2016	3	57.5	2014	Vital Registration	97.2	40.8	
The Bahamas	1980-1984	4	74.6	1980	Vital Registration	100.0	25.4	
The Bahamas	1985-1989	4	79.7	1987	Vital Registration	99.7	20.1	
The Bahamas	1990-1994	3	63.8	1994	Vital Registration	96.0	33.5	
The Bahamas	1995-1999	4	78.0	1999	Vital Registration	87.0	10.3	
The Bahamas	2000-2004	4	80.2	2001	Vital Registration	91.8	12.6	
The Bahamas	2005-2009	4	79.8	2009	Vital Registration	91.9	13.2	
The Bahamas	2010-2016	4	77.6	2011	Vital Registration	89.7	13.4	
The Gambia	1980-1984	1	3.2	1982	Deaths in infancy and early childhood in a well-vaccinated, rural, West African population		20.7	4.1
The Gambia	1985-1989	1	2.6	1989	Changes in the pattern of infant and childhood mortality in upper river division, The Gambia, from 1989 to 1993		29.4	3.6
The Gambia	1990-1994	1	2.5	1991	Changes in the pattern of infant and childhood mortality in upper river division, The Gambia, from 1989 to 1993		30.2	3.6
The Gambia	1995-1999	1	1.1	1999	Reaching millennium development goal 4 - the Gambia		65.1	3.3
The Gambia	2000-2004	1	0.9	2002	Reaching millennium development goal 4 - the Gambia		70.4	3.1

Appendix Table 17. Underlying indicators for percent well-certified for data source with maximum percent well certified in each 5-year time interval for 195 countries, 1980-2016.

Location	Time Window	Stars	Percent Well-Certified [PWC] (%)	Max PWC Data Year	Max PWC Data Source	Completeness (%)	Percent Major Garbage (%)	Verbal Autopsy Adjustment (None for VR) (%)
The Gambia	2005-2009	1	1.3	2006	Preventive measures in infancy to reduce under-five mortality: a case-control study in The Gambia		56.0	2.9
The Gambia	2010-2016	0						
Timor-Leste	1980-1984	0						
Timor-Leste	1985-1989	0						
Timor-Leste	1990-1994	0						
Timor-Leste	1995-1999	0						
Timor-Leste	2000-2004	0						
Timor-Leste	2005-2009	0						
Timor-Leste	2010-2016	0						
Togo	1980-1984	0						
Togo	1985-1989	0						
Togo	1990-1994	0						
Togo	1995-1999	0						
Togo	2000-2004	0						
Togo	2005-2009	0						
Togo	2010-2016	0						
Tonga	1980-1984	0						
Tonga	1985-1989	0						
Tonga	1990-1994	0						
Tonga	1995-1999	0						
Tonga	2000-2004	3	53.6	2003	Vital Registration	100.0	46.4	
Tonga	2005-2009	0						
Tonga	2010-2016	0						
Trinidad and Tobago	1980-1984	4	79.2	1982	Vital Registration	96.9	18.3	
Trinidad and Tobago	1985-1989	4	80.3	1987	Vital Registration	96.9	17.2	
Trinidad and Tobago	1990-1994	4	81.4	1994	Vital Registration	100.0	18.6	
Trinidad and Tobago	1995-1999	5	89.6	1999	Vital Registration	100.0	10.4	
Trinidad and Tobago	2000-2004	5	90.5	2003	Vital Registration	100.0	9.5	
Trinidad and Tobago	2005-2009	5	89.6	2005	Vital Registration	100.0	10.4	
Trinidad and Tobago	2010-2016	5	89.0	2010	Vital Registration	100.0	11.0	
Tunisia	1980-1984	0						
Tunisia	1985-1989	0						
Tunisia	1990-1994	0						
Tunisia	1995-1999	0						
Tunisia	2000-2004	0						
Tunisia	2005-2009	2	28.8	2009	Vital Registration	38.7	25.7	
Tunisia	2010-2016	2	24.7	2013	Vital Registration	38.0	35.0	
Turkey	1980-1984	2	16.9	1984	Vital Registration	38.0	55.6	
Turkey	1985-1989	2	20.7	1989	Vital Registration	45.2	54.2	
Turkey	1990-1994	2	22.1	1993	Vital Registration	48.1	54.1	
Turkey	1995-1999	2	24.9	1998	Vital Registration	55.1	54.9	
Turkey	2000-2004	3	37.4	2002	Turkey Verbal Autopsy Survey 2003		41.5	64.0
Turkey	2005-2009	4	72.8	2009	Vital Registration	90.9	19.9	
Turkey	2010-2016	4	84.4	2013	Vital Registration	99.7	15.4	
Turkmenistan	1980-1984	4	83.9	1981	Vital Registration	95.6	12.2	
Turkmenistan	1985-1989	5	86.0	1986	Vital Registration	98.5	12.6	
Turkmenistan	1990-1994	4	79.7	1993	Vital Registration	95.4	16.5	
Turkmenistan	1995-1999	4	74.1	1995	Vital Registration	92.4	19.8	
Turkmenistan	2000-2004	4	65.5	2003	Vital Registration	77.1	15.1	
Turkmenistan	2005-2009	4	66.8	2005	Vital Registration	80.3	16.9	
Turkmenistan	2010-2016	4	70.6	2014	Vital Registration	93.0	24.1	
Uganda	1980-1984	0						
Uganda	1985-1989	0						
Uganda	1990-1994	0						
Uganda	1995-1999	0						
Uganda	2000-2004	1	0.0	2000	Effect of HIV infection on pregnancy-related mortality in sub-Saharan Africa: secondary analyses of pooled community-based data from the network for Analysing Longitudinal Population-based HIV/AIDS data on Africa (ALPHA)		95.7	0.4
Uganda	2005-2009	1	2.7	2006	Uganda Child Verbal Autopsy Study 2007		12.5	3.1
Uganda	2010-2016	0						
Ukraine	1980-1984	4	84.7	1981	Vital Registration	98.2	13.7	
Ukraine	1985-1989	5	87.8	1985	Vital Registration	100.0	12.2	
Ukraine	1990-1994	4	81.0	1994	Vital Registration	100.0	19.0	
Ukraine	1995-1999	4	83.5	1999	Vital Registration	100.0	16.5	
Ukraine	2000-2004	4	83.8	2000	Vital Registration	100.0	16.2	
Ukraine	2005-2009	5	89.0	2009	Vital Registration	100.0	11.0	
Ukraine	2010-2016	5	90.4	2012	Vital Registration	100.0	9.6	
United Arab Emirates	1980-1984	0						
United Arab Emirates	1985-1989	0						
United Arab Emirates	1990-1994	0						
United Arab Emirates	1995-1999	0						
United Arab Emirates	2000-2004	0						
United Arab Emirates	2005-2009	3	36.5	2007	Vital Registration	71.9	49.3	
United Arab Emirates	2010-2016	0						
United Kingdom	1980-1984	5	93.1	1983	Vital Registration	99.5	6.4	
United Kingdom	1985-1989	5	93.9	1985	Vital Registration	100.0	6.1	
United Kingdom	1990-1994	5	93.9	1991	Vital Registration	99.9	6.1	
United Kingdom	1995-1999	5	91.9	1995	Vital Registration	100.0	8.1	
United Kingdom	2000-2004	5	91.4	2002	Vital Registration	100.0	8.6	
United Kingdom	2005-2009	5	91.4	2007	Vital Registration	100.0	8.6	
United Kingdom	2010-2016	5	91.3	2010	Vital Registration	99.9	8.6	
United States	1980-1984	5	90.3	1980	Vital Registration	100.0	9.7	
United States	1985-1989	5	89.0	1989	Vital Registration	100.0	11.0	
United States	1990-1994	5	89.5	1990	Vital Registration	100.0	10.5	
United States	1995-1999	5	88.8	1995	Vital Registration	100.0	11.2	
United States	2000-2004	5	88.0	2000	Vital Registration	100.0	12.0	
United States	2005-2009	5	87.3	2005	Vital Registration	100.0	12.7	
United States	2010-2016	5	86.9	2010	Vital Registration	100.0	13.1	
Uruguay	1980-1984	4	76.3	1980	Vital Registration	100.0	23.7	
Uruguay	1985-1989	4	75.6	1989	Vital Registration	99.6	24.1	
Uruguay	1990-1994	4	77.2	1991	Vital Registration	98.1	21.3	
Uruguay	1995-1999	4	79.1	1998	Vital Registration	100.0	20.9	
Uruguay	2000-2004	4	79.2	2001	Vital Registration	99.5	20.4	
Uruguay	2005-2009	4	78.6	2005	Vital Registration	100.0	21.4	
Uruguay	2010-2016	4	75.7	2014	Vital Registration	99.0	23.5	
Uzbekistan	1980-1984	4	82.6	1982	Vital Registration	97.8	15.5	
Uzbekistan	1985-1989	5	85.2	1985	Vital Registration	99.7	14.5	
Uzbekistan	1990-1994	4	80.0	1992	Vital Registration	95.0	15.7	
Uzbekistan	1995-1999	4	72.1	1995	Vital Registration	87.3	17.5	
Uzbekistan	2000-2004	3	61.1	2000	Vital Registration	75.7	19.4	
Uzbekistan	2005-2009	3	63.0	2005	Vital Registration	70.9	11.2	
Uzbekistan	2010-2016	4	65.3	2011	Vital Registration	72.2	9.6	
Vanuatu	1980-1984	0						
Vanuatu	1985-1989	0						
Vanuatu	1990-1994	0						
Vanuatu	1995-1999	0						
Vanuatu	2000-2004	0						
Vanuatu	2005-2009	0						
Vanuatu	2010-2016	0						
Venezuela	1980-1984	4	79.2	1983	Vital Registration	98.7	19.7	
Venezuela	1985-1989	4	74.3	1988	Vital Registration	100.0	25.7	
Venezuela	1990-1994	4	81.9	1994	Vital Registration	100.0	18.1	
Venezuela	1995-1999	5	87.8	1999	Vital Registration	100.0	12.2	
Venezuela	2000-2004	5	89.9	2001	Vital Registration	100.0	10.1	
Venezuela	2005-2009	5	89.5	2005	Vital Registration	100.0	10.5	
Venezuela	2010-2016	5	89.0	2013	Vital Registration	100.0	11.0	
Vietnam	1980-1984	0						
Vietnam	1985-1989	1	0.5	1987	Are there social inequities in child morbidity and mortality in rural Vietnam		52.4	1.1

Appendix Table 17. Underlying indicators for percent well-certified for data source with maximum percent well certified in each 5-year time interval for 195 countries, 1980-2016.

Location	Time Window	Stars	Percent Well-Certified [PWC] (%)	Max PWC Data Year	Max PWC Data Source	Completeness (%)	Percent Major Garbage (%)	Verbal Autopsy Adjustment (None for VR) (%)
Vietnam	1990-1994	1	0.1	1994	Maternal mortality in Vietnam in 1994-95		91.9	1.0
Vietnam	1995-1999	1	0.4	1999	Applying verbal autopsy to determine cause of death in rural Vietnam		93.1	6.4
Vietnam	2000-2004	0						
Vietnam	2005-2009	3	44.1	2008	Mortality measures from sample-based surveillance: evidence of the epidemiological transition in Viet Nam, Unpublished data		31.1	64.0
Vietnam	2010-2016	1	3.4	2010	The causes of deaths in Chililab between 2008-2010 based on verbal autopsy method		47.3	6.4
Virgin Islands, U.S.	1980-1984	4	73.2	1980	Vital Registration	95.1	23.1	
Virgin Islands, U.S.	1985-1989	0						
Virgin Islands, U.S.	1990-1994	4	81.6	1994	Vital Registration	90.2	9.6	
Virgin Islands, U.S.	1995-1999	4	84.9	1995	Vital Registration	94.0	9.7	
Virgin Islands, U.S.	2000-2004	4	72.0	2000	Vital Registration	80.8	10.9	
Virgin Islands, U.S.	2005-2009	4	67.9	2007	Vital Registration	75.0	9.4	
Virgin Islands, U.S.	2010-2016	3	60.5	2010	Vital Registration	70.3	13.8	
Yemen	1980-1984	0						
Yemen	1985-1989	0						
Yemen	1990-1994	0						
Yemen	1995-1999	0						
Yemen	2000-2004	0						
Yemen	2005-2009	0						
Yemen	2010-2016	0						
Zambia	1980-1984	0						
Zambia	1985-1989	0						
Zambia	1990-1994	0						
Zambia	1995-1999	0						
Zambia	2000-2004	0						
Zambia	2005-2009	1	5.4	2009	Zambia Sample Vital Registration with Verbal Autopsy (SAVVY) Data 2010		15.6	6.4
Zambia	2010-2016	1	5.5	2010	Zambia Sample Vital Registration with Verbal Autopsy (SAVVY) Data 2010		14.0	6.4
Zimbabwe	1980-1984	0						
Zimbabwe	1985-1989	0						
Zimbabwe	1990-1994	2	32.5	1990	Vital Registration	43.2	24.7	
Zimbabwe	1995-1999	3	35.3	1995	Vital Registration	58.2	39.4	
Zimbabwe	2000-2004	1	0.0	2000	Effect of HIV infection on pregnancy-related mortality in sub-Saharan Africa: secondary analyses of pooled community-based data from the network for Analysing Longitudinal Population-based HIV/AIDS data on Africa (ALPHA)		96.6	0.6
Zimbabwe	2005-2009	2	23.8	2007	Vital Registration	29.6	19.7	
Zimbabwe	2010-2016	0						

Appendix Table 18. Theoretical Minimum Risk Reference Life Table

Age	Life Expectancy
0	86.6
1	85.8
5	81.8
10	76.8
15	71.9
20	66.9
25	62.0
30	57.0
35	52.1
40	47.2
45	42.4
50	37.6
55	32.9
60	28.3
65	23.8
70	19.4
75	15.3
80	11.5
85	8.2
90	5.5
95	3.7
100	2.6
105	1.6
110	1.4

Appendix Table 19. GBD world population age standard		
**Age**	**Percent of Population**	**Rounded**
ENN	0.035667	0.04
LNN	0.106232	0.11
PNN	1.687834	1.69
0-1	1.829733	1.83
1-4	7.182279	7.18
5-9	8.692810	8.69
10-14	8.395204	8.40
15-19	8.098376	8.10
20-24	7.814477	7.81
25-29	7.559828	7.56
30-34	7.248519	7.25
35-39	6.855877	6.86
40-44	6.384231	6.38
45-49	5.849079	5.85
50-54	5.270976	5.27
55-59	4.678016	4.68
60-64	4.057540	4.06
65-69	3.359068	3.36
70-74	2.629266	2.63
75-79	1.896681	1.90
80-84	1.213480	1.21
85-89	0.644468	0.64
90-94	0.257603	0.26
95+	0.082490	0.08
